# A supervised Bayesian method for time (re)annotation of transcriptomics data

**DOI:** 10.1093/nargab/lqaf203

**Published:** 2025-12-31

**Authors:** Elio Nushi, François P Douillard, Katja Selby, Benjamin A Blount, Oliver J Pennington, Nigel P Minton, Miia Lindström, Antti Honkela

**Affiliations:** Department of Computer Science, Faculty of Science, University of Helsinki, 00560, Helsinki, Finland; Department of Food Hygiene and Environmental Health, Faculty of Veterinary Medicine, University of Helsinki, 00790, Helsinki, Finland; Department of Food Hygiene and Environmental Health, Faculty of Veterinary Medicine, University of Helsinki, 00790, Helsinki, Finland; School of Life Sciences, University of Nottingham, NG7 2RD, Nottingham, United Kingdom; BBSRC/EPSRC Synthetic Biology Research Centre (SBRC), University of Nottingham, NG7 2RD, Nottingham, United Kingdom; BBSRC/EPSRC Synthetic Biology Research Centre (SBRC), University of Nottingham, NG7 2RD, Nottingham, United Kingdom; Department of Food Hygiene and Environmental Health, Faculty of Veterinary Medicine, University of Helsinki, 00790, Helsinki, Finland; Department of Computer Science, Faculty of Science, University of Helsinki, 00560, Helsinki, Finland

## Abstract

Transcriptomics experiments are often conducted to capture changes in gene expression over time. However, time annotations may be missing, imprecise, or not reflect the same physiological state of the bacterial culture between different experiments. Assigning accurate time points to these experiments using a reference time course is therefore crucial for identifying differentially expressed genes, and understanding gene regulatory networks for elucidating the studied organism’s physiology and life cycle. This important task, which could enhance the biological interpretation of the transcriptomics experiments, has not been previously addressed. In this work, we propose a novel method to solve the challenge of realigning transcriptomics experiments based on a reference time course. Our method is based on a Bayesian approach that uses Gaussian process regression modeling. We show a use case of applying our method for assigning time annotations in legacy microarray samples of the bacterium *Clostridium botulinum*, which were solely annotated based on the growth phase at the time when the culture aliquots were sampled, utilizing recently collected RNA-Seq time series data comprising multiple replicates as a reference. The method significantly improved the description of the growth phases of the microarray data compared to the original annotations by clearly delineating the microarray samples belonging to different growth phases, as demonstrated by principal component analysis. Consequently, a larger number of differentially expressed genes was detected when comparing experiments belonging to successive growth phases. We compare this innovative approach with a baseline method that uses k-nearest neighbor algorithm and show that our method offers a higher resolution in the description of the data by exposing smaller time changes between samples. We also test the performance of the method on sparse RNA-Seq time series (i.e. sampled every second hour). All the predictions for the samples were within a 30-min margin of their true time.

## Introduction

Time-course transcriptomics experiments have been widely used to explore essential biological processes that exhibit dynamic characteristics such as the cell cycle [1], circadian rhythms [2], and developmental processes [3]. However, collecting time series of high-quality RNA measurements is a financially and time-expensive process. On the other hand, there is a large number of historical transcriptomics experiments (i.e. microarray samples) that have already been performed [4], but their sampling time cannot be directly aligned for reasons such as the differing of lag phase length and changes in inoculum size, for instance. Thus, their use for different comparison analyses is nontrivial. Being able to enrich these experiments with biologically consistent time annotations would increase the number of available transcriptomics time series without additional costs. They can then be used together to complement each other as well as to ensure a stronger statistical significance of the results obtained as compared to using only RNA-Seq data [5]. In a realm where transcriptomics experiments abound, the pressing challenge of inferring precise time points in the absence of established annotations has remained largely uncharted. This crucial task, essential for unraveling differentially expressed genes and comprehending intricate gene regulatory networks, has, until now, eluded comprehensive solutions.

Here, we present a statistical method based on a Bayesian approach to address the time annotation challenge. It can be used to assign time annotations to target omics experiments that are represented as sets of genome-wide values by using reference omics experiments in the form of genome-wide time series. The method can be utilized to annotate historical data archived in repositories, helping to gain a better understanding of the organism under study by uncovering biological pathways, for example, through differential gene expression analysis. Additionally, our approach offers a measure of uncertainty associated with each time annotation assignment.

**Figure 1.**
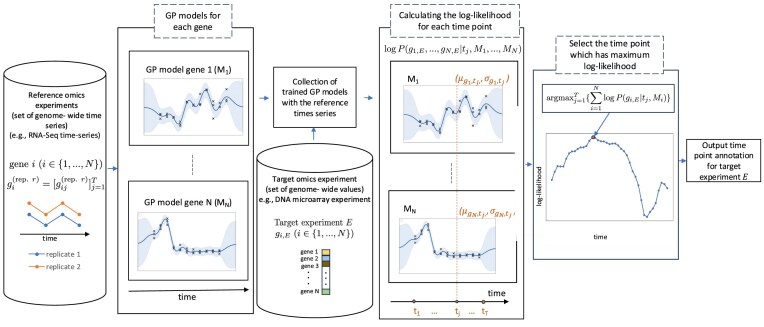
A generic overview of the time annotating method that ends in time annotation. Annotating target omics experiments (e.g. microarray samples) using reference time series (e.g. RNA-Seq samples), process diagram.

In order to make time point predictions for the target experiments, we use Gaussian processes (GPs) to model the behavior of each gene with respect to the time line determined by the reference data. We build the GP models on the basis of the reference data and combine them together through the independence assumption. Then, we model the uncertainty that corresponds to the likelihood that a given target experiment could have been sampled at any chosen time point within the time interval when measurements in the reference time series took place, even at those time points for which no training data exist. Figure 1 gives a general overview of the process that describes our method.

### Related methods

Time-annotated transcriptomics data can provide useful information to understand the dynamic regulation and function of cells [6], and many studies have been performed to reconstruct their temporal structure [7–10]. Magwene *et al.* [7] devised an unsupervised method for ordering bulk microarray samples by knowing the first or the last sample. Trapnell *et al.* [8] extended the former method to accommodate for single-cell variation and to allow for multiple cell fates deriving from a single progenitor cell type. Their unsupervised algorithm is able to reconstruct the sequence of transcriptional changes experienced by cells as they differentiate. Thus, it orders a heterogeneous and unsynchronized group of cells based on their advancement through the acquired knowledge of the differentiation process. Reid and Wernisch [9] presented a principled probabilistic model with an unsupervised Bayesian inference scheme for assigning pseudotime to single-cell time series. In the study, the authors defined pseudotime as a latent dimension that measures the cells’ progress through transitions from state to state. Unlike the previously mentioned methods that estimate orderings of RNA-Seq samples, it explicitly estimates pseudotimes that are on the same scale as the capture time of the experiments, given a set of transcriptomics samples (e.g. single cell RNA-seq). In another study, Campbell and Yau [10] introduced a statistical framework for uncovering pseudotemporal trajectories for both single cell and bulk expression data. Their method accepts gene expression data representing different transcriptomics experiments and maps them to a series of one-dimensional quantities that they call pseudotimes.

Our method differs from the previous works because it uses reference transcriptomics time series to learn the temporal structure of every gene. Given a new experiment, it makes predictions about the actual laboratory time when the experiment was sampled relative to the reference, by providing sensible uncertainties for the predictions. By leveraging a reference time series, our method can more accurately capture the temporal dynamics of the transcriptomics data. The reference time series provides a structured framework that guides the learning process, allowing the model to make more precise predictions about the timing of events in the target data. Additionally, it improves the interpretability of the results by anchoring the analysis to a known temporal structure, making time annotations easier to understand within existing biological knowledge. Thus, it can be used to improve the time annotations of older omics datasets or correct inaccuracies associated with growth phase assignment in the case of transcriptomics data, for instance.

### Contributions

We make four main contributions:

1. **A Bayesian time annotation algorithm** that fits GPs to reference transcriptomics time series and transfers the learned temporal structure to un-timed target data (the “Materials and methods” section).
2. **An integrated Python pipeline** publicly available as described in the “Data availability” section.
3. **A thorough empirical validation** on three scenarios: (i) a hold-out RNA-seq test set mimicking sparse sampling (the “Sparse RNA-Seq hold-out” section), (ii) synthetic noisy data (the “Sensitivity to noise” section), and (iii) a case study re-annotating 80 legacy microarrays from bacterial growth experiments (the “Time re-annotation of microarray samples: a case study” section).
4. **Biological insights** gained after temporal recalibration—e.g. increased detection of differentially expressed genes (the “Sensitivity to noise” section).

#### Road-map

The “Materials and methods” section details the modeling and implementation; the “Data” section introduces all datasets and their pre/post-processing; the “Validation” section validates the method on held-out and synthetic data; the “Time re-annotation of microarray samples: a case study” section presents the microarray case study; and the “Discussion” section discusses results and limitations.

## Materials and methods

Our time annotation method consists of three procedures. First, raw expression profiles are filtered and normalized (the “Filtering and normalization of transcriptomics data” section). Second, each gene is modeled as a GP whose kernel captures temporal dynamics and provides a mechanism for performing time annotation (the “Gaussian process analysis” section). Third, genes whose data are poorly explained by any GP are discarded (the “Model selection and quality control” section). We implement the three steps in an end-to-end Python pipeline described in the “Data availability” section.

### Filtering and normalization of transcriptomics data

Transcriptomics technologies measure the quantity of mRNA and use it to estimate the level of gene expression. However, there is no unique way of quantifying the level of gene expression, and plain comparison between gene expression values coming from different experiments could yield incorrect results. There is often no explicit method for transforming gene expression values to make them comparable, especially when they come from different RNA measurement technologies such as RNA-Seq and DNA microarray. Furthermore, many comparative studies have shown that they can be inconsistent for individual genes [11].

In this work, we took a simple approach to filter and normalize the data in order to increase their comparability. We focused our analysis on those genes that have a maximum value above a certain threshold. The value of the threshold depends on the specifics of the data that we work with. The purpose of this kind of filtering is to remove those genes whose signal-to-noise ratio is very small, because they are likely to contribute more noise than signal in the analysis. Then we log-transformed the data to reduce the difference between extreme values. Subsequently, we apply median-polish normalization [12]. Median polish procedure consists on first finding and then subtracting the median from each experiment and then repeating the same procedure for every gene across different experiments. This procedure is repeated until the proportional reduction in the sum of absolute residuals is less than a value ($\epsilon >0$). This helps to bring the data on the same scale. Below, we define formally the normalization procedure described previously:

Let $G \in \mathbb {R}^{N \times K}$ be a matrix representing the transcriptomics data consisting of $N$ genes and $K$ different measurements for those genes, the procedure is summarized in Algorithm 1.

**Algorithm 1:**
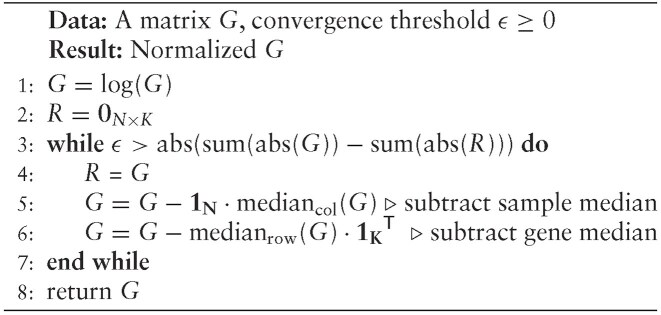


**Figure 2.**
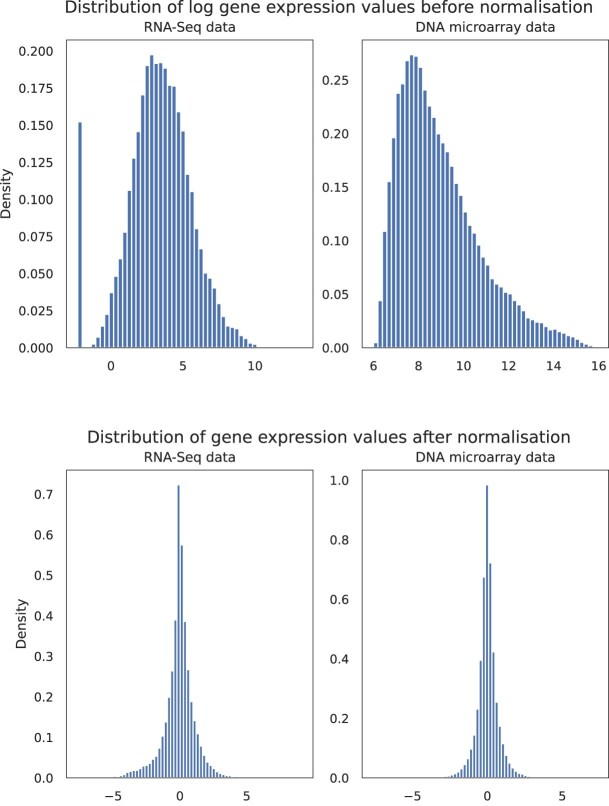
Distribution of transcriptomics values before and after the median normalization.

This approach not only standardizes the data values to a uniform scale but at the same time preserves their original structure as shown in Fig. 2. Thus, it makes them suitable to use for the purpose of our method.

### Gaussian process analysis

GPs are nonparametric methods that are used for building probabilistic models of functional data [13]. In many previous studies, GPs have been widely recognized and adopted for modeling transcriptomics data [6, 9, 14–19]. Below, we are giving the formal definition of a GP:

Definition 1. A GP is a collection of random variables, any finite number of which have a joint Gaussian distribution.

A GP $f(t)$ is completely characterized by a mean function that we write as $m(t)$ and a covariance function $k(t,t^{\prime })$ that is often referred to as the kernel of the GP. We formally write it as follows:


(1) \begin{eqnarray*}
f(t) \sim \mathcal {GP}(m(t), k(t,t^{\prime })).
\end{eqnarray*}

The mean function is assumed to be zero for simplification purposes, thus causing the GP to depend only on the choice of the kernel function. Choosing the kernel function is an important step in the process of modeling with GPs as it determines our assumptions about the function that we want our model to be represented by. The most widely used kernel function for modeling with GPs that we also use in our analysis is the exponentiated quadratic function [13], which is defined as follows:


(2) \begin{eqnarray*}
K_{EQ}(t,t^{\prime }) = \sigma _{f}^{2} ~ \exp \left(- \frac{ \left\Vert t-t^{\prime } \right\Vert ^{2} }{ 2l^{2} }\right).
\end{eqnarray*}

As we can see, the kernel function depends on two parameters: the length-scale $l$ and the variance $\sigma _{f}^{2}$. We will also consider a third parameter $\sigma _n^2$ , which is the Gaussian noise and represents the observation noise in our data. Given a set of inputs $\mathbf {t}$ and a set of outputs $\mathbf {y}$, when modeling with a GP, we try to select the optimal triple $(l,\sigma _f^2,\sigma _n^2)$ that maximizes the marginal likelihood $P(\mathbf {y}|\mathbf {t},l,\sigma _f^2,\sigma _n^2)$ or equivalently maximizes the log marginal likelihood:


(3) \begin{eqnarray*}
\log P(\mathbf {y}|\mathbf {t}) &=& -\frac{1}{2}(\mathbf {y} - m(\mathbf {t}))^T(K(\mathbf {t},\mathbf {t})+\sigma ^2_n I)^{-1}(\mathbf {y} - m(\mathbf {t})) \\&&- \frac{1}{2}\log |K(\mathbf {t},\mathbf {t})+\sigma ^2_n I|-\frac{n}{2}\log 2\pi .
\end{eqnarray*}

We can then estimate the likelihood of $y_*$ at a point $t_*$ in the following way:


(4) \begin{eqnarray*}
P(y_*|t_*)=\frac{1}{\sigma _*\sqrt{2\pi }}\exp \left( -\frac{1}{2}\left(\frac{y_*-\mu _*}{\sigma _*}\right)^{\!2}\, \right),
\end{eqnarray*}

where $\mu _*$ and $\sigma _*$ are the posterior mean and posterior variance at $t_*$ given by


(5) \begin{eqnarray*}
\mu _*= m(t_*) + K(t_*,\mathbf {t})(K(\mathbf {t},\mathbf {t}) + \sigma _n^2I)^{-1}(\mathbf {y} - m(\mathbf {t})),
\end{eqnarray*}

and


(6) \begin{eqnarray*}
\sigma _*^2=K(t_*,t_*)-K(t_*,\mathbf {t})(K(\mathbf {t},\mathbf {t}) +\sigma _n^2I)^{-1}K(t_*,\mathbf {t})^{T}.
\end{eqnarray*}

#### Modeling multiple replicates

Gene expression time series can be collected in multiple replicates to take in consideration the biological variation and measurement errors [14]. In the case of a dataset consisting of $N$ genes and $R$ replicates for each of the genes, which are observed at different time points, the data will be in the form


\begin{eqnarray*}
({t_{j}^{}}, g_{ij}^{(r)}), \: r \in \lbrace 1,\dots ,R\rbrace , \: i \in \lbrace 1,\dots ,N\rbrace , \: {\rm and} \: j \in \lbrace 1,\dots ,T\rbrace ,
\end{eqnarray*}

where $T$ is the number of observations for each gene replicate, $t_j$ is a time point for which an observation about a gene replicate exists, and $g_{ij}^{(r)}$ is the corresponding gene expression value. However, the data do not need to be complete (i.e. not all replicates of a gene must be fully observed at all time points), as the GP can easily handle missing entries. We denote by $f_i(t)$ the latent function that models the gene expression values of gene $g_i$ at every observed time point $t_j$ and replicate $r$. We can thus define a regression problem over functions $f_{i}: {t} \mapsto f_{i}({t})$, the values of which can be samples from the following model:


(7) \begin{eqnarray*}
f_{i}(t) \sim \mathcal {GP}(\mathbf {0}, k_{EQ}(t,t^{\prime })).
\end{eqnarray*}

We denote by $\mu _i=[f_i(t_j)]_{j=1}^{T}$ the vector of values of $f_i(t)$ at the observed time points and let $g_i^{(r)}=[g_{ij}^{(r)}]_{j=1}^{T}$ be the vector of the observed values of $g_i$ in replicate $r$ at the respective time points. We can define the values of $g_i$ as noisy observations of $f_i(t)$ and sample them from the following conditional distribution:


(8) \begin{eqnarray*}
g_{i}^{(r)} | f_{i}(t) \sim N(\mu _{i}, \sigma _{n_i}^{2}\mathbb {I}),
\end{eqnarray*}

where $\sigma _{n_{i}}^{2}$ represents the noise variance between the replicates of gene $g_i$.

We can marginalize out $f_{i}(t)$ and write the model conditioned on the mean of the GP in Equation (7). In the marginalized model, the covariance between two observations $g_{ij}^{(r)}$ and $g_{ij^{\prime }}^{(r^{\prime })}$ will be


(9) \begin{eqnarray*}
\mathrm{Cov}[g_{ij}^{(r)},g_{ij^{\prime }}^{(r^{\prime })}]= K_{EQ}(t_j,t_{j^{\prime }}) + \sigma _{n_i}^{2}\delta (t_j=t_{j^{\prime }})\delta (r=r^{\prime }),
\end{eqnarray*}

where $\delta$ is the Kronecker delta. If we denote by $\mathbf {t}$ the concatenation of the observed time points in all replicates, we can generalize the generative model of the observations of gene $g_i$ on any replicate $r$ and write it as


(10) \begin{eqnarray*}
{\begin{bmatrix}g_{i}^{(1)}\\\vdots \\g_{i}^{(R)} \end{bmatrix}} | \theta \sim \mathcal {N}\left(\mathbf {0}, K\right),
\end{eqnarray*}

where $\theta$ is the set of GP hyperparameters in Equation (7) and $K$ is the covariance matrix obtained by applying the covariance function in Equation (9) on the grid $\mathbf {t}$.

#### Inferring the time of an experiment

Let $\lbrace g_{1,E},g_{2,E},\dots ,g_{N,E}\rbrace$ be gene expression values at a new experiment $E$, and $M_1,M_2,\dots ,M_{N}$ be their GP fitted models fitted on reference time series. In order to select the most probable time point of $E$, for every gene value $g_{i,E}$ we derive a likelihood function over time using its respective GP posterior


(11) \begin{eqnarray*}
\mathcal {L}_{i}(t) = P(g_{i,E}|t,M_i), i \in \lbrace 1,\dots ,N\rbrace .
\end{eqnarray*}

The likelihood of $E$ over time will then be given by


(12) \begin{eqnarray*}
\prod _{i=1}^{N} \mathcal {L}_{i}(t),
\end{eqnarray*}

and we assign the most probable time to $E$ by solving the following optimization problem, which maximizes the likelihood of $E$ over $t$:


(13) \begin{eqnarray*}
\max \limits _{t} \prod _{i=1}^{N} \mathcal {L}_{i}(t).
\end{eqnarray*}

To solve the problem in practice, we restrict the optimization to a discrete grid $\lbrace t_1,t_2,\dots ,t_T\rbrace$. First, we calculate the log-likelihoods


(14) \begin{eqnarray*}
&&\log P(g_{1,E},\dots , g_{N,E}|t_j,M_1,\dots ,M_N)\\&=& \sum _{i=1}^{N} \log P(g_{i,E}|t_j,M_i), j \in \lbrace 1,\dots ,T\rbrace .
\end{eqnarray*}

Then, we assign the most probable time point by solving


(15) \begin{eqnarray*}
\mathrm{argmax}_{j=1}^{T} \left\lbrace \sum _{i=1}^{N} \log P(g_{i,E} |t_j, M_i)\right \rbrace .
\end{eqnarray*}

#### Implementation of GP models

**Figure 3.**
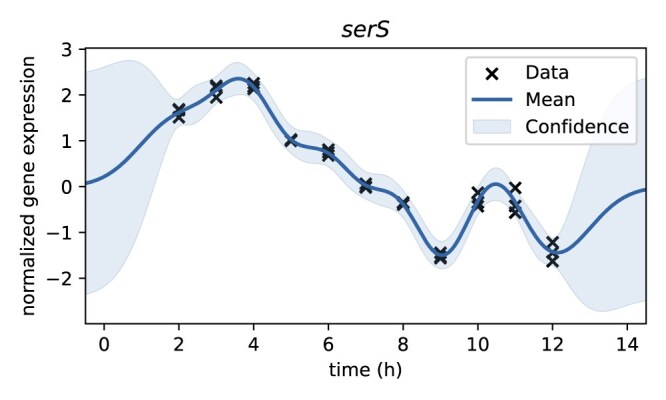
GP plot obtained by fitting the RNA-Seq time series data corresponding to gene *serS*, the blue line is the posterior mean and the light blue region represents the 95% confidence region based on the posterior variance and Gaussian noise. Crosses correspond to the normalized values of the gene for each replicate on different time points.

We used the Python library GPy [20] to build a separate GP model for every gene by following the model described in Equations (7)–(10). Figure 3 gives a visual representation of the GP model for one of the genes.

We used the GP models to generate log-probability distributions $\log P(g_{i,E}|t_j,M_i)$ corresponding to the log-likelihood that the gene $g_i$ , which follows the model $M_i$ can have value $g_{i,E}$ during time $t_j$ as in Equation (14). We calculated the log-likelihoods at equally spaced consecutive time points within the time interval of the training data. After applying this procedure for all the genes in a microarray sample, the most probable time point of the sample was deduced by choosing the one that has the maximum sum of log-likelihoods over all the gene expression values belonging to that sample as shown in Equation (15).

**Figure 4.**
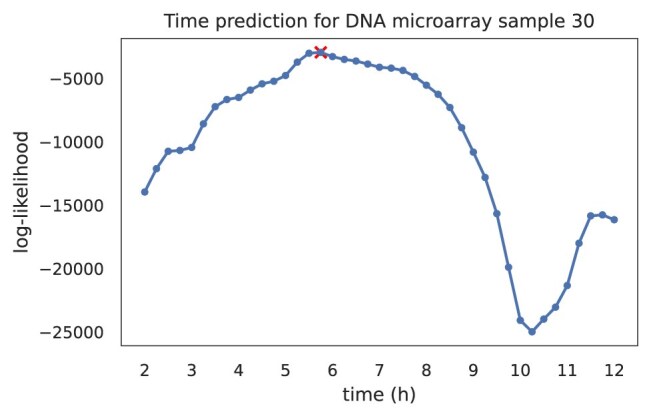
Log-likelihood curve of microarray sample 30 (whose legacy growth phase annotation is mid-exponential phase) for selected time points. The maximum is reached at 5.75 h (marked with cross).

Figure 4 illustrates the log-likelihood curve for one microarray sample, which has its maximum sum over the log-likelihoods of all the gene values at 5.5 h.

### Model selection and quality control

GPs are powerful but sensitive models whose flexibility can lead to issues such as overfitting, underfitting, or spurious extrapolation, particularly in high-dimensional biological data [21]. To ensure reliable time annotations and guard against misleading conclusions, we systematically address potential failure modes of the model through a combination of in-distribution and out-of-distribution quality checks. Below, we enumerate key risks and how our pipeline mitigates them.

#### Quality evaluation in reference data

To avoid overfitting of the GP models in the reference time series, we considered the heuristics of building robust GPs as described in [21] by putting bounding constraints on the length-scale such that $\Delta t_{\min } \le l \le \Delta t_{\max }$, where $\Delta t_{min} = \min (t_i - t_{i-1})$ and $\Delta t_{max} = \max (t_i - t_{j})$.

We address underfitting by performing model comparison against a time-invariant model, similarly to [22]. This is performed by comparing the GP marginal likelihoods with the likelihood obtained from the following time-invariant model:


(16) \begin{eqnarray*}
g_{ij}^{(r)} \sim \mathcal {N}(\mu _i, \sigma _i^{2}), \: r \in \lbrace 1,\dots ,R\rbrace , \: \: j \in \lbrace 1,\dots ,T\rbrace ,
\end{eqnarray*}

where $\mu _i$, $\sigma _i^{2}$ are the mean and variance over all the observations for gene $i$ coming from the reference data. We then set a filter to ignore those gene models whose GP marginal likelihood on the reference data is considerably smaller compared to the invariant model. The rule for filtering the model based on the reference data fits can thus be written as follows:


(17) \begin{eqnarray*}
\log P([g_i^{(r)}]_{r=1}^{R} | t, M_i)+\epsilon <\sum _t\sum _r \log P(g_{i,t}^{(r)} | \mu _i, \sigma _i),
\end{eqnarray*}

where $\epsilon$ is a tolerable difference between the log-likelihoods of the two models.

The removal of the genes for which the flat model substantially outperforms the GP model in terms of marginal likelihood is primarily for computational simplification, as a flat model does not contribute anything to the analysis because it assigns the same likelihood at every time point.

To assess the quality of the GP fits, we performed a leave-one-out cross-validation, which is a standard method for measuring the predictive accuracy after fitting a Bayesian model [23]. This was done by refitting each GP multiple times with the RNA-Seq time series while removing all the observations corresponding to the same time point each time. For a single gene, we denote by $y_{-j}$ the set of all gene expression values, except for those observed at time $t_j$, which we denote by $y_j$. At each iteration, we calculated the log-likelihood of the model $\log P(y_j | t_j, y_{-j})$ to predict the observed gene expression values at the time point that was removed. Then, for each model, we calculate the leave-one-out Bayesian cross-validation estimate $\mathrm{LOO}=\frac{1}{T}\sum _{j=i}^{T}\log P(y_j | t_j, y_{-j})$ (where $T$ is the number of time points) as described in [23]. We applied the same leave-one-out cross-validation procedure in GP models without length-scale regularization.

**Figure 5.**
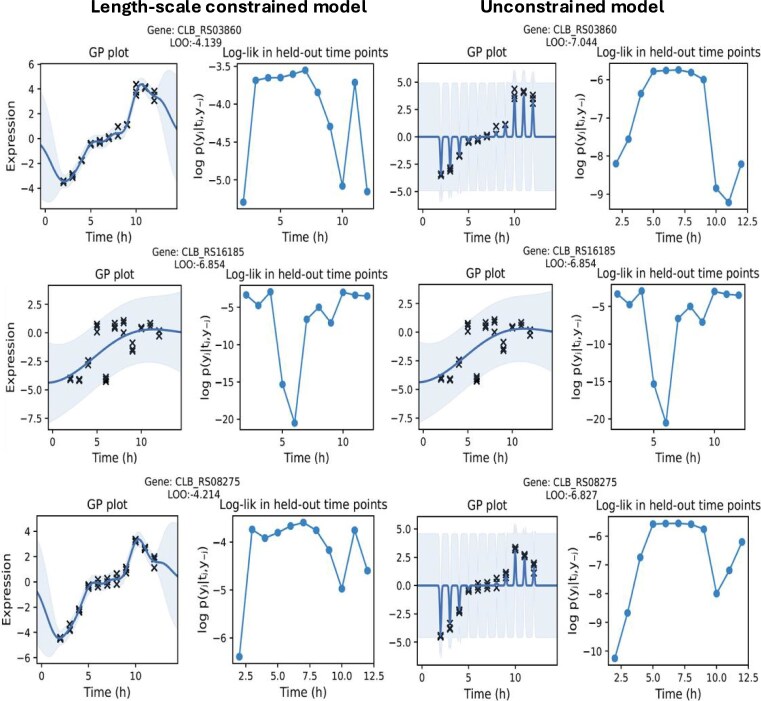
The plots show the GP models that performed worst according to LOO considering both groups (with and without length-scale regularization). The plots indicate that having no boundary constraints on the length-scale results in a smaller length-scale during optimization that leads to overfitting and a worse performance in the leave-one-out cross-validation.

Figure 5 shows the GP plot and its respective LOO of the models (with and without length-scale regularization) that performed worst in the leave-one-out cross-validation. We can clearly see that putting constraints on the length-scale prevents overfitting of the GP model. We include more information about the leave-one-out cross-validation in the Supplementary data (see Section S.6, Supplementary Figs S7 and   S8, and Supplementary LOO plots).

**Figure 6.**
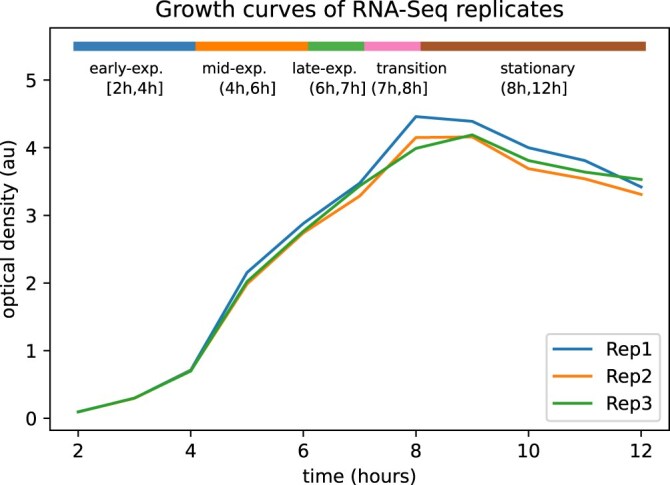
The growth curves monitored during the bacterial growth experiments where the reference RNA-Seq samples were assessed. Each growth curve represents the amount of bacteria at each time point for each experimental replicate. The horizontal bar (at the top of the figure) represents the different growth phases. Each color represents a different growth phase whose length extends within its corresponding time interval.

**Figure 7.**
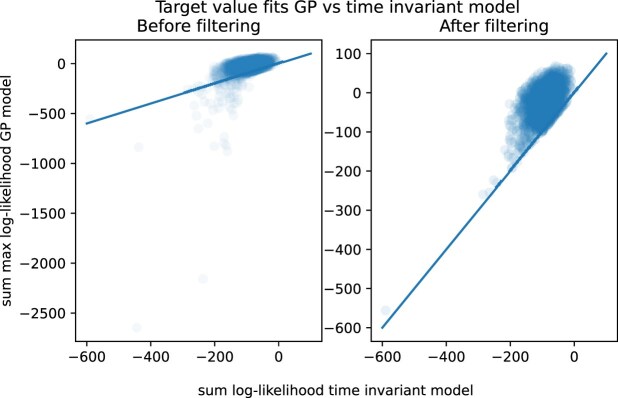
Comparing the compatibility of the microarray data to the fitted GP models versus the compatibility to a time-invariant model, for every gene. To measure the compatibility of the microarray values of a gene to a GP model, we determine the maximum log-likelihood over each time point for each gene value and evaluate their sum. For the time-invariant model, we evaluate the sum of the log-likelihoods of each microarray value of a gene in the model in Equation (18).

**Figure 8.**
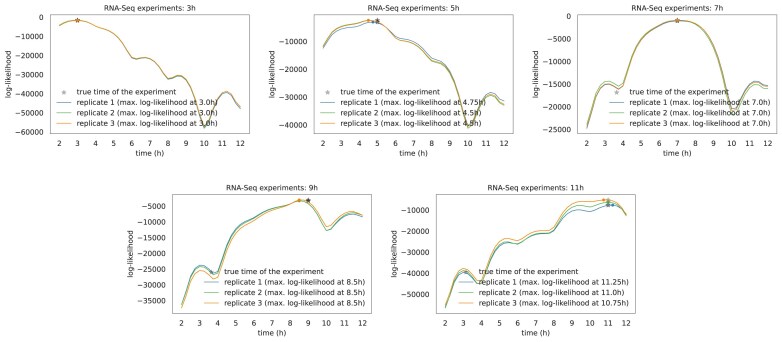
Log-likelihood curves of the RNA-Seq experiments sampled during the odd time points (i.e. 3, 5, 7, 9, and 11 h). The results were obtained by using the even time point RNA-Seq experiments as training data for our method. The maximum likelihood point is found by querying selected time points (e.g. every 15 min within the time interval covered by the training data).

#### Quality evaluation in target data

A gene’s expression values in the target data may lie outside the range observed in the reference time series, resulting in extrapolation where GP predictions are less reliable. To undermine the effect of this discrepancy, we applied the median normalization explained in the “Filtering and normalization of transcriptomicsdata” section.

Furthermore, we evaluate how well the GP models fitted to reference data can fit the target data by comparing the maximum likelihood of each gene’s target value in the corresponding fitted GP model to the baseline time-invariant model in Equation (16) as follows:


(18) \begin{eqnarray*}
g_{i,E}\sim \mathcal {N}(\mu _i, \sigma _i^{2}),
\end{eqnarray*}

where $g_{i,E}$ is the target value of gene $i$ in experiment $E$. We calculate the sum over the target values’ maximum log-likelihoods over each time point in the corresponding fitted GP model, and the sum over the target values’ log-likelihoods evaluated in the time-invariant model. Finally, we filter out from the time annotations method those gene models whose target values fit better in the time-invariant model. The rule for filtering the models based on the compatibility of the target data with the fitted GP models can thus be written as follows:


(19) \begin{eqnarray*}
\sum _j \max _t (\log P(g_{i,E_j}| t, M_i) ) < \sum _j \log P(g_{i,E_j} | \mu _i, \sigma _i),
\end{eqnarray*}

where the log-likelihood in the LHS is based on the posterior distribution of the gene-specific GP model, and the RHS is based on the distribution of the time-independent model defined in Equation (18).

The genes for which target gene expression values were predicted better by a time-invariant model were either those characterized by flat expression dynamics or genes whose target values were significantly different from the reference values (see Section S.4 and Supplementary Fig. S4). Genes whose target values fit better in the time-invariant model are excluded from the annotation method. This step addresses the out-of-distribution generalization problem and ensures that only compatible gene signals are used for annotating target samples.

## Results

The proposed methodology was validated in three complementary settings: a withheld subset of RNA-seq measurements that simulates sparsely sampled data, synthetic data with added noise to test noise sensitivity, and a practical application in which time point annotation is performed for 80 microarray samples. We first describe the datasets and transformations that underpin all empirical work.

### Data

We utilize two types of transcriptomics data from *Clostridium botulinum*. The first type consist of longitudinal RNA-Seq samples, which we use as reference data. The second type consist of legacy microarrays [24] collected at different stages of growth of the bacteria, which are the target data that we want to time annotate. The sources for accessing the data are in the “Data availability” section. A more detailed description of the data can be found in the Supplementary data (see Section S.1).

Terminology: To avoid confusion, we distinguish three related concepts that appear throughout the paper: (i) *legacy growth phase* (LGP) (sometimes also referred to as original annotations)—the categorical phase label (early-exponential, mid-exponential, late-exponential, transition, stationary) assigned by the original microarray samples at the moment of sampling; (ii) *reference time point* (RTP)—the quantitative sampling time (in hours) in the RNA-Seq reference experiment that best matches each microarray sample after recalibration; and (iii) *reference growth phase* (RGP)—the phase implied by that RTP, using the growth curves of the RNA-Seq experiments as reference.

#### Reference data

The reference experiments that we used to fit our method consisted of RNA-Seq samples collected uniformly at different time points (2–12 h) from three replicate cultures of *C. botulinum* strain ATCC 19397 growth in routine laboratory conditions. In parallel with RNA sampling, growth was monitored by optical density measurements as shown in Fig. 6. The RNA samples were collected on the same time points relative to the corresponding growth experiment replicate. The samples were analyzed using paired-end sequencing with Ilumina NextSeq 550 technology. Finally, the RNA-Seq data analysis pipeline ProkSeq [25] was used to perform the respective alignments to the genome, and the gene expression values for every time point were obtained. The gene expression values are expressed in transcripts per million (TPM) units. The final RNA-Seq data accommodate 3491 different genes and their corresponding gene expression values for each time point.

#### Target data

Our target data comprise gene expression values on a logarithmic scale determined during the growth of the *C. botulinum* Group I wild-type strain ATCC 3502, a strain closely related to ATCC 19397 [26], under optimal conditions using microarrays as described [24, 27].

Altogether, 80 microarray samples collected in the past were included in this study. Each sample measured the expression of 3664 genes and was annotated according to the bacterial growth phase at the time it was sampled (i.e. LGP), but not the precise time at which the sample was taken.

The LGP was determined by the original experimenters using a growth curve based on real-time optical density measurements of the culture, which reflect bacterial growth.

Determining the growth phase of a culture by visually interpreting its optical density curve is partly subjective; thus, certain samples might have been assigned to phases that do not truly match the cells’ biological state. One goal of the time annotation is to perform a temporal recalibration of the samples to correct such errors.

Before fitting our statistical models, the data were preprocessed as described in the following section.

#### Data preprocessing

In the data preprocessing, we considered the specific nature of transcriptomics data from bacteria. Of note, different considerations may apply when working with eukaryotic data. In order to simplify the data, we conducted an *operon* identification analysis of the RNA-Seq data using *Rockhopper* software [28]. *Operons* are clusters of co-regulated genes with related functions [29], are usually found in contiguous locations of the DNA, and are commonly co-activated. Assuming that genes within an operon are usually expressed similarly or in a related manner, they were considered to be redundant and to cause bias in the analysis of gene expression over time by putting more weight on the genes that are in large operons. Identifying the operons allowed us to keep only one gene per operon, thus reducing the number of genes considered in the analysis. This simplified the analysis and helped obtain better results.

In order to use the data for our objective goal, we then removed obvious incompatibilities between the microarray and RNA-Seq samples. The following steps summarize the initial preprocessing of the data:

- Remove from the RNA-Seq data all the genes that belong to the same operon except for the one that has the highest median over the set of expression values at each time point to simplify the genes’ set.
- Remove all genes from the RNA-Seq data whose maximum expression value over time is <10 TPM because they introduce unnecessary noise to the data. This is because for small gene expression values the signal to noise ratio is very small [30], thus they are likely to contribute more noise than signal in our analysis
- Consider only the common genes between RNA-Seq and microarrays.
- Log-transform the RNA-seq data (i.e. $G \rightarrow \log (G+\epsilon )$) to bring them to the same order of magnitude as the microarray data (add a small quantity $\epsilon =0.1$TPM to avoid undefined values for logarithm).
- Perform median polish normalization as described in Algorithm 1 to the RNA-Seq and microarray samples respectively. Choose a sensible convergence tolerance $\epsilon$. In the analysis, we set $\epsilon =10^{-5}$ as we observed that in the consecutive iterations, the differences of the residuals were quite negligible and almost 0. This transformation brings the gene expression values from the different datasets on the same scale.

#### Data postprocessing

##### *Evaluating the quality of fitting the GP models to the RNA-Seq data*.

After fitting the GP models with the RNA-Seq data, we evaluated the quality of fitting the models. We compared how well the GPs model the RNA-Seq data, as compared to a time-invariant model. Our analysis showed that GP models are superior to the time-invariant models in terms of representing the RNA-Seq data with the exception of some borderline cases. Thus, we did not filter-out any gene from the time annotation method based on these results. In the Supplementary data (see Section S.3 and Supplementary Fig. S3), we have included a plot that shows the log-likelihood of each gene RNA-Seq time series according to the respective GP model (i.e. log marginal likelihood of the GP), and the sum of the log-likelihoods that they come from the time-invariant models in Equation (16).

##### *Evaluating the compatibility of the microarray values to the fitted GP models*.

Figure 7 shows the quality of the fits of the microarray data between the two models; it shows that a small subset of the genes (i.e. 95 genes from 1780) did not fit better in our model as compared to the time-invariant model, so we ignored those genes when computing the GP time annotation analysis (i.e. following Equation (19)). The issue occurs in those genes where some of the microarray observations are significantly outside the range of the RNA-seq observations, even after normalization. We provide an analysis for the source of incompatibility of those genes’ microarray values with the corresponding fitted GP model in the Supplementary data (see Section S.4 and Supplementary Fig. S4).

### Validation

#### Sparse RNA-Seq hold-out

We tested the efficacy of our method on the RNA-Seq data by fitting the model with the RNA-Seq samples taken at the even time points (i.e. 2, 4, 6, 8, 10, 12 h) and checked how accurately the model would annotate the RNA-Seq samples measured at the odd time points (i.e. 3, 5, 7, 9, 11 h) for all replicates.

**Figure 9.**
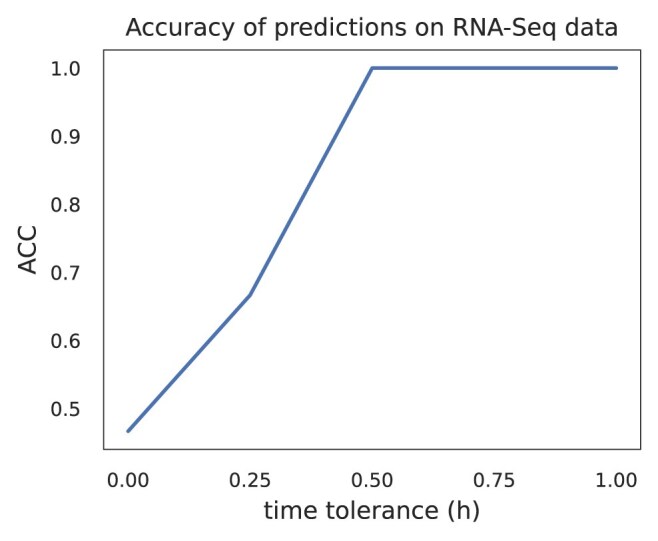
Accuracy of the annotations (i.e. $\mathrm{ACC} =\frac{\mathrm{correct\, predictions}}{\mathrm{total\, predictions}}$) assigned to the odd time point RNA-Seq experiments for different time tolerance intervals. Every annotation is within 0.5 h distance from the true time point of the corresponding experiment.

Figure 8 shows the log-likelihood curves that our model generated for each test data, which were the basis for assigning the time annotations. Figure 9 shows the accuracy of the predictions $\mathrm{ACC} =\frac{\mathrm{correct\, predictions}}{\mathrm{total\, predictions}}$ for different time interval tolerances. All the annotations are within a distance of 30 min from the actual time of the measurement.

We obtained similar accuracy results when training our method on the RNA-Seq time series using two experimental replicates and making predictions for the experiments of the left out replicate. The results of the former analysis can be found in the Supplementary data (see Section S.2 and  Supplementary Figs S1 and S2)

#### Sensitivity to noise

To prove the robustness of our method toward different levels of noise in the data, we simulated five transcriptomics experiments every 15 min in the closed interval (2 and 12 h), by sampling values $g_{i,t_{j}} \sim N(\mu _{g_i, t_{j}},\sigma _{g_i,t_{j}})$, where $g_{i,t_{j}}$ is the value of gene $g_i$ at time $t_j$, and $\mu _{g_i, t_j}, \sigma _{g_i, t_j}$ are the posterior mean, and posterior variance at time $t_j$ of the corresponding GP model. Then, in order to introduce noise in the data, we repeated the procedure by randomly selecting different subsets of genes and sampling their values for each time point from a normal distribution $g_{i,t_{j}} \sim N(\mu _i, \sigma _i)$ , where $\mu _i$ and $\sigma _i$ are the mean and standard deviation of gene $g_i$ over all time points.

**Figure 10.**
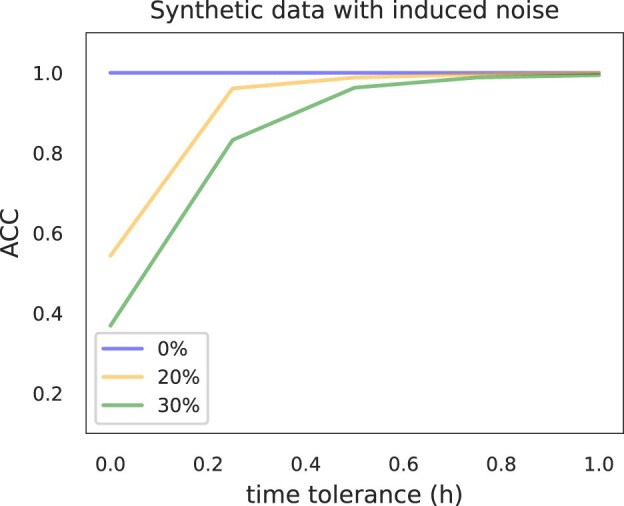
Accuracy of the time annotations (i.e. $\mathrm{ACC} =\frac{\mathrm{correct\, predictions}}{\mathrm{total\, predictions}}$) on simulated transcriptomics experiments with different amounts of noise for different time tolerance intervals. The noise was introduced by randomly selecting different amounts of genes (i.e. 0%, 20%, 30%) and changing their generative models for every time point, when sampling the values for simulating the experiments. Even when 30% of the genes were noisy, 97% of the time annotations were within 0.5 h distance from the true time point of the corresponding experiment.

Figure 10 shows the accuracy of the annotations on simulated transcriptomics experiments with different amounts of noise for 0-, 0.25-, 0.5-, 0.75-, and 1-h time interval tolerances. Even in the case when, we perturbed 30% of the genes, 97% of the annotations were no >0.5 h away from the true time of the experiment. In the case where we did not introduce any noise at all, all the predictions corresponded to the true time of the samples.

### Time re-annotation of microarray samples: a case study

**Figure 11.**
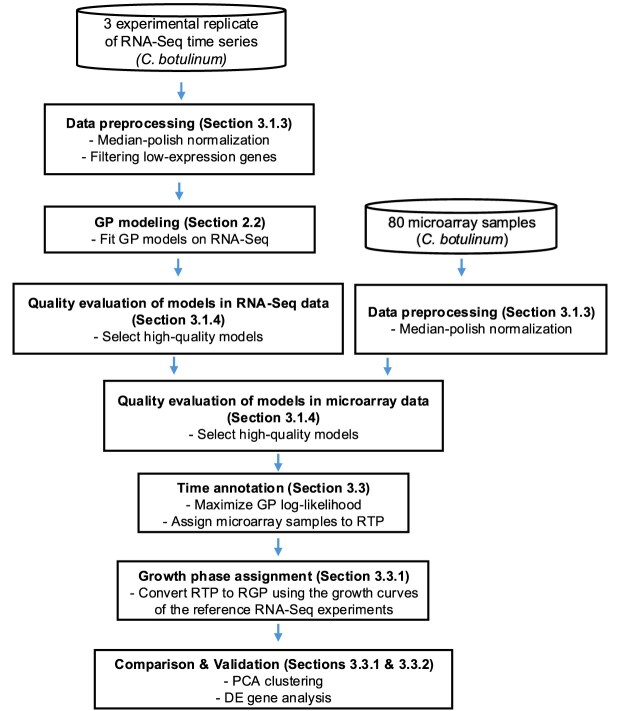
Diagram describing the application of the GP annotation method for the re-annotation of legacy microarray samples. Inside each rectangle it is indicated the relevant section of the paper where the respective process is described.

We describe here the procedure for performing RGP annotation for each of the 80 microarray samples. First, we use the RNA-Seq data to fit the GP models, and assign a RTP to each microarray sample. Then, we use the RTP and the information provided by the growth curves of the reference RNA-Seq experiments to assign a RGP to each microarray sample. Figure 11 represents the steps followed for the re-annotation of the legacy microarray samples.

In a batch culture of bacteria, optical density measurements over time describe a sigmoidal growth curve whose segments (early-, mid-, and late-exponential, transition, and stationary) represent successive physiological states rather than mere clock time. Under constant conditions, replicate cultures advance through these states in a reproducible order, and each state is marked by a characteristic transcriptional programme. This coupling between growth phase and gene-expression profile is the basis of the recalibration performed below: by aligning legacy microarray profiles to the RNA-seq reference time-course, we can assign biologically meaningful timestamps even where the original sampling left temporal gaps.

#### Re-assigning growth phases to the microarray samples

After each microarray sample was matched to its RTP, we used the growth curve that was monitored while collecting the RNA-Seq samples, as shown in Fig. 6, to map that timestamp into an RGP. Based on the shape of this curve, the time intervals were equated with growth phases as follows:

- Early-exponential phase: (2 h, 4 h),
- Mid-exponential phase: (4 h, 6 h),
- Late exponential phase: (6 h, 7 h),
- Transition phase: (7 h, 8 h),
- Stationary phase: (8 h, 12 h).

**Figure 12.**
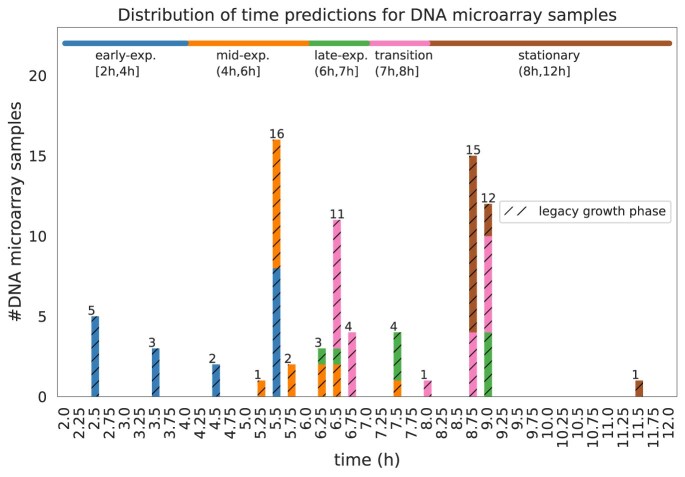
The number of microarray samples for each queried time point. The horizontal bar (at the top of the figure) represents the RGP to which the microarray samples were assigned based on their RTP. Each color represents a different growth phase, whose length extends within its corresponding time interval. The colors of the vertical bars represent the LGP of the microarray samples assigned at the respective time point (notice the hatching to differentiate between the LGP and RGP).

Figure 12 shows the distribution of the RTP for the microarray samples according to our method. By assigning the RGP to the microarray samples based on the RTP, using information provided by the growth curves of the RNA-Seq experiments, we significantly enhanced the description of the microarray data.

A high quality temporal calibration should assign samples with similar expression patterns to the same time point. To assess whether this was achieved, a principal component analysis (PCA) of the expression levels in all microarray samples was performed. The consistency of the grouping of samples from the same time point/growth phase is presented visually and assessed quantitatively. To assess the effectiveness of the GP method, the microarray samples were also aligned with the RNA-Seq timeline using a *k*-nearest neighbors (kNN) classifier. We chose kNN ($k=1$ as it yields the highest accuracy when performing cross validation using the RNA-Seq samples) as a baseline method because it is a very simple method and it works similarly to our method—that is, using the reference time series, it assigns time annotations to our target data. A detailed description of how kNN was used to assign time points to the microarray samples can be found in the Supplementary data (see Section S.5, Supplementary Figs S5 and S6).

**Figure 13.**
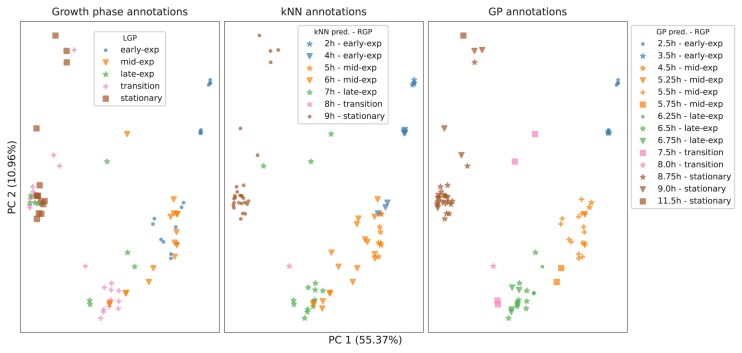
PCA plots of the microarray samples annotated according to their LGP annotations (left), time annotation as assigned by kNN (center), and time annotation as assigned by our method (right). Growth phases are represented by colors. In the case of kNN and GP time annotations, the growth phases of the experiments are determined based on the RGP as defined in the “Re-assigning growth phases to the microarray samples” section.

The improved annotation allowed for clear separation of microarray samples belonging to different growth phases, as demonstrated by the PCA in Fig. 13. Consequently, we successfully identified microarray samples whose LGPs were miss-assigned, as some of the microarray samples appeared likely to have been assigned to incorrect growth phases in the original annotation.

Both methods (kNN- and GP-based) resulted in an improvement compared to the original annotations (i.e. LGP), as shown in Fig. 13. However, our method is considered to be qualitatively better, as it sets a clearer separation between samples belonging to different growth phases. It also filled the sampling gaps: the method interpolated intermediate time points that were never measured experimentally, giving the final annotation a finer-grained temporal resolution.

**Figure 14.**
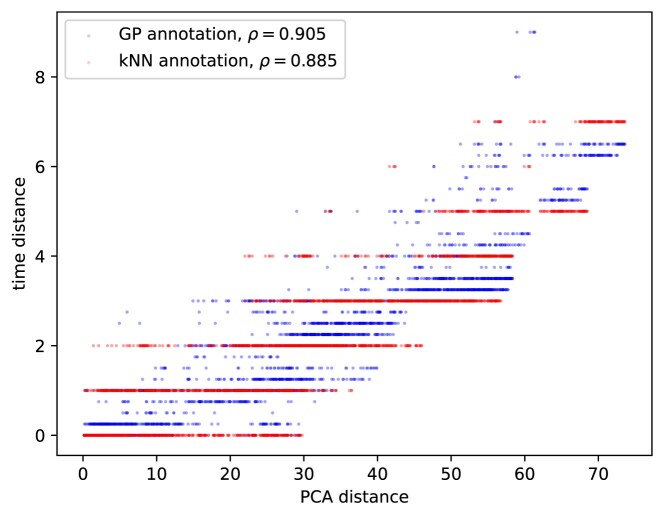
Correlation between the PCA distances of all pairs of microarray samples and their corresponding time annotations distances as assigned by kNN and our GP time annotation method. The Pearson correlation coefficient $\rho$ is higher in the case of our method, which indicates that the microarray samples that appear to be close to each other in the PCA are assigned time annotations that are closer.

In order to quantitatively compare the annotations generated by kNN and our method, we measured the correlation between the distances of the samples in the PCA and their time annotation distances as assigned by each of the methods. Figure 14 shows the PCA distances between every pair of microarray samples and their corresponding time annotation distances as assigned by kNN- and GP-based methods. The Pearson correlation coefficient is higher in the case of the GP annotations, which indicates that the microarray samples that appear to be close to each other in the PCA are assigned time annotations that are closer.

#### Temporal recalibration improves biological interpretation

In order to show the impact of the GP-based time annotations on the downstream analysis, we performed a gene differential expression analysis with LIMMA [31] by comparing the microarray samples assigned to consecutive growth phases (i.e. early-exp. and mid-exp., mid-exp. and late-exp., late-exp. and transition, transition and stationary).

Because gene expression shifts with the growth phase, samples from the same phase should look alike, whereas samples from different phases should diverge. Therefore, a differential-expression test that cleanly contrasts early- versus mid-exponential samples, for instance, should yield more DE genes than a test in which each group contains samples from a mixture of phases.

**Figure 15.**
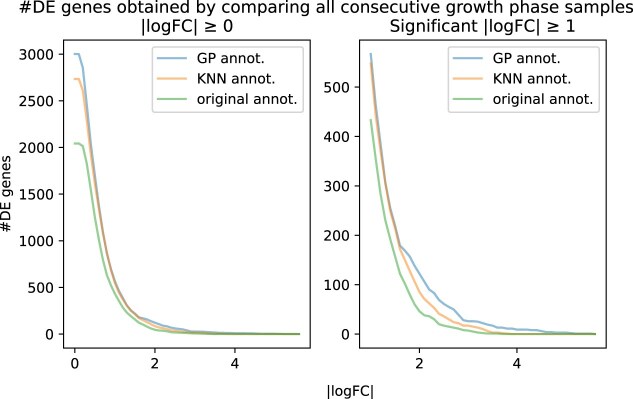
Total number of differentially expressed genes (FDR corrected $P\text{-value} \, {<.05}$) detected by performing DE analysis between all pairs of consecutive growth phases (i.e. early-exp. versus mid-exp., mid-exp. versus late-exp., late-exp. versus transition, transition versus stationary) as a function of the absolute value of $\log _2$-fold change ($|\log$FC$|$) for each of the three different annotations. The plot on the right shows the total number of differentially expressed genes that have $|\log$FC$|\ge 1$ corresponding to a two-fold change in expression.

Figure 15 shows the total number of differentially expressed genes (FDR corrected $P$-value <.05) detected after performing gene differential expression analysis between every pair of consecutive growth phases as annotated by our GP method (RGP), kNN method, and the original LGP annotations.

**Figure 16.**
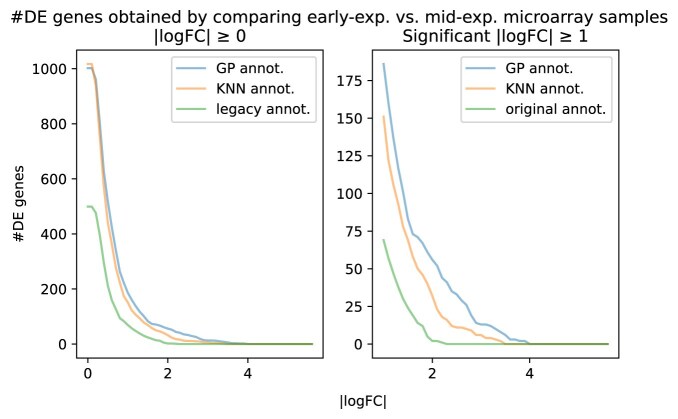
Number of DE genes (FDR-corrected $P\text{-value}<.05$) detected by performing differential expression analysis between early-exp. and mid-exp. microarray samples as a function of the absolute value of $\log _2$-fold change ($|\log \text{FC}|$) for each of the three different annotations. The plot on the right shows the number of DE genes that have $|\log$FC$|\ge 1$ corresponding to a two-fold change in expression.

**Figure 17.**
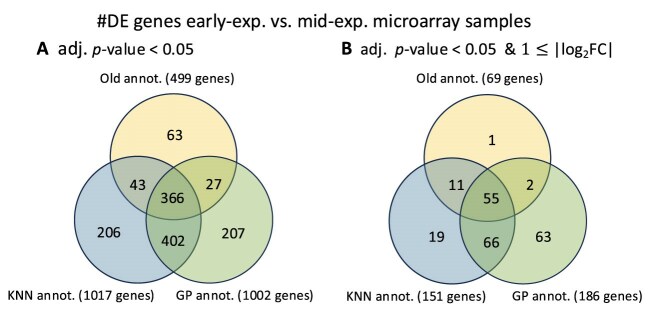
Venn diagrams expressing the number of DE genes (FDR-corrected $P \text{-value}<.05$) detected by performing differential expression analysis between early-exp. and mid-exp. microarray samples for each growth phase annotations (i.e. original annotations, kNN-annotations and GP-annotations) (left). The plot on the right shows the number of DE genes with $|\log$FC$|\ge 1$ corresponding to a two-fold change in expression.

Comparative analysis of the three time-annotation approaches applied to the *C. botulinum* microarray samples revealed significant differences when investigating differentially expressed (DE) genes between growth phases. Here, we specifically compared DE genes between the early logarithmic phase and mid-logarithmic phase using two filtering methods: genes with an adjusted $P$-value (FDR-corrected) of <.05 and/or an absolute logarithmic fold-change ($\log _2$FC) greater than 1.0. As shown in Figs 16 and 17, although all approaches identified a similar set of DE genes, the kNN- and GP-based approaches clearly outperformed the samples with the original annotation. Specifically, when both filtering criteria were applied, the three time annotation approaches did indeed identify DE genes of biological relevance for *C. botulinum* physiology, such as genes encoding components of the botulinum neurotoxin (*BoNT*) protein complex. However, the kNN- and GP-based time annotation approaches further identified 66 DE genes involved in central bacterial processes, such as the sporulation cascade, i.e. genes encoding the sporulation transcription factor *Spo0A*, the anti-sigma F factor, and the stage V sporulation protein, and genes encoding chaperones, i.e. the *Hsp33* family molecular chaperones and *GroEL*. The GP-based time annotation yielded another 63 DE genes with an adjusted (FDR-corrected) $P$-value of <.05, illustrating that the model performed better than the kNN method in this particular example and was able to annotate the samples more accurately, thereby reducing the noise from the whole dataset. We provide the list of all DE genes (early-exp. versus mid-exp.) identified for each growth phase annotation example (FDR-corrected $P\text{-value}<0.05$ and $|\log \text{FC}|\ge 1$) in the Supplementary data (see Supplementary Table DE Genes List).

The fact that the number of DE genes is higher in the case of GP annotation suggests that it does a better job of grouping the experiments into well defined states by fixing the mislabeled samples in the legacy data. In the Supplementary data (see Section S.7 and Supplementary Fig. S9), we provide a more elaborate analysis of how miss-labeling microarray samples affects the number of DE genes.

## Discussion

GPs provide an ideal method for modeling time-course data without making explicit assumptions about the type of function that can best describe them. They can be successfully used to assign time points to target omics experiments by employing a reference time series of experiments from the same level of the omics stack.

We demonstrate this by applying our method for the purpose of assigning time annotations to microarray samples using RNA-Seq time series sampled every hour as training data. The new time annotations not only provided an accurate description of the data from the perspective of growth phases but also indicated samples that appeared likely to have been originally assigned to incorrect growth phases.

We also demonstrate that the method can be successfully applied with sparse time series by inferring predictions for the odd time point RNA-seq samples using the subseries of even RNA-seq samples as a reference. Our method proved to be robust toward noisy data. We show that by generating transcriptomics experiments at different time points using the GP models fit on the RNA-Seq data by altering the values of randomly selected genes. Even in the case where as many as 30% of the genes were altered, 97% of the predicted time annotations were within a 30-min distance from their true time. Our approach can also be applied for proteomics data, metabolomics data, or in general for any data that consists of measurements represented as a set of genome-wide values when a reference time series is provided.

Similar applications of GPs for making exact time annotation on omics experiments have not been explored to the best of our knowledge. We think that there is great potential in applying GPs for solving related problems for determining the time of experiments because of their high modeling flexibility and the possibility that they offer to predict in-between time points, which are left uncovered by the training data.

This method can be used to annotate historical data available in repositories (e.g. GEO) for the sake of improving the biological interpretability of those data. This information can be used to better understand the studied organism by uncovering biological pathways through differential gene expression analysis, for instance. However, in order to perform the annotation, a reference transcriptomics time series of the organisms is needed. Historical data that have time annotation can either be used as reference time series or their correctness can be checked when we have better quality reference measurements.

Our use case of time annotating *C. botulinum* ATCC 3502 microarray samples using *C. botulinum* ATCC 19397 RNA-Seq data suggests that our method is platform agnostic (i.e. it does not discriminate the transcriptomics data based on the technology that was used to generate them: DNA microarrays or RNA-Seq), and it works even in the case when different bacterial strains are used.

### Summary of requirements and assumptions

Certain requirements should be considered when performing time annotation with our GP method:

**General assumptions**: Many genes follow a shared, smoothly evolving temporal pattern, and time is the main driver of variation in gene expression.

**Reference time series**: Enough representative genes should pass the model compatibility filter Equation (17); one experimental replicate time series can be enough, but more replicates are preferable; GPs have been used for modeling short time series of gene expression data [21, 32]. In the “Sparse RNA-Seq hold-out” section, we demonstrate that six time points are enough for making predictions that are no >30 min away from their true time in the holdout observations, provided they are evenly distributed and span the full timeline over which we want to make predictions. However, we need sufficiently dense sampling to have a higher resolution on what is happening in the system.

**Target data**: Ideally, the same organism or closely related organisms having the same physiology as the reference data; same modality as the reference data (e.g. RNA-Seq) or cross-modal (e.g. microarrays) (median-polish normalization should work to reduce platform offset without distorting the overall shape). Enough genes should pass the compatibility filter Equation (19); the method tolerates genes for which the signal is distorted to the extent that, even in cases when 30% of the genes have distorted signals, we obtain predictions for the experiments that are within 1 h of their true time in the synthetic data, as we show in the “Sensitivity to noise” section.

## Supplementary Material

lqaf203_Supplemental_Files

## Data Availability

The RNA-Seq data were deposited in NCBI SRA under GEO project number GSE248529. The DNA microarray data were deposited in GEO under project number GSE261398. The Python implementation of the time annotation method can be accessed through the following DOI link: https://doi.org/10.5281/zenodo.11076487. It can also be accessed on GitHub through the following link: https://github.com/PROBIC/omics-time-annotation.

## References

[1] Spellman PT, Sherlock G, Zhang MQ et al. Comprehensive identification of cell cycle-regulated genes of the yeast *Saccharomyces cerevisiae* by microarray hybridization. Mol biol cell. 1998;9:3273–97. 10.1091/mbc.9.12.3273. 9843569 PMC25624

[2] Straume M . DNA microarray time series analysis: automated statistical assessment of circadian rhythms in gene expression patterning. Methods Enzymol. 2004;383:149–166. 10.1016/S0076-6879(04)83007-6. 15063650

[3] Tomancak P, Beaton A, Weiszmann R et al. Systematic determination of patterns of gene expression during *Drosophila* embryogenesis. Genome Biol. 2002;3:1–14. 10.1186/gb-2002-3-12-research0088. PMC151190 12537577

[4] Thompson JA, Tan J, Greene CS. Cross-platform normalization of microarray and RNA-Seq data for machine learning applications. PeerJ. 2016;4:e1621. 10.7717/peerj.1621. 26844019 PMC4736986

[5] Castillo D, Gálvez JM, Herrera LJ et al. Integration of RNA-Seq data with heterogeneous microarray data for breast cancer profiling. BMC Bioinform. 2017;18:1–15. 10.1186/s12859-017-1925-0. PMC5697344 29157215

[6] Heinonen M, Guipaud O, Milliat F et al. Detecting time periods of differential gene expression using Gaussian processes: an application to endothelial cells exposed to radiotherapy dose fraction. Bioinformatics. 2014;31:728–35. 10.1093/bioinformatics/btu699. 25355790

[7] Magwene PM, Lizardi P, Kim J. Reconstructing the temporal ordering of biological samples using microarray data. Bioinformatics. 2003;19:842–50. 10.1093/bioinformatics/btg081. 12724294

[8] Trapnell C, Cacchiarelli D, Grimsby J et al. The dynamics and regulators of cell fate decisions are revealed by pseudotemporal ordering of single cells. Nature Biotechnol. 2014;32:381–6. 10.1038/nbt.2859. 24658644 PMC4122333

[9] Reid JE, Wernisch L. Pseudotime estimation: deconfounding single cell time series. Bioinformatics. 2016;32:2973–80. 10.1093/bioinformatics/btw372. 27318198 PMC5039927

[10] Campbell KR, Yau C. Uncovering pseudotemporal trajectories with covariates from single cell and bulk expression data. Nat Commun. 2018;9:2442. 10.1038/s41467-018-04696-6. 29934517 PMC6015076

[11] van der Kloet FM, Buurmans J, Jonker MJ et al. Increased comparability between RNA-Seq and microarray data by utilization of gene sets. PLoS. 2020;16:e1008295. 10.1371/journal.pcbi.1008295. PMC7549825 32997685

[12] Tukey J . Exploratory data analysis, Reading, MA, USA: Addison-Wesley Publishing Company, 1977.

[13] Rasmussen C, Williams C. Gaussian processes for machine learning, *Adaptive Computation and Machine Learning*. Cambridge, USA: MIT Press, 2006.

[14] Hensman J, Lawrence ND, Rattray M. Hierarchical Bayesian modelling of gene expression time series across irregularly sampled replicates and clusters. BMC Bioinform. 2013;14:1–12. 10.1186/1471-2105-14-252. PMC3766667 23962281

[15] McDowell IC, Manandhar D, Vockley CM et al. Clustering gene expression time series data using an infinite Gaussian process mixture model. PLoS Comput Biol. 2018;14:e1005896. 10.1371/journal.pcbi.1005896. 29337990 PMC5786324

[16] Lawrence N, Sanguinetti G, Rattray M. Modelling transcriptional regulation using Gaussian processes. In: Schölkopf B, Platt J, Hoffman T. (eds.), Advances in Neural Information Processing Systems, Vol. 19. Cambridge, MA, USA: MIT Press, 2006, 785–92.

[17] Honkela A, Girardot C, Gustafson E et al. Model-based method for transcription factor target identification with limited data. Proc Nat Acad Sci USA. 2010;107:7793–8. 10.1073/pnas.0914285107. 20385836 PMC2867914

[18] Jo K, Sung I, Lee D et al. Inferring transcriptomic cell states and transitions only from time series transcriptome data. Sci Rep. 2021;11:12566. 10.1038/s41598-021-91752-9. 34131182 PMC8206345

[19] Gao P, Honkela A, Rattray M et al. Gaussian process modelling of latent chemical species: applications to inferring transcription factor activities. Bioinformatics. 2008;24:i70–5. 10.1093/bioinformatics/btn278. 18689843

[20] GPy . GPy: a Gaussian process framework in Python. http://github.com/SheffieldML/GPy (3 August 2025, date last accessed).

[21] Topa H, Honkela A. Gaussian process modelling of multiple short time series. Proceedings of the 23rd European Symposium on Artificial Neural Networks, Computational Intelligence and Machine Learning. Bruges, Belgium, 2015, 83–88.

[22] Topa H, Jónás Á, Kofler R et al. Gaussian process test for high-throughput sequencing time series: application to experimental evolution. Bioinformatics. 2015;31:1762–70. 10.1093/bioinformatics/btv014. 25614471 PMC4443671

[23] Vehtari A, Mononen T, Tolvanen V et al. Bayesian leave-one-out cross-validation approximations for Gaussian latent variable models. Machine Learning Res. 2016;17:1–38.

[24] Dahlsten E, Zhang Z, Somervuo P et al. The cold-induced two-component system CBO0366/CBO0365 regulates metabolic pathways with novel roles in Group I *Clostridium botulinum* ATCC 3502 cold tolerance. Appl Environ Microbiol. 2014;80:306–19. 10.1128/AEM.03173-13. 24162575 PMC3910985

[25] Mahmud AKMF, Delhomme N, Nandi S et al. ProkSeq for complete analysis of RNA-Seq data from prokaryotes. Bioinformatics. 2020;37:126–8. 10.1093/bioinformatics/btaa1063. PMC8034529 33367516

[26] Ng V, Lin W-J. Comparison of assembled *Clostridium botulinum* A1 genomes revealed their evolutionary relationship. Genomics. 2014;103:94–106. 10.1016/j.ygeno.2013.12.003. 24369123 PMC3959226

[27] Selby K, Mascher G, Somervuo P et al. Heat shock and prolonged heat stress attenuate neurotoxin and sporulation gene expression in Group I *Clostridium botulinum* strain ATCC 3502. PLoS One. 2017;12:e0176944. 10.1371/journal.pone.0176944. 28464023 PMC5413062

[28] Tjaden B . A computational system for identifying operons based on RNA-Seq data. Methods. 2020;176:62–70. 10.1016/j.ymeth.2019.03.026. 30953757 PMC6776731

[29] Osbourn AE, Field B. Operons. Cell. Mol. Life Sci. 2009;66:3755–75. 10.1007/s00018-009-0114-3. 19662496 PMC2776167

[30] Sha Y, Phan JH, Wang MD. Effect of low-expression gene filtering on detection of differentially expressed genes in RNA-Seq data. In: *2015 37th Annual International Conference of the IEEE Engineering in Medicine and Biology Society (EMBC)*. Piscataway, NJ, USA: IEEE, 2015, 6461–4. 10.1109/EMBC.2015.7319872 PMC4983442 26737772

[31] Ritchie ME, Phipson B, Wu D et al. limma powers differential expression analyses for RNA-sequencing and microarray studies. Nucleic Acids Res. 2015;43:e47 10.1093/nar/gkv007. 25605792 PMC4402510

[32] Liu Q, Lin KK, Andersen B et al. Estimating replicate time shifts using Gaussian process regression. Bioinformatics. 2010;26:770–6. 10.1093/bioinformatics/btq022. 20147305 PMC2832819

