# Supplementary material for: A supervised Bayesian method for time (re)annotation of transcriptomics data
Source: NAR Genom Bioinform. 2025 Dec 31;7(4):lqaf203. doi: 10.1093/nargab/lqaf203 (PMC12754789; doi:10.1093/nargab/lqaf203)
Supplement: lqaf203_Supplemental_Files [file lqaf203_supplemental_files.zip › supplement_to_main_paper.pdf]

Supporting Material

S.1 Detailed description of the experimental data

S.1.1 DNA microarray samples

All 80 DNA microarray experiments described in the study were carried out at the University of Helsinki (Finland) and/or the University of Nottingham (UK) over a spanning of 15 years by different operators, using a similar protocol as described in Selby et al. [2]. All microarray datasets derived from experiments conducted with the same bacterial strain (*C. botulinum* ATCC 3502) under identical growth conditions, including temperature (37°C), anaerobic conditions and growth medium (Tryptone-Peptone-D-Glucose-Yeast broth). The similar set-up allowed us to conduct this work, since we were aiming at characterizing therefore a similar bacterial system. The dataset used in the present study were already deposited in GEO database in the following link `https://www.ncbi.nlm.nih.gov/geo/query/acc.cgi?acc=GSE261398` (access token: khmbeiustpgzbit).

S.1.2 RNA-Seq time series data

S.1.2.1 Bacterial strains, growth conditions and sampling procedures

C. botulinum strain ATCC 19397 was routinely cultured in strict anaerobic conditions at 37 °C in trypticase-peptone-glucose-yeast extract (TPGY) broth. Following two consecutive overnight sub-cultures, three replicate cultures were grown in TPGY broth using a starting inoculum of 1:50. Optical density at 595 nm (OD595) was measured at regular time intervals to monitor the culture growths. From *C. botulinum* cultures, aliquots were taken for further transcriptional, proteomic or metabolomic analysis over time. For transcriptional analysis, three aliquots of 1.5 ml cultures were taken for further RNA extraction. Each aliquot was centrifuged at room temperature at 13,000 x g for 1 min. Then, the supernatants were hastily discarded, and the cell pellets were flash-frozen in liquid nitrogen for 2 min. Frozen cell pellets were then stored at -80 °C until further use.

S.1.2.2 RNA extraction and transcriptional analysis

Total RNA was isolated from the frozen cell pellets using the RNeasy Mini Kit (Qiagen GmbH, Germany) as per manufacturer's instructions. RNA samples were additionally DNase-treated using the Ambion Turbo DNA-Free Kit (Thermo Fischer Scientific, MA, USA). RNA concentration was measured using NanoDrop ND-1000 (Thermo Scientific). Ambion ERCC RNA Spike-In Controls (Thermo Fischer Scientific) were then added to each DNase-treated RNA sample as per manufacturer's instructions. Spiked-in RNA samples were sent for RNA sequencing at the Biomedicum Functional Genomics Unit (FuGU, University of Helsinki, Finland). RNA quality was assessed using Agilent TapeStation (Agilent Technologies) and RNA libraries were prepared using the NEBNext Ultra II Directional RNA Library Prep (New England Biolabs, MA, USA) and were sequenced using Illumina NextSeq (Illumina, CA, USA). Sequencing yielded to 75-bp paired-end sequencing reads. Paired-end raw sequencing reads were processed using RNA-Seq analysis software package ProkSeq [1].

The dataset used in the present study were already deposited in GEO database in the following link `https://www.ncbi.nlm.nih.gov/geo/query/acc.cgi?acc=GSE248529` (access token: qfajmuisbvcxran).

S.2 Annotation accuracy for leave one replicate out

We tested the efficacy of our method by fitting the models with all RNA-Seq samples belonging to two experimental replicates and checked how accurately the model would annotate the RNA-Seq samples of the third replicate.

Supplementary Figure 1 shows the log-likelihood curves that our model generated for each test data, which were the basis for assigning the time annotations. Supplementary Figure 2 shows the accuracy of the predictions $\text{ACC} = \frac{\text{correct predictions}}{\text{total predictions}}$ for different time interval tolerances. All the annotations are within a distance of 30 minutes from the actual time of the measurement.

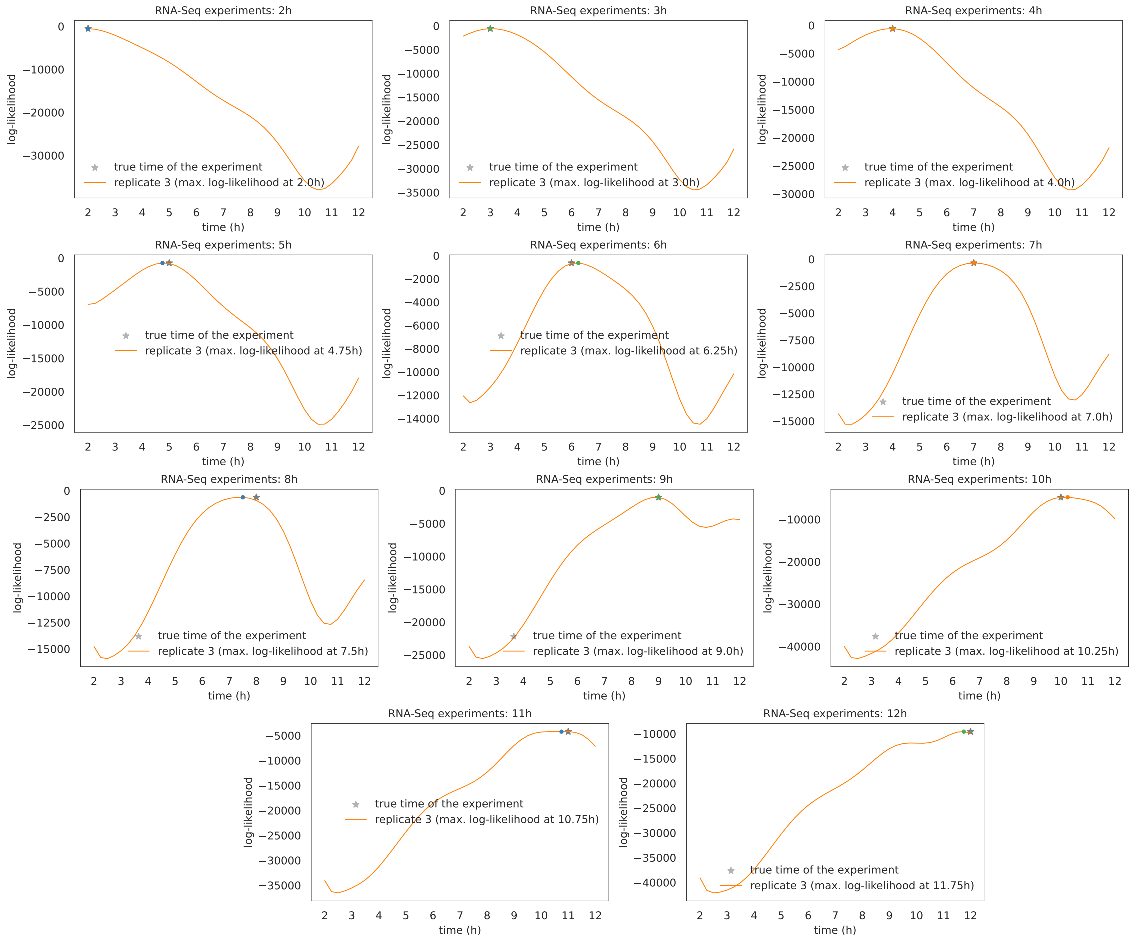

Supplementary Figure 1: Log-likelihood curves of the RNA-Seq experiments of the third experimental replicate. The results were obtained by using two experimental replicate as training data for our method. The maximum likelihood point is found by querying selected time points (e.g., every 15 minutes within the time interval covered by the training data).

S.3 Filtering out GP models that fit poorly on the reference data

Supplementary Figure 3 shows the log-likelihoods of fitting the RNA-Seq time series of each gene to their respective GP model (i.e., log marginal likelihood of the GP) and the sum of the log-likelihoods of fitting them to the time-invariant models. As we can see, the RNA-Seq data fits better on our model with a few borderline cases. Hence, no data were filtered based on this analysis.

S.4 Filtering out GP models that have poor compatibility with the target data

Supplementary Figure 4 shows the fitted GP models of all those genes whose microarray values fit poorly as compared to the time-invariant model (corresponding to the points below the diagonal line in Figure 7 in the main paper). We argue here that ignoring the information provided by those genes does not reduce the quality of our time annotations method. As we can observe from Supplementary Figure 4 the reason for the poor fit of the microarray values is either because the fitted GP model is linear (e.g., first plot from the left in the second row of Supplementary Figure 4), in which case removing the model would not affect the analysis as those models yield constant likelihood for all time point predictions, or because the range of the microarray values is significantly different compared to their RNA-Seq counterpart. In the former case, the microarray values and the RNA-Seq values could not be transformed into a comparable scale by the normalization method,

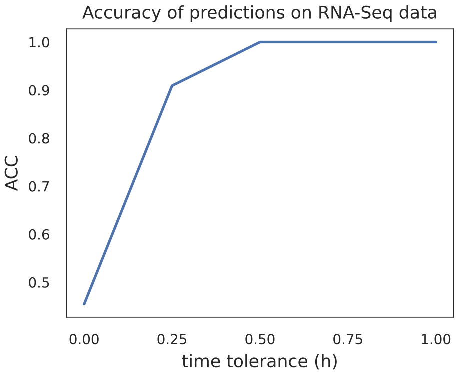

Supplementary Figure 2: Accuracy of the annotations (i.e., ACC $= \frac{\text{correct predictions}}{\text{total predictions}}$) assigned to the samples from the left out experimental replicate for different time tolerance intervals. Every annotation is within 0.5h distance from the true time point of the corresponding experiment.

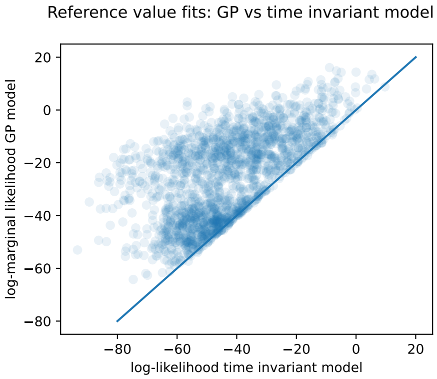

Supplementary Figure 3: Comparing the compatibility of the RNA-Seq data to the GP models versus the time-invariant models for each gene. Every point represents a gene with the x-coordinate being the log-likelihood of the values in the time-invariant model (calculated as the sum of the log-likelihoods of each gene value), and y-coordinate being the log-marginal likelihood of the GP fitting. The blue line is $x = y$.

as such time point predictions for those genes could introduce possible biases given the scale difference between the values of the microarray and RNA-Seq experiments.

S.5 Annotating microarray samples using kNN classifier

In our setting, kNN predicts a microarray sample time point (defined by the gene expression values associated with it) by locating its k closest RNA-Seq samples in the gene expressions space, by calculating the Euclidean distances. After Euclidean distances are computed to every RNA-Seq sample, the k-nearest neighbors are selected. The time point is then assigned by majority voting (e.g., for $k = 3$, if the 3 nearest neighbors consist of two RNA-Seq samples associated with 2 hours and one with 4 hours, then 2 hours is chosen as the prediction for the microarray sample). Then, a growth phase is assigned to the microarray sample according to the procedure described in Section 3.3.1 in the main paper. Considering the way that kNN assigns labels, it is usually recommended that k is an odd number, to avoid situations when there is a tie in the voting of the neighbors to decide the label of the sample under consideration. On the other hand, if we denote by C the labeling class with the smallest number of representatives, then $k < 2 * |C|$ ($|C|$ is the number of elements of class C), otherwise we will always fail to assign to a new sample the label C. In our case $C = 3$, as for each time point we have 3 RNA-Seq samples (one for each experimental replicate). Therefore $k \in \{1, 3, 5\}$. We performed a leave-one-out cross validation in the training set (RNA-Seq samples) and found that $k = 1$ yields the highest accuracy (i.e., ACC $= \frac{\text{correct predictions}}{\text{total predictions}}$) as shown in Supplementary Figure 5. During the leave-one-out cross-validation we remove one sample from the RNA-Seq data, train the kNN classifier in the rest of the data and perform prediction on the held-out sample. After experimenting with all 3 values of k, the correlation values ρ indicated in Supplementary Figure 6 were respectively 0.885, 0.878 and 0.862, that is lower than the correlation in the case of the GP-based method ($\rho = 0.905$).

S.6 Performing leave-one-out cross validation to evaluate the quality of the models

Because a well-behaved Gaussian-process (GP) model should generalize to unseen time points, we quantified predictive performance with leave-one-out cross-validation using leave-one-out Bayesian cross-validation estimate (LOO) [3]. The analysis was carried out both for the regularized GP (by putting constraints on the length-scale) used in the main text ($\Delta t_{\min} \leq l \leq \Delta t_{\max}$, where $\Delta t_{min} = \min(t_i - t_{i-1})$ where $\Delta t_{max} = \max(t_i - t_j)$) and for an otherwise identical GP in which the length-scale was left unconstrained, allowing potential overfitting.

For each RNA-seq time series (n=11 time points) we refitted the GP n times, omitting the observations at one time point t_j at each iteration. After refitting, we evaluated the log-likelihood of the held-out observations $\log p(\mathbf{y}_j | t_j, \mathbf{y}_{-j})$.

Supplementary Figure 7 shows, for both model variants, the top 10 genes with the lowest LOO. We can observe the effect of putting constraints on the length-scale to prevent overfitting of the GP models, thus resulting in higher performance of the leave-one-out cross-validation. We have included the optimized fits of all the GP models from all the variants in the Supporting Material (see Supplementary plots suppl_loo_plots_ordered_constrained_gp.pdf, suppl_loo_plots_ordered_unconstrained_gp.pdf). Supplementary Figure 8 shows the density plot of the LOO for each model. We can observe the longer tail on the left in the case of the density of the unconstrained GP models, which indicates the worst performing models in terms of LOO, as a result of overfitting.

S.7 Effect of growth phase miss-labeling in differential expression (DE) analysis of microarray data

As stated in Section 3.3.2 of the main text, our working hypothesis is that:

1. Genes change expression when cells shift between growth phases.

2. Microarray samples drawn from the same phase should exhibit very similar expression profiles.

If sample labels accurately reflect growth phase, a DE comparison of two pure phases (e.g. early- versus mid-exponential) should therefore yield more statistically significant genes than a comparison in which either group contains a mixture of phases. In the paper we observed exactly this: the Gaussian-process (GP) re-annotation produced a larger set of DE genes (adj. $p \leq 0.05$; $|\log_2 \mathrm{FC}| \geq 1$) than the legacy labels. While a higher DE count is not automatically "better," in this specific context—supported by PCA separation—it indicates that the GP has regrouped previously mis-labeled arrays into coherent physiological states.

S.7.1 Simulation experiment: adding controlled label noise

To verify that the increased number of DE genes arises from cleaner grouping rather than from a hidden bias, we performed the following simulation:

1. Select two clean sets. We took the microarrays that the GP assigned to the early-exponential and mid-exponential phases..

2. Inject label noise. In each trial we randomly swapped the phase label of 35 % and 40 % of the arrays, respectively, thereby creating two noisy groups that mimic mis-annotation

3. Run DE analysis. We applied the identical LIMMA pipeline used in Section 3.3.2.

4. Repeat 100 times. For every trial we recorded the number of DE genes detected at each

S.7.2 Results

Supplementary Figure 9 plots the mean number of DE genes across 100 simulations against the results obtained with (i) the GP labels and (ii) the legacy labels. Introducing 35–40 % label noise reduces the DE genes compared to the GP labels and brings them close to the legacy count across the entire $|\log_2 \mathrm{FC}|$ range. The synthetic mis-labeling confirms that mixed-phase

grouping diminishes statistical power, supporting our claim that the larger DE set obtained with the GP labels reflects a truer biological contrast rather than model over-sensitivity.

Because a 35–40 % shuffle recreates the legacy DE yield, these percentages serve as rough bounds to estimate the fraction of arrays whose growth-phase annotation is incorrect in the original dataset.

References

[1] A. K. M. F. Mahmud, N. Delhomme, S. Nandi, and M. Fällman. ProkSeq for complete analysis of RNA-Seq data from prokaryotes. *Bioinformatics*, 37(1):126–128, 12 2020.

[2] K. Selby, G. Mascher, P. Somervuo, M. Lindström, and H. Korkeala. Heat shock and prolonged heat stress attenuate neurotoxin and sporulation gene expression in Group I Clostridium botulinum strain ATCC 3502. *PLoS One*, 12(5):e0176944, 2017.

[3] A. Vehtari, T. Mononen, V. Tolvanen, T. Sivula, and O. Winther. Bayesian leave-one-out cross-validation approximations for gaussian latent variable models. *Journal of Machine Learning Research*, 17(103):1–38, 2016.

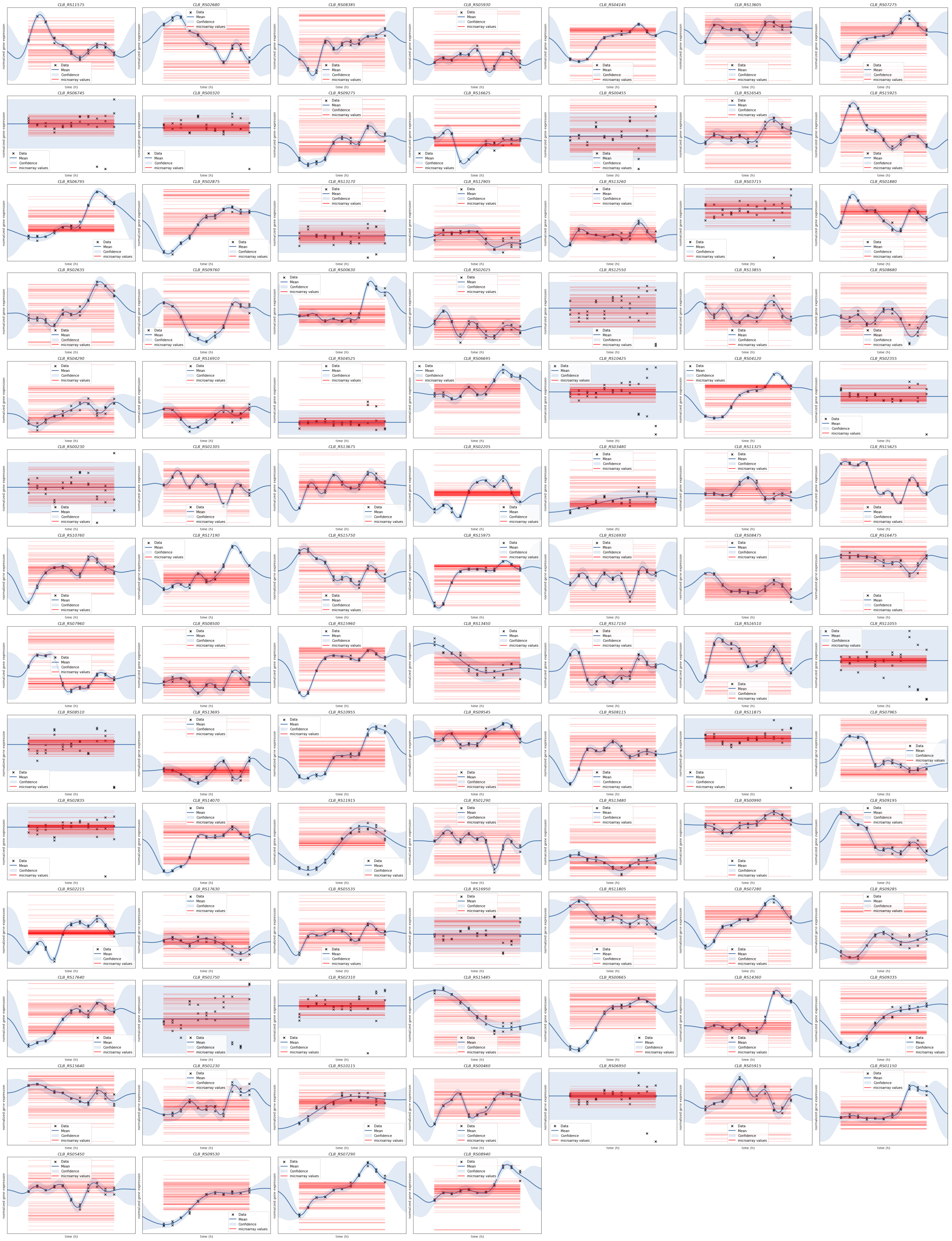

Supplementary Figure 4: GP plots for which the corresponding gene microarray values fit poorly. The intensity of red color represents the frequency of microarray values (occurring at each microarray experiment) of that gene in a particular region of the y-axis.

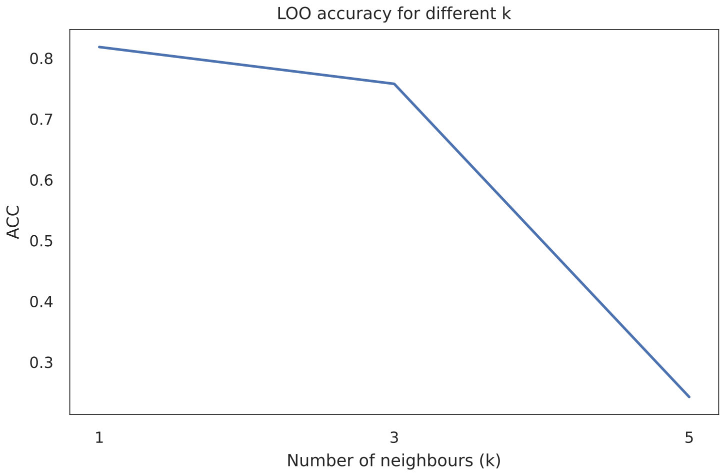

Supplementary Figure 5: Accuracy of the annotations (i.e., ACC $= \frac{\text{correct predictions}}{\text{total predictions}}$) after performing leave-one-out cross validation on the RNA-Seq samples using kNN with $k \in \{1, 2, 3\}$.

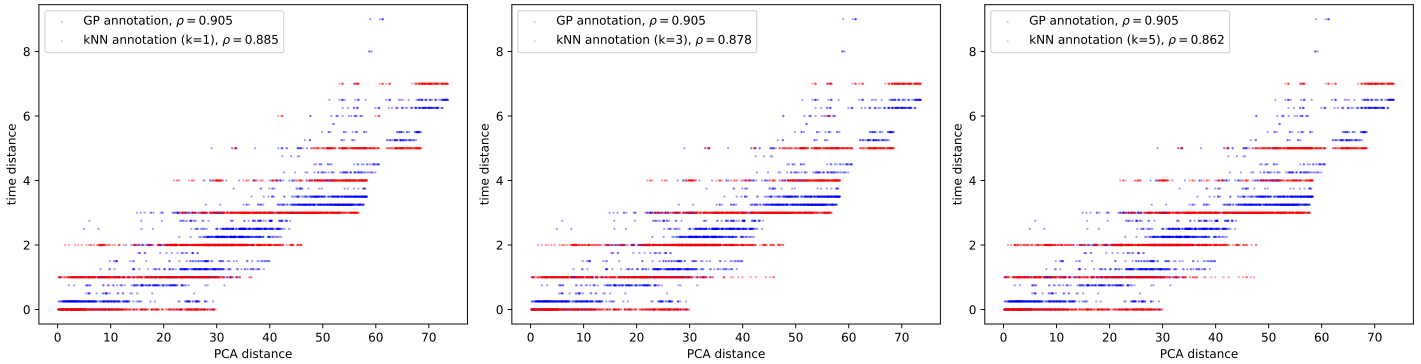

Supplementary Figure 6: Correlation between the PCA distances of all pairs of microarray samples and their corresponding time annotations distances as assigned by kNN and our GP time annotation method, for $k \in \{1, 3, 5\}$. The Pearson correlation coefficient is higher in the case of our method, for all value of k, which indicates that the microarray samples that appear to be close to each other in the PCA are assigned time annotations that are closer.

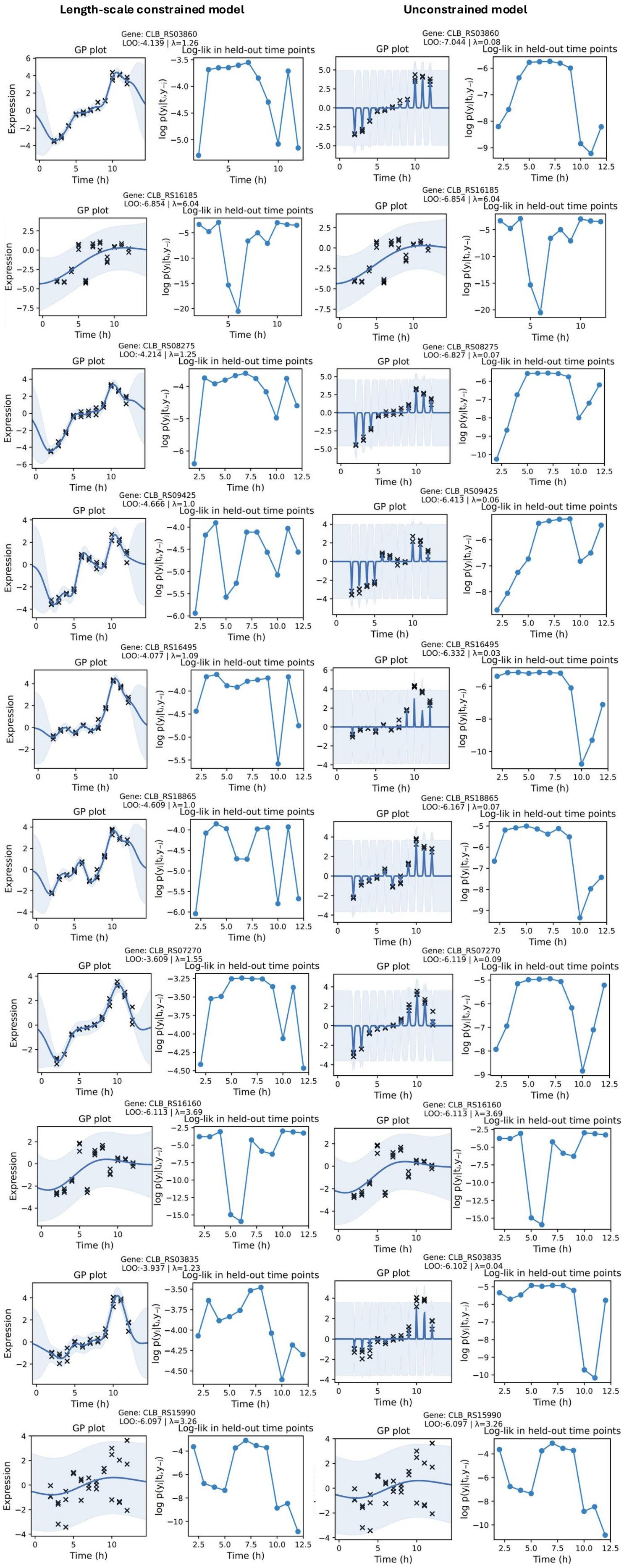

Supplementary Figure 7: Top 10 GP models that have worst LOO from both model groups (i.e., length-scale bounded, and constraints-free). For each model, its GP plot and the log-likelihood of the held-out observations at each time point are shown. The plots indicate that having no boundary constraints on the length-scale results in a smaller length-scale during optimization which leads to overfitting and a worse performance in the leave-one-out cross-validation. Above each plot the values of the optimized length-scale (λ) of the model is indicated.

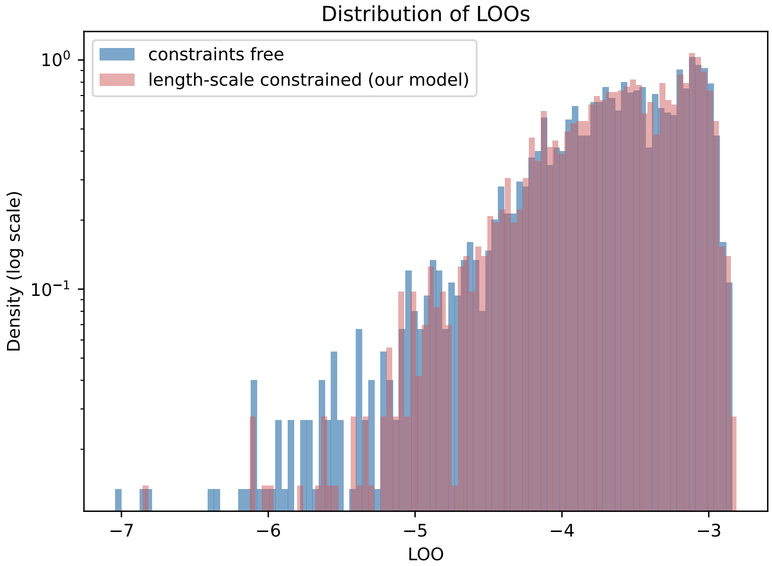

Supplementary Figure 8: Density plot showing the distribution of the LOOs for the regularized GPs and the unconstrained GPs.

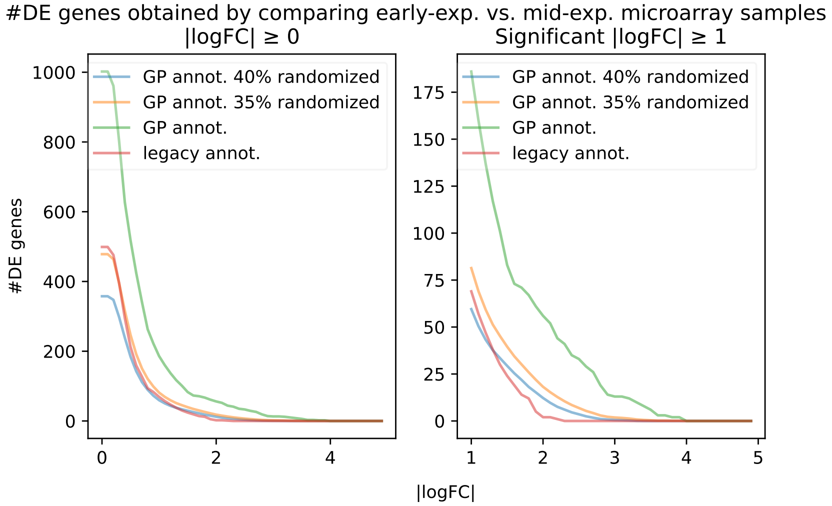

Supplementary Figure 9: Number of differentially expressed genes (adj. $p \leq 0.05$) as a function of $|\log_2 \mathrm{FC}|$ threshold.
