# Supplementary material for: A supervised Bayesian method for time (re)annotation of transcriptomics data
Source: NAR Genom Bioinform. 2025 Dec 31;7(4):lqaf203. doi: 10.1093/nargab/lqaf203 (PMC12754789; doi:10.1093/nargab/lqaf203)
Supplement: lqaf203_Supplemental_Files [file lqaf203_supplemental_files.zip › software-source-code_omics-time-annotation.zip!/software-source-code_omics-time-annotation/validation analyses/box_plot_LOO.pdf]

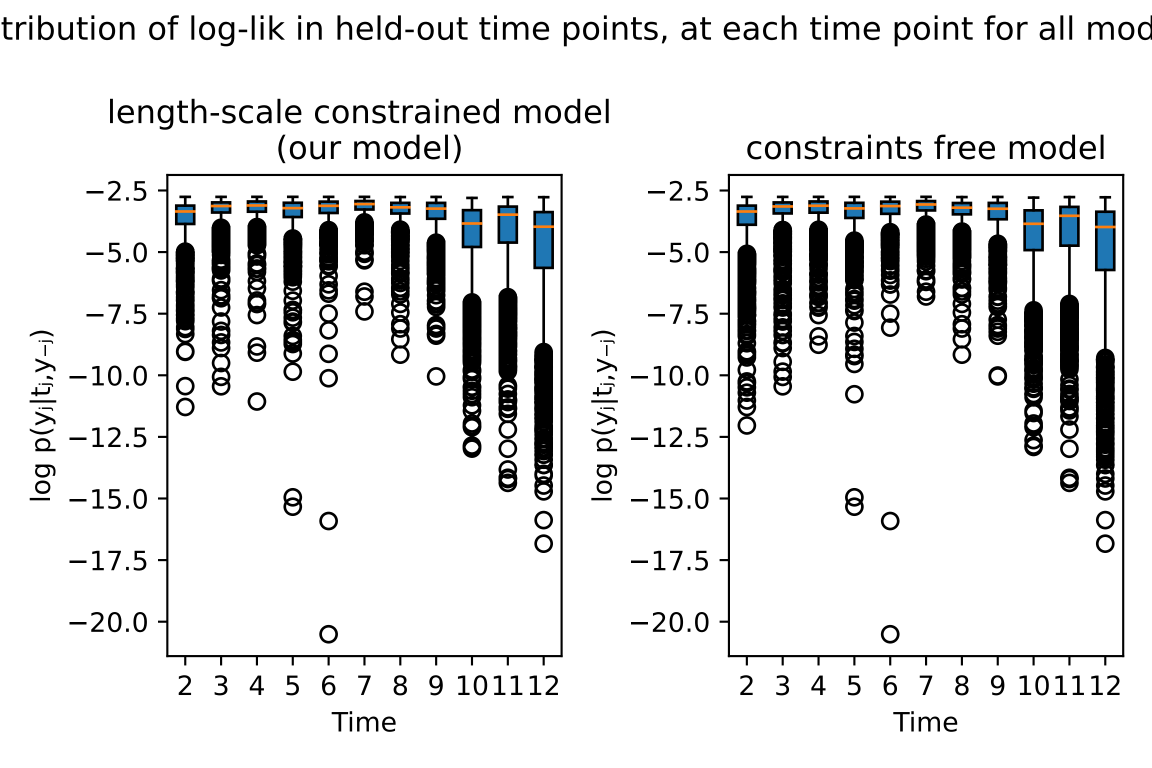
tribution of log-lik in held-out time points, at each time point for all mod
length-scale constrained model
(our model)
constraints free model
log p(y_j|t_j,y_{-j})
Time
−2.5
−5.0
−7.5
−10.0
−12.5
−15.0
−17.5
−20.0
2 3 4 5 6 7 8 9 10 11 12
