# Supplementary material for: A supervised Bayesian method for time (re)annotation of transcriptomics data
Source: NAR Genom Bioinform. 2025 Dec 31;7(4):lqaf203. doi: 10.1093/nargab/lqaf203 (PMC12754789; doi:10.1093/nargab/lqaf203)
Supplement: lqaf203_Supplemental_Files [file lqaf203_supplemental_files.zip › software-source-code_omics-time-annotation.zip!/software-source-code_omics-time-annotation/validation analyses/DE_analysis.pdf]

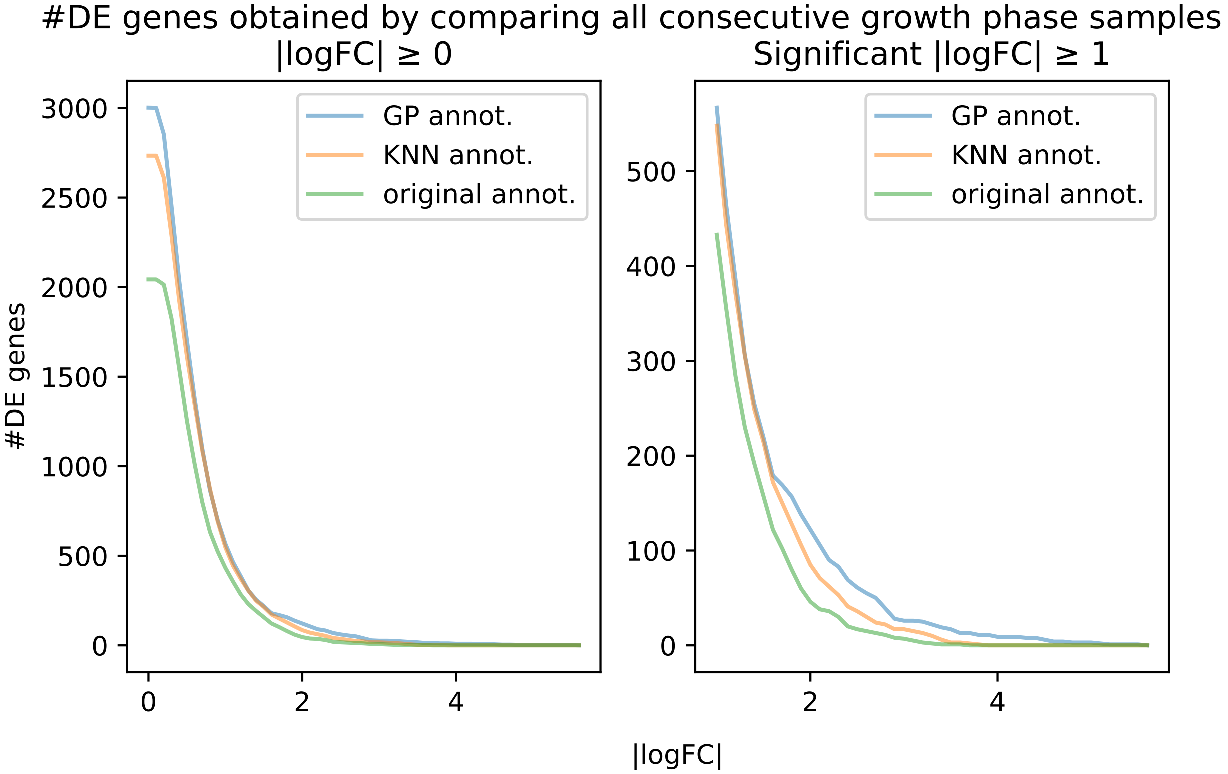

#DE genes obtained by comparing all consecutive growth phase samples
|logFC| ≥ 0
Significant |logFC| ≥ 1
GP annot.
KNN annot.
original annot.
#DE genes
|logFC|
3000
2500
2000
1500
1000
500
0
500
400
300
200
100
0
2
4
