# Supplementary material for: A supervised Bayesian method for time (re)annotation of transcriptomics data
Source: NAR Genom Bioinform. 2025 Dec 31;7(4):lqaf203. doi: 10.1093/nargab/lqaf203 (PMC12754789; doi:10.1093/nargab/lqaf203)
Supplement: lqaf203_Supplemental_Files [file lqaf203_supplemental_files.zip › software-source-code_omics-time-annotation.zip!/software-source-code_omics-time-annotation/validation analyses/synthetic_data_accuracy.pdf]

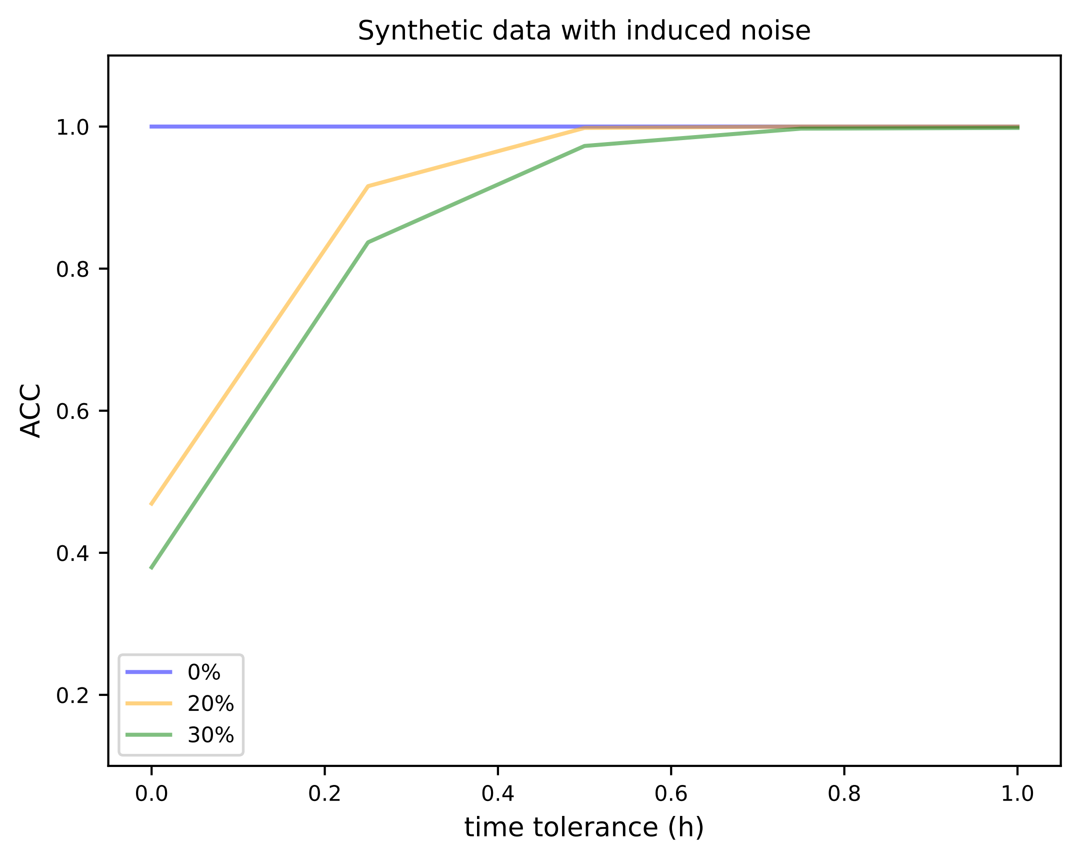

Synthetic data with induced noise
1.0
0.8
0.6
0.4
0.2
ACC
0.0
0.2
0.4
0.6
0.8
1.0
time tolerance (h)
0%
20%
30%
