# Supplementary material for: A supervised Bayesian method for time (re)annotation of transcriptomics data
Source: NAR Genom Bioinform. 2025 Dec 31;7(4):lqaf203. doi: 10.1093/nargab/lqaf203 (PMC12754789; doi:10.1093/nargab/lqaf203)
Supplement: lqaf203_Supplemental_Files [file lqaf203_supplemental_files.zip › software-source-code_omics-time-annotation.zip!/software-source-code_omics-time-annotation/validation analyses/DE_analysis_suppl.pdf]

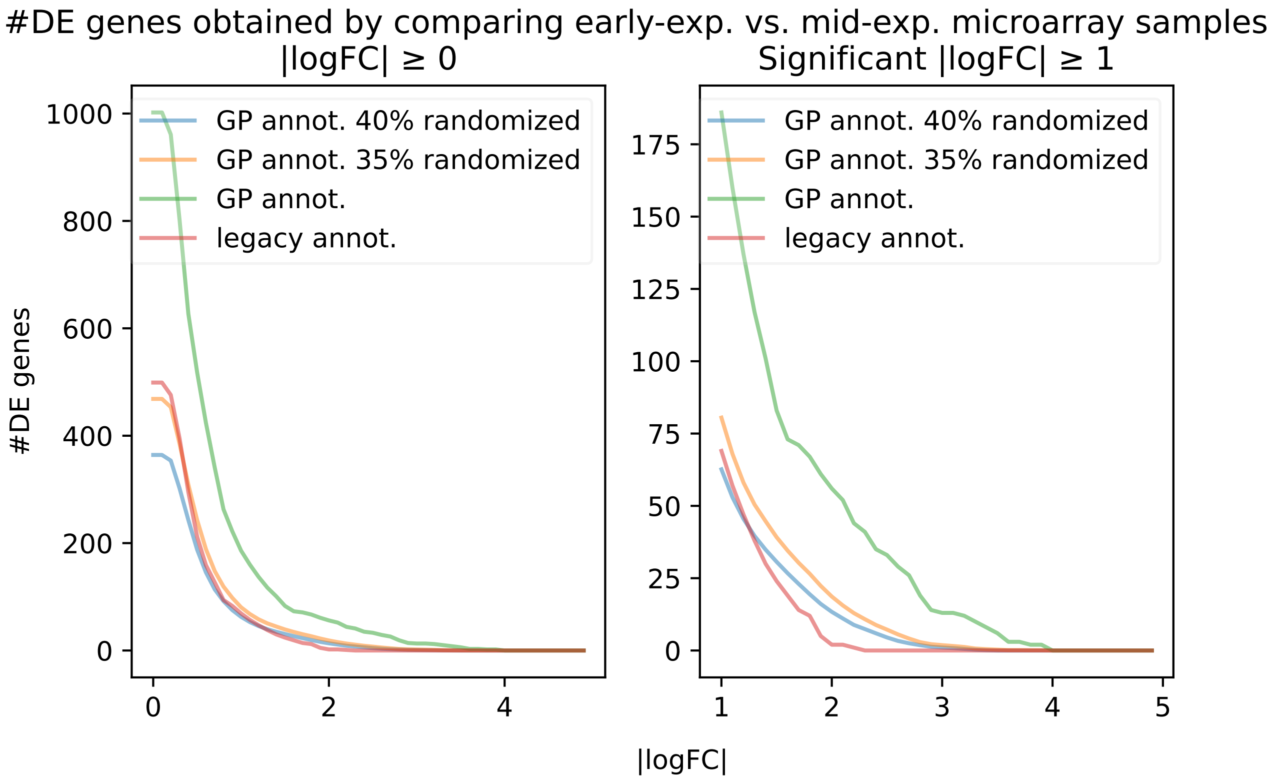
#DE genes obtained by comparing early-exp. vs. mid-exp. microarray samples
|logFC| ≥ 0
Significant |logFC| ≥ 1
GP annot. 40% randomized
GP annot. 35% randomized
GP annot.
legacy annot.
#DE genes
|logFC|
0
200
400
600
800
1000
2
4
25
50
75
100
125
150
175
1
3
5
