# Supplementary material for: A supervised Bayesian method for time (re)annotation of transcriptomics data
Source: NAR Genom Bioinform. 2025 Dec 31;7(4):lqaf203. doi: 10.1093/nargab/lqaf203 (PMC12754789; doi:10.1093/nargab/lqaf203)
Supplement: lqaf203_Supplemental_Files [file lqaf203_supplemental_files.zip › software-source-code_omics-time-annotation.zip!/software-source-code_omics-time-annotation/validation analyses/suppl_loo_plots_ordered_constrained_gp.pdf]

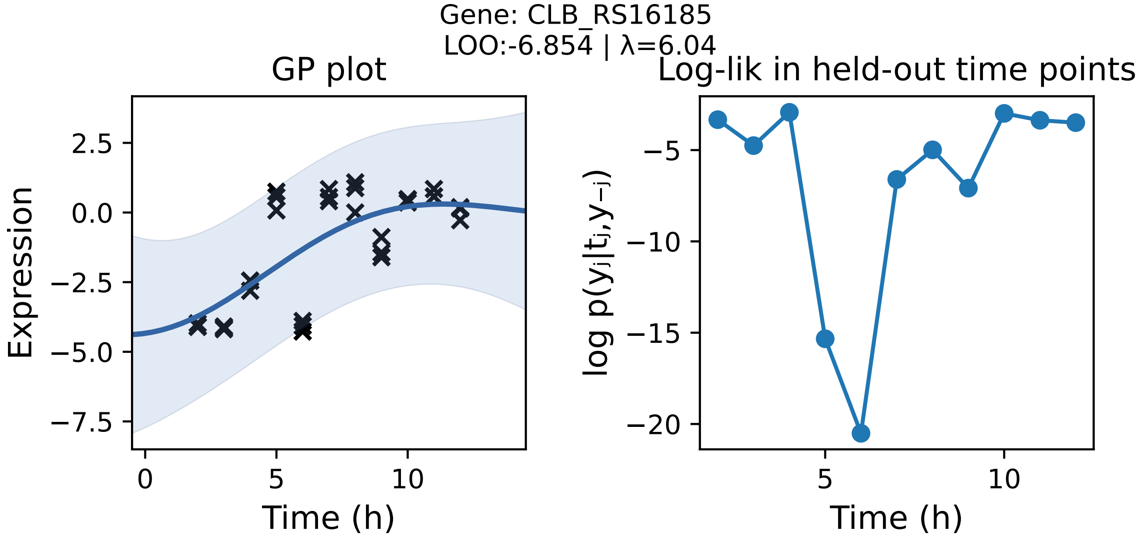
Gene: CLB_RS16185
LOO:-6.854 | λ=6.04
GP plot
Expression
2.5
0.0
−2.5
−5.0
−7.5
0
5
10
Time (h)
Log-lik in held-out time points
log p(y_j|t_j,y_−j)
−5
−10
−15
−20
5
10
Time (h)

Gene: CLB_RS16160
LOO:-6.113 | λ=3.69

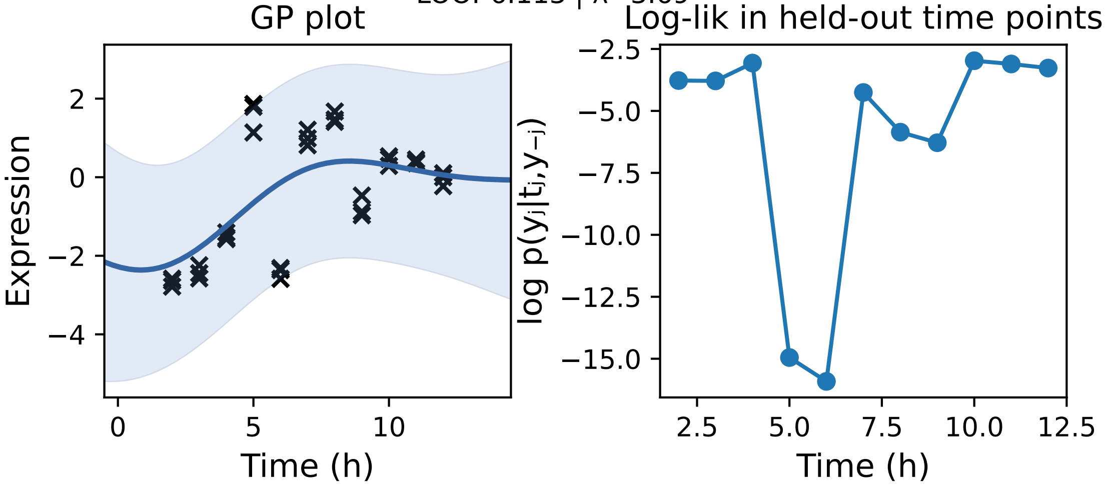

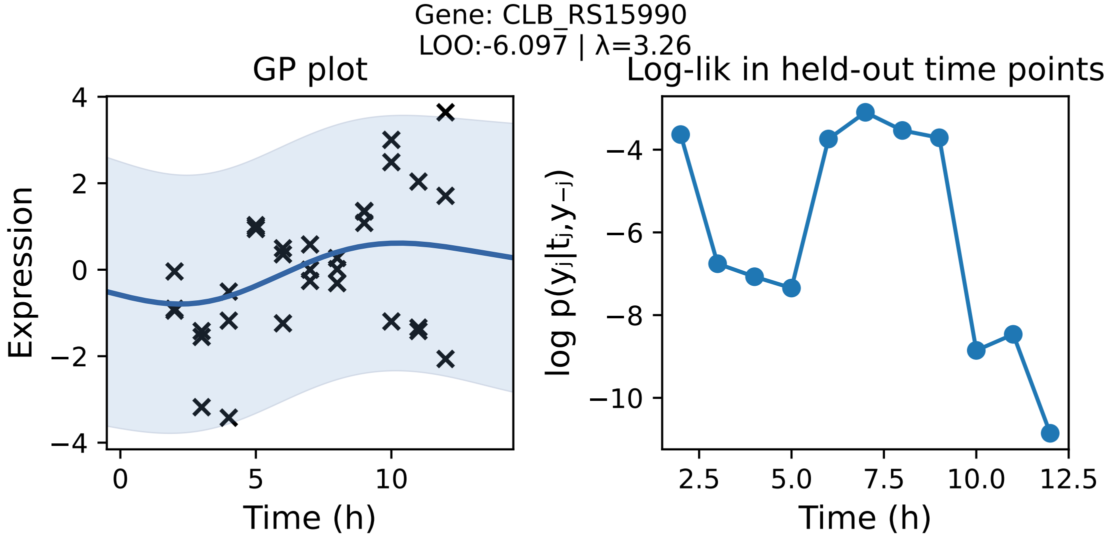
Gene: CLB_RS15990
LOO:-6.097 | λ=3.26
GP plot
Expression
Time (h)
Log-lik in held-out time points
log p(yj|tj,y−j)
Time (h)

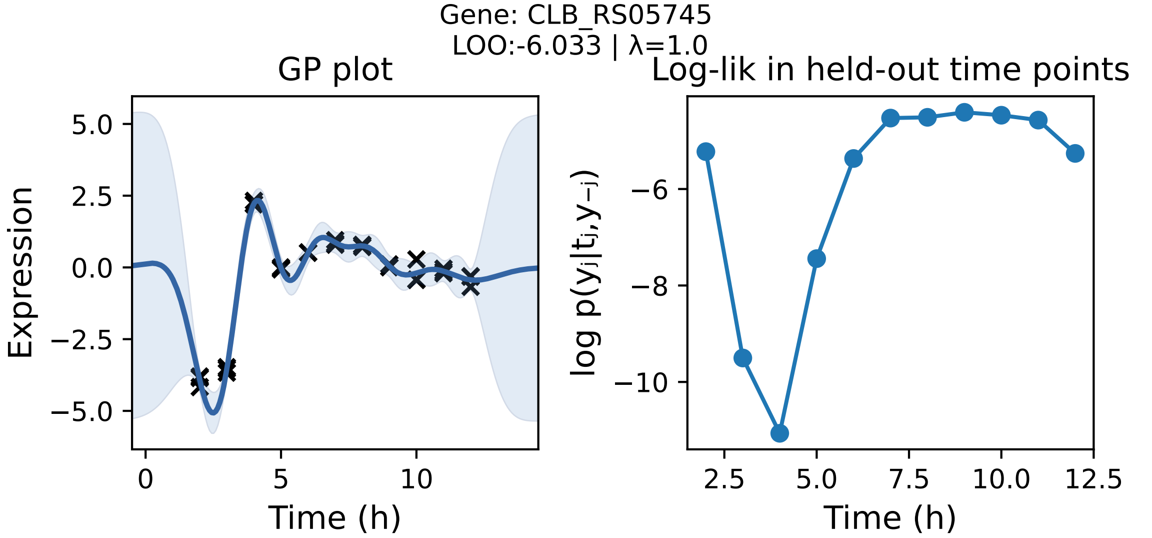
Gene: CLB_RS05745
LOO:-6.033 | λ=1.0
GP plot
Expression
Time (h)
5.0
2.5
0.0
−2.5
−5.0
0
5
10
Log-lik in held-out time points
log p(y_j|t_j,y_−j)
Time (h)
−6
−8
−10
2.5
5.0
7.5
10.0
12.5

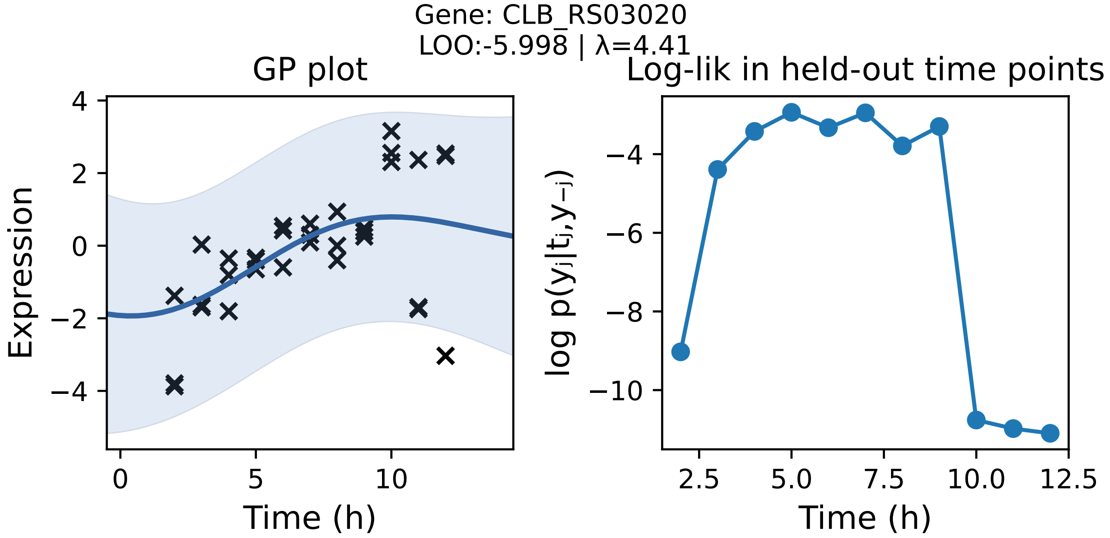
Gene: CLB_RS03020
LOO:-5.998 | λ=4.41
GP plot
Expression
Time (h)
4
2
0
−2
−4
0
5
10
Log-lik in held-out time points
log p(y_j|t_i,y_−j)
Time (h)
−4
−6
−8
−10
2.5
5.0
7.5
10.0
12.5

Gene: CLB_RS10705
LOO:-5.778 | λ=1.0

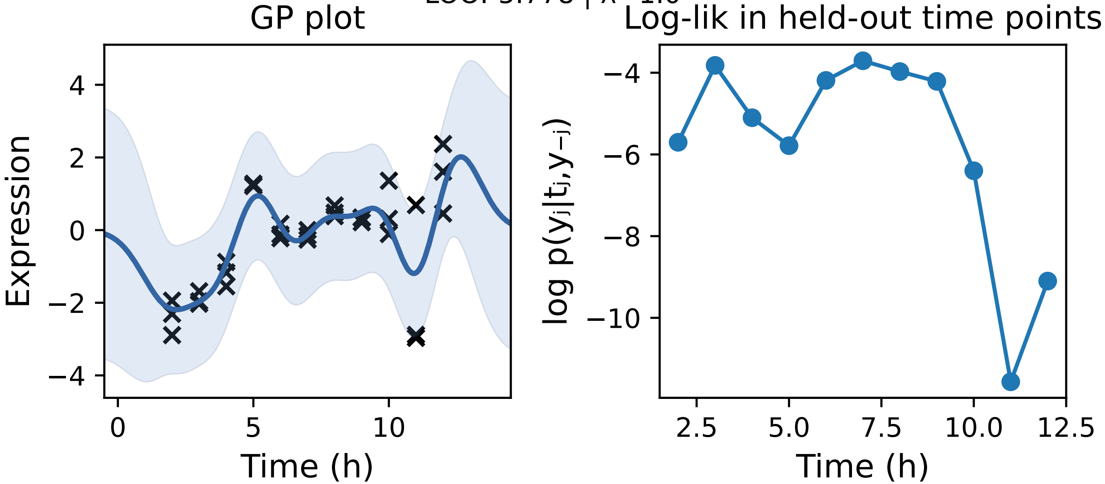

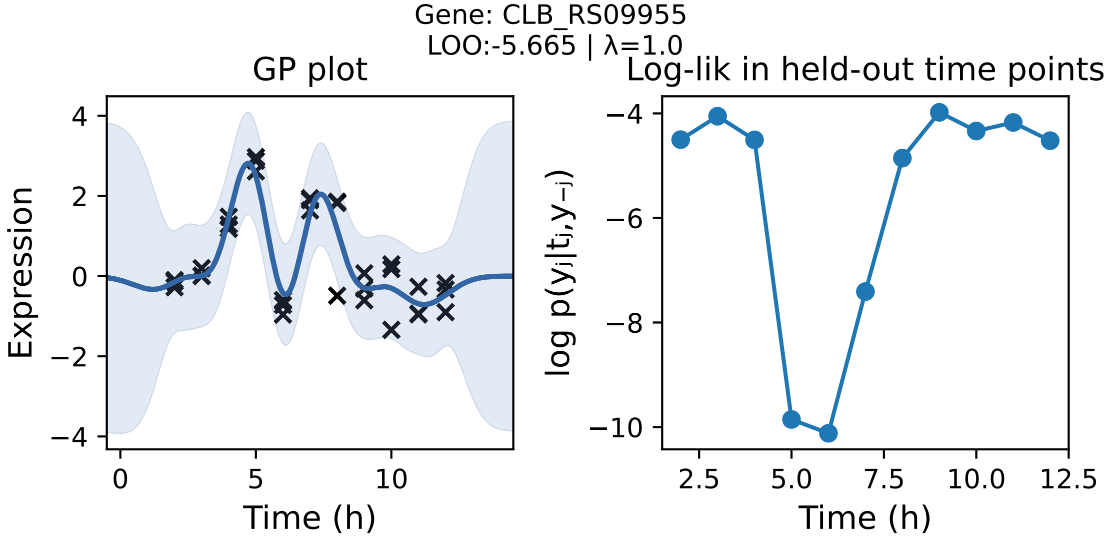
Gene: CLB_RS09955
LOO:-5.665 | λ=1.0
GP plot
Expression
Time (h)
4
2
0
−2
−4
0
5
10
Log-lik in held-out time points
log p(yj|ti,y−j)
Time (h)
−4
−6
−8
−10
2.5
5.0
7.5
10.0
12.5

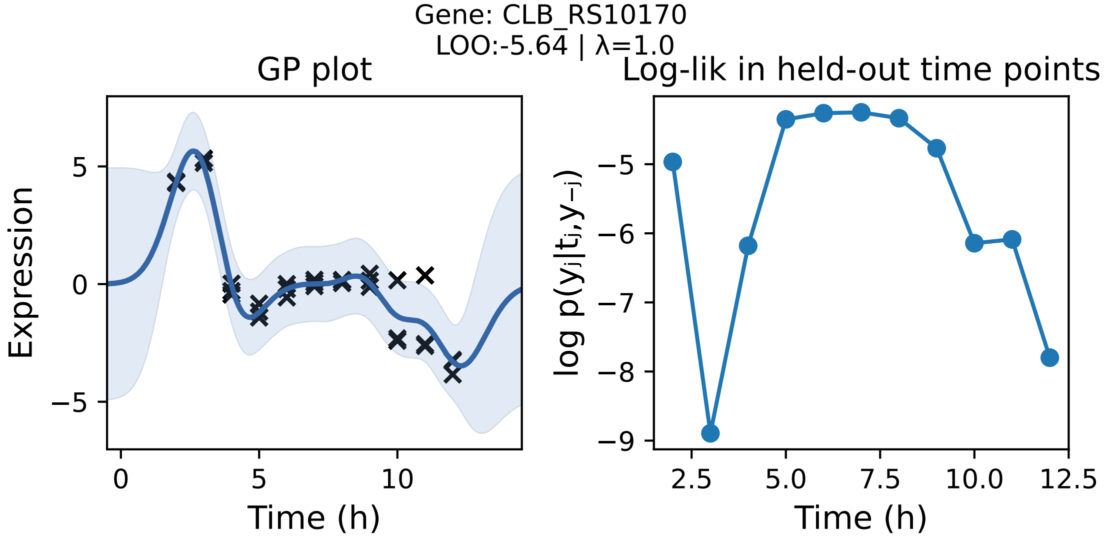

Gene: CLB_RS10170
LOO:-5.64 | λ=1.0
GP plot
Expression
Time (h)
5
0
−5
0
5
10
Log-lik in held-out time points
log p(y_j|t_j,y_−j)
Time (h)
−5
−6
−7
−8
−9
2.5
5.0
7.5
10.0
12.5

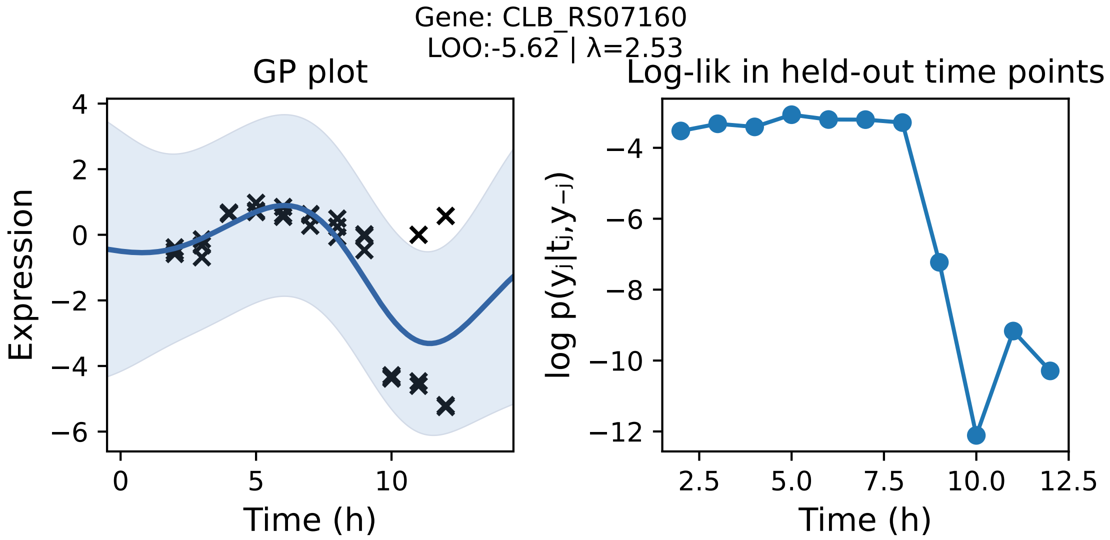

Gene: CLB_RS07160
LOO:-5.62 | λ=2.53
GP plot
Expression
Time (h)
Log-lik in held-out time points
log p(yj|tj,y−j)
Time (h)

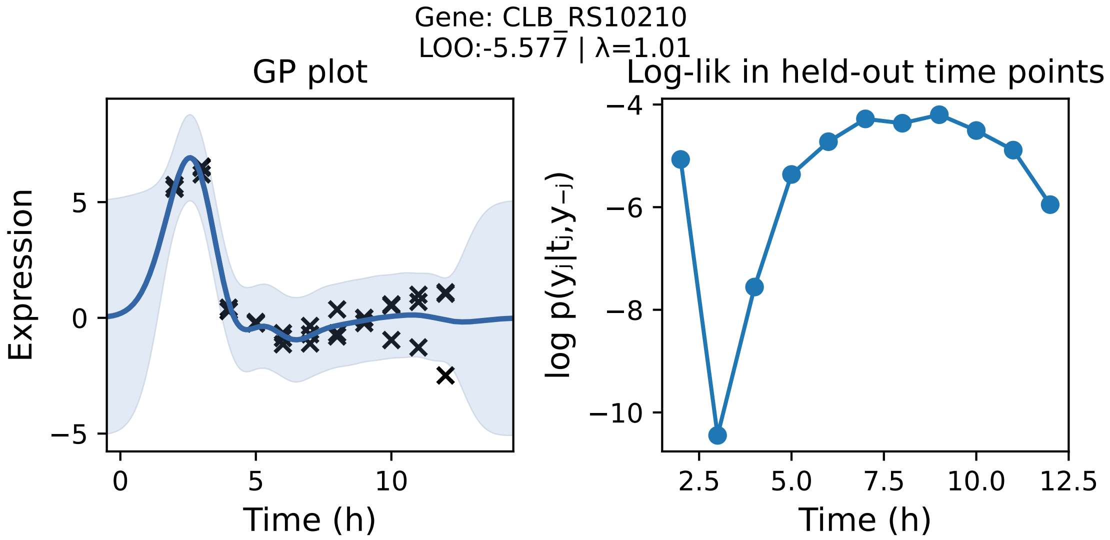

Gene: CLB_RS10210
LOO:-5.577 | λ=1.01
GP plot
Expression
5
0
−5
0
5
10
Time (h)
Log-lik in held-out time points
log p(y_j|t_j,y_−j)
−4
−6
−8
−10
2.5
5.0
7.5
10.0
12.5
Time (h)

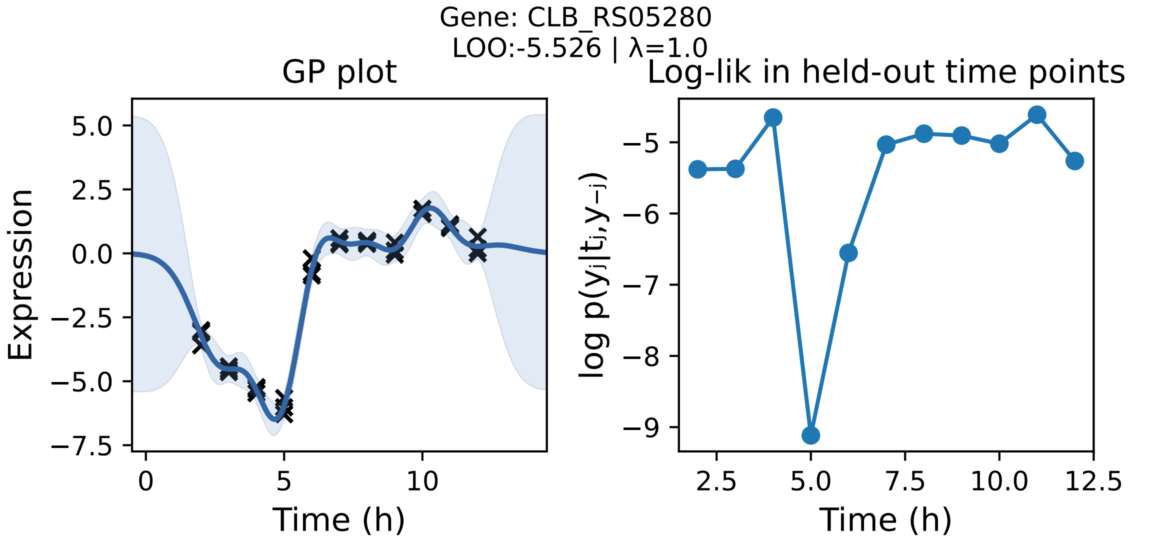

Gene: CLB_RS05280
LOO:-5.526 | λ=1.0
GP plot
Expression
Time (h)
Log-lik in held-out time points
log p(y_j|t_j,y_−j)
Time (h)

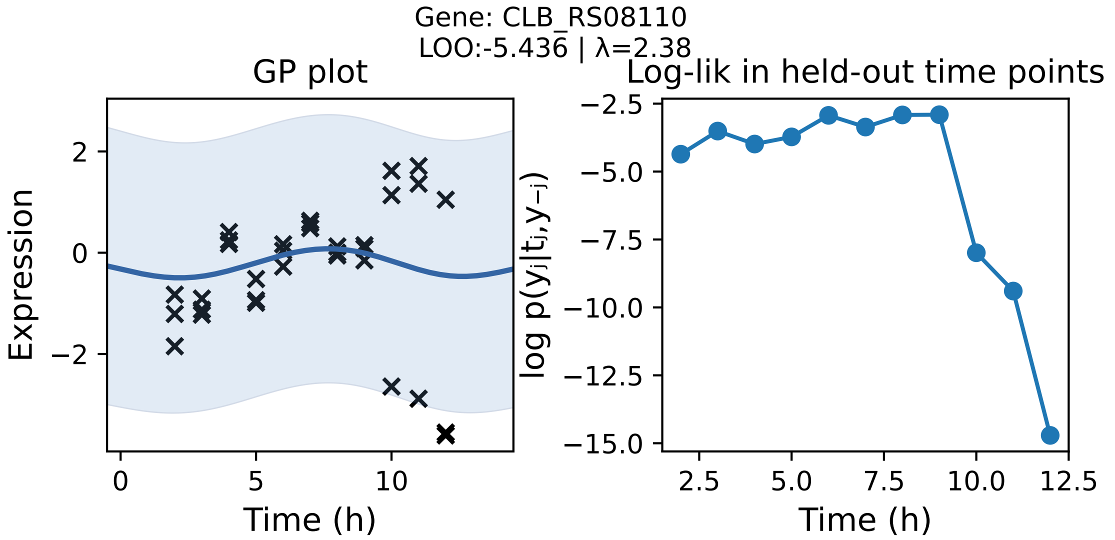

Gene: CLB_RS08110
LOO:-5.436 | λ=2.38
GP plot
Expression
Time (h)
Log-lik in held-out time points
log p(y_j|t_j,y_−j)
Time (h)

Gene: CLB_RS16460
LOO:-5.412 | λ=1.0

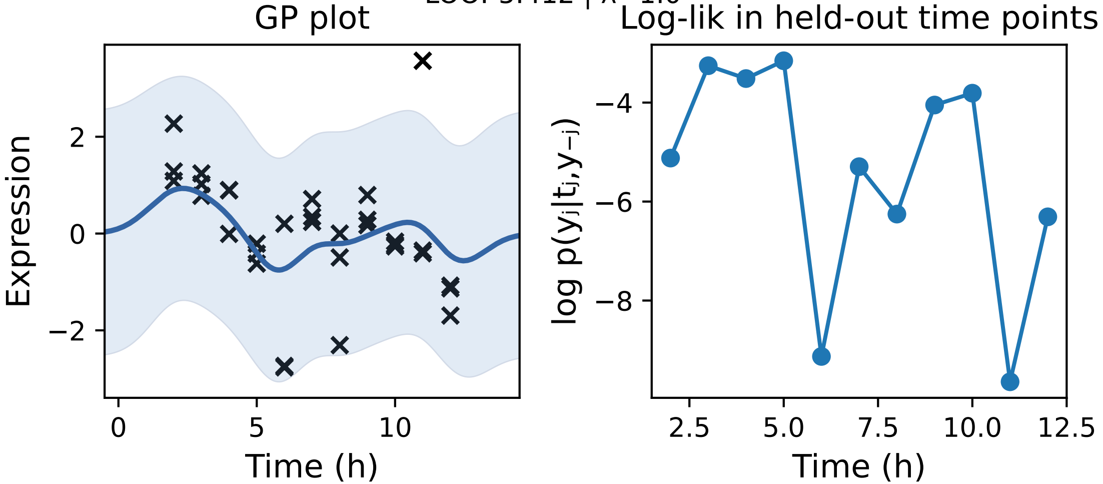

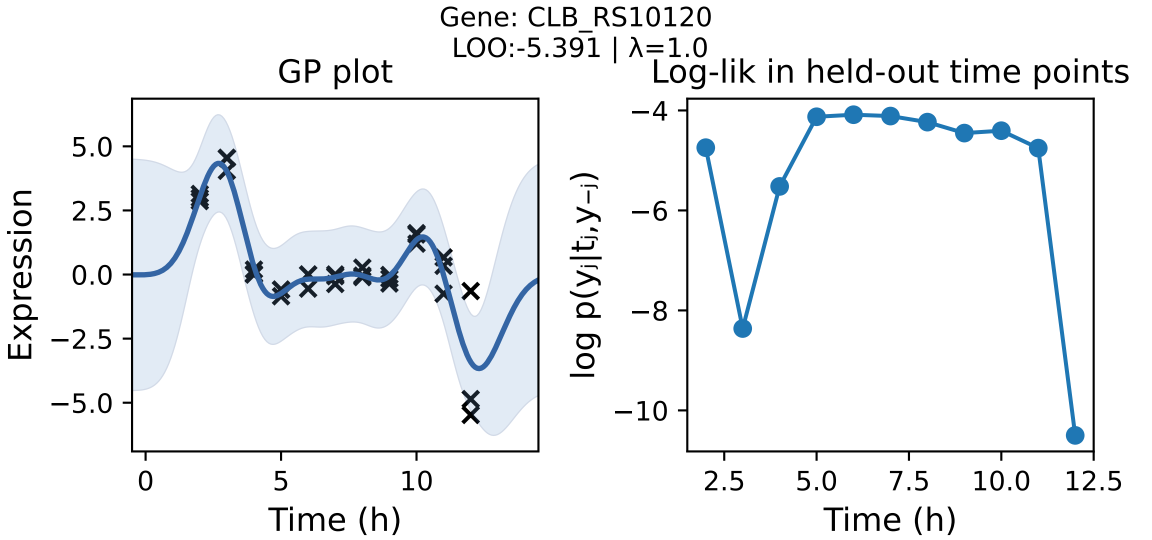
Gene: CLB_RS10120
LOO:-5.391 | λ=1.0
GP plot
Log-lik in held-out time points
Expression
5.0
2.5
0.0
−2.5
−5.0
0
5
10
Time (h)
log p(y_j|t_j,y_−j)
−4
−6
−8
−10
2.5
5.0
7.5
10.0
12.5
Time (h)

Gene: CLB_RS10180
LOO:-5.35 | λ=1.0

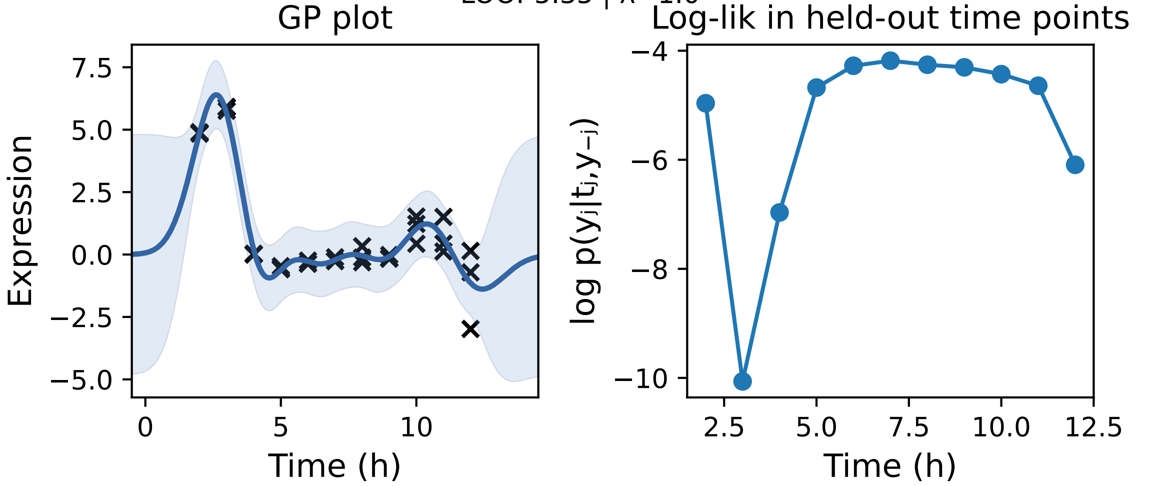

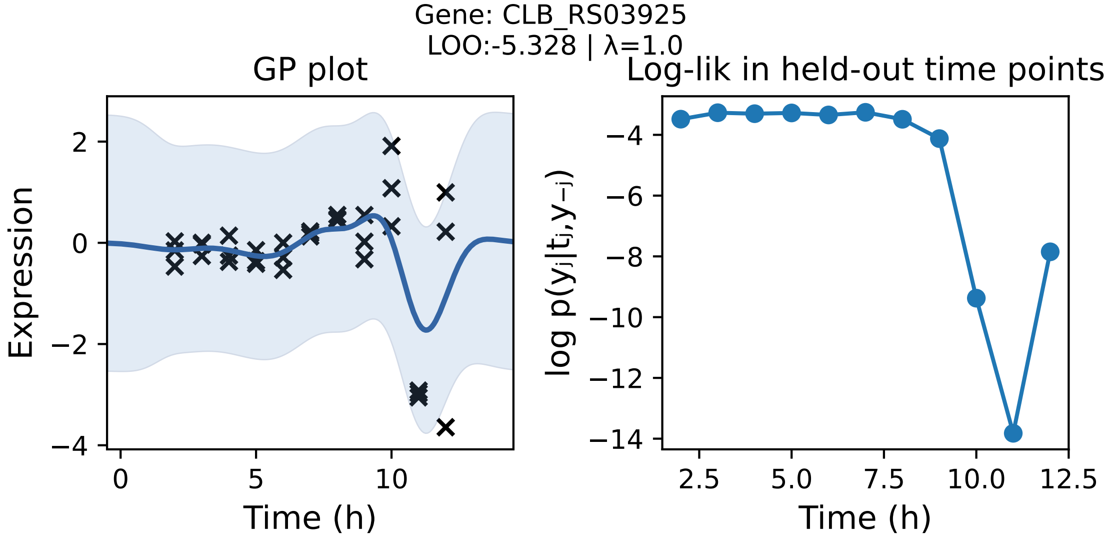

Gene: CLB_RS03925
LOO:-5.328 | λ=1.0
GP plot
Expression
Time (h)
Log-lik in held-out time points
log p(y_j|t_j,y_−j)
Time (h)

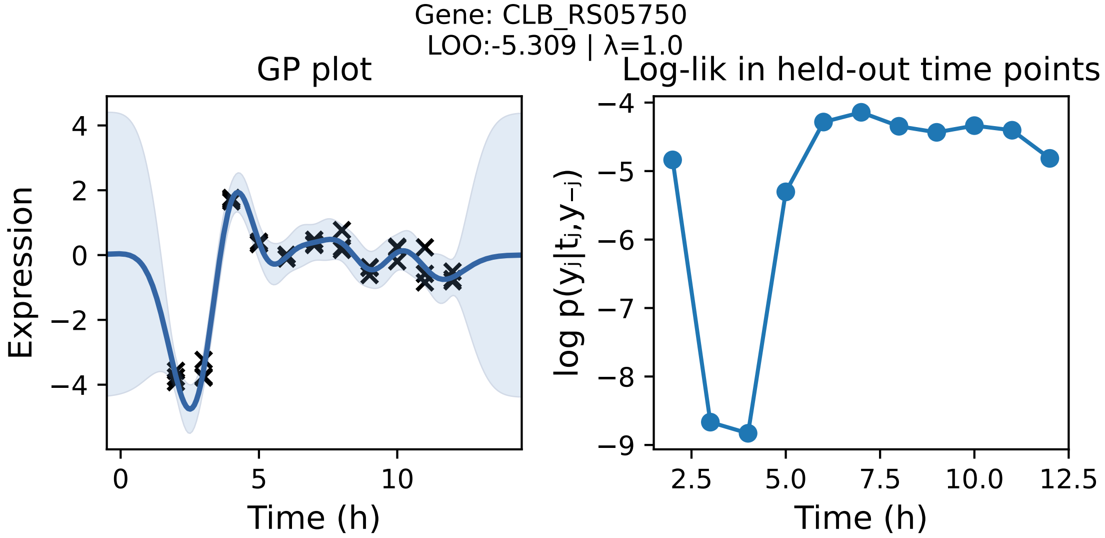

Gene: CLB_RS05750
LOO:-5.309 | λ=1.0
GP plot
Log-lik in held-out time points
Expression
Time (h)
log p(y_j|t_j,y_−j)

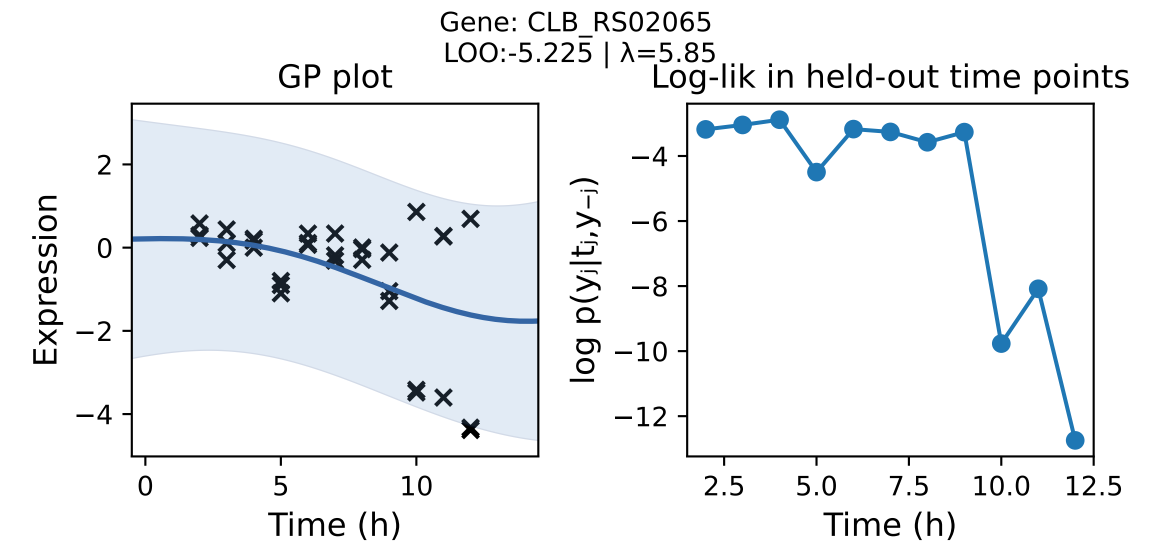

Gene: CLB_RS02065
LOO:-5.225 | λ=5.85
GP plot
Expression
Time (h)
Log-lik in held-out time points
log p(y_j|t_j,y_−j)
Time (h)

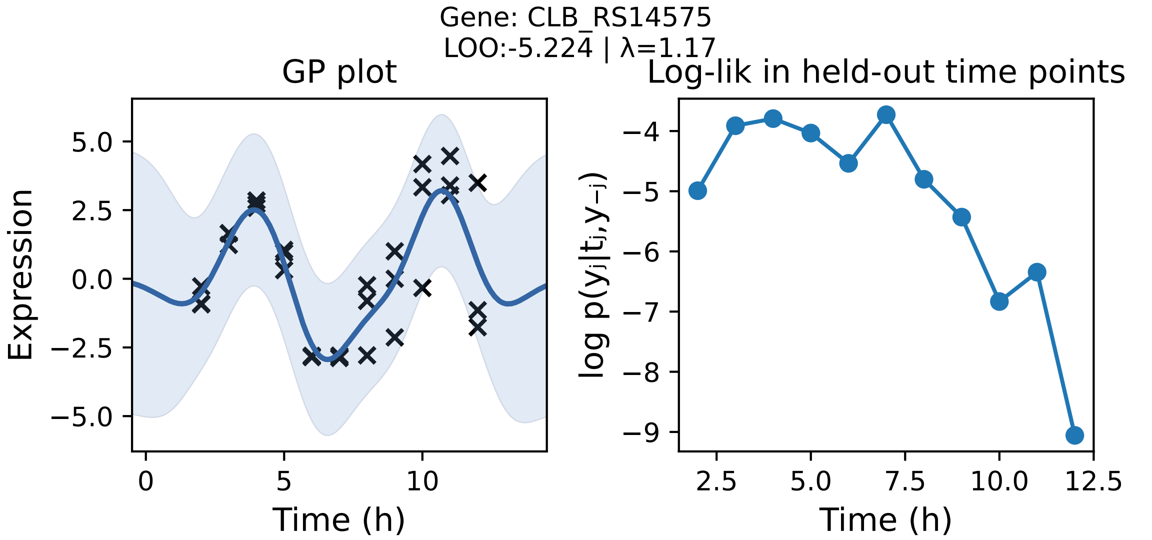

Gene: CLB_RS14575
LOO:-5.224 | λ=1.17
GP plot
Expression
5.0
2.5
0.0
−2.5
−5.0
0
5
10
Time (h)
Log-lik in held-out time points
log p(y_j|t_j,y_−j)
−4
−5
−6
−7
−8
−9
2.5
5.0
7.5
10.0
12.5
Time (h)

Gene: CLB_RS18325
LOO:-5.194 | λ=1.1

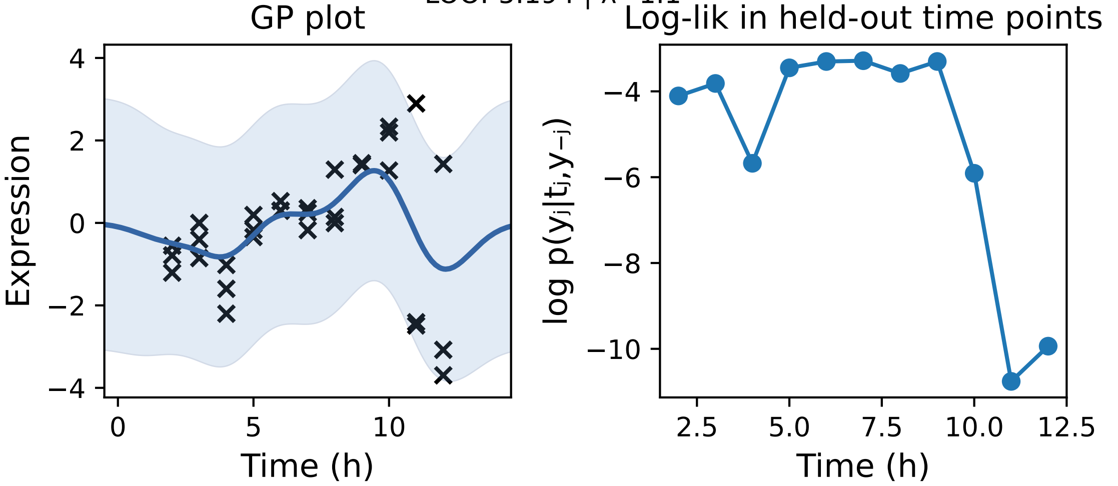

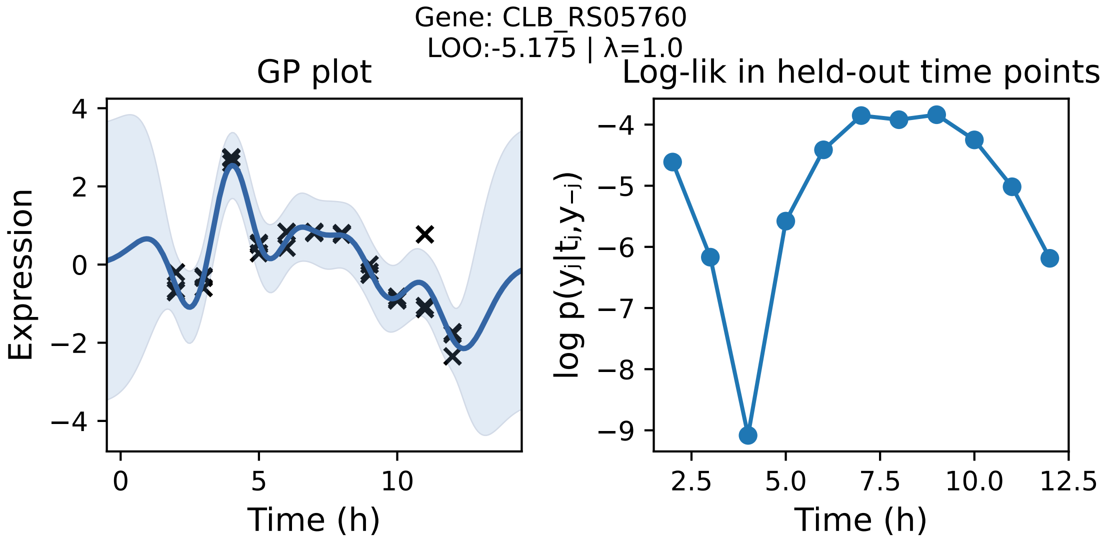
Gene: CLB_RS05760
LOO:-5.175 | λ=1.0
GP plot
Expression
Time (h)
Log-lik in held-out time points
log p(yj|tj,y−j)
Time (h)

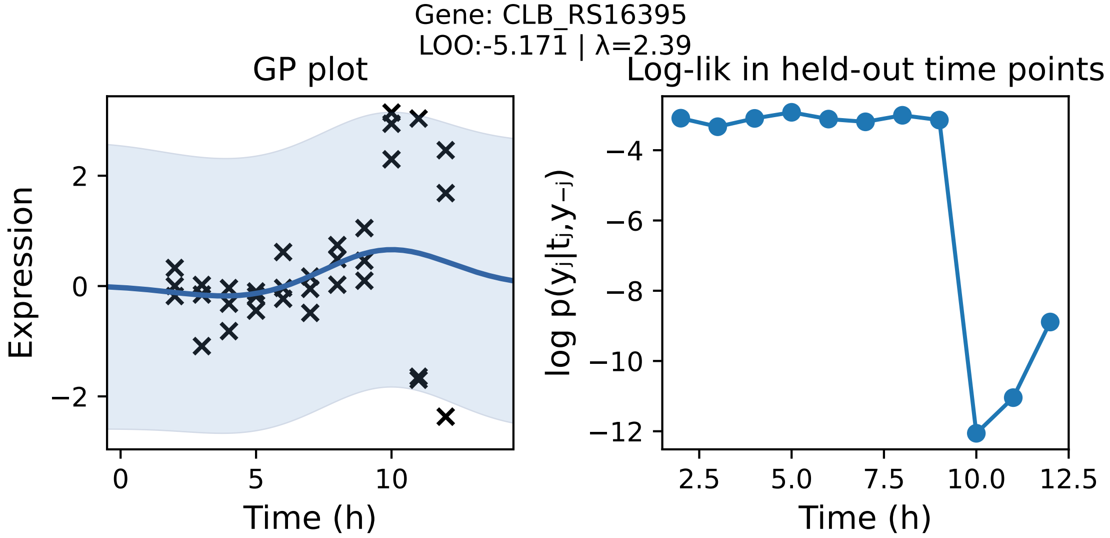
Gene: CLB_RS16395
LOO:-5.171 | λ=2.39
GP plot
Expression
Time (h)
Log-lik in held-out time points
log p(y_j|t_j,y_−j)
Time (h)

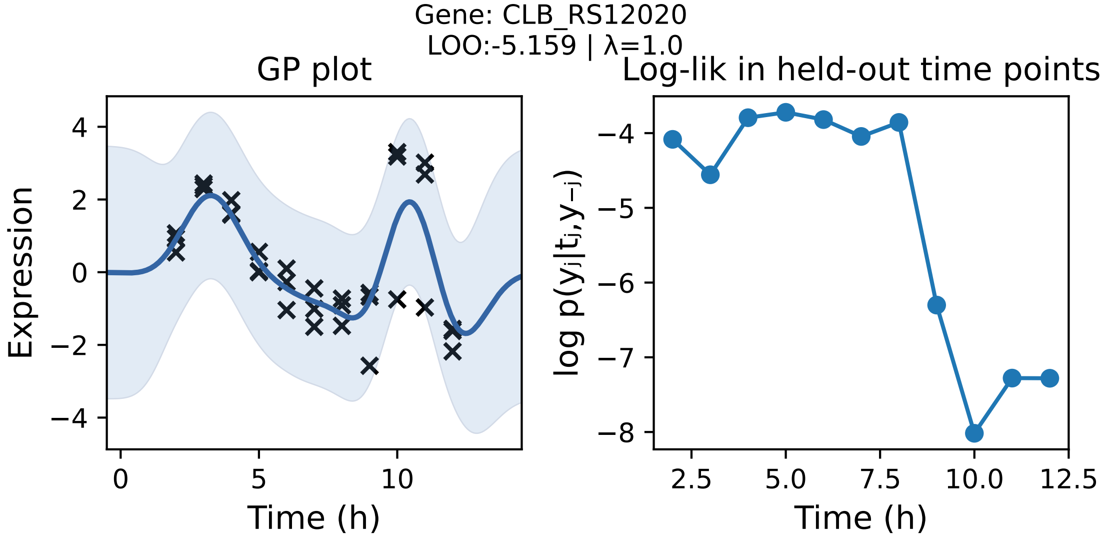

Gene: CLB_RS12020
LOO:-5.159 | λ=1.0
GP plot
Expression
Time (h)
Log-lik in held-out time points
log p(y_j|t_j,y_−j)
Time (h)

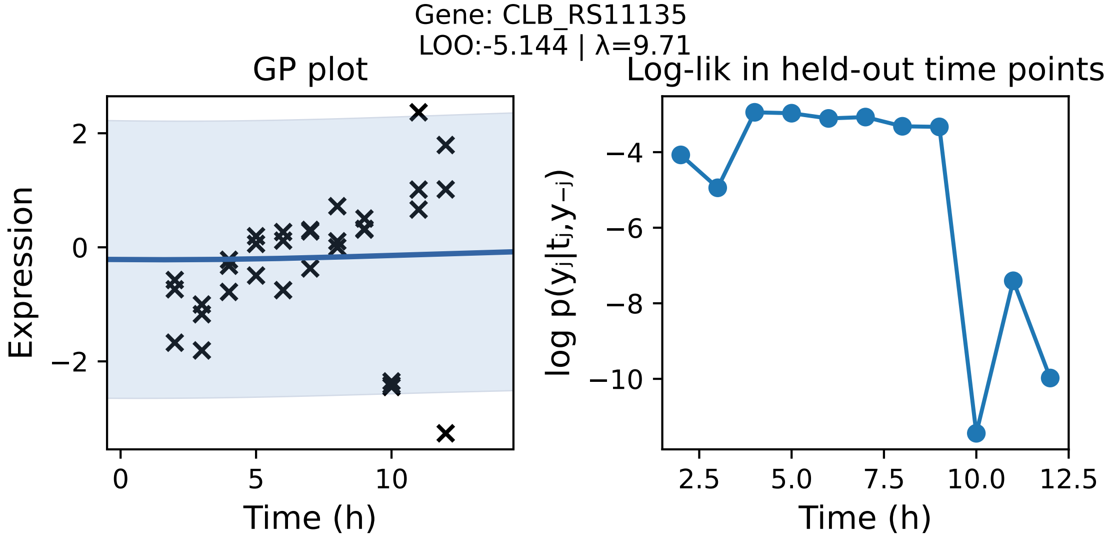

Gene: CLB_RS11135
LOO:-5.144 | λ=9.71
GP plot
Log-lik in held-out time points
Expression
Time (h)
log p(yⱼ|tⱼ,y₋ⱼ)

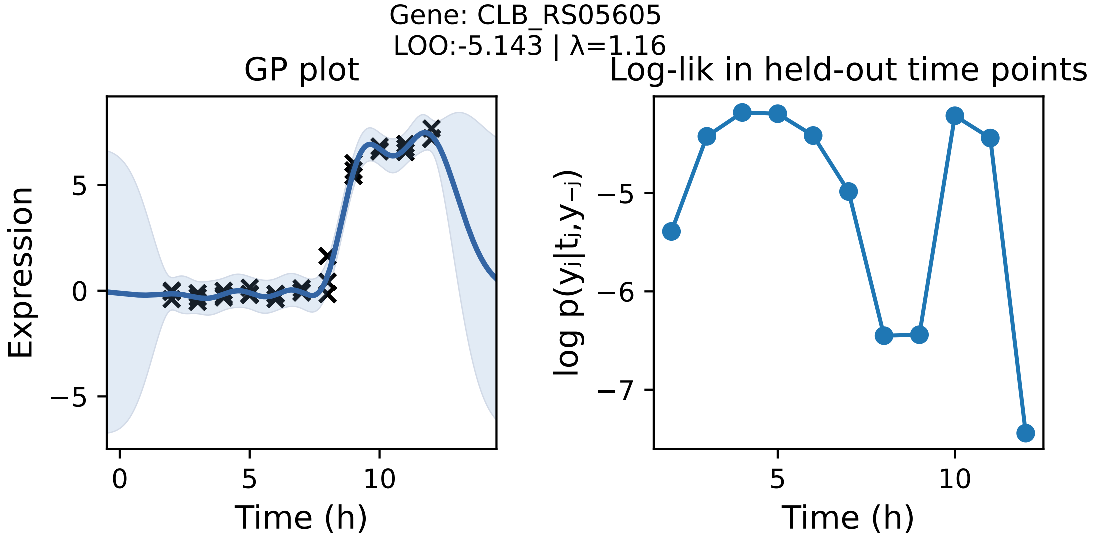
Gene: CLB_RS05605
LOO:-5.143 | λ=1.16
GP plot
Expression
Time (h)
Log-lik in held-out time points
log p(yⱼ|tⱼ,y₋ⱼ)
Time (h)

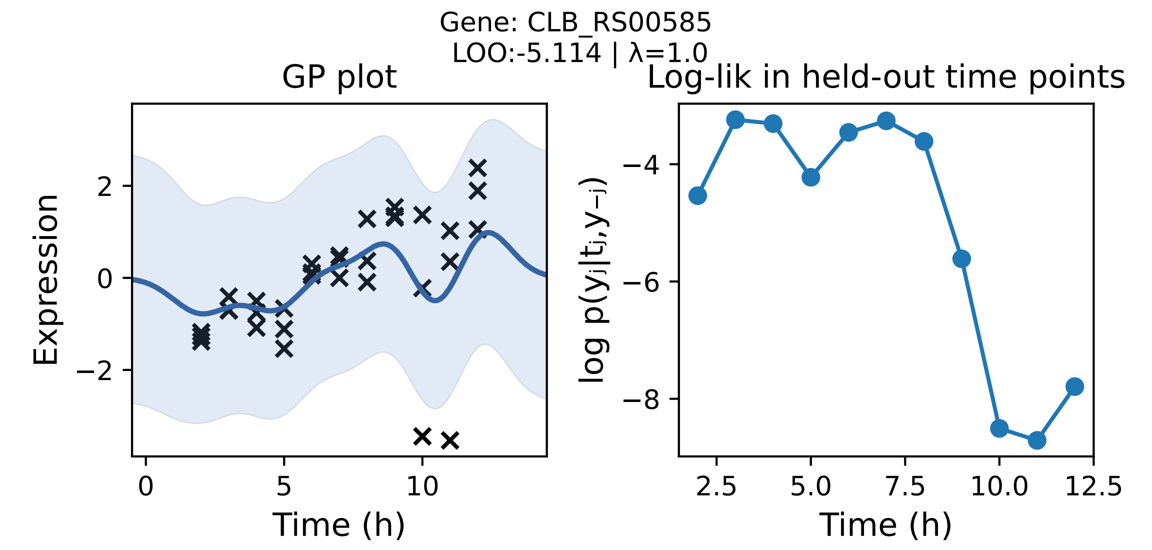
Gene: CLB_RS00585
LOO:-5.114 | λ=1.0
GP plot
Log-lik in held-out time points
Expression
Time (h)
log p(yj|tj,y−j)
Time (h)

Gene: CLB_RS08080
LOO:-5.113 | λ=1.0

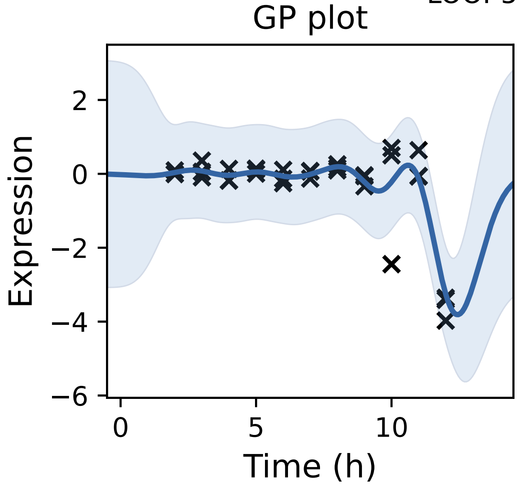

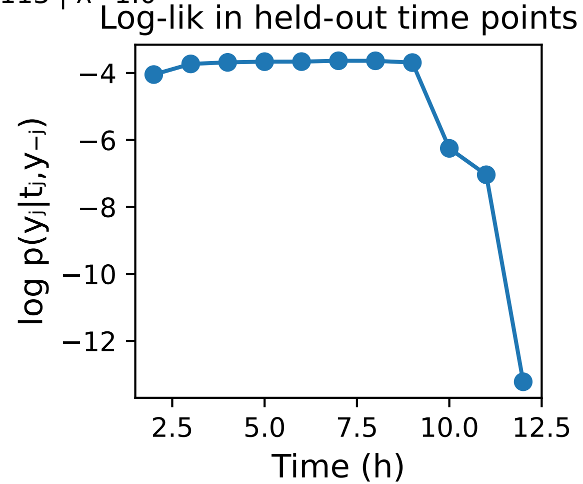

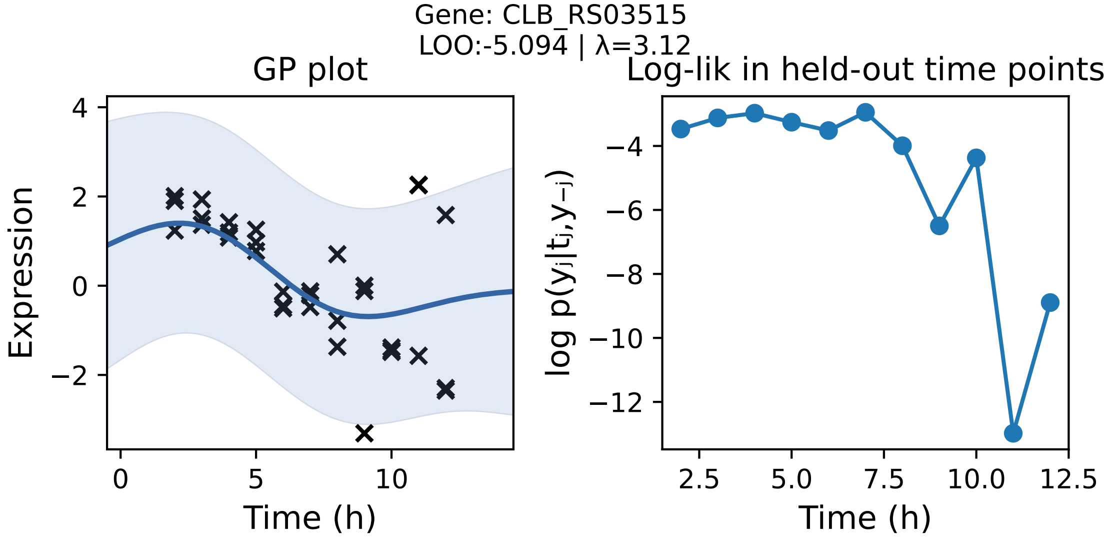

Gene: CLB_RS03515
LOO:-5.094 | λ=3.12
GP plot
Expression
Time (h)
Log-lik in held-out time points
log p(yj|tj,y−j)
Time (h)

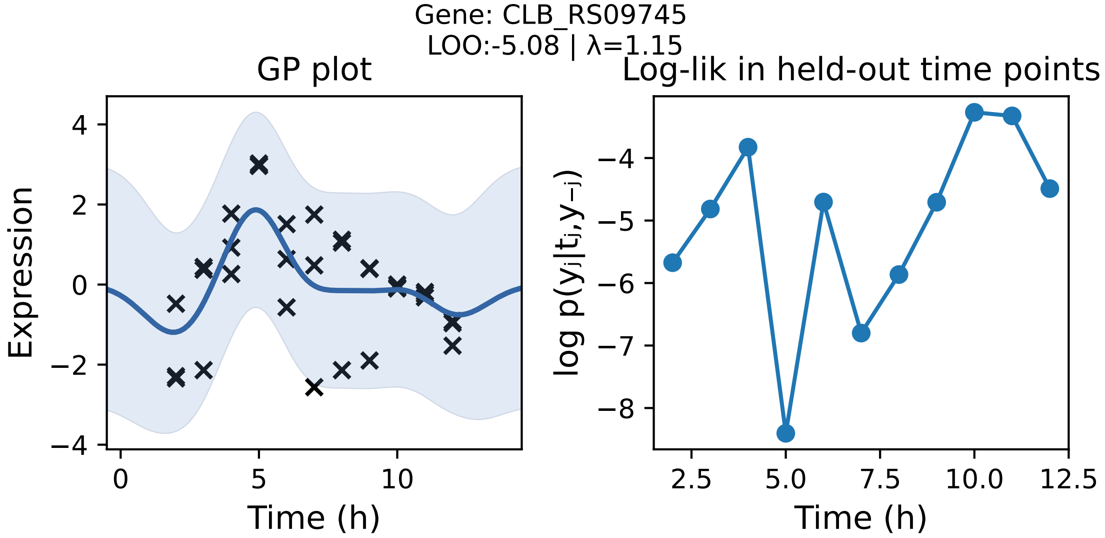

Gene: CLB_RS09745
LOO:-5.08 | λ=1.15
GP plot
Expression
Time (h)
Log-lik in held-out time points
log p(y_j|t_j,y_−j)
Time (h)

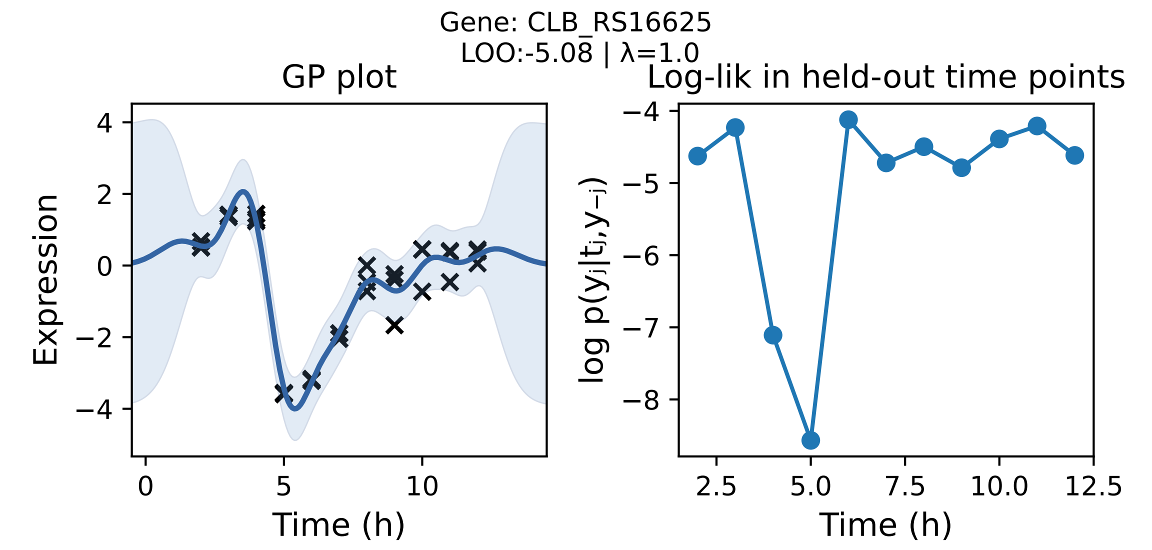
Gene: CLB_RS16625
LOO:-5.08 | λ=1.0
GP plot
Expression
Time (h)
Log-lik in held-out time points
log p(y_j|t_j,y_−j)
Time (h)

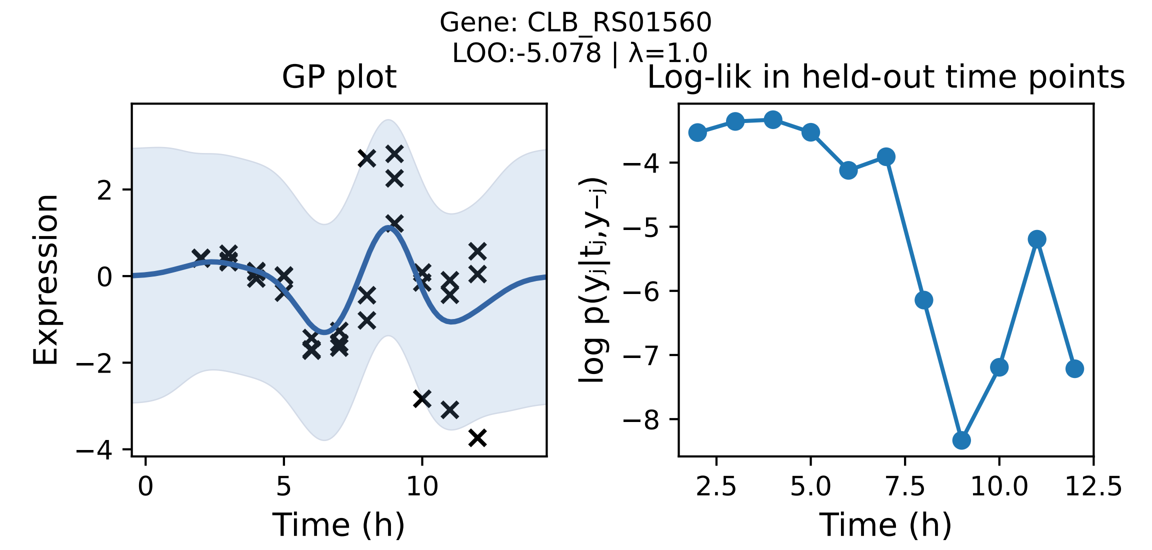
Gene: CLB_RS01560
LOO:-5.078 | λ=1.0
GP plot
Expression
Time (h)
Log-lik in held-out time points
log p(y_j|t_j,y_−j)
Time (h)

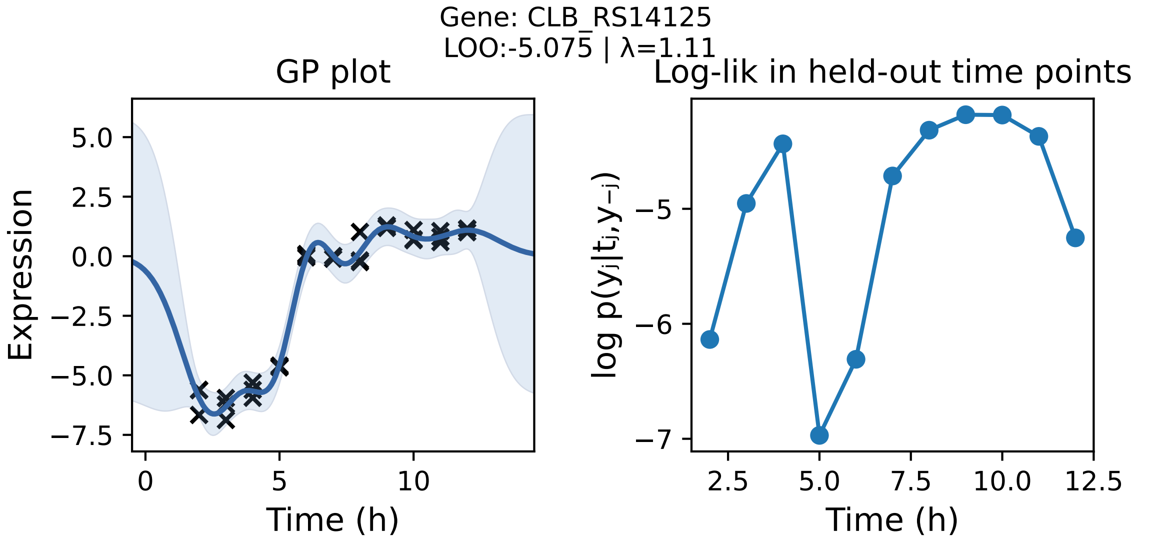

Gene: CLB_RS14125
LOO:-5.075 | λ=1.11
GP plot
Expression
5.0
2.5
0.0
−2.5
−5.0
−7.5
0
5
10
Time (h)
Log-lik in held-out time points
log p(y_j|t_j,y_−j)
−5
−6
−7
2.5
5.0
7.5
10.0
12.5
Time (h)

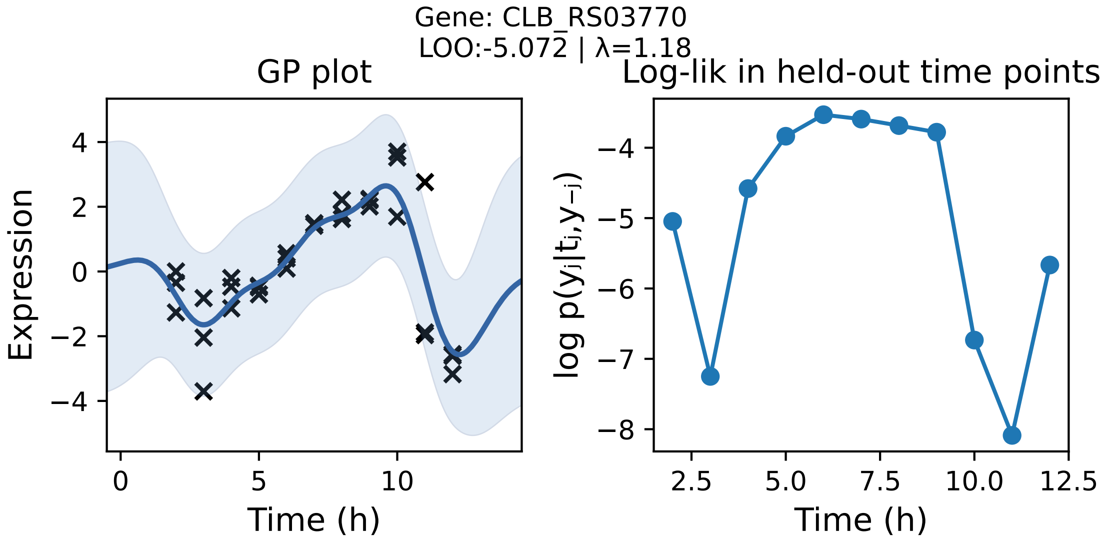

Gene: CLB_RS03770
LOO:-5.072 | λ=1.18
GP plot
Expression
Time (h)
Log-lik in held-out time points
log p(yj|tj,y−j)
Time (h)

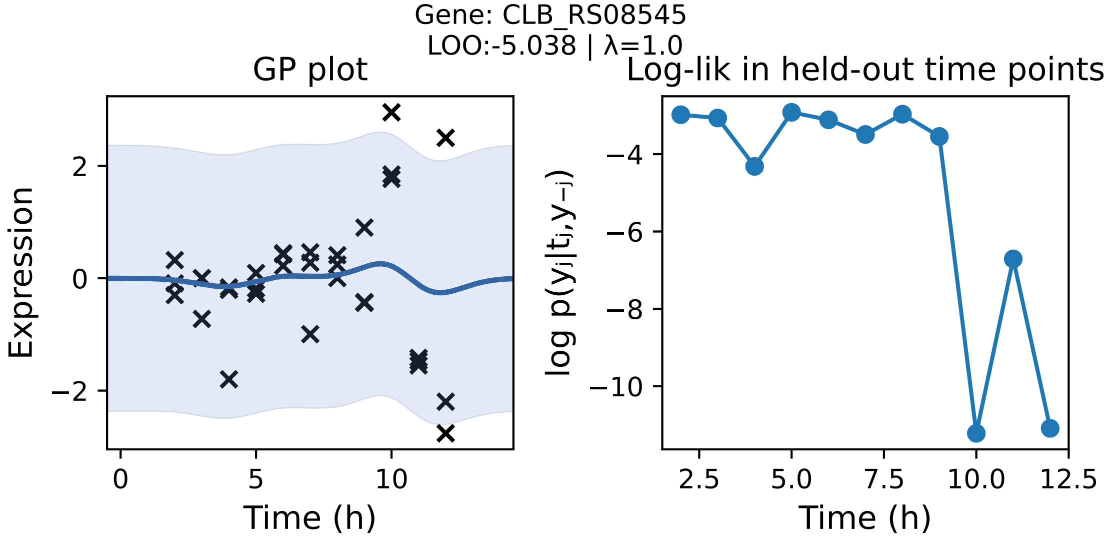
Gene: CLB_RS08545
LOO:-5.038 | λ=1.0
GP plot
Expression
Time (h)
2
0
−2
0
5
10
Log-lik in held-out time points
log p(y_j|t_j,y_−j)
Time (h)
−4
−6
−8
−10
2.5
5.0
7.5
10.0
12.5

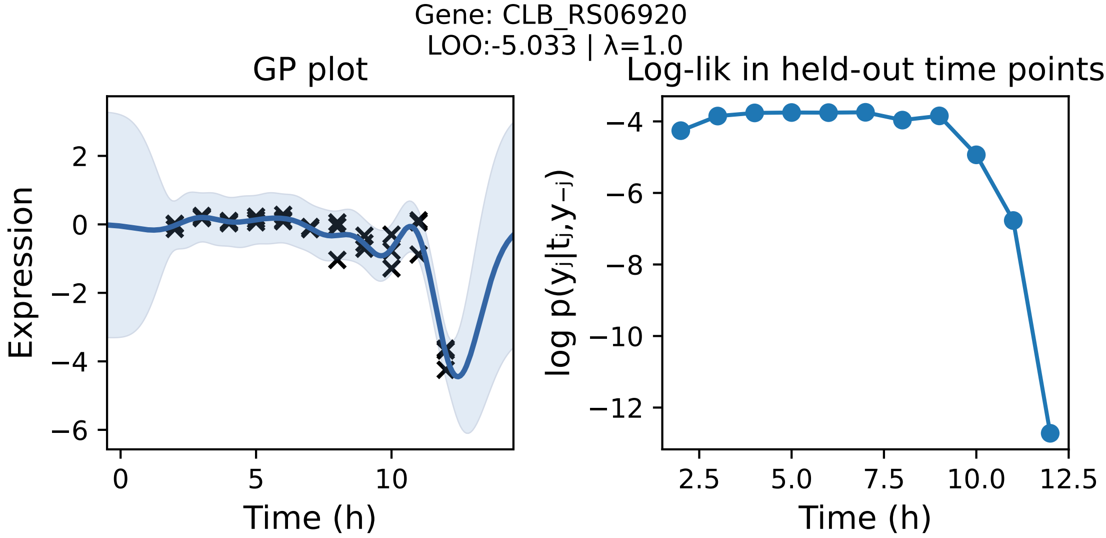

Gene: CLB_RS06920
LOO:-5.033 | λ=1.0
GP plot
Expression
Time (h)
2
0
−2
−4
−6
0
5
10
Log-lik in held-out time points
log p(yj|tj,y−j)
Time (h)
−4
−6
−8
−10
−12
2.5
5.0
7.5
10.0
12.5

Gene: CLB_RS05295
LOO:-5.033 | λ=1.79

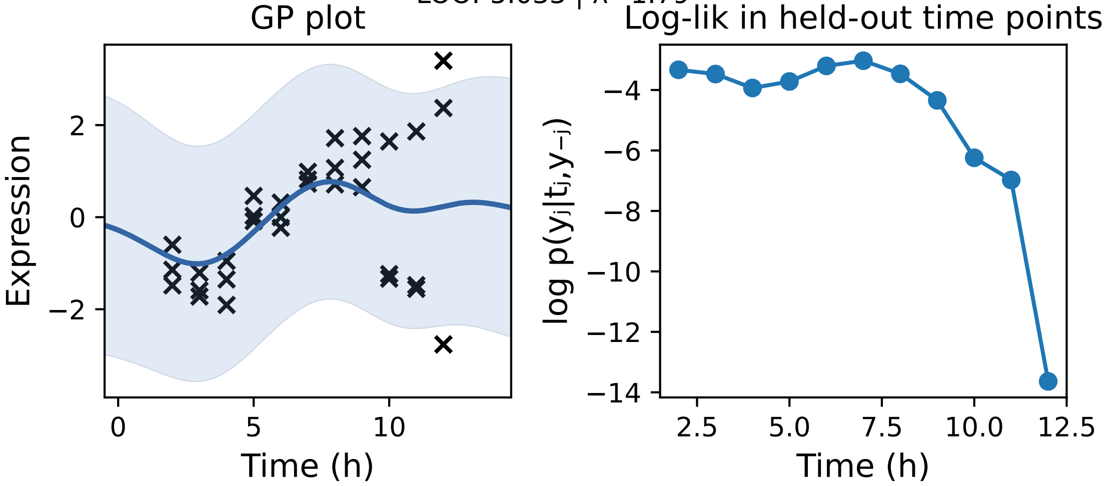

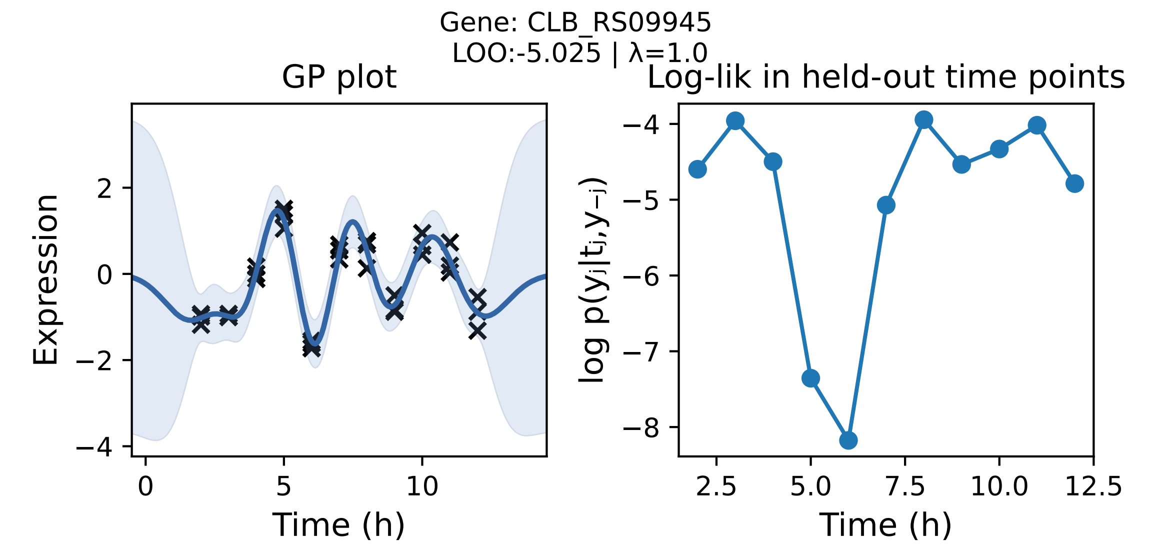

Gene: CLB_RS09945
LOO:-5.025 | λ=1.0
GP plot
Expression
Time (h)
Log-lik in held-out time points
log p(yj|tj,y−j)
Time (h)

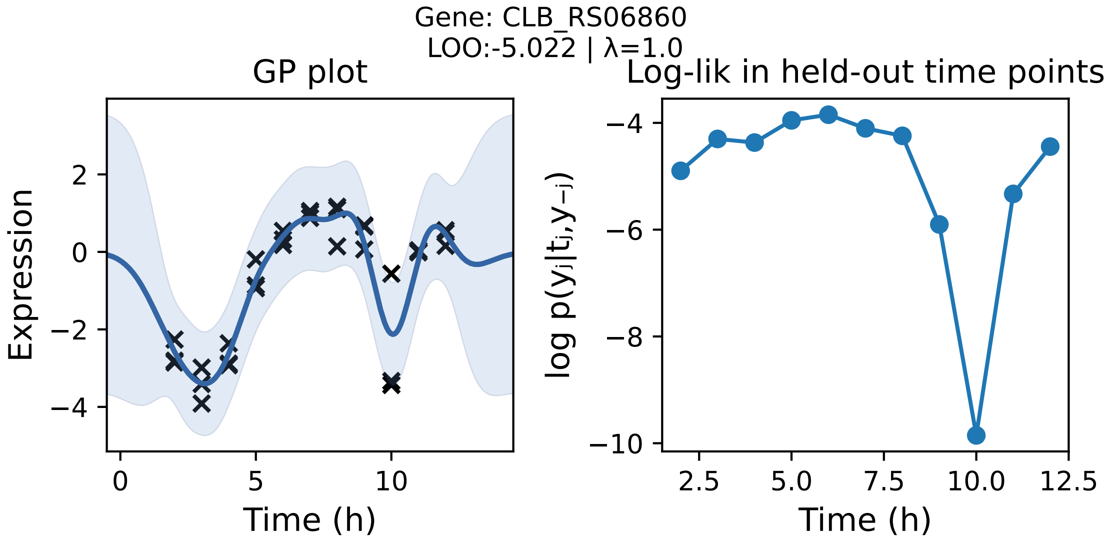
Gene: CLB_RS06860
LOO:-5.022 | λ=1.0
GP plot
Expression
2
0
−2
−4
0
5
10
Time (h)
Log-lik in held-out time points
log p(yⱼ|tⱼ,y₋ⱼ)
−4
−6
−8
−10
2.5
5.0
7.5
10.0
12.5
Time (h)

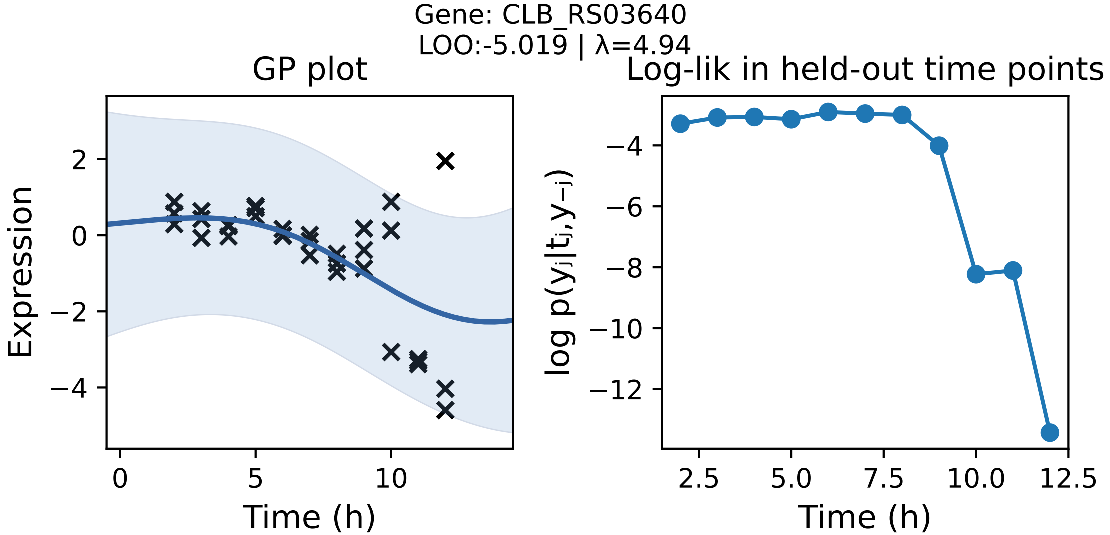
Gene: CLB_RS03640
LOO:-5.019 | λ=4.94
GP plot
Log-lik in held-out time points
Expression
Time (h)
log p(y_j|t_j,y_{-j})
Time (h)
2
0
−2
−4
0
5
10
−4
−6
−8
−10
−12
2.5
5.0
7.5
10.0
12.5

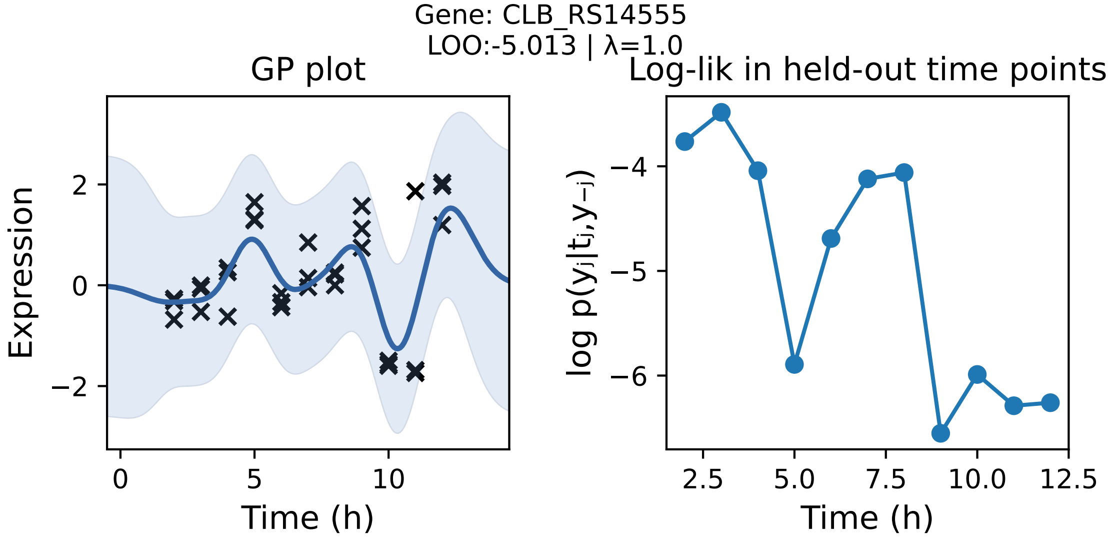
Gene: CLB_RS14555
LOO:-5.013 | λ=1.0
GP plot
Expression
Time (h)
Log-lik in held-out time points
log p(y_j|t_j,y_{-j})
Time (h)

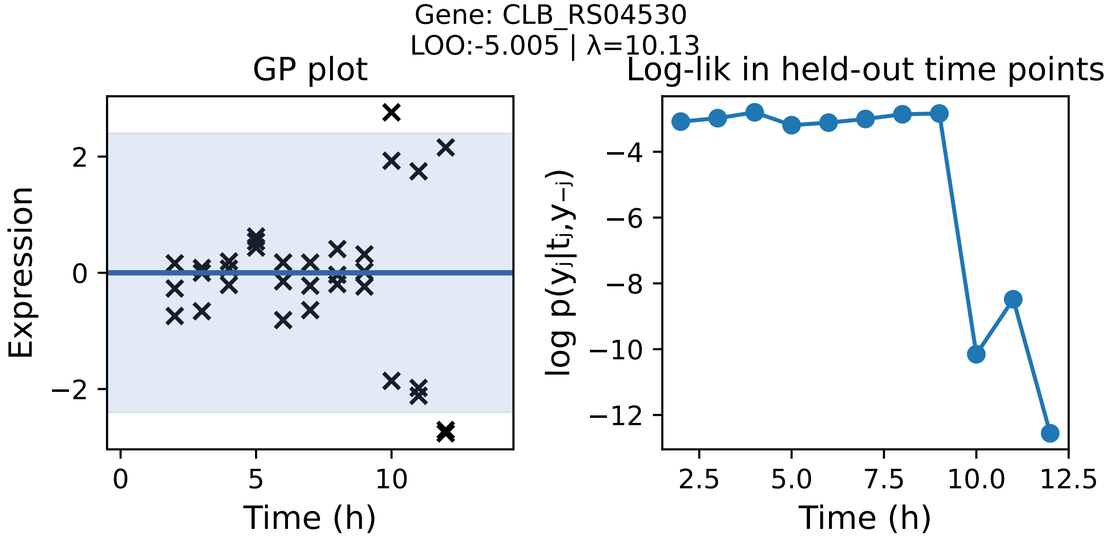

Gene: CLB_RS04530
LOO:-5.005 | λ=10.13
GP plot
Expression
Time (h)
2
0
−2
0
5
10
Log-lik in held-out time points
log p(y_j|t_j,y_−j)
Time (h)
−4
−6
−8
−10
−12
2.5
5.0
7.5
10.0
12.5

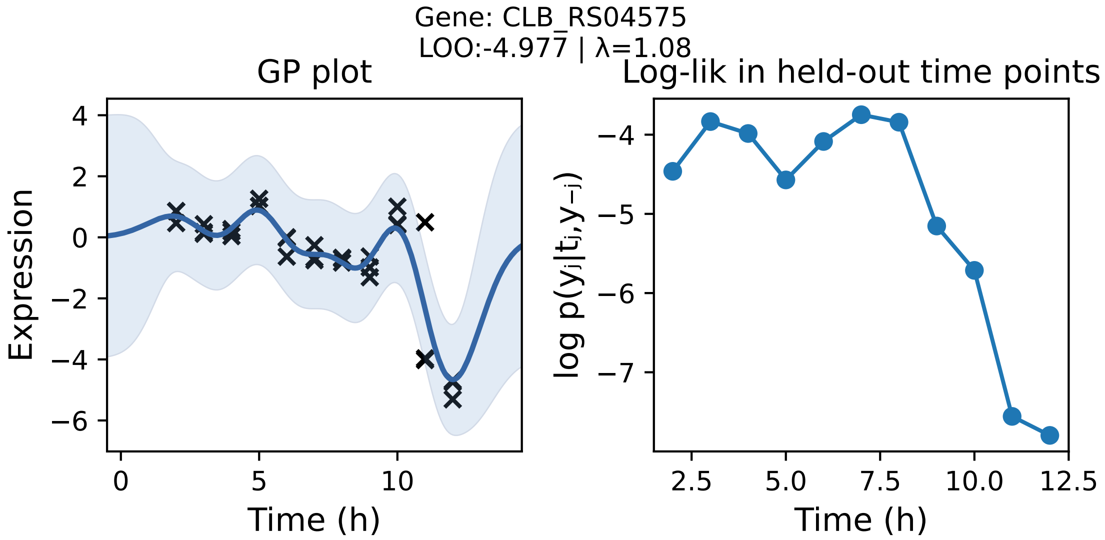

Gene: CLB_RS04575
LOO:-4.977 | λ=1.08
GP plot
Log-lik in held-out time points
Expression
Time (h)
log p(y_j|t_j,y_−j)
Time (h)
4
2
0
−2
−4
−6
0
5
10
−4
−5
−6
−7
2.5
5.0
7.5
10.0
12.5

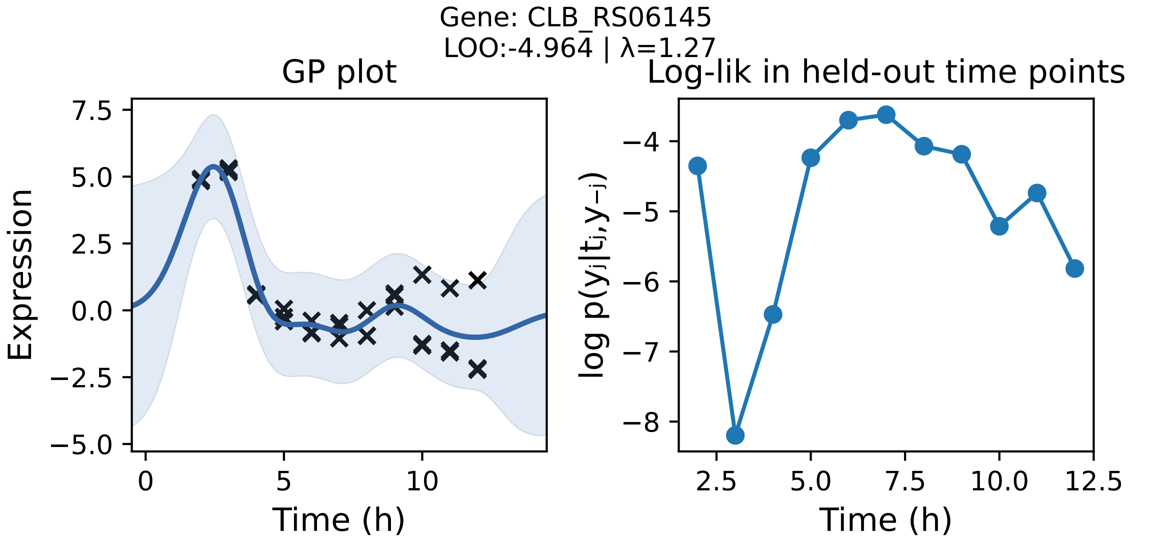

Gene: CLB_RS06145
LOO:-4.964 | λ=1.27
GP plot
Expression
Time (h)
Log-lik in held-out time points
log p(y_j|t_j,y_−j)
Time (h)

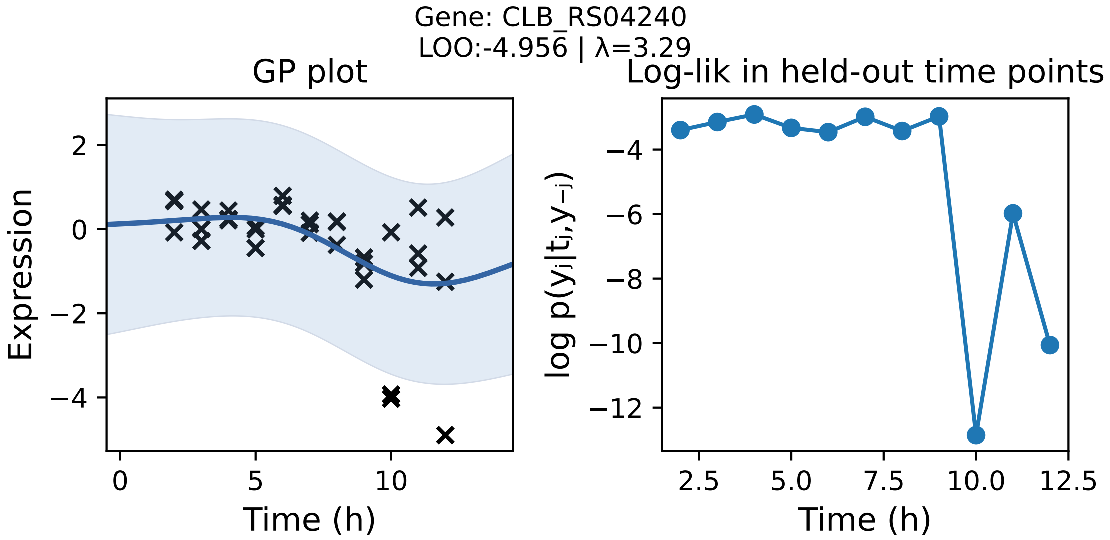
Gene: CLB_RS04240
LOO:-4.956 | λ=3.29
GP plot
Expression
Time (h)
2
0
−2
−4
0
5
10
Log-lik in held-out time points
log p(yⱼ|tⱼ,y₋ⱼ)
Time (h)
−4
−6
−8
−10
−12
2.5
5.0
7.5
10.0
12.5

Gene: CLB_RS16260
LOO:-4.938 | λ=1.0

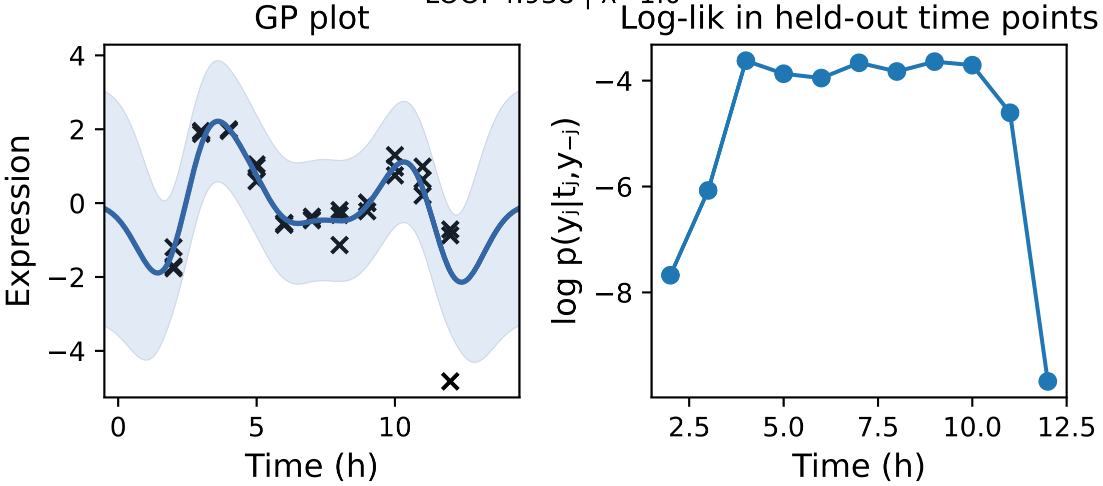

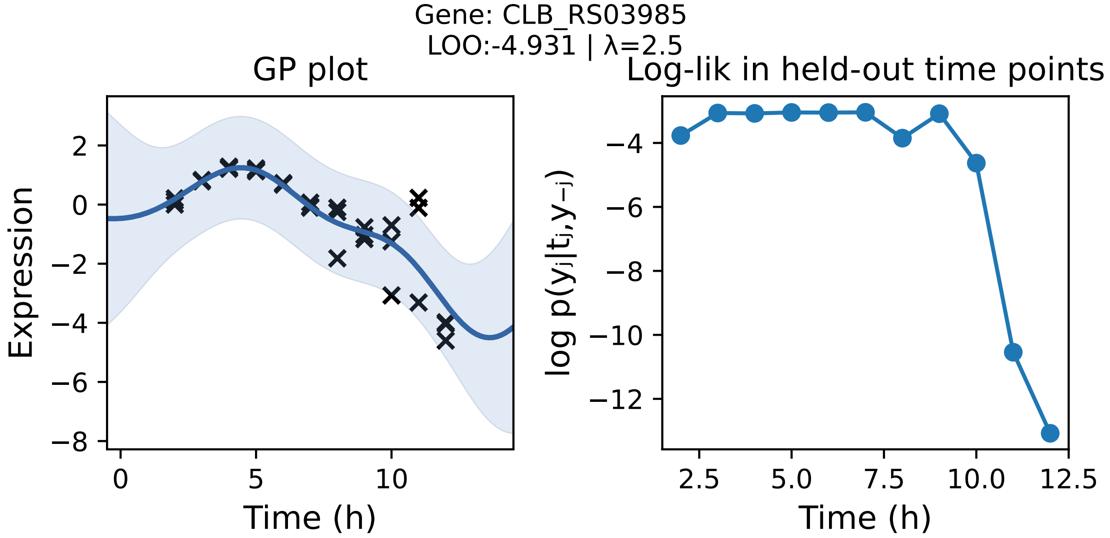
Gene: CLB_RS03985
LOO:-4.931 | λ=2.5
GP plot
Expression
Time (h)
Log-lik in held-out time points
log p(y_j|t_j,y_−j)
Time (h)

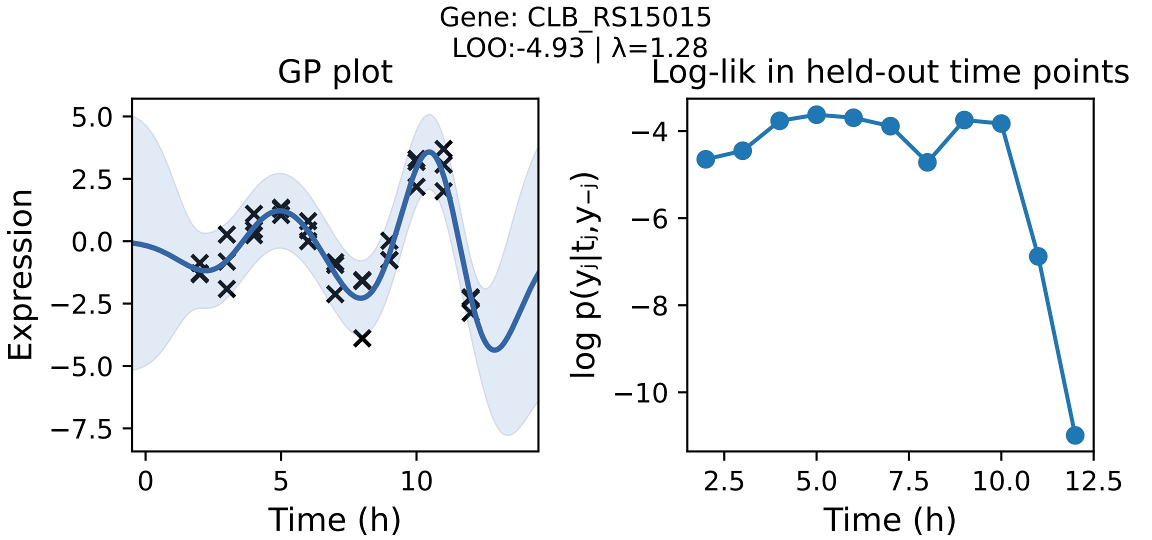

Gene: CLB_RS15015
LOO:-4.93 | λ=1.28
GP plot
Expression
Time (h)
5.0
2.5
0.0
−2.5
−5.0
−7.5
0
5
10
Log-lik in held-out time points
log p(yj|tj,y−j)
Time (h)
−4
−6
−8
−10
2.5
5.0
7.5
10.0
12.5

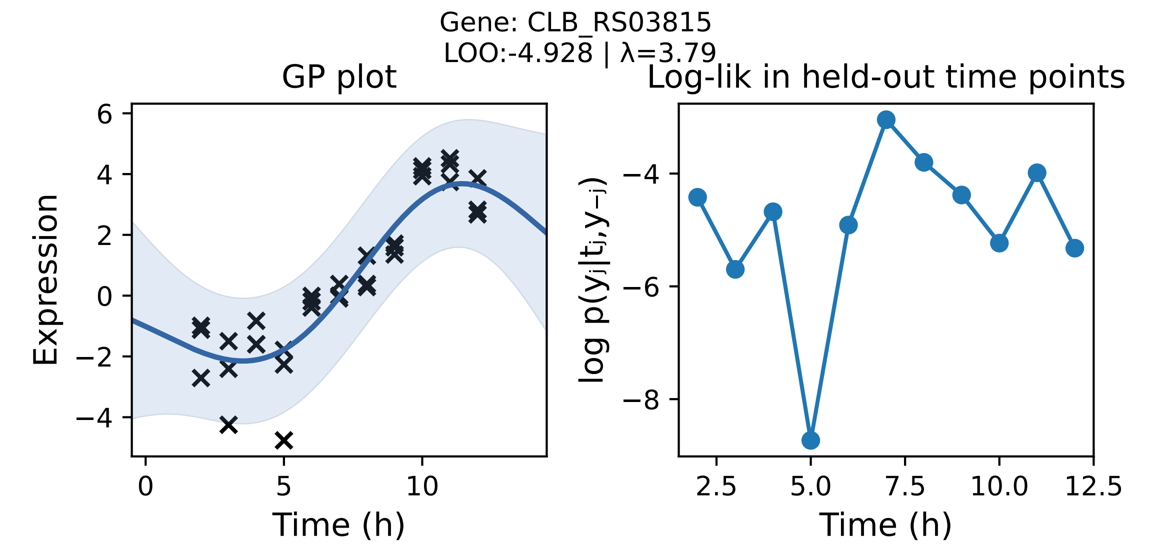

Gene: CLB_RS03815
LOO:-4.928 | λ=3.79
GP plot
Expression
Time (h)
Log-lik in held-out time points
log p(yⱼ|tⱼ,y₋ⱼ)
Time (h)

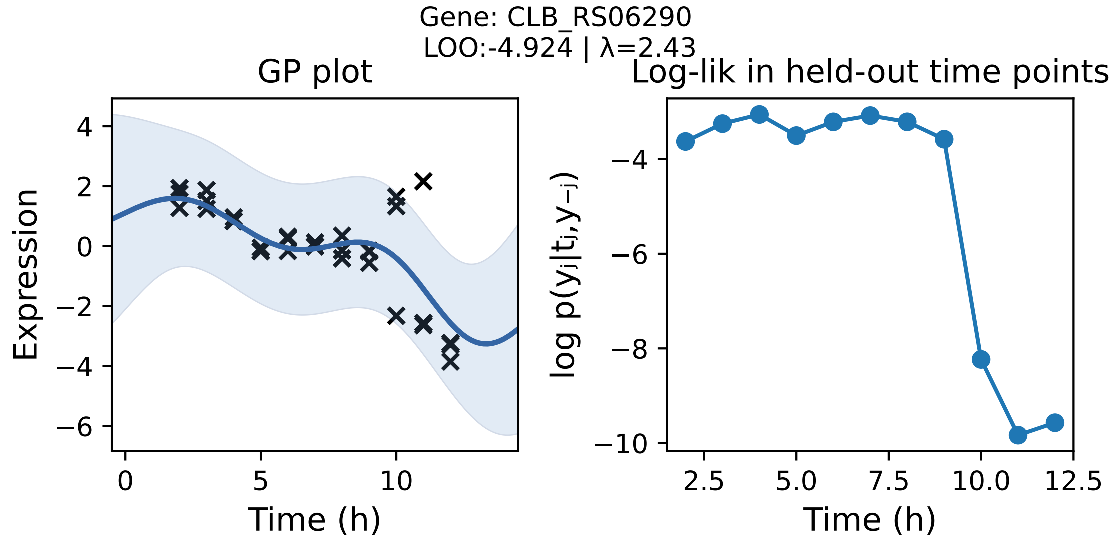

Gene: CLB_RS06290
LOO:-4.924 | λ=2.43
GP plot
Expression
Time (h)
Log-lik in held-out time points
log p(y_j|t_j,y_−j)
Time (h)

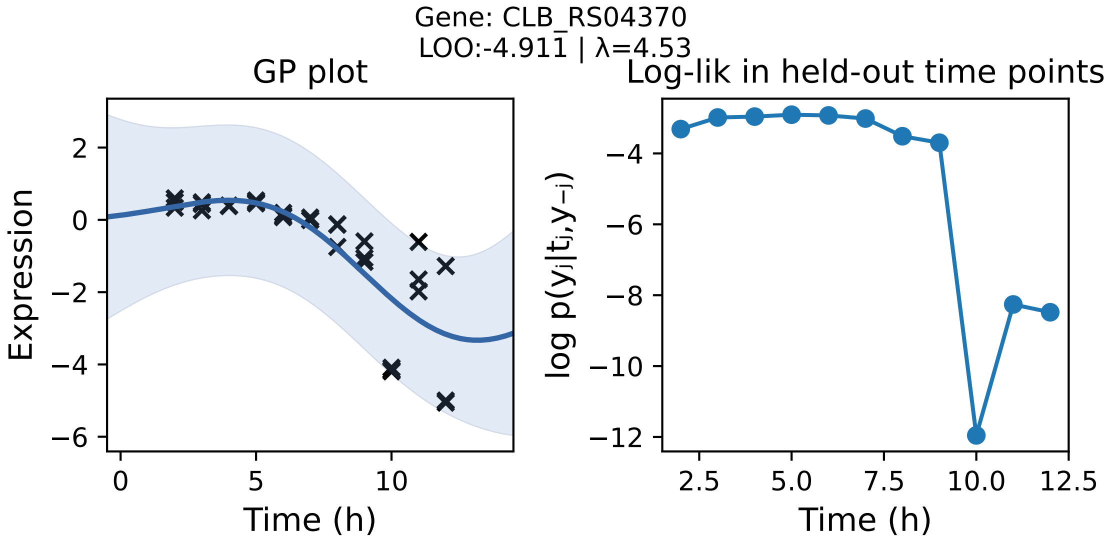

Gene: CLB_RS04370
LOO:-4.911 | λ=4.53
GP plot
Log-lik in held-out time points
Expression
Time (h)
log p(y_j|t_j,y_−j)
Time (h)
2
0
−2
−4
−6
0
5
10
−4
−6
−8
−10
−12
2.5
5.0
7.5
10.0
12.5

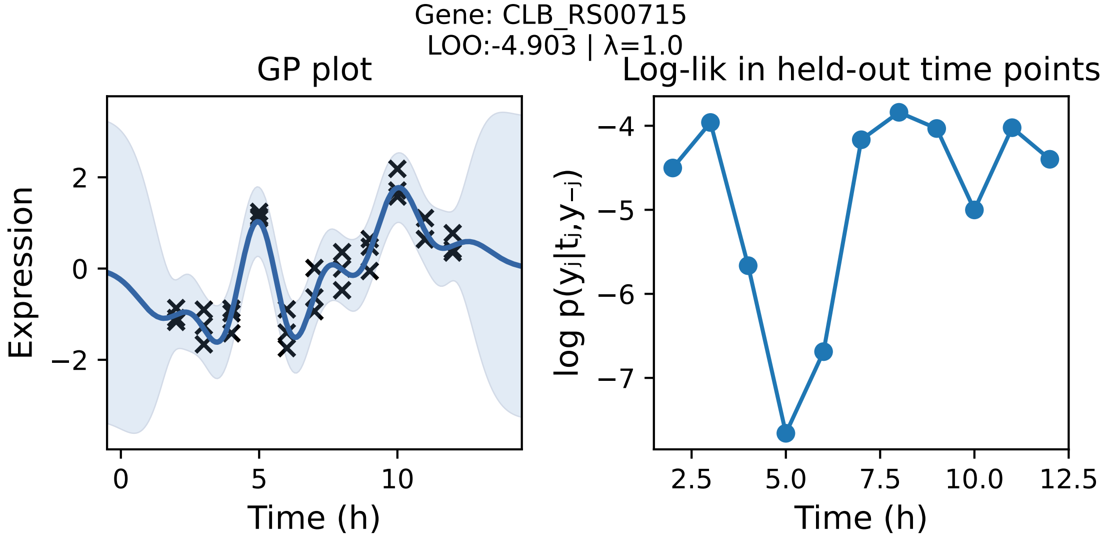

Gene: CLB_RS00715
LOO:-4.903 | λ=1.0
GP plot
Expression
Time (h)
2
0
−2
0
5
10
Log-lik in held-out time points
log p(y_j|t_j,y_−j)
Time (h)
−4
−5
−6
−7
2.5
5.0
7.5
10.0
12.5

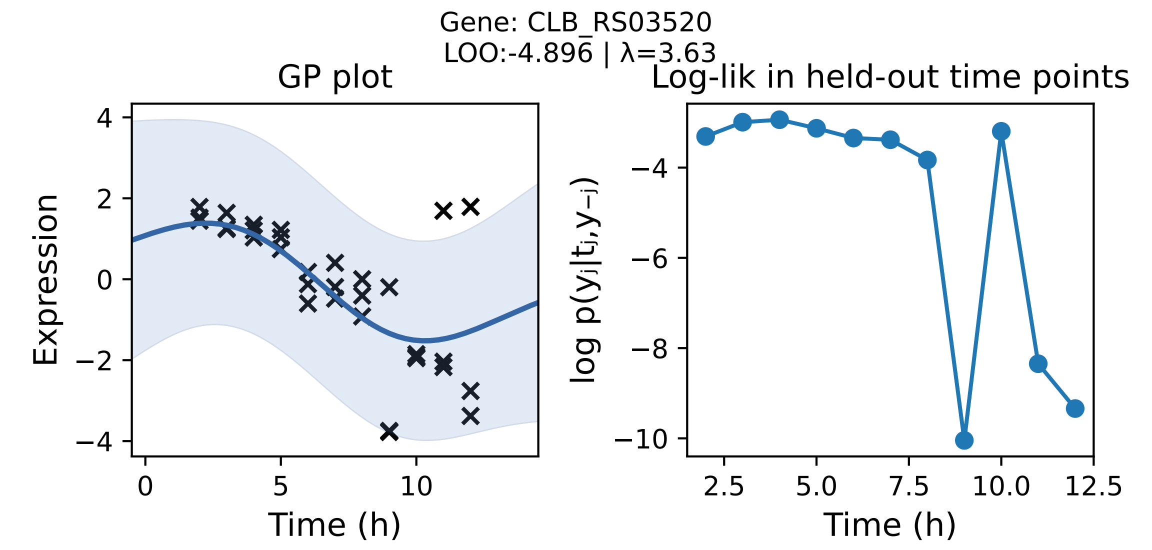

Gene: CLB_RS03520
LOO:-4.896 | λ=3.63
GP plot
Expression
Time (h)
Log-lik in held-out time points
log p(y_j|t_j,y_−j)
Time (h)

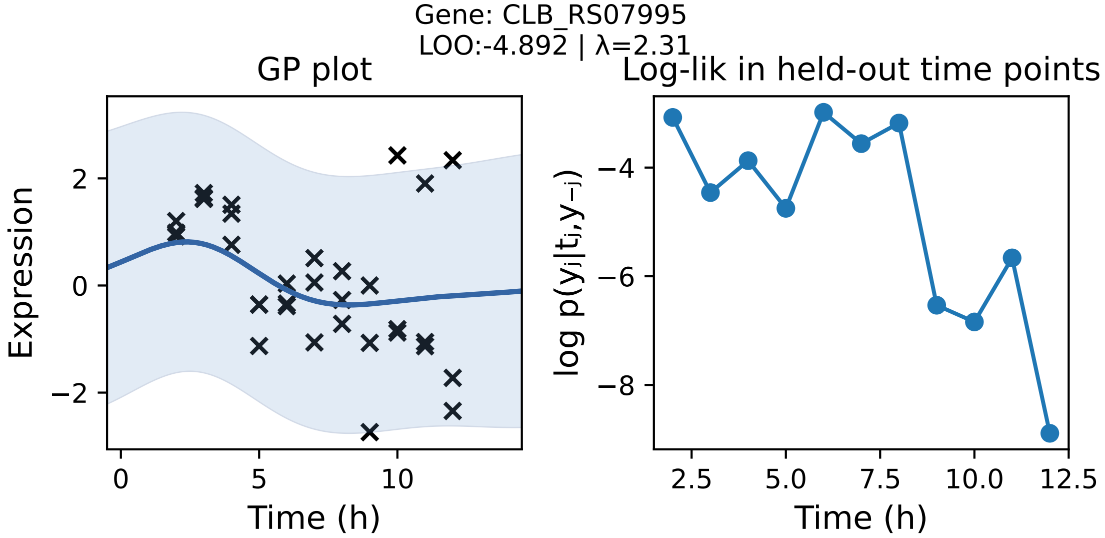

Gene: CLB_RS07995
LOO:-4.892 | λ=2.31
GP plot
Expression
2
0
−2
0
5
10
Time (h)
Log-lik in held-out time points
log p(y_j|t_j,y_−j)
−4
−6
−8
2.5
5.0
7.5
10.0
12.5
Time (h)

Gene: CLB_RS06120
LOO:-4.891 | λ=1.0

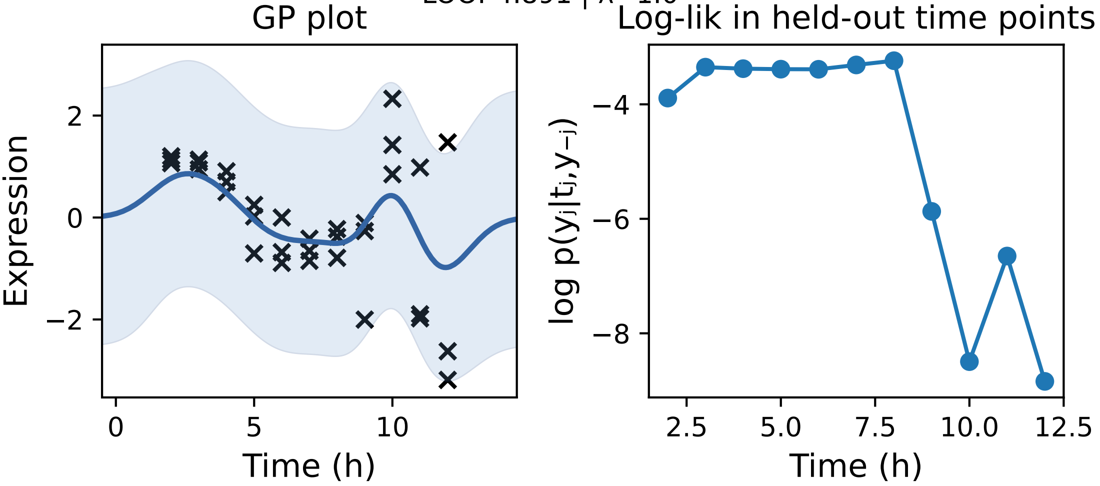

Gene: CLB_RS11170
LOO:-4.889 | λ=1.0

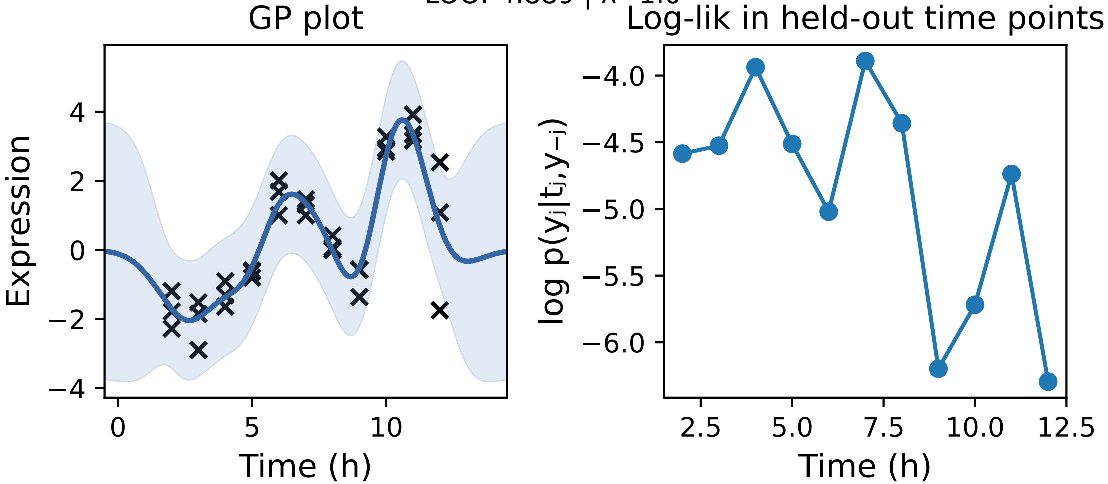

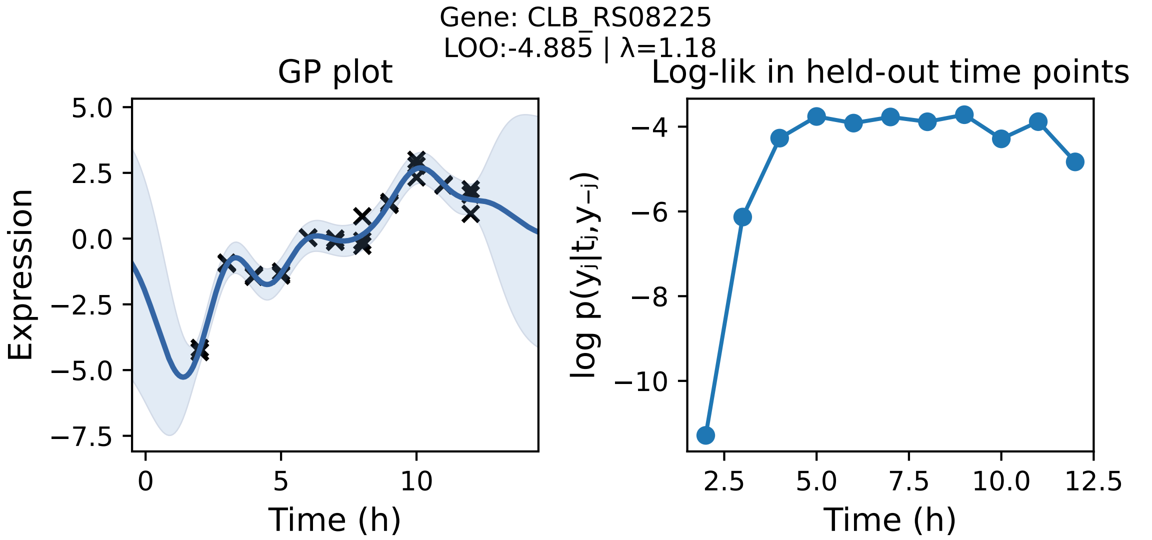

Gene: CLB_RS08225
LOO:-4.885 | λ=1.18
GP plot
Expression
Time (h)
5.0
2.5
0.0
−2.5
−5.0
−7.5
0
5
10
Log-lik in held-out time points
log p(yj|tj,y−j)
Time (h)
−4
−6
−8
−10
2.5
5.0
7.5
10.0
12.5

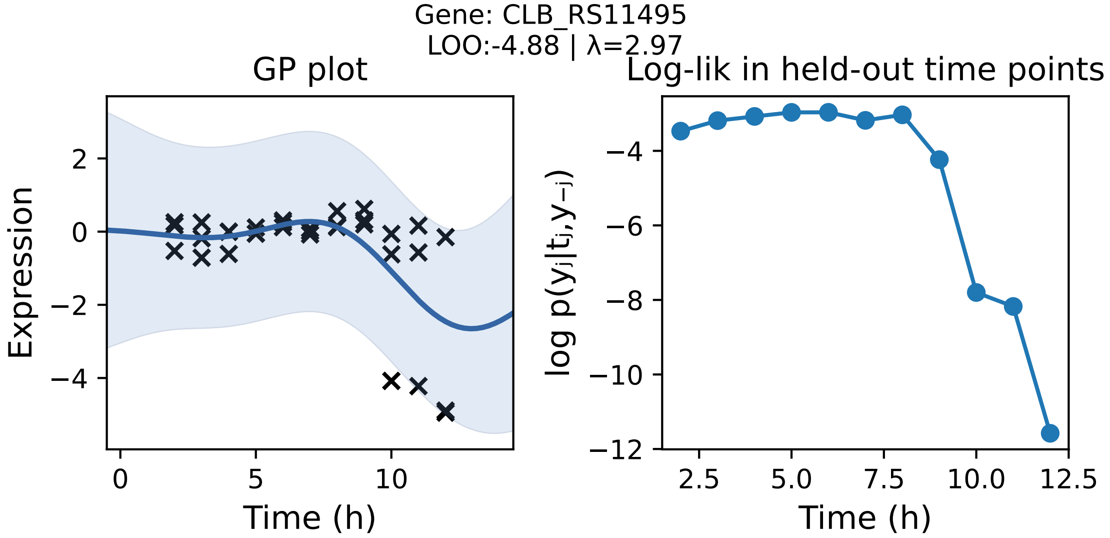

Gene: CLB_RS11495
LOO:-4.88 | λ=2.97
GP plot
Expression
2
0
−2
−4
0
5
10
Time (h)
Log-lik in held-out time points
log p(y_j|t_j,y_−j)
−4
−6
−8
−10
−12
2.5
5.0
7.5
10.0
12.5
Time (h)

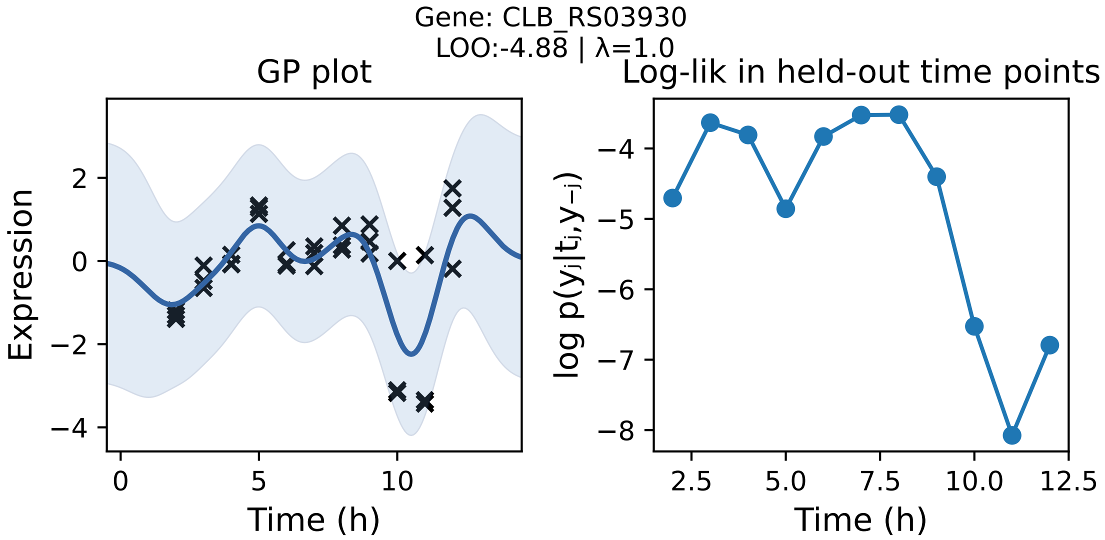

Gene: CLB_RS03930
LOO:-4.88 | λ=1.0
GP plot
Log-lik in held-out time points
Expression
2
0
−2
−4
0
5
10
Time (h)
log p(yj|ti,y−j)
−4
−5
−6
−7
−8
2.5
5.0
7.5
10.0
12.5
Time (h)

Gene: CLB_RS15005
LOO:-4.859 | λ=1.0

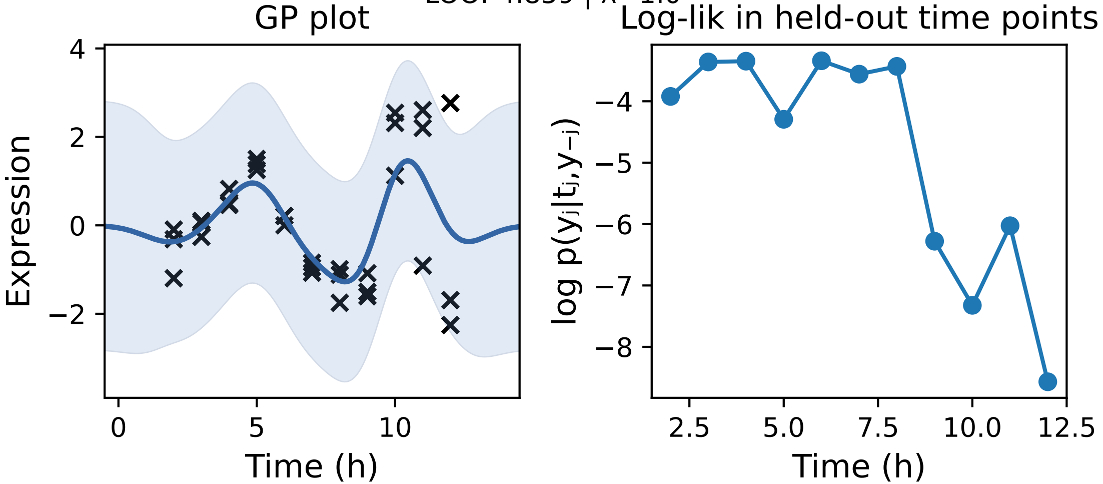

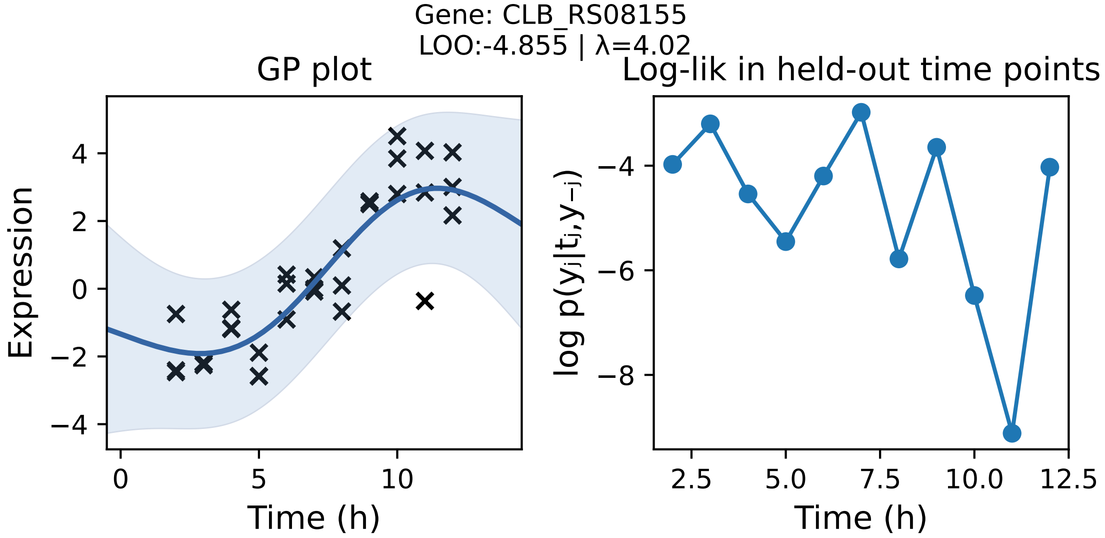

Gene: CLB_RS08155
LOO:-4.855 | λ=4.02
GP plot
Expression
Time (h)
Log-lik in held-out time points
log p(y_j|t_j,y_−j)
Time (h)

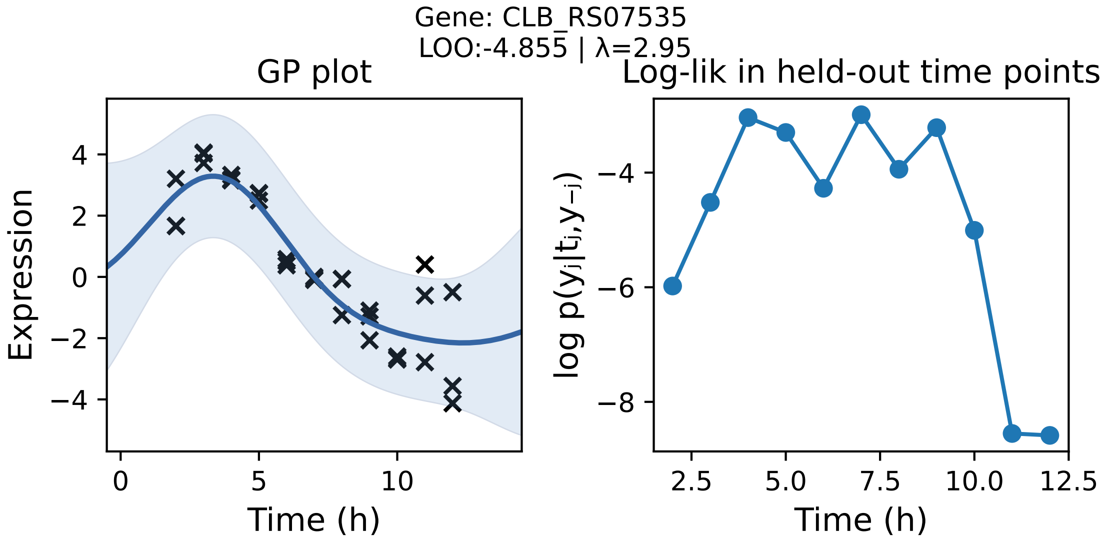

Gene: CLB_RS07535
LOO:-4.855 | λ=2.95
GP plot
Expression
Time (h)
4
2
0
−2
−4
0
5
10
Log-lik in held-out time points
log p(y_j|t_j,y_−j)
−4
−6
−8
2.5
5.0
7.5
10.0
12.5
Time (h)

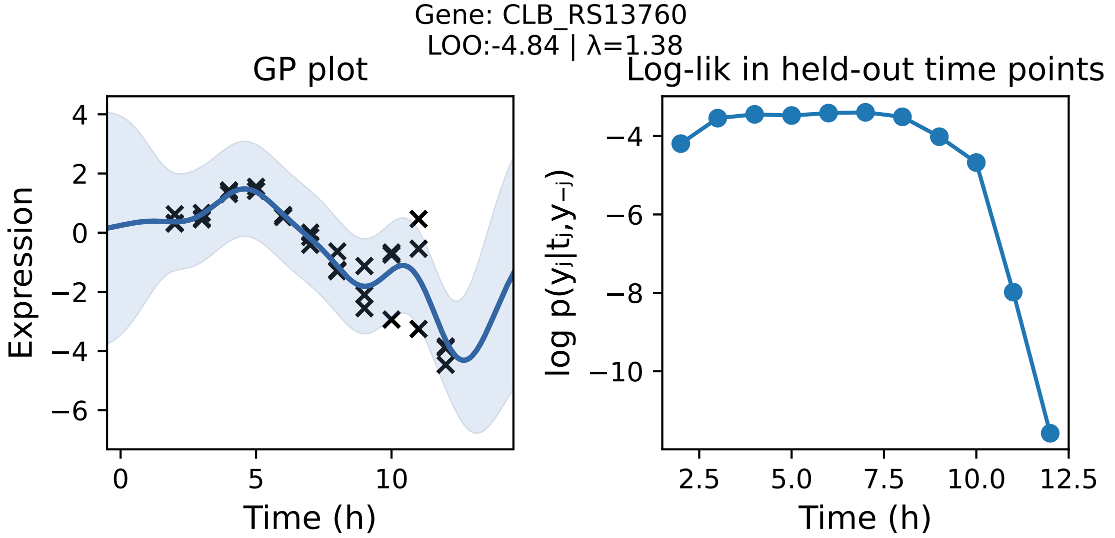

Gene: CLB_RS13760
LOO:-4.84 | λ=1.38
GP plot
Expression
Time (h)
Log-lik in held-out time points
log p(yj|tj,y−j)
Time (h)

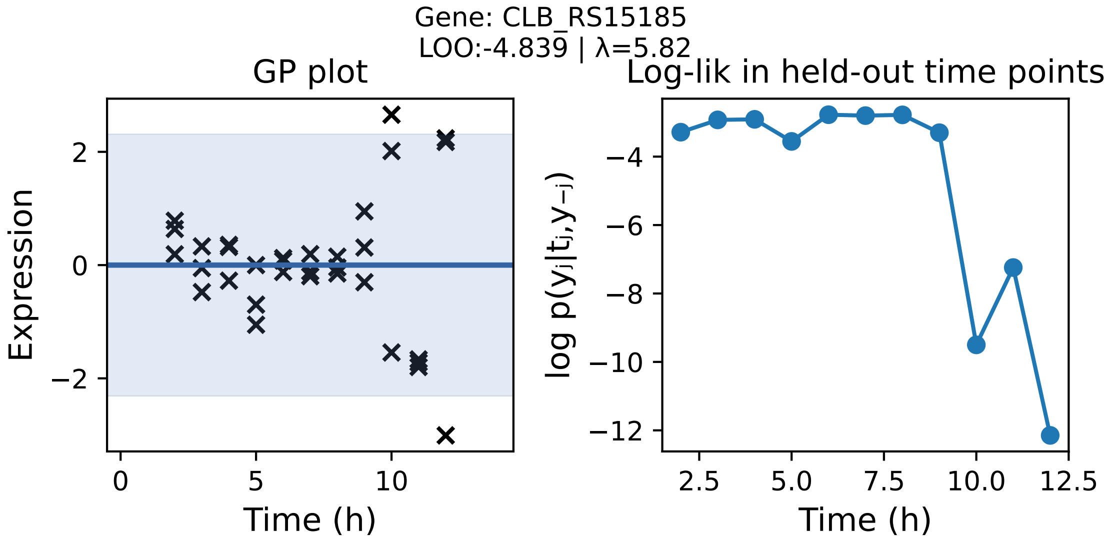
Gene: CLB_RS15185
LOO:-4.839 | λ=5.82
GP plot
Expression
Time (h)
Log-lik in held-out time points
log p(y_j|t_j,y_−j)
Time (h)

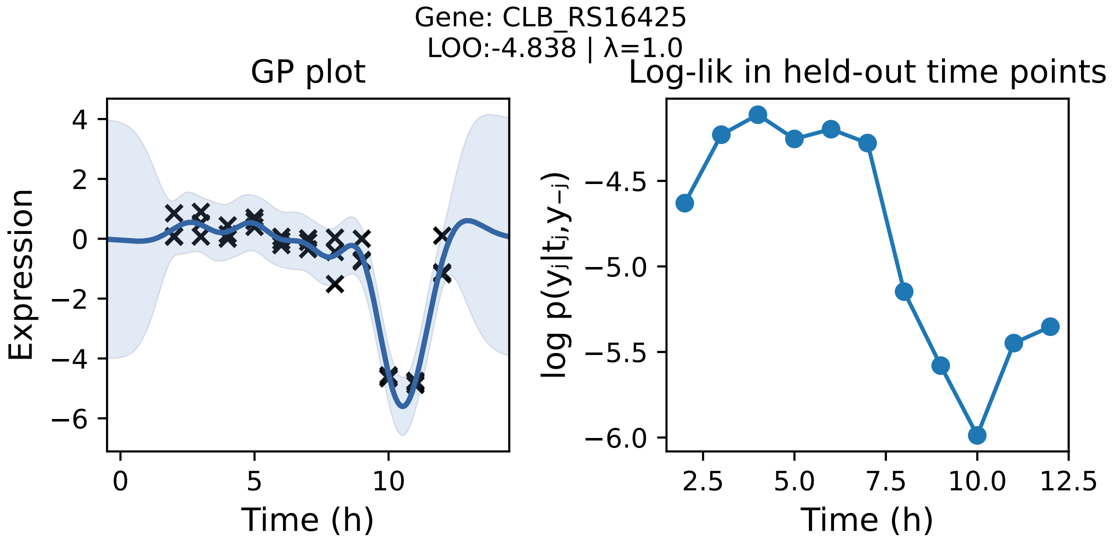

Gene: CLB_RS16425
LOO:-4.838 | λ=1.0
GP plot
Expression
Time (h)
4
2
0
−2
−4
−6
0
5
10
Log-lik in held-out time points
log p(y_j|t_j,y_−j)
Time (h)
−4.5
−5.0
−5.5
−6.0
2.5
5.0
7.5
10.0
12.5

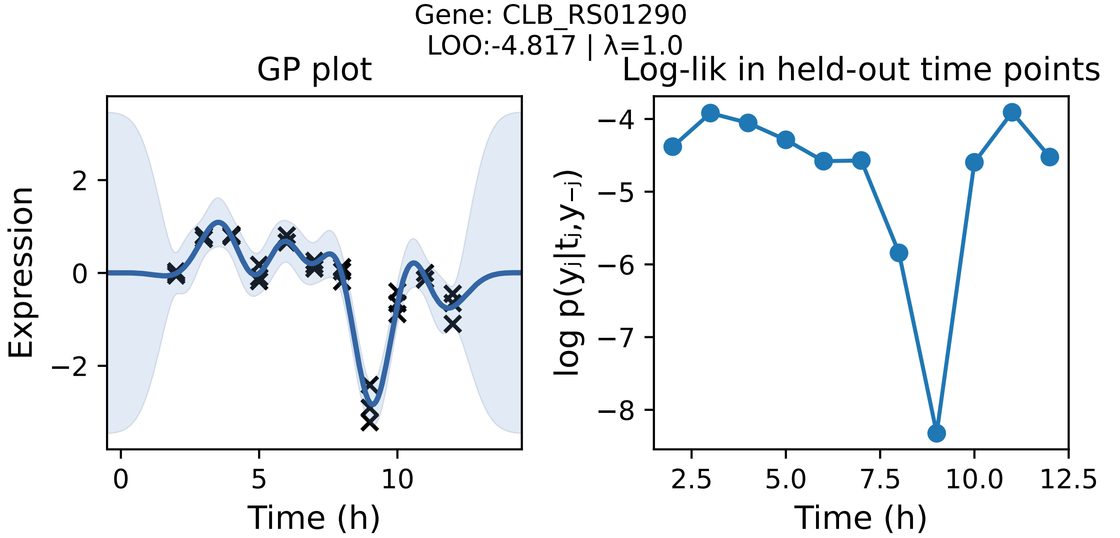

Gene: CLB_RS01290
LOO:-4.817 | λ=1.0
GP plot
Expression
Time (h)
2
0
−2
0
5
10
Log-lik in held-out time points
log p(y_j|t_j,y_−j)
Time (h)
−4
−5
−6
−7
−8
2.5
5.0
7.5
10.0
12.5

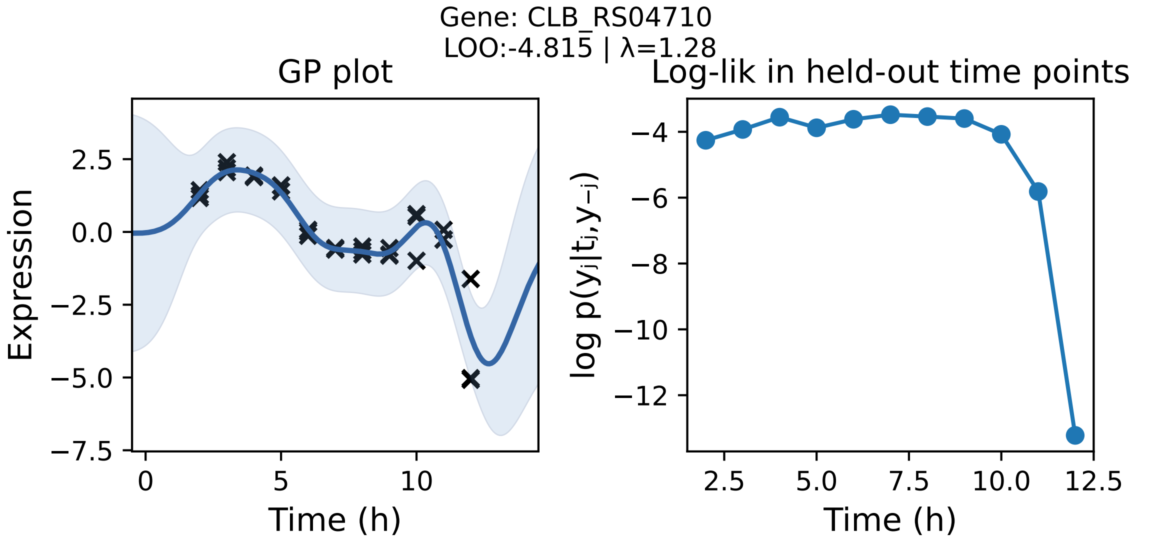

Gene: CLB_RS04710
LOO:-4.815 | λ=1.28
GP plot
Expression
2.5
0.0
−2.5
−5.0
−7.5
0
5
10
Time (h)
Log-lik in held-out time points
log p(yj|tj,y−j)
−4
−6
−8
−10
−12
2.5
5.0
7.5
10.0
12.5
Time (h)

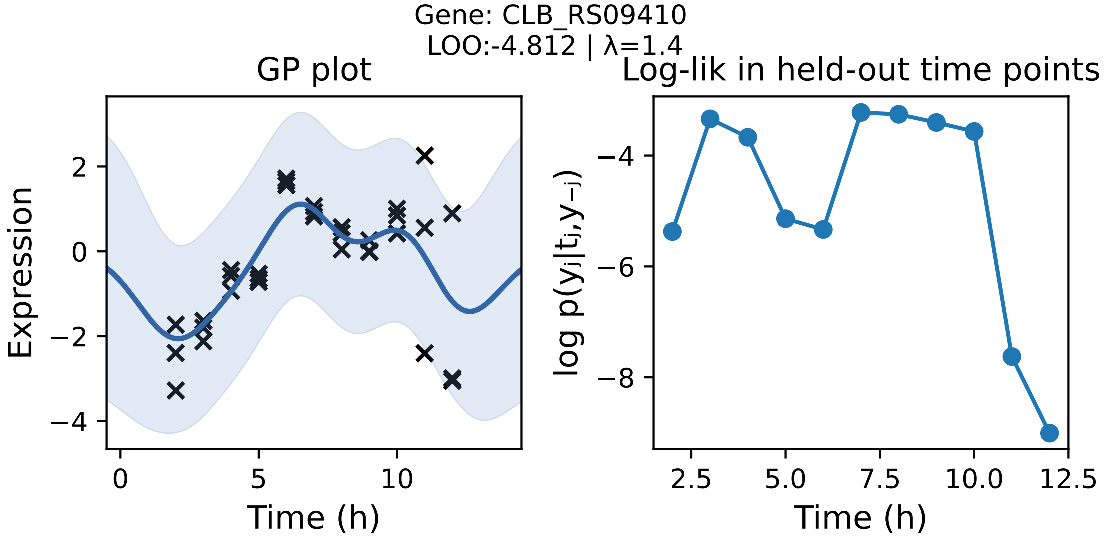

Gene: CLB_RS09410
LOO:-4.812 | λ=1.4
GP plot
Expression
Time (h)
Log-lik in held-out time points
log p(y_j|t_j,y_−j)
Time (h)

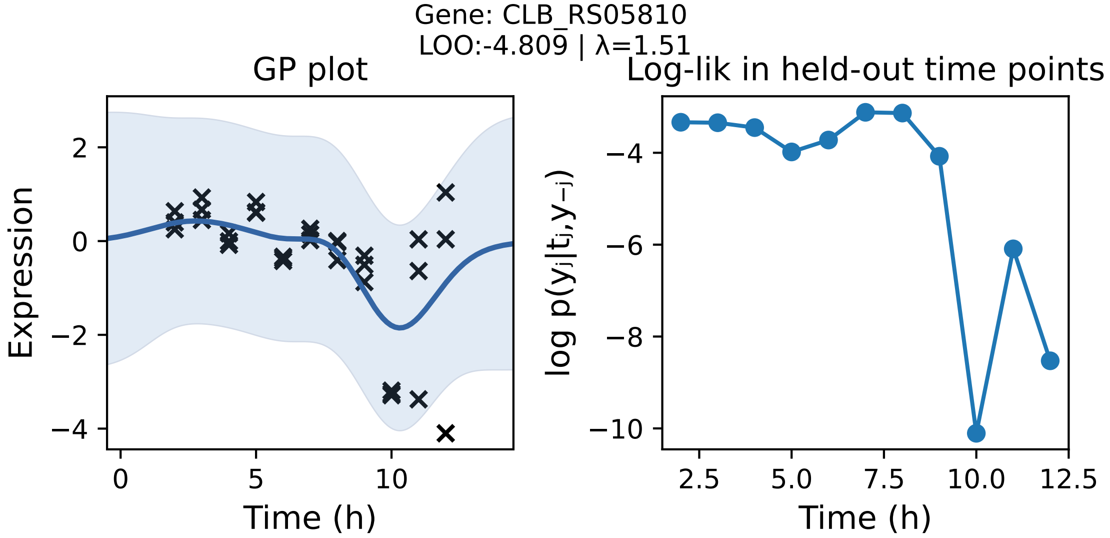

Gene: CLB_RS05810
LOO:-4.809 | λ=1.51
GP plot
Expression
Time (h)
Log-lik in held-out time points
log p(y_j|t_j,y_−j)
Time (h)

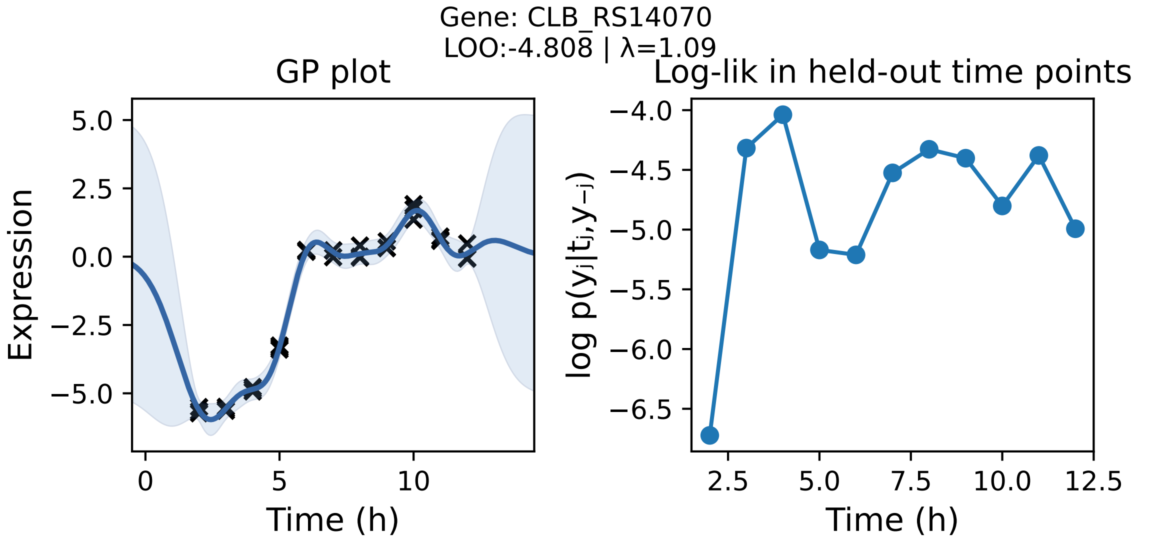

Gene: CLB_RS14070
LOO:-4.808 | λ=1.09
GP plot
Log-lik in held-out time points
Expression
5.0
2.5
0.0
−2.5
−5.0
0
5
10
Time (h)
log p(yj|tj,y−j)
−4.0
−4.5
−5.0
−5.5
−6.0
−6.5
2.5
5.0
7.5
10.0
12.5
Time (h)

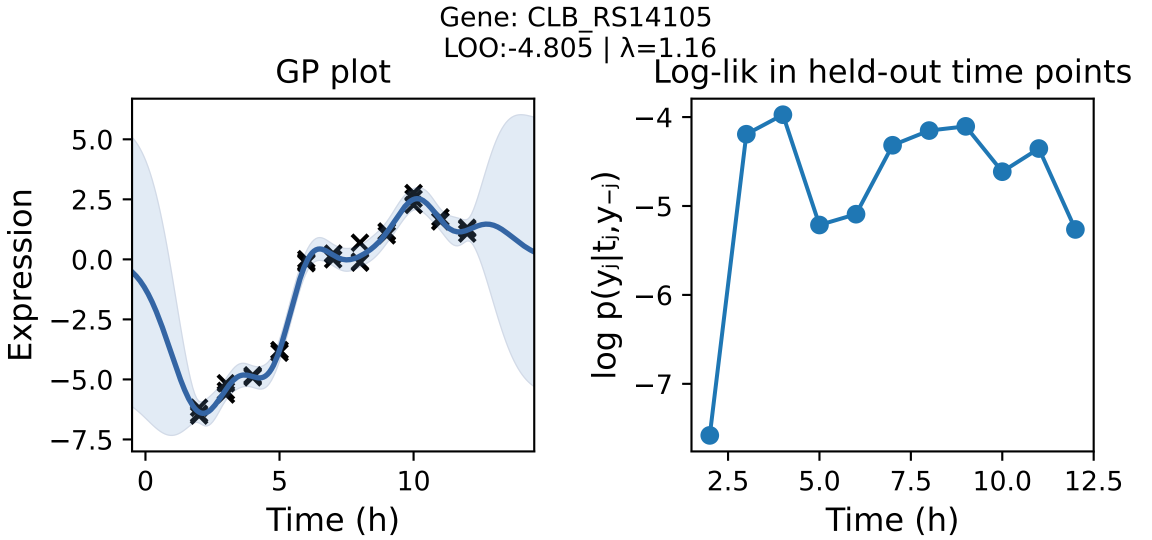

Gene: CLB_RS14105
LOO:-4.805 | λ=1.16
GP plot
Expression
5.0
2.5
0.0
−2.5
−5.0
−7.5
0
5
10
Time (h)
Log-lik in held-out time points
log p(yⱼ|tⱼ,y₋ⱼ)
−4
−5
−6
−7
2.5
5.0
7.5
10.0
12.5
Time (h)

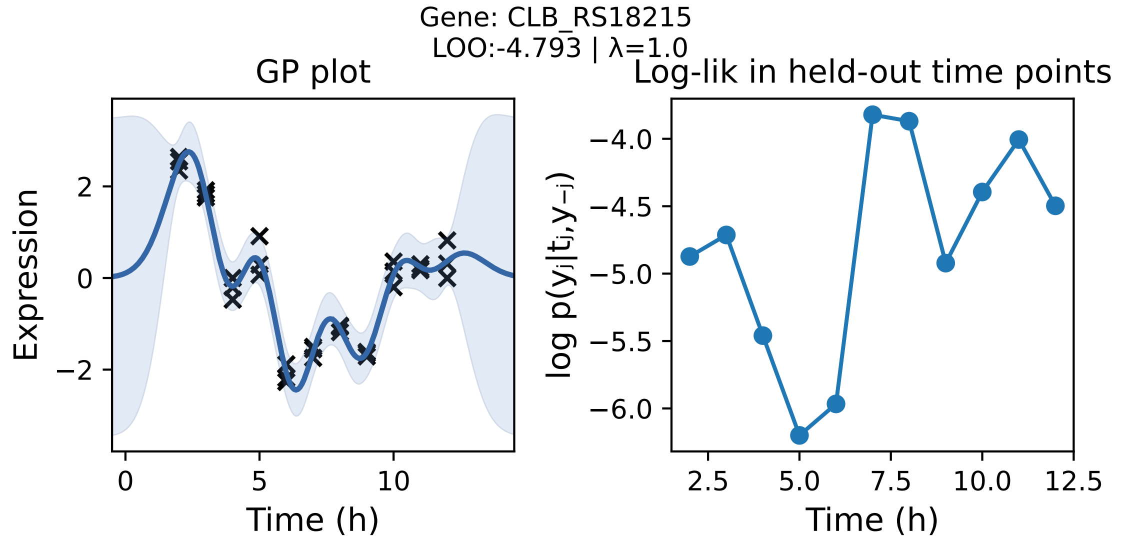
Gene: CLB_RS18215
LOO:-4.793 | λ=1.0
GP plot
Expression
Time (h)
Log-lik in held-out time points
log p(y_j|t_j,y_−j)
Time (h)

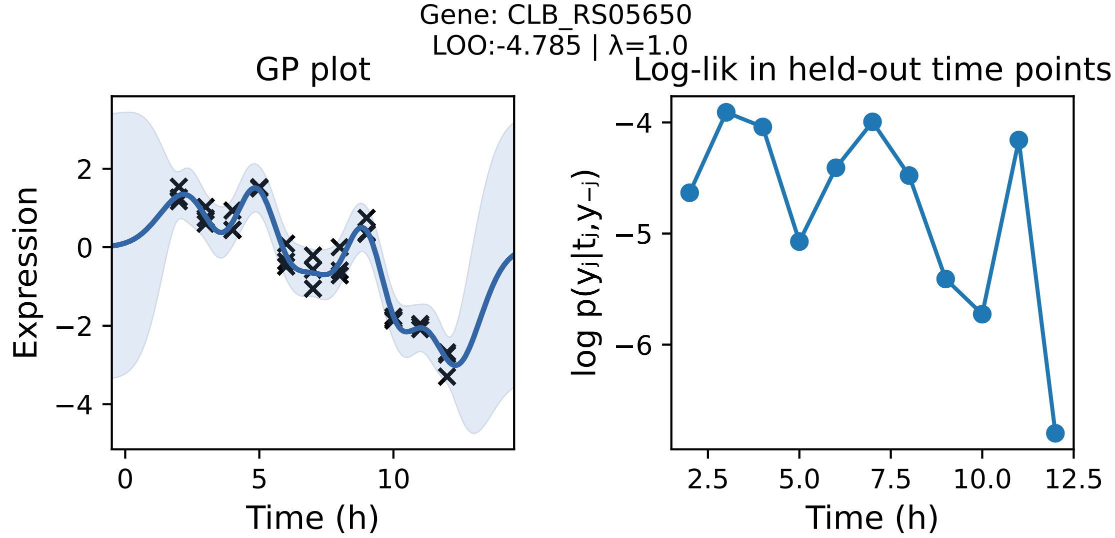

Gene: CLB_RS05650
LOO:-4.785 | λ=1.0
GP plot
Expression
2
0
−2
−4
0
5
10
Time (h)
Log-lik in held-out time points
log p(yj|tj,y−j)
−4
−5
−6
2.5
5.0
7.5
10.0
12.5
Time (h)

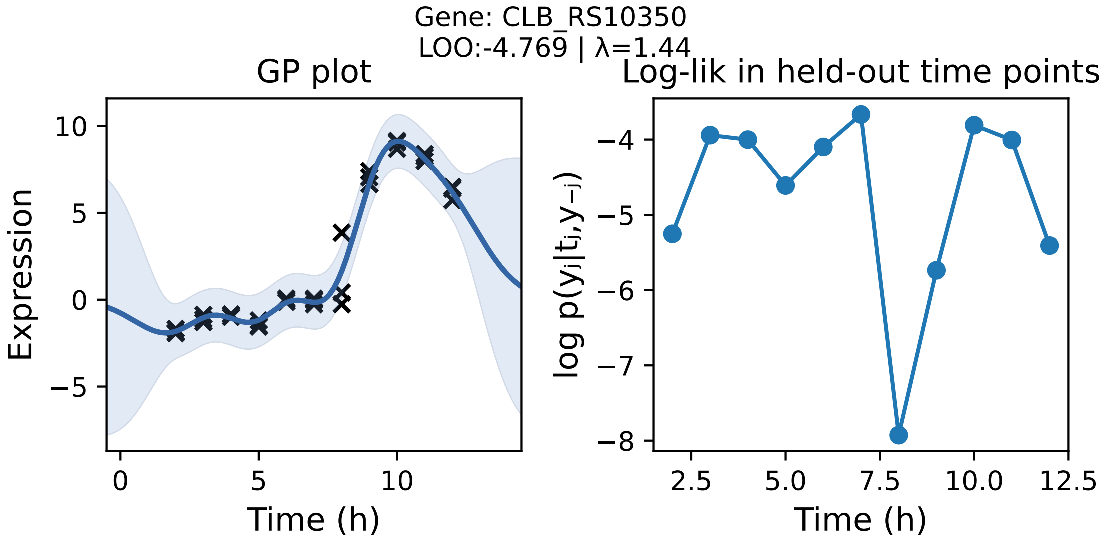

Gene: CLB_RS10350
LOO:-4.769 | λ=1.44
GP plot
Expression
10
5
0
−5
0
5
10
Time (h)
Log-lik in held-out time points
log p(y_j|t_j,y_−j)
−4
−5
−6
−7
−8
2.5
5.0
7.5
10.0
12.5
Time (h)

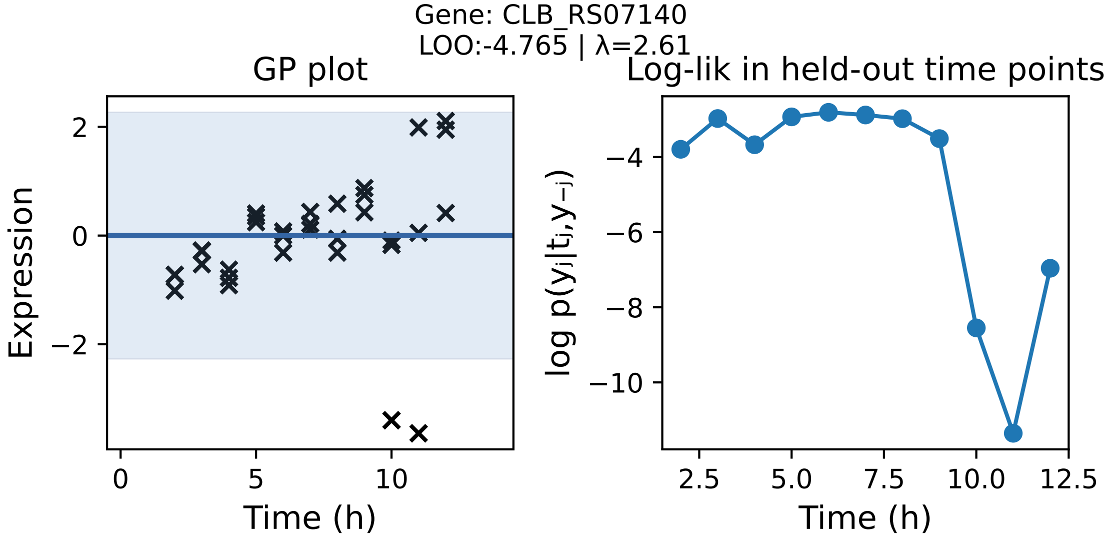
Gene: CLB_RS07140
LOO:-4.765 | λ=2.61
GP plot
Expression
Time (h)
Log-lik in held-out time points
log p(y_j|t_j,y_−j)
Time (h)

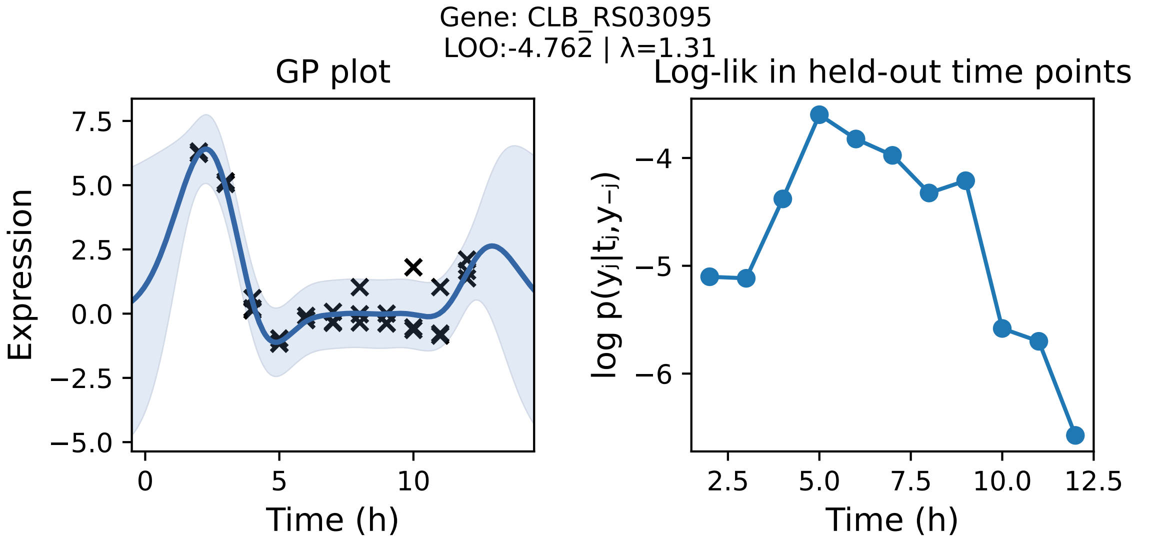

Gene: CLB_RS03095
LOO:-4.762 | λ=1.31
GP plot
Expression
Time (h)
Log-lik in held-out time points
log p(y_j|t_j,y_−j)
Time (h)

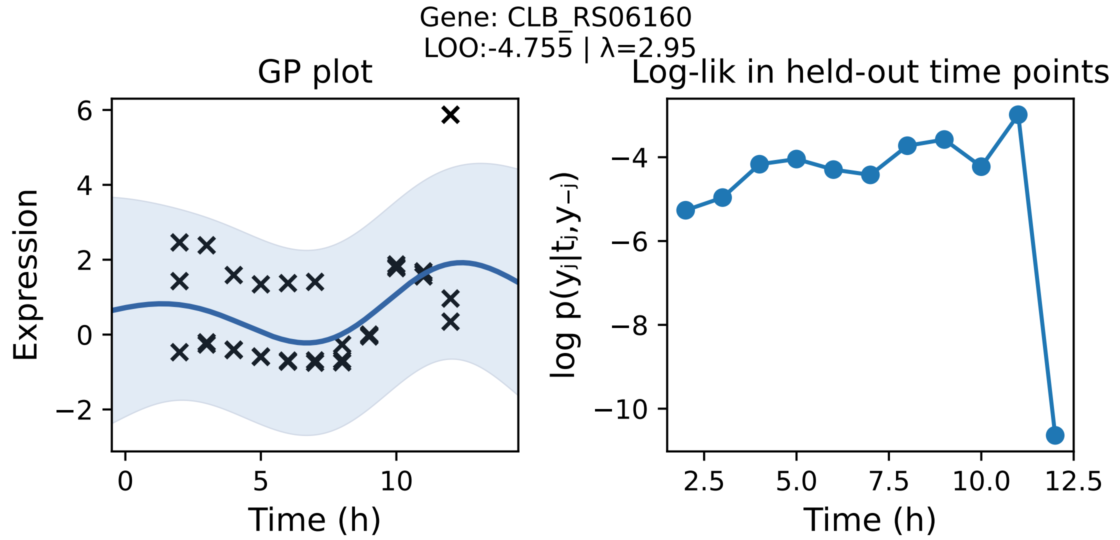

Gene: CLB_RS06160
LOO:-4.755 | λ=2.95
GP plot
Expression
Time (h)
Log-lik in held-out time points
log p(yj|ti,y−j)
Time (h)

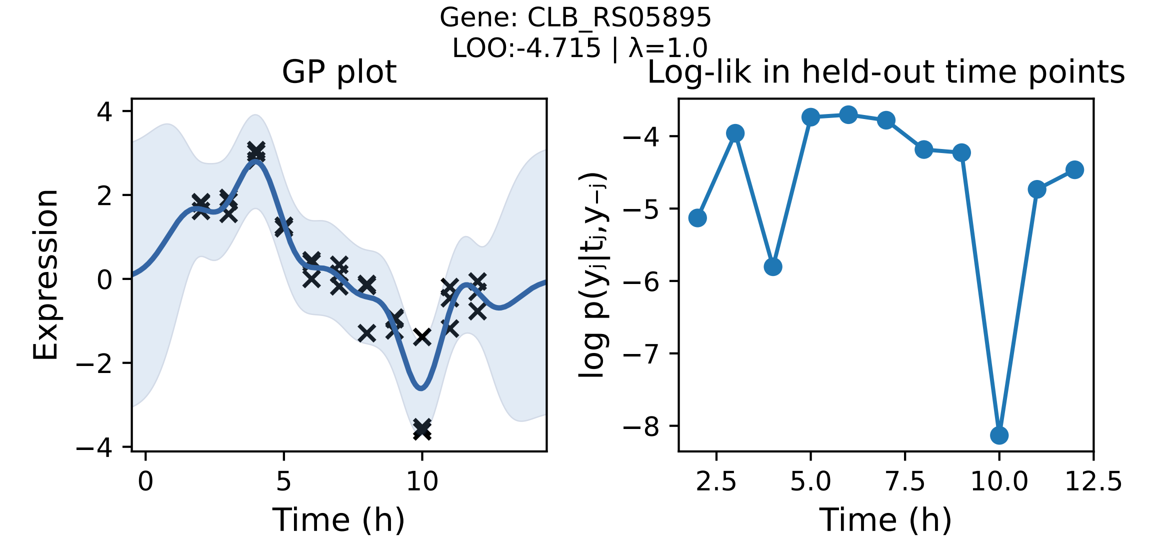

Gene: CLB_RS05895
LOO:-4.715 | λ=1.0
GP plot
Expression
Time (h)
Log-lik in held-out time points
log p(y_j|t_j,y_−j)
Time (h)

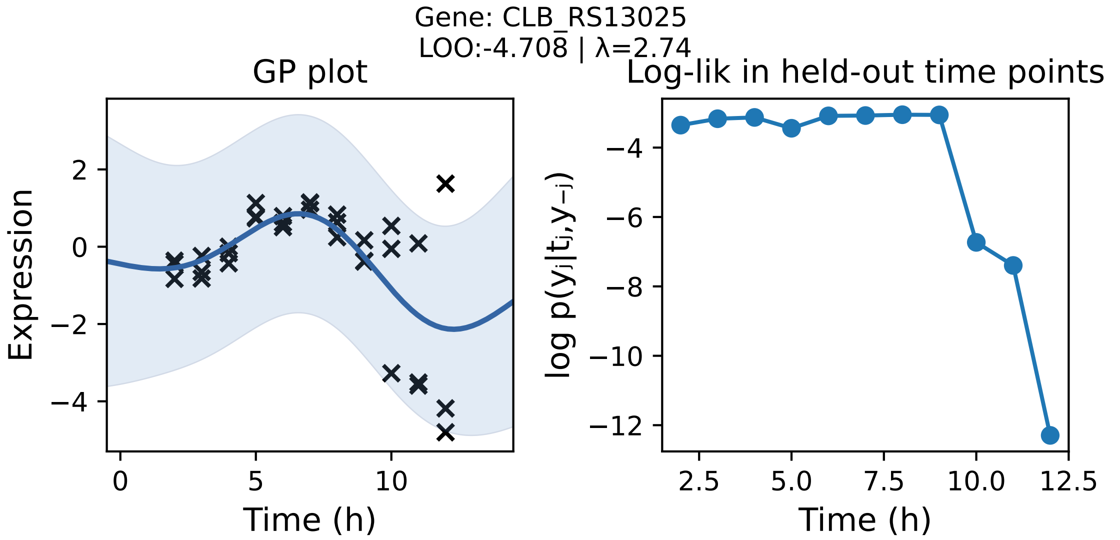

Gene: CLB_RS13025
LOO:-4.708 | λ=2.74
GP plot
Expression
Time (h)
Log-lik in held-out time points
log p(y_j|t_j,y_−j)
Time (h)

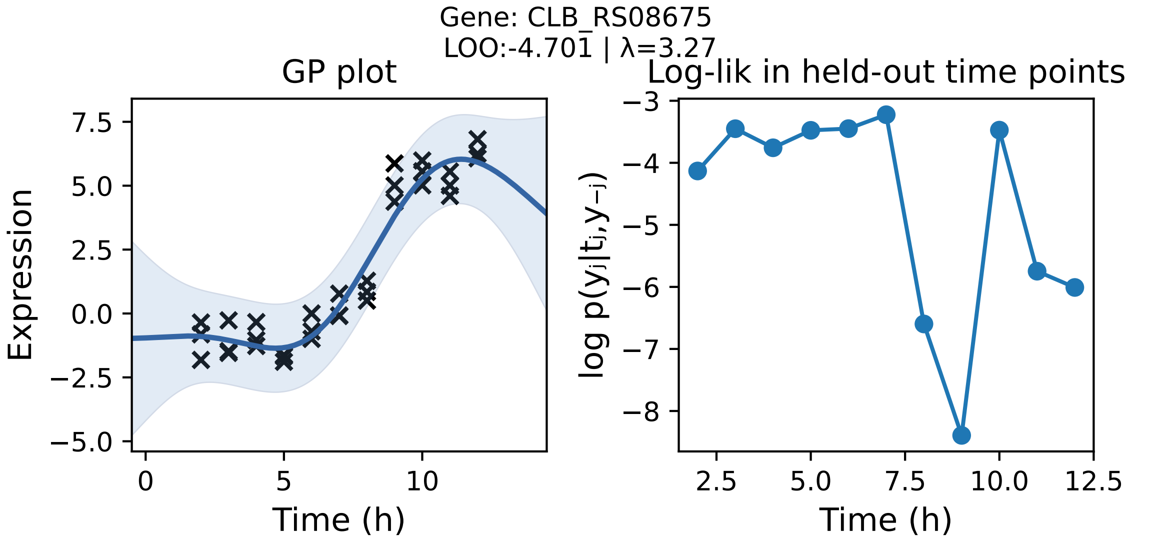

Gene: CLB_RS08675
LOO:-4.701 | λ=3.27
GP plot
Expression
Time (h)
7.5
5.0
2.5
0.0
−2.5
−5.0
0
5
10
Log-lik in held-out time points
log p(y_j|t_j,y_−j)
Time (h)
−3
−4
−5
−6
−7
−8
2.5
5.0
7.5
10.0
12.5

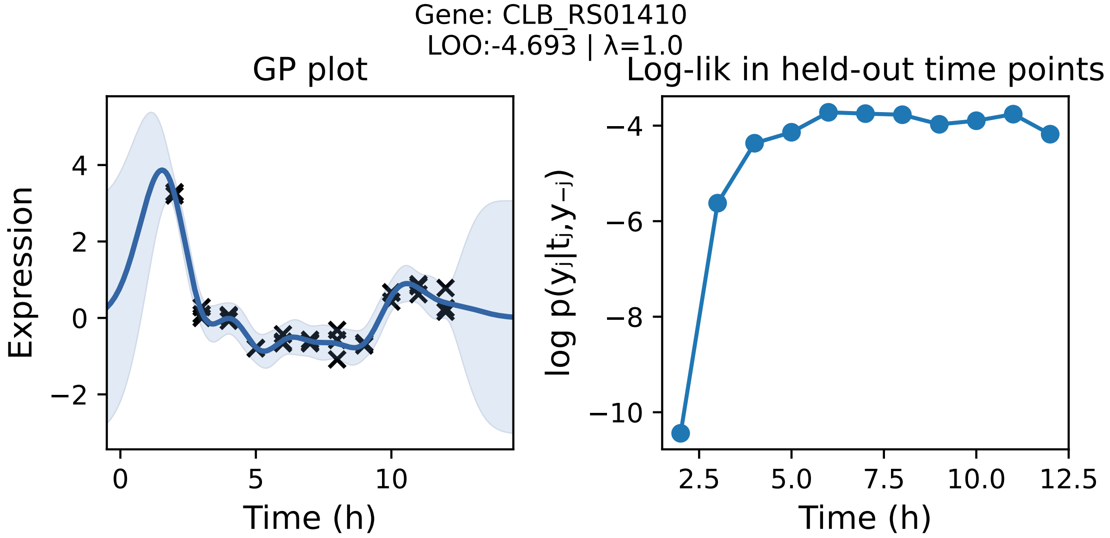

Gene: CLB_RS01410
LOO:-4.693 | λ=1.0
GP plot
Log-lik in held-out time points
Expression
Time (h)
log p(y_j|t_j,y_−j)
Time (h)
4
2
0
−2
0
5
10
−4
−6
−8
−10
2.5
5.0
7.5
10.0
12.5

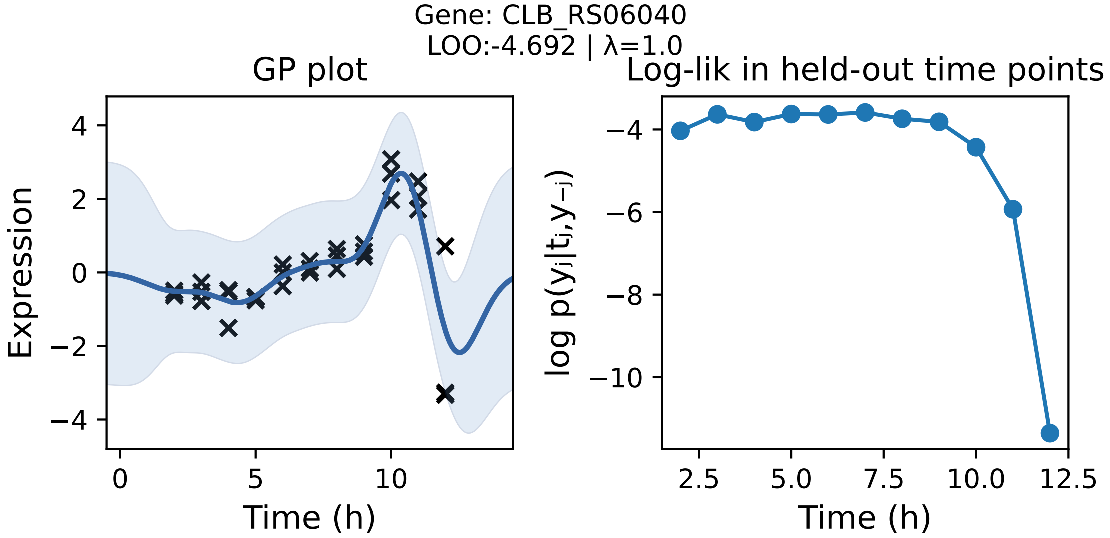

Gene: CLB_RS06040
LOO:-4.692 | λ=1.0
GP plot
Log-lik in held-out time points
Expression
Time (h)
log p(y_j|t_j,y_−j)
Time (h)

Gene: CLB_RS04375
LOO:-4.689 | λ=1.0

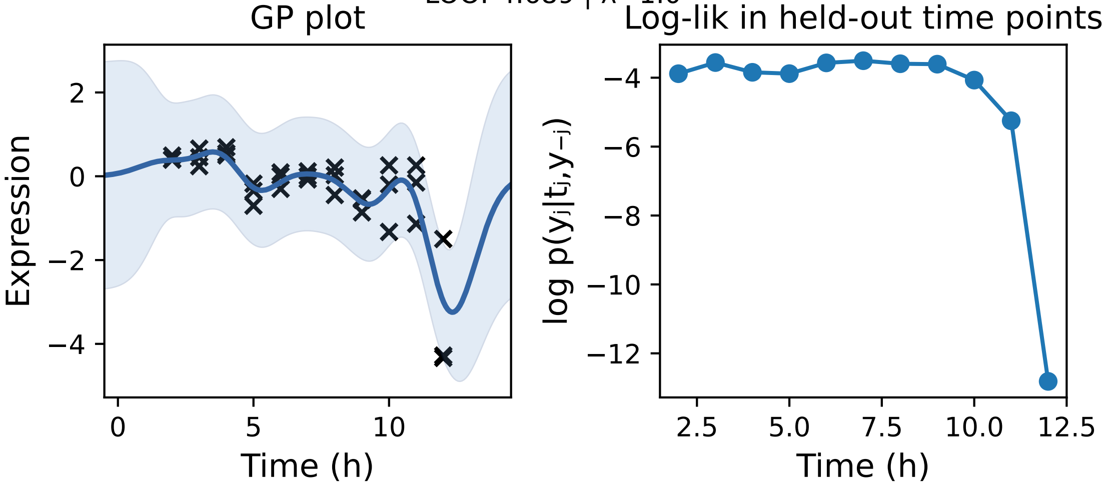

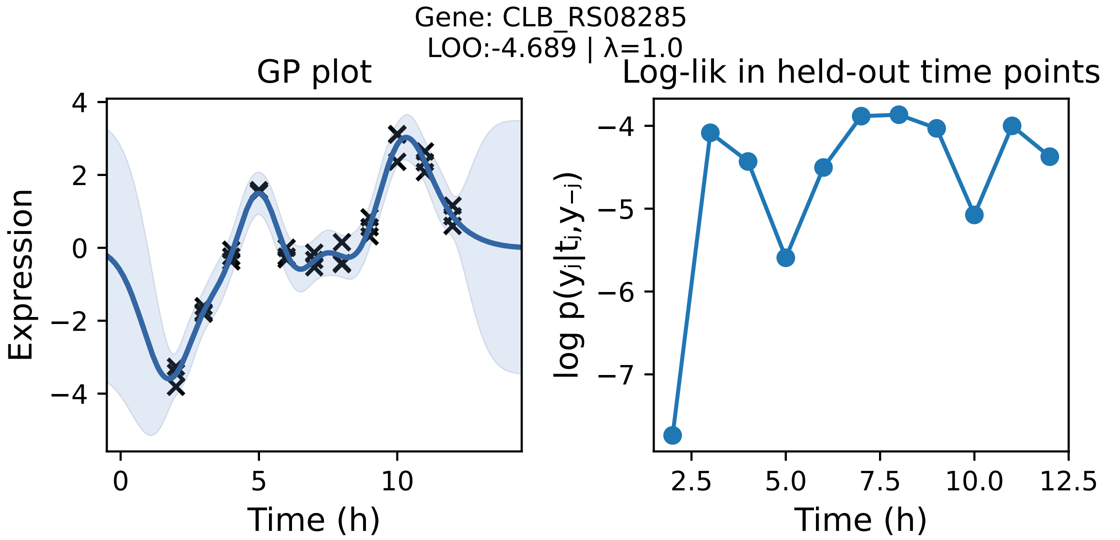

Gene: CLB_RS08285
LOO:-4.689 | λ=1.0
GP plot
Log-lik in held-out time points
Expression
Time (h)
log p(y_j|t_j,y_−j)
Time (h)
4
2
0
−2
−4
0
5
10
−4
−5
−6
−7
2.5
5.0
7.5
10.0
12.5

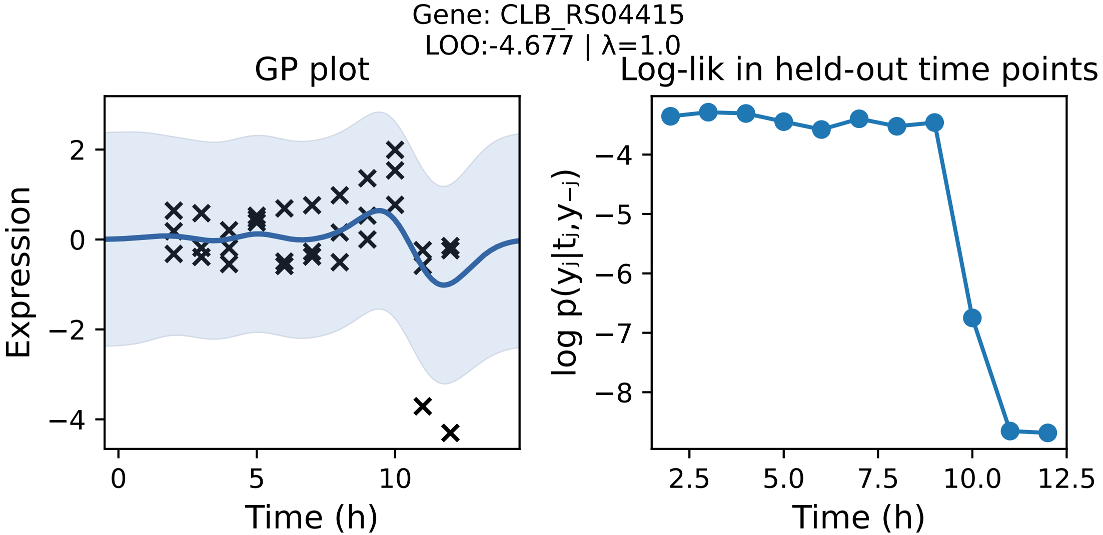
Gene: CLB_RS04415
LOO:-4.677 | λ=1.0
GP plot
Expression
Time (h)
2
0
−2
−4
0
5
10
Log-lik in held-out time points
log p(yj|tj,y−j)
Time (h)
−4
−5
−6
−7
−8
2.5
5.0
7.5
10.0
12.5

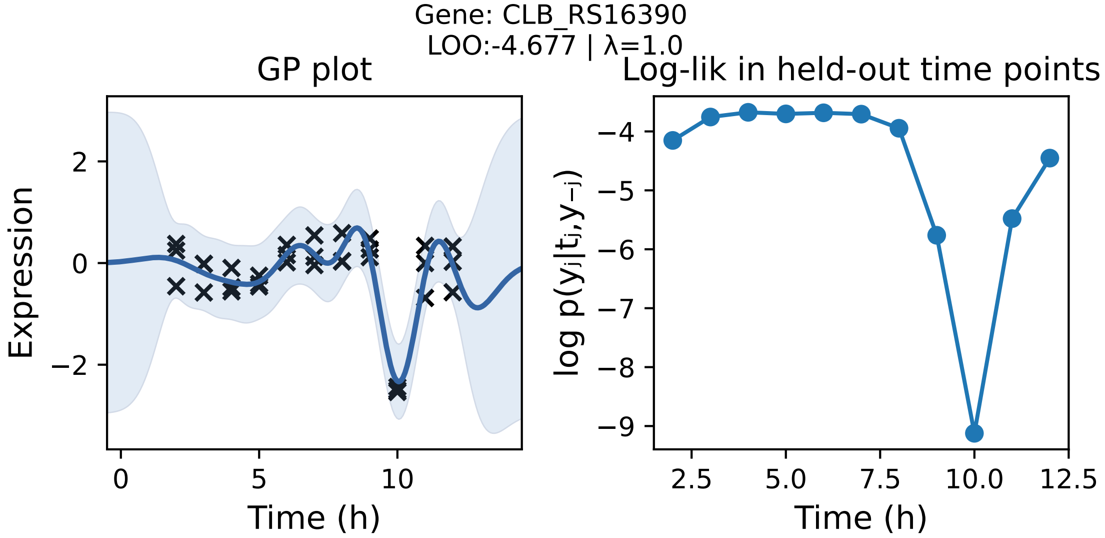

Gene: CLB_RS16390
LOO:-4.677 | λ=1.0
GP plot
Expression
Time (h)
Log-lik in held-out time points
log p(y_j|t_j,y_{−j})
Time (h)

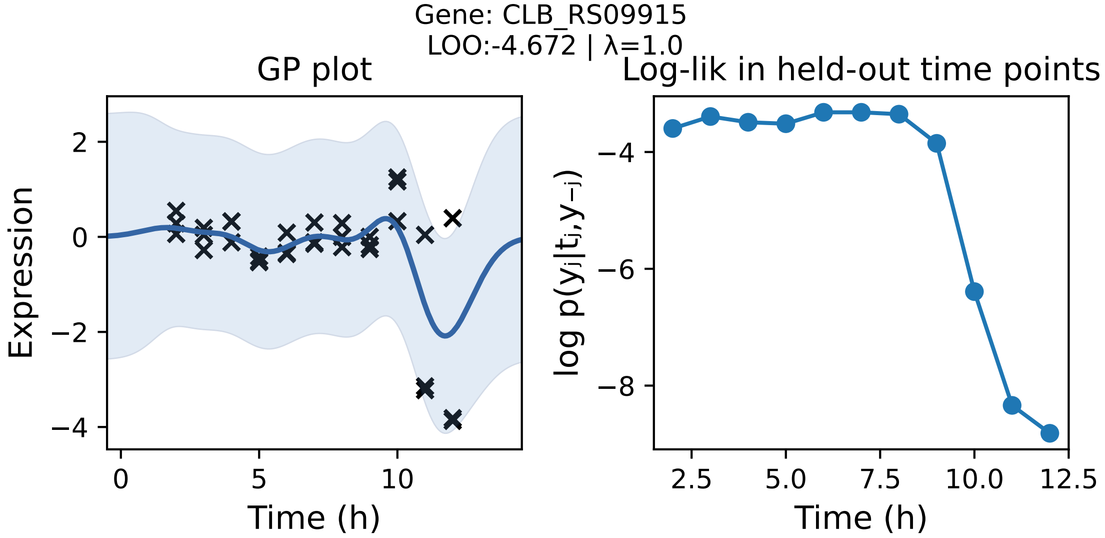
Gene: CLB_RS09915
LOO:-4.672 | λ=1.0
GP plot
Expression
Time (h)
2
0
−2
−4
0
5
10
Log-lik in held-out time points
log p(y_j|t_j,y_−j)
Time (h)
−4
−6
−8
2.5
5.0
7.5
10.0
12.5

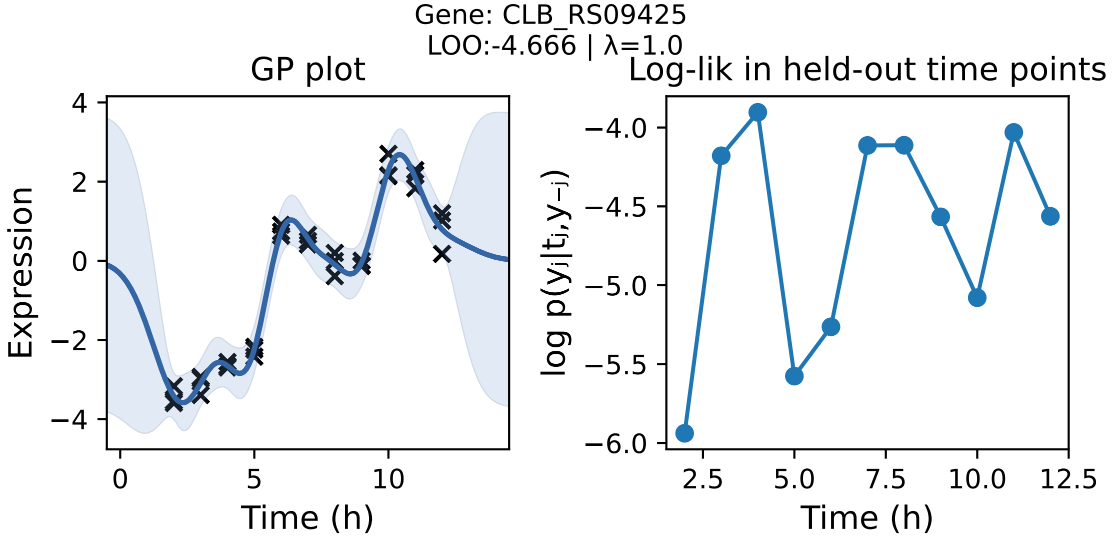
Gene: CLB_RS09425
LOO:-4.666 | λ=1.0
GP plot
Expression
Time (h)
4
2
0
−2
−4
0
5
10
Log-lik in held-out time points
log p(y_j|t_j,y_−j)
−4.0
−4.5
−5.0
−5.5
−6.0
2.5
5.0
7.5
10.0
12.5
Time (h)

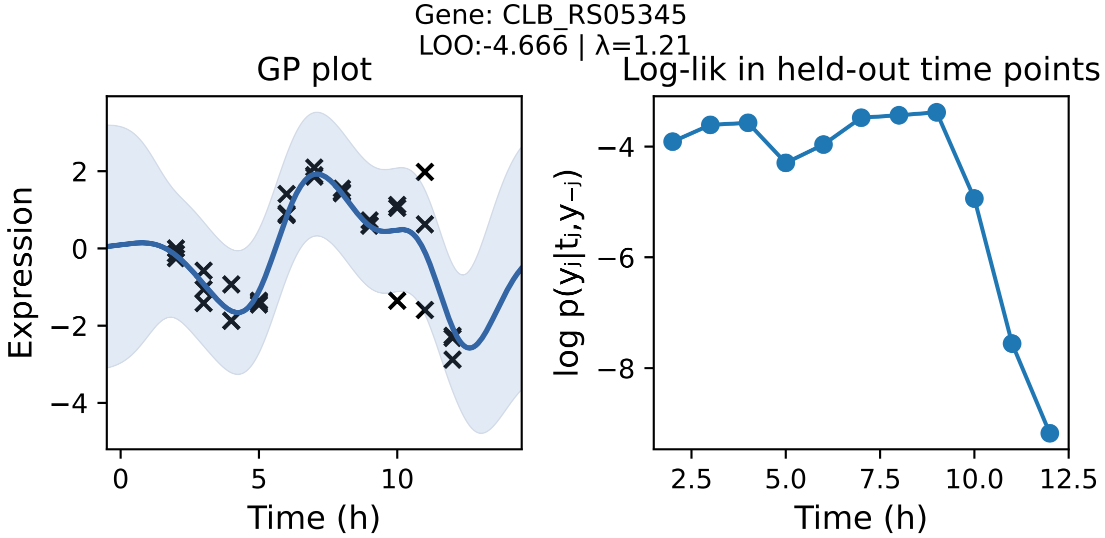

Gene: CLB_RS05345
LOO:-4.666 | λ=1.21
GP plot
Expression
Time (h)
Log-lik in held-out time points
log p(y_j|t_j,y_−j)
Time (h)

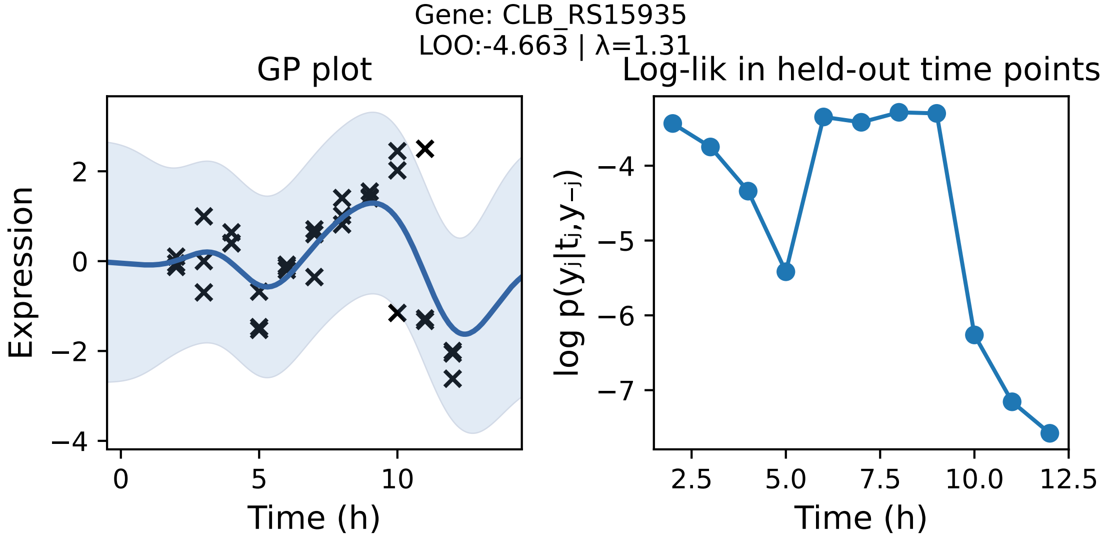

Gene: CLB_RS15935
LOO:-4.663 | λ=1.31
GP plot
Log-lik in held-out time points
Expression
Time (h)
log p(yj|tj,y−j)
Time (h)

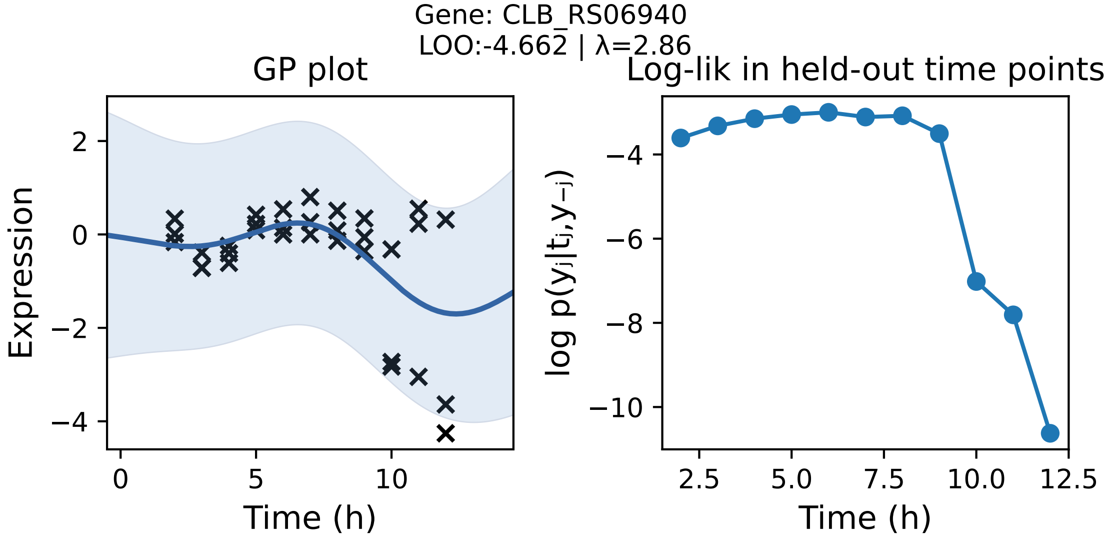

Gene: CLB_RS06940
LOO:-4.662 | λ=2.86
GP plot
Expression
Time (h)
2
0
−2
−4
0
5
10
Log-lik in held-out time points
log p(y_j|t_j,y_−j)
−4
−6
−8
−10
2.5
5.0
7.5
10.0
12.5
Time (h)

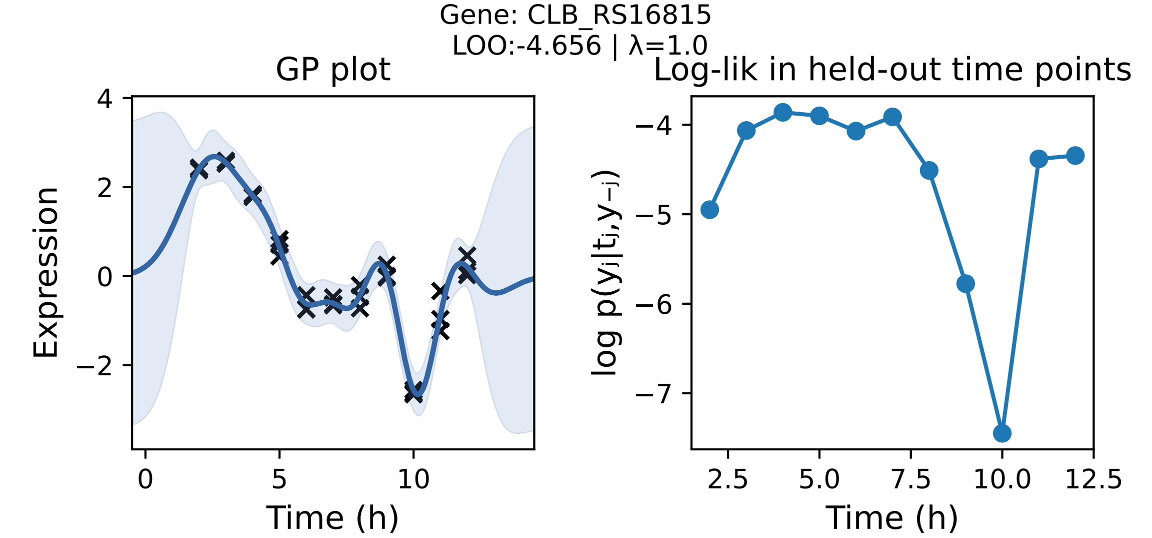

Gene: CLB_RS16815
LOO:-4.656 | λ=1.0
GP plot
Expression
Time (h)
4
2
0
−2
0
5
10
Log-lik in held-out time points
log p(y_j|t_j,y_−j)
Time (h)
−4
−5
−6
−7
2.5
5.0
7.5
10.0
12.5

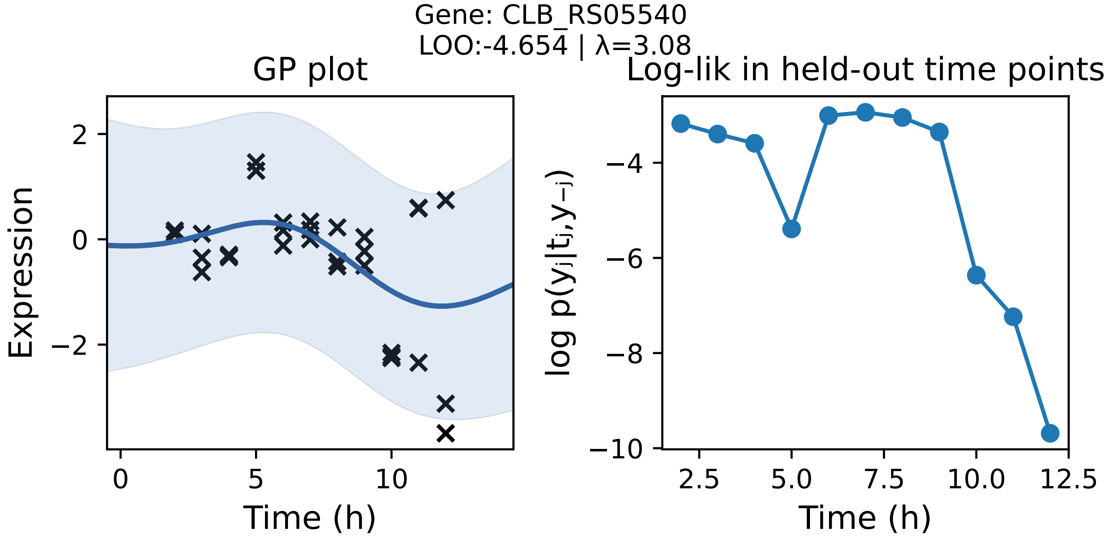

Gene: CLB_RS05540
LOO:-4.654 | λ=3.08
GP plot
Expression
Time (h)
Log-lik in held-out time points
log p(y_j|t_j,y_−j)
Time (h)

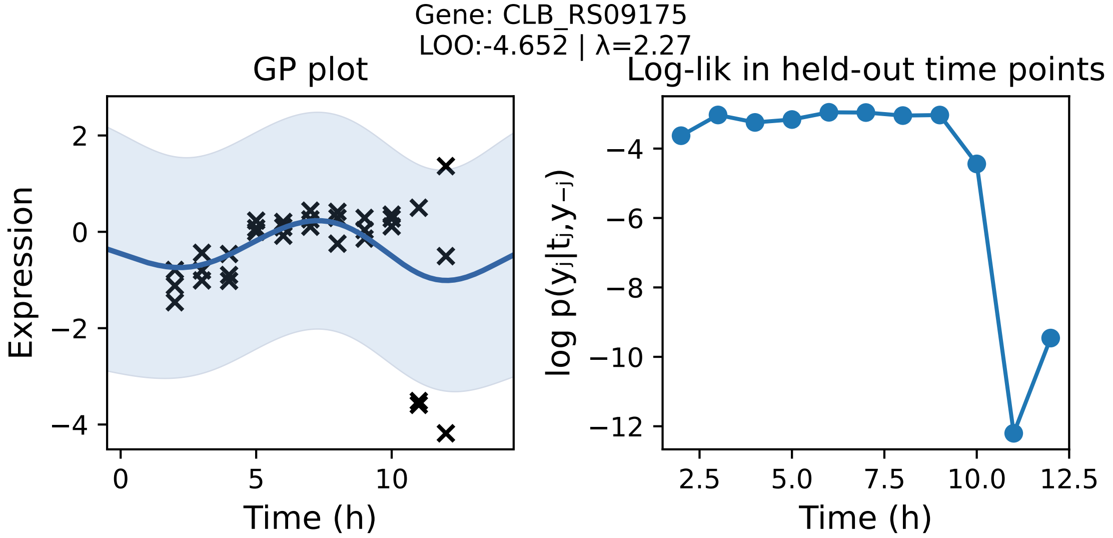
Gene: CLB_RS09175
LOO:-4.652 | λ=2.27
GP plot
Expression
Time (h)
2
0
−2
−4
0
5
10
Log-lik in held-out time points
log p(y_j|t_j,y_−j)
Time (h)
−4
−6
−8
−10
−12
2.5
5.0
7.5
10.0
12.5

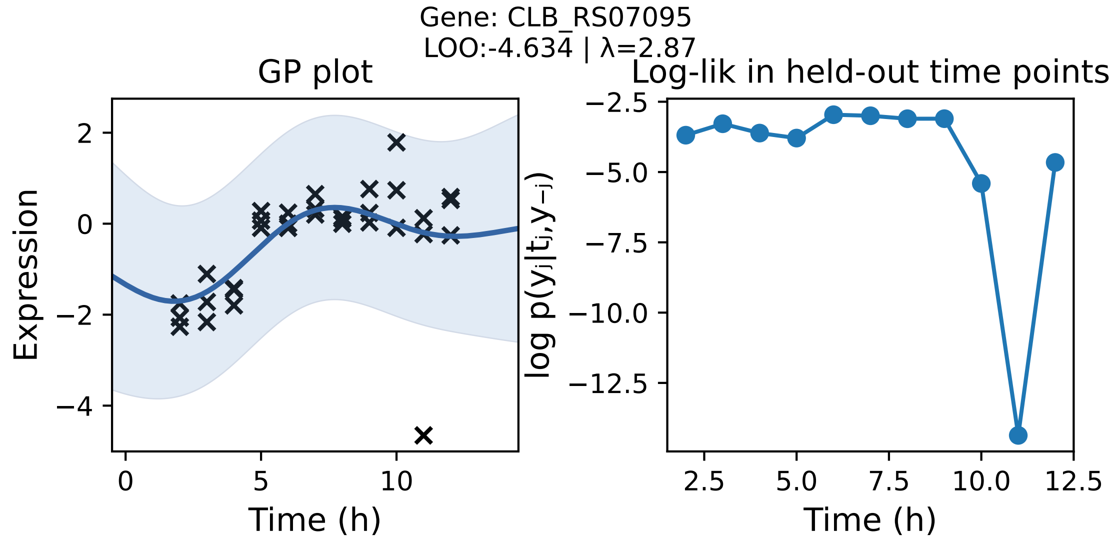

Gene: CLB_RS07095
LOO:-4.634 | λ=2.87
GP plot
Expression
Time (h)
Log-lik in held-out time points
log p(yj|tj,y−j)
Time (h)

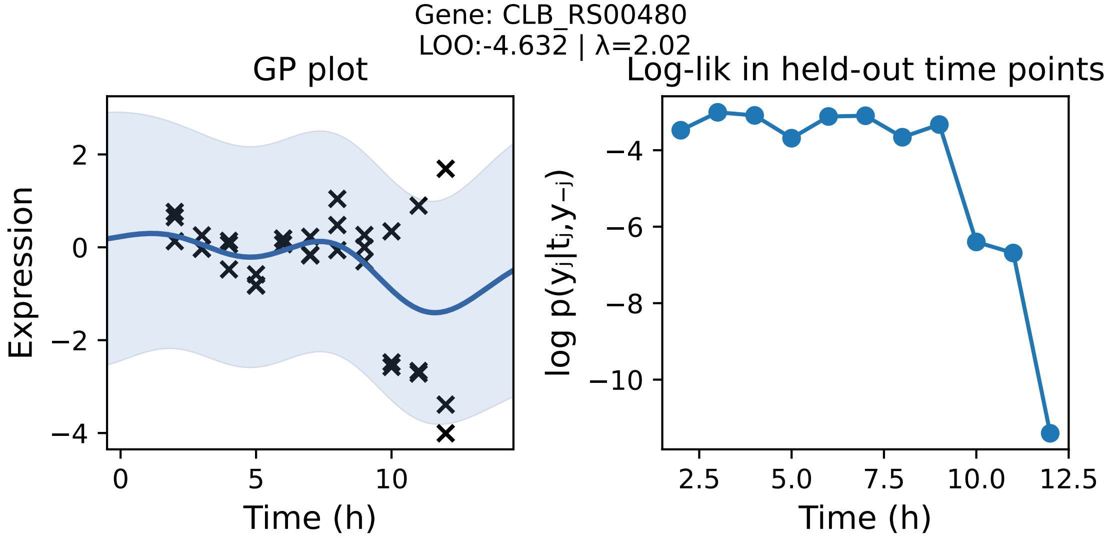

Gene: CLB_RS00480
LOO:-4.632 | λ=2.02
GP plot
Expression
Time (h)
2
0
−2
−4
0
5
10
Log-lik in held-out time points
log p(y_j|t_j,y_−j)
Time (h)
−4
−6
−8
−10
2.5
5.0
7.5
10.0
12.5

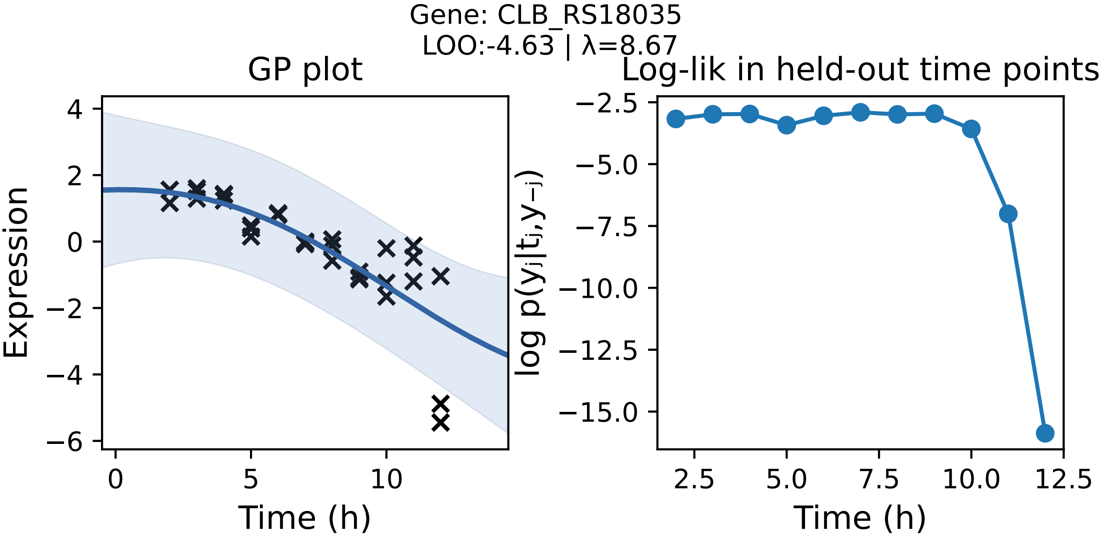

Gene: CLB_RS18035
LOO:-4.63 | λ=8.67
GP plot
Expression
Time (h)
4
2
0
−2
−4
−6
0
5
10
Log-lik in held-out time points
log p(yj|tj,y−j)
Time (h)
−2.5
−5.0
−7.5
−10.0
−12.5
−15.0
2.5
5.0
7.5
10.0
12.5

Gene: CLB_RS05455
LOO:-4.619 | λ=4.4

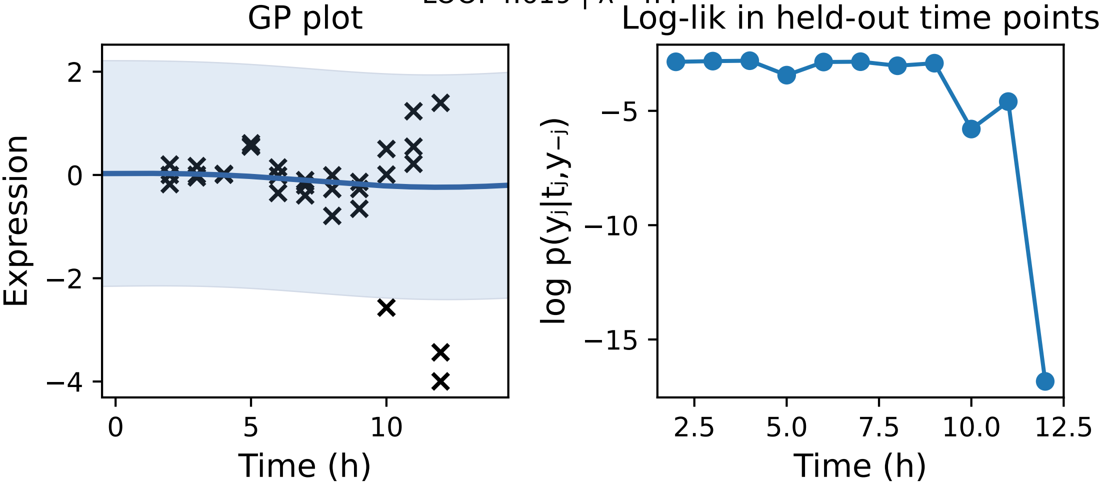

Gene: CLB_RS06235
LOO:-4.615 | λ=1.0

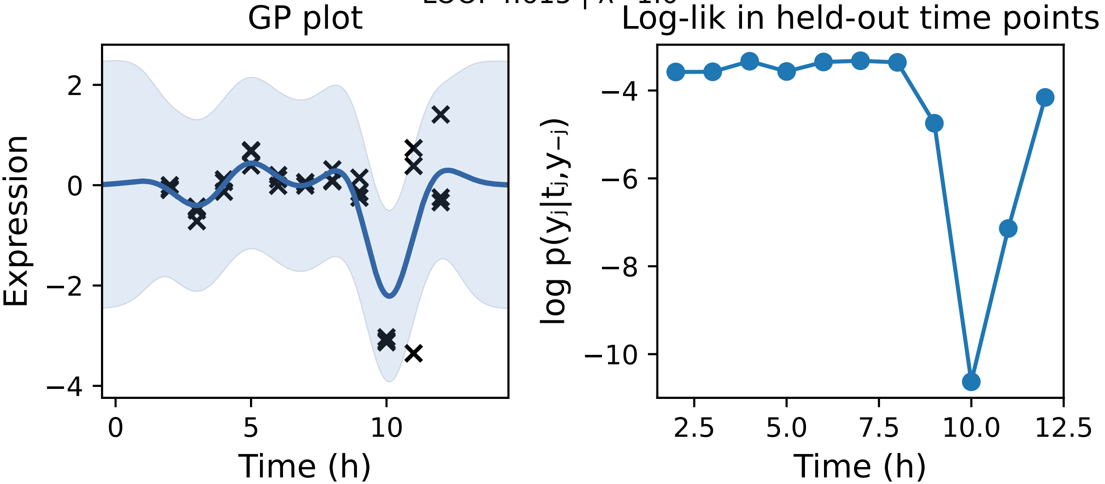

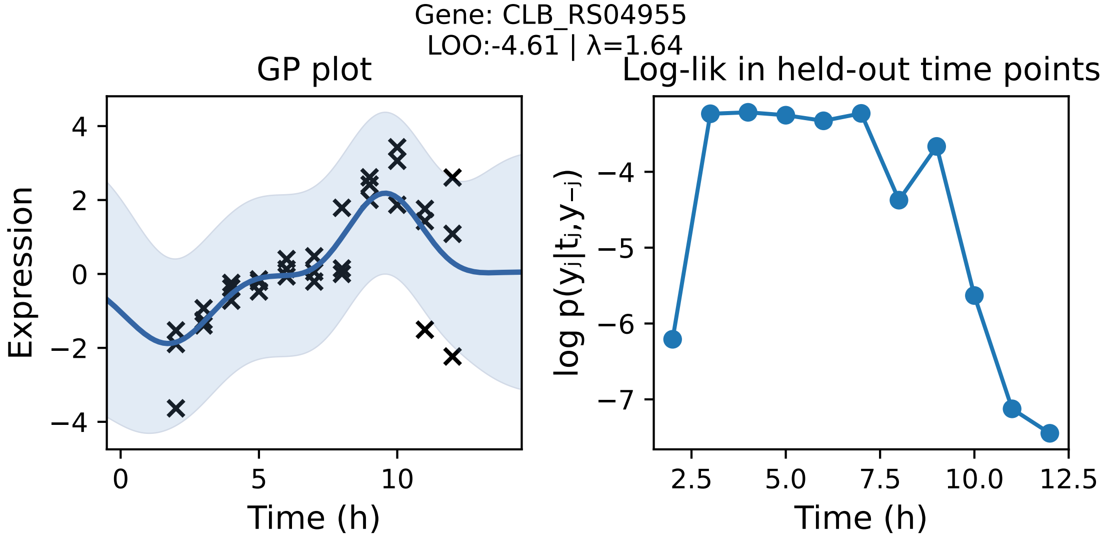

Gene: CLB_RS04955
LOO:-4.61 | λ=1.64
GP plot
Expression
Time (h)
4
2
0
−2
−4
0
5
10
Log-lik in held-out time points
log p(y_j|t_j,y_−j)
Time (h)
−4
−5
−6
−7
2.5
5.0
7.5
10.0
12.5

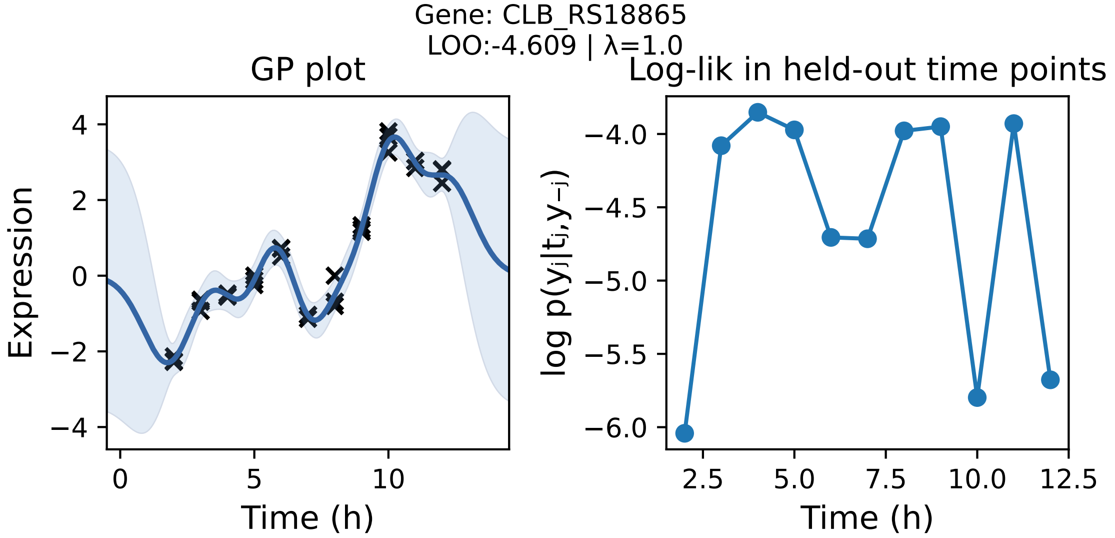

Gene: CLB_RS18865
LOO:-4.609 | λ=1.0
GP plot
Expression
Time (h)
Log-lik in held-out time points
log p(y_j|t_j,y_{-j})
Time (h)

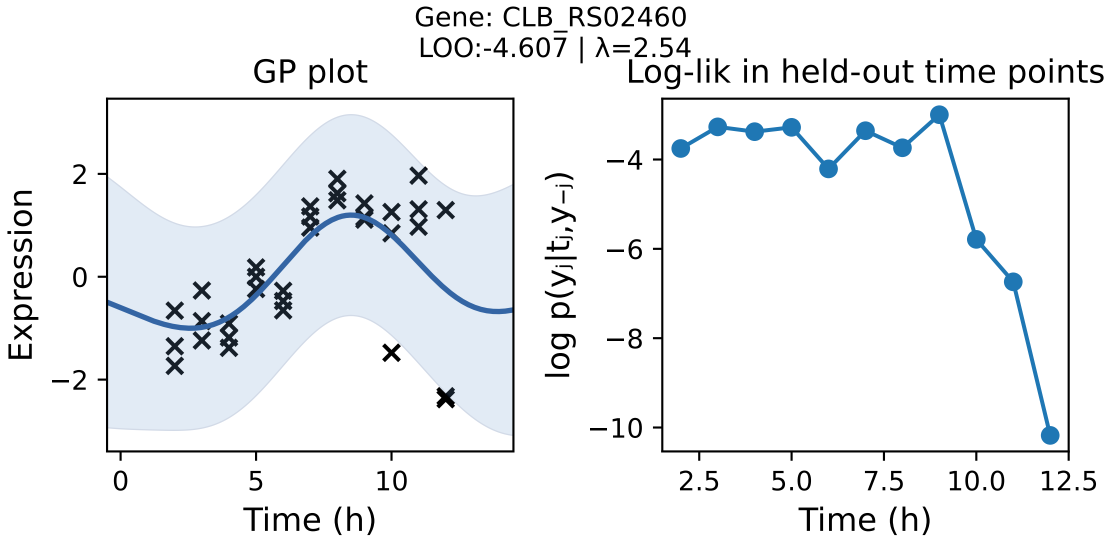
Gene: CLB_RS02460
LOO:-4.607 | λ=2.54
GP plot
Expression
Time (h)
Log-lik in held-out time points
log p(y_j|t_j,y_−j)
Time (h)

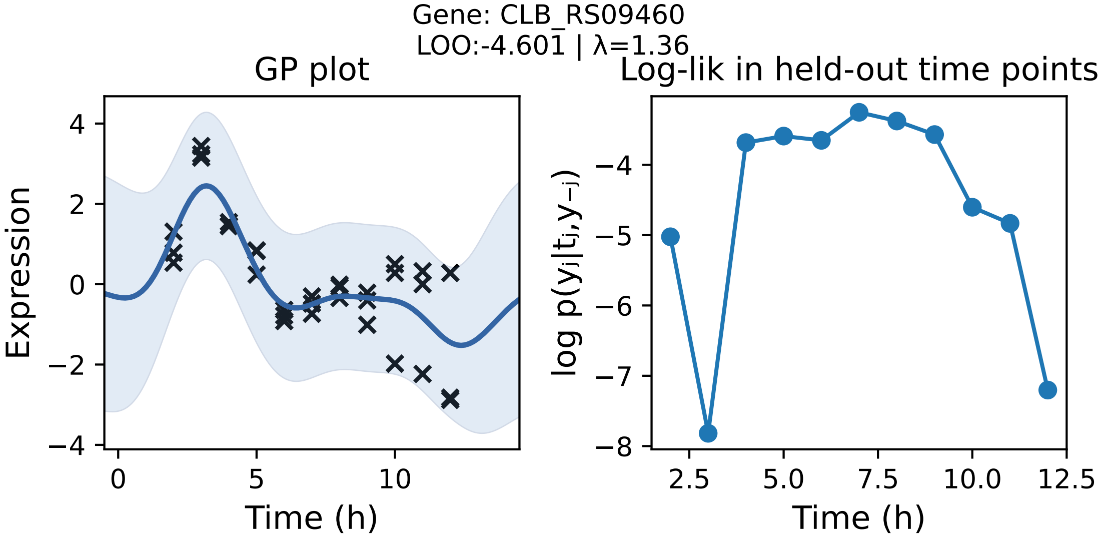

Gene: CLB_RS09460
LOO:-4.601 | λ=1.36
GP plot
Expression
Time (h)
4
2
0
−2
−4
0
5
10
Log-lik in held-out time points
log p(y_j|t_j,y_−j)
Time (h)
−4
−5
−6
−7
−8
2.5
5.0
7.5
10.0
12.5

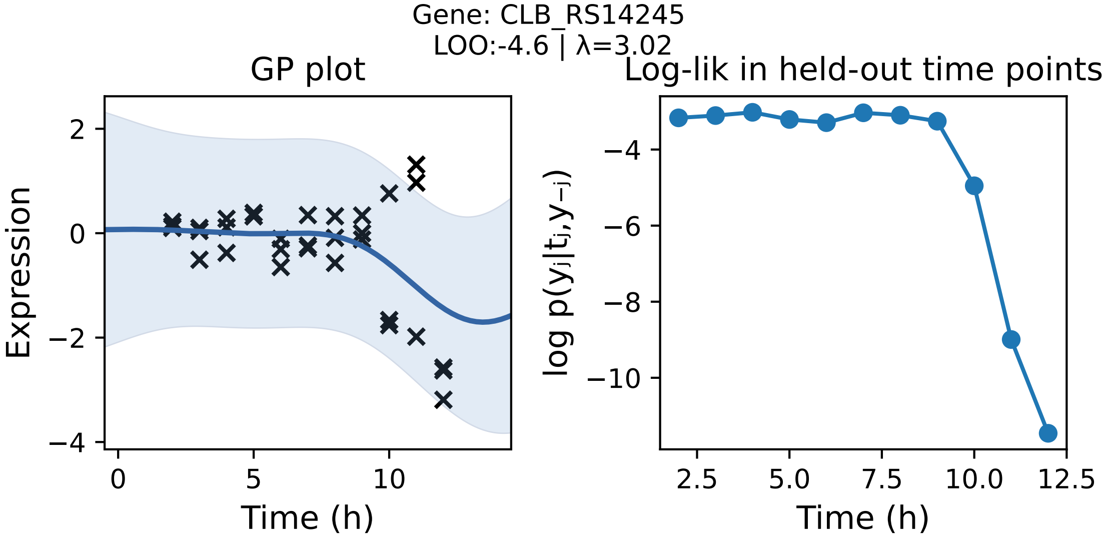
Gene: CLB_RS14245
LOO:-4.6 | λ=3.02
GP plot
Expression
Time (h)
2
0
−2
−4
0
5
10
Log-lik in held-out time points
log p(y_j|t_j,y_−j)
−4
−6
−8
−10
2.5
5.0
7.5
10.0
12.5
Time (h)

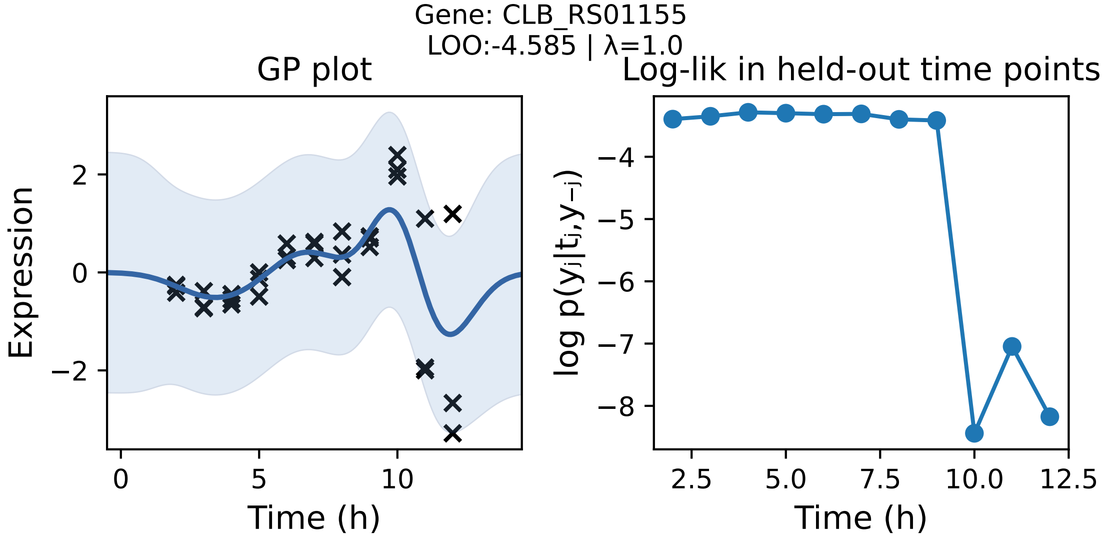

Gene: CLB_RS01155
LOO:-4.585 | λ=1.0
GP plot
Expression
Time (h)
Log-lik in held-out time points
log p(y_j|t_j,y_−j)
Time (h)

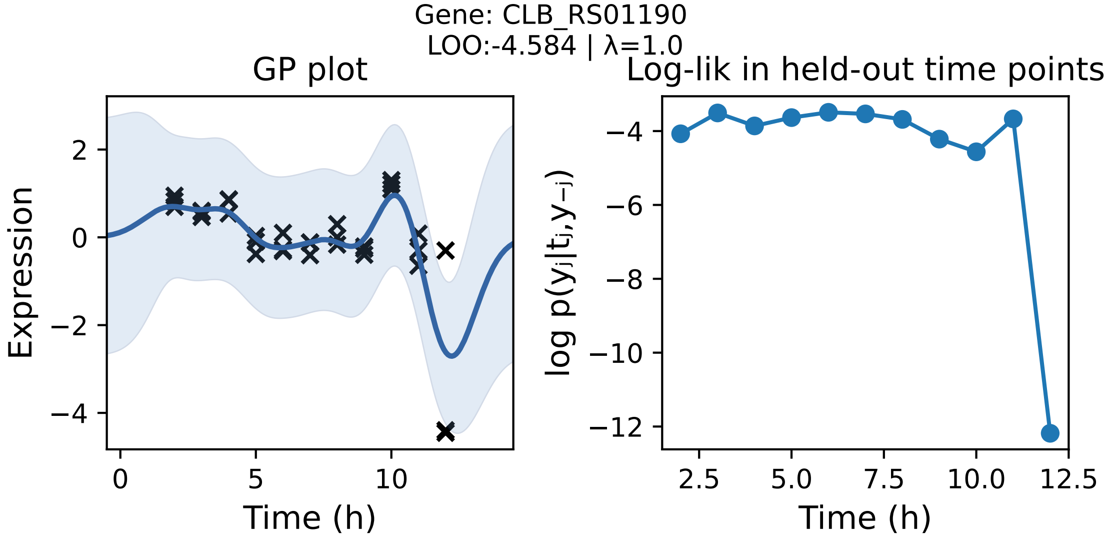

Gene: CLB_RS01190
LOO:-4.584 | λ=1.0
GP plot
Expression
Time (h)
2
0
−2
−4
0
5
10
Log-lik in held-out time points
log p(y_j|t_j,y_−j)
Time (h)
−4
−6
−8
−10
−12
2.5
5.0
7.5
10.0
12.5

Gene: CLB_RS00385
LOO:-4.581 | λ=3.02

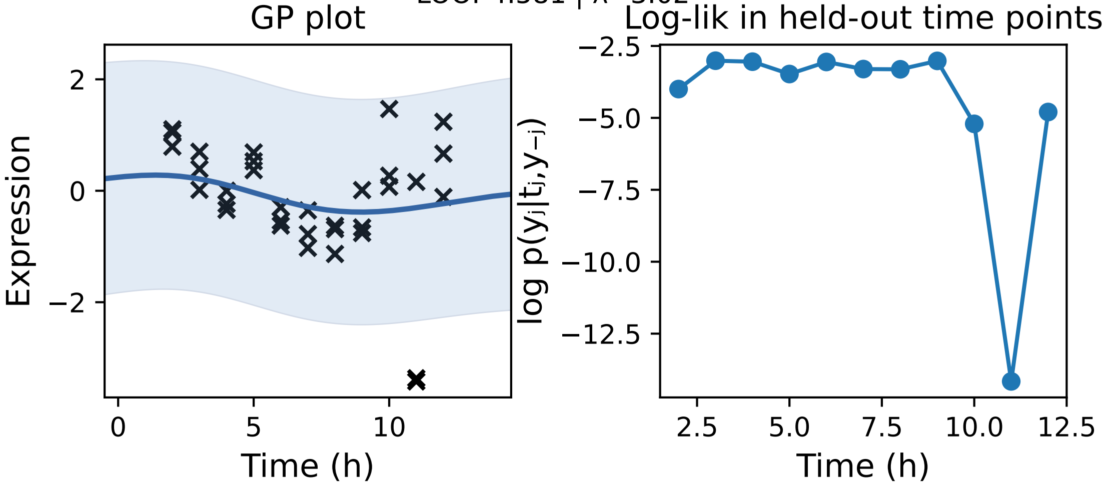

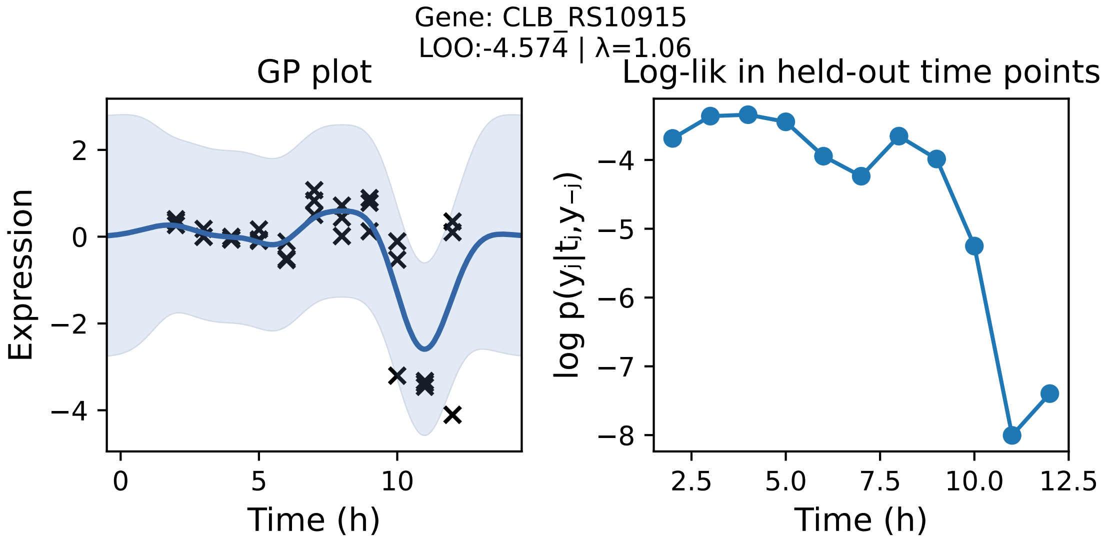

Gene: CLB_RS10915
LOO:-4.574 | λ=1.06
GP plot
Expression
Time (h)
2
0
−2
−4
0
5
10
Log-lik in held-out time points
log p(y_j|t_j,y_−j)
Time (h)
−4
−5
−6
−7
−8
2.5
5.0
7.5
10.0
12.5

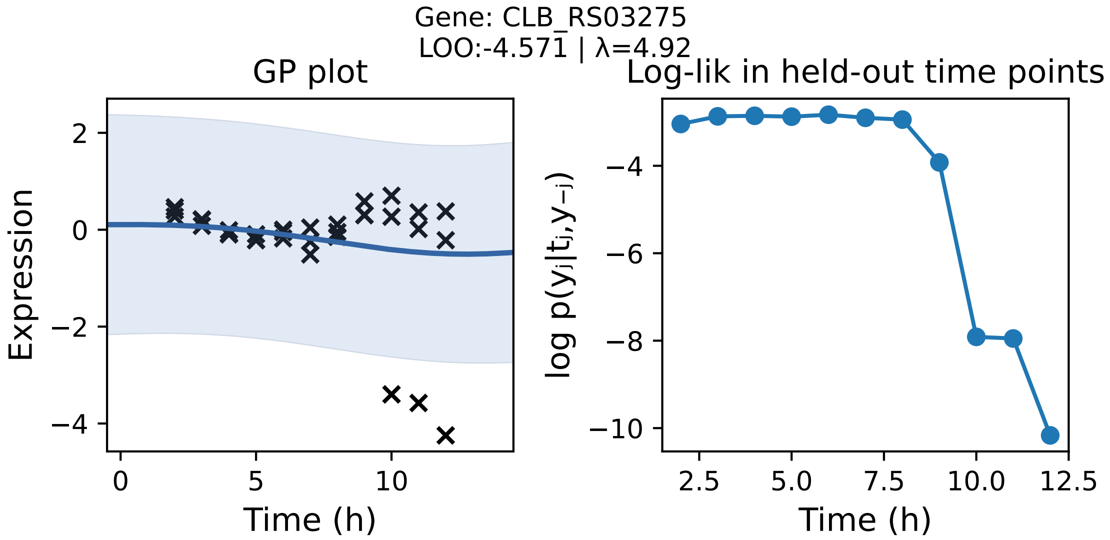

Gene: CLB_RS03275
LOO:-4.571 | λ=4.92
GP plot
Expression
Time (h)
Log-lik in held-out time points
log p(y_j|t_j,y_−j)
Time (h)

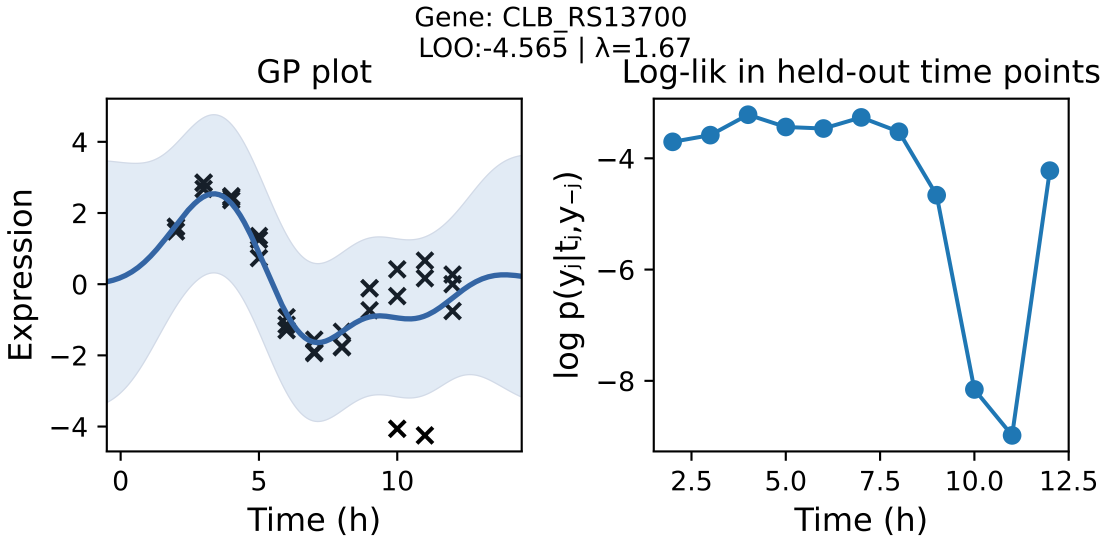
Gene: CLB_RS13700
LOO:-4.565 | λ=1.67
GP plot
Expression
Time (h)
Log-lik in held-out time points
log p(y_j|t_j,y_−j)
Time (h)

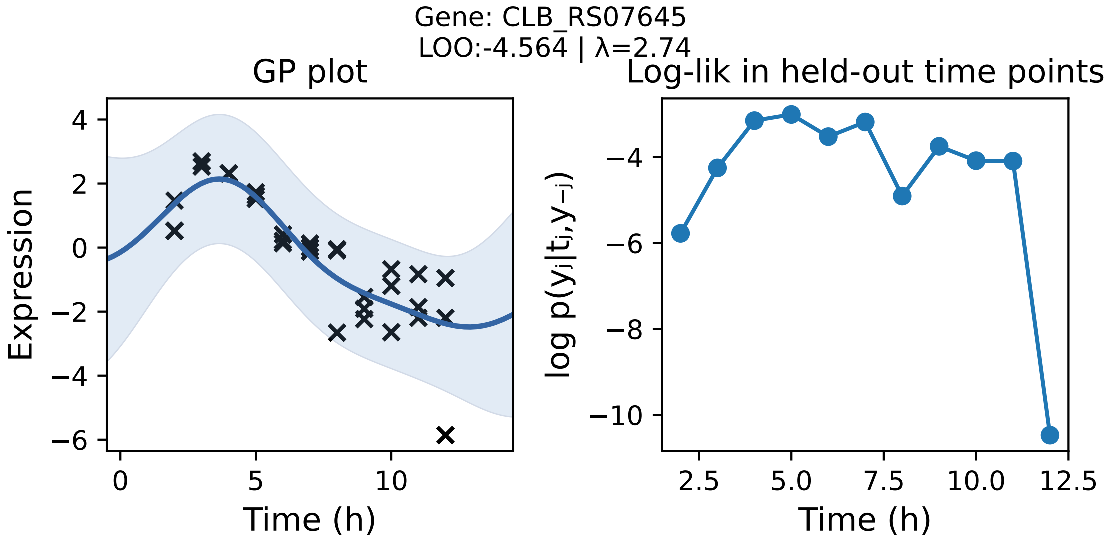

Gene: CLB_RS07645
LOO:-4.564 | λ=2.74
GP plot
Expression
Time (h)
4
2
0
−2
−4
−6
0
5
10
Log-lik in held-out time points
log p(y_j|t_i,y_−j)
Time (h)
−4
−6
−8
−10
2.5
5.0
7.5
10.0
12.5

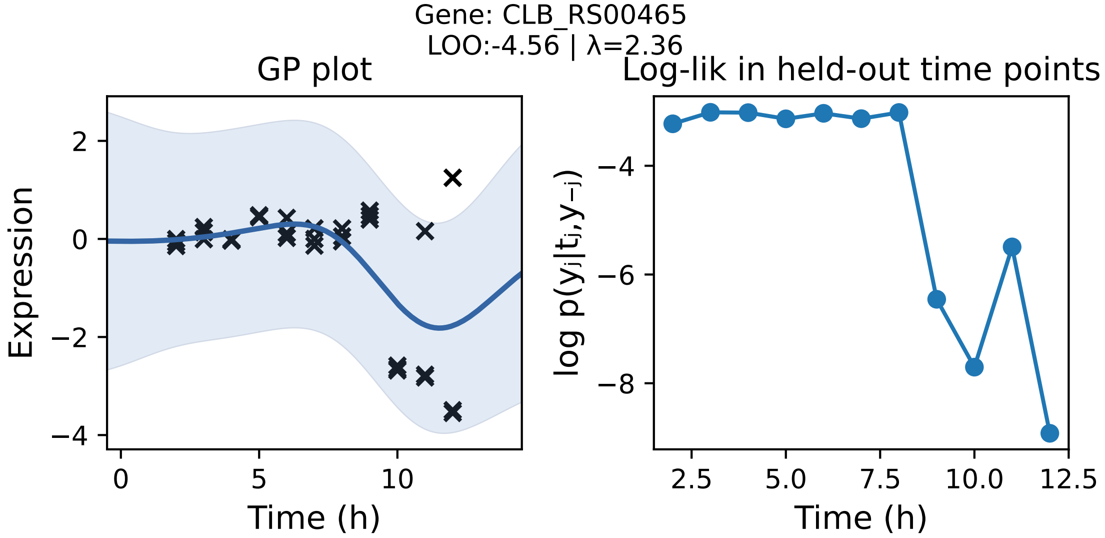

Gene: CLB_RS00465
LOO:-4.56 | λ=2.36
GP plot
Expression
2
0
−2
−4
0
5
10
Time (h)
Log-lik in held-out time points
log p(y_j|t_j,y_−j)
−4
−6
−8
2.5
5.0
7.5
10.0
12.5
Time (h)

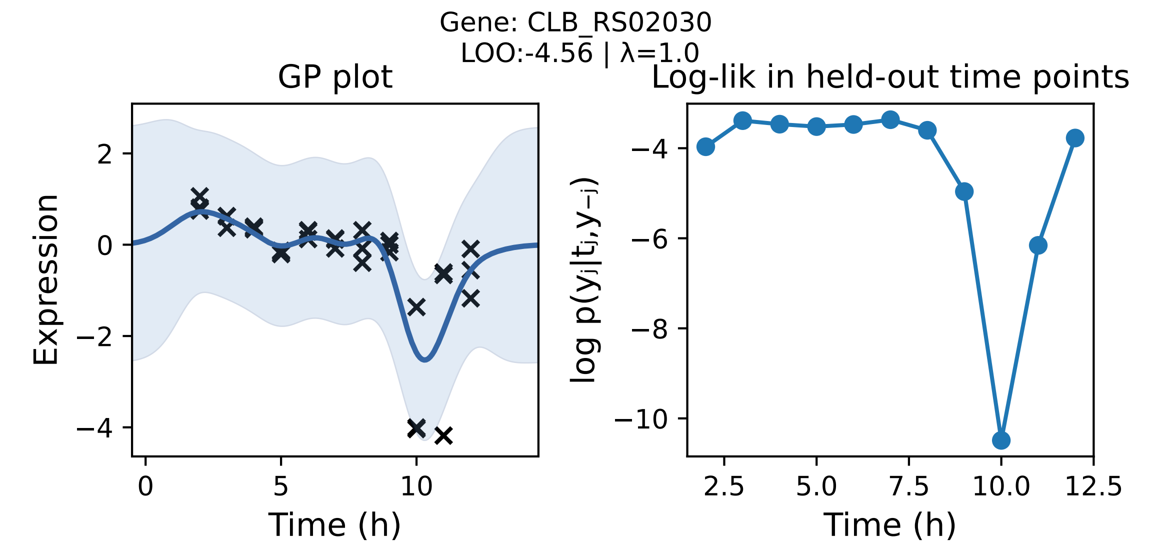

Gene: CLB_RS02030
LOO:-4.56 | λ=1.0
GP plot
Expression
Time (h)
2
0
−2
−4
0
5
10
Log-lik in held-out time points
log p(yⱼ|tⱼ,y₋ⱼ)
Time (h)
−4
−6
−8
−10
2.5
5.0
7.5
10.0
12.5

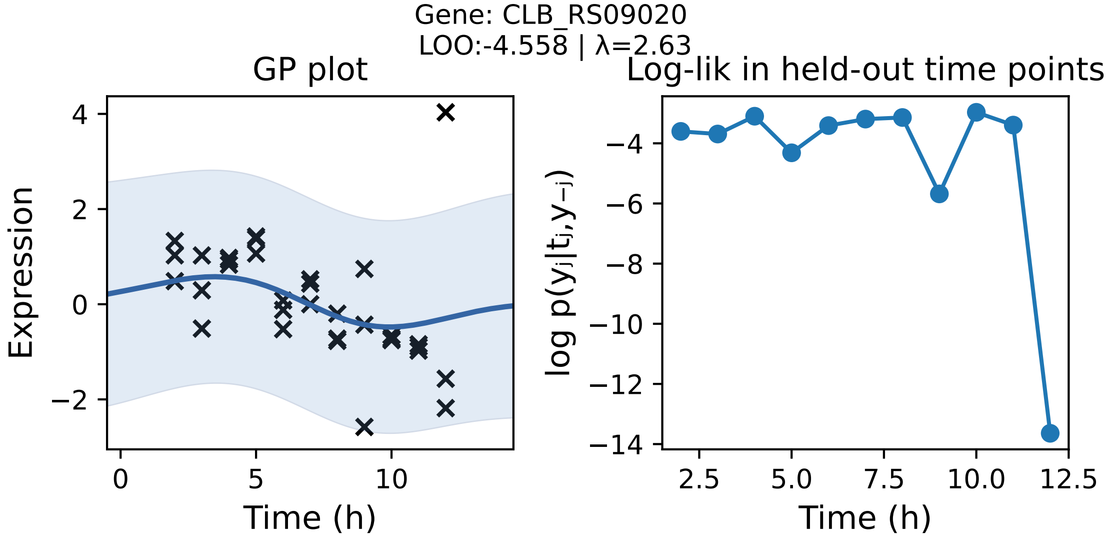

Gene: CLB_RS09020
LOO:-4.558 | λ=2.63
GP plot
Expression
Time (h)
4
2
0
−2
0
5
10
Log-lik in held-out time points
log p(y_j|t_j,y_−j)
Time (h)
−4
−6
−8
−10
−12
−14
2.5
5.0
7.5
10.0
12.5

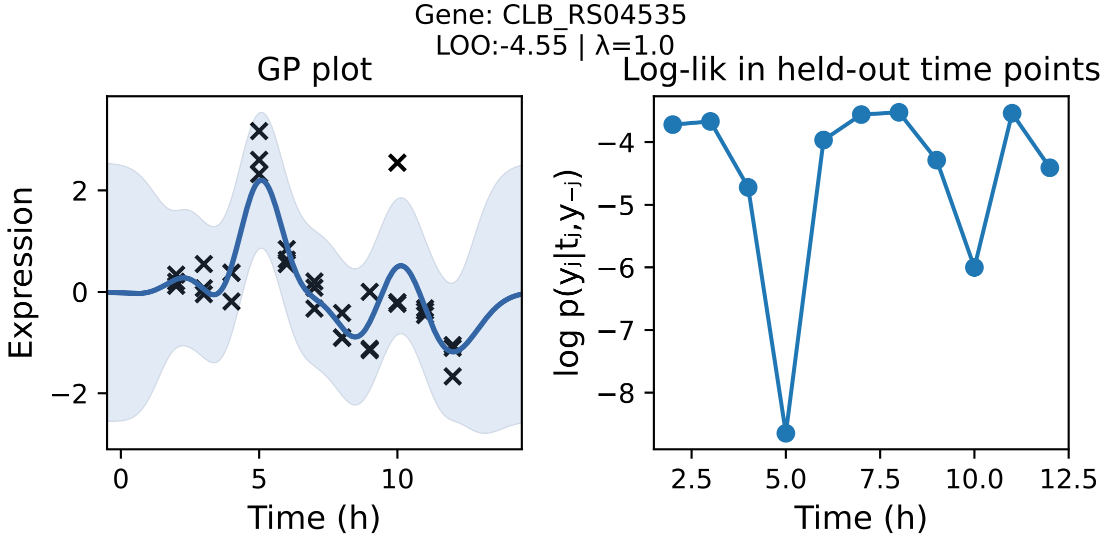
Gene: CLB_RS04535
LOO:-4.55 | λ=1.0
GP plot
Expression
Time (h)
Log-lik in held-out time points
log p(yj|tj,y−j)
Time (h)

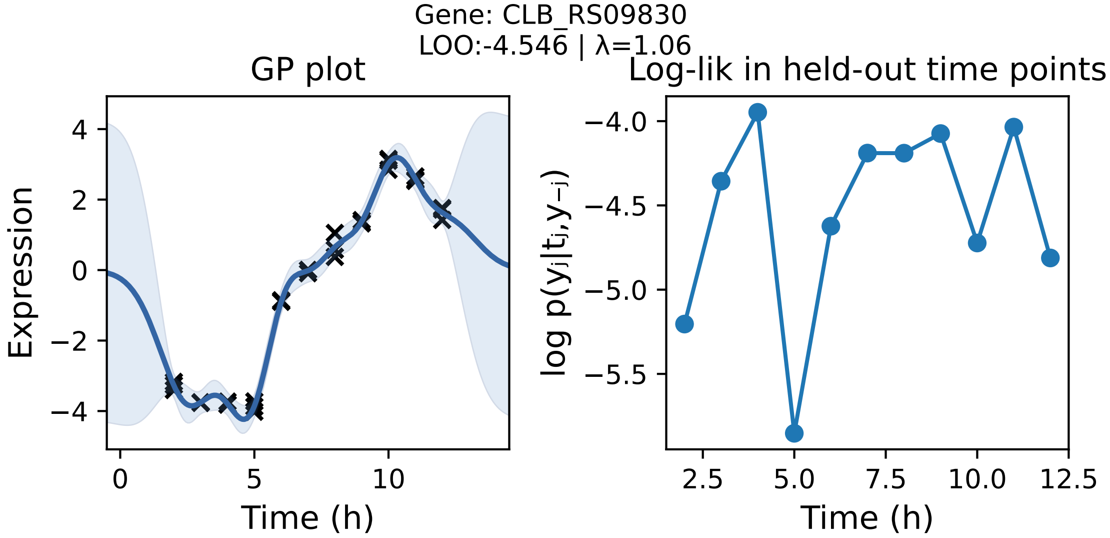
Gene: CLB_RS09830
LOO:-4.546 | λ=1.06
GP plot
Log-lik in held-out time points
Expression
Time (h)
log p(y_j|t_j,y_−j)
4
2
0
−2
−4
0
5
10
−4.0
−4.5
−5.0
−5.5
2.5
5.0
7.5
10.0
12.5

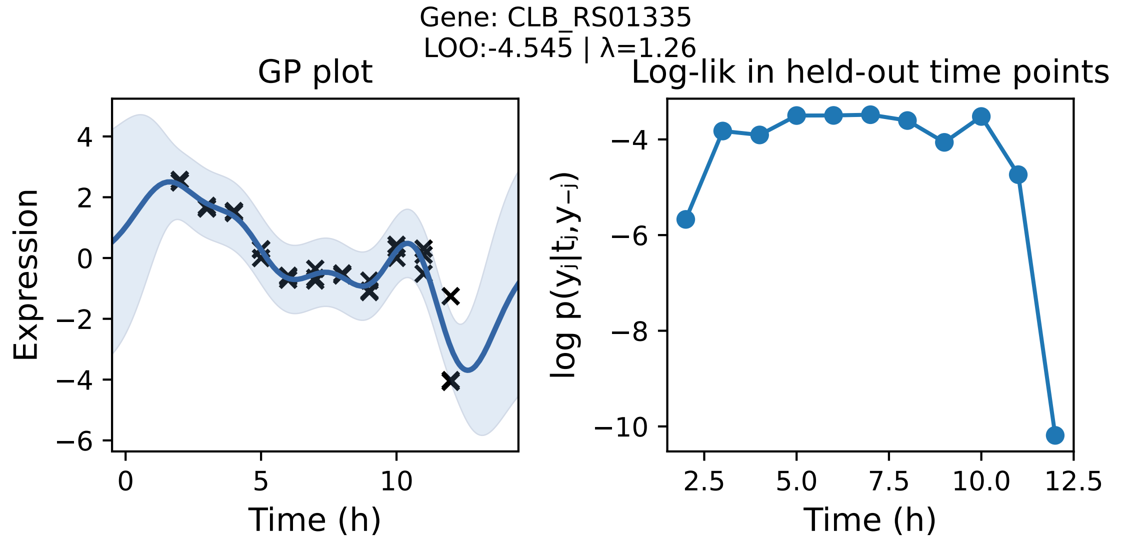

Gene: CLB_RS01335
LOO:-4.545 | λ=1.26
GP plot
Expression
Time (h)
Log-lik in held-out time points
log p(y_j|t_j,y_-j)
Time (h)

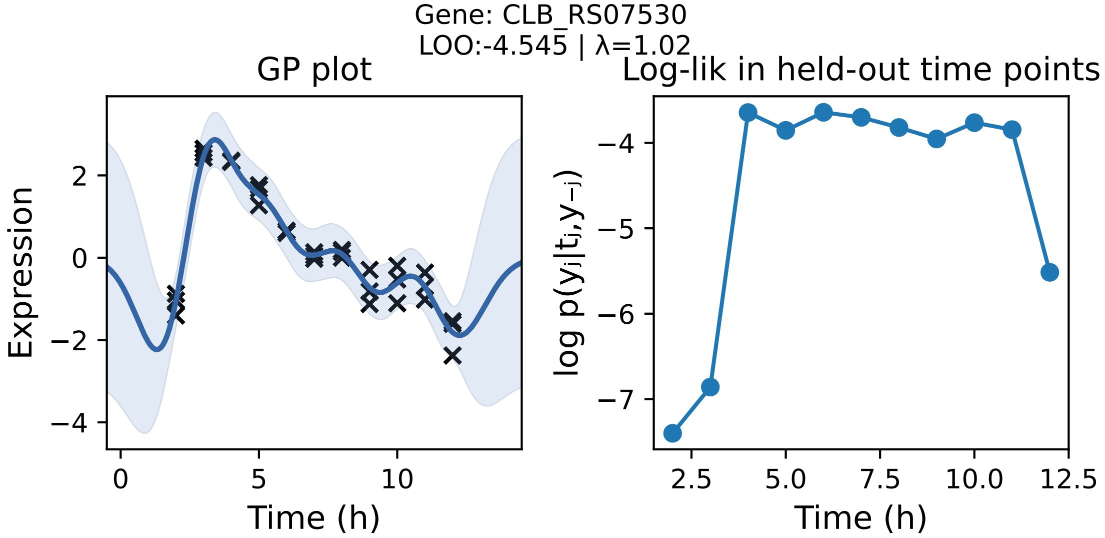
Gene: CLB_RS07530
LOO:-4.545 | λ=1.02
GP plot
Expression
2
0
−2
−4
0
5
10
Time (h)
Log-lik in held-out time points
log p(y_j|t_j,y_−j)
−4
−5
−6
−7
2.5
5.0
7.5
10.0
12.5
Time (h)

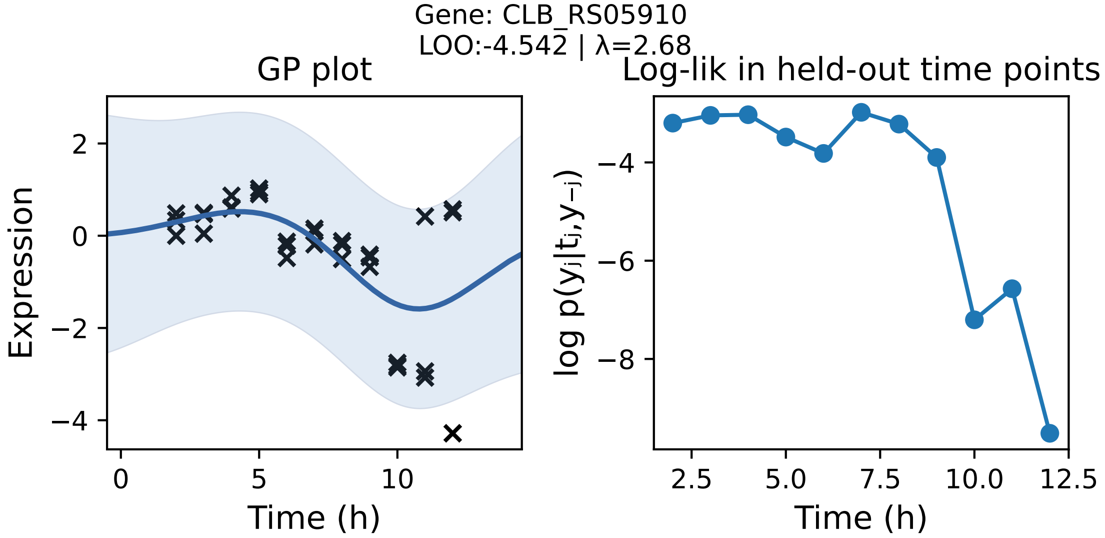
Gene: CLB_RS05910
LOO:-4.542 | λ=2.68
GP plot
Expression
Time (h)
Log-lik in held-out time points
log p(y_j|t_j,y_−j)
Time (h)
2
0
−2
−4
0
5
10
−4
−6
−8
2.5
5.0
7.5
10.0
12.5

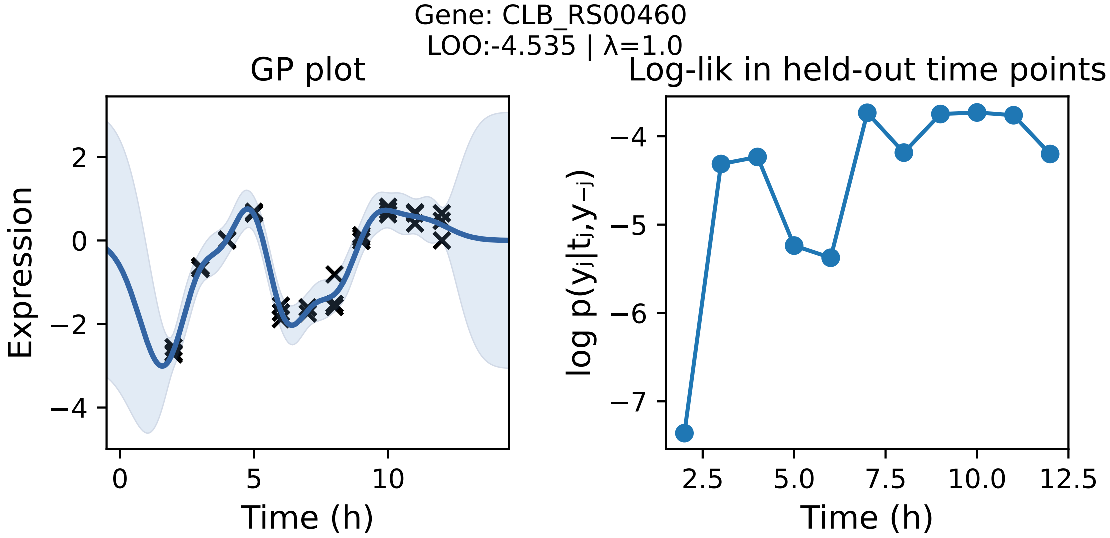

Gene: CLB_RS00460
LOO:-4.535 | λ=1.0
GP plot
Expression
Time (h)
Log-lik in held-out time points
log p(y_j|t_j,y_−j)
Time (h)

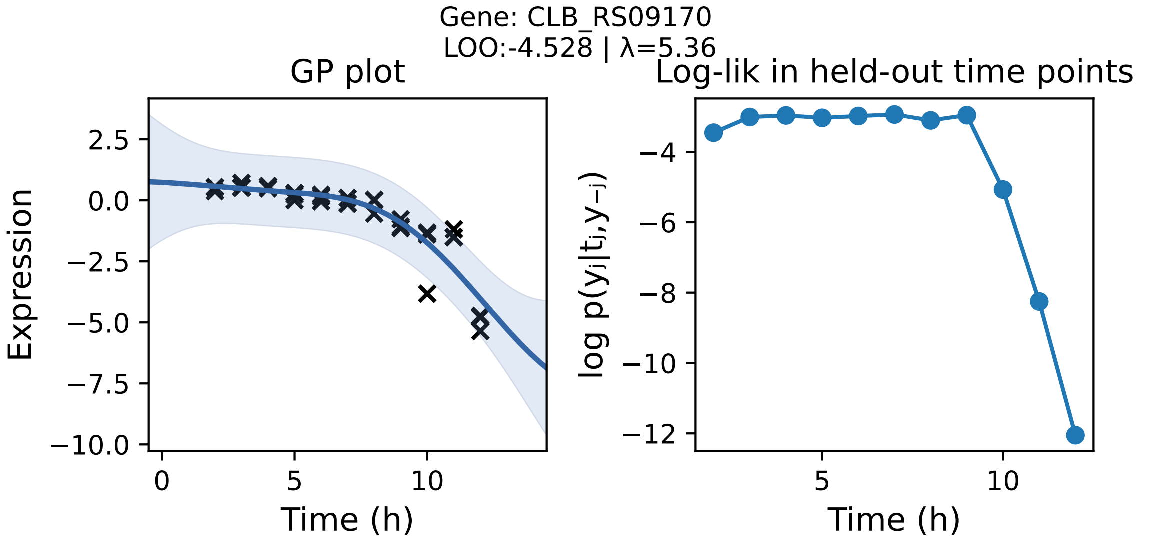
Gene: CLB_RS09170
LOO:-4.528 | λ=5.36
GP plot
Log-lik in held-out time points
Expression
2.5
0.0
−2.5
−5.0
−7.5
−10.0
0
5
10
Time (h)
log p(y_j|t_j,y_−j)
−4
−6
−8
−10
−12
5
10
Time (h)

Gene: CLB_RS08405
LOO:-4.523 | λ=3.82

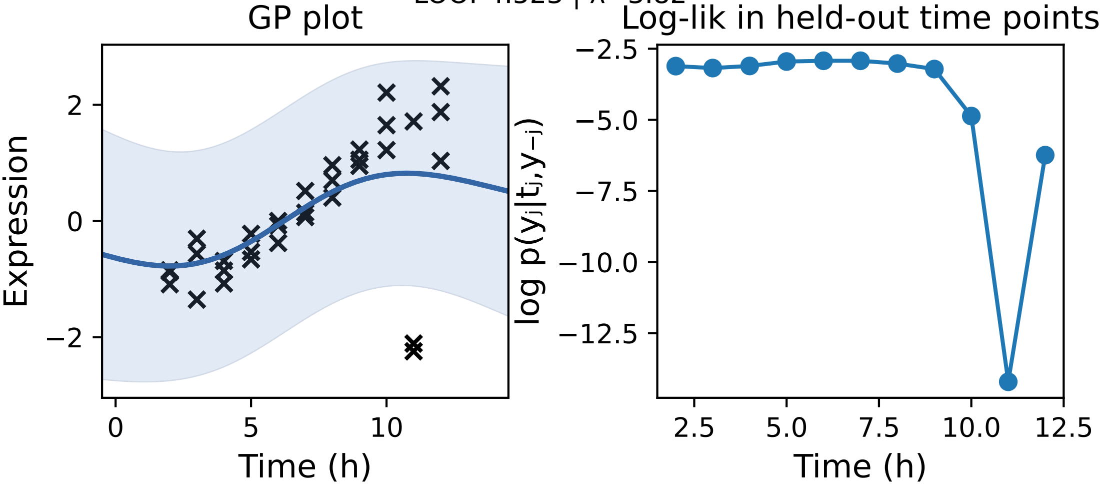

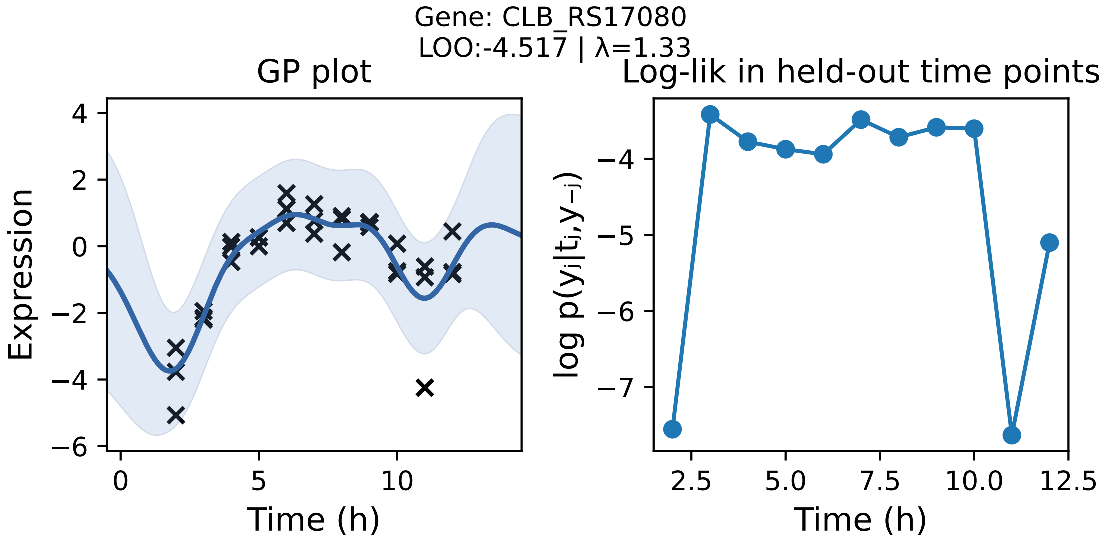

Gene: CLB_RS17080
LOO:-4.517 | λ=1.33
GP plot
Expression
Time (h)
4
2
0
−2
−4
−6
0
5
10
Log-lik in held-out time points
log p(y_j|t_j,y_−j)
−4
−5
−6
−7
2.5
5.0
7.5
10.0
12.5
Time (h)

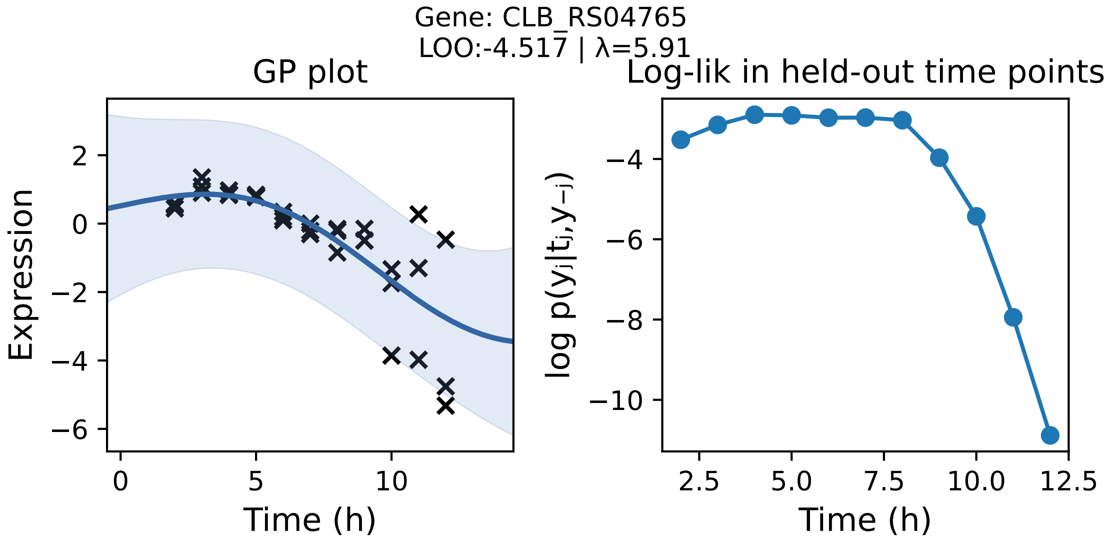
Gene: CLB_RS04765
LOO:-4.517 | λ=5.91
GP plot
Expression
Time (h)
2
0
−2
−4
−6
0
5
10
Log-lik in held-out time points
log p(y_j|t_j,y_−j)
Time (h)
−4
−6
−8
−10
2.5
5.0
7.5
10.0
12.5

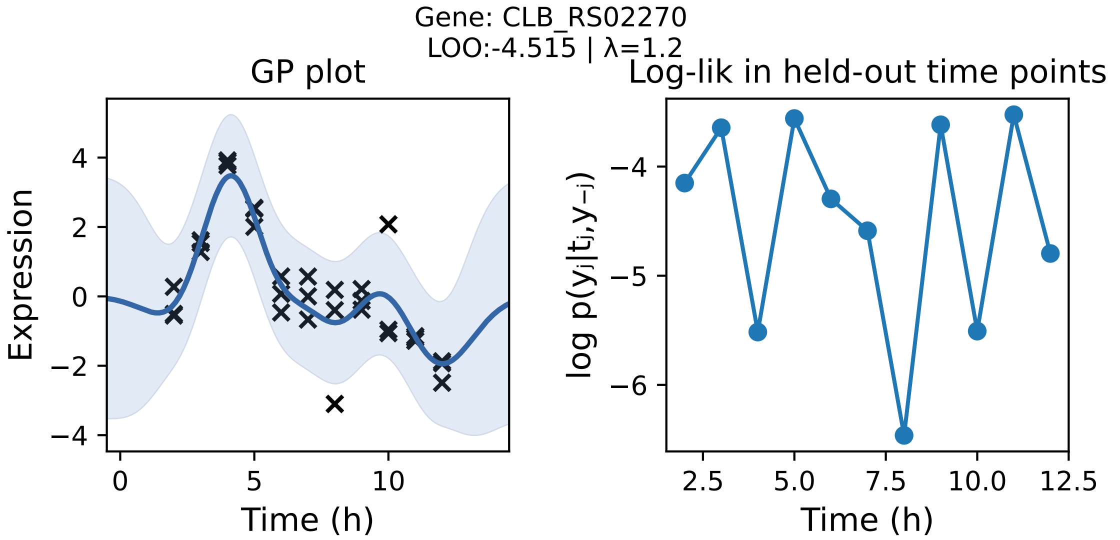
Gene: CLB_RS02270
LOO:-4.515 | λ=1.2
GP plot
Expression
Time (h)
4
2
0
−2
−4
0
5
10
Log-lik in held-out time points
log p(y_j|t_j,y_−j)
Time (h)
−4
−5
−6
2.5
5.0
7.5
10.0
12.5

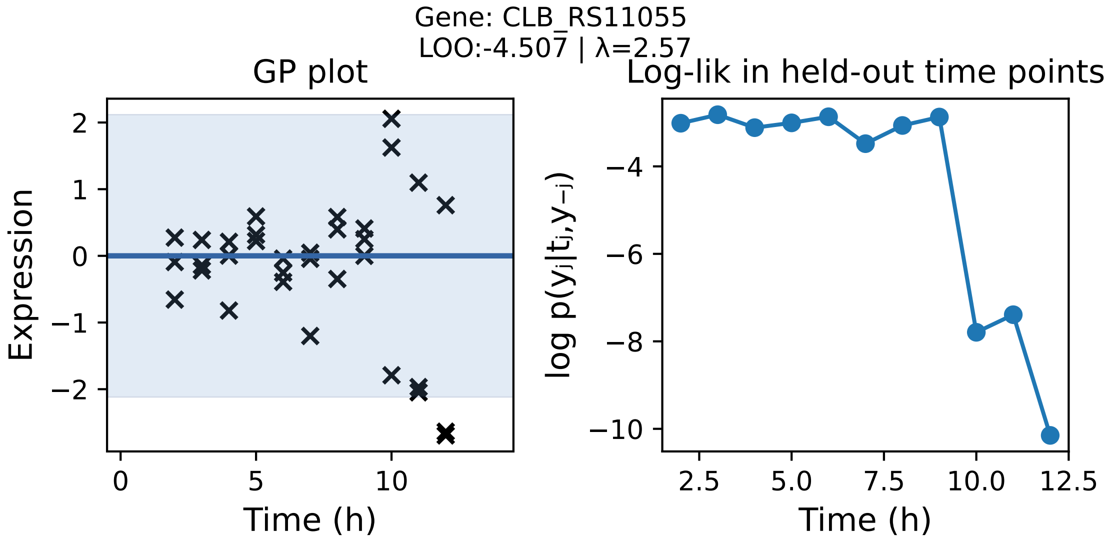
Gene: CLB_RS11055
LOO:-4.507 | λ=2.57
GP plot
Expression
Time (h)
Log-lik in held-out time points
log p(yj|tj,y−j)
Time (h)

Gene: CLB_RS09270
LOO:-4.502 | λ=1.58

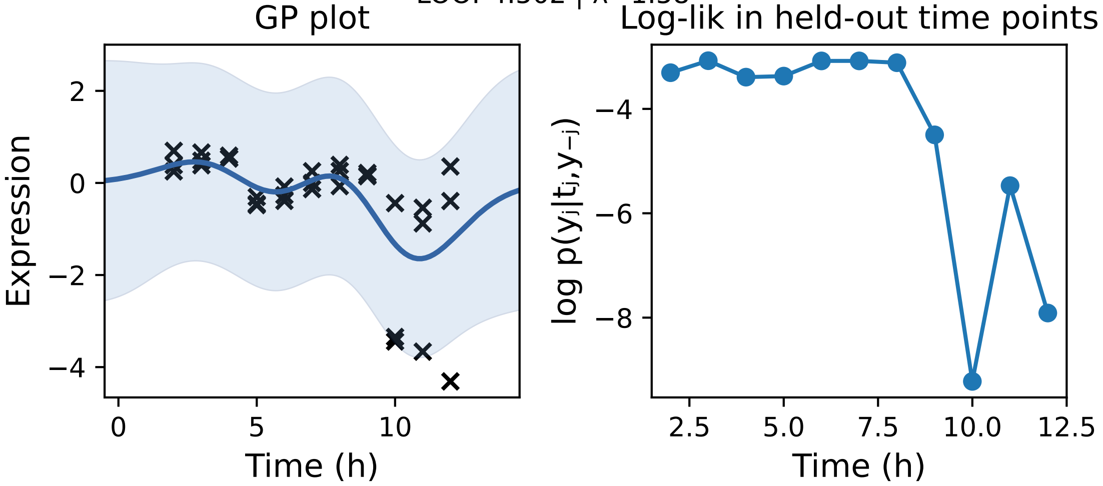

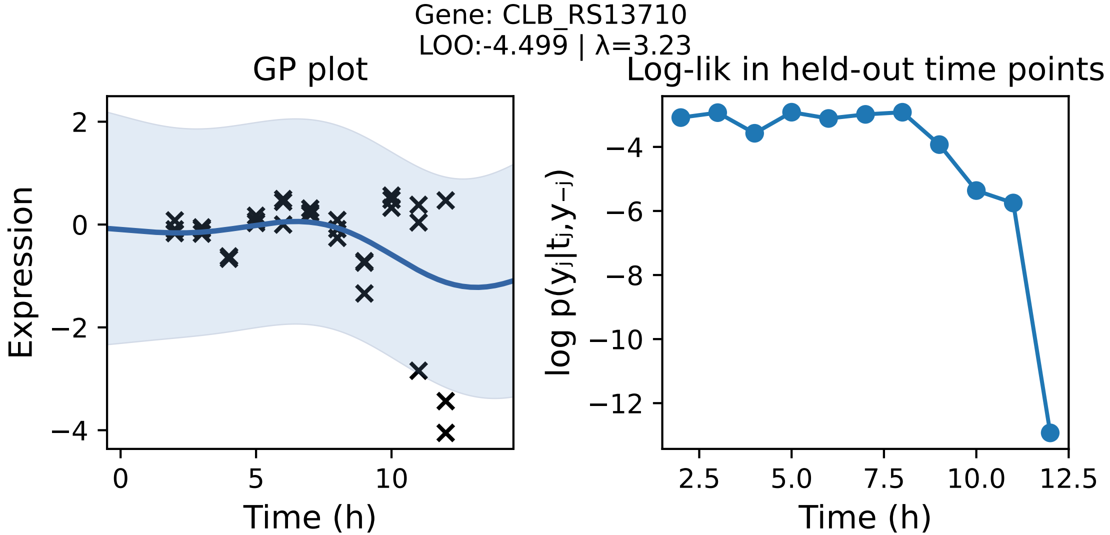

Gene: CLB_RS13710
LOO:-4.499 | λ=3.23
GP plot
Expression
Time (h)
Log-lik in held-out time points
log p(y_j|t_j,y_−j)
Time (h)

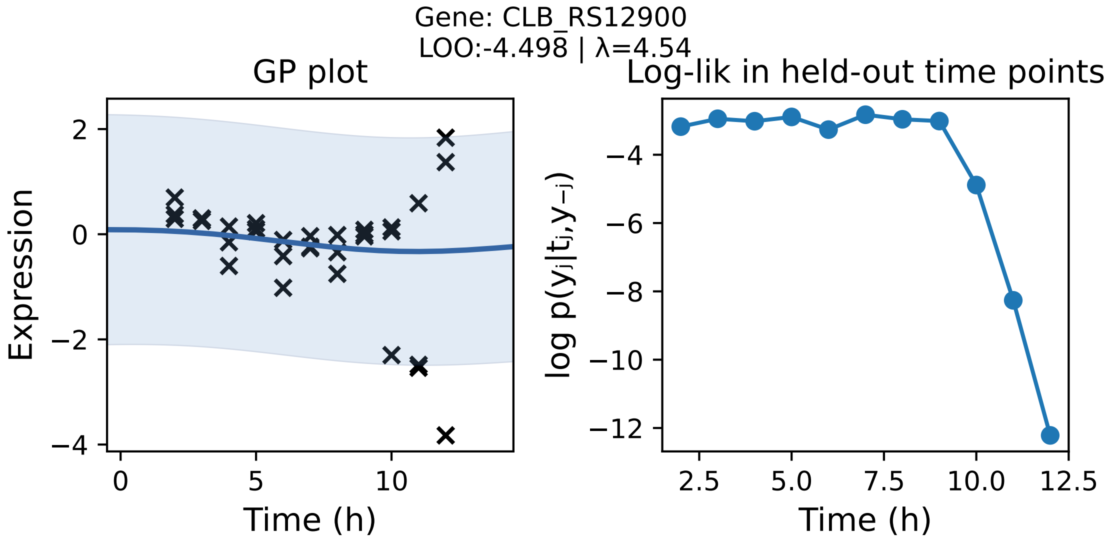
Gene: CLB_RS12900
LOO:-4.498 | λ=4.54
GP plot
Log-lik in held-out time points
Expression
Time (h)
log p(y_j|t_j,y_−j)
Time (h)

Gene: CLB_RS02195
LOO:-4.496 | λ=1.0

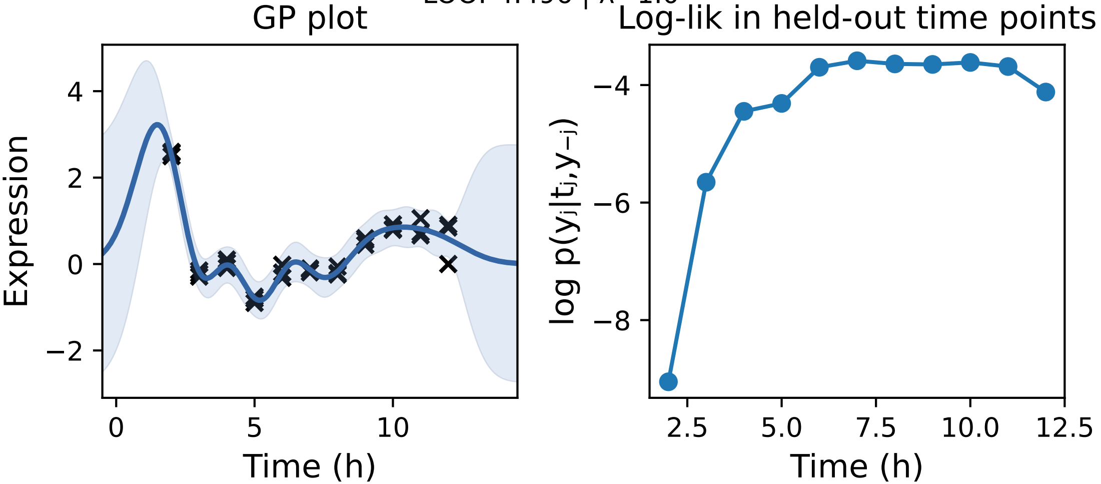

Gene: CLB_RS12610
LOO:-4.494 | λ=4.01

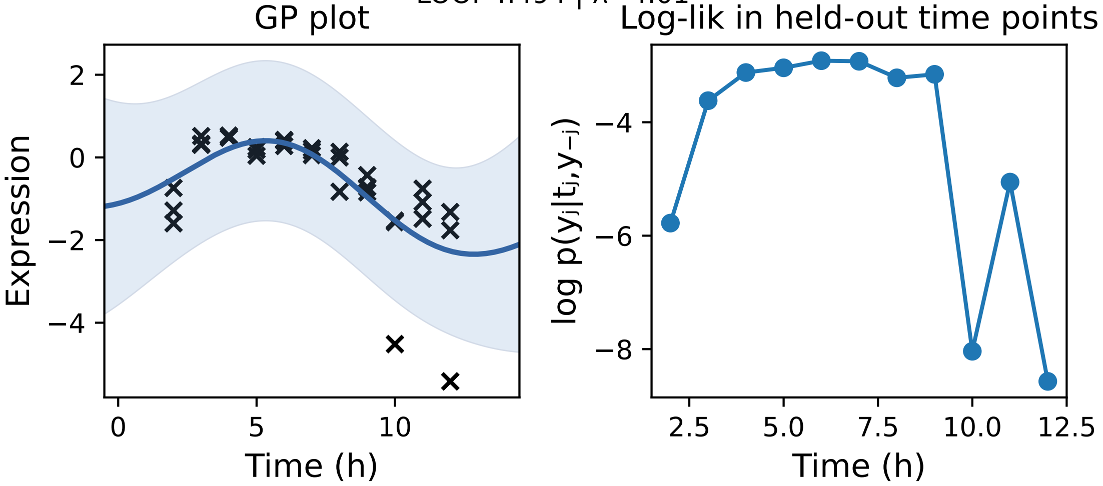

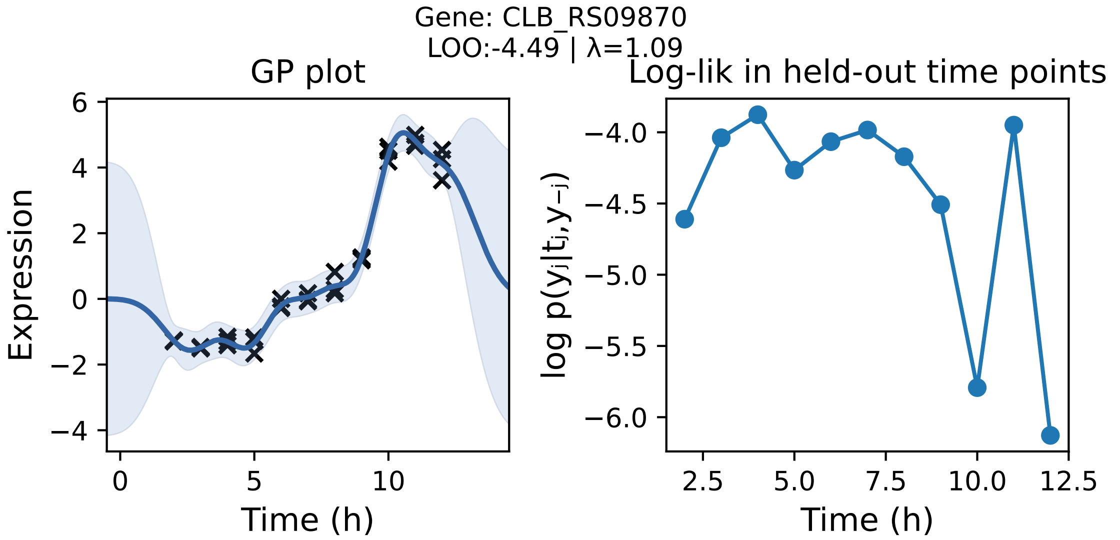

Gene: CLB_RS09870
LOO:-4.49 | λ=1.09
GP plot
Expression
Time (h)
6
4
2
0
−2
−4
0
5
10
Log-lik in held-out time points
log p(y_j|t_j,y_−j)
Time (h)
−4.0
−4.5
−5.0
−5.5
−6.0
2.5
5.0
7.5
10.0
12.5

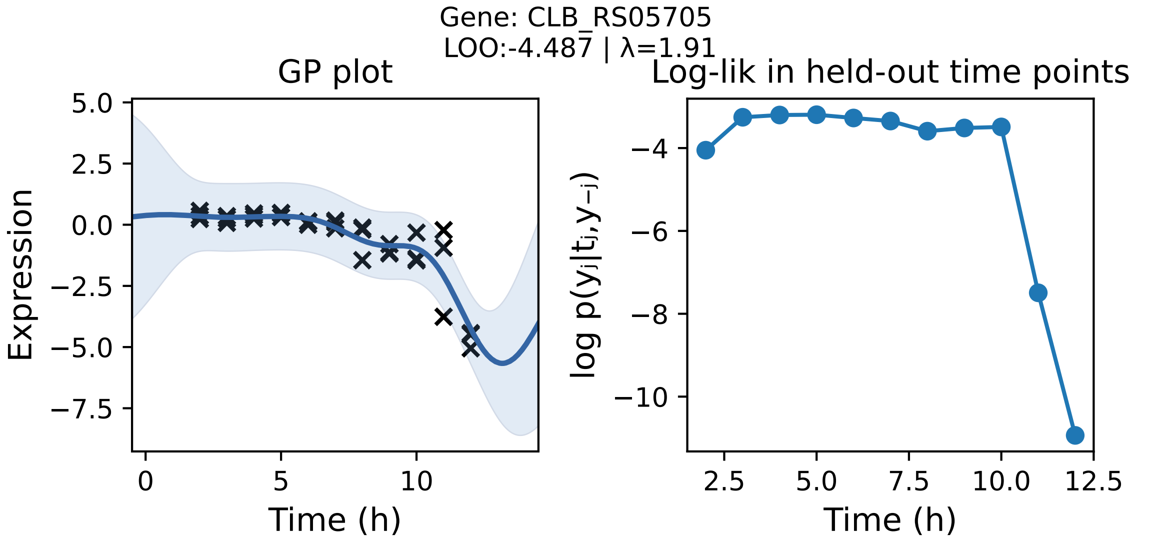

Gene: CLB_RS05705
LOO:-4.487 | λ=1.91
GP plot
Log-lik in held-out time points
Expression
Time (h)
log p(y_j|t_j,y_−j)
5.0
2.5
0.0
−2.5
−5.0
−7.5
0
5
10
−4
−6
−8
−10
2.5
5.0
7.5
10.0
12.5

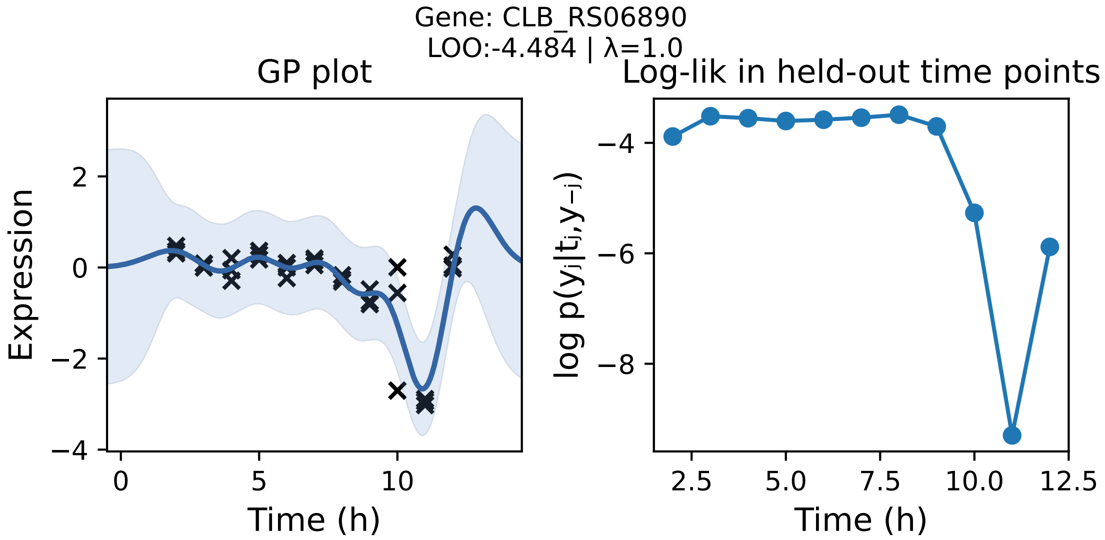

Gene: CLB_RS06890
LOO:-4.484 | λ=1.0
GP plot
Expression
2
0
−2
−4
0
5
10
Time (h)
Log-lik in held-out time points
log p(yj|tj,y−j)
−4
−6
−8
2.5
5.0
7.5
10.0
12.5
Time (h)

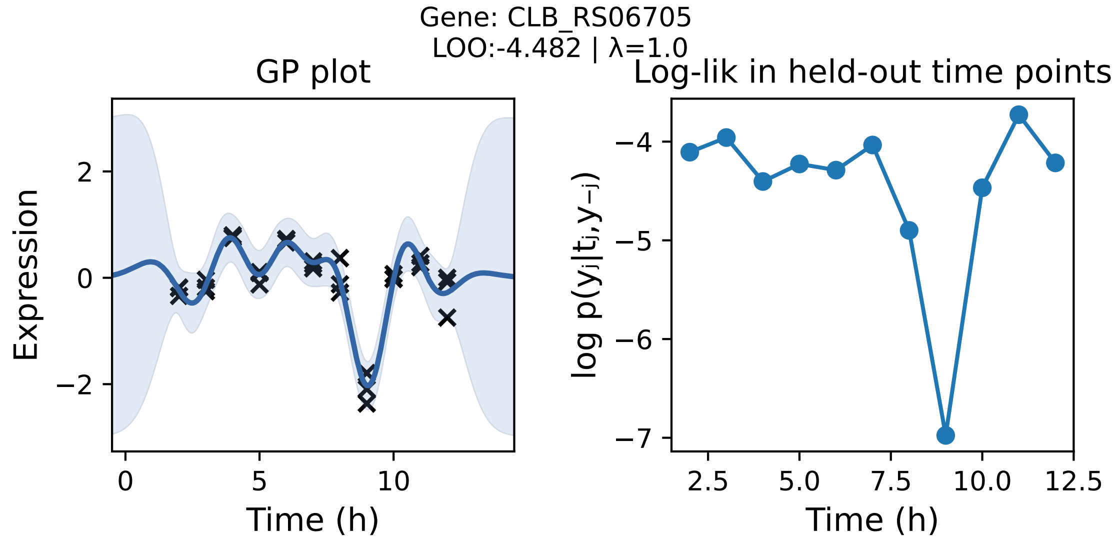

Gene: CLB_RS06705
LOO:-4.482 | λ=1.0
GP plot
Log-lik in held-out time points
Expression
2
0
−2
0
5
10
Time (h)
log p(yj|tj,y−j)
−4
−5
−6
−7
2.5
5.0
7.5
10.0
12.5
Time (h)

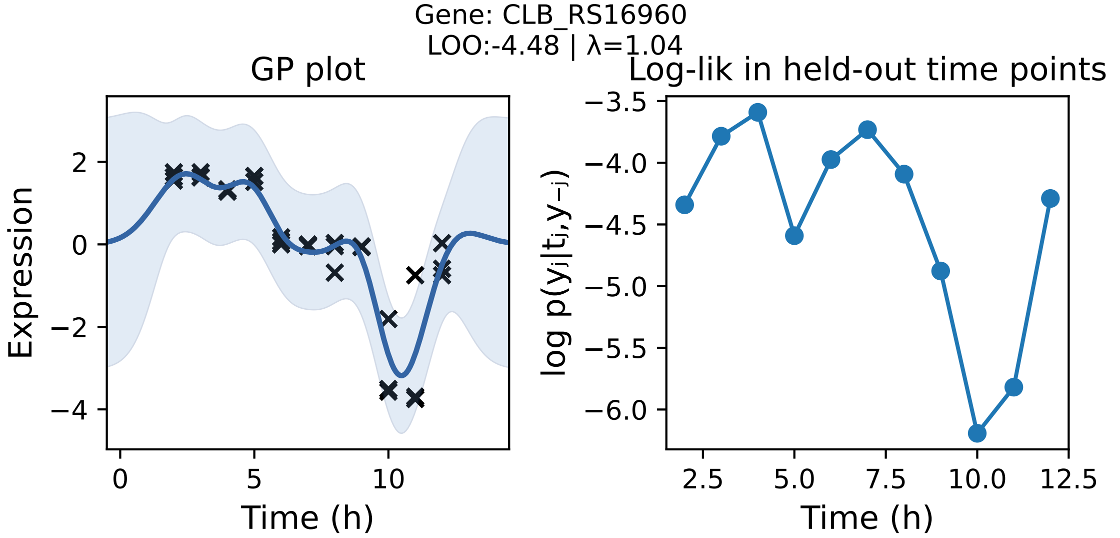

Gene: CLB_RS16960
LOO:-4.48 | λ=1.04
GP plot
Expression
Time (h)
Log-lik in held-out time points
log p(y_j|t_j,y_−j)
Time (h)

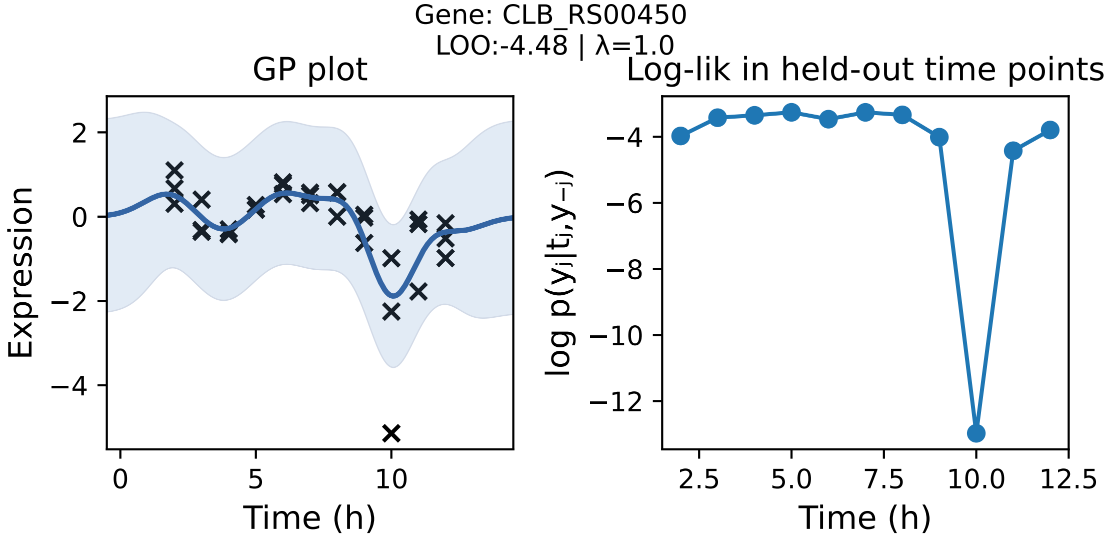

Gene: CLB_RS00450
LOO:-4.48 | λ=1.0
GP plot
Expression
Time (h)
2
0
−2
−4
0
5
10
Log-lik in held-out time points
log p(y_j|t_j,y_−j)
−4
−6
−8
−10
−12
2.5
5.0
7.5
10.0
12.5
Time (h)

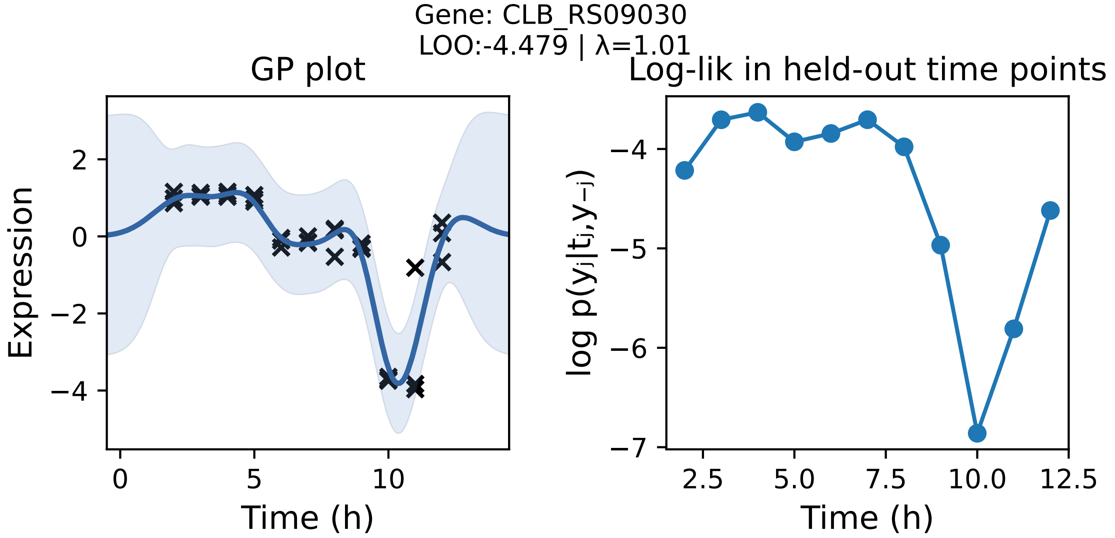
Gene: CLB_RS09030
LOO:-4.479 | λ=1.01
GP plot
Expression
Time (h)
2
0
−2
−4
0
5
10
Log-lik in held-out time points
log p(yj|tj,y−j)
Time (h)
−4
−5
−6
−7
2.5
5.0
7.5
10.0
12.5

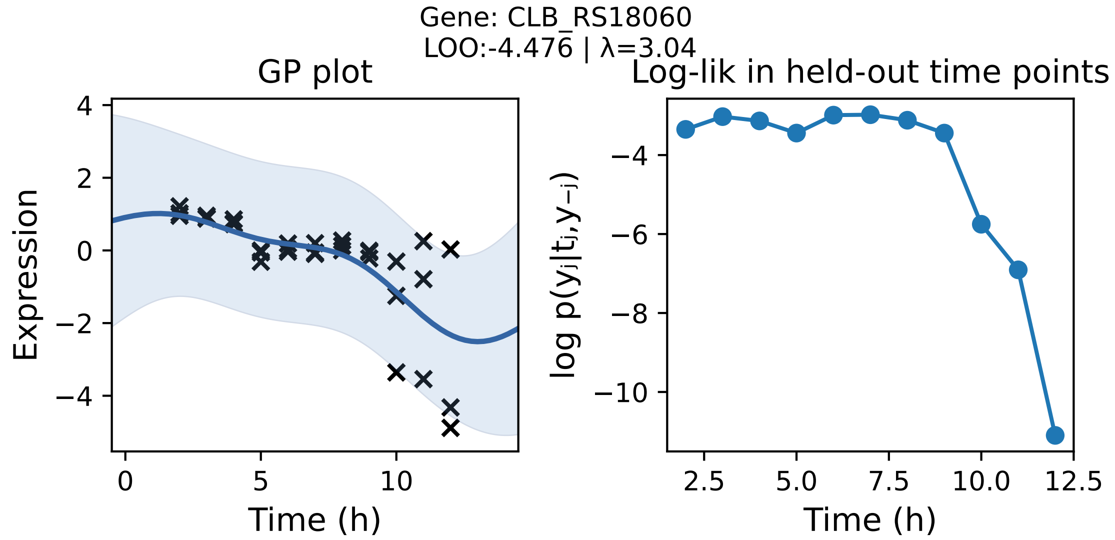
Gene: CLB_RS18060
LOO:-4.476 | λ=3.04
GP plot
Log-lik in held-out time points
Expression
Time (h)
log p(yj|tj,y−j)
Time (h)

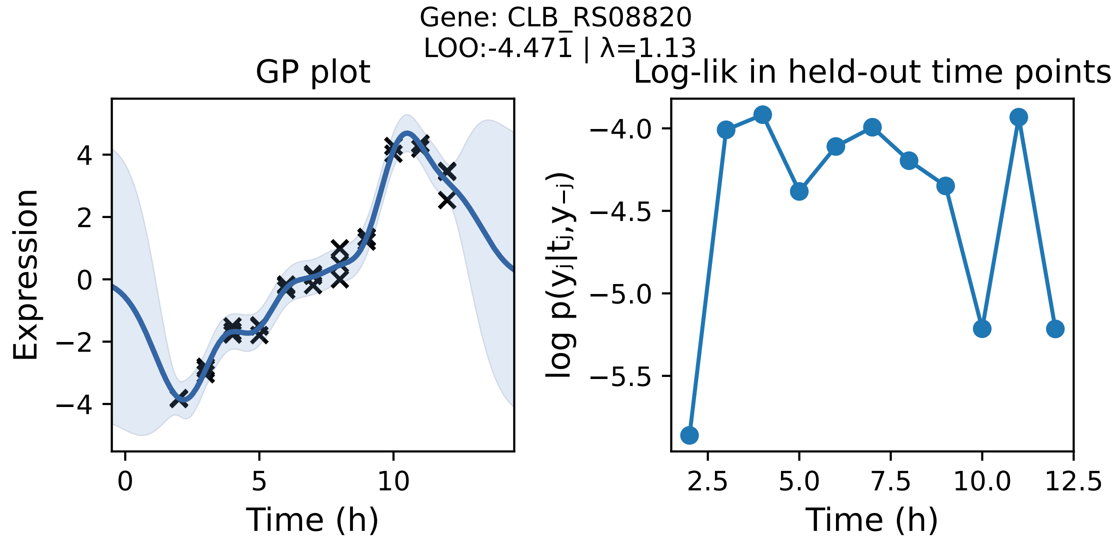

Gene: CLB_RS08820
LOO:-4.471 | λ=1.13
GP plot
Expression
Time (h)
Log-lik in held-out time points
log p(y_j|t_j,y_−j)
Time (h)

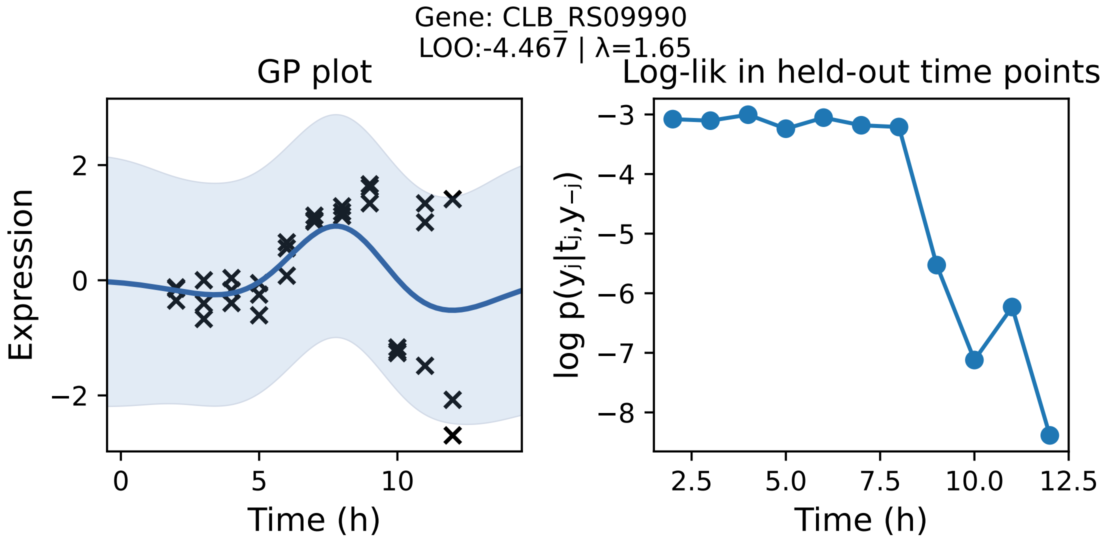
Gene: CLB_RS09990
LOO:-4.467 | λ=1.65
GP plot
Expression
Time (h)
2
0
−2
0
5
10
Log-lik in held-out time points
log p(yj|tj,y−j)
−3
−4
−5
−6
−7
−8
2.5
5.0
7.5
10.0
12.5
Time (h)

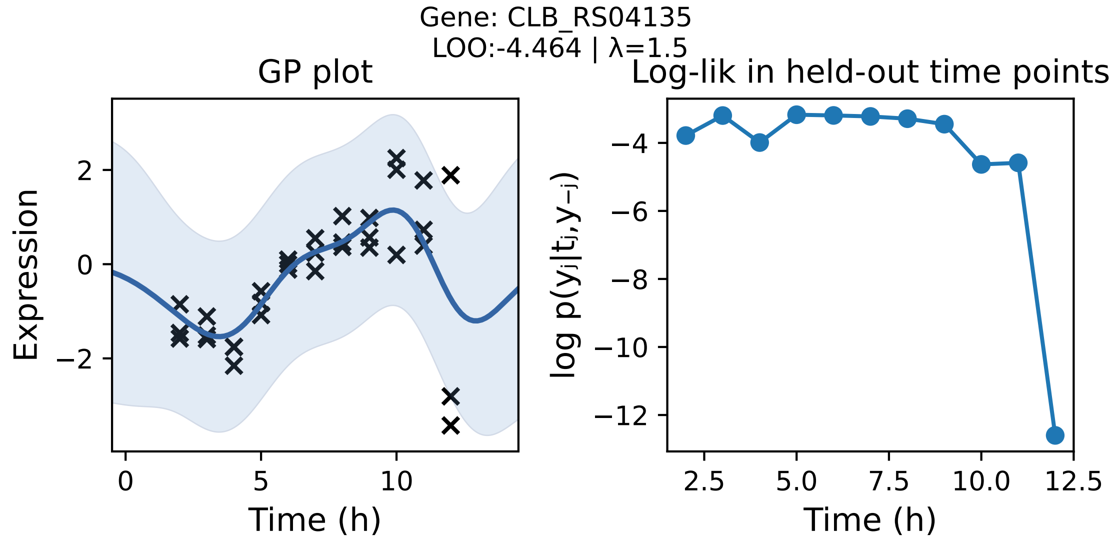
Gene: CLB_RS04135
LOO:-4.464 | λ=1.5
GP plot
Log-lik in held-out time points
Expression
Time (h)
0
5
10
2
0
−2
log p(yj|ti,y−j)
−4
−6
−8
−10
−12
2.5
5.0
7.5
10.0
12.5
Time (h)

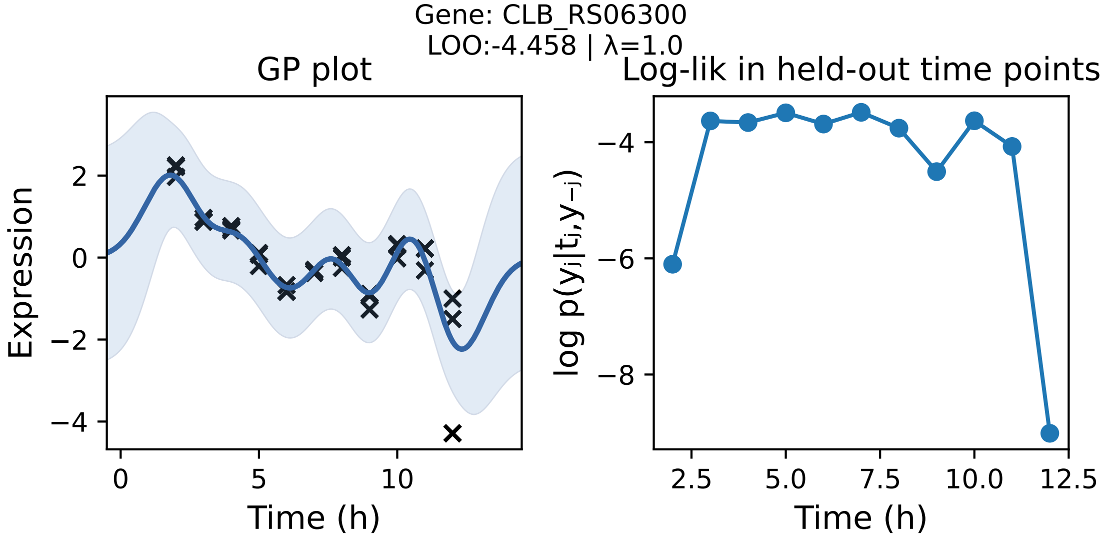

Gene: CLB_RS06300
LOO:-4.458 | λ=1.0
GP plot
Expression
Time (h)
Log-lik in held-out time points
log p(y_j|t_j,y_−j)
Time (h)

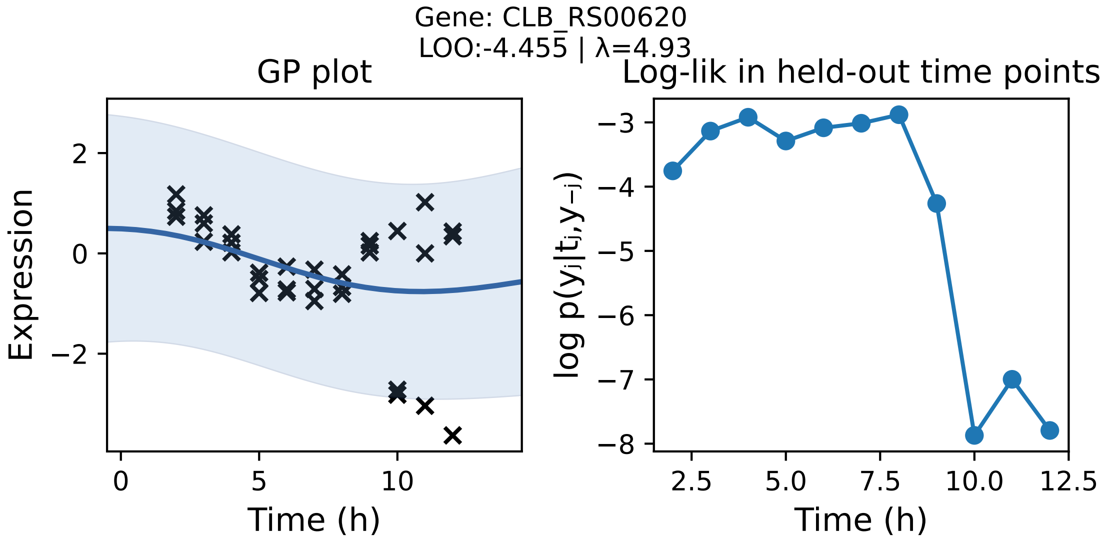
Gene: CLB_RS00620
LOO:-4.455 | λ=4.93
GP plot
Log-lik in held-out time points
Expression
Time (h)
log p(y_j|t_j,y_−j)
Time (h)

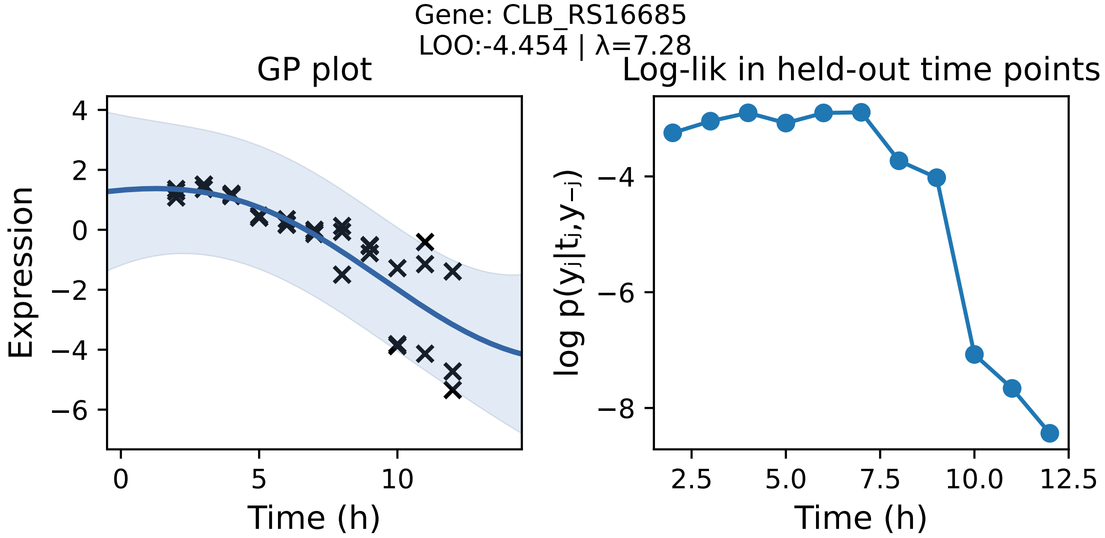

Gene: CLB_RS16685
LOO:-4.454 | λ=7.28
GP plot
Log-lik in held-out time points
Expression
Time (h)
log p(y_j|t_j,y_−j)
Time (h)
4
2
0
−2
−4
−6
0
5
10
−4
−6
−8
2.5
5.0
7.5
10.0
12.5

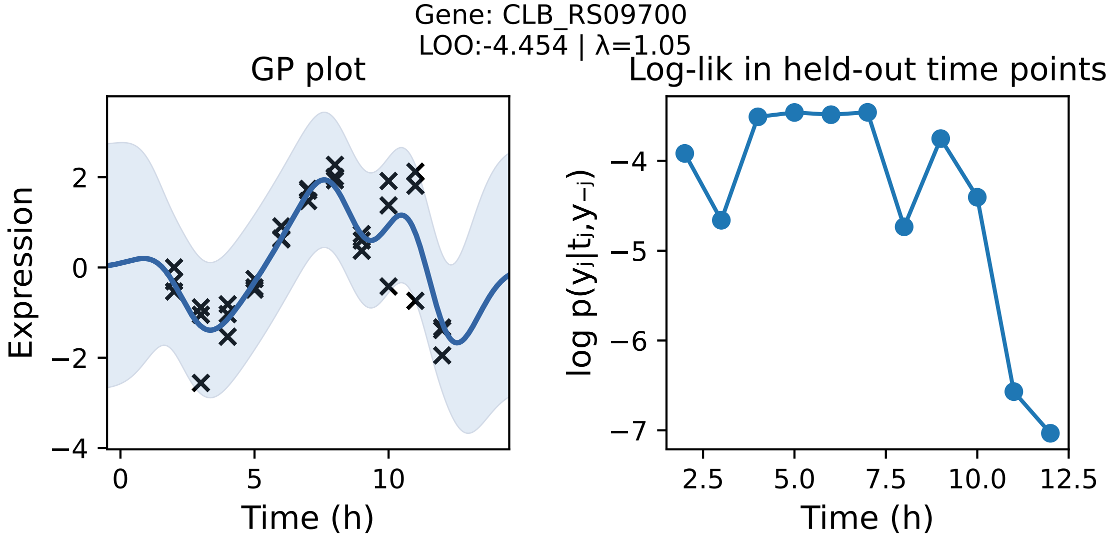
Gene: CLB_RS09700
LOO:-4.454 | λ=1.05
GP plot
Expression
Time (h)
Log-lik in held-out time points
log p(y_j|t_j,y_−j)
Time (h)

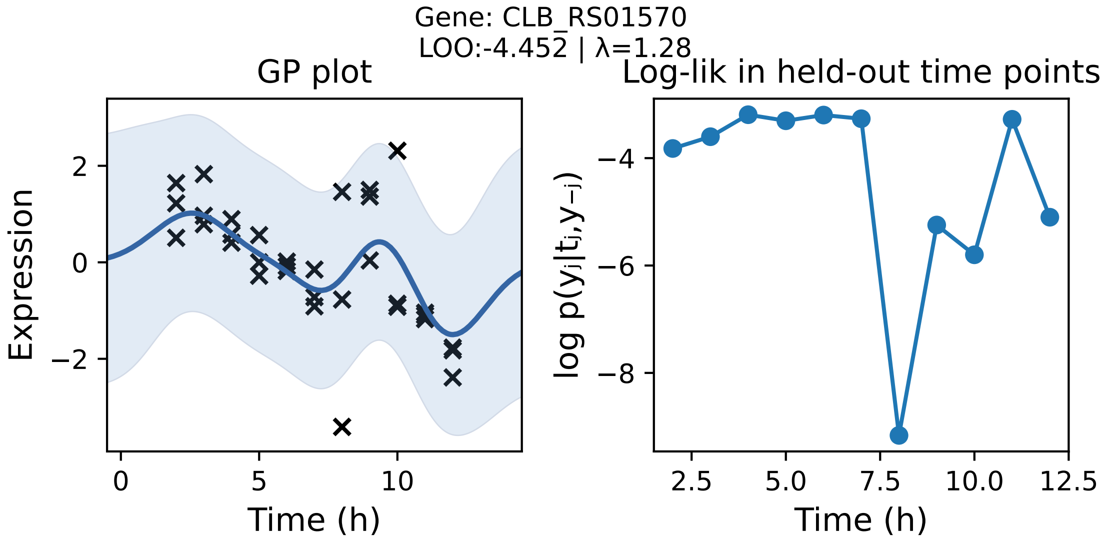

Gene: CLB_RS01570
LOO:-4.452 | λ=1.28
GP plot
Expression
Time (h)
Log-lik in held-out time points
log p(yj|tj,y−j)
Time (h)

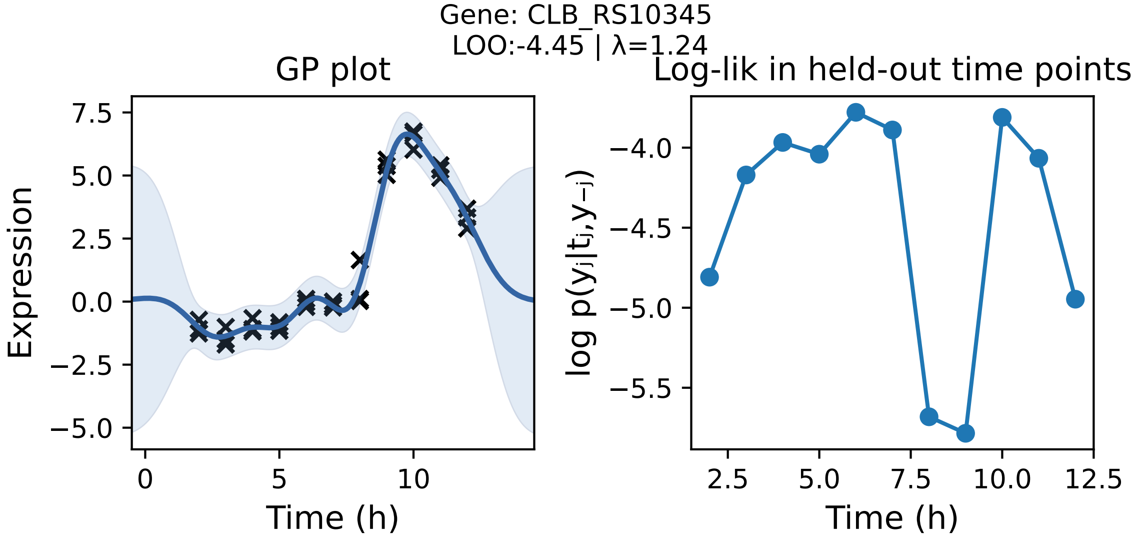

Gene: CLB_RS10345
LOO:-4.45 | λ=1.24
GP plot
Expression
Time (h)
7.5
5.0
2.5
0.0
−2.5
−5.0
0
5
10
Log-lik in held-out time points
log p(y_j|t_j,y_−j)
−4.0
−4.5
−5.0
−5.5
2.5
5.0
7.5
10.0
12.5
Time (h)

Gene: CLB_RS15960
LOO:-4.449 | λ=1.19

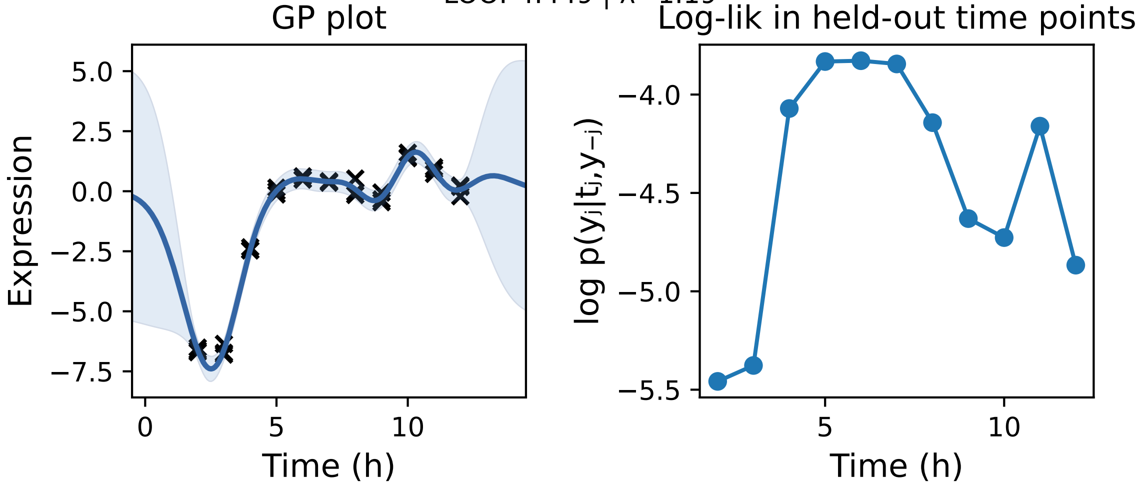

Gene: CLB_RS18855
LOO:-4.448 | λ=1.18

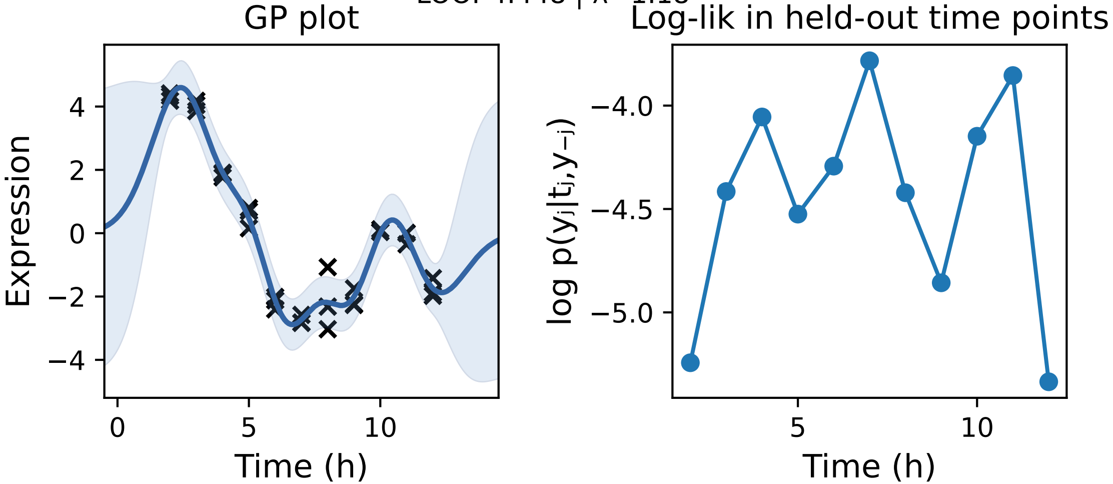

Gene: CLB_RS10640
LOO:-4.442 | λ=1.02

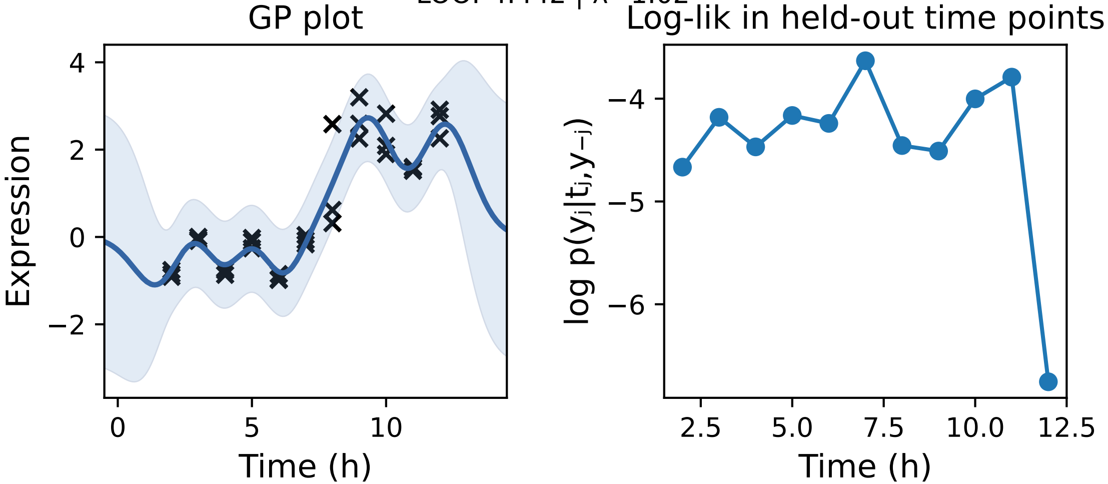

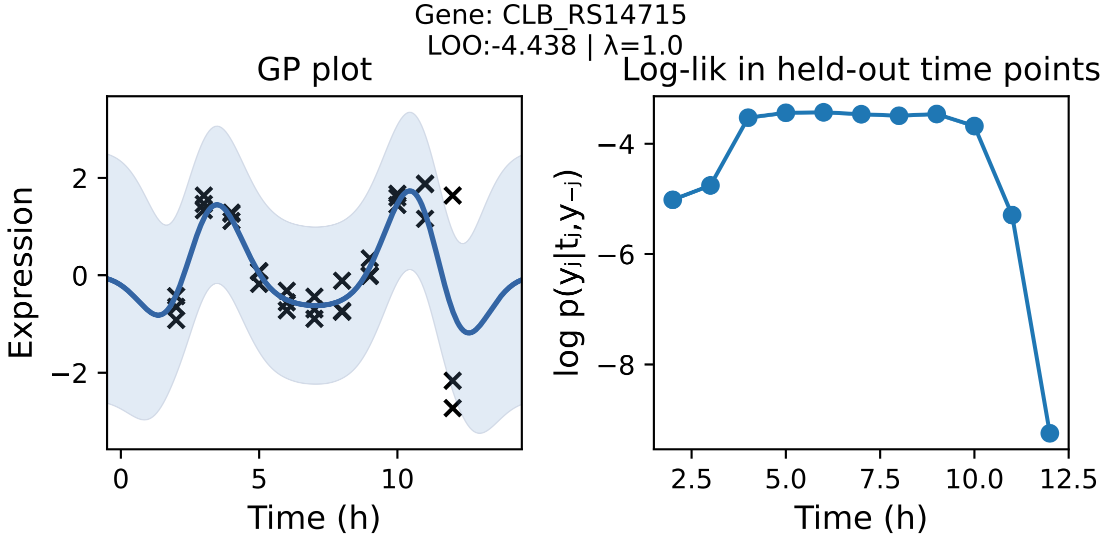

Gene: CLB_RS14715
LOO:-4.438 | λ=1.0
GP plot
Expression
Time (h)
Log-lik in held-out time points
log p(y_j|t_j,y_−j)
Time (h)
2
0
−2
0
5
10
−4
−6
−8
2.5
5.0
7.5
10.0
12.5

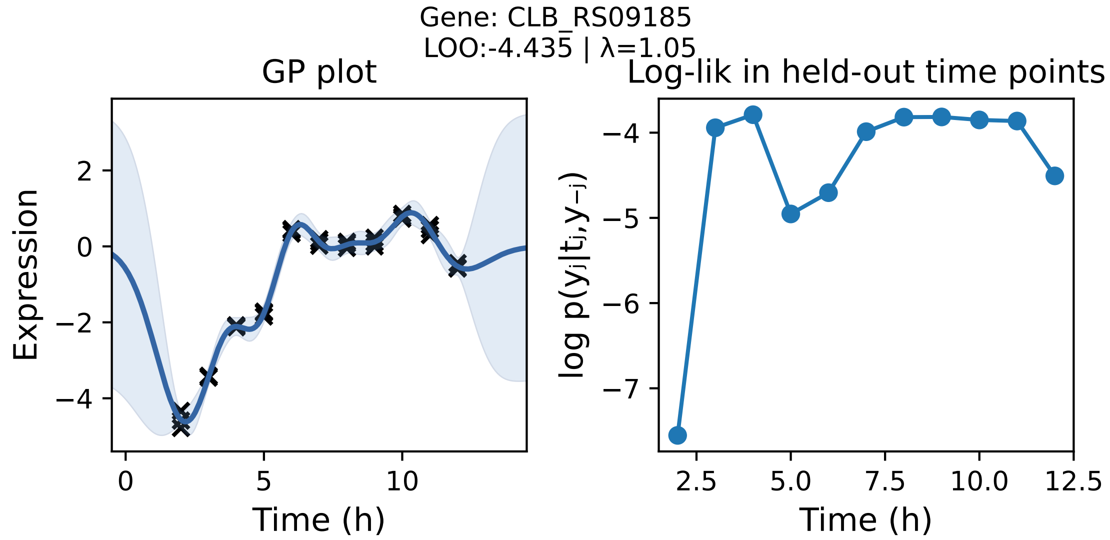

Gene: CLB_RS09185
LOO:-4.435 | λ=1.05
GP plot
Expression
Time (h)
Log-lik in held-out time points
log p(y_j|t_j,y_−j)
Time (h)

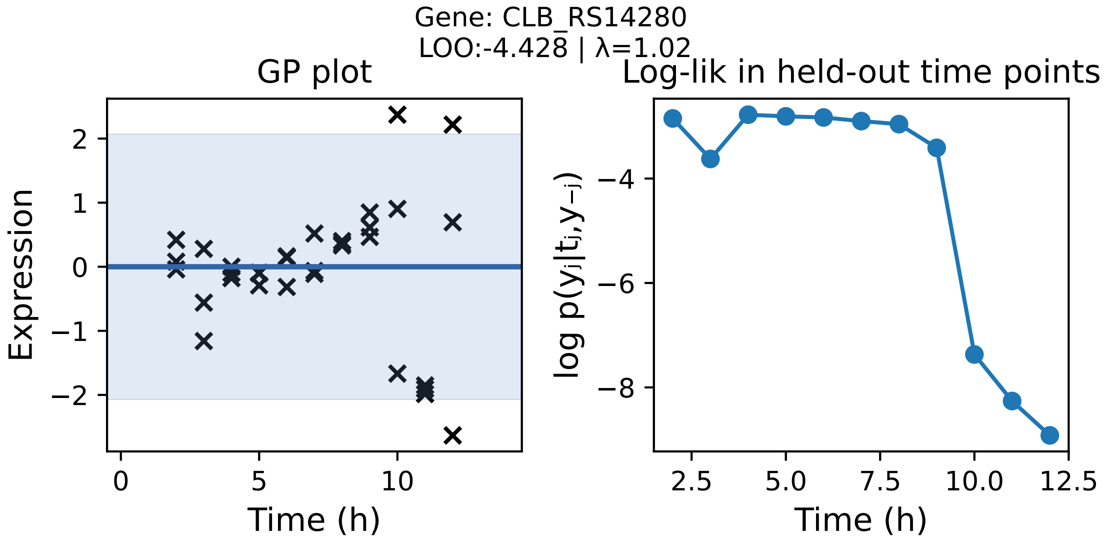

Gene: CLB_RS14280
LOO:-4.428 | λ=1.02
GP plot
Expression
Time (h)
Log-lik in held-out time points
log p(y_j|t_j,y_−j)
Time (h)

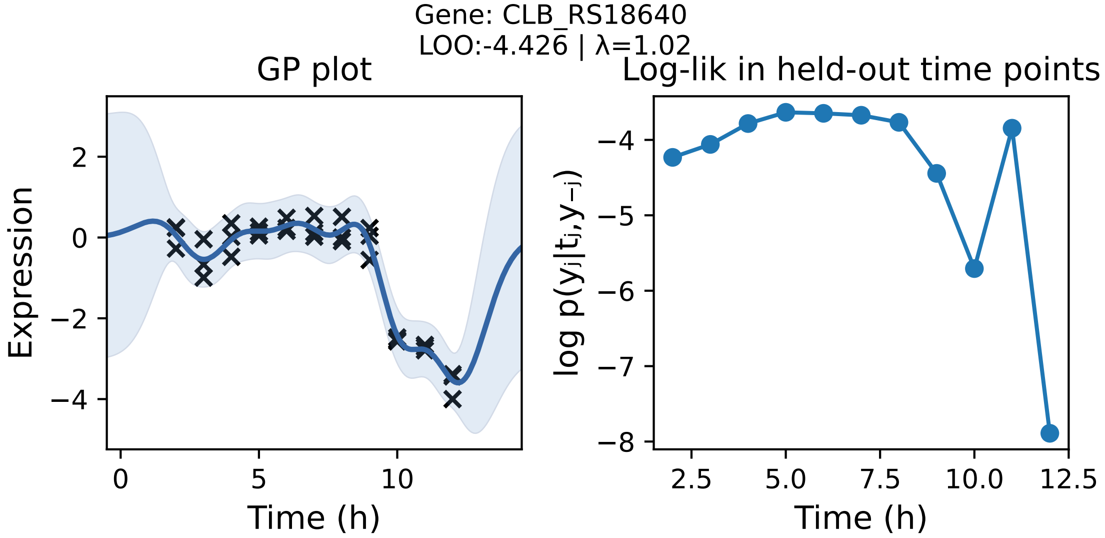

Gene: CLB_RS18640
LOO:-4.426 | λ=1.02
GP plot
Expression
Time (h)
Log-lik in held-out time points
log p(y_j|t_j,y_−j)
Time (h)

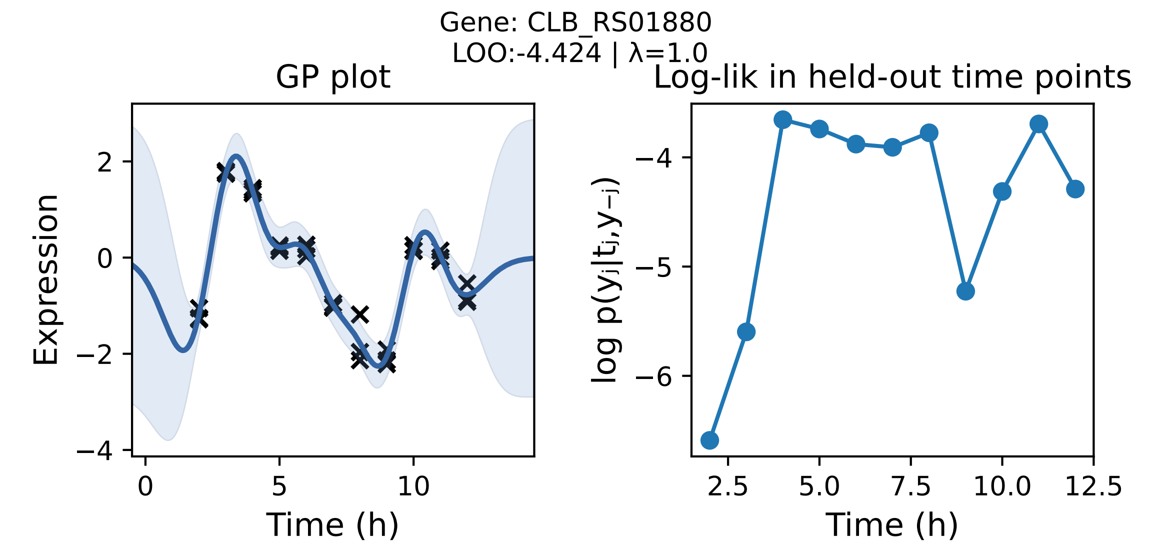
Gene: CLB_RS01880
LOO:-4.424 | λ=1.0
GP plot
Expression
Time (h)
Log-lik in held-out time points
log p(y_j|t_j,y_−j)
Time (h)

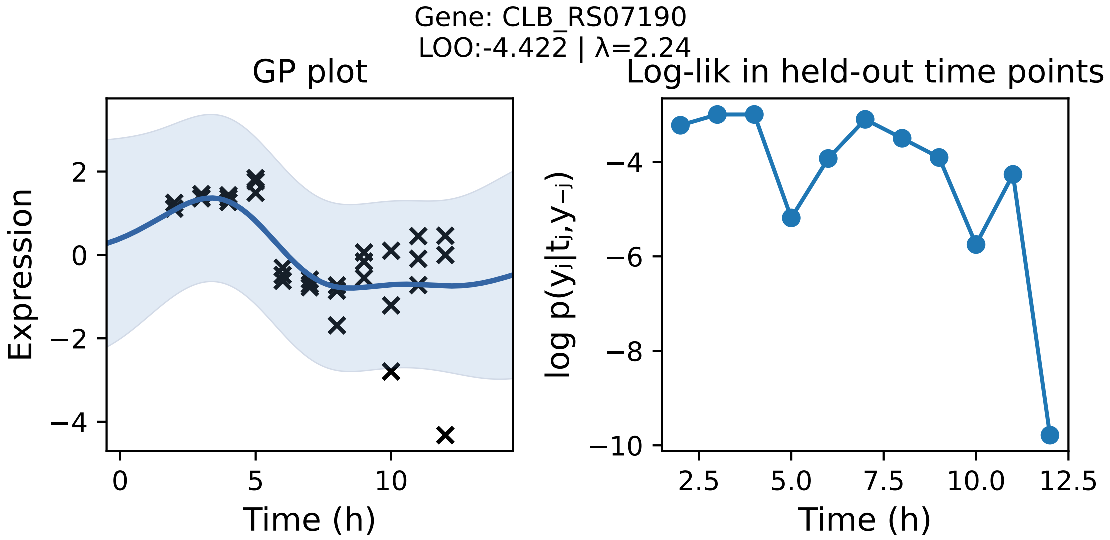
Gene: CLB_RS07190
LOO:-4.422 | λ=2.24
GP plot
Expression
Time (h)
Log-lik in held-out time points
log p(y_j|t_j,y_−j)
Time (h)

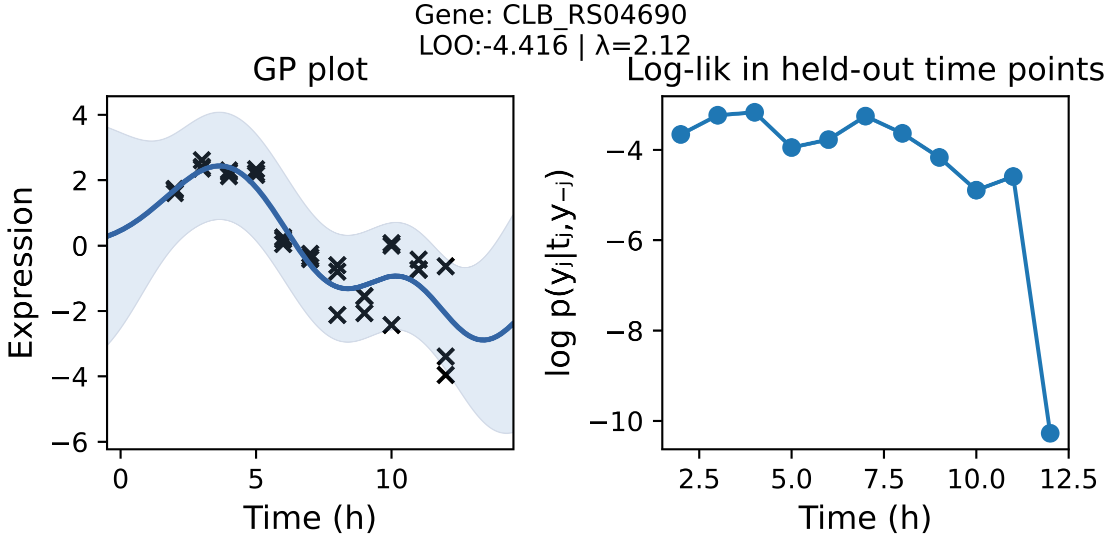
Gene: CLB_RS04690
LOO:-4.416 | λ=2.12
GP plot
Expression
Time (h)
4
2
0
−2
−4
−6
0
5
10
Log-lik in held-out time points
log p(y_j|t_j,y_−j)
Time (h)
−4
−6
−8
−10
2.5
5.0
7.5
10.0
12.5

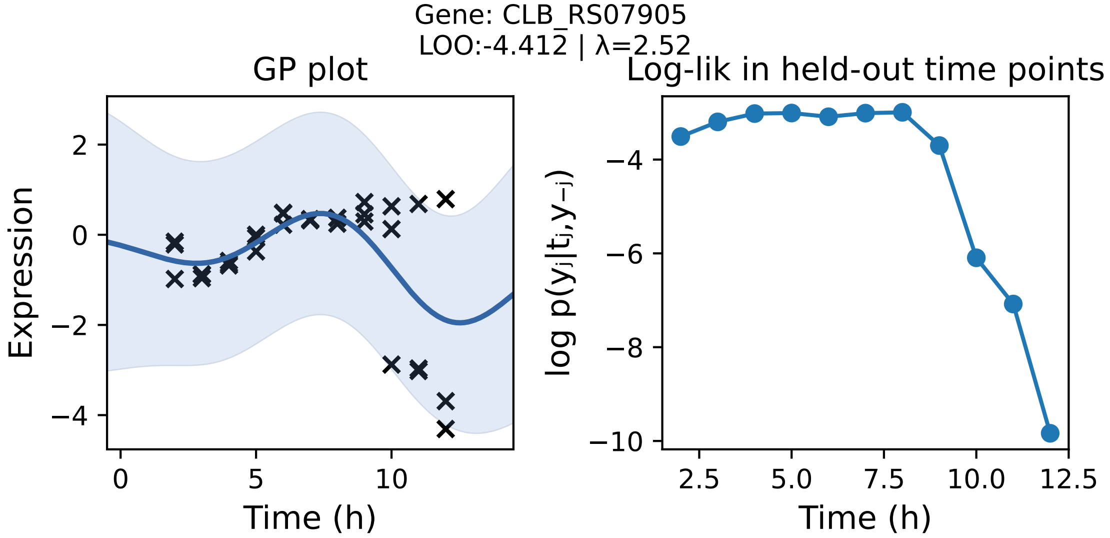

Gene: CLB_RS07905
LOO:-4.412 | λ=2.52
GP plot
Expression
Time (h)
Log-lik in held-out time points
log p(y_j|t_j,y_−j)
Time (h)

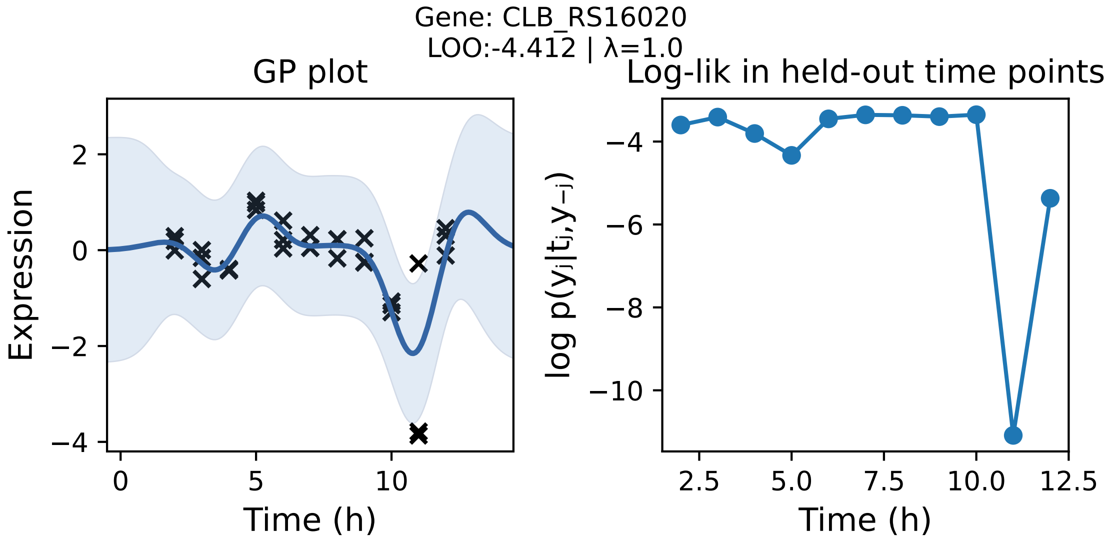

Gene: CLB_RS16020
LOO:-4.412 | λ=1.0
GP plot
Expression
Time (h)
2
0
−2
−4
0
5
10
Log-lik in held-out time points
log p(yj|ti,y−j)
Time (h)
−4
−6
−8
−10
2.5
5.0
7.5
10.0
12.5

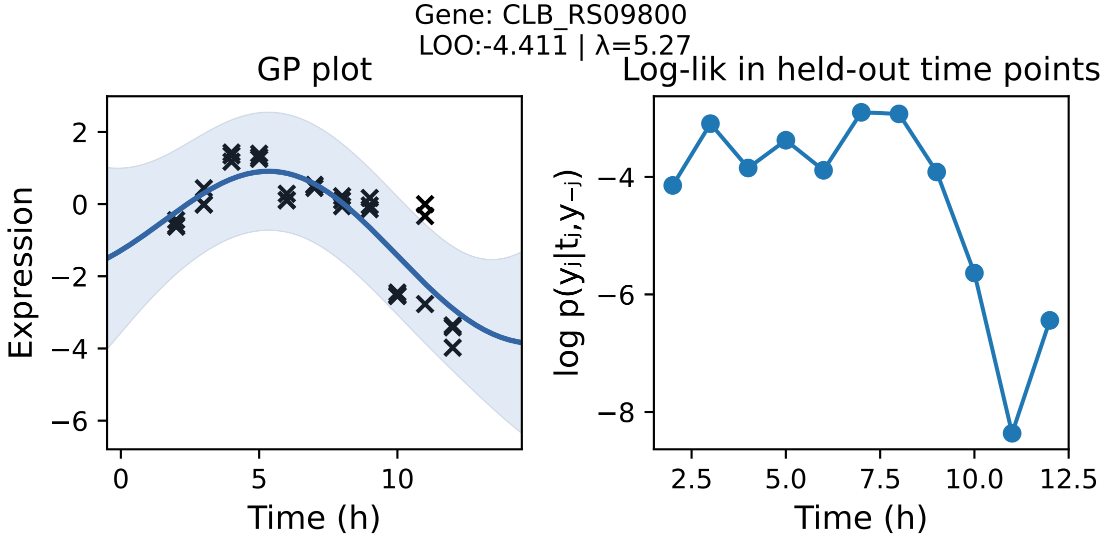

Gene: CLB_RS09800
LOO:-4.411 | λ=5.27
GP plot
Expression
Time (h)
2
0
−2
−4
−6
0
5
10
Log-lik in held-out time points
log p(y_j|t_j,y_−j)
Time (h)
−4
−6
−8
2.5
5.0
7.5
10.0
12.5

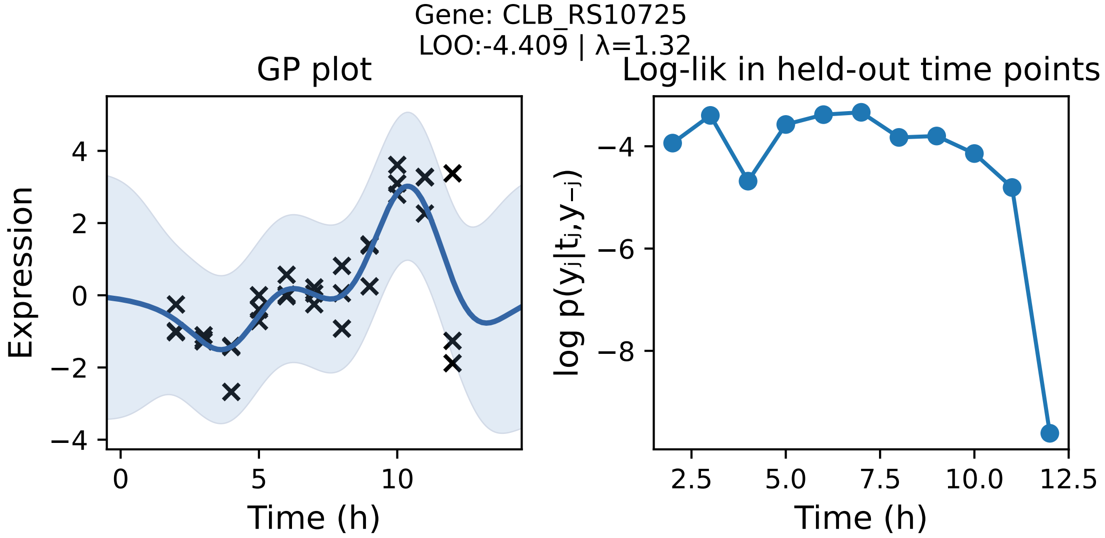

Gene: CLB_RS10725
LOO:-4.409 | λ=1.32
GP plot
Expression
Time (h)
4
2
0
−2
−4
0
5
10
Log-lik in held-out time points
log p(y_j|t_j,y_−j)
Time (h)
−4
−6
−8
2.5
5.0
7.5
10.0
12.5

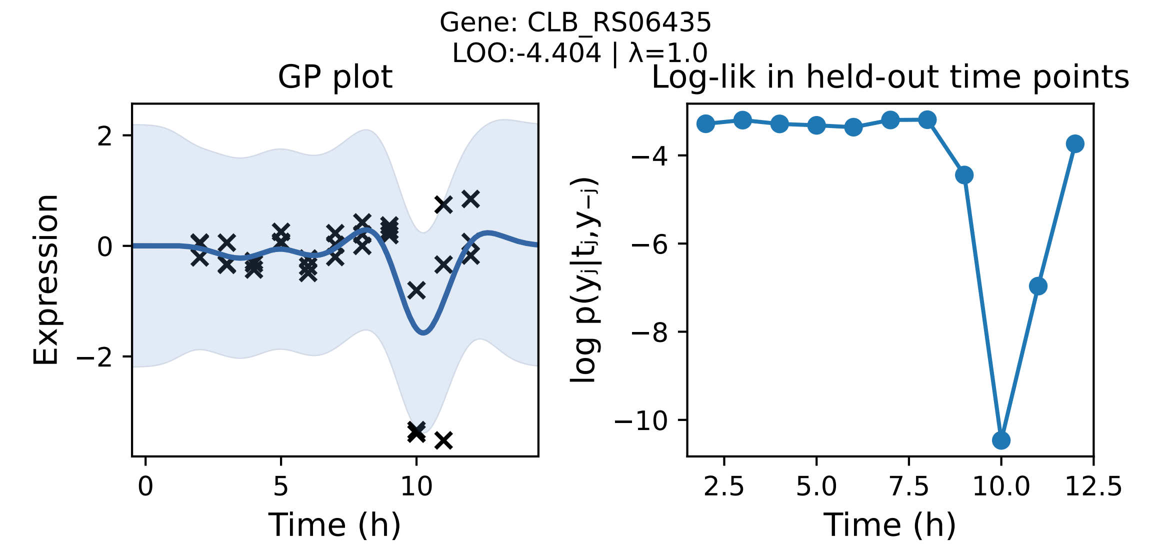
Gene: CLB_RS06435
LOO:-4.404 | λ=1.0
GP plot
Expression
Time (h)
2
0
−2
0
5
10
Log-lik in held-out time points
log p(yⱼ|tⱼ,y₋ⱼ)
Time (h)
−4
−6
−8
−10
2.5
5.0
7.5
10.0
12.5

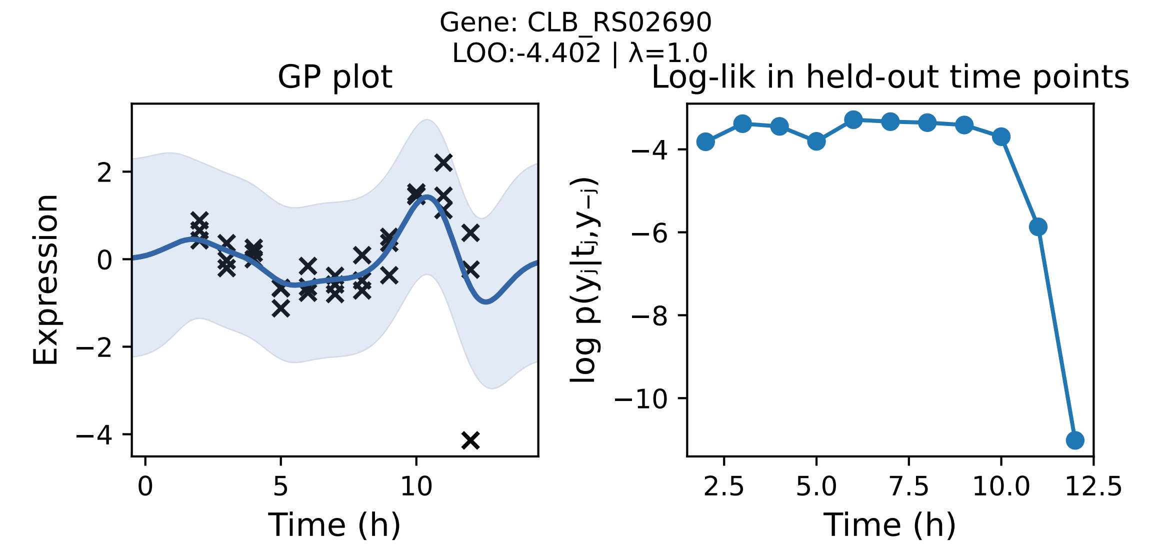
Gene: CLB_RS02690
LOO:-4.402 | λ=1.0
GP plot
Expression
Time (h)
Log-lik in held-out time points
log p(yj|ti,y−j)
Time (h)

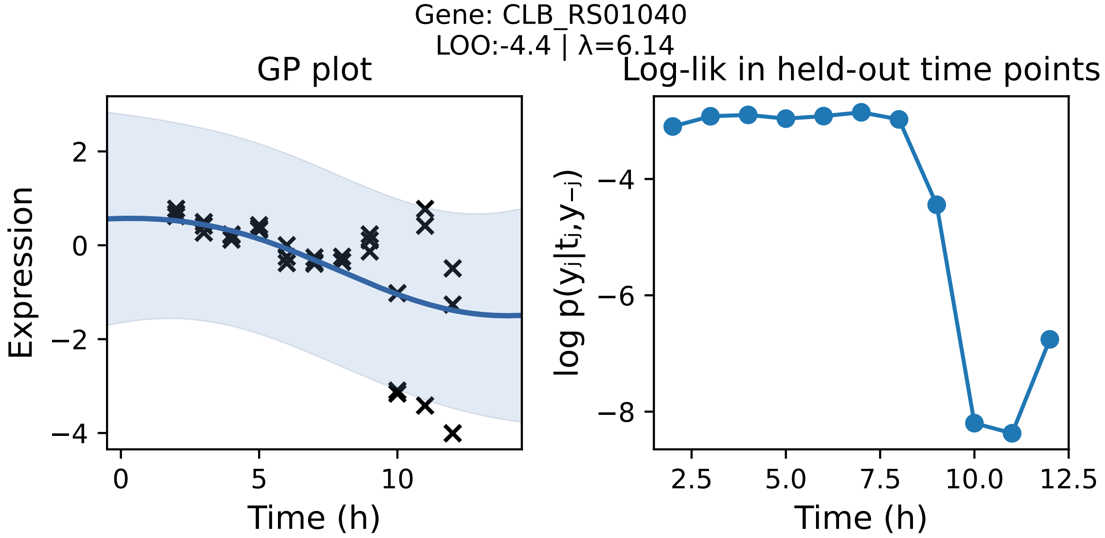

Gene: CLB_RS01040
LOO:-4.4 | λ=6.14
GP plot
Expression
Time (h)
0
5
10
2
0
−2
−4
Log-lik in held-out time points
log p(y_j|t_j,y_−j)
Time (h)
2.5
5.0
7.5
10.0
12.5
−4
−6
−8

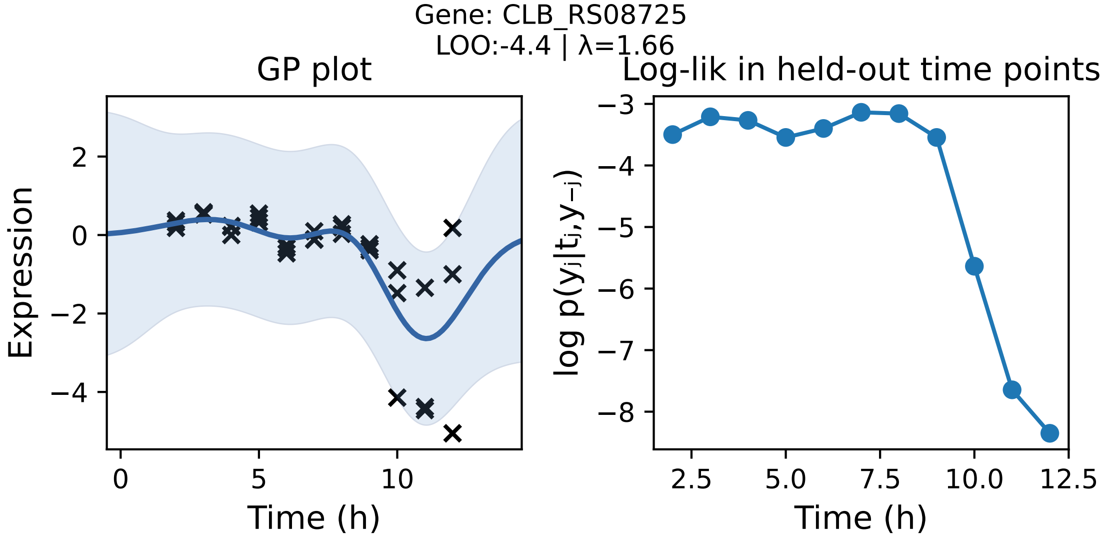
Gene: CLB_RS08725
LOO:-4.4 | λ=1.66
GP plot
Expression
Time (h)
Log-lik in held-out time points
log p(y_j|t_j,y_−j)
Time (h)

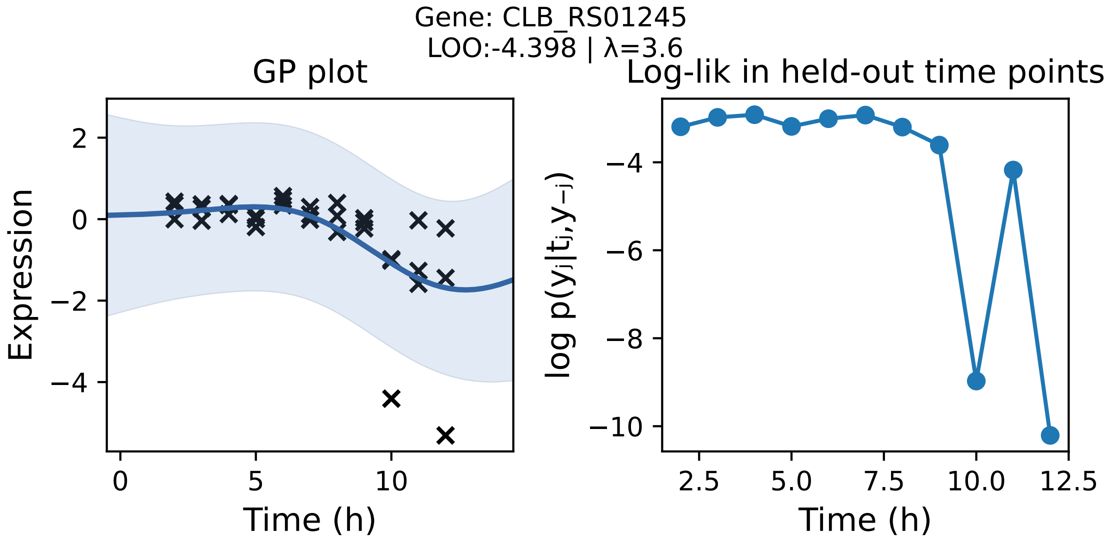

Gene: CLB_RS01245
LOO:-4.398 | λ=3.6
GP plot
Expression
Time (h)
Log-lik in held-out time points
log p(y_j|t_j,y_−j)
Time (h)

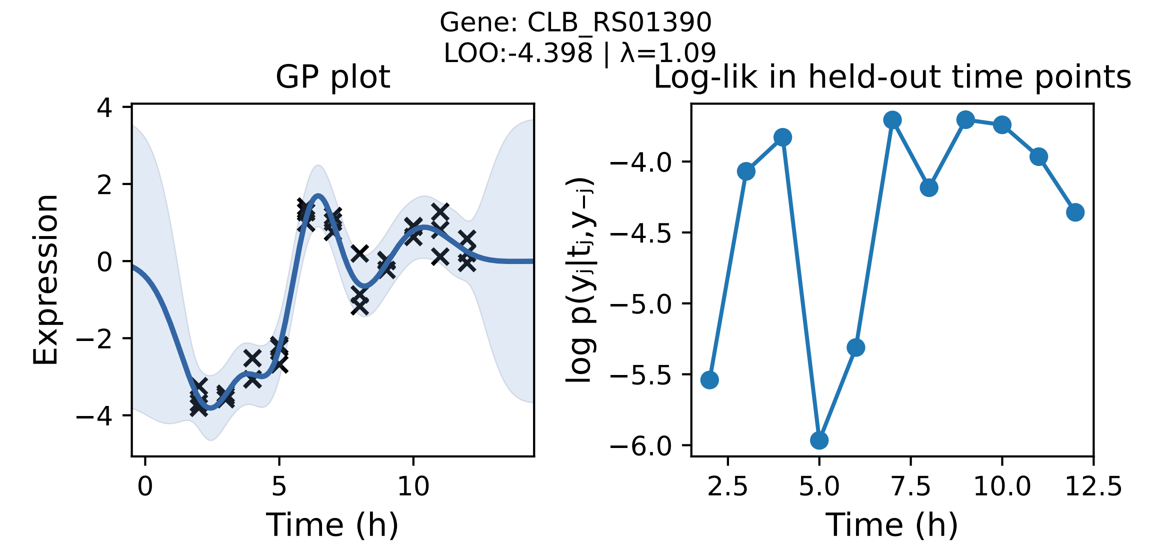

Gene: CLB_RS01390
LOO:-4.398 | λ=1.09
GP plot
Log-lik in held-out time points
Expression
Time (h)
log p(yj|tj,y−j)
Time (h)
4
2
0
−2
−4
0
5
10
−4.0
−4.5
−5.0
−5.5
−6.0
2.5
5.0
7.5
10.0
12.5

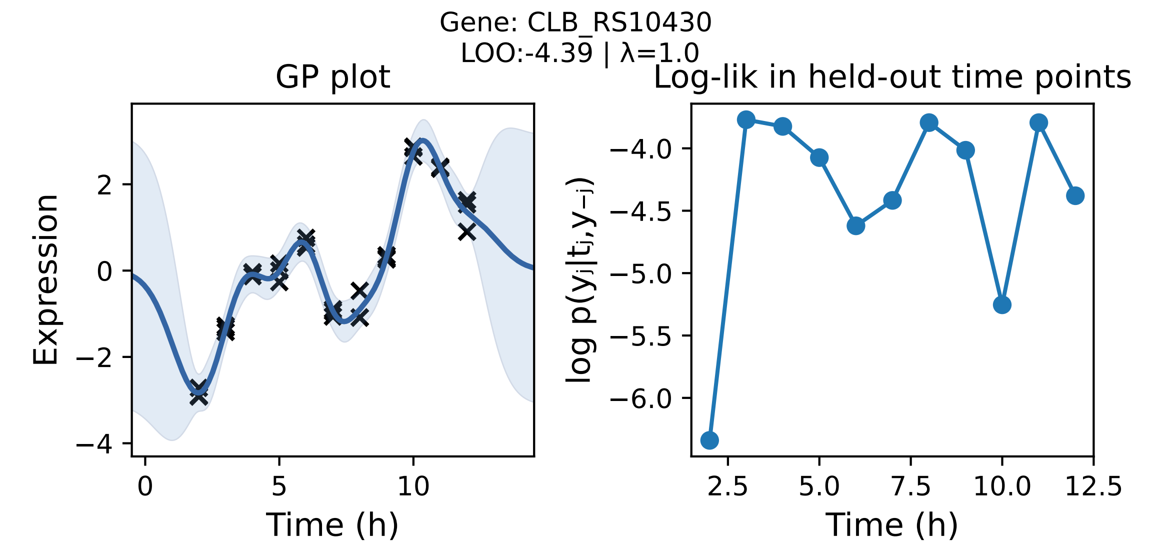

Gene: CLB_RS10430
LOO:-4.39 | λ=1.0
GP plot
Expression
Time (h)
Log-lik in held-out time points
log p(y_j|t_j,y_−j)
Time (h)

Gene: CLB_RS09890
LOO:-4.389 | λ=1.56

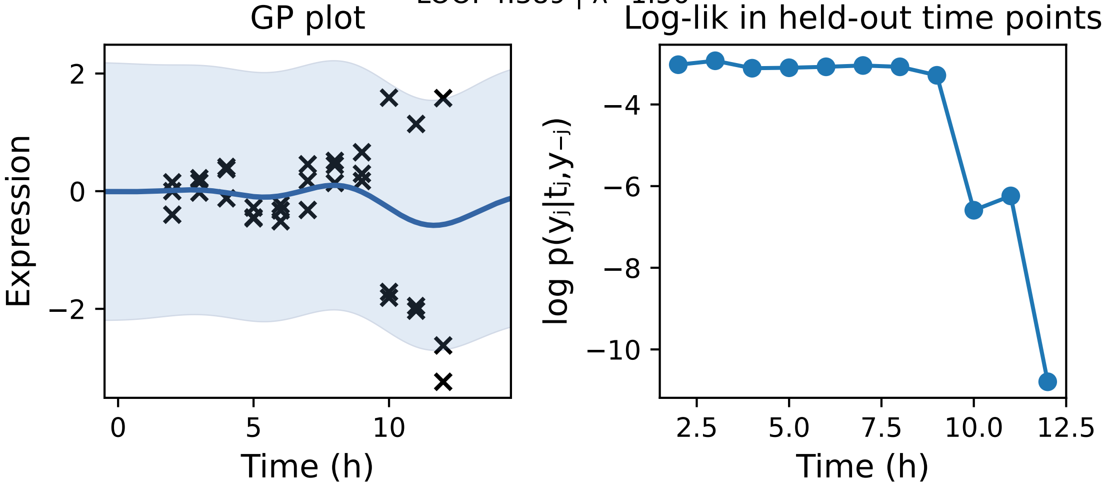

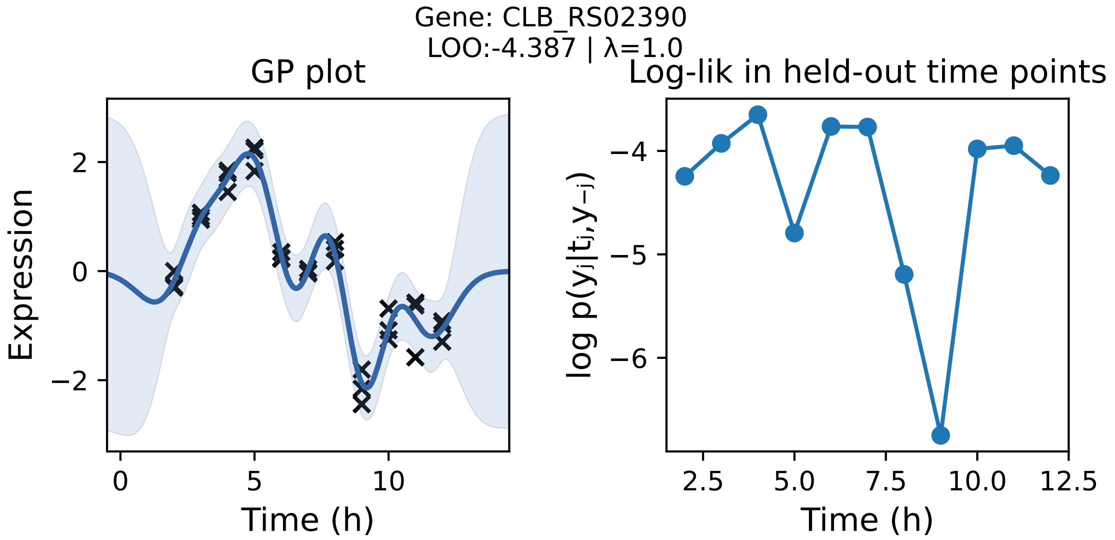
Gene: CLB_RS02390
LOO:-4.387 | λ=1.0
GP plot
Expression
Time (h)
Log-lik in held-out time points
log p(y_j|t_j,y_−j)
Time (h)

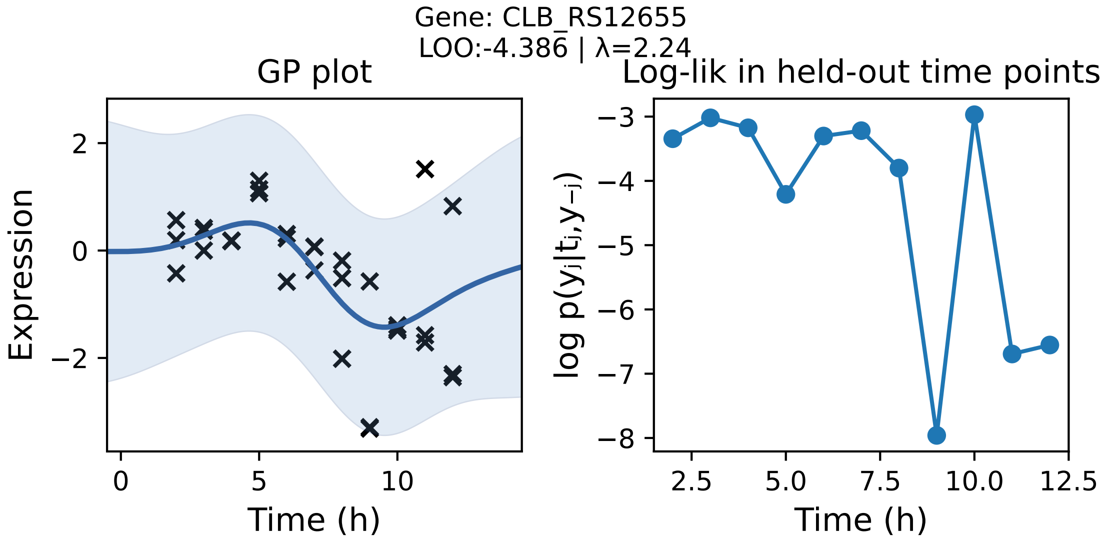

Gene: CLB_RS12655
LOO:-4.386 | λ=2.24
GP plot
Expression
Time (h)
0
5
10
2
0
−2
Log-lik in held-out time points
log p(y_j|t_j,y_−j)
Time (h)
2.5
5.0
7.5
10.0
12.5
−3
−4
−5
−6
−7
−8

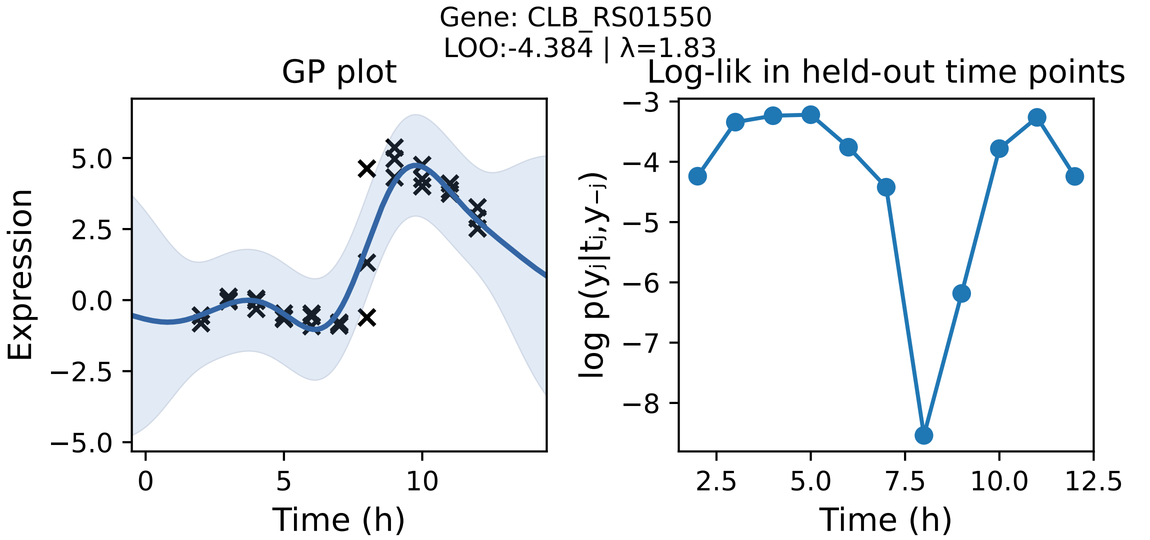
Gene: CLB_RS01550
LOO:-4.384 | λ=1.83
GP plot
Expression
Time (h)
5.0
2.5
0.0
−2.5
−5.0
0
5
10
Log-lik in held-out time points
log p(y_j|t_j,y_−j)
Time (h)
−3
−4
−5
−6
−7
−8
2.5
5.0
7.5
10.0
12.5

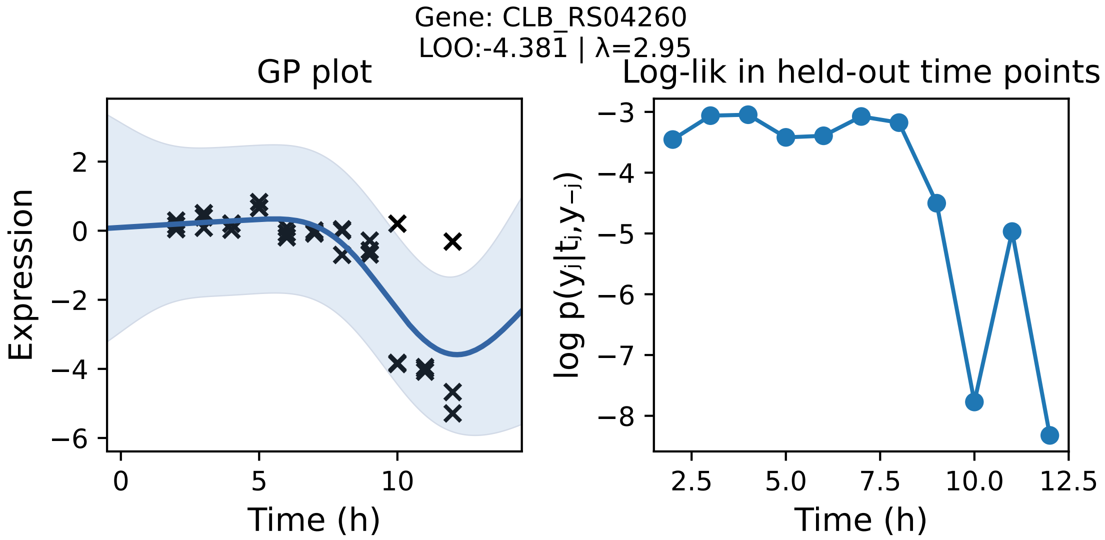

Gene: CLB_RS04260
LOO:-4.381 | λ=2.95
GP plot
Expression
Time (h)
Log-lik in held-out time points
log p(y_j|t_j,y_−j)
Time (h)

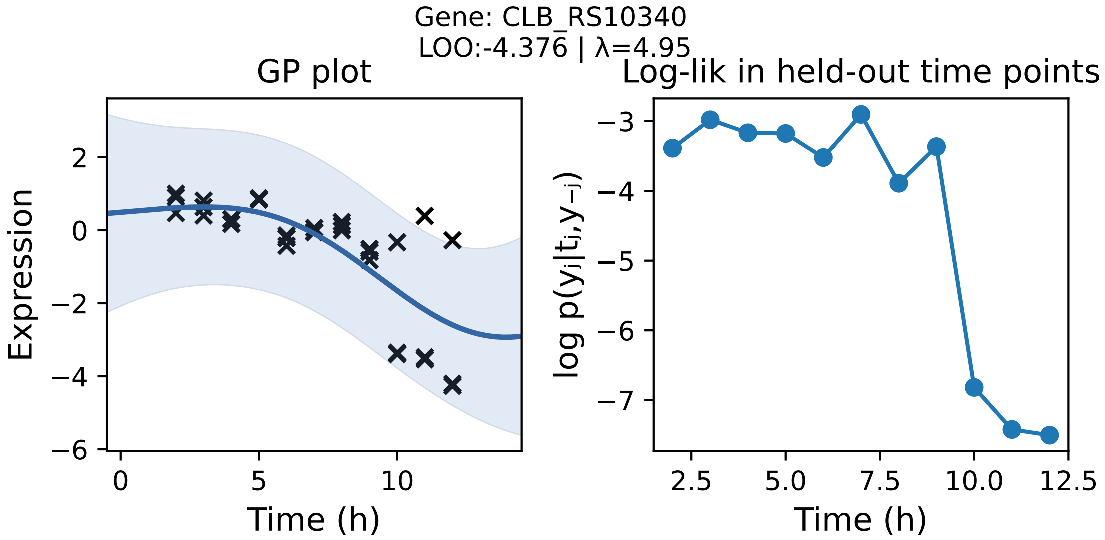

Gene: CLB_RS10340
LOO:-4.376 | λ=4.95
GP plot
Log-lik in held-out time points
Expression
Time (h)
log p(yj|tj,y−j)
Time (h)

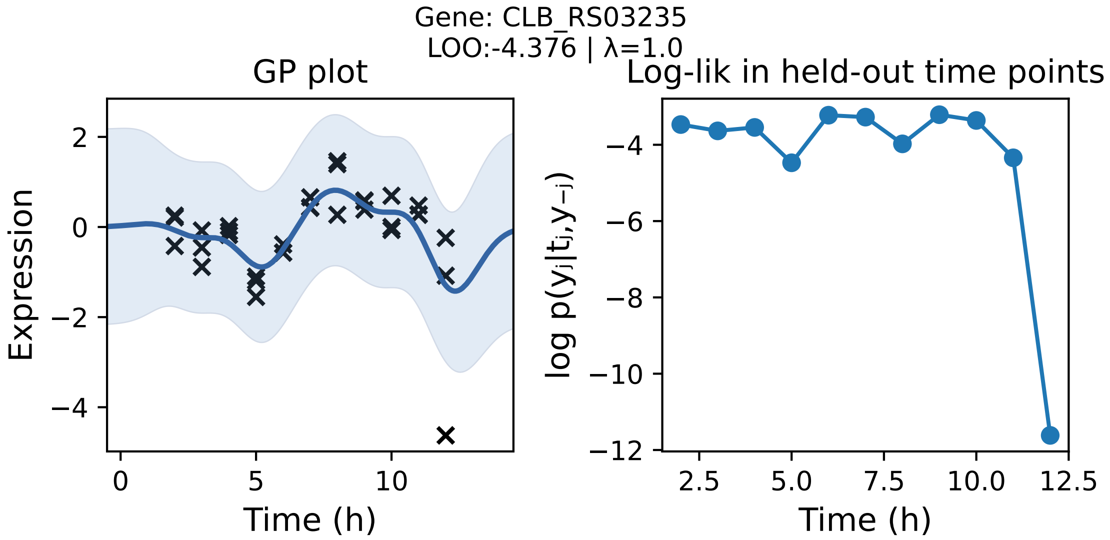

Gene: CLB_RS03235
LOO:-4.376 | λ=1.0
GP plot
Expression
Time (h)
2
0
−2
−4
0
5
10
Log-lik in held-out time points
log p(y_j|t_j,y_−j)
Time (h)
−4
−6
−8
−10
−12
2.5
5.0
7.5
10.0
12.5

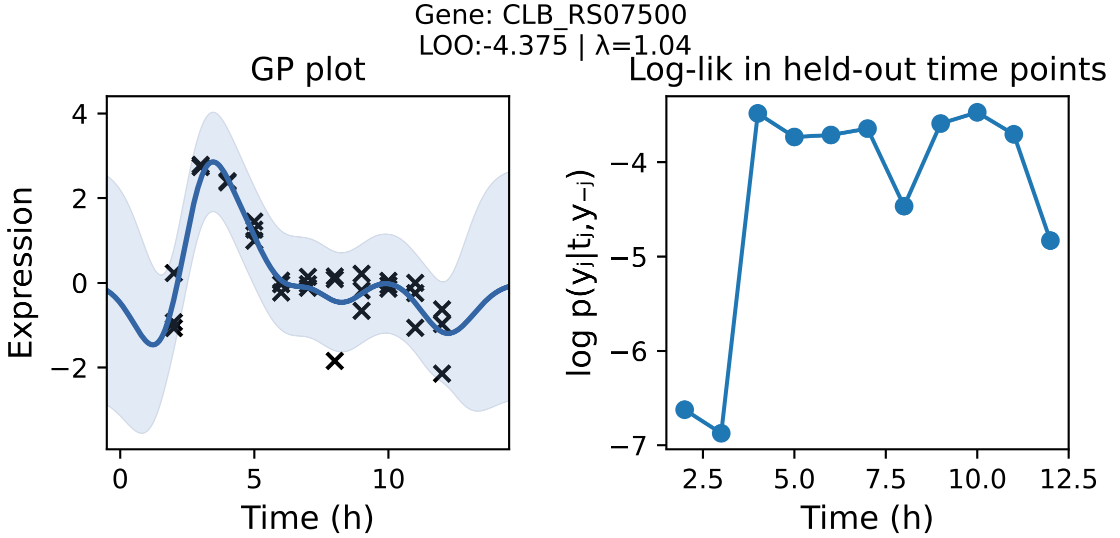
Gene: CLB_RS07500
LOO:-4.375 | λ=1.04
GP plot
Expression
Time (h)
4
2
0
−2
0
5
10
Log-lik in held-out time points
log p(yj|tj,y−j)
Time (h)
−4
−5
−6
−7
2.5
5.0
7.5
10.0
12.5

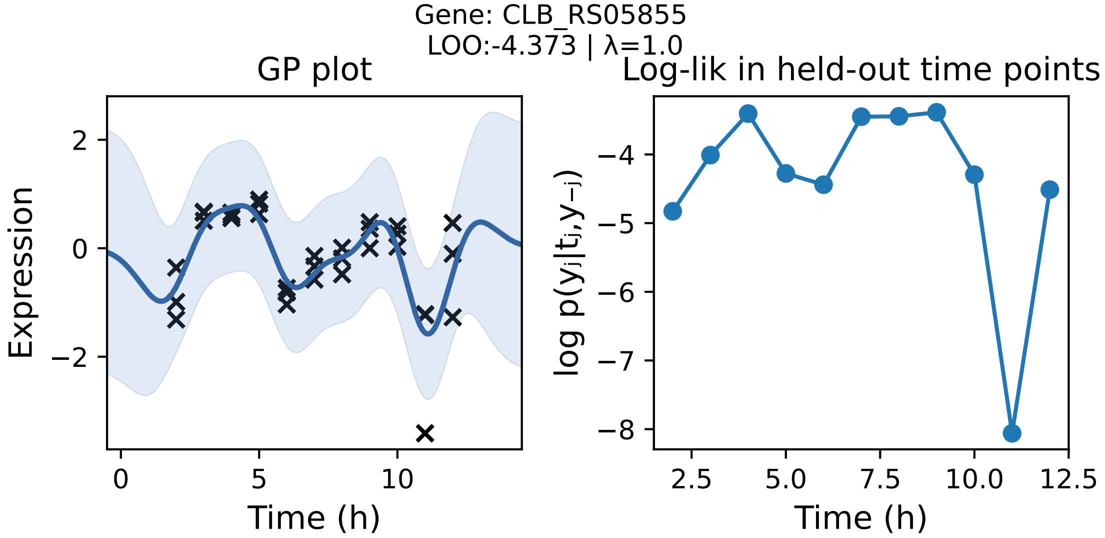

Gene: CLB_RS05855
LOO:-4.373 | λ=1.0
GP plot
Expression
Time (h)
2
0
−2
0
5
10
Log-lik in held-out time points
log p(y_j|t_j,y_−j)
Time (h)
−4
−5
−6
−7
−8
2.5
5.0
7.5
10.0
12.5

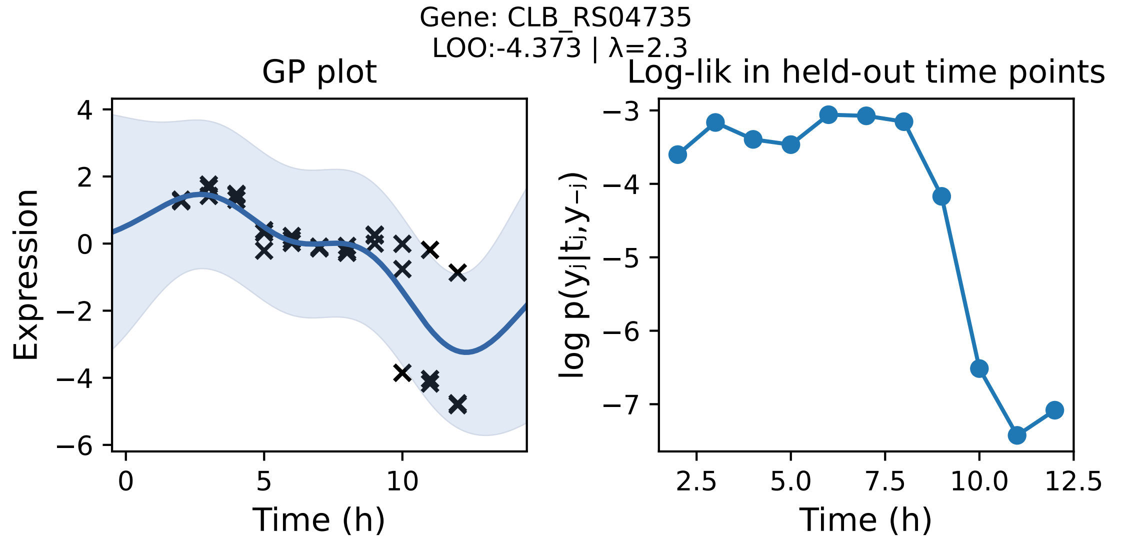

Gene: CLB_RS04735
LOO:-4.373 | λ=2.3
GP plot
Log-lik in held-out time points
Expression
Time (h)
log p(y_j|t_j,y_−j)
Time (h)

Gene: CLB_RS15405
LOO:-4.37 | λ=5.1

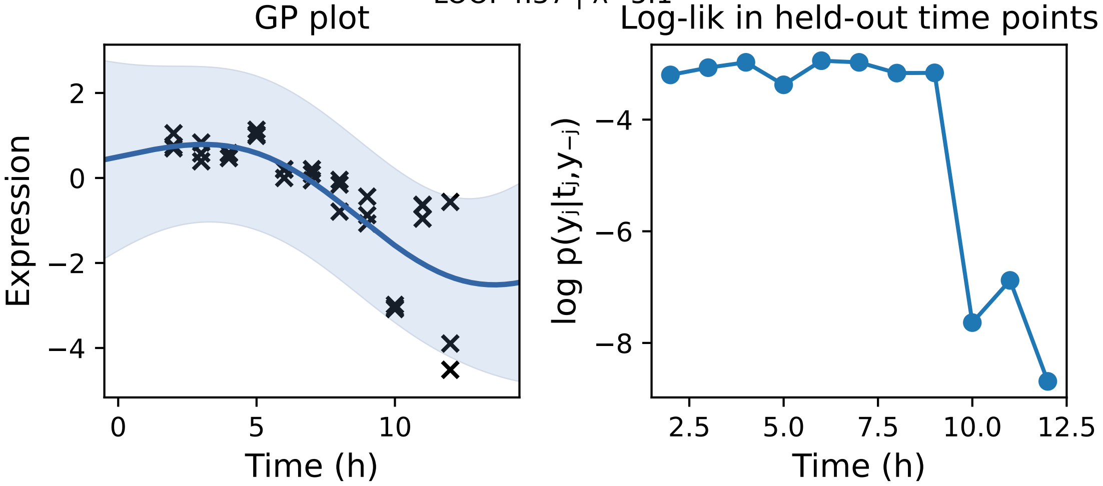

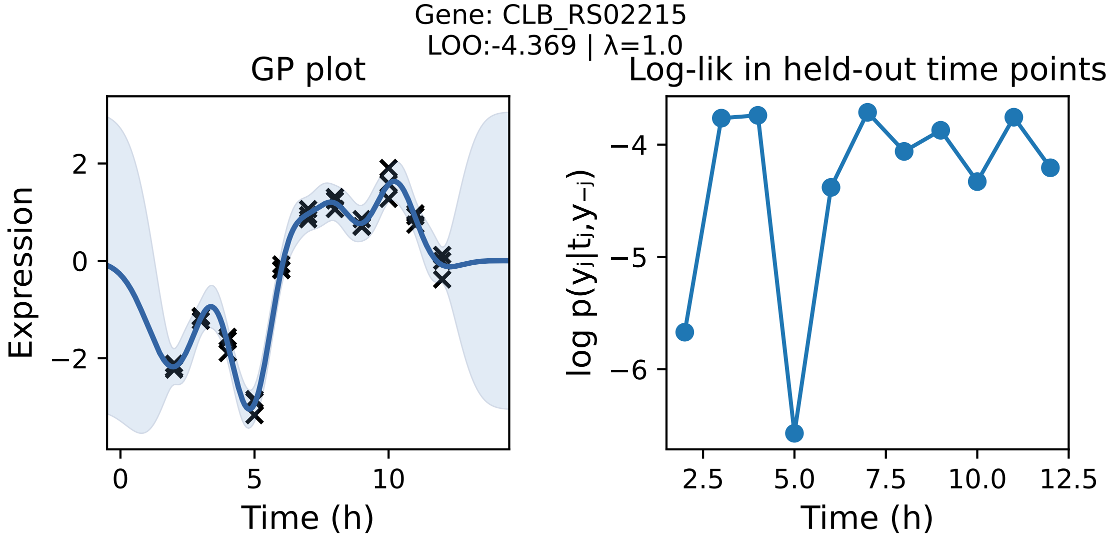
Gene: CLB_RS02215
LOO:-4.369 | λ=1.0
GP plot
Log-lik in held-out time points
Expression
Time (h)
2
0
−2
0
5
10
log p(yj|tj,y−j)
−4
−5
−6
2.5
5.0
7.5
10.0
12.5
Time (h)

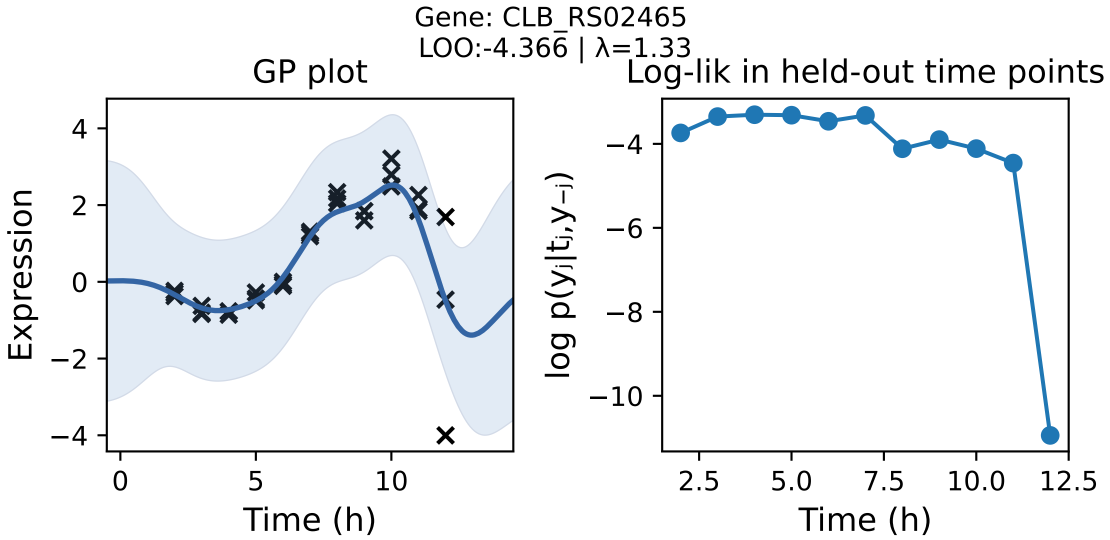

Gene: CLB_RS02465
LOO:-4.366 | λ=1.33
GP plot
Expression
Time (h)
4
2
0
−2
−4
0
5
10
Log-lik in held-out time points
log p(y_j|t_j,y_−j)
Time (h)
−4
−6
−8
−10
2.5
5.0
7.5
10.0
12.5

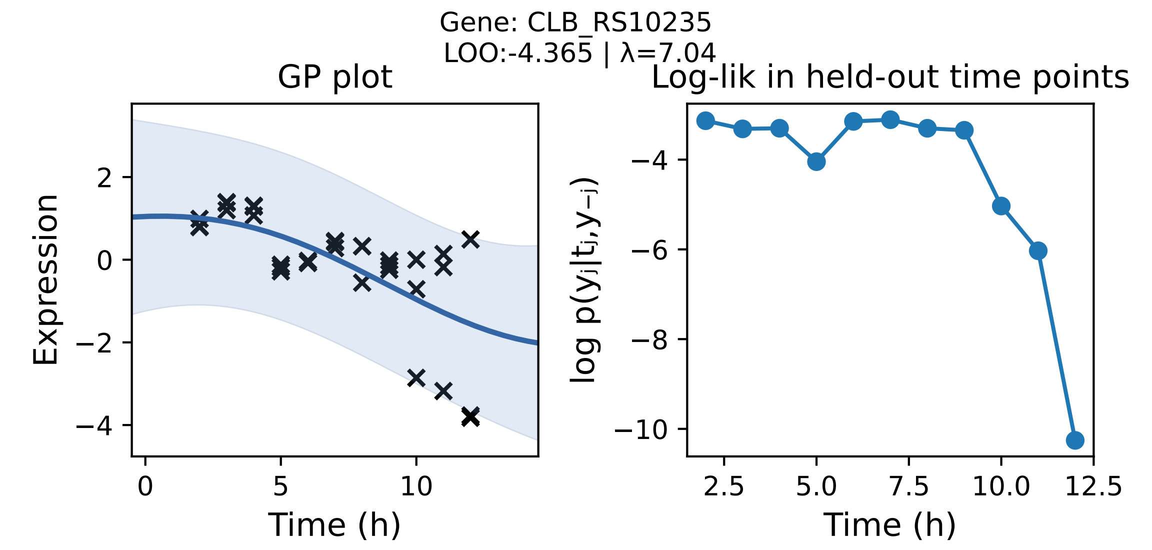

Gene: CLB_RS10235
LOO:-4.365 | λ=7.04
GP plot
Expression
Time (h)
2
0
−2
−4
0
5
10
Log-lik in held-out time points
log p(y_j|t_j,y_−j)
Time (h)
−4
−6
−8
−10
2.5
5.0
7.5
10.0
12.5

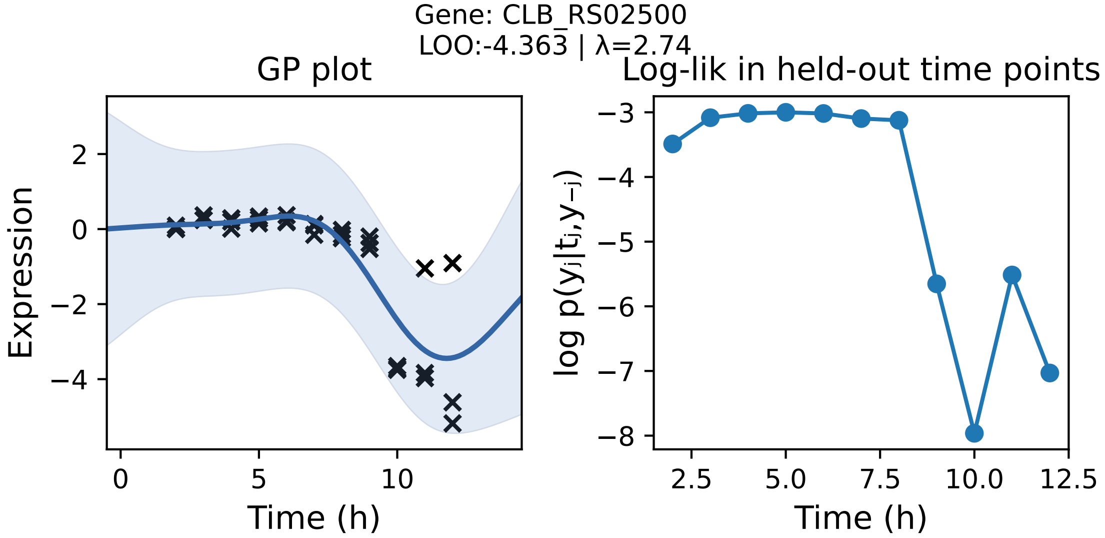

Gene: CLB_RS02500
LOO:-4.363 | λ=2.74
GP plot
Expression
Time (h)
Log-lik in held-out time points
log p(y_j|t_j,y_−j)
Time (h)

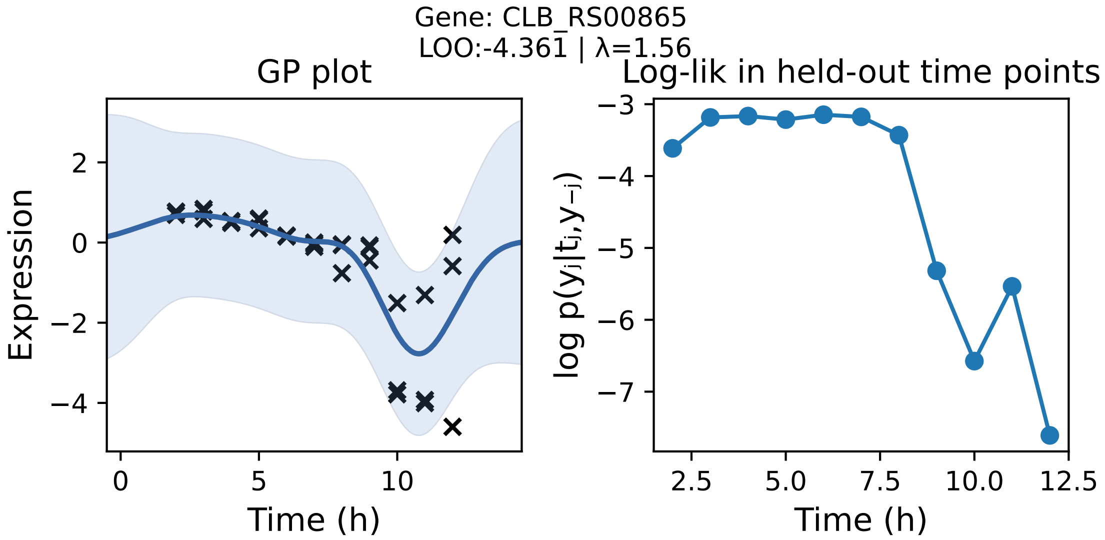

Gene: CLB_RS00865
LOO:-4.361 | λ=1.56
GP plot
Expression
Time (h)
Log-lik in held-out time points
log p(y_j|t_j,y_−j)
Time (h)

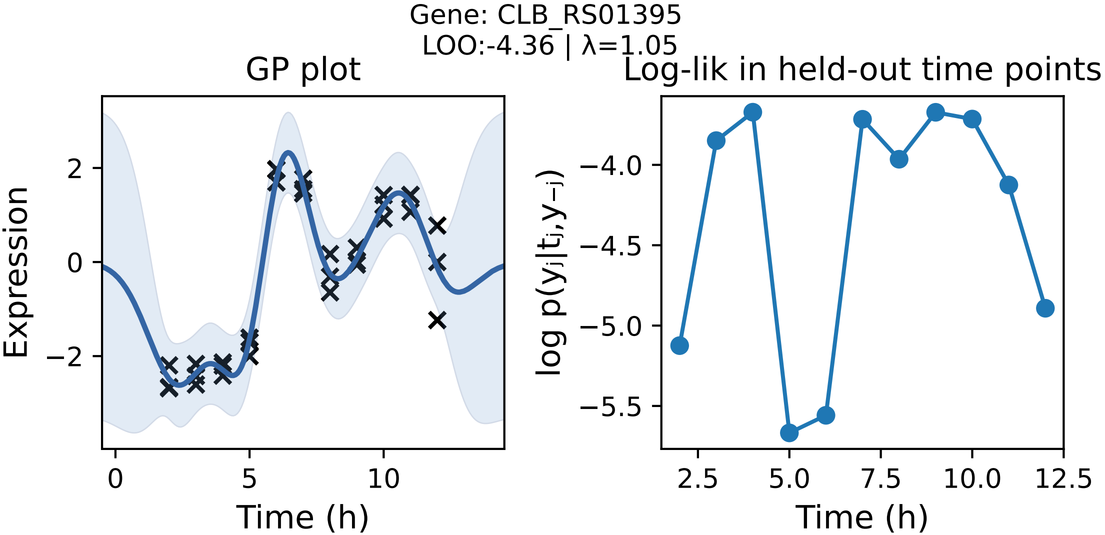

Gene: CLB_RS01395
LOO:-4.36 | λ=1.05
GP plot
Expression
Time (h)
Log-lik in held-out time points
log p(y_j|t_j,y_−j)
Time (h)

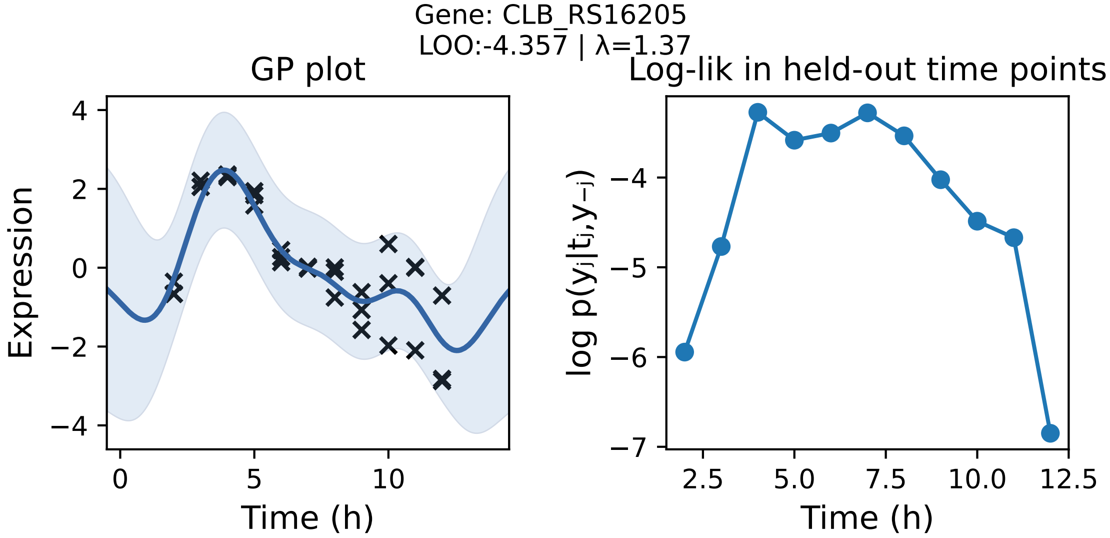

Gene: CLB_RS16205
LOO:-4.357 | λ=1.37
GP plot
Expression
Time (h)
Log-lik in held-out time points
log p(y_j|t_j,y_−j)
Time (h)

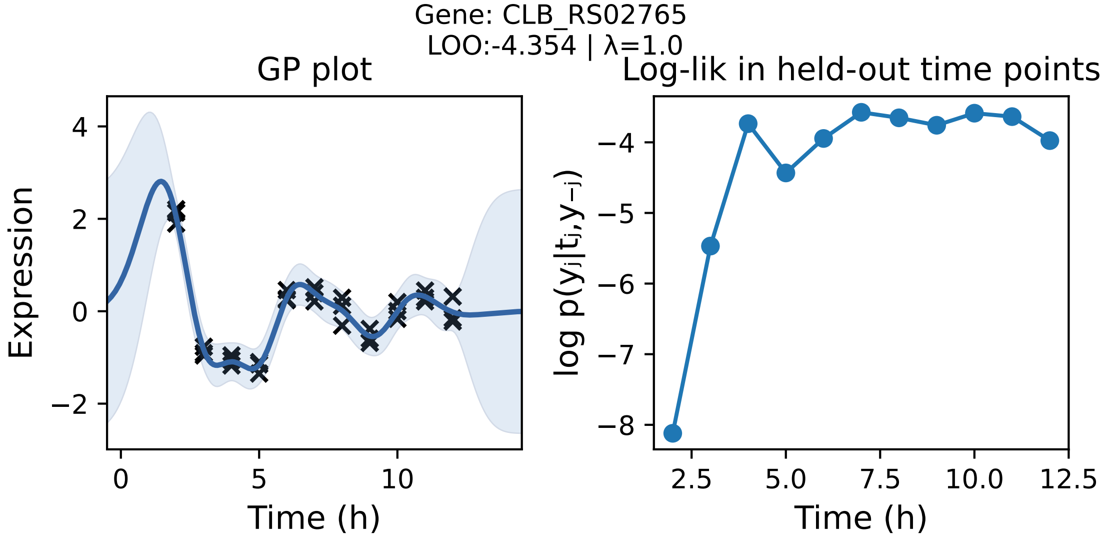

Gene: CLB_RS02765
LOO:-4.354 | λ=1.0
GP plot
Expression
Time (h)
4
2
0
−2
0
5
10
Log-lik in held-out time points
log p(yj|tj,y−j)
Time (h)
−4
−5
−6
−7
−8
2.5
5.0
7.5
10.0
12.5

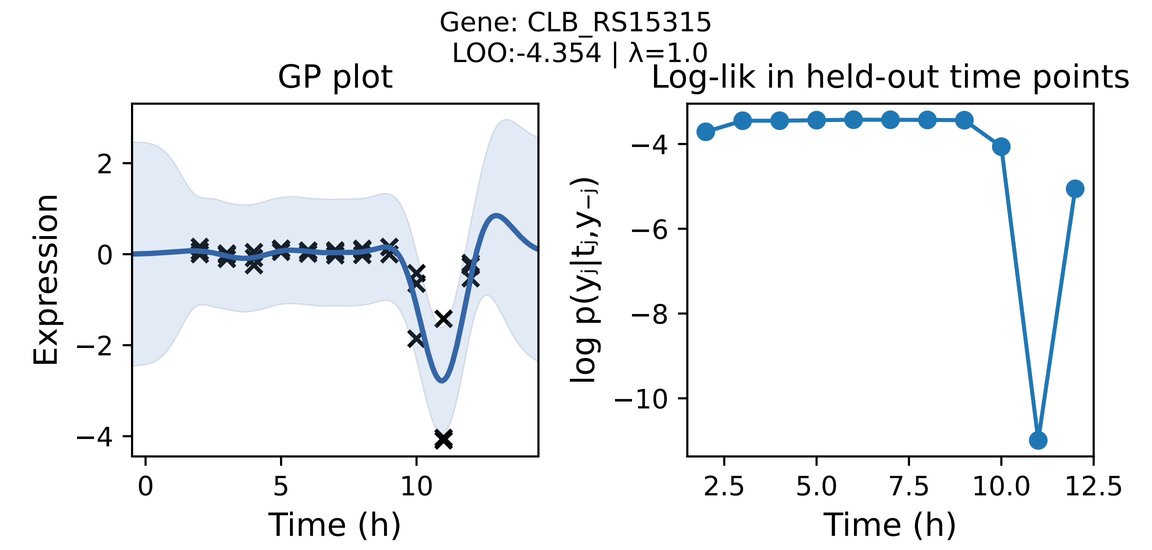

Gene: CLB_RS15315
LOO:-4.354 | λ=1.0
GP plot
Expression
Time (h)
2
0
−2
−4
0
5
10
Log-lik in held-out time points
log p(y_j|t_j,y_−j)
Time (h)
−4
−6
−8
−10
2.5
5.0
7.5
10.0
12.5

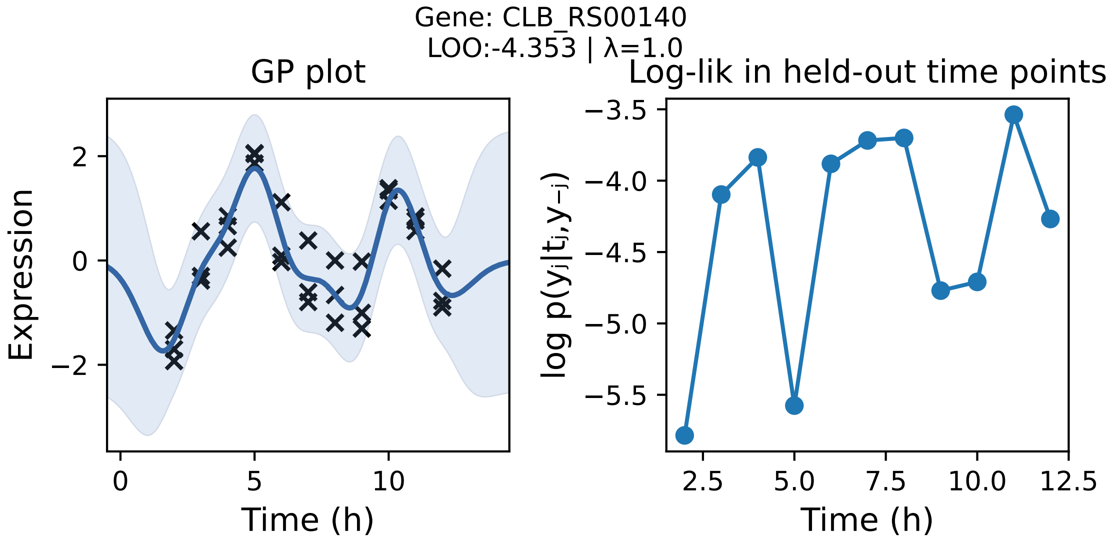
Gene: CLB_RS00140
LOO:-4.353 | λ=1.0
GP plot
Expression
Time (h)
Log-lik in held-out time points
log p(y_j|t_j,y_−j)
Time (h)
2
0
−2
0
5
10
−3.5
−4.0
−4.5
−5.0
−5.5
2.5
5.0
7.5
10.0
12.5

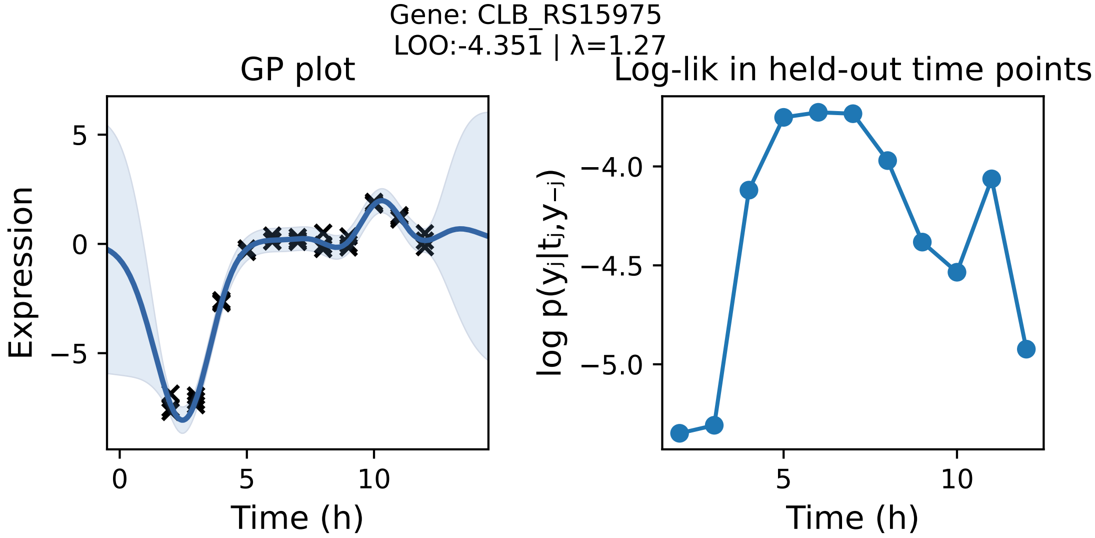
Gene: CLB_RS15975
LOO:-4.351 | λ=1.27
GP plot
Expression
Time (h)
5
0
−5
0
5
10
Log-lik in held-out time points
log p(y_j|t_j,y_−j)
Time (h)
−4.0
−4.5
−5.0
5
10

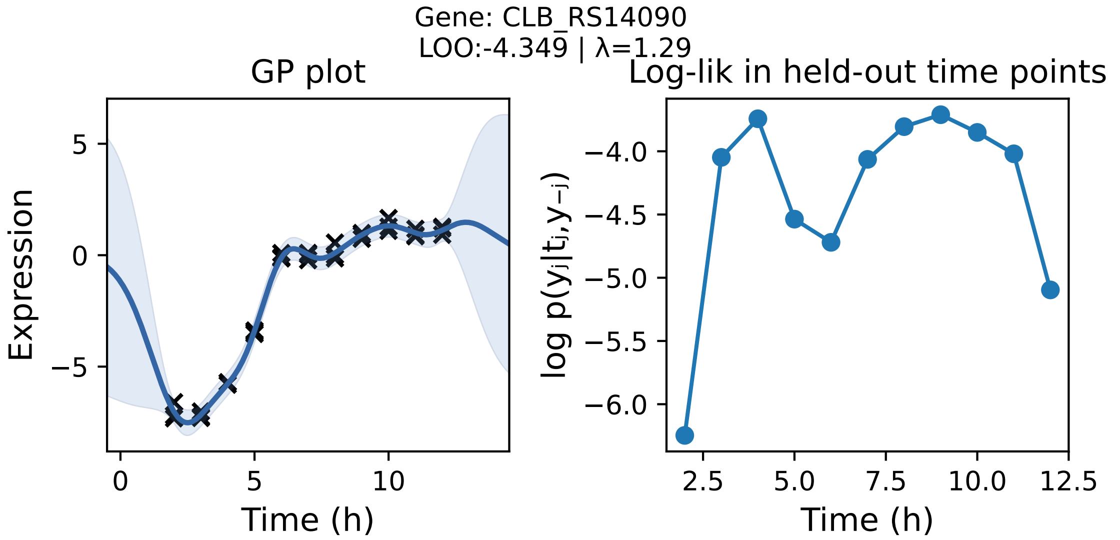

Gene: CLB_RS14090
LOO:-4.349 | λ=1.29
GP plot
Expression
Time (h)
Log-lik in held-out time points
log p(y_j|t_j,y_−j)
Time (h)

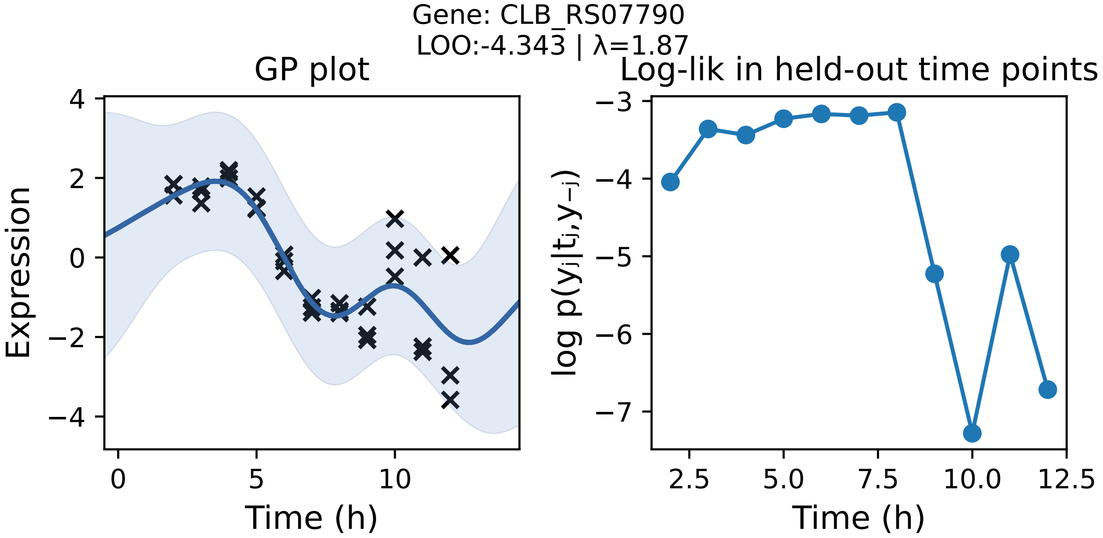

Gene: CLB_RS07790
LOO:-4.343 | λ=1.87
GP plot
Expression
Time (h)
4
2
0
−2
−4
0
5
10
Log-lik in held-out time points
log p(y_j|t_j,y_−j)
Time (h)
−3
−4
−5
−6
−7
2.5
5.0
7.5
10.0
12.5

Gene: CLB_RS10425
LOO:-4.337 | λ=2.01

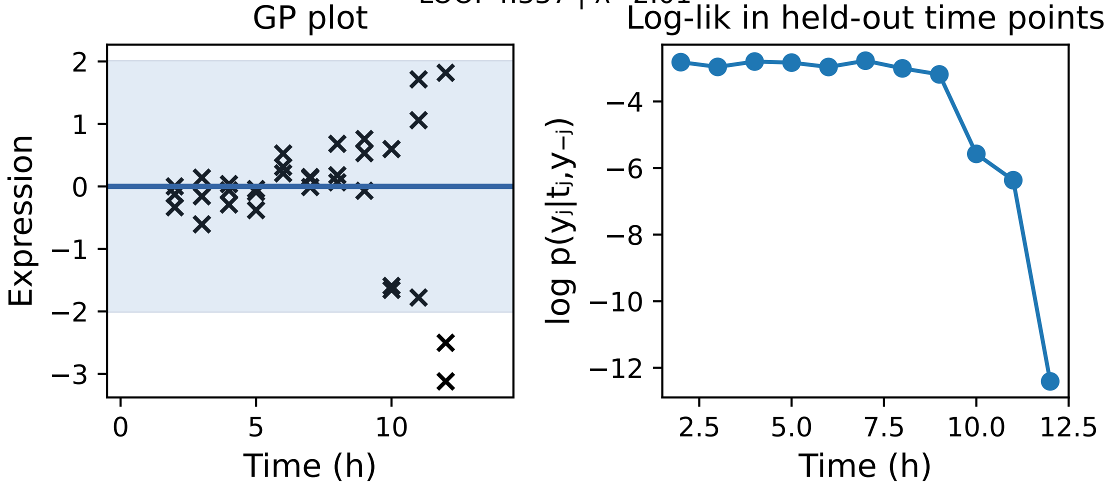

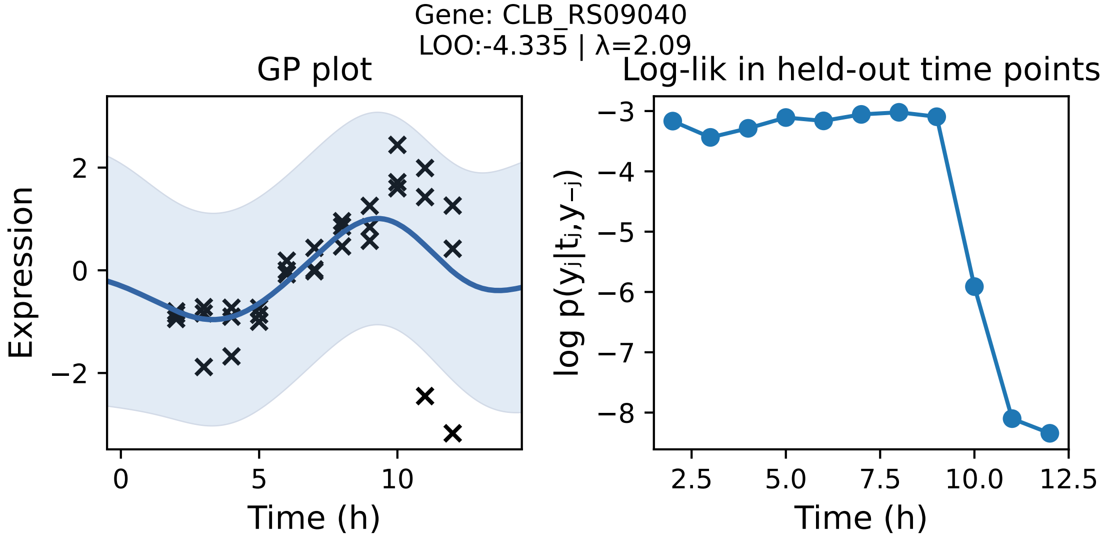

Gene: CLB_RS09040
LOO:-4.335 | λ=2.09
GP plot
Expression
Time (h)
Log-lik in held-out time points
log p(y_j|t_j,y_−j)
Time (h)

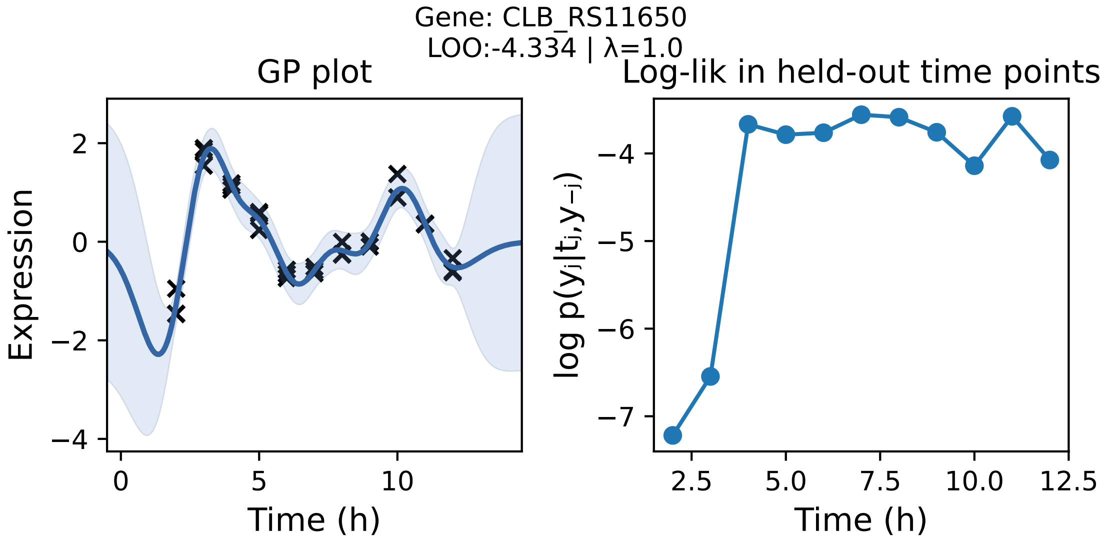

Gene: CLB_RS11650
LOO:-4.334 | λ=1.0
GP plot
Expression
Time (h)
2
0
−2
−4
0
5
10
Log-lik in held-out time points
log p(y_j|t_j,y_−j)
Time (h)
−4
−5
−6
−7
2.5
5.0
7.5
10.0
12.5

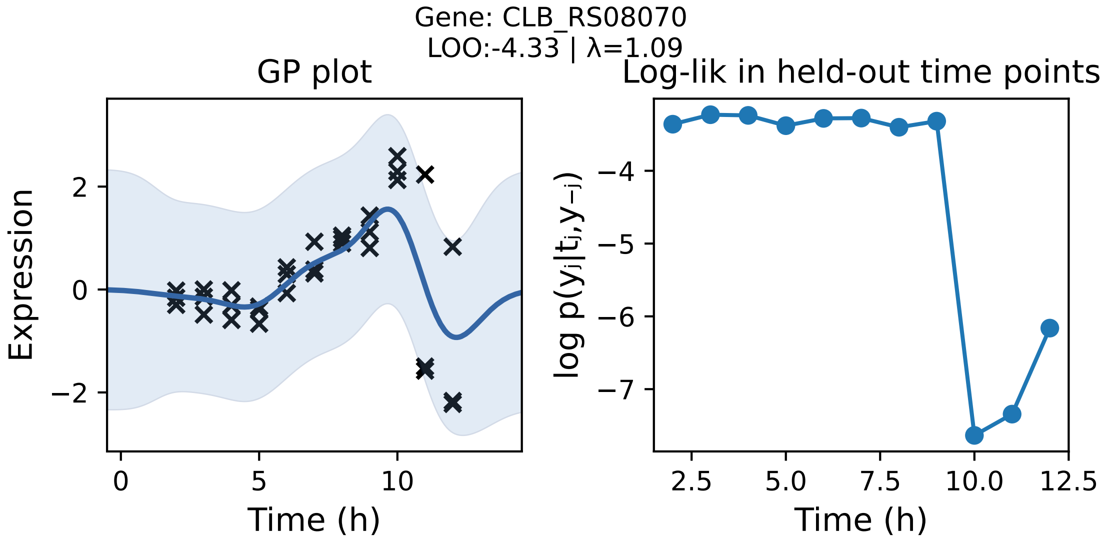

Gene: CLB_RS08070
LOO:-4.33 | λ=1.09
GP plot
Expression
Time (h)
Log-lik in held-out time points
log p(y_j|t_j,y_−j)
Time (h)

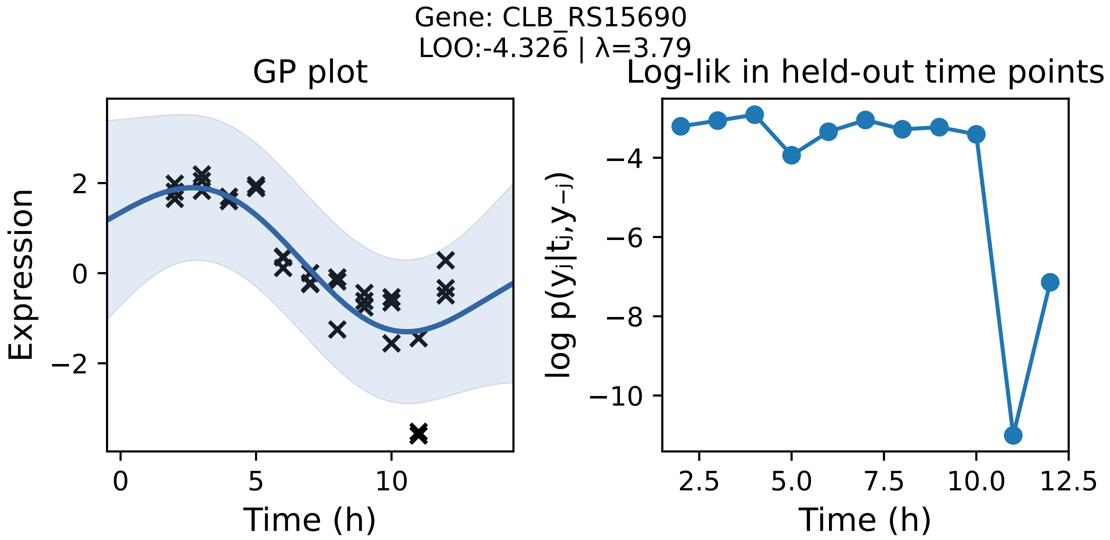
Gene: CLB_RS15690
LOO:-4.326 | λ=3.79
GP plot
Expression
Time (h)
Log-lik in held-out time points
log p(y_j|t_j,y_−j)
Time (h)

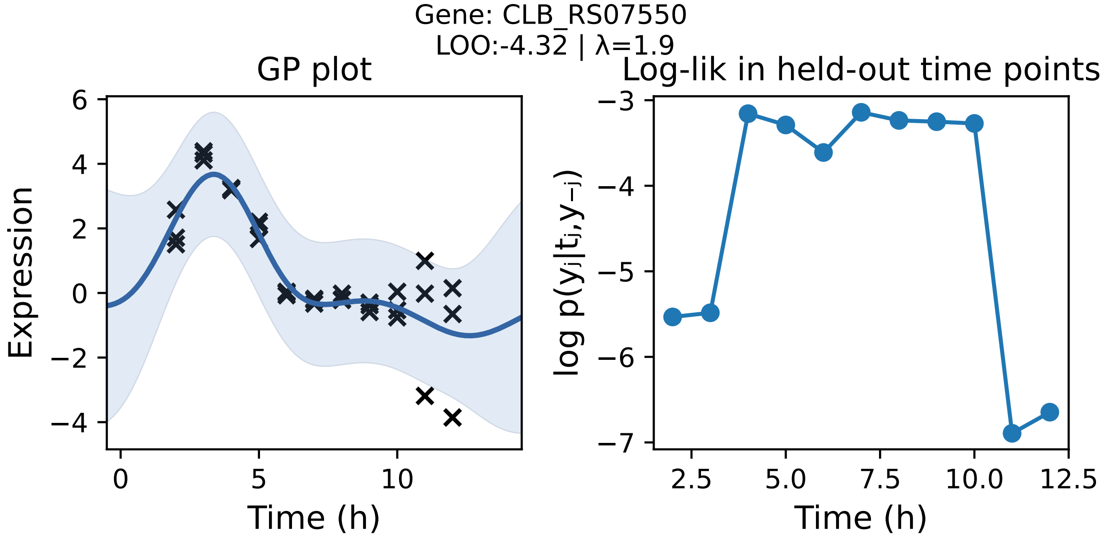

Gene: CLB_RS07550
LOO:-4.32 | λ=1.9
GP plot
Log-lik in held-out time points
Expression
Time (h)
log p(y_j|t_j,y_−j)
Time (h)

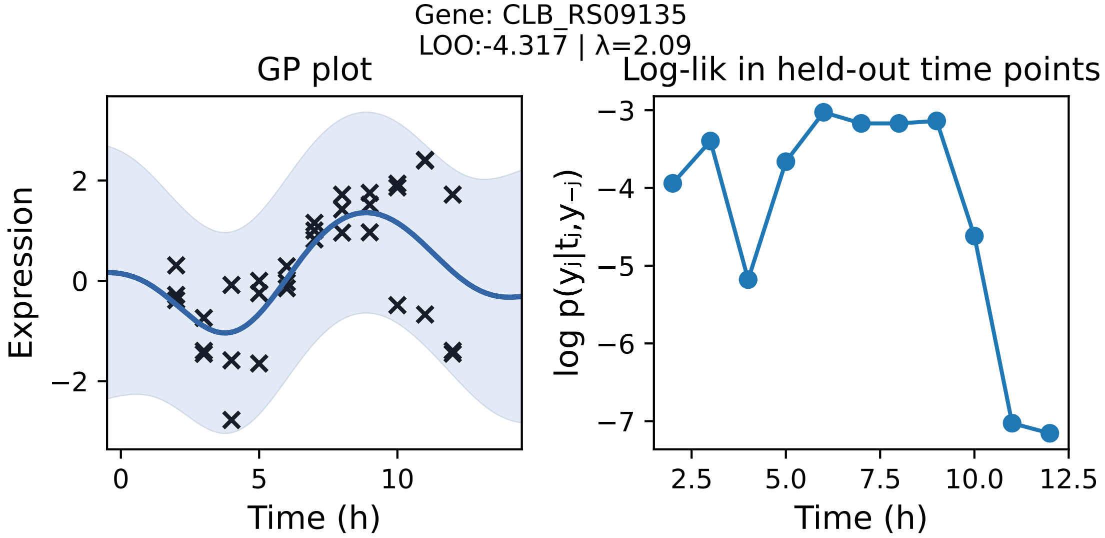
Gene: CLB_RS09135
LOO:-4.317 | λ=2.09
GP plot
Log-lik in held-out time points
Expression
Time (h)
log p(y_j|t_j,y_−j)
2
0
−2
0
5
10
−3
−4
−5
−6
−7
2.5
5.0
7.5
10.0
12.5

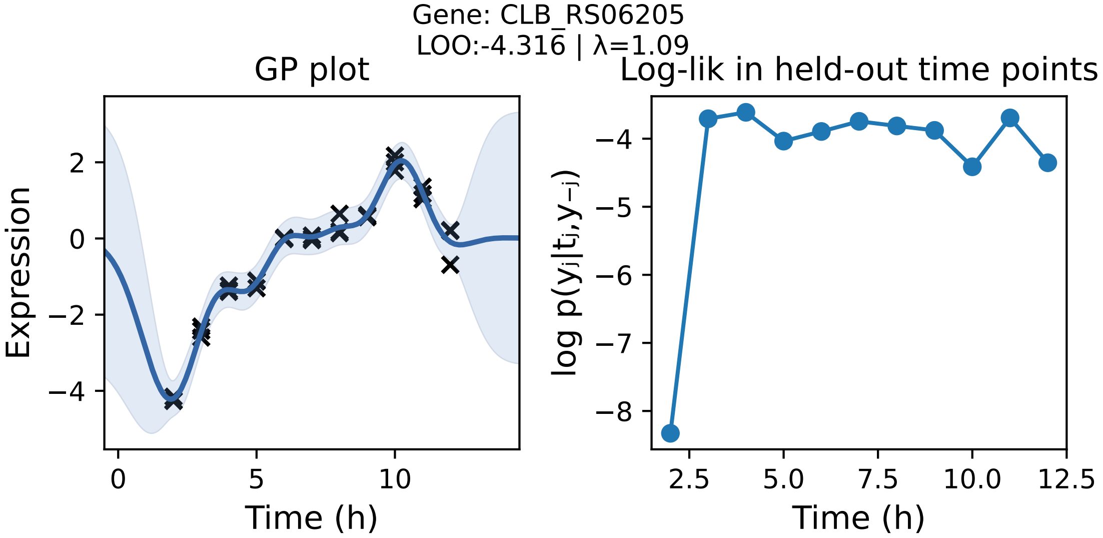
Gene: CLB_RS06205
LOO:-4.316 | λ=1.09
GP plot
Expression
Time (h)
Log-lik in held-out time points
log p(y_j|t_j,y_−j)
Time (h)

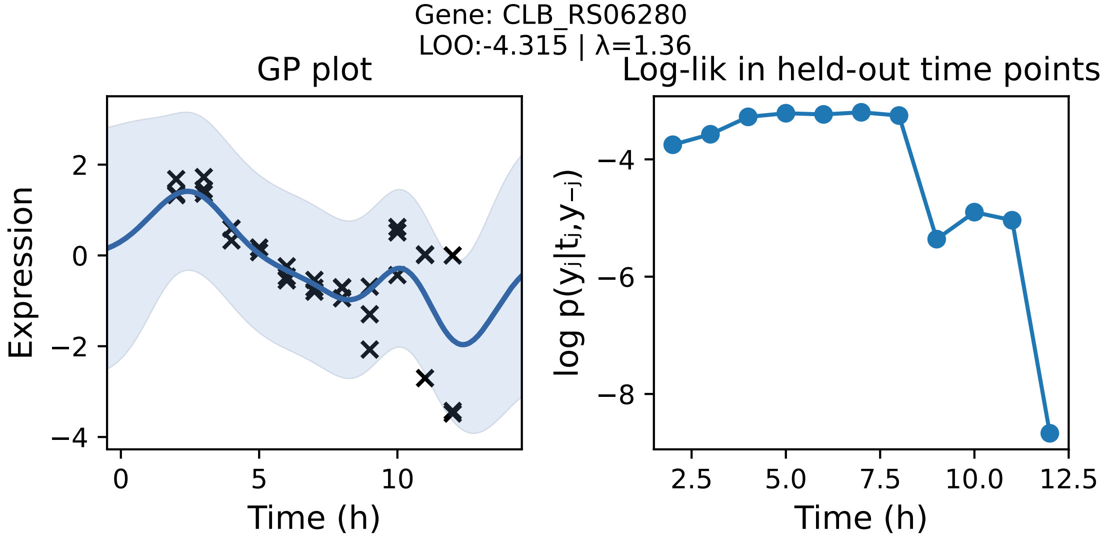

Gene: CLB_RS06280
LOO:-4.315 | λ=1.36
GP plot
Expression
Time (h)
Log-lik in held-out time points
log p(y_j|t_j,y_−j)
Time (h)

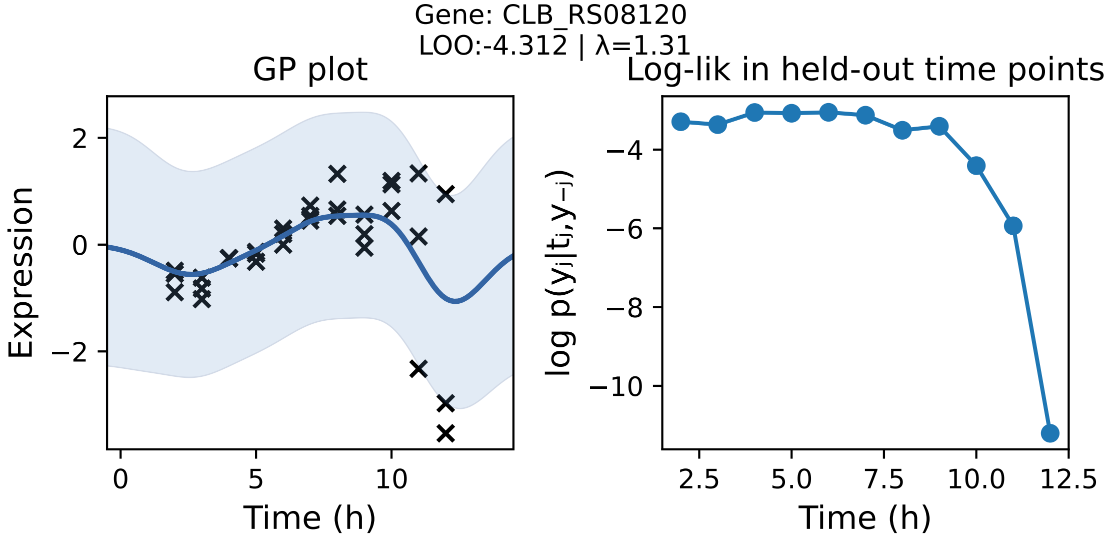

Gene: CLB_RS08120
LOO:-4.312 | λ=1.31
GP plot
Expression
Time (h)
2
0
−2
0
5
10
Log-lik in held-out time points
log p(y_j|t_j,y_−j)
Time (h)
−4
−6
−8
−10
2.5
5.0
7.5
10.0
12.5

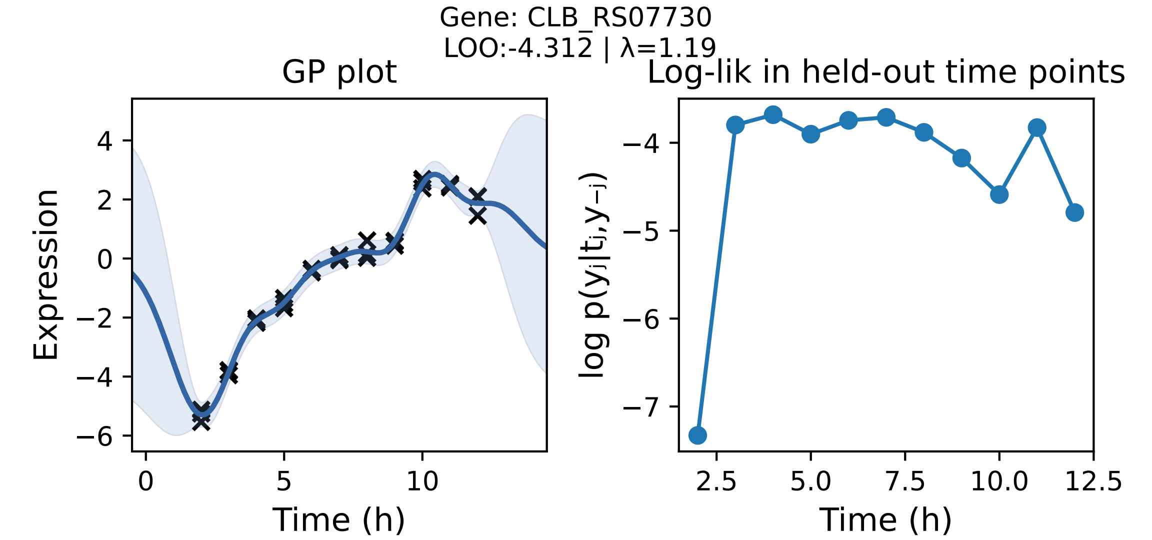
Gene: CLB_RS07730
LOO:-4.312 | λ=1.19
GP plot
Log-lik in held-out time points
Expression
Time (h)
log p(yj|tj,y−j)
Time (h)

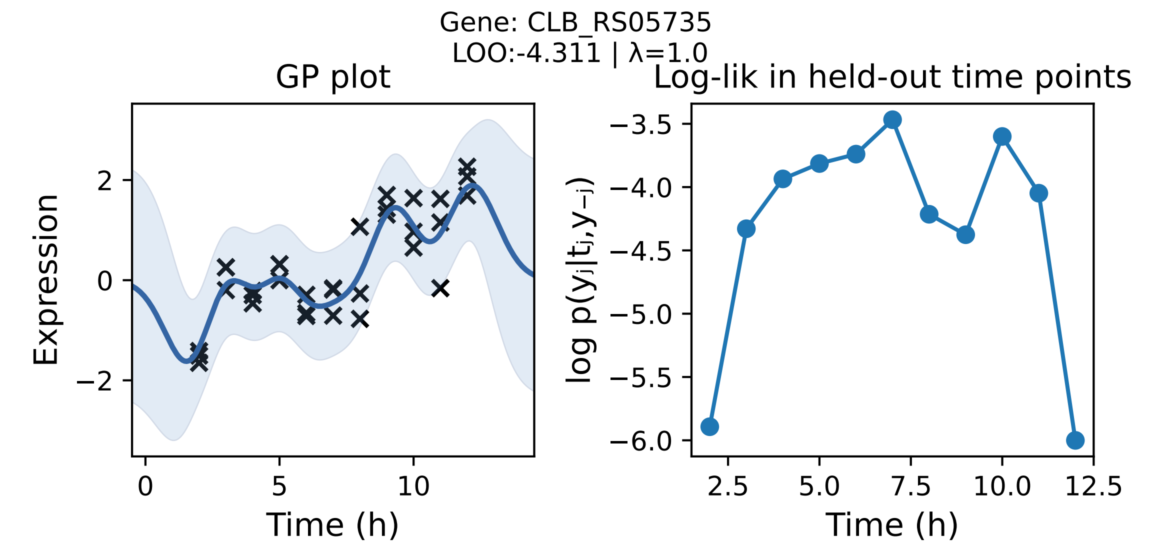
Gene: CLB_RS05735
LOO:-4.311 | λ=1.0
GP plot
Log-lik in held-out time points
Expression
Time (h)
log p(y_j|t_j,y_−j)
Time (h)
2
0
−2
0
5
10
−3.5
−4.0
−4.5
−5.0
−5.5
−6.0
2.5
5.0
7.5
10.0
12.5

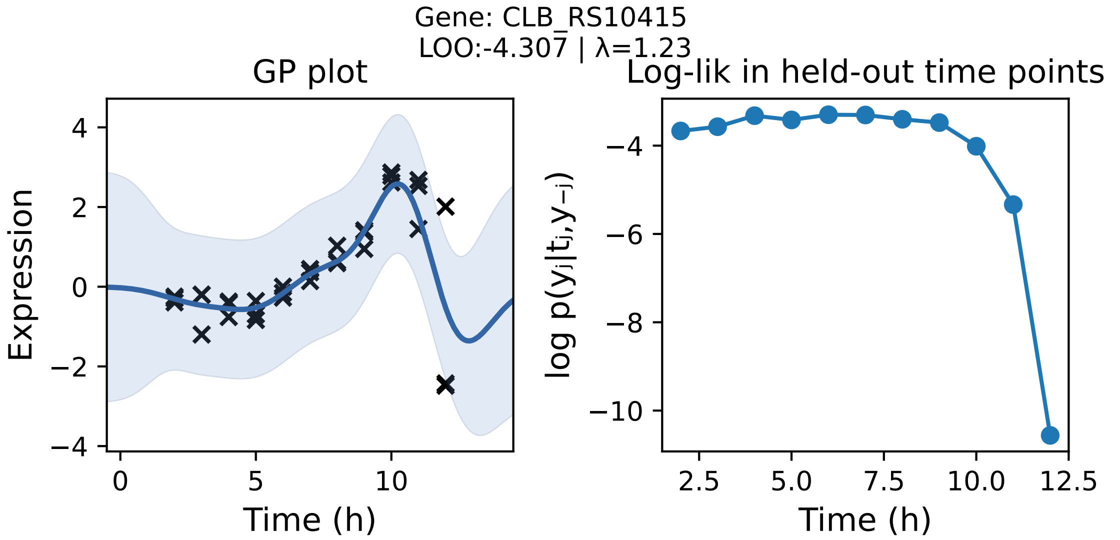

Gene: CLB_RS10415
LOO:-4.307 | λ=1.23
GP plot
Expression
Time (h)
4
2
0
−2
−4
0
5
10
Log-lik in held-out time points
log p(y_j|t_j,y_−j)
Time (h)
−4
−6
−8
−10
2.5
5.0
7.5
10.0
12.5

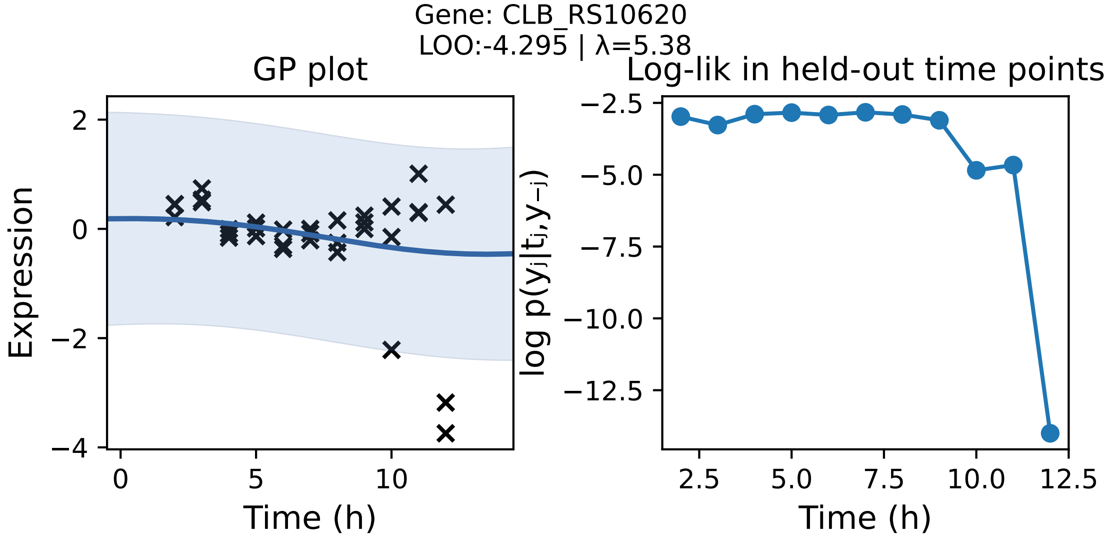
Gene: CLB_RS10620
LOO:-4.295 | λ=5.38
GP plot
Log-lik in held-out time points
Expression
Time (h)
log p(y_j|t_j,y_{-j})
2
0
−2
−4
0
5
10
−2.5
−5.0
−7.5
−10.0
−12.5
2.5
5.0
7.5
10.0
12.5

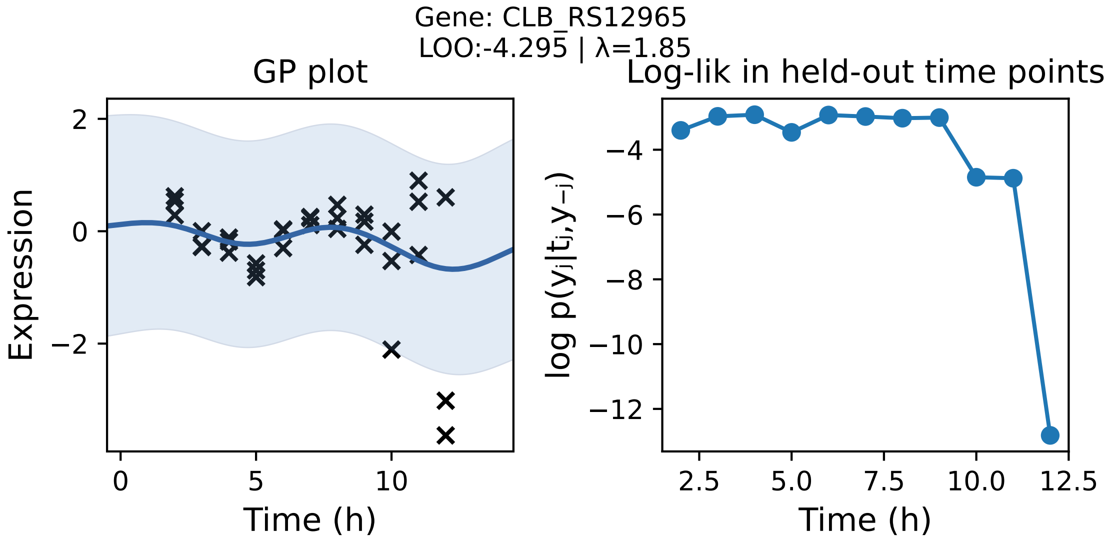
Gene: CLB_RS12965
LOO:-4.295 | λ=1.85
GP plot
Expression
Time (h)
Log-lik in held-out time points
log p(y_j|t_j,y_−j)
Time (h)

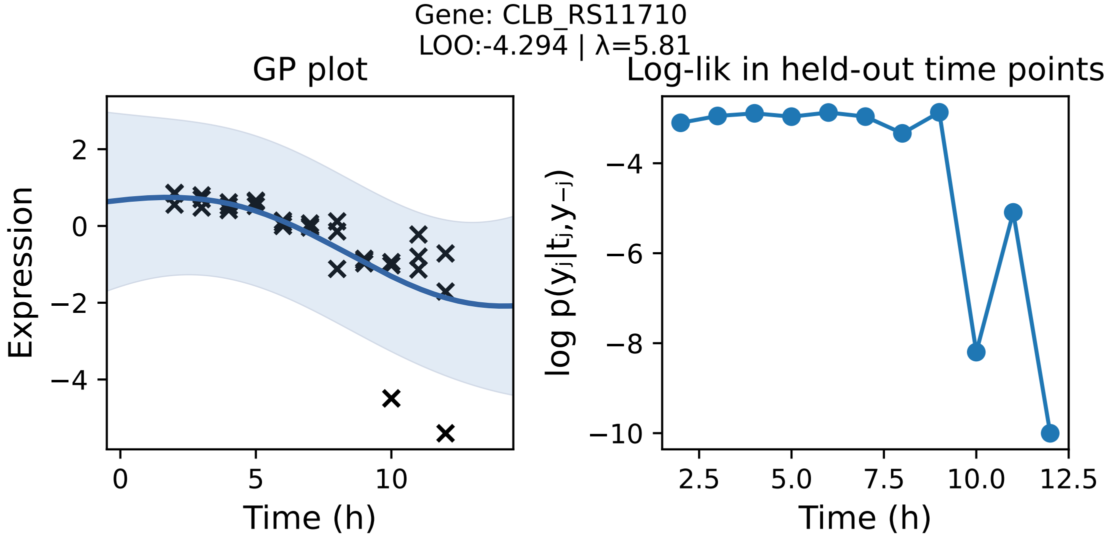
Gene: CLB_RS11710
LOO:-4.294 | λ=5.81
GP plot
Expression
Time (h)
Log-lik in held-out time points
log p(y_j|t_j,y_−j)
Time (h)

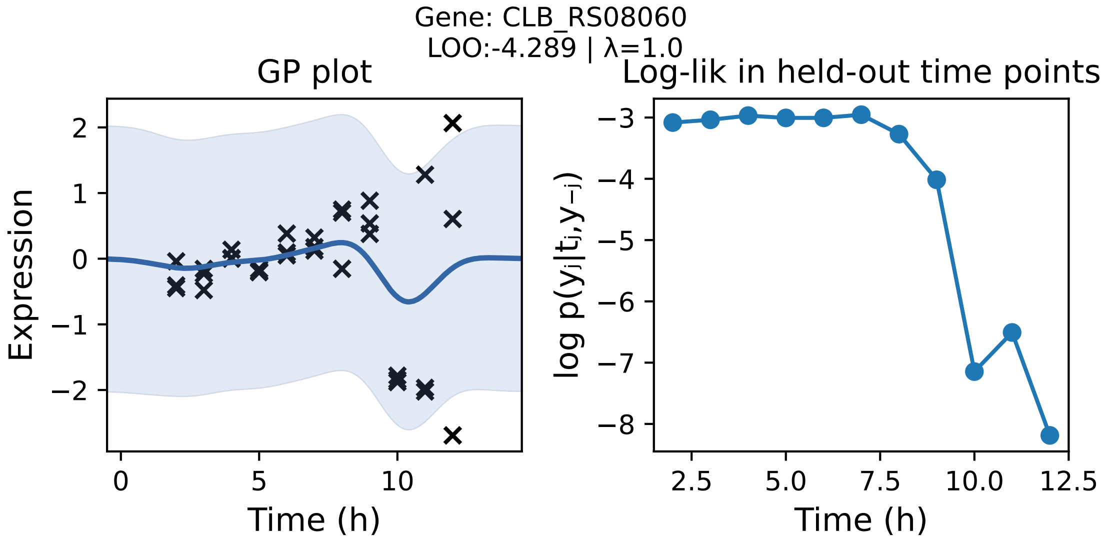

Gene: CLB_RS08060
LOO:-4.289 | λ=1.0
GP plot
Expression
Time (h)
Log-lik in held-out time points
log p(y_j|t_j,y_{-j})
Time (h)

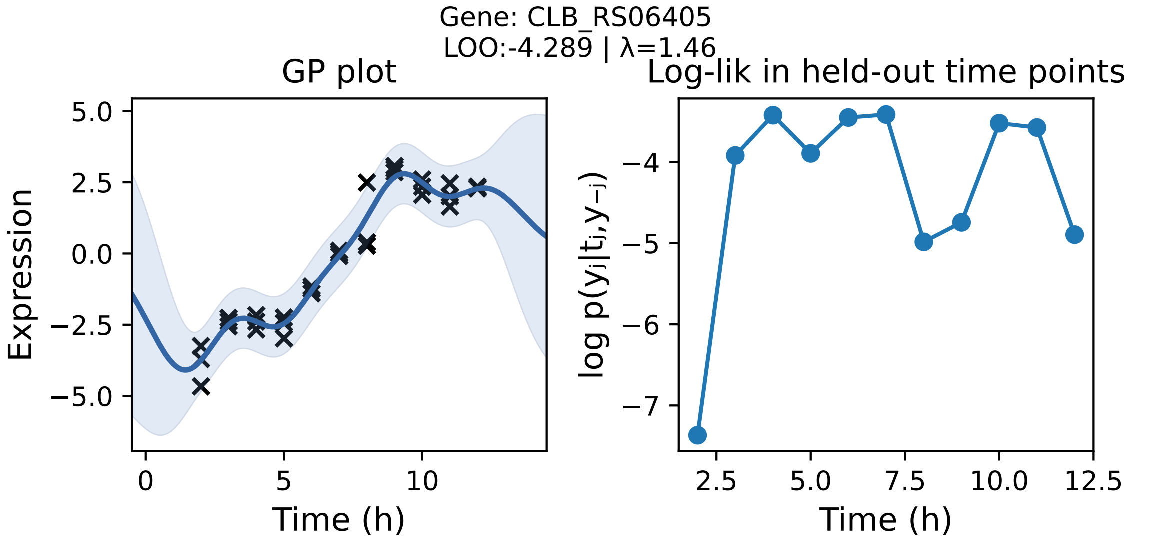
Gene: CLB_RS06405
LOO:-4.289 | λ=1.46
GP plot
Expression
5.0
2.5
0.0
−2.5
−5.0
0
5
10
Time (h)
Log-lik in held-out time points
log p(y_j|t_j,y_−j)
−4
−5
−6
−7
2.5
5.0
7.5
10.0
12.5
Time (h)

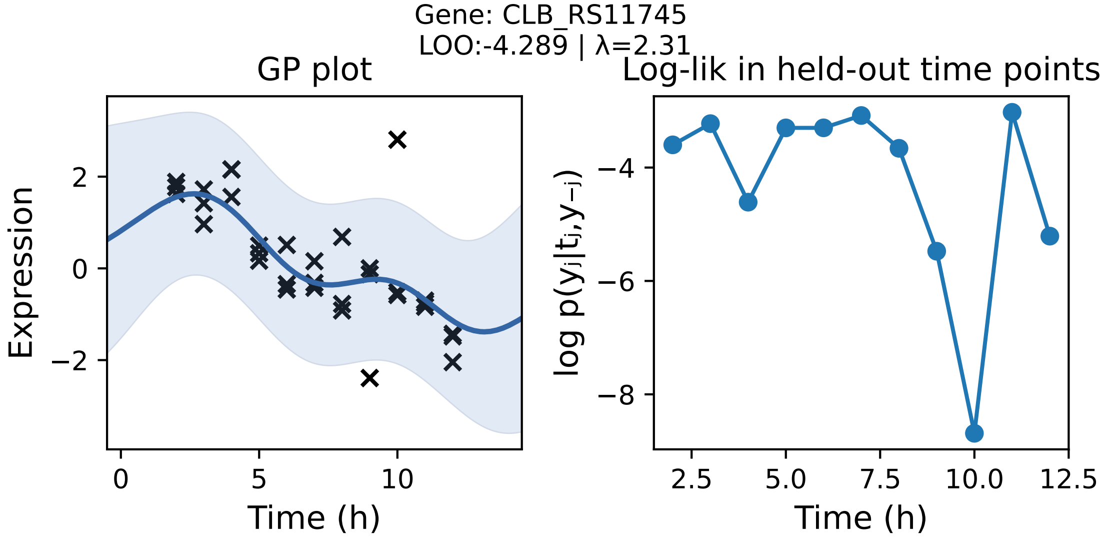

Gene: CLB_RS11745
LOO:-4.289 | λ=2.31
GP plot
Expression
Time (h)
Log-lik in held-out time points
log p(y_j|t_j,y_−j)
Time (h)

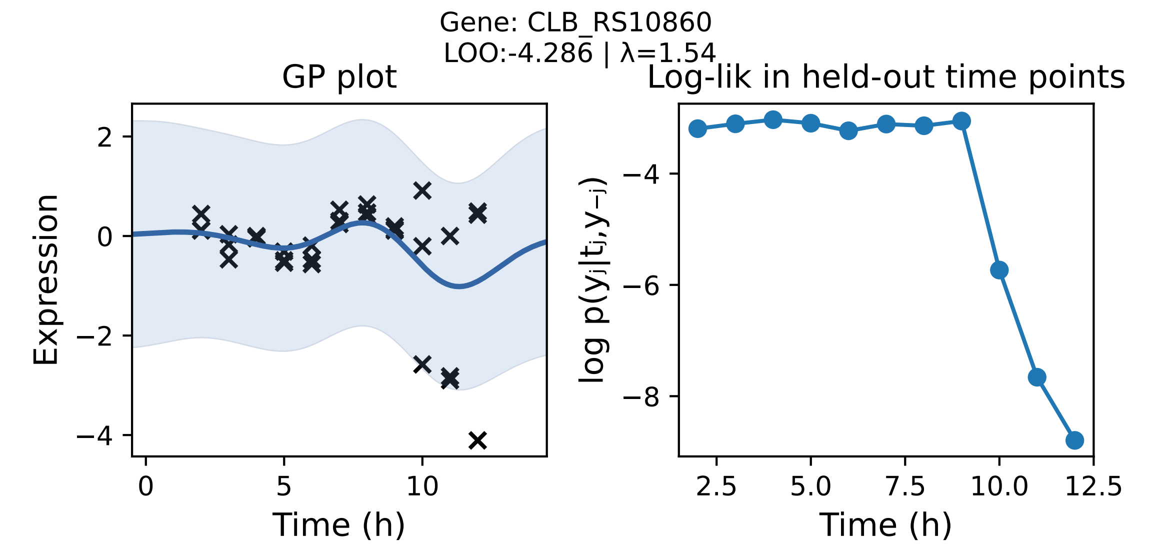

Gene: CLB_RS10860
LOO:-4.286 | λ=1.54
GP plot
Expression
Time (h)
Log-lik in held-out time points
log p(yj|tj,y−j)
Time (h)

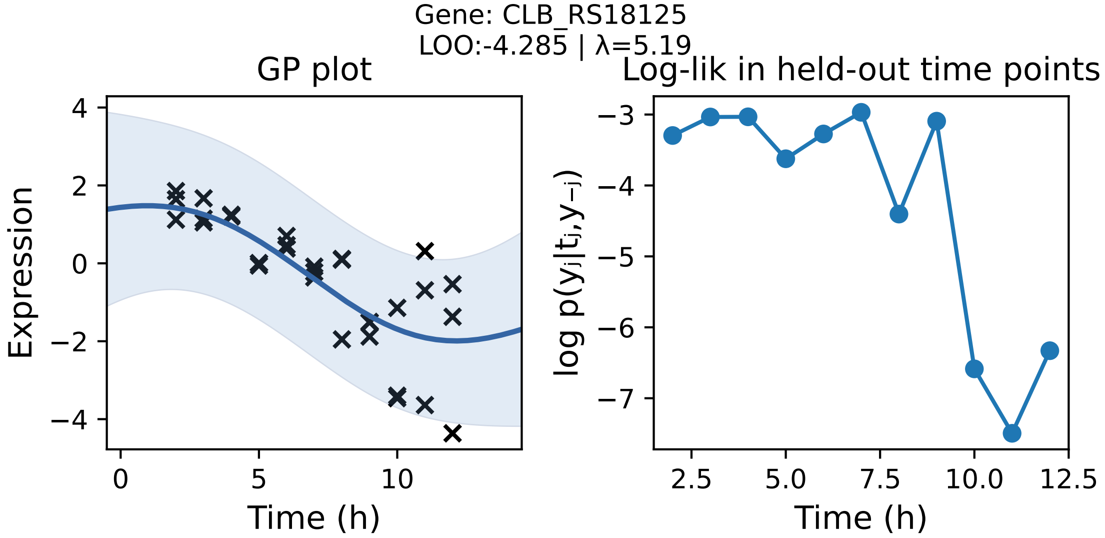

Gene: CLB_RS18125
LOO:-4.285 | λ=5.19
GP plot
Expression
Time (h)
Log-lik in held-out time points
log p(y_j|t_j,y_−j)
Time (h)

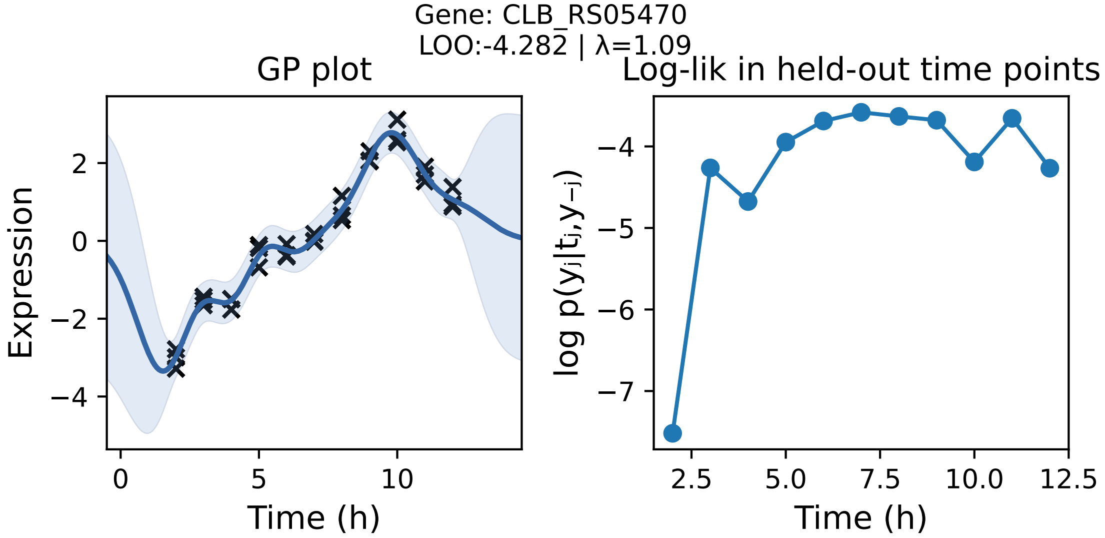

Gene: CLB_RS05470
LOO:-4.282 | λ=1.09
GP plot
Log-lik in held-out time points
Expression
Time (h)
log p(yj|tj,y−j)
Time (h)

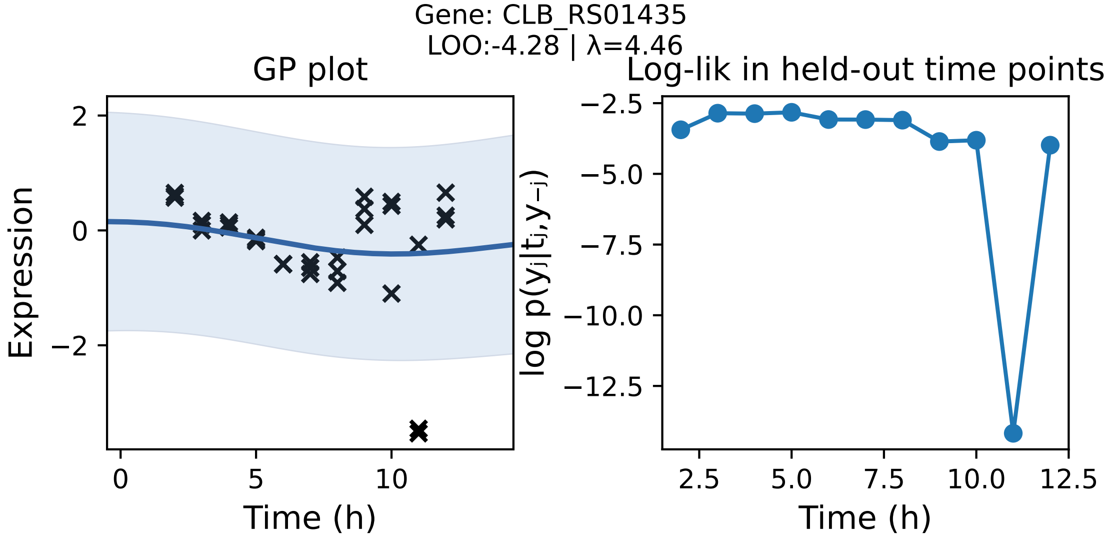

Gene: CLB_RS01435
LOO:-4.28 | λ=4.46
GP plot
Expression
Time (h)
2
0
−2
0
5
10
Log-lik in held-out time points
log p(y_j|t_j,y_−j)
Time (h)
−2.5
−5.0
−7.5
−10.0
−12.5
2.5
5.0
7.5
10.0
12.5

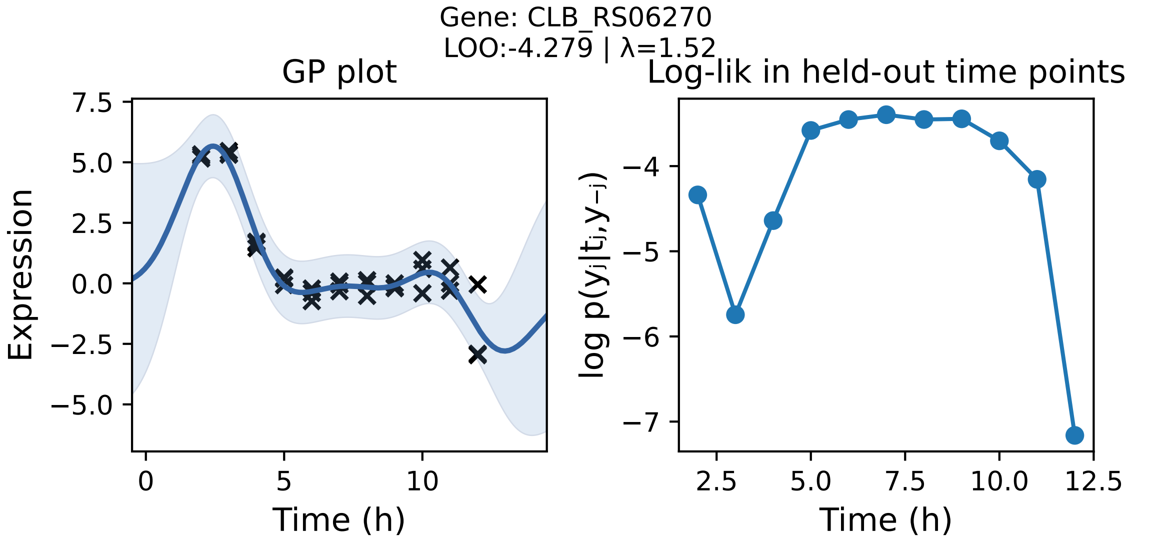

Gene: CLB_RS06270
LOO:-4.279 | λ=1.52
GP plot
Log-lik in held-out time points
Expression
Time (h)
log p(yj|tj,y−j)
Time (h)
7.5
5.0
2.5
0.0
−2.5
−5.0
0
5
10
−4
−5
−6
−7
2.5
5.0
7.5
10.0
12.5

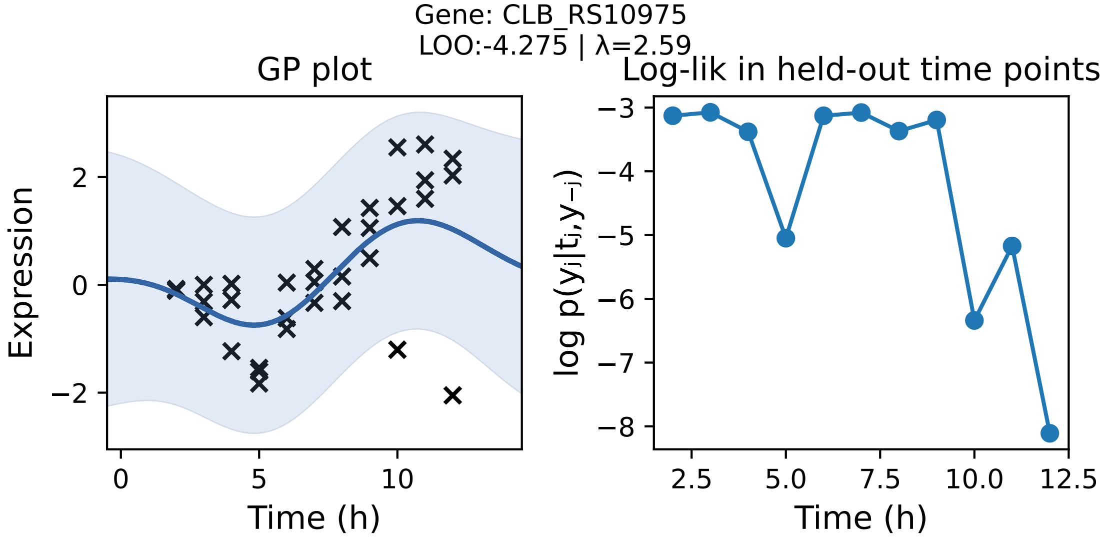

Gene: CLB_RS10975
LOO:-4.275 | λ=2.59
GP plot
Expression
Time (h)
Log-lik in held-out time points
log p(y_j|t_j,y_−j)
Time (h)

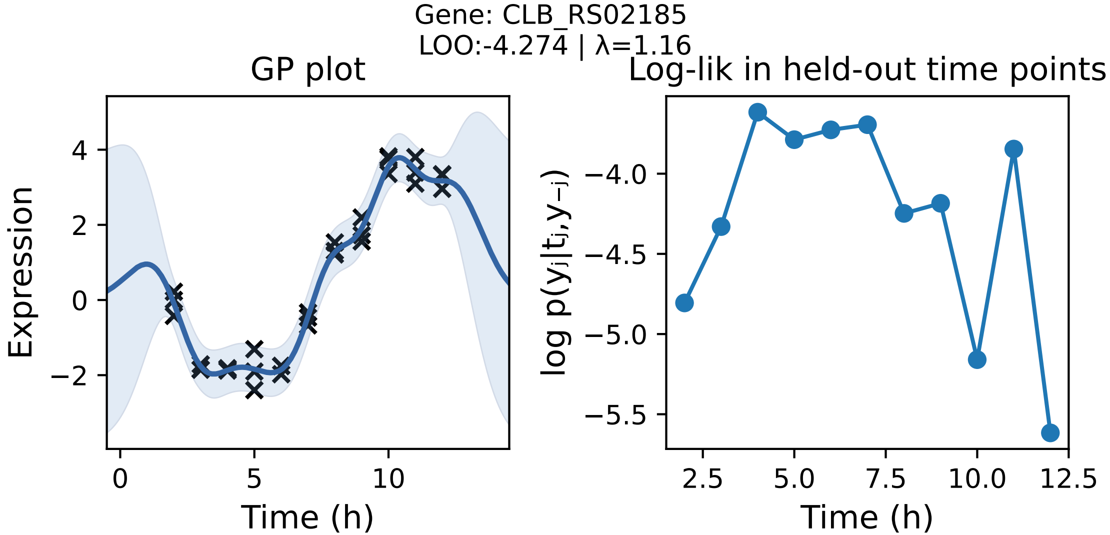
Gene: CLB_RS02185
LOO:-4.274 | λ=1.16
GP plot
Log-lik in held-out time points
Expression
Time (h)
log p(y_j|t_j,y_−j)
Time (h)
4
2
0
−2
0
5
10
−4.0
−4.5
−5.0
−5.5
2.5
5.0
7.5
10.0
12.5

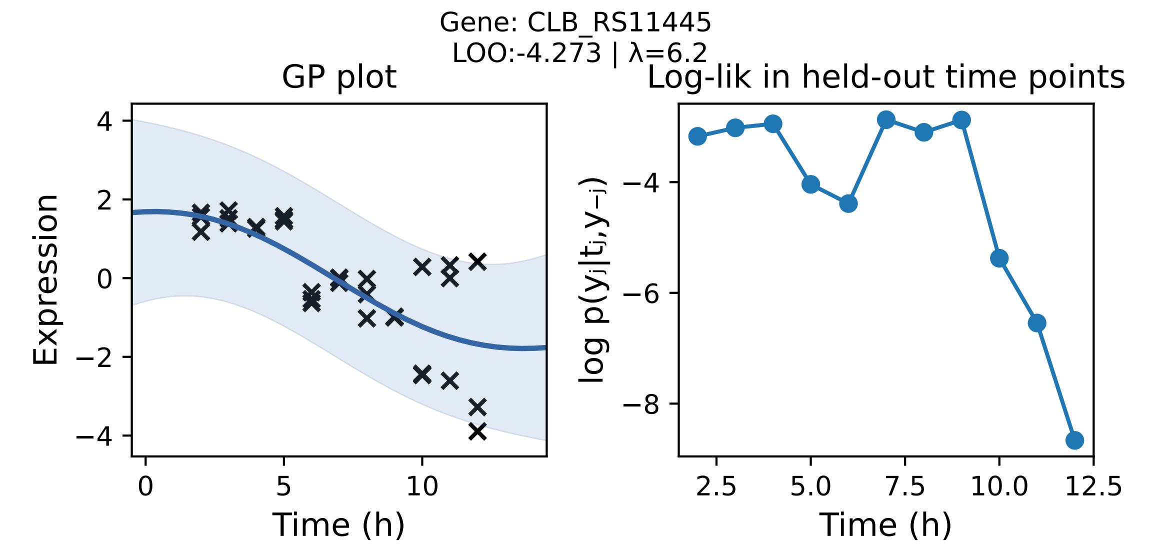
Gene: CLB_RS11445
LOO:-4.273 | λ=6.2
GP plot
Expression
Time (h)
Log-lik in held-out time points
log p(yj|tj,y−j)
Time (h)

Gene: CLB_RS15555
LOO:-4.271 | λ=2.06

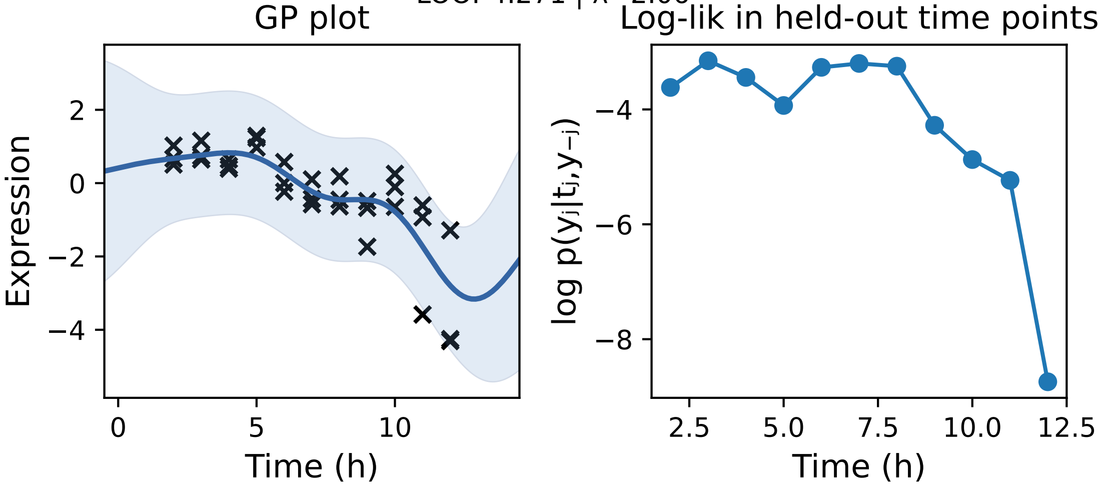

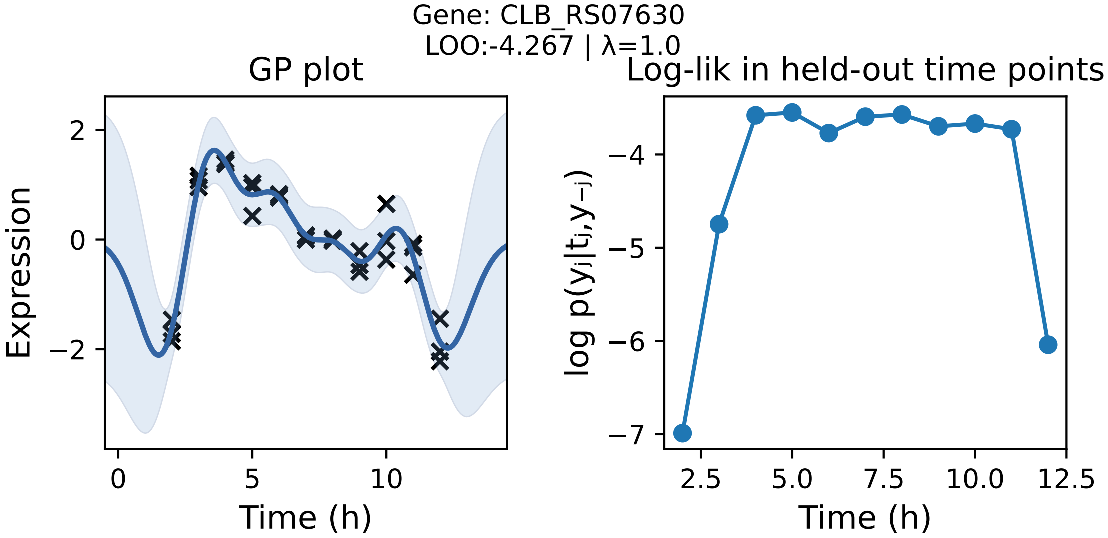
Gene: CLB_RS07630
LOO:-4.267 | λ=1.0
GP plot
Expression
Time (h)
Log-lik in held-out time points
log p(yj|tj,y−j)
Time (h)

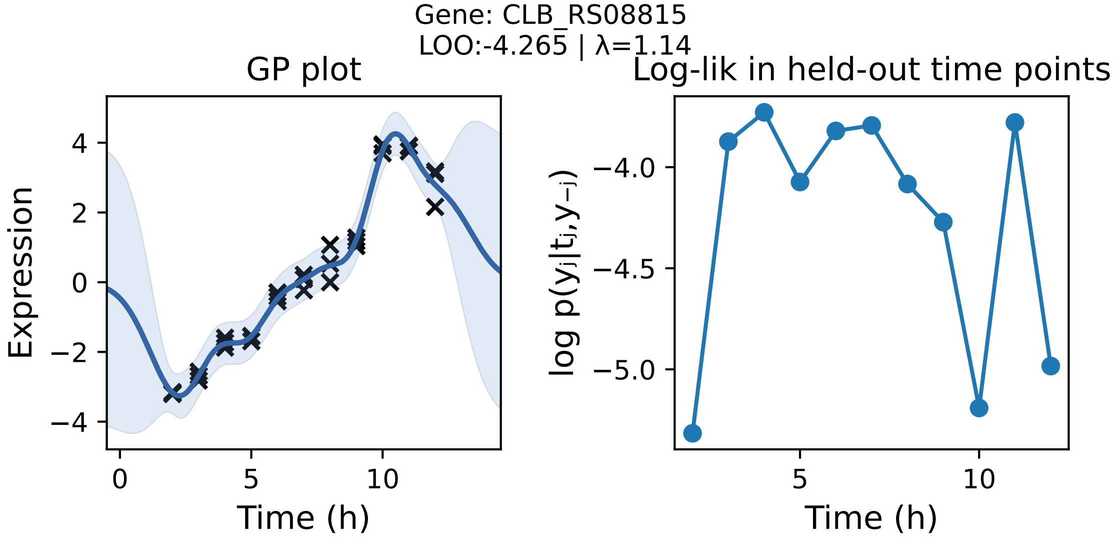

Gene: CLB_RS08815
LOO:-4.265 | λ=1.14
GP plot
Expression
Time (h)
4
2
0
−2
−4
0
5
10
Log-lik in held-out time points
log p(y_j|t_j,y_−j)
−4.0
−4.5
−5.0
5
10
Time (h)

Gene: CLB_RS07465
LOO:-4.264 | λ=1.95

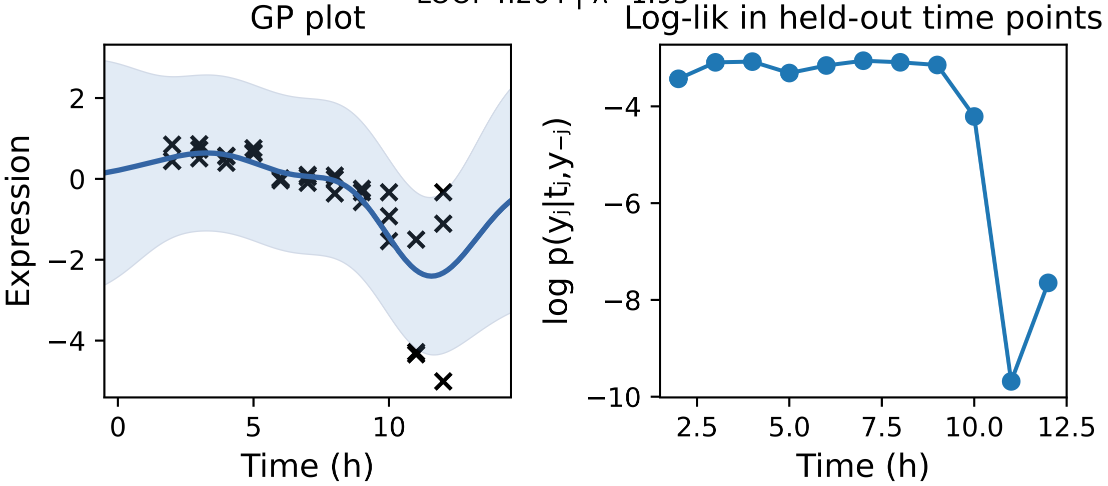

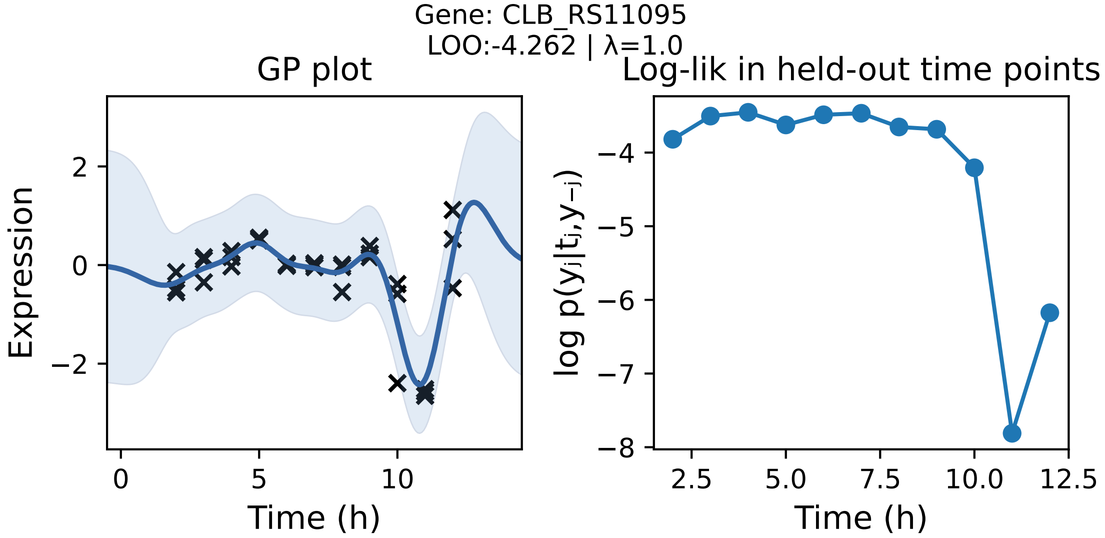

Gene: CLB_RS11095
LOO:-4.262 | λ=1.0
GP plot
Log-lik in held-out time points
Expression
2
0
−2
0
5
10
Time (h)
log p(y_j|t_j,y_−j)
−4
−5
−6
−7
−8
2.5
5.0
7.5
10.0
12.5
Time (h)

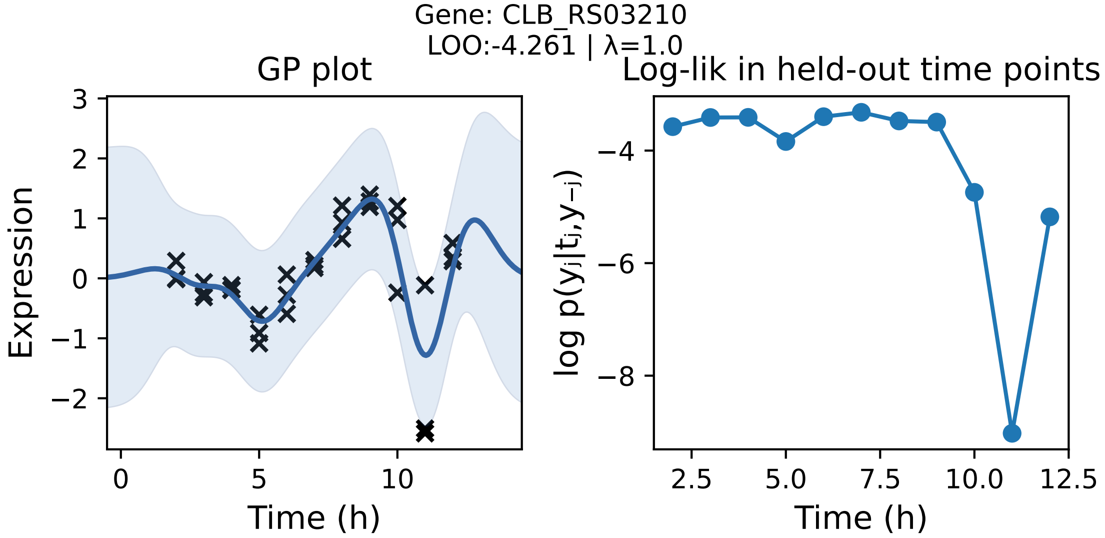

Gene: CLB_RS03210
LOO:-4.261 | λ=1.0
GP plot
Expression
Time (h)
Log-lik in held-out time points
log p(y_j|t_j,y_−j)
Time (h)

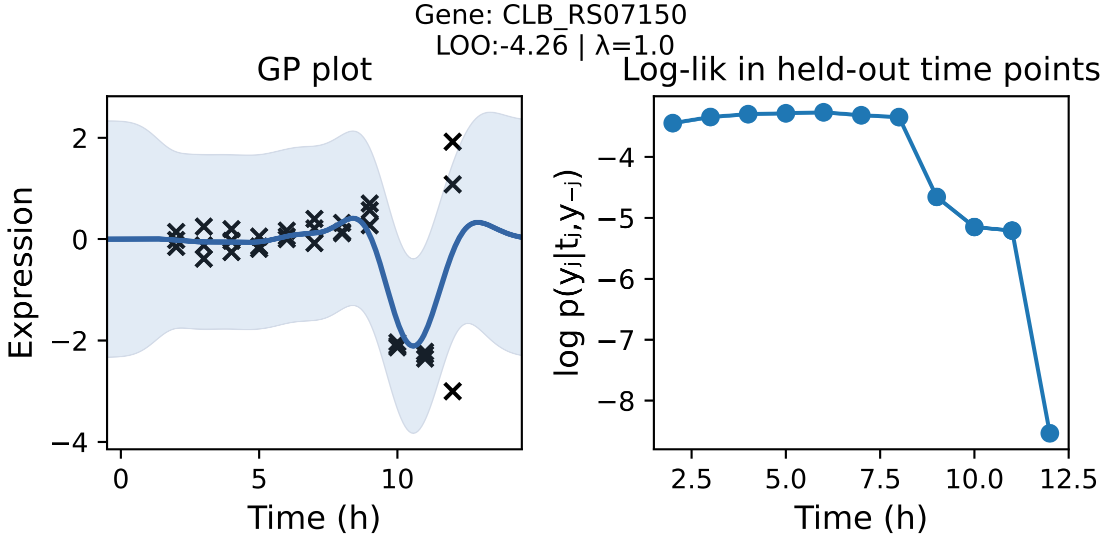

Gene: CLB_RS07150
LOO:-4.26 | λ=1.0
GP plot
Expression
Time (h)
Log-lik in held-out time points
log p(y_j|t_j,y_−j)
Time (h)

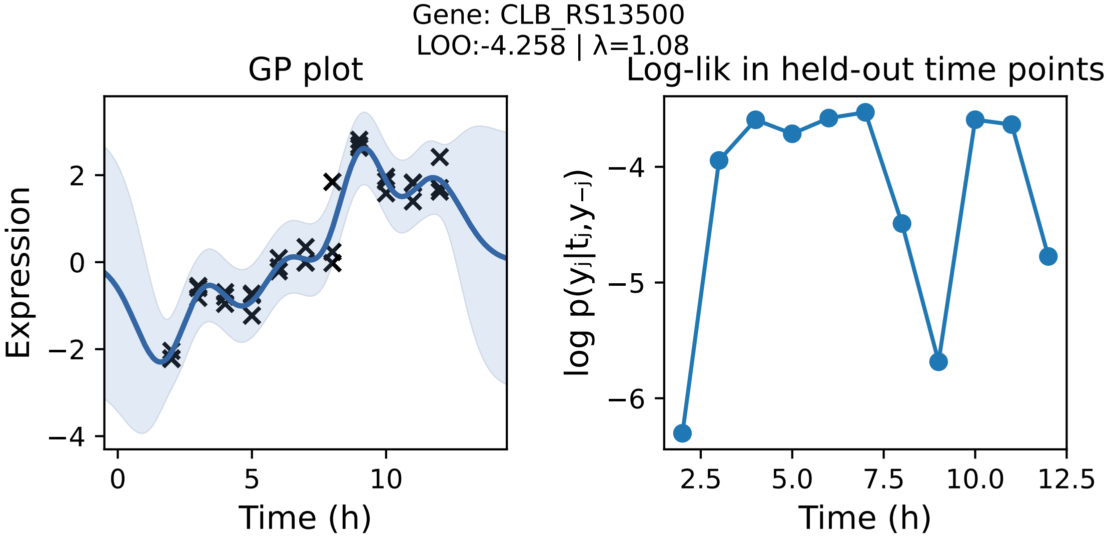

Gene: CLB_RS13500
LOO:-4.258 | λ=1.08
GP plot
Expression
Time (h)
Log-lik in held-out time points
log p(y_j|t_j,y_−j)
Time (h)

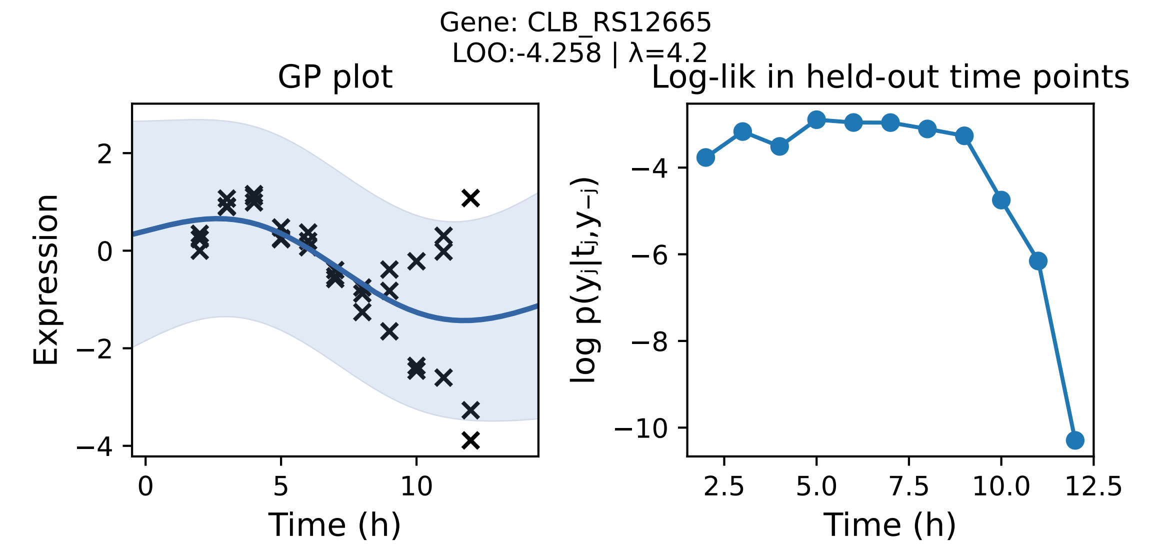
Gene: CLB_RS12665
LOO:-4.258 | λ=4.2
GP plot
Expression
Time (h)
2
0
−2
−4
0
5
10
Log-lik in held-out time points
log p(y_j|t_j,y_−j)
Time (h)
−4
−6
−8
−10
2.5
5.0
7.5
10.0
12.5

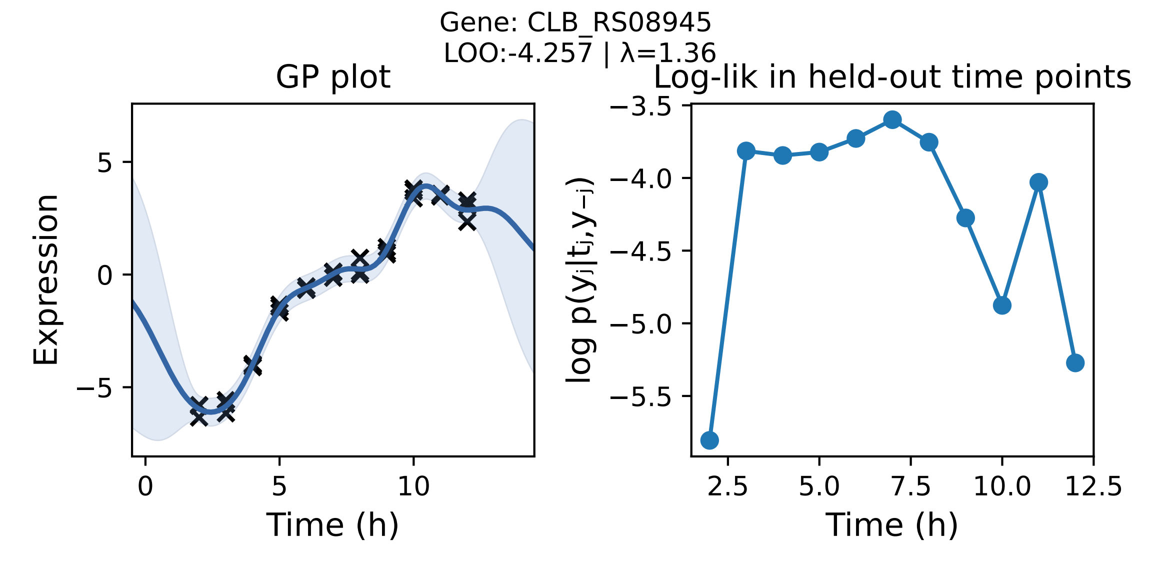

Gene: CLB_RS08945
LOO:-4.257 | λ=1.36
GP plot
Expression
Time (h)
Log-lik in held-out time points
log p(y_j|t_j,y_−j)
Time (h)
5
0
−5
0
5
10
−3.5
−4.0
−4.5
−5.0
−5.5
2.5
5.0
7.5
10.0
12.5

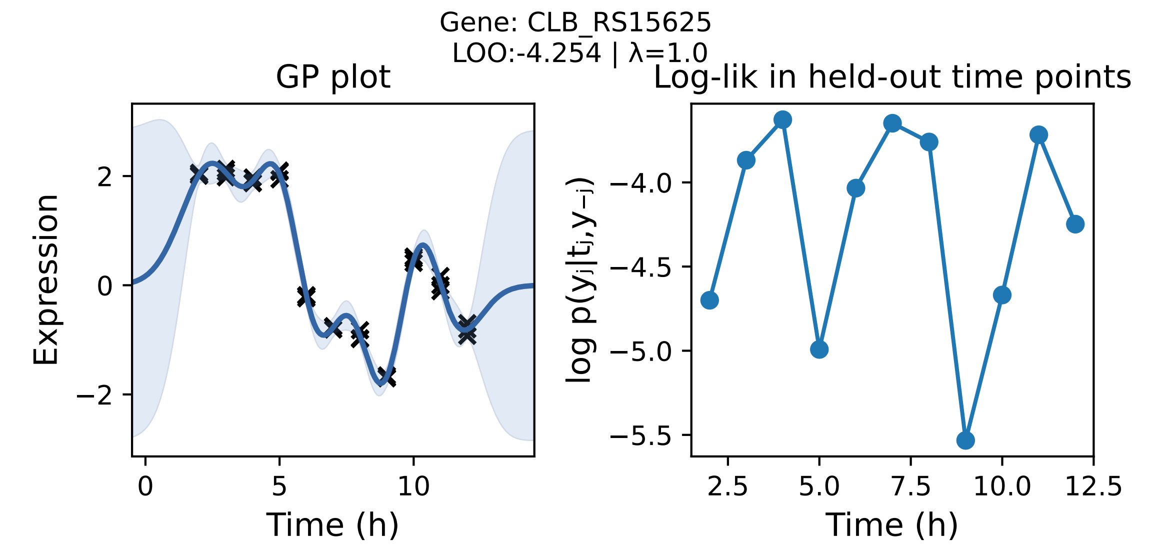

Gene: CLB_RS15625
LOO:-4.254 | λ=1.0
GP plot
Expression
Time (h)
2
0
−2
0
5
10
Log-lik in held-out time points
log p(y_j|t_j,y_−j)
Time (h)
−4.0
−4.5
−5.0
−5.5
2.5
5.0
7.5
10.0
12.5

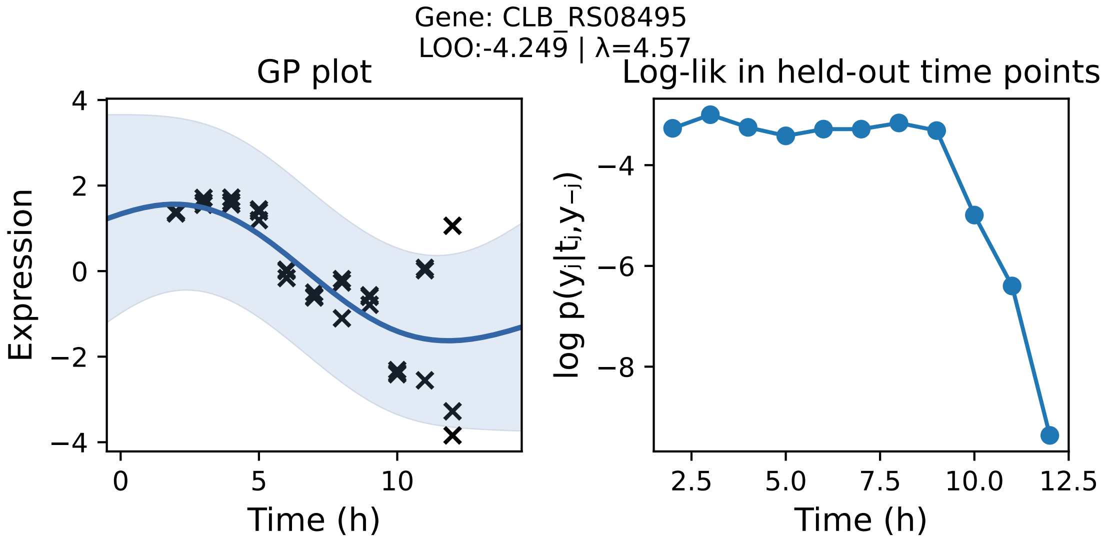
Gene: CLB_RS08495
LOO:-4.249 | λ=4.57
GP plot
Expression
Time (h)
Log-lik in held-out time points
log p(yj|tj,y−j)
Time (h)

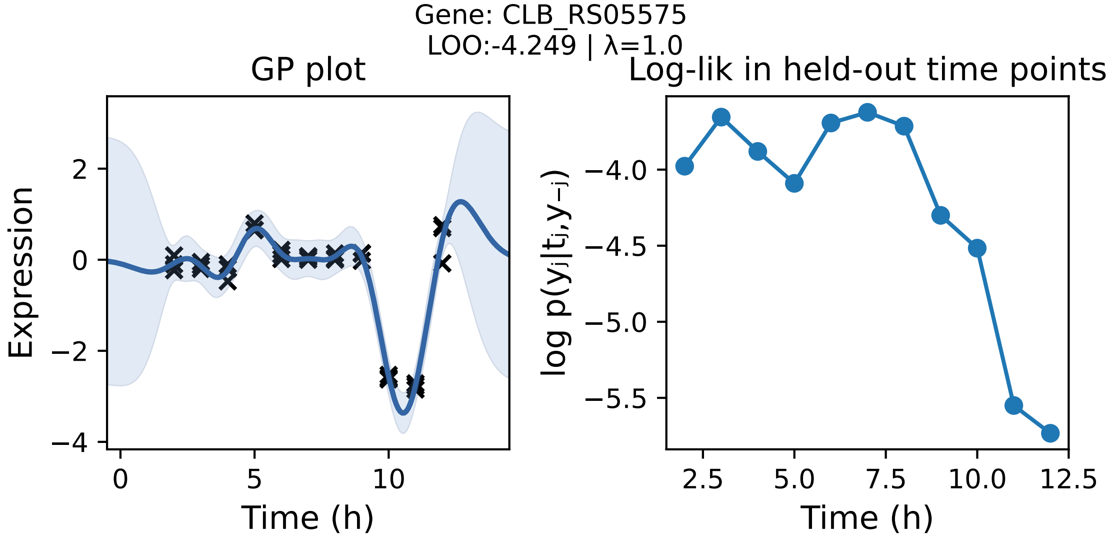
Gene: CLB_RS05575
LOO:-4.249 | λ=1.0
GP plot
Expression
Time (h)
Log-lik in held-out time points
log p(y_j|t_j,y_−j)
Time (h)

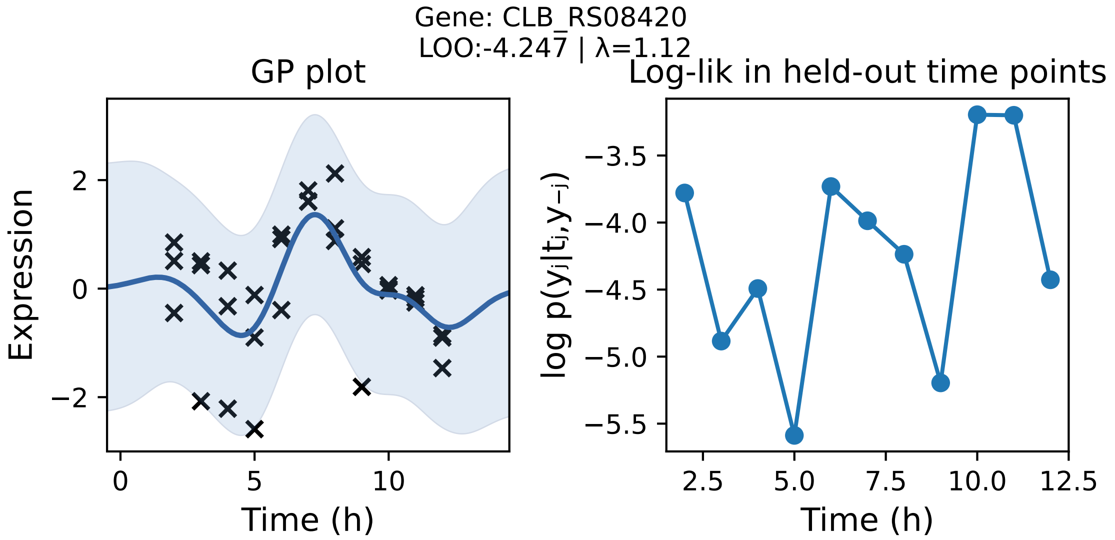

Gene: CLB_RS08420
LOO:-4.247 | λ=1.12
GP plot
Expression
Time (h)
Log-lik in held-out time points
log p(y_j|t_j,y_−j)
Time (h)

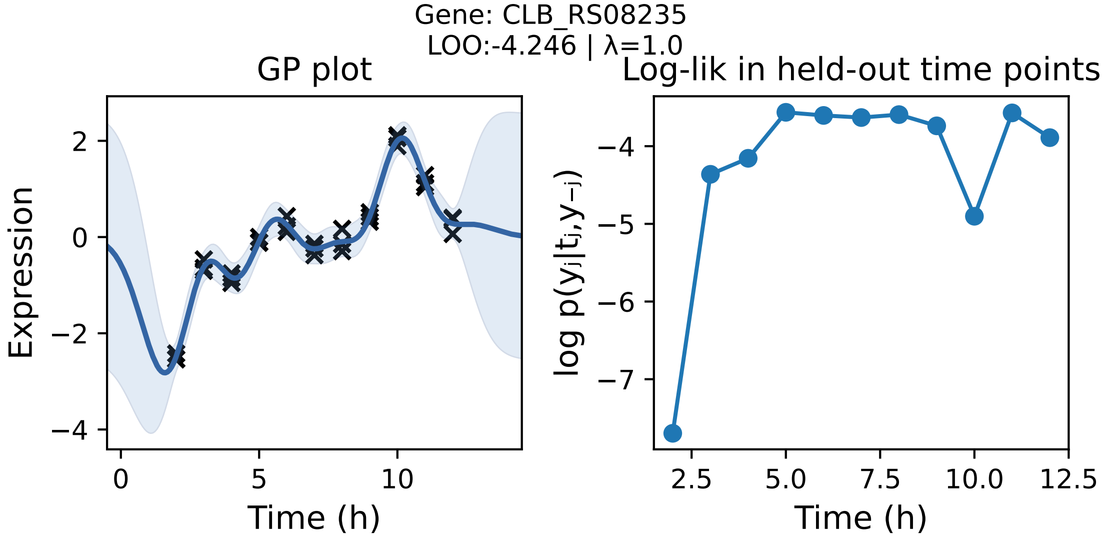

Gene: CLB_RS08235
LOO:-4.246 | λ=1.0
GP plot
Expression
Time (h)
Log-lik in held-out time points
log p(yj|tj,y−j)
Time (h)

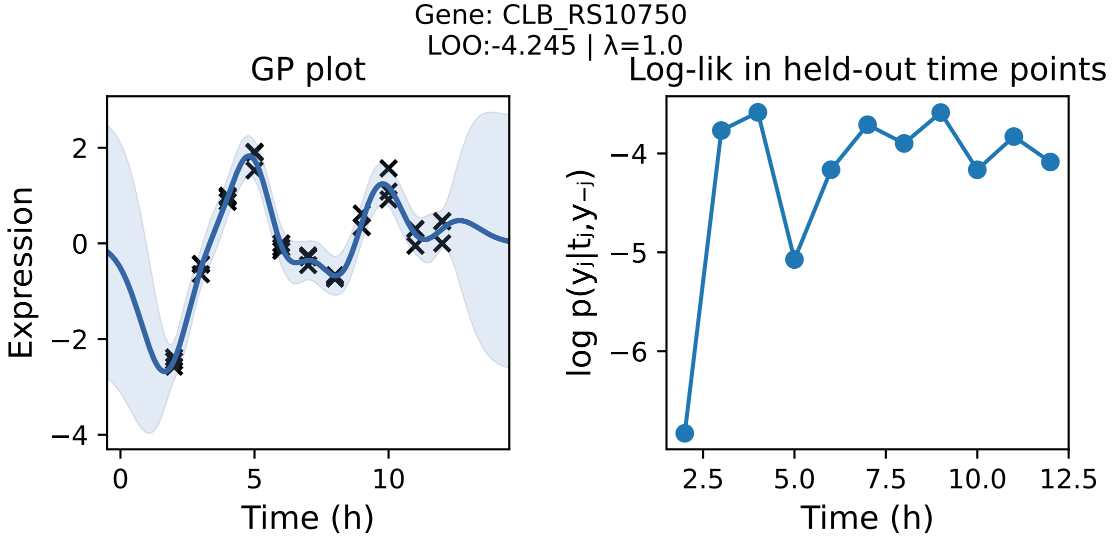

Gene: CLB_RS10750
LOO:-4.245 | λ=1.0
GP plot
Expression
Time (h)
Log-lik in held-out time points
log p(y_j|t_j,y_−j)
Time (h)

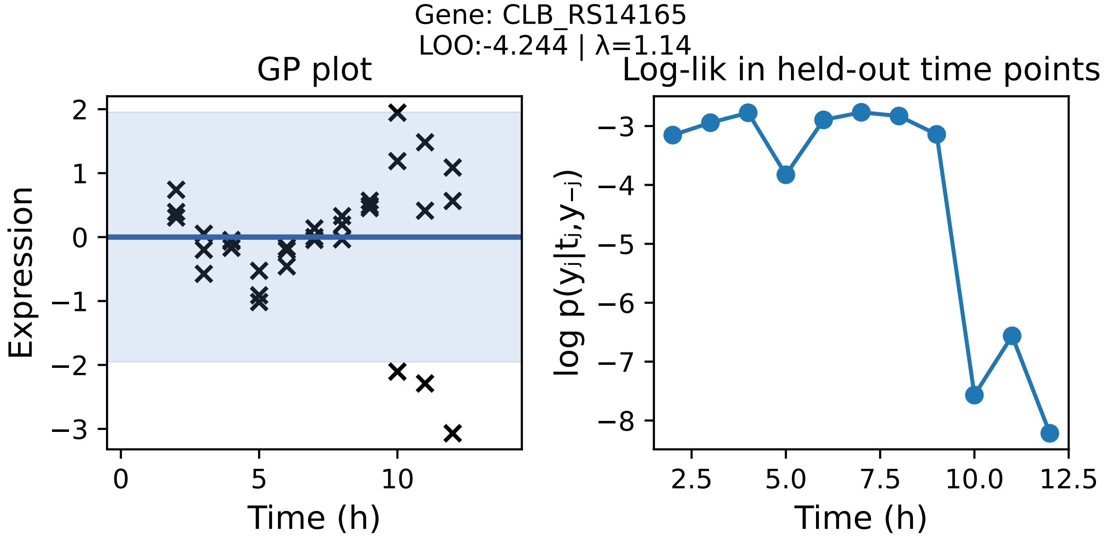

Gene: CLB_RS14165
LOO:-4.244 | λ=1.14
GP plot
Log-lik in held-out time points
Expression
Time (h)
log p(y_j|t_j,y_−j)
Time (h)
2
1
0
−1
−2
−3
0
5
10
−3
−4
−5
−6
−7
−8
2.5
5.0
7.5
10.0
12.5

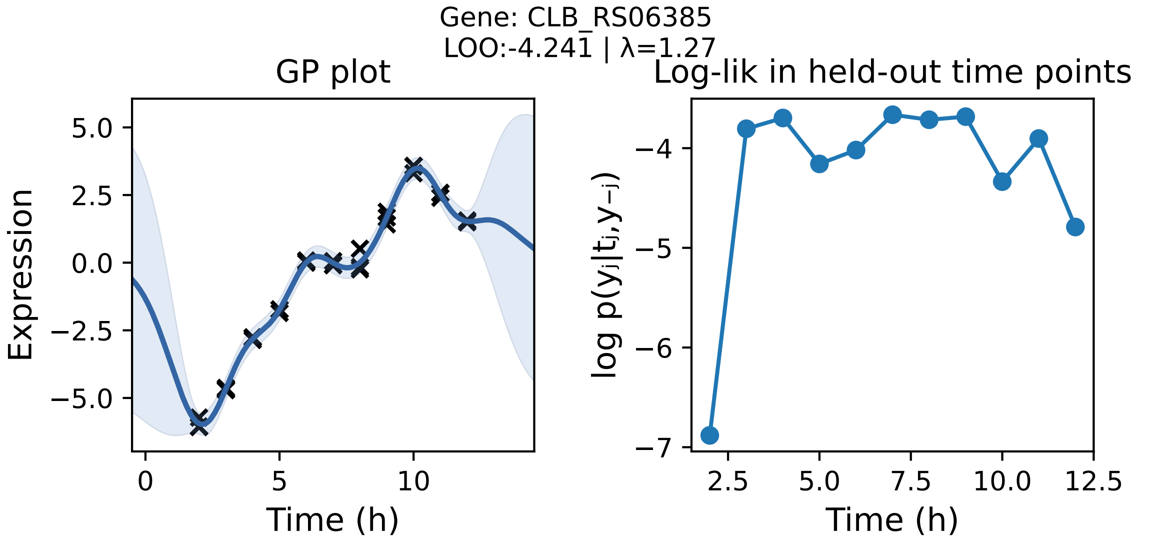

Gene: CLB_RS06385
LOO:-4.241 | λ=1.27
GP plot
Expression
Time (h)
5.0
2.5
0.0
−2.5
−5.0
0
5
10
Log-lik in held-out time points
log p(y_j|t_j,y_−j)
−4
−5
−6
−7
2.5
5.0
7.5
10.0
12.5
Time (h)

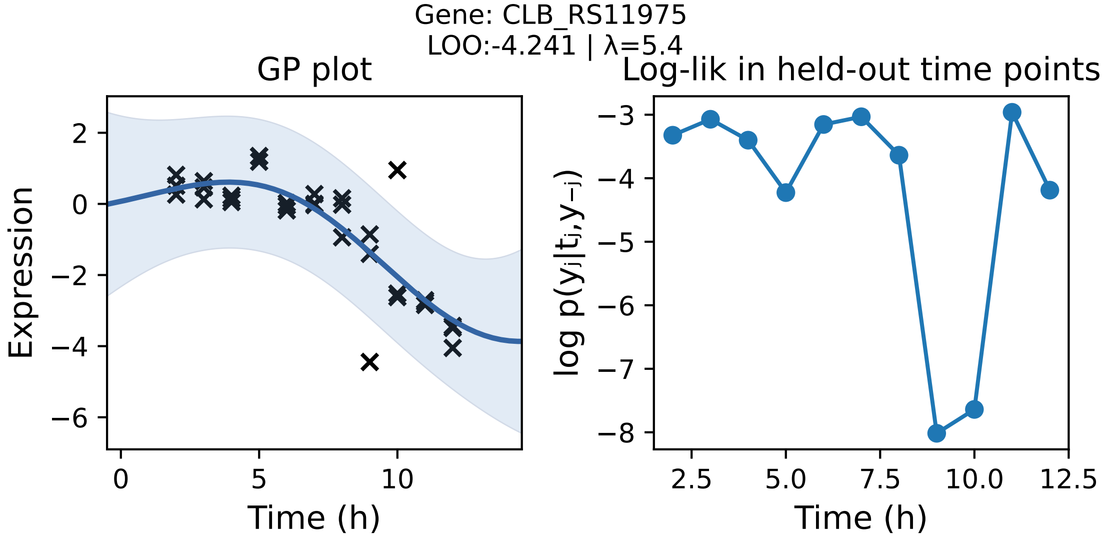

Gene: CLB_RS11975
LOO:-4.241 | λ=5.4
GP plot
Expression
Time (h)
Log-lik in held-out time points
log p(y_j|t_j,y_−j)
Time (h)

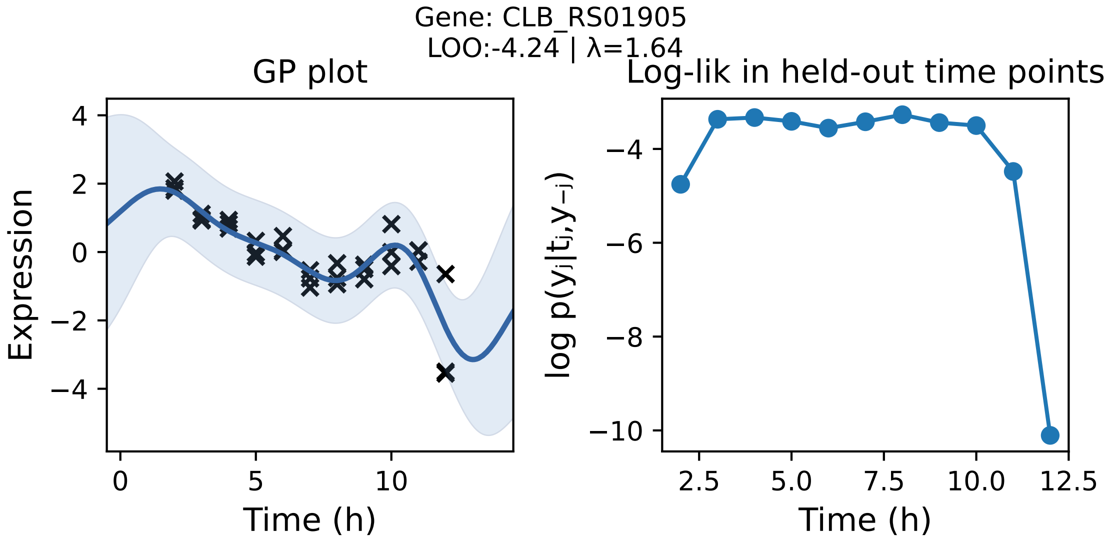

Gene: CLB_RS01905
LOO:-4.24 | λ=1.64
GP plot
Expression
Time (h)
Log-lik in held-out time points
log p(yj|tj,y−j)
Time (h)

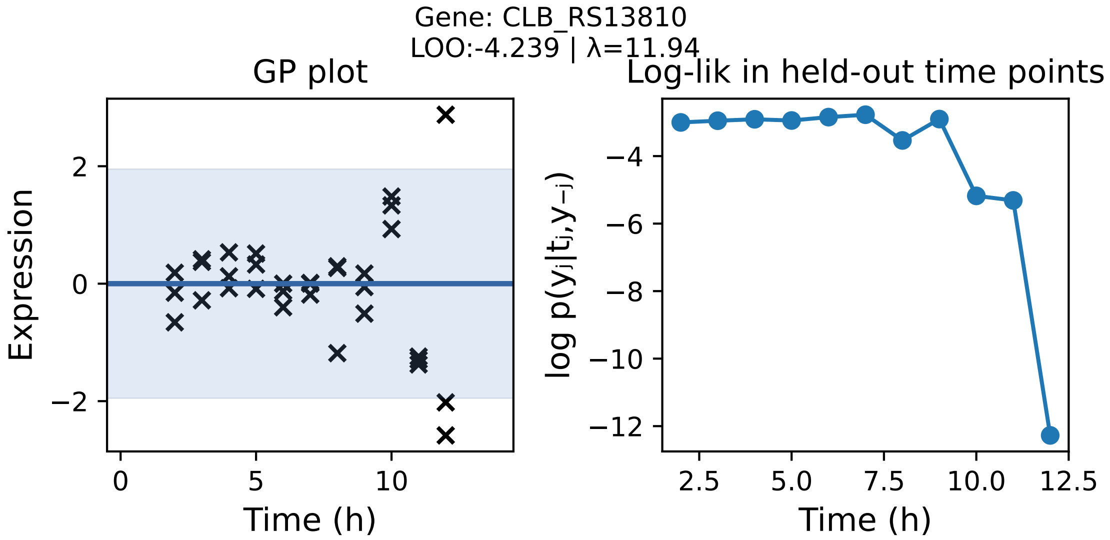

Gene: CLB_RS13810
LOO:-4.239 | λ=11.94
GP plot
Expression
Time (h)
0
5
10
2
0
−2
Log-lik in held-out time points
log p(y_j|t_j,y_−j)
Time (h)
2.5
5.0
7.5
10.0
12.5
−4
−6
−8
−10
−12

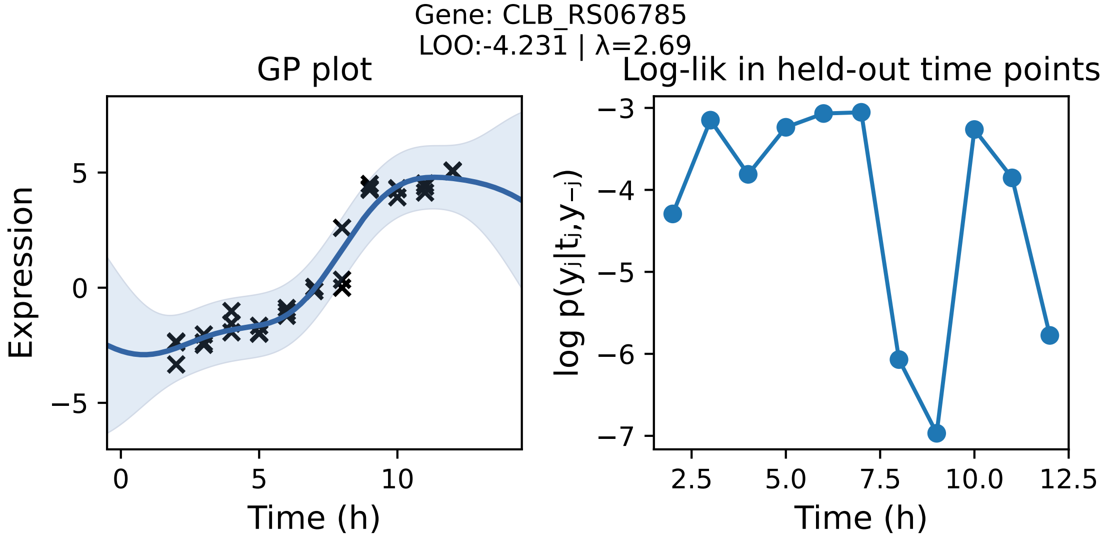
Gene: CLB_RS06785
LOO:-4.231 | λ=2.69
GP plot
Expression
5
0
−5
0
5
10
Time (h)
Log-lik in held-out time points
log p(y_j|t_j,y_−j)
−3
−4
−5
−6
−7
2.5
5.0
7.5
10.0
12.5
Time (h)

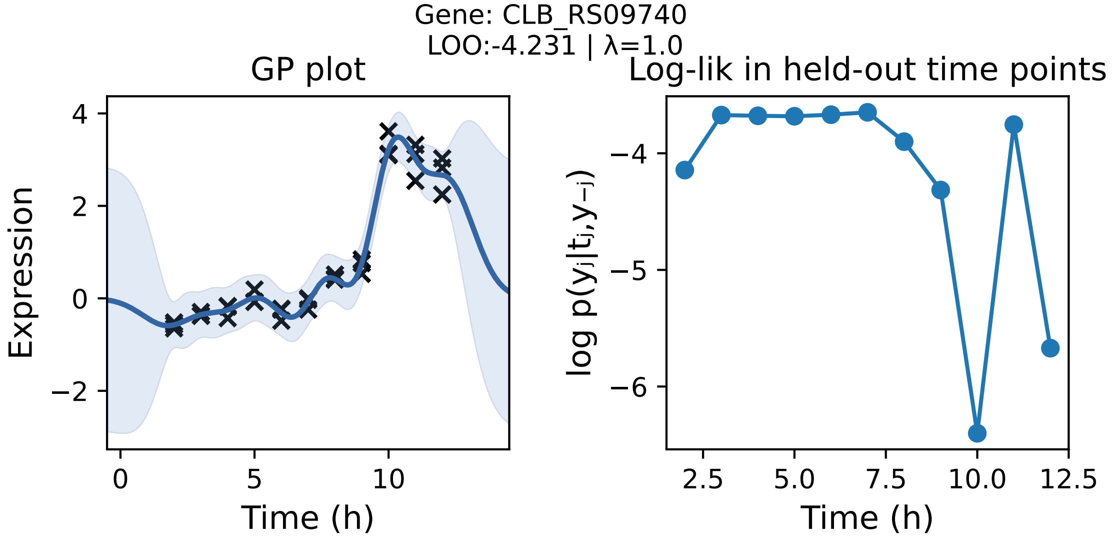
Gene: CLB_RS09740
LOO:-4.231 | λ=1.0
GP plot
Expression
Time (h)
Log-lik in held-out time points
log p(y_j|t_j,y_-j)
Time (h)

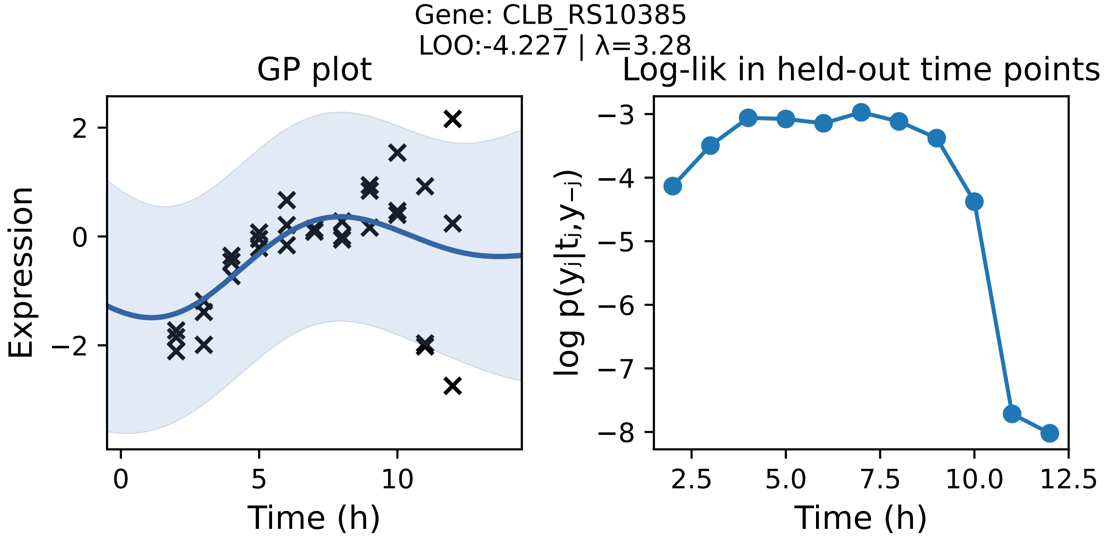

Gene: CLB_RS10385
LOO:-4.227 | λ=3.28
GP plot
Expression
Time (h)
Log-lik in held-out time points
log p(y_j|t_j,y_−j)
Time (h)

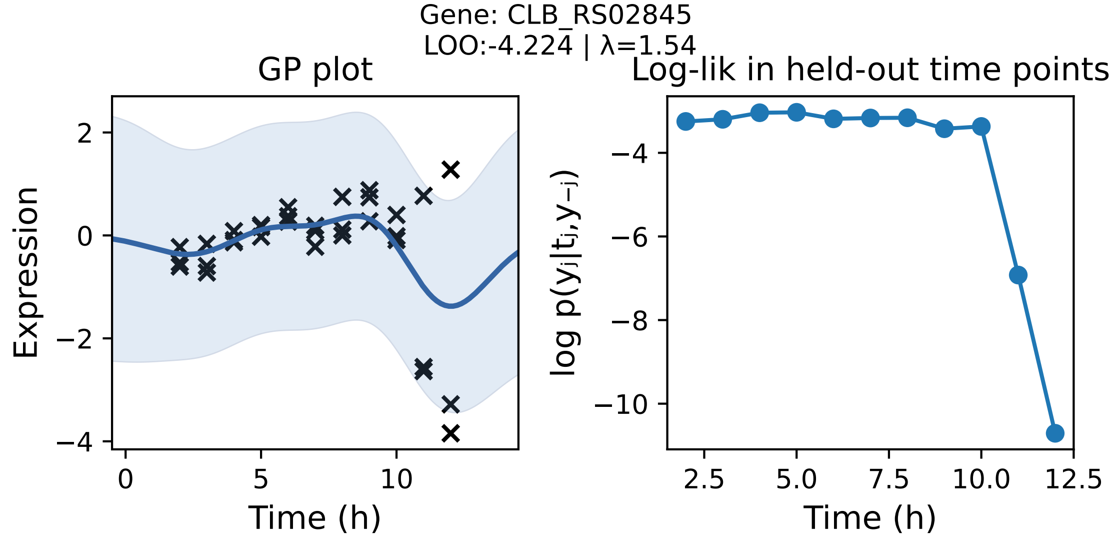

Gene: CLB_RS02845
LOO:-4.224 | λ=1.54
GP plot
Expression
Time (h)
2
0
−2
−4
0
5
10
Log-lik in held-out time points
log p(yⱼ|tⱼ,y₋ⱼ)
Time (h)
−4
−6
−8
−10
2.5
5.0
7.5
10.0
12.5

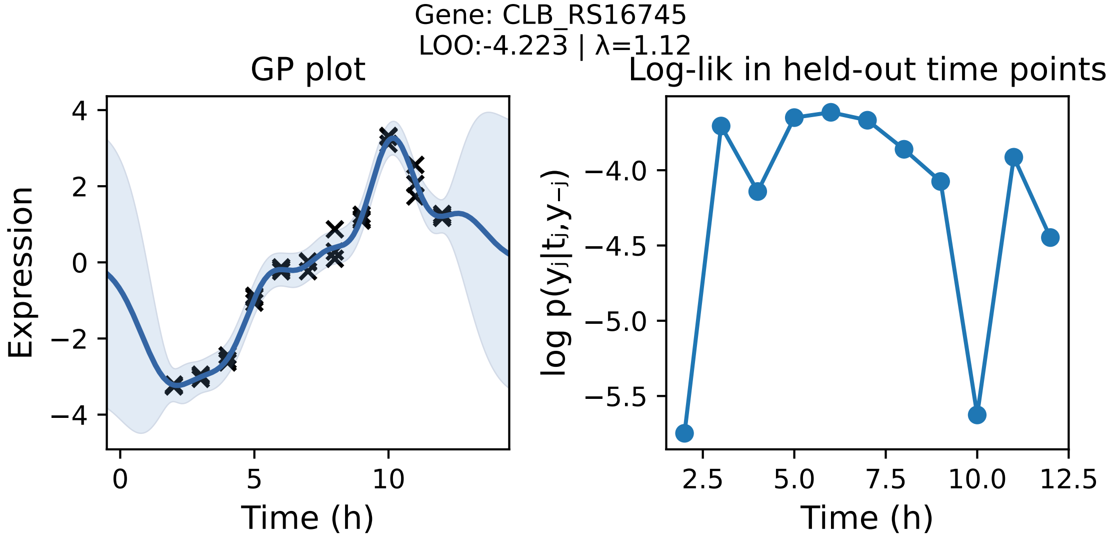

Gene: CLB_RS16745
LOO:-4.223 | λ=1.12
GP plot
Expression
Time (h)
Log-lik in held-out time points
log p(y_j|t_j,y_−j)
Time (h)

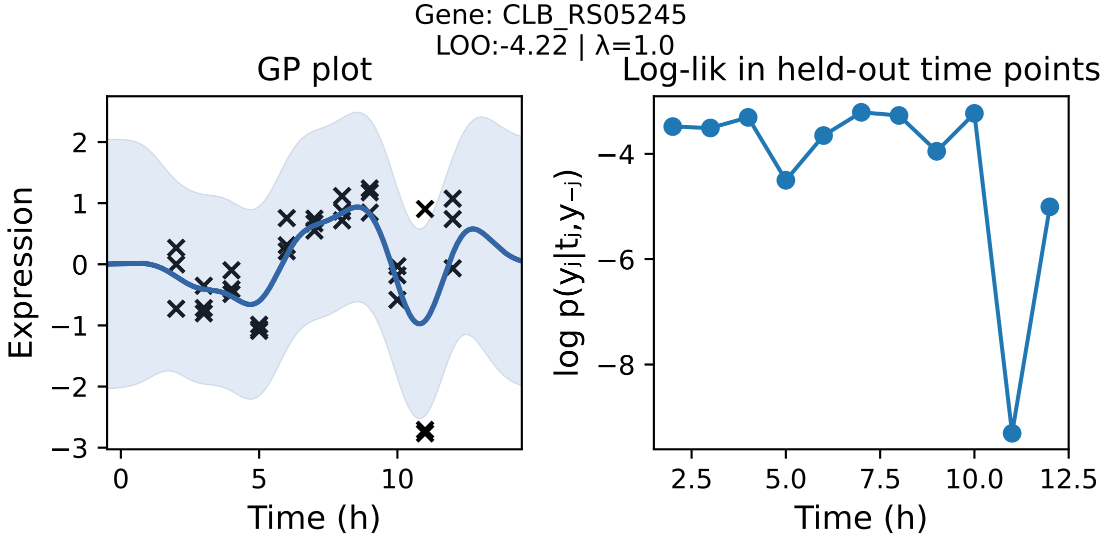

Gene: CLB_RS05245
LOO:-4.22 | λ=1.0
GP plot
Log-lik in held-out time points
Expression
Time (h)
2
1
0
−1
−2
−3
0
5
10
log p(yj|tj,y−j)
−4
−6
−8
2.5
5.0
7.5
10.0
12.5
Time (h)

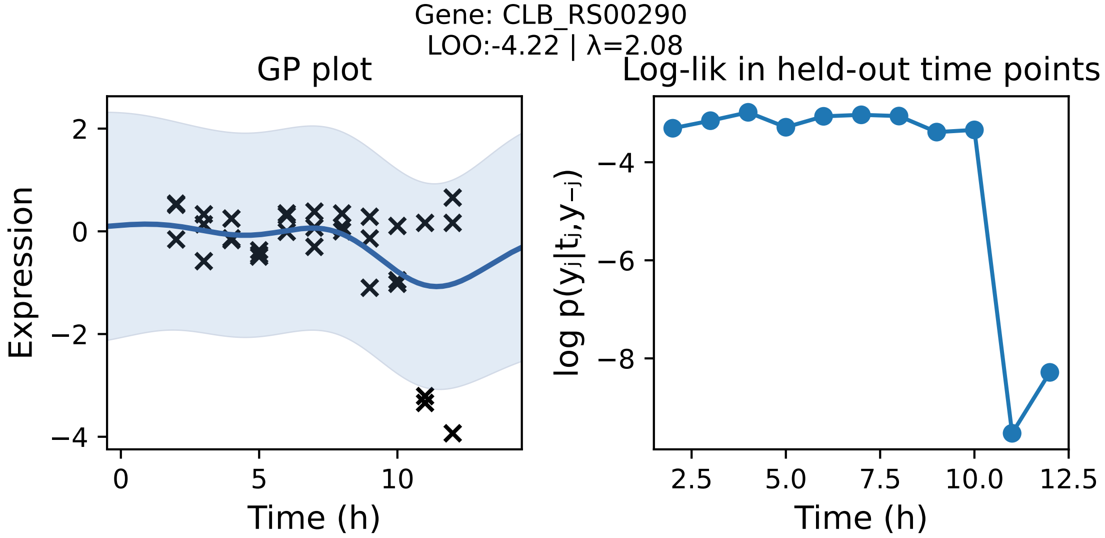

Gene: CLB_RS00290
LOO:-4.22 | λ=2.08
GP plot
Expression
Time (h)
2
0
−2
−4
0
5
10
Log-lik in held-out time points
$\log p(y_j|t_j, y_{-j})$
Time (h)
−4
−6
−8
2.5
5.0
7.5
10.0
12.5

Gene: CLB_RS02285
LOO:-4.215 | λ=1.0

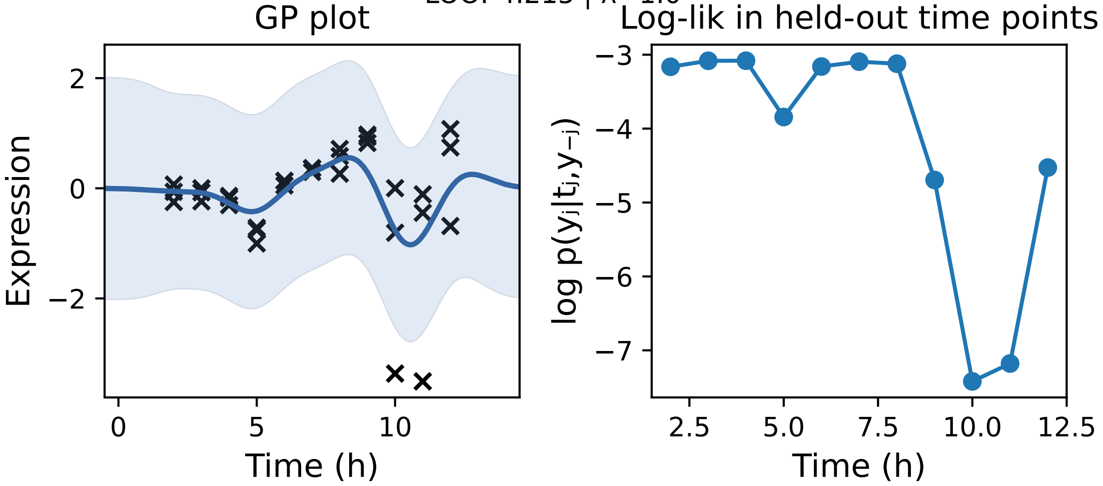

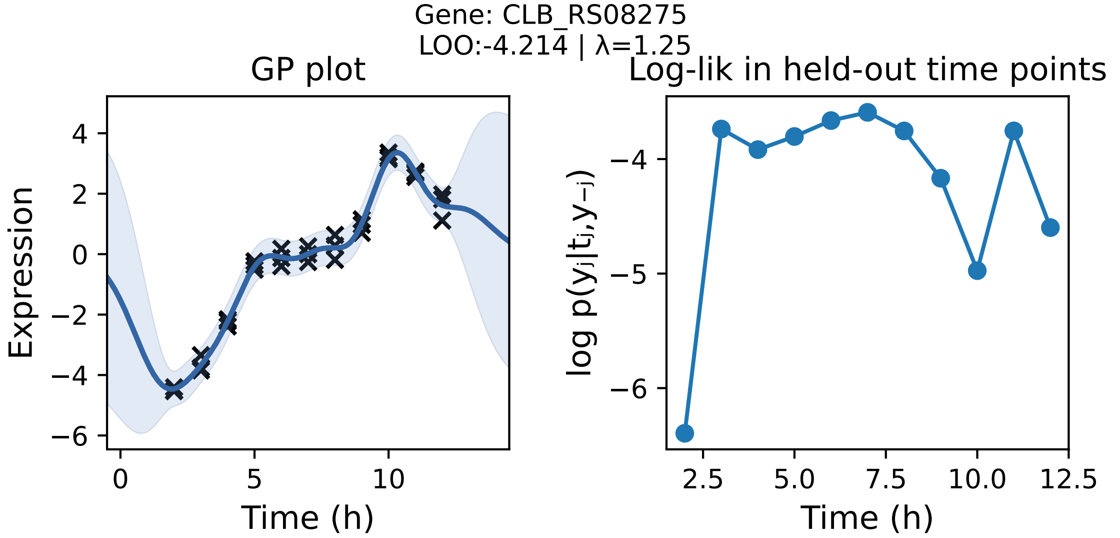

Gene: CLB_RS08275
LOO:-4.214 | λ=1.25
GP plot
Expression
Time (h)
Log-lik in held-out time points
log p(y_j|t_j,y_−j)
Time (h)

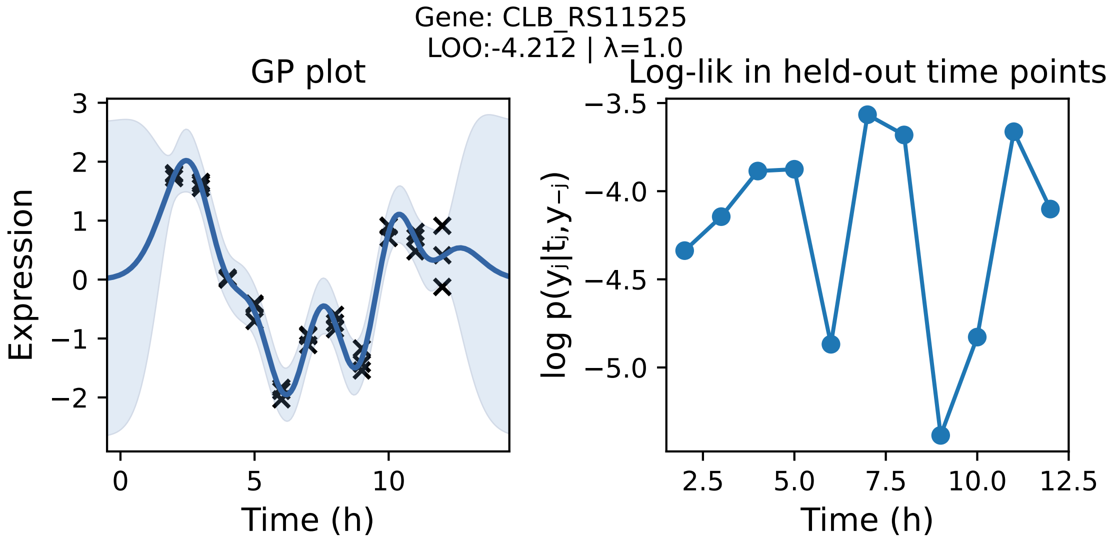
Gene: CLB_RS11525
LOO:-4.212 | λ=1.0
GP plot
Expression
Time (h)
3
2
1
0
−1
−2
0
5
10
Log-lik in held-out time points
log p(y_j|t_j,y_−j)
Time (h)
−3.5
−4.0
−4.5
−5.0
2.5
5.0
7.5
10.0
12.5

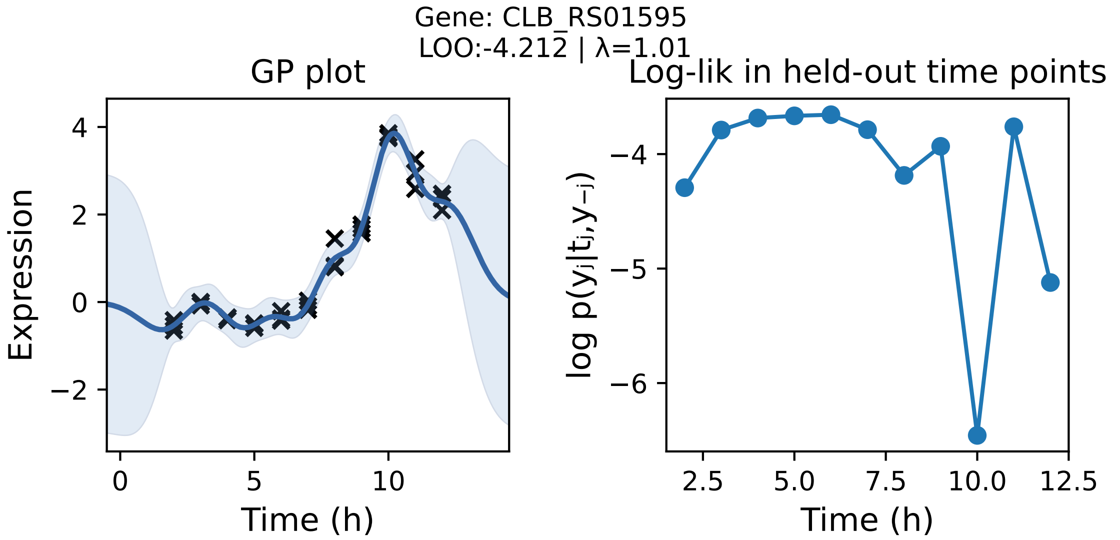

Gene: CLB_RS01595
LOO:-4.212 | λ=1.01
GP plot
Expression
Time (h)
Log-lik in held-out time points
log p(yj|tj,y−j)
Time (h)

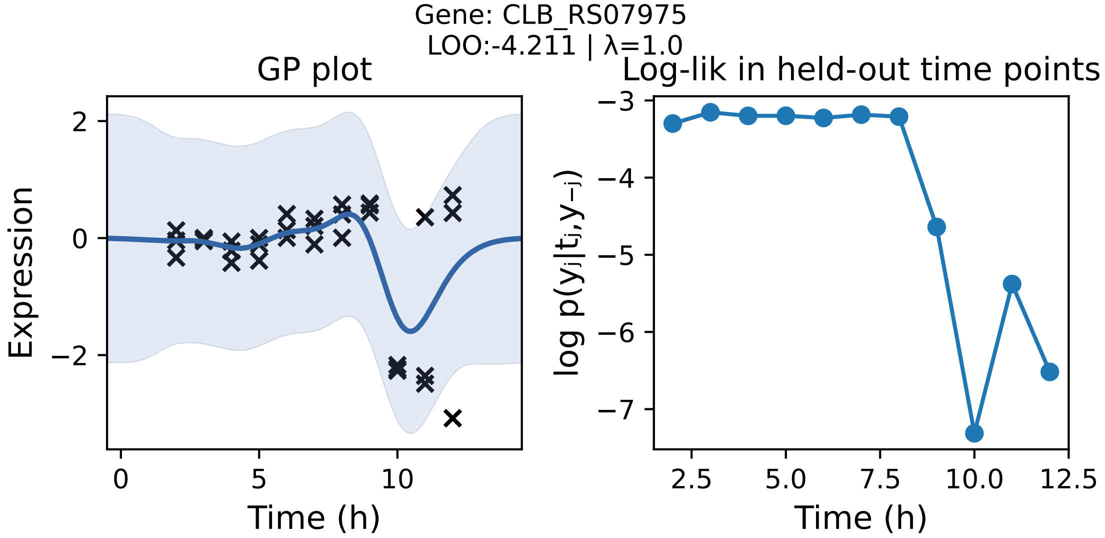

Gene: CLB_RS07975
LOO:-4.211 | λ=1.0
GP plot
Expression
Time (h)
Log-lik in held-out time points
log p(y_j|t_j,y_−j)
Time (h)

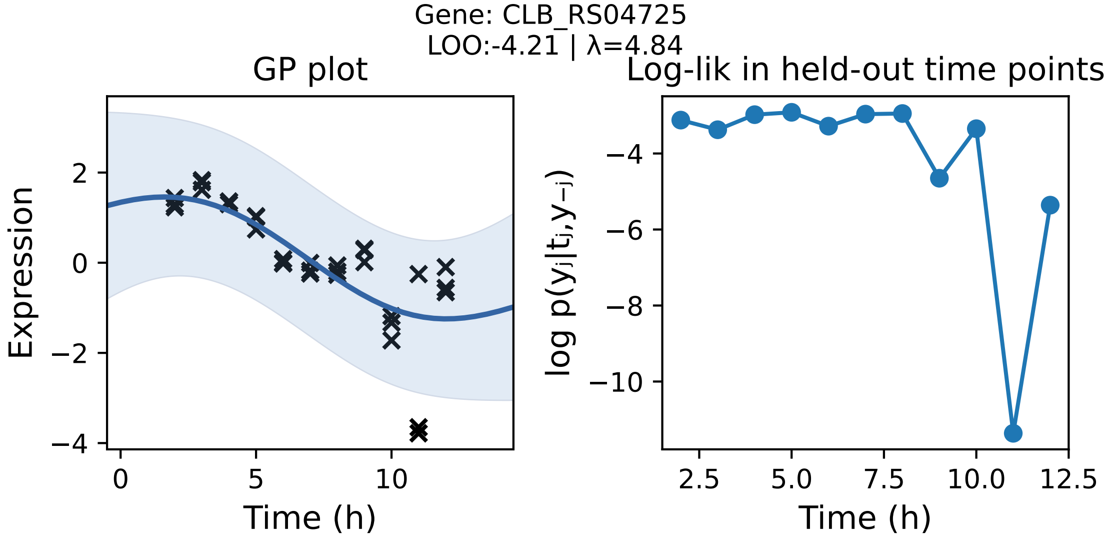
Gene: CLB_RS04725
LOO:-4.21 | λ=4.84
GP plot
Log-lik in held-out time points
Expression
Time (h)
log p(yⱼ|tⱼ,y₋ⱼ)
Time (h)
2
0
−2
−4
0
5
10
−4
−6
−8
−10
2.5
5.0
7.5
10.0
12.5

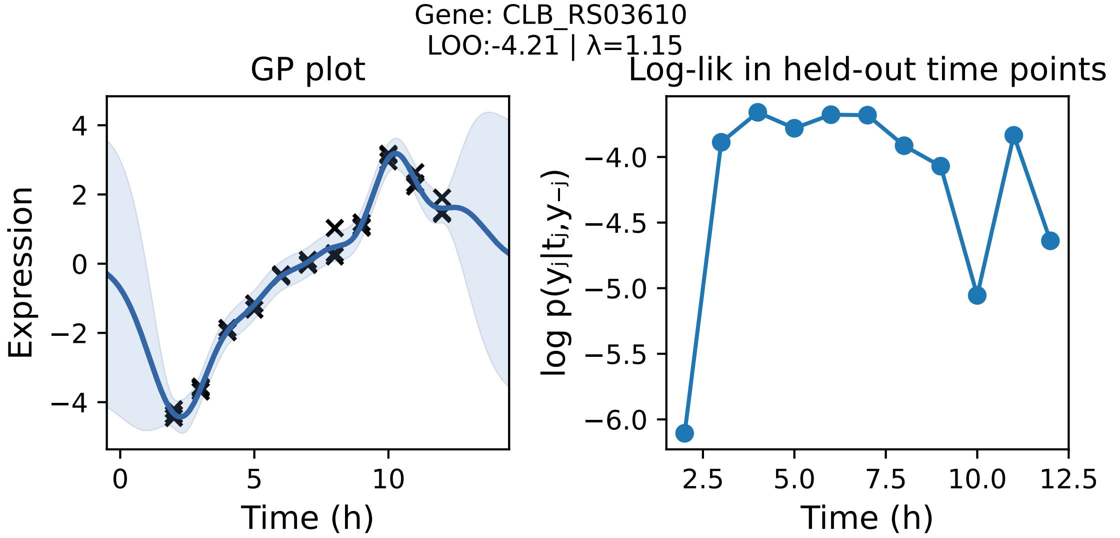

Gene: CLB_RS03610
LOO:-4.21 | λ=1.15
GP plot
Expression
Time (h)
4
2
0
−2
−4
0
5
10
Log-lik in held-out time points
log p(yj|tj,y−j)
−4.0
−4.5
−5.0
−5.5
−6.0
2.5
5.0
7.5
10.0
12.5
Time (h)

Gene: CLB_RS00490
LOO:-4.208 | λ=1.04

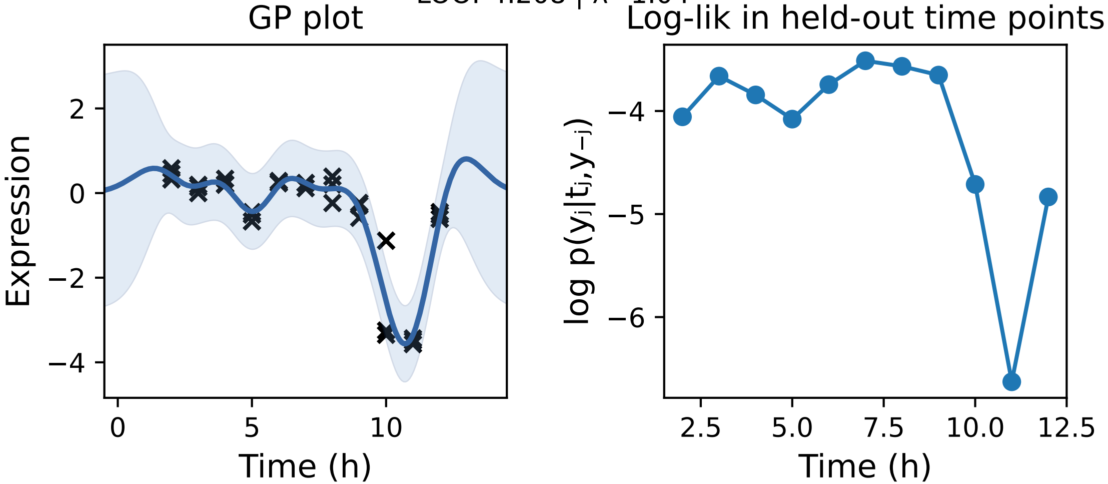

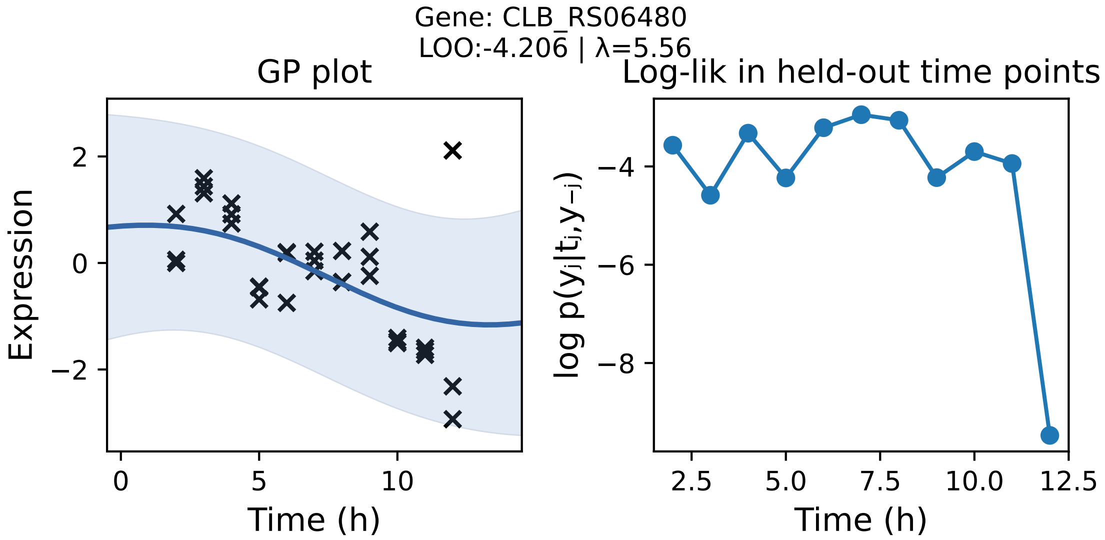

Gene: CLB_RS06480
LOO:-4.206 | λ=5.56
GP plot
Expression
Time (h)
2
0
−2
0
5
10
Log-lik in held-out time points
log p(y_j|t_j,y_−j)
Time (h)
−4
−6
−8
2.5
5.0
7.5
10.0
12.5

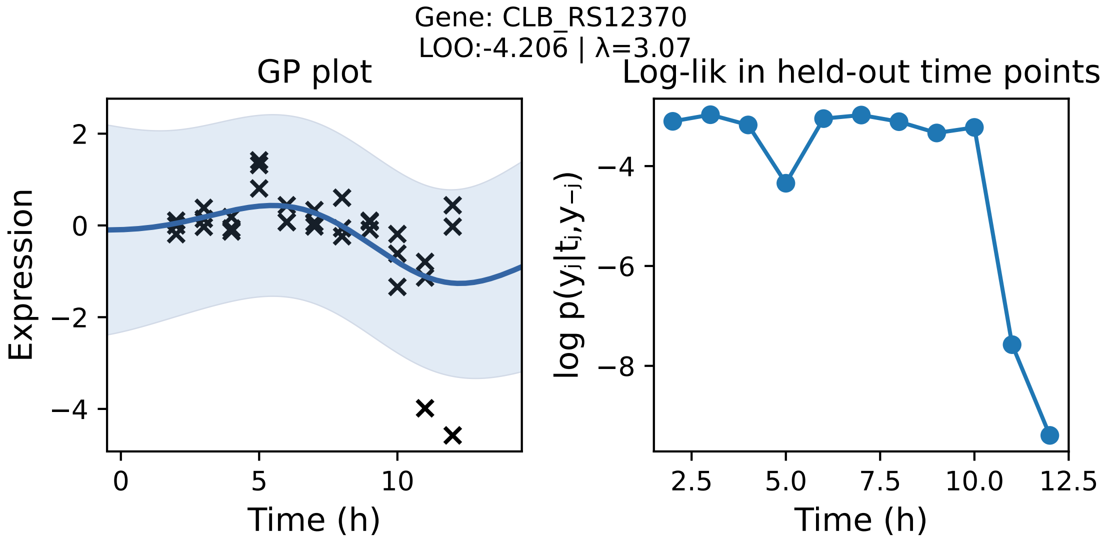

Gene: CLB_RS12370
LOO:-4.206 | λ=3.07
GP plot
Expression
Time (h)
2
0
−2
−4
0
5
10
Log-lik in held-out time points
log p(yj|tj,y−j)
Time (h)
−4
−6
−8
2.5
5.0
7.5
10.0
12.5

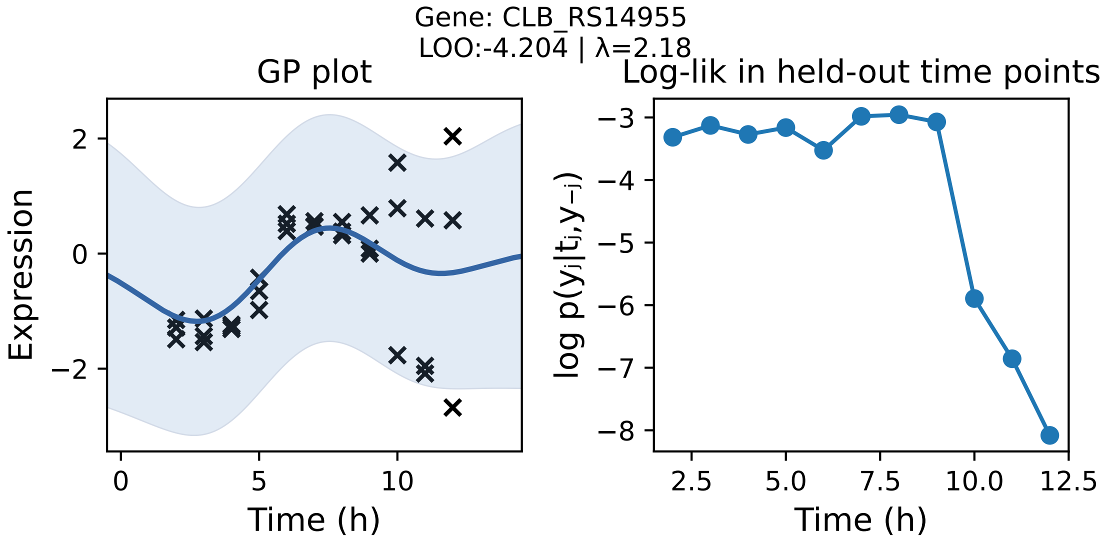
Gene: CLB_RS14955
LOO:-4.204 | λ=2.18
GP plot
Expression
Time (h)
Log-lik in held-out time points
log p(y_j|t_j,y_−j)
Time (h)

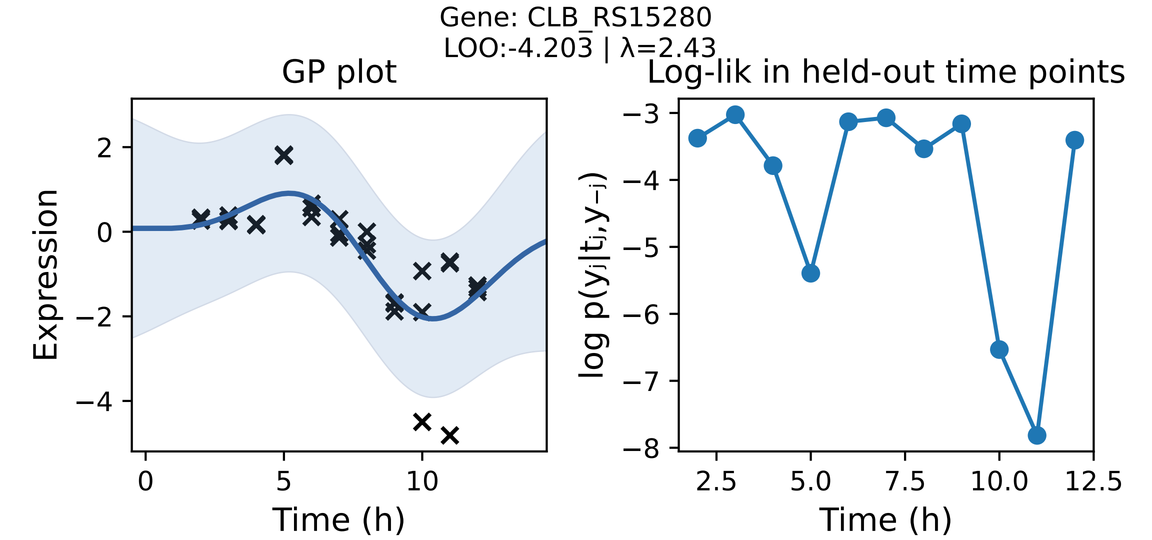

Gene: CLB_RS15280
LOO:-4.203 | λ=2.43
GP plot
Expression
Time (h)
Log-lik in held-out time points
log p(y_j|t_j,y_−j)
Time (h)

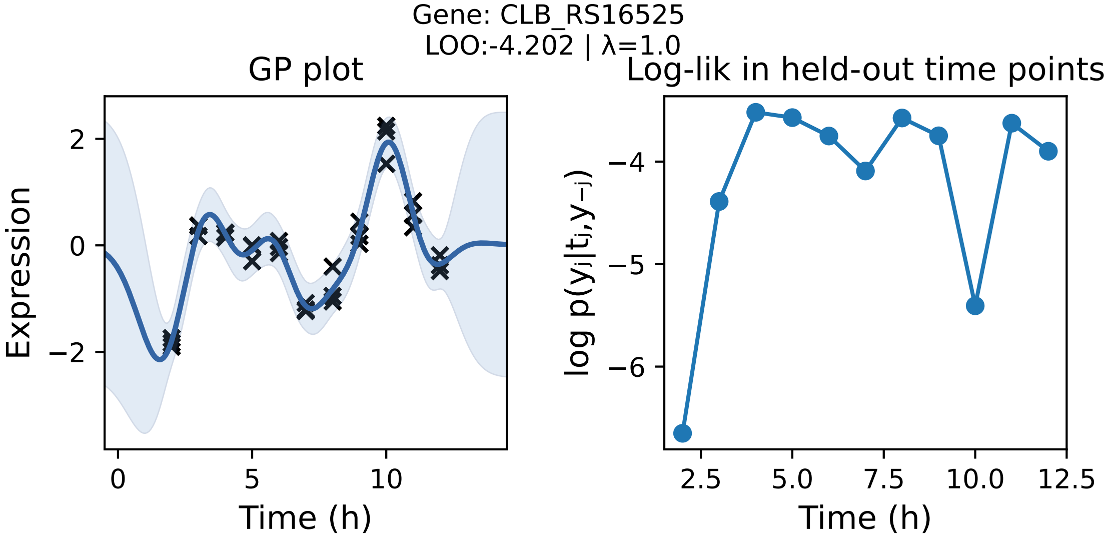

Gene: CLB_RS16525
LOO:-4.202 | λ=1.0
GP plot
Expression
Time (h)
2
0
−2
0
5
10
Log-lik in held-out time points
log p(y_j|t_j,y_−j)
Time (h)
−4
−5
−6
2.5
5.0
7.5
10.0
12.5

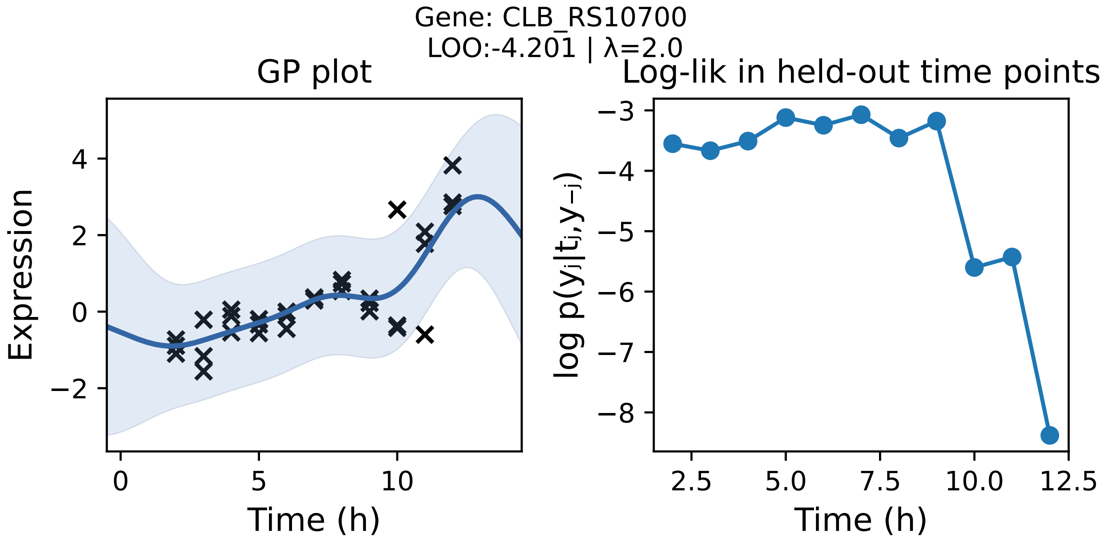
Gene: CLB_RS10700
LOO:-4.201 | λ=2.0
GP plot
Expression
Time (h)
Log-lik in held-out time points
log p(y_j|t_j,y_−j)
Time (h)

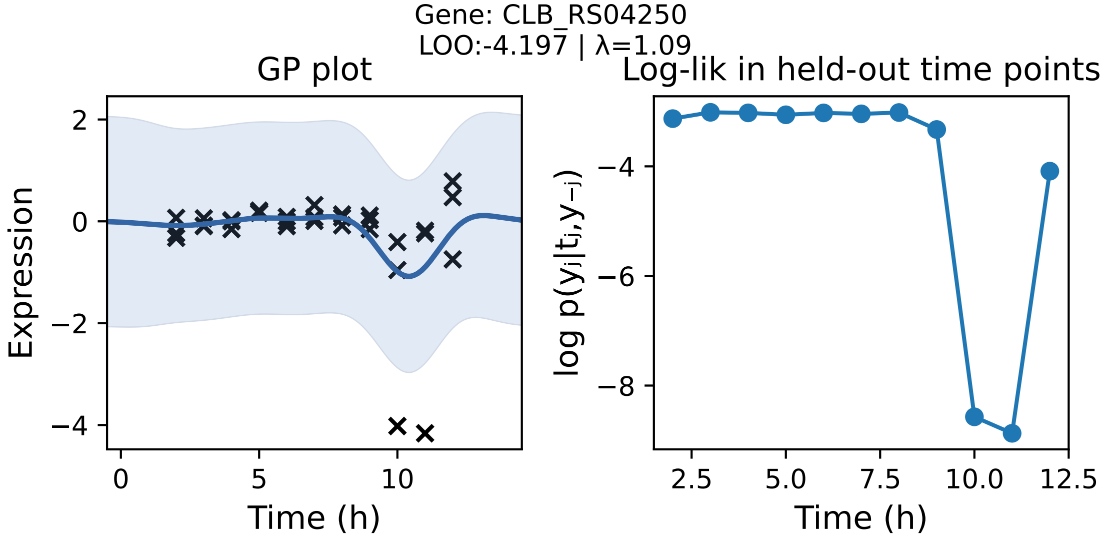

Gene: CLB_RS04250
LOO:-4.197 | λ=1.09
GP plot
Expression
Time (h)
Log-lik in held-out time points
log p(y_j|t_j,y_−j)
Time (h)

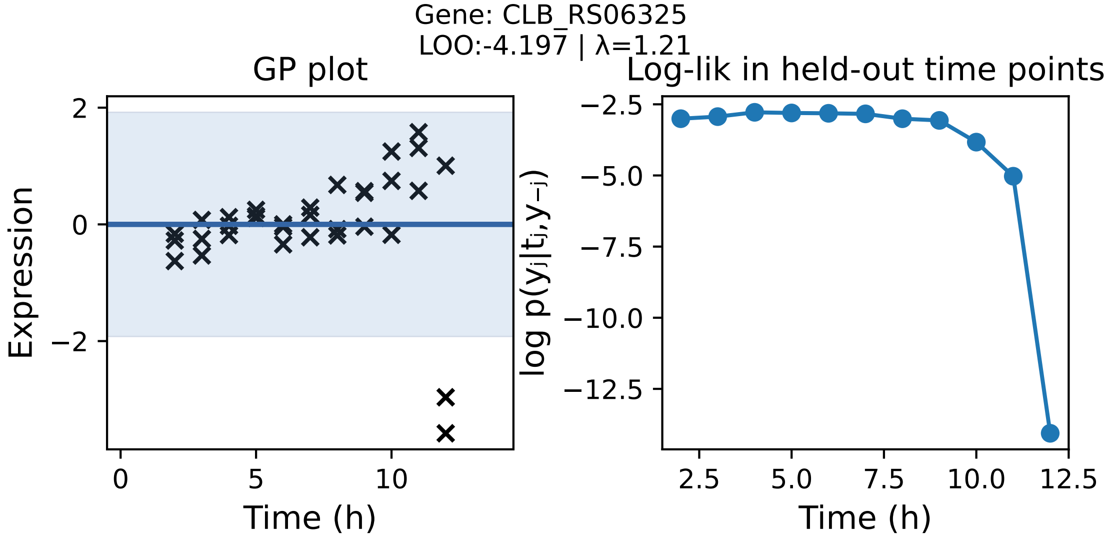

Gene: CLB_RS06325
LOO:-4.197 | λ=1.21
GP plot
Expression
Time (h)
Log-lik in held-out time points
log p(y_j|t_j,y_-j)
Time (h)
2
0
−2
0
5
10
−2.5
−5.0
−7.5
−10.0
−12.5
2.5
5.0
7.5
10.0
12.5

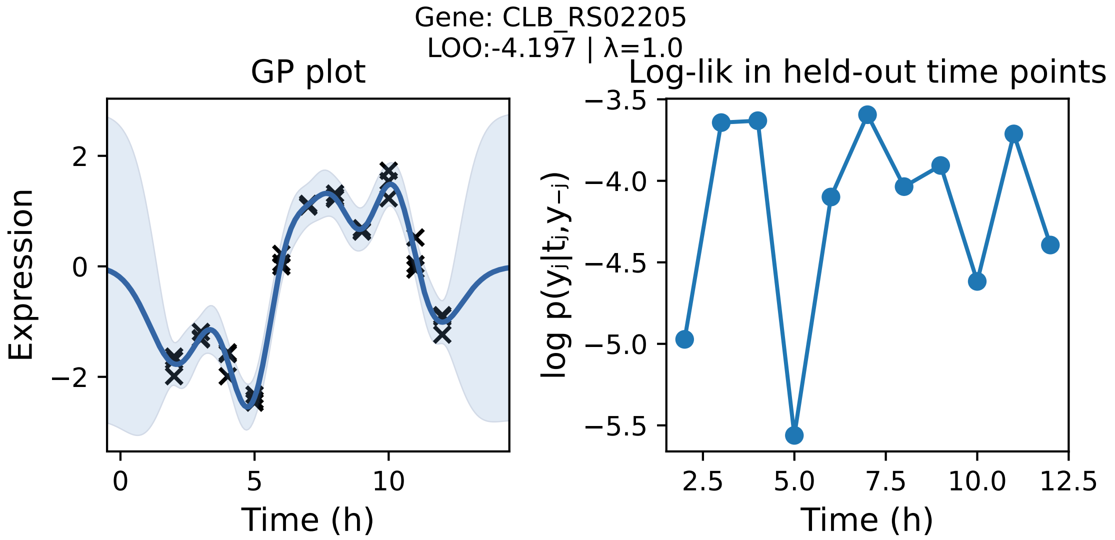

Gene: CLB_RS02205
LOO:-4.197 | λ=1.0
GP plot
Expression
Time (h)
2
0
−2
0
5
10
Log-lik in held-out time points
log p(y_j|t_j,y_−j)
Time (h)
−3.5
−4.0
−4.5
−5.0
−5.5
2.5
5.0
7.5
10.0
12.5

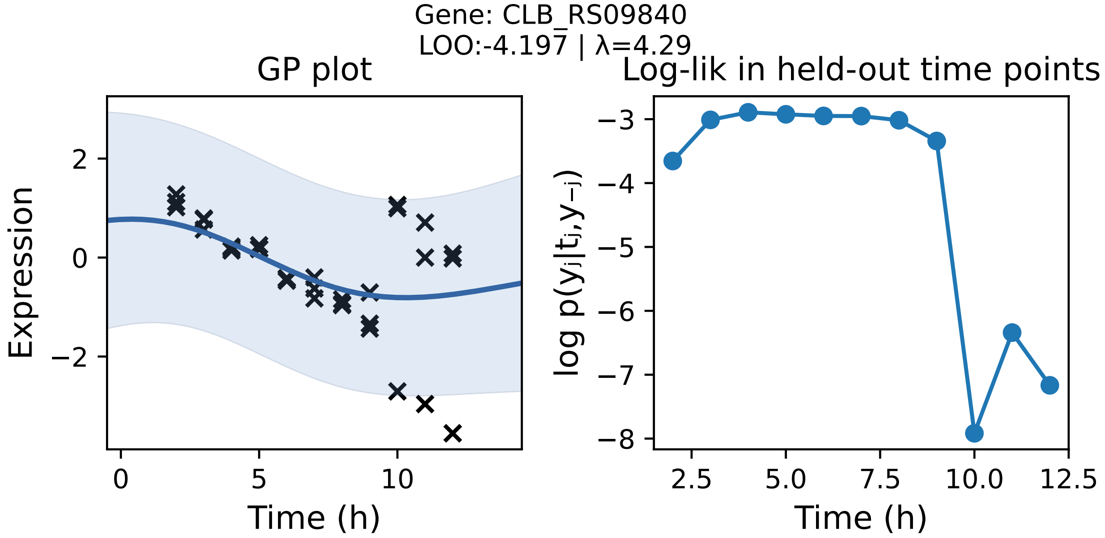
Gene: CLB_RS09840
LOO:-4.197 | λ=4.29
GP plot
Log-lik in held-out time points
Expression
Time (h)
log p(yj|tj,y−j)
2
0
−2
0
5
10
−3
−4
−5
−6
−7
−8
2.5
5.0
7.5
10.0
12.5

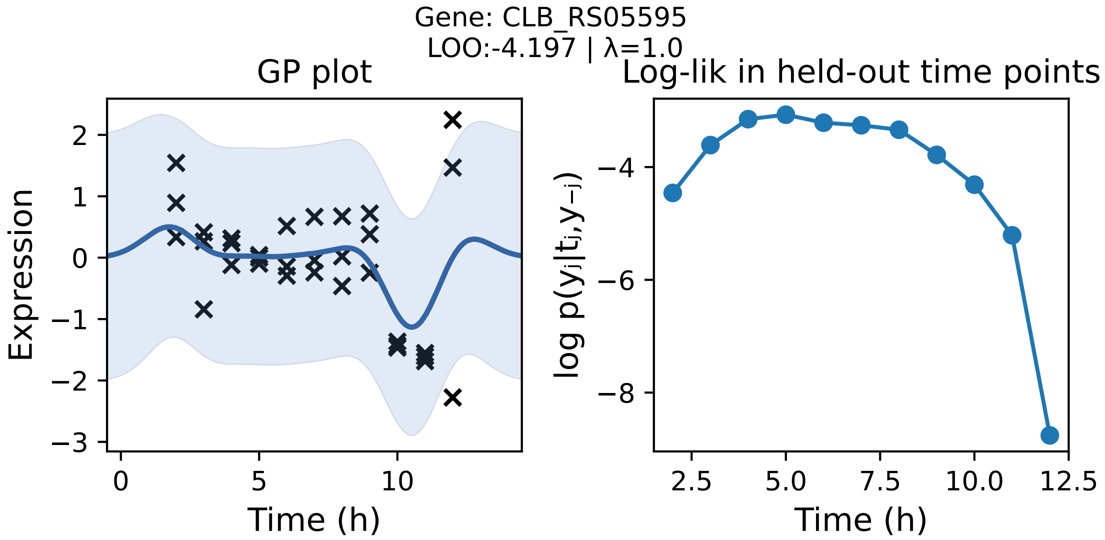
Gene: CLB_RS05595
LOO:-4.197 | λ=1.0
GP plot
Log-lik in held-out time points
Expression
Time (h)
log p(yj|tj,y−j)
Time (h)
2
1
0
−1
−2
−3
0
5
10
−4
−6
−8
2.5
5.0
7.5
10.0
12.5

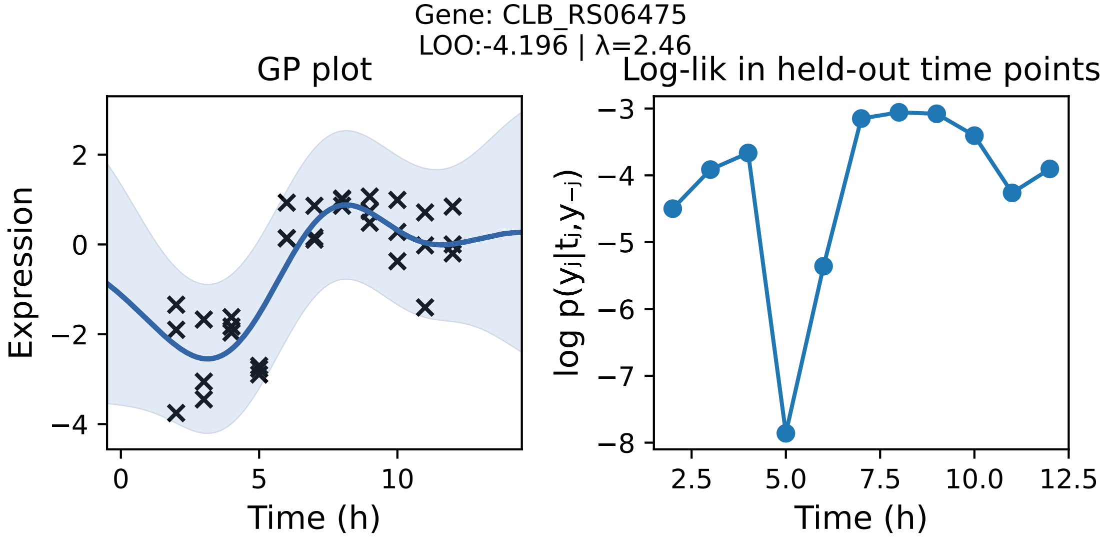
Gene: CLB_RS06475
LOO:-4.196 | λ=2.46
GP plot
Expression
Time (h)
Log-lik in held-out time points
log p(y_j|t_j,y_−j)
Time (h)

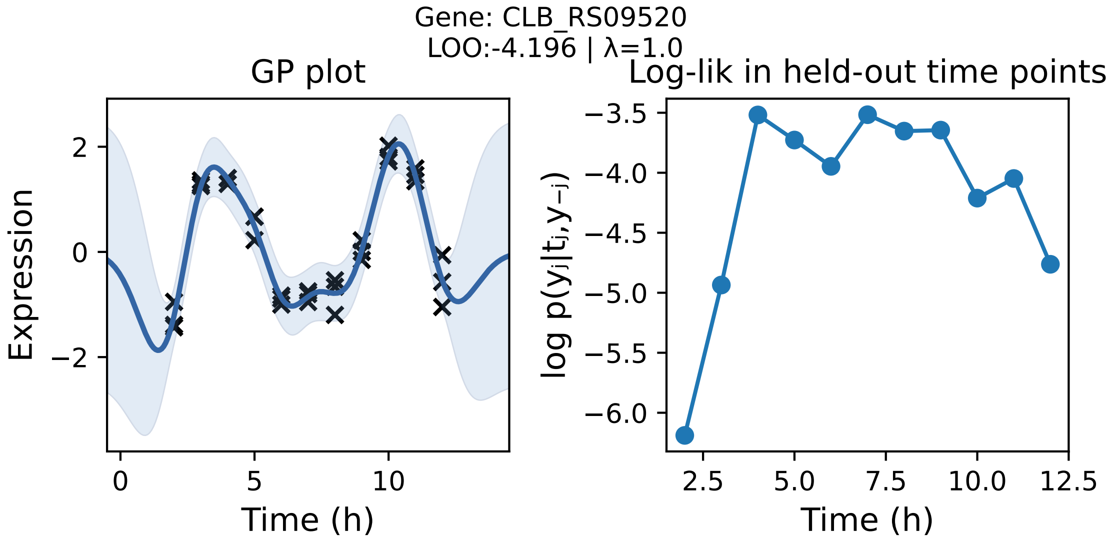
Gene: CLB_RS09520
LOO:-4.196 | λ=1.0
GP plot
Expression
Time (h)
2
0
−2
0
5
10
Log-lik in held-out time points
log p(y_j|t_j,y_−j)
Time (h)
−3.5
−4.0
−4.5
−5.0
−5.5
−6.0
2.5
5.0
7.5
10.0
12.5

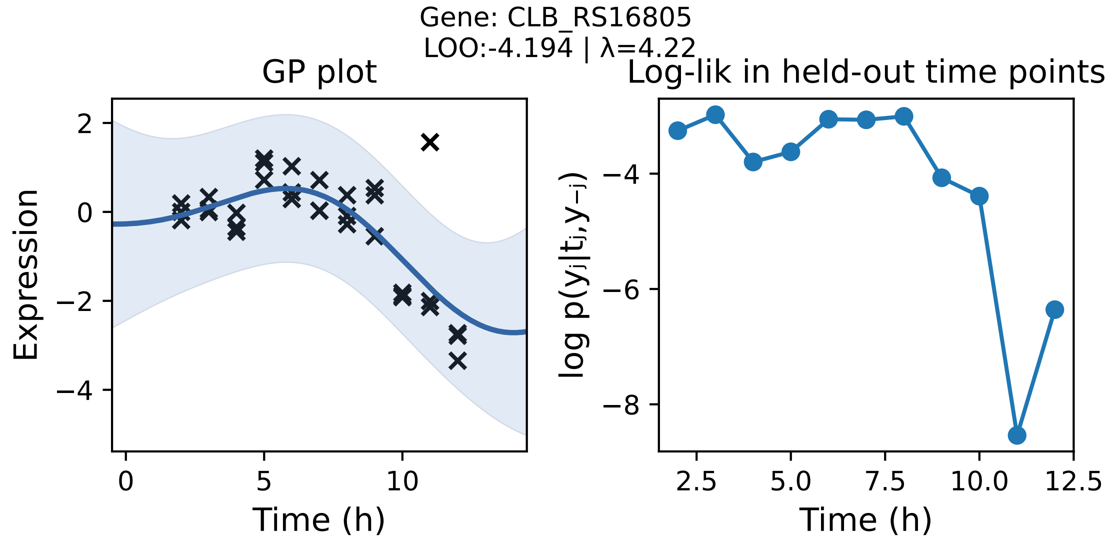

Gene: CLB_RS16805
LOO:-4.194 | λ=4.22
GP plot
Expression
Time (h)
Log-lik in held-out time points
log p(y_j|t_j,y_−j)
Time (h)

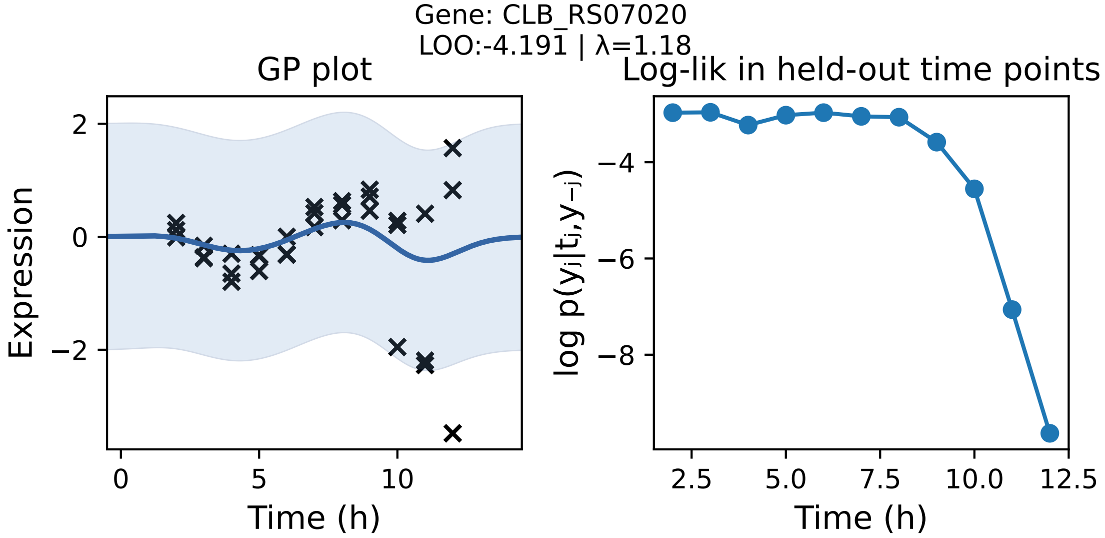

Gene: CLB_RS07020
LOO:-4.191 | λ=1.18
GP plot
Log-lik in held-out time points
Expression
Time (h)
log p(yj|tj,y−j)
2
0
−2
0
5
10
−4
−6
−8
2.5
5.0
7.5
10.0
12.5

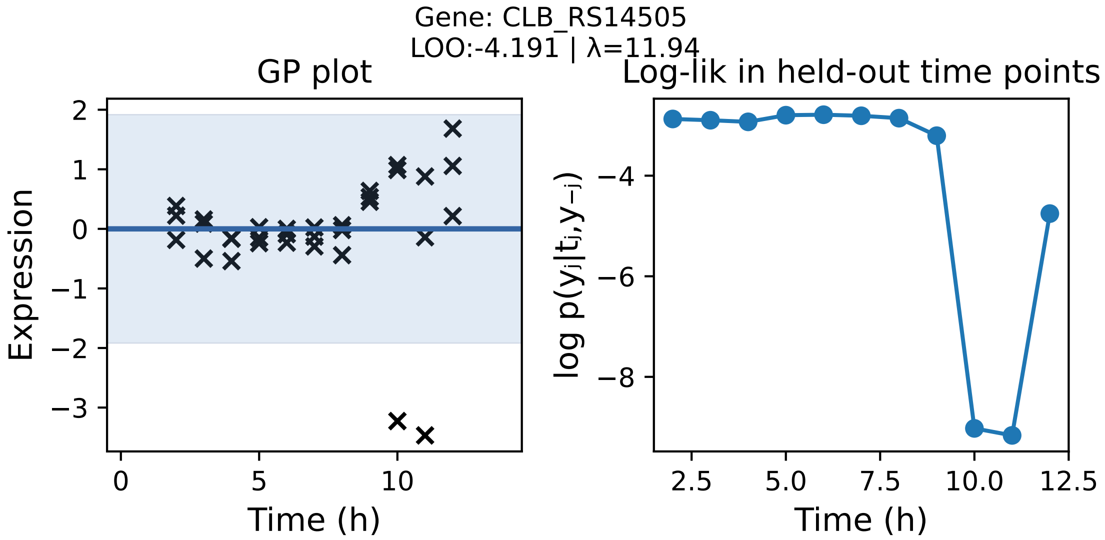
Gene: CLB_RS14505
LOO:-4.191 | λ=11.94
GP plot
Expression
Time (h)
Log-lik in held-out time points
log p(y_j|t_j,y_−j)
Time (h)

Gene: CLB_RS03230
LOO:-4.189 | λ=1.0

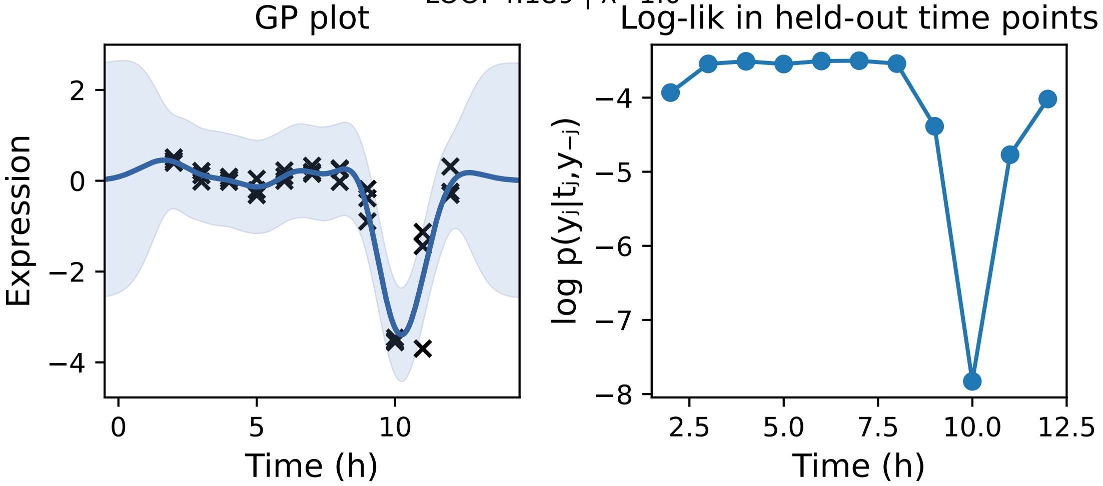

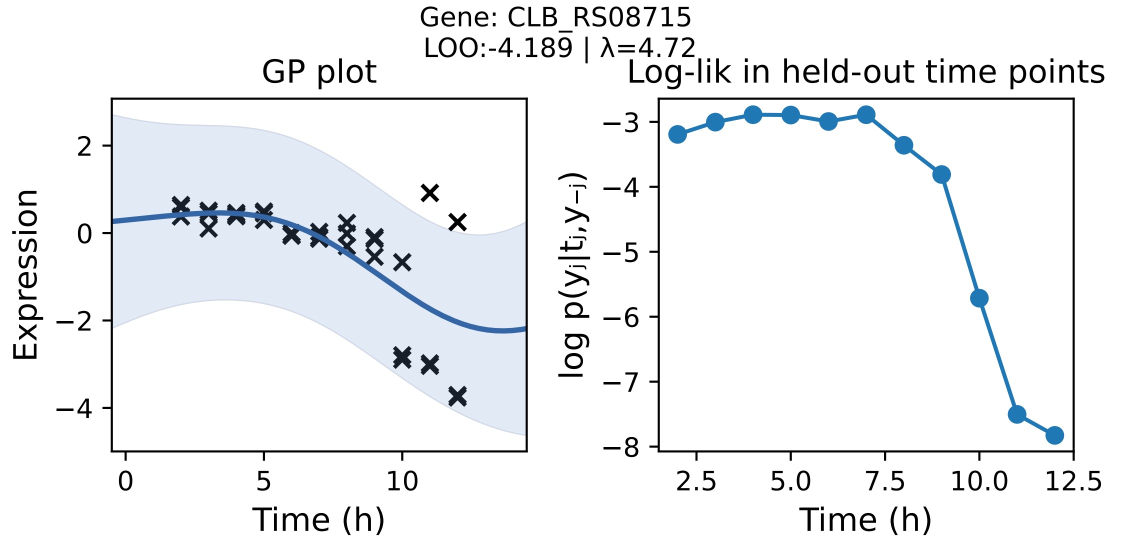
Gene: CLB_RS08715
LOO:-4.189 | λ=4.72
GP plot
Expression
Time (h)
Log-lik in held-out time points
log p(y_j|t_j,y_−j)
Time (h)
2
0
−2
−4
0
5
10
−3
−4
−5
−6
−7
−8
2.5
5.0
7.5
10.0
12.5

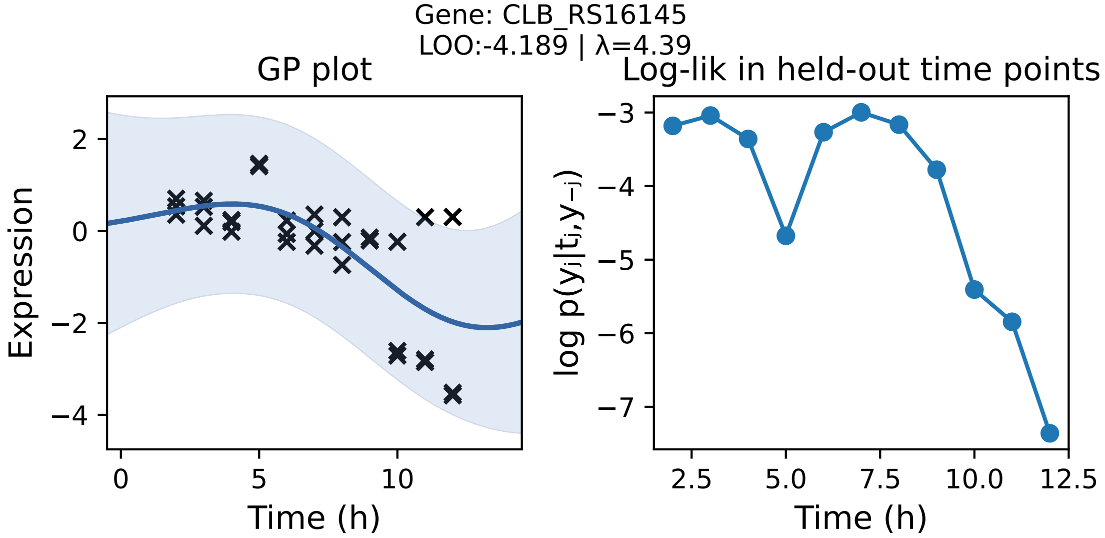

Gene: CLB_RS16145
LOO:-4.189 | λ=4.39
GP plot
Expression
Time (h)
Log-lik in held-out time points
log p(y_j|t_j,y_−j)
Time (h)

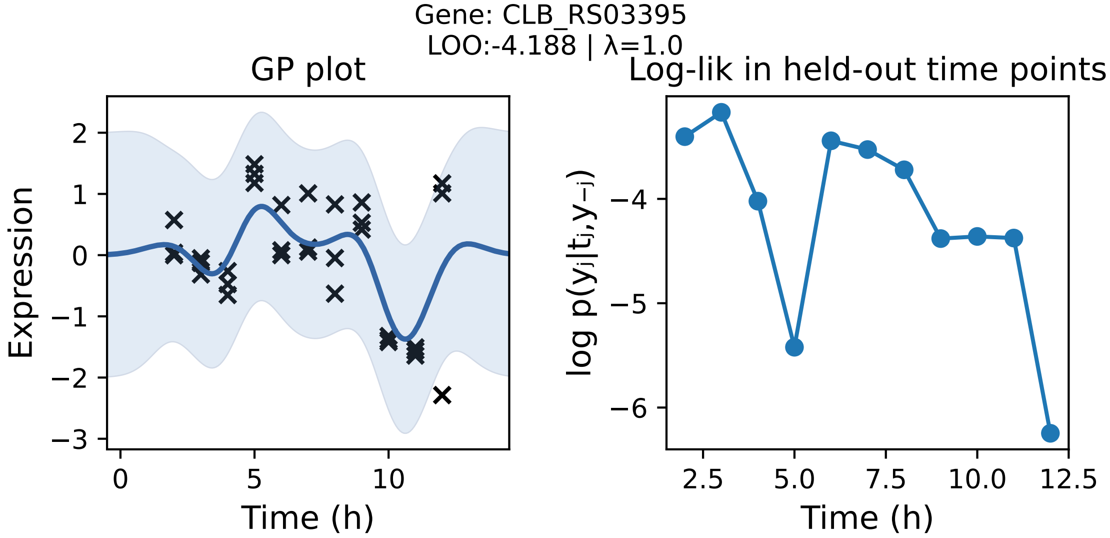
Gene: CLB_RS03395
LOO:-4.188 | λ=1.0
GP plot
Expression
Time (h)
Log-lik in held-out time points
log p(yⱼ|tⱼ,y₋ⱼ)
Time (h)

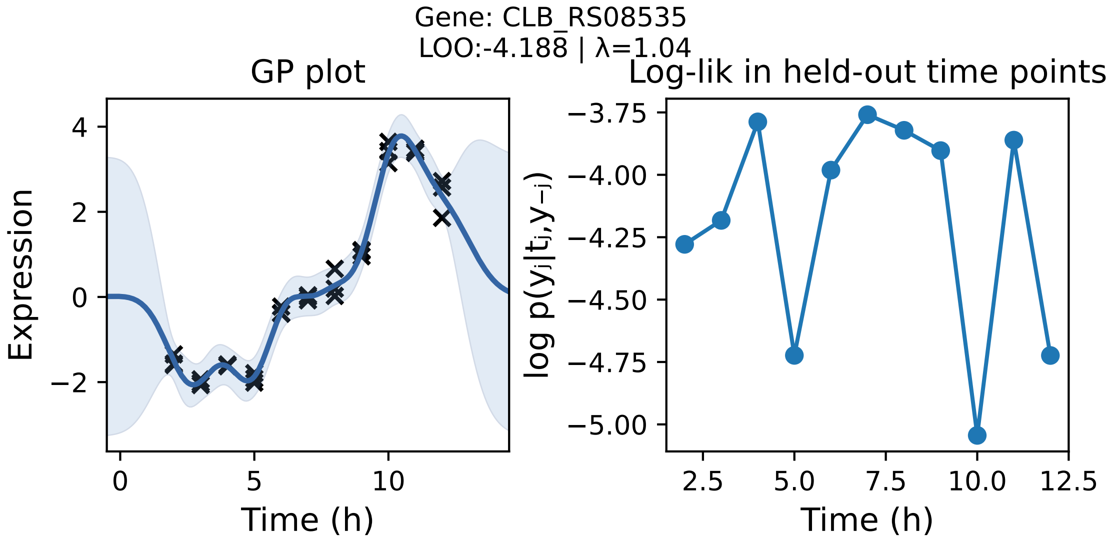

Gene: CLB_RS08535
LOO:-4.188 | λ=1.04
GP plot
Expression
Time (h)
Log-lik in held-out time points
log p(yj|tj,y−j)
Time (h)

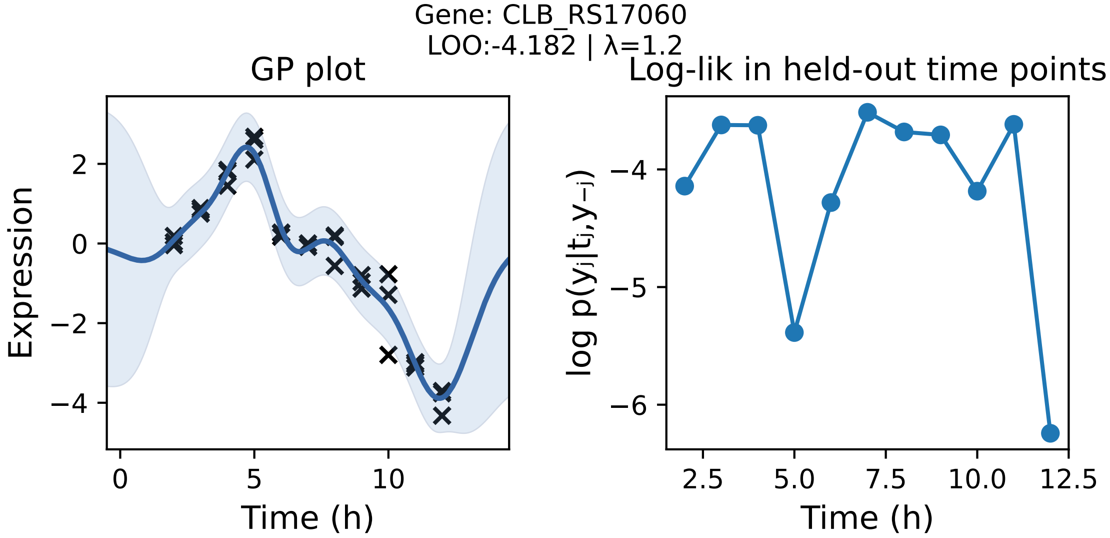

Gene: CLB_RS17060
LOO:-4.182 | λ=1.2
GP plot
Expression
Time (h)
Log-lik in held-out time points
log p(yj|tj,y−j)
Time (h)

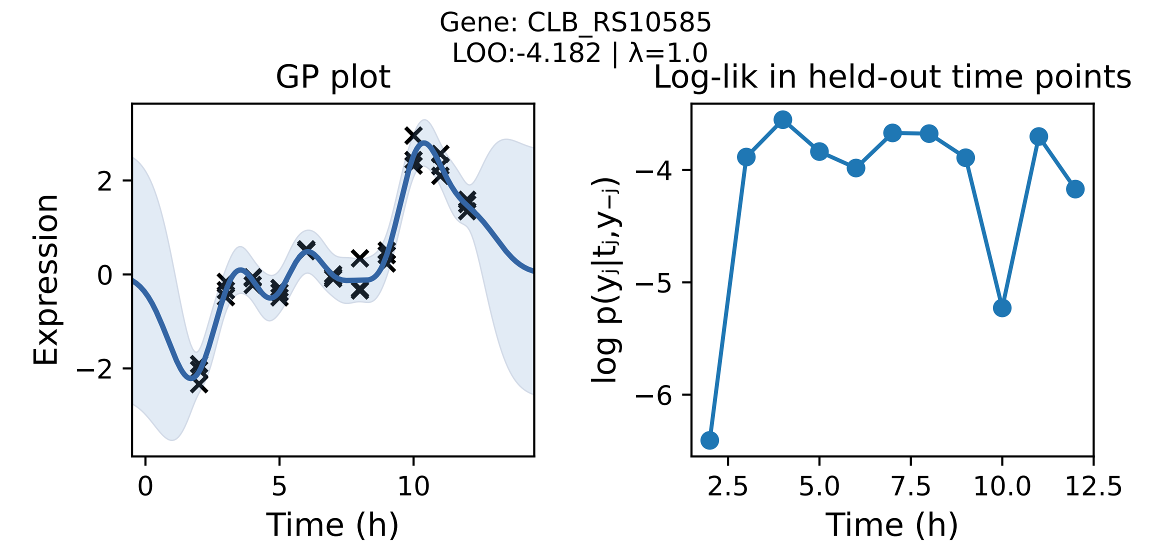

Gene: CLB_RS10585
LOO:-4.182 | λ=1.0
GP plot
Expression
Time (h)
Log-lik in held-out time points
log p(y_j|t_j,y_−j)
Time (h)

Gene: CLB_RS02695
LOO:-4.182 | λ=4.96

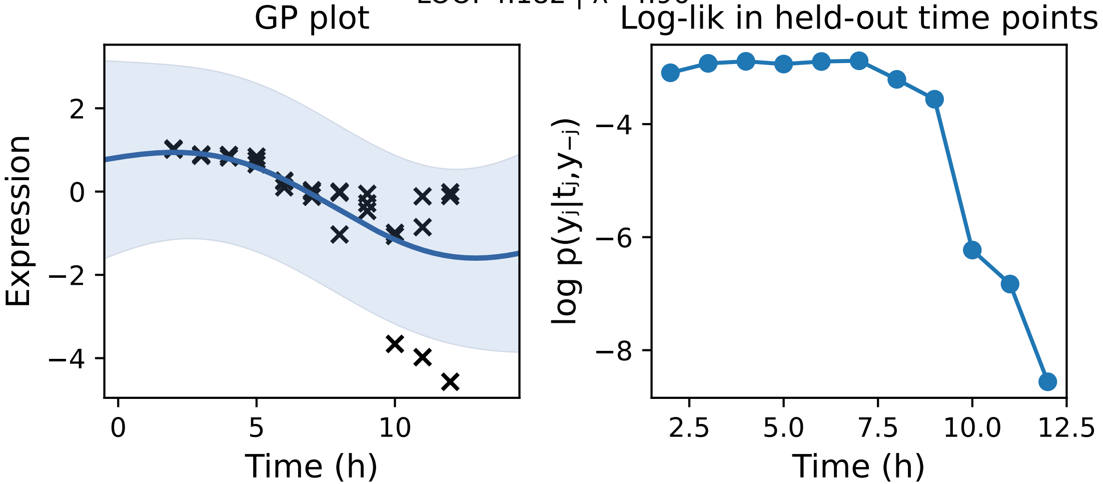

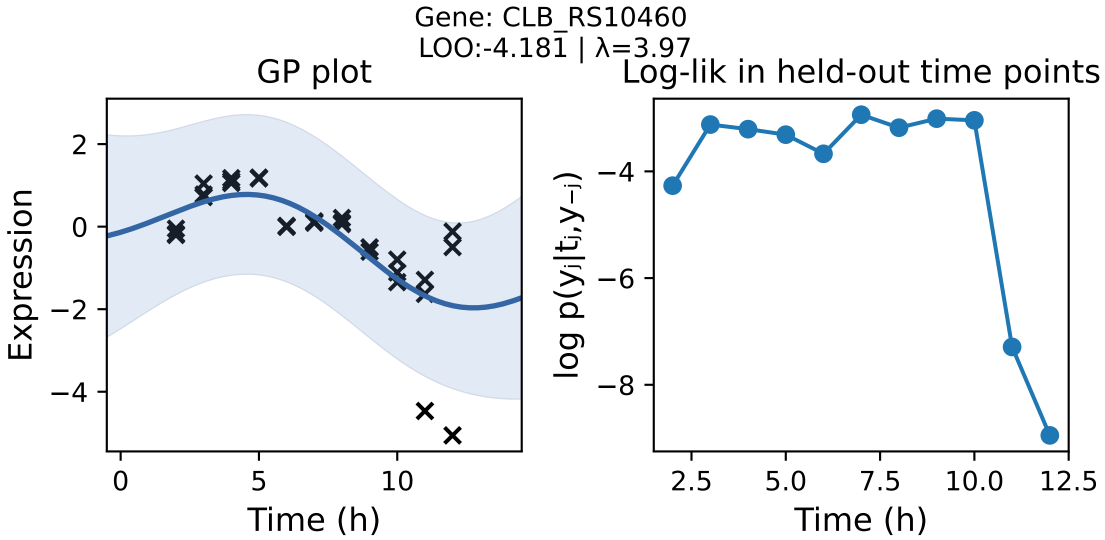

Gene: CLB_RS10460
LOO:-4.181 | λ=3.97
GP plot
Log-lik in held-out time points
Expression
Time (h)
log p(y_j|t_j,y_-j)
Time (h)

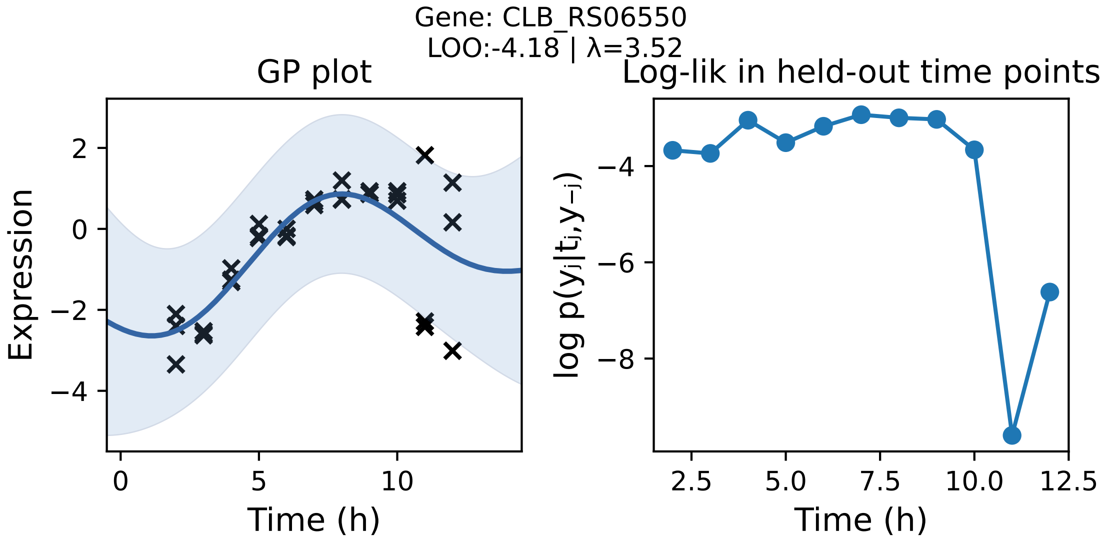
Gene: CLB_RS06550
LOO:-4.18 | λ=3.52
GP plot
Expression
Time (h)
Log-lik in held-out time points
log p(y_j|t_j,y_−j)
Time (h)

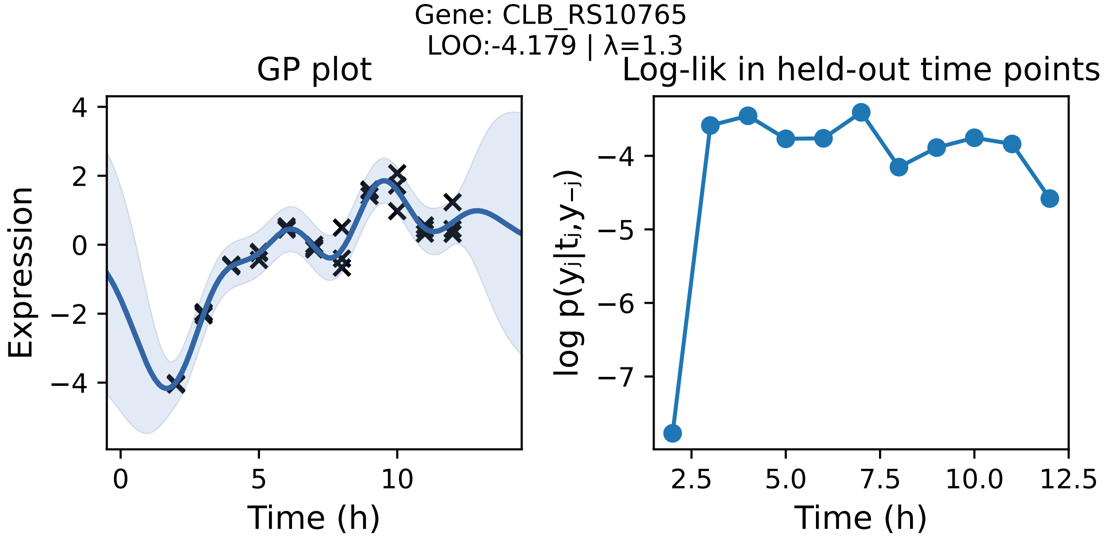

Gene: CLB_RS10765
LOO:-4.179 | λ=1.3
GP plot
Log-lik in held-out time points
Expression
Time (h)
log p(y_j|t_j,y_−j)
Time (h)

Gene: CLB_RS00190
LOO:-4.178 | λ=1.13

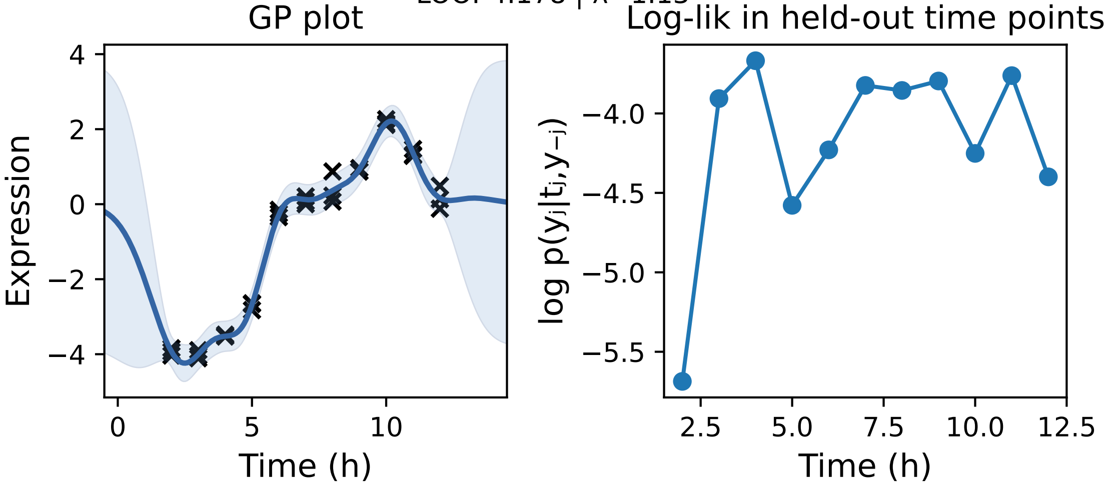

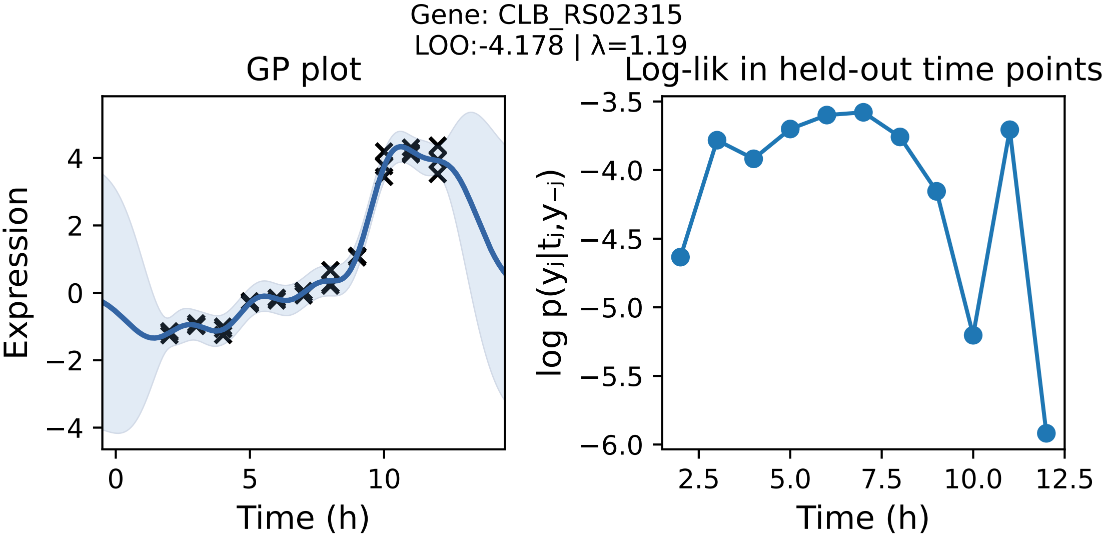
Gene: CLB_RS02315
LOO:-4.178 | λ=1.19
GP plot
Expression
4
2
0
−2
−4
0
5
10
Time (h)
Log-lik in held-out time points
log p(y_j|t_j,y_−j)
−3.5
−4.0
−4.5
−5.0
−5.5
−6.0
2.5
5.0
7.5
10.0
12.5
Time (h)

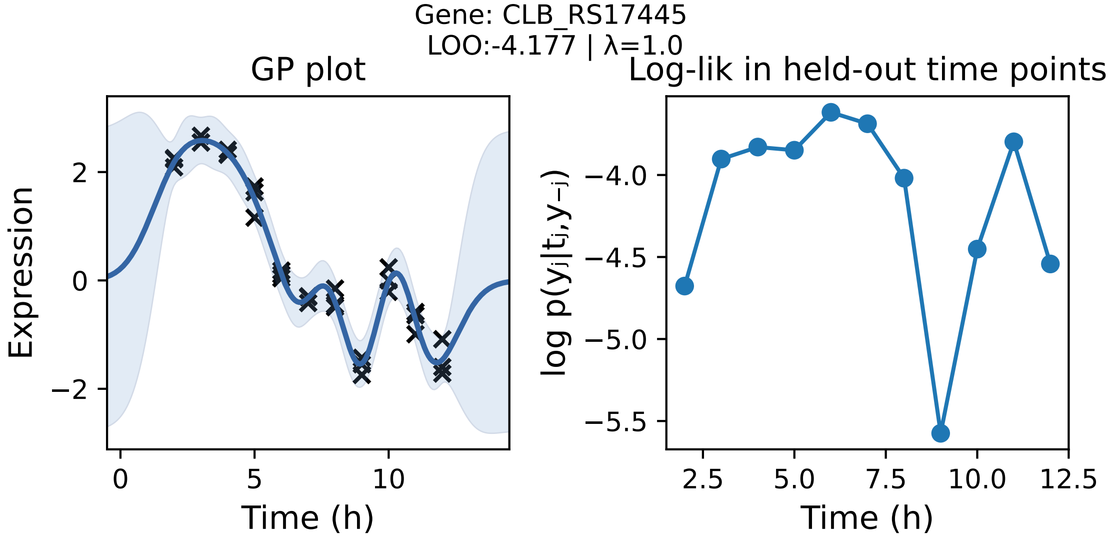

Gene: CLB_RS17445
LOO:-4.177 | λ=1.0
GP plot
Expression
Time (h)
2
0
−2
0
5
10
Log-lik in held-out time points
log p(y_j|t_j,y_−j)
Time (h)
−4.0
−4.5
−5.0
−5.5
2.5
5.0
7.5
10.0
12.5

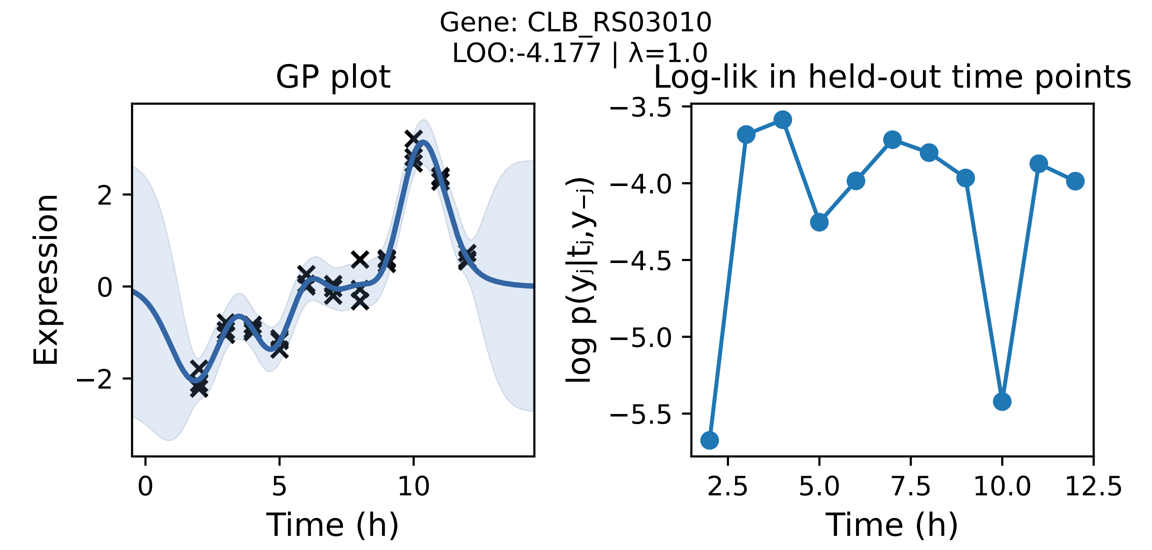

Gene: CLB_RS03010
LOO:-4.177 | λ=1.0
GP plot
Expression
Time (h)
Log-lik in held-out time points
log p(y_j|t_j,y_−j)
Time (h)

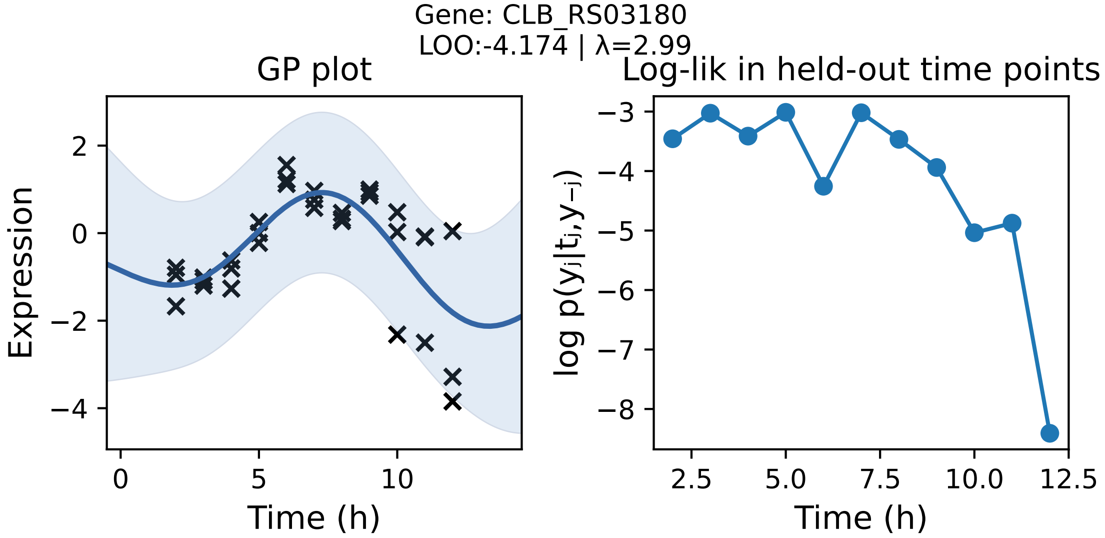
Gene: CLB_RS03180
LOO:-4.174 | λ=2.99
GP plot
Expression
Time (h)
Log-lik in held-out time points
log p(yj|tj,y−j)
Time (h)

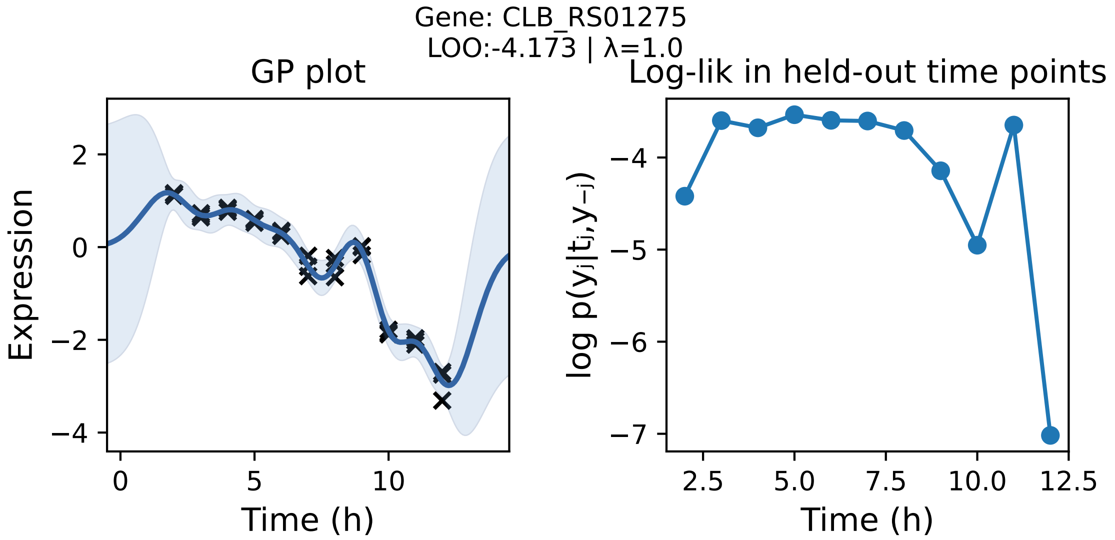

Gene: CLB_RS01275
LOO:-4.173 | λ=1.0
GP plot
Expression
Time (h)
2
0
−2
−4
0
5
10
Log-lik in held-out time points
log p(y_j|t_j,y_−j)
Time (h)
−4
−5
−6
−7
2.5
5.0
7.5
10.0
12.5

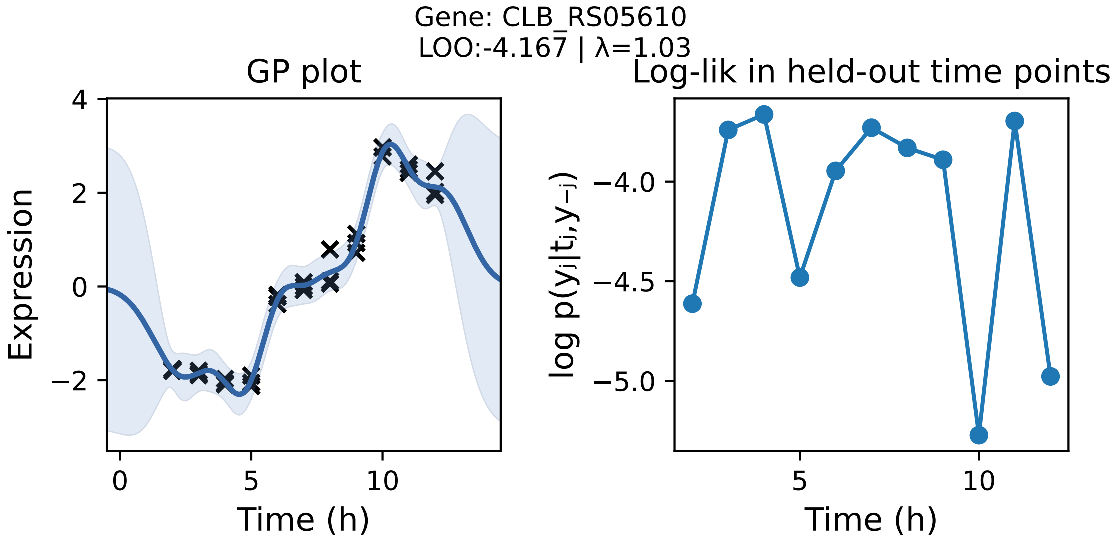

Gene: CLB_RS05610
LOO:-4.167 | λ=1.03
GP plot
Expression
Time (h)
4
2
0
−2
0
5
10
Log-lik in held-out time points
log p(y_j|t_j,y_−j)
−4.0
−4.5
−5.0
5
10
Time (h)

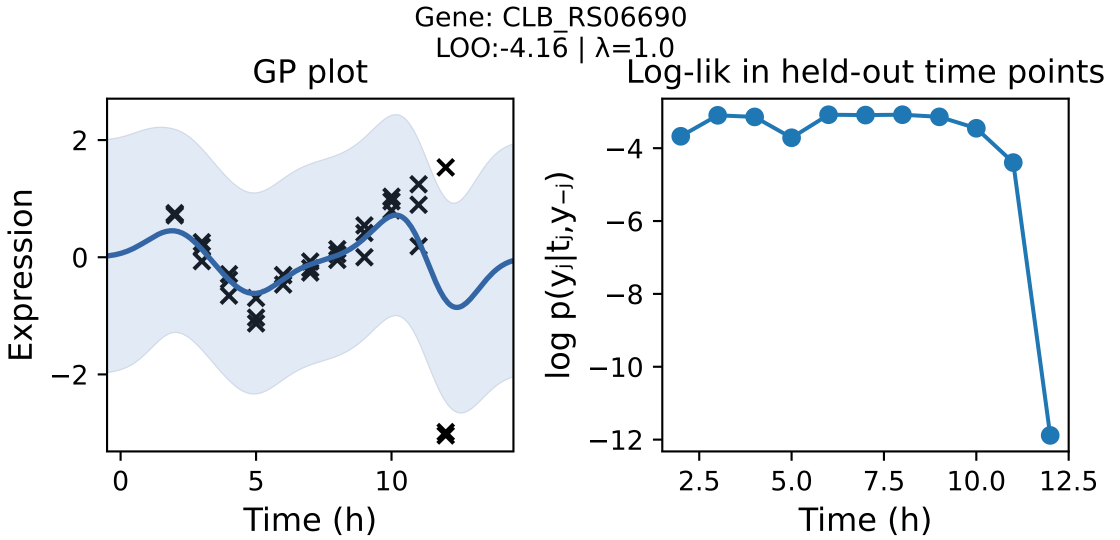

Gene: CLB_RS06690
LOO:-4.16 | λ=1.0
GP plot
Expression
Time (h)
2
0
−2
0
5
10
Log-lik in held-out time points
log p(y_j|t_j,y_−j)
Time (h)
−4
−6
−8
−10
−12
2.5
5.0
7.5
10.0
12.5

Gene: CLB_RS07960
LOO:-4.159 | λ=1.0

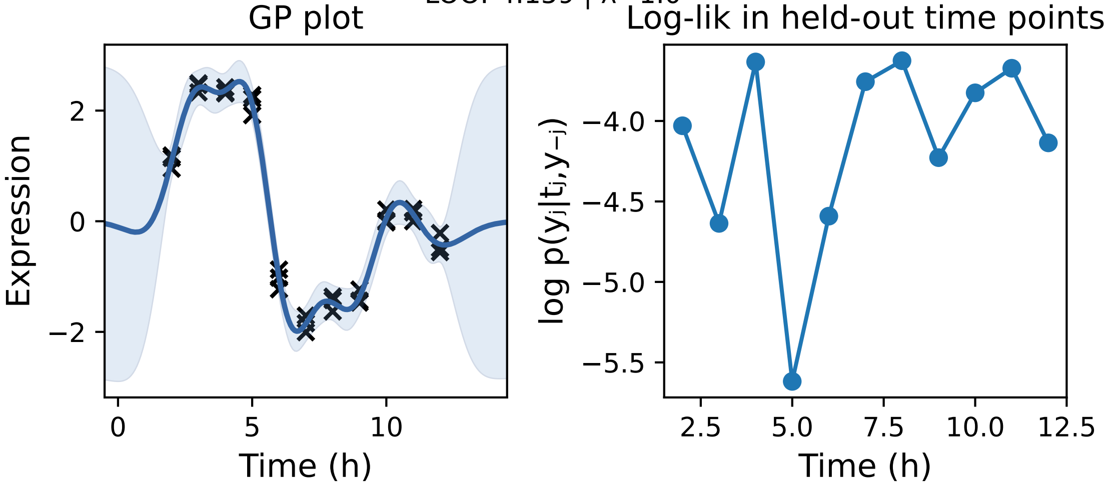

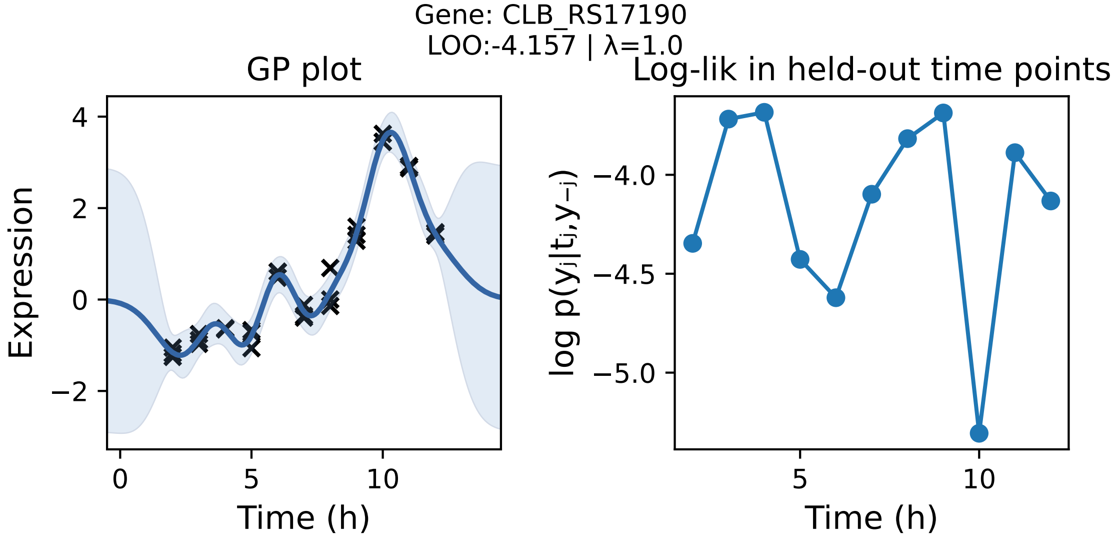
Gene: CLB_RS17190
LOO:-4.157 | λ=1.0
GP plot
Expression
Time (h)
Log-lik in held-out time points
log p(y_j|t_j,y_−j)
Time (h)

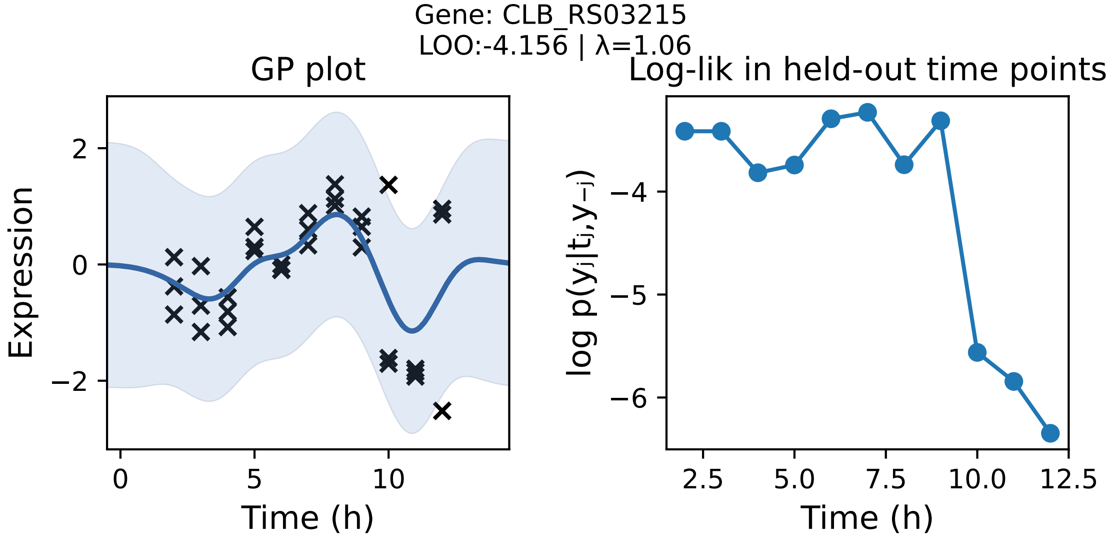

Gene: CLB_RS03215
LOO:-4.156 | λ=1.06
GP plot
Expression
Time (h)
Log-lik in held-out time points
log p(y_j|t_j,y_−j)
Time (h)

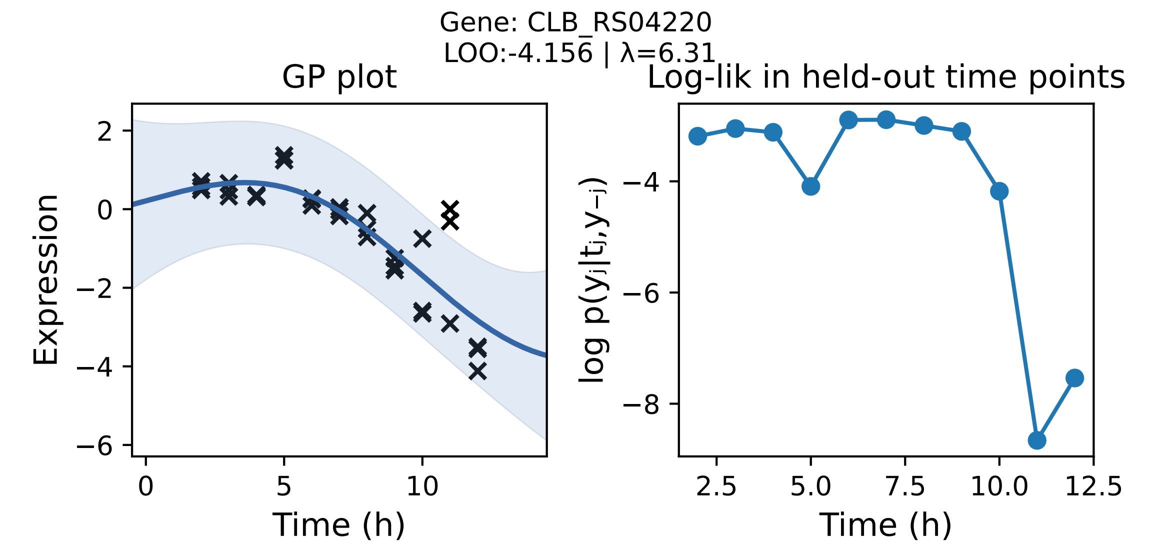

Gene: CLB_RS04220
LOO:-4.156 | λ=6.31
GP plot
Expression
Time (h)
Log-lik in held-out time points
log p(y_j|t_j,y_−j)
Time (h)

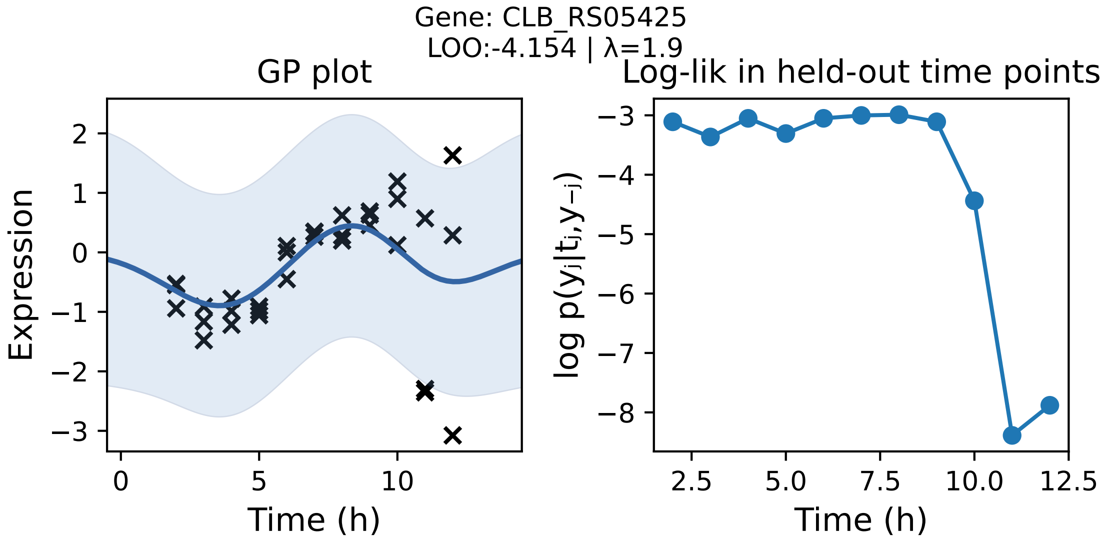
Gene: CLB_RS05425
LOO:-4.154 | λ=1.9
GP plot
Log-lik in held-out time points
Expression
Time (h)
log p(yj|tj,y−j)
Time (h)
2
1
0
−1
−2
−3
0
5
10
−3
−4
−5
−6
−7
−8
2.5
5.0
7.5
10.0
12.5

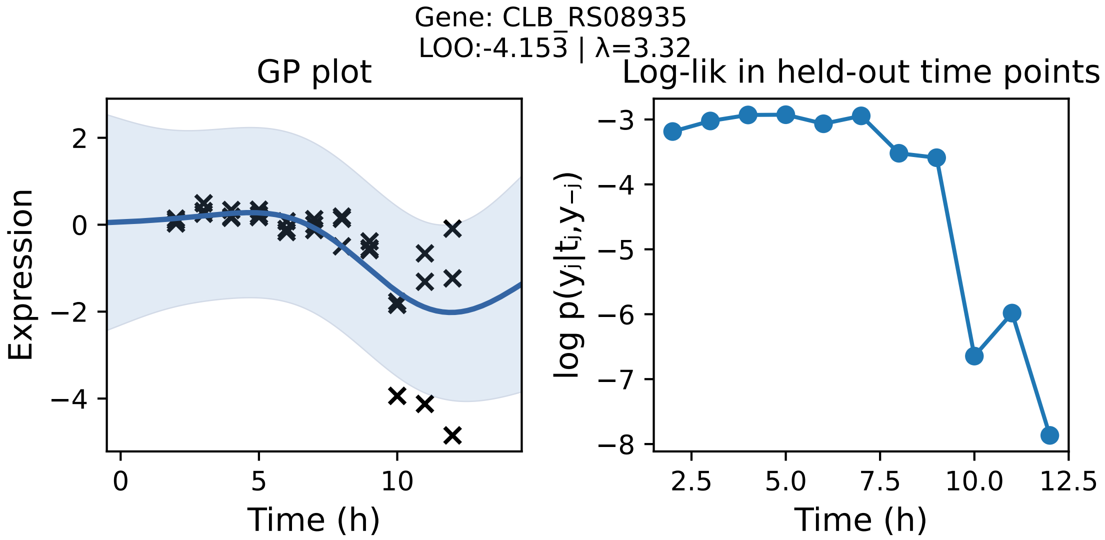

Gene: CLB_RS08935
LOO:-4.153 | λ=3.32
GP plot
Log-lik in held-out time points
Expression
Time (h)
log p(y_j|t_j,y_−j)
2
0
−2
−4
0
5
10
−3
−4
−5
−6
−7
−8
2.5
5.0
7.5
10.0
12.5

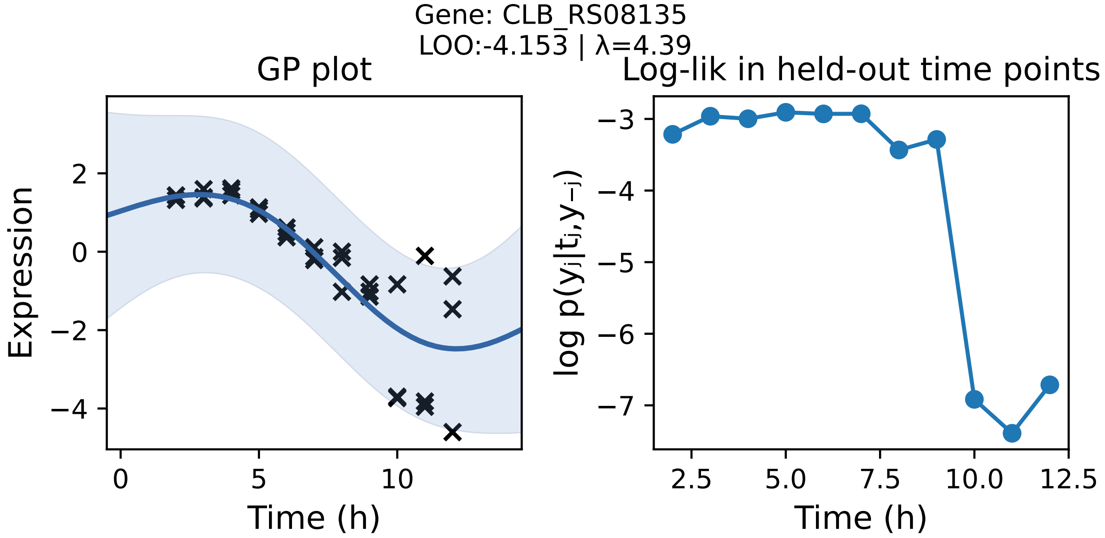

Gene: CLB_RS08135
LOO:-4.153 | λ=4.39
GP plot
Log-lik in held-out time points
Expression
Time (h)
Time (h)
$\log p(y_j|t_j,y_{-j})$
2
0
−2
−4
0
5
10
−3
−4
−5
−6
−7
2.5
5.0
7.5
10.0
12.5

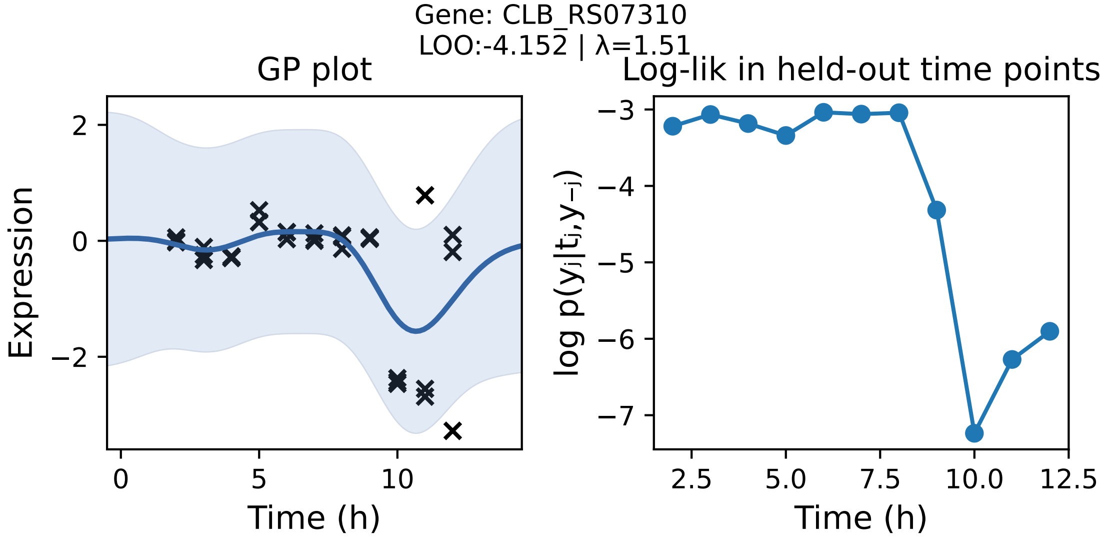

Gene: CLB_RS07310
LOO:-4.152 | λ=1.51
GP plot
Log-lik in held-out time points
Expression
Time (h)
2
0
−2
0
5
10
log p(y_j|t_j,y_−j)
−3
−4
−5
−6
−7
2.5
5.0
7.5
10.0
12.5
Time (h)

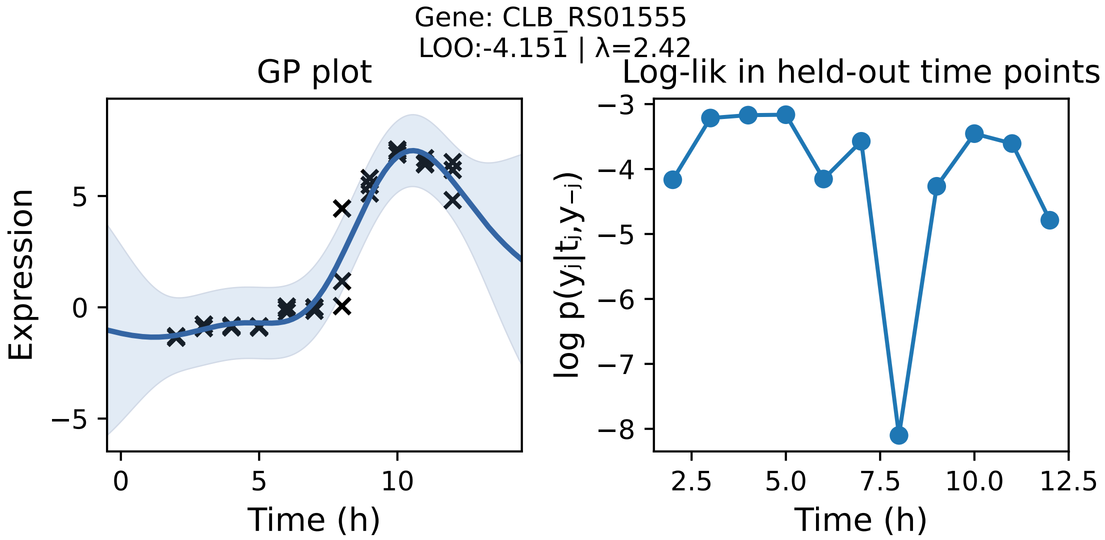

Gene: CLB_RS01555
LOO:-4.151 | λ=2.42
GP plot
Expression
Time (h)
Log-lik in held-out time points
log p(y_j|t_j,y_−j)
Time (h)

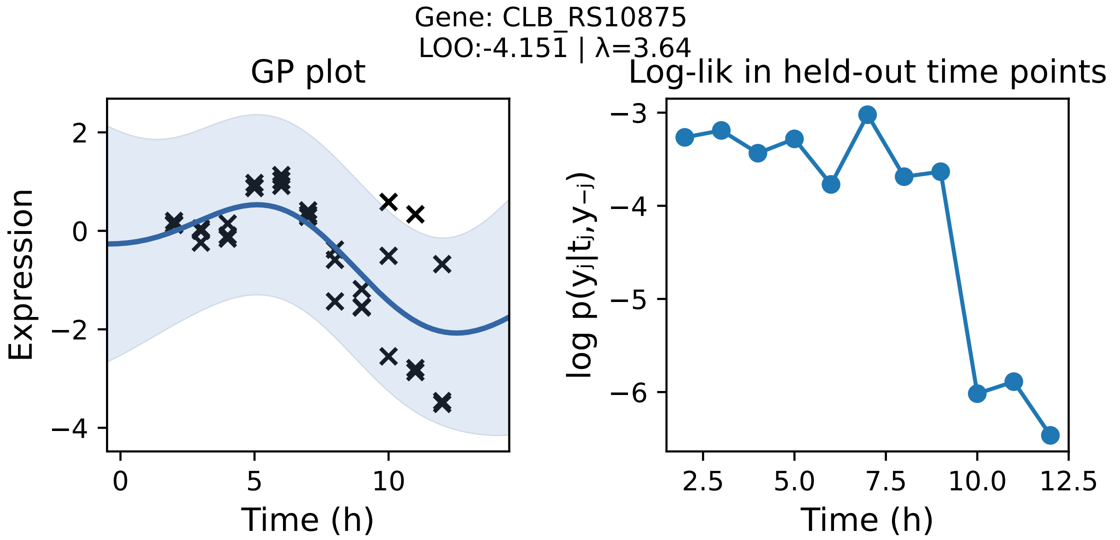

Gene: CLB_RS10875
LOO:-4.151 | λ=3.64
GP plot
Expression
Time (h)
2
0
−2
−4
0
5
10
Log-lik in held-out time points
log p(y_j|t_j,y_−j)
Time (h)
−3
−4
−5
−6
2.5
5.0
7.5
10.0
12.5

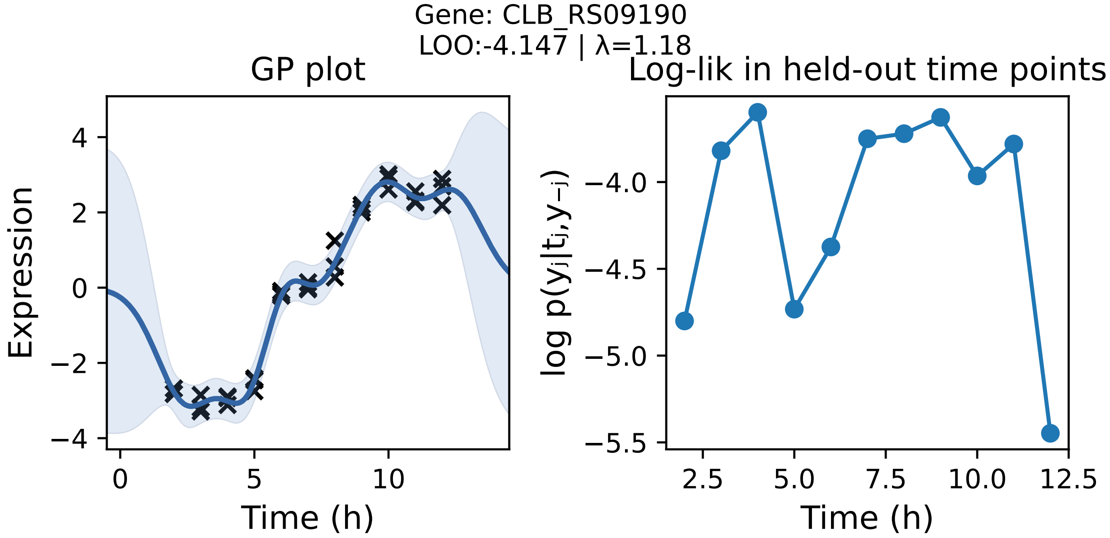

Gene: CLB_RS09190
LOO:-4.147 | λ=1.18
GP plot
Log-lik in held-out time points
Expression
4
2
0
−2
−4
0
5
10
Time (h)
log p(y_j|t_j,y_−j)
−4.0
−4.5
−5.0
−5.5
2.5
5.0
7.5
10.0
12.5
Time (h)

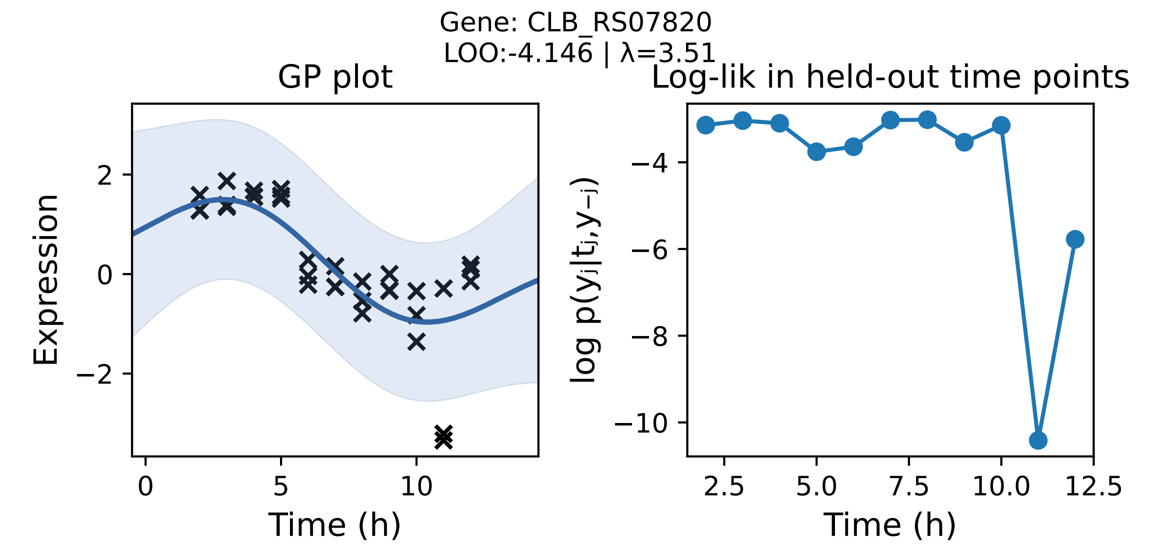

Gene: CLB_RS07820
LOO:-4.146 | λ=3.51
GP plot
Expression
Time (h)
2
0
−2
0
5
10
Log-lik in held-out time points
log p(y_j|t_j,y_−j)
Time (h)
−4
−6
−8
−10
2.5
5.0
7.5
10.0
12.5

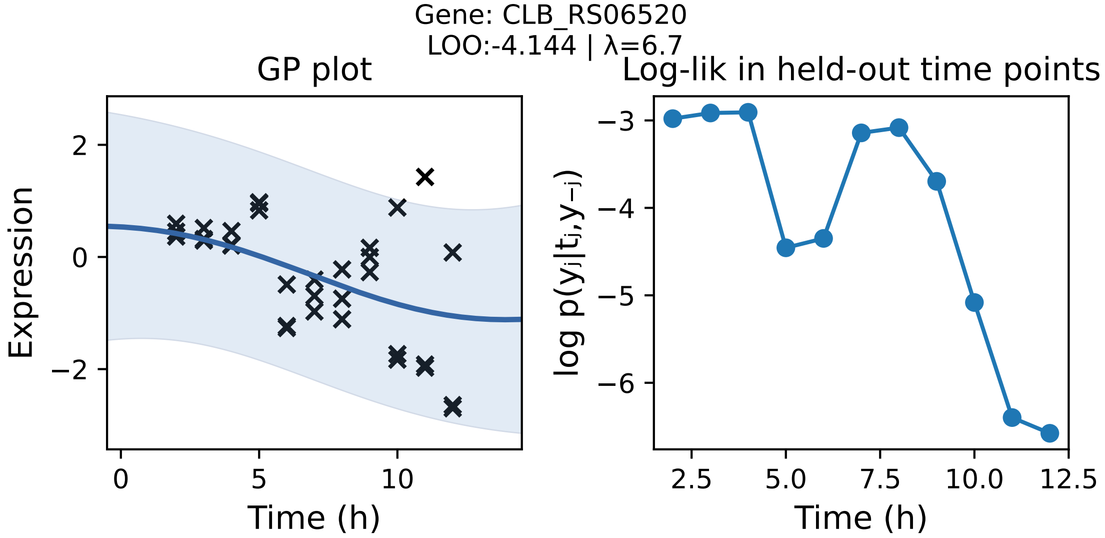

Gene: CLB_RS06520
LOO:-4.144 | λ=6.7
GP plot
Expression
Time (h)
2
0
−2
0
5
10
Log-lik in held-out time points
log p(yj|tj,y−j)
−3
−4
−5
−6
2.5
5.0
7.5
10.0
12.5
Time (h)

Gene: CLB_RS10315
LOO:-4.144 | λ=1.15

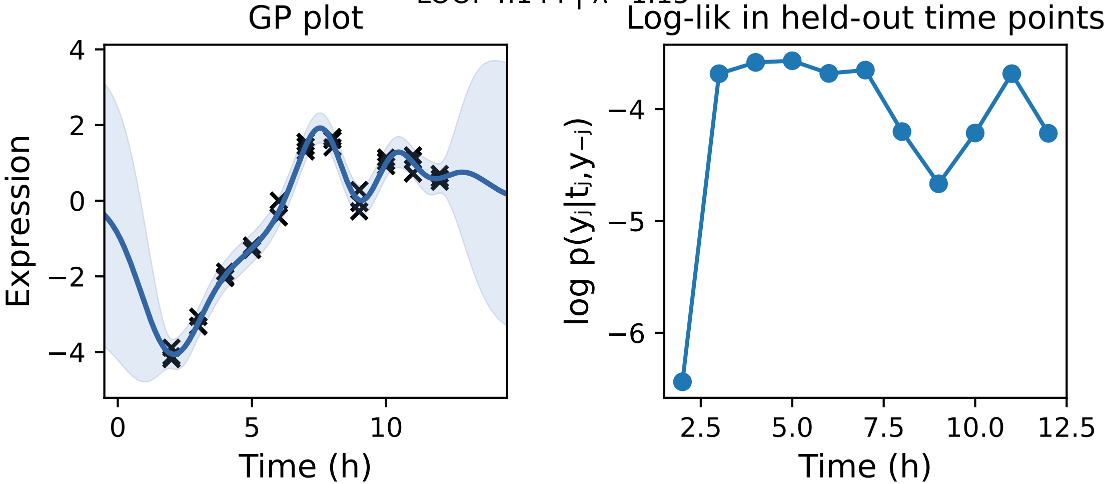

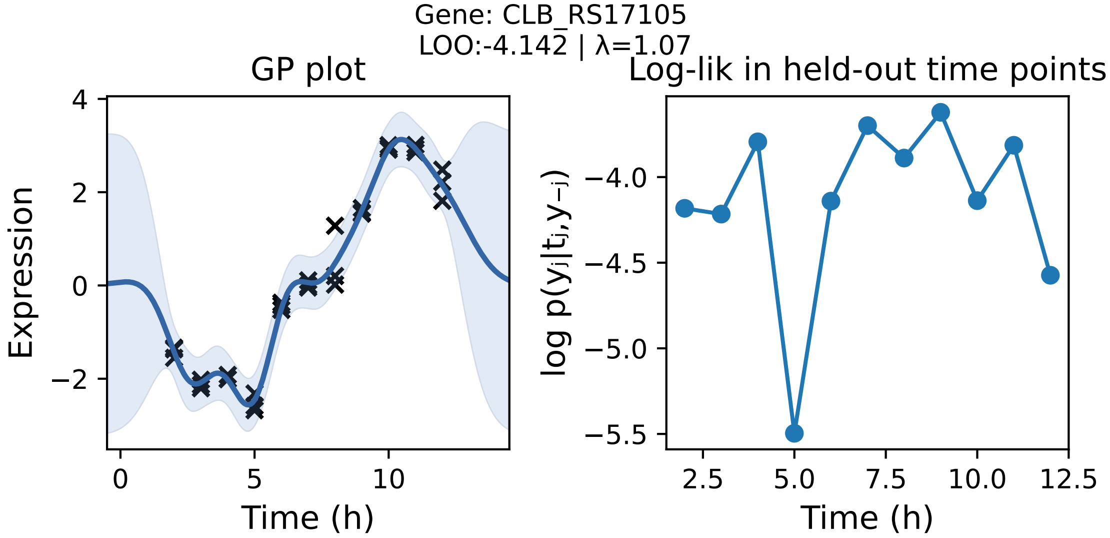

Gene: CLB_RS17105
LOO:-4.142 | λ=1.07
GP plot
Expression
Time (h)
Log-lik in held-out time points
log p(y_j|t_j,y_−j)
Time (h)

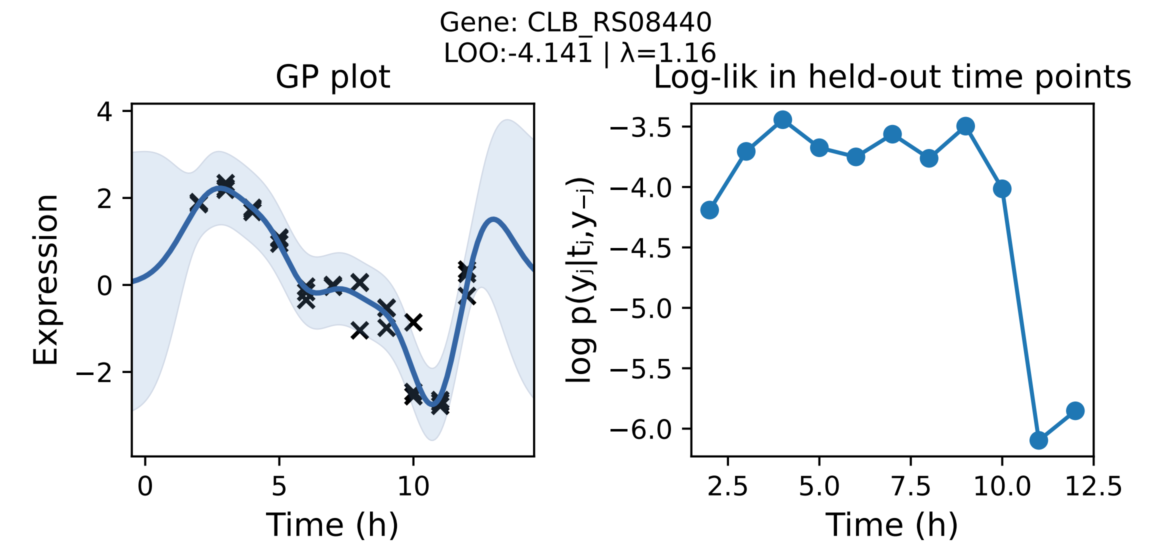

Gene: CLB_RS08440
LOO:-4.141 | λ=1.16
GP plot
Expression
Time (h)
4
2
0
−2
0
5
10
Log-lik in held-out time points
log p(y_j|t_j,y_−j)
−3.5
−4.0
−4.5
−5.0
−5.5
−6.0
2.5
5.0
7.5
10.0
12.5
Time (h)

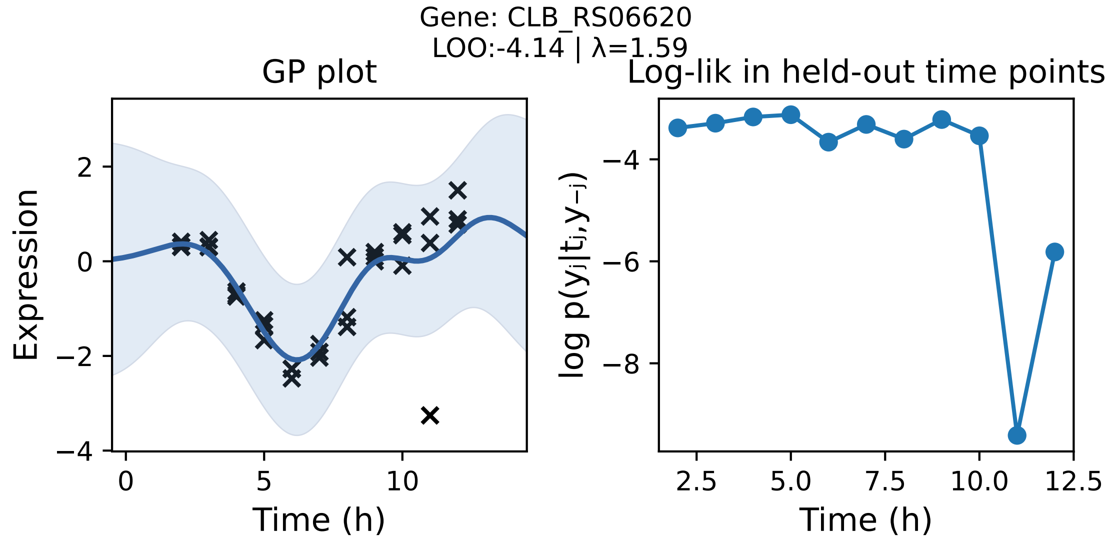

Gene: CLB_RS06620
LOO:-4.14 | λ=1.59
GP plot
Expression
Time (h)
2
0
−2
−4
0
5
10
Log-lik in held-out time points
log p(y_j|t_j,y_−j)
−4
−6
−8
2.5
5.0
7.5
10.0
12.5
Time (h)

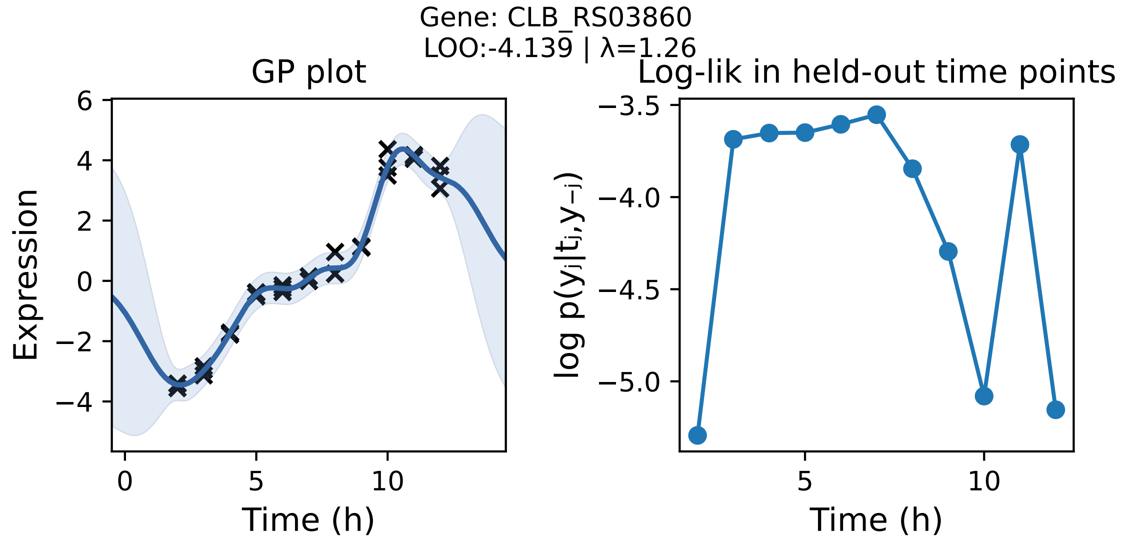

Gene: CLB_RS03860
LOO:-4.139 | λ=1.26
GP plot
Expression
Time (h)
Log-lik in held-out time points
log p(y_j|t_j,y_−j)
Time (h)

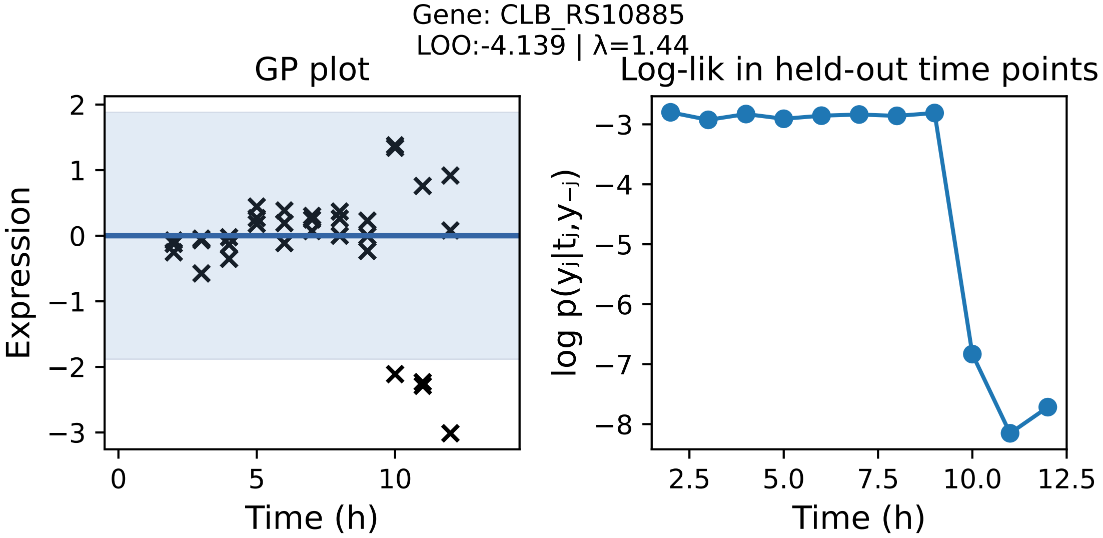

Gene: CLB_RS10885
LOO:-4.139 | λ=1.44
GP plot
Log-lik in held-out time points
Expression
Time (h)
log p(yj|tj,y−j)
Time (h)

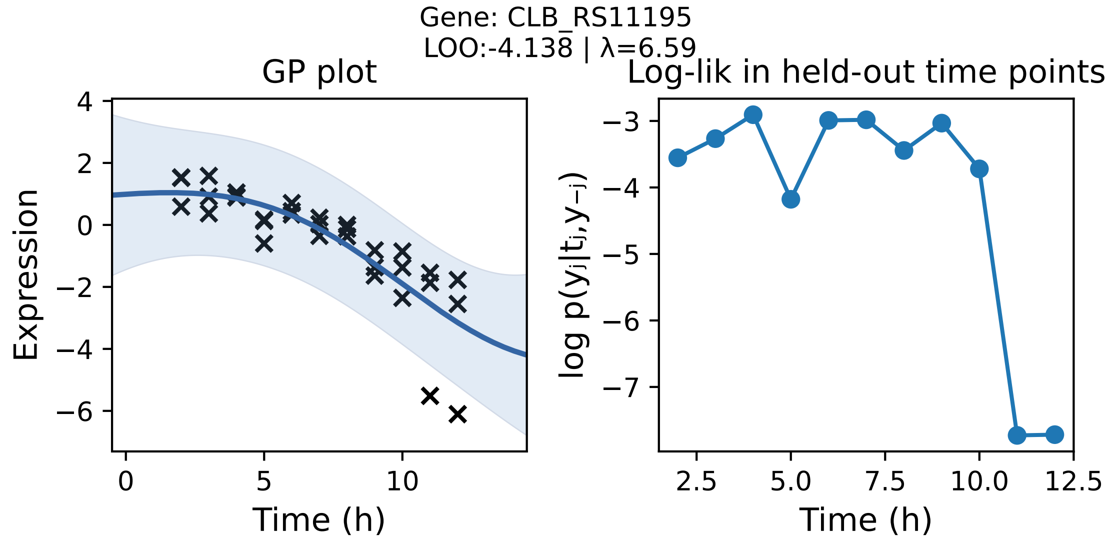

Gene: CLB_RS11195
LOO:-4.138 | λ=6.59
GP plot
Log-lik in held-out time points
Expression
Time (h)
log p(y_j|t_j,y_−j)
4
2
0
−2
−4
−6
0
5
10
−3
−4
−5
−6
−7
2.5
5.0
7.5
10.0
12.5

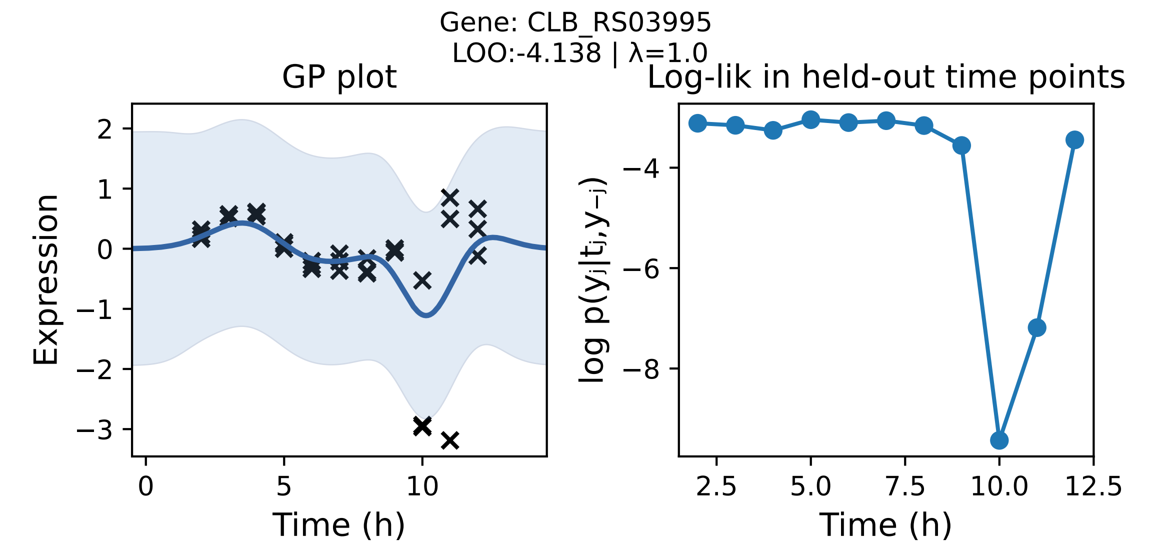

Gene: CLB_RS03995
LOO:-4.138 | λ=1.0
GP plot
Log-lik in held-out time points
Expression
2
1
0
−1
−2
−3
0
5
10
Time (h)
log p(y_j|t_j,y_−j)
−4
−6
−8
2.5
5.0
7.5
10.0
12.5
Time (h)

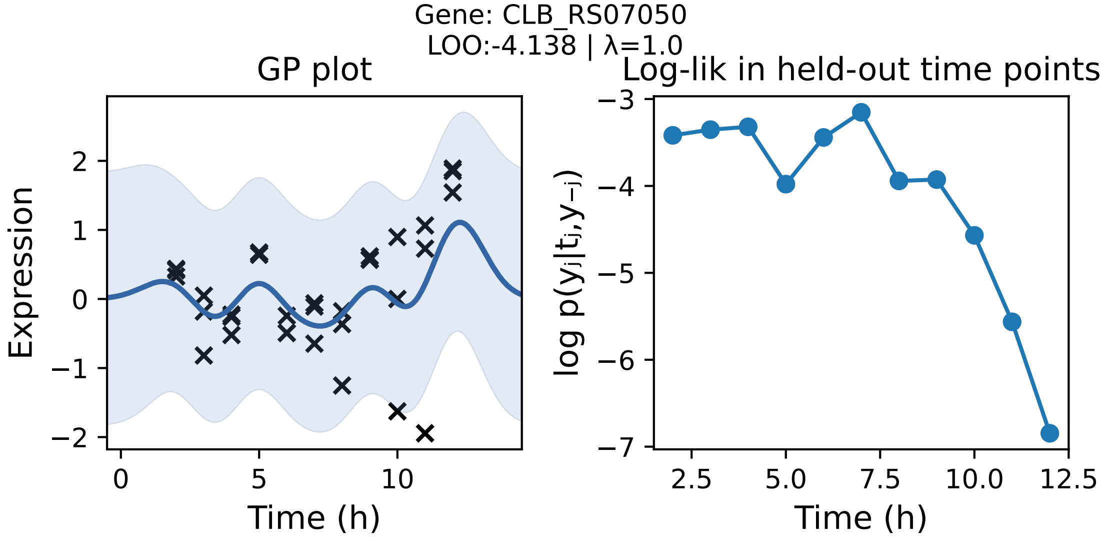
Gene: CLB_RS07050
LOO:-4.138 | λ=1.0
GP plot
Expression
Time (h)
Log-lik in held-out time points
log p(y_j|t_j,y_−j)
Time (h)

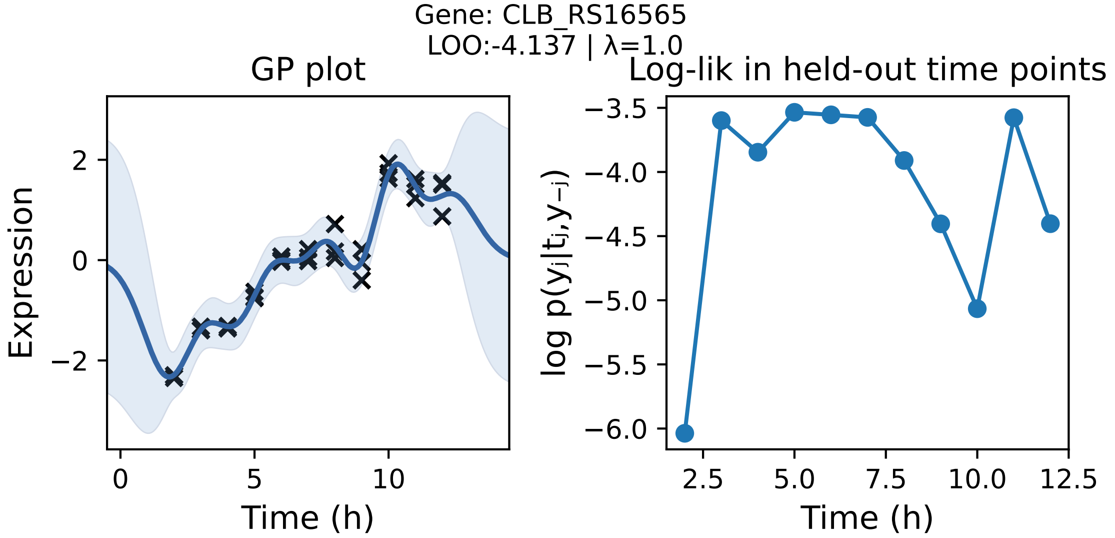

Gene: CLB_RS16565
LOO:-4.137 | λ=1.0
GP plot
Expression
Time (h)
Log-lik in held-out time points
log p(y_j|t_j,y_−j)
Time (h)

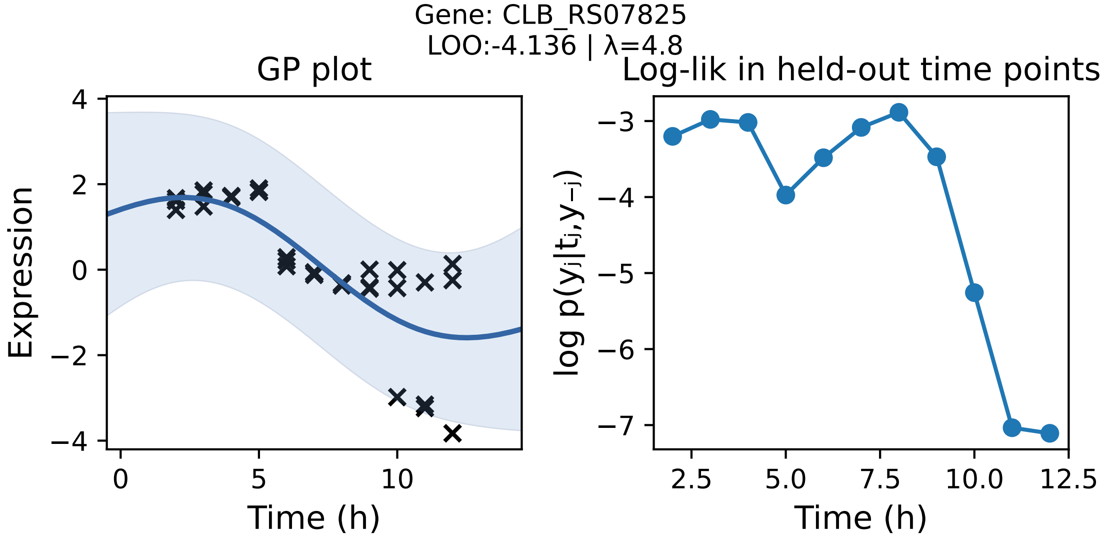

Gene: CLB_RS07825
LOO:-4.136 | λ=4.8
GP plot
Expression
Time (h)
Log-lik in held-out time points
log p(y_j|t_j,y_−j)
Time (h)

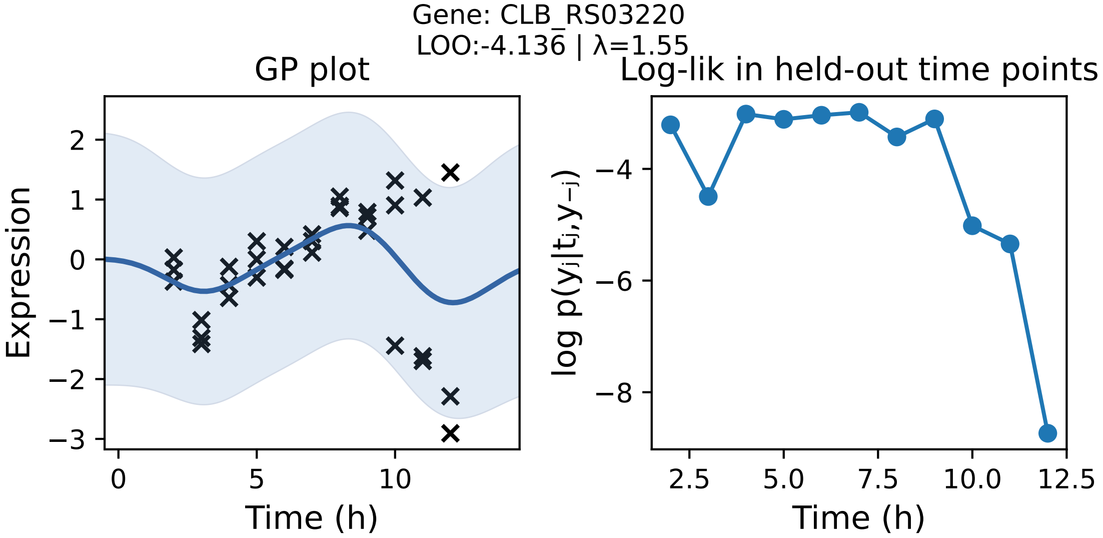

Gene: CLB_RS03220
LOO:-4.136 | λ=1.55
GP plot
Expression
Time (h)
Log-lik in held-out time points
log p(y_j|t_j,y_−j)
Time (h)

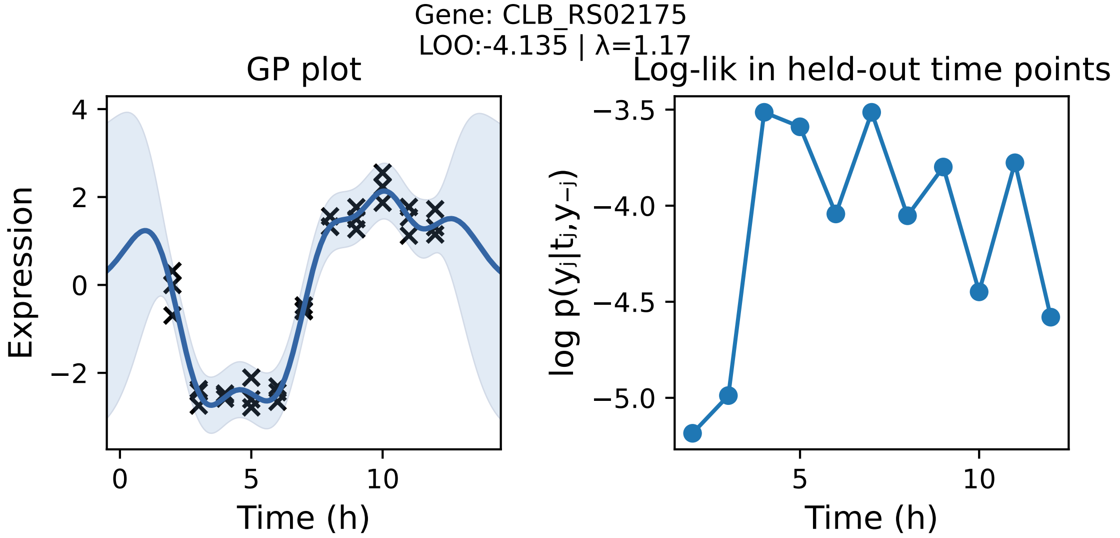

Gene: CLB_RS02175
LOO:-4.135 | λ=1.17
GP plot
Expression
4
2
0
−2
0
5
10
Time (h)
Log-lik in held-out time points
log p(y_j|t_j,y_−j)
−3.5
−4.0
−4.5
−5.0
5
10
Time (h)

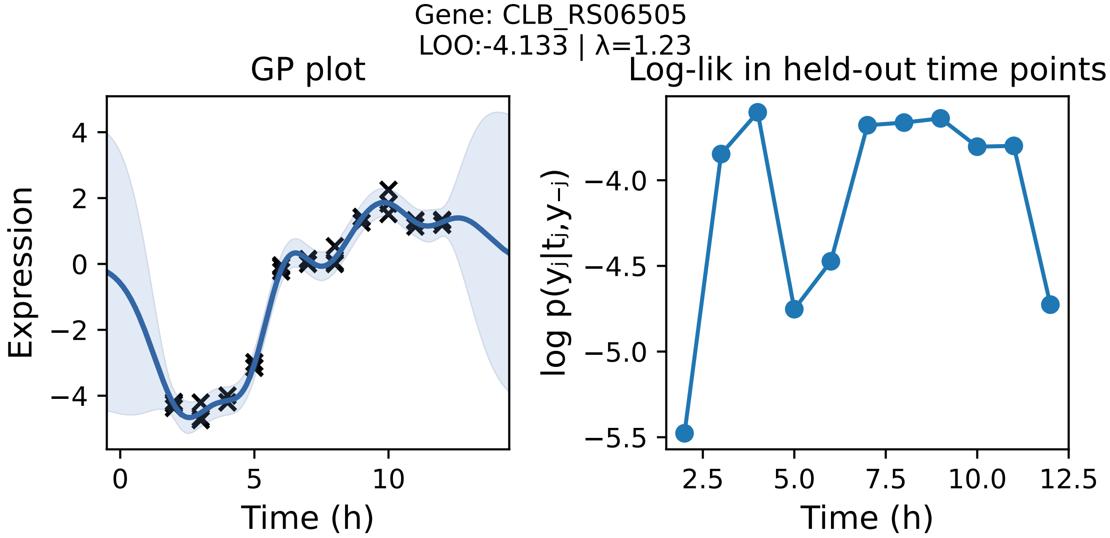

Gene: CLB_RS06505
LOO:-4.133 | λ=1.23
GP plot
Expression
Time (h)
Log-lik in held-out time points
log p(y_j|t_j,y_−j)
Time (h)

Gene: CLB_RS02560
LOO:-4.13 | λ=1.28

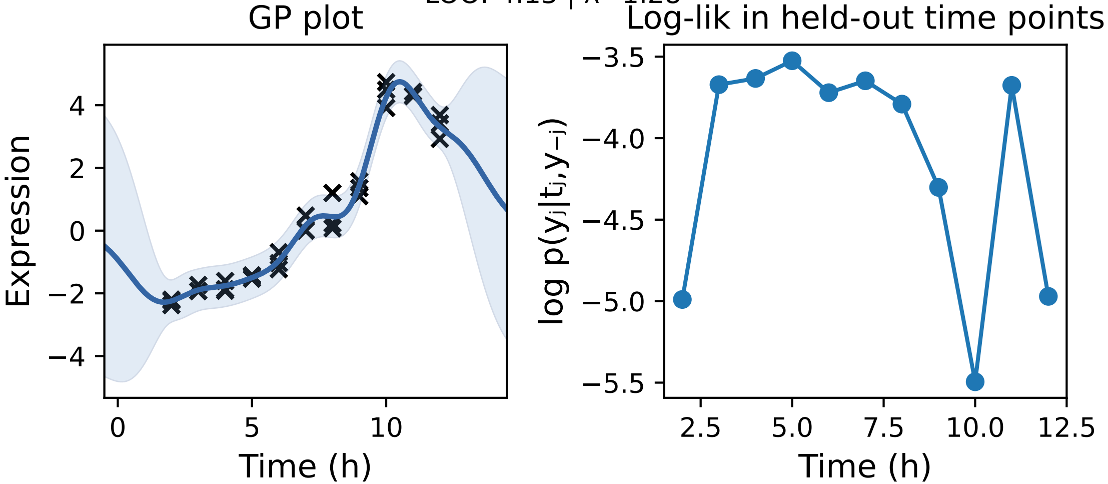

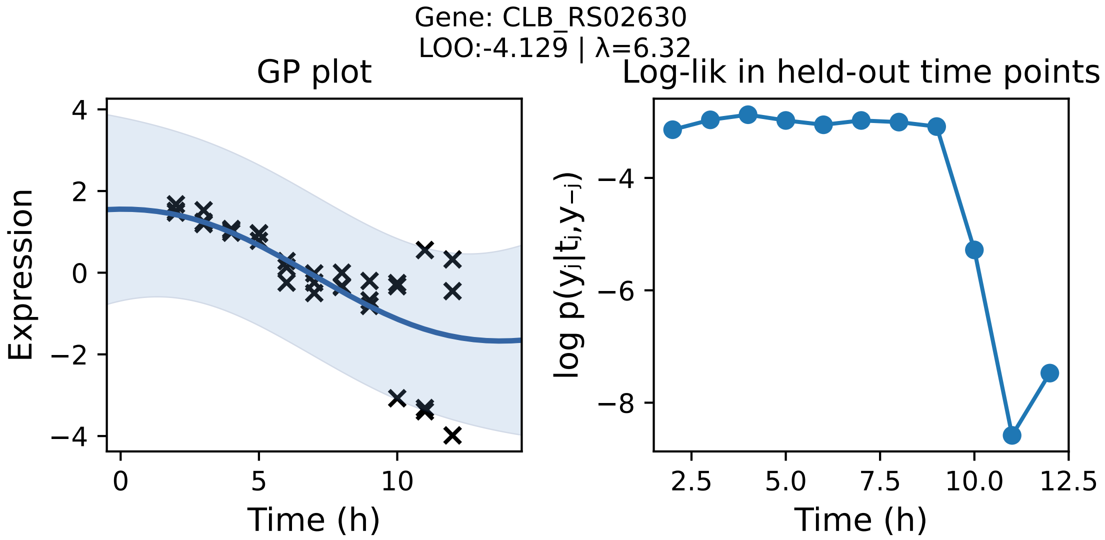

Gene: CLB_RS02630
LOO:-4.129 | λ=6.32
GP plot
Log-lik in held-out time points
Expression
Time (h)
log p(yj|tj,y−j)
Time (h)
4
2
0
−2
−4
0
5
10
−4
−6
−8
2.5
5.0
7.5
10.0
12.5

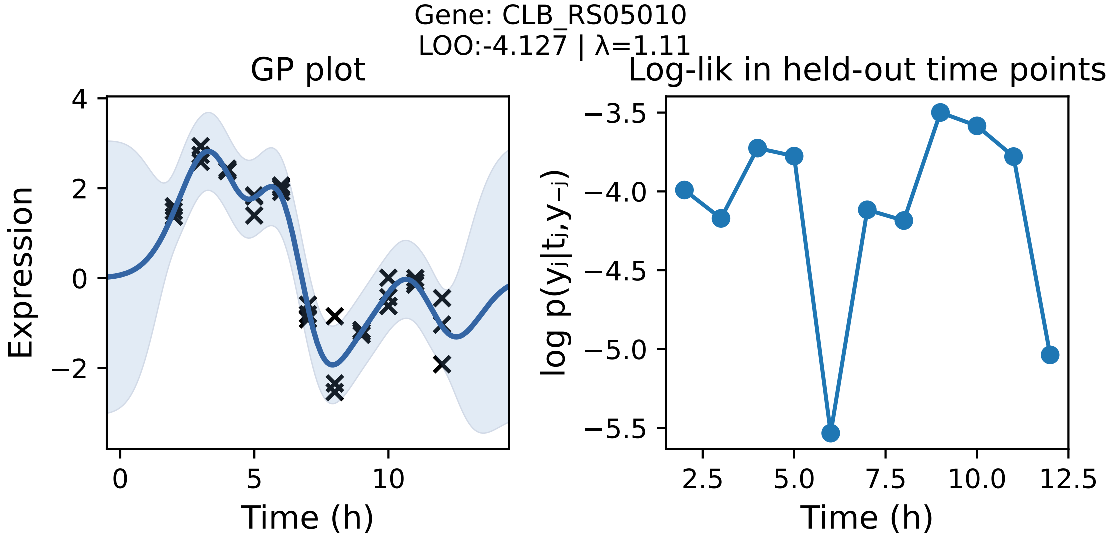

Gene: CLB_RS05010
LOO:-4.127 | λ=1.11
GP plot
Expression
Time (h)
Log-lik in held-out time points
log p(y_j|t_j,y_{-j})
Time (h)

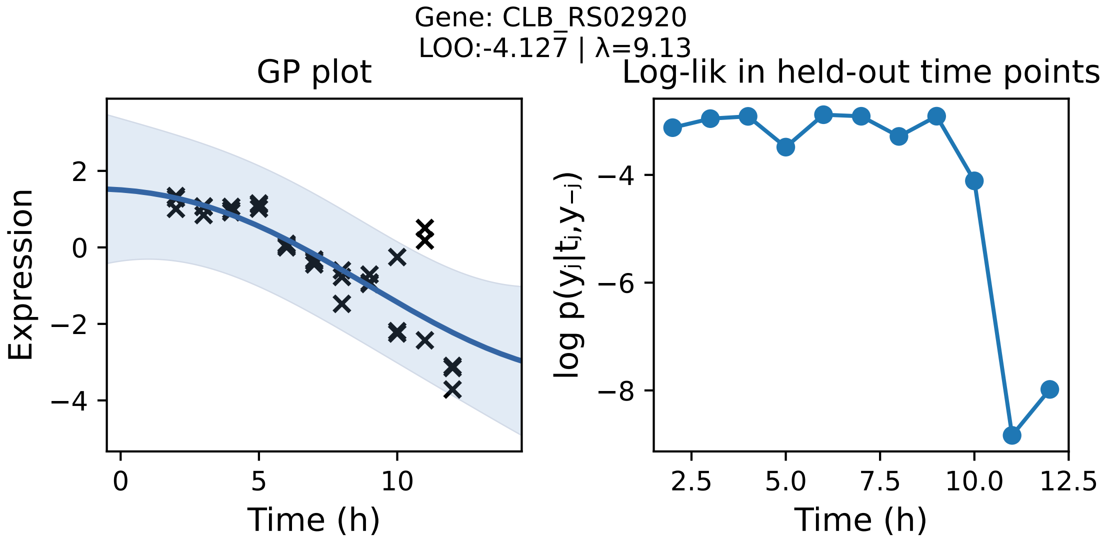

Gene: CLB_RS02920
LOO:-4.127 | λ=9.13
GP plot
Expression
Time (h)
2
0
−2
−4
0
5
10
Log-lik in held-out time points
log p(y_j|t_j,y_−j)
Time (h)
−4
−6
−8
2.5
5.0
7.5
10.0
12.5

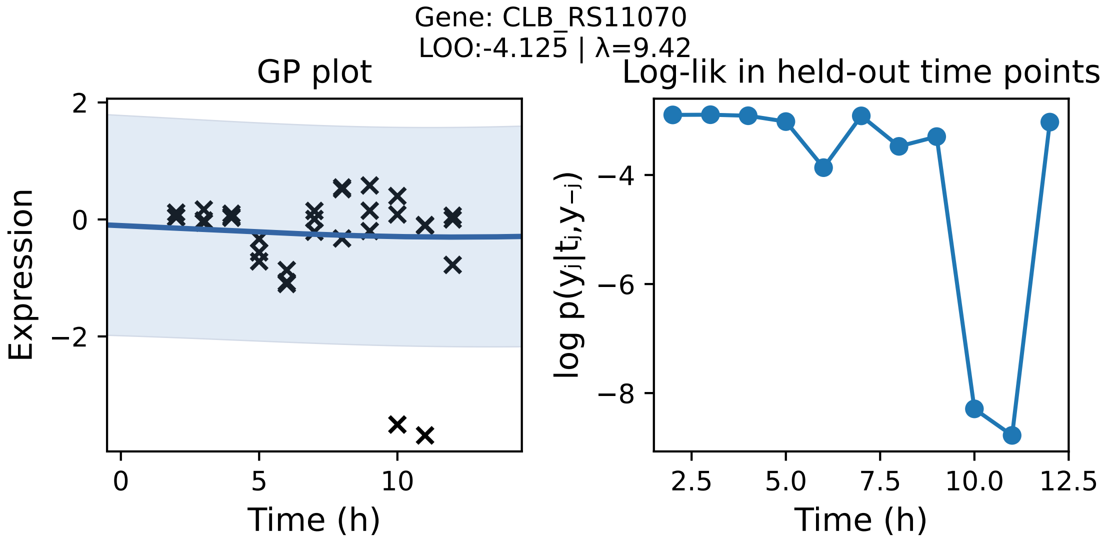

Gene: CLB_RS11070
LOO:-4.125 | λ=9.42
GP plot
Expression
Time (h)
Log-lik in held-out time points
log p(y_j|t_j,y_−j)
Time (h)

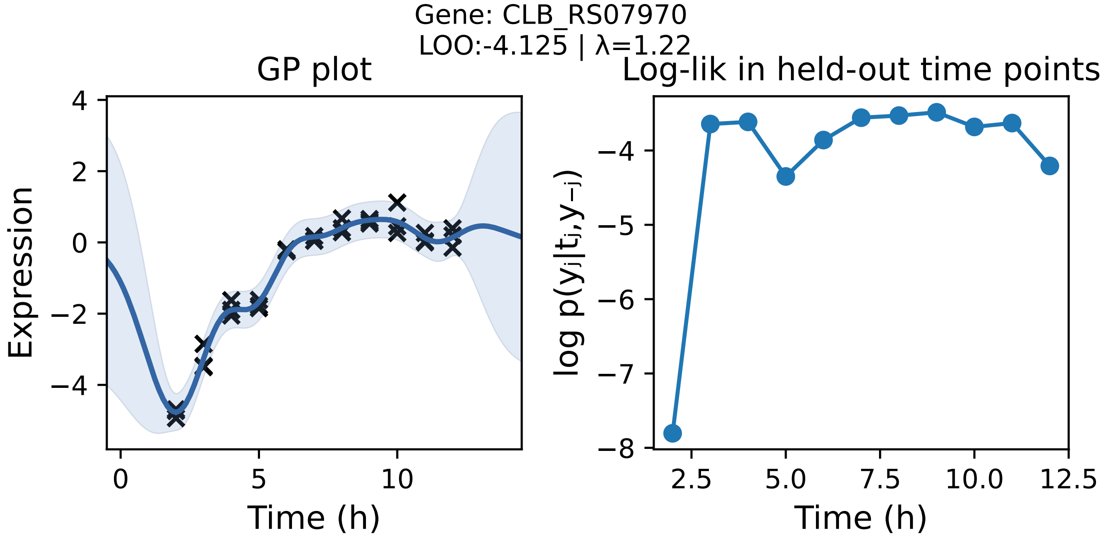

Gene: CLB_RS07970
LOO:-4.125 | λ=1.22
GP plot
Log-lik in held-out time points
Expression
Time (h)
log p(y_j|t_j,y_−j)
Time (h)
4
2
0
−2
−4
0
5
10
−4
−5
−6
−7
−8
2.5
5.0
7.5
10.0
12.5

Gene: CLB_RS06565
LOO:-4.125 | λ=2.12

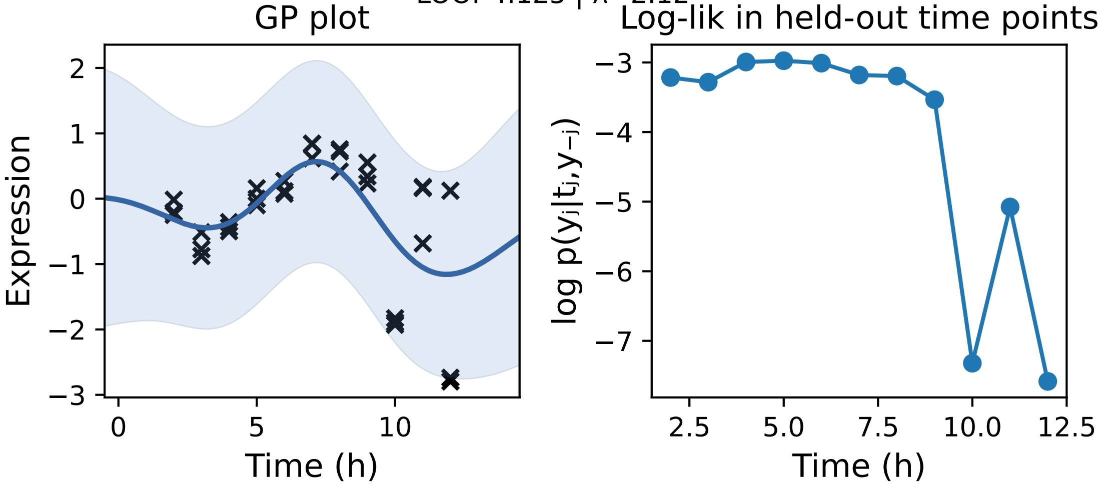

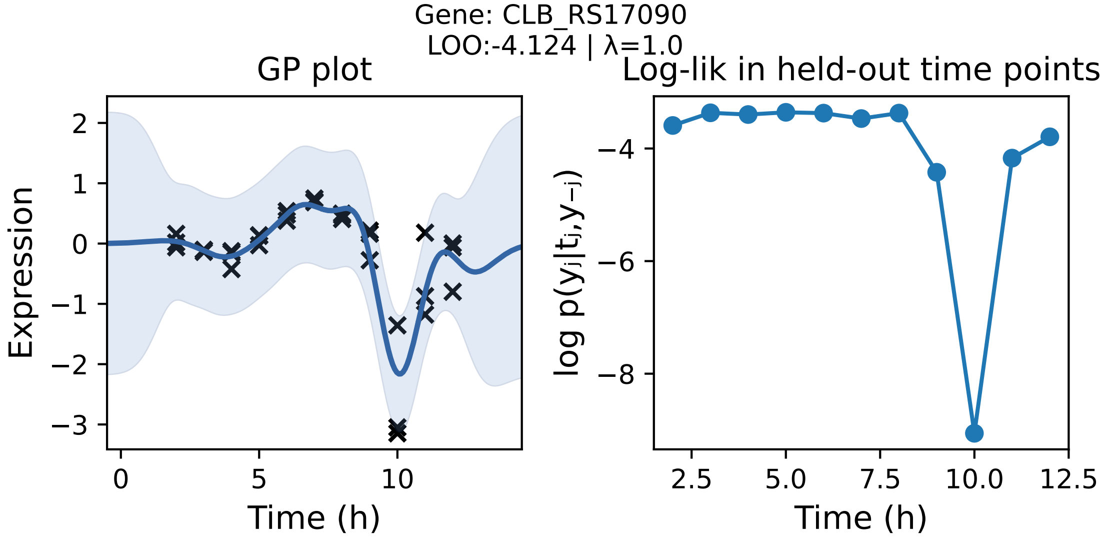

Gene: CLB_RS17090
LOO:-4.124 | λ=1.0
GP plot
Expression
Time (h)
2
1
0
−1
−2
−3
0
5
10
Log-lik in held-out time points
log p(y_j|t_j,y_−j)
Time (h)
−4
−6
−8
2.5
5.0
7.5
10.0
12.5

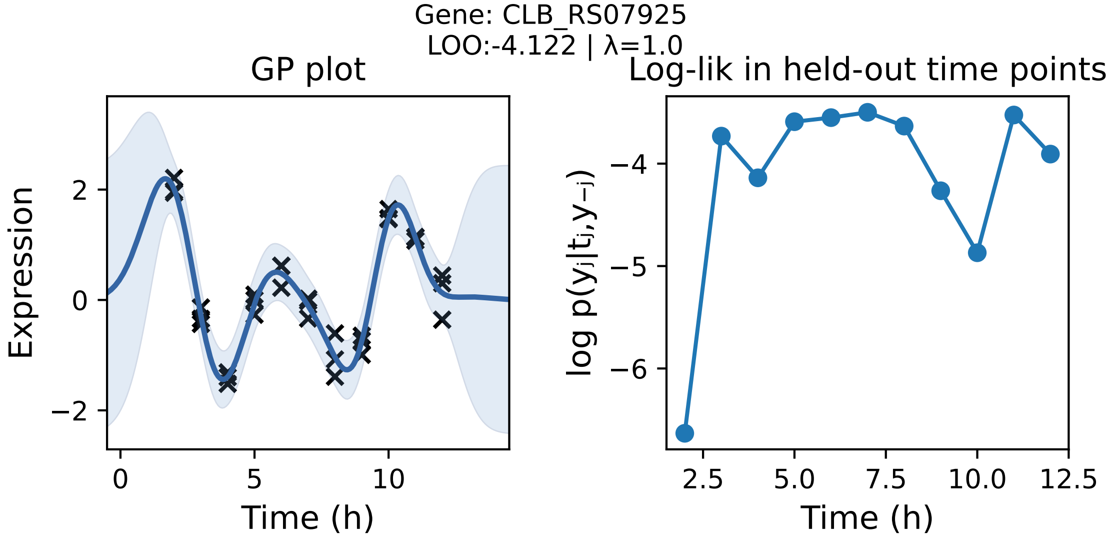

Gene: CLB_RS07925
LOO:-4.122 | λ=1.0
GP plot
Expression
2
0
−2
0
5
10
Time (h)
Log-lik in held-out time points
log p(yj|tj,y−j)
−4
−5
−6
2.5
5.0
7.5
10.0
12.5
Time (h)

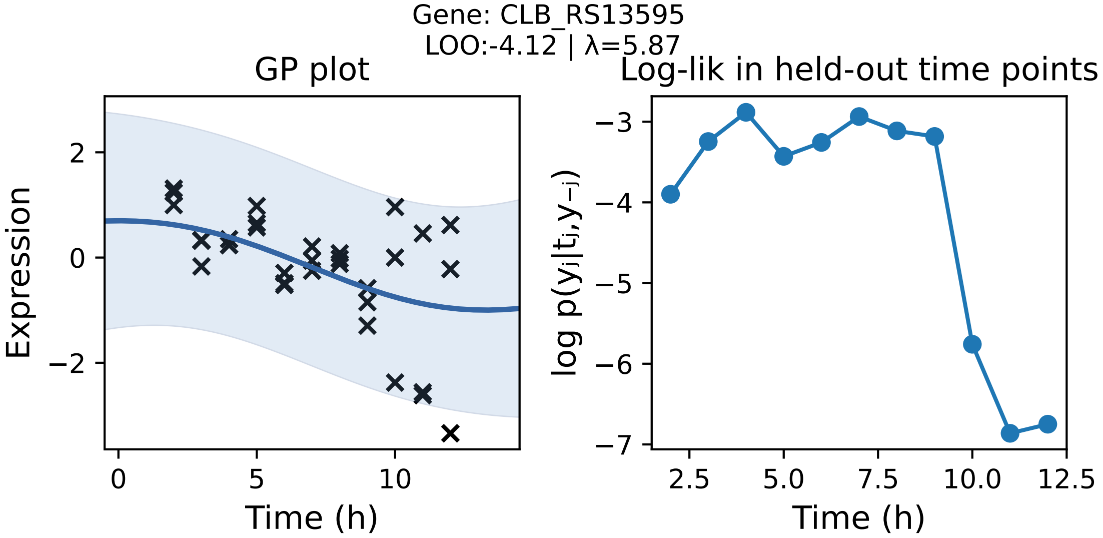
Gene: CLB_RS13595
LOO:-4.12 | λ=5.87
GP plot
Expression
2
0
−2
0
5
10
Time (h)
Log-lik in held-out time points
log p(y_j|t_j,y_−j)
−3
−4
−5
−6
−7
2.5
5.0
7.5
10.0
12.5
Time (h)

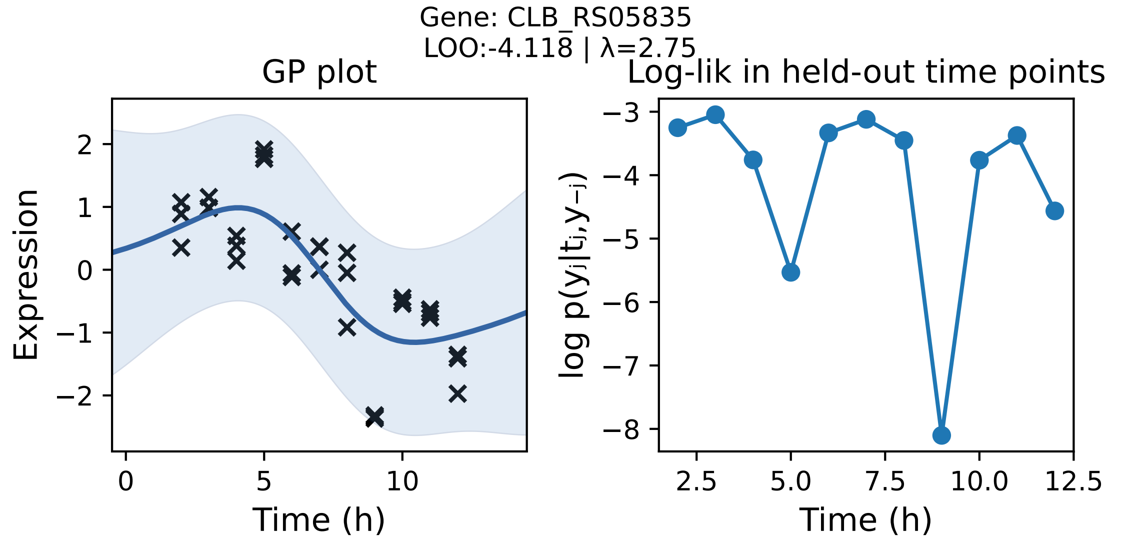

Gene: CLB_RS05835
LOO:-4.118 | λ=2.75
GP plot
Expression
Time (h)
Log-lik in held-out time points
log p(y_j|t_j,y_−j)
Time (h)

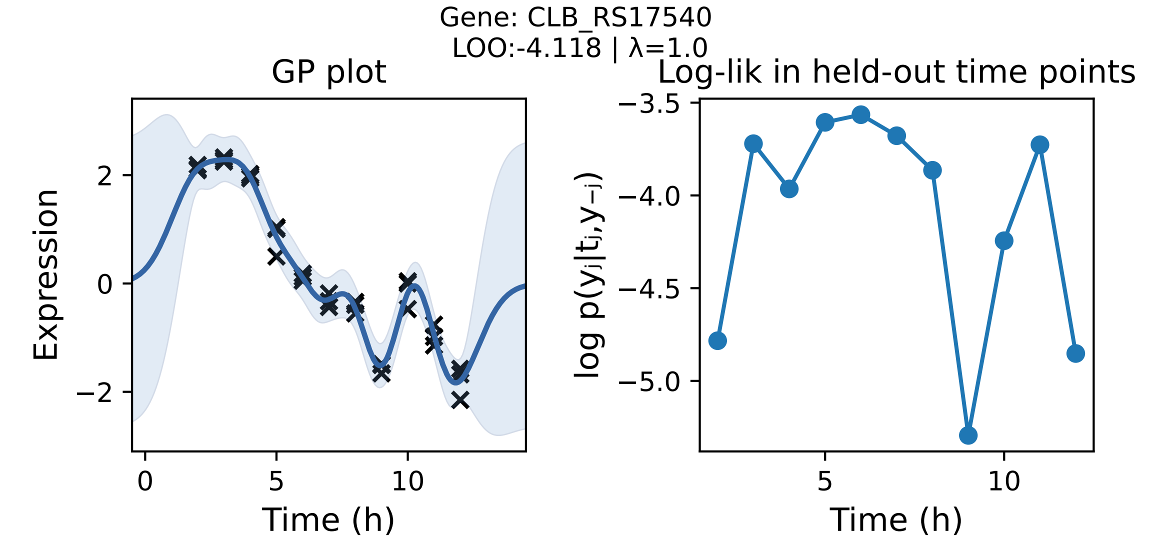

Gene: CLB_RS17540
LOO:-4.118 | λ=1.0
GP plot
Expression
Time (h)
Log-lik in held-out time points
log p(y_j|t_j,y_−j)
Time (h)

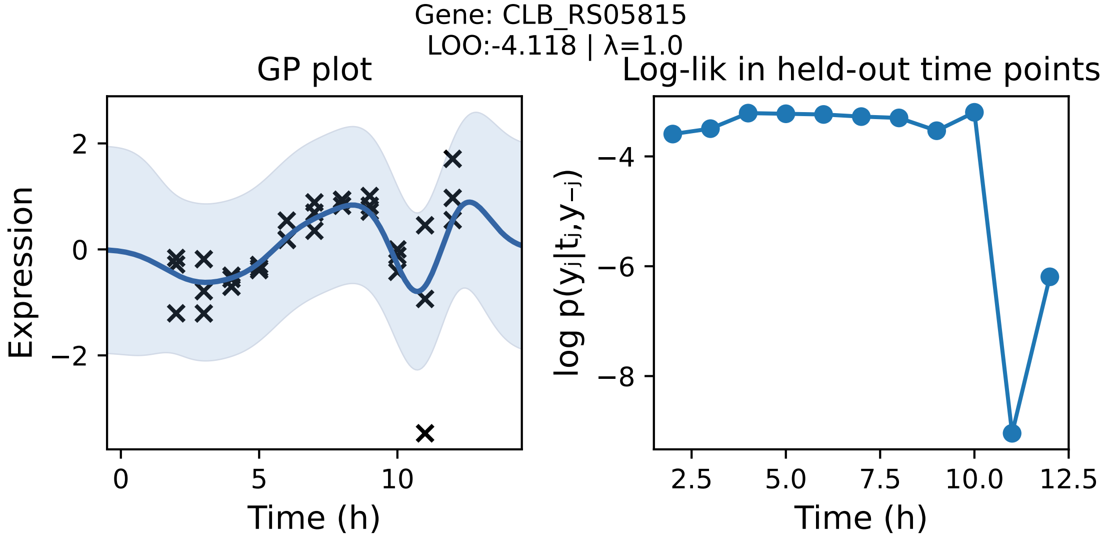
Gene: CLB_RS05815
LOO:-4.118 | λ=1.0
GP plot
Expression
2
0
−2
0
5
10
Time (h)
Log-lik in held-out time points
log p(y_j|t_j,y_−j)
−4
−6
−8
2.5
5.0
7.5
10.0
12.5
Time (h)

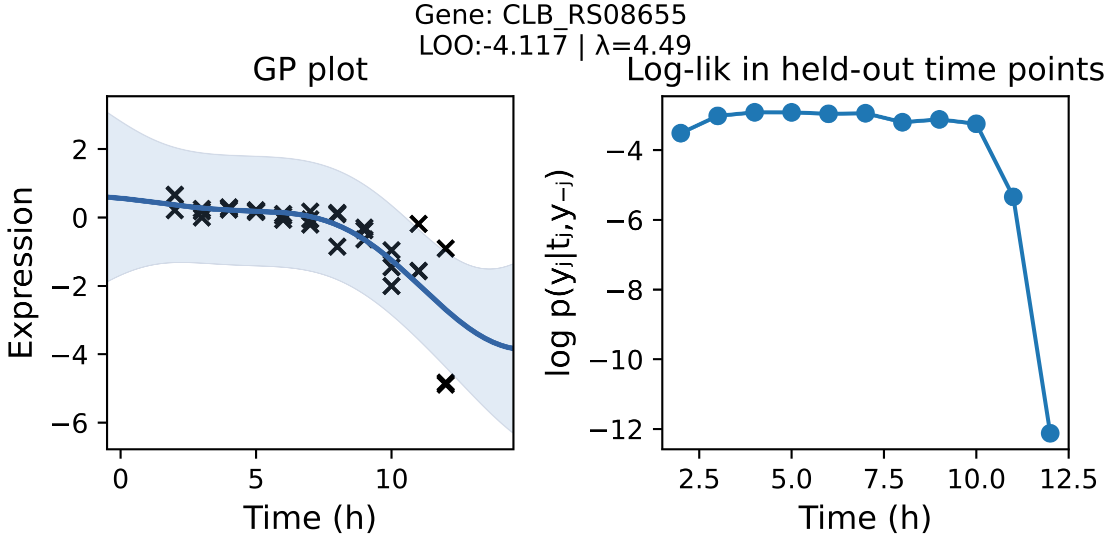
Gene: CLB_RS08655
LOO:-4.117 | λ=4.49
GP plot
Expression
Time (h)
Log-lik in held-out time points
log p(yj|tj,y−j)
Time (h)

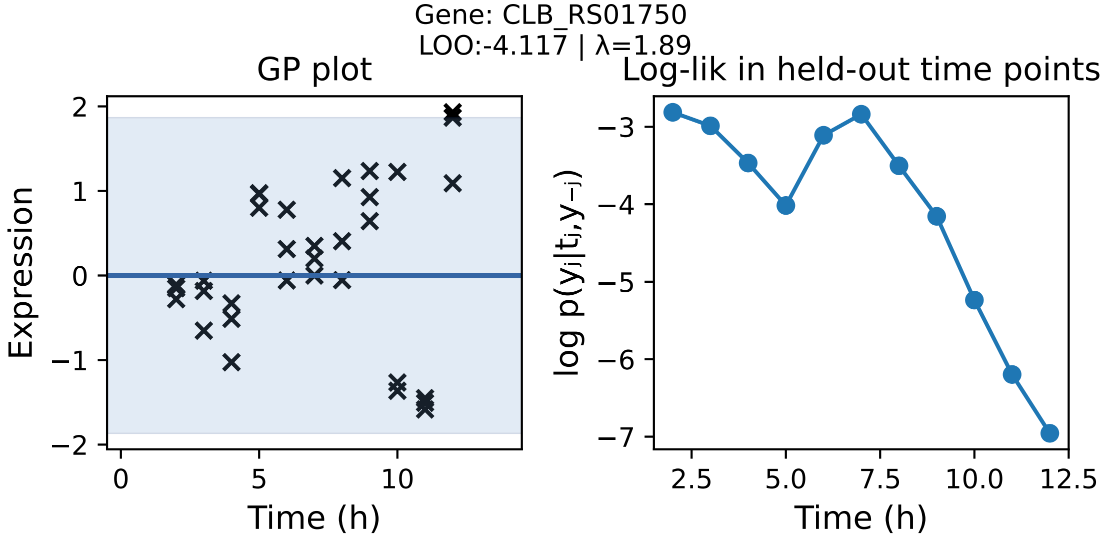

Gene: CLB_RS01750
LOO:-4.117 | λ=1.89
GP plot
Expression
Time (h)
2
1
0
−1
−2
0
5
10
Log-lik in held-out time points
log p(y_j|t_j,y_−j)
Time (h)
−3
−4
−5
−6
−7
2.5
5.0
7.5
10.0
12.5

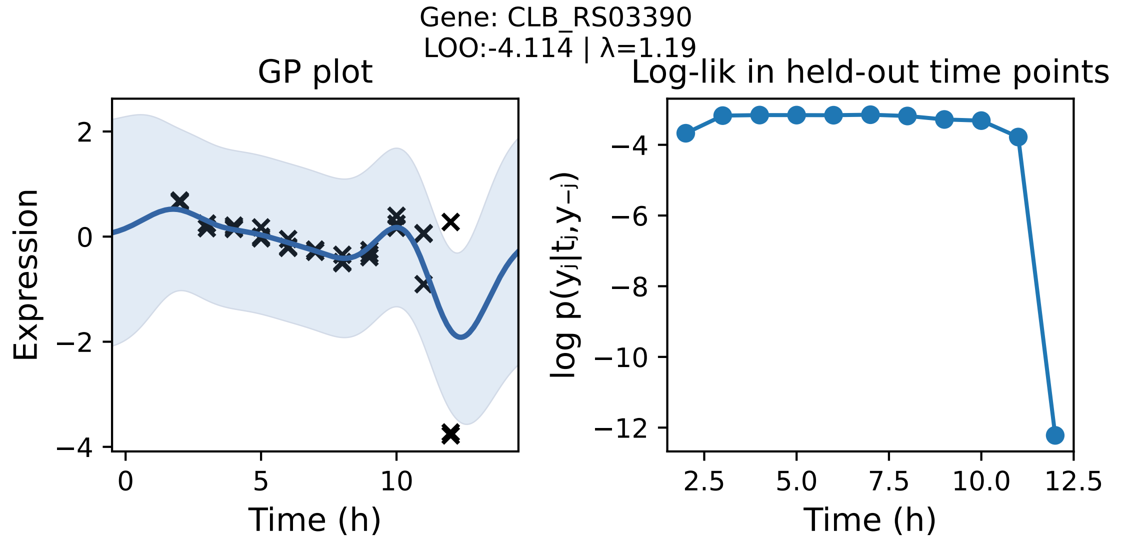
Gene: CLB_RS03390
LOO:-4.114 | λ=1.19
GP plot
Expression
Time (h)
2
0
−2
−4
0
5
10
Log-lik in held-out time points
log p(yⱼ|tⱼ,y₋ⱼ)
Time (h)
−4
−6
−8
−10
−12
2.5
5.0
7.5
10.0
12.5

Gene: CLB_RS13505
LOO:-4.114 | λ=1.16

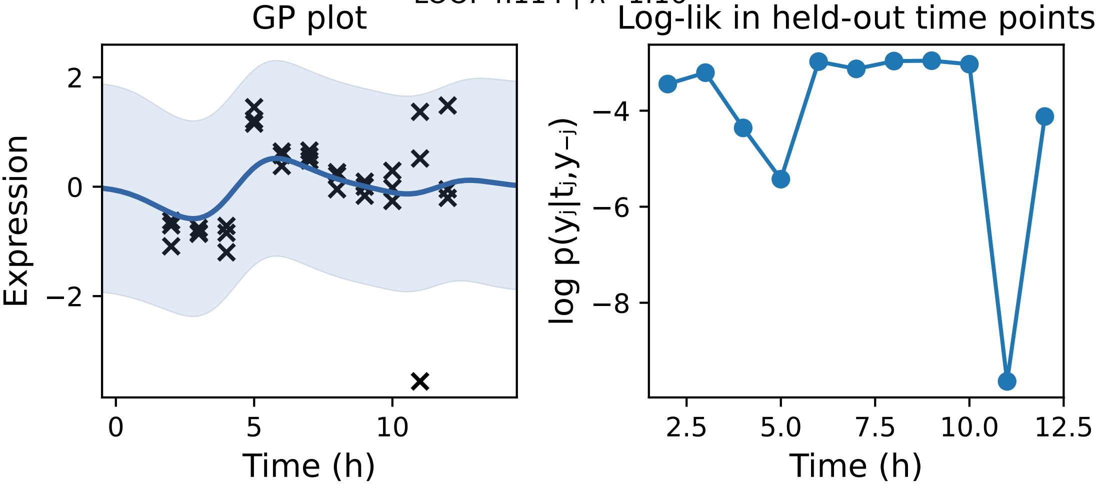

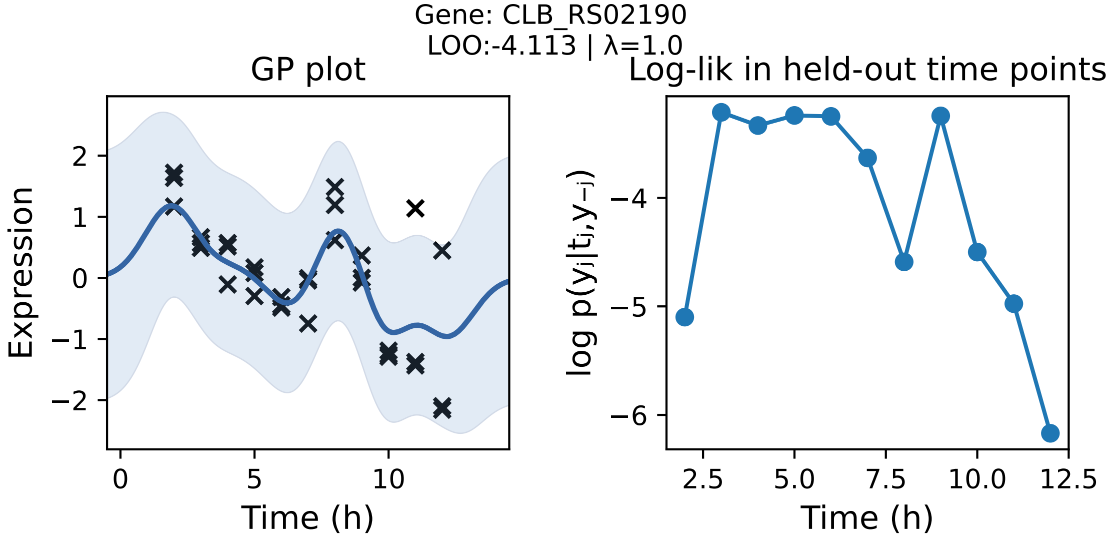

Gene: CLB_RS02190
LOO:-4.113 | λ=1.0
GP plot
Expression
Time (h)
2
1
0
−1
−2
0
5
10
Log-lik in held-out time points
log p(y_j|t_j,y_−j)
−4
−5
−6
2.5
5.0
7.5
10.0
12.5
Time (h)

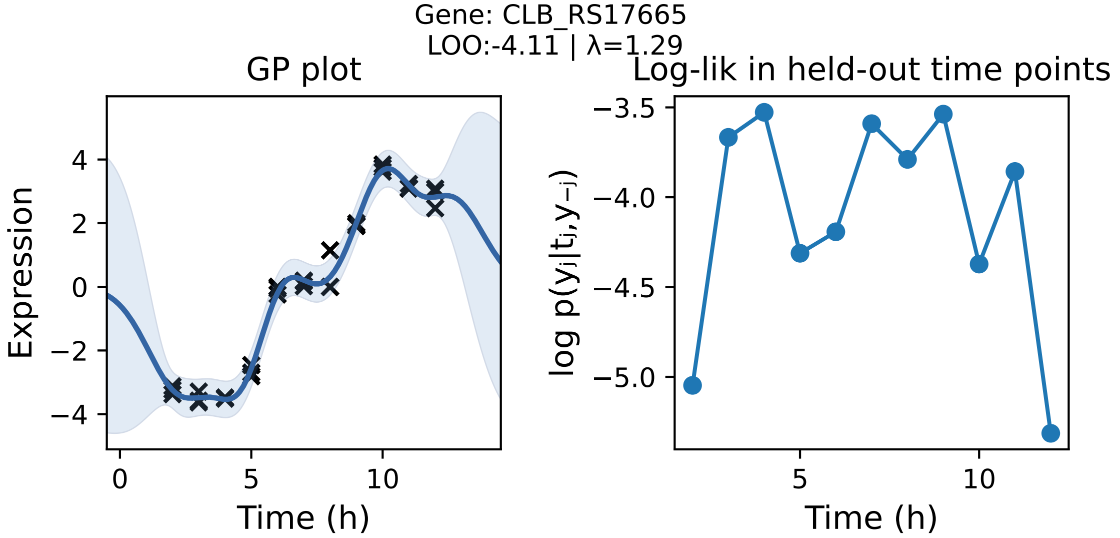

Gene: CLB_RS17665
LOO:-4.11 | λ=1.29
GP plot
Expression
Time (h)
Log-lik in held-out time points
log p(y_j|t_j,y_−j)
Time (h)

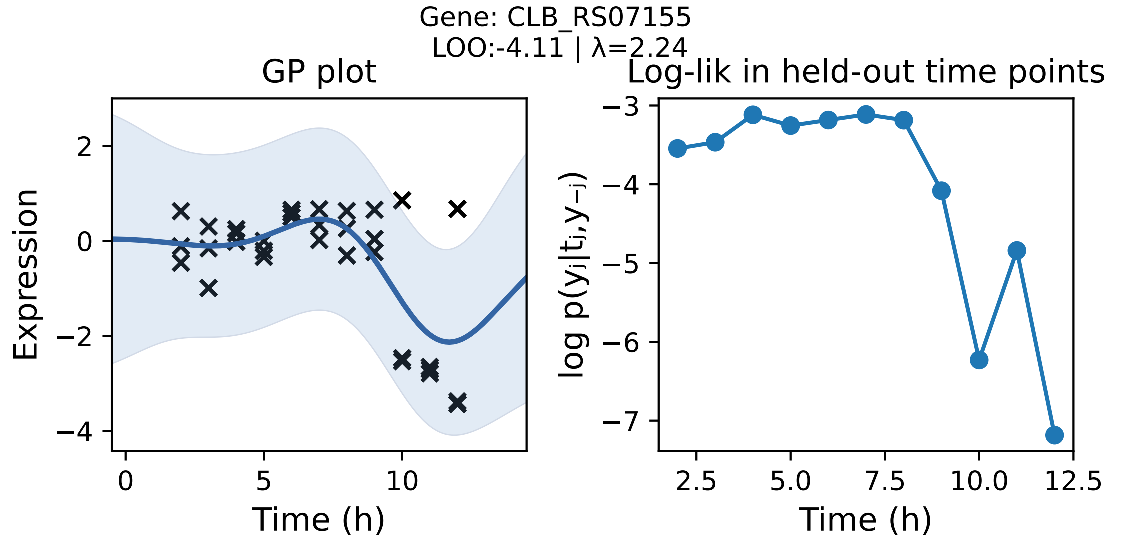

Gene: CLB_RS07155
LOO:-4.11 | λ=2.24
GP plot
Expression
Time (h)
Log-lik in held-out time points
log p(yj|tj,y−j)
Time (h)

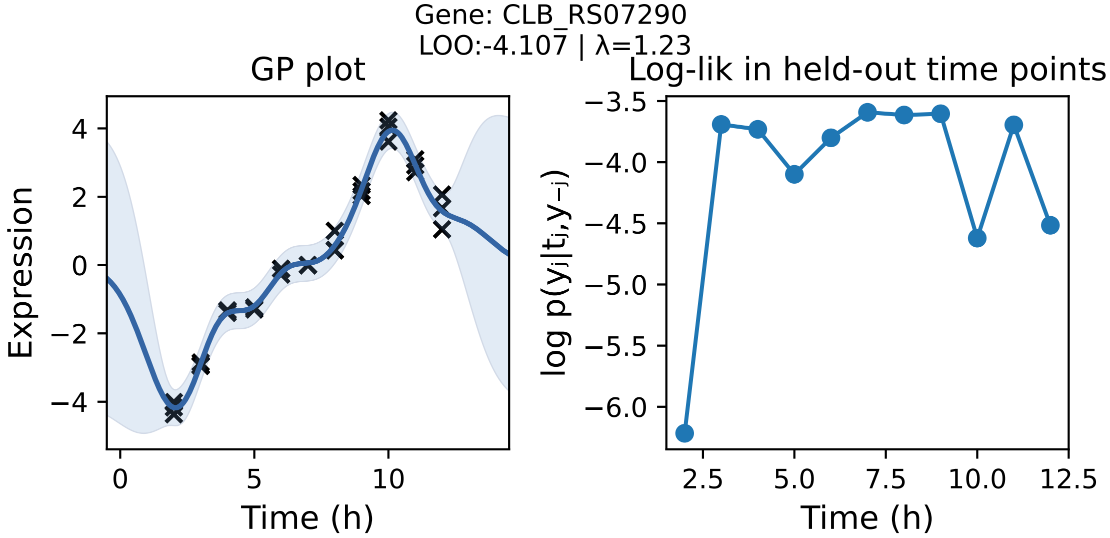

Gene: CLB_RS07290
LOO:-4.107 | λ=1.23
GP plot
Expression
Time (h)
Log-lik in held-out time points
log p(y_j|t_j,y_−j)
Time (h)

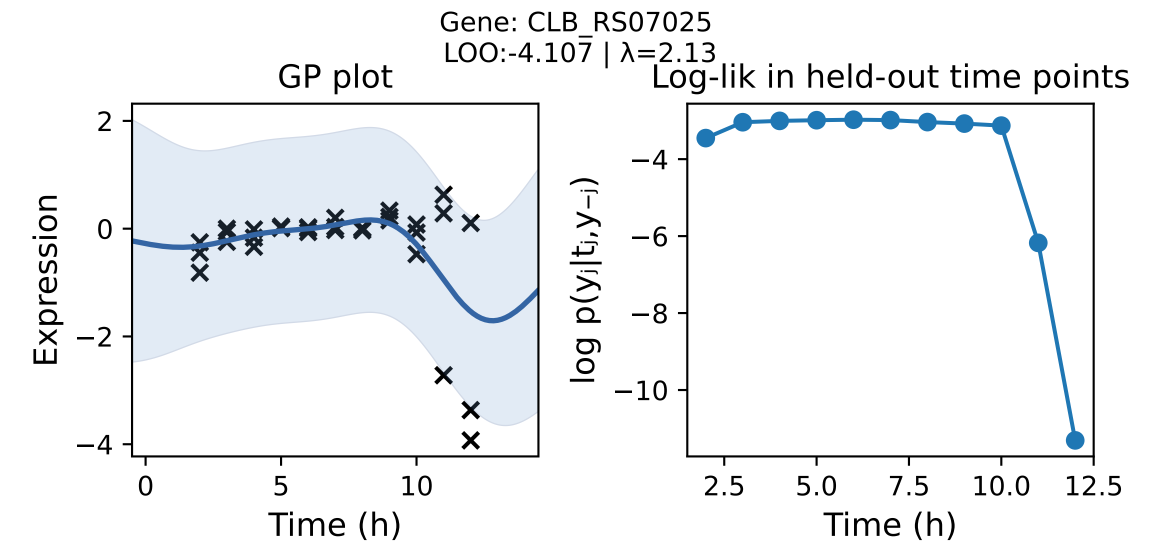

Gene: CLB_RS07025
LOO:-4.107 | λ=2.13
GP plot
Log-lik in held-out time points
Expression
Time (h)
log p(yj|tj,y−j)
Time (h)

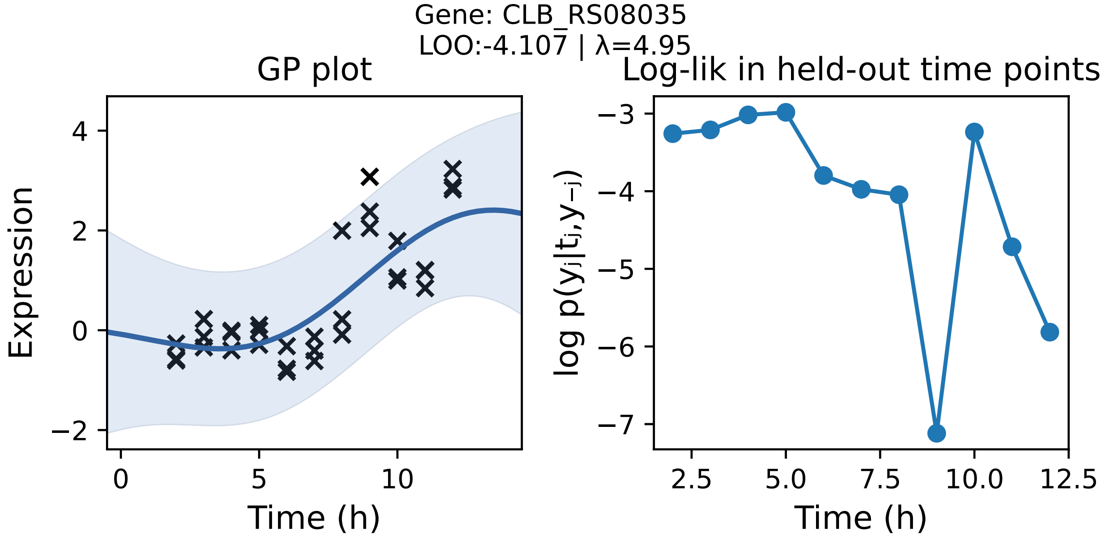

Gene: CLB_RS08035
LOO:-4.107 | λ=4.95
GP plot
Expression
Time (h)
Log-lik in held-out time points
log p(y_j|t_j,y_−j)
Time (h)

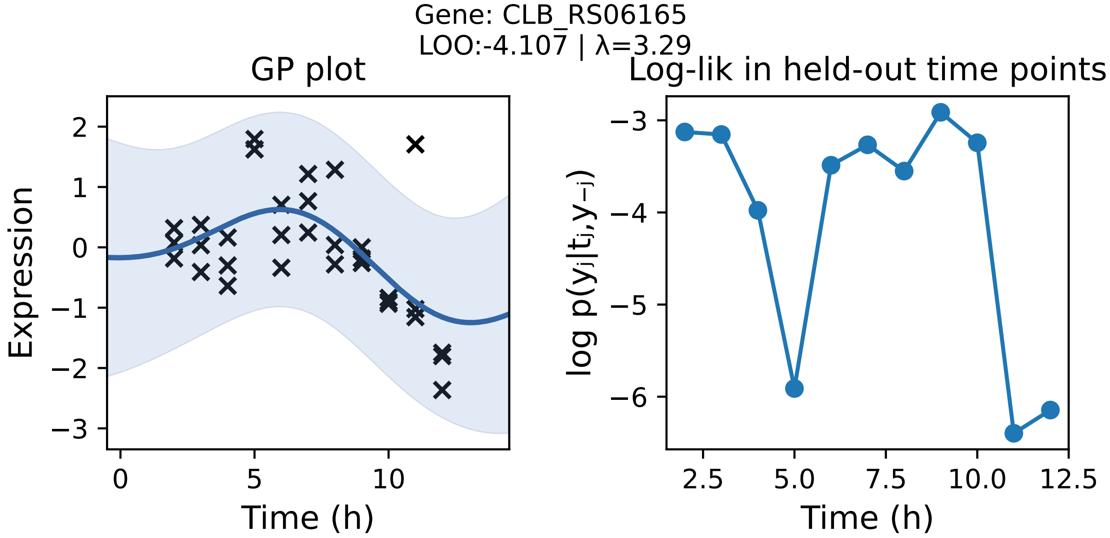
Gene: CLB_RS06165
LOO:-4.107 | λ=3.29
GP plot
Expression
Time (h)
Log-lik in held-out time points
log p(y_j|t_j,y_−j)
Time (h)

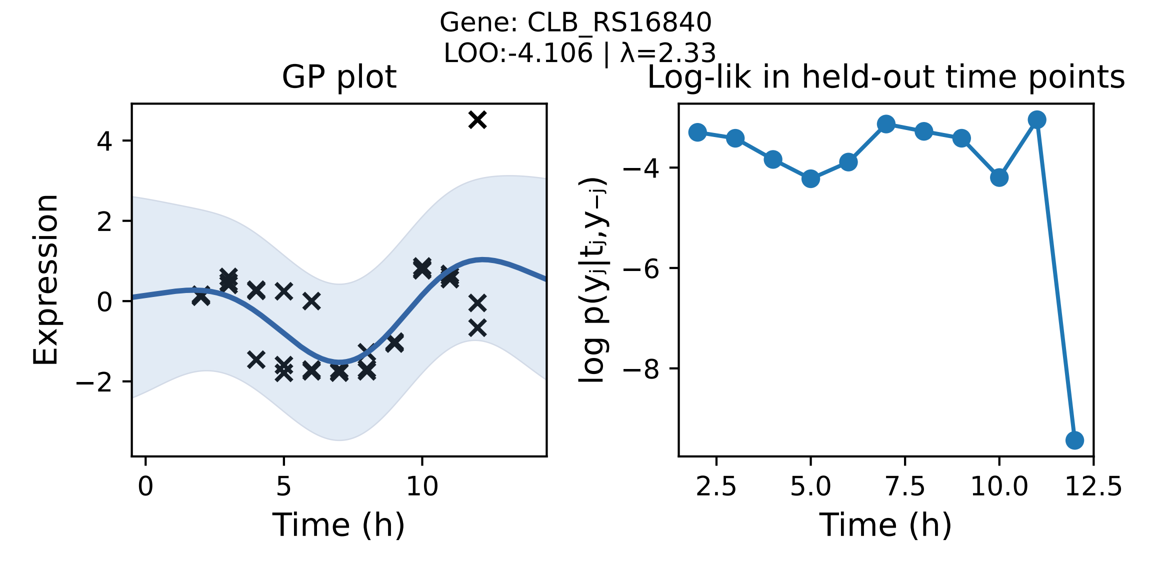

Gene: CLB_RS16840
LOO:-4.106 | λ=2.33
GP plot
Log-lik in held-out time points
Expression
4
2
0
−2
0
5
10
Time (h)
log p(y_j|t_j,y_−j)
−4
−6
−8
2.5
5.0
7.5
10.0
12.5
Time (h)

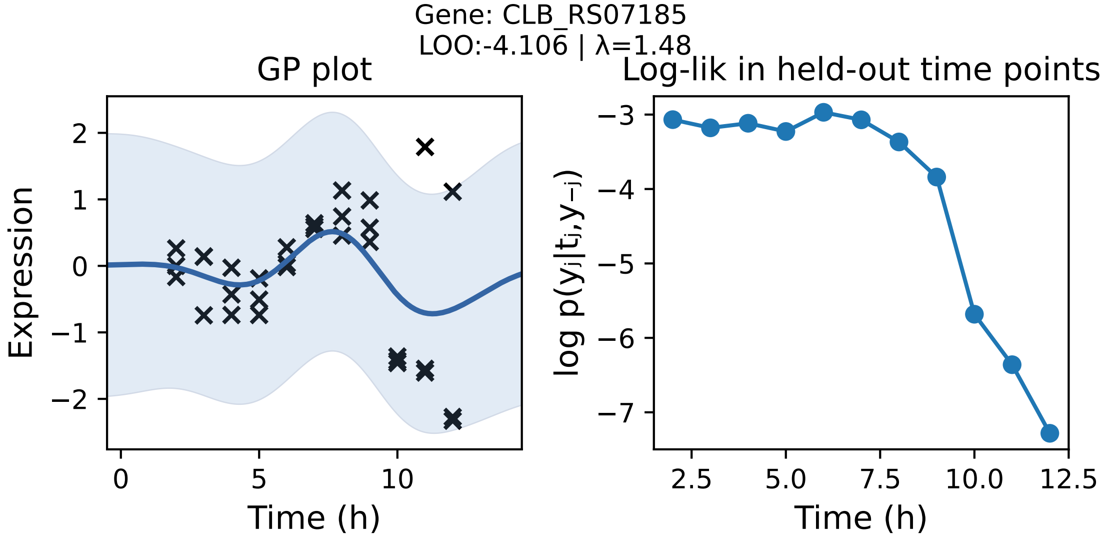

Gene: CLB_RS07185
LOO:-4.106 | λ=1.48
GP plot
Expression
Time (h)
Log-lik in held-out time points
log p(y_j|t_j,y_−j)
Time (h)

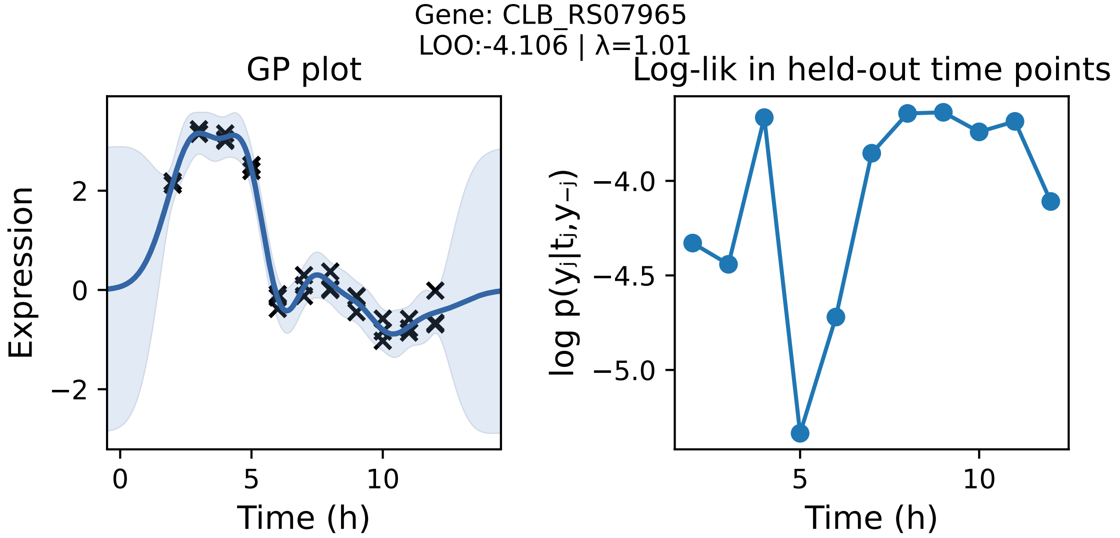
Gene: CLB_RS07965
LOO:-4.106 | λ=1.01
GP plot
Log-lik in held-out time points
Expression
Time (h)
log p(y_j|t_j,y_−j)
Time (h)
2
0
−2
0
5
10
−4.0
−4.5
−5.0
5
10

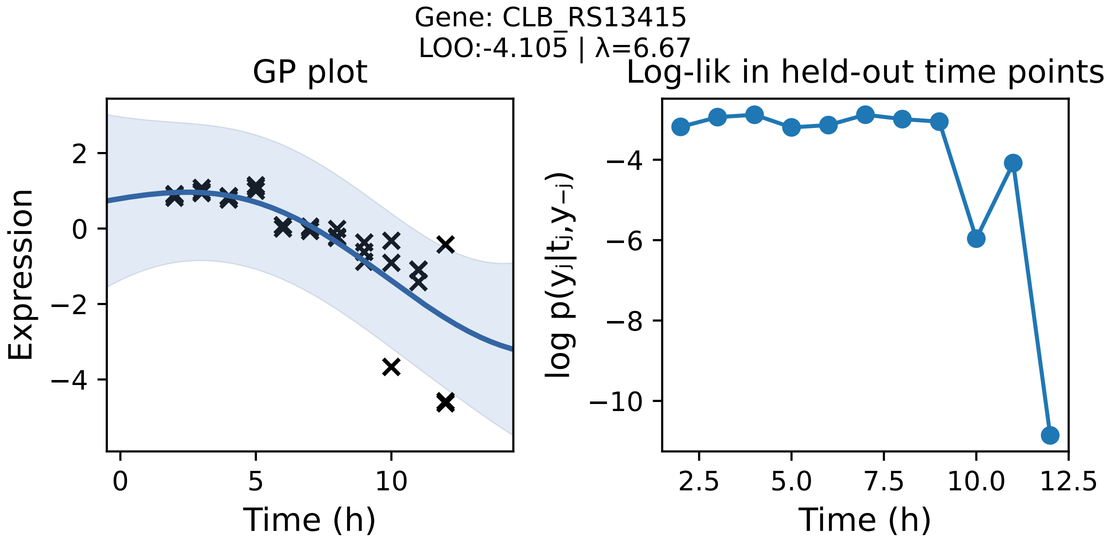

Gene: CLB_RS13415
LOO:-4.105 | λ=6.67
GP plot
Expression
2
0
−2
−4
0
5
10
Time (h)
Log-lik in held-out time points
log p(y_j|t_j,y_−j)
−4
−6
−8
−10
2.5
5.0
7.5
10.0
12.5
Time (h)

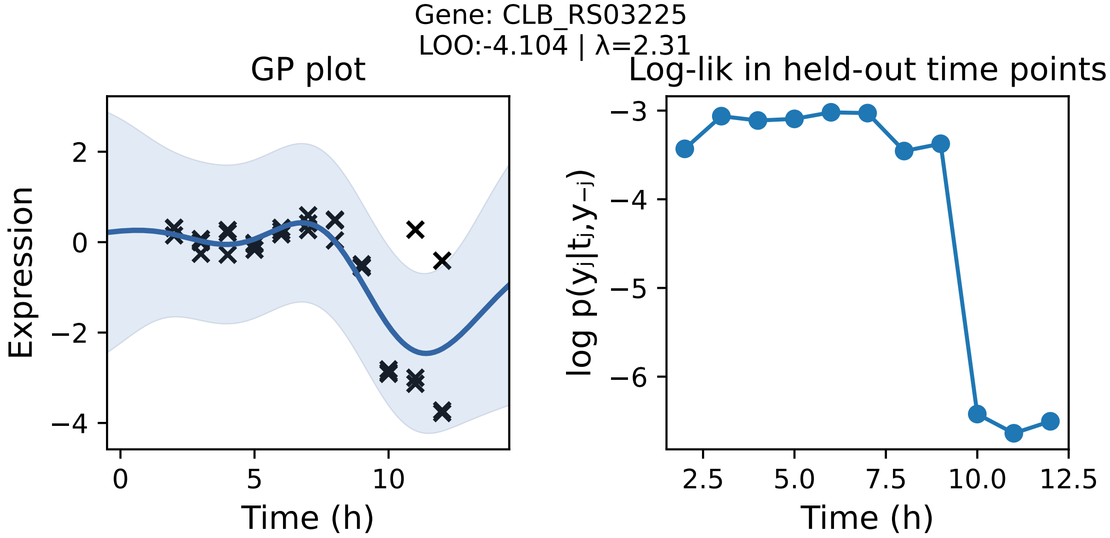

Gene: CLB_RS03225
LOO:-4.104 | λ=2.31
GP plot
Expression
Time (h)
Log-lik in held-out time points
log p(y_j|t_j,y_−j)
Time (h)

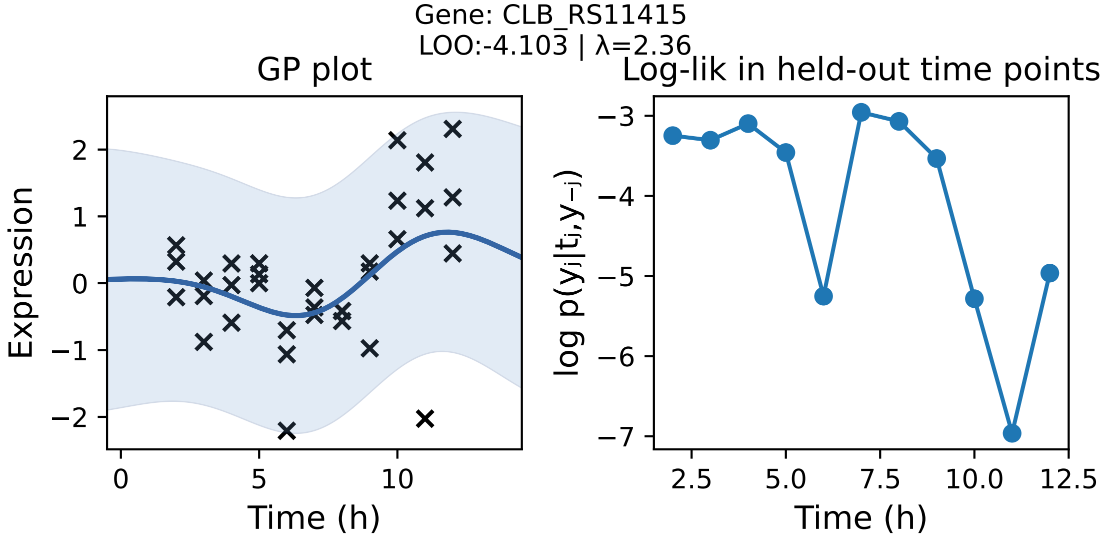
Gene: CLB_RS11415
LOO:-4.103 | λ=2.36
GP plot
Expression
Time (h)
Log-lik in held-out time points
log p(y_j|t_j,y_−j)
Time (h)

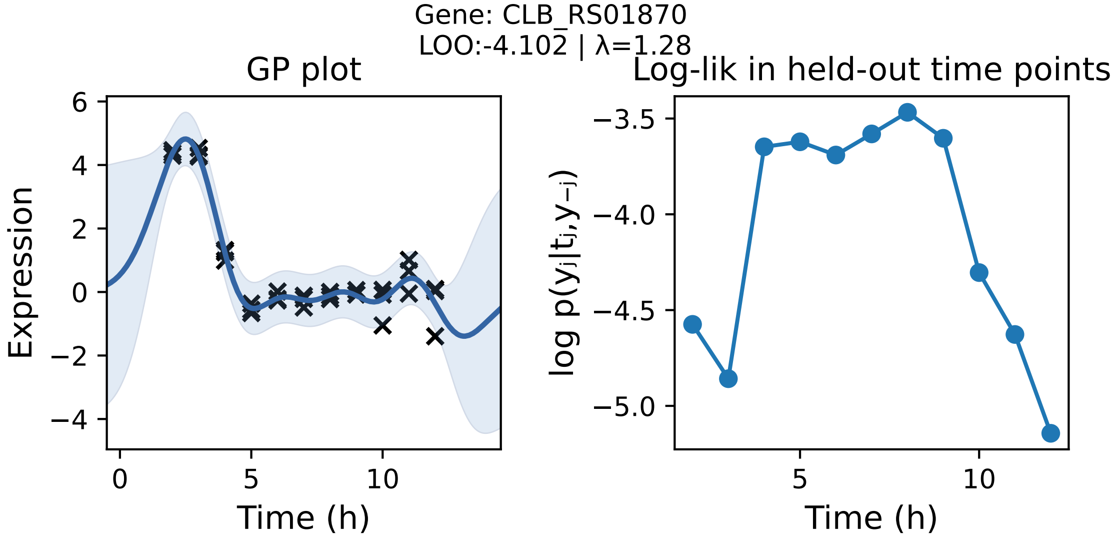

Gene: CLB_RS01870
LOO:-4.102 | λ=1.28
GP plot
Expression
Time (h)
Log-lik in held-out time points
log p(y_j|t_j,y_−j)
Time (h)

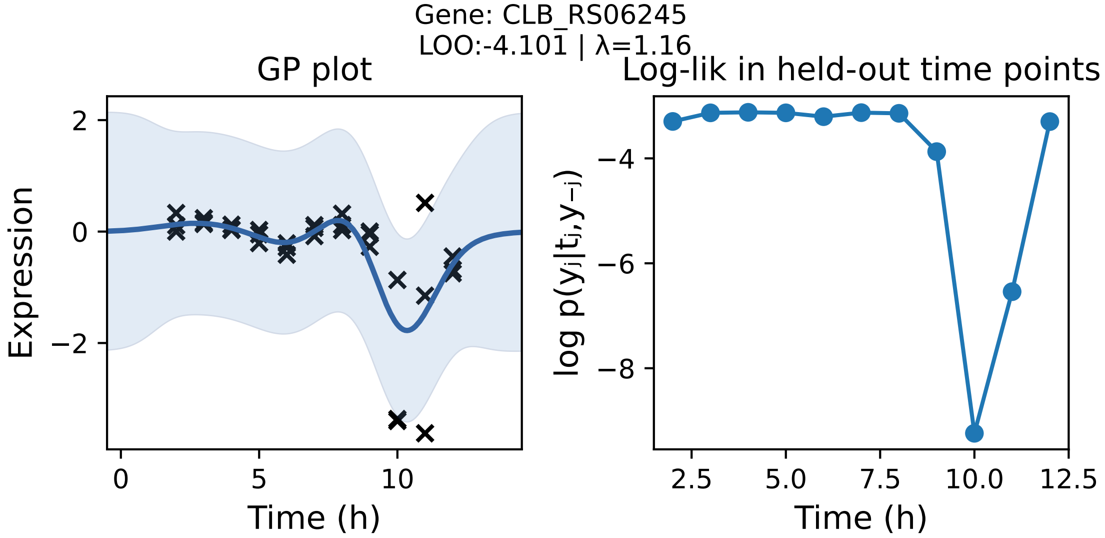

Gene: CLB_RS06245
LOO:-4.101 | λ=1.16
GP plot
Log-lik in held-out time points
Expression
2
0
−2
0
5
10
Time (h)
log p(yj|tj,y−j)
−4
−6
−8
2.5
5.0
7.5
10.0
12.5
Time (h)

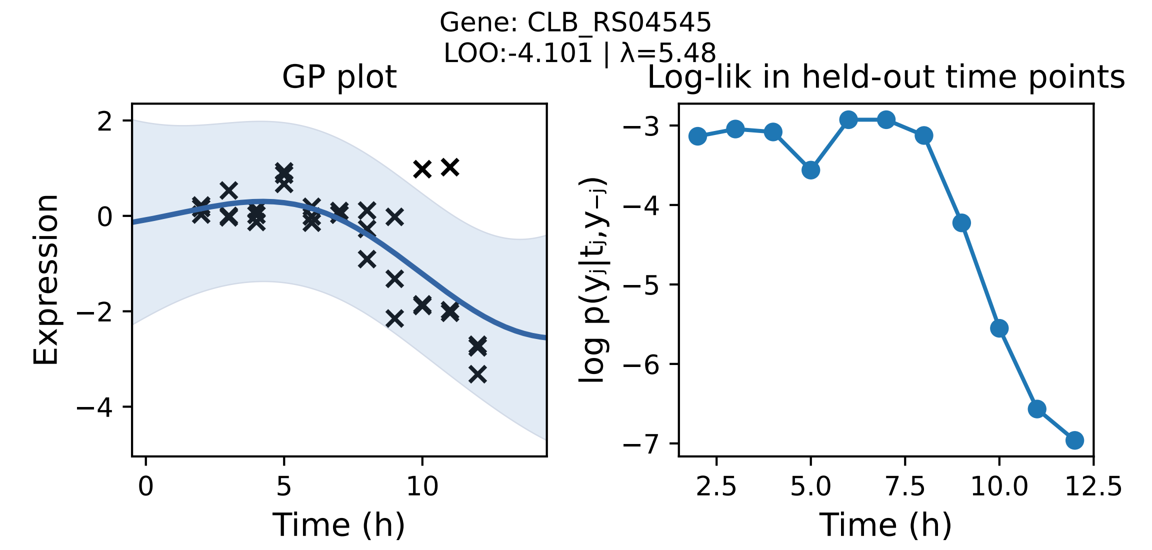

Gene: CLB_RS04545
LOO:-4.101 | λ=5.48
GP plot
Log-lik in held-out time points
Expression
Time (h)
log p(yj|tj,y−j)
Time (h)
2
0
−2
−4
0
5
10
−3
−4
−5
−6
−7
2.5
5.0
7.5
10.0
12.5

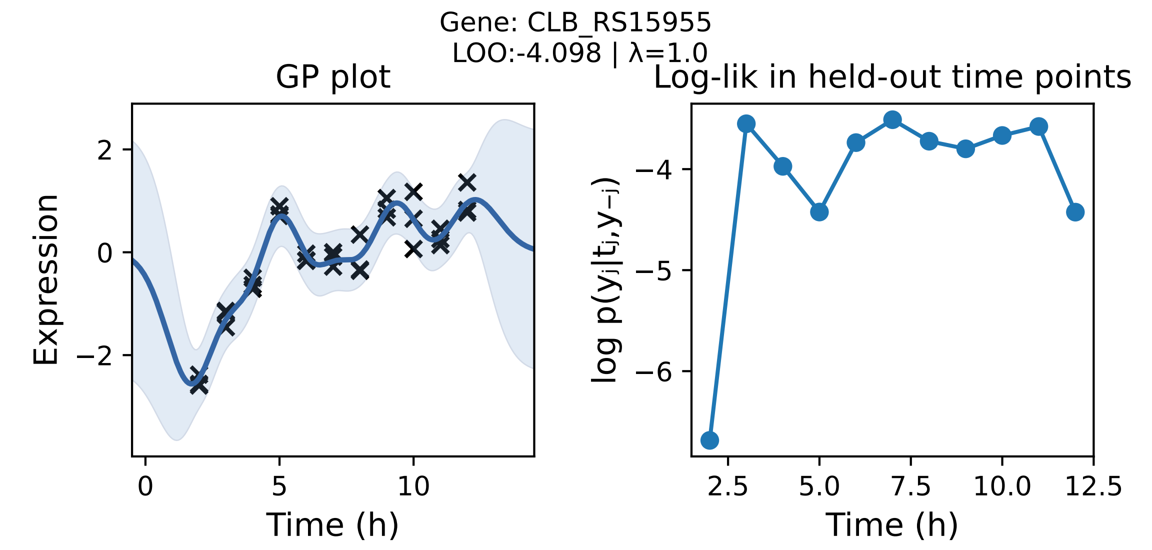
Gene: CLB_RS15955
LOO:-4.098 | λ=1.0
GP plot
Expression
Time (h)
Log-lik in held-out time points
log p(y_j|t_j,y_−j)
Time (h)

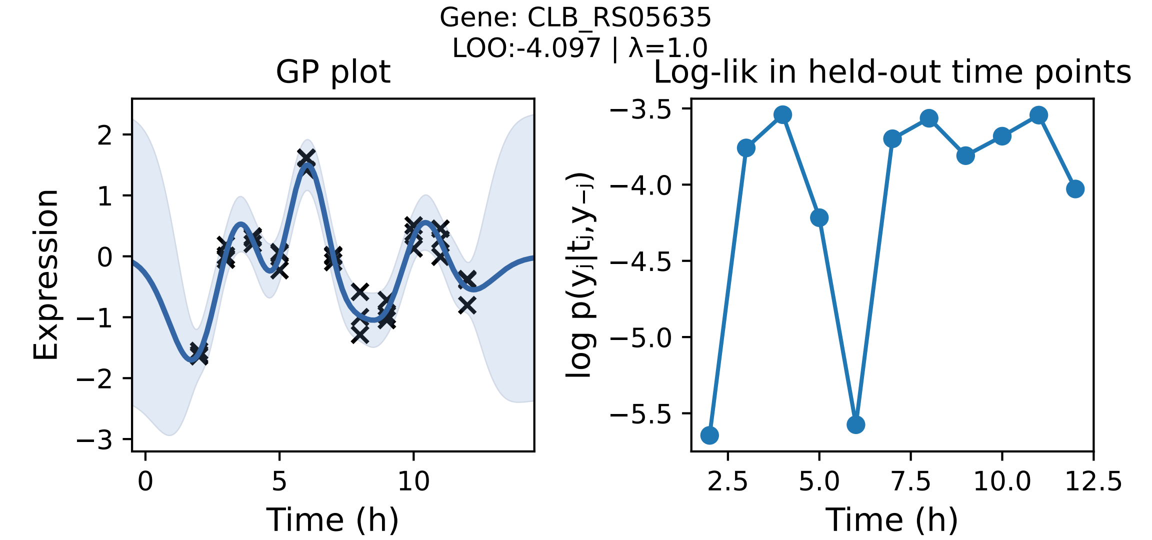
Gene: CLB_RS05635
LOO:-4.097 | λ=1.0
GP plot
Expression
Time (h)
Log-lik in held-out time points
log p(y_j|t_j,y_−j)
Time (h)

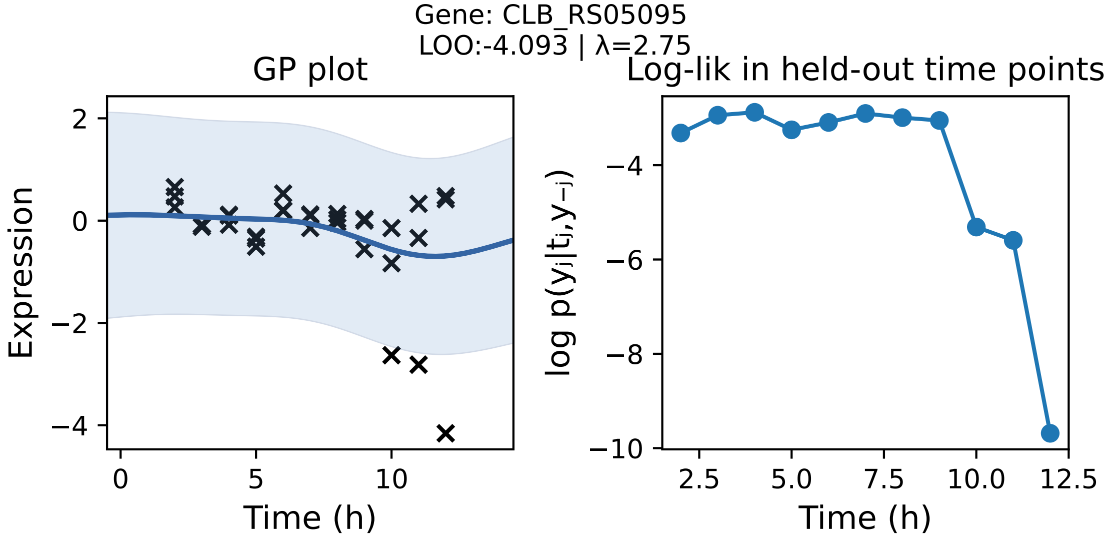

Gene: CLB_RS05095
LOO:-4.093 | λ=2.75
GP plot
Expression
Time (h)
2
0
−2
−4
0
5
10
Log-lik in held-out time points
log p(y_j|t_j,y_−j)
Time (h)
−4
−6
−8
−10
2.5
5.0
7.5
10.0
12.5

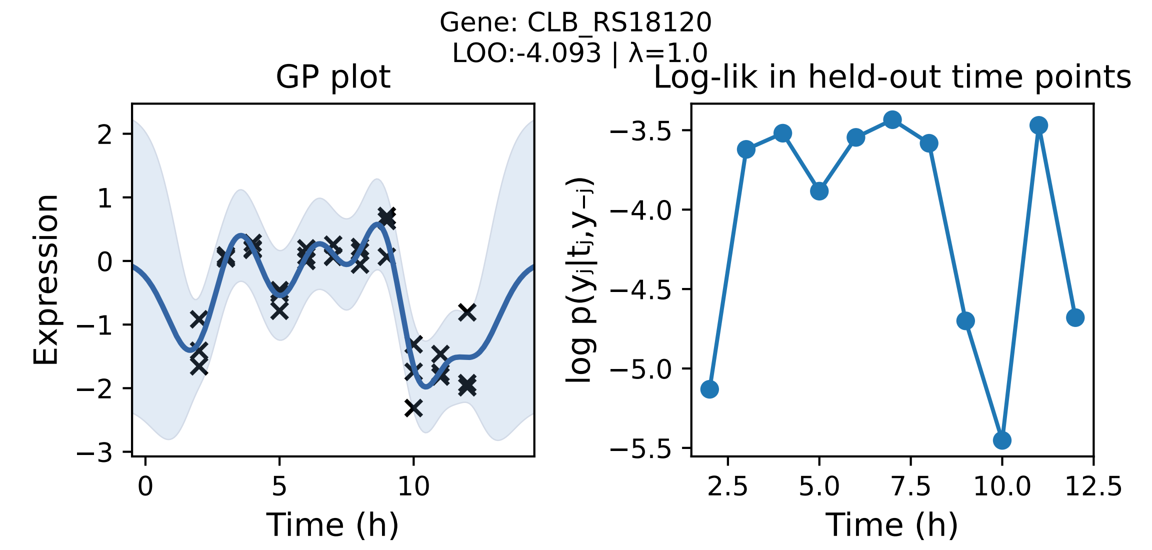
Gene: CLB_RS18120
LOO:-4.093 | λ=1.0
GP plot
Expression
Time (h)
Log-lik in held-out time points
log p(y_j|t_j,y_{-j})
Time (h)

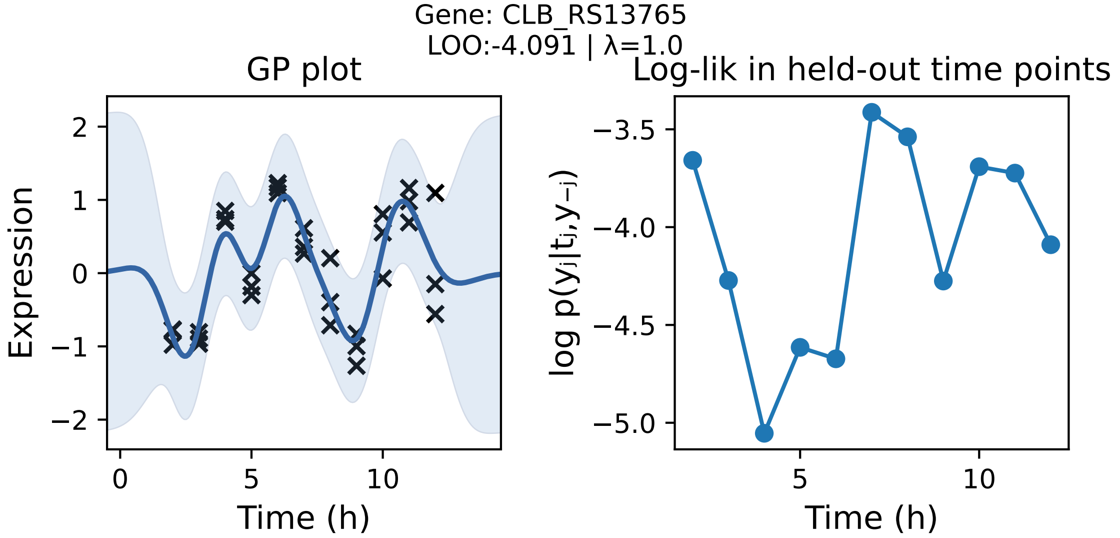

Gene: CLB_RS13765
LOO:-4.091 | λ=1.0
GP plot
Expression
Time (h)
Log-lik in held-out time points
log p(y_j|t_j,y_{-j})
Time (h)

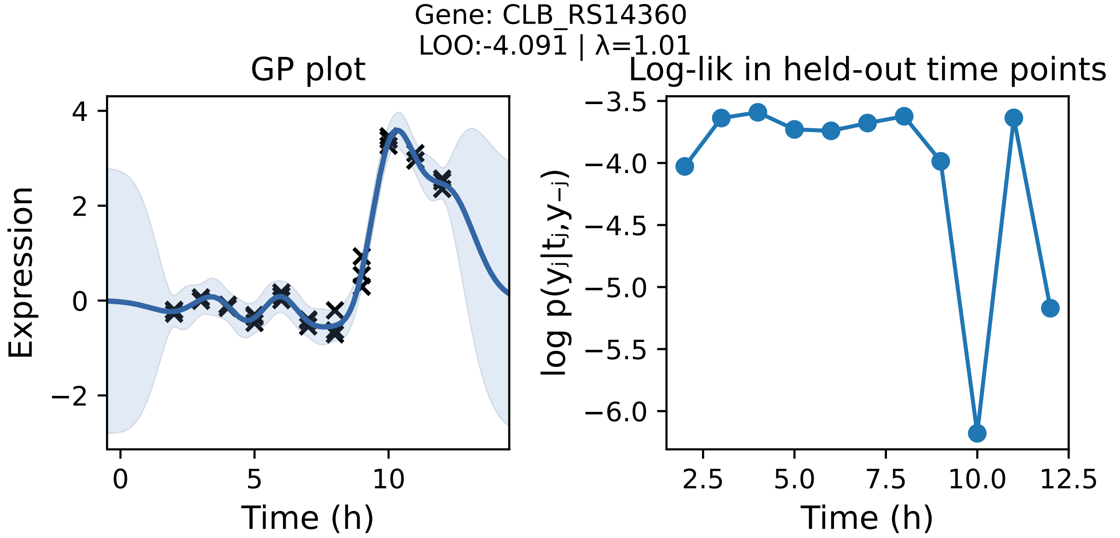

Gene: CLB_RS14360
LOO:-4.091 | λ=1.01
GP plot
Log-lik in held-out time points
Expression
Time (h)
log p(y_j|t_j,y_−j)
Time (h)
4
2
0
−2
0
5
10
−3.5
−4.0
−4.5
−5.0
−5.5
−6.0
2.5
5.0
7.5
10.0
12.5

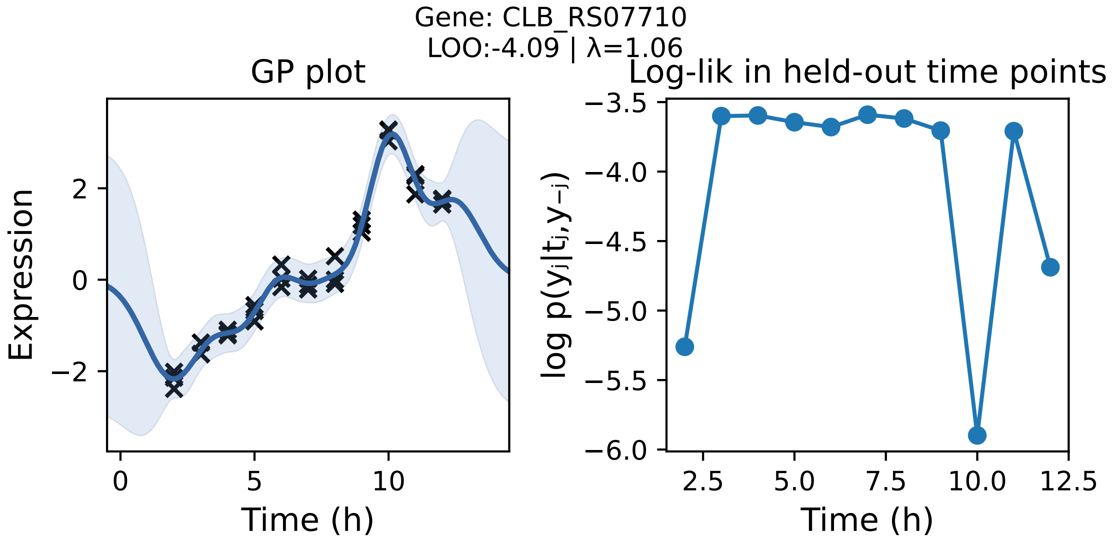

Gene: CLB_RS07710
LOO:-4.09 | λ=1.06
GP plot
Expression
Time (h)
Log-lik in held-out time points
log p(y_j|t_j,y_−j)
Time (h)

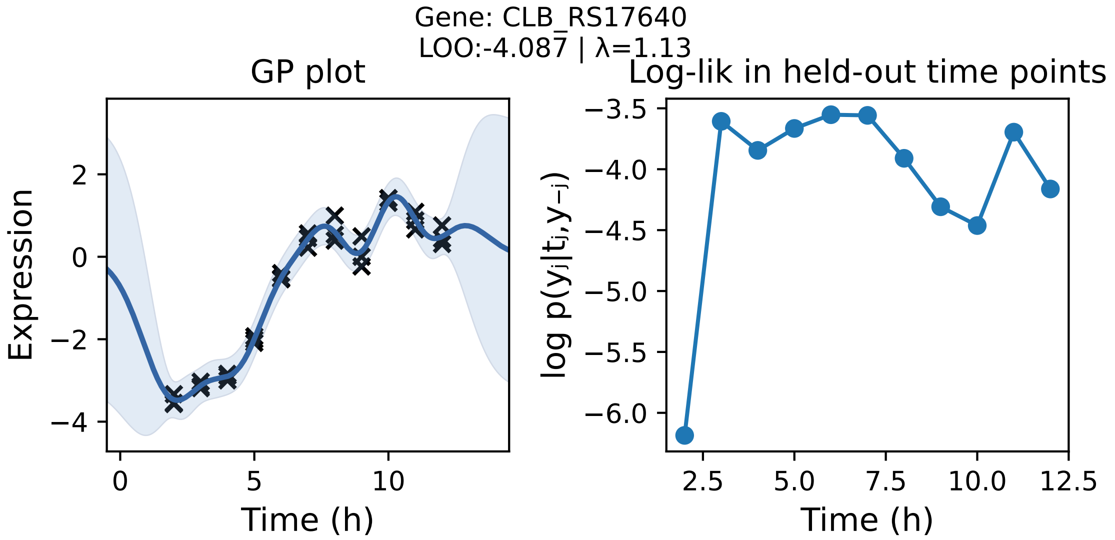
Gene: CLB_RS17640
LOO:-4.087 | λ=1.13
GP plot
Expression
Time (h)
Log-lik in held-out time points
log p(y_j|t_j,y_−j)
Time (h)

Gene: CLB_RS02340
LOO:-4.086 | λ=1.0

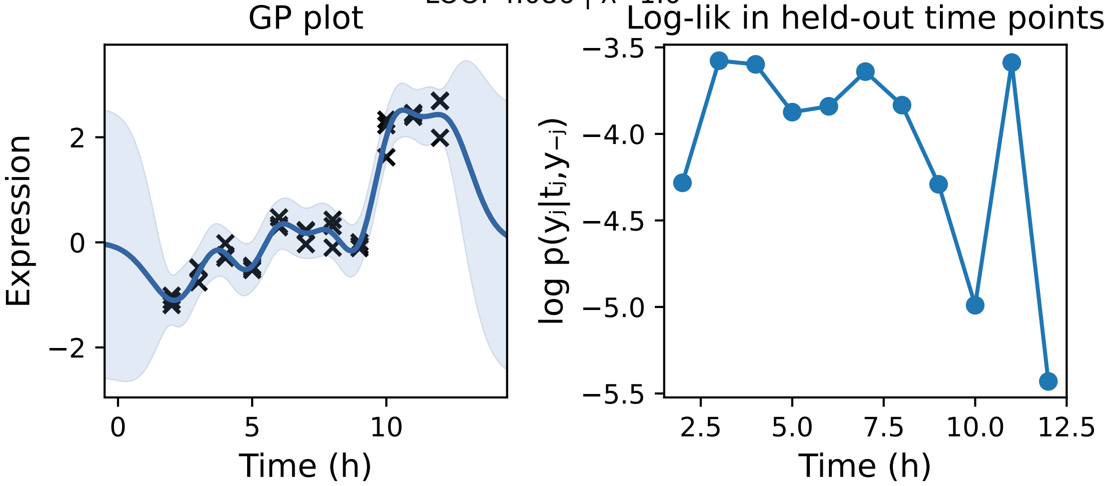

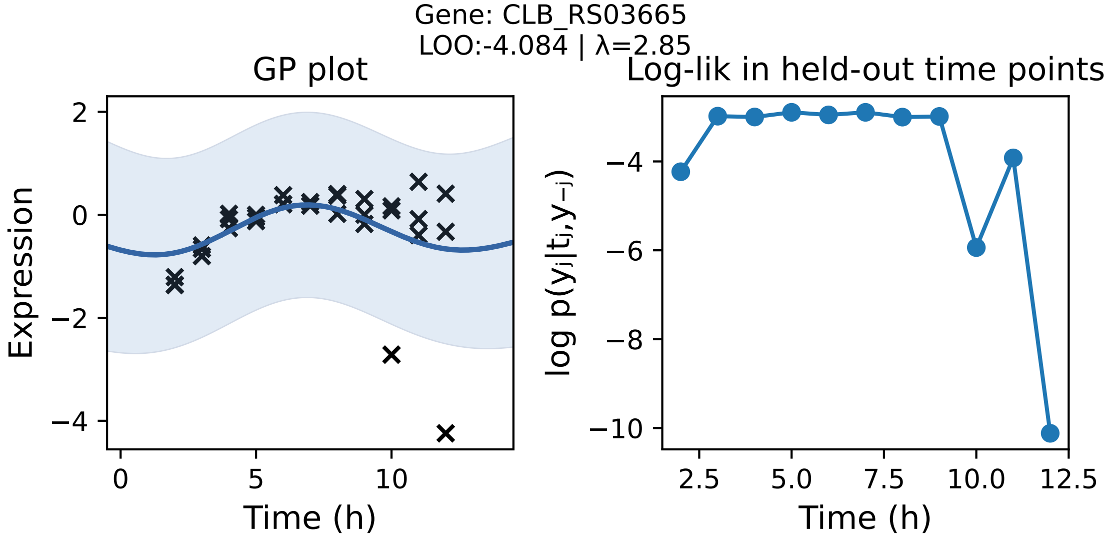

Gene: CLB_RS03665
LOO:-4.084 | λ=2.85
GP plot
Expression
Time (h)
Log-lik in held-out time points
log p(y_j|t_j,y_−j)
Time (h)

Gene: CLB_RS04300
LOO:-4.084 | λ=1.0

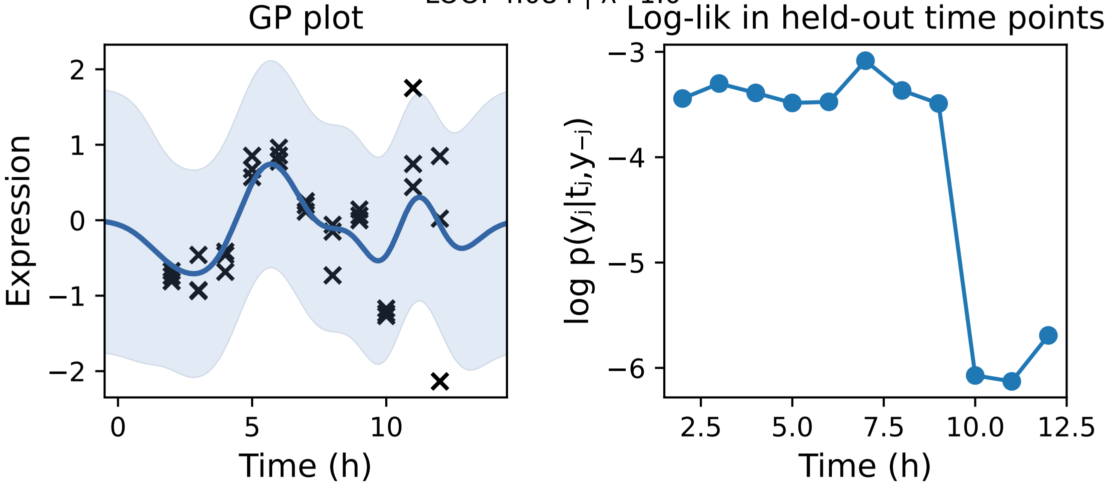

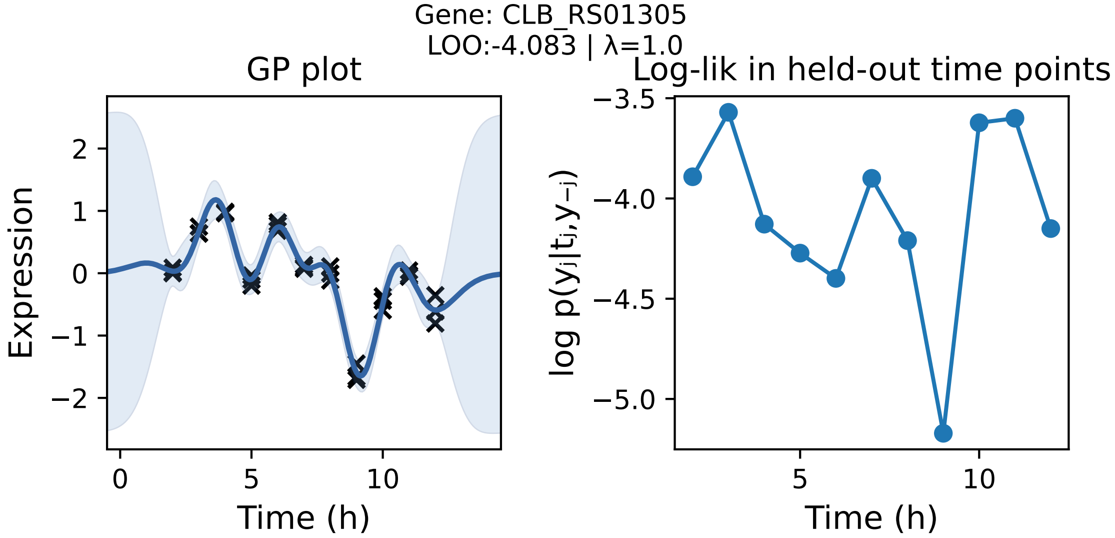

Gene: CLB_RS01305
LOO:-4.083 | λ=1.0
GP plot
Log-lik in held-out time points
Expression
Time (h)
log p(yj|tj,y−j)
Time (h)

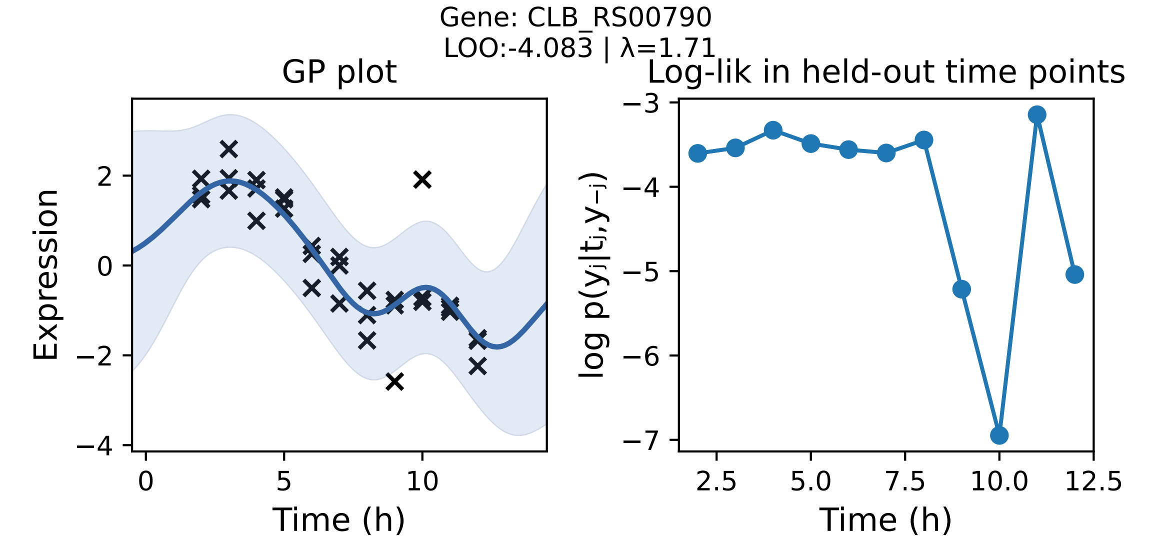

Gene: CLB_RS00790
LOO:-4.083 | λ=1.71
GP plot
Expression
Time (h)
2
0
−2
−4
0
5
10
Log-lik in held-out time points
log p(y_j|t_j,y_−j)
Time (h)
−3
−4
−5
−6
−7
2.5
5.0
7.5
10.0
12.5

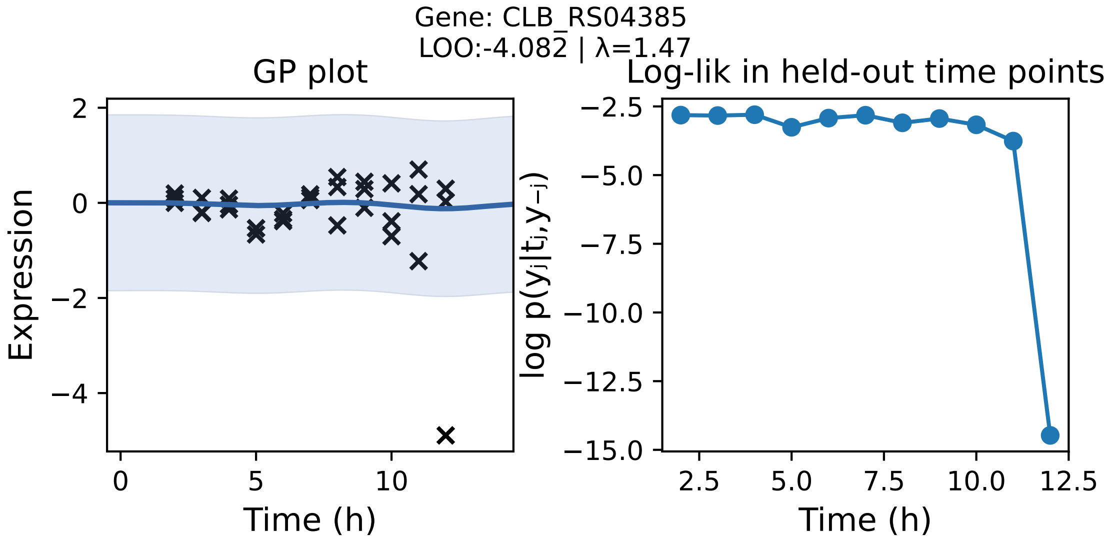

Gene: CLB_RS04385
LOO:-4.082 | λ=1.47
GP plot
Log-lik in held-out time points
Expression
Time (h)
log p(y_j|t_j,y_−j)
Time (h)

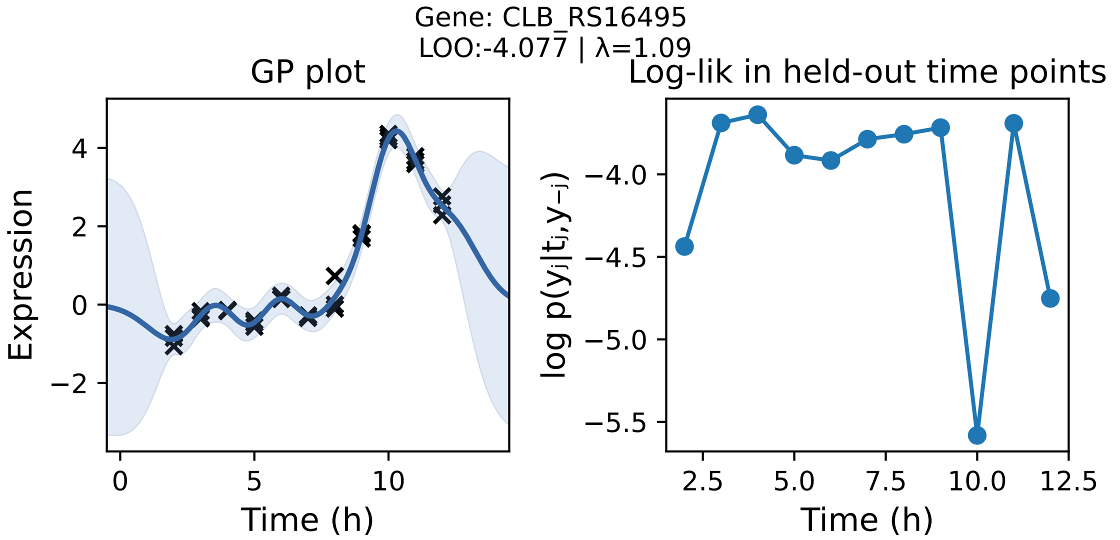

Gene: CLB_RS16495
LOO:-4.077 | λ=1.09
GP plot
Expression
Time (h)
Log-lik in held-out time points
log p(y_j|t_j,y_−j)
Time (h)

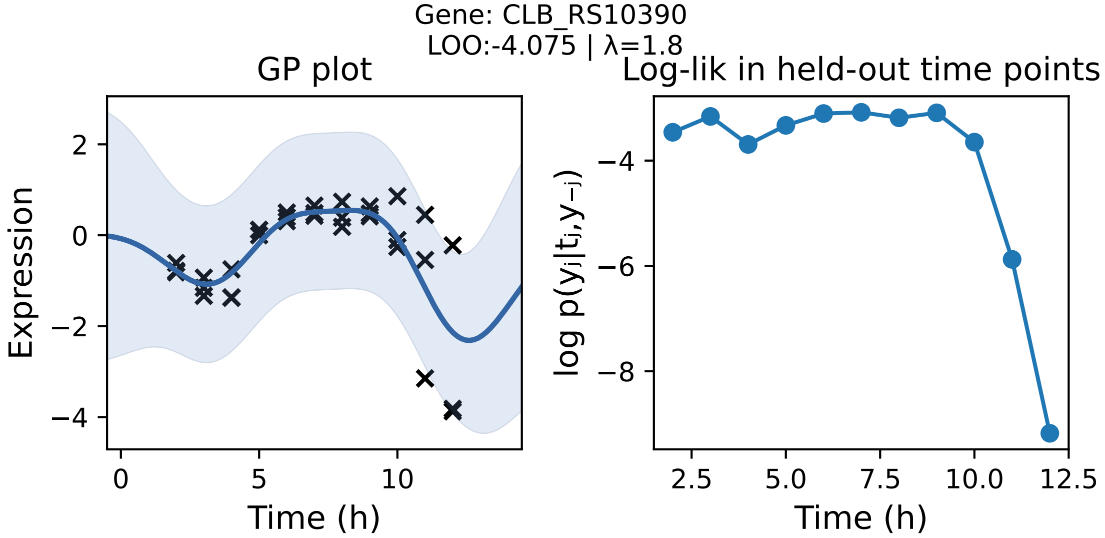

Gene: CLB_RS10390
LOO:-4.075 | λ=1.8
GP plot
Log-lik in held-out time points
Expression
Time (h)
log p(y_j|t_j,y_−j)
Time (h)
2
0
−2
−4
0
5
10
−4
−6
−8
2.5
5.0
7.5
10.0
12.5

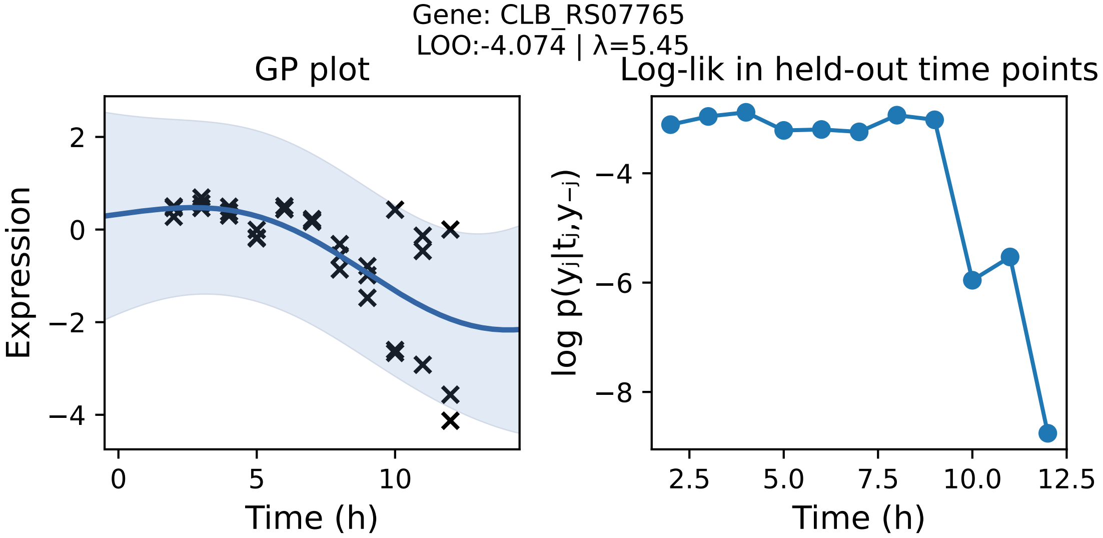
Gene: CLB_RS07765
LOO:-4.074 | λ=5.45
GP plot
Expression
Time (h)
Log-lik in held-out time points
log p(y_j|t_j,y_−j)
Time (h)

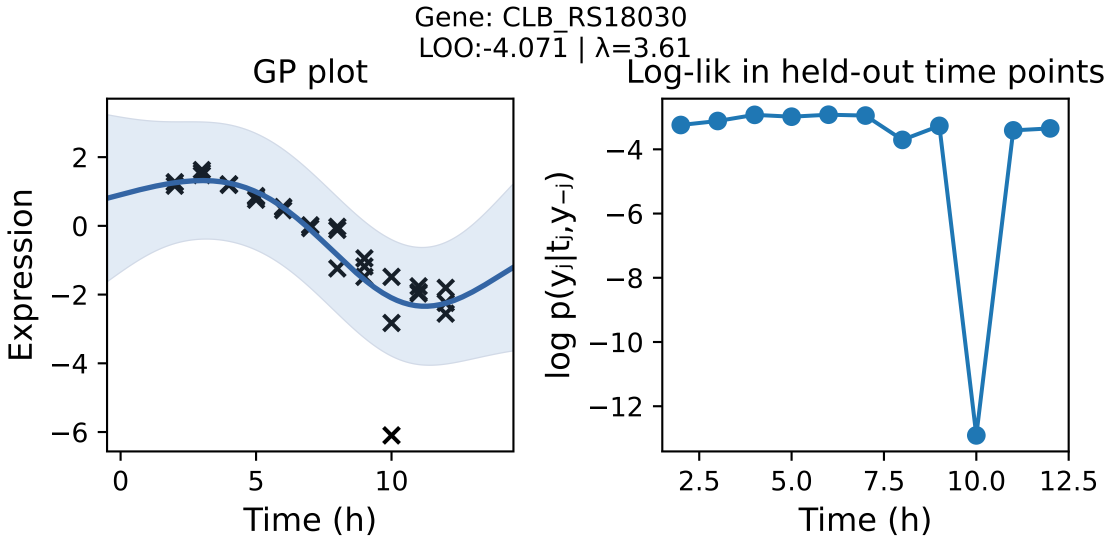
Gene: CLB_RS18030
LOO:-4.071 | λ=3.61
GP plot
Expression
Time (h)
Log-lik in held-out time points
log p(y_j|t_j,y_−j)
Time (h)

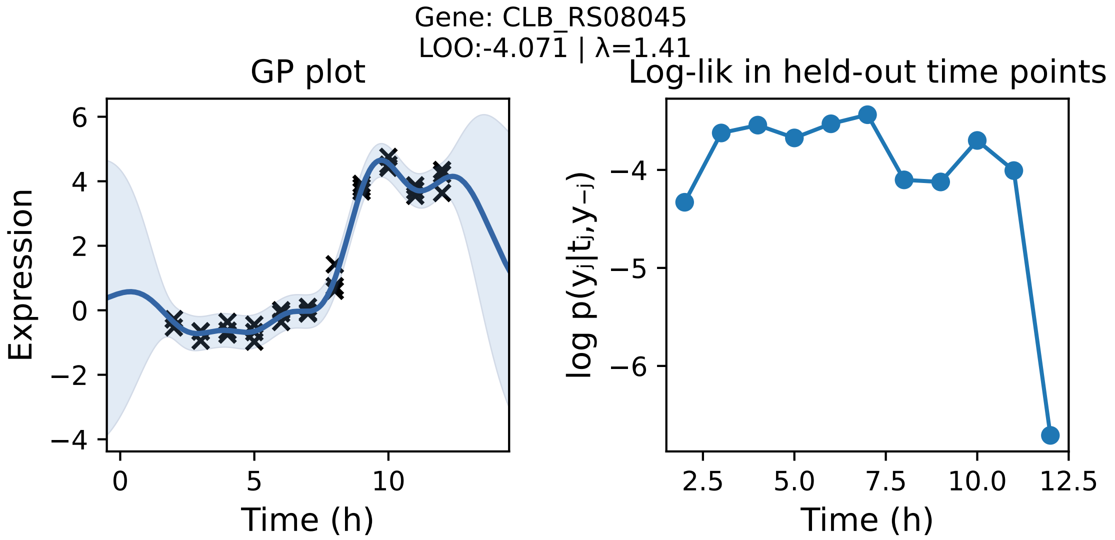

Gene: CLB_RS08045
LOO:-4.071 | λ=1.41
GP plot
Expression
6
4
2
0
−2
−4
0
5
10
Time (h)
Log-lik in held-out time points
log p(y_j|t_j,y_−j)
−4
−5
−6
2.5
5.0
7.5
10.0
12.5
Time (h)

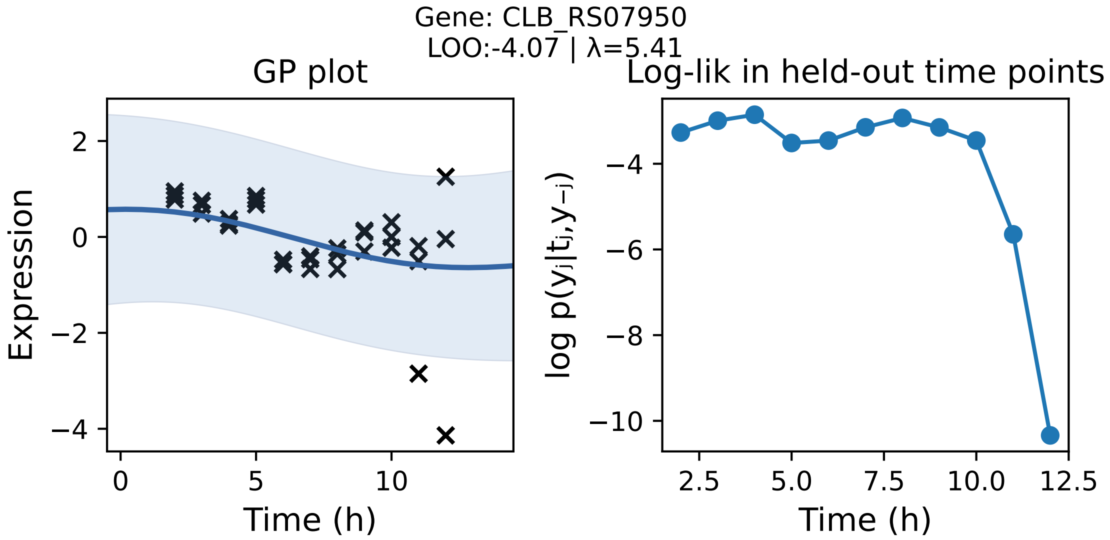

Gene: CLB_RS07950
LOO:-4.07 | λ=5.41
GP plot
Expression
Time (h)
2
0
−2
−4
0
5
10
Log-lik in held-out time points
log p(yⱼ|tⱼ,y₋ⱼ)
−4
−6
−8
−10
2.5
5.0
7.5
10.0
12.5
Time (h)

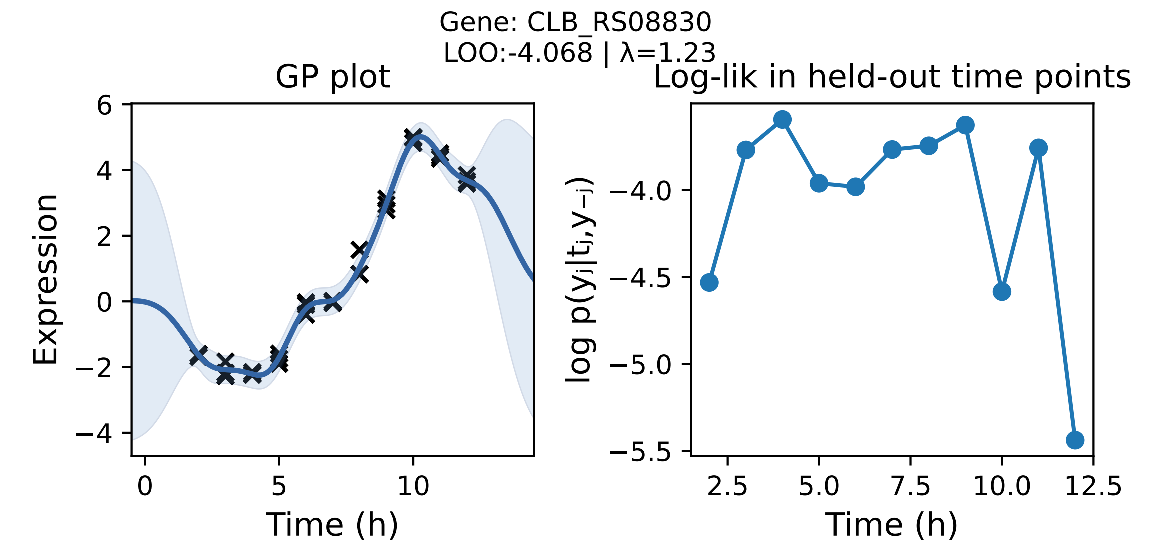
Gene: CLB_RS08830
LOO:-4.068 | λ=1.23
GP plot
Expression
Time (h)
Log-lik in held-out time points
log p(yj|tj,y−j)
Time (h)

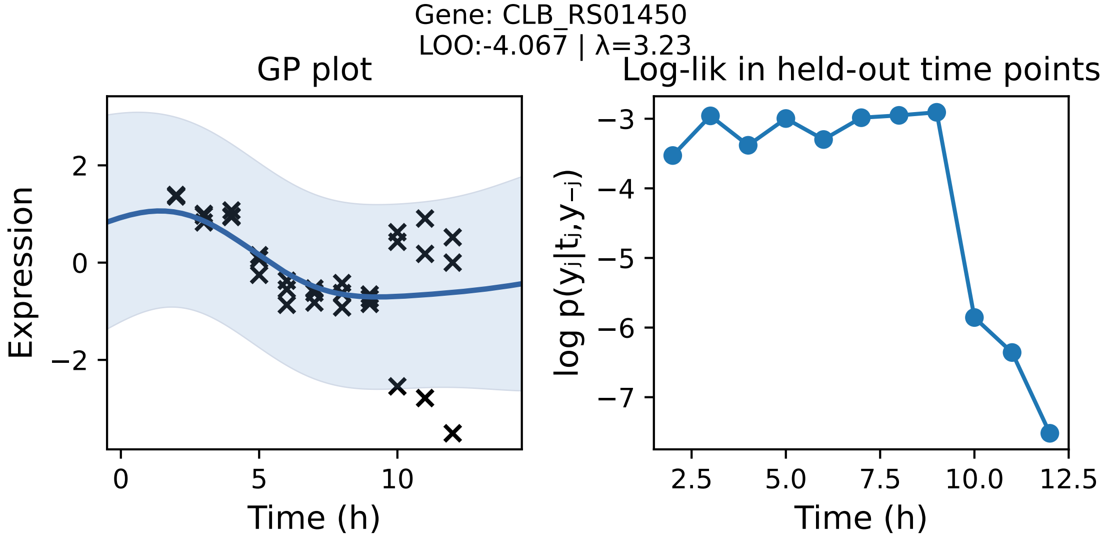
Gene: CLB_RS01450
LOO:-4.067 | λ=3.23
GP plot
Expression
Time (h)
2
0
−2
0
5
10
Log-lik in held-out time points
log p(y_j|t_j,y_−j)
Time (h)
−3
−4
−5
−6
−7
2.5
5.0
7.5
10.0
12.5

Gene: CLB_RS17950
LOO:-4.066 | λ=2.45

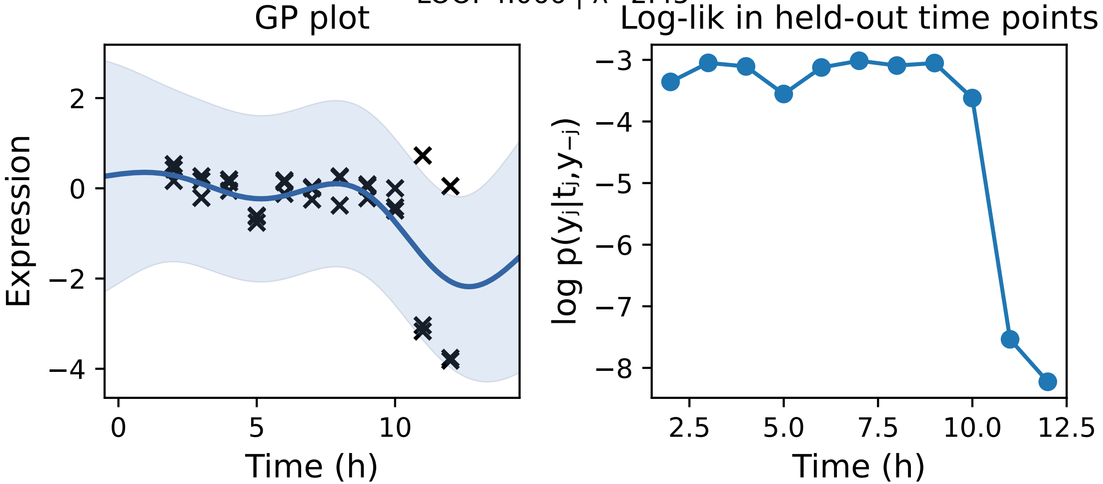

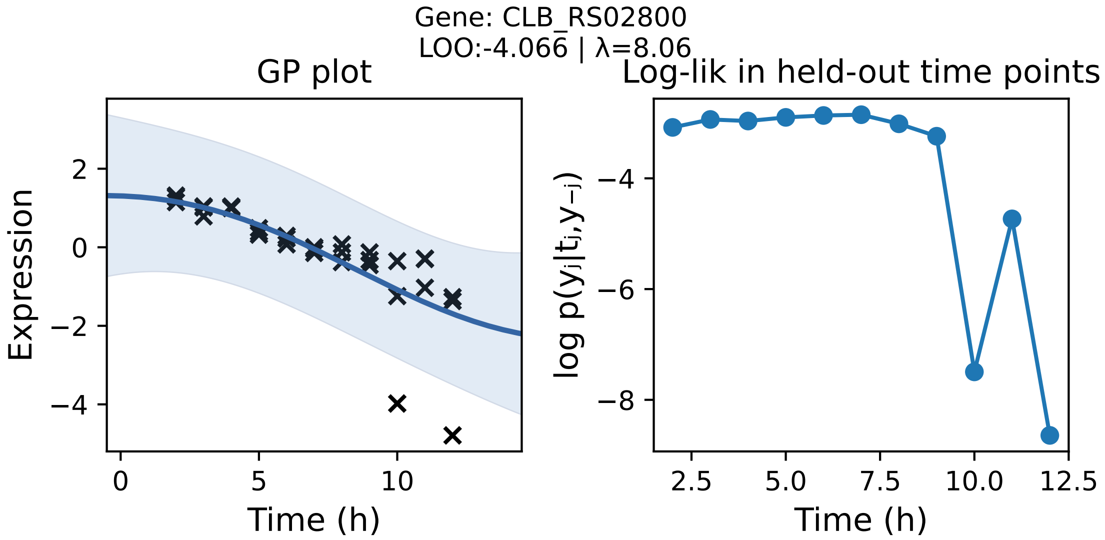

Gene: CLB_RS02800
LOO:-4.066 | λ=8.06
GP plot
Expression
Time (h)
Log-lik in held-out time points
log p(y_j|t_j,y_−j)
Time (h)

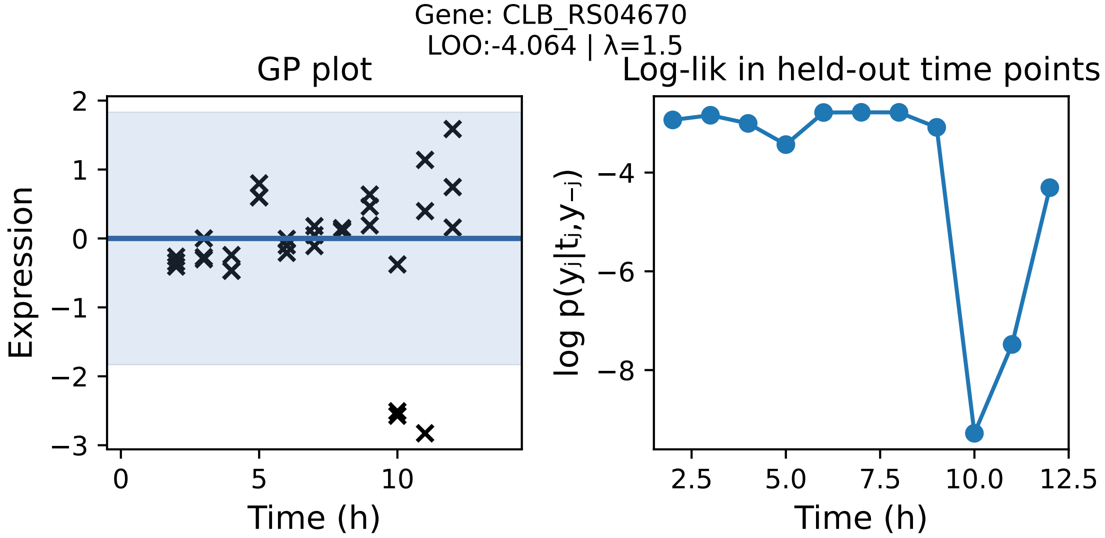

Gene: CLB_RS04670
LOO:-4.064 | λ=1.5
GP plot
Log-lik in held-out time points
Expression
2
1
0
−1
−2
−3
0
5
10
Time (h)
log p(yⱼ|tⱼ,y₋ⱼ)
−4
−6
−8
2.5
5.0
7.5
10.0
12.5
Time (h)

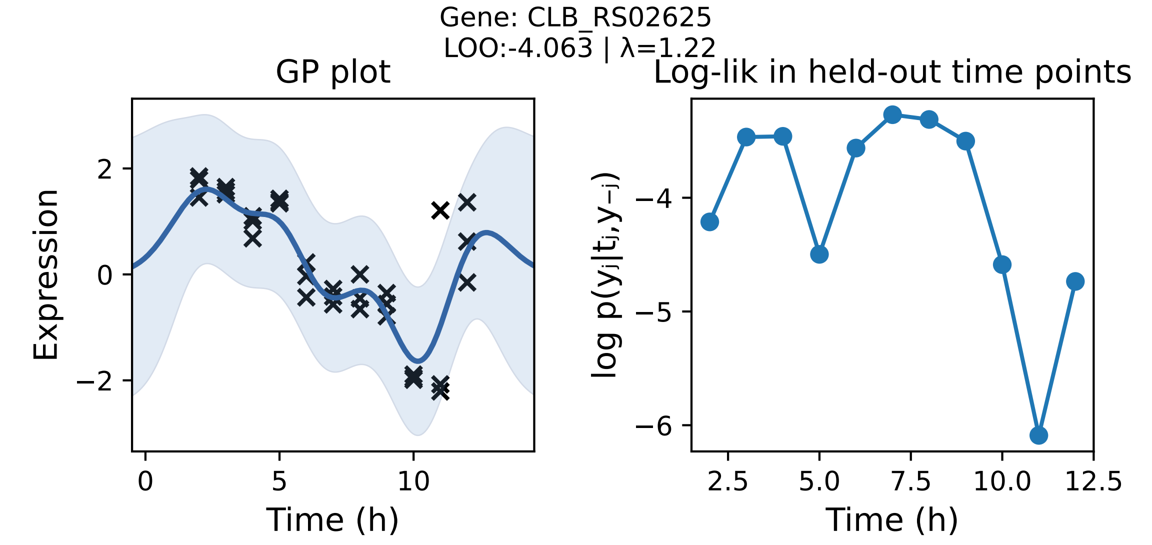

Gene: CLB_RS02625
LOO:-4.063 | λ=1.22
GP plot
Expression
Time (h)
Log-lik in held-out time points
log p(y_j|t_j,y_−j)
Time (h)

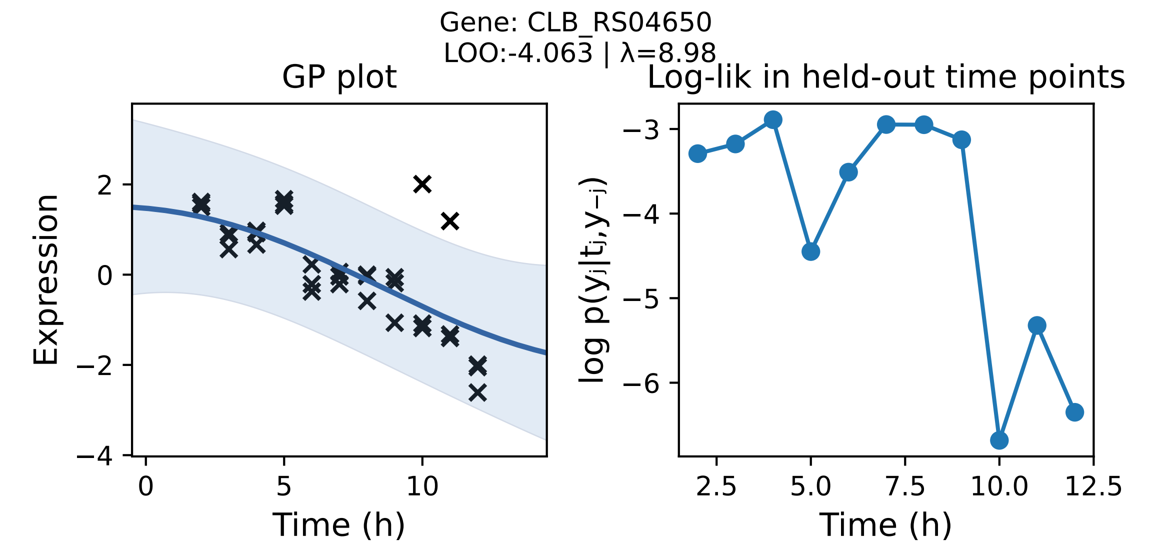
Gene: CLB_RS04650
LOO:-4.063 | λ=8.98
GP plot
Log-lik in held-out time points
Expression
Time (h)
log p(y_j|t_j,y_−j)
Time (h)
2
0
−2
−4
0
5
10
−3
−4
−5
−6
2.5
5.0
7.5
10.0
12.5

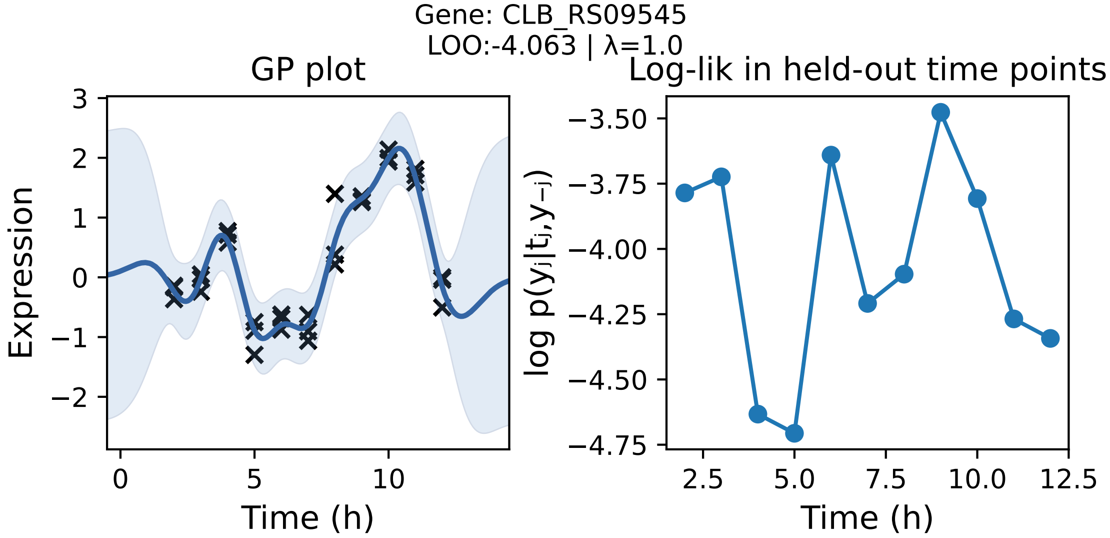

Gene: CLB_RS09545
LOO:-4.063 | λ=1.0
GP plot
Log-lik in held-out time points
Expression
Time (h)
log p(y_j|t_j,y_−j)
Time (h)

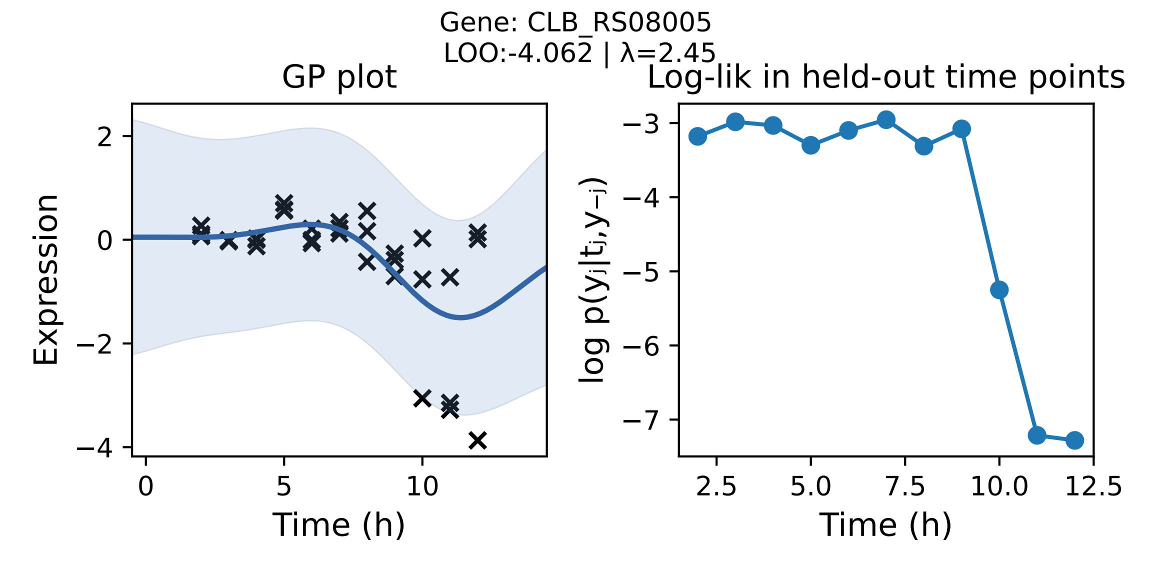
Gene: CLB_RS08005
LOO:-4.062 | λ=2.45
GP plot
Expression
Time (h)
2
0
−2
−4
0
5
10
Log-lik in held-out time points
log p(y_j|t_j,y_−j)
Time (h)
−3
−4
−5
−6
−7
2.5
5.0
7.5
10.0
12.5

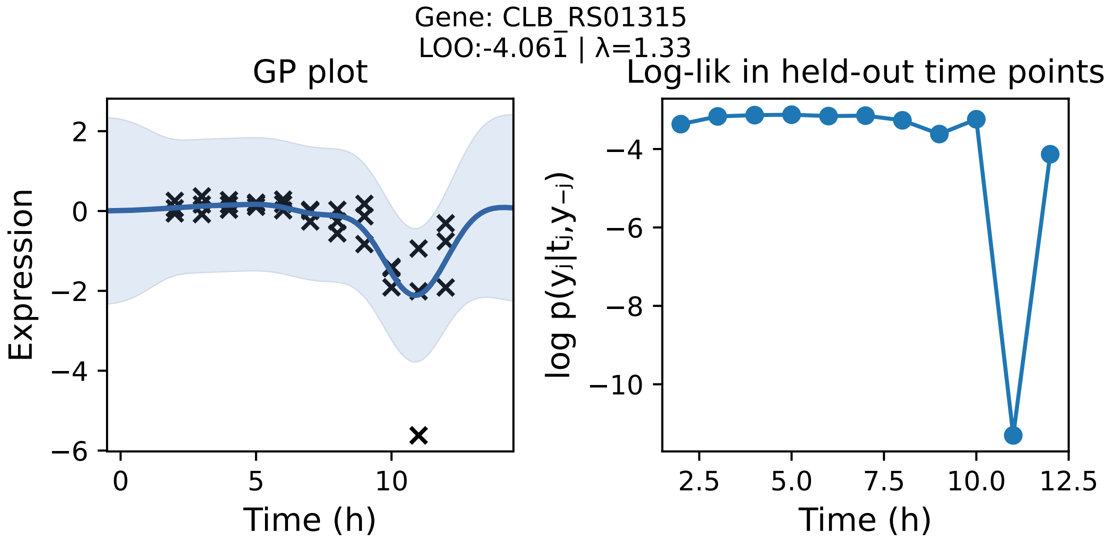

Gene: CLB_RS01315
LOO:-4.061 | λ=1.33
GP plot
Expression
Time (h)
2
0
−2
−4
−6
0
5
10
Log-lik in held-out time points
log p(y_j|t_j,y_−j)
Time (h)
−4
−6
−8
−10
2.5
5.0
7.5
10.0
12.5

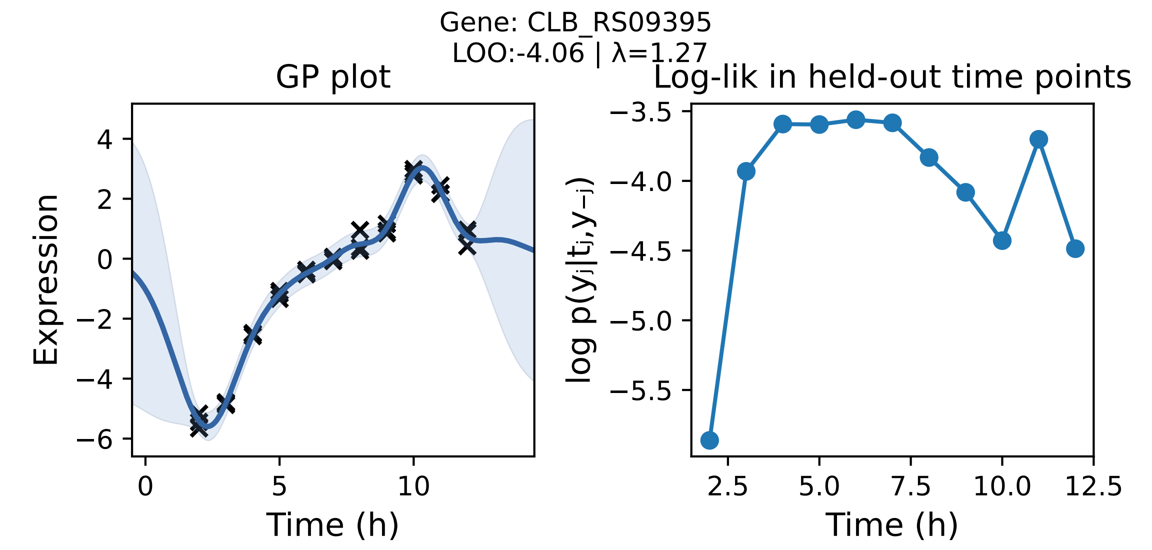

Gene: CLB_RS09395
LOO:-4.06 | λ=1.27
GP plot
Expression
Time (h)
4
2
0
−2
−4
−6
0
5
10
Log-lik in held-out time points
log p(y_j|t_j,y_−j)
Time (h)
−3.5
−4.0
−4.5
−5.0
−5.5
2.5
5.0
7.5
10.0
12.5

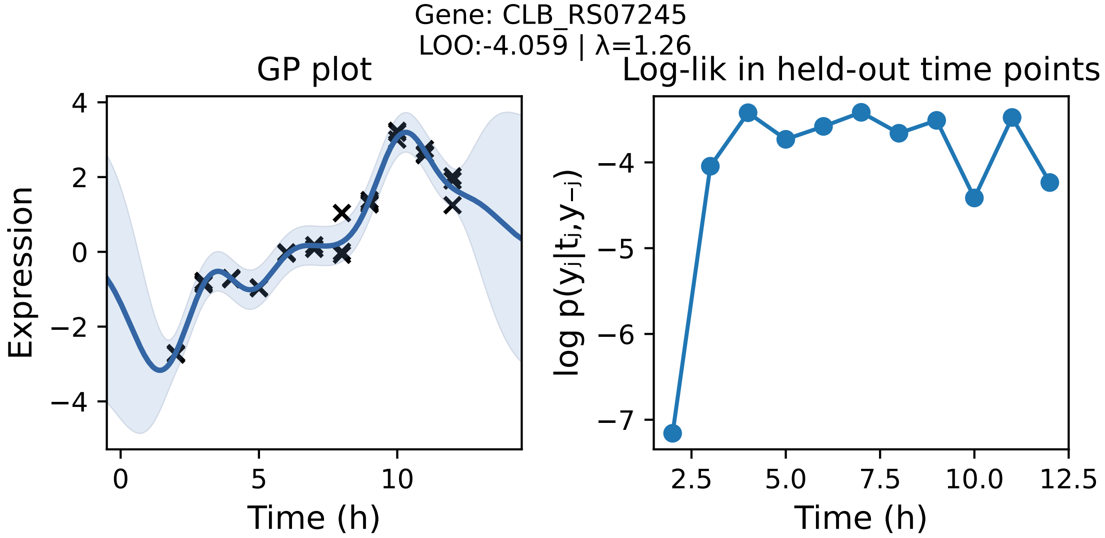

Gene: CLB_RS07245
LOO:-4.059 | λ=1.26
GP plot
Log-lik in held-out time points
Expression
Time (h)
log p(y_j|t_j,y_−j)
Time (h)

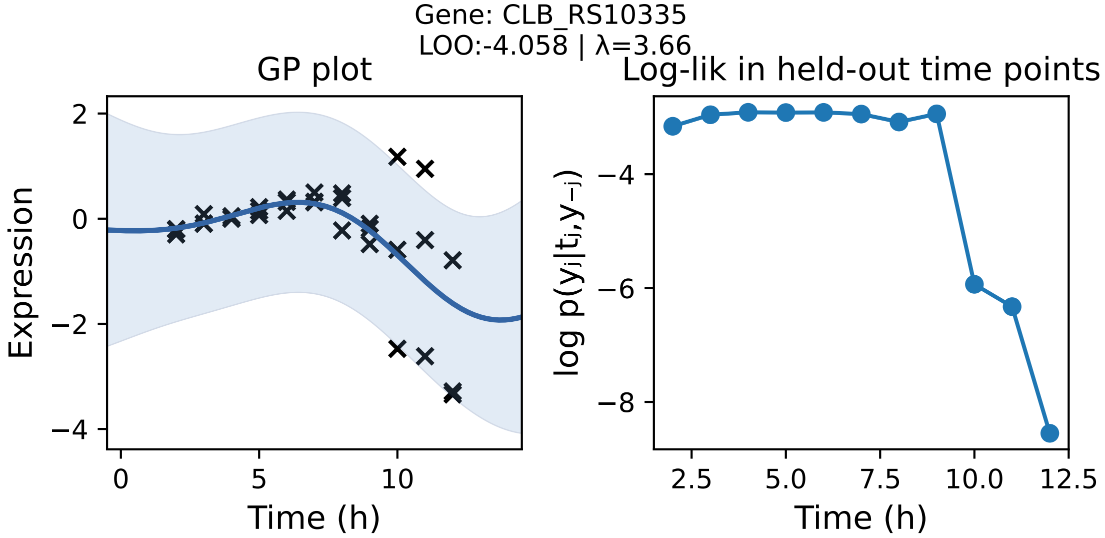

Gene: CLB_RS10335
LOO:-4.058 | λ=3.66
GP plot
Expression
Time (h)
2
0
−2
−4
0
5
10
Log-lik in held-out time points
log p(yj|tj,y−j)
Time (h)
−4
−6
−8
2.5
5.0
7.5
10.0
12.5

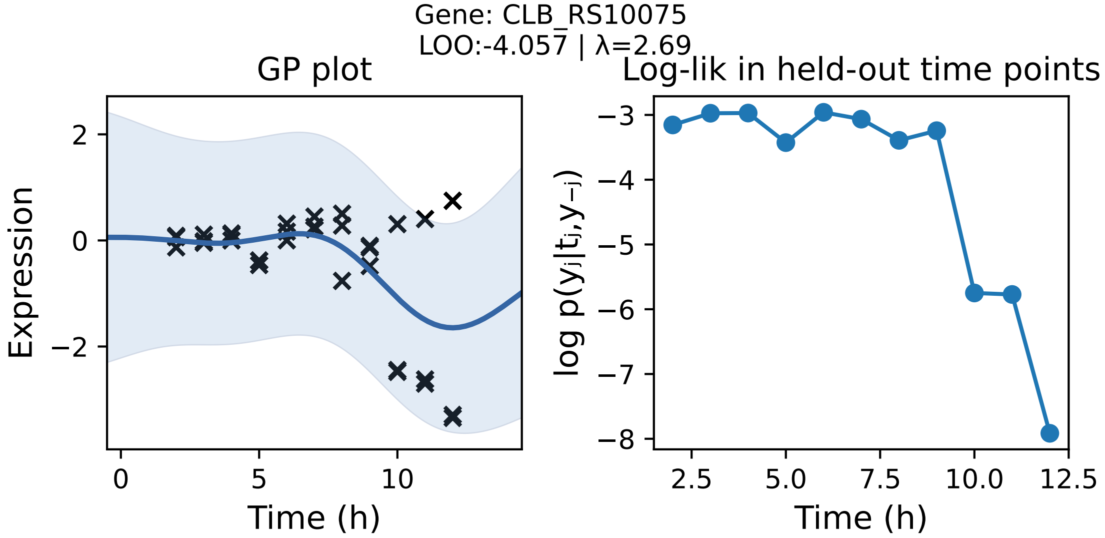

Gene: CLB_RS10075
LOO:-4.057 | λ=2.69
GP plot
Expression
Time (h)
Log-lik in held-out time points
log p(yj|tj,y−j)
Time (h)

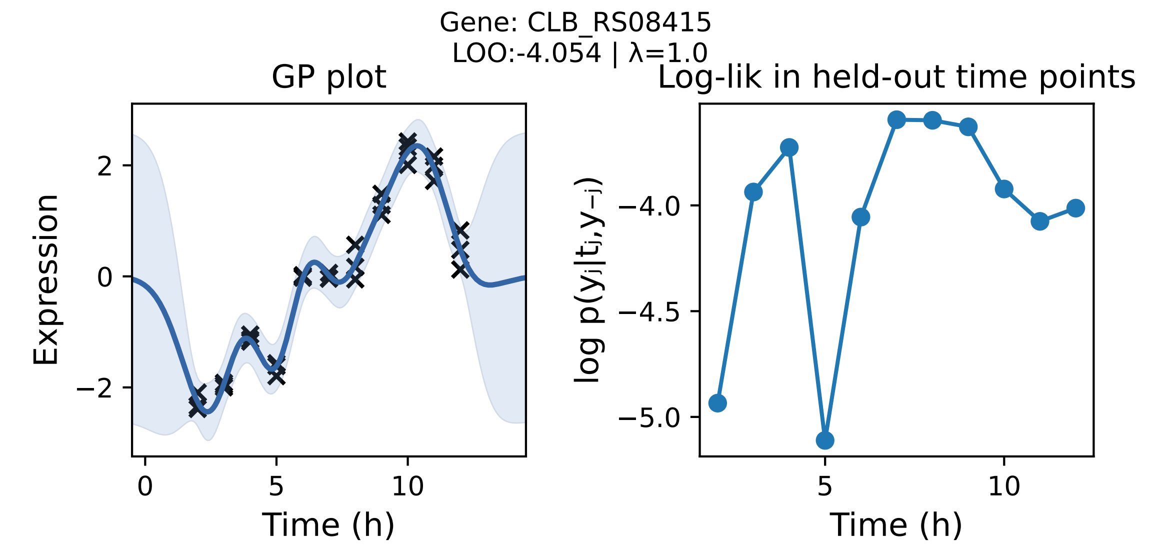
Gene: CLB_RS08415
LOO:-4.054 | λ=1.0
GP plot
Expression
Time (h)
Log-lik in held-out time points
log p(y_j|t_j,y_−j)
Time (h)

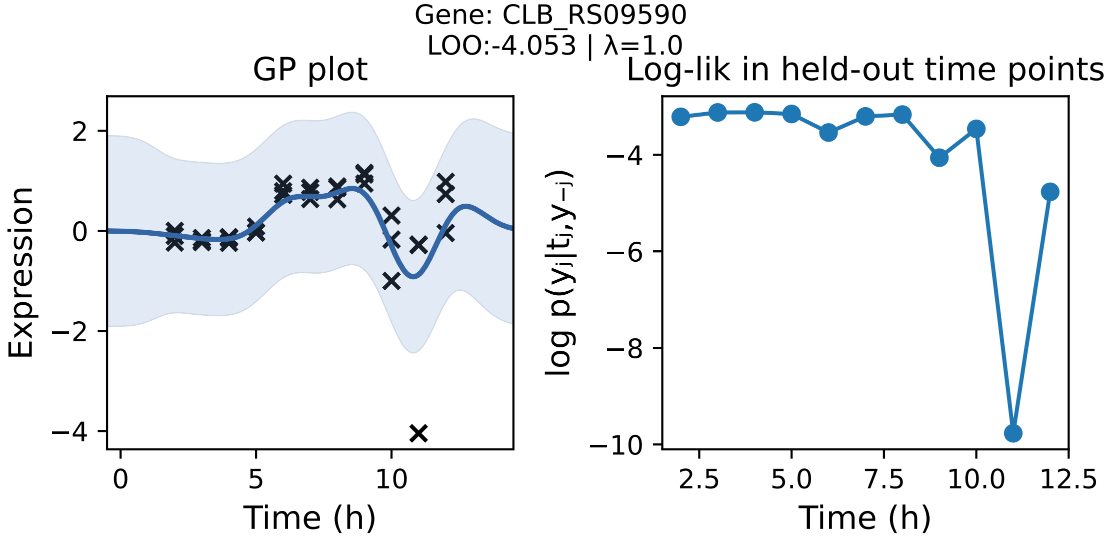
Gene: CLB_RS09590
LOO:-4.053 | λ=1.0
GP plot
Expression
Time (h)
Log-lik in held-out time points
log p(yj|tj,y−j)
Time (h)
2
0
−2
−4
0
5
10
−4
−6
−8
−10
2.5
5.0
7.5
10.0
12.5

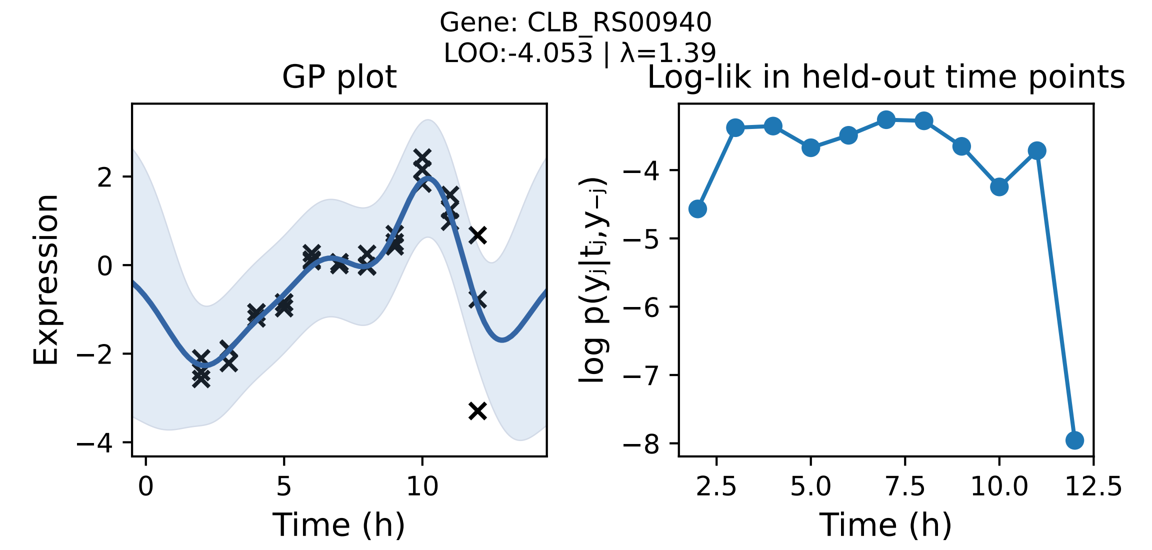
Gene: CLB_RS00940
LOO:-4.053 | λ=1.39
GP plot
Expression
Time (h)
0
5
10
2
0
−2
−4
Log-lik in held-out time points
log p(yj|tj,y−j)
Time (h)
2.5
5.0
7.5
10.0
12.5
−4
−5
−6
−7
−8

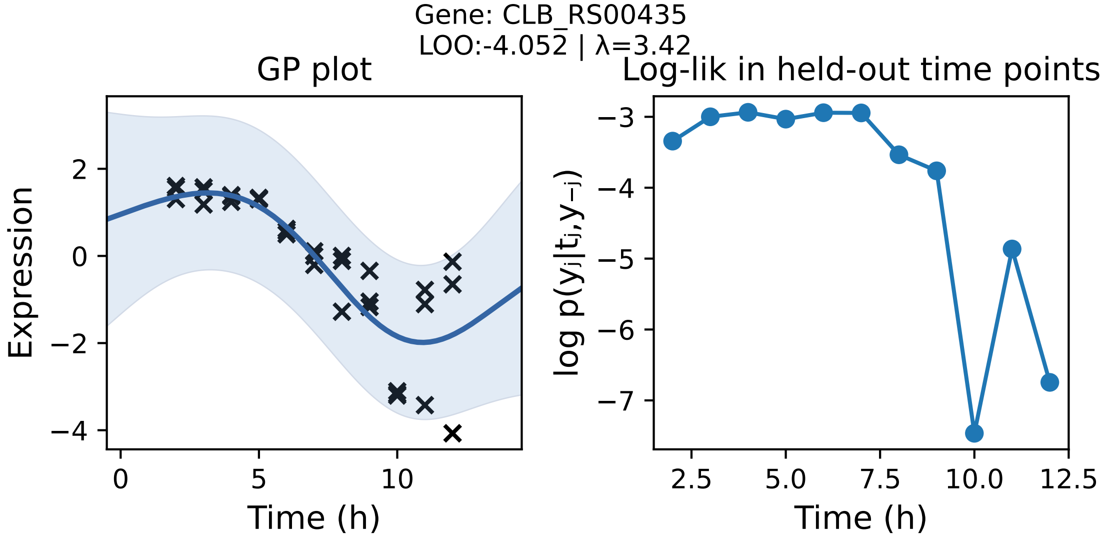
Gene: CLB_RS00435
LOO:-4.052 | λ=3.42
GP plot
Expression
Time (h)
Log-lik in held-out time points
log p(y_j|t_j,y_−j)
Time (h)

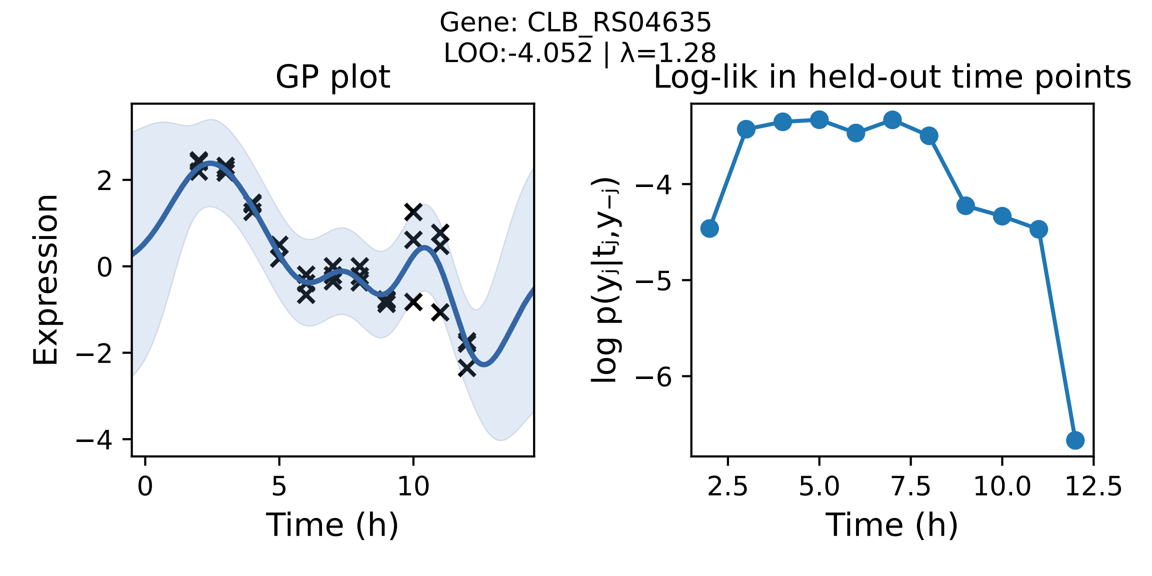

Gene: CLB_RS04635
LOO:-4.052 | λ=1.28
GP plot
Expression
Time (h)
Log-lik in held-out time points
log p(y_j|t_j,y_−j)
Time (h)

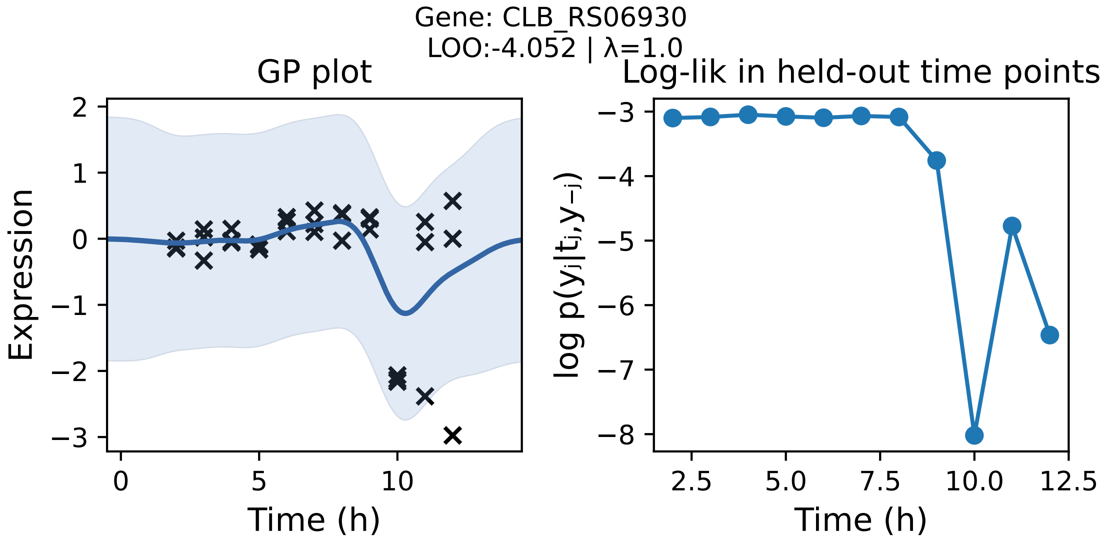

Gene: CLB_RS06930
LOO:-4.052 | λ=1.0
GP plot
Expression
Time (h)
Log-lik in held-out time points
log p(y_j|t_j,y_−j)
Time (h)

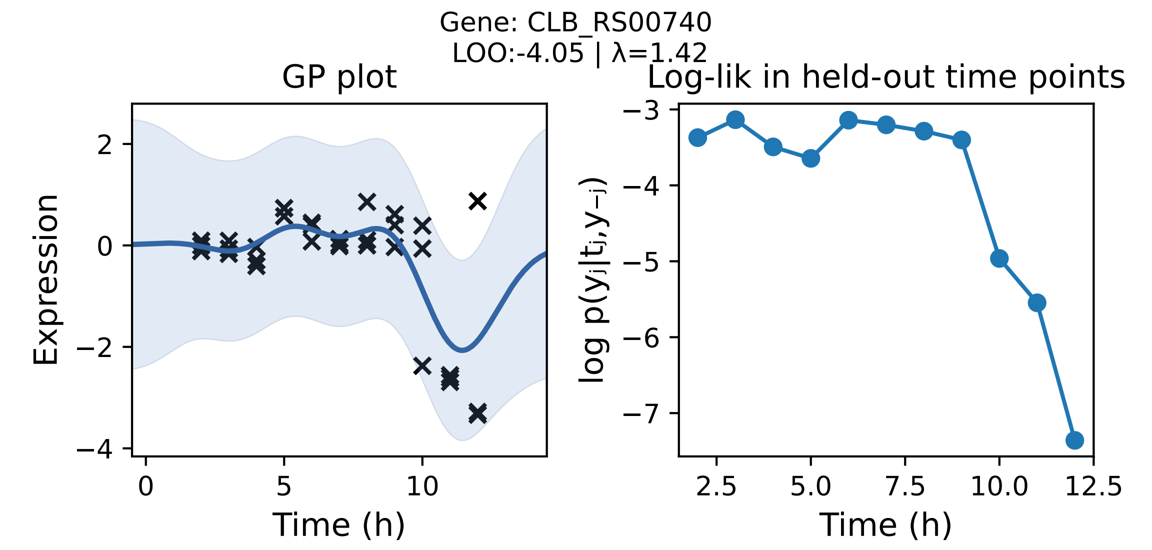
Gene: CLB_RS00740
LOO:-4.05 | λ=1.42
GP plot
Log-lik in held-out time points
Expression
Time (h)
log p(y_j|t_j,y_−j)
Time (h)
2
0
−2
−4
0
5
10
−3
−4
−5
−6
−7
2.5
5.0
7.5
10.0
12.5

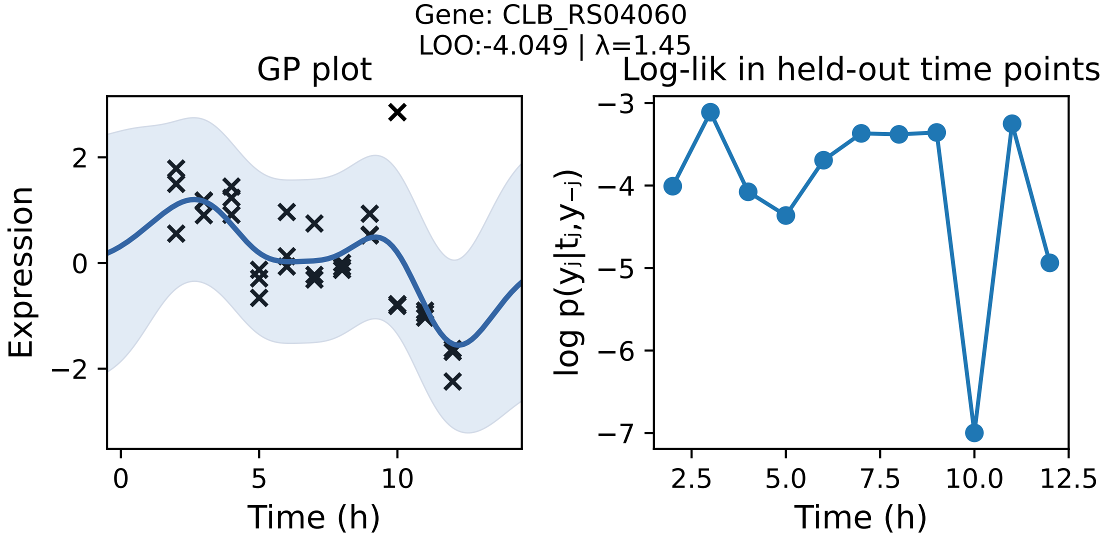
Gene: CLB_RS04060
LOO:-4.049 | λ=1.45
GP plot
Expression
Time (h)
2
0
−2
0
5
10
Log-lik in held-out time points
log p(yj|tj,y−j)
−3
−4
−5
−6
−7
2.5
5.0
7.5
10.0
12.5
Time (h)

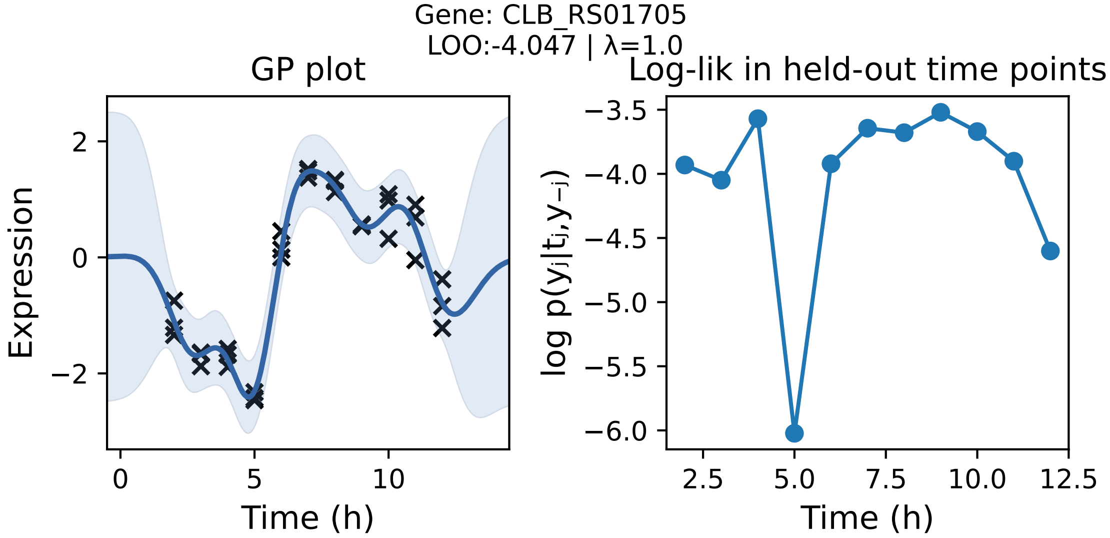

Gene: CLB_RS01705
LOO:-4.047 | λ=1.0
GP plot
Expression
Time (h)
Log-lik in held-out time points
log p(y_j|t_j,y_−j)
Time (h)

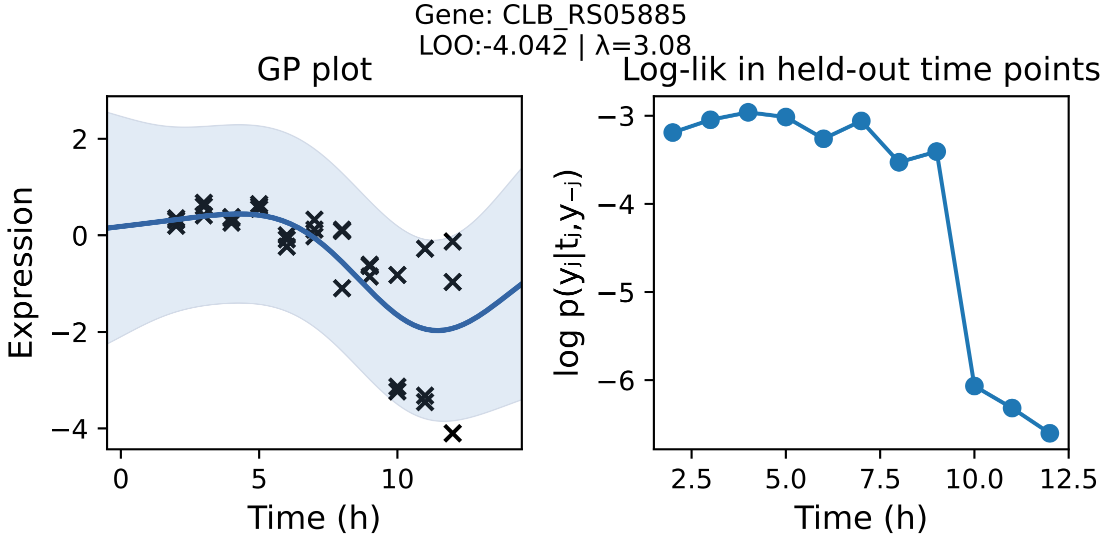

Gene: CLB_RS05885
LOO:-4.042 | λ=3.08
GP plot
Expression
Time (h)
Log-lik in held-out time points
log p(y_j|t_j,y_−j)
Time (h)

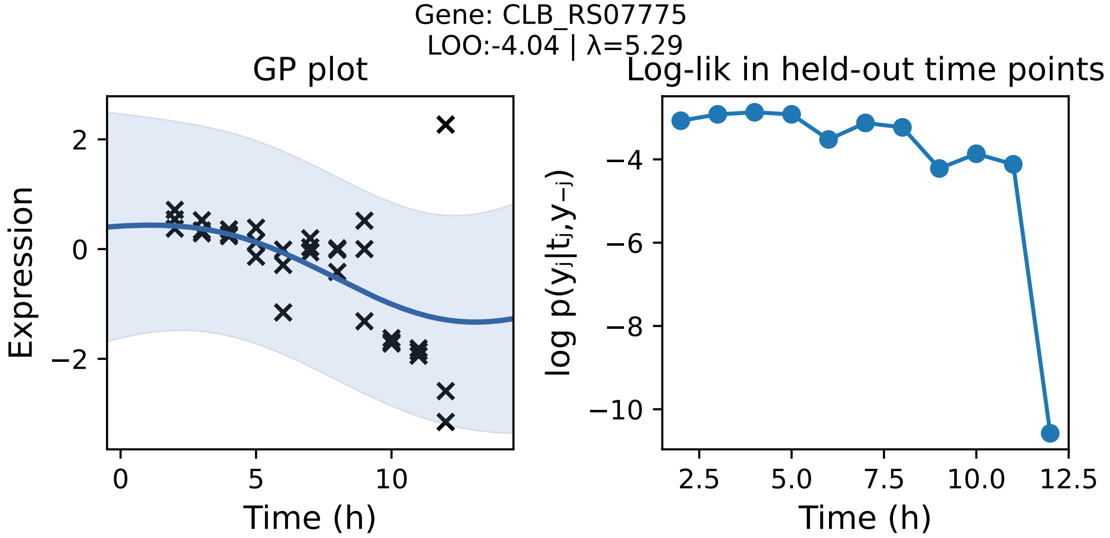

Gene: CLB_RS07775
LOO:-4.04 | λ=5.29
GP plot
Expression
Time (h)
Log-lik in held-out time points
log p(y_j|t_j,y_−j)
Time (h)

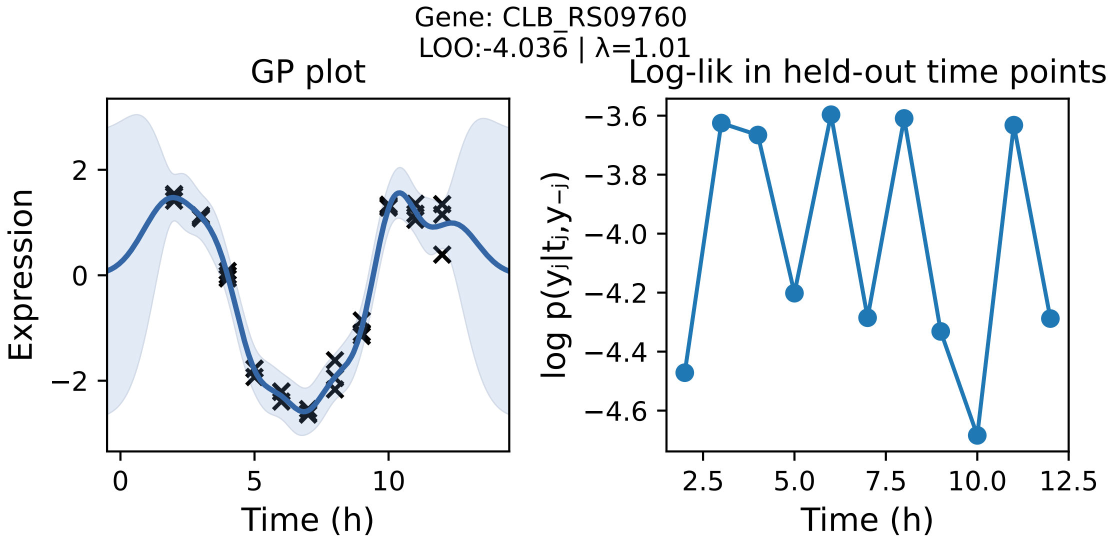
Gene: CLB_RS09760
LOO:-4.036 | λ=1.01
GP plot
Expression
Time (h)
Log-lik in held-out time points
log p(y_j|t_j,y_−j)
Time (h)

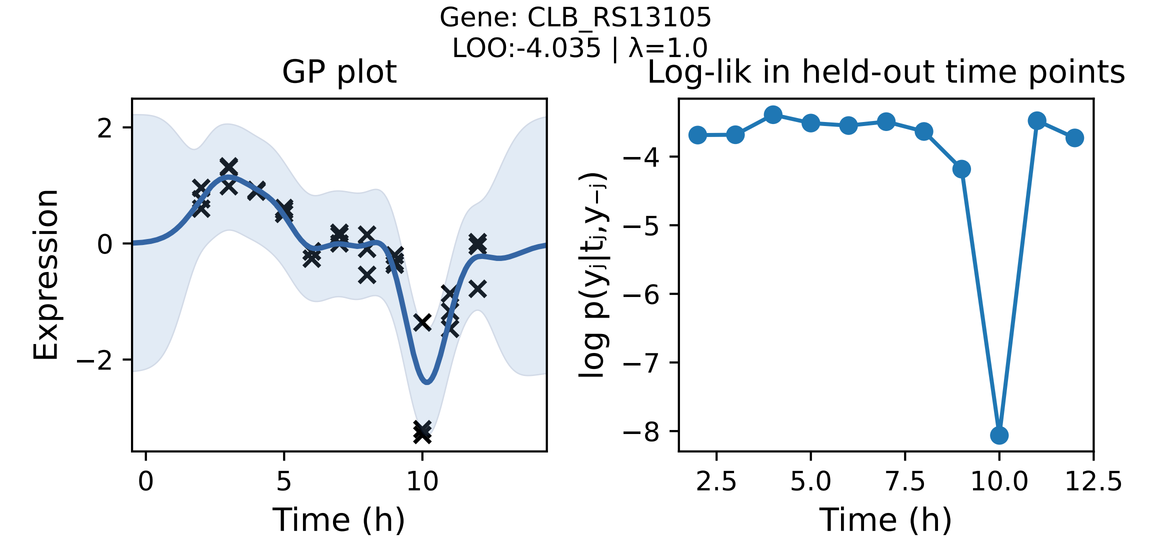

Gene: CLB_RS13105
LOO:-4.035 | λ=1.0
GP plot
Expression
Time (h)
Log-lik in held-out time points
log p(y_j|t_j,y_−j)
Time (h)

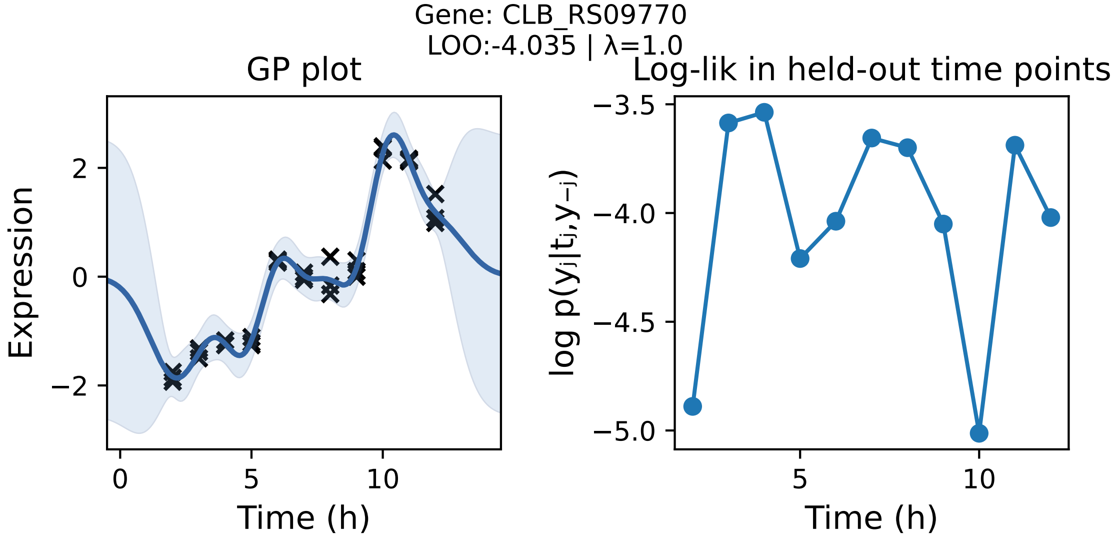
Gene: CLB_RS09770
LOO:-4.035 | λ=1.0
GP plot
Log-lik in held-out time points
Expression
Time (h)
log p(y_j|t_j,y_−j)
2
0
−2
0
5
10
−3.5
−4.0
−4.5
−5.0

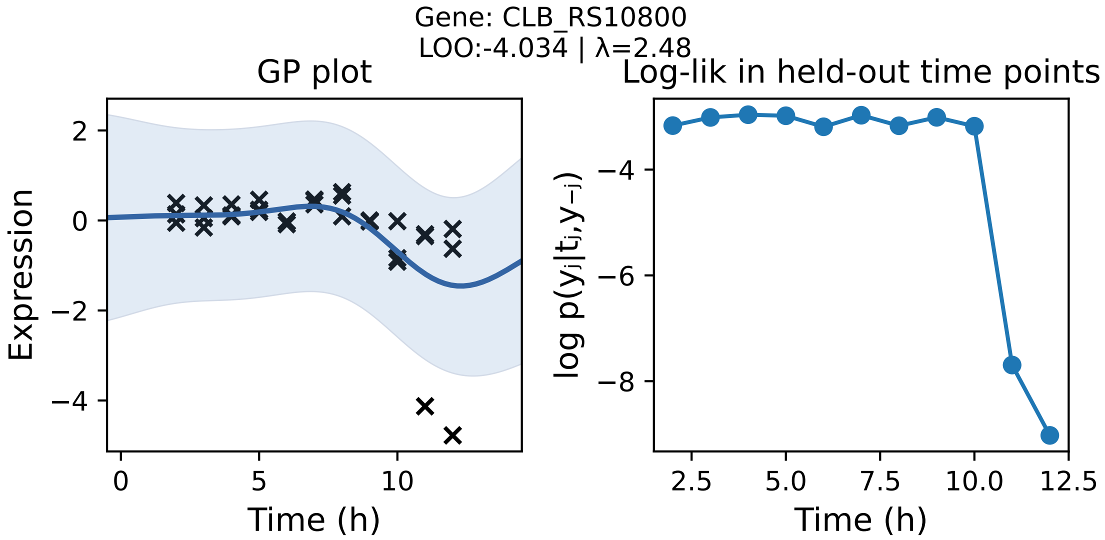
Gene: CLB_RS10800
LOO:-4.034 | λ=2.48
GP plot
Log-lik in held-out time points
Expression
Time (h)
log p(yj|tj,y−j)
Time (h)
2
0
−2
−4
0
5
10
−4
−6
−8
2.5
5.0
7.5
10.0
12.5

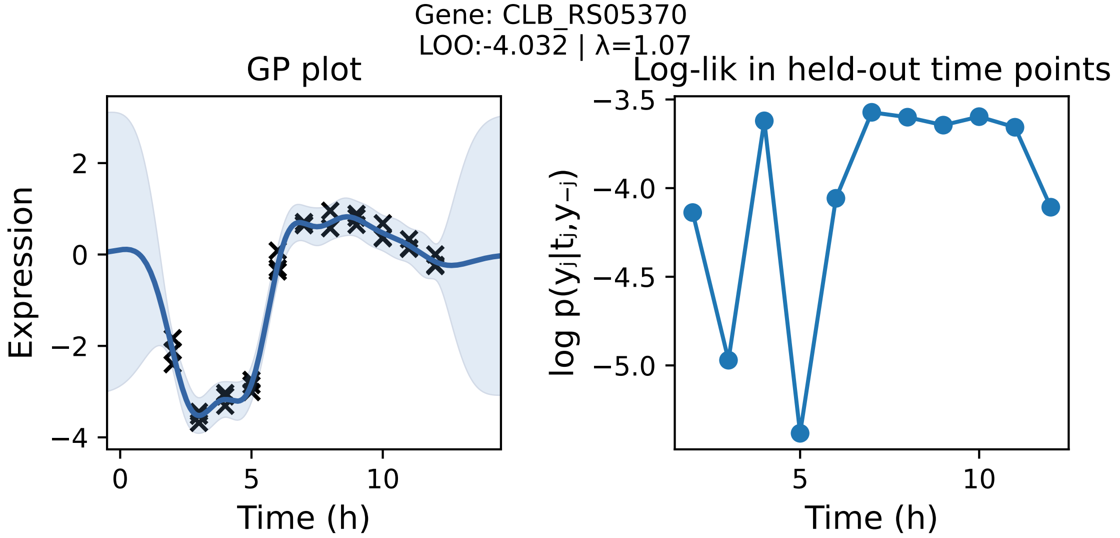

Gene: CLB_RS05370
LOO:-4.032 | λ=1.07
GP plot
Expression
Time (h)
Log-lik in held-out time points
log p(y_j|t_j,y_−j)
Time (h)

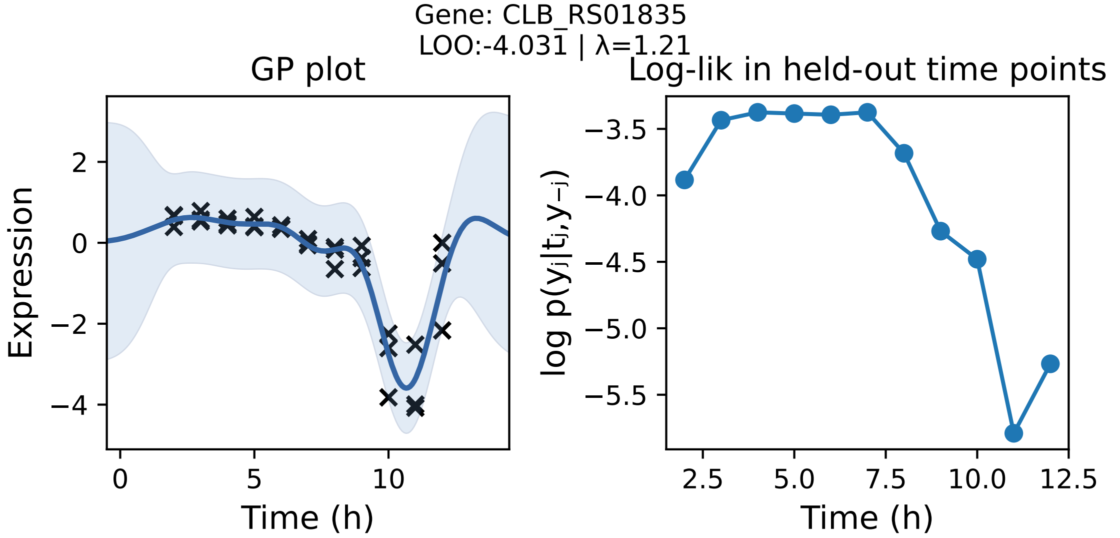

Gene: CLB_RS01835
LOO:-4.031 | λ=1.21
GP plot
Expression
Time (h)
Log-lik in held-out time points
log p(y_j|t_j,y_−j)
Time (h)

Gene: CLB_RS13475
LOO:-4.03 | λ=4.4

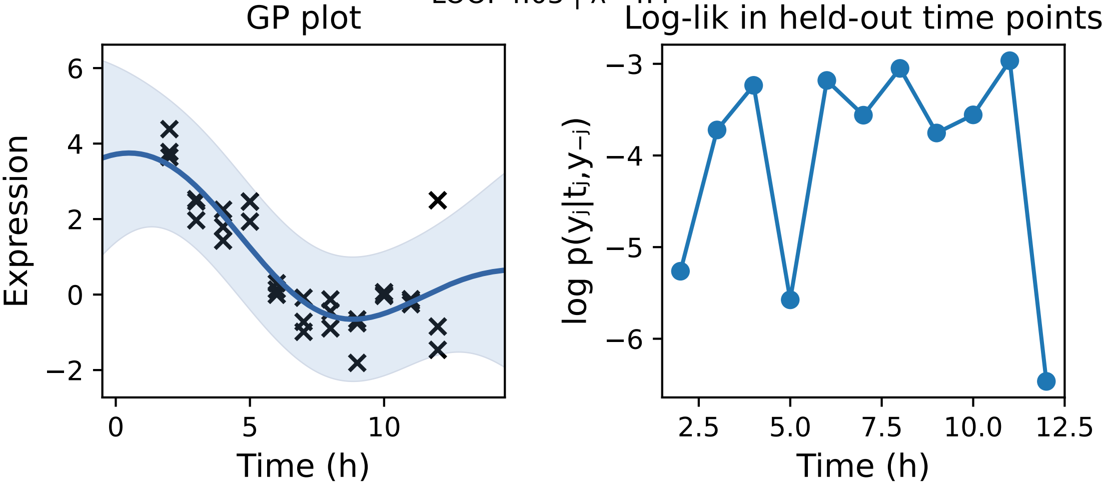

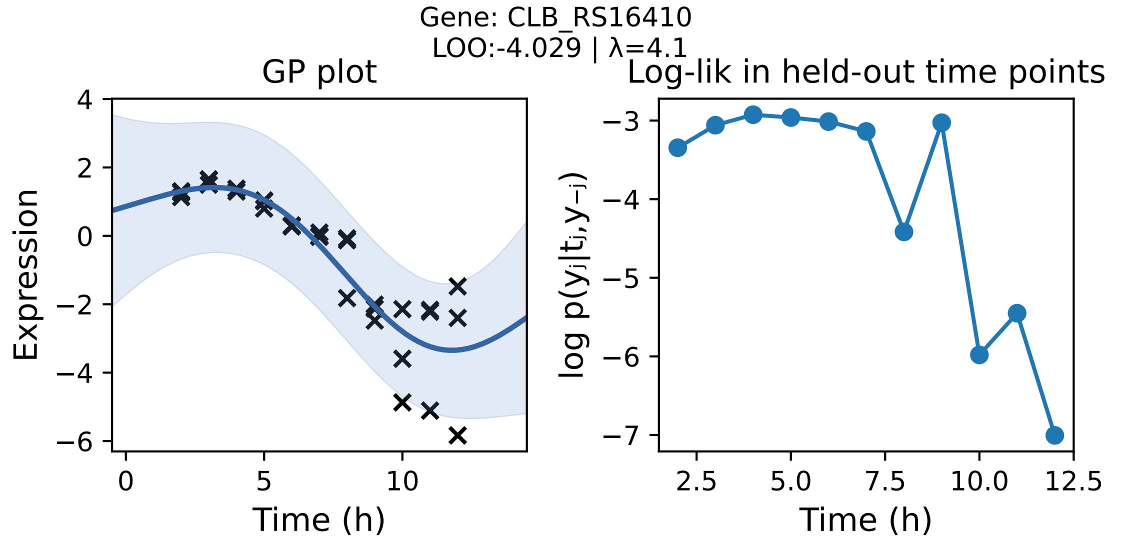

Gene: CLB_RS16410
LOO:-4.029 | λ=4.1
GP plot
Expression
Time (h)
4
2
0
−2
−4
−6
0
5
10
Log-lik in held-out time points
log p(y_j|t_j,y_−j)
Time (h)
−3
−4
−5
−6
−7
2.5
5.0
7.5
10.0
12.5

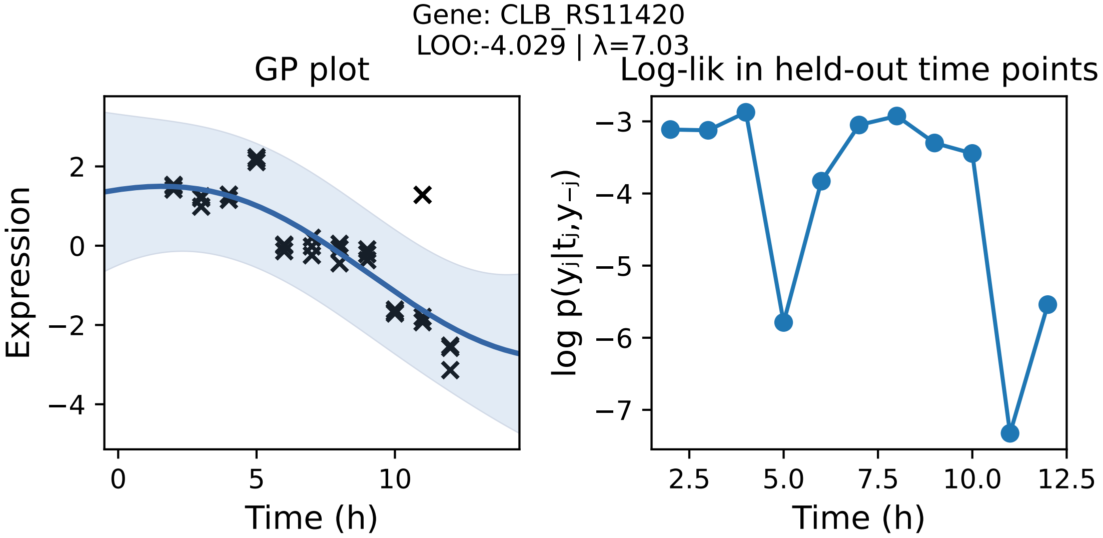

Gene: CLB_RS11420
LOO:-4.029 | λ=7.03
GP plot
Expression
2
0
−2
−4
0
5
10
Time (h)
Log-lik in held-out time points
log p(y_j|t_j,y_−j)
−3
−4
−5
−6
−7
2.5
5.0
7.5
10.0
12.5
Time (h)

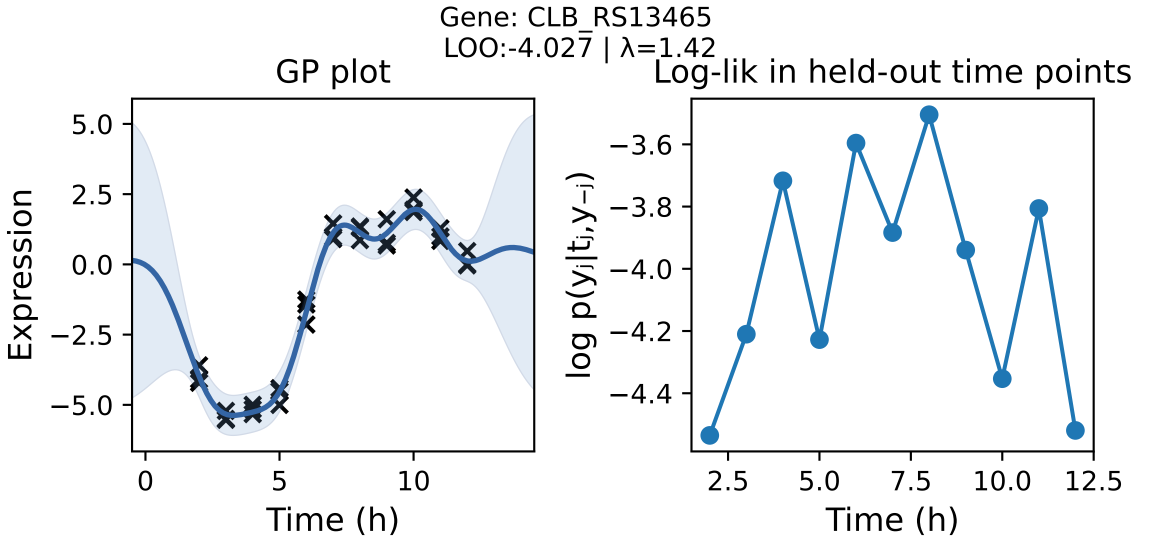
Gene: CLB_RS13465
LOO:-4.027 | λ=1.42
GP plot
Expression
5.0
2.5
0.0
−2.5
−5.0
0
5
10
Time (h)
Log-lik in held-out time points
log p(yj|tj,y−j)
−3.6
−3.8
−4.0
−4.2
−4.4
2.5
5.0
7.5
10.0
12.5
Time (h)

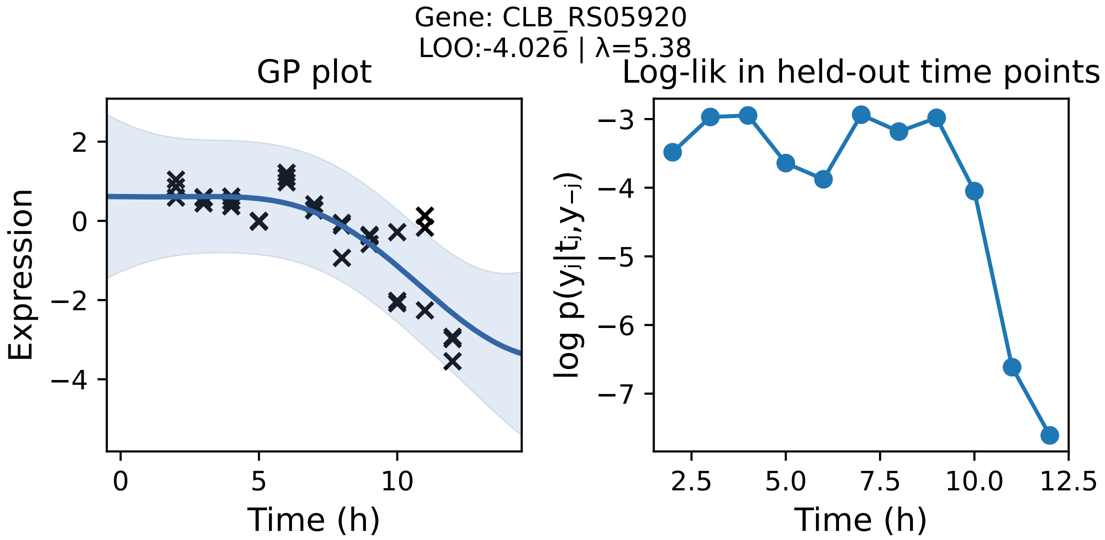

Gene: CLB_RS05920
LOO:-4.026 | λ=5.38
GP plot
Log-lik in held-out time points
Expression
Time (h)
log p(yⱼ|tⱼ,y₋ⱼ)
Time (h)
2
0
−2
−4
0
5
10
−3
−4
−5
−6
−7
2.5
5.0
7.5
10.0
12.5

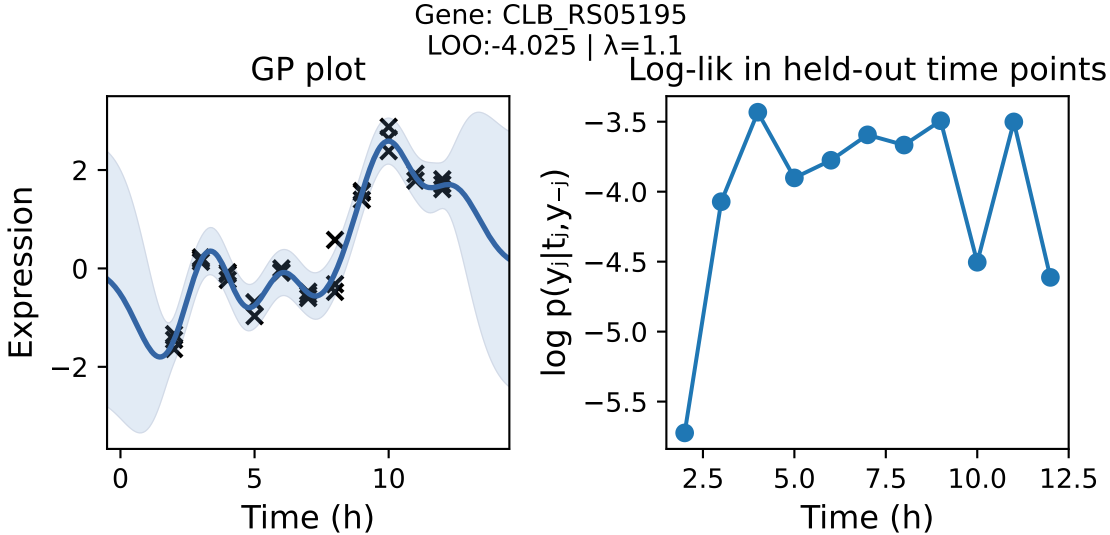

Gene: CLB_RS05195
LOO:-4.025 | λ=1.1
GP plot
Expression
Time (h)
Log-lik in held-out time points
log p(y_j|t_j,y_−j)
Time (h)

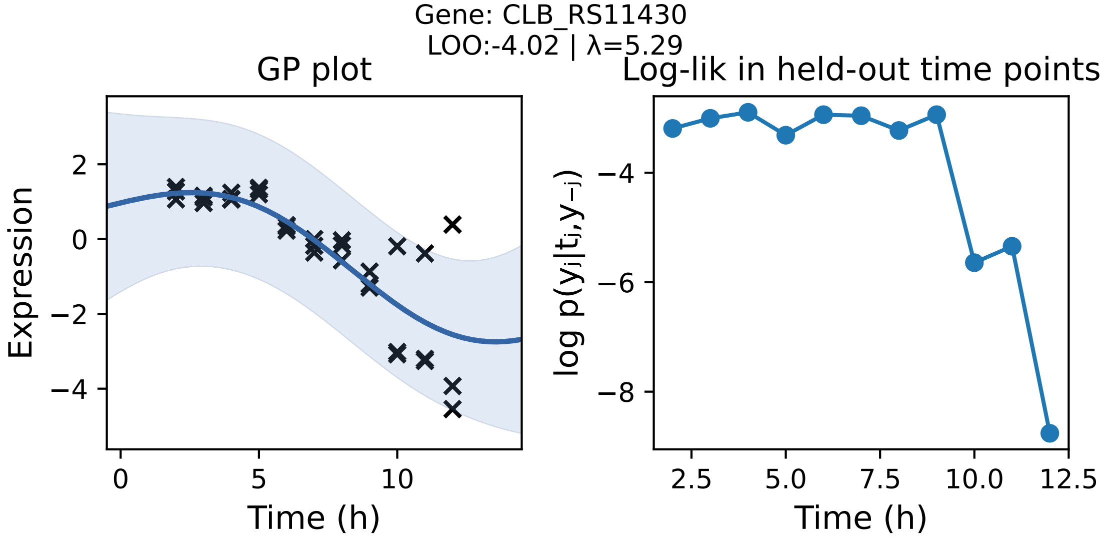

Gene: CLB_RS11430
LOO:-4.02 | λ=5.29
GP plot
Expression
Time (h)
2
0
−2
−4
0
5
10
Log-lik in held-out time points
log p(y_j|t_j,y_−j)
Time (h)
−4
−6
−8
2.5
5.0
7.5
10.0
12.5

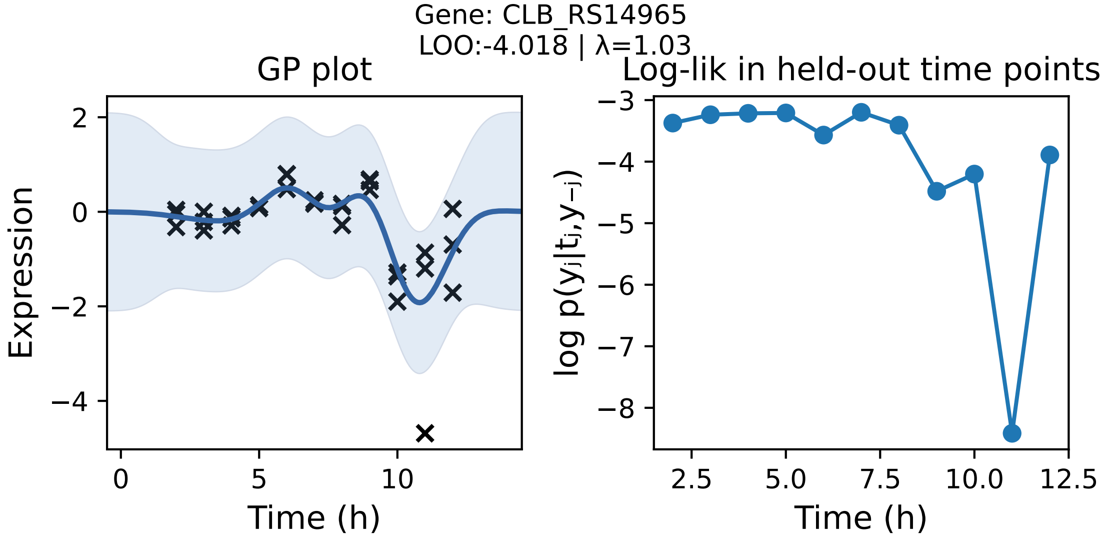

Gene: CLB_RS14965
LOO:-4.018 | λ=1.03
GP plot
Log-lik in held-out time points
Expression
Time (h)
log p(y_j|t_j,y_−j)
Time (h)

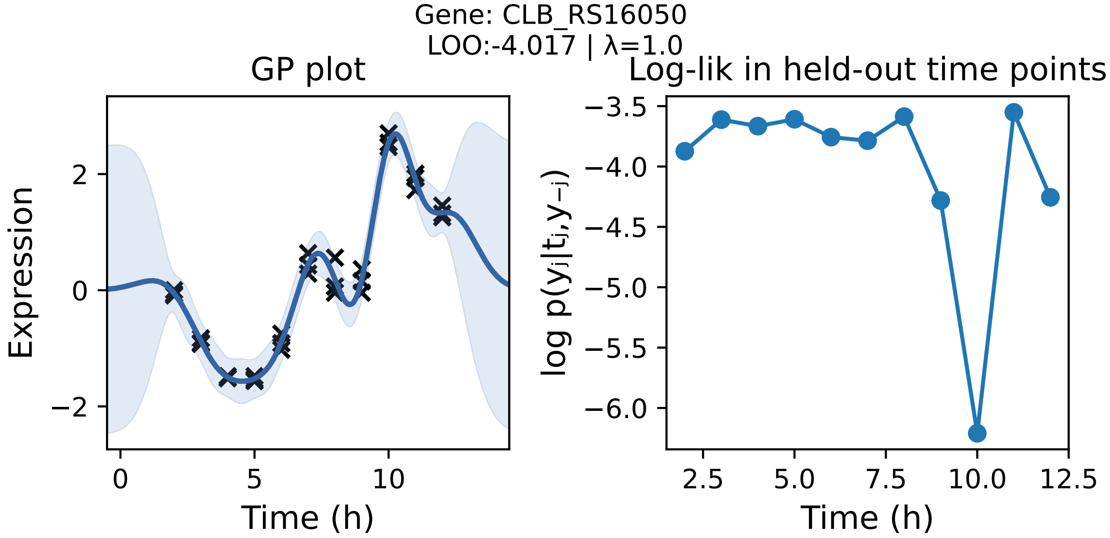

Gene: CLB_RS16050
LOO:-4.017 | λ=1.0
GP plot
Expression
Time (h)
2
0
−2
0
5
10
Log-lik in held-out time points
log p(yj|tj,y−j)
Time (h)
−3.5
−4.0
−4.5
−5.0
−5.5
−6.0
2.5
5.0
7.5
10.0
12.5

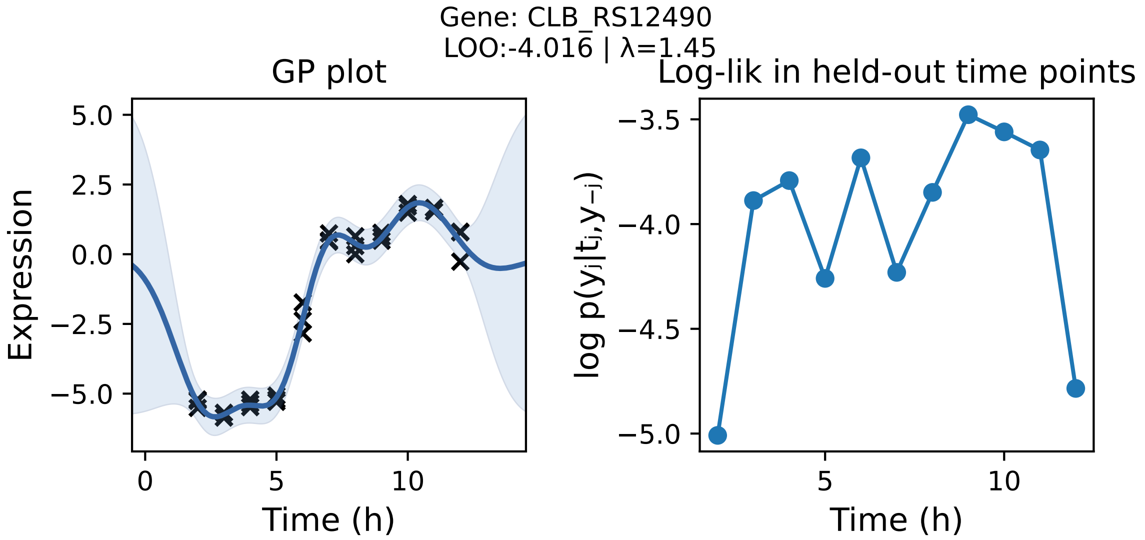
Gene: CLB_RS12490
LOO:-4.016 | λ=1.45
GP plot
Expression
Time (h)
5.0
2.5
0.0
−2.5
−5.0
0
5
10
Log-lik in held-out time points
log p(y_j|t_j,y_−j)
Time (h)
−3.5
−4.0
−4.5
−5.0
5
10

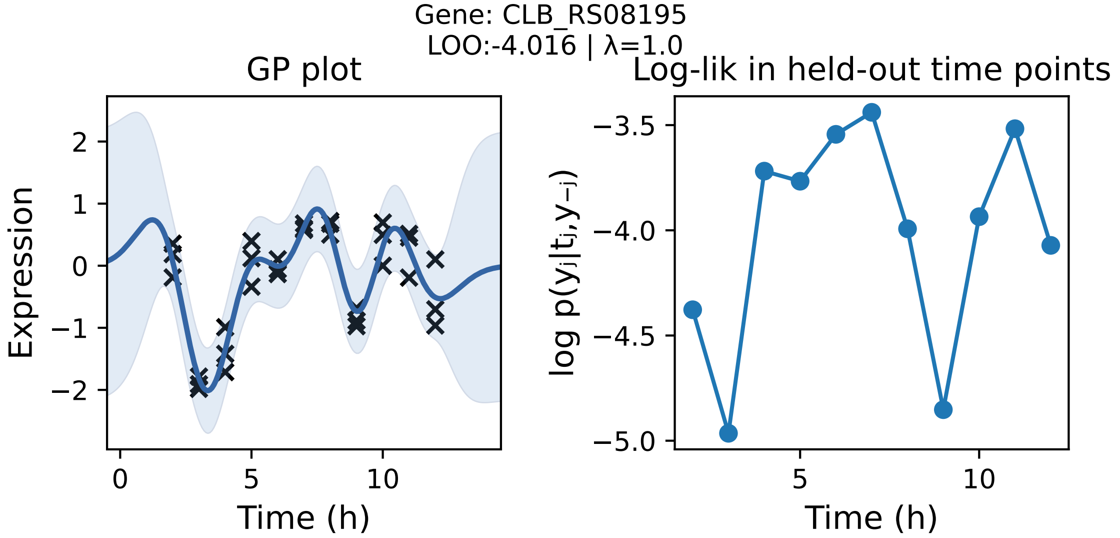

Gene: CLB_RS08195
LOO:-4.016 | λ=1.0
GP plot
Log-lik in held-out time points
Expression
2
1
0
−1
−2
0
5
10
Time (h)
log p(yj|tj,y−j)
−3.5
−4.0
−4.5
−5.0
5
10
Time (h)

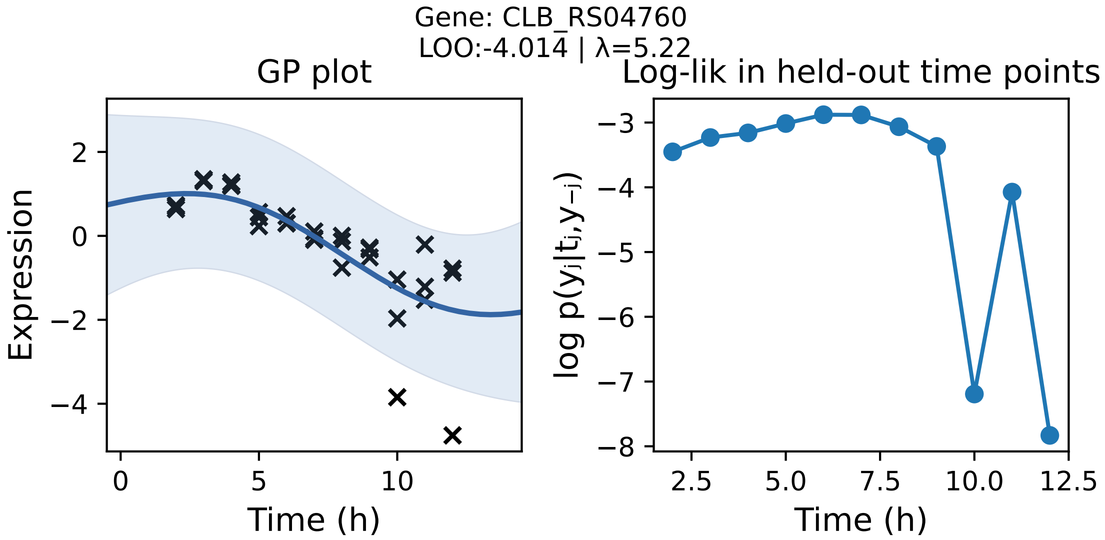

Gene: CLB_RS04760
LOO:-4.014 | λ=5.22
GP plot
Expression
Time (h)
Log-lik in held-out time points
log p(y_j|t_j,y_−j)
Time (h)

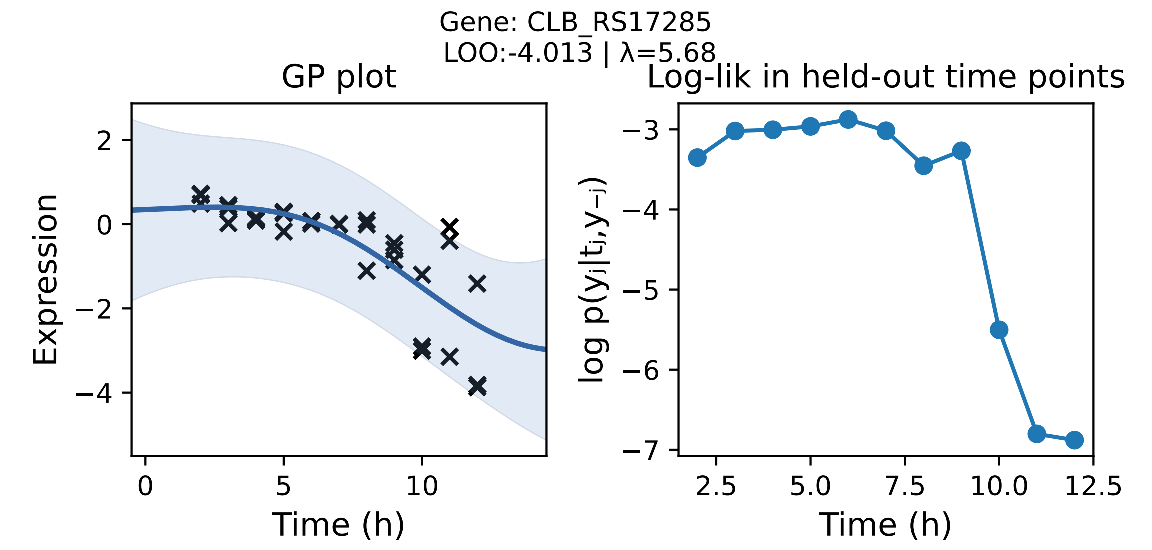

Gene: CLB_RS17285
LOO:-4.013 | λ=5.68
GP plot
Expression
Time (h)
Log-lik in held-out time points
log p(y_j|t_j,y_−j)
Time (h)

Gene: CLB_RS05985
LOO:-4.012 | λ=1.08

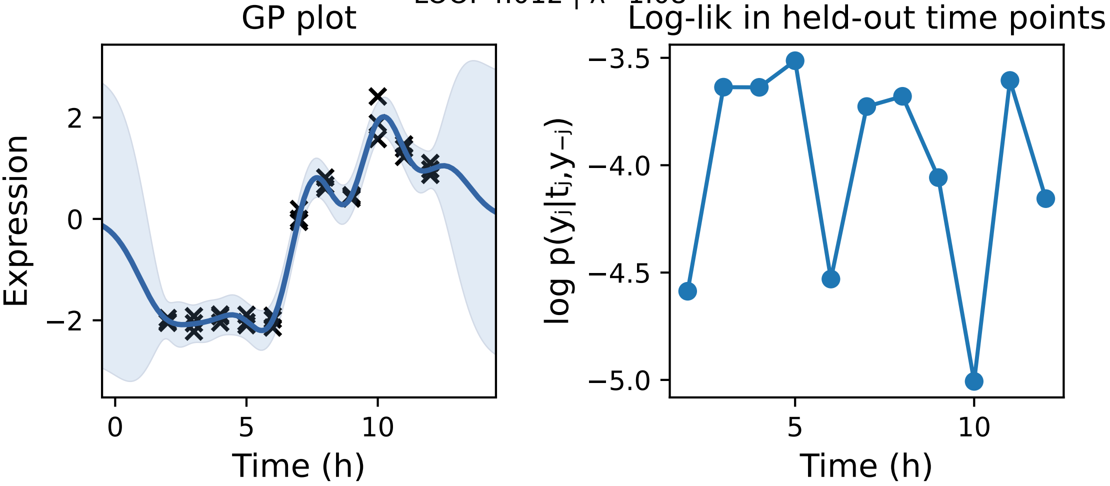

Gene: CLB_RS04855
LOO:-4.011 | λ=4.91

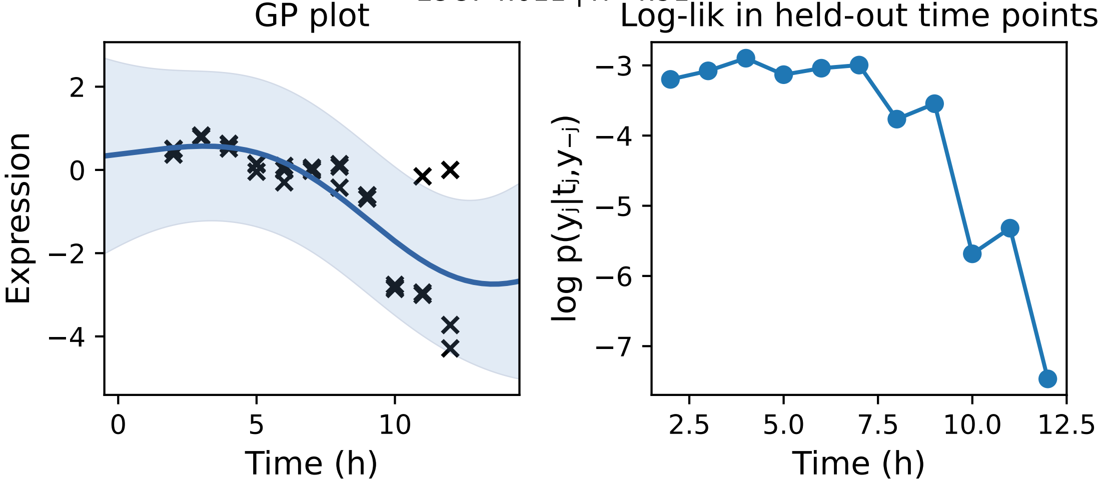

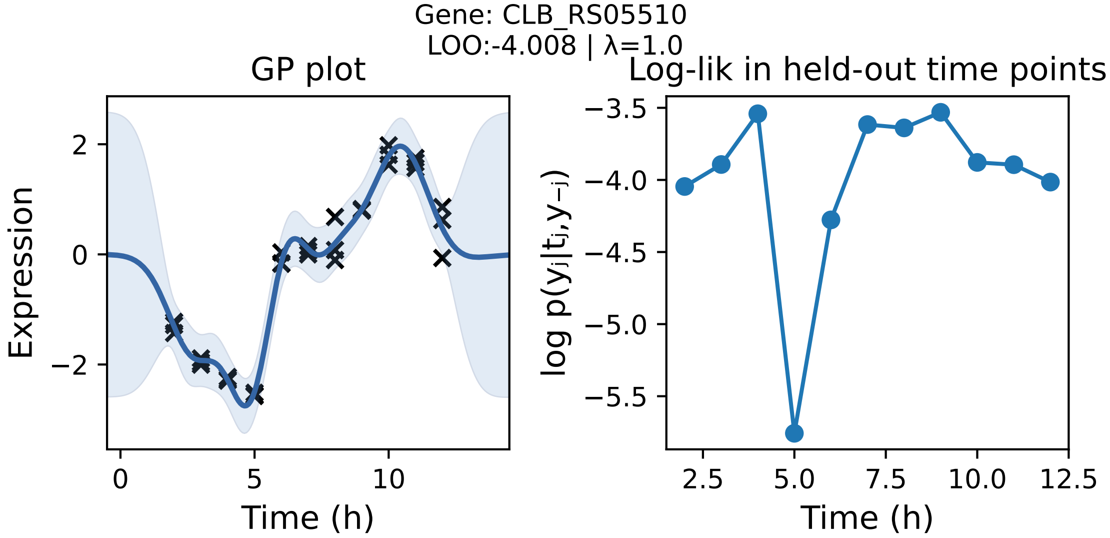

Gene: CLB_RS05510
LOO:-4.008 | λ=1.0
GP plot
Expression
Time (h)
Log-lik in held-out time points
log p(y_j|t_j,y_−j)
Time (h)

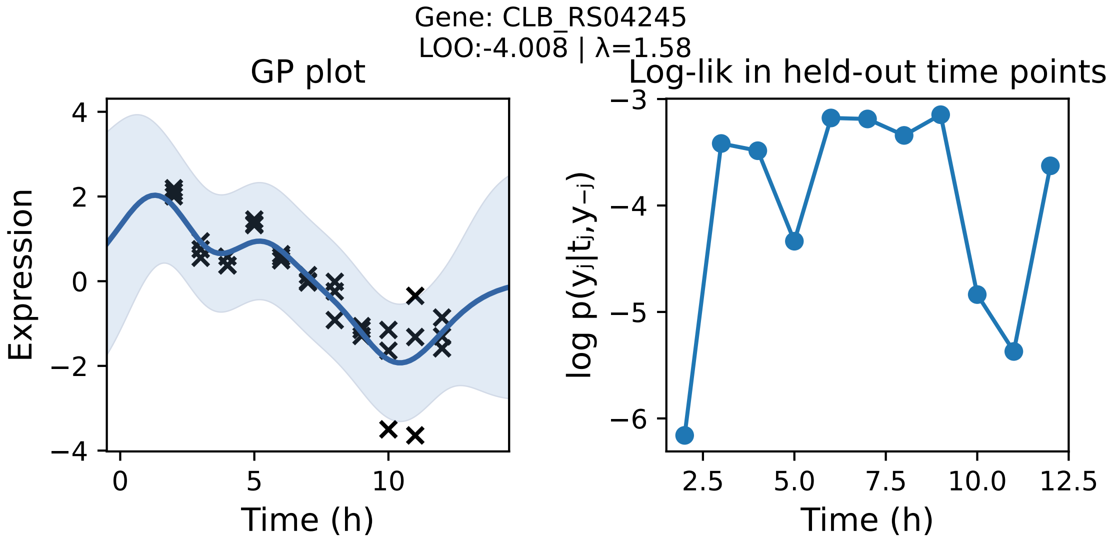

Gene: CLB_RS04245
LOO:-4.008 | λ=1.58
GP plot
Expression
Time (h)
4
2
0
−2
−4
0
5
10
Log-lik in held-out time points
log p(y_j|t_j,y_−j)
Time (h)
−3
−4
−5
−6
2.5
5.0
7.5
10.0
12.5

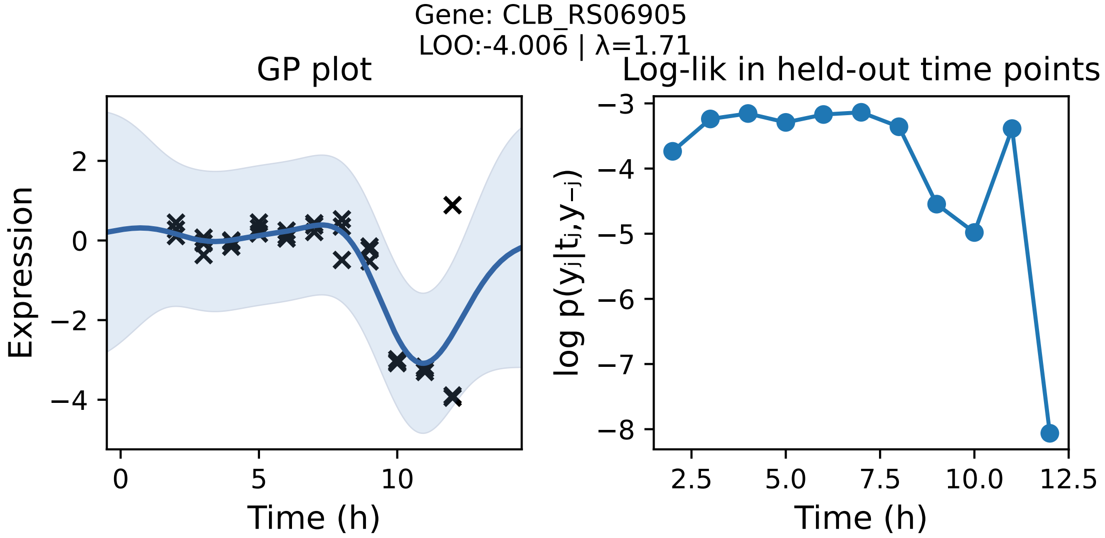
Gene: CLB_RS06905
LOO:-4.006 | λ=1.71
GP plot
Expression
Time (h)
Log-lik in held-out time points
log p(y_j|t_j,y_−j)
Time (h)
2
0
−2
−4
0
5
10
−3
−4
−5
−6
−7
−8
2.5
5.0
7.5
10.0
12.5

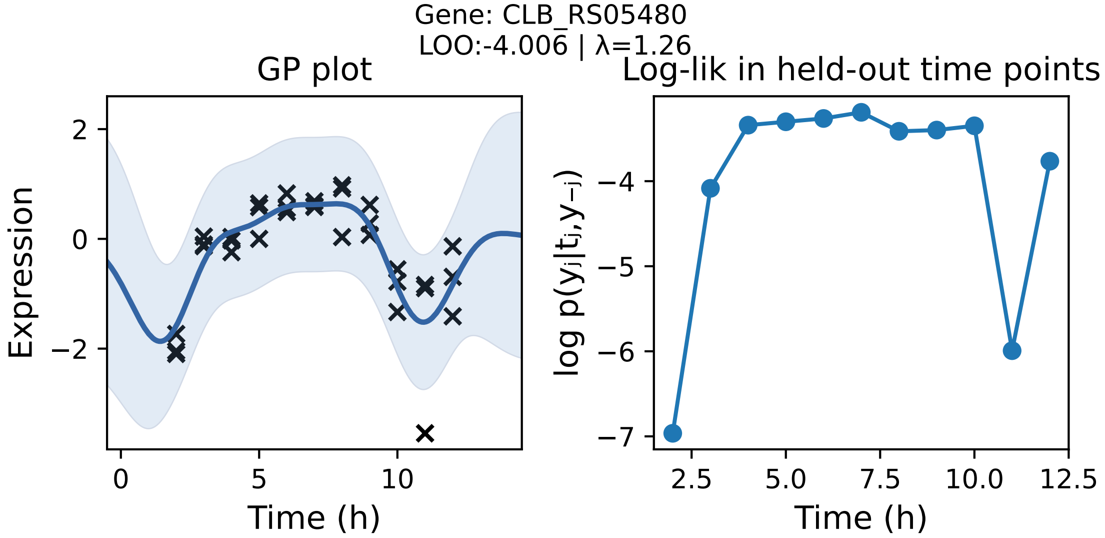

Gene: CLB_RS05480
LOO:-4.006 | λ=1.26
GP plot
Expression
Time (h)
2
0
−2
0
5
10
Log-lik in held-out time points
log p(y_j|t_j,y_{−j})
Time (h)
−4
−5
−6
−7
2.5
5.0
7.5
10.0
12.5

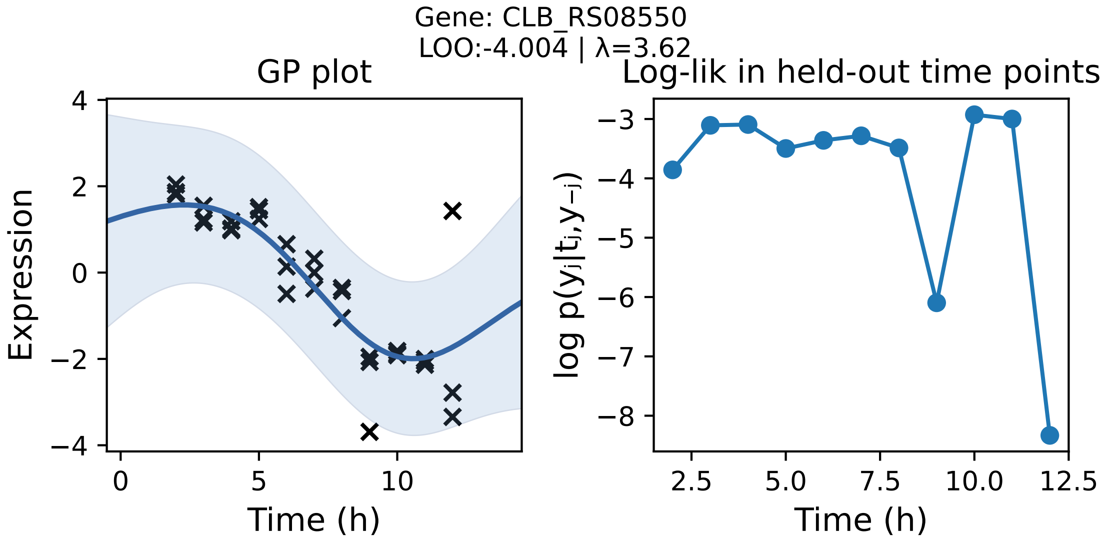

Gene: CLB_RS08550
LOO:-4.004 | λ=3.62
GP plot
Log-lik in held-out time points
Expression
Time (h)
log p(y_j|t_j,y_−j)
4
2
0
−2
−4
0
5
10
−3
−4
−5
−6
−7
−8
2.5
5.0
7.5
10.0
12.5

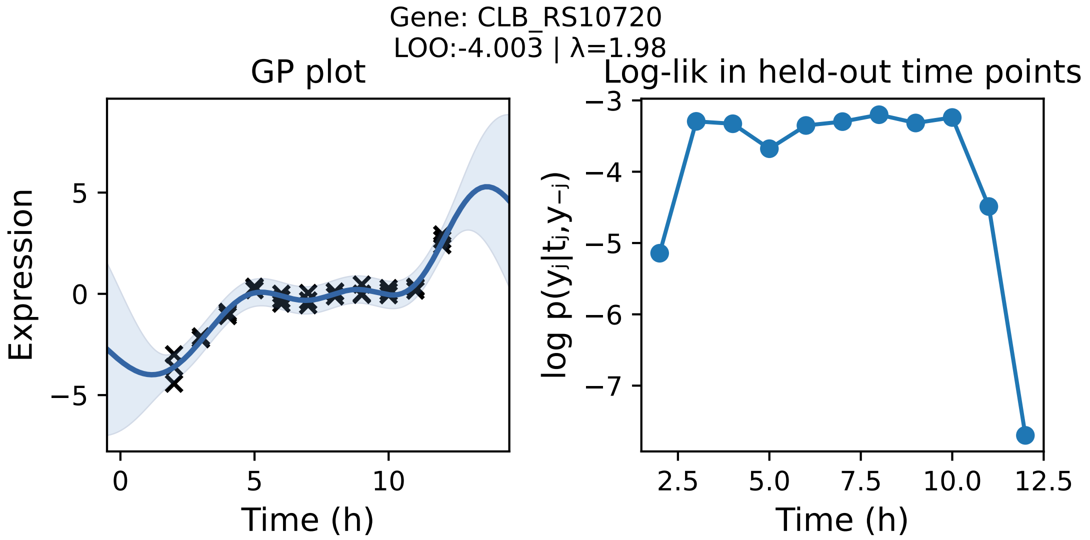

Gene: CLB_RS10720
LOO:-4.003 | λ=1.98
GP plot
Expression
5
0
−5
0
5
10
Time (h)
Log-lik in held-out time points
log p(y_j|t_j,y_−j)
−3
−4
−5
−6
−7
2.5
5.0
7.5
10.0
12.5
Time (h)

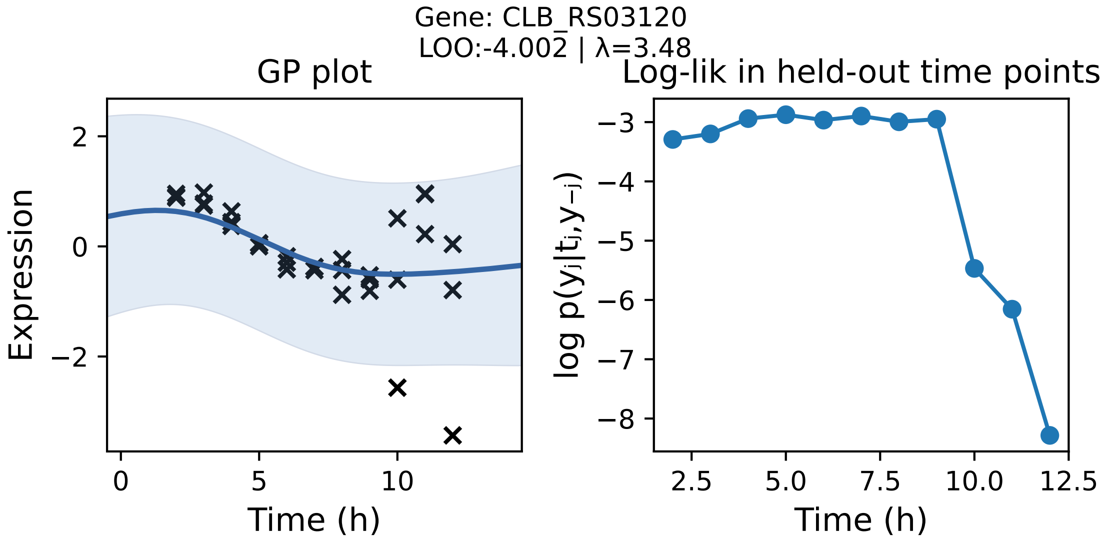
Gene: CLB_RS03120
LOO:-4.002 | λ=3.48
GP plot
Expression
Time (h)
Log-lik in held-out time points
log p(y_j|t_j,y_−j)
Time (h)

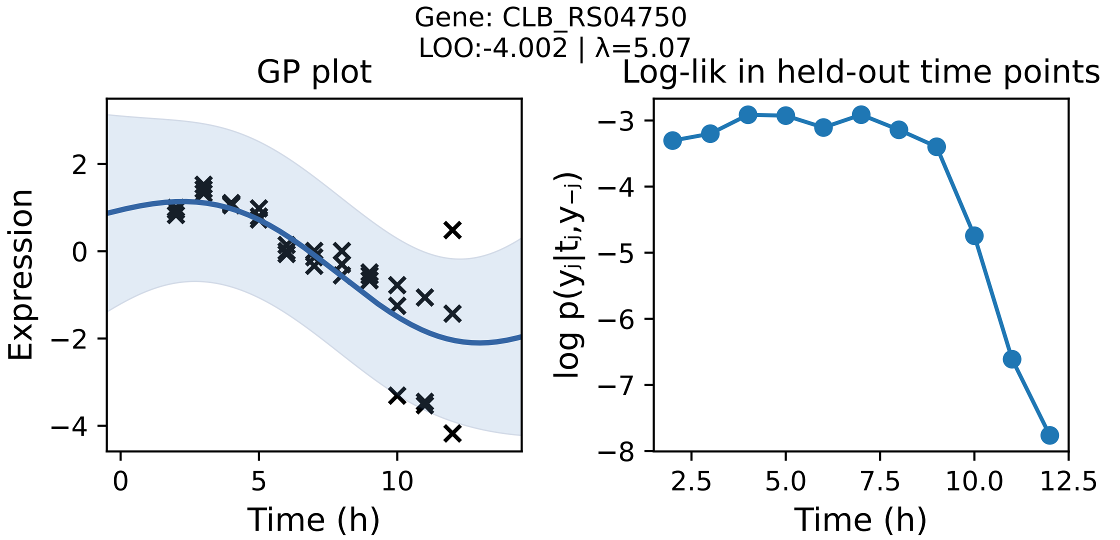

Gene: CLB_RS04750
LOO:-4.002 | λ=5.07
GP plot
Expression
Time (h)
Log-lik in held-out time points
log p(yj|tj,y−j)
Time (h)

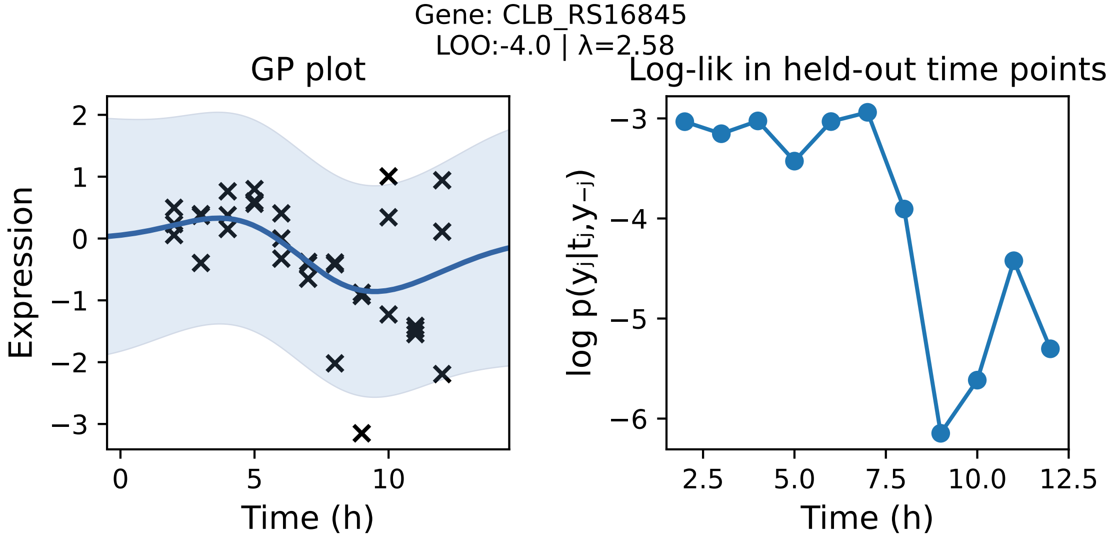

Gene: CLB_RS16845
LOO:-4.0 | λ=2.58
GP plot
Log-lik in held-out time points
Expression
Time (h)
log p(y_j|t_j,y_−j)
Time (h)
2
1
0
−1
−2
−3
0
5
10
−3
−4
−5
−6
2.5
5.0
7.5
10.0
12.5

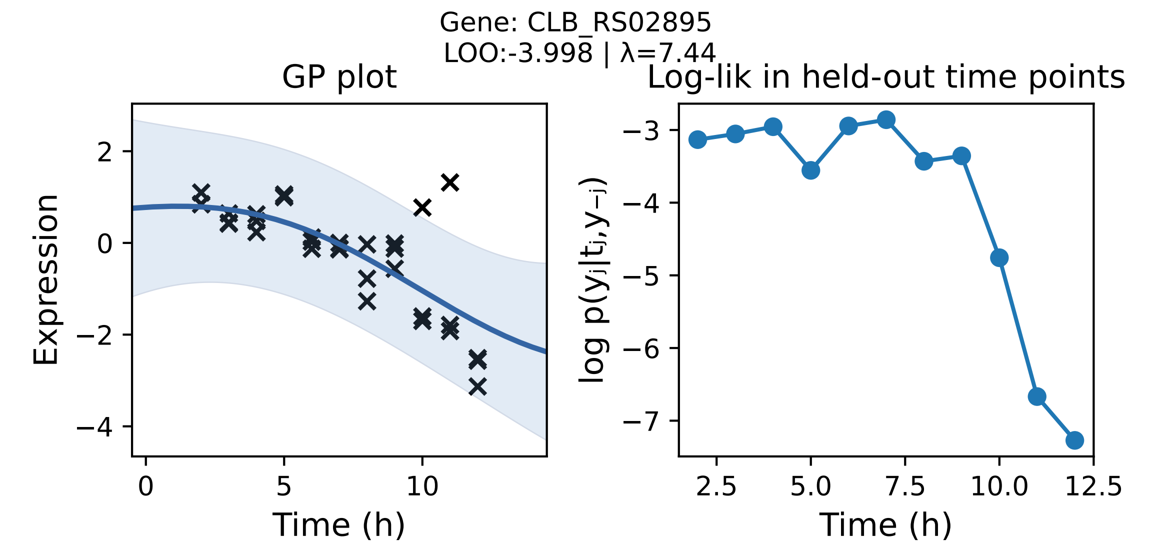
Gene: CLB_RS02895
LOO:-3.998 | λ=7.44
GP plot
Log-lik in held-out time points
Expression
Time (h)
log p(y_j|t_j,y_−j)
2
0
−2
−4
5
10
−3
−4
−5
−6
−7
2.5
5.0
7.5
10.0
12.5

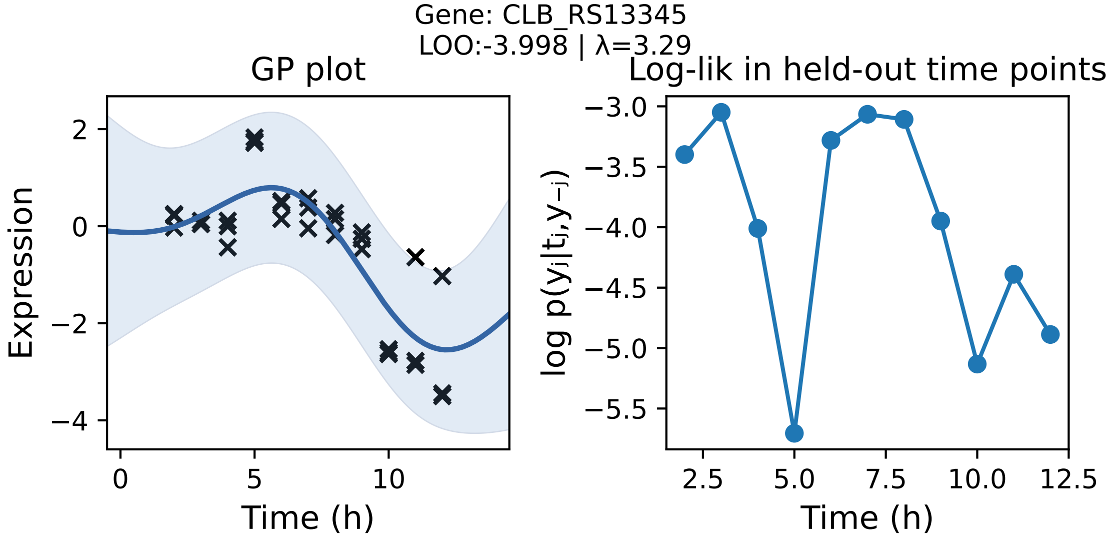

Gene: CLB_RS13345
LOO:-3.998 | λ=3.29
GP plot
Expression
Time (h)
2
0
−2
−4
0
5
10
Log-lik in held-out time points
log p(y_j|t_j,y_−j)
Time (h)
−3.0
−3.5
−4.0
−4.5
−5.0
−5.5
2.5
5.0
7.5
10.0
12.5

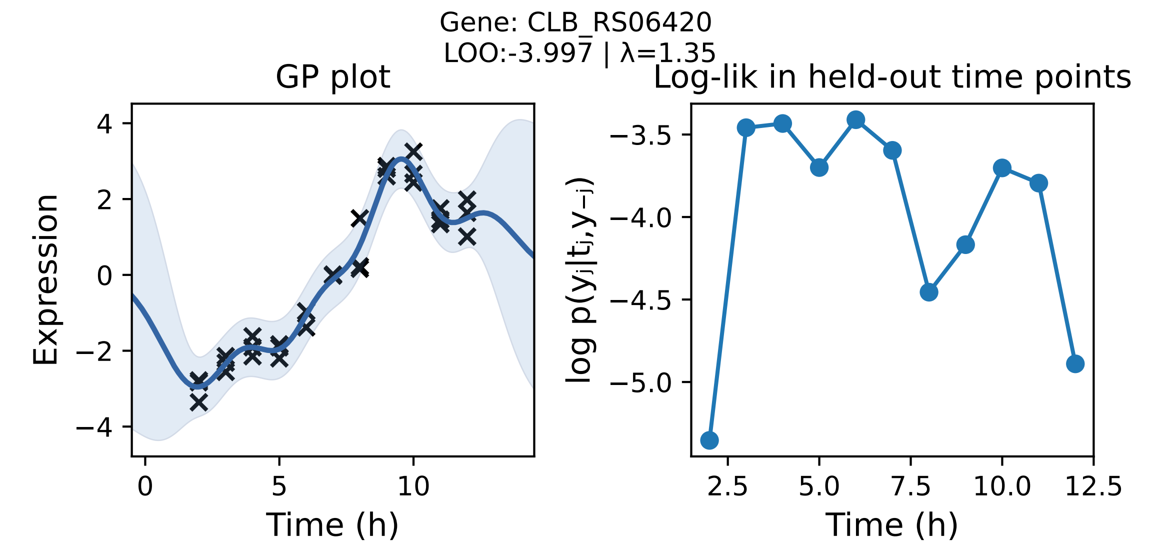
Gene: CLB_RS06420
LOO:-3.997 | λ=1.35
GP plot
Expression
Time (h)
Log-lik in held-out time points
log p(y_j|t_j,y_−j)
Time (h)

Gene: CLB_RS10930
LOO:-3.996 | λ=1.0

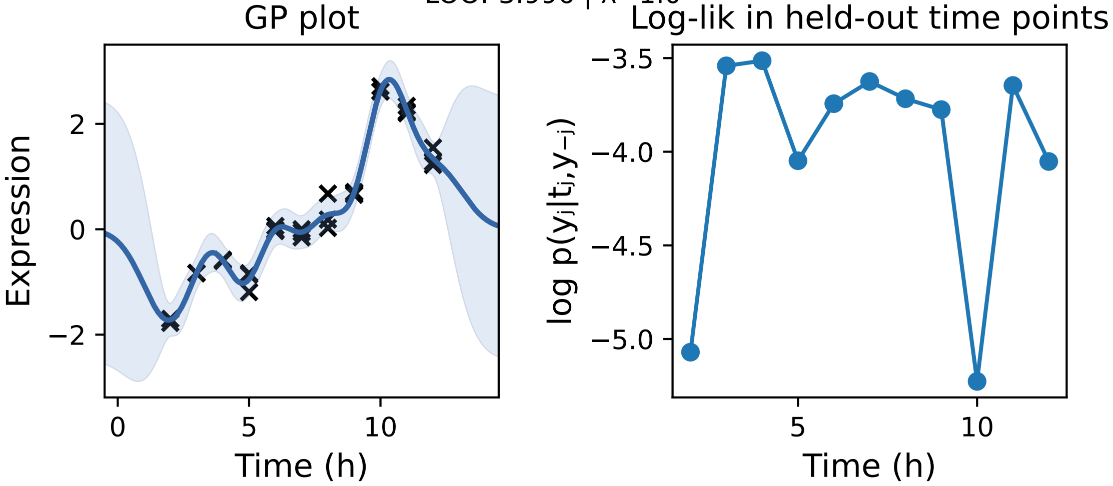

Gene: CLB_RS05440
LOO:-3.994 | λ=1.28

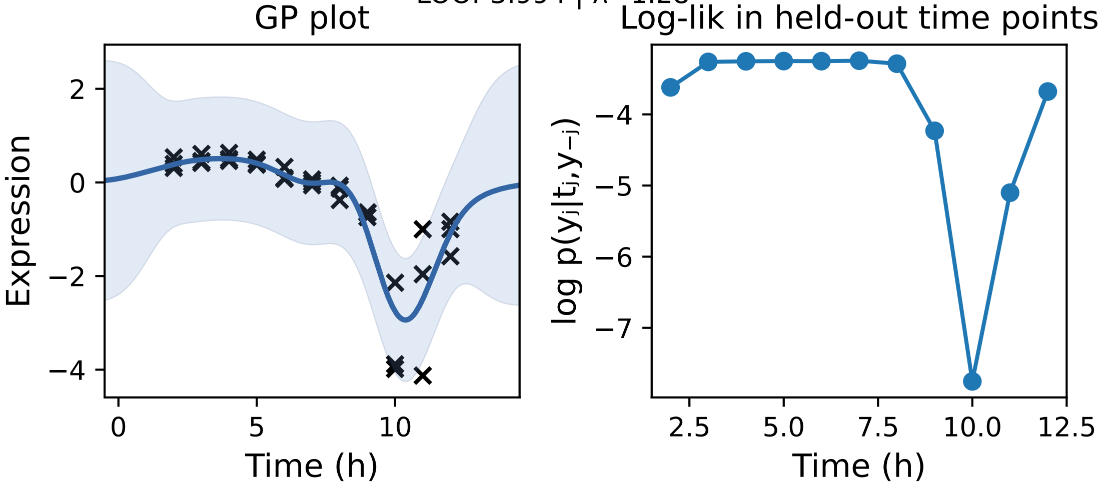

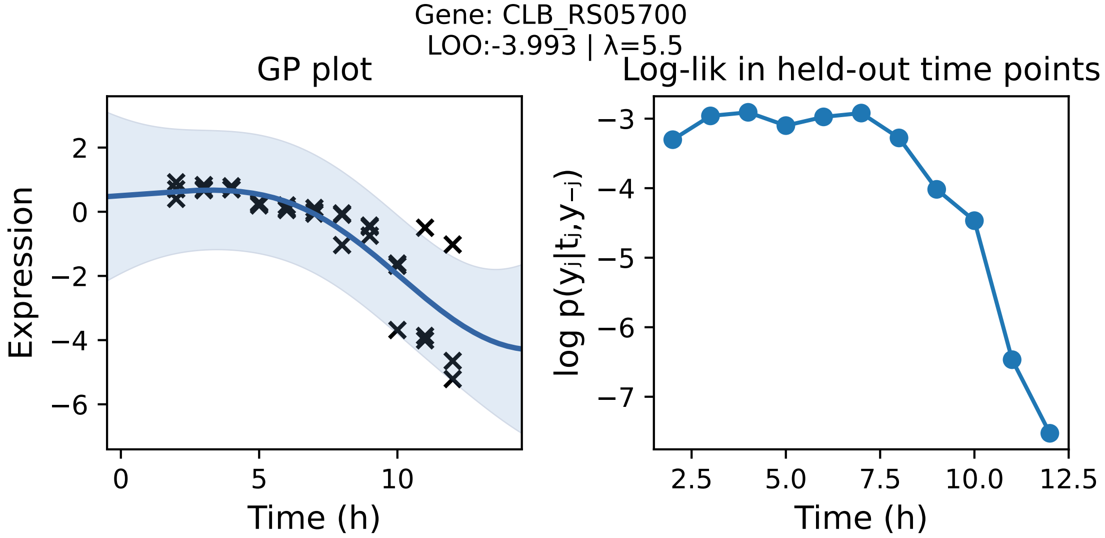

Gene: CLB_RS05700
LOO:-3.993 | λ=5.5
GP plot
Log-lik in held-out time points
Expression
2
0
−2
−4
−6
0
5
10
Time (h)
log p(yj|tj,y−j)
−3
−4
−5
−6
−7
2.5
5.0
7.5
10.0
12.5
Time (h)

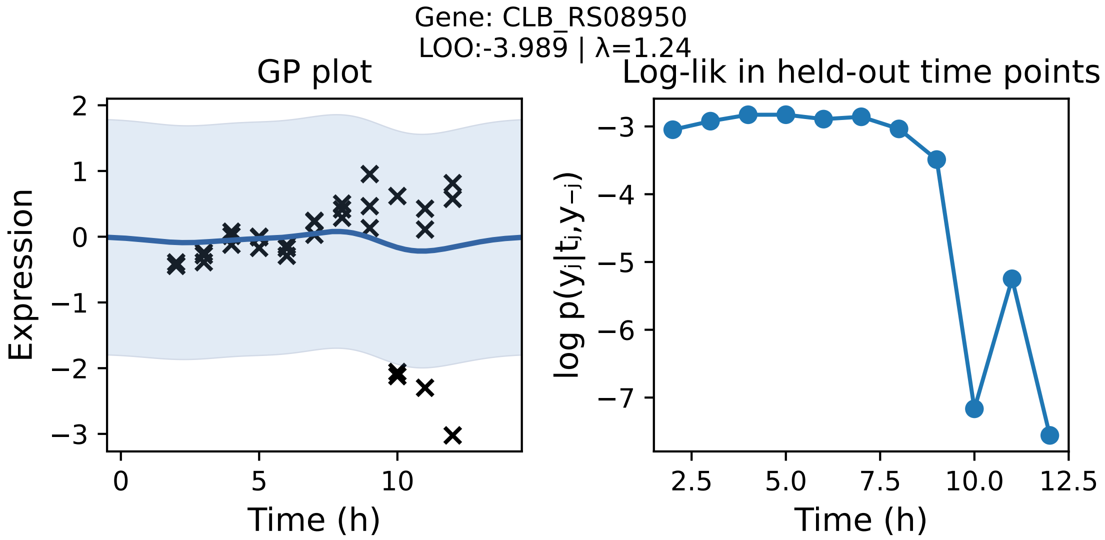

Gene: CLB_RS08950
LOO:-3.989 | λ=1.24
GP plot
Log-lik in held-out time points
Expression
Time (h)
log p(yj|tj,y−j)
Time (h)

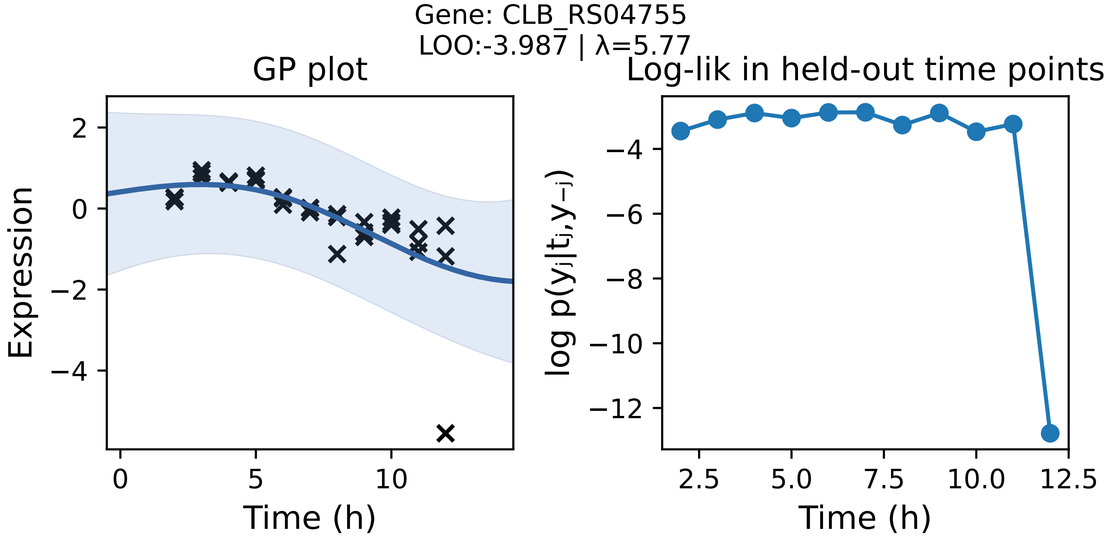
Gene: CLB_RS04755
LOO:-3.987 | λ=5.77
GP plot
Expression
Time (h)
2
0
−2
−4
0
5
10
Log-lik in held-out time points
log p(y_j|t_j,y_−j)
Time (h)
−4
−6
−8
−10
−12
2.5
5.0
7.5
10.0
12.5

Gene: CLB_RS08615
LOO:-3.984 | λ=1.0

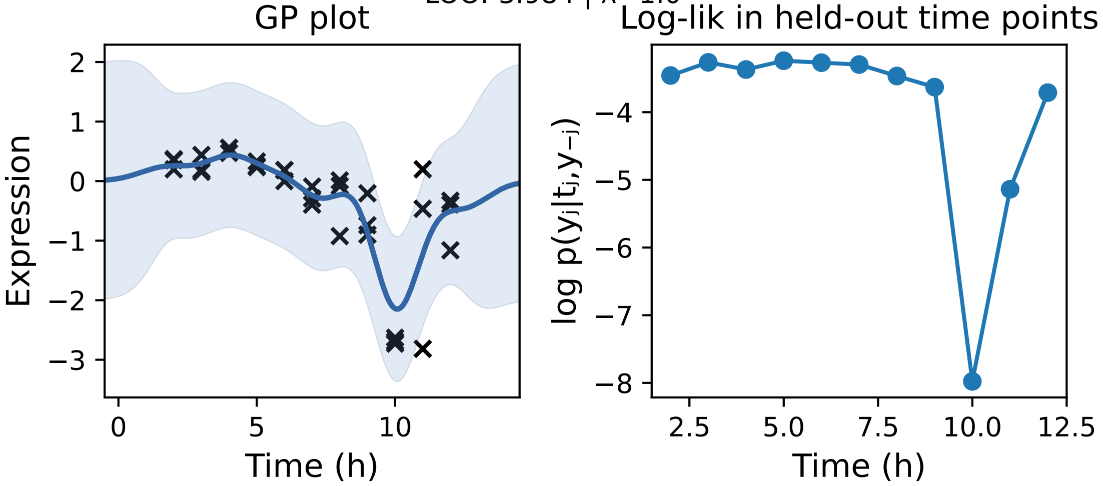

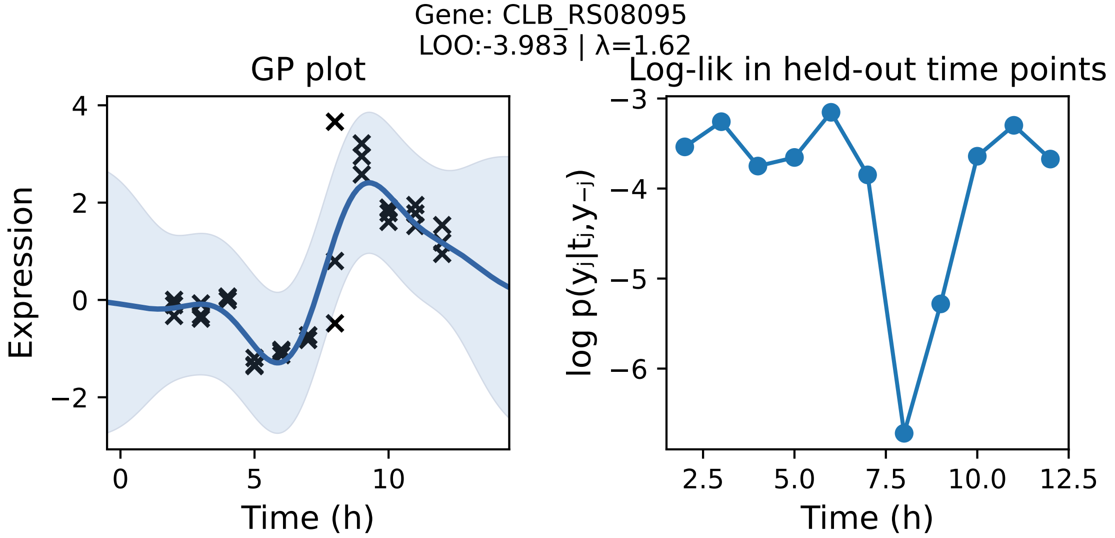
Gene: CLB_RS08095
LOO:-3.983 | λ=1.62
GP plot
Expression
Time (h)
Log-lik in held-out time points
log p(yj|tj,y−j)
Time (h)

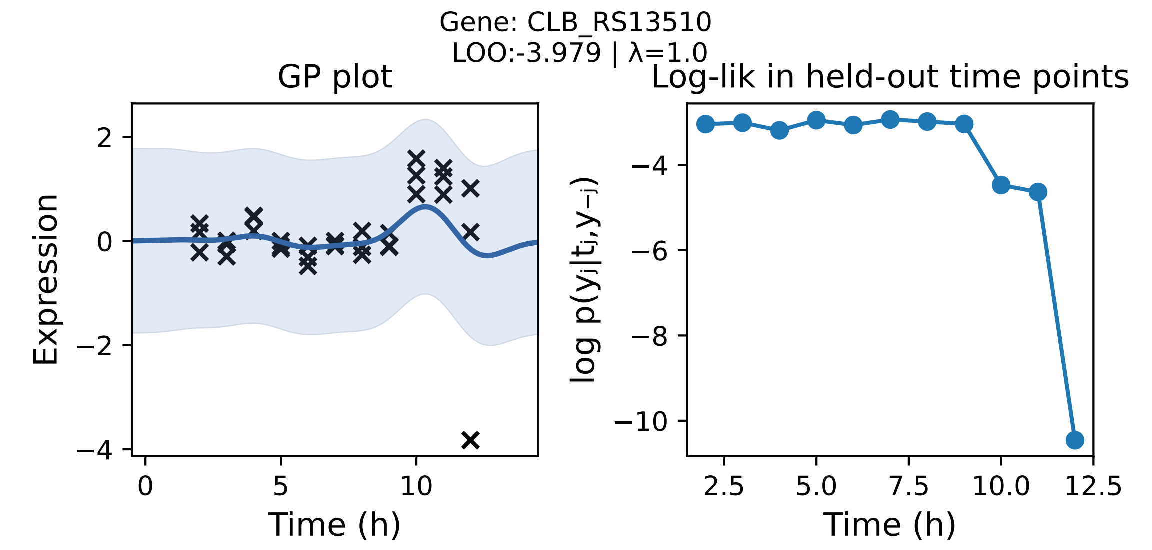

Gene: CLB_RS13510
LOO:-3.979 | λ=1.0
GP plot
Expression
Time (h)
Log-lik in held-out time points
log p(yⱼ|tⱼ,y₋ⱼ)
Time (h)

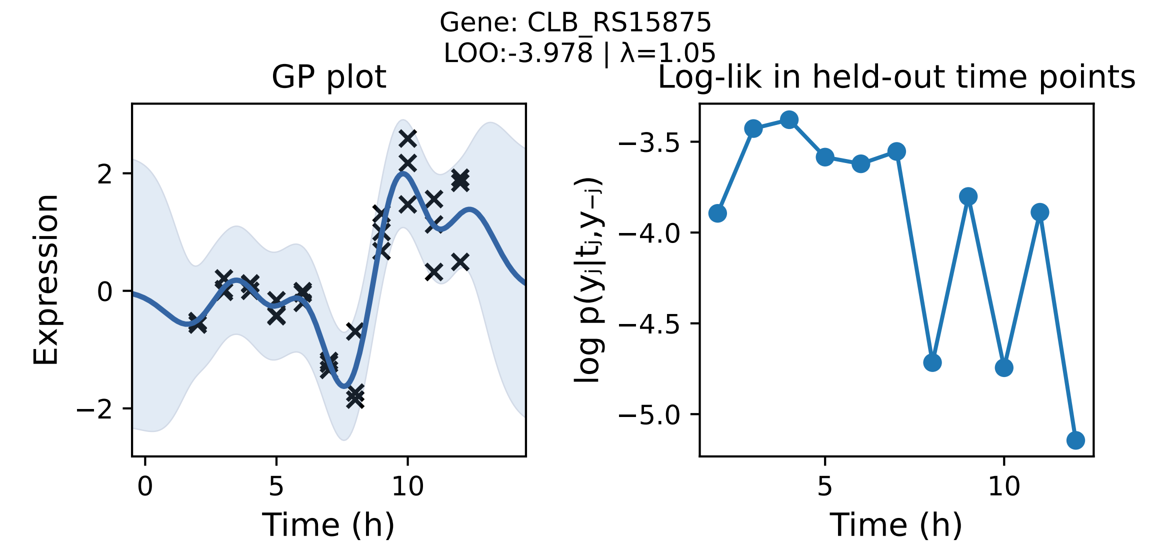
Gene: CLB_RS15875
LOO:-3.978 | λ=1.05
GP plot
Expression
Time (h)
Log-lik in held-out time points
log p(y_j|t_j,y_−j)
Time (h)

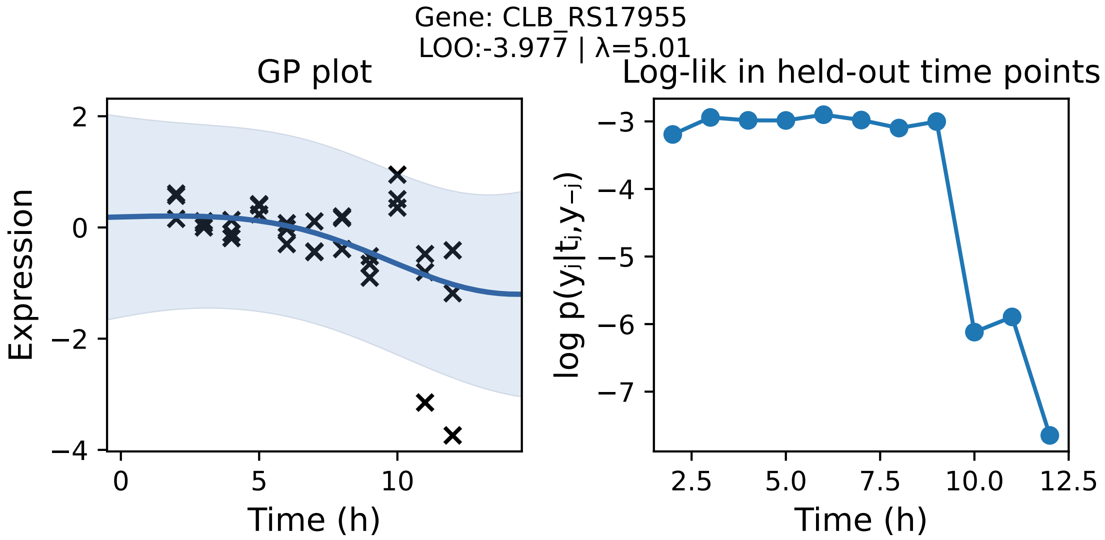

Gene: CLB_RS17955
LOO:-3.977 | λ=5.01
GP plot
Log-lik in held-out time points
Expression
Time (h)
log p(y_j|t_j,y_−j)
2
0
−2
−4
0
5
10
−3
−4
−5
−6
−7
2.5
5.0
7.5
10.0
12.5

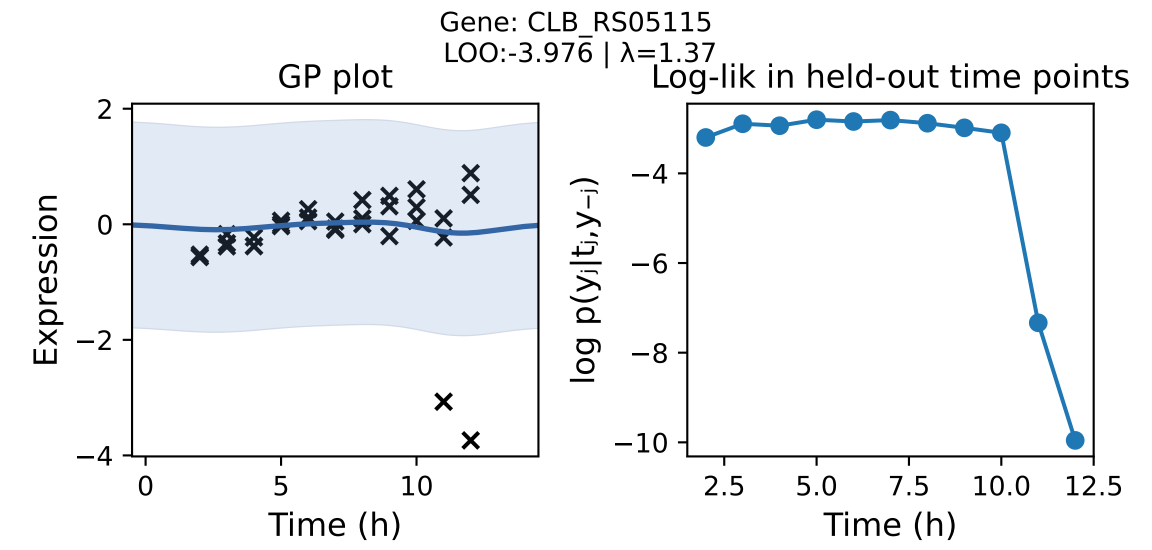
Gene: CLB_RS05115
LOO:-3.976 | λ=1.37
GP plot
Expression
Time (h)
Log-lik in held-out time points
log p(y_j|t_j,y_−j)
Time (h)

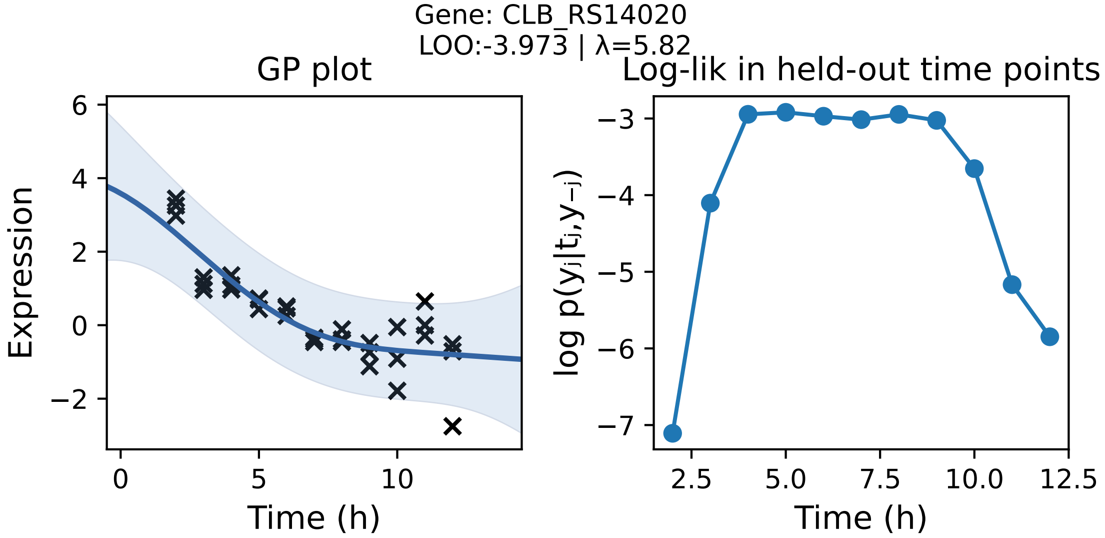
Gene: CLB_RS14020
LOO:-3.973 | λ=5.82
GP plot
Expression
Time (h)
Log-lik in held-out time points
log p(y_j|t_j,y_−j)
Time (h)

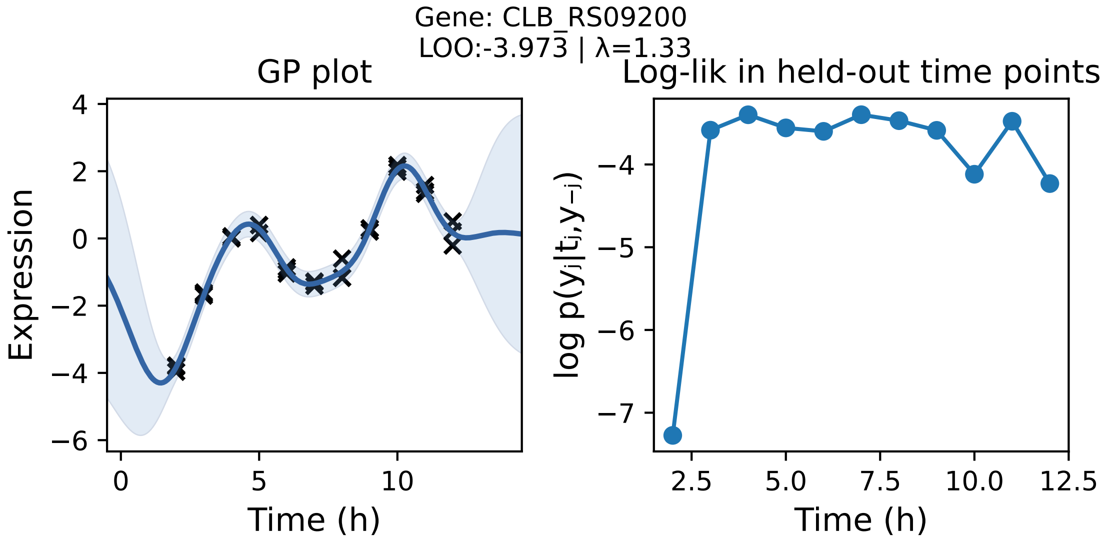

Gene: CLB_RS09200
LOO:-3.973 | λ=1.33
GP plot
Log-lik in held-out time points
Expression
Time (h)
log p(y_j|t_j,y_−j)
4
2
0
−2
−4
−6
0
5
10
−4
−5
−6
−7
2.5
5.0
7.5
10.0
12.5

Gene: CLB_RS17150
LOO:-3.972 | λ=1.0

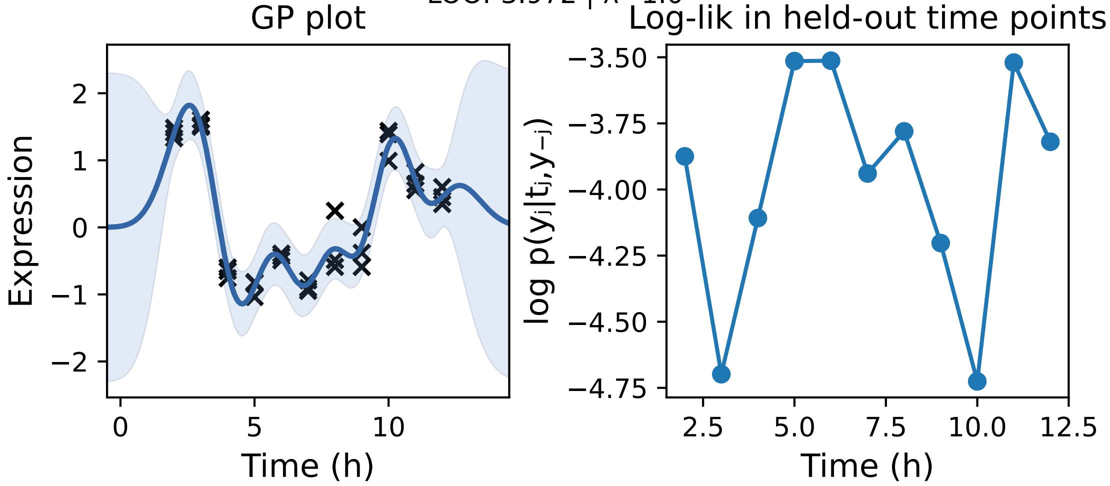

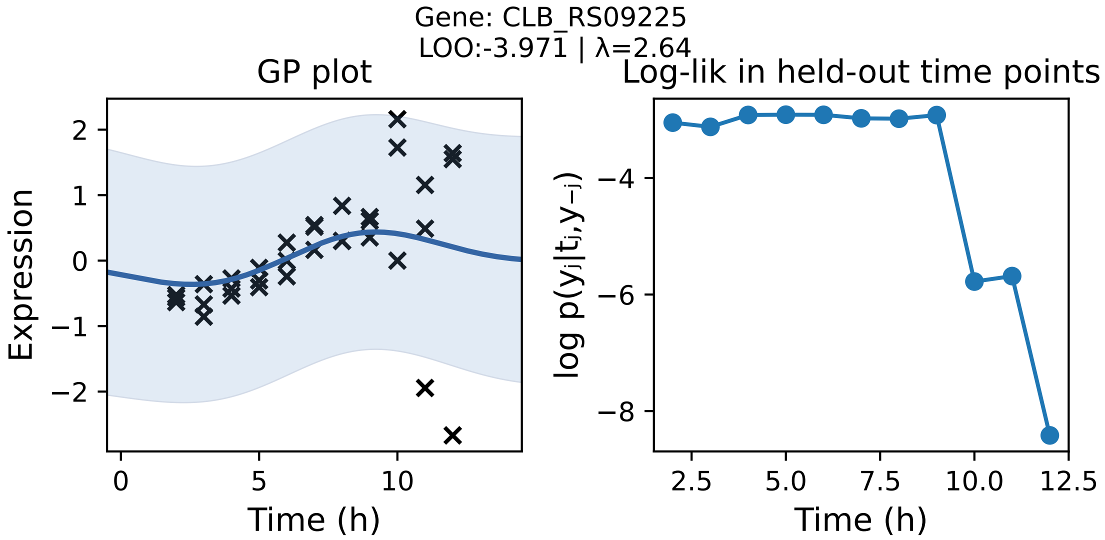

Gene: CLB_RS09225
LOO:-3.971 | λ=2.64
GP plot
Expression
Time (h)
Log-lik in held-out time points
log p(y_j|t_j,y_−j)
Time (h)

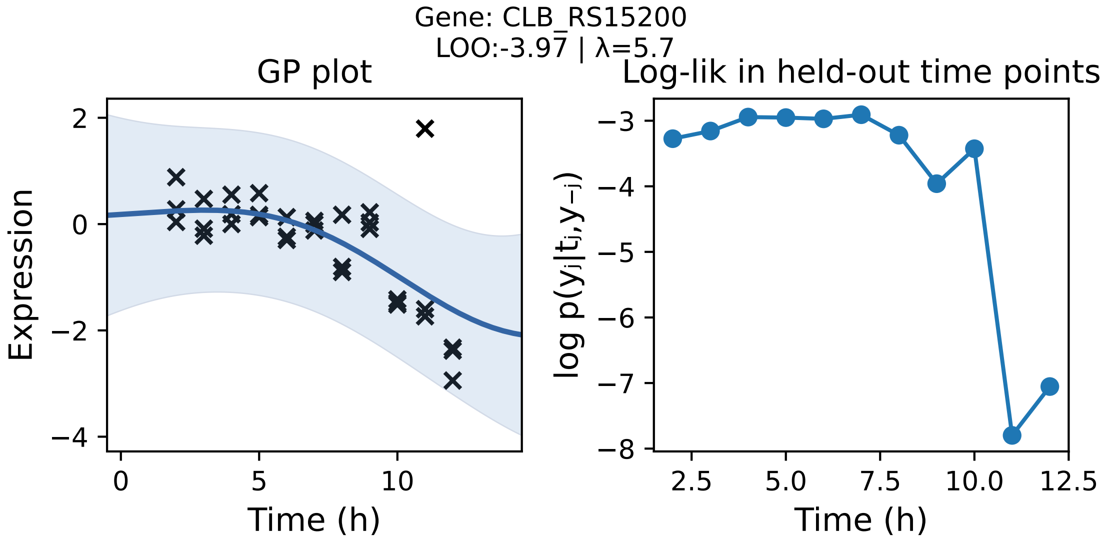

Gene: CLB_RS15200
LOO:-3.97 | λ=5.7
GP plot
Expression
2
0
−2
−4
0
5
10
Time (h)
Log-lik in held-out time points
log p(y_j|t_j,y_−j)
−3
−4
−5
−6
−7
−8
2.5
5.0
7.5
10.0
12.5
Time (h)

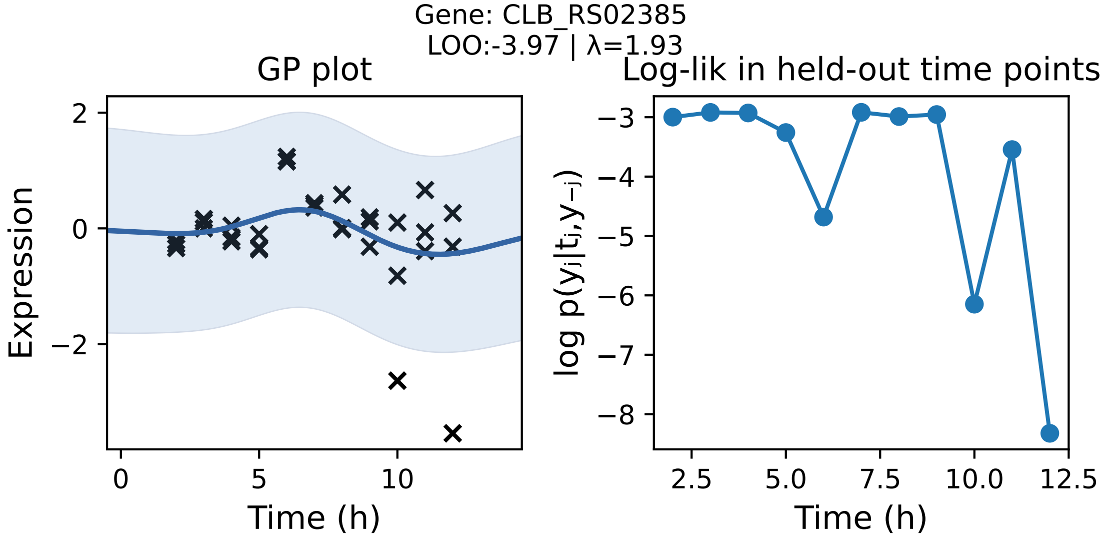

Gene: CLB_RS02385
LOO:-3.97 | λ=1.93
GP plot
Expression
2
0
−2
0
5
10
Time (h)
Log-lik in held-out time points
log p(y_j|t_j,y_−j)
−3
−4
−5
−6
−7
−8
2.5
5.0
7.5
10.0
12.5
Time (h)

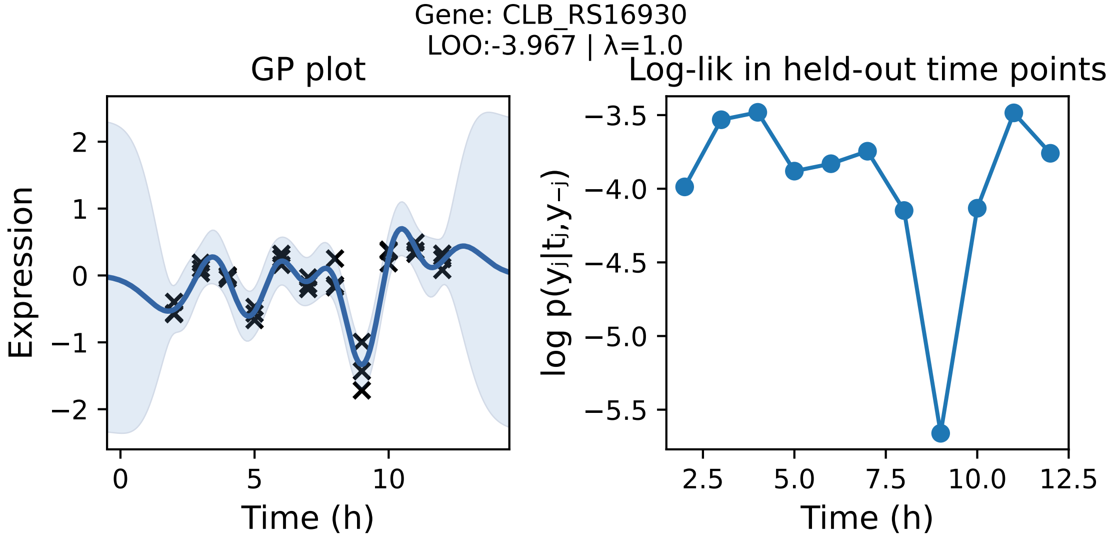

Gene: CLB_RS16930
LOO:-3.967 | λ=1.0
GP plot
Expression
Time (h)
2
1
0
−1
−2
0
5
10
Log-lik in held-out time points
log p(y_j|t_j,y_−j)
−3.5
−4.0
−4.5
−5.0
−5.5
2.5
5.0
7.5
10.0
12.5
Time (h)

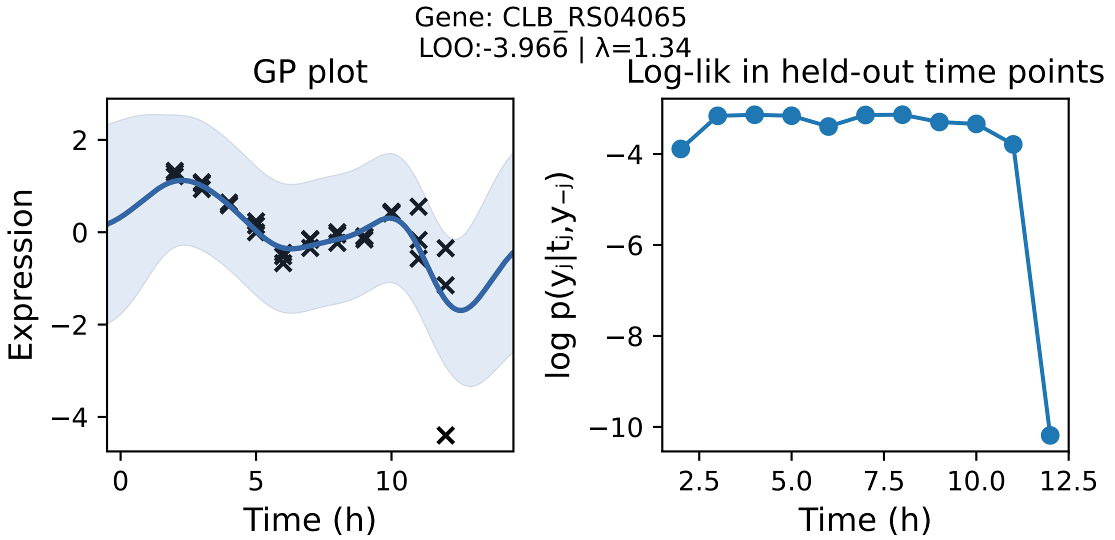
Gene: CLB_RS04065
LOO:-3.966 | λ=1.34
GP plot
Expression
Time (h)
Log-lik in held-out time points
log p(y_j|t_j,y_−j)
Time (h)

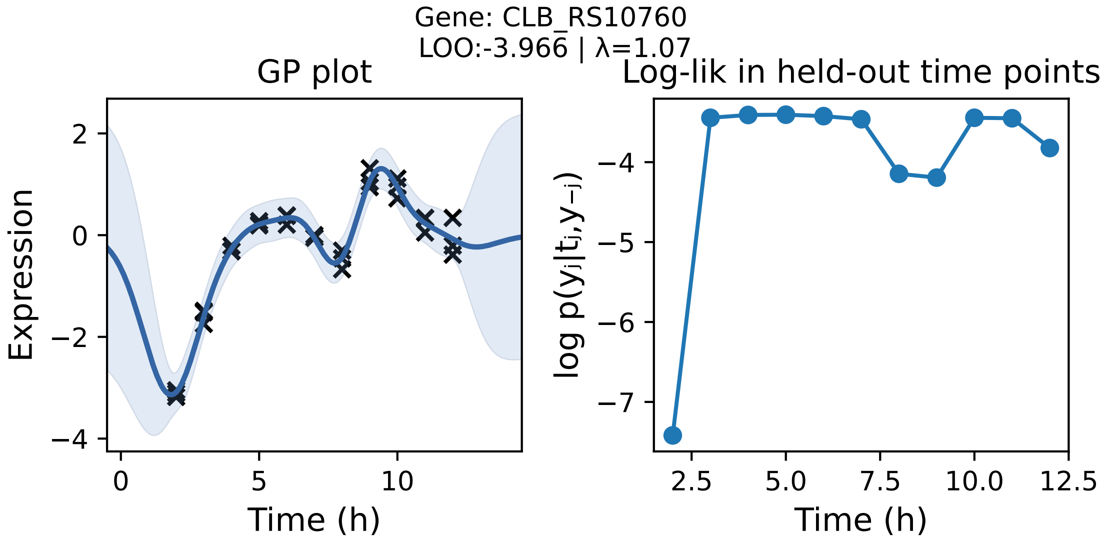
Gene: CLB_RS10760
LOO:-3.966 | λ=1.07
GP plot
Expression
Time (h)
2
0
−2
−4
0
5
10
Log-lik in held-out time points
log p(y_j|t_j,y_−j)
Time (h)
−4
−5
−6
−7
2.5
5.0
7.5
10.0
12.5

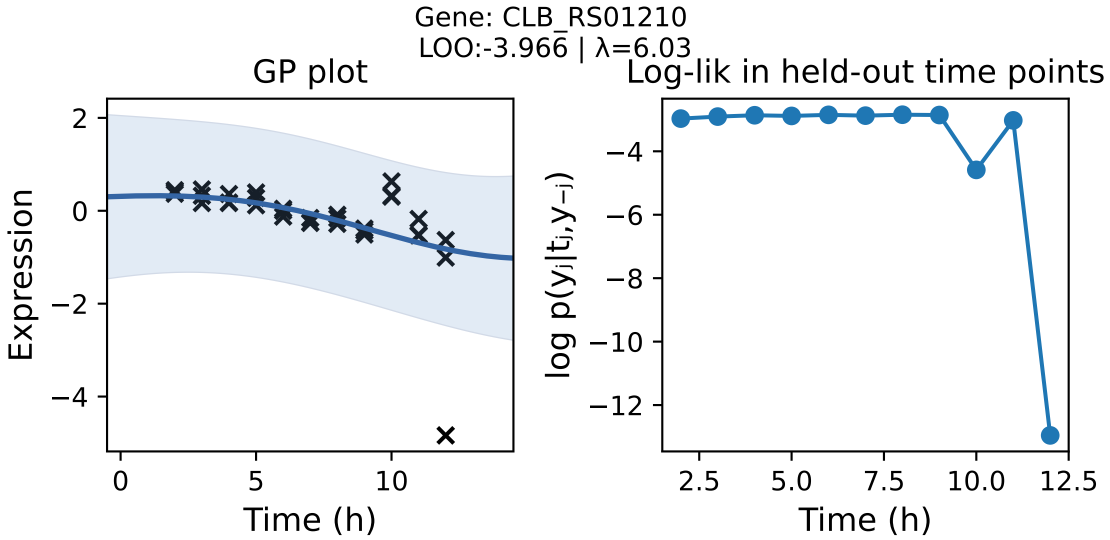
Gene: CLB_RS01210
LOO:-3.966 | λ=6.03
GP plot
Expression
Time (h)
0
5
10
2
0
−2
−4
Log-lik in held-out time points
log p(y_j|t_j,y_−j)
Time (h)
2.5
5.0
7.5
10.0
12.5
−4
−6
−8
−10
−12

Gene: CLB_RS02675
LOO:-3.965 | λ=1.24

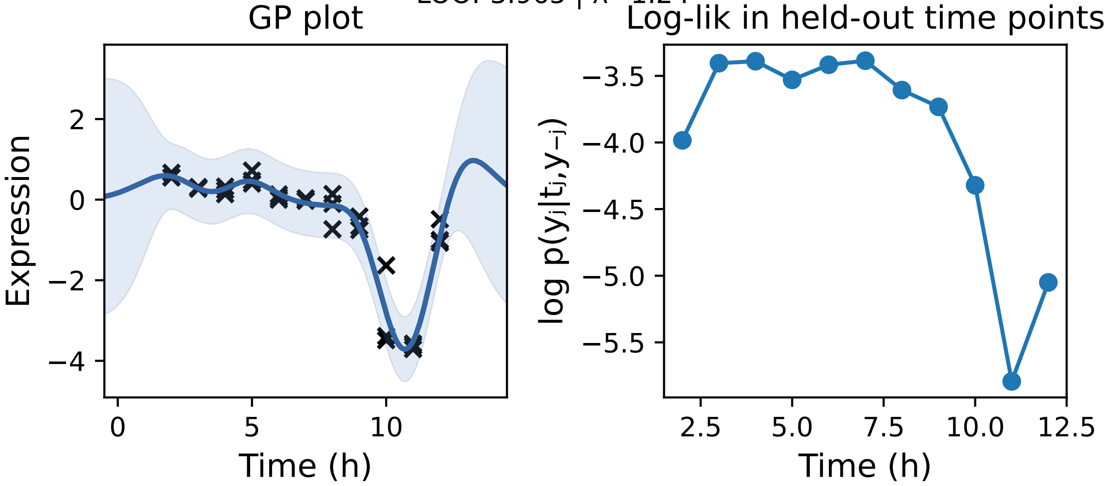

Gene: CLB_RS06350
LOO:-3.965 | λ=1.59

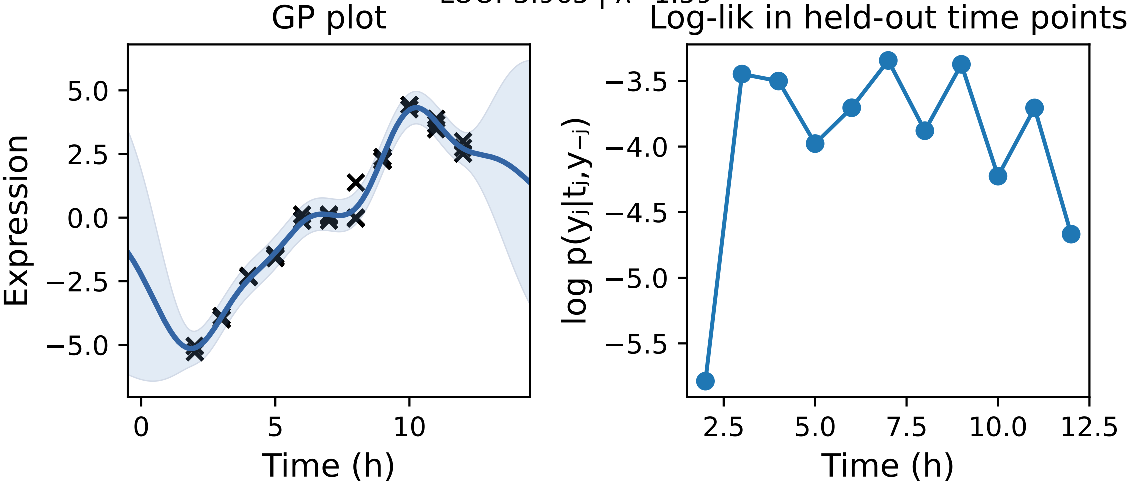

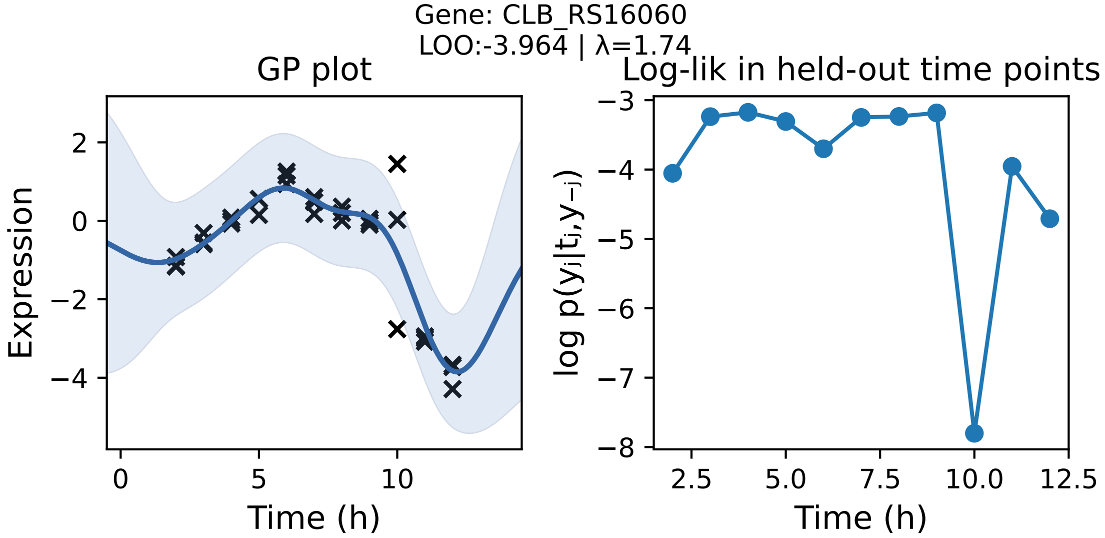

Gene: CLB_RS16060
LOO:-3.964 | λ=1.74
GP plot
Log-lik in held-out time points
Expression
Time (h)
log p(yj|tj,y−j)
Time (h)
2
0
−2
−4
0
5
10
−3
−4
−5
−6
−7
−8
2.5
5.0
7.5
10.0
12.5

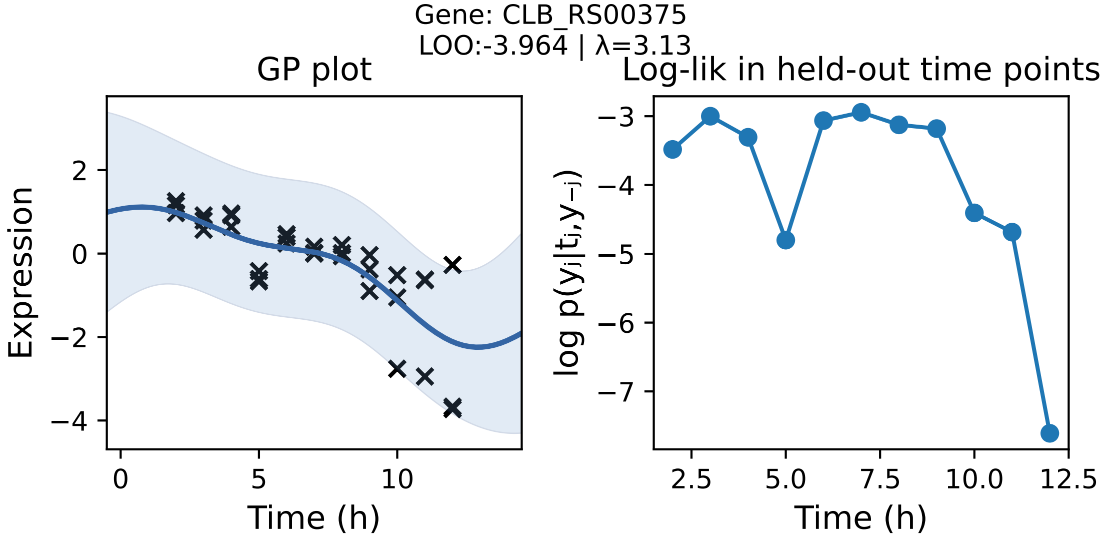

Gene: CLB_RS00375
LOO:-3.964 | λ=3.13
GP plot
Expression
Time (h)
Log-lik in held-out time points
log p(y_j|t_j,y_−j)
Time (h)

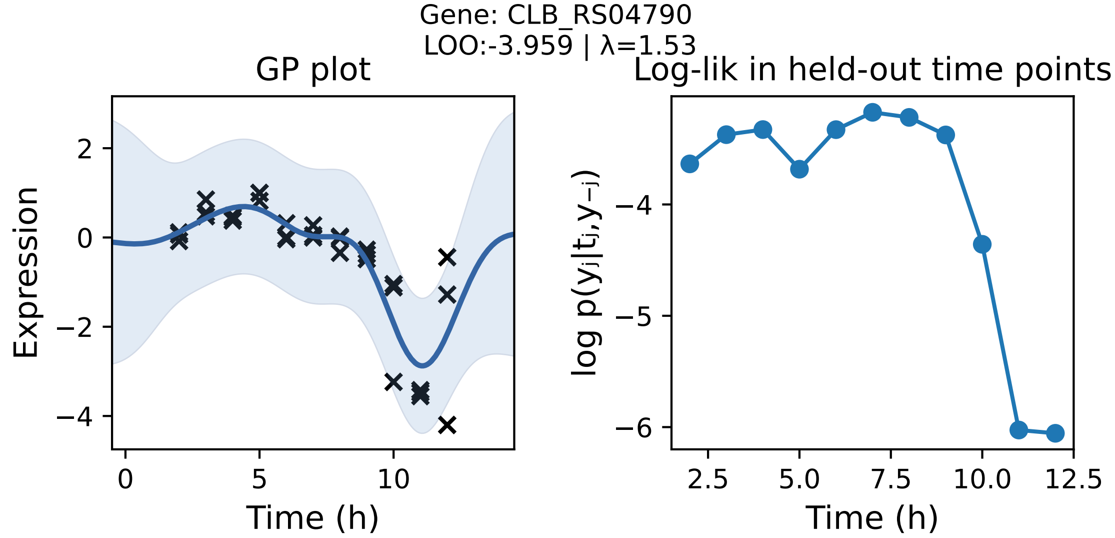
Gene: CLB_RS04790
LOO:-3.959 | λ=1.53
GP plot
Log-lik in held-out time points
Expression
Time (h)
log p(yj|tj,y−j)
Time (h)
2
0
−2
−4
0
5
10
−4
−5
−6
2.5
5.0
7.5
10.0
12.5

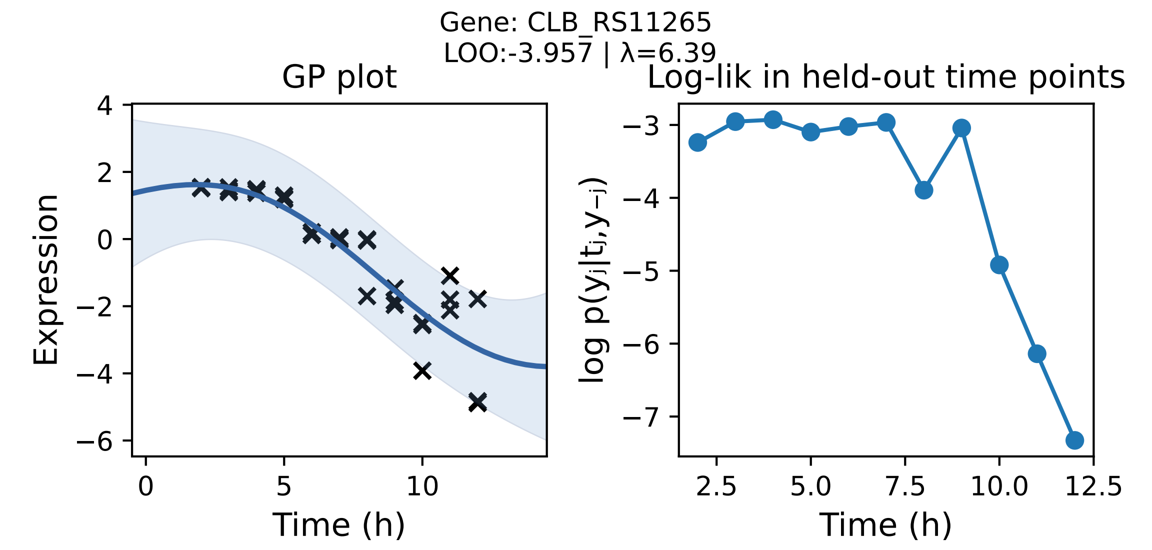

Gene: CLB_RS11265
LOO:-3.957 | λ=6.39
GP plot
Expression
Time (h)
4
2
0
−2
−4
−6
0
5
10
Log-lik in held-out time points
log p(y_j|t_j,y_−j)
Time (h)
−3
−4
−5
−6
−7
2.5
5.0
7.5
10.0
12.5

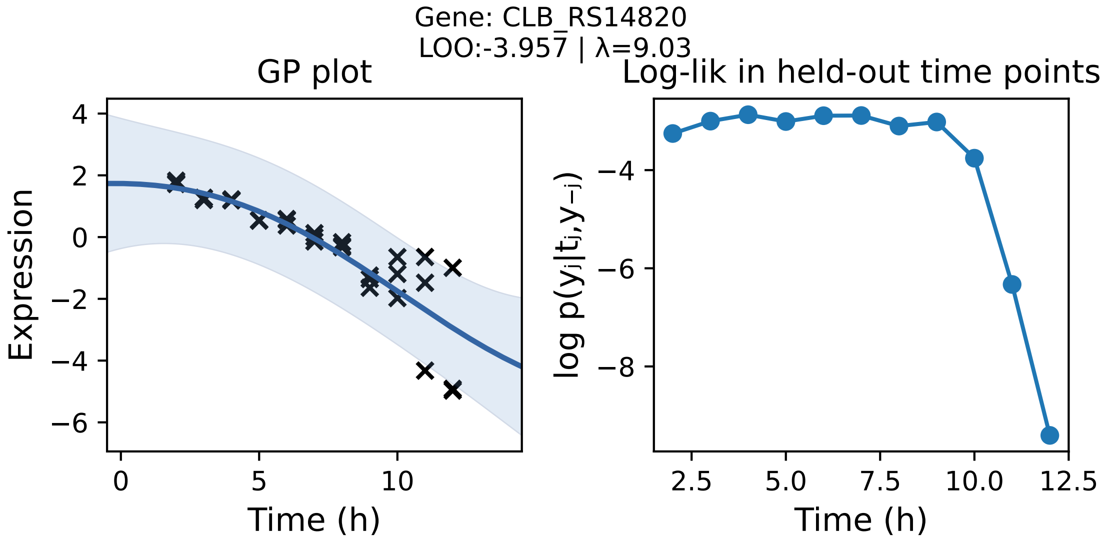

Gene: CLB_RS14820
LOO:-3.957 | λ=9.03
GP plot
Expression
Time (h)
4
2
0
−2
−4
−6
0
5
10
Log-lik in held-out time points
log p(y_j|t_j,y_−j)
−4
−6
−8
2.5
5.0
7.5
10.0
12.5
Time (h)

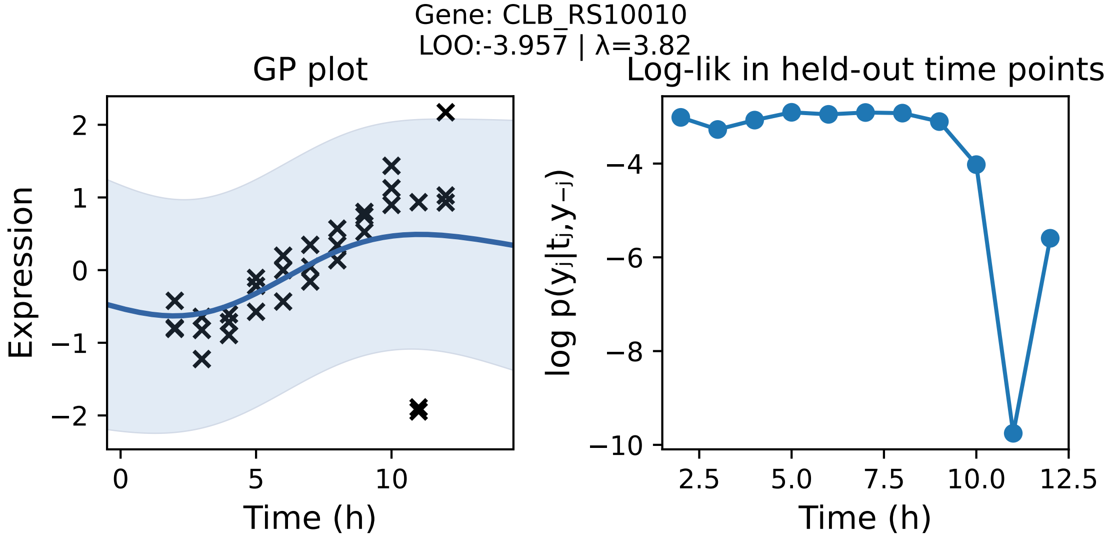
Gene: CLB_RS10010
LOO:-3.957 | λ=3.82
GP plot
Expression
Time (h)
Log-lik in held-out time points
log p(y_j|t_j,y_−j)
Time (h)
2
1
0
−1
−2
0
5
10
−4
−6
−8
−10
2.5
5.0
7.5
10.0
12.5

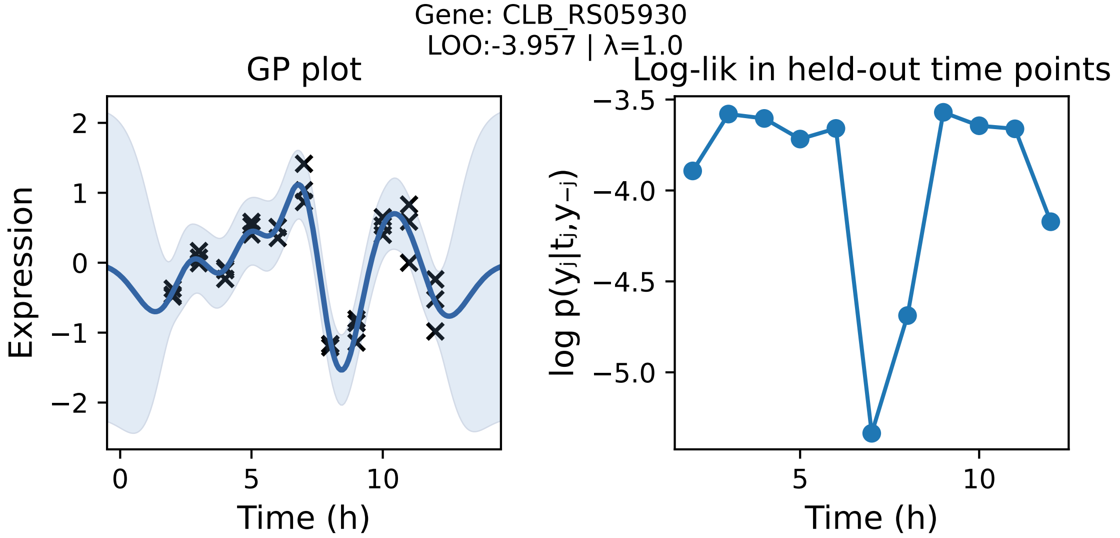

Gene: CLB_RS05930
LOO:-3.957 | λ=1.0
GP plot
Expression
Time (h)
2
1
0
−1
−2
0
5
10
Log-lik in held-out time points
log p(y_j|t_j,y_−j)
Time (h)
−3.5
−4.0
−4.5
−5.0
5
10

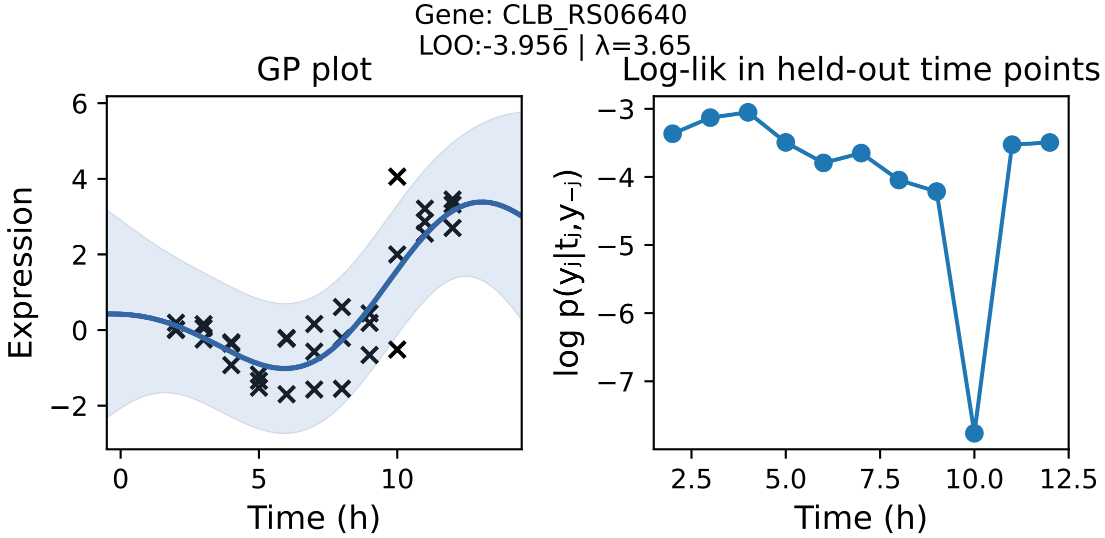

Gene: CLB_RS06640
LOO:-3.956 | λ=3.65
GP plot
Expression
Time (h)
Log-lik in held-out time points
log p(y_j|t_j,y_−j)
Time (h)

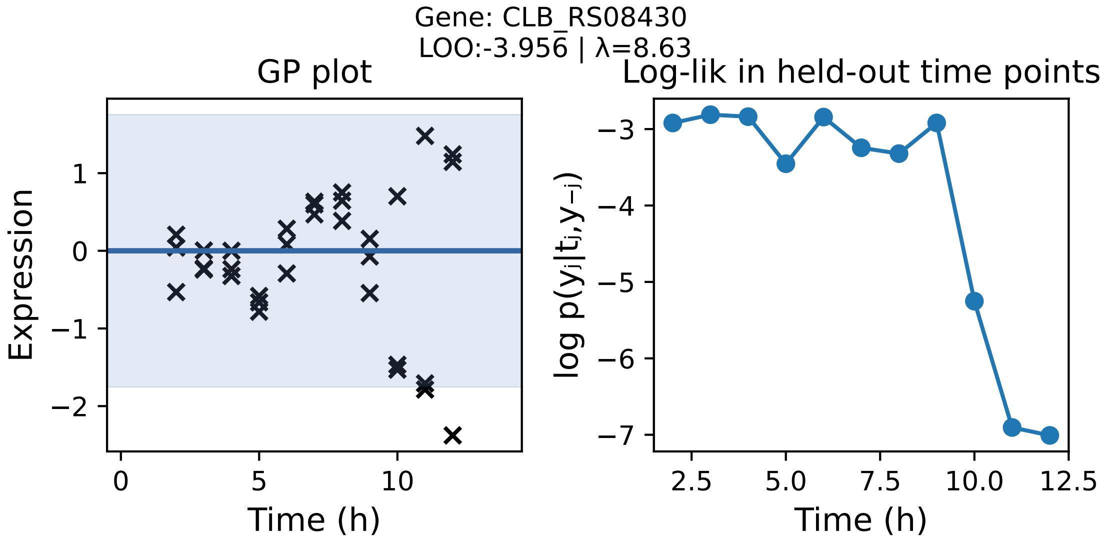

Gene: CLB_RS08430
LOO:-3.956 | λ=8.63
GP plot
Log-lik in held-out time points
Expression
Time (h)
log p(y_j|t_j,y_−j)
1
0
−1
−2
0
5
10
−3
−4
−5
−6
−7
2.5
5.0
7.5
10.0
12.5

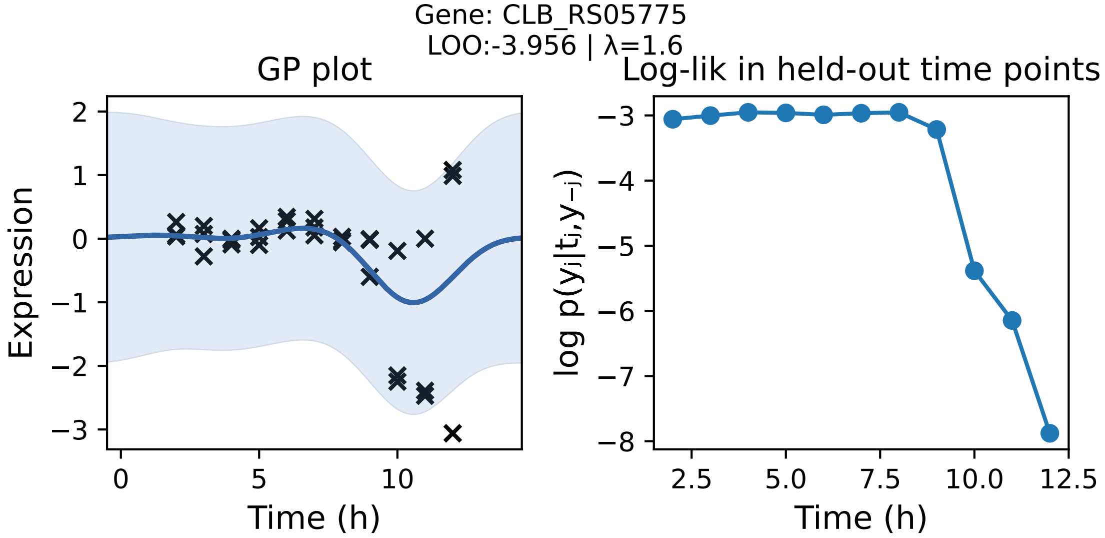

Gene: CLB_RS05775
LOO:-3.956 | λ=1.6
GP plot
Expression
Time (h)
Log-lik in held-out time points
log p(y_j|t_j,y_−j)
Time (h)

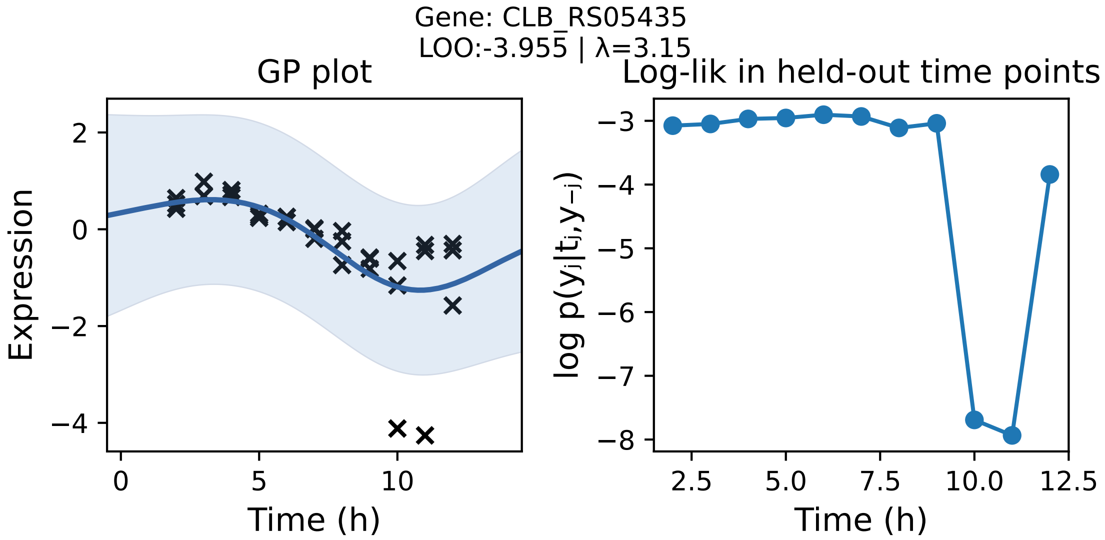
Gene: CLB_RS05435
LOO:-3.955 | λ=3.15
GP plot
Expression
Time (h)
Log-lik in held-out time points
log p(y_j|t_j,y_−j)
Time (h)

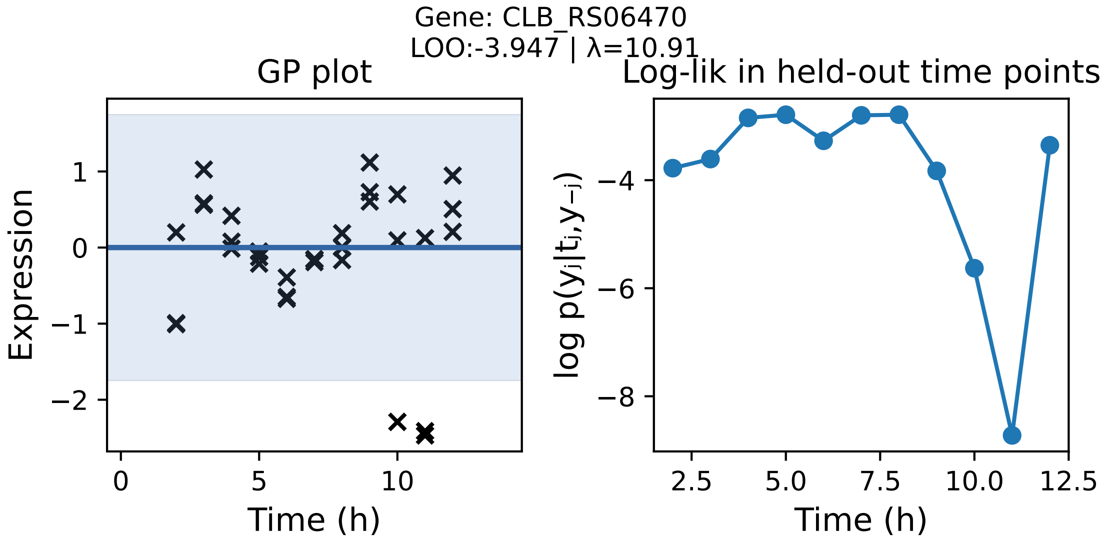

Gene: CLB_RS06470
LOO:-3.947 | λ=10.91
GP plot
Expression
Time (h)
1
0
−1
−2
0
5
10
Log-lik in held-out time points
log p(y_j|t_j,y_−j)
Time (h)
−4
−6
−8
2.5
5.0
7.5
10.0
12.5

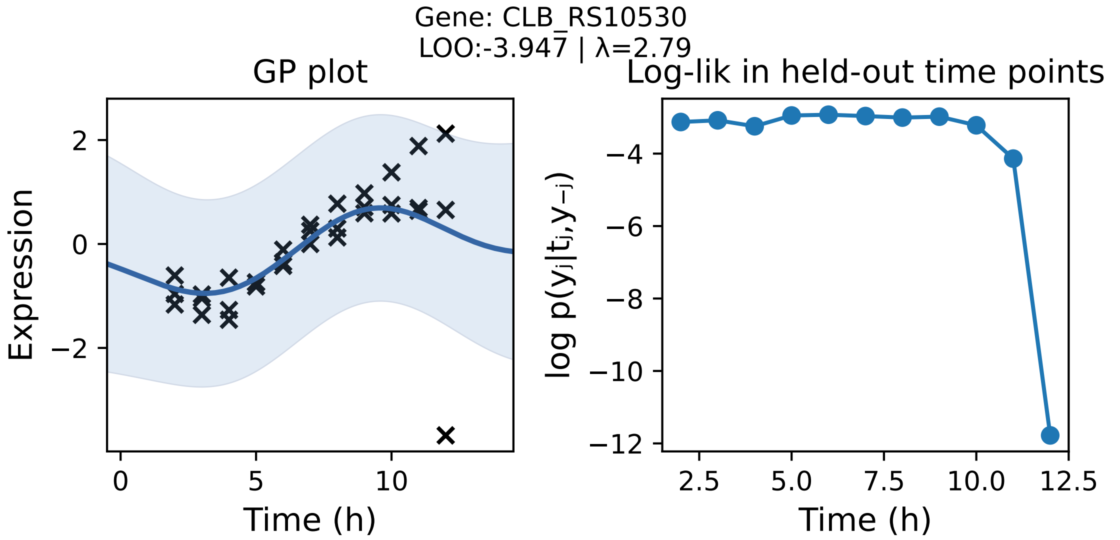

Gene: CLB_RS10530
LOO:-3.947 | λ=2.79
GP plot
Expression
Time (h)
Log-lik in held-out time points
log p(y_j|t_i,y_-j)
Time (h)

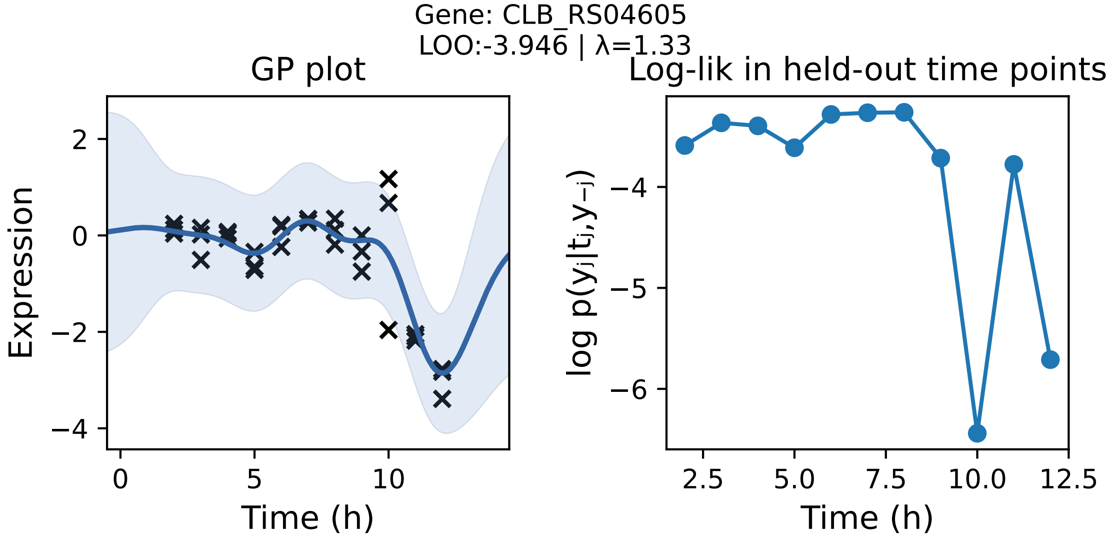

Gene: CLB_RS04605
LOO:-3.946 | λ=1.33
GP plot
Expression
Time (h)
2
0
−2
−4
0
5
10
Log-lik in held-out time points
log p(y_j|t_j,y_−j)
Time (h)
−4
−5
−6
2.5
5.0
7.5
10.0
12.5

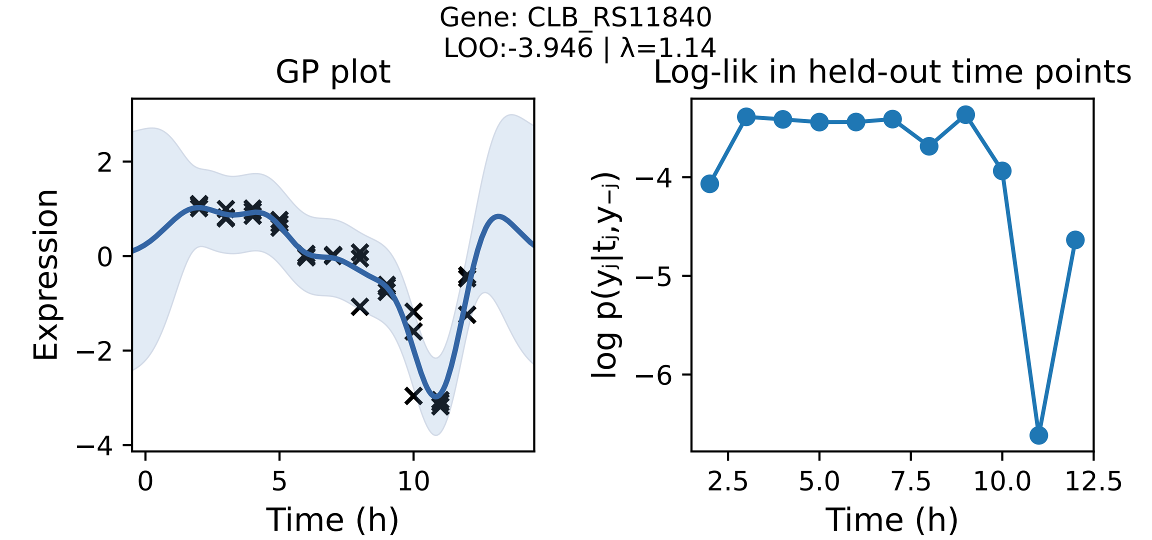

Gene: CLB_RS11840
LOO:-3.946 | λ=1.14
GP plot
Expression
2
0
−2
−4
0
5
10
Time (h)
Log-lik in held-out time points
log p(y_j|t_j,y_−j)
−4
−5
−6
2.5
5.0
7.5
10.0
12.5
Time (h)

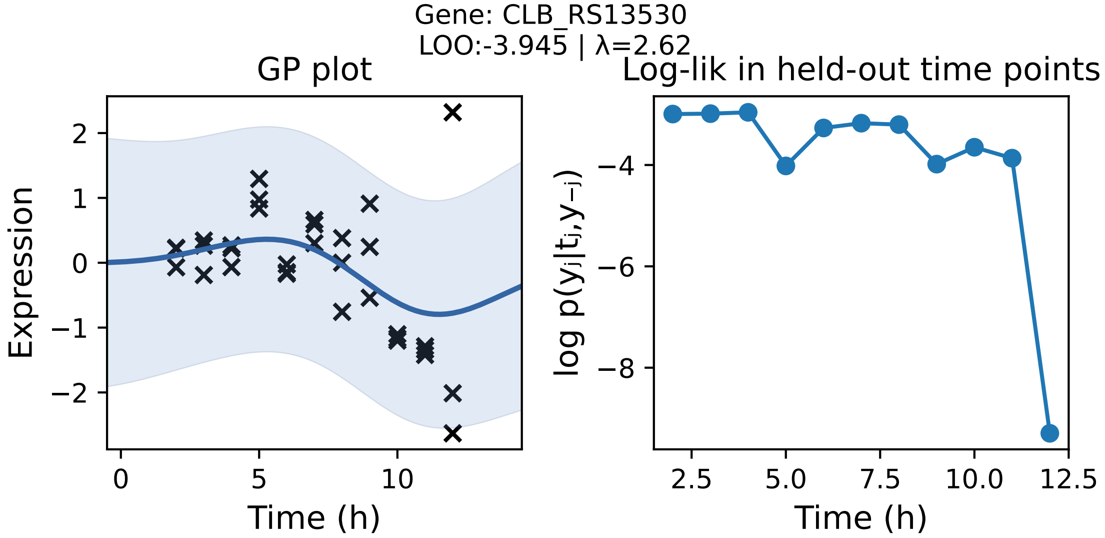

Gene: CLB_RS13530
LOO:-3.945 | λ=2.62
GP plot
Expression
Time (h)
Log-lik in held-out time points
log p(y_j|t_j,y_−j)
Time (h)

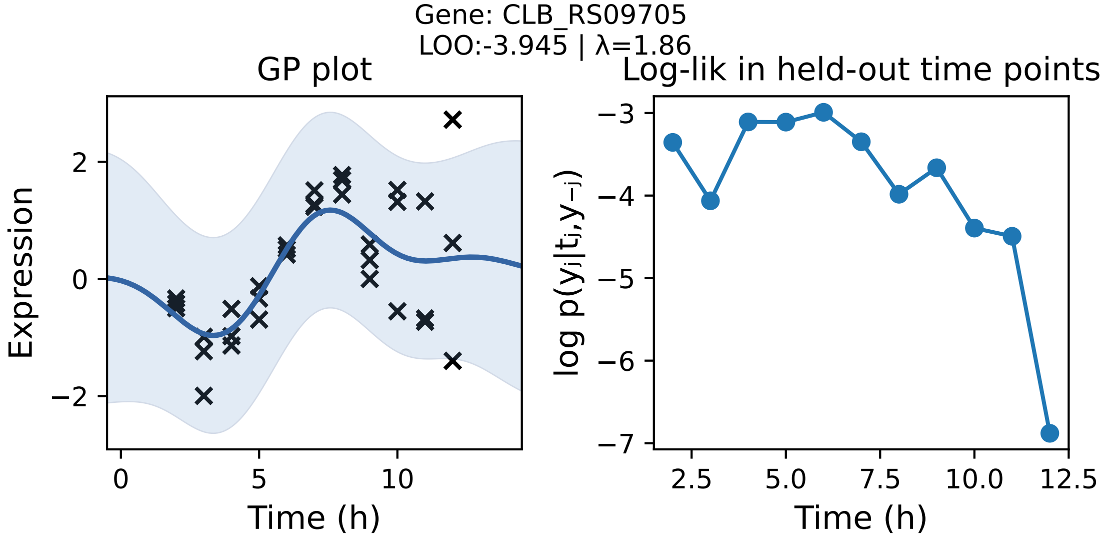

Gene: CLB_RS09705
LOO:-3.945 | λ=1.86
GP plot
Expression
Time (h)
Log-lik in held-out time points
log p(y_j|t_j,y_−j)
Time (h)

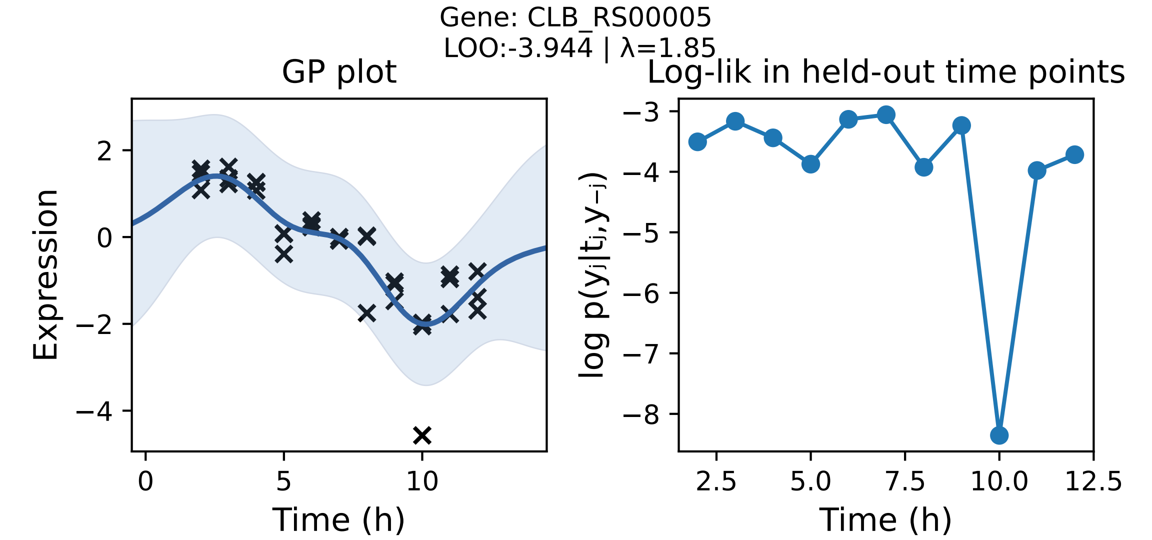

Gene: CLB_RS00005
LOO:-3.944 | λ=1.85
GP plot
Expression
Time (h)
Log-lik in held-out time points
log p(y_j|t_j,y_−j)
Time (h)

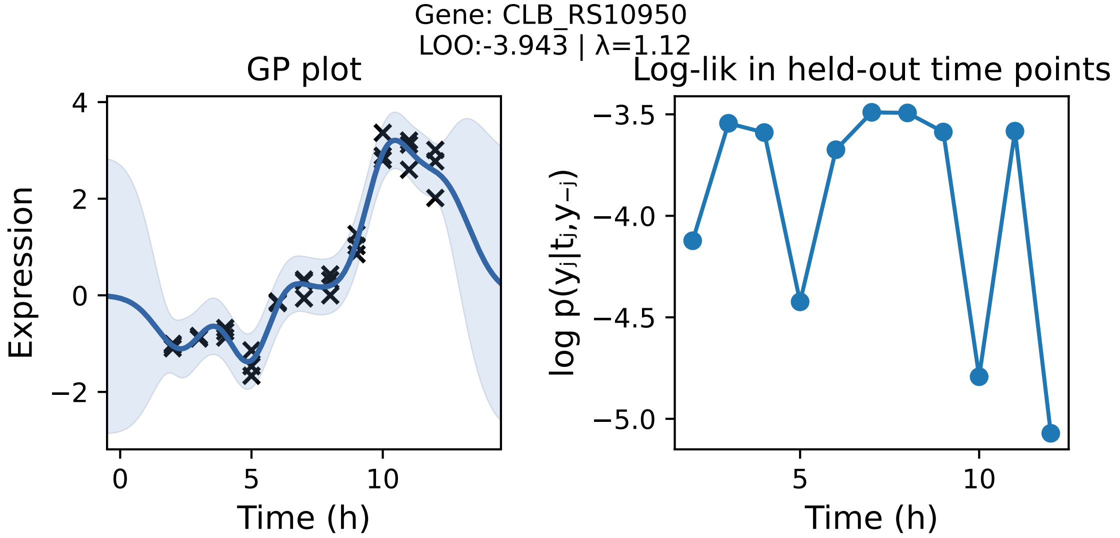
Gene: CLB_RS10950
LOO:-3.943 | λ=1.12
GP plot
Expression
Time (h)
Log-lik in held-out time points
log p(yj|tj,y−j)
Time (h)

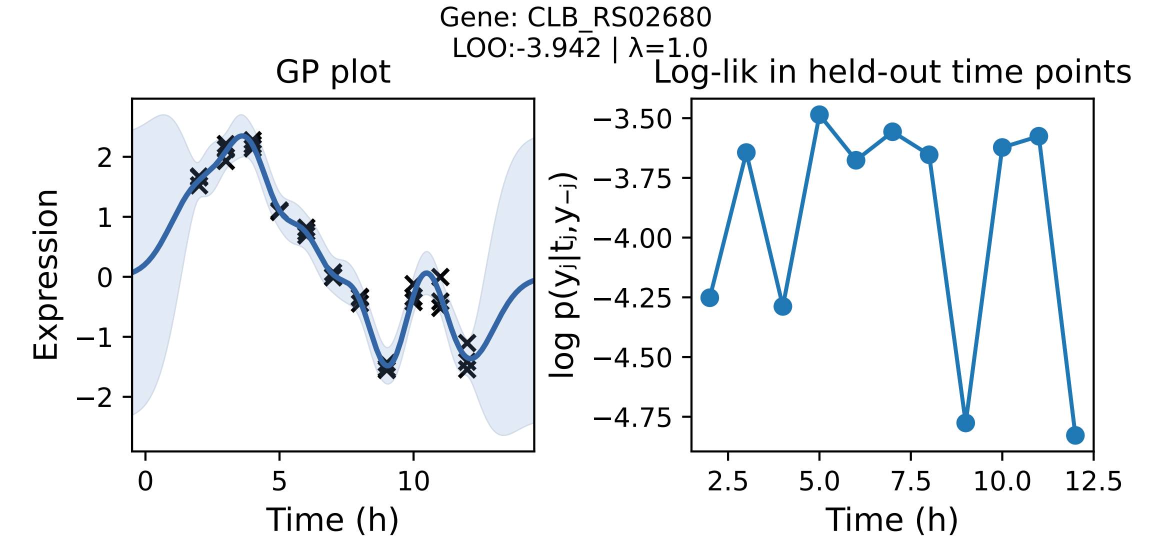
Gene: CLB_RS02680
LOO:-3.942 | λ=1.0
GP plot
Expression
Time (h)
Log-lik in held-out time points
log p(y_j|t_j,y_{-j})
Time (h)

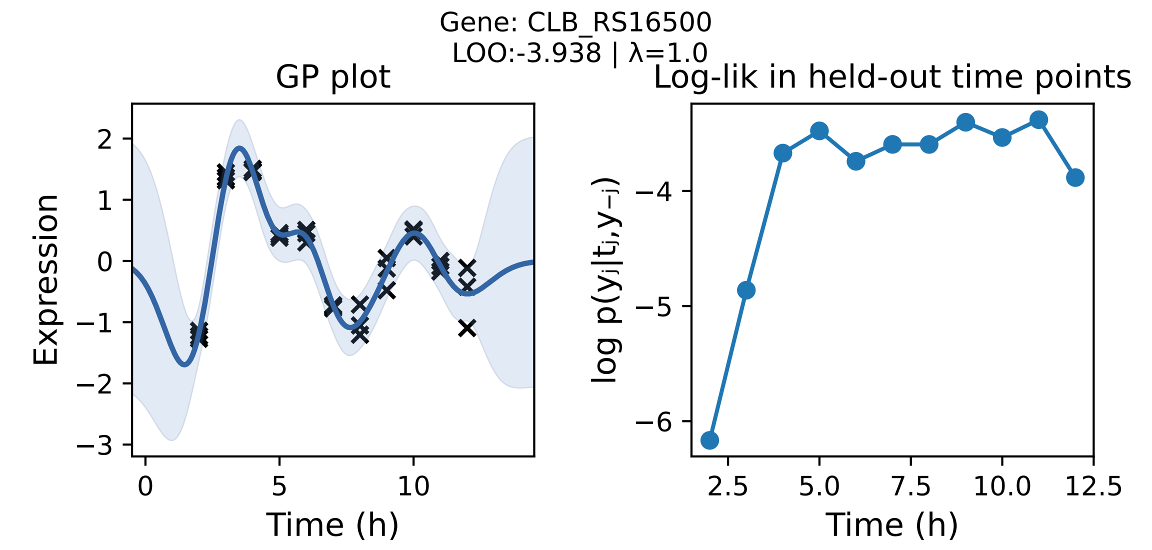

Gene: CLB_RS16500
LOO:-3.938 | λ=1.0
GP plot
Log-lik in held-out time points
Expression
Time (h)
log p(yj|tj,y−j)
2
1
0
−1
−2
−3
0
5
10
−4
−5
−6
2.5
5.0
7.5
10.0
12.5

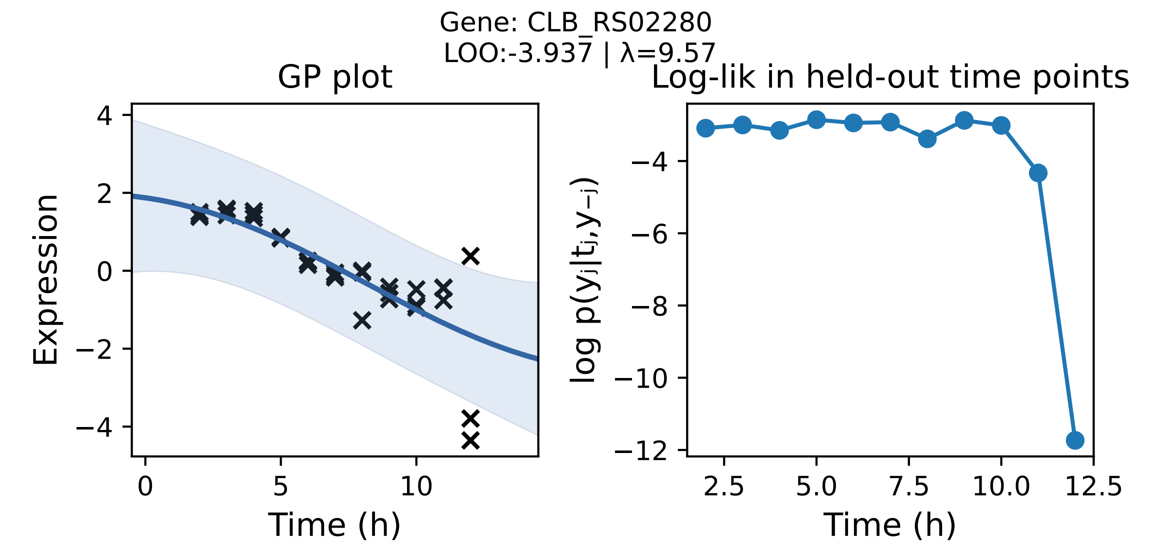
Gene: CLB_RS02280
LOO:-3.937 | λ=9.57
GP plot
Expression
Time (h)
4
2
0
−2
−4
0
5
10
Log-lik in held-out time points
log p(yⱼ|tⱼ,y₋ⱼ)
Time (h)
−4
−6
−8
−10
−12
2.5
5.0
7.5
10.0
12.5

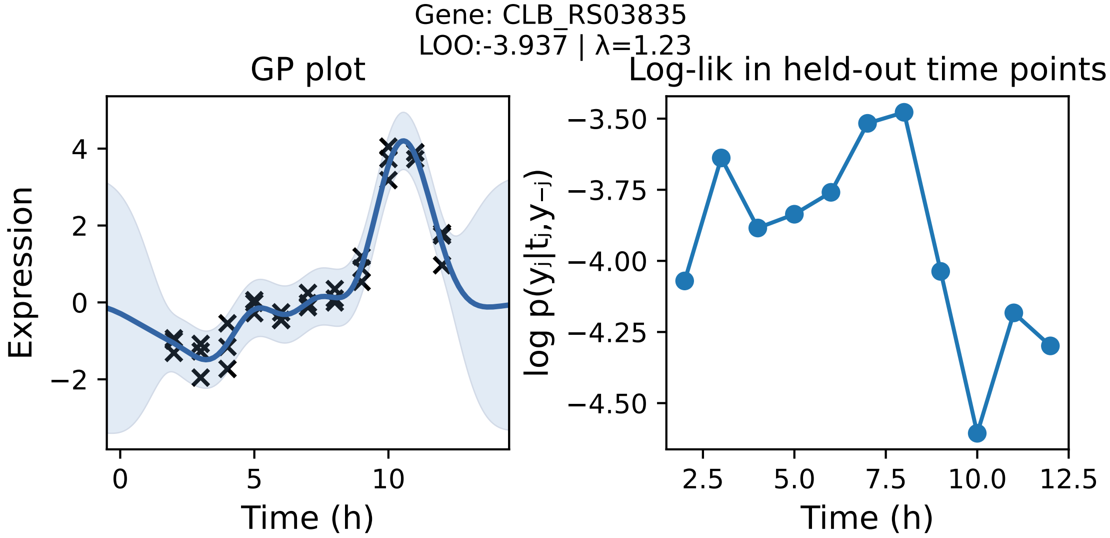
Gene: CLB_RS03835
LOO:-3.937 | λ=1.23
GP plot
Expression
Time (h)
Log-lik in held-out time points
log p(y_j|t_j,y_{-j})
Time (h)

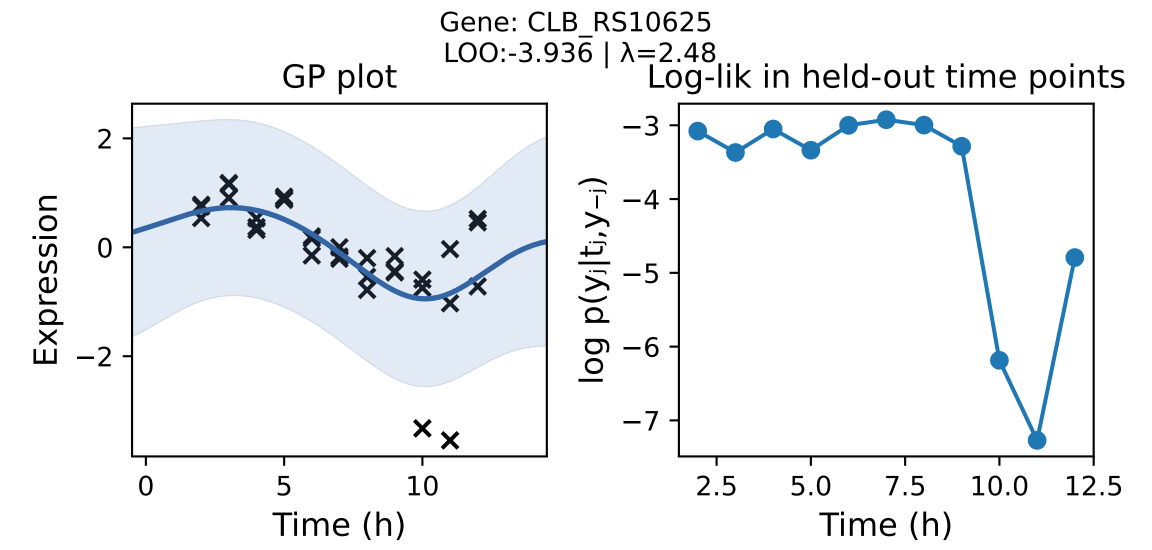
Gene: CLB_RS10625
LOO:-3.936 | λ=2.48
GP plot
Expression
Time (h)
Log-lik in held-out time points
log p(yj|tj,y−j)
Time (h)

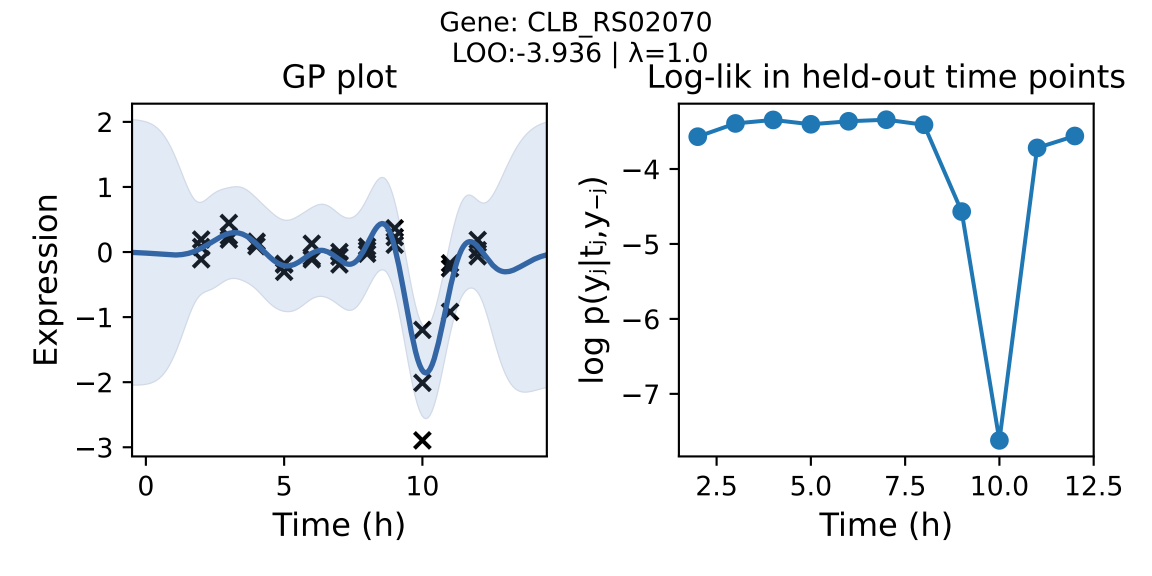
Gene: CLB_RS02070
LOO:-3.936 | λ=1.0
GP plot
Log-lik in held-out time points
Expression
Time (h)
log p(y_j|t_j,y_−j)
Time (h)

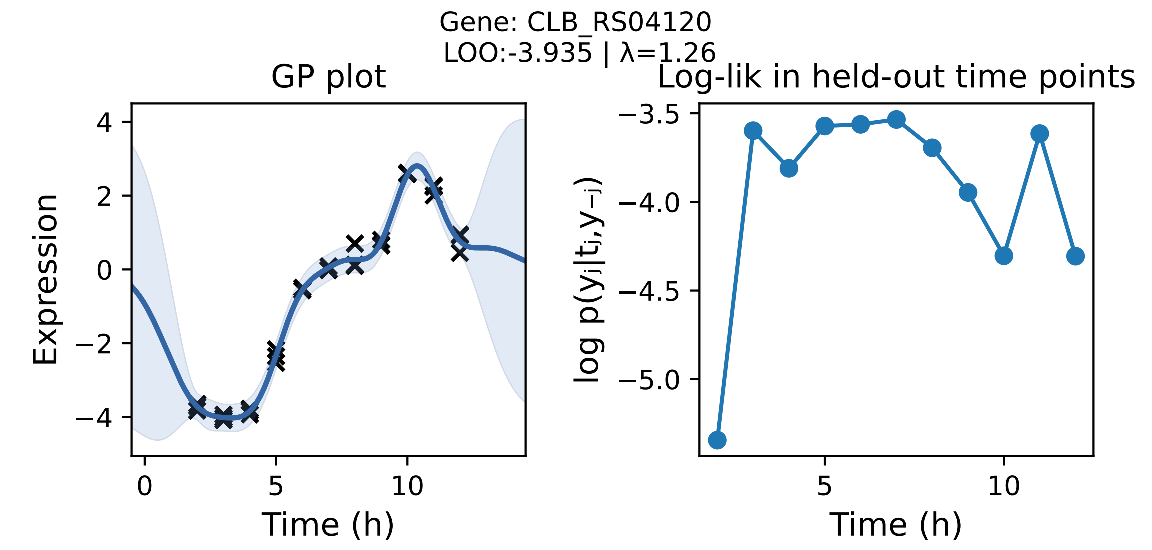
Gene: CLB_RS04120
LOO:-3.935 | λ=1.26
GP plot
Expression
Time (h)
Log-lik in held-out time points
log p(y_j|t_j,y_−j)
Time (h)

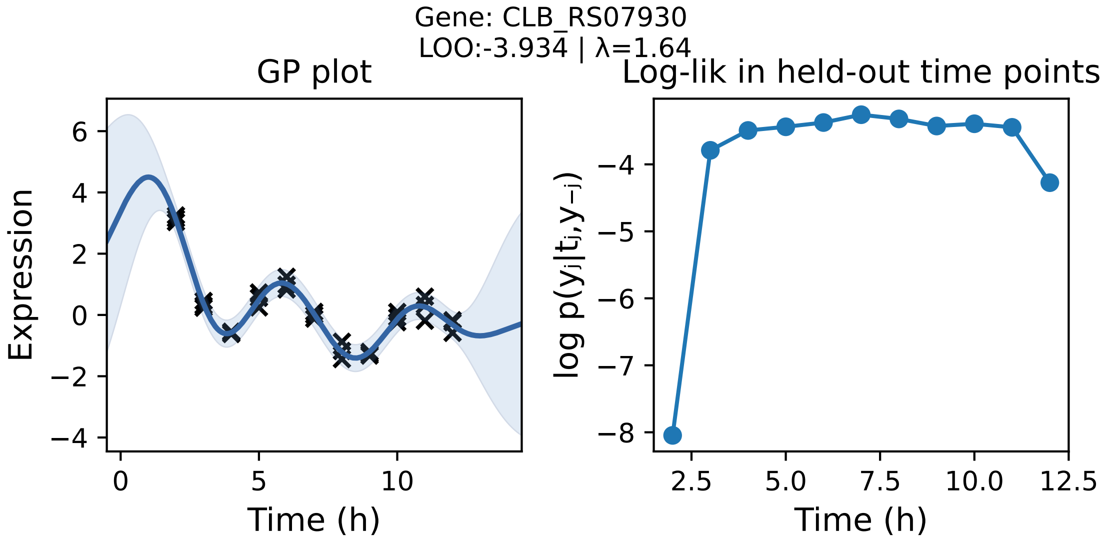
Gene: CLB_RS07930
LOO:-3.934 | λ=1.64
GP plot
Expression
Time (h)
6
4
2
0
−2
−4
0
5
10
Log-lik in held-out time points
log p(y_j|t_j,y_−j)
Time (h)
−4
−5
−6
−7
−8
2.5
5.0
7.5
10.0
12.5

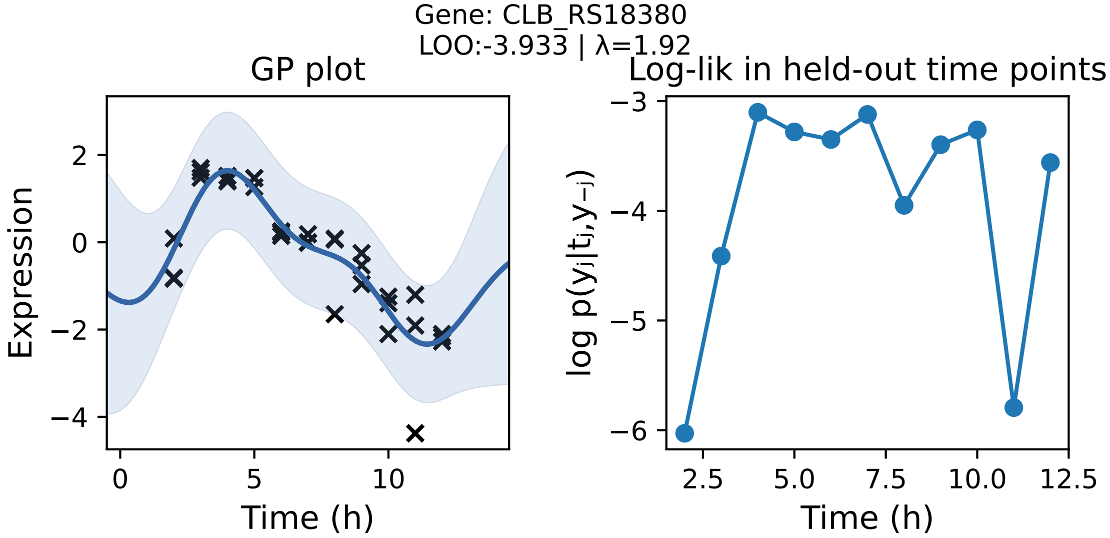

Gene: CLB_RS18380
LOO:-3.933 | λ=1.92
GP plot
Expression
Time (h)
2
0
−2
−4
0
5
10
Log-lik in held-out time points
log p(yᵢ|tᵢ,y₋ᵢ)
Time (h)
−3
−4
−5
−6
2.5
5.0
7.5
10.0
12.5

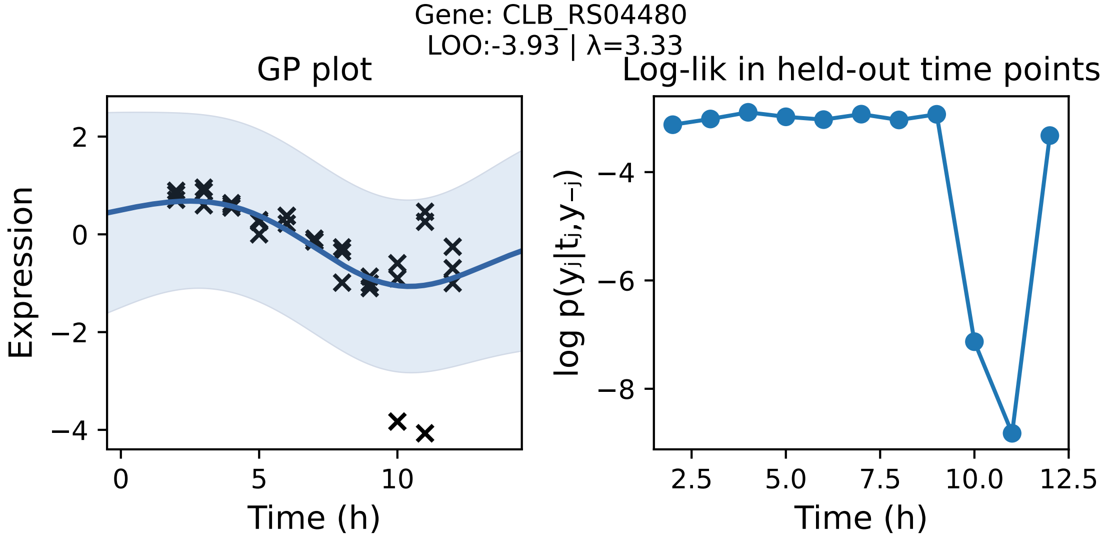
Gene: CLB_RS04480
LOO:-3.93 | λ=3.33
GP plot
Expression
Time (h)
2
0
−2
−4
0
5
10
Log-lik in held-out time points
log p(y_j|t_j,y_−j)
Time (h)
−4
−6
−8
2.5
5.0
7.5
10.0
12.5

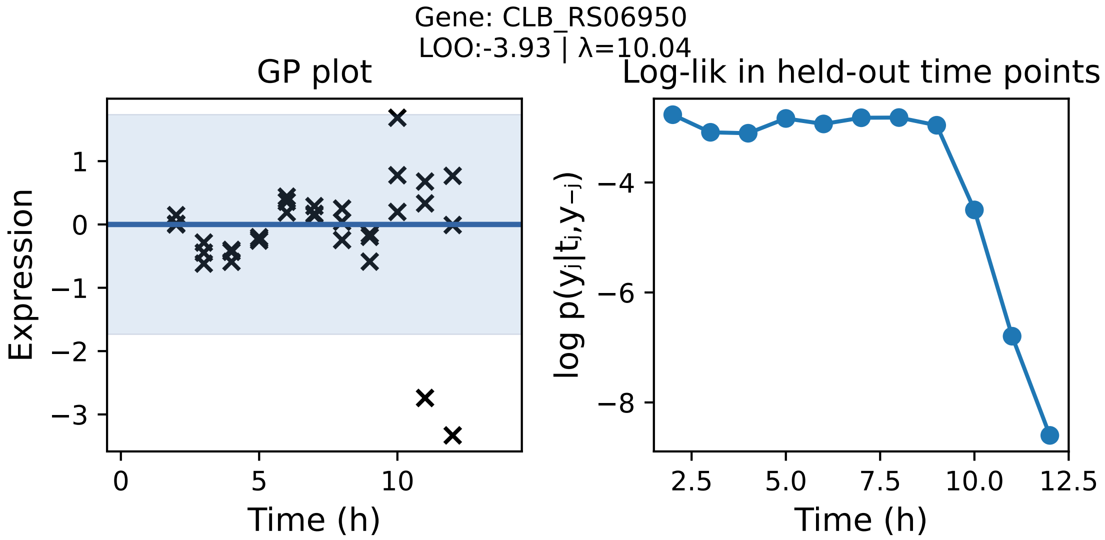
Gene: CLB_RS06950
LOO:-3.93 | λ=10.04
GP plot
Expression
Time (h)
Log-lik in held-out time points
log p(y_j|t_j,y_-j)
Time (h)

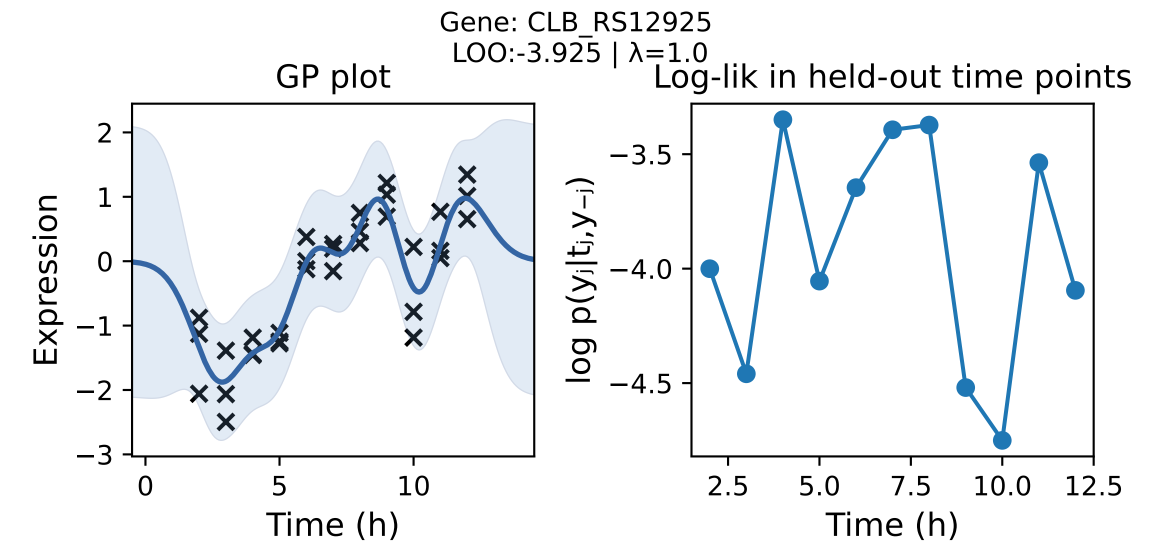

Gene: CLB_RS12925
LOO:-3.925 | λ=1.0
GP plot
Expression
Time (h)
Log-lik in held-out time points
log p(y_j|t_j,y_−j)
Time (h)

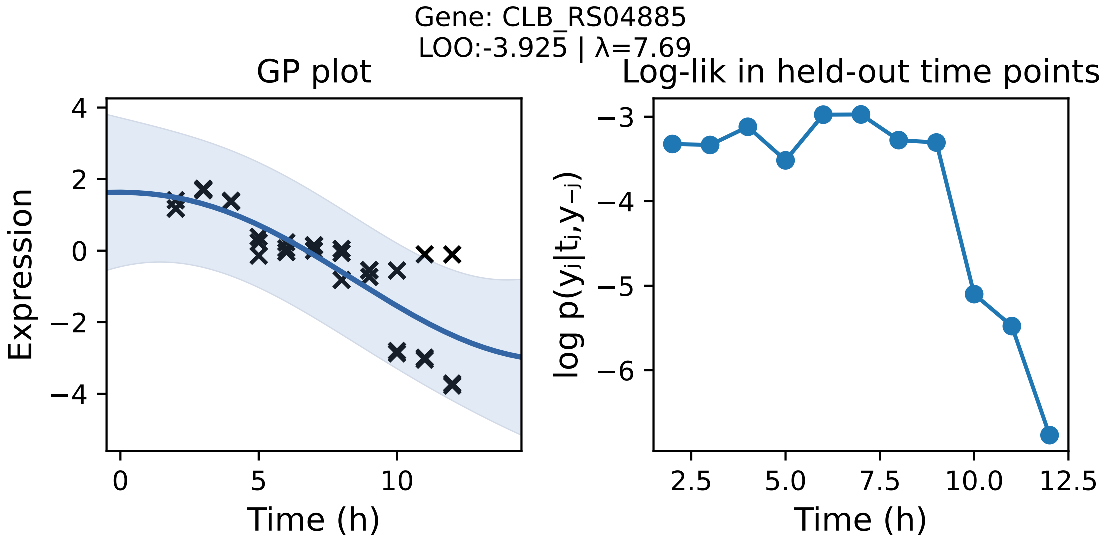

Gene: CLB_RS04885
LOO:-3.925 | λ=7.69
GP plot
Expression
Time (h)
4
2
0
−2
−4
0
5
10
Log-lik in held-out time points
log p(yj|tj,y−j)
Time (h)
−3
−4
−5
−6
2.5
5.0
7.5
10.0
12.5

Gene: CLB_RS18185
LOO:-3.922 | λ=4.51

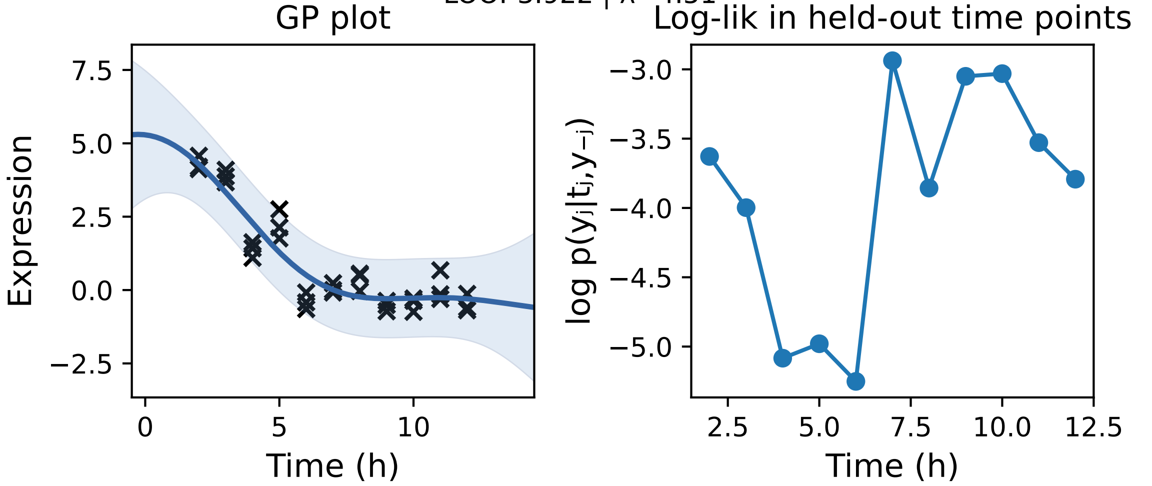

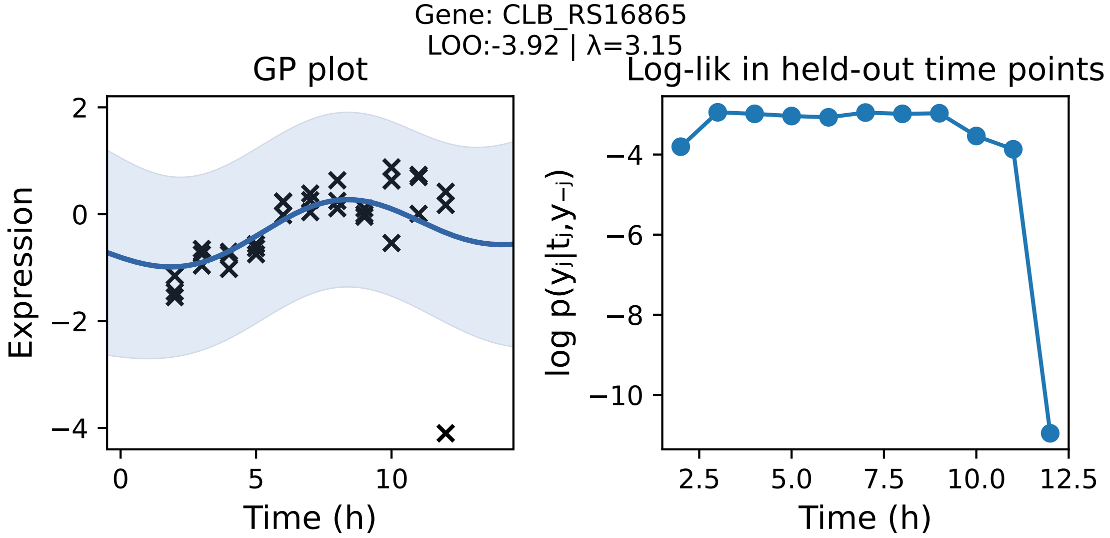

Gene: CLB_RS16865
LOO:-3.92 | λ=3.15
GP plot
Expression
Time (h)
2
0
−2
−4
0
5
10
Log-lik in held-out time points
log p(y_j|t_j,y_−j)
Time (h)
−4
−6
−8
−10
2.5
5.0
7.5
10.0
12.5

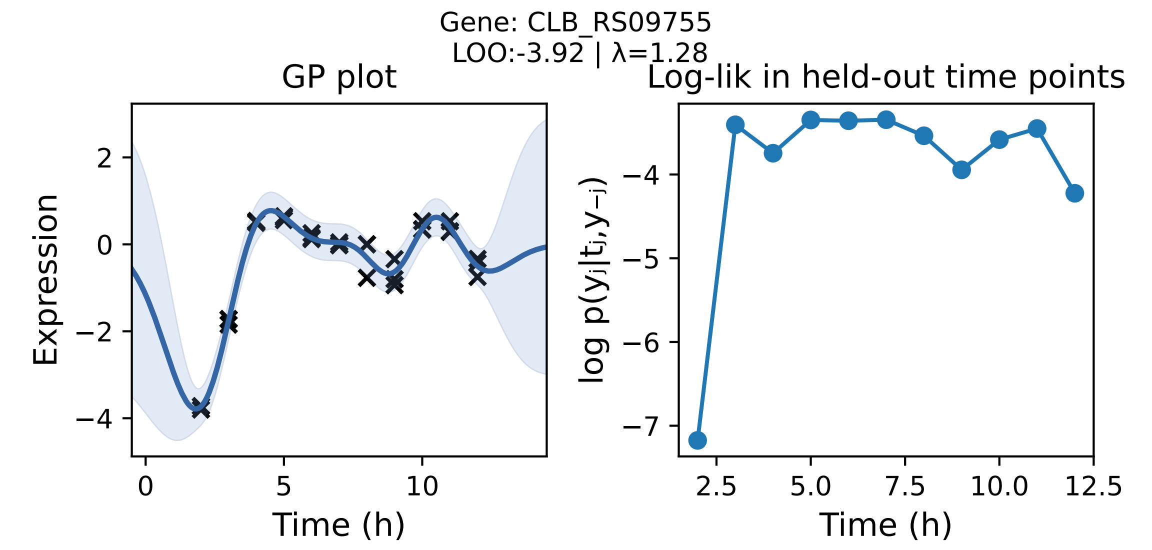

Gene: CLB_RS09755
LOO:-3.92 | λ=1.28
GP plot
Expression
Time (h)
2
0
−2
−4
0
5
10
Log-lik in held-out time points
log p(y_j|t_j,y_−j)
Time (h)
−4
−5
−6
−7
2.5
5.0
7.5
10.0
12.5

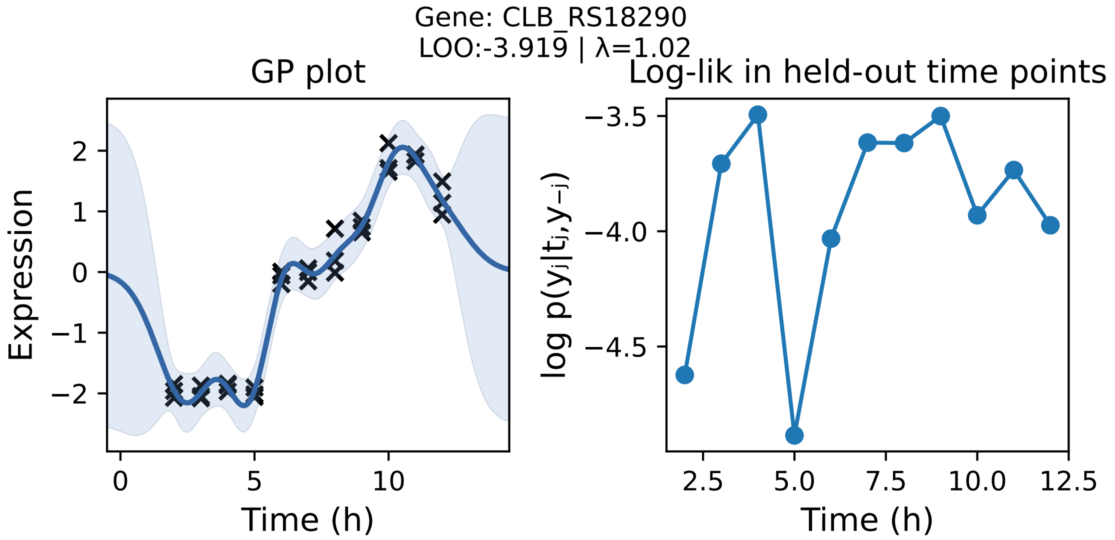

Gene: CLB_RS18290
LOO:-3.919 | λ=1.02
GP plot
Log-lik in held-out time points
Expression
Time (h)
log p(y_j|t_j,y_−j)
2
1
0
−1
−2
0
5
10
−3.5
−4.0
−4.5
2.5
5.0
7.5
10.0
12.5

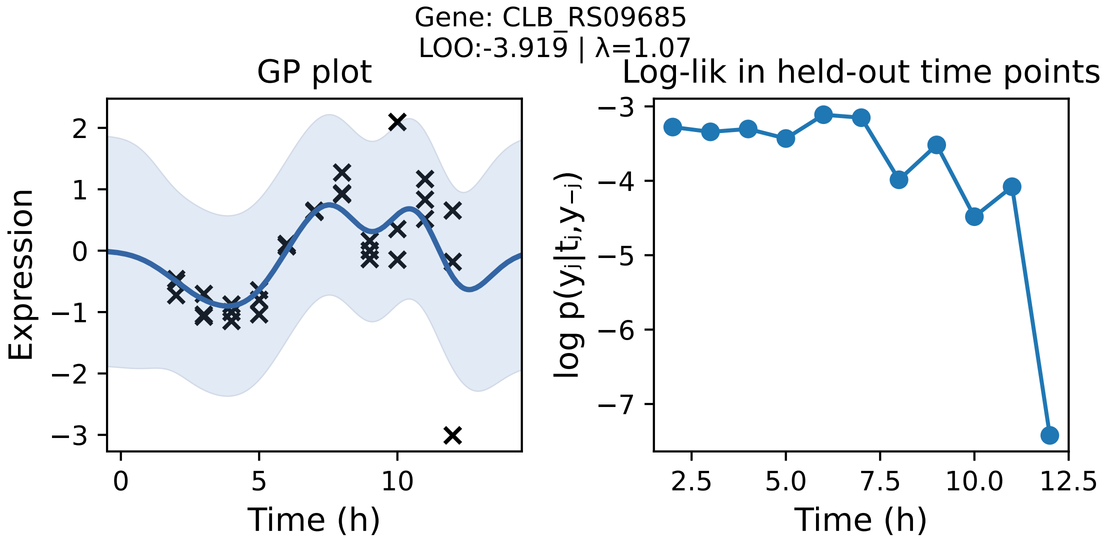

Gene: CLB_RS09685
LOO:-3.919 | λ=1.07
GP plot
Expression
Time (h)
2
1
0
−1
−2
−3
0
5
10
Log-lik in held-out time points
log p(y_j|t_j,y_−j)
Time (h)
−3
−4
−5
−6
−7
2.5
5.0
7.5
10.0
12.5

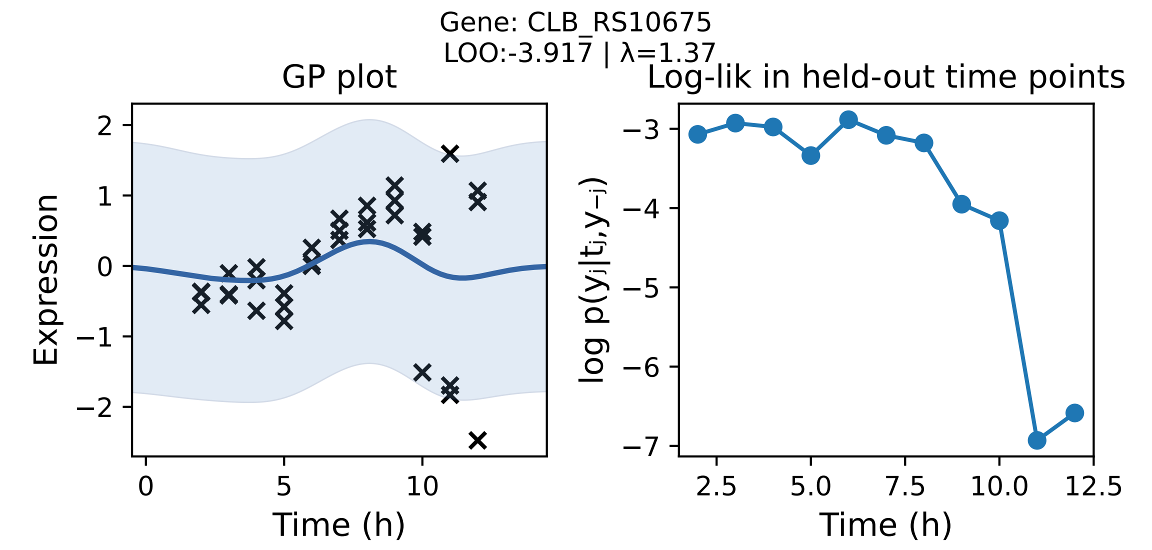
Gene: CLB_RS10675
LOO:-3.917 | λ=1.37
GP plot
Expression
Time (h)
Log-lik in held-out time points
log p(y_j|t_j,y_{-j})
Time (h)
2
1
0
−1
−2
0
5
10
−3
−4
−5
−6
−7
2.5
5.0
7.5
10.0
12.5

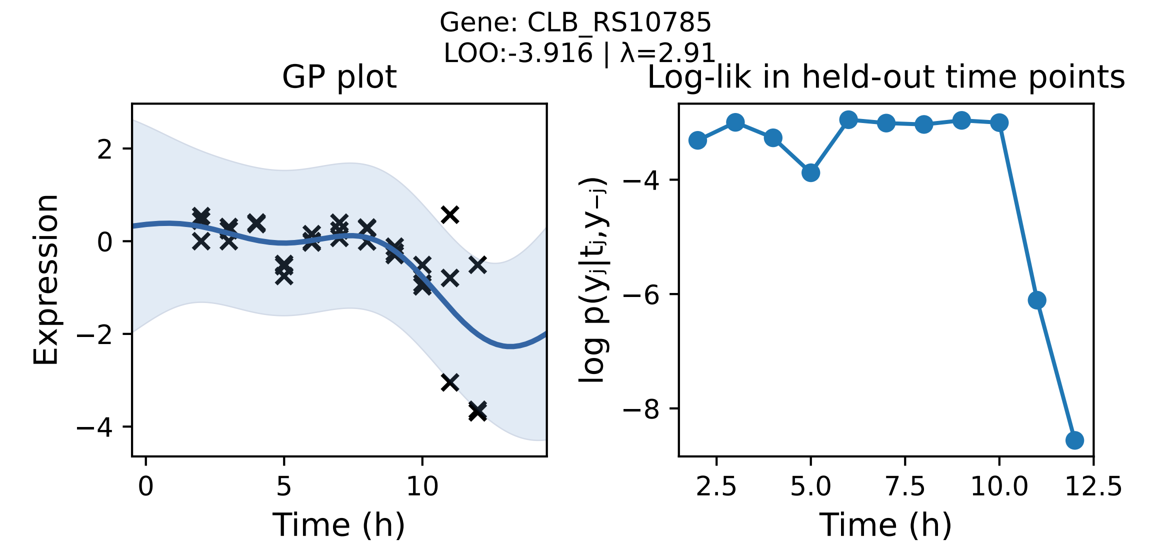
Gene: CLB_RS10785
LOO:-3.916 | λ=2.91
GP plot
Expression
Time (h)
Log-lik in held-out time points
log p(y_j|t_j,y_−j)
Time (h)

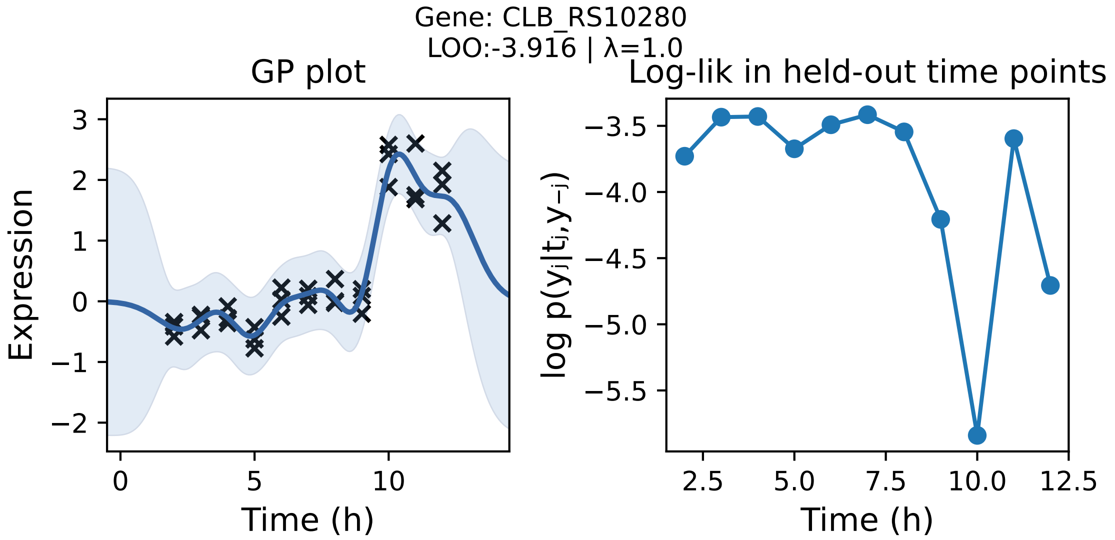

Gene: CLB_RS10280
LOO:-3.916 | λ=1.0
GP plot
Log-lik in held-out time points
Expression
Time (h)
log p(y_j|t_j,y_{-j})
3
2
1
0
−1
−2
0
5
10
−3.5
−4.0
−4.5
−5.0
−5.5
2.5
5.0
7.5
10.0
12.5

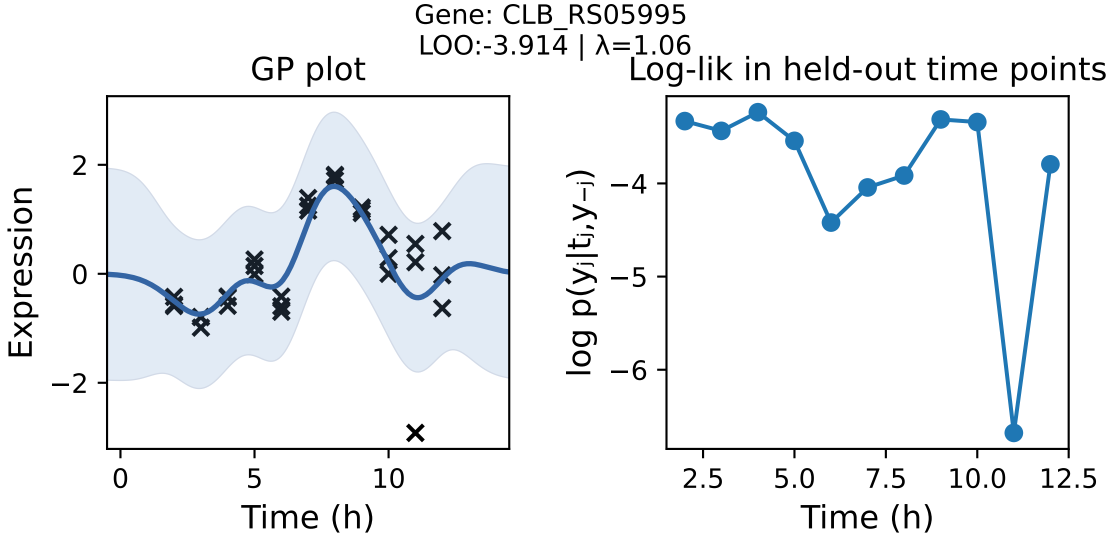
Gene: CLB_RS05995
LOO:-3.914 | λ=1.06
GP plot
Expression
Time (h)
Log-lik in held-out time points
log p(y_j|t_j,y_−j)
Time (h)

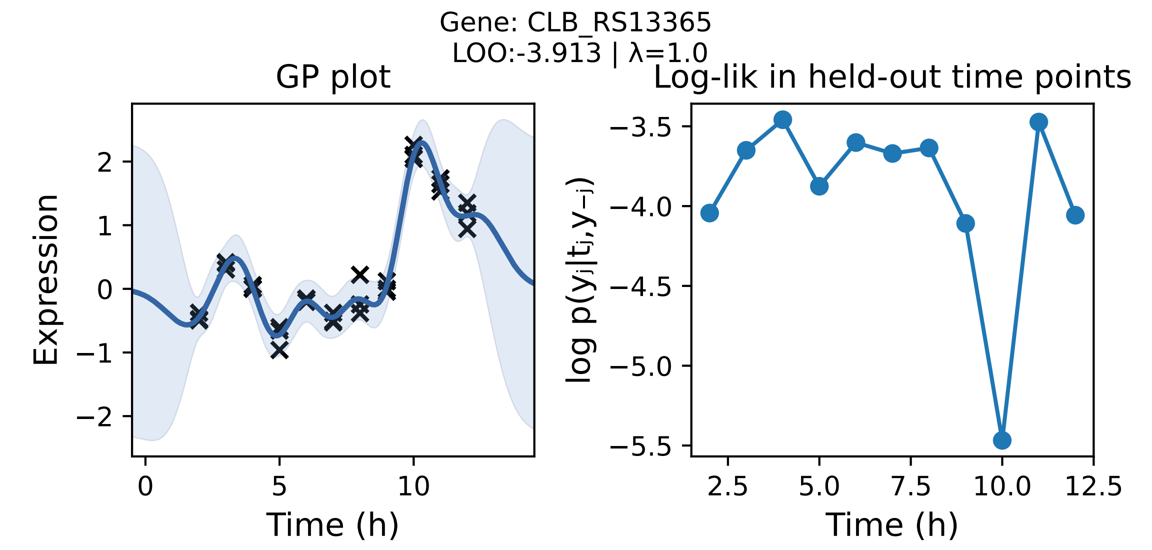

Gene: CLB_RS13365
LOO:-3.913 | λ=1.0
GP plot
Log-lik in held-out time points
Expression
Time (h)
log p(y_j|t_j,y_−j)
Time (h)

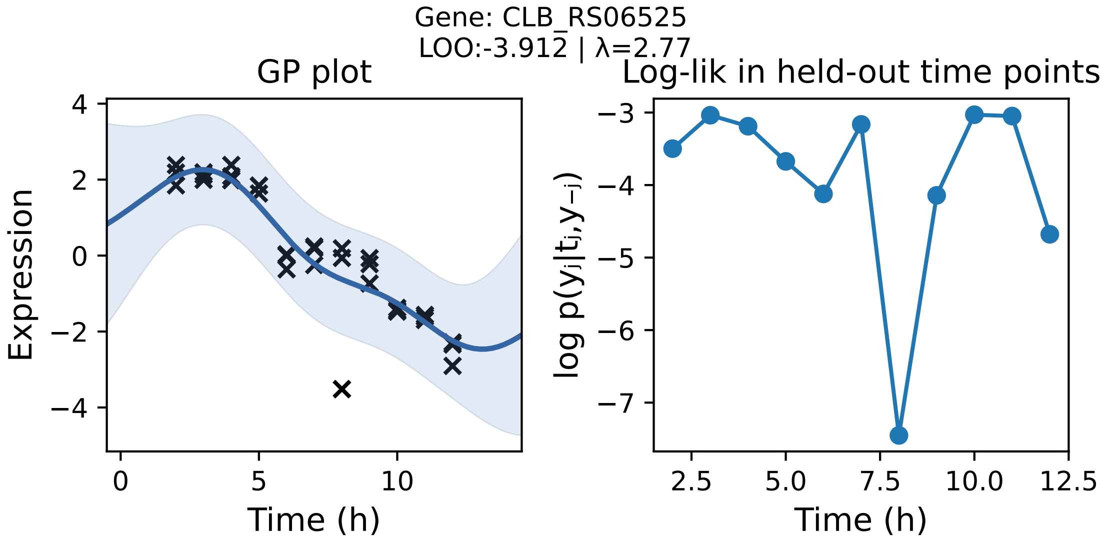

Gene: CLB_RS06525
LOO:-3.912 | λ=2.77
GP plot
Log-lik in held-out time points
Expression
Time (h)
log p(y_j|t_j,y_−j)
4
2
0
−2
−4
0
5
10
−3
−4
−5
−6
−7
2.5
5.0
7.5
10.0
12.5

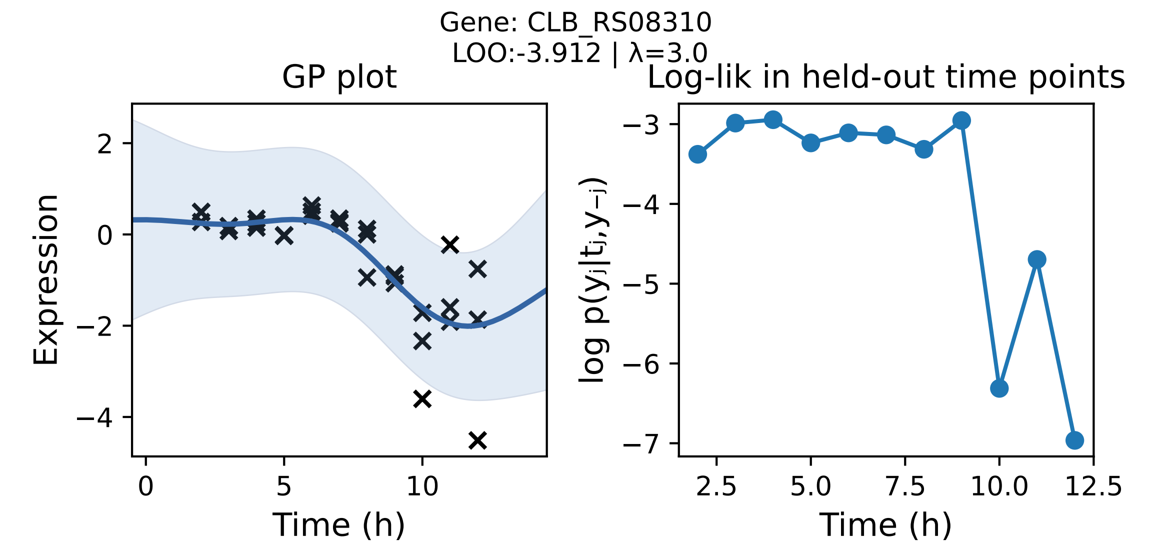
Gene: CLB_RS08310
LOO:-3.912 | λ=3.0
GP plot
Expression
Time (h)
Log-lik in held-out time points
log p(y_j|t_j,y_−j)
Time (h)

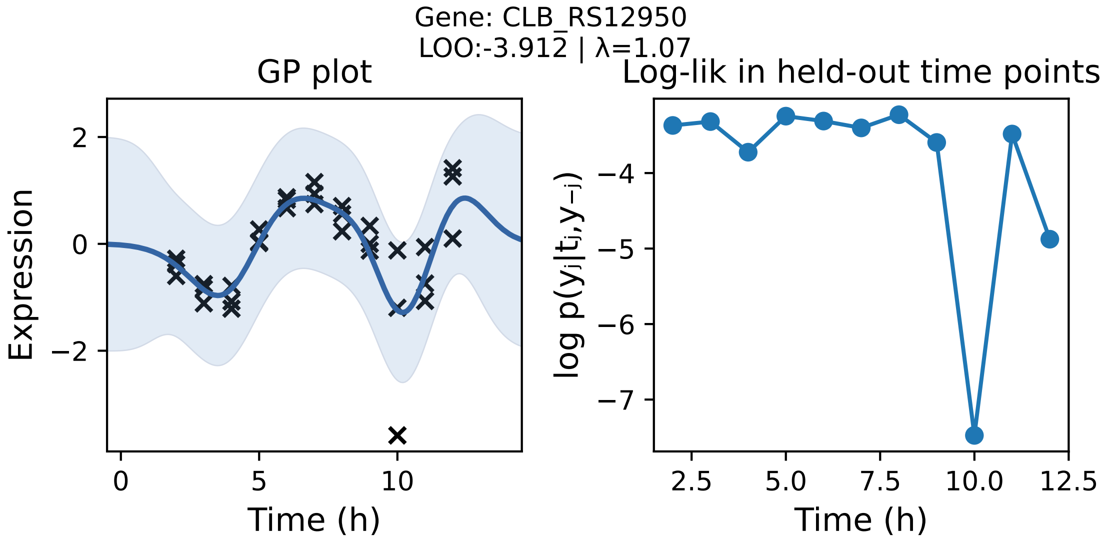

Gene: CLB_RS12950
LOO:-3.912 | λ=1.07
GP plot
Expression
2
0
−2
0
5
10
Time (h)
Log-lik in held-out time points
log p(y_j|t_j,y_−j)
−4
−5
−6
−7
2.5
5.0
7.5
10.0
12.5
Time (h)

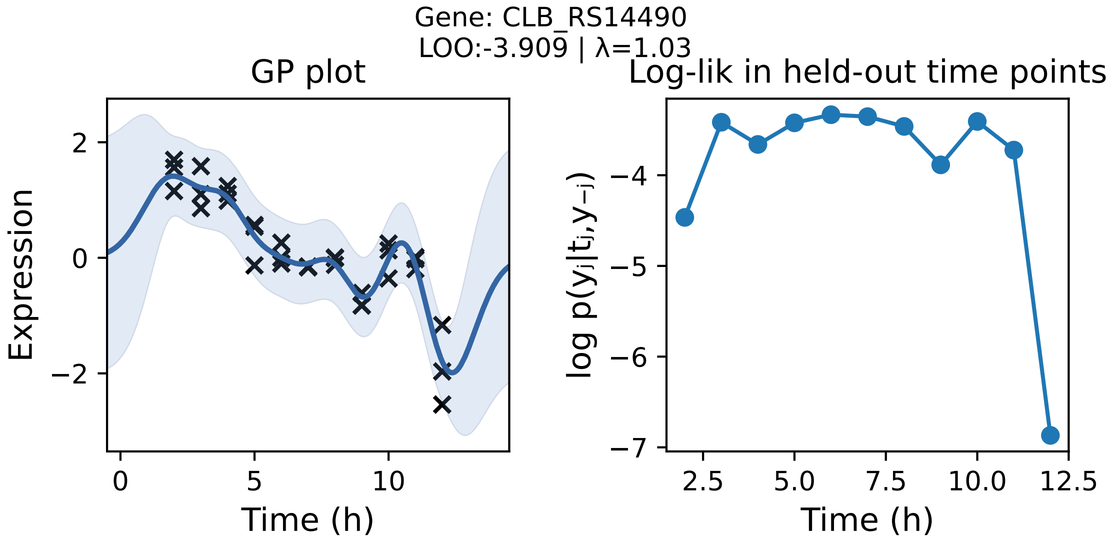
Gene: CLB_RS14490
LOO:-3.909 | λ=1.03
GP plot
Expression
Time (h)
Log-lik in held-out time points
log p(y_j|t_j,y_−j)
Time (h)

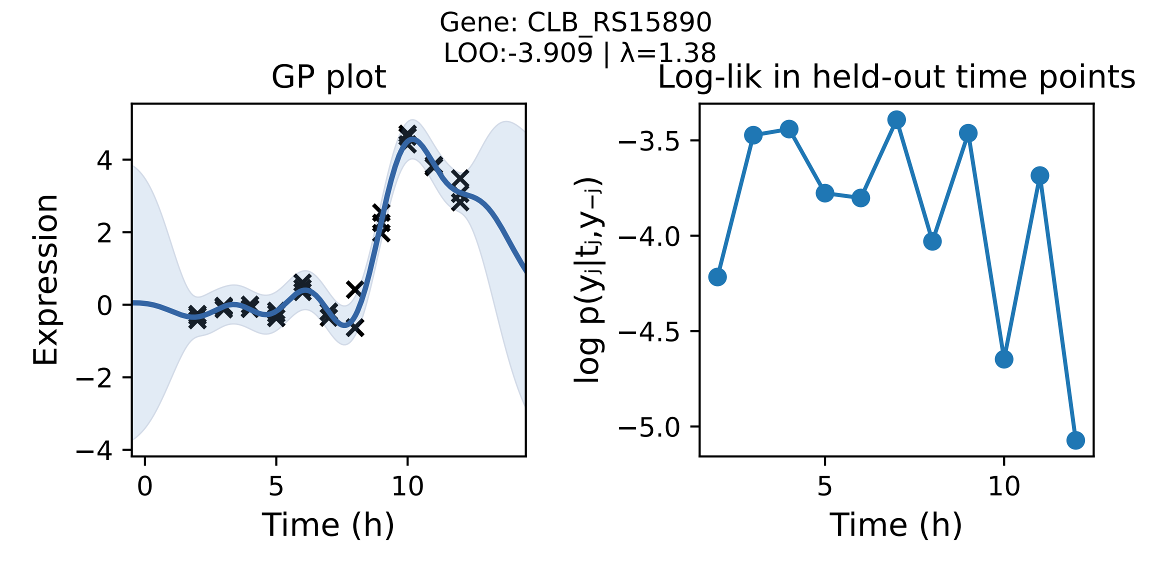
Gene: CLB_RS15890
LOO:-3.909 | λ=1.38
GP plot
Expression
Time (h)
Log-lik in held-out time points
log p(y_j|t_j,y_−j)
Time (h)

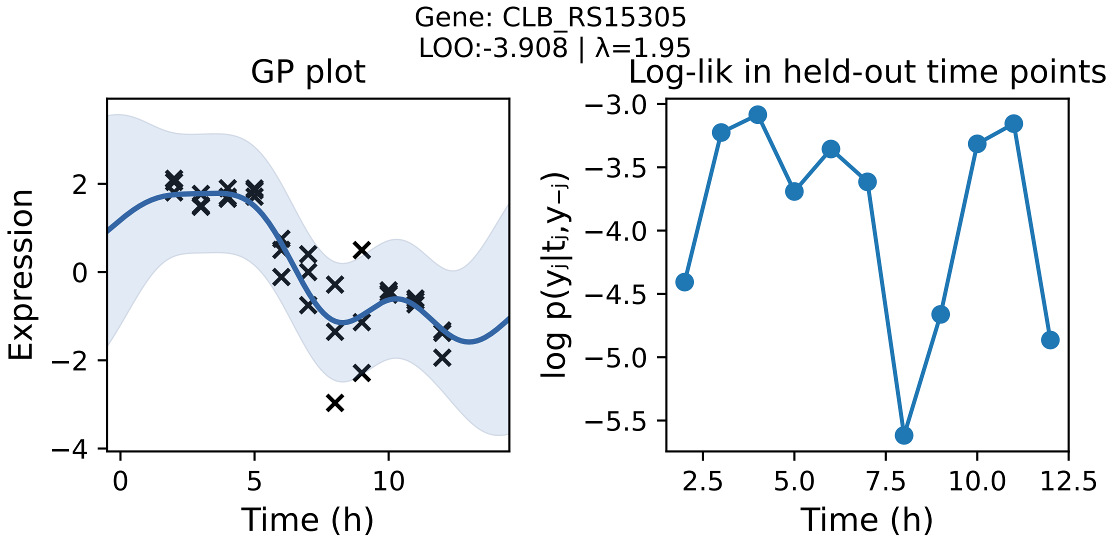
Gene: CLB_RS15305
LOO:-3.908 | λ=1.95
GP plot
Expression
Time (h)
Log-lik in held-out time points
log p(y_j|t_j,y_−j)
Time (h)

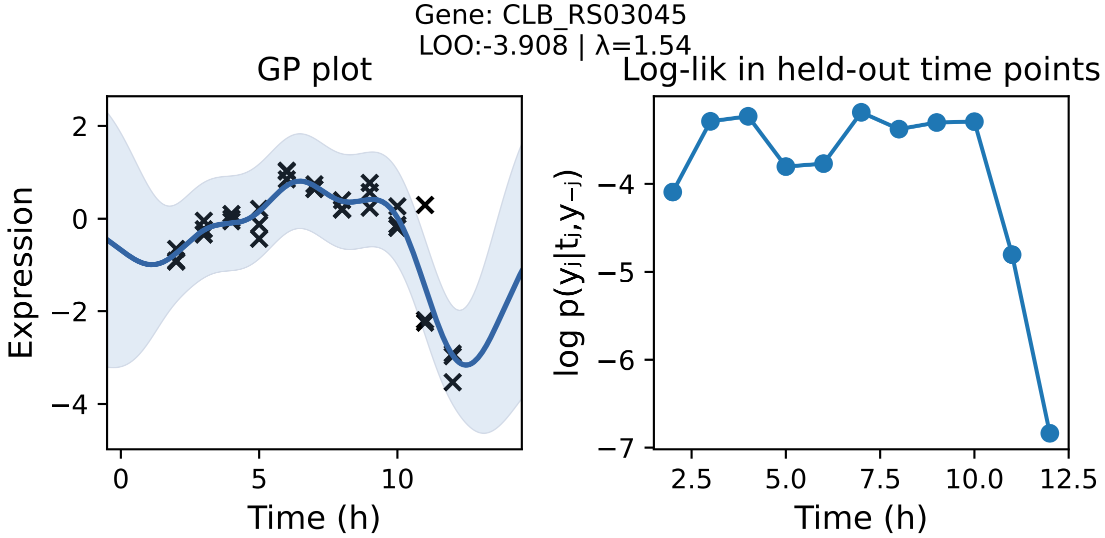

Gene: CLB_RS03045
LOO:-3.908 | λ=1.54
GP plot
Expression
Time (h)
Log-lik in held-out time points
log p(y_j|t_j,y_−j)
Time (h)

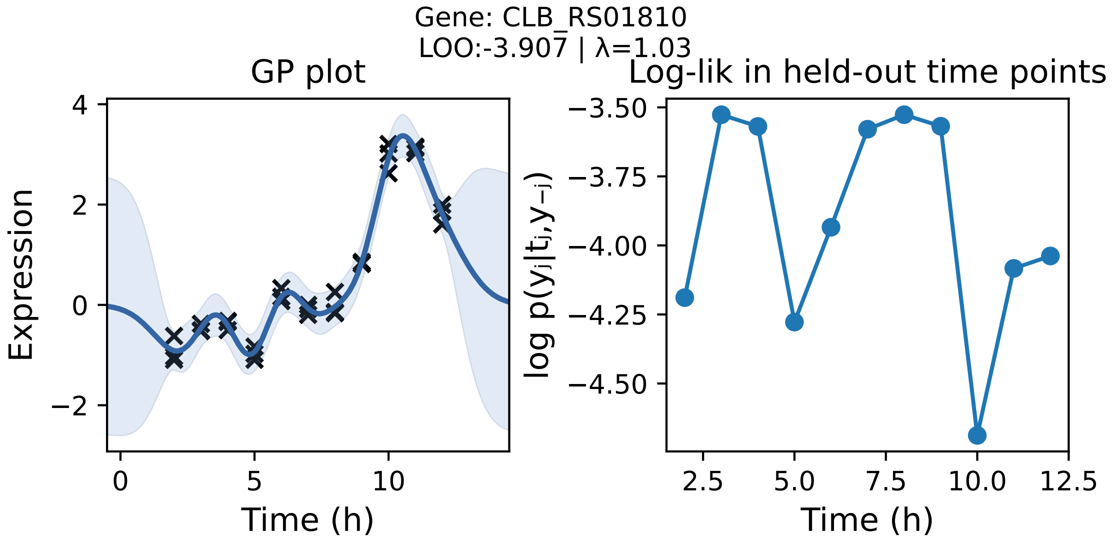
Gene: CLB_RS01810
LOO:-3.907 | λ=1.03
GP plot
Expression
Time (h)
Log-lik in held-out time points
log p(y_j|t_j,y_−j)
Time (h)

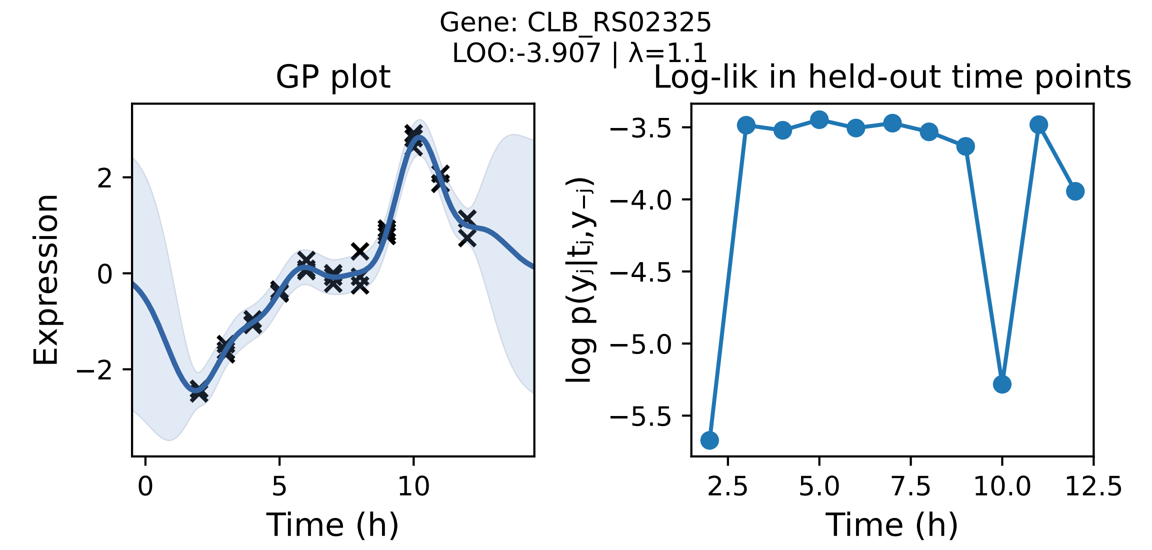
Gene: CLB_RS02325
LOO:-3.907 | λ=1.1
GP plot
Expression
Time (h)
2
0
−2
0
5
10
Log-lik in held-out time points
log p(y_j|t_j,y_−j)
Time (h)
−3.5
−4.0
−4.5
−5.0
−5.5
2.5
5.0
7.5
10.0
12.5

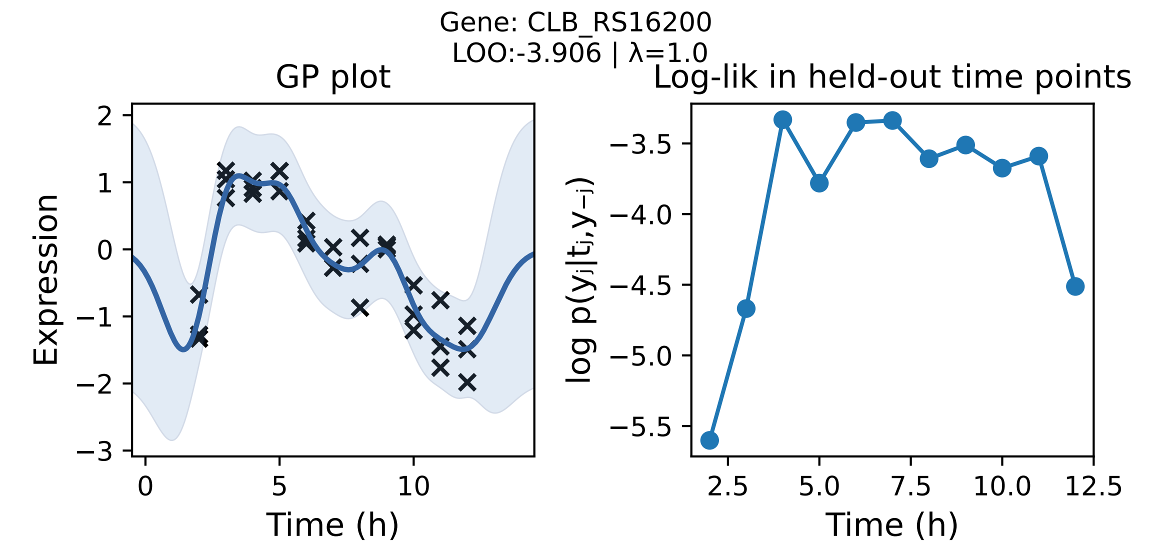

Gene: CLB_RS16200
LOO:-3.906 | λ=1.0
GP plot
Expression
Time (h)
Log-lik in held-out time points
log p(yj|tj,y−j)
Time (h)

Gene: CLB_RS06790
LOO:-3.906 | λ=1.59

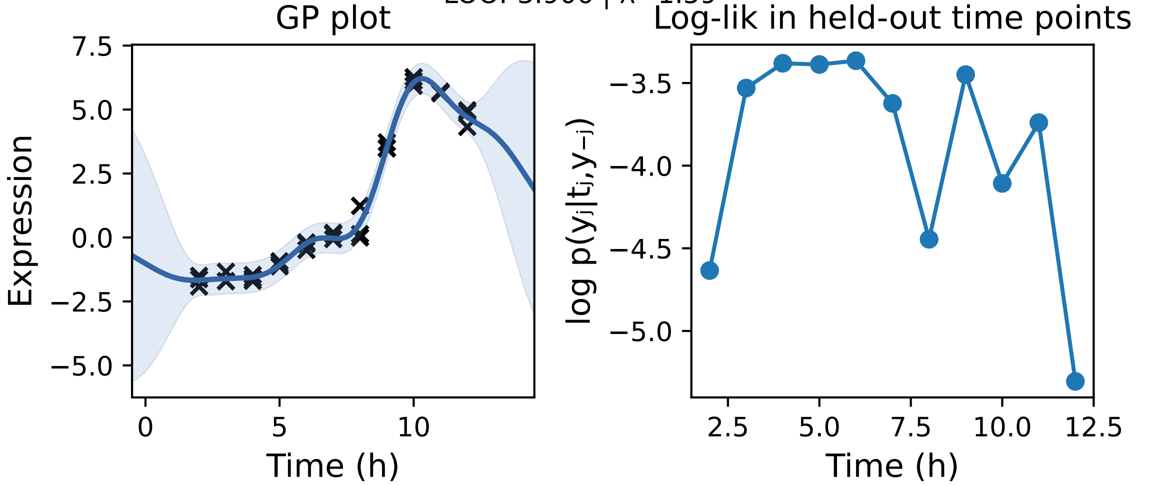

Gene: CLB_RS10825
LOO:-3.906 | λ=1.08

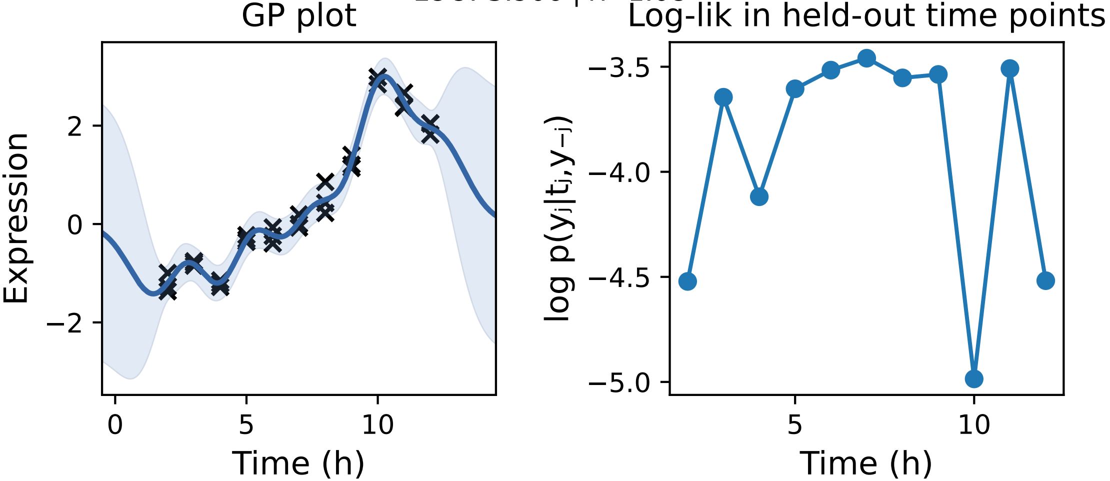

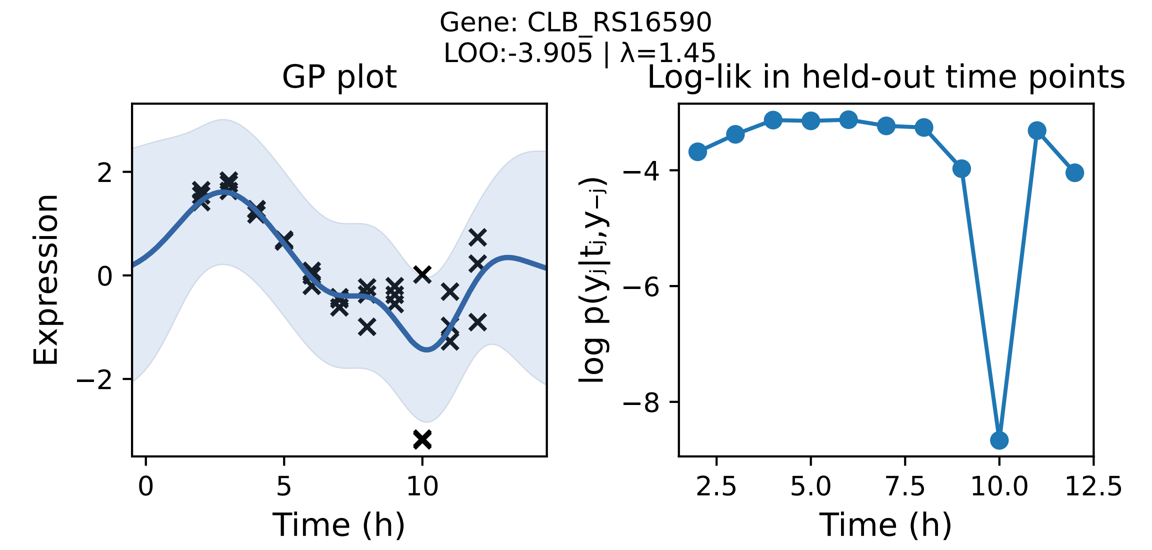
Gene: CLB_RS16590
LOO:-3.905 | λ=1.45
GP plot
Log-lik in held-out time points
Expression
Time (h)
log p(yj|tj,y−j)
Time (h)

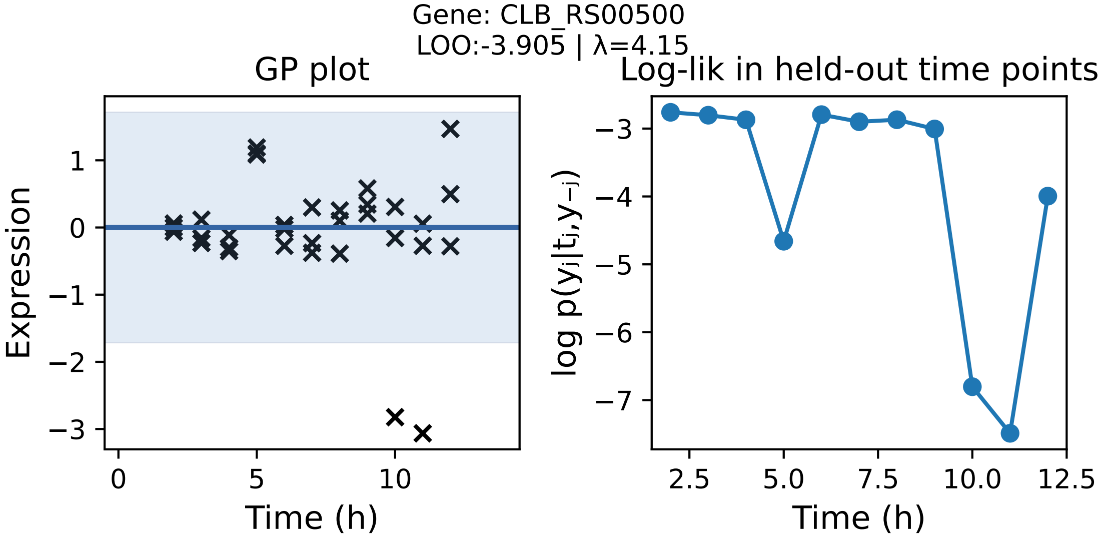

Gene: CLB_RS00500
LOO:-3.905 | λ=4.15
GP plot
Expression
Time (h)
Log-lik in held-out time points
log p(yj|tj,y−j)
Time (h)

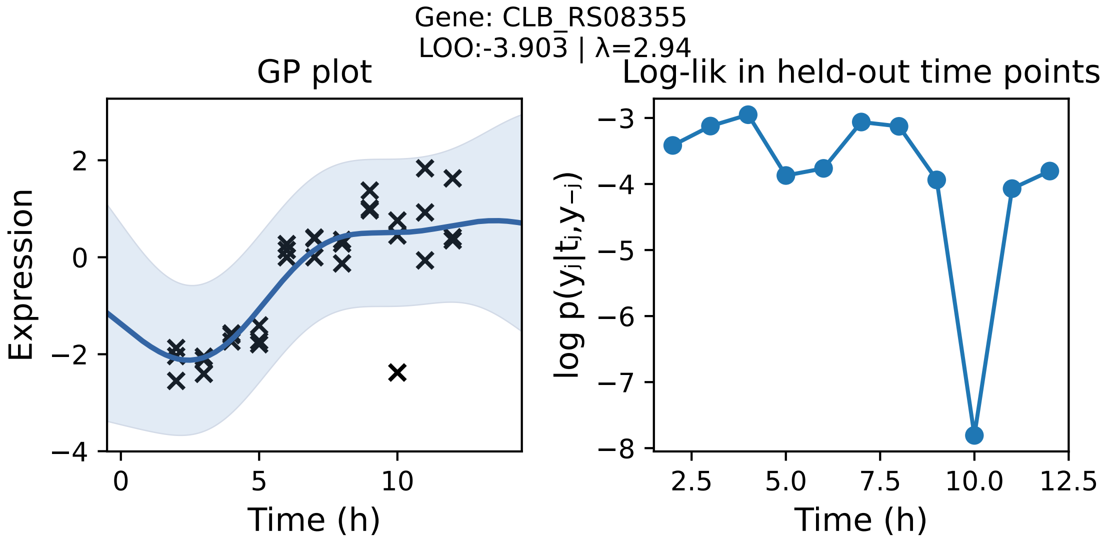

Gene: CLB_RS08355
LOO:-3.903 | λ=2.94
GP plot
Log-lik in held-out time points
Expression
2
0
−2
−4
0
5
10
Time (h)
log p(yⱼ|tⱼ,y₋ⱼ)
−3
−4
−5
−6
−7
−8
2.5
5.0
7.5
10.0
12.5
Time (h)

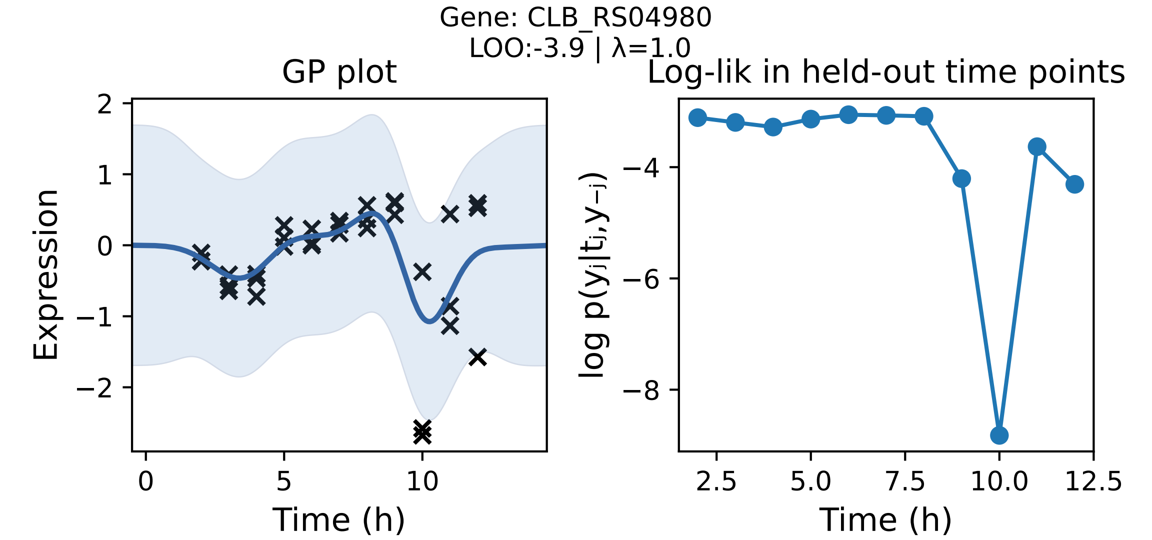

Gene: CLB_RS04980
LOO:-3.9 | λ=1.0
GP plot
Expression
Time (h)
Log-lik in held-out time points
log p(y_j|t_j,y_−j)
Time (h)

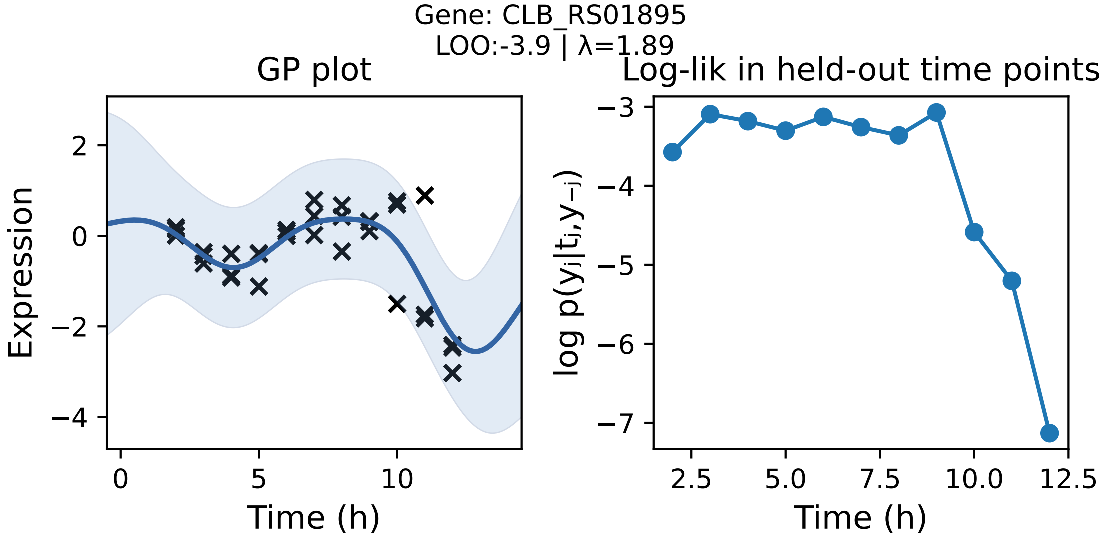

Gene: CLB_RS01895
LOO:-3.9 | λ=1.89
GP plot
Expression
Time (h)
Log-lik in held-out time points
log p(y_j|t_j,y_−j)
Time (h)

Gene: CLB_RS15100
LOO:-3.898 | λ=1.37

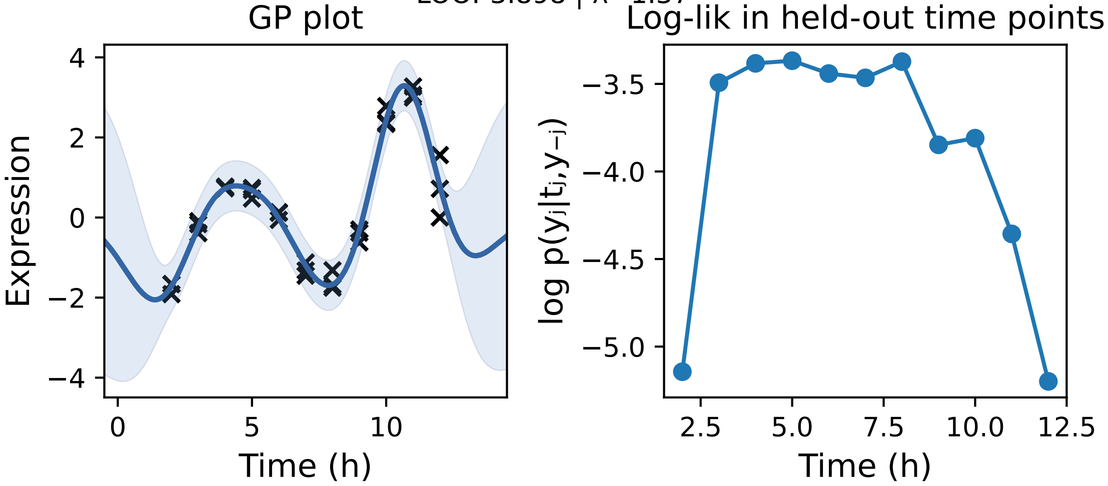

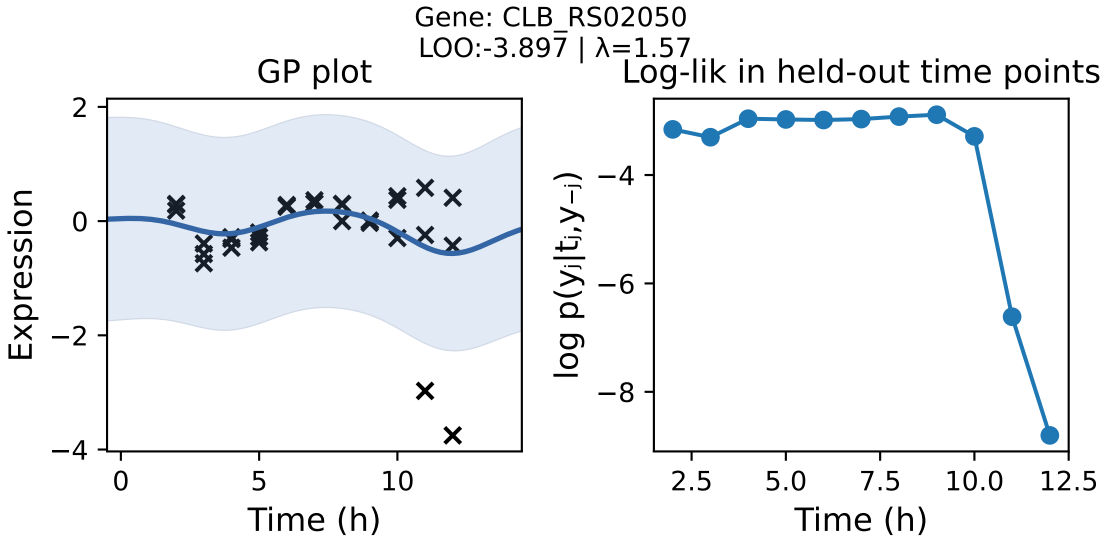
Gene: CLB_RS02050
LOO:-3.897 | λ=1.57
GP plot
Expression
Time (h)
2
0
−2
−4
0
5
10
Log-lik in held-out time points
log p(y_j|t_j,y_−j)
Time (h)
−4
−6
−8
2.5
5.0
7.5
10.0
12.5

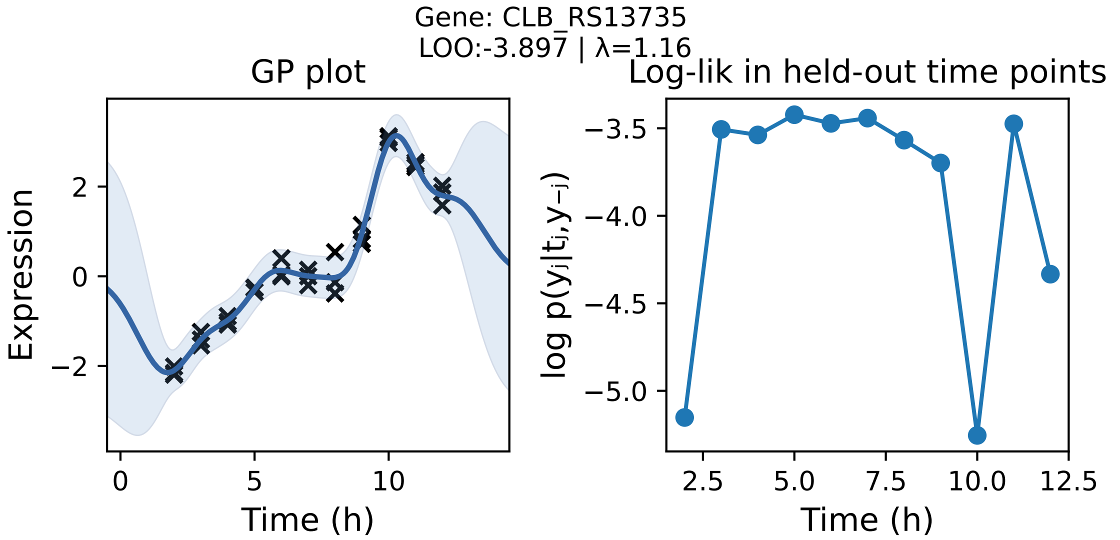
Gene: CLB_RS13735
LOO:-3.897 | λ=1.16
GP plot
Expression
2
0
−2
0
5
10
Time (h)
Log-lik in held-out time points
log p(yj|tj,y−j)
−3.5
−4.0
−4.5
−5.0
2.5
5.0
7.5
10.0
12.5
Time (h)

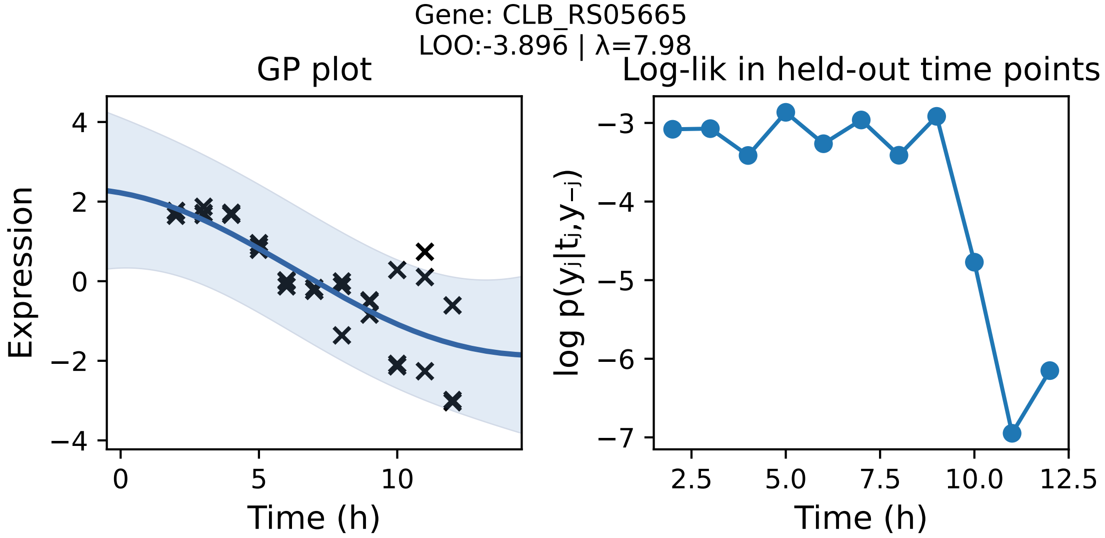
Gene: CLB_RS05665
LOO:-3.896 | λ=7.98
GP plot
Expression
Time (h)
Log-lik in held-out time points
log p(y_j|t_j,y_−j)
Time (h)

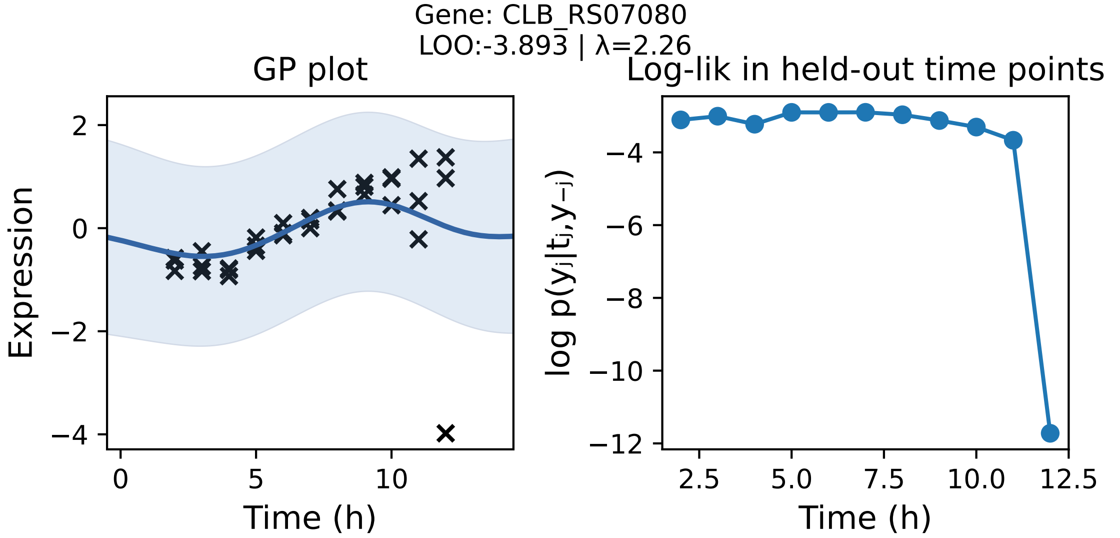

Gene: CLB_RS07080
LOO:-3.893 | λ=2.26
GP plot
Expression
Time (h)
Log-lik in held-out time points
log p(yj|tj,y−j)
Time (h)

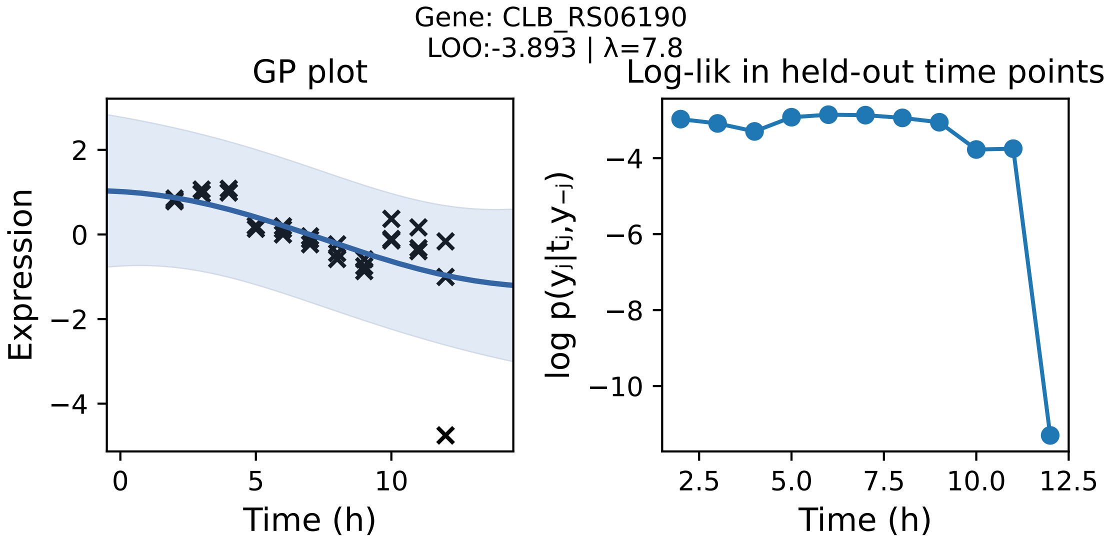

Gene: CLB_RS06190
LOO:-3.893 | λ=7.8
GP plot
Expression
Time (h)
Log-lik in held-out time points
log p(yj|tj,y−j)
Time (h)

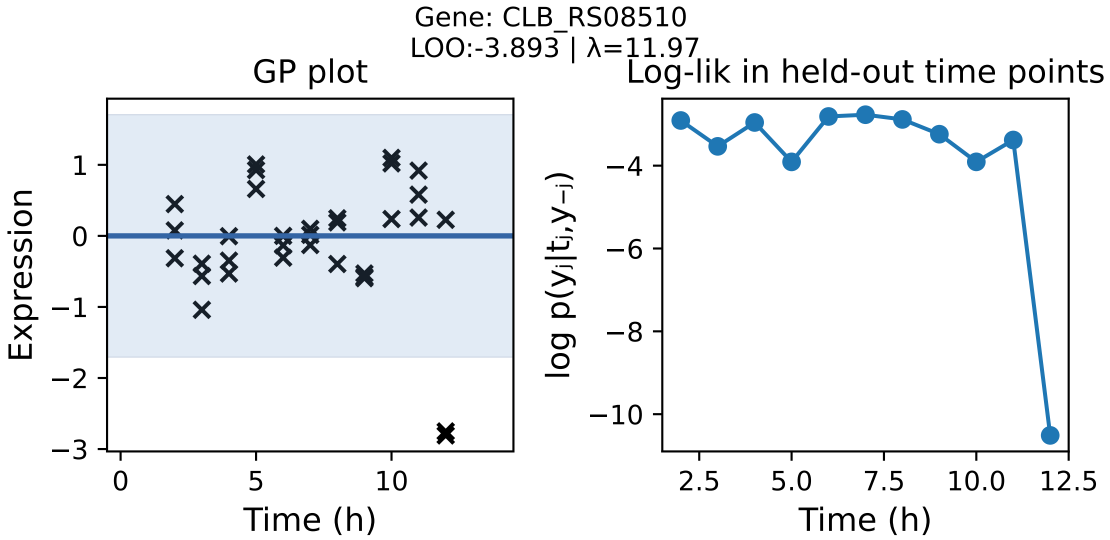
Gene: CLB_RS08510
LOO:-3.893 | λ=11.97
GP plot
Expression
Time (h)
Log-lik in held-out time points
log p(yj|tj,y−j)
Time (h)

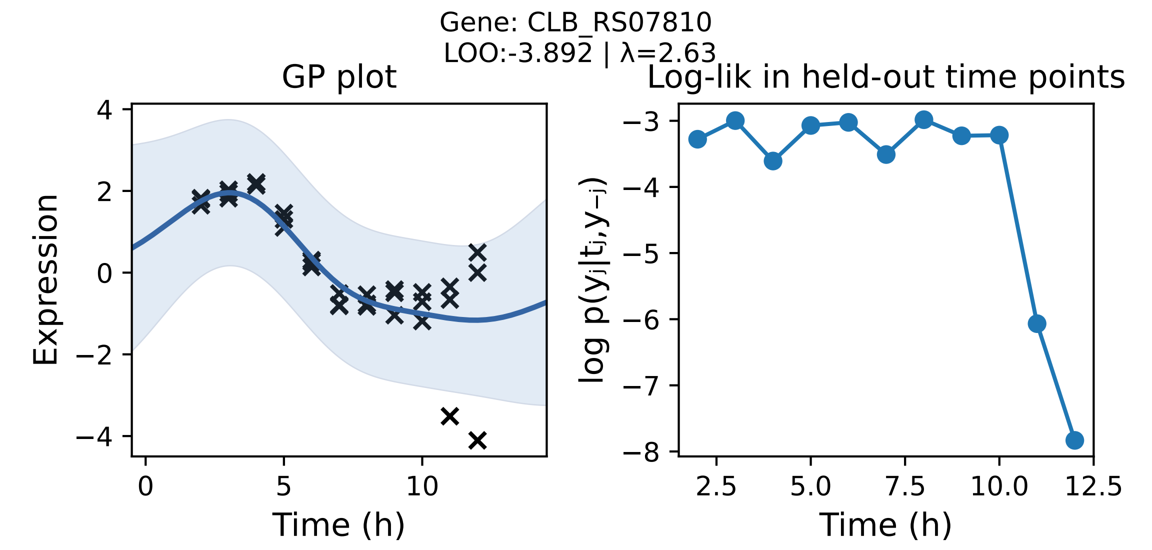
Gene: CLB_RS07810
LOO:-3.892 | λ=2.63
GP plot
Log-lik in held-out time points
Expression
Time (h)
log p(y_j|t_j,y_{-j})
Time (h)

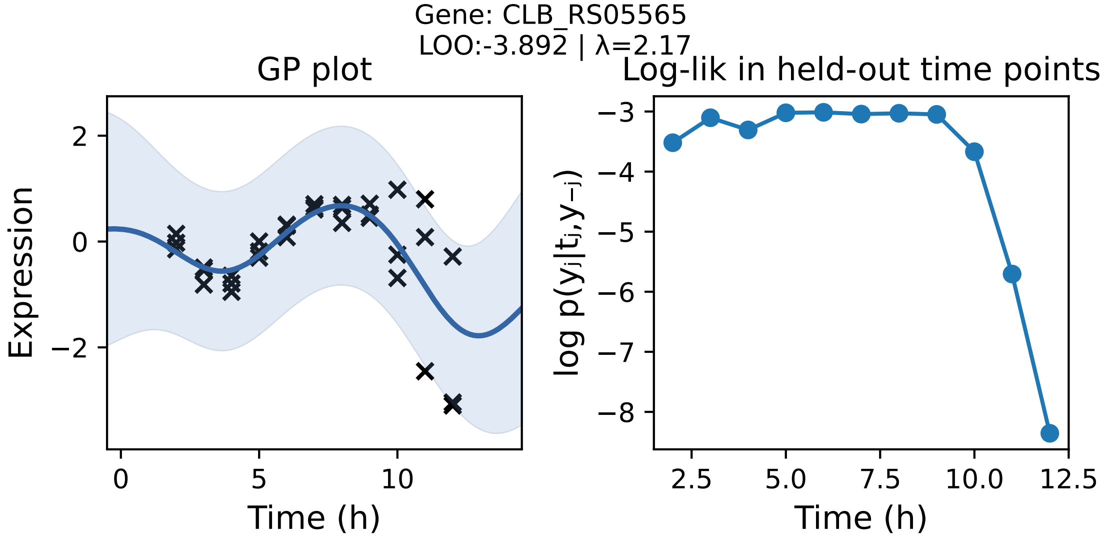

Gene: CLB_RS05565
LOO:-3.892 | λ=2.17
GP plot
Expression
Time (h)
Log-lik in held-out time points
log p(y_j|t_j,y_−j)
Time (h)

Gene: CLB_RS15340
LOO:-3.892 | λ=1.09

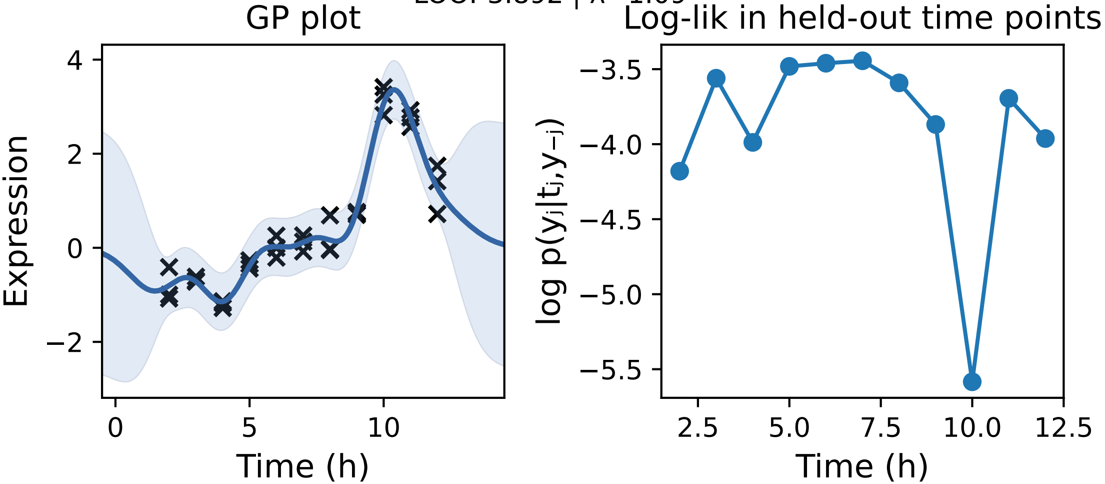

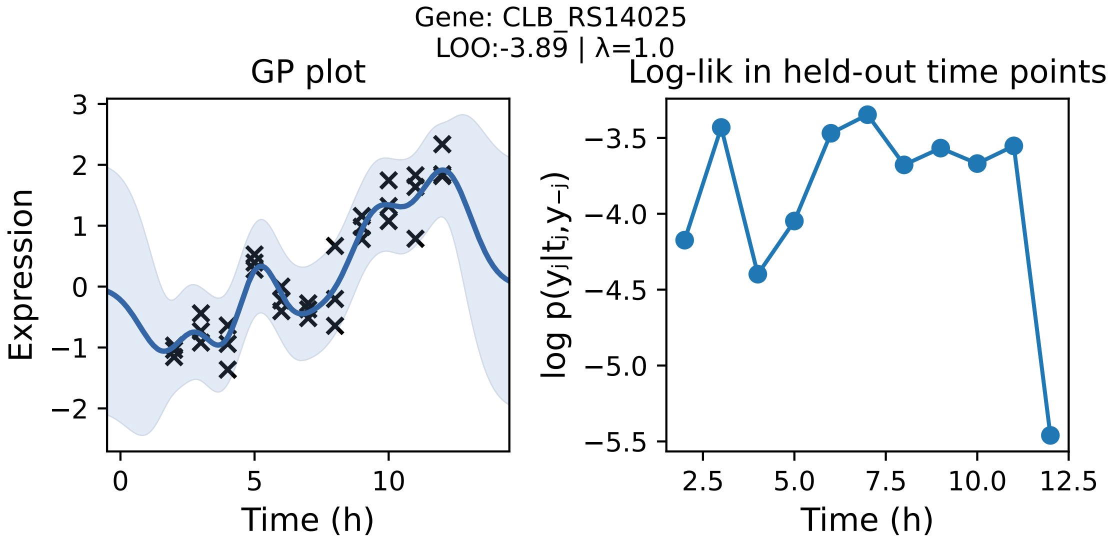

Gene: CLB_RS14025
LOO:-3.89 | λ=1.0
GP plot
Log-lik in held-out time points
Expression
Time (h)
log p(y_j|t_j,y_−j)
Time (h)

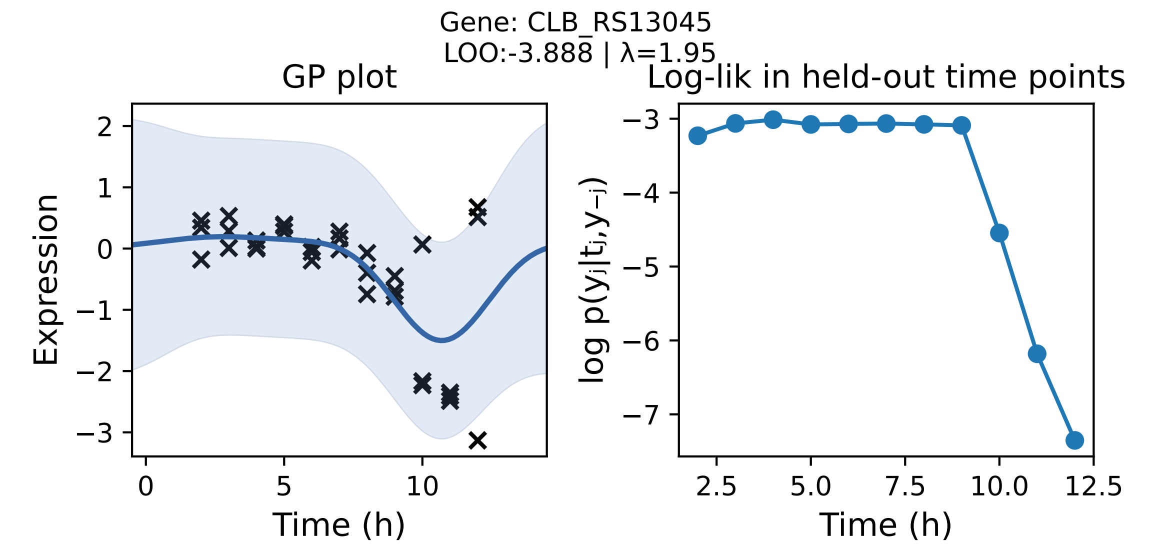

Gene: CLB_RS13045
LOO:-3.888 | λ=1.95
GP plot
Expression
Time (h)
Log-lik in held-out time points
log p(y_j|t_j,y_−j)
Time (h)

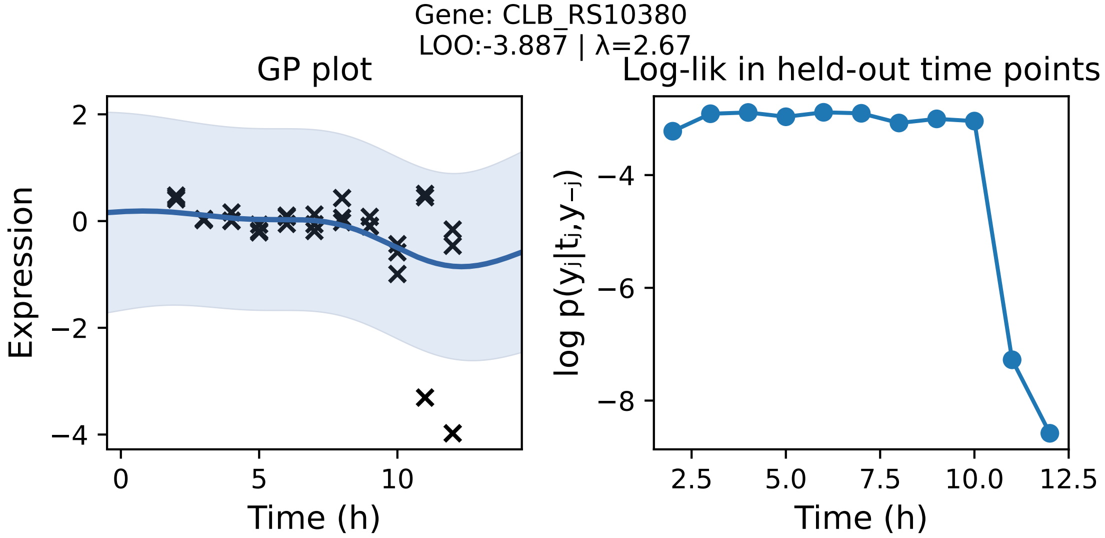

Gene: CLB_RS10380
LOO:-3.887 | λ=2.67
GP plot
Log-lik in held-out time points
Expression
2
0
−2
−4
0
5
10
Time (h)
log p(y_j|t_j,y_−j)
−4
−6
−8
2.5
5.0
7.5
10.0
12.5
Time (h)

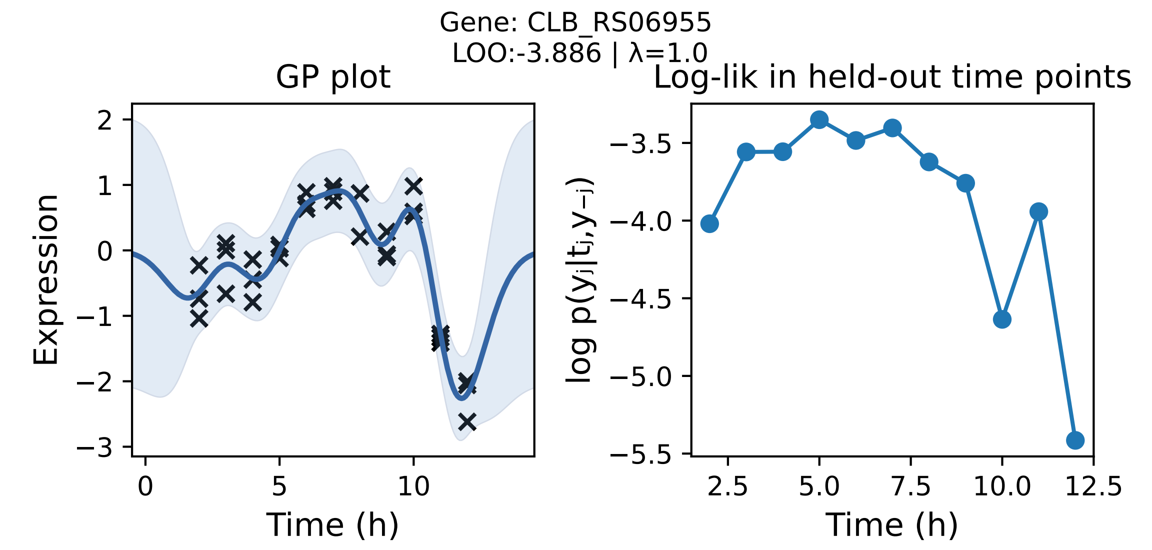
Gene: CLB_RS06955
LOO:-3.886 | λ=1.0
GP plot
Expression
Time (h)
Log-lik in held-out time points
log p(y_j|t_j,y_−j)
Time (h)

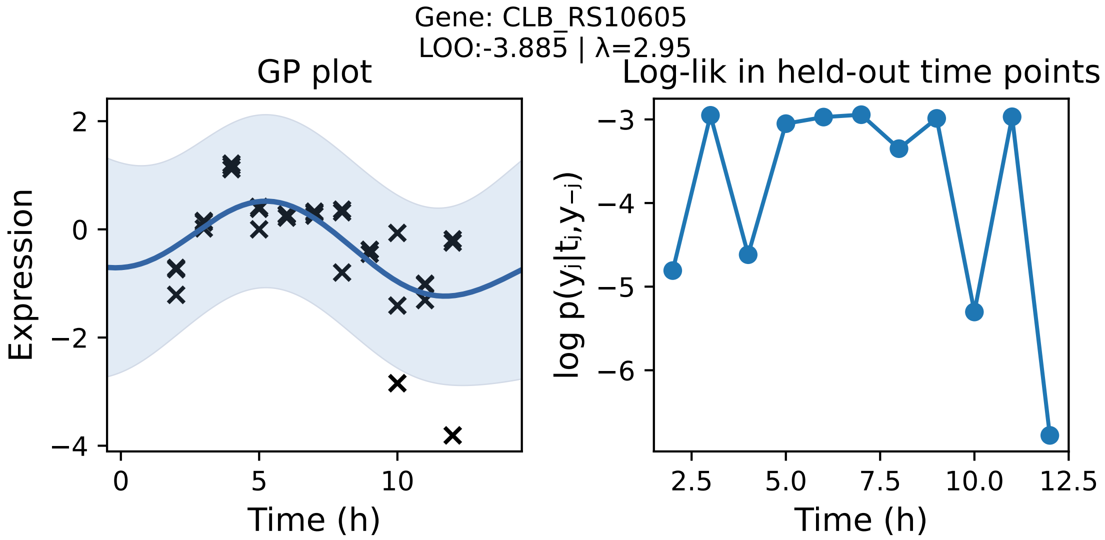

Gene: CLB_RS10605
LOO:-3.885 | λ=2.95
GP plot
Log-lik in held-out time points
Expression
2
0
−2
−4
0
5
10
Time (h)
log p(y_j|t_j,y_−j)
−3
−4
−5
−6
2.5
5.0
7.5
10.0
12.5
Time (h)

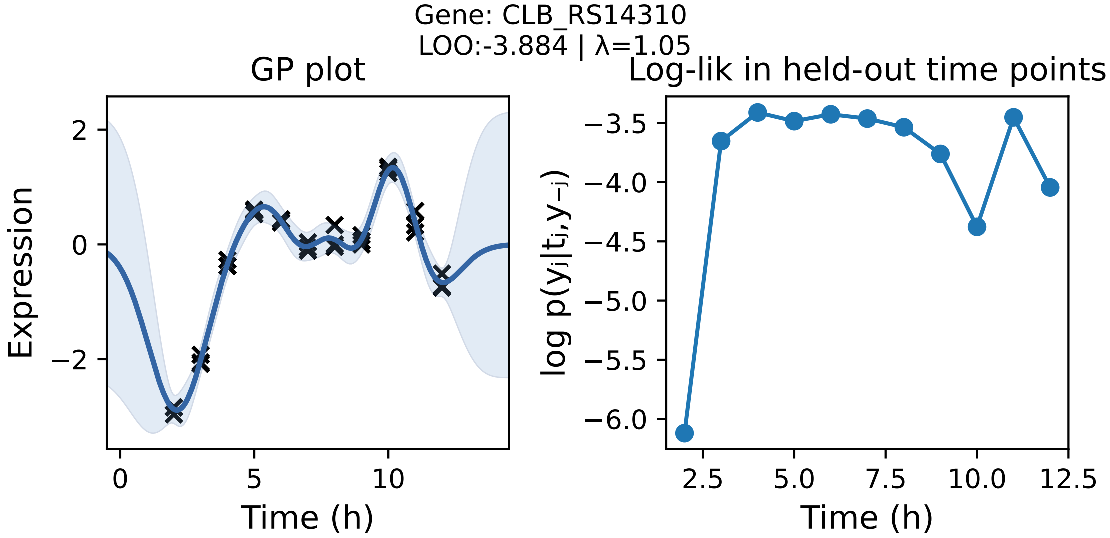

Gene: CLB_RS14310
LOO:-3.884 | λ=1.05
GP plot
Expression
Time (h)
Log-lik in held-out time points
log p(y_j|t_j,y_−j)
Time (h)

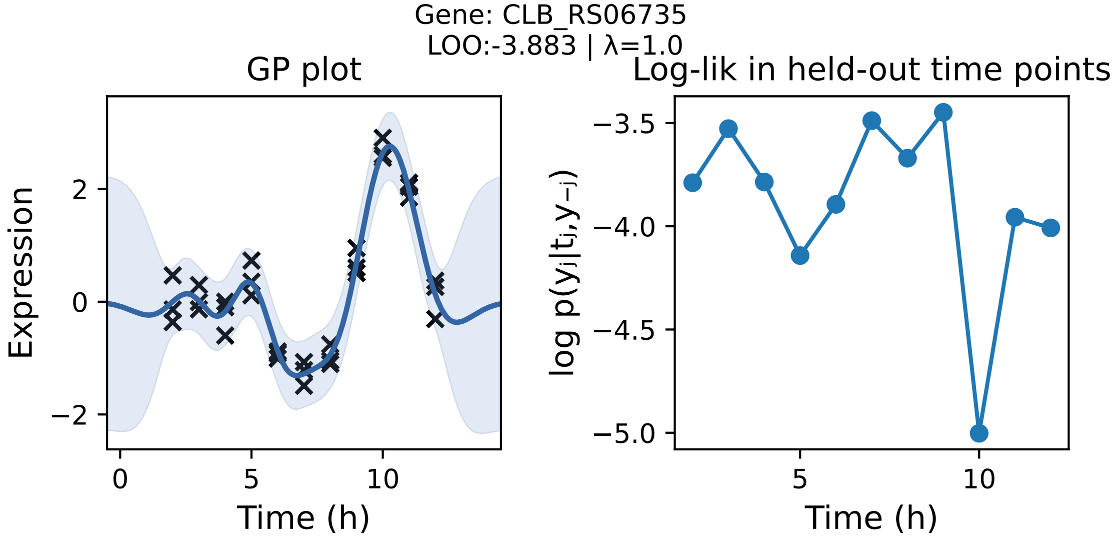

Gene: CLB_RS06735
LOO:-3.883 | λ=1.0
GP plot
Expression
Time (h)
Log-lik in held-out time points
log p(yj|tj,y−j)
Time (h)

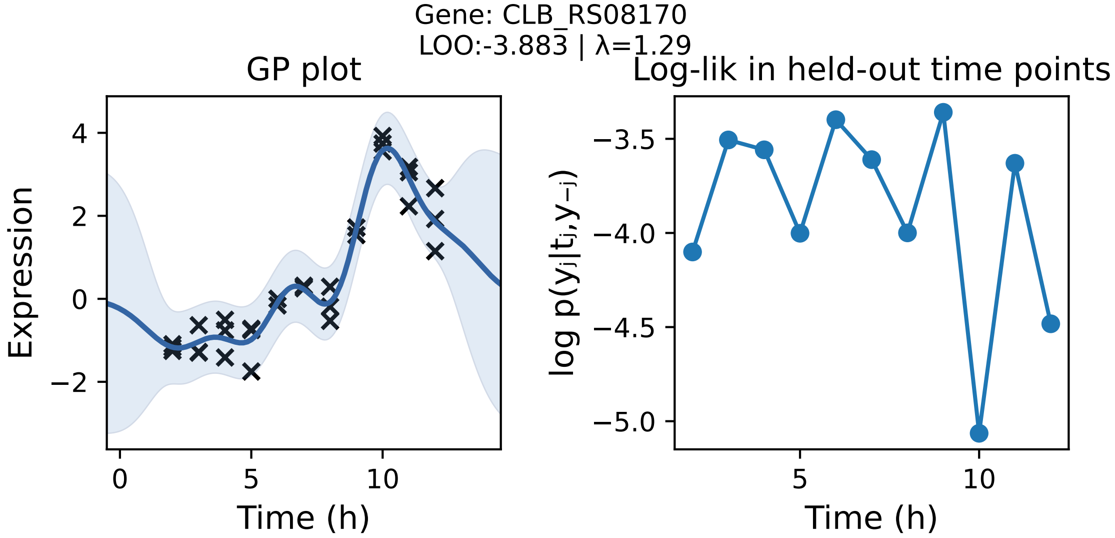
Gene: CLB_RS08170
LOO:-3.883 | λ=1.29
GP plot
Expression
Time (h)
Log-lik in held-out time points
log p(y_j|t_j,y_−j)
Time (h)

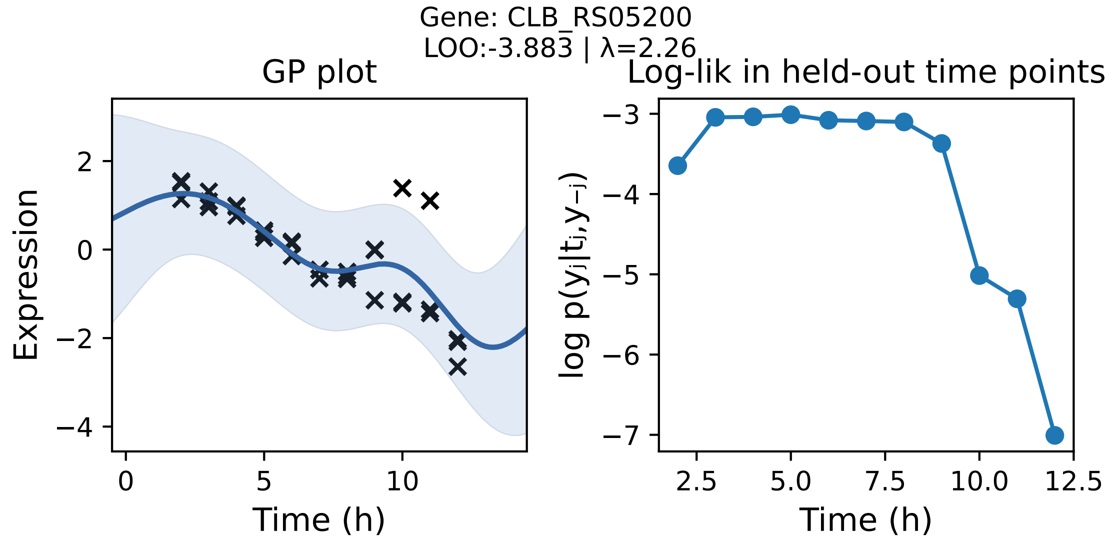

Gene: CLB_RS05200
LOO:-3.883 | λ=2.26
GP plot
Log-lik in held-out time points
Expression
Time (h)
log p(y_j|t_j,y_{-j})
Time (h)

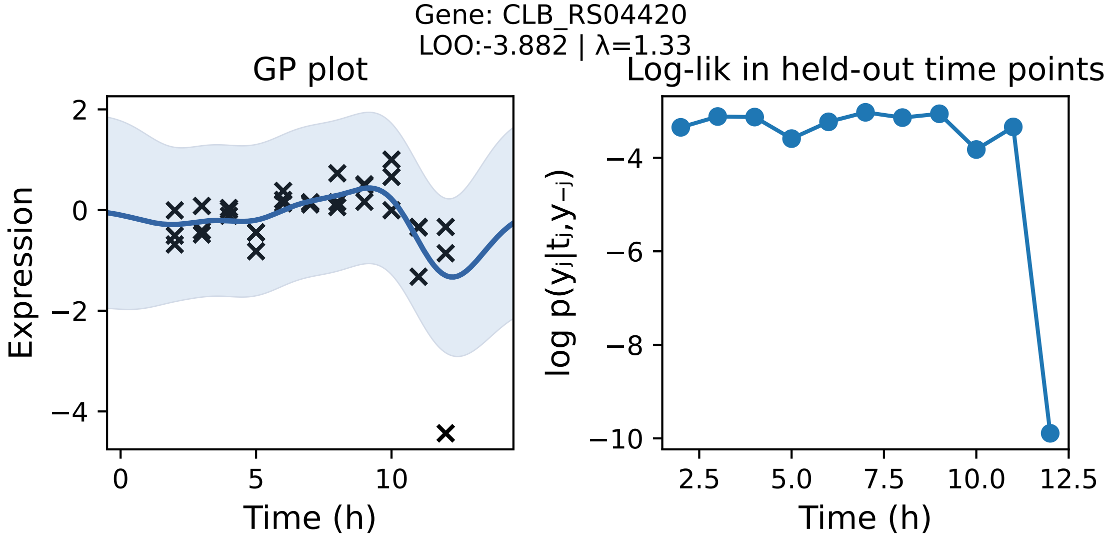

Gene: CLB_RS04420
LOO:-3.882 | λ=1.33
GP plot
Expression
Time (h)
Log-lik in held-out time points
log p(y_j|t_j,y_−j)
Time (h)

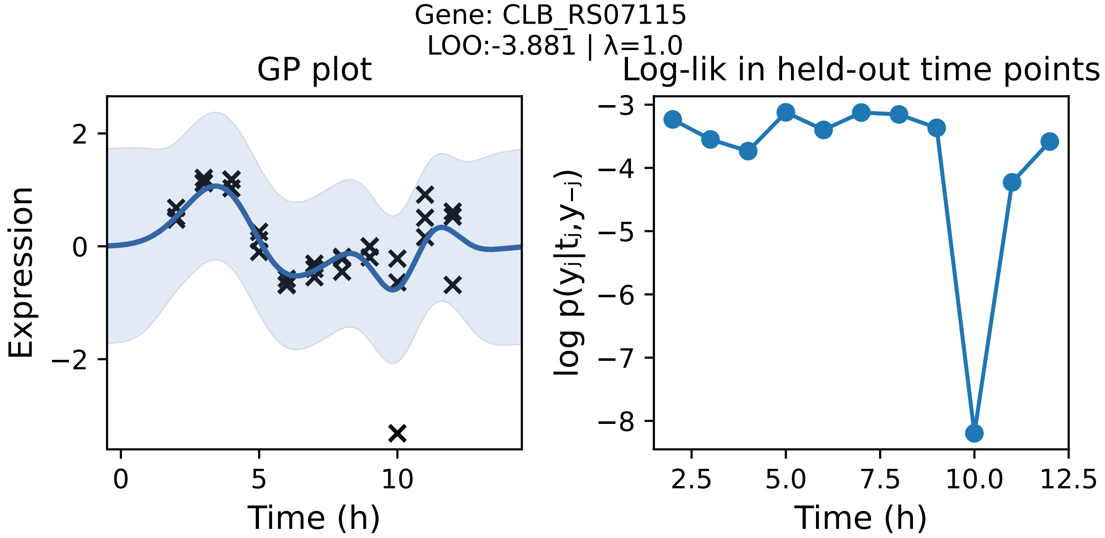

Gene: CLB_RS07115
LOO:-3.881 | λ=1.0
GP plot
Expression
Time (h)
2
0
−2
0
5
10
Log-lik in held-out time points
log p(yj|tj,y−j)
Time (h)
−3
−4
−5
−6
−7
−8
2.5
5.0
7.5
10.0
12.5

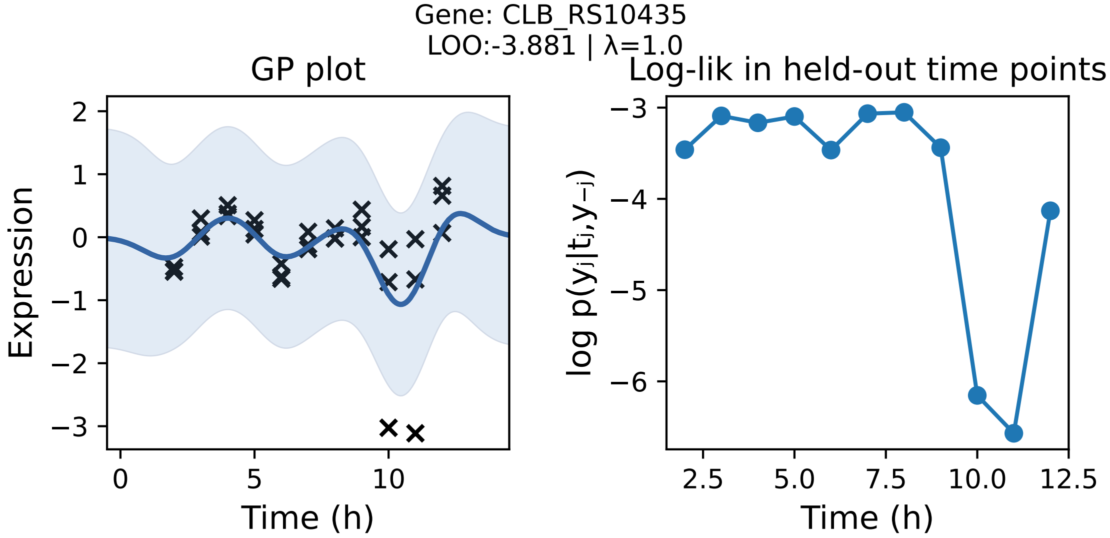
Gene: CLB_RS10435
LOO:-3.881 | λ=1.0
GP plot
Expression
Time (h)
Log-lik in held-out time points
log p(y_j|t_i,y_−j)
Time (h)

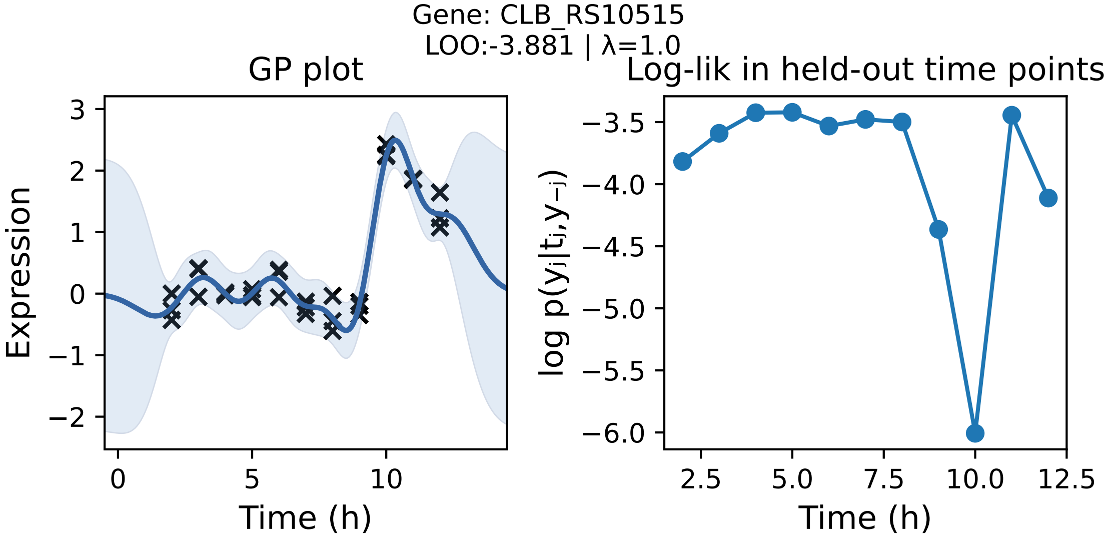

Gene: CLB_RS10515
LOO:-3.881 | λ=1.0
GP plot
Expression
Time (h)
3
2
1
0
−1
−2
0
5
10
Log-lik in held-out time points
$\log p(y_j|t_j, y_{-j})$
−3.5
−4.0
−4.5
−5.0
−5.5
−6.0
2.5
5.0
7.5
10.0
12.5
Time (h)

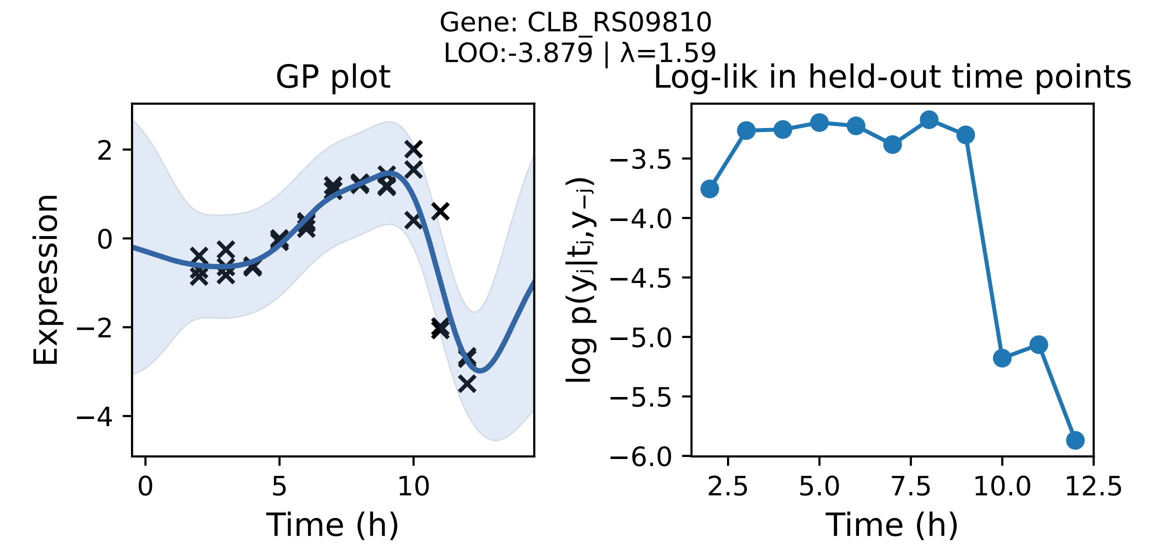

Gene: CLB_RS09810
LOO:-3.879 | λ=1.59
GP plot
Expression
Time (h)
Log-lik in held-out time points
log p(y_j|t_j,y_−j)
Time (h)

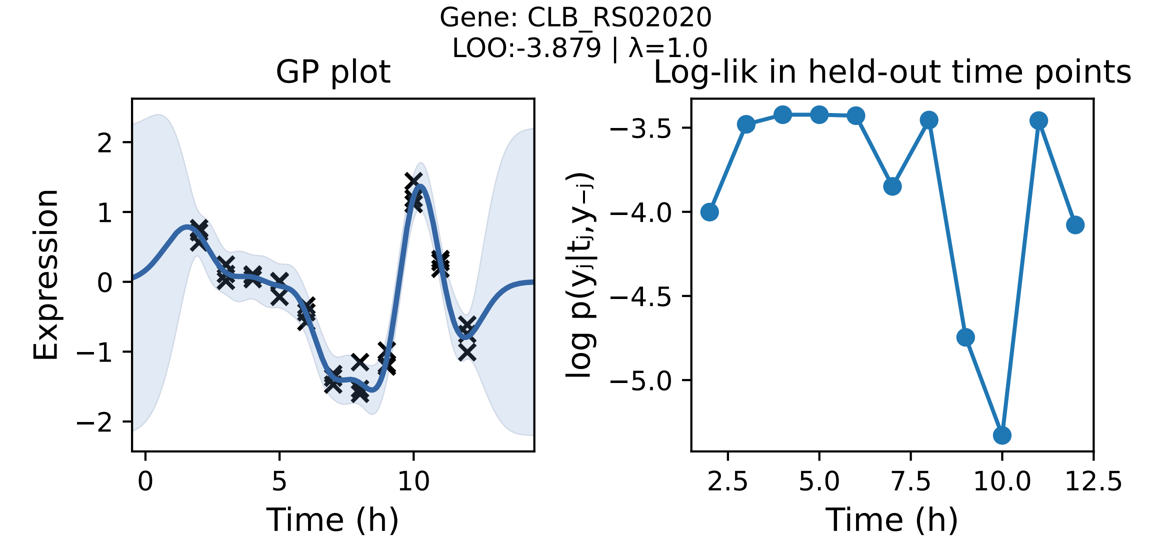

Gene: CLB_RS02020
LOO:-3.879 | λ=1.0
GP plot
Expression
Time (h)
Log-lik in held-out time points
log p(y_j|t_j,y_−j)
Time (h)

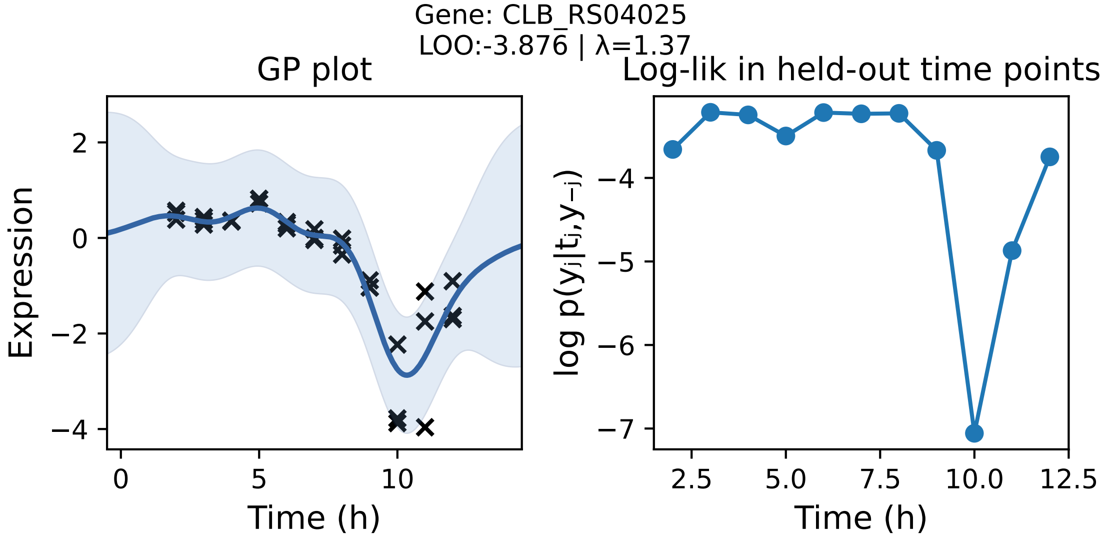

Gene: CLB_RS04025
LOO:-3.876 | λ=1.37
GP plot
Log-lik in held-out time points
Expression
Time (h)
log p(y_j|t_j,y_−j)
Time (h)
2
0
−2
−4
0
5
10
−4
−5
−6
−7
2.5
5.0
7.5
10.0
12.5

Gene: CLB_RS16510
LOO:-3.873 | λ=1.0

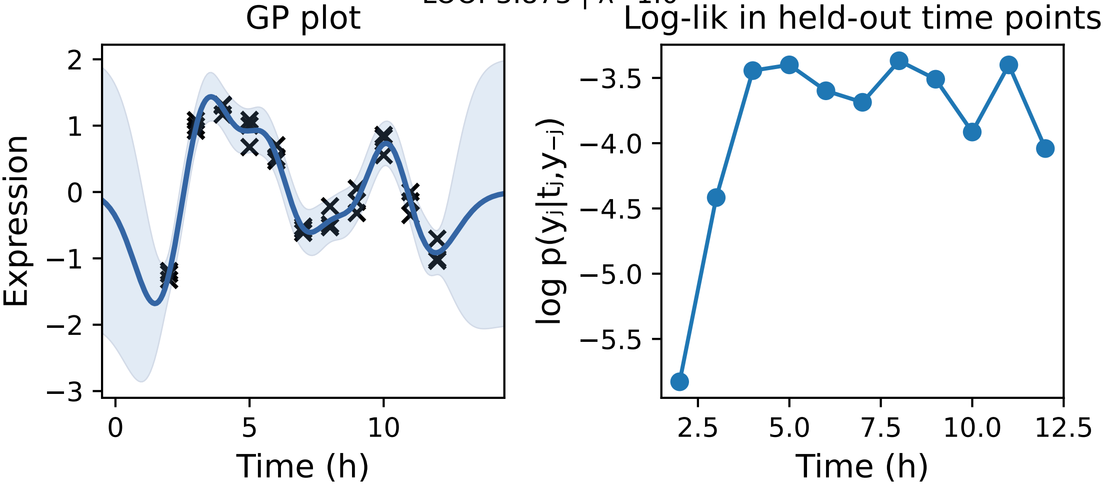

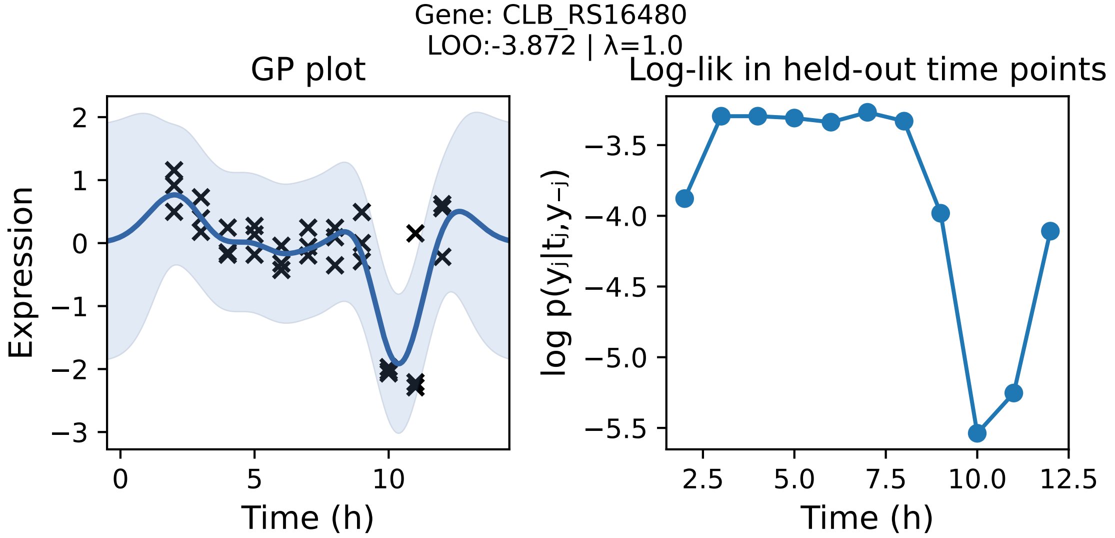
Gene: CLB_RS16480
LOO:-3.872 | λ=1.0
GP plot
Log-lik in held-out time points
Expression
Time (h)
log p(y_j|t_j,y_−j)
2
1
0
−1
−2
−3
0
5
10
−3.5
−4.0
−4.5
−5.0
−5.5
2.5
5.0
7.5
10.0
12.5

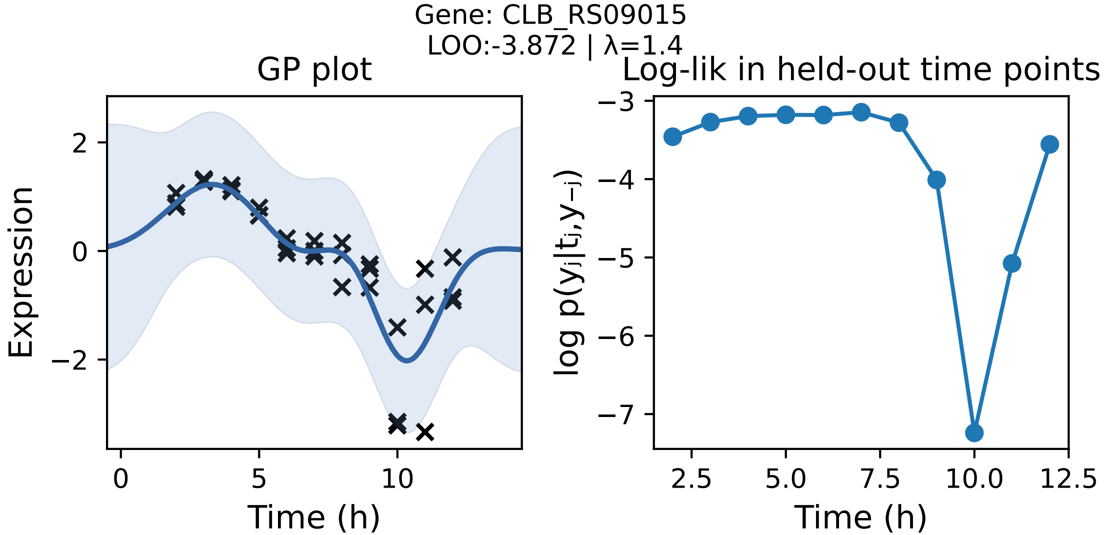
Gene: CLB_RS09015
LOO:-3.872 | λ=1.4
GP plot
Log-lik in held-out time points
Expression
Time (h)
log p(y_j|t_j,y_−j)
Time (h)
2
0
−2
0
5
10
−3
−4
−5
−6
−7
2.5
5.0
7.5
10.0
12.5

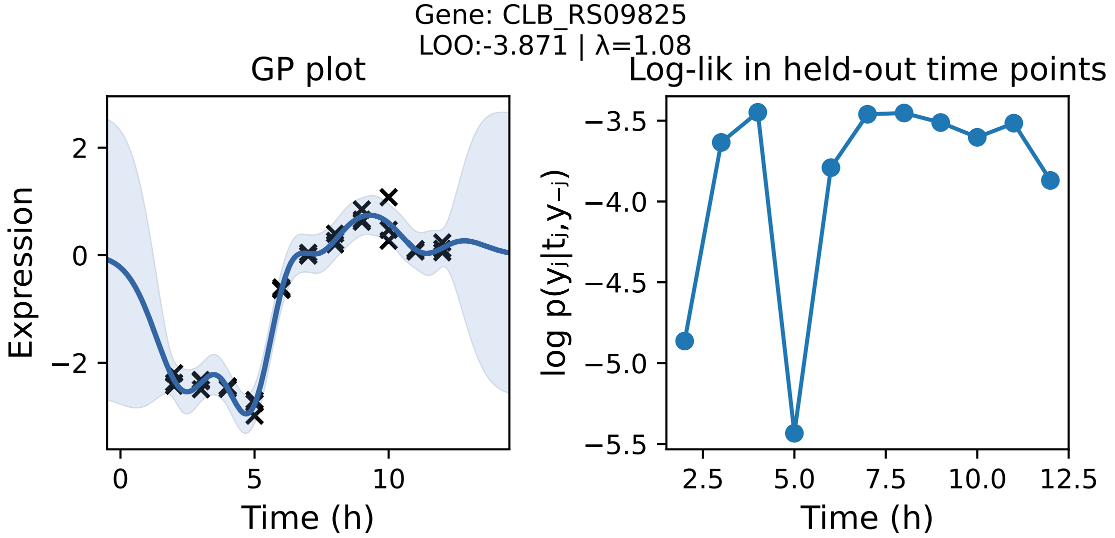

Gene: CLB_RS09825
LOO:-3.871 | λ=1.08
GP plot
Expression
Time (h)
2
0
−2
0
5
10
Log-lik in held-out time points
log p(y_j|t_j,y_−j)
Time (h)
−3.5
−4.0
−4.5
−5.0
−5.5
2.5
5.0
7.5
10.0
12.5

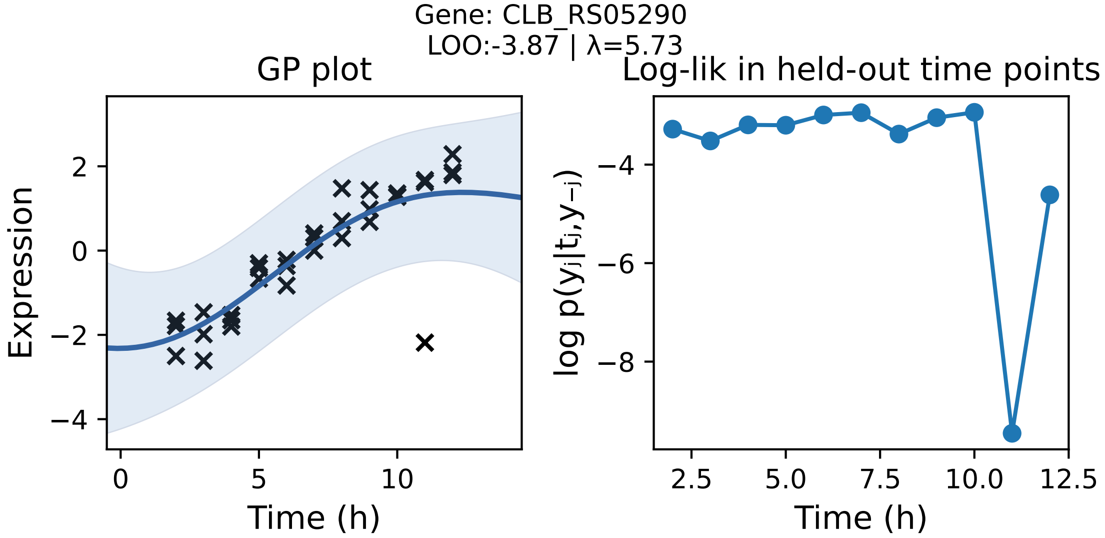

Gene: CLB_RS05290
LOO:-3.87 | λ=5.73
GP plot
Expression
Time (h)
Log-lik in held-out time points
log p(y_j|t_j,y_−j)
Time (h)

Gene: CLB_RS16825
LOO:-3.869 | λ=1.51

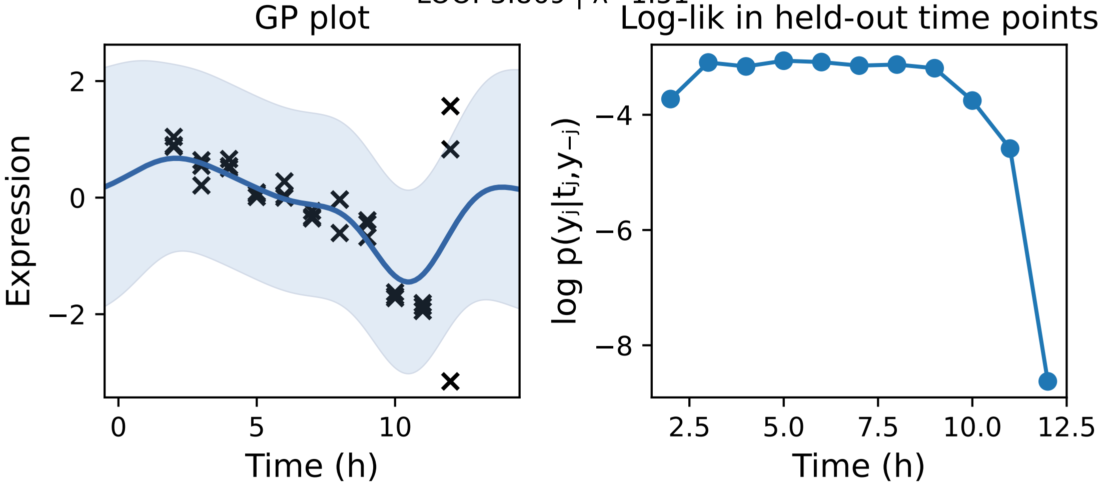

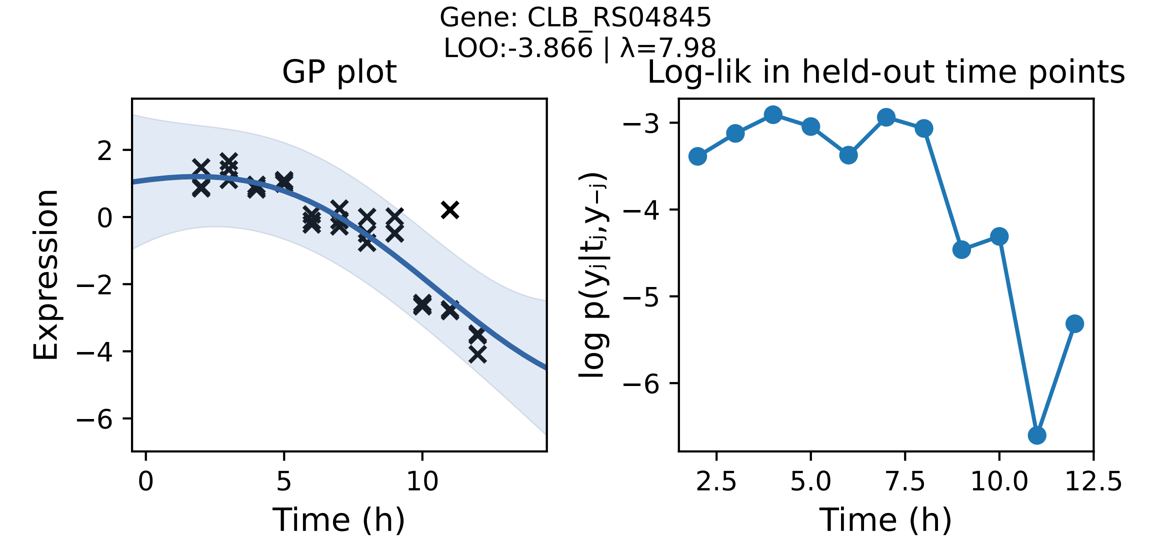
Gene: CLB_RS04845
LOO:-3.866 | λ=7.98
GP plot
Expression
Time (h)
Log-lik in held-out time points
log p(y_j|t_j,y_−j)
Time (h)

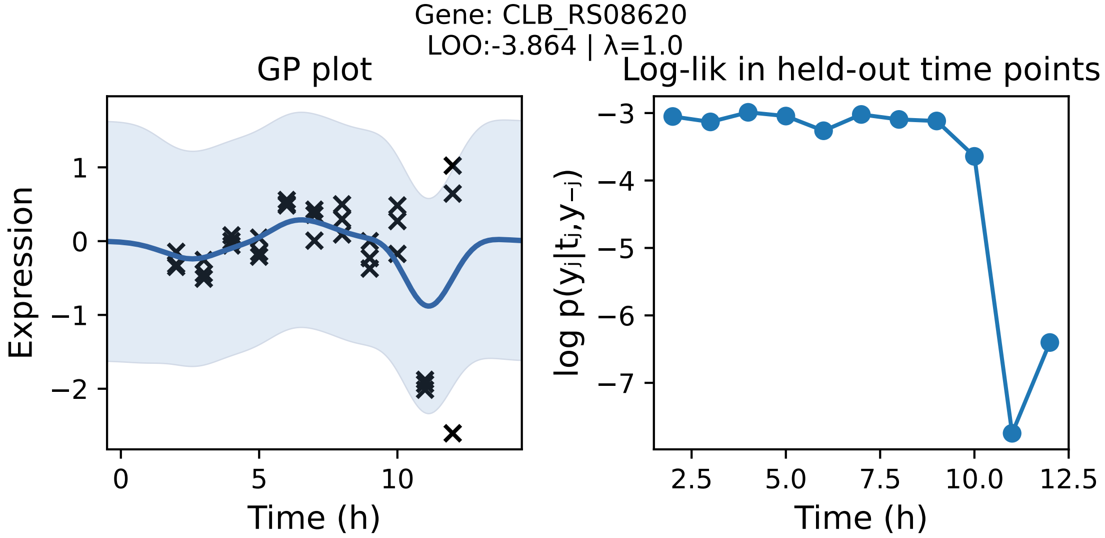

Gene: CLB_RS08620
LOO:-3.864 | λ=1.0
GP plot
Expression
Time (h)
Log-lik in held-out time points
log p(yj|tj,y−j)
Time (h)

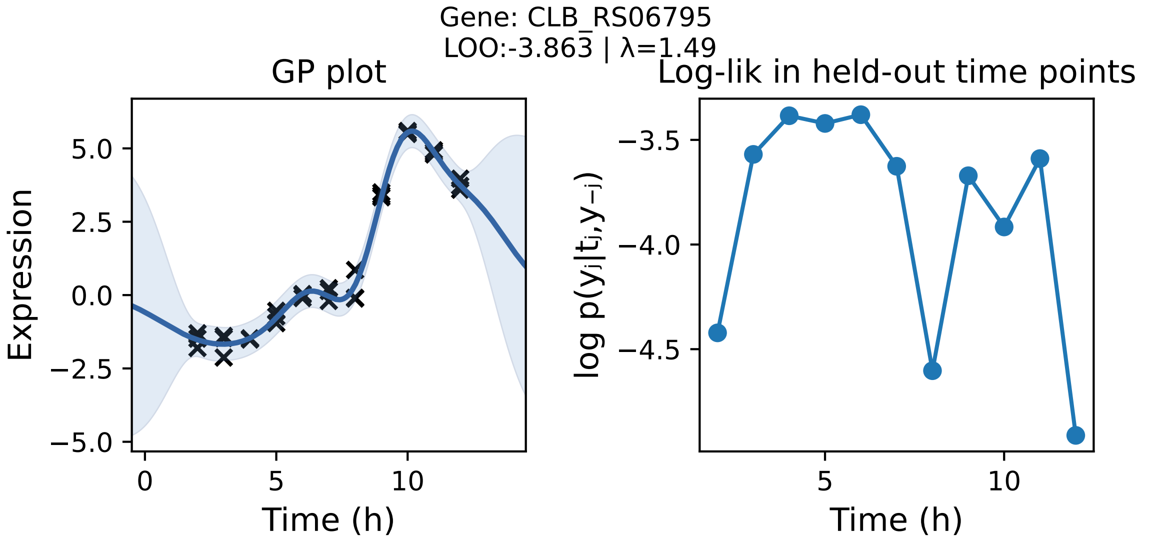

Gene: CLB_RS06795
LOO:-3.863 | λ=1.49
GP plot
Expression
Time (h)
Log-lik in held-out time points
log p(y_j|t_j,y_−j)
Time (h)

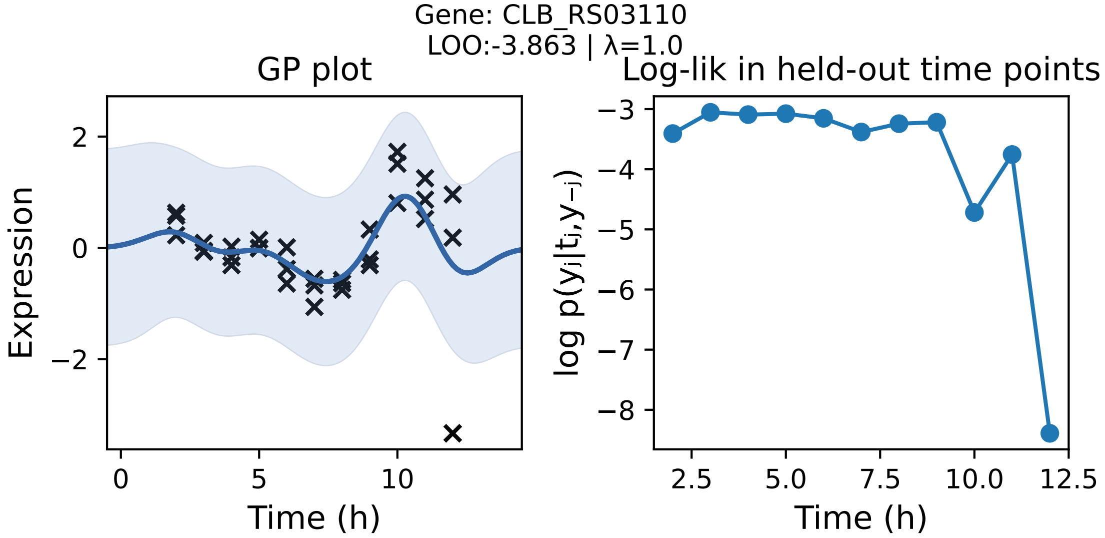

Gene: CLB_RS03110
LOO:-3.863 | λ=1.0
GP plot
Log-lik in held-out time points
Expression
Time (h)
log p(y_j|t_j,y_−j)
Time (h)

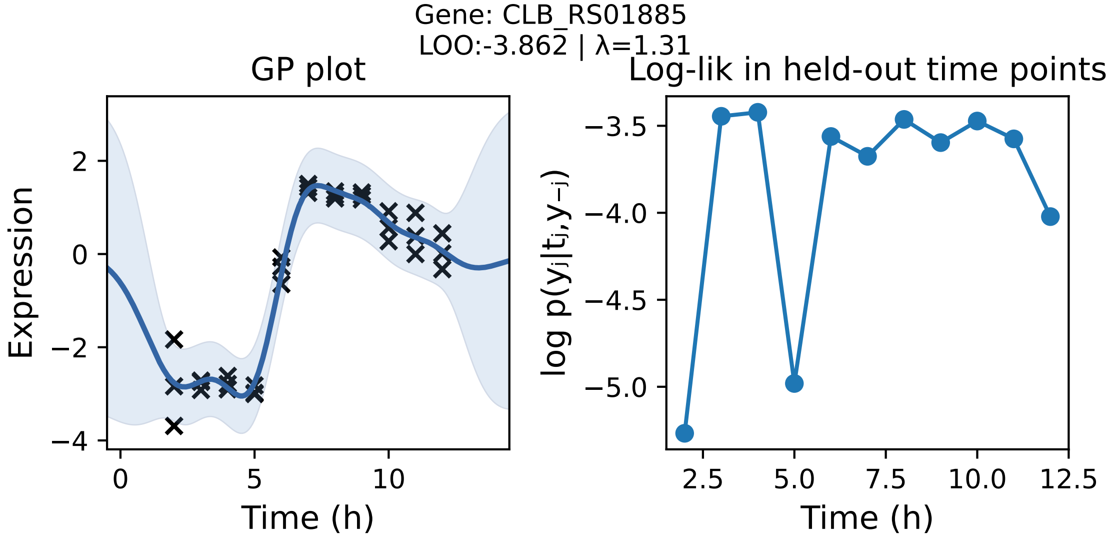

Gene: CLB_RS01885
LOO:-3.862 | λ=1.31
GP plot
Expression
Time (h)
Log-lik in held-out time points
log p(y_j|t_j,y_{-j})
Time (h)

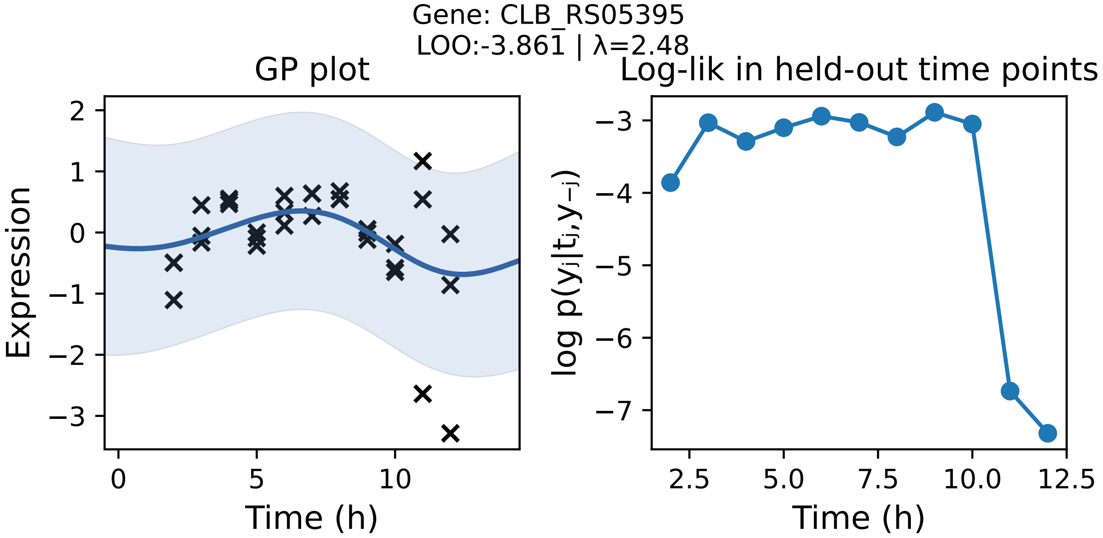
Gene: CLB_RS05395
LOO:-3.861 | λ=2.48
GP plot
Expression
Time (h)
Log-lik in held-out time points
log p(y_j|t_j,y_−j)
Time (h)

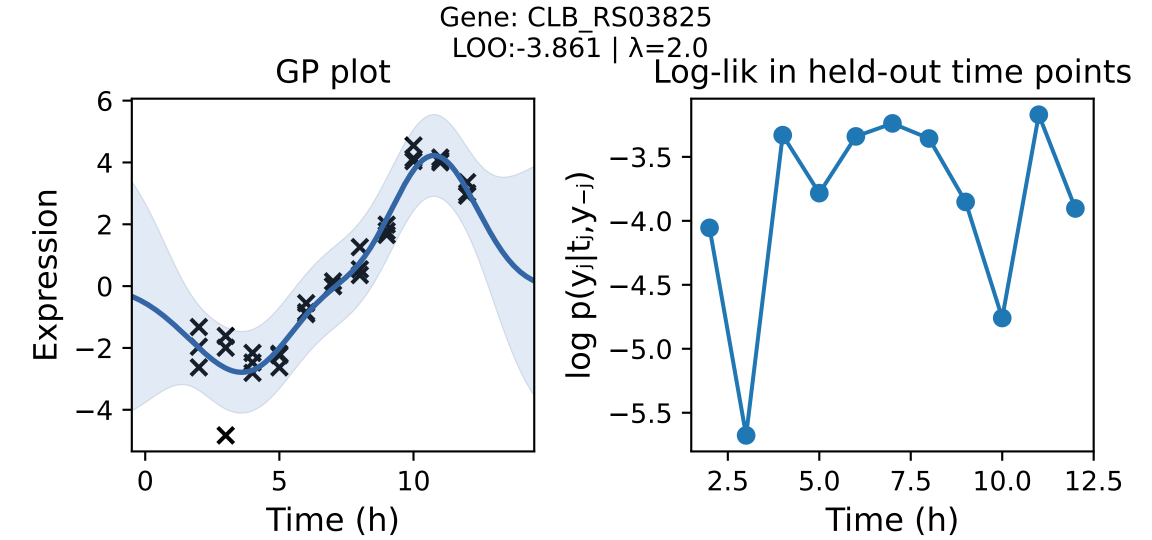

Gene: CLB_RS03825
LOO:-3.861 | λ=2.0
GP plot
Expression
Time (h)
6
4
2
0
−2
−4
0
5
10
Log-lik in held-out time points
log p(y_j|t_j,y_−j)
Time (h)
−3.5
−4.0
−4.5
−5.0
−5.5
2.5
5.0
7.5
10.0
12.5

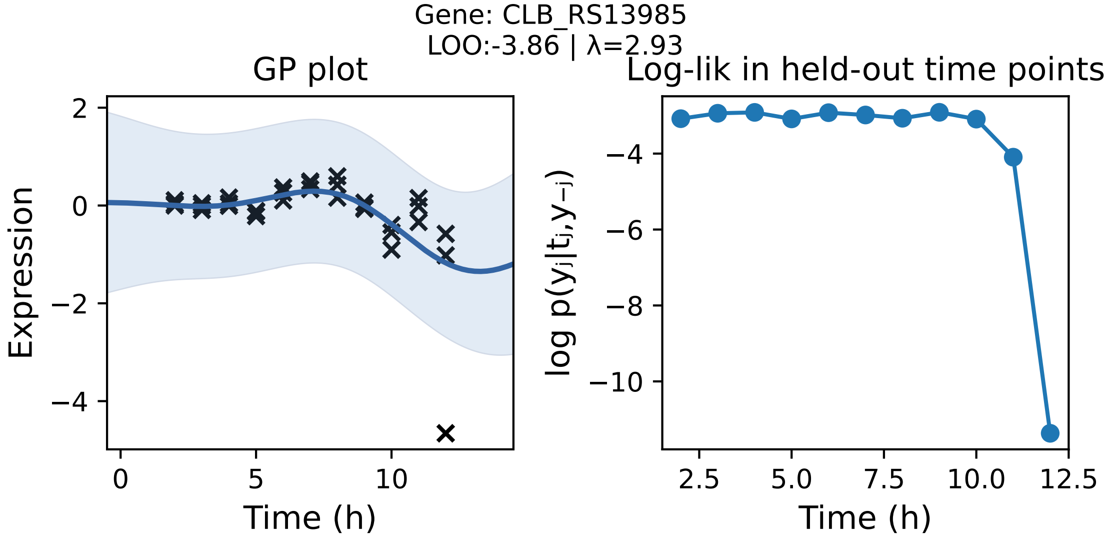

Gene: CLB_RS13985
LOO:-3.86 | λ=2.93
GP plot
Expression
Time (h)
2
0
−2
−4
0
5
10
Log-lik in held-out time points
log p(y_j|t_j,y_−j)
Time (h)
−4
−6
−8
−10
2.5
5.0
7.5
10.0
12.5

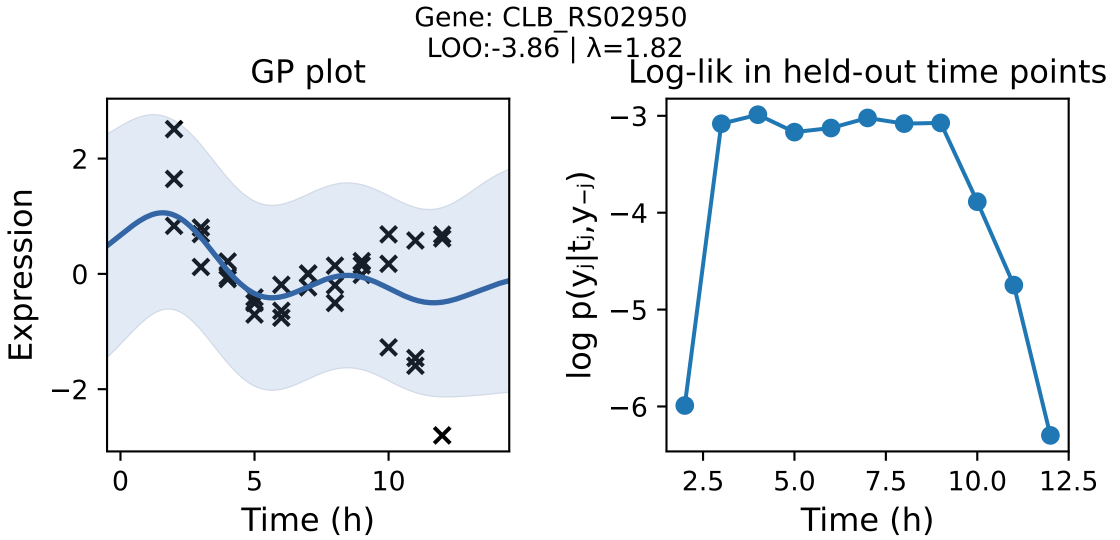

Gene: CLB_RS02950
LOO:-3.86 | λ=1.82
GP plot
Expression
Time (h)
2
0
−2
0
5
10
Log-lik in held-out time points
log p(y_j|t_j,y_−j)
Time (h)
−3
−4
−5
−6
2.5
5.0
7.5
10.0
12.5

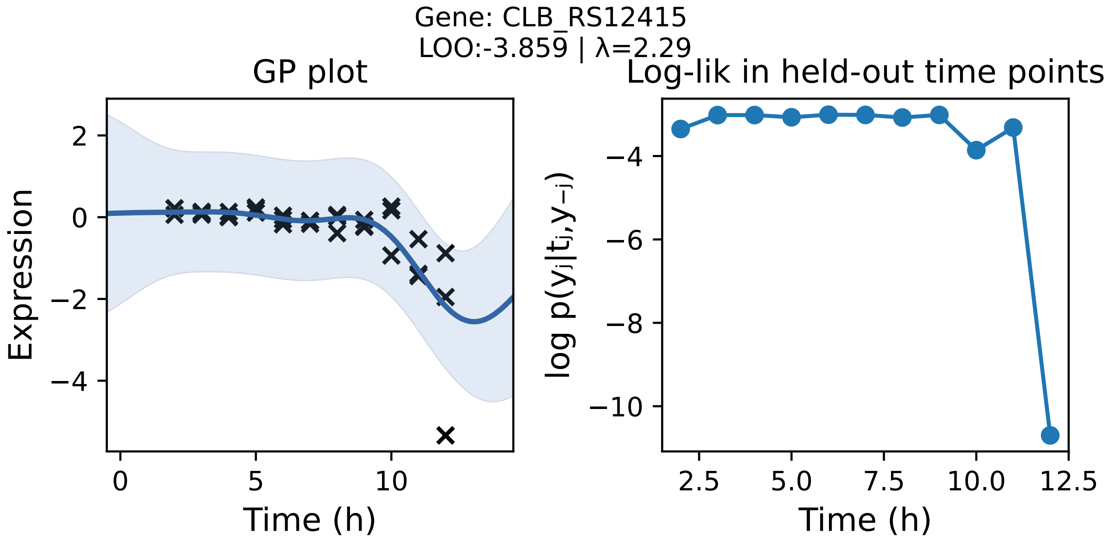

Gene: CLB_RS12415
LOO:-3.859 | λ=2.29
GP plot
Expression
Time (h)
2
0
−2
−4
0
5
10
Log-lik in held-out time points
log p(y_j|t_j,y_−j)
Time (h)
−4
−6
−8
−10
2.5
5.0
7.5
10.0
12.5

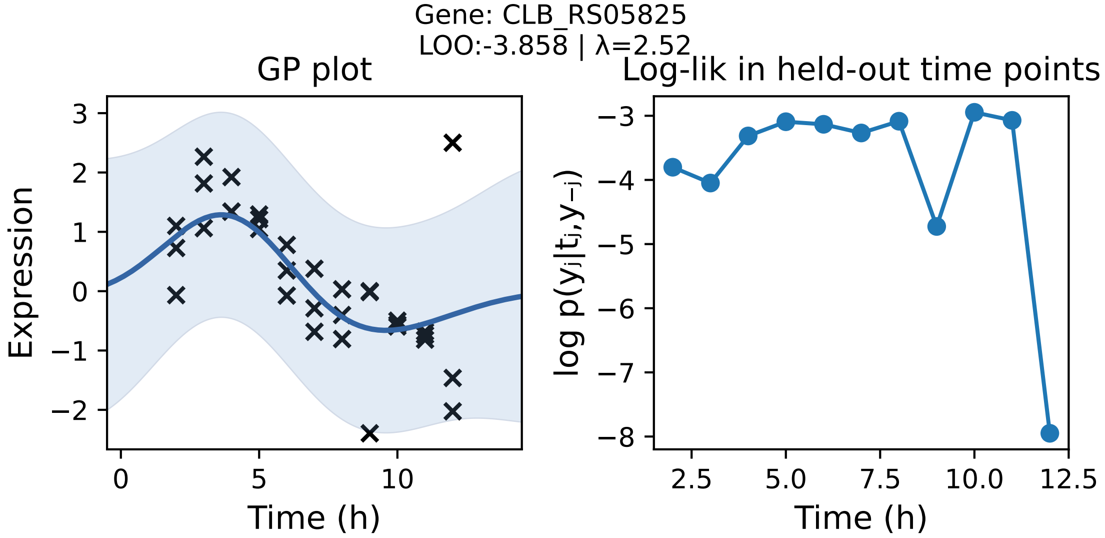

Gene: CLB_RS05825
LOO:-3.858 | λ=2.52
GP plot
Expression
Time (h)
Log-lik in held-out time points
log p(yj|tj,y−j)
Time (h)

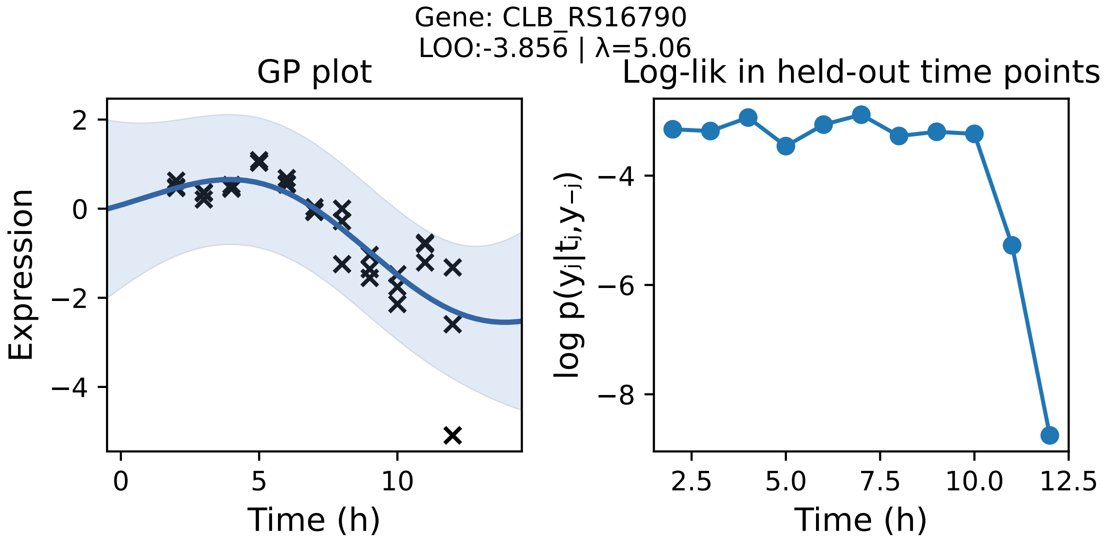
Gene: CLB_RS16790
LOO:-3.856 | λ=5.06
GP plot
Expression
Time (h)
Log-lik in held-out time points
log p(y_j|t_j,y_-j)
Time (h)
2
0
−2
−4
0
5
10
−4
−6
−8
2.5
5.0
7.5
10.0
12.5

Gene: CLB_RS03170
LOO:-3.856 | λ=3.17

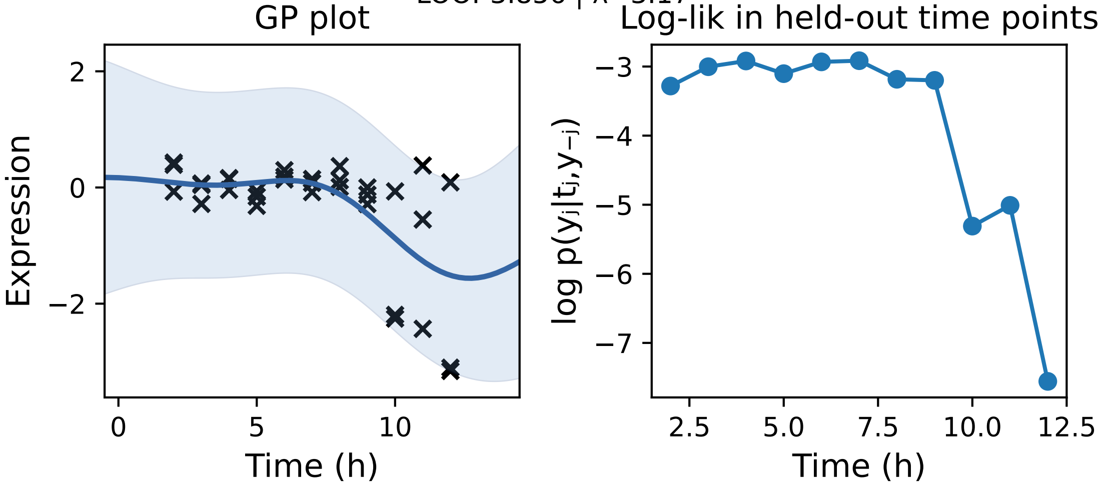

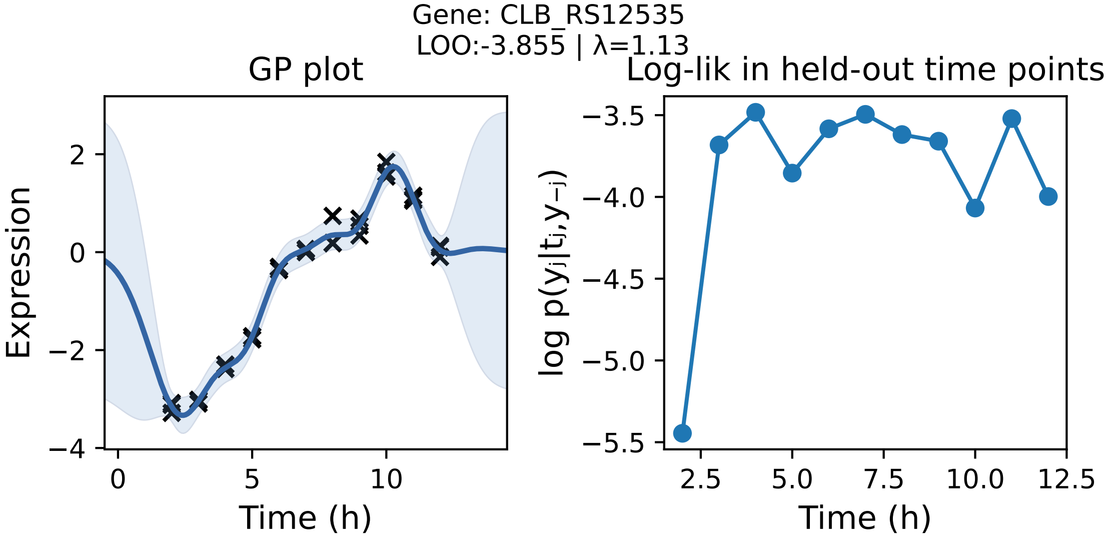

Gene: CLB_RS12535
LOO:-3.855 | λ=1.13
GP plot
Expression
Time (h)
2
0
−2
−4
0
5
10
Log-lik in held-out time points
log p(y_j|t_j,y_−j)
Time (h)
−3.5
−4.0
−4.5
−5.0
−5.5
2.5
5.0
7.5
10.0
12.5

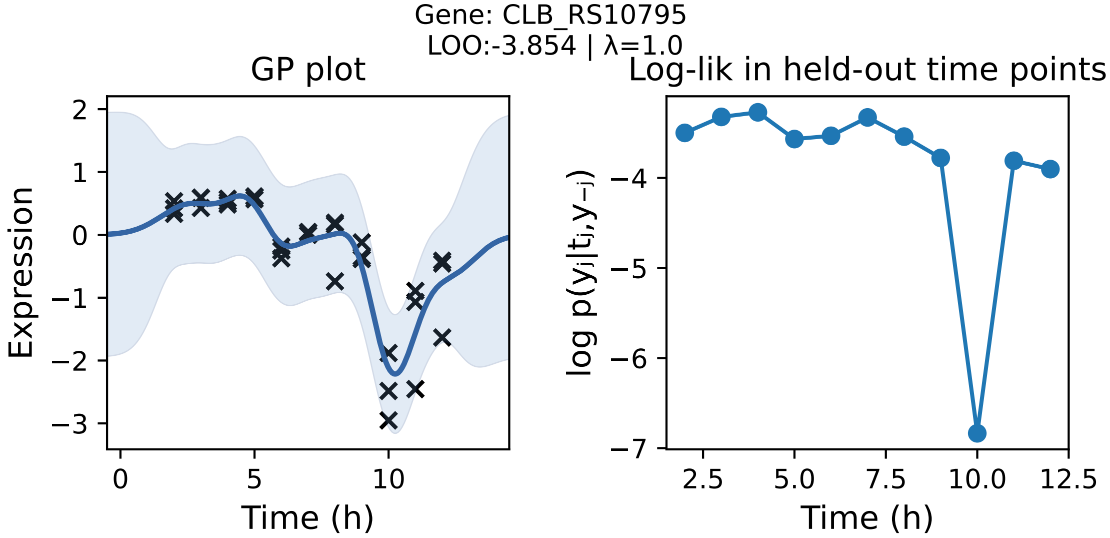
Gene: CLB_RS10795
LOO:-3.854 | λ=1.0
GP plot
Expression
2
1
0
−1
−2
−3
0
5
10
Time (h)
Log-lik in held-out time points
log p(y_j|t_j,y_−j)
−4
−5
−6
−7
2.5
5.0
7.5
10.0
12.5
Time (h)

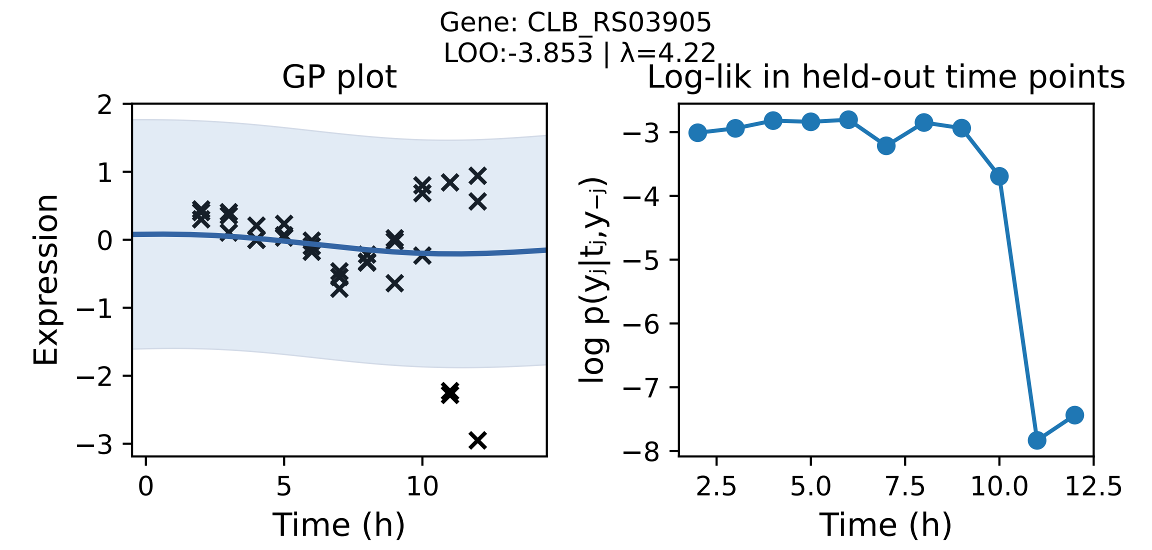

Gene: CLB_RS03905
LOO:-3.853 | λ=4.22
GP plot
Log-lik in held-out time points
Expression
Time (h)
log p(y_j|t_j,y_−j)
2
1
0
−1
−2
−3
0
5
10
−3
−4
−5
−6
−7
−8
2.5
5.0
7.5
10.0
12.5

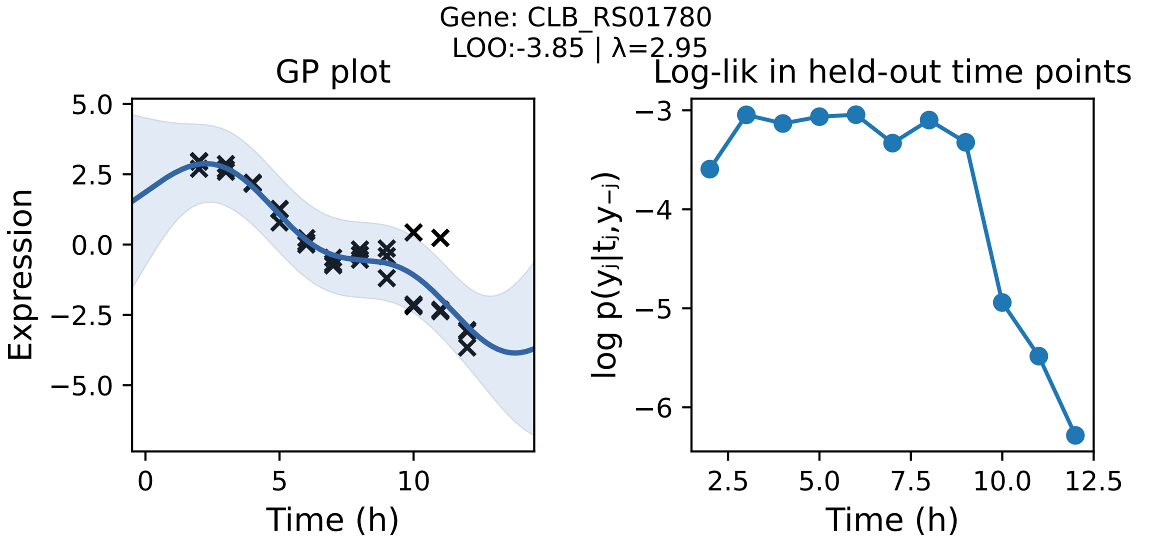
Gene: CLB_RS01780
LOO:-3.85 | λ=2.95
GP plot
Expression
Time (h)
5.0
2.5
0.0
−2.5
−5.0
0
5
10
Log-lik in held-out time points
log p(y_j|t_j,y_−j)
Time (h)
−3
−4
−5
−6
2.5
5.0
7.5
10.0
12.5

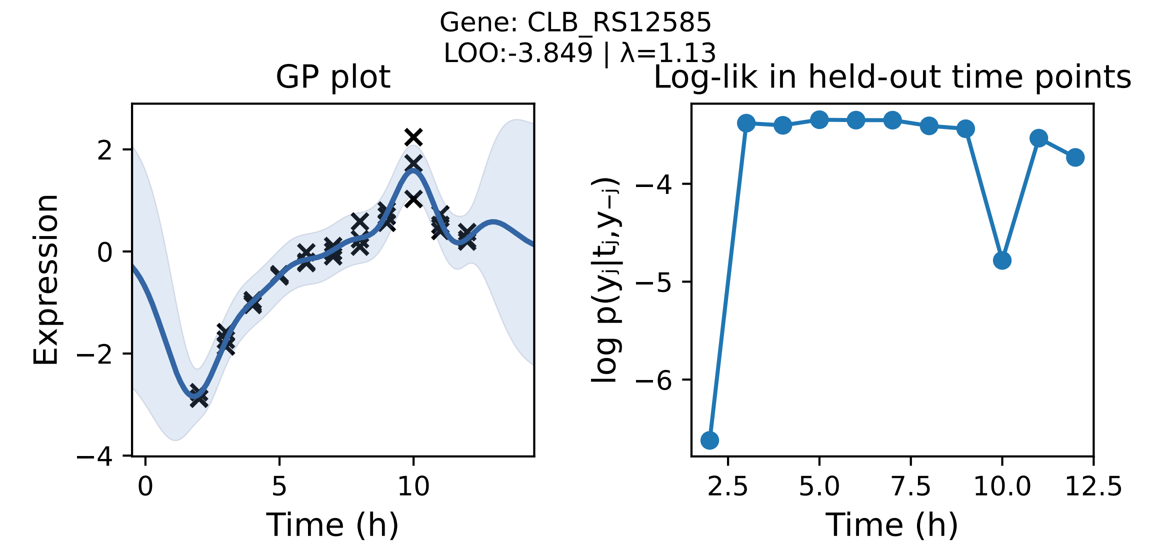

Gene: CLB_RS12585
LOO:-3.849 | λ=1.13
GP plot
Expression
Time (h)
Log-lik in held-out time points
log p(y_j|t_j,y_−j)
Time (h)

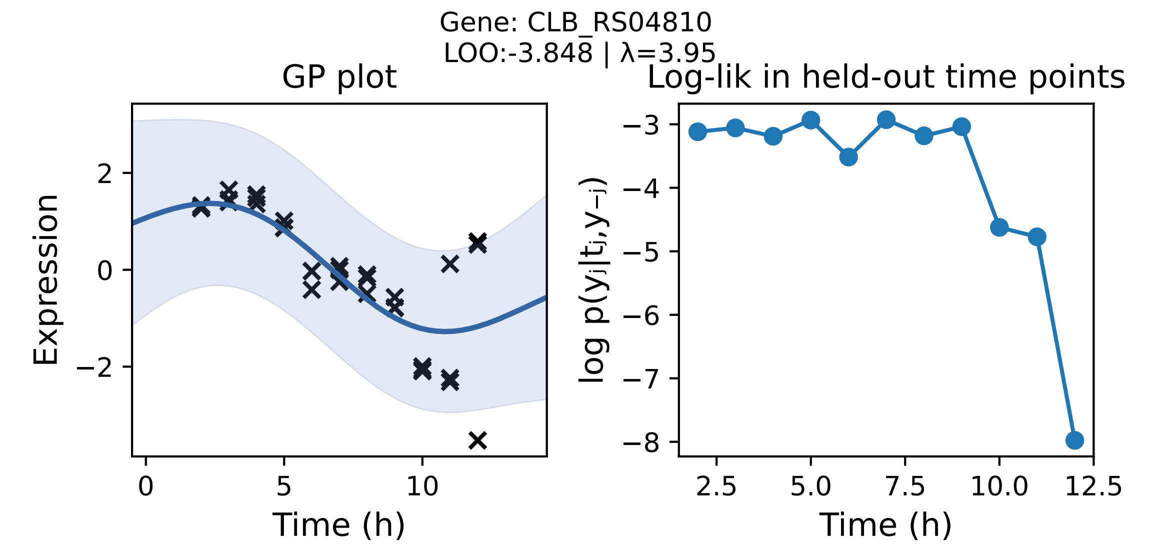

Gene: CLB_RS04810
LOO:-3.848 | λ=3.95
GP plot
Expression
Time (h)
Log-lik in held-out time points
log p(y_j|t_j,y_−j)
Time (h)

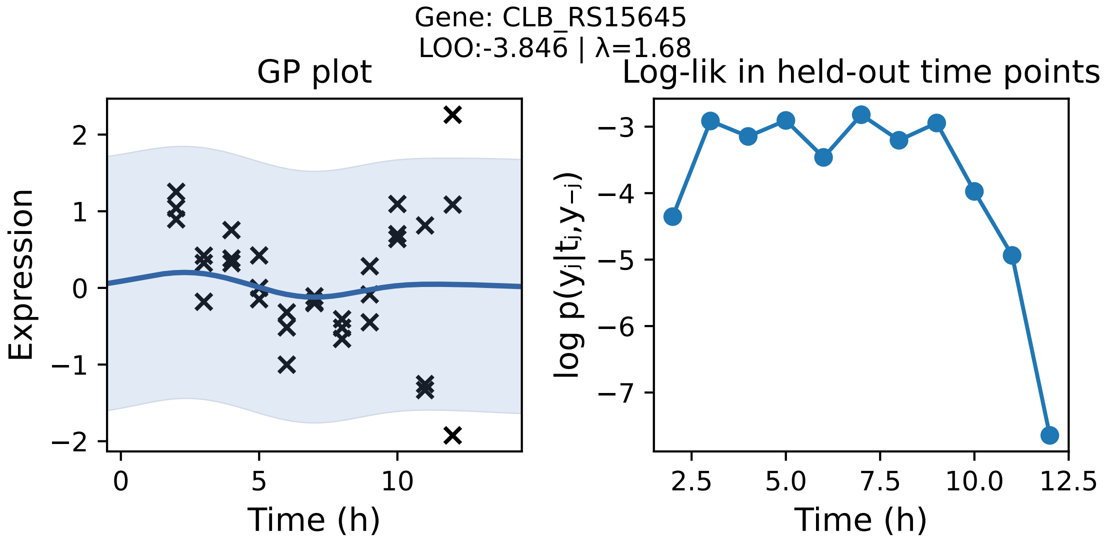

Gene: CLB_RS15645
LOO:-3.846 | λ=1.68
GP plot
Expression
Time (h)
Log-lik in held-out time points
$\log p(y_j|t_j, y_{-j})$
Time (h)

Gene: CLB_RS03025
LOO:-3.846 | λ=2.04

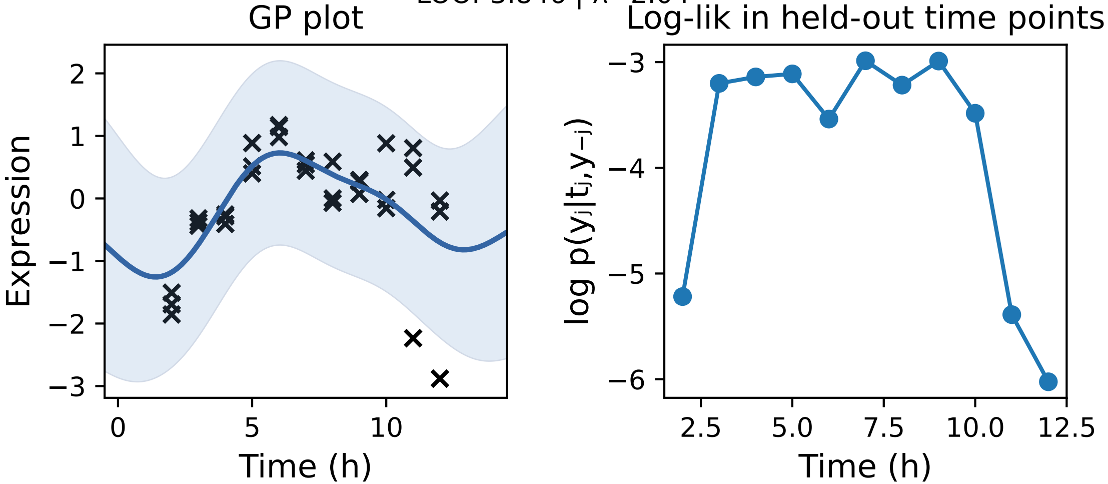

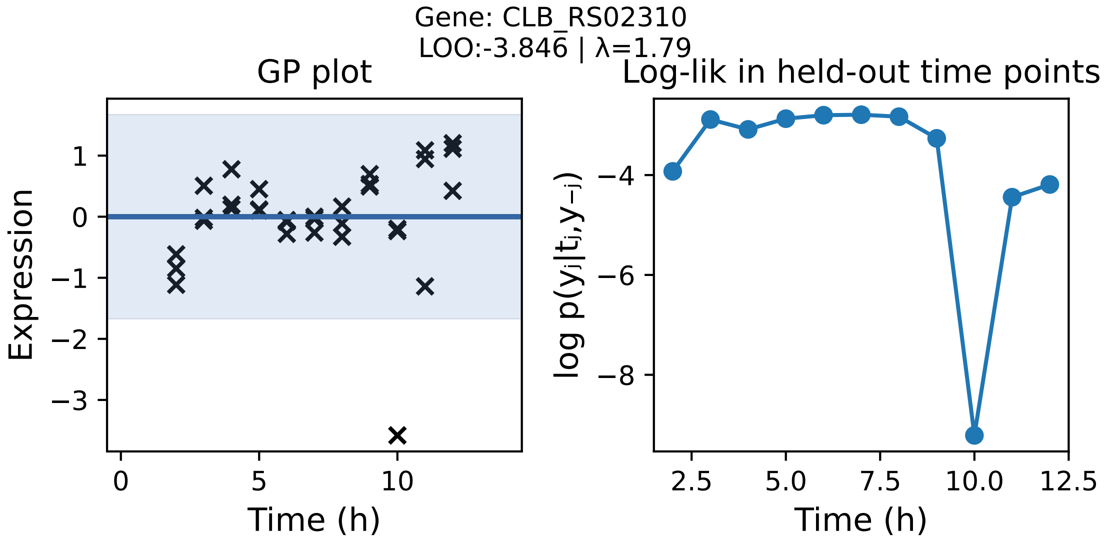

Gene: CLB_RS02310
LOO:-3.846 | λ=1.79
GP plot
Log-lik in held-out time points
Expression
Time (h)
log p(yj|tj,y−j)
1
0
−1
−2
−3
0
5
10
−4
−6
−8
2.5
5.0
7.5
10.0
12.5

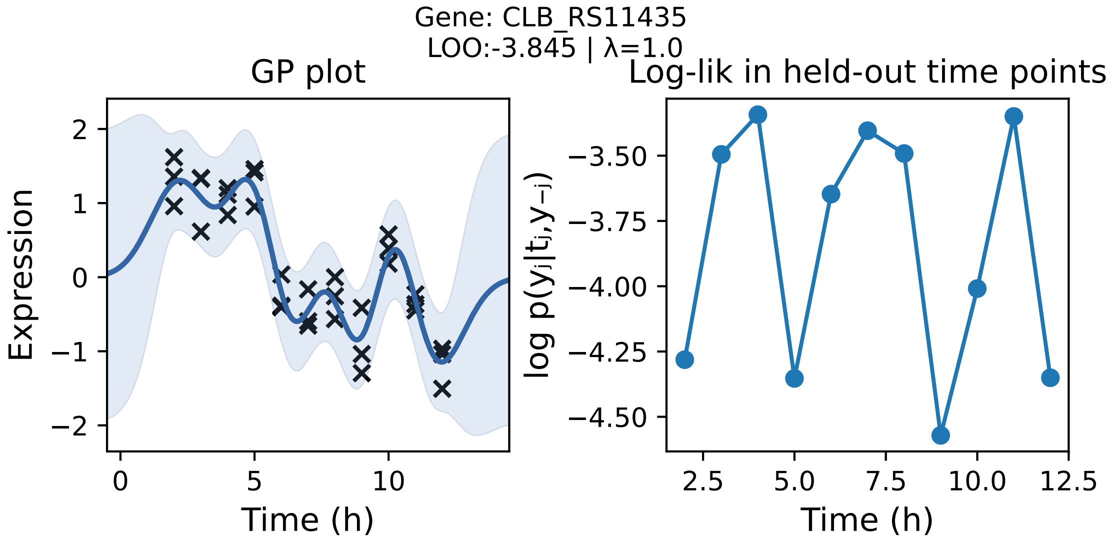

Gene: CLB_RS11435
LOO:-3.845 | λ=1.0
GP plot
Expression
Time (h)
Log-lik in held-out time points
log p(y_j|t_j,y_−j)
Time (h)

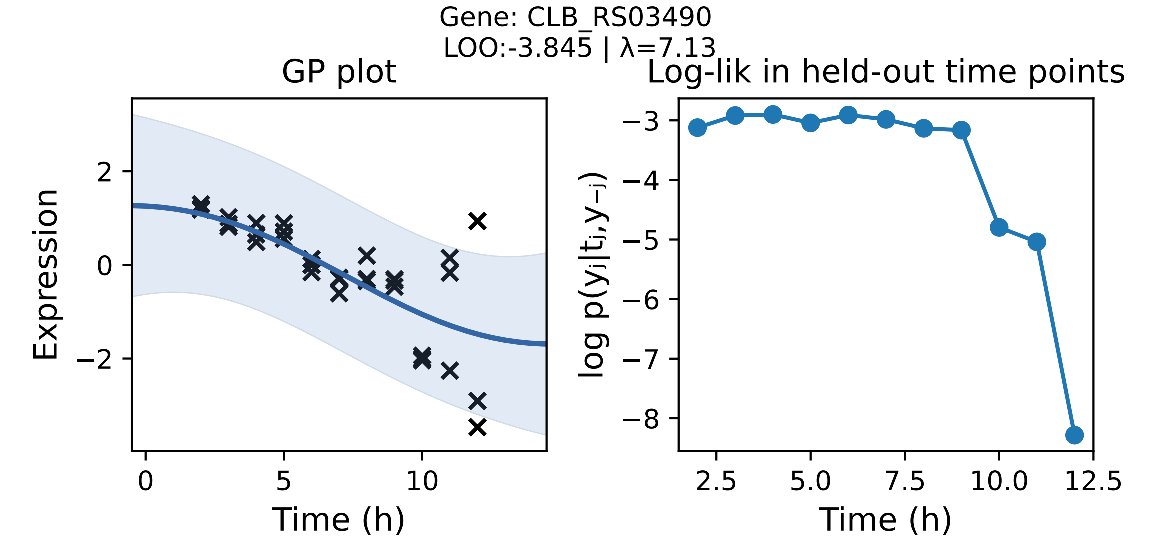
Gene: CLB_RS03490
LOO:-3.845 | λ=7.13
GP plot
Expression
Time (h)
Log-lik in held-out time points
log p(y_j|t_j,y_−j)
Time (h)

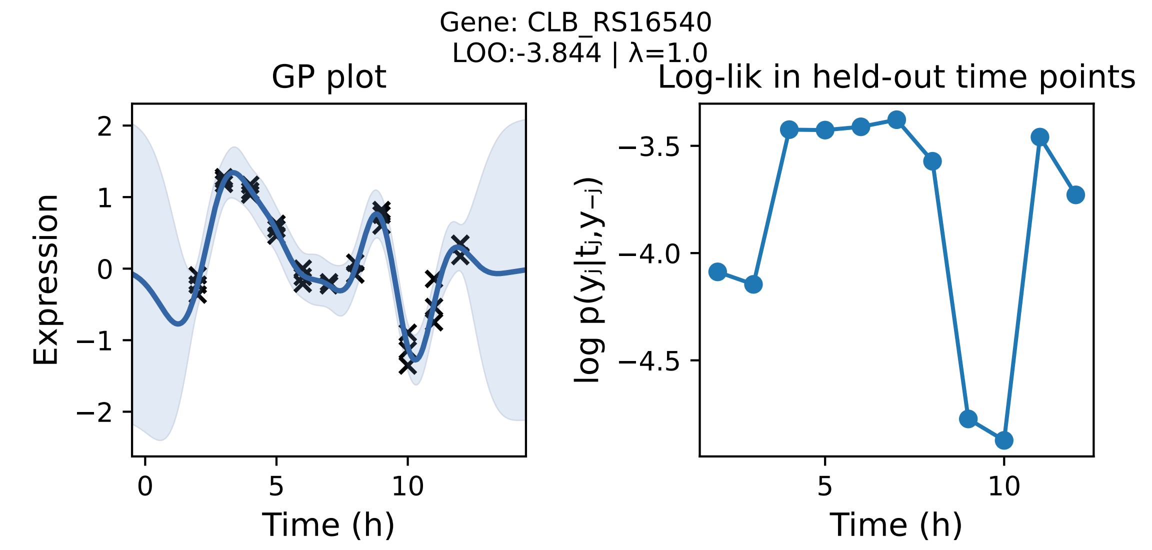

Gene: CLB_RS16540
LOO:-3.844 | λ=1.0
GP plot
Expression
Time (h)
Log-lik in held-out time points
log p(yj|tj,y−j)
Time (h)

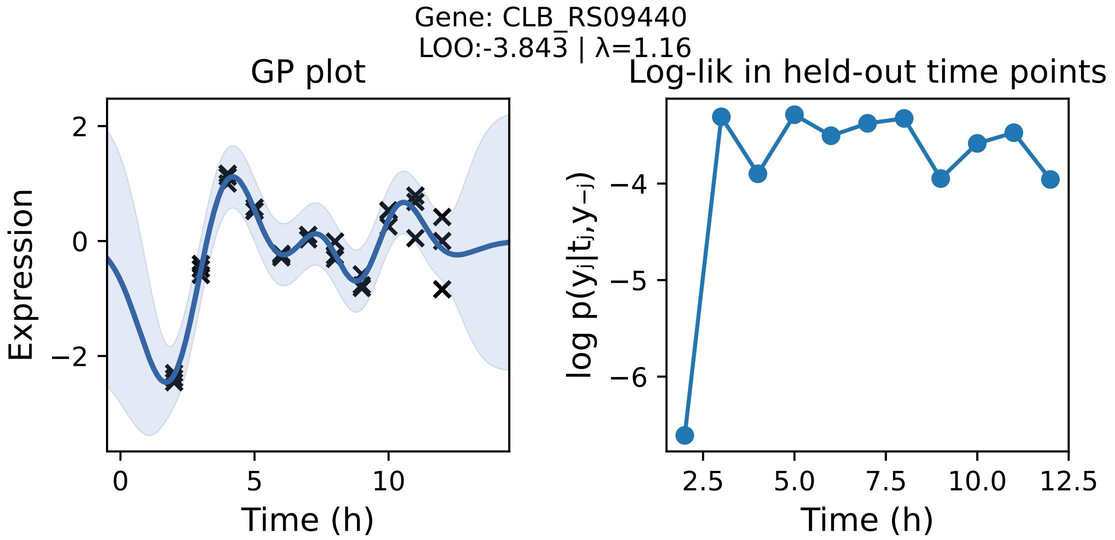

Gene: CLB_RS09440
LOO:-3.843 | λ=1.16
GP plot
Expression
Time (h)
2
0
−2
0
5
10
Log-lik in held-out time points
log p(y_j|t_j,y_−j)
−4
−5
−6
2.5
5.0
7.5
10.0
12.5
Time (h)

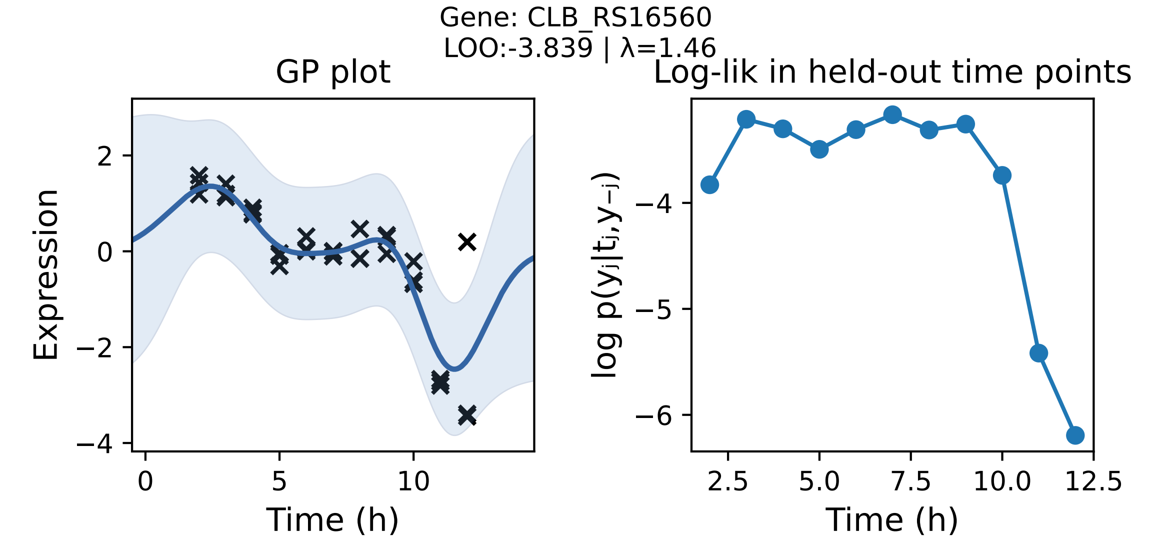

Gene: CLB_RS16560
LOO:-3.839 | λ=1.46
GP plot
Expression
Time (h)
2
0
−2
−4
0
5
10
Log-lik in held-out time points
log p(yj|tj,y−j)
Time (h)
−4
−5
−6
2.5
5.0
7.5
10.0
12.5

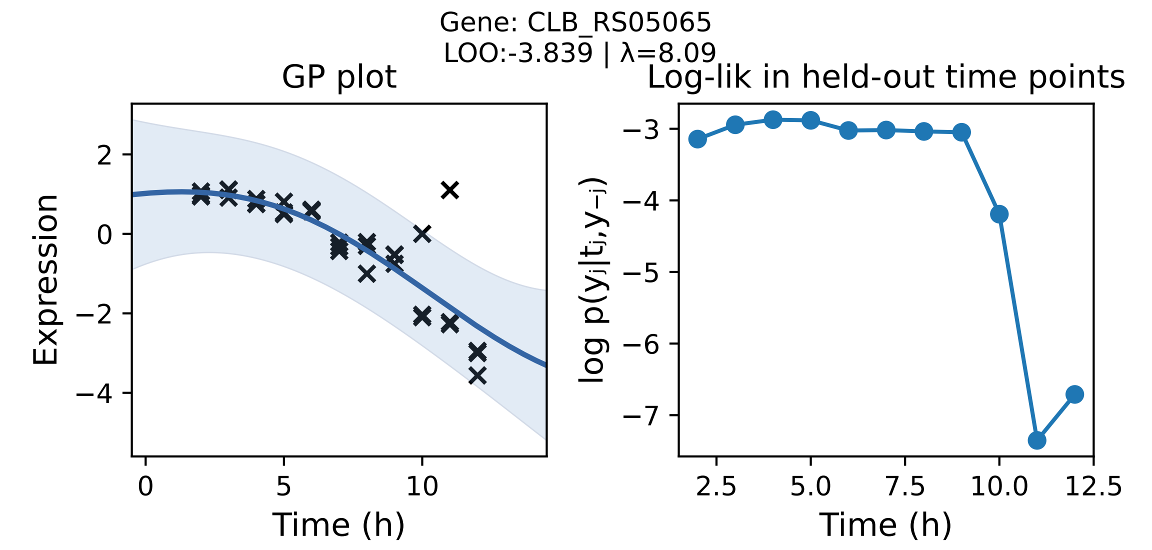
Gene: CLB_RS05065
LOO:-3.839 | λ=8.09
GP plot
Log-lik in held-out time points
Expression
Time (h)
log p(y_j|t_j,y_-j)
Time (h)
2
0
−2
−4
0
5
10
−3
−4
−5
−6
−7
2.5
5.0
7.5
10.0
12.5

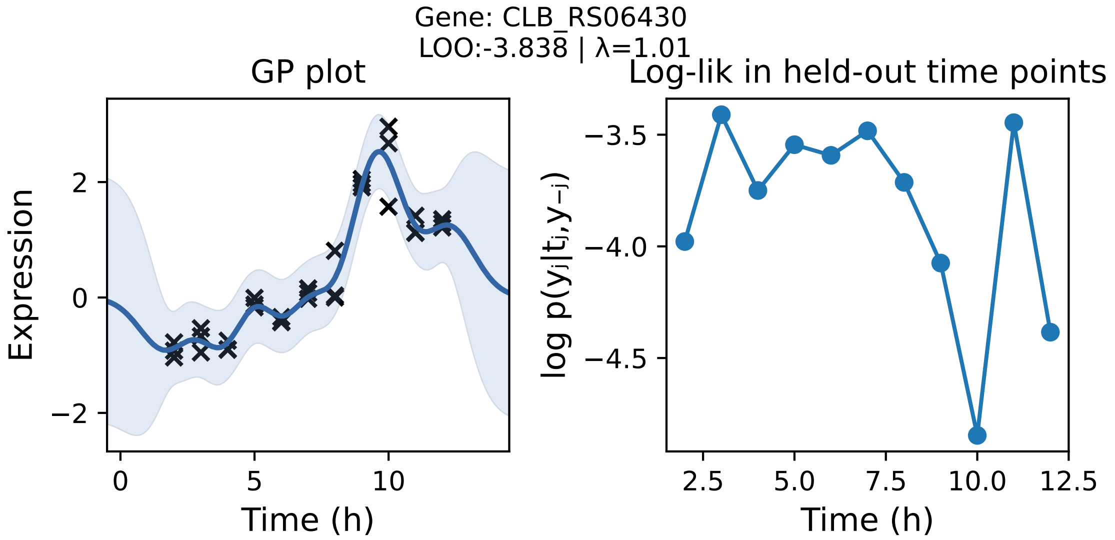

Gene: CLB_RS06430
LOO:-3.838 | λ=1.01
GP plot
Expression
Time (h)
2
0
−2
0
5
10
Log-lik in held-out time points
log p(yj|tj,y−j)
Time (h)
−3.5
−4.0
−4.5
2.5
5.0
7.5
10.0
12.5

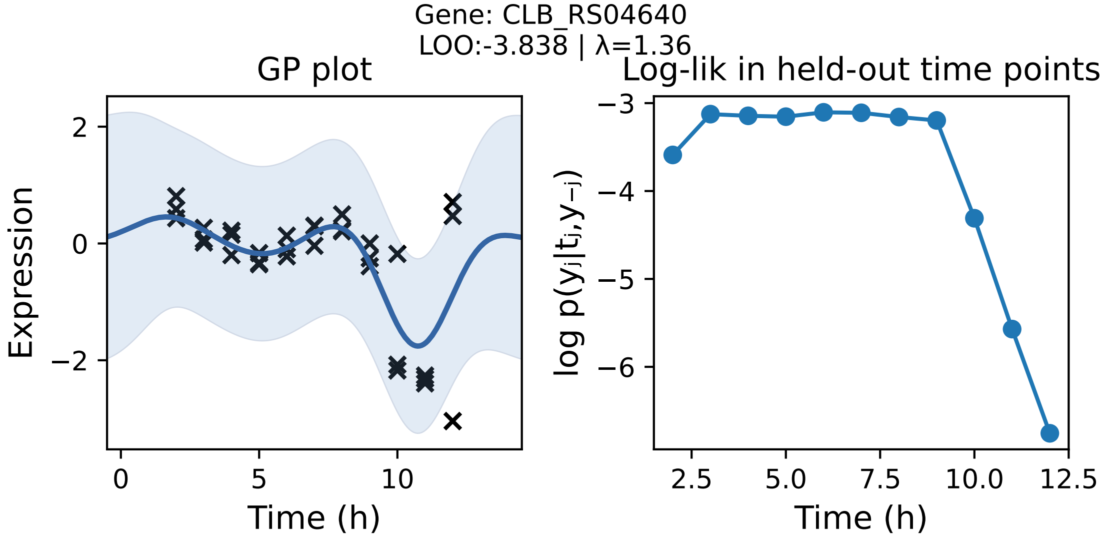

Gene: CLB_RS04640
LOO:-3.838 | λ=1.36
GP plot
Log-lik in held-out time points
Expression
Time (h)
log p(y_j|t_j,y_−j)
2
0
−2
0
5
10
−3
−4
−5
−6
2.5
5.0
7.5
10.0
12.5

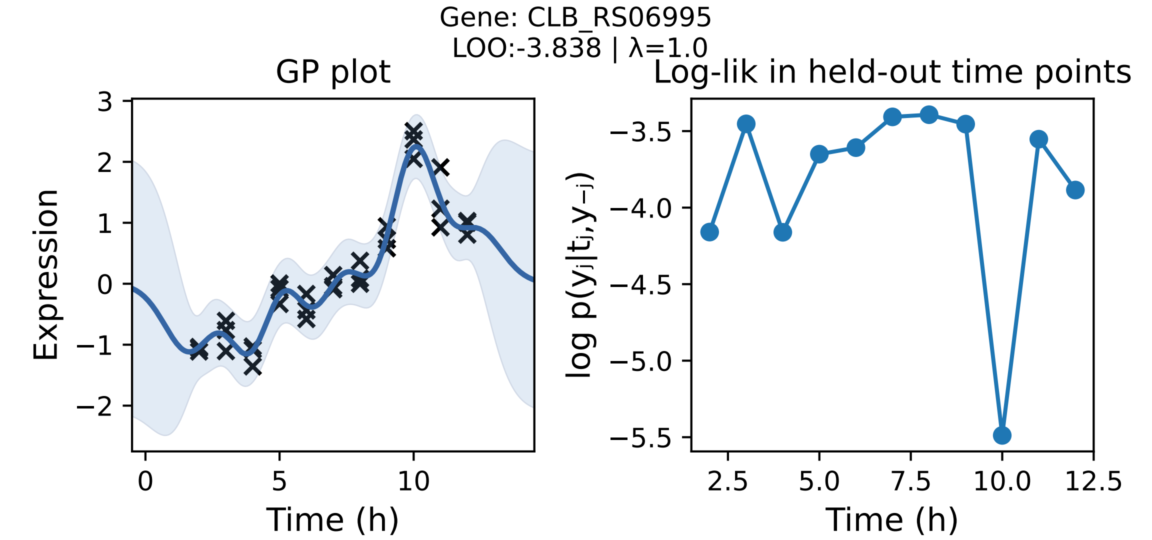
Gene: CLB_RS06995
LOO:-3.838 | λ=1.0
GP plot
Expression
Time (h)
Log-lik in held-out time points
log p(y_j|t_j,y_−j)
Time (h)

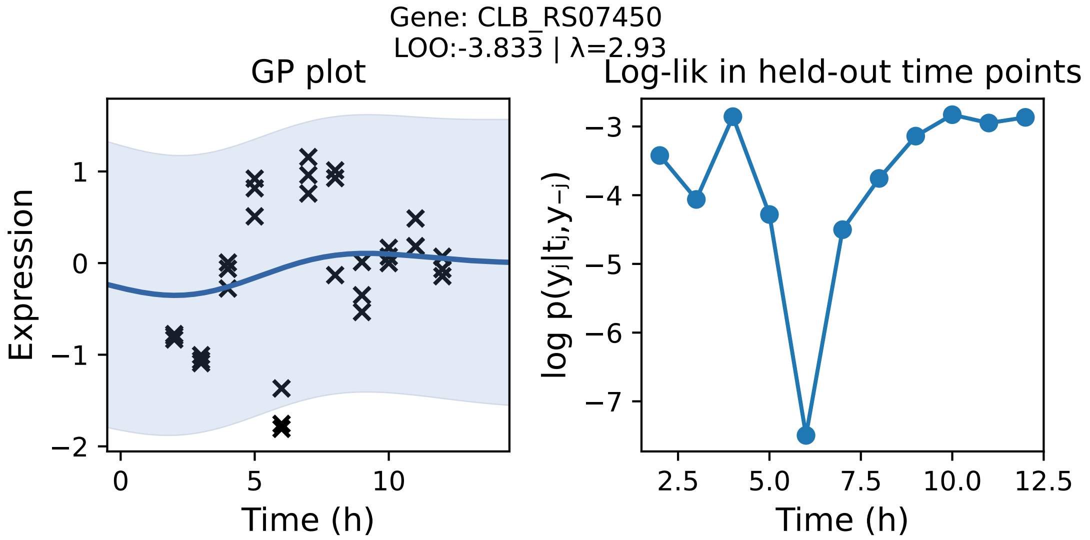
Gene: CLB_RS07450
LOO:-3.833 | λ=2.93
GP plot
Expression
Time (h)
0
5
10
1
0
−1
−2
Log-lik in held-out time points
log p(y_j|t_j,y_−j)
Time (h)
2.5
5.0
7.5
10.0
12.5
−3
−4
−5
−6
−7

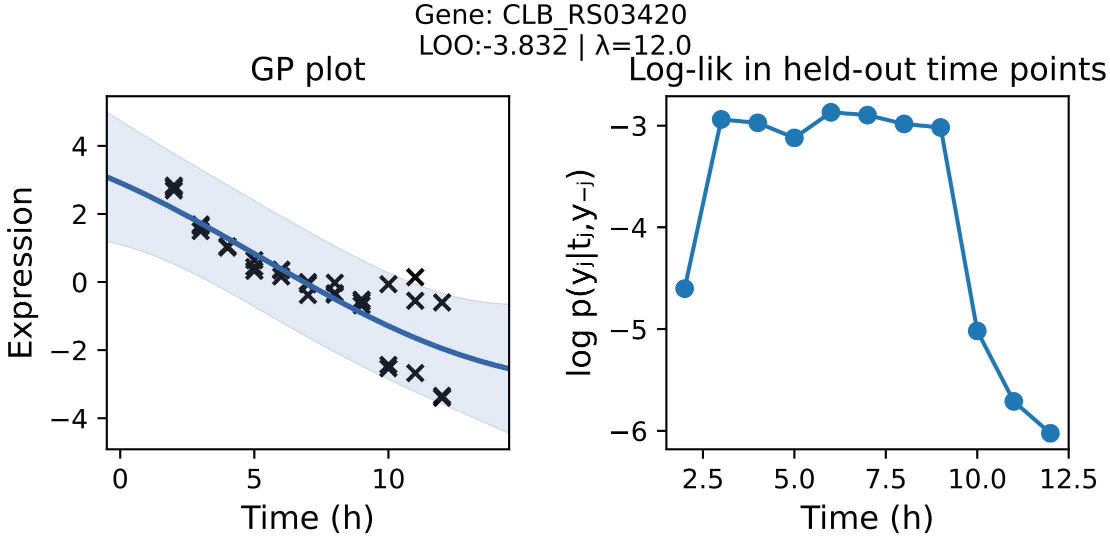
Gene: CLB_RS03420
LOO:-3.832 | λ=12.0
GP plot
Expression
Time (h)
Log-lik in held-out time points
log p(y_j|t_j,y_−j)
Time (h)

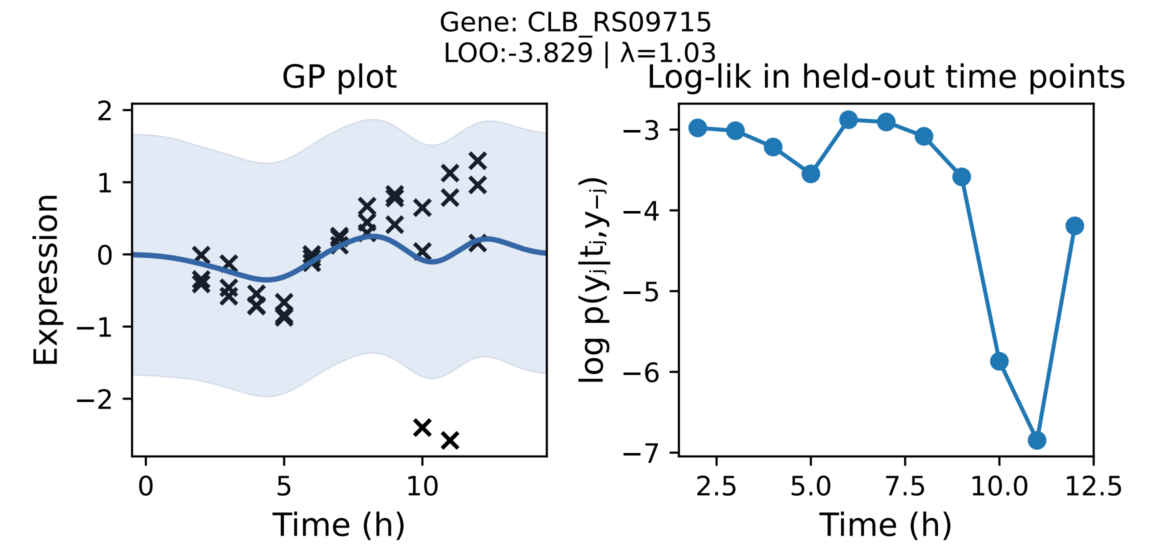
Gene: CLB_RS09715
LOO:-3.829 | λ=1.03
GP plot
Log-lik in held-out time points
Expression
Time (h)
log p(yj|tj,y−j)
Time (h)
2
1
0
−1
−2
0
5
10
−3
−4
−5
−6
−7
2.5
5.0
7.5
10.0
12.5

Gene: CLB_RS05255
LOO:-3.828 | λ=1.35

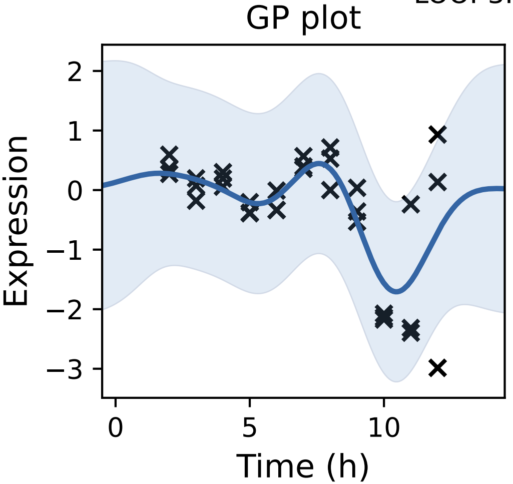

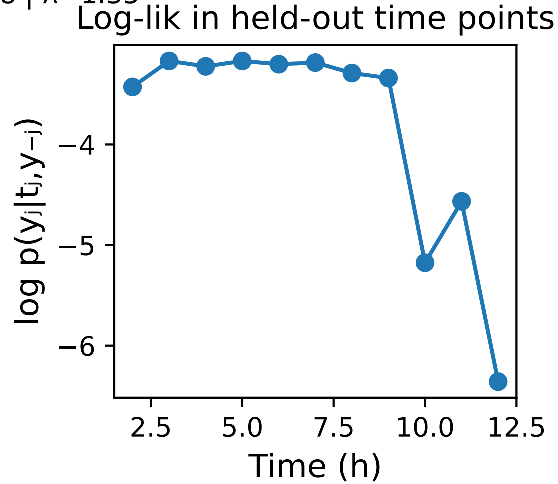

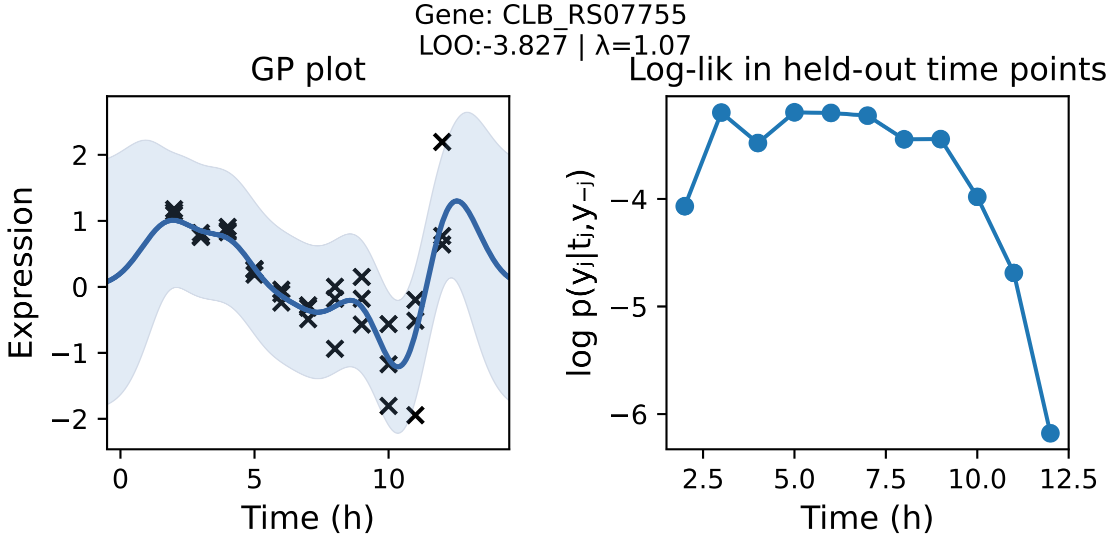
Gene: CLB_RS07755
LOO:-3.827 | λ=1.07
GP plot
Expression
Time (h)
Log-lik in held-out time points
log p(yⱼ|tⱼ,y₋ⱼ)
Time (h)
2
1
0
−1
−2
0
5
10
−4
−5
−6
2.5
5.0
7.5
10.0
12.5

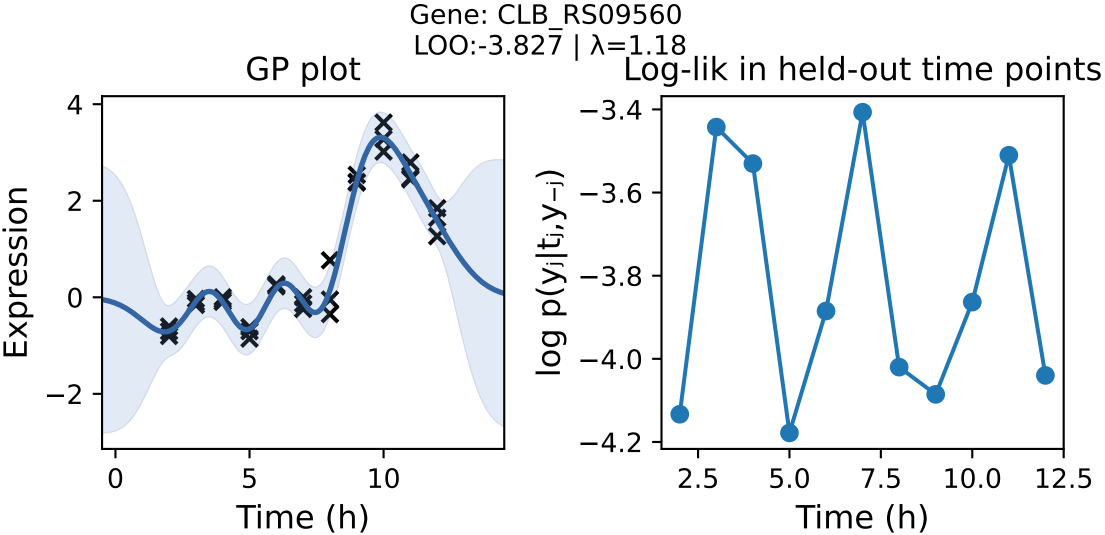

Gene: CLB_RS09560
LOO:-3.827 | λ=1.18
GP plot
Expression
Time (h)
Log-lik in held-out time points
log p(y_j|t_j,y_−j)
Time (h)

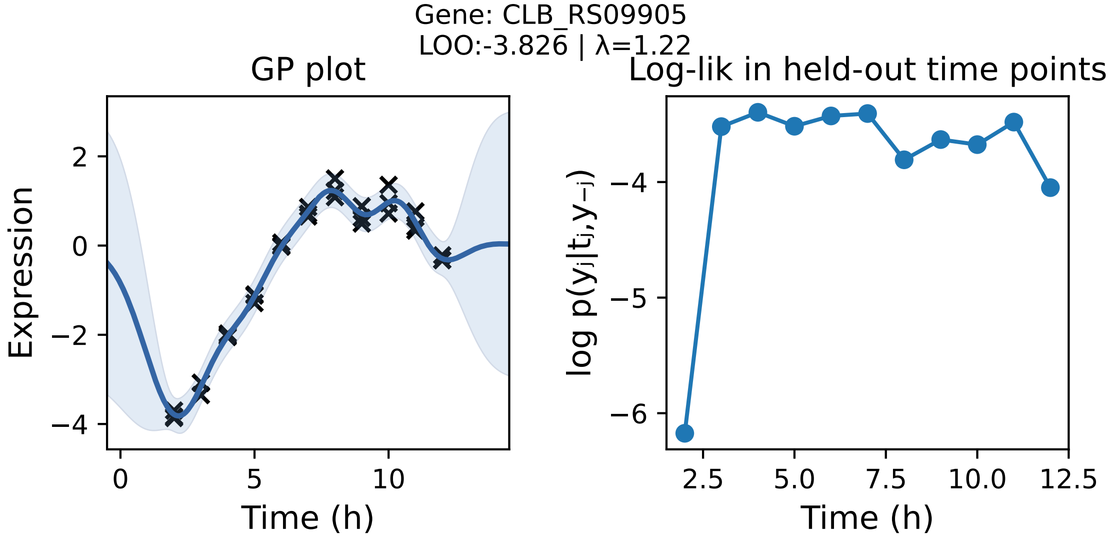
Gene: CLB_RS09905
LOO:-3.826 | λ=1.22
GP plot
Expression
Time (h)
Log-lik in held-out time points
log p(yj|tj,y−j)
Time (h)

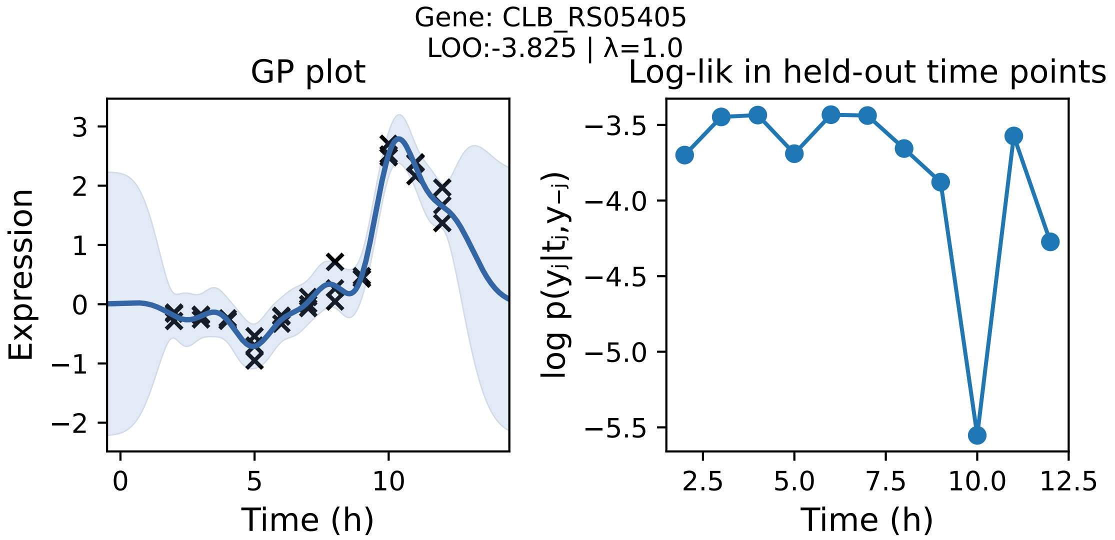

Gene: CLB_RS05405
LOO:-3.825 | λ=1.0
GP plot
Log-lik in held-out time points
Expression
Time (h)
log p(yj|tj,y−j)
Time (h)

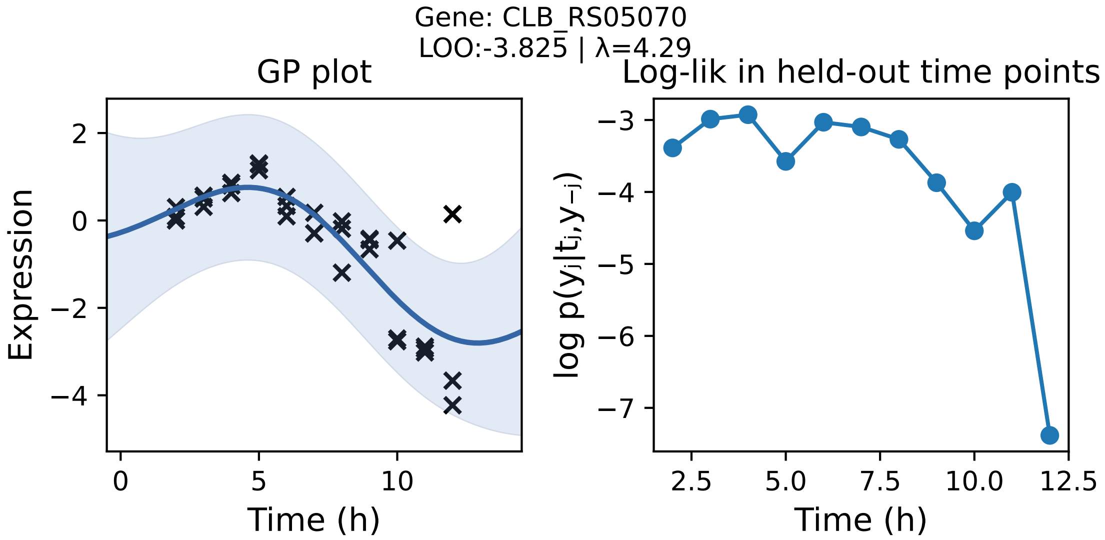

Gene: CLB_RS05070
LOO:-3.825 | λ=4.29
GP plot
Expression
Time (h)
2
0
−2
−4
0
5
10
Log-lik in held-out time points
log p(y_j|t_j,y_−j)
Time (h)
−3
−4
−5
−6
−7
2.5
5.0
7.5
10.0
12.5

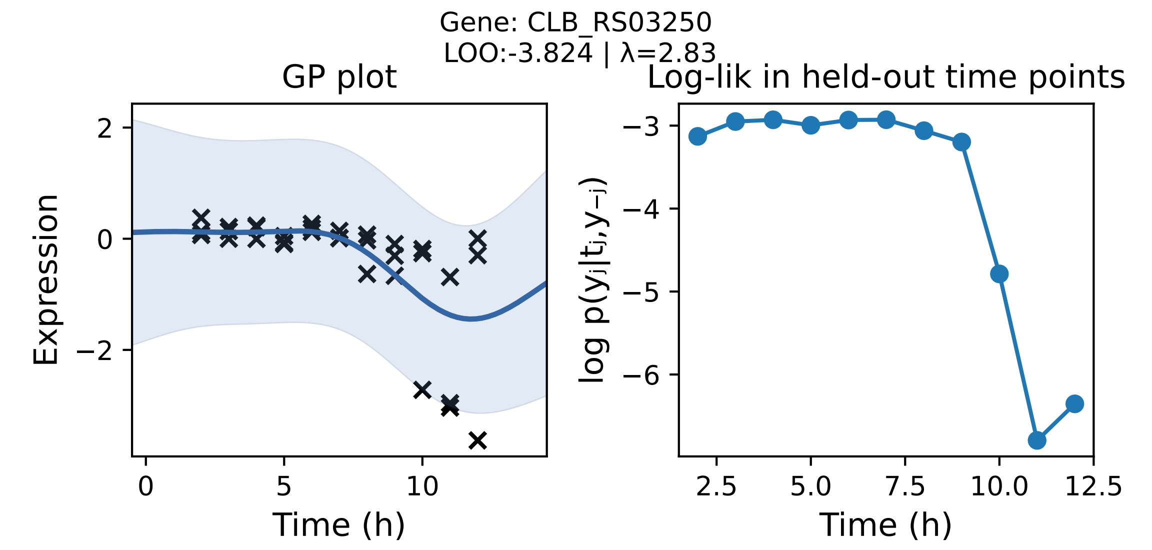

Gene: CLB_RS03250
LOO:-3.824 | λ=2.83
GP plot
Expression
Time (h)
2
0
−2
0
5
10
Log-lik in held-out time points
log p(yj|tj,y−j)
Time (h)
−3
−4
−5
−6
2.5
5.0
7.5
10.0
12.5

Gene: CLB_RS00630
LOO:-3.823 | λ=1.0

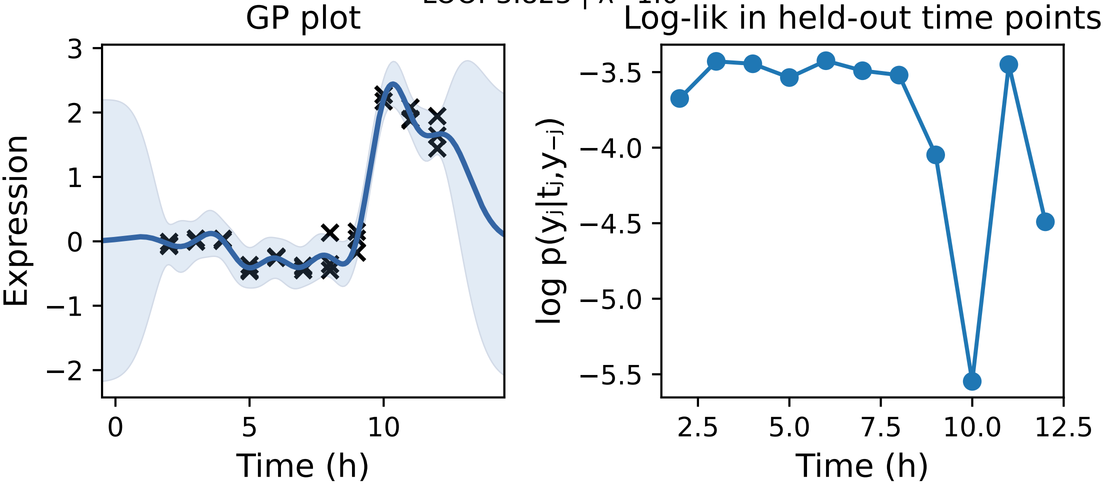

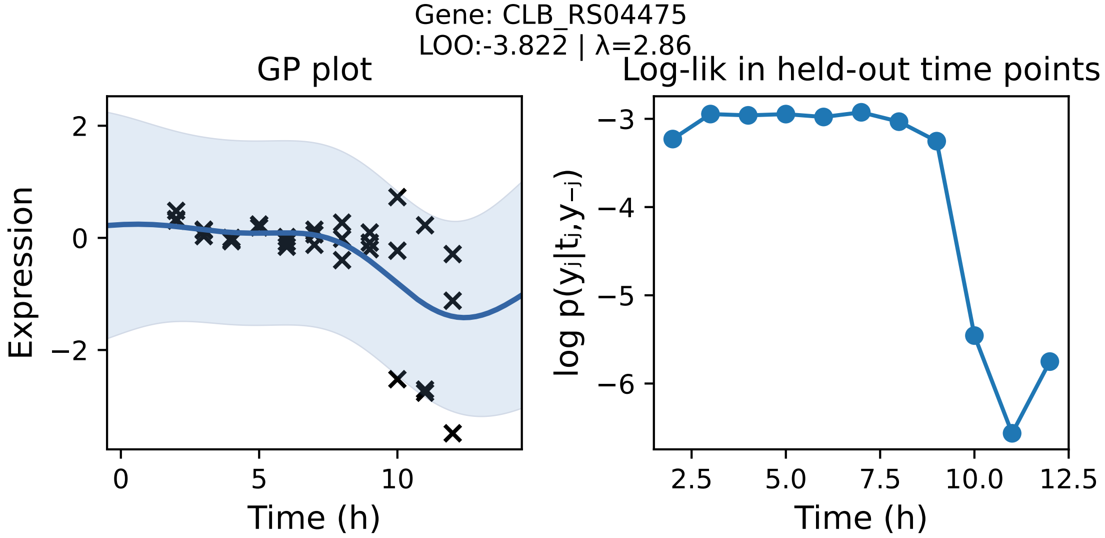

Gene: CLB_RS04475
LOO:-3.822 | λ=2.86
GP plot
Expression
Time (h)
2
0
−2
0
5
10
Log-lik in held-out time points
log p(y_j|t_j,y_−j)
Time (h)
−3
−4
−5
−6
2.5
5.0
7.5
10.0
12.5

Gene: CLB_RS04825
LOO:-3.821 | λ=2.58

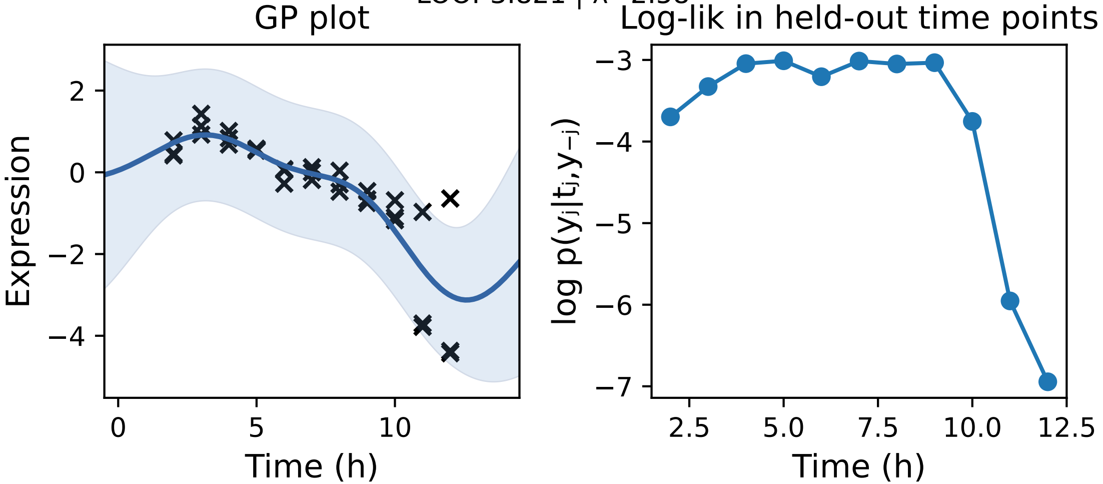

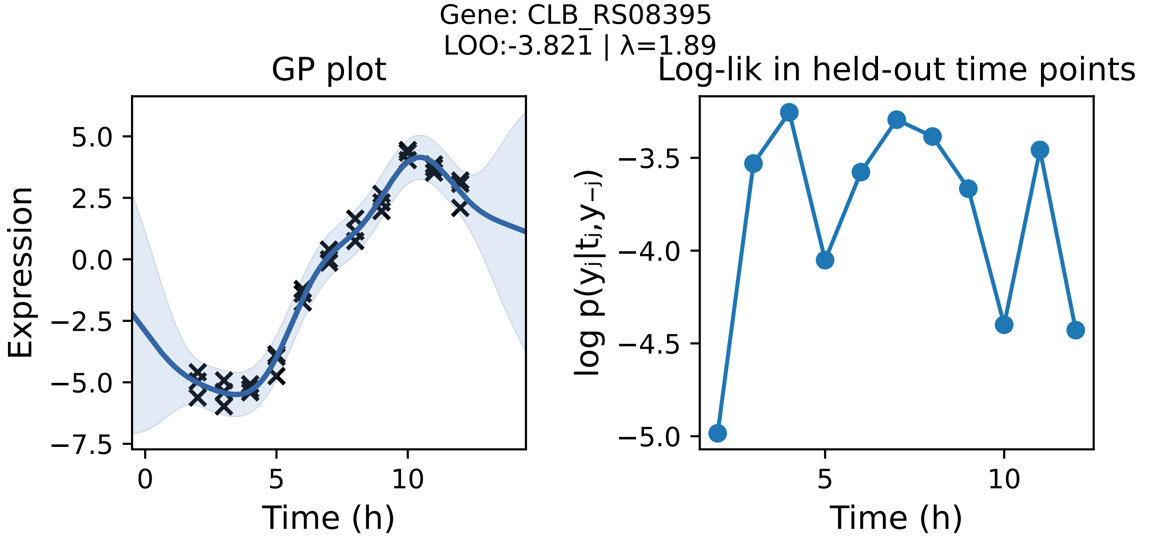

Gene: CLB_RS08395
LOO:-3.821 | λ=1.89
GP plot
Expression
Time (h)
5.0
2.5
0.0
−2.5
−5.0
−7.5
0
5
10
Log-lik in held-out time points
log p(y_j|t_j,y_−j)
Time (h)
−3.5
−4.0
−4.5
−5.0
5
10

Gene: CLB_RS06095
LOO:-3.821 | λ=1.82

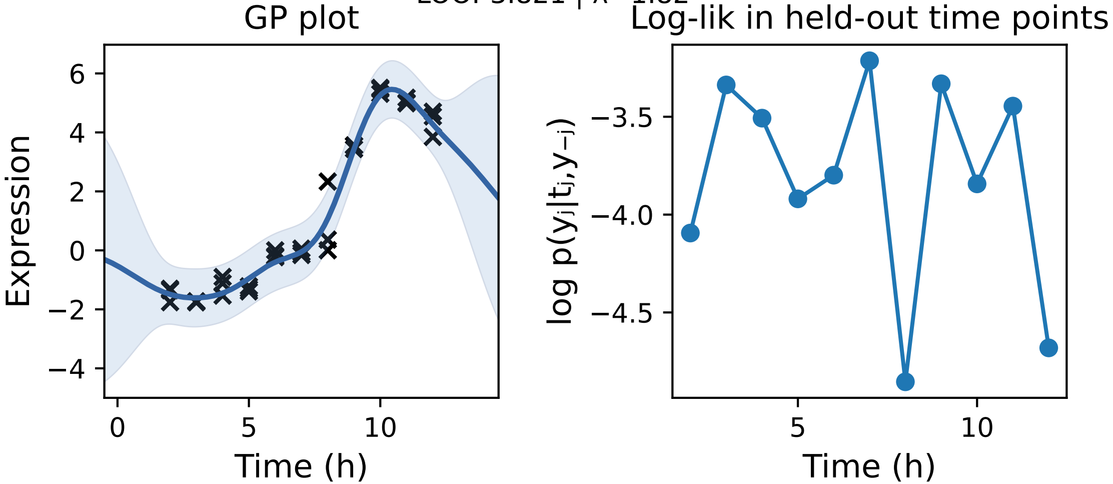

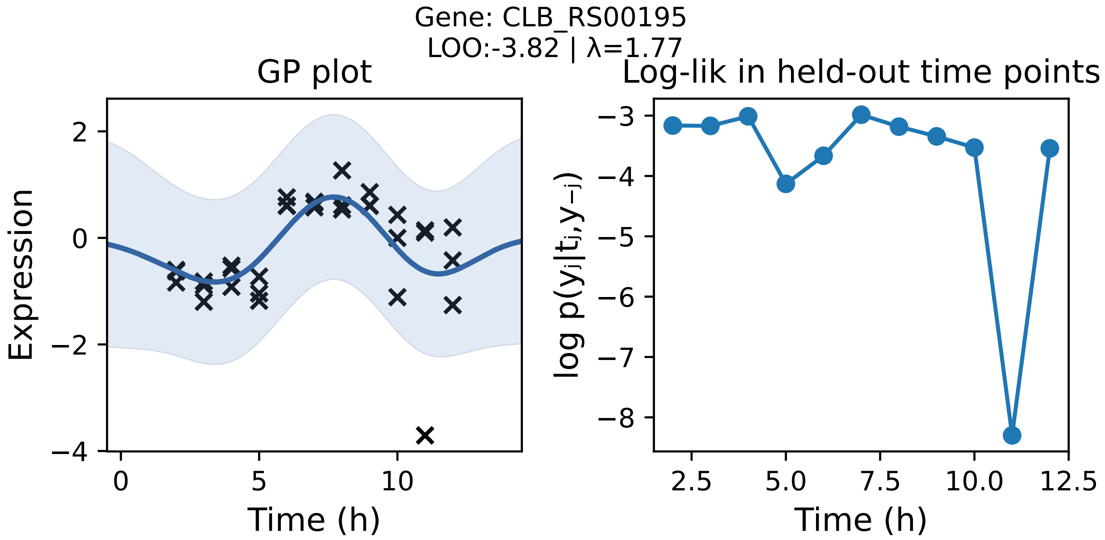
Gene: CLB_RS00195
LOO:-3.82 | λ=1.77
GP plot
Log-lik in held-out time points
Expression
Time (h)
log p(y_j|t_j,y_−j)
Time (h)
2
0
−2
−4
0
5
10
−3
−4
−5
−6
−7
−8
2.5
5.0
7.5
10.0
12.5

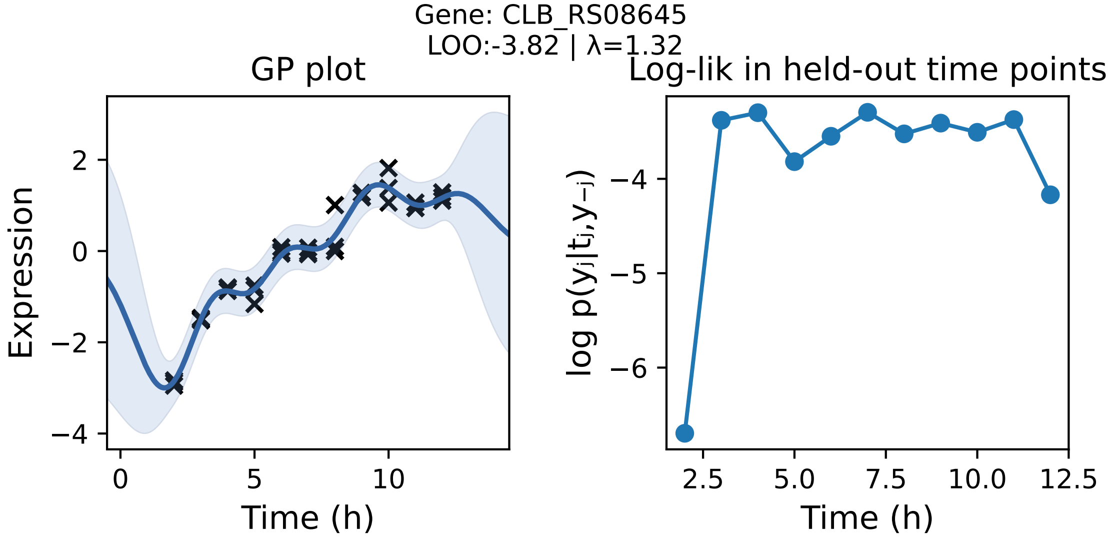
Gene: CLB_RS08645
LOO:-3.82 | λ=1.32
GP plot
Expression
Time (h)
Log-lik in held-out time points
log p(y_j|t_j,y_−j)
Time (h)

Gene: CLB_RS15785
LOO:-3.819 | λ=7.24

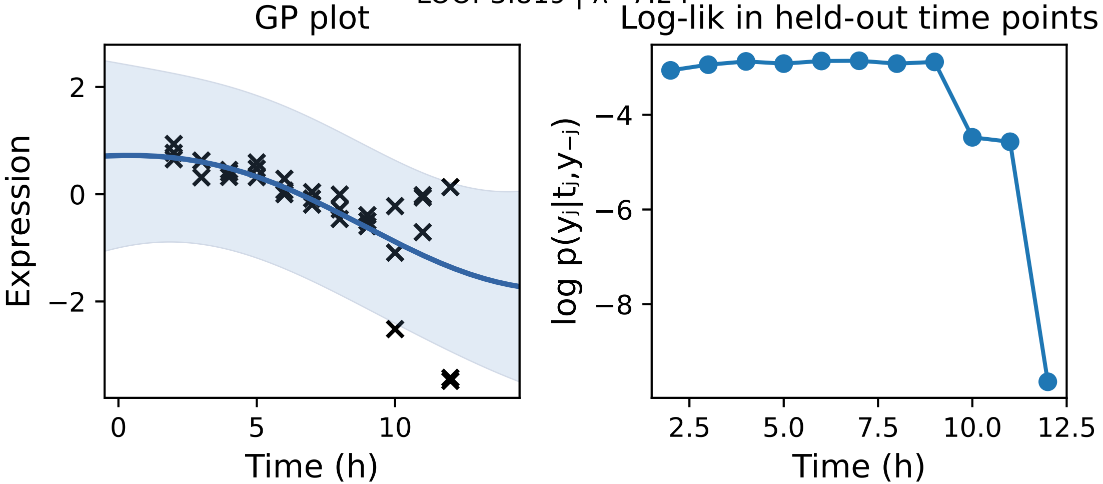

Gene: CLB_RS09645
LOO:-3.819 | λ=1.43

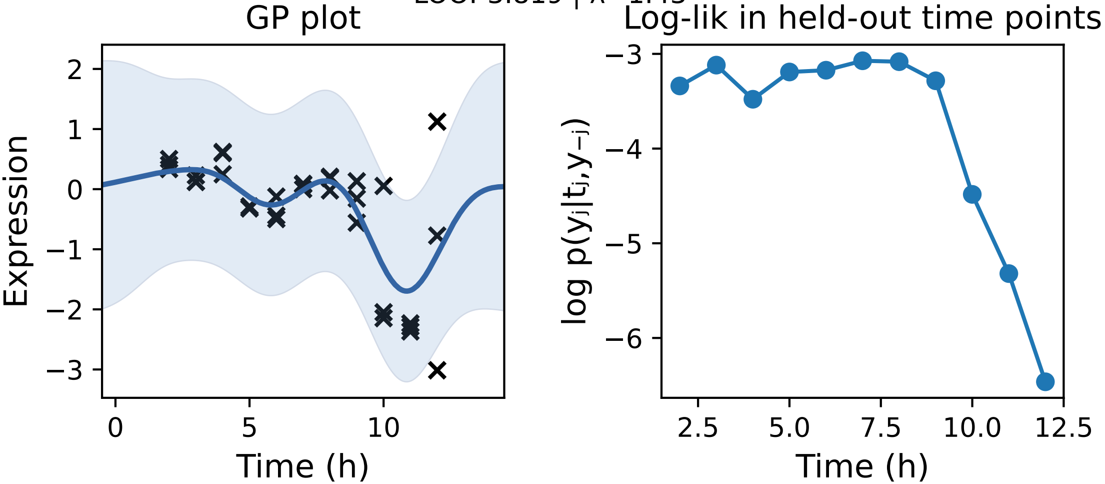

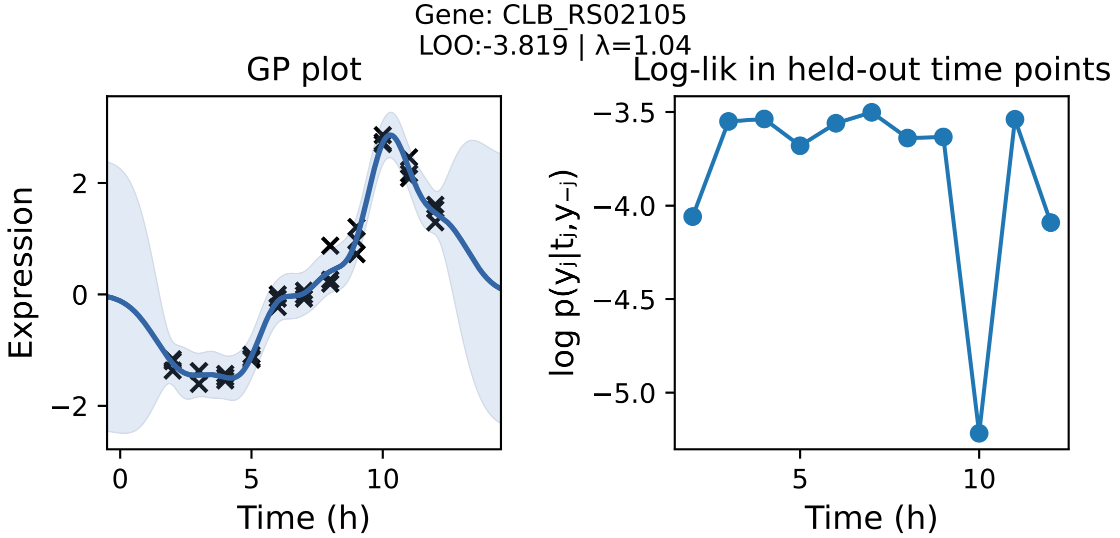

Gene: CLB_RS02105
LOO:-3.819 | λ=1.04
GP plot
Expression
Time (h)
Log-lik in held-out time points
log p(y_j|t_j,y_−j)
Time (h)

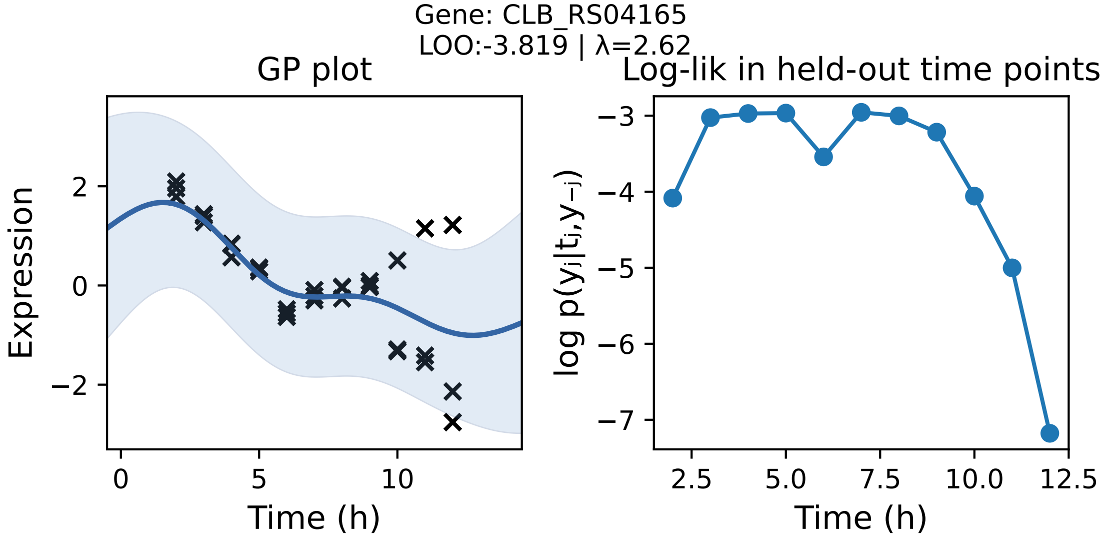
Gene: CLB_RS04165
LOO:-3.819 | λ=2.62
GP plot
Expression
2
0
−2
0
5
10
Time (h)
Log-lik in held-out time points
log p(y_j|t_j,y_−j)
−3
−4
−5
−6
−7
2.5
5.0
7.5
10.0
12.5
Time (h)

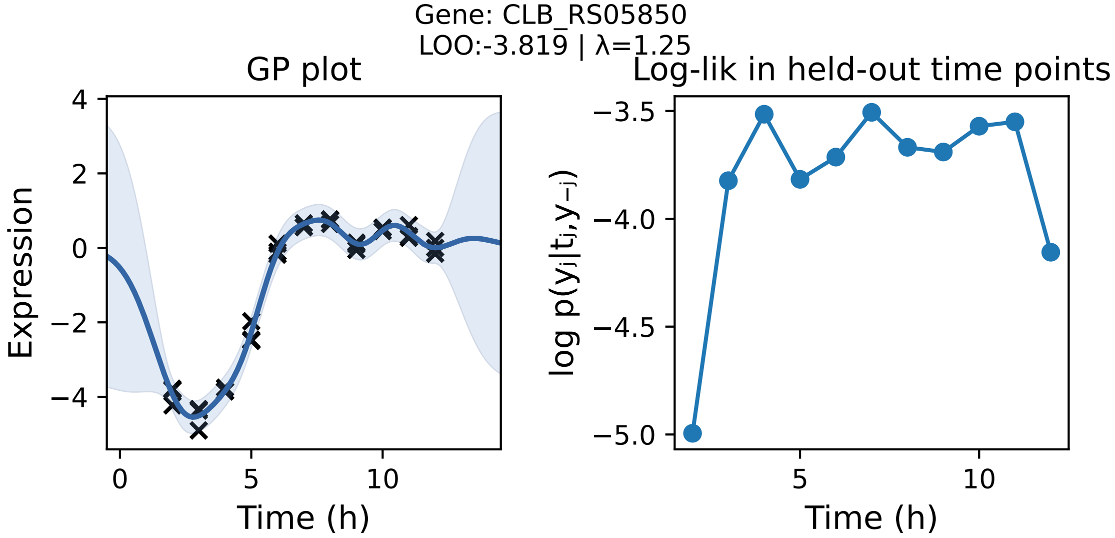

Gene: CLB_RS05850
LOO:-3.819 | λ=1.25
GP plot
Log-lik in held-out time points
Expression
Time (h)
log p(y_j|t_j,y_−j)
4
2
0
−2
−4
0
5
10
−3.5
−4.0
−4.5
−5.0

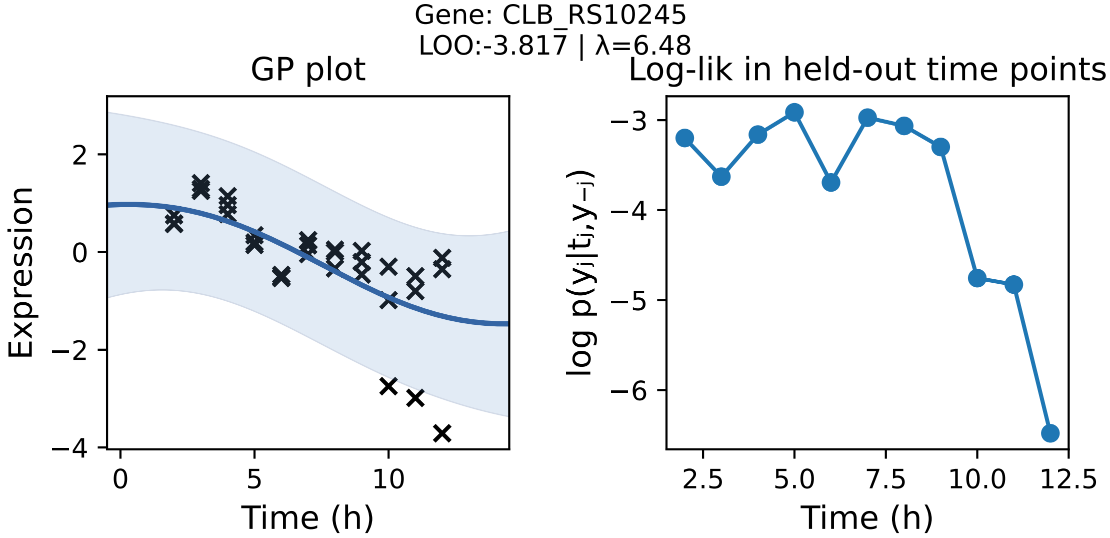

Gene: CLB_RS10245
LOO:-3.817 | λ=6.48
GP plot
Expression
Time (h)
2
0
−2
−4
0
5
10
Log-lik in held-out time points
log p(yj|tj,y−j)
−3
−4
−5
−6
2.5
5.0
7.5
10.0
12.5

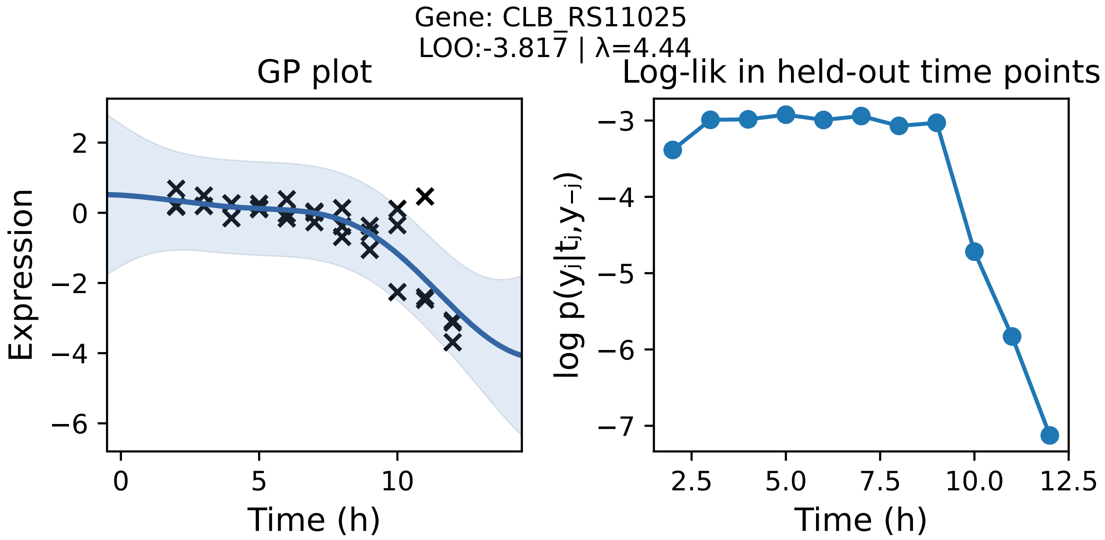
Gene: CLB_RS11025
LOO:-3.817 | λ=4.44
GP plot
Expression
Time (h)
Log-lik in held-out time points
log p(yj|tj,y−j)
Time (h)

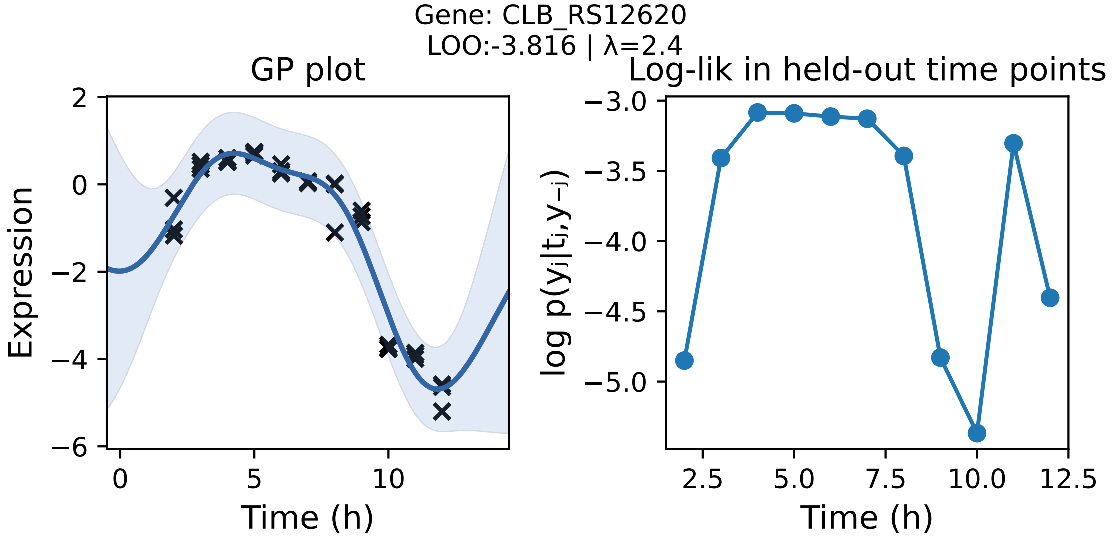

Gene: CLB_RS12620
LOO:-3.816 | λ=2.4
GP plot
Expression
Time (h)
2
0
−2
−4
−6
0
5
10
Log-lik in held-out time points
log p(y_j|t_j,y_−j)
Time (h)
−3.0
−3.5
−4.0
−4.5
−5.0
2.5
5.0
7.5
10.0
12.5

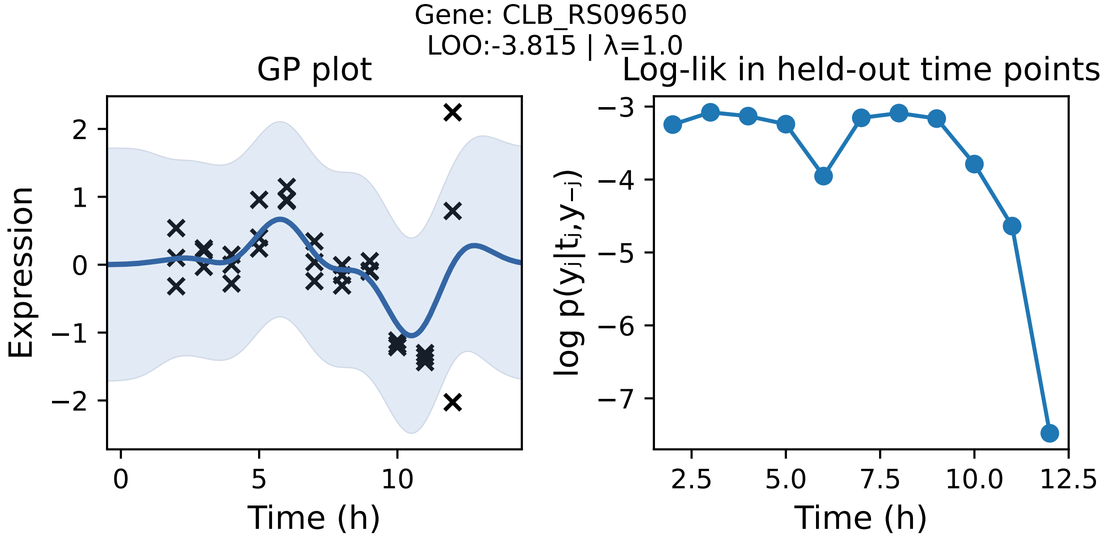

Gene: CLB_RS09650
LOO:-3.815 | λ=1.0
GP plot
Expression
Time (h)
Log-lik in held-out time points
log p(y_j|t_j,y_−j)
Time (h)

Gene: CLB_RS04930
LOO:-3.812 | λ=1.13

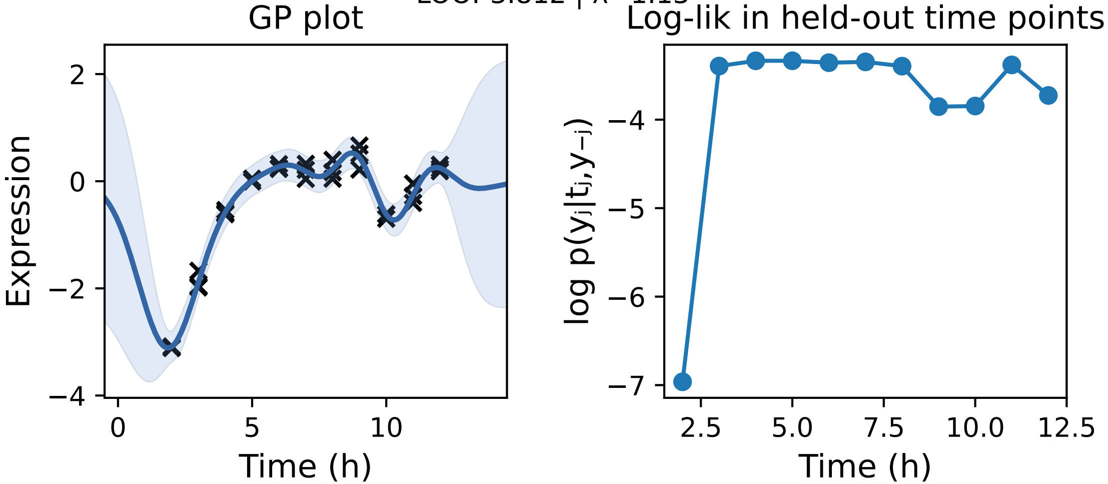

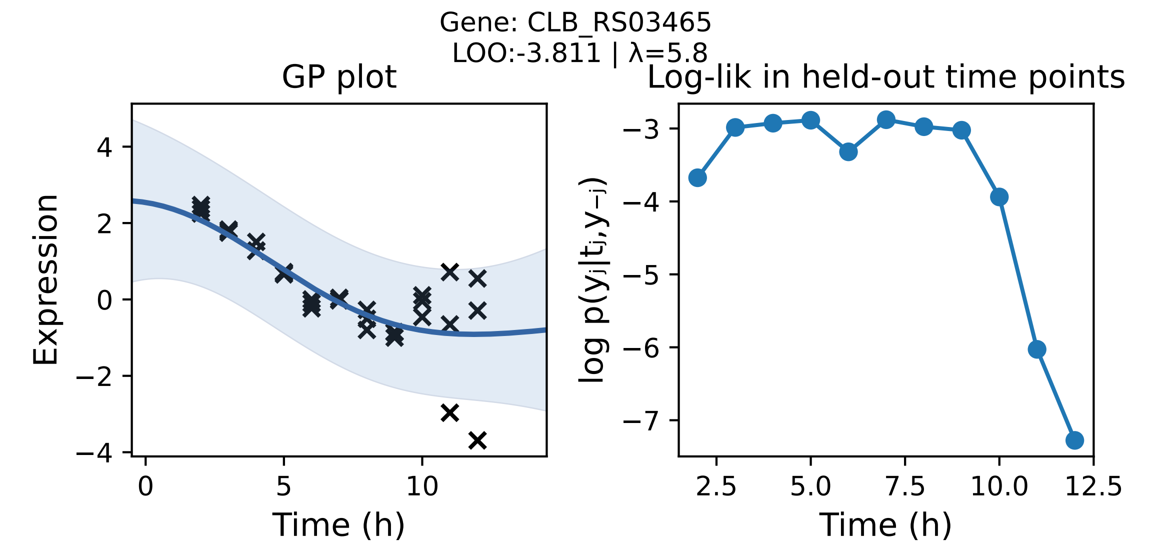
Gene: CLB_RS03465
LOO:-3.811 | λ=5.8
GP plot
Expression
Time (h)
Log-lik in held-out time points
log p(yj|tj,y−j)
Time (h)

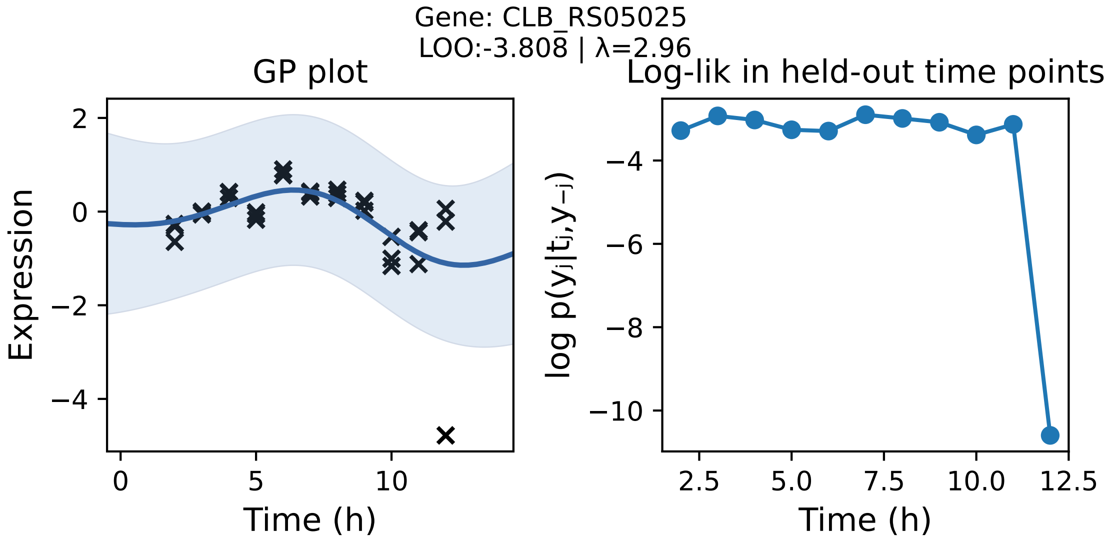

Gene: CLB_RS05025
LOO:-3.808 | λ=2.96
GP plot
Expression
Time (h)
Log-lik in held-out time points
log p(y_j|t_j,y_−j)
Time (h)
2
0
−2
−4
0
5
10
−4
−6
−8
−10
2.5
5.0
7.5
10.0
12.5

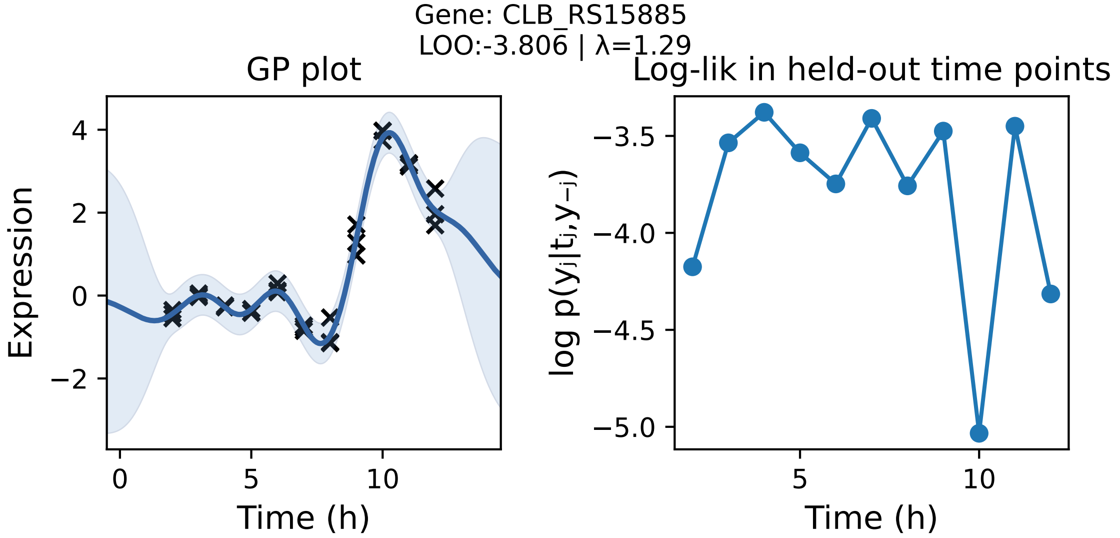

Gene: CLB_RS15885
LOO:-3.806 | λ=1.29
GP plot
Expression
Time (h)
4
2
0
−2
0
5
10
Log-lik in held-out time points
log p(y_j|t_j,y_−j)
Time (h)
−3.5
−4.0
−4.5
−5.0
5
10

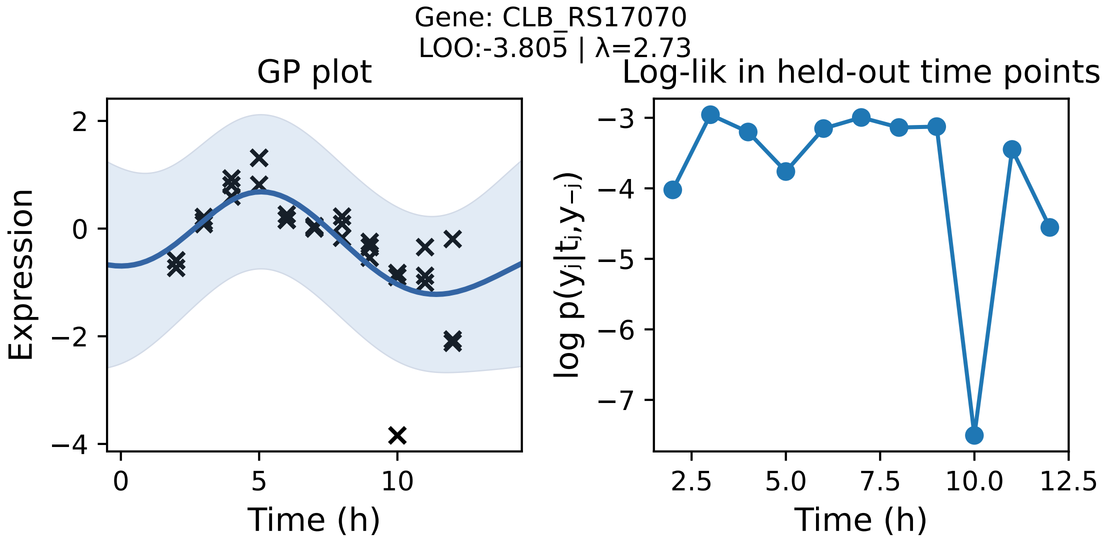
Gene: CLB_RS17070
LOO:-3.805 | λ=2.73
GP plot
Expression
Time (h)
2
0
−2
−4
0
5
10
Log-lik in held-out time points
log p(yj|tj,y−j)
Time (h)
−3
−4
−5
−6
−7
2.5
5.0
7.5
10.0
12.5

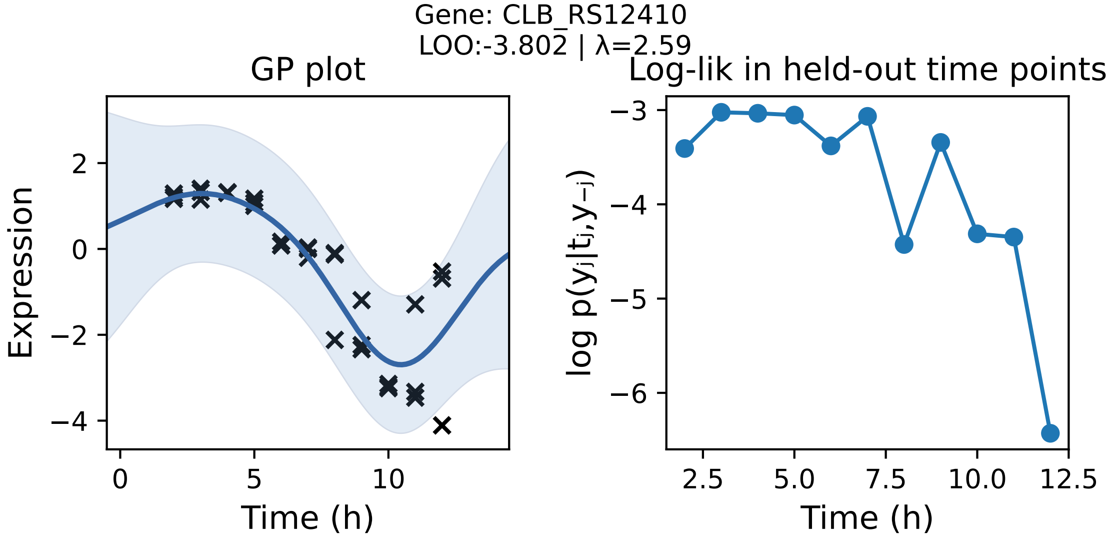
Gene: CLB_RS12410
LOO:-3.802 | λ=2.59
GP plot
Expression
Time (h)
Log-lik in held-out time points
log p(yⱼ|tⱼ,y₋ⱼ)
Time (h)

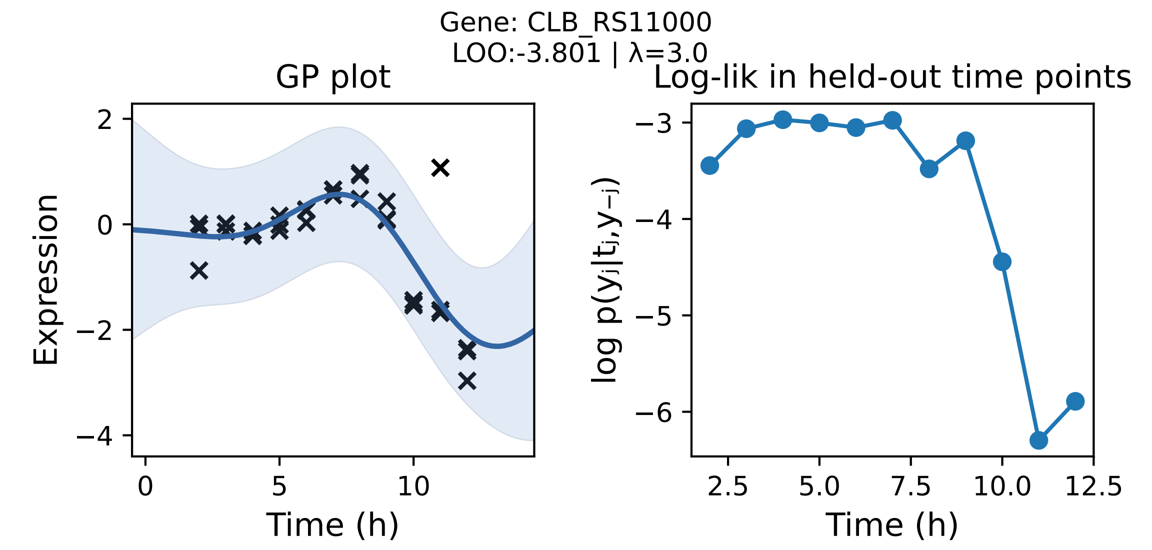

Gene: CLB_RS11000
LOO:-3.801 | λ=3.0
GP plot
Log-lik in held-out time points
Expression
Time (h)
log p(yj|tj,y−j)
Time (h)

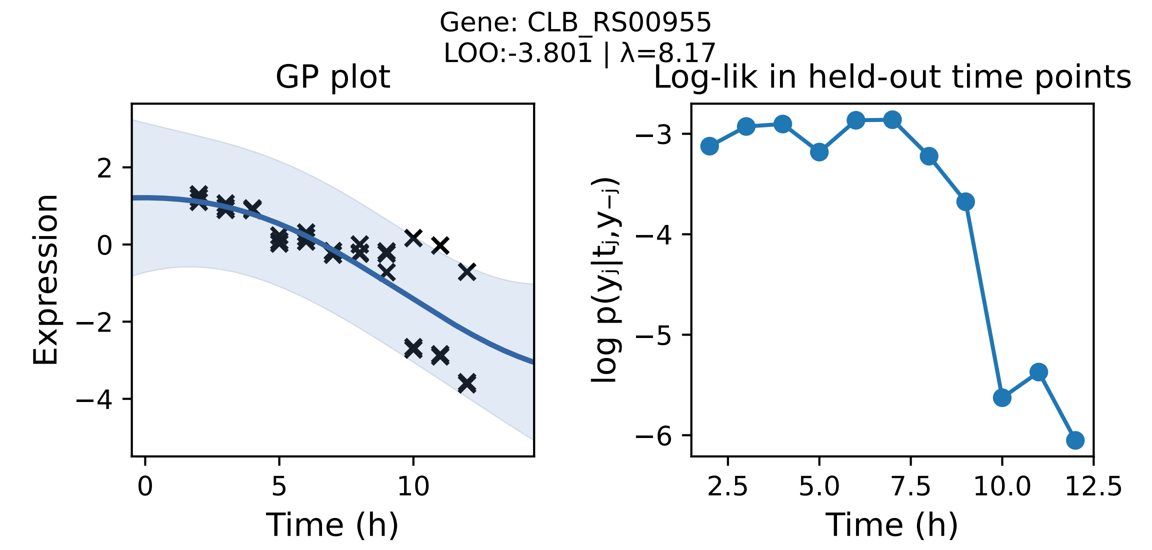

Gene: CLB_RS00955
LOO:-3.801 | λ=8.17
GP plot
Log-lik in held-out time points
Expression
Time (h)
log p(yj|tj,y−j)
2
0
−2
−4
0
5
10
−3
−4
−5
−6
2.5
5.0
7.5
10.0
12.5

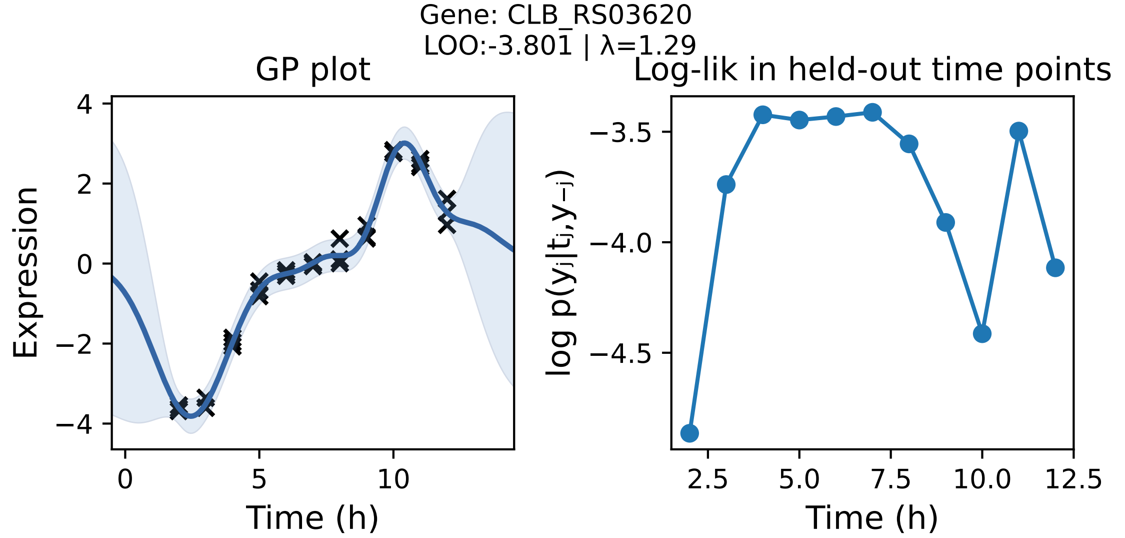

Gene: CLB_RS03620
LOO:-3.801 | λ=1.29
GP plot
Expression
Time (h)
Log-lik in held-out time points
log p(y_j|t_j,y_−j)
Time (h)

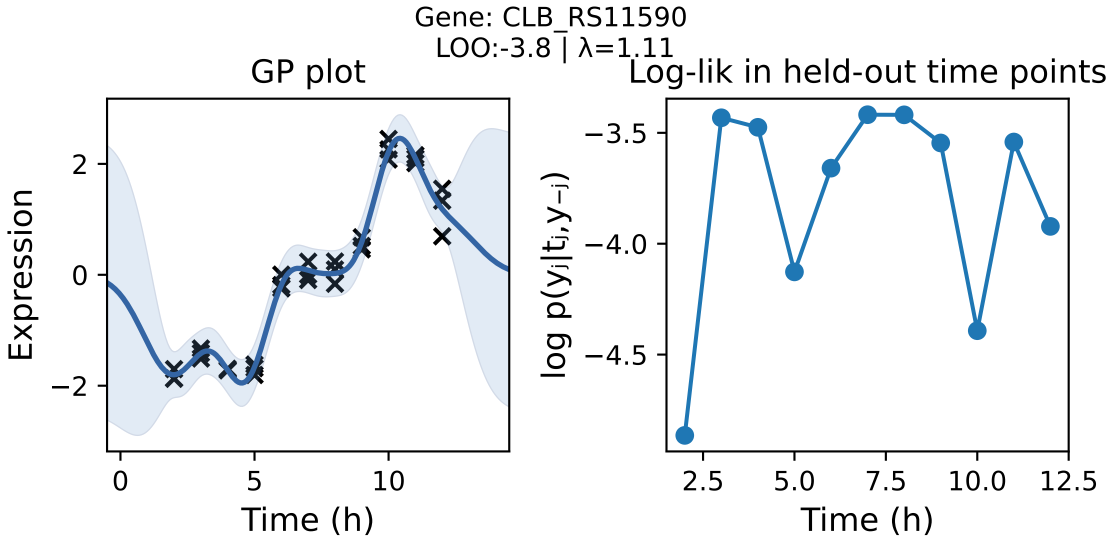
Gene: CLB_RS11590
LOO:-3.8 | λ=1.11
GP plot
Expression
Time (h)
Log-lik in held-out time points
log p(y_j|t_j,y_−j)
Time (h)

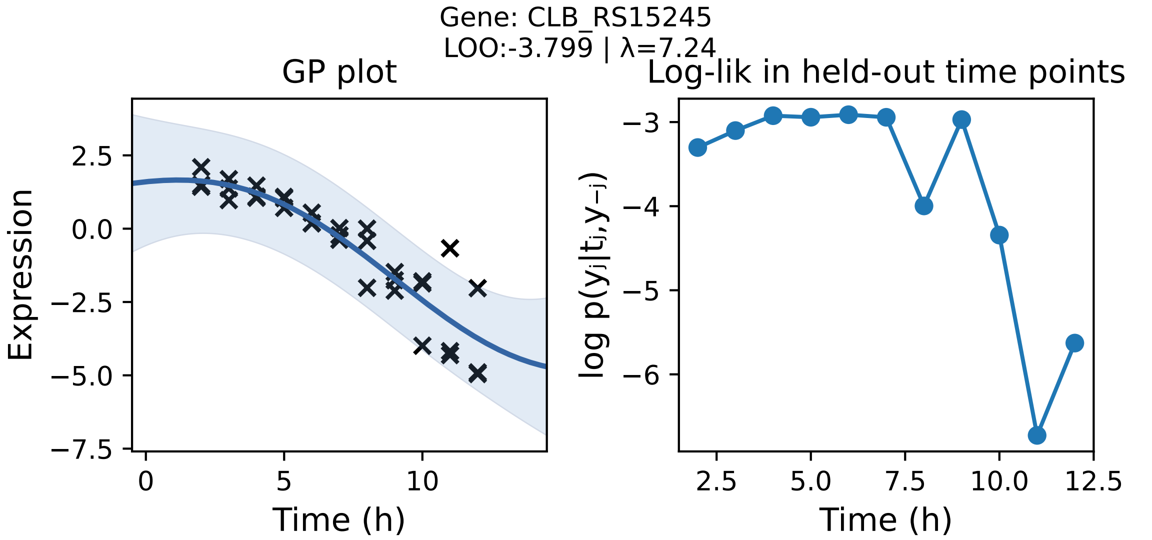

Gene: CLB_RS15245
LOO:-3.799 | λ=7.24
GP plot
Expression
Time (h)
2.5
0.0
−2.5
−5.0
−7.5
0
5
10
Log-lik in held-out time points
log p(y_j|t_j,y_−j)
Time (h)
−3
−4
−5
−6
2.5
5.0
7.5
10.0
12.5

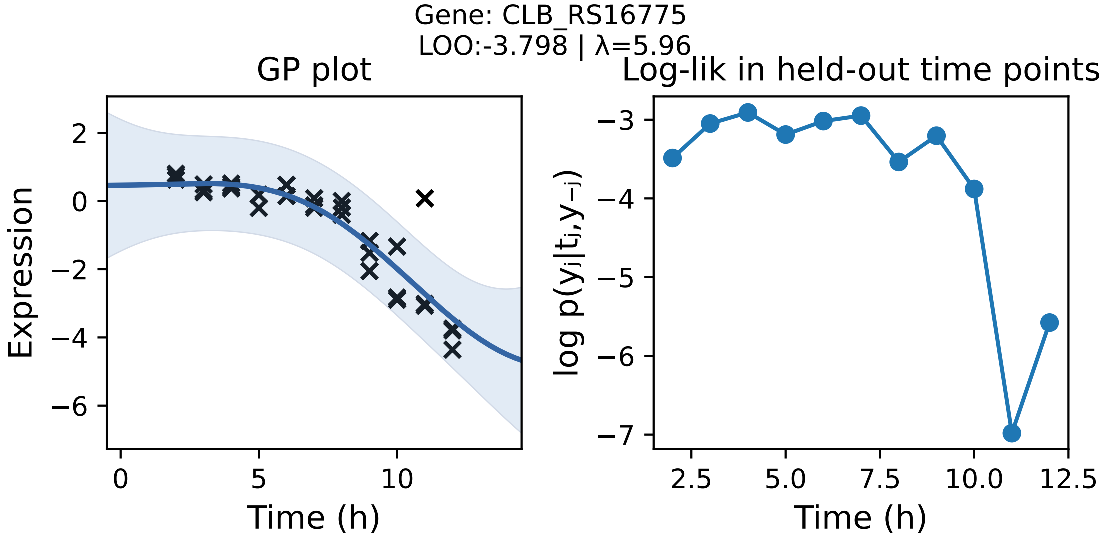

Gene: CLB_RS16775
LOO:-3.798 | λ=5.96
GP plot
Expression
Time (h)
Log-lik in held-out time points
log p(y_j|t_j,y_−j)
Time (h)

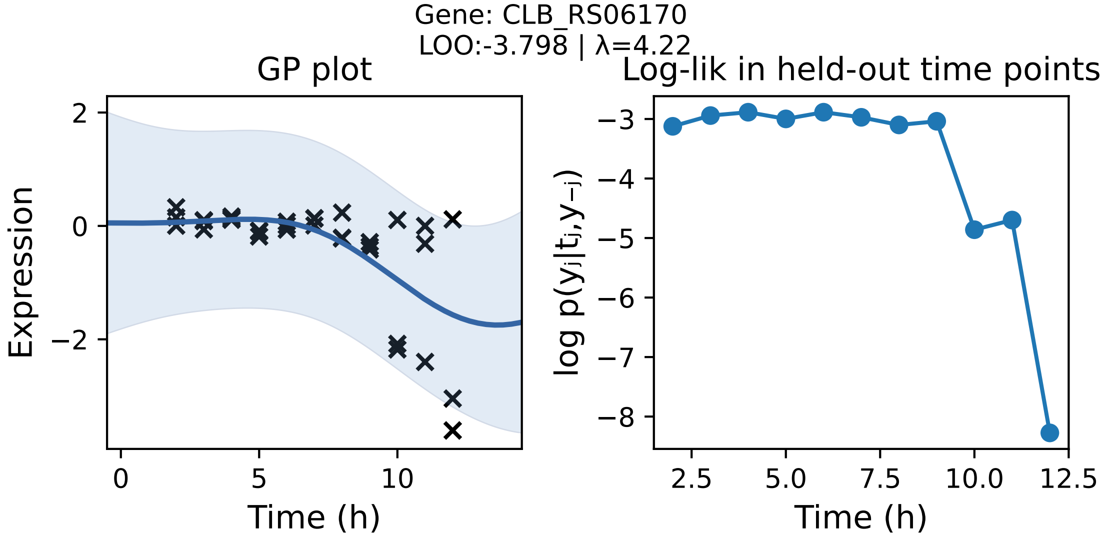
Gene: CLB_RS06170
LOO:-3.798 | λ=4.22
GP plot
Log-lik in held-out time points
Expression
Time (h)
log p(y_j|t_j,y_−j)
2
0
−2
0
5
10
−3
−4
−5
−6
−7
−8
2.5
5.0
7.5
10.0
12.5

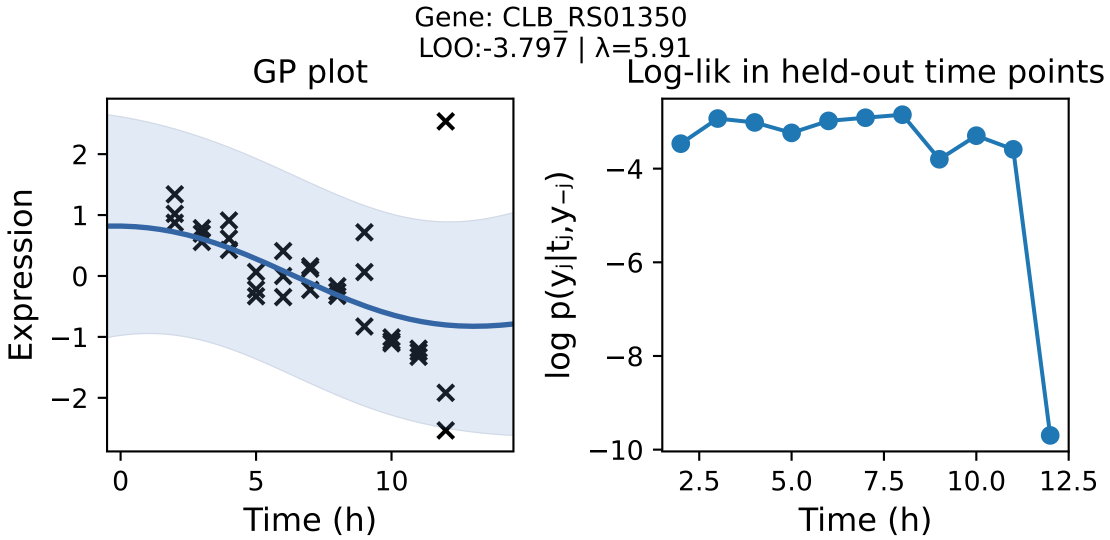
Gene: CLB_RS01350
LOO:-3.797 | λ=5.91
GP plot
Expression
Time (h)
Log-lik in held-out time points
log p(y_j|t_j,y_-j)
Time (h)
2
1
0
−1
−2
0
5
10
−4
−6
−8
−10
2.5
5.0
7.5
10.0
12.5

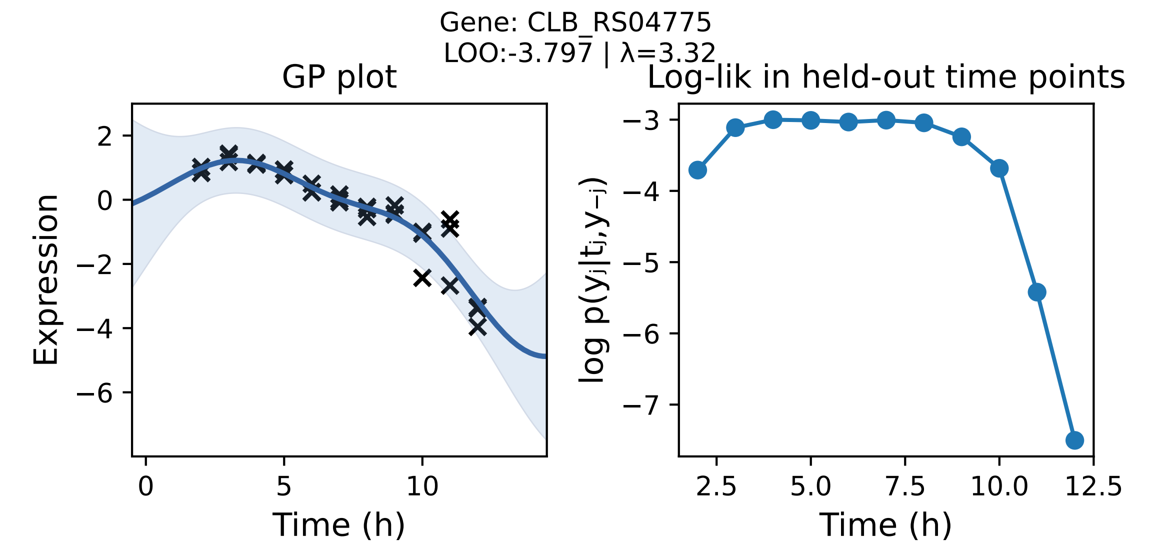

Gene: CLB_RS04775
LOO:-3.797 | λ=3.32
GP plot
Expression
Time (h)
Log-lik in held-out time points
log p(yj|tj,y−j)
Time (h)

Gene: CLB_RS08215
LOO:-3.796 | λ=4.9

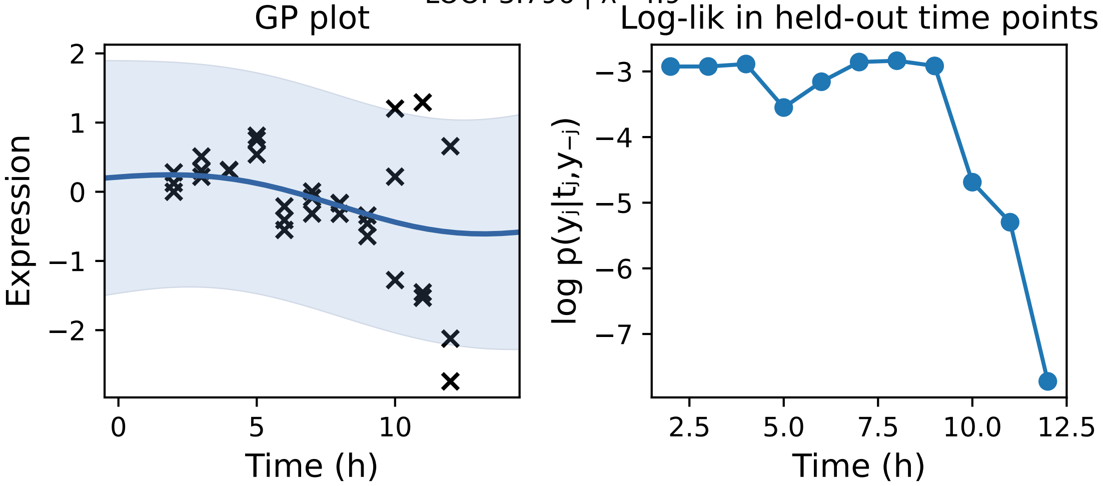

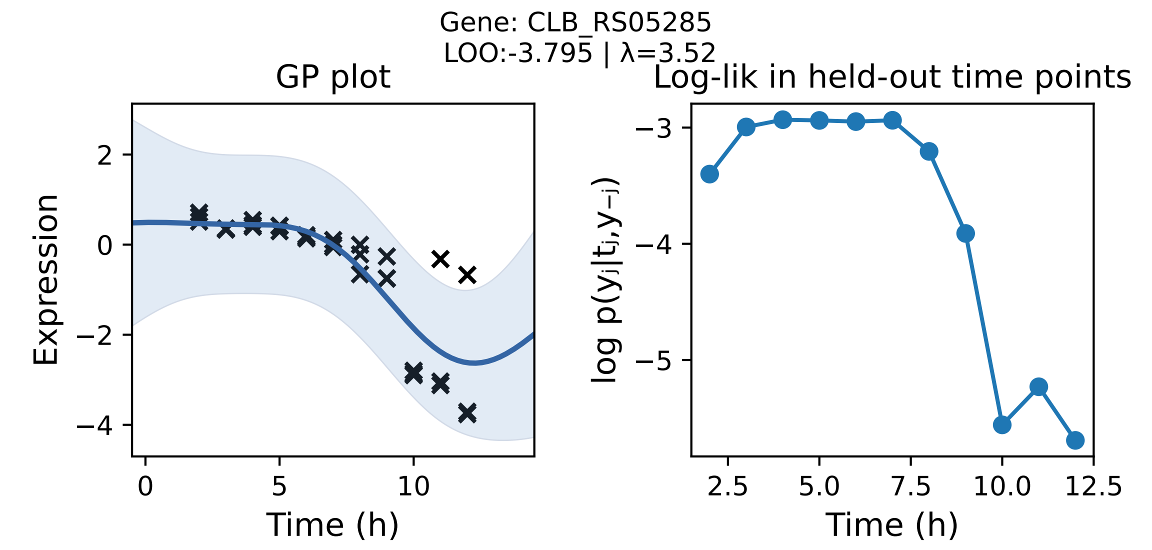

Gene: CLB_RS05285
LOO:-3.795 | λ=3.52
GP plot
Expression
Time (h)
2
0
−2
−4
0
5
10
Log-lik in held-out time points
log p(y_j|t_j,y_−j)
Time (h)
−3
−4
−5
2.5
5.0
7.5
10.0
12.5

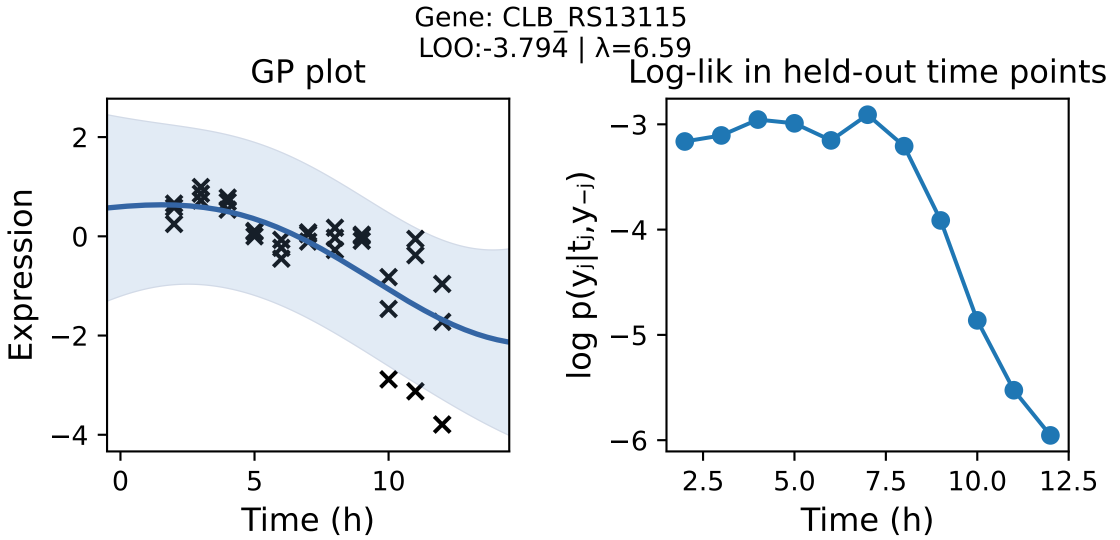
Gene: CLB_RS13115
LOO:-3.794 | λ=6.59
GP plot
Expression
2
0
−2
−4
0
5
10
Time (h)
Log-lik in held-out time points
log p(y_j|t_j,y_−j)
−3
−4
−5
−6
2.5
5.0
7.5
10.0
12.5
Time (h)

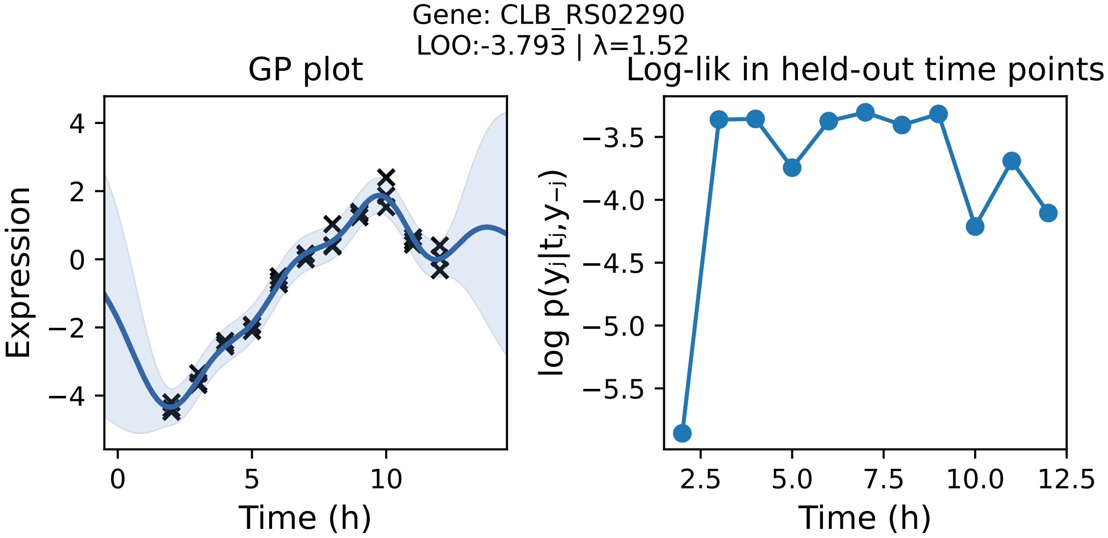
Gene: CLB_RS02290
LOO:-3.793 | λ=1.52
GP plot
Expression
4
2
0
−2
−4
0
5
10
Time (h)
Log-lik in held-out time points
log p(yⱼ|tⱼ,y₋ⱼ)
−3.5
−4.0
−4.5
−5.0
−5.5
2.5
5.0
7.5
10.0
12.5
Time (h)

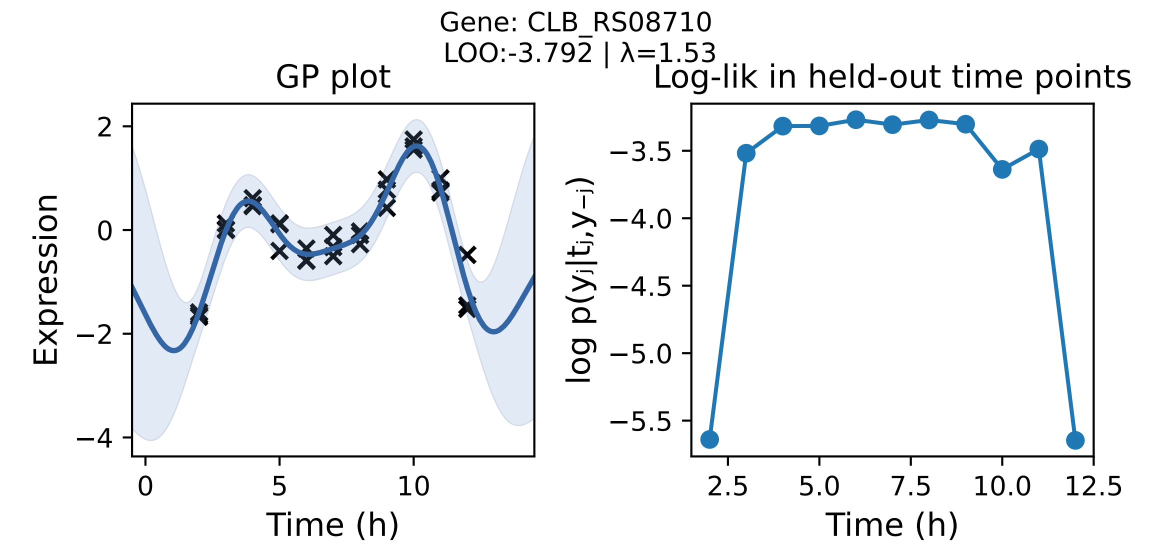
Gene: CLB_RS08710
LOO:-3.792 | λ=1.53
GP plot
Expression
Time (h)
Log-lik in held-out time points
log p(y_j|t_j,y_−j)
Time (h)

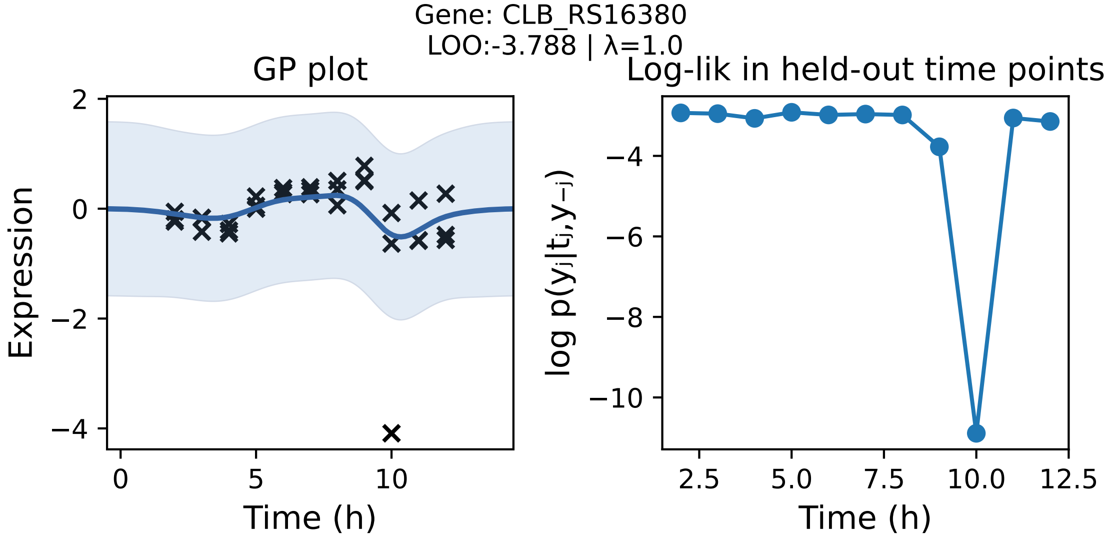
Gene: CLB_RS16380
LOO:-3.788 | λ=1.0
GP plot
Expression
Time (h)
Log-lik in held-out time points
log p(y_j|t_j,y_−j)
Time (h)

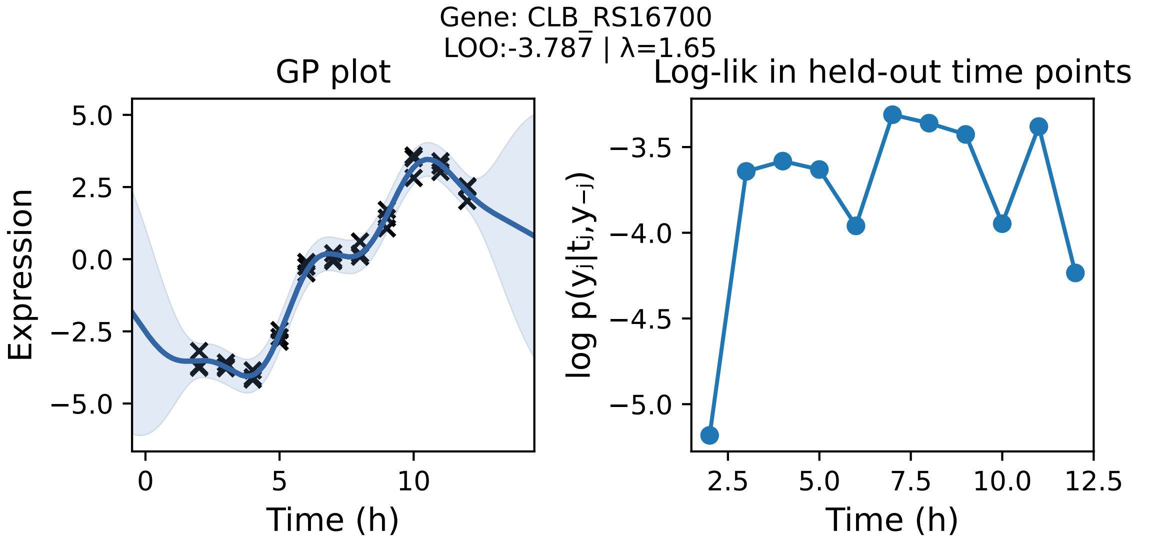

Gene: CLB_RS16700
LOO:-3.787 | λ=1.65
GP plot
Log-lik in held-out time points
Expression
Time (h)
log p(y_j|t_j,y_−j)
5.0
2.5
0.0
−2.5
−5.0
0
5
10
−3.5
−4.0
−4.5
−5.0
2.5
5.0
7.5
10.0
12.5

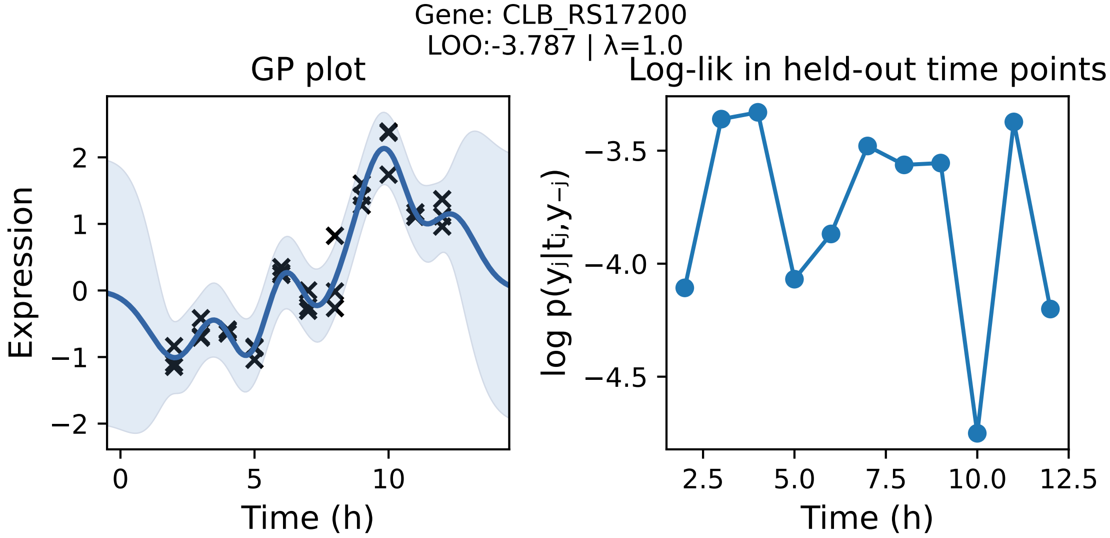

Gene: CLB_RS17200
LOO:-3.787 | λ=1.0
GP plot
Expression
Time (h)
Log-lik in held-out time points
log p(y_j|t_j,y_−j)
Time (h)

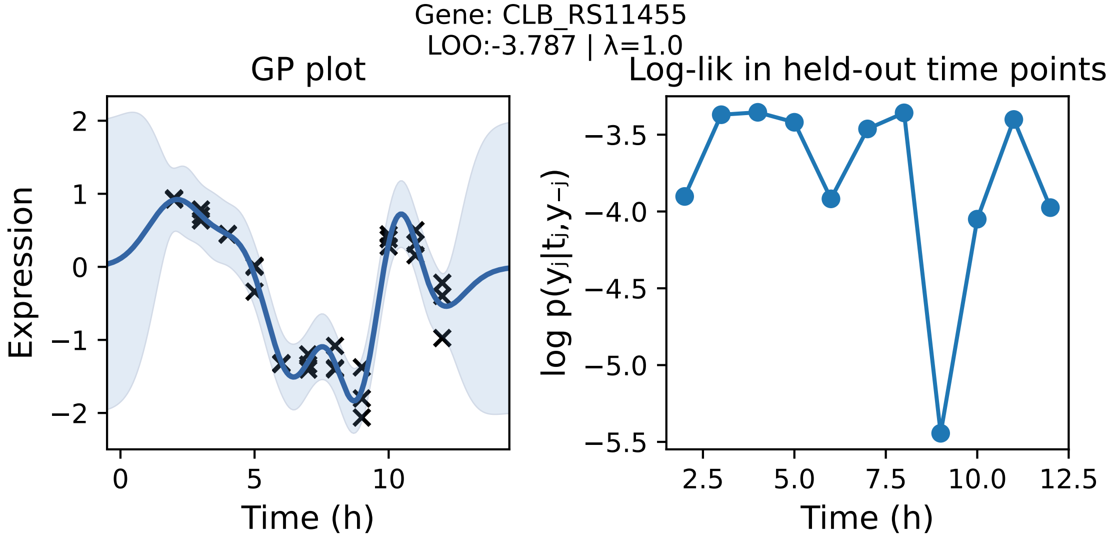

Gene: CLB_RS11455
LOO:-3.787 | λ=1.0
GP plot
Expression
Time (h)
2
1
0
−1
−2
0
5
10
Log-lik in held-out time points
log p(y_j|t_j,y_−j)
−3.5
−4.0
−4.5
−5.0
−5.5
2.5
5.0
7.5
10.0
12.5
Time (h)

Gene: CLB_RS07320
LOO:-3.787 | λ=2.94

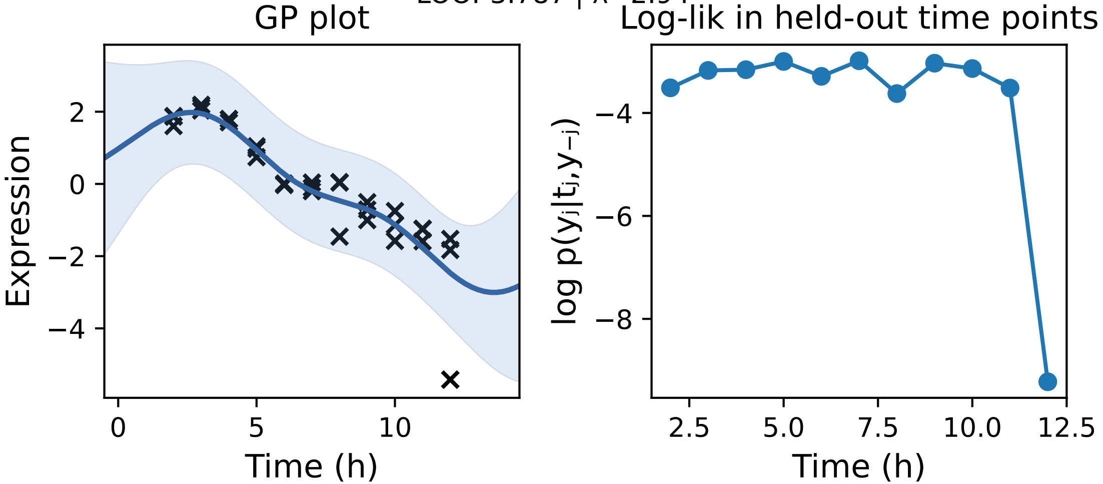

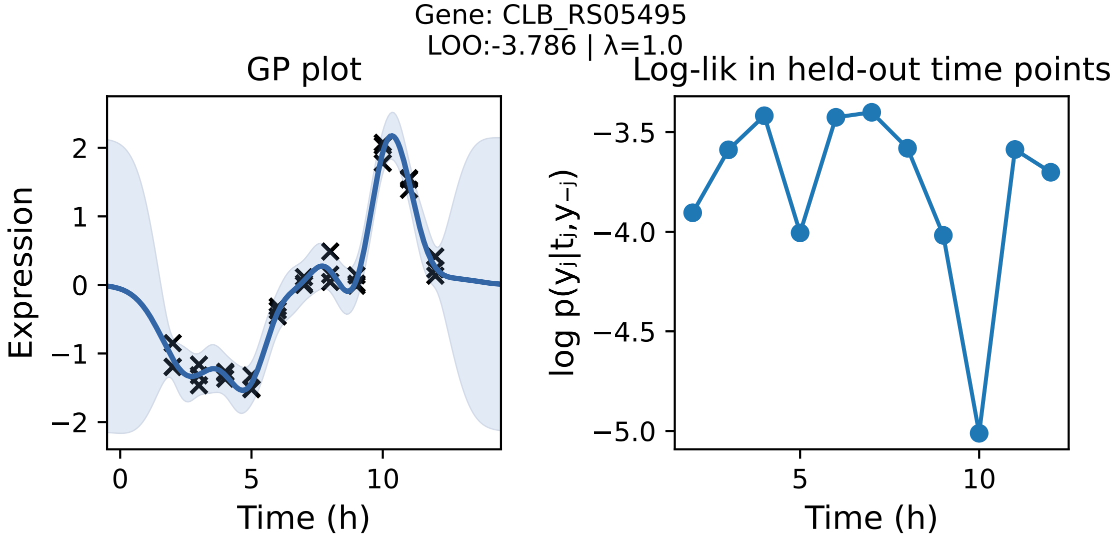

Gene: CLB_RS05495
LOO:-3.786 | λ=1.0
GP plot
Expression
Time (h)
Log-lik in held-out time points
log p(y_j|t_j,y_−j)
Time (h)

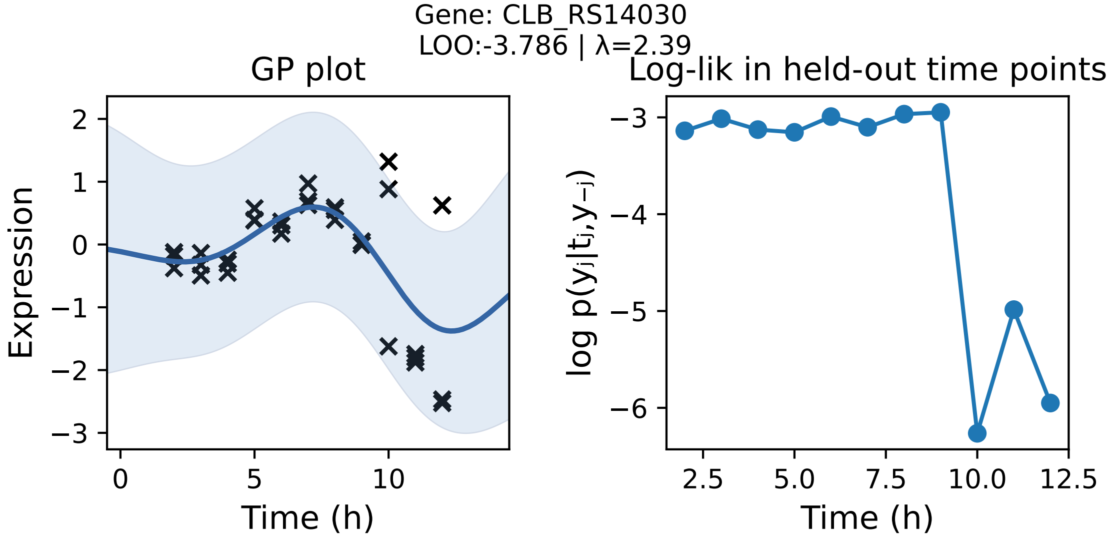
Gene: CLB_RS14030
LOO:-3.786 | λ=2.39
GP plot
Expression
Time (h)
2
1
0
−1
−2
−3
0
5
10
Log-lik in held-out time points
log p(y_j|t_j,y_−j)
−3
−4
−5
−6
2.5
5.0
7.5
10.0
12.5
Time (h)

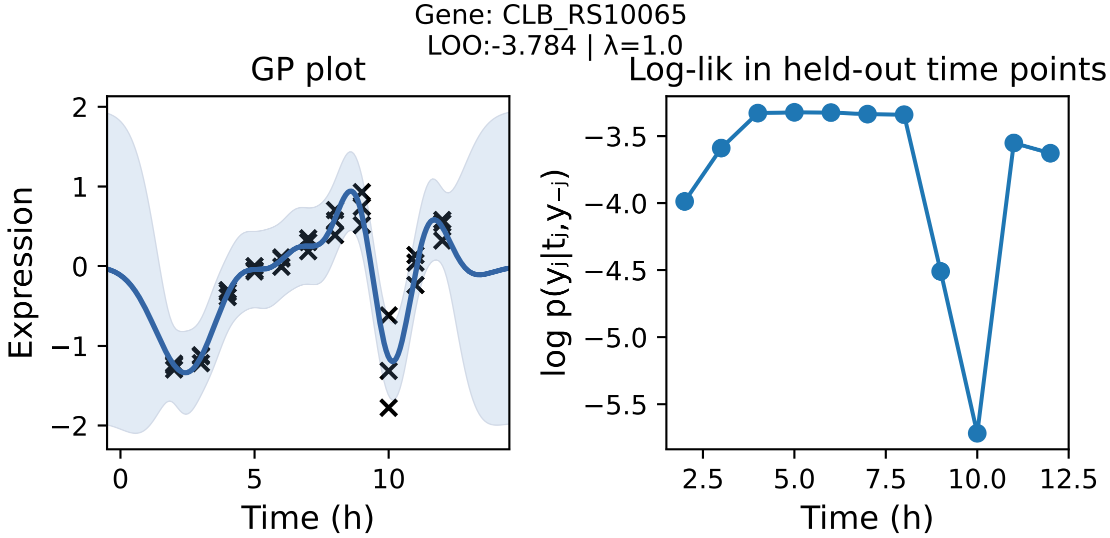

Gene: CLB_RS10065
LOO:-3.784 | λ=1.0
GP plot
Log-lik in held-out time points
Expression
Time (h)
log p(y_j|t_j,y_−j)
Time (h)
2
1
0
−1
−2
0
5
10
−3.5
−4.0
−4.5
−5.0
−5.5
2.5
5.0
7.5
10.0
12.5

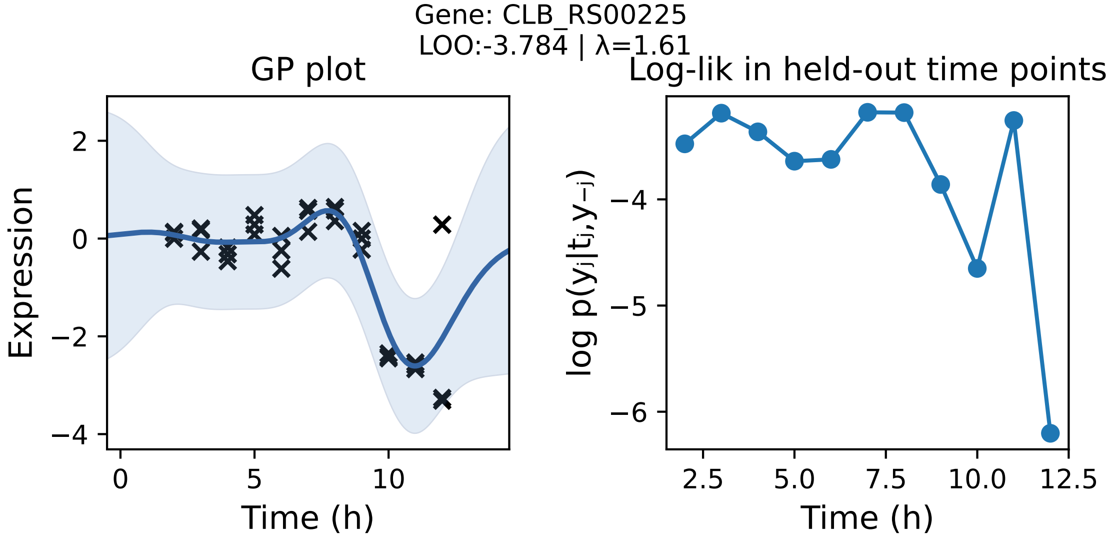
Gene: CLB_RS00225
LOO:-3.784 | λ=1.61
GP plot
Log-lik in held-out time points
Expression
Time (h)
log p(y_j|t_j,y_-j)
Time (h)
2
0
−2
−4
0
5
10
−4
−5
−6
2.5
5.0
7.5
10.0
12.5

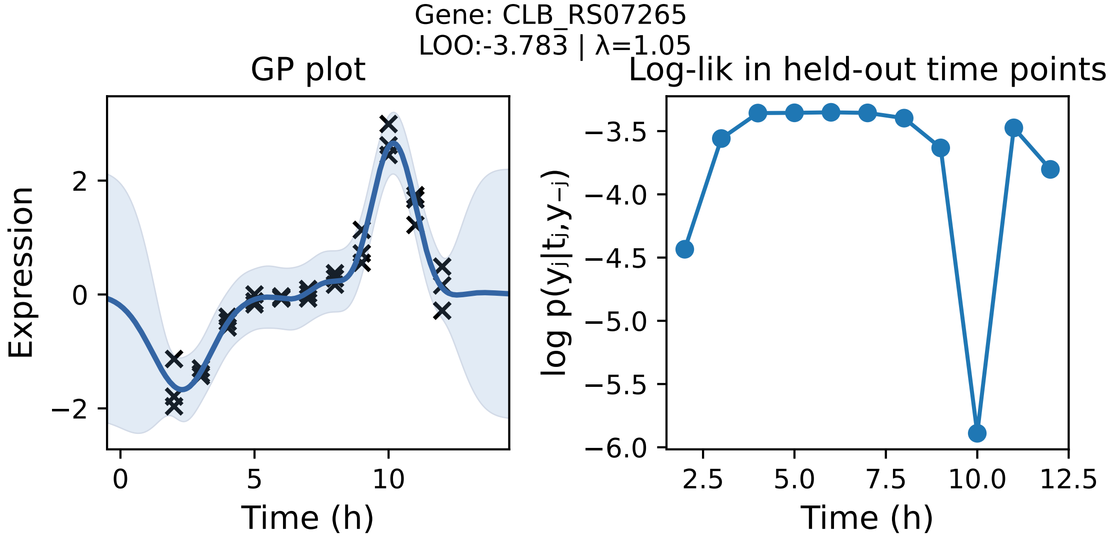
Gene: CLB_RS07265
LOO:-3.783 | λ=1.05
GP plot
Expression
Time (h)
Log-lik in held-out time points
log p(y_j|t_j,y_-j)
Time (h)

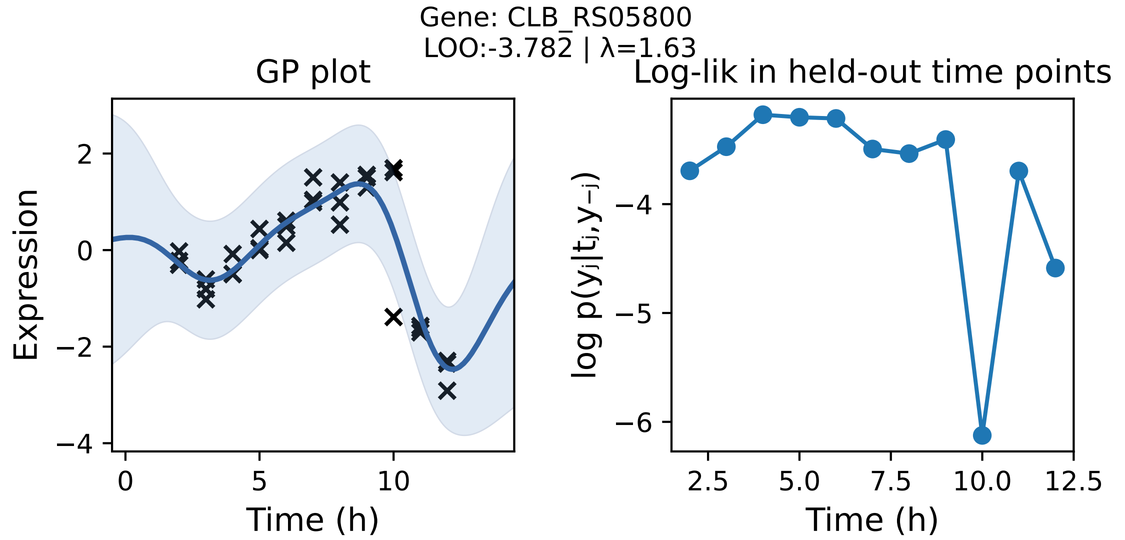

Gene: CLB_RS05800
LOO:-3.782 | λ=1.63
GP plot
Expression
Time (h)
2
0
−2
−4
0
5
10
Log-lik in held-out time points
log p(y_j|t_j,y_−j)
Time (h)
−4
−5
−6
2.5
5.0
7.5
10.0
12.5

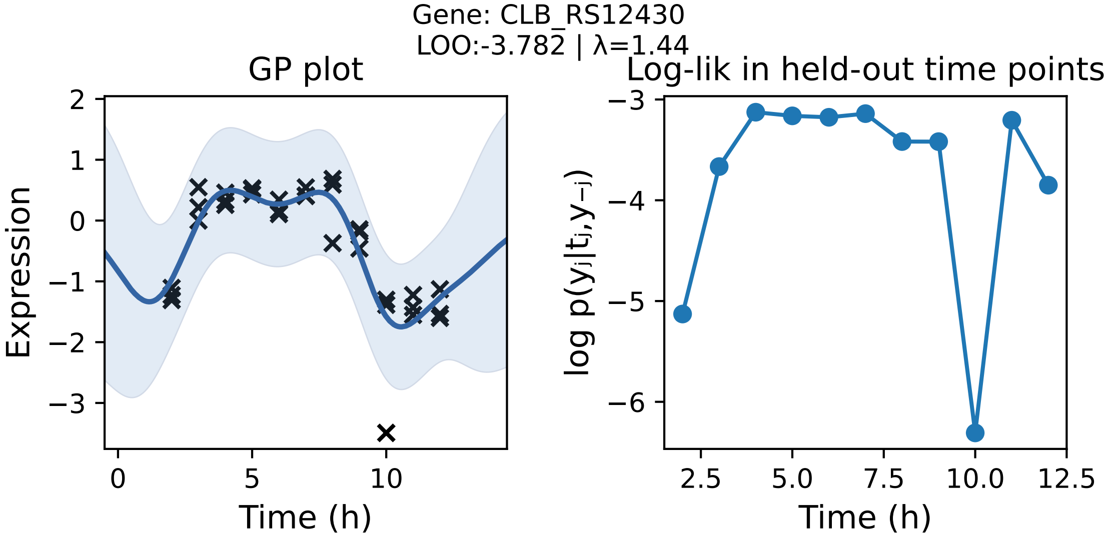

Gene: CLB_RS12430
LOO:-3.782 | λ=1.44
GP plot
Expression
Time (h)
2
1
0
−1
−2
−3
0
5
10
Log-lik in held-out time points
log p(y_j|t_j,y_−j)
Time (h)
−3
−4
−5
−6
2.5
5.0
7.5
10.0
12.5

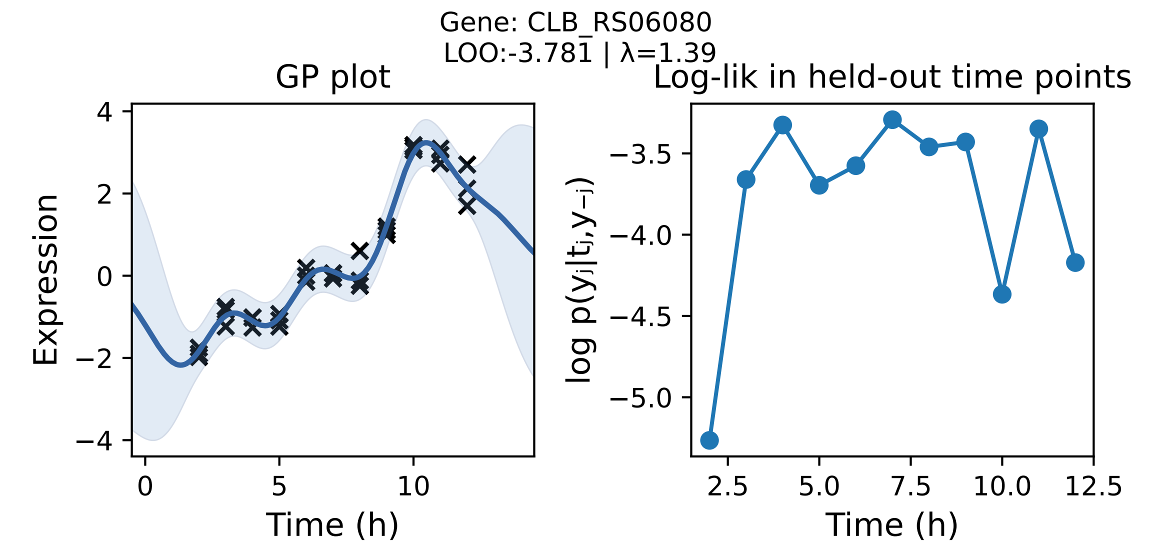

Gene: CLB_RS06080
LOO:-3.781 | λ=1.39
GP plot
Expression
Time (h)
4
2
0
−2
−4
0
5
10
Log-lik in held-out time points
log p(y_j|t_j,y_−j)
Time (h)
−3.5
−4.0
−4.5
−5.0
2.5
5.0
7.5
10.0
12.5

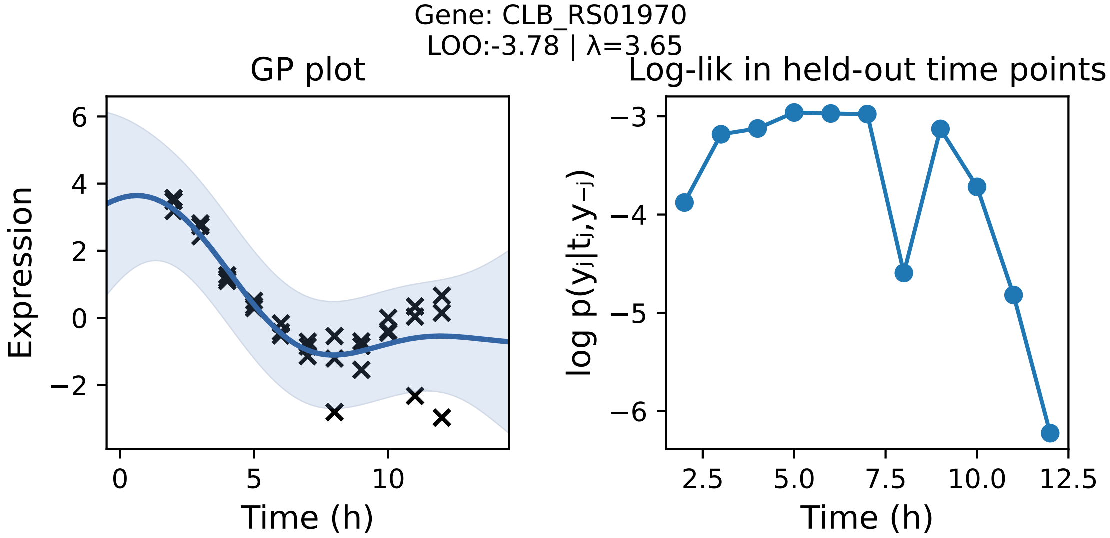

Gene: CLB_RS01970
LOO:-3.78 | λ=3.65
GP plot
Log-lik in held-out time points
Expression
Time (h)
log p(y_j|t_j,y_−j)
Time (h)
6
4
2
0
−2
0
5
10
−3
−4
−5
−6
2.5
5.0
7.5
10.0
12.5

Gene: CLB_RS16040
LOO:-3.78 | λ=1.19

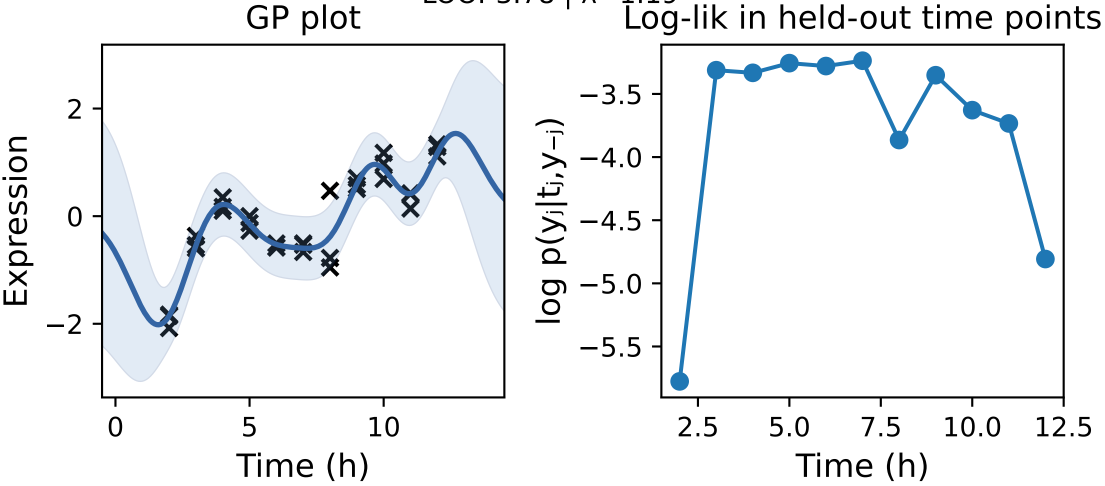

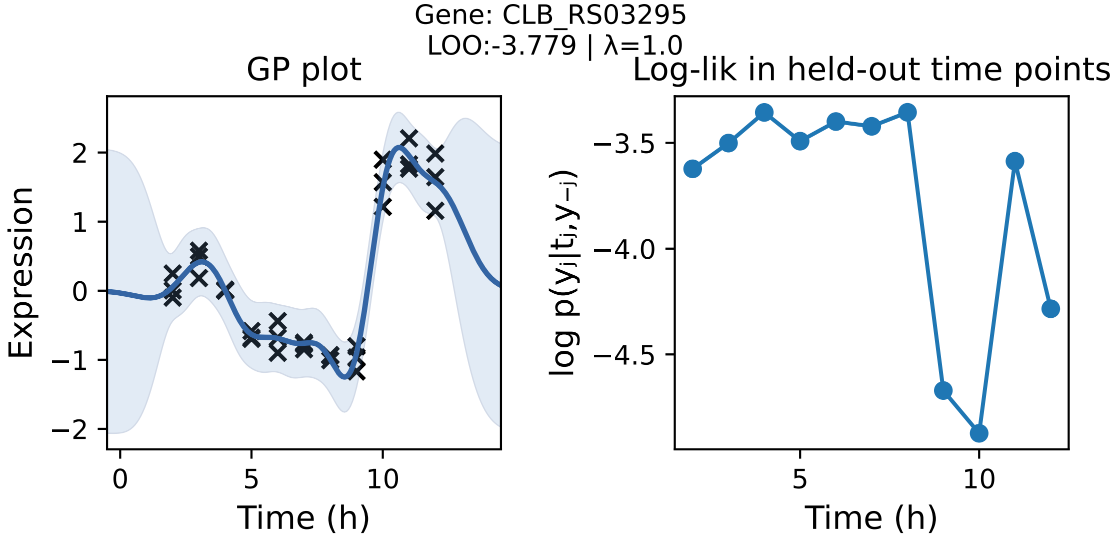

Gene: CLB_RS03295
LOO:-3.779 | λ=1.0
GP plot
Log-lik in held-out time points
Expression
log p(y_j|t_j,y_−j)
Time (h)
Time (h)
2
1
0
−1
−2
0
5
10
−3.5
−4.0
−4.5
5
10

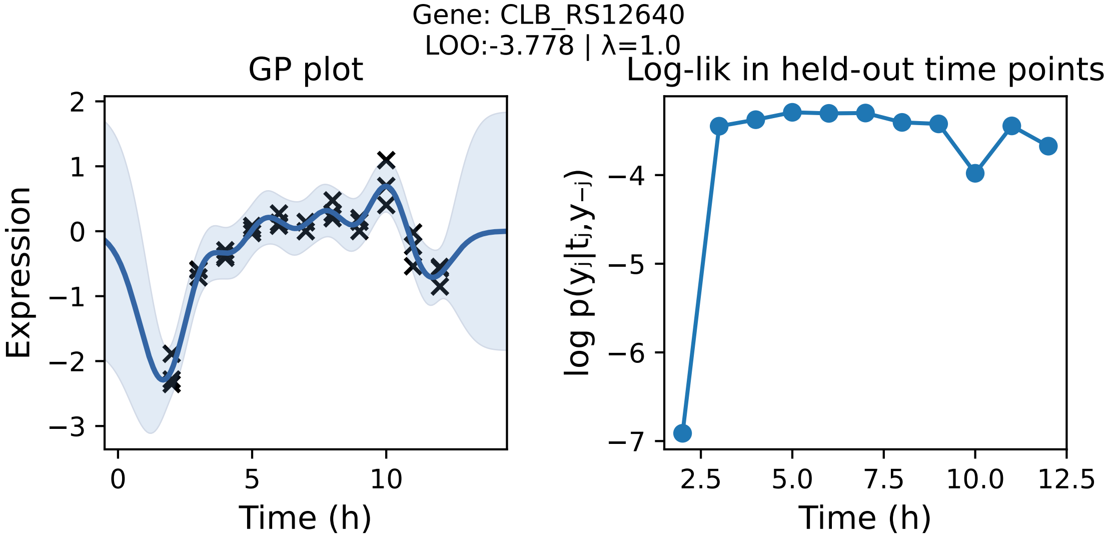
Gene: CLB_RS12640
LOO:-3.778 | λ=1.0
GP plot
Expression
Time (h)
Log-lik in held-out time points
log p(y_j|t_j,y_−j)
Time (h)

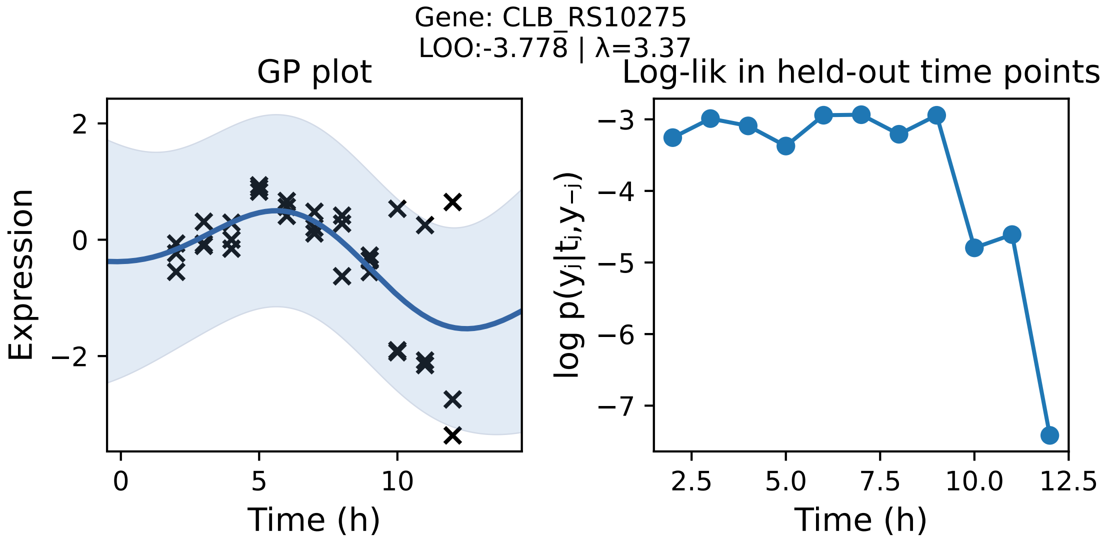
Gene: CLB_RS10275
LOO:-3.778 | λ=3.37
GP plot
Expression
Time (h)
Log-lik in held-out time points
log p(yj|tj,y−j)
Time (h)

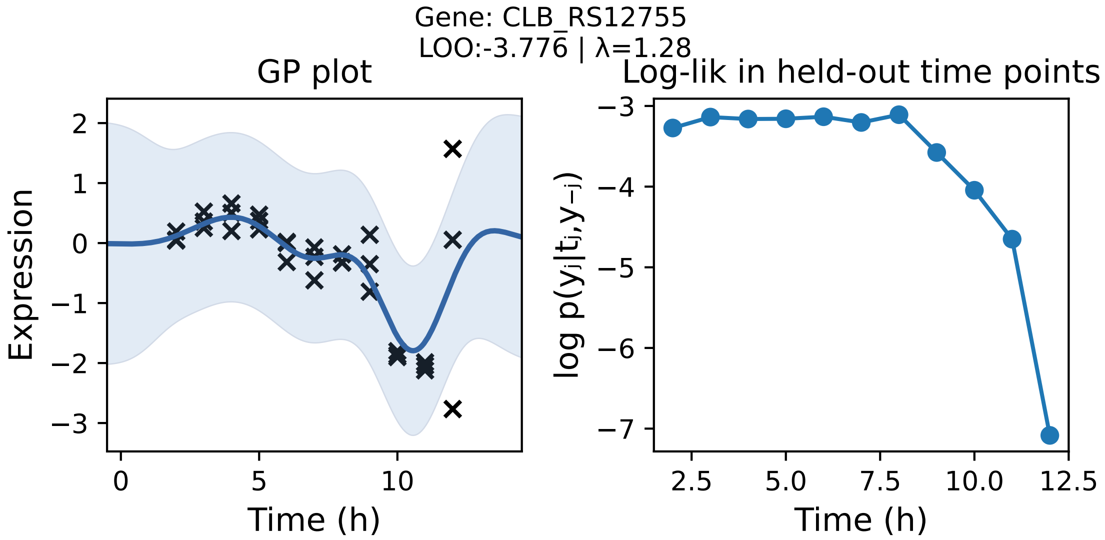

Gene: CLB_RS12755
LOO:-3.776 | λ=1.28
GP plot
Expression
Time (h)
Log-lik in held-out time points
log p(y_j|t_j,y_−j)
Time (h)

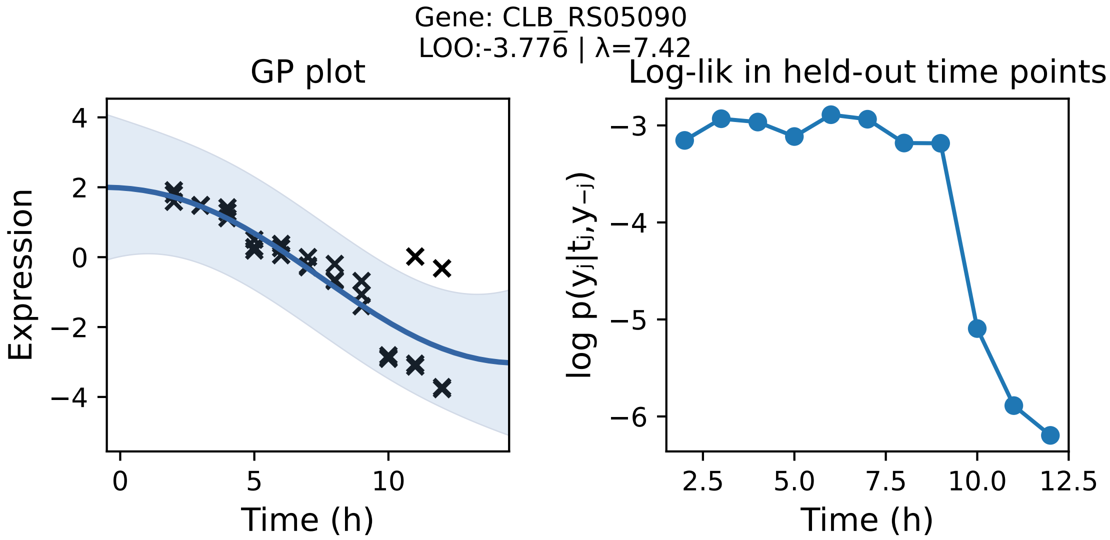
Gene: CLB_RS05090
LOO:-3.776 | λ=7.42
GP plot
Expression
Time (h)
Log-lik in held-out time points
log p(y_j|t_j,y_−j)
Time (h)

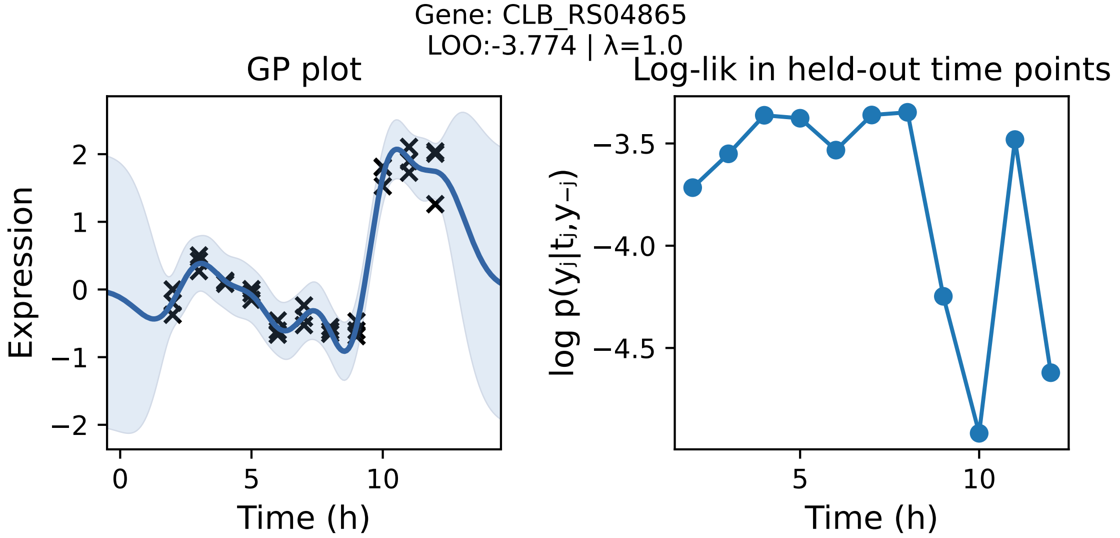

Gene: CLB_RS04865
LOO:-3.774 | λ=1.0
GP plot
Expression
Time (h)
2
1
0
−1
−2
0
5
10
Log-lik in held-out time points
log p(y_j|t_j,y_−j)
Time (h)
−3.5
−4.0
−4.5
5
10

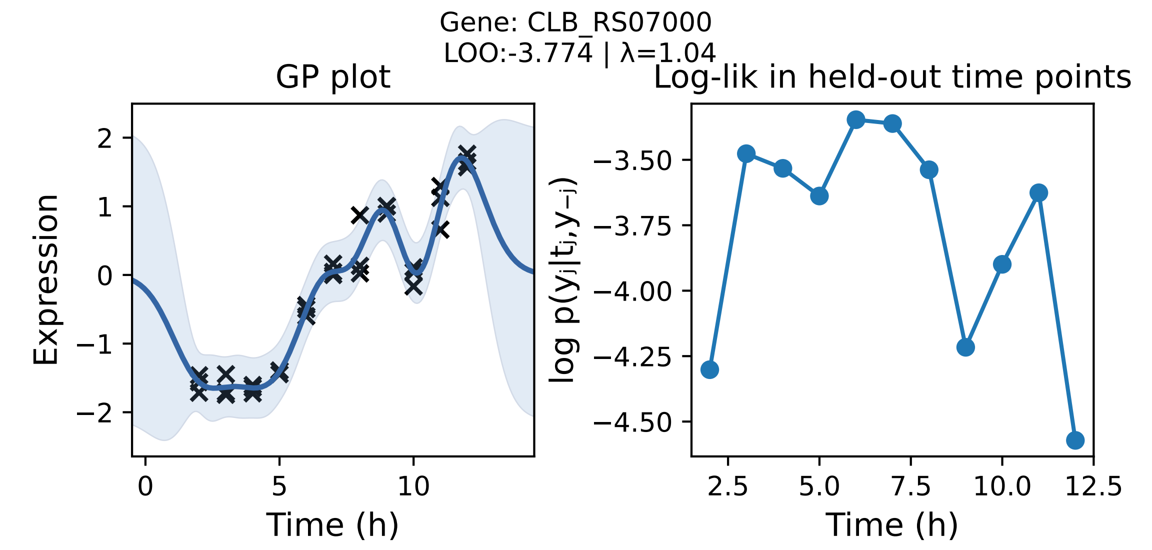

Gene: CLB_RS07000
LOO:-3.774 | λ=1.04
GP plot
Expression
Time (h)
Log-lik in held-out time points
log p(yj|tj,y−j)
Time (h)

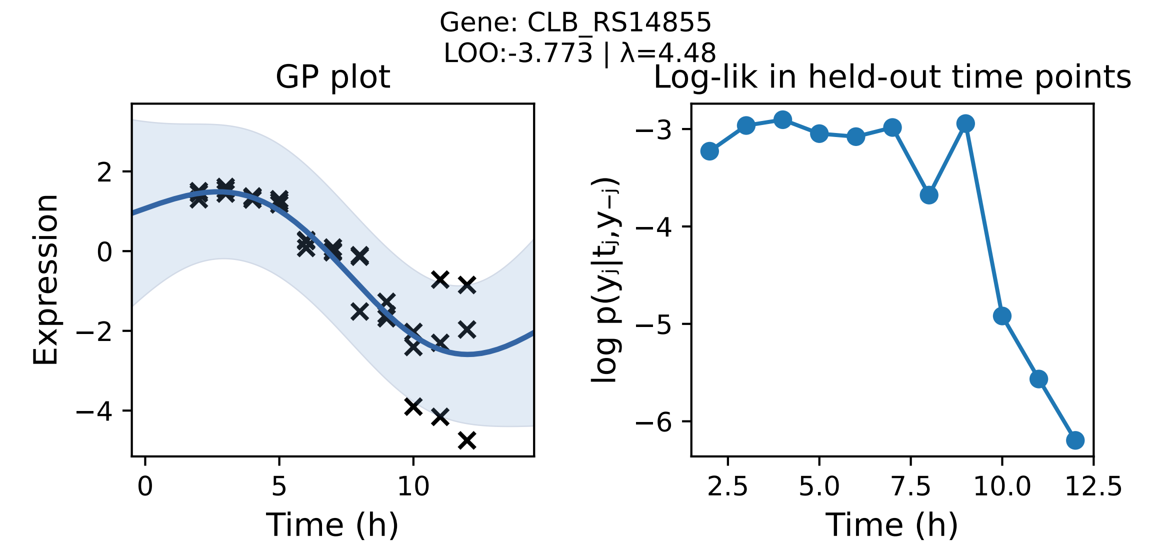
Gene: CLB_RS14855
LOO:-3.773 | λ=4.48
GP plot
Expression
Time (h)
Log-lik in held-out time points
log p(yj|tj,y−j)
Time (h)

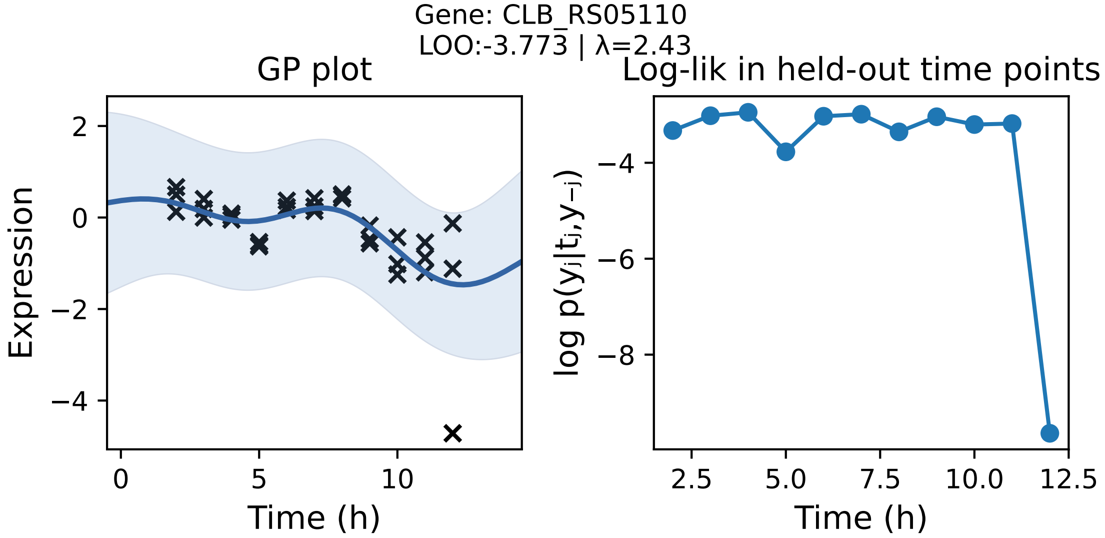

Gene: CLB_RS05110
LOO:-3.773 | λ=2.43
GP plot
Log-lik in held-out time points
Expression
Time (h)
log p(y_j|t_j,y_−j)
Time (h)
2
0
−2
−4
0
5
10
−4
−6
−8
2.5
5.0
7.5
10.0
12.5

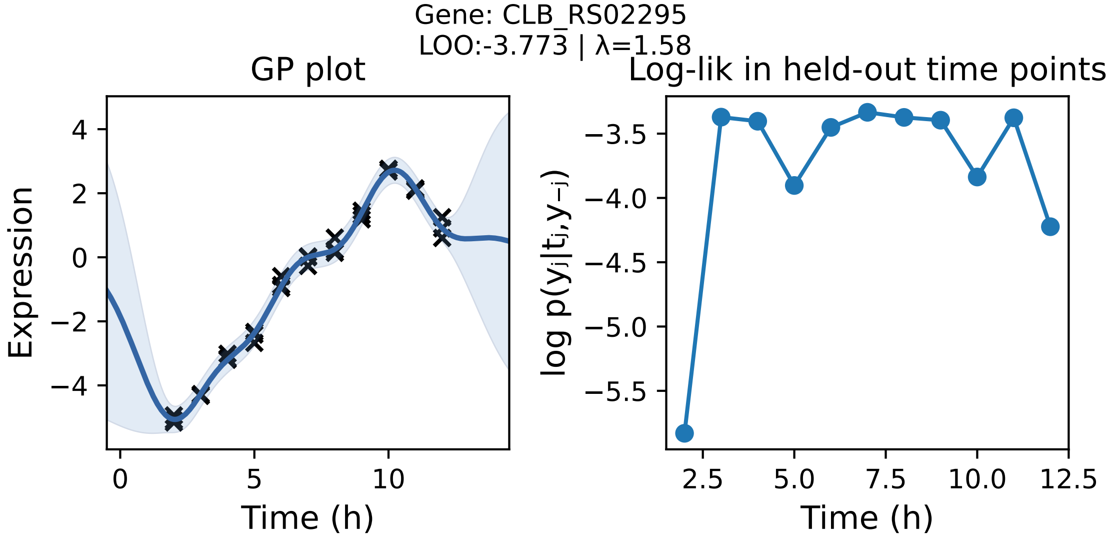

Gene: CLB_RS02295
LOO:-3.773 | λ=1.58
GP plot
Expression
Time (h)
Log-lik in held-out time points
log p(y_j|t_j,y_−j)
Time (h)

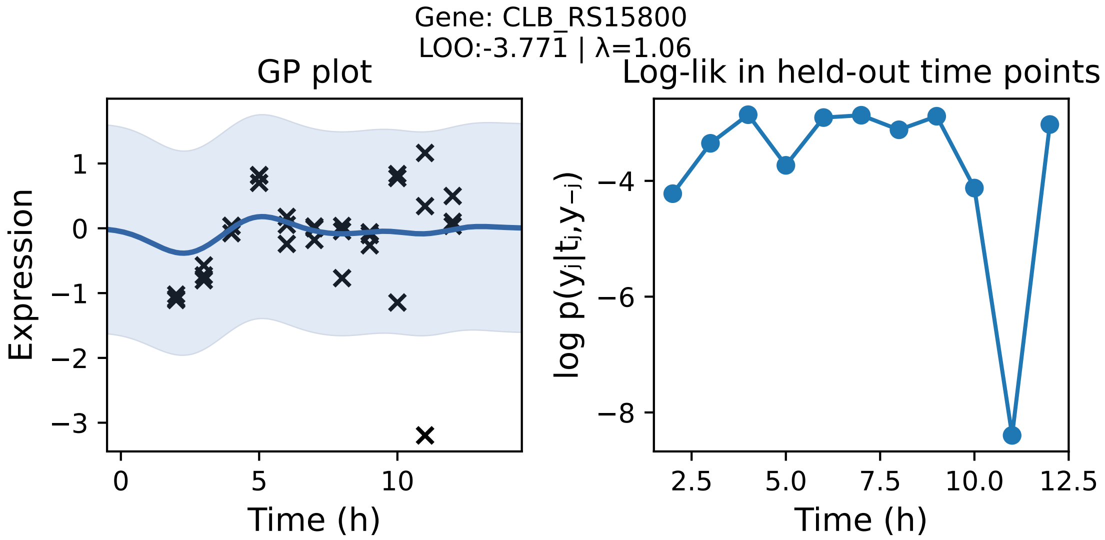

Gene: CLB_RS15800
LOO:-3.771 | λ=1.06
GP plot
Expression
Time (h)
Log-lik in held-out time points
log p(yj|tj,y−j)
Time (h)

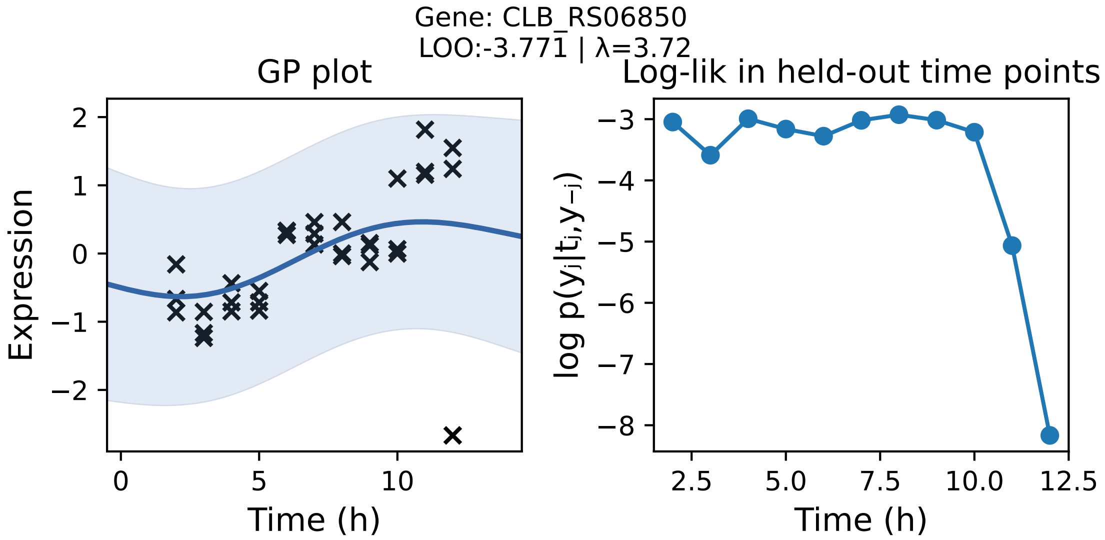

Gene: CLB_RS06850
LOO:-3.771 | λ=3.72
GP plot
Expression
Time (h)
2
1
0
−1
−2
0
5
10
Log-lik in held-out time points
log p(y_j|t_j,y_−j)
Time (h)
−3
−4
−5
−6
−7
−8
2.5
5.0
7.5
10.0
12.5

Gene: CLB_RS15090
LOO:-3.77 | λ=1.24

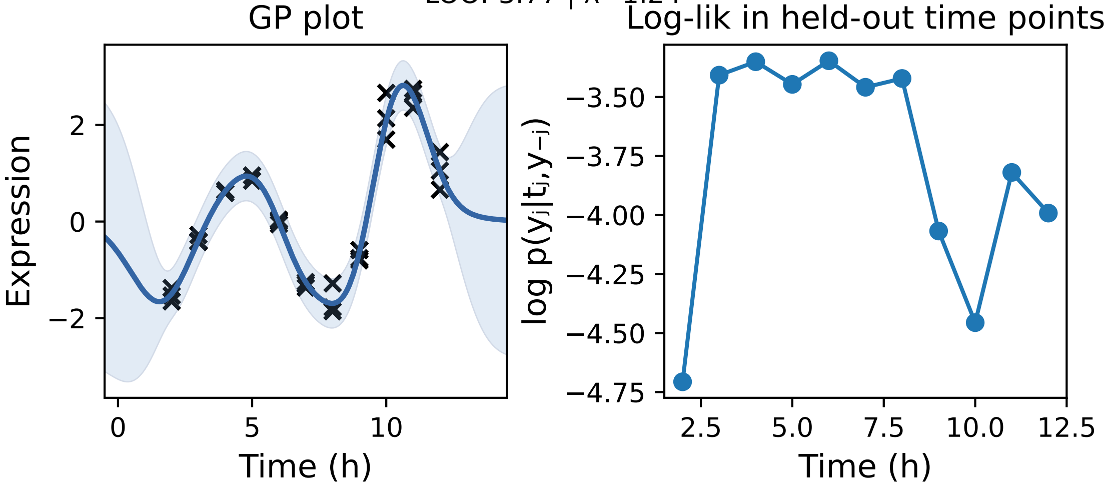

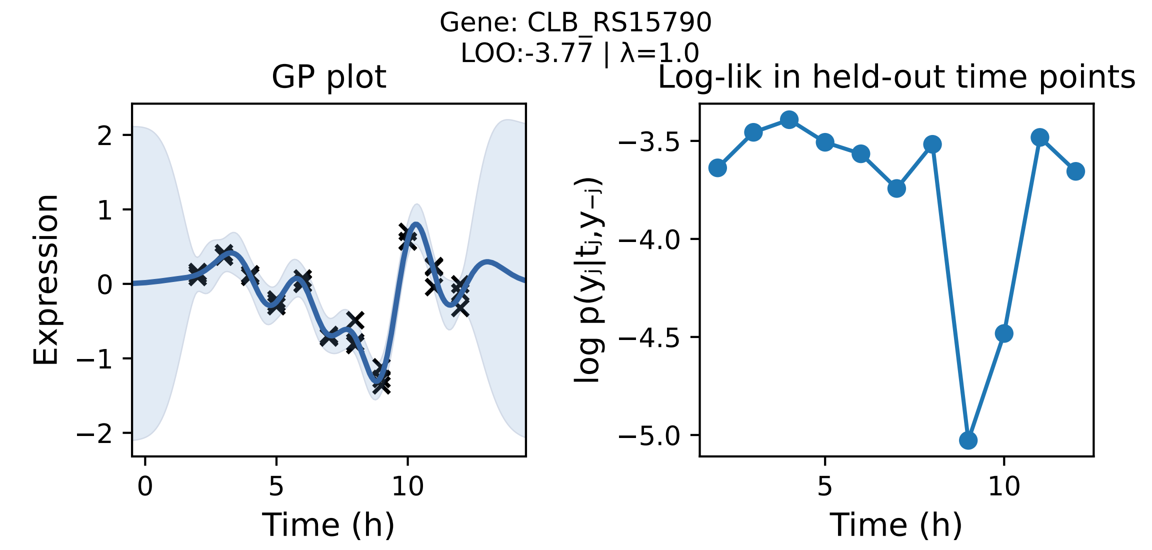

Gene: CLB_RS15790
LOO:-3.77 | λ=1.0
GP plot
Expression
2
1
0
−1
−2
0
5
10
Time (h)
Log-lik in held-out time points
log p(y_j|t_j,y_−j)
−3.5
−4.0
−4.5
−5.0
5
10
Time (h)

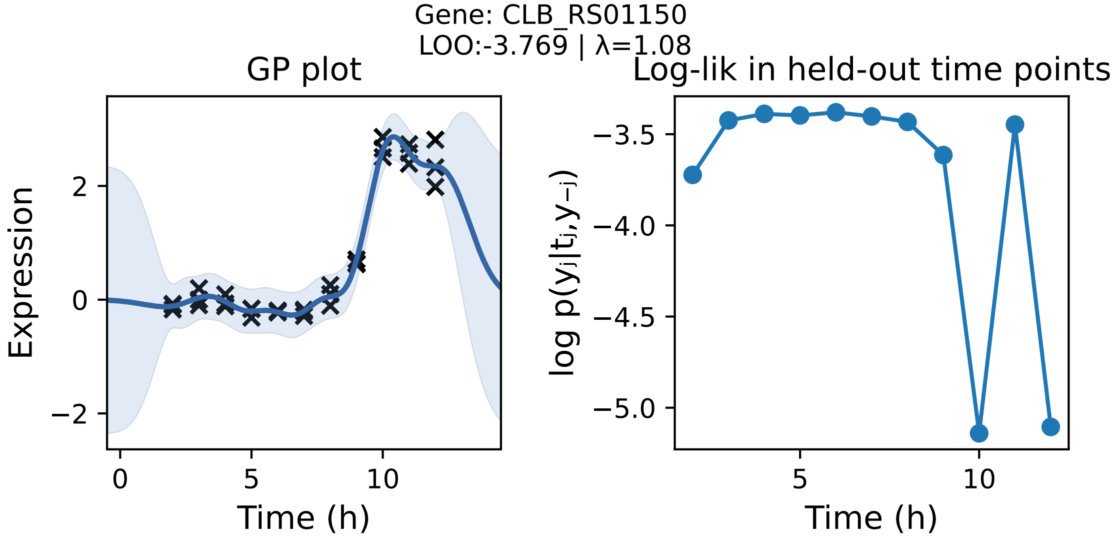
Gene: CLB_RS01150
LOO:-3.769 | λ=1.08
GP plot
Expression
Time (h)
2
0
−2
0
5
10
Log-lik in held-out time points
log p(y_j|t_j,y_−j)
Time (h)
−3.5
−4.0
−4.5
−5.0
5
10

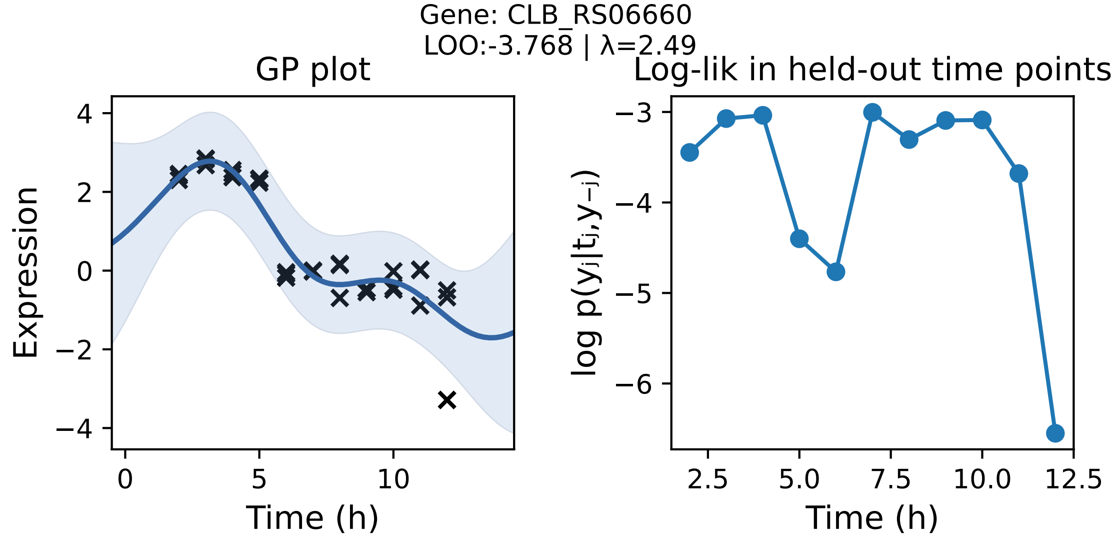

Gene: CLB_RS06660
LOO:-3.768 | λ=2.49
GP plot
Expression
Time (h)
Log-lik in held-out time points
log p(y_j|t_j,y_−j)
Time (h)

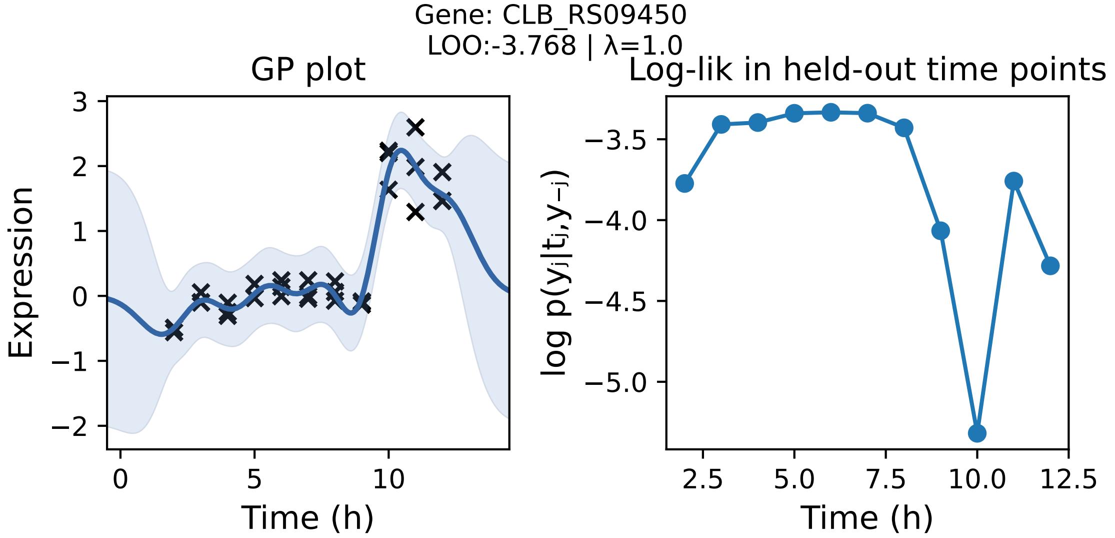

Gene: CLB_RS09450
LOO:-3.768 | λ=1.0
GP plot
Expression
Time (h)
Log-lik in held-out time points
log p(yj|tj,y−j)
Time (h)

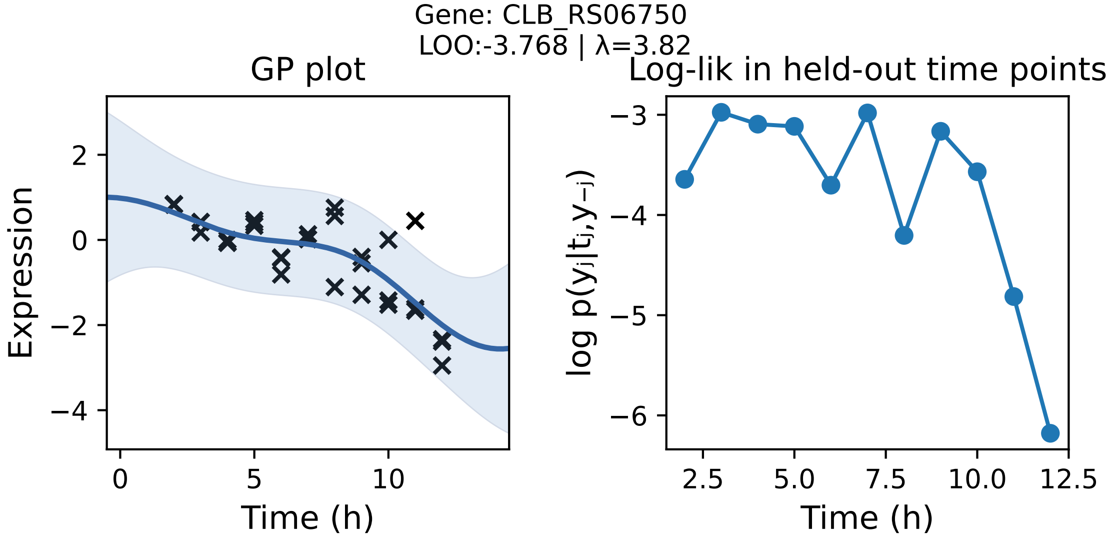

Gene: CLB_RS06750
LOO:-3.768 | λ=3.82
GP plot
Expression
Time (h)
Log-lik in held-out time points
log p(y_j|t_j,y_−j)
Time (h)

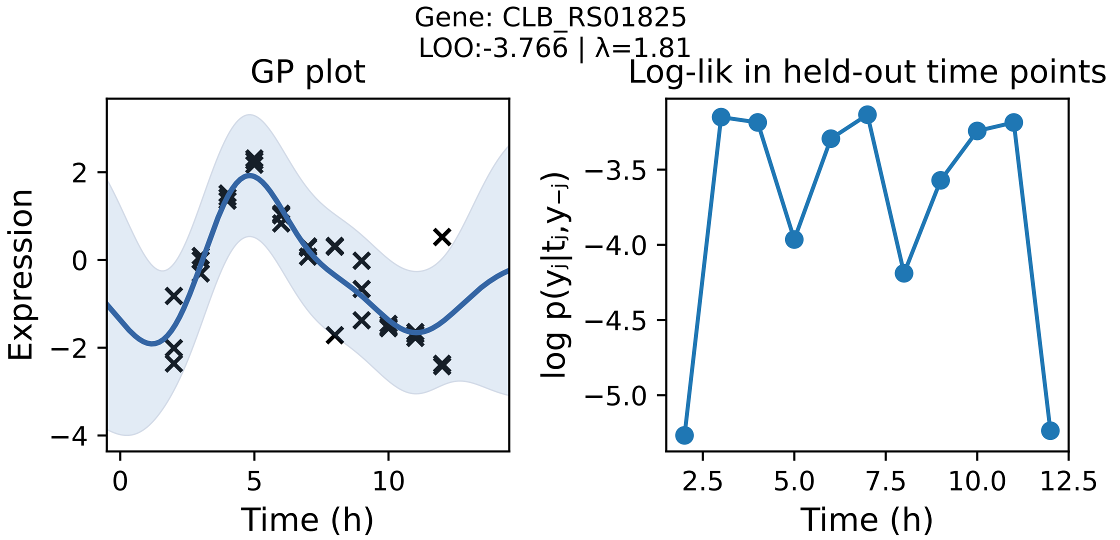

Gene: CLB_RS01825
LOO:-3.766 | λ=1.81
GP plot
Log-lik in held-out time points
Expression
Time (h)
log p(y_j|t_j,y_−j)
2
0
−2
−4
0
5
10
−3.5
−4.0
−4.5
−5.0
2.5
5.0
7.5
10.0
12.5

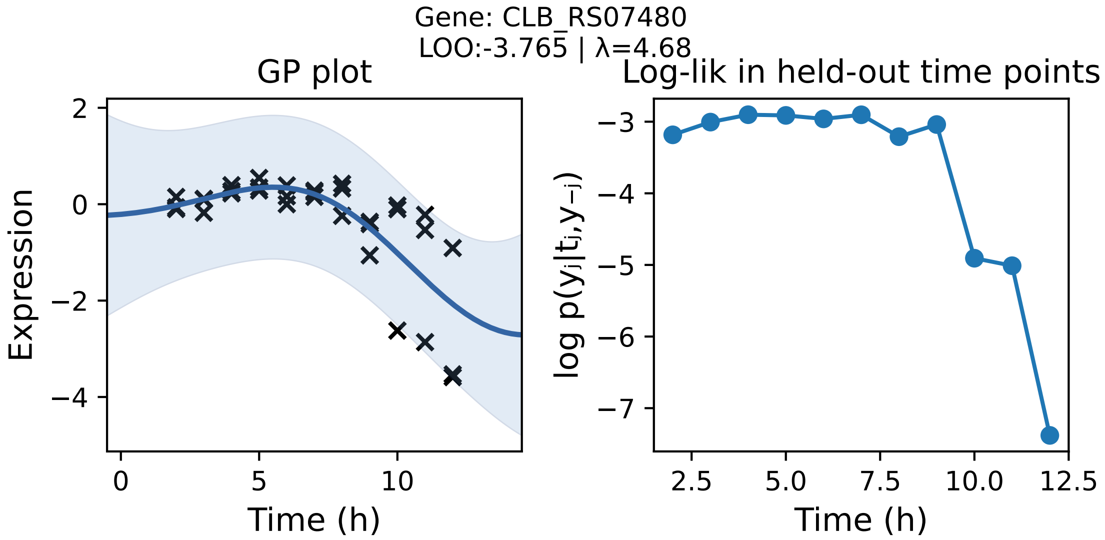

Gene: CLB_RS07480
LOO:-3.765 | λ=4.68
GP plot
Log-lik in held-out time points
Expression
Time (h)
log p(yj|tj,y−j)
Time (h)

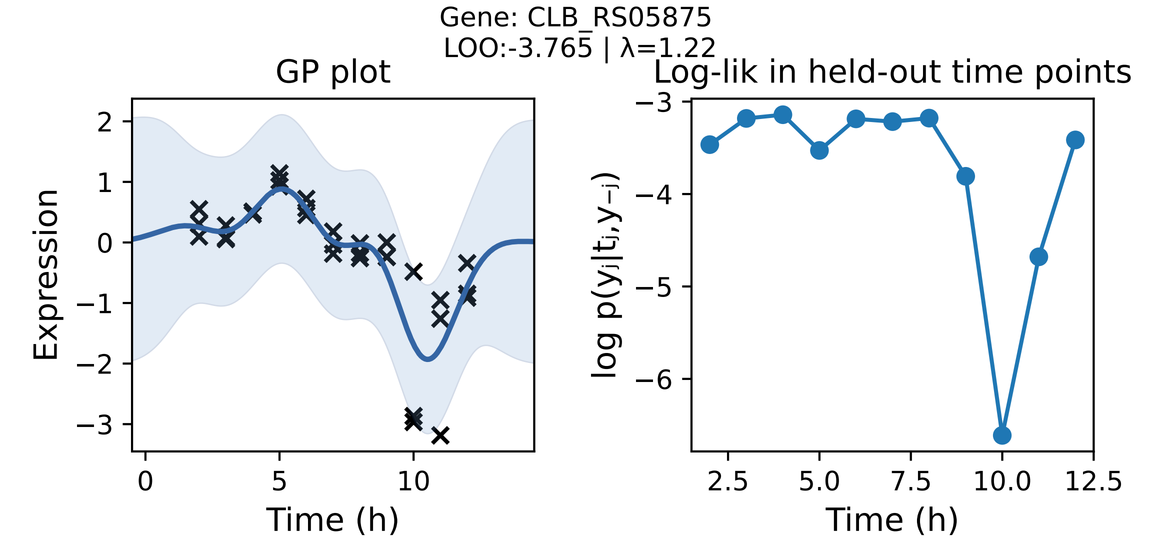
Gene: CLB_RS05875
LOO:-3.765 | λ=1.22
GP plot
Expression
Time (h)
Log-lik in held-out time points
log p(y_j|t_j,y_−j)
Time (h)

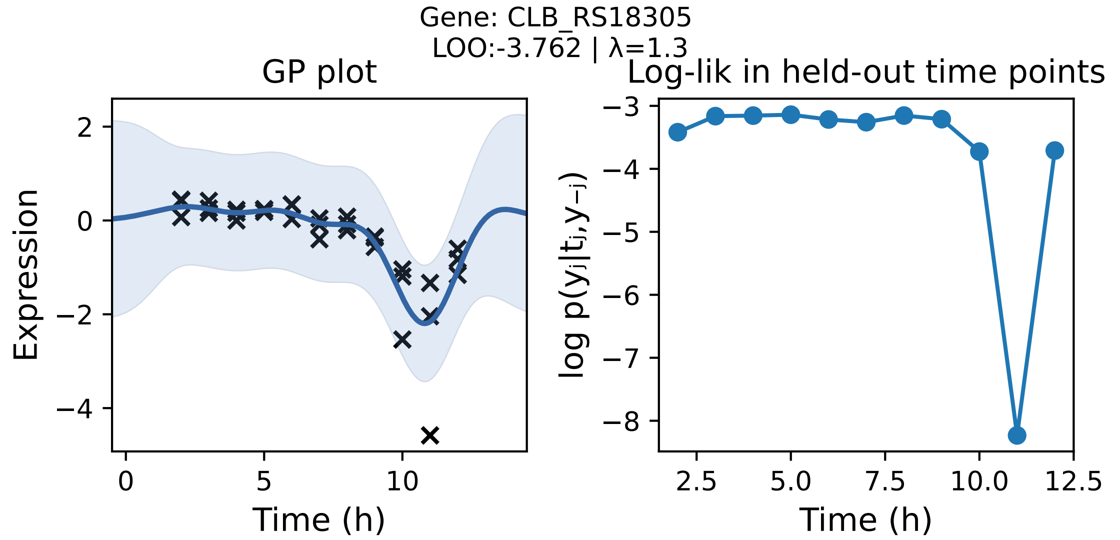

Gene: CLB_RS18305
LOO:-3.762 | λ=1.3
GP plot
Log-lik in held-out time points
Expression
2
0
−2
−4
0
5
10
Time (h)
log p(yⱼ|tⱼ,y₋ⱼ)
−3
−4
−5
−6
−7
−8
2.5
5.0
7.5
10.0
12.5
Time (h)

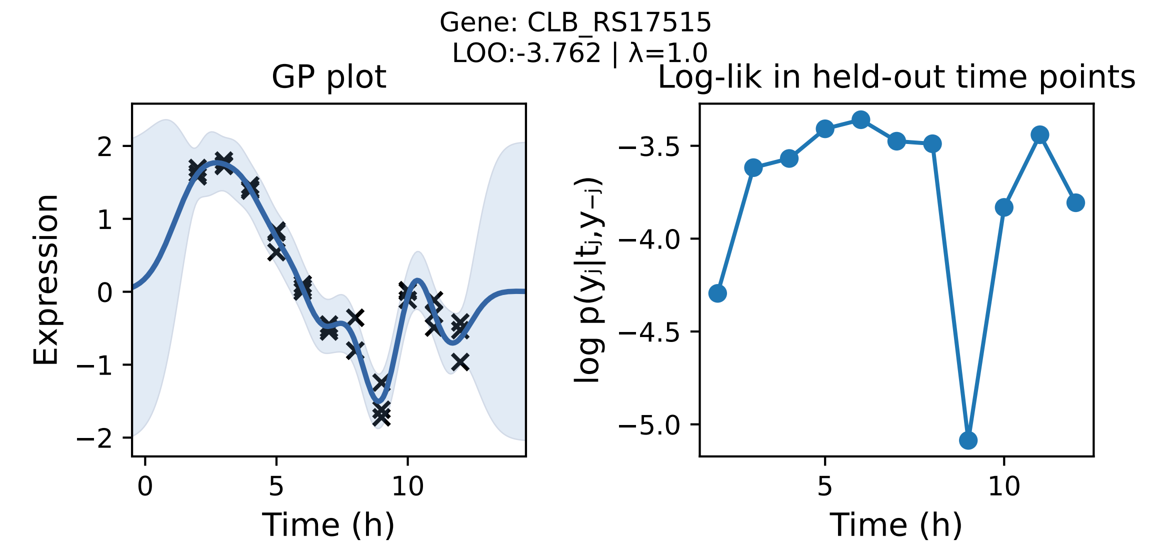
Gene: CLB_RS17515
LOO:-3.762 | λ=1.0
GP plot
Expression
Time (h)
Log-lik in held-out time points
log p(y_j|t_j,y_−j)
Time (h)

Gene: CLB_RS04230
LOO:-3.761 | λ=4.4

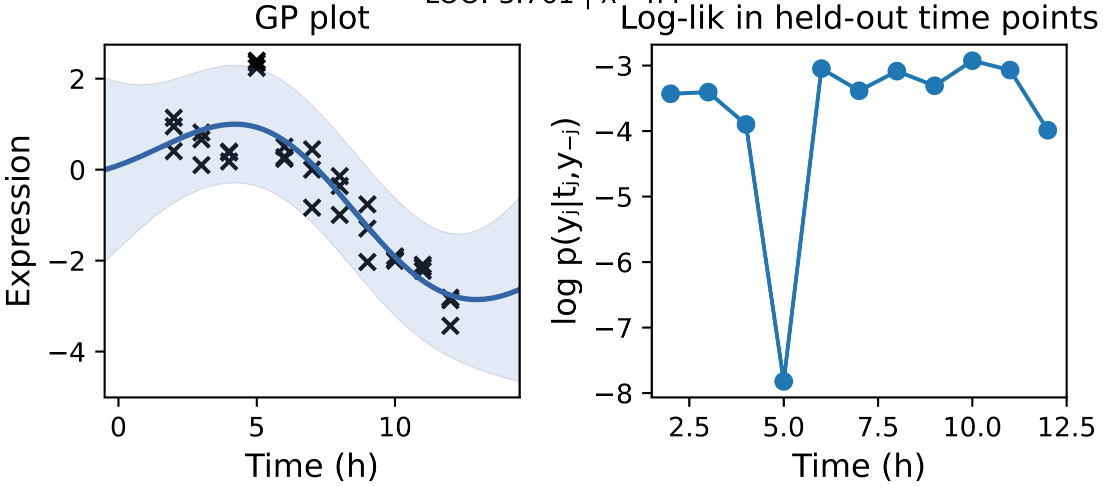

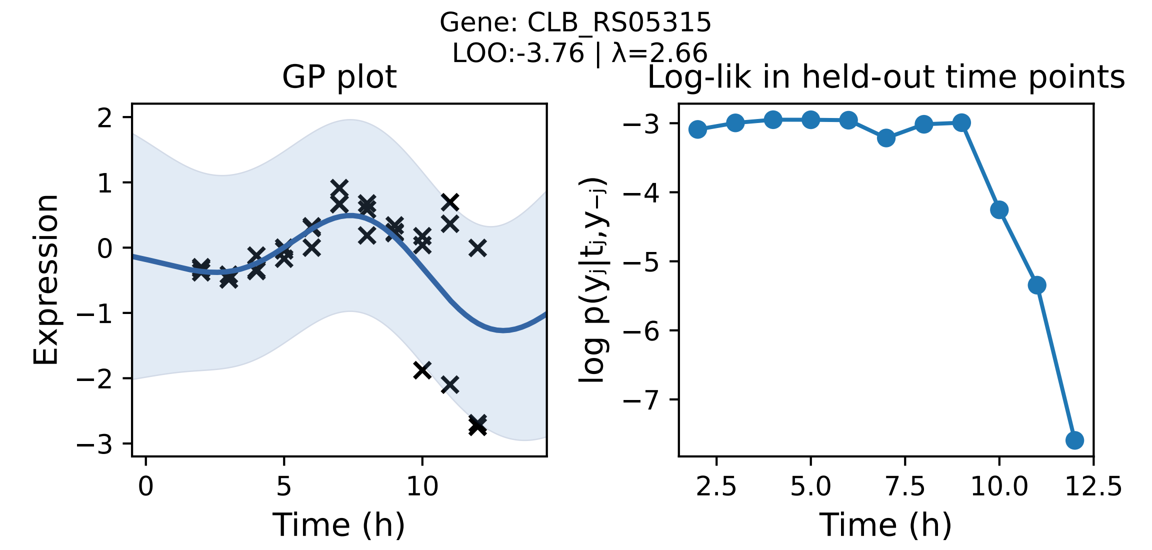

Gene: CLB_RS05315
LOO:-3.76 | λ=2.66
GP plot
Log-lik in held-out time points
Expression
Time (h)
log p(y_j|t_j,y_−j)
Time (h)

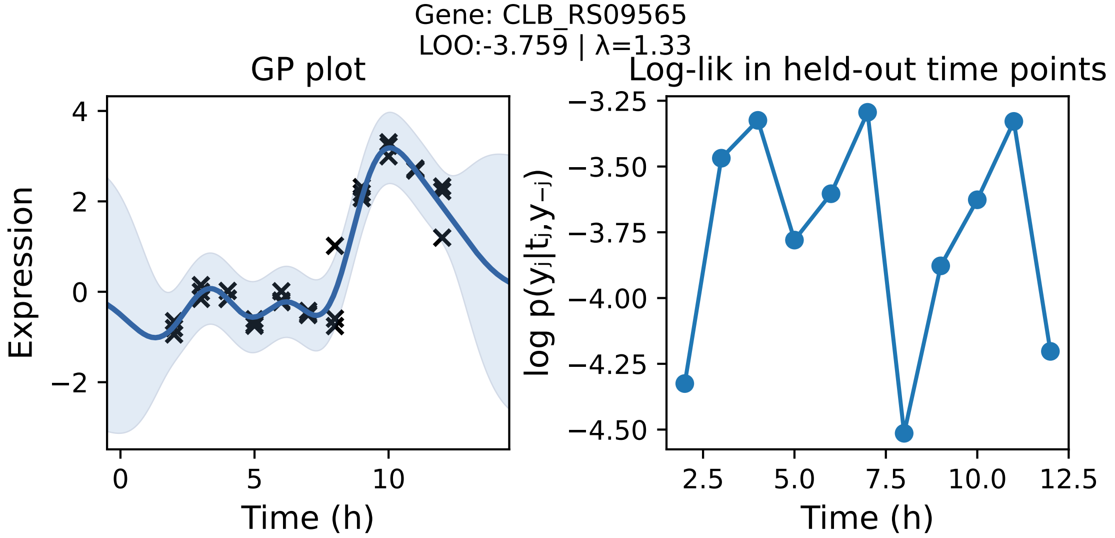
Gene: CLB_RS09565
LOO:-3.759 | λ=1.33
GP plot
Expression
Time (h)
Log-lik in held-out time points
log p(y_j|t_j,y_−j)
Time (h)
4
2
0
−2
0
5
10
−3.25
−3.50
−3.75
−4.00
−4.25
−4.50
2.5
5.0
7.5
10.0
12.5

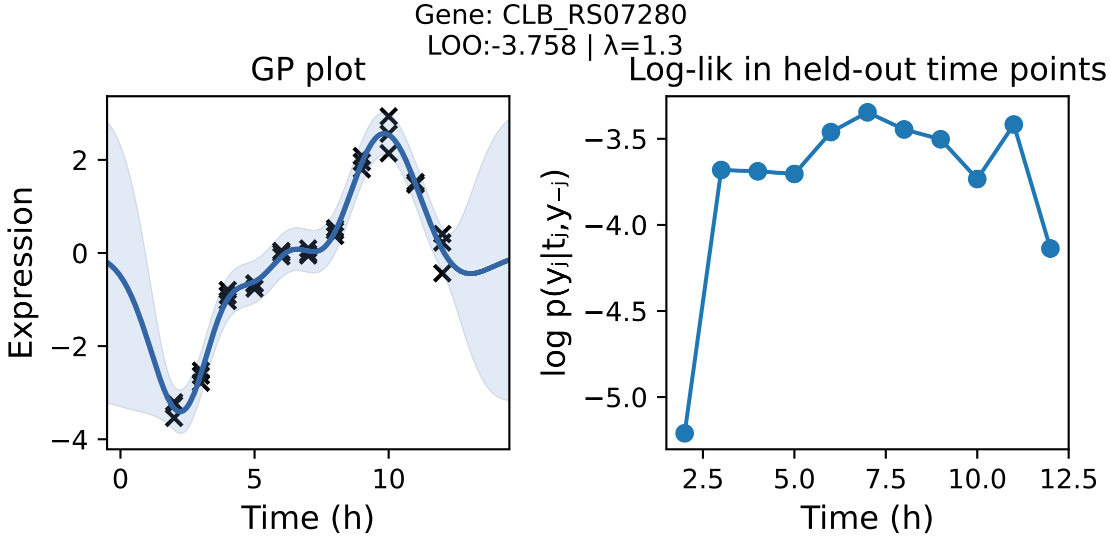

Gene: CLB_RS07280
LOO:-3.758 | λ=1.3
GP plot
Expression
Time (h)
Log-lik in held-out time points
log p(y_j|t_j,y_−j)
Time (h)

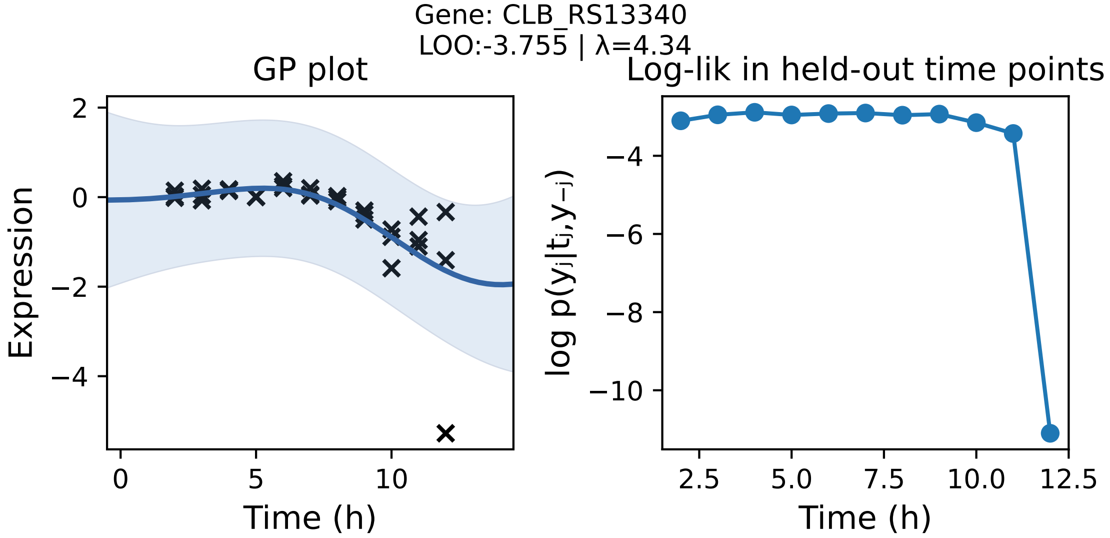

Gene: CLB_RS13340
LOO:-3.755 | λ=4.34
GP plot
Expression
Time (h)
2
0
−2
−4
0
5
10
Log-lik in held-out time points
log p(y_j|t_j,y_−j)
Time (h)
−4
−6
−8
−10
2.5
5.0
7.5
10.0
12.5

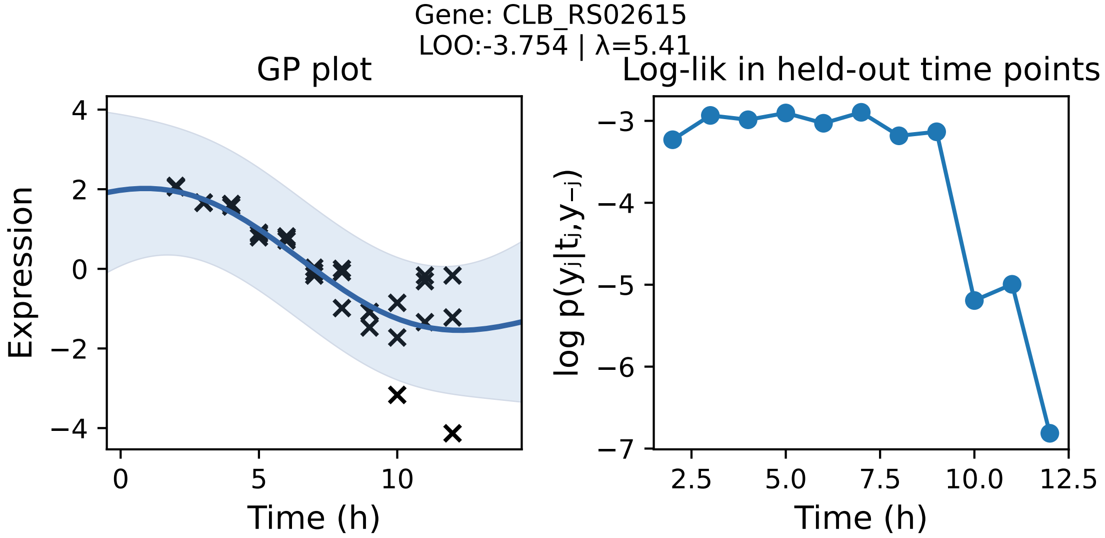

Gene: CLB_RS02615
LOO:-3.754 | λ=5.41
GP plot
Expression
Time (h)
4
2
0
−2
−4
0
5
10
Log-lik in held-out time points
log p(yj|tj,y−j)
Time (h)
−3
−4
−5
−6
−7
2.5
5.0
7.5
10.0
12.5

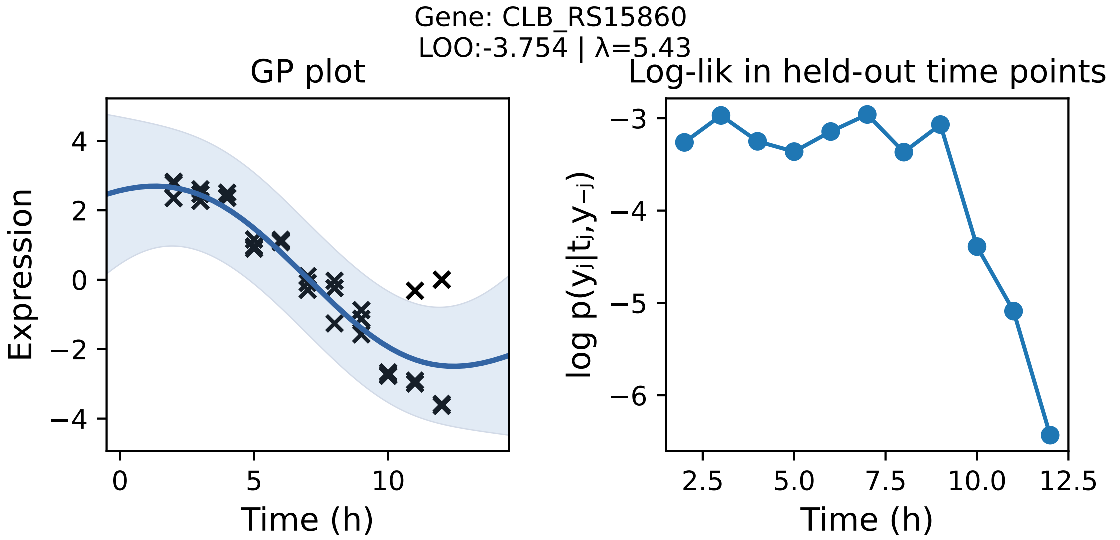

Gene: CLB_RS15860
LOO:-3.754 | λ=5.43
GP plot
Expression
Time (h)
Log-lik in held-out time points
log p(y_j|t_j,y_−j)
Time (h)

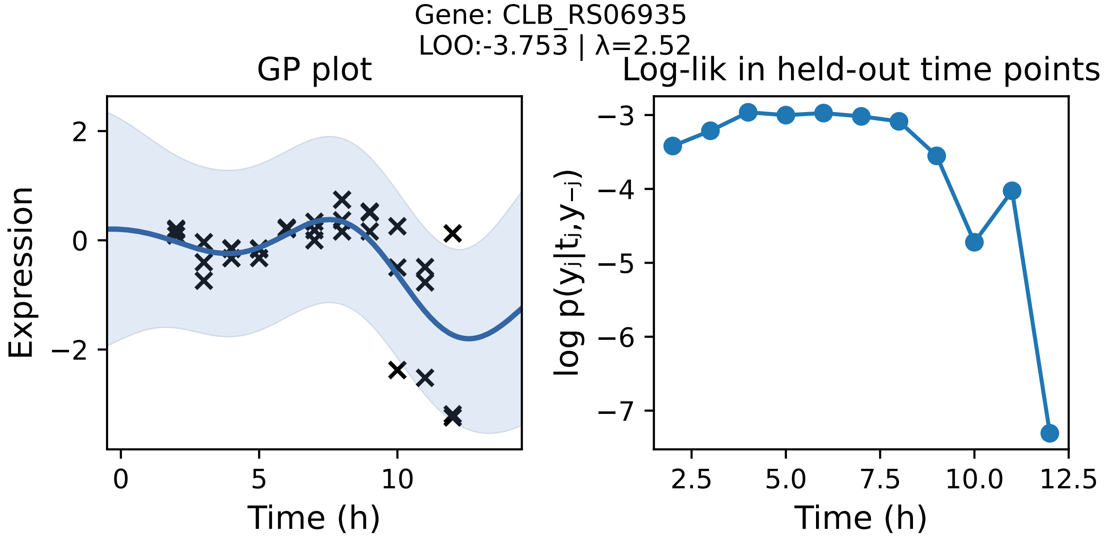

Gene: CLB_RS06935
LOO:-3.753 | λ=2.52
GP plot
Expression
Time (h)
Log-lik in held-out time points
log p(y_j|t_j,y_−j)
Time (h)

Gene: CLB_RS12910
LOO:-3.752 | λ=4.06

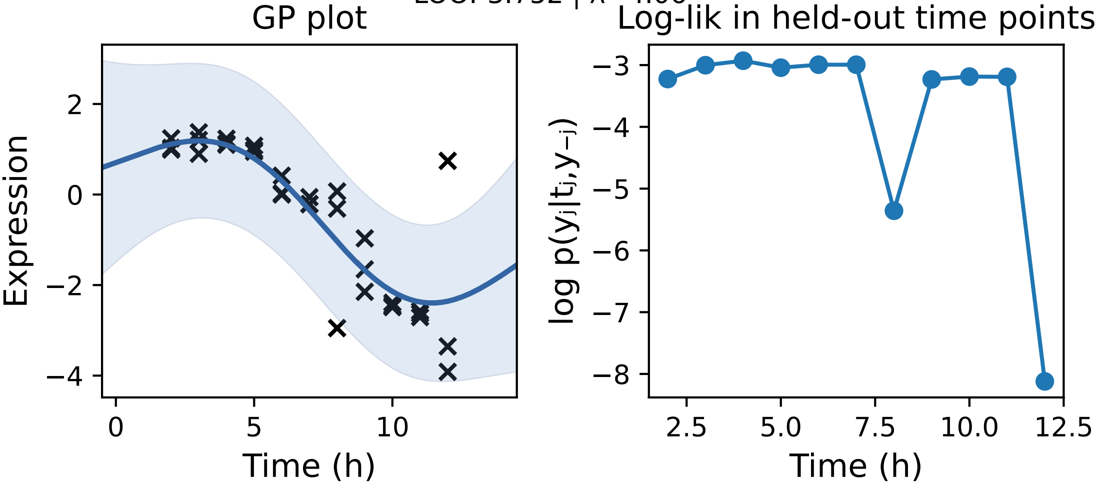

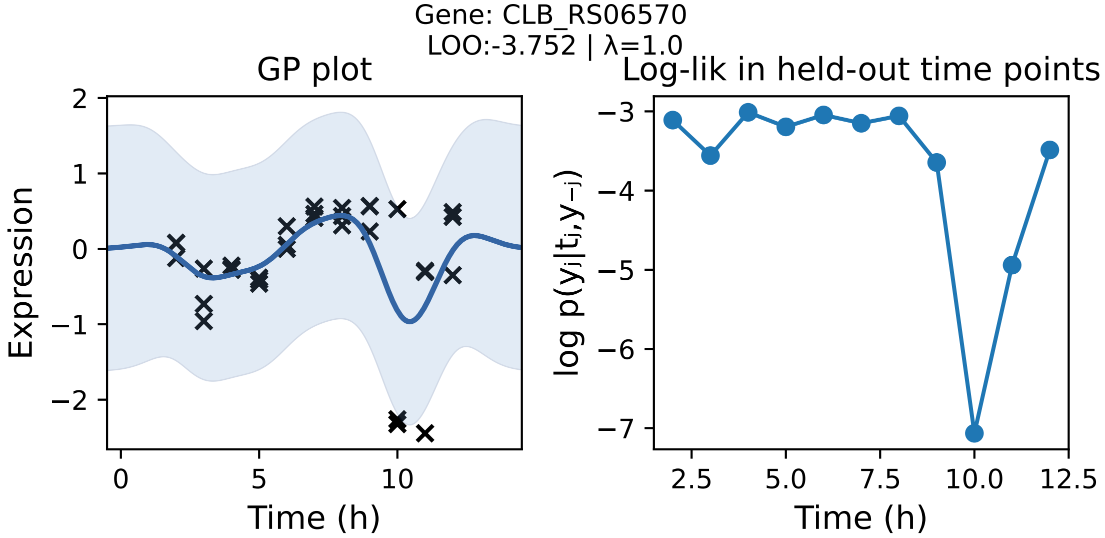

Gene: CLB_RS06570
LOO:-3.752 | λ=1.0
GP plot
Expression
Time (h)
2
1
0
−1
−2
0
5
10
Log-lik in held-out time points
log p(y_j|t_j,y_−j)
Time (h)
−3
−4
−5
−6
−7
2.5
5.0
7.5
10.0
12.5

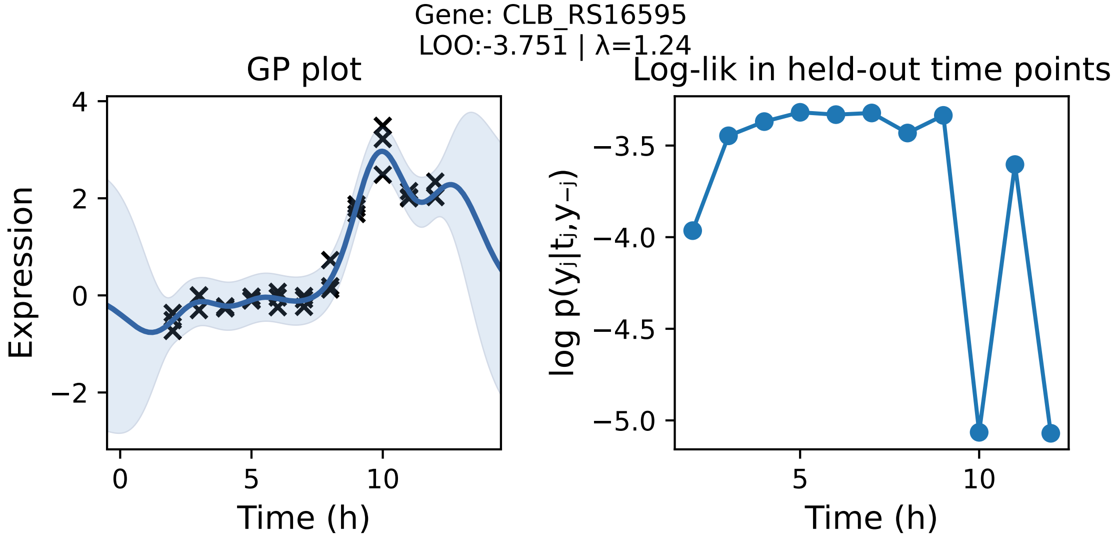
Gene: CLB_RS16595
LOO:-3.751 | λ=1.24
GP plot
Expression
Time (h)
Log-lik in held-out time points
log p(y_j|t_j,y_−j)
Time (h)

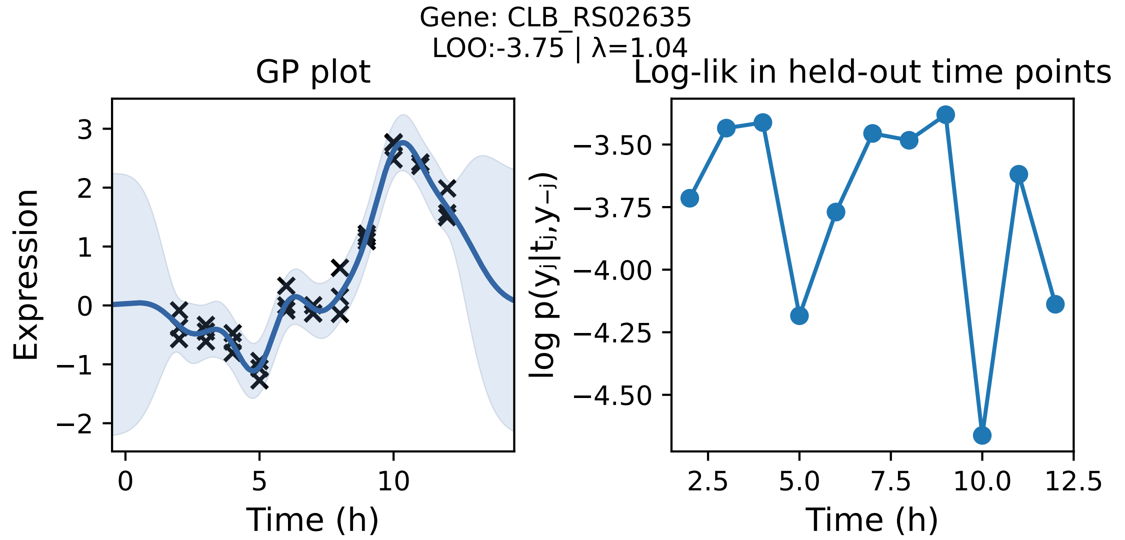
Gene: CLB_RS02635
LOO:-3.75 | λ=1.04
GP plot
Expression
Time (h)
3
2
1
0
−1
−2
0
5
10
Log-lik in held-out time points
log p(y_j|t_j,y_−j)
Time (h)
−3.50
−3.75
−4.00
−4.25
−4.50
2.5
5.0
7.5
10.0
12.5

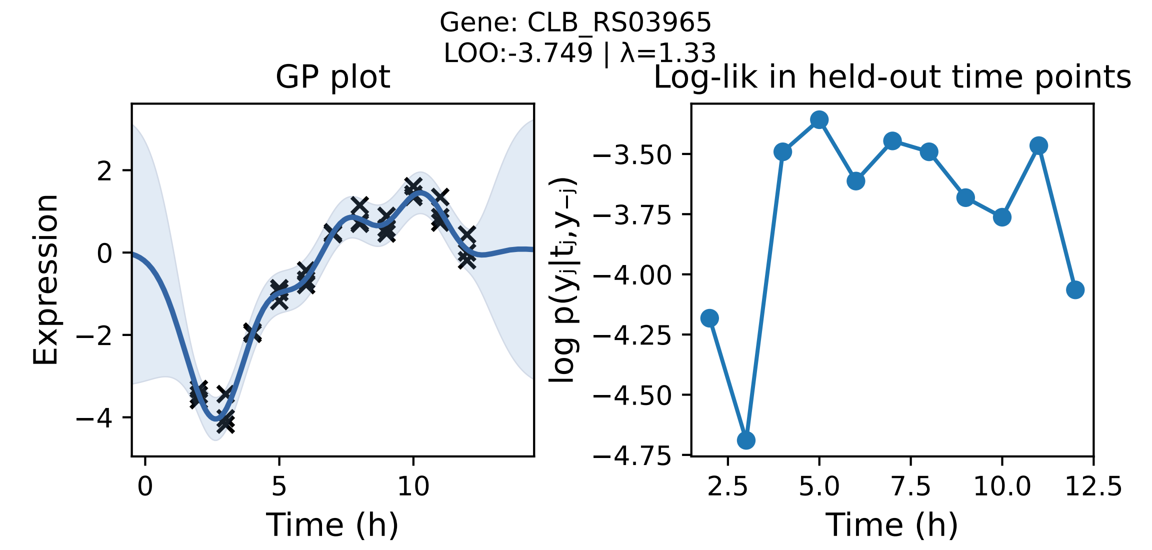

Gene: CLB_RS03965
LOO:-3.749 | λ=1.33
GP plot
Expression
Time (h)
Log-lik in held-out time points
log p(yⱼ|tⱼ,y₋ⱼ)
Time (h)

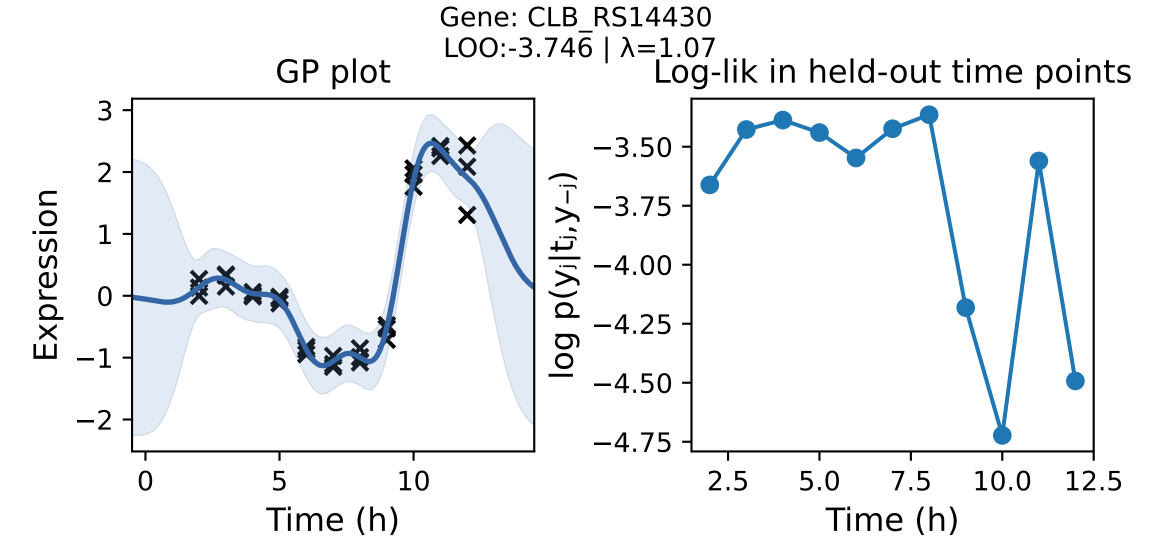

Gene: CLB_RS14430
LOO:-3.746 | λ=1.07
GP plot
Expression
Time (h)
Log-lik in held-out time points
log p(y_j|t_j,y_−j)
Time (h)

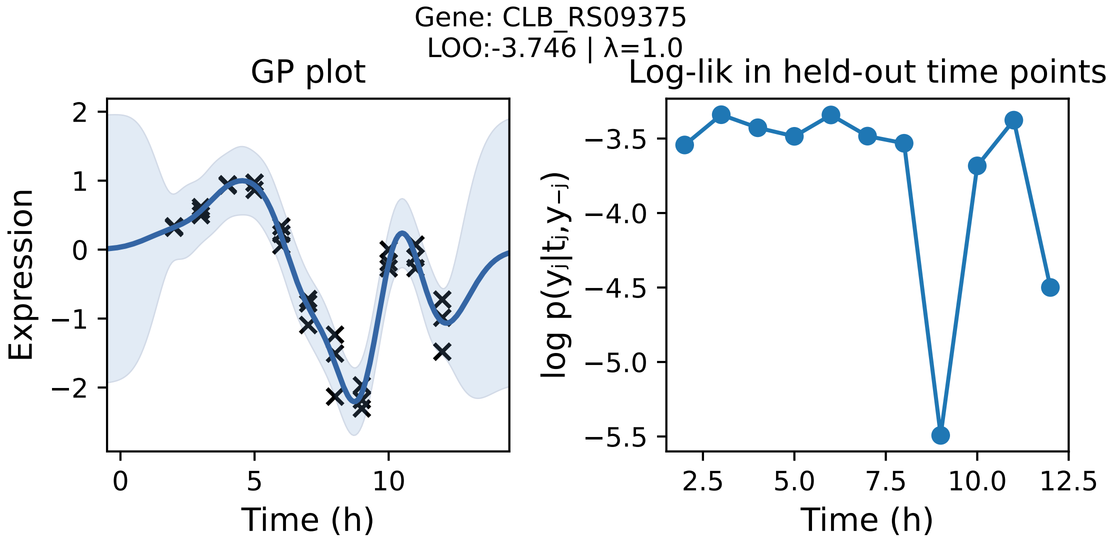

Gene: CLB_RS09375
LOO:-3.746 | λ=1.0
GP plot
Log-lik in held-out time points
Expression
Time (h)
log p(y_j|t_j,y_−j)
Time (h)
2
1
0
−1
−2
0
5
10
−3.5
−4.0
−4.5
−5.0
−5.5
2.5
5.0
7.5
10.0
12.5

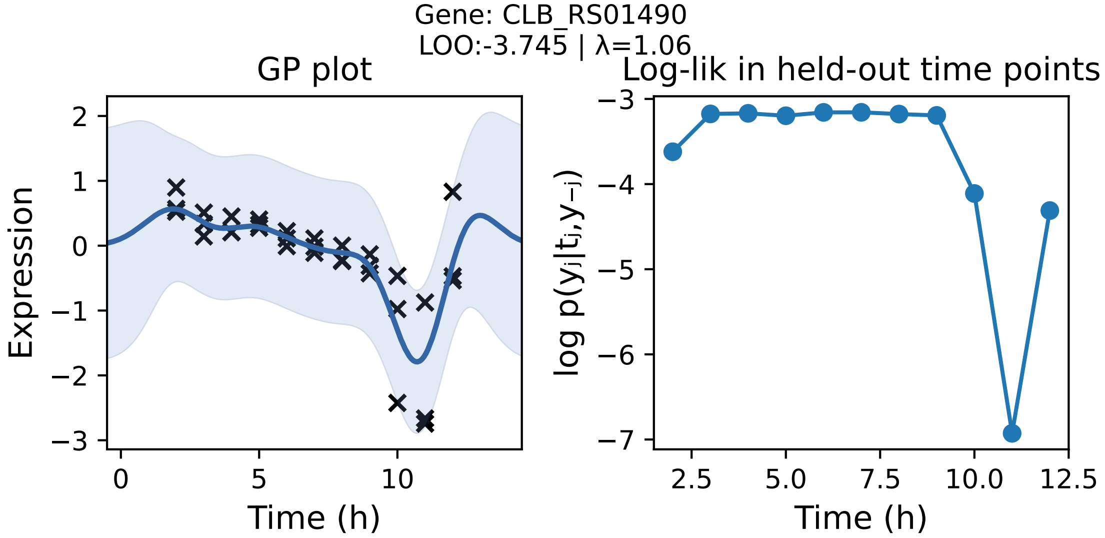

Gene: CLB_RS01490
LOO:-3.745 | λ=1.06
GP plot
Expression
Time (h)
Log-lik in held-out time points
log p(y_j|t_j,y_−j)
Time (h)

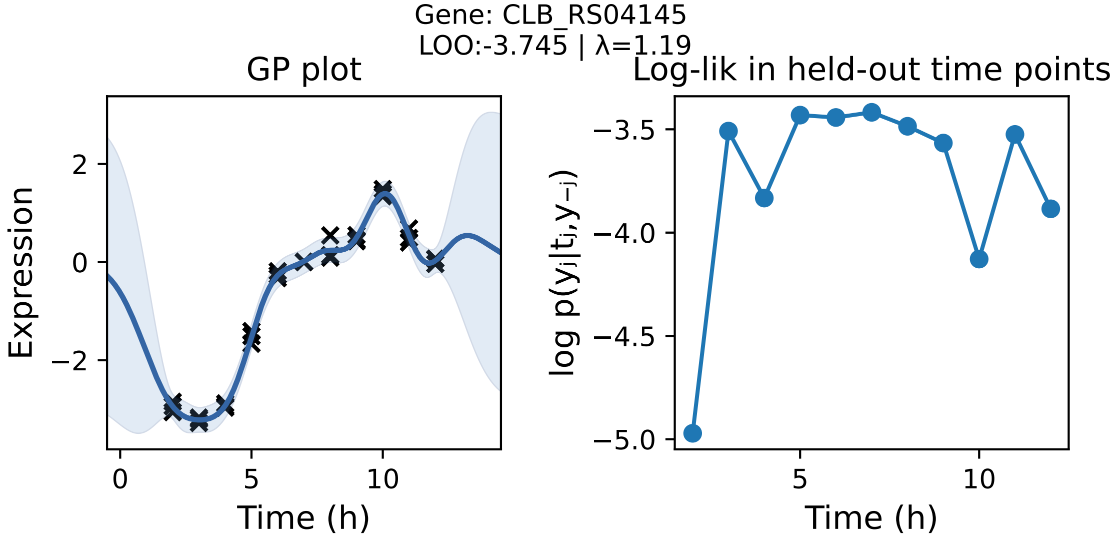

Gene: CLB_RS04145
LOO:-3.745 | λ=1.19
GP plot
Expression
Time (h)
Log-lik in held-out time points
log p(y_j|t_j,y_−j)
Time (h)

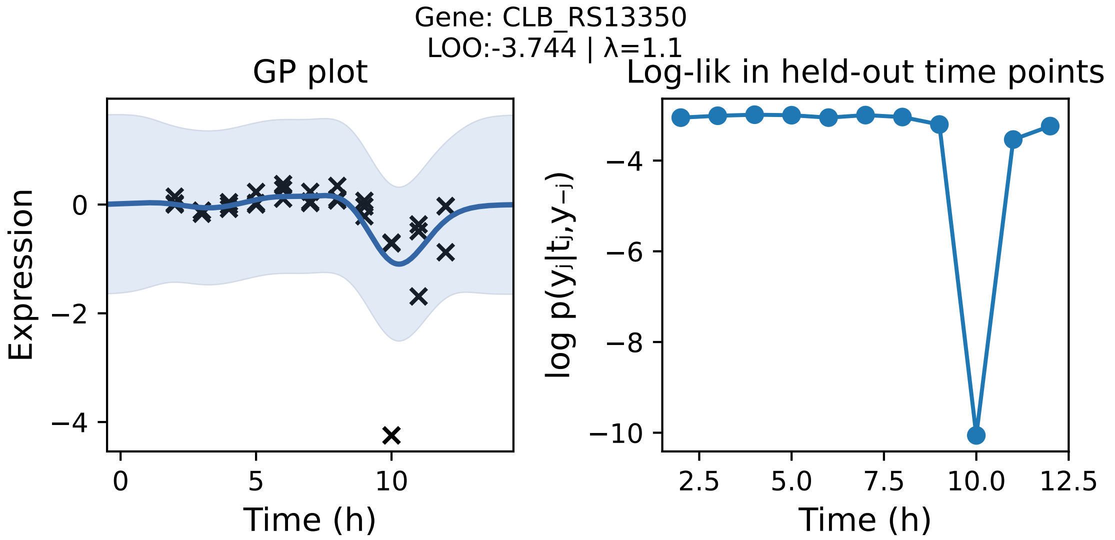
Gene: CLB_RS13350
LOO:-3.744 | λ=1.1
GP plot
Expression
Time (h)
0
−2
−4
0
5
10
Log-lik in held-out time points
log p(y_j|t_j,y_−j)
Time (h)
−4
−6
−8
−10
2.5
5.0
7.5
10.0
12.5

Gene: CLB_RS05535
LOO:-3.743 | λ=1.0

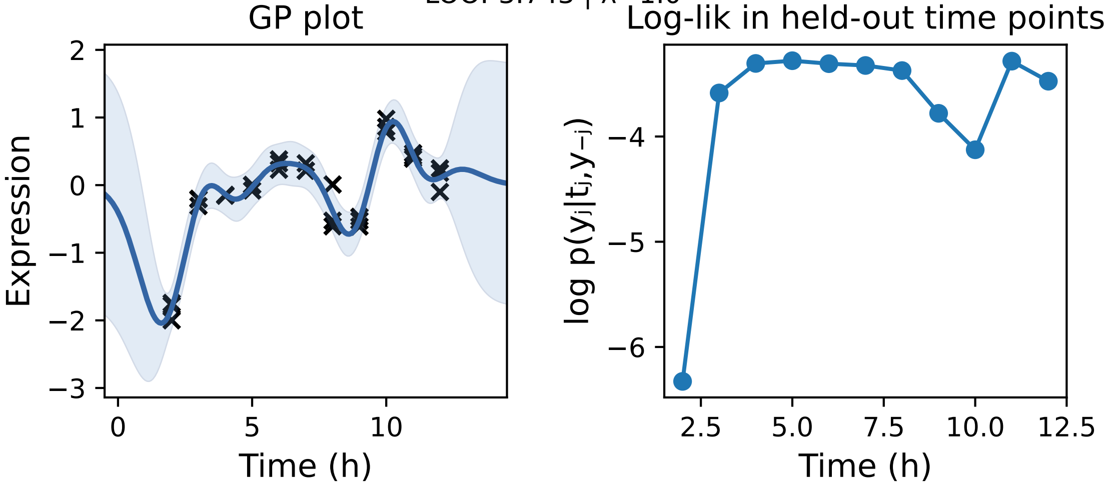

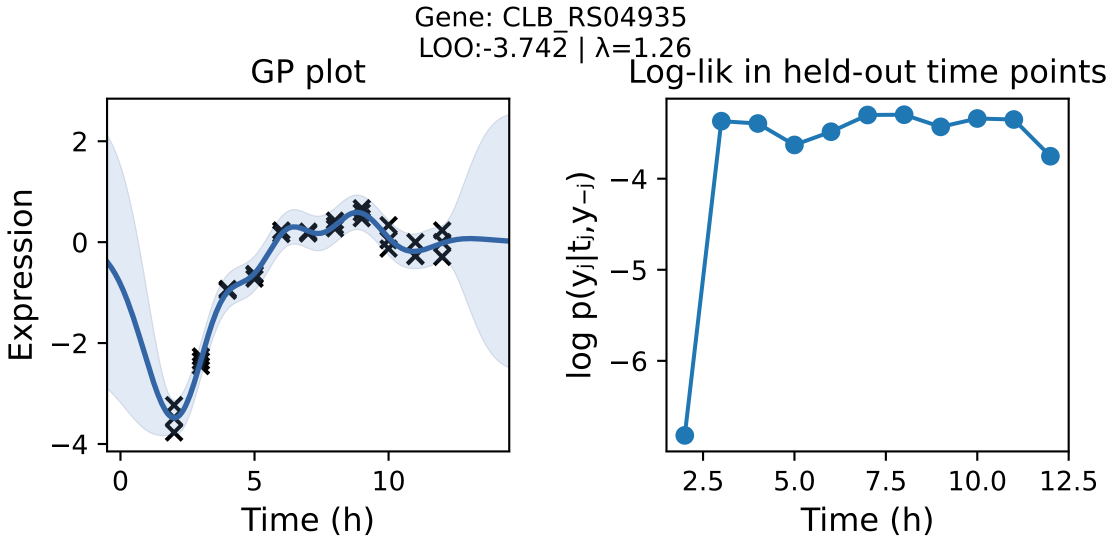

Gene: CLB_RS04935
LOO:-3.742 | λ=1.26
GP plot
Expression
2
0
−2
−4
0
5
10
Time (h)
Log-lik in held-out time points
log p(y_j|t_j,y_−j)
−4
−5
−6
2.5
5.0
7.5
10.0
12.5
Time (h)

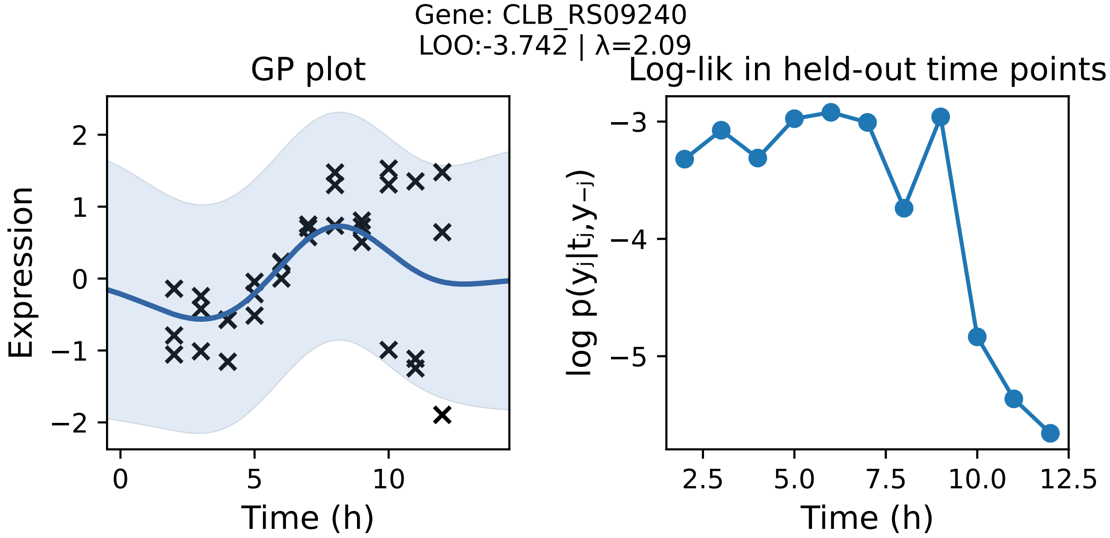
Gene: CLB_RS09240
LOO:-3.742 | λ=2.09
GP plot
Expression
Time (h)
Log-lik in held-out time points
log p(y_j|t_j,y_−j)
Time (h)

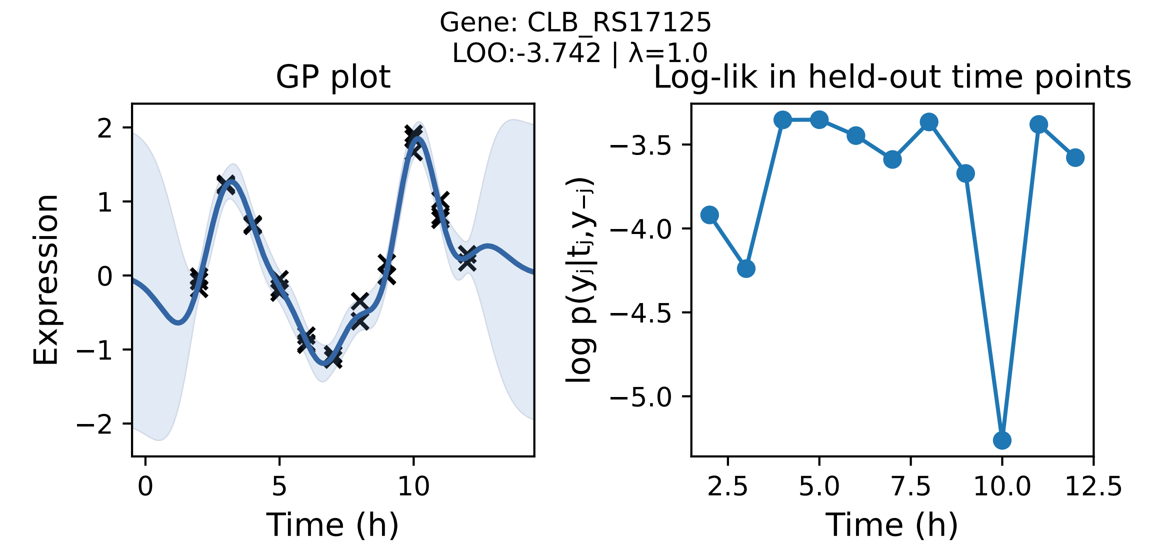

Gene: CLB_RS17125
LOO:-3.742 | λ=1.0
GP plot
Log-lik in held-out time points
Expression
Time (h)
log p(y_j|t_j,y_−j)
Time (h)

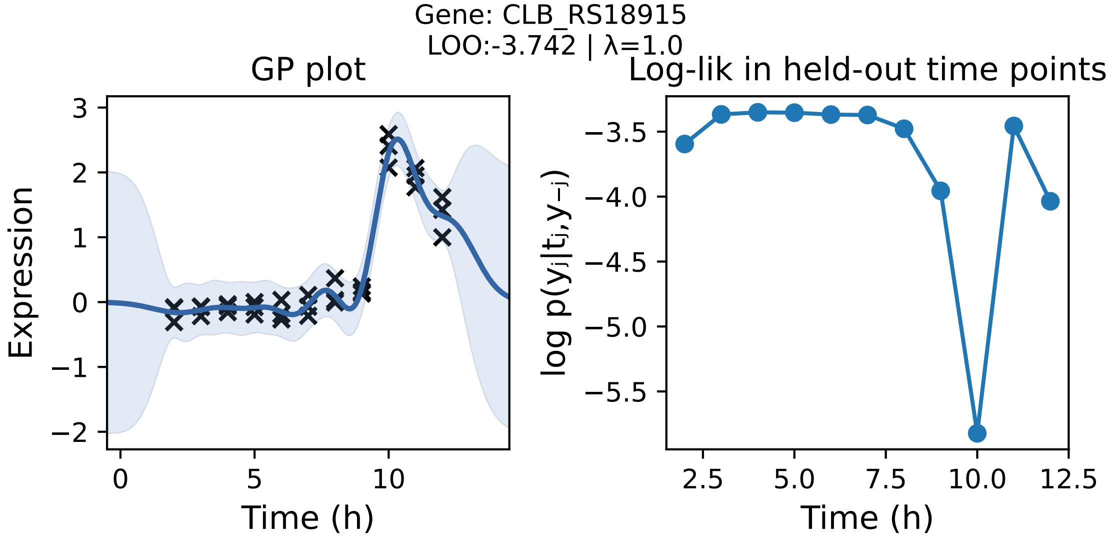

Gene: CLB_RS18915
LOO:-3.742 | λ=1.0
GP plot
Expression
Time (h)
Log-lik in held-out time points
log p(y_j|t_j,y_−j)
Time (h)

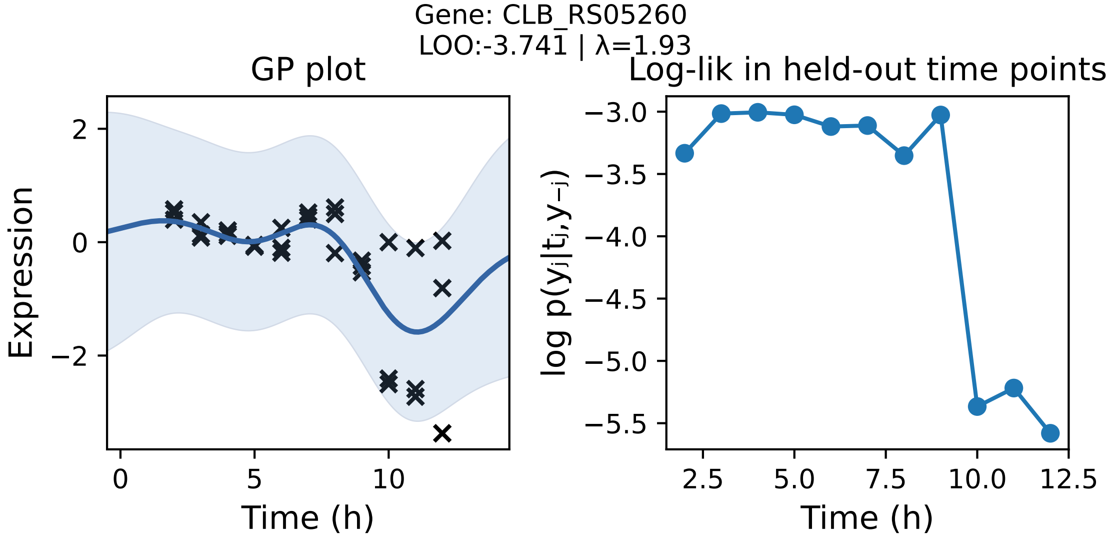

Gene: CLB_RS05260
LOO:-3.741 | λ=1.93
GP plot
Expression
Time (h)
Log-lik in held-out time points
log p(y_j|t_j,y_−j)
Time (h)

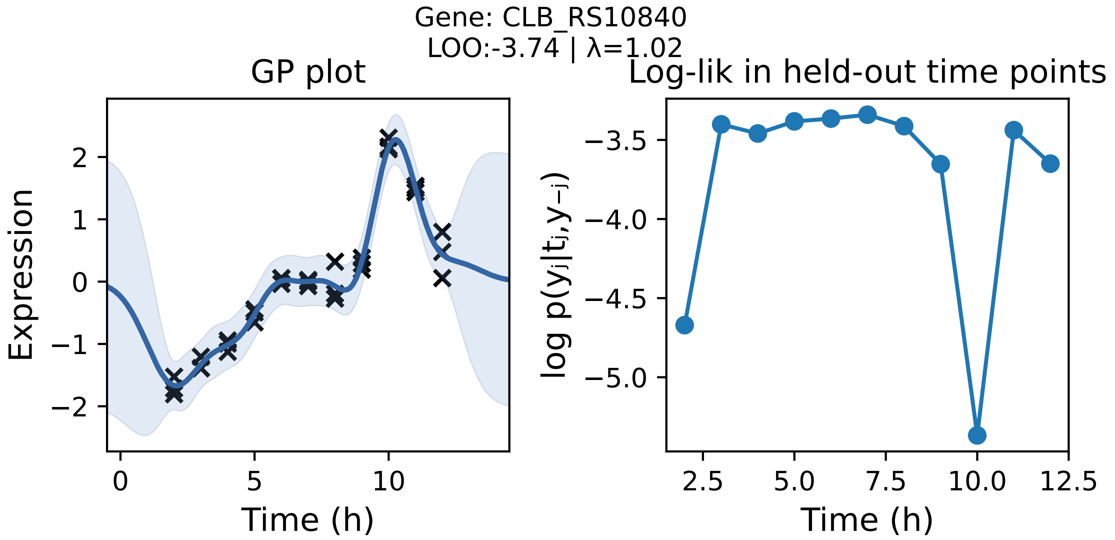
Gene: CLB_RS10840
LOO:-3.74 | λ=1.02
GP plot
Log-lik in held-out time points
Expression
Time (h)
log p(y_j|t_j,y_−j)
Time (h)
2
1
0
−1
−2
0
5
10
−3.5
−4.0
−4.5
−5.0
2.5
5.0
7.5
10.0
12.5

Gene: CLB_RS13990
LOO:-3.739 | λ=1.05

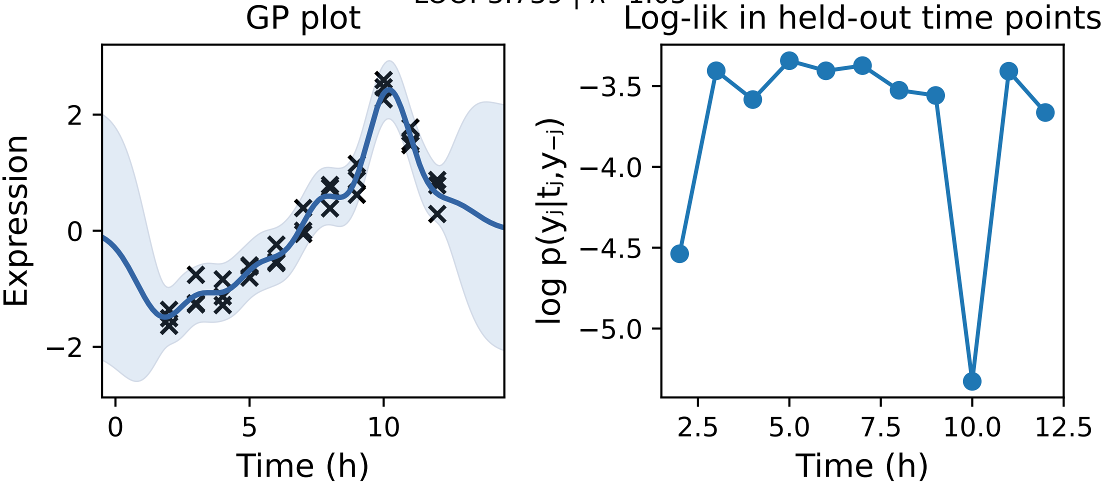

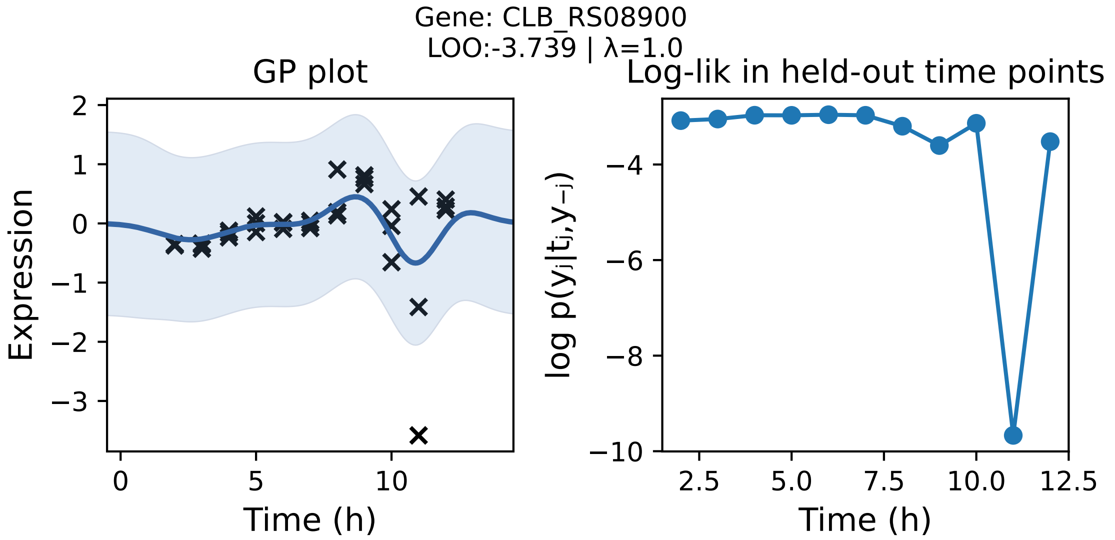

Gene: CLB_RS08900
LOO:-3.739 | λ=1.0
GP plot
Expression
Time (h)
2
1
0
−1
−2
−3
0
5
10
Log-lik in held-out time points
log p(yj|tj,y−j)
−4
−6
−8
−10
2.5
5.0
7.5
10.0
12.5
Time (h)

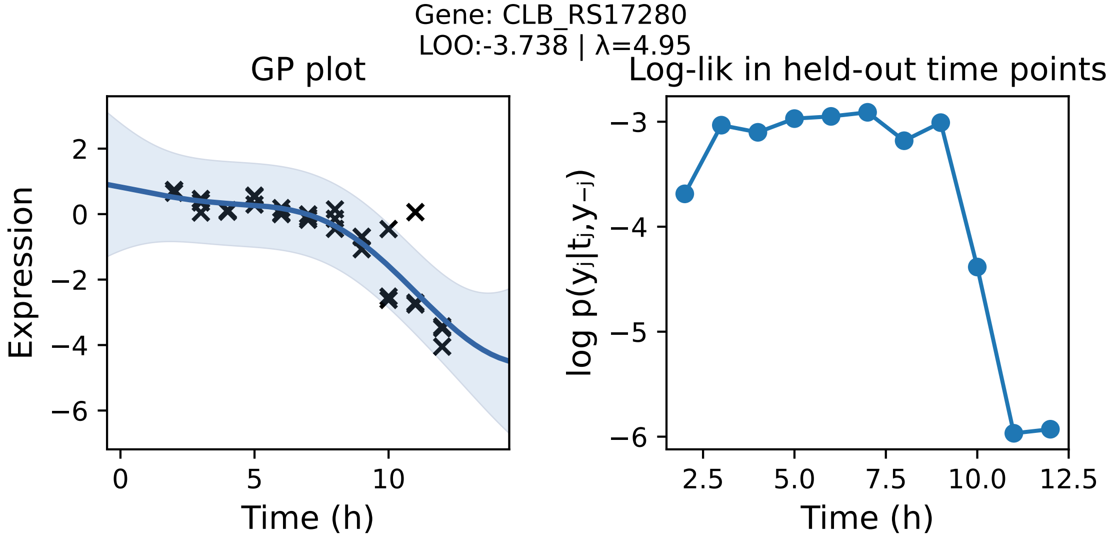

Gene: CLB_RS17280
LOO:-3.738 | λ=4.95
GP plot
Expression
Time (h)
2
0
−2
−4
−6
0
5
10
Log-lik in held-out time points
log p(y_j|t_j,y_−j)
Time (h)
−3
−4
−5
−6
2.5
5.0
7.5
10.0
12.5

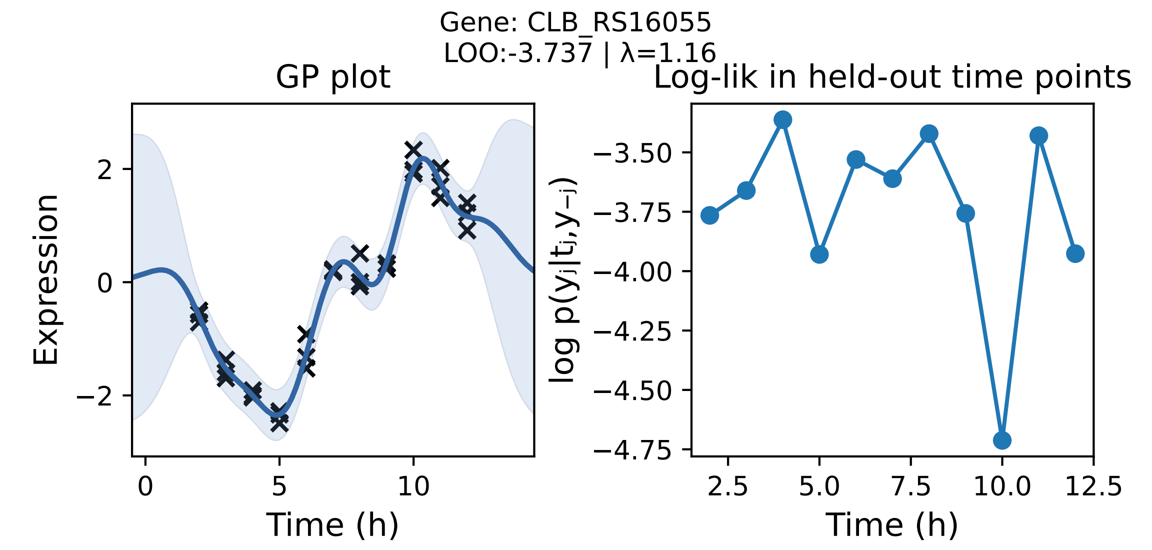

Gene: CLB_RS16055
LOO:-3.737 | λ=1.16
GP plot
Expression
Time (h)
Log-lik in held-out time points
log p(y_j|t_j,y_−j)
Time (h)

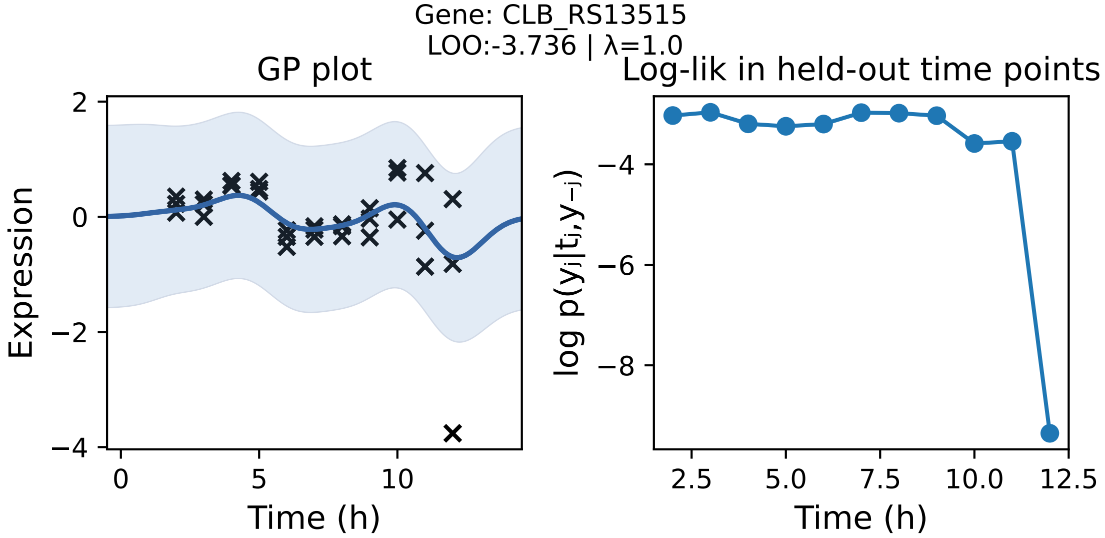
Gene: CLB_RS13515
LOO:-3.736 | λ=1.0
GP plot
Log-lik in held-out time points
Expression
Time (h)
log p(yⱼ|tⱼ,y₋ⱼ)
2
0
−2
−4
5
10
−4
−6
−8
2.5
5.0
7.5
10.0
12.5

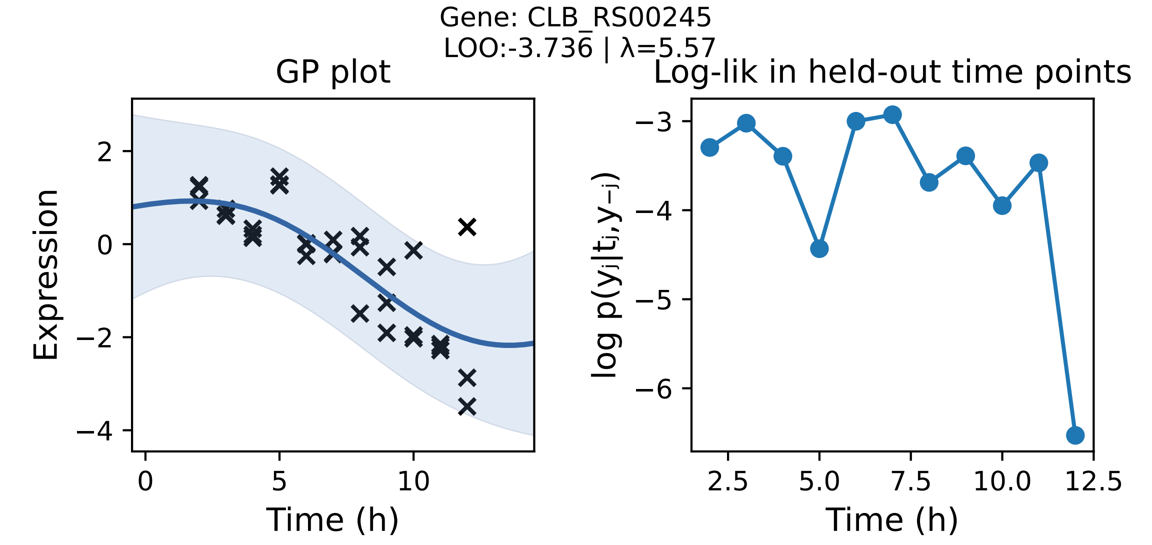

Gene: CLB_RS00245
LOO:-3.736 | λ=5.57
GP plot
Expression
Time (h)
Log-lik in held-out time points
log p(y_j|t_j,y_−j)
Time (h)

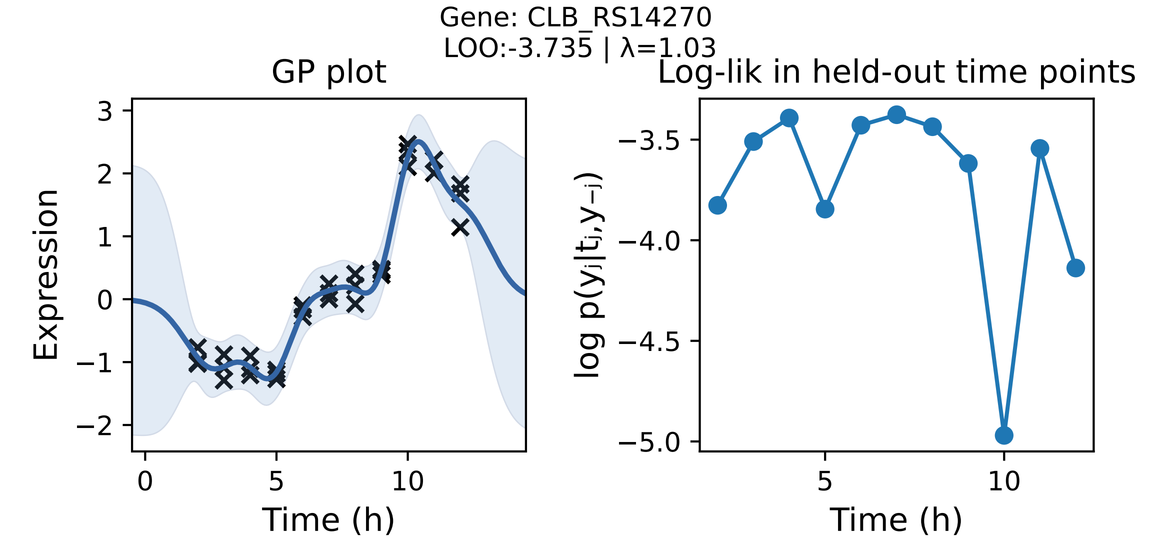

Gene: CLB_RS14270
LOO:-3.735 | λ=1.03
GP plot
Expression
Time (h)
Log-lik in held-out time points
log p(y_j|t_j,y_−j)
Time (h)

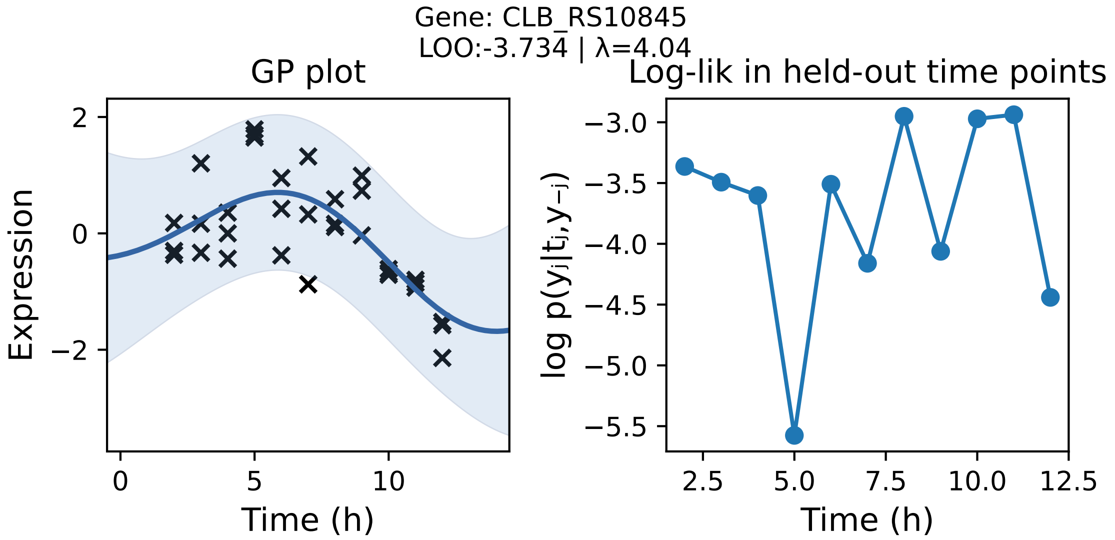

Gene: CLB_RS10845
LOO:-3.734 | λ=4.04
GP plot
Log-lik in held-out time points
Expression
Time (h)
log p(y_j|t_j,y_−j)
Time (h)
2
0
−2
0
5
10
−3.0
−3.5
−4.0
−4.5
−5.0
−5.5
2.5
5.0
7.5
10.0
12.5

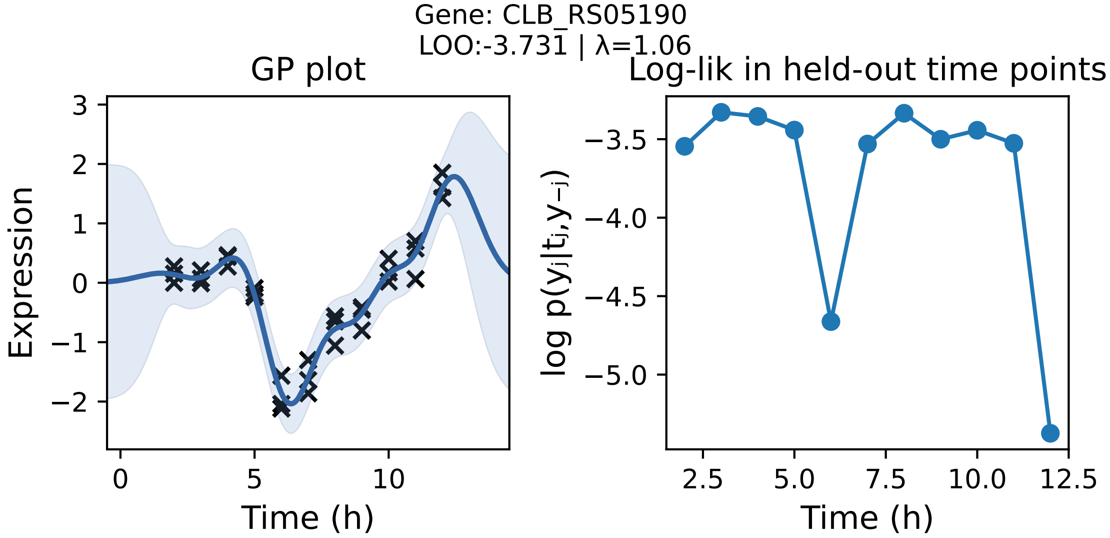

Gene: CLB_RS05190
LOO:-3.731 | λ=1.06
GP plot
Expression
Time (h)
3
2
1
0
−1
−2
0
5
10
Log-lik in held-out time points
log p(y_j|t_j,y_−j)
Time (h)
−3.5
−4.0
−4.5
−5.0
2.5
5.0
7.5
10.0
12.5

Gene: CLB_RS15835
LOO:-3.731 | λ=2.29

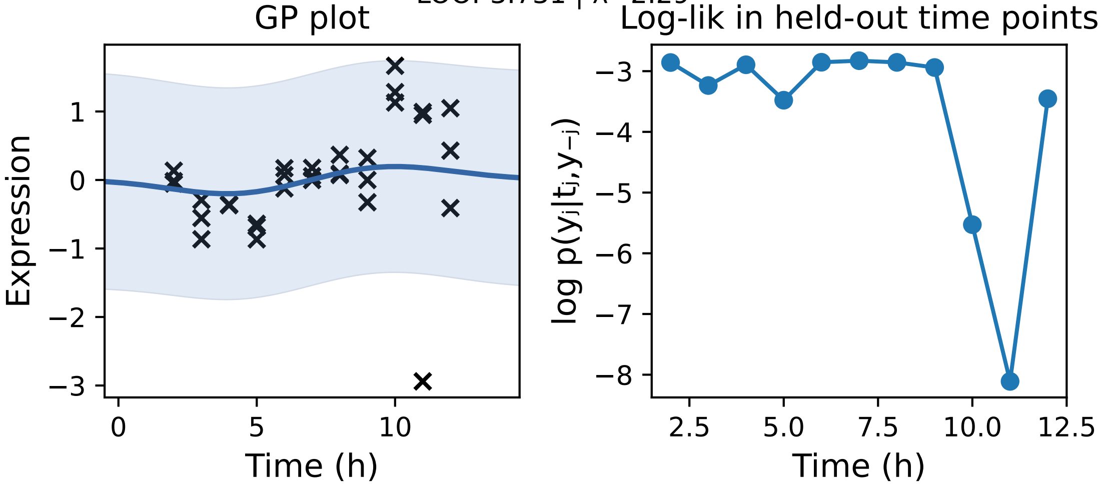

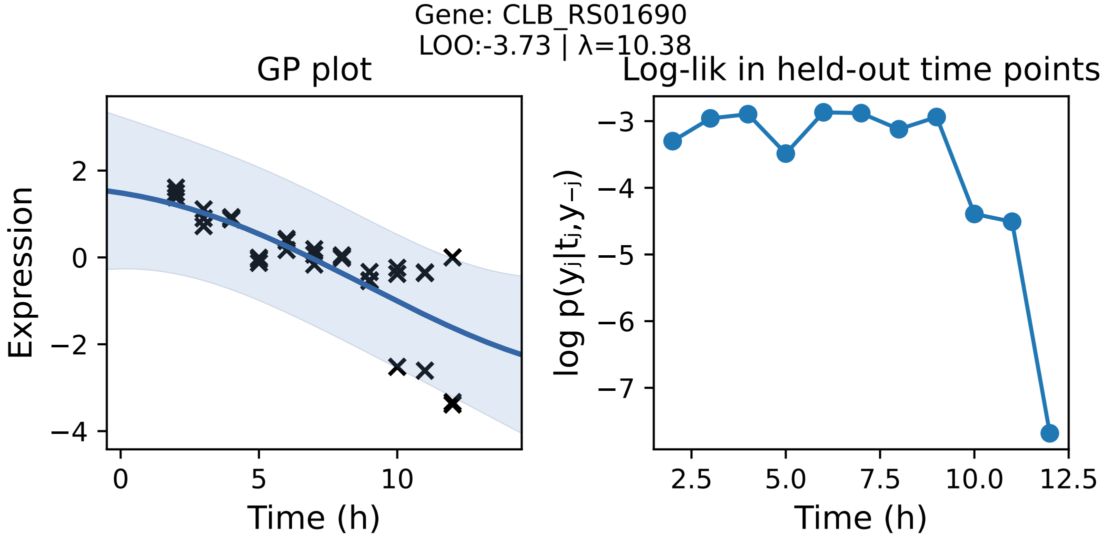

Gene: CLB_RS01690
LOO:-3.73 | λ=10.38
GP plot
Expression
Time (h)
2
0
−2
−4
0
5
10
Log-lik in held-out time points
log p(yj|tj,y−j)
−3
−4
−5
−6
−7
2.5
5.0
7.5
10.0
12.5
Time (h)

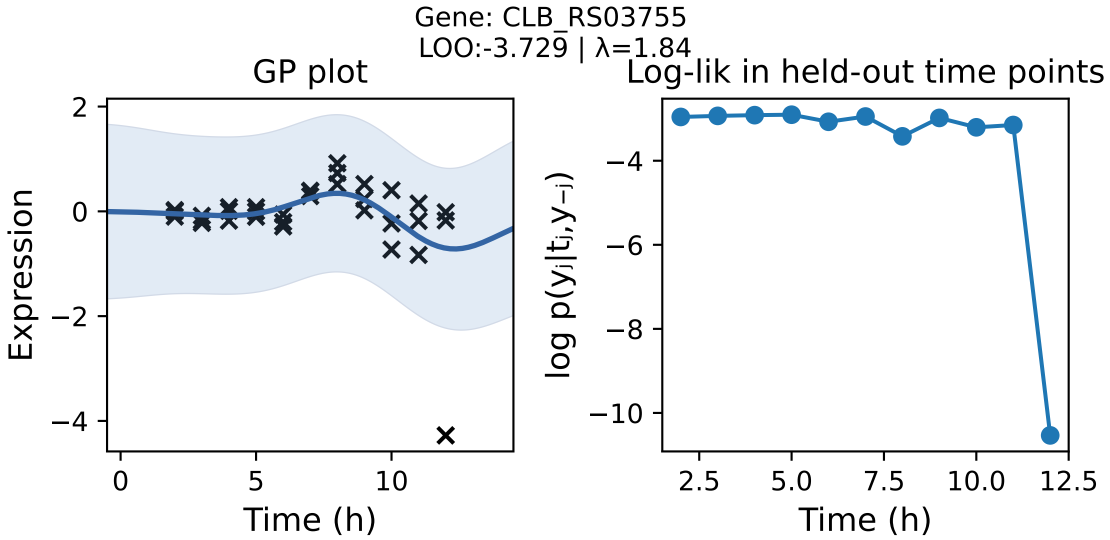
Gene: CLB_RS03755
LOO:-3.729 | λ=1.84
GP plot
Expression
Time (h)
2
0
−2
−4
0
5
10
Log-lik in held-out time points
log p(y_j|t_j,y_−j)
Time (h)
−4
−6
−8
−10
2.5
5.0
7.5
10.0
12.5

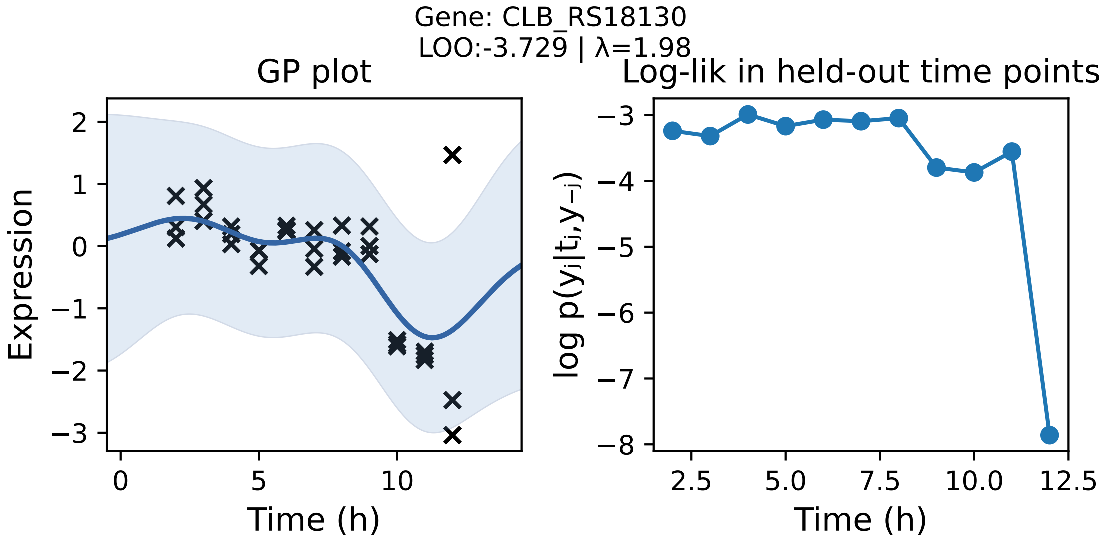

Gene: CLB_RS18130
LOO:-3.729 | λ=1.98
GP plot
Log-lik in held-out time points
Expression
Time (h)
log p(yj|tj,y−j)
Time (h)

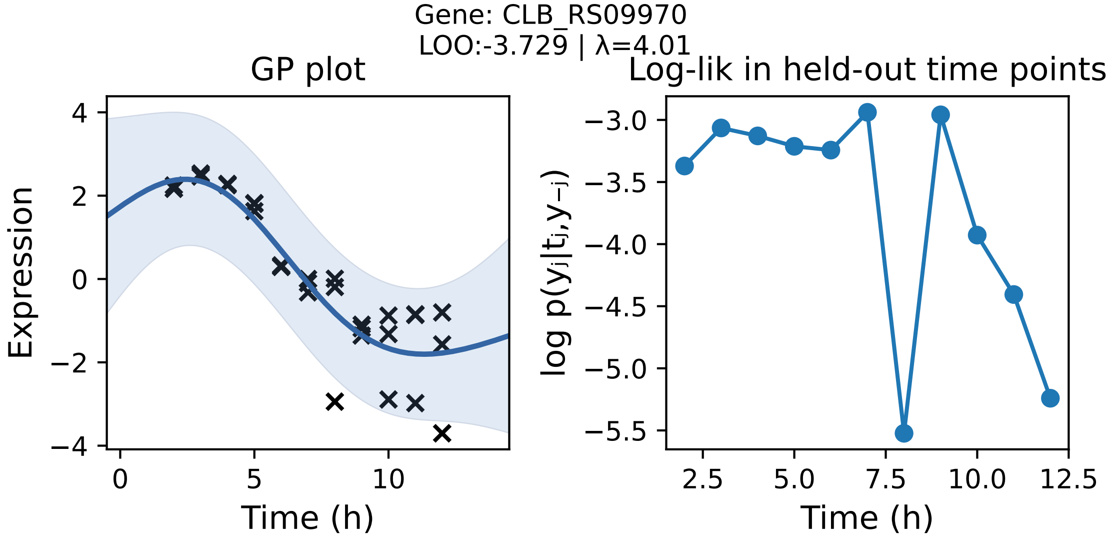

Gene: CLB_RS09970
LOO:-3.729 | λ=4.01
GP plot
Expression
Time (h)
Log-lik in held-out time points
log p(y_j|t_j,y_−j)
Time (h)

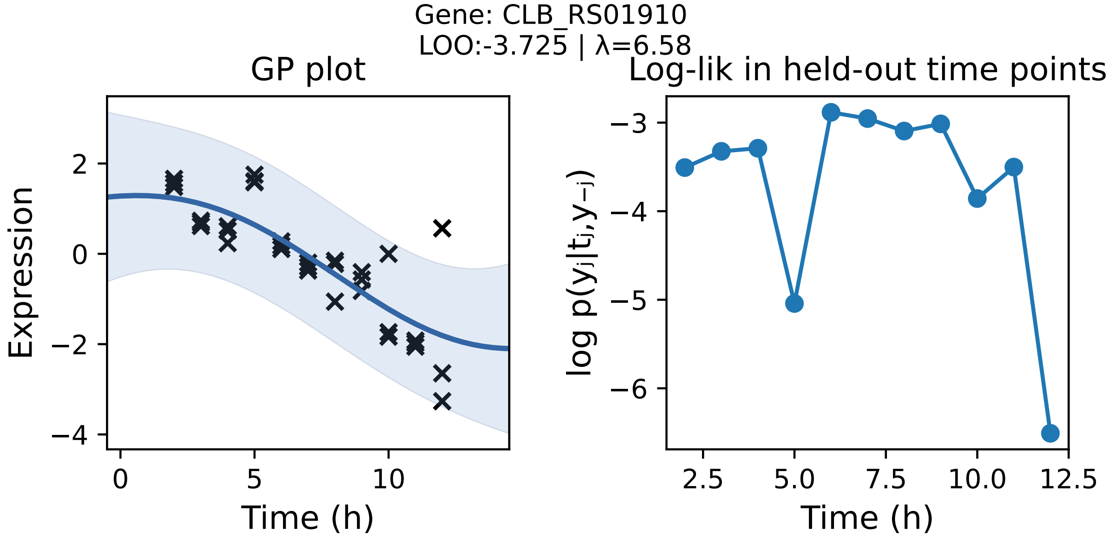
Gene: CLB_RS01910
LOO:-3.725 | λ=6.58
GP plot
Expression
Time (h)
2
0
−2
−4
0
5
10
Log-lik in held-out time points
log p(y_j|t_j,y_−j)
Time (h)
−3
−4
−5
−6
2.5
5.0
7.5
10.0
12.5

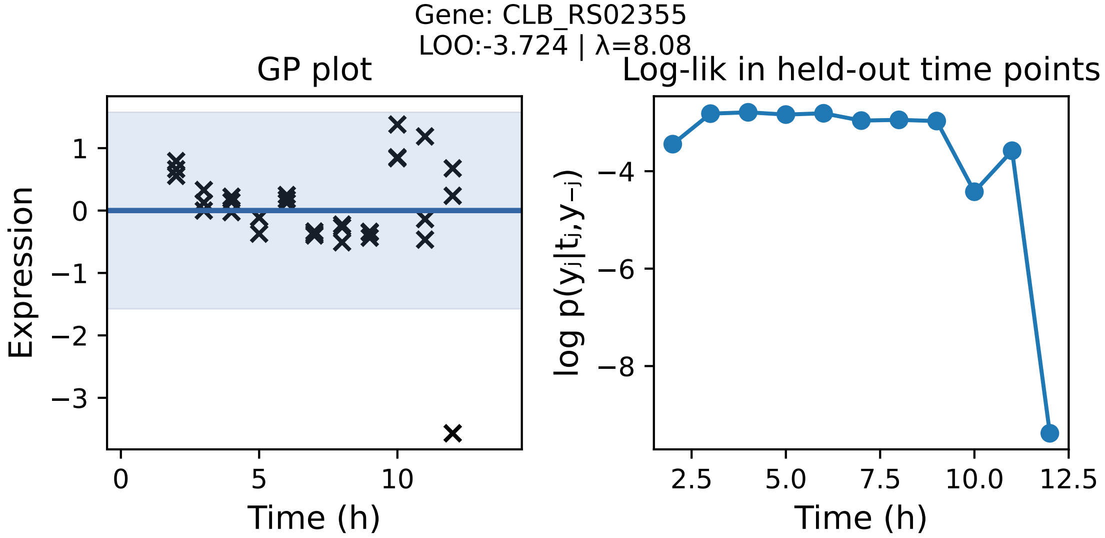
Gene: CLB_RS02355
LOO:-3.724 | λ=8.08
GP plot
Expression
Time (h)
1
0
−1
−2
−3
0
5
10
Log-lik in held-out time points
log p(y_j|t_j,y_−j)
Time (h)
−4
−6
−8
2.5
5.0
7.5
10.0
12.5

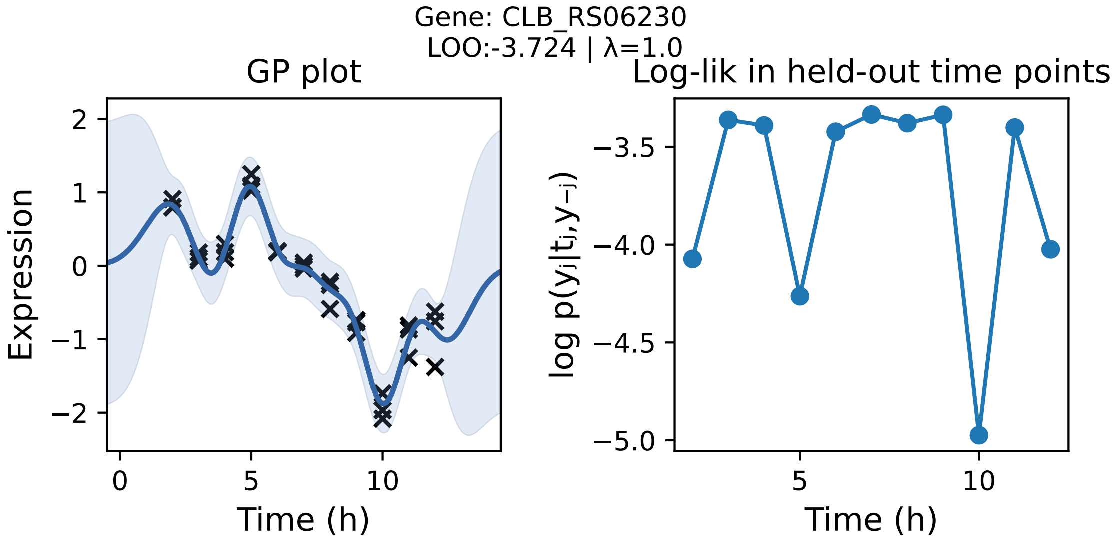

Gene: CLB_RS06230
LOO:-3.724 | λ=1.0
GP plot
Expression
Time (h)
Log-lik in held-out time points
log p(yj|tj,y−j)
Time (h)

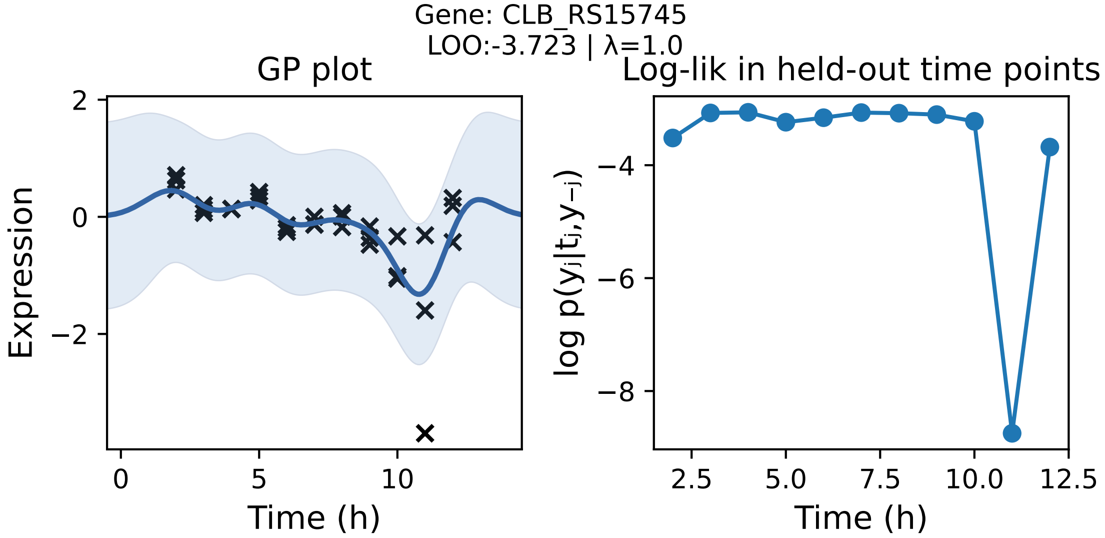
Gene: CLB_RS15745
LOO:-3.723 | λ=1.0
GP plot
Log-lik in held-out time points
Expression
Time (h)
log p(y_j|t_j,y_−j)
Time (h)
2
0
−2
0
5
10
−4
−6
−8
2.5
5.0
7.5
10.0
12.5

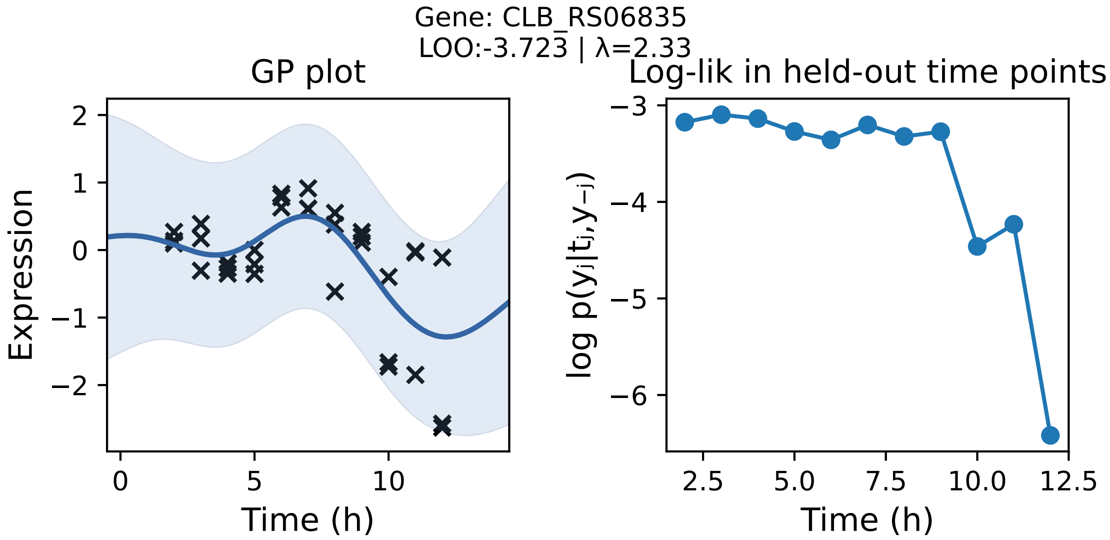
Gene: CLB_RS06835
LOO:-3.723 | λ=2.33
GP plot
Expression
Time (h)
Log-lik in held-out time points
log p(y_j|t_j,y_−j)
Time (h)

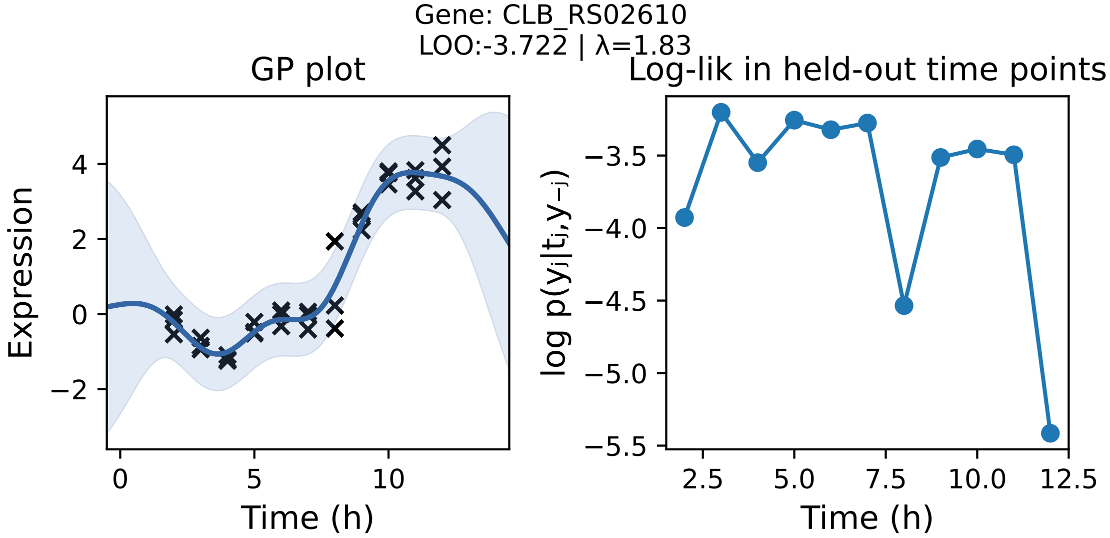
Gene: CLB_RS02610
LOO:-3.722 | λ=1.83
GP plot
Expression
Time (h)
Log-lik in held-out time points
log p(y_j|t_j,y_−j)
Time (h)
4
2
0
−2
0
5
10
−3.5
−4.0
−4.5
−5.0
−5.5
2.5
5.0
7.5
10.0
12.5

Gene: CLB_RS06195
LOO:-3.722 | λ=2.97

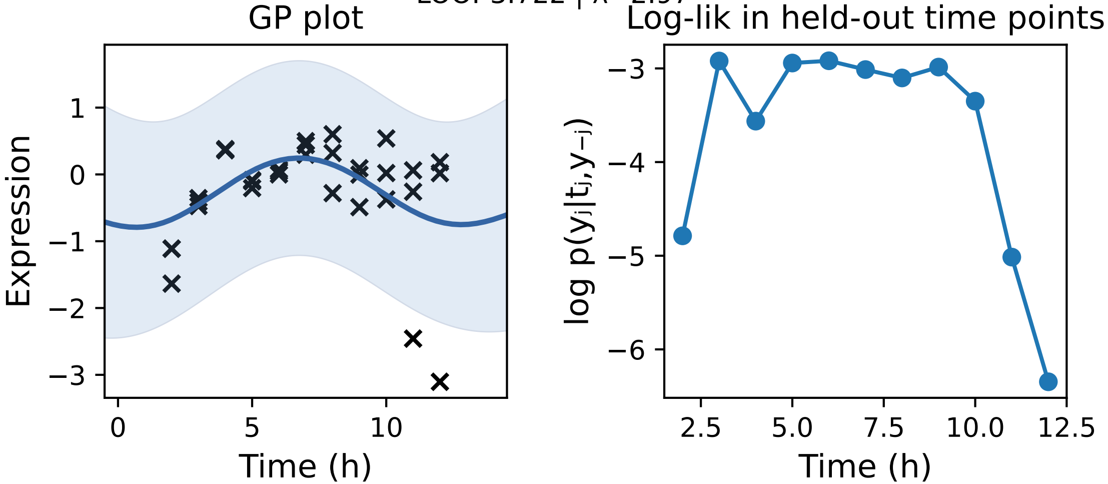

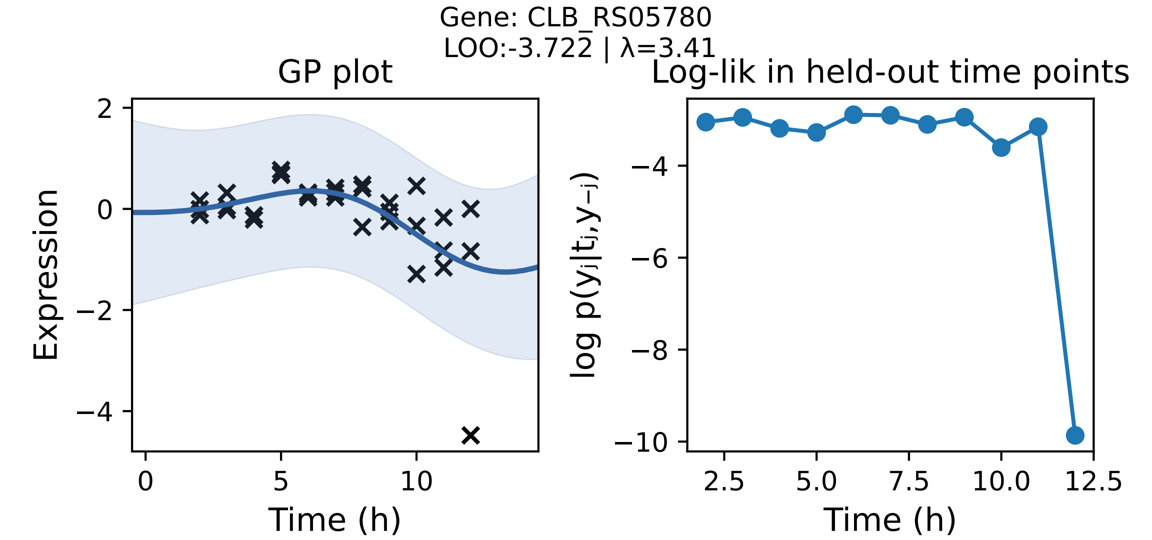

Gene: CLB_RS05780
LOO:-3.722 | λ=3.41
GP plot
Expression
Time (h)
Log-lik in held-out time points
log p(yj|tj,y−j)
Time (h)

Gene: CLB_RS05180
LOO:-3.721 | λ=2.6

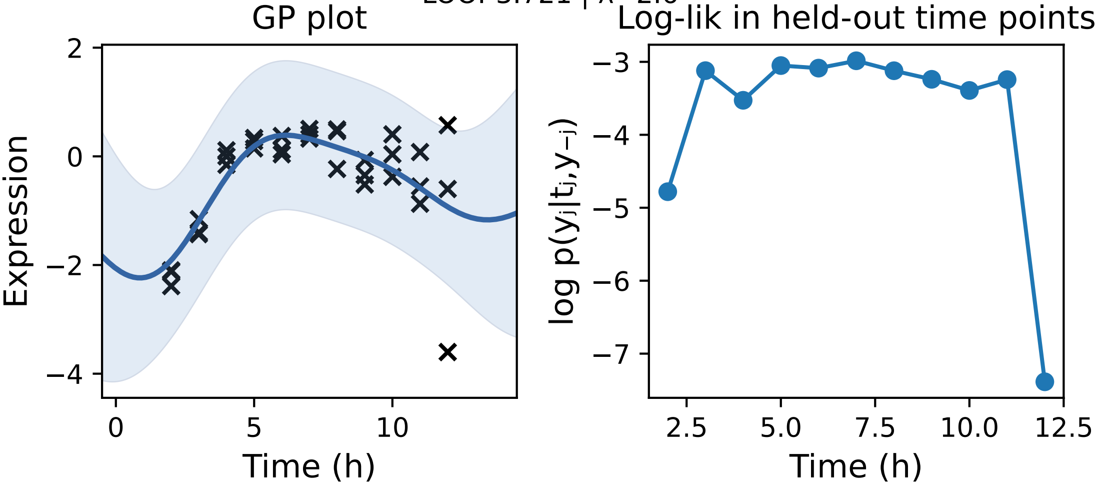

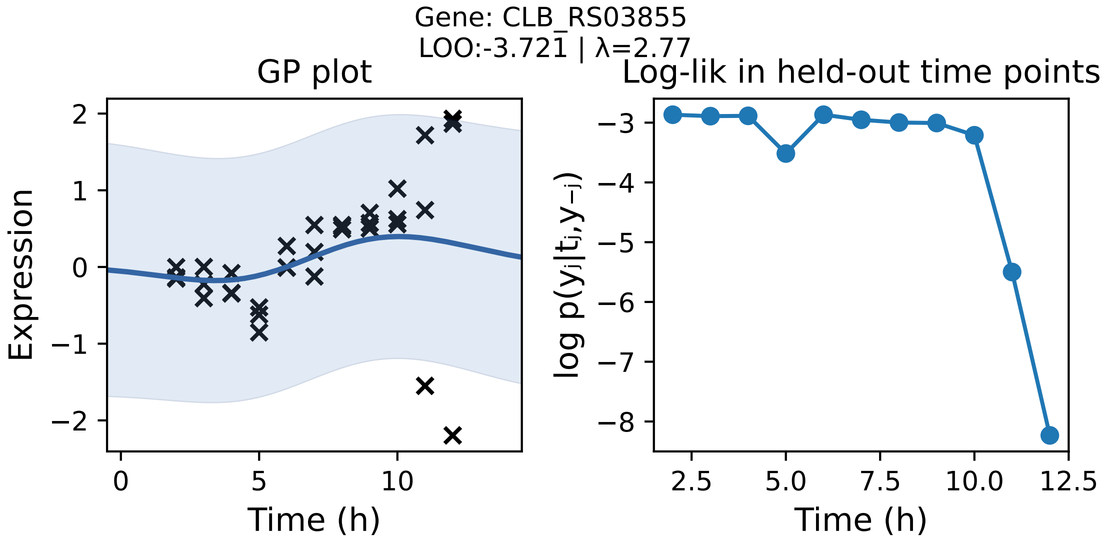
Gene: CLB_RS03855
LOO:-3.721 | λ=2.77
GP plot
Expression
Time (h)
Log-lik in held-out time points
log p(y_j|t_j,y_−j)
Time (h)

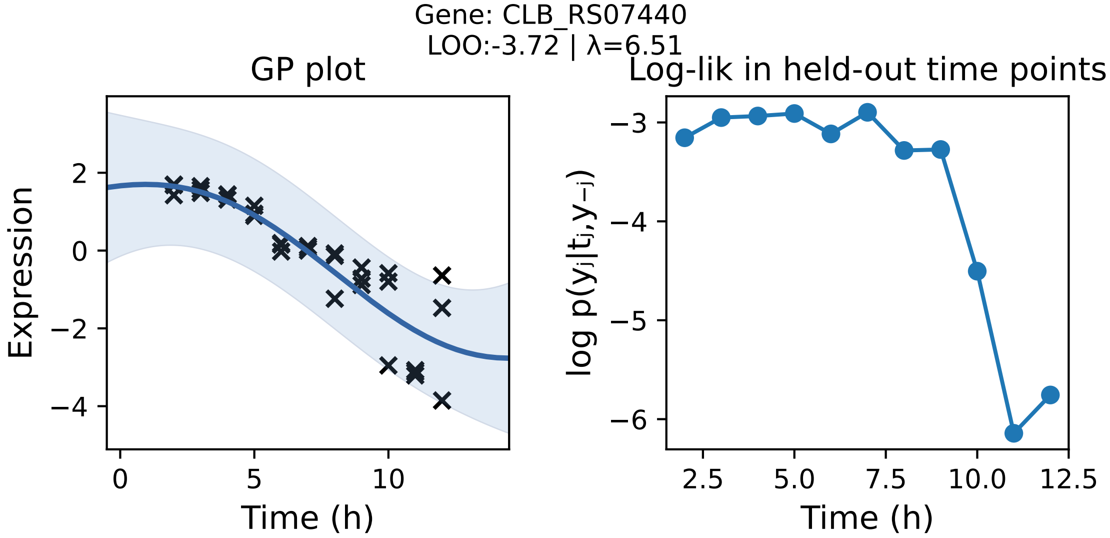

Gene: CLB_RS07440
LOO:-3.72 | λ=6.51
GP plot
Log-lik in held-out time points
Expression
2
0
−2
−4
0
5
10
Time (h)
log p(yⱼ|tⱼ,y₋ⱼ)
−3
−4
−5
−6
2.5
5.0
7.5
10.0
12.5
Time (h)

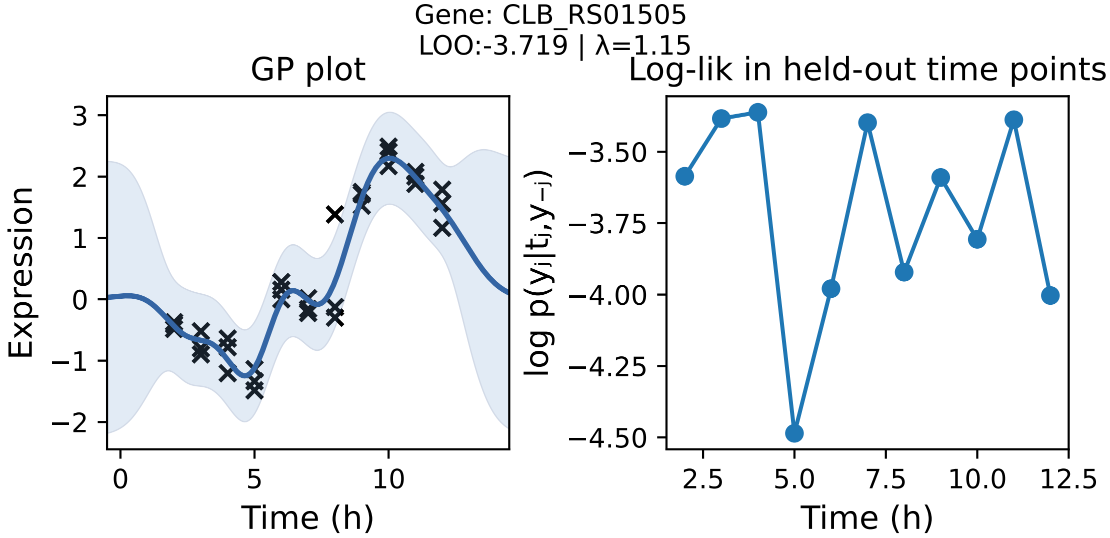

Gene: CLB_RS01505
LOO:-3.719 | λ=1.15
GP plot
Log-lik in held-out time points
Expression
Time (h)
log p(yj|tj,y−j)
3
2
1
0
−1
−2
0
5
10
−3.50
−3.75
−4.00
−4.25
−4.50
2.5
5.0
7.5
10.0
12.5

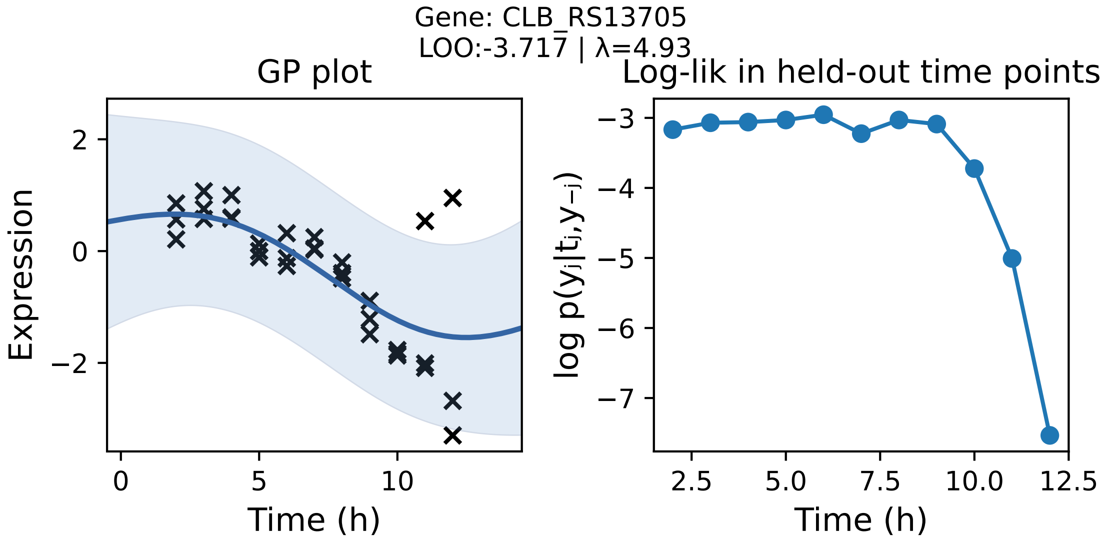

Gene: CLB_RS13705
LOO:-3.717 | λ=4.93
GP plot
Expression
Time (h)
2
0
−2
0
5
10
Log-lik in held-out time points
log p(y_j|t_j,y_−j)
Time (h)
−3
−4
−5
−6
−7
2.5
5.0
7.5
10.0
12.5

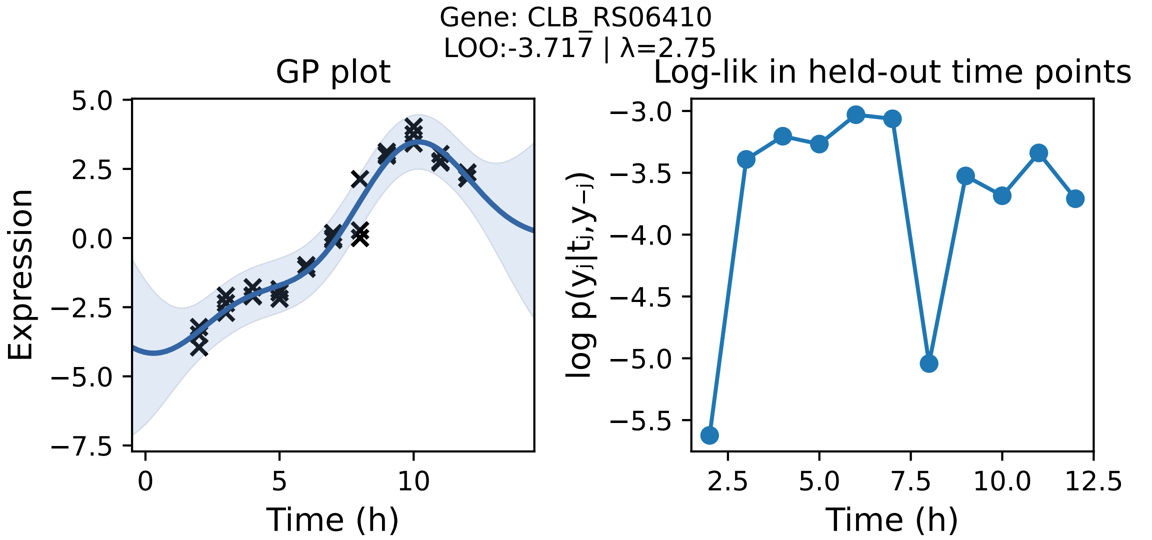
Gene: CLB_RS06410
LOO:-3.717 | λ=2.75
GP plot
Log-lik in held-out time points
Expression
Time (h)
log p(yj|tj,y−j)
5.0
2.5
0.0
−2.5
−5.0
−7.5
0
5
10
−3.0
−3.5
−4.0
−4.5
−5.0
−5.5
2.5
5.0
7.5
10.0
12.5

Gene: CLB_RS02820
LOO:-3.716 | λ=1.0

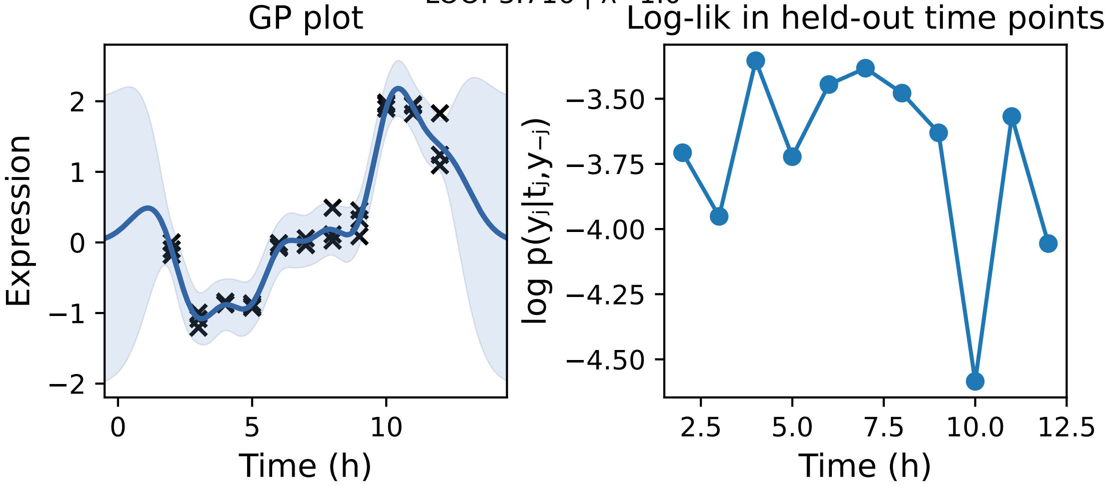

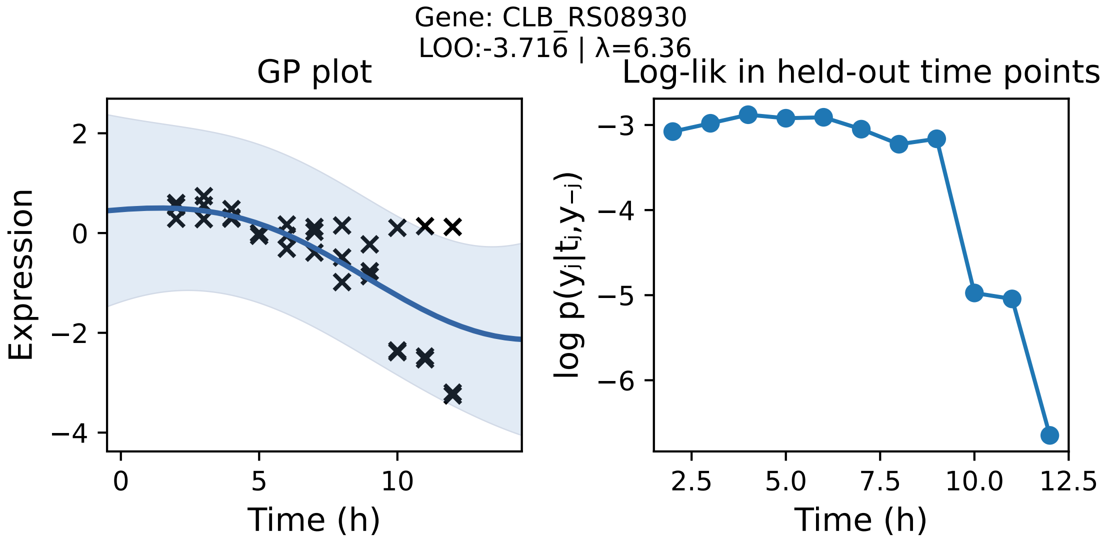
Gene: CLB_RS08930
LOO:-3.716 | λ=6.36
GP plot
Log-lik in held-out time points
Expression
Time (h)
log p(y_j|t_j,y_−j)
Time (h)

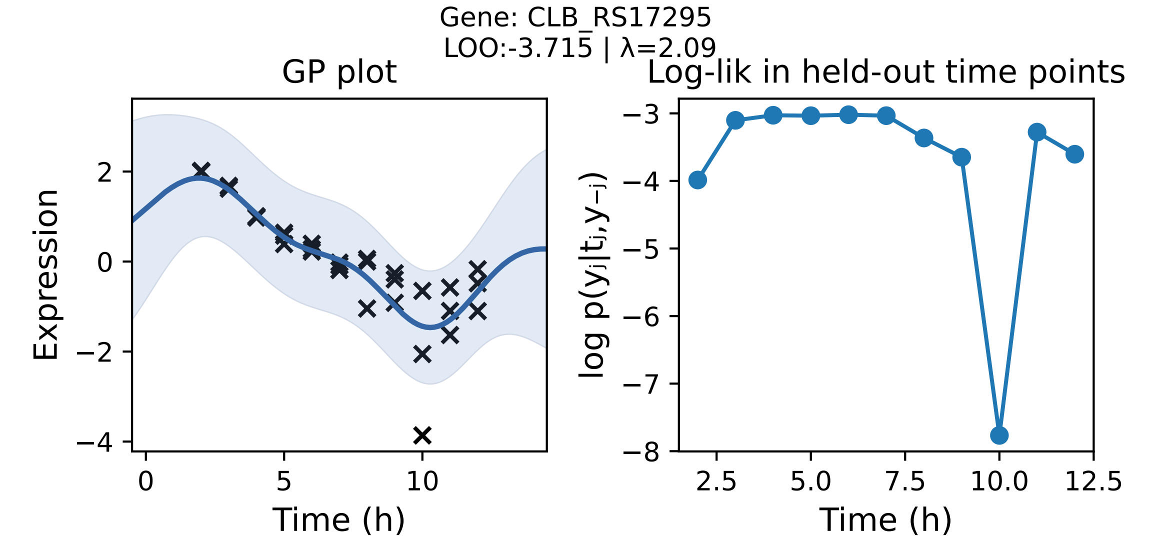

Gene: CLB_RS17295
LOO:-3.715 | λ=2.09
GP plot
Expression
Time (h)
Log-lik in held-out time points
log p(y_j|t_j,y_−j)
Time (h)

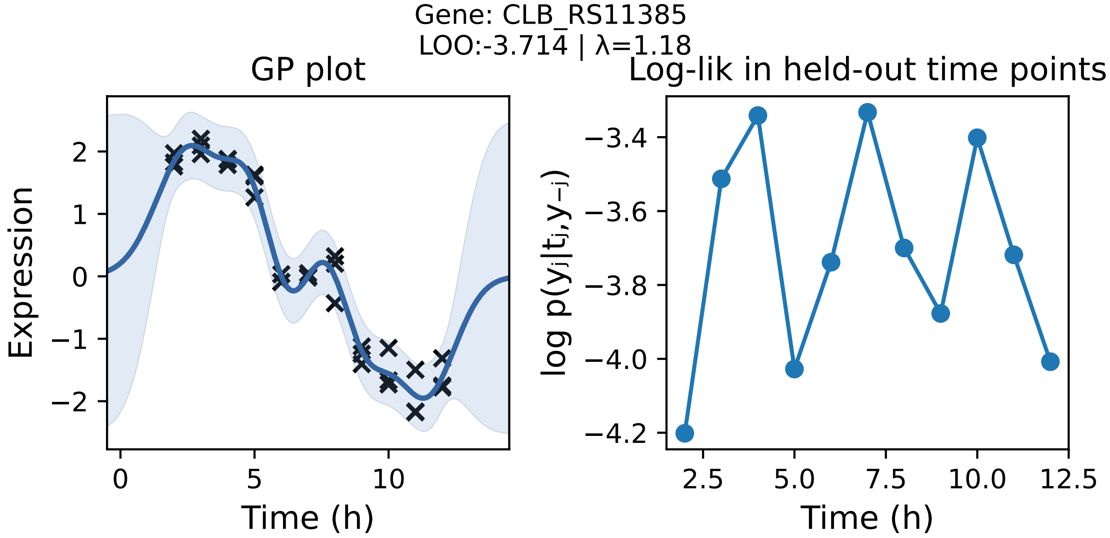

Gene: CLB_RS11385
LOO:-3.714 | λ=1.18
GP plot
Expression
Time (h)
Log-lik in held-out time points
log p(y_j|t_j,y_−j)
Time (h)

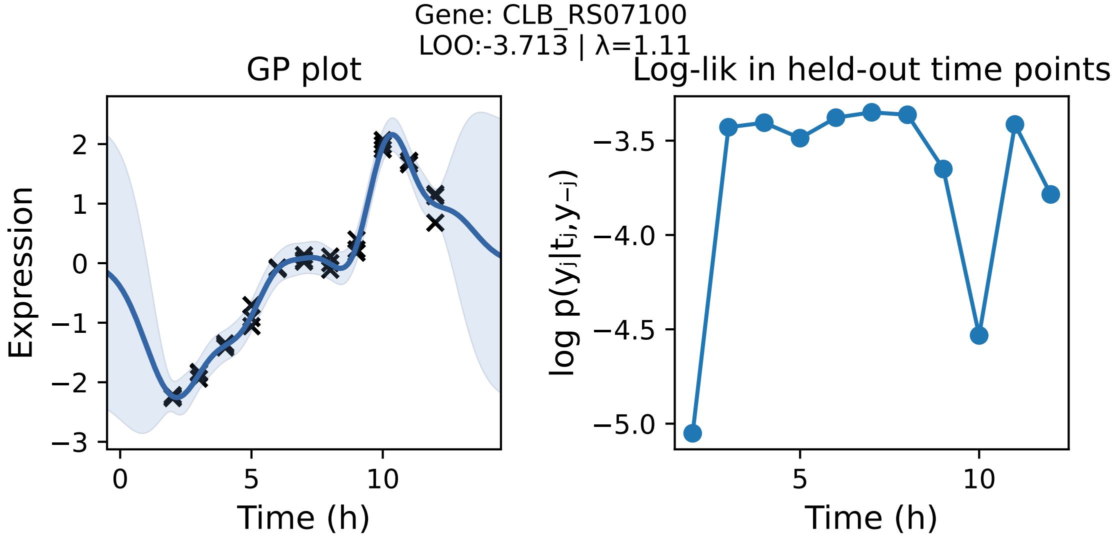

Gene: CLB_RS07100
LOO:-3.713 | λ=1.11
GP plot
Expression
Time (h)
Log-lik in held-out time points
log p(y_j|t_j,y_−j)
Time (h)

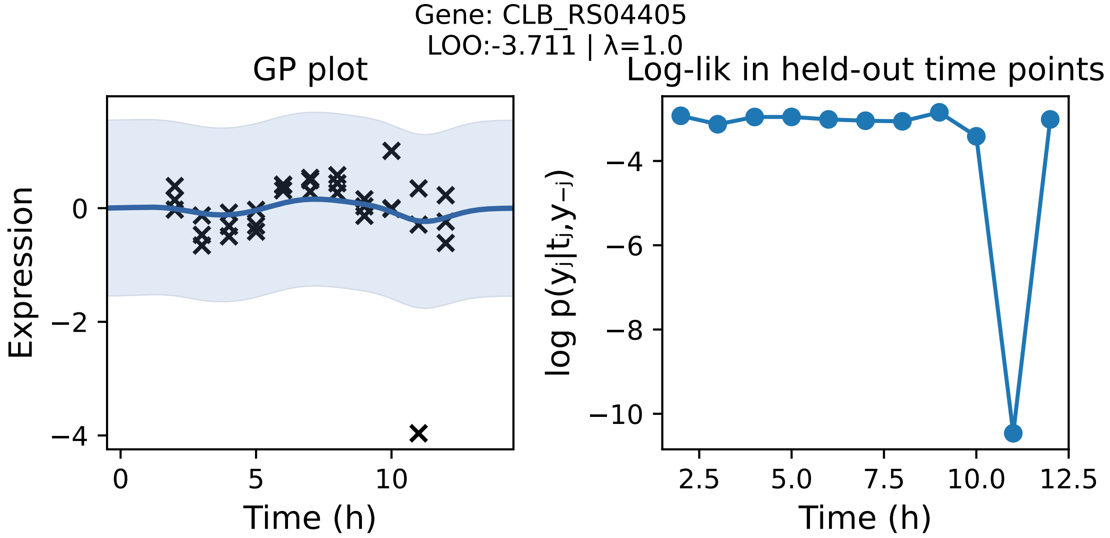

Gene: CLB_RS04405
LOO:-3.711 | λ=1.0
GP plot
Expression
Time (h)
0
−2
−4
0
5
10
Log-lik in held-out time points
log p(y_j|t_j,y_−j)
Time (h)
−4
−6
−8
−10
2.5
5.0
7.5
10.0
12.5

Gene: CLB_RS07680
LOO:-3.711 | λ=1.5

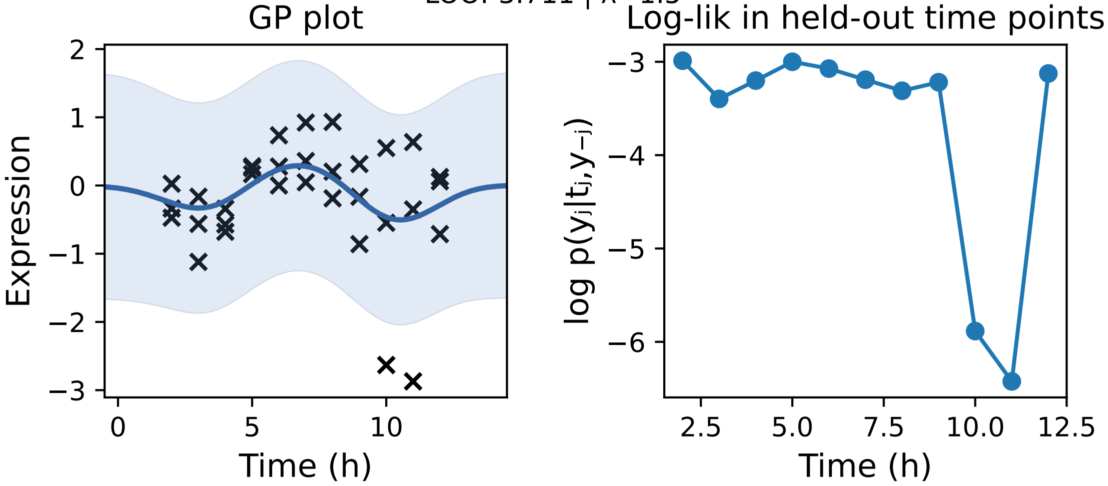

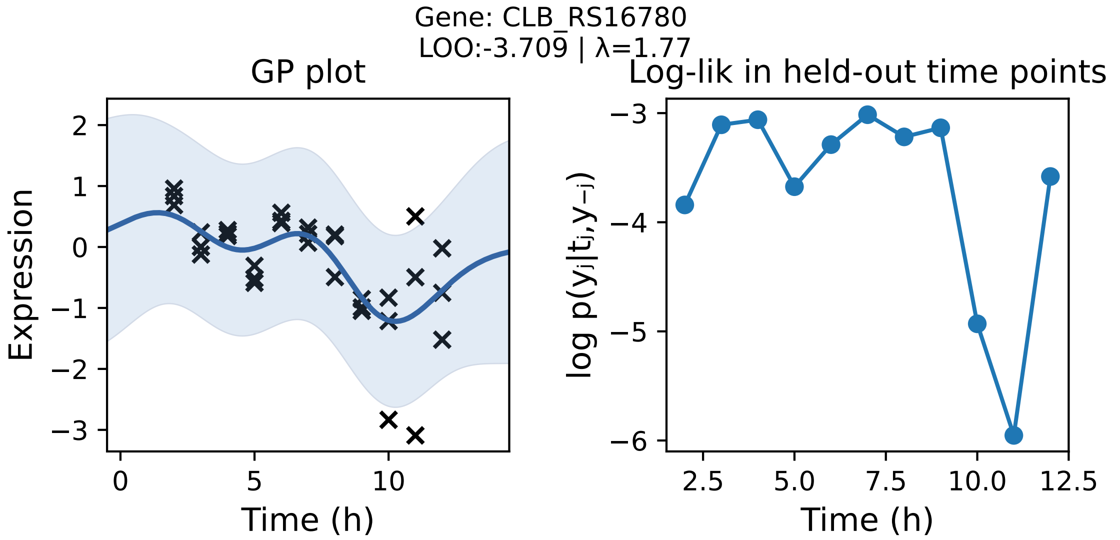

Gene: CLB_RS16780
LOO:-3.709 | λ=1.77
GP plot
Expression
Time (h)
Log-lik in held-out time points
log p(y_j|t_j,y_−j)
Time (h)

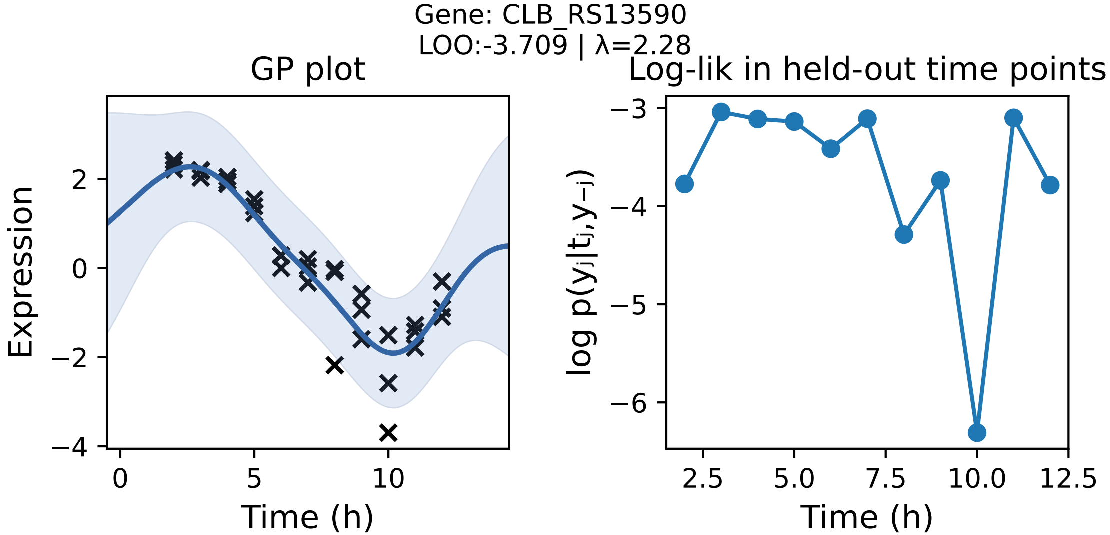
Gene: CLB_RS13590
LOO:-3.709 | λ=2.28
GP plot
Expression
Time (h)
Log-lik in held-out time points
log p(y_j|t_j,y_−j)
Time (h)

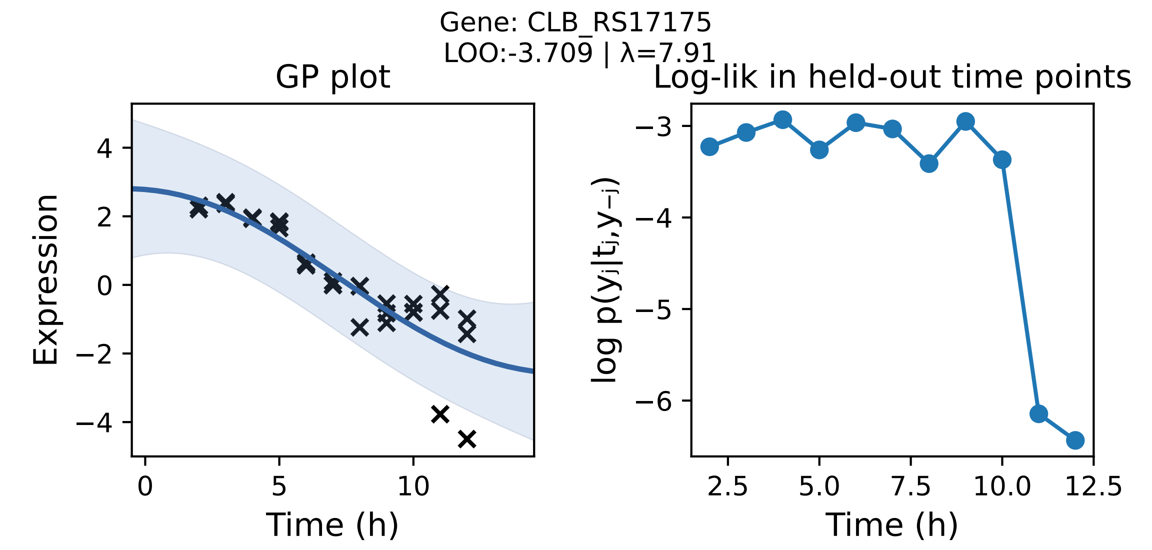

Gene: CLB_RS17175
LOO:-3.709 | λ=7.91
GP plot
Expression
Time (h)
4
2
0
−2
−4
0
5
10
Log-lik in held-out time points
log p(yⱼ|tⱼ,y₋ⱼ)
Time (h)
−3
−4
−5
−6
2.5
5.0
7.5
10.0
12.5

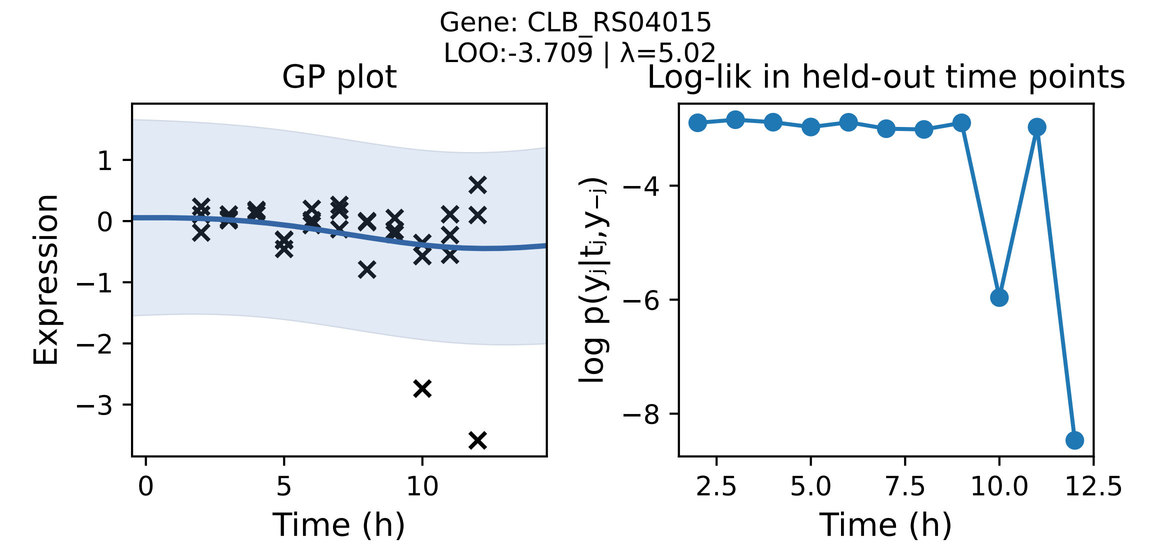
Gene: CLB_RS04015
LOO:-3.709 | λ=5.02
GP plot
Log-lik in held-out time points
Expression
Time (h)
log p(y_j|t_j,y_−j)
1
0
−1
−2
−3
0
5
10
−4
−6
−8
2.5
5.0
7.5
10.0
12.5

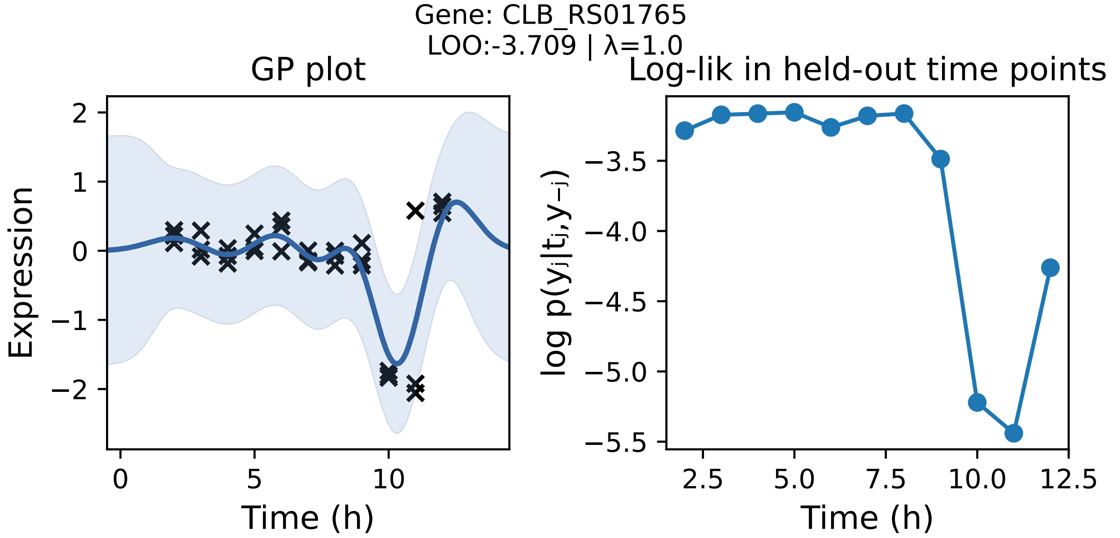
Gene: CLB_RS01765
LOO:-3.709 | λ=1.0
GP plot
Expression
Time (h)
2
1
0
−1
−2
0
5
10
Log-lik in held-out time points
log p(y_j|t_j,y_−j)
−3.5
−4.0
−4.5
−5.0
−5.5
2.5
5.0
7.5
10.0
12.5
Time (h)

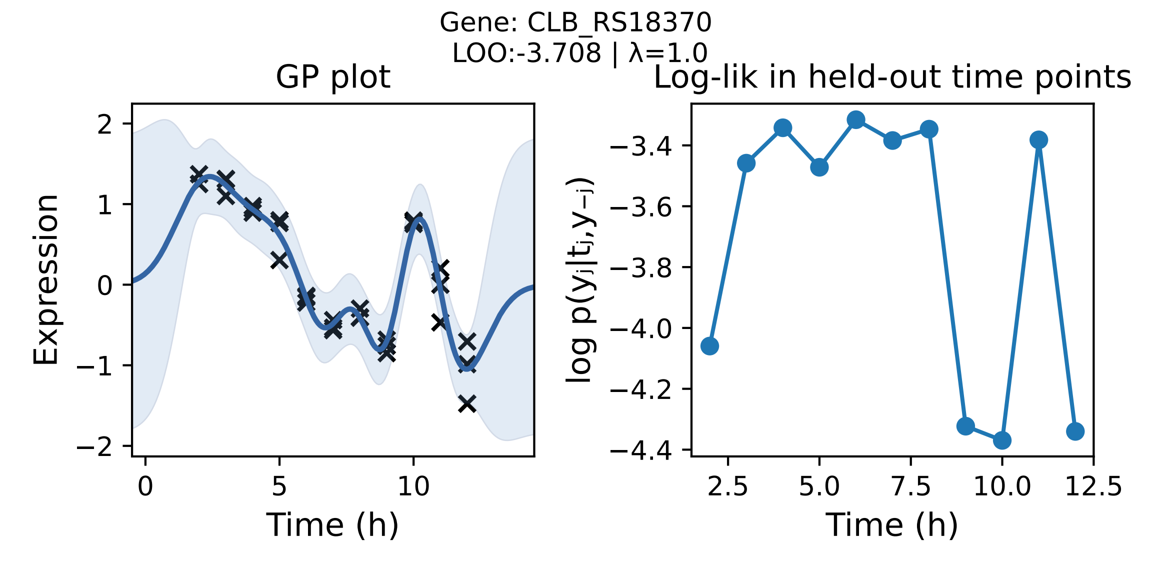
Gene: CLB_RS18370
LOO:-3.708 | λ=1.0
GP plot
Expression
Time (h)
Log-lik in held-out time points
log p(y_j|t_j,y_−j)
Time (h)

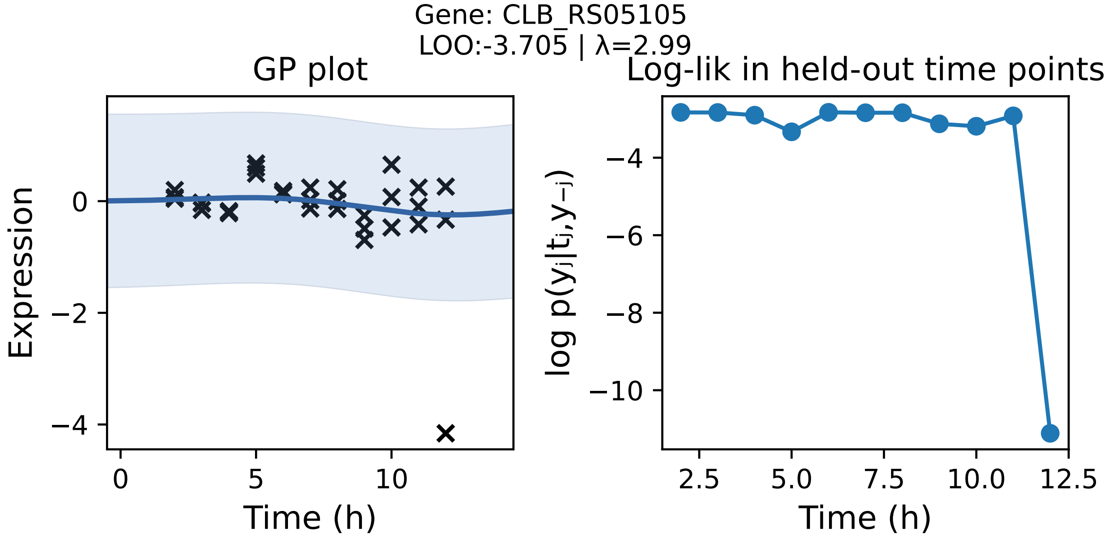

Gene: CLB_RS05105
LOO:-3.705 | λ=2.99
GP plot
Expression
0
−2
−4
0
5
10
Time (h)
Log-lik in held-out time points
log p(y_j|t_j,y_−j)
−4
−6
−8
−10
2.5
5.0
7.5
10.0
12.5
Time (h)

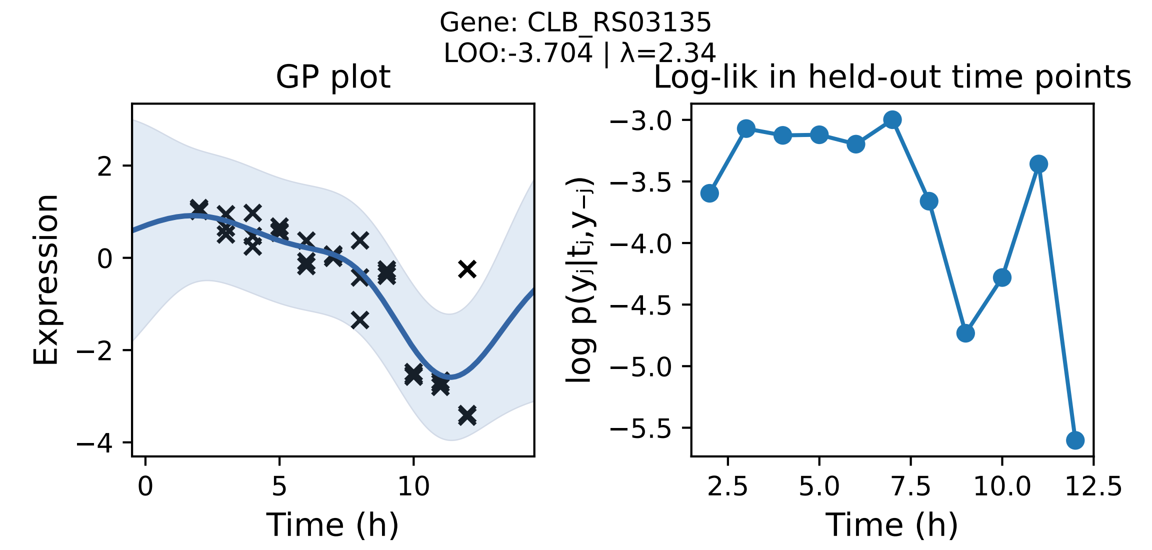
Gene: CLB_RS03135
LOO:-3.704 | λ=2.34
GP plot
Log-lik in held-out time points
Expression
Time (h)
log p(y_j|t_j,y_−j)
Time (h)

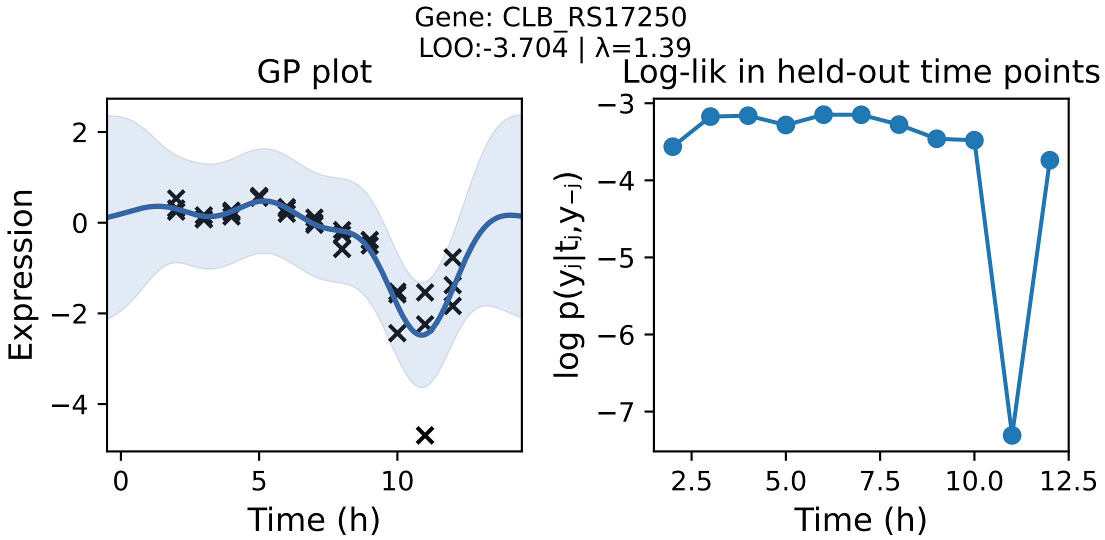

Gene: CLB_RS17250
LOO:-3.704 | λ=1.39
GP plot
Expression
Time (h)
Log-lik in held-out time points
log p(y_j|t_j,y_−j)
Time (h)

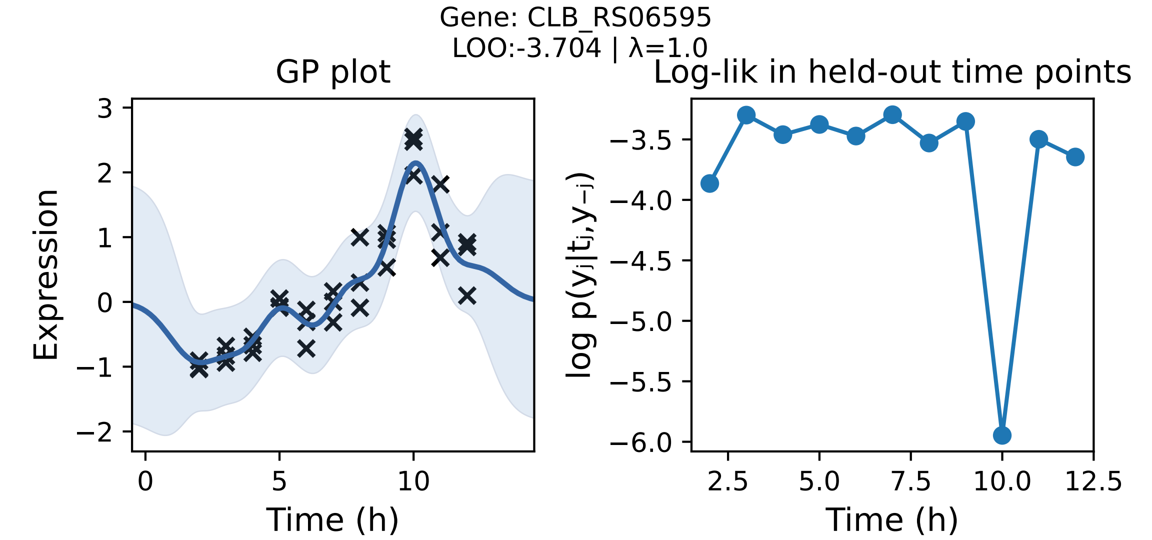
Gene: CLB_RS06595
LOO:-3.704 | λ=1.0
GP plot
Expression
Time (h)
Log-lik in held-out time points
log p(y_j|t_j,y_−j)
Time (h)

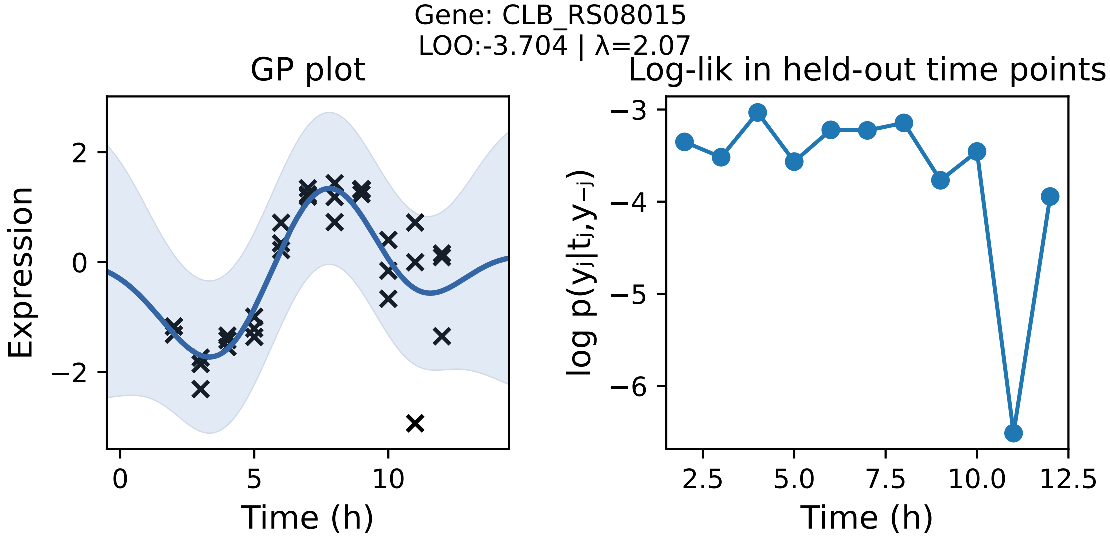

Gene: CLB_RS08015
LOO:-3.704 | λ=2.07
GP plot
Expression
Time (h)
Log-lik in held-out time points
log p(y_j|t_j,y_−j)
Time (h)

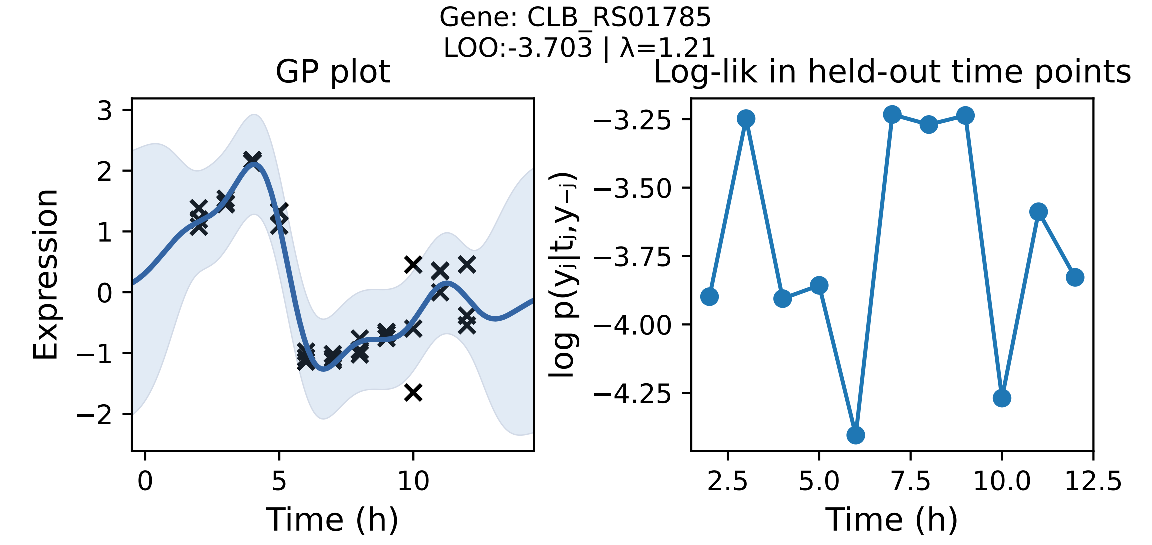

Gene: CLB_RS01785
LOO:-3.703 | λ=1.21
GP plot
Expression
Time (h)
Log-lik in held-out time points
log p(y_j|t_j,y_-j)
Time (h)

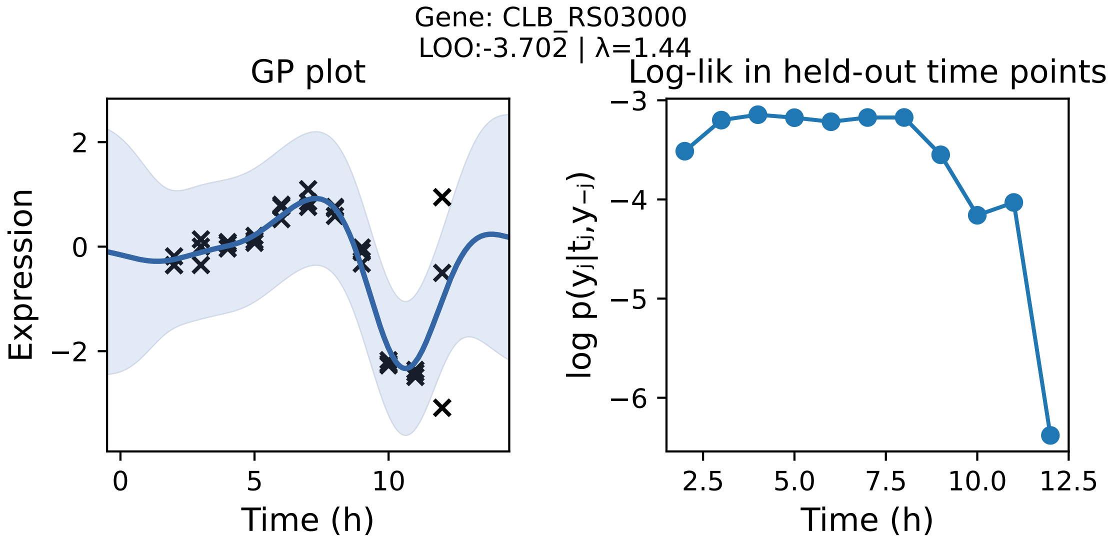

Gene: CLB_RS03000
LOO:-3.702 | λ=1.44
GP plot
Expression
Time (h)
2
0
−2
0
5
10
Log-lik in held-out time points
log p(y_j|t_j,y_−j)
Time (h)
−3
−4
−5
−6
2.5
5.0
7.5
10.0
12.5

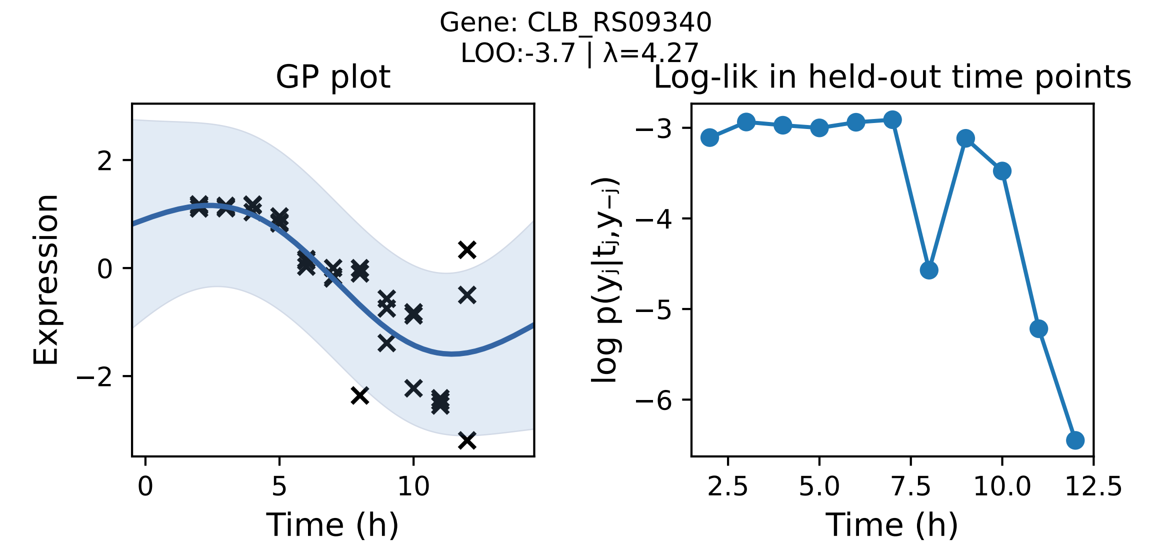

Gene: CLB_RS09340
LOO:-3.7 | λ=4.27
GP plot
Expression
Time (h)
2
0
−2
0
5
10
Log-lik in held-out time points
log p(y_j|t_j,y_−j)
Time (h)
−3
−4
−5
−6
2.5
5.0
7.5
10.0
12.5

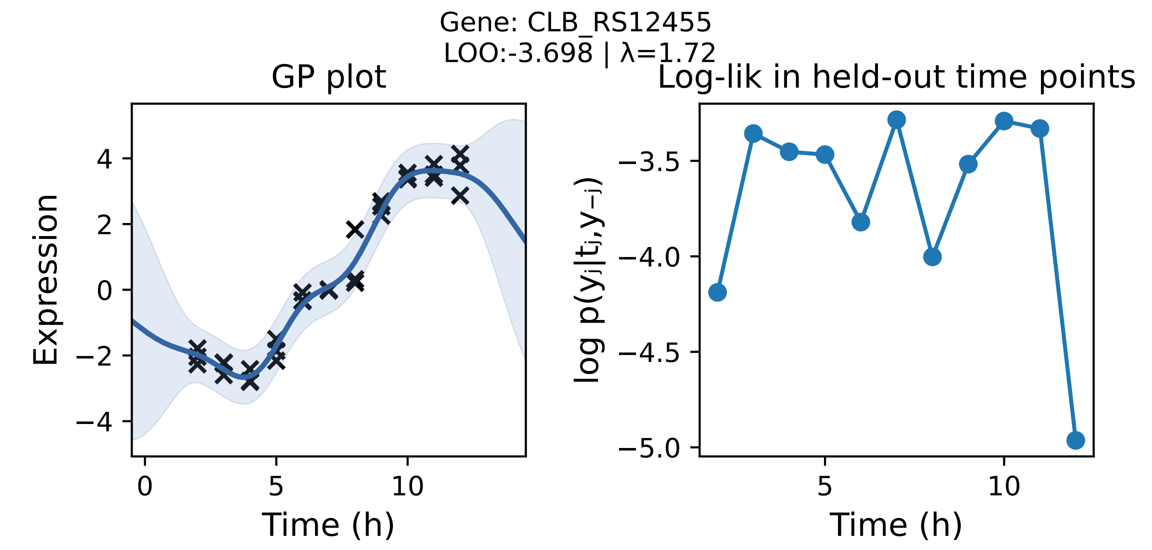

Gene: CLB_RS12455
LOO:-3.698 | λ=1.72
GP plot
Expression
Time (h)
4
2
0
−2
−4
0
5
10
Log-lik in held-out time points
log p(y_j|t_j,y_−j)
−3.5
−4.0
−4.5
−5.0
5
10
Time (h)

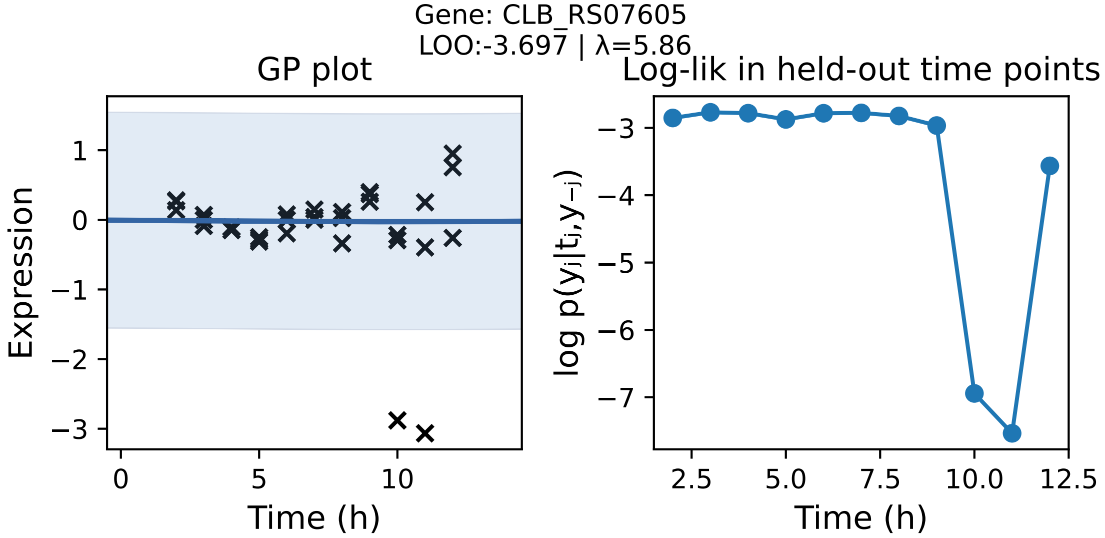
Gene: CLB_RS07605
LOO:-3.697 | λ=5.86
GP plot
Log-lik in held-out time points
Expression
Time (h)
log p(yj|tj,y−j)
Time (h)
1
0
−1
−2
−3
0
5
10
−3
−4
−5
−6
−7
2.5
5.0
7.5
10.0
12.5

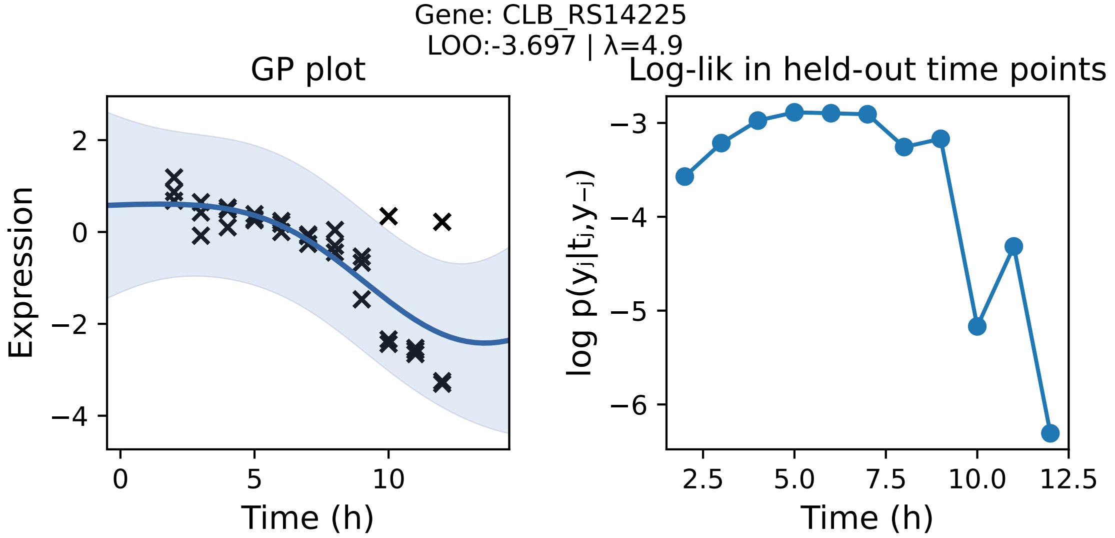
Gene: CLB_RS14225
LOO:-3.697 | λ=4.9
GP plot
Expression
Time (h)
2
0
−2
−4
0
5
10
Log-lik in held-out time points
log p(yⱼ|tⱼ,y₋ⱼ)
Time (h)
−3
−4
−5
−6
2.5
5.0
7.5
10.0
12.5

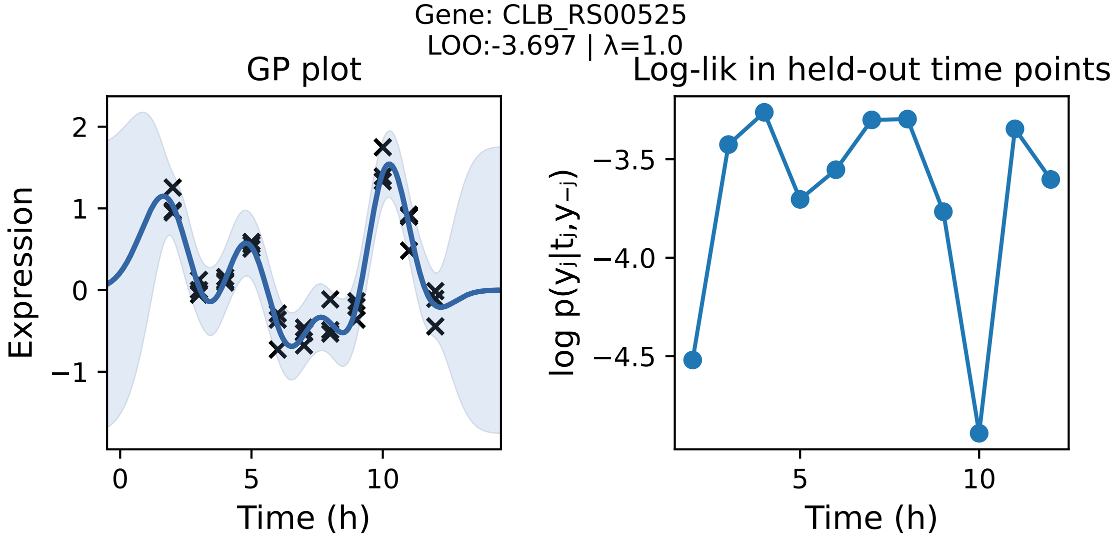

Gene: CLB_RS00525
LOO:-3.697 | λ=1.0
GP plot
Expression
Time (h)
Log-lik in held-out time points
log p(y_j|t_j,y_−j)
Time (h)

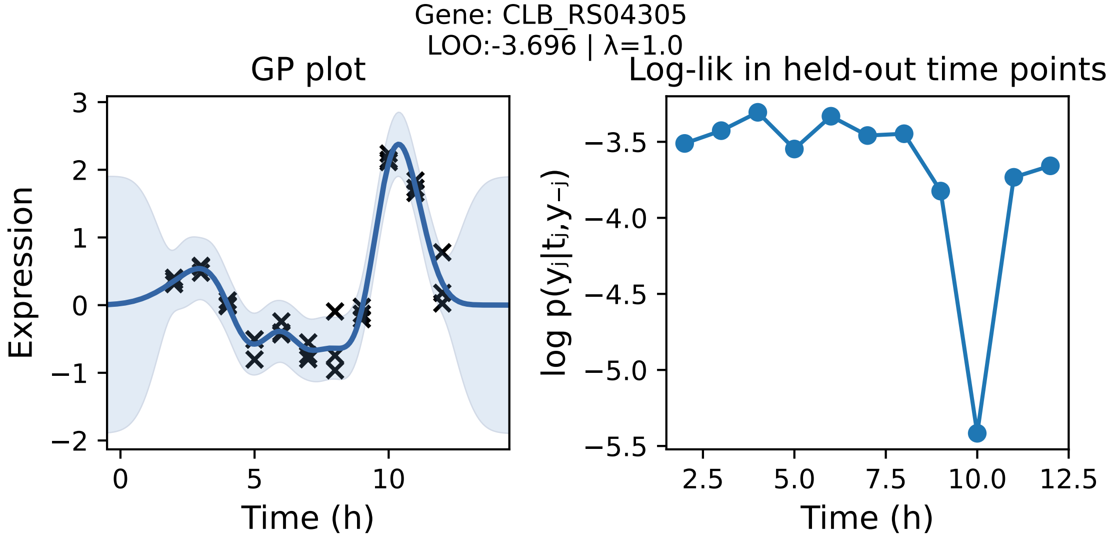

Gene: CLB_RS04305
LOO:-3.696 | λ=1.0
GP plot
Expression
Time (h)
Log-lik in held-out time points
log p(y_j|t_j,y_−j)
Time (h)

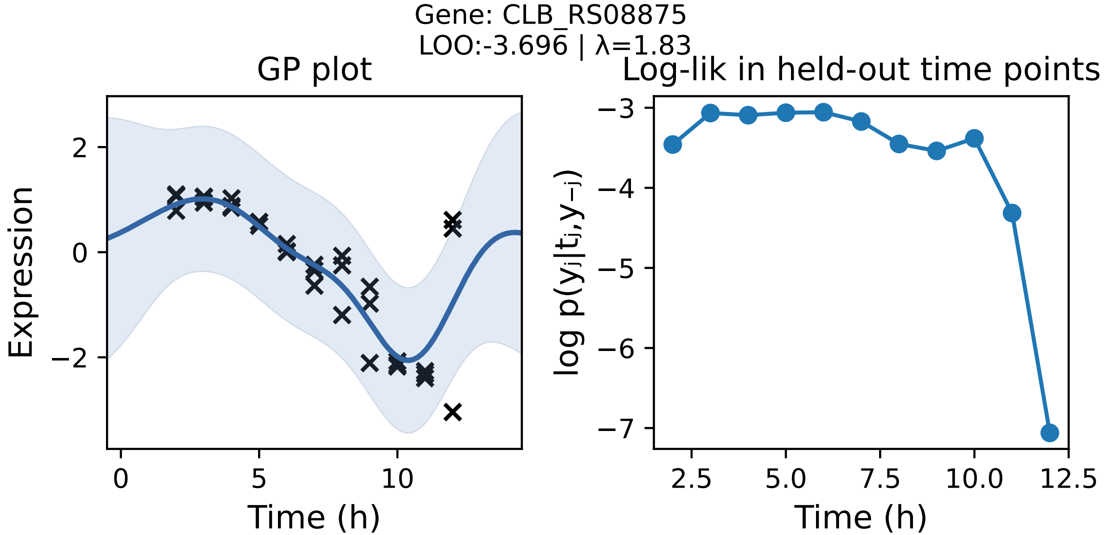

Gene: CLB_RS08875
LOO:-3.696 | λ=1.83
GP plot
Expression
Time (h)
2
0
−2
0
5
10
Log-lik in held-out time points
log p(y_j|t_j,y_−j)
−3
−4
−5
−6
−7
2.5
5.0
7.5
10.0
12.5
Time (h)

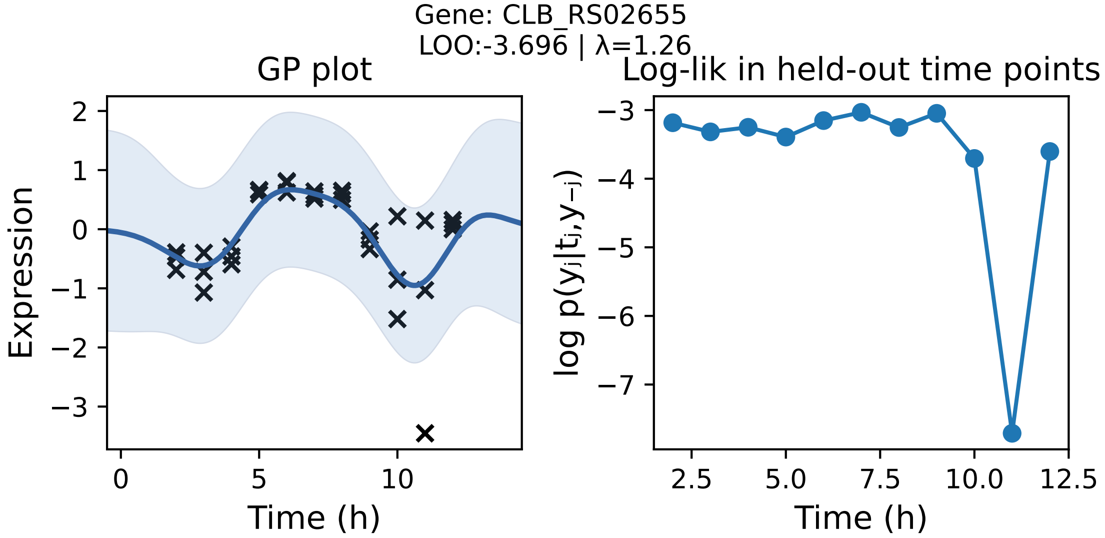

Gene: CLB_RS02655
LOO:-3.696 | λ=1.26
GP plot
Log-lik in held-out time points
Expression
2
1
0
−1
−2
−3
0
5
10
Time (h)
log p(yj|tj,y−j)
−3
−4
−5
−6
−7
2.5
5.0
7.5
10.0
12.5
Time (h)

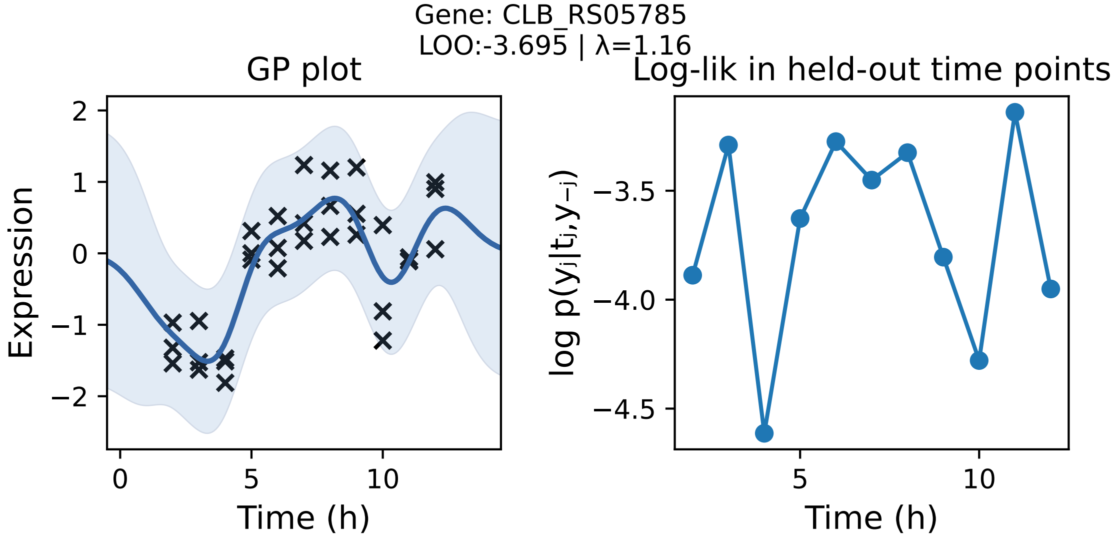

Gene: CLB_RS05785
LOO:-3.695 | λ=1.16
GP plot
Expression
Time (h)
Log-lik in held-out time points
log p(y_j|t_j,y_−j)
Time (h)

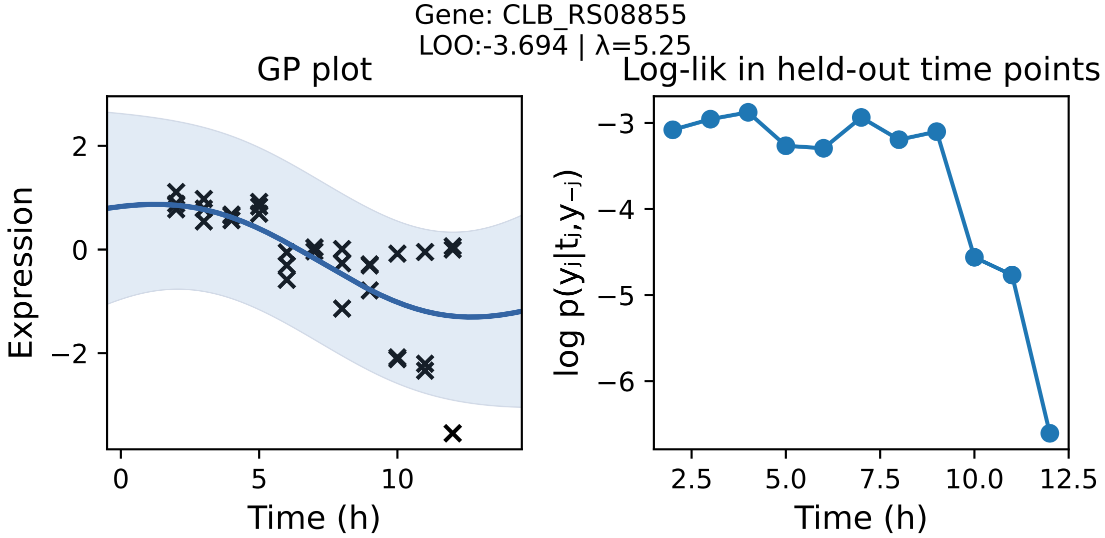
Gene: CLB_RS08855
LOO:-3.694 | λ=5.25
GP plot
Expression
Time (h)
Log-lik in held-out time points
log p(y_j|t_j,y_−j)
Time (h)

Gene: CLB_RS02380
LOO:-3.693 | λ=1.0

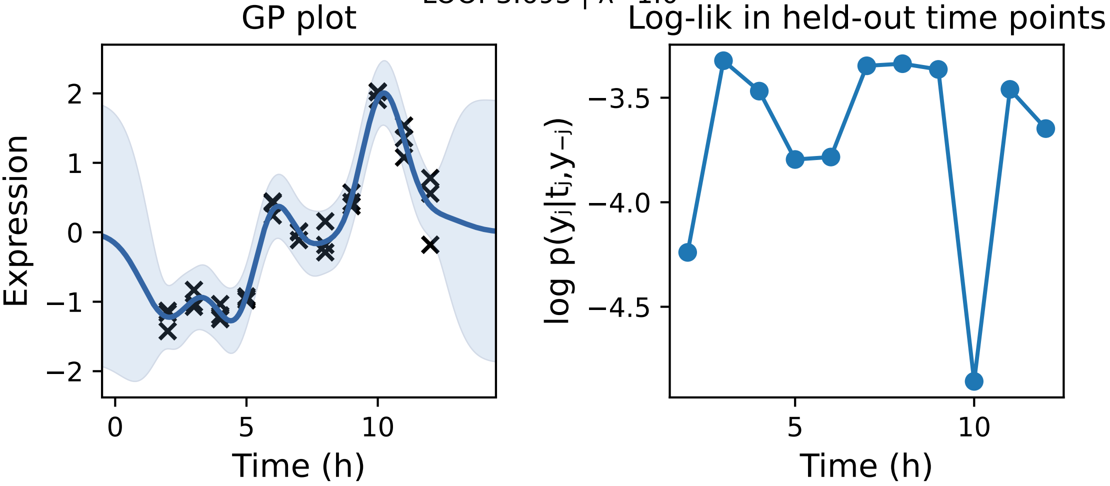

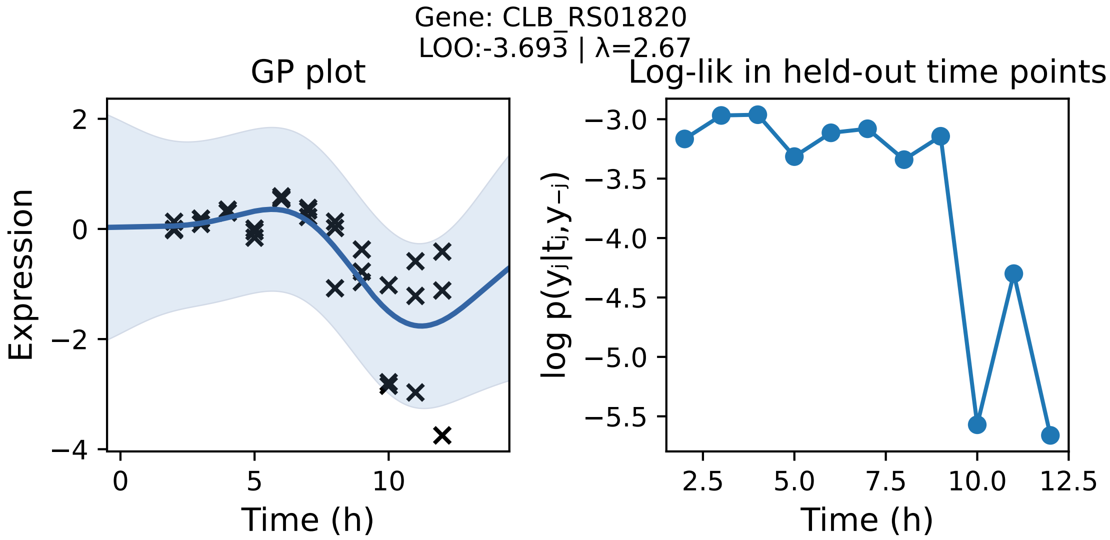

Gene: CLB_RS01820
LOO:-3.693 | λ=2.67
GP plot
Expression
Time (h)
2
0
−2
−4
0
5
10
Log-lik in held-out time points
log p(y_j|t_j,y_−j)
−3.0
−3.5
−4.0
−4.5
−5.0
−5.5
2.5
5.0
7.5
10.0
12.5
Time (h)

Gene: CLB_RS00430
LOO:-3.693 | λ=1.0

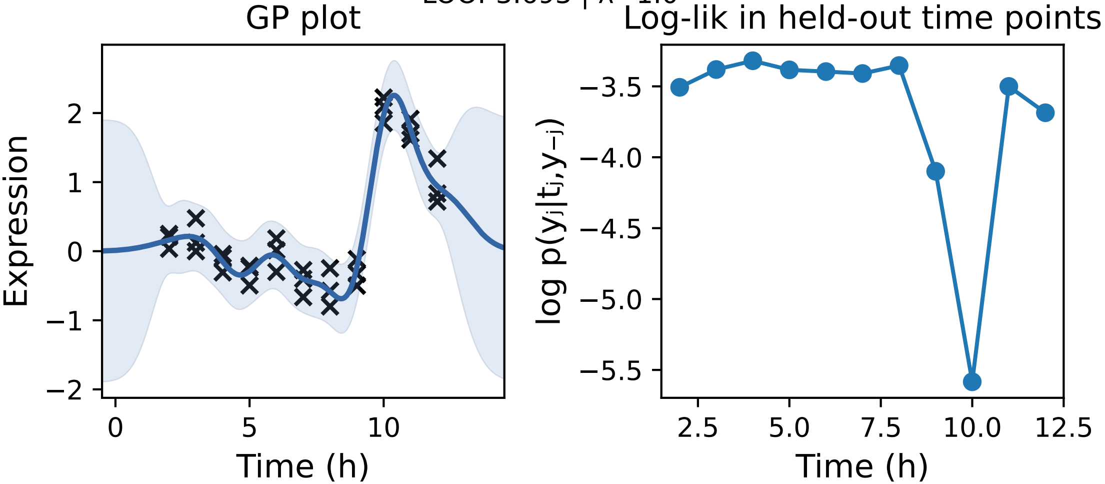

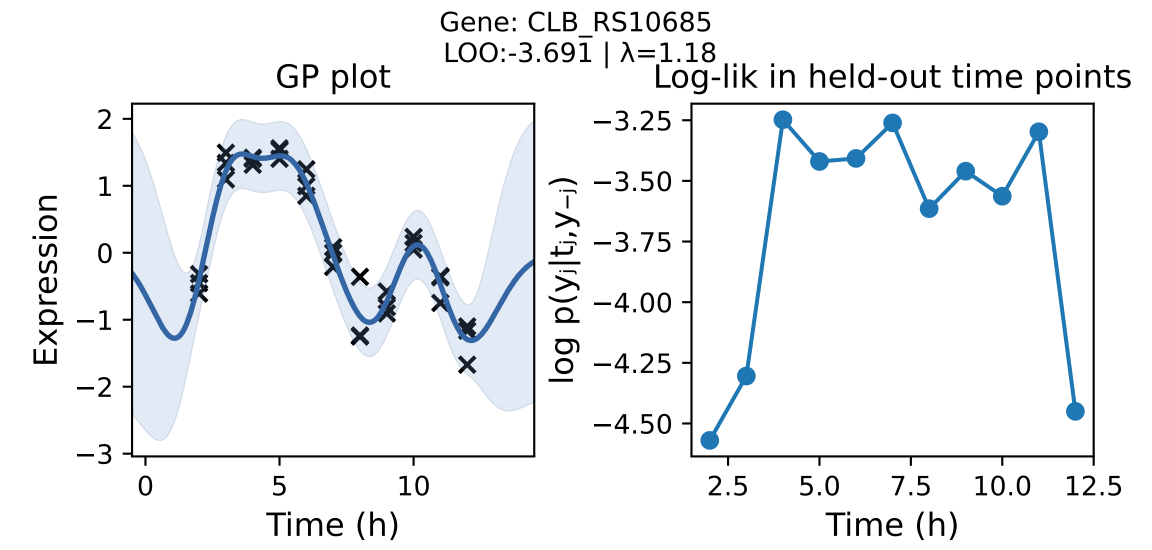

Gene: CLB_RS10685
LOO:-3.691 | λ=1.18
GP plot
Expression
Time (h)
Log-lik in held-out time points
log p(y_j|t_j,y_−j)
Time (h)

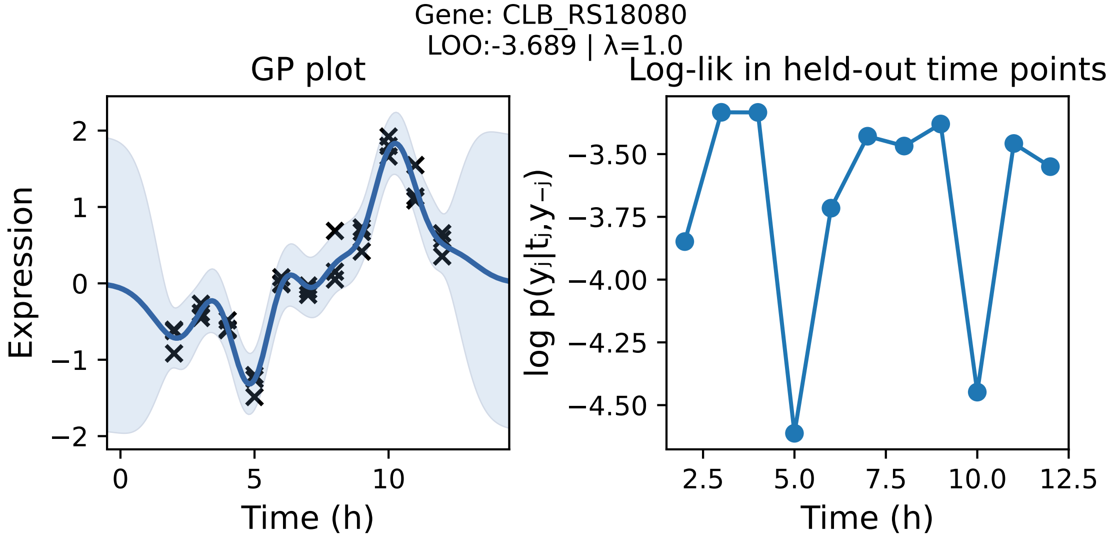

Gene: CLB_RS18080
LOO:-3.689 | λ=1.0
GP plot
Expression
Time (h)
0
5
10
2
1
0
−1
−2
Log-lik in held-out time points
log p(y_j|t_j,y_−j)
Time (h)
2.5
5.0
7.5
10.0
12.5
−3.50
−3.75
−4.00
−4.25
−4.50

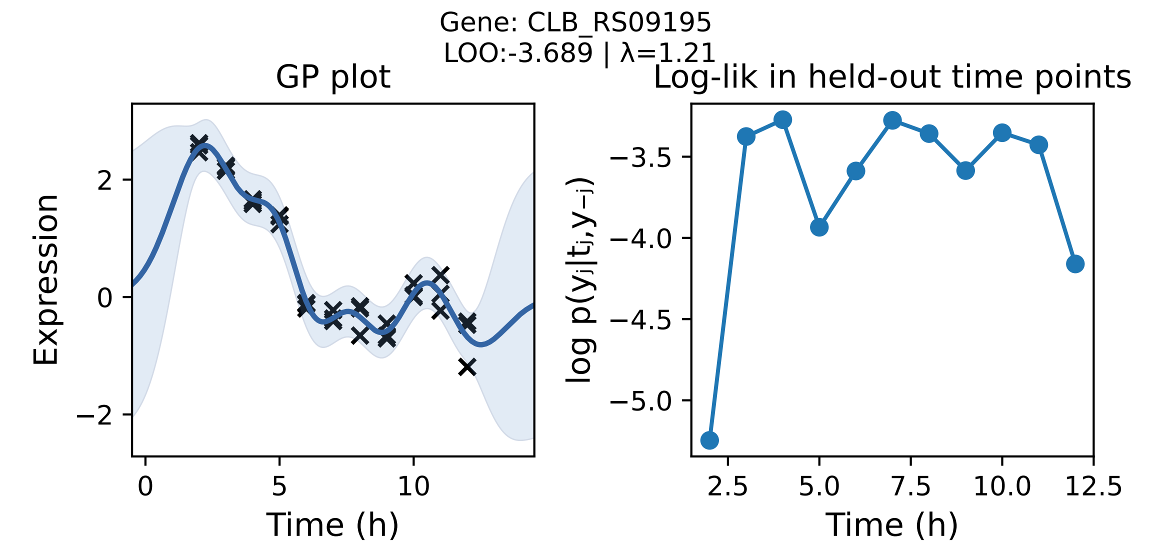
Gene: CLB_RS09195
LOO:-3.689 | λ=1.21
GP plot
Expression
Time (h)
2
0
−2
0
5
10
Log-lik in held-out time points
log p(y_j|t_j,y_−j)
Time (h)
−3.5
−4.0
−4.5
−5.0
2.5
5.0
7.5
10.0
12.5

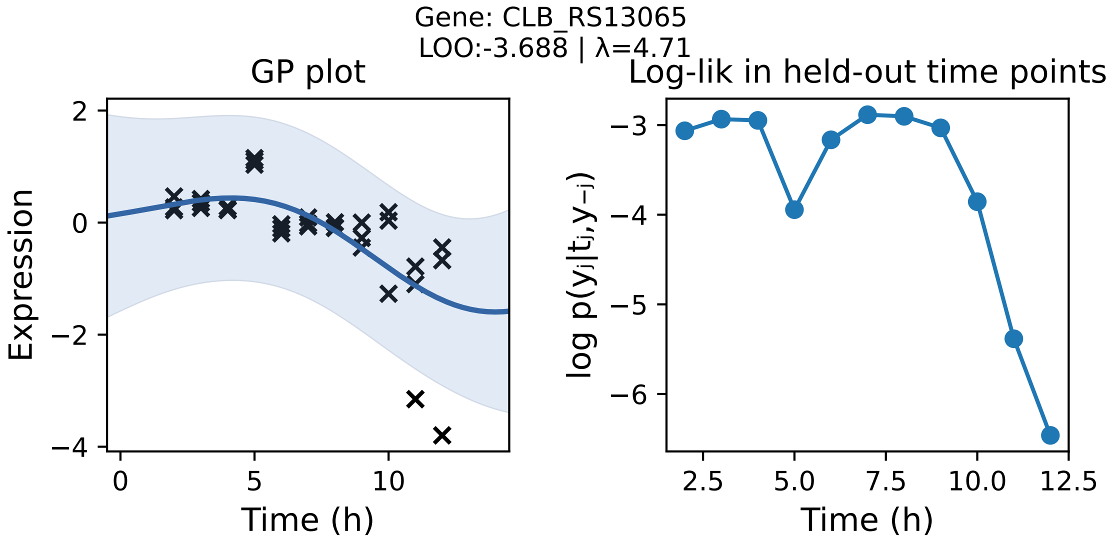
Gene: CLB_RS13065
LOO:-3.688 | λ=4.71
GP plot
Expression
Time (h)
Log-lik in held-out time points
log p(y_j|t_j,y_−j)
Time (h)

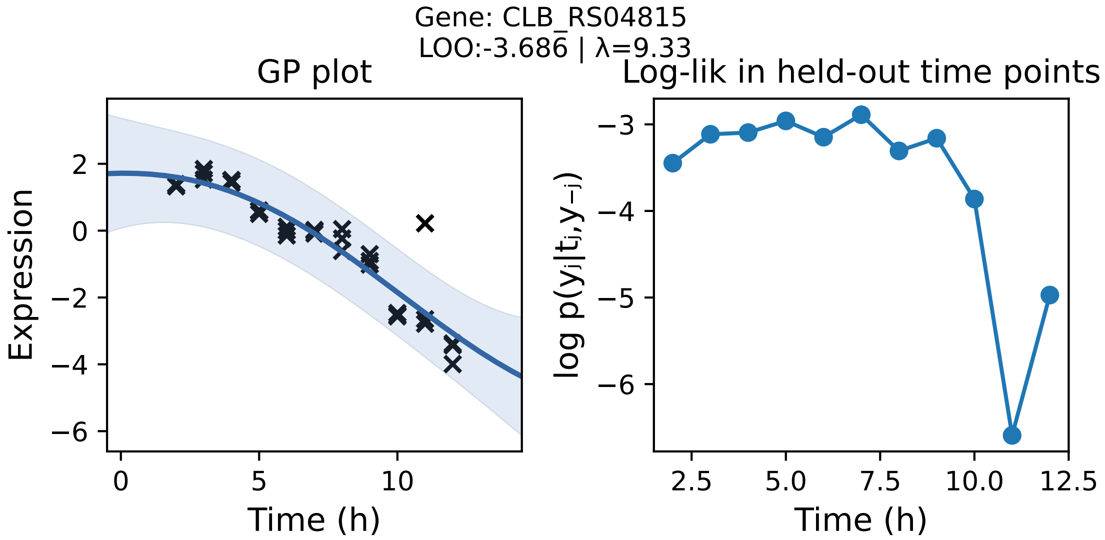
Gene: CLB_RS04815
LOO:-3.686 | λ=9.33
GP plot
Expression
Time (h)
Log-lik in held-out time points
log p(y_j|t_j,y_−j)
Time (h)
2
0
−2
−4
−6
0
5
10
−3
−4
−5
−6
2.5
5.0
7.5
10.0
12.5

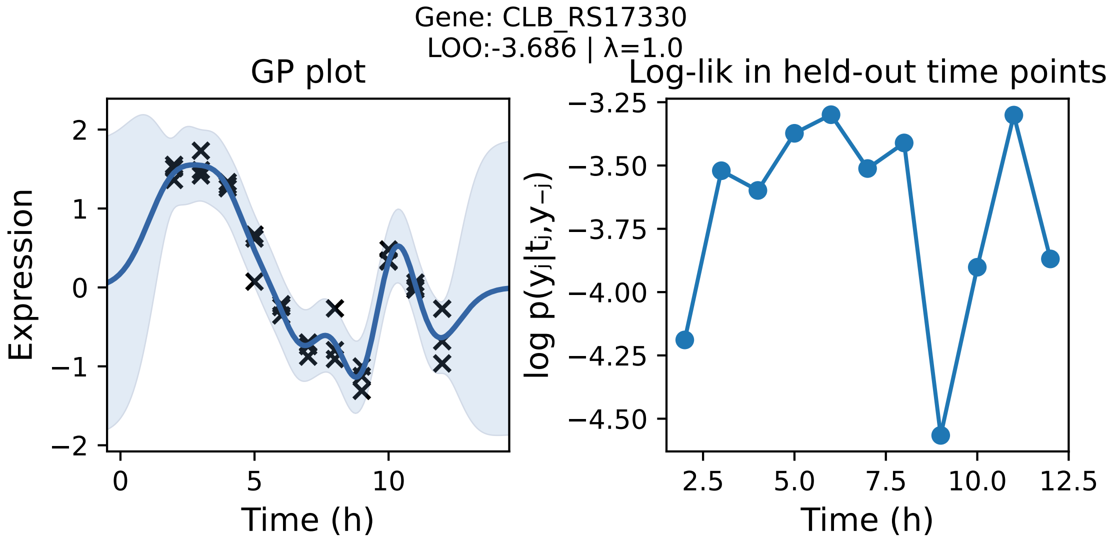

Gene: CLB_RS17330
LOO:-3.686 | λ=1.0
GP plot
Expression
Time (h)
Log-lik in held-out time points
log p(y_j|t_j,y_−j)
Time (h)

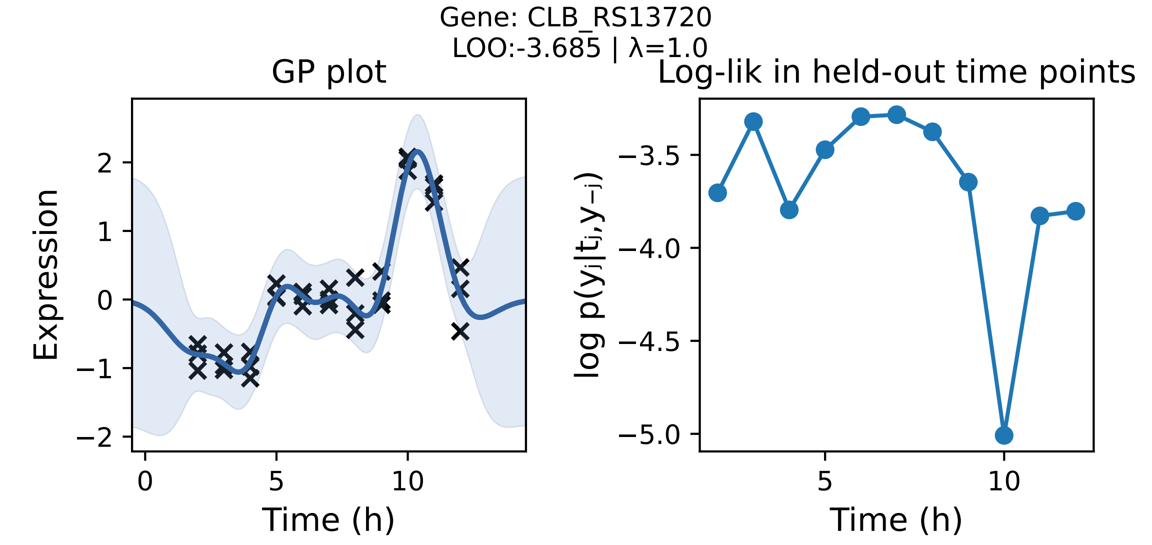

Gene: CLB_RS13720
LOO:-3.685 | λ=1.0
GP plot
Expression
Time (h)
Log-lik in held-out time points
log p(y_j|t_j,y_−j)
Time (h)

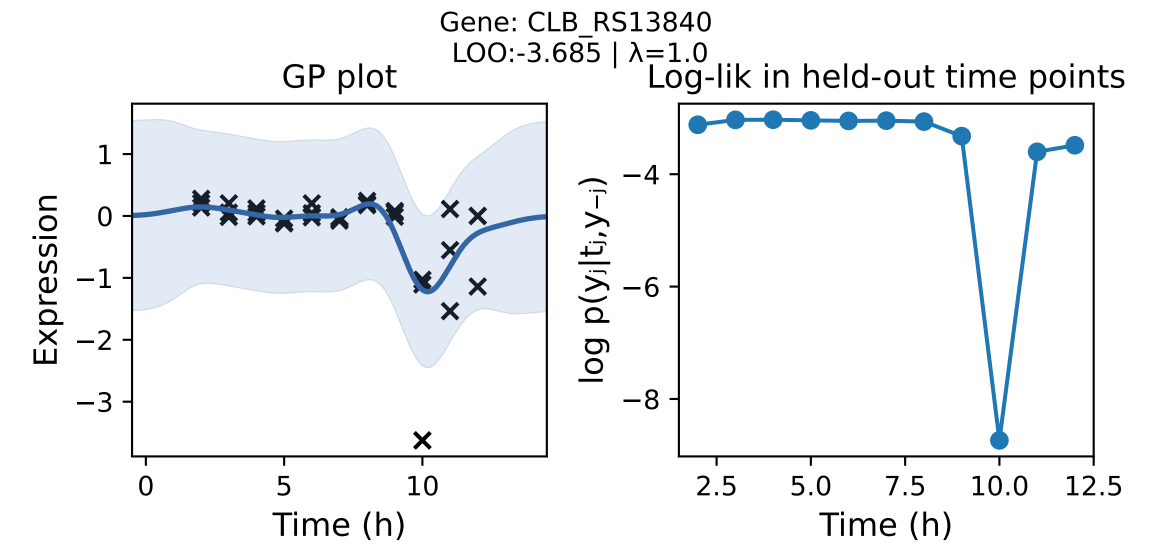

Gene: CLB_RS13840
LOO:-3.685 | λ=1.0
GP plot
Log-lik in held-out time points
Expression
1
0
−1
−2
−3
0
5
10
Time (h)
log p(yⱼ|tⱼ,y₋ⱼ)
−4
−6
−8
2.5
5.0
7.5
10.0
12.5
Time (h)

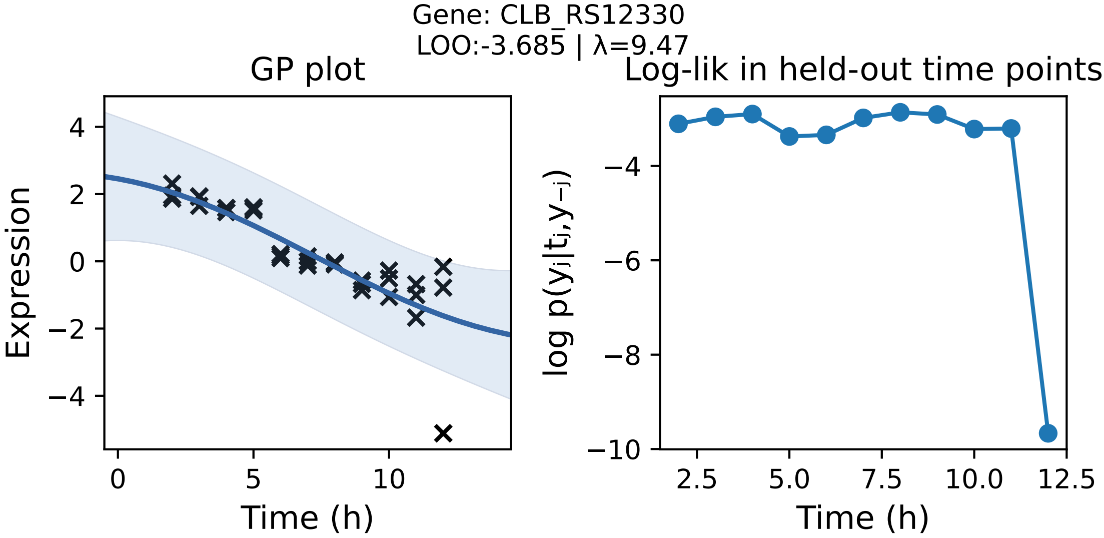
Gene: CLB_RS12330
LOO:-3.685 | λ=9.47
GP plot
Expression
Time (h)
Log-lik in held-out time points
log p(y_j|t_j,y_−j)
Time (h)

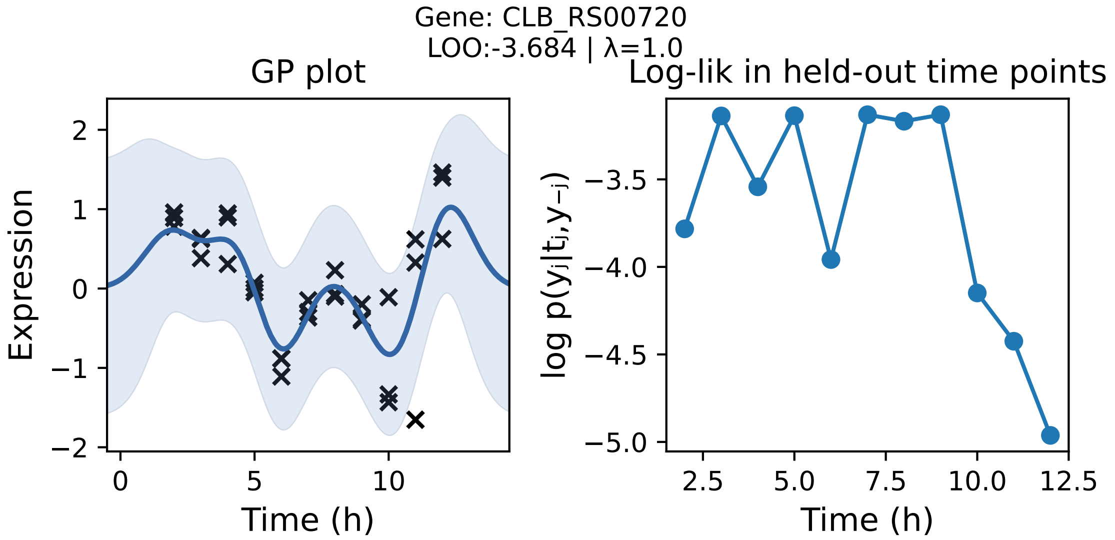

Gene: CLB_RS00720
LOO:-3.684 | λ=1.0
GP plot
Expression
2
1
0
−1
−2
0
5
10
Time (h)
Log-lik in held-out time points
log p(y_j|t_j,y_−j)
−3.5
−4.0
−4.5
−5.0
2.5
5.0
7.5
10.0
12.5
Time (h)

Gene: CLB_RS13185
LOO:-3.683 | λ=5.78

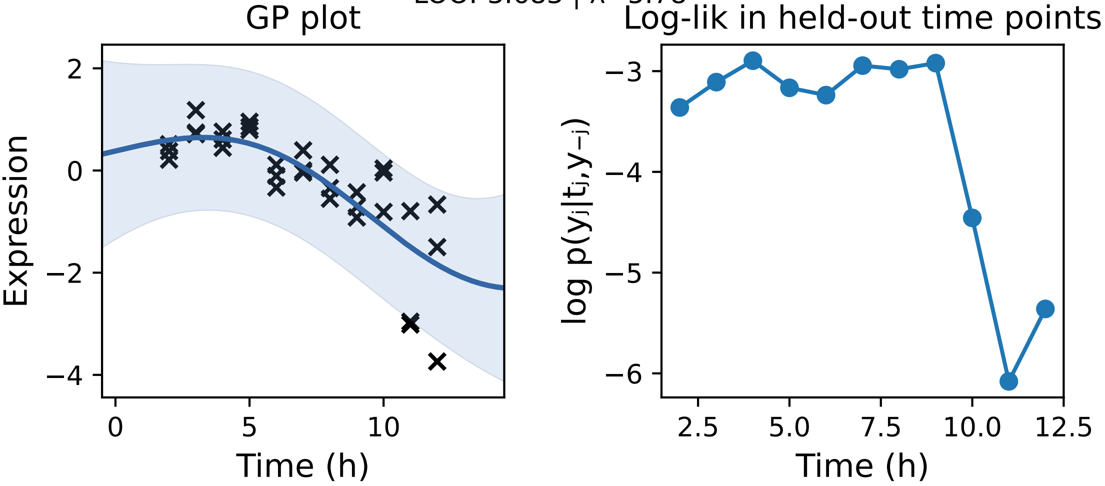

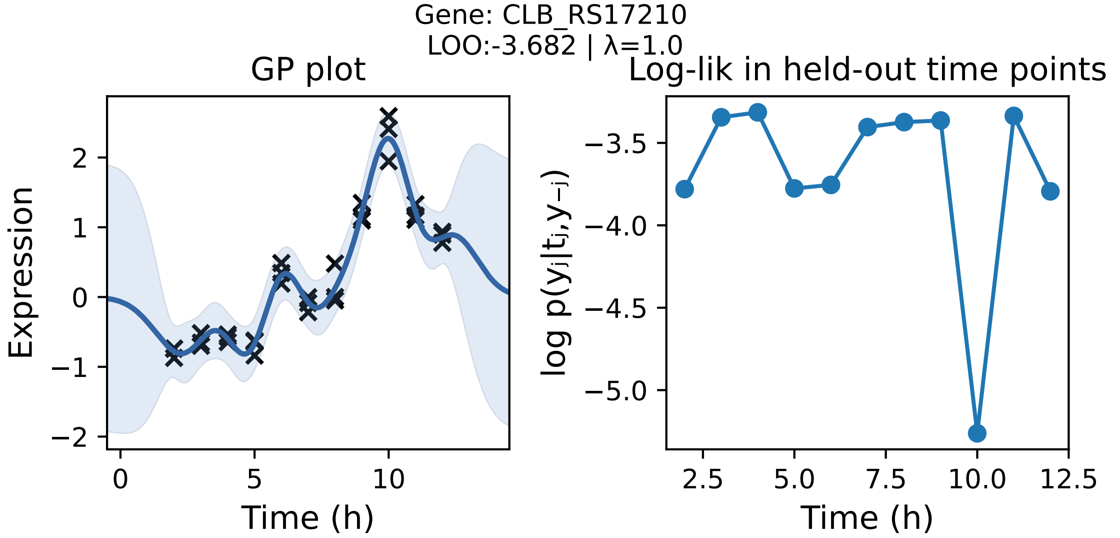

Gene: CLB_RS17210
LOO:-3.682 | λ=1.0
GP plot
Expression
Time (h)
2
1
0
−1
−2
0
5
10
Log-lik in held-out time points
log p(y_j|t_j,y_{−j})
Time (h)
−3.5
−4.0
−4.5
−5.0
2.5
5.0
7.5
10.0
12.5

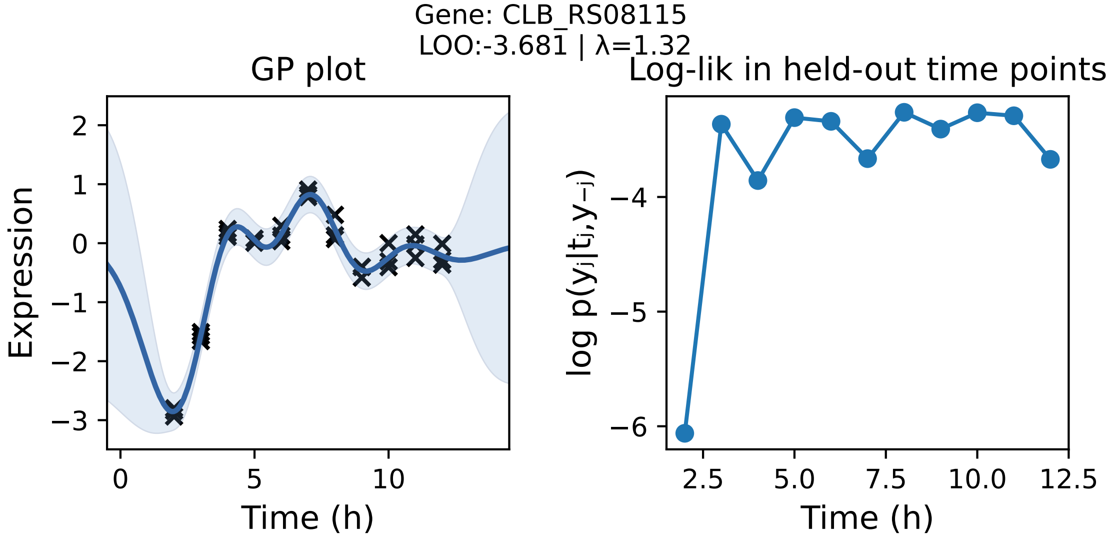

Gene: CLB_RS08115
LOO:-3.681 | λ=1.32
GP plot
Expression
Time (h)
Log-lik in held-out time points
log p(y_j|t_j,y_−j)
Time (h)

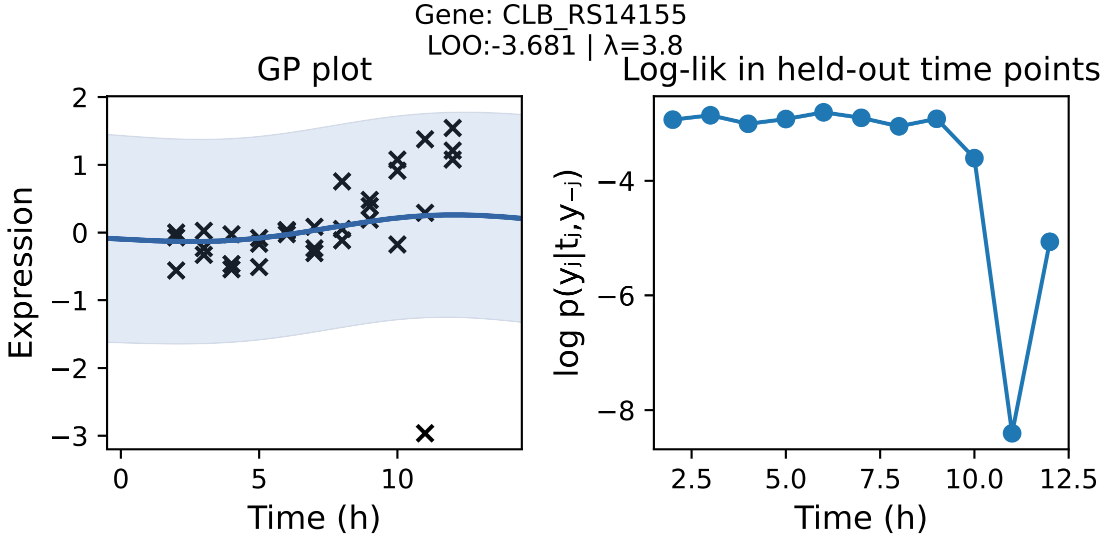

Gene: CLB_RS14155
LOO:-3.681 | λ=3.8
GP plot
Log-lik in held-out time points
Expression
Time (h)
log p(y_j|t_j,y_{-j})
2
1
0
−1
−2
−3
0
5
10
−4
−6
−8
2.5
5.0
7.5
10.0
12.5

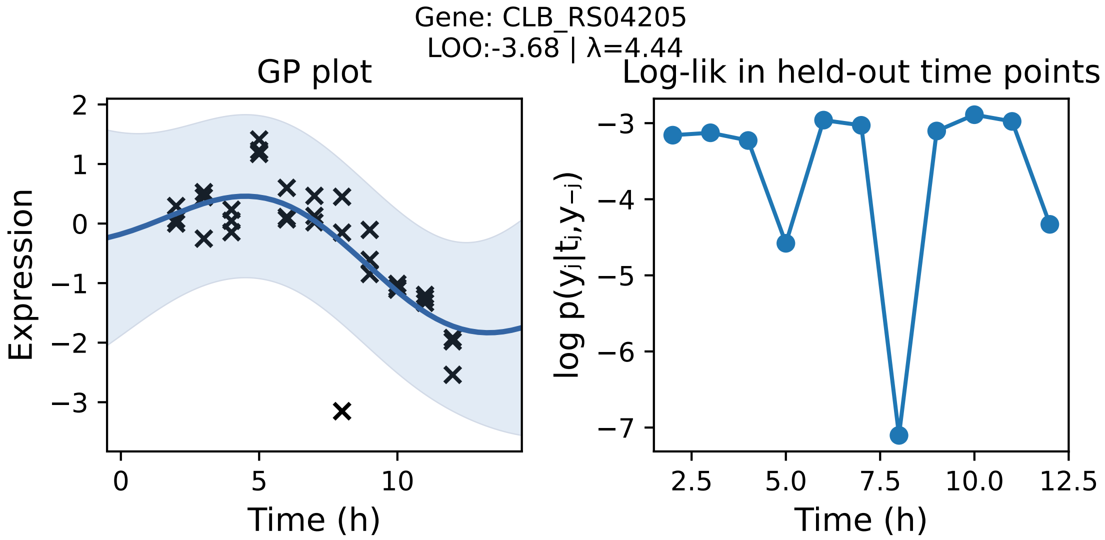

Gene: CLB_RS04205
LOO:-3.68 | λ=4.44
GP plot
Log-lik in held-out time points
Expression
Time (h)
log p(y_j|t_j,y_−j)
Time (h)

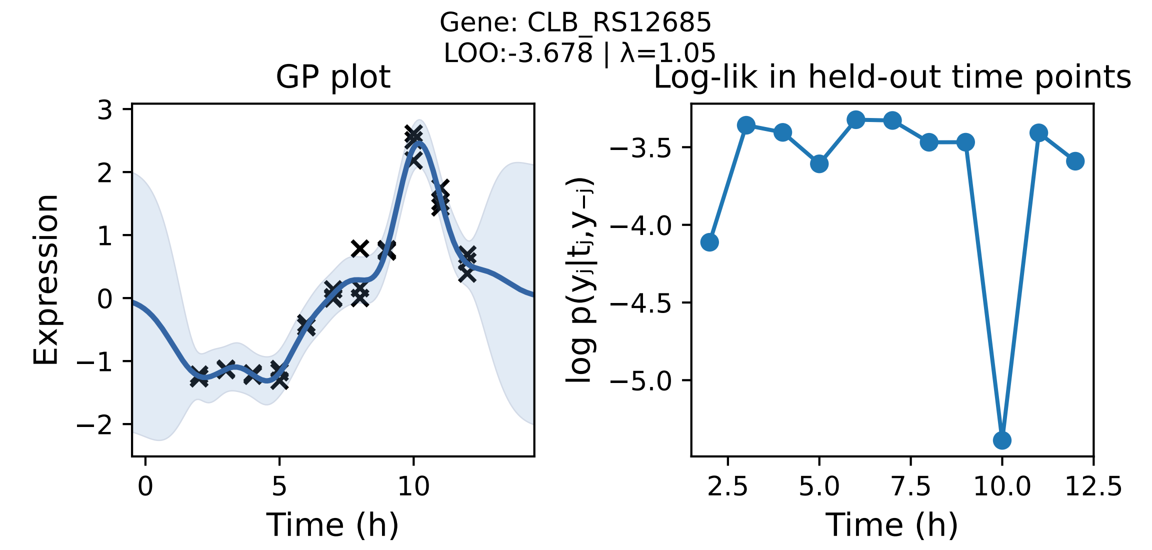

Gene: CLB_RS12685
LOO:-3.678 | λ=1.05
GP plot
Expression
Time (h)
Log-lik in held-out time points
log p(y_j|t_j,y_−j)
Time (h)

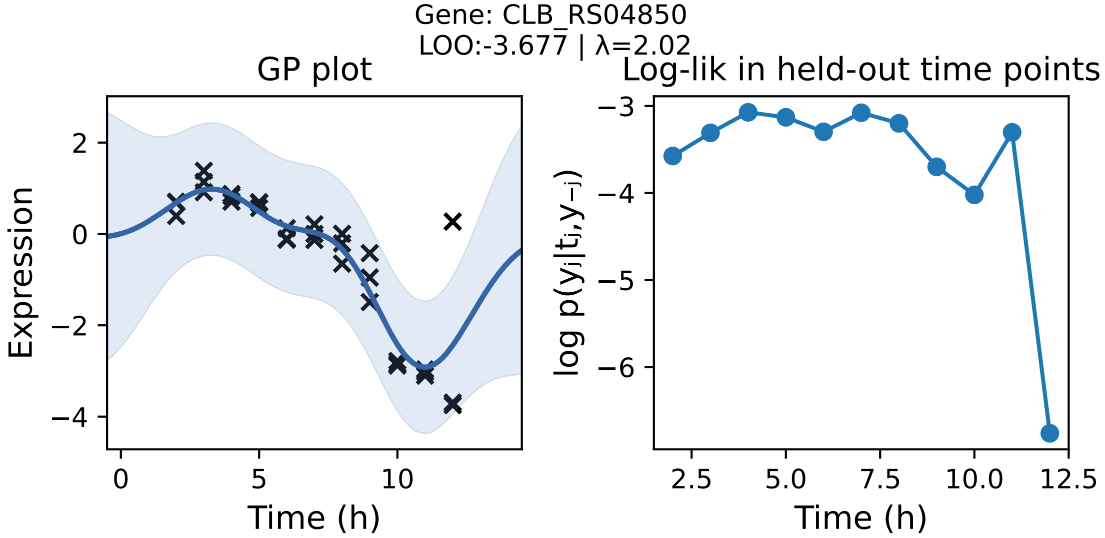
Gene: CLB_RS04850
LOO:-3.677 | λ=2.02
GP plot
Log-lik in held-out time points
Expression
Time (h)
log p(yj|tj,y−j)
2
0
−2
−4
0
5
10
−3
−4
−5
−6
2.5
5.0
7.5
10.0
12.5

Gene: CLB_RS00795
LOO:-3.676 | λ=3.42

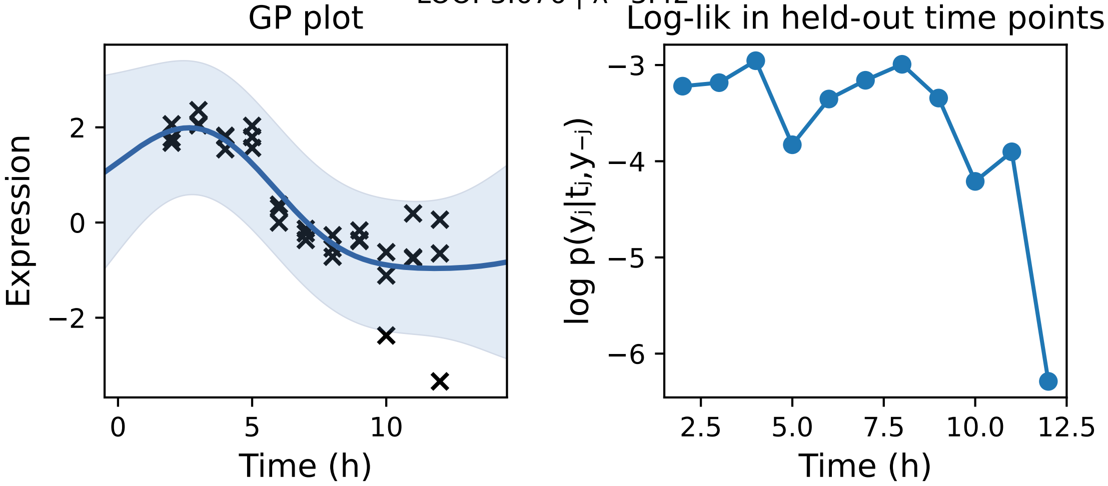

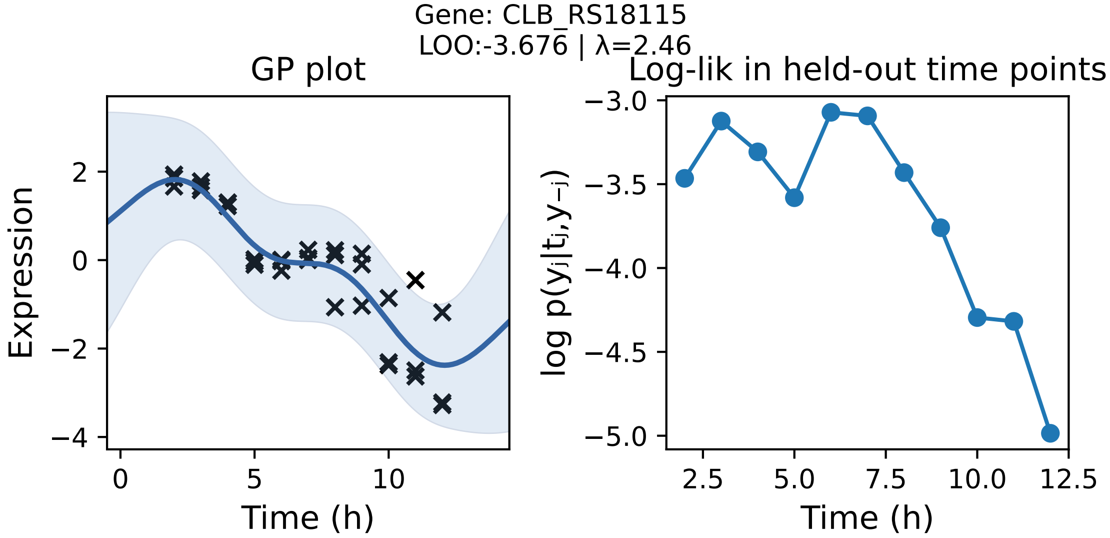

Gene: CLB_RS18115
LOO:-3.676 | λ=2.46
GP plot
Expression
Time (h)
2
0
−2
−4
0
5
10
Log-lik in held-out time points
log p(yⱼ|tⱼ,y₋ⱼ)
Time (h)
−3.0
−3.5
−4.0
−4.5
−5.0
2.5
5.0
7.5
10.0
12.5

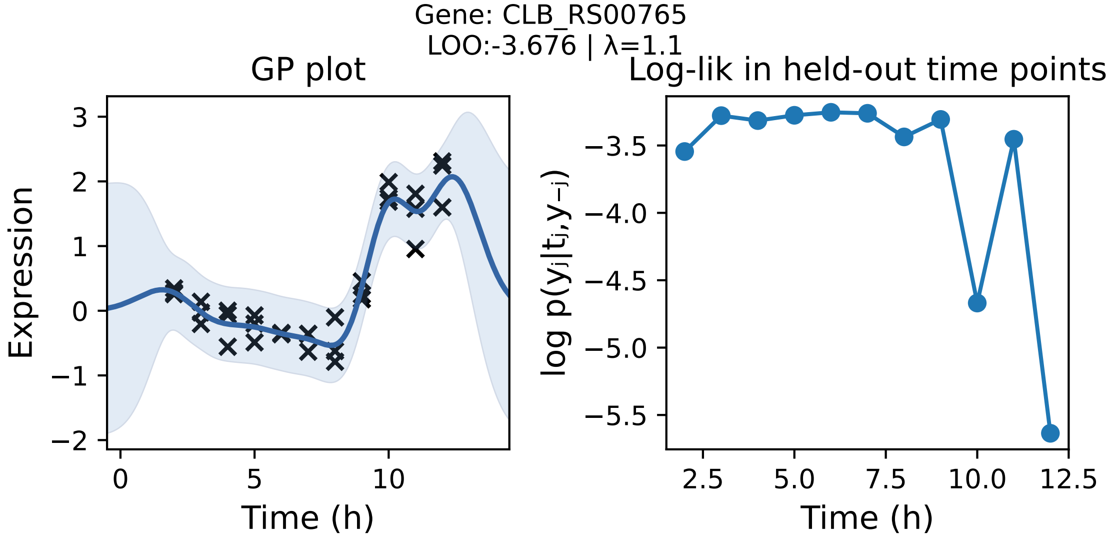
Gene: CLB_RS00765
LOO:-3.676 | λ=1.1
GP plot
Expression
Time (h)
3
2
1
0
−1
−2
0
5
10
Log-lik in held-out time points
log p(y_j|t_j,y_−j)
−3.5
−4.0
−4.5
−5.0
−5.5
2.5
5.0
7.5
10.0
12.5
Time (h)

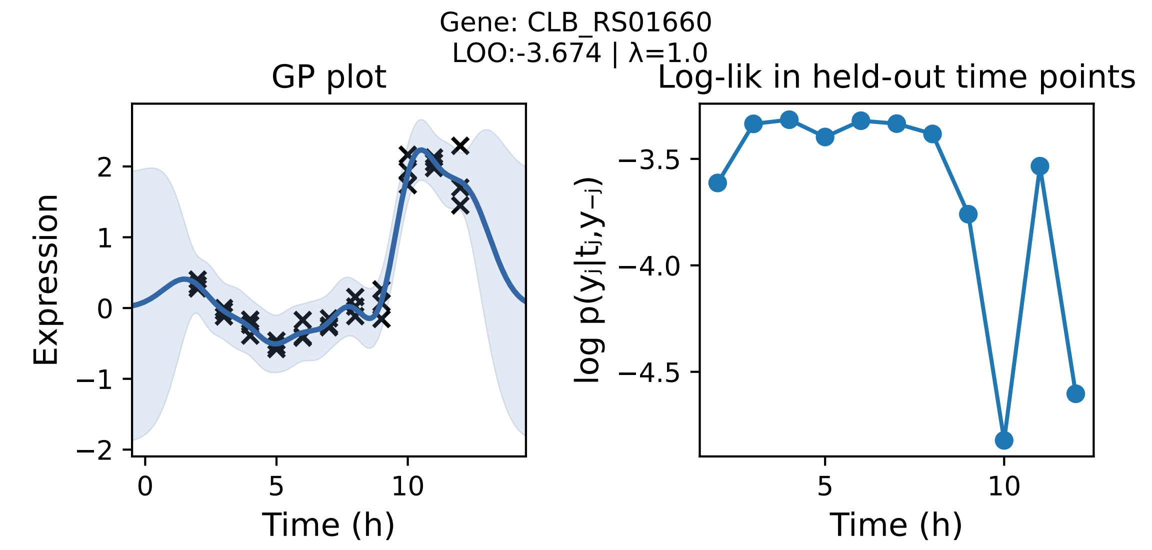
Gene: CLB_RS01660
LOO:-3.674 | λ=1.0
GP plot
Expression
Time (h)
Log-lik in held-out time points
log p(y_j|t_j,y_−j)
Time (h)

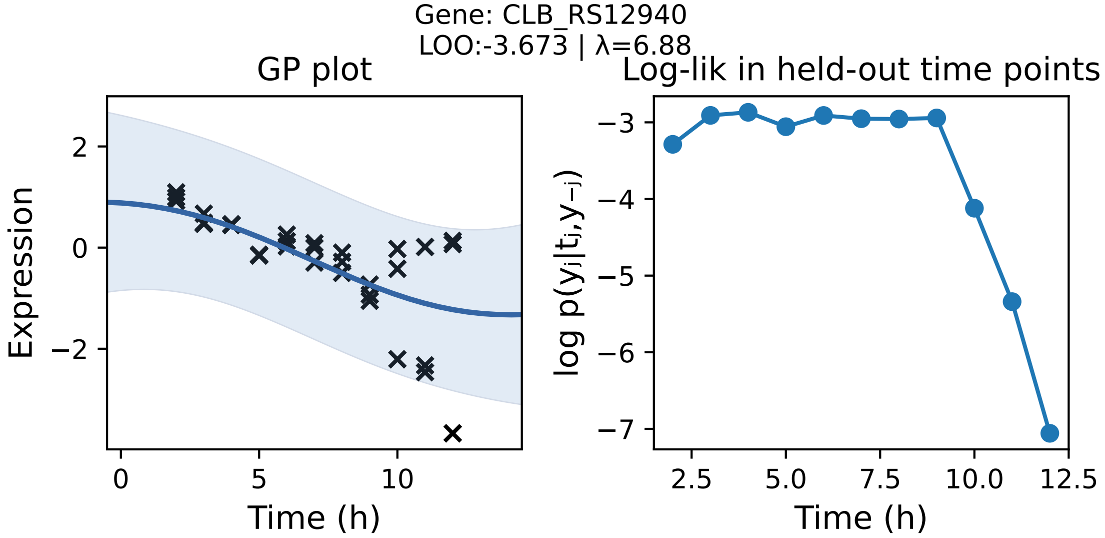
Gene: CLB_RS12940
LOO:-3.673 | λ=6.88
GP plot
Expression
Time (h)
Log-lik in held-out time points
log p(y_j|t_j,y_−j)
Time (h)
2
0
−2
0
5
10
−3
−4
−5
−6
−7
2.5
5.0
7.5
10.0
12.5

Gene: CLB_RS09250
LOO:-3.673 | λ=3.06

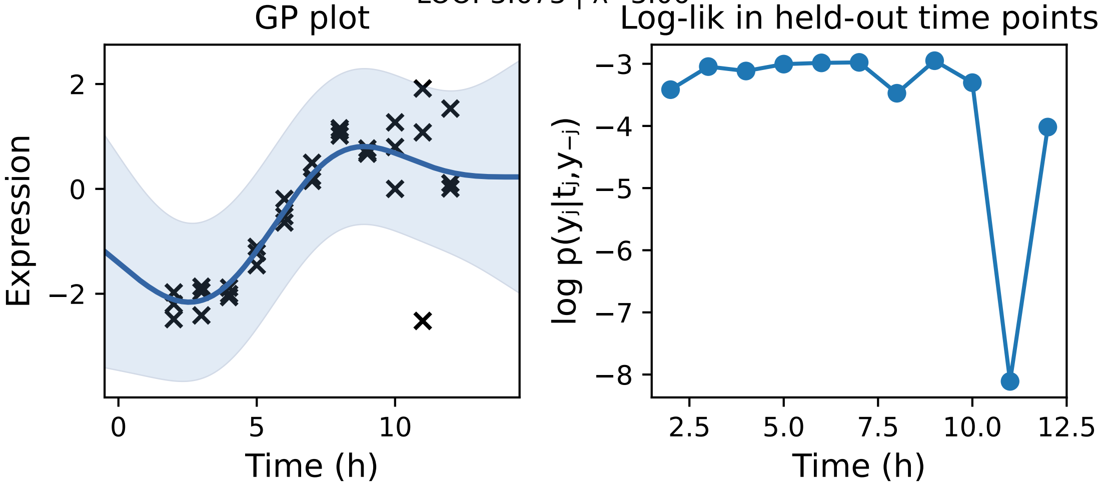

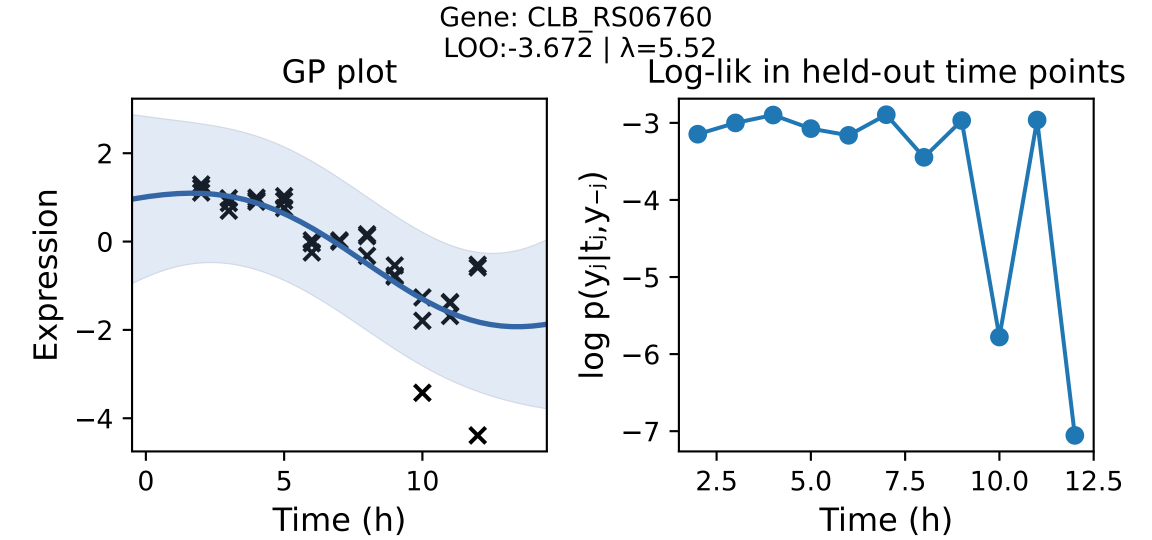
Gene: CLB_RS06760
LOO:-3.672 | λ=5.52
GP plot
Log-lik in held-out time points
Expression
Time (h)
log p(yj|tj,y−j)
Time (h)

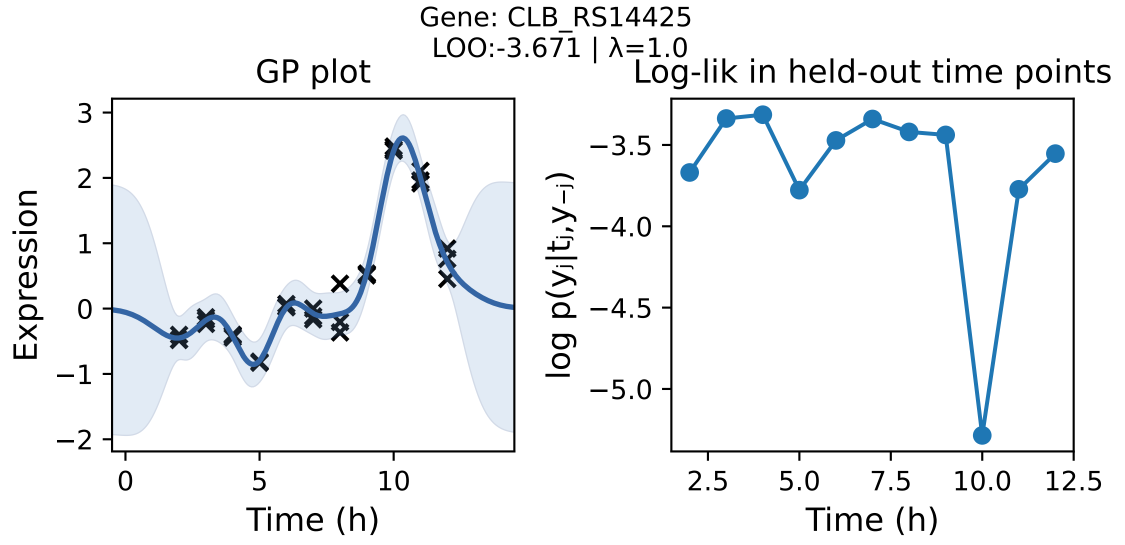

Gene: CLB_RS14425
LOO:-3.671 | λ=1.0
GP plot
Expression
Time (h)
Log-lik in held-out time points
log p(y_j|t_j,y_−j)
Time (h)

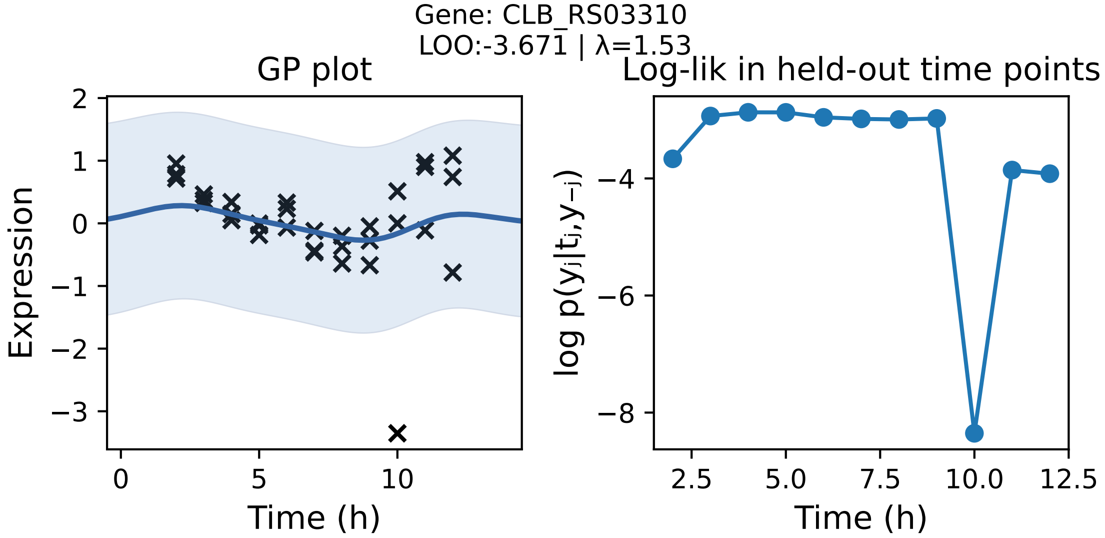

Gene: CLB_RS03310
LOO:-3.671 | λ=1.53
GP plot
Expression
Time (h)
Log-lik in held-out time points
log p(y_j|t_j,y_−j)
Time (h)

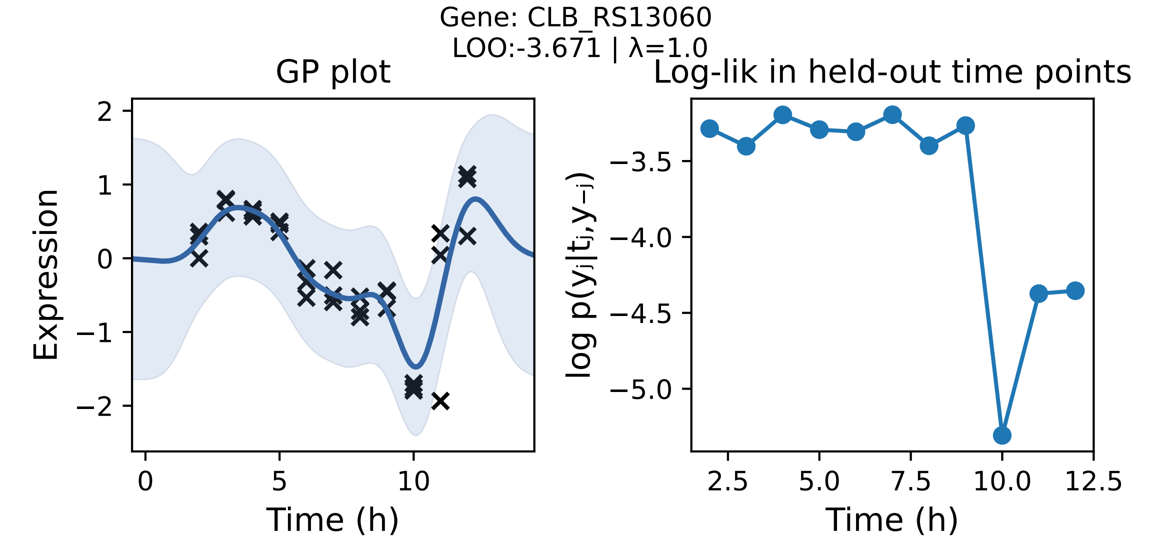
Gene: CLB_RS13060
LOO:-3.671 | λ=1.0
GP plot
Expression
Time (h)
Log-lik in held-out time points
log p(y_j|t_j,y_−j)
Time (h)

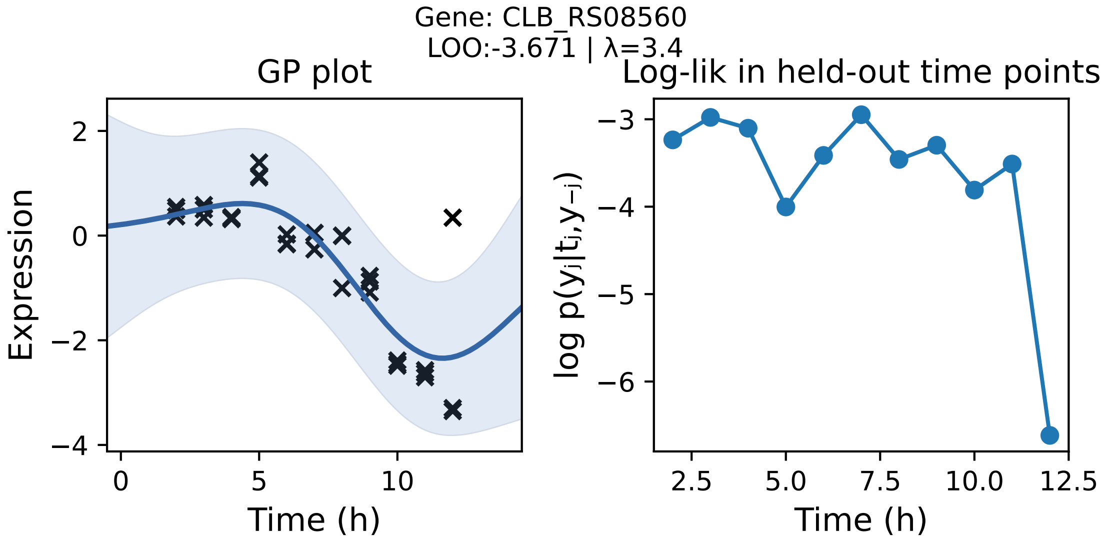

Gene: CLB_RS08560
LOO:-3.671 | λ=3.4
GP plot
Expression
Time (h)
Log-lik in held-out time points
log p(y_j|t_j,y_−j)
Time (h)

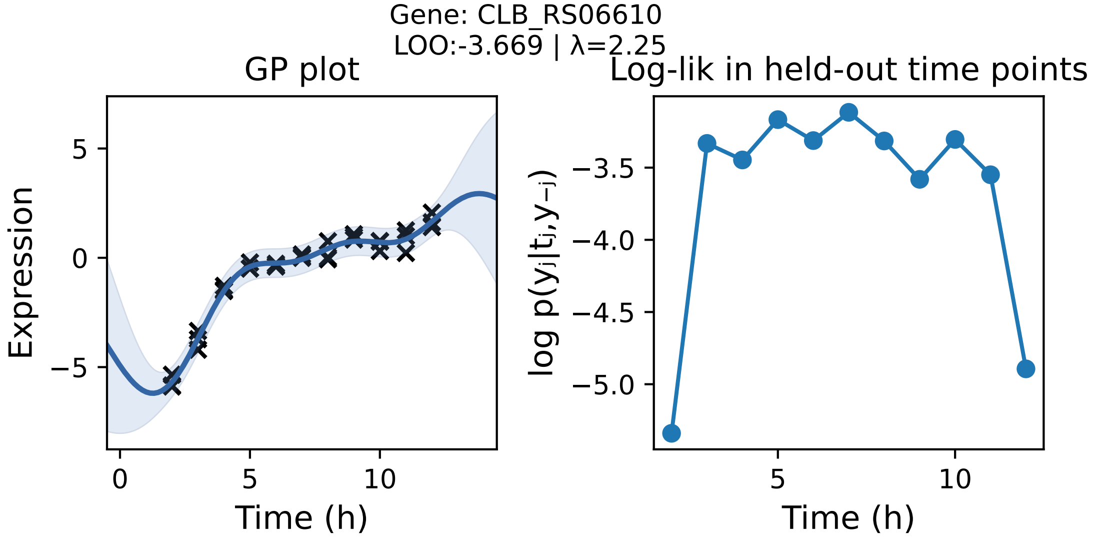

Gene: CLB_RS06610
LOO:-3.669 | λ=2.25
GP plot
Expression
Time (h)
5
0
−5
0
5
10
Log-lik in held-out time points
log p(yj|tj,y−j)
−3.5
−4.0
−4.5
−5.0
5
10
Time (h)

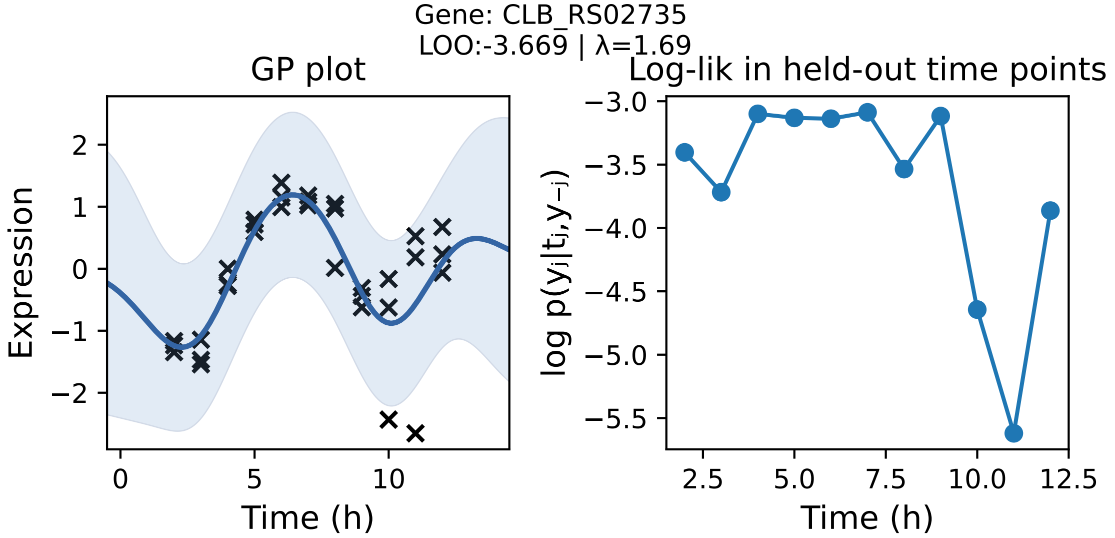
Gene: CLB_RS02735
LOO:-3.669 | λ=1.69
GP plot
Log-lik in held-out time points
Expression
Time (h)
log p(yj|tj,y−j)
Time (h)
2
1
0
−1
−2
0
5
10
−3.0
−3.5
−4.0
−4.5
−5.0
−5.5
2.5
5.0
7.5
10.0
12.5

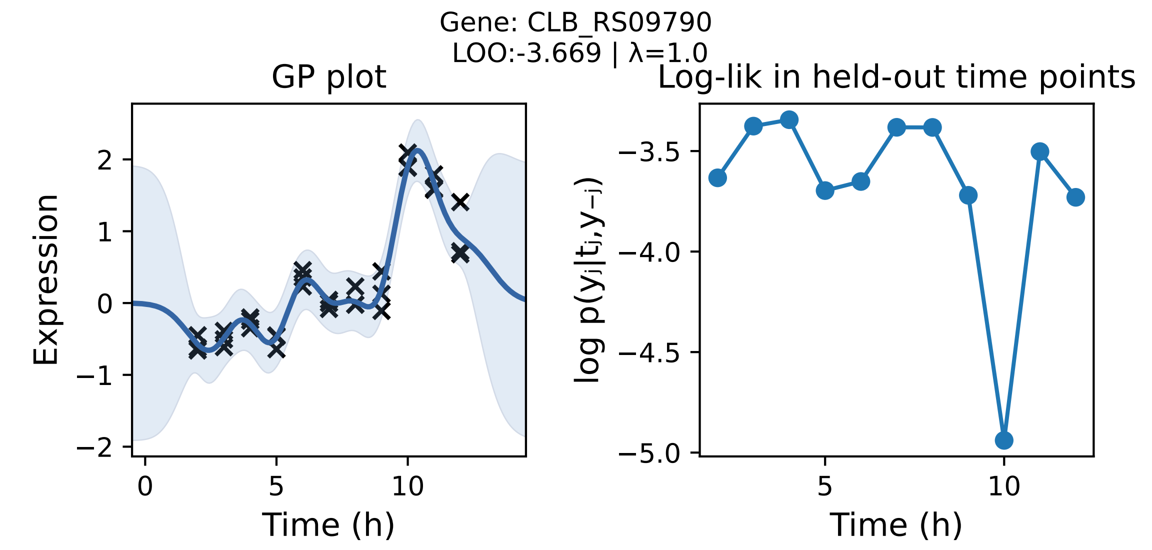
Gene: CLB_RS09790
LOO:-3.669 | λ=1.0
GP plot
Expression
Time (h)
Log-lik in held-out time points
log p(y_j|t_j,y_−j)
Time (h)

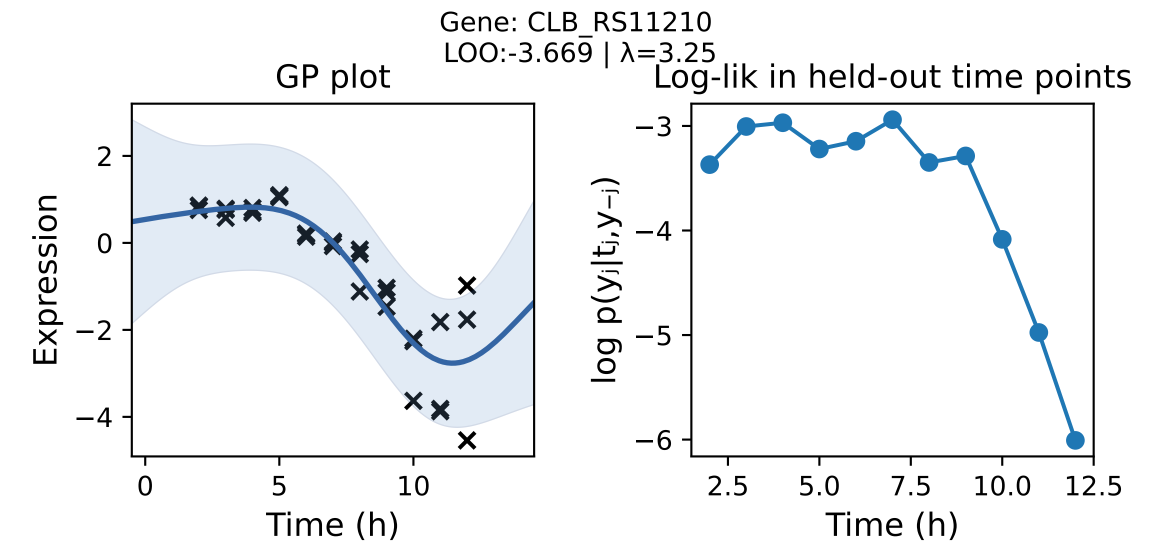
Gene: CLB_RS11210
LOO:-3.669 | λ=3.25
GP plot
Expression
Time (h)
2
0
−2
−4
0
5
10
Log-lik in held-out time points
log $p(y_j|t_j, y_{-j})$
Time (h)
−3
−4
−5
−6
2.5
5.0
7.5
10.0
12.5

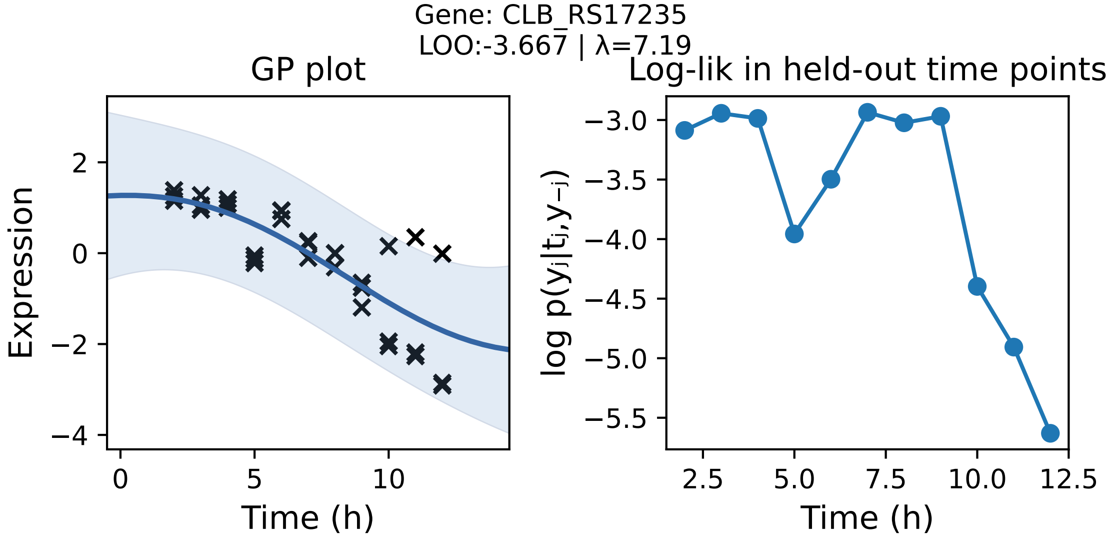

Gene: CLB_RS17235
LOO:-3.667 | λ=7.19
GP plot
Expression
Time (h)
2
0
−2
−4
0
5
10
Log-lik in held-out time points
log p(y_j|t_j,y_−j)
Time (h)
−3.0
−3.5
−4.0
−4.5
−5.0
−5.5
2.5
5.0
7.5
10.0
12.5

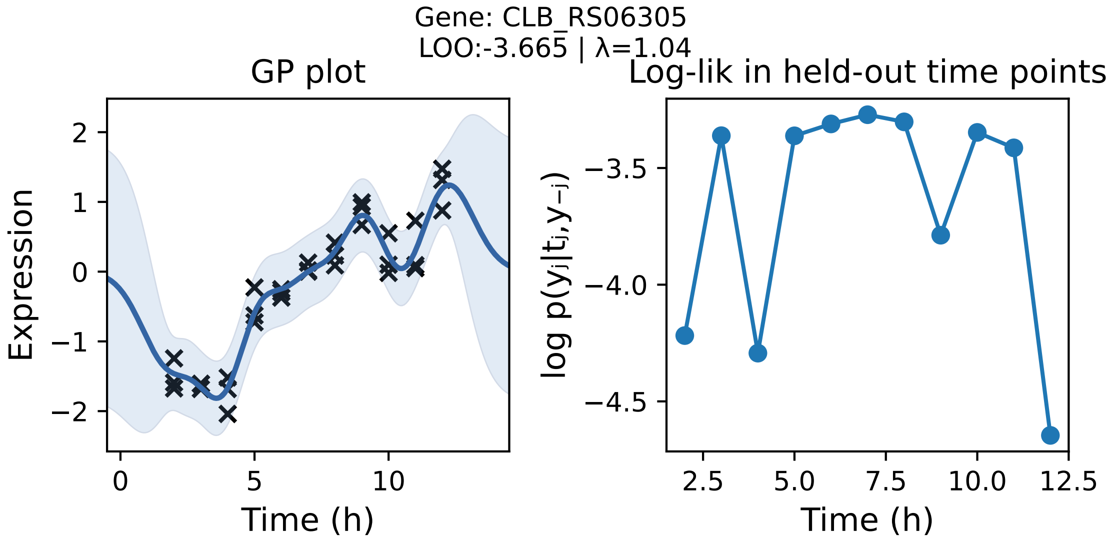

Gene: CLB_RS06305
LOO:-3.665 | λ=1.04
GP plot
Expression
Time (h)
Log-lik in held-out time points
log p(y_j|t_j,y_−j)
Time (h)

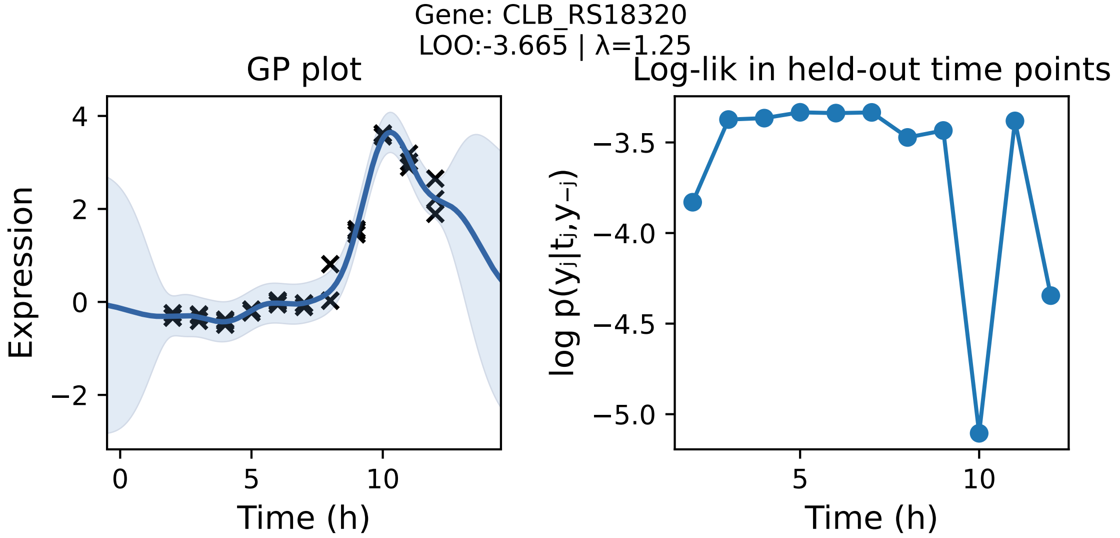

Gene: CLB_RS18320
LOO:-3.665 | λ=1.25
GP plot
Expression
Time (h)
4
2
0
−2
0
5
10
Log-lik in held-out time points
log p(y_j|t_j,y_−j)
Time (h)
−3.5
−4.0
−4.5
−5.0
5
10

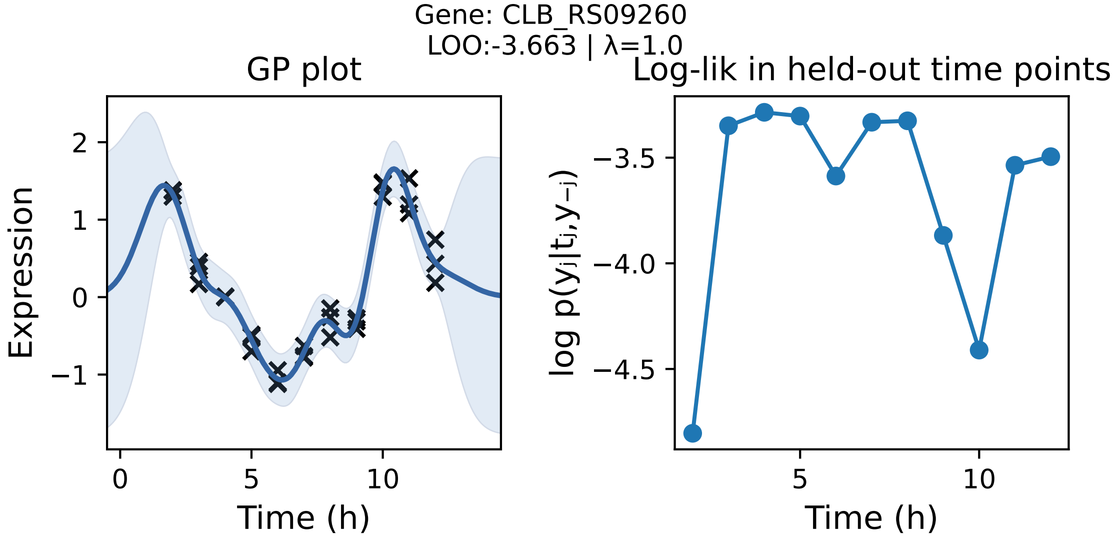

Gene: CLB_RS09260
LOO:-3.663 | λ=1.0
GP plot
Expression
Time (h)
Log-lik in held-out time points
log p(y_j|t_j,y_−j)
Time (h)

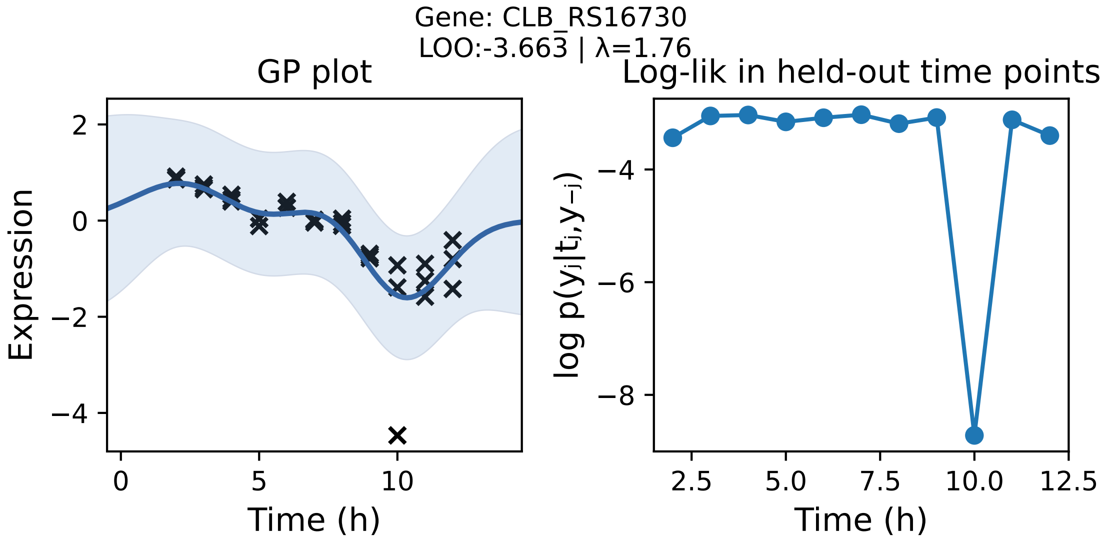

Gene: CLB_RS16730
LOO:-3.663 | λ=1.76
GP plot
Log-lik in held-out time points
Expression
Time (h)
log p(y_j|t_j,y_−j)
Time (h)
2
0
−2
−4
0
5
10
−4
−6
−8
2.5
5.0
7.5
10.0
12.5

Gene: CLB_RS12765
LOO:-3.661 | λ=1.0

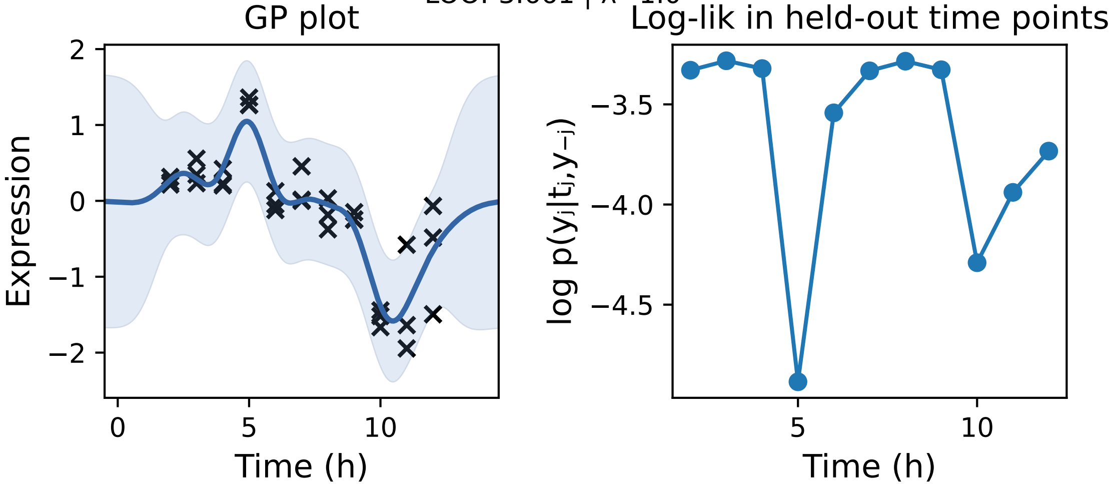

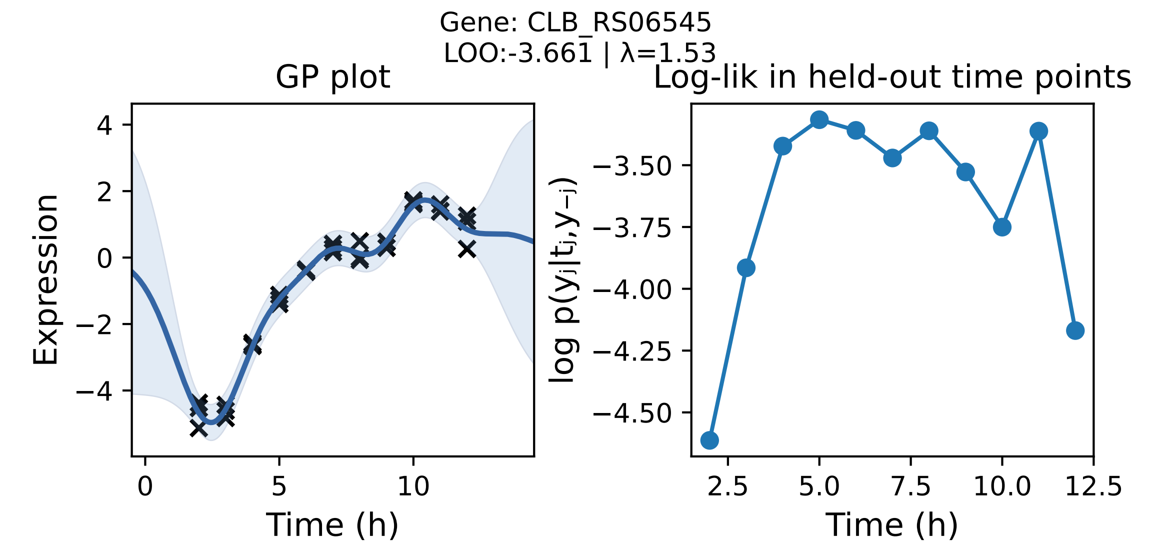

Gene: CLB_RS06545
LOO:-3.661 | λ=1.53
GP plot
Expression
Time (h)
4
2
0
−2
−4
0
5
10
Log-lik in held-out time points
log p(yj|tj,y−j)
Time (h)
−3.50
−3.75
−4.00
−4.25
−4.50
2.5
5.0
7.5
10.0
12.5

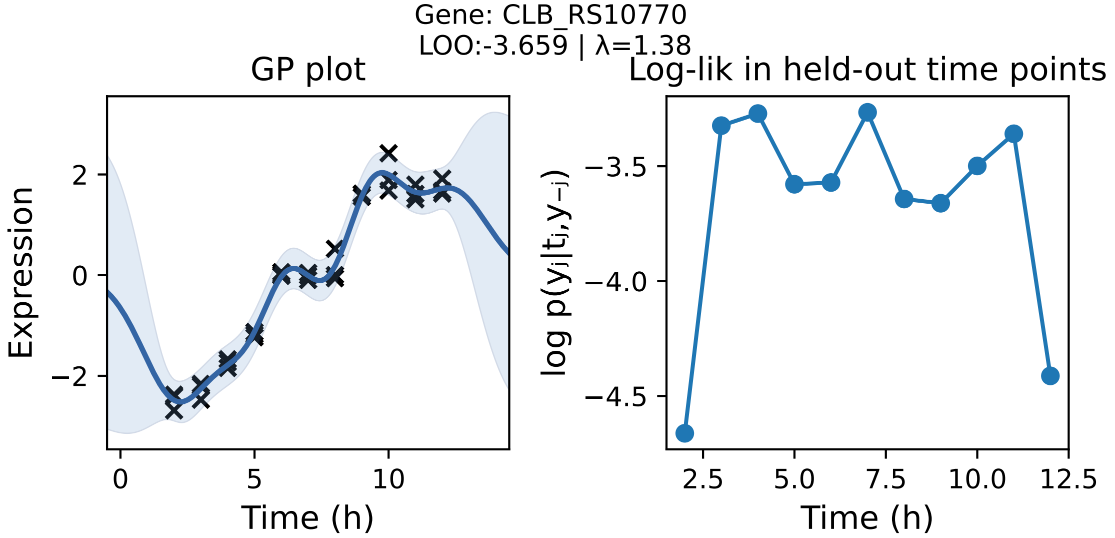

Gene: CLB_RS10770
LOO:-3.659 | λ=1.38
GP plot
Log-lik in held-out time points
Expression
Time (h)
log p(y_j|t_j,y_−j)
Time (h)
2
0
−2
0
5
10
−3.5
−4.0
−4.5
2.5
5.0
7.5
10.0
12.5

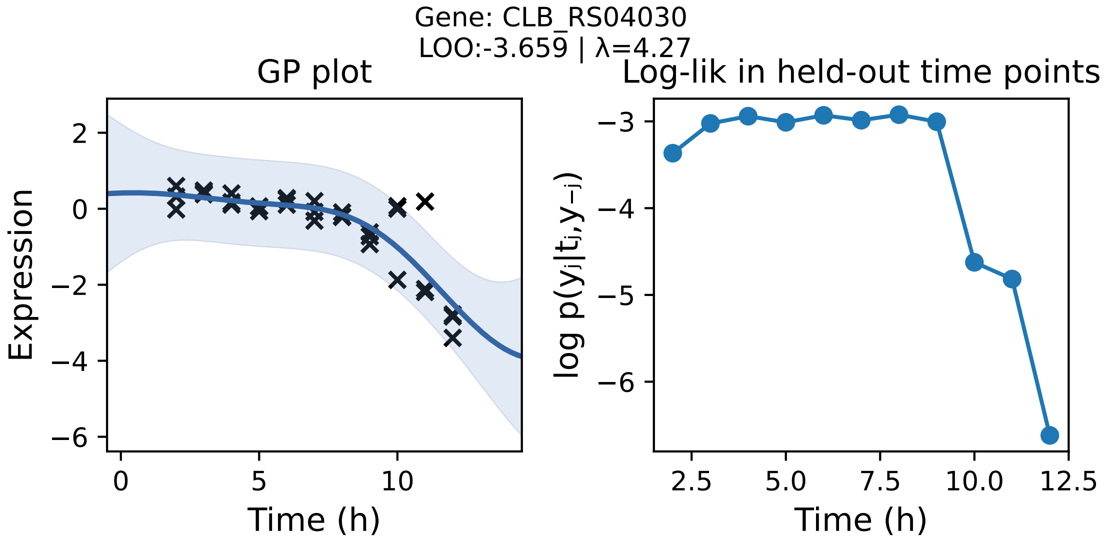

Gene: CLB_RS04030
LOO:-3.659 | λ=4.27
GP plot
Log-lik in held-out time points
Expression
Time (h)
log p(y_j|t_j,y_−j)
Time (h)
2
0
−2
−4
−6
0
5
10
−3
−4
−5
−6
2.5
5.0
7.5
10.0
12.5

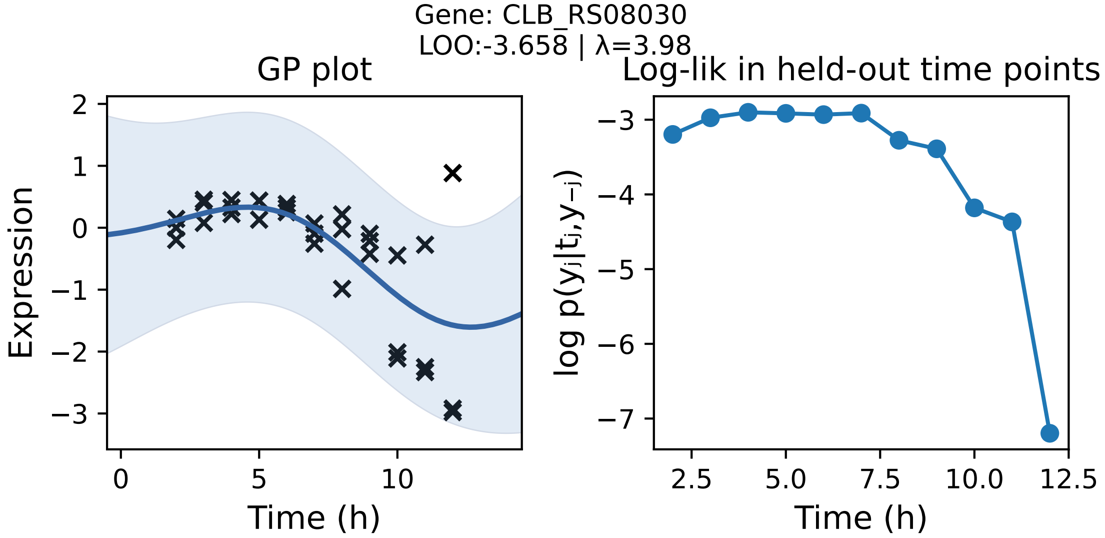

Gene: CLB_RS08030
LOO:-3.658 | λ=3.98
GP plot
Expression
Time (h)
2
1
0
−1
−2
−3
0
5
10
Log-lik in held-out time points
log p(yj|tj,y−j)
Time (h)
−3
−4
−5
−6
−7
2.5
5.0
7.5
10.0
12.5

Gene: CLB_RS03715
LOO:-3.657 | λ=11.53

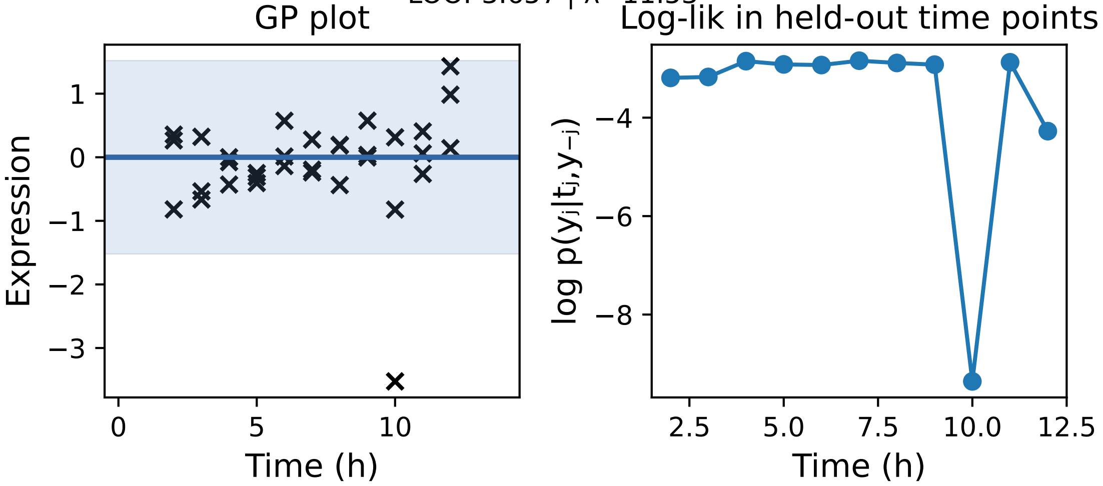

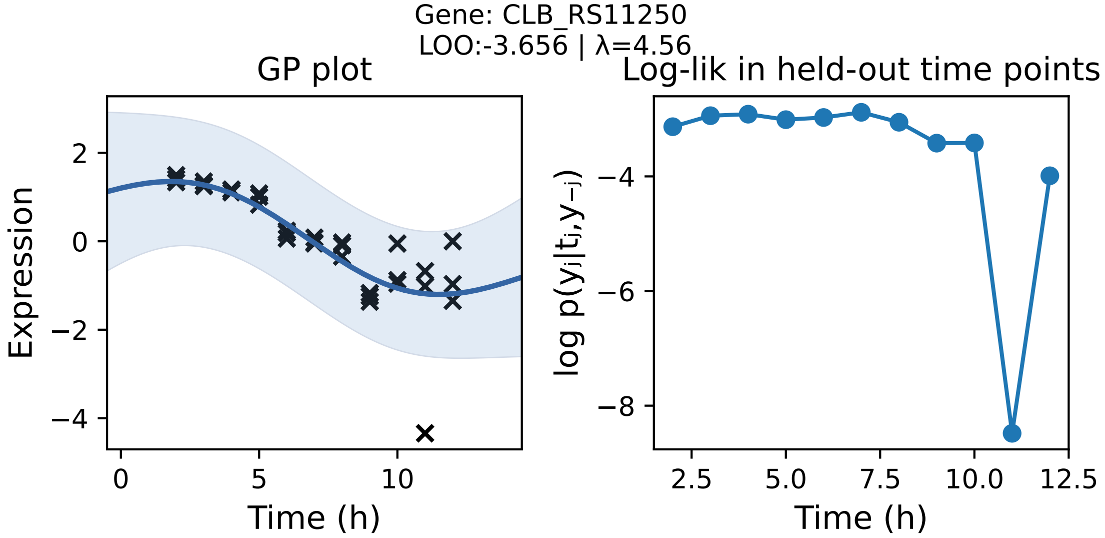

Gene: CLB_RS11250
LOO:-3.656 | λ=4.56
GP plot
Log-lik in held-out time points
Expression
Time (h)
log p(y_j|t_j,y_−j)
2
0
−2
−4
0
5
10
−4
−6
−8
2.5
5.0
7.5
10.0
12.5

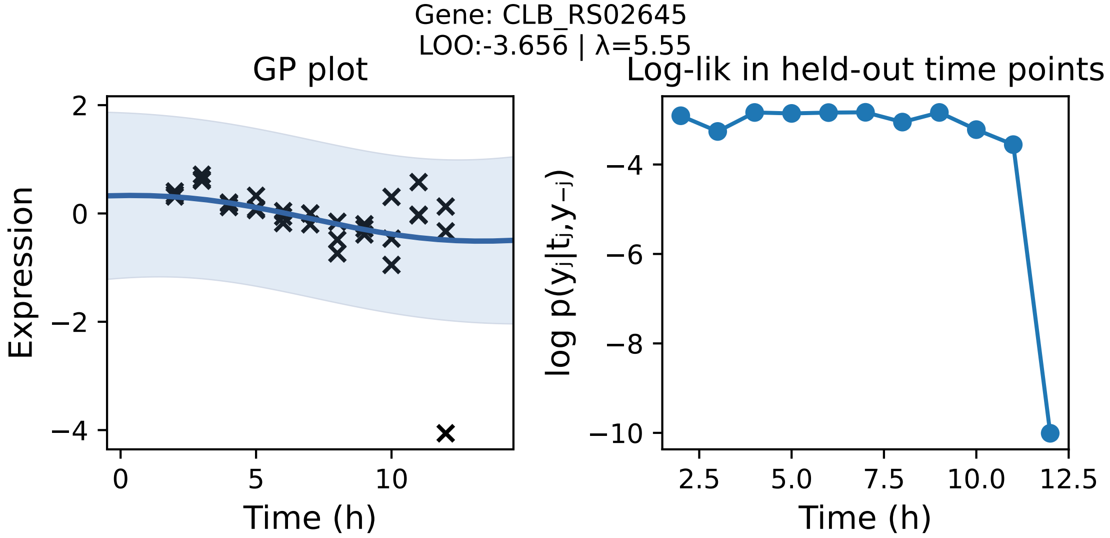
Gene: CLB_RS02645
LOO:-3.656 | λ=5.55
GP plot
Expression
Time (h)
Log-lik in held-out time points
log p(y_j|t_j,y_−j)
Time (h)

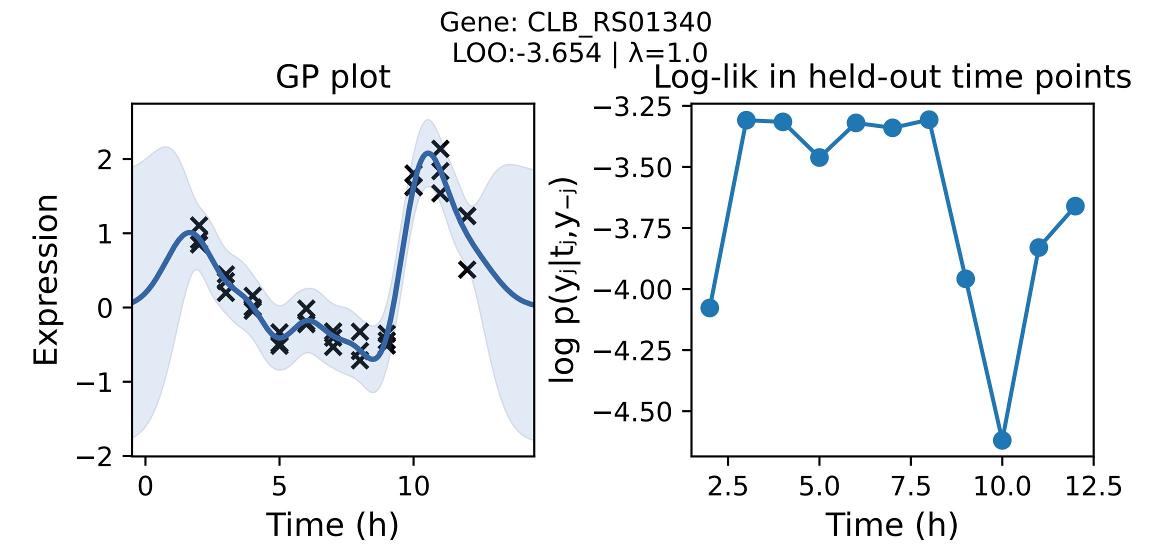

Gene: CLB_RS01340
LOO:-3.654 | λ=1.0
GP plot
Expression
Time (h)
Log-lik in held-out time points
log p(y_j|t_j,y_−j)
Time (h)

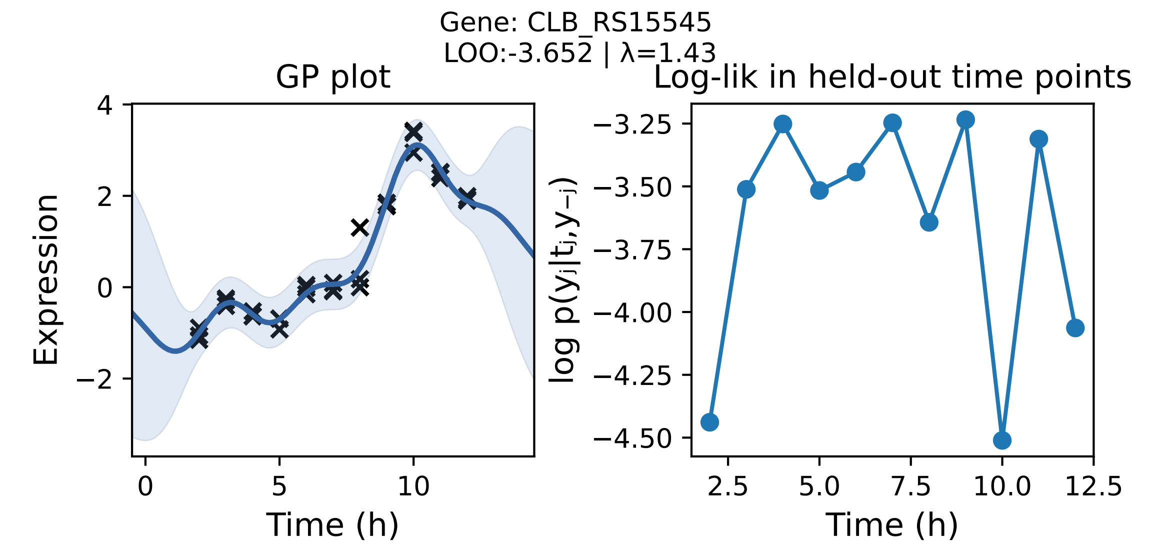

Gene: CLB_RS15545
LOO:-3.652 | λ=1.43
GP plot
Expression
Time (h)
Log-lik in held-out time points
log p(yj|tj,y−j)
Time (h)

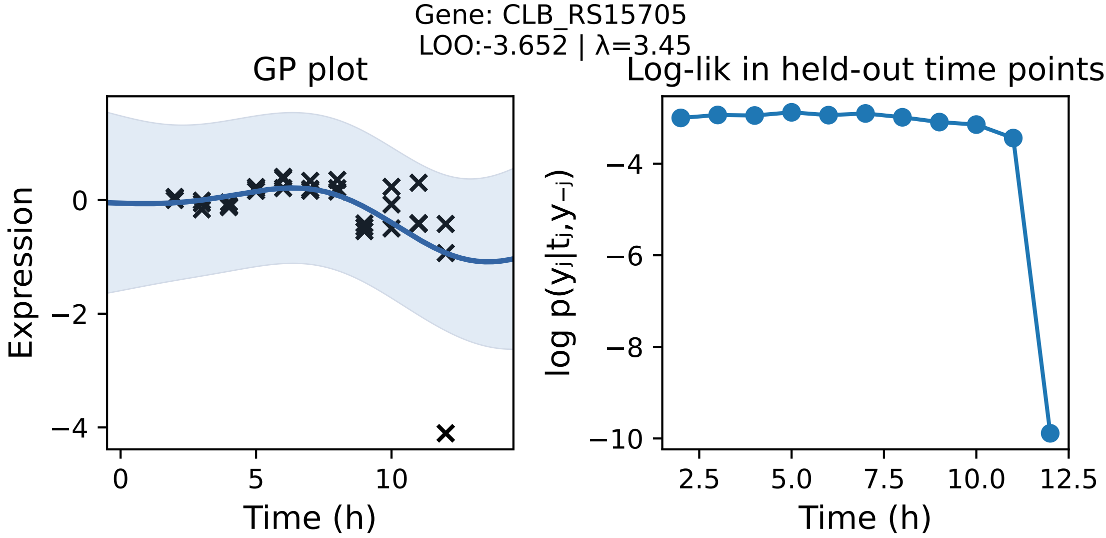
Gene: CLB_RS15705
LOO:-3.652 | λ=3.45
GP plot
Expression
Time (h)
0
−2
−4
0
5
10
Log-lik in held-out time points
log p(y_j|t_j,y_−j)
Time (h)
−4
−6
−8
−10
2.5
5.0
7.5
10.0
12.5

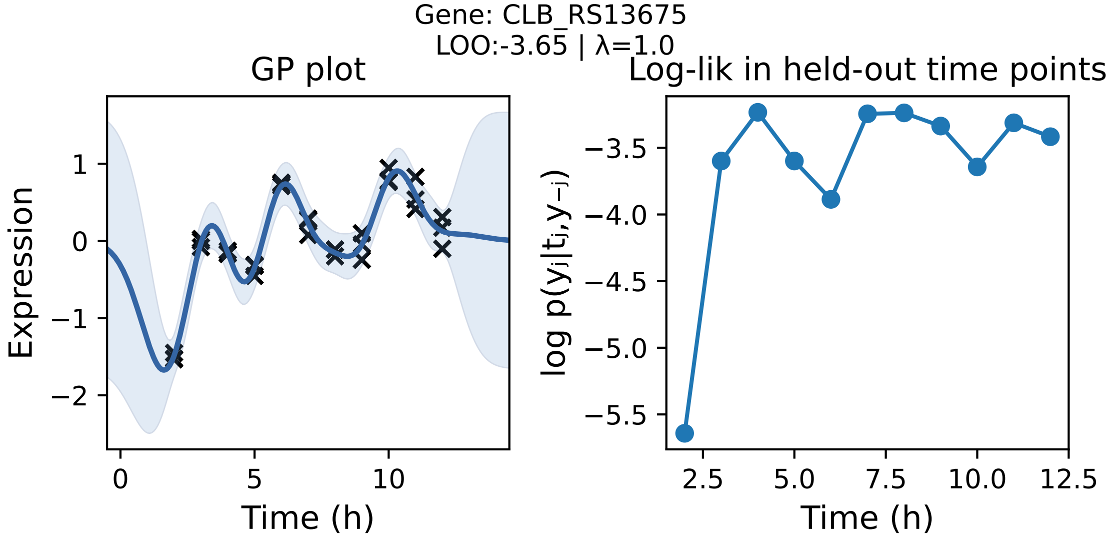

Gene: CLB_RS13675
LOO:-3.65 | λ=1.0
GP plot
Expression
Time (h)
Log-lik in held-out time points
log p(yj|tj,y−j)
Time (h)

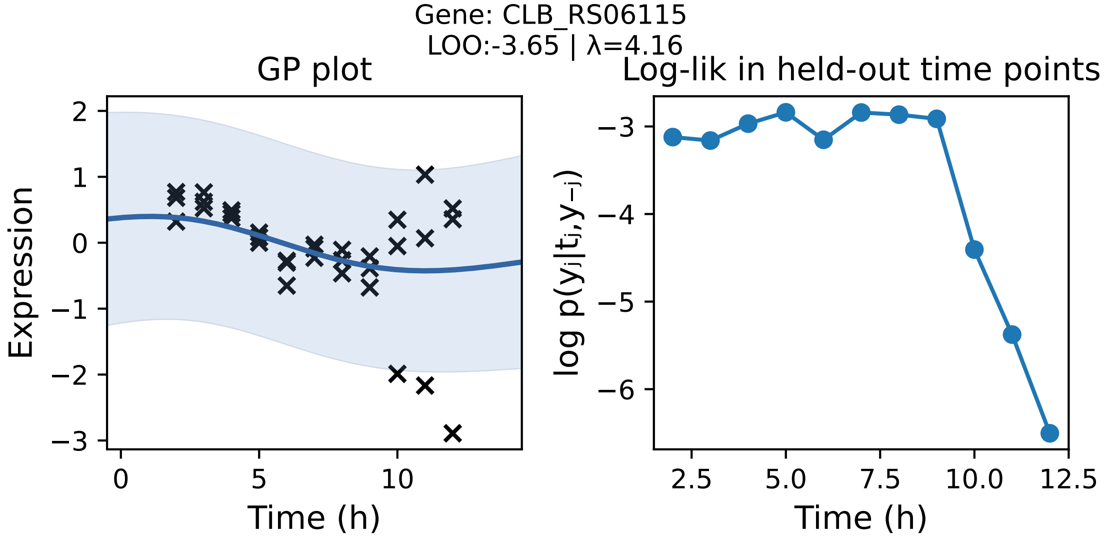

Gene: CLB_RS06115
LOO:-3.65 | λ=4.16
GP plot
Log-lik in held-out time points
Expression
Time (h)
log p(y_j|t_j,y_{-j})
Time (h)
2
1
0
−1
−2
−3
0
5
10
−3
−4
−5
−6
2.5
5.0
7.5
10.0
12.5

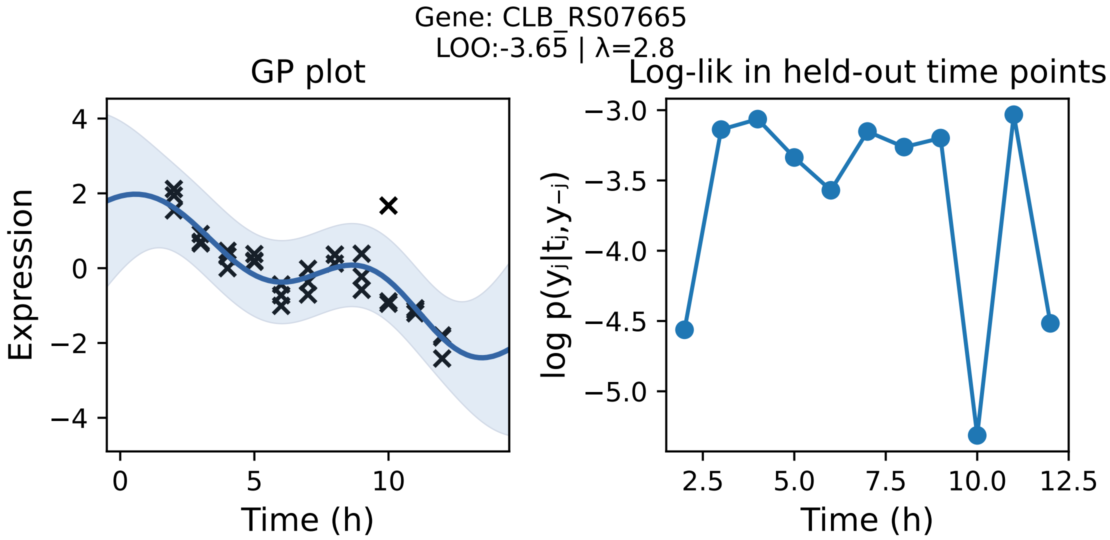
Gene: CLB_RS07665
LOO:-3.65 | λ=2.8
GP plot
Expression
Time (h)
Log-lik in held-out time points
log p(y_j|t_j,y_−j)
Time (h)

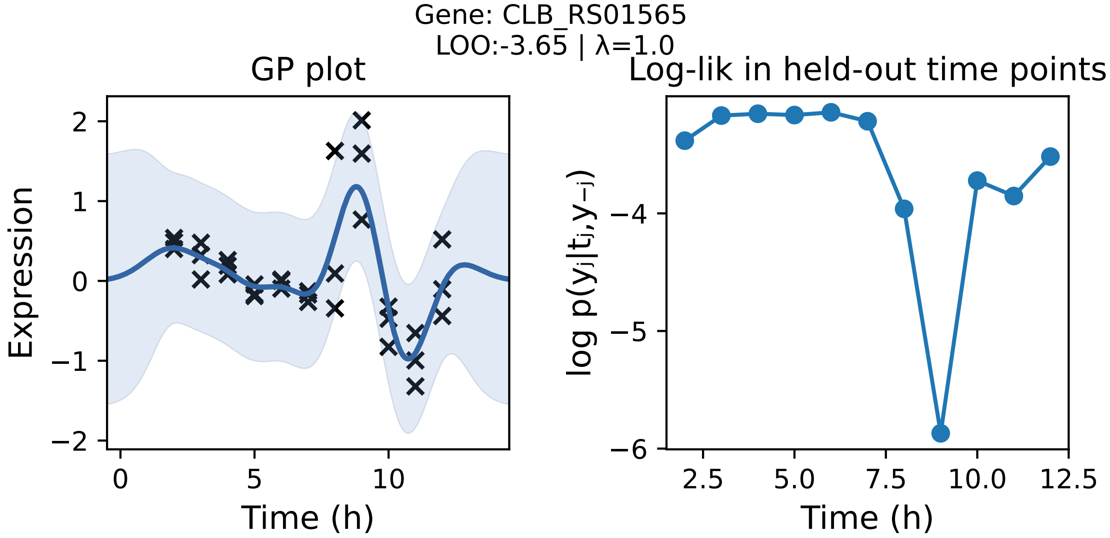

Gene: CLB_RS01565
LOO:-3.65 | λ=1.0
GP plot
Expression
Time (h)
2
1
0
−1
−2
0
5
10
Log-lik in held-out time points
log p(yj|tj,y−j)
Time (h)
−4
−5
−6
2.5
5.0
7.5
10.0
12.5

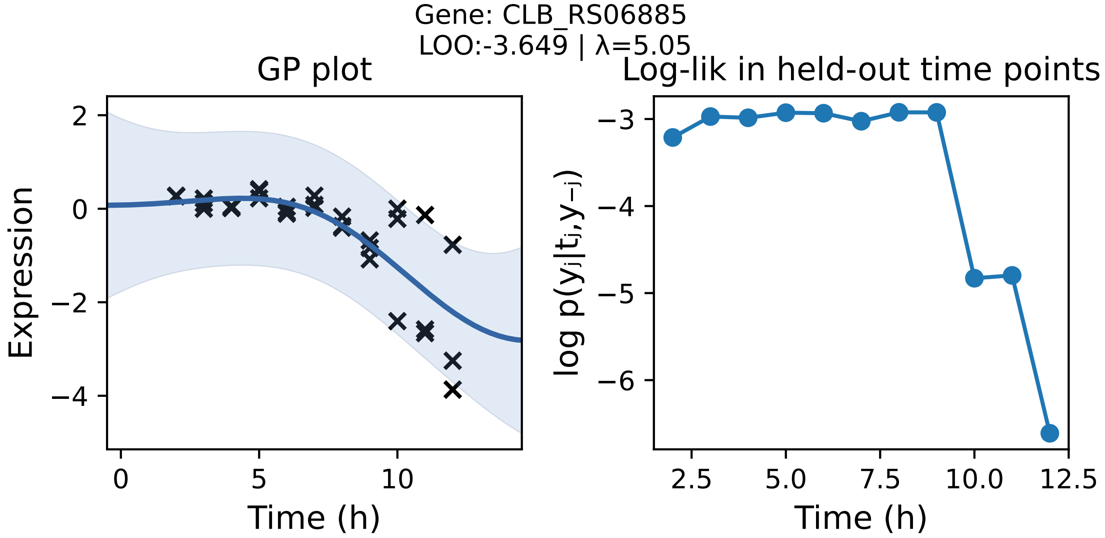

Gene: CLB_RS06885
LOO:-3.649 | λ=5.05
GP plot
Expression
Time (h)
Log-lik in held-out time points
log p(y_j|t_j,y_−j)
Time (h)

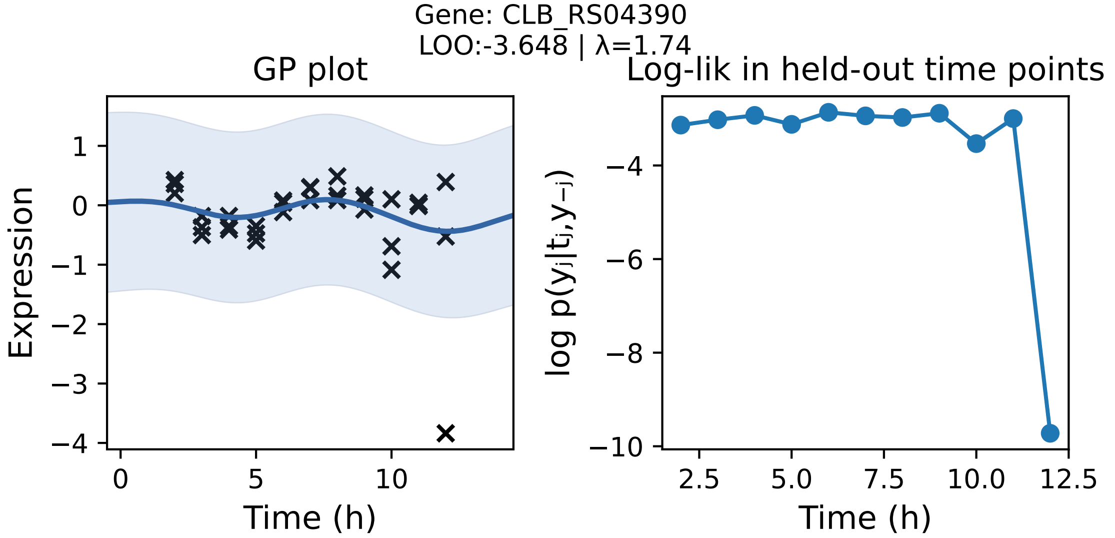

Gene: CLB_RS04390
LOO:-3.648 | λ=1.74
GP plot
Expression
Time (h)
1
0
−1
−2
−3
−4
0
5
10
Log-lik in held-out time points
log p(y_j|t_j,y_−j)
−4
−6
−8
−10
2.5
5.0
7.5
10.0
12.5
Time (h)

Gene: CLB_RS01120
LOO:-3.648 | λ=7.43

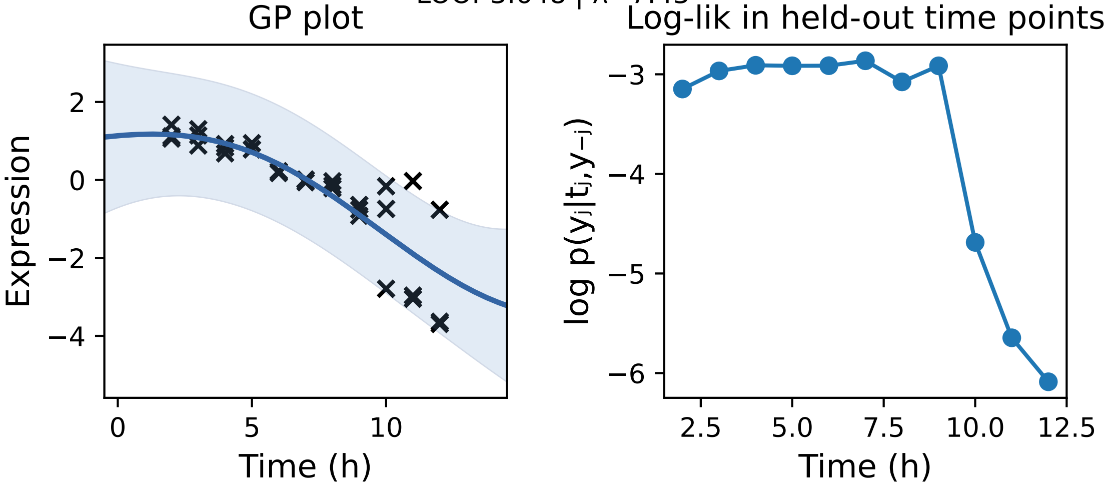

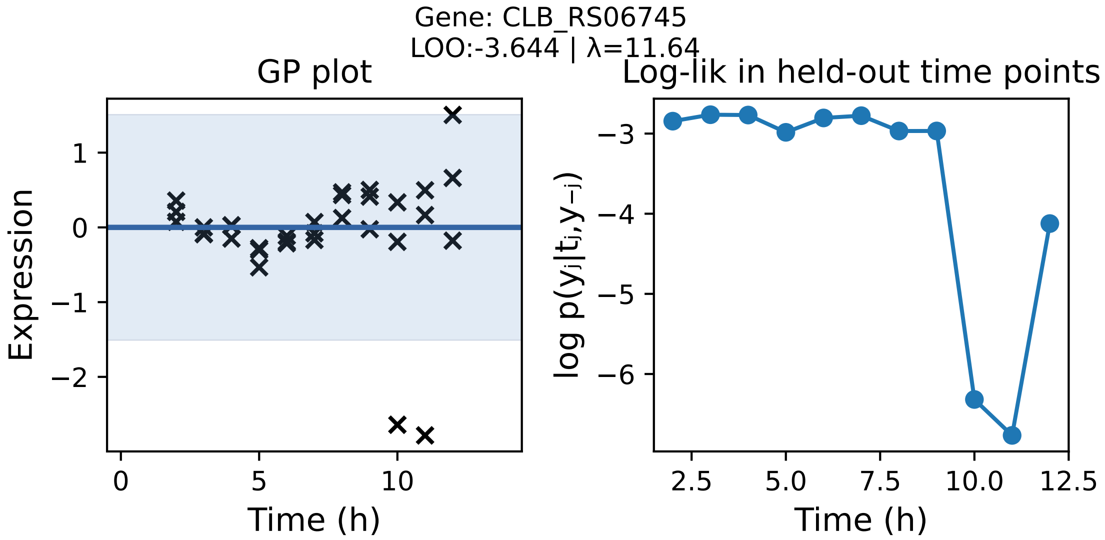

Gene: CLB_RS06745
LOO:-3.644 | λ=11.64
GP plot
Expression
Time (h)
Log-lik in held-out time points
log p(y_j|t_j,y_−j)
Time (h)

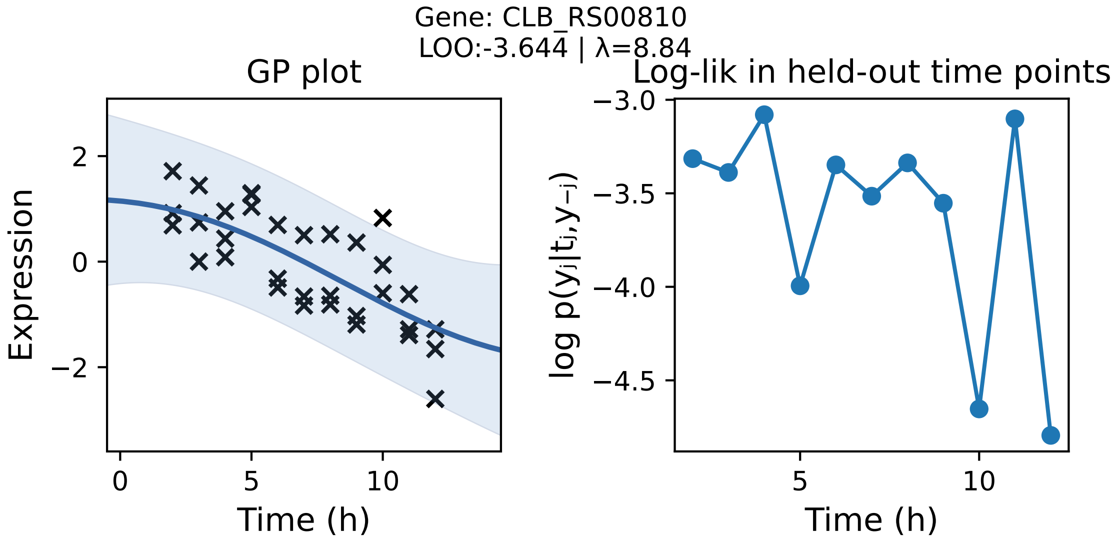

Gene: CLB_RS00810
LOO:-3.644 | λ=8.84
GP plot
Expression
Time (h)
Log-lik in held-out time points
log p(y_j|t_j,y_−j)
Time (h)

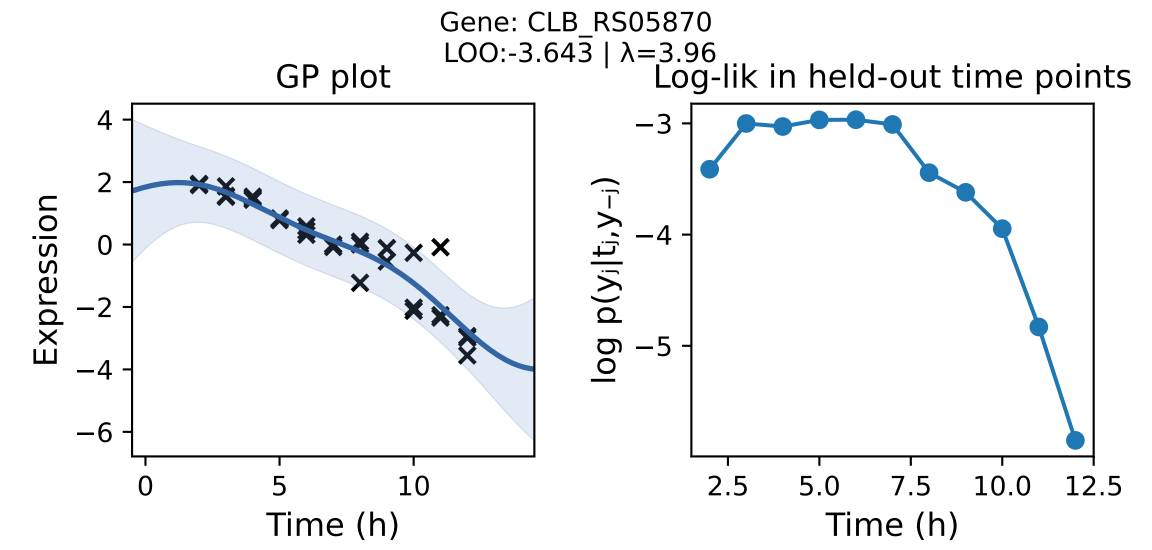

Gene: CLB_RS05870
LOO:-3.643 | λ=3.96
GP plot
Log-lik in held-out time points
Expression
Time (h)
log p(y_j|t_j,y_−j)
4
2
0
−2
−4
−6
0
5
10
−3
−4
−5
2.5
5.0
7.5
10.0
12.5

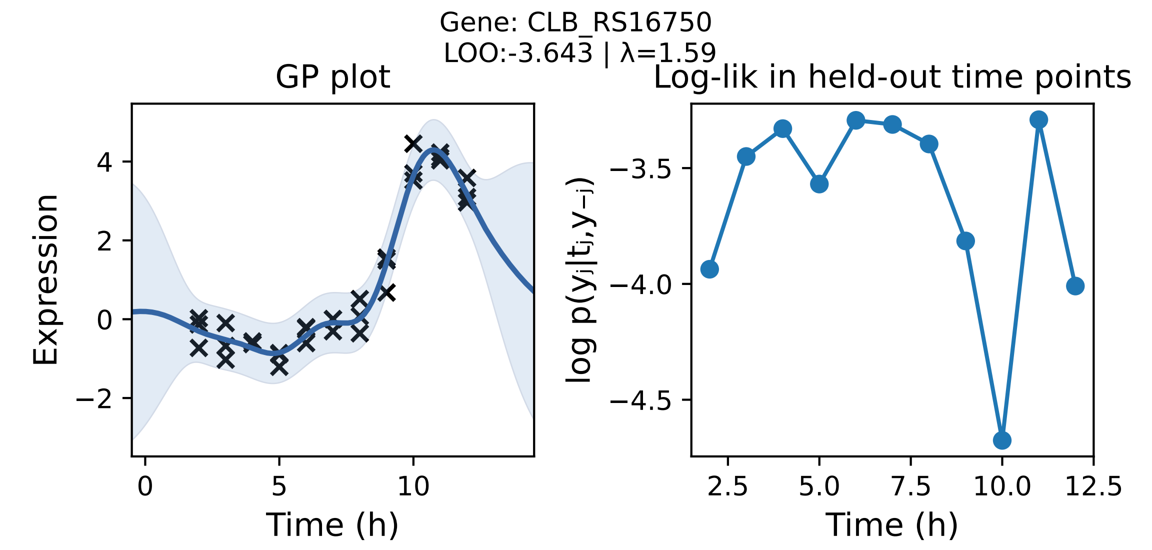
Gene: CLB_RS16750
LOO:-3.643 | λ=1.59
GP plot
Expression
Time (h)
Log-lik in held-out time points
log p(y_j|t_j,y_−j)
Time (h)

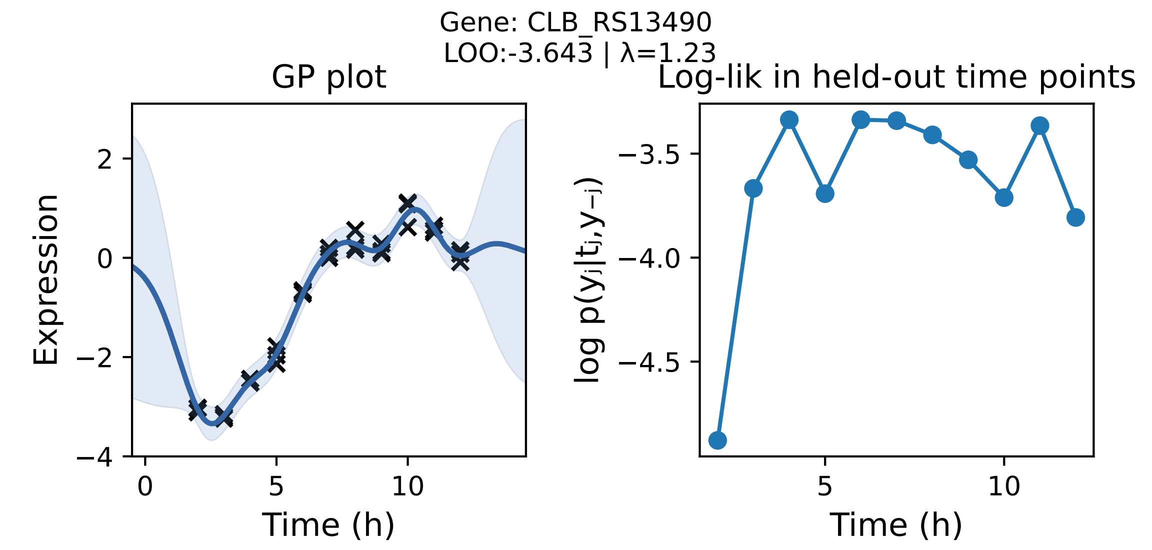

Gene: CLB_RS13490
LOO:-3.643 | λ=1.23
GP plot
Log-lik in held-out time points
Expression
2
0
−2
−4
0
5
10
Time (h)
log p(y_j|t_j,y_−j)
−3.5
−4.0
−4.5
5
10
Time (h)

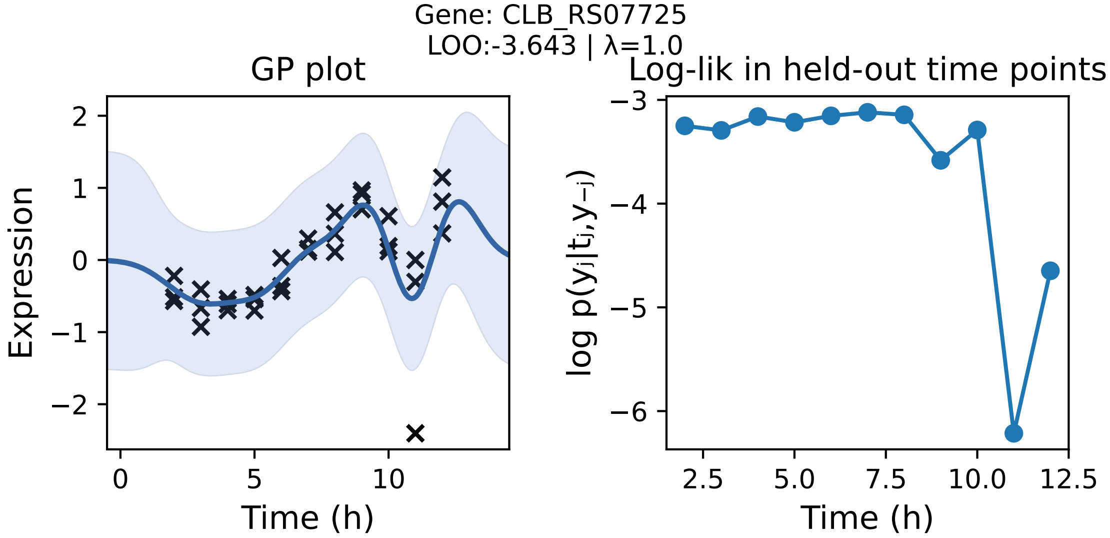

Gene: CLB_RS07725
LOO:-3.643 | λ=1.0
GP plot
Expression
Time (h)
2
1
0
−1
−2
0
5
10
Log-lik in held-out time points
log p(yⱼ|tⱼ,y₋ⱼ)
Time (h)
−3
−4
−5
−6
2.5
5.0
7.5
10.0
12.5

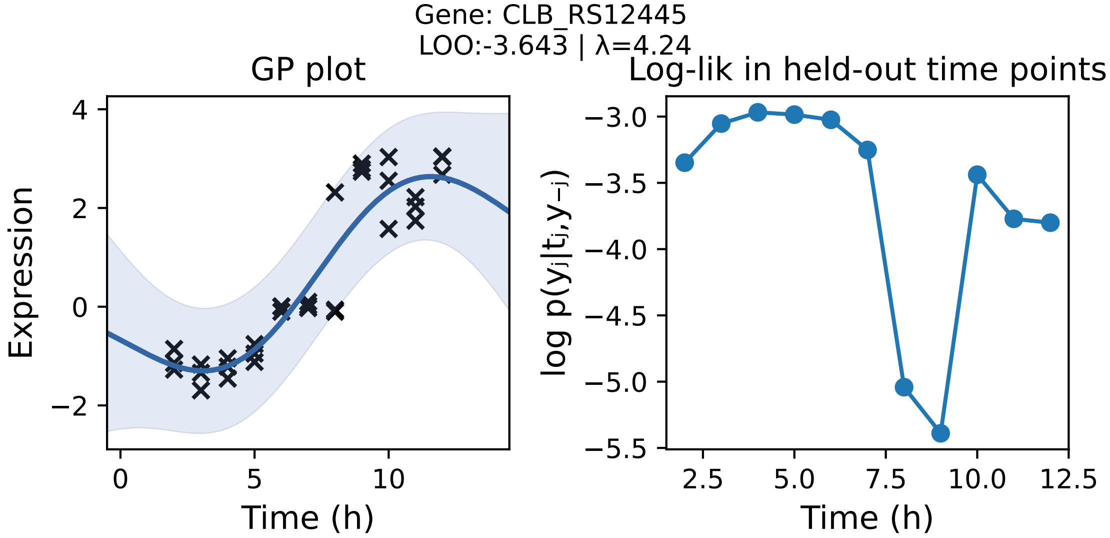
Gene: CLB_RS12445
LOO:-3.643 | λ=4.24
GP plot
Expression
Time (h)
4
2
0
−2
0
5
10
Log-lik in held-out time points
log p(y_j|t_j,y_−j)
Time (h)
−3.0
−3.5
−4.0
−4.5
−5.0
−5.5
2.5
5.0
7.5
10.0
12.5

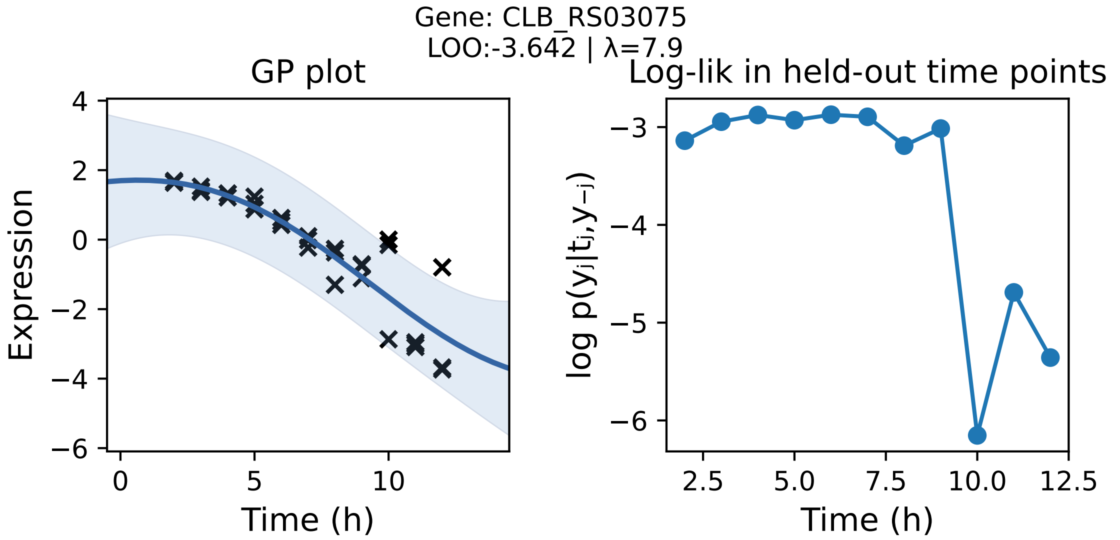

Gene: CLB_RS03075
LOO:-3.642 | λ=7.9
GP plot
Expression
Time (h)
4
2
0
−2
−4
−6
0
5
10
Log-lik in held-out time points
log p(y_j|t_j,y_−j)
Time (h)
−3
−4
−5
−6
2.5
5.0
7.5
10.0
12.5

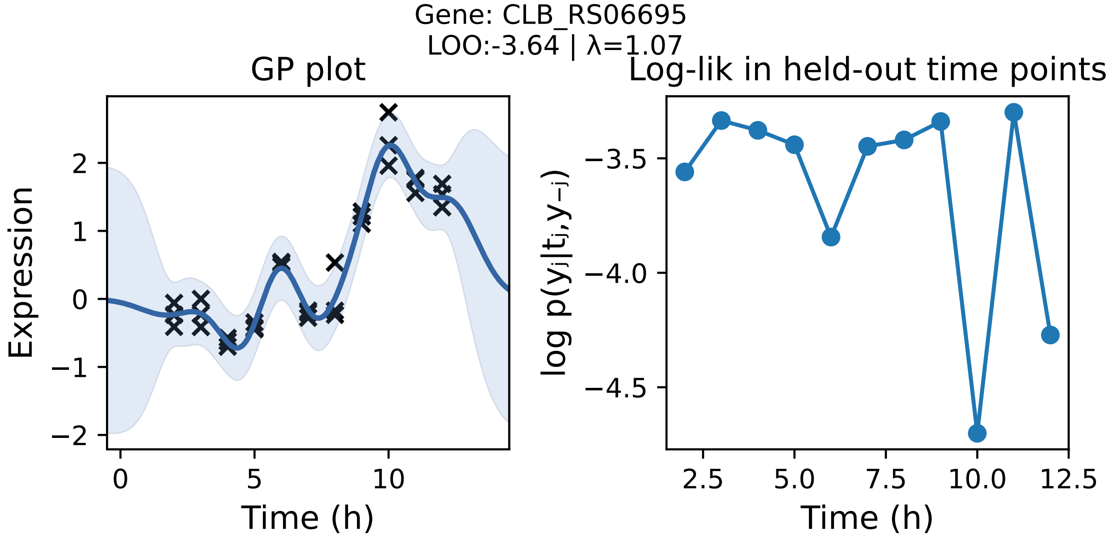

Gene: CLB_RS06695
LOO:-3.64 | λ=1.07
GP plot
Log-lik in held-out time points
Expression
Time (h)
log p(y_j|t_j,y_−j)
Time (h)

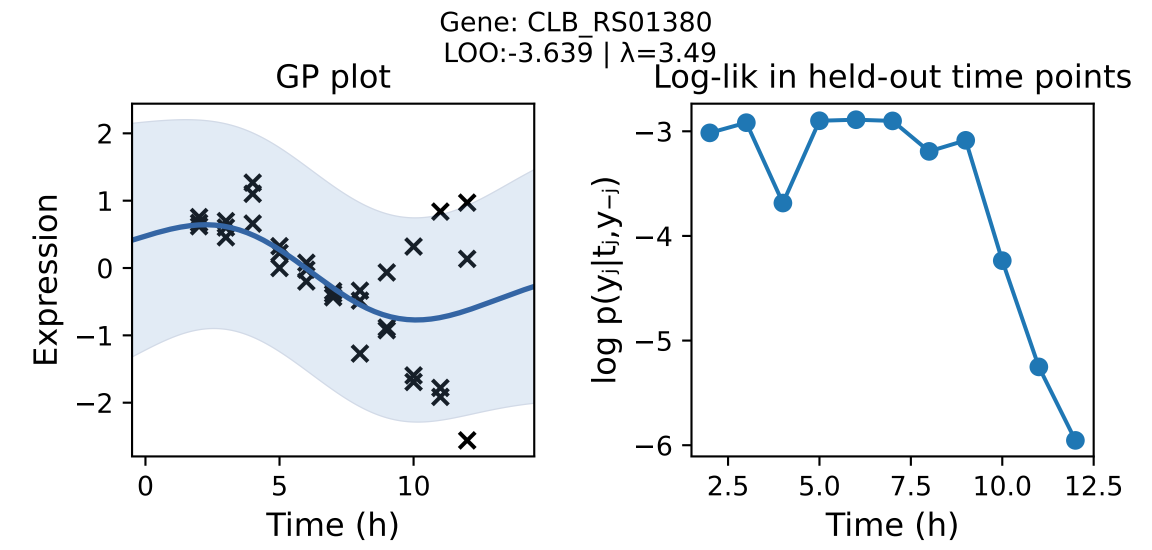

Gene: CLB_RS01380
LOO:-3.639 | λ=3.49
GP plot
Expression
Time (h)
Log-lik in held-out time points
log p(y_j|t_j,y_−j)
Time (h)

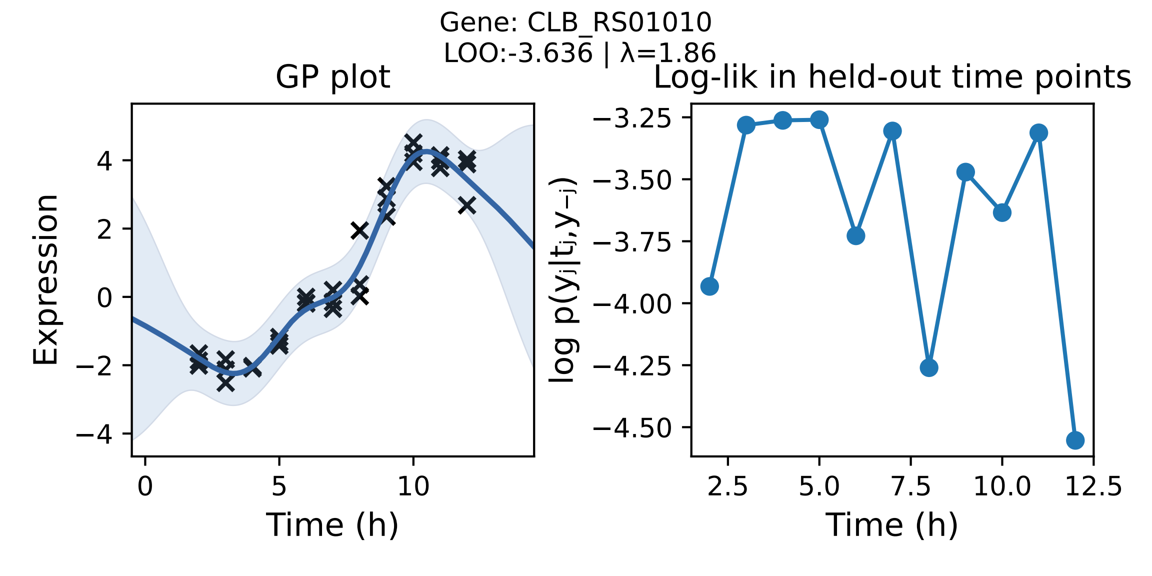
Gene: CLB_RS01010
LOO:-3.636 | λ=1.86
GP plot
Expression
Time (h)
Log-lik in held-out time points
log p(yⱼ|tⱼ,y₋ⱼ)
Time (h)

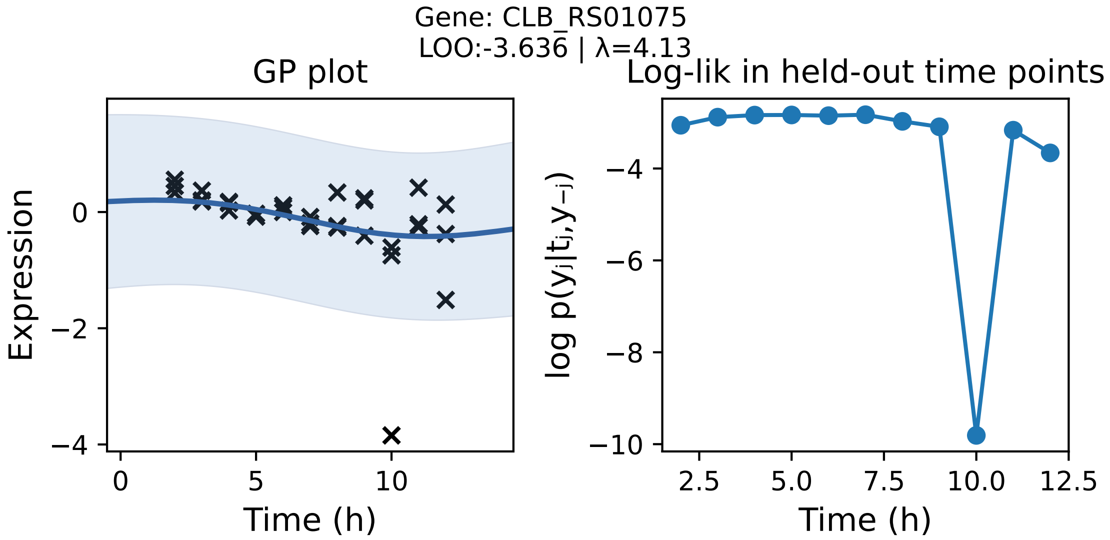

Gene: CLB_RS01075
LOO:-3.636 | λ=4.13
GP plot
Expression
Time (h)
0
−2
−4
0
5
10
Log-lik in held-out time points
log p(yj|tj,y−j)
−4
−6
−8
−10
2.5
5.0
7.5
10.0
12.5

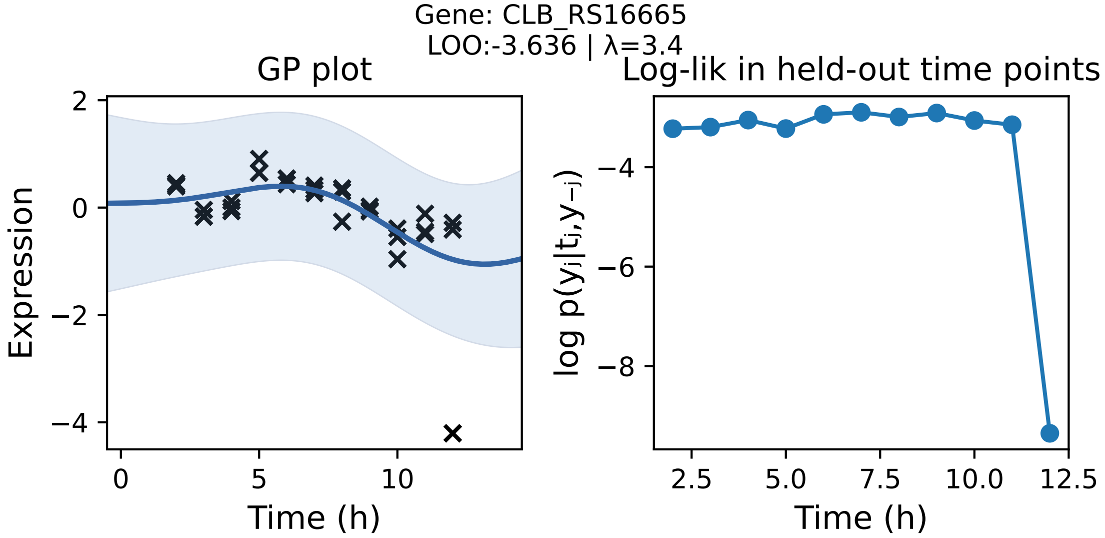

Gene: CLB_RS16665
LOO:-3.636 | λ=3.4
GP plot
Expression
Time (h)
2
0
−2
−4
0
5
10
Log-lik in held-out time points
log p(y_j|t_j,y_−j)
−4
−6
−8
2.5
5.0
7.5
10.0
12.5
Time (h)

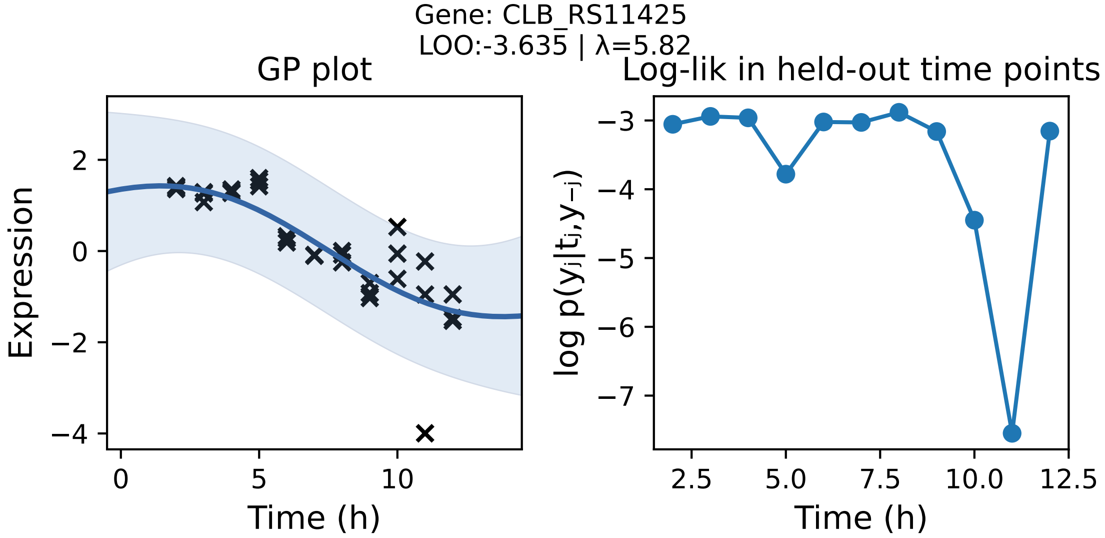

Gene: CLB_RS11425
LOO:-3.635 | λ=5.82
GP plot
Expression
Time (h)
Log-lik in held-out time points
log p(y_j|t_j,y_−j)
Time (h)

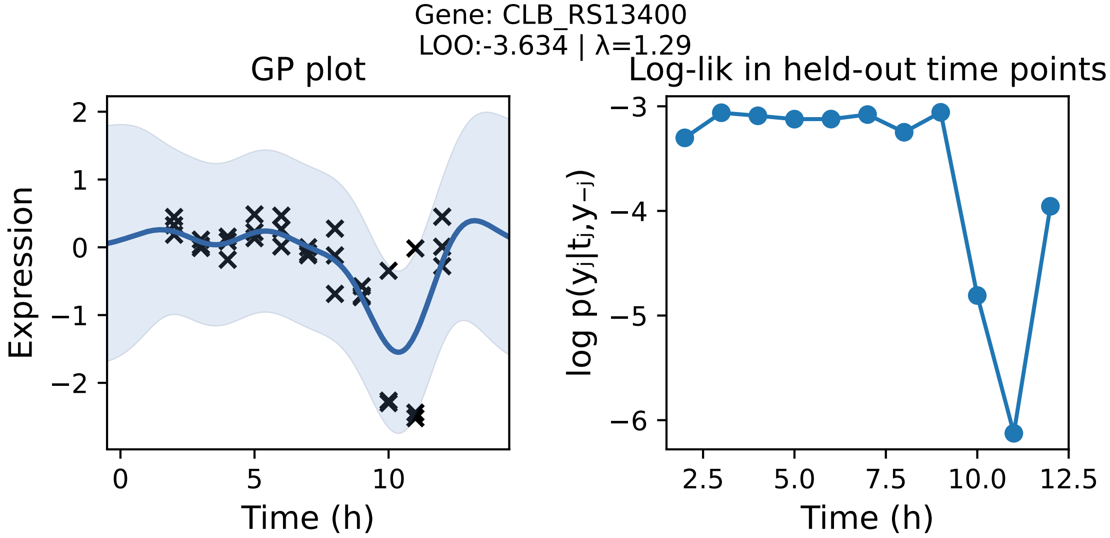

Gene: CLB_RS13400
LOO:-3.634 | λ=1.29
GP plot
Expression
Time (h)
2
1
0
−1
−2
0
5
10
Log-lik in held-out time points
log p(y_j|t_j,y_−j)
Time (h)
−3
−4
−5
−6
2.5
5.0
7.5
10.0
12.5

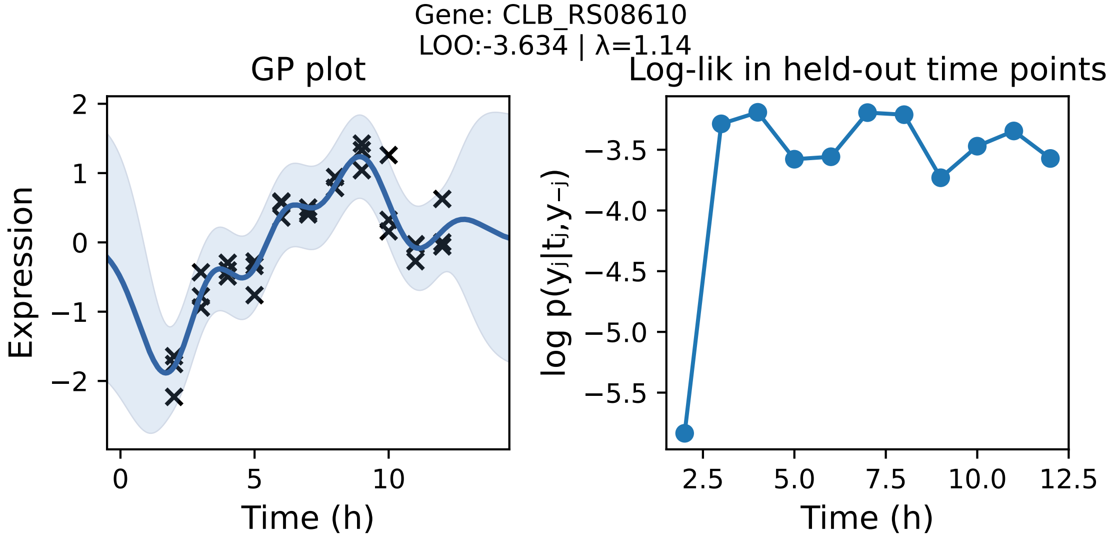

Gene: CLB_RS08610
LOO:-3.634 | λ=1.14
GP plot
Expression
Time (h)
Log-lik in held-out time points
log p(y_j|t_j,y_−j)
Time (h)

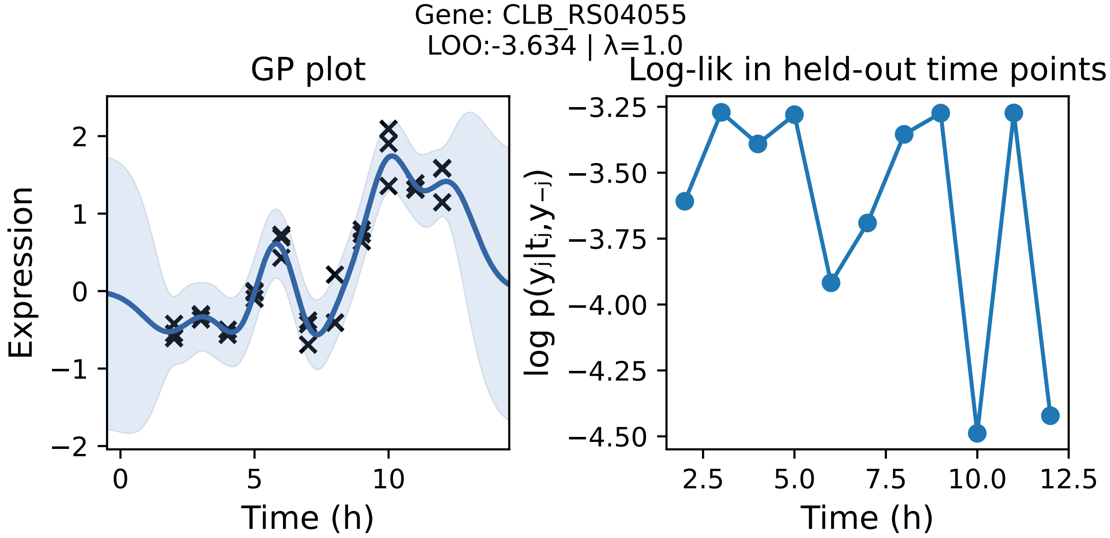
Gene: CLB_RS04055
LOO:-3.634 | λ=1.0
GP plot
Expression
Time (h)
Log-lik in held-out time points
log p(yj|tj,y−j)
Time (h)
−3.25
−3.50
−3.75
−4.00
−4.25
−4.50
2.5
5.0
7.5
10.0
12.5
2
1
0
−1
−2
0
5
10

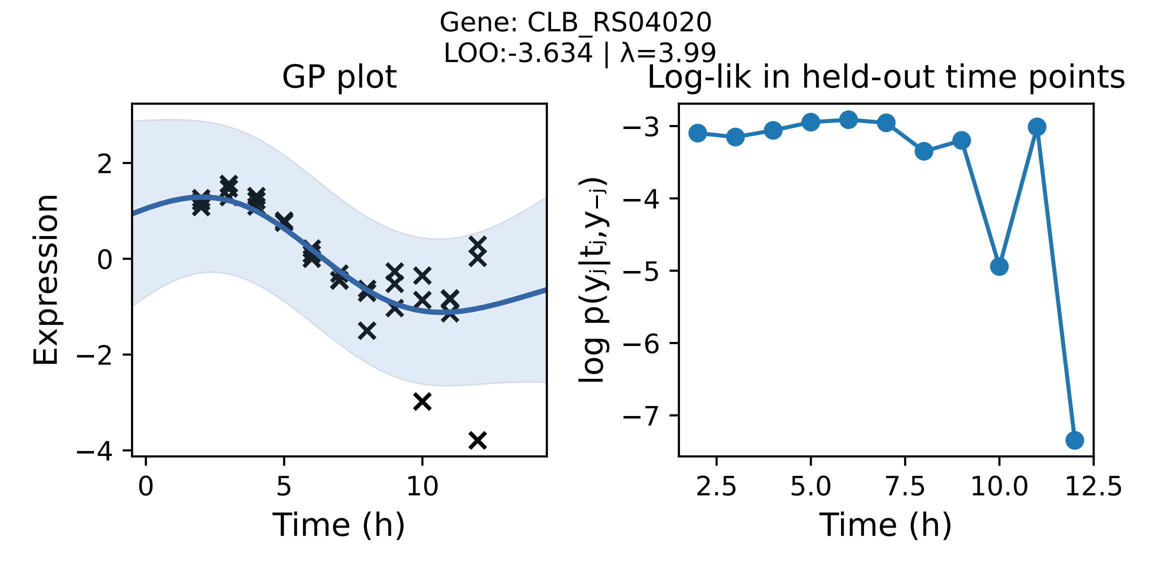

Gene: CLB_RS04020
LOO:-3.634 | λ=3.99
GP plot
Expression
Time (h)
Log-lik in held-out time points
log p(y_j|t_j,y_−j)
Time (h)

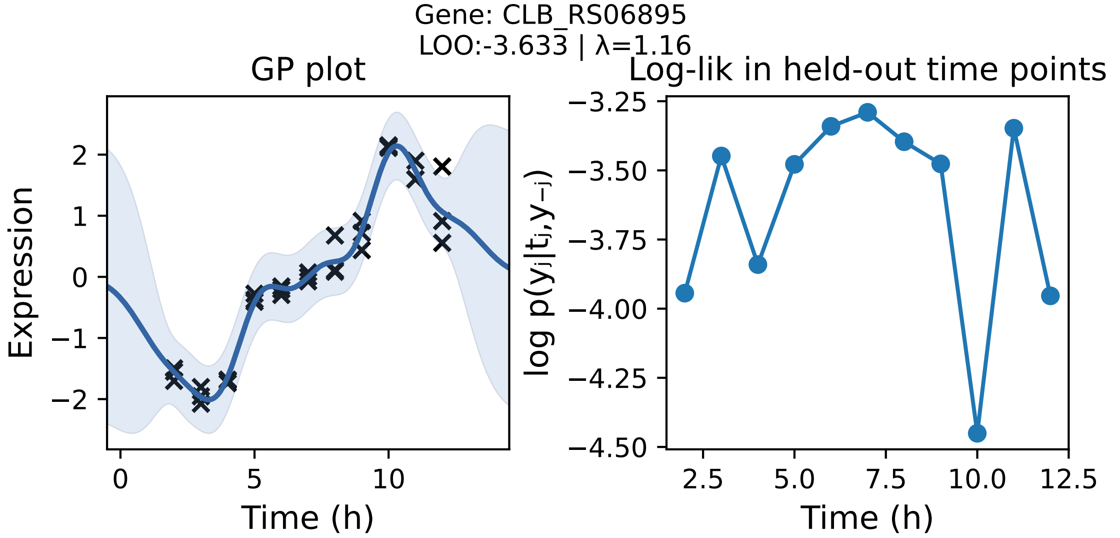

Gene: CLB_RS06895
LOO:-3.633 | λ=1.16
GP plot
Expression
Time (h)
Log-lik in held-out time points
log p(yj|tj,y−j)
Time (h)

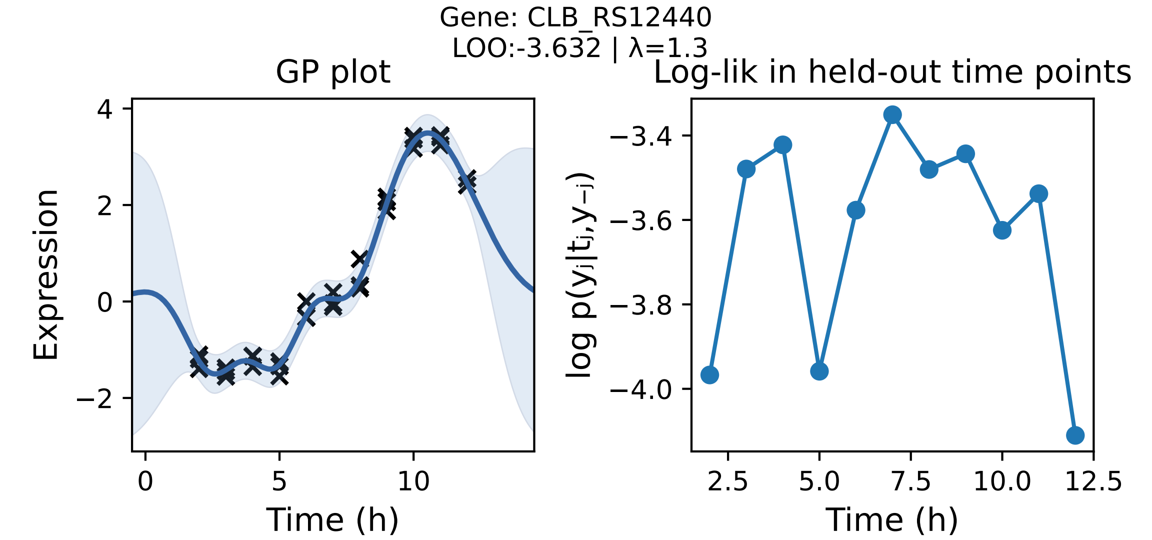
Gene: CLB_RS12440
LOO:-3.632 | λ=1.3
GP plot
Expression
Time (h)
Log-lik in held-out time points
log p(y_j|t_j,y_−j)
Time (h)

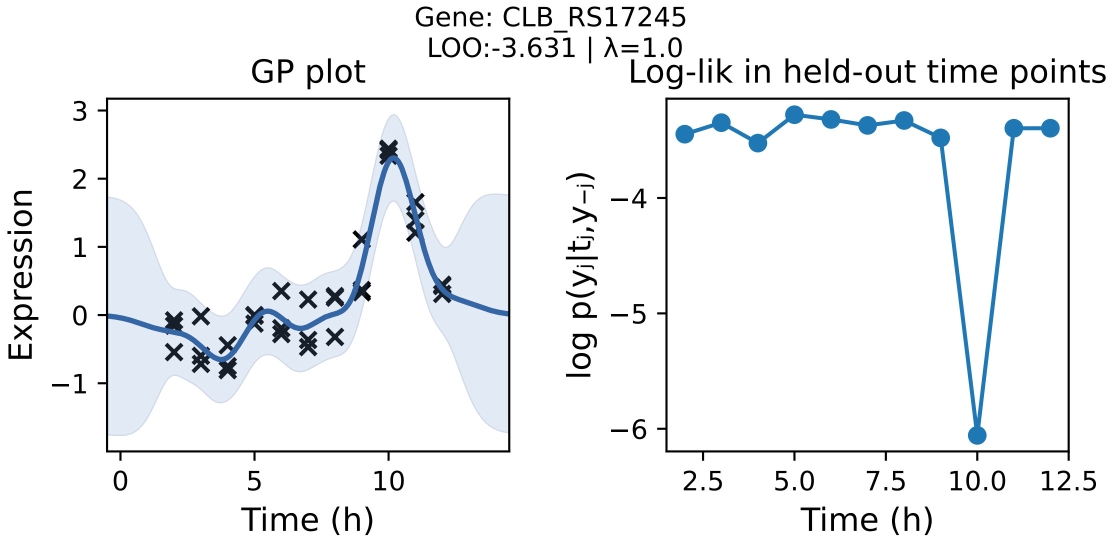

Gene: CLB_RS17245
LOO:-3.631 | λ=1.0
GP plot
Log-lik in held-out time points
Expression
Time (h)
log p(y_j|t_j,y_−j)
3
2
1
0
−1
0
5
10
−4
−5
−6
2.5
5.0
7.5
10.0
12.5

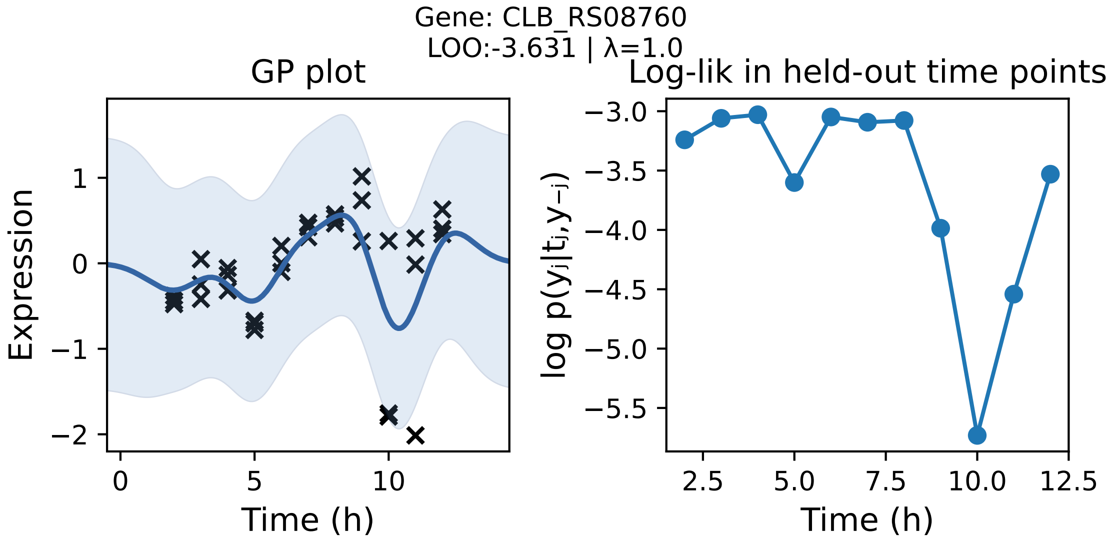

Gene: CLB_RS08760
LOO:-3.631 | λ=1.0
GP plot
Log-lik in held-out time points
Expression
Time (h)
log p(y_j|t_j,y_−j)
Time (h)
1
0
−1
−2
0
5
10
−3.0
−3.5
−4.0
−4.5
−5.0
−5.5
2.5
5.0
7.5
10.0
12.5

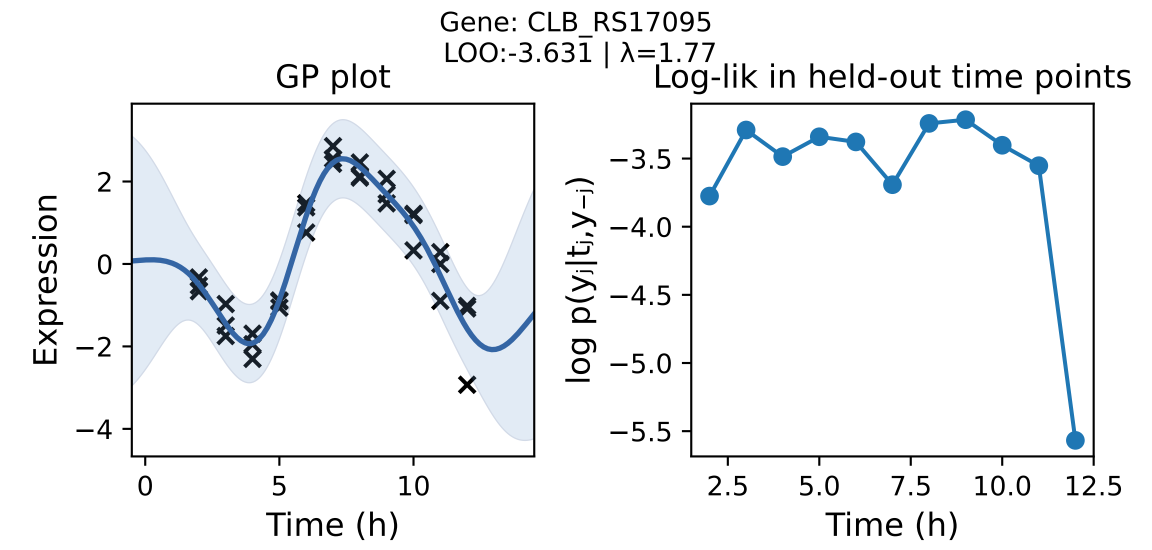
Gene: CLB_RS17095
LOO:-3.631 | λ=1.77
GP plot
Expression
Time (h)
Log-lik in held-out time points
log p(y_j|t_j,y_−j)
Time (h)

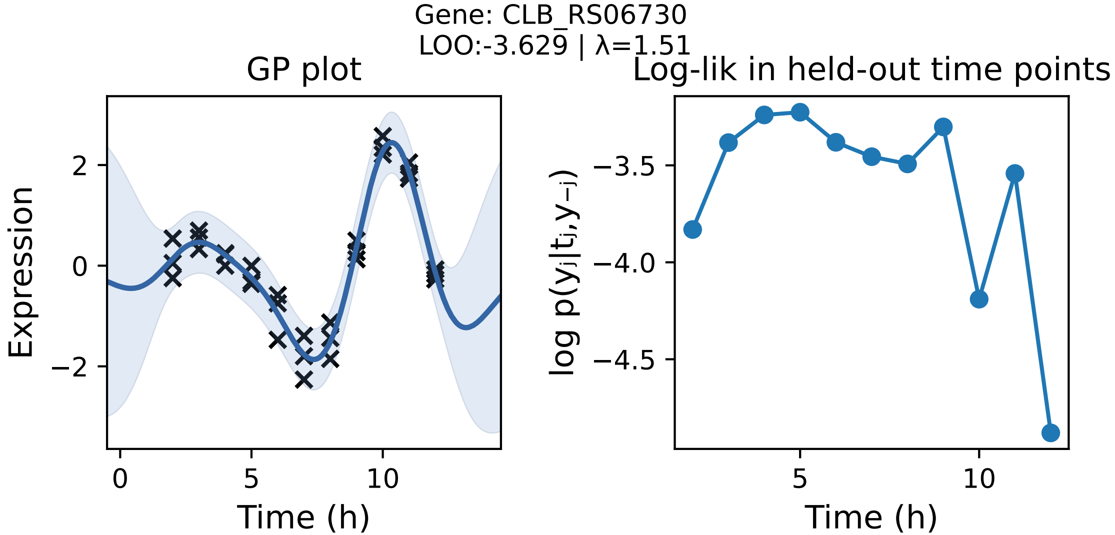
Gene: CLB_RS06730
LOO:-3.629 | λ=1.51
GP plot
Expression
Time (h)
2
0
−2
0
5
10
Log-lik in held-out time points
log p(y_j|t_j,y_−j)
−3.5
−4.0
−4.5
5
10
Time (h)

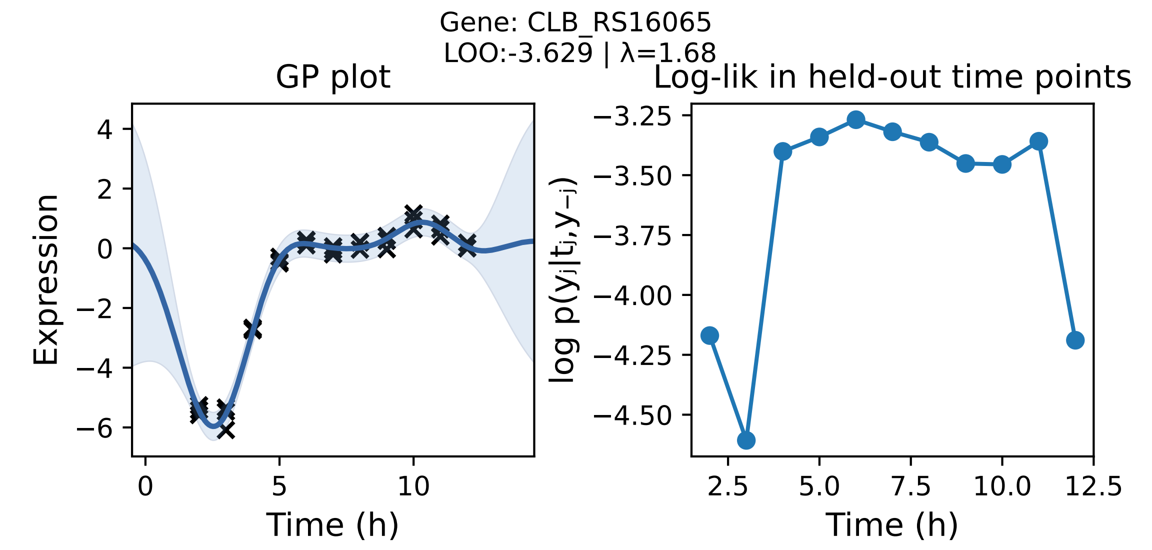

Gene: CLB_RS16065
LOO:-3.629 | λ=1.68
GP plot
Log-lik in held-out time points
Expression
Time (h)
log p(yⱼ|tⱼ,y₋ⱼ)
Time (h)
4
2
0
−2
−4
−6
0
5
10
−3.25
−3.50
−3.75
−4.00
−4.25
−4.50
2.5
5.0
7.5
10.0
12.5

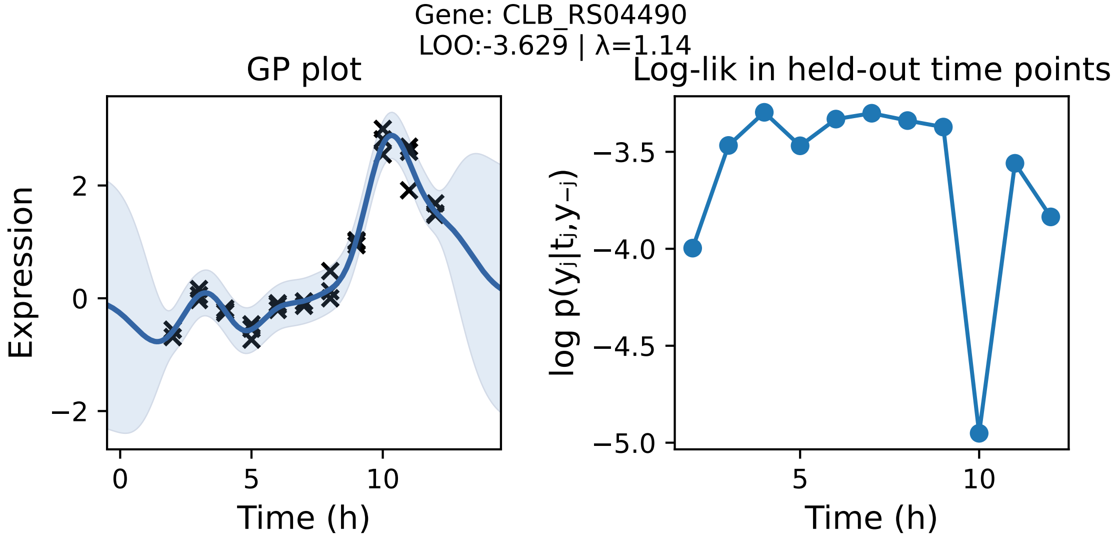
Gene: CLB_RS04490
LOO:-3.629 | λ=1.14
GP plot
Expression
Time (h)
Log-lik in held-out time points
log p(y_j|t_j,y_−j)
Time (h)

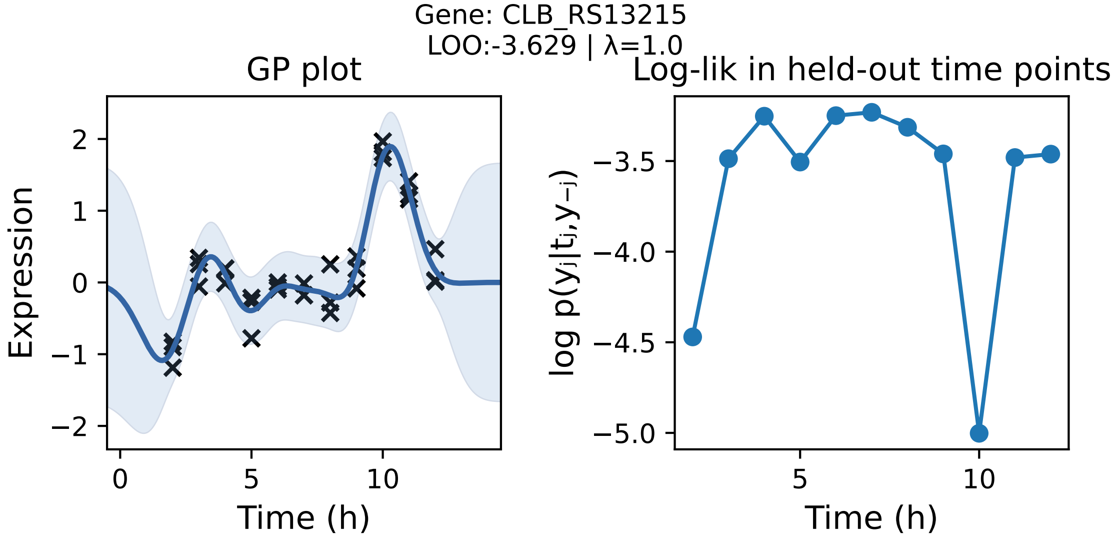
Gene: CLB_RS13215
LOO:-3.629 | λ=1.0
GP plot
Expression
Time (h)
2
1
0
−1
−2
0
5
10
Log-lik in held-out time points
log p(yj|tj,y−j)
−3.5
−4.0
−4.5
−5.0
5
10
Time (h)

Gene: CLB_RS11875
LOO:-3.628 | λ=1.07

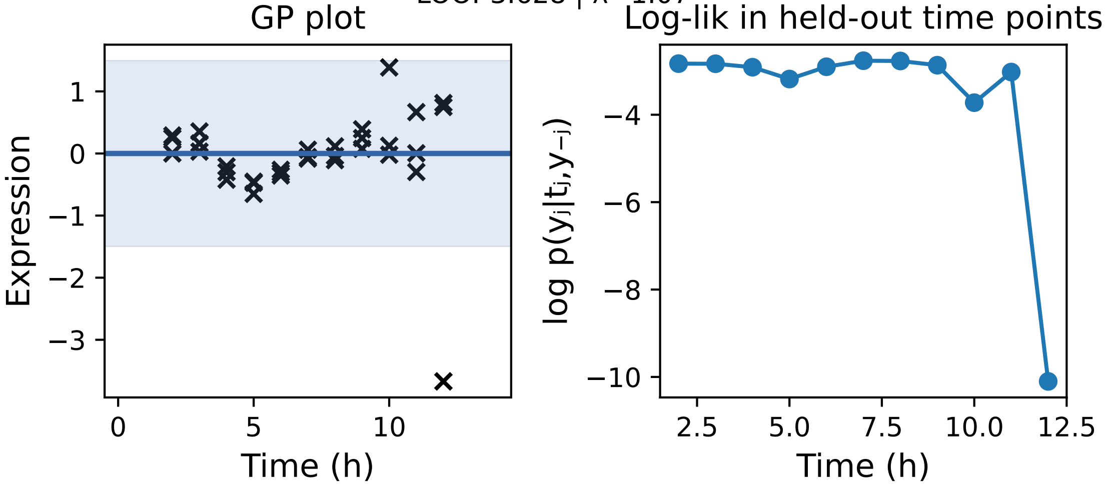

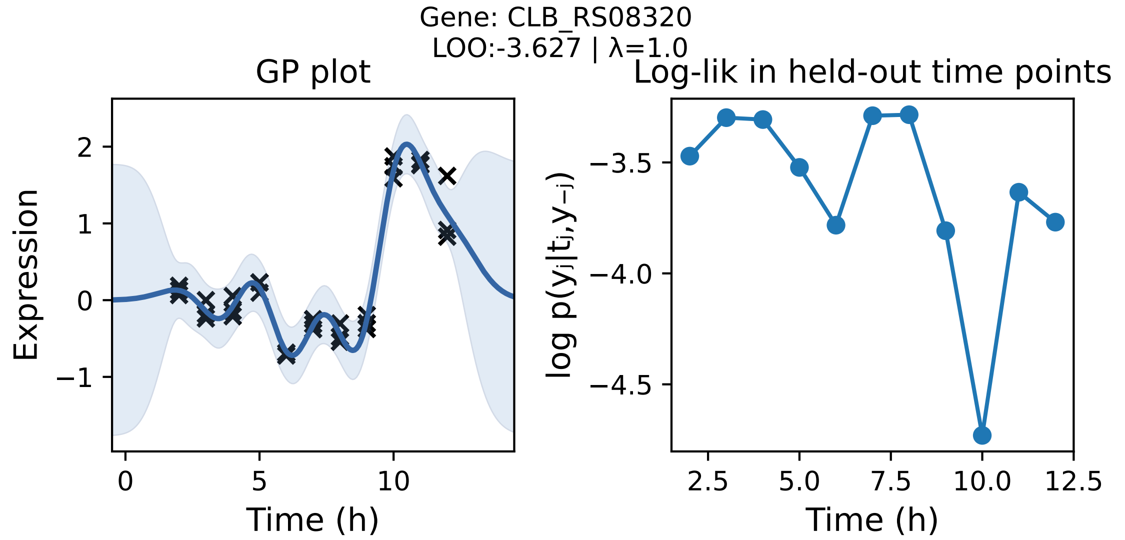
Gene: CLB_RS08320
LOO:-3.627 | λ=1.0
GP plot
Expression
Time (h)
Log-lik in held-out time points
log p(y_j|t_j,y_−j)
Time (h)

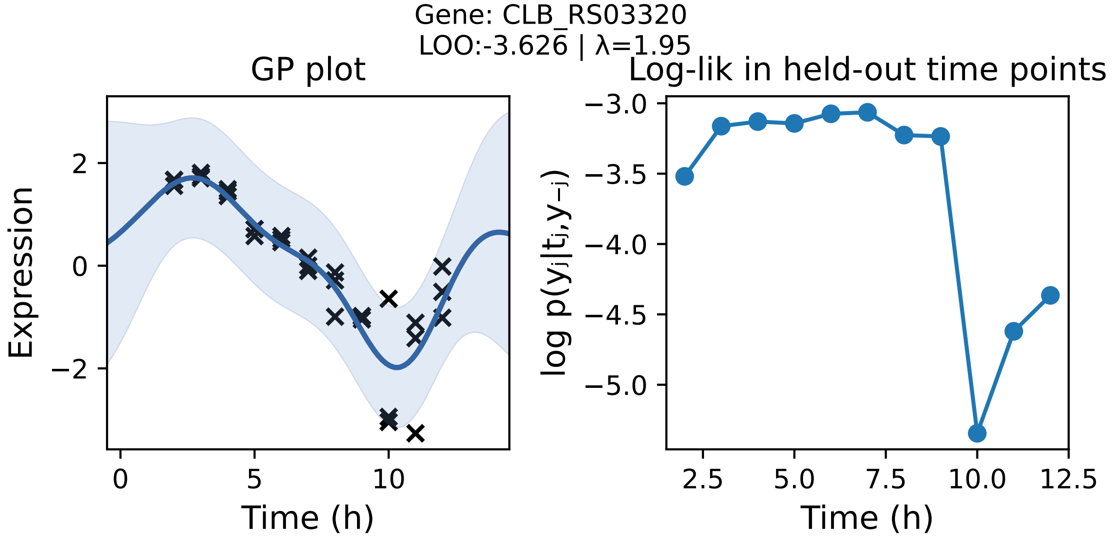

Gene: CLB_RS03320
LOO:-3.626 | λ=1.95
GP plot
Expression
Time (h)
Log-lik in held-out time points
log p(y_j|t_j,y_−j)
Time (h)

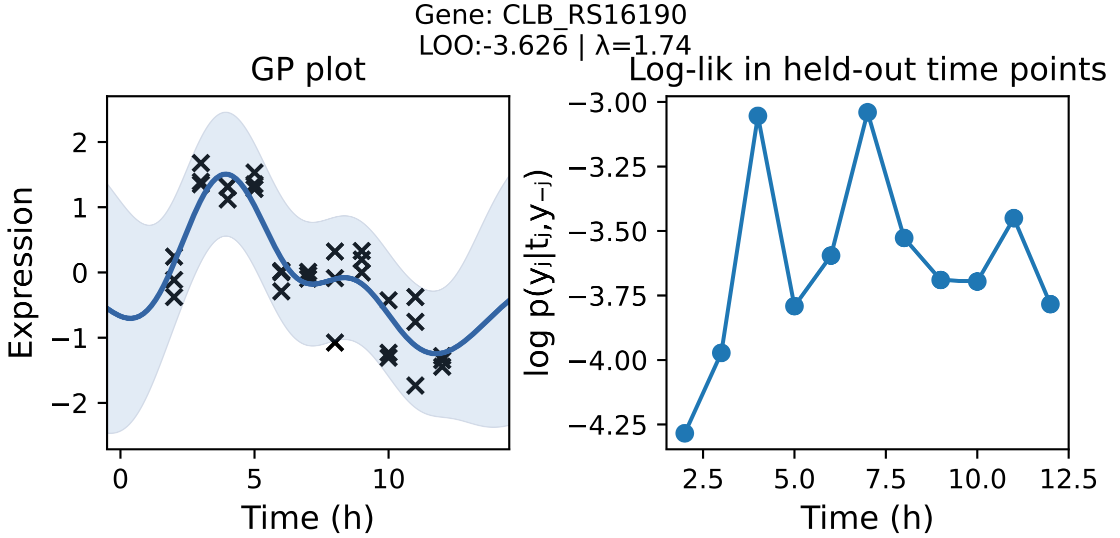

Gene: CLB_RS16190
LOO:-3.626 | λ=1.74
GP plot
Expression
Time (h)
Log-lik in held-out time points
log p(yj|tj,y−j)
Time (h)

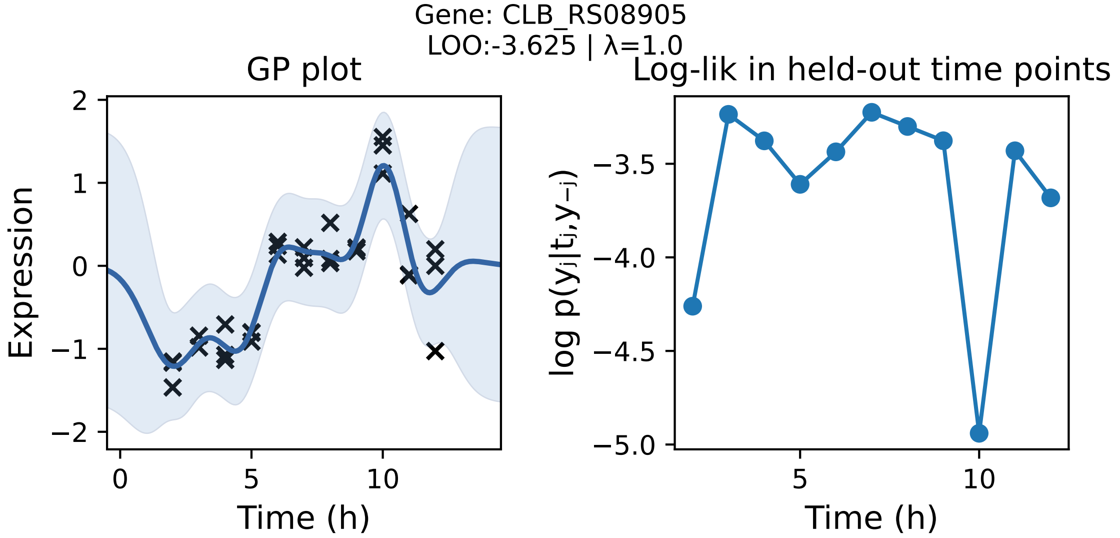

Gene: CLB_RS08905
LOO:-3.625 | λ=1.0
GP plot
Expression
Time (h)
Log-lik in held-out time points
log p(y_j|t_j,y_−j)
Time (h)

Gene: CLB_RS05880
LOO:-3.623 | λ=4.59

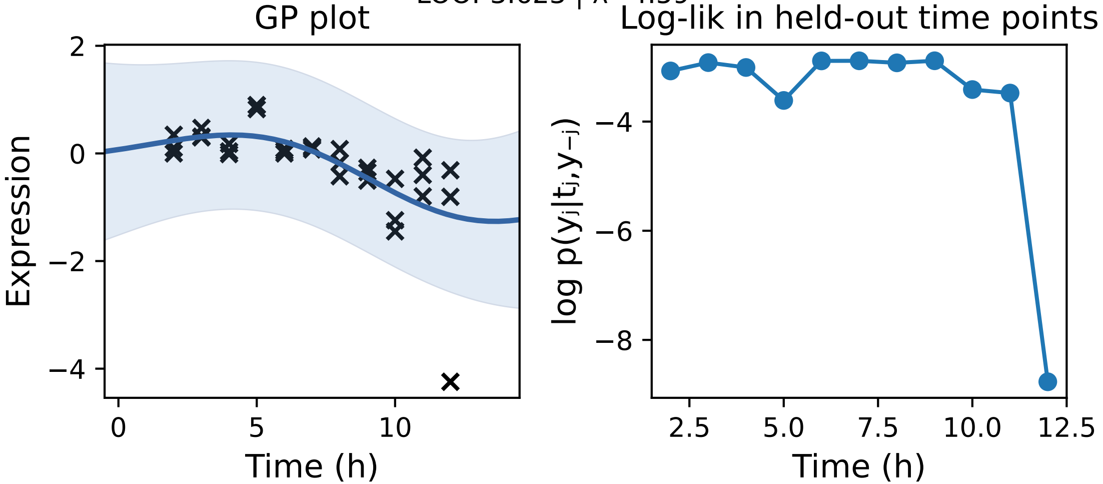

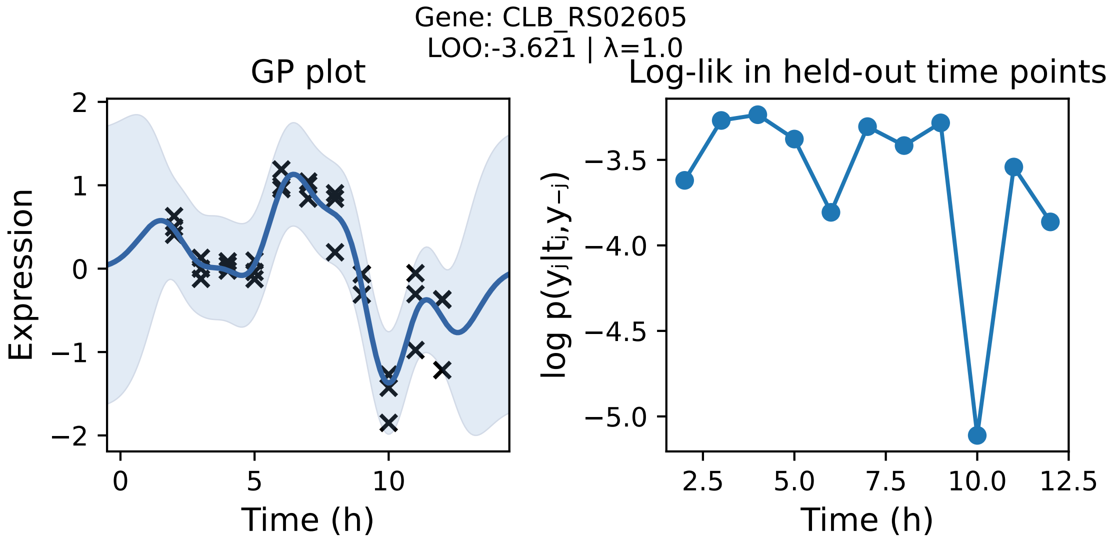

Gene: CLB_RS02605
LOO:-3.621 | λ=1.0
GP plot
Expression
Time (h)
2
1
0
−1
−2
0
5
10
Log-lik in held-out time points
log p(y_j|t_j,y_−j)
−3.5
−4.0
−4.5
−5.0
2.5
5.0
7.5
10.0
12.5
Time (h)

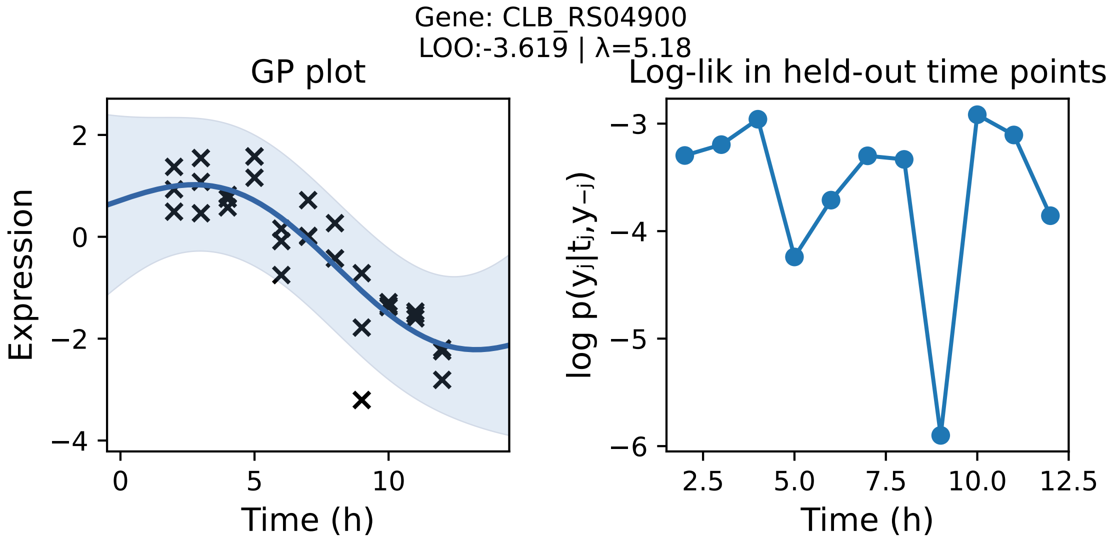

Gene: CLB_RS04900
LOO:-3.619 | λ=5.18
GP plot
Expression
Time (h)
2
0
−2
−4
0
5
10
Log-lik in held-out time points
log p(y_j|t_j,y_−j)
Time (h)
−3
−4
−5
−6
2.5
5.0
7.5
10.0
12.5

Gene: CLB_RS04425
LOO:-3.619 | λ=1.92

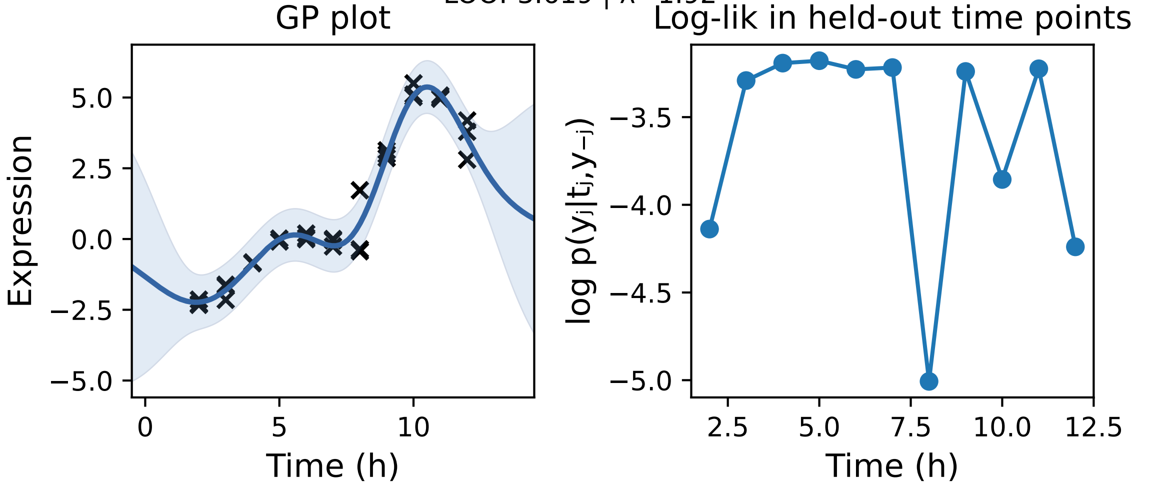

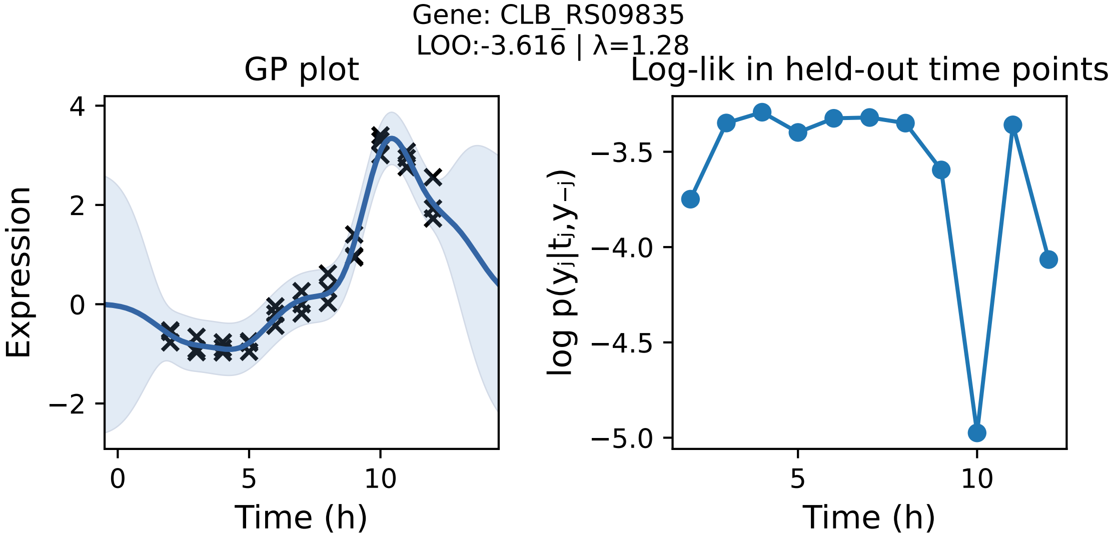

Gene: CLB_RS09835
LOO:-3.616 | λ=1.28
GP plot
Log-lik in held-out time points
Expression
4
2
0
−2
0
5
10
Time (h)
log p(y_j|t_j,y_−j)
−3.5
−4.0
−4.5
−5.0
5
10
Time (h)

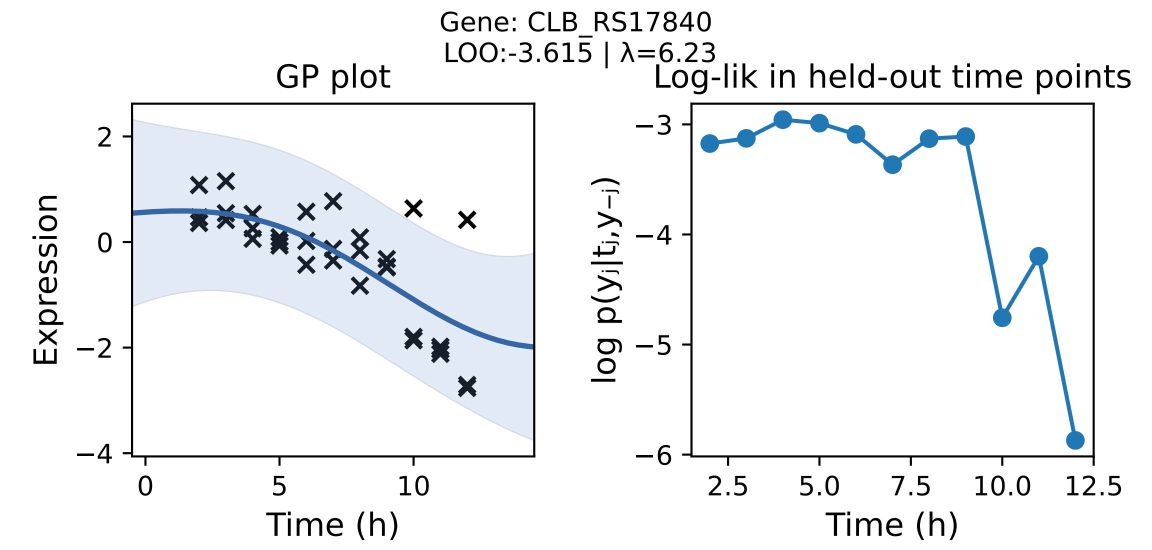

Gene: CLB_RS17840
LOO:-3.615 | λ=6.23
GP plot
Expression
Time (h)
Log-lik in held-out time points
log p(yj|tj,y−j)
Time (h)

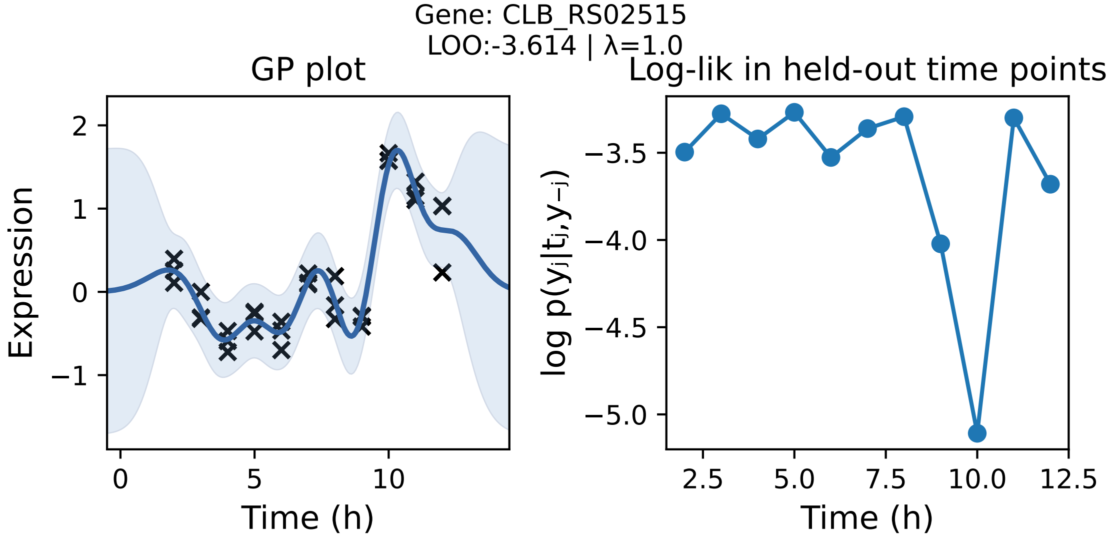

Gene: CLB_RS02515
LOO:-3.614 | λ=1.0
GP plot
Expression
Time (h)
Log-lik in held-out time points
log p(yj|tj,y−j)
Time (h)

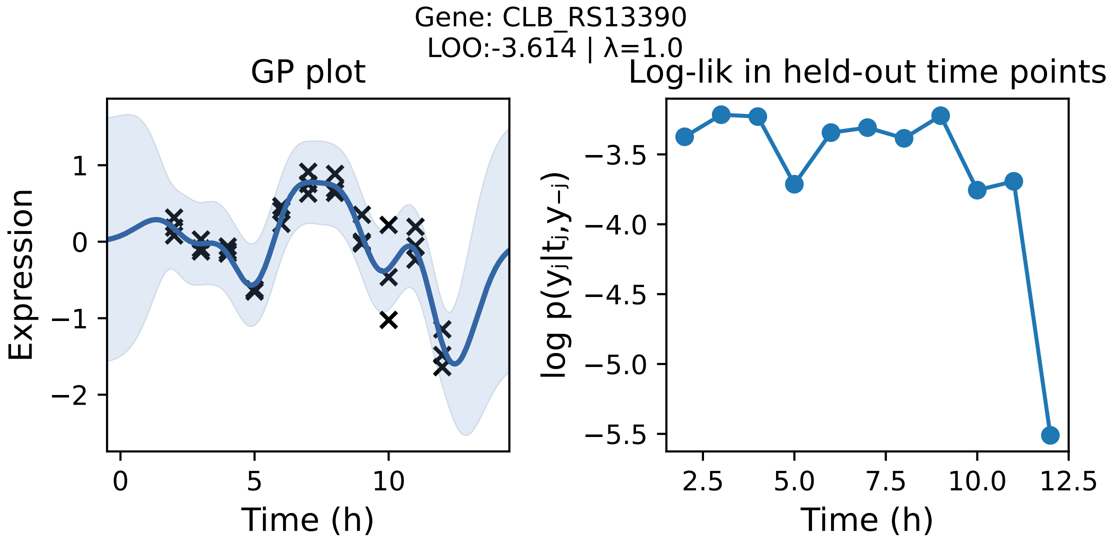

Gene: CLB_RS13390
LOO:-3.614 | λ=1.0
GP plot
Expression
Time (h)
1
0
−1
−2
0
5
10
Log-lik in held-out time points
log p(y_j|t_j,y_−j)
Time (h)
−3.5
−4.0
−4.5
−5.0
−5.5
2.5
5.0
7.5
10.0
12.5

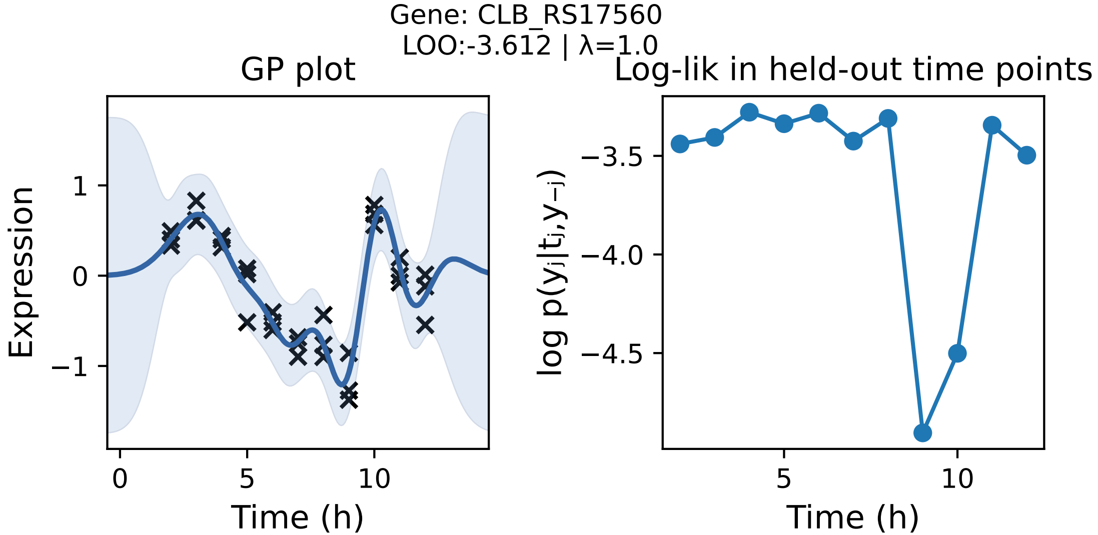

Gene: CLB_RS17560
LOO:-3.612 | λ=1.0
GP plot
Expression
Time (h)
Log-lik in held-out time points
log p(y_j|t_j,y_−j)
Time (h)

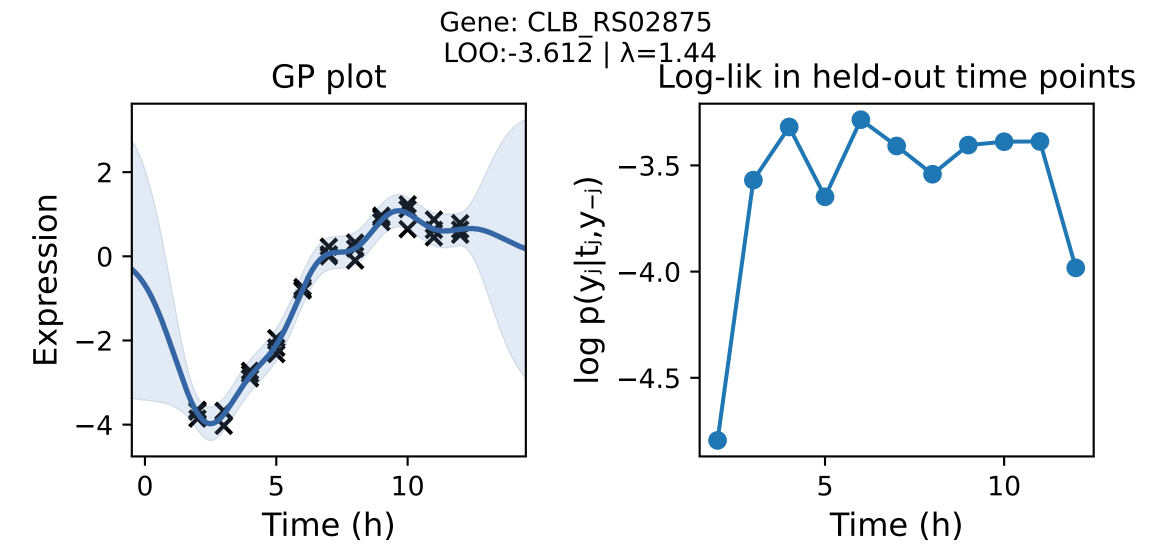
Gene: CLB_RS02875
LOO:-3.612 | λ=1.44
GP plot
Expression
Time (h)
Log-lik in held-out time points
log p(y_j|t_j,y_−j)
Time (h)

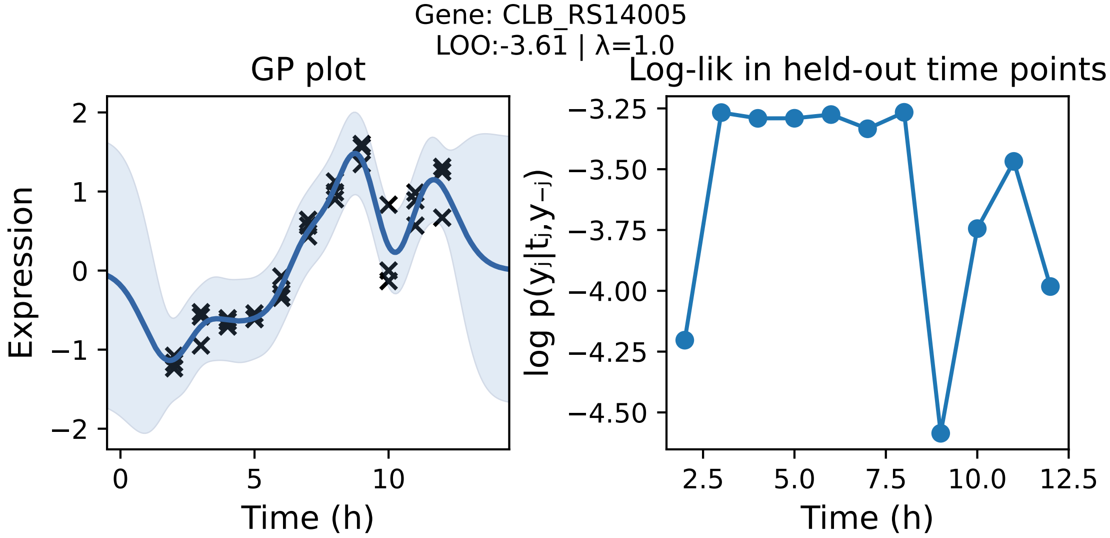

Gene: CLB_RS14005
LOO:-3.61 | λ=1.0
GP plot
Expression
Time (h)
Log-lik in held-out time points
log p(y_j|t_j,y_−j)
Time (h)

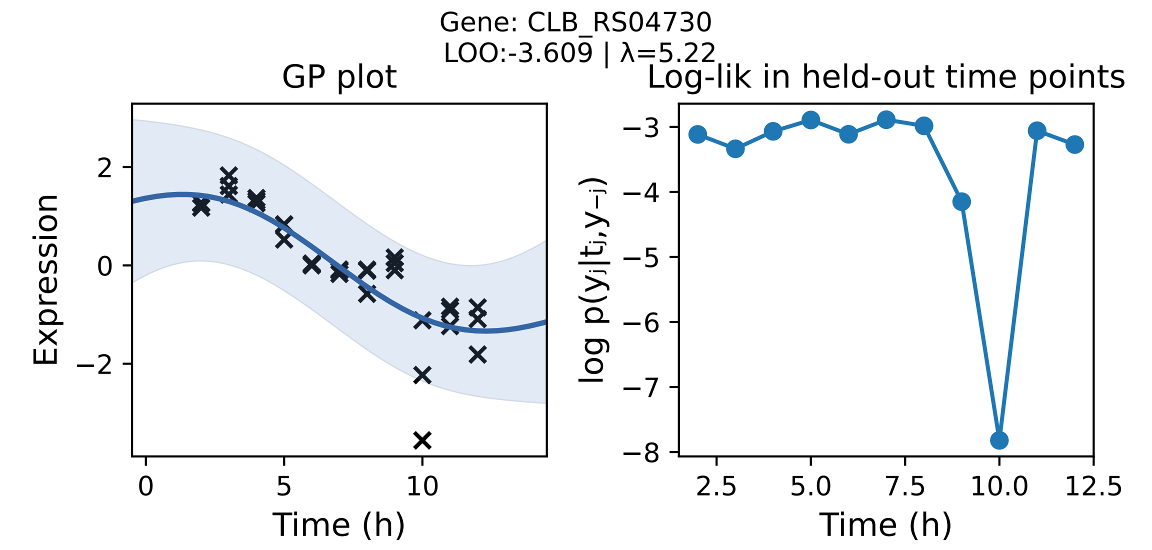

Gene: CLB_RS04730
LOO:-3.609 | λ=5.22
GP plot
Expression
Time (h)
0
5
10
2
0
−2
Log-lik in held-out time points
log p(y_j|t_j,y_−j)
Time (h)
2.5
5.0
7.5
10.0
12.5
−3
−4
−5
−6
−7
−8

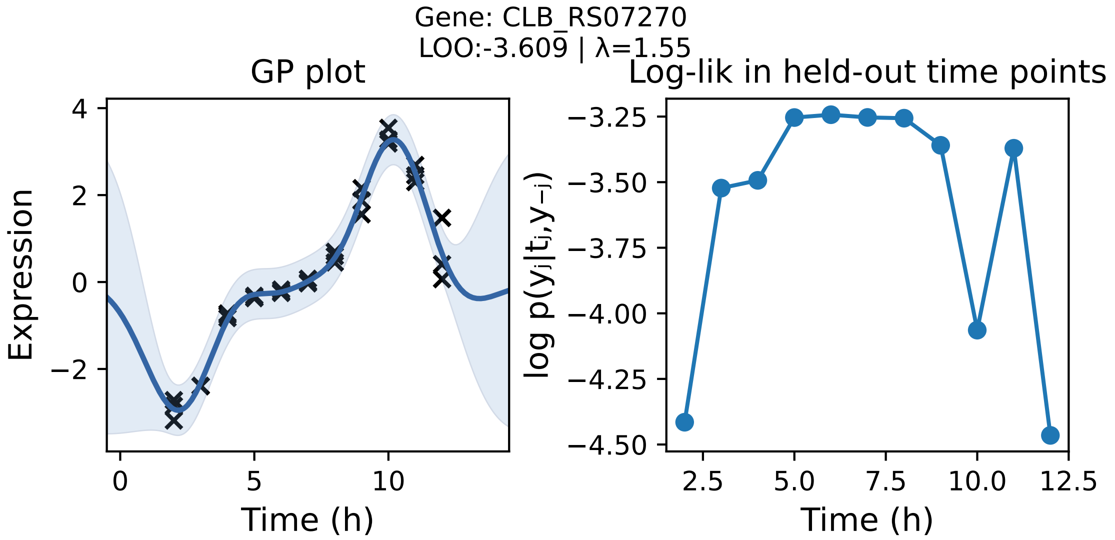

Gene: CLB_RS07270
LOO:-3.609 | λ=1.55
GP plot
Expression
Time (h)
4
2
0
−2
0
5
10
Log-lik in held-out time points
log p(y_j|t_j,y_−j)
Time (h)
−3.25
−3.50
−3.75
−4.00
−4.25
−4.50
2.5
5.0
7.5
10.0
12.5

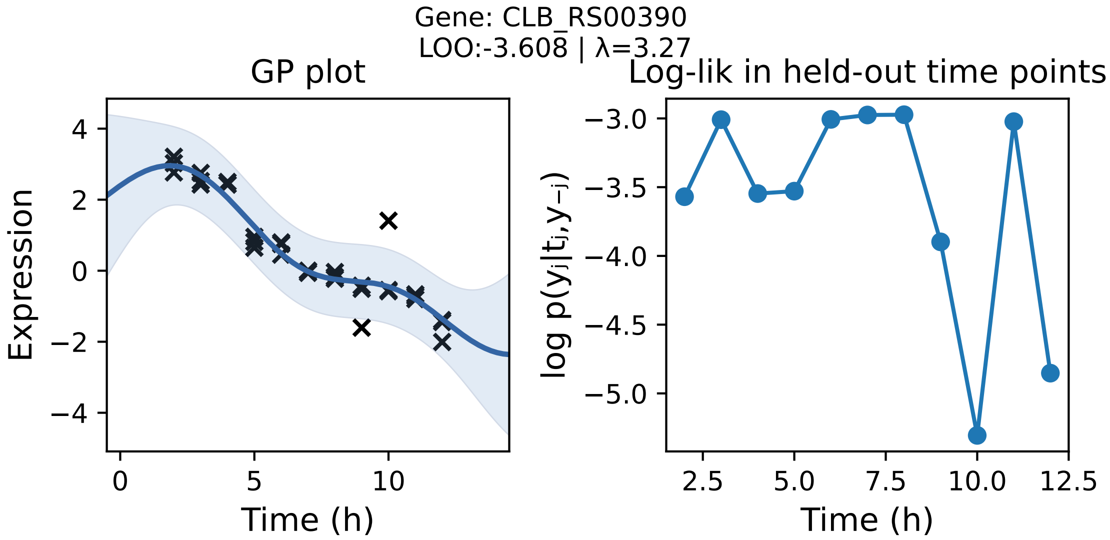

Gene: CLB_RS00390
LOO:-3.608 | λ=3.27
GP plot
Expression
Time (h)
Log-lik in held-out time points
log p(y_j|t_j,y_−j)
Time (h)

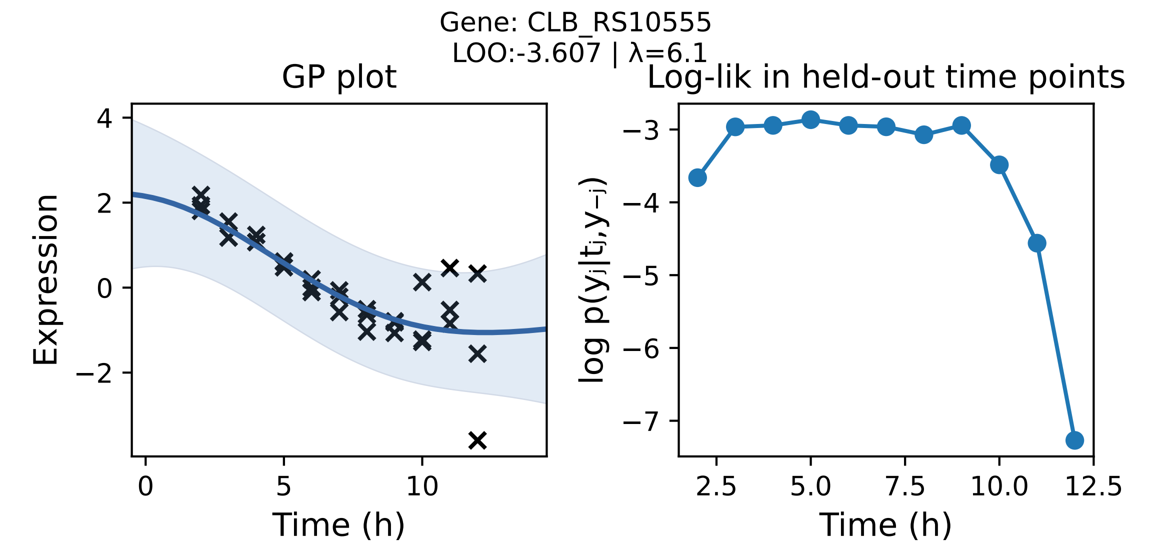
Gene: CLB_RS10555
LOO:-3.607 | λ=6.1
GP plot
Expression
Time (h)
Log-lik in held-out time points
log p(y_j|t_j,y_−j)
Time (h)

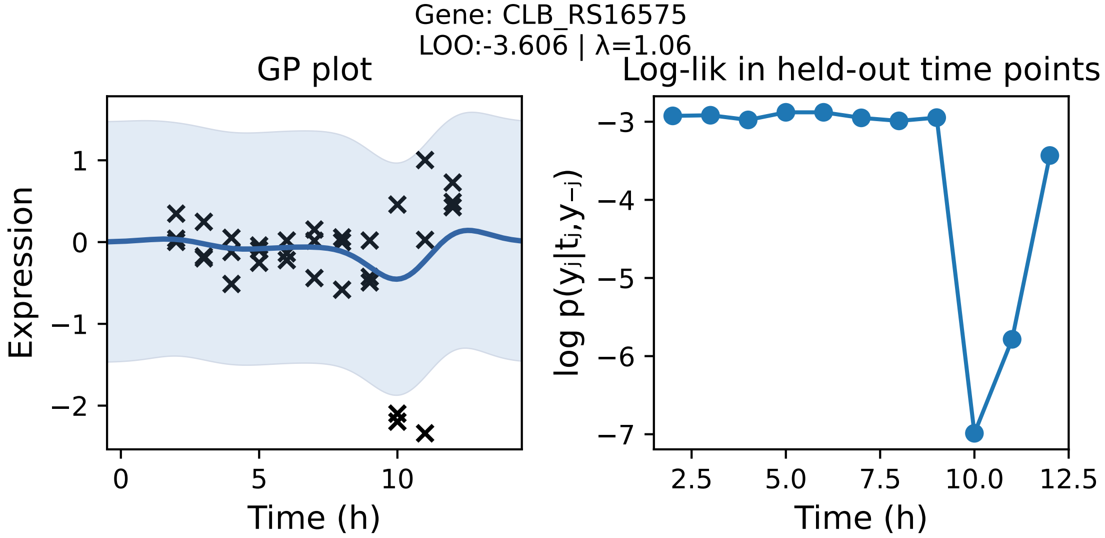

Gene: CLB_RS16575
LOO:-3.606 | λ=1.06
GP plot
Expression
Time (h)
1
0
−1
−2
0
5
10
Log-lik in held-out time points
log p(yj|tj,y−j)
Time (h)
−3
−4
−5
−6
−7
2.5
5.0
7.5
10.0
12.5

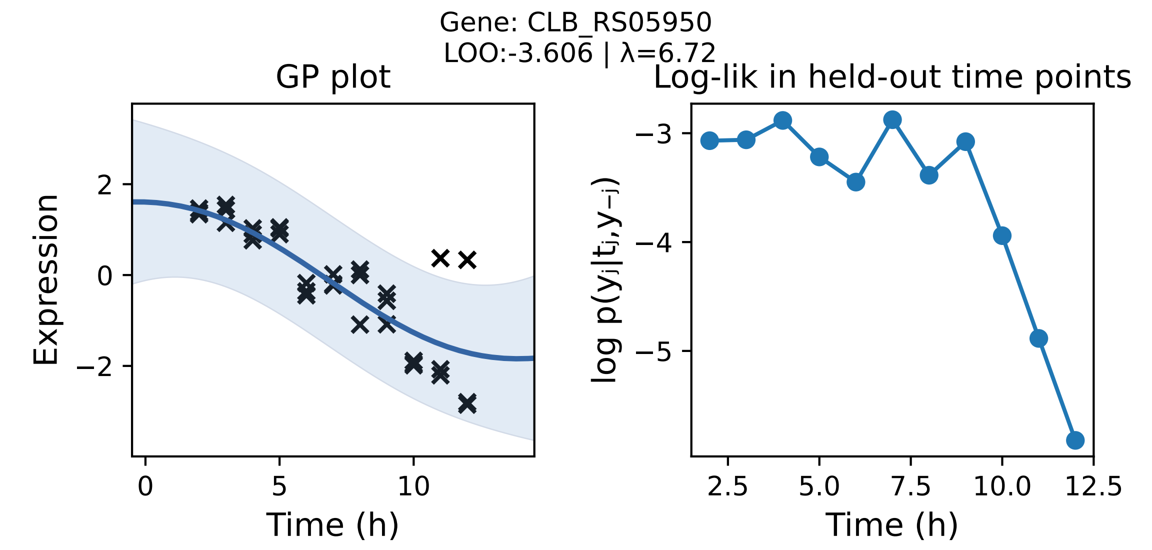

Gene: CLB_RS05950
LOO:-3.606 | λ=6.72
GP plot
Expression
Time (h)
2
0
−2
0
5
10
Log-lik in held-out time points
log p(yj|tj,y−j)
Time (h)
−3
−4
−5
2.5
5.0
7.5
10.0
12.5

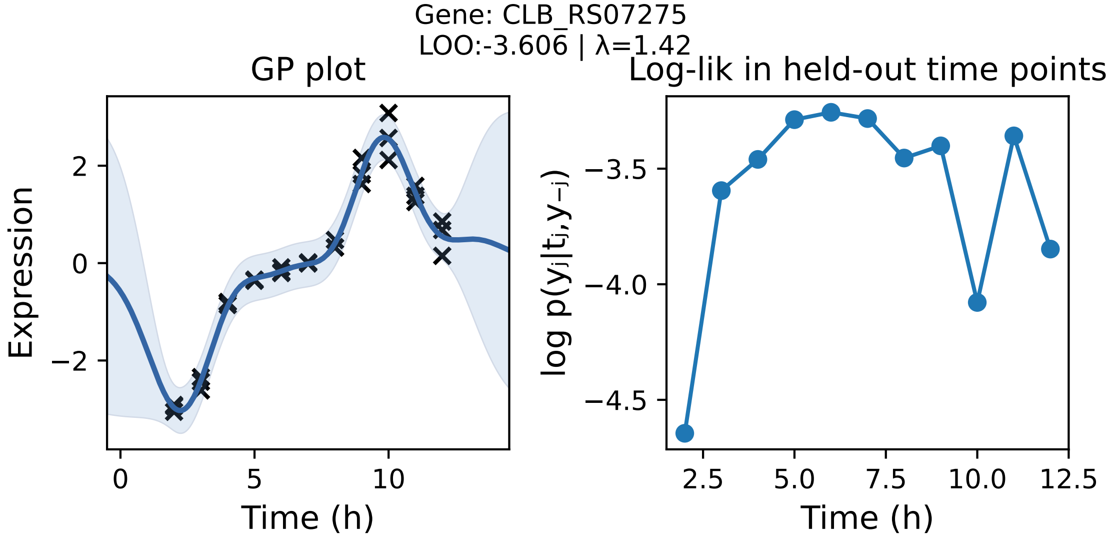

Gene: CLB_RS07275
LOO:-3.606 | λ=1.42
GP plot
Expression
Time (h)
2
0
−2
0
5
10
Log-lik in held-out time points
log p(y_j|t_j,y_−j)
Time (h)
−3.5
−4.0
−4.5
2.5
5.0
7.5
10.0
12.5

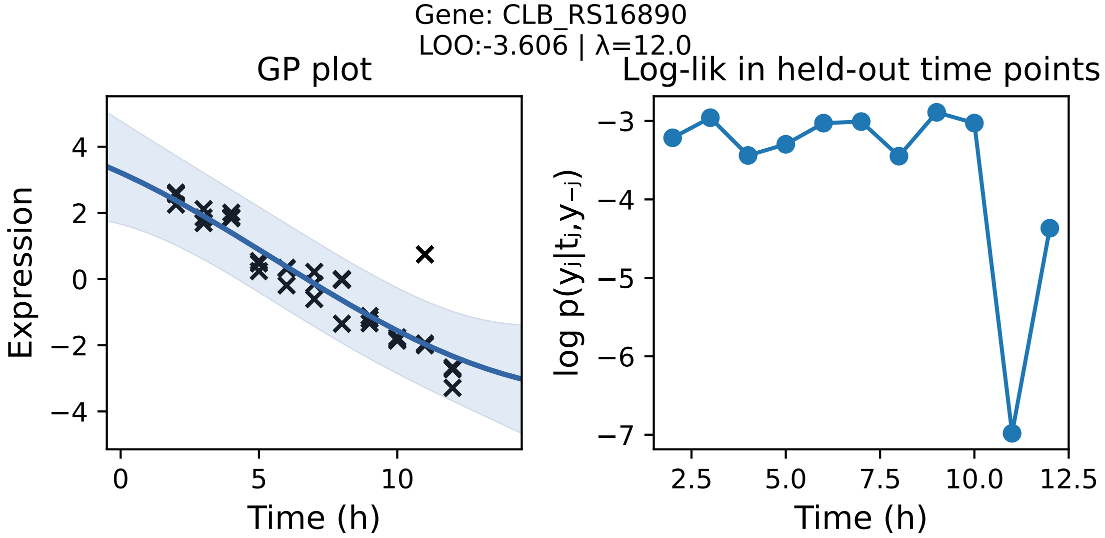

Gene: CLB_RS16890
LOO:-3.606 | λ=12.0
GP plot
Expression
Time (h)
Log-lik in held-out time points
log p(y_j|t_j,y_−j)
Time (h)

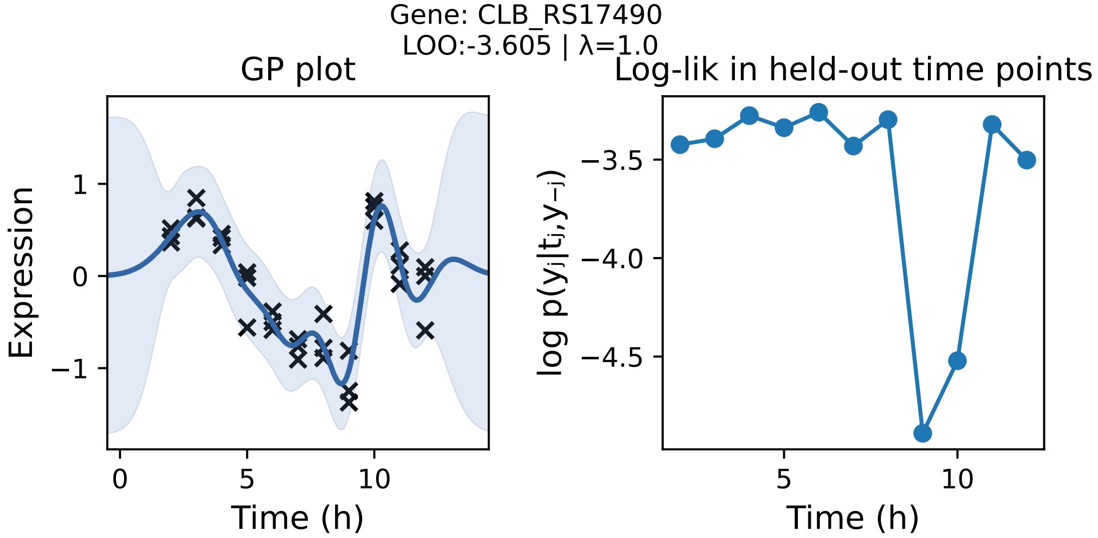
Gene: CLB_RS17490
LOO:-3.605 | λ=1.0
GP plot
Expression
Time (h)
1
0
−1
0
5
10
Log-lik in held-out time points
log p(y_j|t_j,y_−j)
Time (h)
−3.5
−4.0
−4.5
5
10

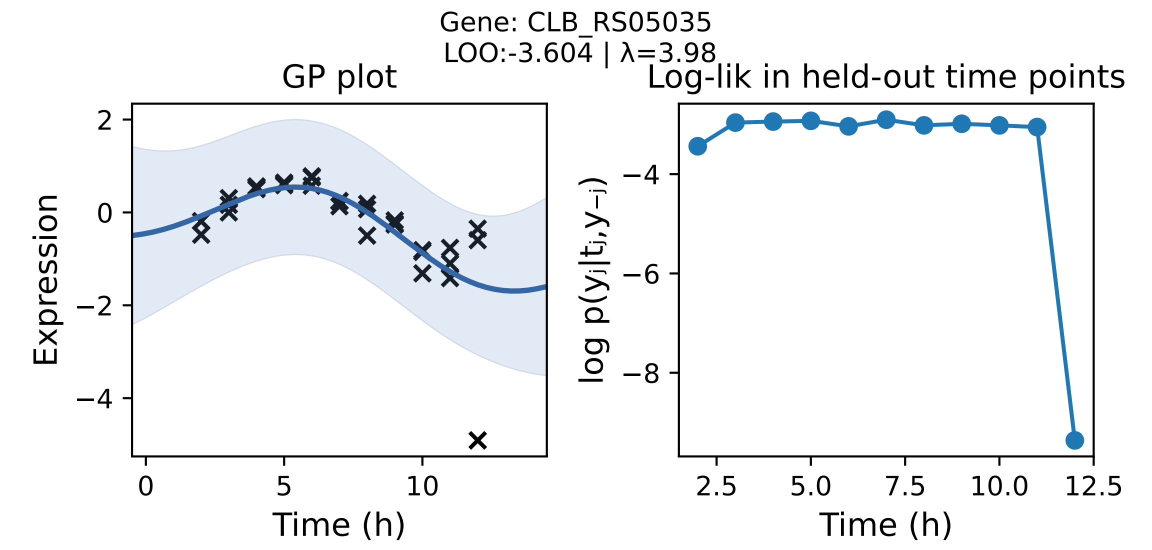

Gene: CLB_RS05035
LOO:-3.604 | λ=3.98
GP plot
Expression
Time (h)
2
0
−2
−4
0
5
10
Log-lik in held-out time points
log p(y_j|t_j,y_−j)
Time (h)
−4
−6
−8
2.5
5.0
7.5
10.0
12.5

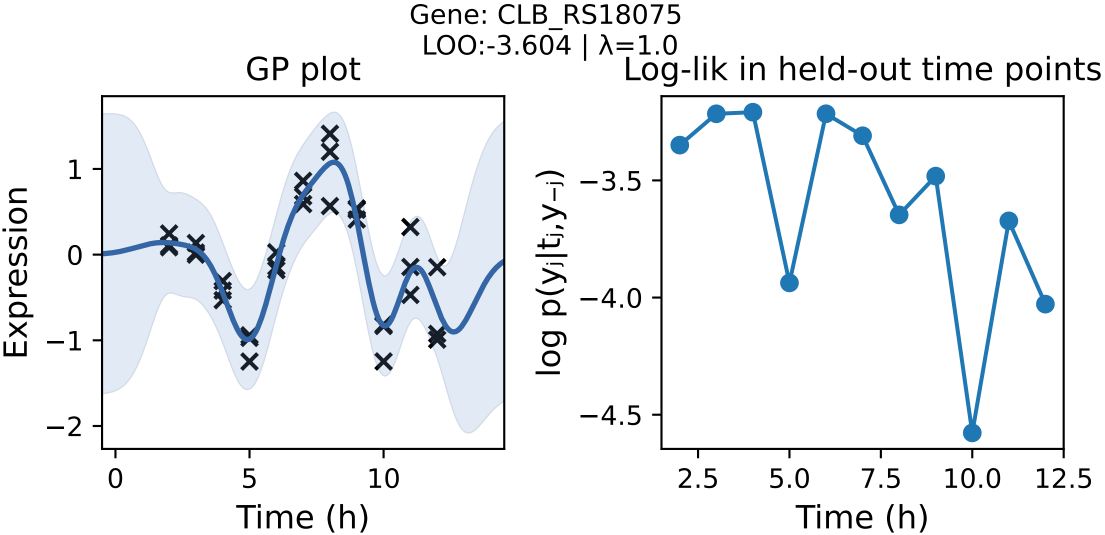
Gene: CLB_RS18075
LOO:-3.604 | λ=1.0
GP plot
Expression
Time (h)
1
0
−1
−2
0
5
10
Log-lik in held-out time points
log p(y_j|t_j,y_−j)
−3.5
−4.0
−4.5
2.5
5.0
7.5
10.0
12.5
Time (h)

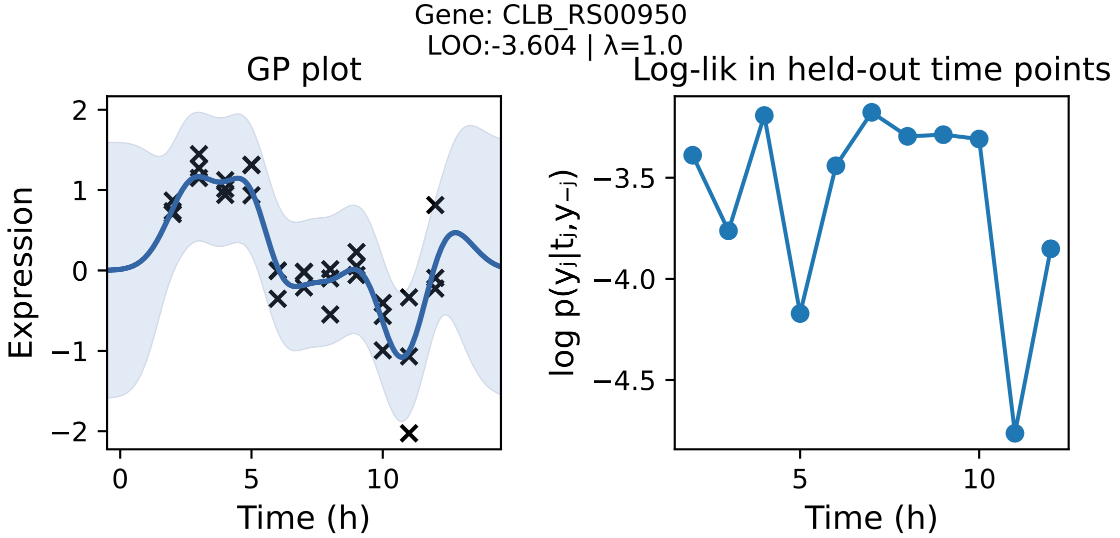
Gene: CLB_RS00950
LOO:-3.604 | λ=1.0
GP plot
Expression
Time (h)
Log-lik in held-out time points
log p(y_j|t_j,y_−j)
Time (h)

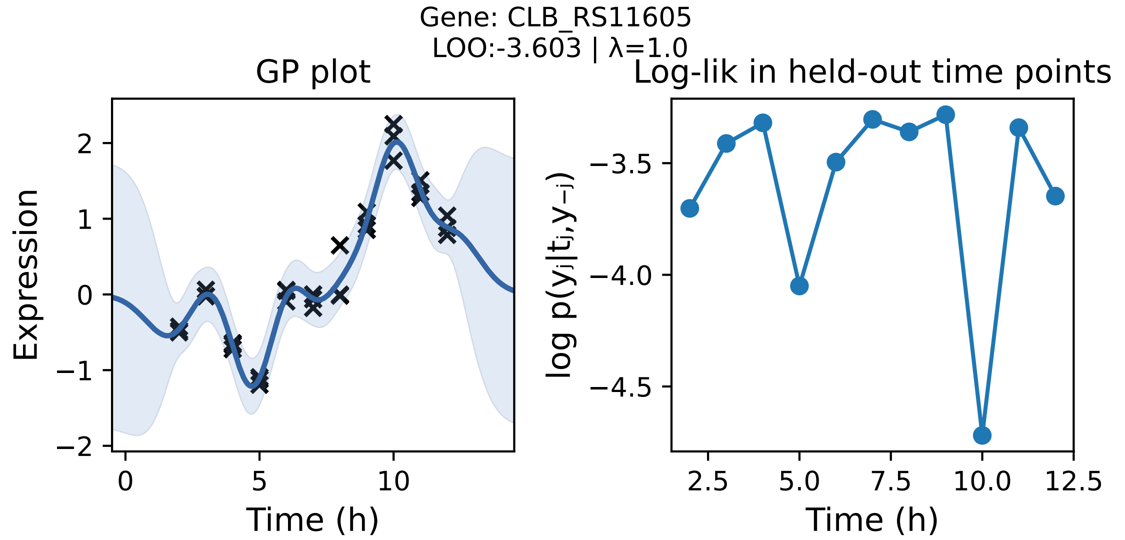

Gene: CLB_RS11605
LOO:-3.603 | λ=1.0
GP plot
Expression
Time (h)
2
1
0
−1
−2
0
5
10
Log-lik in held-out time points
log p(y_j|t_j,y_−j)
−3.5
−4.0
−4.5
2.5
5.0
7.5
10.0
12.5
Time (h)

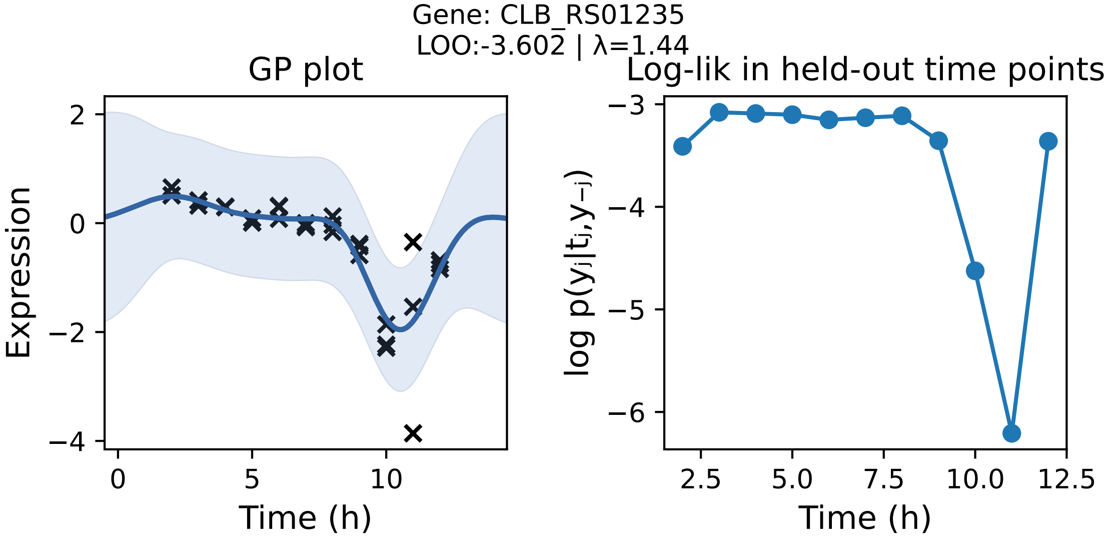

Gene: CLB_RS01235
LOO:-3.602 | λ=1.44
GP plot
Expression
Time (h)
2
0
−2
−4
0
5
10
Log-lik in held-out time points
log p(y_j|t_j,y_−j)
Time (h)
−3
−4
−5
−6
2.5
5.0
7.5
10.0
12.5

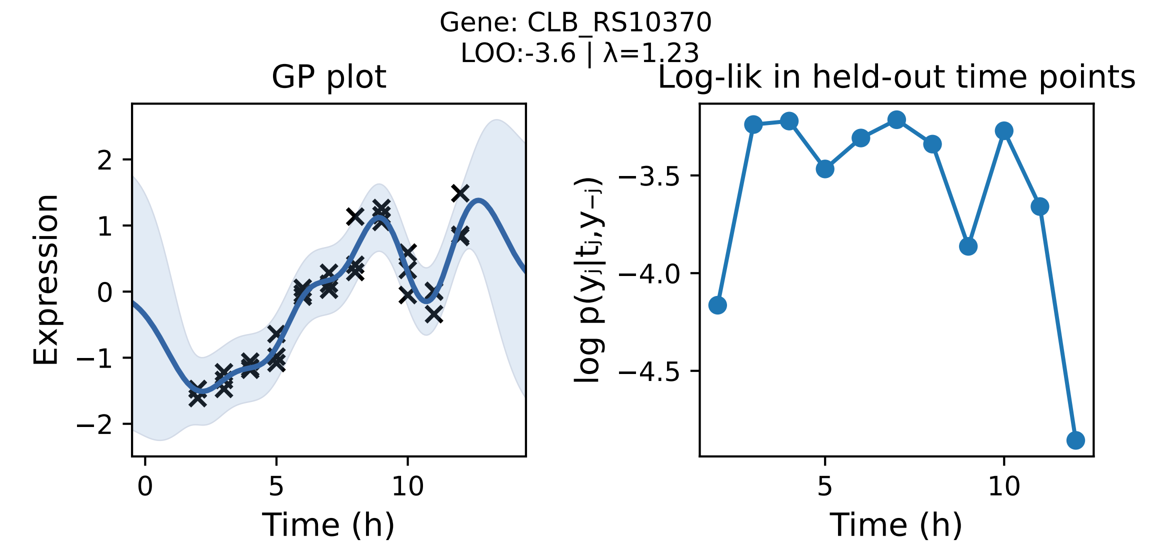

Gene: CLB_RS10370
LOO:-3.6 | λ=1.23
GP plot
Expression
Time (h)
Log-lik in held-out time points
log p(y_j|t_j,y_−j)
Time (h)

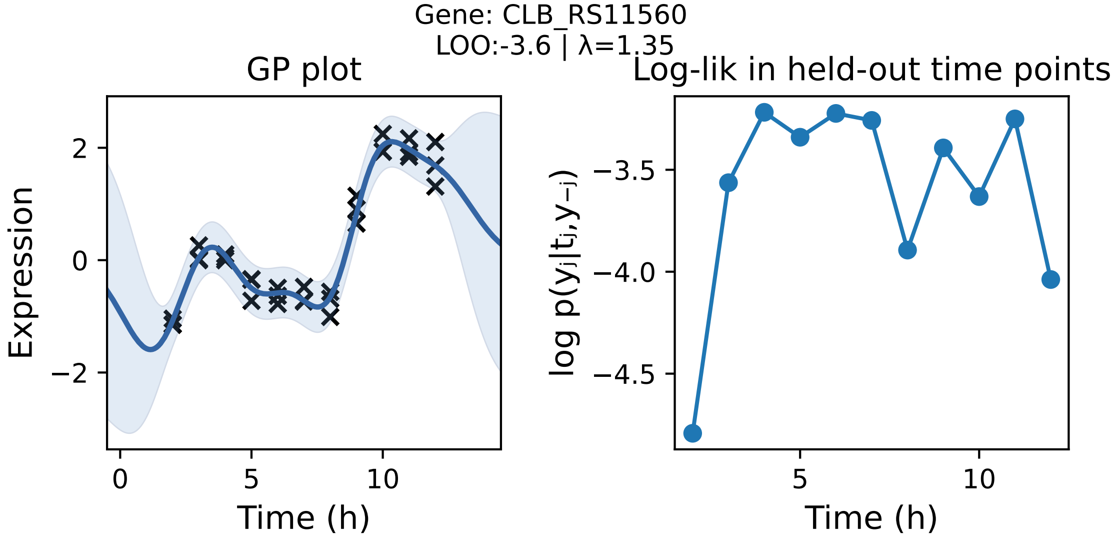

Gene: CLB_RS11560
LOO:-3.6 | λ=1.35
GP plot
Expression
2
0
−2
0
5
10
Time (h)
Log-lik in held-out time points
log p(y_j|t_j,y_−j)
−3.5
−4.0
−4.5
5
10
Time (h)

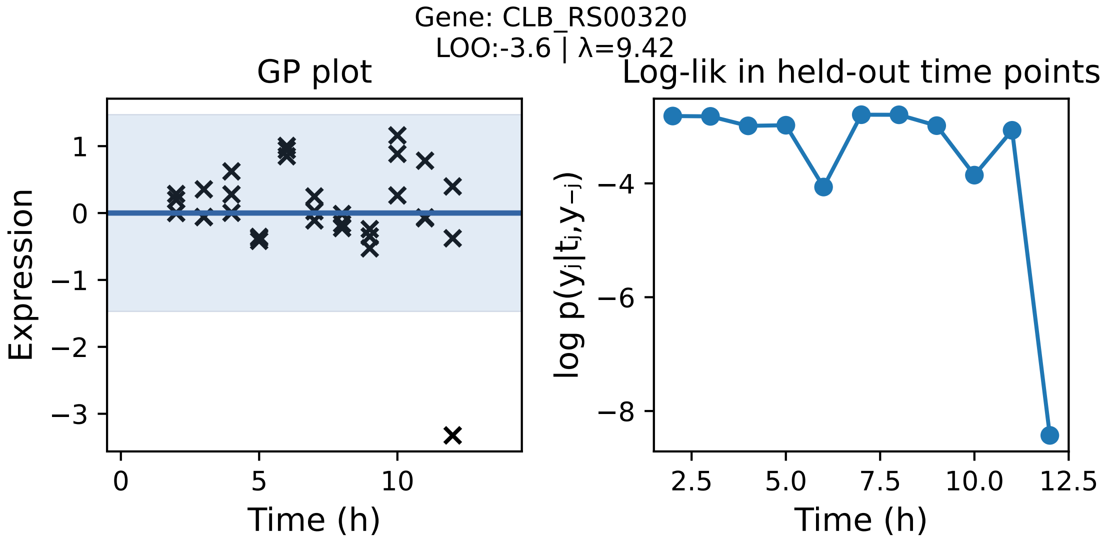
Gene: CLB_RS00320
LOO:-3.6 | λ=9.42
GP plot
Log-lik in held-out time points
Expression
Time (h)
log p(y_j|t_j,y_−j)
Time (h)
1
0
−1
−2
−3
0
5
10
−4
−6
−8
2.5
5.0
7.5
10.0
12.5

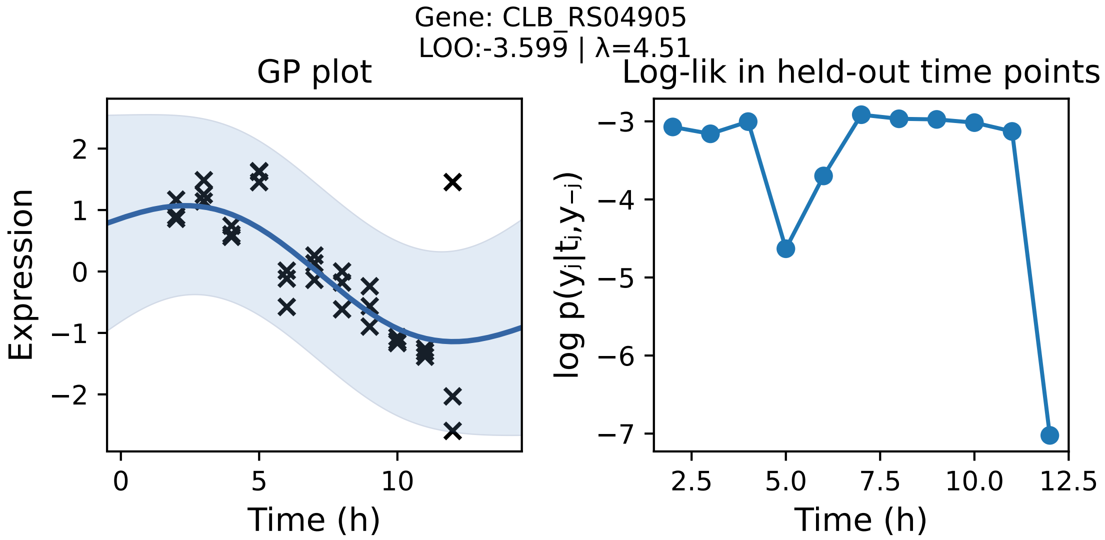

Gene: CLB_RS04905
LOO:-3.599 | λ=4.51
GP plot
Log-lik in held-out time points
Expression
Time (h)
log p(yj|tj,y−j)
Time (h)
2
1
0
−1
−2
0
5
10
−3
−4
−5
−6
−7
2.5
5.0
7.5
10.0
12.5

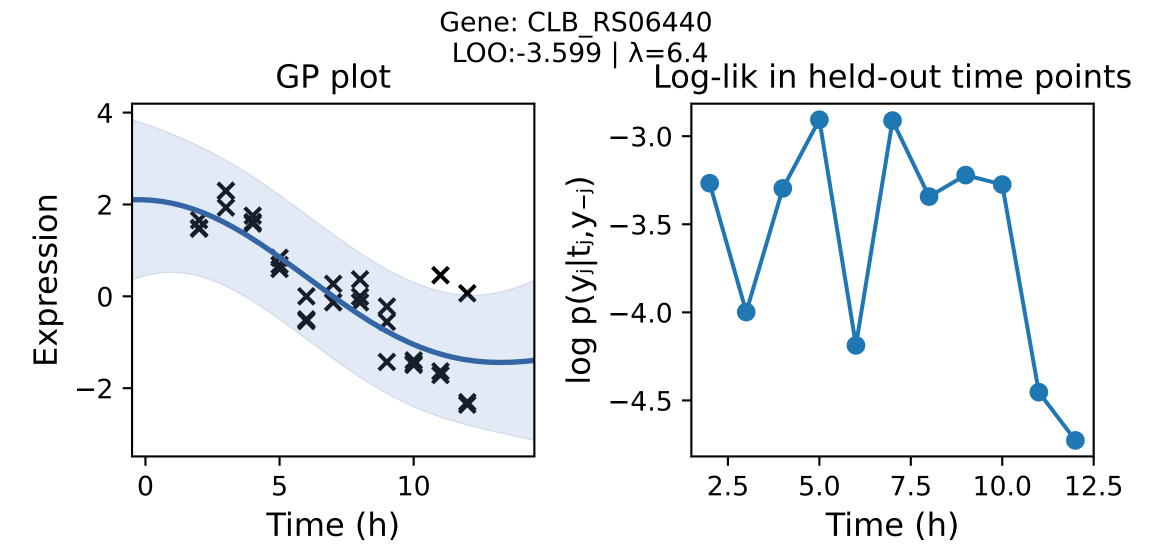

Gene: CLB_RS06440
LOO:-3.599 | λ=6.4
GP plot
Expression
Time (h)
Log-lik in held-out time points
log p(y_j|t_j,y_−j)
Time (h)

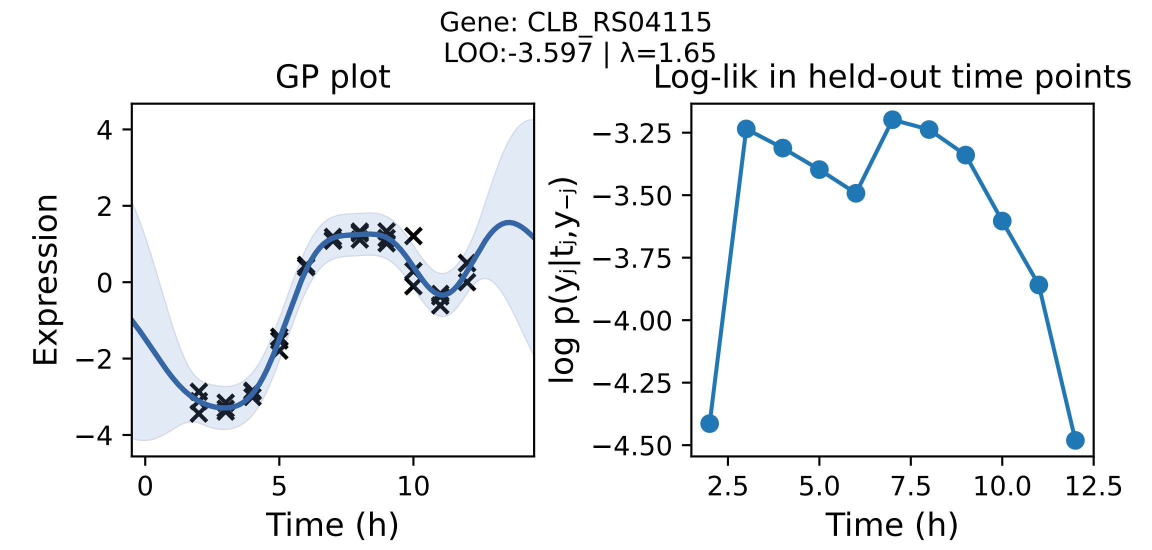
Gene: CLB_RS04115
LOO:-3.597 | λ=1.65
GP plot
Log-lik in held-out time points
Expression
4
2
0
−2
−4
0
5
10
Time (h)
log p(yⱼ|tⱼ,y₋ⱼ)
−3.25
−3.50
−3.75
−4.00
−4.25
−4.50
2.5
5.0
7.5
10.0
12.5
Time (h)

Gene: CLB_RS13630
LOO:-3.595 | λ=1.0

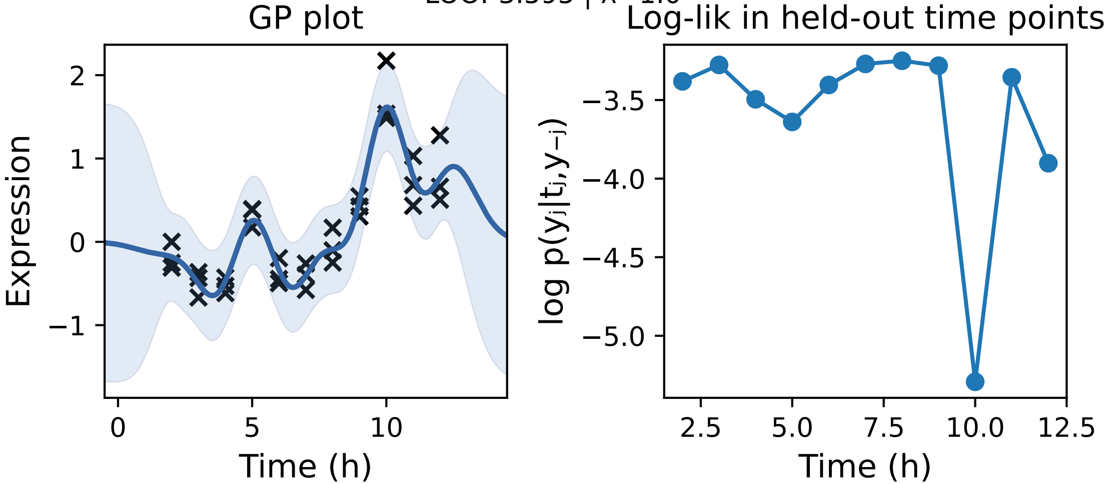

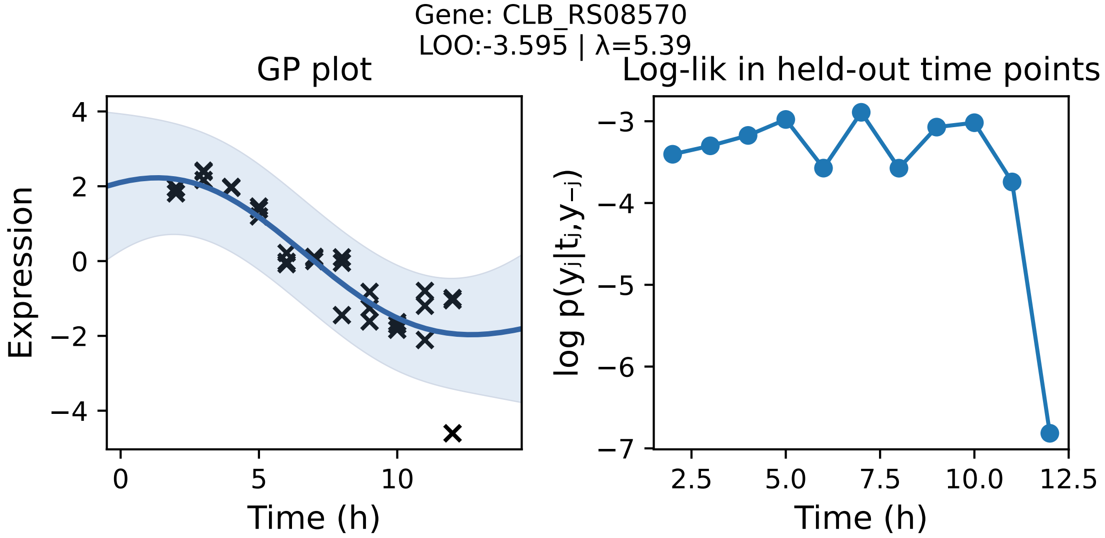

Gene: CLB_RS08570
LOO:-3.595 | λ=5.39
GP plot
Expression
Time (h)
Log-lik in held-out time points
log p(y_j|t_j,y_−j)
Time (h)
4
2
0
−2
−4
0
5
10
−3
−4
−5
−6
−7
2.5
5.0
7.5
10.0
12.5

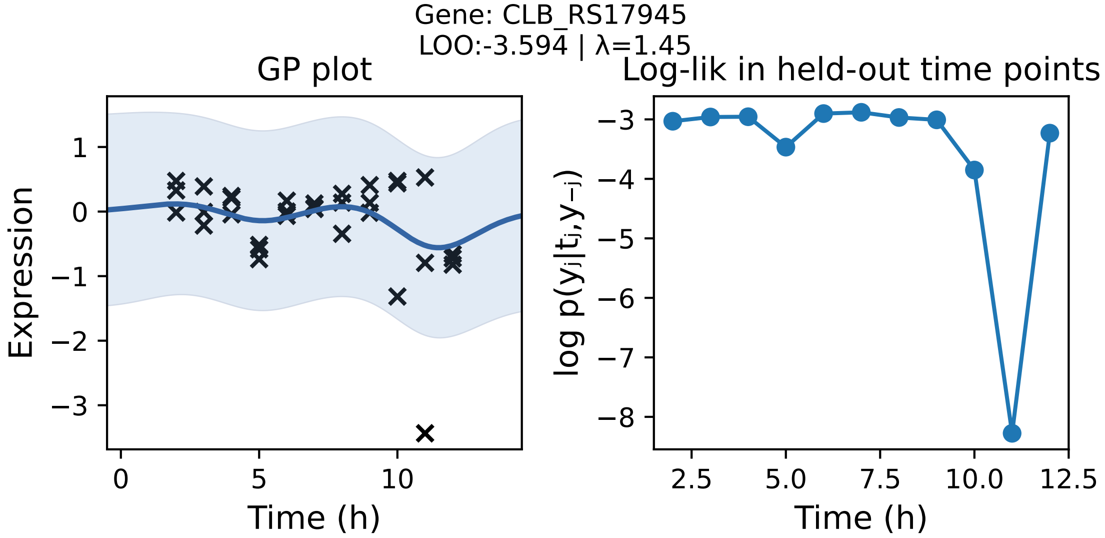

Gene: CLB_RS17945
LOO:-3.594 | λ=1.45
GP plot
Expression
1
0
−1
−2
−3
0
5
10
Time (h)
Log-lik in held-out time points
log p(y_j|t_j,y_−j)
−3
−4
−5
−6
−7
−8
2.5
5.0
7.5
10.0
12.5
Time (h)

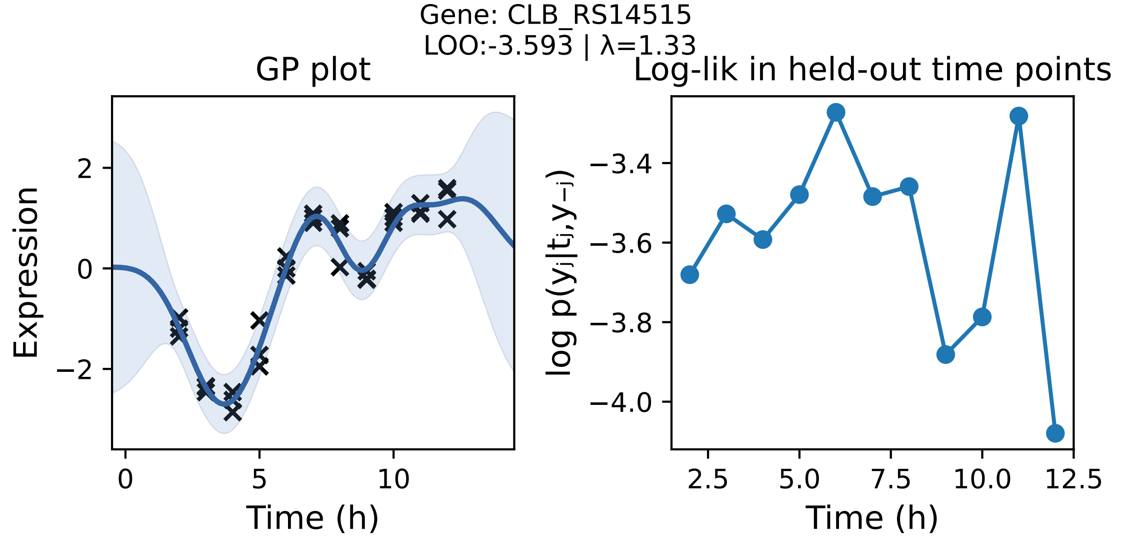

Gene: CLB_RS14515
LOO:-3.593 | λ=1.33
GP plot
Expression
Time (h)
2
0
−2
0
5
10
Log-lik in held-out time points
log p(y_j|t_j,y_−j)
−3.4
−3.6
−3.8
−4.0
2.5
5.0
7.5
10.0
12.5
Time (h)

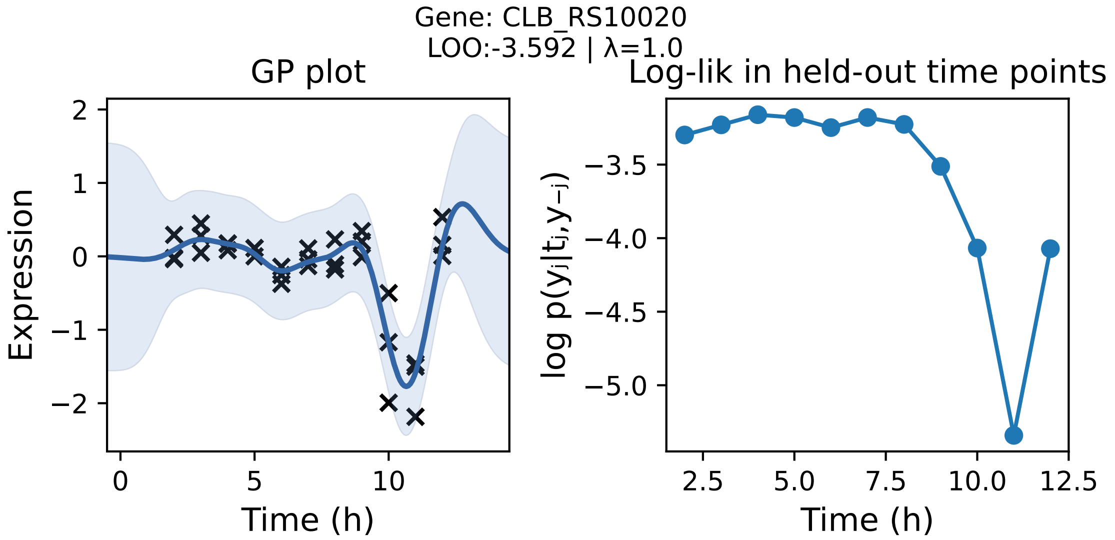
Gene: CLB_RS10020
LOO:-3.592 | λ=1.0
GP plot
Expression
Time (h)
2
1
0
−1
−2
0
5
10
Log-lik in held-out time points
log p(y_j|t_j,y_−j)
Time (h)
−3.5
−4.0
−4.5
−5.0
2.5
5.0
7.5
10.0
12.5

Gene: CLB_RS01140
LOO:-3.592 | λ=1.0

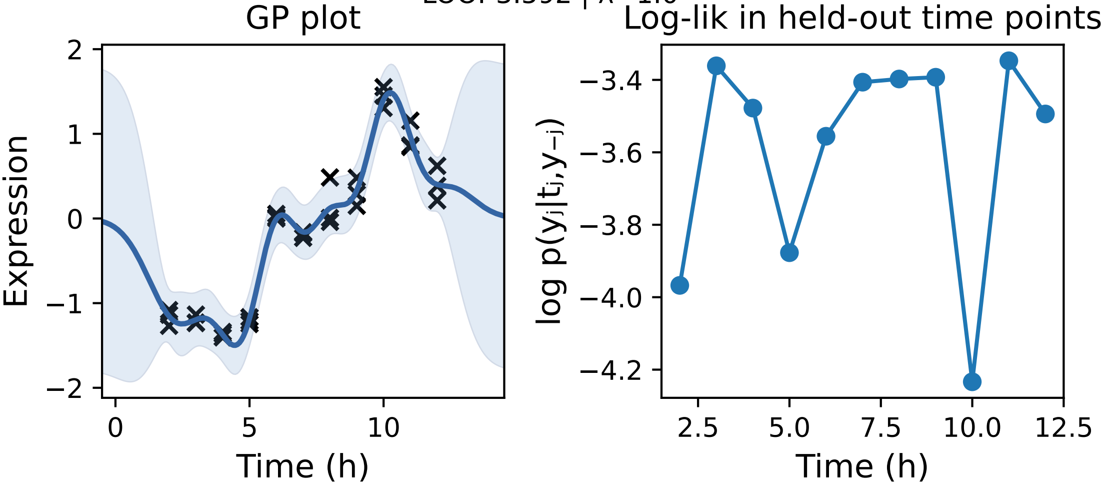

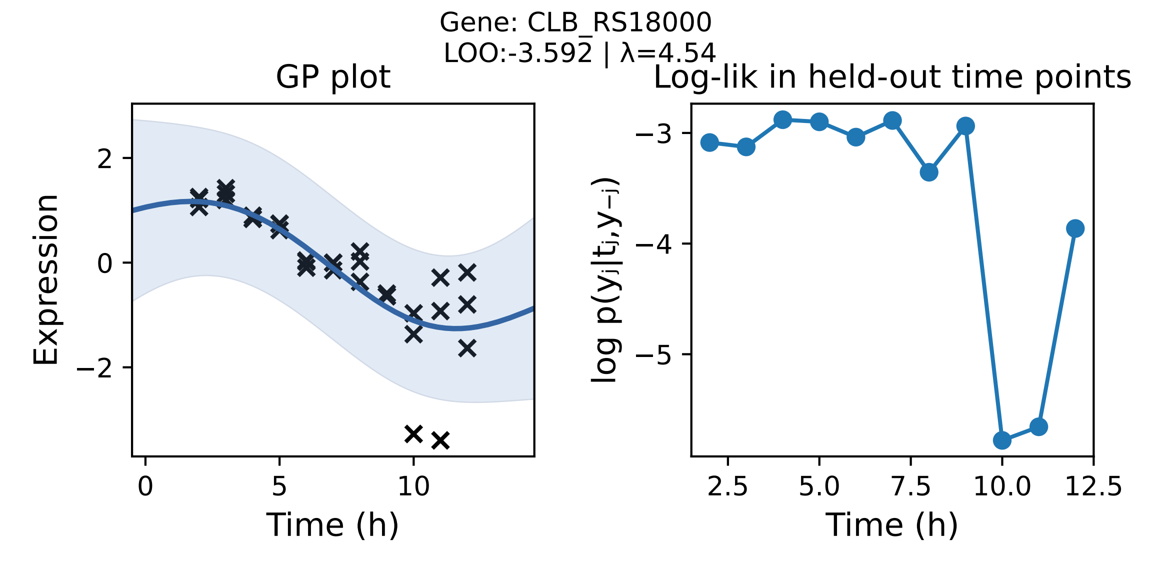
Gene: CLB_RS18000
LOO:-3.592 | λ=4.54
GP plot
Expression
Time (h)
Log-lik in held-out time points
log p(y_j|t_j,y_−j)
Time (h)

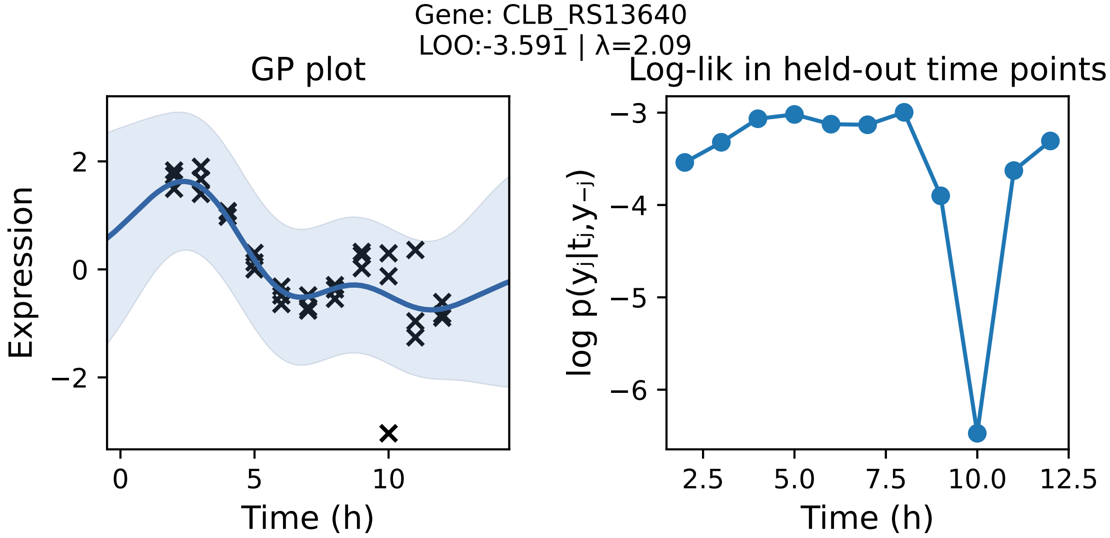
Gene: CLB_RS13640
LOO:-3.591 | λ=2.09
GP plot
Expression
Time (h)
Log-lik in held-out time points
log p(y_i|t_i,y_-j)
Time (h)

Gene: CLB_RS08940
LOO:-3.591 | λ=1.19

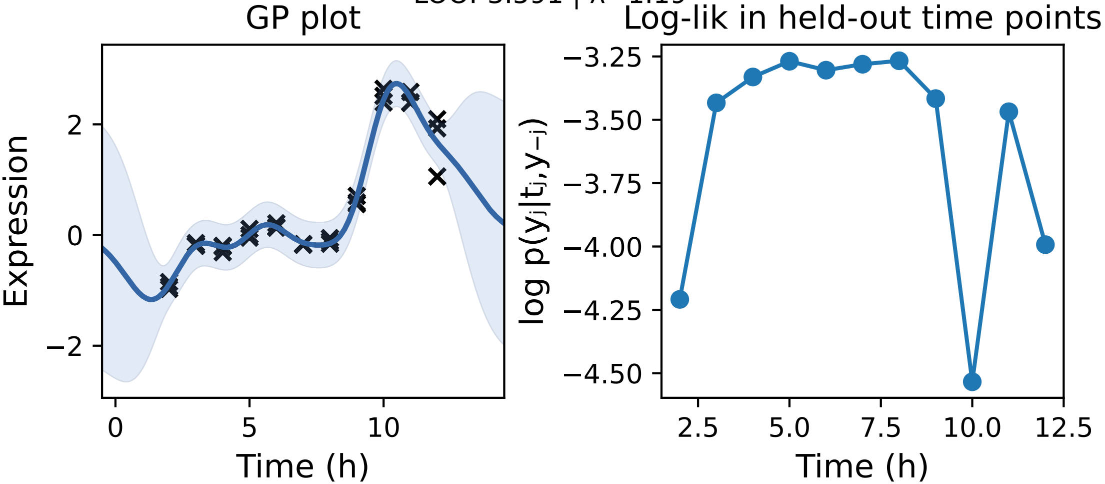

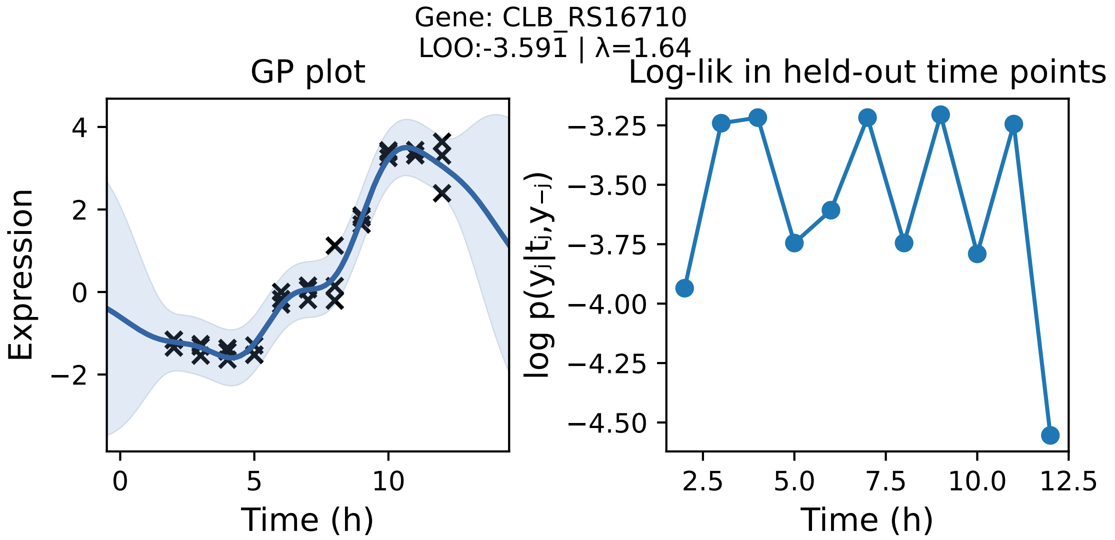
Gene: CLB_RS16710
LOO:-3.591 | λ=1.64
GP plot
Expression
Time (h)
Log-lik in held-out time points
log p(y_j|t_j,y_−j)
Time (h)

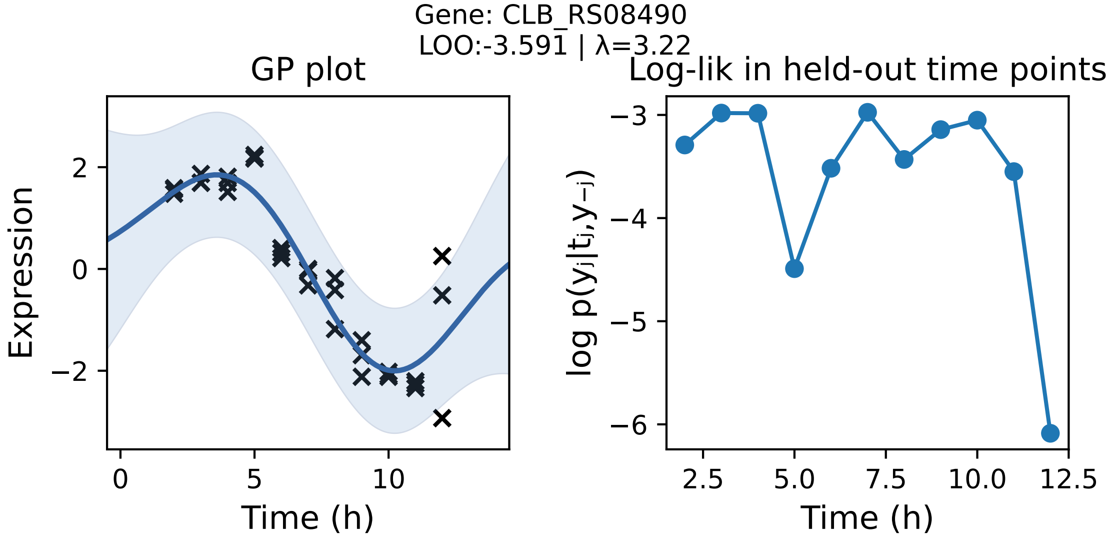

Gene: CLB_RS08490
LOO:-3.591 | λ=3.22
GP plot
Expression
Time (h)
Log-lik in held-out time points
log p(y_j|t_j,y_−j)
Time (h)

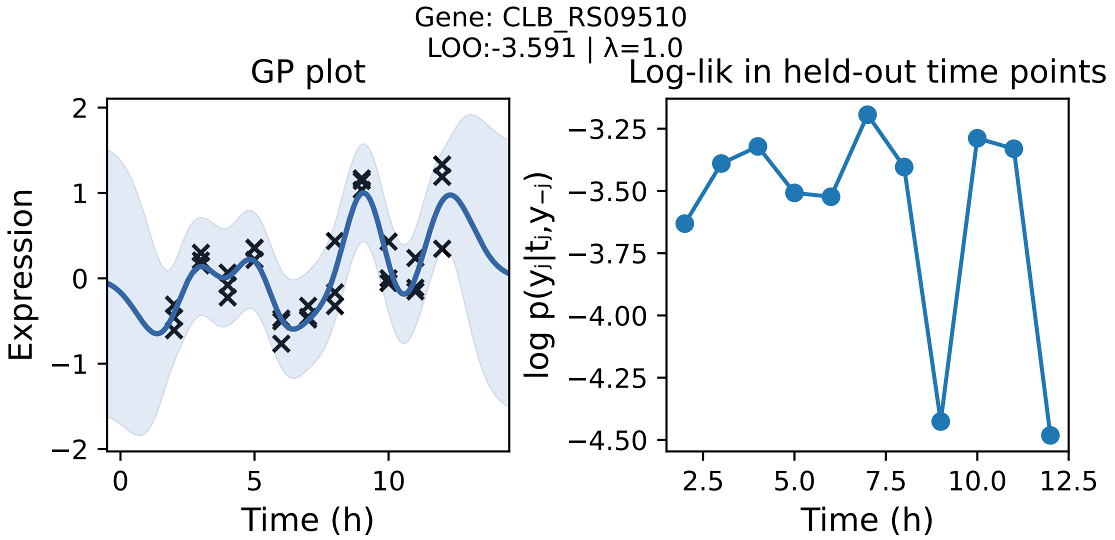
Gene: CLB_RS09510
LOO:-3.591 | λ=1.0
GP plot
Log-lik in held-out time points
Expression
Time (h)
log p(y_j|t_j,y_−j)
2
1
0
−1
−2
0
5
10
−3.25
−3.50
−3.75
−4.00
−4.25
−4.50
2.5
5.0
7.5
10.0
12.5

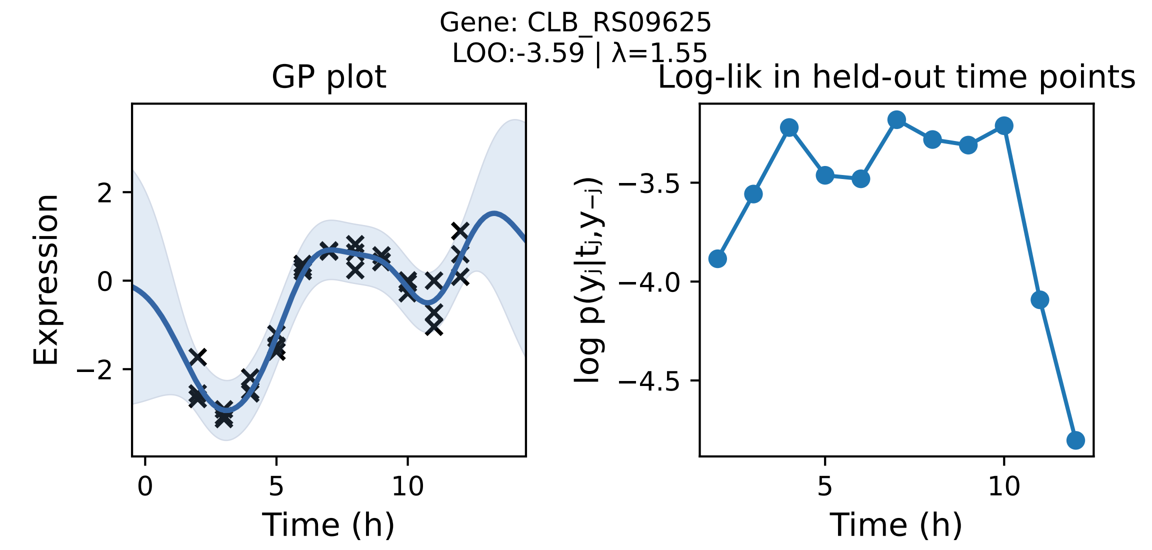
Gene: CLB_RS09625
LOO:-3.59 | λ=1.55
GP plot
Expression
2
0
−2
0
5
10
Time (h)
Log-lik in held-out time points
log p(y_j|t_j,y_−j)
−3.5
−4.0
−4.5
5
10
Time (h)

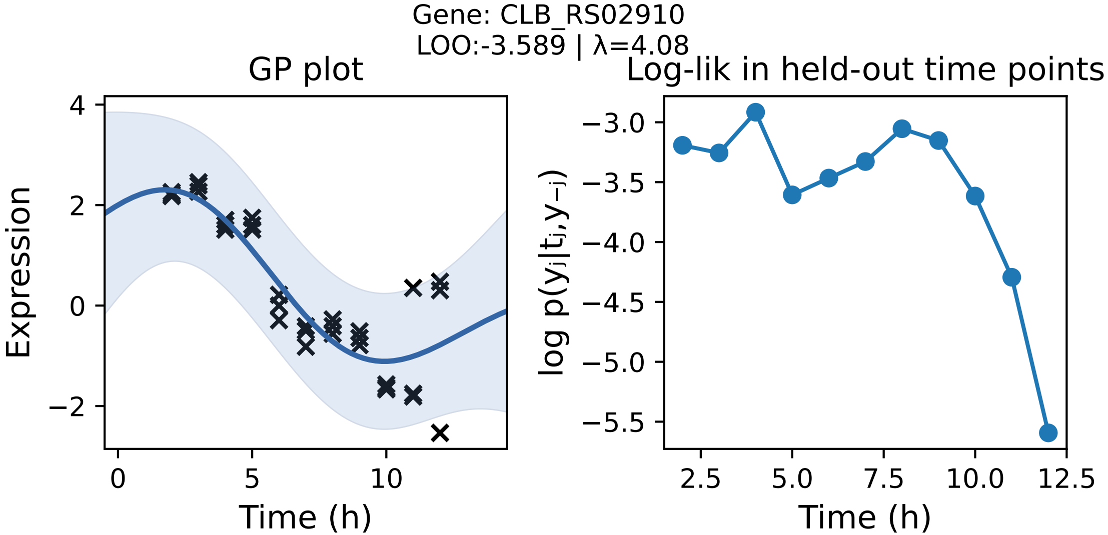

Gene: CLB_RS02910
LOO:-3.589 | λ=4.08
GP plot
Expression
Time (h)
4
2
0
−2
0
5
10
Log-lik in held-out time points
log p(y_j|t_j,y_−j)
Time (h)
−3.0
−3.5
−4.0
−4.5
−5.0
−5.5
2.5
5.0
7.5
10.0
12.5

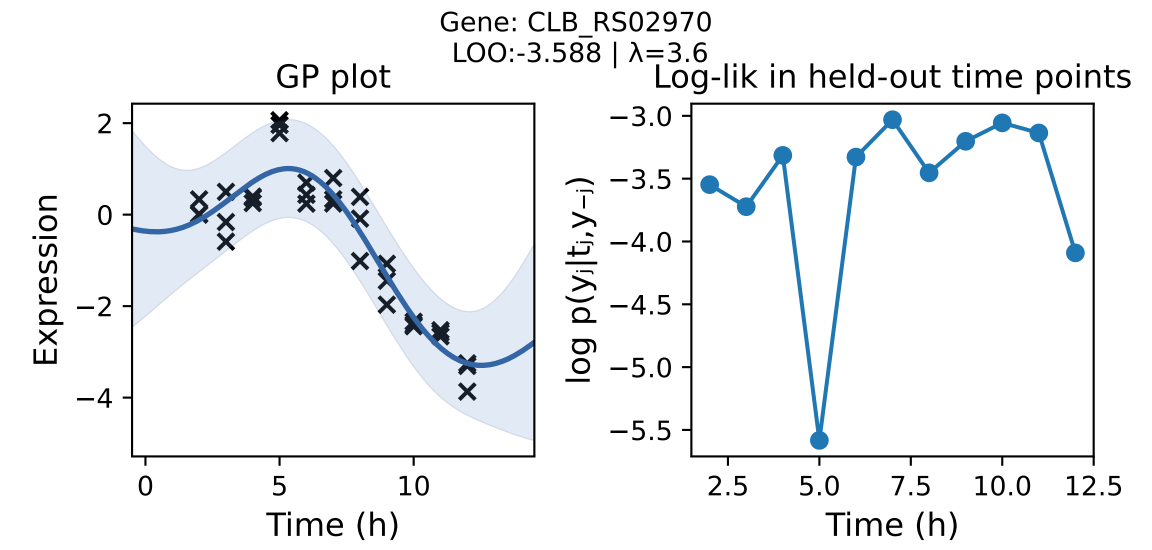

Gene: CLB_RS02970
LOO:-3.588 | λ=3.6
GP plot
Expression
Time (h)
Log-lik in held-out time points
log p(y_j|t_j,y_−j)
Time (h)

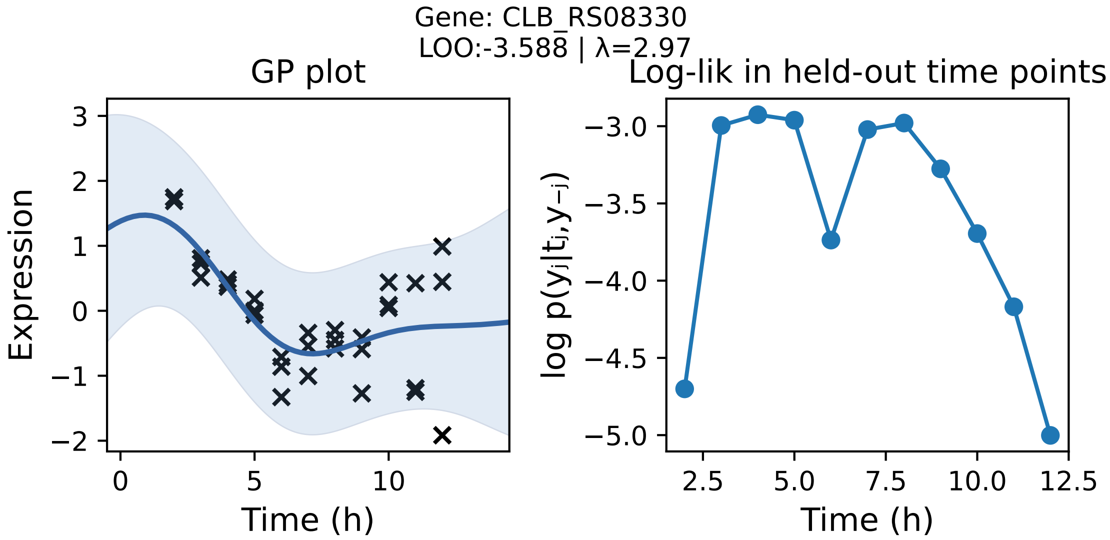

Gene: CLB_RS08330
LOO:-3.588 | λ=2.97
GP plot
Expression
Time (h)
Log-lik in held-out time points
log p(y_j|t_j,y_−j)
Time (h)

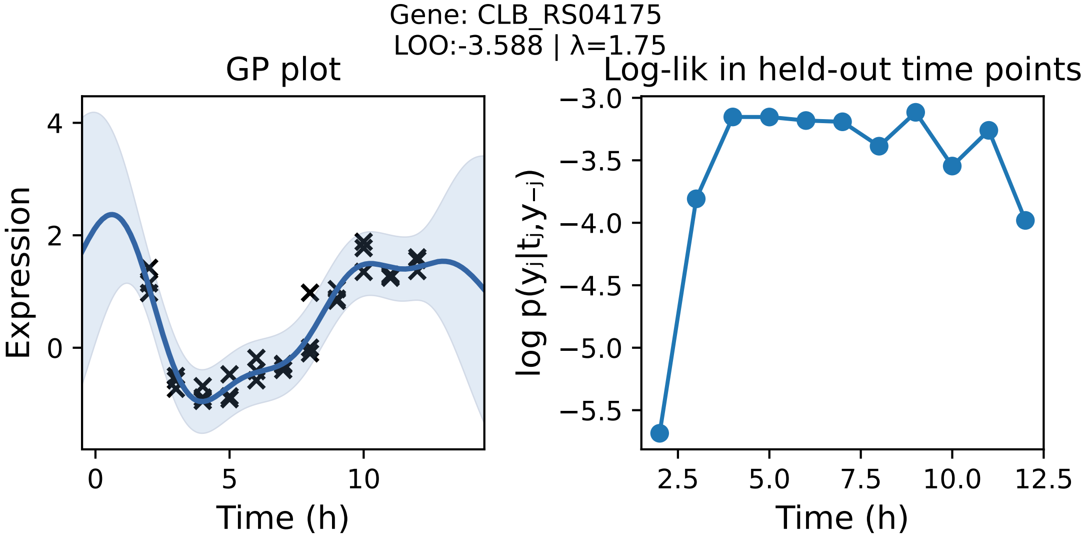

Gene: CLB_RS04175
LOO:-3.588 | λ=1.75
GP plot
Expression
Time (h)
0
2
4
0
5
10
Log-lik in held-out time points
log p(y_j|t_j,y_−j)
Time (h)
−3.0
−3.5
−4.0
−4.5
−5.0
−5.5
2.5
5.0
7.5
10.0
12.5

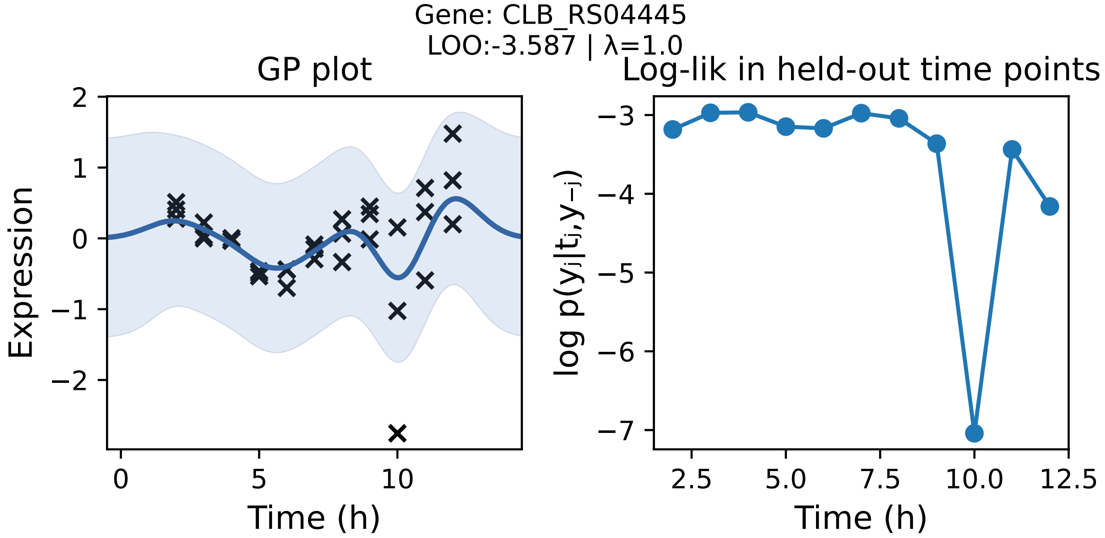

Gene: CLB_RS04445
LOO:-3.587 | λ=1.0
GP plot
Expression
Time (h)
2
1
0
−1
−2
0
5
10
Log-lik in held-out time points
log p(y_j|t_j,y_−j)
Time (h)
−3
−4
−5
−6
−7
2.5
5.0
7.5
10.0
12.5

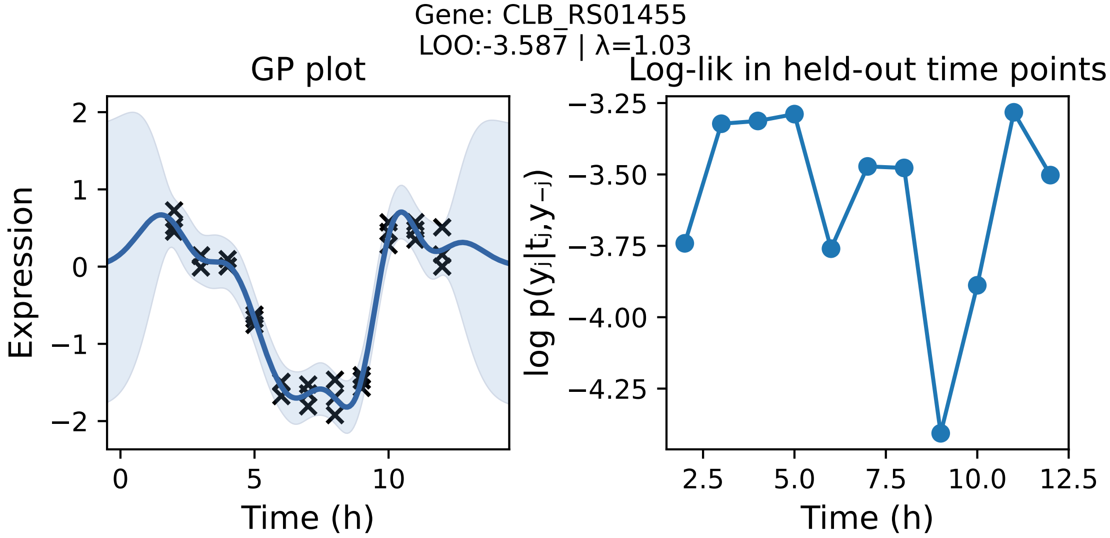
Gene: CLB_RS01455
LOO:-3.587 | λ=1.03
GP plot
Log-lik in held-out time points
Expression
Time (h)
log p(y_j|t_j,y_−j)
Time (h)

Gene: CLB_RS11275
LOO:-3.585 | λ=1.0

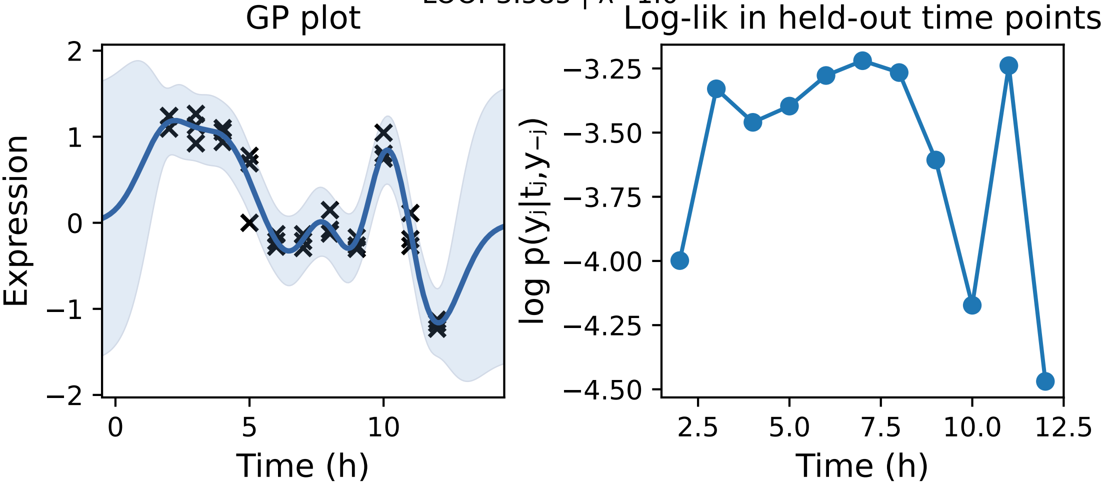

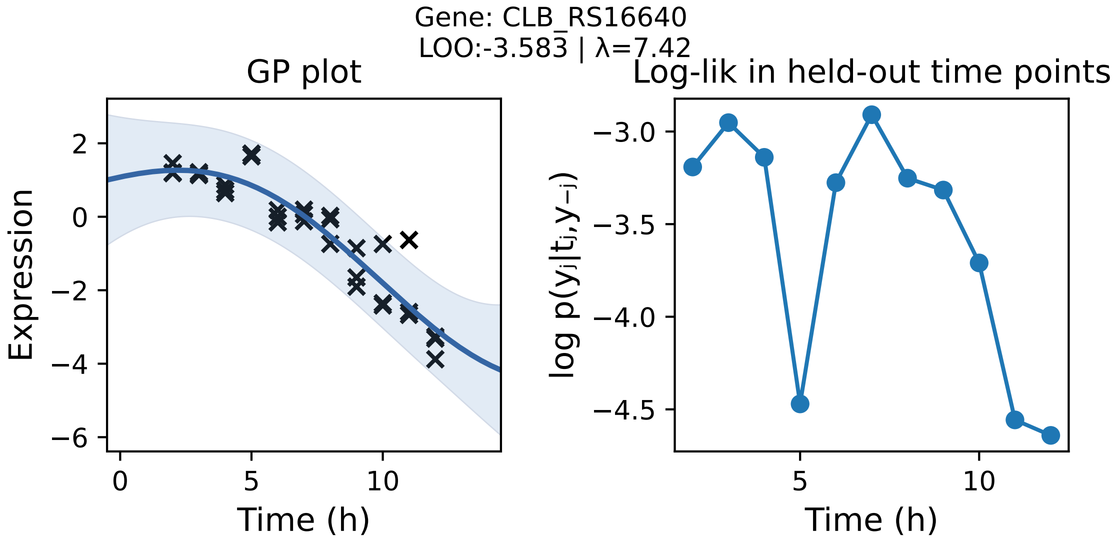

Gene: CLB_RS16640
LOO:-3.583 | λ=7.42
GP plot
Expression
Time (h)
Log-lik in held-out time points
log p(y_j|t_j,y_−j)
Time (h)

Gene: CLB_RS15565
LOO:-3.582 | λ=1.0

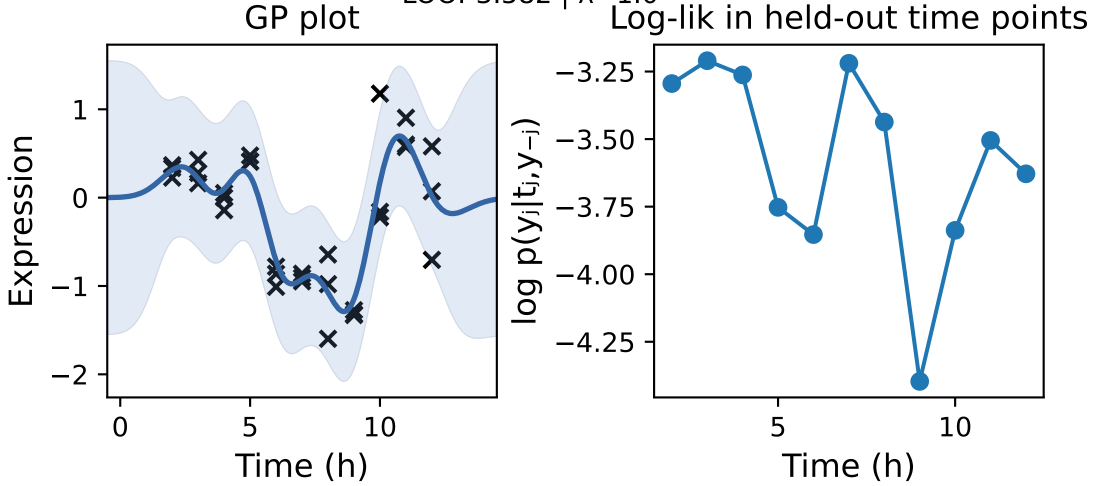

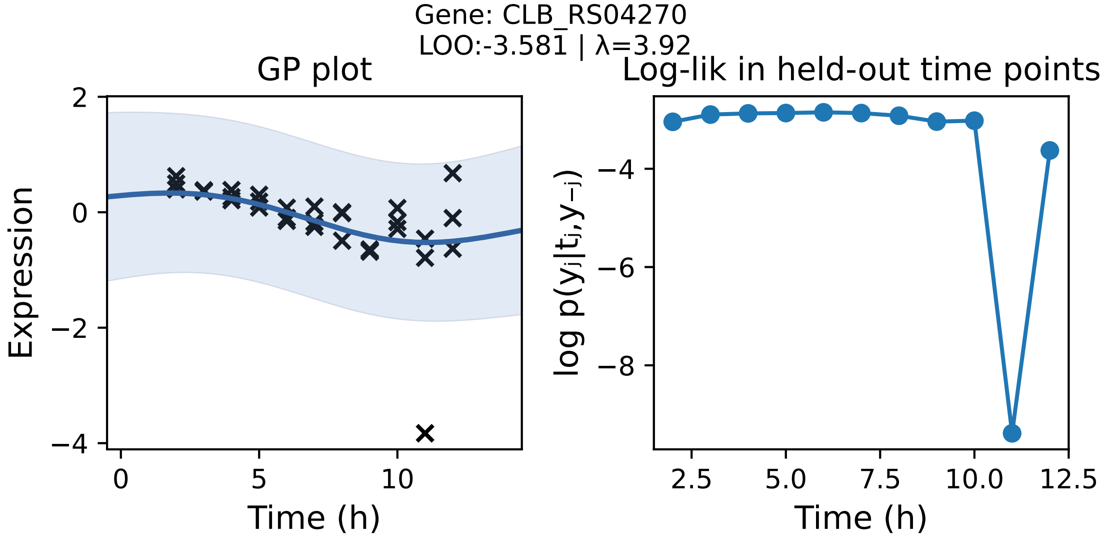

Gene: CLB_RS04270
LOO:-3.581 | λ=3.92
GP plot
Expression
Time (h)
2
0
−2
−4
0
5
10
Log-lik in held-out time points
log p(y_j|t_j,y_−j)
Time (h)
−4
−6
−8
2.5
5.0
7.5
10.0
12.5

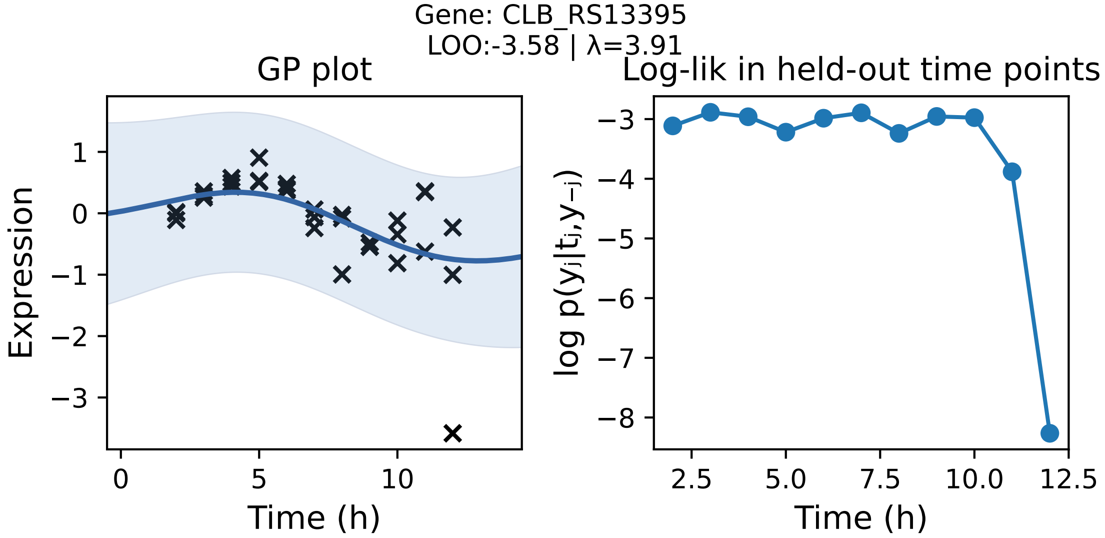
Gene: CLB_RS13395
LOO:-3.58 | λ=3.91
GP plot
Expression
Time (h)
Log-lik in held-out time points
log p(yj|tj,y−j)
Time (h)

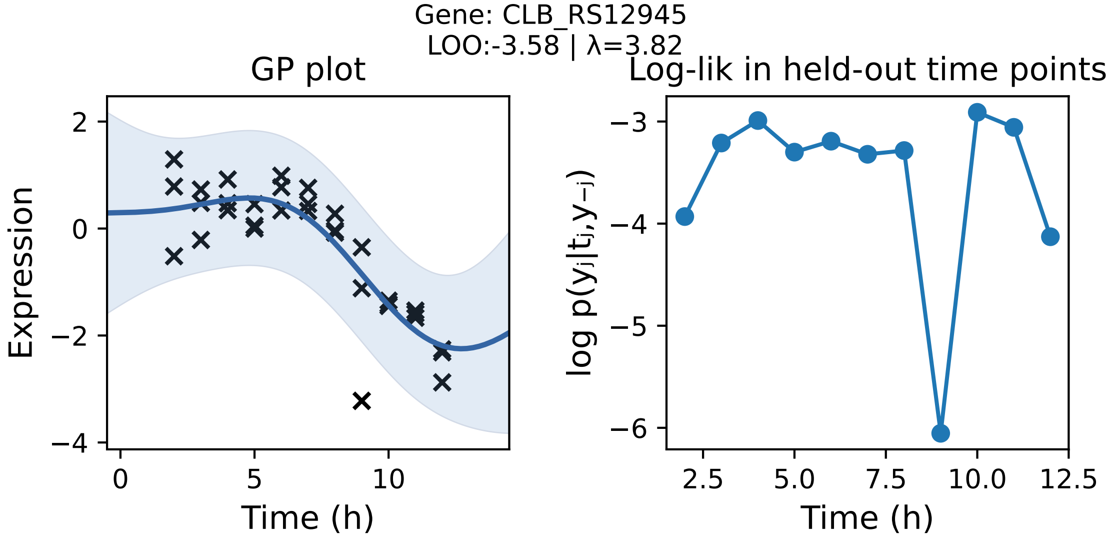

Gene: CLB_RS12945
LOO:-3.58 | λ=3.82
GP plot
Expression
Time (h)
2
0
−2
−4
0
5
10
Log-lik in held-out time points
log p(y_j|t_j,y_−j)
Time (h)
−3
−4
−5
−6
2.5
5.0
7.5
10.0
12.5

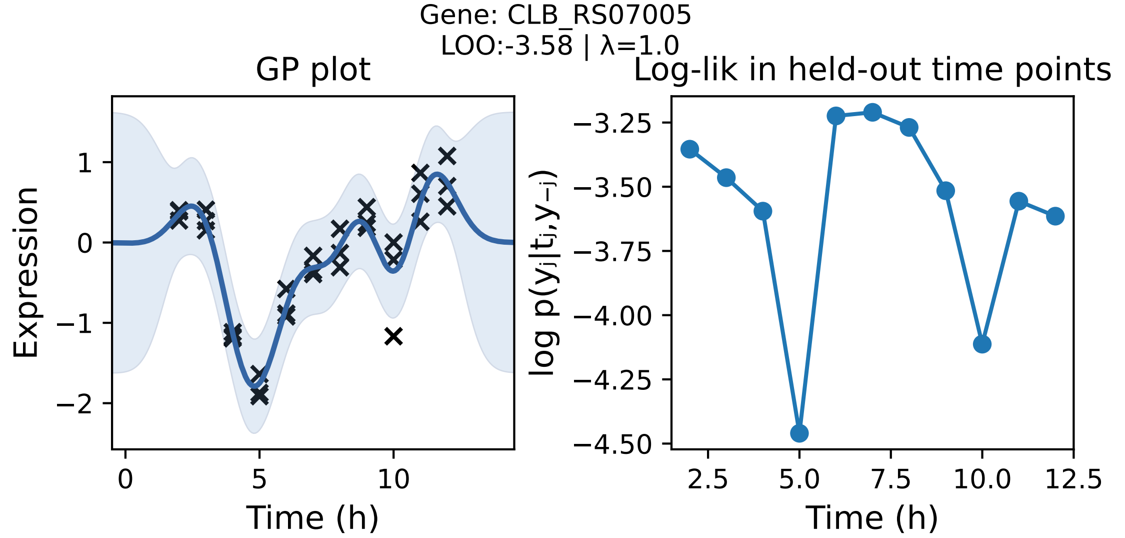

Gene: CLB_RS07005
LOO:-3.58 | λ=1.0
GP plot
Expression
Time (h)
1
0
−1
−2
0
5
10
Log-lik in held-out time points
log p(y_j|t_j,y_−j)
Time (h)
−3.25
−3.50
−3.75
−4.00
−4.25
−4.50
2.5
5.0
7.5
10.0
12.5

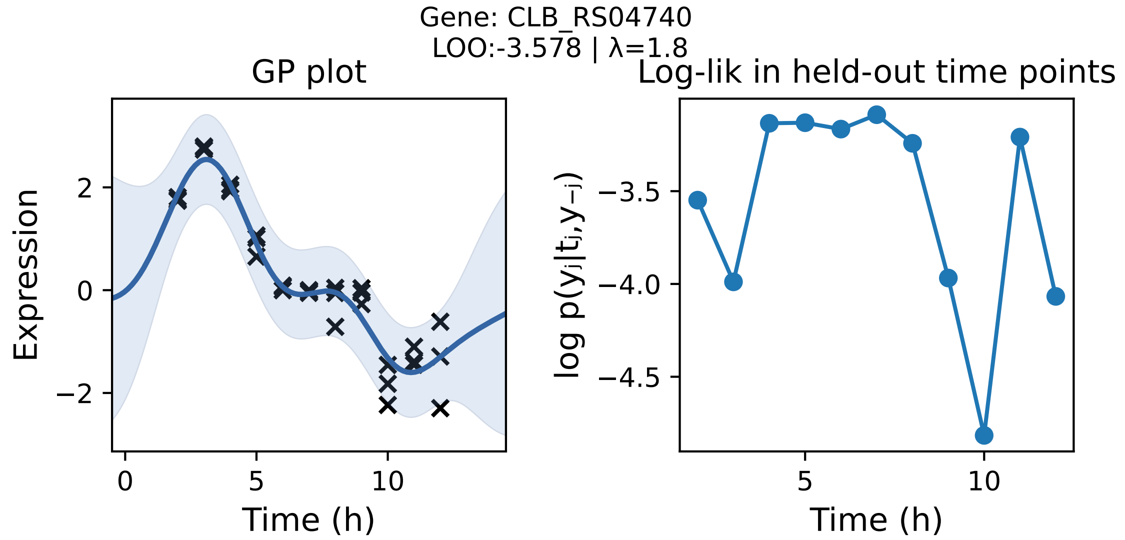

Gene: CLB_RS04740
LOO:-3.578 | λ=1.8
GP plot
Expression
Time (h)
2
0
−2
0
5
10
Log-lik in held-out time points
log p(y_j|t_j,y_−j)
−3.5
−4.0
−4.5
5
10
Time (h)

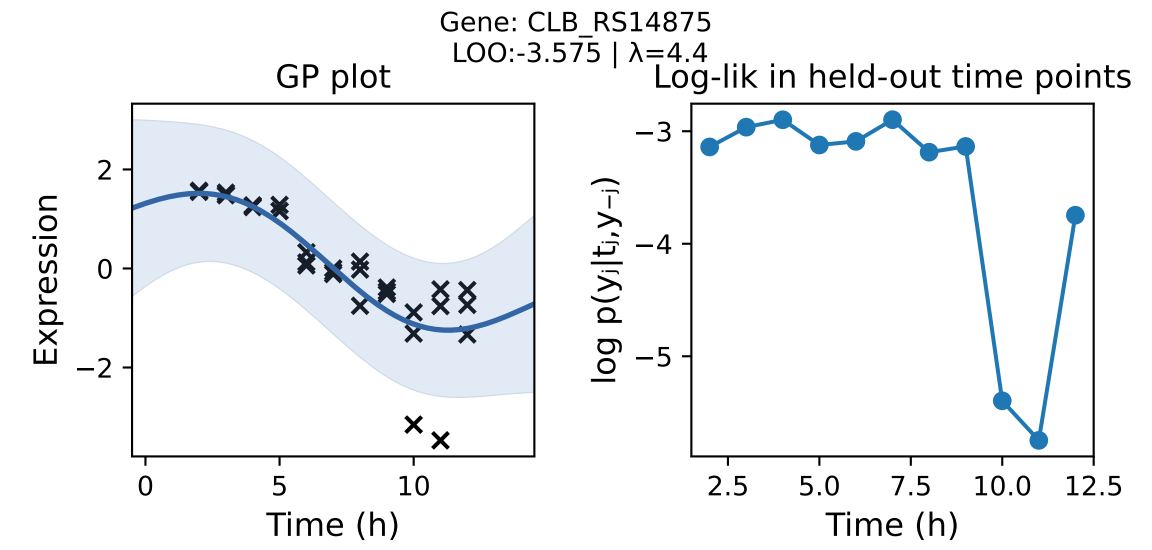
Gene: CLB_RS14875
LOO:-3.575 | λ=4.4
GP plot
Expression
Time (h)
Log-lik in held-out time points
log p(y_j|t_j,y_−j)
Time (h)

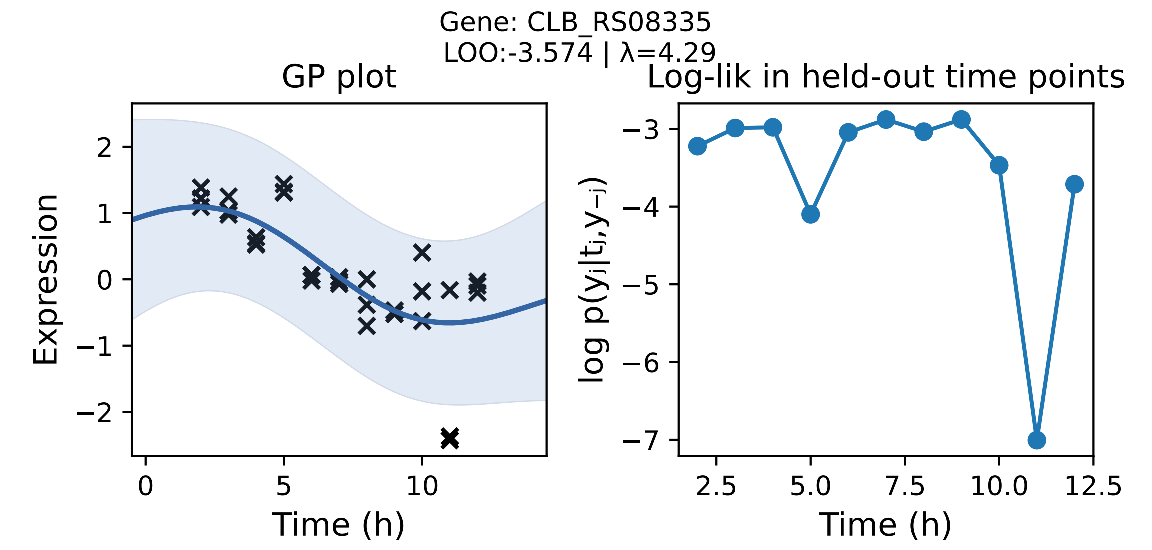

Gene: CLB_RS08335
LOO:-3.574 | λ=4.29
GP plot
Expression
Time (h)
2
1
0
−1
−2
0
5
10
Log-lik in held-out time points
log p(yⱼ|tⱼ,y₋ⱼ)
−3
−4
−5
−6
−7
2.5
5.0
7.5
10.0
12.5
Time (h)

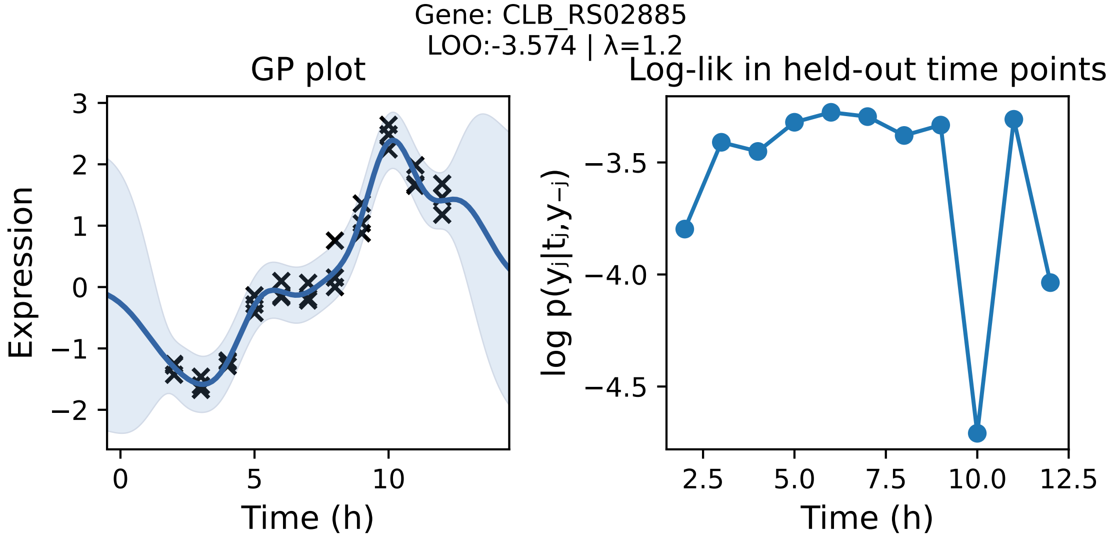

Gene: CLB_RS02885
LOO:-3.574 | λ=1.2
GP plot
Expression
Time (h)
Log-lik in held-out time points
log p(y_j|t_j,y_−j)
Time (h)

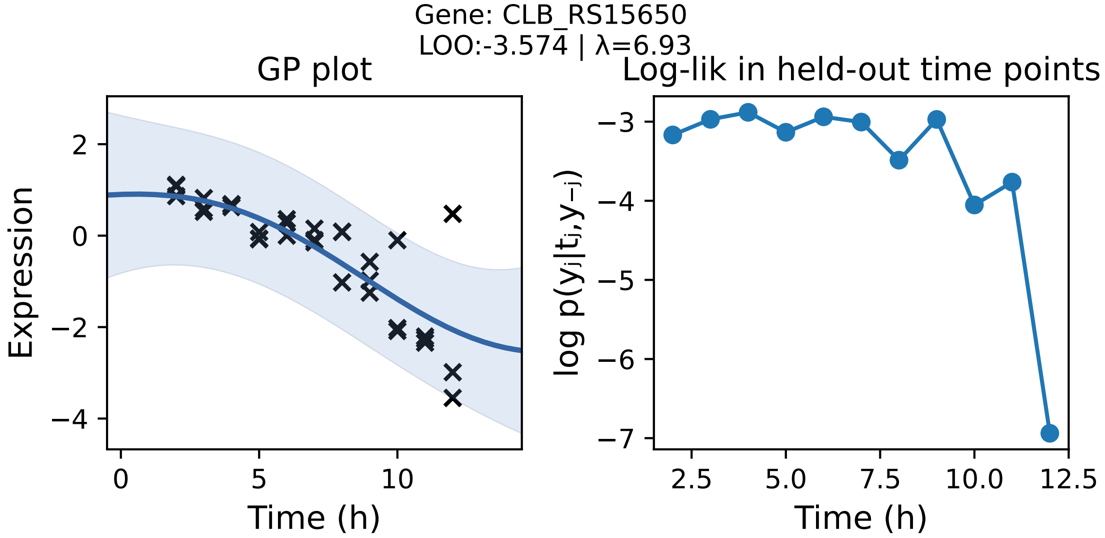

Gene: CLB_RS15650
LOO:-3.574 | λ=6.93
GP plot
Expression
Time (h)
Log-lik in held-out time points
log p(y_j|t_j,y_−j)
Time (h)

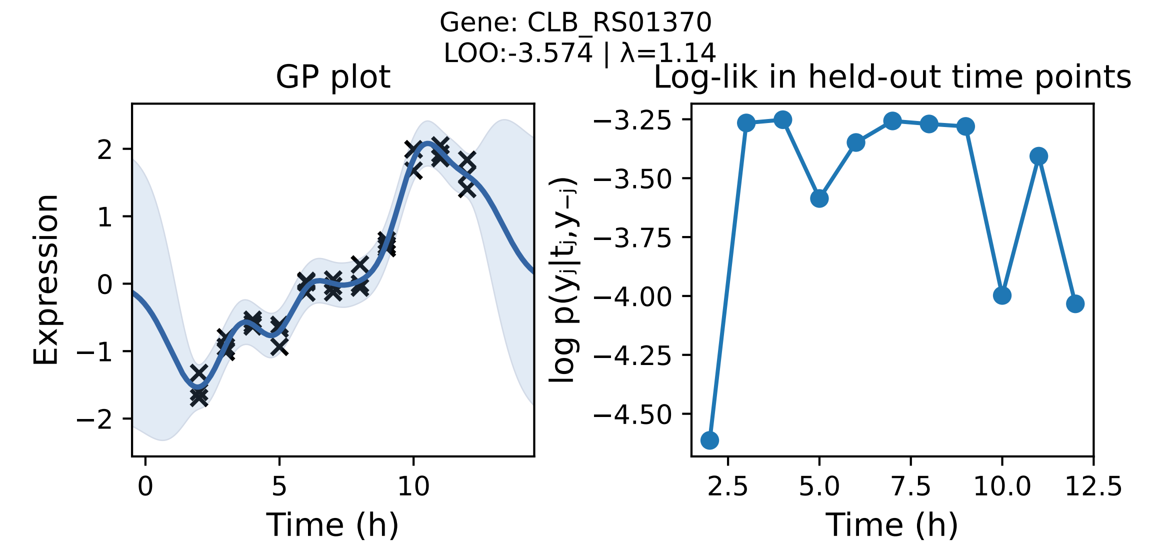

Gene: CLB_RS01370
LOO:-3.574 | λ=1.14
GP plot
Expression
Time (h)
Log-lik in held-out time points
log p(y_j|t_j,y_−j)
Time (h)

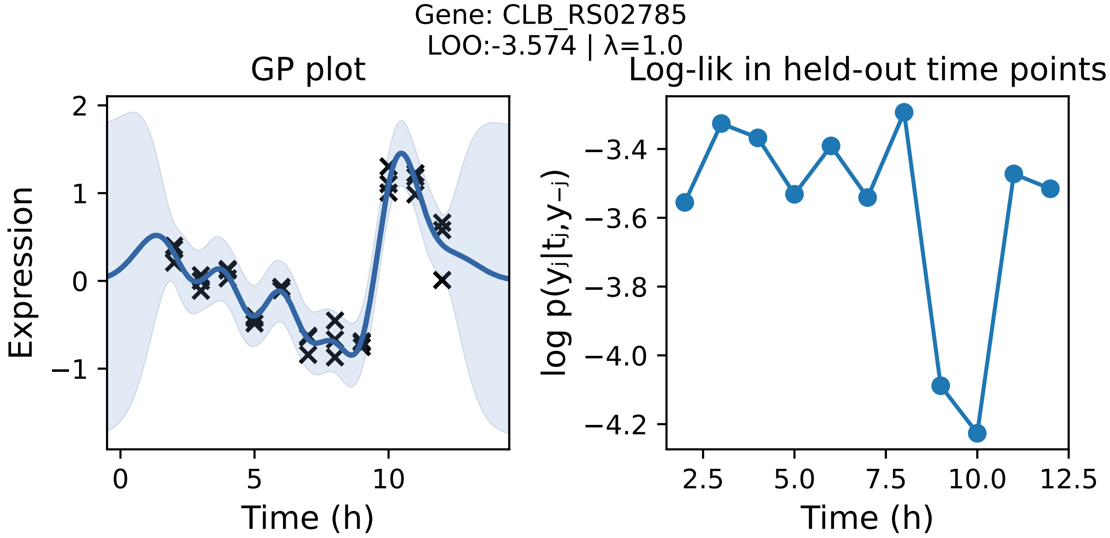

Gene: CLB_RS02785
LOO:-3.574 | λ=1.0
GP plot
Expression
Time (h)
2
1
0
−1
0
5
10
Log-lik in held-out time points
log p(yj|tj,y−j)
Time (h)
−3.4
−3.6
−3.8
−4.0
−4.2
2.5
5.0
7.5
10.0
12.5

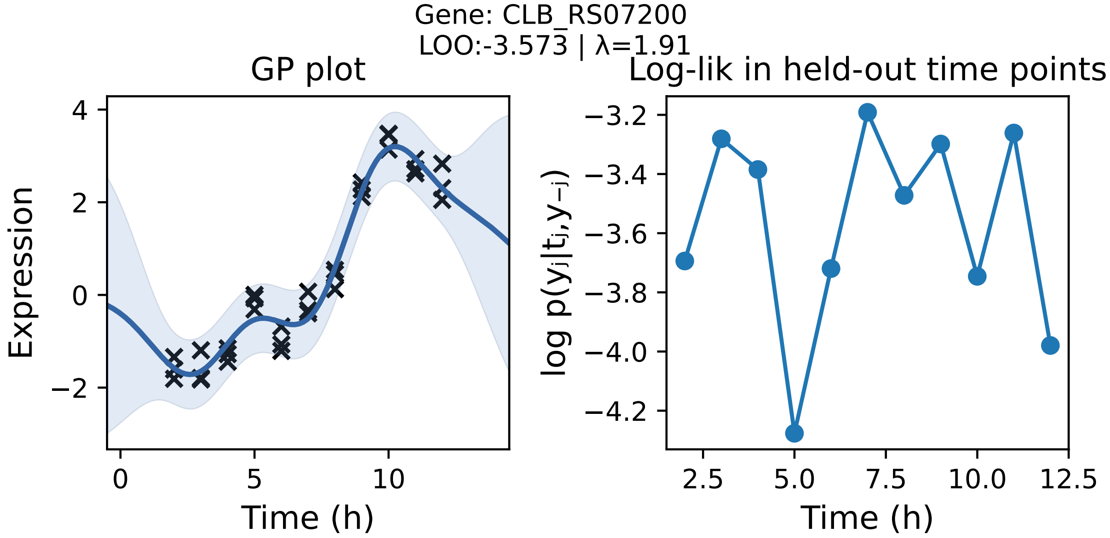

Gene: CLB_RS07200
LOO:-3.573 | λ=1.91
GP plot
Expression
Time (h)
Log-lik in held-out time points
log p(y_j|t_j,y_−j)
Time (h)

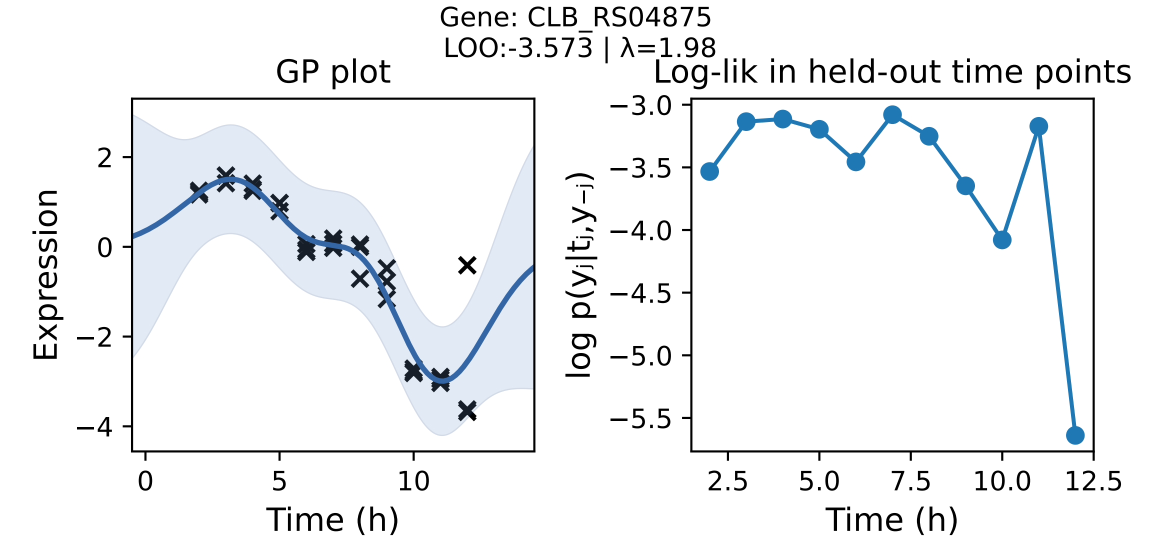

Gene: CLB_RS04875
LOO:-3.573 | λ=1.98
GP plot
Log-lik in held-out time points
Expression
2
0
−2
−4
0
5
10
Time (h)
log p(yⱼ|tⱼ,y₋ⱼ)
−3.0
−3.5
−4.0
−4.5
−5.0
−5.5
2.5
5.0
7.5
10.0
12.5
Time (h)

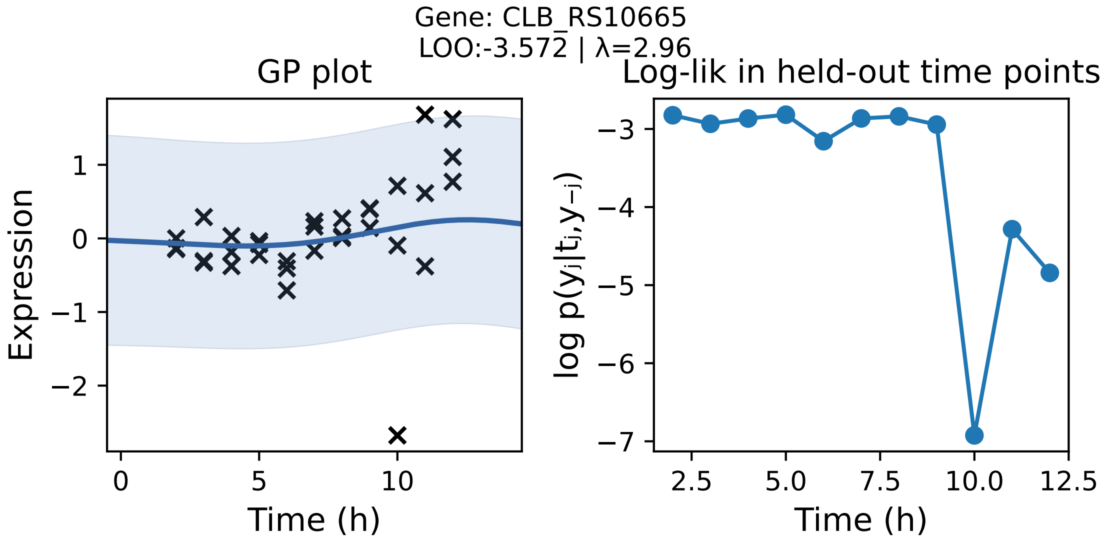

Gene: CLB_RS10665
LOO:-3.572 | λ=2.96
GP plot
Log-lik in held-out time points
Expression
Time (h)
log p(y_j|t_j,y_−j)
Time (h)
1
0
−1
−2
0
5
10
−3
−4
−5
−6
−7
2.5
5.0
7.5
10.0
12.5

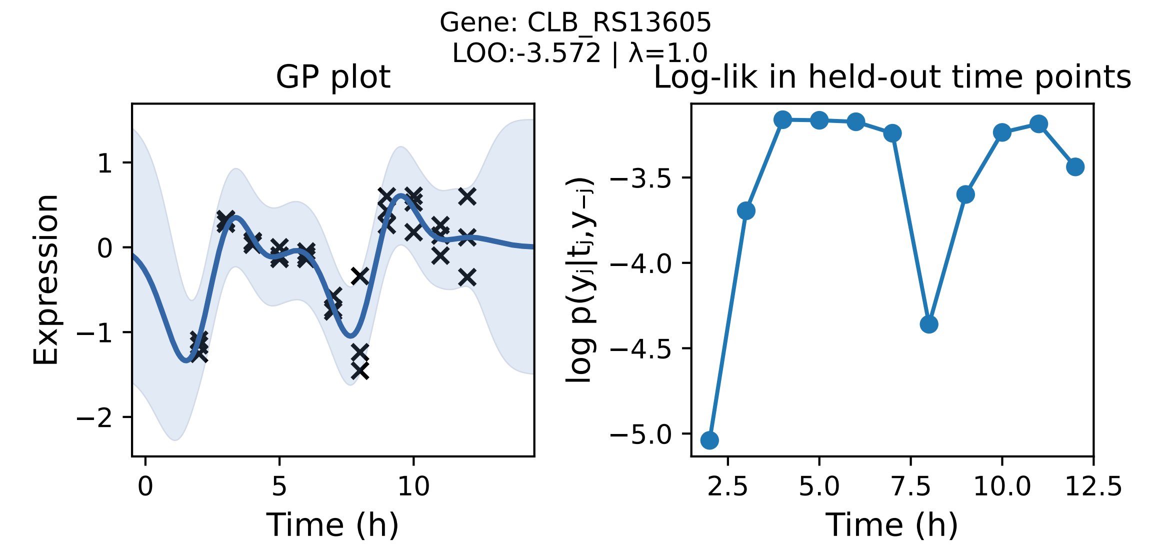

Gene: CLB_RS13605
LOO:-3.572 | λ=1.0
GP plot
Expression
Time (h)
Log-lik in held-out time points
log p(y_j|t_j,y_−j)
Time (h)

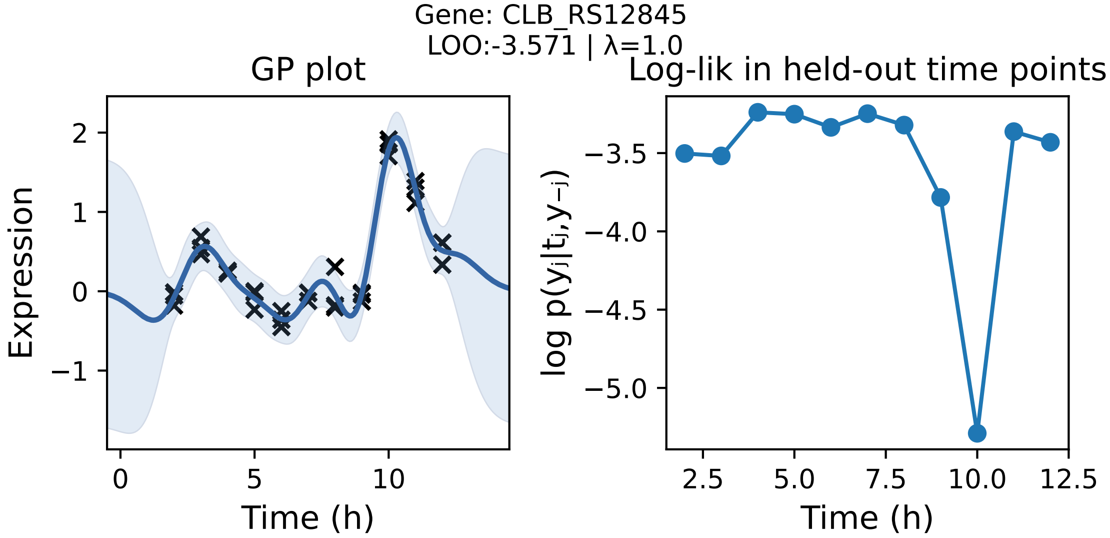

Gene: CLB_RS12845
LOO:-3.571 | λ=1.0
GP plot
Expression
Time (h)
Log-lik in held-out time points
log p(y_j|t_j,y_{-j})
Time (h)

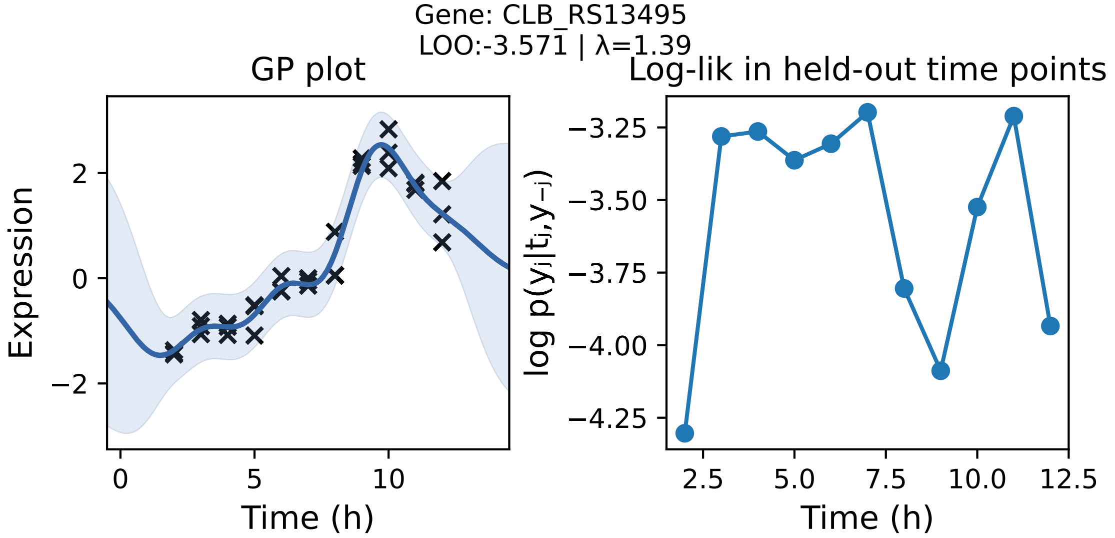

Gene: CLB_RS13495
LOO:-3.571 | λ=1.39
GP plot
Expression
Time (h)
Log-lik in held-out time points
log p(y_j|t_j,y_−j)
Time (h)

Gene: CLB_RS18095
LOO:-3.57 | λ=3.31

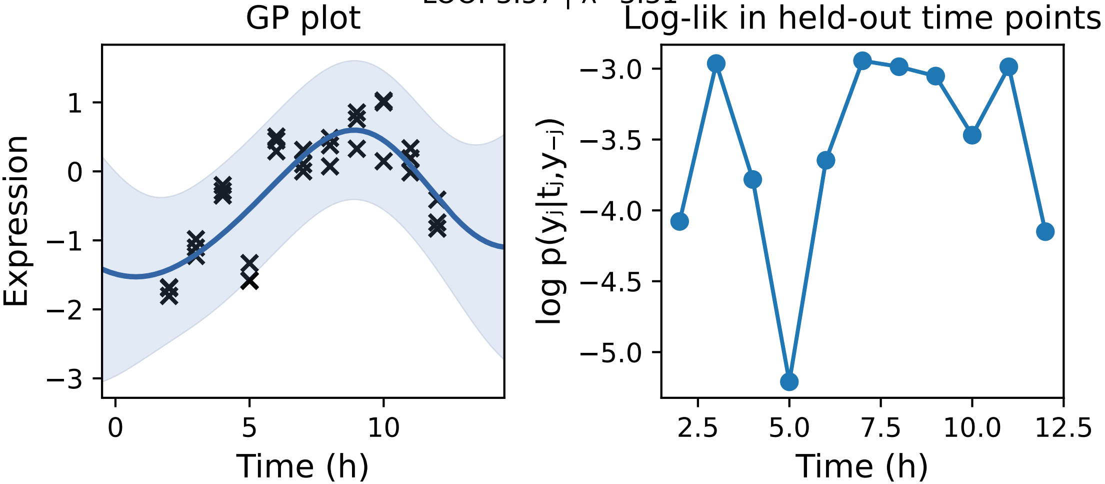

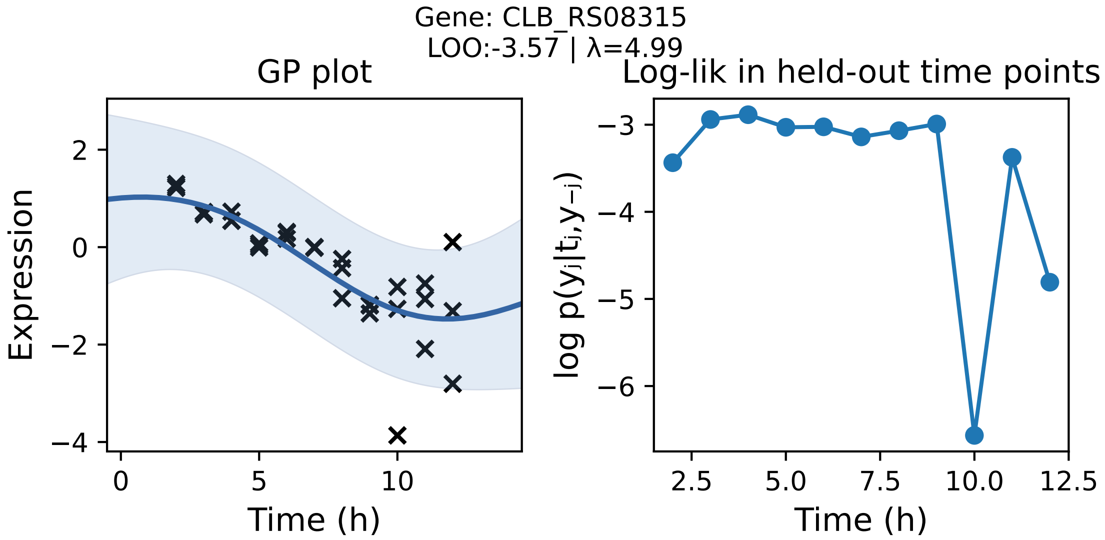
Gene: CLB_RS08315
LOO:-3.57 | λ=4.99
GP plot
Expression
Time (h)
Log-lik in held-out time points
log p(y_j|t_j,y_−j)
Time (h)

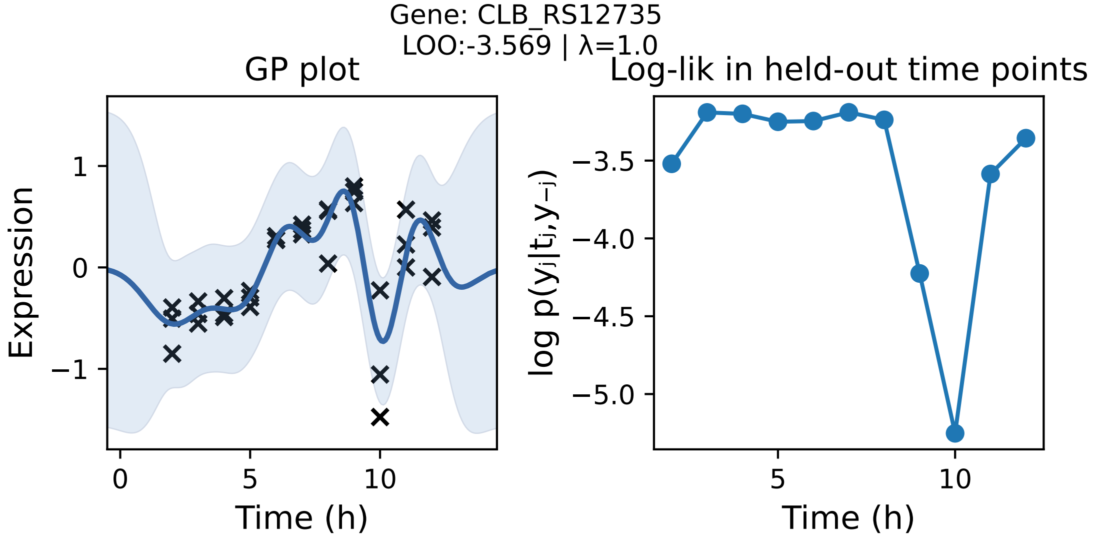
Gene: CLB_RS12735
LOO:-3.569 | λ=1.0
GP plot
Expression
Time (h)
Log-lik in held-out time points
log p(y_j|t_j,y_−j)
Time (h)

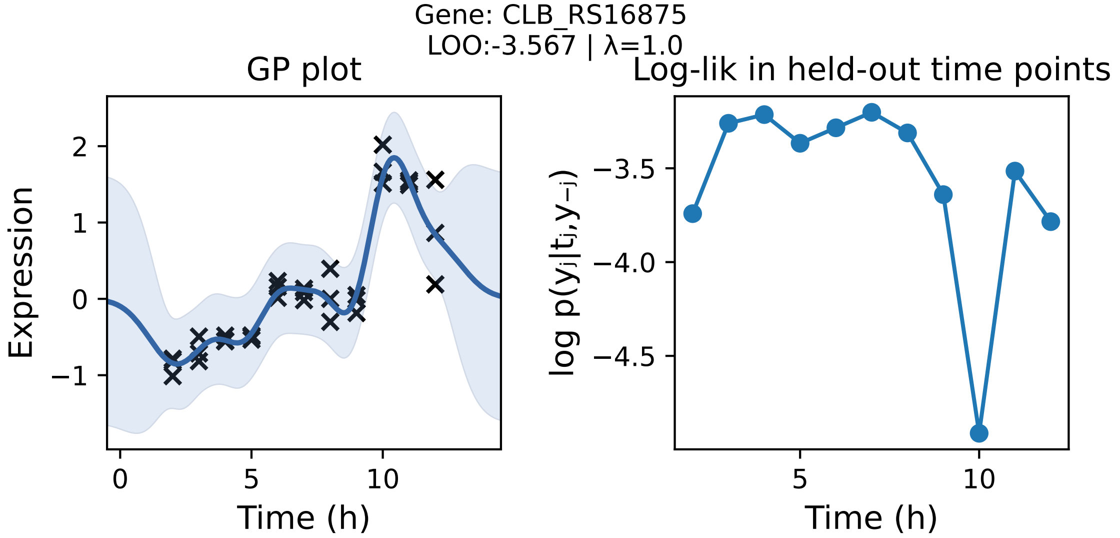

Gene: CLB_RS16875
LOO:-3.567 | λ=1.0
GP plot
Expression
Time (h)
Log-lik in held-out time points
log p(y_j|t_j,y_−j)
Time (h)

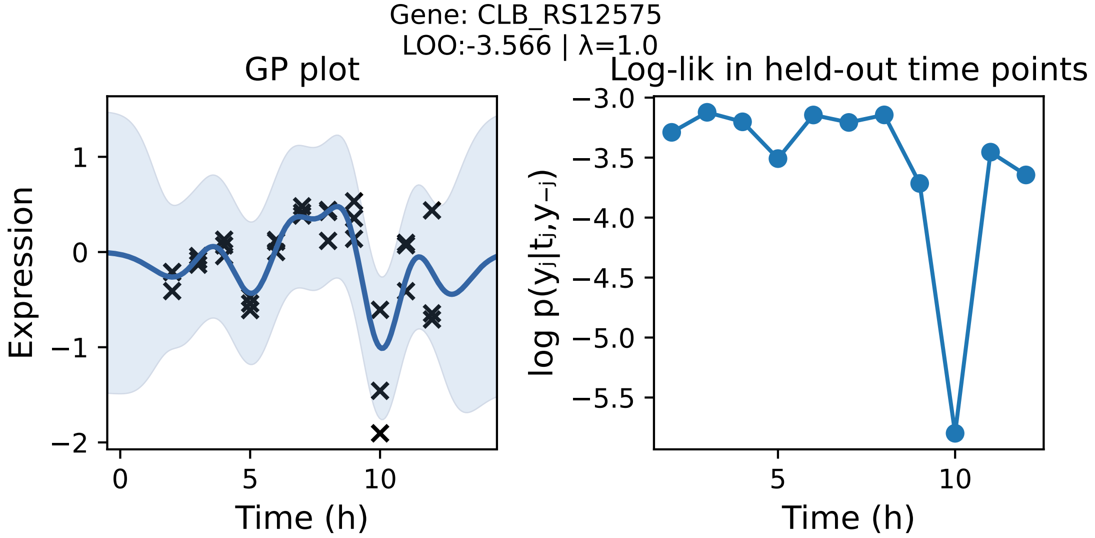
Gene: CLB_RS12575
LOO:-3.566 | λ=1.0
GP plot
Expression
Time (h)
1
0
−1
−2
0
5
10
Log-lik in held-out time points
log p(yⱼ|tⱼ,y₋ⱼ)
−3.0
−3.5
−4.0
−4.5
−5.0
−5.5
5
10
Time (h)

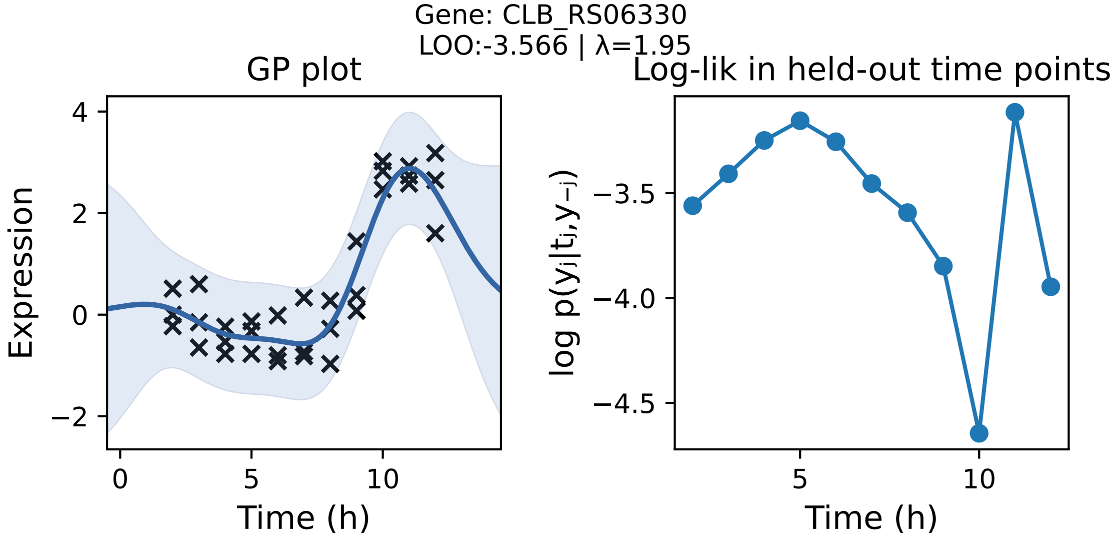
Gene: CLB_RS06330
LOO:-3.566 | λ=1.95
GP plot
Expression
Time (h)
4
2
0
−2
0
5
10
Log-lik in held-out time points
log p(y_j|t_j,y_−j)
−3.5
−4.0
−4.5
5
10
Time (h)

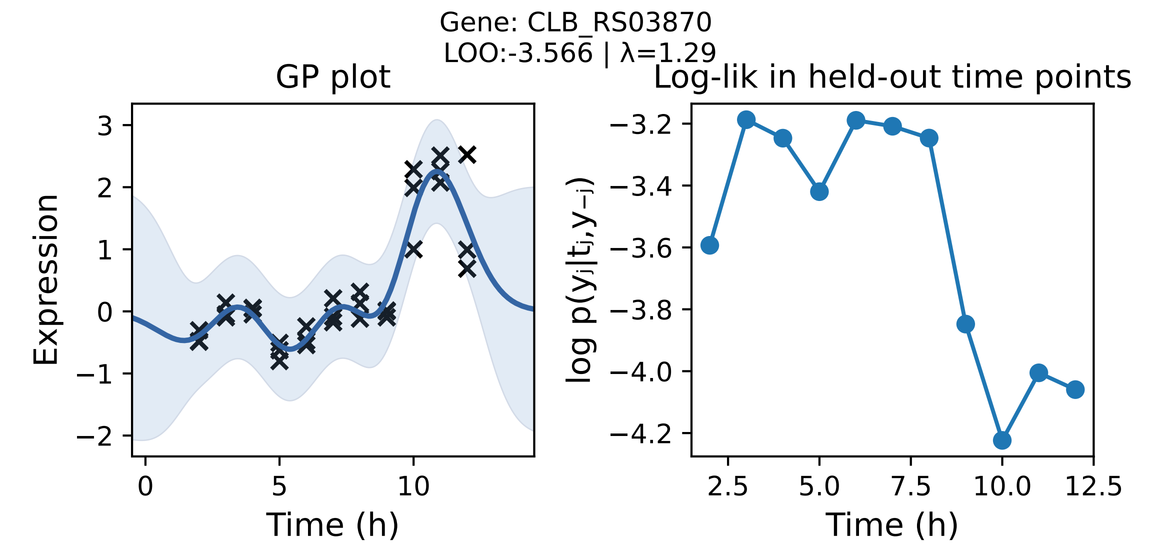
Gene: CLB_RS03870
LOO:-3.566 | λ=1.29
GP plot
Expression
Time (h)
Log-lik in held-out time points
log p(y_j|t_j,y_−j)
Time (h)

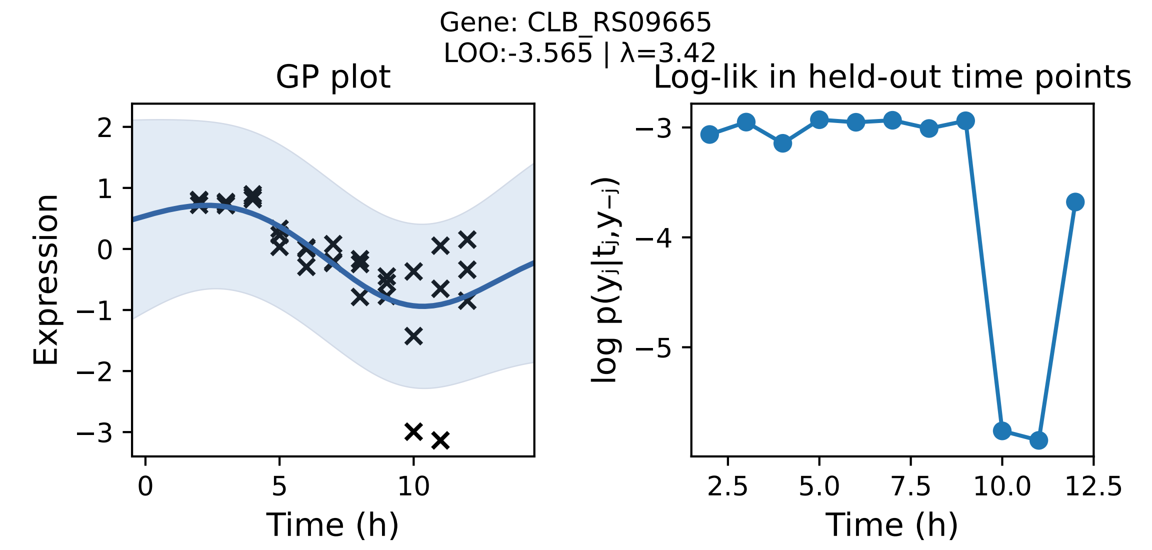

Gene: CLB_RS09665
LOO:-3.565 | λ=3.42
GP plot
Expression
Time (h)
Log-lik in held-out time points
log p(y_j|t_j,y_−j)
Time (h)

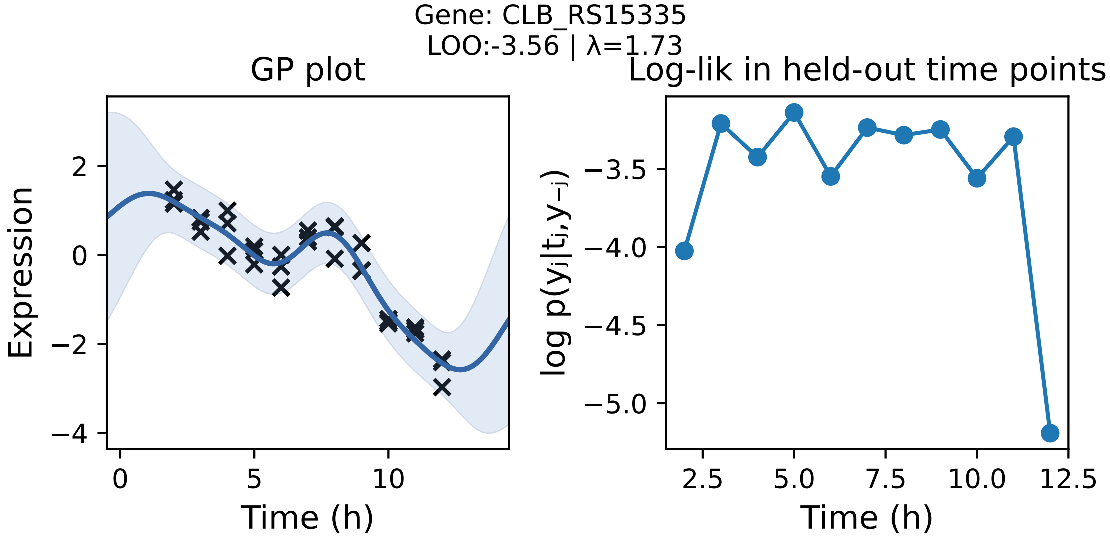
Gene: CLB_RS15335
LOO:-3.56 | λ=1.73
GP plot
Log-lik in held-out time points
Expression
Time (h)
log p(y_j|t_j,y_−j)
2
0
−2
−4
0
5
10
−3.5
−4.0
−4.5
−5.0
2.5
5.0
7.5
10.0
12.5

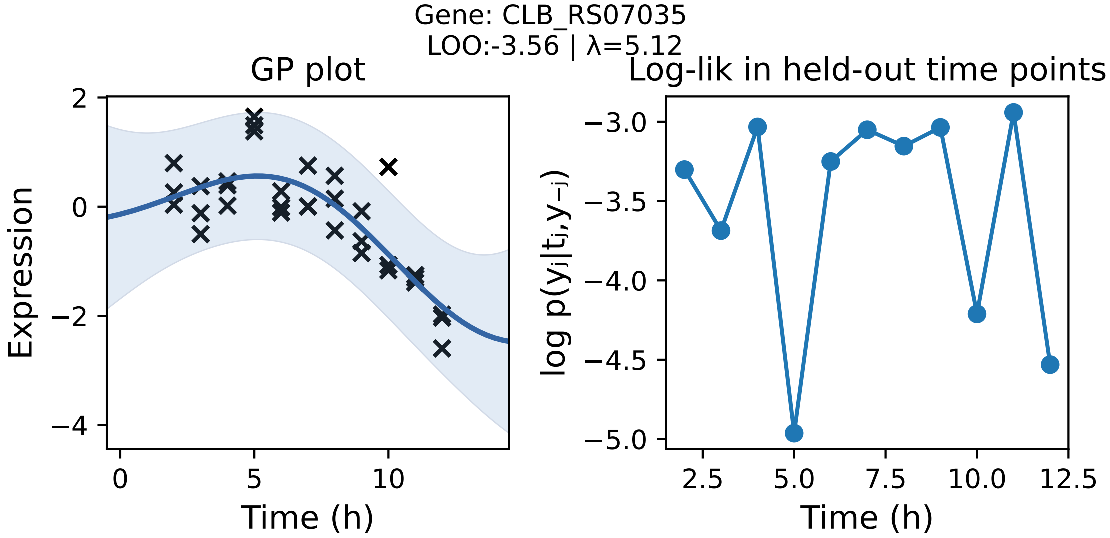
Gene: CLB_RS07035
LOO:-3.56 | λ=5.12
GP plot
Expression
Time (h)
Log-lik in held-out time points
log p(y_j|t_j,y_−j)
Time (h)

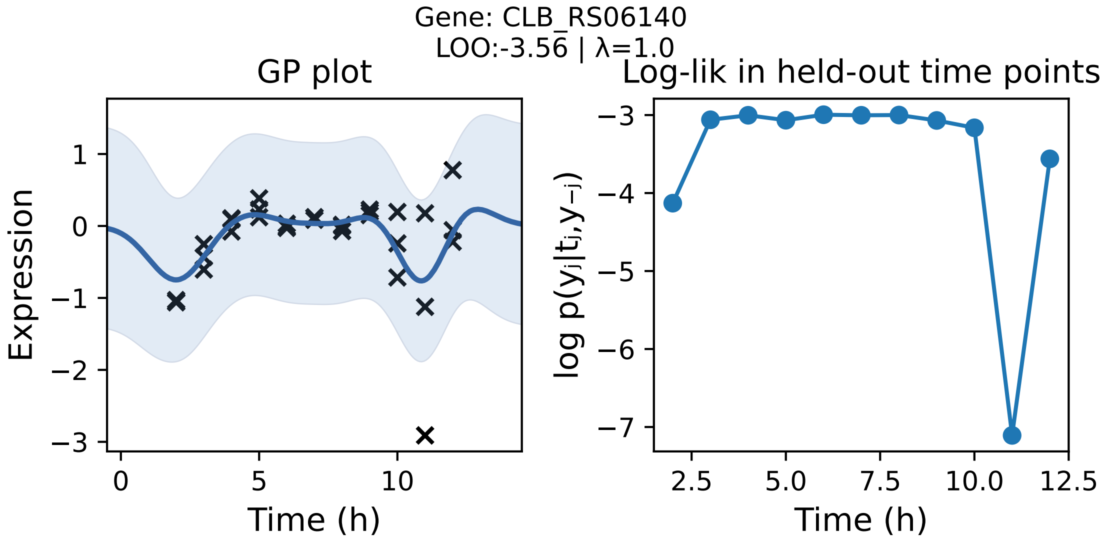
Gene: CLB_RS06140
LOO:-3.56 | λ=1.0
GP plot
Expression
Time (h)
Log-lik in held-out time points
log p(yj|tj,y−j)
Time (h)

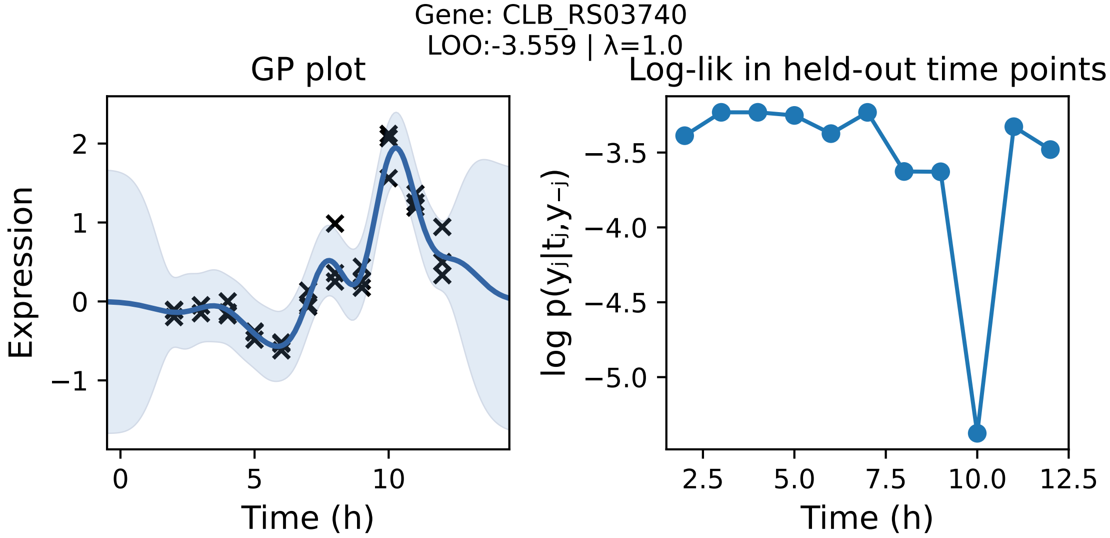

Gene: CLB_RS03740
LOO:-3.559 | λ=1.0
GP plot
Expression
Time (h)
Log-lik in held-out time points
log p(y_j|t_j,y_−j)
Time (h)

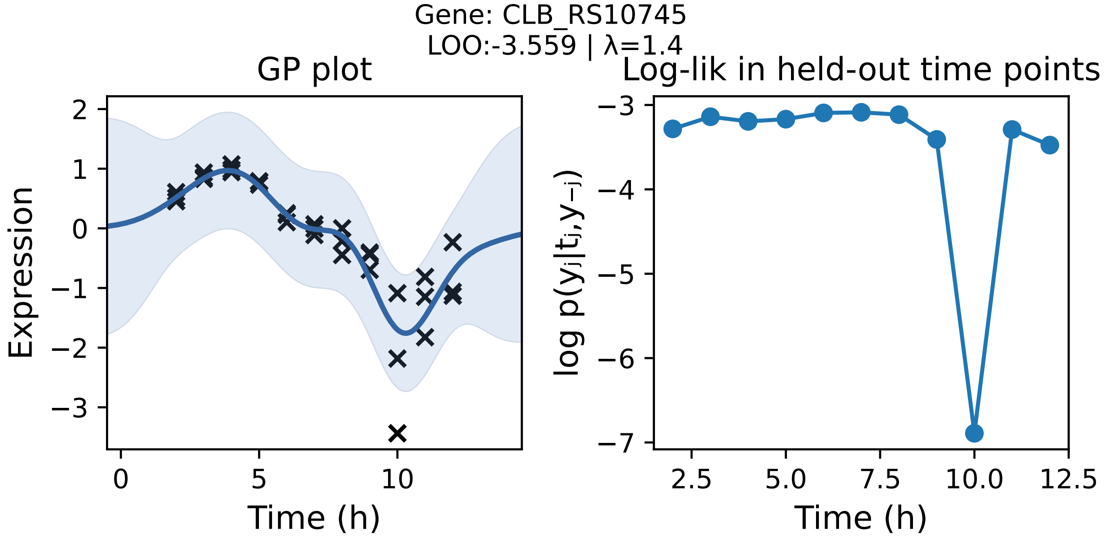

Gene: CLB_RS10745
LOO:-3.559 | λ=1.4
GP plot
Expression
Time (h)
Log-lik in held-out time points
log p(y_j|t_j,y_−j)
Time (h)

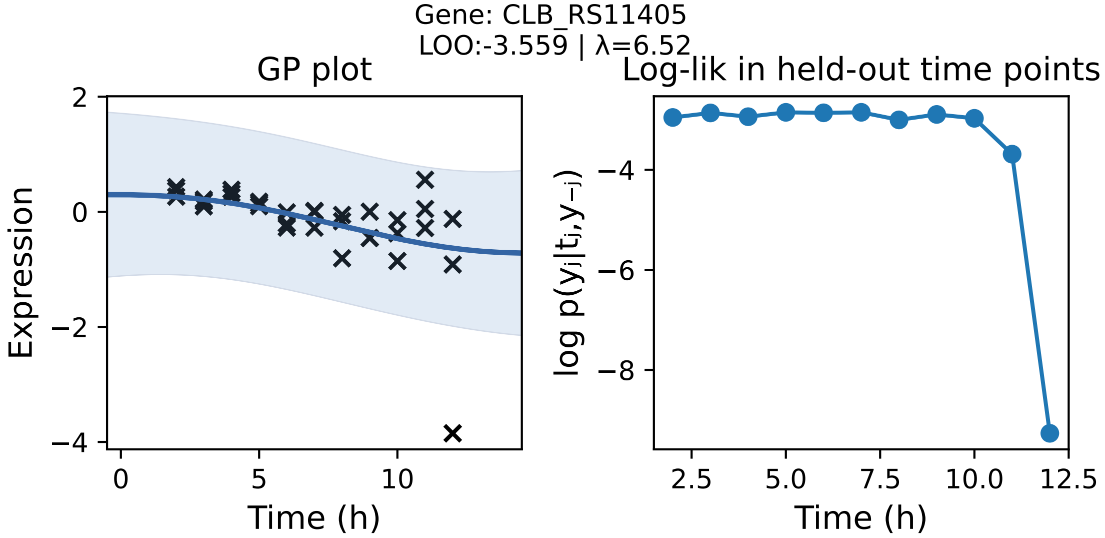

Gene: CLB_RS11405
LOO:-3.559 | λ=6.52
GP plot
Expression
Time (h)
Log-lik in held-out time points
log p(yj|tj,y−j)
Time (h)

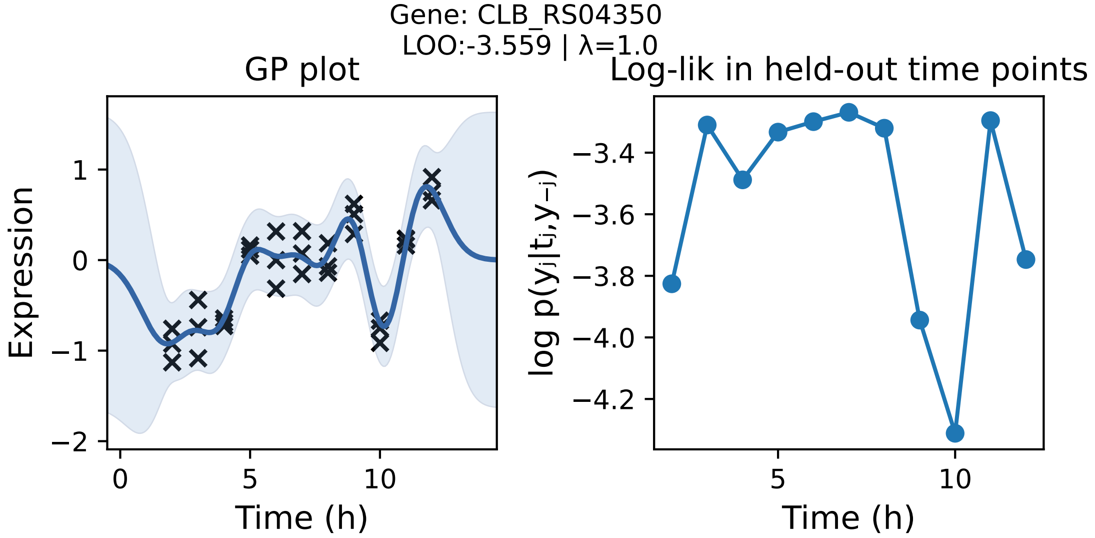

Gene: CLB_RS04350
LOO:-3.559 | λ=1.0
GP plot
Expression
Time (h)
Log-lik in held-out time points
log p(yⱼ|tⱼ,y₋ⱼ)
Time (h)

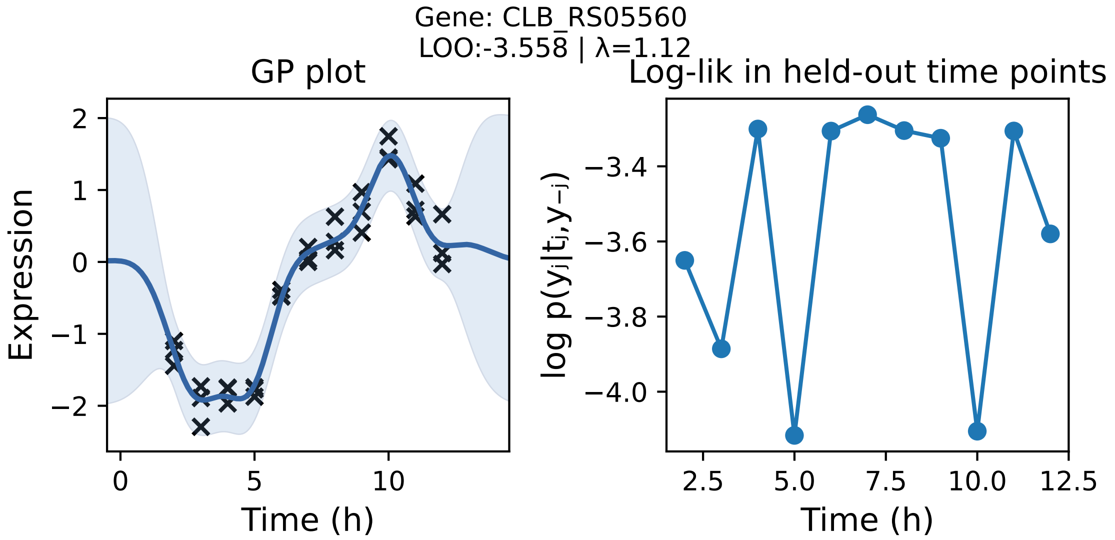

Gene: CLB_RS05560
LOO:-3.558 | λ=1.12
GP plot
Log-lik in held-out time points
Expression
Time (h)
log p(y_j|t_j,y_{-j})
Time (h)

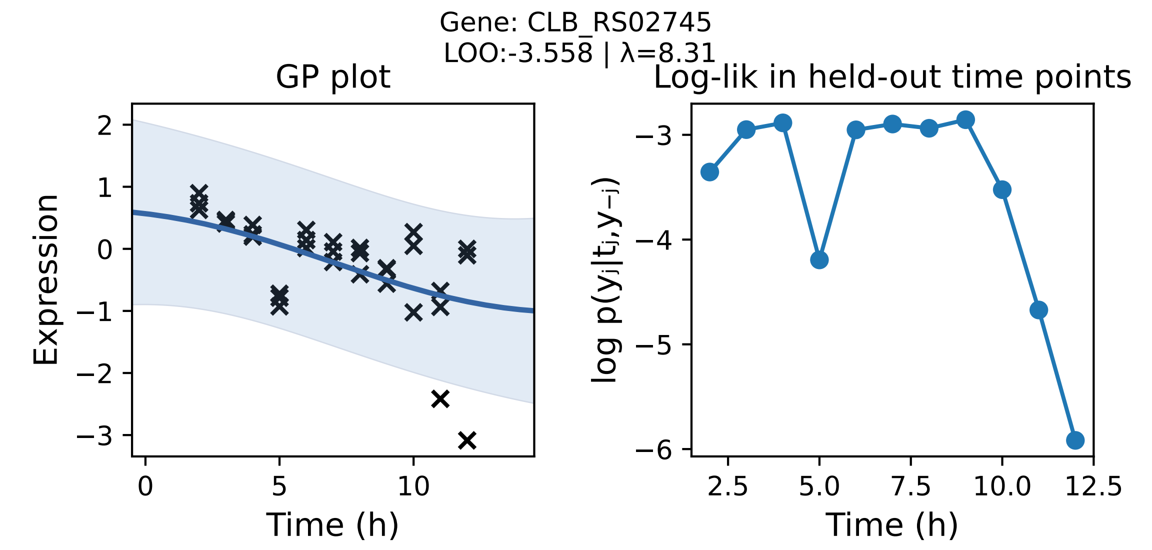
Gene: CLB_RS02745
LOO:-3.558 | λ=8.31
GP plot
Expression
Time (h)
Log-lik in held-out time points
log p(y_j|t_j,y_−j)
Time (h)

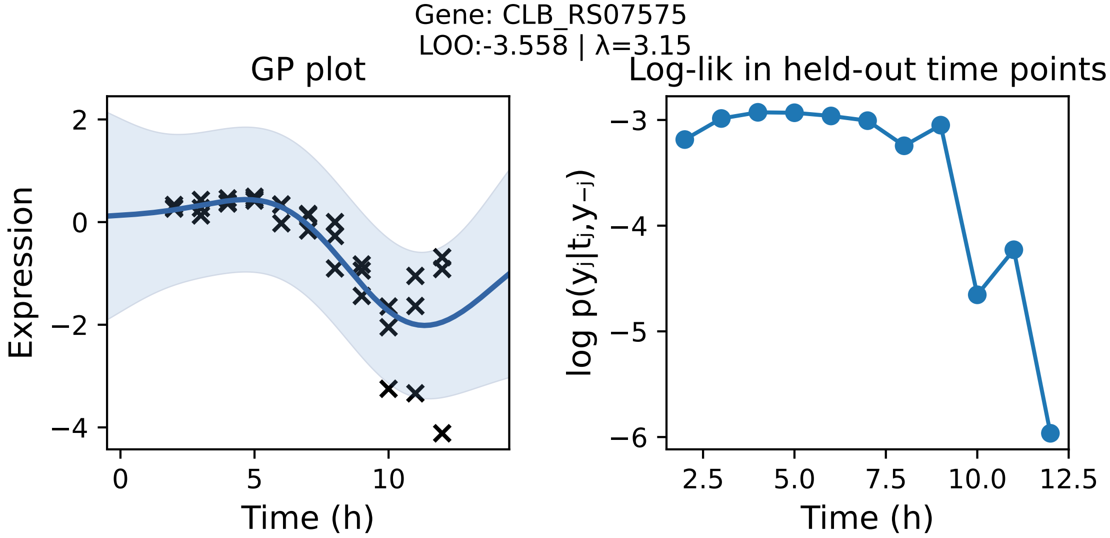

Gene: CLB_RS07575
LOO:-3.558 | λ=3.15
GP plot
Expression
Time (h)
Log-lik in held-out time points
log p(y_j|t_j,y_{-j})
Time (h)

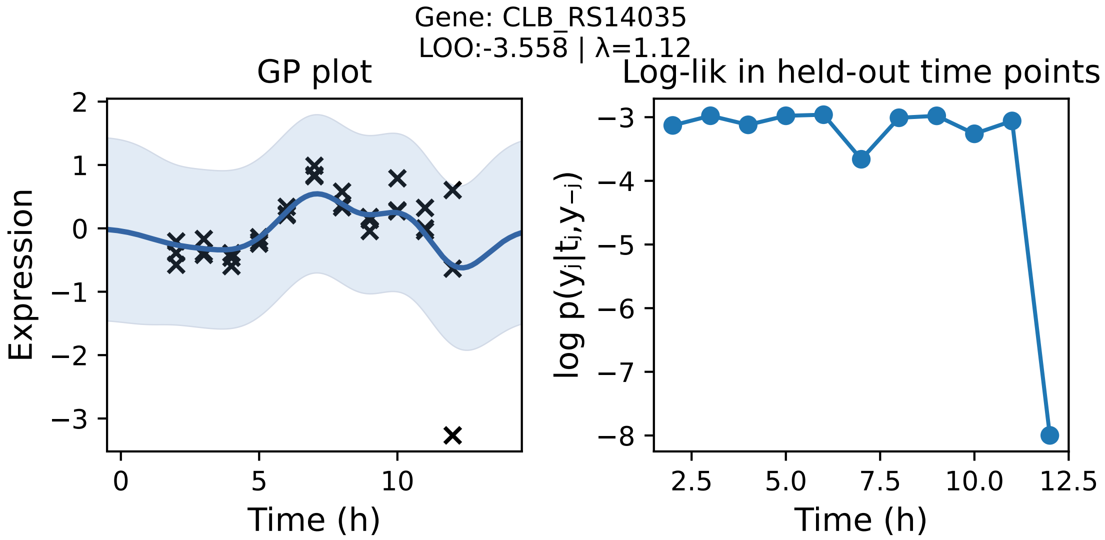

Gene: CLB_RS14035
LOO:-3.558 | λ=1.12
GP plot
Expression
Time (h)
Log-lik in held-out time points
log p(y_j|t_j,y_−j)
Time (h)

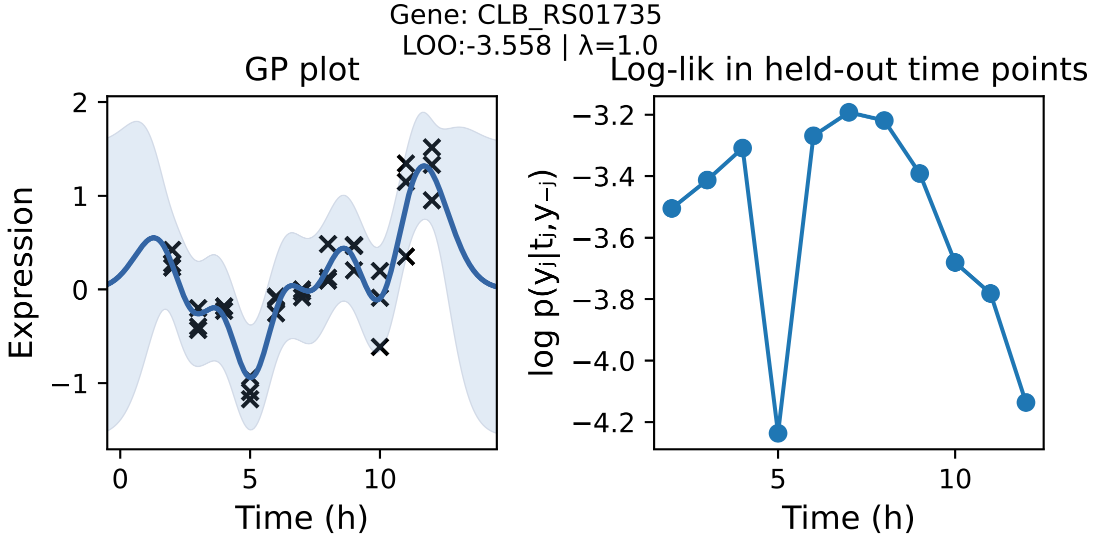
Gene: CLB_RS01735
LOO:-3.558 | λ=1.0
GP plot
Expression
Time (h)
Log-lik in held-out time points
log p(y_j|t_j,y_−j)
Time (h)

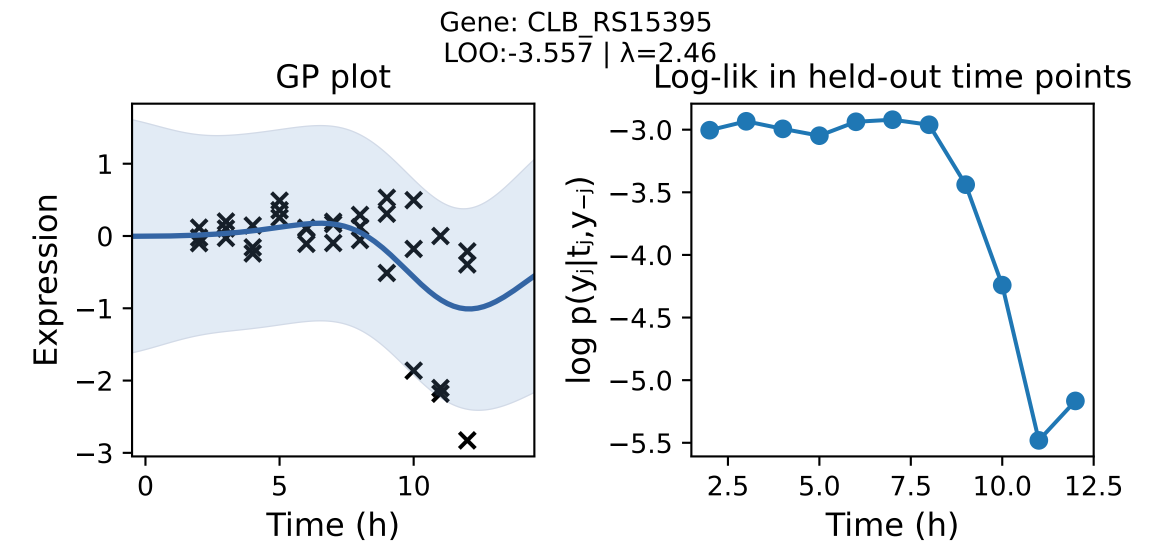

Gene: CLB_RS15395
LOO:-3.557 | λ=2.46
GP plot
Log-lik in held-out time points
Expression
Time (h)
log p(yj|tj,y−j)
Time (h)
1
0
−1
−2
−3
0
5
10
−3.0
−3.5
−4.0
−4.5
−5.0
−5.5
2.5
5.0
7.5
10.0
12.5

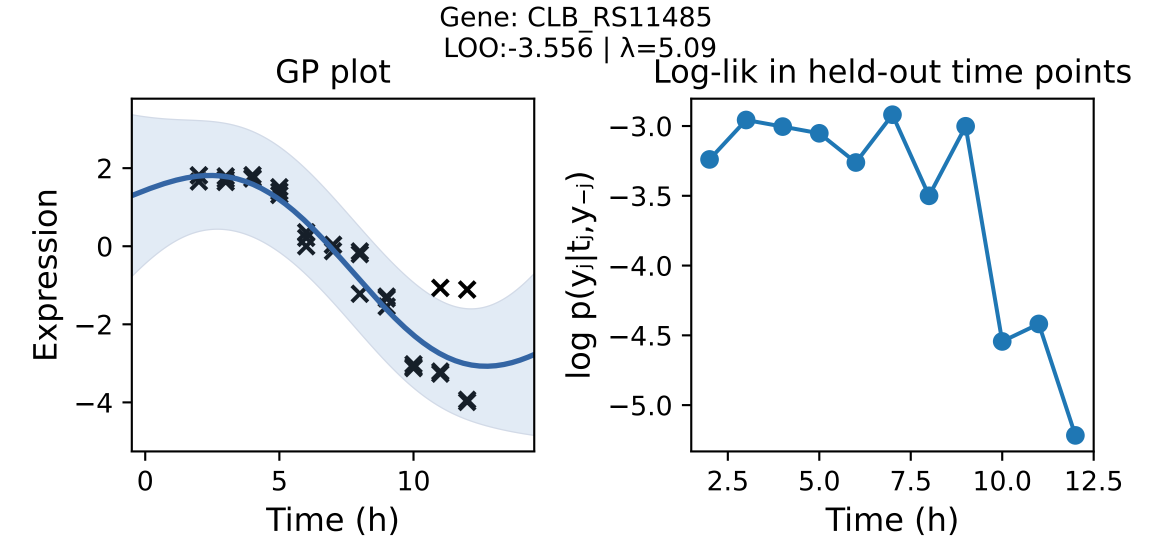

Gene: CLB_RS11485
LOO:-3.556 | λ=5.09
GP plot
Expression
Time (h)
Log-lik in held-out time points
log p(y_j|t_j,y_−j)
Time (h)

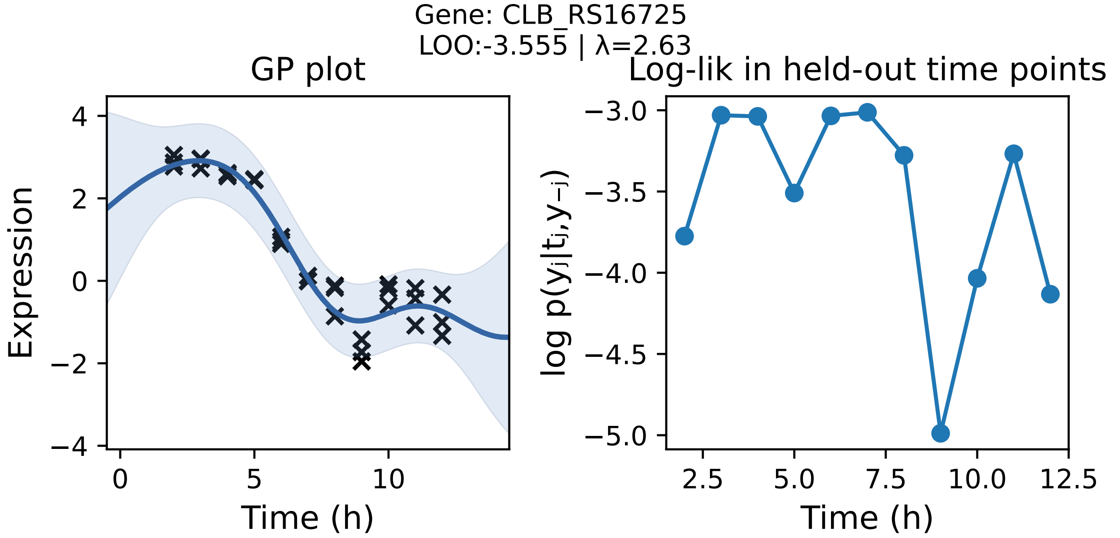

Gene: CLB_RS16725
LOO:-3.555 | λ=2.63
GP plot
Expression
Time (h)
4
2
0
−2
−4
0
5
10
Log-lik in held-out time points
log p(y_j|t_j,y_−j)
Time (h)
−3.0
−3.5
−4.0
−4.5
−5.0
2.5
5.0
7.5
10.0
12.5

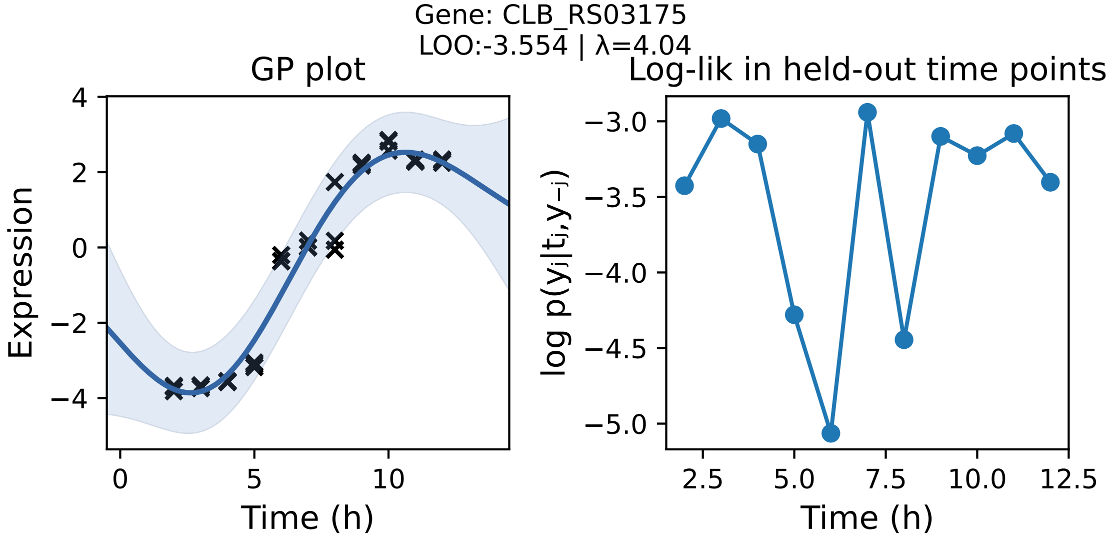

Gene: CLB_RS03175
LOO:-3.554 | λ=4.04
GP plot
Expression
Time (h)
Log-lik in held-out time points
log p(y_j|t_j,y_−j)
Time (h)

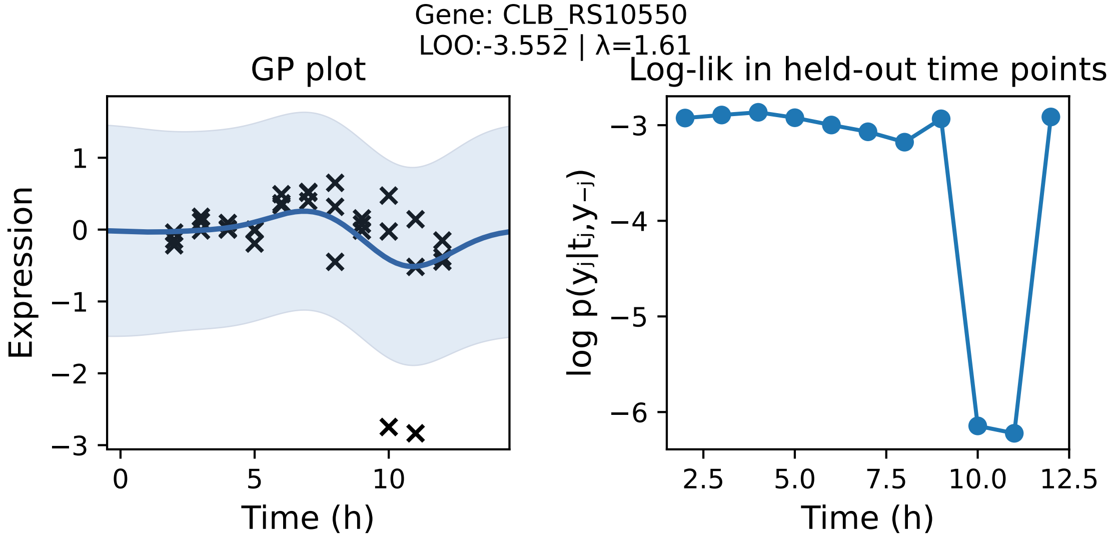

Gene: CLB_RS10550
LOO:-3.552 | λ=1.61
GP plot
Expression
Time (h)
Log-lik in held-out time points
log p(y_j|t_j,y_−j)
Time (h)

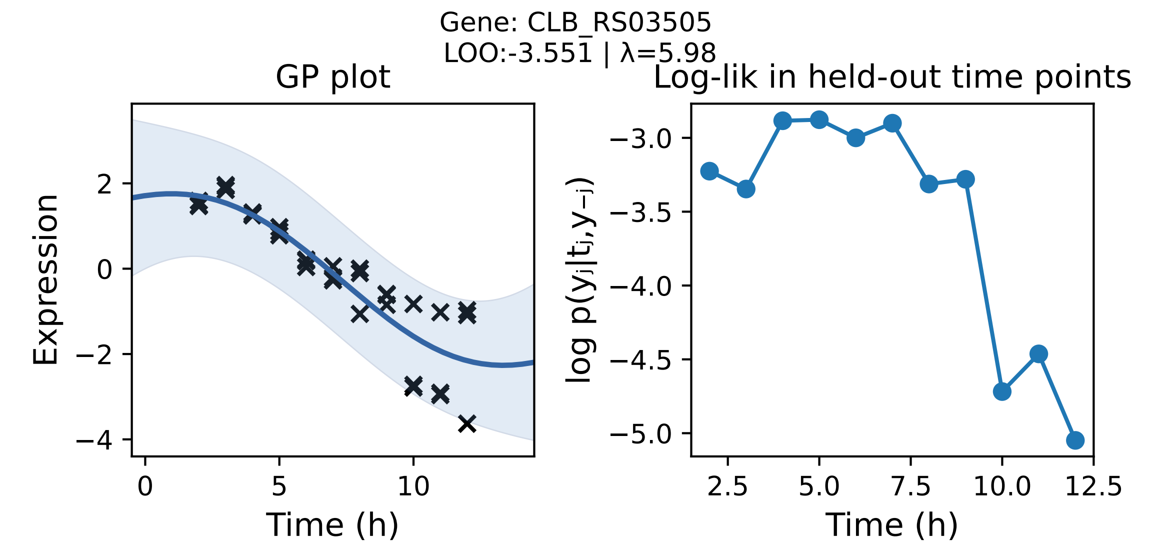

Gene: CLB_RS03505
LOO:-3.551 | λ=5.98
GP plot
Expression
Time (h)
2
0
−2
−4
0
5
10
Log-lik in held-out time points
log p(y_j|t_j,y_−j)
Time (h)
−3.0
−3.5
−4.0
−4.5
−5.0
2.5
5.0
7.5
10.0
12.5

Gene: CLB_RS08670
LOO:-3.551 | λ=1.95

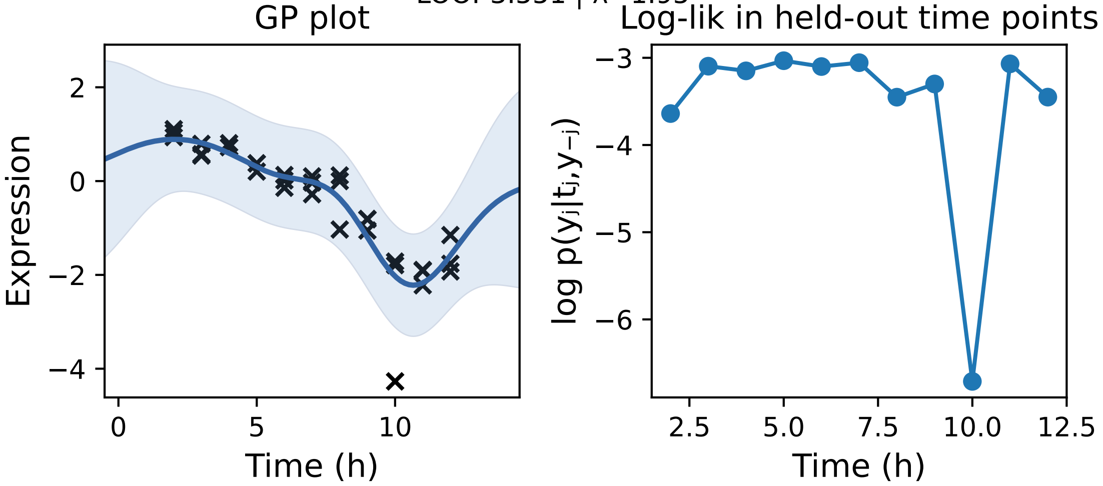

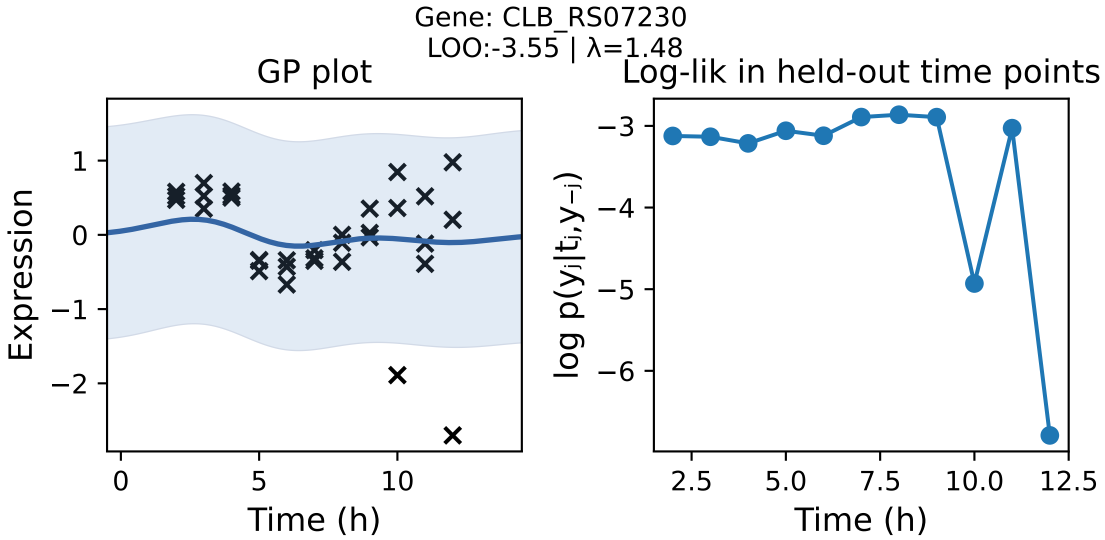

Gene: CLB_RS07230
LOO:-3.55 | λ=1.48
GP plot
Expression
Time (h)
Log-lik in held-out time points
log p(y_j|t_j,y_−j)
Time (h)

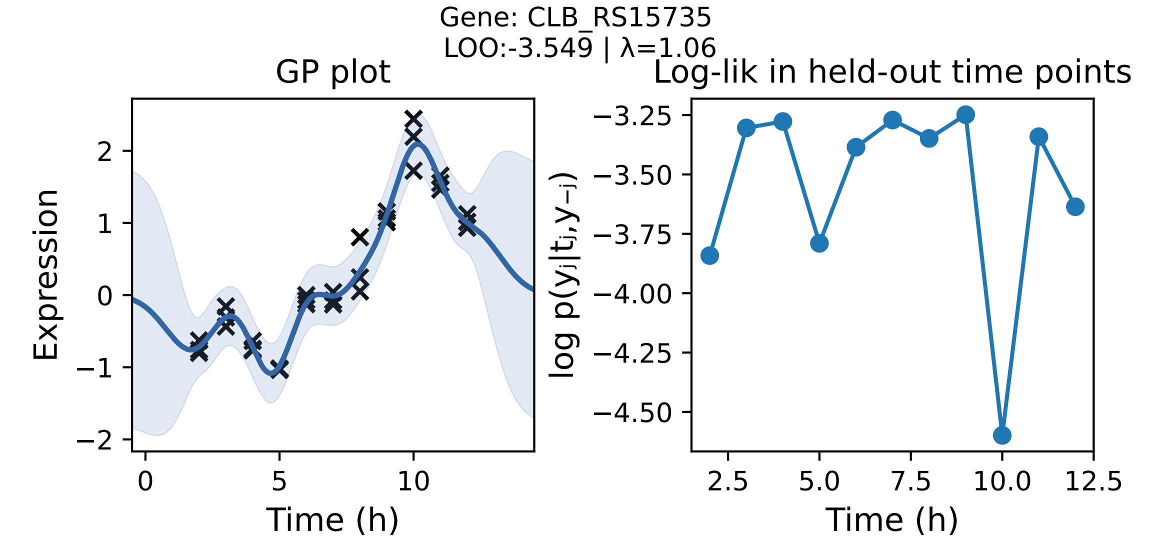

Gene: CLB_RS15735
LOO:-3.549 | λ=1.06
GP plot
Expression
Time (h)
Log-lik in held-out time points
log p(y_j|t_j,y_-j)
Time (h)

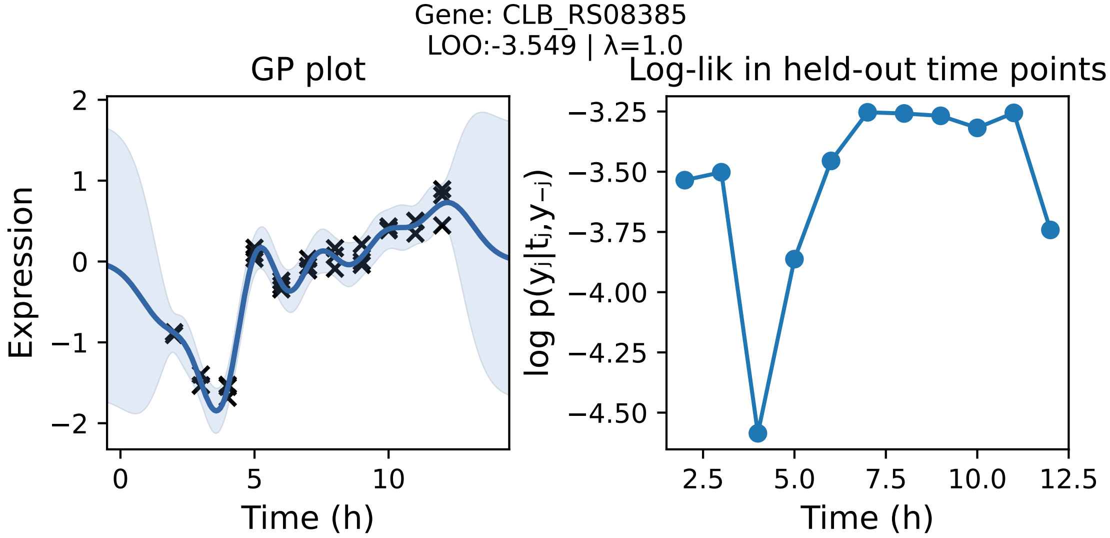

Gene: CLB_RS08385
LOO:-3.549 | λ=1.0
GP plot
Log-lik in held-out time points
Expression
Time (h)
log p(y_j|t_j,y_−j)
2
1
0
−1
−2
0
5
10
−3.25
−3.50
−3.75
−4.00
−4.25
−4.50
2.5
5.0
7.5
10.0
12.5

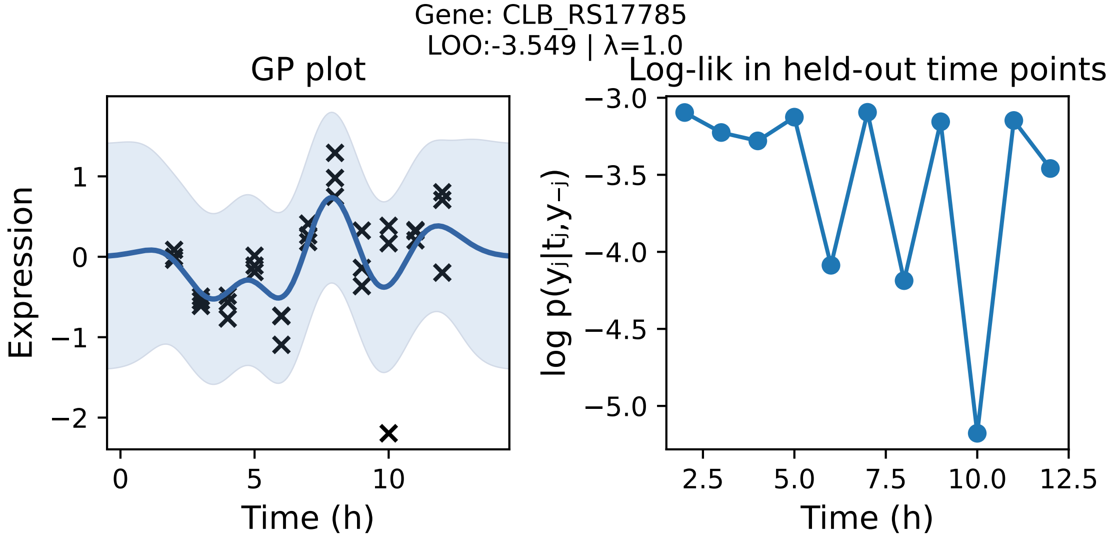

Gene: CLB_RS17785
LOO:-3.549 | λ=1.0
GP plot
Expression
Time (h)
Log-lik in held-out time points
log p(y_j|t_j,y_−j)
Time (h)

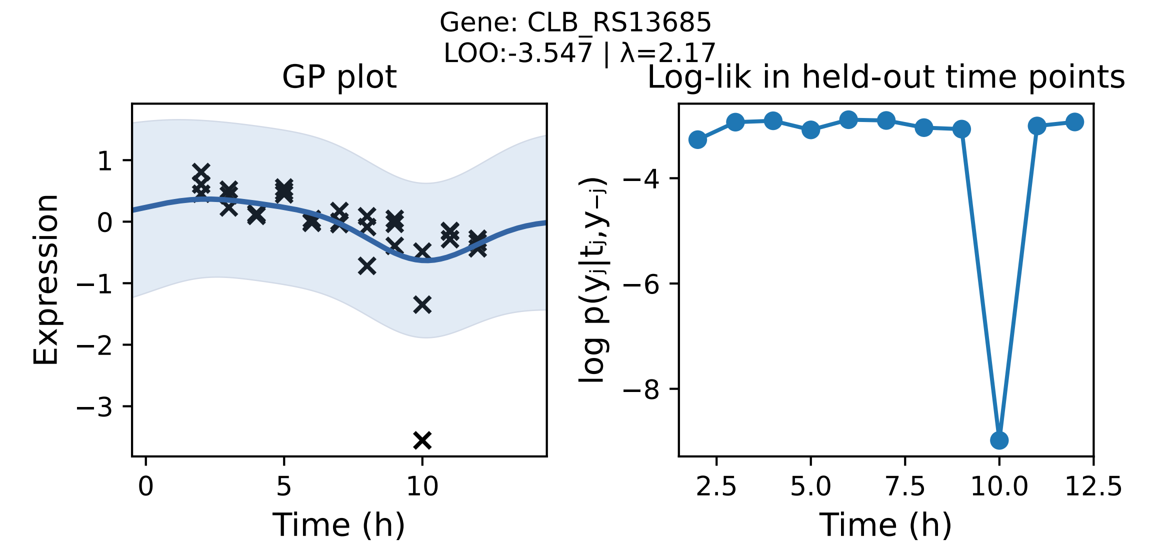

Gene: CLB_RS13685
LOO:-3.547 | λ=2.17
GP plot
Expression
Time (h)
Log-lik in held-out time points
log p(y_j|t_j,y_−j)
Time (h)

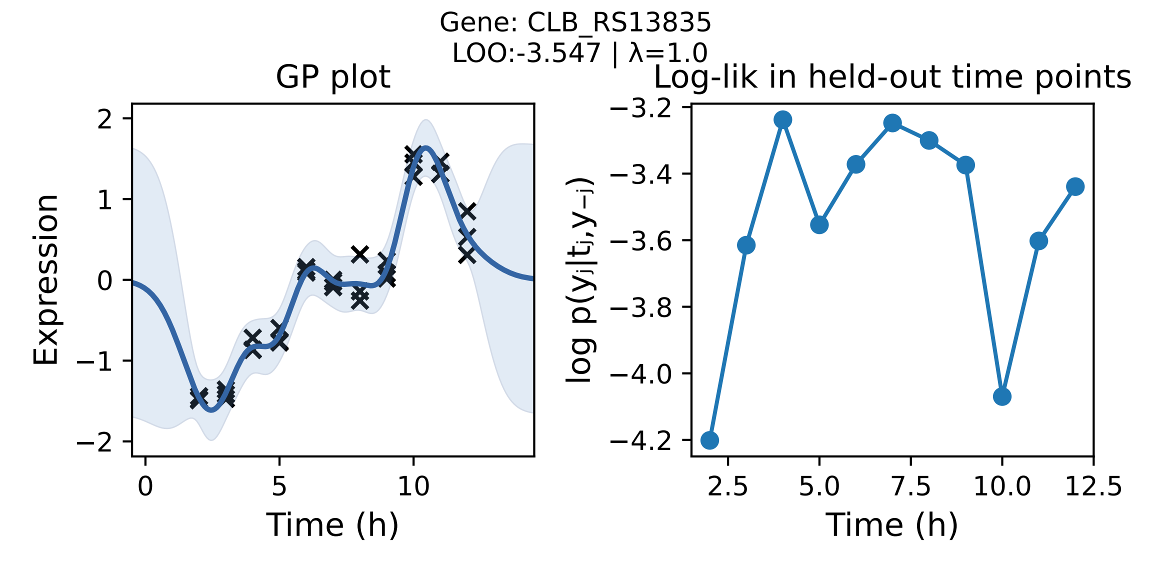
Gene: CLB_RS13835
LOO:-3.547 | λ=1.0
GP plot
Expression
Time (h)
Log-lik in held-out time points
log p(y_j|t_j,y_−j)
Time (h)

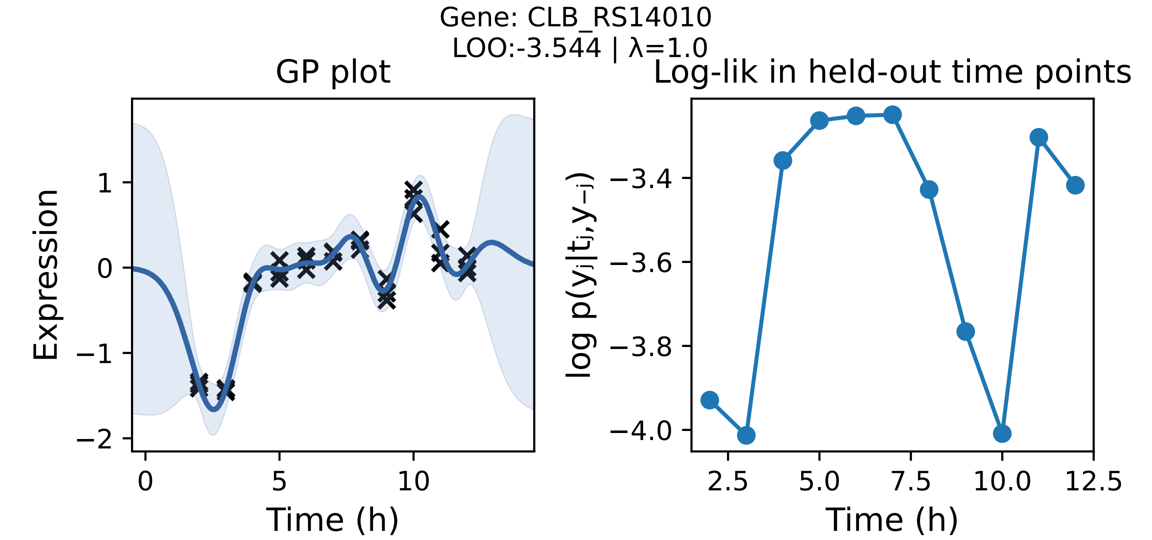
Gene: CLB_RS14010
LOO:-3.544 | λ=1.0
GP plot
Expression
Time (h)
Log-lik in held-out time points
log p(y_j|t_j,y_−j)
Time (h)

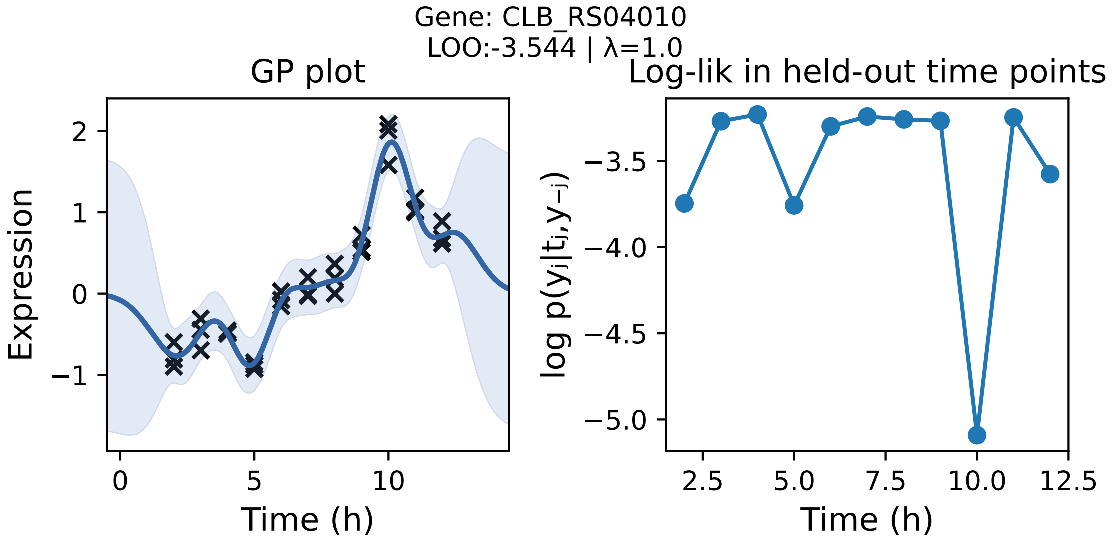

Gene: CLB_RS04010
LOO:-3.544 | λ=1.0
GP plot
Expression
Time (h)
2
1
0
−1
0
5
10
Log-lik in held-out time points
log p(y_j|t_j,y_−j)
Time (h)
−3.5
−4.0
−4.5
−5.0
2.5
5.0
7.5
10.0
12.5

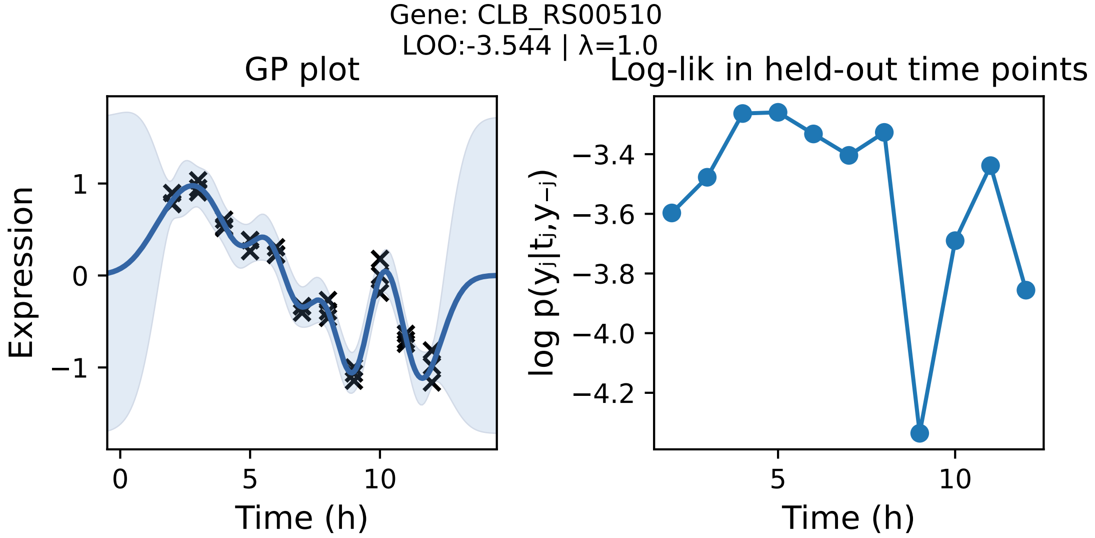

Gene: CLB_RS00510
LOO:-3.544 | λ=1.0
GP plot
Log-lik in held-out time points
Expression
Time (h)
log p(yj|tj,y−j)
Time (h)

Gene: CLB_RS05645
LOO:-3.544 | λ=5.51

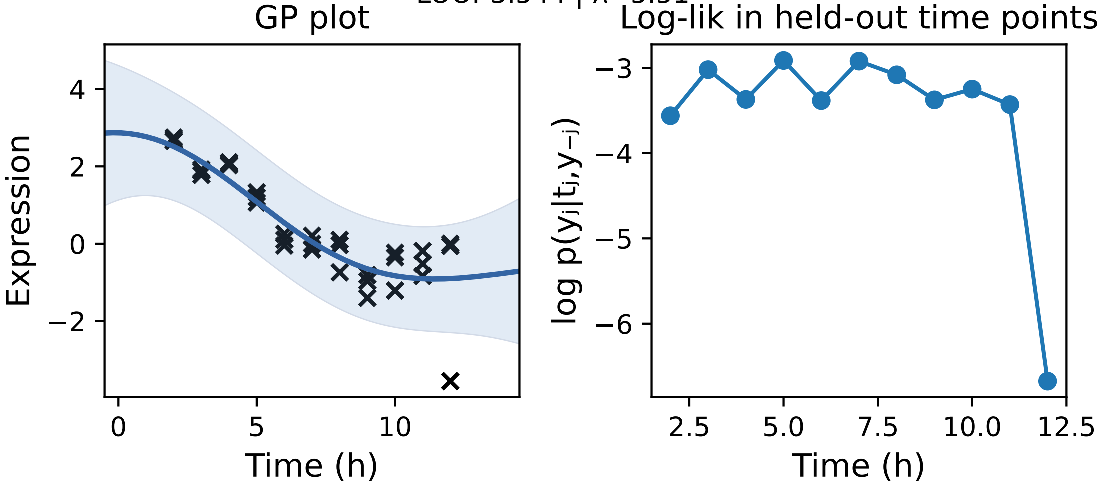

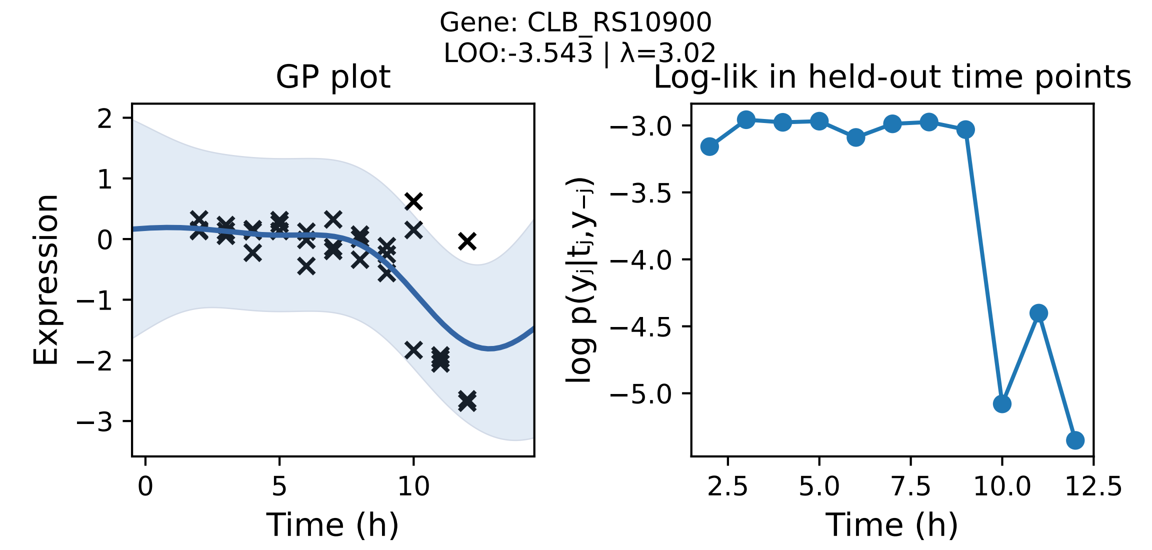
Gene: CLB_RS10900
LOO:-3.543 | λ=3.02
GP plot
Expression
Time (h)
Log-lik in held-out time points
log p(y_j|t_j,y_−j)
Time (h)

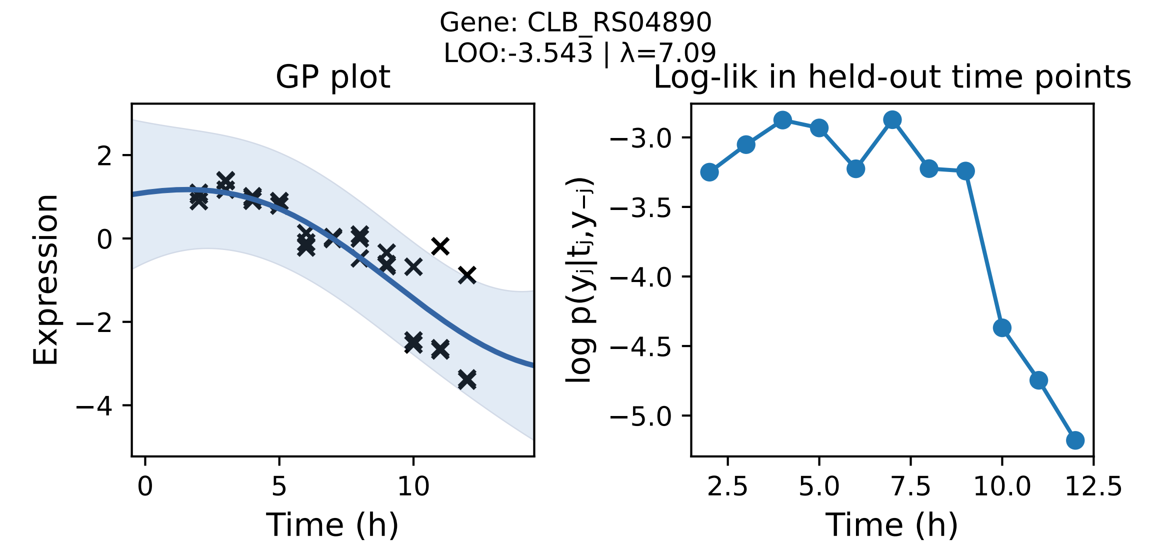
Gene: CLB_RS04890
LOO:-3.543 | λ=7.09
GP plot
Expression
Time (h)
2
0
−2
−4
0
5
10
Log-lik in held-out time points
log p(y_j|t_j,y_−j)
−3.0
−3.5
−4.0
−4.5
−5.0
2.5
5.0
7.5
10.0
12.5
Time (h)

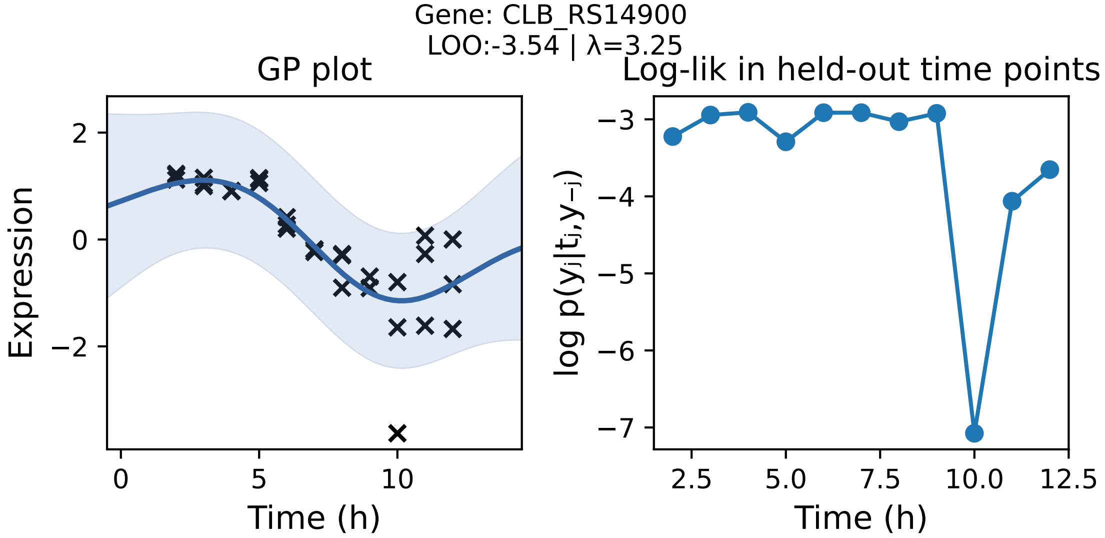
Gene: CLB_RS14900
LOO:-3.54 | λ=3.25
GP plot
Expression
Time (h)
Log-lik in held-out time points
log p(y_j|t_j,y_−j)
Time (h)

Gene: CLB_RS00075
LOO:-3.54 | λ=1.0

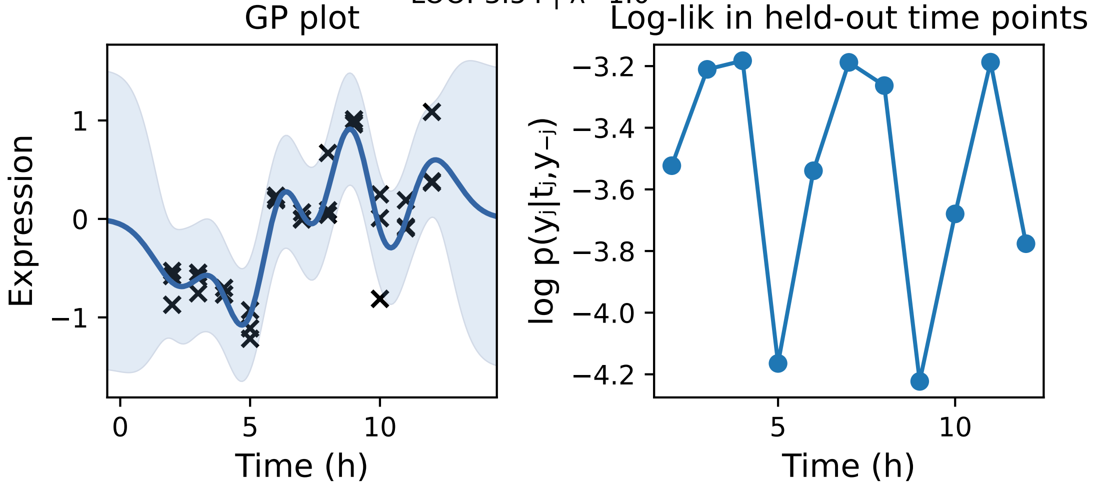

Gene: CLB_RS10955
LOO:-3.538 | λ=1.05

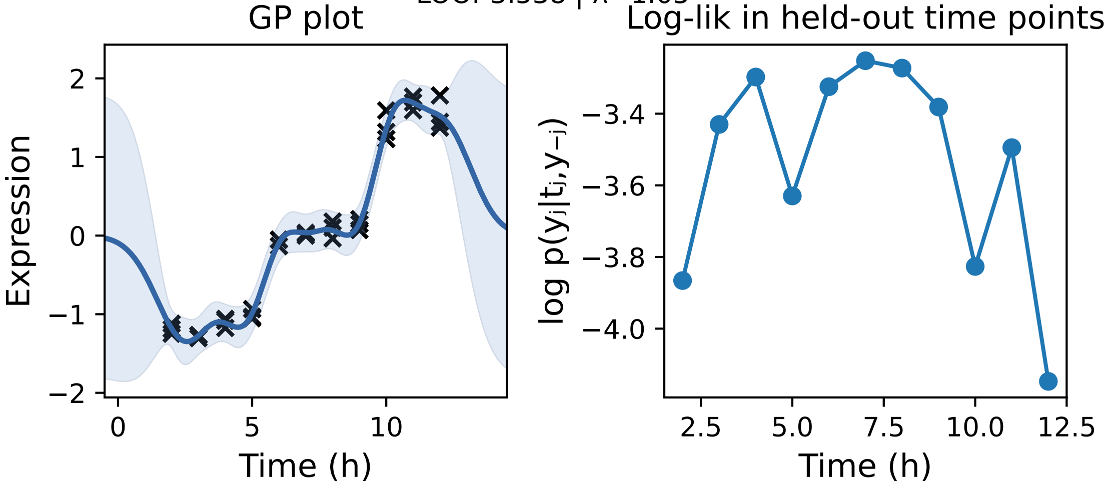

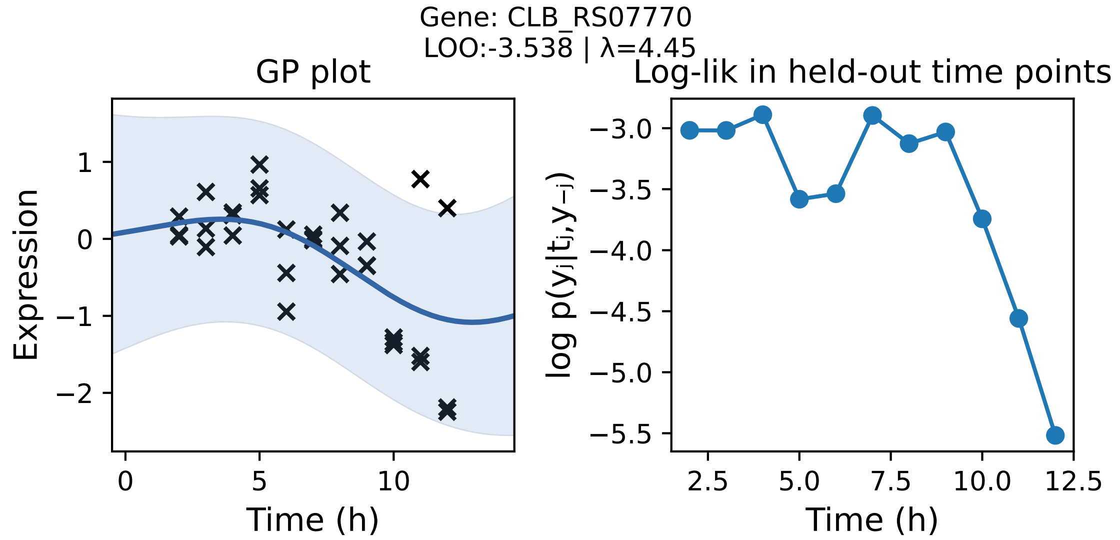

Gene: CLB_RS07770
LOO:-3.538 | λ=4.45
GP plot
Expression
Time (h)
1
0
−1
−2
0
5
10
Log-lik in held-out time points
log p(y_j|t_j,y_−j)
−3.0
−3.5
−4.0
−4.5
−5.0
−5.5
2.5
5.0
7.5
10.0
12.5
Time (h)

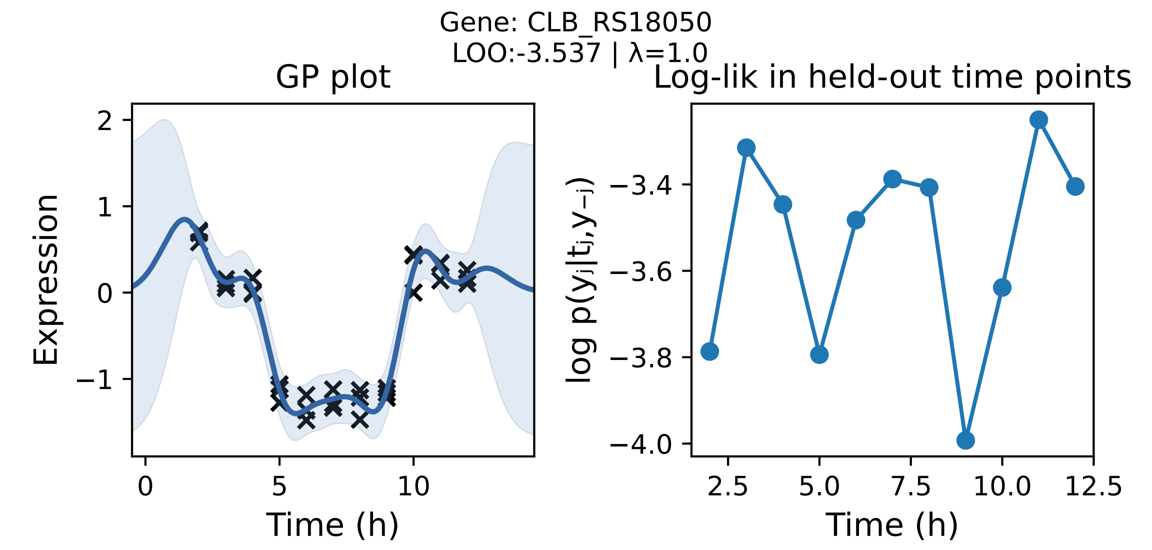
Gene: CLB_RS18050
LOO:-3.537 | λ=1.0
GP plot
Log-lik in held-out time points
Expression
Time (h)
log p(y_j|t_j,y_−j)
Time (h)
2
1
0
−1
0
5
10
−3.4
−3.6
−3.8
−4.0
2.5
5.0
7.5
10.0
12.5

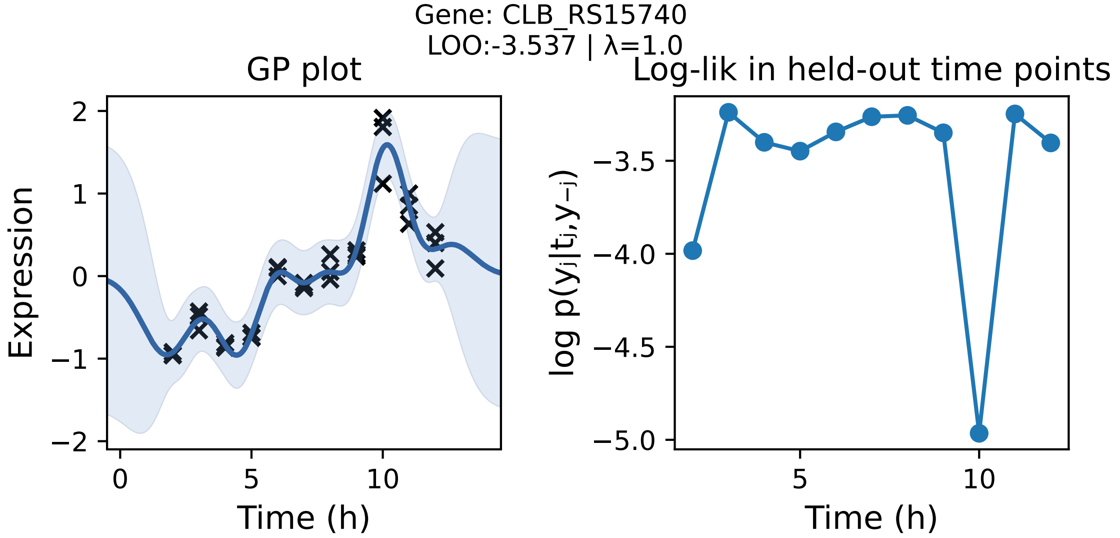

Gene: CLB_RS15740
LOO:-3.537 | λ=1.0
GP plot
Expression
Time (h)
Log-lik in held-out time points
log p(y_j|t_j,y_−j)
Time (h)

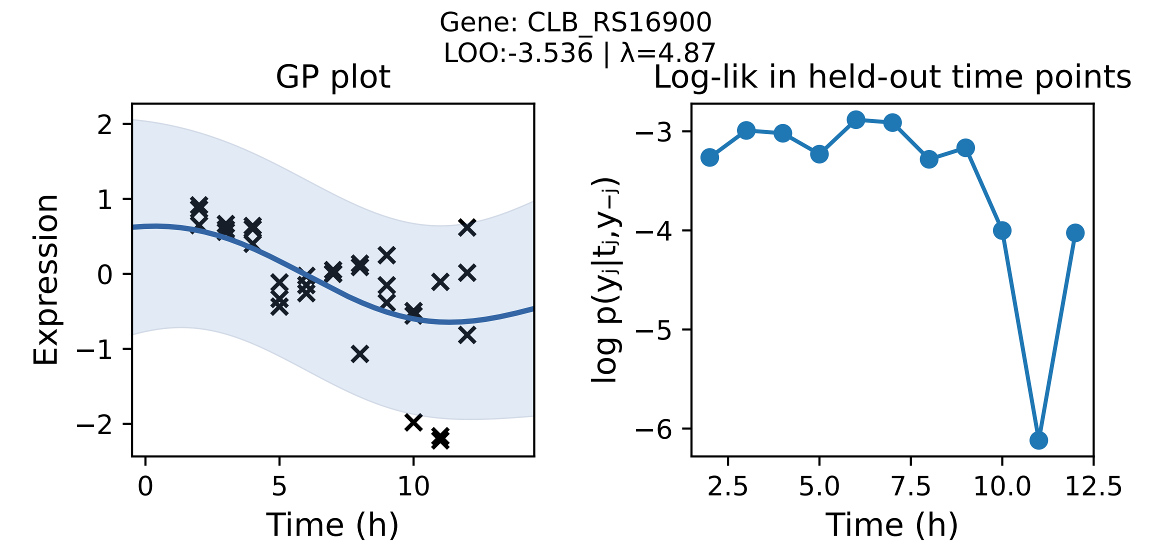

Gene: CLB_RS16900
LOO:-3.536 | λ=4.87
GP plot
Expression
Time (h)
Log-lik in held-out time points
log p(y_j|t_j,y_−j)
Time (h)

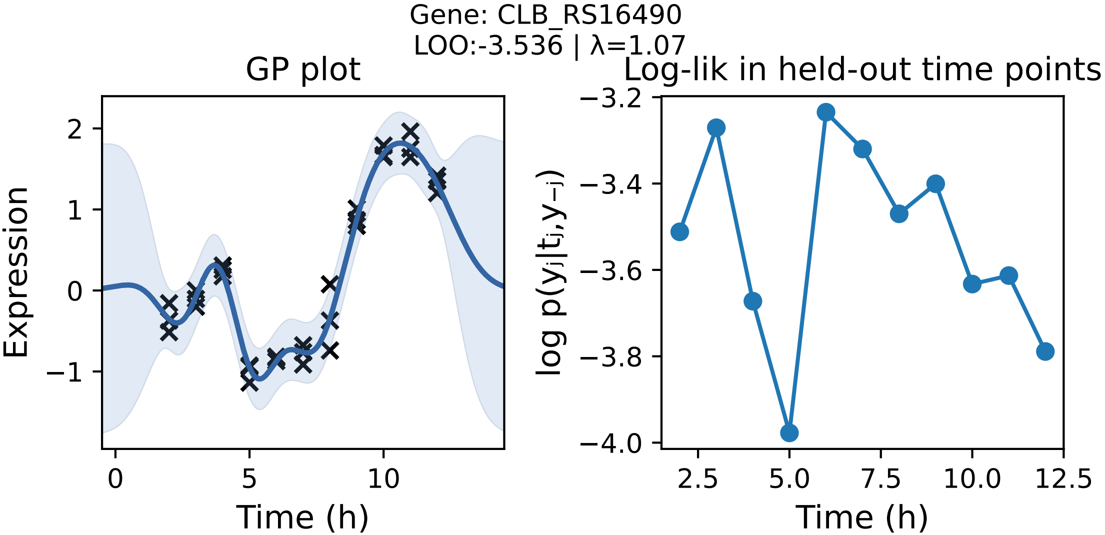
Gene: CLB_RS16490
LOO:-3.536 | λ=1.07
GP plot
Expression
Time (h)
2
1
0
−1
0
5
10
Log-lik in held-out time points
log p(y_j|t_j,y_−j)
−3.2
−3.4
−3.6
−3.8
−4.0
2.5
5.0
7.5
10.0
12.5
Time (h)

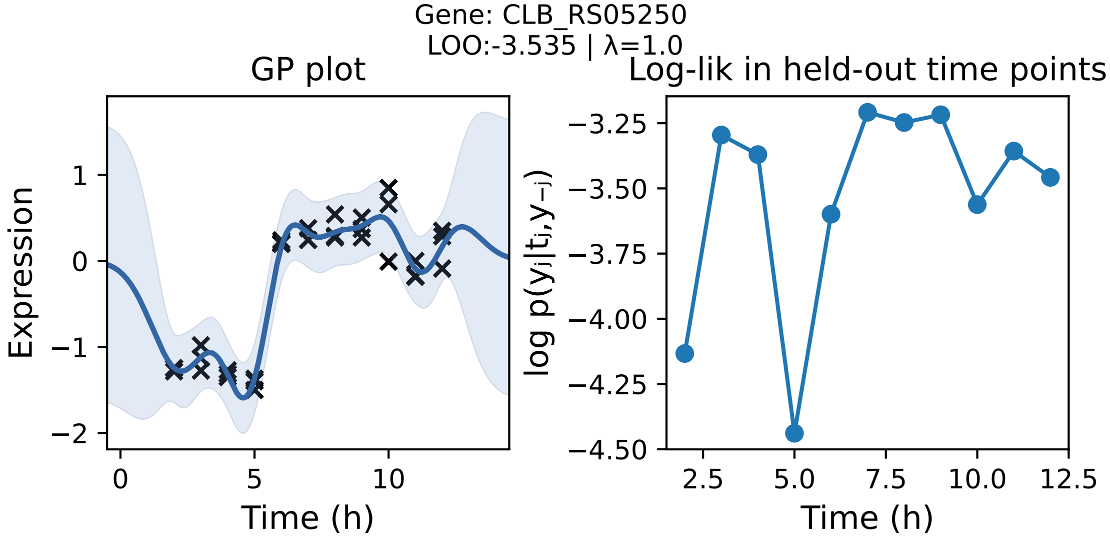

Gene: CLB_RS05250
LOO:-3.535 | λ=1.0
GP plot
Expression
1
0
−1
−2
0
5
10
Time (h)
Log-lik in held-out time points
log p(y_j|t_j,y_−j)
−3.25
−3.50
−3.75
−4.00
−4.25
−4.50
2.5
5.0
7.5
10.0
12.5
Time (h)

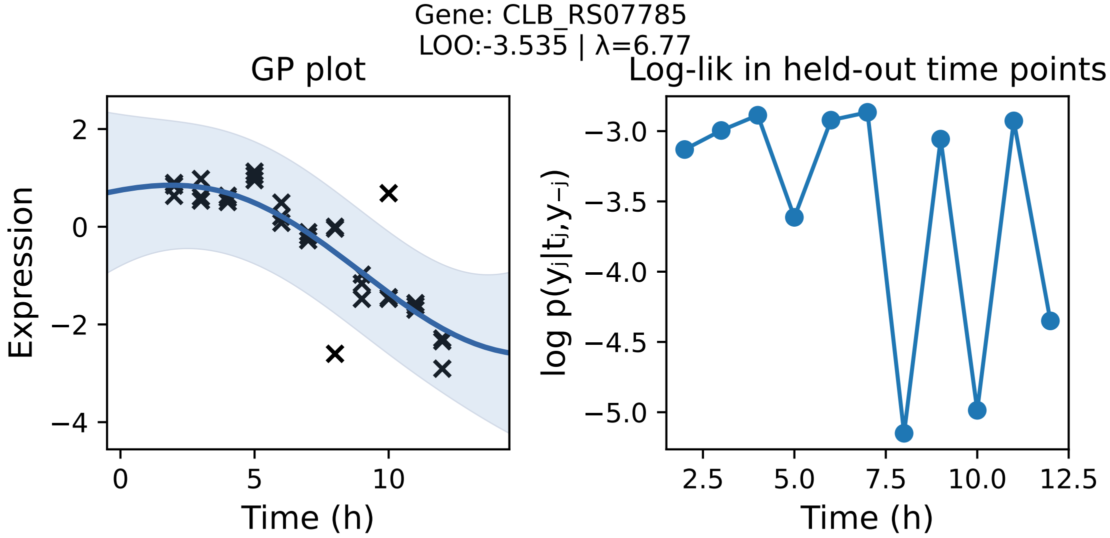

Gene: CLB_RS07785
LOO:-3.535 | λ=6.77
GP plot
Expression
Time (h)
Log-lik in held-out time points
log p(y_j|t_j,y_−j)
Time (h)

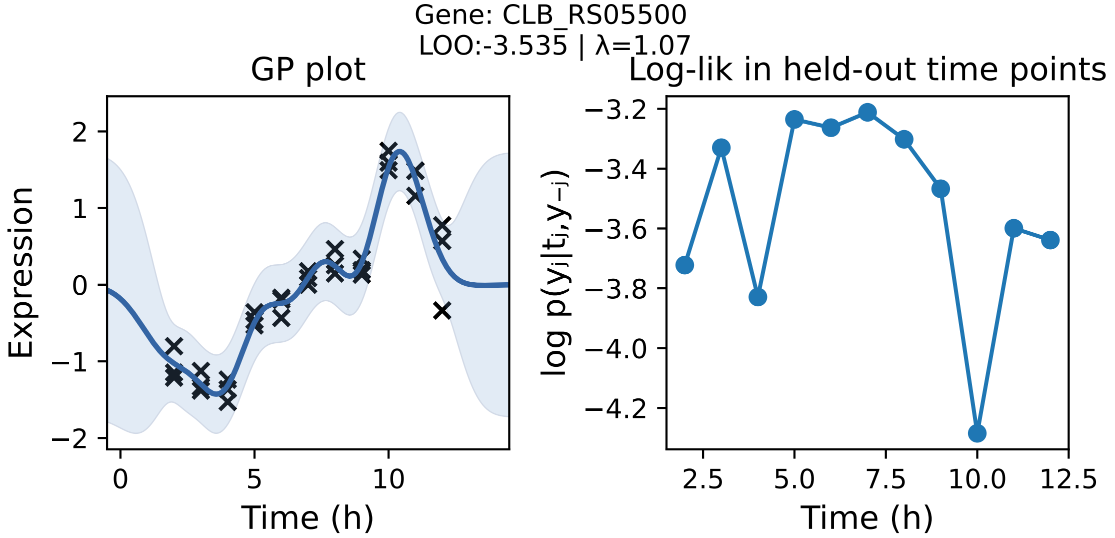

Gene: CLB_RS05500
LOO:-3.535 | λ=1.07
GP plot
Expression
Time (h)
2
1
0
−1
−2
0
5
10
Log-lik in held-out time points
log p(y_j|t_j,y_−j)
Time (h)
−3.2
−3.4
−3.6
−3.8
−4.0
−4.2
2.5
5.0
7.5
10.0
12.5

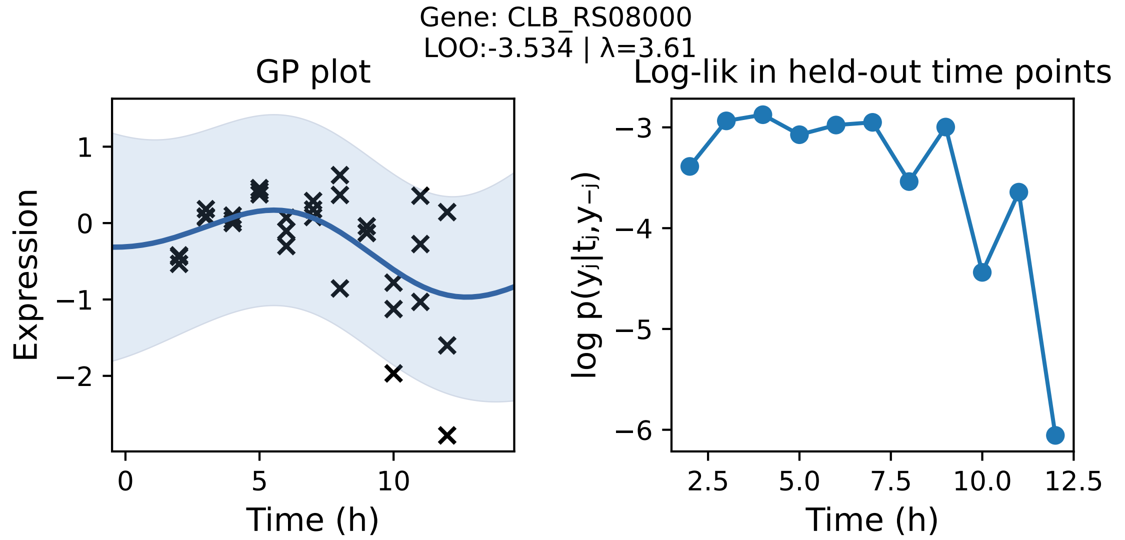
Gene: CLB_RS08000
LOO:-3.534 | λ=3.61
GP plot
Expression
Time (h)
1
0
−1
−2
0
5
10
Log-lik in held-out time points
log p(yⱼ|tⱼ,y₋ⱼ)
Time (h)
−3
−4
−5
−6
2.5
5.0
7.5
10.0
12.5

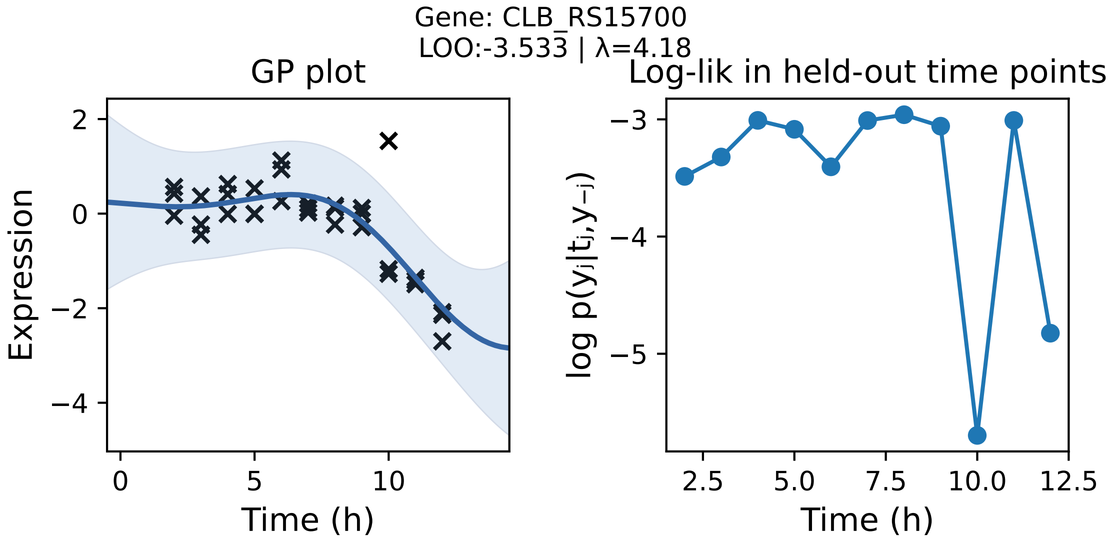
Gene: CLB_RS15700
LOO:-3.533 | λ=4.18
GP plot
Log-lik in held-out time points
Expression
Time (h)
2
0
−2
−4
0
5
10
log p(yⱼ|tⱼ,y₋ⱼ)
−3
−4
−5
2.5
5.0
7.5
10.0
12.5
Time (h)

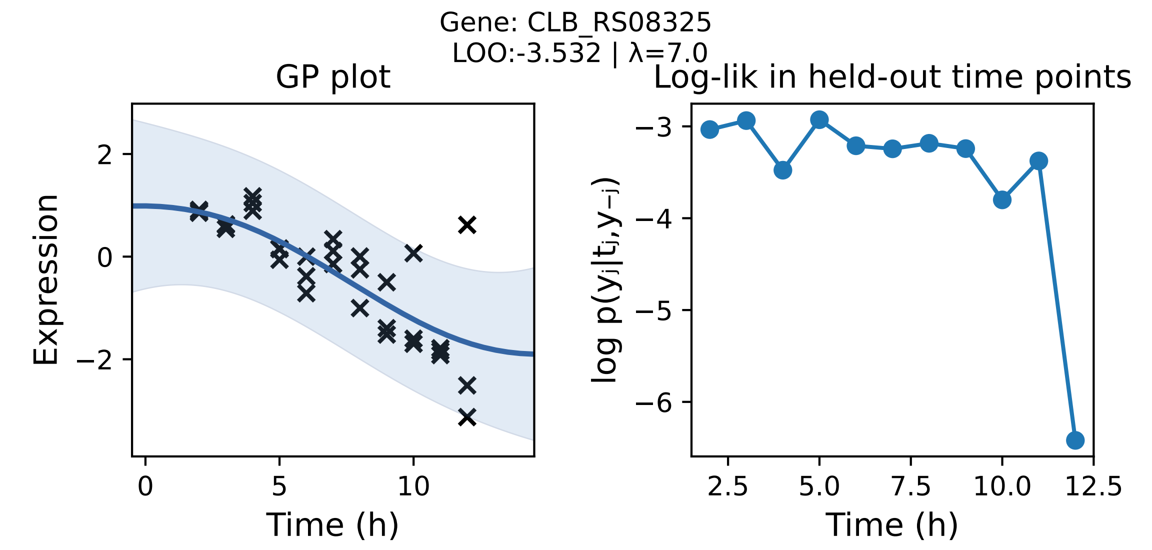

Gene: CLB_RS08325
LOO:-3.532 | λ=7.0
GP plot
Expression
Time (h)
Log-lik in held-out time points
log p(yj|tj,y−j)
Time (h)

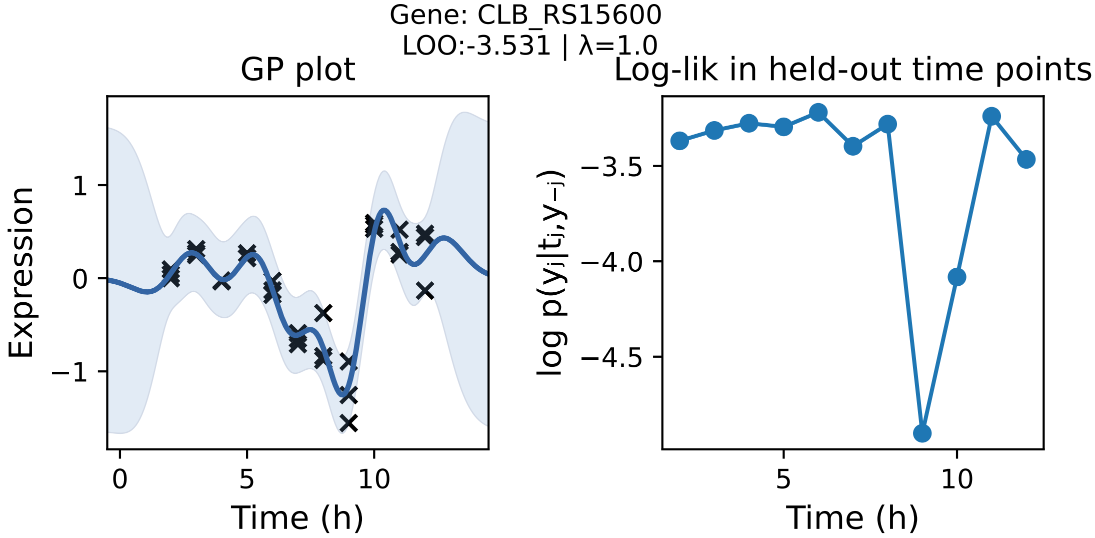

Gene: CLB_RS15600
LOO:-3.531 | λ=1.0
GP plot
Expression
Time (h)
Log-lik in held-out time points
log p(y_j|t_j,y_−j)
Time (h)

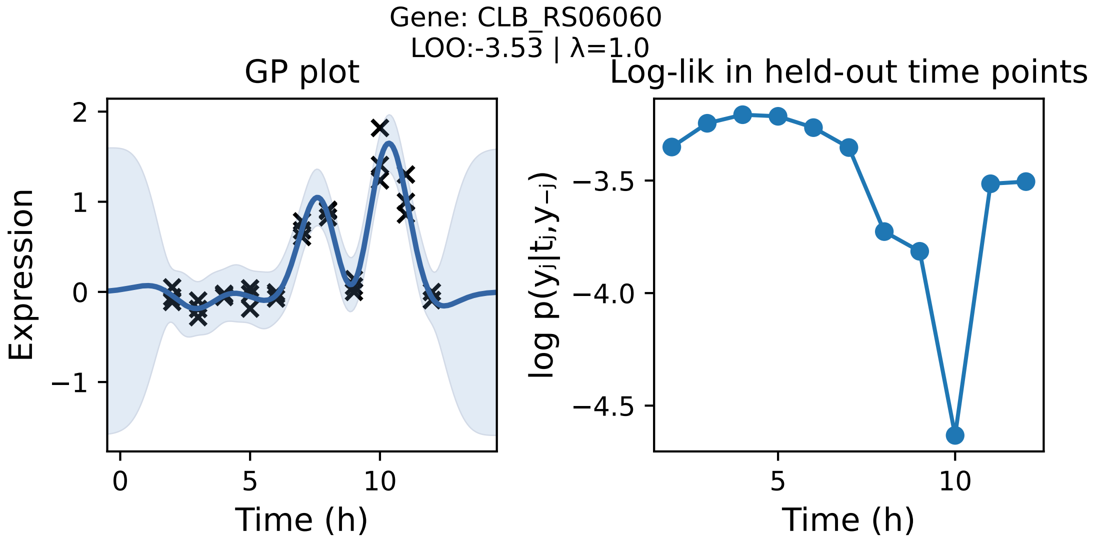

Gene: CLB_RS06060
LOO:-3.53 | λ=1.0
GP plot
Log-lik in held-out time points
Expression
Time (h)
log p(y_j|t_j,y_−j)
2
1
0
−1
0
5
10
−3.5
−4.0
−4.5
5
10
Time (h)

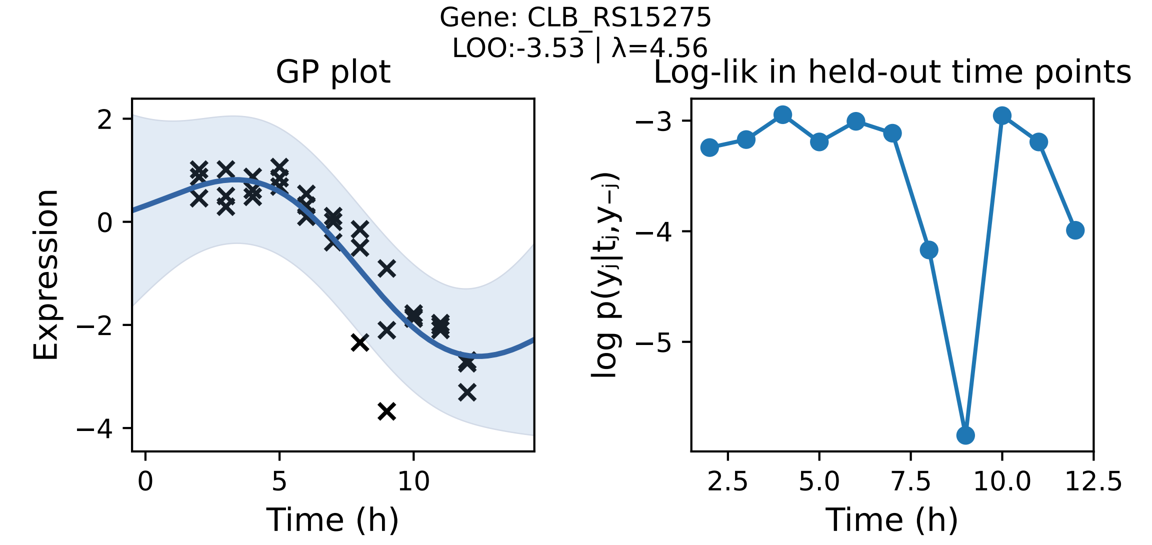
Gene: CLB_RS15275
LOO:-3.53 | λ=4.56
GP plot
Expression
Time (h)
Log-lik in held-out time points
log p(y_j|t_j,y_−j)
Time (h)

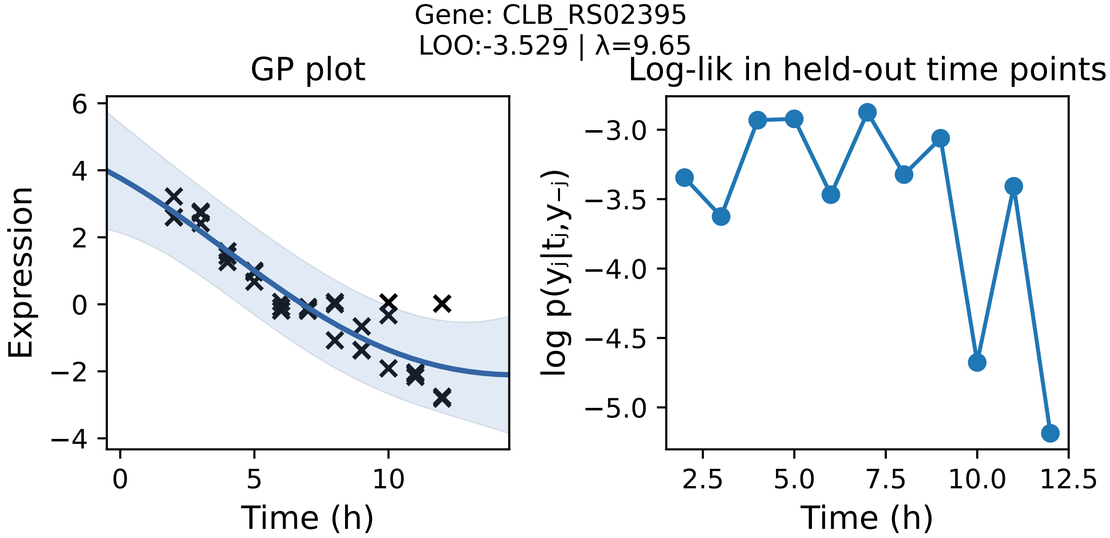
Gene: CLB_RS02395
LOO:-3.529 | λ=9.65
GP plot
Expression
Time (h)
Log-lik in held-out time points
log p(y_j|t_j,y_−j)
Time (h)

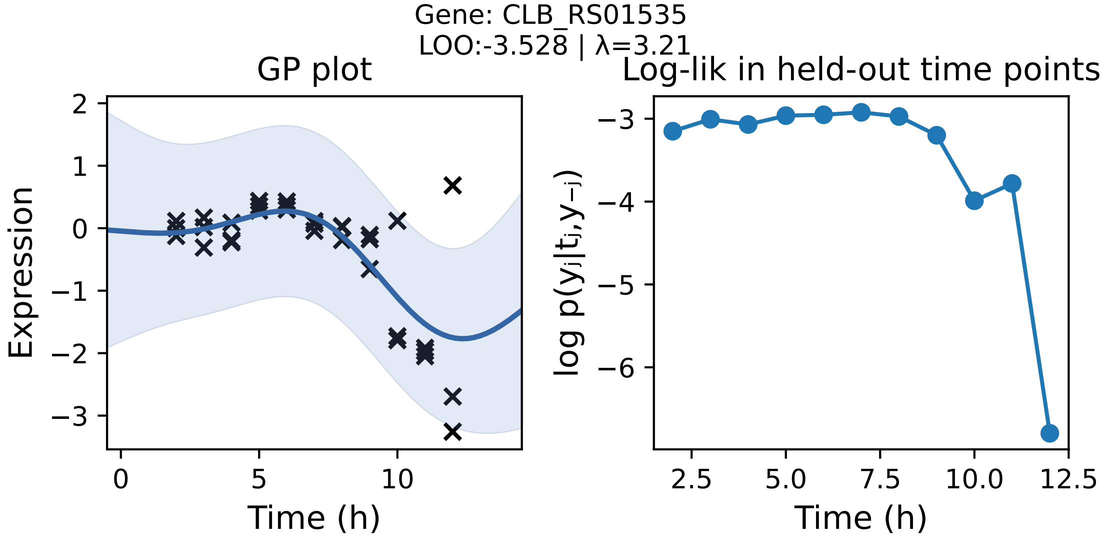

Gene: CLB_RS01535
LOO:-3.528 | λ=3.21
GP plot
Expression
Time (h)
Log-lik in held-out time points
log p(y_j|t_j,y_−j)
Time (h)

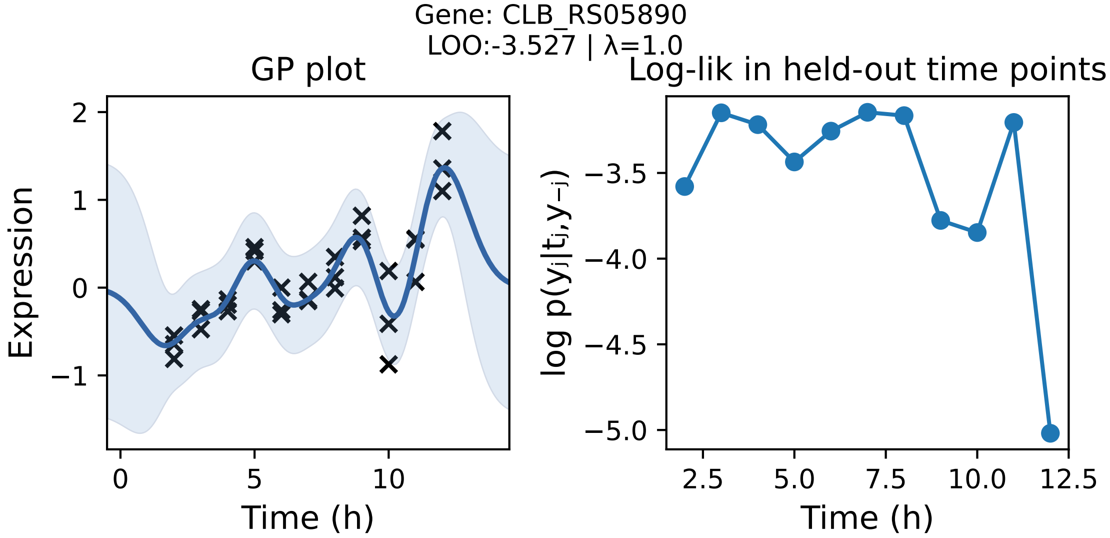

Gene: CLB_RS05890
LOO:-3.527 | λ=1.0
GP plot
Expression
Time (h)
2
1
0
−1
0
5
10
Log-lik in held-out time points
log p(y_j|t_j,y_−j)
Time (h)
−3.5
−4.0
−4.5
−5.0
2.5
5.0
7.5
10.0
12.5

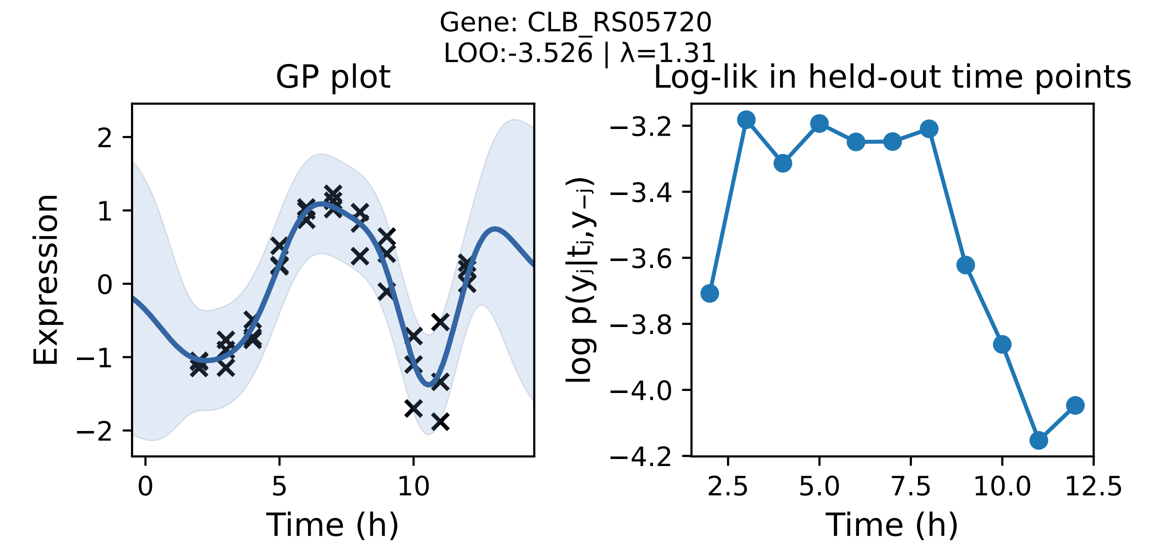
Gene: CLB_RS05720
LOO:-3.526 | λ=1.31
GP plot
Expression
Time (h)
Log-lik in held-out time points
log p(y_j|t_j,y_−j)
Time (h)

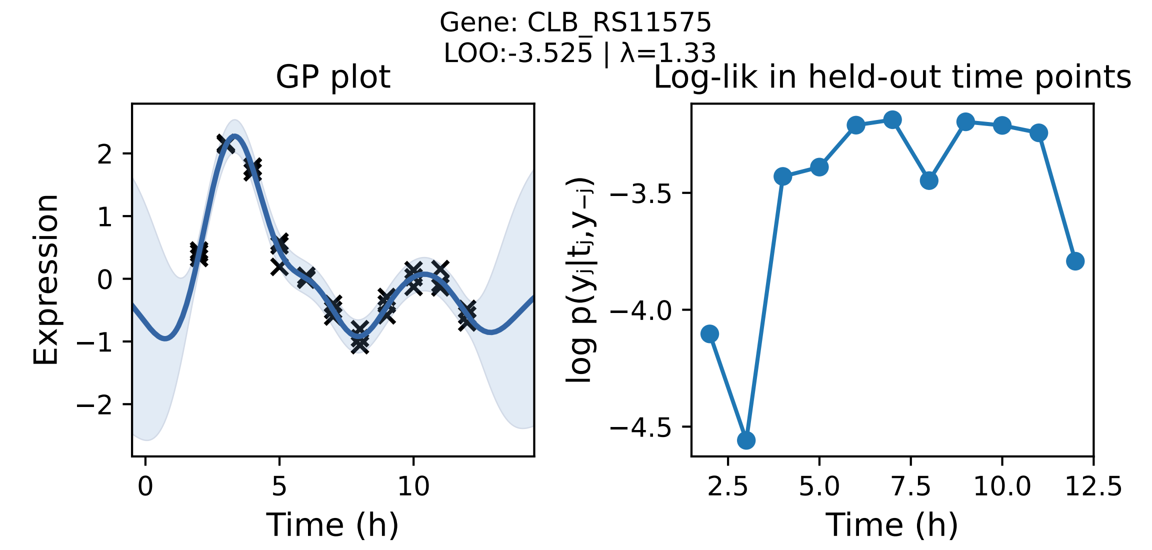
Gene: CLB_RS11575
LOO:-3.525 | λ=1.33
GP plot
Expression
Time (h)
Log-lik in held-out time points
log p(y_j|t_j,y_{-j})
Time (h)

Gene: CLB_RS07740
LOO:-3.524 | λ=1.0

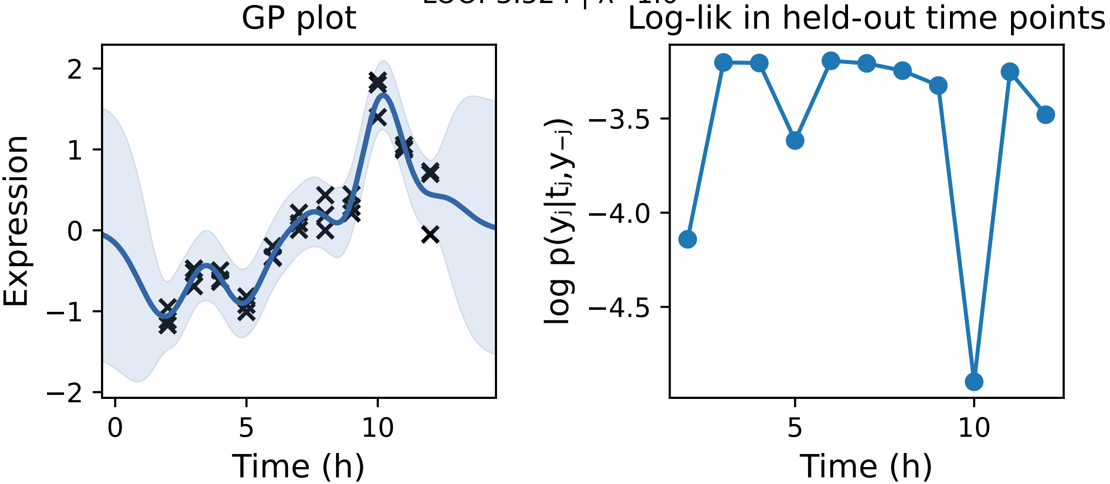

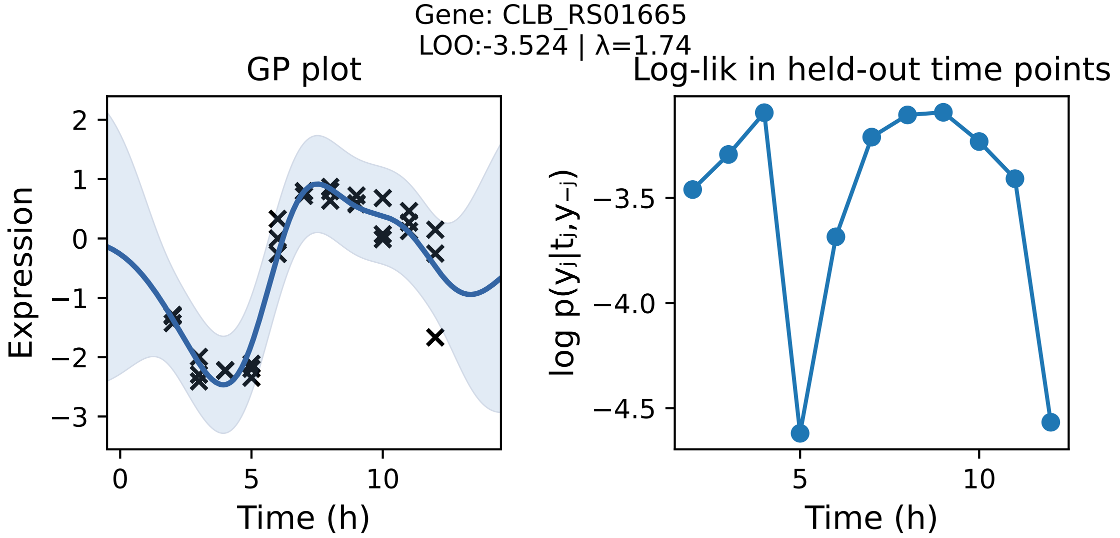

Gene: CLB_RS01665
LOO:-3.524 | λ=1.74
GP plot
Expression
Time (h)
2
1
0
−1
−2
−3
0
5
10
Log-lik in held-out time points
log p(y_j|t_j,y_−j)
−3.5
−4.0
−4.5
5
10
Time (h)

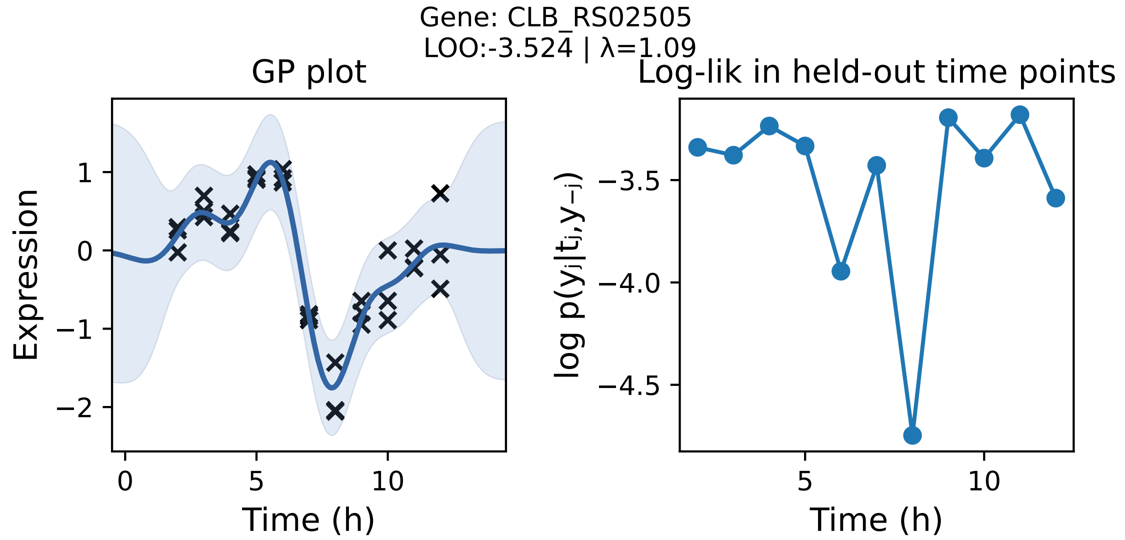
Gene: CLB_RS02505
LOO:-3.524 | λ=1.09
GP plot
Expression
Time (h)
Log-lik in held-out time points
log p(y_j|t_j,y_−j)
Time (h)

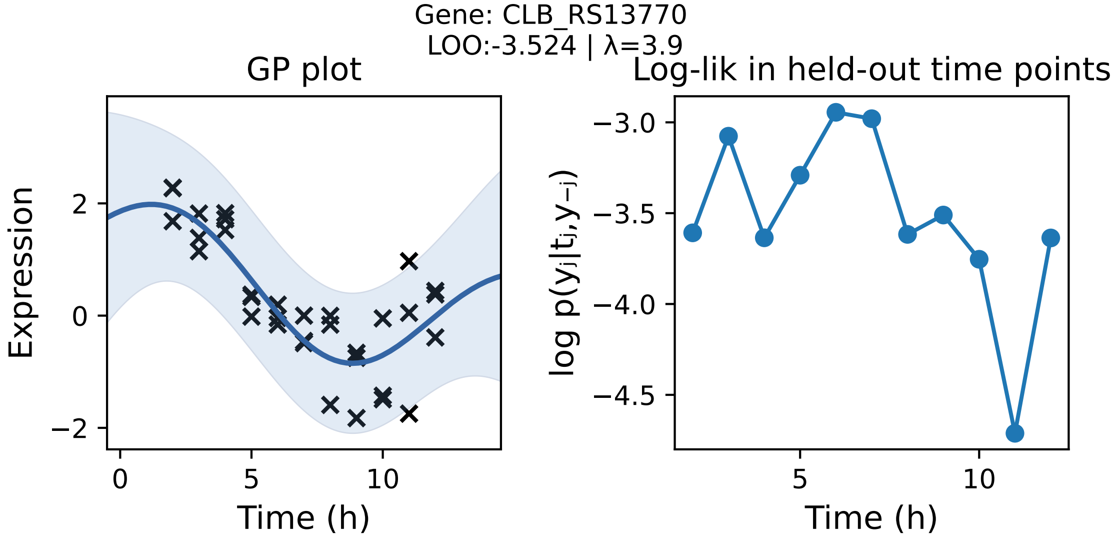

Gene: CLB_RS13770
LOO:-3.524 | λ=3.9
GP plot
Expression
Time (h)
Log-lik in held-out time points
log p(y_j|t_j,y_−j)
Time (h)

Gene: CLB_RS13580
LOO:-3.524 | λ=2.78

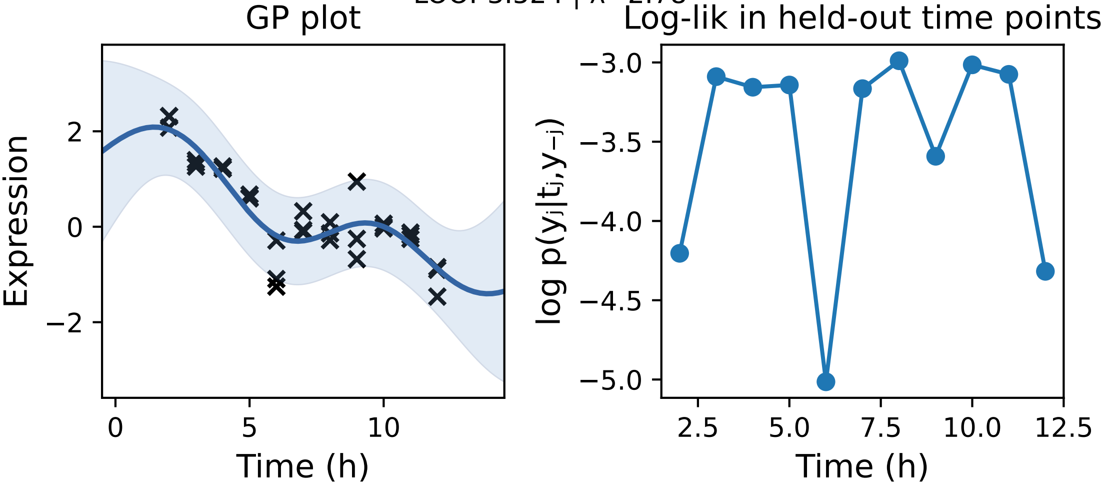

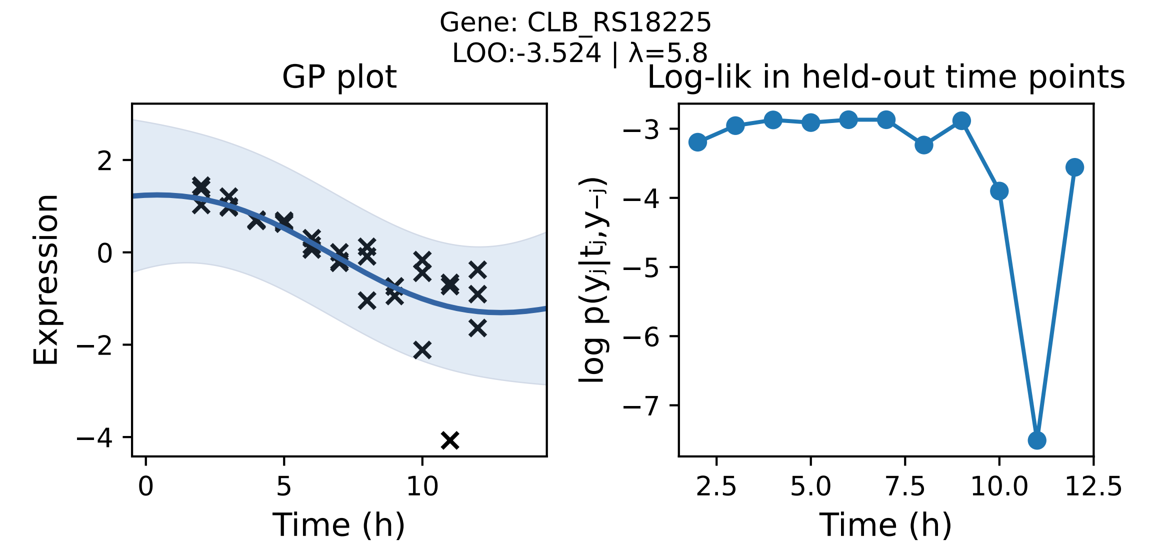

Gene: CLB_RS18225
LOO:-3.524 | λ=5.8
GP plot
Expression
Time (h)
Log-lik in held-out time points
log p(yⱼ|tⱼ,y₋ⱼ)
Time (h)

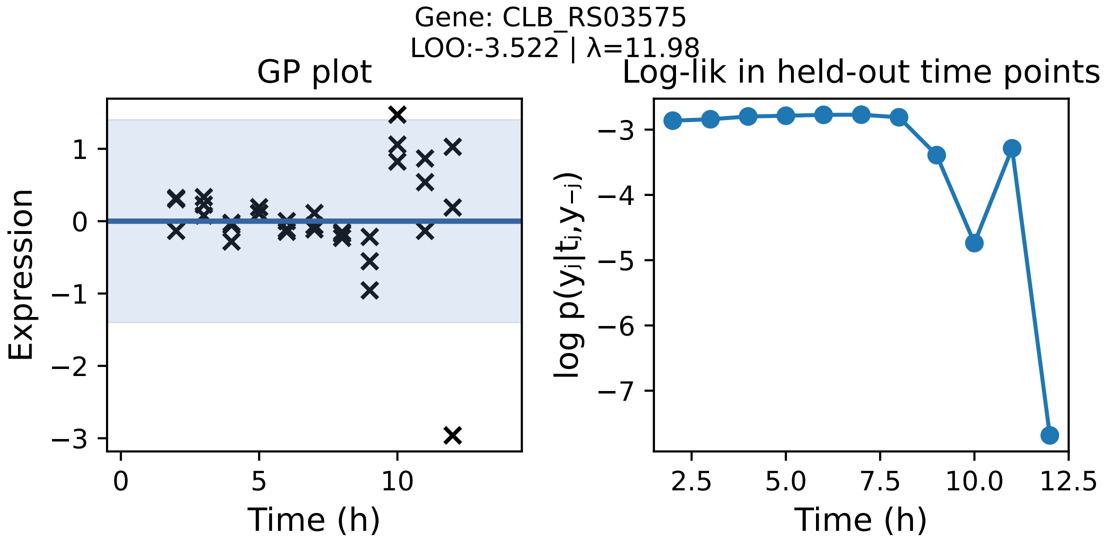
Gene: CLB_RS03575
LOO:-3.522 | λ=11.98
GP plot
Log-lik in held-out time points
Expression
Time (h)
log p(y_j|t_j,y_−j)
Time (h)
1
0
−1
−2
−3
0
5
10
−3
−4
−5
−6
−7
2.5
5.0
7.5
10.0
12.5

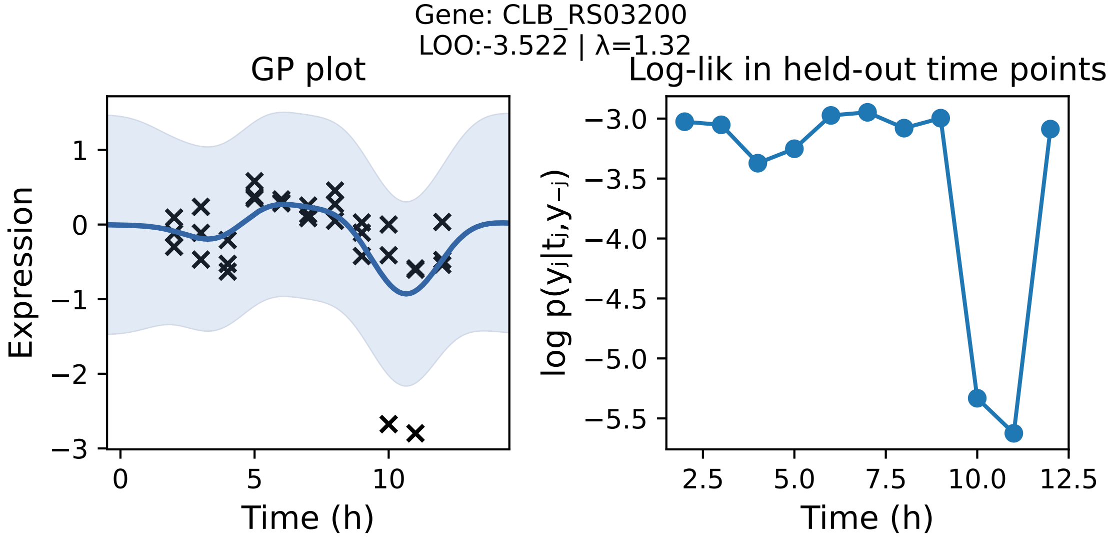

Gene: CLB_RS03200
LOO:-3.522 | λ=1.32
GP plot
Log-lik in held-out time points
Expression
Time (h)
log p(y_j|t_j,y_−j)
1
0
−1
−2
−3
0
5
10
−3.0
−3.5
−4.0
−4.5
−5.0
−5.5
2.5
5.0
7.5
10.0
12.5

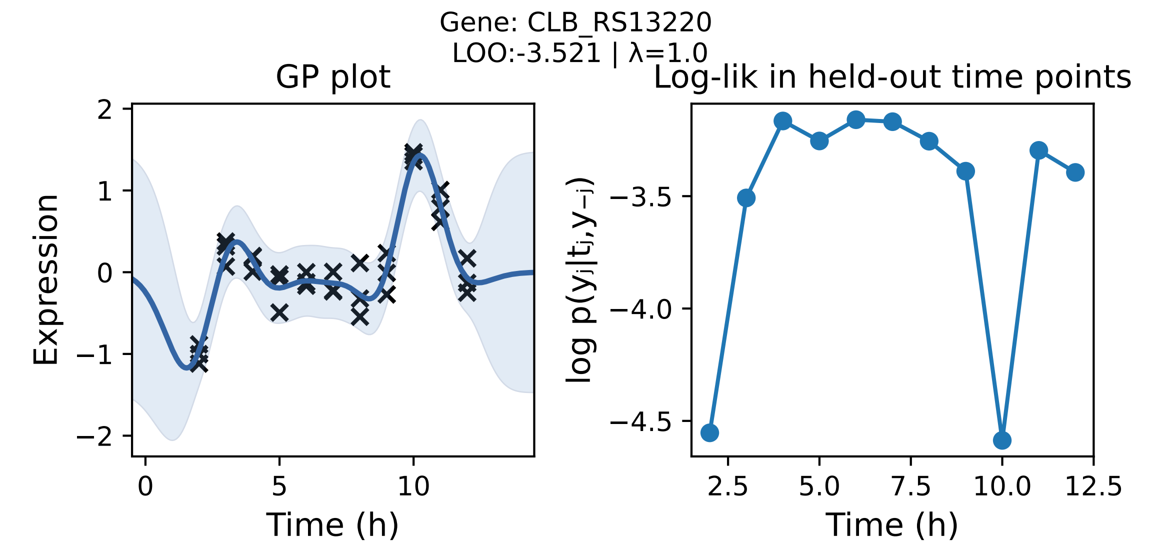

Gene: CLB_RS13220
LOO:-3.521 | λ=1.0
GP plot
Expression
Time (h)
2
1
0
−1
−2
0
5
10
Log-lik in held-out time points
log p(y_j|t_j,y_−j)
Time (h)
−3.5
−4.0
−4.5
2.5
5.0
7.5
10.0
12.5

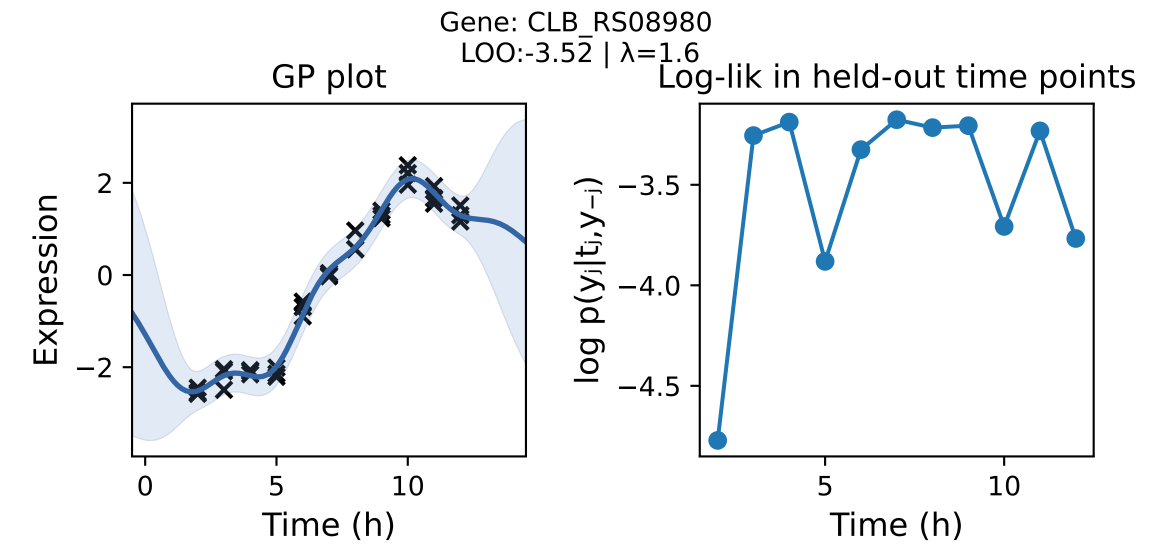
Gene: CLB_RS08980
LOO:-3.52 | λ=1.6
GP plot
Expression
Time (h)
2
0
−2
0
5
10
Log-lik in held-out time points
log p(y_j|t_j,y_−j)
−3.5
−4.0
−4.5
5
10
Time (h)

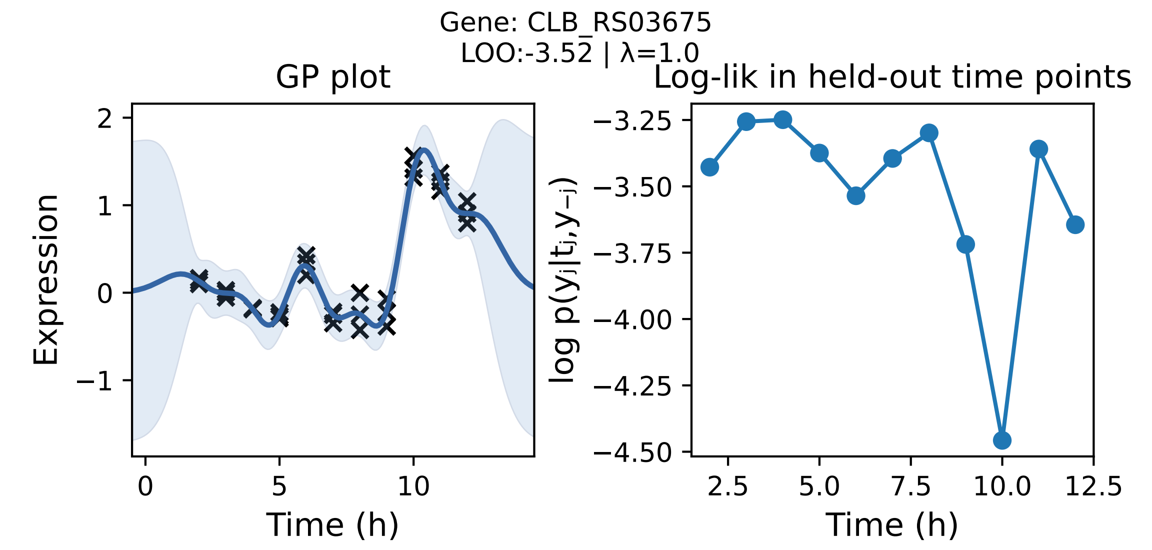
Gene: CLB_RS03675
LOO:-3.52 | λ=1.0
GP plot
Expression
Time (h)
Log-lik in held-out time points
log p(y_j|t_j,y_−j)
Time (h)

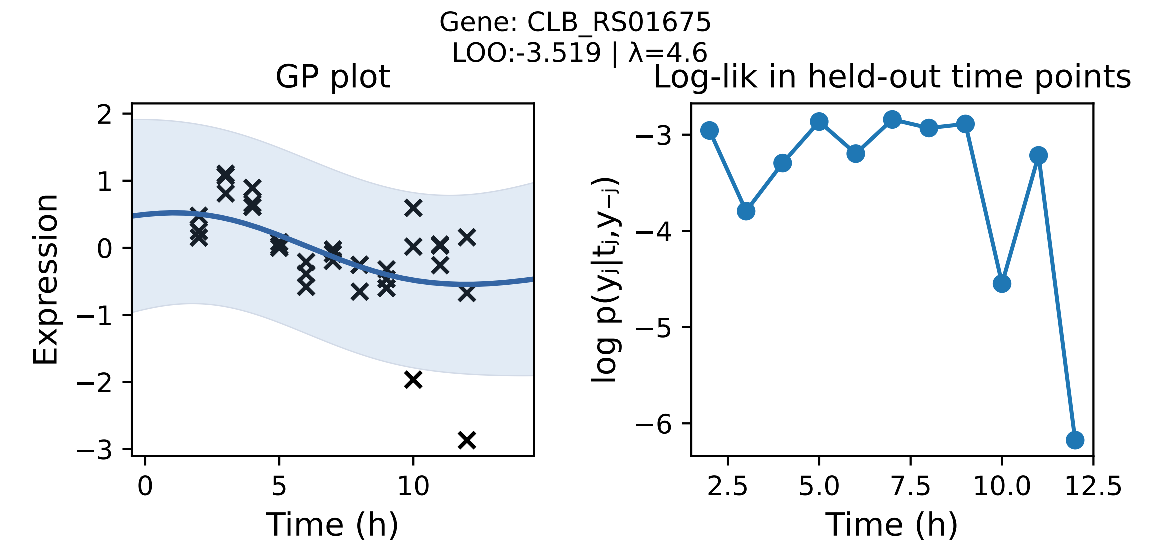

Gene: CLB_RS01675
LOO:-3.519 | λ=4.6
GP plot
Expression
Time (h)
Log-lik in held-out time points
log p(y_j|t_j,y_−j)
Time (h)

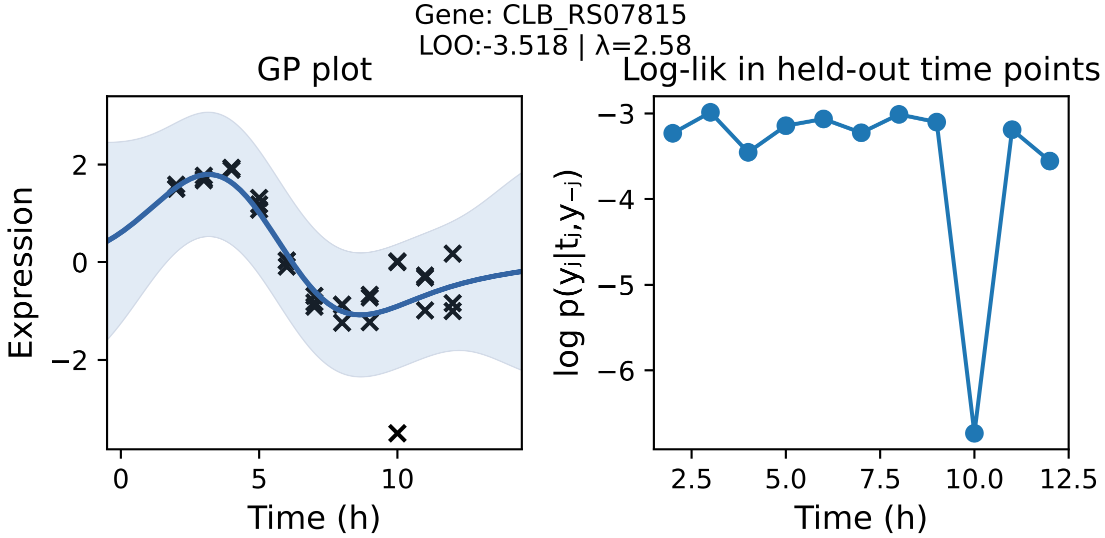
Gene: CLB_RS07815
LOO:-3.518 | λ=2.58
GP plot
Expression
Time (h)
Log-lik in held-out time points
log p(y_j|t_j,y_−j)
Time (h)

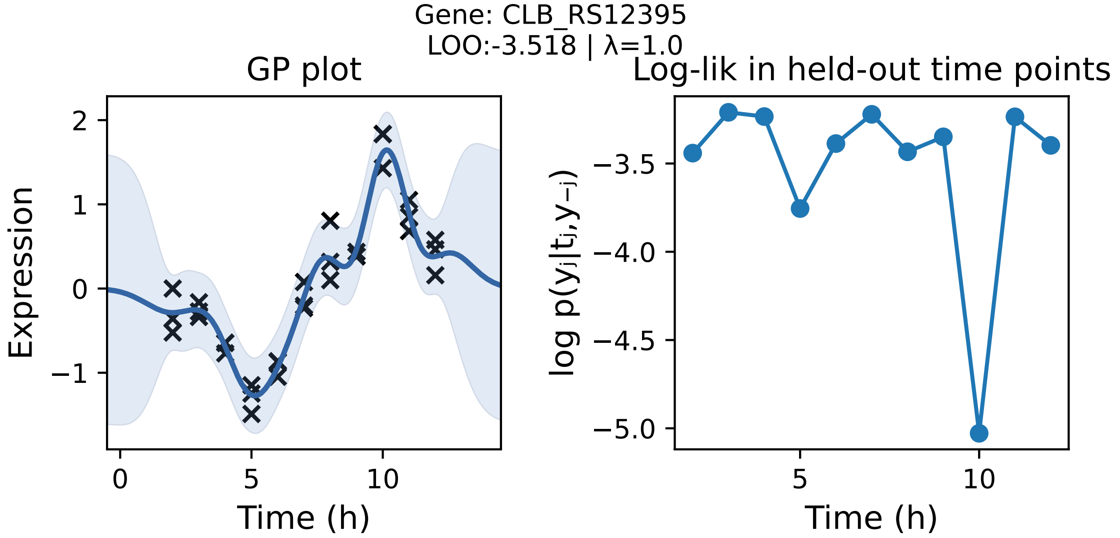
Gene: CLB_RS12395
LOO:-3.518 | λ=1.0
GP plot
Expression
Time (h)
Log-lik in held-out time points
log p(y_j|t_j,y_−j)
Time (h)
2
1
0
−1
0
5
10
−3.5
−4.0
−4.5
−5.0
5
10

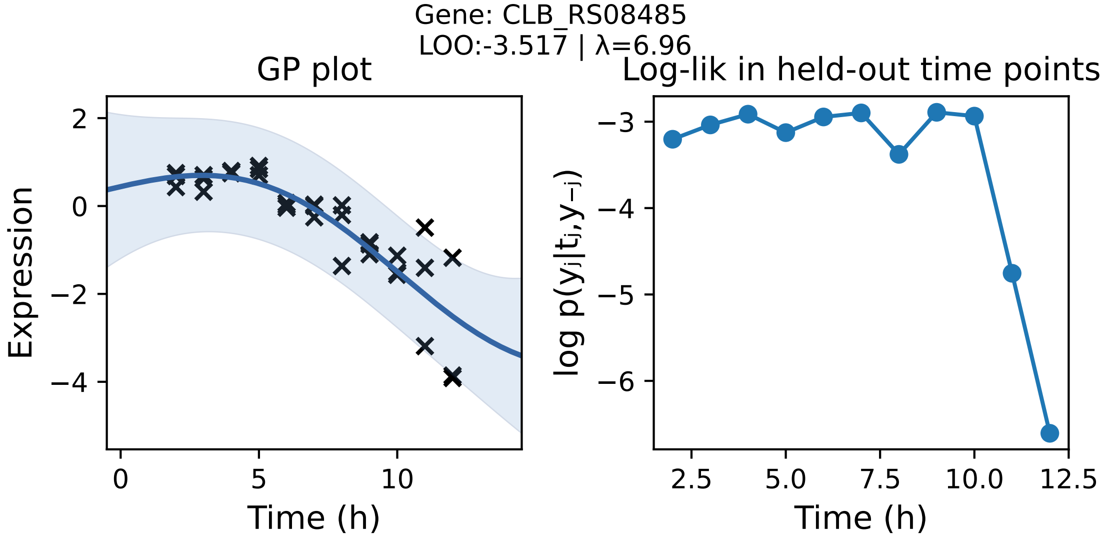
Gene: CLB_RS08485
LOO:-3.517 | λ=6.96
GP plot
Expression
Time (h)
2
0
−2
−4
0
5
10
Log-lik in held-out time points
log p(yj|tj,y−j)
Time (h)
−3
−4
−5
−6
2.5
5.0
7.5
10.0
12.5

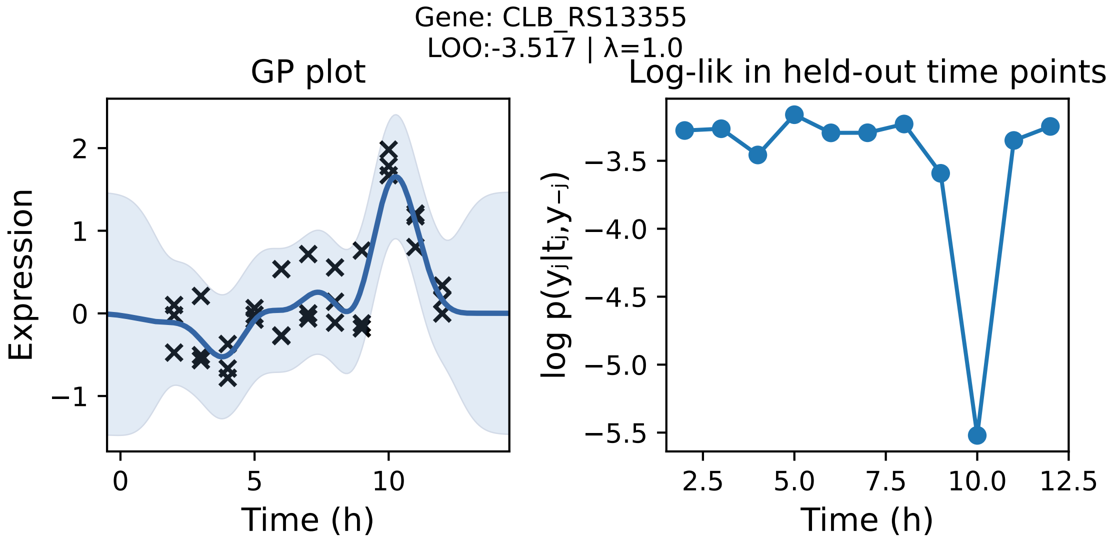
Gene: CLB_RS13355
LOO:-3.517 | λ=1.0
GP plot
Expression
Time (h)
2
1
0
−1
0
5
10
Log-lik in held-out time points
log p(y_j|t_j,y_−j)
−3.5
−4.0
−4.5
−5.0
−5.5
2.5
5.0
7.5
10.0
12.5
Time (h)

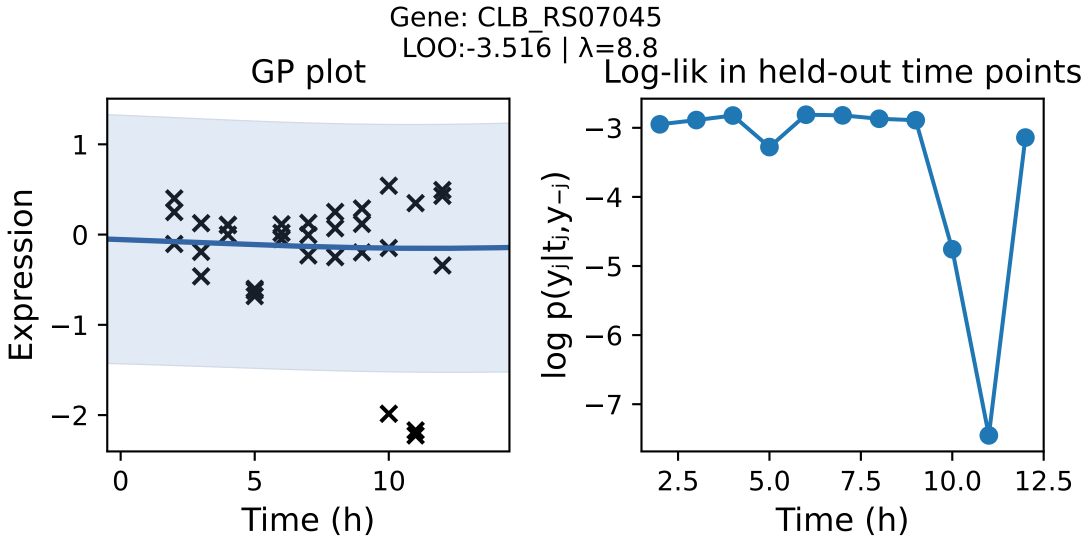

Gene: CLB_RS07045
LOO:-3.516 | λ=8.8
GP plot
Expression
Time (h)
Log-lik in held-out time points
log p(y_j|t_j,y_−j)
Time (h)

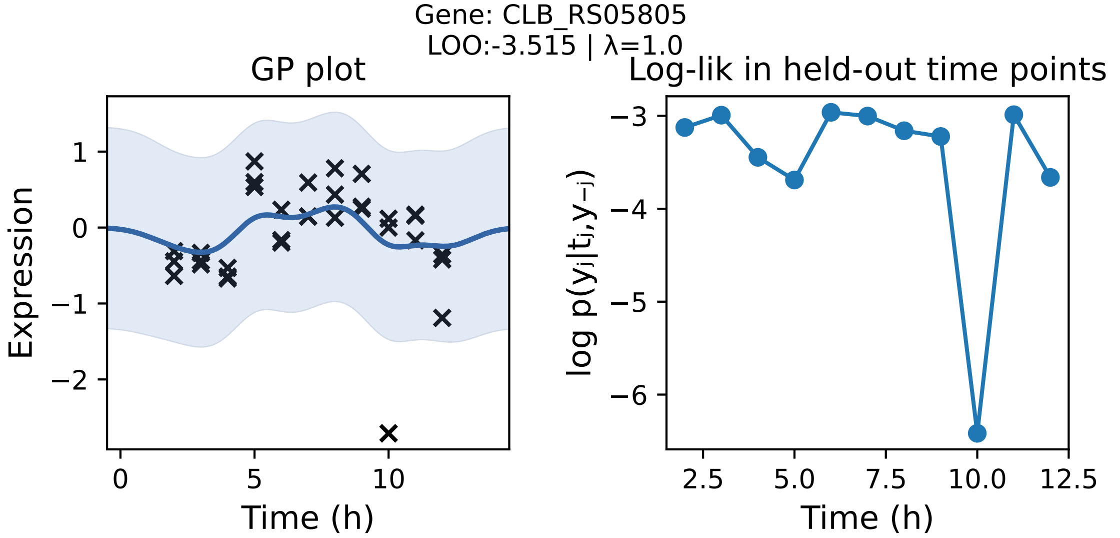
Gene: CLB_RS05805
LOO:-3.515 | λ=1.0
GP plot
Expression
Time (h)
1
0
−1
−2
0
5
10
Log-lik in held-out time points
log p(yⱼ|tⱼ,y₋ⱼ)
Time (h)
−3
−4
−5
−6
2.5
5.0
7.5
10.0
12.5

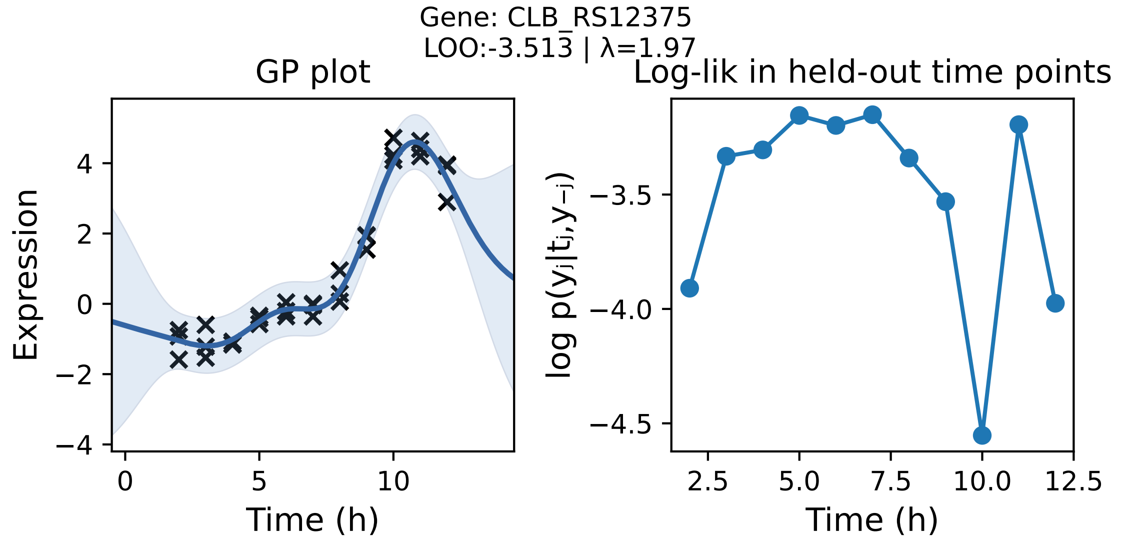
Gene: CLB_RS12375
LOO:-3.513 | λ=1.97
GP plot
Expression
Time (h)
Log-lik in held-out time points
log p(y_j|t_j,y_−j)
Time (h)

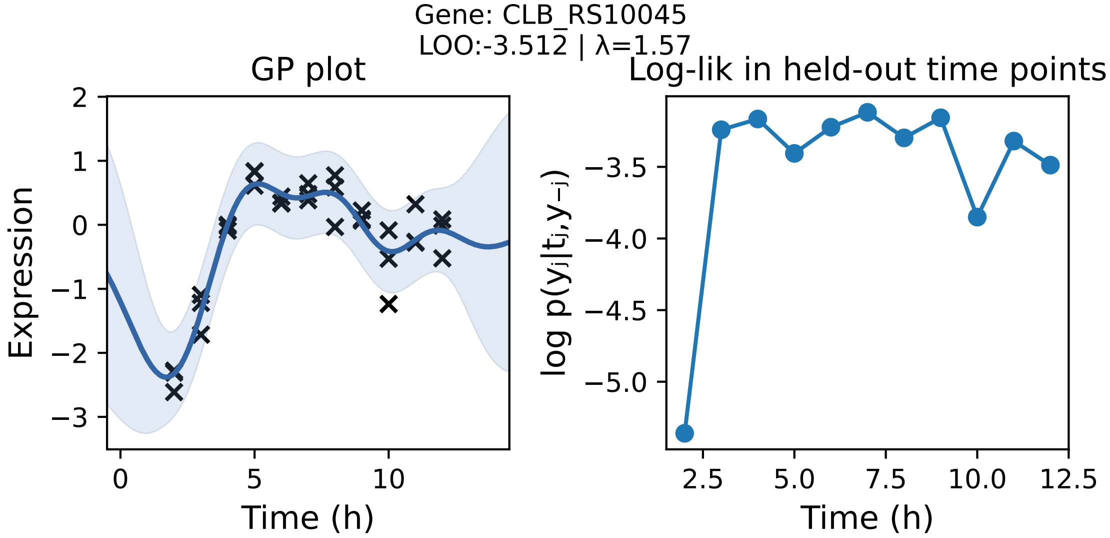
Gene: CLB_RS10045
LOO:-3.512 | λ=1.57
GP plot
Expression
Time (h)
Log-lik in held-out time points
log p(y_j|t_j,y_−j)
Time (h)

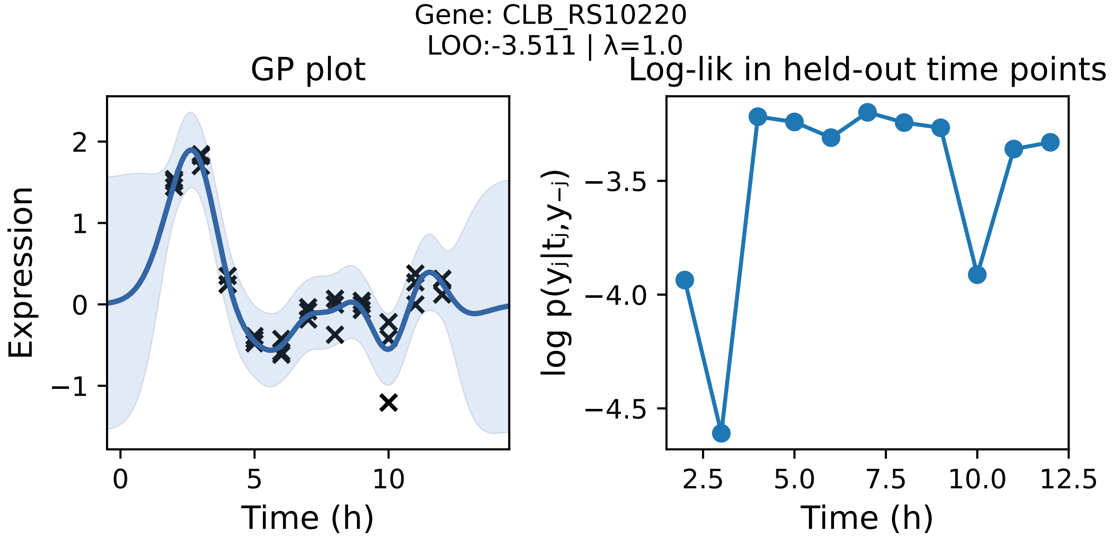
Gene: CLB_RS10220
LOO:-3.511 | λ=1.0
GP plot
Expression
Time (h)
Log-lik in held-out time points
log p(y_j|t_j,y_−j)
Time (h)

Gene: CLB_RS00175
LOO:-3.511 | λ=1.54

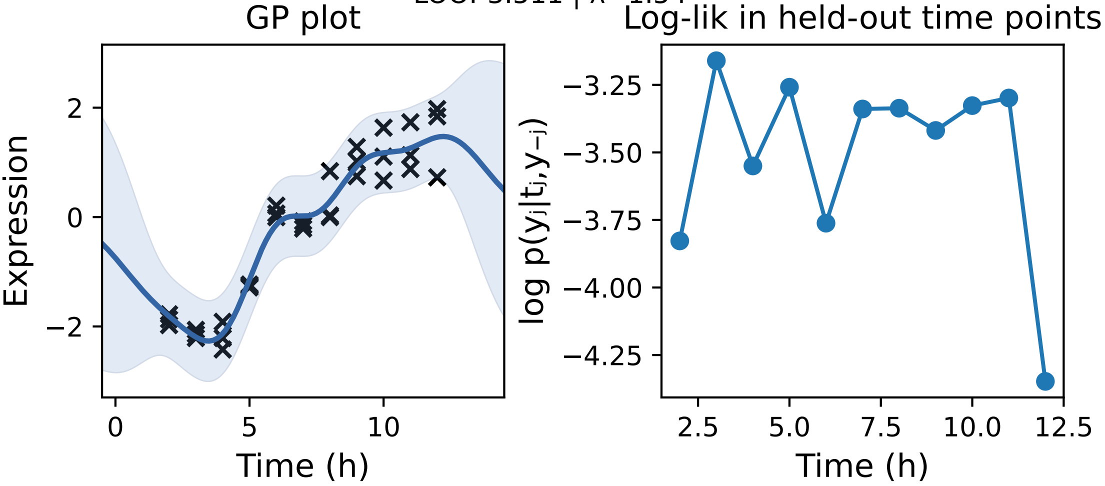

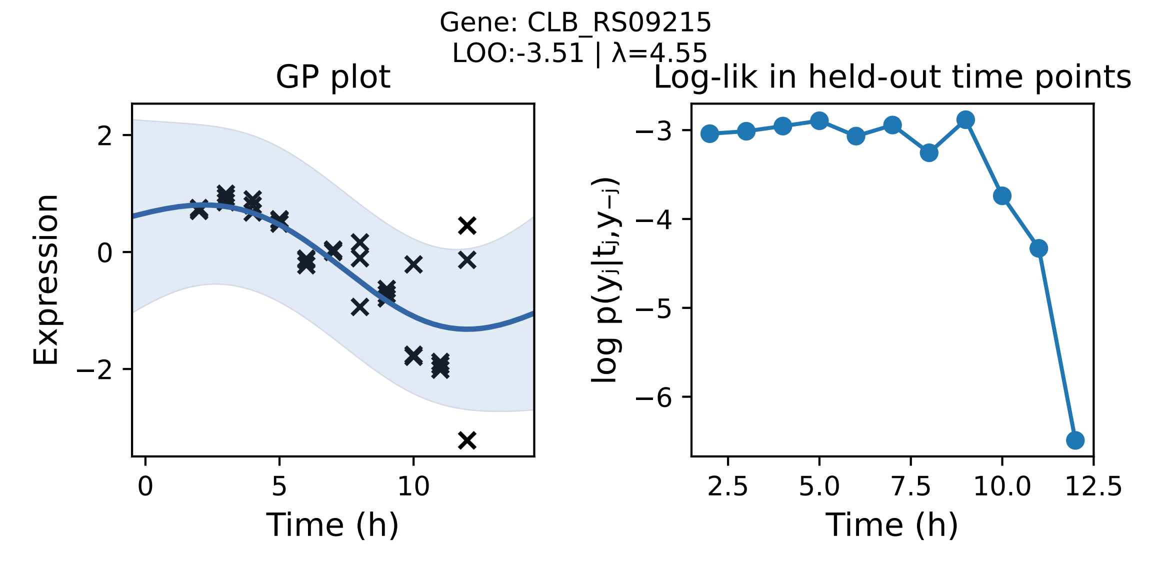
Gene: CLB_RS09215
LOO:-3.51 | λ=4.55
GP plot
Expression
Time (h)
Log-lik in held-out time points
log p(y_j|t_j,y_−j)
Time (h)

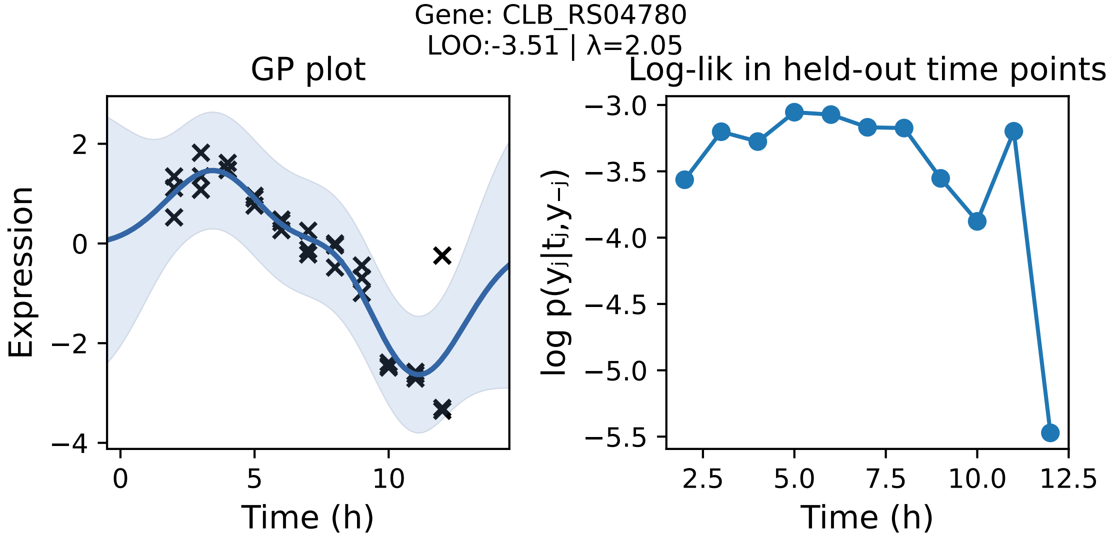

Gene: CLB_RS04780
LOO:-3.51 | λ=2.05
GP plot
Expression
Time (h)
2
0
−2
−4
0
5
10
Log-lik in held-out time points
log p(y_j|t_j,y_−j)
Time (h)
−3.0
−3.5
−4.0
−4.5
−5.0
−5.5
2.5
5.0
7.5
10.0
12.5

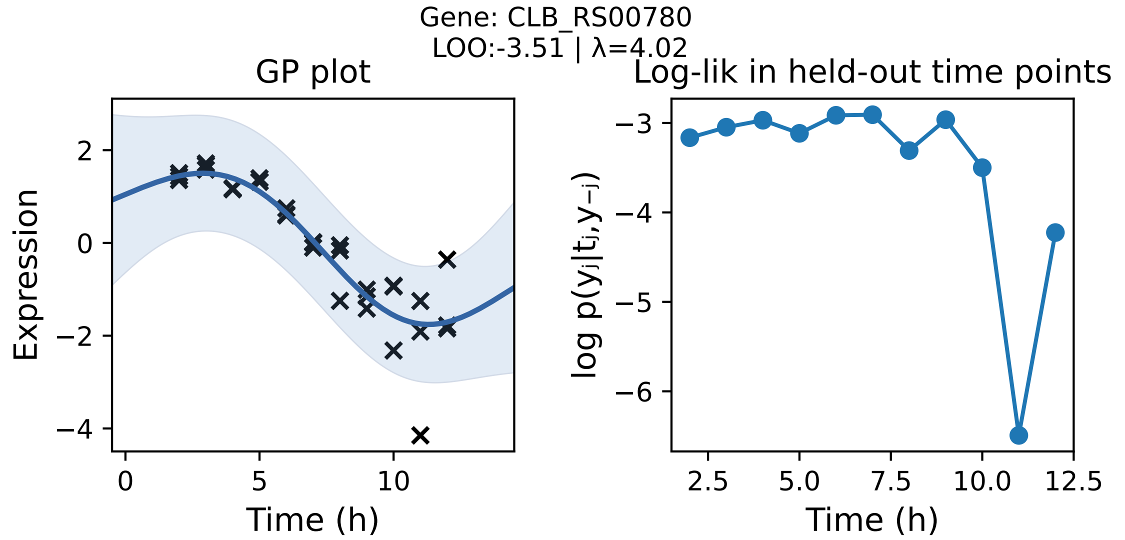

Gene: CLB_RS00780
LOO:-3.51 | λ=4.02
GP plot
Expression
Time (h)
Log-lik in held-out time points
log p(y_j|t_j,y_−j)
Time (h)

Gene: CLB_RS07120
LOO:-3.508 | λ=1.84

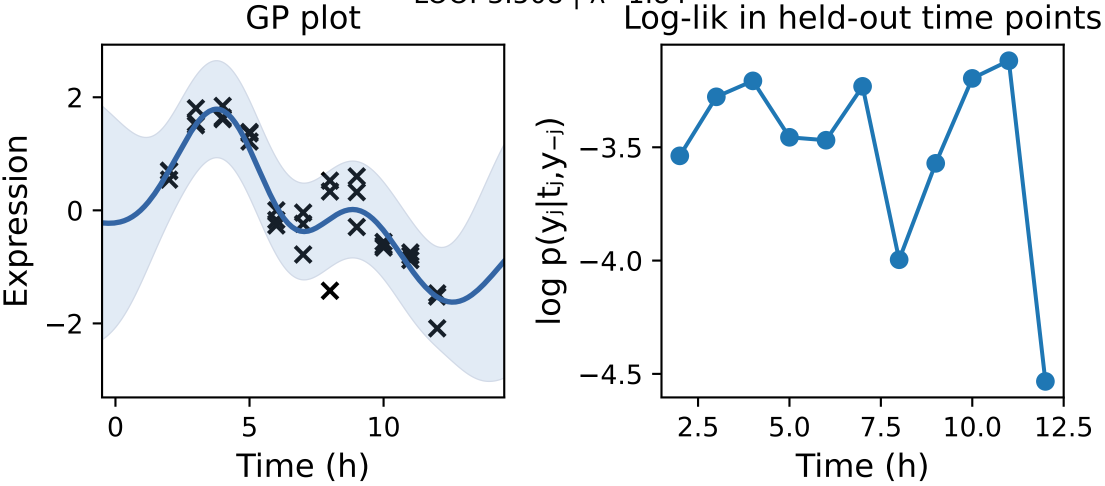

Gene: CLB_RS01580
LOO:-3.508 | λ=1.09

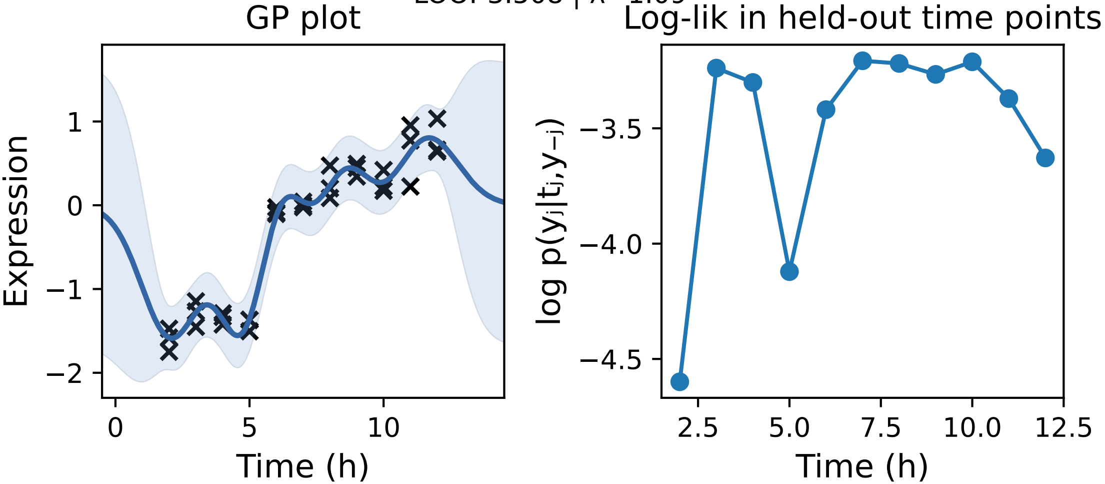

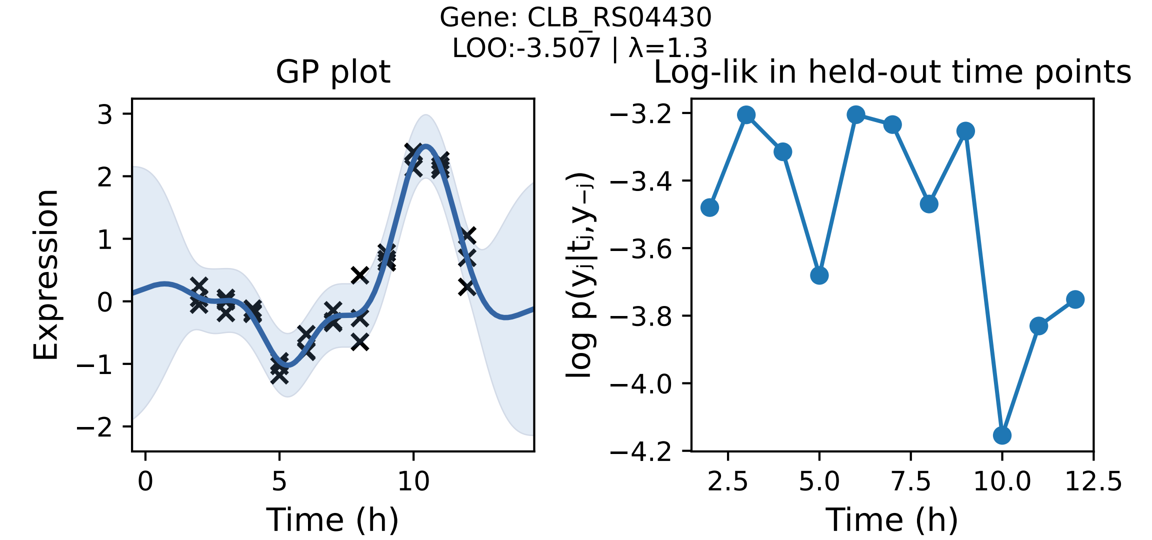
Gene: CLB_RS04430
LOO:-3.507 | λ=1.3
GP plot
Expression
Time (h)
Log-lik in held-out time points
log p(y_j|t_j,y_−j)
Time (h)

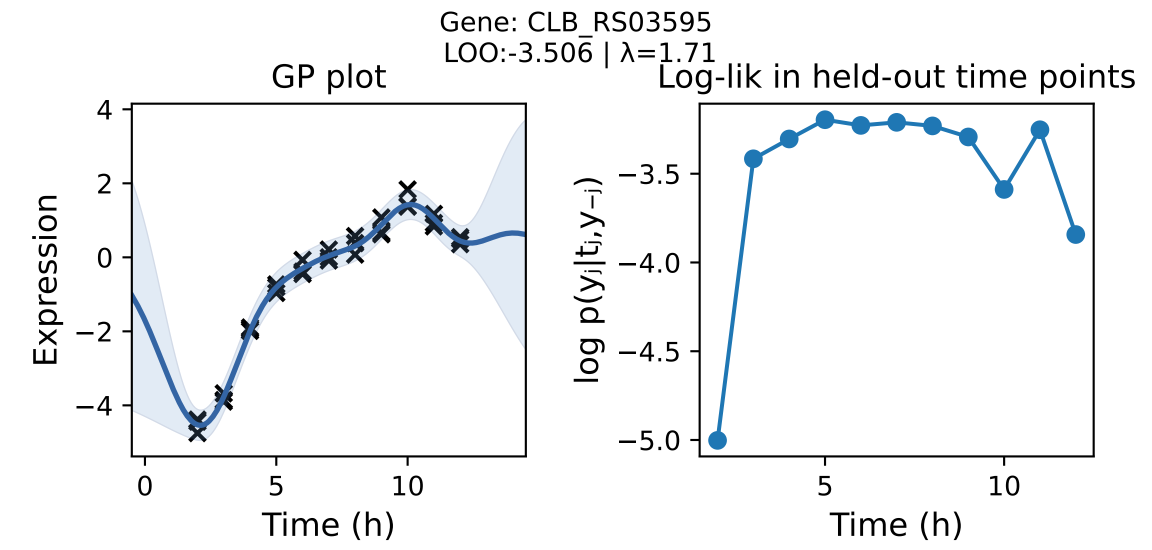

Gene: CLB_RS03595
LOO:-3.506 | λ=1.71
GP plot
Expression
Time (h)
4
2
0
−2
−4
0
5
10
Log-lik in held-out time points
log p(y_j|t_j,y_−j)
Time (h)
−3.5
−4.0
−4.5
−5.0
5
10

Gene: CLB_RS02775
LOO:-3.506 | λ=6.85

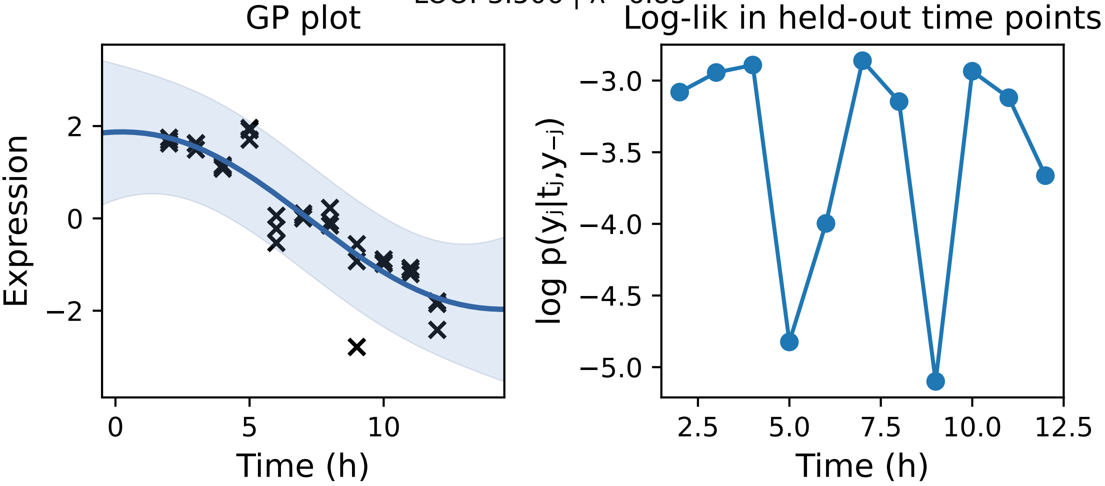

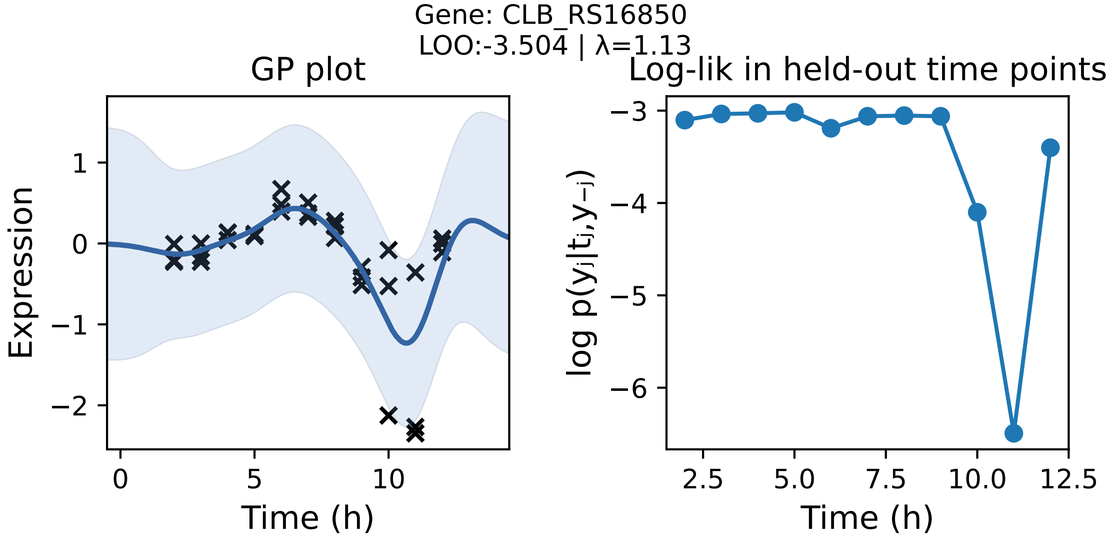

Gene: CLB_RS16850
LOO:-3.504 | λ=1.13
GP plot
Expression
Time (h)
Log-lik in held-out time points
log p(y_j|t_j,y_−j)
Time (h)

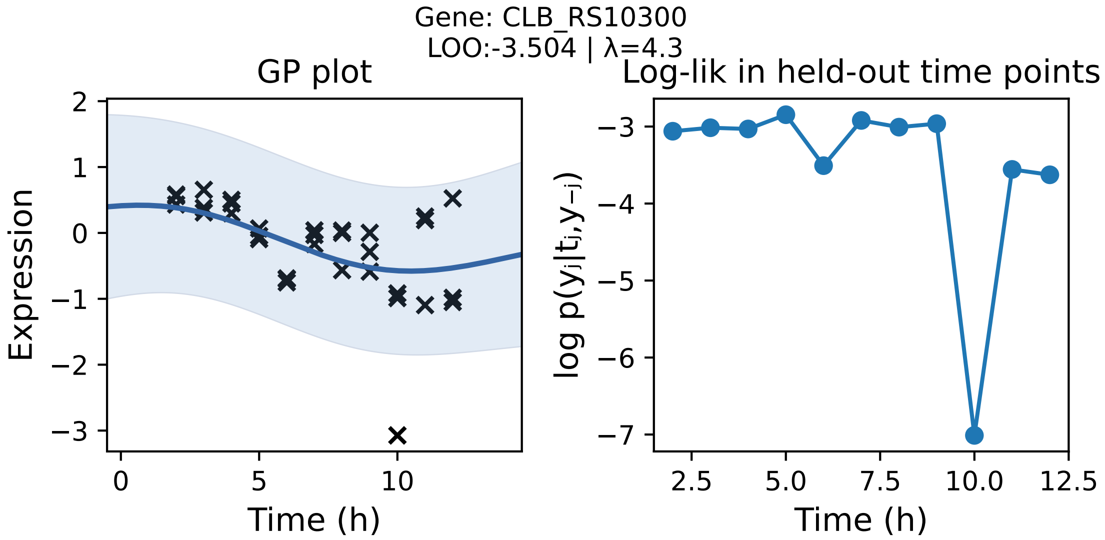

Gene: CLB_RS10300
LOO:-3.504 | λ=4.3
GP plot
Log-lik in held-out time points
Expression
Time (h)
log p(y_j|t_j,y_{−j})
Time (h)

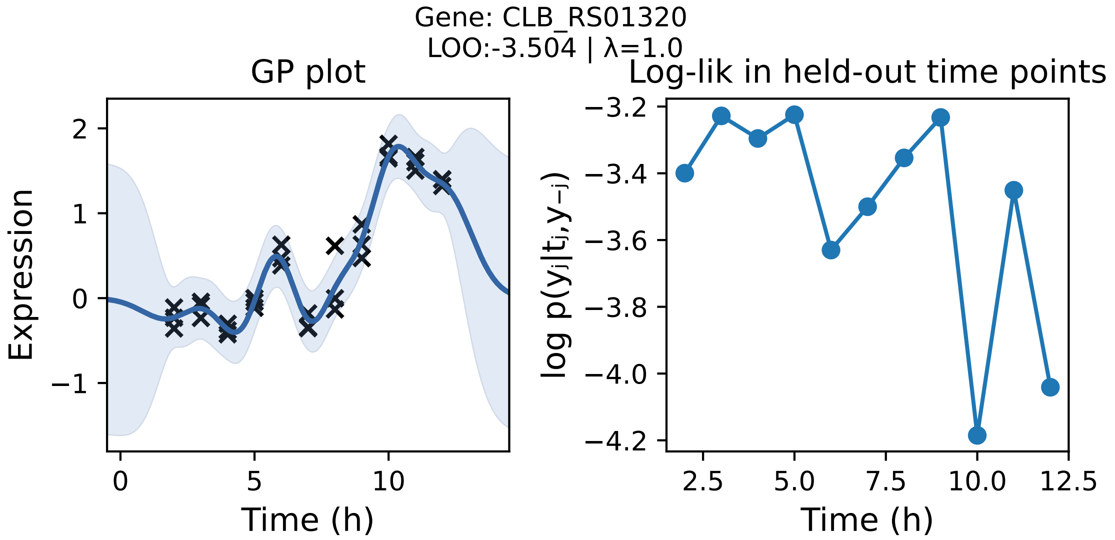
Gene: CLB_RS01320
LOO:-3.504 | λ=1.0
GP plot
Expression
Time (h)
2
1
0
−1
0
5
10
Log-lik in held-out time points
log p(y_j|t_j,y_−j)
Time (h)
−3.2
−3.4
−3.6
−3.8
−4.0
−4.2
2.5
5.0
7.5
10.0
12.5

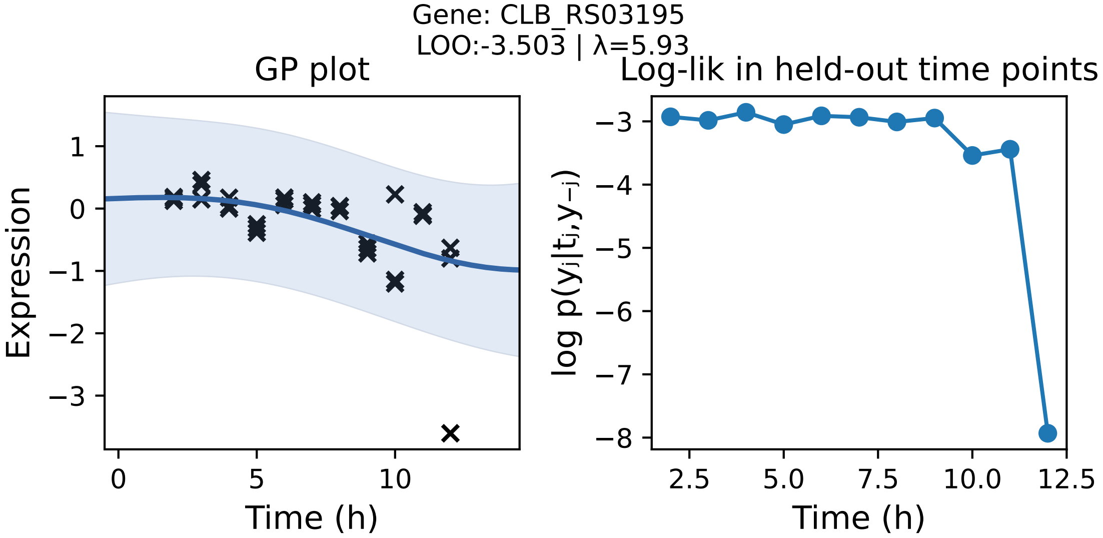
Gene: CLB_RS03195
LOO:-3.503 | λ=5.93
GP plot
Log-lik in held-out time points
Expression
Time (h)
log p(y_j|t_j,y_−j)
Time (h)

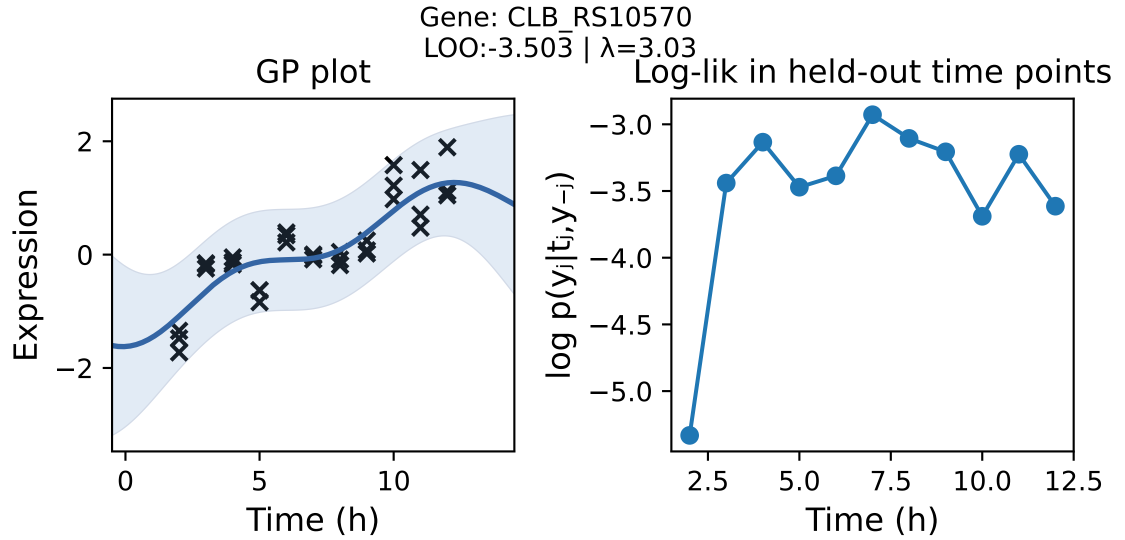

Gene: CLB_RS10570
LOO:-3.503 | λ=3.03
GP plot
Expression
Time (h)
2
0
−2
0
5
10
Log-lik in held-out time points
log p(y_j|t_j,y_−j)
−3.0
−3.5
−4.0
−4.5
−5.0
2.5
5.0
7.5
10.0
12.5
Time (h)

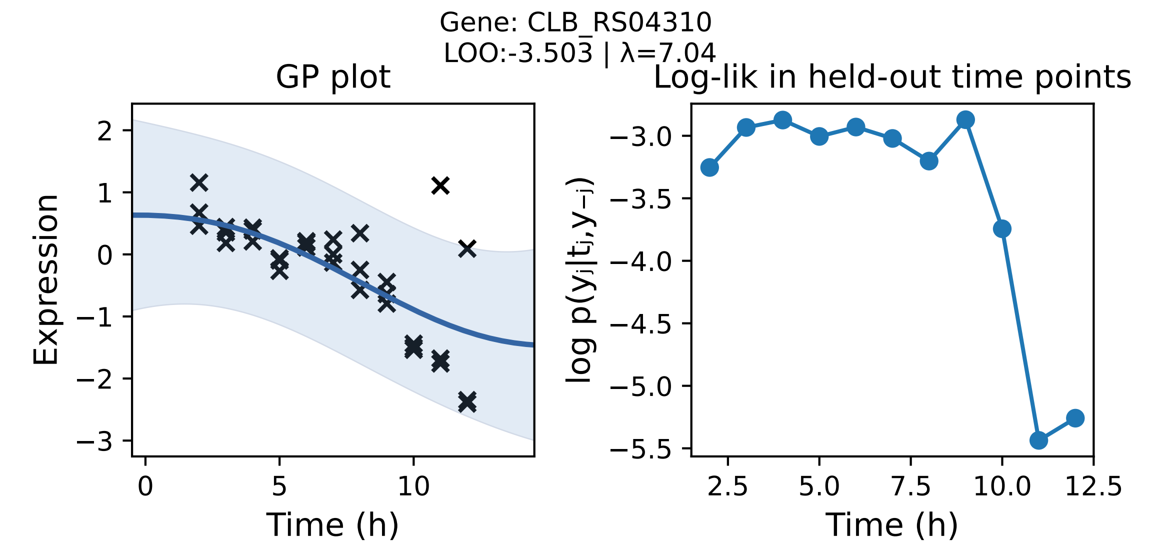

Gene: CLB_RS04310
LOO:-3.503 | λ=7.04
GP plot
Expression
Time (h)
Log-lik in held-out time points
log p(y_j|t_j,y_−j)
Time (h)
2
1
0
−1
−2
−3
0
5
10
−3.0
−3.5
−4.0
−4.5
−5.0
−5.5
2.5
5.0
7.5
10.0
12.5

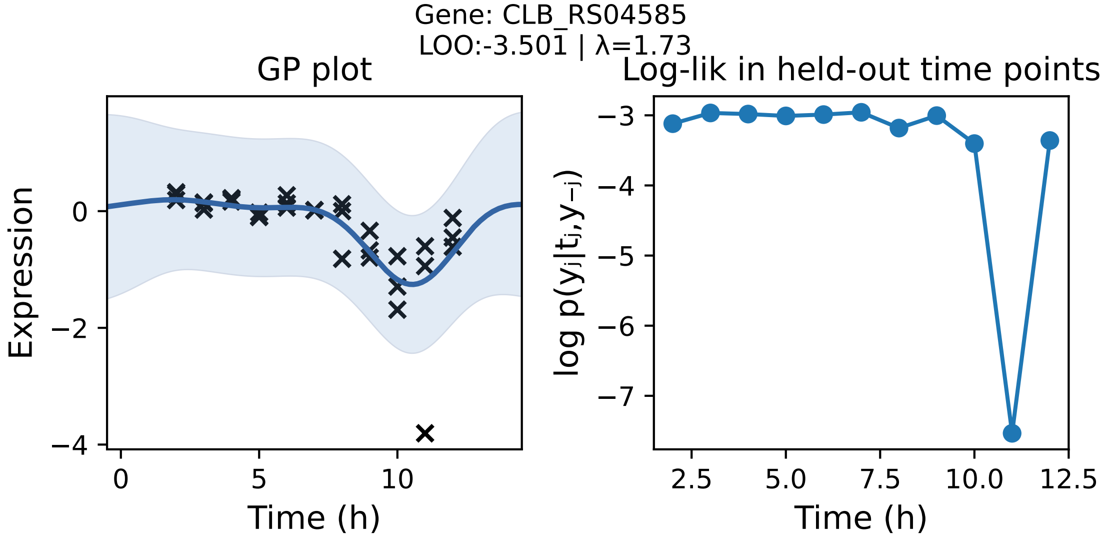
Gene: CLB_RS04585
LOO:-3.501 | λ=1.73
GP plot
Expression
0
−2
−4
0
5
10
Time (h)
Log-lik in held-out time points
log p(y_j|t_j,y_−j)
−3
−4
−5
−6
−7
2.5
5.0
7.5
10.0
12.5
Time (h)

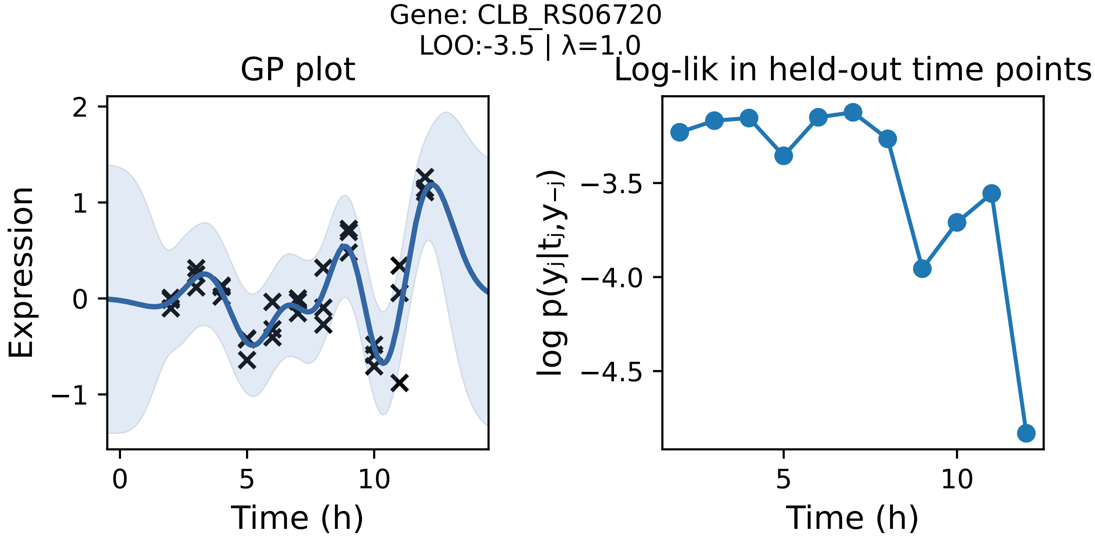
Gene: CLB_RS06720
LOO:-3.5 | λ=1.0
GP plot
Expression
Time (h)
Log-lik in held-out time points
log p(y_j|t_j,y_−j)
Time (h)

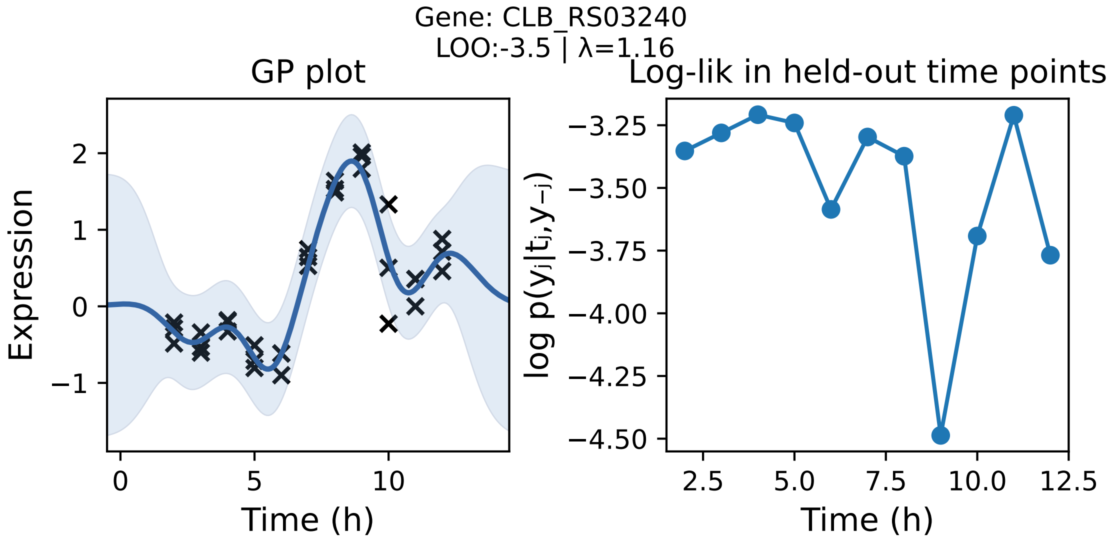

Gene: CLB_RS03240
LOO:-3.5 | λ=1.16
GP plot
Expression
Time (h)
Log-lik in held-out time points
log p(y_j|t_j,y_−j)
Time (h)

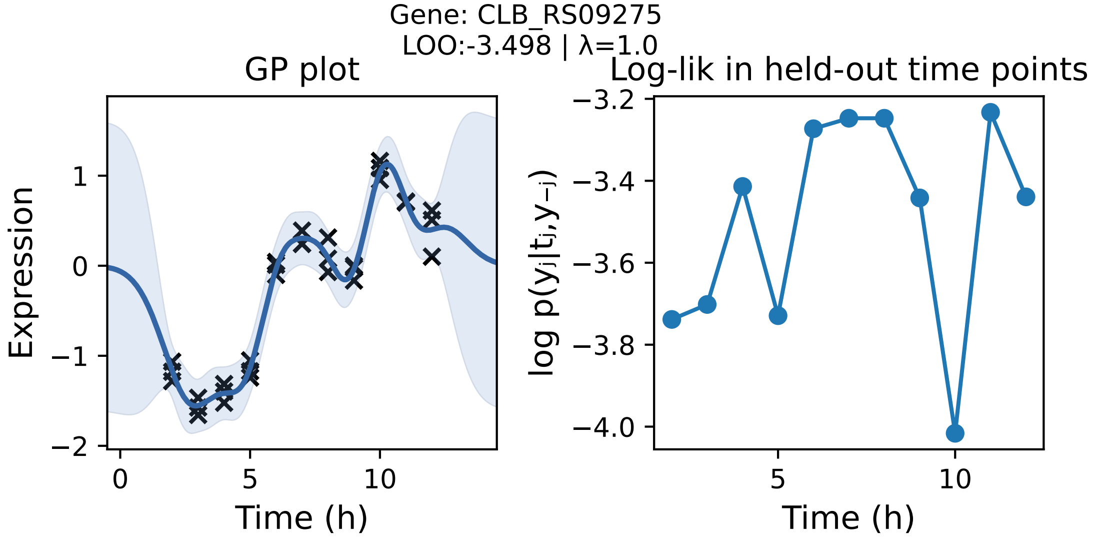
Gene: CLB_RS09275
LOO:-3.498 | λ=1.0
GP plot
Expression
Time (h)
1
0
−1
−2
0
5
10
Log-lik in held-out time points
log p(yⱼ|tⱼ,y₋ⱼ)
Time (h)
−3.2
−3.4
−3.6
−3.8
−4.0
5
10

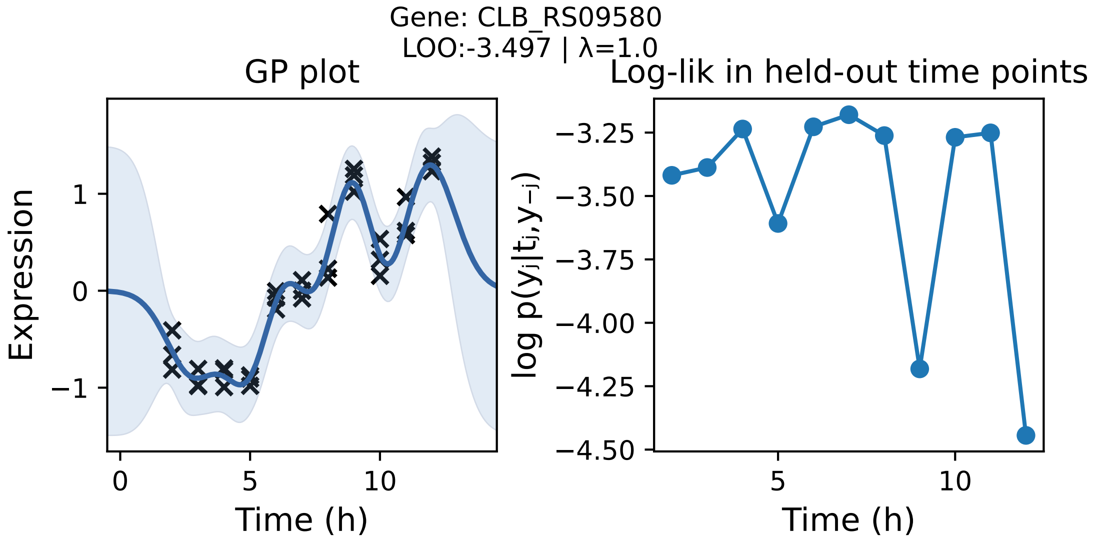

Gene: CLB_RS09580
LOO:-3.497 | λ=1.0
GP plot
Expression
Time (h)
Log-lik in held-out time points
log p(y_j|t_j,y_−j)
Time (h)

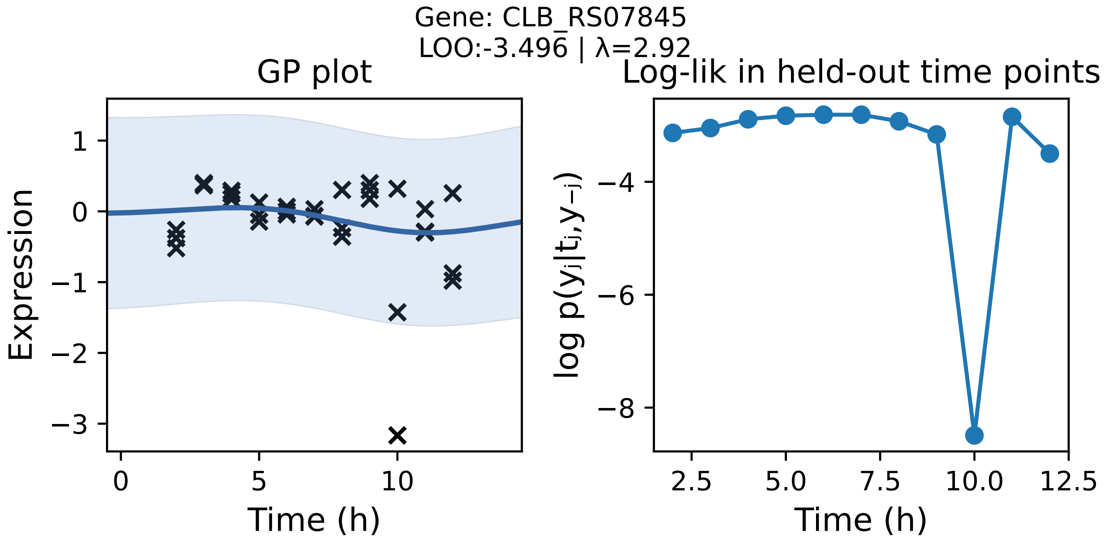

Gene: CLB_RS07845
LOO:-3.496 | λ=2.92
GP plot
Expression
Time (h)
1
0
−1
−2
−3
0
5
10
Log-lik in held-out time points
log p(y_j|t_j,y_−j)
Time (h)
−4
−6
−8
2.5
5.0
7.5
10.0
12.5

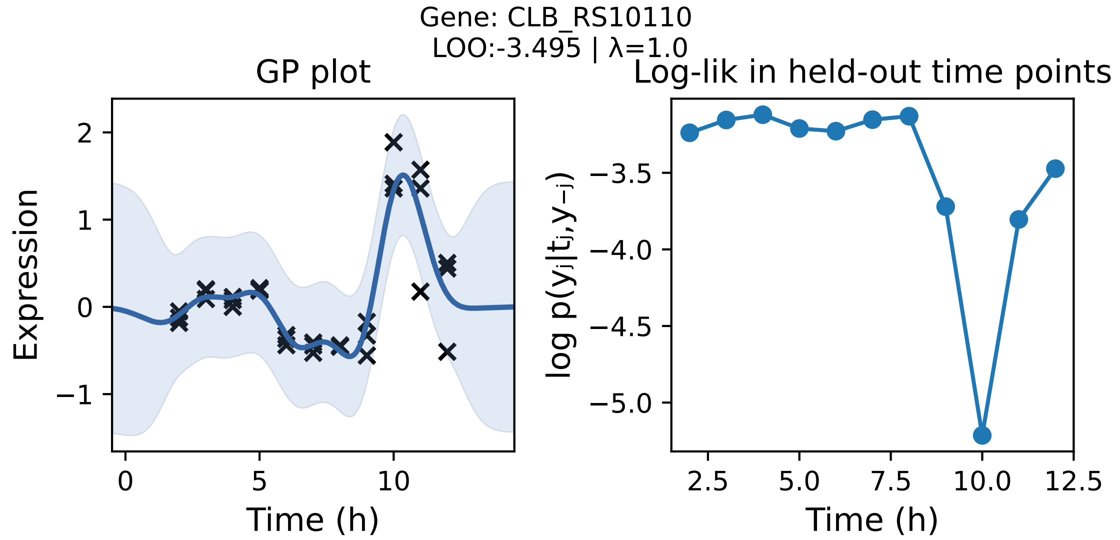

Gene: CLB_RS10110
LOO:-3.495 | λ=1.0
GP plot
Expression
Time (h)
Log-lik in held-out time points
log p(y_j|t_j,y_−j)
Time (h)

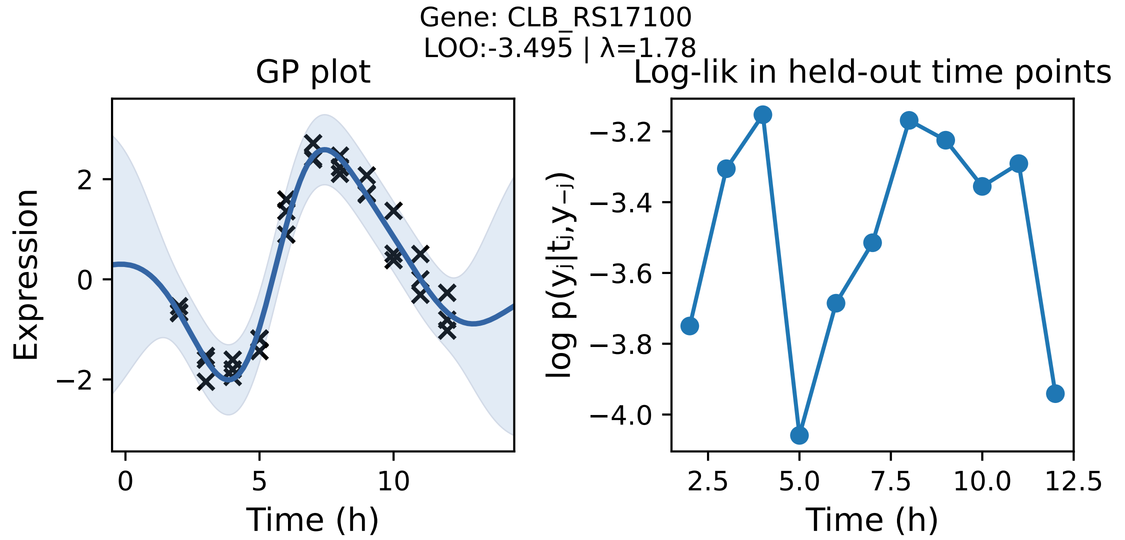

Gene: CLB_RS17100
LOO:-3.495 | λ=1.78
GP plot
Expression
Time (h)
Log-lik in held-out time points
log p(y_j|t_j,y_−j)
Time (h)

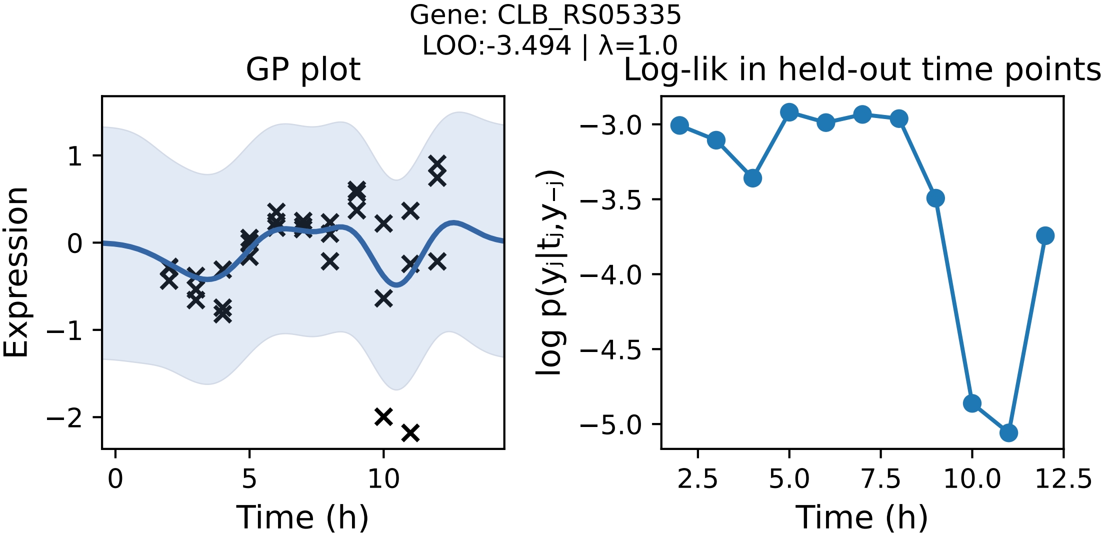

Gene: CLB_RS05335
LOO:-3.494 | λ=1.0
GP plot
Expression
Time (h)
Log-lik in held-out time points
log p(y_j|t_j,y_−j)
Time (h)

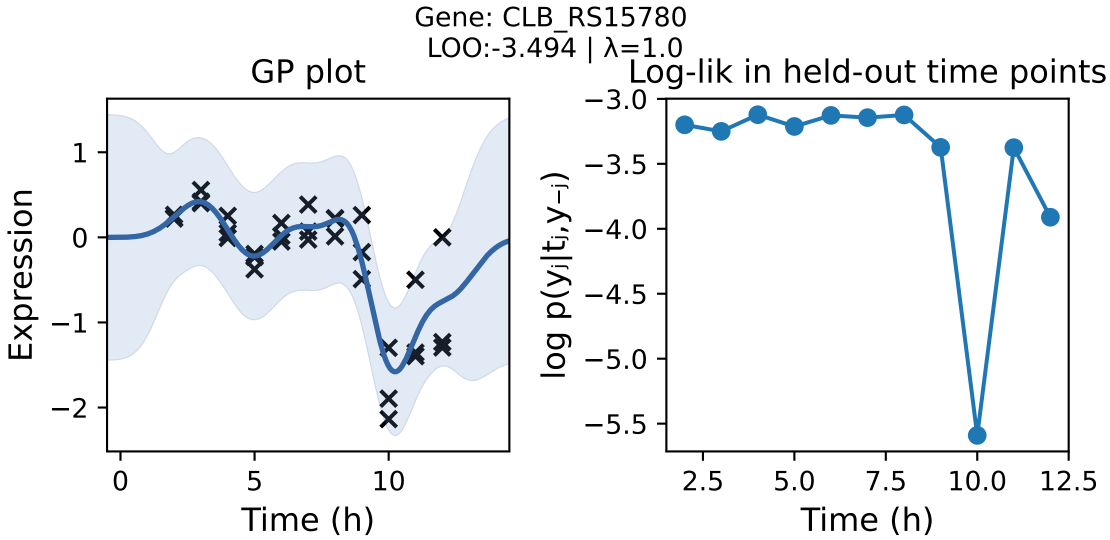

Gene: CLB_RS15780
LOO:-3.494 | λ=1.0
GP plot
Expression
1
0
−1
−2
0
5
10
Time (h)
Log-lik in held-out time points
log p(yⱼ|tⱼ,y₋ⱼ)
−3.0
−3.5
−4.0
−4.5
−5.0
−5.5
2.5
5.0
7.5
10.0
12.5
Time (h)

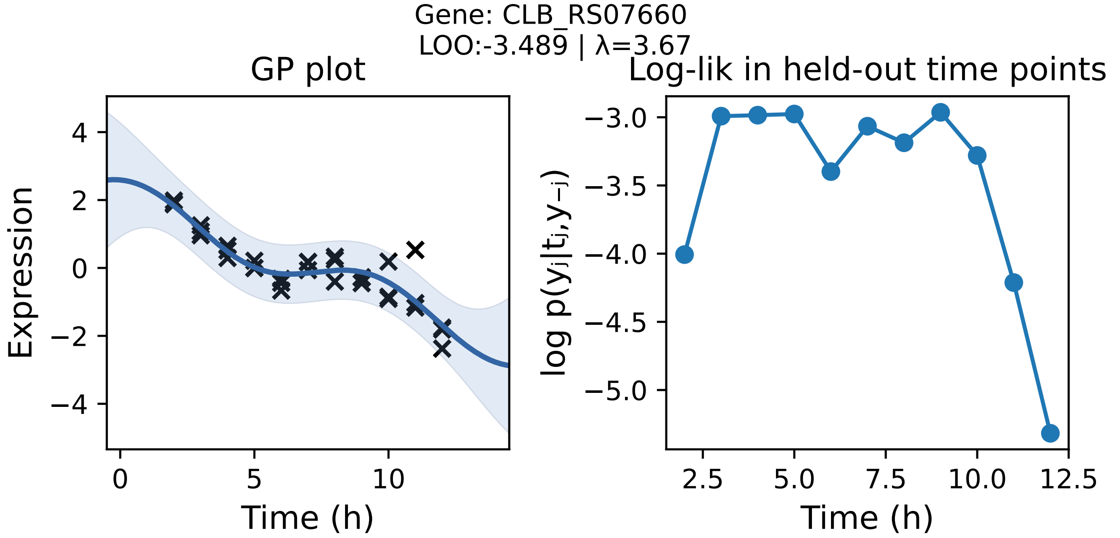

Gene: CLB_RS07660
LOO:-3.489 | λ=3.67
GP plot
Expression
Time (h)
Log-lik in held-out time points
log p(y_j|t_j,y_−j)
Time (h)

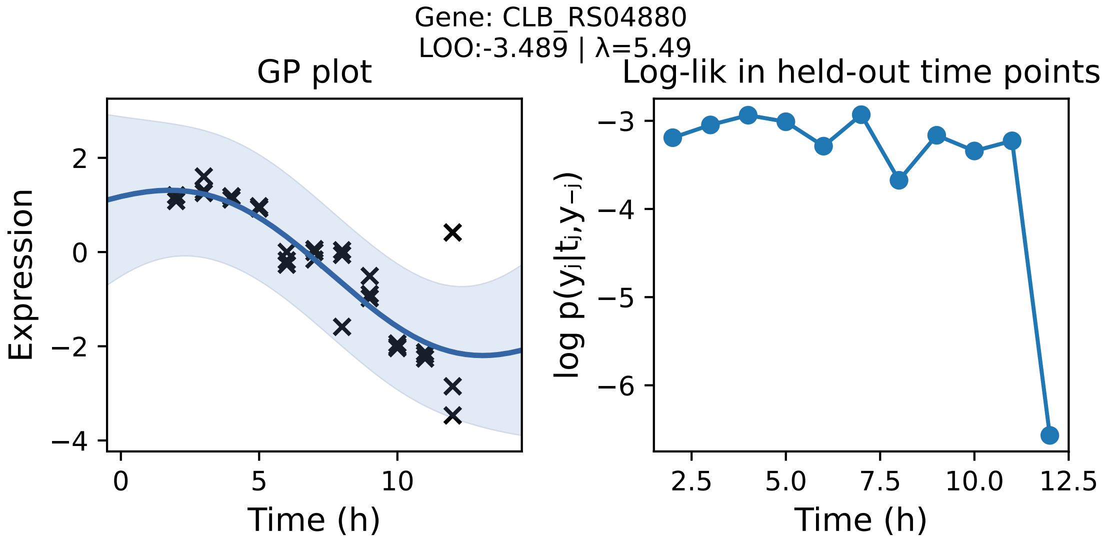

Gene: CLB_RS04880
LOO:-3.489 | λ=5.49
GP plot
Expression
Time (h)
Log-lik in held-out time points
log p(yⱼ|tⱼ,y₋ⱼ)
Time (h)

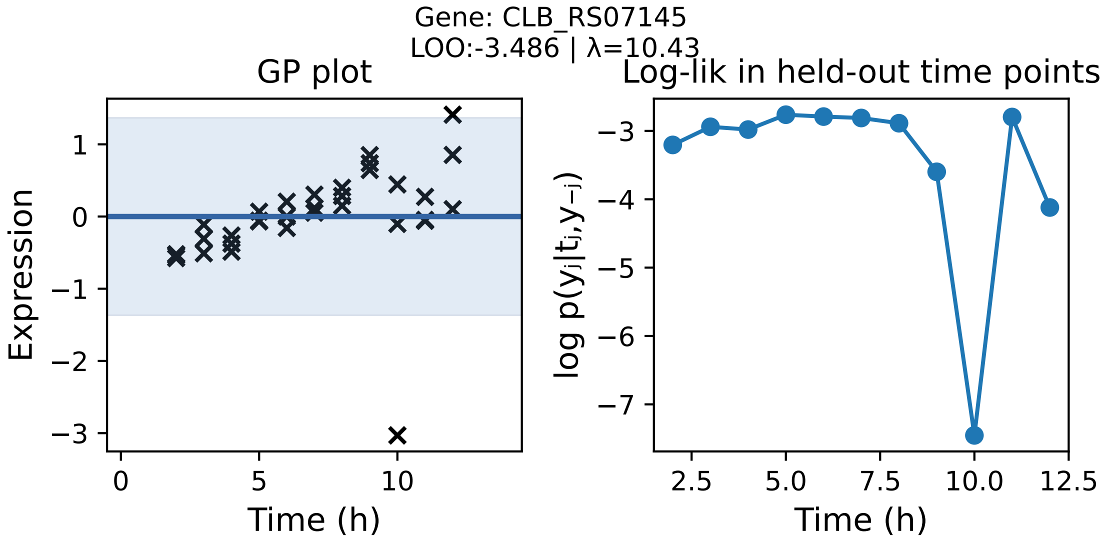
Gene: CLB_RS07145
LOO:-3.486 | λ=10.43
GP plot
Expression
Time (h)
Log-lik in held-out time points
log p(yj|tj,y−j)
Time (h)

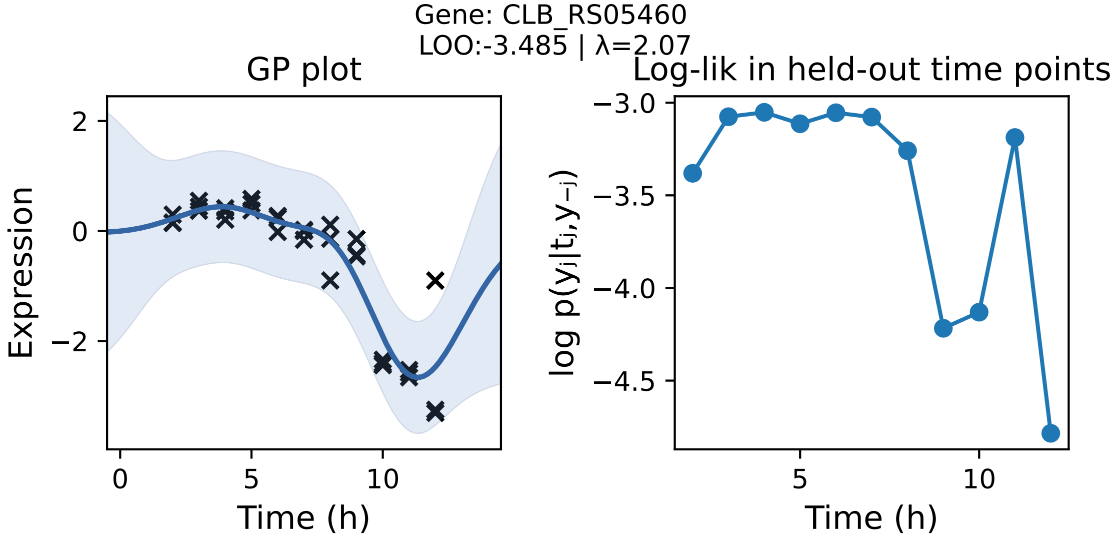
Gene: CLB_RS05460
LOO:-3.485 | λ=2.07
GP plot
Expression
Time (h)
2
0
−2
0
5
10
Log-lik in held-out time points
log p(y_j|t_j,y_−j)
Time (h)
−3.0
−3.5
−4.0
−4.5
5
10

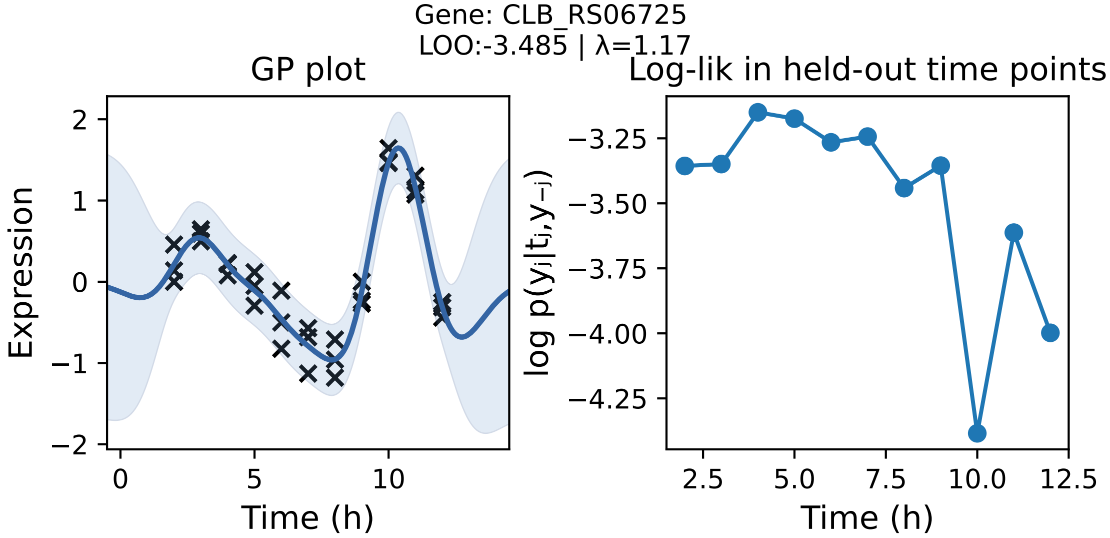

Gene: CLB_RS06725
LOO:-3.485 | λ=1.17
GP plot
Expression
Time (h)
Log-lik in held-out time points
log p(y_j|t_j,y_−j)
Time (h)

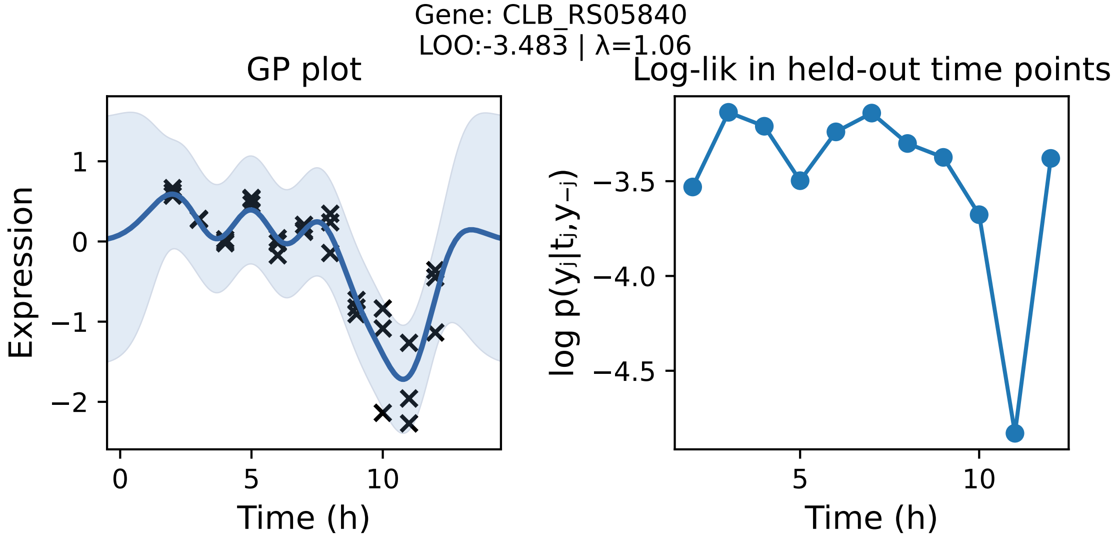

Gene: CLB_RS05840
LOO:-3.483 | λ=1.06
GP plot
Expression
Time (h)
Log-lik in held-out time points
log p(y_j|t_j,y_−j)
Time (h)

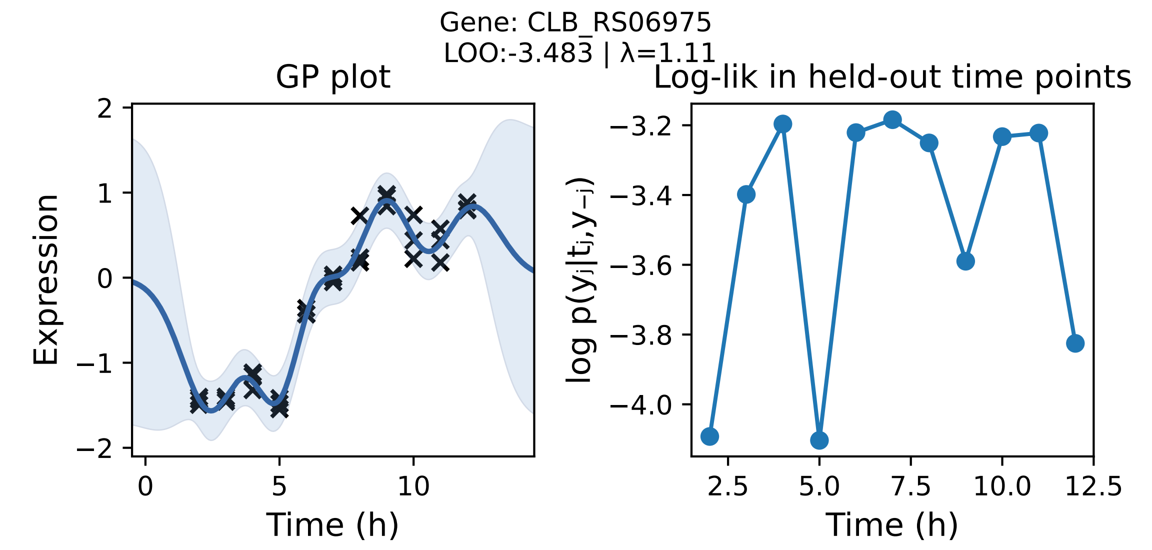

Gene: CLB_RS06975
LOO:-3.483 | λ=1.11
GP plot
Expression
Time (h)
2
1
0
−1
−2
0
5
10
Log-lik in held-out time points
log p(y_j|t_j,y_−j)
Time (h)
−3.2
−3.4
−3.6
−3.8
−4.0
2.5
5.0
7.5
10.0
12.5

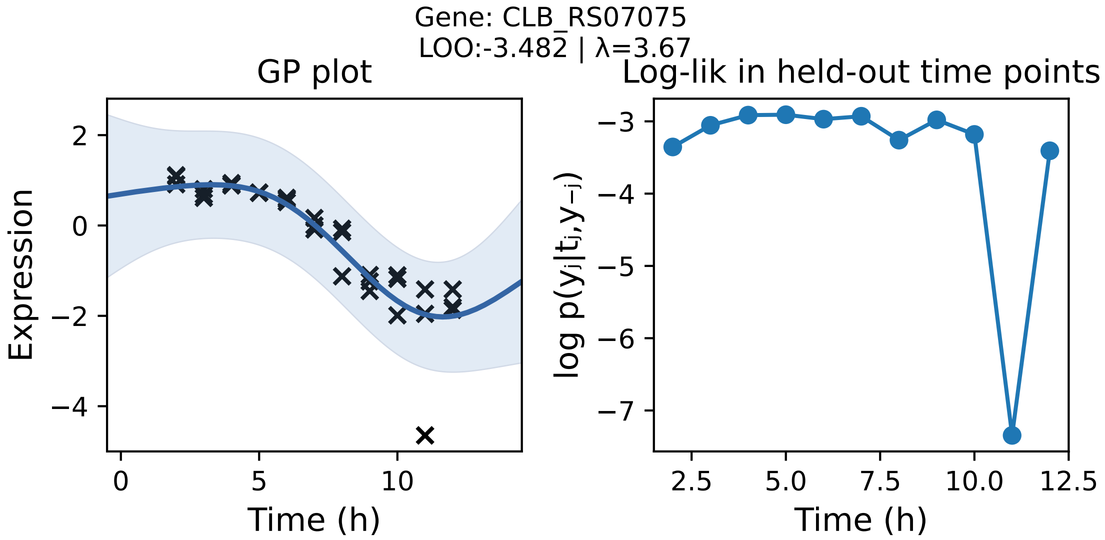

Gene: CLB_RS07075
LOO:-3.482 | λ=3.67
GP plot
Log-lik in held-out time points
Expression
Time (h)
log p(yⱼ|tⱼ,y₋ⱼ)
Time (h)

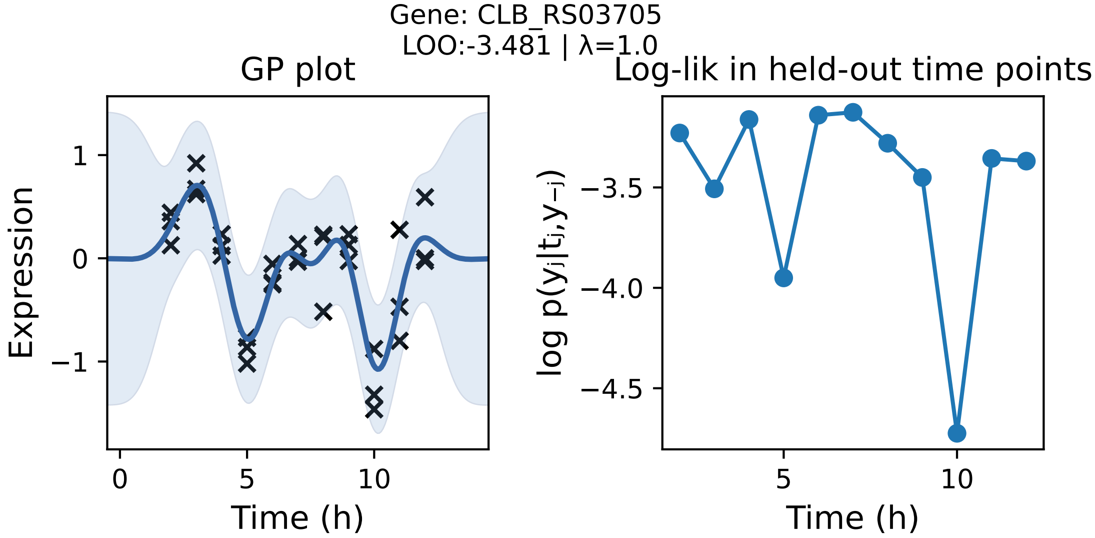

Gene: CLB_RS03705
LOO:-3.481 | λ=1.0
GP plot
Expression
1
0
−1
0
5
10
Time (h)
Log-lik in held-out time points
log p(y_j|t_j,y_−j)
−3.5
−4.0
−4.5
5
10
Time (h)

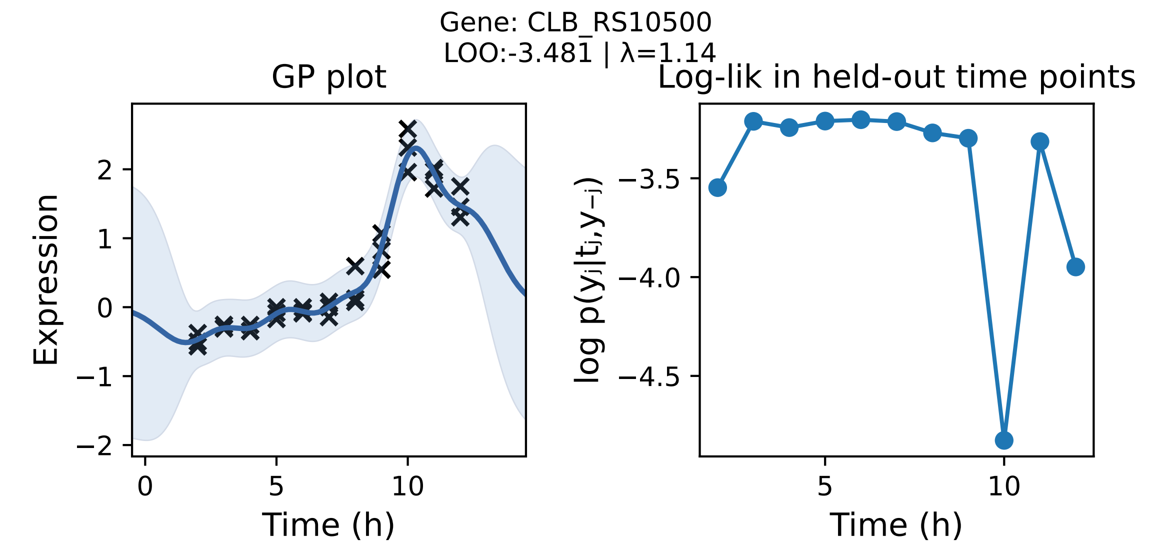

Gene: CLB_RS10500
LOO:-3.481 | λ=1.14
GP plot
Expression
Time (h)
Log-lik in held-out time points
log p(y_j|t_j,y_−j)
Time (h)

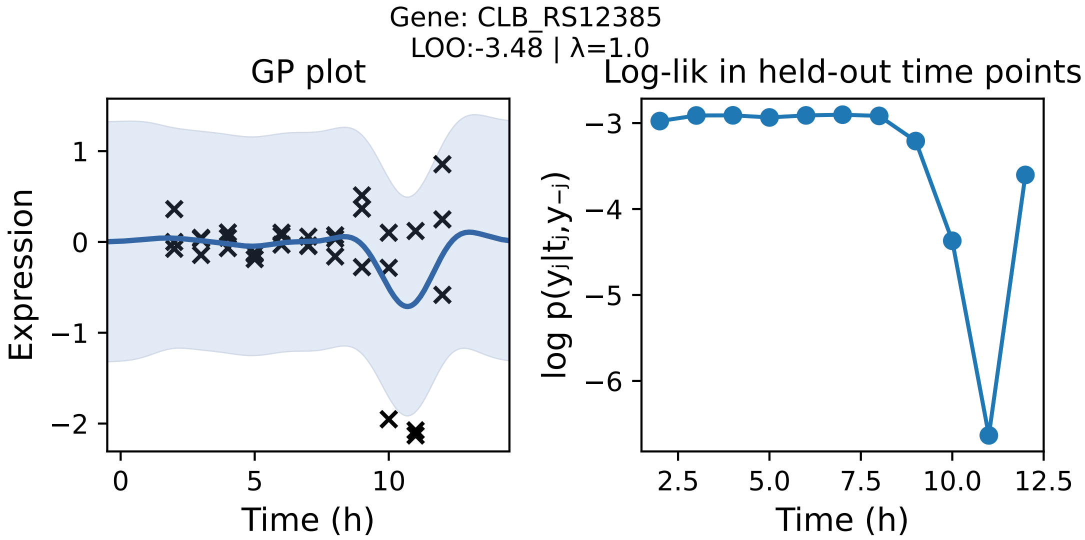
Gene: CLB_RS12385
LOO:-3.48 | λ=1.0
GP plot
Expression
Time (h)
Log-lik in held-out time points
log p(y_j|t_j,y_−j)
Time (h)

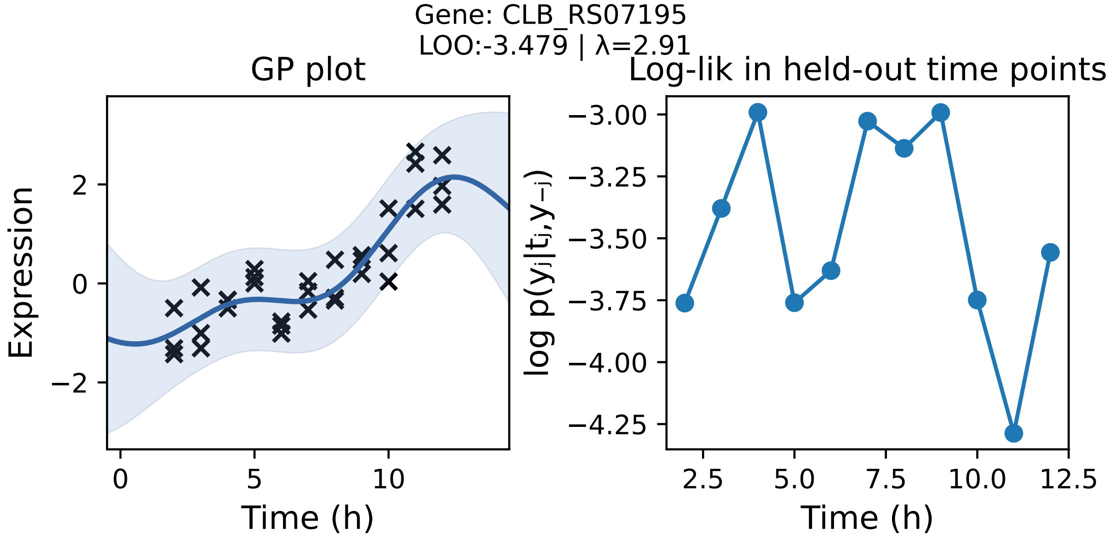

Gene: CLB_RS07195
LOO:-3.479 | λ=2.91
GP plot
Expression
Time (h)
Log-lik in held-out time points
log p(y_j|t_j,y_{-j})
Time (h)

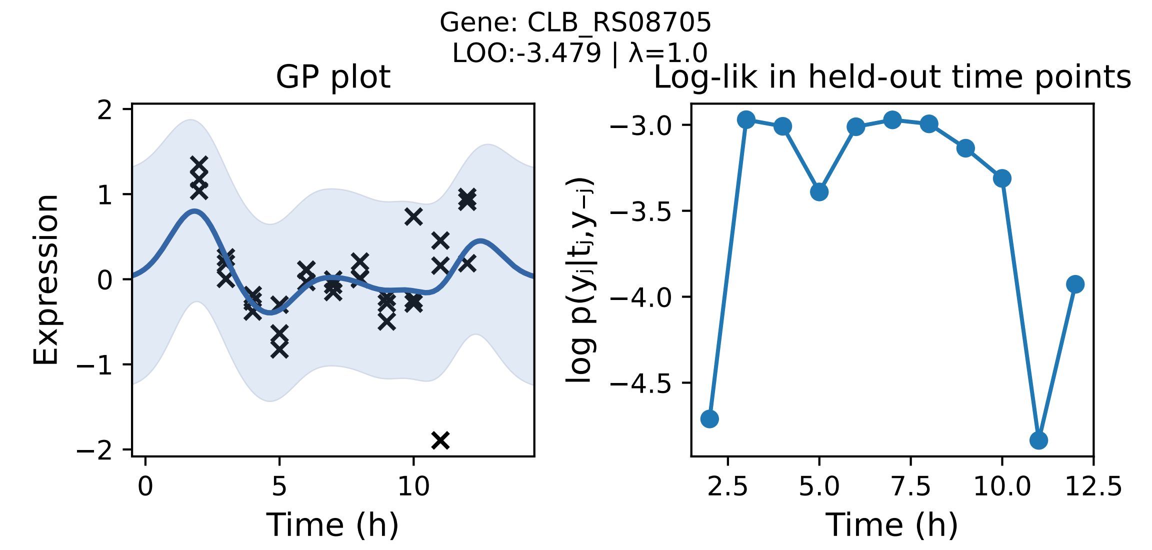

Gene: CLB_RS08705
LOO:-3.479 | λ=1.0
GP plot
Expression
Time (h)
Log-lik in held-out time points
log p(y_j|t_j,y_−j)
Time (h)

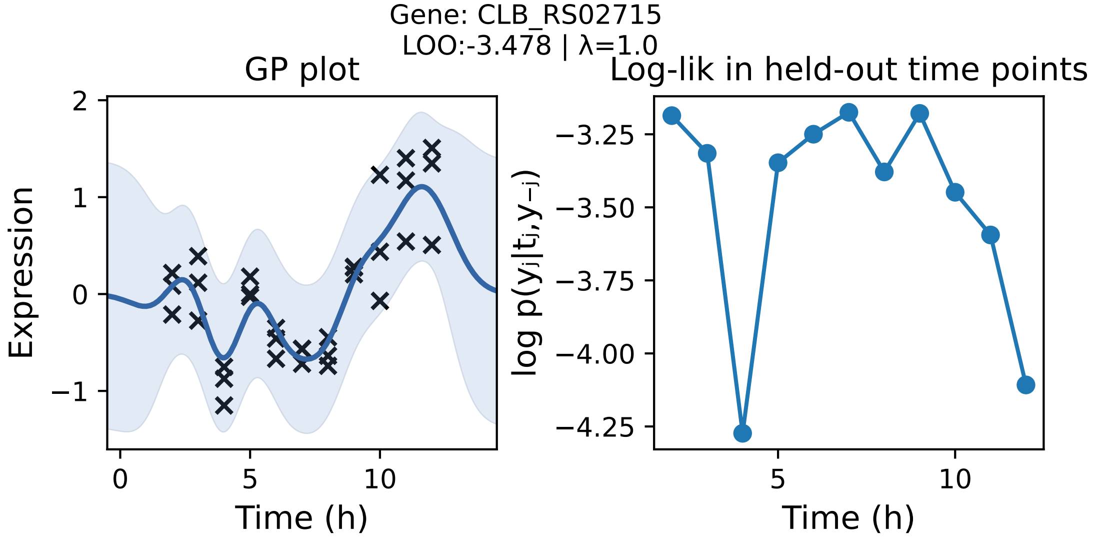

Gene: CLB_RS02715
LOO:-3.478 | λ=1.0
GP plot
Expression
Time (h)
Log-lik in held-out time points
log p(yj|tj,y−j)
Time (h)

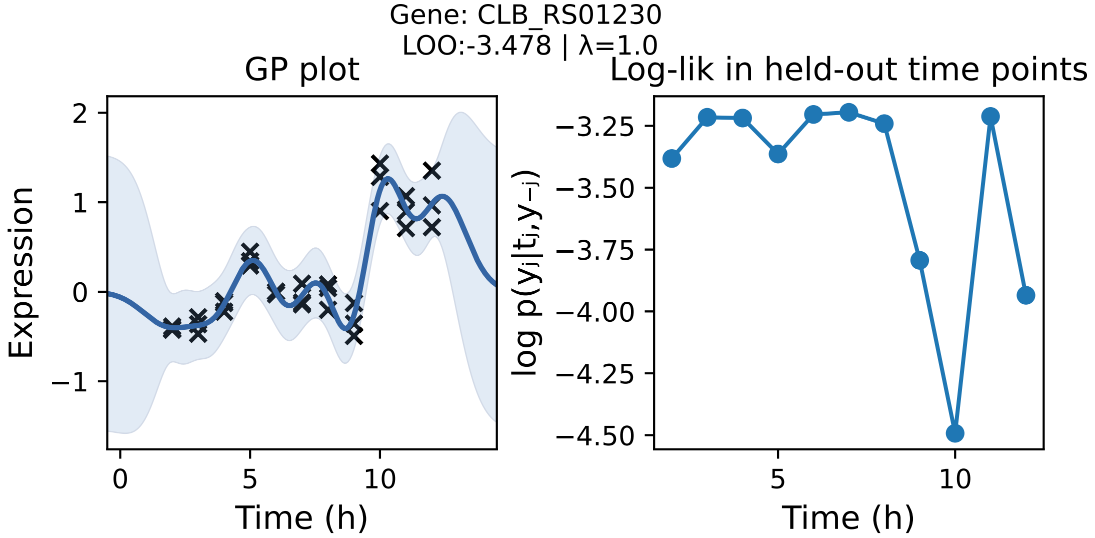

Gene: CLB_RS01230
LOO:-3.478 | λ=1.0
GP plot
Expression
Time (h)
Log-lik in held-out time points
log p(y_j|t_j,y_−j)
Time (h)

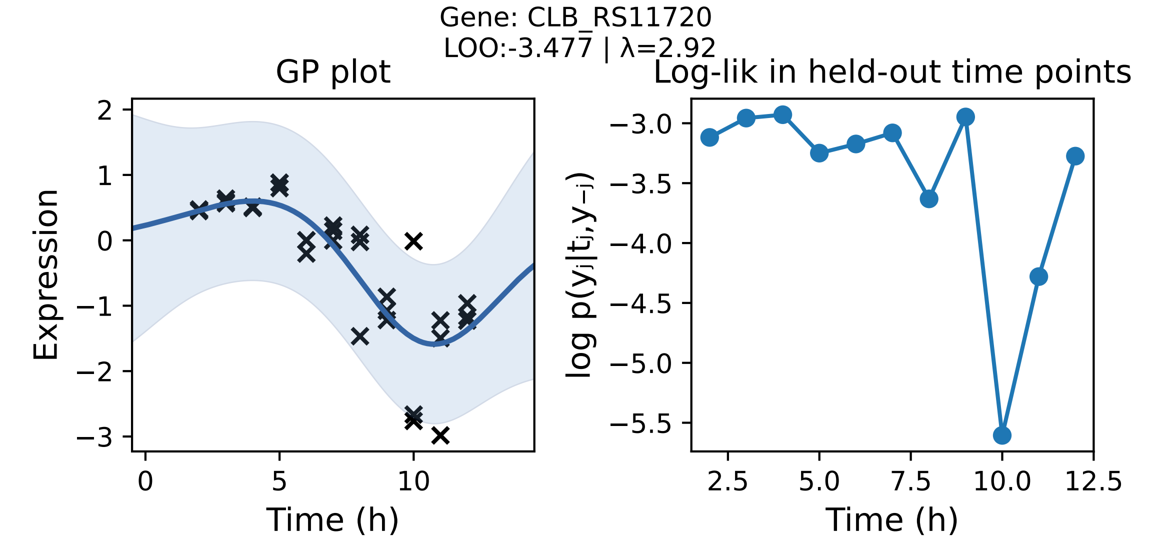
Gene: CLB_RS11720
LOO:-3.477 | λ=2.92
GP plot
Expression
Time (h)
Log-lik in held-out time points
log p(y_j|t_j,y_−j)
Time (h)

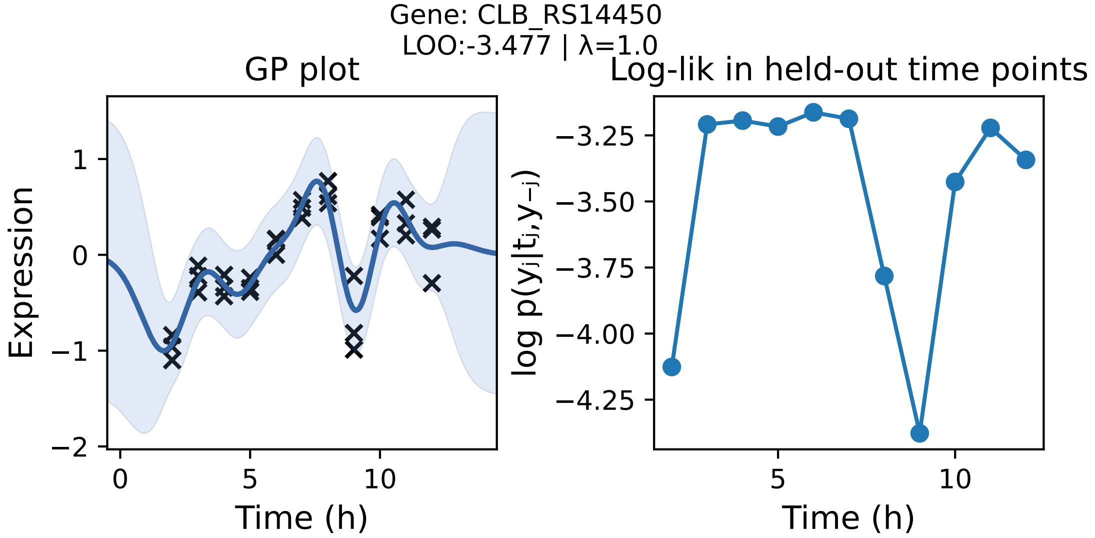

Gene: CLB_RS14450
LOO:-3.477 | λ=1.0
GP plot
Expression
1
0
−1
−2
0
5
10
Time (h)
Log-lik in held-out time points
log p(y_j|t_j,y_−j)
−3.25
−3.50
−3.75
−4.00
−4.25
5
10
Time (h)

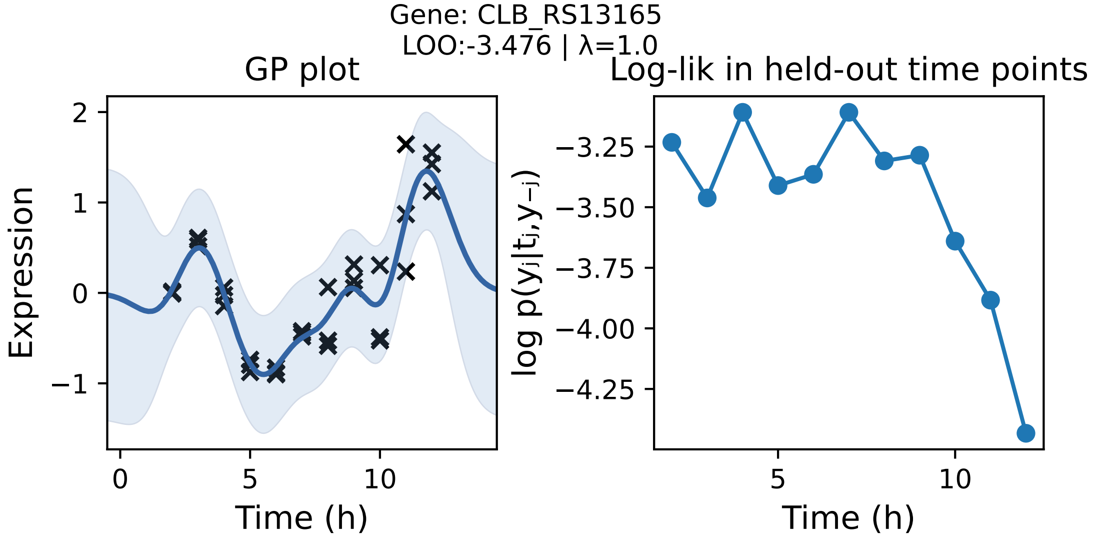
Gene: CLB_RS13165
LOO:-3.476 | λ=1.0
GP plot
Expression
Time (h)
Log-lik in held-out time points
log p(yj|tj,y−j)
Time (h)

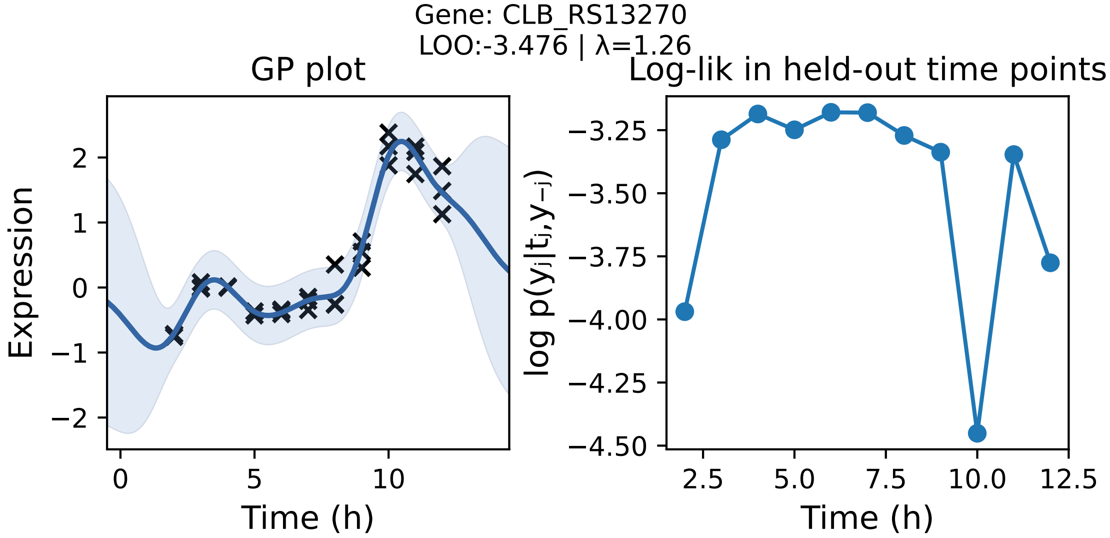
Gene: CLB_RS13270
LOO:-3.476 | λ=1.26
GP plot
Expression
Time (h)
2
1
0
−1
−2
0
5
10
Log-lik in held-out time points
log p(y_j|t_j,y_−j)
−3.25
−3.50
−3.75
−4.00
−4.25
−4.50
2.5
5.0
7.5
10.0
12.5
Time (h)

Gene: CLB_RS16630
LOO:-3.476 | λ=1.0

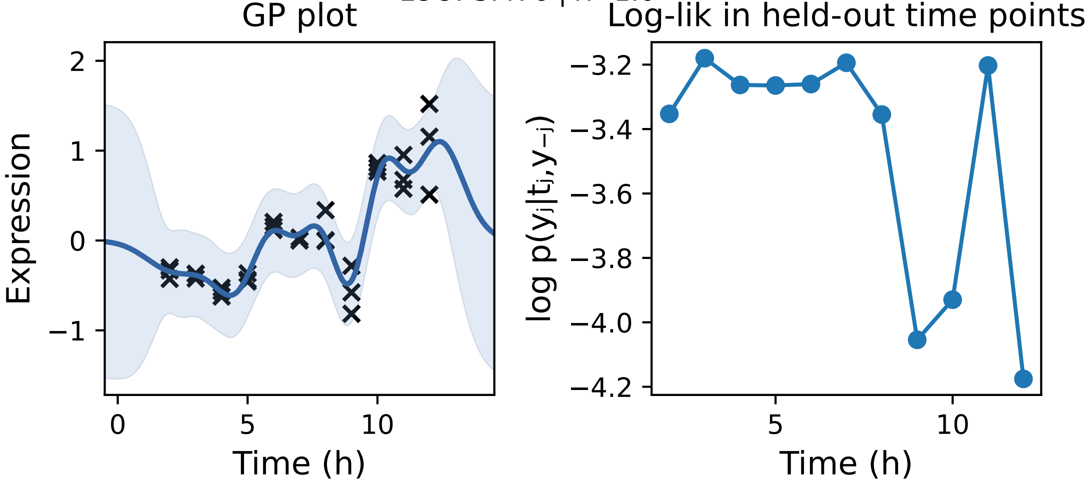

Gene: CLB_RS05225
LOO:-3.474 | λ=1.0

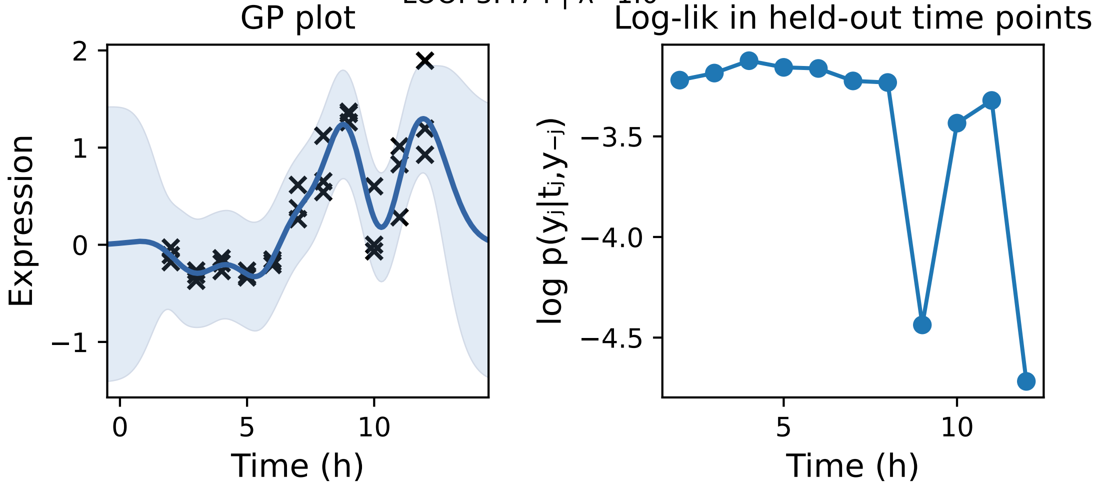

Gene: CLB_RS03540
LOO:-3.474 | λ=1.14

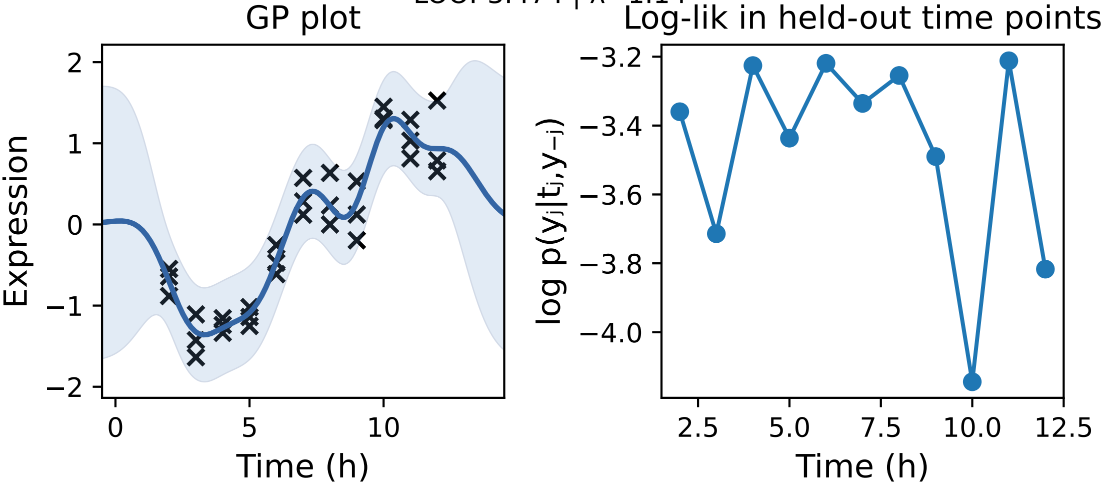

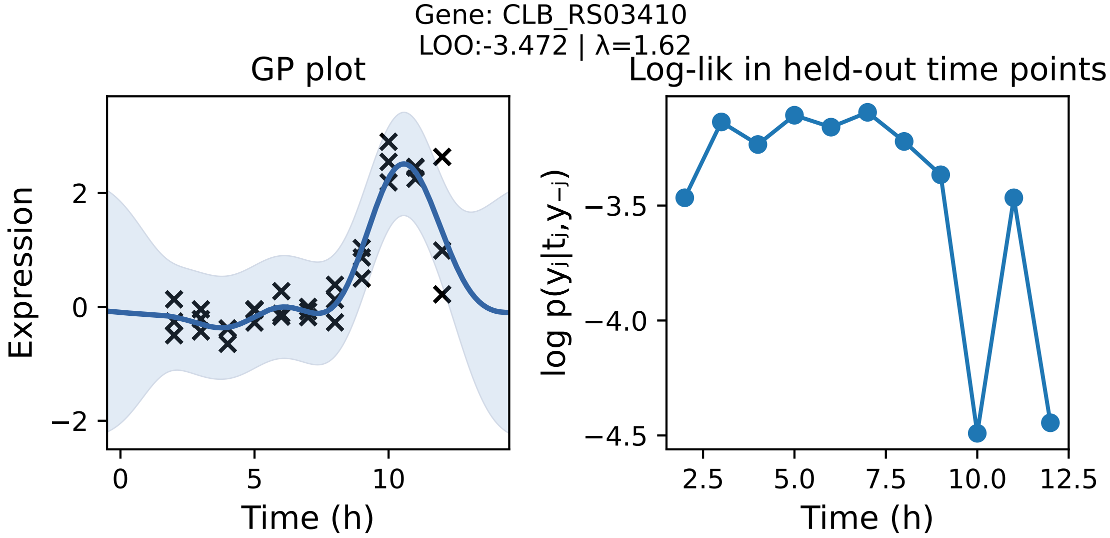
Gene: CLB_RS03410
LOO:-3.472 | λ=1.62
GP plot
Expression
Time (h)
Log-lik in held-out time points
log p(y_j|t_j,y_−j)
Time (h)

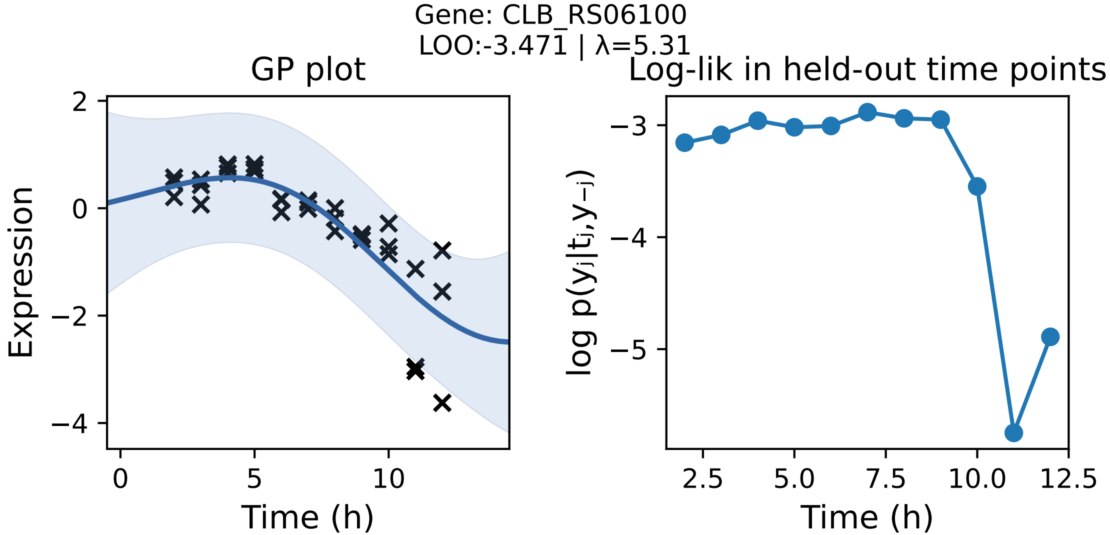

Gene: CLB_RS06100
LOO:-3.471 | λ=5.31
GP plot
Expression
Time (h)
2
0
−2
−4
0
5
10
Log-lik in held-out time points
log p(y_j|t_j,y_−j)
Time (h)
−3
−4
−5
2.5
5.0
7.5
10.0
12.5

Gene: CLB_RS09605
LOO:-3.469 | λ=9.29

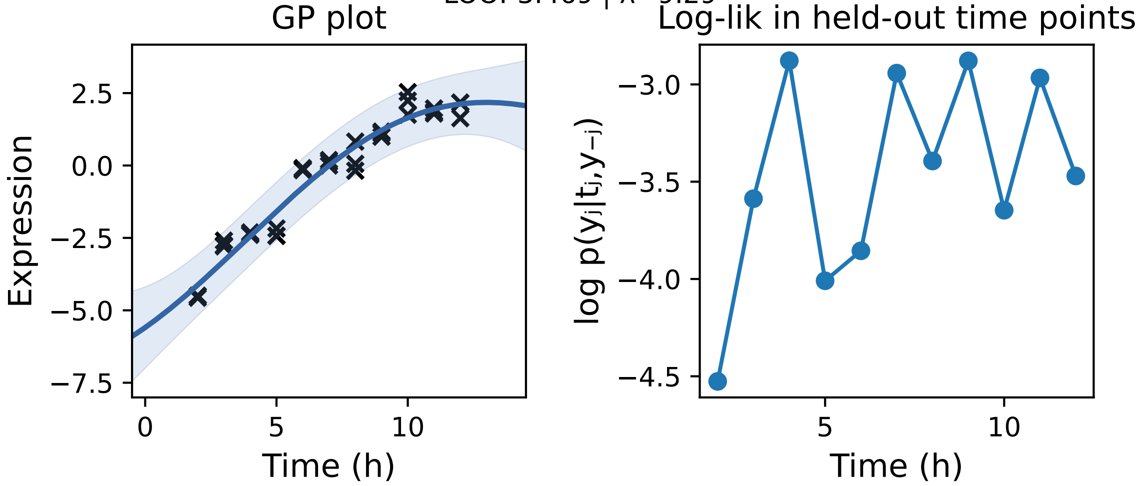

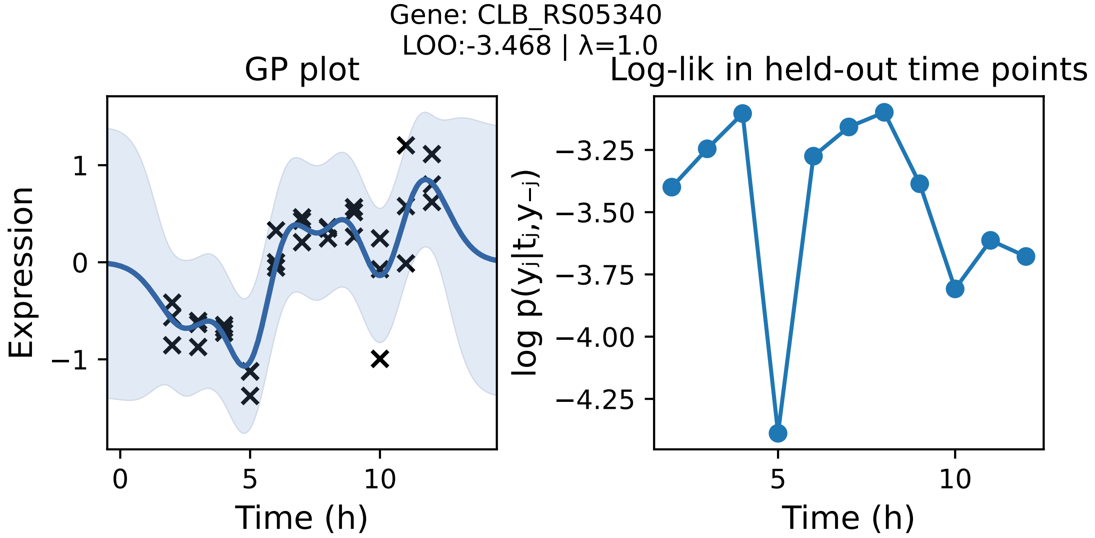

Gene: CLB_RS05340
LOO:-3.468 | λ=1.0
GP plot
Log-lik in held-out time points
Expression
Time (h)
log p(y_j|t_j,y_−j)
1
0
−1
0
5
10
−3.25
−3.50
−3.75
−4.00
−4.25
5
10
Time (h)

Gene: CLB_RS10635
LOO:-3.468 | λ=2.75

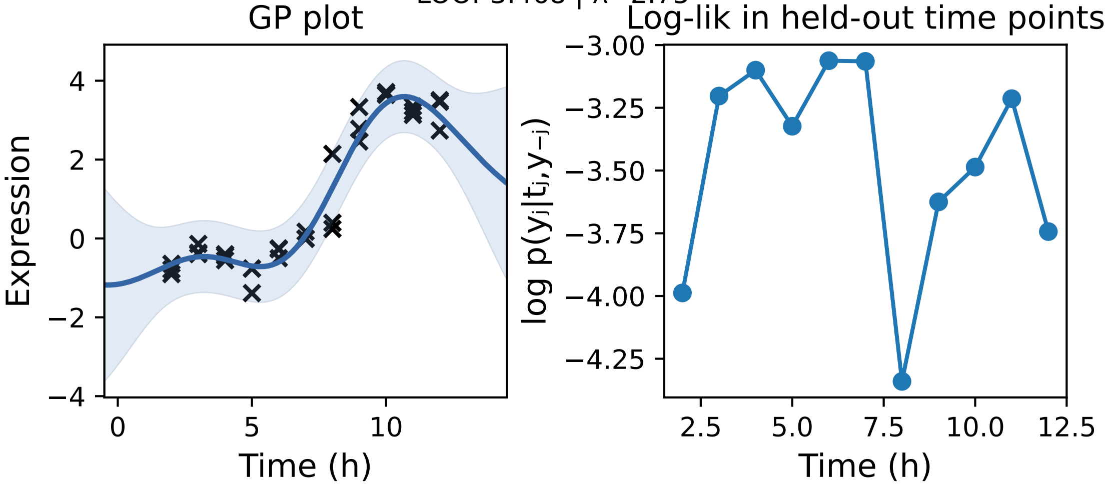

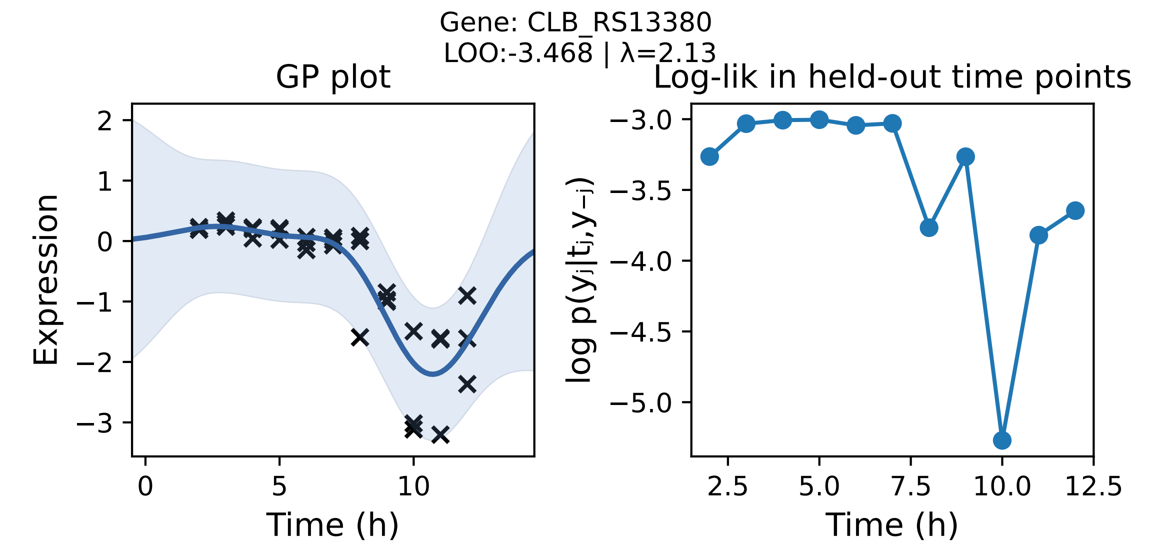

Gene: CLB_RS13380
LOO:-3.468 | λ=2.13
GP plot
Expression
Time (h)
Log-lik in held-out time points
log p(y_j|t_j,y_−j)
Time (h)

Gene: CLB_RS01030
LOO:-3.468 | λ=3.17

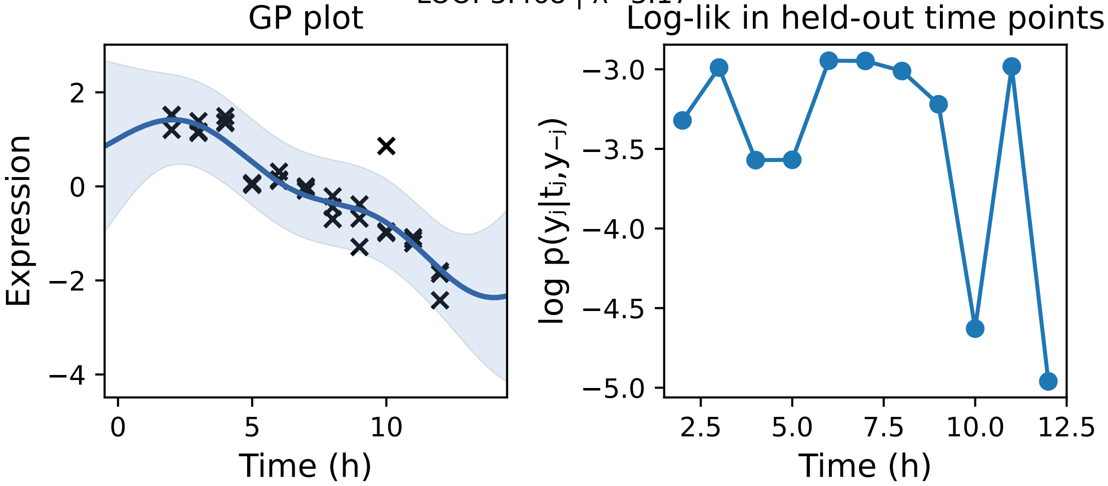

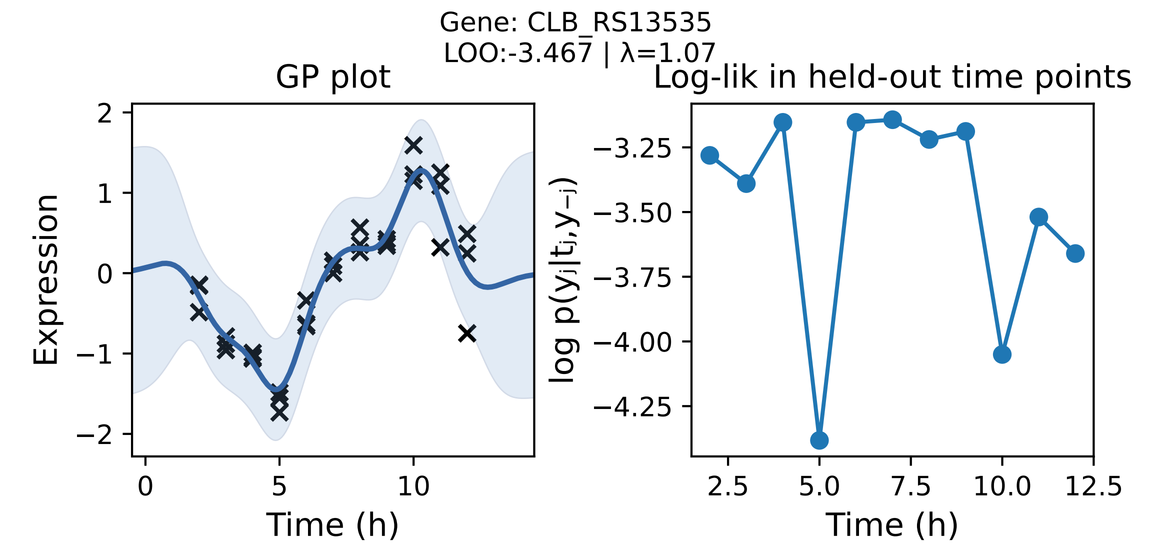

Gene: CLB_RS13535
LOO:-3.467 | λ=1.07
GP plot
Log-lik in held-out time points
Expression
Time (h)
log p(y_j|t_j,y_−j)
Time (h)
2
1
0
−1
−2
0
5
10
−3.25
−3.50
−3.75
−4.00
−4.25
2.5
5.0
7.5
10.0
12.5

Gene: CLB_RS02990
LOO:-3.467 | λ=1.19

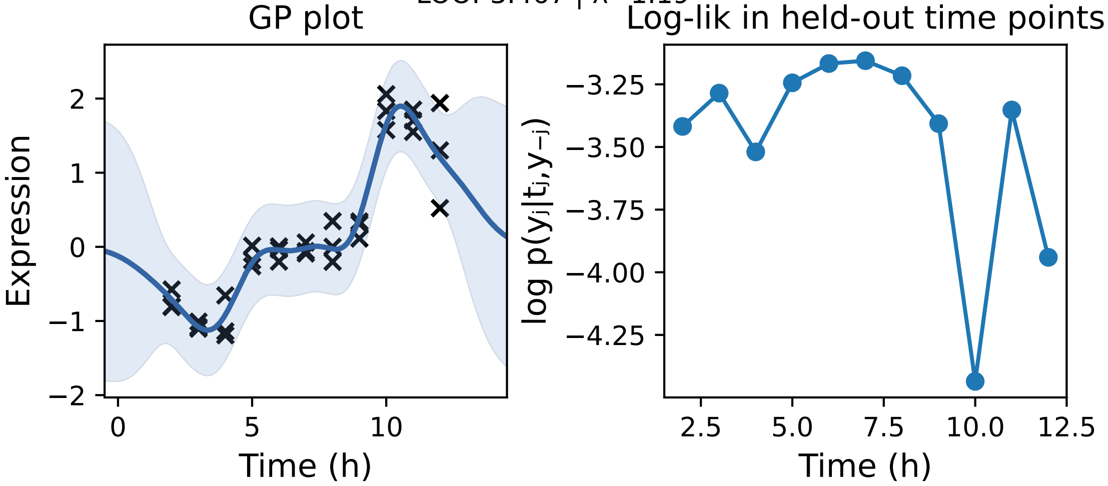

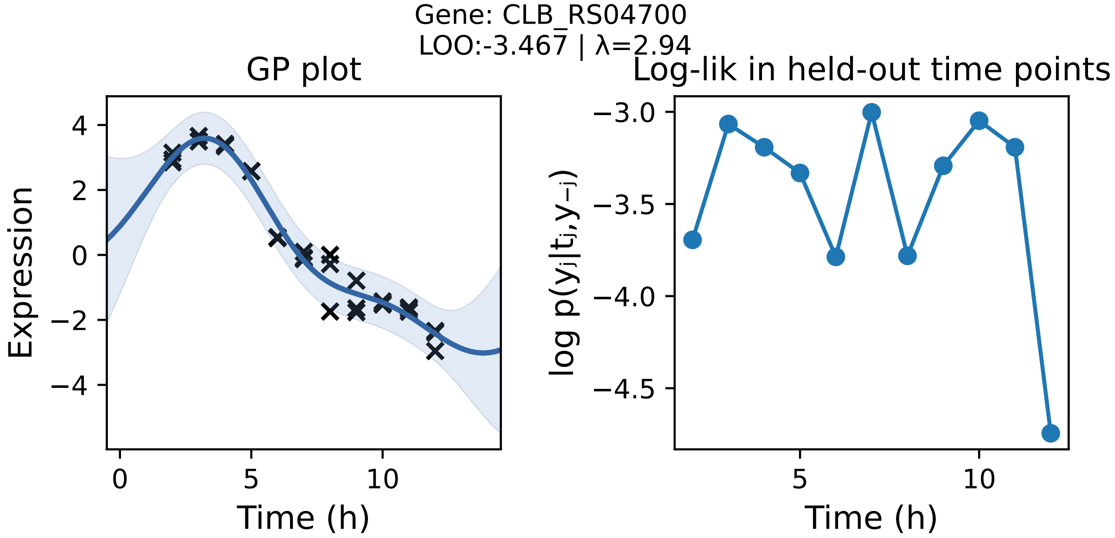

Gene: CLB_RS04700
LOO:-3.467 | λ=2.94
GP plot
Expression
Time (h)
4
2
0
−2
−4
0
5
10
Log-lik in held-out time points
log p(y_j|t_j,y_−j)
Time (h)
−3.0
−3.5
−4.0
−4.5
5
10

Gene: CLB_RS15605
LOO:-3.465 | λ=1.0

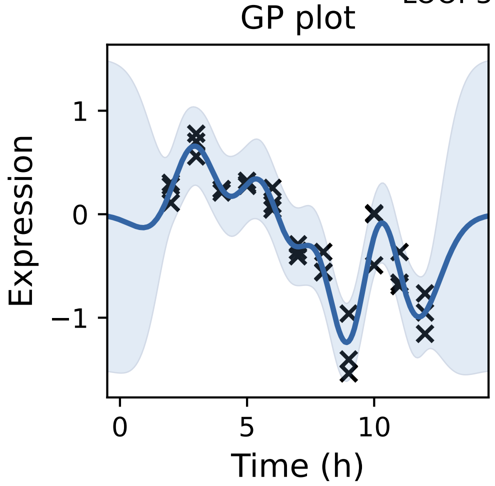

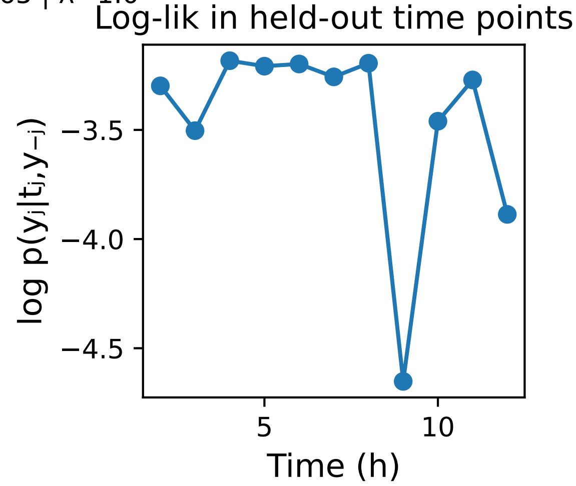

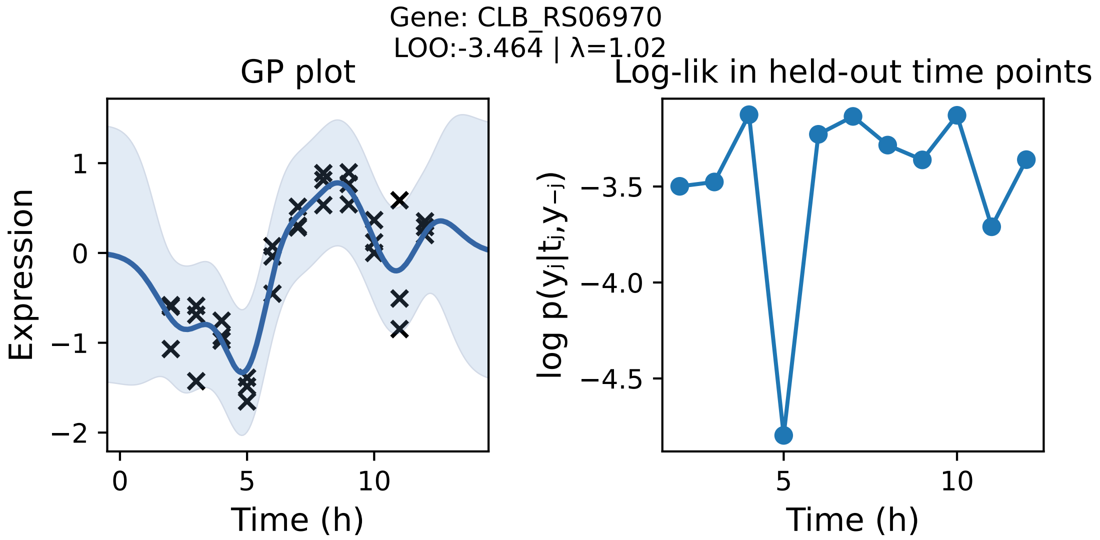

Gene: CLB_RS06970
LOO:-3.464 | λ=1.02
GP plot
Expression
Time (h)
Log-lik in held-out time points
log p(y_j|t_j,y_−j)
Time (h)

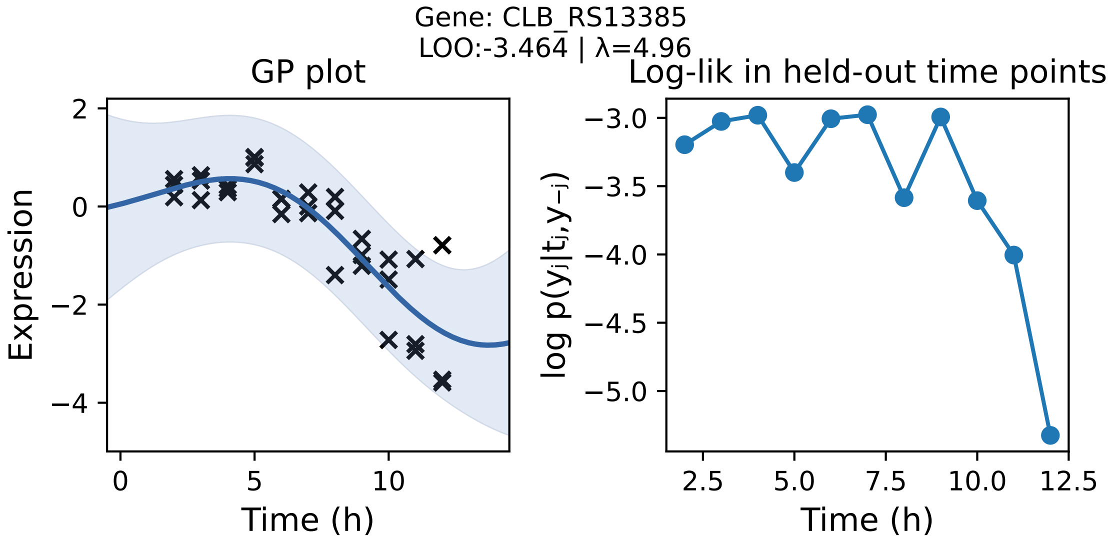
Gene: CLB_RS13385
LOO:-3.464 | λ=4.96
GP plot
Expression
Time (h)
Log-lik in held-out time points
log p(yj|tj,y−j)
Time (h)

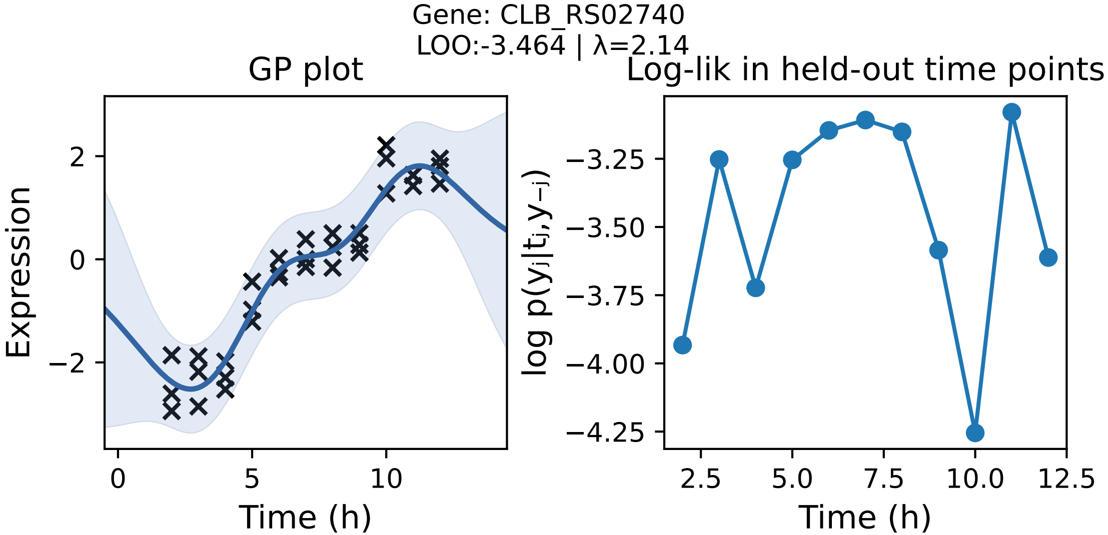
Gene: CLB_RS02740
LOO:-3.464 | λ=2.14
GP plot
Expression
Time (h)
2
0
−2
0
5
10
Log-lik in held-out time points
log p(yj|tj,y−j)
−3.25
−3.50
−3.75
−4.00
−4.25
2.5
5.0
7.5
10.0
12.5
Time (h)

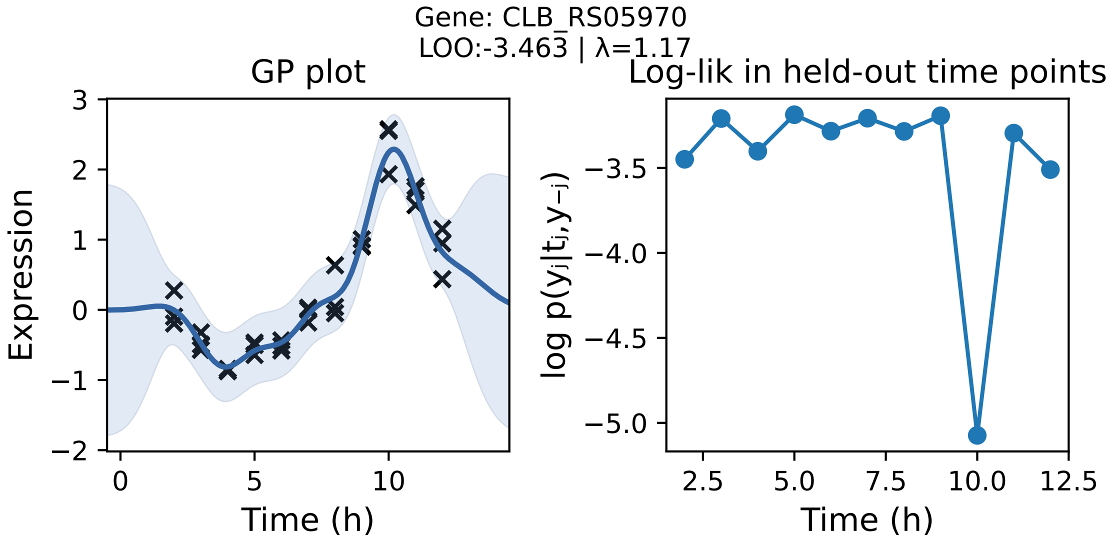

Gene: CLB_RS05970
LOO:-3.463 | λ=1.17
GP plot
Expression
Time (h)
3
2
1
0
−1
−2
0
5
10
Log-lik in held-out time points
log p(y_j|t_j,y_−j)
Time (h)
−3.5
−4.0
−4.5
−5.0
2.5
5.0
7.5
10.0
12.5

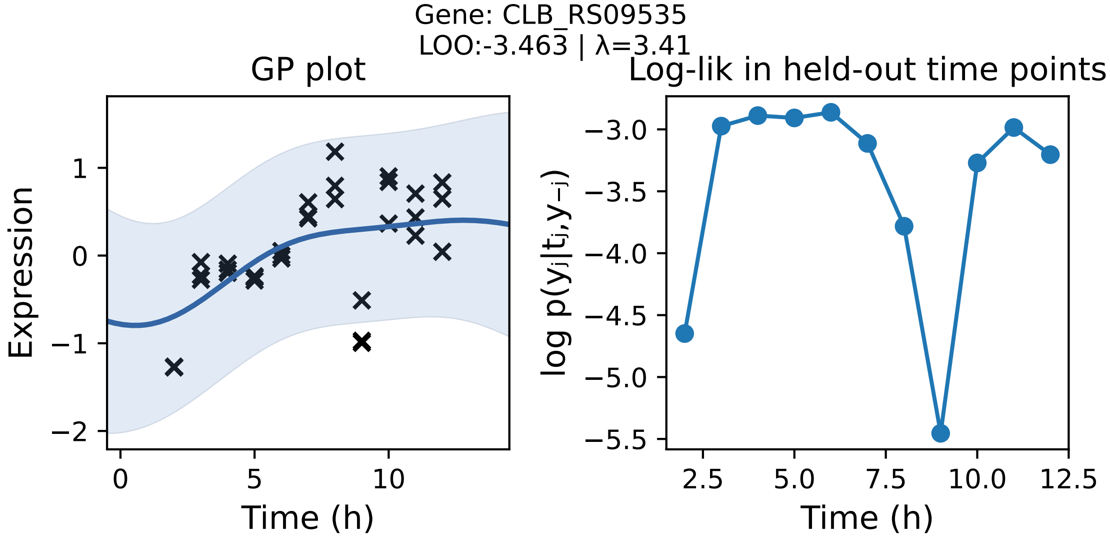

Gene: CLB_RS09535
LOO:-3.463 | λ=3.41
GP plot
Expression
Time (h)
1
0
−1
−2
0
5
10
Log-lik in held-out time points
log p(y_j|t_j,y_−j)
−3.0
−3.5
−4.0
−4.5
−5.0
−5.5
2.5
5.0
7.5
10.0
12.5
Time (h)

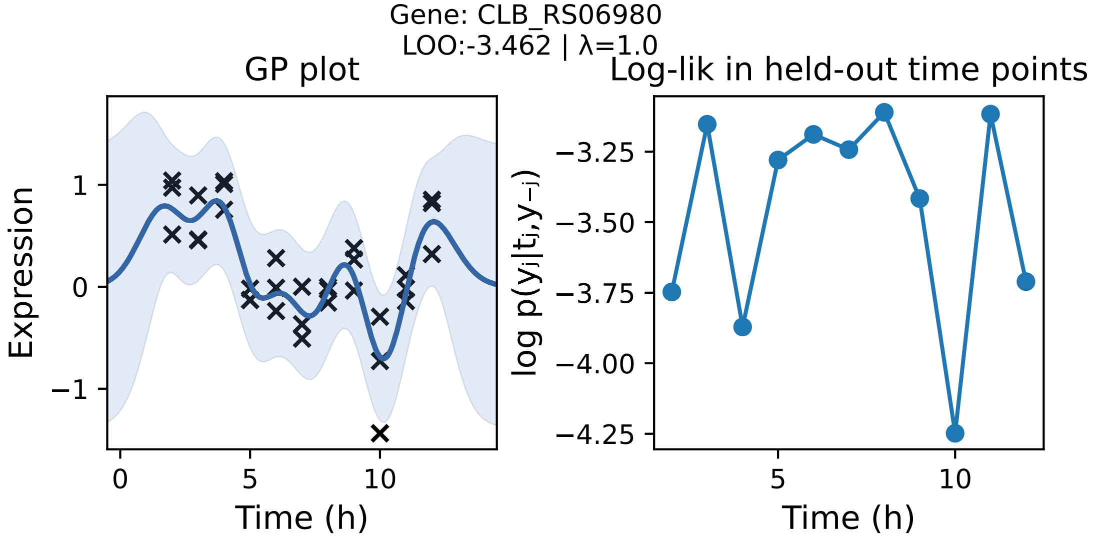

Gene: CLB_RS06980
LOO:-3.462 | λ=1.0
GP plot
Expression
Time (h)
Log-lik in held-out time points
log p(yⱼ|tⱼ,y₋ⱼ)
Time (h)

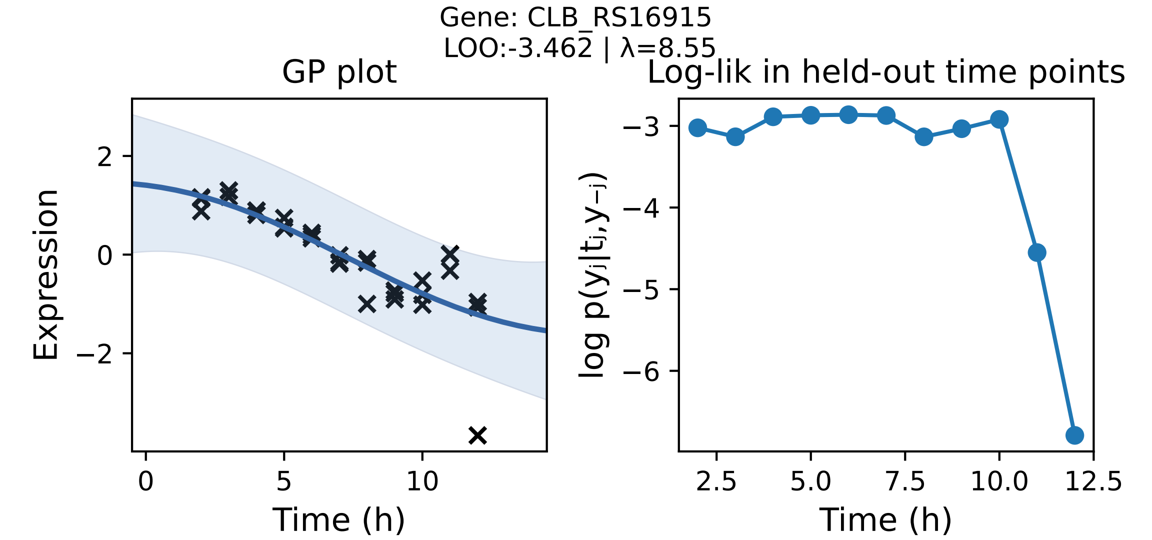

Gene: CLB_RS16915
LOO:-3.462 | λ=8.55
GP plot
Expression
Time (h)
Log-lik in held-out time points
log p(y_j|t_j,y_−j)
Time (h)

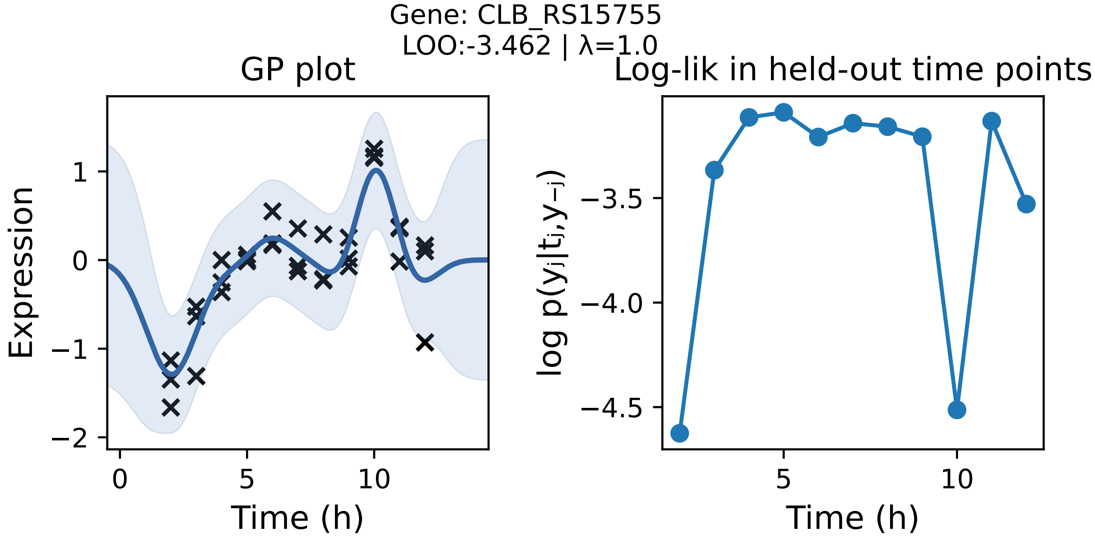

Gene: CLB_RS15755
LOO:-3.462 | λ=1.0
GP plot
Expression
Time (h)
Log-lik in held-out time points
log p(y_j|t_j,y_−j)
Time (h)

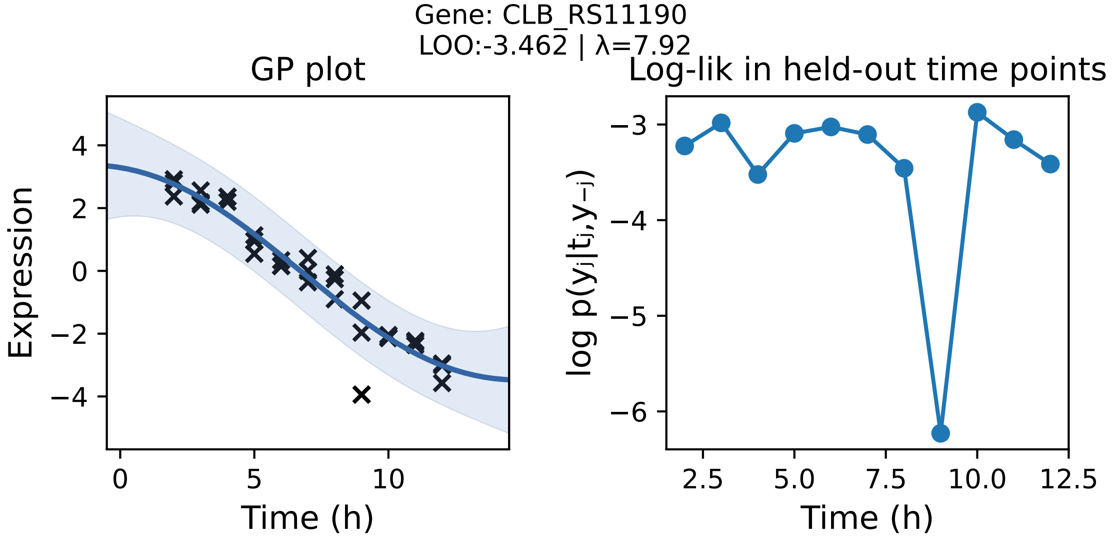
Gene: CLB_RS11190
LOO:-3.462 | λ=7.92
GP plot
Expression
Time (h)
Log-lik in held-out time points
log p(y_j|t_j,y_−j)
Time (h)
4
2
0
−2
−4
0
5
10
−3
−4
−5
−6
2.5
5.0
7.5
10.0
12.5

Gene: CLB_RS14415
LOO:-3.462 | λ=1.0

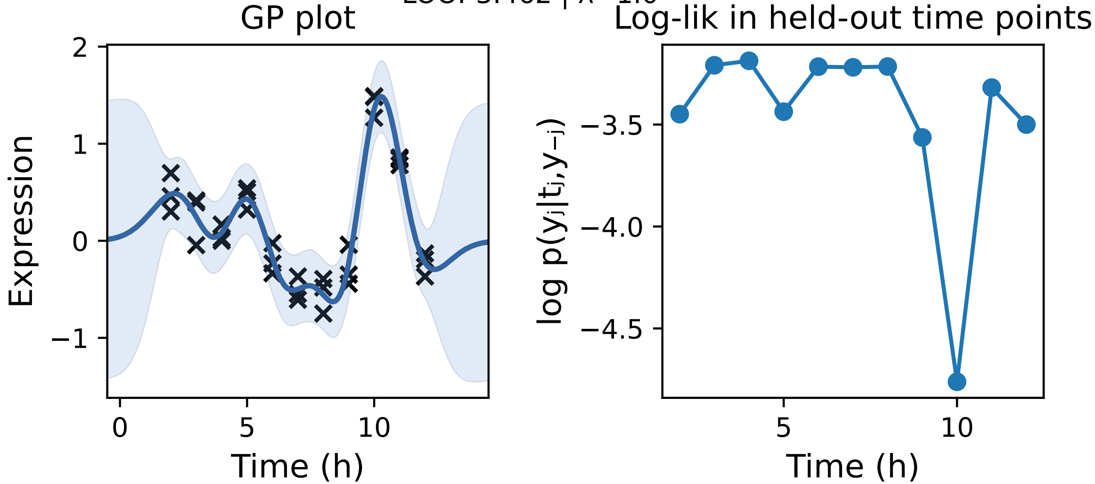

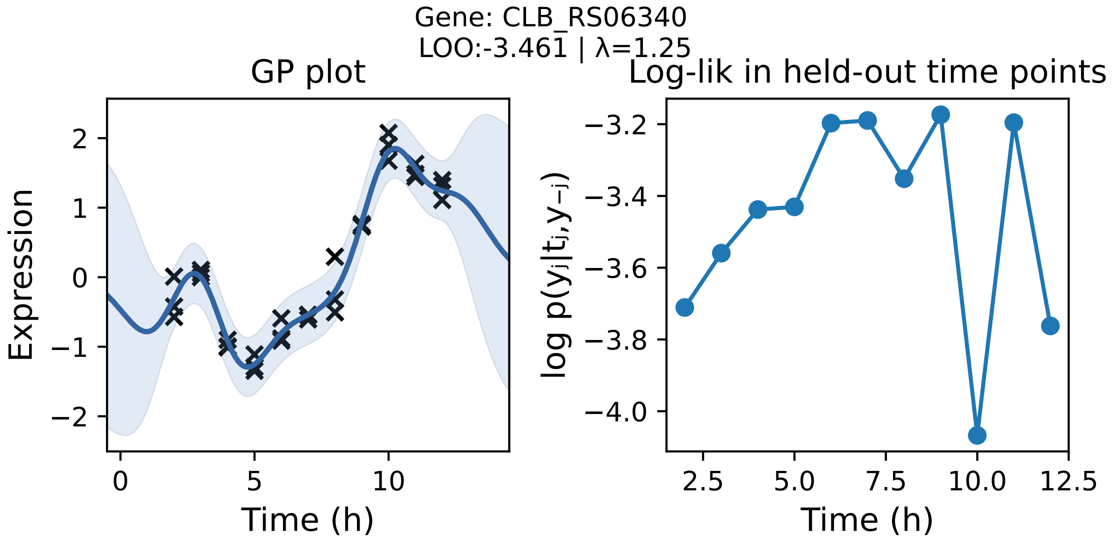
Gene: CLB_RS06340
LOO:-3.461 | λ=1.25
GP plot
Expression
Time (h)
2
1
0
−1
−2
0
5
10
Log-lik in held-out time points
log p(y_j|t_j,y_−j)
Time (h)
−3.2
−3.4
−3.6
−3.8
−4.0
2.5
5.0
7.5
10.0
12.5

Gene: CLB_RS17140
LOO:-3.461 | λ=6.05

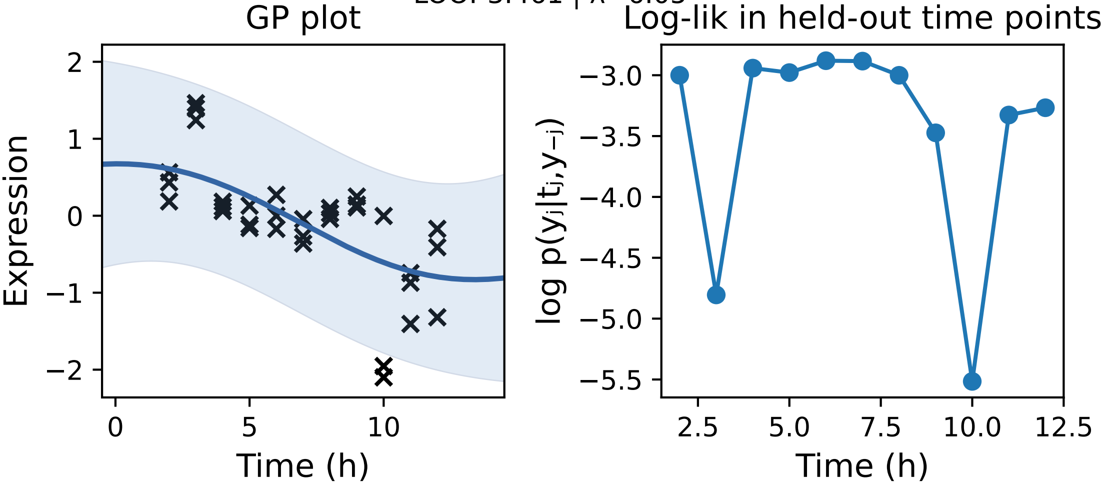

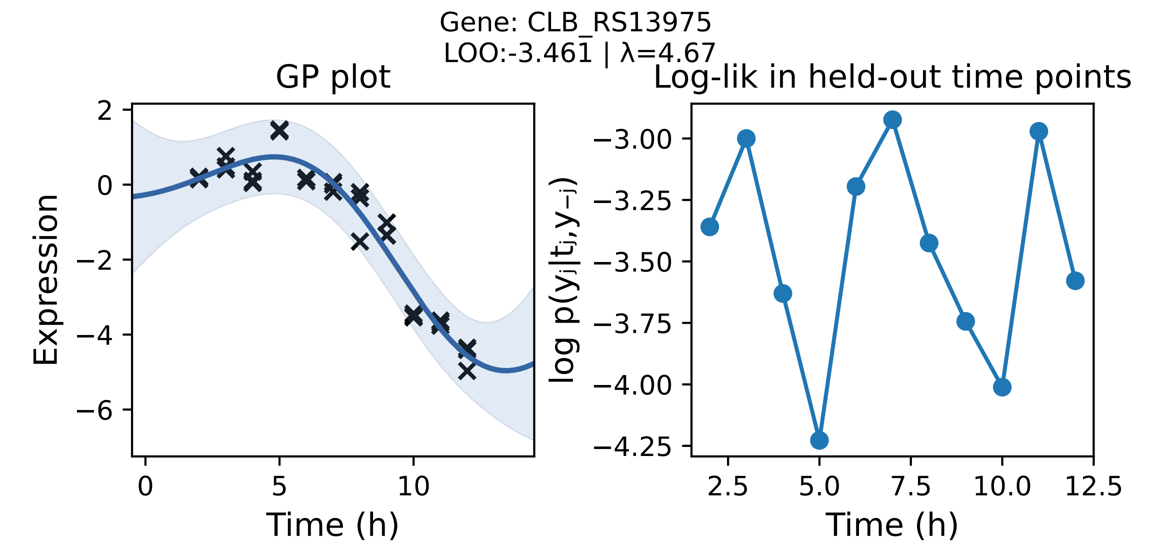
Gene: CLB_RS13975
LOO:-3.461 | λ=4.67
GP plot
Expression
Time (h)
Log-lik in held-out time points
log p(y_j|t_j,y_−j)
Time (h)

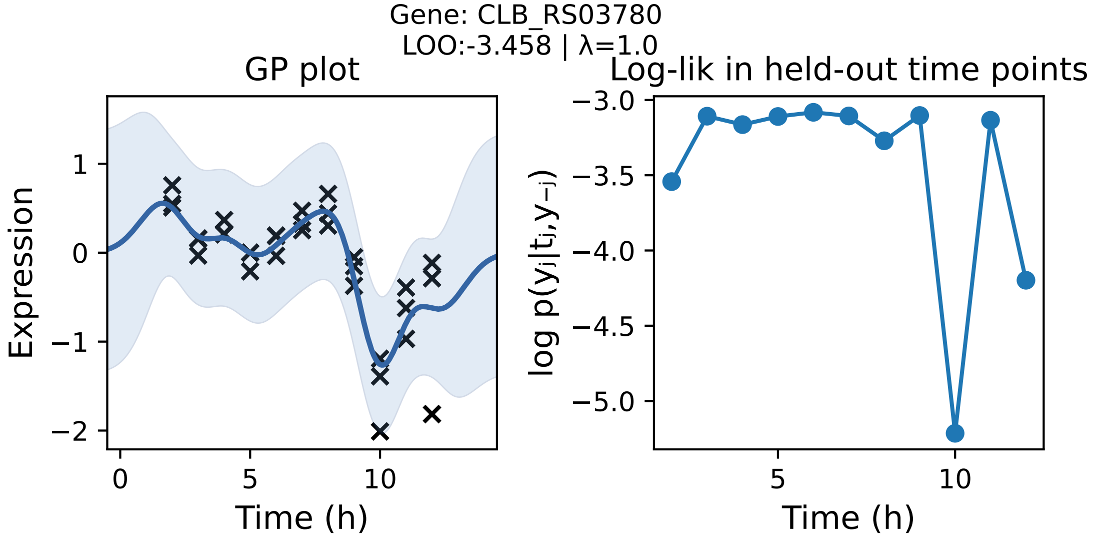
Gene: CLB_RS03780
LOO:-3.458 | λ=1.0
GP plot
Expression
Time (h)
1
0
−1
−2
0
5
10
Log-lik in held-out time points
log p(y_j|t_j,y_−j)
−3.0
−3.5
−4.0
−4.5
−5.0
5
10
Time (h)

Gene: CLB_RS03625
LOO:-3.457 | λ=1.82

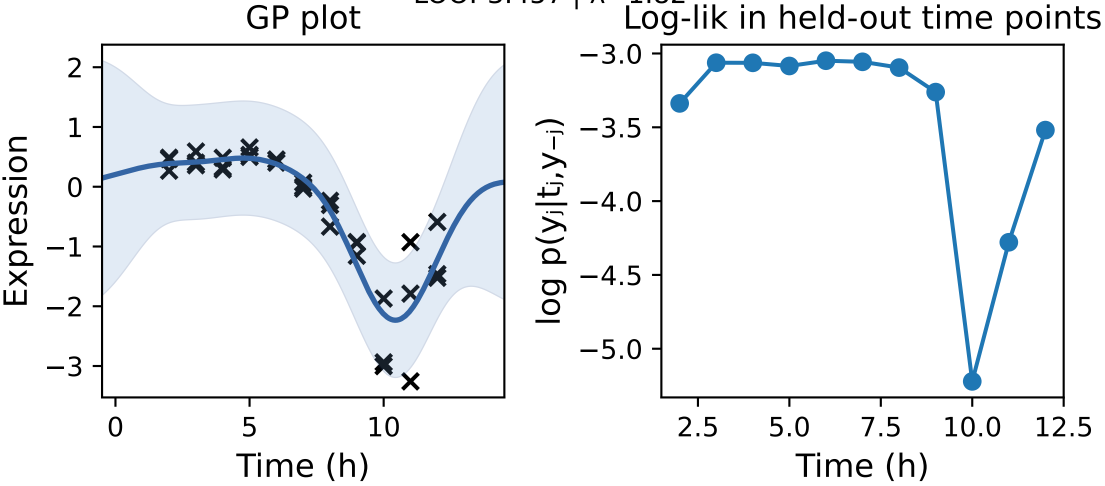

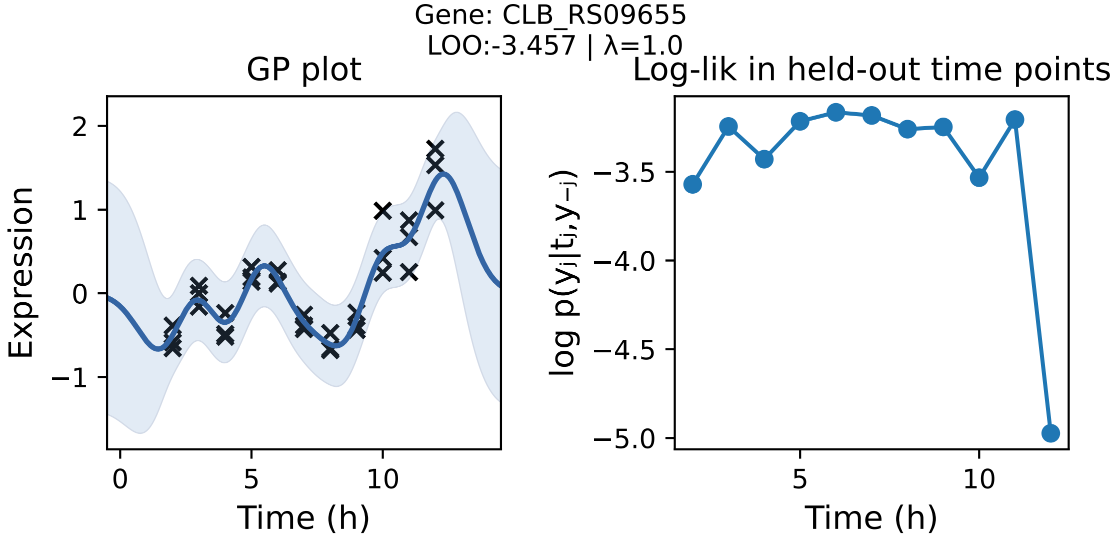
Gene: CLB_RS09655
LOO:-3.457 | λ=1.0
GP plot
Expression
Time (h)
2
1
0
−1
0
5
10
Log-lik in held-out time points
log p(y_j|t_j,y_−j)
Time (h)
−3.5
−4.0
−4.5
−5.0
5
10

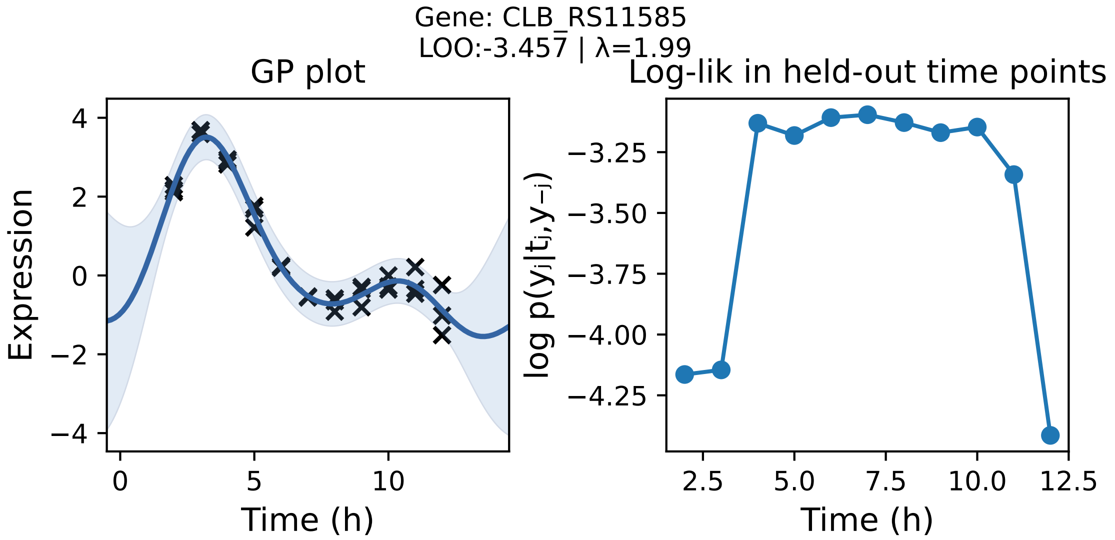

Gene: CLB_RS11585
LOO:-3.457 | λ=1.99
GP plot
Log-lik in held-out time points
Expression
4
2
0
−2
−4
0
5
10
Time (h)
log p(yⱼ|tⱼ,y₋ⱼ)
−3.25
−3.50
−3.75
−4.00
−4.25
2.5
5.0
7.5
10.0
12.5
Time (h)

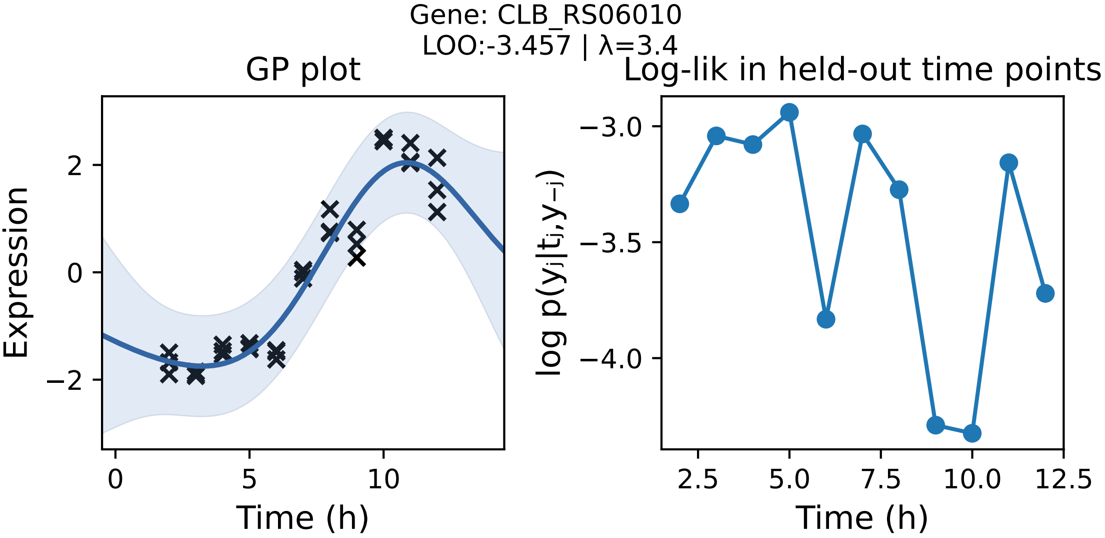

Gene: CLB_RS06010
LOO:-3.457 | λ=3.4
GP plot
Expression
Time (h)
Log-lik in held-out time points
log p(y_j|t_j,y_−j)
Time (h)

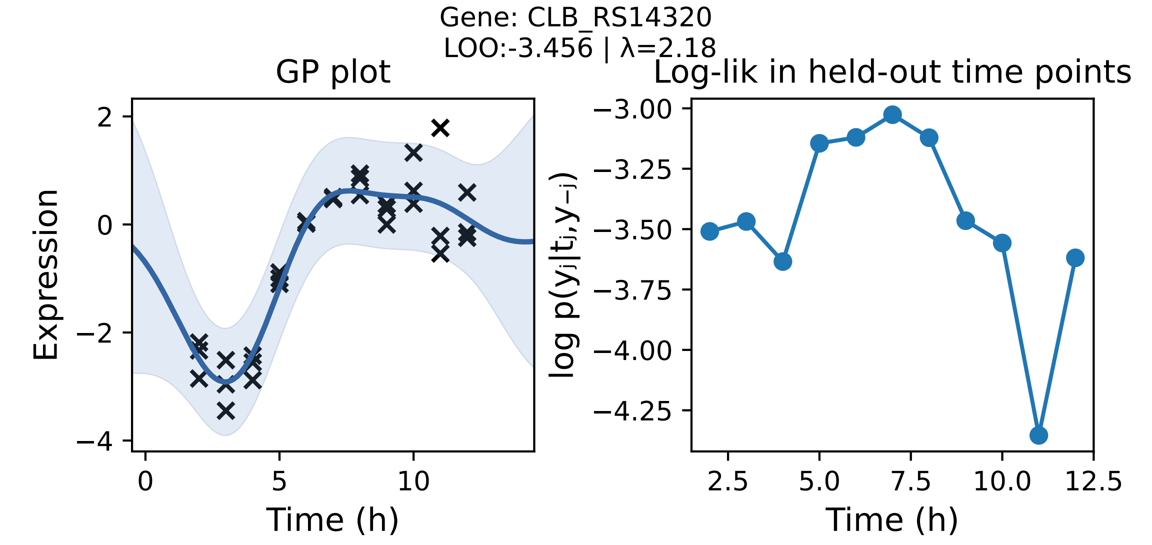

Gene: CLB_RS14320
LOO:-3.456 | λ=2.18
GP plot
Expression
Time (h)
2
0
−2
−4
0
5
10
Log-lik in held-out time points
log p(y_j|t_j,y_−j)
Time (h)
−3.00
−3.25
−3.50
−3.75
−4.00
−4.25
2.5
5.0
7.5
10.0
12.5

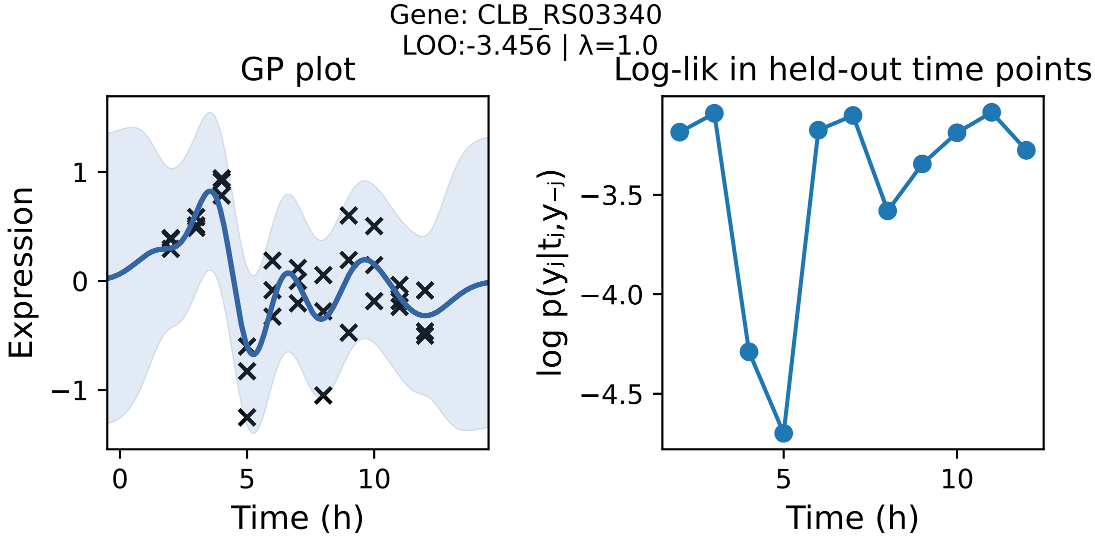

Gene: CLB_RS03340
LOO:-3.456 | λ=1.0
GP plot
Expression
Time (h)
Log-lik in held-out time points
log p(y_j|t_j,y_−j)
Time (h)

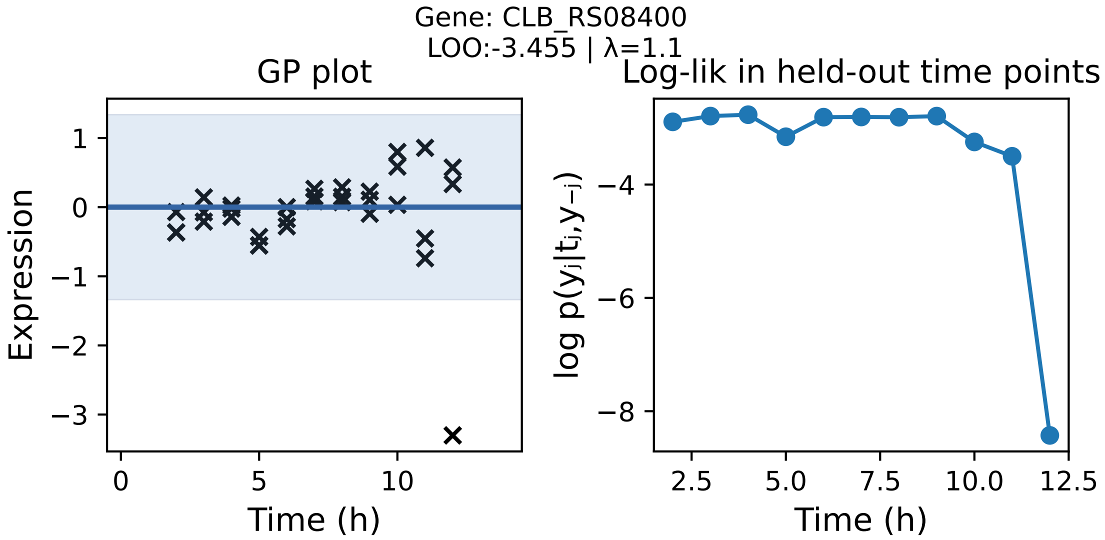

Gene: CLB_RS08400
LOO:-3.455 | λ=1.1
GP plot
Expression
Time (h)
Log-lik in held-out time points
log p(y_j|t_j,y_−j)
Time (h)

Gene: CLB_RS11075
LOO:-3.454 | λ=1.69

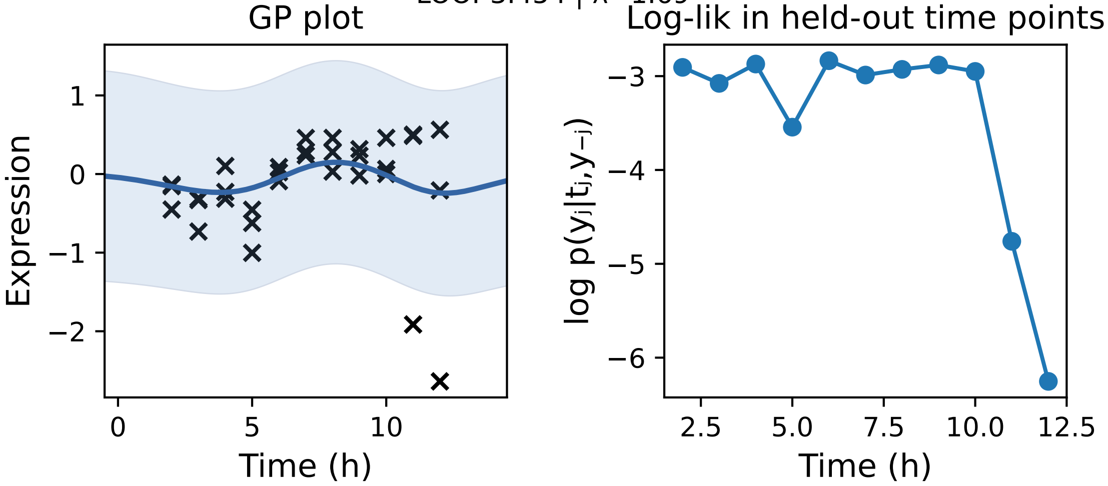

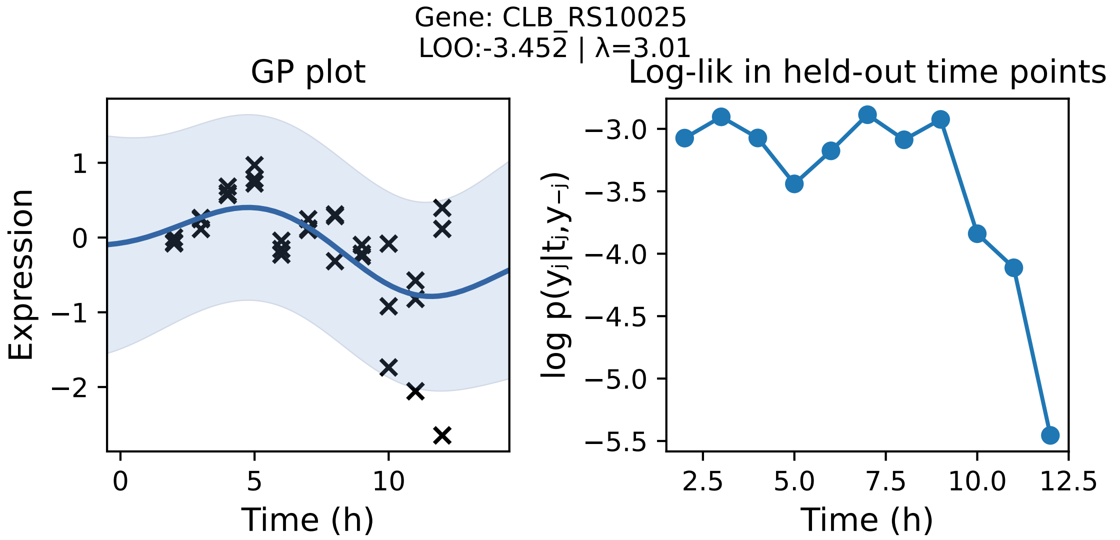

Gene: CLB_RS10025
LOO:-3.452 | λ=3.01
GP plot
Expression
Time (h)
1
0
−1
−2
0
5
10
Log-lik in held-out time points
log p(y_j|t_j,y_−j)
−3.0
−3.5
−4.0
−4.5
−5.0
−5.5
2.5
5.0
7.5
10.0
12.5
Time (h)

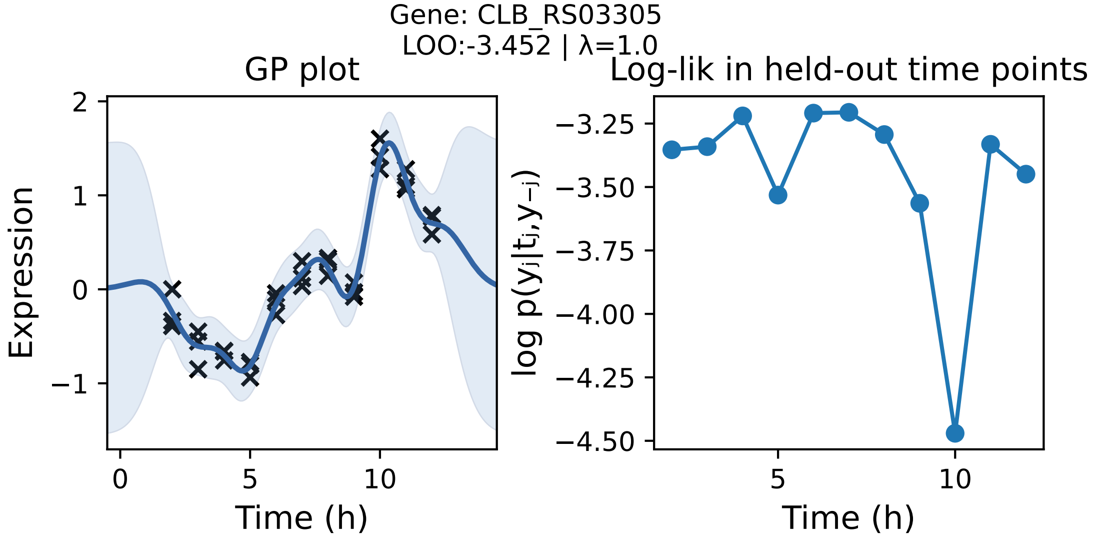

Gene: CLB_RS03305
LOO:-3.452 | λ=1.0
GP plot
Expression
Time (h)
Log-lik in held-out time points
log p(yj|tj,y−j)
Time (h)
−3.25
−3.50
−3.75
−4.00
−4.25
−4.50

Gene: CLB_RS13050
LOO:-3.451 | λ=5.11

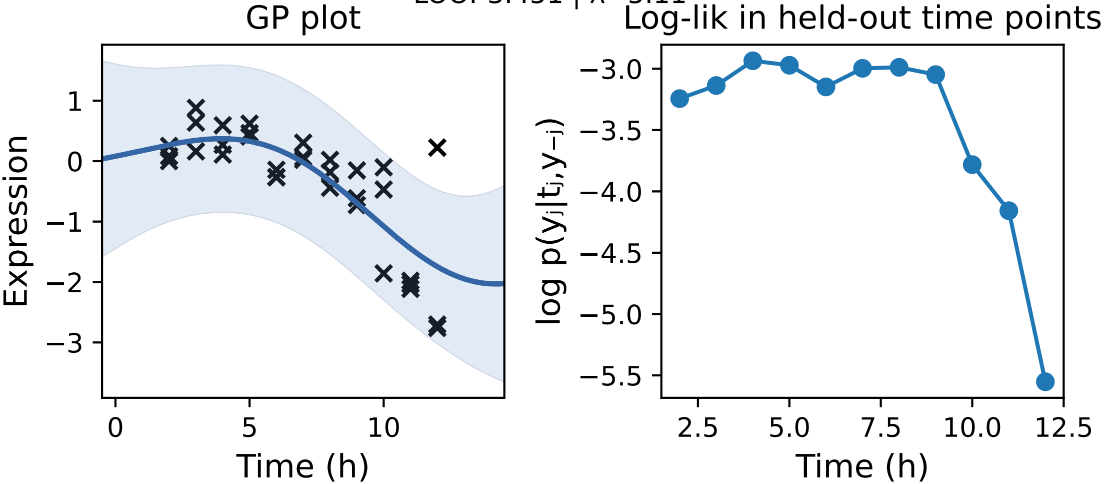

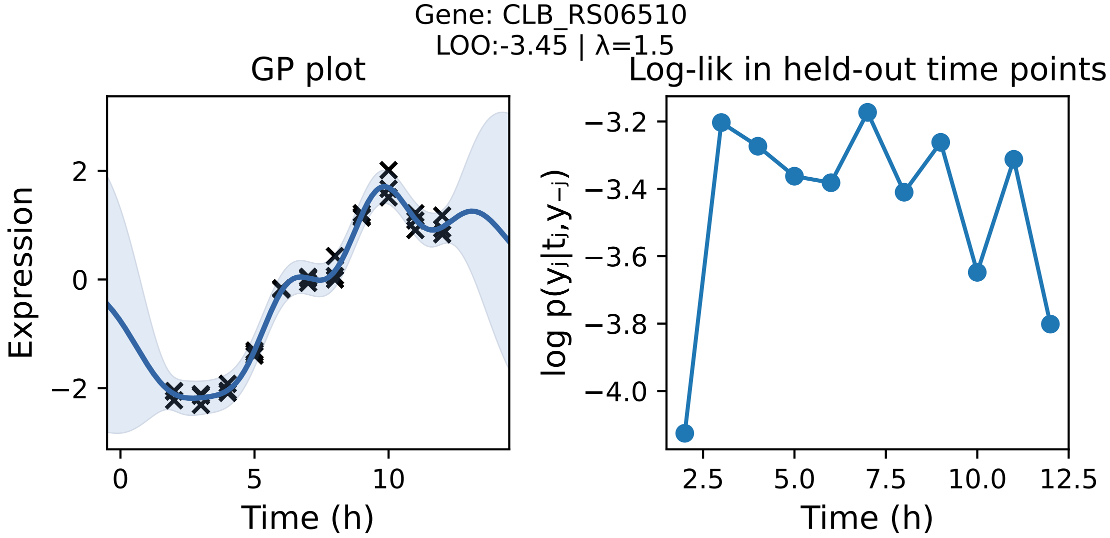

Gene: CLB_RS06510
LOO:-3.45 | λ=1.5
GP plot
Expression
Time (h)
Log-lik in held-out time points
log p(y_j|t_j,y_−j)
Time (h)

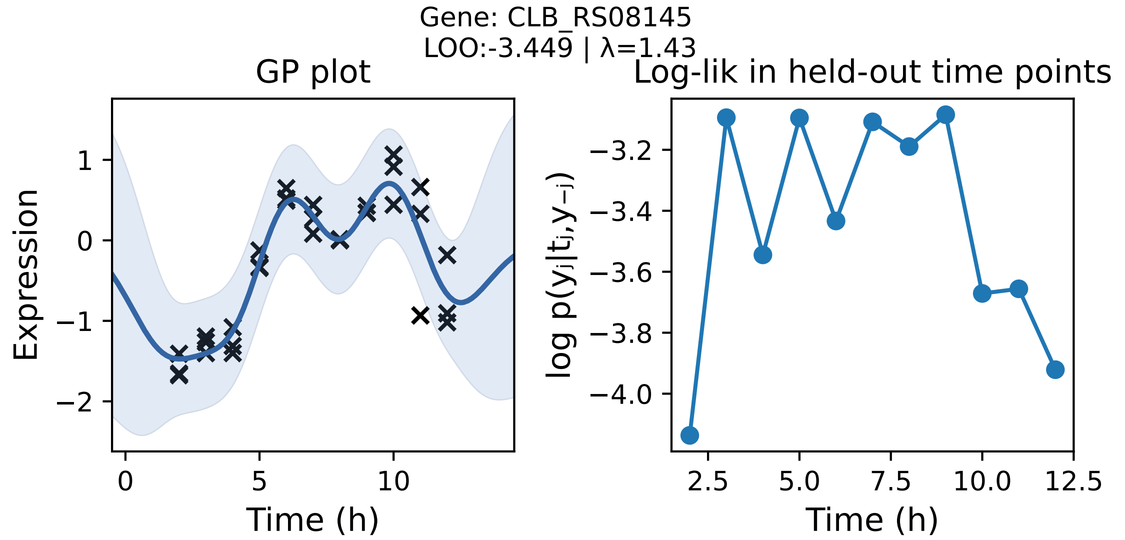

Gene: CLB_RS08145
LOO:-3.449 | λ=1.43
GP plot
Expression
Time (h)
Log-lik in held-out time points
log p(y_j|t_j,y_−j)
Time (h)

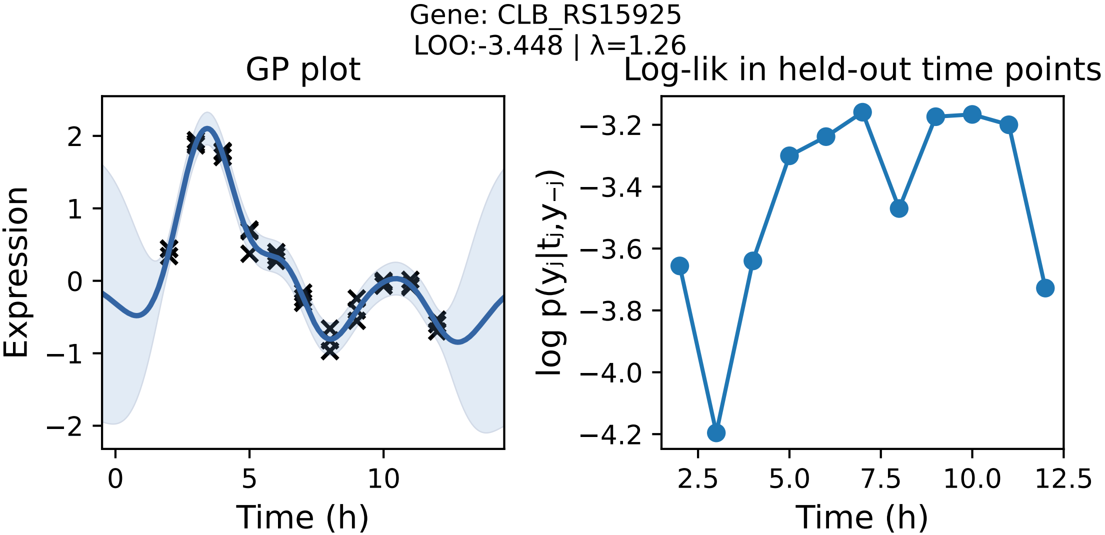

Gene: CLB_RS15925
LOO:-3.448 | λ=1.26
GP plot
Expression
2
1
0
−1
−2
0
5
10
Time (h)
Log-lik in held-out time points
log p(y_j|t_j,y_−j)
−3.2
−3.4
−3.6
−3.8
−4.0
−4.2
2.5
5.0
7.5
10.0
12.5
Time (h)

Gene: CLB_RS01365
LOO:-3.447 | λ=5.8

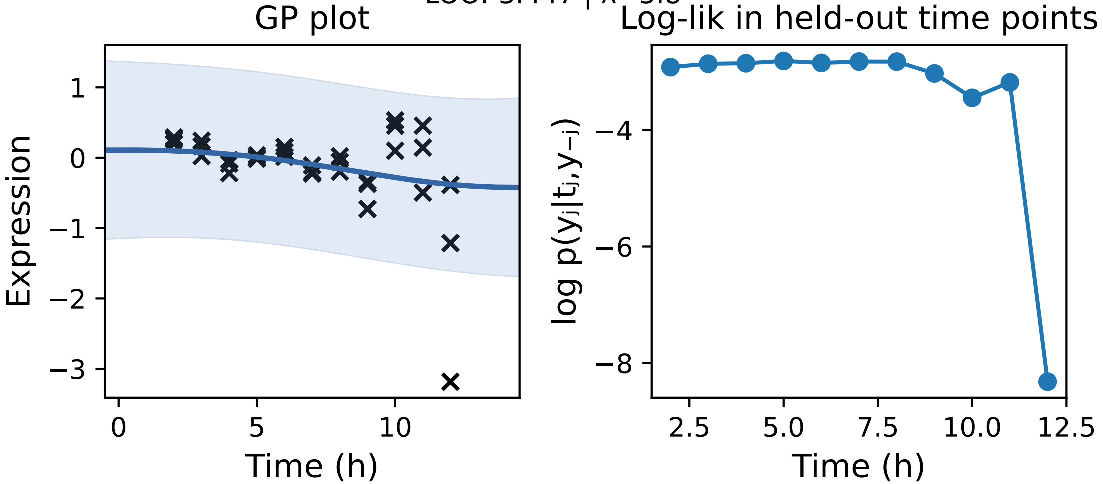

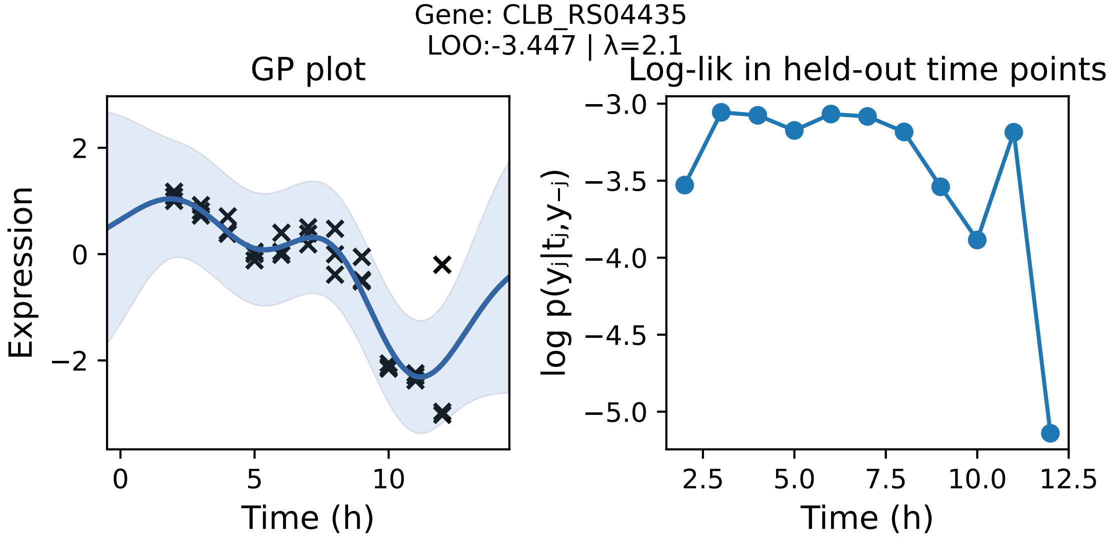

Gene: CLB_RS04435
LOO:-3.447 | λ=2.1
GP plot
Expression
Time (h)
Log-lik in held-out time points
log p(y_j|t_j,y_−j)
Time (h)
−3.0
−3.5
−4.0
−4.5
−5.0
2.5
5.0
7.5
10.0
12.5
2
0
−2
0
5
10

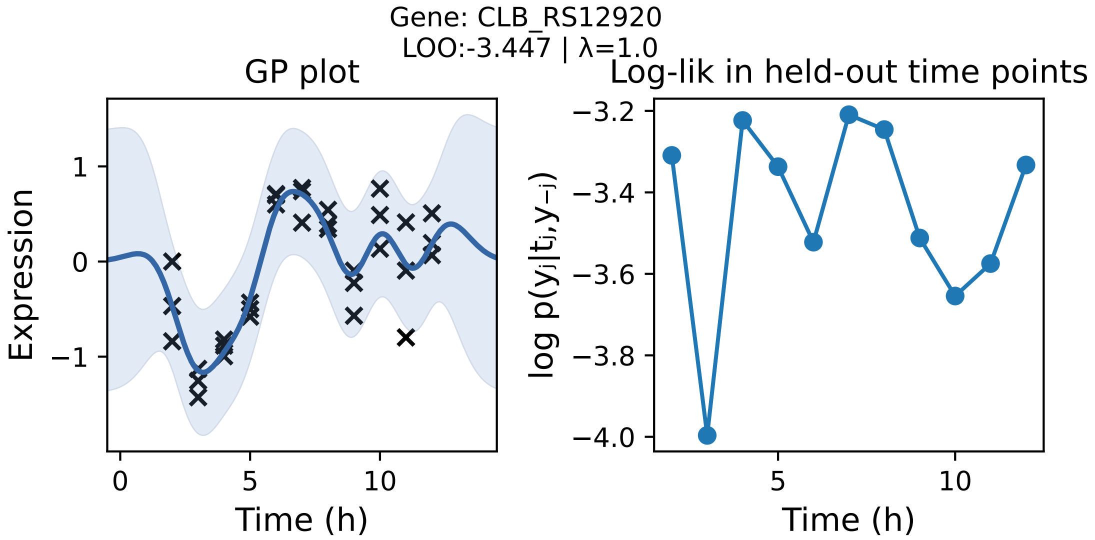

Gene: CLB_RS12920
LOO:-3.447 | λ=1.0
GP plot
Expression
Time (h)
0
5
10
1
0
−1
Log-lik in held-out time points
log p(y_j|t_j,y_−j)
Time (h)
−3.2
−3.4
−3.6
−3.8
−4.0
5
10

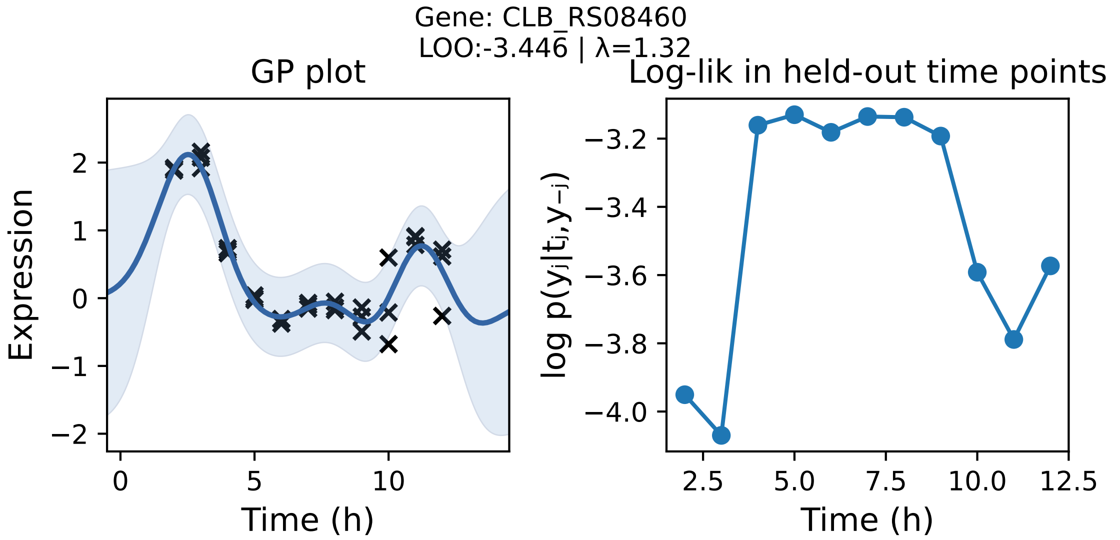

Gene: CLB_RS08460
LOO:-3.446 | λ=1.32
GP plot
Expression
Time (h)
Log-lik in held-out time points
log p(y_j|t_j,y_−j)
Time (h)

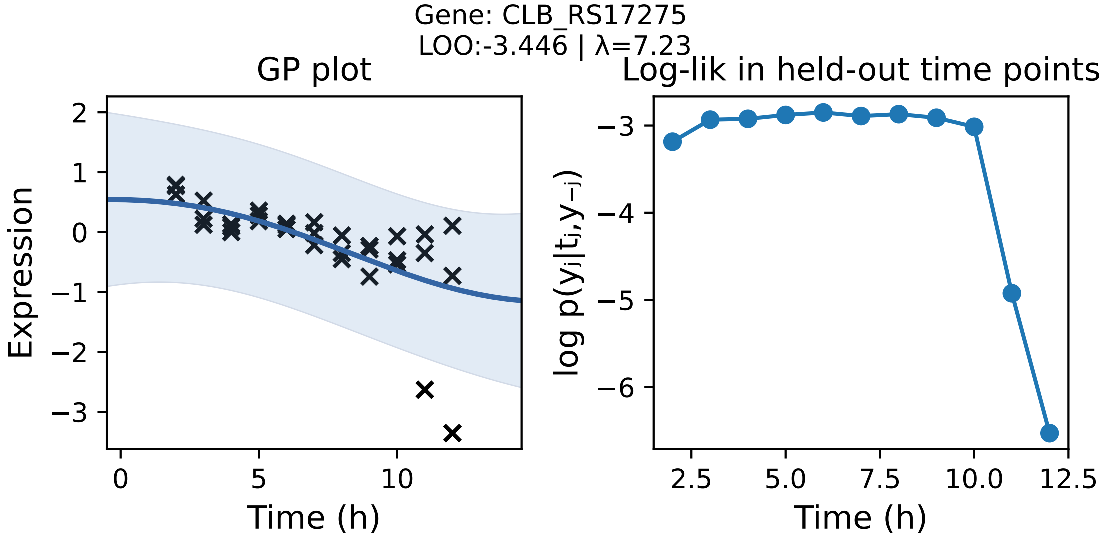

Gene: CLB_RS17275
LOO:-3.446 | λ=7.23
GP plot
Expression
Time (h)
Log-lik in held-out time points
log p(y_j|t_j,y_−j)
Time (h)

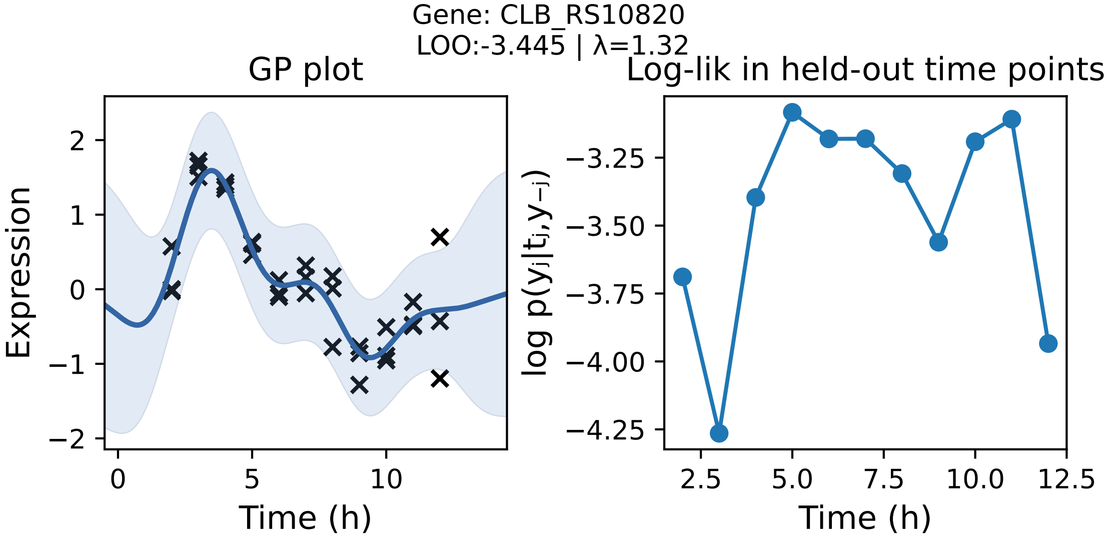

Gene: CLB_RS10820
LOO:-3.445 | λ=1.32
GP plot
Expression
Time (h)
2
1
0
−1
−2
0
5
10
Log-lik in held-out time points
log p(yj|ti,y−j)
−3.25
−3.50
−3.75
−4.00
−4.25
2.5
5.0
7.5
10.0
12.5
Time (h)

Gene: CLB_RS15210
LOO:-3.444 | λ=1.13

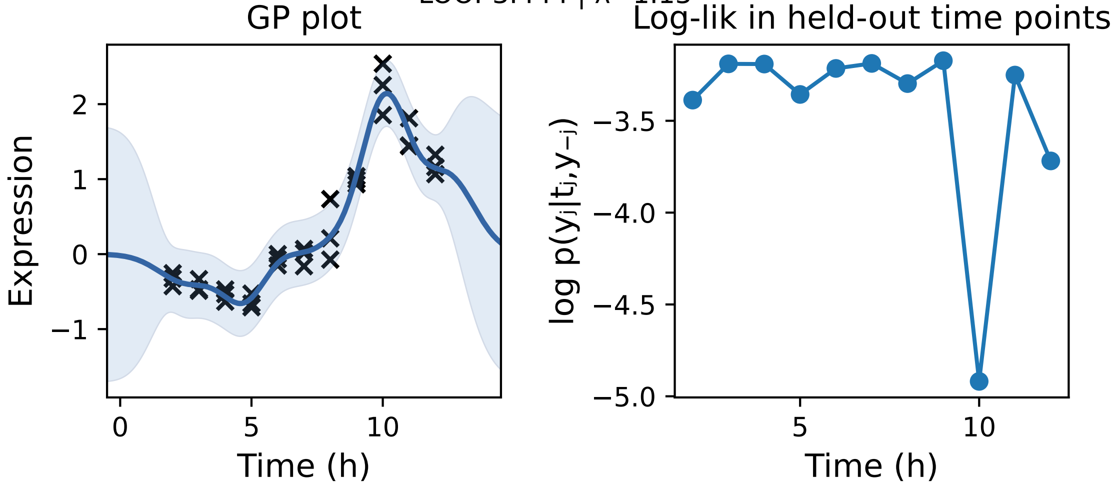

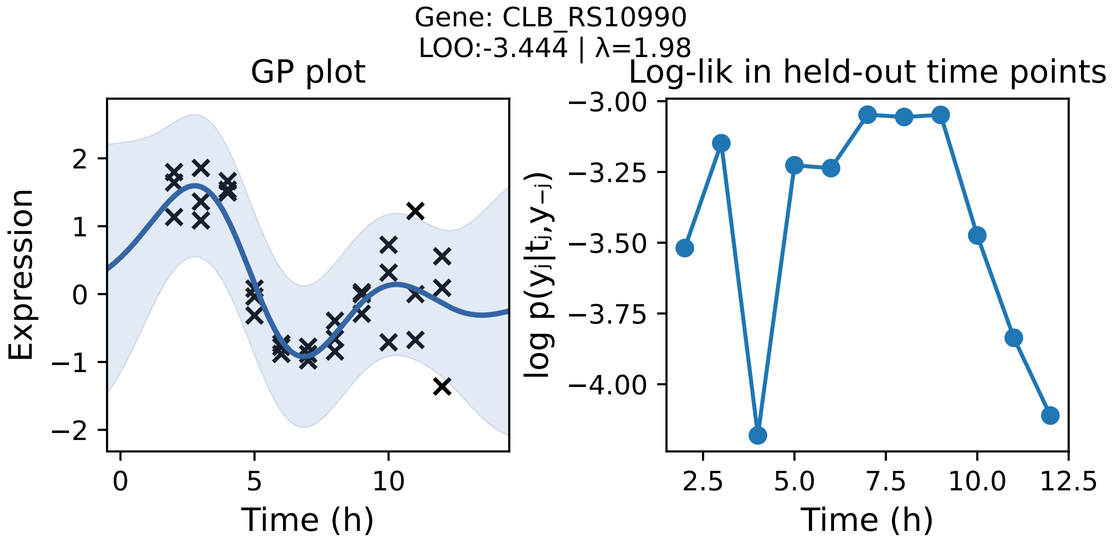

Gene: CLB_RS10990
LOO:-3.444 | λ=1.98
GP plot
Expression
Time (h)
Log-lik in held-out time points
log p(y_j|t_j,y_{−j})
Time (h)

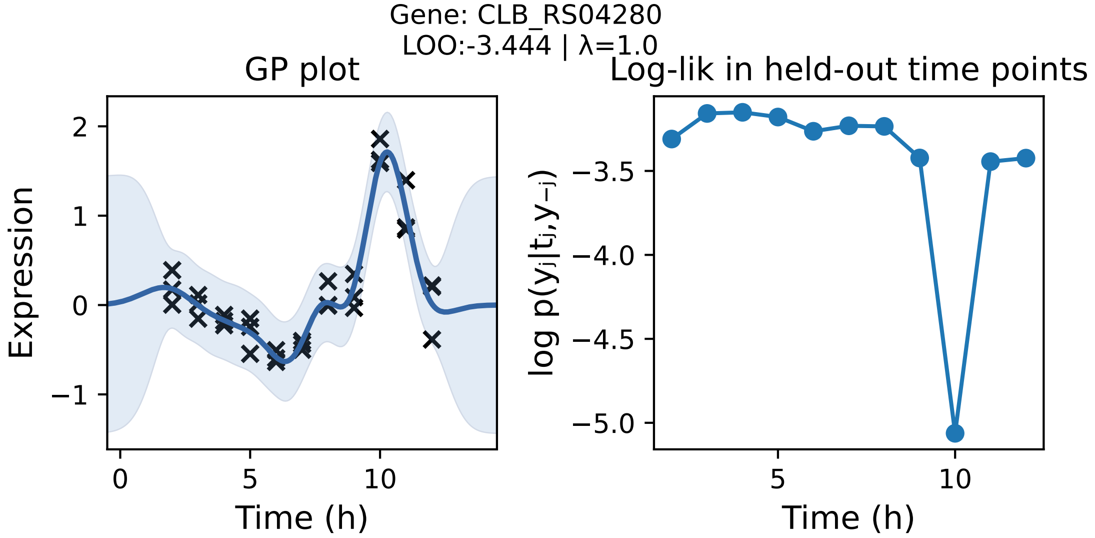

Gene: CLB_RS04280
LOO:-3.444 | λ=1.0
GP plot
Expression
Time (h)
2
1
0
−1
0
5
10
Log-lik in held-out time points
log p(y_j|t_j,y_−j)
−3.5
−4.0
−4.5
−5.0
5
10
Time (h)

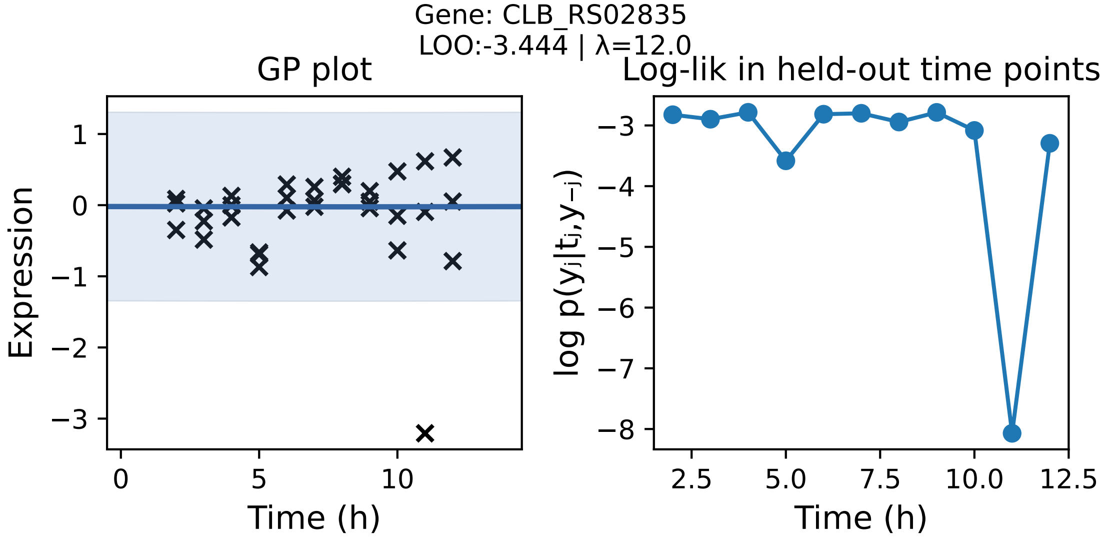

Gene: CLB_RS02835
LOO:-3.444 | λ=12.0
GP plot
Log-lik in held-out time points
Expression
Time (h)
log p(y_j|t_j,y_−j)
Time (h)
1
0
−1
−2
−3
0
5
10
−3
−4
−5
−6
−7
−8
2.5
5.0
7.5
10.0
12.5

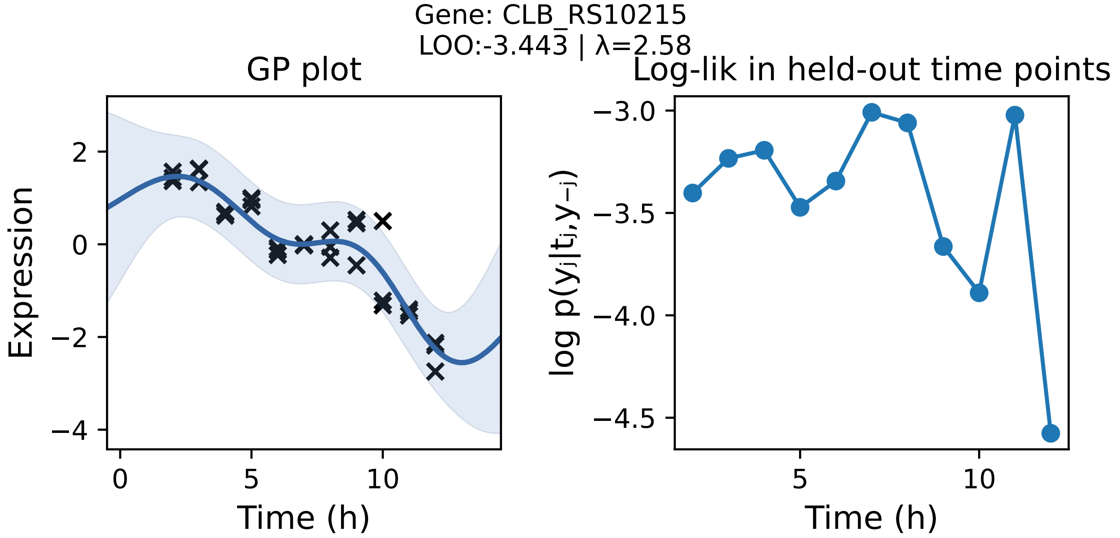

Gene: CLB_RS10215
LOO:-3.443 | λ=2.58
GP plot
Expression
Time (h)
2
0
−2
−4
0
5
10
Log-lik in held-out time points
log p(y_j|t_j,y_−j)
−3.0
−3.5
−4.0
−4.5
5
10
Time (h)

Gene: CLB_RS16150
LOO:-3.442 | λ=1.0

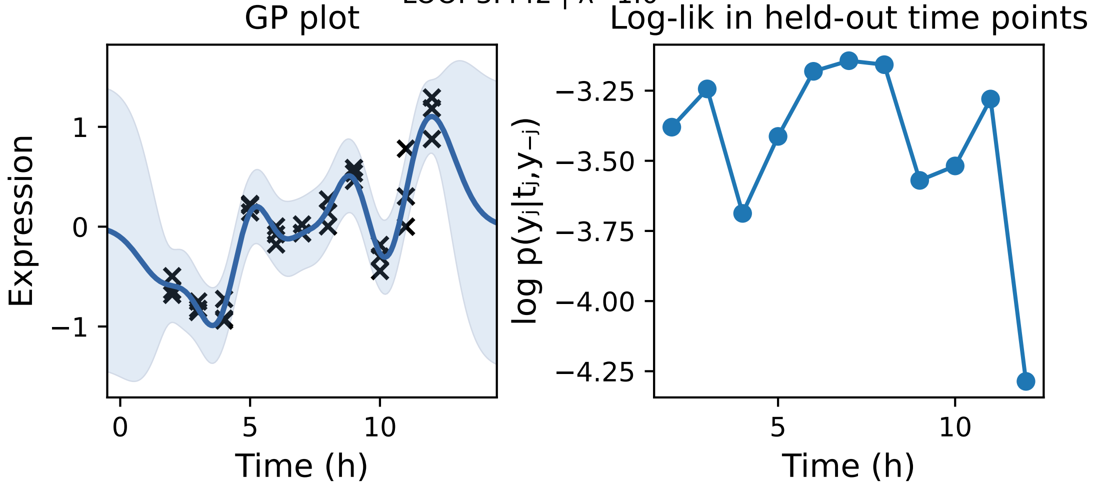

Gene: CLB_RS10510
LOO:-3.441 | λ=1.0

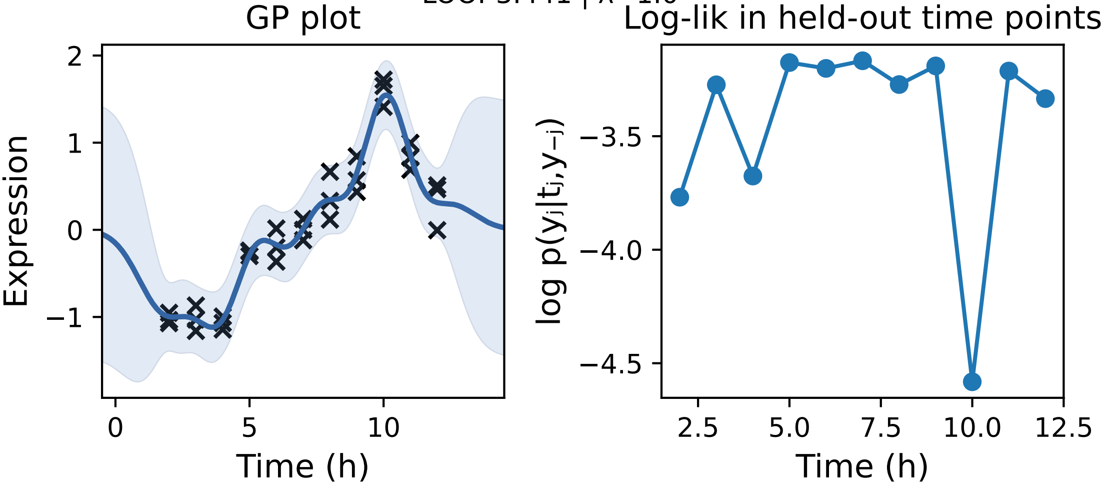

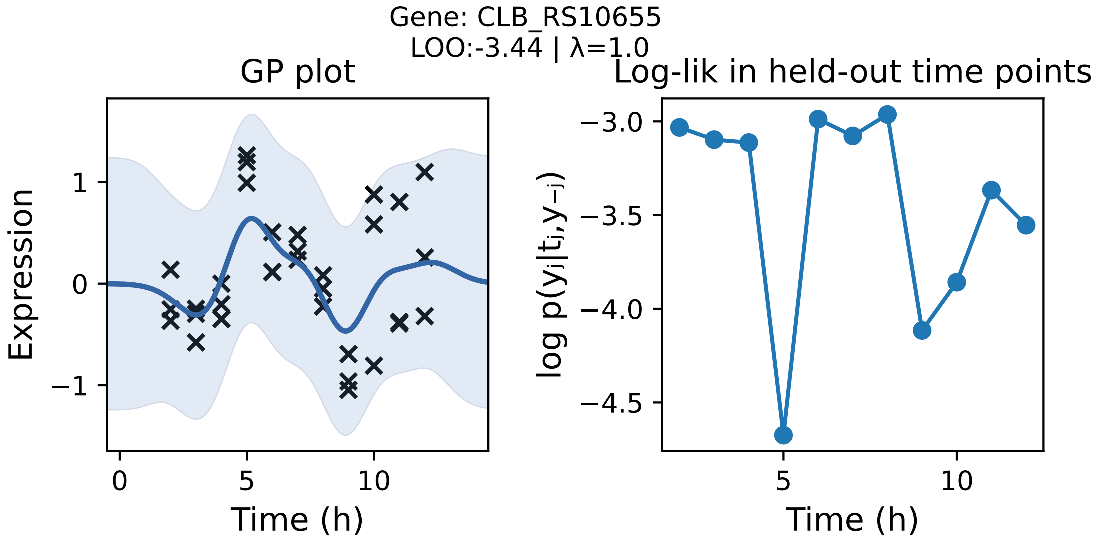
Gene: CLB_RS10655
LOO:-3.44 | λ=1.0
GP plot
Expression
Time (h)
Log-lik in held-out time points
log p(yj|tj,y−j)
Time (h)
−3.0
−3.5
−4.0
−4.5
5
10
1
0
−1
0
5
10

Gene: CLB_RS02350
LOO:-3.439 | λ=2.54

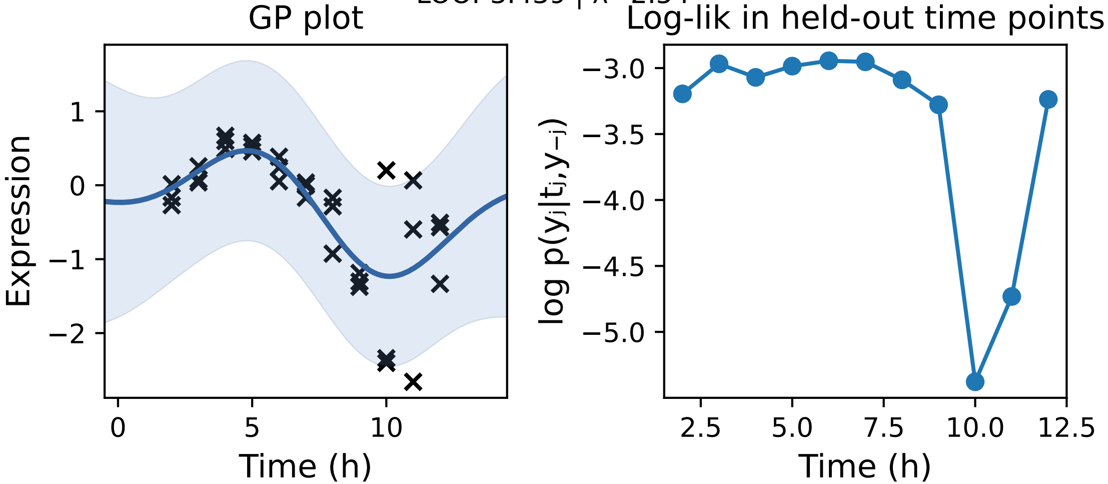

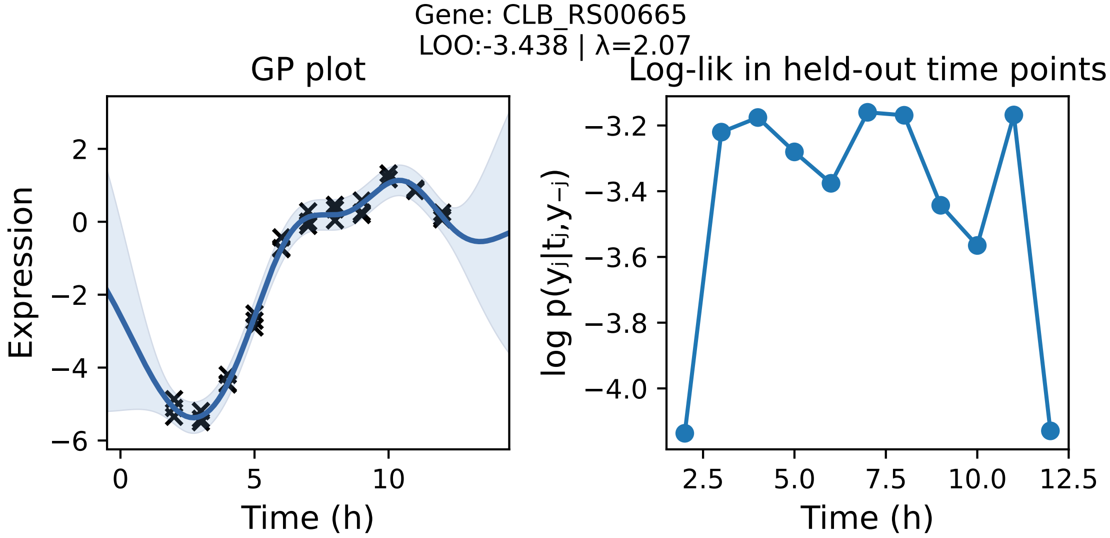
Gene: CLB_RS00665
LOO:-3.438 | λ=2.07
GP plot
Expression
Time (h)
Log-lik in held-out time points
log p(y_j|t_j,y_−j)
Time (h)

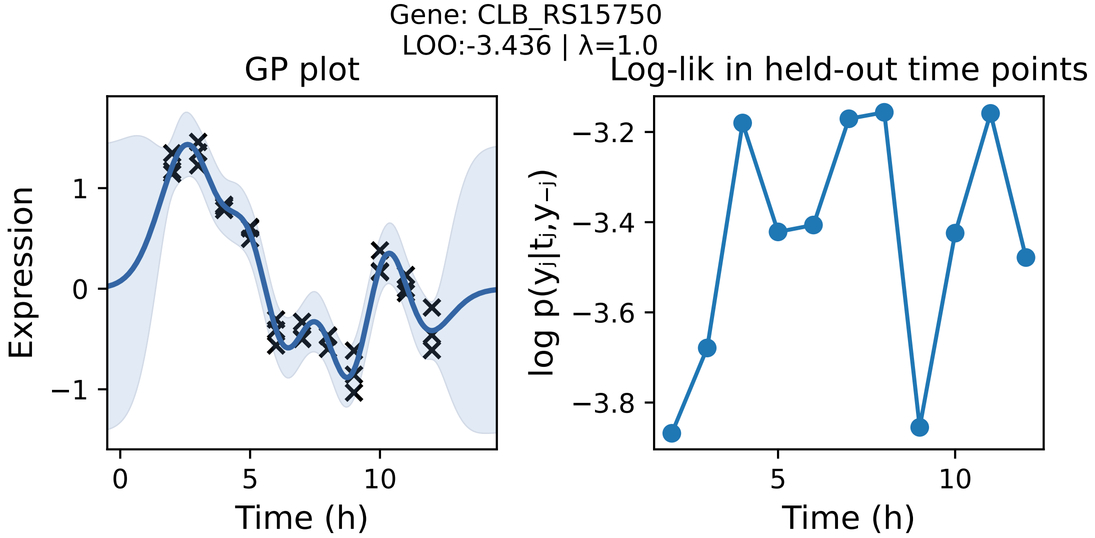

Gene: CLB_RS15750
LOO:-3.436 | λ=1.0
GP plot
Expression
1
0
−1
0
5
10
Time (h)
Log-lik in held-out time points
log p(y_j|t_j,y_−j)
−3.2
−3.4
−3.6
−3.8
5
10
Time (h)

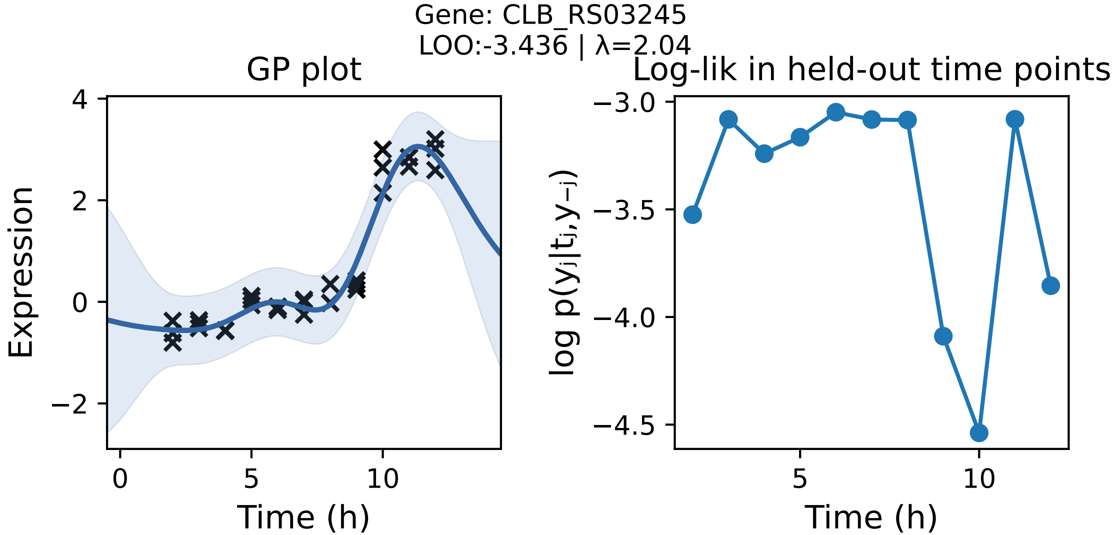

Gene: CLB_RS03245
LOO:-3.436 | λ=2.04
GP plot
Expression
Time (h)
4
2
0
−2
0
5
10
Log-lik in held-out time points
log p(y_j|t_j,y_−j)
Time (h)
−3.0
−3.5
−4.0
−4.5
5
10

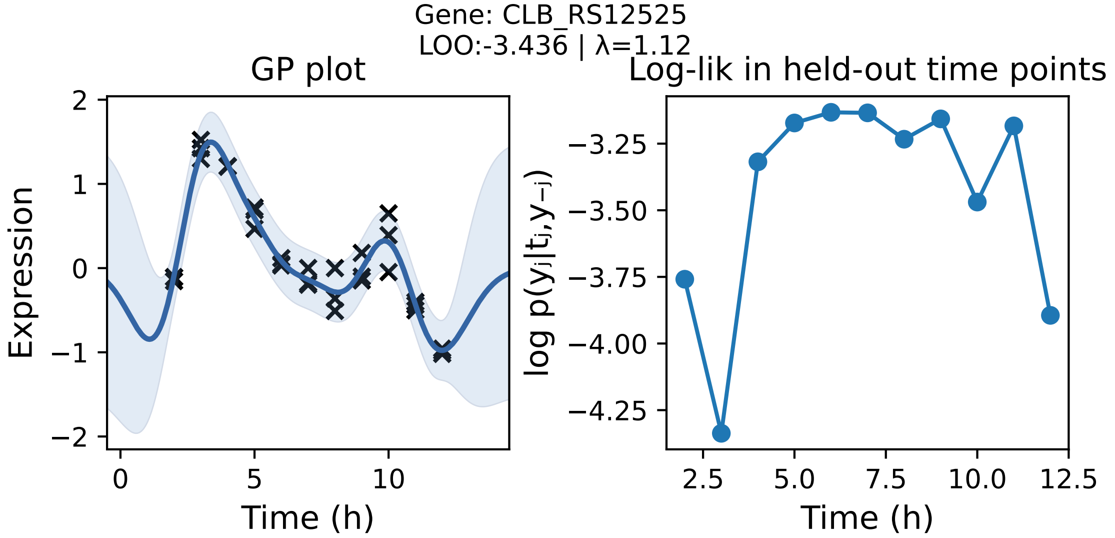

Gene: CLB_RS12525
LOO:-3.436 | λ=1.12
GP plot
Expression
Time (h)
2
1
0
−1
−2
0
5
10
Log-lik in held-out time points
log p(y_j|t_j,y_−j)
Time (h)
−3.25
−3.50
−3.75
−4.00
−4.25
2.5
5.0
7.5
10.0
12.5

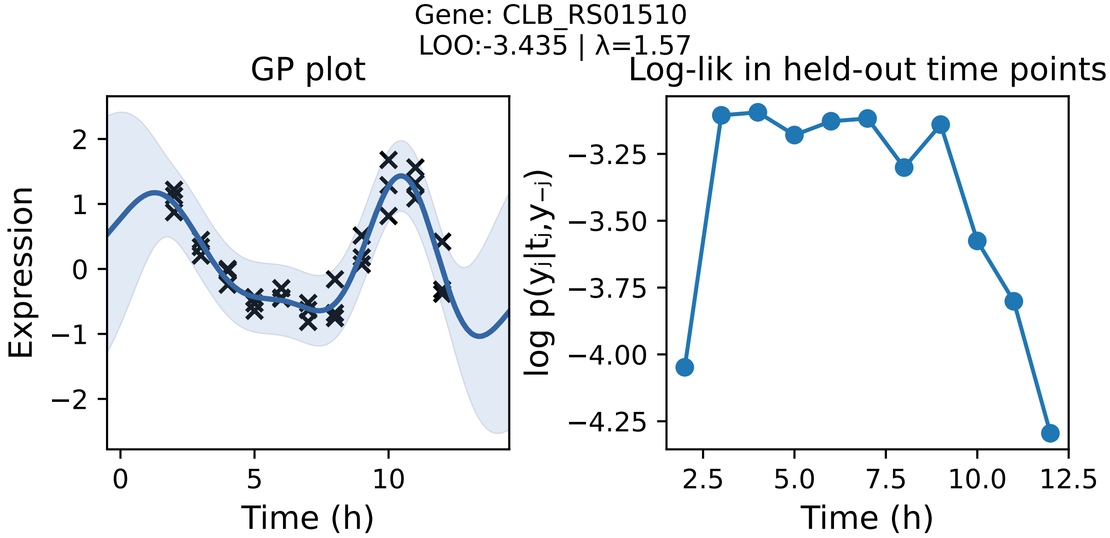

Gene: CLB_RS01510
LOO:-3.435 | λ=1.57
GP plot
Expression
Time (h)
Log-lik in held-out time points
log p(yⱼ|tⱼ,y₋ⱼ)
Time (h)

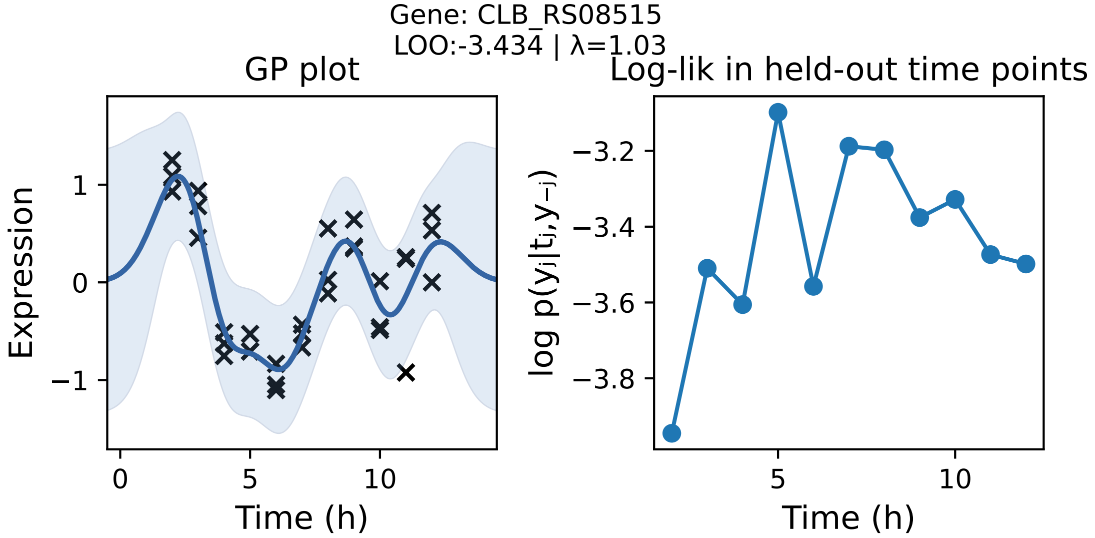

Gene: CLB_RS08515
LOO:-3.434 | λ=1.03
GP plot
Expression
Time (h)
Log-lik in held-out time points
log p(yj|tj,y−j)
Time (h)

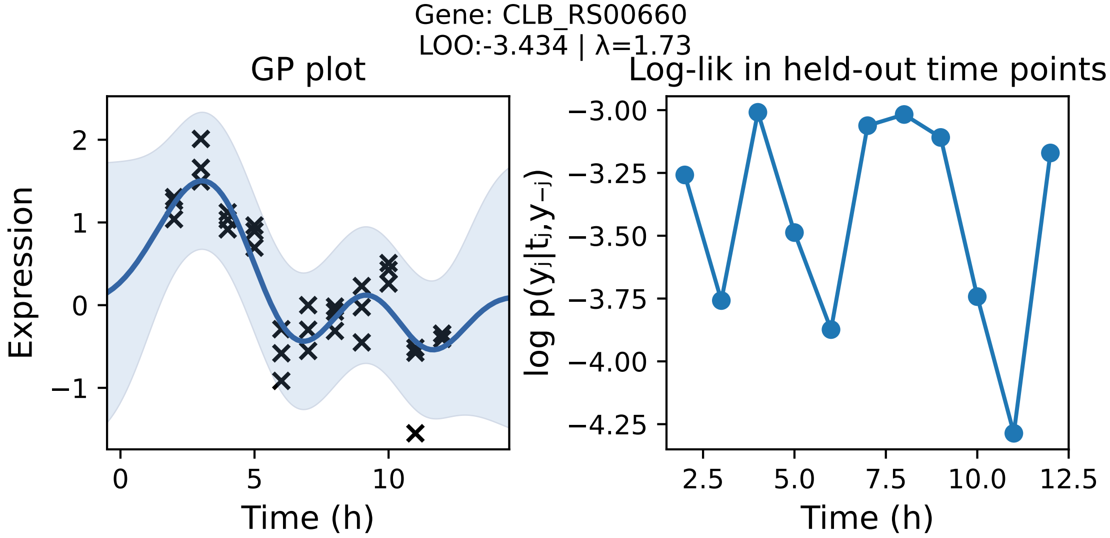

Gene: CLB_RS00660
LOO:-3.434 | λ=1.73
GP plot
Expression
Time (h)
2
1
0
−1
0
5
10
Log-lik in held-out time points
log p(y_j|t_j,y_−j)
−3.00
−3.25
−3.50
−3.75
−4.00
−4.25
2.5
5.0
7.5
10.0
12.5
Time (h)

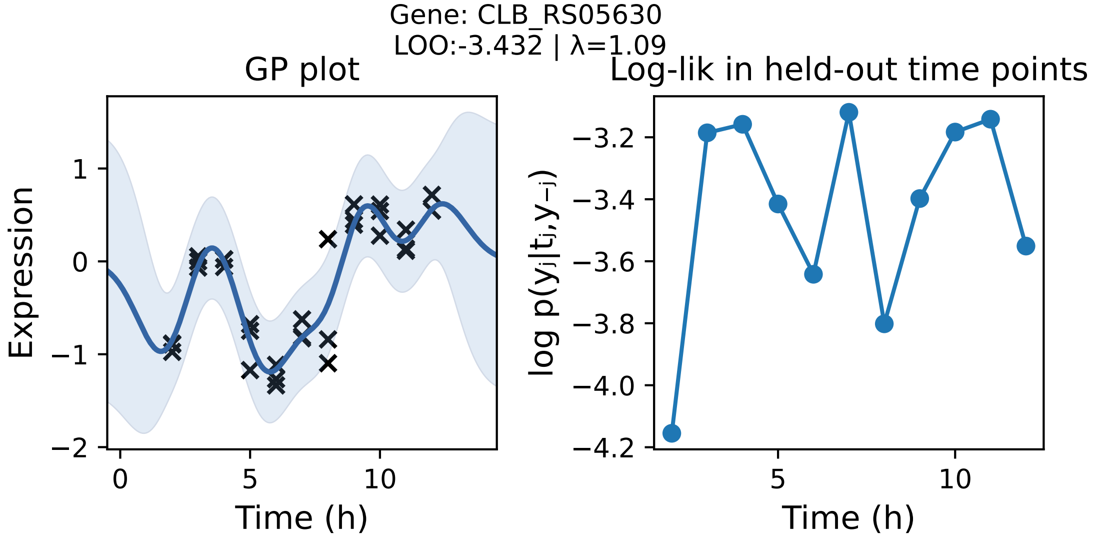

Gene: CLB_RS05630
LOO:-3.432 | λ=1.09
GP plot
Expression
1
0
−1
−2
0
5
10
Time (h)
Log-lik in held-out time points
log p(yj|tj,y−j)
−3.2
−3.4
−3.6
−3.8
−4.0
−4.2
5
10
Time (h)

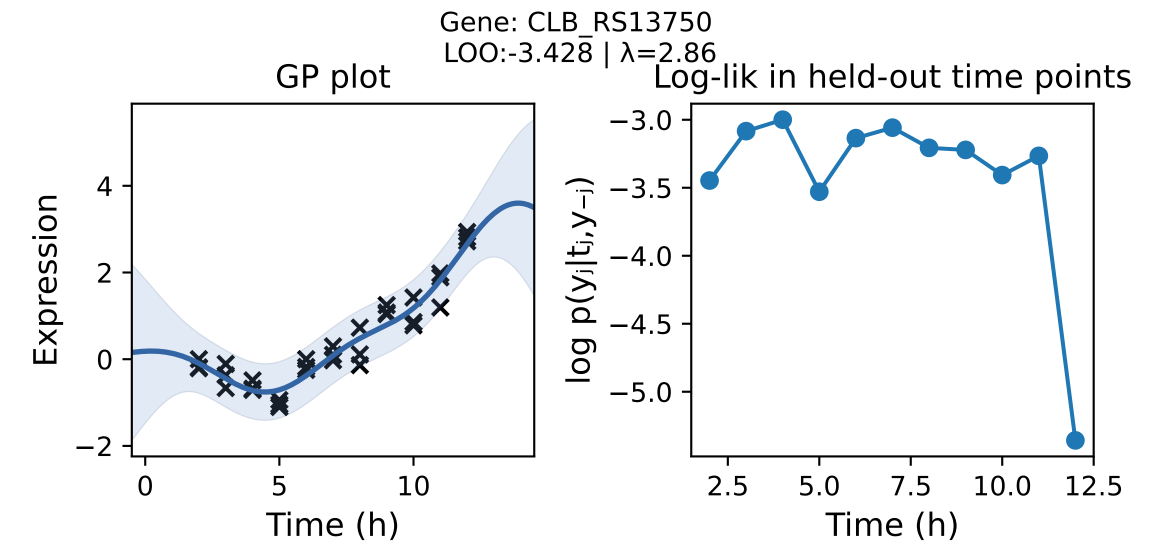

Gene: CLB_RS13750
LOO:-3.428 | λ=2.86
GP plot
Expression
Time (h)
0
5
10
−2
0
2
4
Log-lik in held-out time points
log p(y_j|t_j,y_−j)
Time (h)
2.5
5.0
7.5
10.0
12.5
−3.0
−3.5
−4.0
−4.5
−5.0

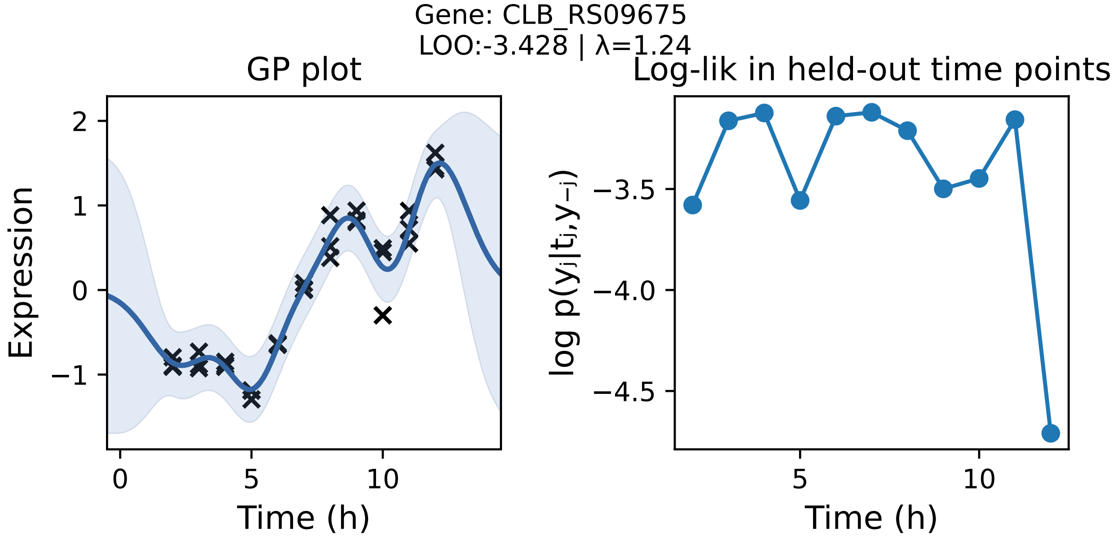

Gene: CLB_RS09675
LOO:-3.428 | λ=1.24
GP plot
Expression
Time (h)
2
1
0
−1
0
5
10
Log-lik in held-out time points
log p(y_j|t_j,y_−j)
−3.5
−4.0
−4.5
5
10
Time (h)

Gene: CLB_RS01405
LOO:-3.427 | λ=1.0

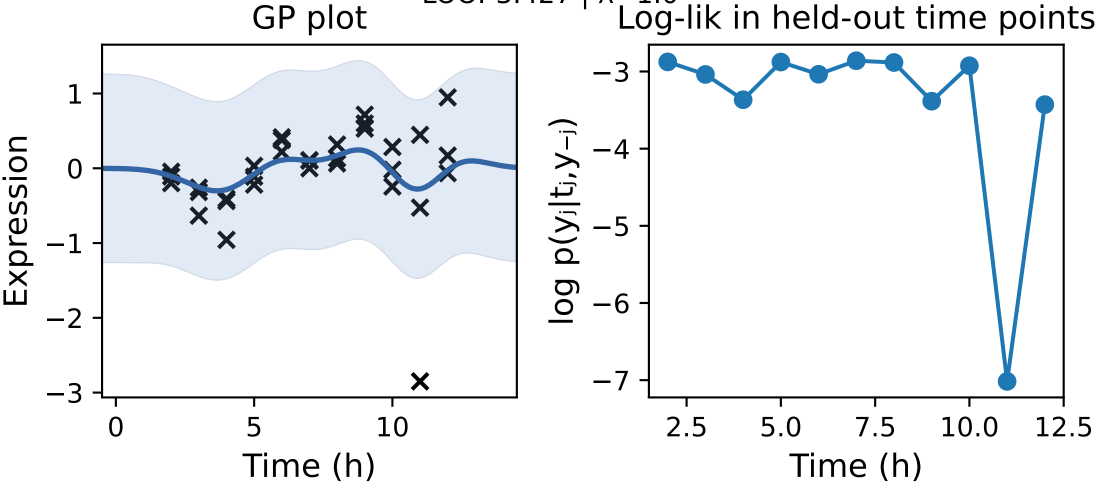

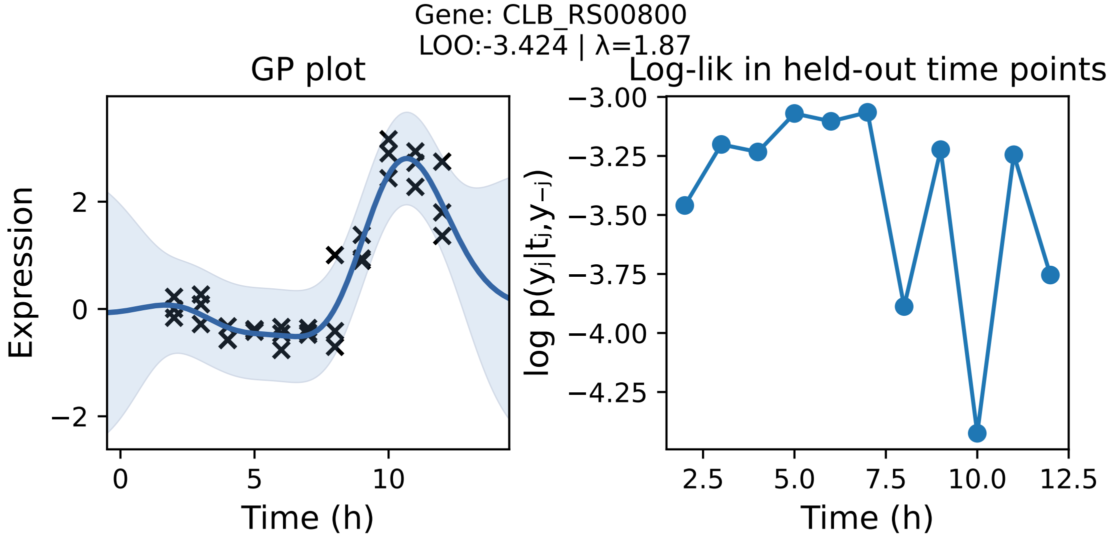

Gene: CLB_RS00800
LOO:-3.424 | λ=1.87
GP plot
Expression
Time (h)
2
0
−2
0
5
10
Log-lik in held-out time points
log p(y_j|t_j,y_−j)
−3.00
−3.25
−3.50
−3.75
−4.00
−4.25
2.5
5.0
7.5
10.0
12.5
Time (h)

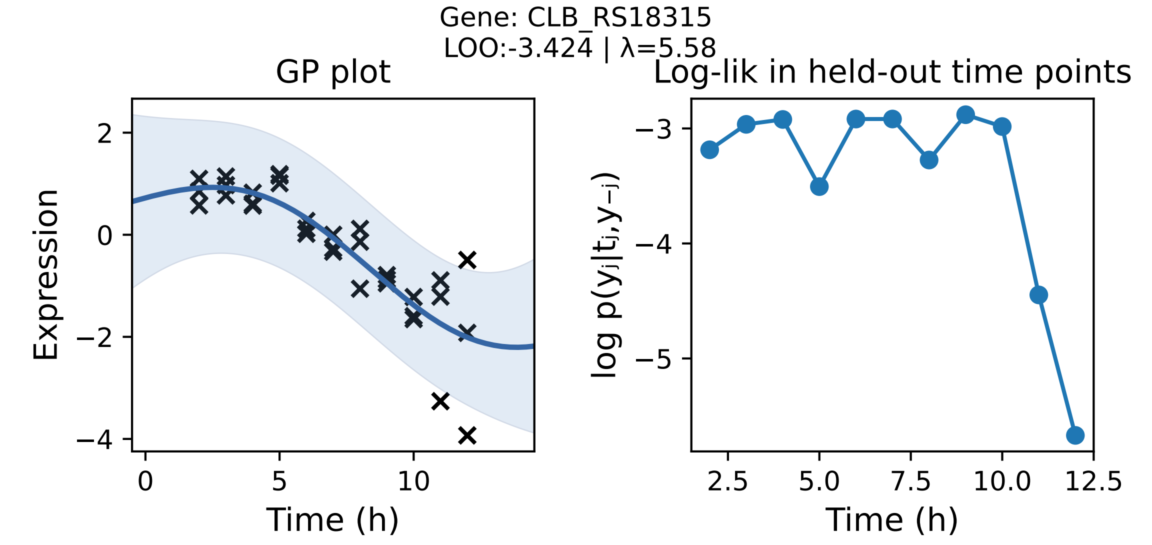

Gene: CLB_RS18315
LOO:-3.424 | λ=5.58
GP plot
Expression
Time (h)
Log-lik in held-out time points
log p(y_j|t_j,y_−j)
Time (h)

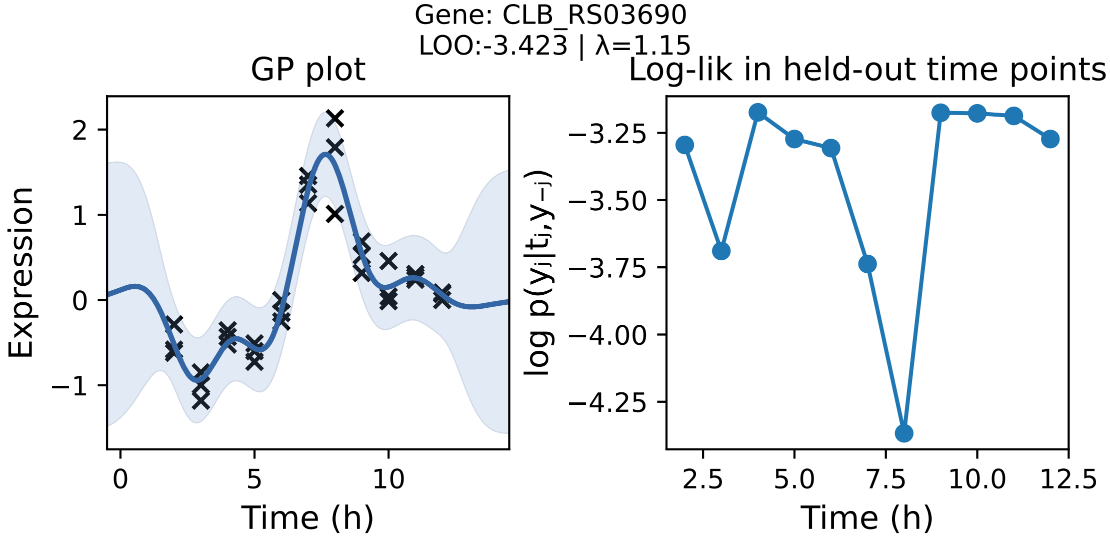

Gene: CLB_RS03690
LOO:-3.423 | λ=1.15
GP plot
Expression
Time (h)
Log-lik in held-out time points
log p(y_j|t_j,y_−j)
Time (h)

Gene: CLB_RS12355
LOO:-3.423 | λ=1.0

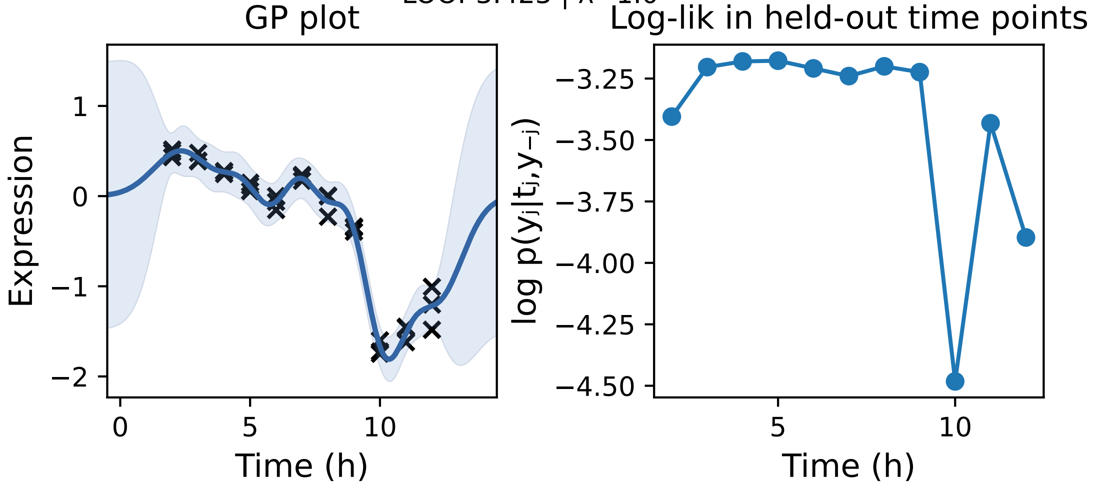

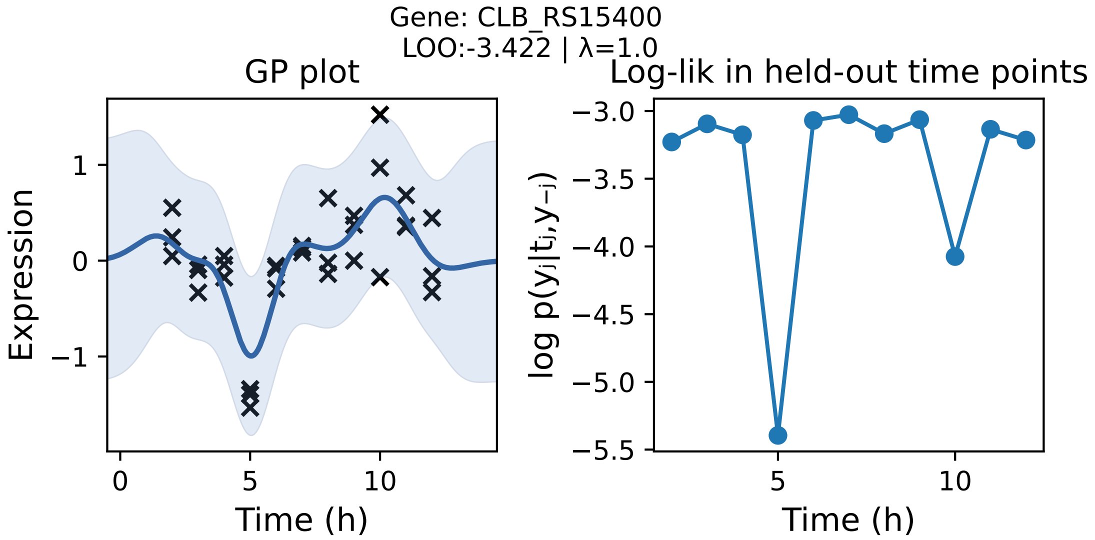
Gene: CLB_RS15400
LOO:-3.422 | λ=1.0
GP plot
Expression
1
0
−1
0
5
10
Time (h)
Log-lik in held-out time points
log p(yj|tj,y−j)
−3.0
−3.5
−4.0
−4.5
−5.0
−5.5
5
10
Time (h)

Gene: CLB_RS06650
LOO:-3.416 | λ=5.13

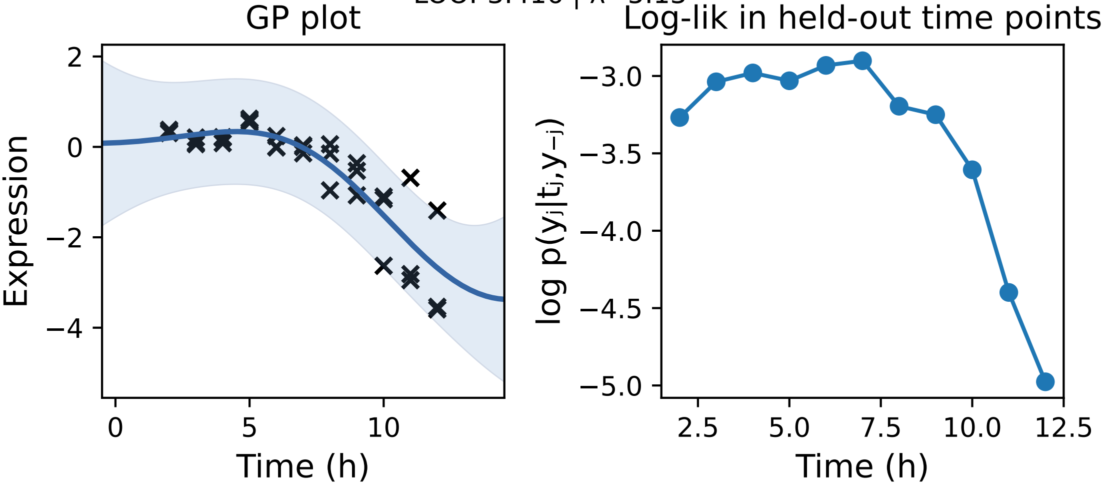

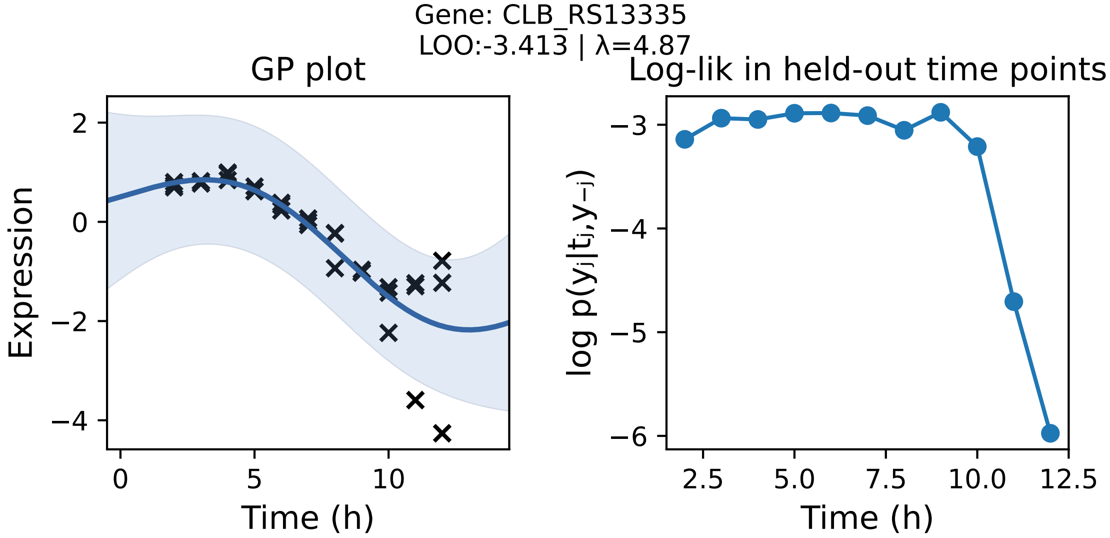
Gene: CLB_RS13335
LOO:-3.413 | λ=4.87
GP plot
Expression
Time (h)
2
0
−2
−4
0
5
10
Log-lik in held-out time points
log p(y_j|t_j,y_−j)
Time (h)
−3
−4
−5
−6
2.5
5.0
7.5
10.0
12.5

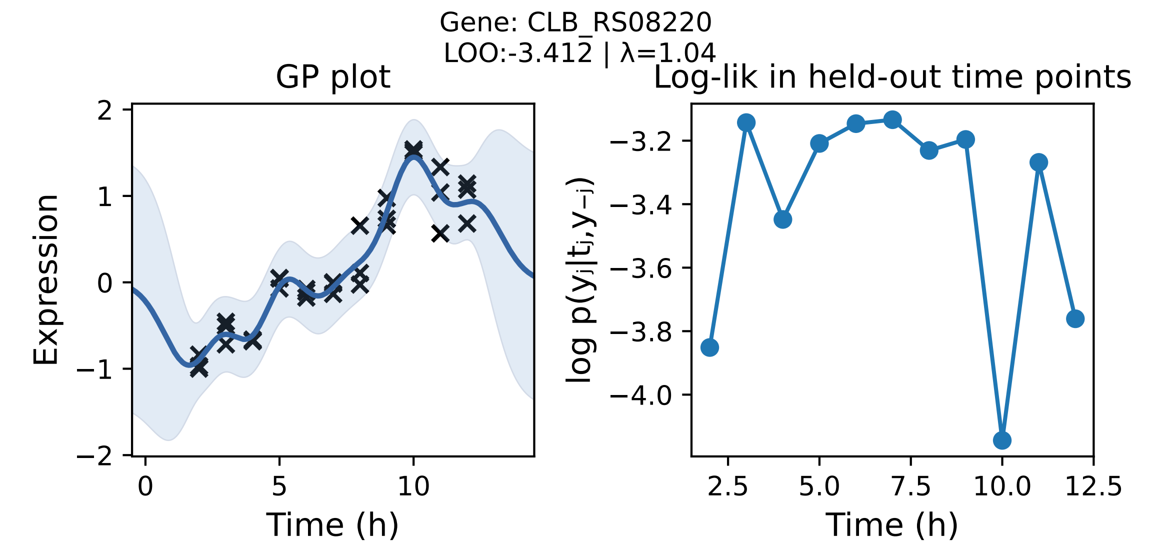

Gene: CLB_RS08220
LOO:-3.412 | λ=1.04
GP plot
Expression
Time (h)
2
1
0
−1
−2
0
5
10
Log-lik in held-out time points
log p(y_j|t_j,y_−j)
−3.2
−3.4
−3.6
−3.8
−4.0
2.5
5.0
7.5
10.0
12.5
Time (h)

Gene: CLB_RS03480
LOO:-3.412 | λ=5.74

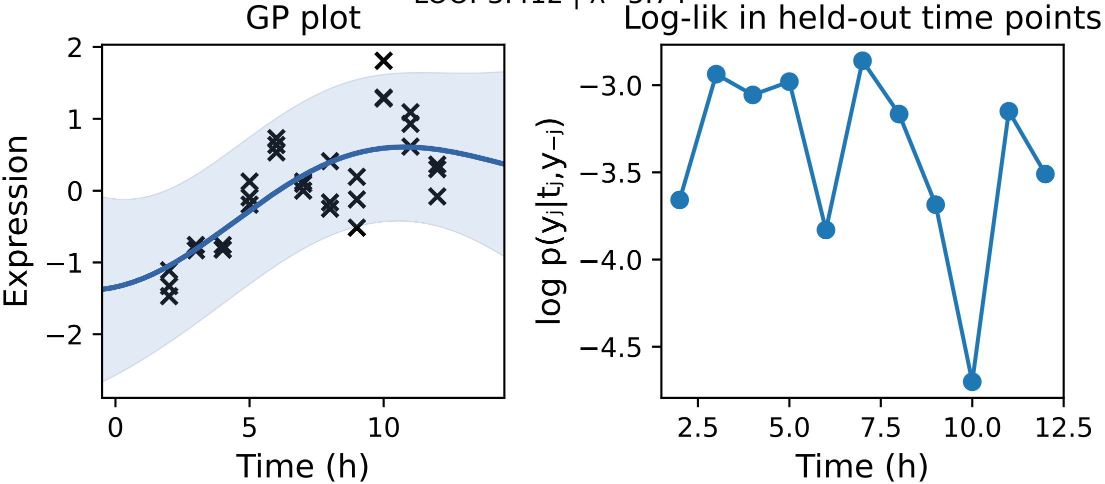

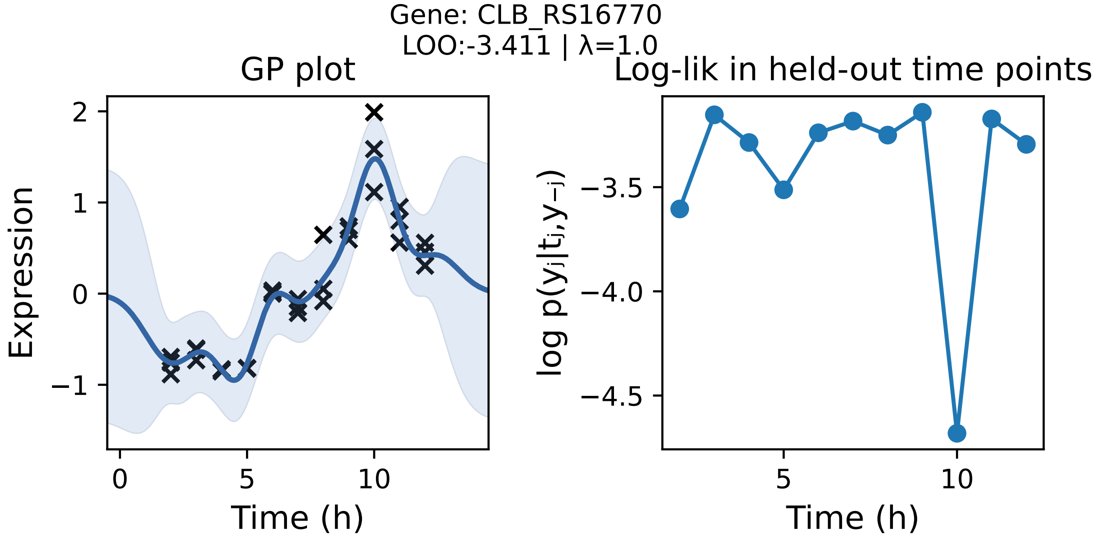

Gene: CLB_RS16770
LOO:-3.411 | λ=1.0
GP plot
Expression
Time (h)
Log-lik in held-out time points
log p(y_j|t_j,y_−j)
Time (h)
2
1
0
−1
0
5
10
−3.5
−4.0
−4.5
5
10

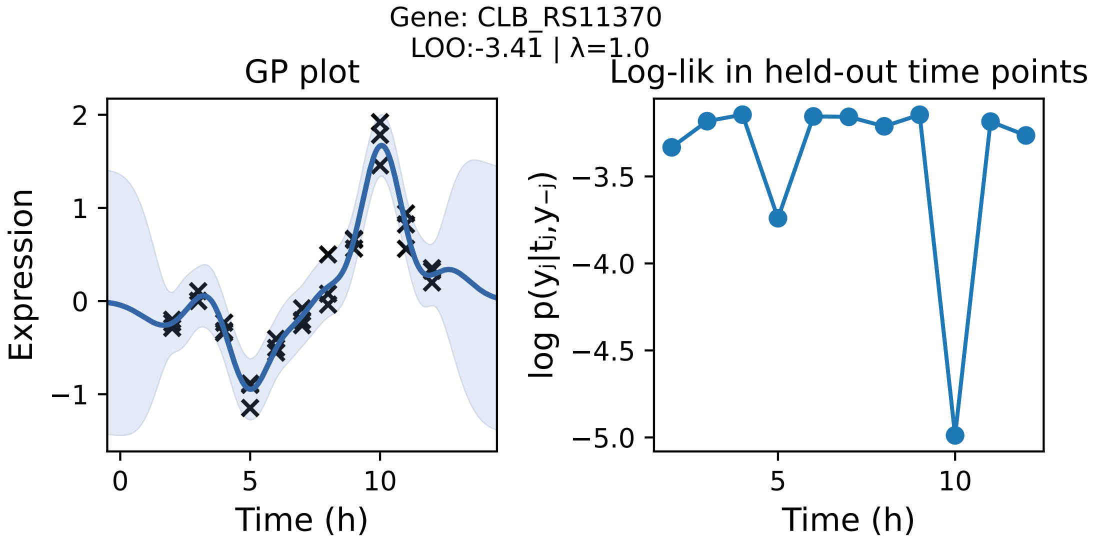

Gene: CLB_RS11370
LOO:-3.41 | λ=1.0
GP plot
Expression
Time (h)
Log-lik in held-out time points
log p(y_j|t_j,y_−j)
Time (h)

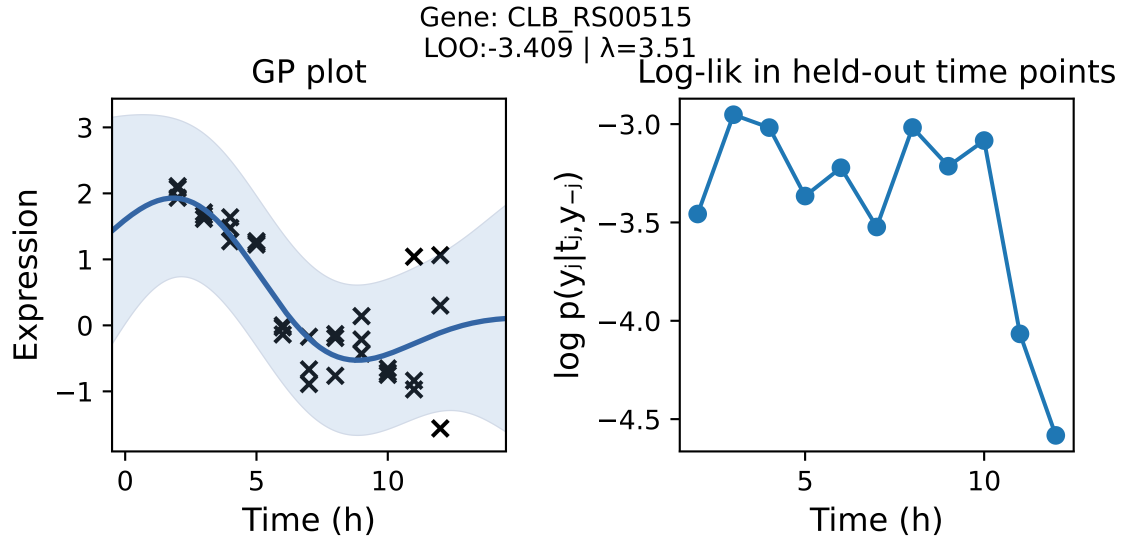
Gene: CLB_RS00515
LOO:-3.409 | λ=3.51
GP plot
Expression
Time (h)
Log-lik in held-out time points
log p(y_j|t_j,y_−j)
Time (h)

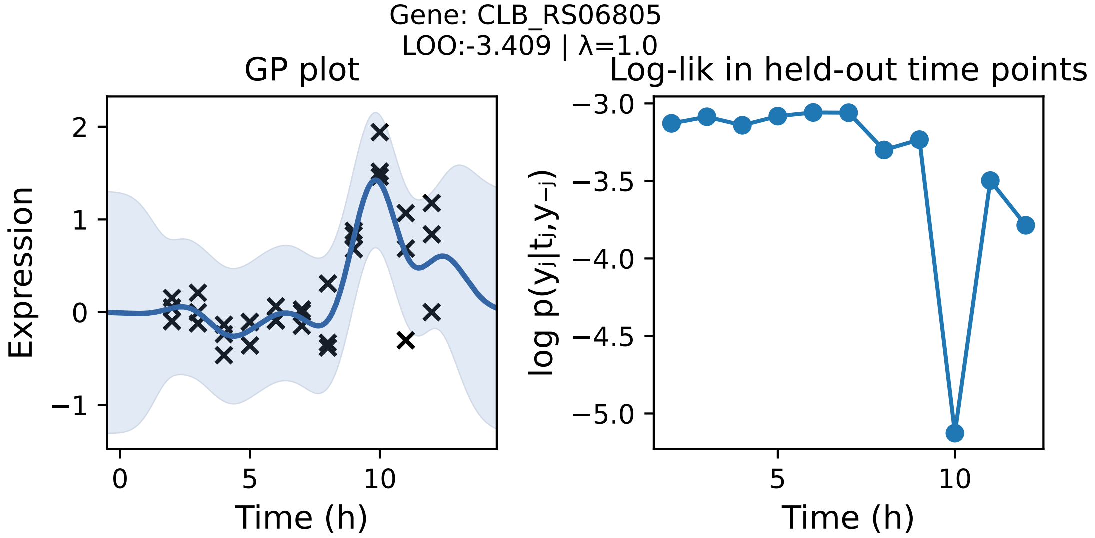

Gene: CLB_RS06805
LOO:-3.409 | λ=1.0
GP plot
Expression
Time (h)
Log-lik in held-out time points
log p(yj|tj,y−j)
Time (h)

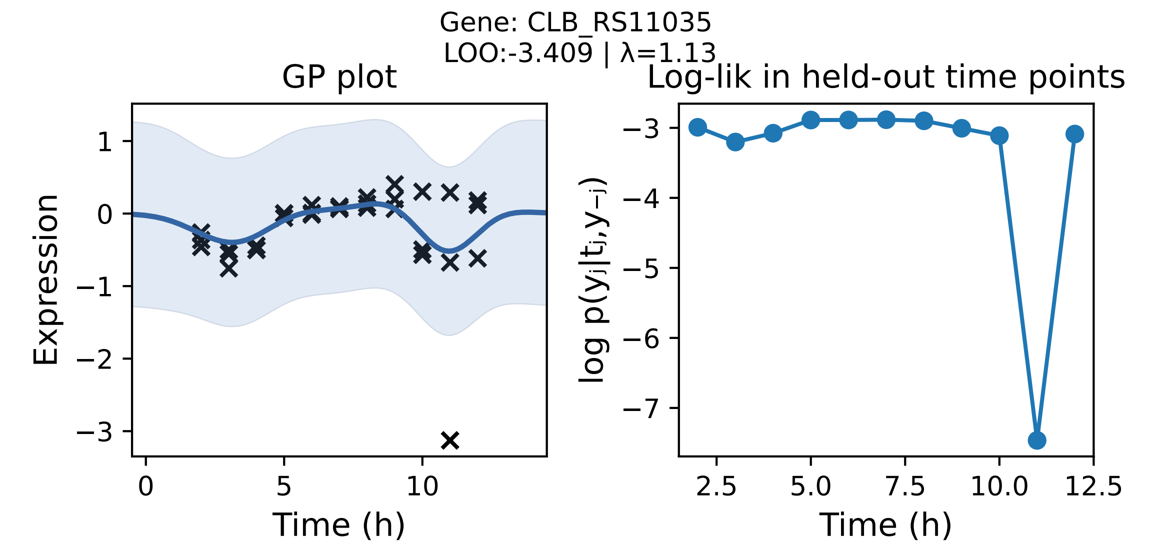

Gene: CLB_RS11035
LOO:-3.409 | λ=1.13
GP plot
Expression
Time (h)
Log-lik in held-out time points
log p(yj|tj,y−j)
Time (h)

Gene: CLB_RS02960
LOO:-3.409 | λ=1.0

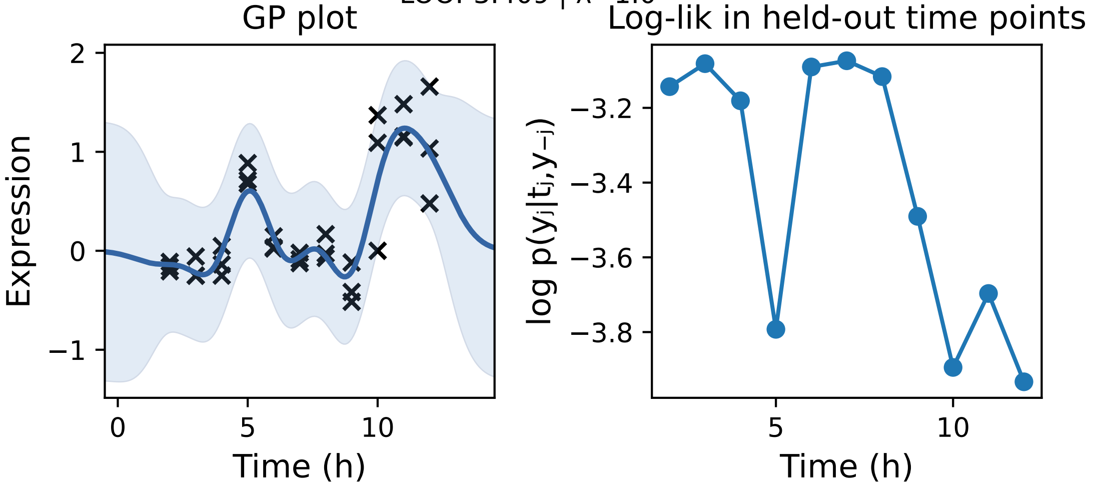

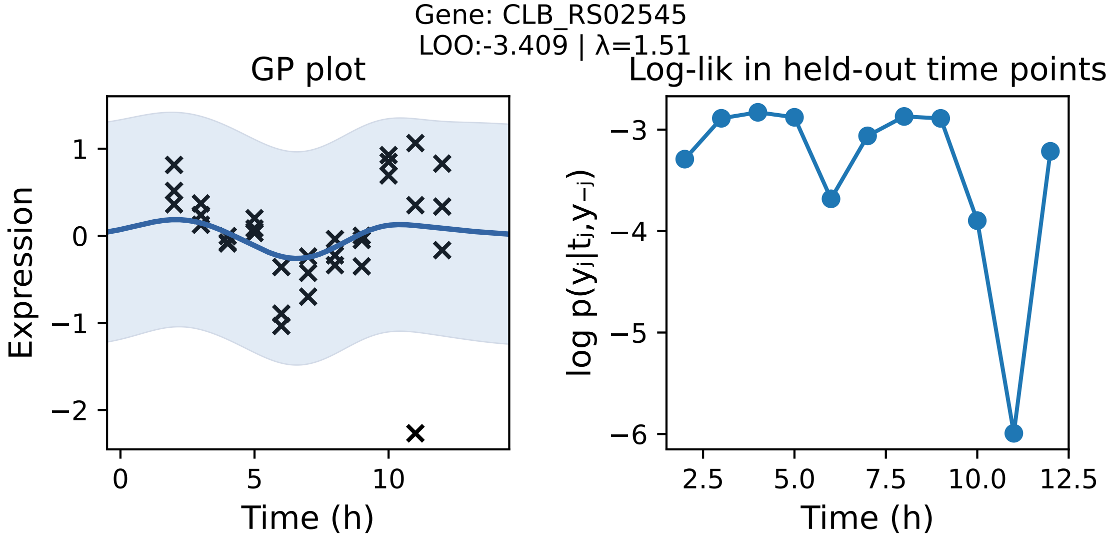

Gene: CLB_RS02545
LOO:-3.409 | λ=1.51
GP plot
Expression
Time (h)
Log-lik in held-out time points
log p(yj|tj,y−j)
Time (h)

Gene: CLB_RS11150
LOO:-3.408 | λ=1.0

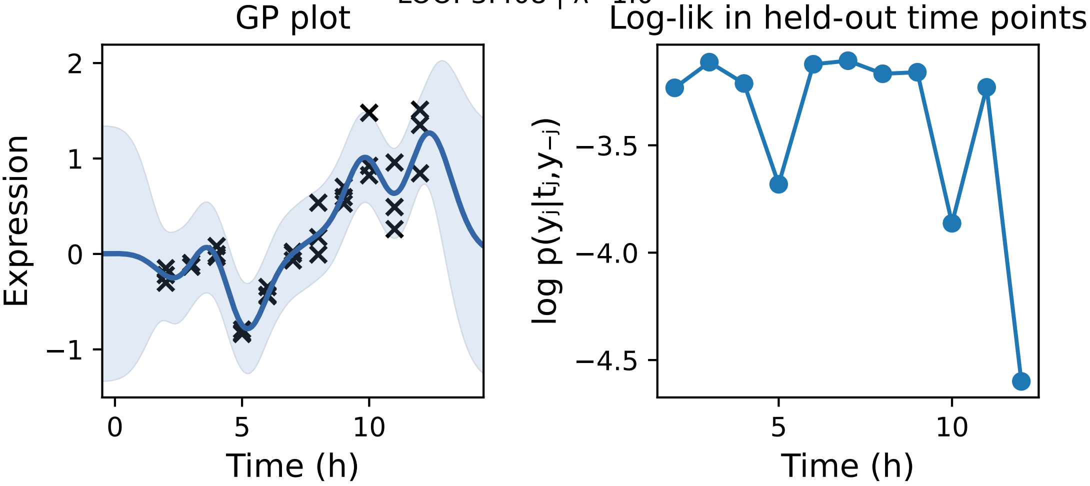

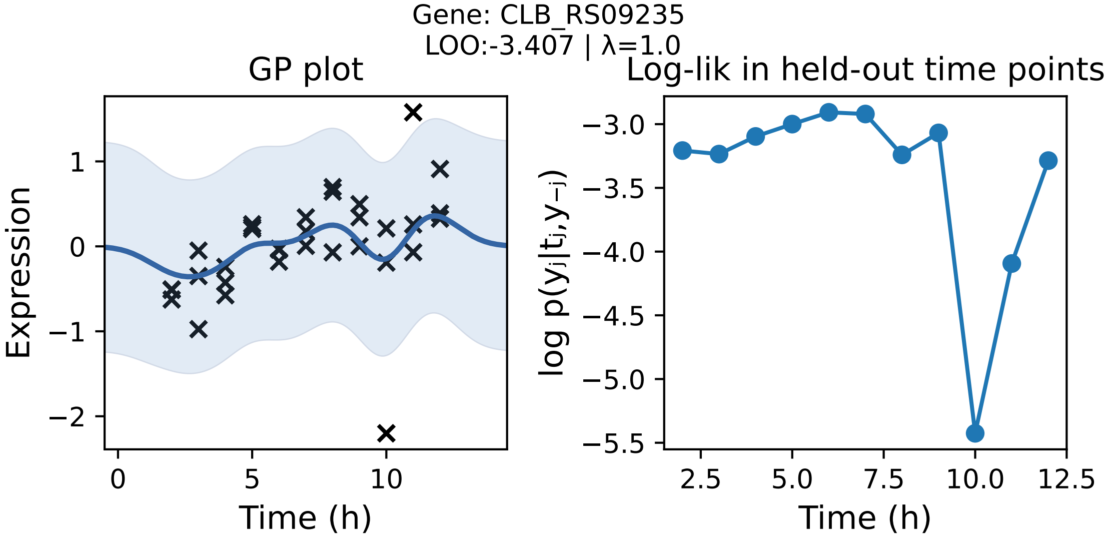

Gene: CLB_RS09235
LOO:-3.407 | λ=1.0
GP plot
Expression
Time (h)
1
0
−1
−2
0
5
10
Log-lik in held-out time points
log p(y_j|t_j,y_−j)
−3.0
−3.5
−4.0
−4.5
−5.0
−5.5
2.5
5.0
7.5
10.0
12.5
Time (h)

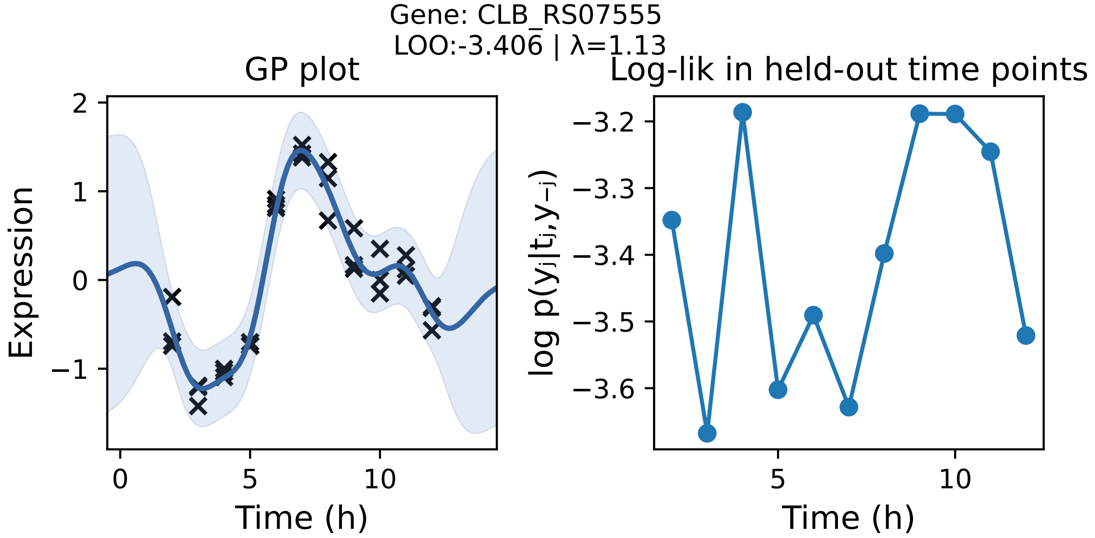

Gene: CLB_RS07555
LOO:-3.406 | λ=1.13
GP plot
Log-lik in held-out time points
Expression
Time (h)
log p(y_j|t_j,y_−j)
Time (h)
2
1
0
−1
0
5
10
−3.2
−3.3
−3.4
−3.5
−3.6
5
10

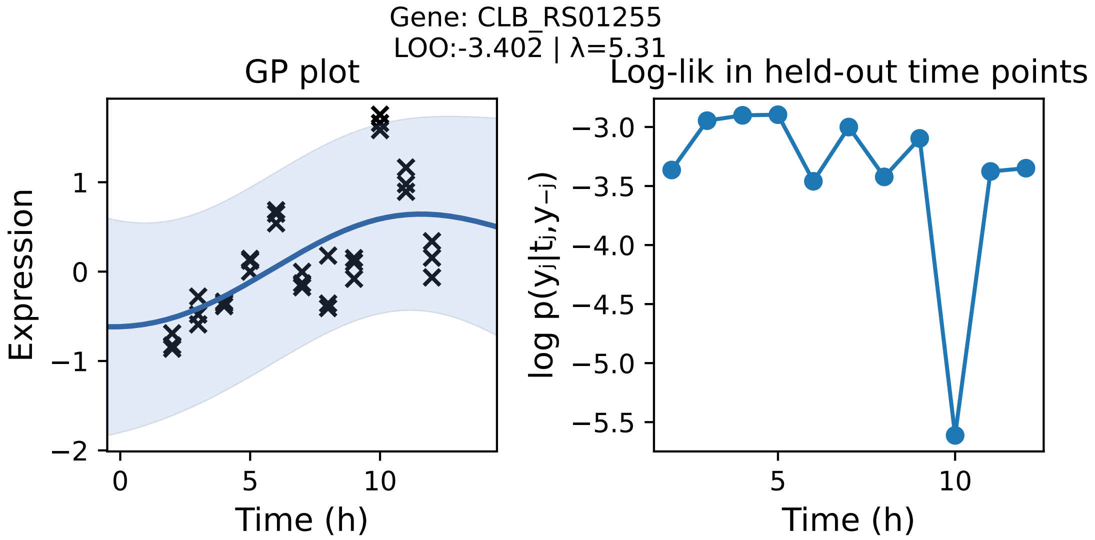

Gene: CLB_RS01255
LOO:-3.402 | λ=5.31
GP plot
Expression
Time (h)
Log-lik in held-out time points
log p(y_j|t_j,y_−j)
Time (h)

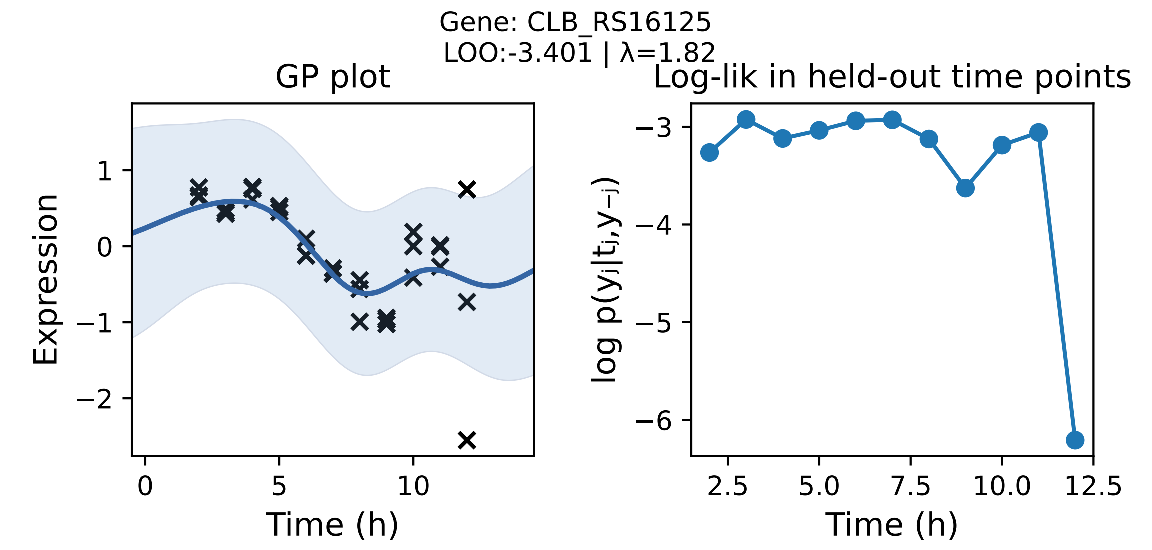

Gene: CLB_RS16125
LOO:-3.401 | λ=1.82
GP plot
Expression
Time (h)
1
0
−1
−2
0
5
10
Log-lik in held-out time points
log p(y_j|t_j,y_−j)
Time (h)
−3
−4
−5
−6
2.5
5.0
7.5
10.0
12.5

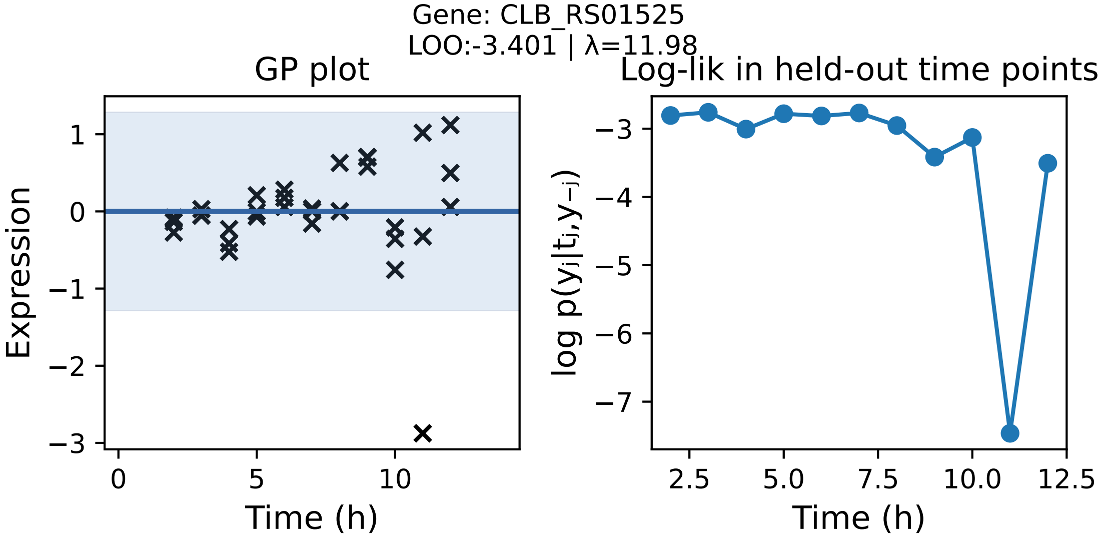

Gene: CLB_RS01525
LOO:-3.401 | λ=11.98
GP plot
Expression
Time (h)
Log-lik in held-out time points
log p(y_j|t_j,y_−j)
Time (h)

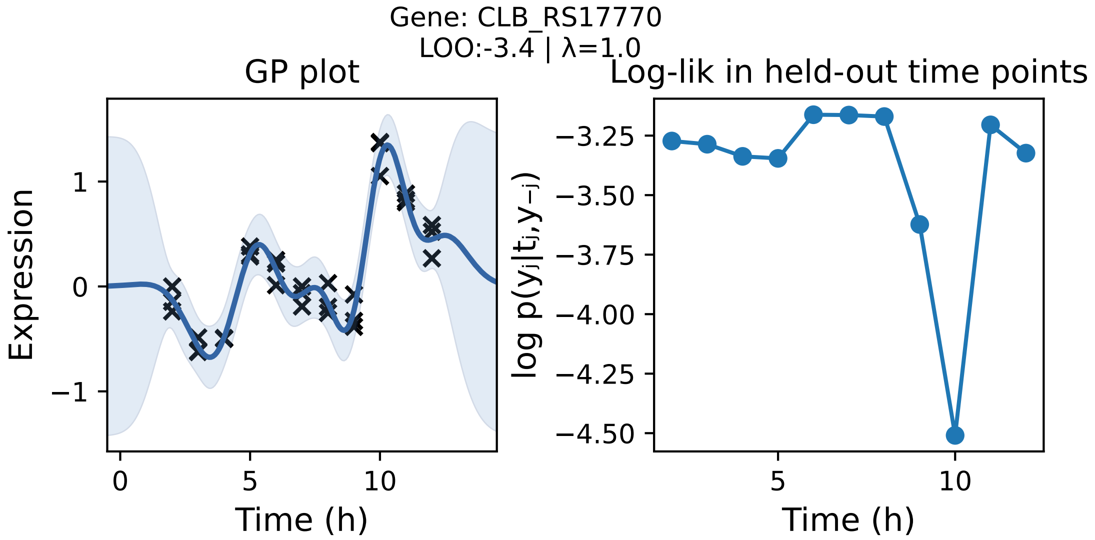

Gene: CLB_RS17770
LOO:-3.4 | λ=1.0
GP plot
Log-lik in held-out time points
Expression
1
0
−1
0
5
10
Time (h)
log p(y_j|t_j,y_{−j})
−3.25
−3.50
−3.75
−4.00
−4.25
−4.50
5
10
Time (h)

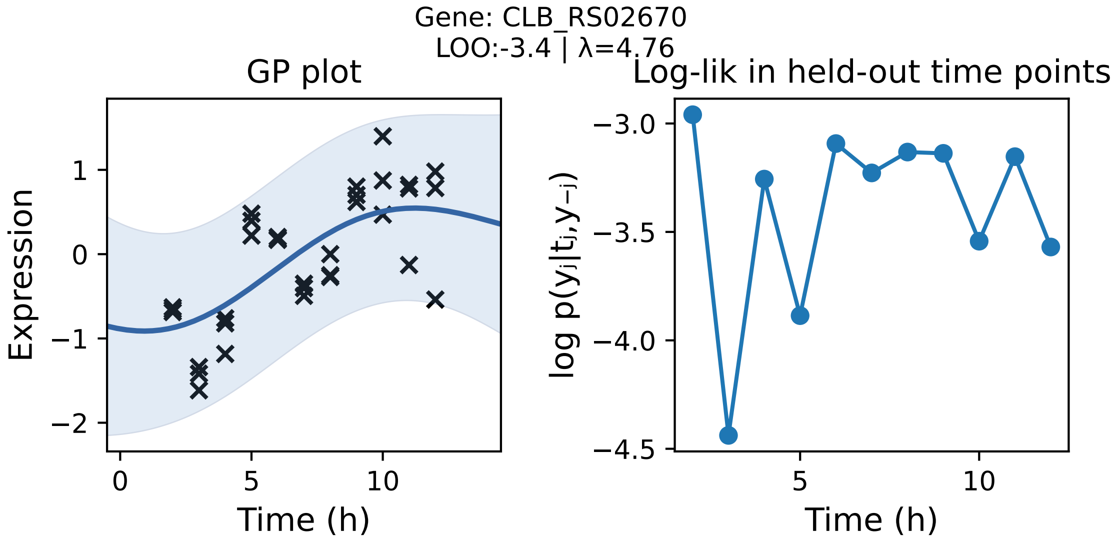

Gene: CLB_RS02670
LOO:-3.4 | λ=4.76
GP plot
Expression
Time (h)
Log-lik in held-out time points
log p(y_j|t_j,y_−j)
Time (h)

Gene: CLB_RS03475
LOO:-3.398 | λ=1.0

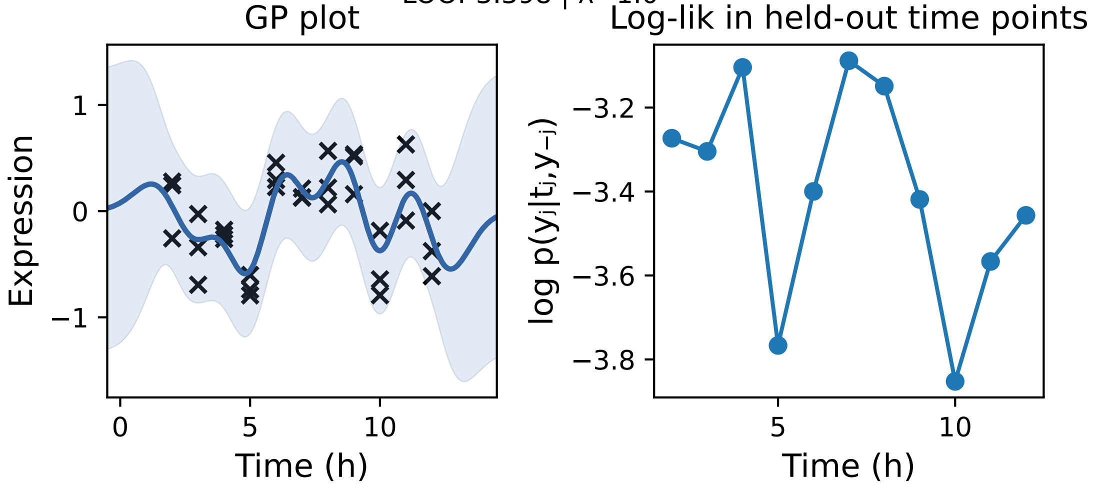

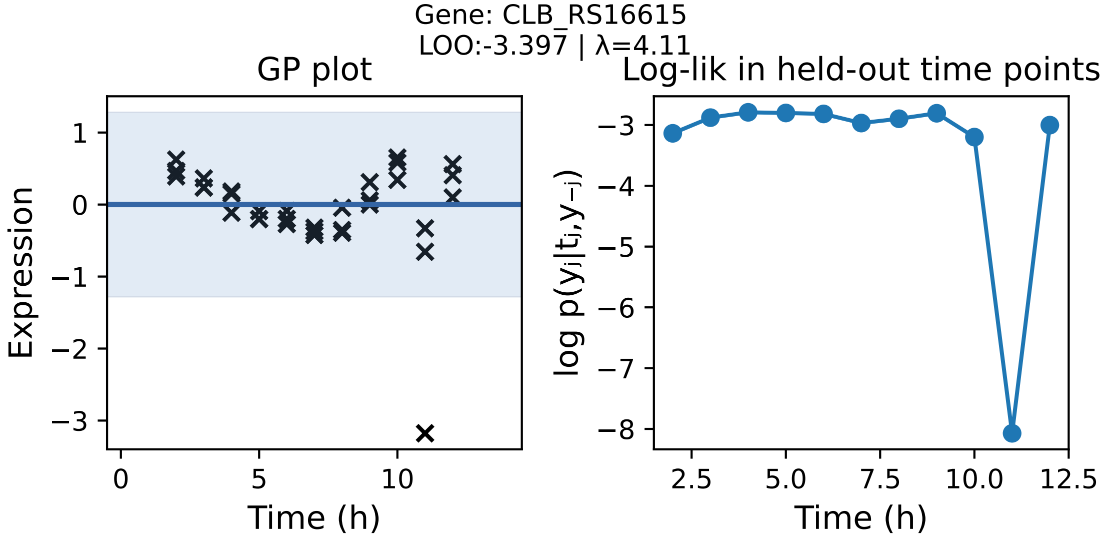
Gene: CLB_RS16615
LOO:-3.397 | λ=4.11
GP plot
Expression
1
0
−1
−2
−3
0
5
10
Time (h)
Log-lik in held-out time points
log p(y_j|t_j,y_−j)
−3
−4
−5
−6
−7
−8
2.5
5.0
7.5
10.0
12.5
Time (h)

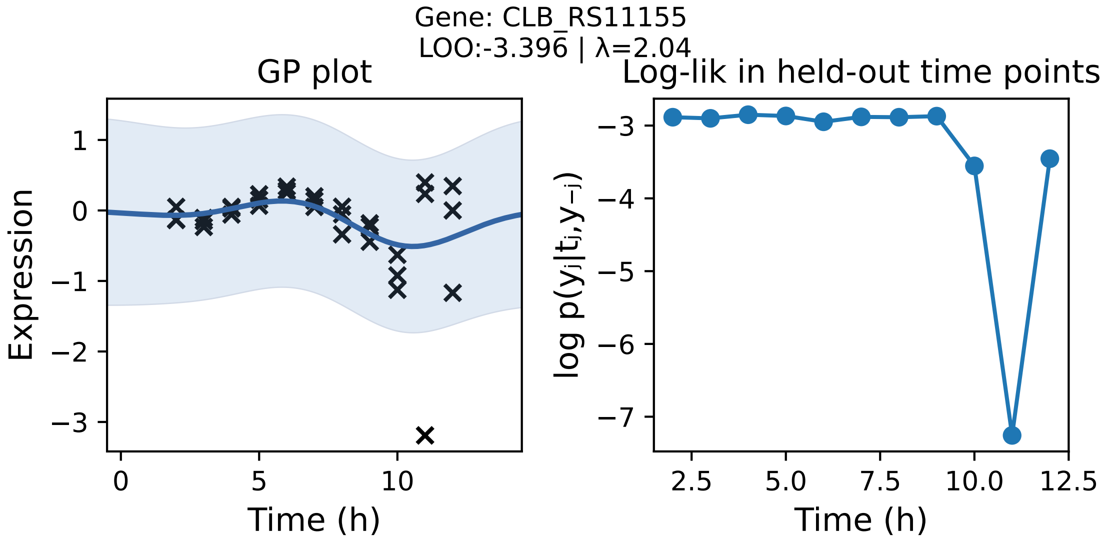
Gene: CLB_RS11155
LOO:-3.396 | λ=2.04
GP plot
Expression
Time (h)
1
0
−1
−2
−3
0
5
10
Log-lik in held-out time points
log p(y_j|t_j,y_−j)
Time (h)
−3
−4
−5
−6
−7
2.5
5.0
7.5
10.0
12.5

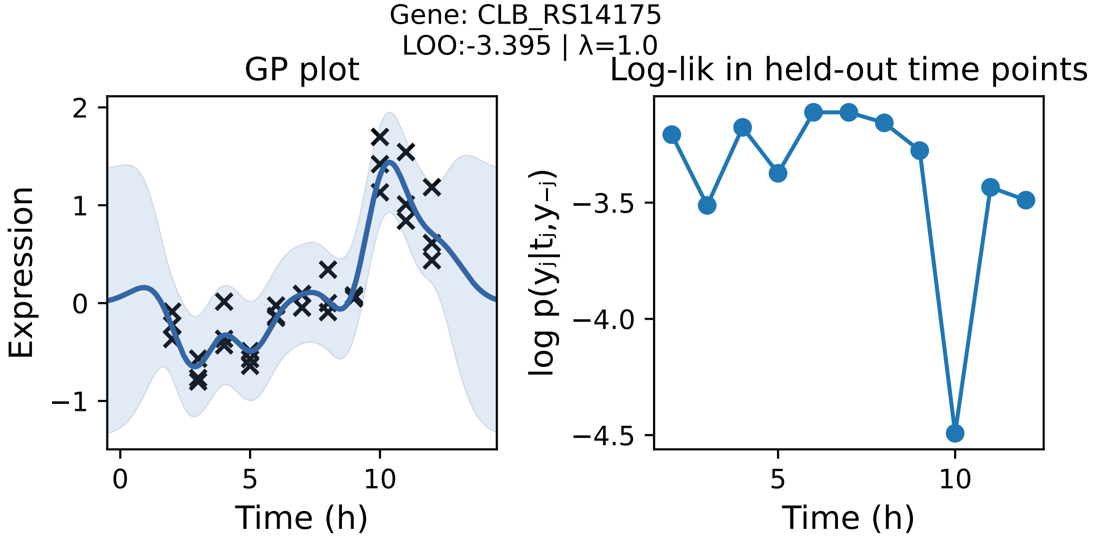

Gene: CLB_RS14175
LOO:-3.395 | λ=1.0
GP plot
Expression
Time (h)
2
1
0
−1
0
5
10
Log-lik in held-out time points
log p(yⱼ|tⱼ,y₋ⱼ)
−3.5
−4.0
−4.5
5
10
Time (h)

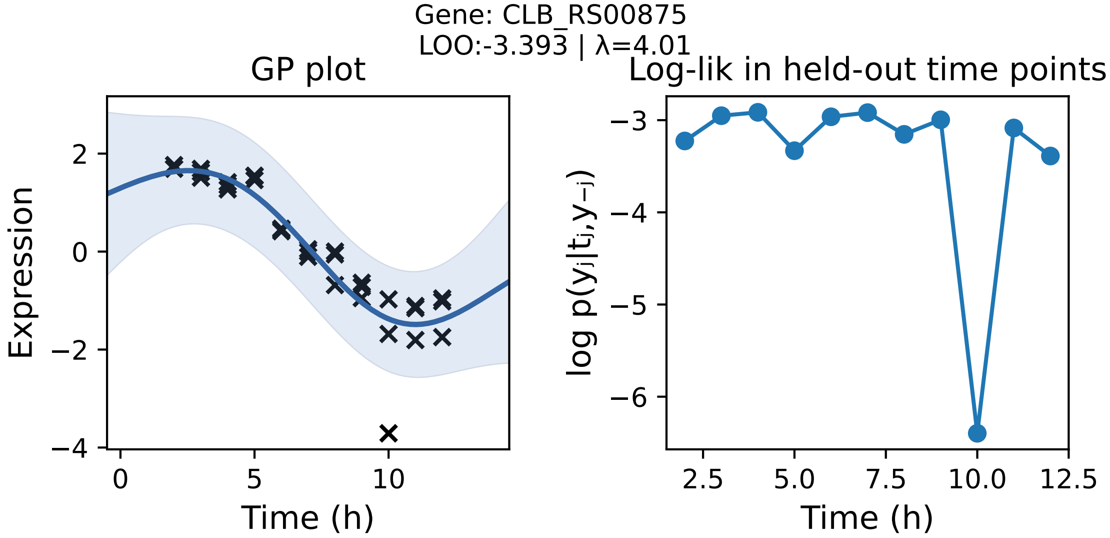

Gene: CLB_RS00875
LOO:-3.393 | λ=4.01
GP plot
Expression
Time (h)
Log-lik in held-out time points
log p(y_j|t_j,y_−j)
Time (h)

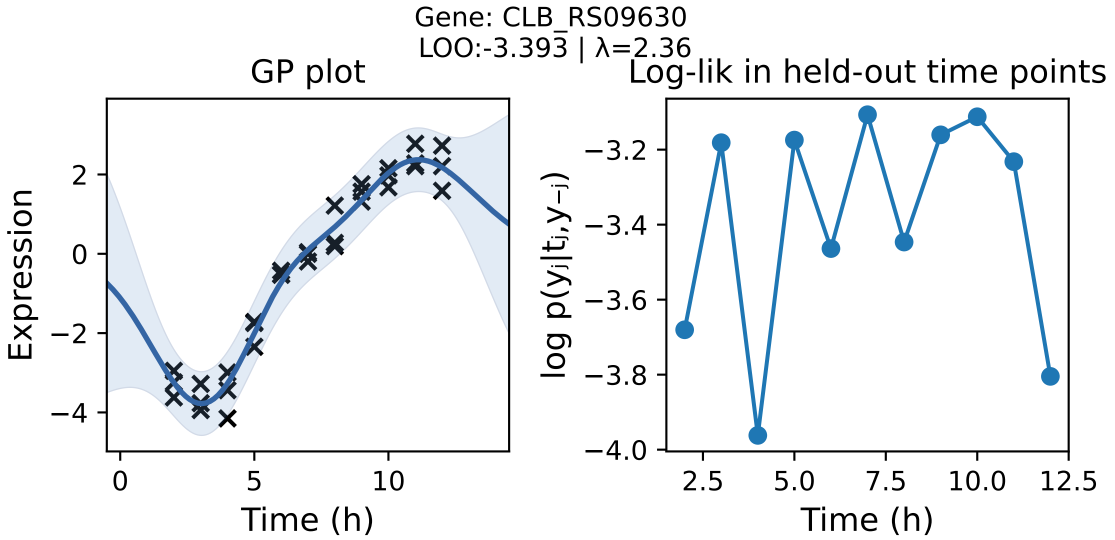

Gene: CLB_RS09630
LOO:-3.393 | λ=2.36
GP plot
Expression
Time (h)
Log-lik in held-out time points
log p(y_j|t_j,y_−j)
Time (h)

Gene: CLB_RS16895
LOO:-3.393 | λ=2.93

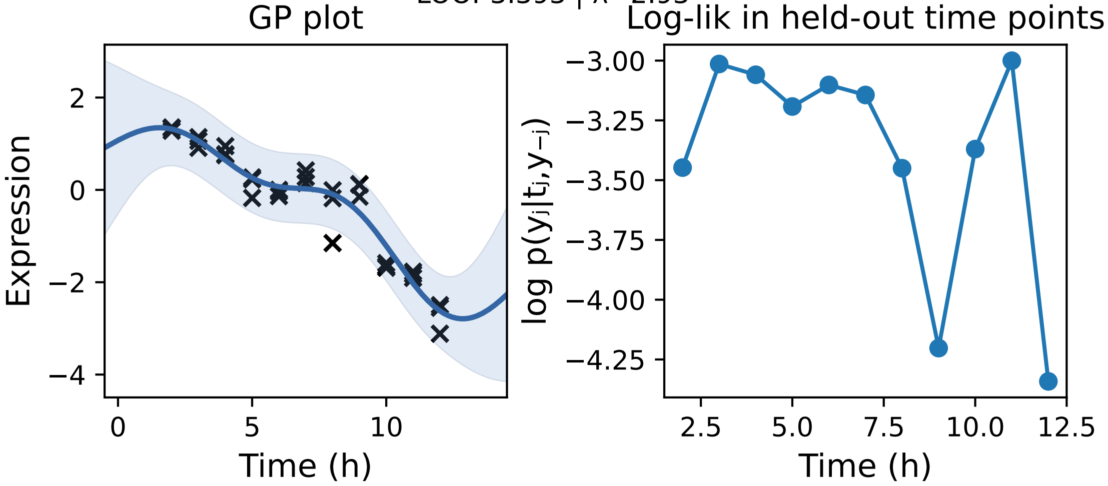

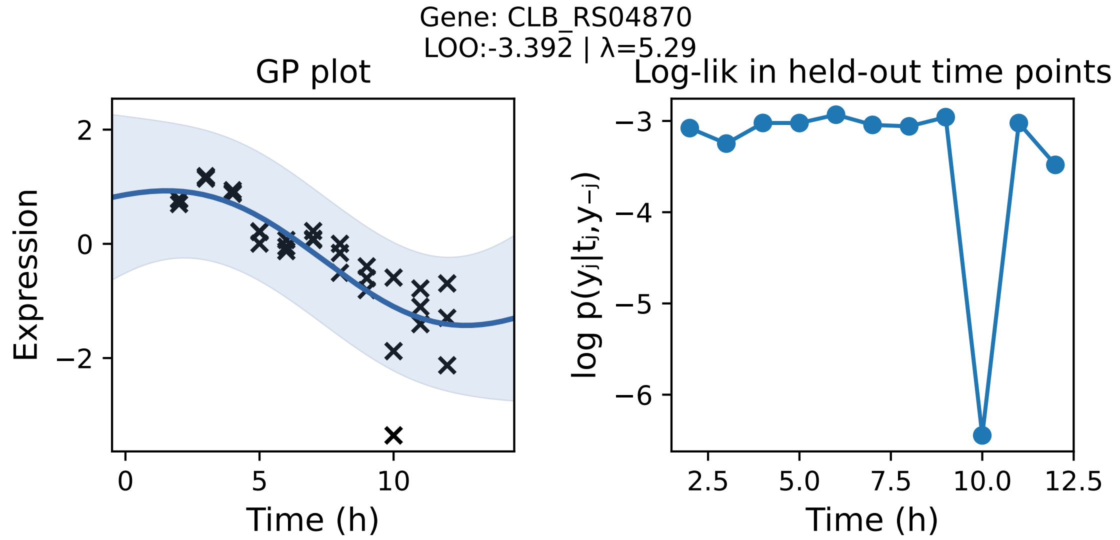

Gene: CLB_RS04870
LOO:-3.392 | λ=5.29
GP plot
Expression
Time (h)
2
0
−2
0
5
10
Log-lik in held-out time points
log p(y_j|t_j,y_−j)
Time (h)
−3
−4
−5
−6
2.5
5.0
7.5
10.0
12.5

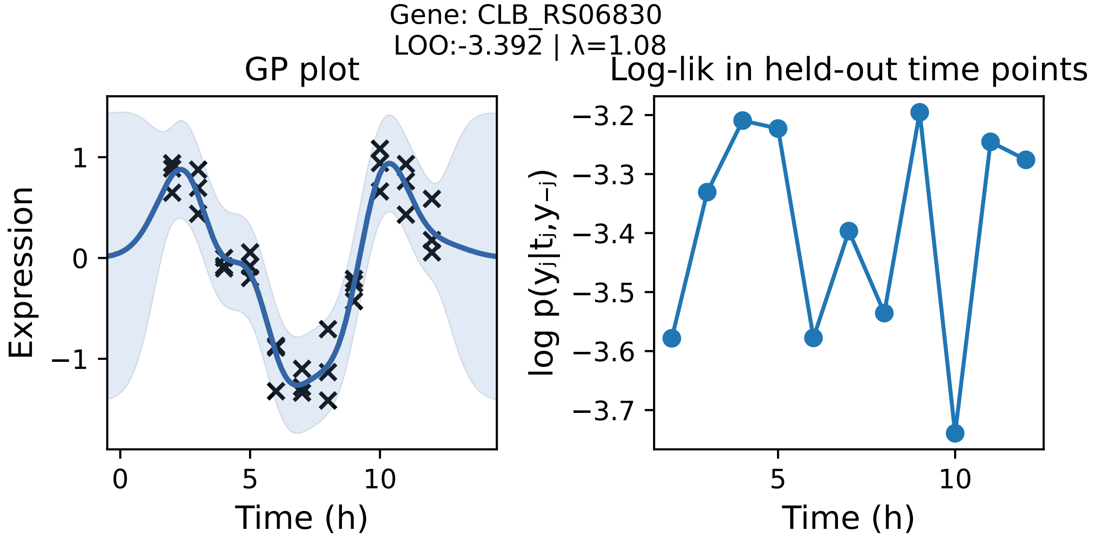
Gene: CLB_RS06830
LOO:-3.392 | λ=1.08
GP plot
Expression
Time (h)
Log-lik in held-out time points
log p(y_j|t_j,y_−j)
Time (h)

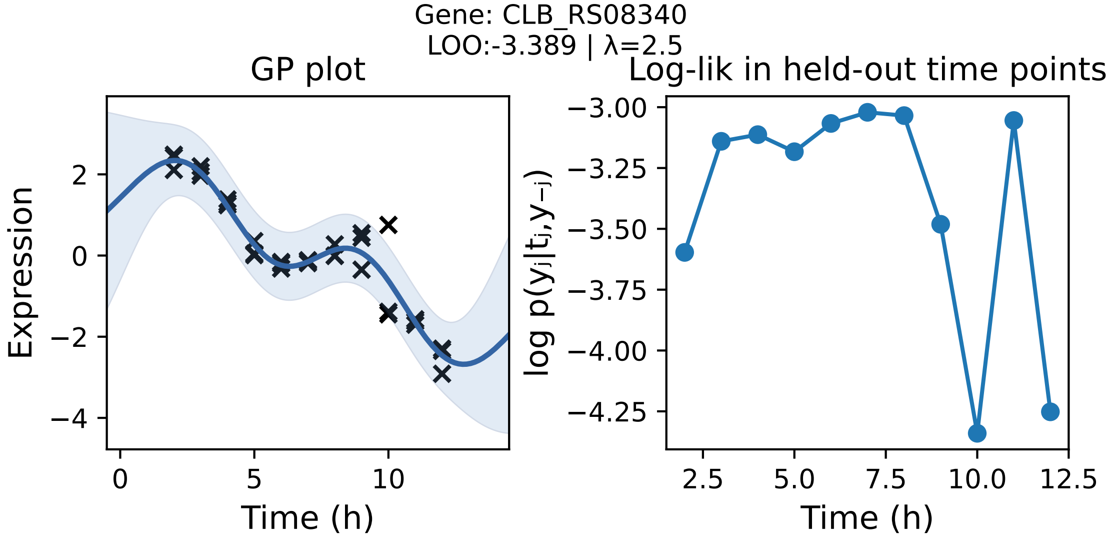

Gene: CLB_RS08340
LOO:-3.389 | λ=2.5
GP plot
Expression
Time (h)
Log-lik in held-out time points
log p(y_j|t_j,y_−j)
Time (h)

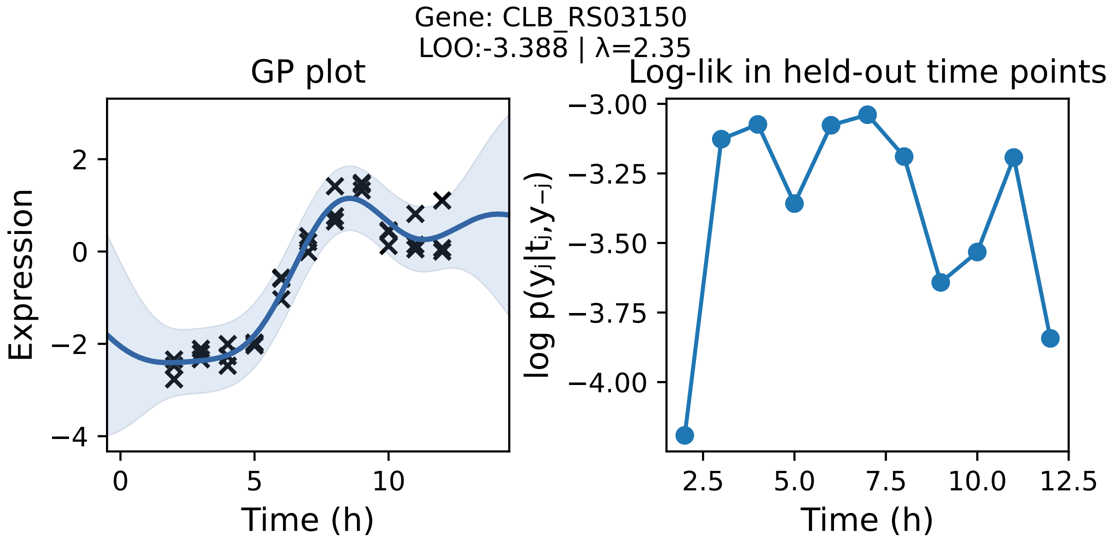

Gene: CLB_RS03150
LOO:-3.388 | λ=2.35
GP plot
Expression
Time (h)
2
0
−2
−4
0
5
10
Log-lik in held-out time points
log p(y_j|t_j,y_−j)
−3.00
−3.25
−3.50
−3.75
−4.00
2.5
5.0
7.5
10.0
12.5
Time (h)

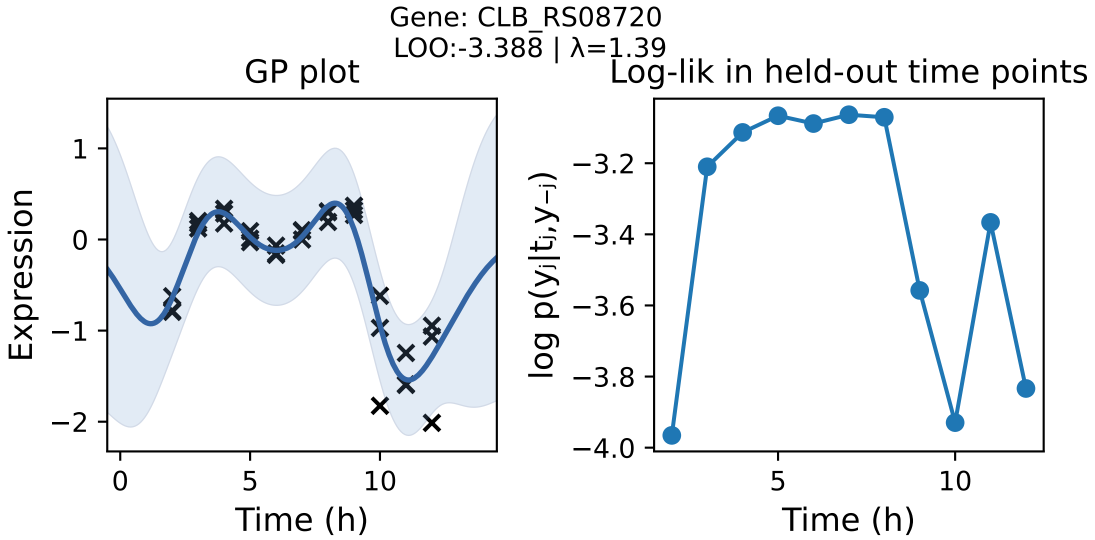
Gene: CLB_RS08720
LOO:-3.388 | λ=1.39
GP plot
Expression
Time (h)
Log-lik in held-out time points
log p(yj|tj,y−j)
Time (h)

Gene: CLB_RS03090
LOO:-3.388 | λ=1.0

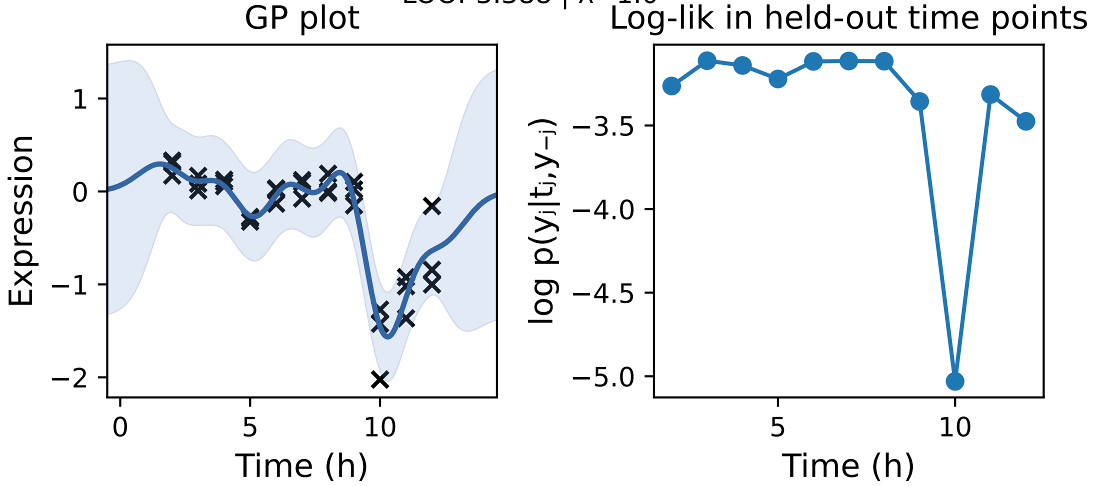

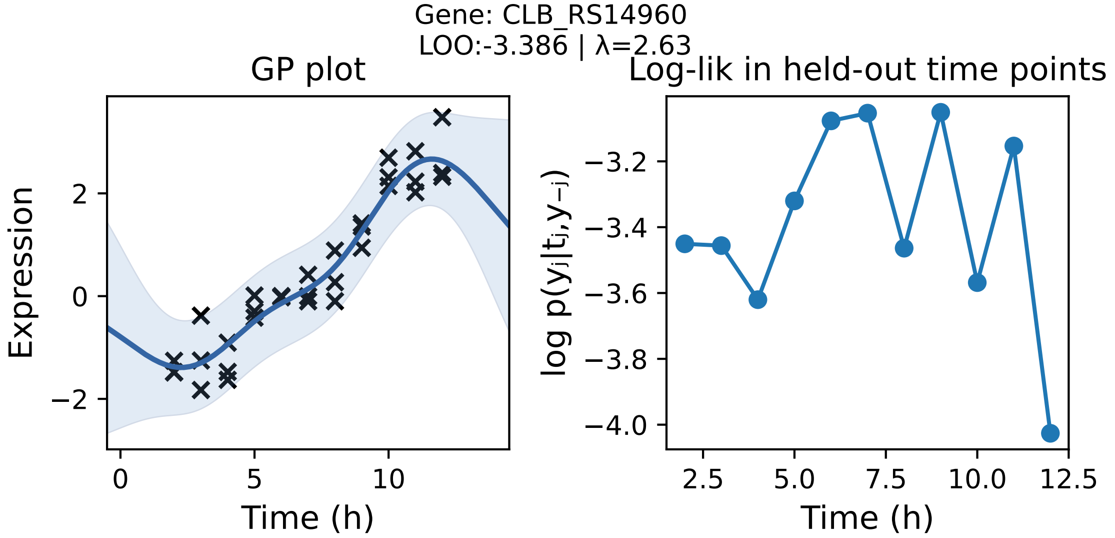

Gene: CLB_RS14960
LOO:-3.386 | λ=2.63
GP plot
Expression
Time (h)
0
5
10
2
0
−2
Log-lik in held-out time points
log p(y_j|t_j,y_−j)
−3.2
−3.4
−3.6
−3.8
−4.0
2.5
5.0
7.5
10.0
12.5
Time (h)

Gene: CLB_RS10265
LOO:-3.385 | λ=1.43

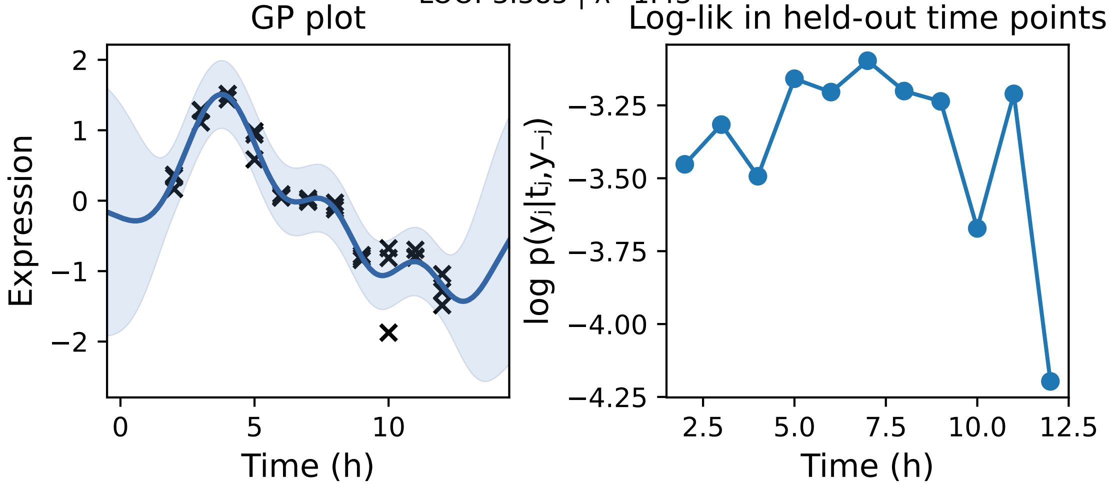

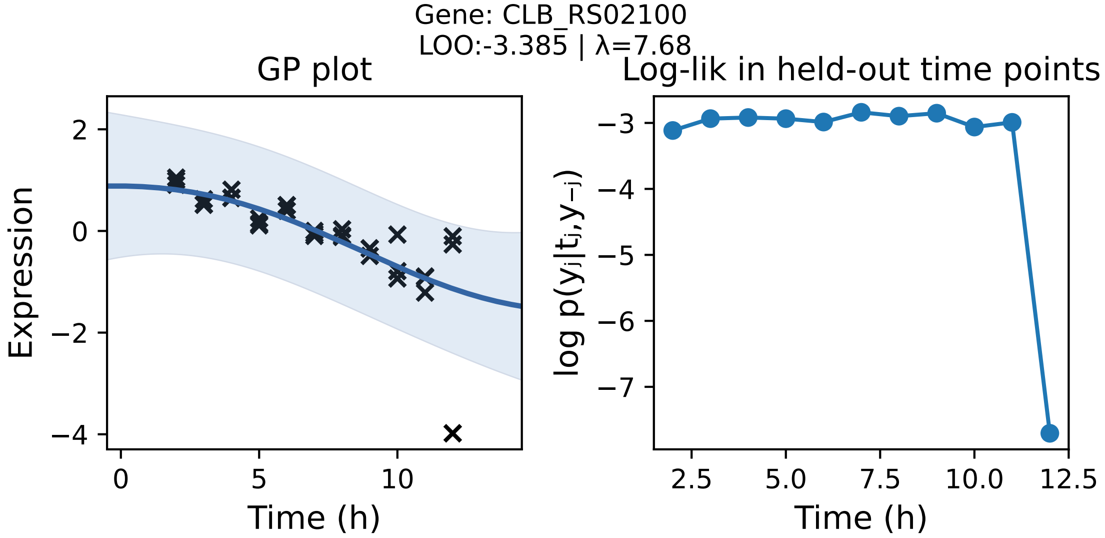

Gene: CLB_RS02100
LOO:-3.385 | λ=7.68
GP plot
Expression
Time (h)
2
0
−2
−4
0
5
10
Log-lik in held-out time points
log p(y_j|t_j,y_−j)
Time (h)
−3
−4
−5
−6
−7
2.5
5.0
7.5
10.0
12.5

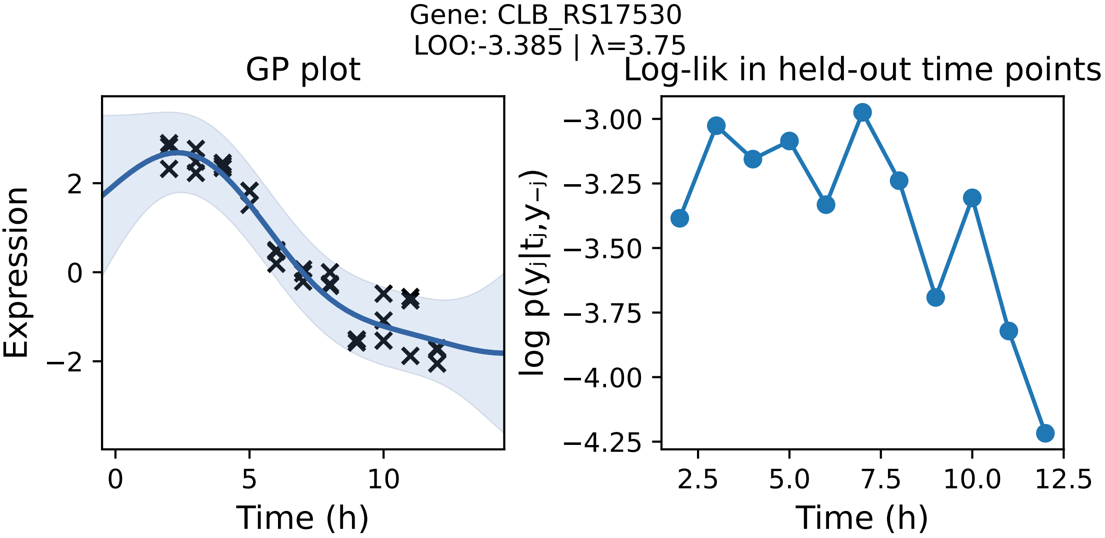

Gene: CLB_RS17530
LOO:-3.385 | λ=3.75
GP plot
Expression
Time (h)
2
0
−2
0
5
10
Log-lik in held-out time points
log p(y_j|t_j,y_−j)
Time (h)
−3.00
−3.25
−3.50
−3.75
−4.00
−4.25
2.5
5.0
7.5
10.0
12.5

Gene: CLB_RS06685
LOO:-3.384 | λ=2.49

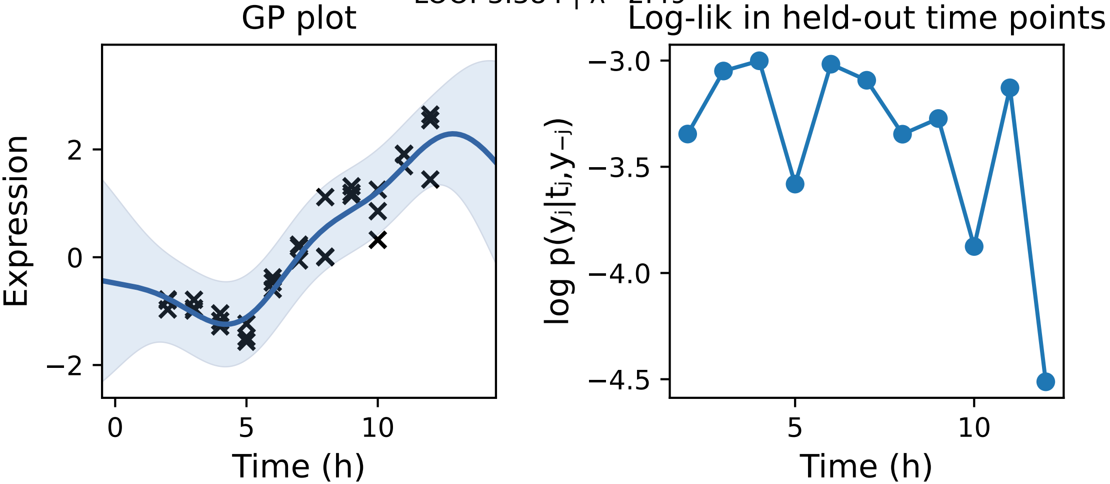

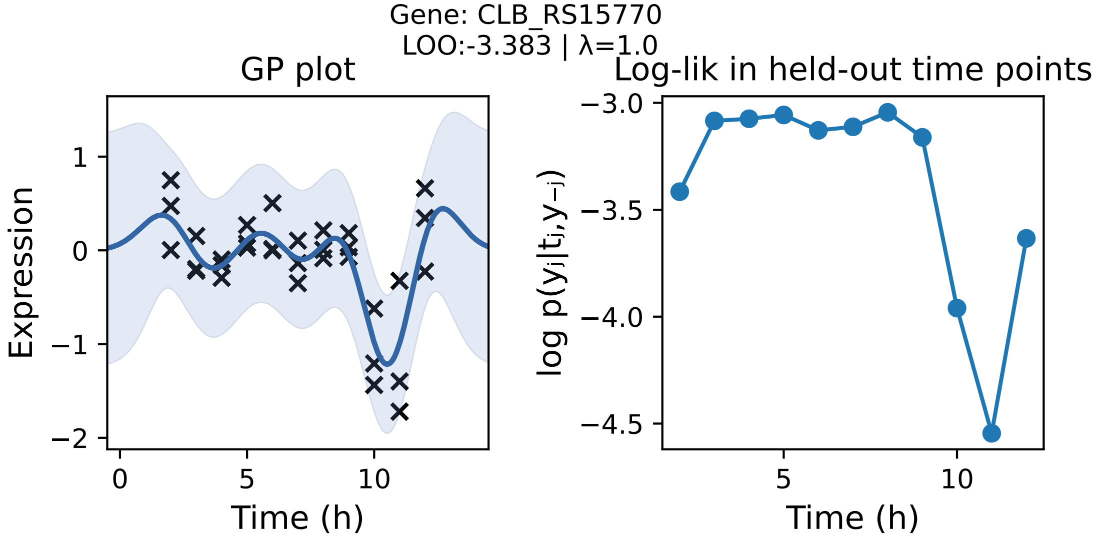

Gene: CLB_RS15770
LOO:-3.383 | λ=1.0
GP plot
Expression
Time (h)
Log-lik in held-out time points
log p(y_j|t_j,y_−j)
Time (h)

Gene: CLB_RS10830
LOO:-3.382 | λ=1.07

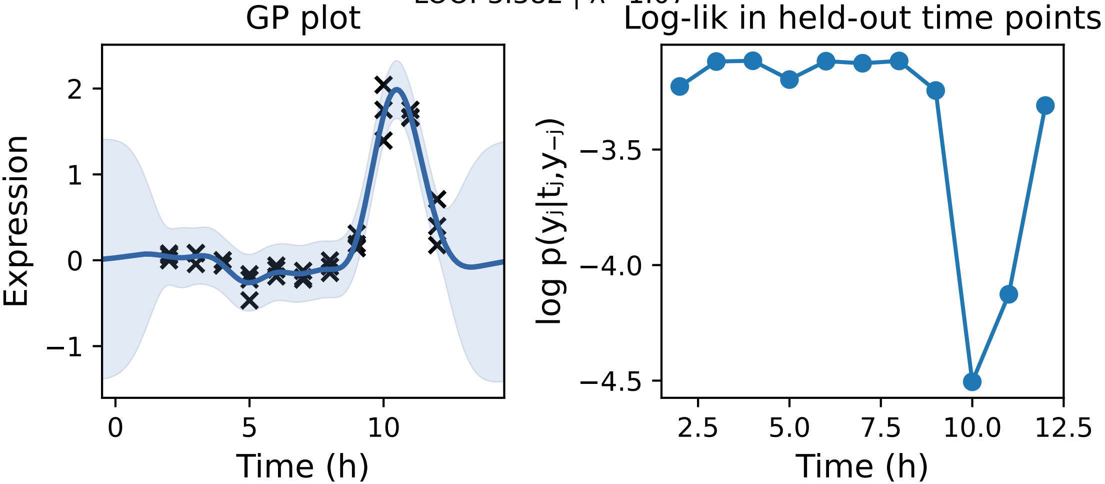

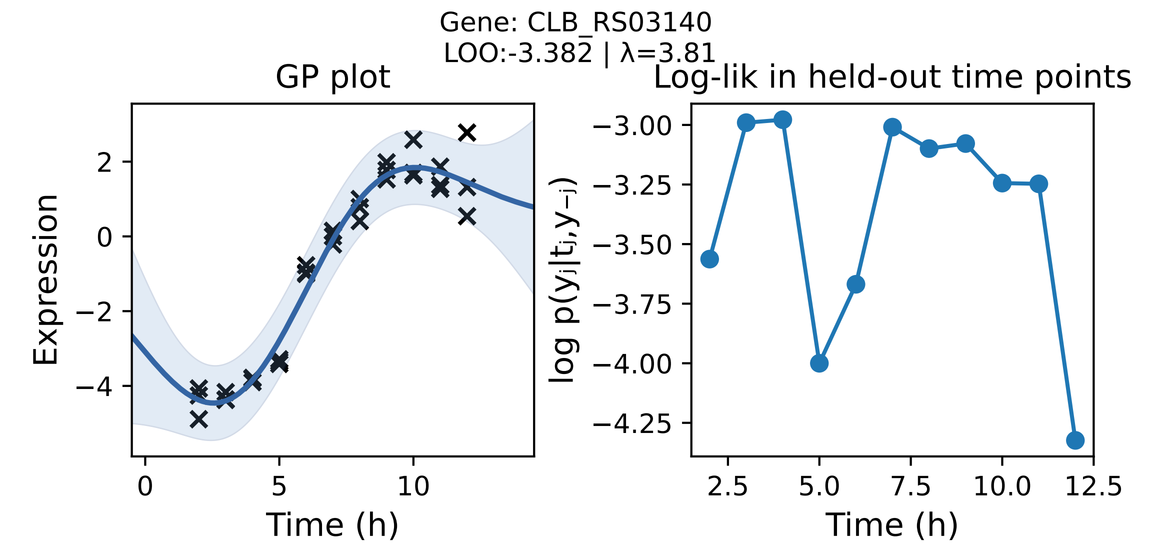

Gene: CLB_RS03140
LOO:-3.382 | λ=3.81
GP plot
Log-lik in held-out time points
Expression
Time (h)
log p(y_j|t_j,y_−j)
Time (h)

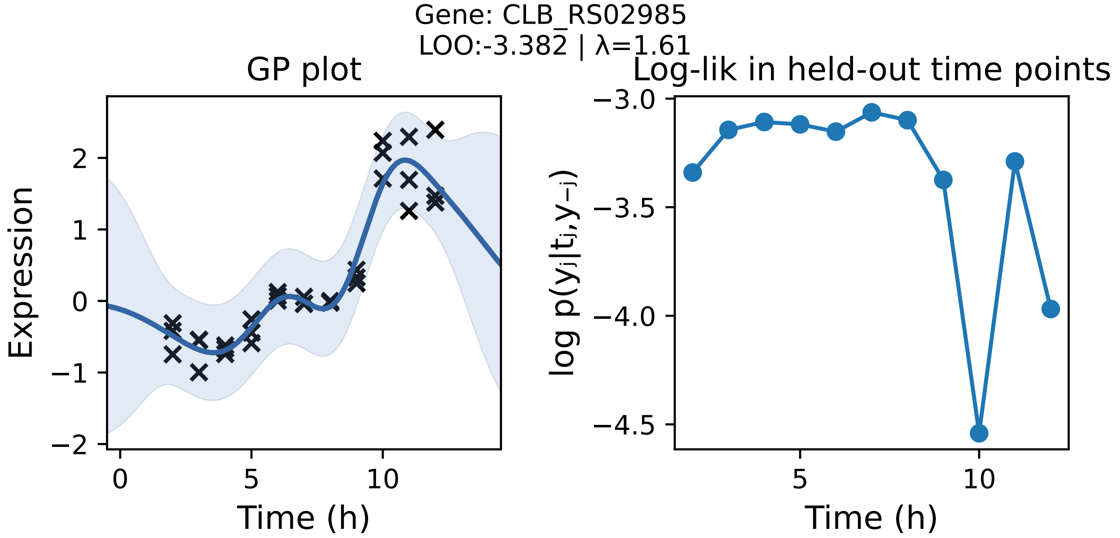
Gene: CLB_RS02985
LOO:-3.382 | λ=1.61
GP plot
Expression
Time (h)
Log-lik in held-out time points
log p(y_j|t_j,y_−j)
Time (h)
2
1
0
−1
−2
0
5
10
−3.0
−3.5
−4.0
−4.5
5
10

Gene: CLB_RS09805
LOO:-3.382 | λ=1.03

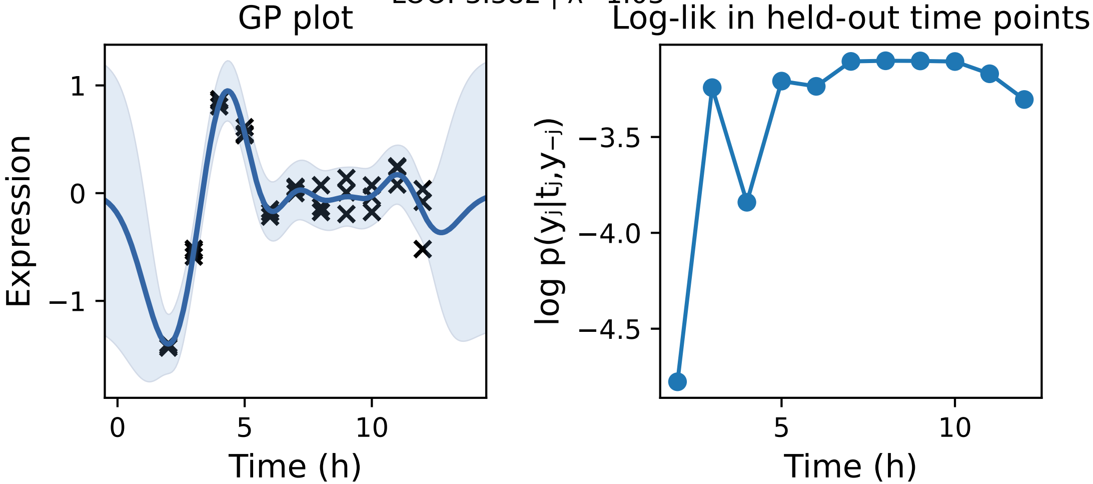

Gene: CLB_RS13755
LOO:-3.38 | λ=1.68

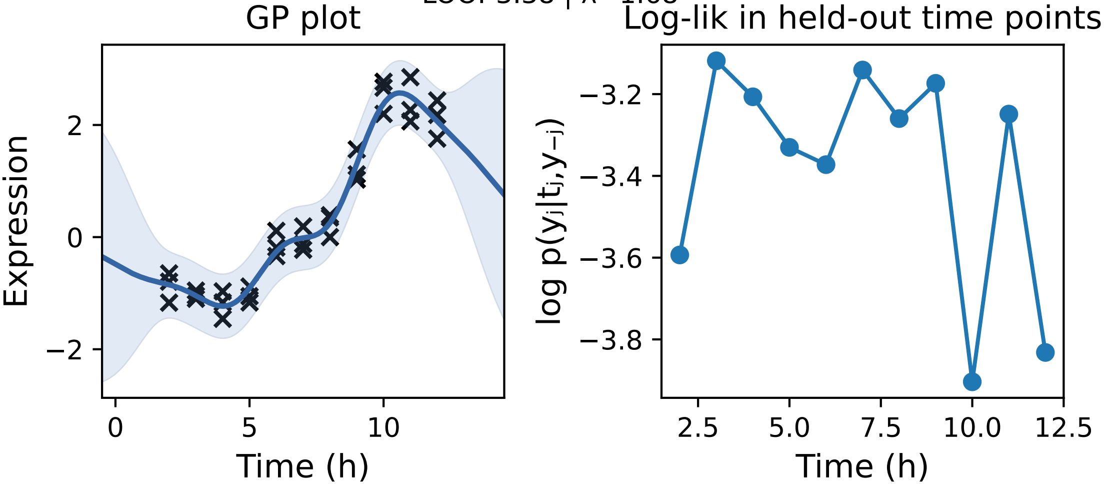

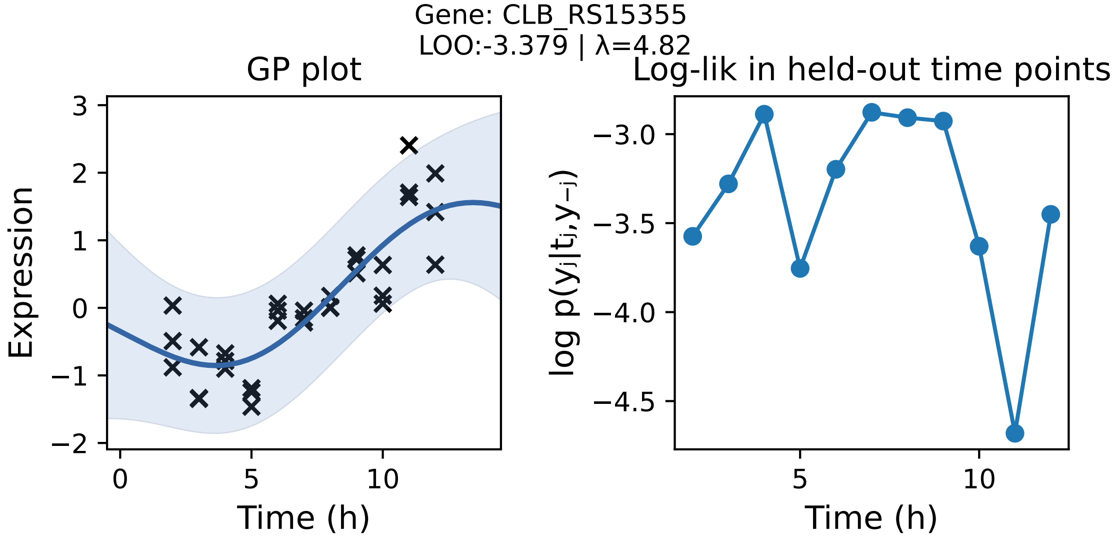

Gene: CLB_RS15355
LOO:-3.379 | λ=4.82
GP plot
Expression
Time (h)
Log-lik in held-out time points
log p(y_j|t_j,y_−j)
Time (h)

Gene: CLB_RS12350
LOO:-3.378 | λ=2.92

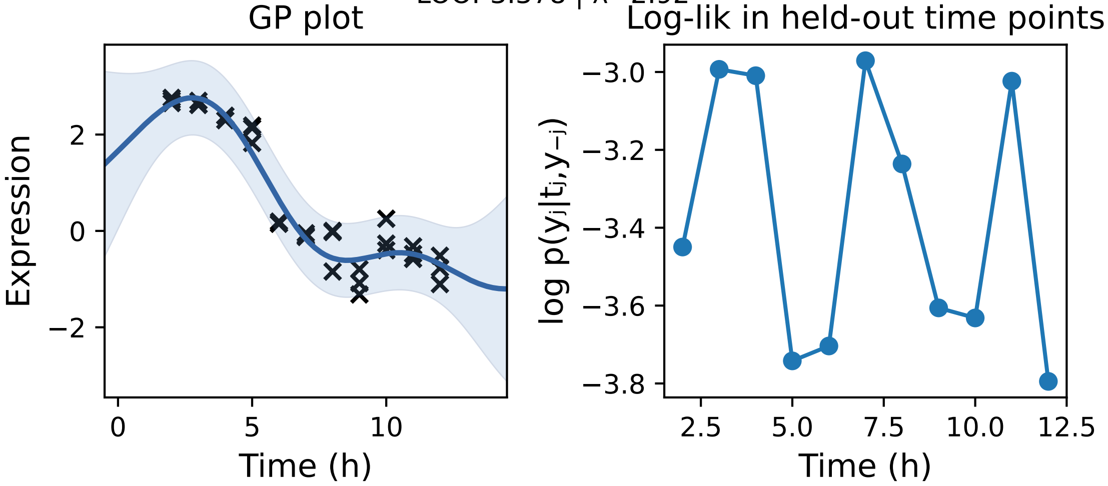

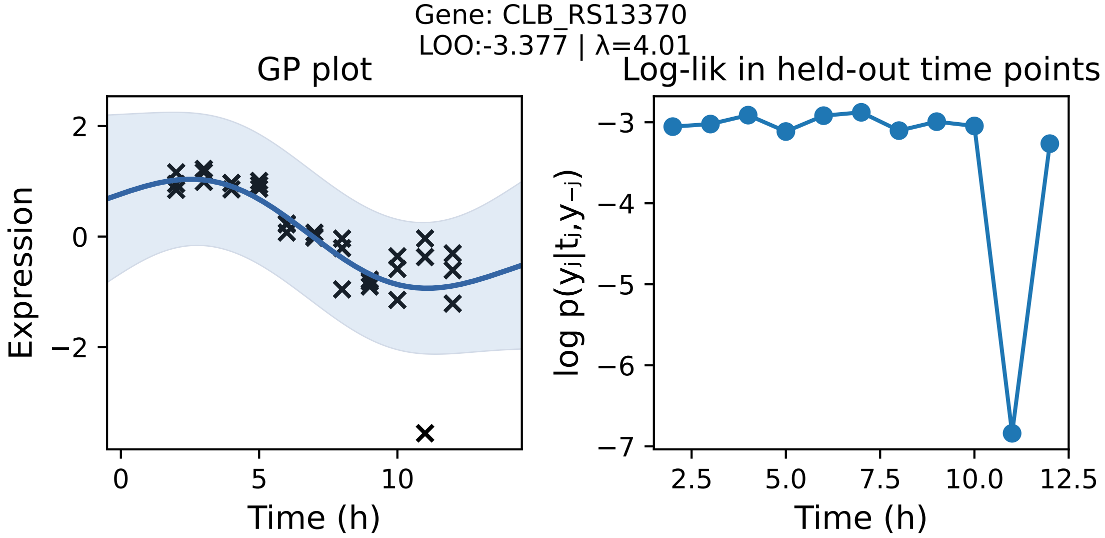

Gene: CLB_RS13370
LOO:-3.377 | λ=4.01
GP plot
Expression
Time (h)
Log-lik in held-out time points
log p(y_j|t_j,y_−j)
Time (h)

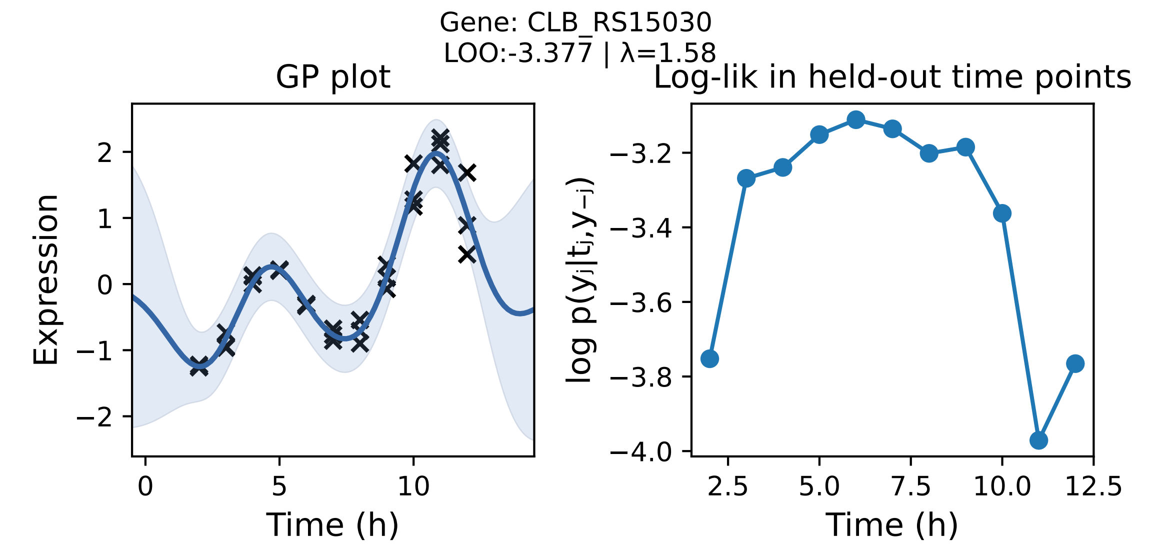

Gene: CLB_RS15030
LOO:-3.377 | λ=1.58
GP plot
Expression
Time (h)
Log-lik in held-out time points
log p(y_j|t_j,y_−j)
Time (h)

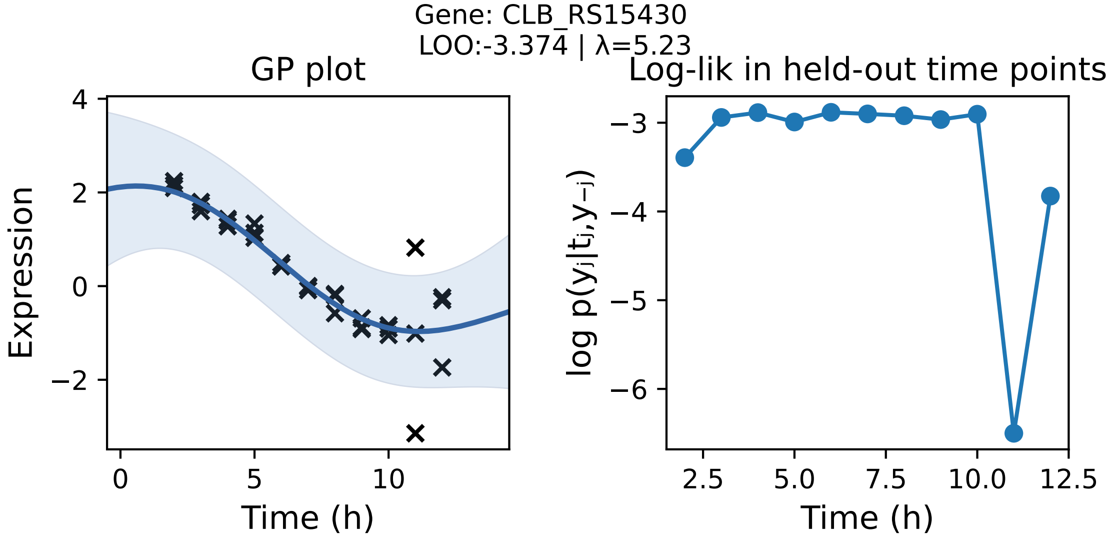
Gene: CLB_RS15430
LOO:-3.374 | λ=5.23
GP plot
Expression
Time (h)
Log-lik in held-out time points
log p(yj|tj,y−j)
Time (h)

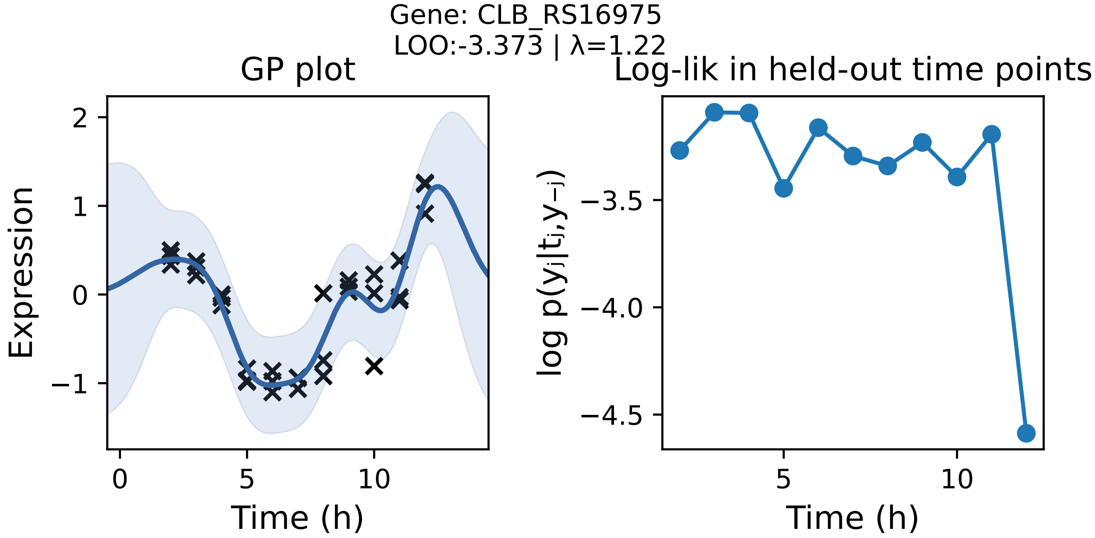

Gene: CLB_RS16975
LOO:-3.373 | λ=1.22
GP plot
Expression
Time (h)
Log-lik in held-out time points
log p(y_j|t_j,y_−j)
Time (h)

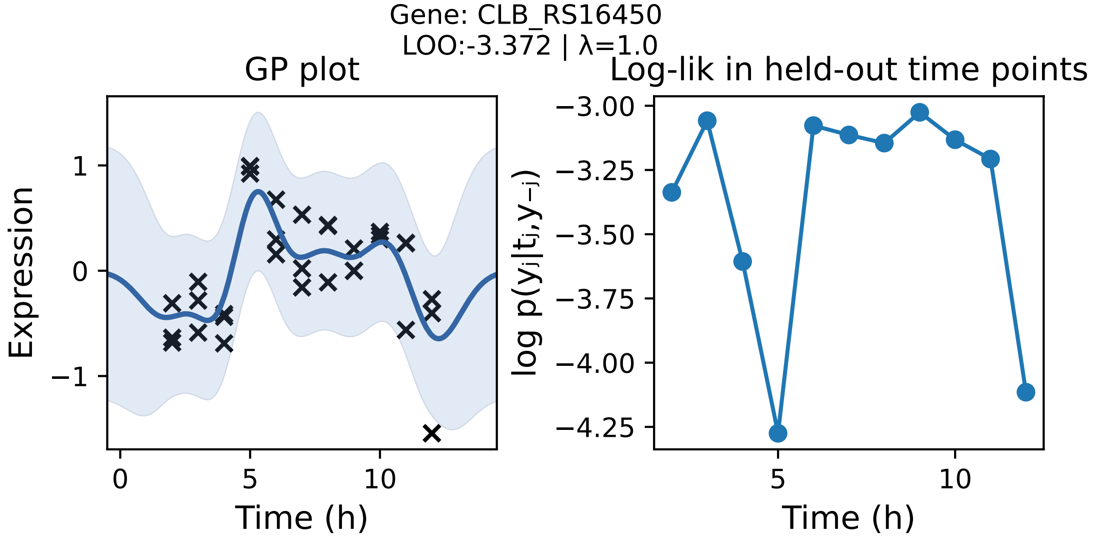

Gene: CLB_RS16450
LOO:-3.372 | λ=1.0
GP plot
Expression
Time (h)
Log-lik in held-out time points
log p(y_j|t_j,y_−j)
Time (h)

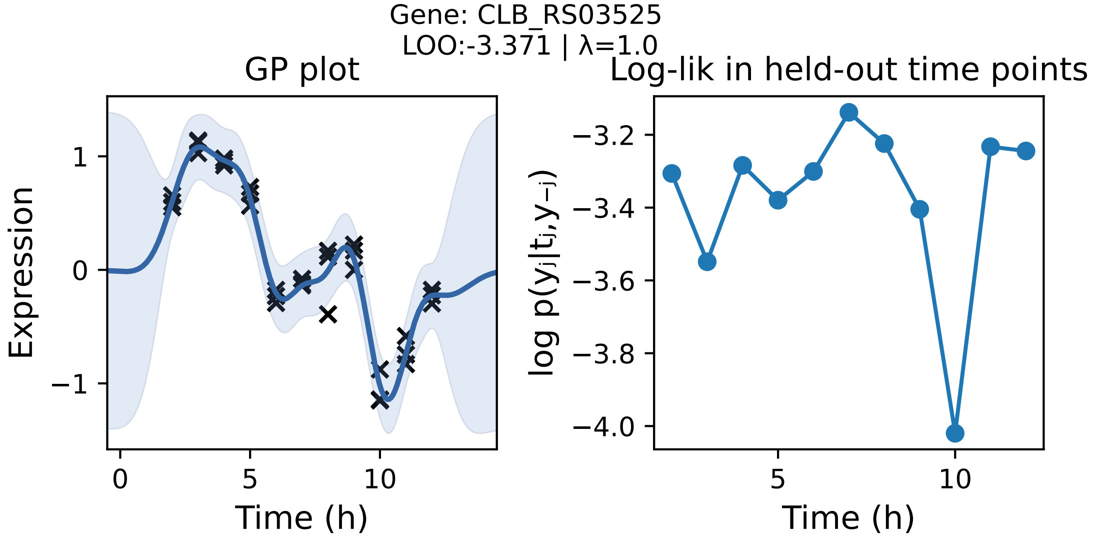

Gene: CLB_RS03525
LOO:-3.371 | λ=1.0
GP plot
Expression
Time (h)
1
0
−1
0
5
10
Log-lik in held-out time points
log p(yj|tj,y−j)
−3.2
−3.4
−3.6
−3.8
−4.0
5
10
Time (h)

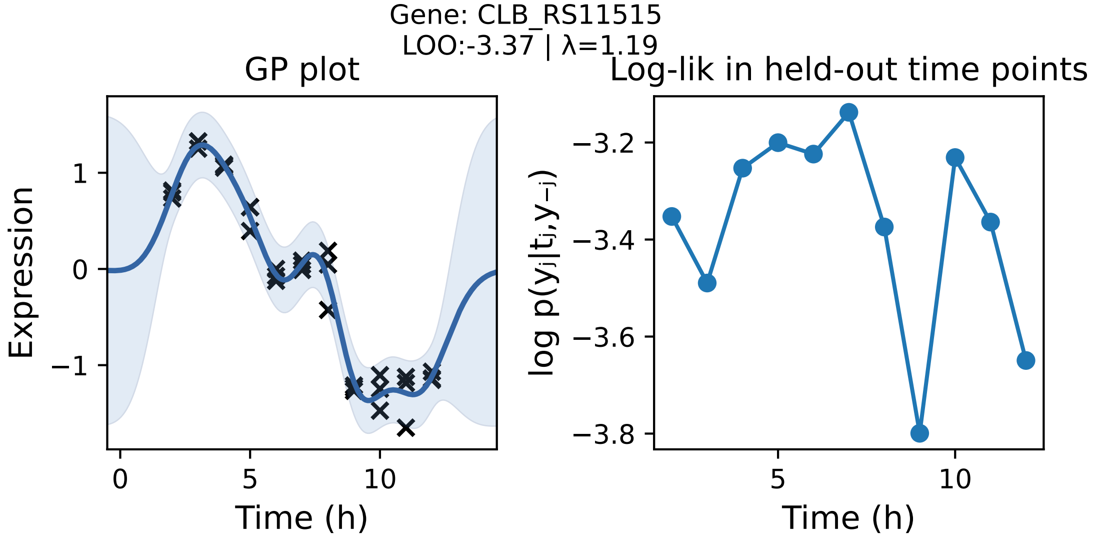
Gene: CLB_RS11515
LOO:-3.37 | λ=1.19
GP plot
Expression
1
0
−1
0
5
10
Time (h)
Log-lik in held-out time points
log p(yj|tj,y−j)
−3.2
−3.4
−3.6
−3.8
5
10
Time (h)

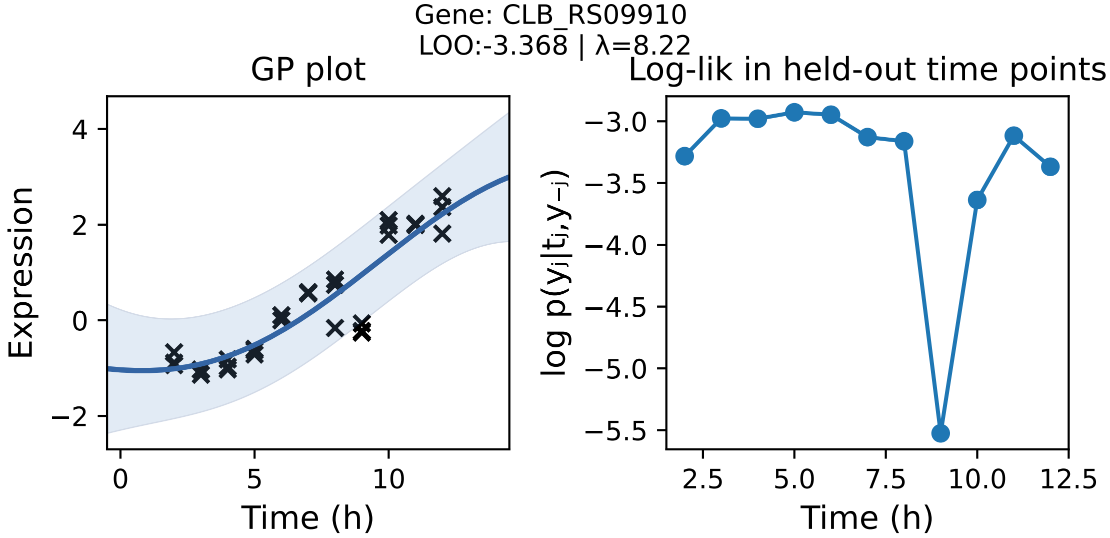

Gene: CLB_RS09910
LOO:-3.368 | λ=8.22
GP plot
Expression
Time (h)
Log-lik in held-out time points
log p(y_j|t_j,y_−j)
Time (h)

Gene: CLB_RS04620
LOO:-3.367 | λ=2.73

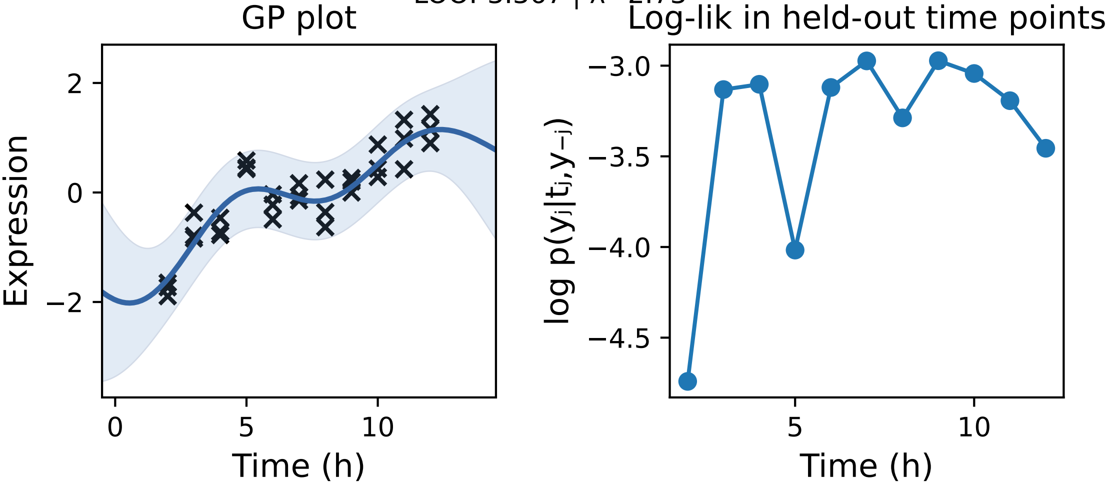

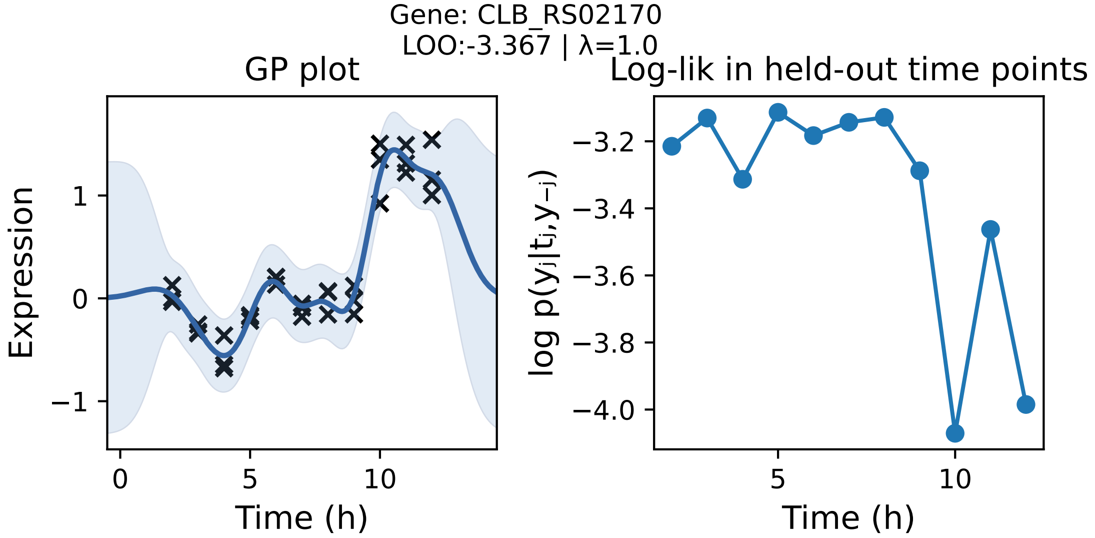

Gene: CLB_RS02170
LOO:-3.367 | λ=1.0
GP plot
Expression
1
0
−1
0
5
10
Time (h)
Log-lik in held-out time points
log p(y_j|t_j,y_−j)
−3.2
−3.4
−3.6
−3.8
−4.0
5
10
Time (h)

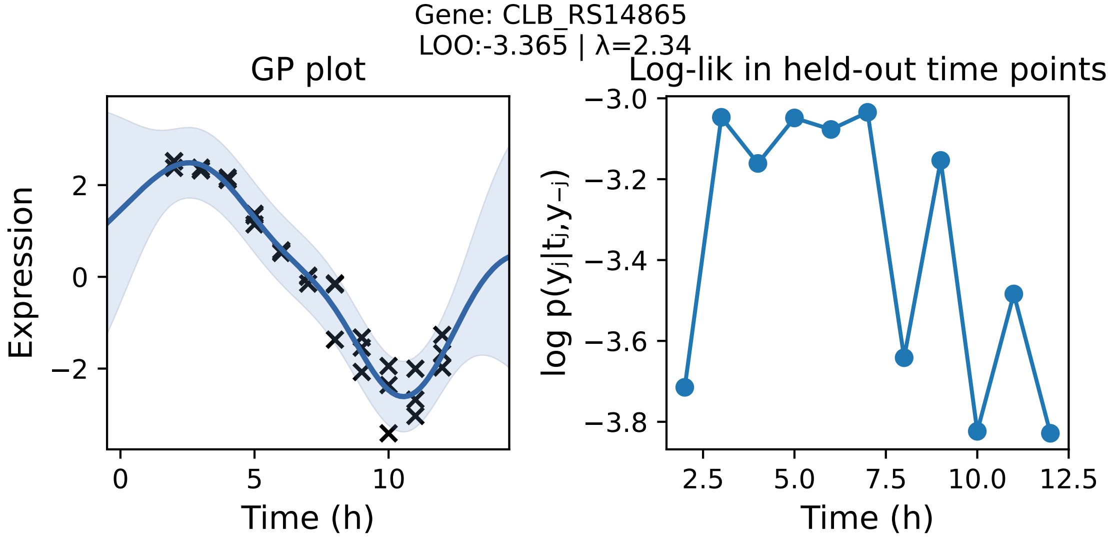

Gene: CLB_RS14865
LOO:-3.365 | λ=2.34
GP plot
Log-lik in held-out time points
Expression
Time (h)
log p(y_j|t_j,y_−j)
Time (h)
2
0
−2
0
5
10
−3.0
−3.2
−3.4
−3.6
−3.8
2.5
5.0
7.5
10.0
12.5

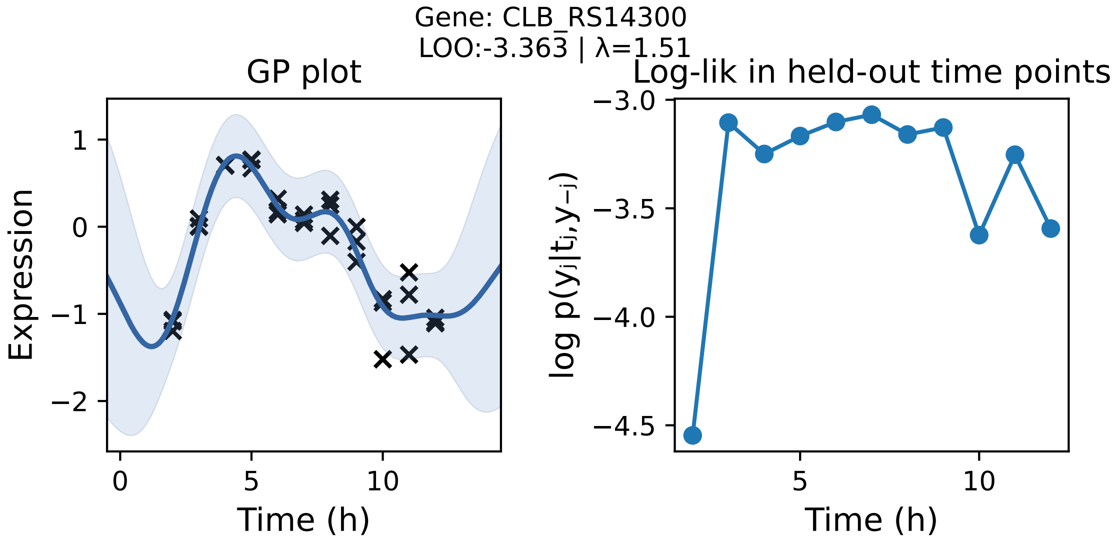

Gene: CLB_RS14300
LOO:-3.363 | λ=1.51
GP plot
Expression
Time (h)
Log-lik in held-out time points
log p(y_j|t_j,y_−j)
Time (h)

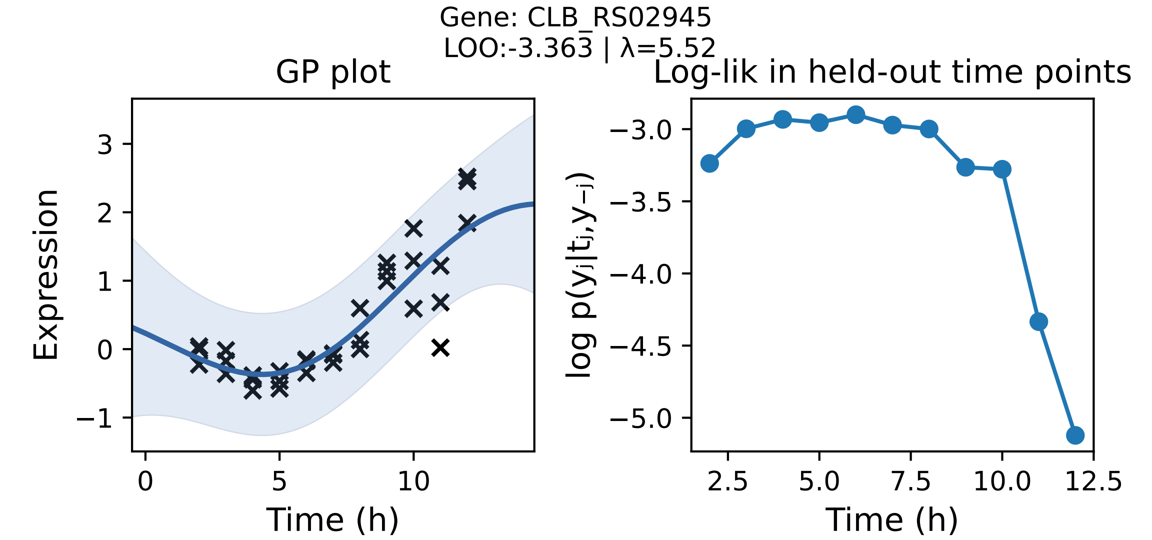
Gene: CLB_RS02945
LOO:-3.363 | λ=5.52
GP plot
Expression
Time (h)
Log-lik in held-out time points
log p(y_j|t_j,y_−j)
Time (h)

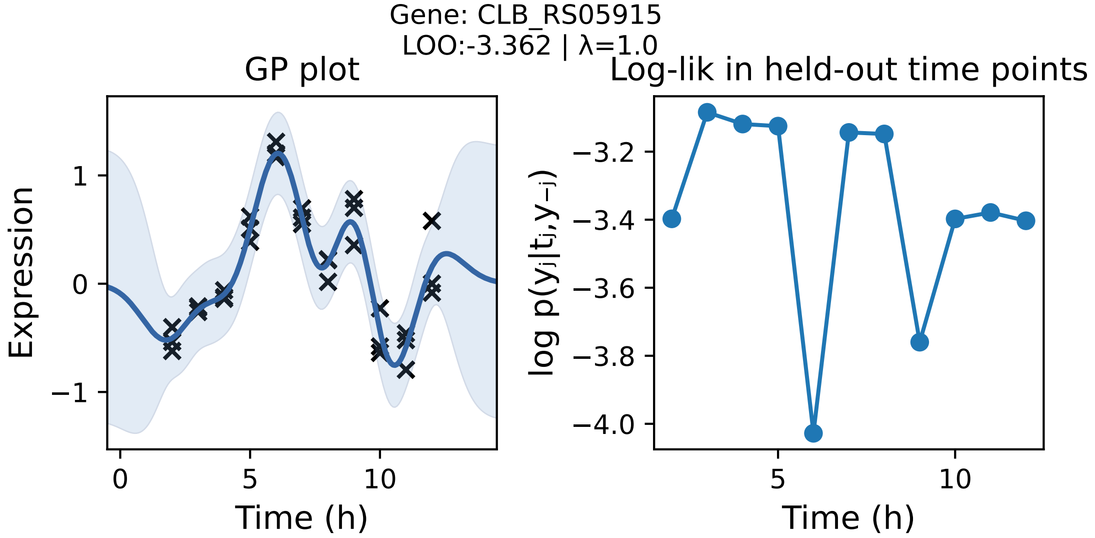

Gene: CLB_RS05915
LOO:-3.362 | λ=1.0
GP plot
Expression
1
0
−1
0
5
10
Time (h)
Log-lik in held-out time points
log p(yj|tj,y−j)
−3.2
−3.4
−3.6
−3.8
−4.0
5
10
Time (h)

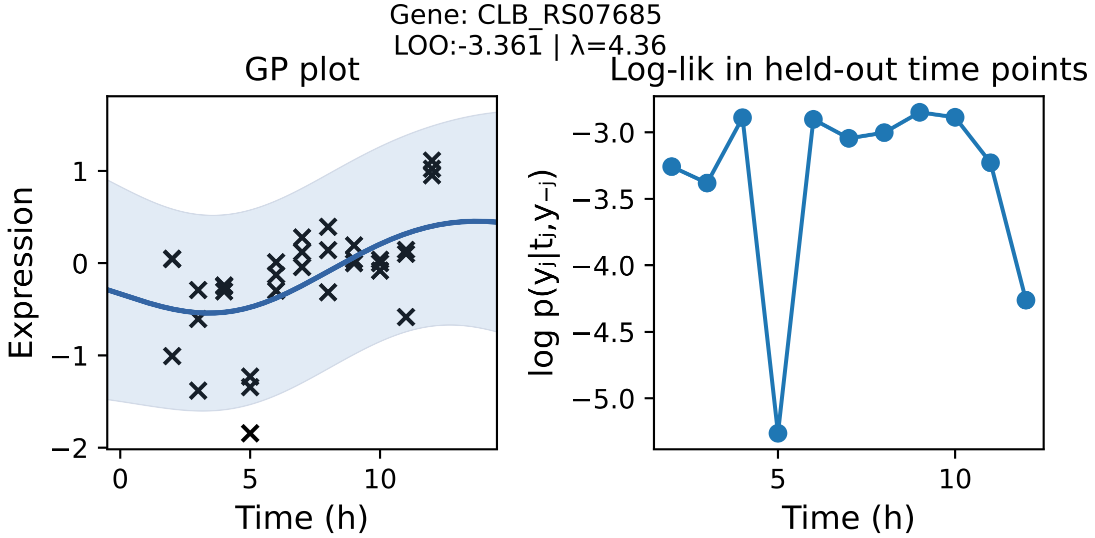

Gene: CLB_RS07685
LOO:-3.361 | λ=4.36
GP plot
Expression
Time (h)
Log-lik in held-out time points
log p(y_j|t_j,y_−j)
Time (h)

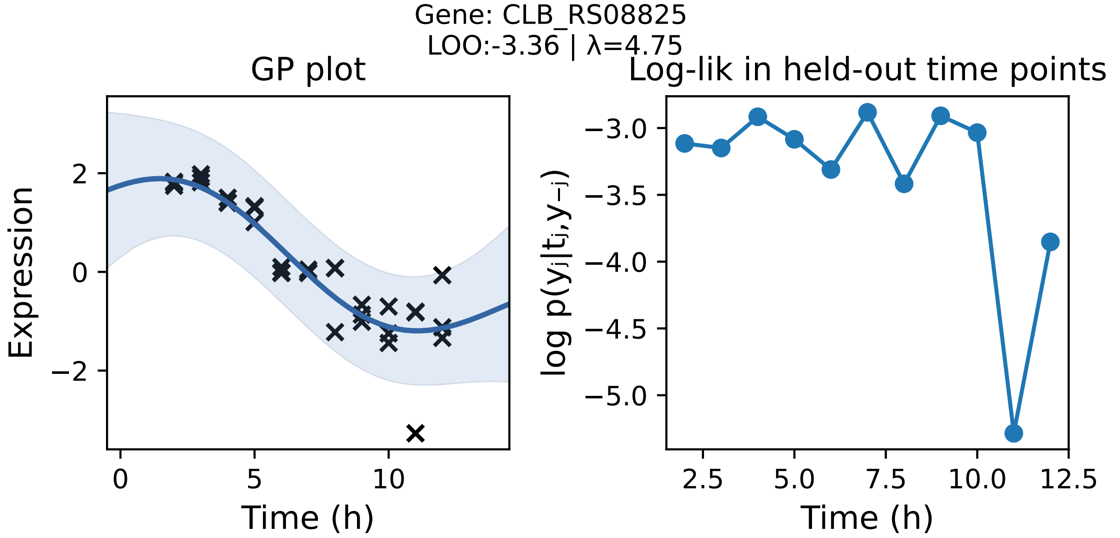

Gene: CLB_RS08825
LOO:-3.36 | λ=4.75
GP plot
Expression
Time (h)
Log-lik in held-out time points
log p(y_j|t_j,y_−j)
Time (h)

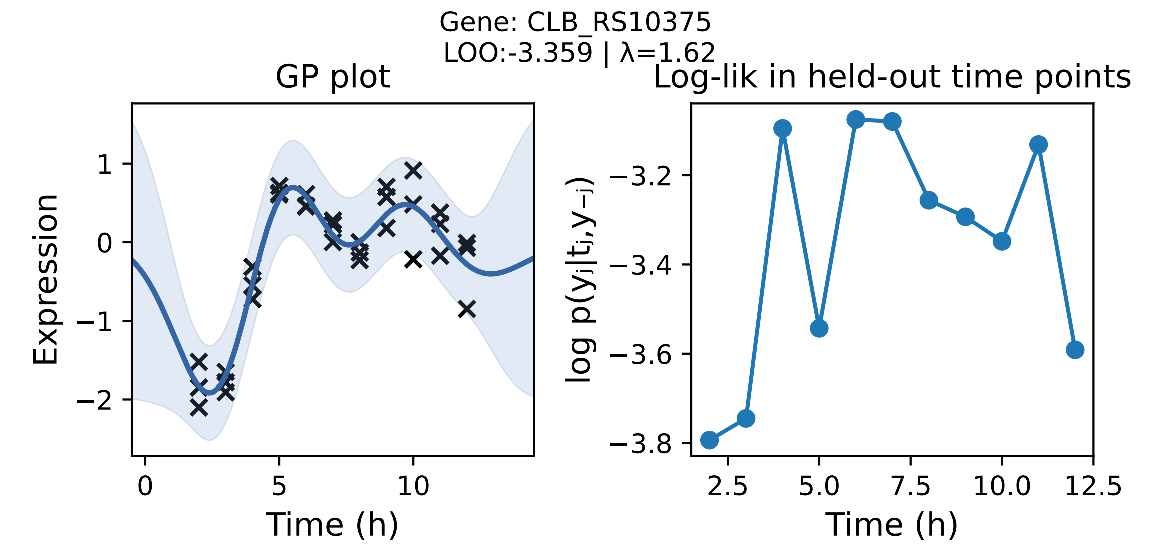

Gene: CLB_RS10375
LOO:-3.359 | λ=1.62
GP plot
Expression
Time (h)
Log-lik in held-out time points
log p(y_j|t_j,y_−j)
Time (h)

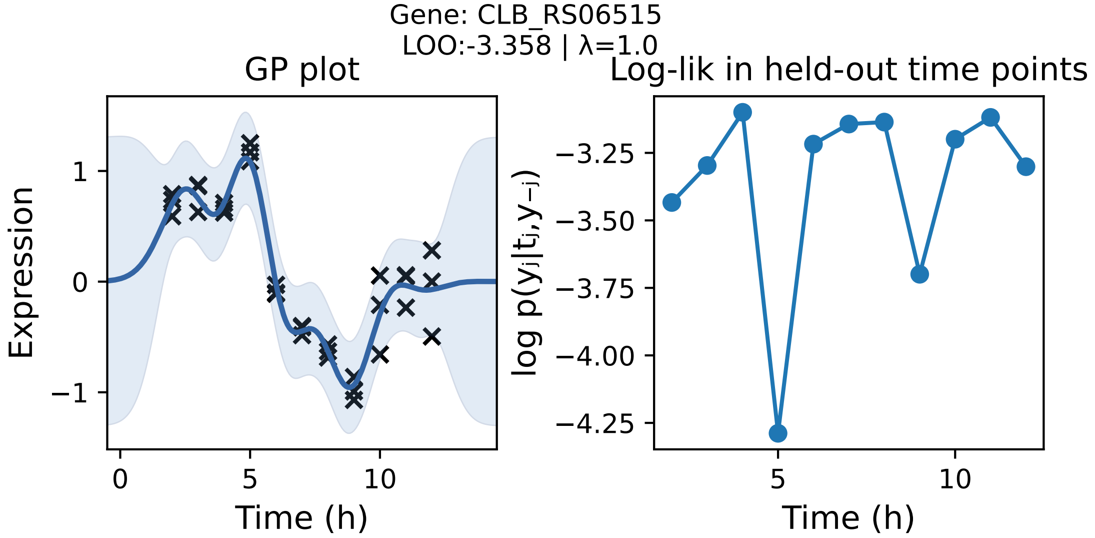

Gene: CLB_RS06515
LOO:-3.358 | λ=1.0
GP plot
Expression
Time (h)
Log-lik in held-out time points
log p(y_j|t_j,y_{-j})
−3.25
−3.50
−3.75
−4.00
−4.25
Time (h)

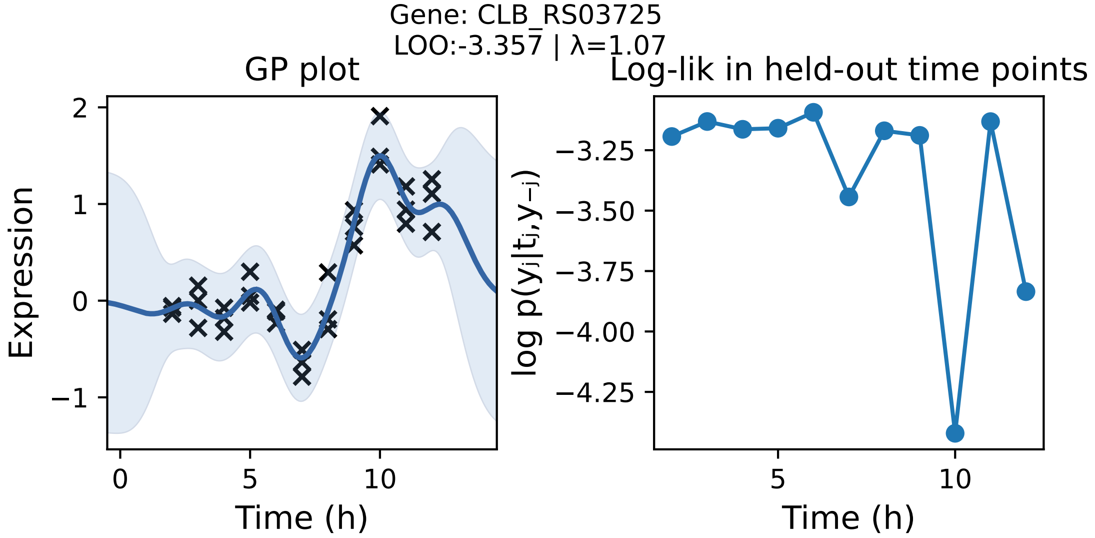

Gene: CLB_RS03725
LOO:-3.357 | λ=1.07
GP plot
Expression
Time (h)
Log-lik in held-out time points
log p(y_j|t_j,y_−j)
Time (h)
2
1
0
−1
0
5
10
−3.25
−3.50
−3.75
−4.00
−4.25
5
10

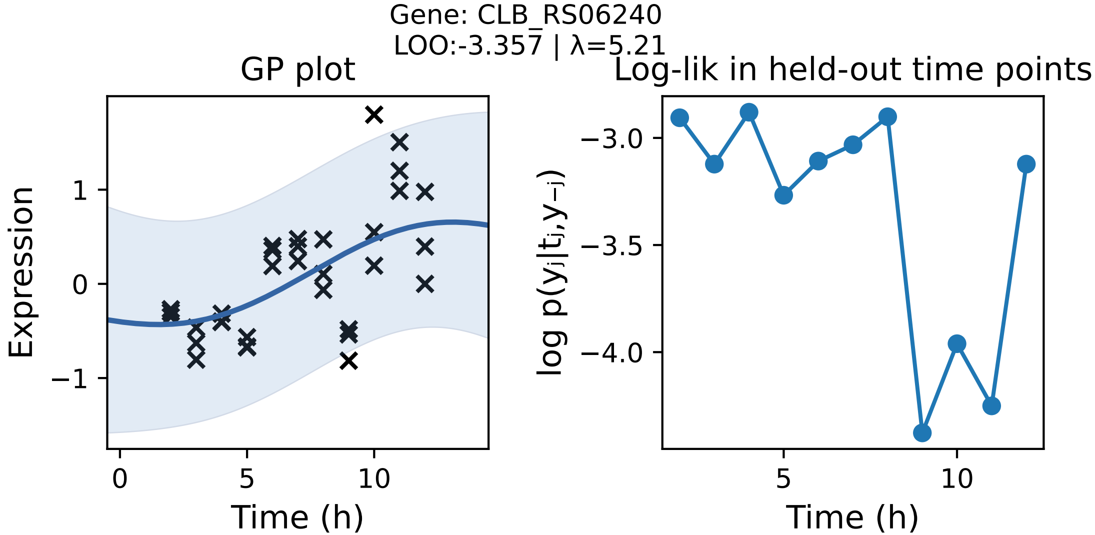

Gene: CLB_RS06240
LOO:-3.357 | λ=5.21
GP plot
Expression
Time (h)
1
0
−1
0
5
10
Log-lik in held-out time points
log p(y_j|t_j,y_−j)
Time (h)
−3.0
−3.5
−4.0
5
10

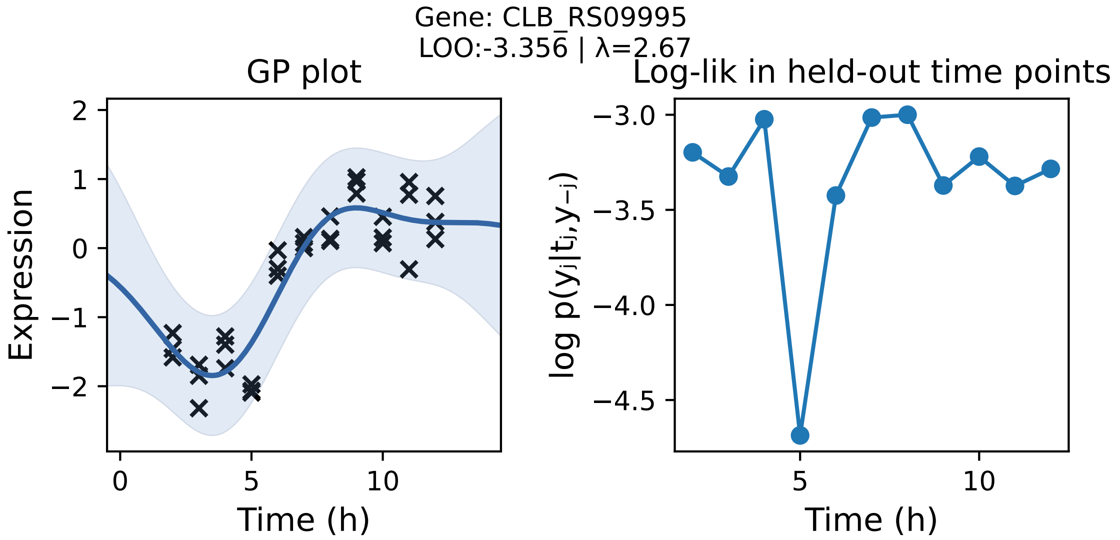

Gene: CLB_RS09995
LOO:-3.356 | λ=2.67
GP plot
Expression
Time (h)
2
1
0
−1
−2
0
5
10
Log-lik in held-out time points
log p(y_j|t_j,y_−j)
Time (h)
−3.0
−3.5
−4.0
−4.5
5
10

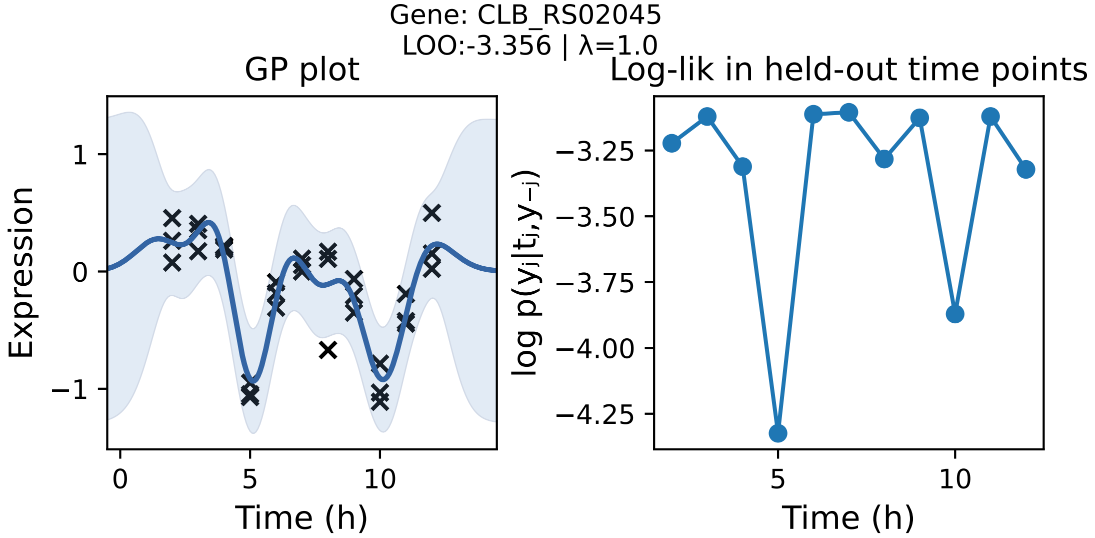

Gene: CLB_RS02045
LOO:-3.356 | λ=1.0
GP plot
Log-lik in held-out time points
Expression
Time (h)
log p(yj|tj,y−j)
1
0
−1
0
5
10
−3.25
−3.50
−3.75
−4.00
−4.25

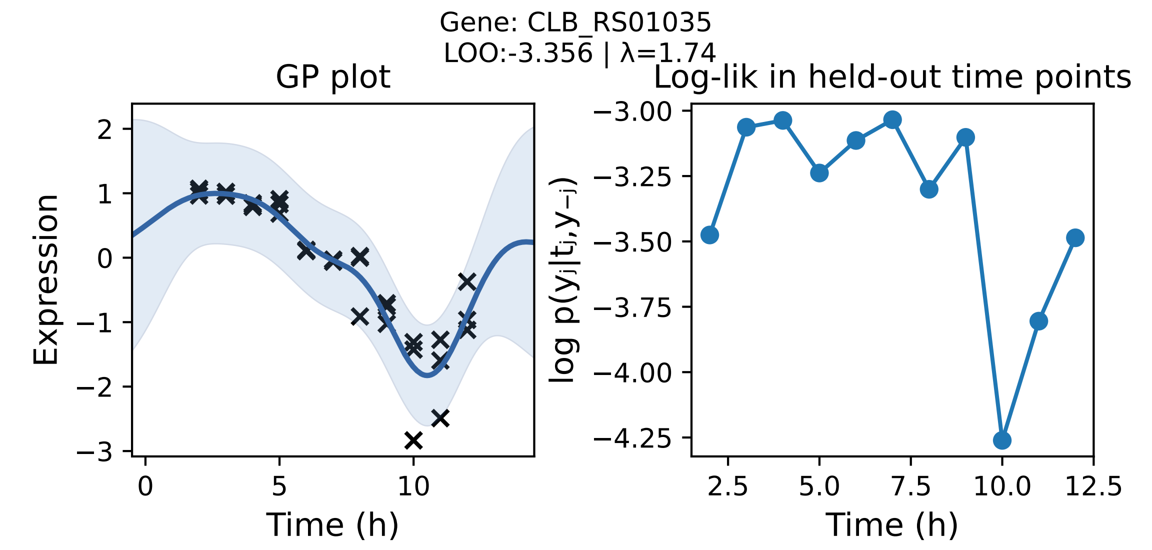

Gene: CLB_RS01035
LOO:-3.356 | λ=1.74
GP plot
Expression
Time (h)
Log-lik in held-out time points
log p(y_j|t_j,y_−j)
Time (h)

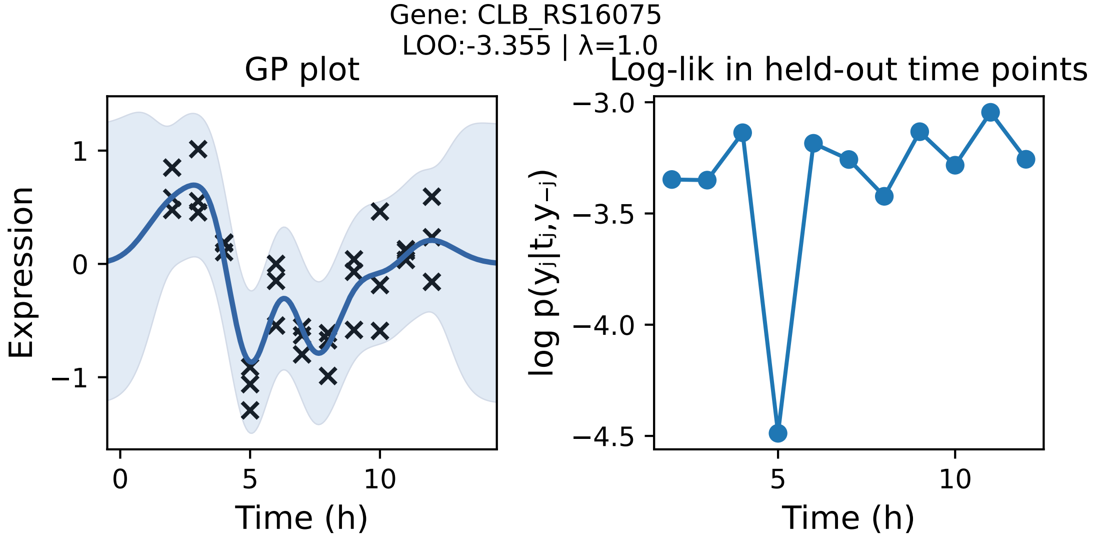

Gene: CLB_RS16075
LOO:-3.355 | λ=1.0
GP plot
Expression
1
0
−1
0
5
10
Time (h)
Log-lik in held-out time points
log p(yj|tj,y−j)
−3.0
−3.5
−4.0
−4.5
5
10
Time (h)

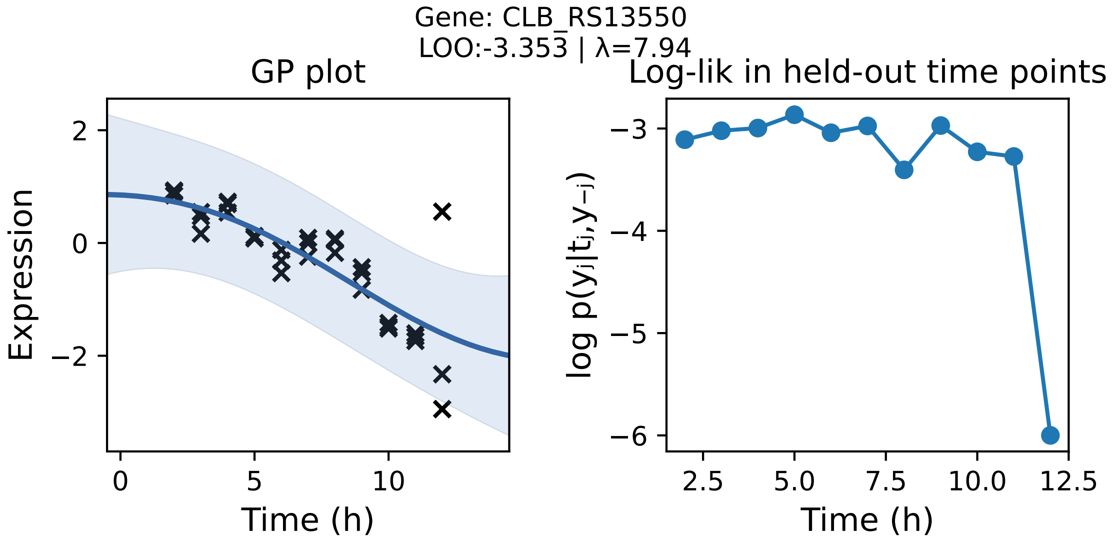
Gene: CLB_RS13550
LOO:-3.353 | λ=7.94
GP plot
Expression
Time (h)
Log-lik in held-out time points
log p(yⱼ|tⱼ,y₋ⱼ)
Time (h)

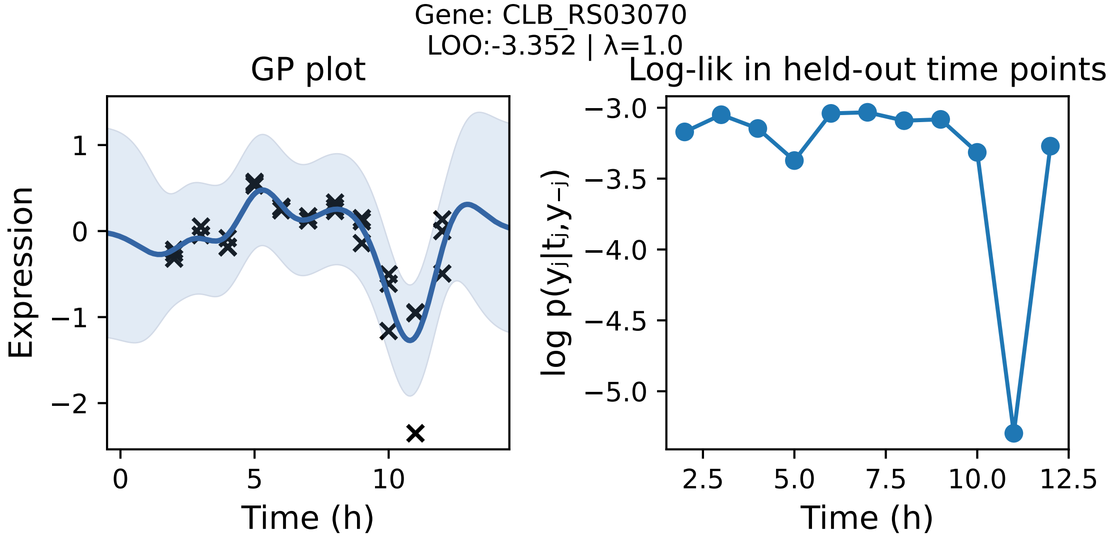
Gene: CLB_RS03070
LOO:-3.352 | λ=1.0
GP plot
Expression
Time (h)
1
0
−1
−2
0
5
10
Log-lik in held-out time points
log p(y_j|t_j,y_−j)
Time (h)
−3.0
−3.5
−4.0
−4.5
−5.0
2.5
5.0
7.5
10.0
12.5

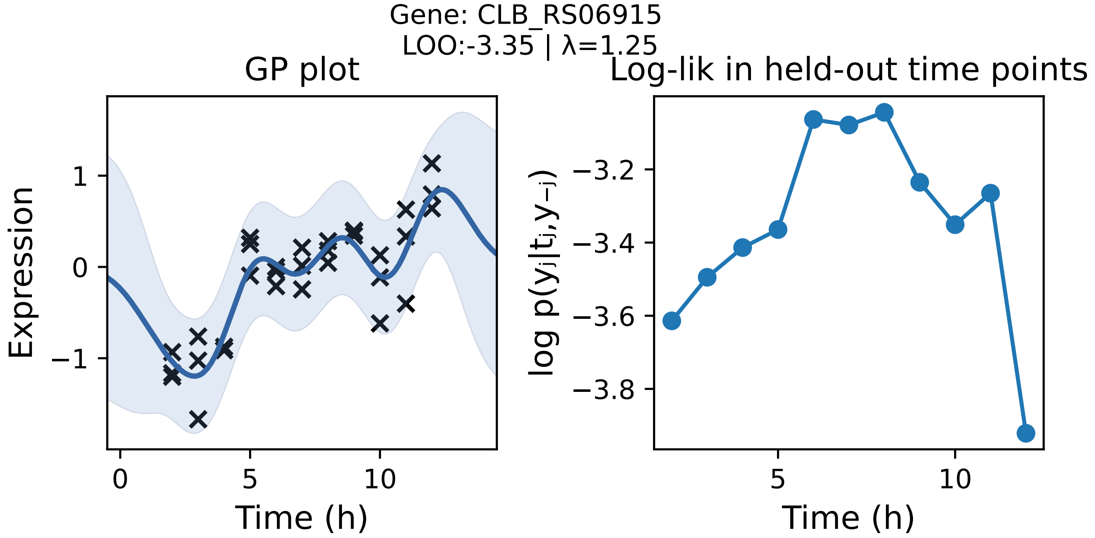

Gene: CLB_RS06915
LOO:-3.35 | λ=1.25
GP plot
Expression
Time (h)
Log-lik in held-out time points
log p(y_j|t_j,y_−j)
Time (h)

Gene: CLB_RS03335
LOO:-3.349 | λ=5.05

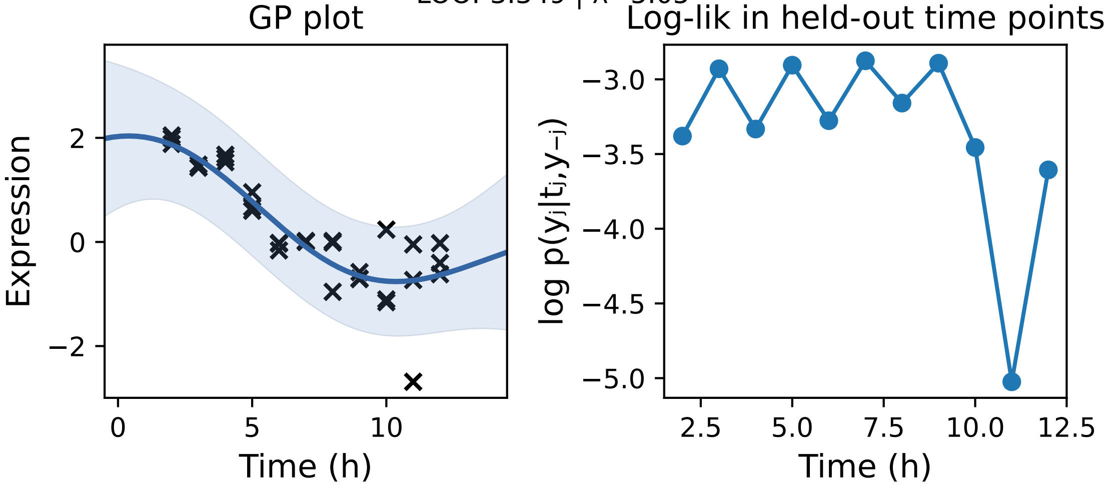

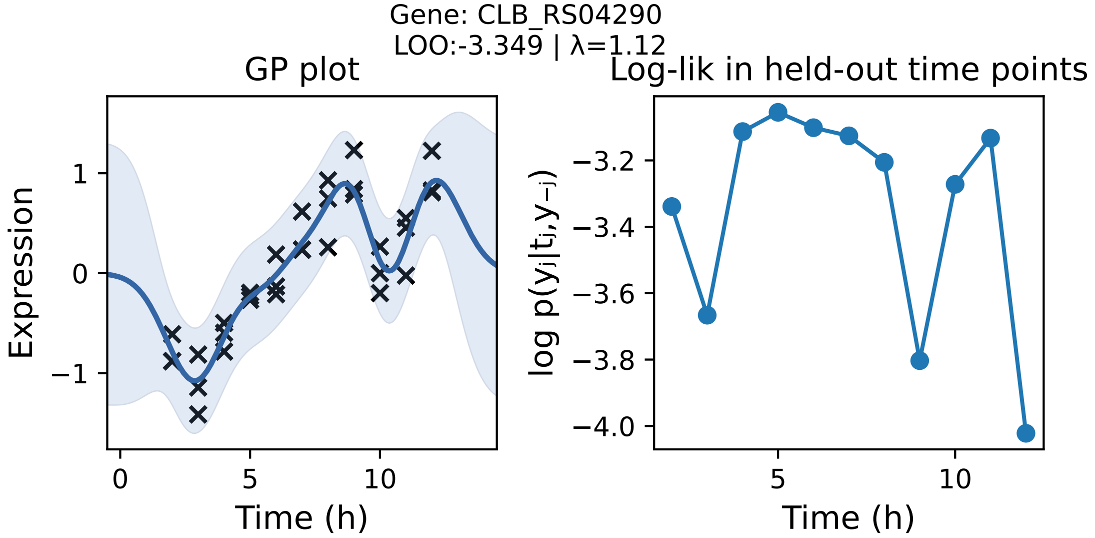

Gene: CLB_RS04290
LOO:-3.349 | λ=1.12
GP plot
Expression
Time (h)
Log-lik in held-out time points
log p(yj|tj,y−j)
Time (h)

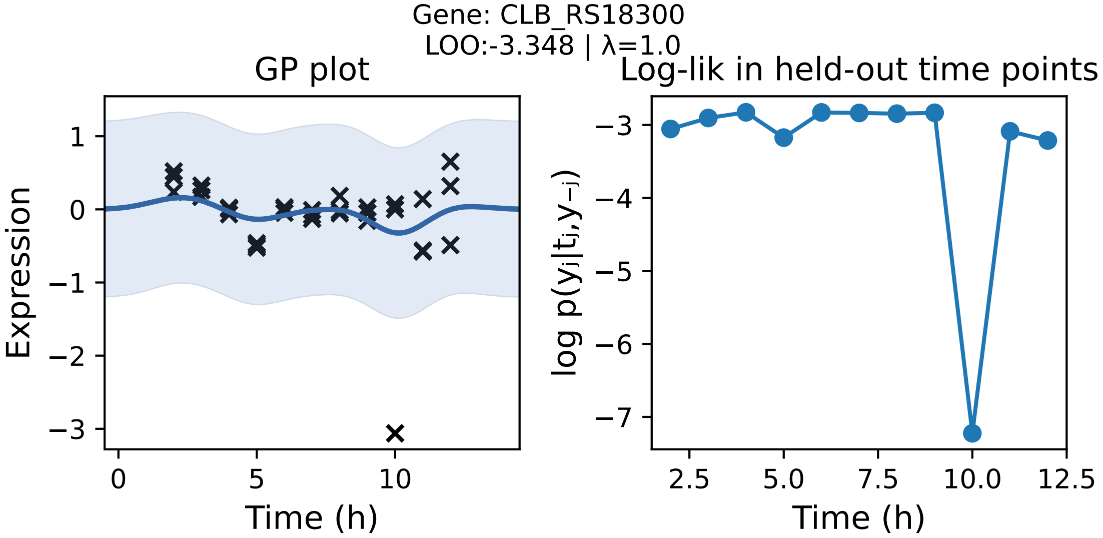

Gene: CLB_RS18300
LOO:-3.348 | λ=1.0
GP plot
Log-lik in held-out time points
Expression
Time (h)
log p(yj|tj,y−j)

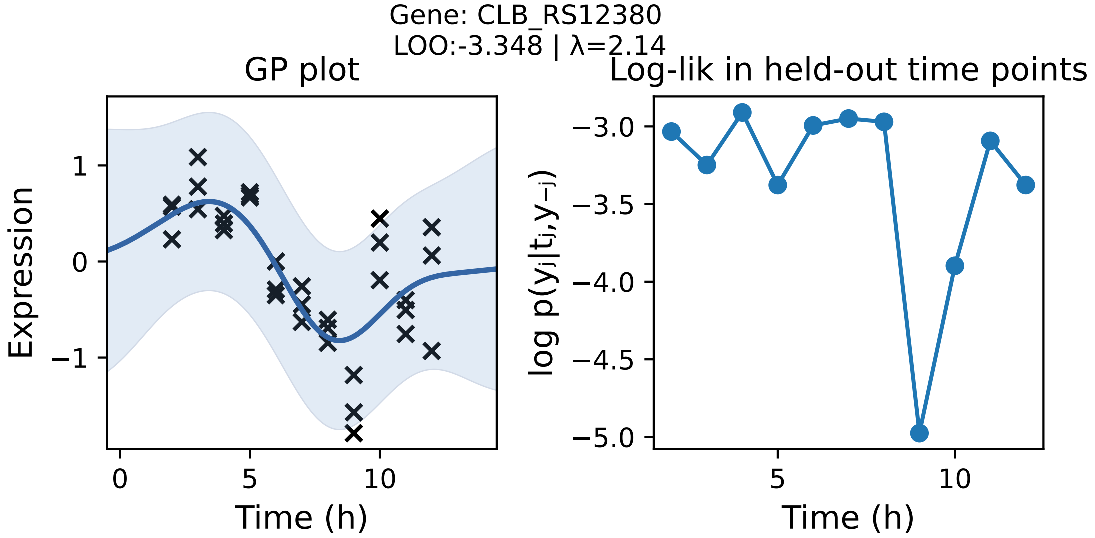

Gene: CLB_RS12380
LOO:-3.348 | λ=2.14
GP plot
Expression
Time (h)
Log-lik in held-out time points
log p(y_j|t_j,y_−j)
Time (h)

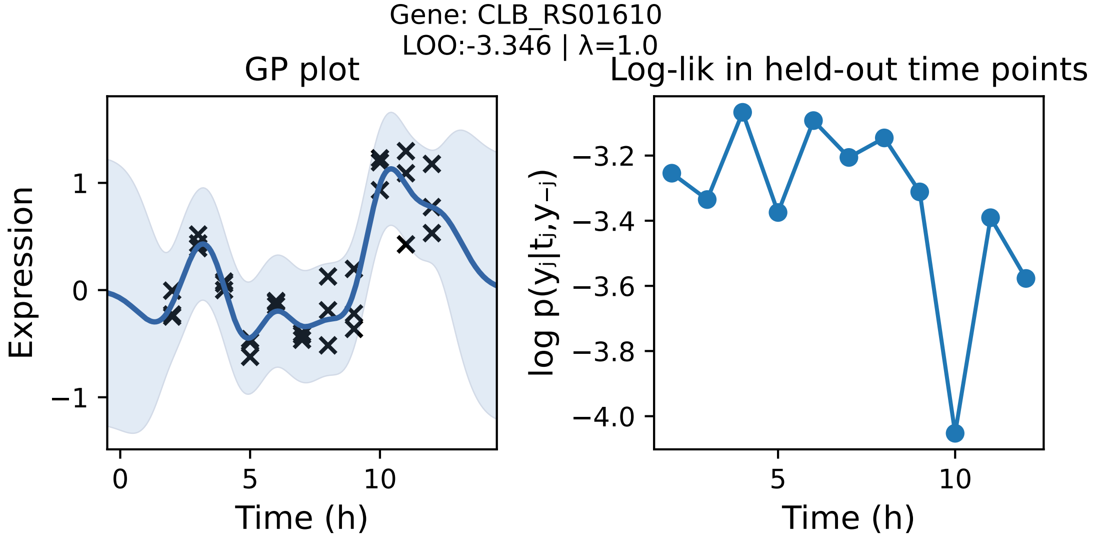

Gene: CLB_RS01610
LOO:-3.346 | λ=1.0
GP plot
Expression
Time (h)
Log-lik in held-out time points
log p(y_j|t_j,y_−j)
Time (h)

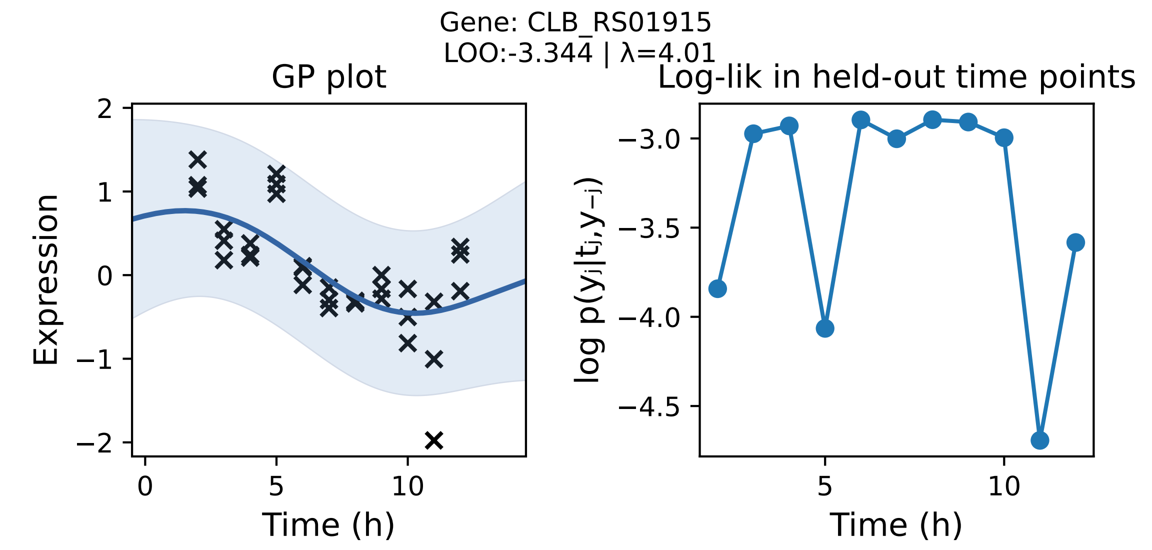

Gene: CLB_RS01915
LOO:-3.344 | λ=4.01
GP plot
Expression
Time (h)
Log-lik in held-out time points
log p(yj|tj,y−j)
Time (h)

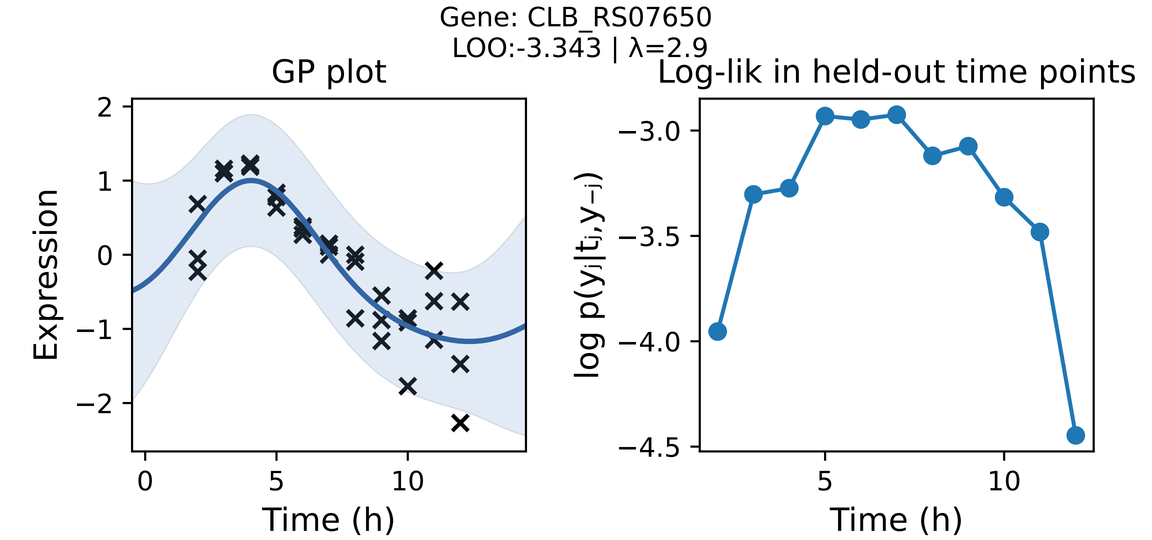

Gene: CLB_RS07650
LOO:-3.343 | λ=2.9
GP plot
Expression
Time (h)
Log-lik in held-out time points
log p(y_j|t_j,y_−j)
Time (h)

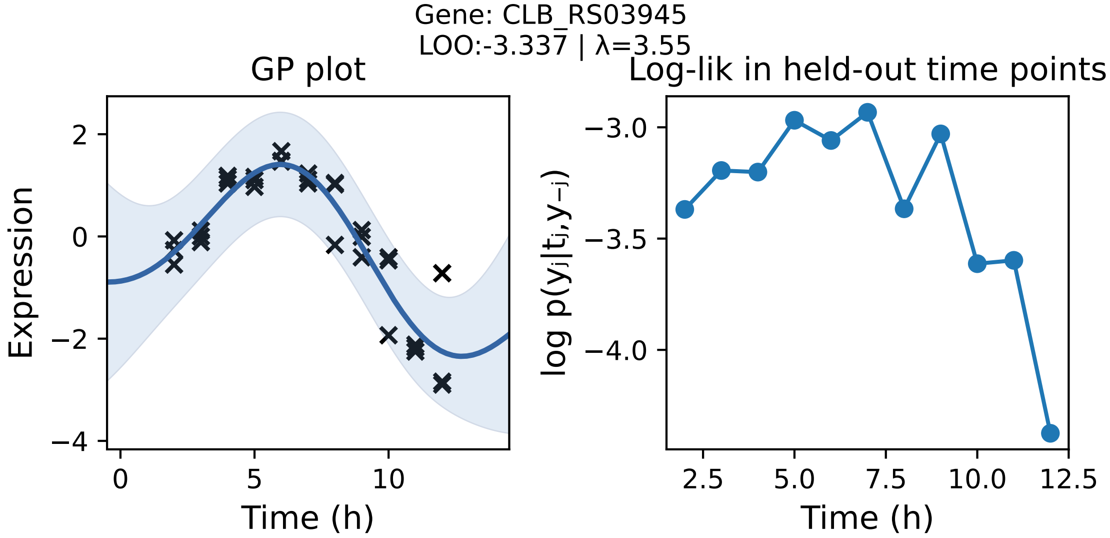
Gene: CLB_RS03945
LOO:-3.337 | λ=3.55
GP plot
Log-lik in held-out time points
Expression
Time (h)
log p(y_j|t_j,y_−j)
2
0
−2
−4
0
5
10
−3.0
−3.5
−4.0
2.5
5.0
7.5
10.0
12.5

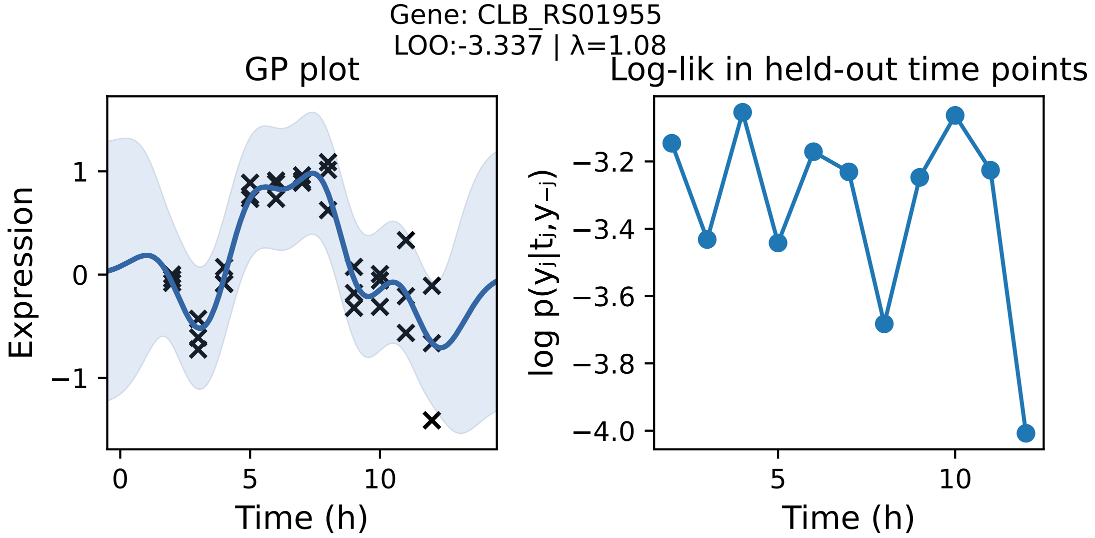
Gene: CLB_RS01955
LOO:-3.337 | λ=1.08
GP plot
Expression
1
0
−1
0
5
10
Time (h)
Log-lik in held-out time points
log p(y_j|t_j,y_−j)
−3.2
−3.4
−3.6
−3.8
−4.0
5
10
Time (h)

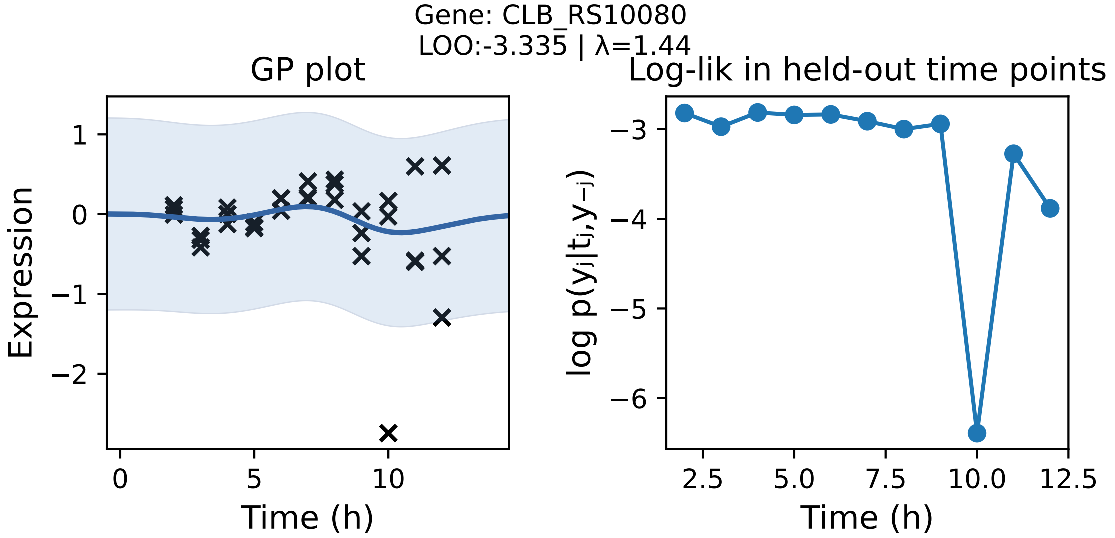

Gene: CLB_RS10080
LOO:-3.335 | λ=1.44
GP plot
Expression
Time (h)
Log-lik in held-out time points
log p(yⱼ|tⱼ,y₋ⱼ)
Time (h)

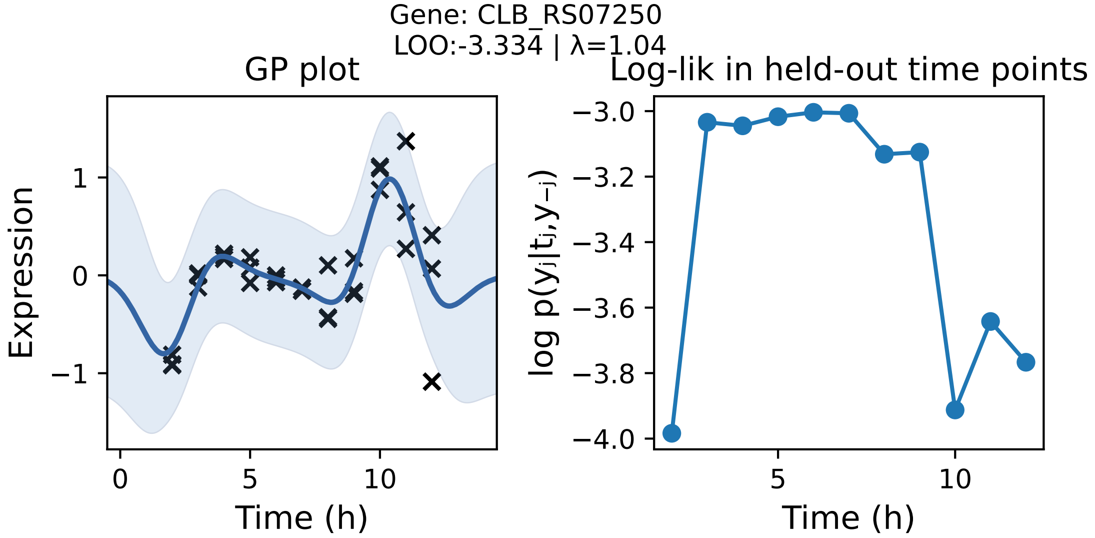

Gene: CLB_RS07250
LOO:-3.334 | λ=1.04
GP plot
Expression
Time (h)
Log-lik in held-out time points
log p(y_j|t_j,y_−j)
Time (h)

Gene: CLB_RS04285
LOO:-3.333 | λ=1.0

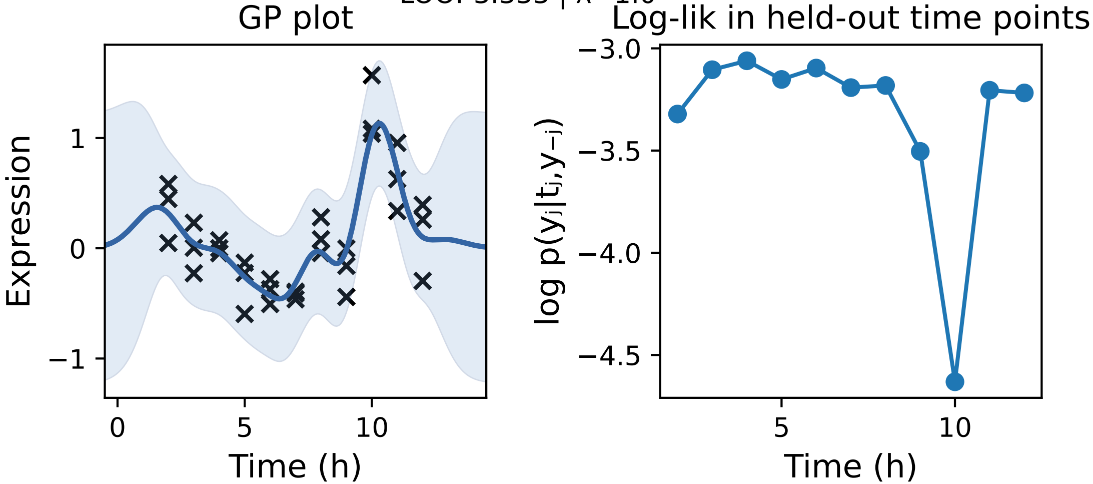

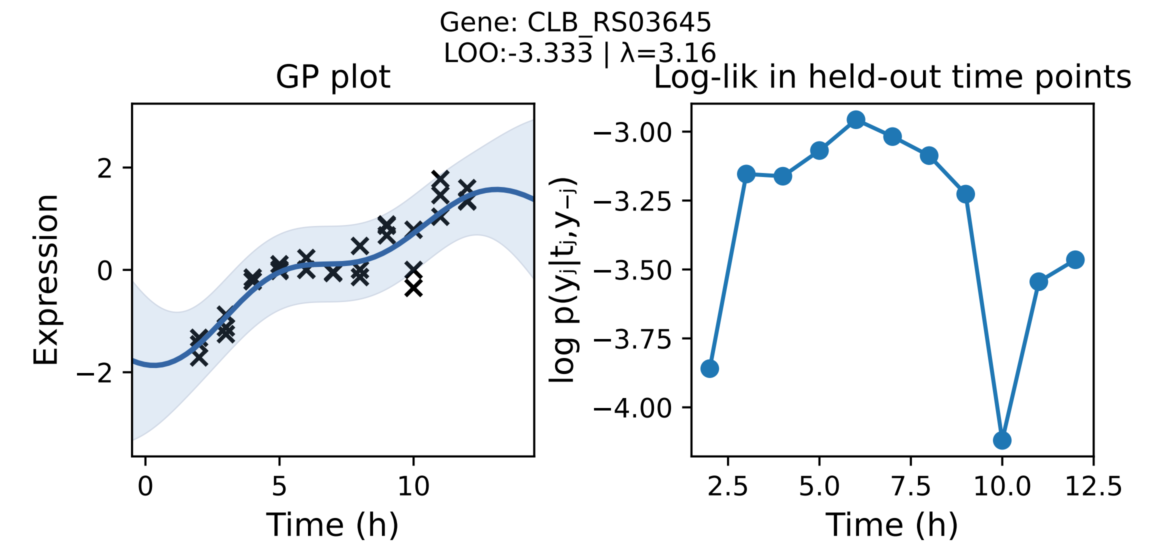
Gene: CLB_RS03645
LOO:-3.333 | λ=3.16
GP plot
Expression
Time (h)
Log-lik in held-out time points
log p(yj|tj,y−j)
Time (h)

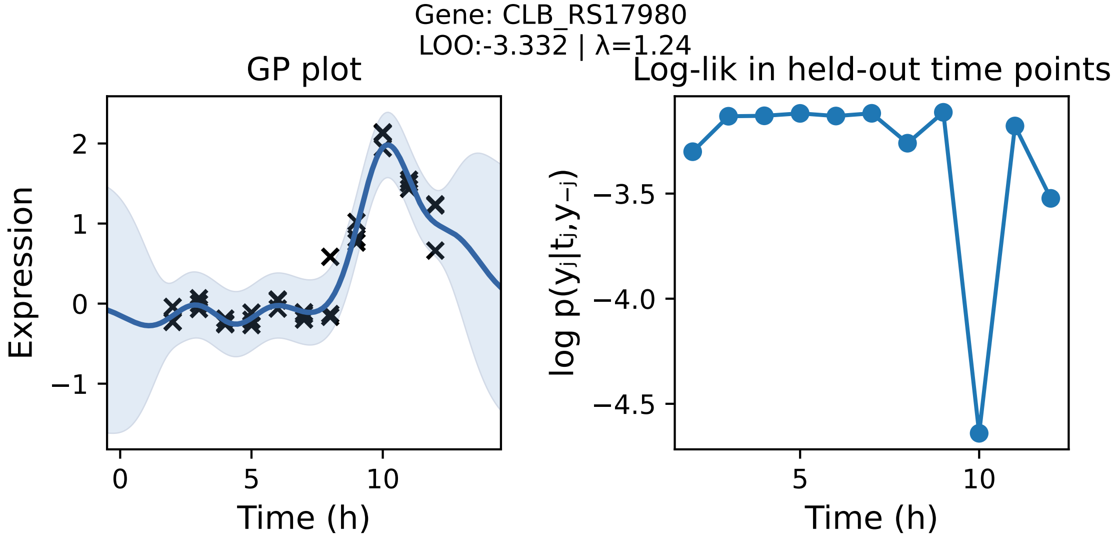

Gene: CLB_RS17980
LOO:-3.332 | λ=1.24
GP plot
Expression
Time (h)
Log-lik in held-out time points
log p(y_j|t_j,y_−j)
Time (h)

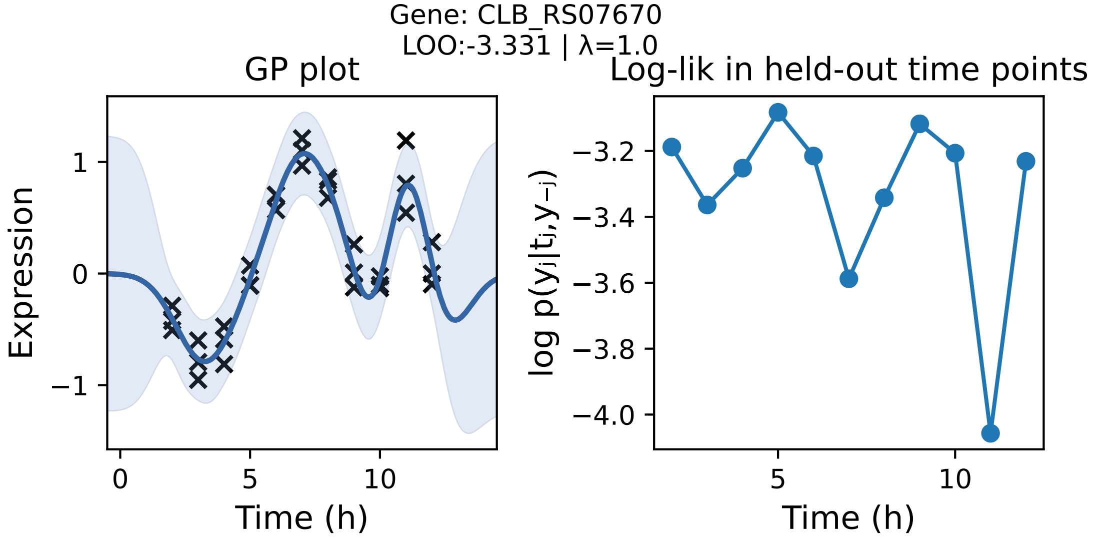

Gene: CLB_RS07670
LOO:-3.331 | λ=1.0
GP plot
Expression
Time (h)
1
0
−1
0
5
10
Log-lik in held-out time points
log p(yj|tj,y−j)
Time (h)
−3.2
−3.4
−3.6
−3.8
−4.0
5
10

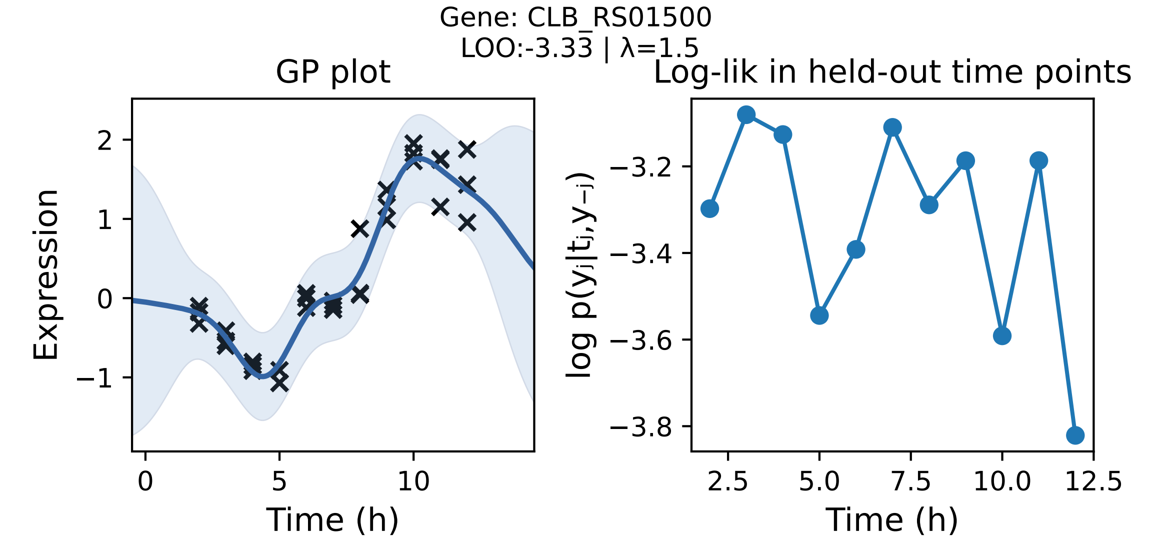

Gene: CLB_RS01500
LOO:-3.33 | λ=1.5
GP plot
Expression
Time (h)
Log-lik in held-out time points
log p(y_j|t_j,y_−j)
Time (h)

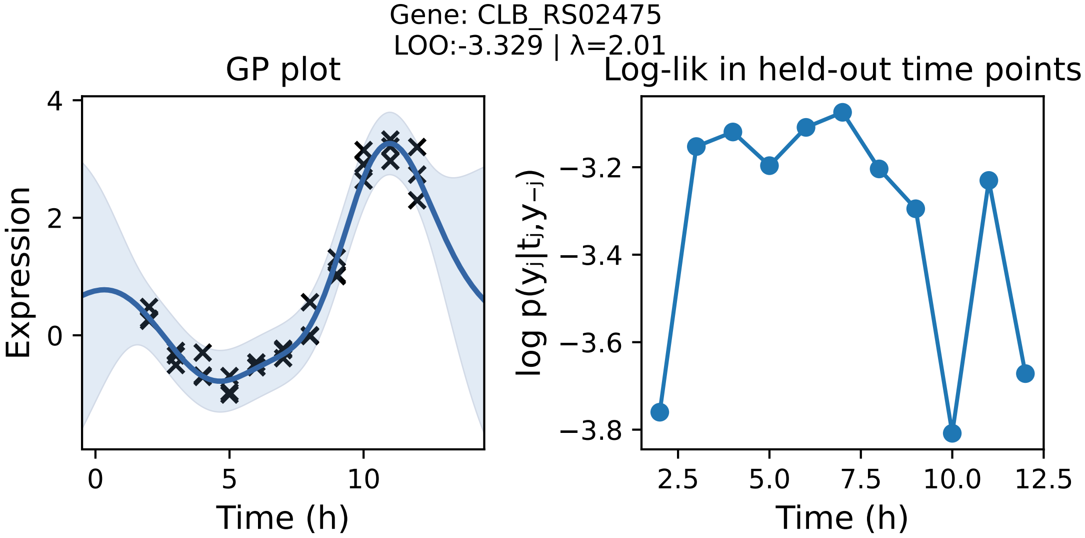

Gene: CLB_RS02475
LOO:-3.329 | λ=2.01
GP plot
Expression
Time (h)
0
2
4
0
5
10
Log-lik in held-out time points
log p(y_j|t_j,y_-j)
−3.2
−3.4
−3.6
−3.8
2.5
5.0
7.5
10.0
12.5
Time (h)

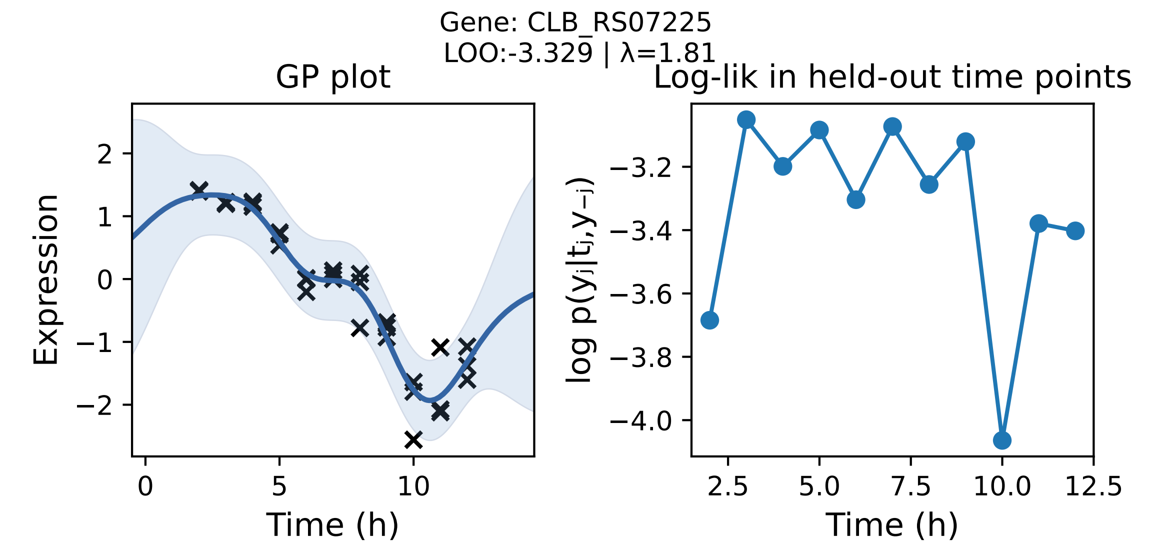

Gene: CLB_RS07225
LOO:-3.329 | λ=1.81
GP plot
Expression
Time (h)
Log-lik in held-out time points
log p(y_j|t_j,y_−j)
Time (h)

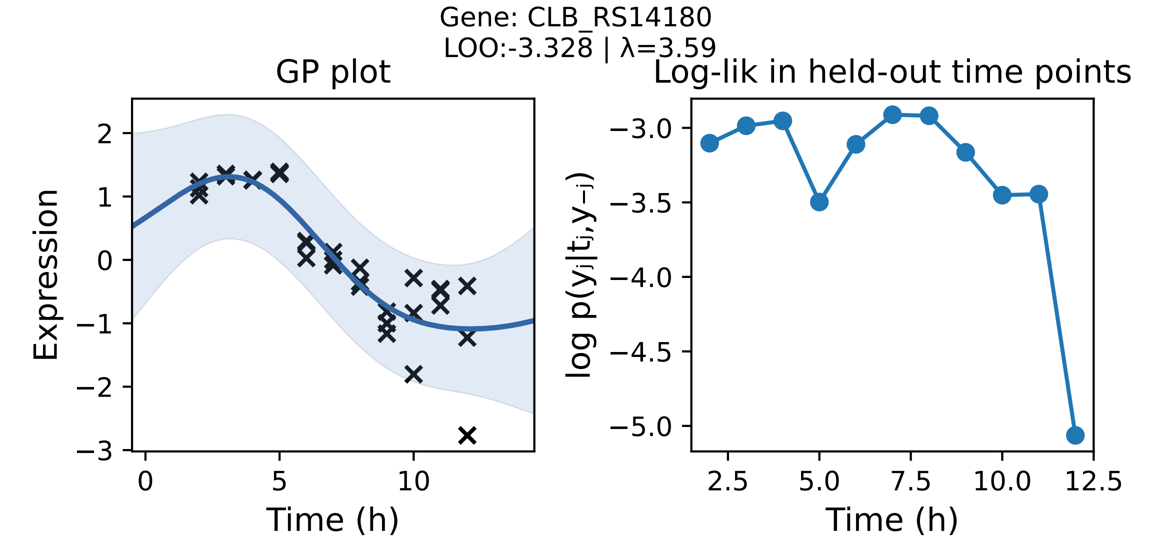

Gene: CLB_RS14180
LOO:-3.328 | λ=3.59
GP plot
Expression
Time (h)
2
1
0
−1
−2
−3
0
5
10
Log-lik in held-out time points
log p(y_j|t_j,y_−j)
Time (h)
−3.0
−3.5
−4.0
−4.5
−5.0
2.5
5.0
7.5
10.0
12.5

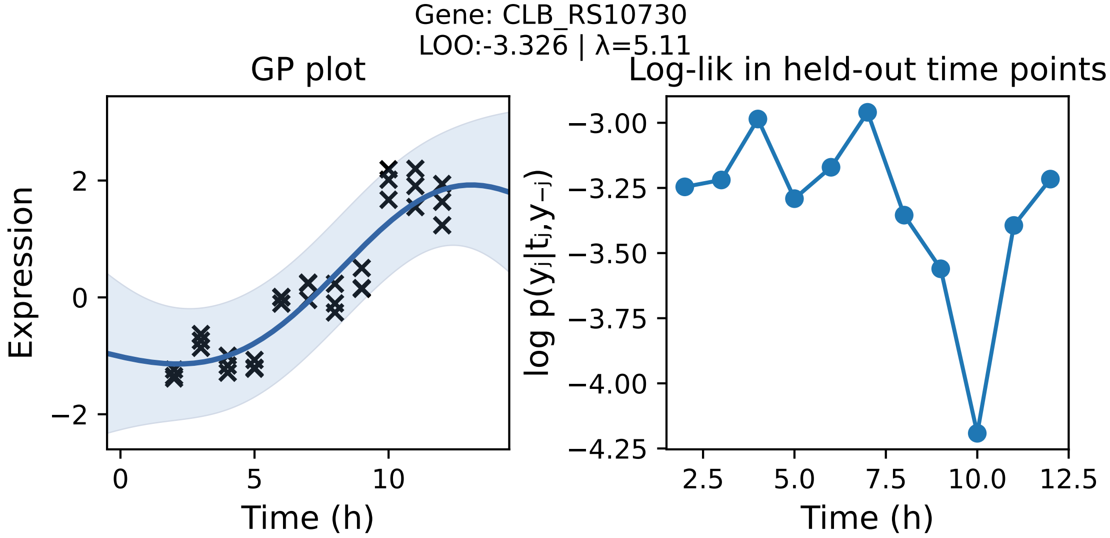

Gene: CLB_RS10730
LOO:-3.326 | λ=5.11
GP plot
Expression
Time (h)
Log-lik in held-out time points
log p(y_j|t_j,y_{−j})
Time (h)

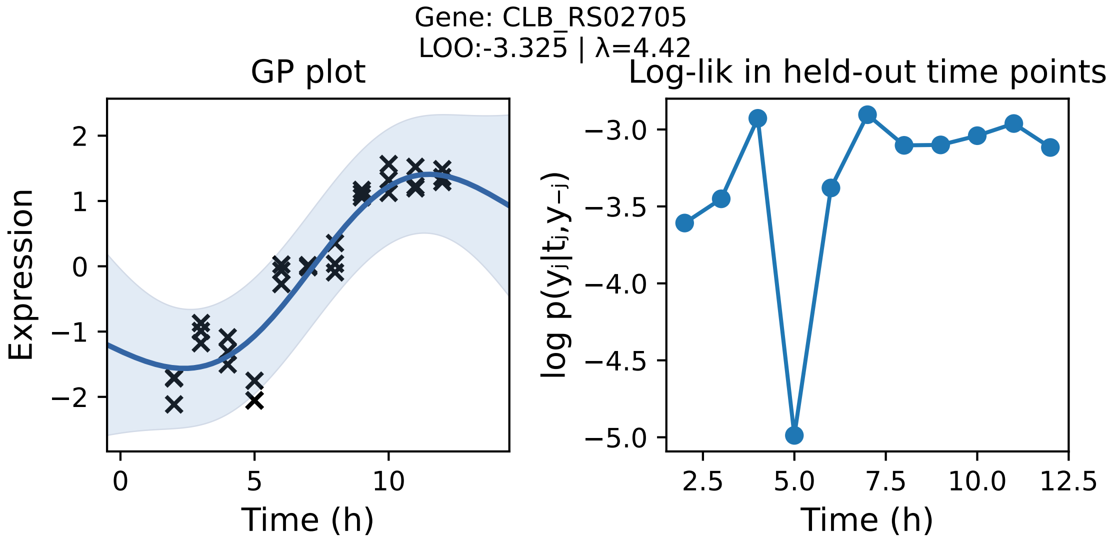

Gene: CLB_RS02705
LOO:-3.325 | λ=4.42
GP plot
Expression
Time (h)
2
1
0
−1
−2
0
5
10
Log-lik in held-out time points
log p(yj|tj,y−j)
Time (h)
−3.0
−3.5
−4.0
−4.5
−5.0
2.5
5.0
7.5
10.0
12.5

Gene: CLB_RS07485
LOO:-3.325 | λ=1.0

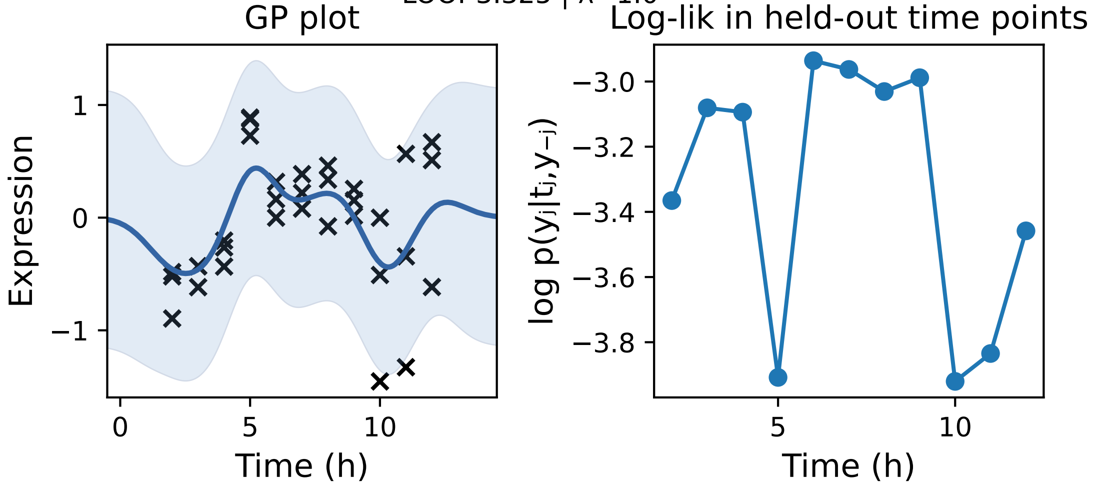

Gene: CLB_RS17505
LOO:-3.325 | λ=1.97

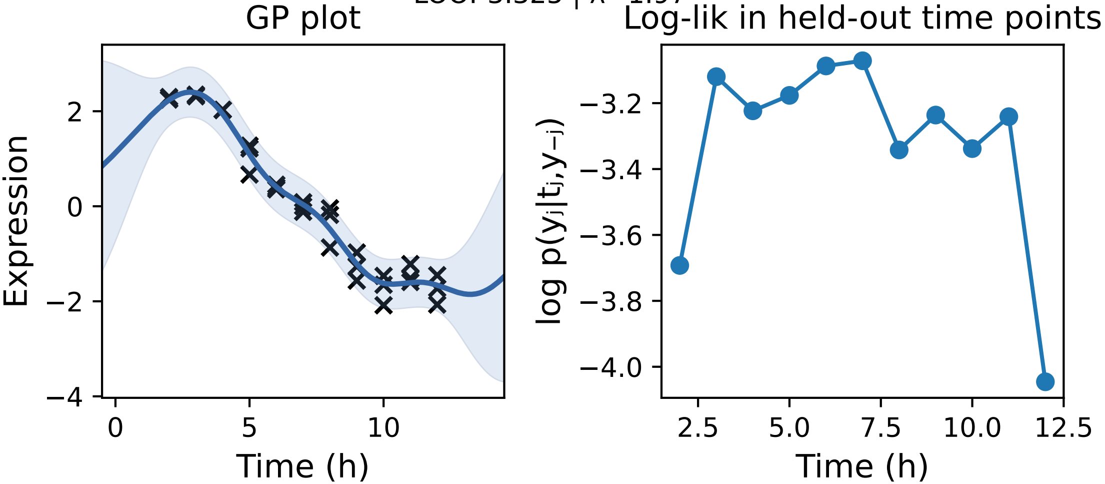

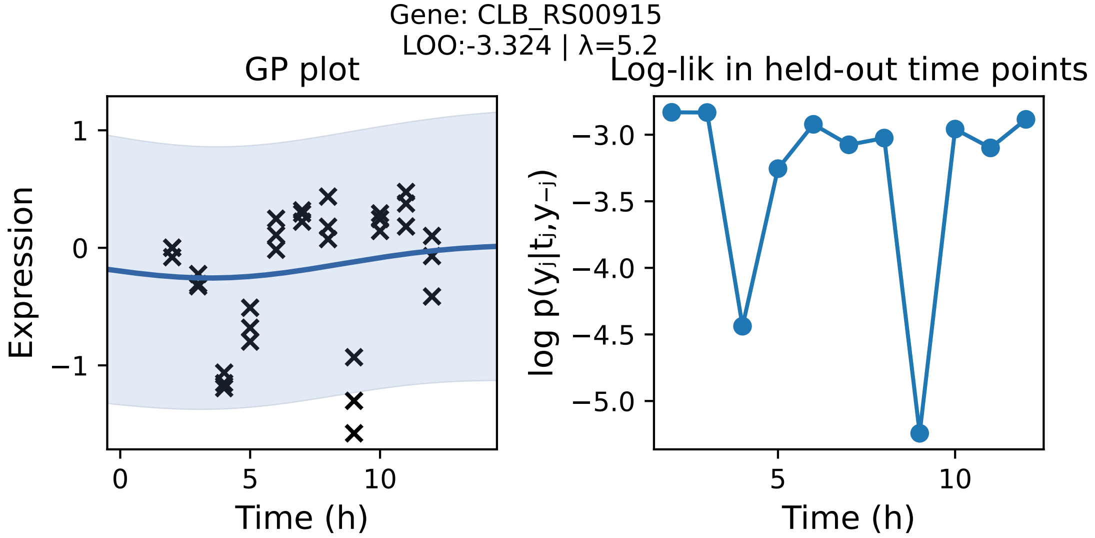

Gene: CLB_RS00915
LOO:-3.324 | λ=5.2
GP plot
Expression
Time (h)
Log-lik in held-out time points
log p(yj|tj,y−j)
Time (h)

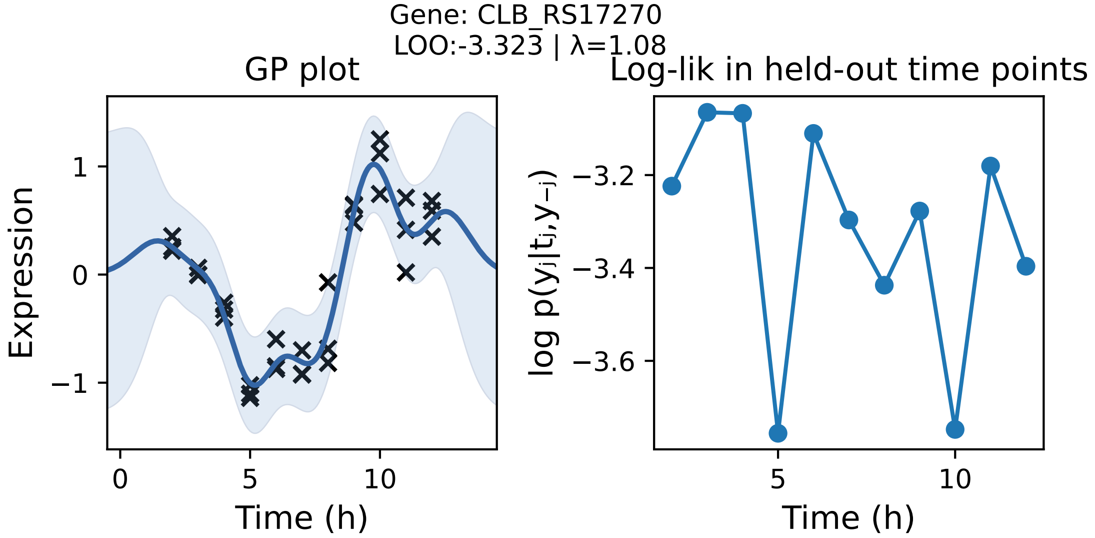
Gene: CLB_RS17270
LOO:-3.323 | λ=1.08
GP plot
Expression
1
0
−1
0
5
10
Time (h)
Log-lik in held-out time points
log p(yⱼ|tⱼ,y₋ⱼ)
−3.2
−3.4
−3.6
5
10
Time (h)

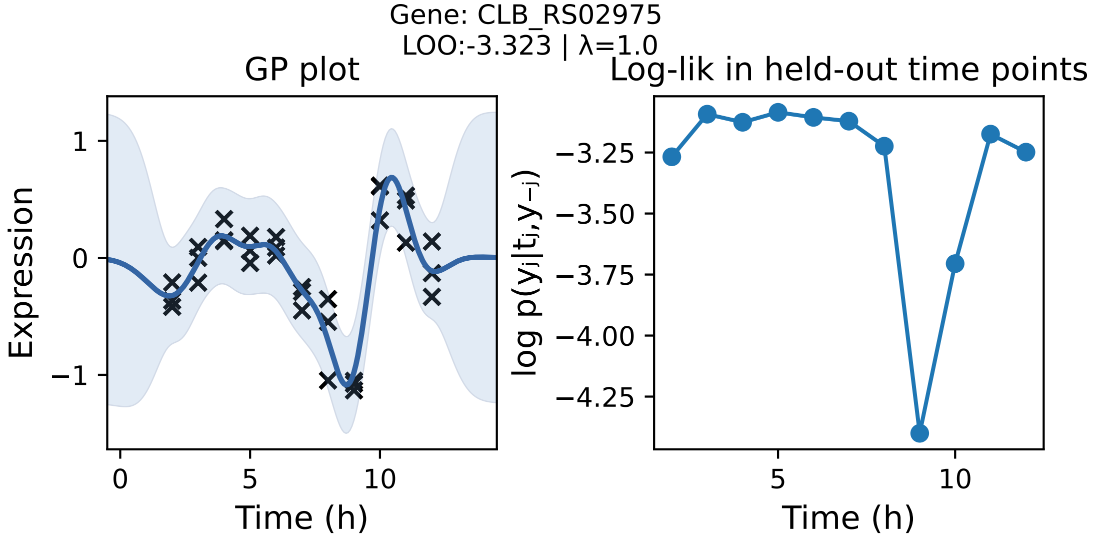

Gene: CLB_RS02975
LOO:-3.323 | λ=1.0
GP plot
Expression
Time (h)
1
0
−1
0
5
10
Log-lik in held-out time points
log p(y_j|t_j,y_−j)
Time (h)
−3.25
−3.50
−3.75
−4.00
−4.25
5
10

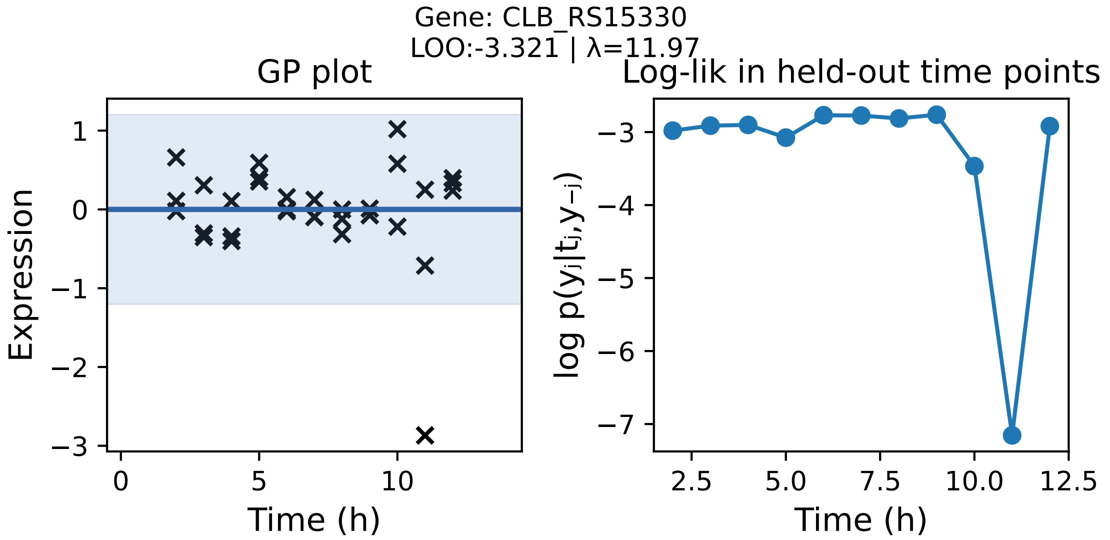

Gene: CLB_RS15330
LOO:-3.321 | λ=11.97
GP plot
Expression
Time (h)
Log-lik in held-out time points
log p(yj|tj,y−j)
Time (h)

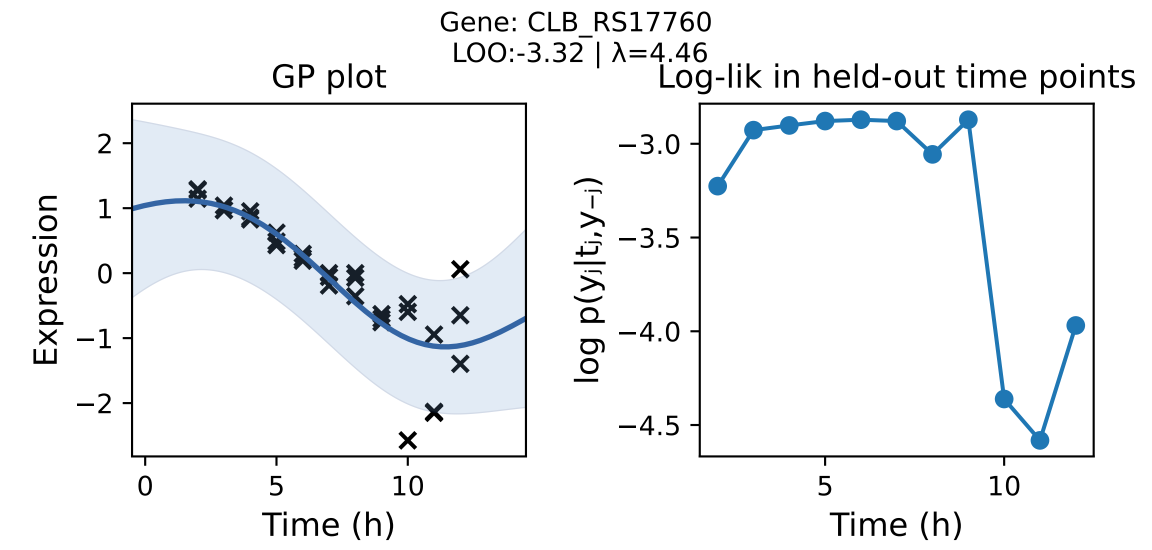

Gene: CLB_RS17760
LOO:-3.32 | λ=4.46
GP plot
Expression
Time (h)
Log-lik in held-out time points
log p(y_j|t_j,y_−j)
Time (h)

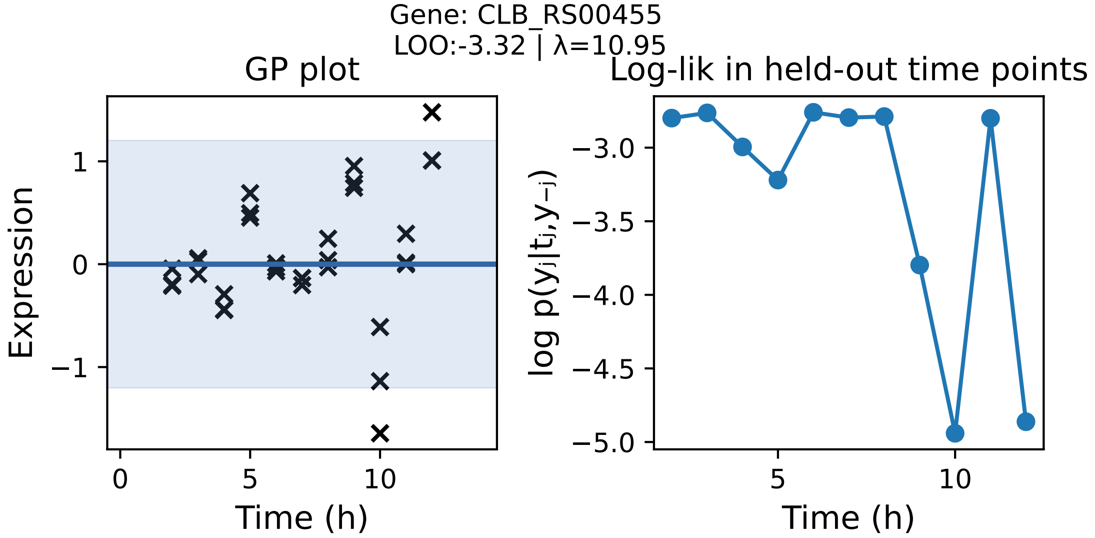

Gene: CLB_RS00455
LOO:-3.32 | λ=10.95
GP plot
Expression
Time (h)
Log-lik in held-out time points
log p(y_j|t_j,y_−j)
Time (h)

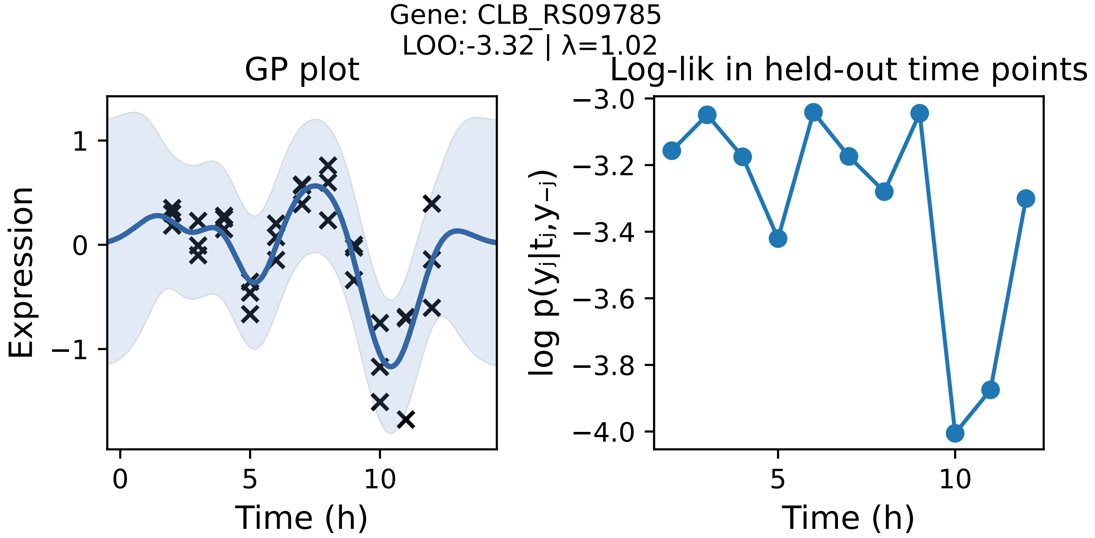

Gene: CLB_RS09785
LOO:-3.32 | λ=1.02
GP plot
Expression
1
0
−1
0
5
10
Time (h)
Log-lik in held-out time points
log p(y_j|t_j,y_−j)
−3.0
−3.2
−3.4
−3.6
−3.8
−4.0
5
10
Time (h)

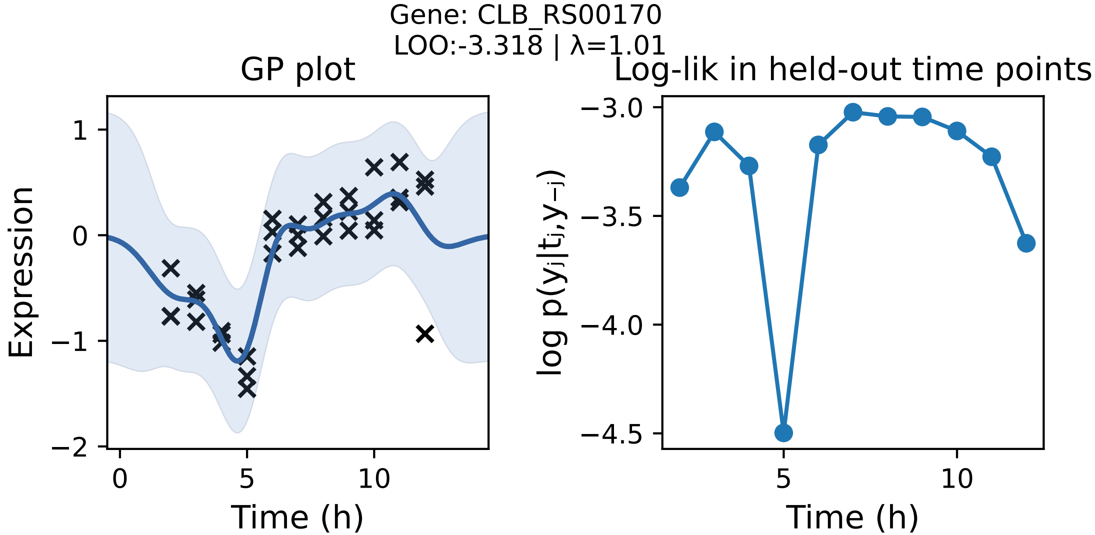

Gene: CLB_RS00170
LOO:-3.318 | λ=1.01
GP plot
Expression
Time (h)
Log-lik in held-out time points
log p(y_j|t_j,y_−j)
Time (h)

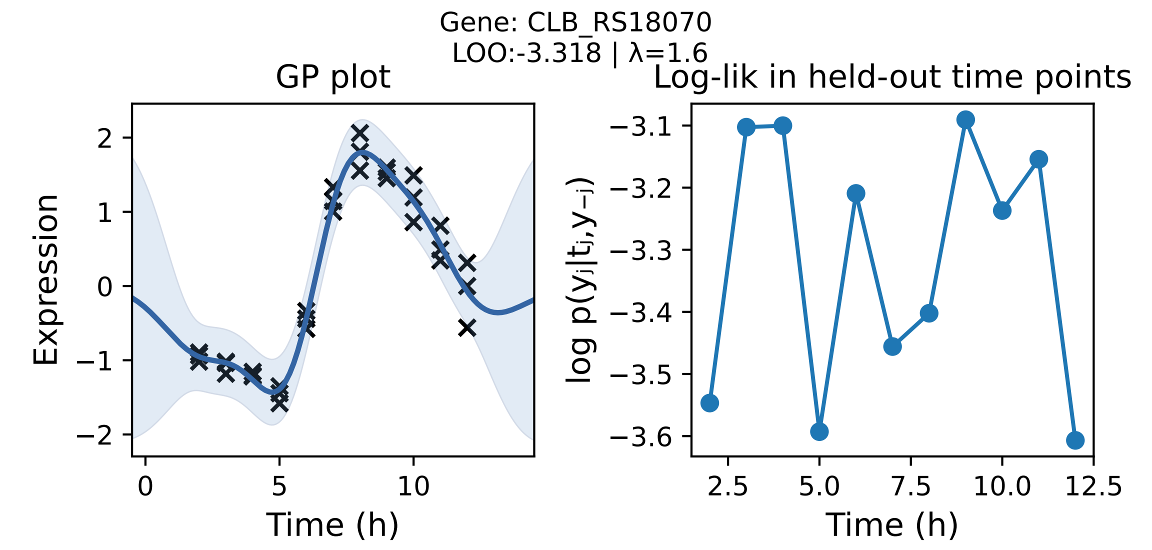

Gene: CLB_RS18070
LOO:-3.318 | λ=1.6
GP plot
Expression
Time (h)
Log-lik in held-out time points
log p(y_j|t_j,y_−j)
Time (h)

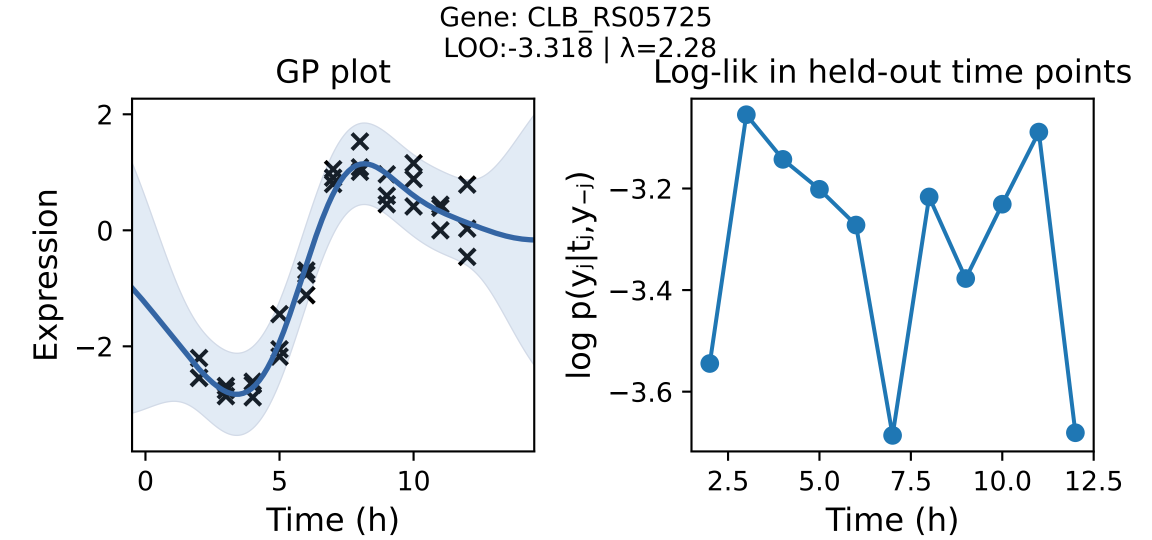

Gene: CLB_RS05725
LOO:-3.318 | λ=2.28
GP plot
Expression
Time (h)
Log-lik in held-out time points
log p(yj|tj,y−j)
Time (h)

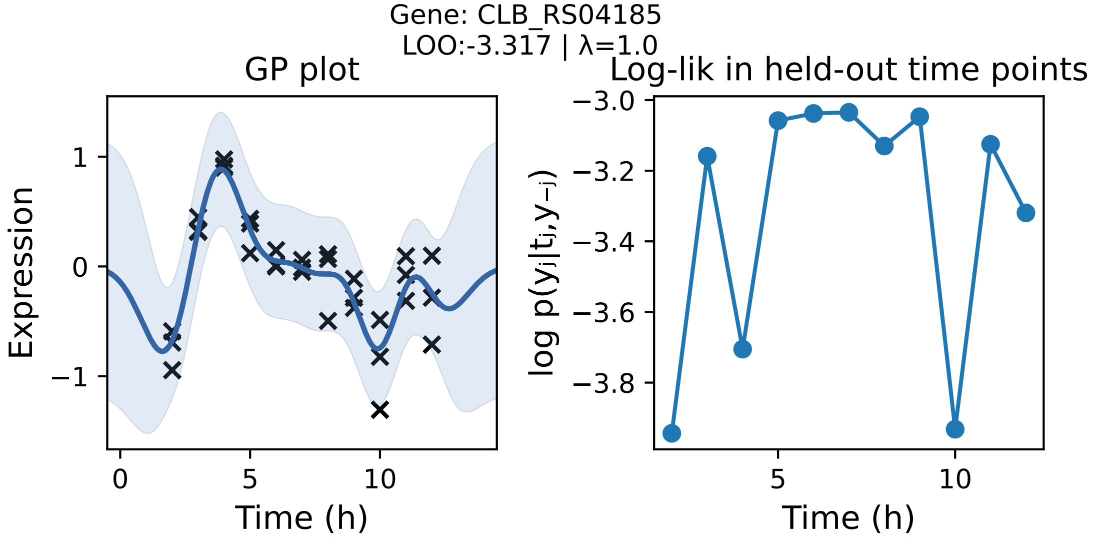

Gene: CLB_RS04185
LOO:-3.317 | λ=1.0
GP plot
Expression
Time (h)
Log-lik in held-out time points
log p(yj|tj,y−j)
Time (h)

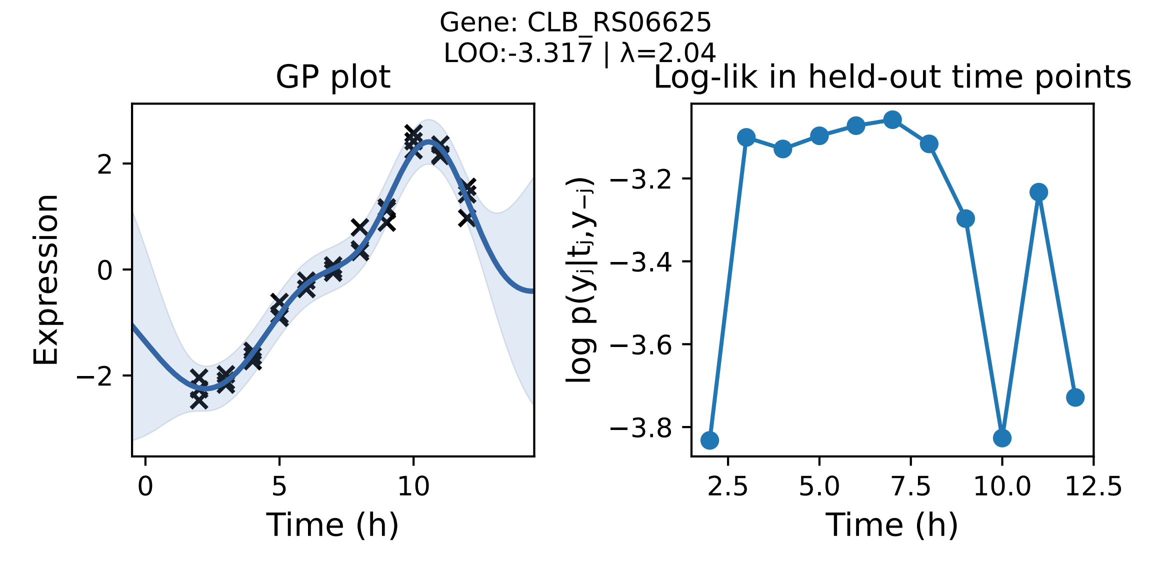
Gene: CLB_RS06625
LOO:-3.317 | λ=2.04
GP plot
Expression
Time (h)
Log-lik in held-out time points
log p(y_j|t_j,y_−j)
Time (h)

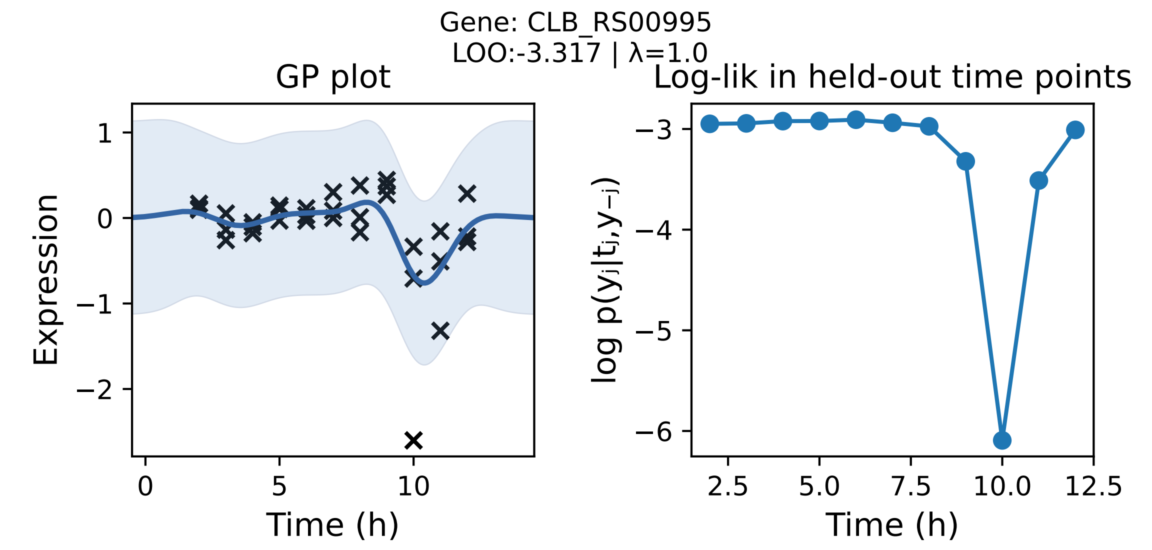

Gene: CLB_RS00995
LOO:-3.317 | λ=1.0
GP plot
Expression
Time (h)
Log-lik in held-out time points
log p(y_j|t_j,y_−j)
Time (h)

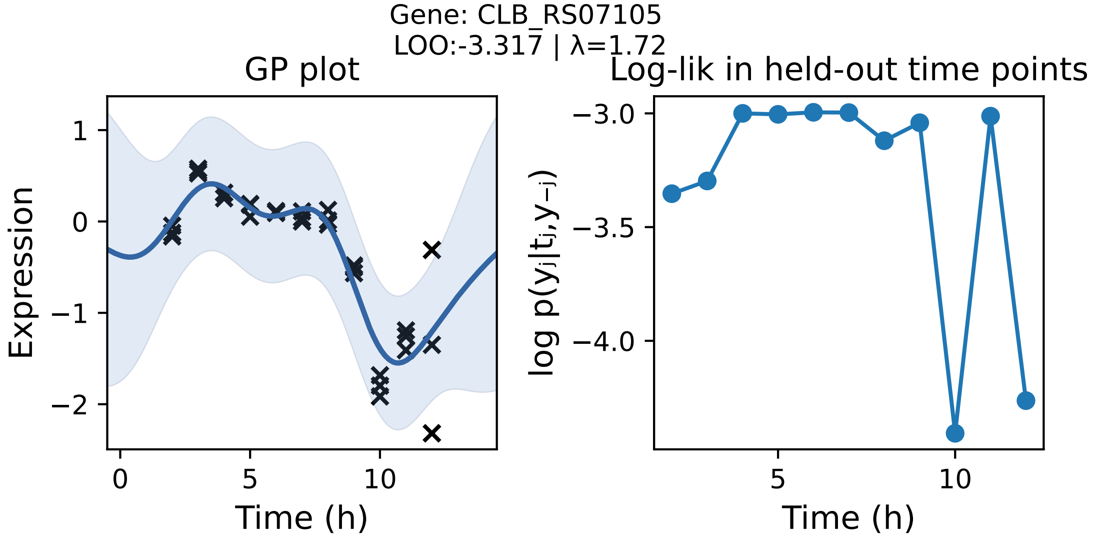
Gene: CLB_RS07105
LOO:-3.317 | λ=1.72
GP plot
Expression
Time (h)
1
0
−1
−2
0
5
10
Log-lik in held-out time points
log p(y_j|t_j,y_−j)
Time (h)
−3.0
−3.5
−4.0
5
10

Gene: CLB_RS08025
LOO:-3.317 | λ=2.47

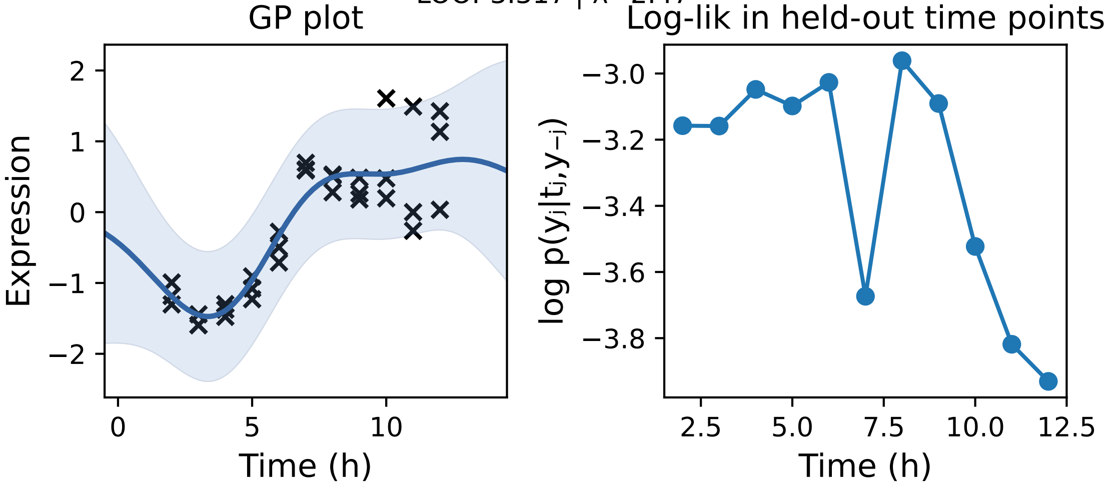

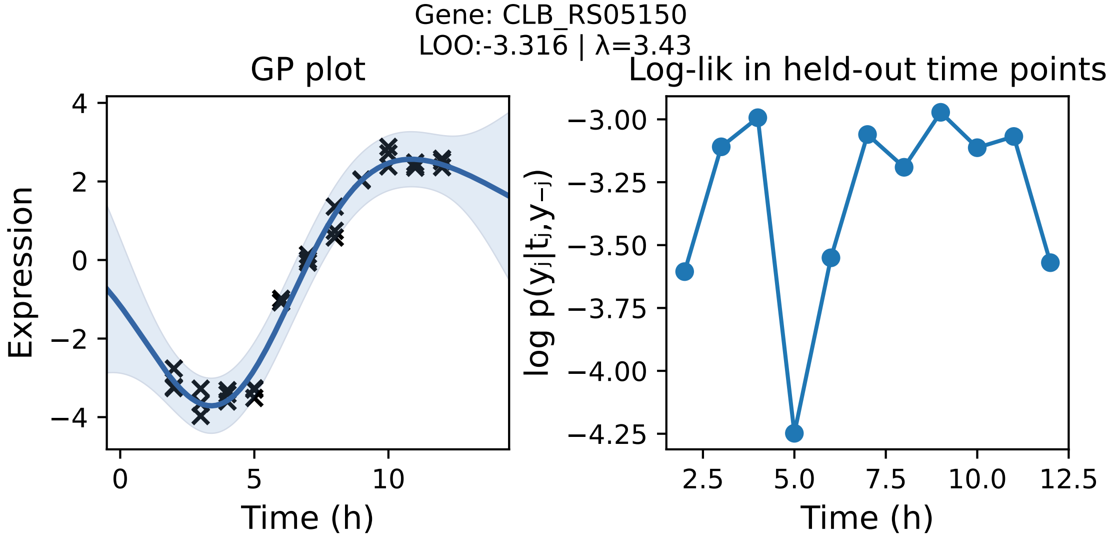
Gene: CLB_RS05150
LOO:-3.316 | λ=3.43
GP plot
Expression
Time (h)
4
2
0
−2
−4
0
5
10
Log-lik in held-out time points
log p(y_j|t_j,y_−j)
−3.00
−3.25
−3.50
−3.75
−4.00
−4.25
2.5
5.0
7.5
10.0
12.5
Time (h)

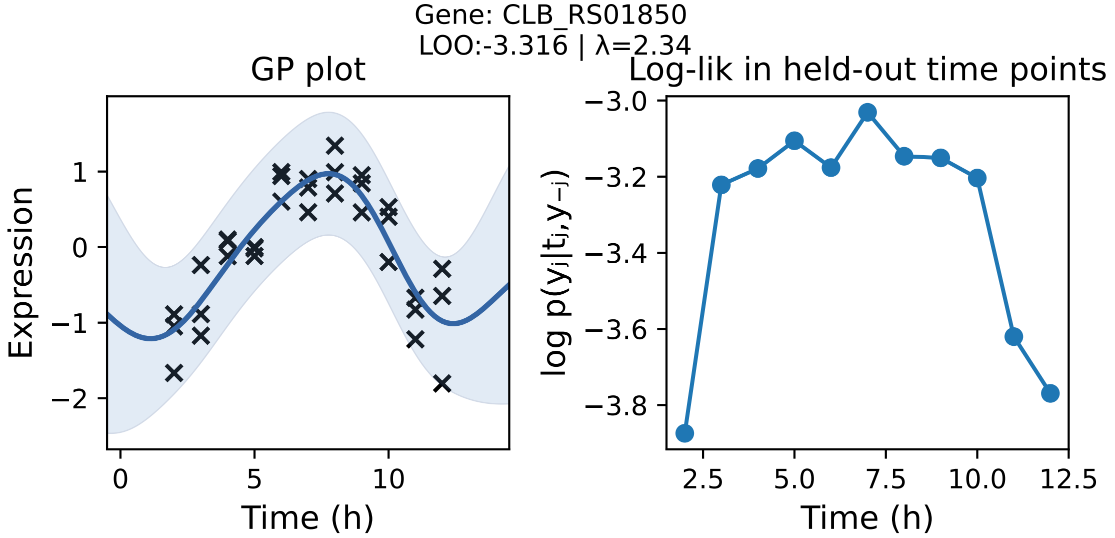

Gene: CLB_RS01850
LOO:-3.316 | λ=2.34
GP plot
Expression
Time (h)
1
0
−1
−2
0
5
10
Log-lik in held-out time points
log p(y_j|t_j,y_−j)
Time (h)
−3.0
−3.2
−3.4
−3.6
−3.8
2.5
5.0
7.5
10.0
12.5

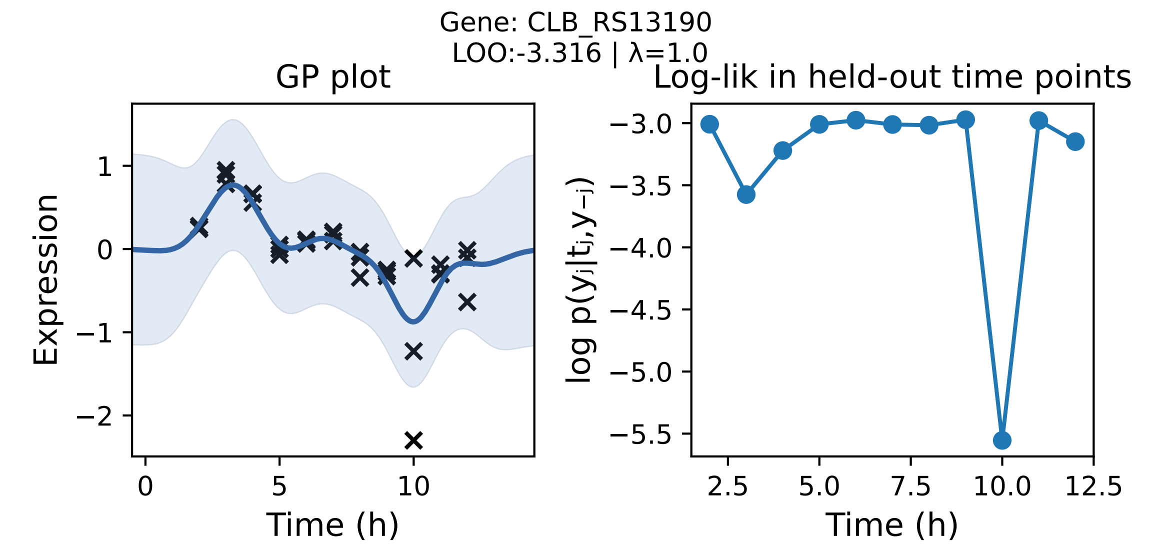
Gene: CLB_RS13190
LOO:-3.316 | λ=1.0
GP plot
Expression
Time (h)
Log-lik in held-out time points
log p(y_j|t_j,y_−j)
Time (h)

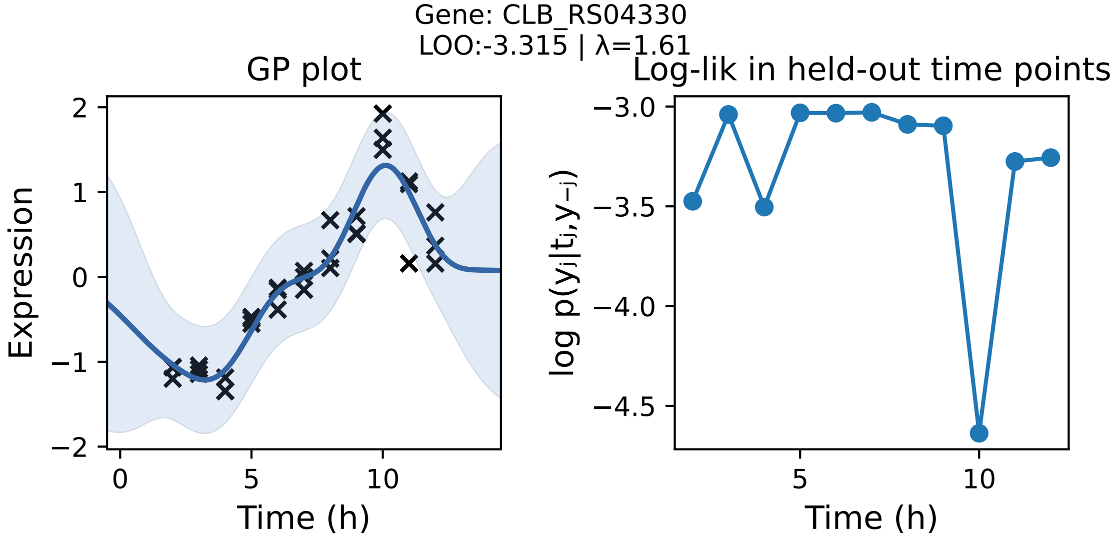

Gene: CLB_RS04330
LOO:-3.315 | λ=1.61
GP plot
Expression
Time (h)
2
1
0
−1
−2
0
5
10
Log-lik in held-out time points
log p(y_j|t_j,y_−j)
Time (h)
−3.0
−3.5
−4.0
−4.5
5
10

Gene: CLB_RS02220
LOO:-3.312 | λ=1.8

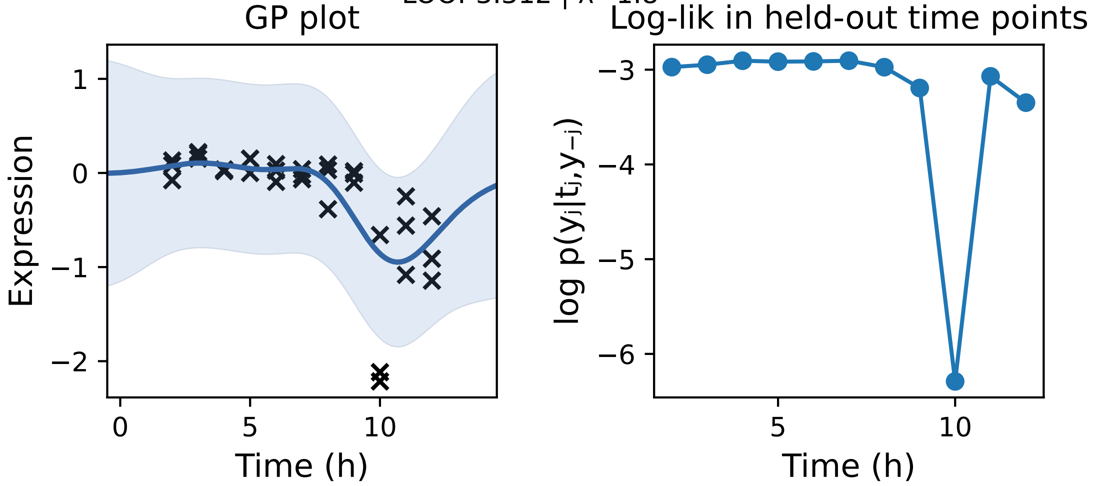

Gene: CLB_RS02550
LOO:-3.312 | λ=2.09

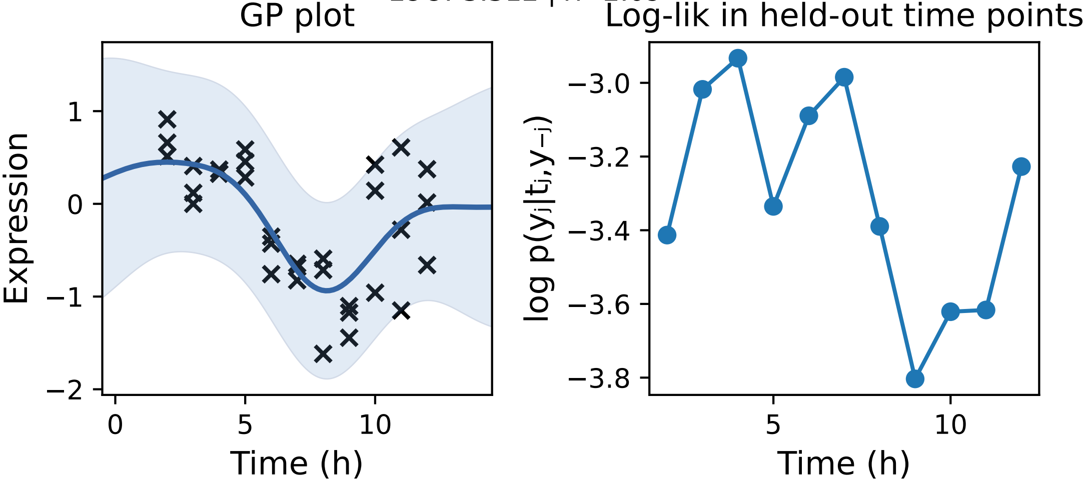

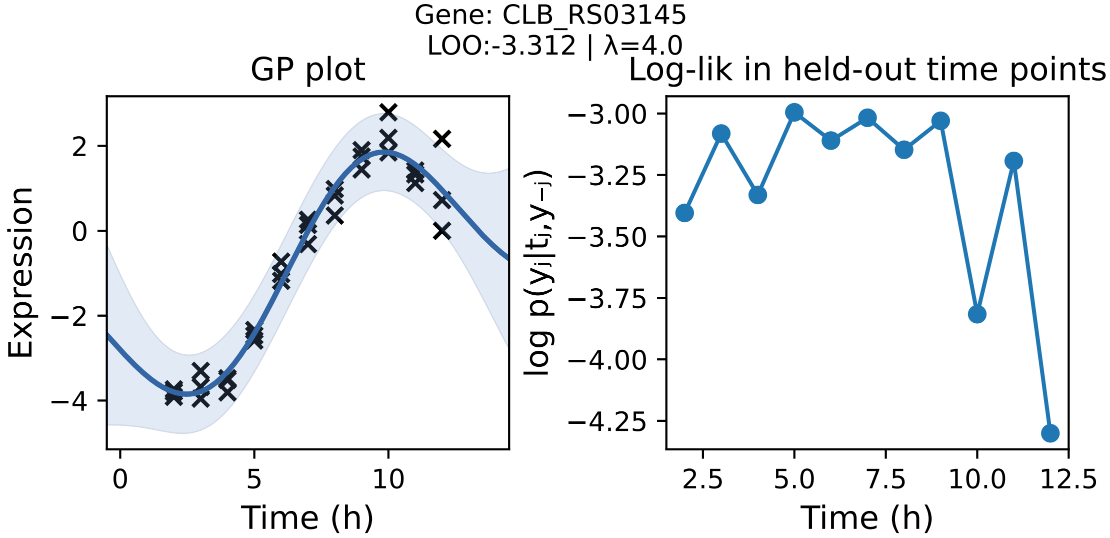
Gene: CLB_RS03145
LOO:-3.312 | λ=4.0
GP plot
Expression
Time (h)
Log-lik in held-out time points
log p(yj|tj,y−j)
Time (h)

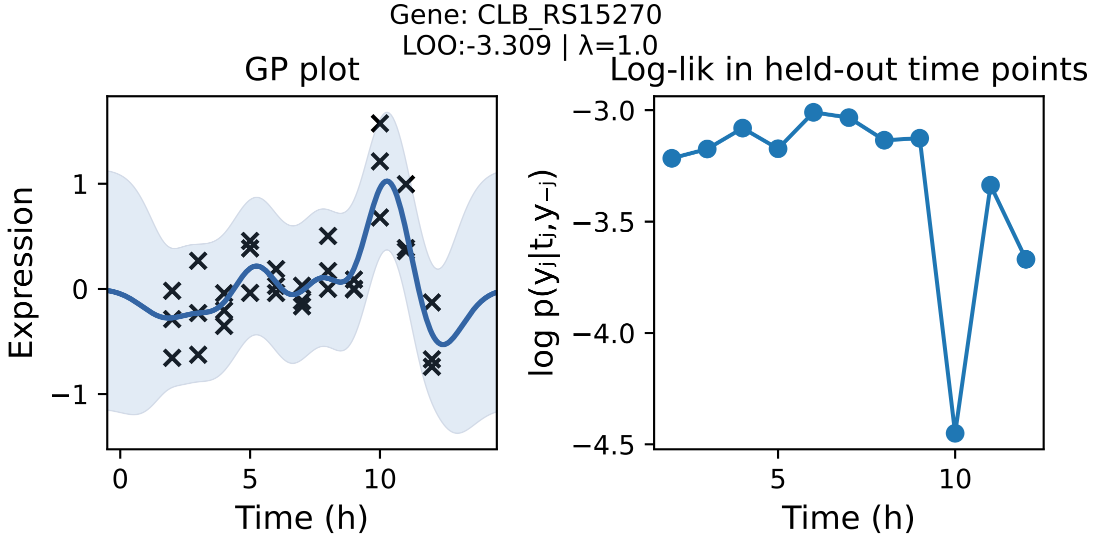

Gene: CLB_RS15270
LOO:-3.309 | λ=1.0
GP plot
Expression
1
0
−1
0
5
10
Time (h)
Log-lik in held-out time points
log p(yj|tj,y−j)
−3.0
−3.5
−4.0
−4.5
5
10
Time (h)

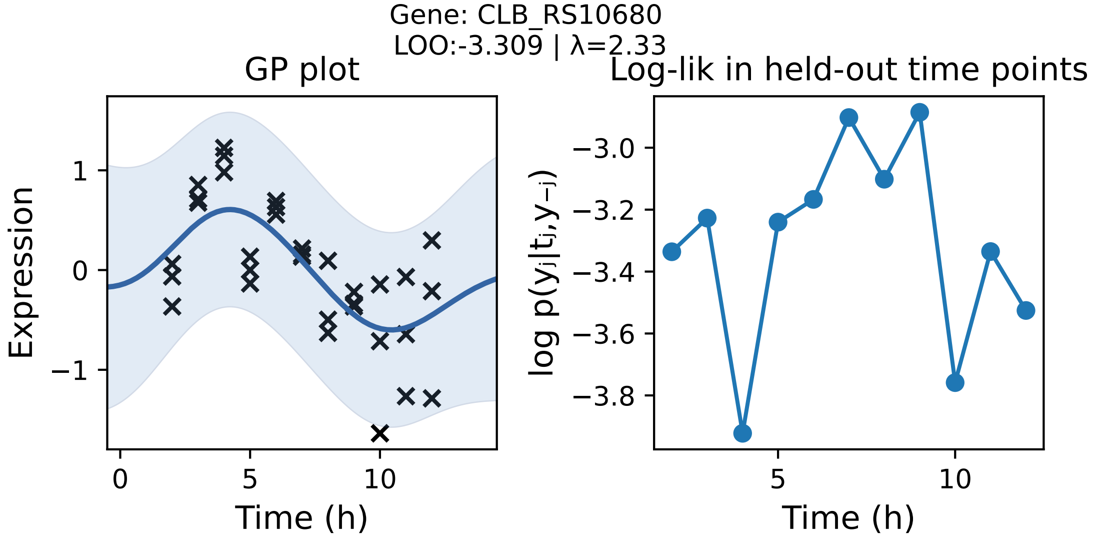

Gene: CLB_RS10680
LOO:-3.309 | λ=2.33
GP plot
Expression
1
0
−1
0
5
10
Time (h)
Log-lik in held-out time points
log p(y_j|t_j,y_−j)
−3.0
−3.2
−3.4
−3.6
−3.8
5
10
Time (h)

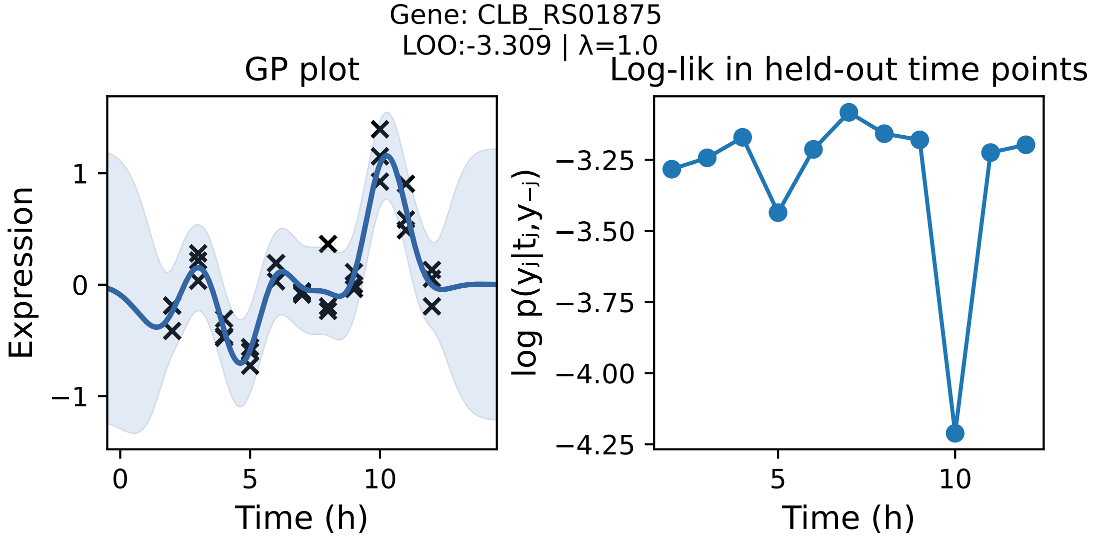

Gene: CLB_RS01875
LOO:-3.309 | λ=1.0
GP plot
Log-lik in held-out time points
Expression
1
0
−1
0
5
10
Time (h)
log p(yⱼ|tⱼ,y₋ⱼ)
−3.25
−3.50
−3.75
−4.00
−4.25
5
10
Time (h)

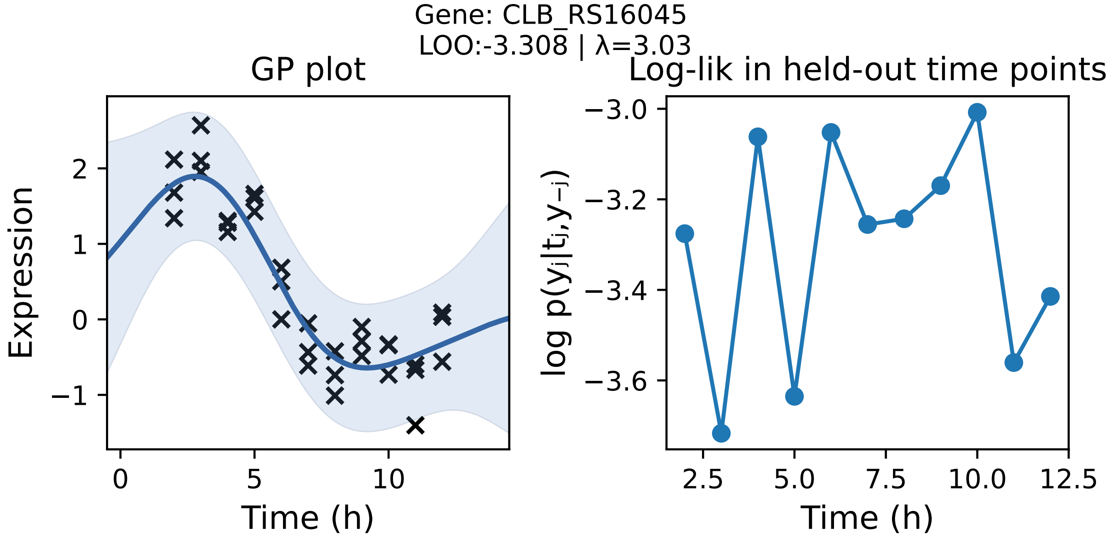

Gene: CLB_RS16045
LOO:-3.308 | λ=3.03
GP plot
Expression
Time (h)
Log-lik in held-out time points
log p(y_j|t_j,y_−j)
Time (h)

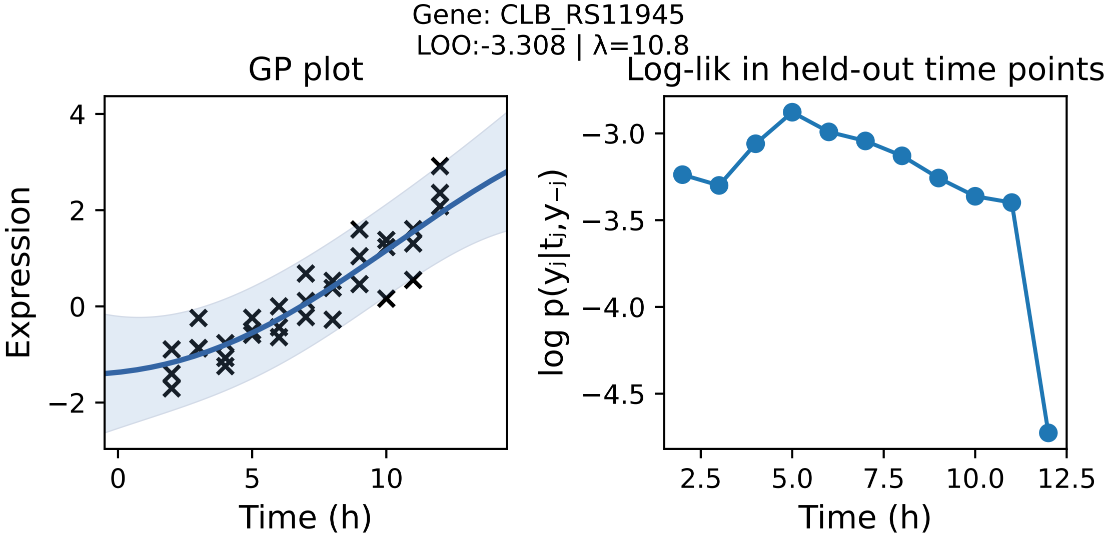
Gene: CLB_RS11945
LOO:-3.308 | λ=10.8
GP plot
Expression
Time (h)
4
2
0
−2
0
5
10
Log-lik in held-out time points
log p(y_j|t_j,y_−j)
Time (h)
−3.0
−3.5
−4.0
−4.5
2.5
5.0
7.5
10.0
12.5

Gene: CLB_RS03190
LOO:-3.306 | λ=1.0

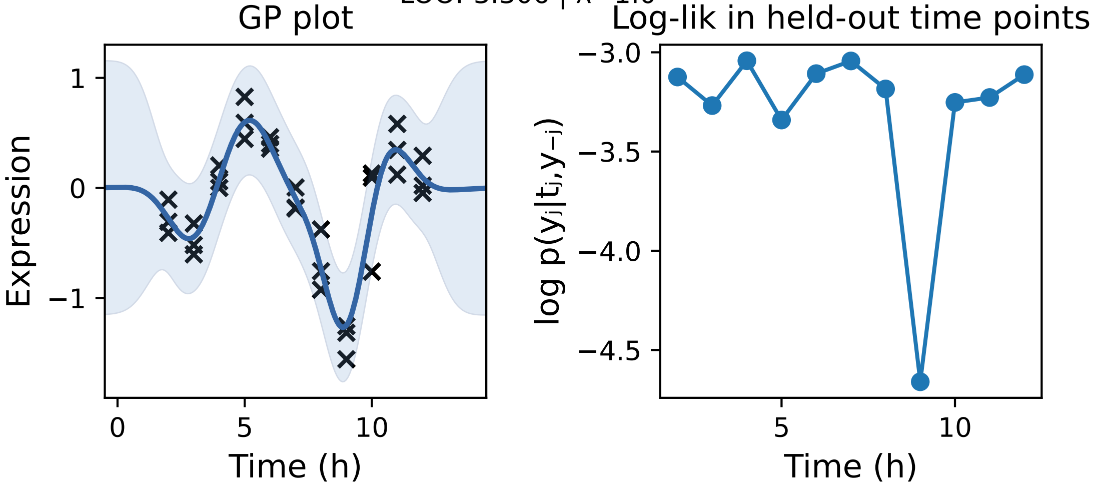

Gene: CLB_RS00215
LOO:-3.306 | λ=1.0

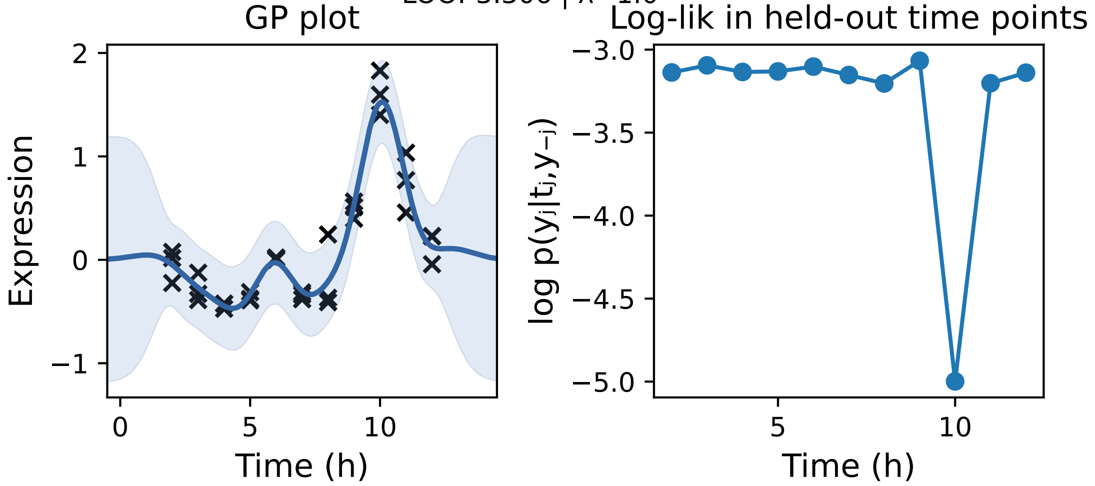

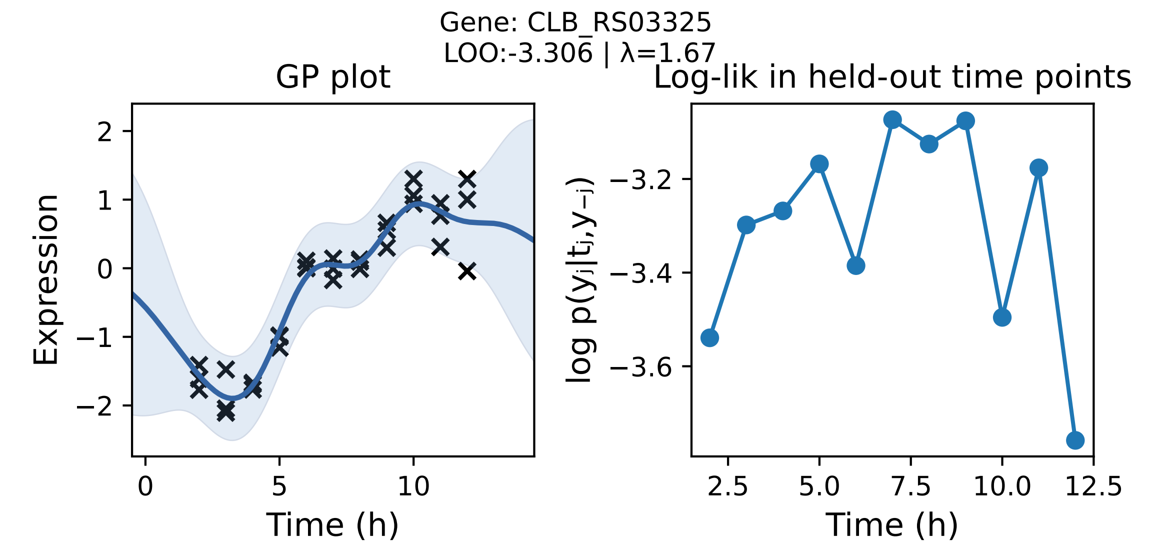

Gene: CLB_RS03325
LOO:-3.306 | λ=1.67
GP plot
Expression
Time (h)
Log-lik in held-out time points
log p(yj|tj,y−j)
Time (h)

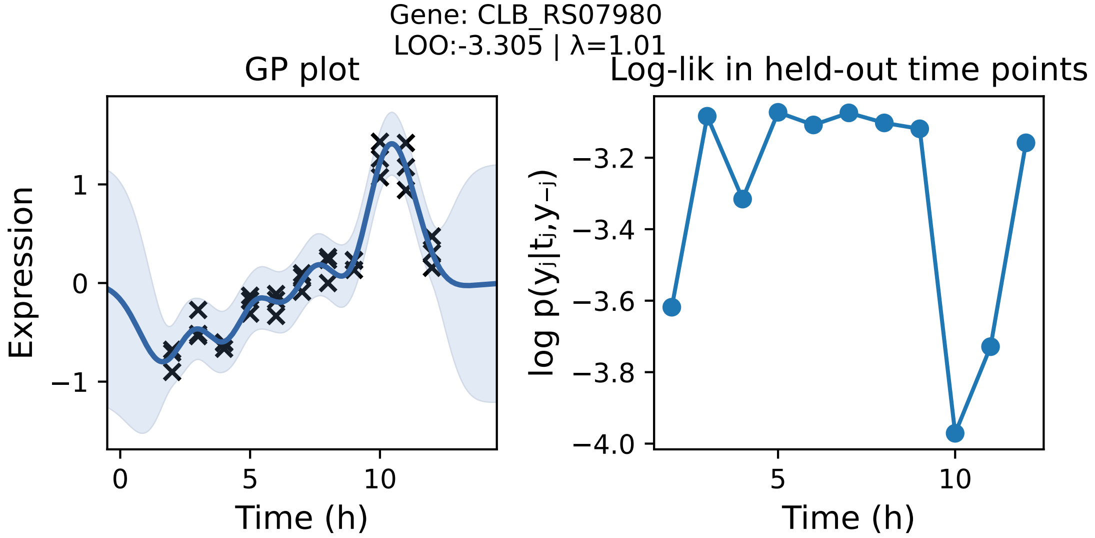

Gene: CLB_RS07980
LOO:-3.305 | λ=1.01
GP plot
Expression
Time (h)
Log-lik in held-out time points
log p(y_j|t_j,y_−j)
Time (h)

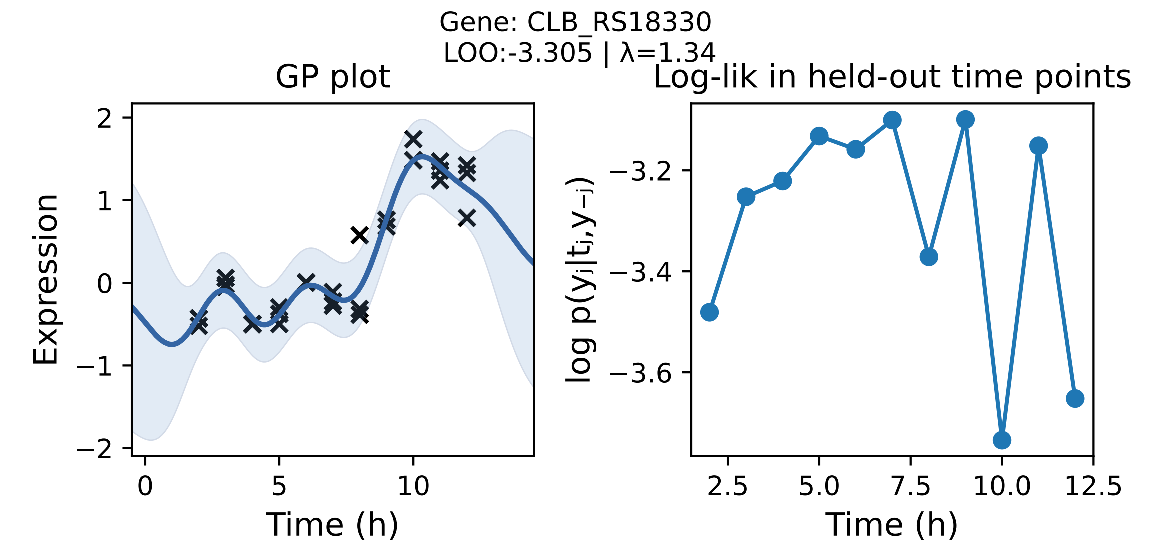

Gene: CLB_RS18330
LOO:-3.305 | λ=1.34
GP plot
Expression
Time (h)
Log-lik in held-out time points
log p(y_j|t_j,y_−j)
Time (h)

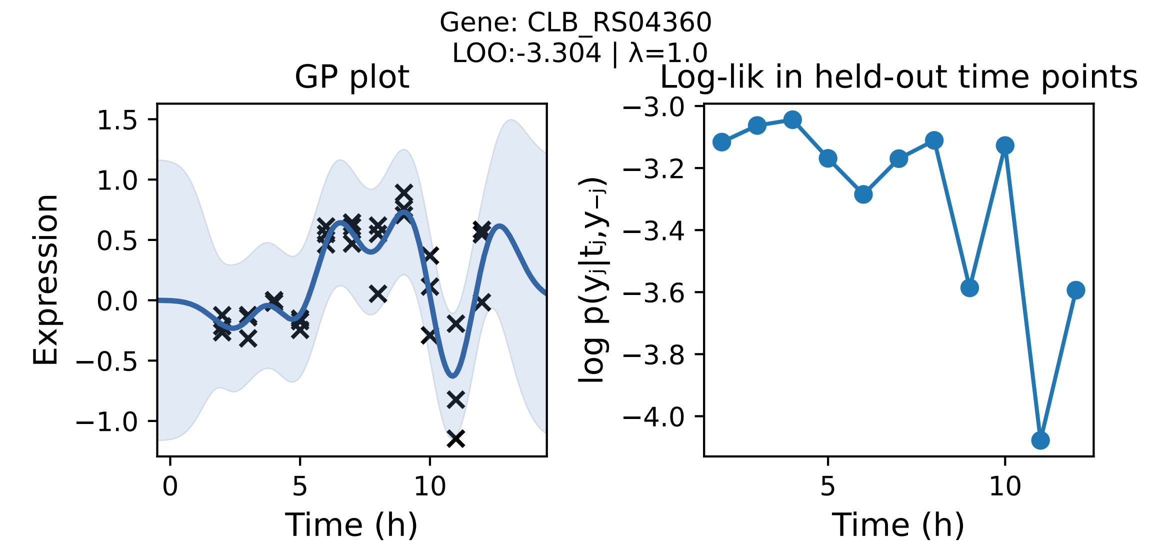

Gene: CLB_RS04360
LOO:-3.304 | λ=1.0
GP plot
Expression
Time (h)
Log-lik in held-out time points
log p(y_j|t_j,y_−j)
Time (h)

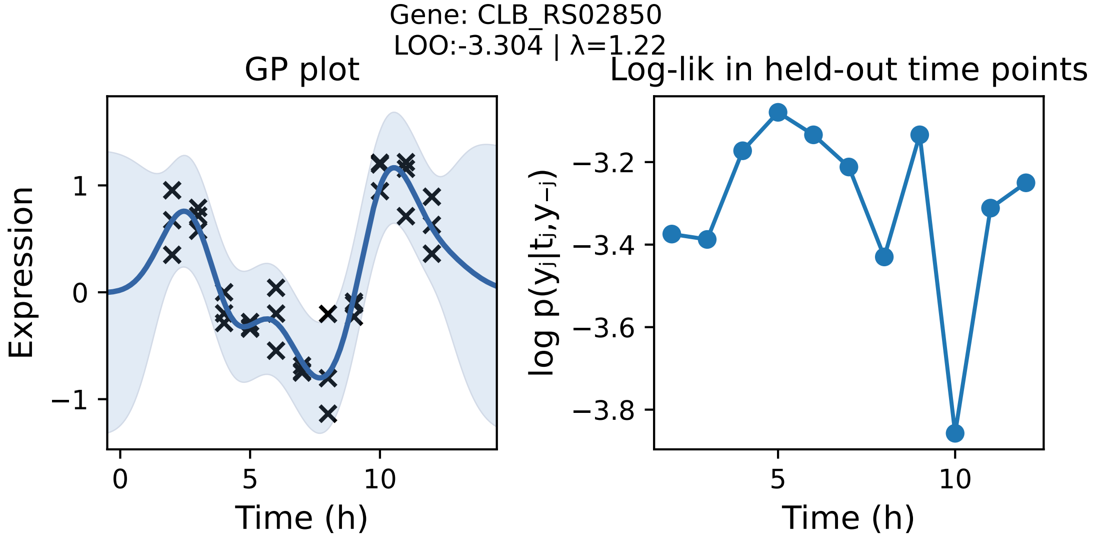

Gene: CLB_RS02850
LOO:-3.304 | λ=1.22
GP plot
Log-lik in held-out time points
Expression
1
0
−1
0
5
10
Time (h)
log p(yj|tj,y−j)
−3.2
−3.4
−3.6
−3.8
5
10
Time (h)

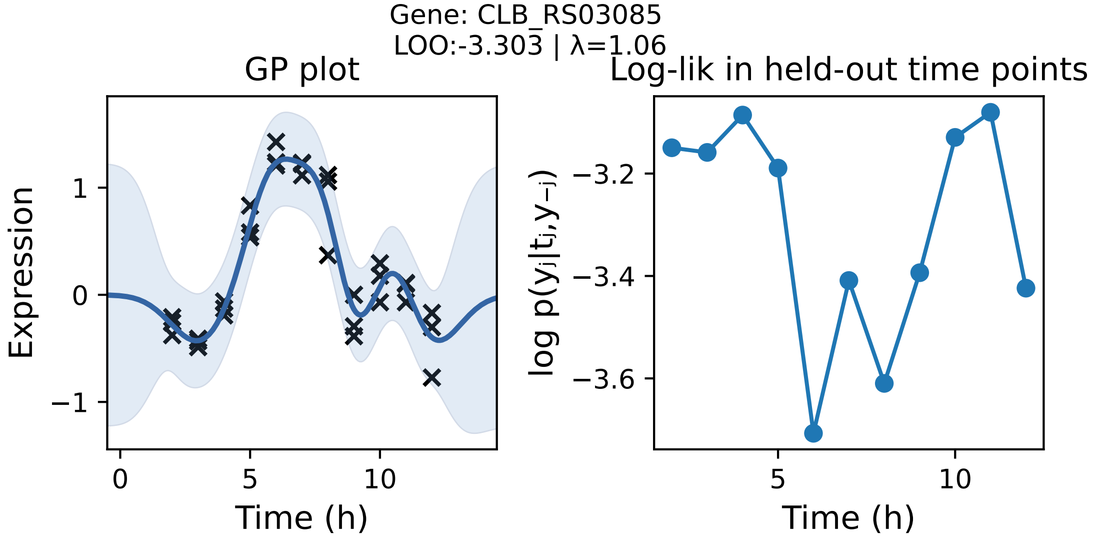

Gene: CLB_RS03085
LOO:-3.303 | λ=1.06
GP plot
Expression
Time (h)
Log-lik in held-out time points
log p(y_j|t_j,y_−j)
Time (h)

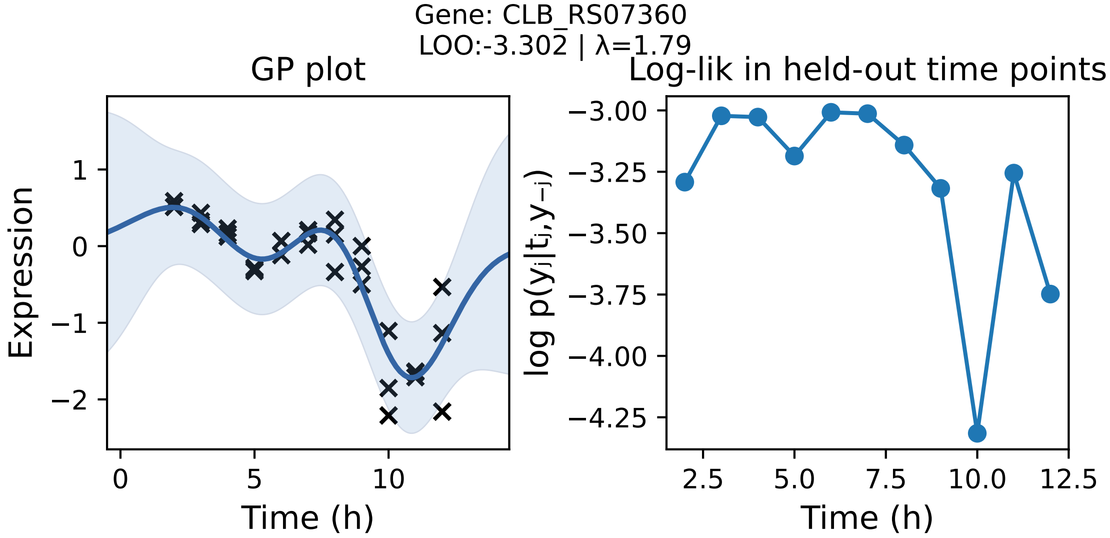

Gene: CLB_RS07360
LOO:-3.302 | λ=1.79
GP plot
Expression
Time (h)
Log-lik in held-out time points
log p(y_j|t_j,y_−j)
Time (h)

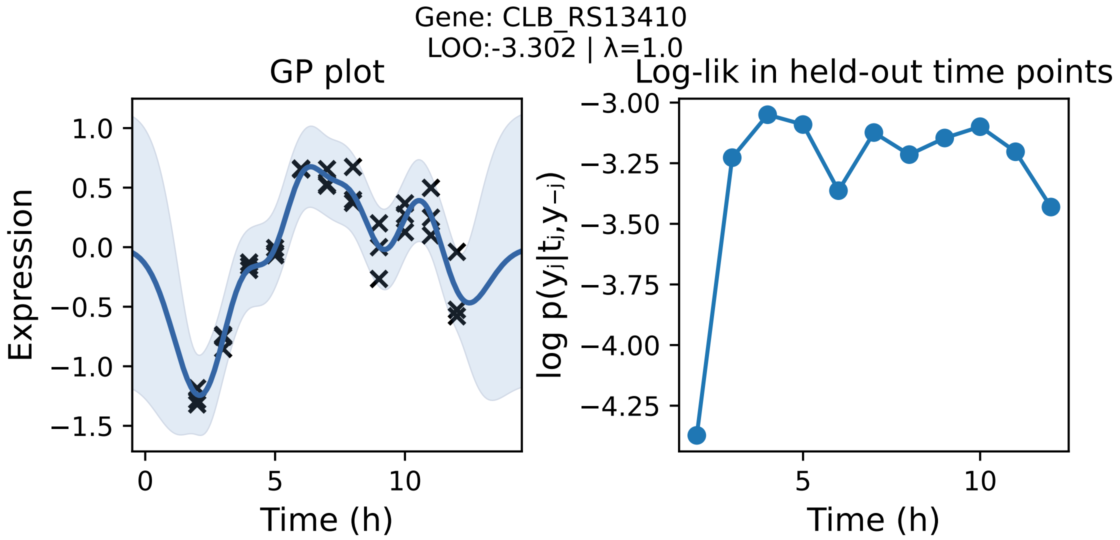

Gene: CLB_RS13410
LOO:-3.302 | λ=1.0
GP plot
Expression
Time (h)
1.0
0.5
0.0
−0.5
−1.0
−1.5
0
5
10
Log-lik in held-out time points
log p(y_j|t_j,y_−j)
Time (h)
−3.00
−3.25
−3.50
−3.75
−4.00
−4.25
5
10

Gene: CLB_RS05210
LOO:-3.299 | λ=2.31

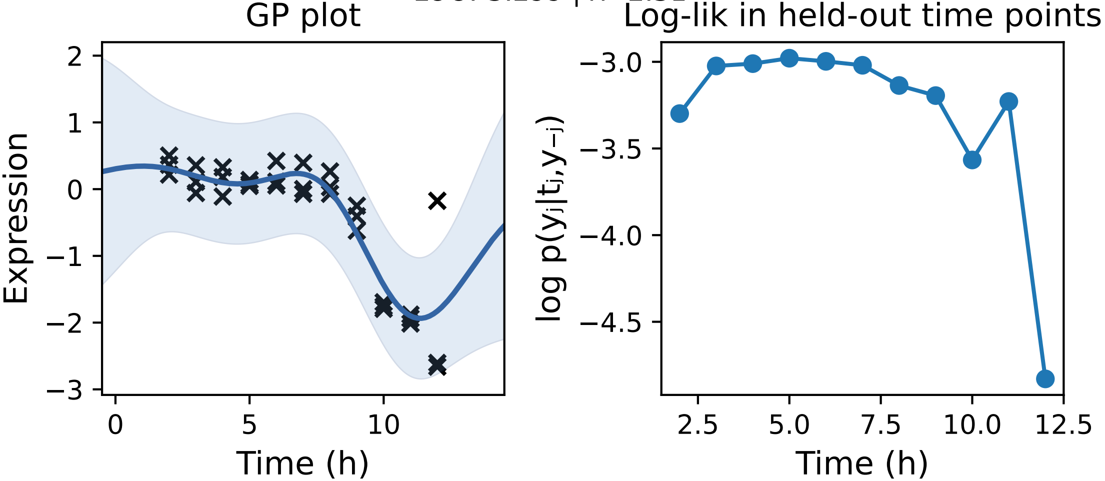

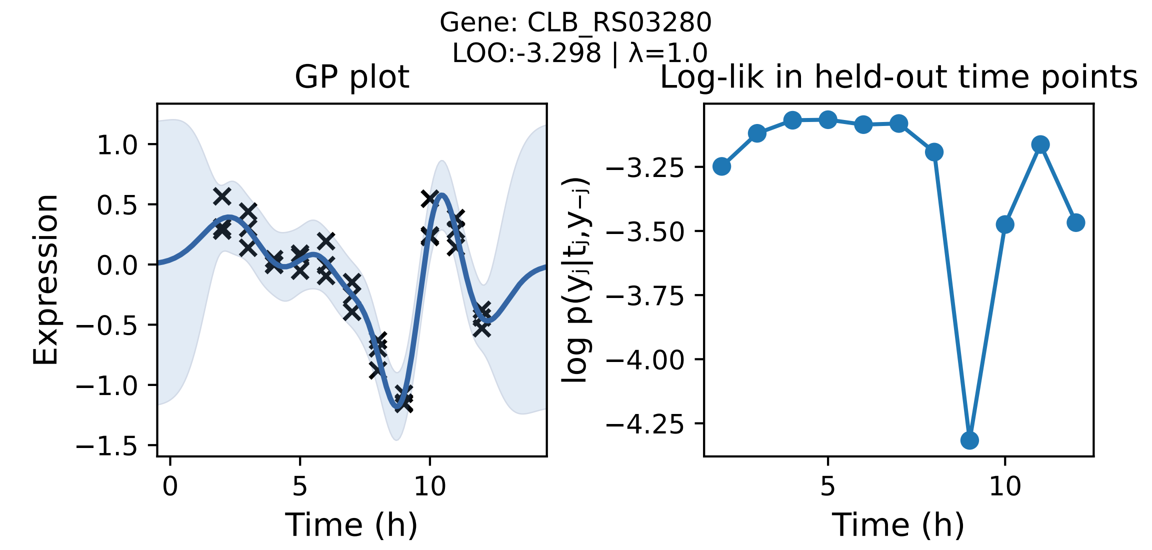

Gene: CLB_RS03280
LOO:-3.298 | λ=1.0
GP plot
Expression
1.0
0.5
0.0
−0.5
−1.0
−1.5
0
5
10
Time (h)
Log-lik in held-out time points
log p(yⱼ|tⱼ,y₋ⱼ)
−3.25
−3.50
−3.75
−4.00
−4.25
5
10
Time (h)

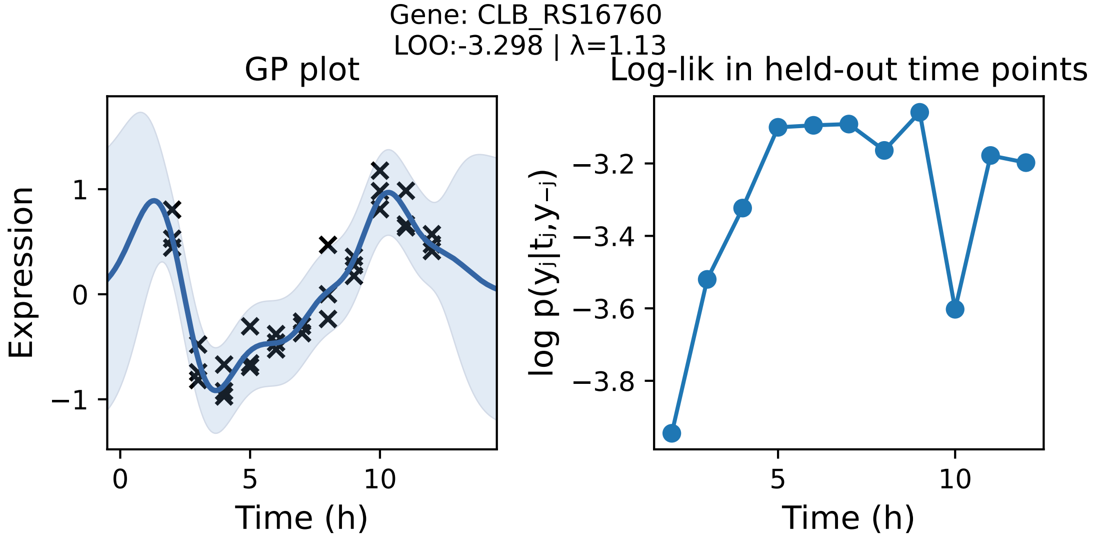

Gene: CLB_RS16760
LOO:-3.298 | λ=1.13
GP plot
Expression
Time (h)
Log-lik in held-out time points
log p(y_j|t_j,y_−j)
Time (h)

Gene: CLB_RS14340
LOO:-3.297 | λ=1.0

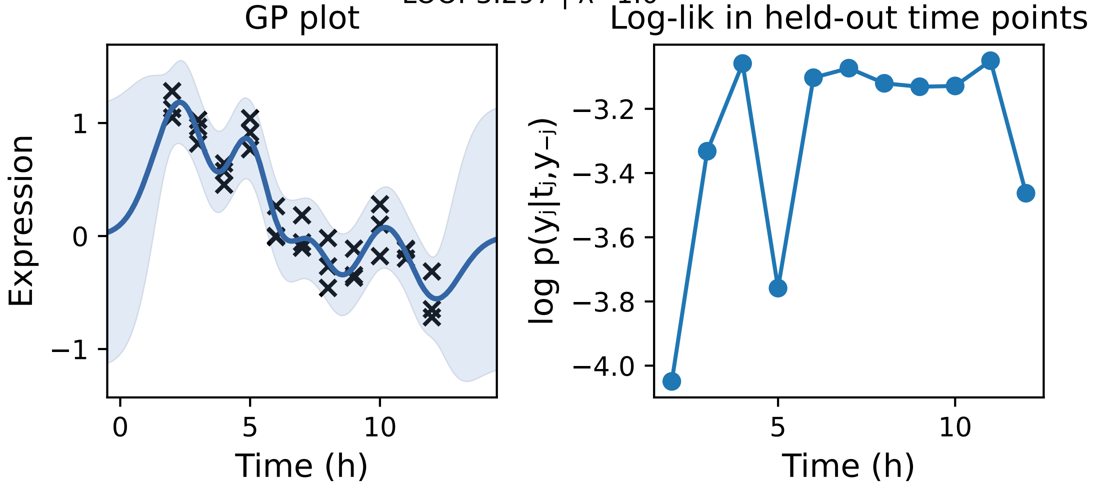

Gene: CLB_RS02335
LOO:-3.297 | λ=2.39

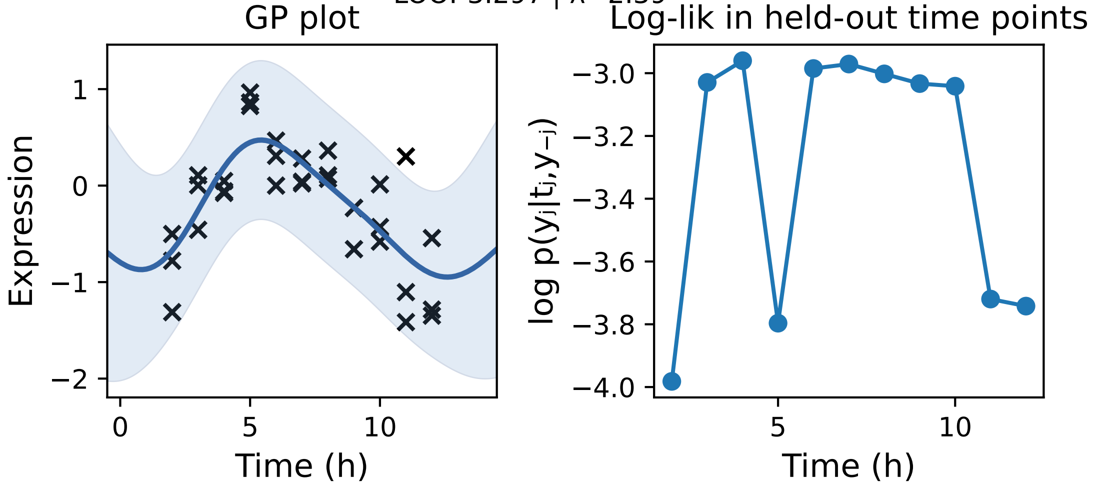

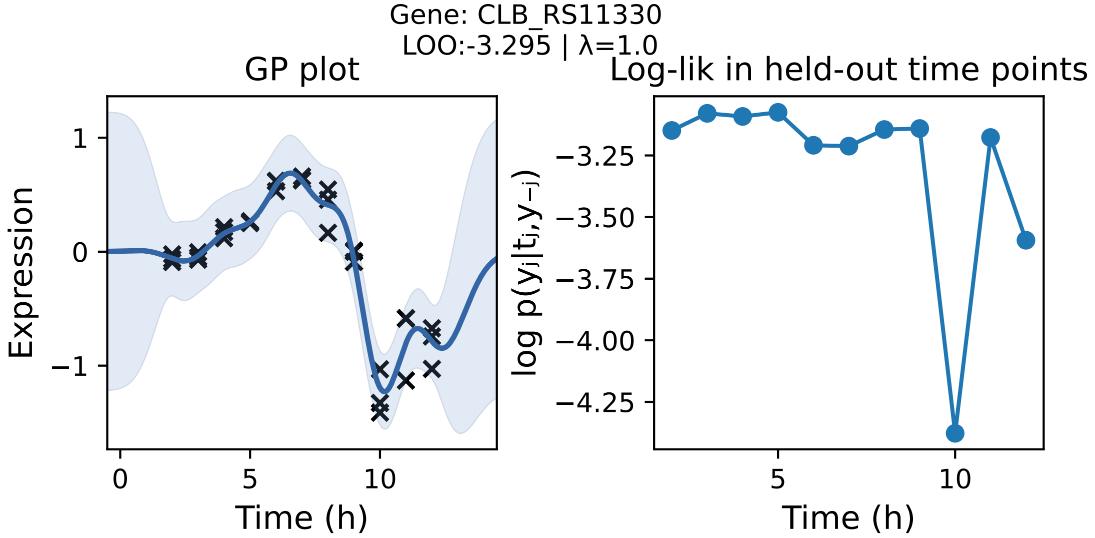
Gene: CLB_RS11330
LOO:-3.295 | λ=1.0
GP plot
Log-lik in held-out time points
Expression
1
0
−1
0
5
10
Time (h)
log p(yⱼ|tⱼ,y₋ⱼ)
−3.25
−3.50
−3.75
−4.00
−4.25
5
10
Time (h)

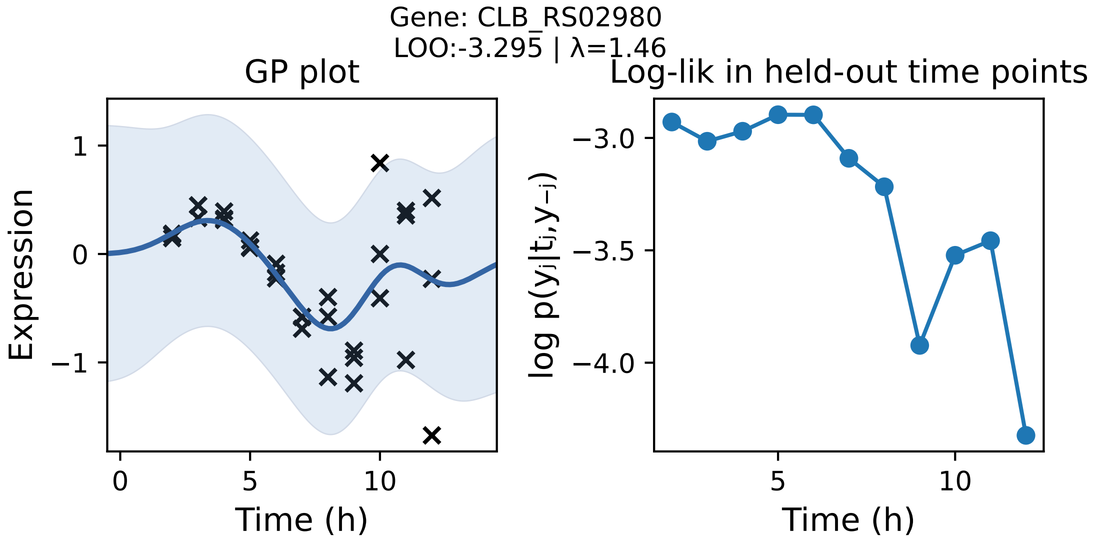

Gene: CLB_RS02980
LOO:-3.295 | λ=1.46
GP plot
Log-lik in held-out time points
Expression
Time (h)
log p(y_j|t_j,y_−j)
1
0
−1
0
5
10
−3.0
−3.5
−4.0

Gene: CLB_RS08470
LOO:-3.292 | λ=1.82

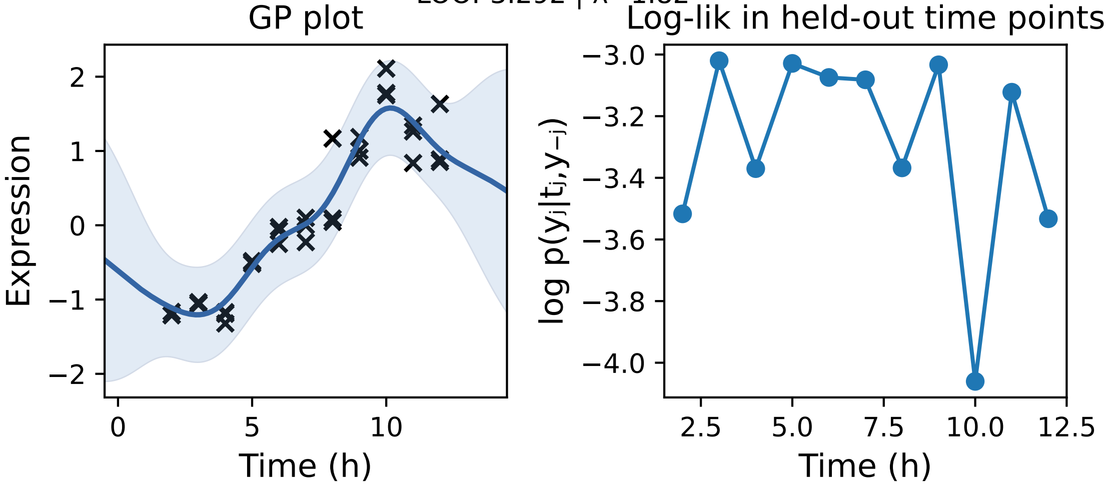

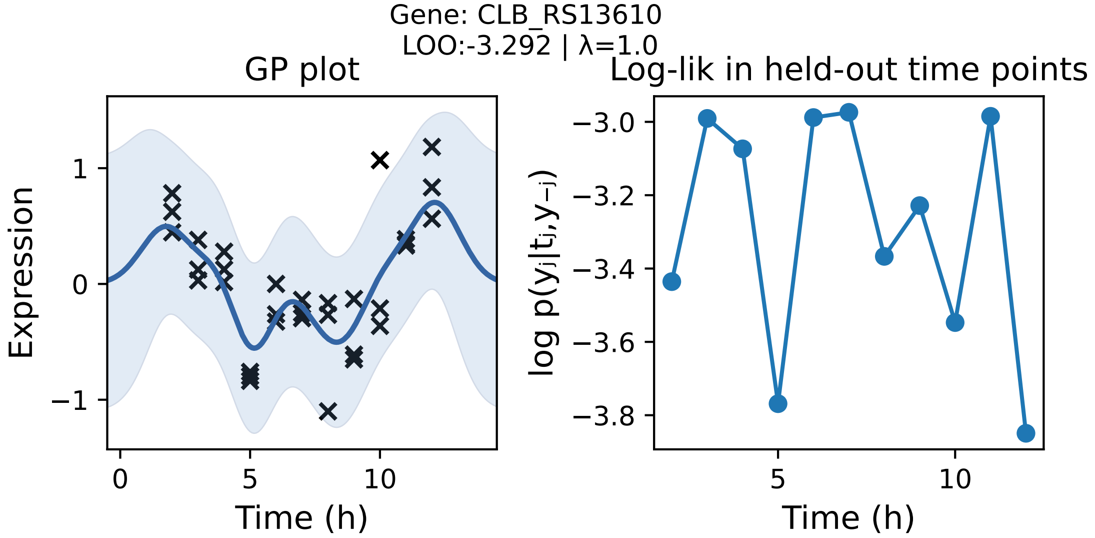

Gene: CLB_RS13610
LOO:-3.292 | λ=1.0
GP plot
Log-lik in held-out time points
Expression
Time (h)
log p(y_j|t_j,y_−j)
1
0
−1
0
5
10
−3.0
−3.2
−3.4
−3.6
−3.8

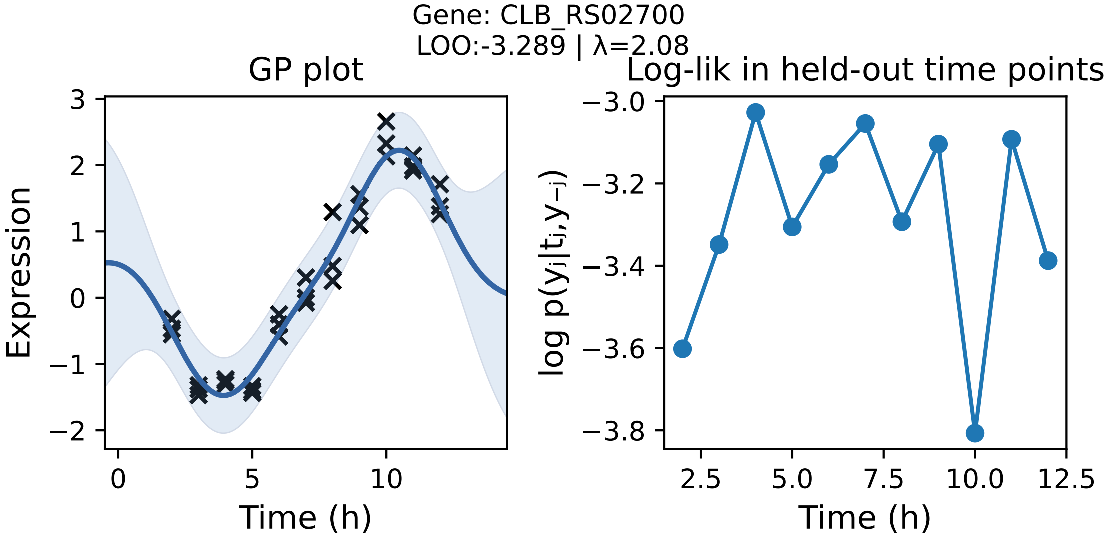

Gene: CLB_RS02700
LOO:-3.289 | λ=2.08
GP plot
Expression
Time (h)
3
2
1
0
−1
−2
0
5
10
Log-lik in held-out time points
$\log p(y_j|t_j, y_{-j})$
−3.0
−3.2
−3.4
−3.6
−3.8
2.5
5.0
7.5
10.0
12.5
Time (h)

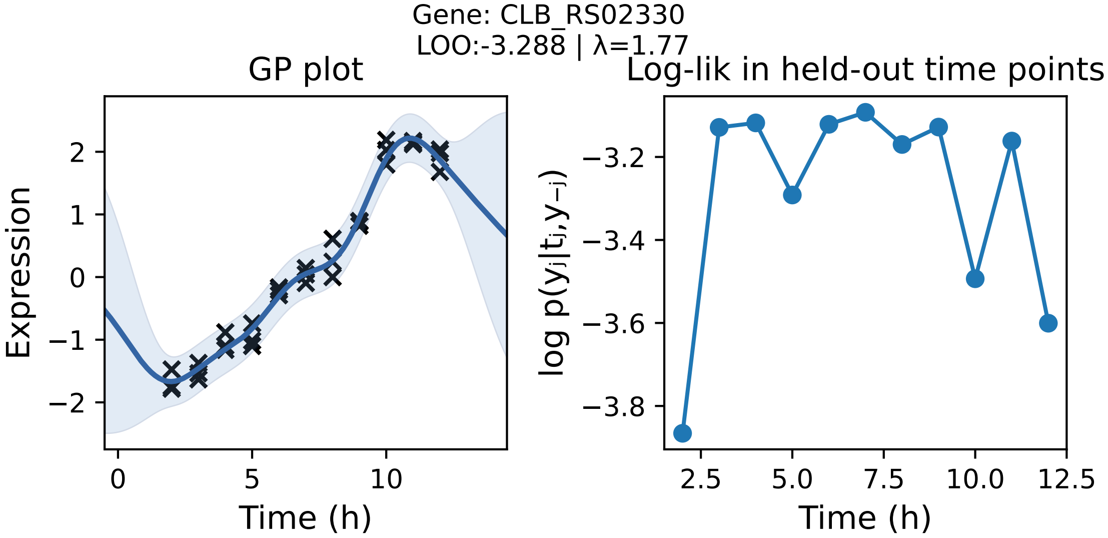
Gene: CLB_RS02330
LOO:-3.288 | λ=1.77
GP plot
Expression
Time (h)
Log-lik in held-out time points
log p(y_j|t_j,y_{-j})
Time (h)

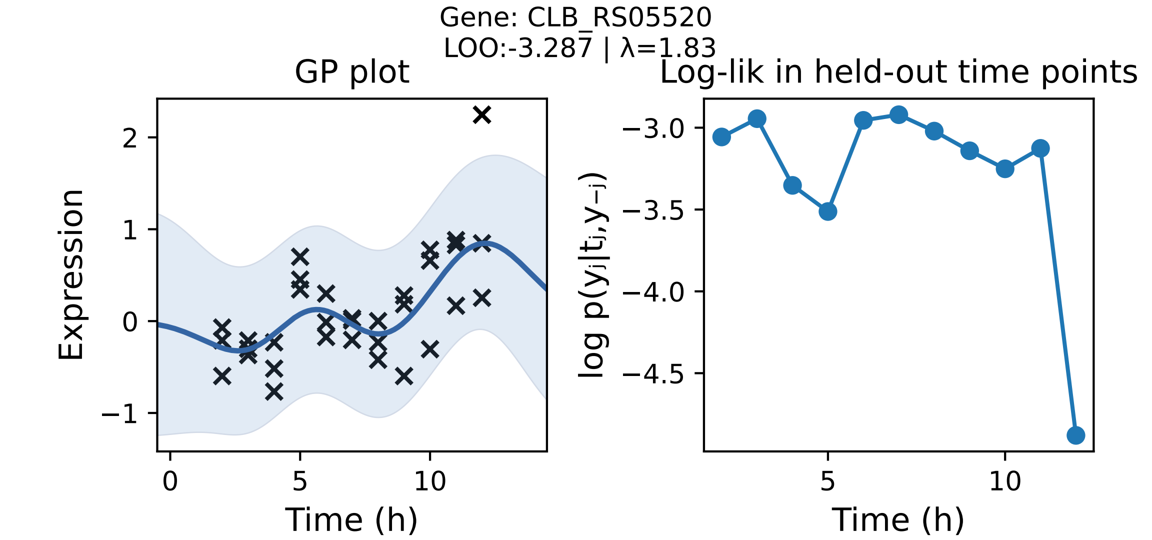

Gene: CLB_RS05520
LOO:-3.287 | λ=1.83
GP plot
Expression
Time (h)
Log-lik in held-out time points
log p(yj|tj,y−j)
Time (h)

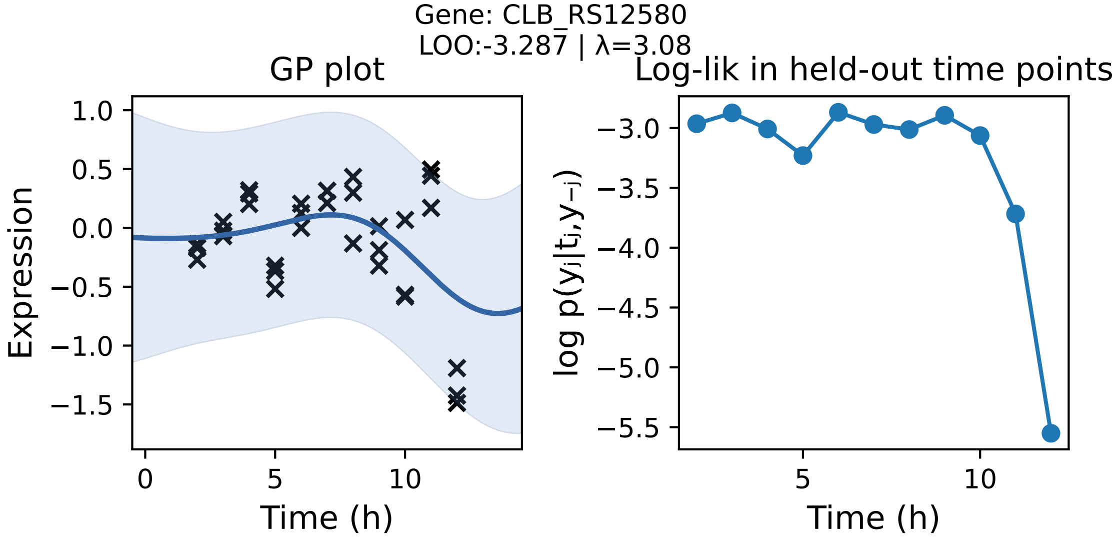

Gene: CLB_RS12580
LOO:-3.287 | λ=3.08
GP plot
Expression
Time (h)
Log-lik in held-out time points
log p(y_j|t_j,y_−j)
Time (h)

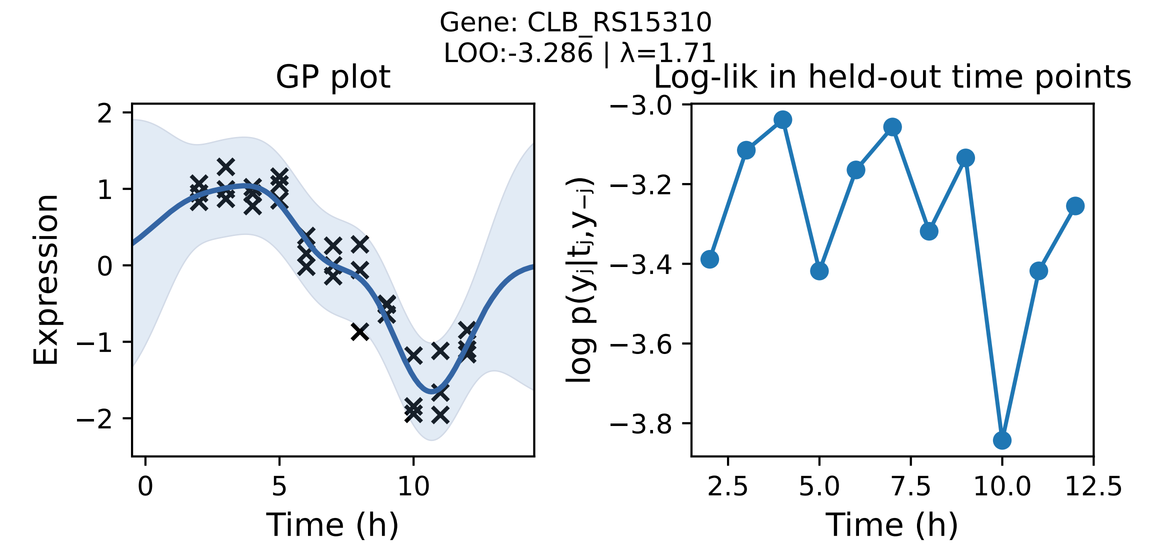

Gene: CLB_RS15310
LOO:-3.286 | λ=1.71
GP plot
Expression
Time (h)
2
1
0
−1
−2
0
5
10
Log-lik in held-out time points
log p(y_j|t_j,y_−j)
Time (h)
−3.0
−3.2
−3.4
−3.6
−3.8
2.5
5.0
7.5
10.0
12.5

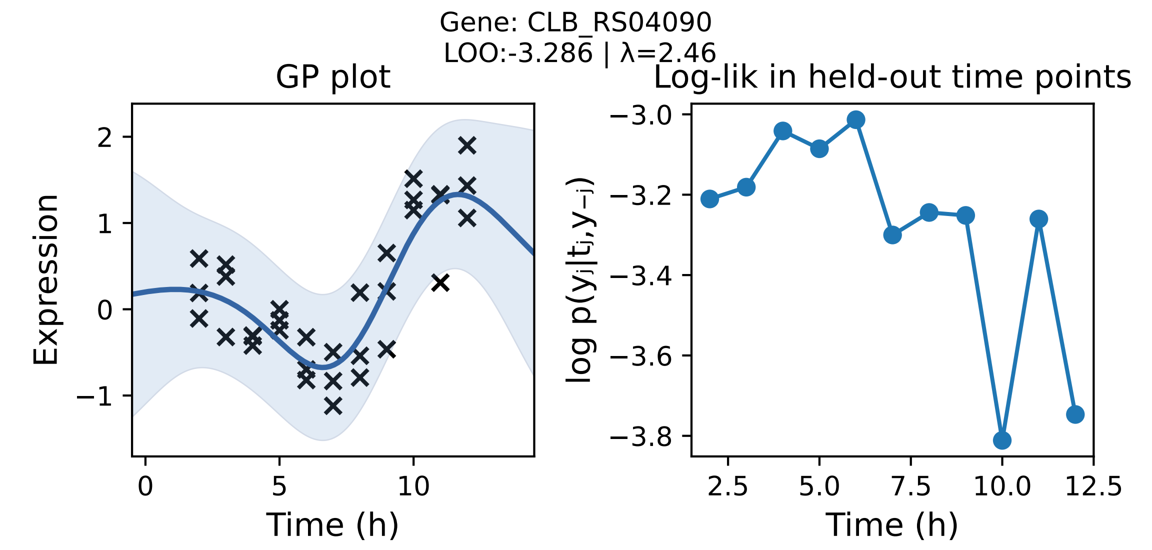
Gene: CLB_RS04090
LOO:-3.286 | λ=2.46
GP plot
Expression
Time (h)
2
1
0
−1
0
5
10
Log-lik in held-out time points
log p(y_j|t_j,y_−j)
Time (h)
−3.0
−3.2
−3.4
−3.6
−3.8
2.5
5.0
7.5
10.0
12.5

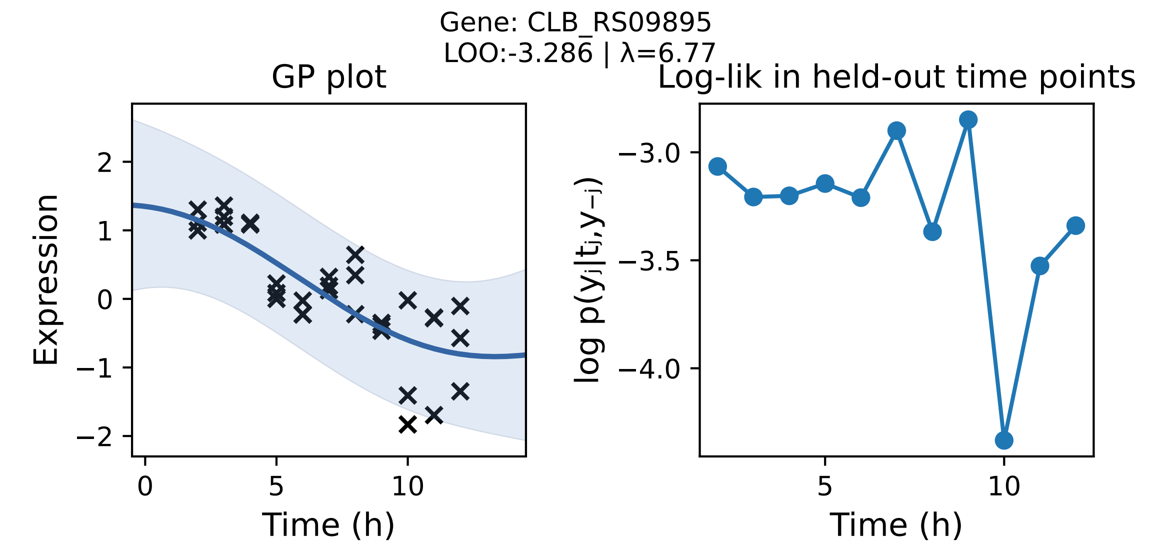

Gene: CLB_RS09895
LOO:-3.286 | λ=6.77
GP plot
Expression
Time (h)
Log-lik in held-out time points
log p(y_j|t_j,y_−j)
Time (h)

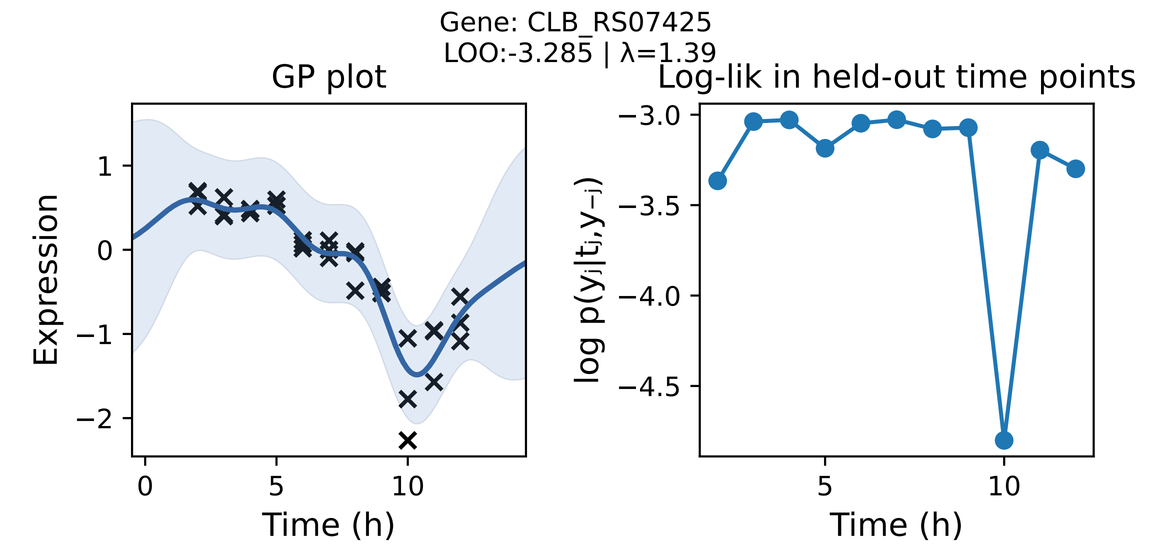
Gene: CLB_RS07425
LOO:-3.285 | λ=1.39
GP plot
Expression
Time (h)
Log-lik in held-out time points
log p(y_j|t_j,y_−j)
Time (h)

Gene: CLB_RS11100
LOO:-3.285 | λ=3.69

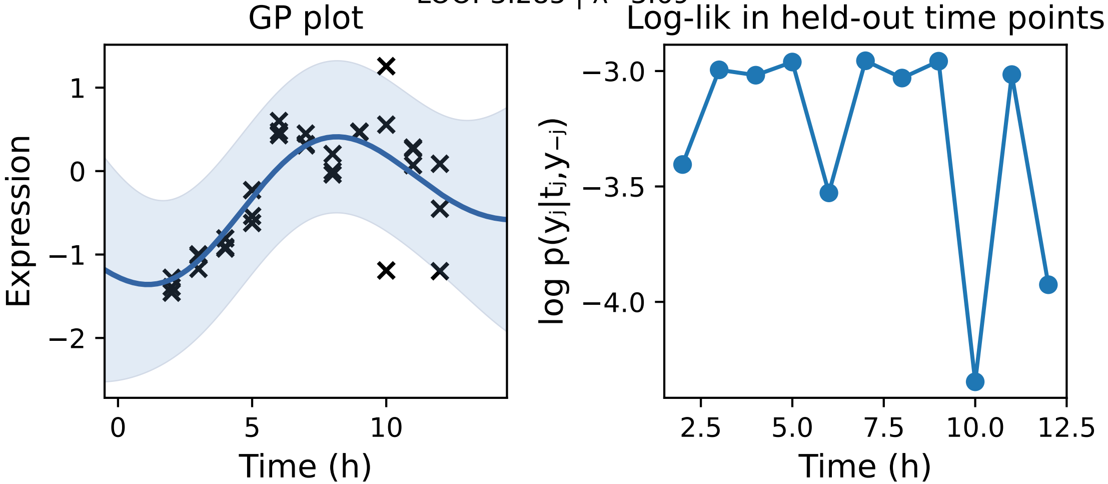

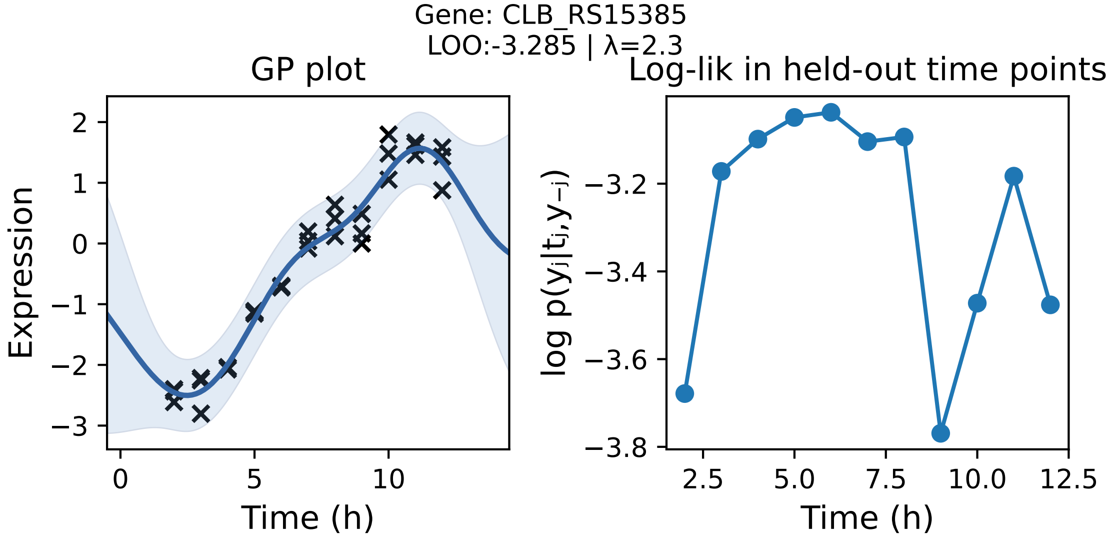
Gene: CLB_RS15385
LOO:-3.285 | λ=2.3
GP plot
Expression
Time (h)
Log-lik in held-out time points
log p(y_j|t_j,y_−j)
Time (h)

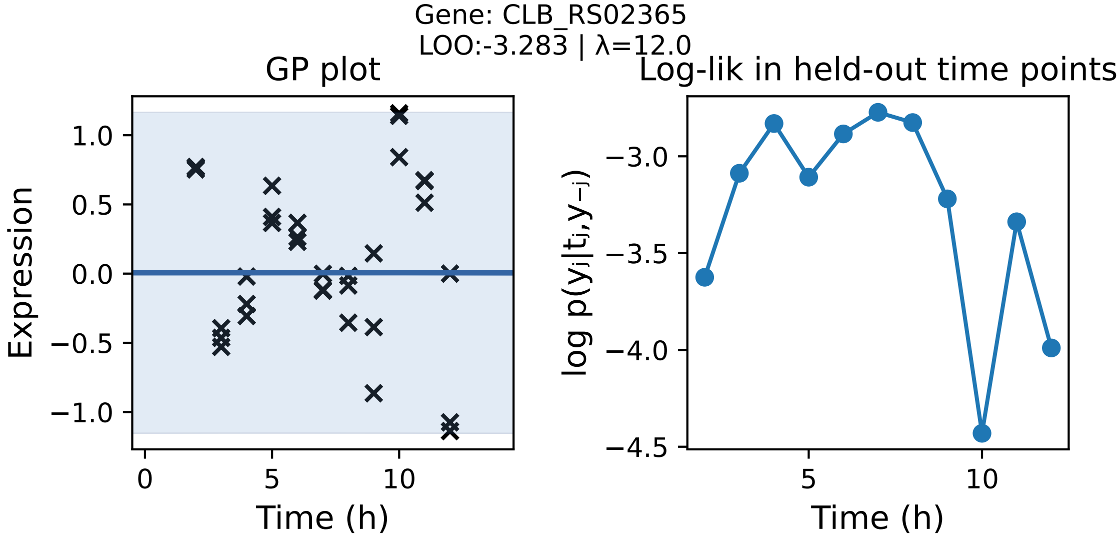
Gene: CLB_RS02365
LOO:-3.283 | λ=12.0
GP plot
Expression
Time (h)
Log-lik in held-out time points
log p(y_j|t_j,y_−j)
Time (h)

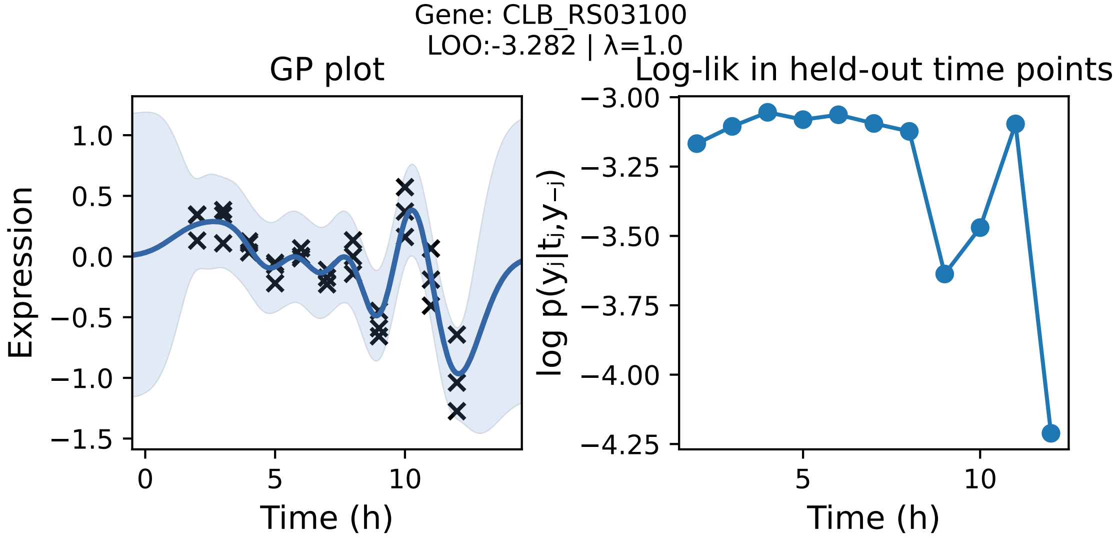
Gene: CLB_RS03100
LOO:-3.282 | λ=1.0
GP plot
Expression
Time (h)
1.0
0.5
0.0
−0.5
−1.0
−1.5
0
5
10
Log-lik in held-out time points
log p(y_j|t_j,y_−j)
−3.00
−3.25
−3.50
−3.75
−4.00
−4.25
5
10
Time (h)

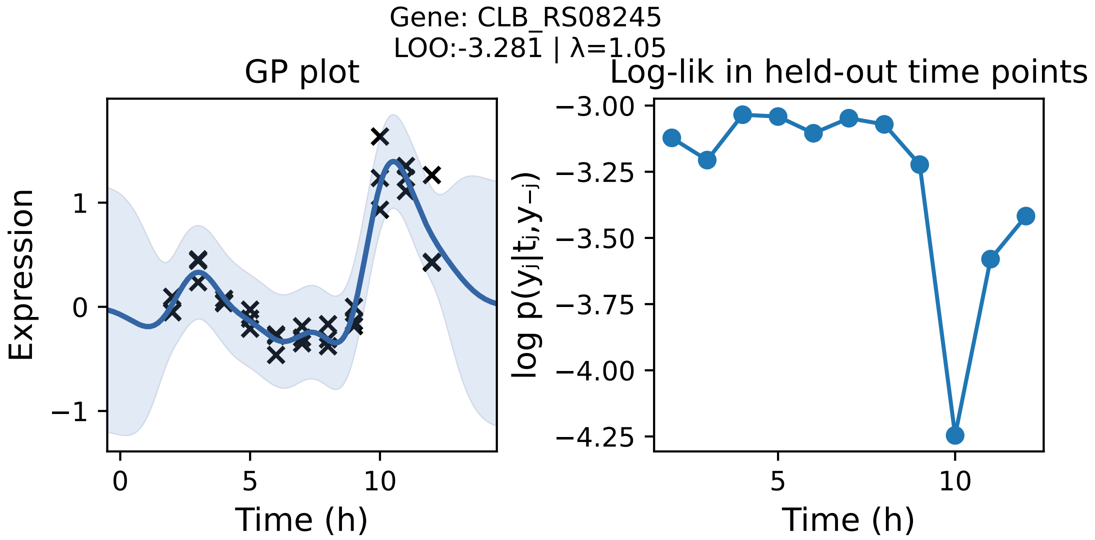

Gene: CLB_RS08245
LOO:-3.281 | λ=1.05
GP plot
Expression
Time (h)
Log-lik in held-out time points
log p(y_j|t_j,y_−j)
Time (h)

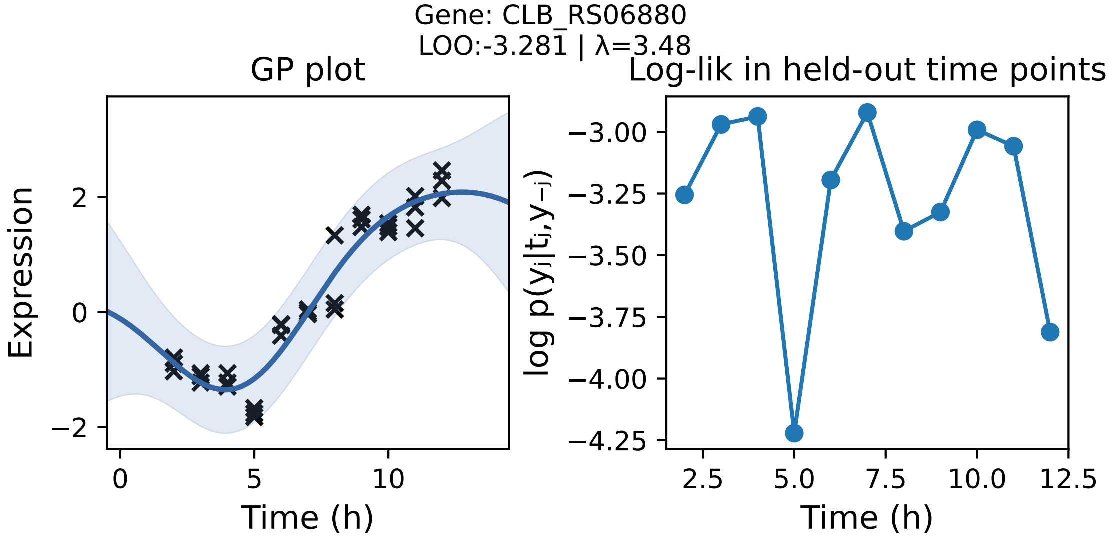

Gene: CLB_RS06880
LOO:-3.281 | λ=3.48
GP plot
Expression
Time (h)
Log-lik in held-out time points
log p(y_j|t_j,y_−j)
Time (h)

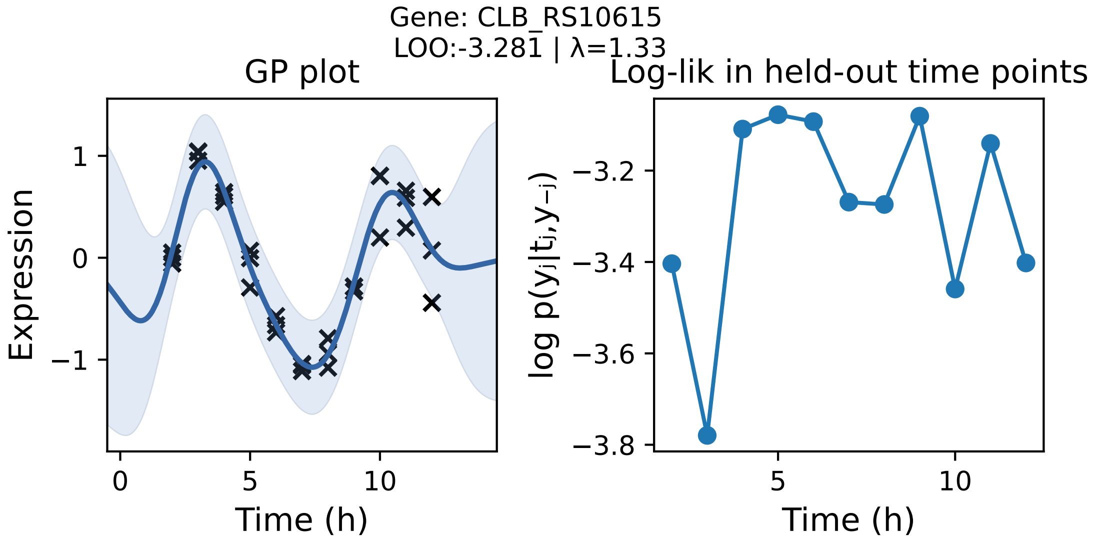

Gene: CLB_RS10615
LOO:-3.281 | λ=1.33
GP plot
Expression
Time (h)
Log-lik in held-out time points
log p(y_j|t_j,y_−j)
Time (h)

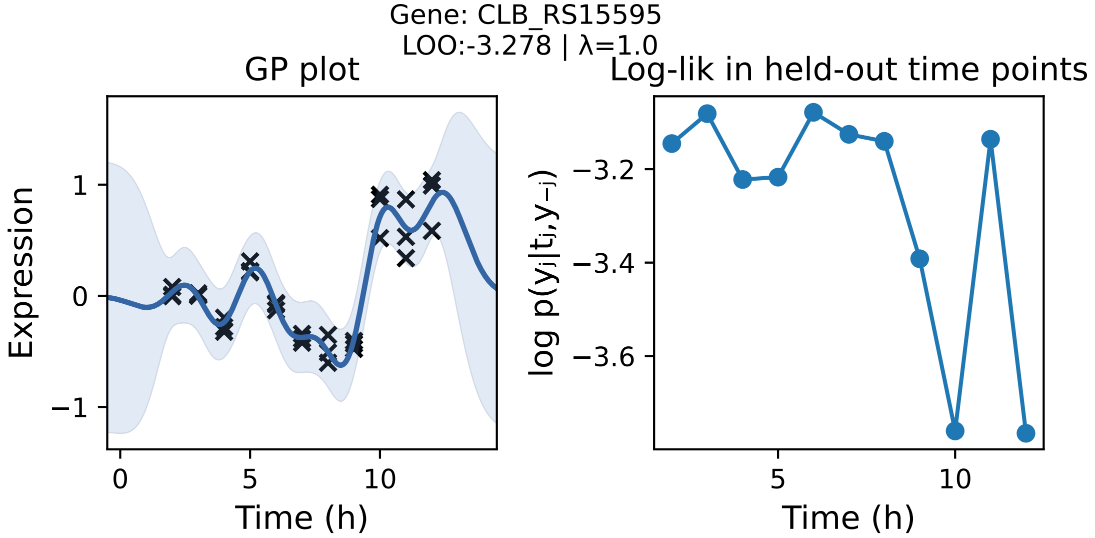

Gene: CLB_RS15595
LOO:-3.278 | λ=1.0
GP plot
Expression
1
0
−1
0
5
10
Time (h)
Log-lik in held-out time points
log p(y_j|t_j,y_−j)
−3.2
−3.4
−3.6
5
10
Time (h)

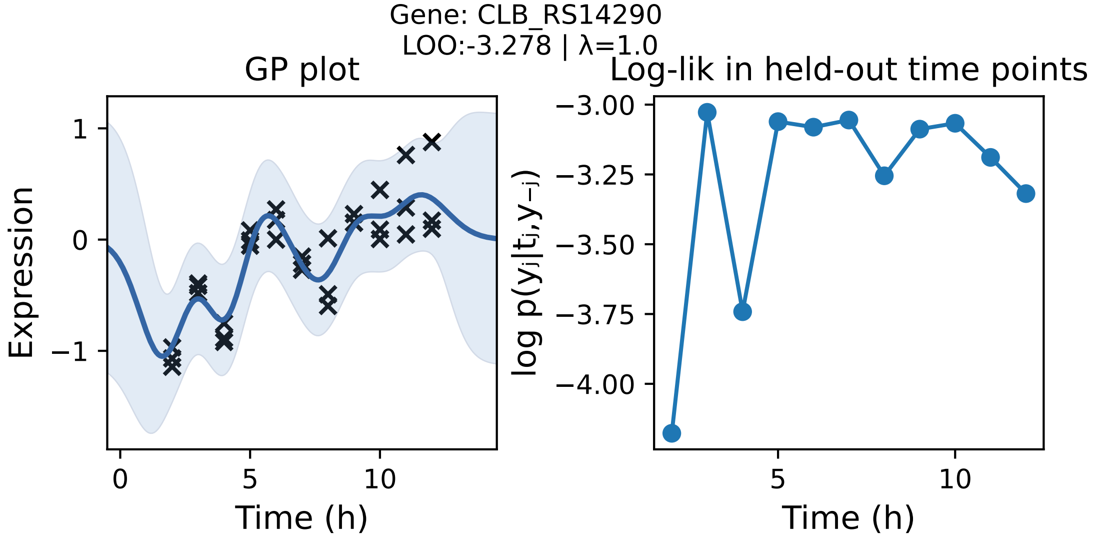

Gene: CLB_RS14290
LOO:-3.278 | λ=1.0
GP plot
Expression
1
0
−1
0
5
10
Time (h)
Log-lik in held-out time points
log p(y_j|t_j,y_−j)
−3.00
−3.25
−3.50
−3.75
−4.00
5
10
Time (h)

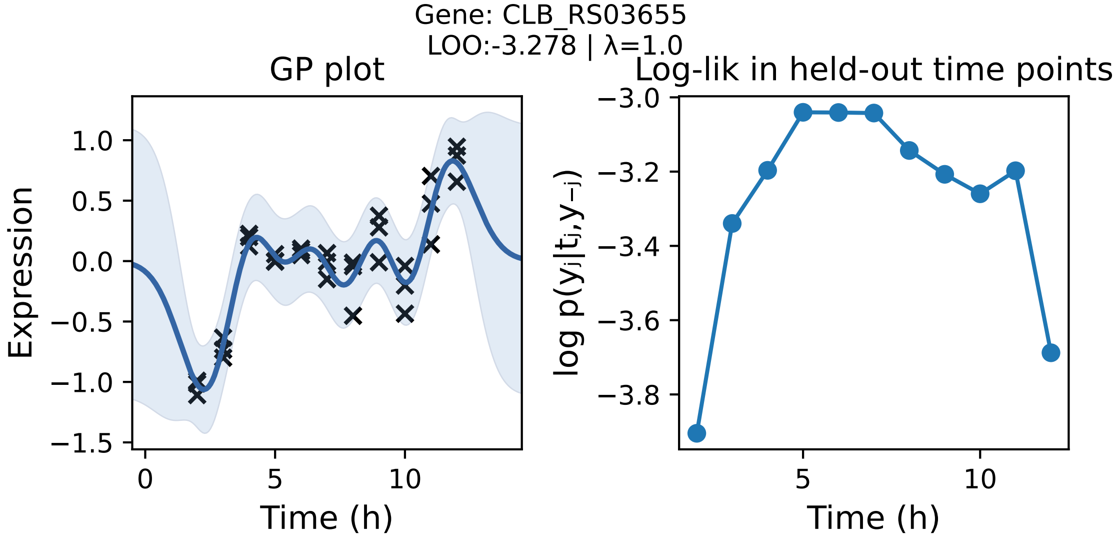

Gene: CLB_RS03655
LOO:-3.278 | λ=1.0
GP plot
Expression
Time (h)
1.0
0.5
0.0
−0.5
−1.0
−1.5
0
5
10
Log-lik in held-out time points
log p(yⱼ|tⱼ,y₋ⱼ)
Time (h)
−3.0
−3.2
−3.4
−3.6
−3.8
5
10

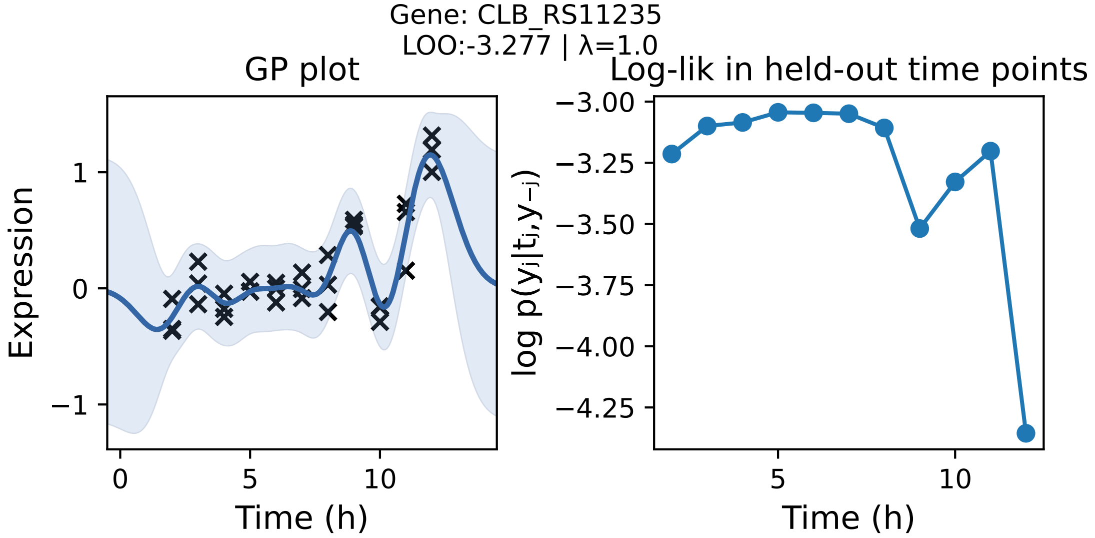

Gene: CLB_RS11235
LOO:-3.277 | λ=1.0
GP plot
Expression
Time (h)
Log-lik in held-out time points
log p(y_j|t_j,y_−j)
Time (h)

Gene: CLB_RS00645
LOO:-3.276 | λ=2.06

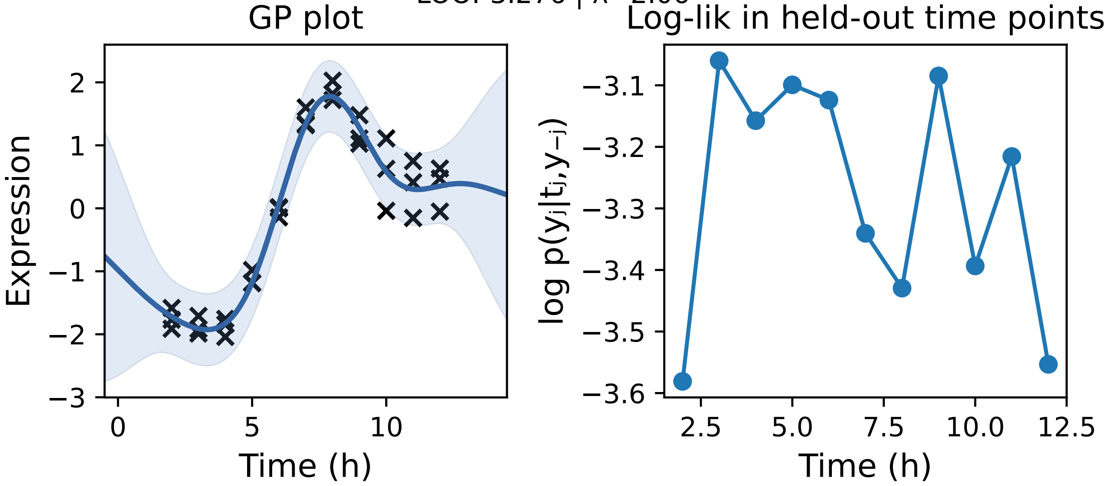

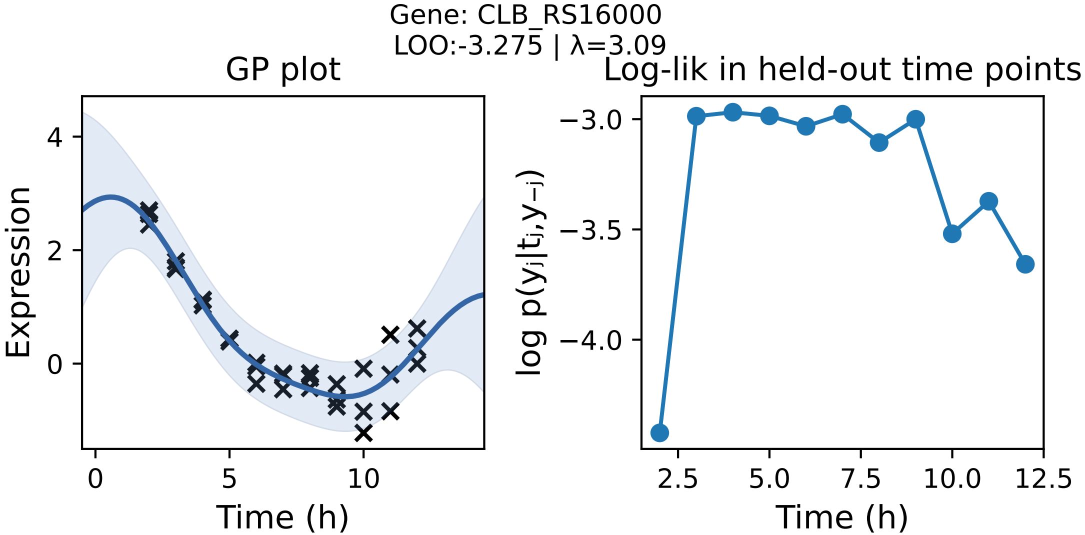

Gene: CLB_RS16000
LOO:-3.275 | λ=3.09
GP plot
Expression
Time (h)
0
2
4
0
5
10
Log-lik in held-out time points
log p(y_j|t_j,y_−j)
Time (h)
−3.0
−3.5
−4.0
2.5
5.0
7.5
10.0
12.5

Gene: CLB_RS08530
LOO:-3.273 | λ=1.97

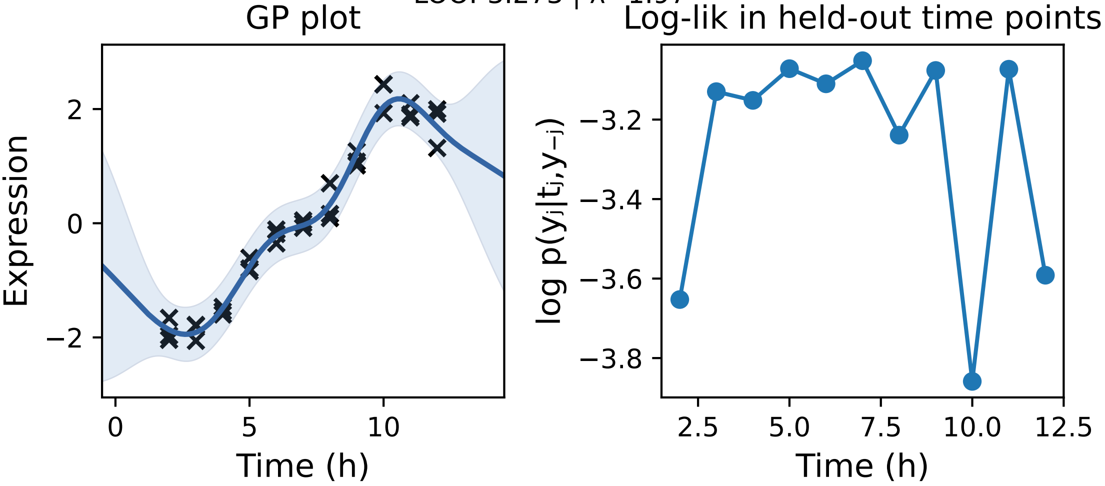

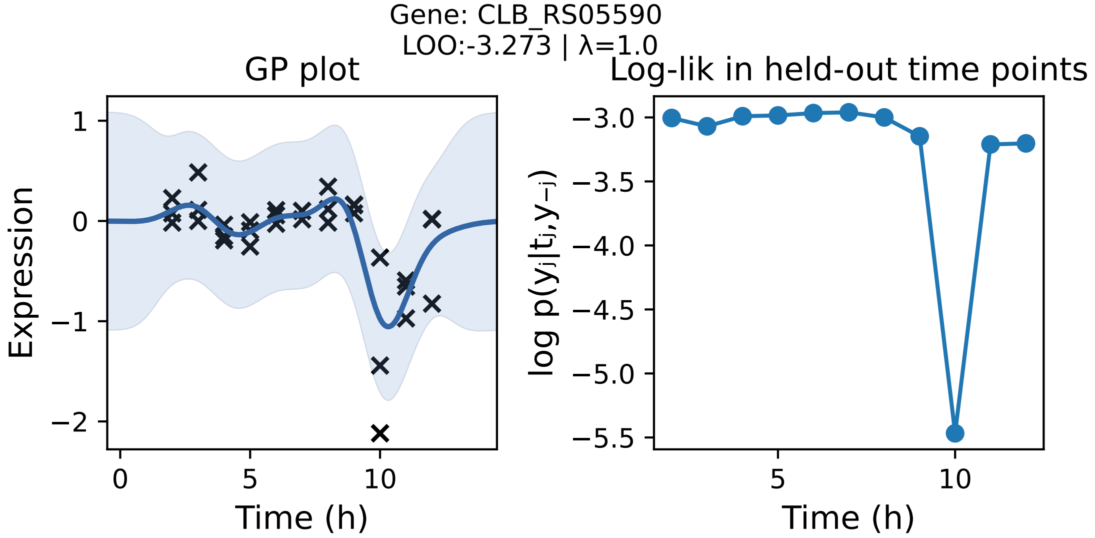

Gene: CLB_RS05590
LOO:-3.273 | λ=1.0
GP plot
Expression
Time (h)
1
0
−1
−2
0
5
10
Log-lik in held-out time points
log p(y_j|t_j,y_−j)
Time (h)
−3.0
−3.5
−4.0
−4.5
−5.0
−5.5
5
10

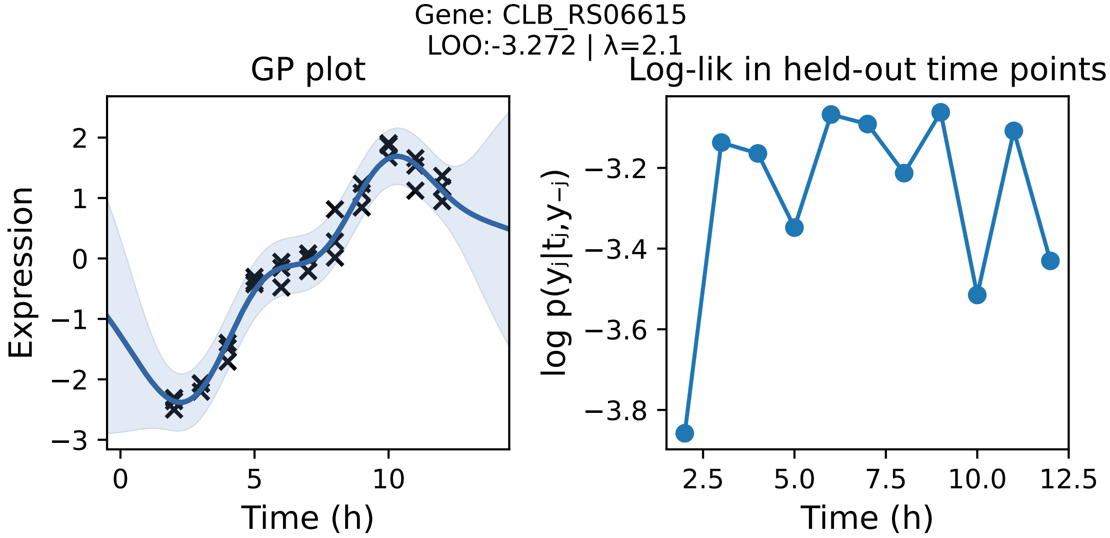

Gene: CLB_RS06615
LOO:-3.272 | λ=2.1
GP plot
Expression
Time (h)
2
1
0
−1
−2
−3
0
5
10
Log-lik in held-out time points
log p(y_j|t_j,y_−j)
Time (h)
−3.2
−3.4
−3.6
−3.8
2.5
5.0
7.5
10.0
12.5

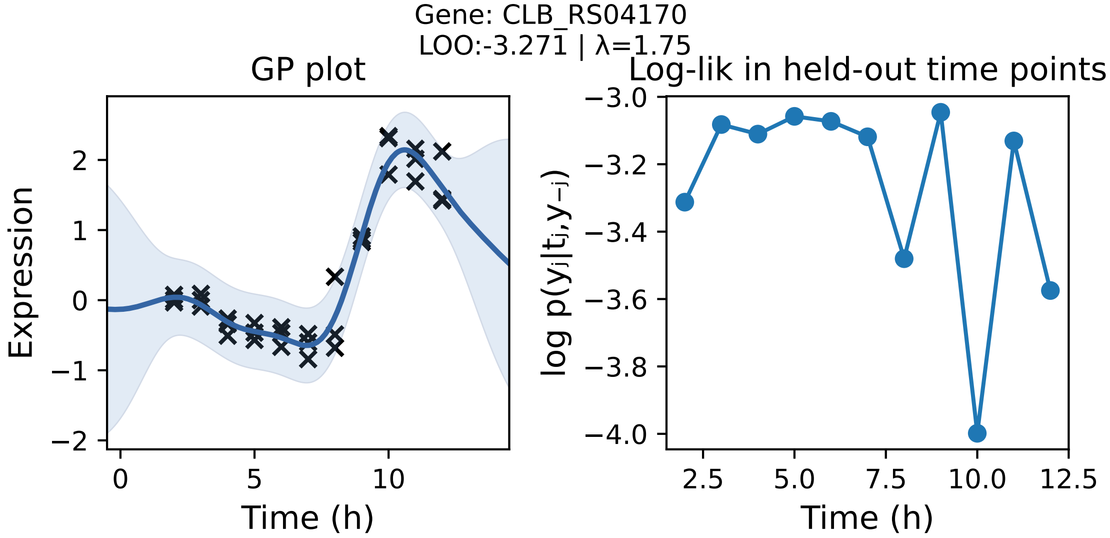

Gene: CLB_RS04170
LOO:-3.271 | λ=1.75
GP plot
Expression
Time (h)
0
5
10
−2
−1
0
1
2
Log-lik in held-out time points
log p(y_j|t_j,y_−j)
Time (h)
2.5
5.0
7.5
10.0
12.5
−3.0
−3.2
−3.4
−3.6
−3.8
−4.0

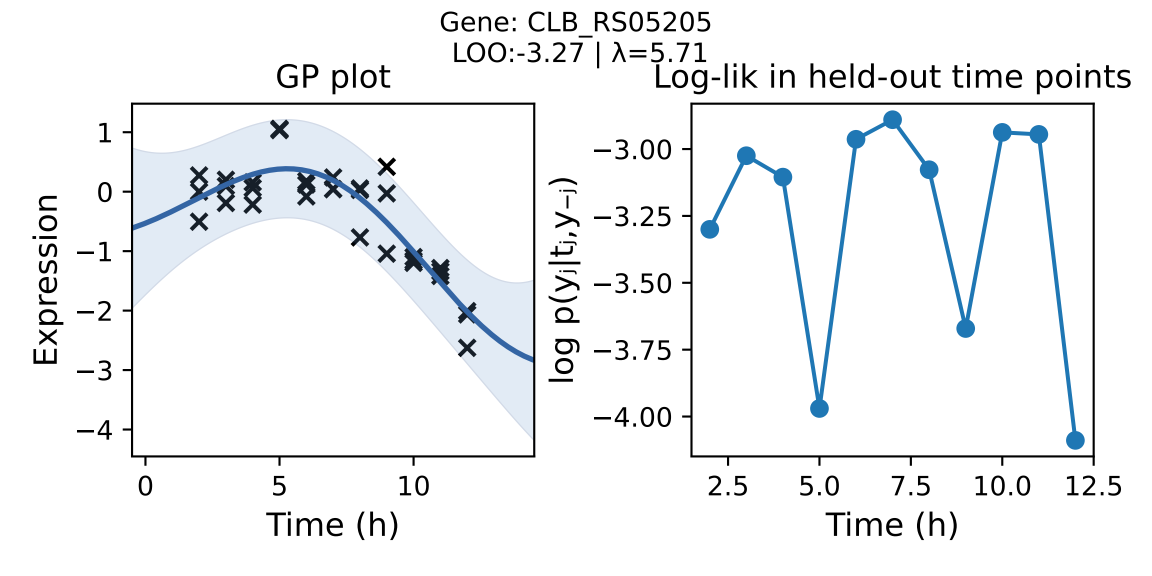

Gene: CLB_RS05205
LOO:-3.27 | λ=5.71
GP plot
Expression
Time (h)
1
0
−1
−2
−3
−4
0
5
10
Log-lik in held-out time points
log p(y_j|t_j,y_−j)
Time (h)
−3.00
−3.25
−3.50
−3.75
−4.00
2.5
5.0
7.5
10.0
12.5

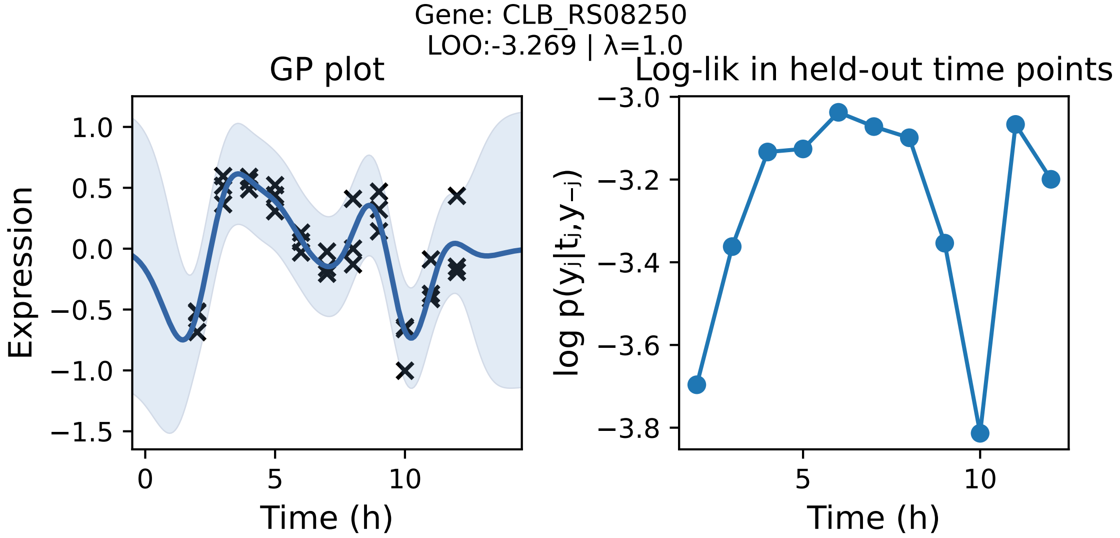

Gene: CLB_RS08250
LOO:-3.269 | λ=1.0
GP plot
Log-lik in held-out time points
Expression
Time (h)
log p(yj|tj,y−j)
Time (h)

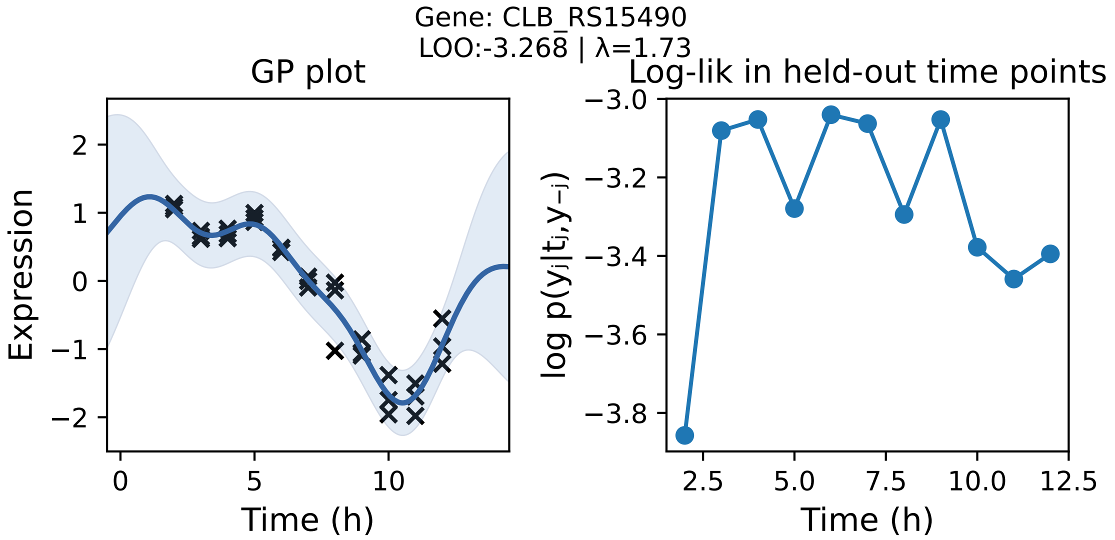

Gene: CLB_RS15490
LOO:-3.268 | λ=1.73
GP plot
Expression
Time (h)
2
1
0
−1
−2
0
5
10
Log-lik in held-out time points
log p(y_j|t_j,y_−j)
Time (h)
−3.0
−3.2
−3.4
−3.6
−3.8
2.5
5.0
7.5
10.0
12.5

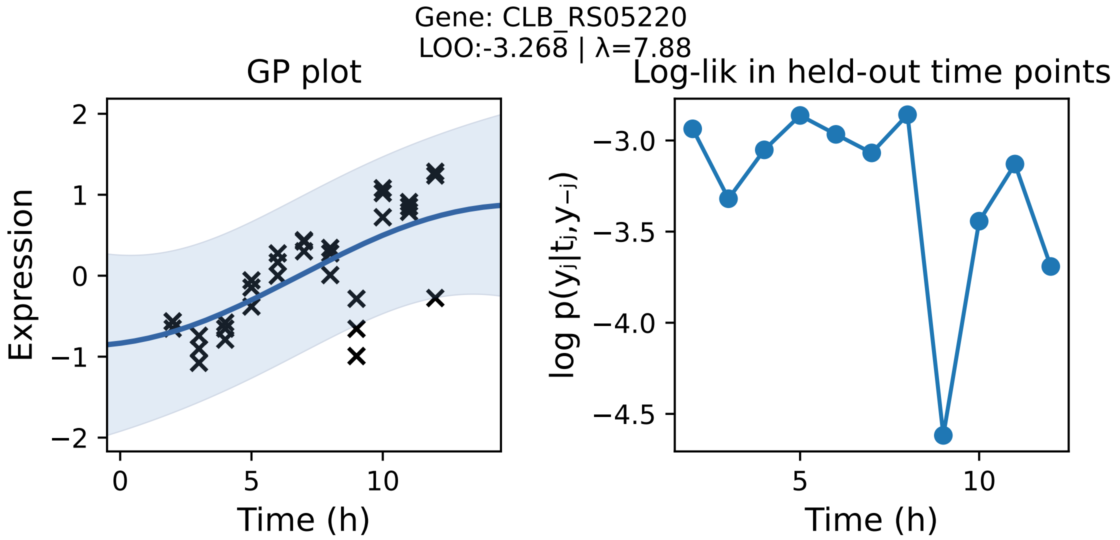

Gene: CLB_RS05220
LOO:-3.268 | λ=7.88
GP plot
Expression
2
1
0
−1
−2
0
5
10
Time (h)
Log-lik in held-out time points
$\log p(y_j|t_j, y_{-j})$
−3.0
−3.5
−4.0
−4.5
5
10
Time (h)

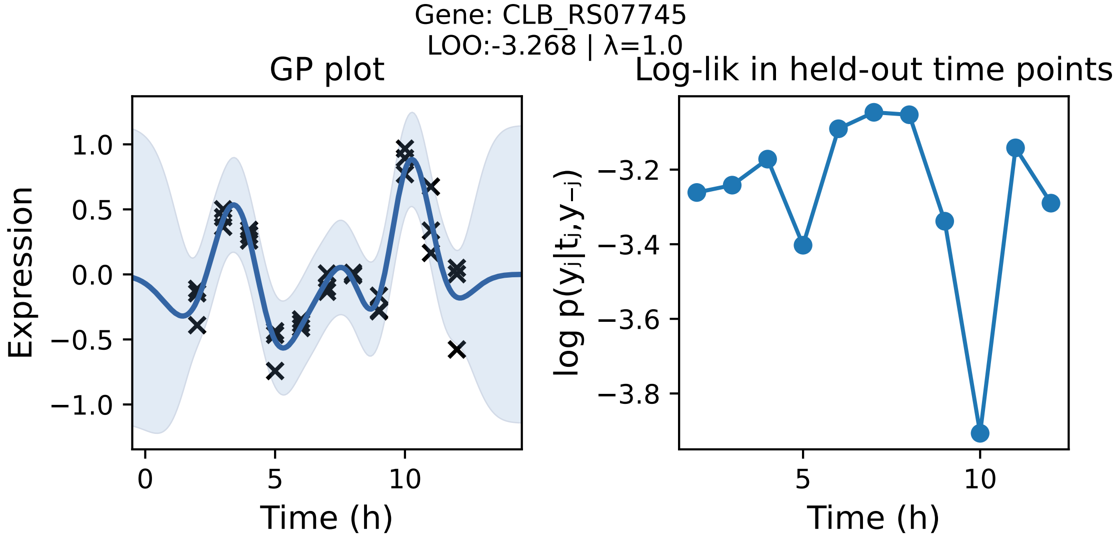
Gene: CLB_RS07745
LOO:-3.268 | λ=1.0
GP plot
Expression
Time (h)
1.0
0.5
0.0
−0.5
−1.0
0
5
10
Log-lik in held-out time points
log p(y_j|t_j,y_−j)
−3.2
−3.4
−3.6
−3.8
5
10
Time (h)

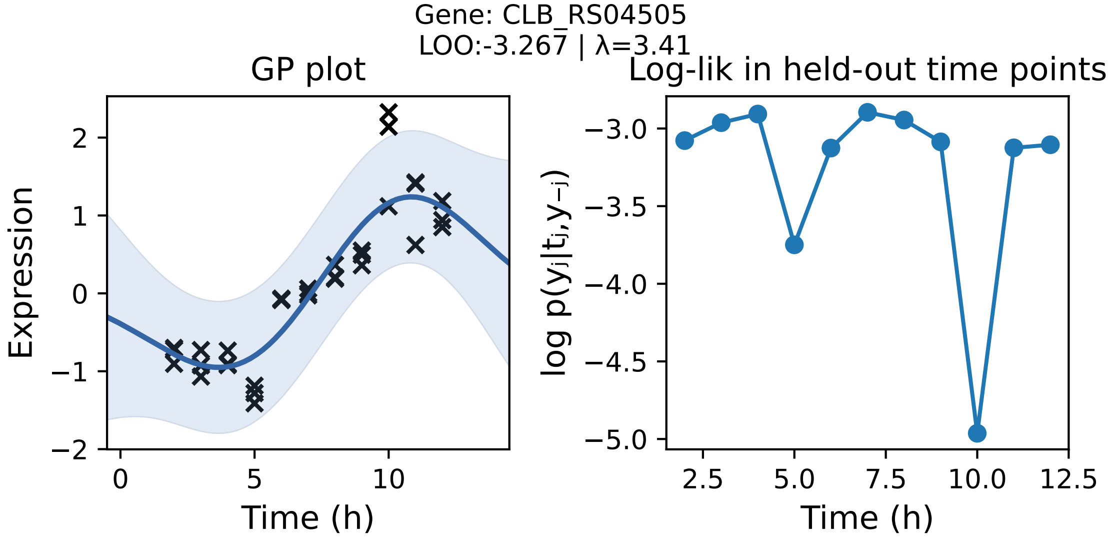

Gene: CLB_RS04505
LOO:-3.267 | λ=3.41
GP plot
Expression
Time (h)
Log-lik in held-out time points
log p(y_j|t_j,y_−j)
Time (h)

Gene: CLB_RS15660
LOO:-3.267 | λ=3.3

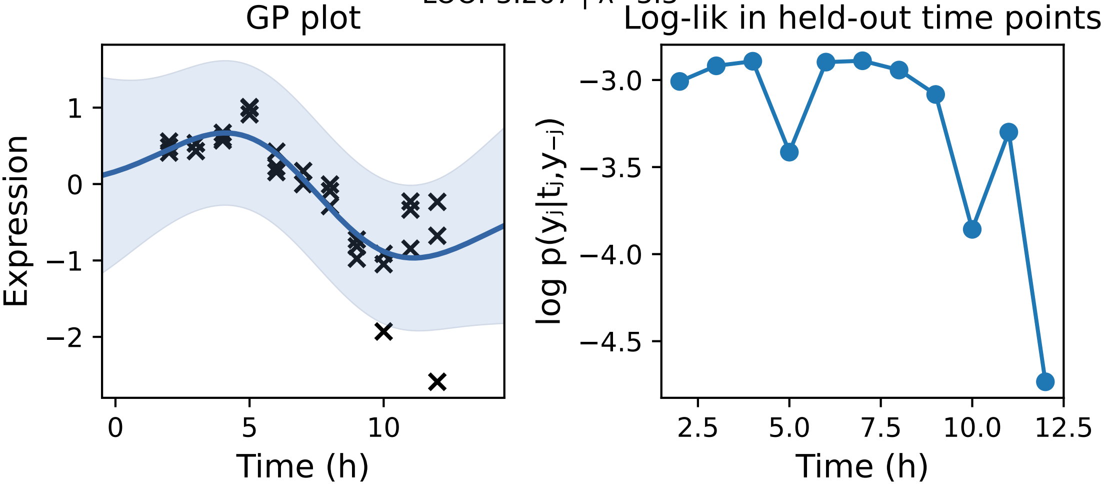

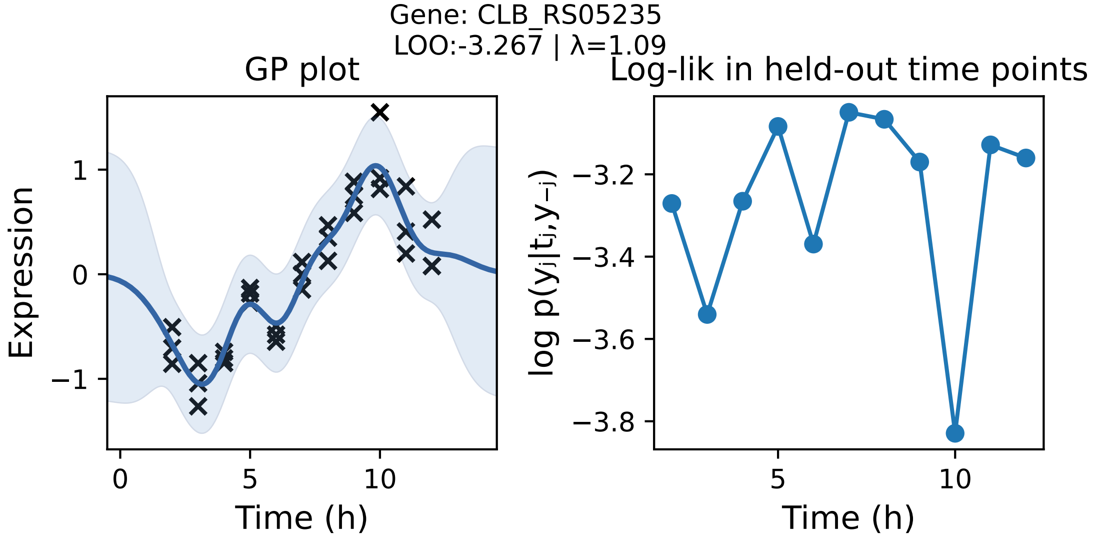

Gene: CLB_RS05235
LOO:-3.267 | λ=1.09
GP plot
Expression
Time (h)
1
0
−1
0
5
10
Log-lik in held-out time points
log p(y_j|t_j,y_−j)
Time (h)
−3.2
−3.4
−3.6
−3.8
5
10

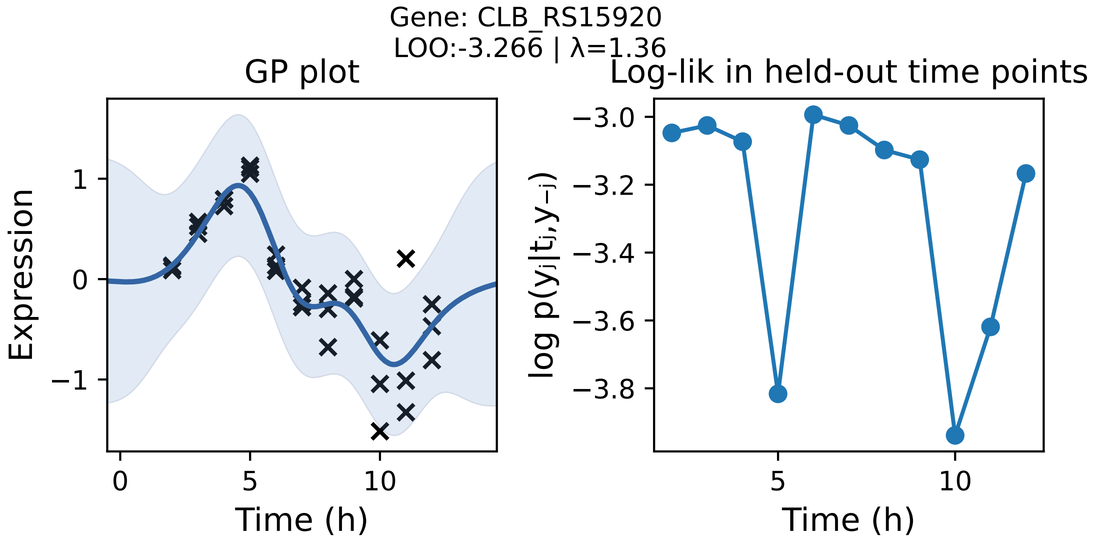

Gene: CLB_RS15920
LOO:-3.266 | λ=1.36
GP plot
Log-lik in held-out time points
Expression
1
0
−1
0
5
10
Time (h)
log p(yⱼ|tⱼ,y₋ⱼ)
−3.0
−3.2
−3.4
−3.6
−3.8
5
10
Time (h)

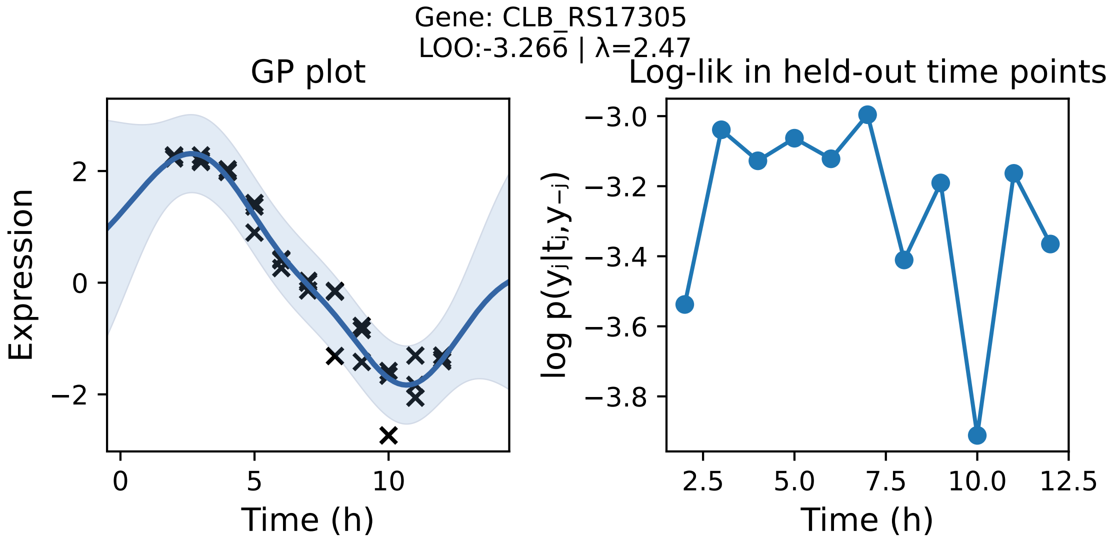

Gene: CLB_RS17305
LOO:-3.266 | λ=2.47
GP plot
Expression
2
0
−2
0
5
10
Time (h)
Log-lik in held-out time points
log p(y_j|t_j,y_−j)
−3.0
−3.2
−3.4
−3.6
−3.8
2.5
5.0
7.5
10.0
12.5
Time (h)

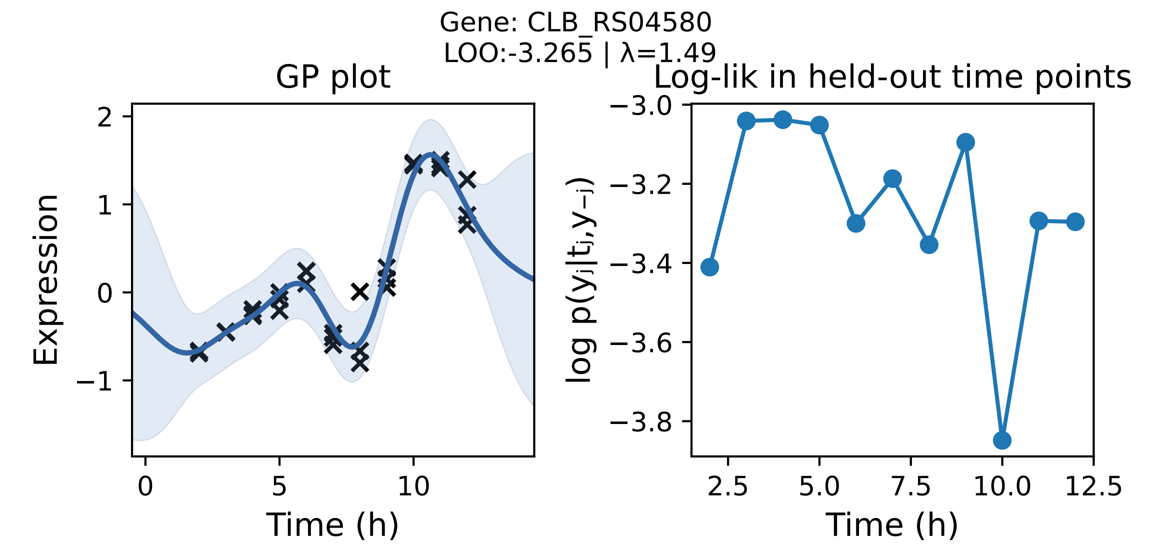

Gene: CLB_RS04580
LOO:-3.265 | λ=1.49
GP plot
Expression
Time (h)
2
1
0
−1
0
5
10
Log-lik in held-out time points
log p(y_j|t_j,y_−j)
Time (h)
−3.0
−3.2
−3.4
−3.6
−3.8
2.5
5.0
7.5
10.0
12.5

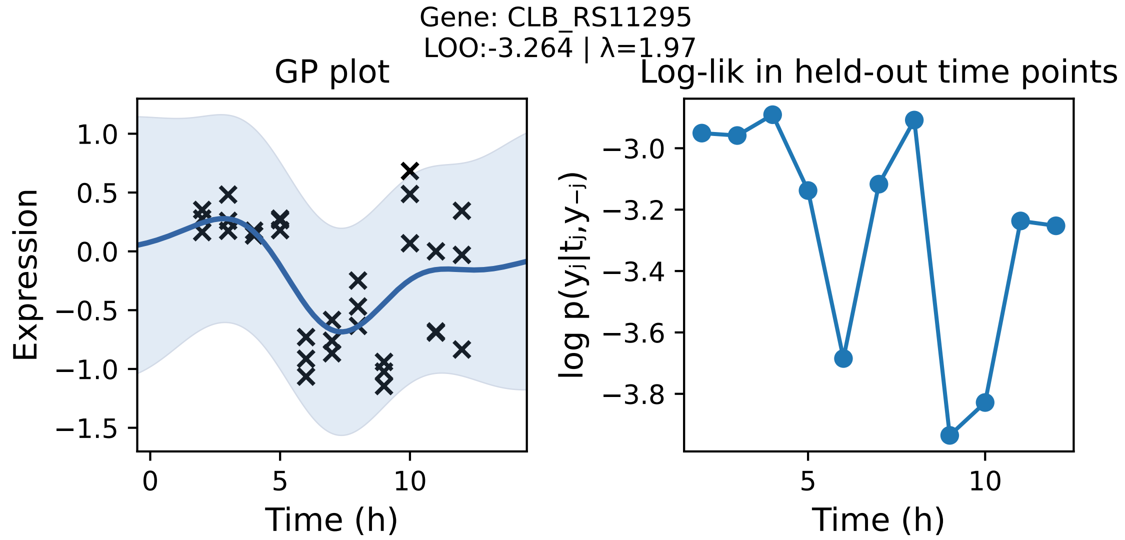

Gene: CLB_RS11295
LOO:-3.264 | λ=1.97
GP plot
Expression
1.0
0.5
0.0
−0.5
−1.0
−1.5
0
5
10
Time (h)
Log-lik in held-out time points
log p(y_j|t_j,y_−j)
−3.0
−3.2
−3.4
−3.6
−3.8
5
10
Time (h)

Gene: CLB_RS16910
LOO:-3.263 | λ=1.2

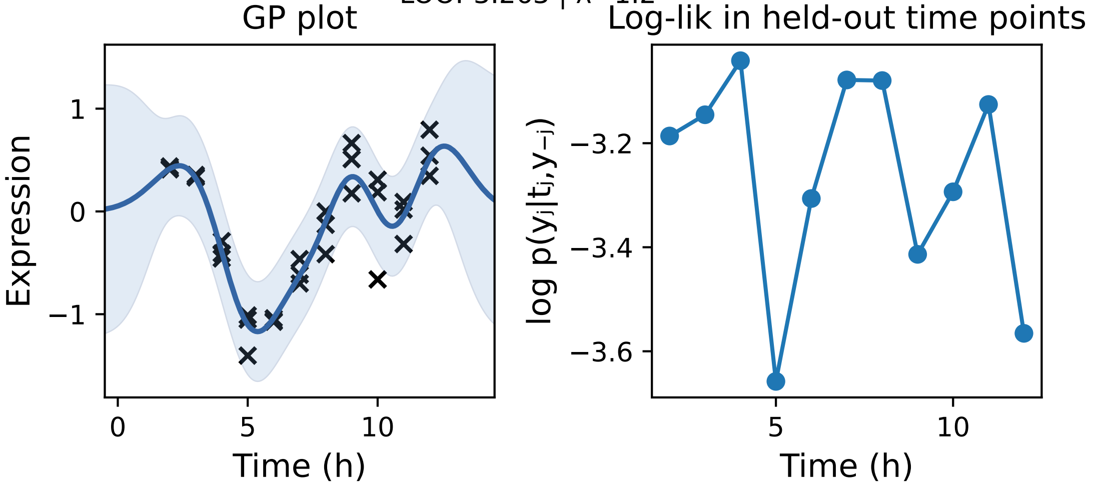

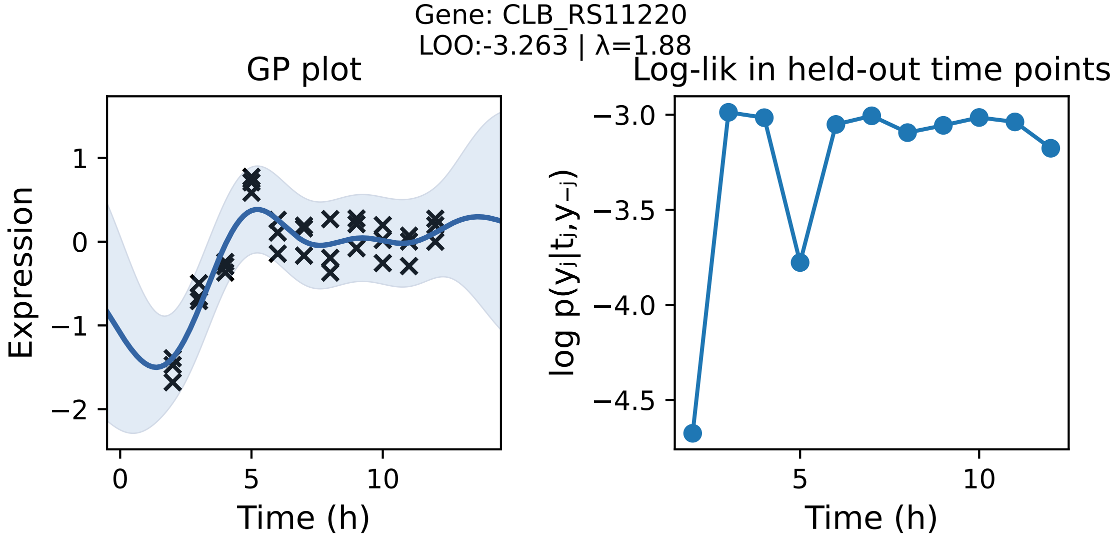

Gene: CLB_RS11220
LOO:-3.263 | λ=1.88
GP plot
Expression
1
0
−1
−2
0
5
10
Time (h)
Log-lik in held-out time points
log p(y_j|t_j,y_−j)
−3.0
−3.5
−4.0
−4.5
5
10
Time (h)

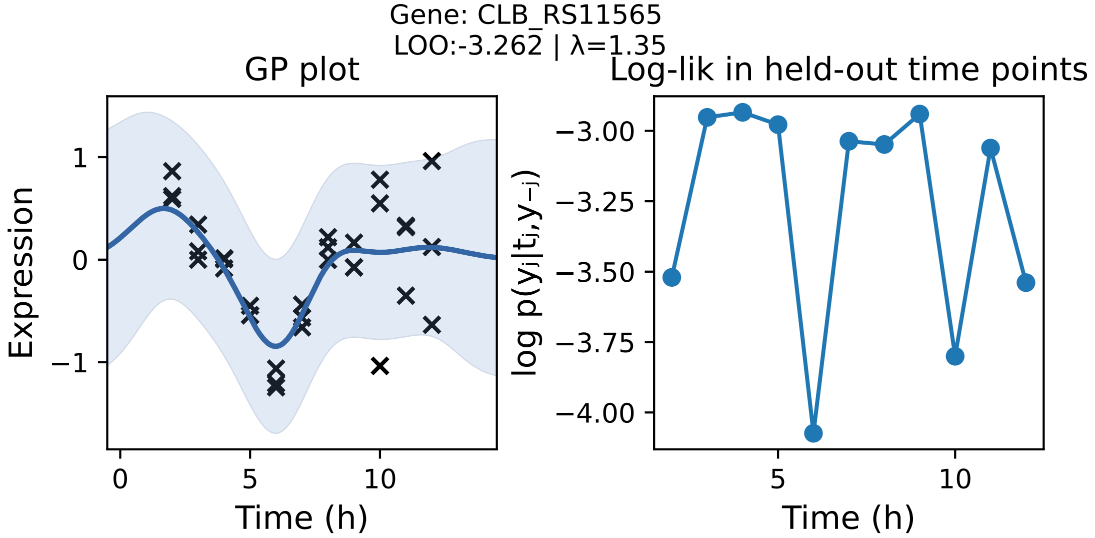

Gene: CLB_RS11565
LOO:-3.262 | λ=1.35
GP plot
Expression
Time (h)
Log-lik in held-out time points
log p(y_j|t_j,y_−j)
Time (h)

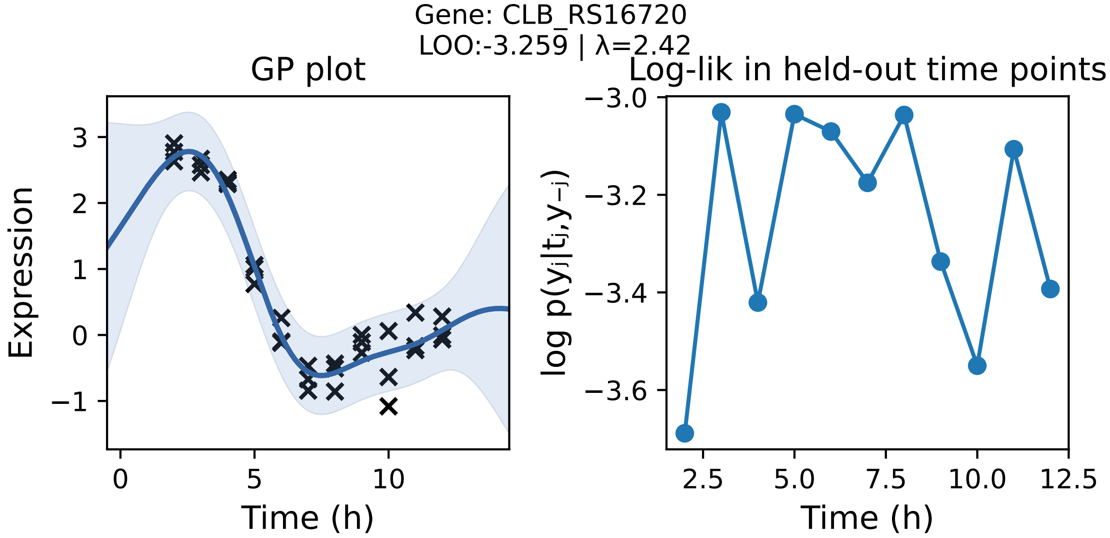

Gene: CLB_RS16720
LOO:-3.259 | λ=2.42
GP plot
Expression
Time (h)
Log-lik in held-out time points
log p(y_j|t_j,y_−j)
Time (h)

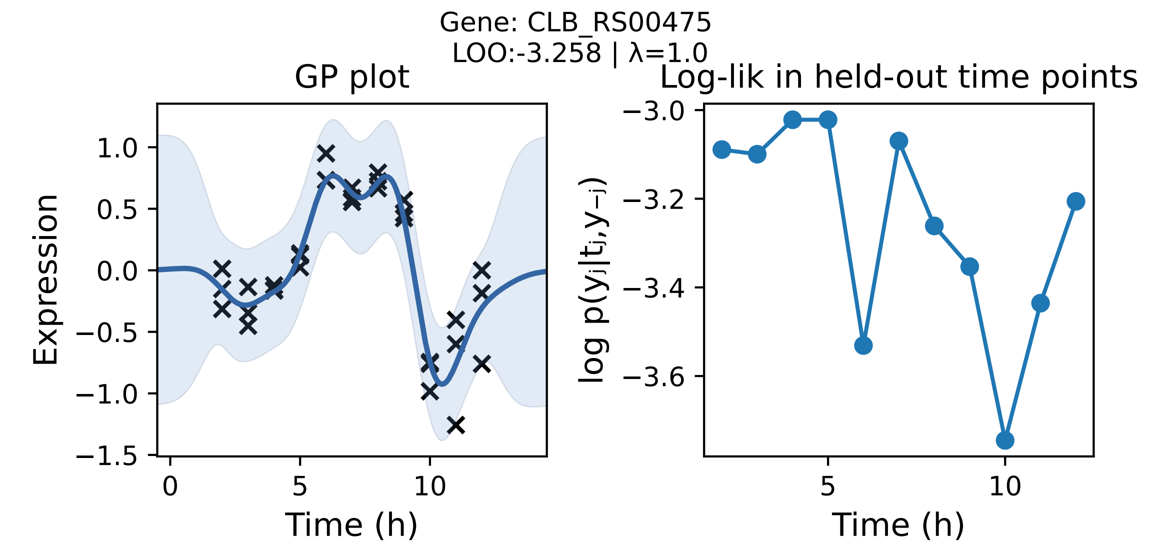
Gene: CLB_RS00475
LOO:-3.258 | λ=1.0
GP plot
Expression
Time (h)
1.0
0.5
0.0
−0.5
−1.0
−1.5
0
5
10
Log-lik in held-out time points
log p(y_j|t_j,y_−j)
−3.0
−3.2
−3.4
−3.6
5
10
Time (h)

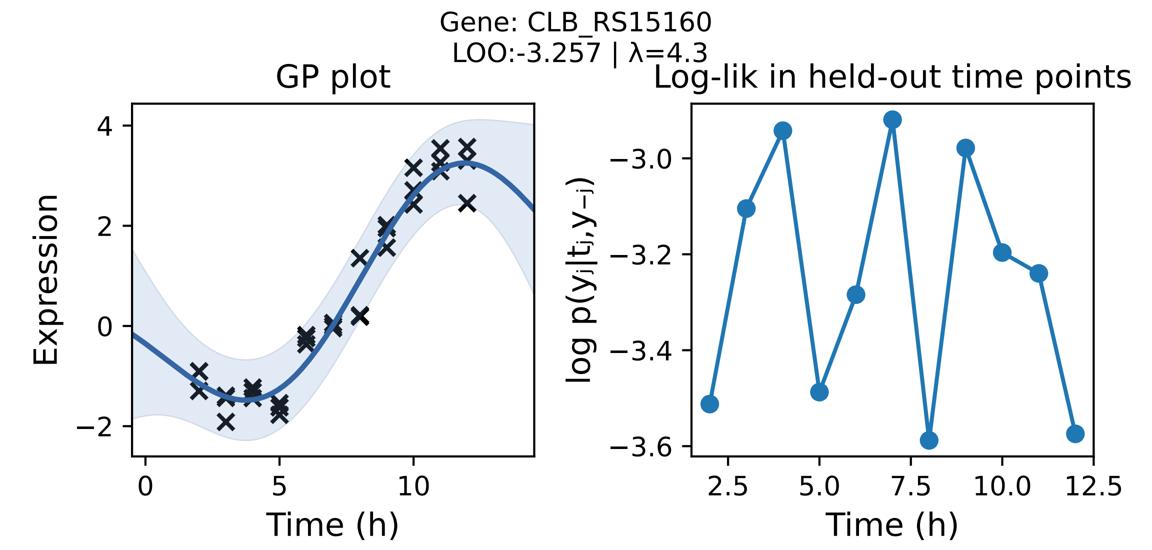
Gene: CLB_RS15160
LOO:-3.257 | λ=4.3
GP plot
Expression
Time (h)
Log-lik in held-out time points
log p(y_j|t_j,y_−j)
Time (h)
4
2
0
−2
0
5
10
−3.0
−3.2
−3.4
−3.6
2.5
5.0
7.5
10.0
12.5

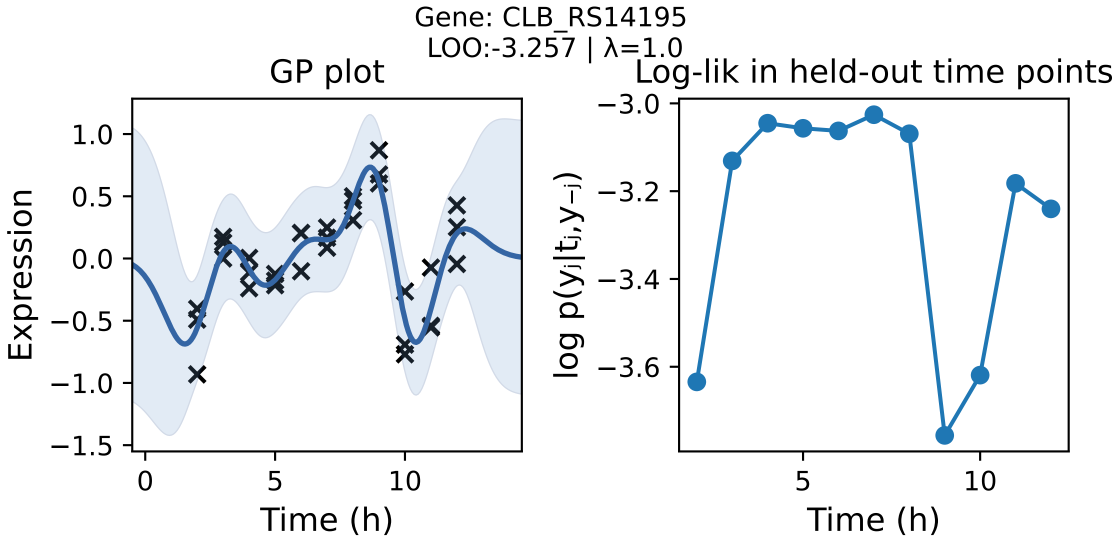
Gene: CLB_RS14195
LOO:-3.257 | λ=1.0
GP plot
Expression
Time (h)
1.0
0.5
0.0
−0.5
−1.0
−1.5
0
5
10
Log-lik in held-out time points
log p(y_j|t_j,y_−j)
−3.0
−3.2
−3.4
−3.6
5
10
Time (h)

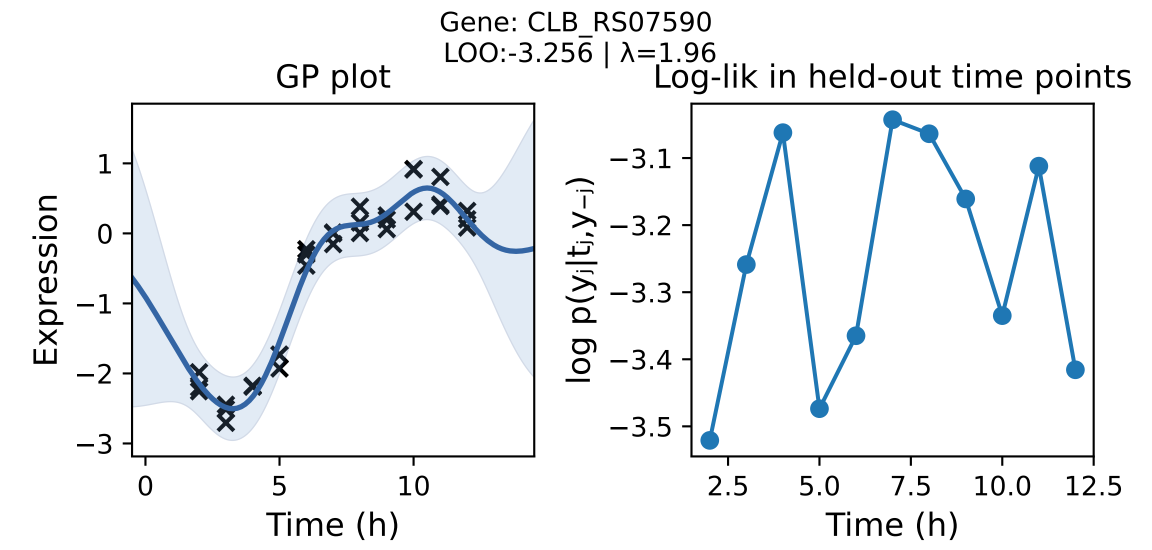
Gene: CLB_RS07590
LOO:-3.256 | λ=1.96
GP plot
Expression
Time (h)
0
5
10
1
0
−1
−2
−3
Log-lik in held-out time points
log p(y_j|t_j,y_−j)
Time (h)
2.5
5.0
7.5
10.0
12.5
−3.1
−3.2
−3.3
−3.4
−3.5

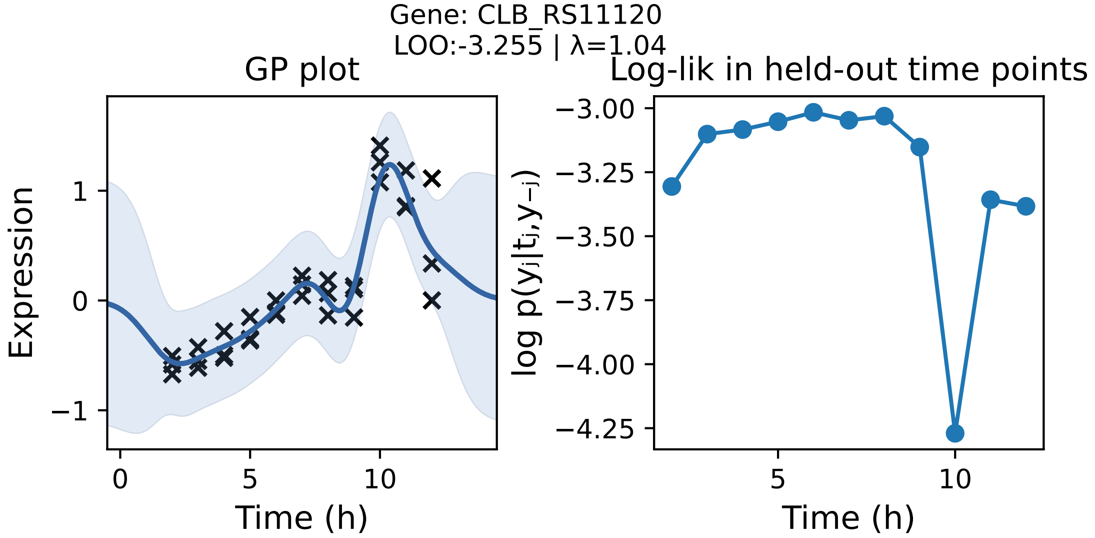
Gene: CLB_RS11120
LOO:-3.255 | λ=1.04
GP plot
Expression
Time (h)
Log-lik in held-out time points
log p(y_j|t_j,y_−j)
Time (h)
1
0
−1
0
5
10
−3.00
−3.25
−3.50
−3.75
−4.00
−4.25
5
10

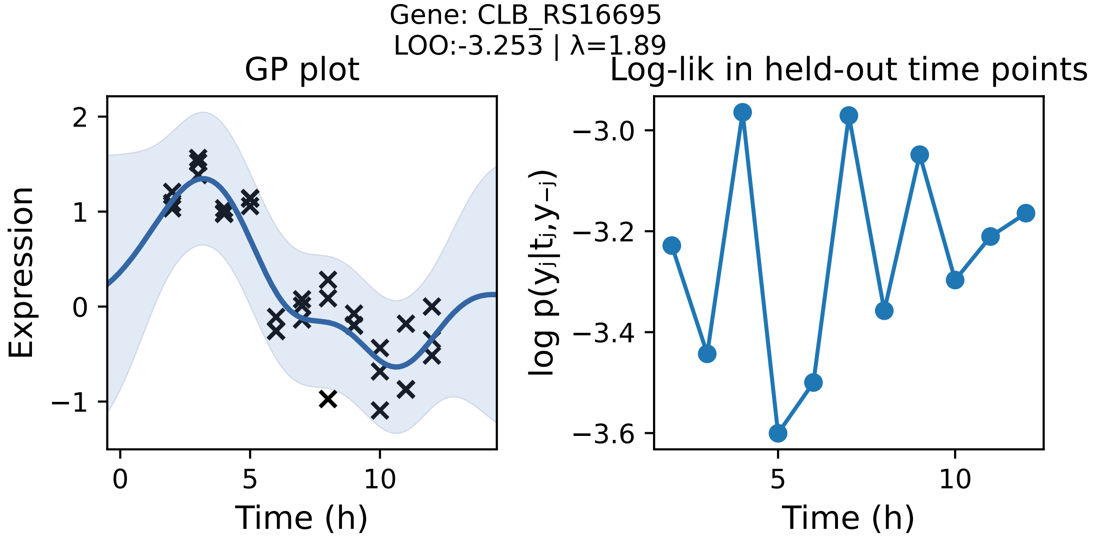

Gene: CLB_RS16695
LOO:-3.253 | λ=1.89
GP plot
Expression
Time (h)
Log-lik in held-out time points
log p(y_j|t_j,y_−j)
Time (h)

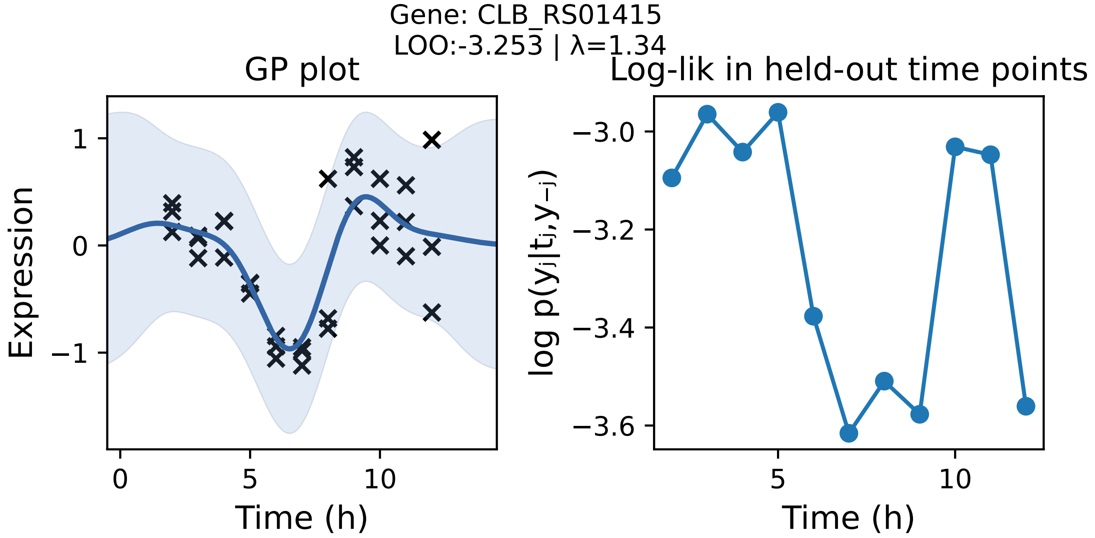

Gene: CLB_RS01415
LOO:-3.253 | λ=1.34
GP plot
Log-lik in held-out time points
Expression
Time (h)
log p(yj|tj,y−j)
Time (h)

Gene: CLB_RS11735
LOO:-3.252 | λ=3.69

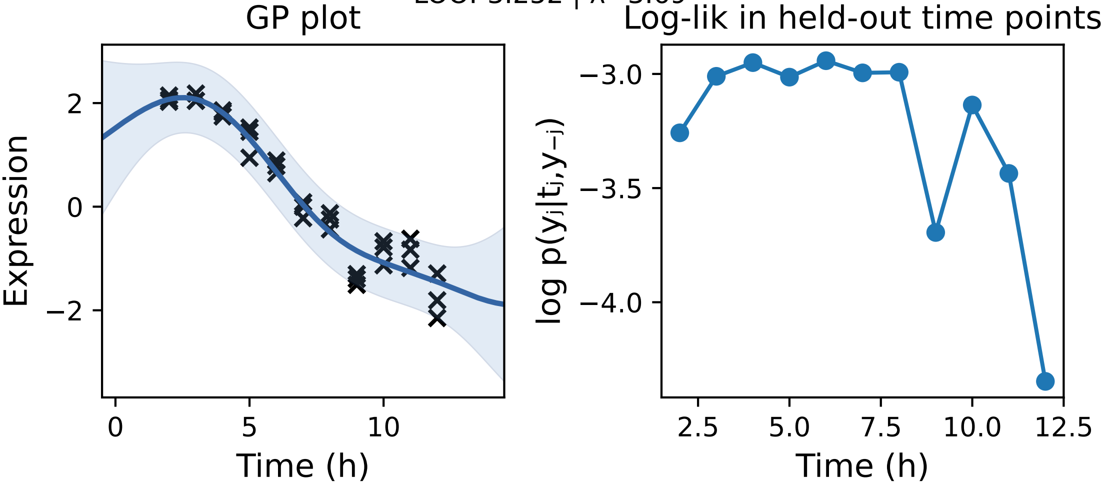

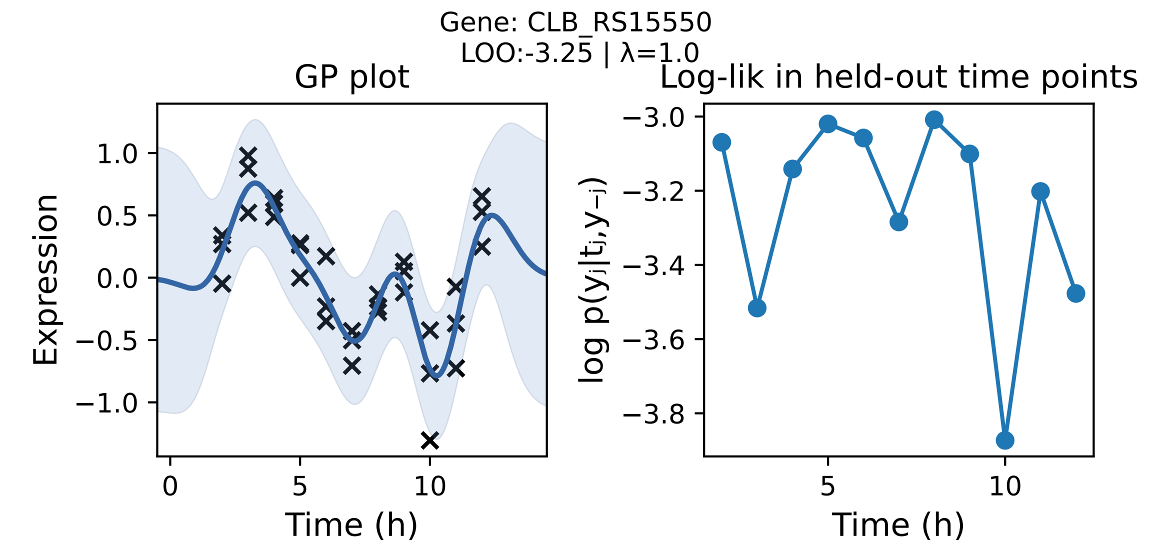

Gene: CLB_RS15550
LOO:-3.25 | λ=1.0
GP plot
Log-lik in held-out time points
Expression
1.0
0.5
0.0
−0.5
−1.0
0
5
10
Time (h)
log p(y_j|t_j,y_−j)
−3.0
−3.2
−3.4
−3.6
−3.8
5
10
Time (h)

Gene: CLB_RS01575
LOO:-3.25 | λ=3.39

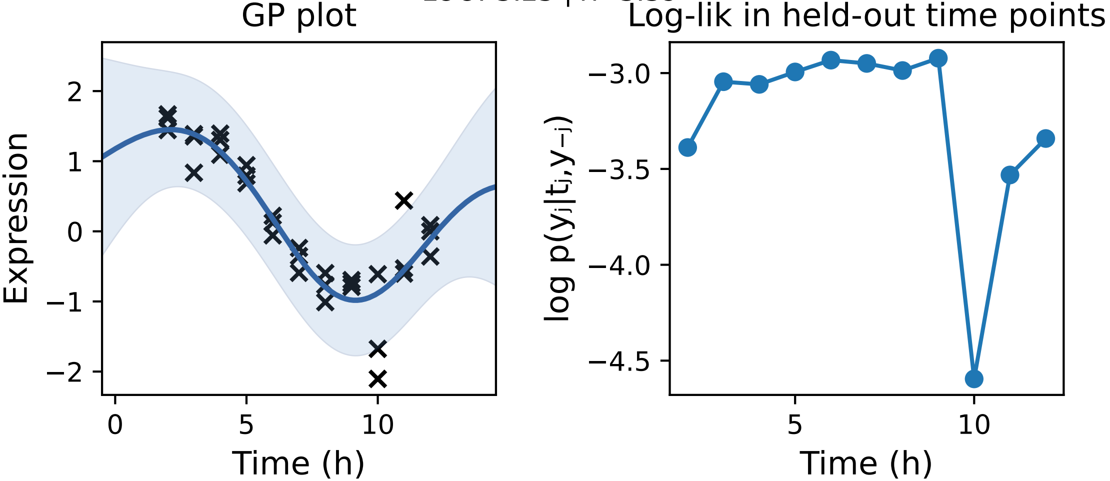

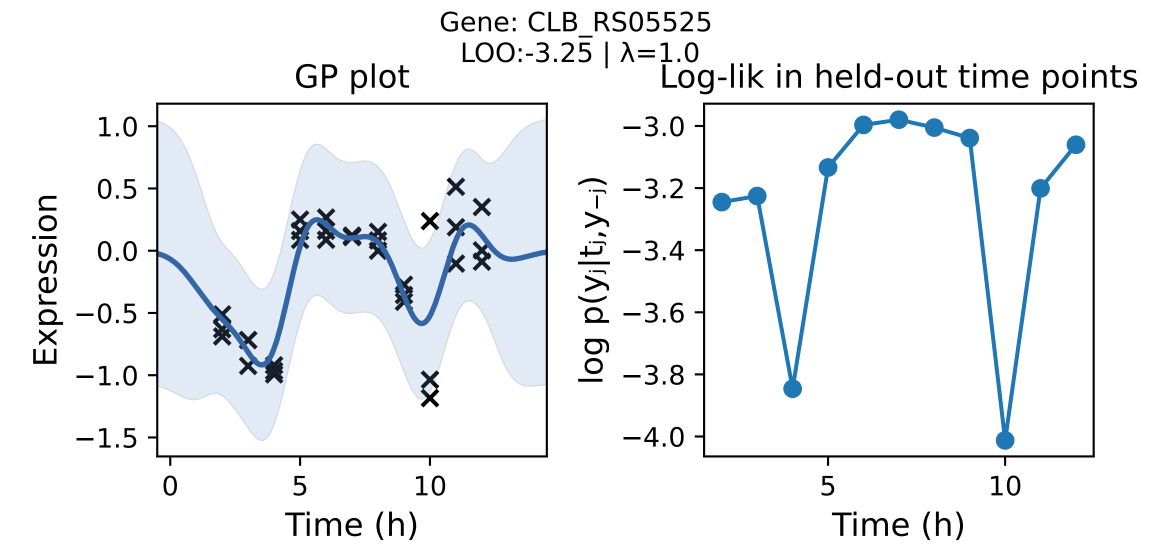

Gene: CLB_RS05525
LOO:-3.25 | λ=1.0
GP plot
Expression
1.0
0.5
0.0
−0.5
−1.0
−1.5
0
5
10
Time (h)
Log-lik in held-out time points
log p(y_j|t_j,y_−j)
−3.0
−3.2
−3.4
−3.6
−3.8
−4.0
5
10
Time (h)

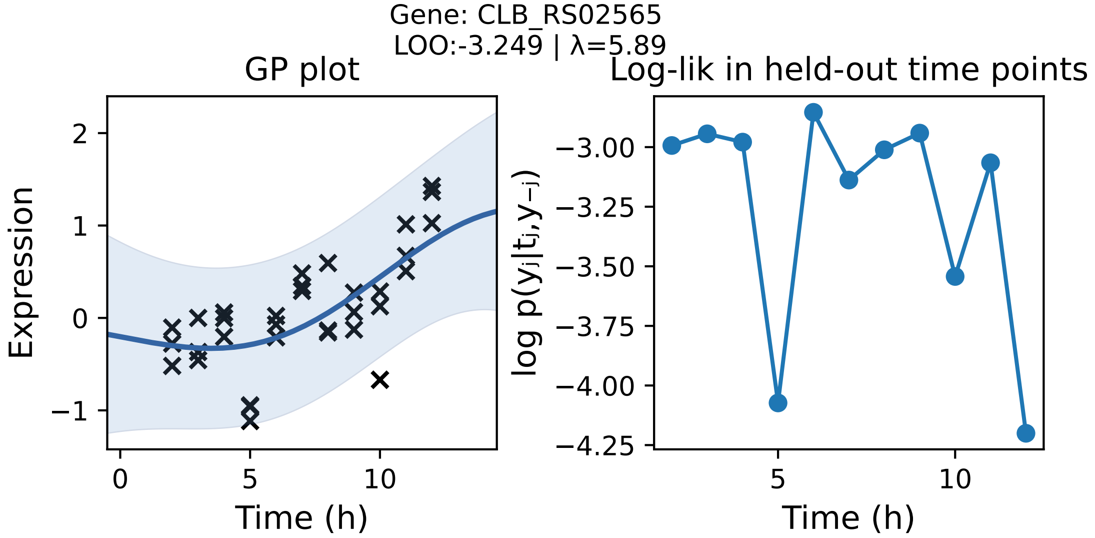

Gene: CLB_RS02565
LOO:-3.249 | λ=5.89
GP plot
Expression
Time (h)
2
1
0
−1
0
5
10
Log-lik in held-out time points
log p(y_j|t_j,y_−j)
−3.00
−3.25
−3.50
−3.75
−4.00
−4.25
5
10
Time (h)

Gene: CLB_RS16795
LOO:-3.248 | λ=2.36

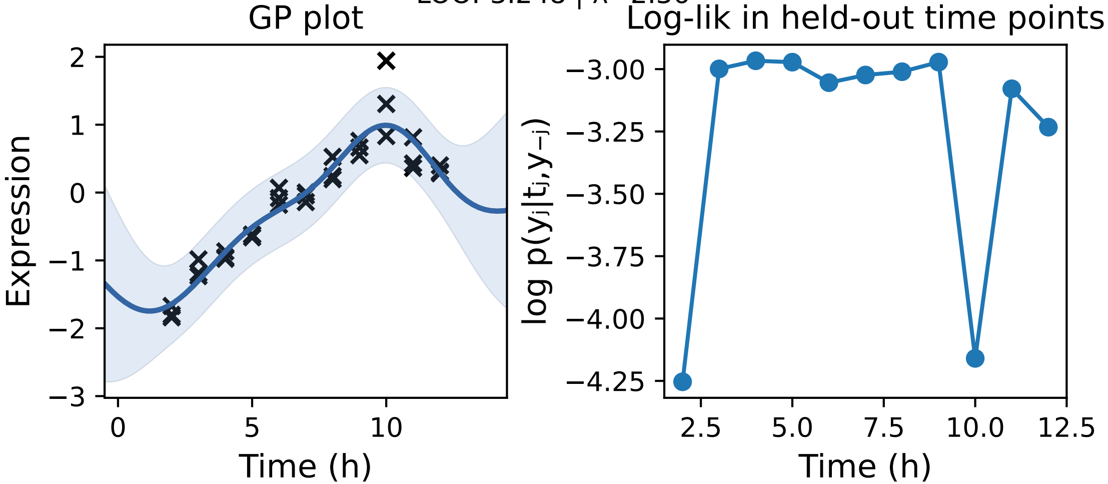

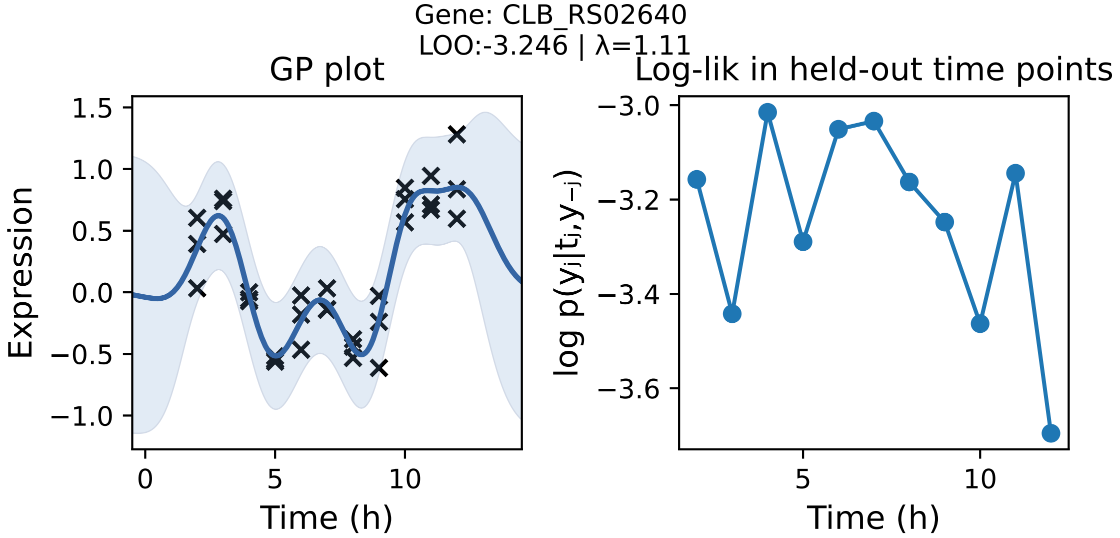

Gene: CLB_RS02640
LOO:-3.246 | λ=1.11
GP plot
Log-lik in held-out time points
Expression
1.5
1.0
0.5
0.0
−0.5
−1.0
0
5
10
Time (h)
log p(yj|tj,y−j)
−3.0
−3.2
−3.4
−3.6
5
10
Time (h)

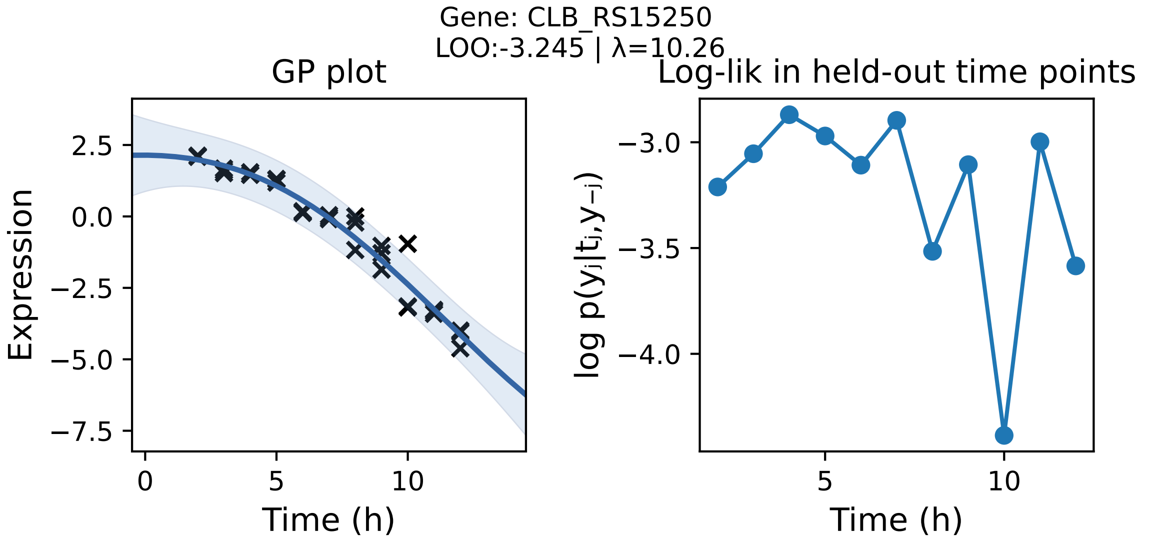
Gene: CLB_RS15250
LOO:-3.245 | λ=10.26
GP plot
Log-lik in held-out time points
Expression
Time (h)
log p(y_j|t_j,y_−j)
2.5
0.0
−2.5
−5.0
−7.5
0
5
10
−3.0
−3.5
−4.0

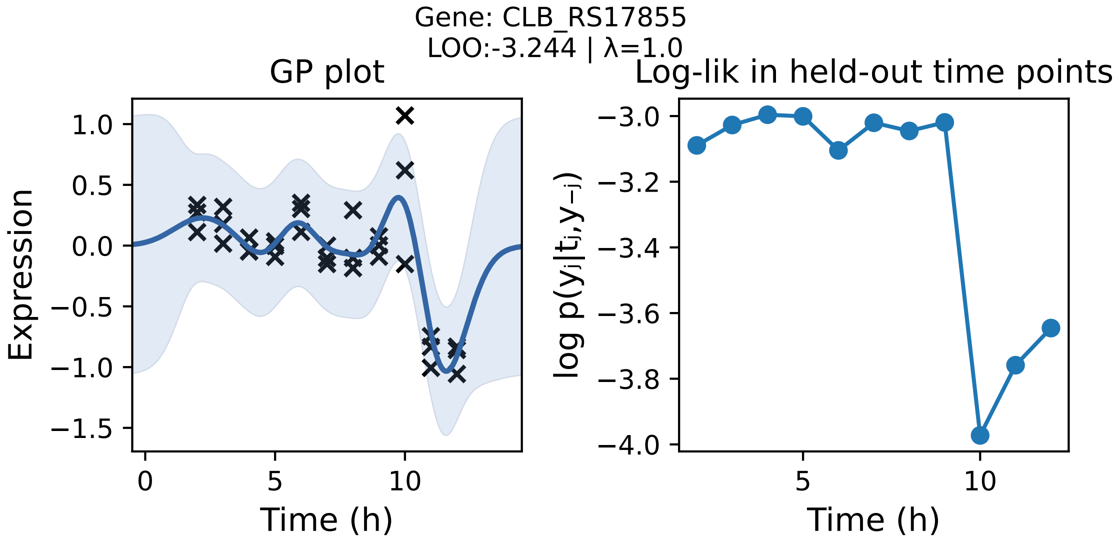

Gene: CLB_RS17855
LOO:-3.244 | λ=1.0
GP plot
Expression
Time (h)
1.0
0.5
0.0
−0.5
−1.0
−1.5
0
5
10
Log-lik in held-out time points
log p(y_j|t_j,y_−j)
Time (h)
−3.0
−3.2
−3.4
−3.6
−3.8
−4.0
5
10

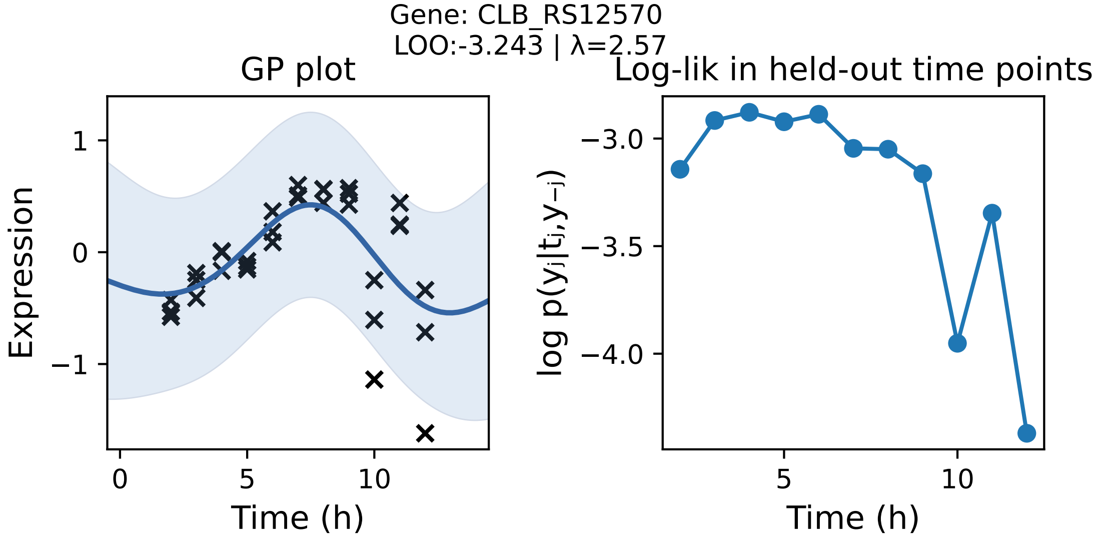
Gene: CLB_RS12570
LOO:-3.243 | λ=2.57
GP plot
Expression
Time (h)
1
0
−1
0
5
10
Log-lik in held-out time points
log p(y_j|t_j,y_−j)
−3.0
−3.5
−4.0
5
10
Time (h)

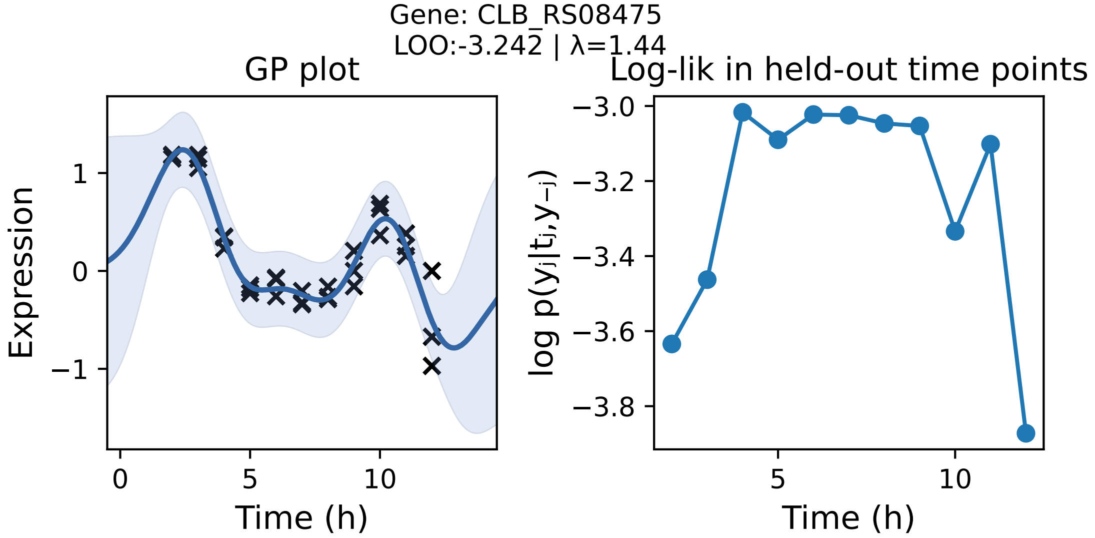
Gene: CLB_RS08475
LOO:-3.242 | λ=1.44
GP plot
Expression
Time (h)
Log-lik in held-out time points
log p(y_j|t_j,y_−j)
Time (h)

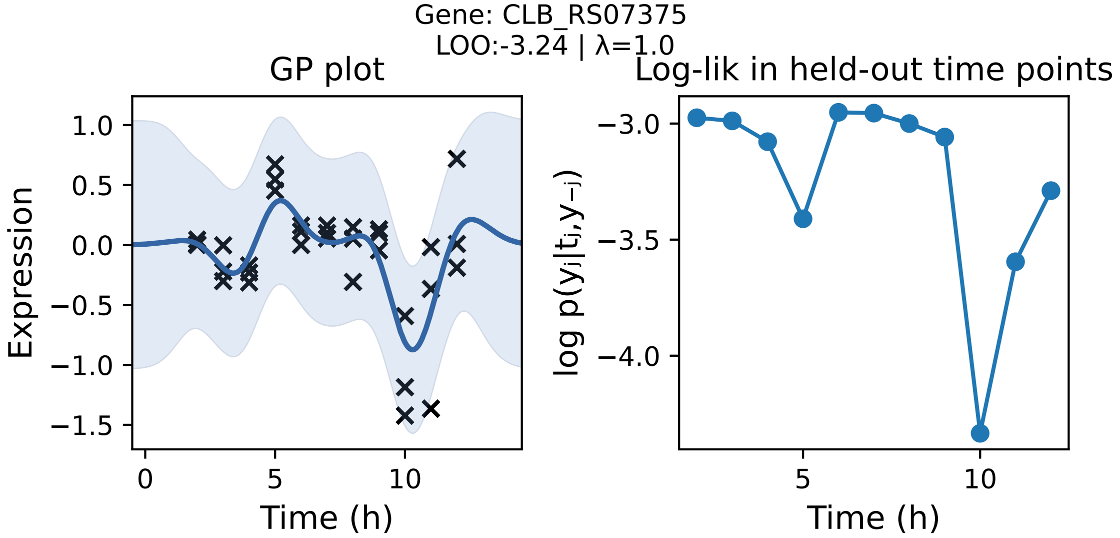
Gene: CLB_RS07375
LOO:-3.24 | λ=1.0
GP plot
Log-lik in held-out time points
Expression
Time (h)
log p(yj|tj,y−j)
1.0
0.5
0.0
−0.5
−1.0
−1.5
0
5
10
−3.0
−3.5
−4.0

Gene: CLB_RS13600
LOO:-3.239 | λ=1.0

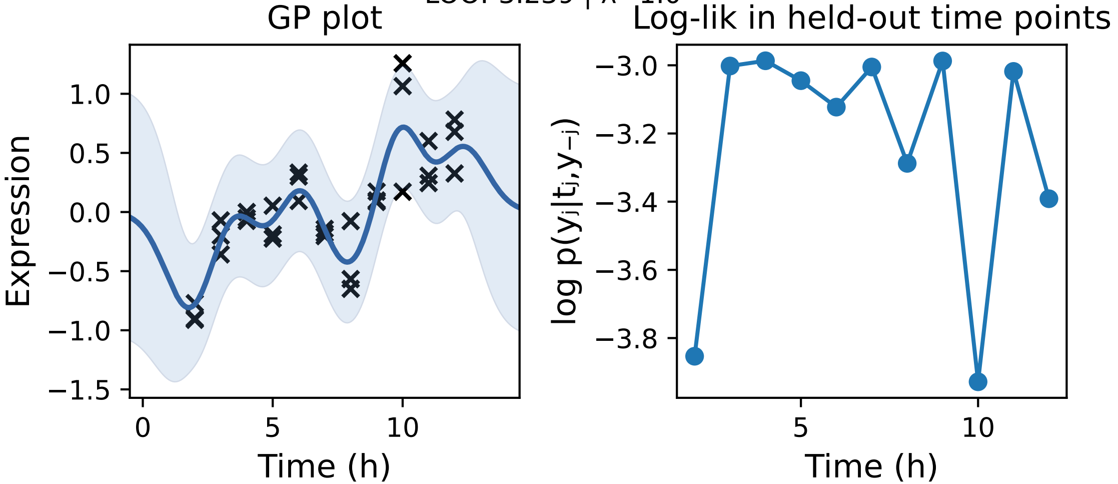

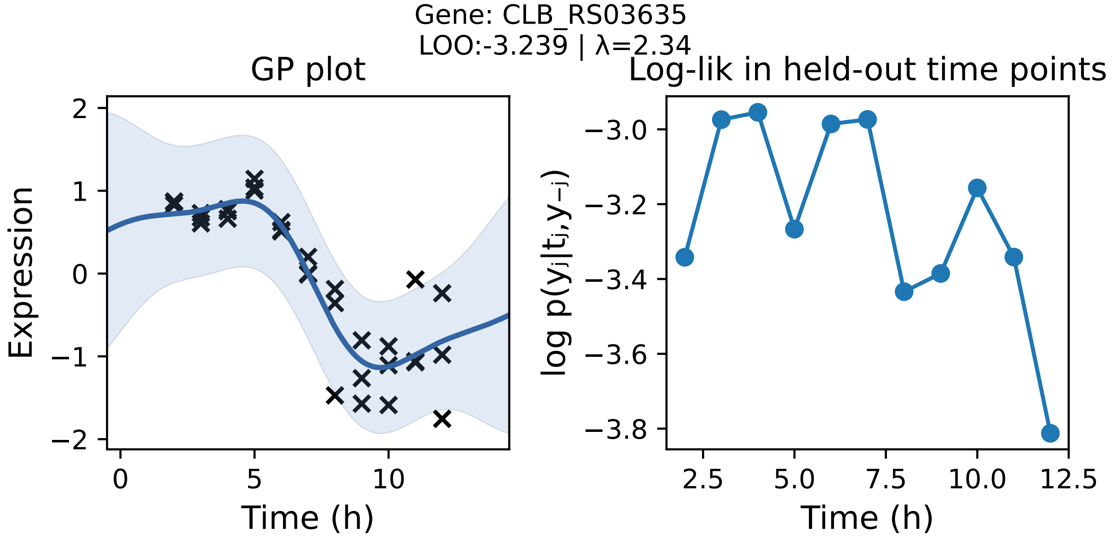

Gene: CLB_RS03635
LOO:-3.239 | λ=2.34
GP plot
Expression
Time (h)
2
1
0
−1
−2
0
5
10
Log-lik in held-out time points
log p(y_j|t_j,y_−j)
Time (h)
−3.0
−3.2
−3.4
−3.6
−3.8
2.5
5.0
7.5
10.0
12.5

Gene: CLB_RS02370
LOO:-3.238 | λ=1.0

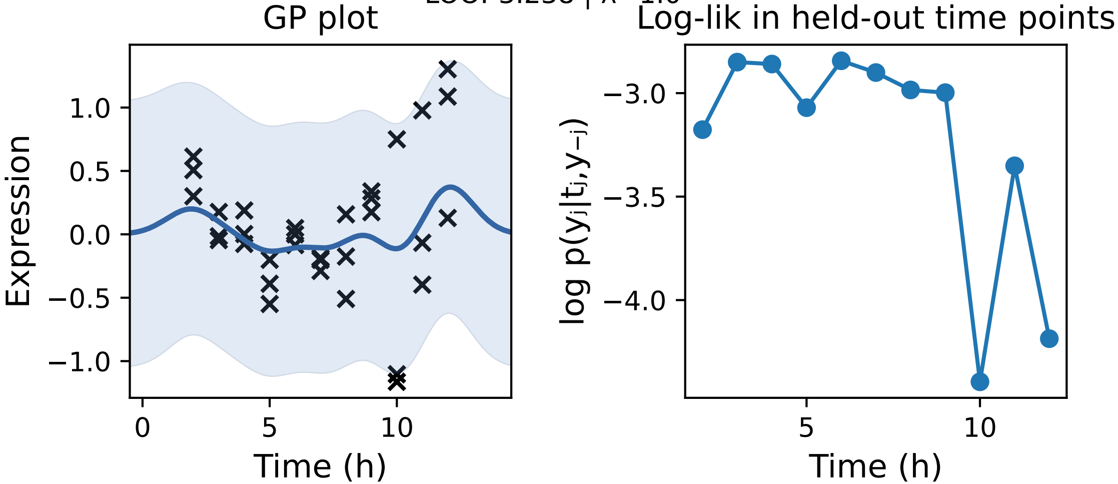

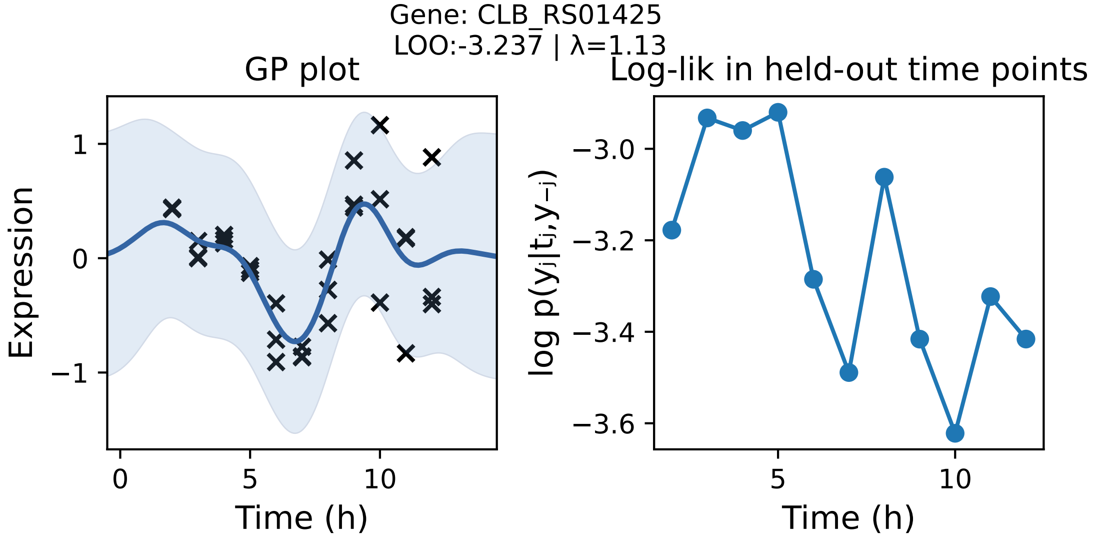

Gene: CLB_RS01425
LOO:-3.237 | λ=1.13
GP plot
Expression
1
0
−1
0
5
10
Time (h)
Log-lik in held-out time points
log p(yj|tj,y−j)
−3.0
−3.2
−3.4
−3.6
5
10
Time (h)

Gene: CLB_RS11085
LOO:-3.236 | λ=3.34

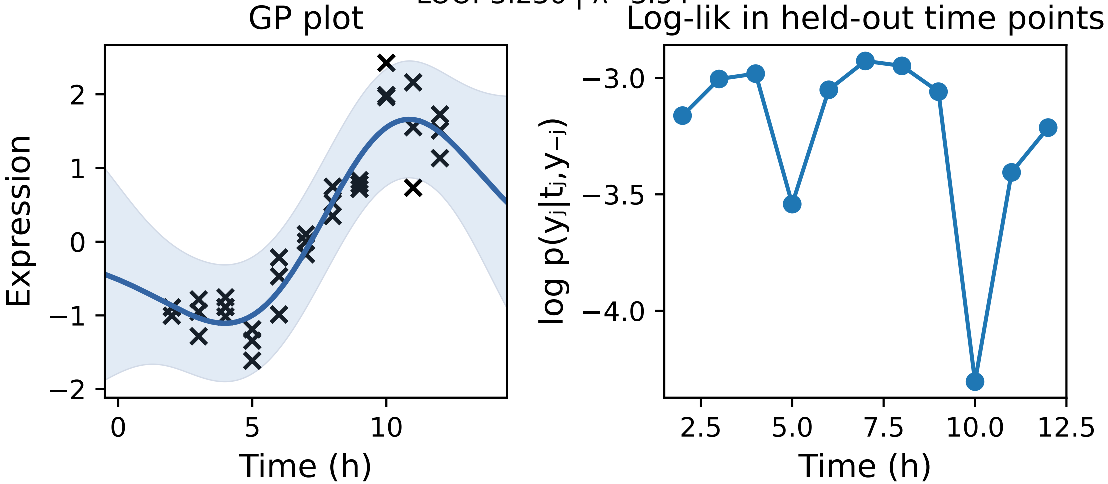

Gene: CLB_RS16545
LOO:-3.236 | λ=1.14

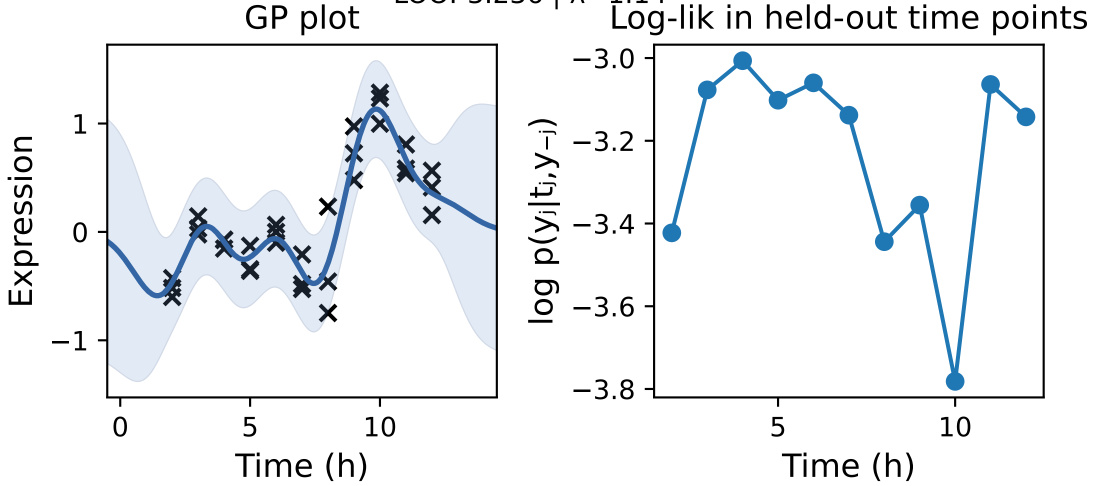

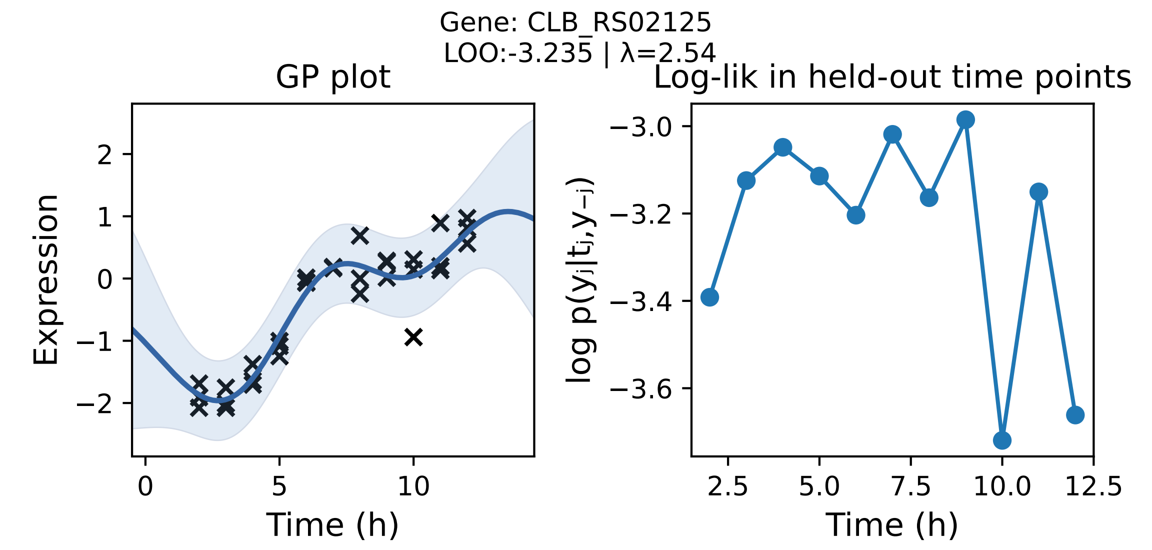

Gene: CLB_RS02125
LOO:-3.235 | λ=2.54
GP plot
Expression
Time (h)
Log-lik in held-out time points
log p(y_j|t_j,y_−j)
Time (h)

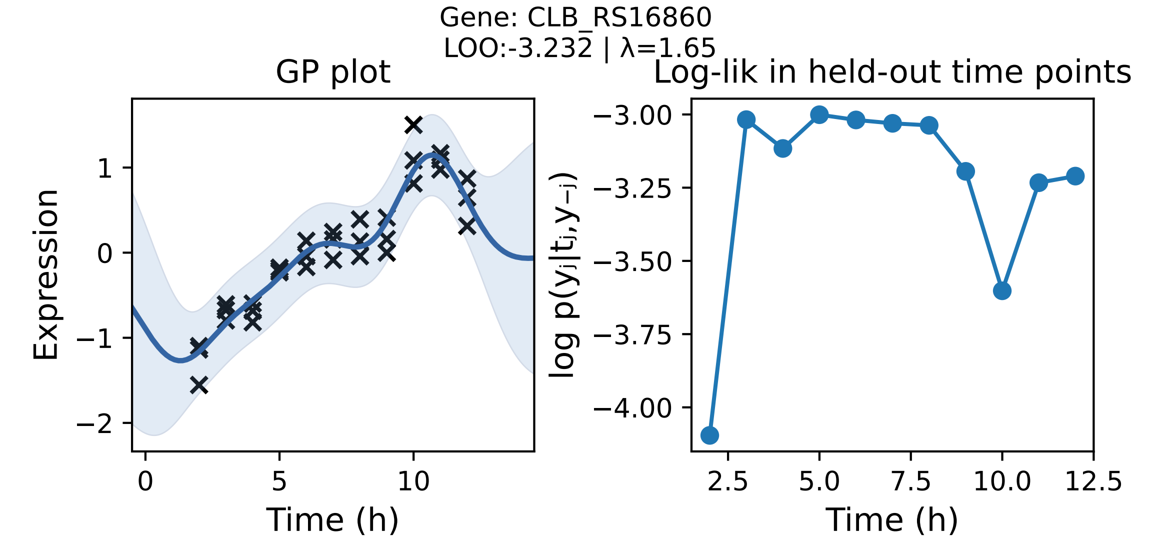

Gene: CLB_RS16860
LOO:-3.232 | λ=1.65
GP plot
Expression
Time (h)
Log-lik in held-out time points
log p(y_j|t_j,y_−j)
Time (h)

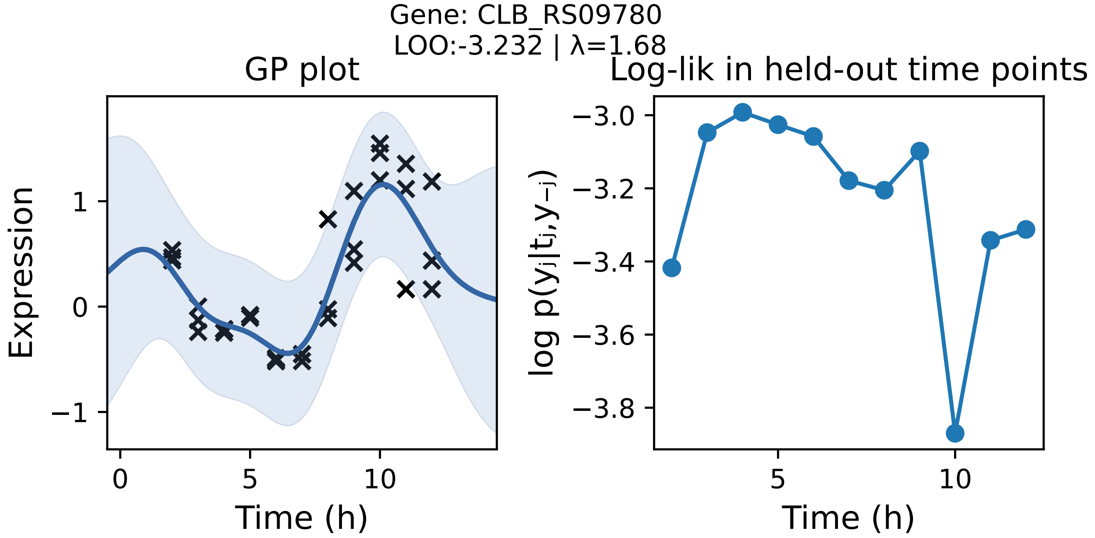

Gene: CLB_RS09780
LOO:-3.232 | λ=1.68
GP plot
Log-lik in held-out time points
Expression
Time (h)
log p(y_j|t_j,y_−j)
1
0
−1
0
5
10
−3.0
−3.2
−3.4
−3.6
−3.8

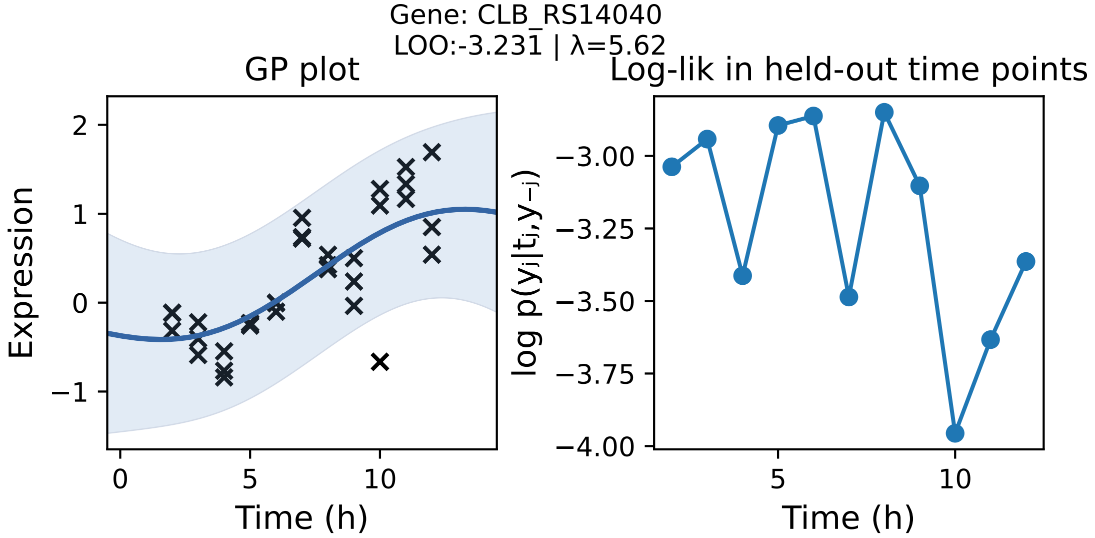
Gene: CLB_RS14040
LOO:-3.231 | λ=5.62
GP plot
Log-lik in held-out time points
Expression
Time (h)
log p(yj|tj,y−j)
Time (h)
2
1
0
−1
0
5
10
−3.00
−3.25
−3.50
−3.75
−4.00
5
10

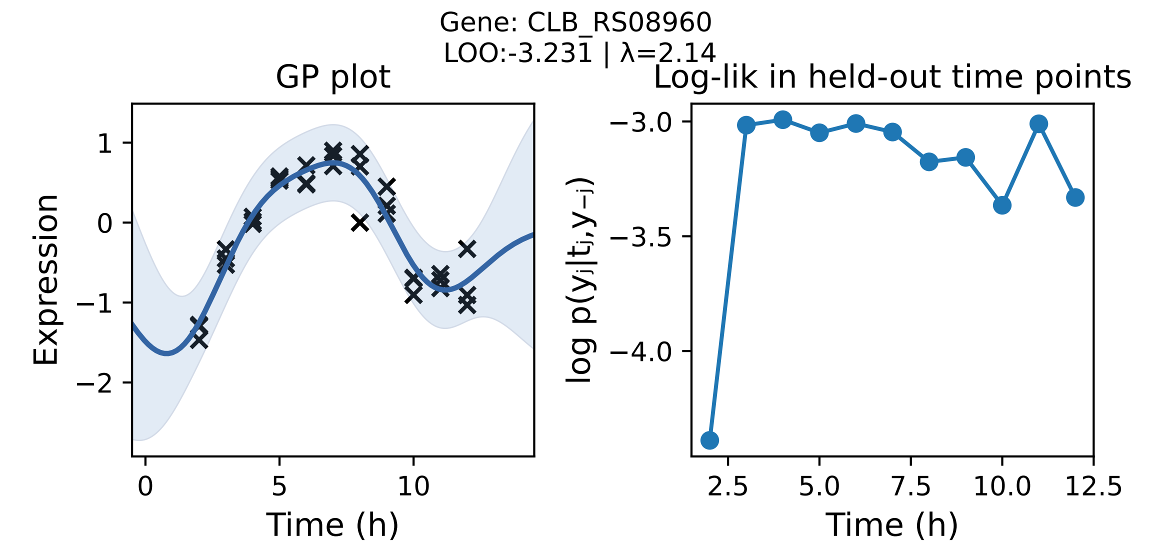

Gene: CLB_RS08960
LOO:-3.231 | λ=2.14
GP plot
Expression
Time (h)
1
0
−1
−2
0
5
10
Log-lik in held-out time points
log p(y_j|t_j,y_−j)
−3.0
−3.5
−4.0
2.5
5.0
7.5
10.0
12.5
Time (h)

Gene: CLB_RS05820
LOO:-3.231 | λ=4.32

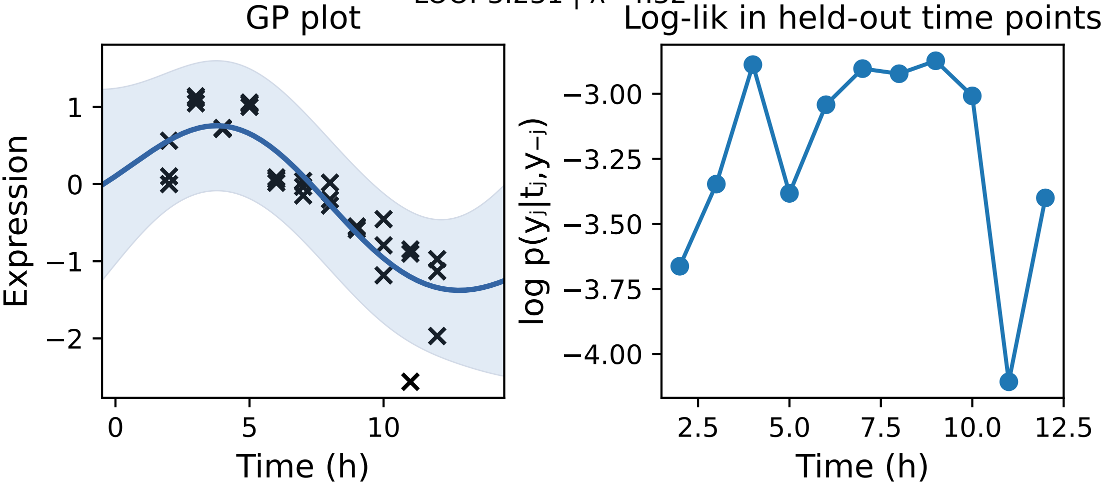

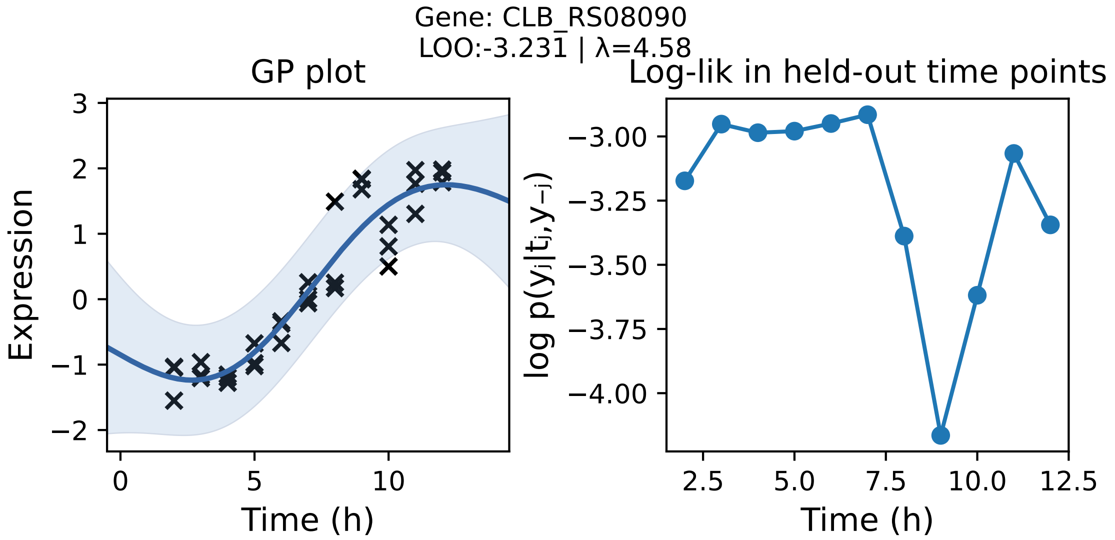
Gene: CLB_RS08090
LOO:-3.231 | λ=4.58
GP plot
Expression
Time (h)
3
2
1
0
−1
−2
0
5
10
Log-lik in held-out time points
log p(y_j|t_j,y_{−j})
Time (h)
−3.00
−3.25
−3.50
−3.75
−4.00
2.5
5.0
7.5
10.0
12.5

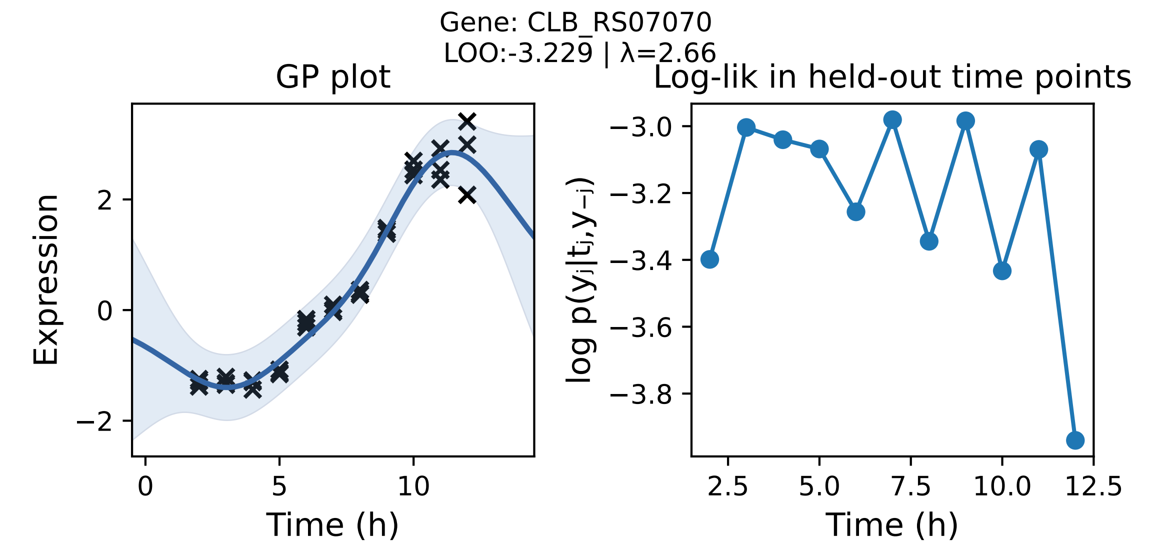

Gene: CLB_RS07070
LOO:-3.229 | λ=2.66
GP plot
Expression
Time (h)
Log-lik in held-out time points
log p(y_j|t_j,y_−j)
Time (h)

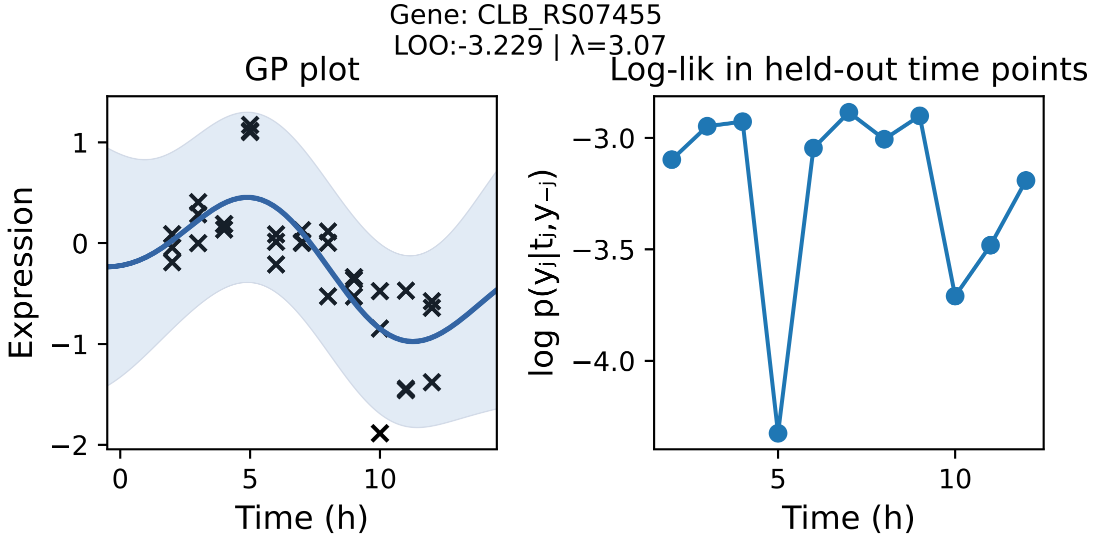

Gene: CLB_RS07455
LOO:-3.229 | λ=3.07
GP plot
Expression
1
0
−1
−2
0
5
10
Time (h)
Log-lik in held-out time points
log p(y_j|t_j,y_−j)
−3.0
−3.5
−4.0
5
10
Time (h)

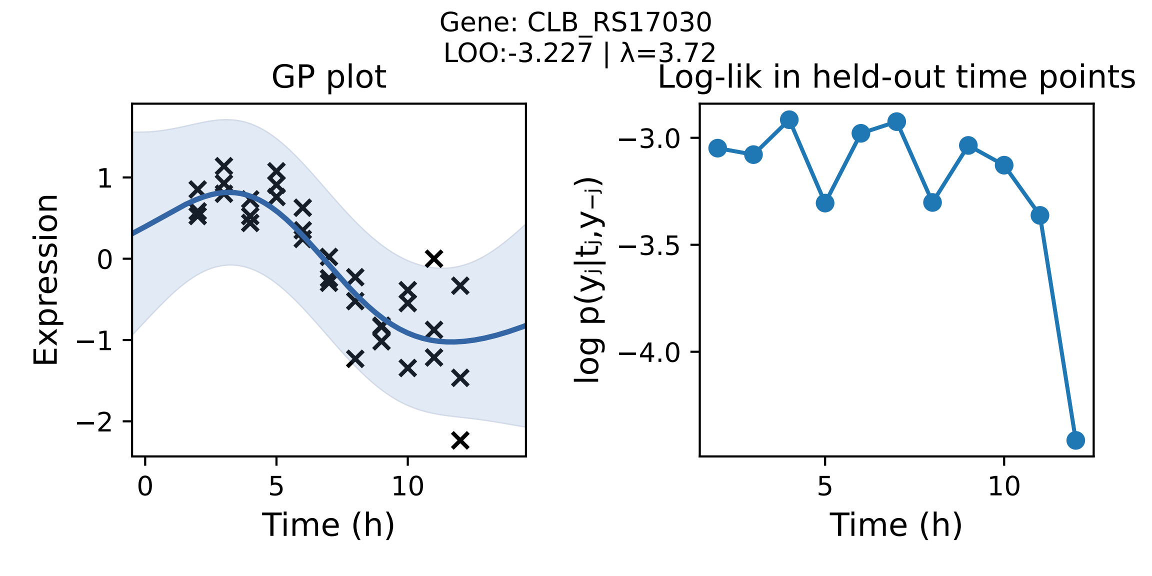

Gene: CLB_RS17030
LOO:-3.227 | λ=3.72
GP plot
Expression
Time (h)
Log-lik in held-out time points
log p(y_j|t_j,y_−j)
Time (h)

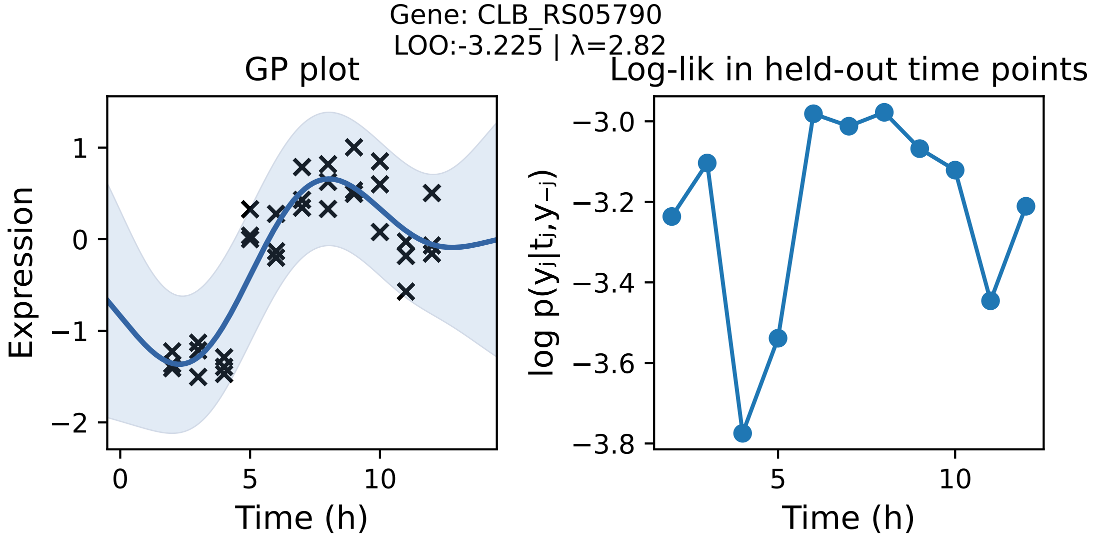

Gene: CLB_RS05790
LOO:-3.225 | λ=2.82
GP plot
Expression
Time (h)
1
0
−1
−2
0
5
10
Log-lik in held-out time points
log p(y_j|t_j,y_−j)
Time (h)
−3.0
−3.2
−3.4
−3.6
−3.8
5
10

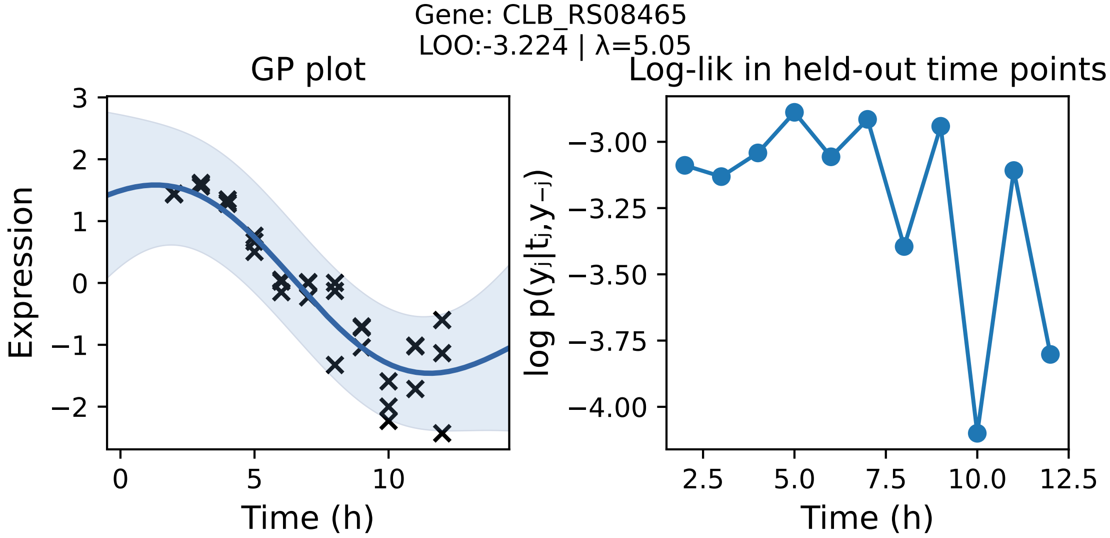

Gene: CLB_RS08465
LOO:-3.224 | λ=5.05
GP plot
Expression
Time (h)
Log-lik in held-out time points
log p(y_j|t_j,y_−j)
Time (h)

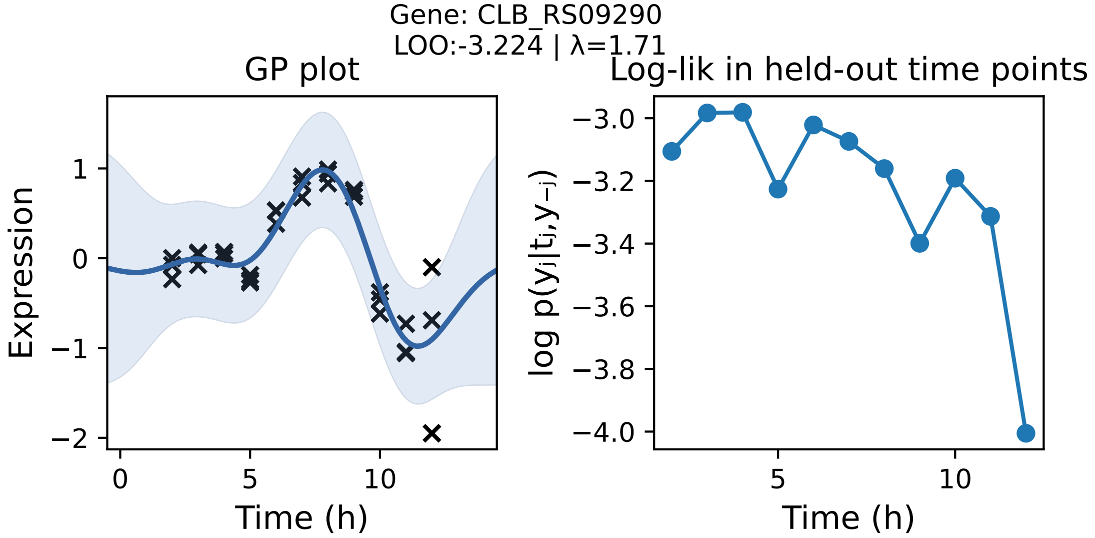

Gene: CLB_RS09290
LOO:-3.224 | λ=1.71
GP plot
Expression
Time (h)
Log-lik in held-out time points
log p(yj|tj,y−j)
Time (h)

Gene: CLB_RS14215
LOO:-3.222 | λ=1.0

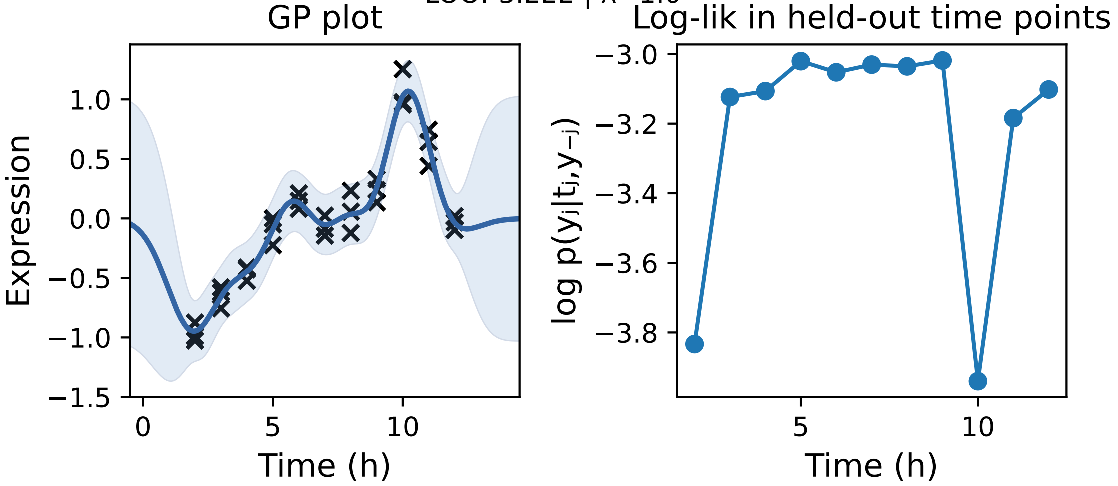

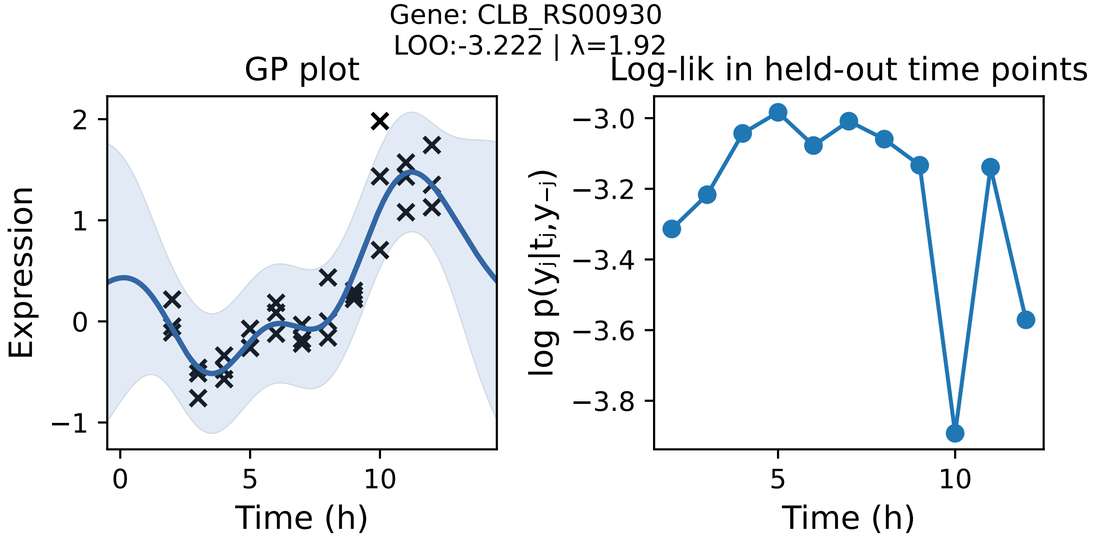

Gene: CLB_RS00930
LOO:-3.222 | λ=1.92
GP plot
Expression
Time (h)
2
1
0
−1
0
5
10
Log-lik in held-out time points
log p(y_j|t_j,y_−j)
−3.0
−3.2
−3.4
−3.6
−3.8
5
10
Time (h)

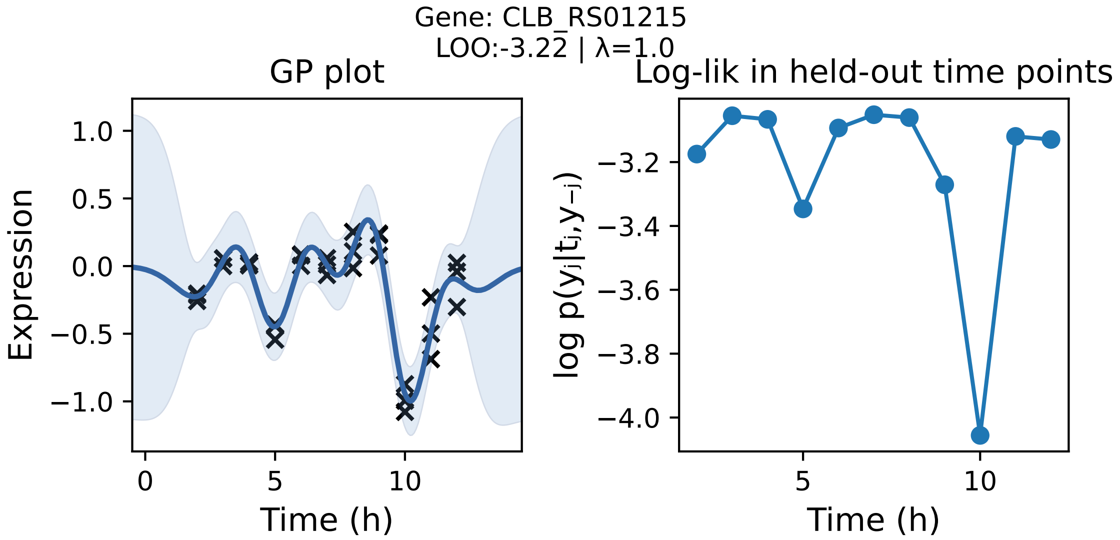

Gene: CLB_RS01215
LOO:-3.22 | λ=1.0
GP plot
Expression
1.0
0.5
0.0
−0.5
−1.0
0
5
10
Time (h)
Log-lik in held-out time points
log p(y_j|t_j,y_−j)
−3.2
−3.4
−3.6
−3.8
−4.0
5
10
Time (h)

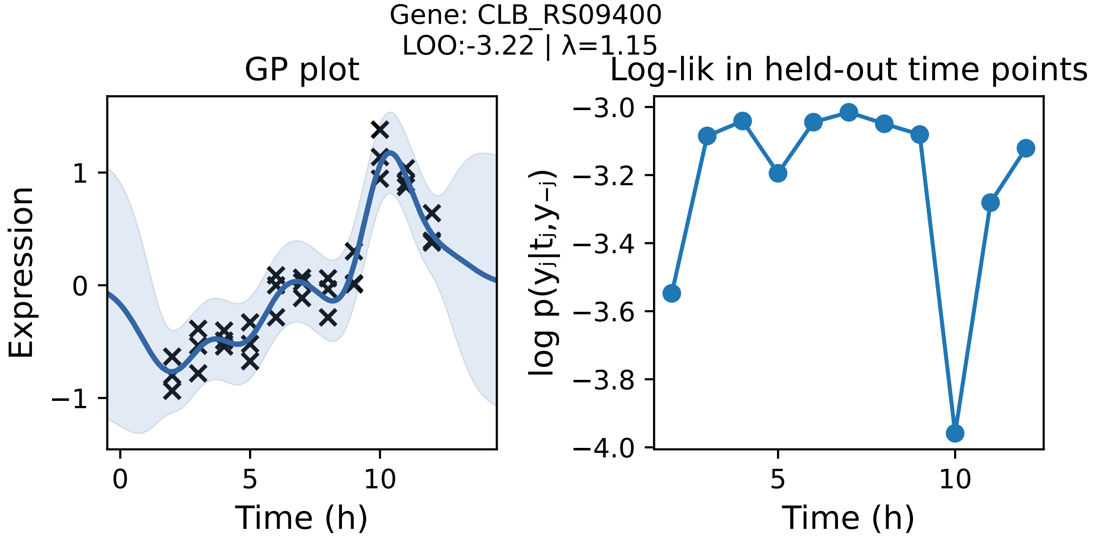

Gene: CLB_RS09400
LOO:-3.22 | λ=1.15
GP plot
Expression
Time (h)
Log-lik in held-out time points
log p(y_j|t_j,y_−j)
Time (h)

Gene: CLB_RS08600
LOO:-3.22 | λ=3.68

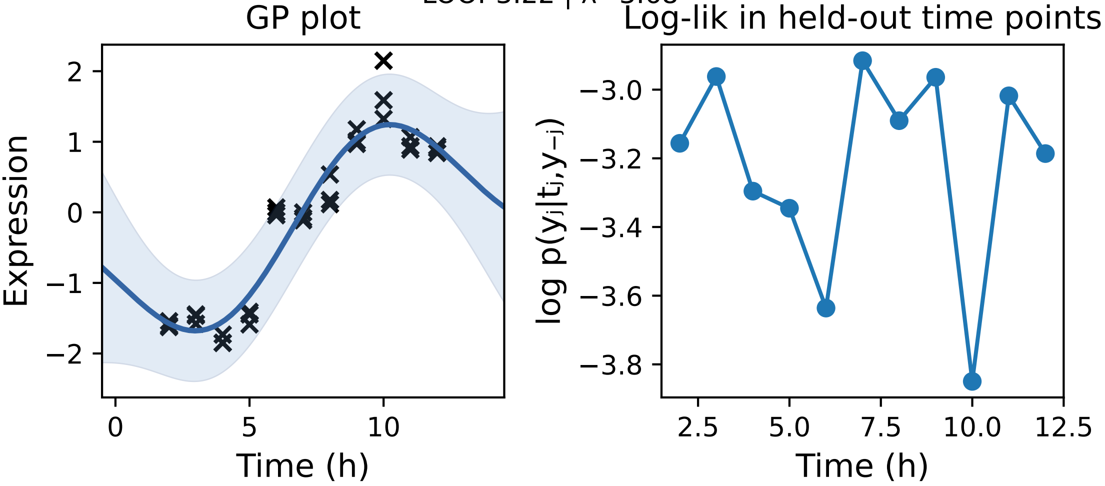

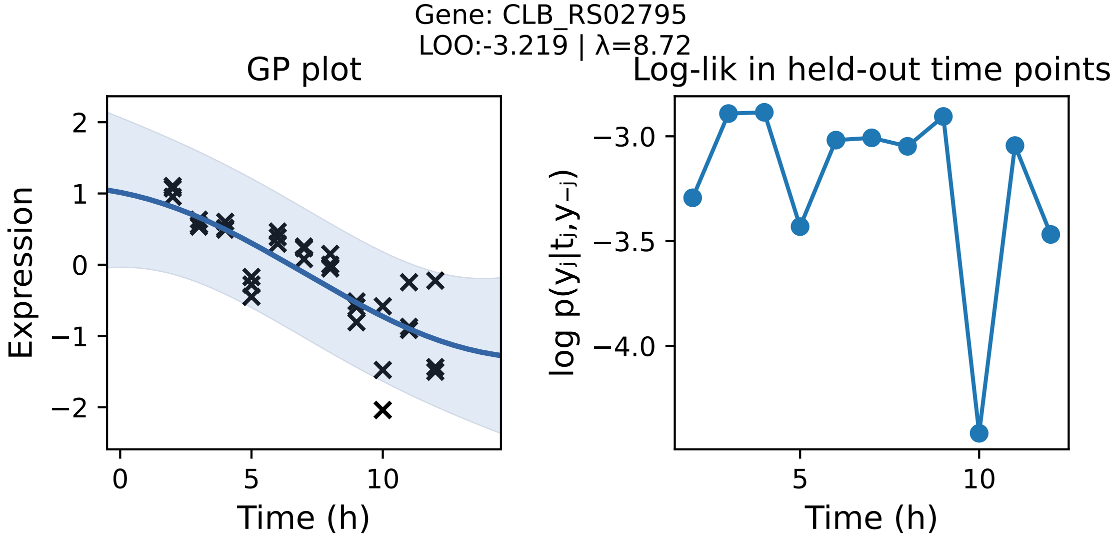

Gene: CLB_RS02795
LOO:-3.219 | λ=8.72
GP plot
Expression
Time (h)
Log-lik in held-out time points
log p(y_j|t_j,y_−j)
Time (h)

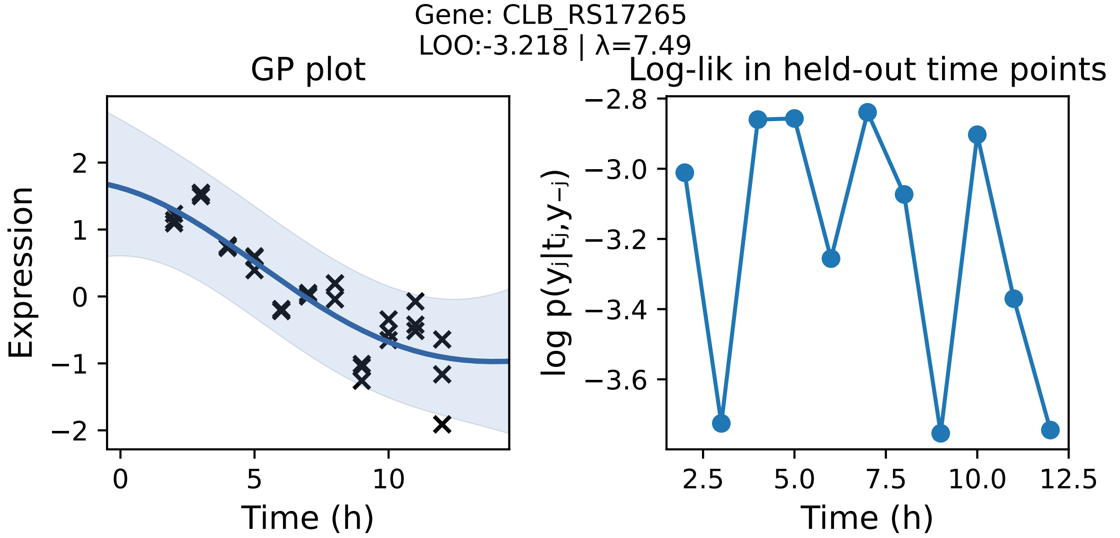

Gene: CLB_RS17265
LOO:-3.218 | λ=7.49
GP plot
Expression
2
1
0
−1
−2
0
5
10
Time (h)
Log-lik in held-out time points
log p(y_j|t_j,y_−j)
−2.8
−3.0
−3.2
−3.4
−3.6
2.5
5.0
7.5
10.0
12.5
Time (h)

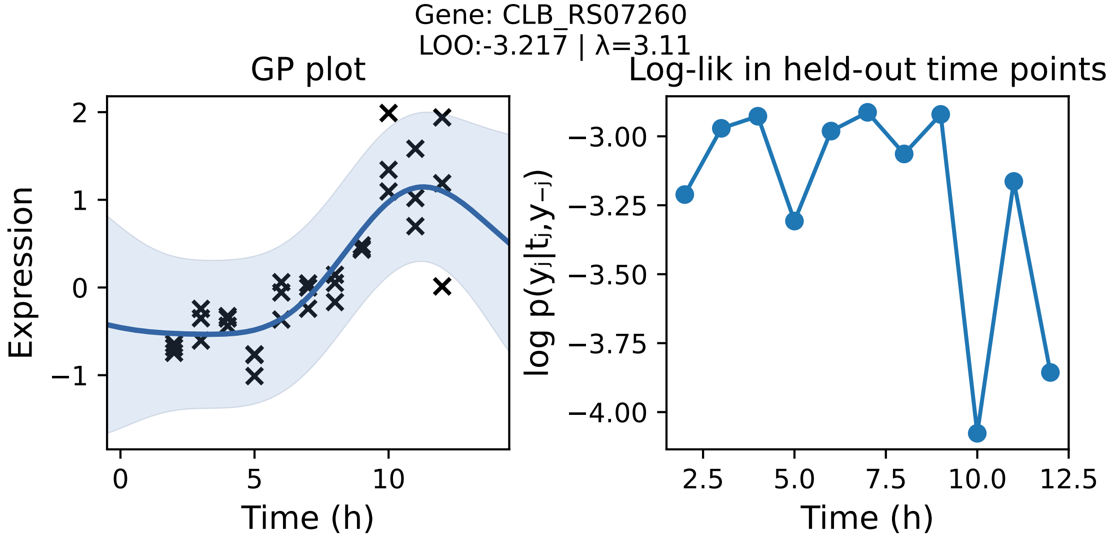
Gene: CLB_RS07260
LOO:-3.217 | λ=3.11
GP plot
Expression
Time (h)
Log-lik in held-out time points
log p(y_j|t_j,y_−j)
Time (h)

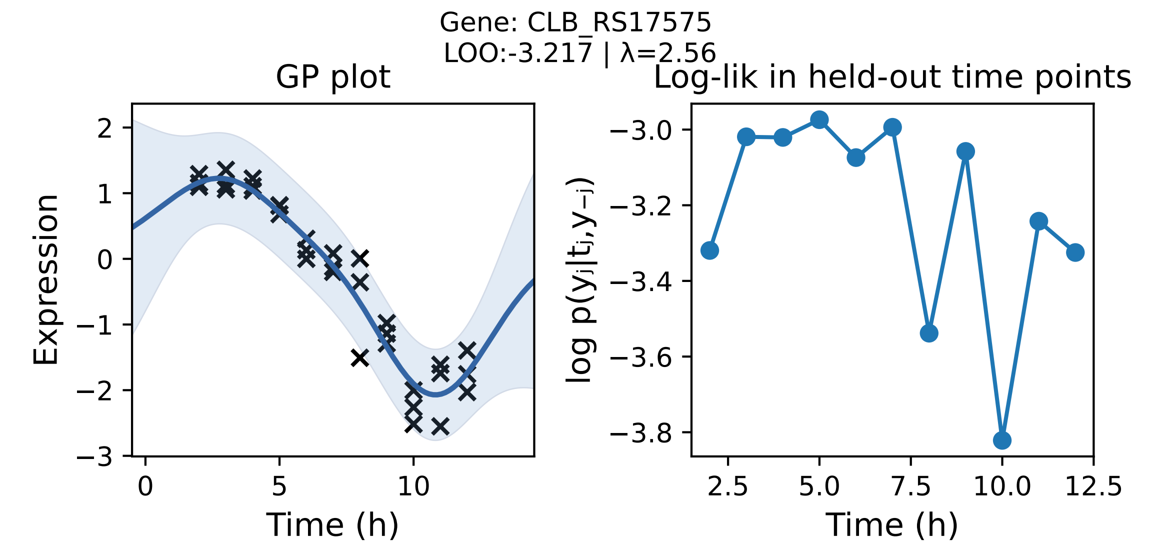

Gene: CLB_RS17575
LOO:-3.217 | λ=2.56
GP plot
Expression
Time (h)
Log-lik in held-out time points
log p(y_j|t_j,y_−j)
Time (h)

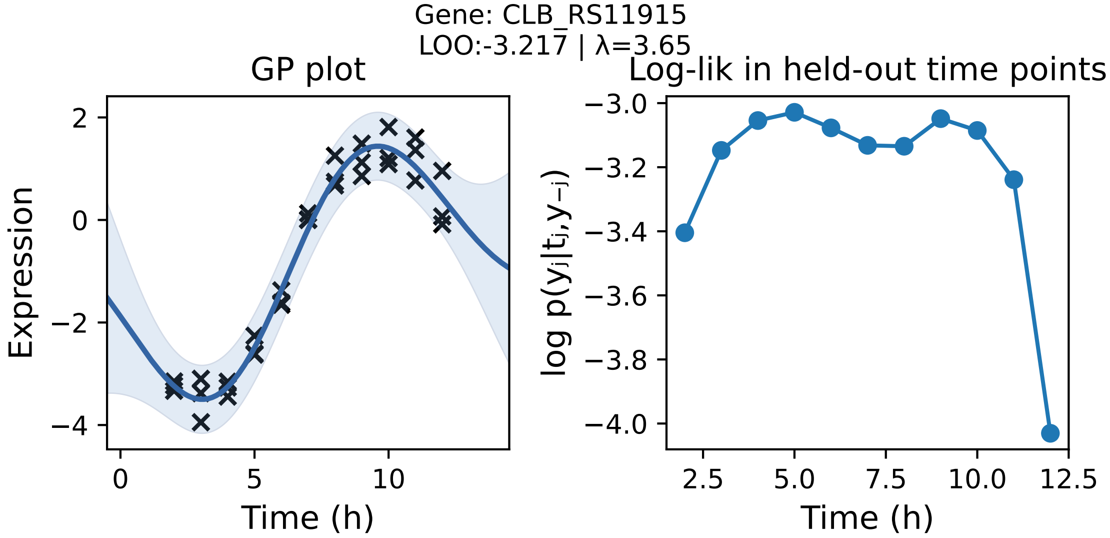

Gene: CLB_RS11915
LOO:-3.217 | λ=3.65
GP plot
Expression
Time (h)
2
0
−2
−4
0
5
10
Log-lik in held-out time points
log p(y_j|t_j,y_−j)
Time (h)
−3.0
−3.2
−3.4
−3.6
−3.8
−4.0
2.5
5.0
7.5
10.0
12.5

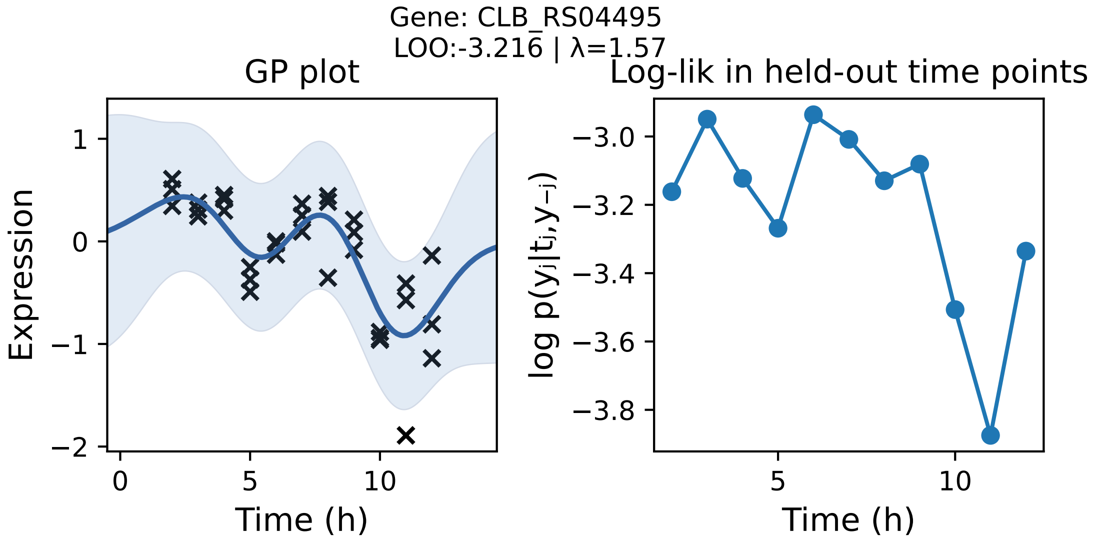

Gene: CLB_RS04495
LOO:-3.216 | λ=1.57
GP plot
Expression
Time (h)
Log-lik in held-out time points
log p(yj|tj,y−j)
Time (h)

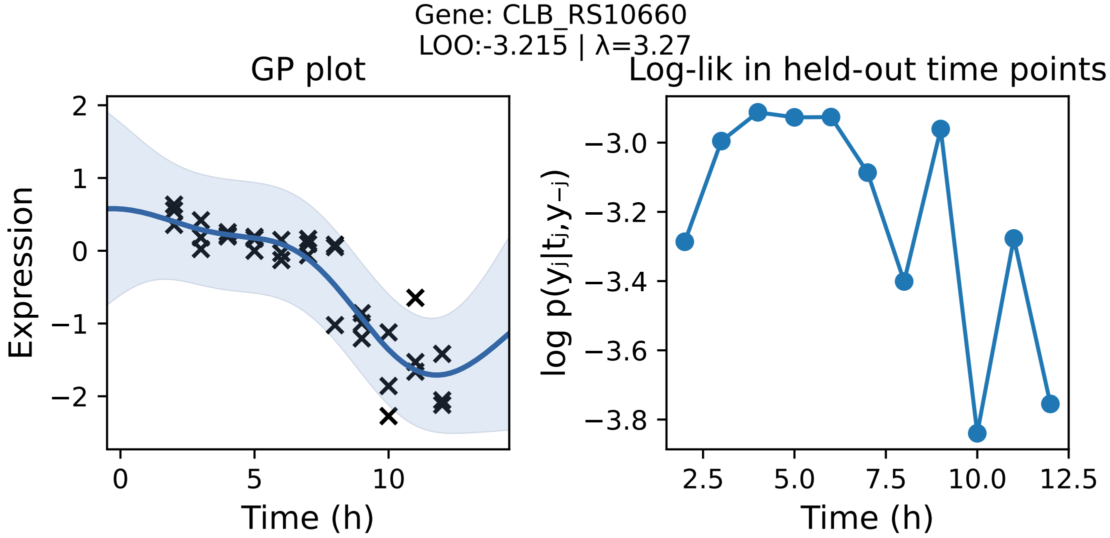

Gene: CLB_RS10660
LOO:-3.215 | λ=3.27
GP plot
Expression
Time (h)
Log-lik in held-out time points
log p(y_j|t_j,y_−j)
Time (h)

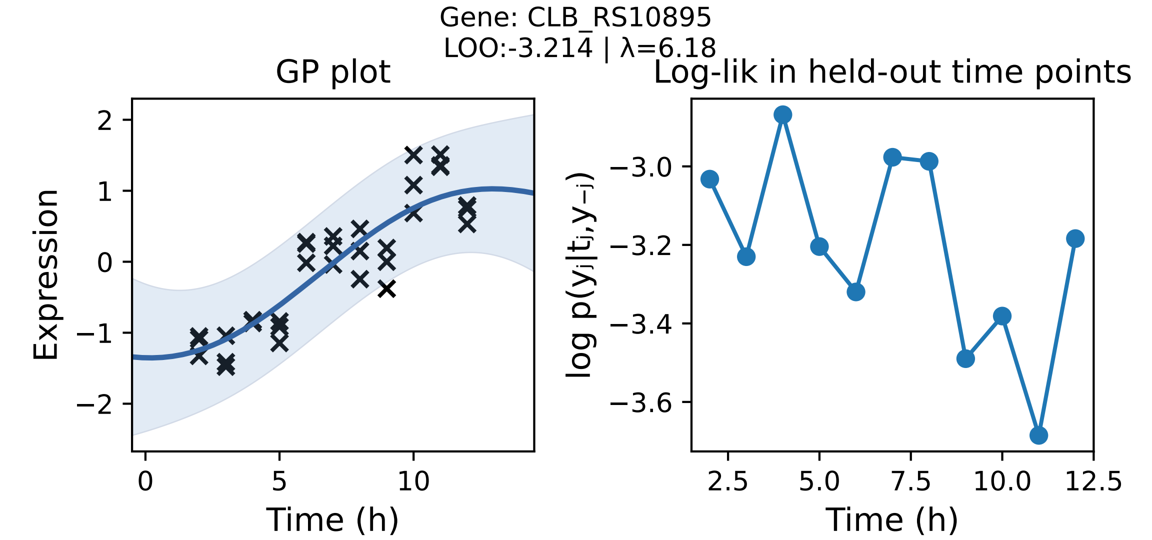

Gene: CLB_RS10895
LOO:-3.214 | λ=6.18
GP plot
Expression
Time (h)
Log-lik in held-out time points
log p(y_j|t_j,y_−j)
Time (h)

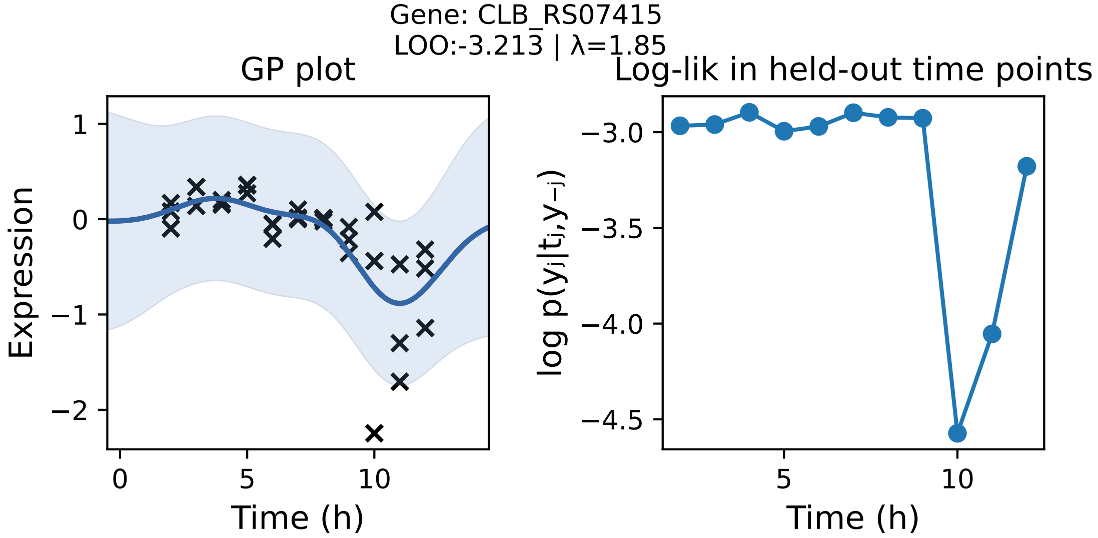

Gene: CLB_RS07415
LOO:-3.213 | λ=1.85
GP plot
Expression
Time (h)
Log-lik in held-out time points
log p(y_j|t_j,y_−j)
Time (h)

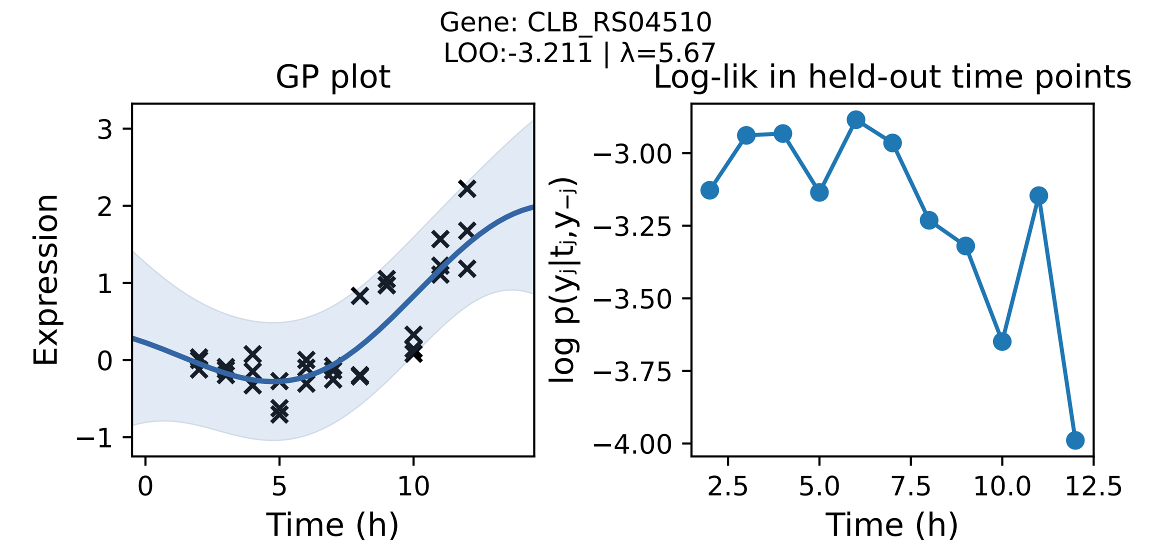

Gene: CLB_RS04510
LOO:-3.211 | λ=5.67
GP plot
Expression
Time (h)
Log-lik in held-out time points
log p(y_j|t_j,y_−j)
Time (h)

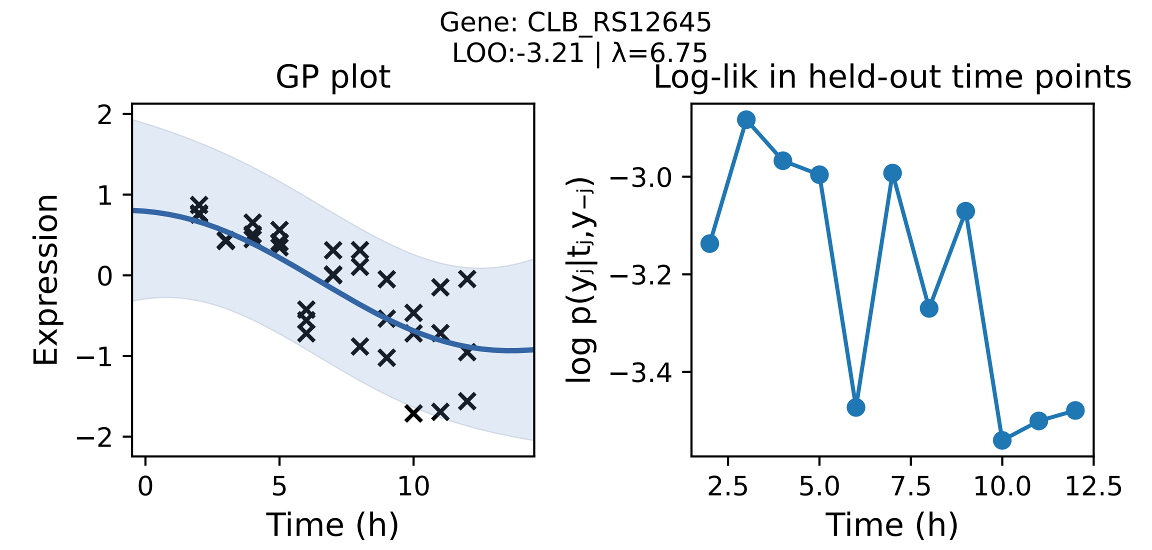
Gene: CLB_RS12645
LOO:-3.21 | λ=6.75
GP plot
Expression
Time (h)
Log-lik in held-out time points
log p(y_j|t_j,y_−j)
Time (h)
2
1
0
−1
−2
0
5
10
−3.0
−3.2
−3.4
2.5
5.0
7.5
10.0
12.5

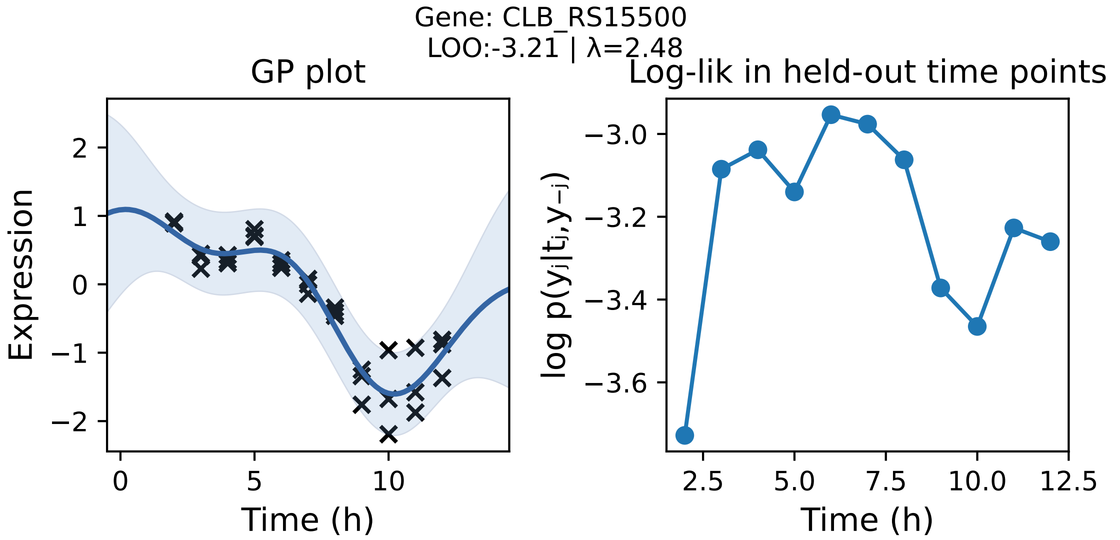
Gene: CLB_RS15500
LOO:-3.21 | λ=2.48
GP plot
Expression
Time (h)
2
1
0
−1
−2
0
5
10
Log-lik in held-out time points
log p(y_j|t_j,y_−j)
Time (h)
−3.0
−3.2
−3.4
−3.6
2.5
5.0
7.5
10.0
12.5

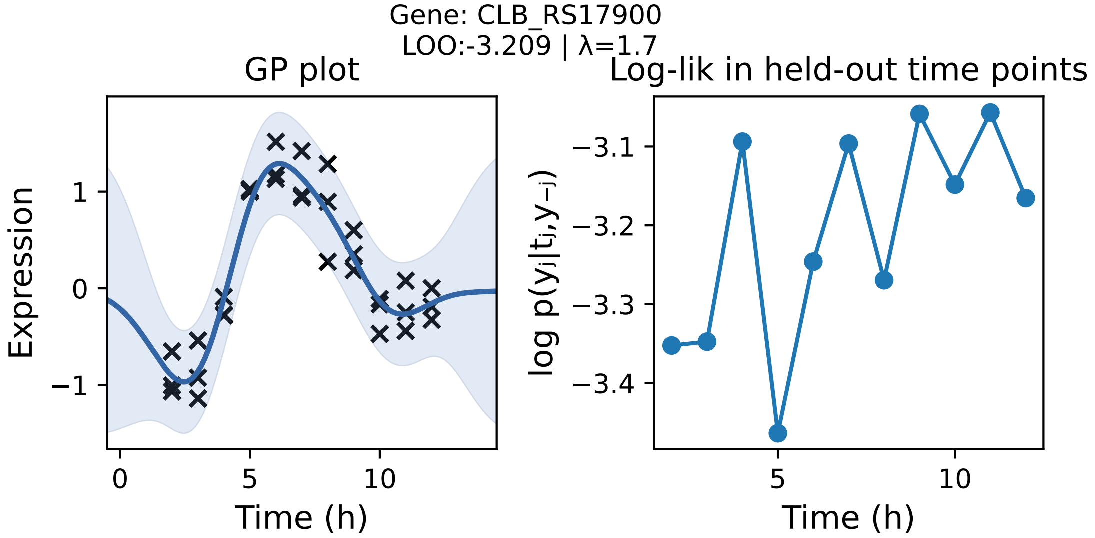

Gene: CLB_RS17900
LOO:-3.209 | λ=1.7
GP plot
Expression
1
0
−1
0
5
10
Time (h)
Log-lik in held-out time points
log p(yⱼ|tⱼ,y₋ⱼ)
−3.1
−3.2
−3.3
−3.4
5
10
Time (h)

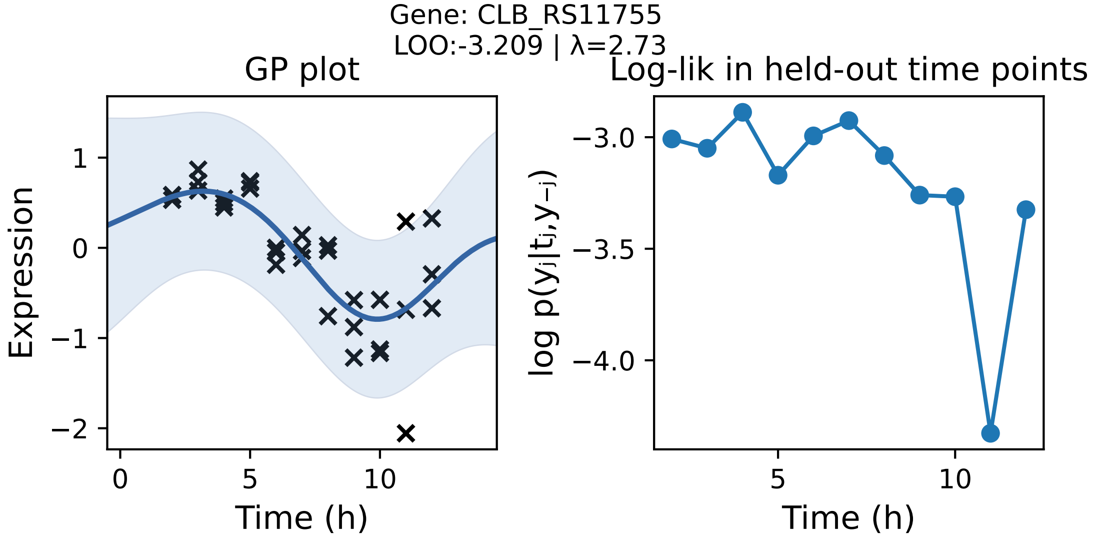

Gene: CLB_RS11755
LOO:-3.209 | λ=2.73
GP plot
Expression
Time (h)
Log-lik in held-out time points
log p(yj|tj,y−j)
Time (h)

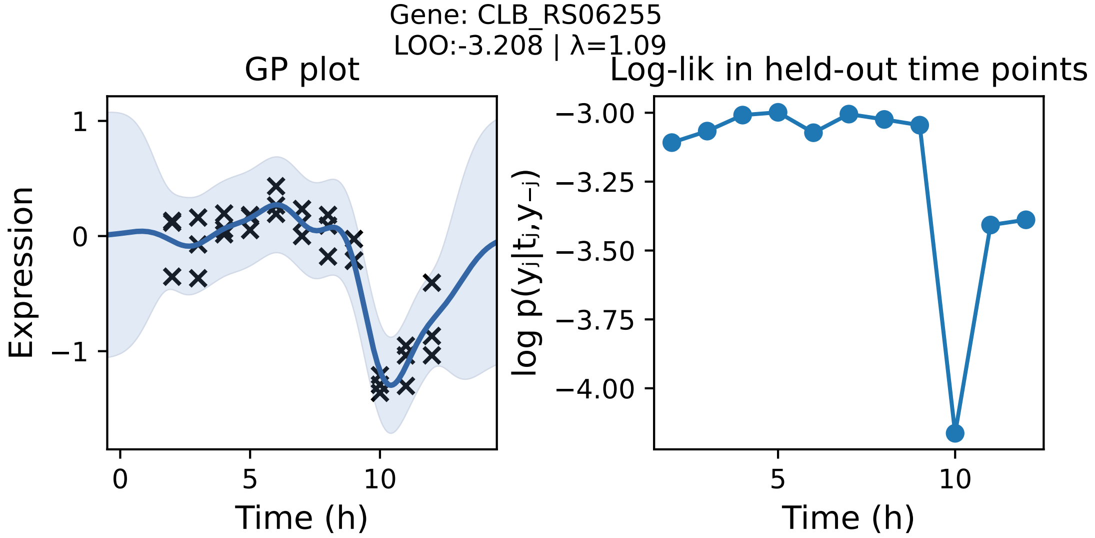

Gene: CLB_RS06255
LOO:-3.208 | λ=1.09
GP plot
Expression
Time (h)
Log-lik in held-out time points
log p(y_j|t_j,y_−j)
Time (h)

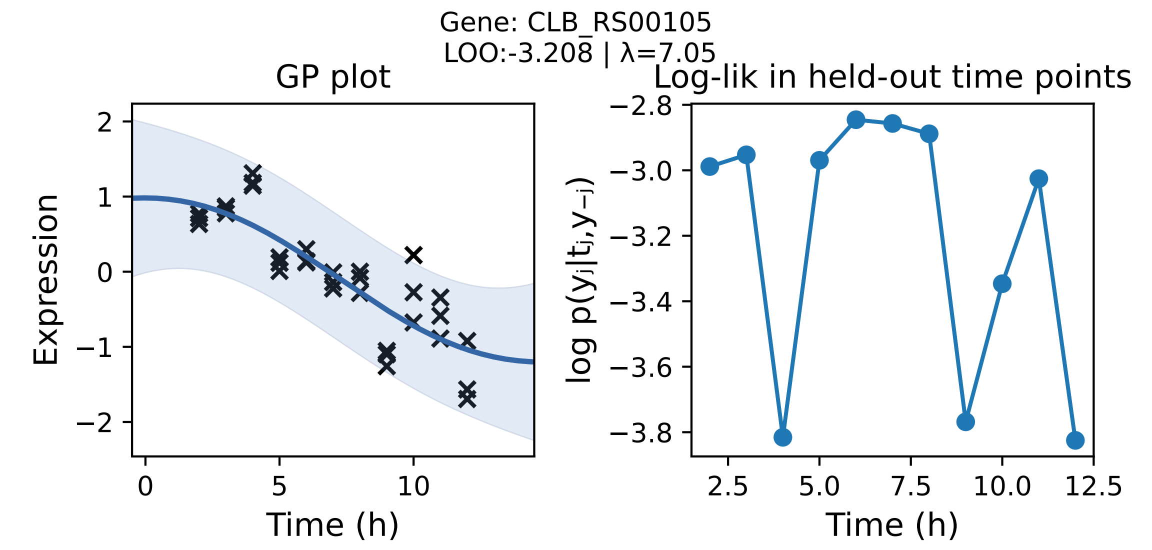

Gene: CLB_RS00105
LOO:-3.208 | λ=7.05
GP plot
Expression
Time (h)
Log-lik in held-out time points
log p(y_j|t_j,y_−j)
Time (h)

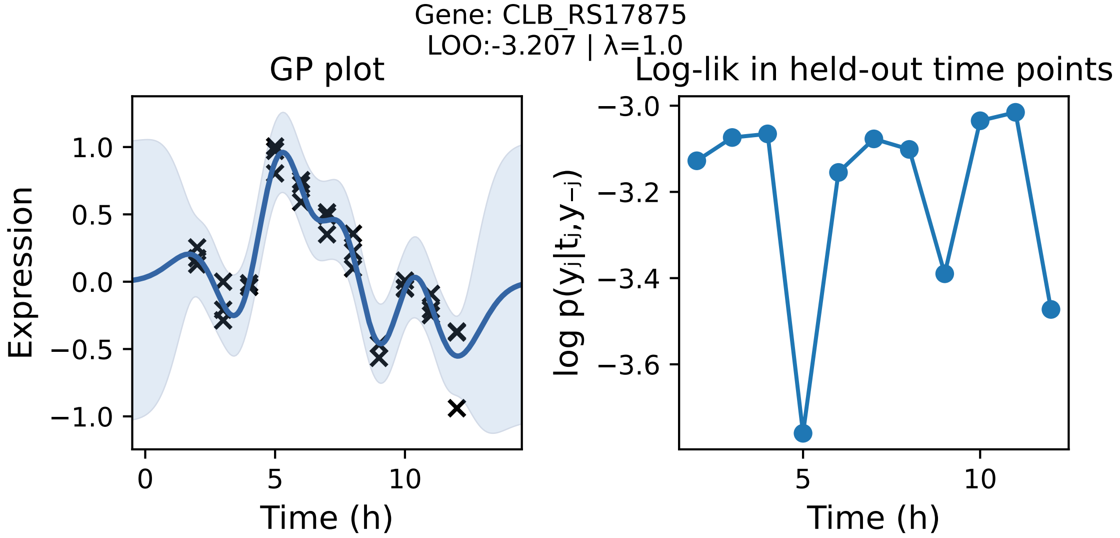

Gene: CLB_RS17875
LOO:-3.207 | λ=1.0
GP plot
Log-lik in held-out time points
Expression
Time (h)
log p(y_j|t_j,y_−j)
Time (h)

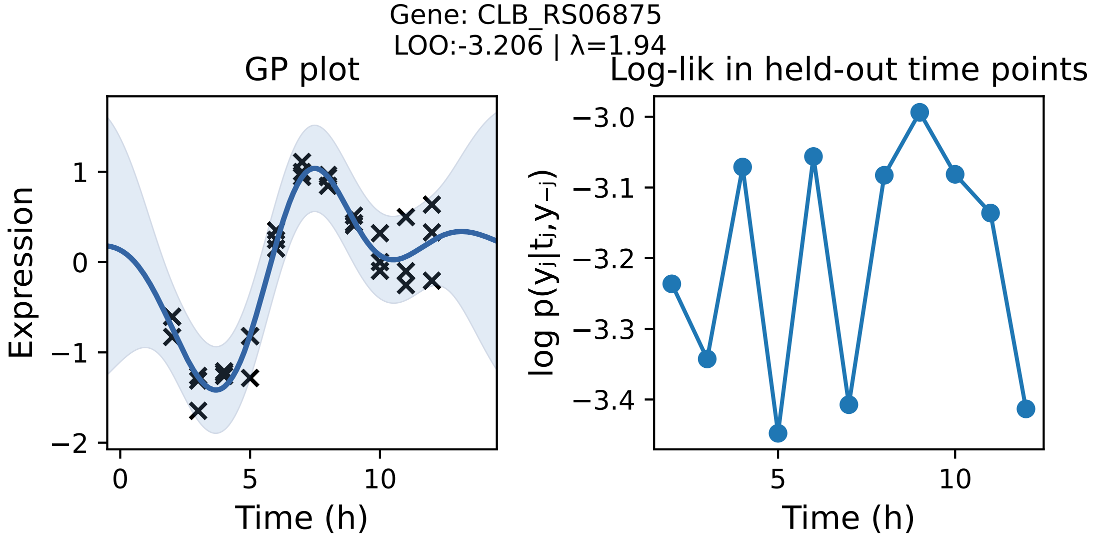

Gene: CLB_RS06875
LOO:-3.206 | λ=1.94
GP plot
Expression
Time (h)
Log-lik in held-out time points
log p(y_j|t_j,y_−j)
Time (h)

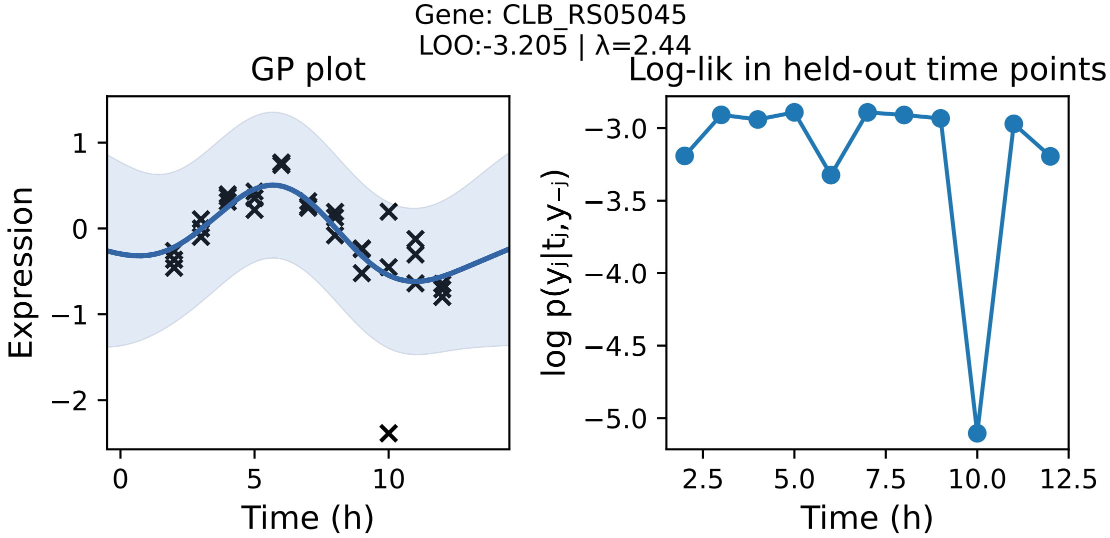
Gene: CLB_RS05045
LOO:-3.205 | λ=2.44
GP plot
Expression
Time (h)
Log-lik in held-out time points
log p(y_j|t_j,y_−j)
Time (h)

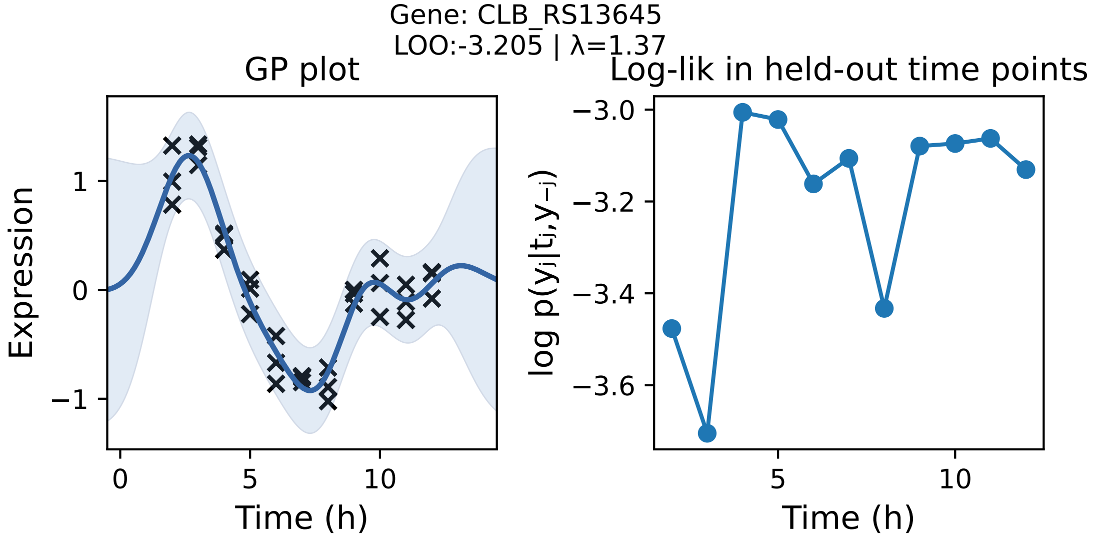

Gene: CLB_RS13645
LOO:-3.205 | λ=1.37
GP plot
Expression
Time (h)
Log-lik in held-out time points
log p(y_j|t_j,y_−j)
Time (h)

Gene: CLB_RS11950
LOO:-3.205 | λ=3.57

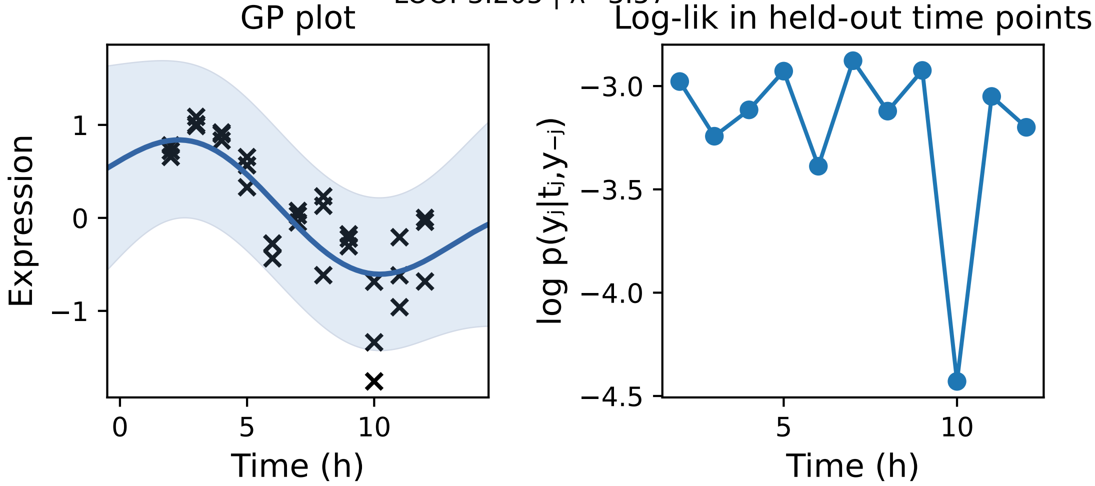

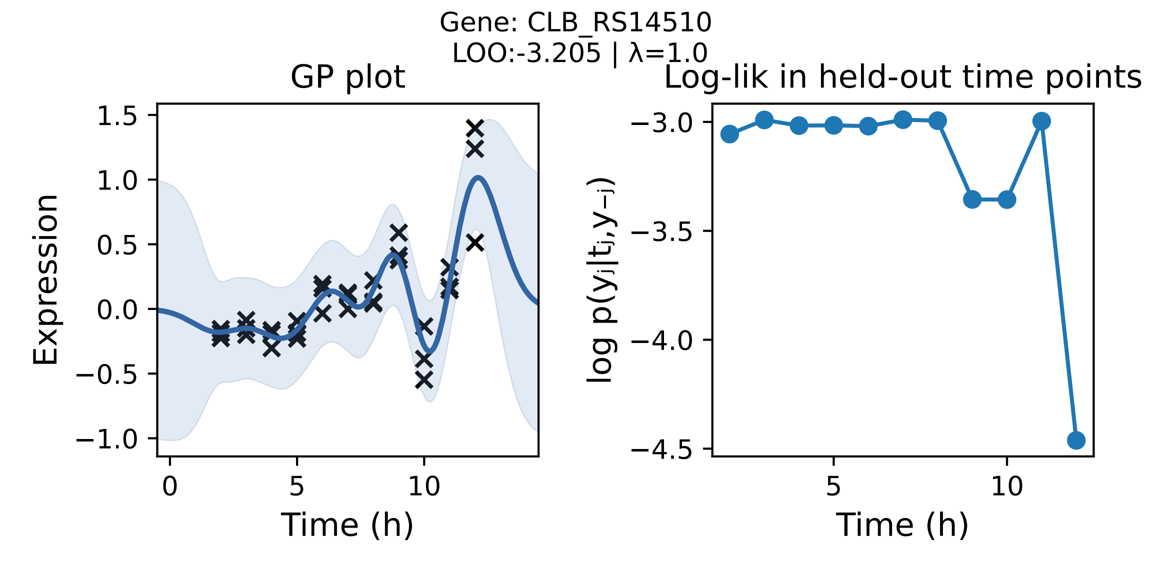

Gene: CLB_RS14510
LOO:-3.205 | λ=1.0
GP plot
Log-lik in held-out time points
Expression
1.5
1.0
0.5
0.0
−0.5
−1.0
0
5
10
Time (h)
log p(y_j|t_j,y_−j)
−3.0
−3.5
−4.0
−4.5
5
10
Time (h)

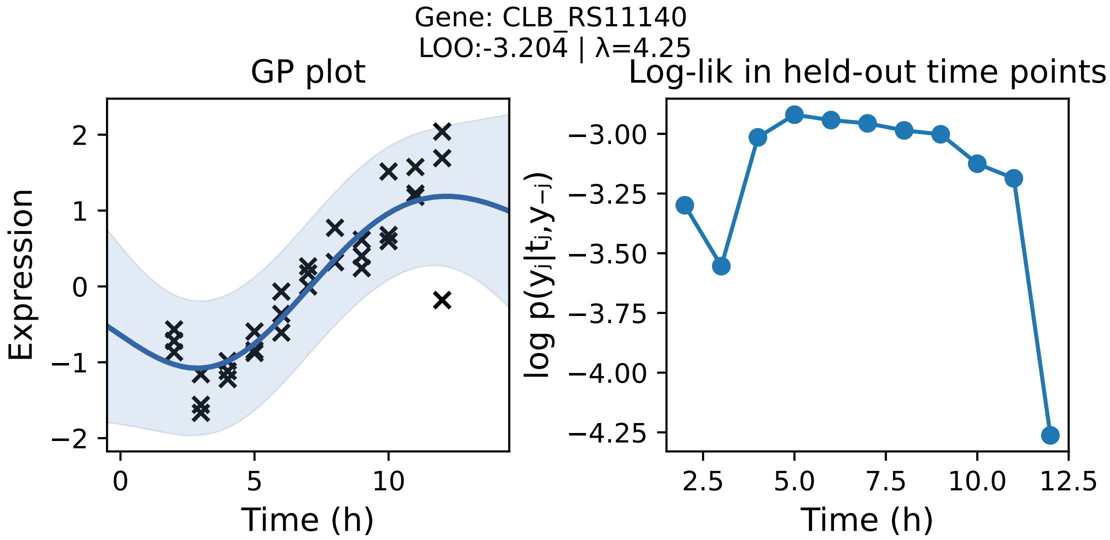

Gene: CLB_RS11140
LOO:-3.204 | λ=4.25
GP plot
Expression
Time (h)
2
1
0
−1
−2
0
5
10
Log-lik in held-out time points
log p(y_j|t_j,y_−j)
Time (h)
−3.00
−3.25
−3.50
−3.75
−4.00
−4.25
2.5
5.0
7.5
10.0
12.5

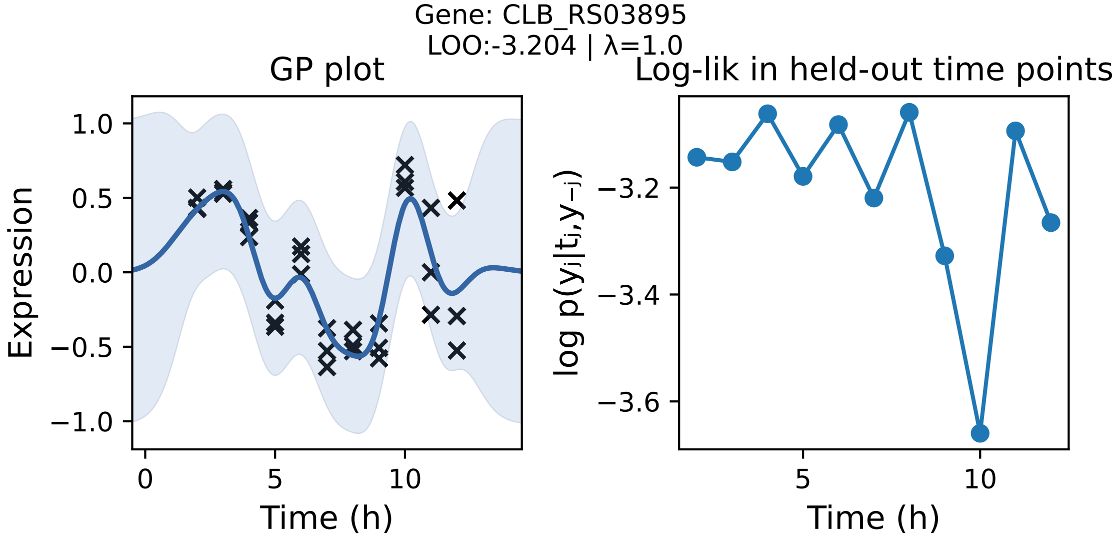

Gene: CLB_RS03895
LOO:-3.204 | λ=1.0
GP plot
Expression
1.0
0.5
0.0
−0.5
−1.0
0
5
10
Time (h)
Log-lik in held-out time points
log p(yj|tj,y−j)
−3.2
−3.4
−3.6
5
10
Time (h)

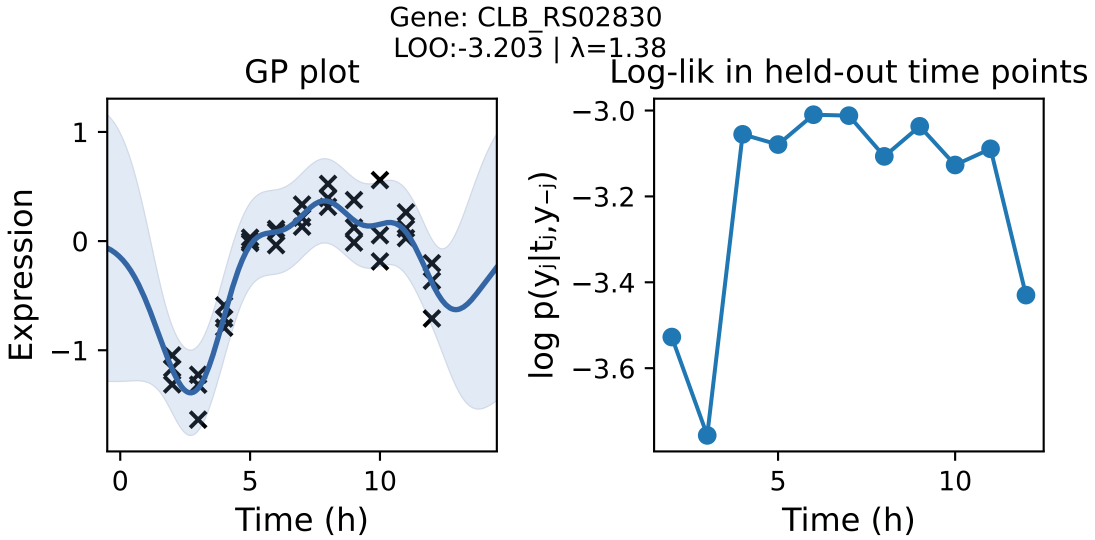

Gene: CLB_RS02830
LOO:-3.203 | λ=1.38
GP plot
Expression
Time (h)
Log-lik in held-out time points
log p(yj|tj,y−j)
Time (h)

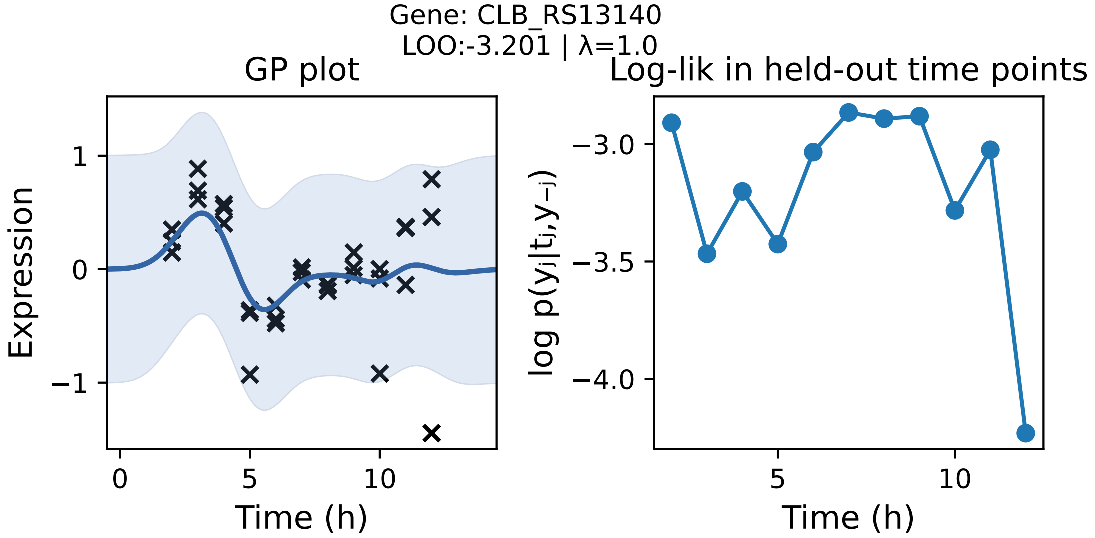
Gene: CLB_RS13140
LOO:-3.201 | λ=1.0
GP plot
Expression
Time (h)
1
0
−1
0
5
10
Log-lik in held-out time points
log p(y_j|t_j,y_−j)
Time (h)
−3.0
−3.5
−4.0
5
10

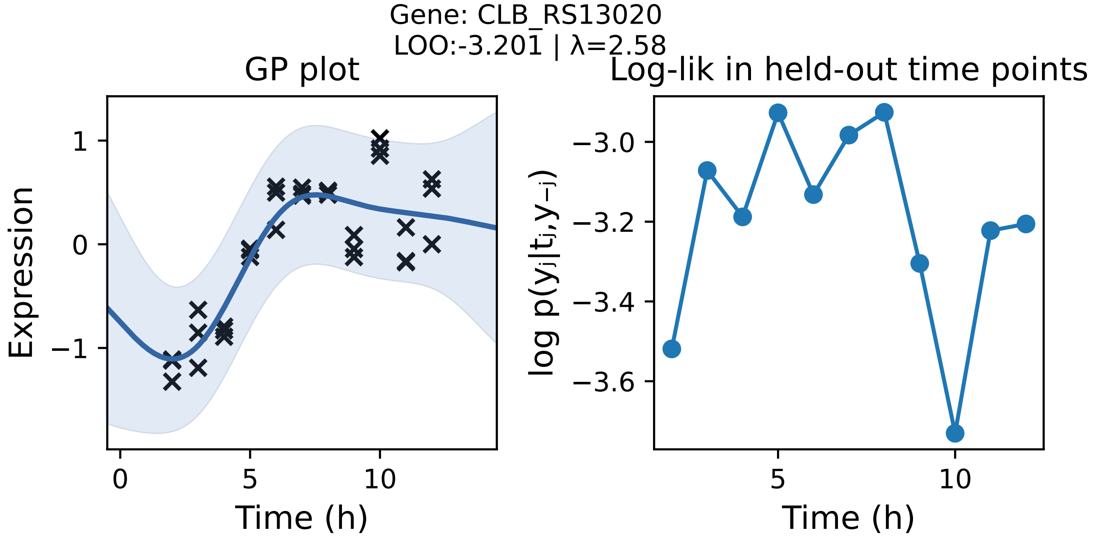
Gene: CLB_RS13020
LOO:-3.201 | λ=2.58
GP plot
Expression
Time (h)
Log-lik in held-out time points
log p(yⱼ|tⱼ,y₋ⱼ)
Time (h)

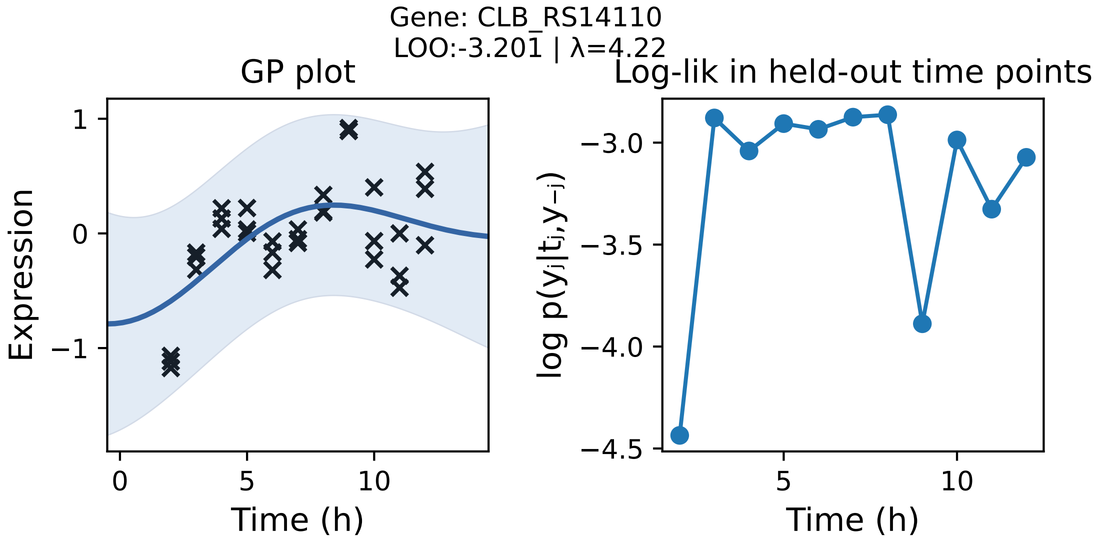

Gene: CLB_RS14110
LOO:-3.201 | λ=4.22
GP plot
Expression
1
0
−1
0
5
10
Time (h)
Log-lik in held-out time points
log p(yj|tj,y−j)
−3.0
−3.5
−4.0
−4.5
5
10
Time (h)

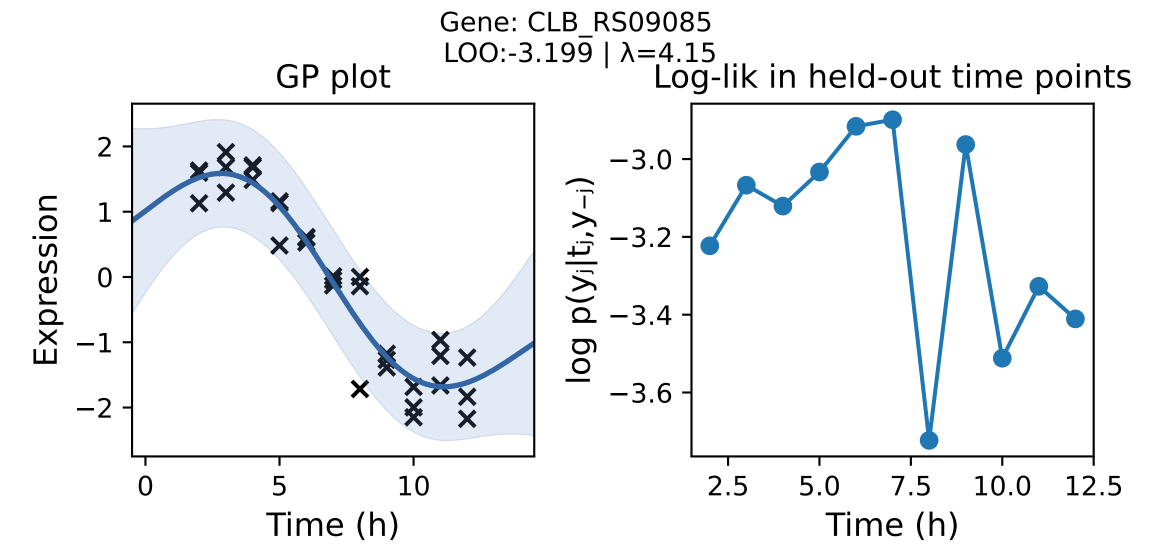
Gene: CLB_RS09085
LOO:-3.199 | λ=4.15
GP plot
Expression
Time (h)
Log-lik in held-out time points
log p(y_j|t_j,y_−j)
Time (h)

Gene: CLB_RS13970
LOO:-3.199 | λ=3.11

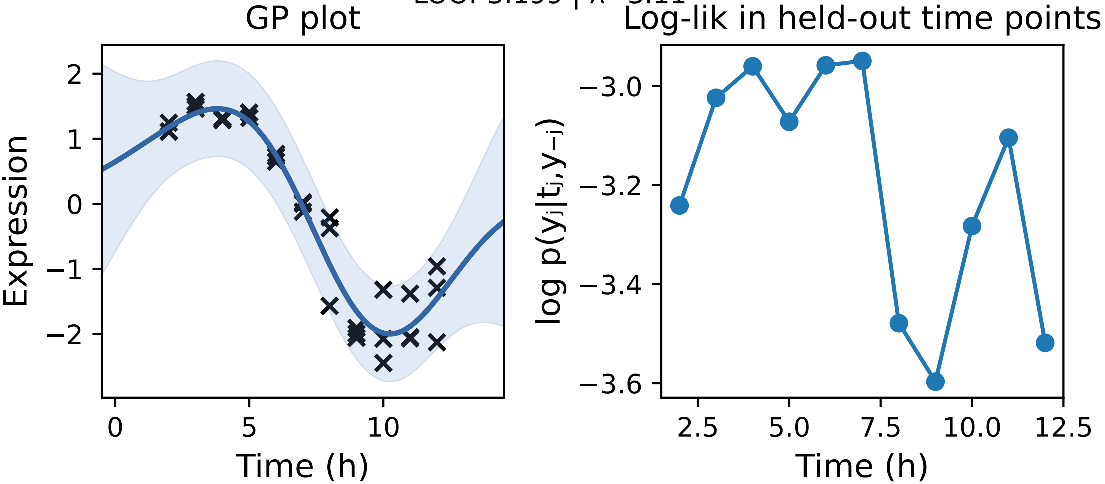

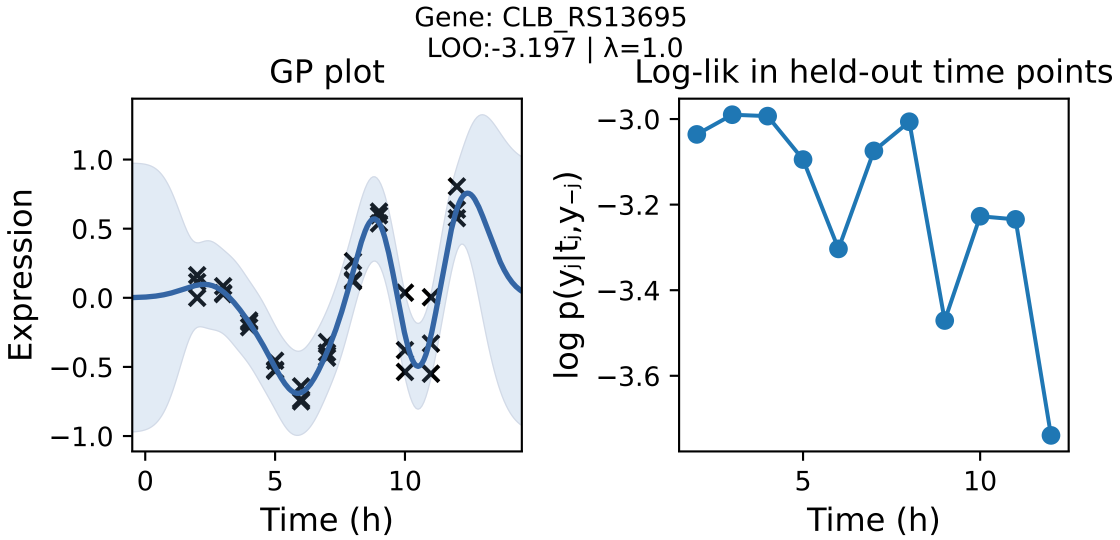

Gene: CLB_RS13695
LOO:-3.197 | λ=1.0
GP plot
Expression
Time (h)
1.0
0.5
0.0
−0.5
−1.0
0
5
10
Log-lik in held-out time points
log p(y_j|t_j,y_−j)
−3.0
−3.2
−3.4
−3.6
5
10
Time (h)

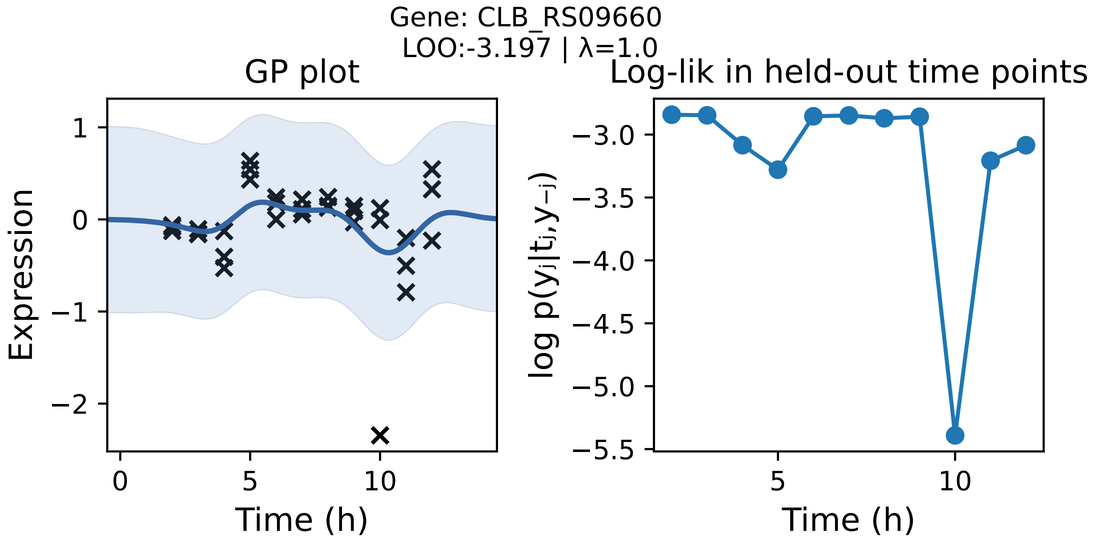

Gene: CLB_RS09660
LOO:-3.197 | λ=1.0
GP plot
Expression
Time (h)
Log-lik in held-out time points
log p(yj|tj,y−j)
Time (h)

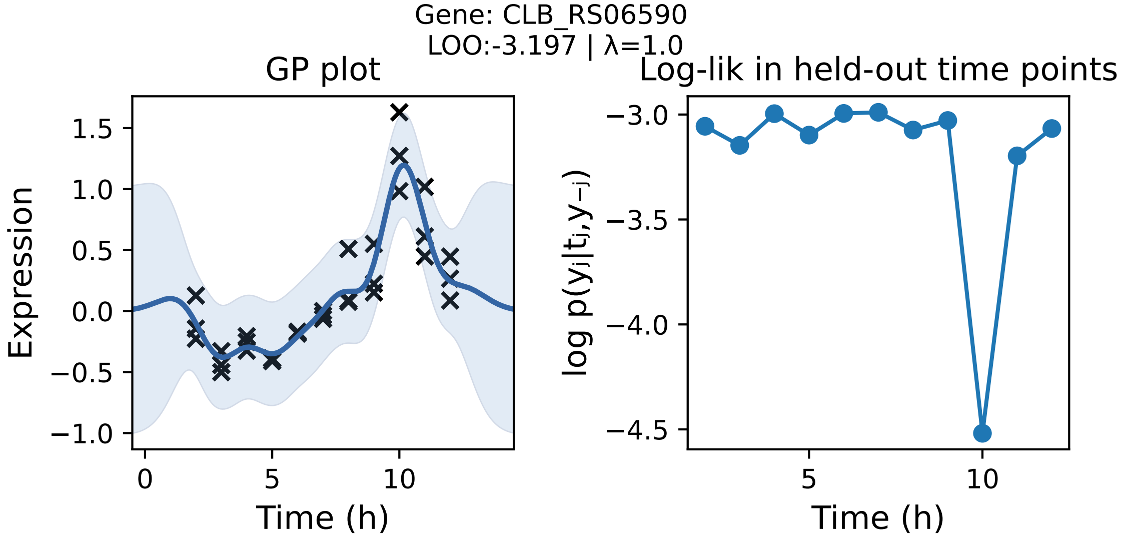
Gene: CLB_RS06590
LOO:-3.197 | λ=1.0
GP plot
Expression
Time (h)
1.5
1.0
0.5
0.0
−0.5
−1.0
0
5
10
Log-lik in held-out time points
log p(y_j|t_j,y_−j)
−3.0
−3.5
−4.0
−4.5
5
10
Time (h)

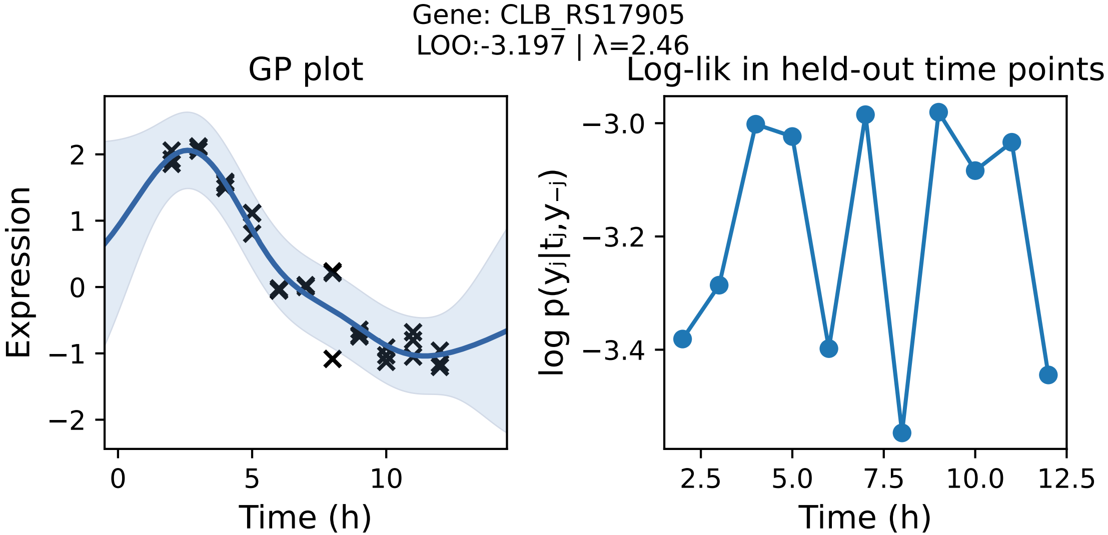

Gene: CLB_RS17905
LOO:-3.197 | λ=2.46
GP plot
Expression
Time (h)
Log-lik in held-out time points
log p(y_j|t_j,y_−j)
Time (h)

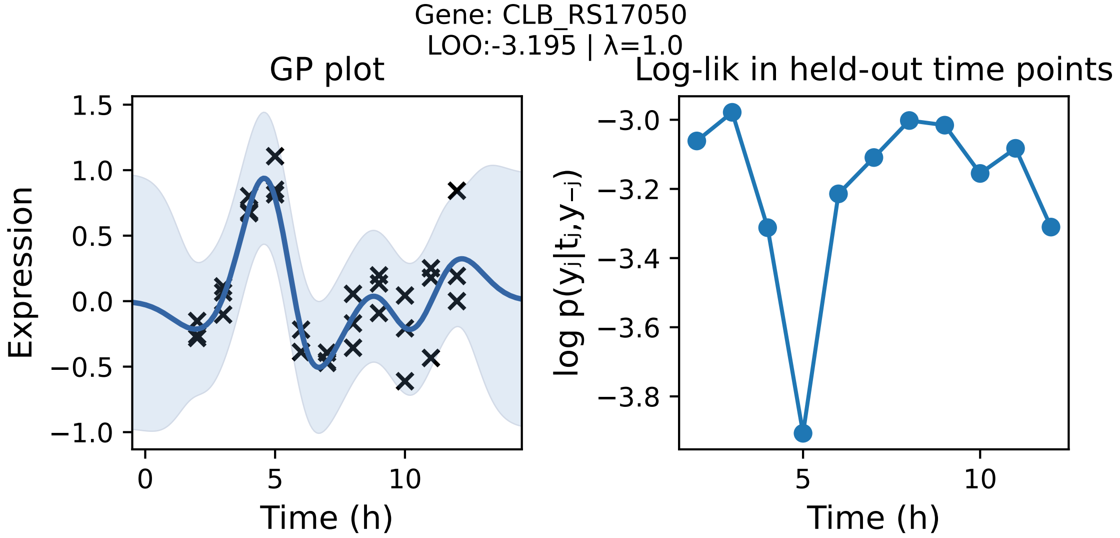

Gene: CLB_RS17050
LOO:-3.195 | λ=1.0
GP plot
Expression
Time (h)
Log-lik in held-out time points
log p(yj|tj,y−j)
Time (h)

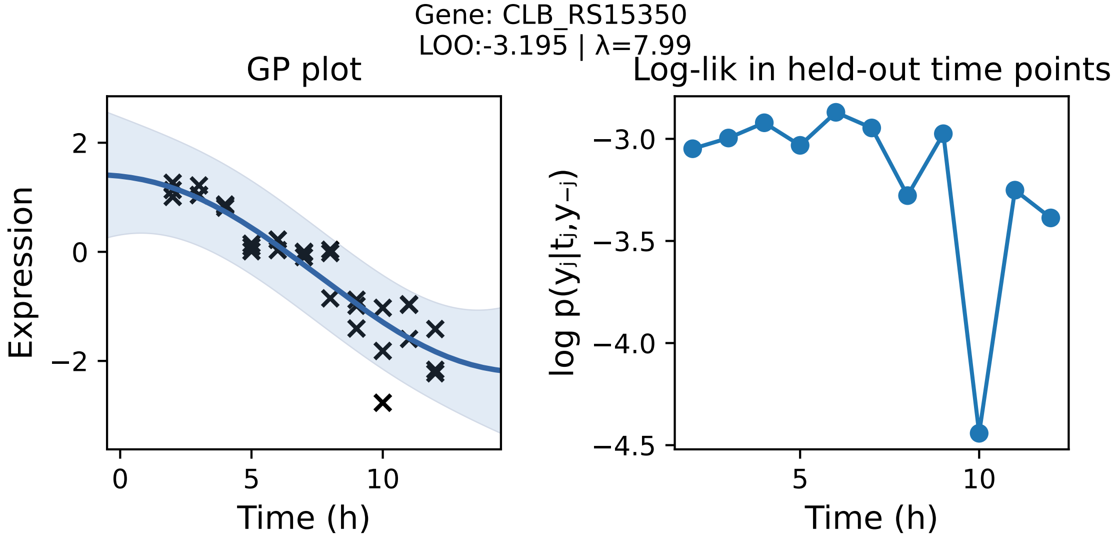

Gene: CLB_RS15350
LOO:-3.195 | λ=7.99
GP plot
Expression
Time (h)
Log-lik in held-out time points
log p(y_j|t_j,y_−j)
Time (h)

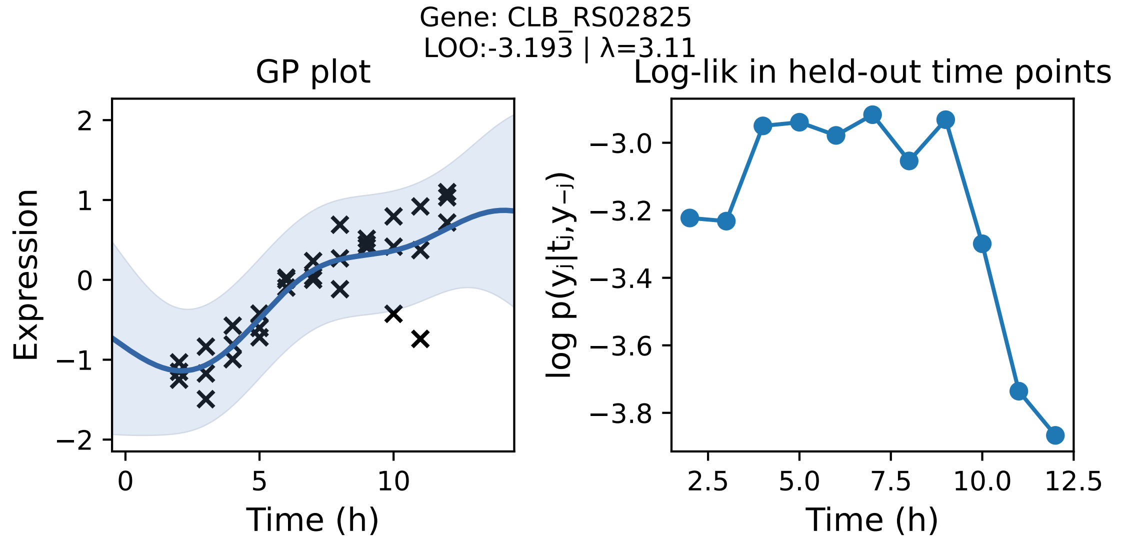

Gene: CLB_RS02825
LOO:-3.193 | λ=3.11
GP plot
Expression
Time (h)
2
1
0
−1
−2
0
5
10
Log-lik in held-out time points
log p(y_j|t_j,y_−j)
Time (h)
−3.0
−3.2
−3.4
−3.6
−3.8
2.5
5.0
7.5
10.0
12.5

Gene: CLB_RS11185
LOO:-3.193 | λ=2.28

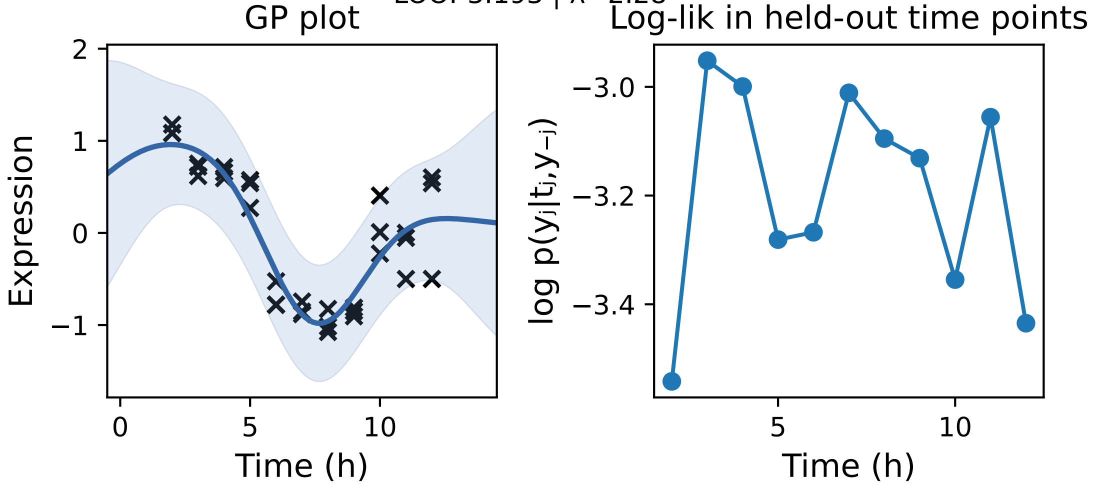

Gene: CLB_RS05975
LOO:-3.193 | λ=5.82

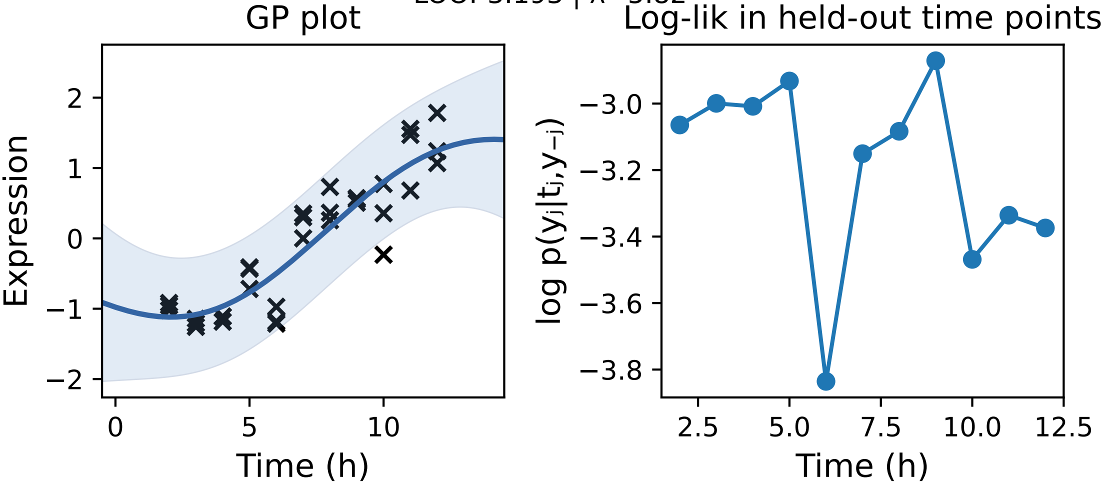

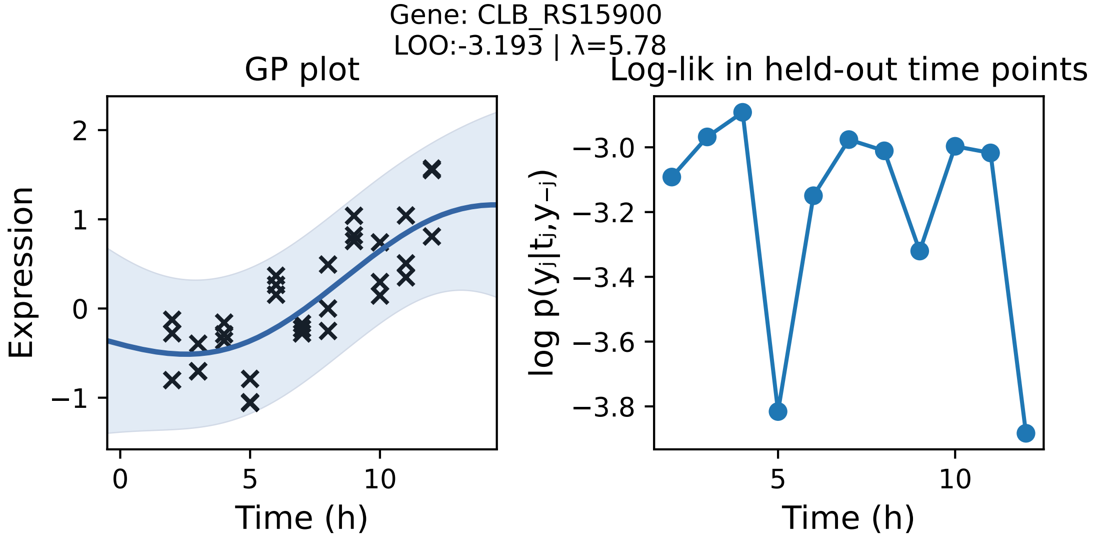
Gene: CLB_RS15900
LOO:-3.193 | λ=5.78
GP plot
Expression
Time (h)
Log-lik in held-out time points
log p(yj|tj,y−j)
Time (h)

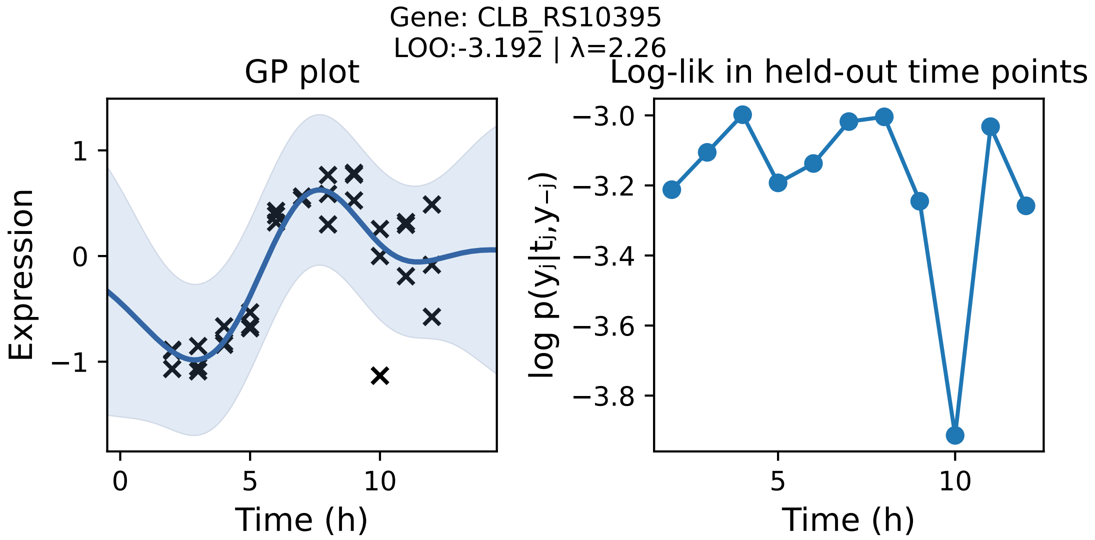

Gene: CLB_RS10395
LOO:-3.192 | λ=2.26
GP plot
Log-lik in held-out time points
Expression
Time (h)
log p(yj|tj,y−j)

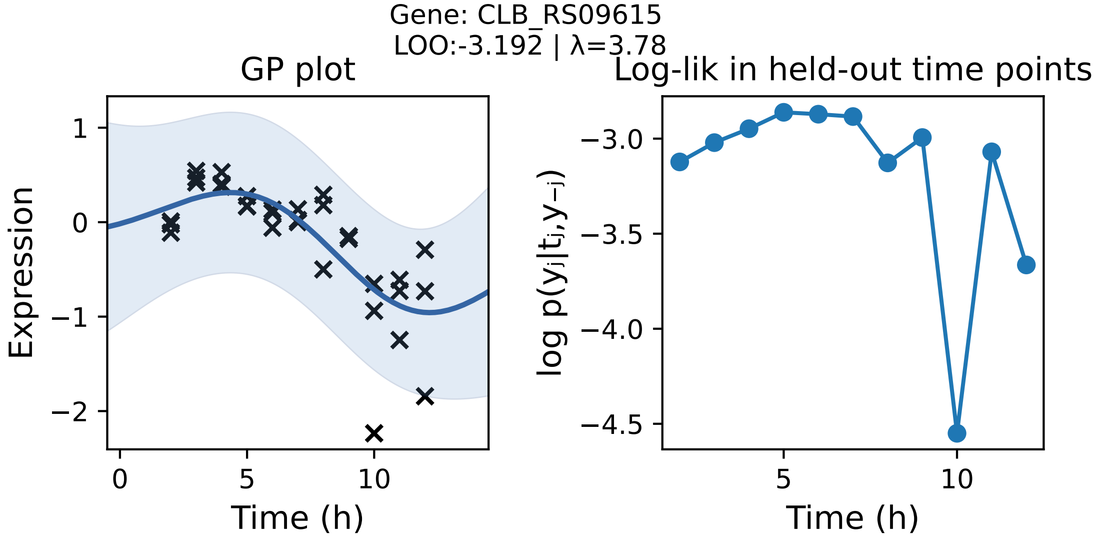

Gene: CLB_RS09615
LOO:-3.192 | λ=3.78
GP plot
Expression
Time (h)
1
0
−1
−2
0
5
10
Log-lik in held-out time points
log p(y_j|t_j,y_−j)
Time (h)
−3.0
−3.5
−4.0
−4.5
5
10

Gene: CLB_RS06540
LOO:-3.192 | λ=3.05

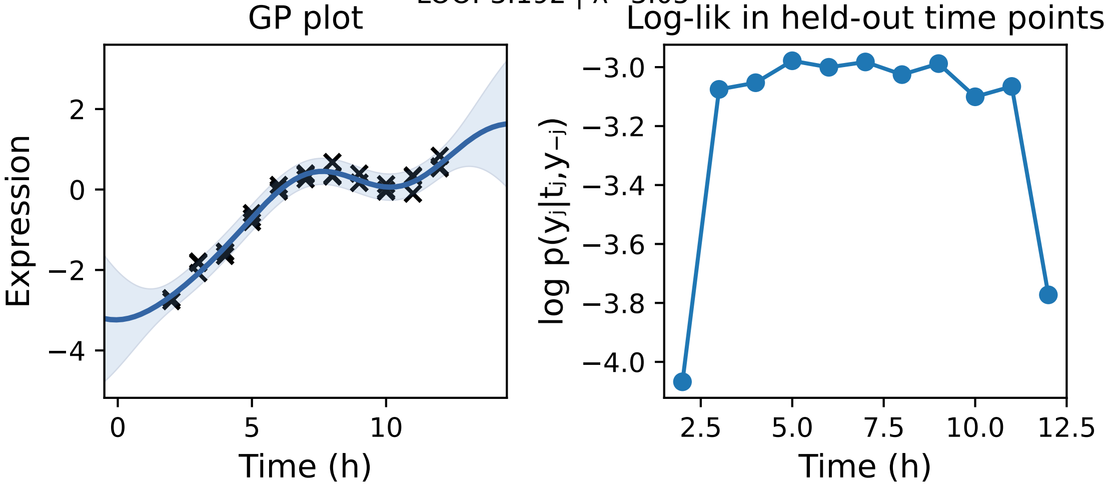

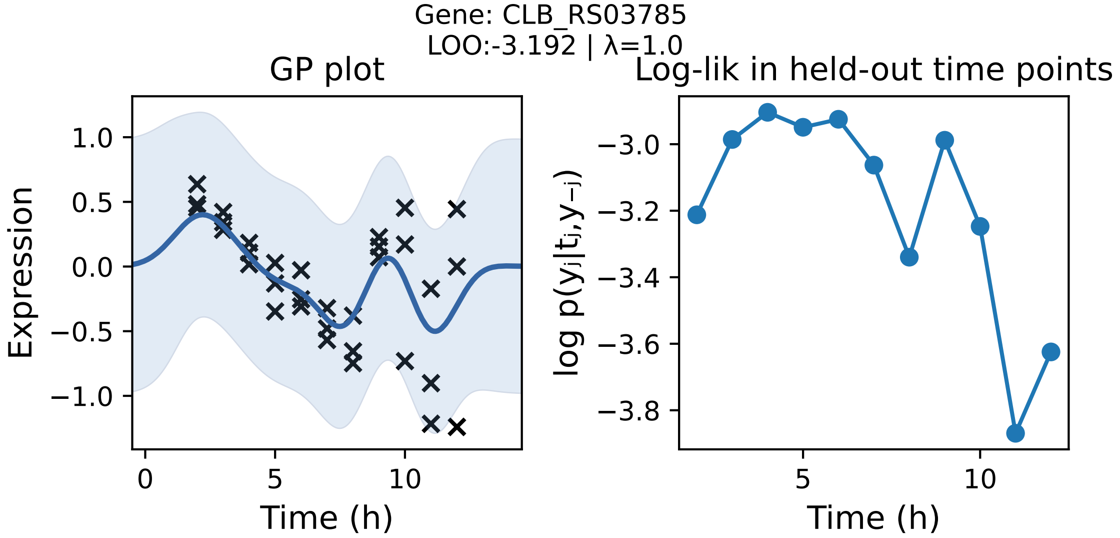

Gene: CLB_RS03785
LOO:-3.192 | λ=1.0
GP plot
Log-lik in held-out time points
Expression
Time (h)
log p(y_j|t_j,y_−j)
Time (h)

Gene: CLB_RS15615
LOO:-3.188 | λ=1.0

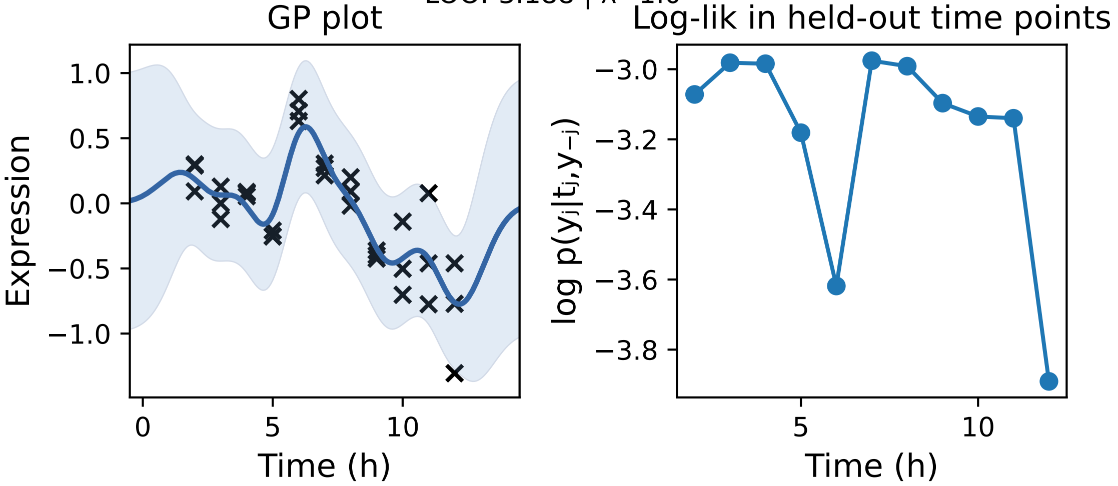

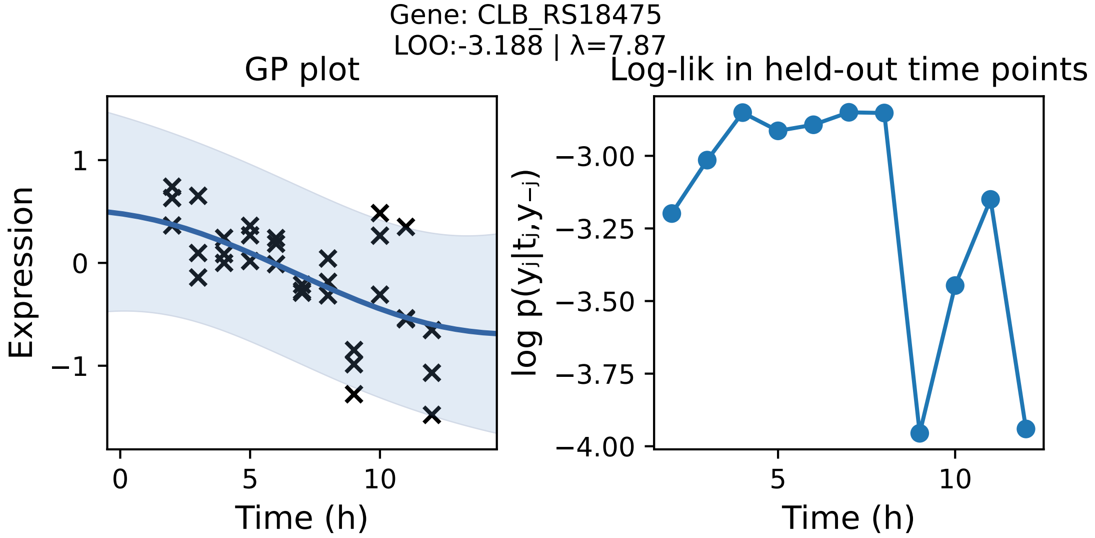

Gene: CLB_RS18475
LOO:-3.188 | λ=7.87
GP plot
Log-lik in held-out time points
Expression
1
0
−1
0
5
10
Time (h)
log p(yj|tj,y−j)
−3.00
−3.25
−3.50
−3.75
−4.00
5
10
Time (h)

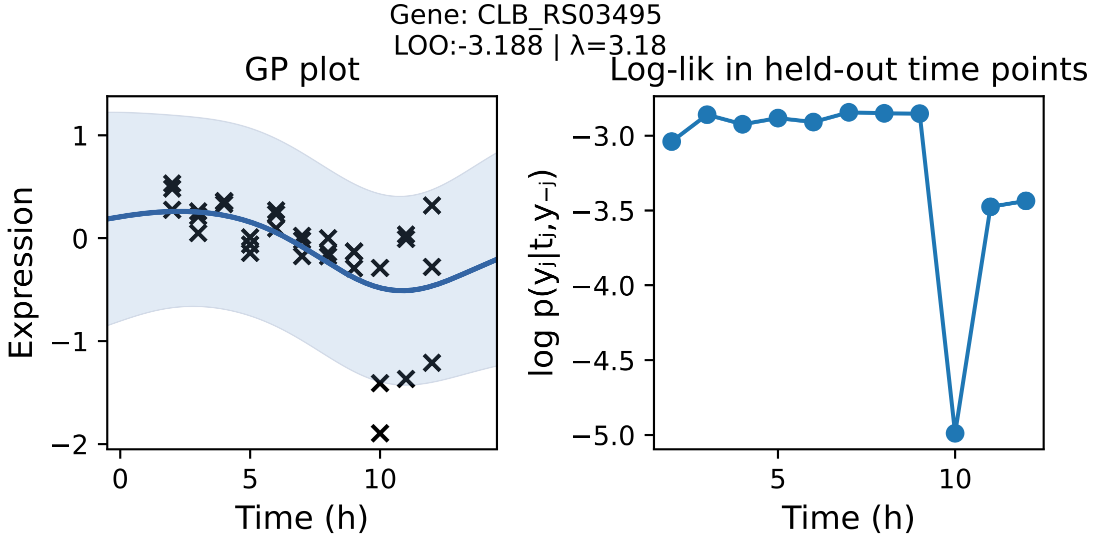

Gene: CLB_RS03495
LOO:-3.188 | λ=3.18
GP plot
Log-lik in held-out time points
Expression
Time (h)
log p(yj|ti,y−j)
1
0
−1
−2
0
5
10
−3.0
−3.5
−4.0
−4.5
−5.0

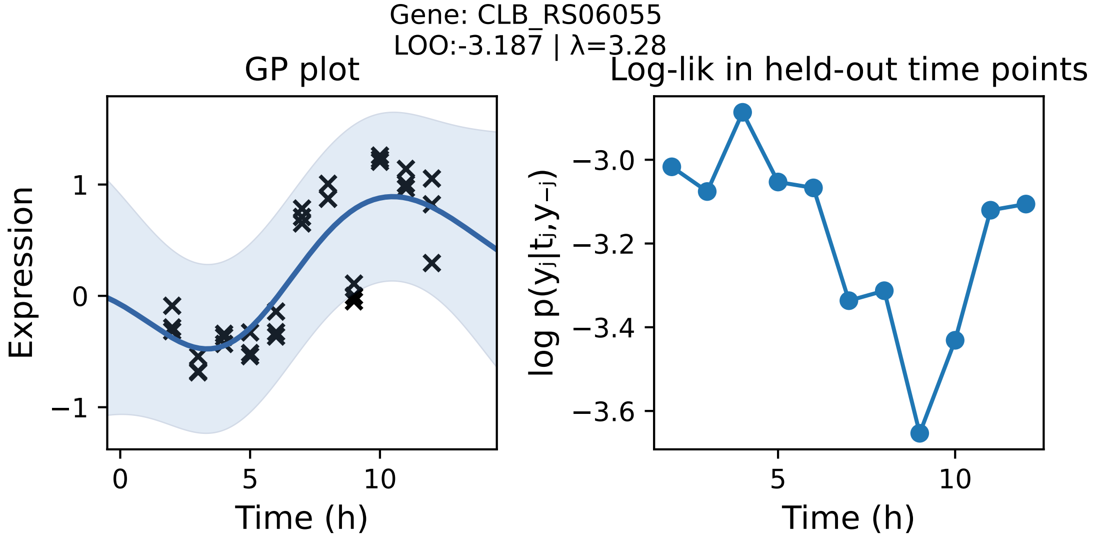

Gene: CLB_RS06055
LOO:-3.187 | λ=3.28
GP plot
Log-lik in held-out time points
Expression
Time (h)
log p(y_j|t_j,y_−j)
Time (h)
1
0
−1
0
5
10
−3.0
−3.2
−3.4
−3.6
5
10

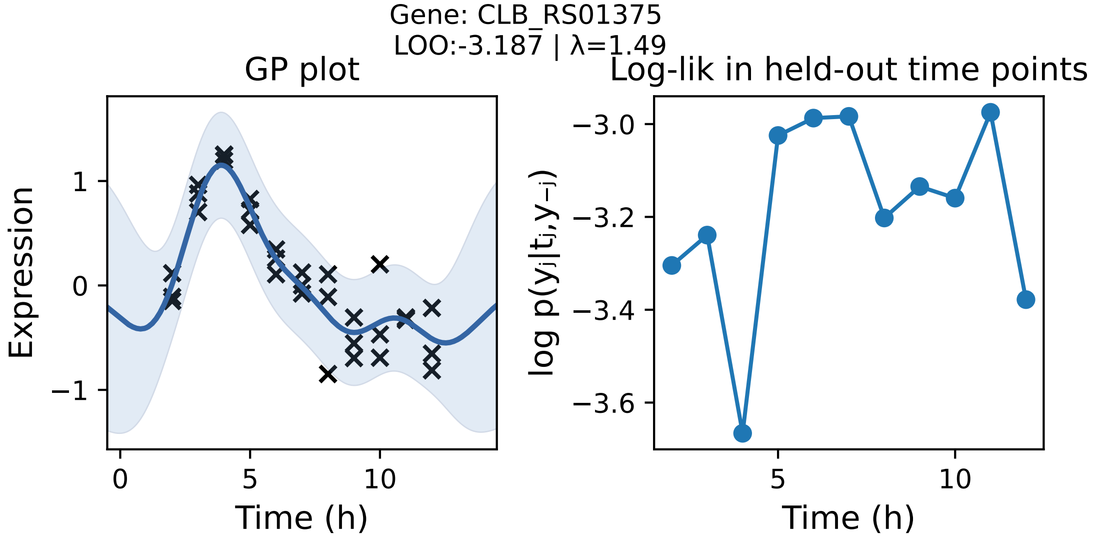

Gene: CLB_RS01375
LOO:-3.187 | λ=1.49
GP plot
Expression
Time (h)
1
0
−1
0
5
10
Log-lik in held-out time points
log p(yⱼ|tⱼ,y₋ⱼ)
Time (h)
−3.0
−3.2
−3.4
−3.6
5
10

Gene: CLB_RS05770
LOO:-3.187 | λ=5.12

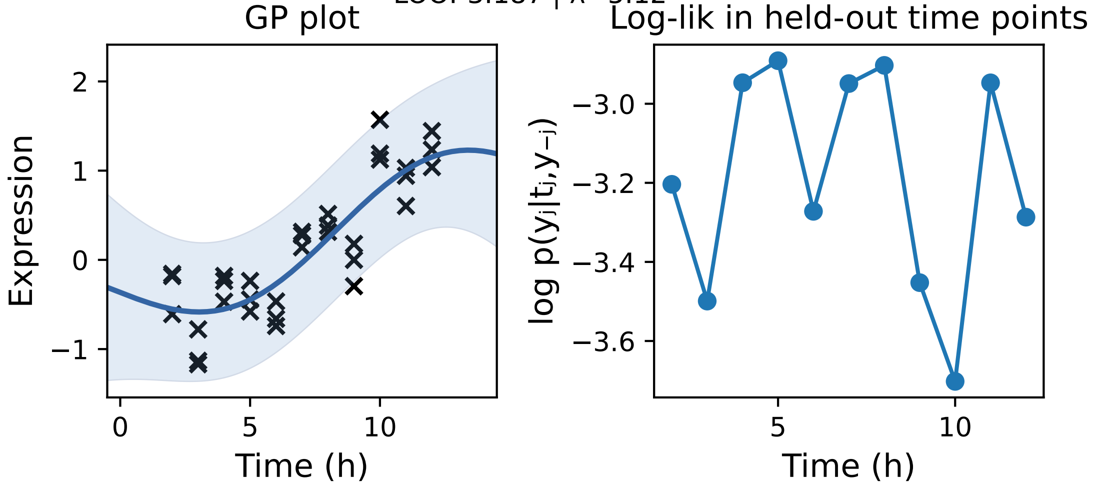

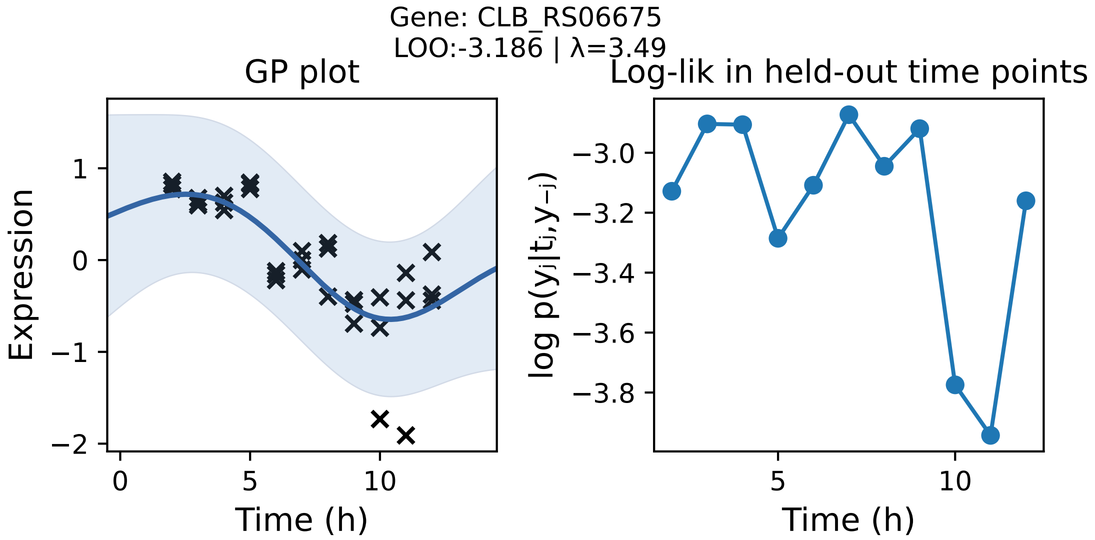

Gene: CLB_RS06675
LOO:-3.186 | λ=3.49
GP plot
Expression
Time (h)
1
0
−1
−2
0
5
10
Log-lik in held-out time points
log p(yj|tj,y−j)
−3.0
−3.2
−3.4
−3.6
−3.8
5
10
Time (h)

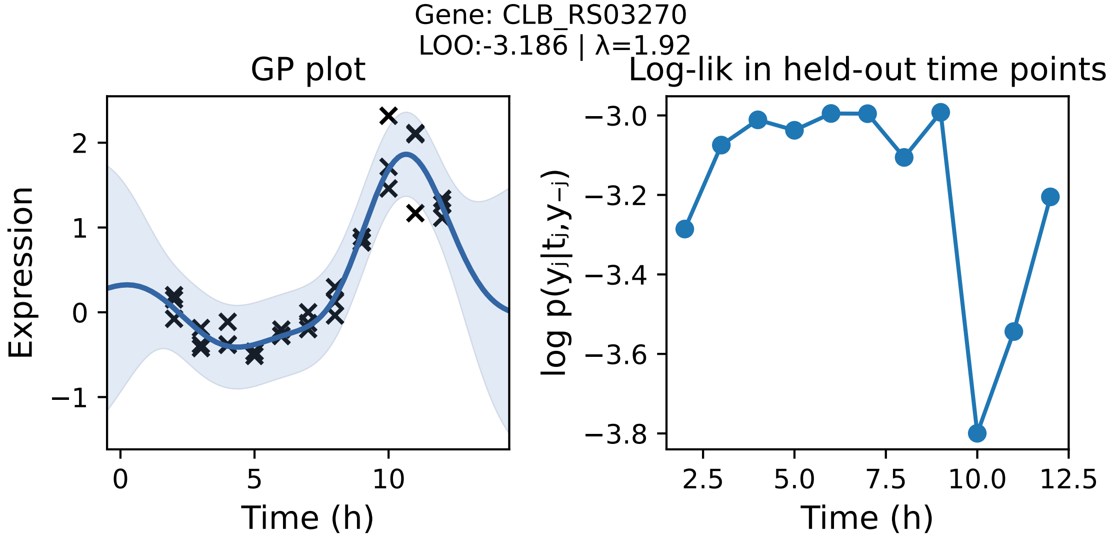

Gene: CLB_RS03270
LOO:-3.186 | λ=1.92
GP plot
Expression
Time (h)
Log-lik in held-out time points
log p(y_j|t_j,y_−j)
Time (h)

Gene: CLB_RS04215
LOO:-3.186 | λ=4.31

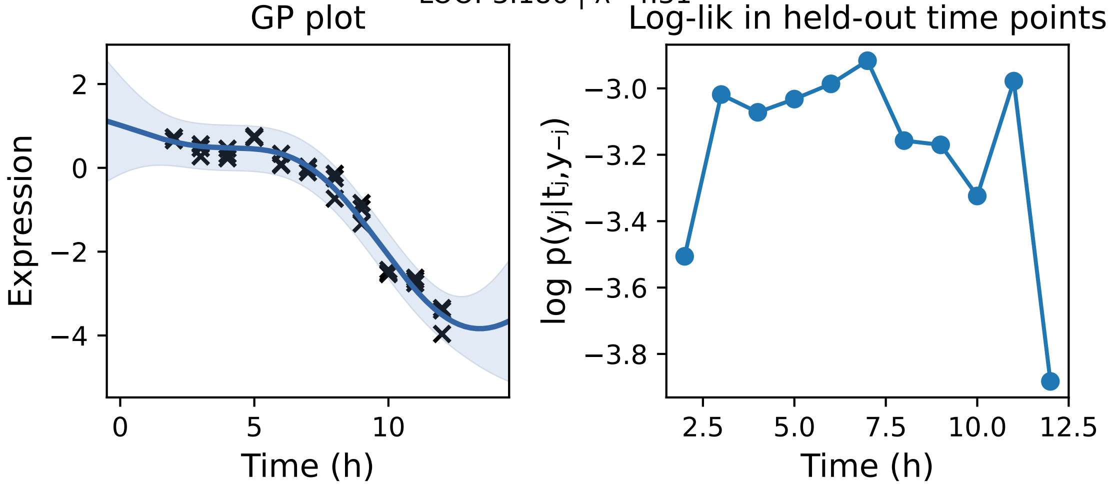

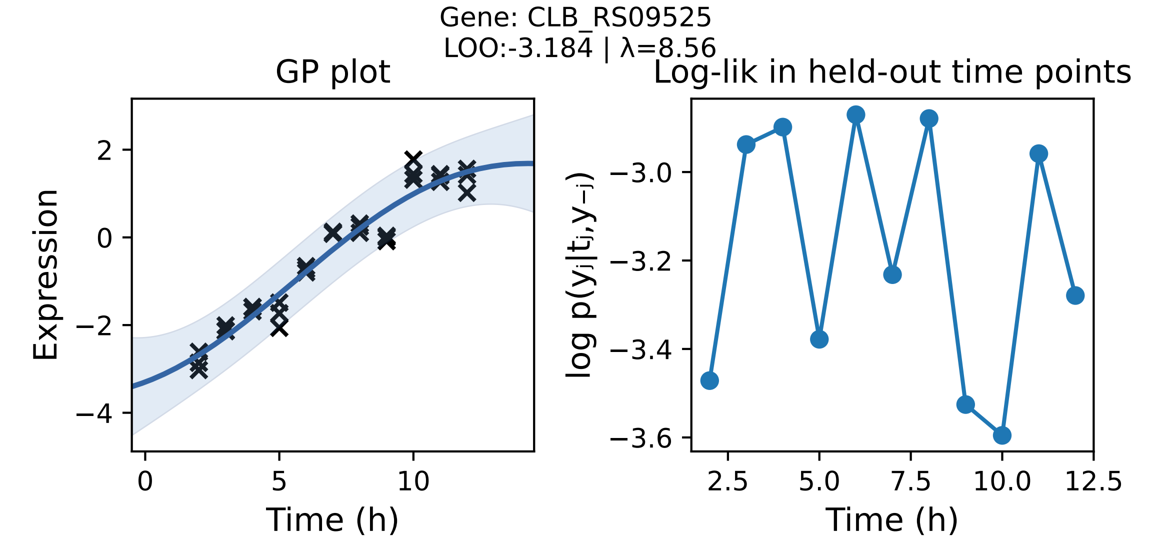

Gene: CLB_RS09525
LOO:-3.184 | λ=8.56
GP plot
Expression
Time (h)
Log-lik in held-out time points
log p(y_j|t_j,y_−j)
Time (h)

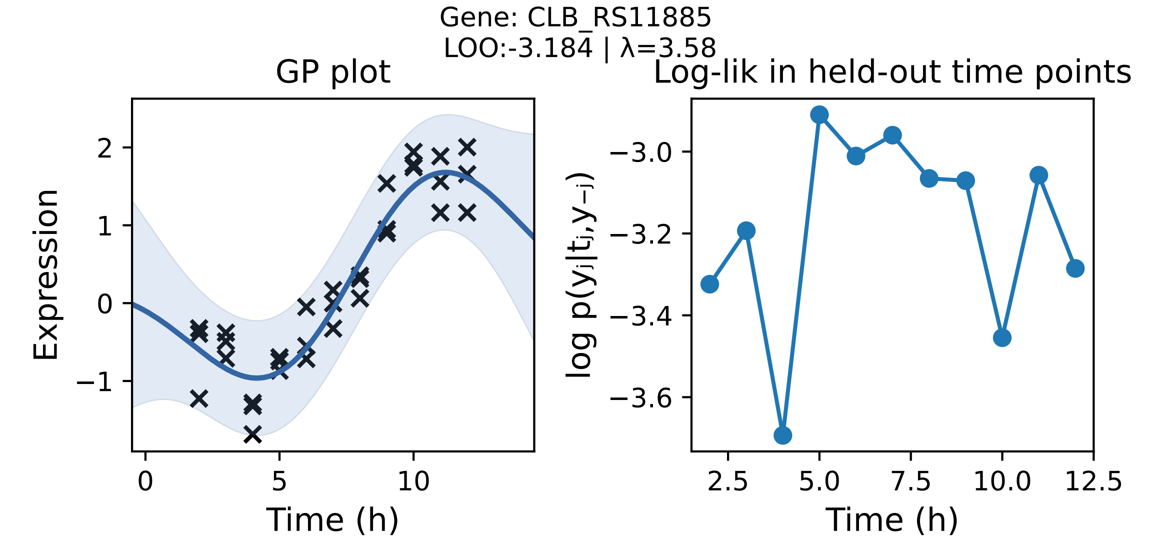

Gene: CLB_RS11885
LOO:-3.184 | λ=3.58
GP plot
Expression
Time (h)
Log-lik in held-out time points
log p(y_j|t_j,y_−j)
Time (h)

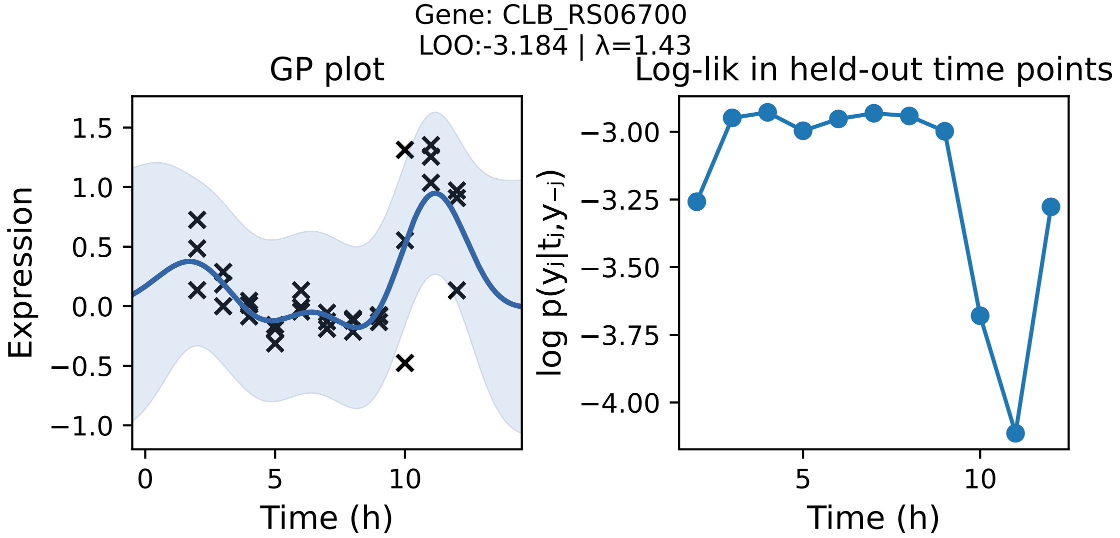

Gene: CLB_RS06700
LOO:-3.184 | λ=1.43
GP plot
Expression
Time (h)
1.5
1.0
0.5
0.0
−0.5
−1.0
0
5
10
Log-lik in held-out time points
log p(y_j|t_j,y_−j)
−3.00
−3.25
−3.50
−3.75
−4.00
5
10
Time (h)

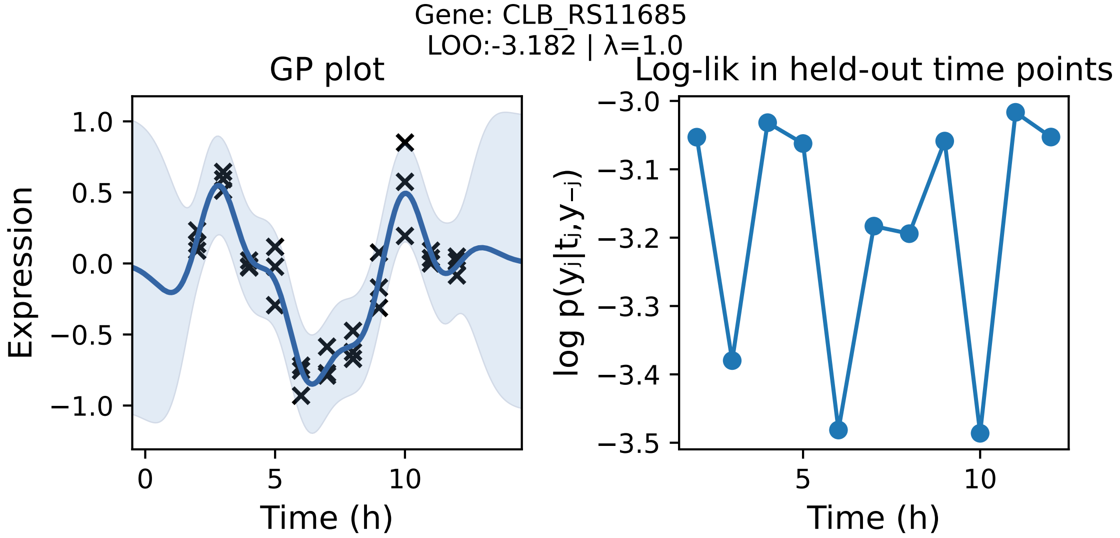

Gene: CLB_RS11685
LOO:-3.182 | λ=1.0
GP plot
Expression
1.0
0.5
0.0
−0.5
−1.0
0
5
10
Time (h)
Log-lik in held-out time points
log p(yj|tj,y−j)
−3.0
−3.1
−3.2
−3.3
−3.4
−3.5
5
10
Time (h)

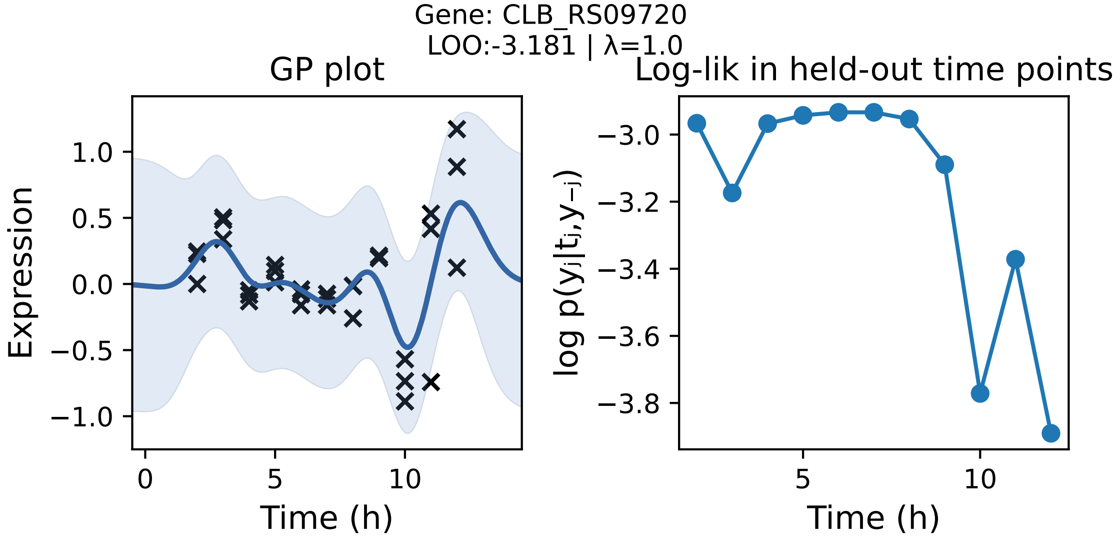
Gene: CLB_RS09720
LOO:-3.181 | λ=1.0
GP plot
Expression
Time (h)
1.0
0.5
0.0
−0.5
−1.0
0
5
10
Log-lik in held-out time points
log p(yⱼ|tⱼ,y₋ⱼ)
−3.0
−3.2
−3.4
−3.6
−3.8
5
10
Time (h)

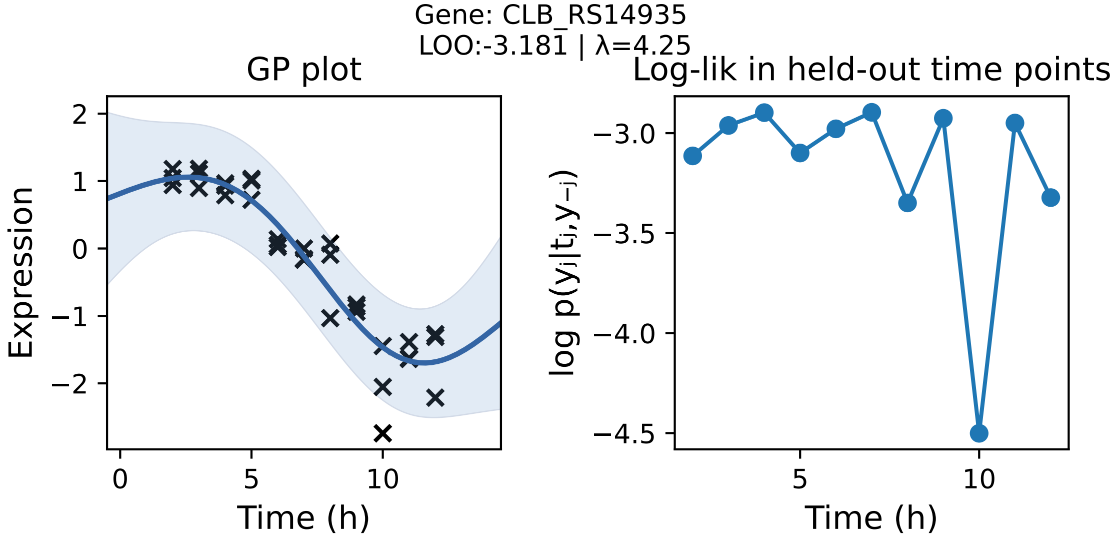

Gene: CLB_RS14935
LOO:-3.181 | λ=4.25
GP plot
Expression
Time (h)
Log-lik in held-out time points
log p(y_j|t_j,y_−j)
Time (h)

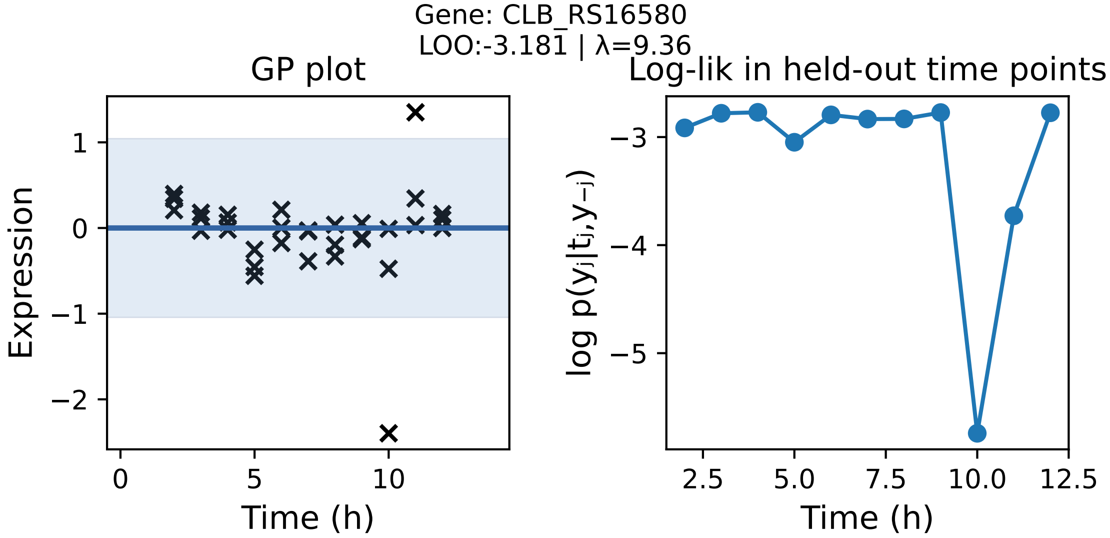

Gene: CLB_RS16580
LOO:-3.181 | λ=9.36
GP plot
Expression
Time (h)
Log-lik in held-out time points
log p(y_j|t_j,y_−j)
Time (h)

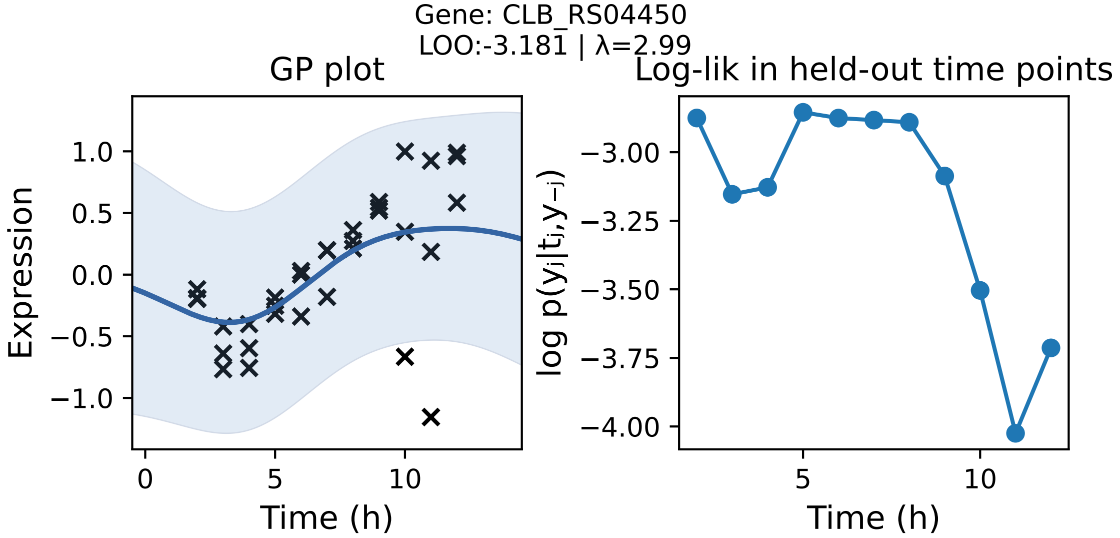

Gene: CLB_RS04450
LOO:-3.181 | λ=2.99
GP plot
Expression
1.0
0.5
0.0
−0.5
−1.0
0
5
10
Time (h)
Log-lik in held-out time points
log p(y_j|t_j,y_−j)
−3.00
−3.25
−3.50
−3.75
−4.00
5
10
Time (h)

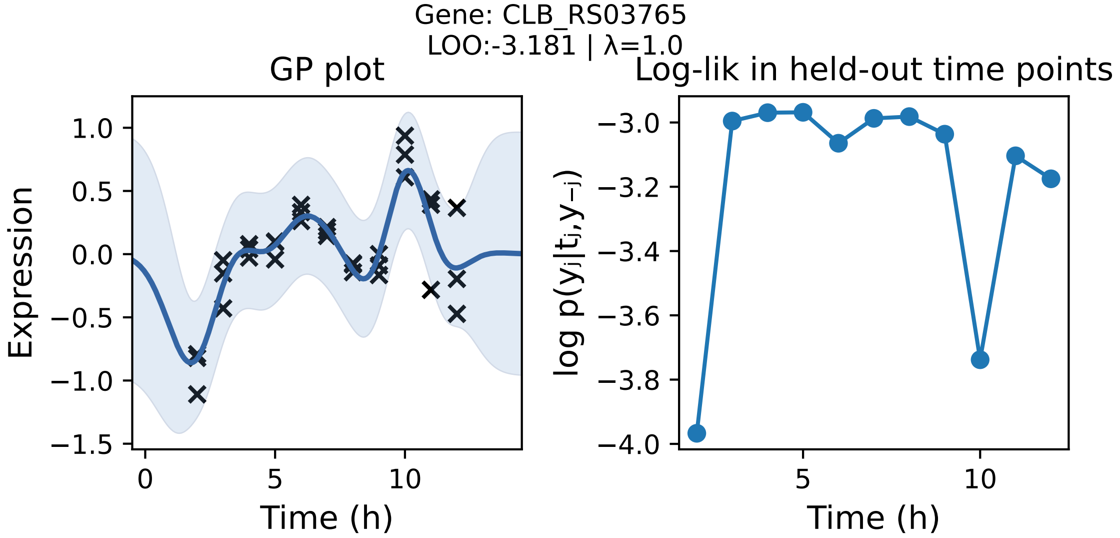

Gene: CLB_RS03765
LOO:-3.181 | λ=1.0
GP plot
Expression
Time (h)
1.0
0.5
0.0
−0.5
−1.0
−1.5
0
5
10
Log-lik in held-out time points
log p(yj|tj,y−j)
Time (h)
−3.0
−3.2
−3.4
−3.6
−3.8
−4.0
5
10

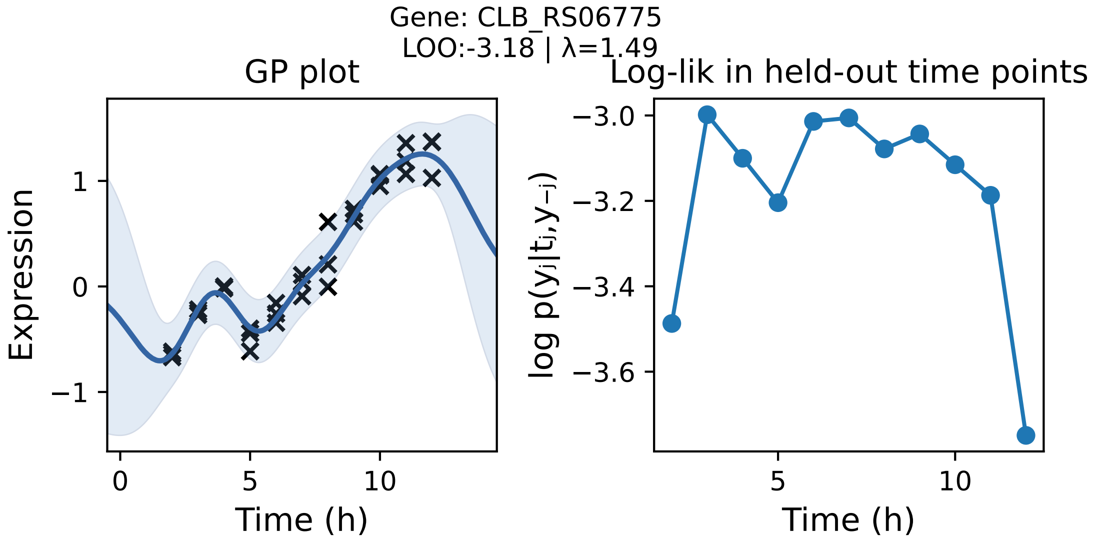

Gene: CLB_RS06775
LOO:-3.18 | λ=1.49
GP plot
Expression
Time (h)
Log-lik in held-out time points
log p(y_j|t_j,y_−j)
Time (h)

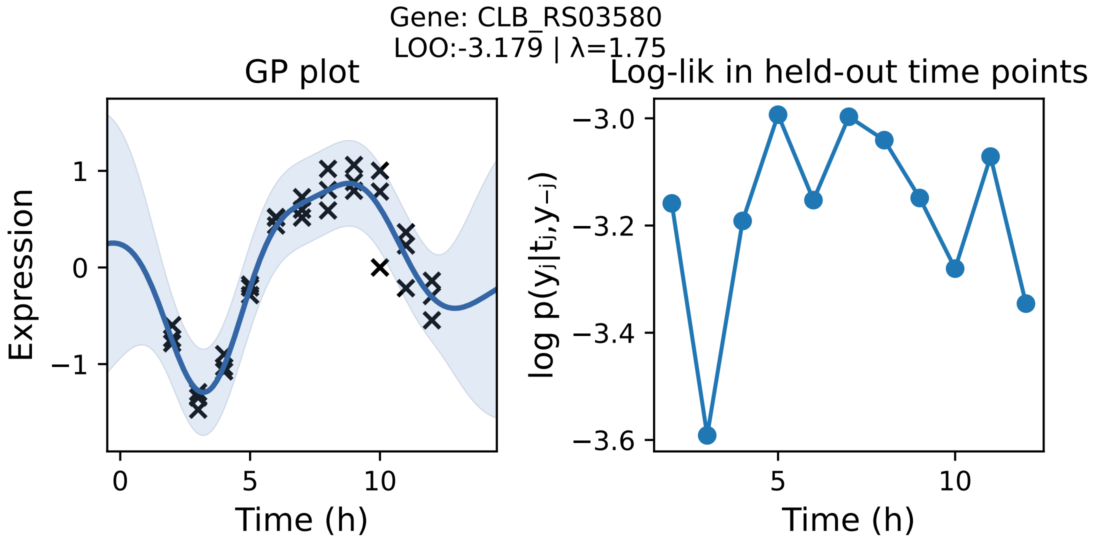
Gene: CLB_RS03580
LOO:-3.179 | λ=1.75
GP plot
Expression
Time (h)
1
0
−1
0
5
10
Log-lik in held-out time points
log p(yj|tj,y−j)
−3.0
−3.2
−3.4
−3.6
5
10
Time (h)

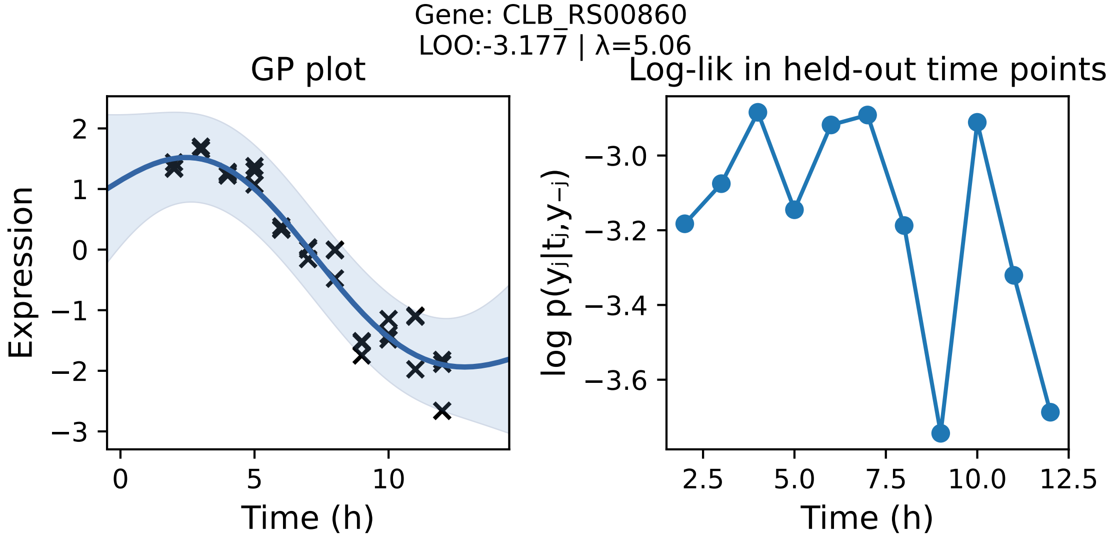

Gene: CLB_RS00860
LOO:-3.177 | λ=5.06
GP plot
Expression
Time (h)
Log-lik in held-out time points
log p(y_j|t_j,y_−j)
Time (h)

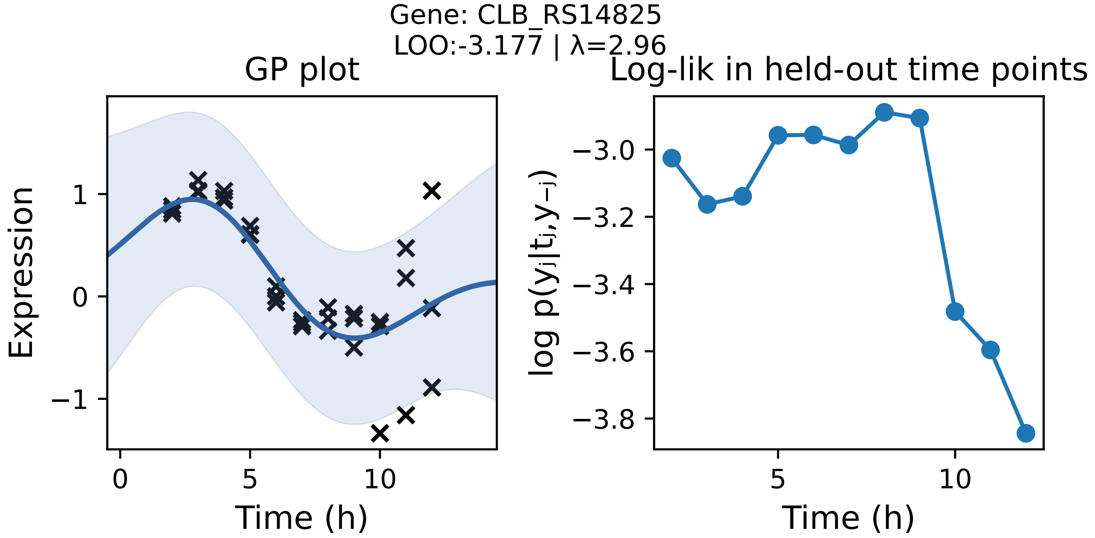

Gene: CLB_RS14825
LOO:-3.177 | λ=2.96
GP plot
Expression
Time (h)
Log-lik in held-out time points
log p(y_j|t_j,y_−j)
Time (h)
0
5
10
1
0
−1
−3.0
−3.2
−3.4
−3.6
−3.8
5
10

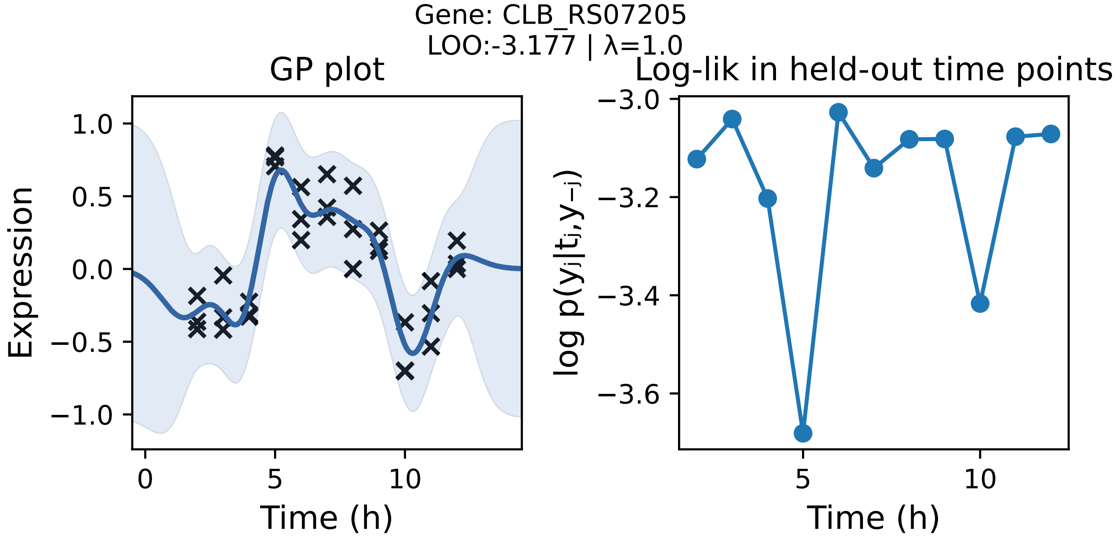
Gene: CLB_RS07205
LOO:-3.177 | λ=1.0
GP plot
Expression
Time (h)
Log-lik in held-out time points
log p(y_j|t_j,y_−j)
Time (h)

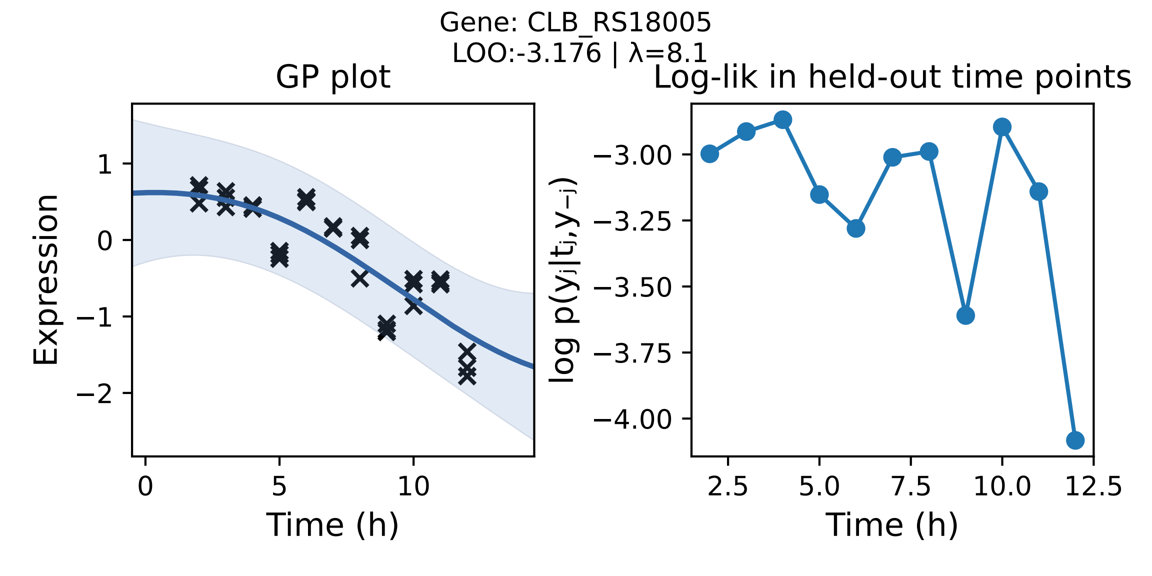

Gene: CLB_RS18005
LOO:-3.176 | λ=8.1
GP plot
Expression
Time (h)
Log-lik in held-out time points
log p(y_j|t_j,y_−j)
Time (h)

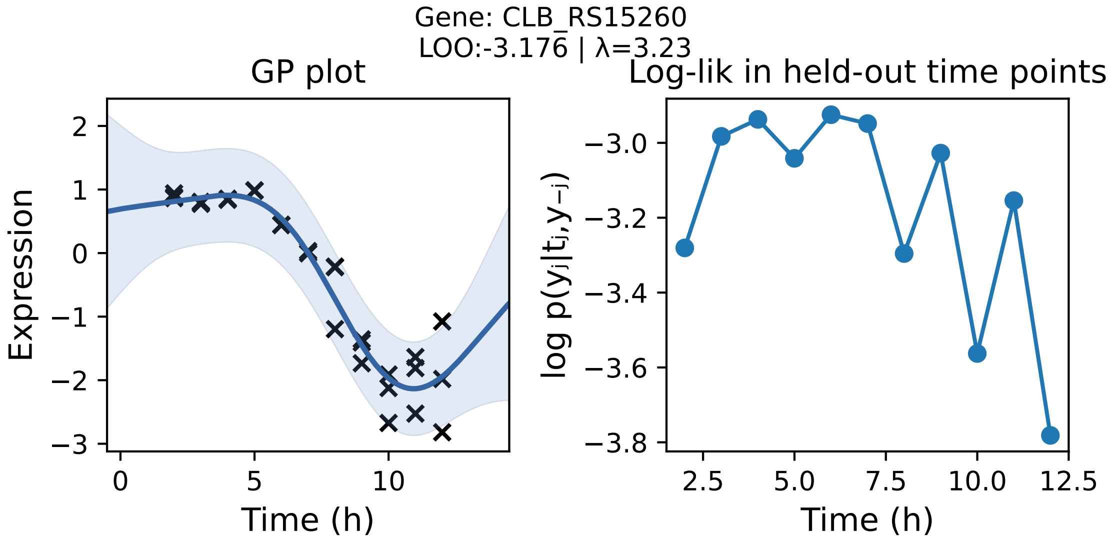

Gene: CLB_RS15260
LOO:-3.176 | λ=3.23
GP plot
Expression
Time (h)
Log-lik in held-out time points
log p(y_j|t_j,y_−j)
Time (h)

Gene: CLB_RS17615
LOO:-3.174 | λ=1.76

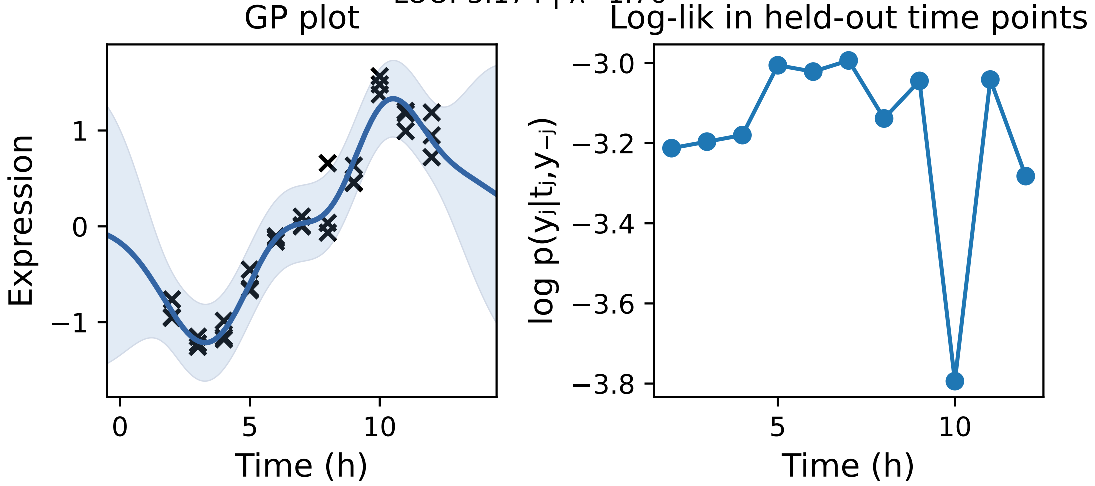

Gene: CLB_RS13775
LOO:-3.173 | λ=4.05

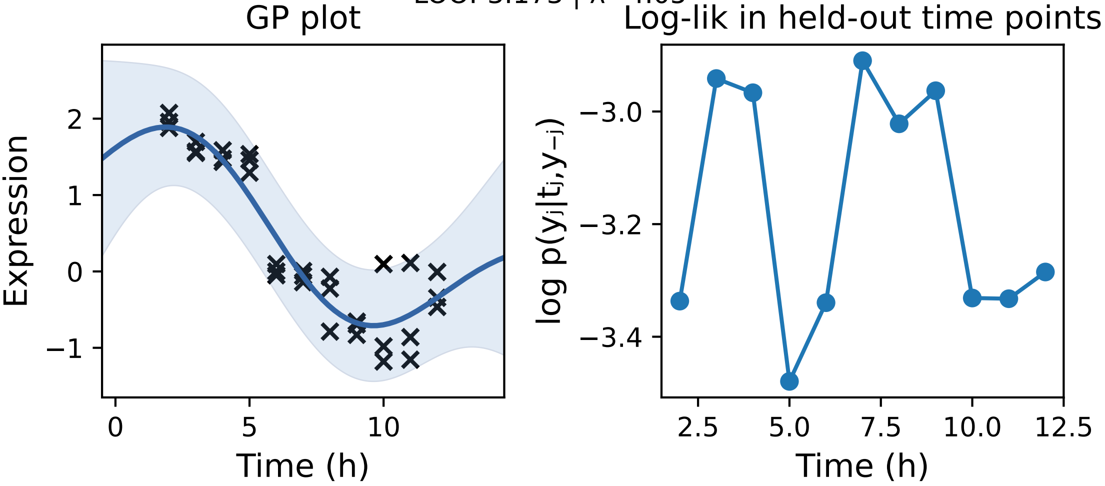

Gene: CLB_RS17975
LOO:-3.173 | λ=1.0

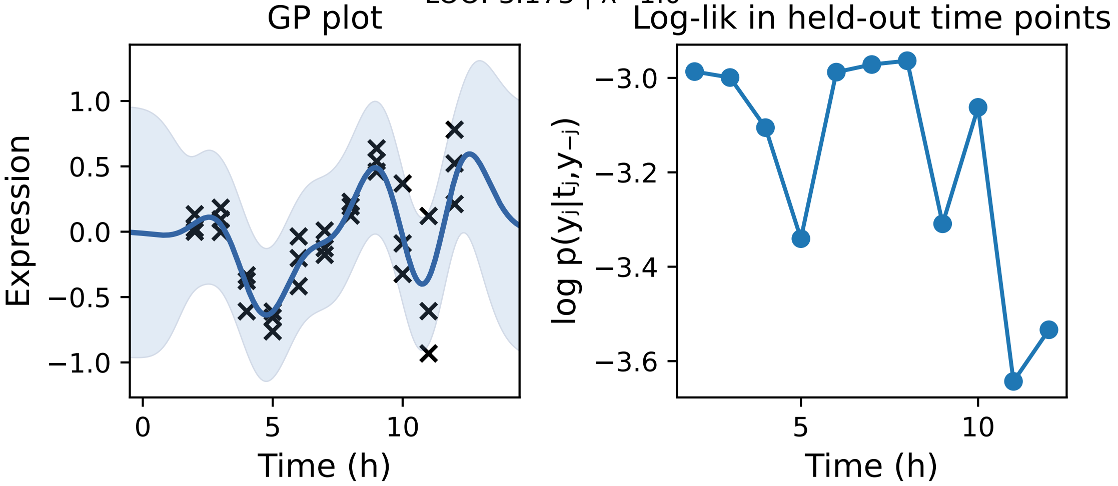

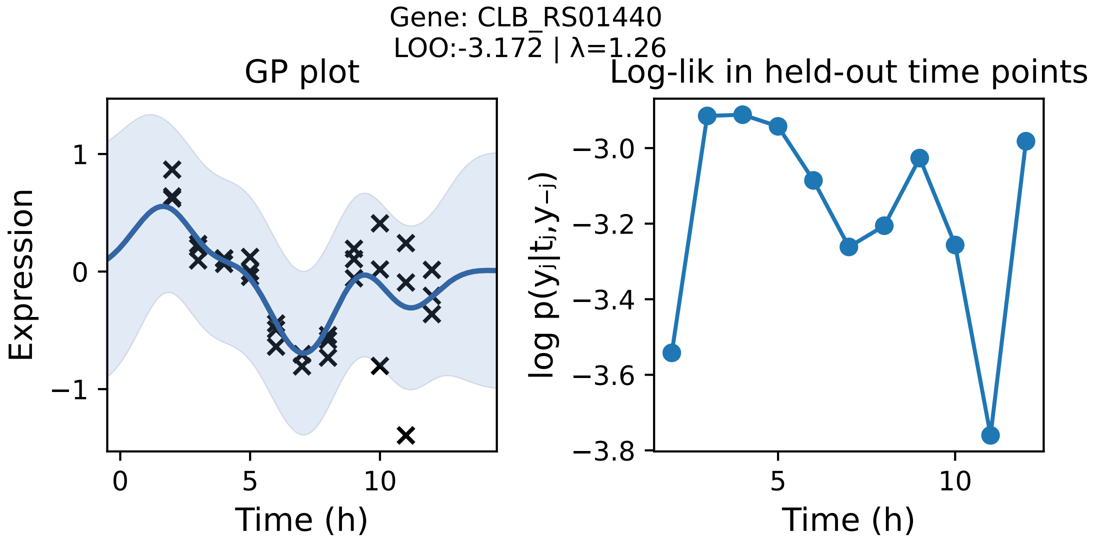

Gene: CLB_RS01440
LOO:-3.172 | λ=1.26
GP plot
Expression
Time (h)
Log-lik in held-out time points
log p(y_j|t_j,y_−j)
Time (h)

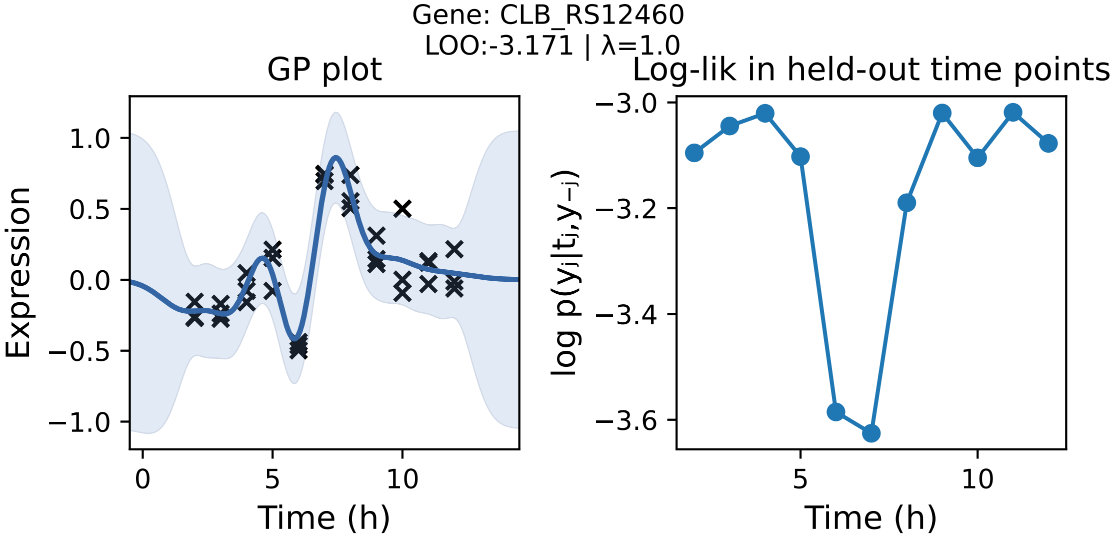

Gene: CLB_RS12460
LOO:-3.171 | λ=1.0
GP plot
Expression
1.0
0.5
0.0
−0.5
−1.0
0
5
10
Time (h)
Log-lik in held-out time points
log p(yj|tj,y−j)
−3.0
−3.2
−3.4
−3.6
5
10
Time (h)

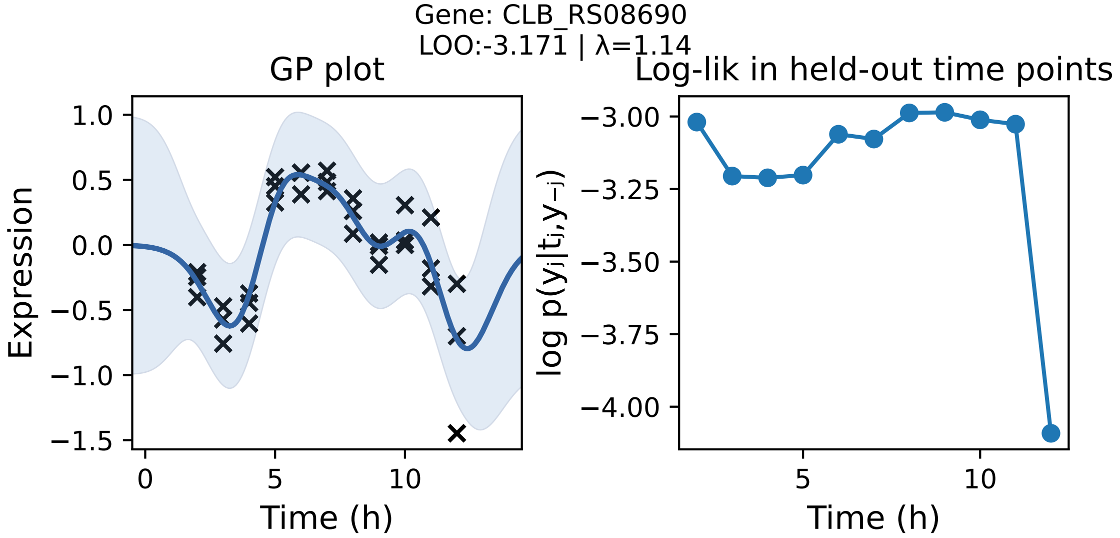

Gene: CLB_RS08690
LOO:-3.171 | λ=1.14
GP plot
Log-lik in held-out time points
Expression
1.0
0.5
0.0
−0.5
−1.0
−1.5
0
5
10
Time (h)
log p(y_j|t_j,y_−j)
−3.00
−3.25
−3.50
−3.75
−4.00
5
10
Time (h)

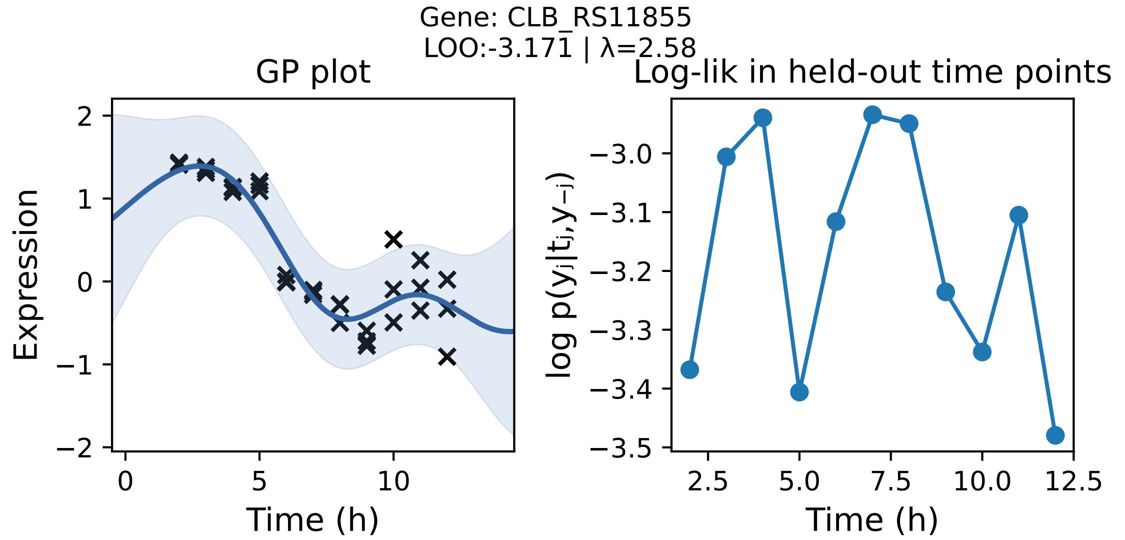
Gene: CLB_RS11855
LOO:-3.171 | λ=2.58
GP plot
Expression
Time (h)
Log-lik in held-out time points
log p(y_j|t_j,y_−j)
Time (h)

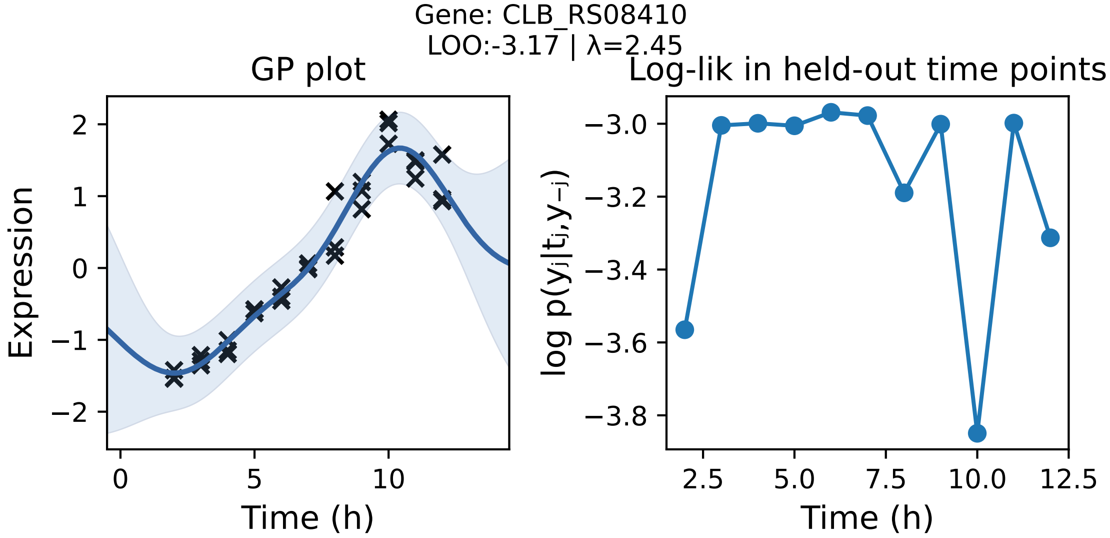

Gene: CLB_RS08410
LOO:-3.17 | λ=2.45
GP plot
Expression
Time (h)
Log-lik in held-out time points
log p(y_j|t_j,y_−j)
Time (h)

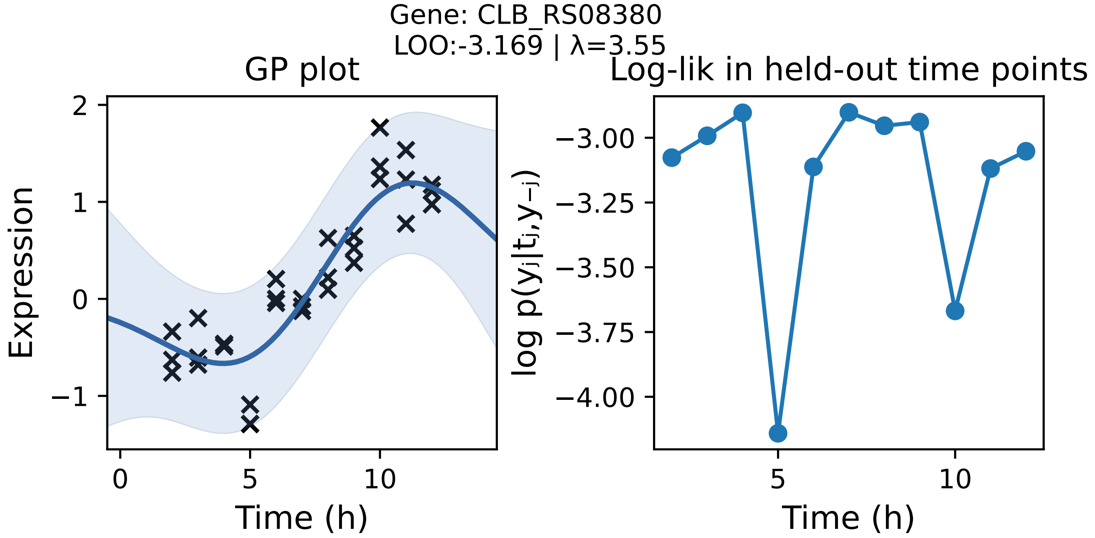

Gene: CLB_RS08380
LOO:-3.169 | λ=3.55
GP plot
Expression
Time (h)
2
1
0
−1
0
5
10
Log-lik in held-out time points
log p(y_j|t_j,y_−j)
−3.00
−3.25
−3.50
−3.75
−4.00
5
10
Time (h)

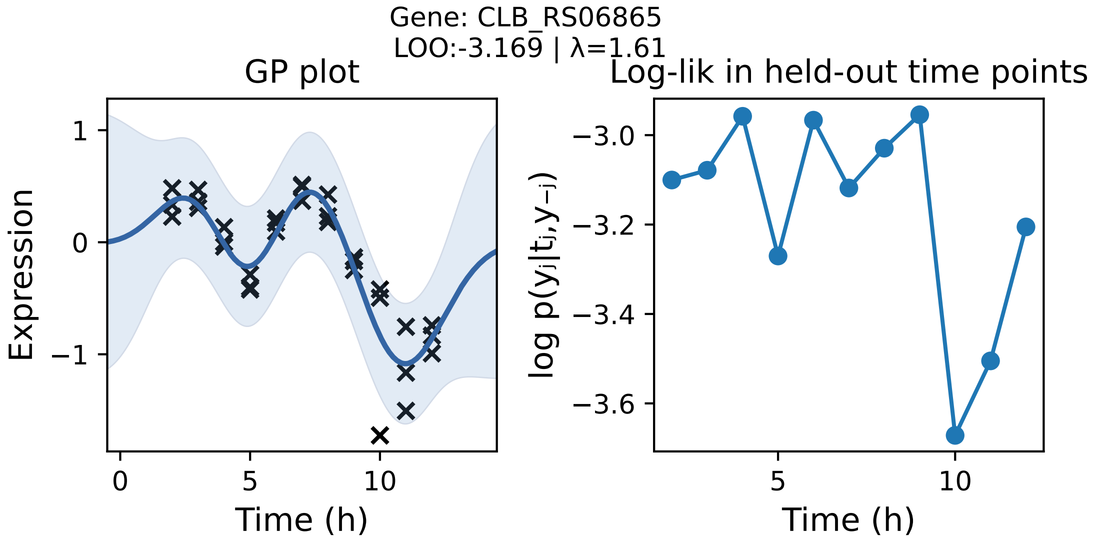

Gene: CLB_RS06865
LOO:-3.169 | λ=1.61
GP plot
Expression
Time (h)
1
0
−1
0
5
10
Log-lik in held-out time points
log p(yj|tj,y−j)
−3.0
−3.2
−3.4
−3.6
5
10
Time (h)

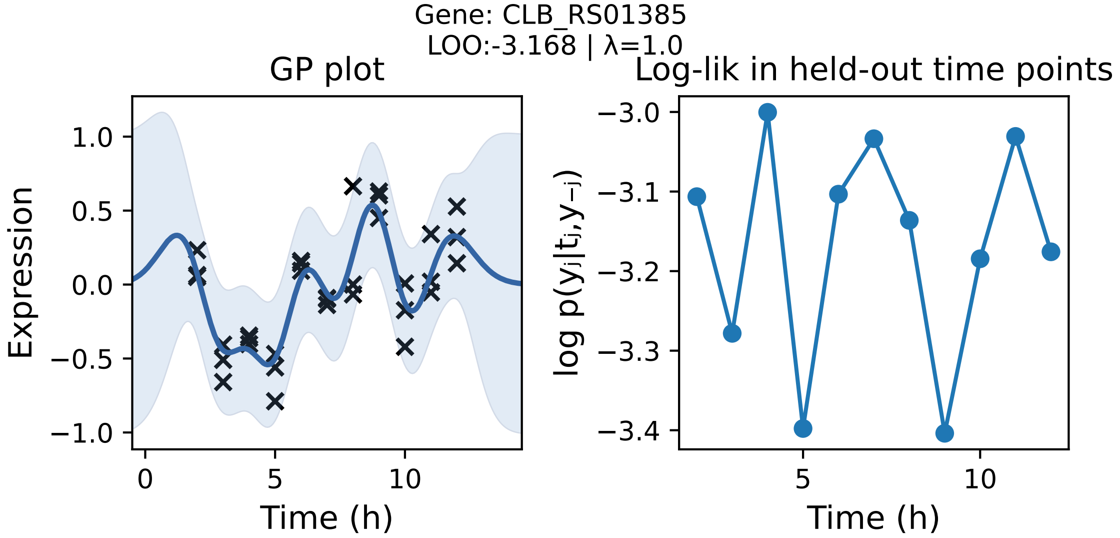

Gene: CLB_RS01385
LOO:-3.168 | λ=1.0
GP plot
Log-lik in held-out time points
Expression
Time (h)
log p(yⱼ|tⱼ,y₋ⱼ)
1.0
0.5
0.0
−0.5
−1.0
0
5
10
−3.0
−3.1
−3.2
−3.3
−3.4

Gene: CLB_RS07565
LOO:-3.167 | λ=1.98

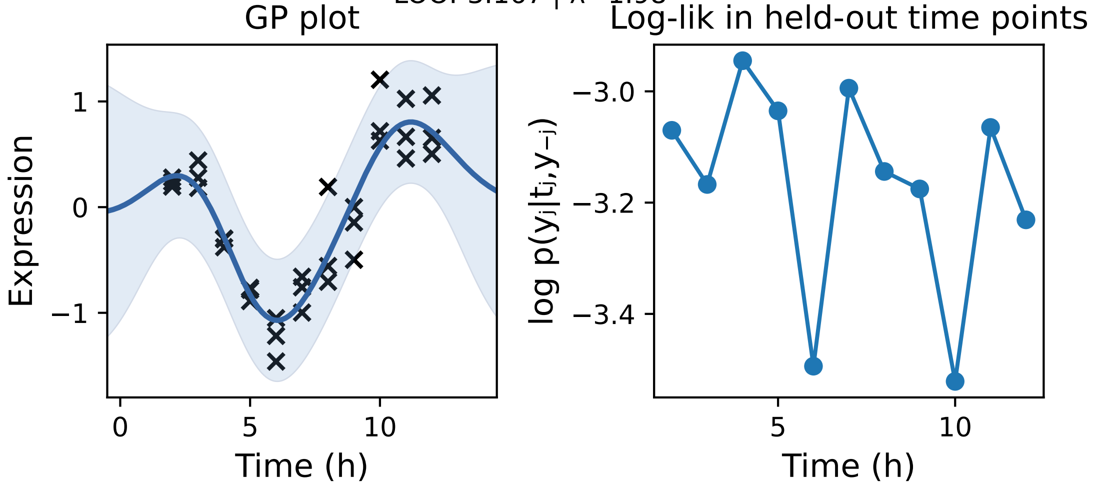

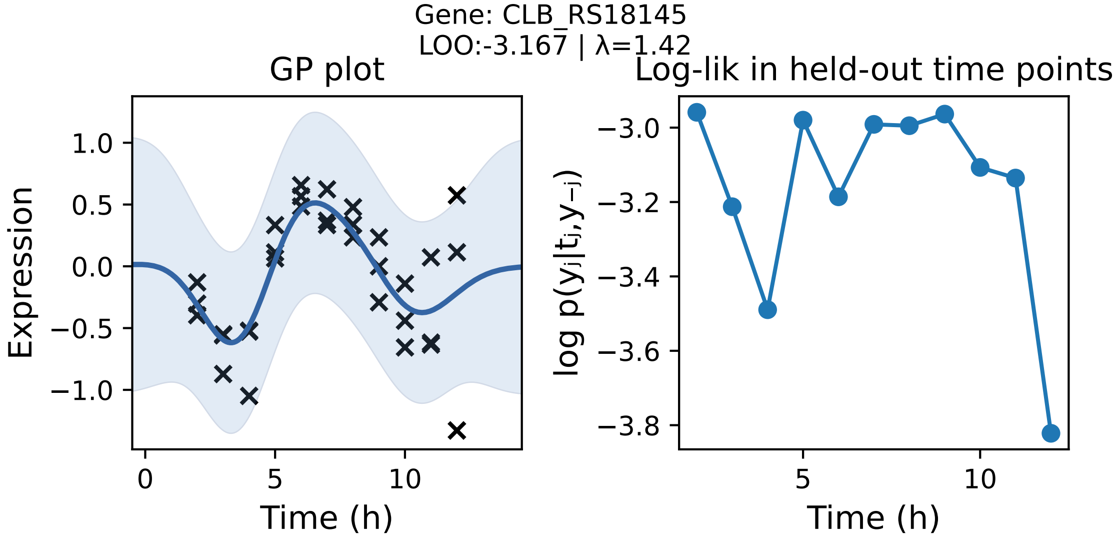
Gene: CLB_RS18145
LOO:-3.167 | λ=1.42
GP plot
Expression
Time (h)
Log-lik in held-out time points
log p(y_j|t_j,y_−j)
Time (h)

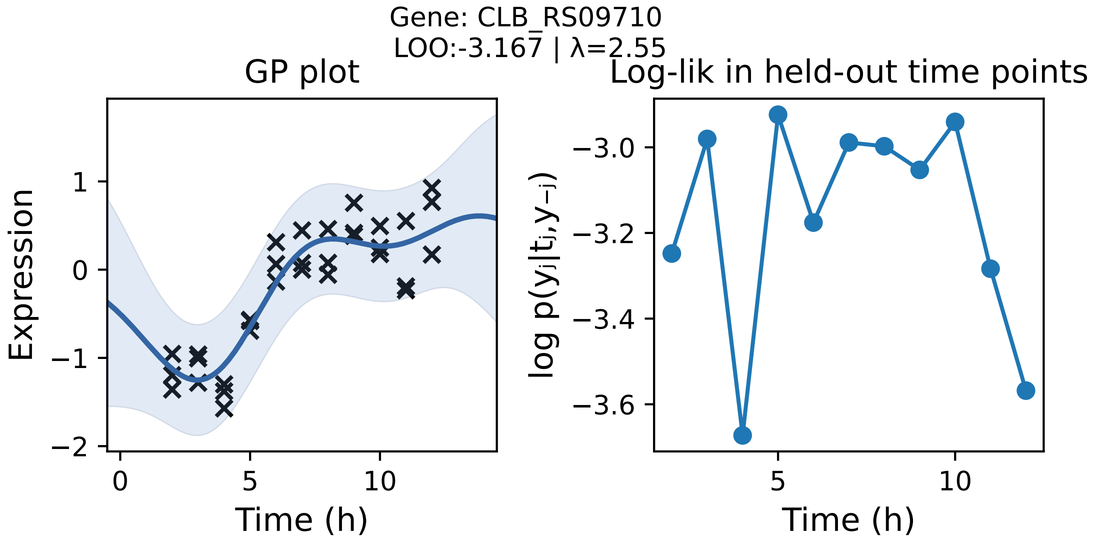

Gene: CLB_RS09710
LOO:-3.167 | λ=2.55
GP plot
Expression
Time (h)
1
0
−1
−2
0
5
10
Log-lik in held-out time points
log p(y_j|t_j,y_−j)
Time (h)
−3.0
−3.2
−3.4
−3.6
5
10

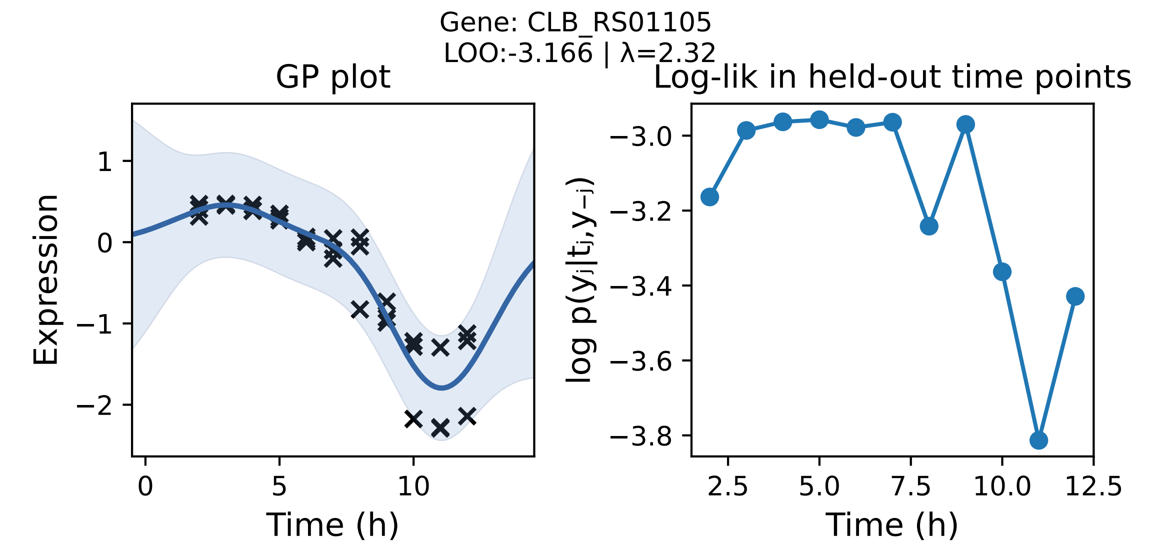

Gene: CLB_RS01105
LOO:-3.166 | λ=2.32
GP plot
Expression
Time (h)
Log-lik in held-out time points
log p(y_j|t_j,y_−j)
Time (h)

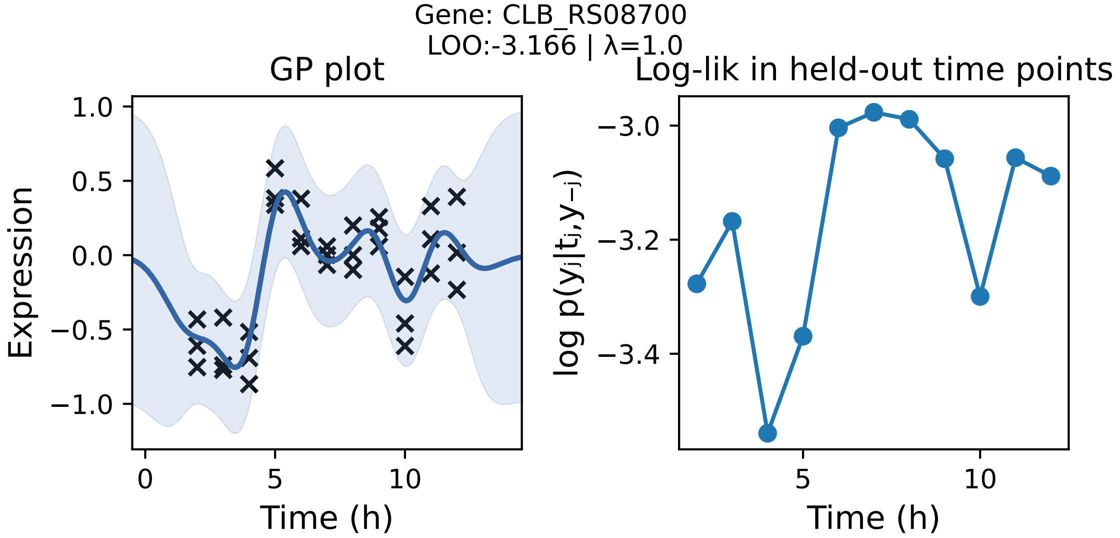
Gene: CLB_RS08700
LOO:-3.166 | λ=1.0
GP plot
Expression
Time (h)
1.0
0.5
0.0
−0.5
−1.0
0
5
10
Log-lik in held-out time points
log p(yj|tj,y−j)
−3.0
−3.2
−3.4
5
10
Time (h)

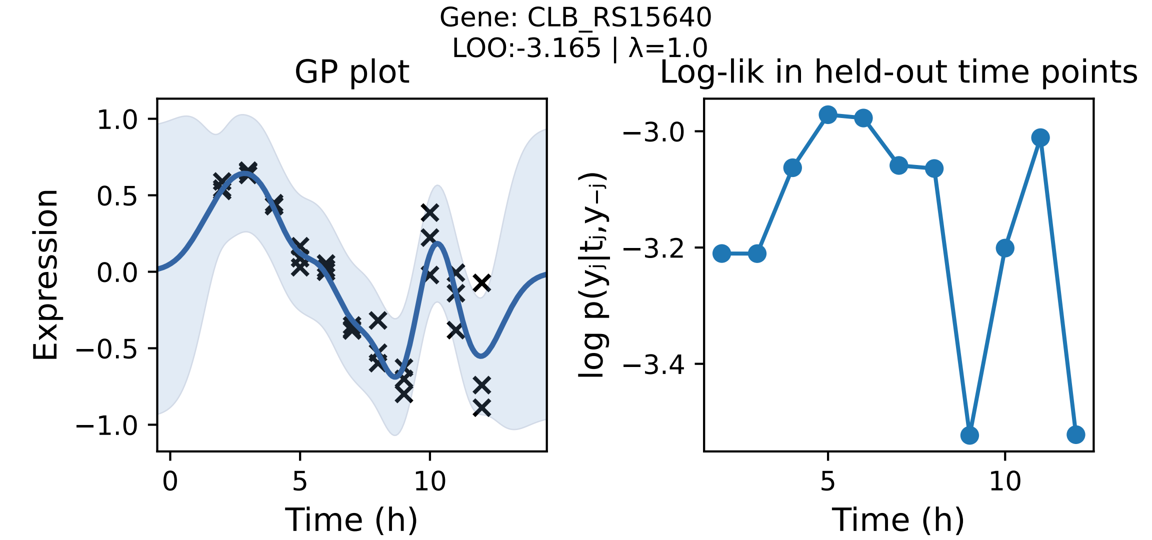
Gene: CLB_RS15640
LOO:-3.165 | λ=1.0
GP plot
Expression
1.0
0.5
0.0
−0.5
−1.0
0
5
10
Time (h)
Log-lik in held-out time points
log p(yj|tj,y−j)
−3.0
−3.2
−3.4
5
10
Time (h)

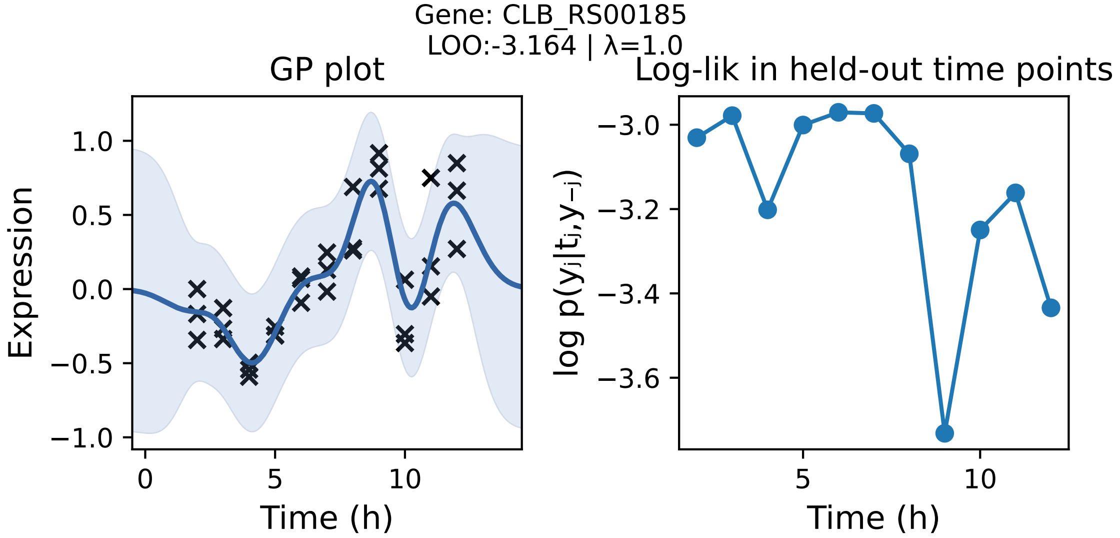

Gene: CLB_RS00185
LOO:-3.164 | λ=1.0
GP plot
Log-lik in held-out time points
Expression
1.0
0.5
0.0
−0.5
−1.0
0
5
10
Time (h)
log p(yj|tj,y−j)
−3.0
−3.2
−3.4
−3.6
5
10
Time (h)

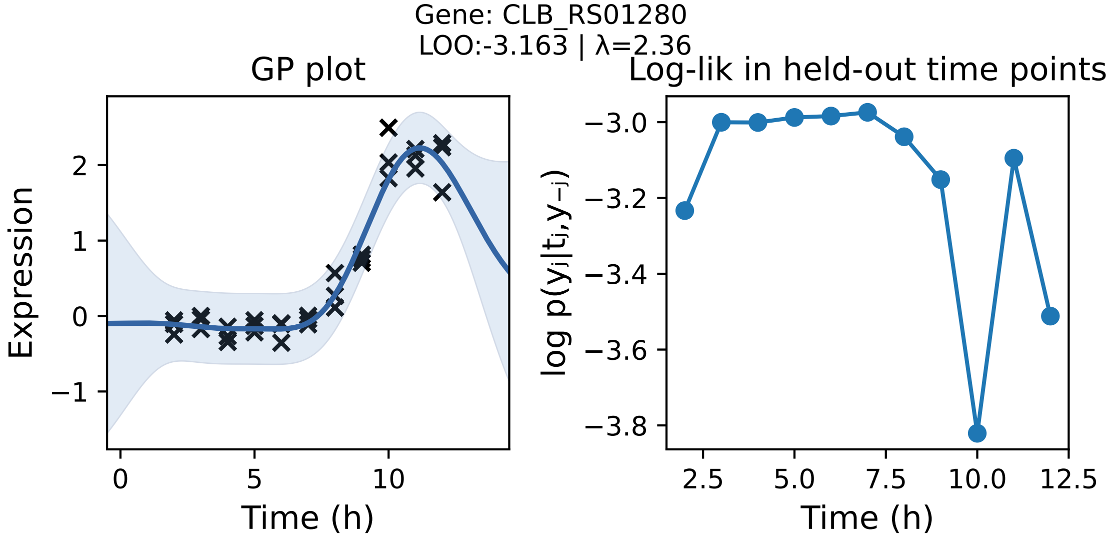

Gene: CLB_RS01280
LOO:-3.163 | λ=2.36
GP plot
Expression
Time (h)
Log-lik in held-out time points
log p(y_j|t_j,y_−j)
Time (h)

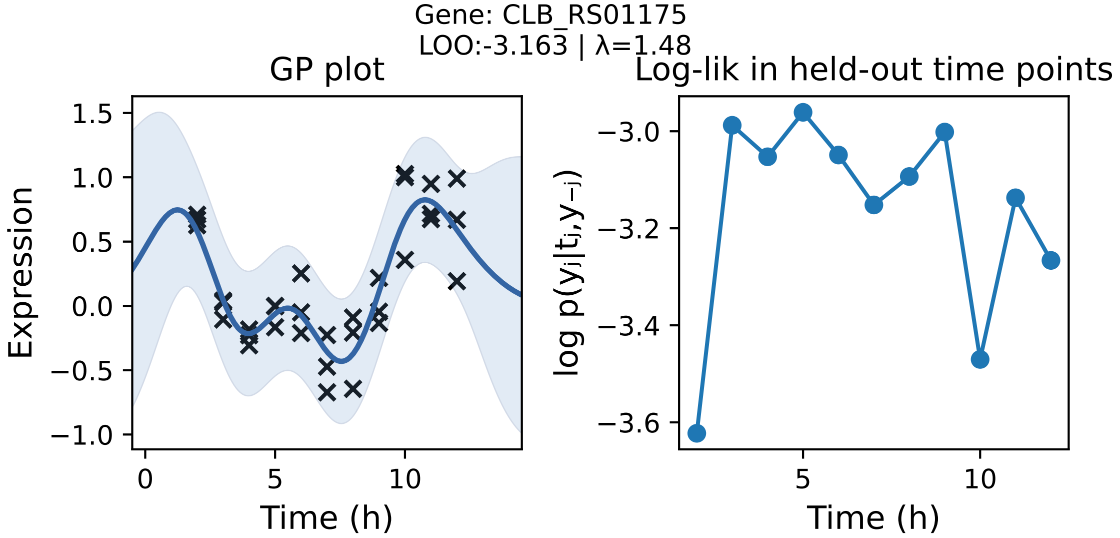

Gene: CLB_RS01175
LOO:-3.163 | λ=1.48
GP plot
Expression
Time (h)
1.5
1.0
0.5
0.0
−0.5
−1.0
0
5
10
Log-lik in held-out time points
log p(y_j|t_j,y_−j)
−3.0
−3.2
−3.4
−3.6
5
10
Time (h)

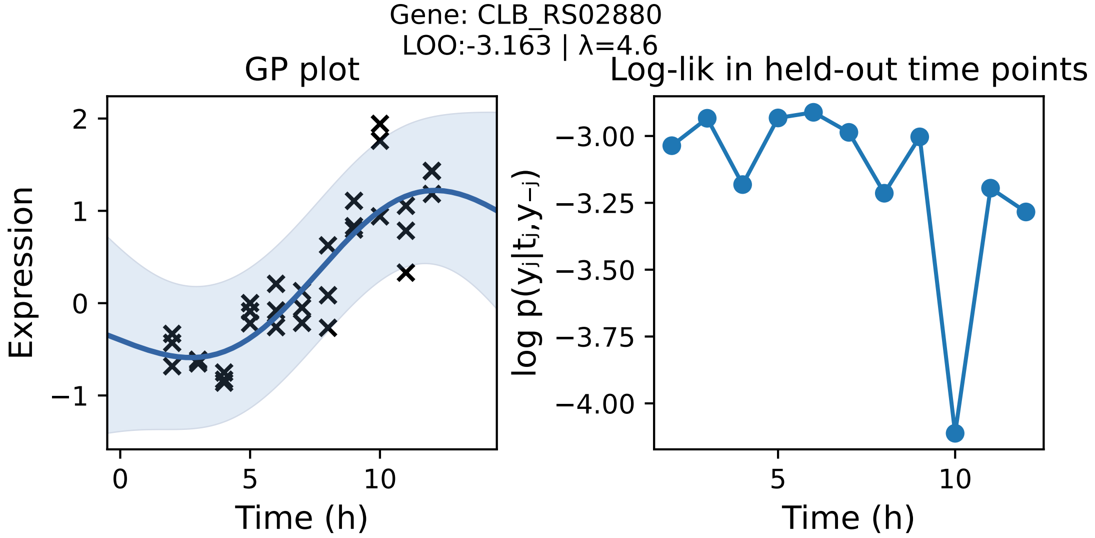
Gene: CLB_RS02880
LOO:-3.163 | λ=4.6
GP plot
Log-lik in held-out time points
Expression
Time (h)
log p(y_j|t_j,y_−j)
Time (h)
2
1
0
−1
0
5
10
−3.00
−3.25
−3.50
−3.75
−4.00
5
10

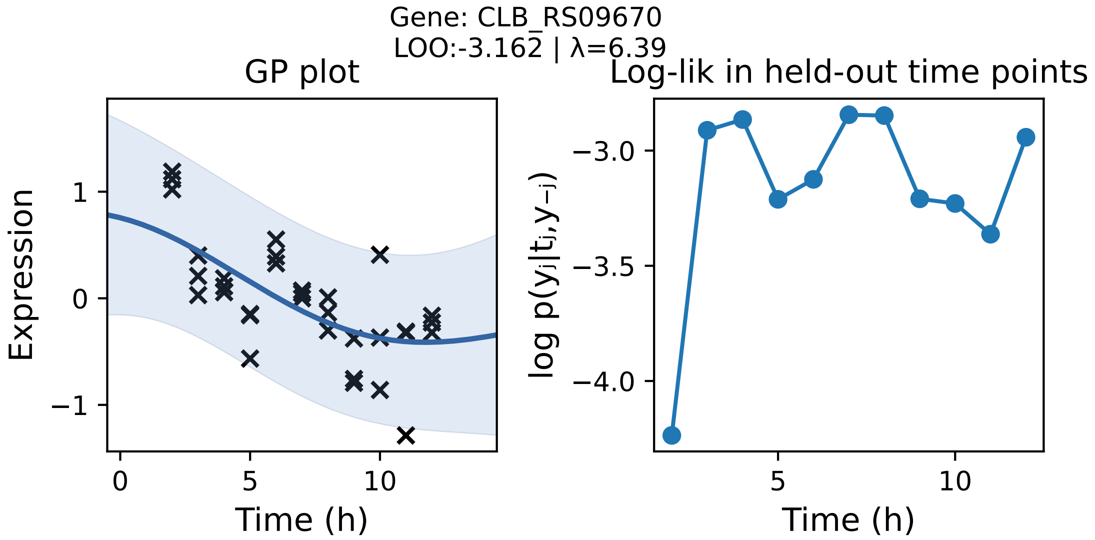
Gene: CLB_RS09670
LOO:-3.162 | λ=6.39
GP plot
Expression
Time (h)
Log-lik in held-out time points
log p(yj|tj,y−j)
Time (h)
1
0
−1
0
5
10
−3.0
−3.5
−4.0
5
10

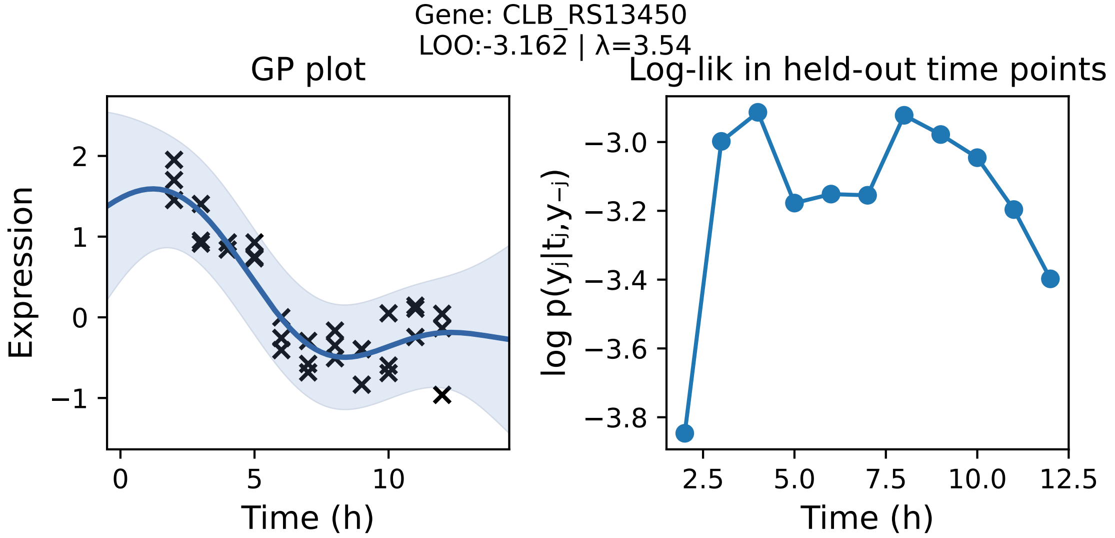

Gene: CLB_RS13450
LOO:-3.162 | λ=3.54
GP plot
Expression
Time (h)
Log-lik in held-out time points
log p(yj|tj,y−j)
Time (h)

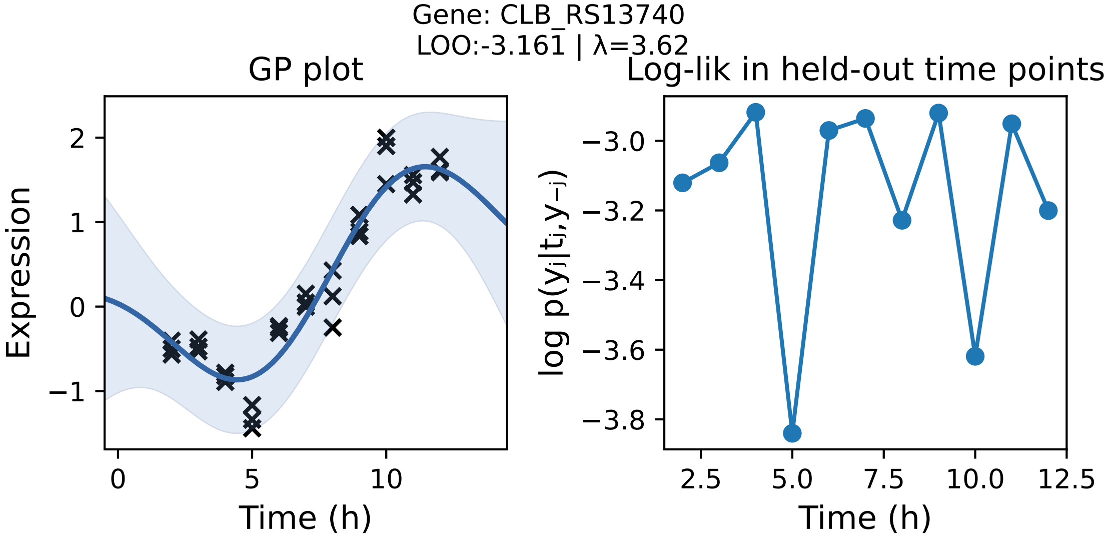

Gene: CLB_RS13740
LOO:-3.161 | λ=3.62
GP plot
Expression
Time (h)
Log-lik in held-out time points
log p(y_j|t_j,y_−j)
Time (h)

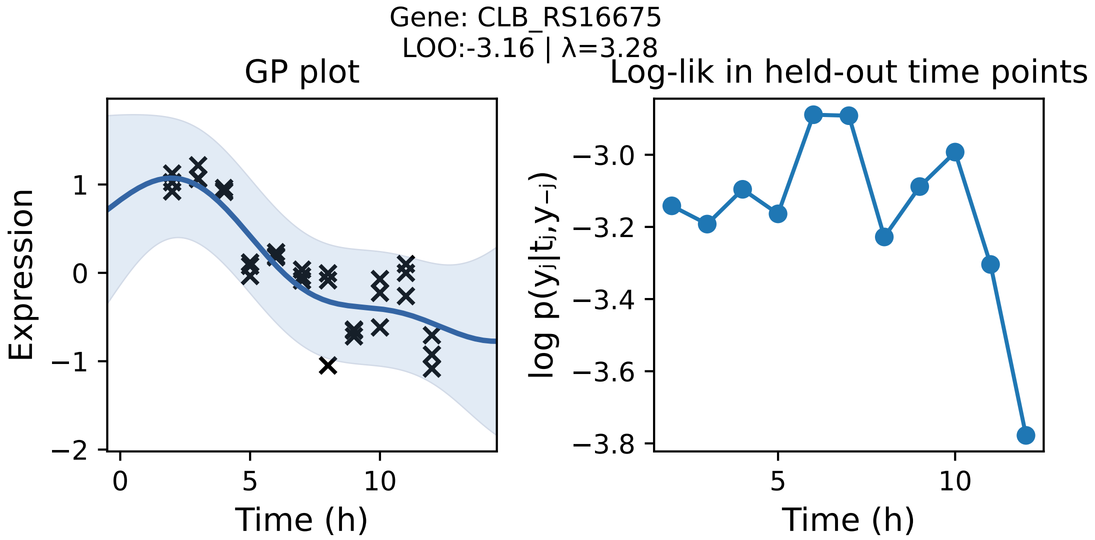

Gene: CLB_RS16675
LOO:-3.16 | λ=3.28
GP plot
Expression
Time (h)
Log-lik in held-out time points
log p(y_j|t_j,y_−j)
Time (h)

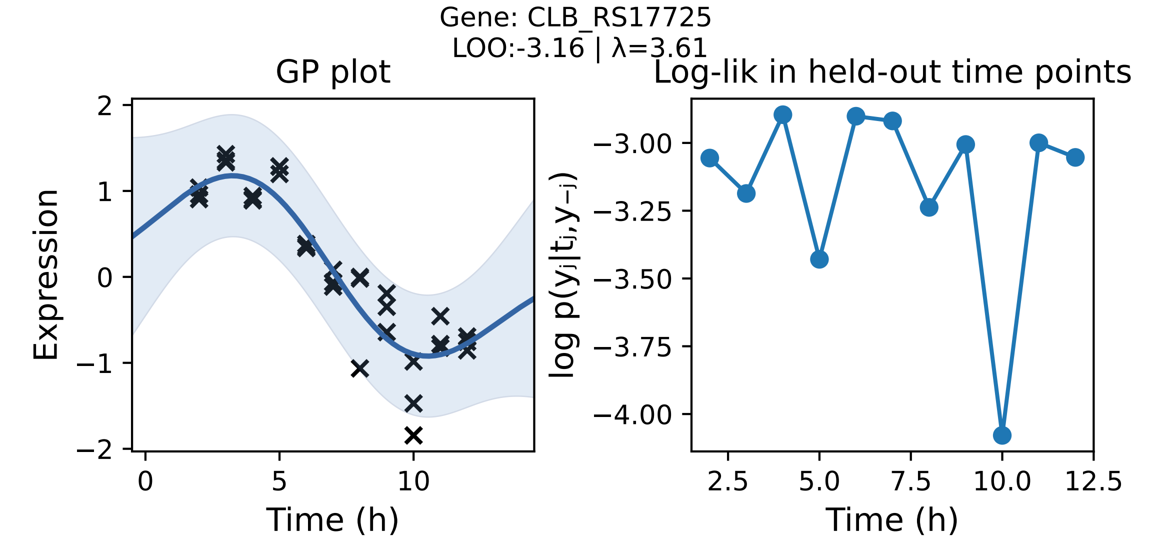

Gene: CLB_RS17725
LOO:-3.16 | λ=3.61
GP plot
Expression
Time (h)
2
1
0
−1
−2
0
5
10
Log-lik in held-out time points
$\log p(y_j|t_j, y_{-j})$
−3.00
−3.25
−3.50
−3.75
−4.00
2.5
5.0
7.5
10.0
12.5
Time (h)

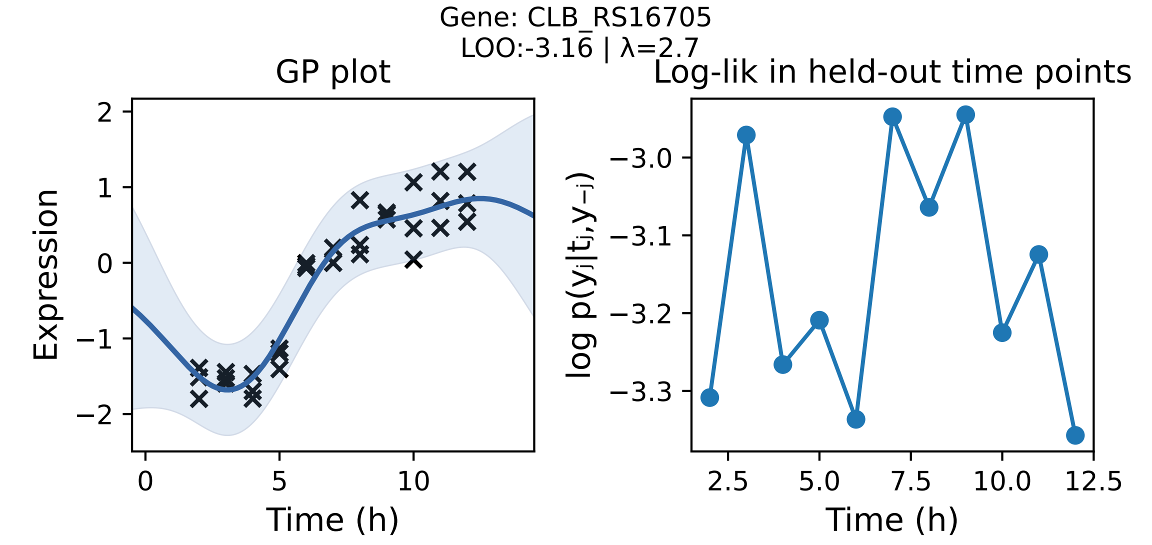

Gene: CLB_RS16705
LOO:-3.16 | λ=2.7
GP plot
Expression
Time (h)
2
1
0
−1
−2
0
5
10
Log-lik in held-out time points
log p(y_j|t_j,y_−j)
−3.0
−3.1
−3.2
−3.3
2.5
5.0
7.5
10.0
12.5
Time (h)

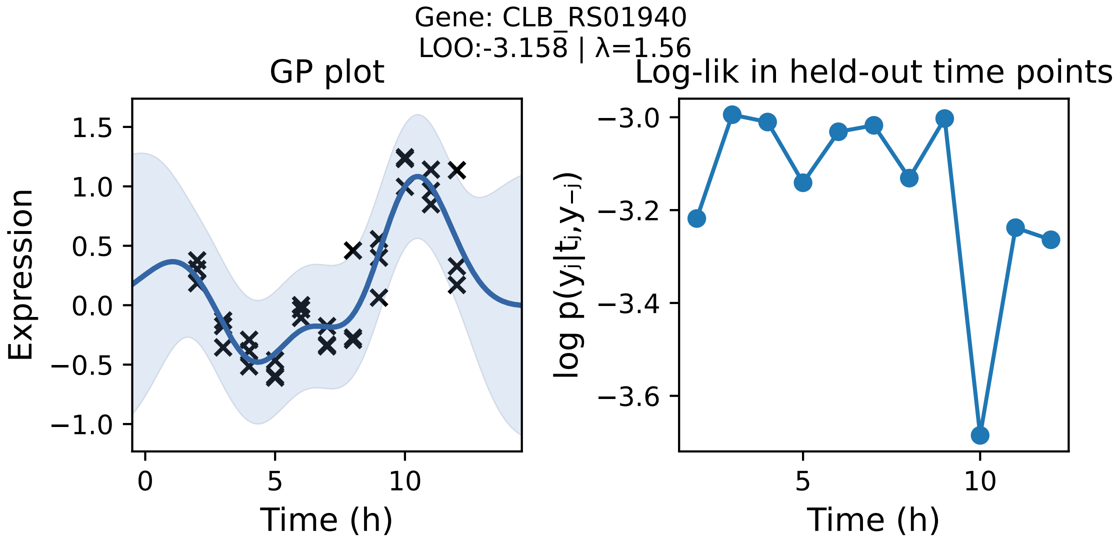

Gene: CLB_RS01940
LOO:-3.158 | λ=1.56
GP plot
Log-lik in held-out time points
Expression
1.5
1.0
0.5
0.0
−0.5
−1.0
0
5
10
Time (h)
log p(y_j|t_j,y_−j)
−3.0
−3.2
−3.4
−3.6
5
10
Time (h)

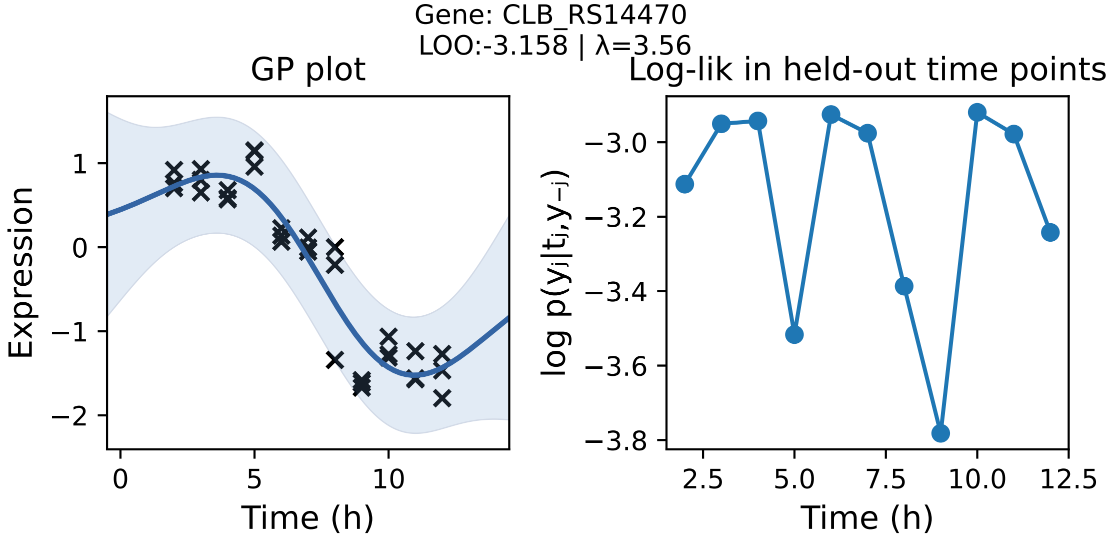

Gene: CLB_RS14470
LOO:-3.158 | λ=3.56
GP plot
Expression
Time (h)
Log-lik in held-out time points
log p(y_j|t_j,y_−j)
Time (h)

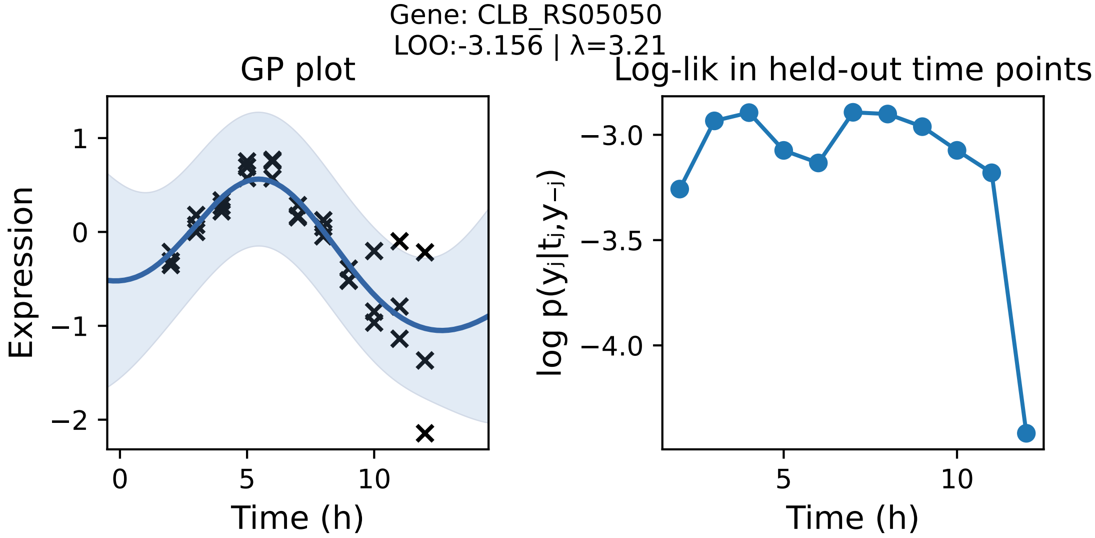

Gene: CLB_RS05050
LOO:-3.156 | λ=3.21
GP plot
Expression
Time (h)
Log-lik in held-out time points
log p(y_j|t_j,y_−j)
Time (h)

Gene: CLB_RS09285
LOO:-3.155 | λ=1.77

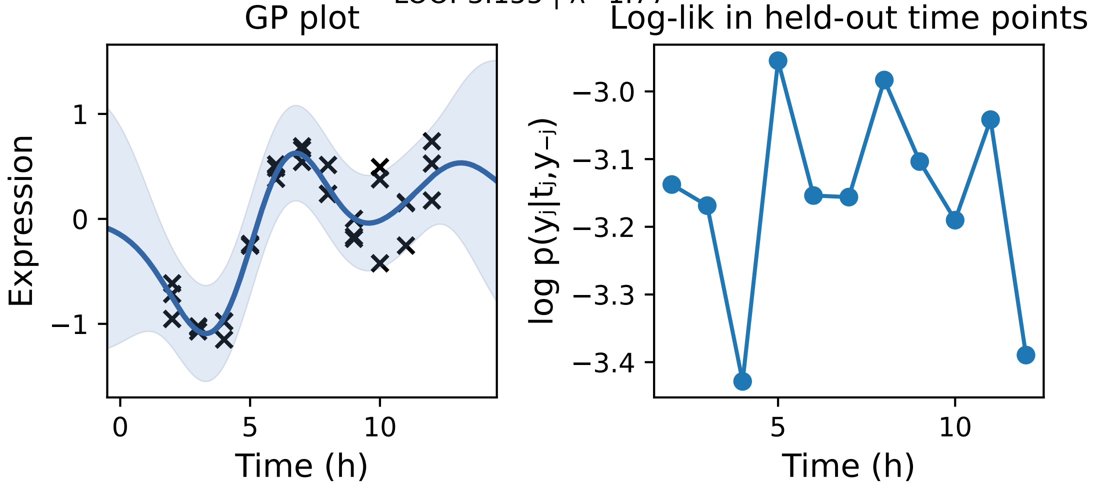

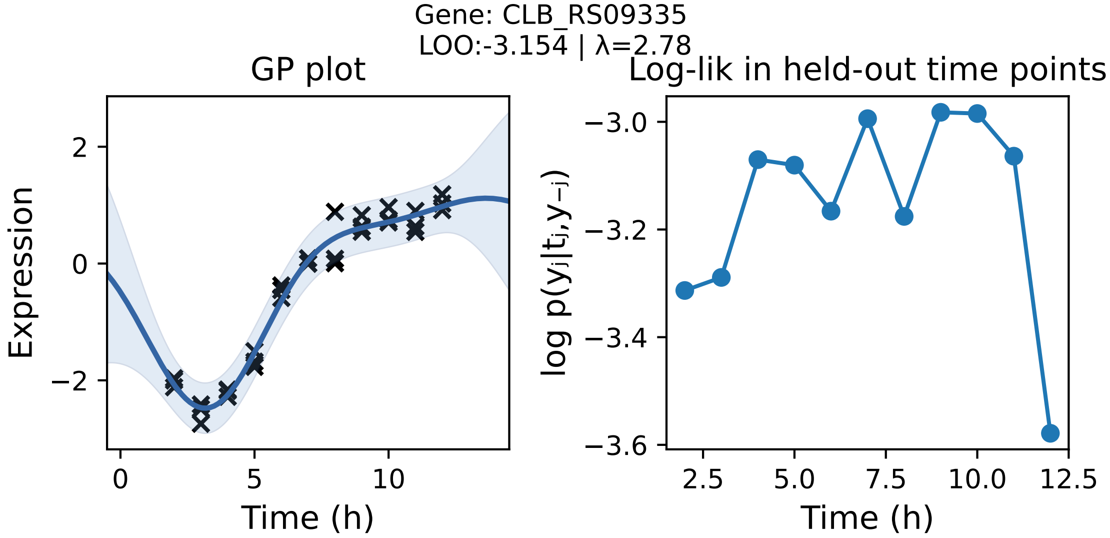

Gene: CLB_RS09335
LOO:-3.154 | λ=2.78
GP plot
Expression
Time (h)
2
0
−2
0
5
10
Log-lik in held-out time points
log p(y_j|t_j,y_−j)
Time (h)
−3.0
−3.2
−3.4
−3.6
2.5
5.0
7.5
10.0
12.5

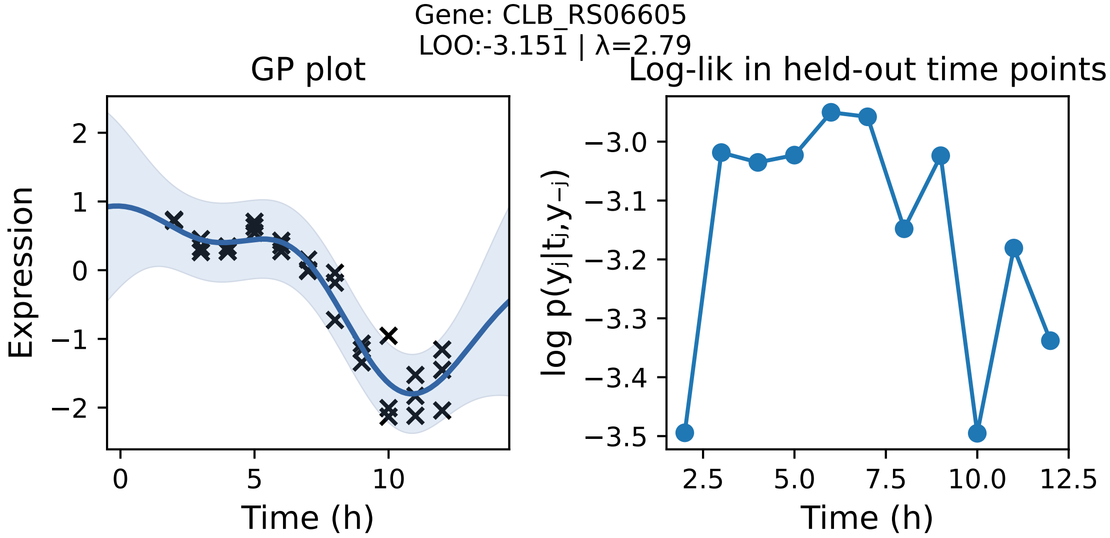

Gene: CLB_RS06605
LOO:-3.151 | λ=2.79
GP plot
Expression
Time (h)
Log-lik in held-out time points
log p(y_j|t_j,y_−j)
Time (h)

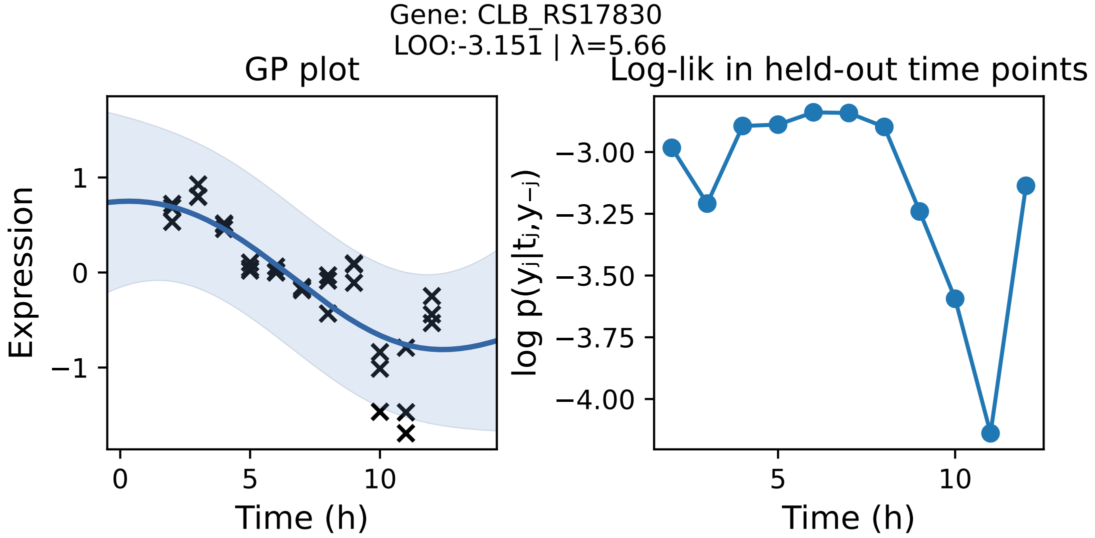

Gene: CLB_RS17830
LOO:-3.151 | λ=5.66
GP plot
Expression
Time (h)
Log-lik in held-out time points
log p(yⱼ|tⱼ,y₋ⱼ)
Time (h)

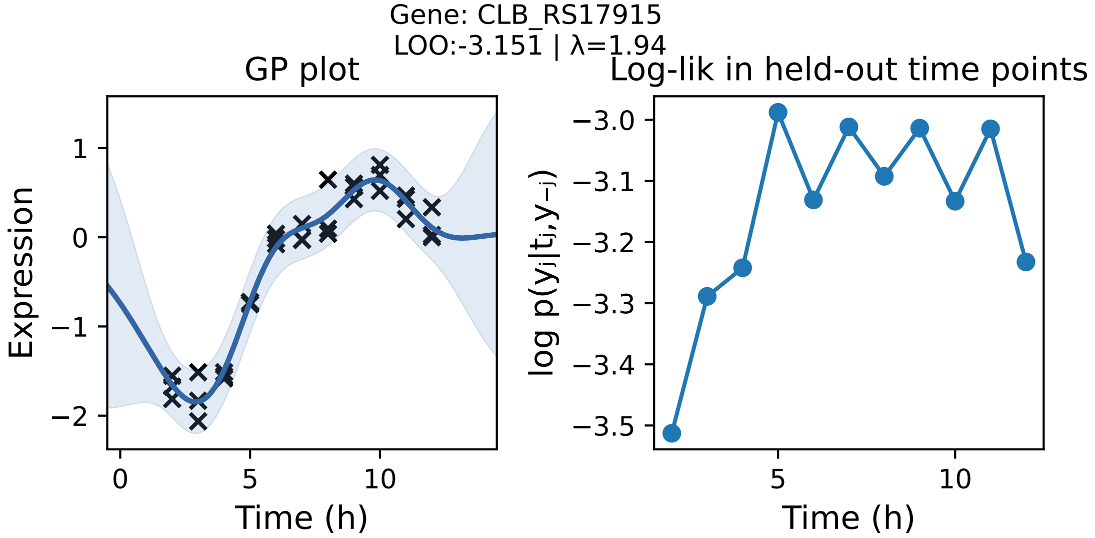

Gene: CLB_RS17915
LOO:-3.151 | λ=1.94
GP plot
Expression
1
0
−1
−2
0
5
10
Time (h)
Log-lik in held-out time points
log p(y_j|t_j,y_−j)
−3.0
−3.1
−3.2
−3.3
−3.4
−3.5
5
10
Time (h)

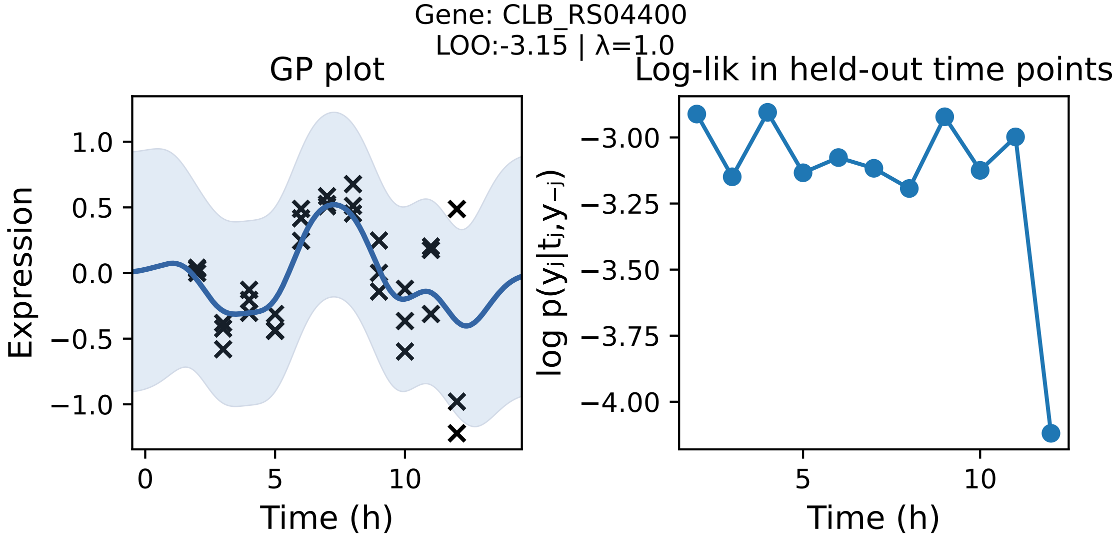

Gene: CLB_RS04400
LOO:-3.15 | λ=1.0
GP plot
Expression
1.0
0.5
0.0
−0.5
−1.0
0
5
10
Time (h)
Log-lik in held-out time points
log p(yj|tj,y−j)
−3.00
−3.25
−3.50
−3.75
−4.00
5
10
Time (h)

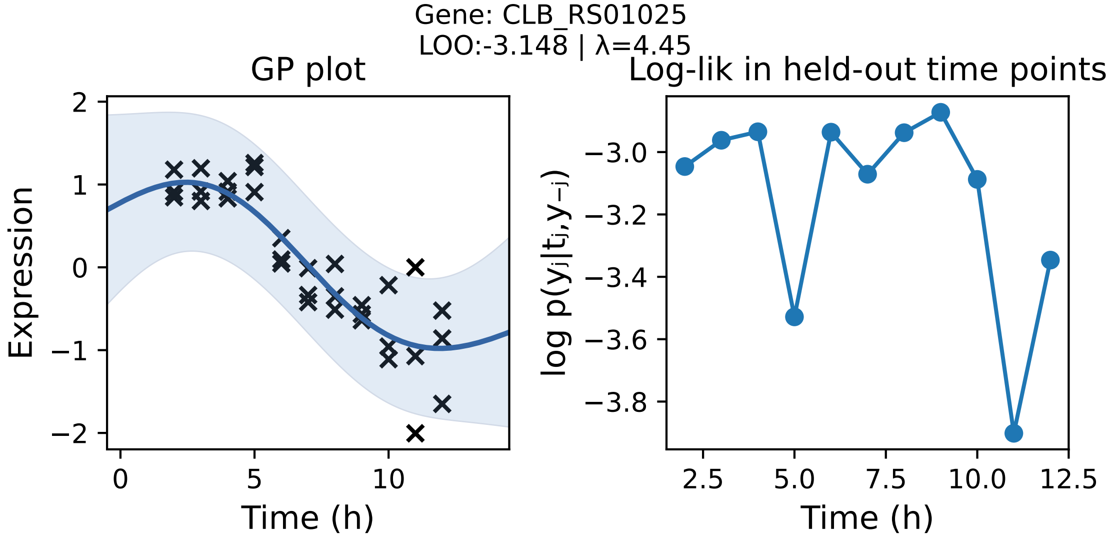
Gene: CLB_RS01025
LOO:-3.148 | λ=4.45
GP plot
Expression
Time (h)
Log-lik in held-out time points
log p(y_j|t_j,y_−j)
Time (h)

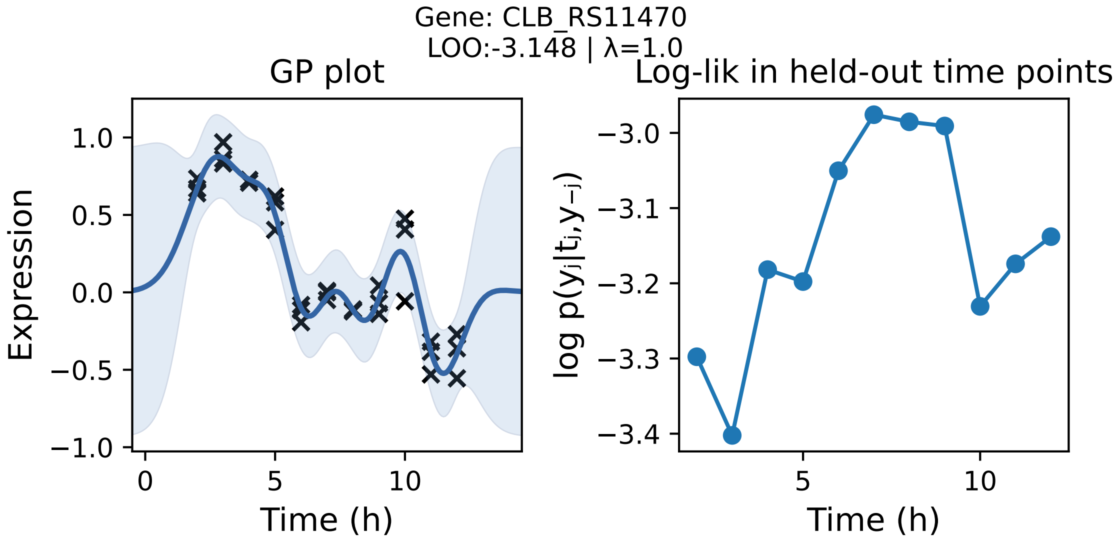

Gene: CLB_RS11470
LOO:-3.148 | λ=1.0
GP plot
Expression
1.0
0.5
0.0
−0.5
−1.0
0
5
10
Time (h)
Log-lik in held-out time points
log p(yj|tj,y−j)
−3.0
−3.1
−3.2
−3.3
−3.4
5
10
Time (h)

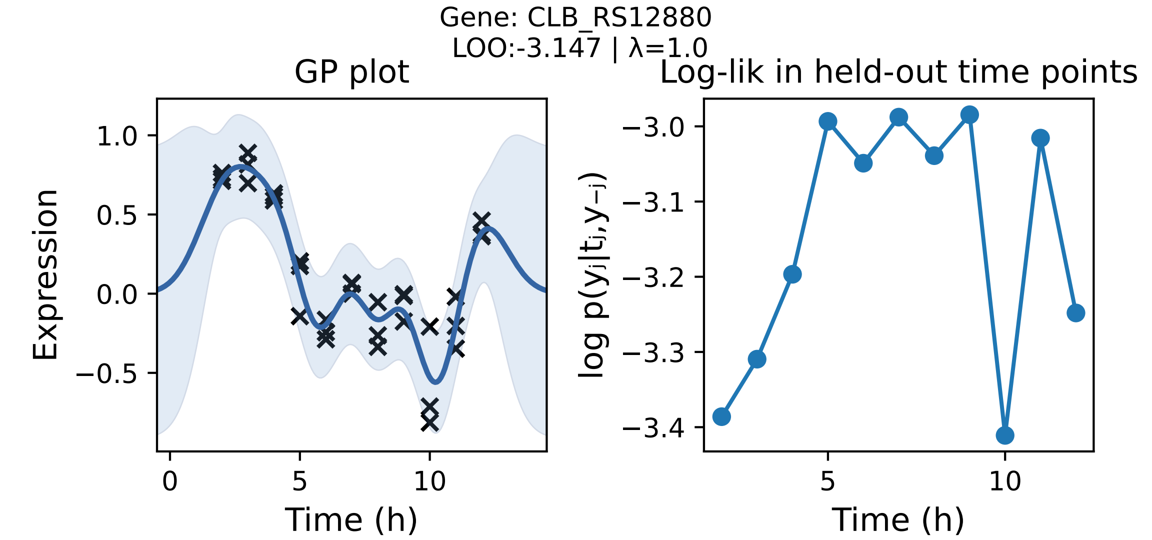
Gene: CLB_RS12880
LOO:-3.147 | λ=1.0
GP plot
Expression
1.0
0.5
0.0
−0.5
0
5
10
Time (h)
Log-lik in held-out time points
log p(yj|tj,y−j)
−3.0
−3.1
−3.2
−3.3
−3.4
5
10
Time (h)

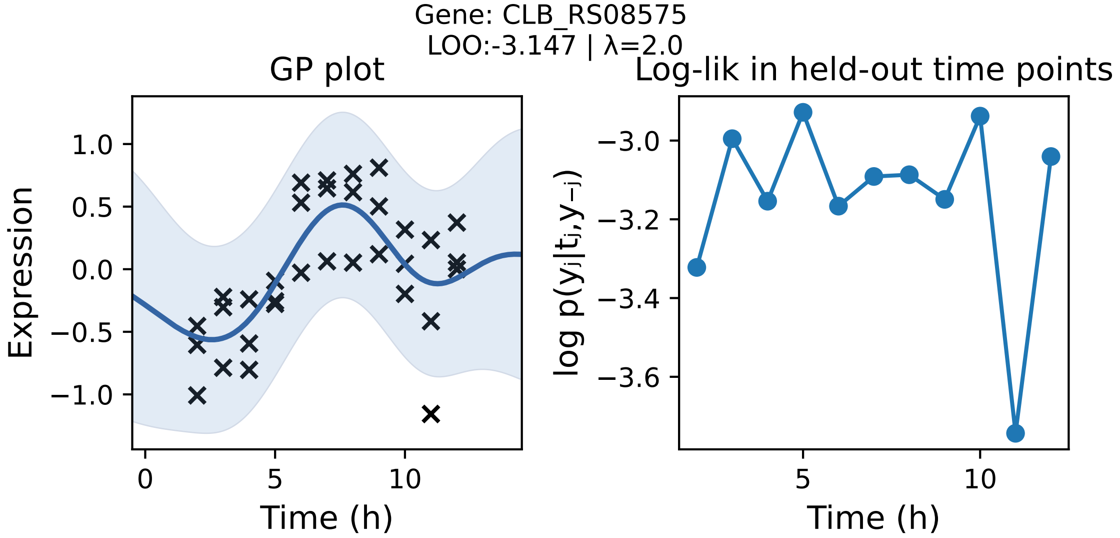

Gene: CLB_RS08575
LOO:-3.147 | λ=2.0
GP plot
Expression
1.0
0.5
0.0
−0.5
−1.0
0
5
10
Time (h)
Log-lik in held-out time points
log p(yj|tj,y−j)
−3.0
−3.2
−3.4
−3.6
5
10
Time (h)

Gene: CLB_RS13260
LOO:-3.147 | λ=1.0

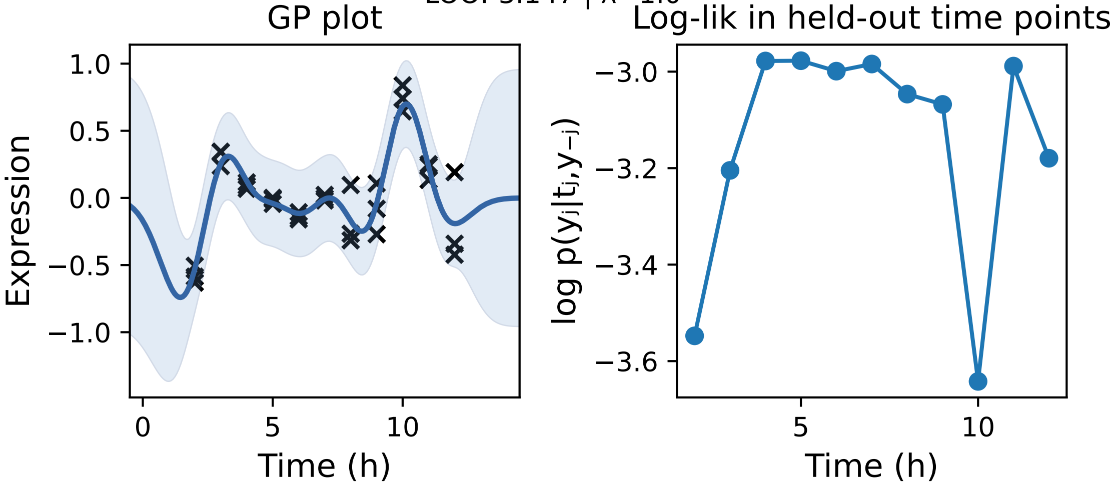

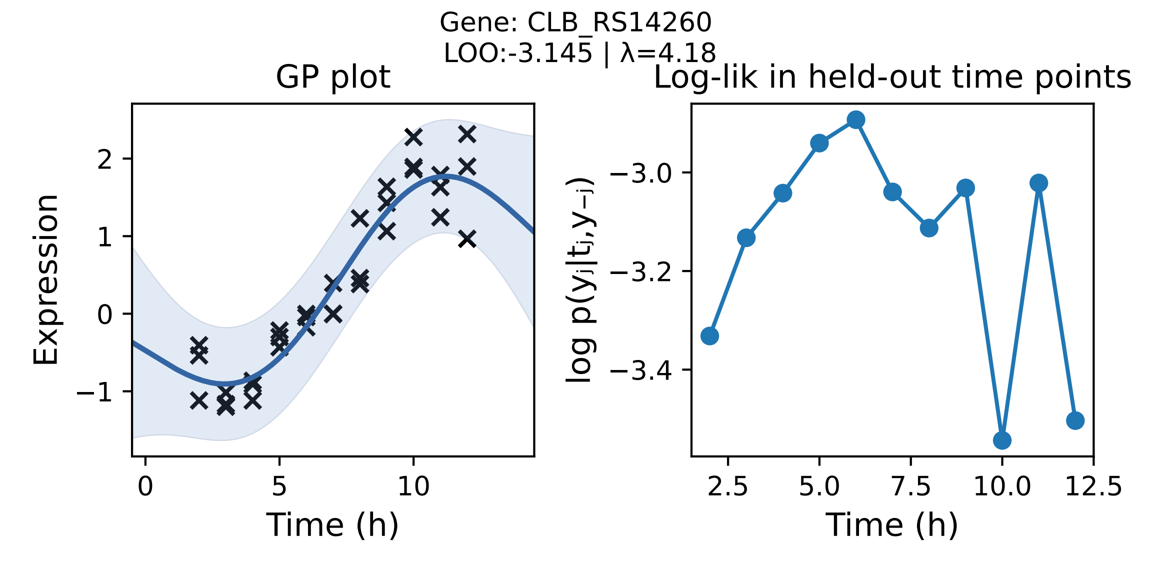

Gene: CLB_RS14260
LOO:-3.145 | λ=4.18
GP plot
Expression
Time (h)
Log-lik in held-out time points
log p(y_j|t_j,y_−j)
Time (h)

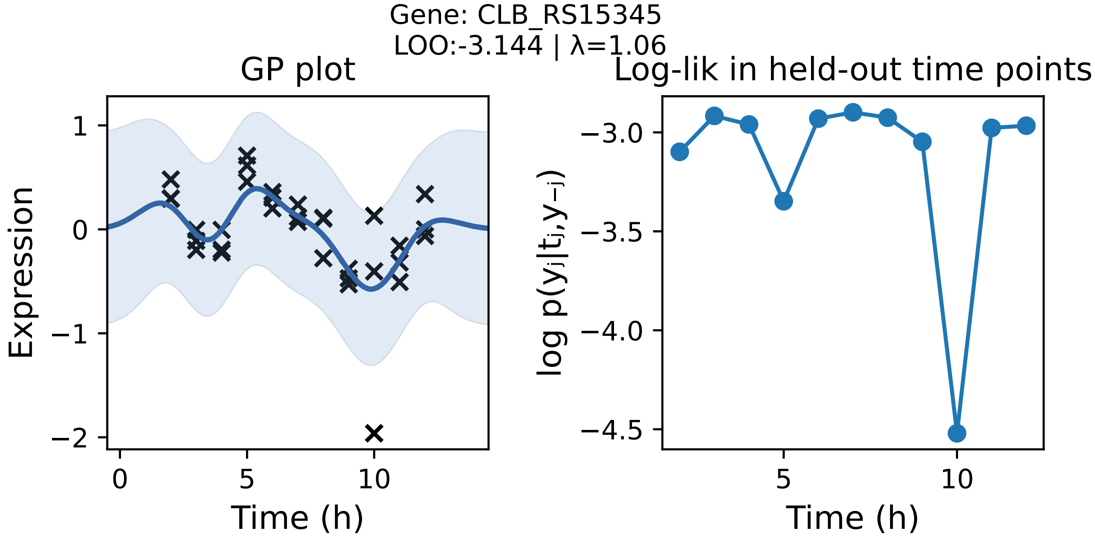

Gene: CLB_RS15345
LOO:-3.144 | λ=1.06
GP plot
Expression
Time (h)
Log-lik in held-out time points
log p(y_j|t_j,y_−j)
Time (h)

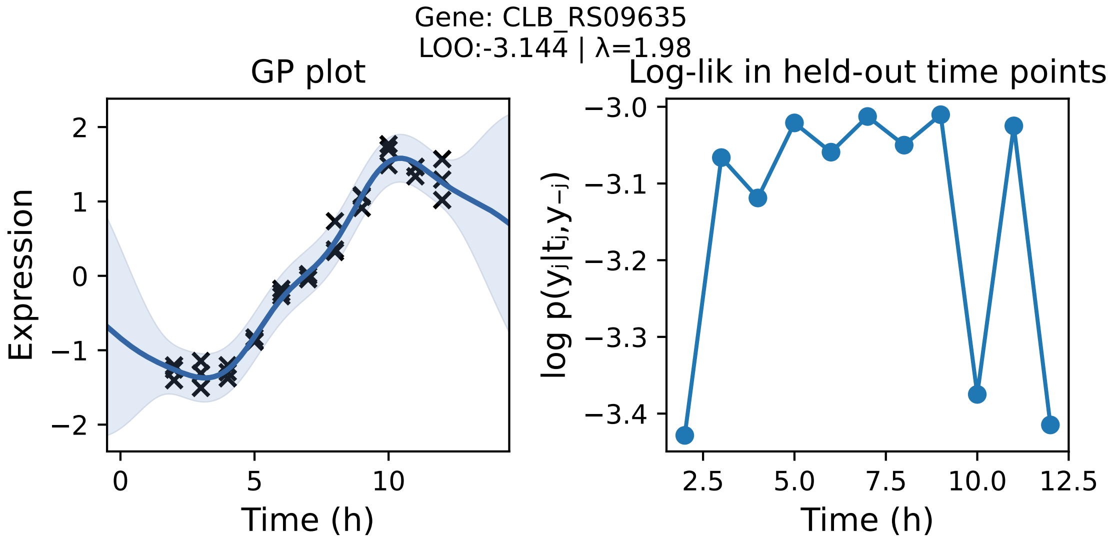

Gene: CLB_RS09635
LOO:-3.144 | λ=1.98
GP plot
Expression
Time (h)
Log-lik in held-out time points
log p(yj|tj,y−j)
Time (h)

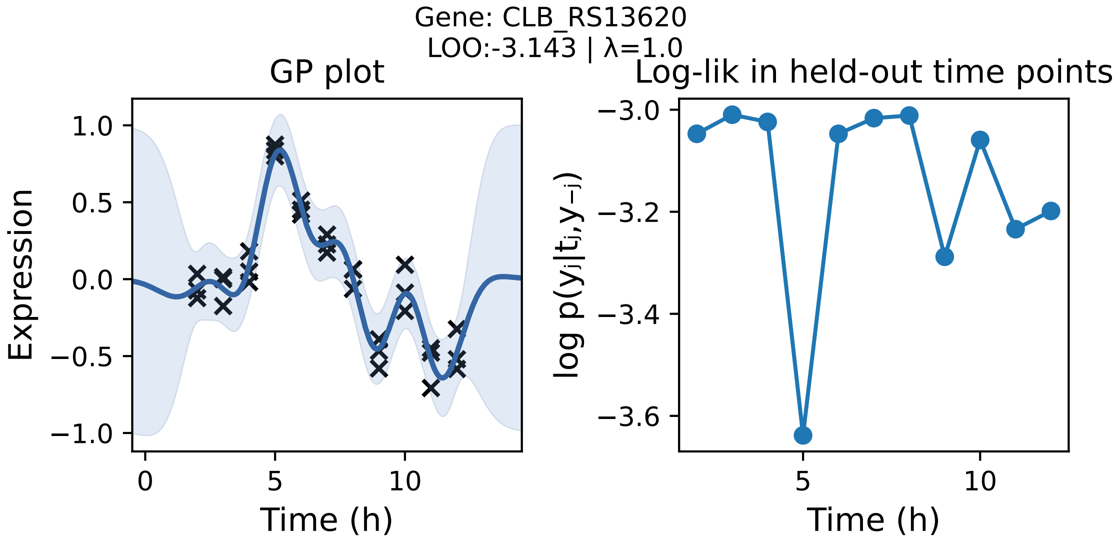

Gene: CLB_RS13620
LOO:-3.143 | λ=1.0
GP plot
Expression
1.0
0.5
0.0
−0.5
−1.0
0
5
10
Time (h)
Log-lik in held-out time points
log p(yj|tj,y−j)
−3.0
−3.2
−3.4
−3.6
5
10
Time (h)

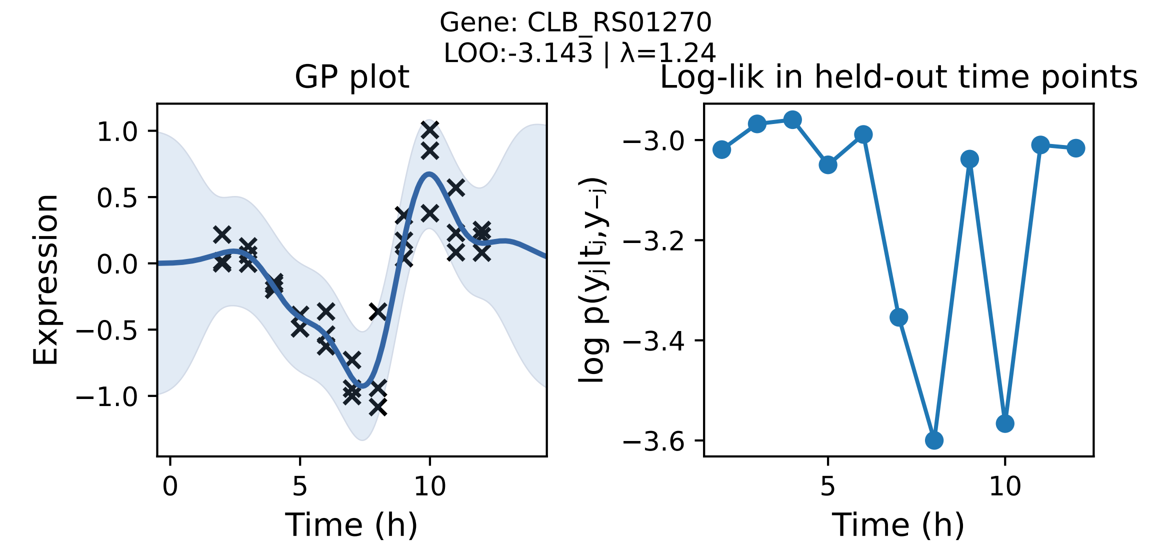

Gene: CLB_RS01270
LOO:-3.143 | λ=1.24
GP plot
Log-lik in held-out time points
Expression
Time (h)
log p(y_j|t_j,y_−j)
1.0
0.5
0.0
−0.5
−1.0
0
5
10
−3.0
−3.2
−3.4
−3.6

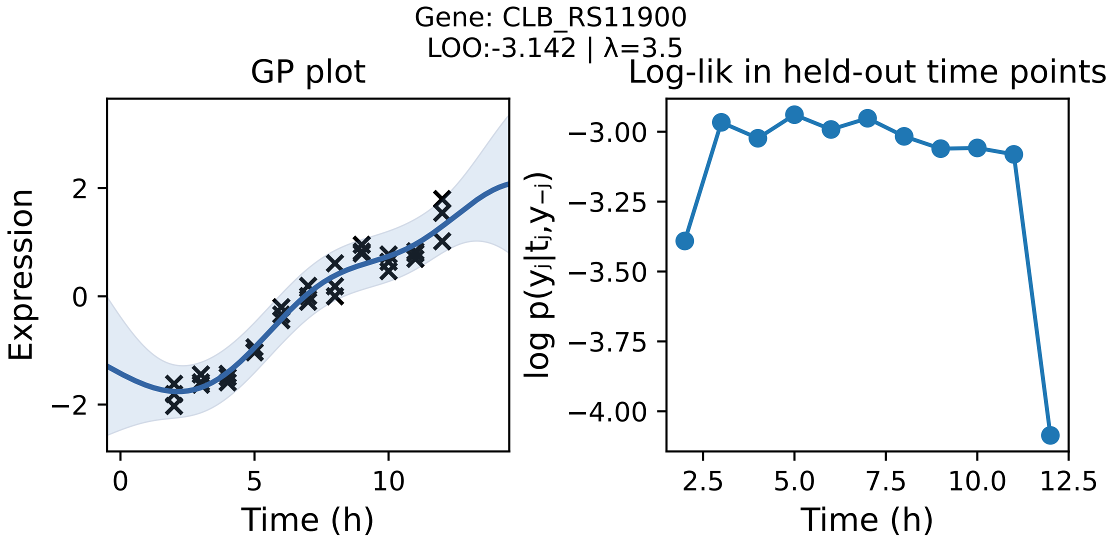
Gene: CLB_RS11900
LOO:-3.142 | λ=3.5
GP plot
Expression
Time (h)
2
0
−2
0
5
10
Log-lik in held-out time points
log p(y_j|t_j,y_−j)
−3.00
−3.25
−3.50
−3.75
−4.00
2.5
5.0
7.5
10.0
12.5
Time (h)

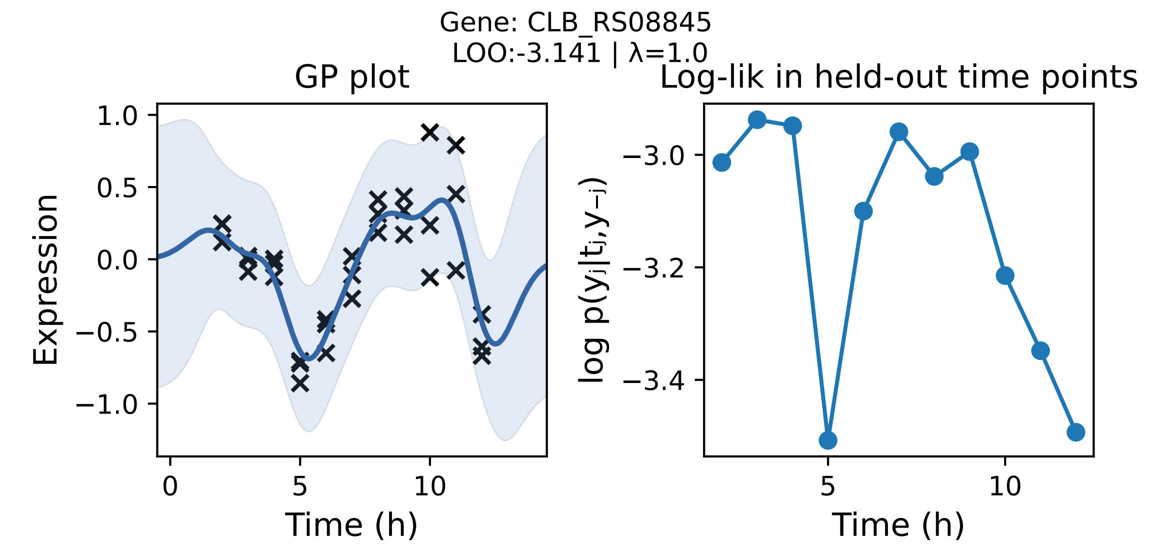

Gene: CLB_RS08845
LOO:-3.141 | λ=1.0
GP plot
Expression
Time (h)
Log-lik in held-out time points
log p(y_j|t_j,y_−j)
Time (h)

Gene: CLB_RS10230
LOO:-3.141 | λ=2.34

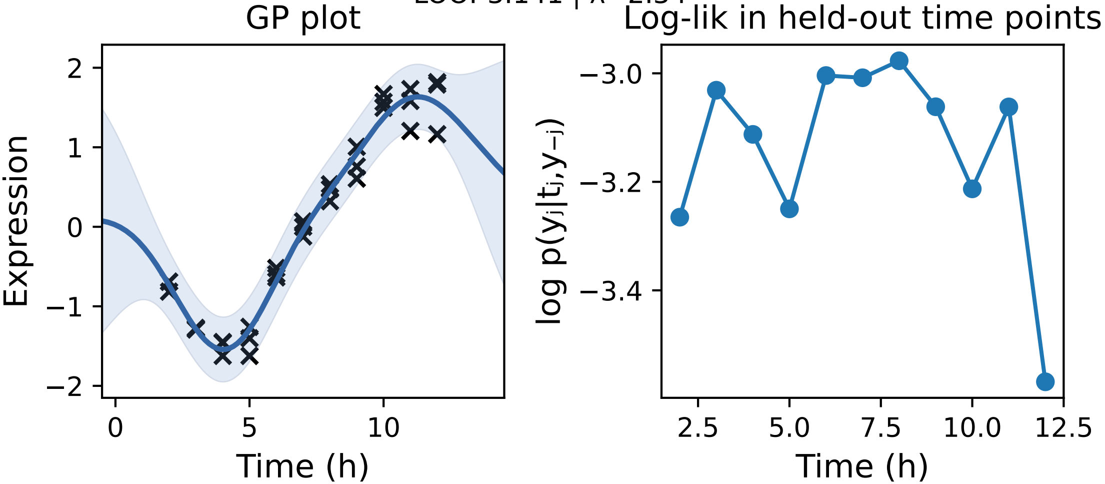

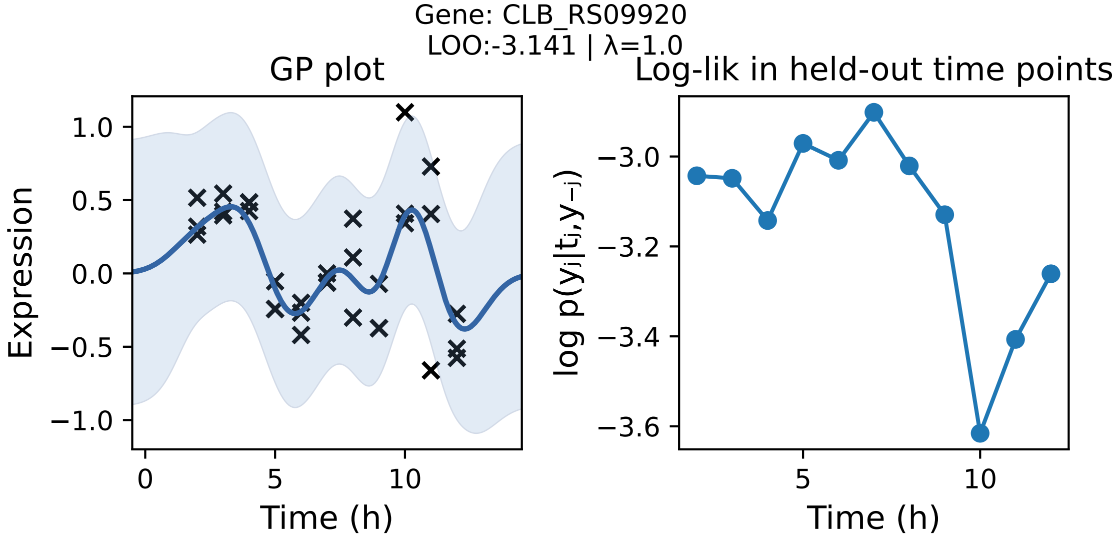

Gene: CLB_RS09920
LOO:-3.141 | λ=1.0
GP plot
Log-lik in held-out time points
Expression
1.0
0.5
0.0
−0.5
−1.0
0
5
10
Time (h)
log p(y_j|t_j,y_−j)
−3.0
−3.2
−3.4
−3.6
5
10
Time (h)

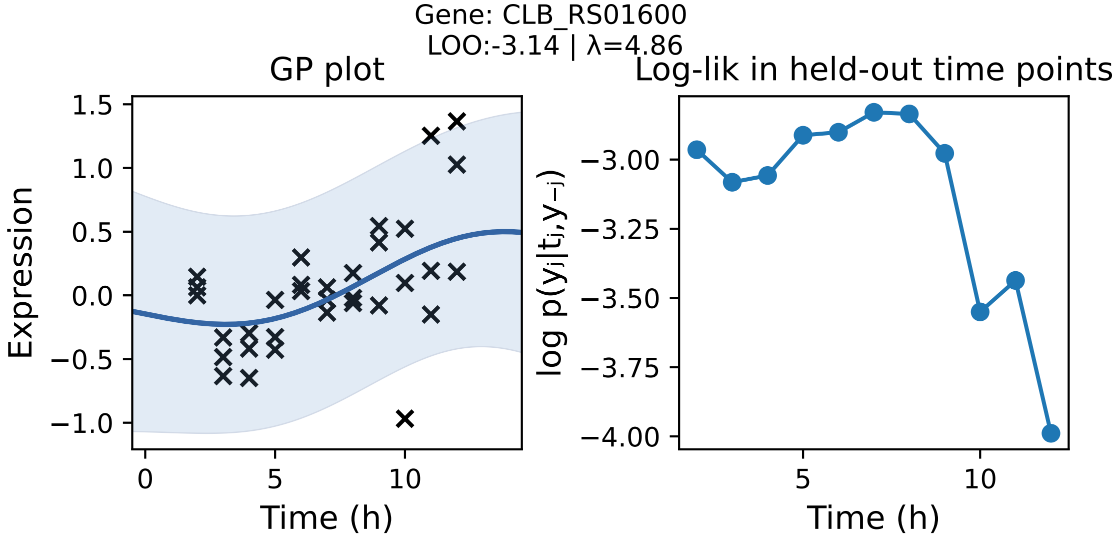

Gene: CLB_RS01600
LOO:-3.14 | λ=4.86
GP plot
Expression
1.5
1.0
0.5
0.0
−0.5
−1.0
0
5
10
Time (h)
Log-lik in held-out time points
log p(y_j|t_j,y_−j)
−3.00
−3.25
−3.50
−3.75
−4.00
5
10
Time (h)

Gene: CLB_RS15295
LOO:-3.139 | λ=2.94

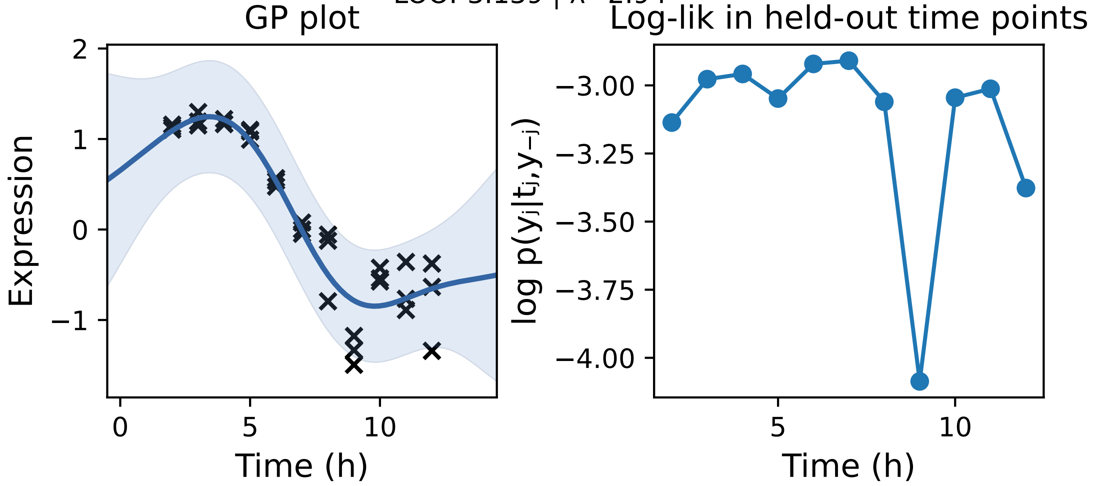

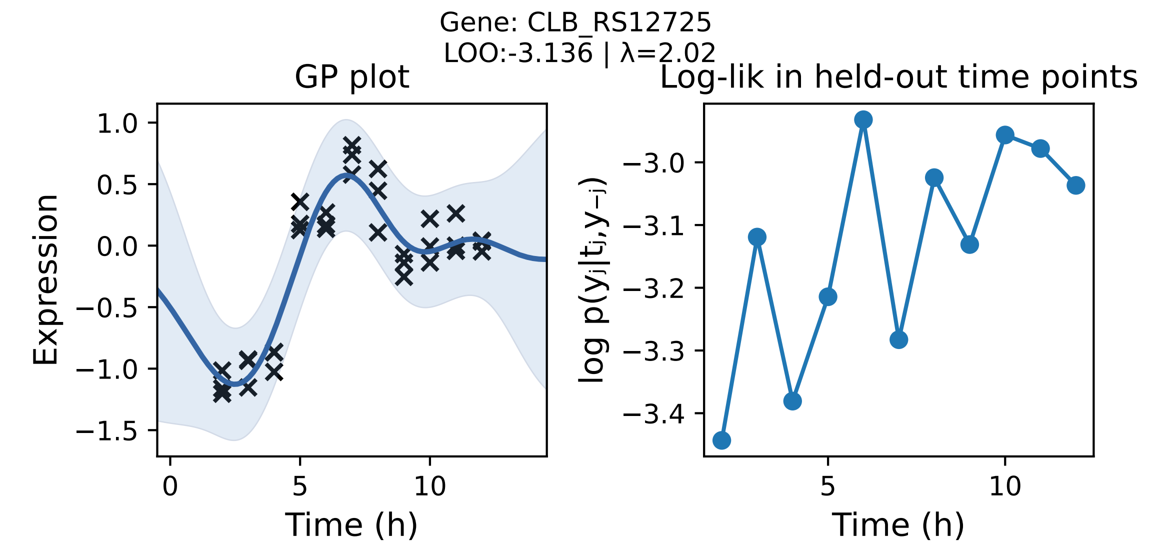

Gene: CLB_RS12725
LOO:-3.136 | λ=2.02
GP plot
Log-lik in held-out time points
Expression
1.0
0.5
0.0
−0.5
−1.0
−1.5
0
5
10
Time (h)
log p(yj|tj,y−j)
−3.0
−3.1
−3.2
−3.3
−3.4
5
10
Time (h)

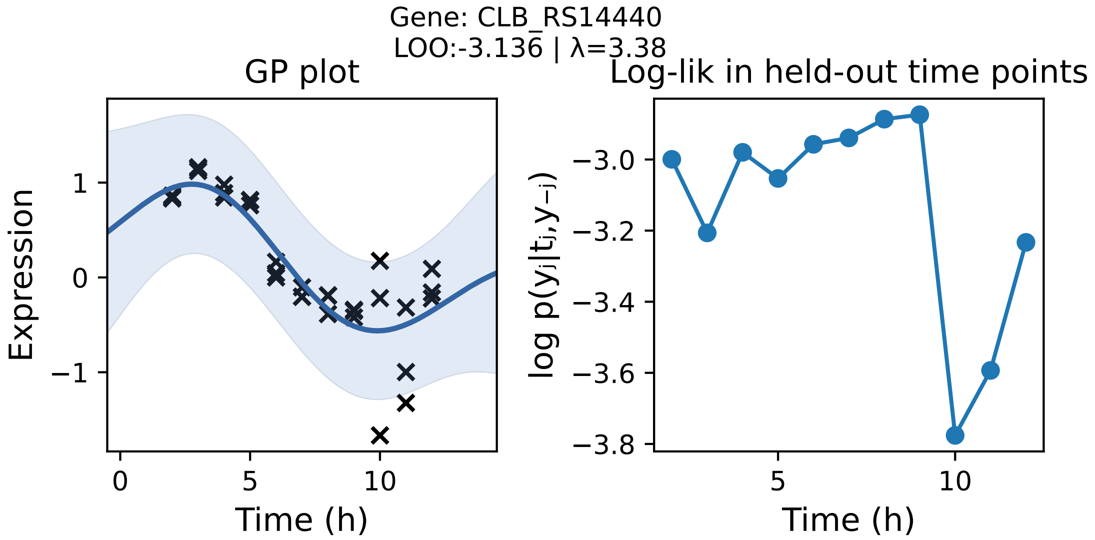

Gene: CLB_RS14440
LOO:-3.136 | λ=3.38
GP plot
Expression
Time (h)
Log-lik in held-out time points
log p(y_j|t_j,y_−j)
Time (h)

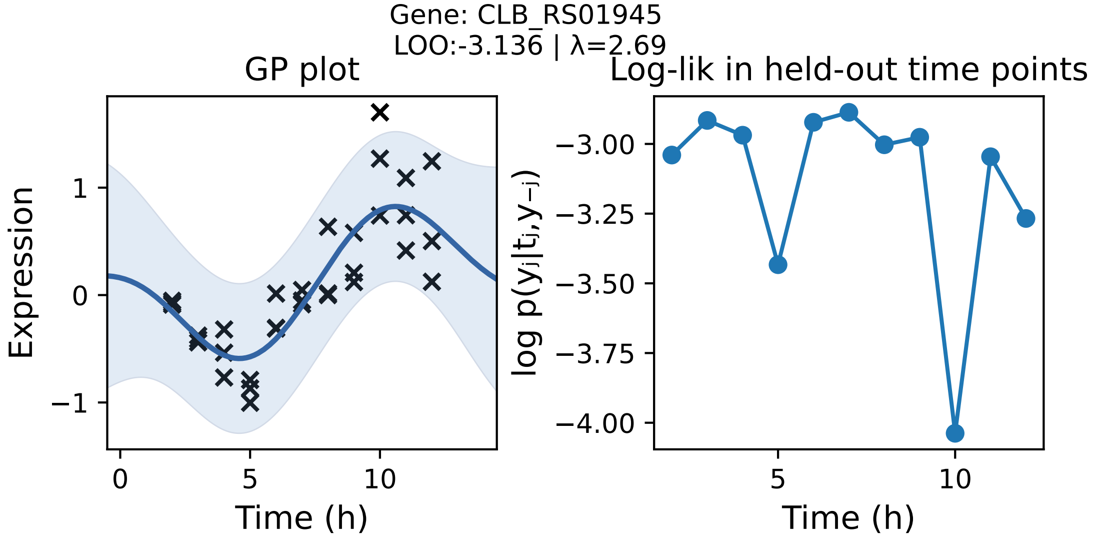

Gene: CLB_RS01945
LOO:-3.136 | λ=2.69
GP plot
Expression
1
0
−1
0
5
10
Time (h)
Log-lik in held-out time points
log p(y_j|t_j,y_−j)
−3.00
−3.25
−3.50
−3.75
−4.00
5
10
Time (h)

Gene: CLB_RS03685
LOO:-3.135 | λ=6.03

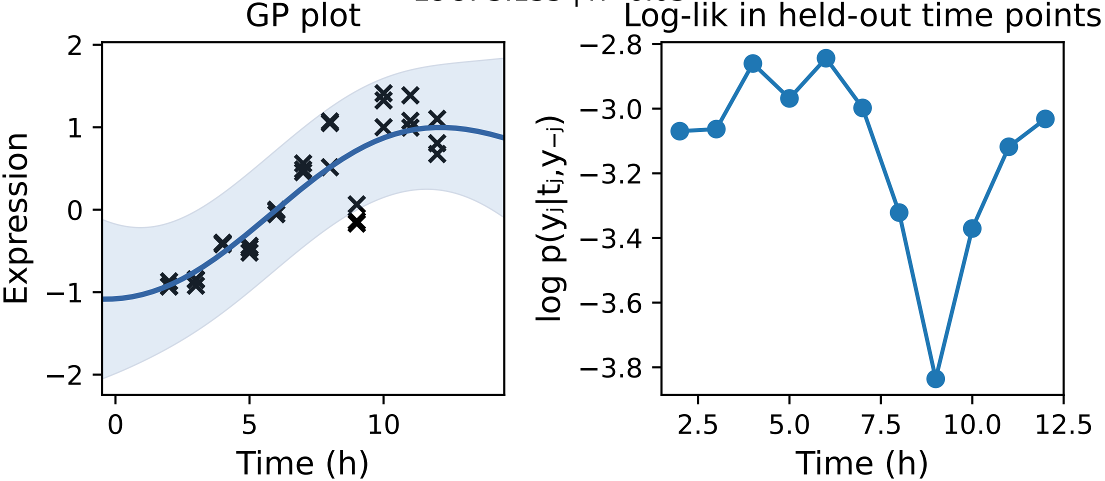

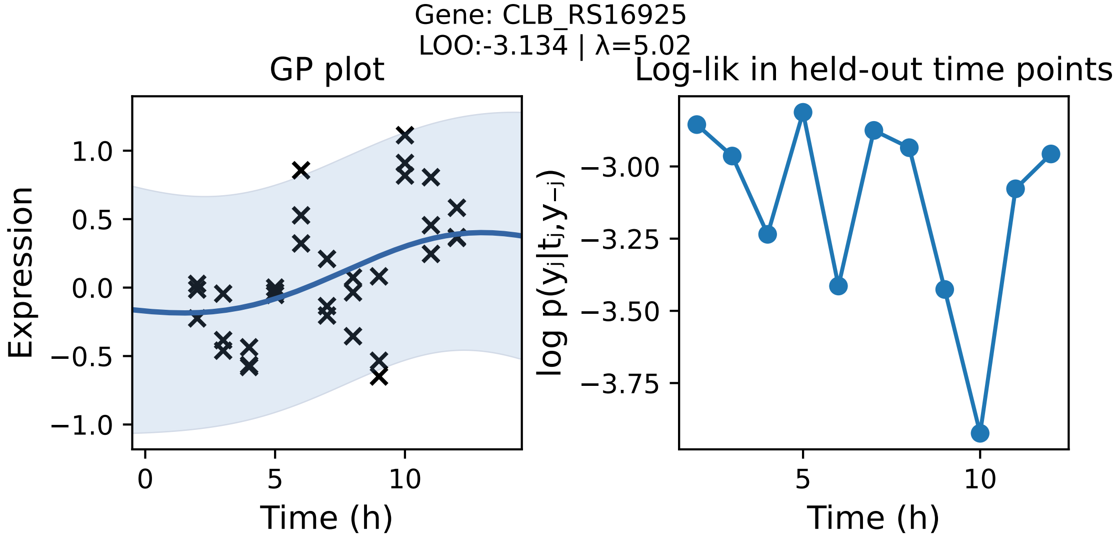

Gene: CLB_RS16925
LOO:-3.134 | λ=5.02
GP plot
Expression
Time (h)
Log-lik in held-out time points
log p(y_j|t_j,y_−j)
Time (h)
1.0
0.5
0.0
−0.5
−1.0
0
5
10
−3.00
−3.25
−3.50
−3.75
5
10

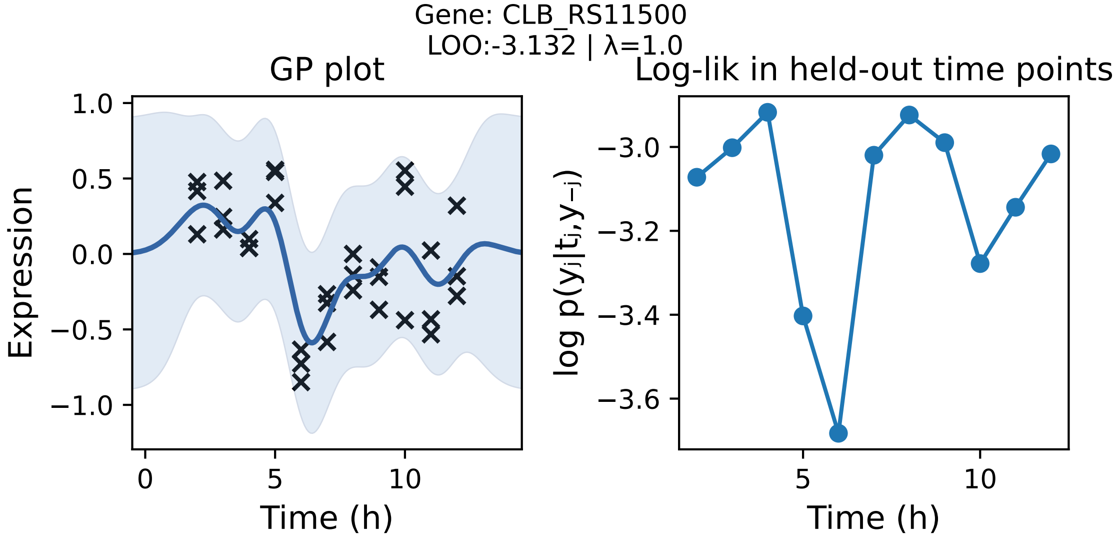

Gene: CLB_RS11500
LOO:-3.132 | λ=1.0
GP plot
Expression
Time (h)
1.0
0.5
0.0
−0.5
−1.0
0
5
10
Log-lik in held-out time points
log p(y_j|t_j,y_−j)
Time (h)
−3.0
−3.2
−3.4
−3.6
5
10

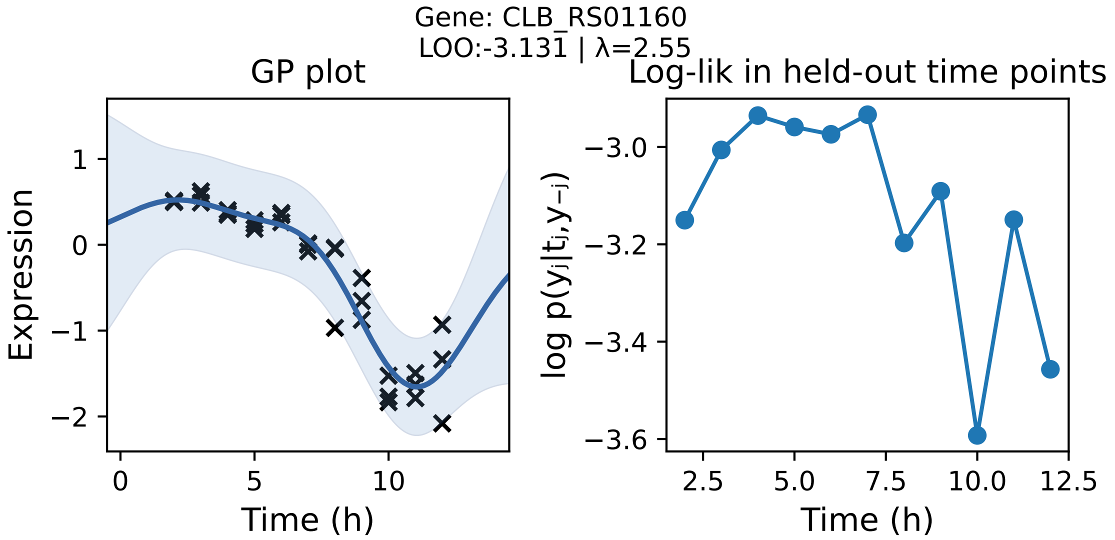
Gene: CLB_RS01160
LOO:-3.131 | λ=2.55
GP plot
Expression
Time (h)
1
0
−1
−2
0
5
10
Log-lik in held-out time points
log p(yj|tj,y−j)
Time (h)
−3.0
−3.2
−3.4
−3.6
2.5
5.0
7.5
10.0
12.5

Gene: CLB_RS00520
LOO:-3.131 | λ=7.72

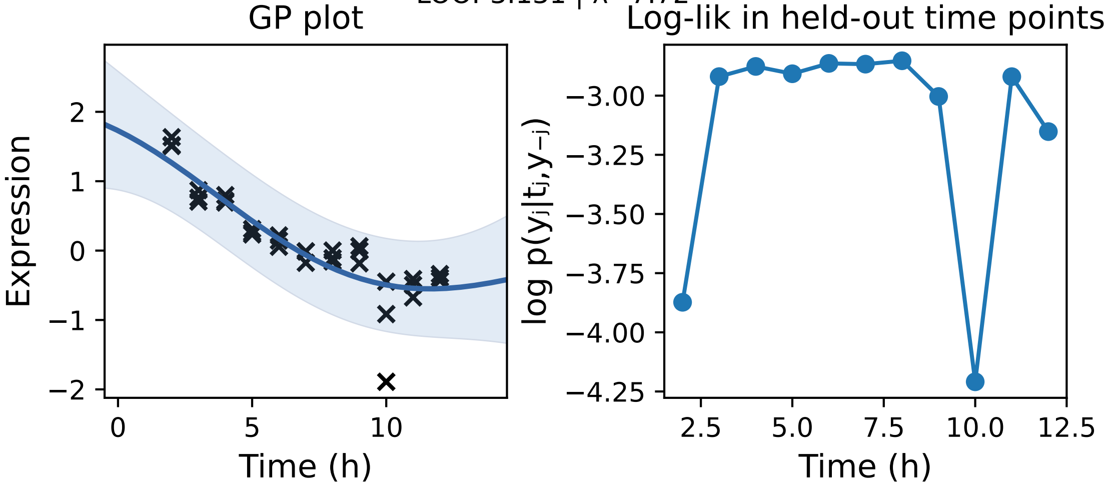

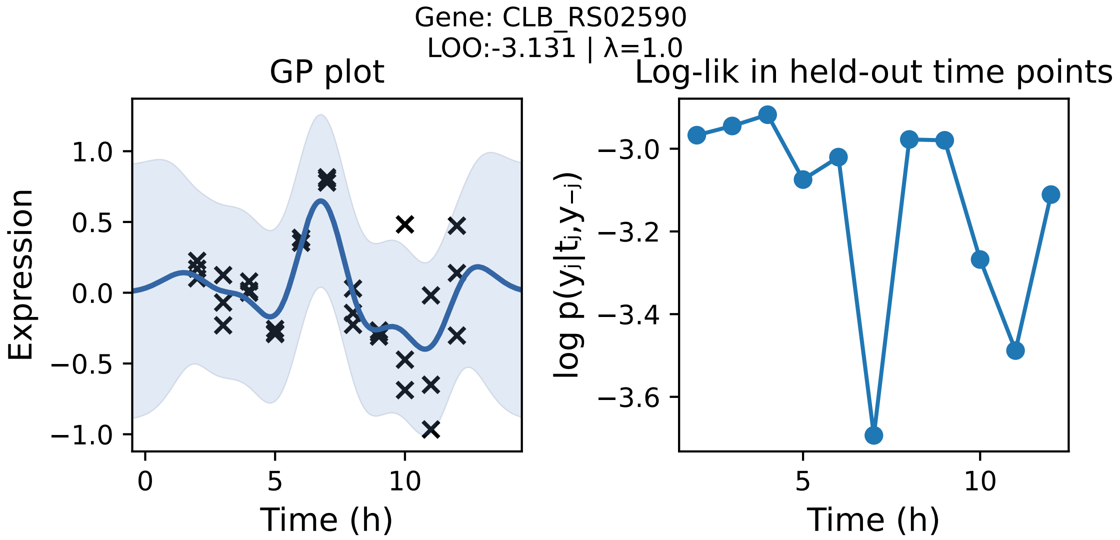
Gene: CLB_RS02590
LOO:-3.131 | λ=1.0
GP plot
Expression
Time (h)
1.0
0.5
0.0
−0.5
−1.0
0
5
10
Log-lik in held-out time points
log p(y_j|t_j,y_−j)
−3.0
−3.2
−3.4
−3.6
5
10
Time (h)

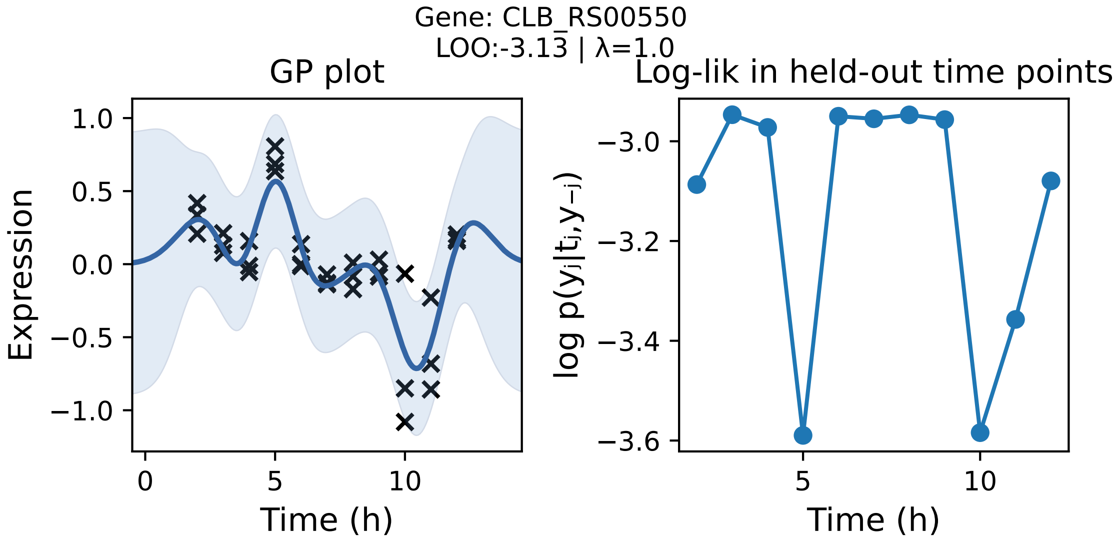

Gene: CLB_RS00550
LOO:-3.13 | λ=1.0
GP plot
Log-lik in held-out time points
Expression
1.0
0.5
0.0
−0.5
−1.0
0
5
10
Time (h)
log p(yj|tj,y−j)
−3.0
−3.2
−3.4
−3.6
5
10
Time (h)

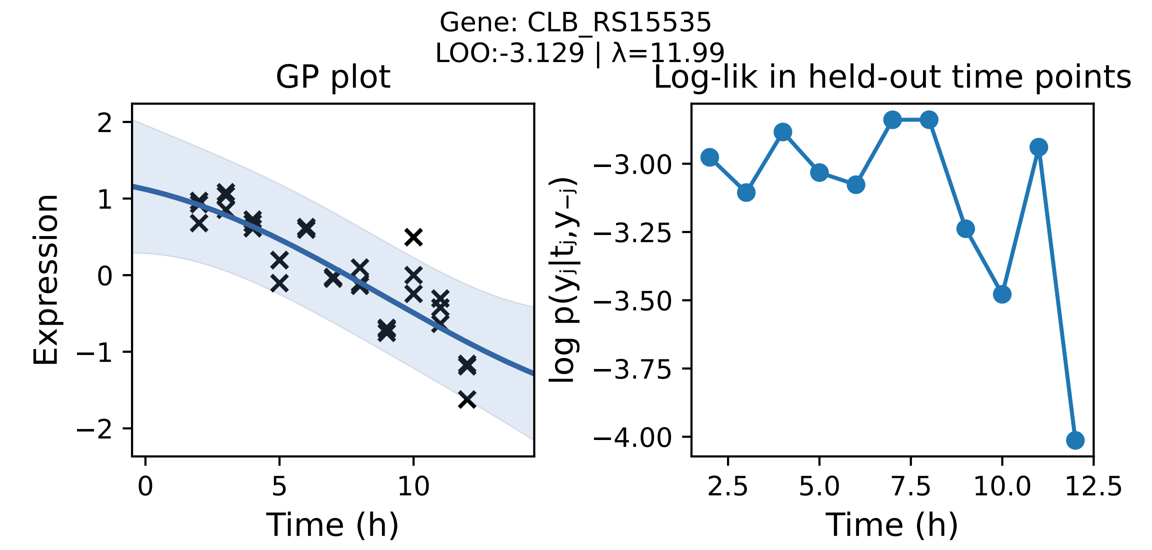
Gene: CLB_RS15535
LOO:-3.129 | λ=11.99
GP plot
Expression
Time (h)
Log-lik in held-out time points
log p(y_j|t_j,y_−j)
Time (h)
2
1
0
−1
−2
0
5
10
−3.00
−3.25
−3.50
−3.75
−4.00
2.5
5.0
7.5
10.0
12.5

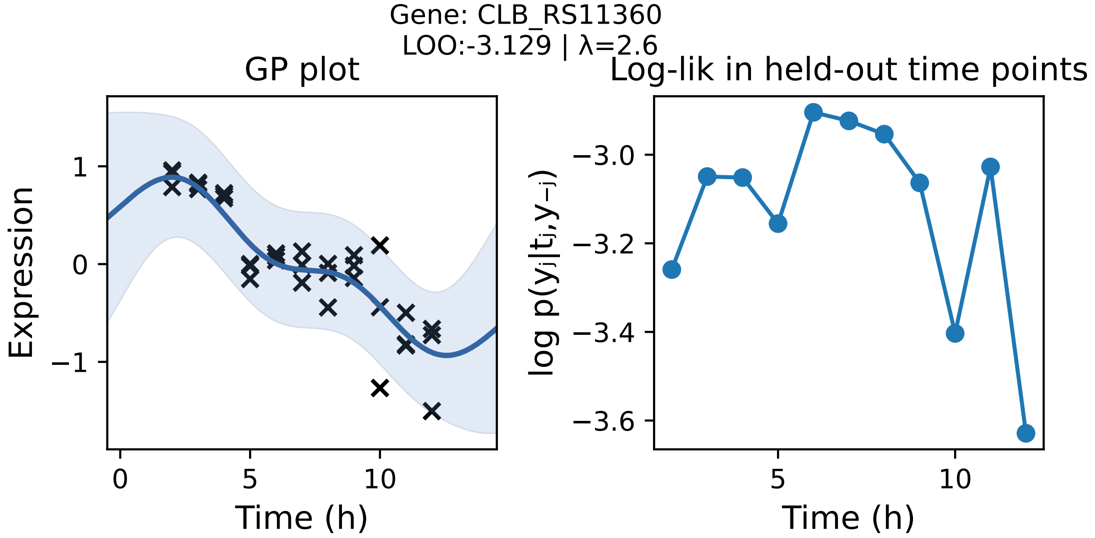
Gene: CLB_RS11360
LOO:-3.129 | λ=2.6
GP plot
Log-lik in held-out time points
Expression
1
0
−1
0
5
10
Time (h)
log p(yⱼ|tⱼ,y₋ⱼ)
−3.0
−3.2
−3.4
−3.6
5
10
Time (h)

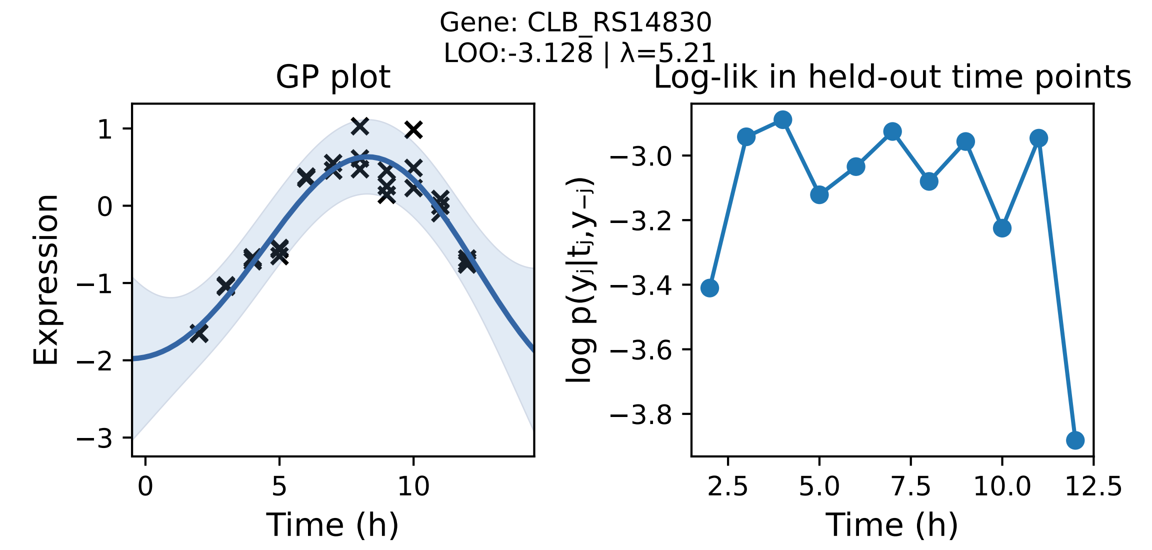

Gene: CLB_RS14830
LOO:-3.128 | λ=5.21
GP plot
Expression
Time (h)
1
0
−1
−2
−3
0
5
10
Log-lik in held-out time points
log p(y_j|t_j,y_−j)
−3.0
−3.2
−3.4
−3.6
−3.8
2.5
5.0
7.5
10.0
12.5
Time (h)

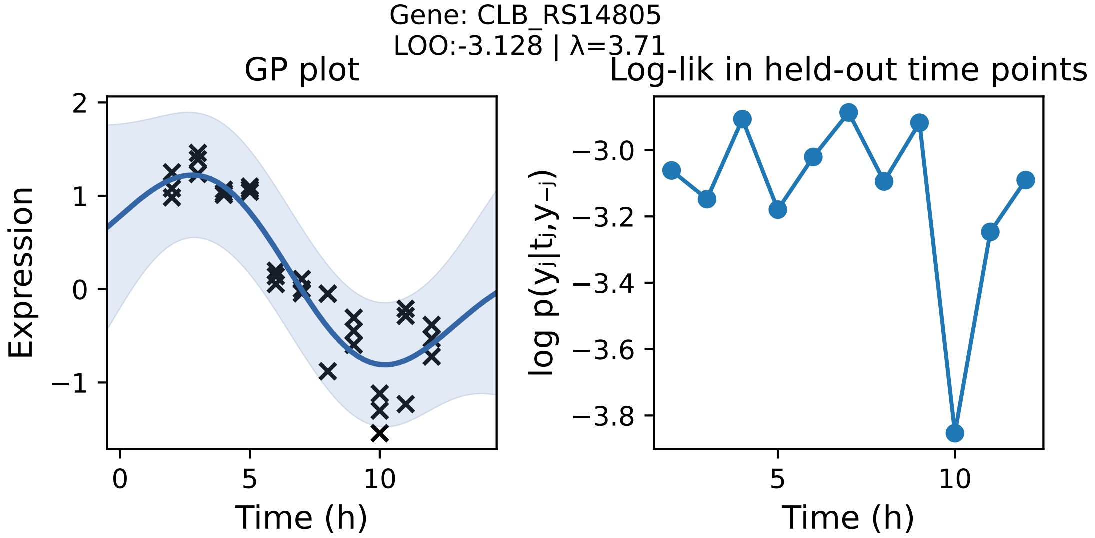
Gene: CLB_RS14805
LOO:-3.128 | λ=3.71
GP plot
Expression
Time (h)
Log-lik in held-out time points
log p(y_j|t_j,y_−j)
Time (h)

Gene: CLB_RS18310
LOO:-3.127 | λ=8.76

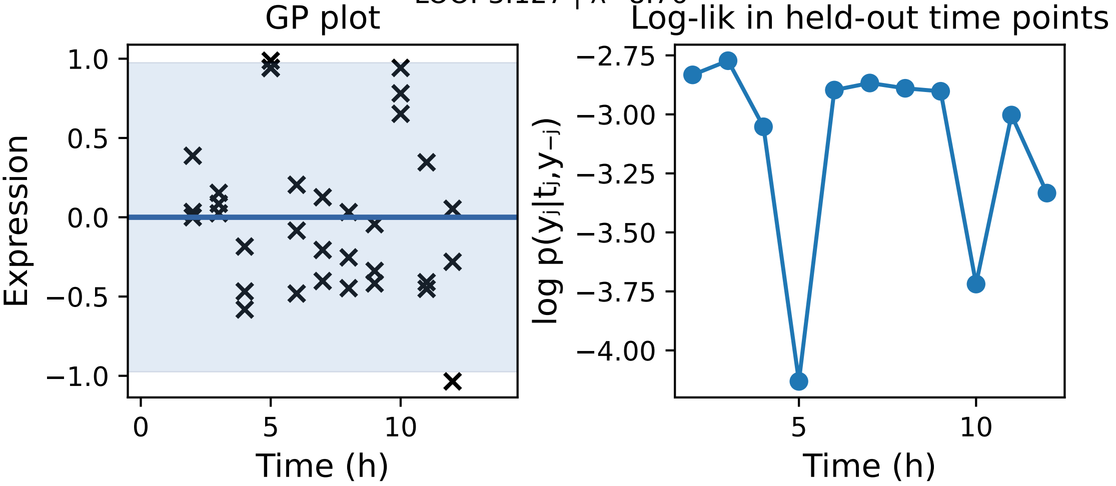

Gene: CLB_RS14185
LOO:-3.127 | λ=3.05

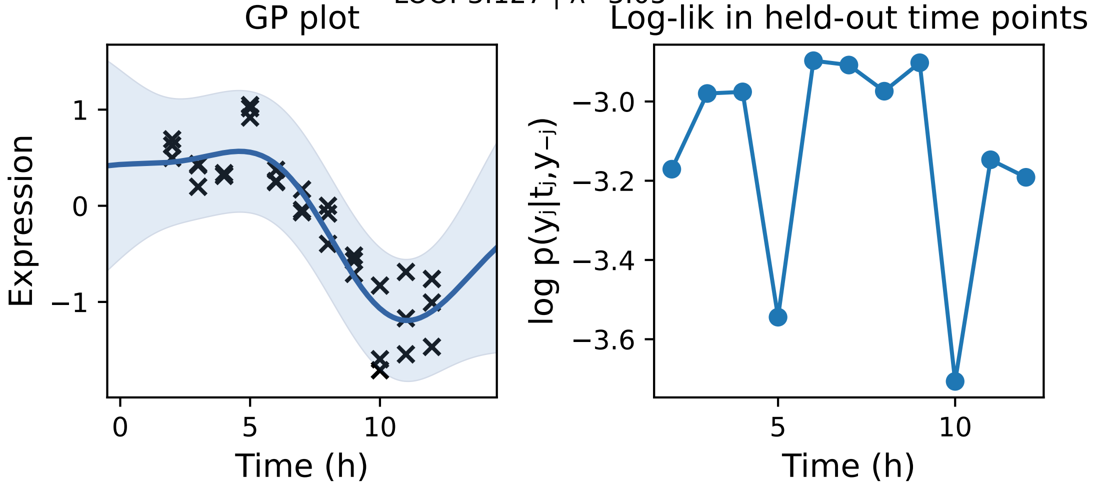

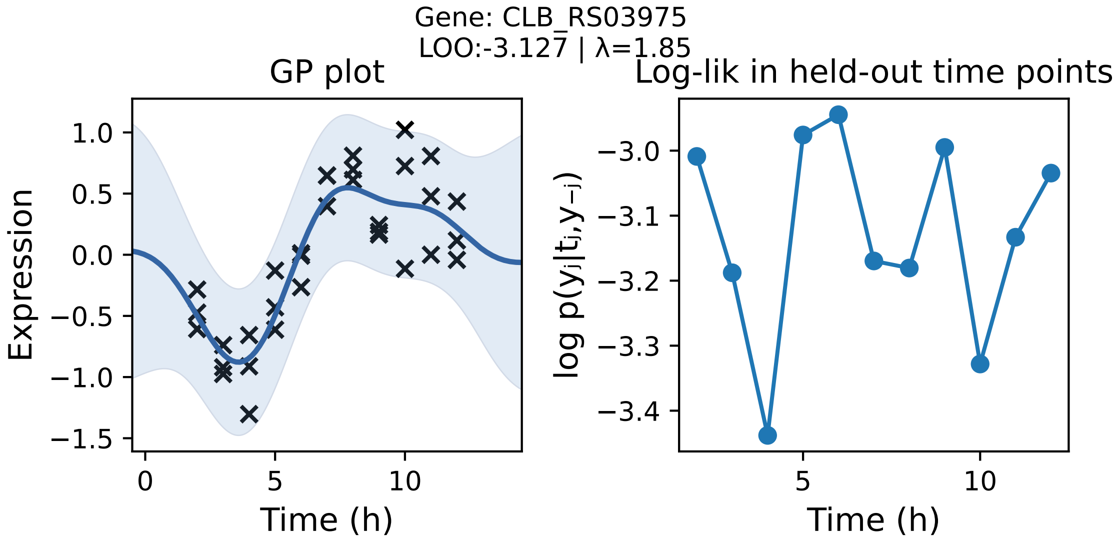

Gene: CLB_RS03975
LOO:-3.127 | λ=1.85
GP plot
Expression
Time (h)
1.0
0.5
0.0
−0.5
−1.0
−1.5
0
5
10
Log-lik in held-out time points
log p(y_j|t_j,y_−j)
−3.0
−3.1
−3.2
−3.3
−3.4
5
10
Time (h)

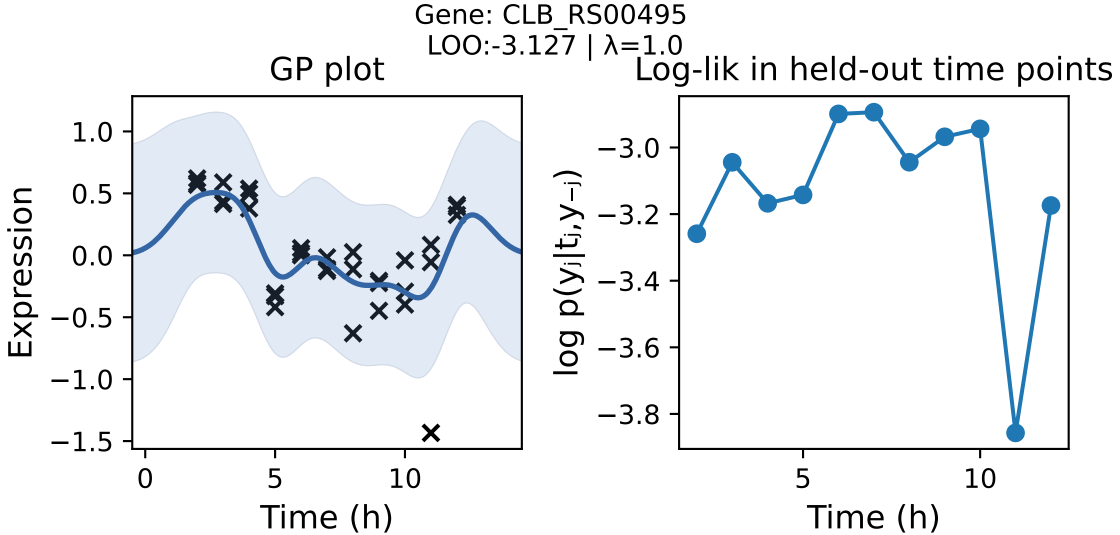

Gene: CLB_RS00495
LOO:-3.127 | λ=1.0
GP plot
Expression
Time (h)
1.0
0.5
0.0
−0.5
−1.0
−1.5
0
5
10
Log-lik in held-out time points
log p(yj|tj,y−j)
Time (h)
−3.0
−3.2
−3.4
−3.6
−3.8
5
10

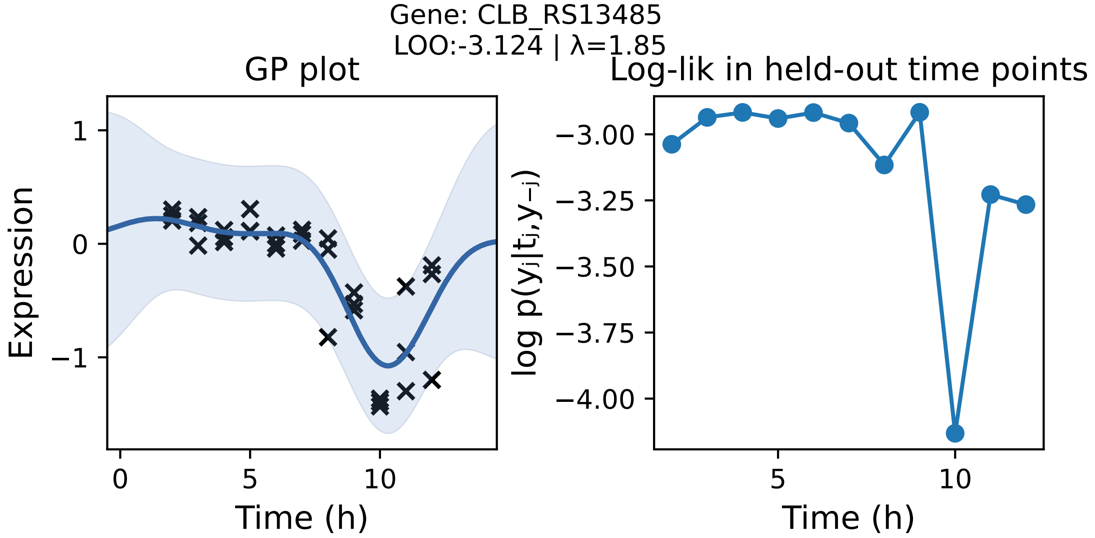

Gene: CLB_RS13485
LOO:-3.124 | λ=1.85
GP plot
Expression
1
0
−1
0
5
10
Time (h)
Log-lik in held-out time points
log p(y_j|t_j,y_−j)
−3.00
−3.25
−3.50
−3.75
−4.00
5
10
Time (h)

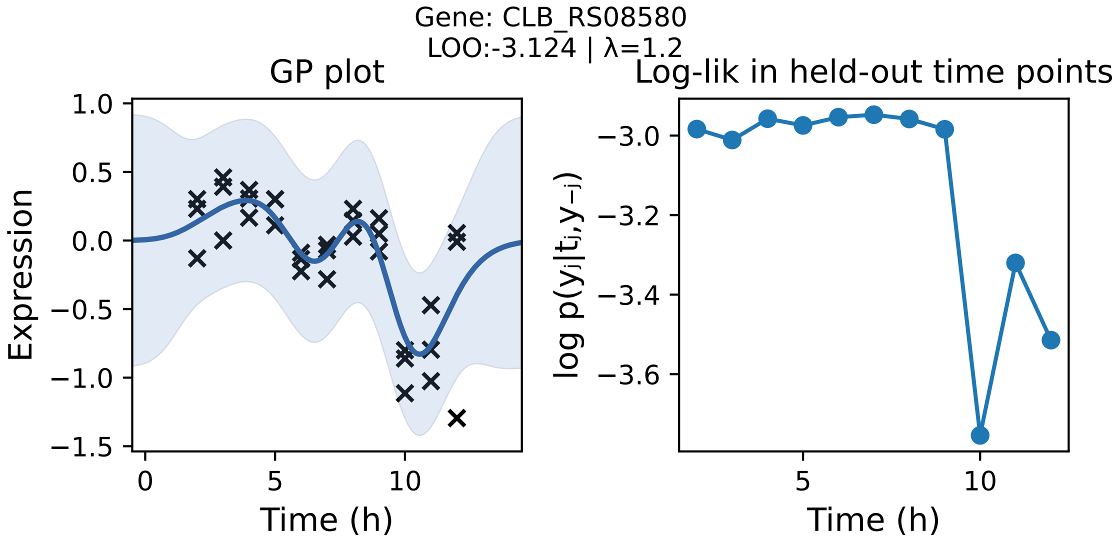

Gene: CLB_RS08580
LOO:-3.124 | λ=1.2
GP plot
Expression
1.0
0.5
0.0
−0.5
−1.0
−1.5
0
5
10
Time (h)
Log-lik in held-out time points
log p(yj|tj,y−j)
−3.0
−3.2
−3.4
−3.6
5
10
Time (h)

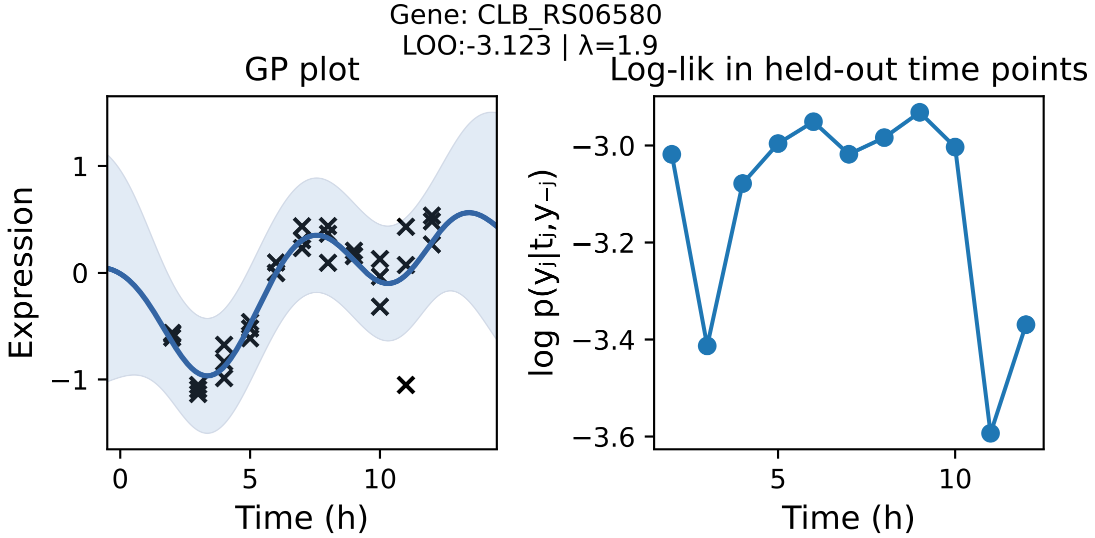
Gene: CLB_RS06580
LOO:-3.123 | λ=1.9
GP plot
Expression
1
0
−1
0
5
10
Time (h)
Log-lik in held-out time points
log p(yj|tj,y−j)
−3.0
−3.2
−3.4
−3.6
5
10
Time (h)

Gene: CLB_RS07340
LOO:-3.123 | λ=1.23

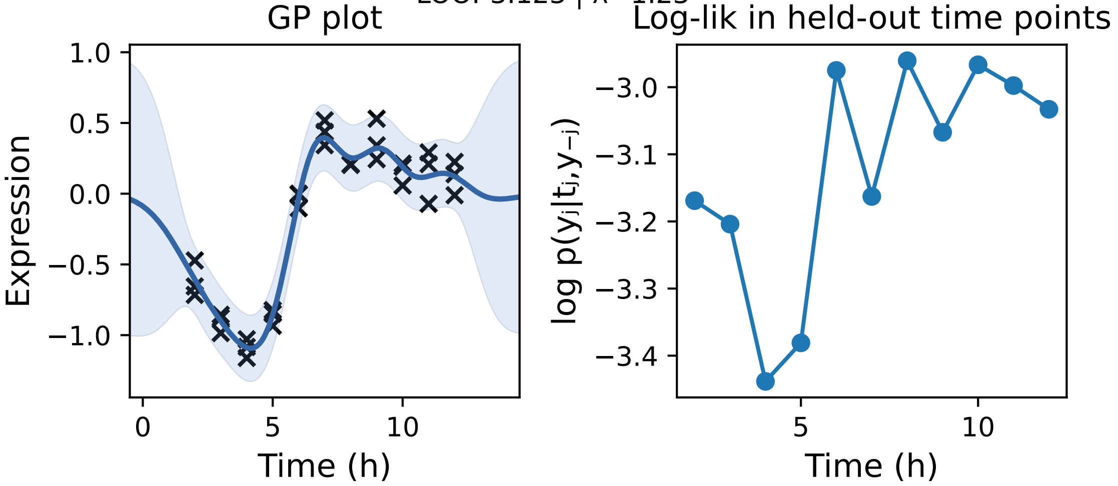

Gene: CLB_RS13460
LOO:-3.122 | λ=1.7

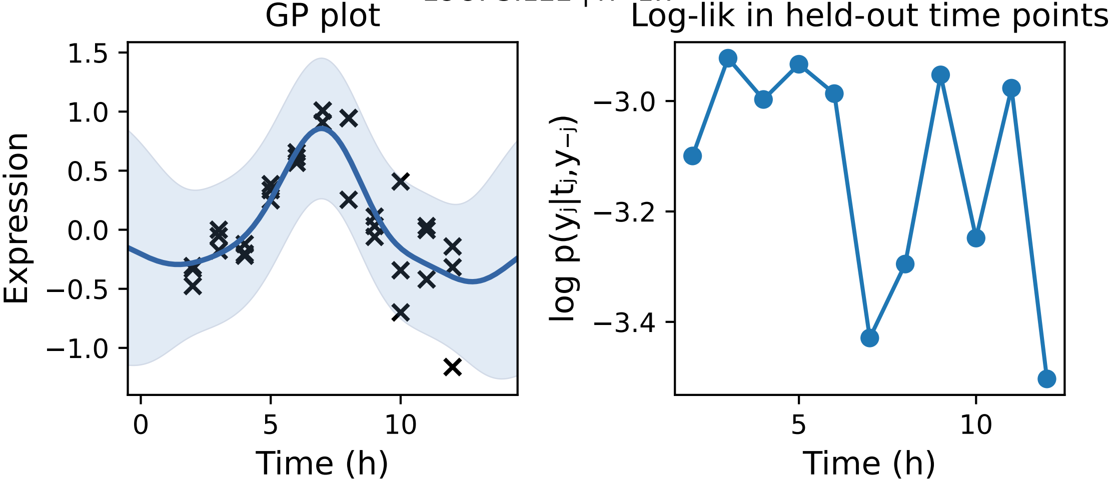

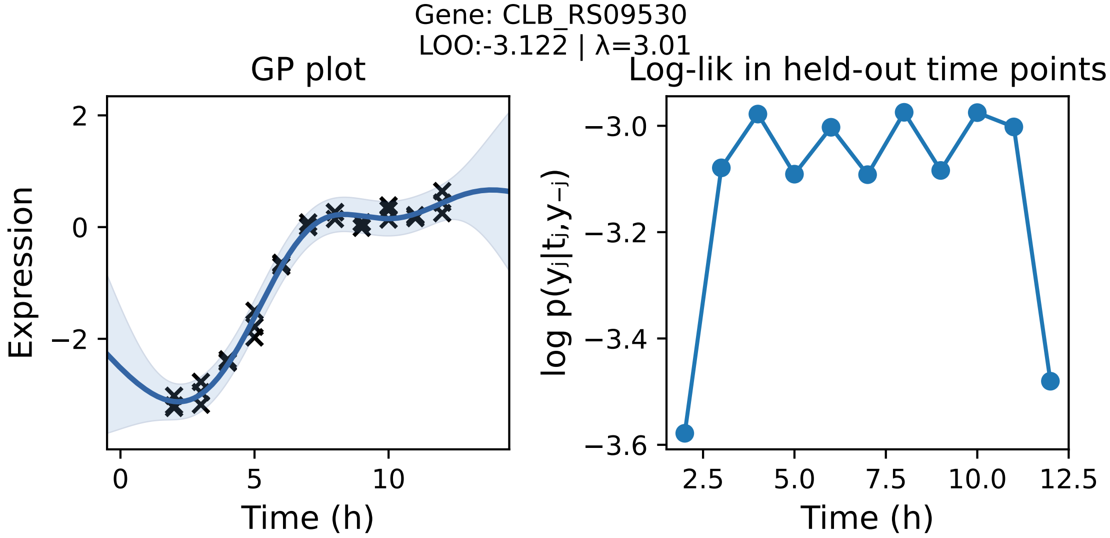
Gene: CLB_RS09530
LOO:-3.122 | λ=3.01
GP plot
Expression
Time (h)
2
0
−2
0
5
10
Log-lik in held-out time points
log p(y_j|t_j,y_−j)
Time (h)
−3.0
−3.2
−3.4
−3.6
2.5
5.0
7.5
10.0
12.5

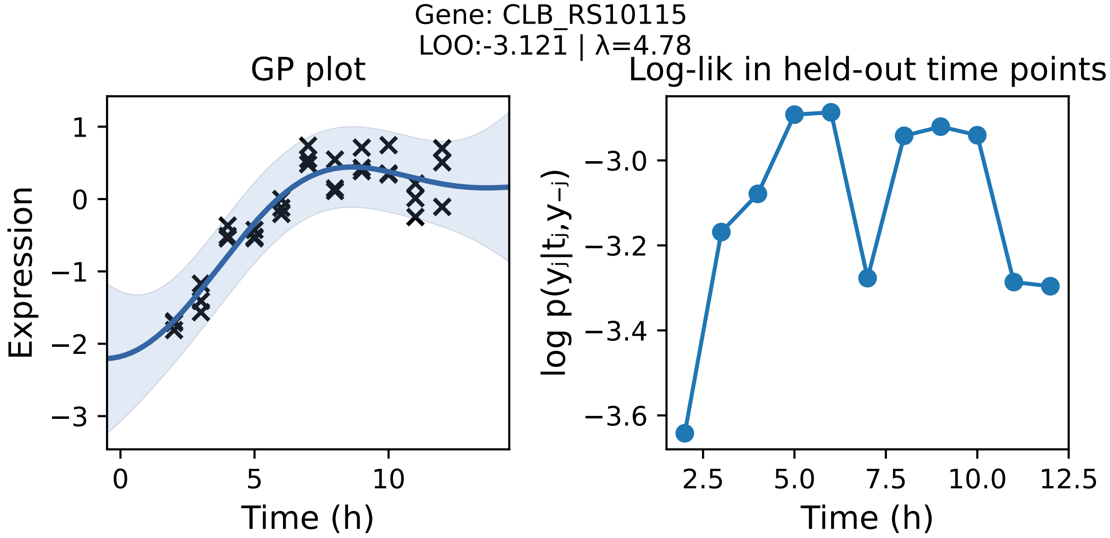

Gene: CLB_RS10115
LOO:-3.121 | λ=4.78
GP plot
Expression
Time (h)
1
0
−1
−2
−3
0
5
10
Log-lik in held-out time points
log p(y_j|t_j,y_−j)
Time (h)
−3.0
−3.2
−3.4
−3.6
2.5
5.0
7.5
10.0
12.5

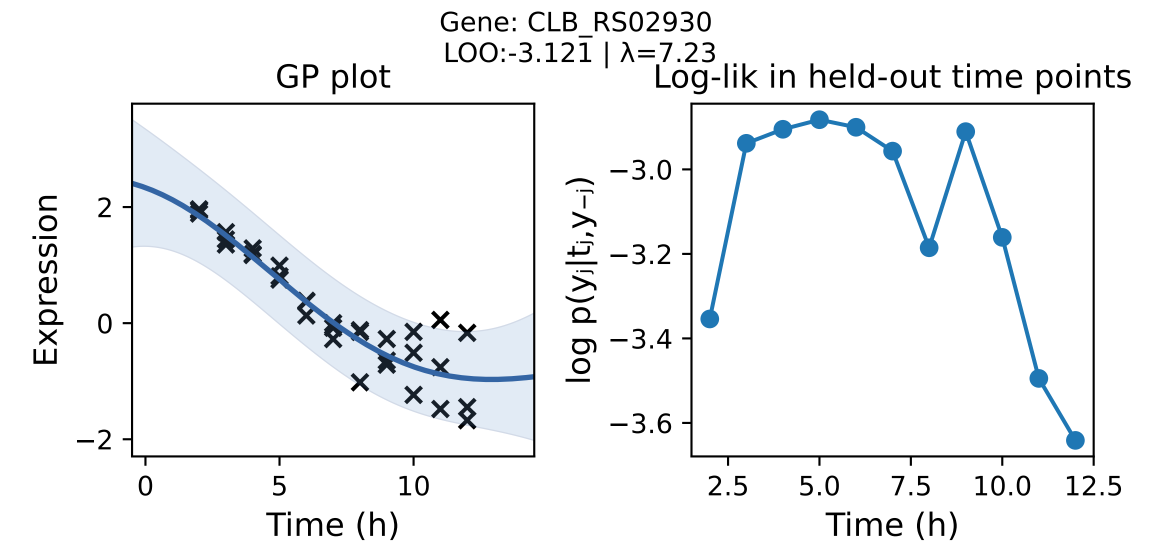
Gene: CLB_RS02930
LOO:-3.121 | λ=7.23
GP plot
Expression
2
0
−2
0
5
10
Time (h)
Log-lik in held-out time points
log p(y_j|t_j,y_−j)
−3.0
−3.2
−3.4
−3.6
2.5
5.0
7.5
10.0
12.5
Time (h)

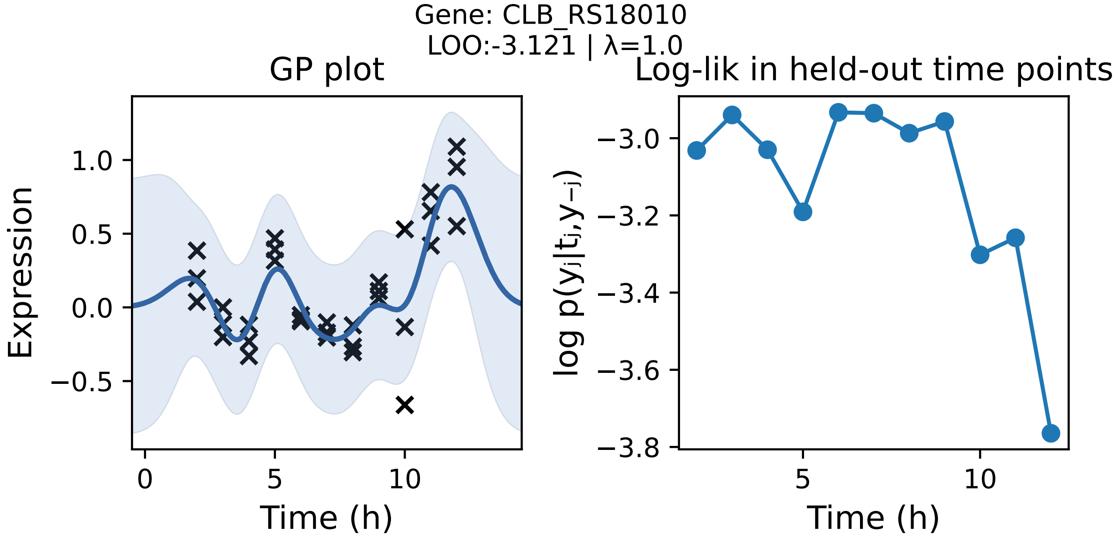

Gene: CLB_RS18010
LOO:-3.121 | λ=1.0
GP plot
Expression
Time (h)
1.0
0.5
0.0
−0.5
0
5
10
Log-lik in held-out time points
log p(y_j|t_j,y_−j)
Time (h)
−3.0
−3.2
−3.4
−3.6
−3.8
5
10

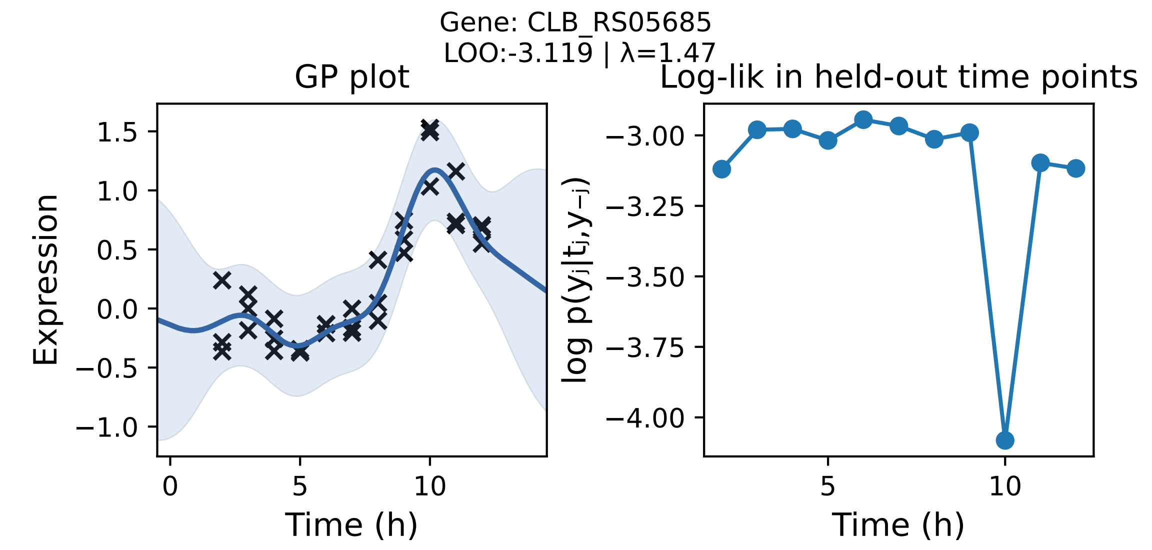

Gene: CLB_RS05685
LOO:-3.119 | λ=1.47
GP plot
Log-lik in held-out time points
Expression
1.5
1.0
0.5
0.0
−0.5
−1.0
0
5
10
Time (h)
log p(y_j|t_j,y_−j)
−3.00
−3.25
−3.50
−3.75
−4.00
5
10
Time (h)

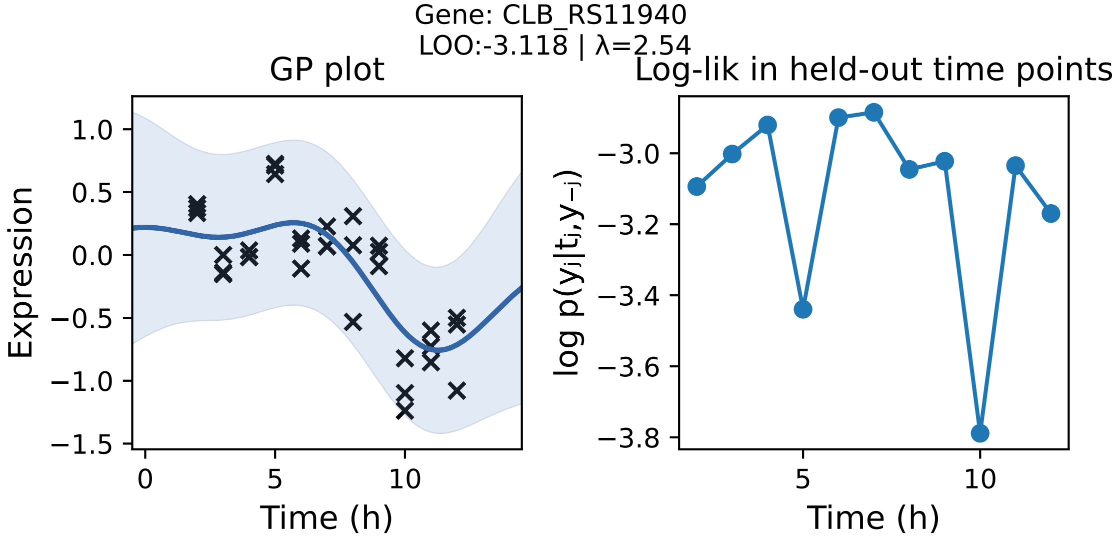

Gene: CLB_RS11940
LOO:-3.118 | λ=2.54
GP plot
Log-lik in held-out time points
Expression
1.0
0.5
0.0
−0.5
−1.0
−1.5
0
5
10
Time (h)
log p(yj|tj,y−j)
−3.0
−3.2
−3.4
−3.6
−3.8
5
10
Time (h)

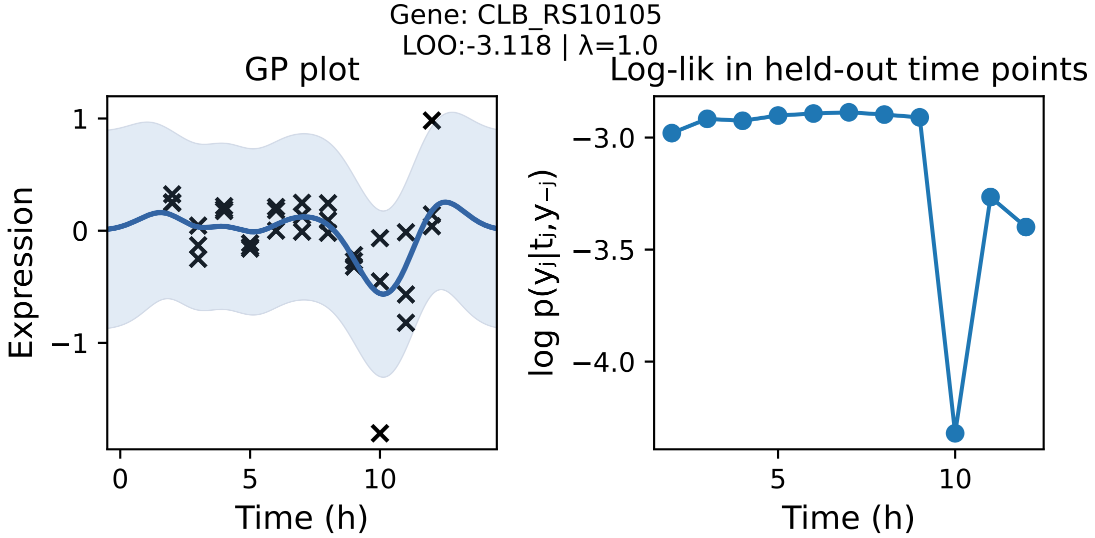
Gene: CLB_RS10105
LOO:-3.118 | λ=1.0
GP plot
Expression
Time (h)
Log-lik in held-out time points
log p(y_j|t_j,y_−j)
Time (h)
1
0
−1
0
5
10
−3.0
−3.5
−4.0
5
10

Gene: CLB_RS03375
LOO:-3.118 | λ=5.4

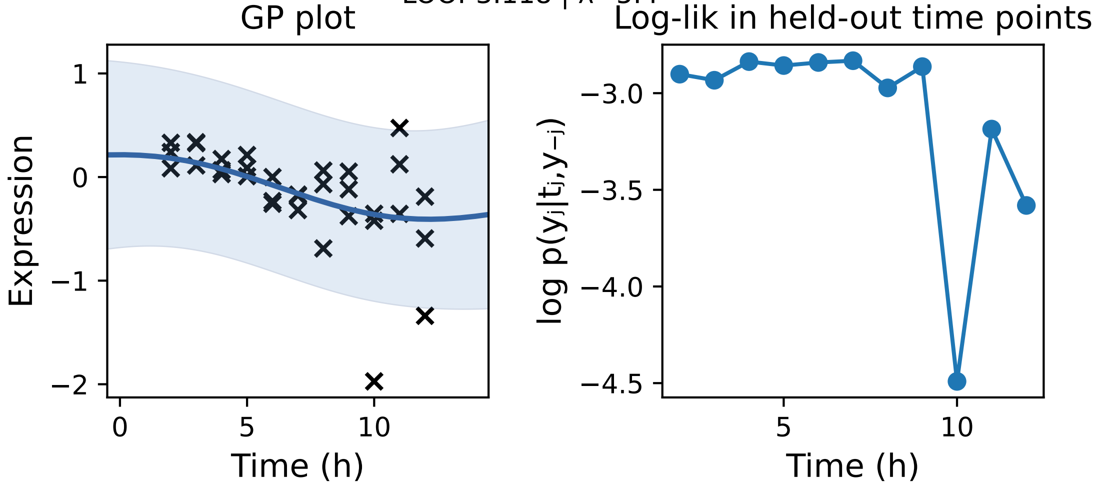

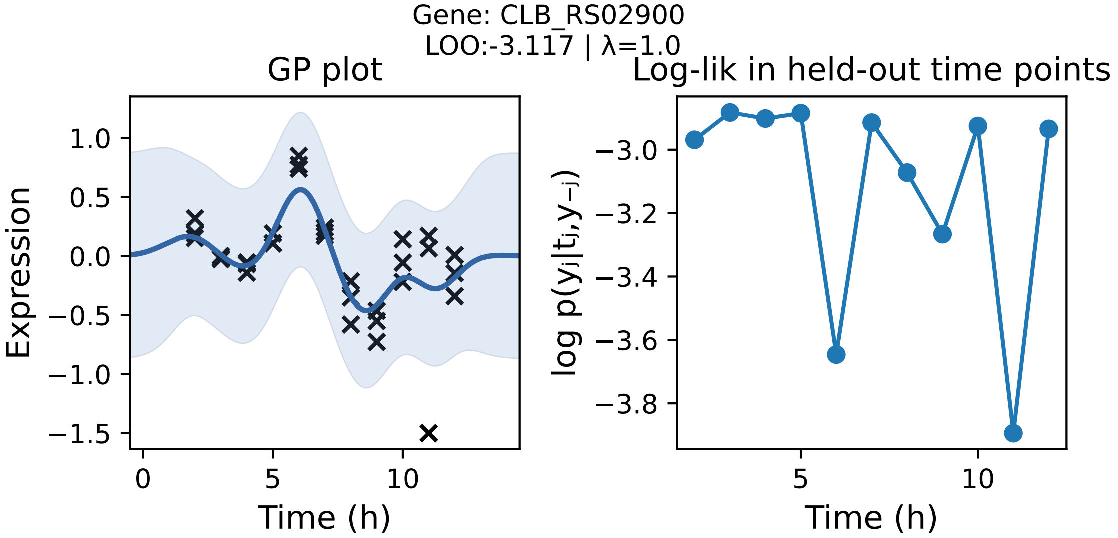

Gene: CLB_RS02900
LOO:-3.117 | λ=1.0
GP plot
Expression
1.0
0.5
0.0
−0.5
−1.0
−1.5
0
5
10
Time (h)
Log-lik in held-out time points
log p(y_j|t_j,y_−j)
−3.0
−3.2
−3.4
−3.6
−3.8
5
10
Time (h)

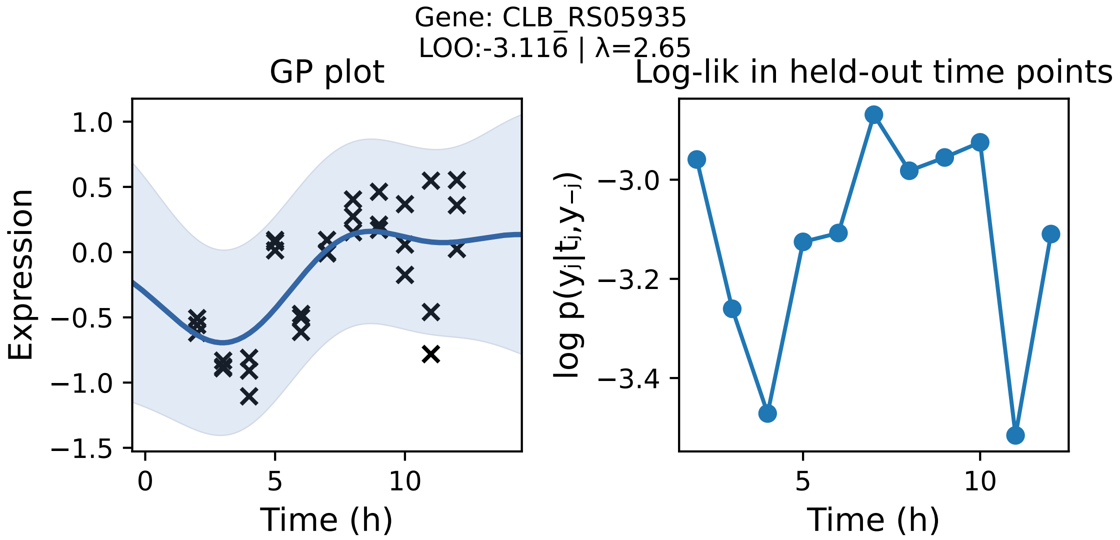

Gene: CLB_RS05935
LOO:-3.116 | λ=2.65
GP plot
Expression
Time (h)
1.0
0.5
0.0
−0.5
−1.0
−1.5
0
5
10
Log-lik in held-out time points
log p(y_j|t_j,y_−j)
−3.0
−3.2
−3.4
5
10
Time (h)

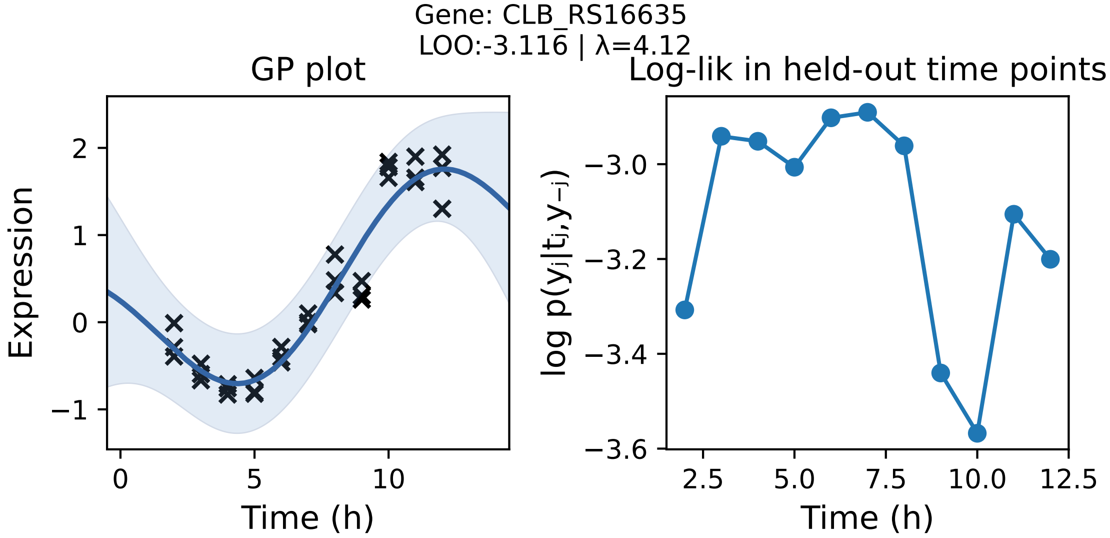
Gene: CLB_RS16635
LOO:-3.116 | λ=4.12
GP plot
Expression
Time (h)
2
1
0
−1
0
5
10
Log-lik in held-out time points
log p(y_j|t_j,y_−j)
Time (h)
−3.0
−3.2
−3.4
−3.6
2.5
5.0
7.5
10.0
12.5

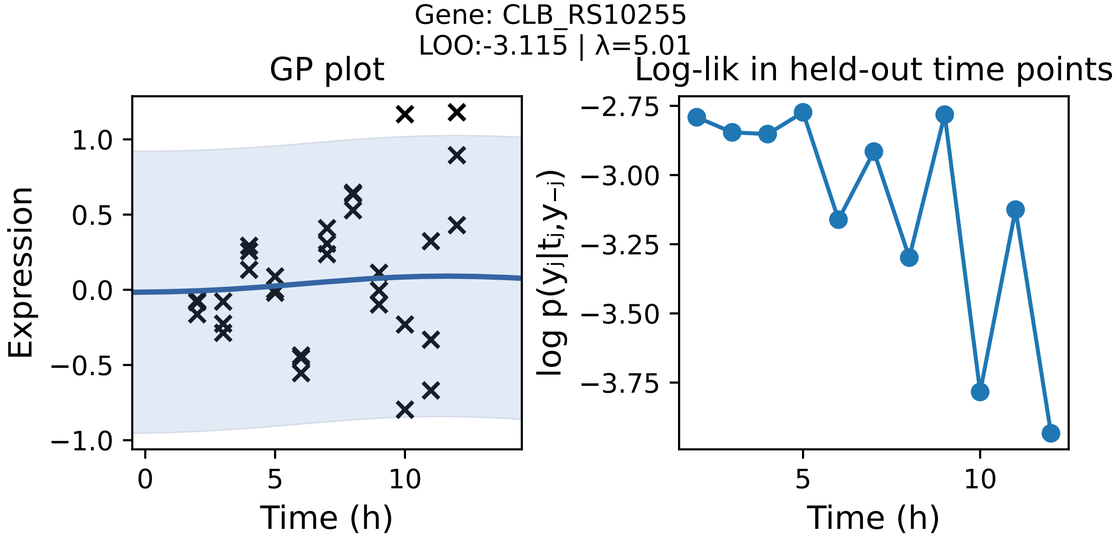
Gene: CLB_RS10255
LOO:-3.115 | λ=5.01
GP plot
Expression
Time (h)
Log-lik in held-out time points
log p(yj|tj,y−j)
Time (h)

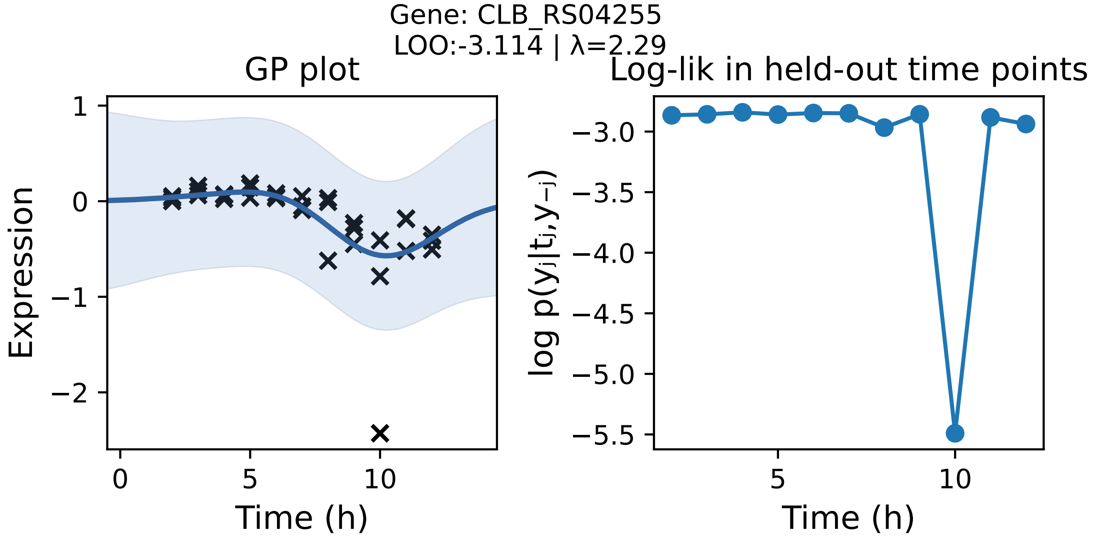

Gene: CLB_RS04255
LOO:-3.114 | λ=2.29
GP plot
Expression
Time (h)
Log-lik in held-out time points
log p(yj|tj,y−j)
Time (h)

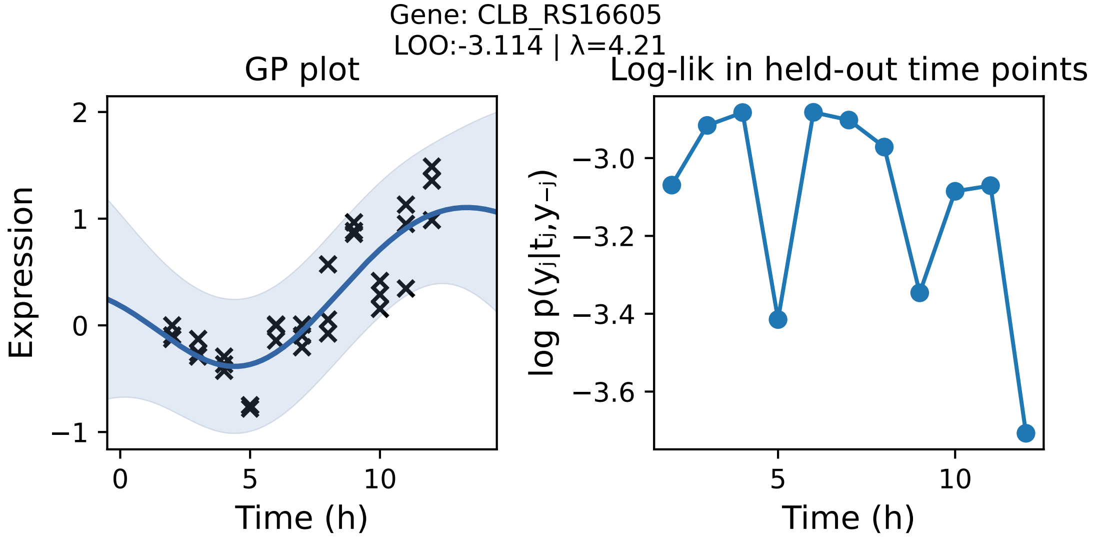

Gene: CLB_RS16605
LOO:-3.114 | λ=4.21
GP plot
Expression
Time (h)
2
1
0
−1
0
5
10
Log-lik in held-out time points
log p(y_j|t_j,y_−j)
−3.0
−3.2
−3.4
−3.6
5
10
Time (h)

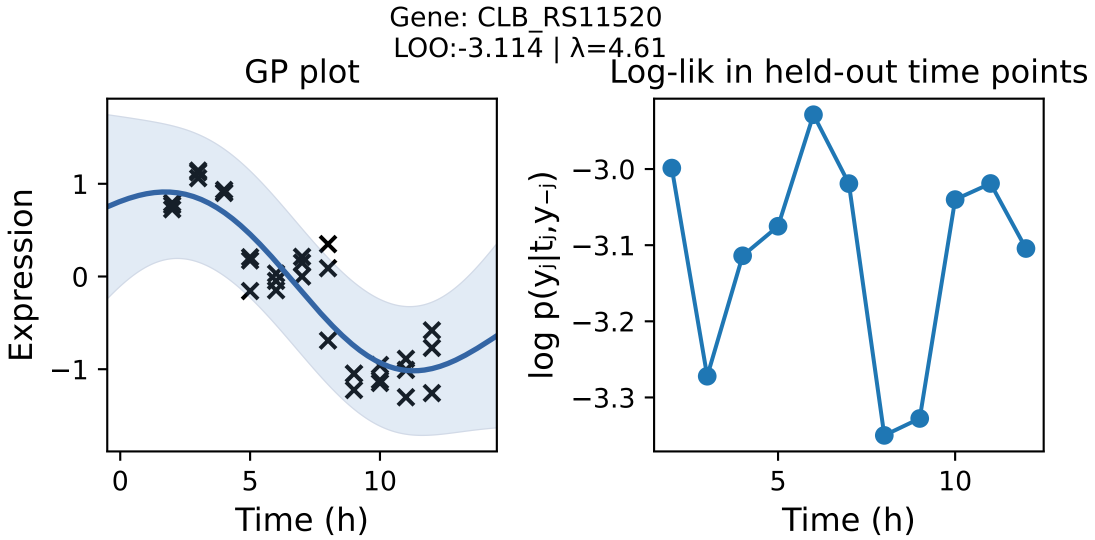

Gene: CLB_RS11520
LOO:-3.114 | λ=4.61
GP plot
Log-lik in held-out time points
Expression
1
0
−1
0
5
10
Time (h)
log p(y_j|t_j,y_{−j})
−3.0
−3.1
−3.2
−3.3
5
10
Time (h)

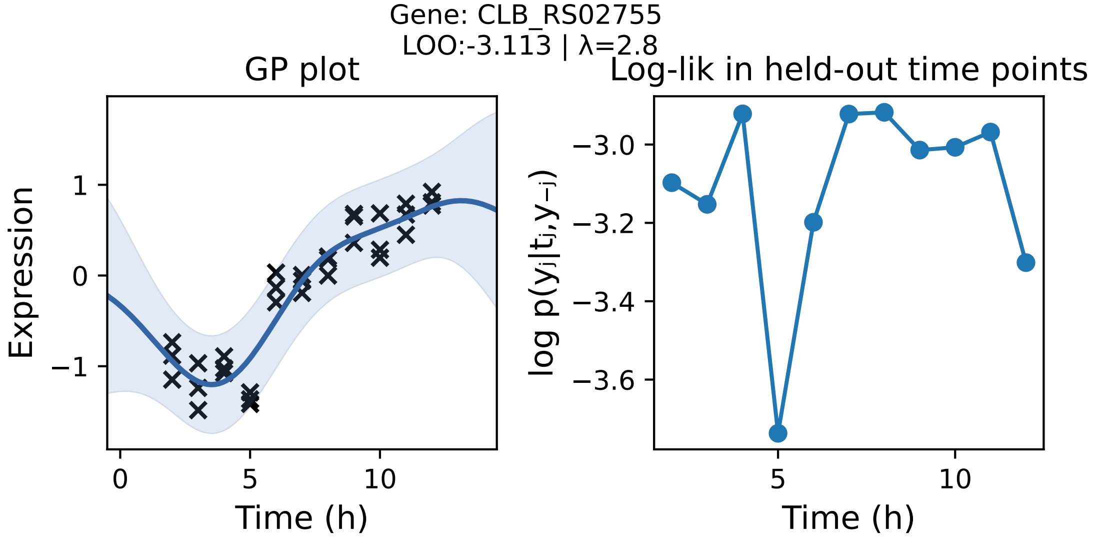
Gene: CLB_RS02755
LOO:-3.113 | λ=2.8
GP plot
Expression
Time (h)
1
0
−1
0
5
10
Log-lik in held-out time points
log p(yj|tj,y−j)
Time (h)
−3.0
−3.2
−3.4
−3.6
5
10

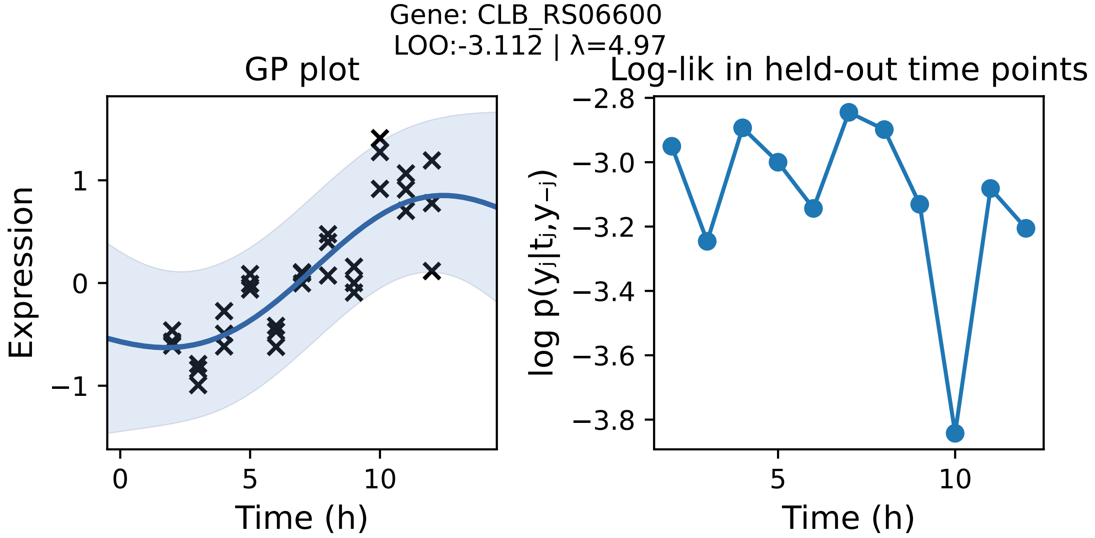
Gene: CLB_RS06600
LOO:-3.112 | λ=4.97
GP plot
Expression
Time (h)
Log-lik in held-out time points
log p(y_j|t_j,y_−j)
Time (h)

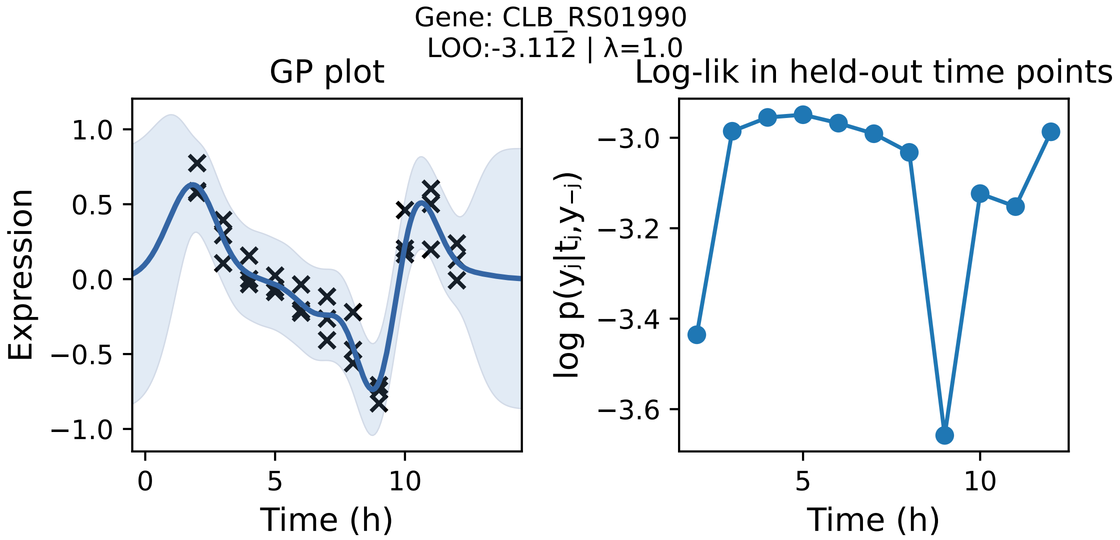

Gene: CLB_RS01990
LOO:-3.112 | λ=1.0
GP plot
Expression
1.0
0.5
0.0
−0.5
−1.0
0
5
10
Time (h)
Log-lik in held-out time points
log p(yj|tj,y−j)
−3.0
−3.2
−3.4
−3.6
5
10
Time (h)

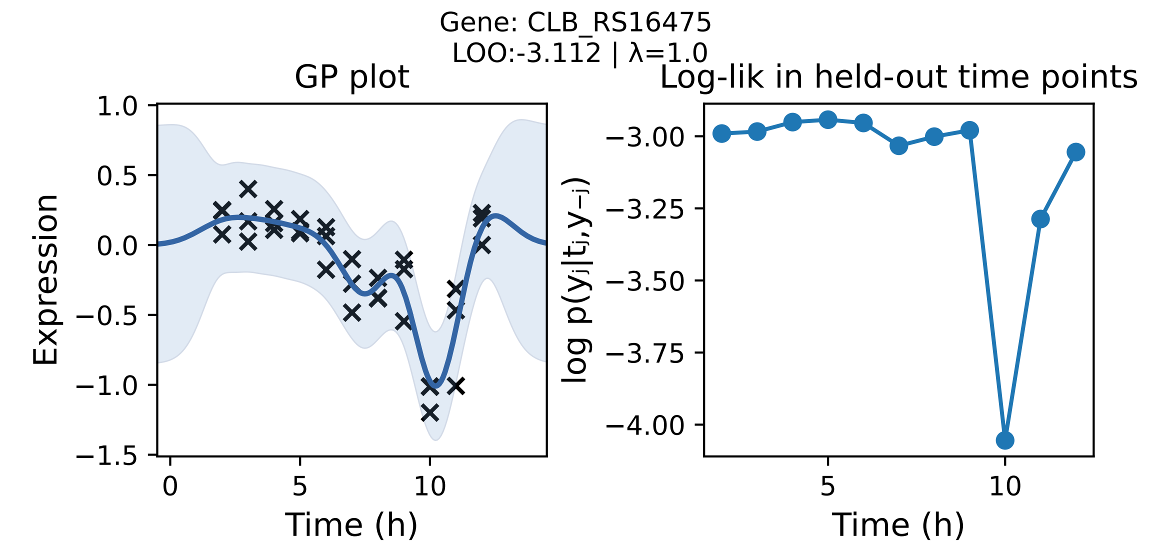

Gene: CLB_RS16475
LOO:-3.112 | λ=1.0
GP plot
Expression
Time (h)
1.0
0.5
0.0
−0.5
−1.0
−1.5
0
5
10
Log-lik in held-out time points
log p(y_j|t_j,y_−j)
−3.00
−3.25
−3.50
−3.75
−4.00
5
10
Time (h)

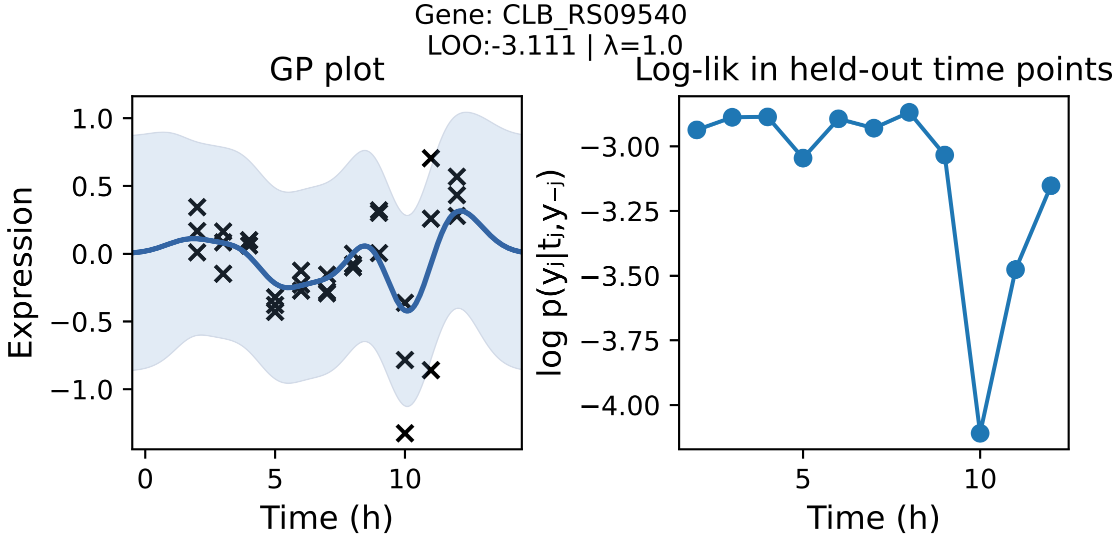

Gene: CLB_RS09540
LOO:-3.111 | λ=1.0
GP plot
Expression
1.0
0.5
0.0
−0.5
−1.0
0
5
10
Time (h)
Log-lik in held-out time points
log p(yj|tj,y−j)
−3.00
−3.25
−3.50
−3.75
−4.00
5
10
Time (h)

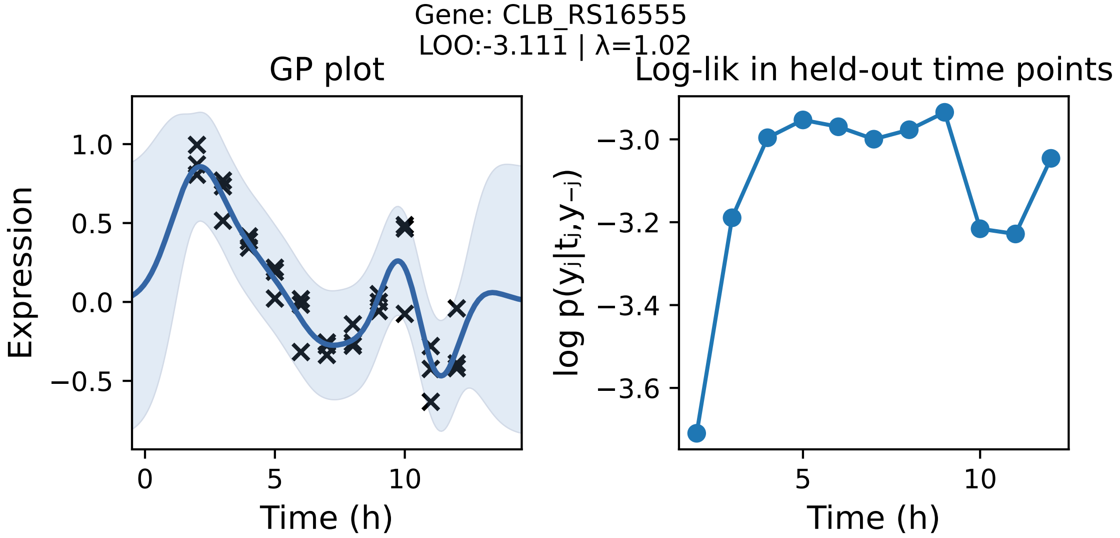

Gene: CLB_RS16555
LOO:-3.111 | λ=1.02
GP plot
Expression
1.0
0.5
0.0
−0.5
0
5
10
Time (h)
Log-lik in held-out time points
log p(y_j|t_j,y_−j)
−3.0
−3.2
−3.4
−3.6
5
10
Time (h)

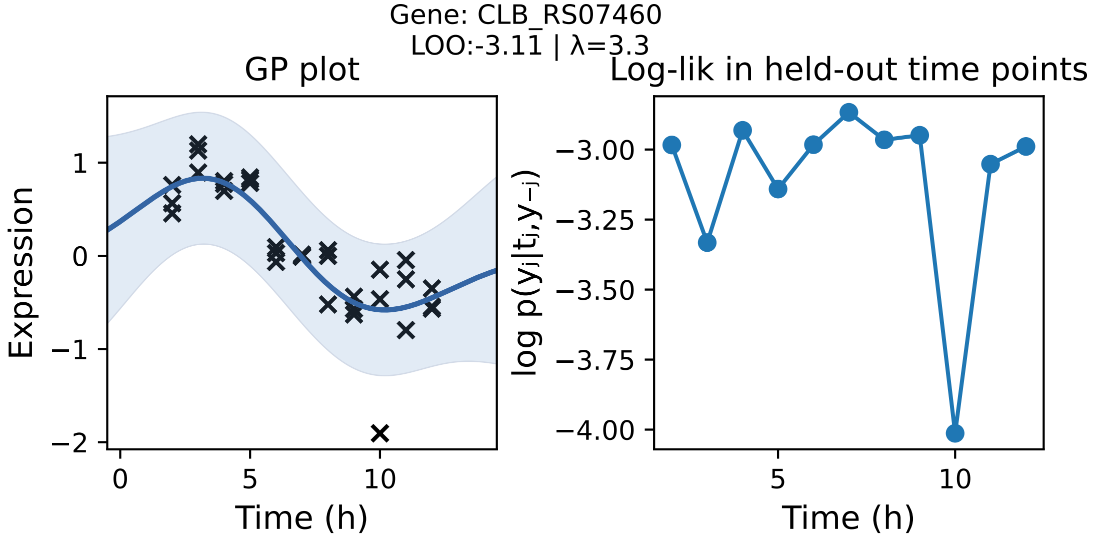

Gene: CLB_RS07460
LOO:-3.11 | λ=3.3
GP plot
Expression
Time (h)
1
0
−1
−2
0
5
10
Log-lik in held-out time points
log p(y_j|t_j,y_−j)
Time (h)
−3.00
−3.25
−3.50
−3.75
−4.00
5
10

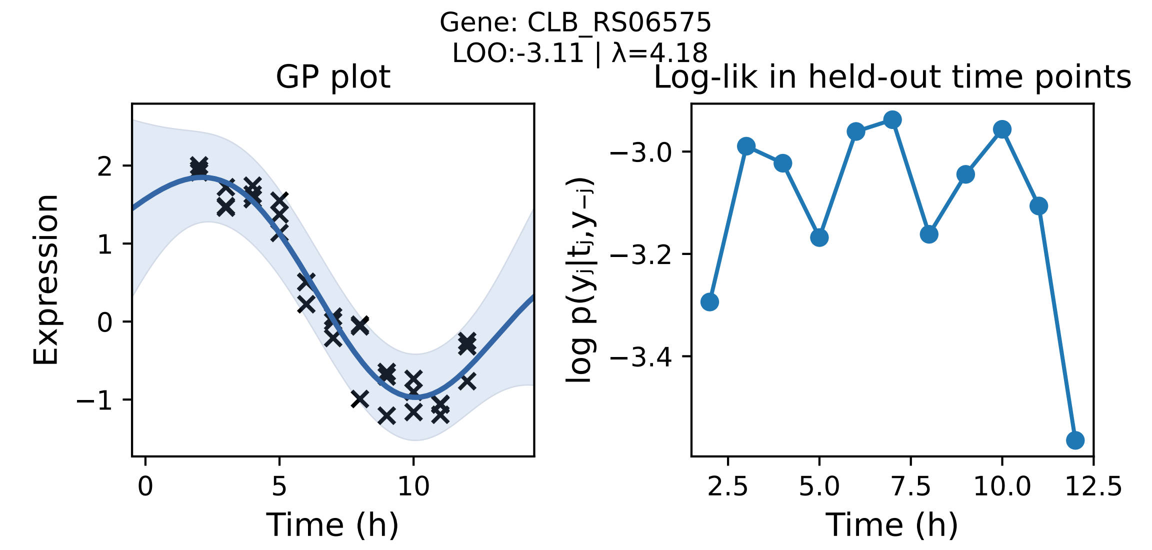
Gene: CLB_RS06575
LOO:-3.11 | λ=4.18
GP plot
Expression
Time (h)
Log-lik in held-out time points
log p(y_j|t_j,y_−j)
Time (h)

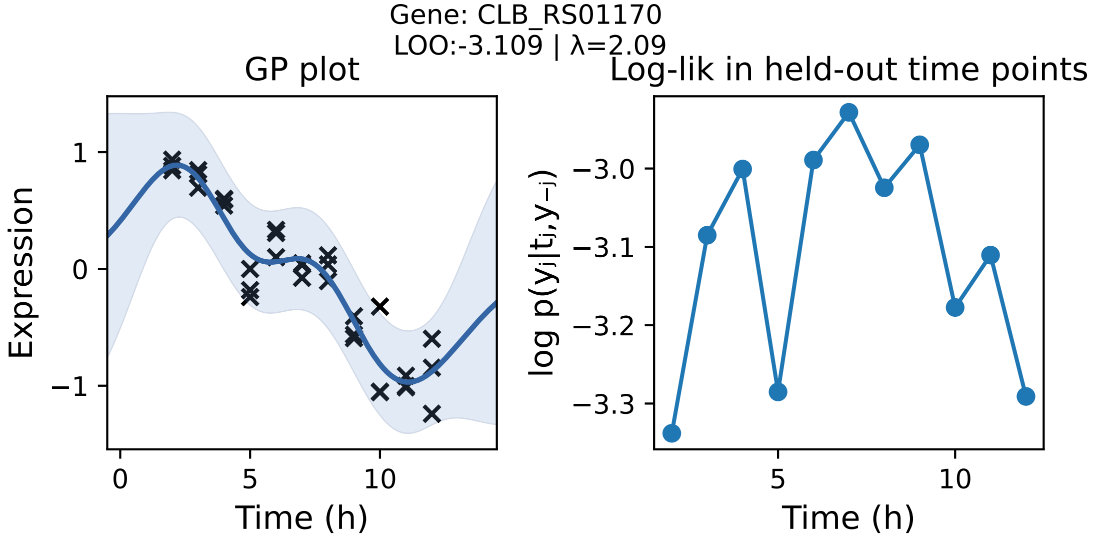

Gene: CLB_RS01170
LOO:-3.109 | λ=2.09
GP plot
Expression
Time (h)
1
0
−1
0
5
10
Log-lik in held-out time points
log p(y_j|t_j,y_−j)
−3.0
−3.1
−3.2
−3.3
5
10
Time (h)

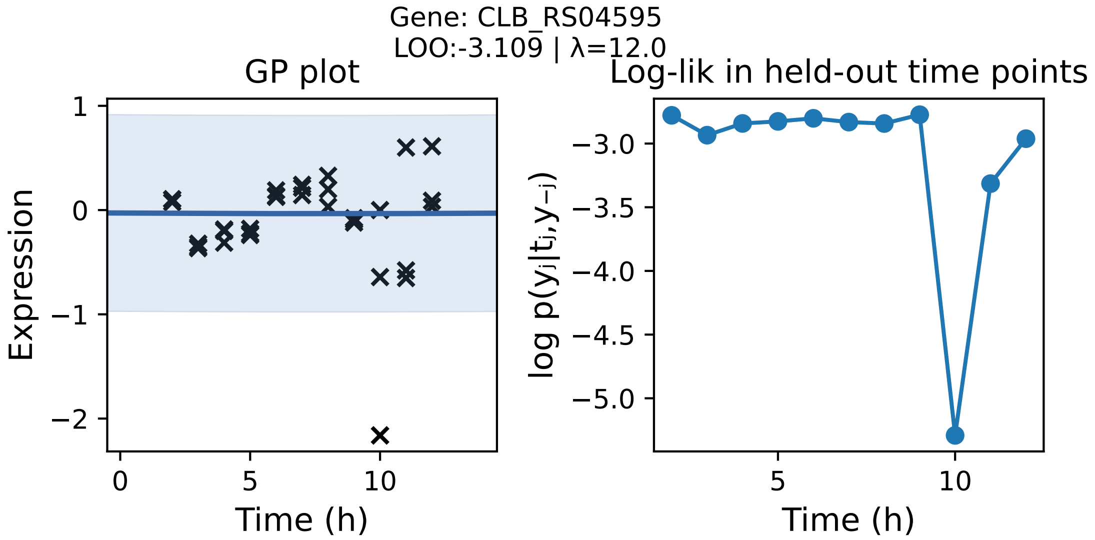
Gene: CLB_RS04595
LOO:-3.109 | λ=12.0
GP plot
Expression
Time (h)
1
0
−1
−2
0
5
10
Log-lik in held-out time points
log p(y_j|t_j,y_−j)
−3.0
−3.5
−4.0
−4.5
−5.0
5
10
Time (h)

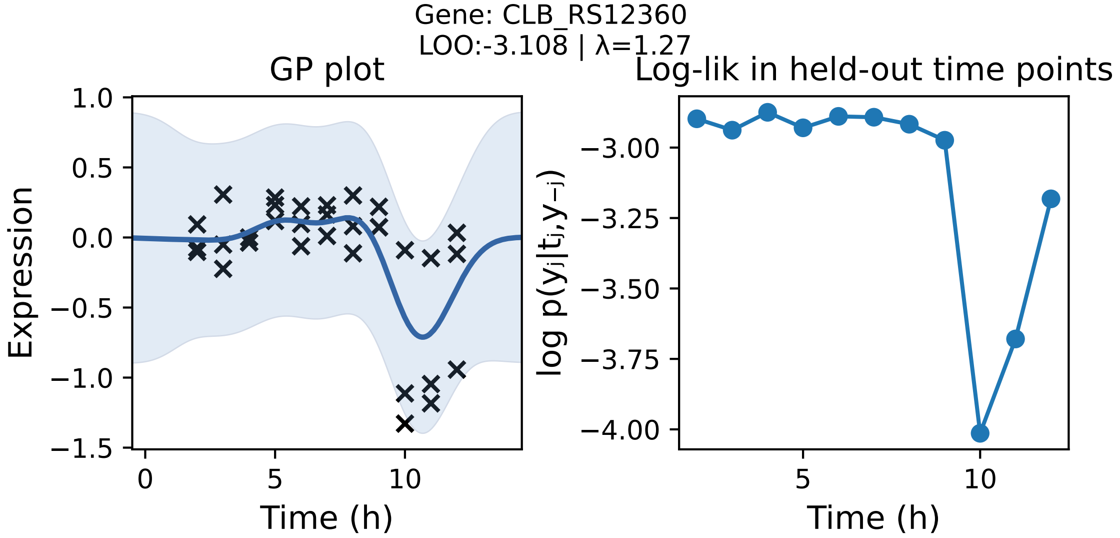

Gene: CLB_RS12360
LOO:-3.108 | λ=1.27
GP plot
Expression
Time (h)
Log-lik in held-out time points
log p(y_j|t_j,y_−j)
Time (h)

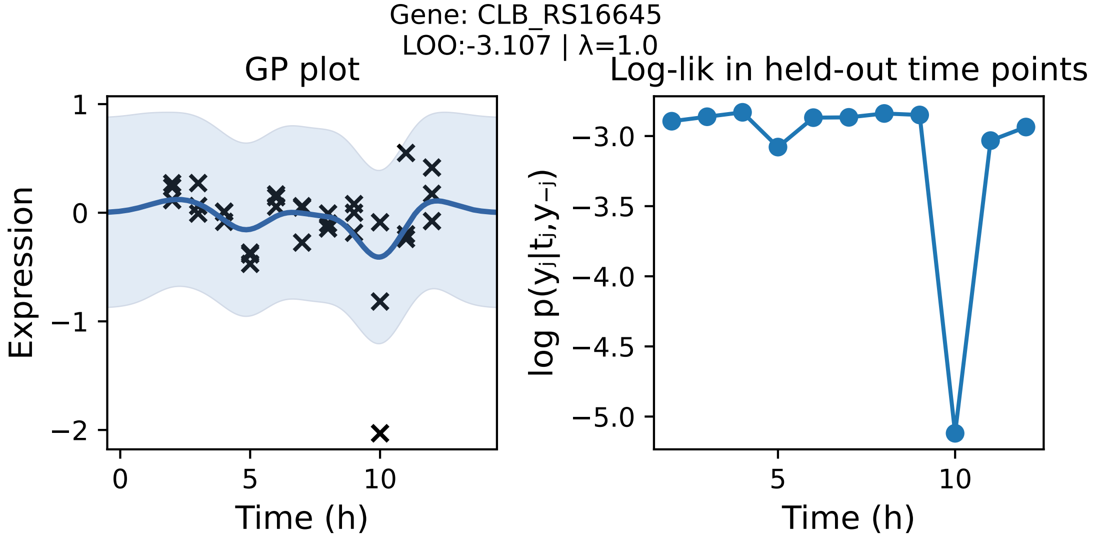

Gene: CLB_RS16645
LOO:-3.107 | λ=1.0
GP plot
Expression
Time (h)
Log-lik in held-out time points
log p(y_j|t_j,y_−j)
Time (h)

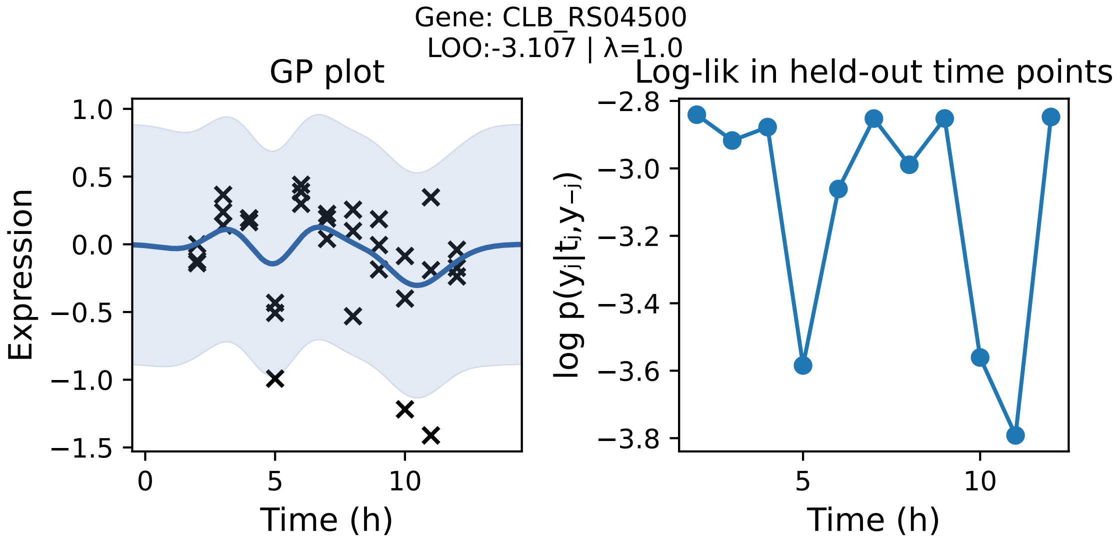

Gene: CLB_RS04500
LOO:-3.107 | λ=1.0
GP plot
Expression
Time (h)
1.0
0.5
0.0
−0.5
−1.0
−1.5
0
5
10
Log-lik in held-out time points
log p(y_j|t_j,y_−j)
−2.8
−3.0
−3.2
−3.4
−3.6
−3.8
5
10
Time (h)

Gene: CLB_RS07700
LOO:-3.105 | λ=1.22

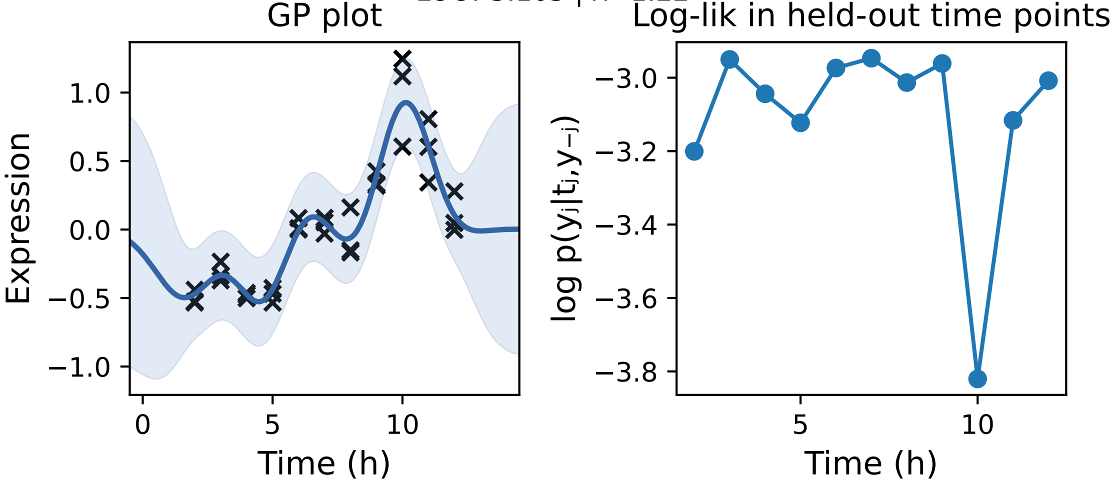

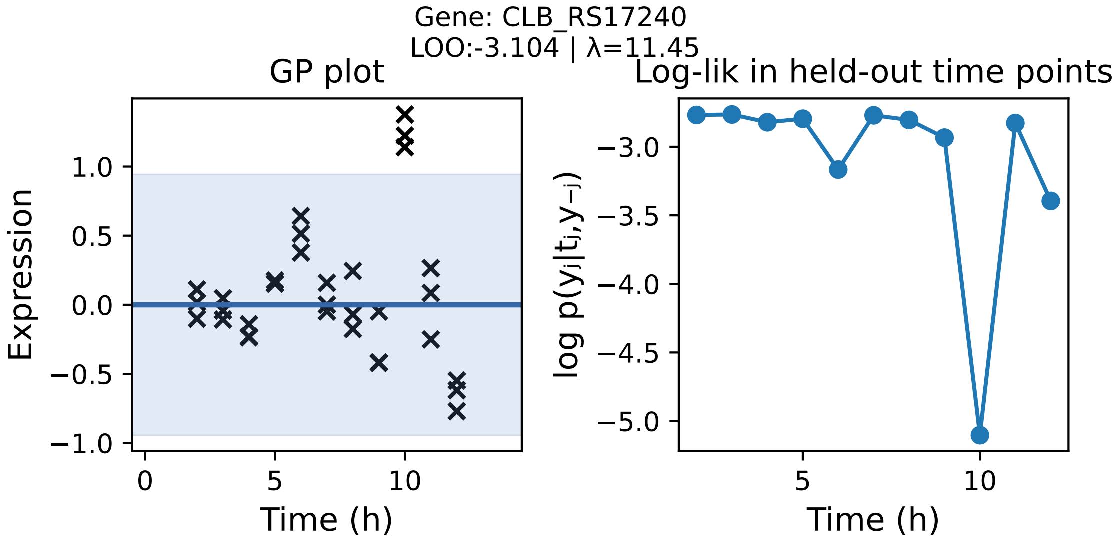

Gene: CLB_RS17240
LOO:-3.104 | λ=11.45
GP plot
Expression
Time (h)
1.0
0.5
0.0
−0.5
−1.0
0
5
10
Log-lik in held-out time points
log p(y_j|t_j,y_−j)
Time (h)
−3.0
−3.5
−4.0
−4.5
−5.0
5
10

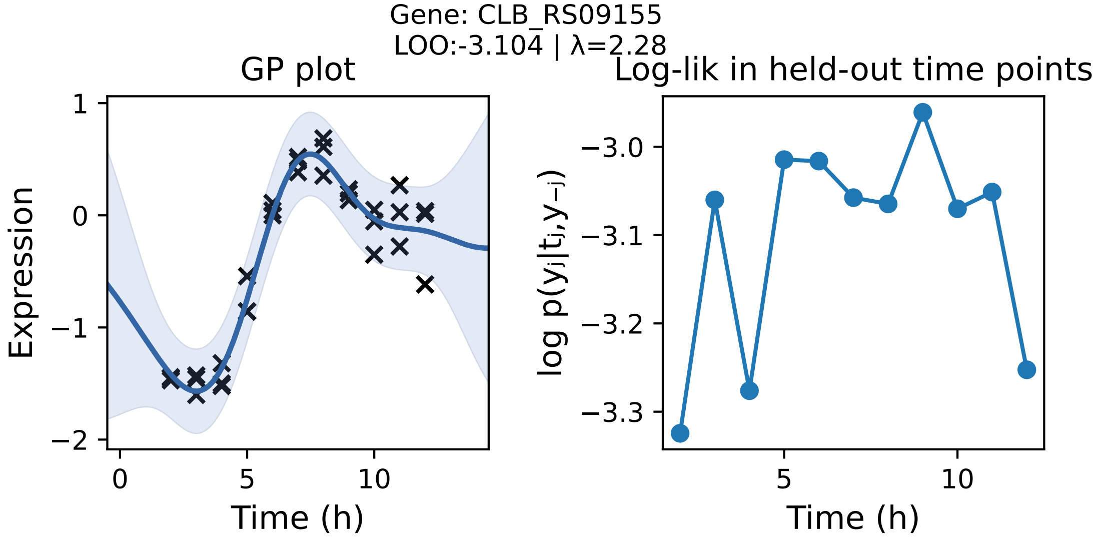

Gene: CLB_RS09155
LOO:-3.104 | λ=2.28
GP plot
Expression
Time (h)
Log-lik in held-out time points
log p(y_j|t_j,y_−j)
Time (h)

Gene: CLB_RS07835
LOO:-3.104 | λ=1.0

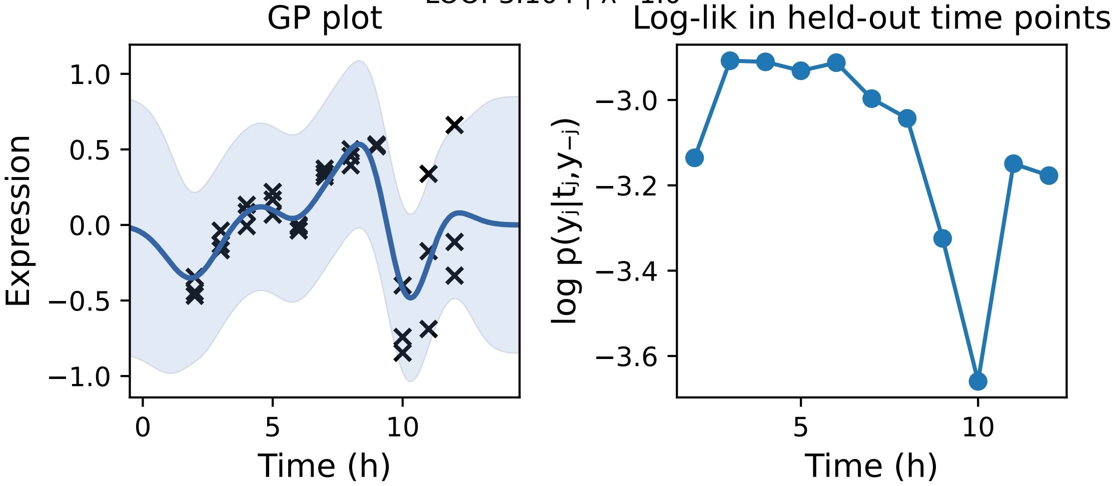

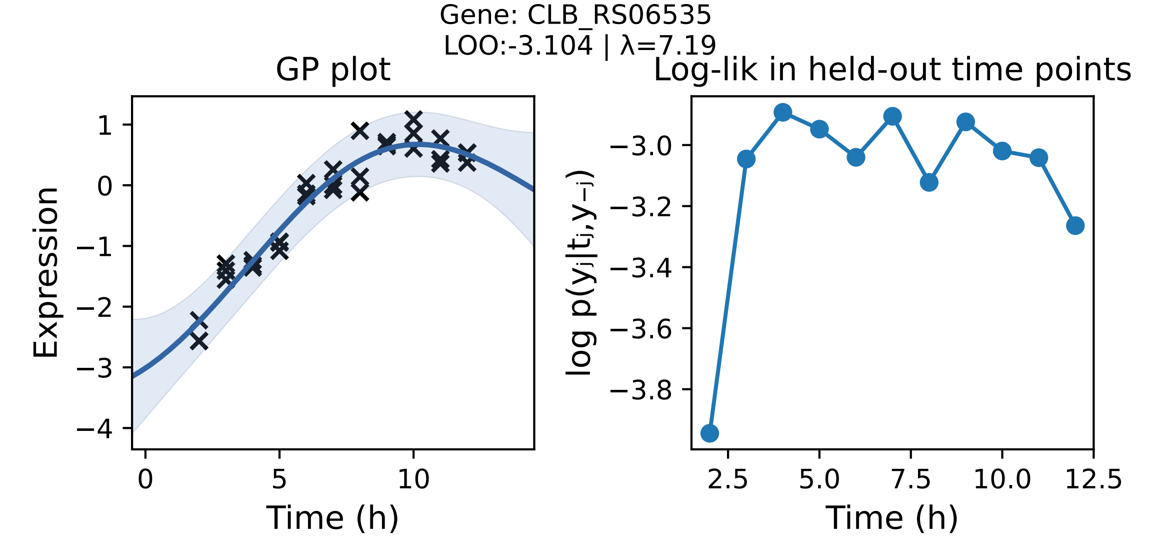
Gene: CLB_RS06535
LOO:-3.104 | λ=7.19
GP plot
Expression
Time (h)
1
0
−1
−2
−3
−4
0
5
10
Log-lik in held-out time points
log p(y_j|t_j,y_−j)
Time (h)
−3.0
−3.2
−3.4
−3.6
−3.8
2.5
5.0
7.5
10.0
12.5

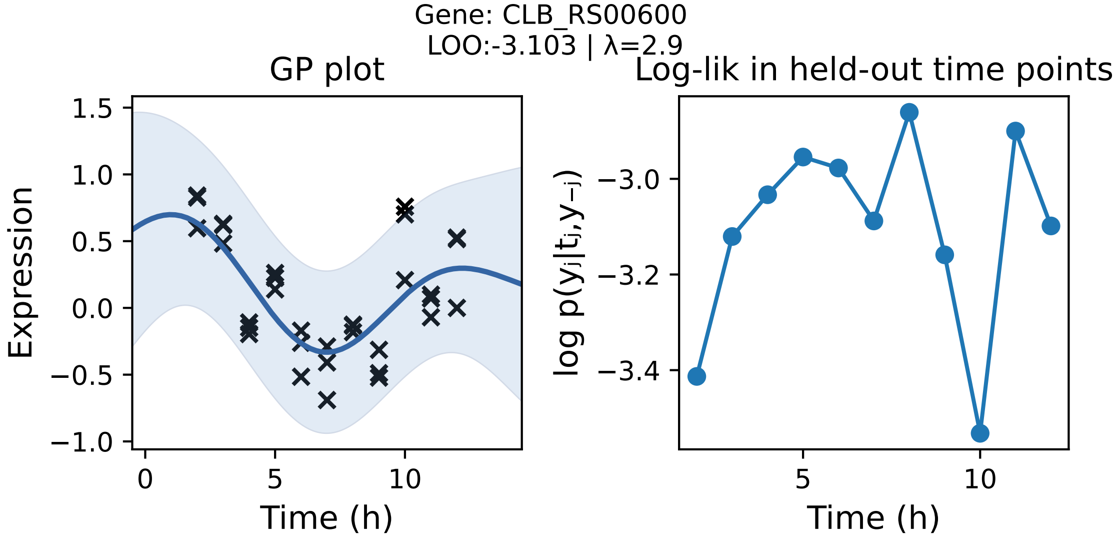

Gene: CLB_RS00600
LOO:-3.103 | λ=2.9
GP plot
Expression
1.5
1.0
0.5
0.0
−0.5
−1.0
0
5
10
Time (h)
Log-lik in held-out time points
log p(yj|tj,y−j)
−3.0
−3.2
−3.4
5
10
Time (h)

Gene: CLB_RS04805
LOO:-3.103 | λ=1.79

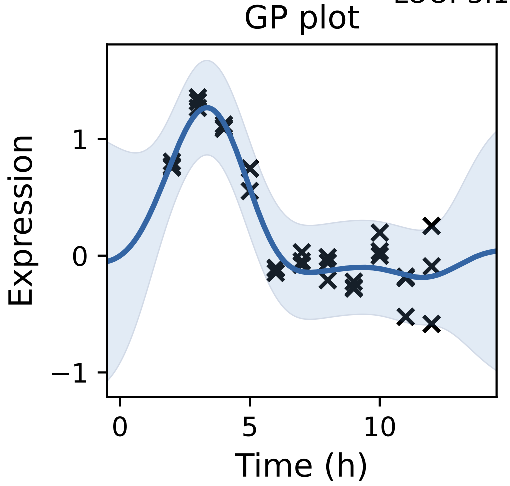

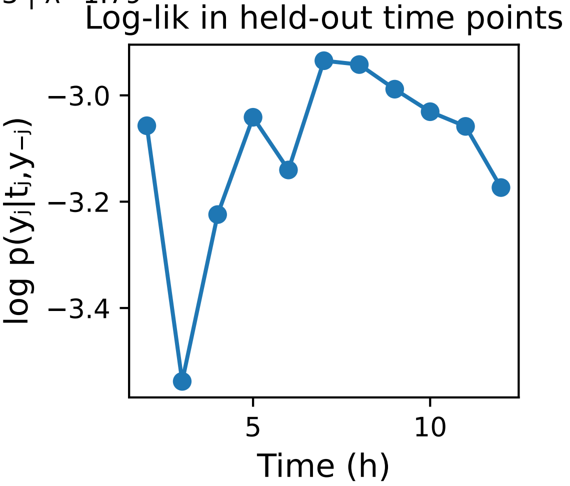

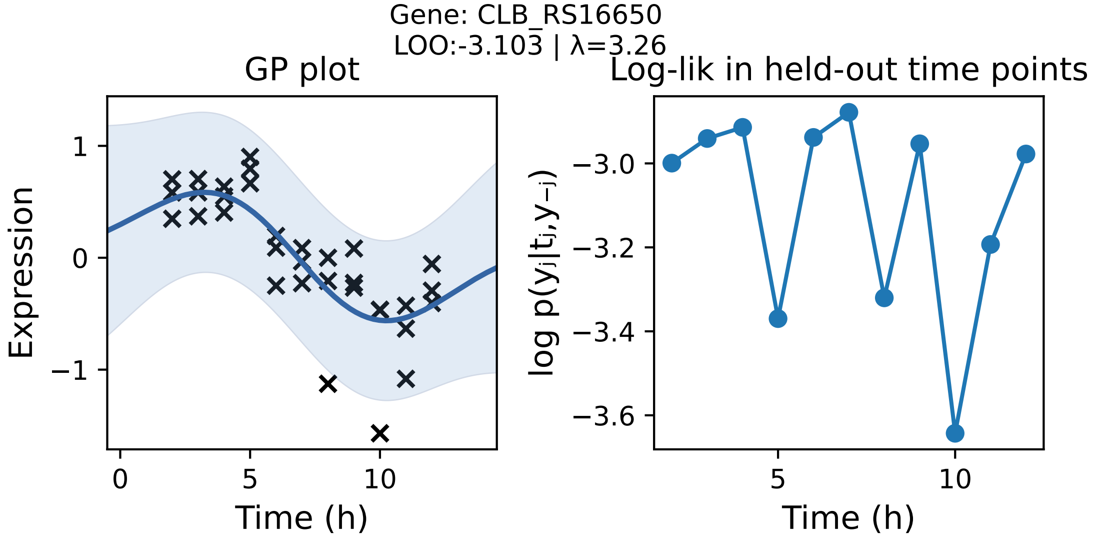

Gene: CLB_RS16650
LOO:-3.103 | λ=3.26
GP plot
Expression
Time (h)
Log-lik in held-out time points
log p(yj|tj,y−j)
Time (h)

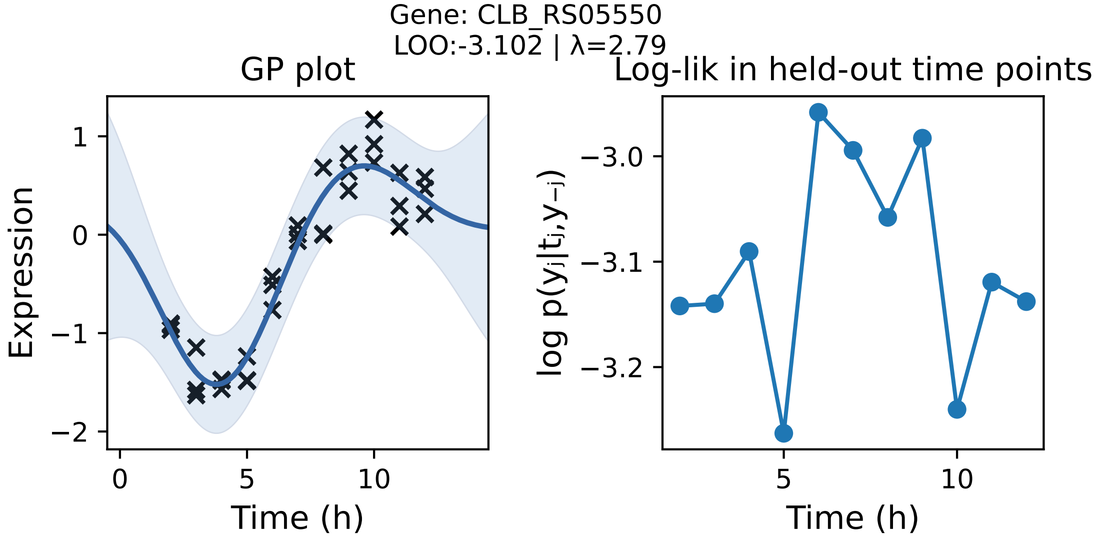

Gene: CLB_RS05550
LOO:-3.102 | λ=2.79
GP plot
Expression
Time (h)
Log-lik in held-out time points
log p(y_j|t_j,y_−j)
Time (h)

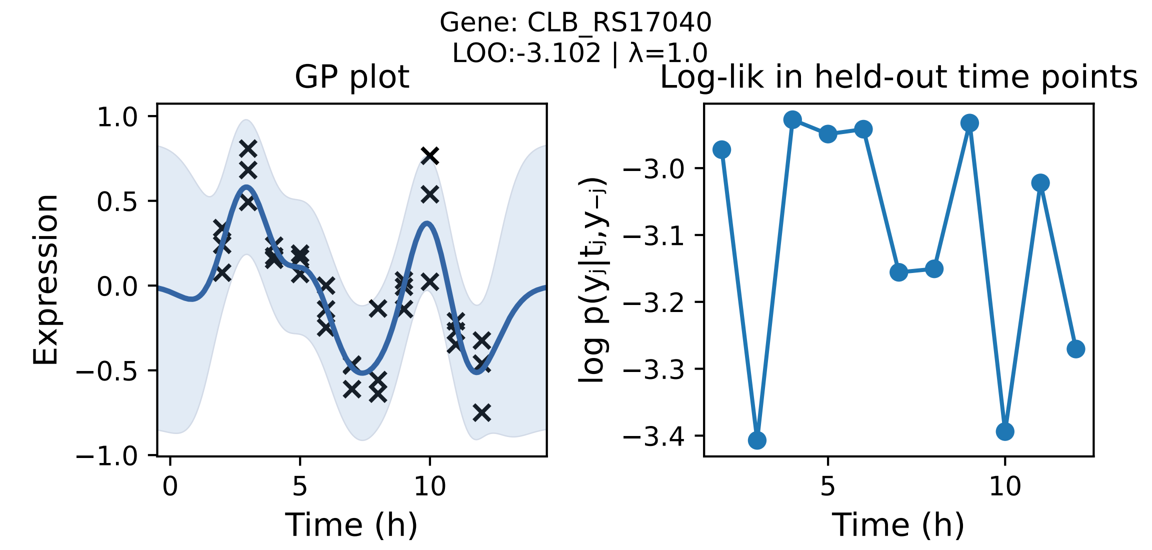

Gene: CLB_RS17040
LOO:-3.102 | λ=1.0
GP plot
Log-lik in held-out time points
Expression
1.0
0.5
0.0
−0.5
−1.0
0
5
10
Time (h)
log p(y_j|t_j,y_−j)
−3.0
−3.1
−3.2
−3.3
−3.4
5
10
Time (h)

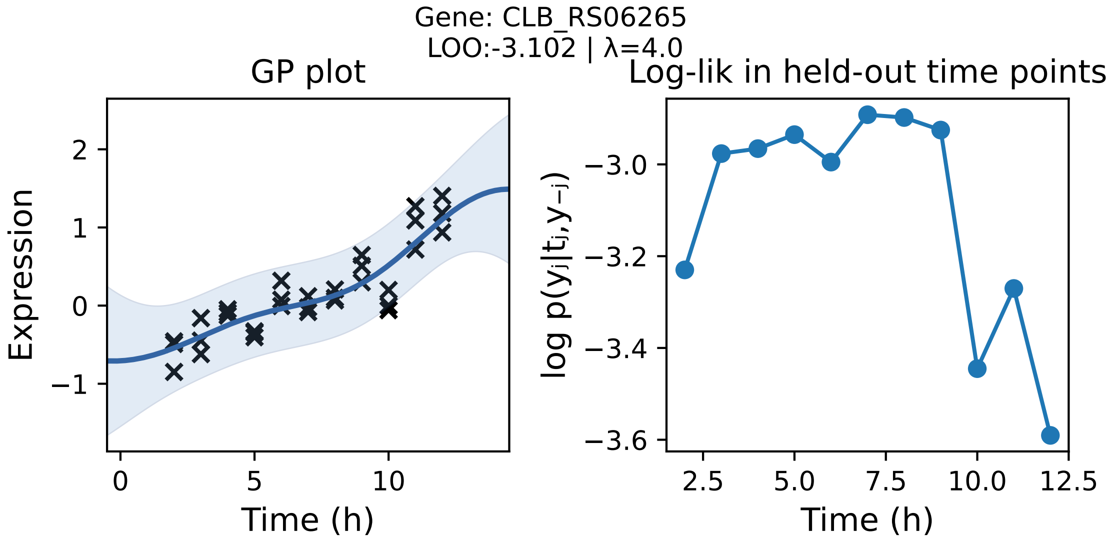

Gene: CLB_RS06265
LOO:-3.102 | λ=4.0
GP plot
Expression
Time (h)
2
1
0
−1
0
5
10
Log-lik in held-out time points
log p(yj|tj,y−j)
Time (h)
−3.0
−3.2
−3.4
−3.6
2.5
5.0
7.5
10.0
12.5

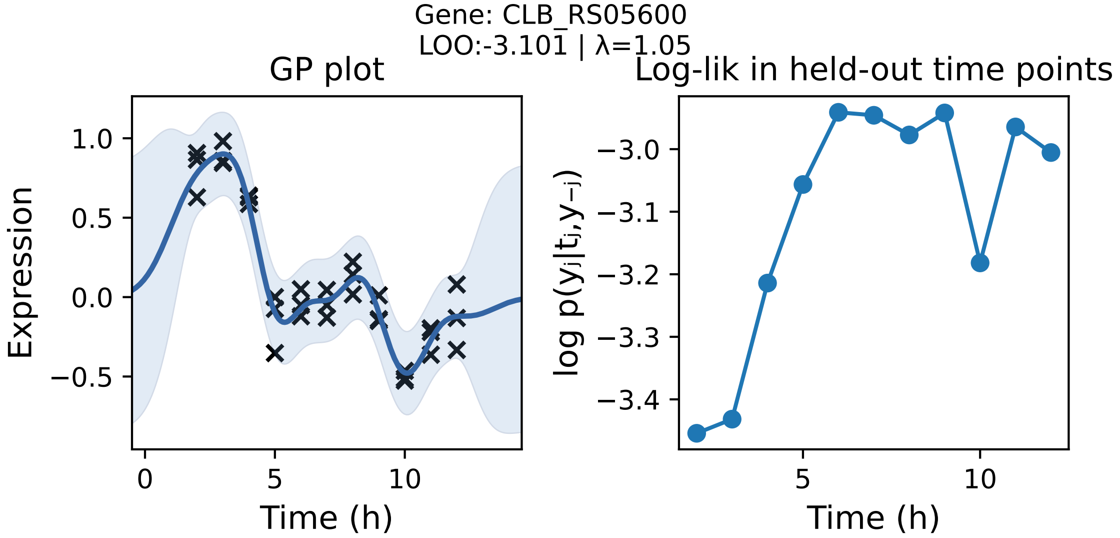

Gene: CLB_RS05600
LOO:-3.101 | λ=1.05
GP plot
Expression
Time (h)
1.0
0.5
0.0
−0.5
0
5
10
Log-lik in held-out time points
log p(yj|tj,y−j)
−3.0
−3.1
−3.2
−3.3
−3.4
5
10
Time (h)

Gene: CLB_RS07420
LOO:-3.1 | λ=1.76

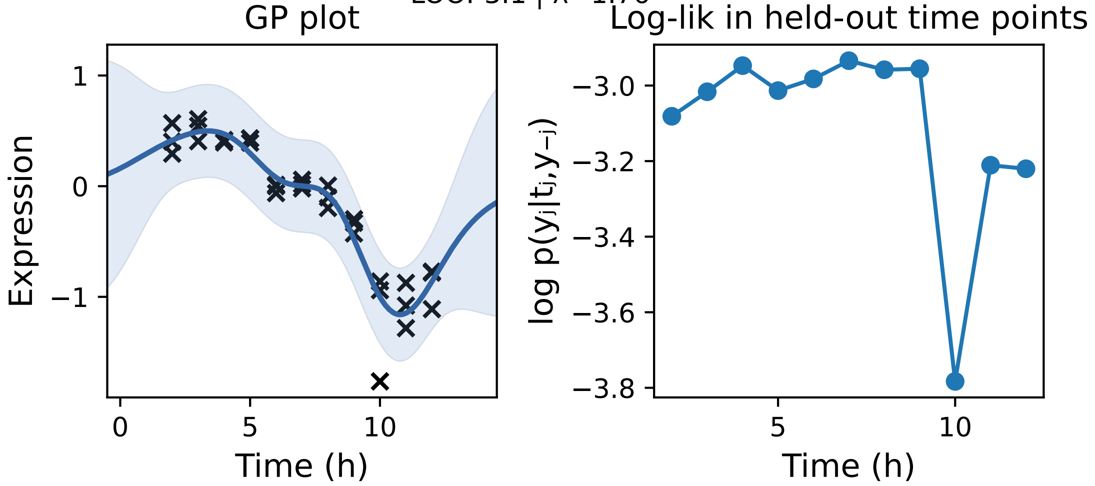

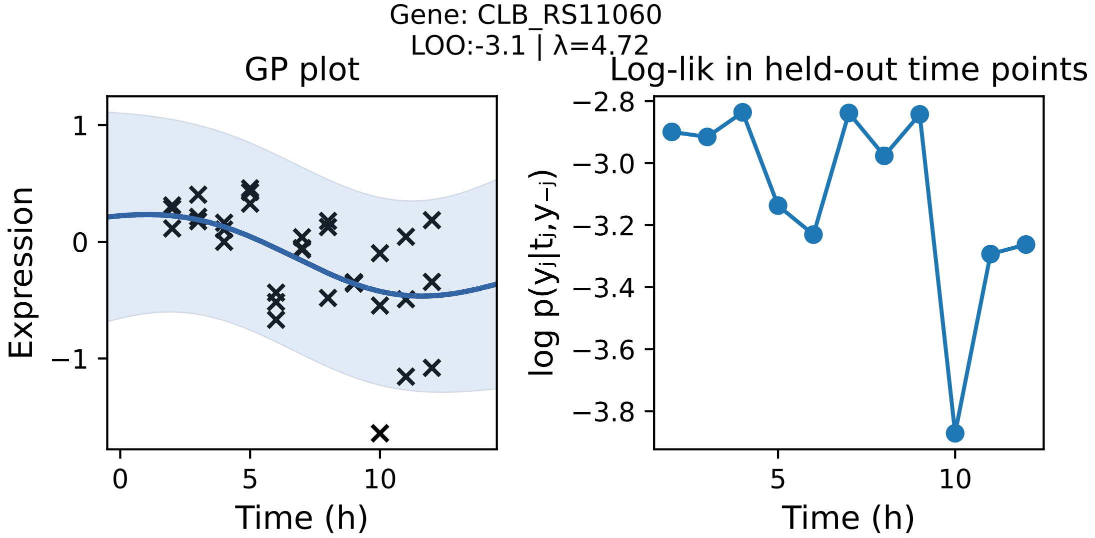

Gene: CLB_RS11060
LOO:-3.1 | λ=4.72
GP plot
Expression
1
0
−1
0
5
10
Time (h)
Log-lik in held-out time points
log p(y_j|t_j,y_−j)
−2.8
−3.0
−3.2
−3.4
−3.6
−3.8
5
10
Time (h)

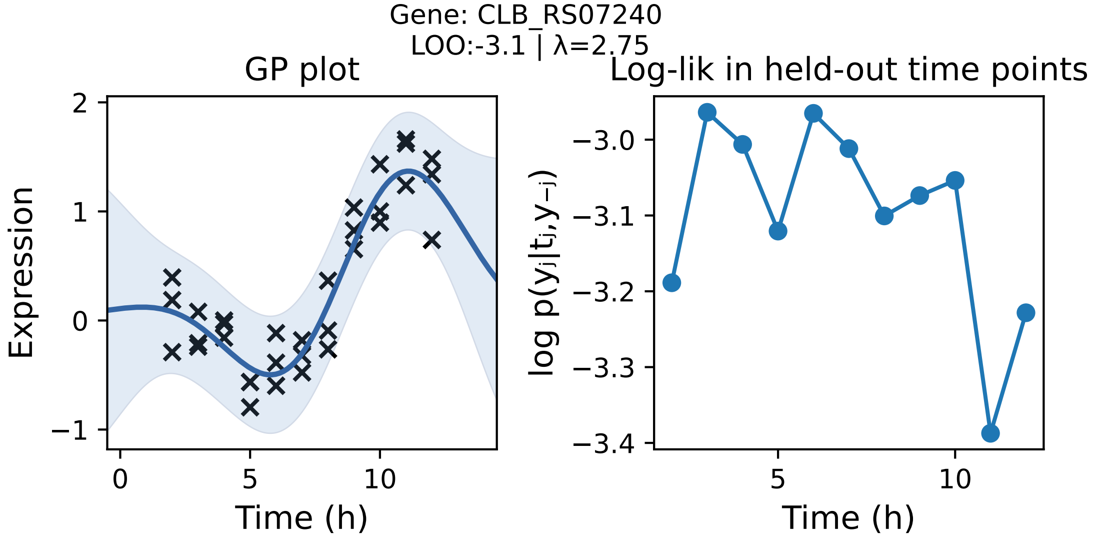

Gene: CLB_RS07240
LOO:-3.1 | λ=2.75
GP plot
Expression
Time (h)
Log-lik in held-out time points
log p(y_j|t_j,y_−j)
Time (h)

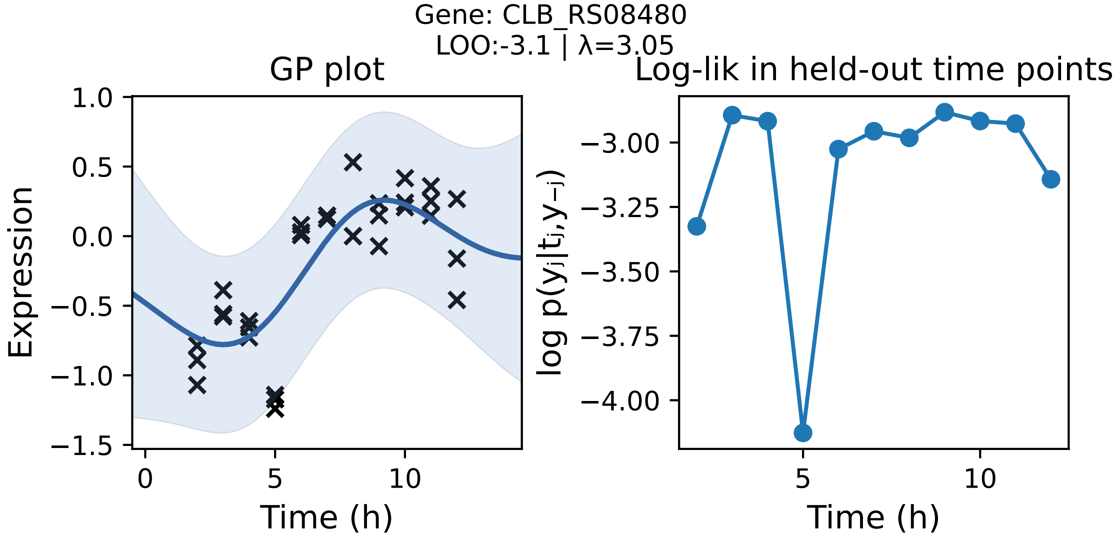

Gene: CLB_RS08480
LOO:-3.1 | λ=3.05
GP plot
Expression
Time (h)
1.0
0.5
0.0
−0.5
−1.0
−1.5
0
5
10
Log-lik in held-out time points
log p(yj|tj,y−j)
−3.00
−3.25
−3.50
−3.75
−4.00
5
10
Time (h)

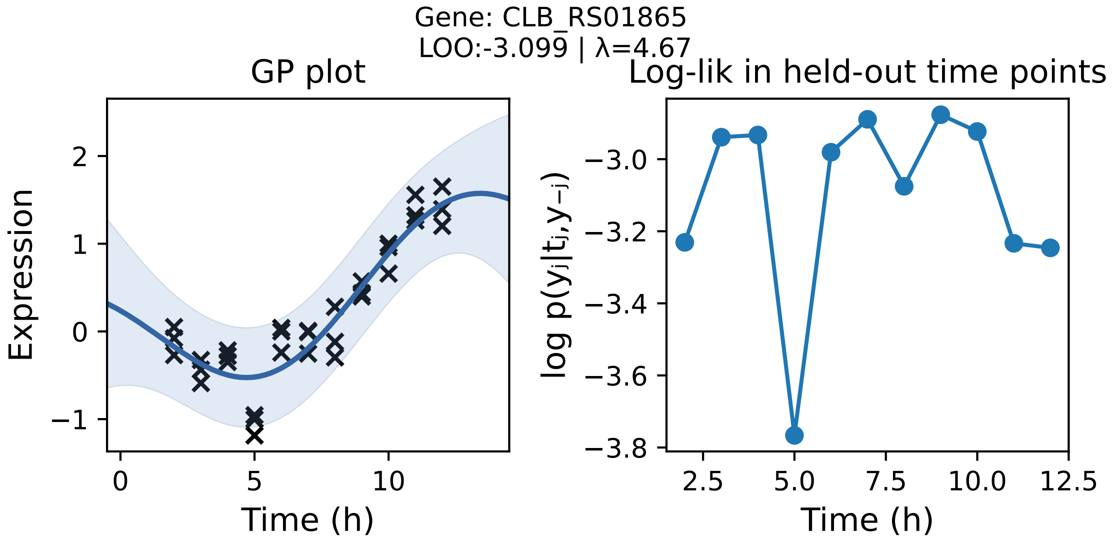

Gene: CLB_RS01865
LOO:-3.099 | λ=4.67
GP plot
Expression
Time (h)
Log-lik in held-out time points
log p(y_j|t_j,y_−j)
Time (h)

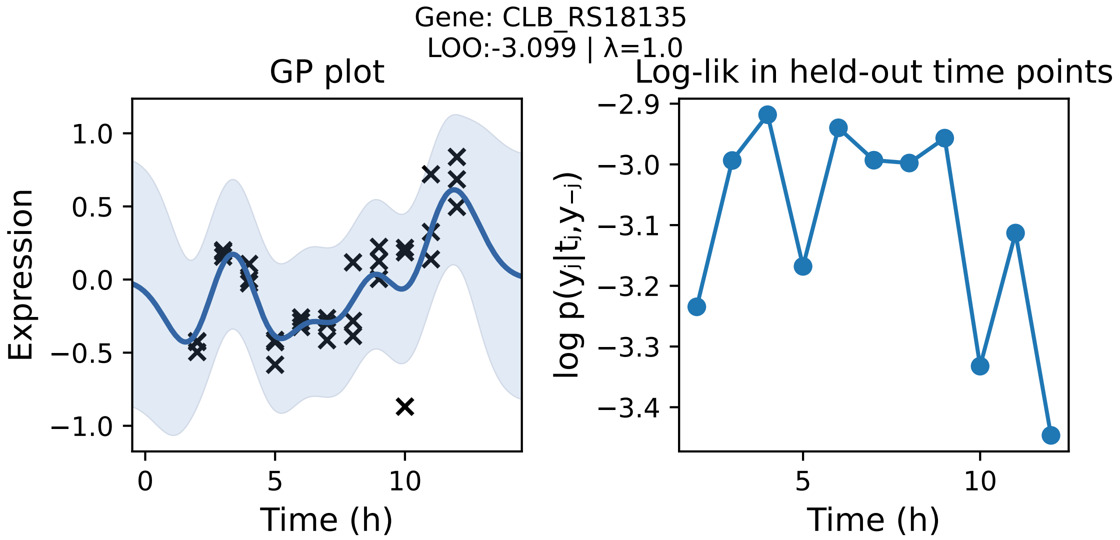

Gene: CLB_RS18135
LOO:-3.099 | λ=1.0
GP plot
Log-lik in held-out time points
Expression
Time (h)
log p(yj|tj,y−j)
1.0
0.5
0.0
−0.5
−1.0
0
5
10
−2.9
−3.0
−3.1
−3.2
−3.3
−3.4

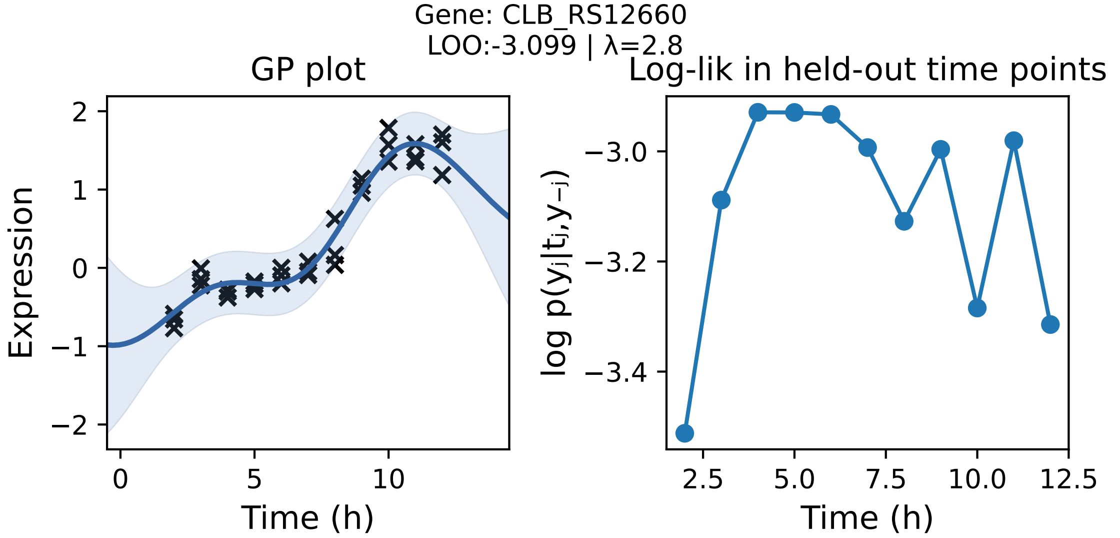

Gene: CLB_RS12660
LOO:-3.099 | λ=2.8
GP plot
Expression
Time (h)
2
1
0
−1
−2
0
5
10
Log-lik in held-out time points
log p(y_j|t_j,y_−j)
Time (h)
−3.0
−3.2
−3.4
2.5
5.0
7.5
10.0
12.5

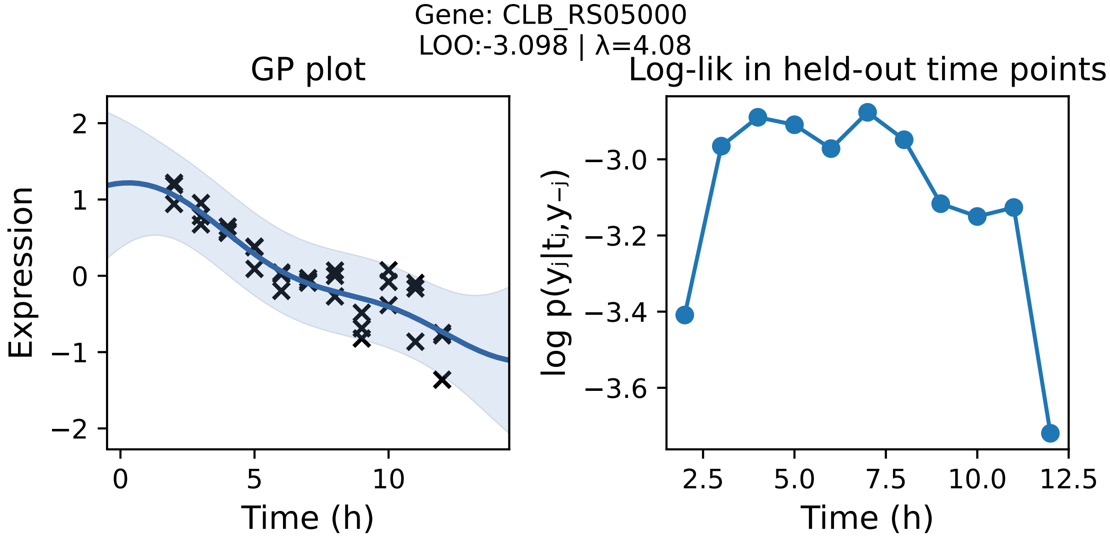
Gene: CLB_RS05000
LOO:-3.098 | λ=4.08
GP plot
Expression
Time (h)
Log-lik in held-out time points
log p(y_j|t_j,y_−j)
Time (h)

Gene: CLB_RS13425
LOO:-3.098 | λ=3.82

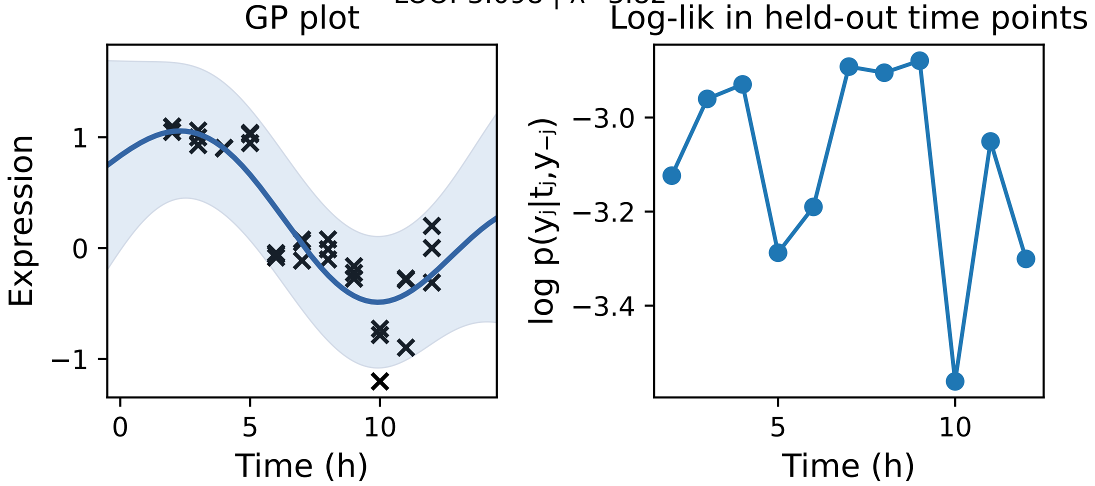

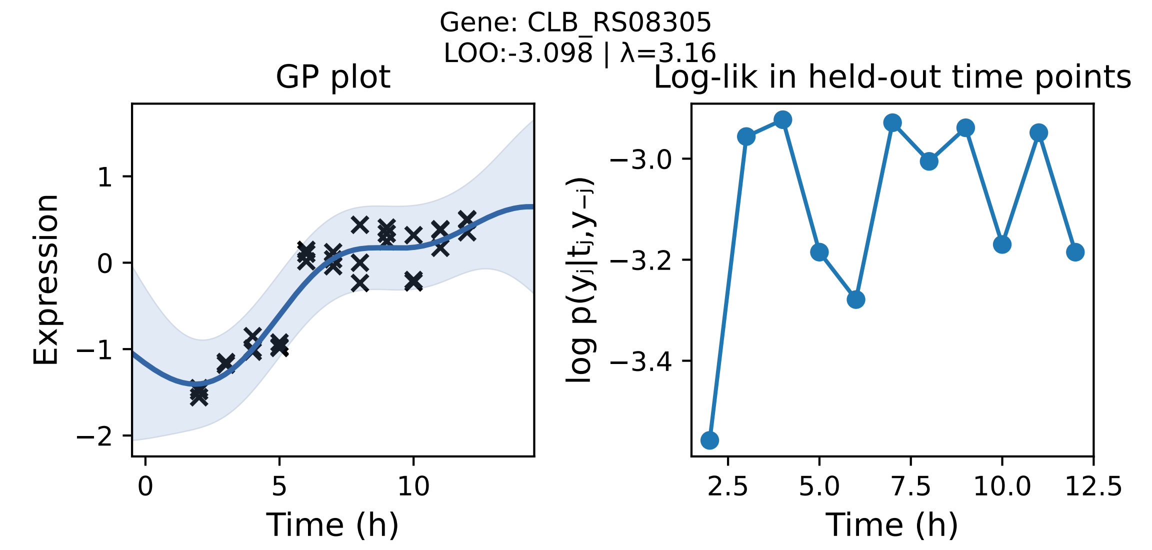

Gene: CLB_RS08305
LOO:-3.098 | λ=3.16
GP plot
Expression
Time (h)
Log-lik in held-out time points
log p(y_j|t_j,y_−j)
Time (h)

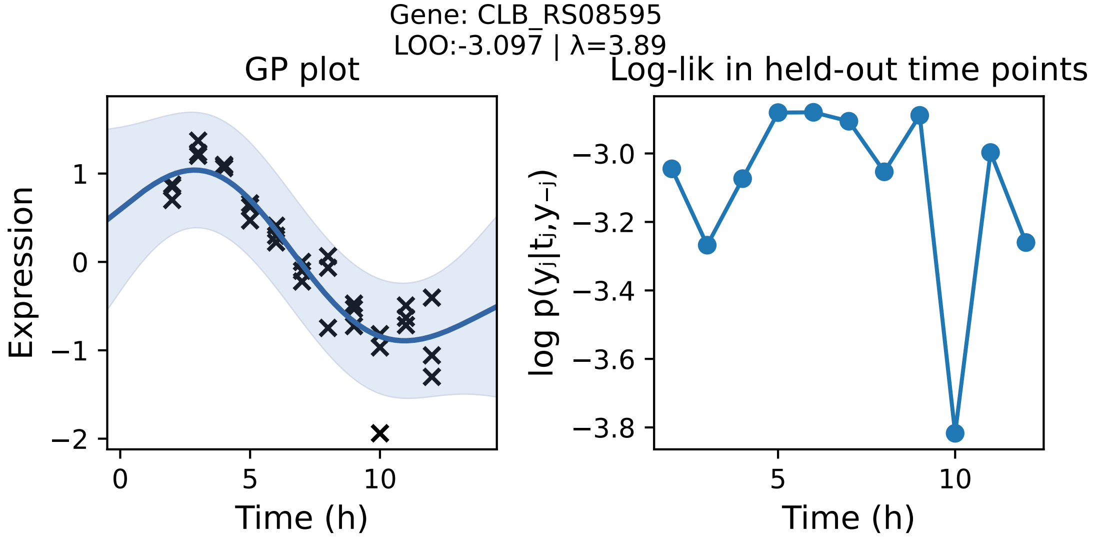

Gene: CLB_RS08595
LOO:-3.097 | λ=3.89
GP plot
Log-lik in held-out time points
Expression
Time (h)
log p(y_j|t_j,y_−j)
Time (h)

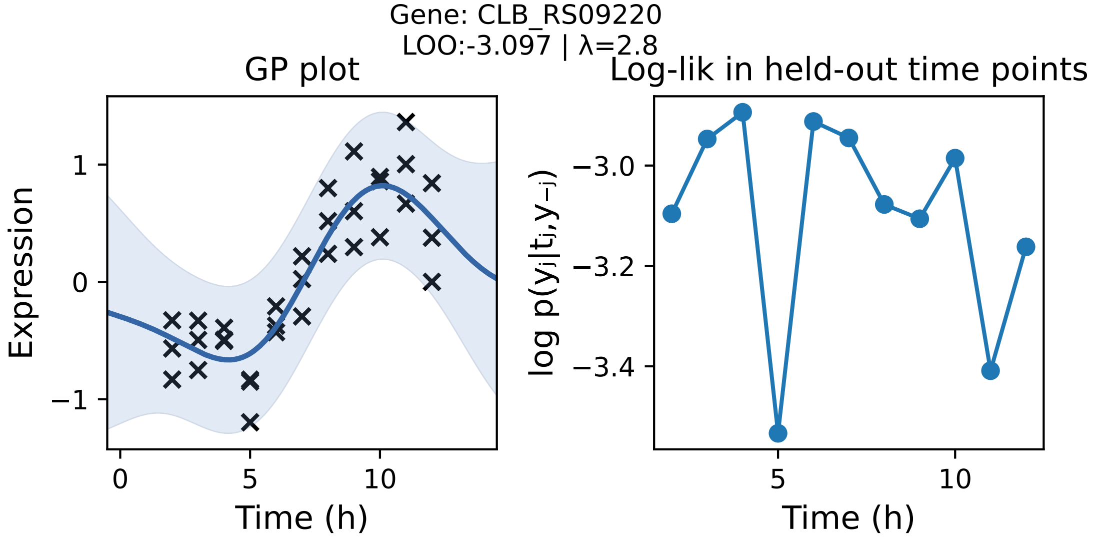

Gene: CLB_RS09220
LOO:-3.097 | λ=2.8
GP plot
Expression
Time (h)
Log-lik in held-out time points
log p(yj|tj,y−j)
Time (h)

Gene: CLB_RS07055
LOO:-3.097 | λ=4.21

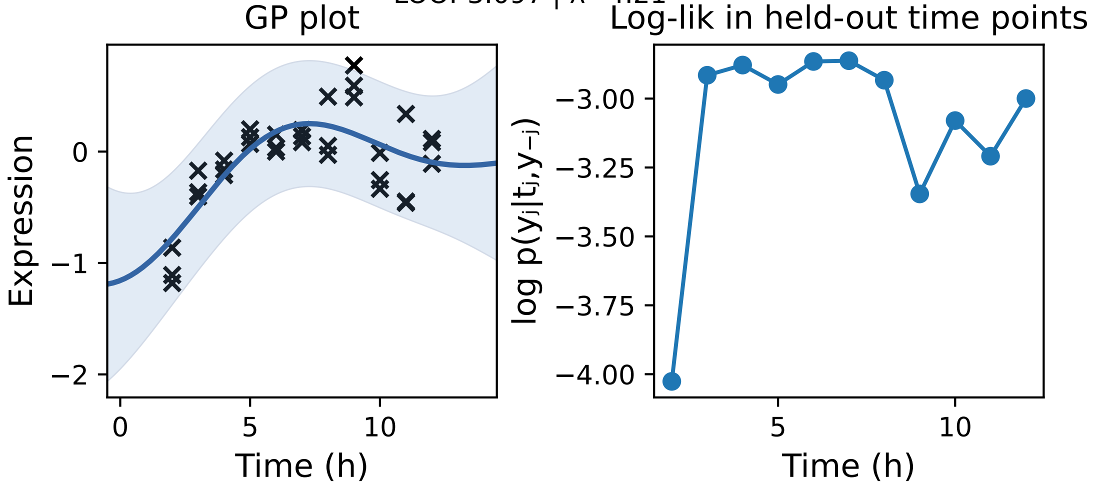

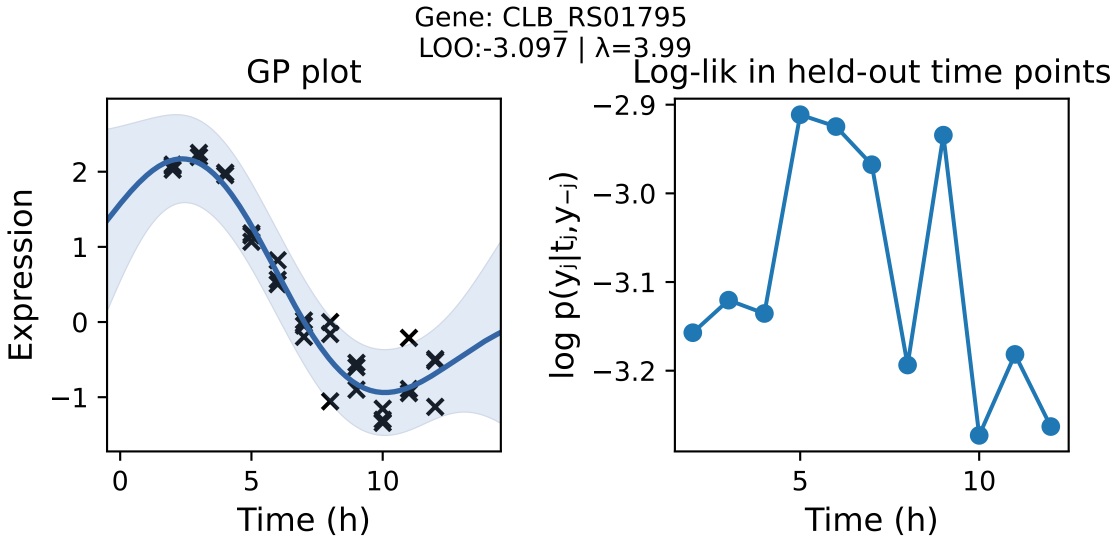

Gene: CLB_RS01795
LOO:-3.097 | λ=3.99
GP plot
Expression
Time (h)
Log-lik in held-out time points
log p(y_j|t_j,y_−j)
Time (h)

Gene: CLB_RS03360
LOO:-3.096 | λ=3.19

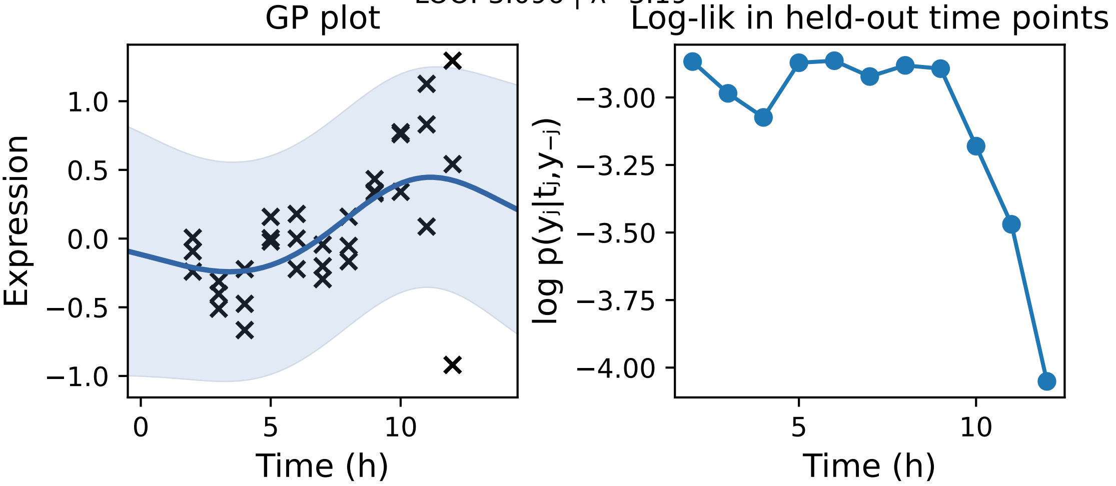

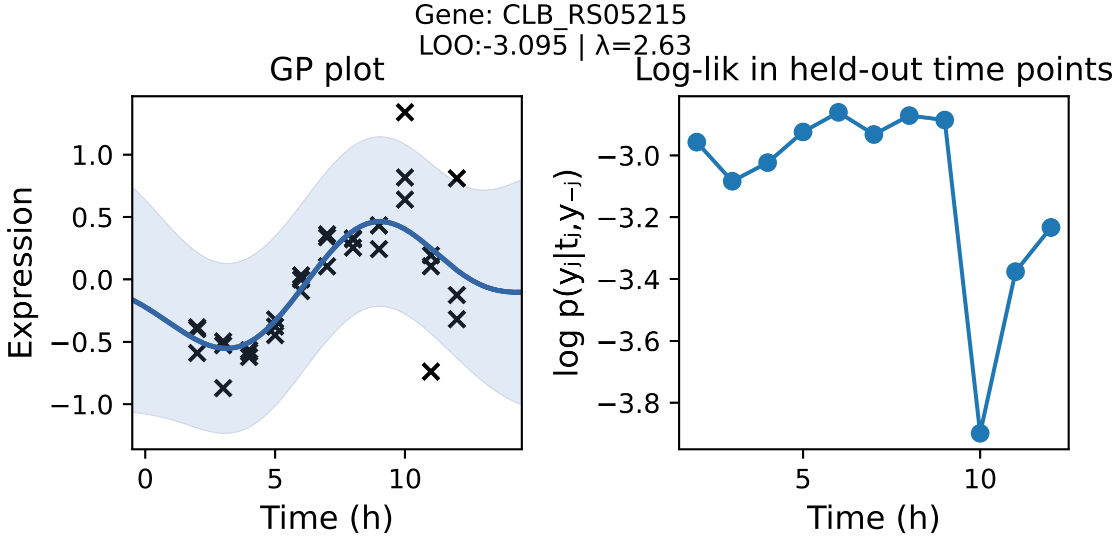

Gene: CLB_RS05215
LOO:-3.095 | λ=2.63
GP plot
Expression
Time (h)
1.0
0.5
0.0
−0.5
−1.0
0
5
10
Log-lik in held-out time points
log p(y_j|t_j,y_−j)
Time (h)
−3.0
−3.2
−3.4
−3.6
−3.8
5
10

Gene: CLB_RS05955
LOO:-3.095 | λ=2.58

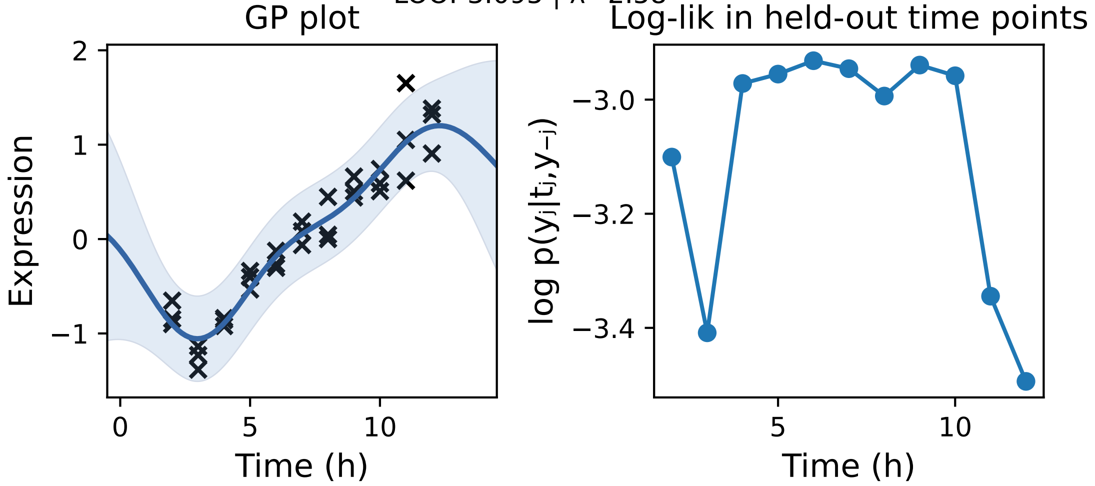

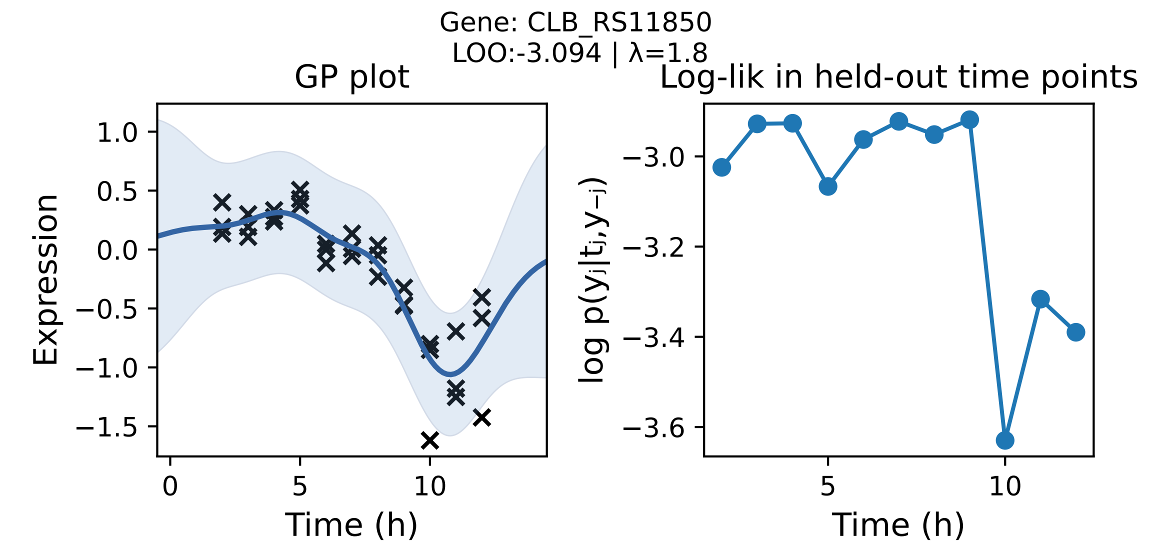

Gene: CLB_RS11850
LOO:-3.094 | λ=1.8
GP plot
Expression
Time (h)
1.0
0.5
0.0
−0.5
−1.0
−1.5
0
5
10
Log-lik in held-out time points
log p(y_j|t_j,y_{−j})
−3.0
−3.2
−3.4
−3.6
5
10
Time (h)

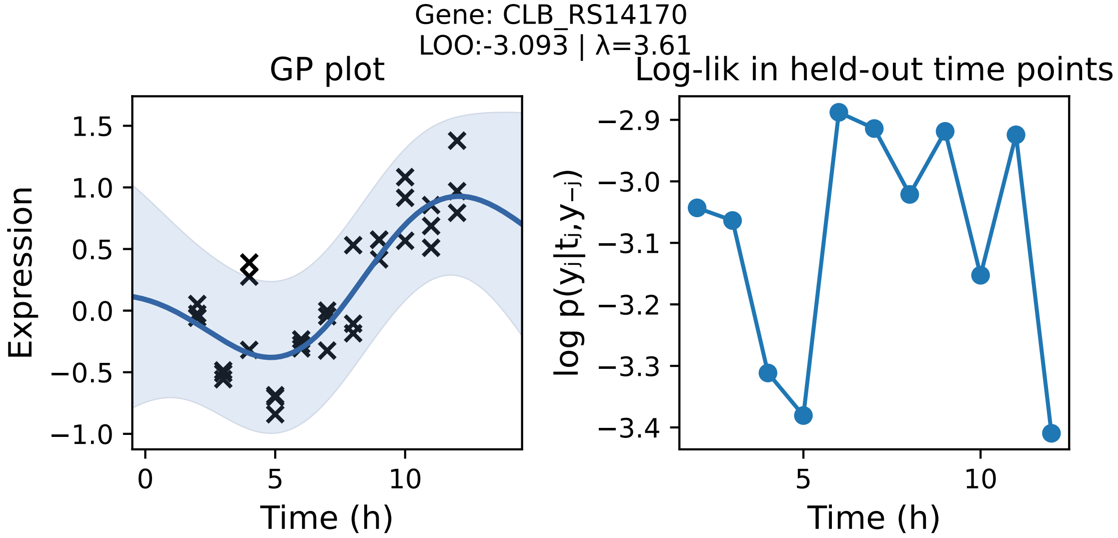
Gene: CLB_RS14170
LOO:-3.093 | λ=3.61
GP plot
Log-lik in held-out time points
1.5
1.0
0.5
0.0
−0.5
−1.0
Expression
0
5
10
Time (h)
−2.9
−3.0
−3.1
−3.2
−3.3
−3.4
log p(yj|tj,y−j)
5
10
Time (h)

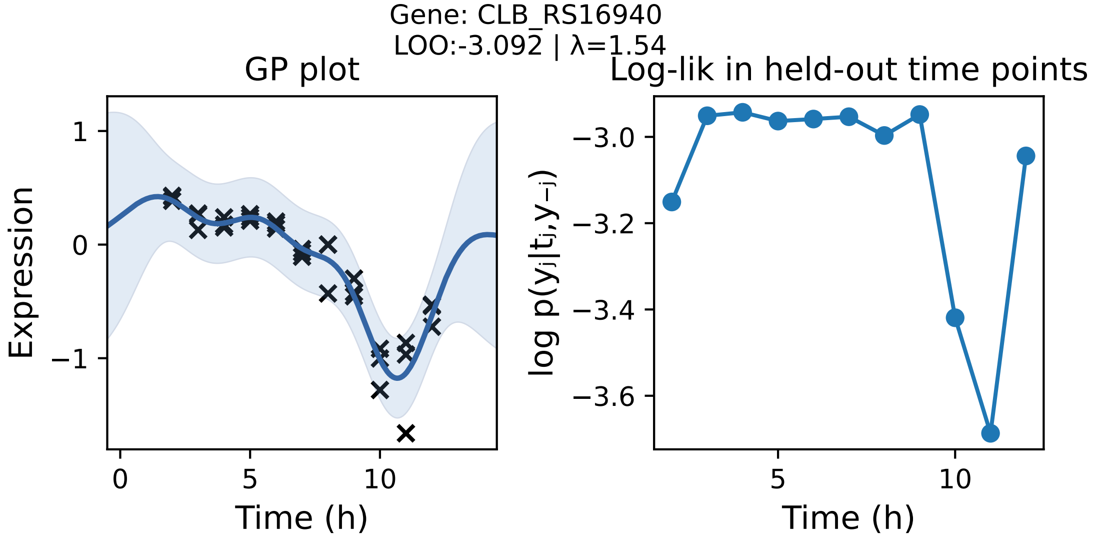

Gene: CLB_RS16940
LOO:-3.092 | λ=1.54
GP plot
Expression
Time (h)
Log-lik in held-out time points
log p(y_j|t_j,y_−j)
Time (h)

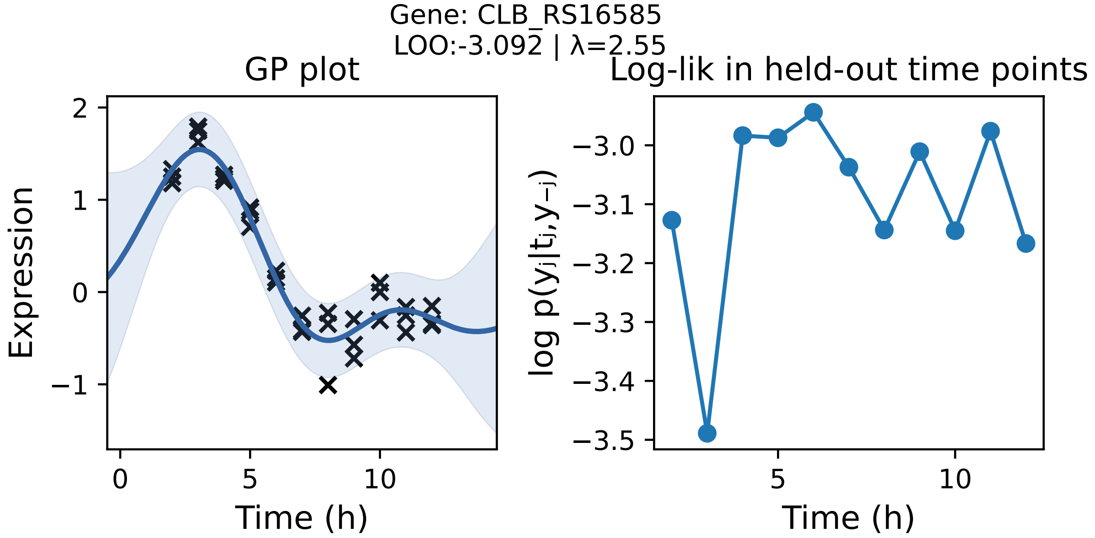

Gene: CLB_RS16585
LOO:-3.092 | λ=2.55
GP plot
Expression
Time (h)
Log-lik in held-out time points
log p(y_j|t_j,y_−j)
Time (h)

Gene: CLB_RS16440
LOO:-3.09 | λ=6.57

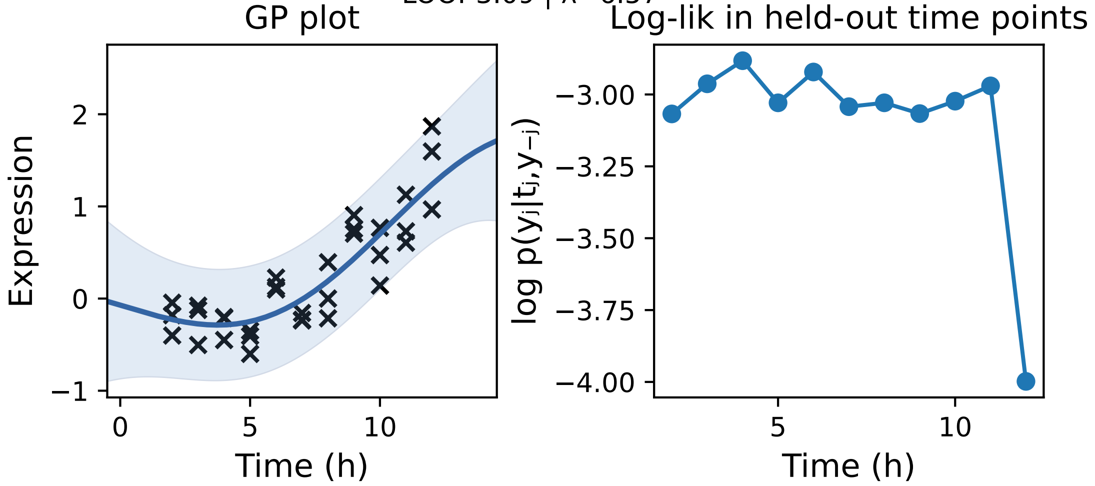

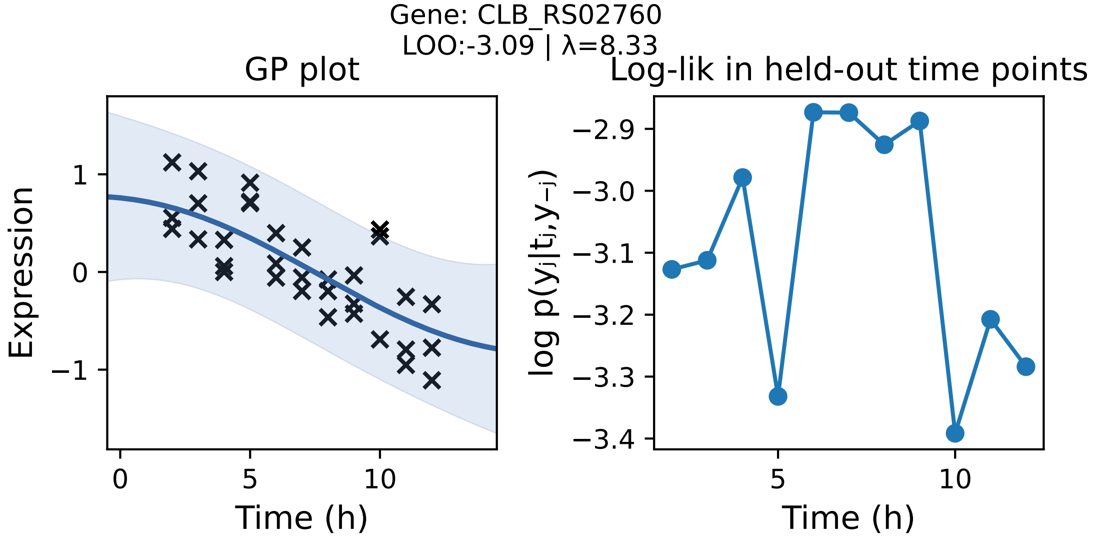

Gene: CLB_RS02760
LOO:-3.09 | λ=8.33
GP plot
Log-lik in held-out time points
Expression
1
0
−1
0
5
10
Time (h)
log p(y_j|t_j,y_−j)
−2.9
−3.0
−3.1
−3.2
−3.3
−3.4
5
10
Time (h)

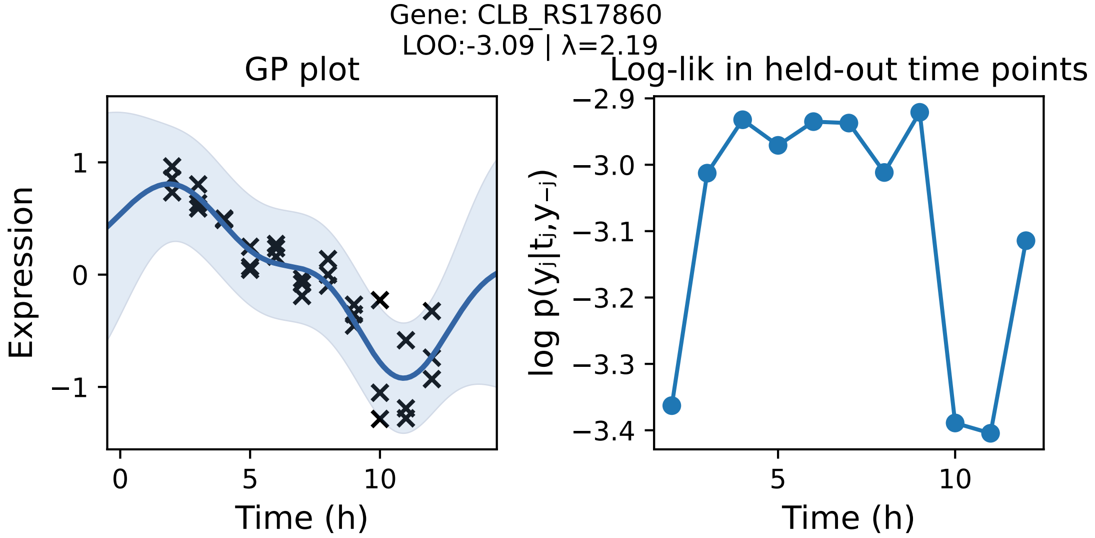
Gene: CLB_RS17860
LOO:-3.09 | λ=2.19
GP plot
Log-lik in held-out time points
Expression
Time (h)
log p(y_j|t_j,y_−j)
Time (h)
1
0
−1
0
5
10
−2.9
−3.0
−3.1
−3.2
−3.3
−3.4
5
10

Gene: CLB_RS05740
LOO:-3.089 | λ=1.8

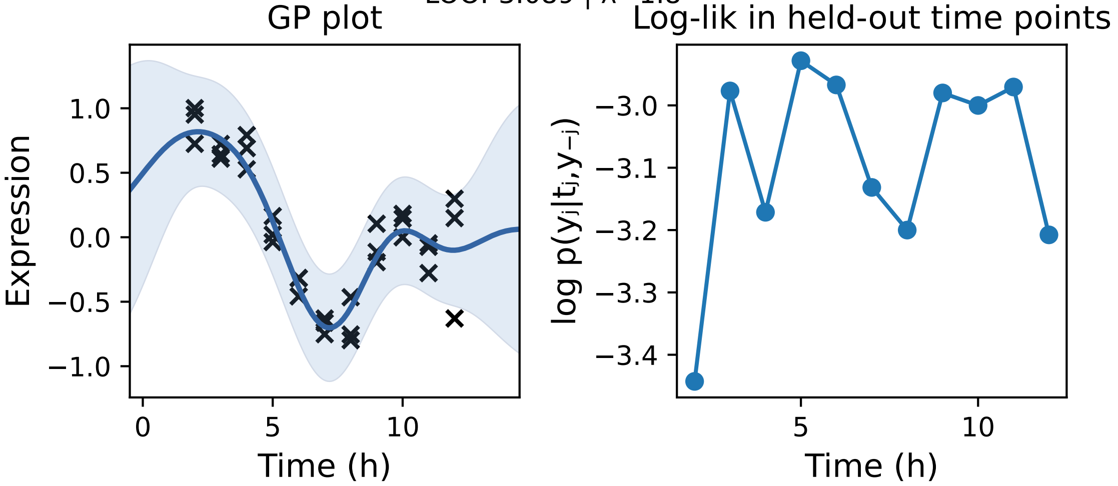

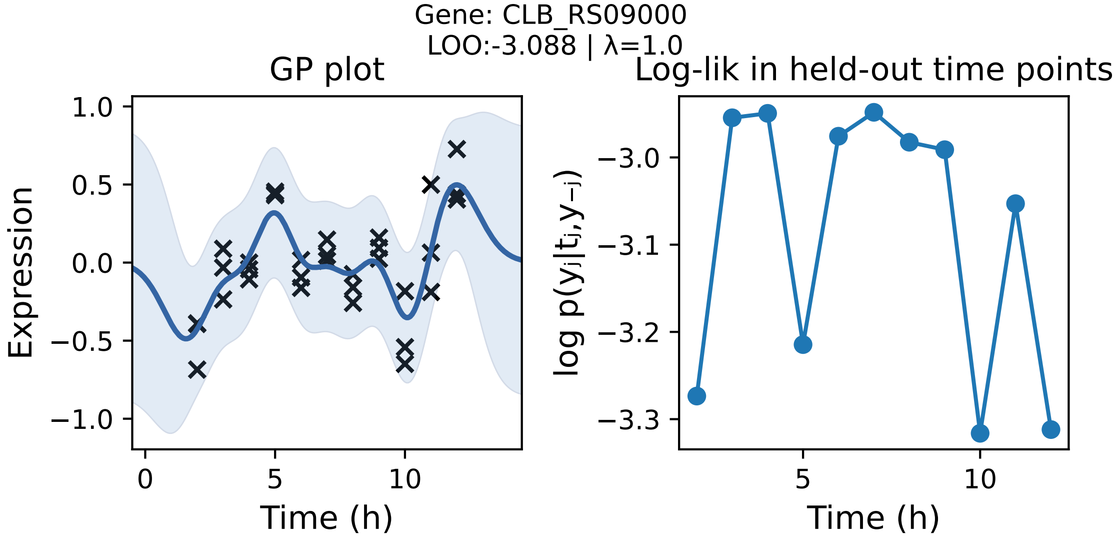
Gene: CLB_RS09000
LOO:-3.088 | λ=1.0
GP plot
Expression
1.0
0.5
0.0
−0.5
−1.0
0
5
10
Time (h)
Log-lik in held-out time points
log p(y_j|t_j,y_−j)
−3.0
−3.1
−3.2
−3.3
5
10
Time (h)

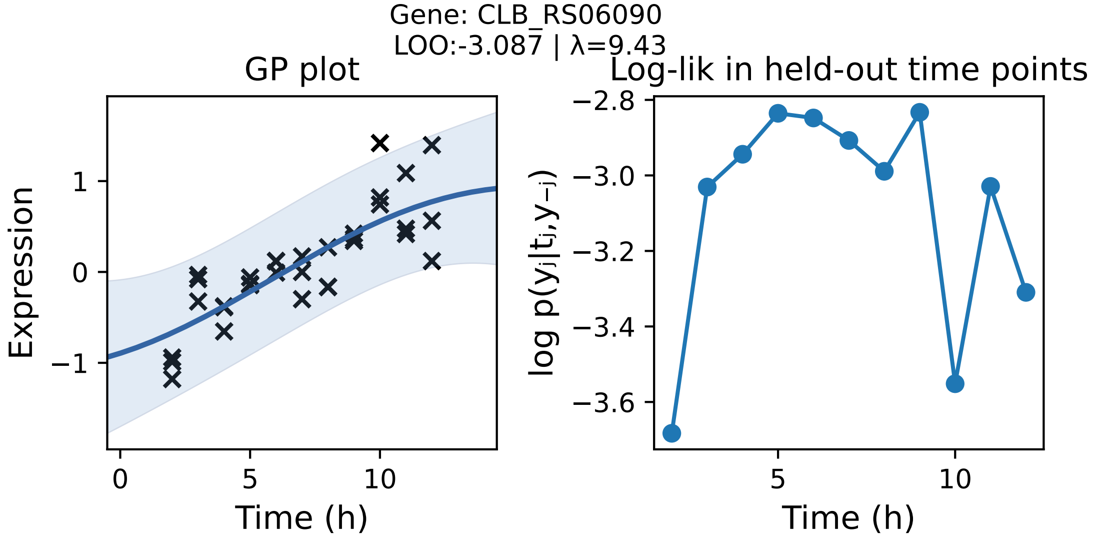

Gene: CLB_RS06090
LOO:-3.087 | λ=9.43
GP plot
Expression
Time (h)
1
0
−1
0
5
10
Log-lik in held-out time points
log p(yj|tj,y−j)
Time (h)
−2.8
−3.0
−3.2
−3.4
−3.6
5
10

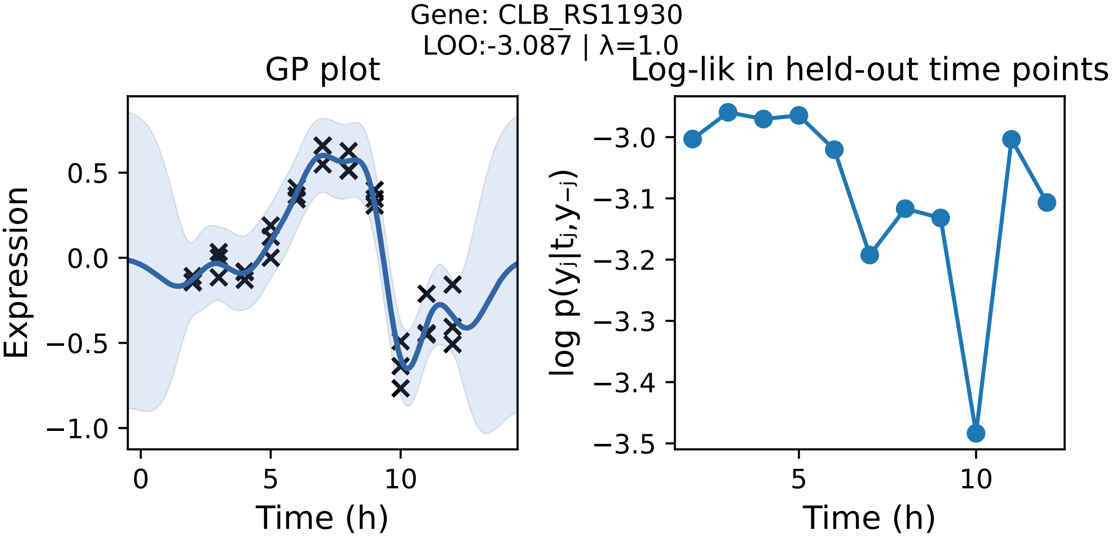

Gene: CLB_RS11930
LOO:-3.087 | λ=1.0
GP plot
Expression
Time (h)
Log-lik in held-out time points
log p(y_j|t_j,y_−j)
Time (h)

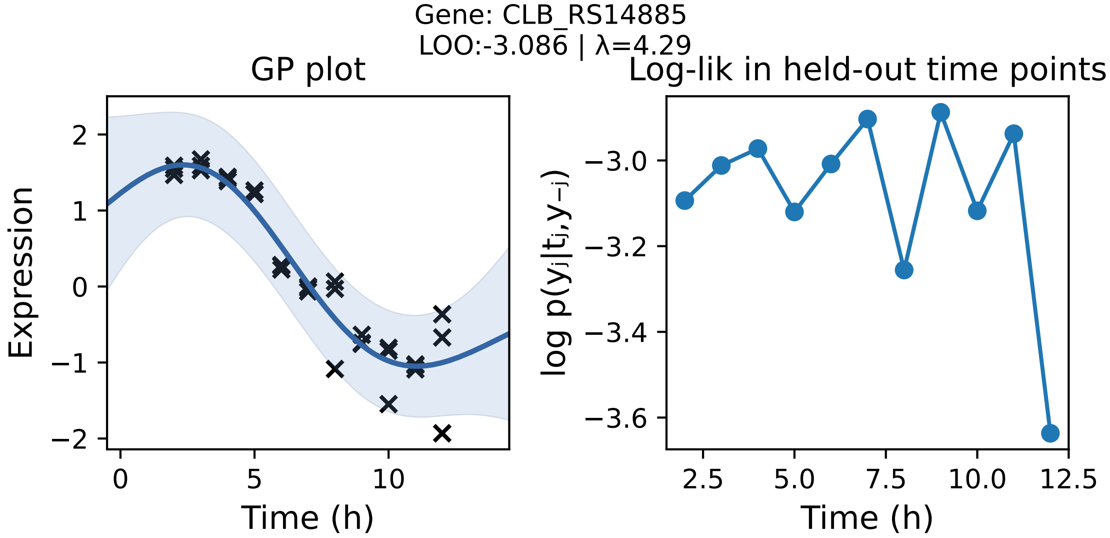

Gene: CLB_RS14885
LOO:-3.086 | λ=4.29
GP plot
Expression
Time (h)
2
1
0
−1
−2
0
5
10
Log-lik in held-out time points
log p(y_j|t_j,y_−j)
Time (h)
−3.0
−3.2
−3.4
−3.6
2.5
5.0
7.5
10.0
12.5

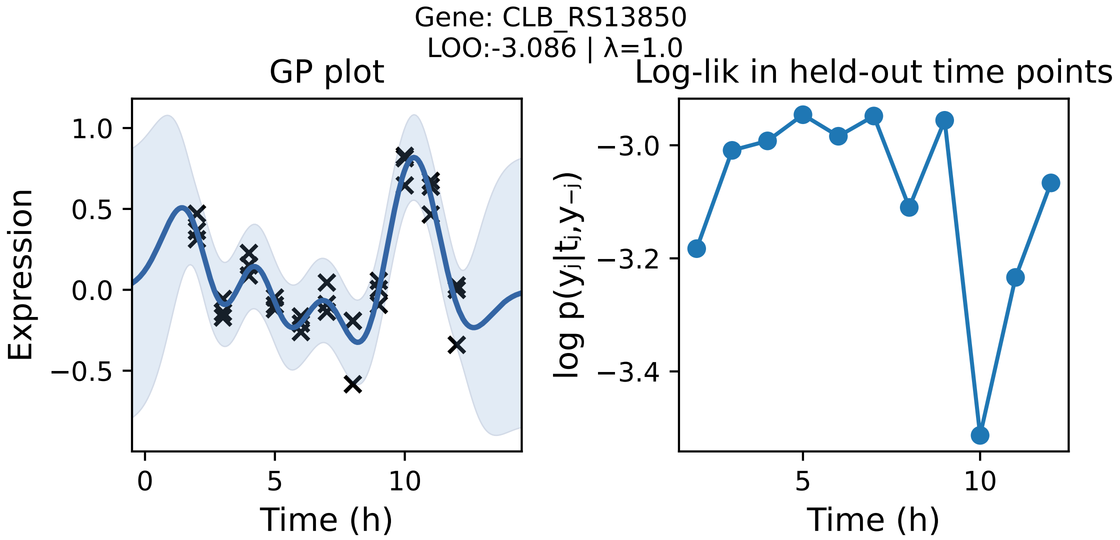

Gene: CLB_RS13850
LOO:-3.086 | λ=1.0
GP plot
Expression
Time (h)
1.0
0.5
0.0
−0.5
0
5
10
Log-lik in held-out time points
log p(y_j|t_j,y_−j)
Time (h)
−3.0
−3.2
−3.4
5
10

Gene: CLB_RS05485
LOO:-3.085 | λ=1.0

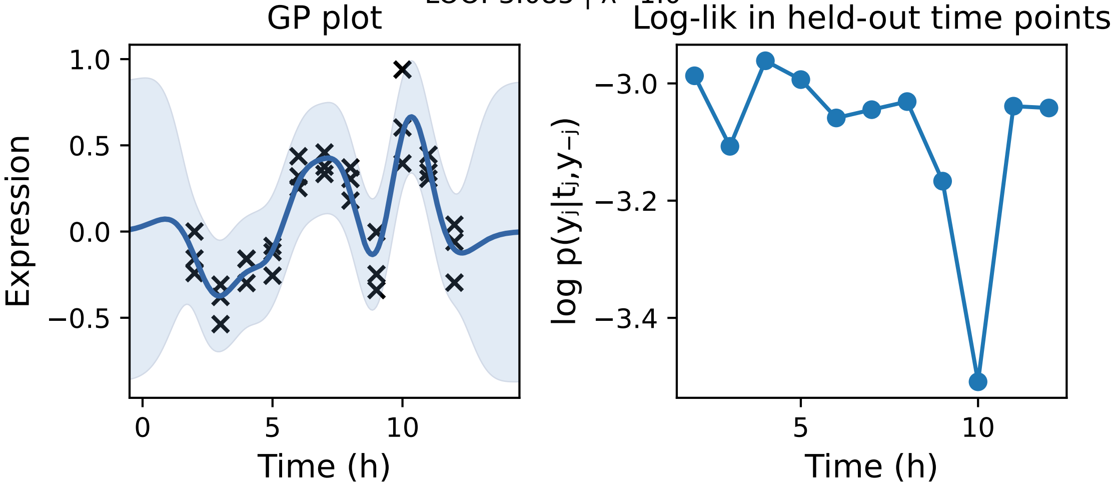

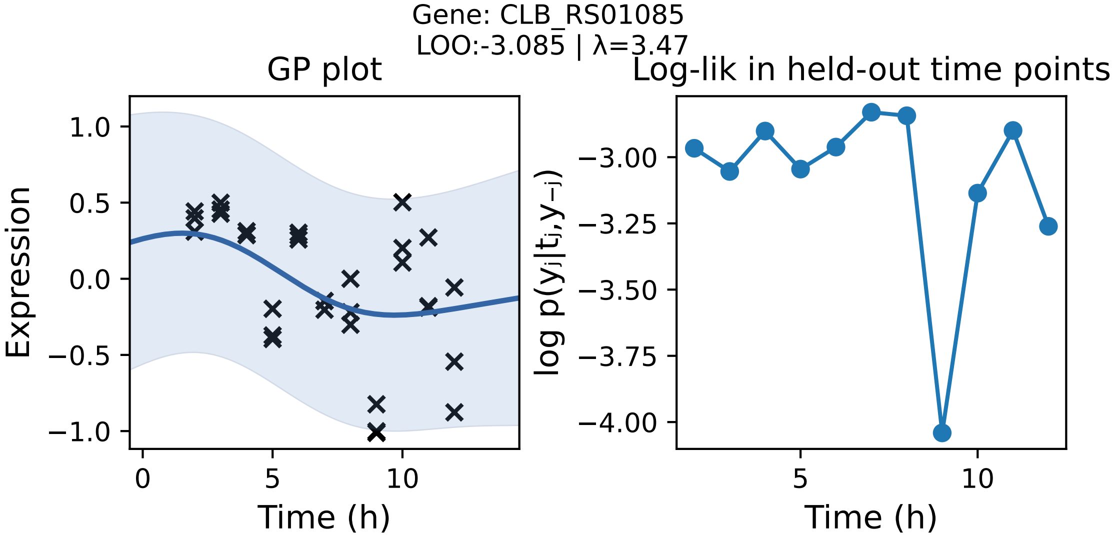

Gene: CLB_RS01085
LOO:-3.085 | λ=3.47
GP plot
Expression
1.0
0.5
0.0
−0.5
−1.0
0
5
10
Time (h)
Log-lik in held-out time points
log p(y_j|t_j,y_−j)
−3.00
−3.25
−3.50
−3.75
−4.00
5
10
Time (h)

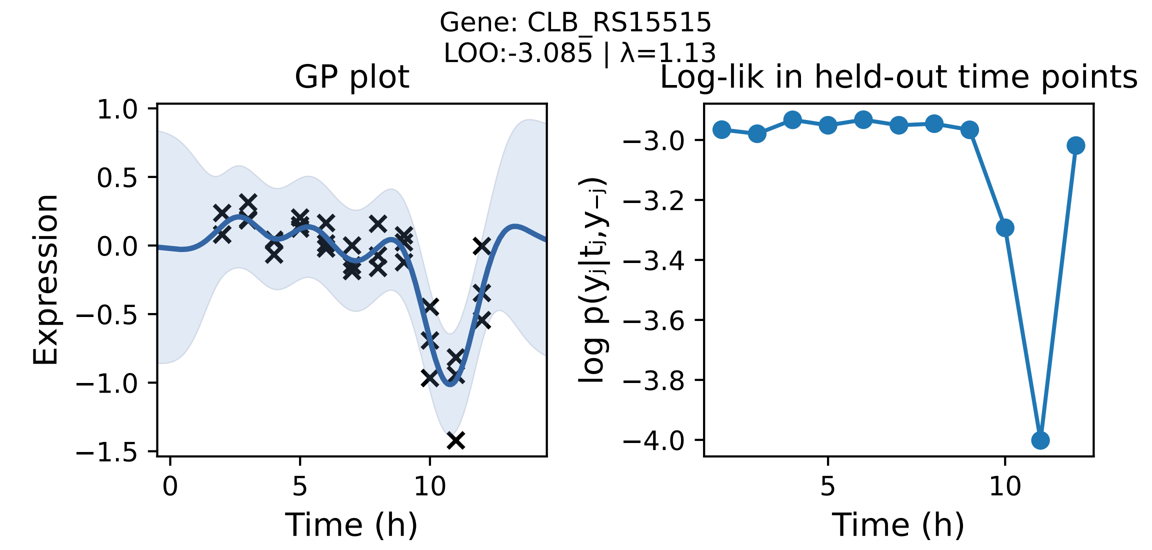

Gene: CLB_RS15515
LOO:-3.085 | λ=1.13
GP plot
Expression
1.0
0.5
0.0
−0.5
−1.0
−1.5
0
5
10
Time (h)
Log-lik in held-out time points
log p(y_j|t_j,y_−j)
−3.0
−3.2
−3.4
−3.6
−3.8
−4.0
5
10
Time (h)

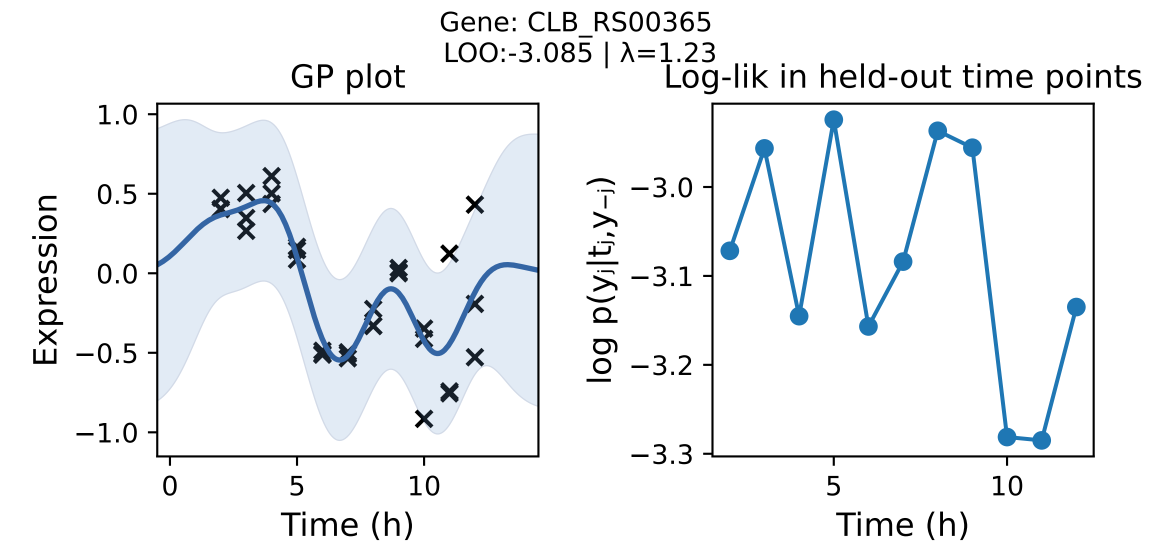

Gene: CLB_RS00365
LOO:-3.085 | λ=1.23
GP plot
Expression
1.0
0.5
0.0
−0.5
−1.0
0
5
10
Time (h)
Log-lik in held-out time points
log p(y_j|t_j,y_−j)
−3.0
−3.1
−3.2
−3.3
5
10
Time (h)

Gene: CLB_RS02810
LOO:-3.085 | λ=7.44

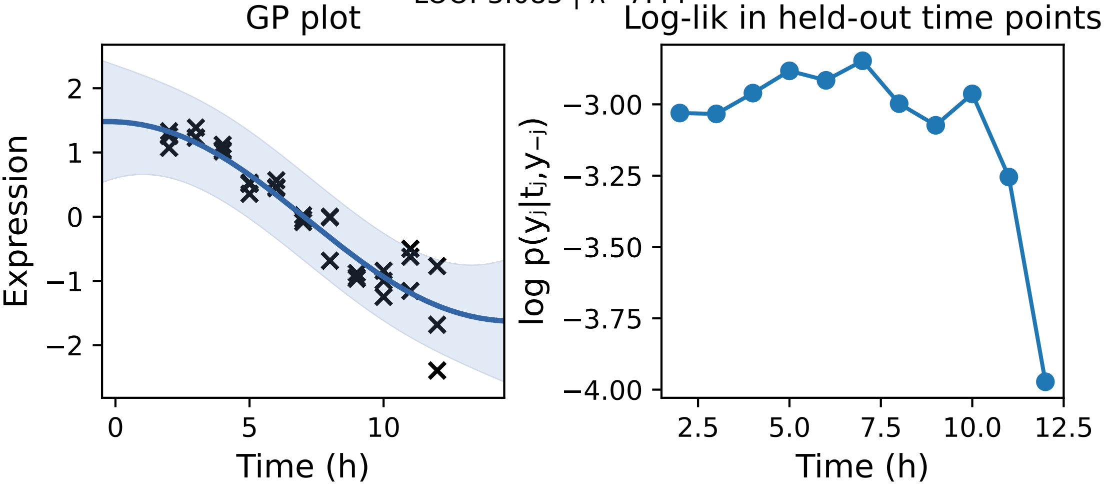

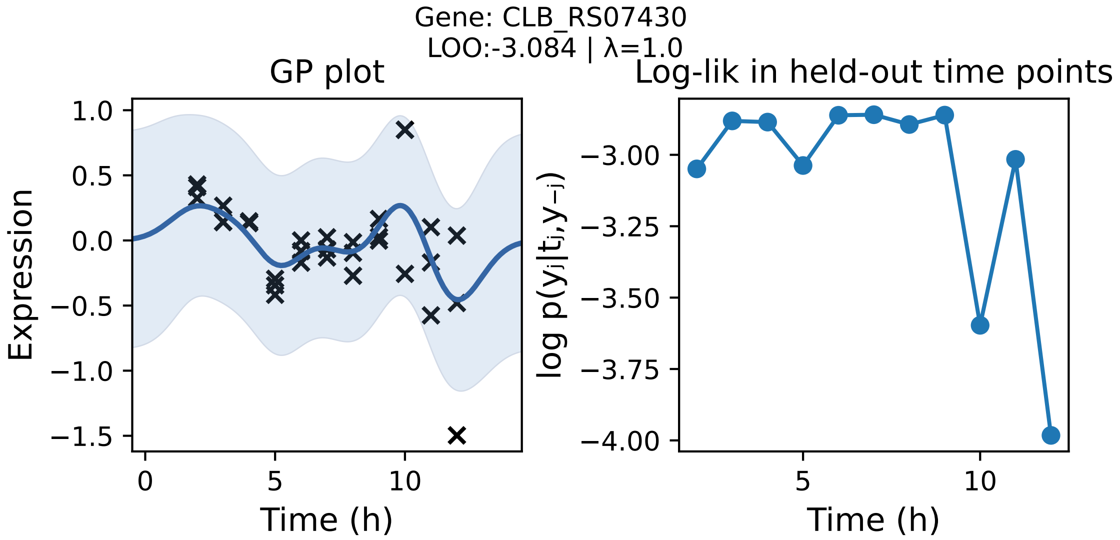

Gene: CLB_RS07430
LOO:-3.084 | λ=1.0
GP plot
Log-lik in held-out time points
Expression
1.0
0.5
0.0
−0.5
−1.0
−1.5
0
5
10
Time (h)
log p(y_j|t_j,y_−j)
−3.00
−3.25
−3.50
−3.75
−4.00
5
10
Time (h)

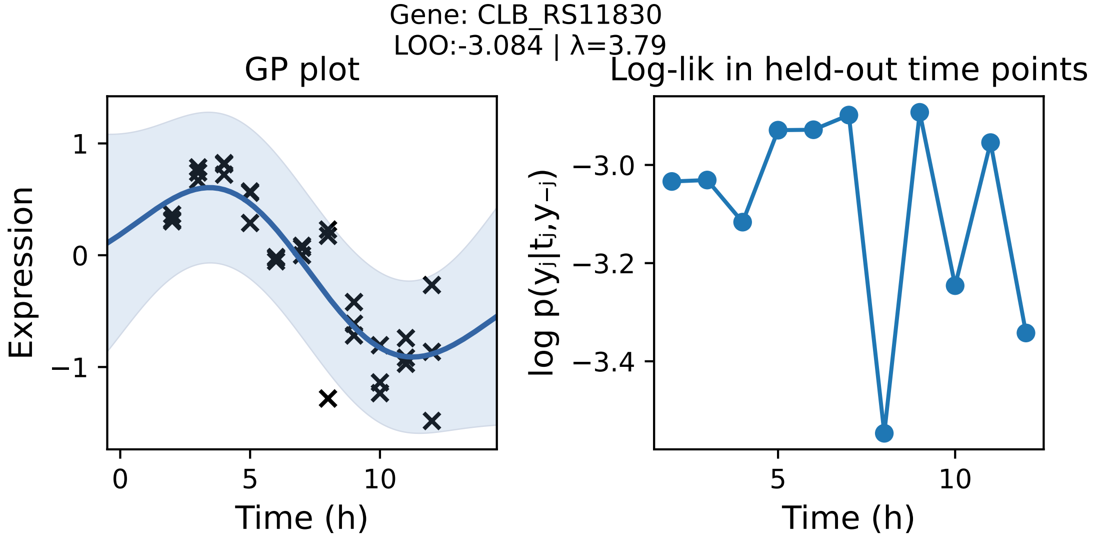

Gene: CLB_RS11830
LOO:-3.084 | λ=3.79
GP plot
Expression
1
0
−1
0
5
10
Time (h)
Log-lik in held-out time points
log p(yj|tj,y−j)
−3.0
−3.2
−3.4
5
10
Time (h)

Gene: CLB_RS07090
LOO:-3.083 | λ=4.27

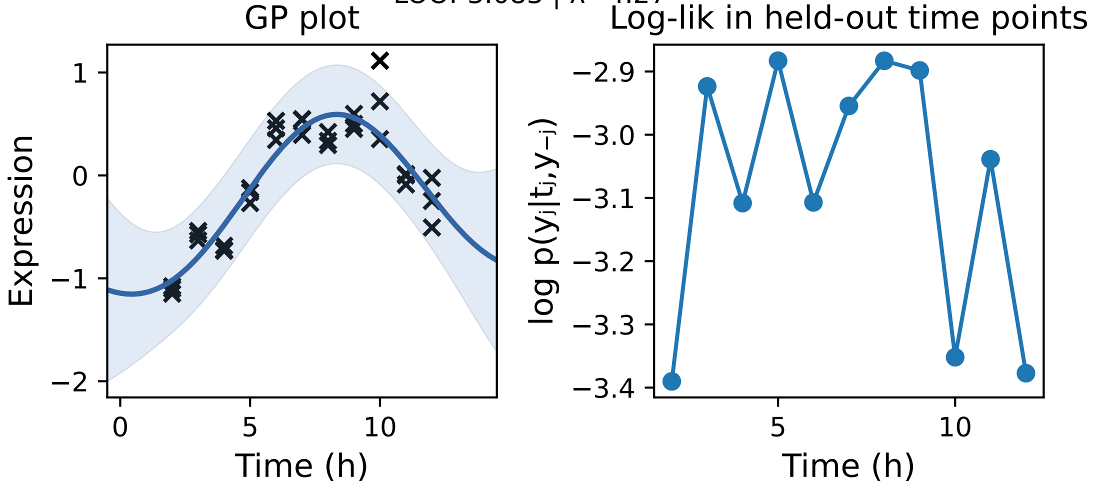

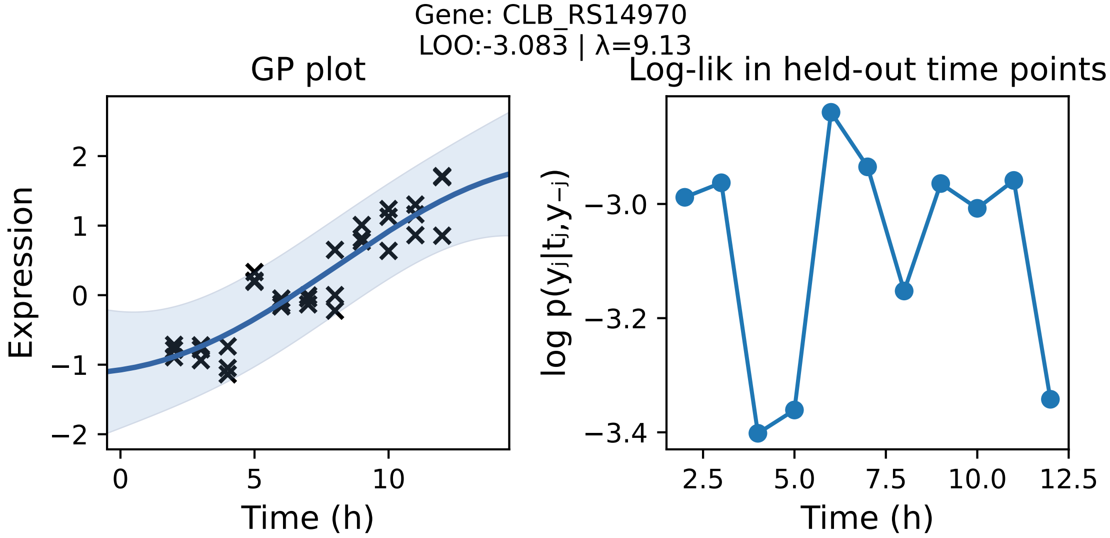

Gene: CLB_RS14970
LOO:-3.083 | λ=9.13
GP plot
Expression
Time (h)
Log-lik in held-out time points
log p(y_j|t_j,y_−j)
Time (h)

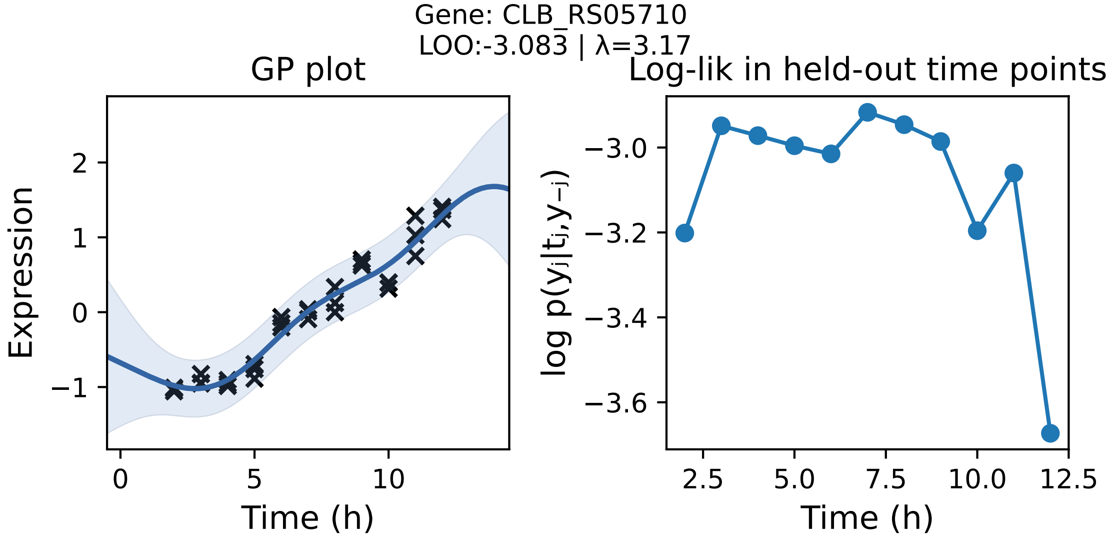

Gene: CLB_RS05710
LOO:-3.083 | λ=3.17
GP plot
Expression
Time (h)
Log-lik in held-out time points
log p(y_j|t_j,y_−j)
Time (h)

Gene: CLB_RS05450
LOO:-3.083 | λ=1.0

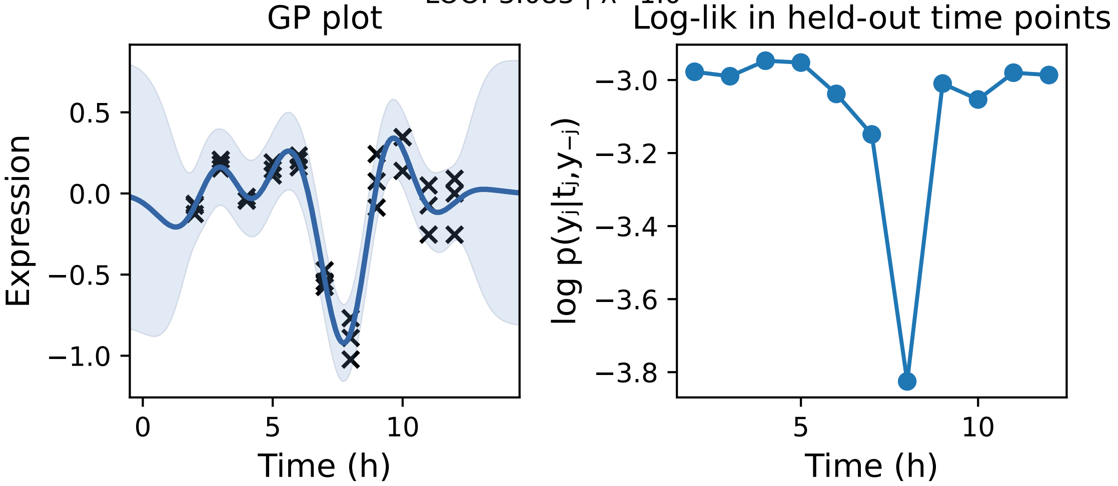

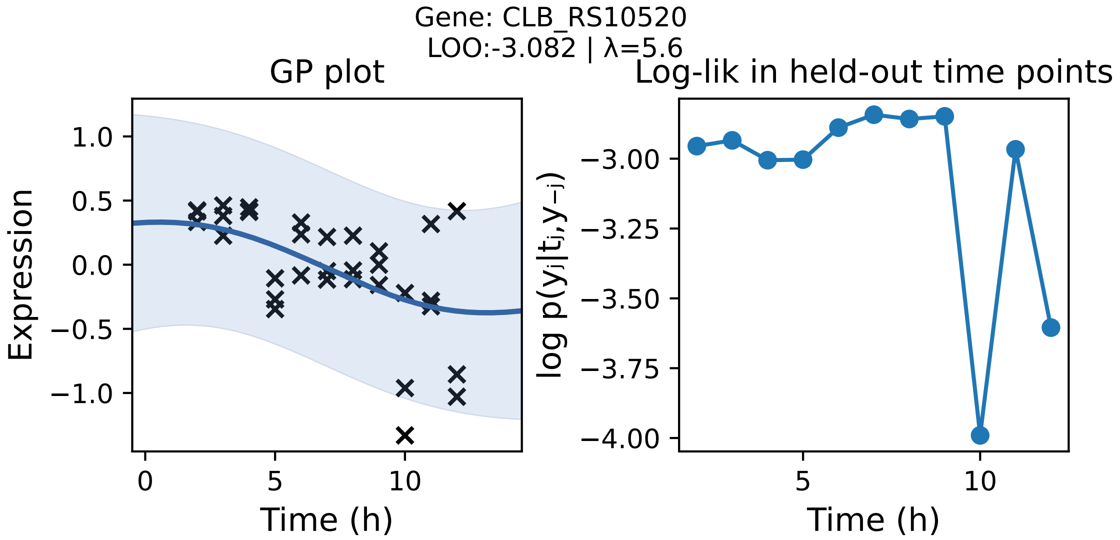

Gene: CLB_RS10520
LOO:-3.082 | λ=5.6
GP plot
Expression
1.0
0.5
0.0
−0.5
−1.0
0
5
10
Time (h)
Log-lik in held-out time points
log p(y_j|t_j,y_−j)
−3.00
−3.25
−3.50
−3.75
−4.00
5
10
Time (h)

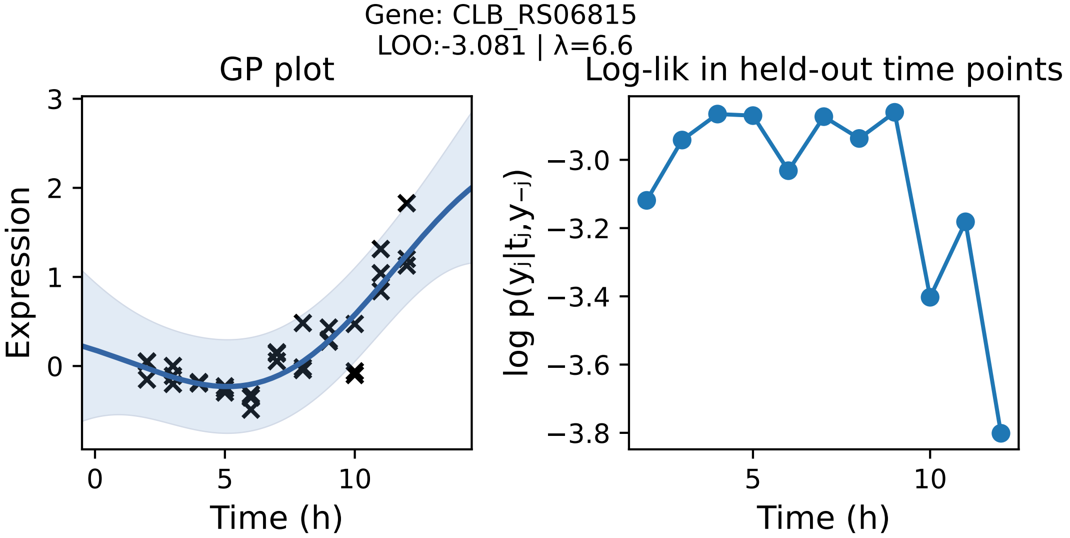

Gene: CLB_RS06815
LOO:-3.081 | λ=6.6
GP plot
Expression
Time (h)
Log-lik in held-out time points
log p(y_j|t_j,y_−j)
Time (h)

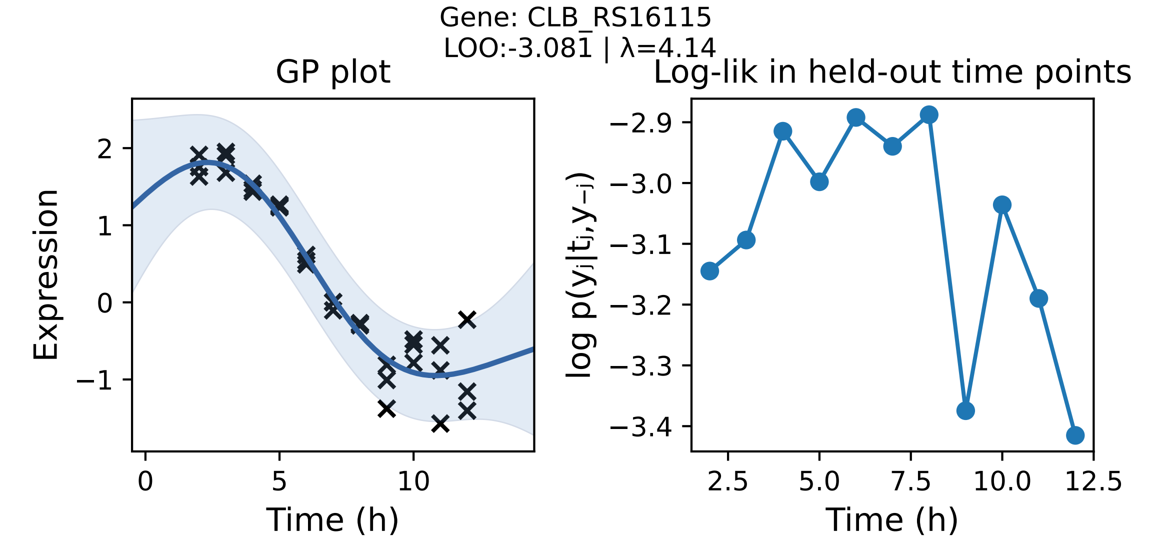

Gene: CLB_RS16115
LOO:-3.081 | λ=4.14
GP plot
Expression
Time (h)
Log-lik in held-out time points
log p(yj|tj,y−j)
Time (h)

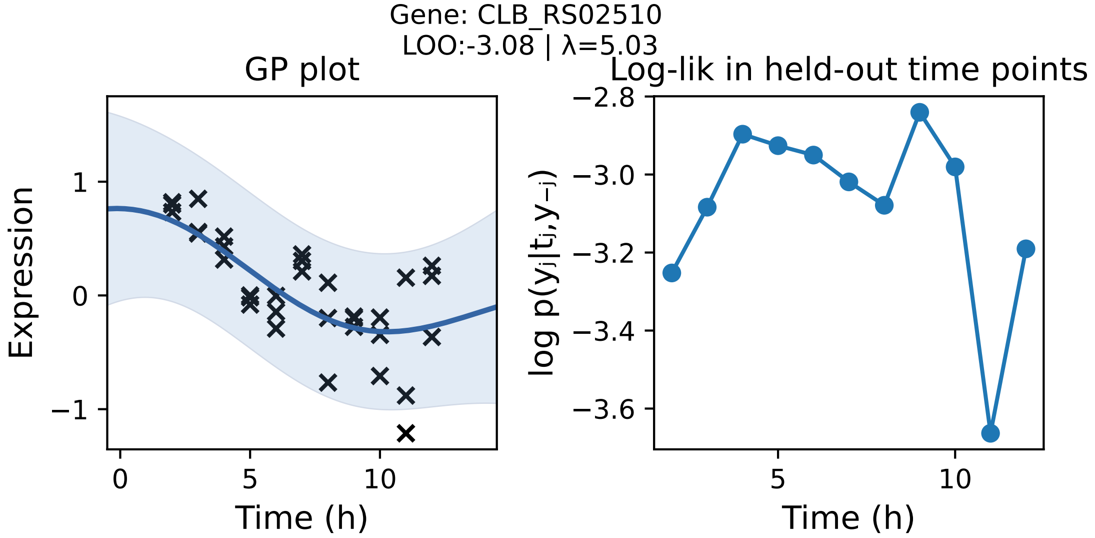
Gene: CLB_RS02510
LOO:-3.08 | λ=5.03
GP plot
Expression
Time (h)
Log-lik in held-out time points
log p(y_j|t_j,y_−j)
Time (h)
1
0
−1
0
5
10
−2.8
−3.0
−3.2
−3.4
−3.6
5
10

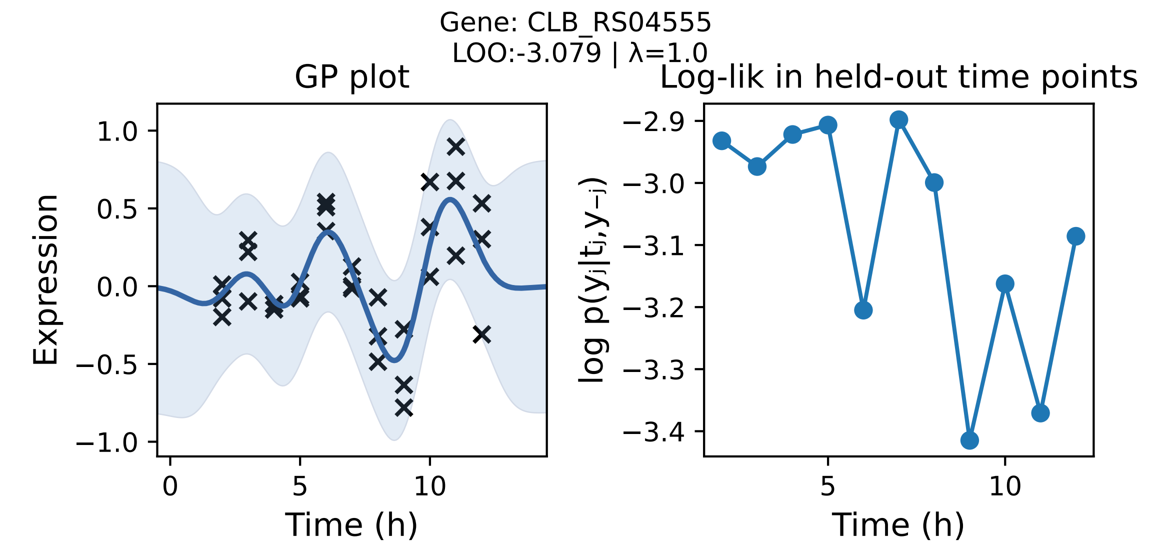

Gene: CLB_RS04555
LOO:-3.079 | λ=1.0
GP plot
Expression
1.0
0.5
0.0
−0.5
−1.0
0
5
10
Time (h)
Log-lik in held-out time points
log p(y_j|t_j,y_−j)
−2.9
−3.0
−3.1
−3.2
−3.3
−3.4
5
10
Time (h)

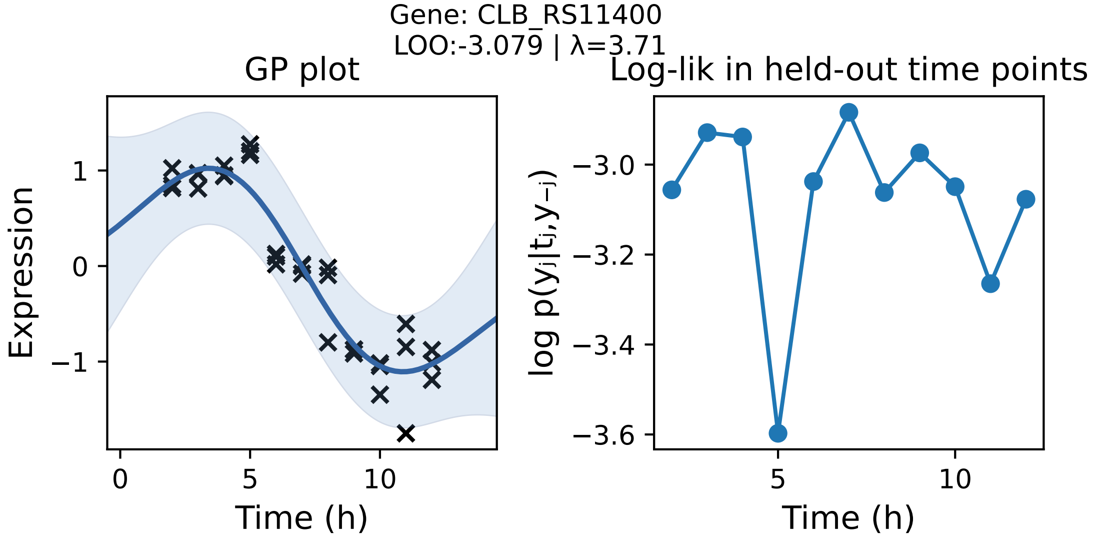

Gene: CLB_RS11400
LOO:-3.079 | λ=3.71
GP plot
Expression
1
0
−1
0
5
10
Time (h)
Log-lik in held-out time points
log p(y_j|t_j,y_−j)
−3.0
−3.2
−3.4
−3.6
5
10
Time (h)

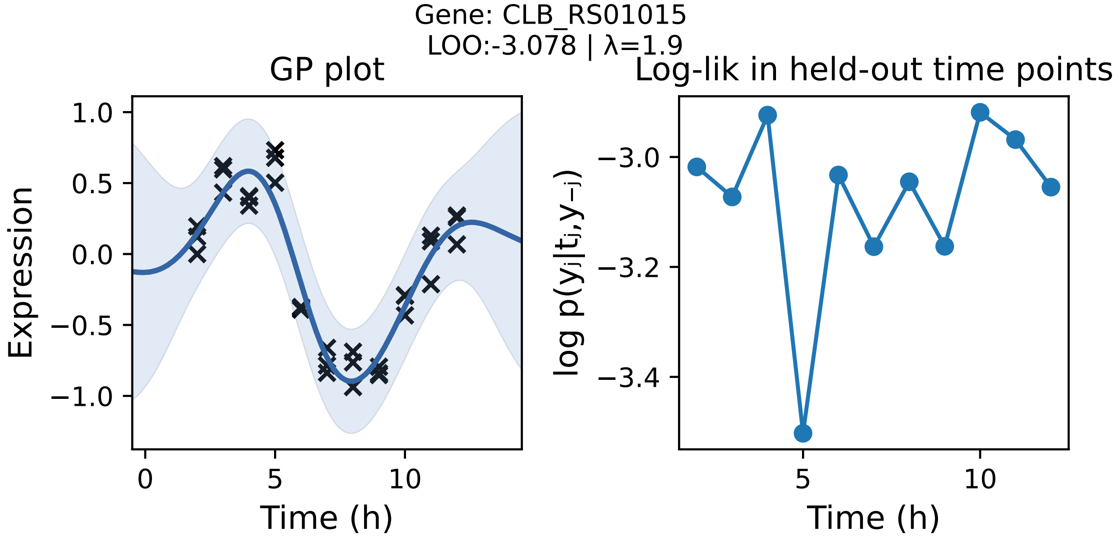
Gene: CLB_RS01015
LOO:-3.078 | λ=1.9
GP plot
Expression
Time (h)
Log-lik in held-out time points
log p(y_j|t_j,y_−j)
Time (h)

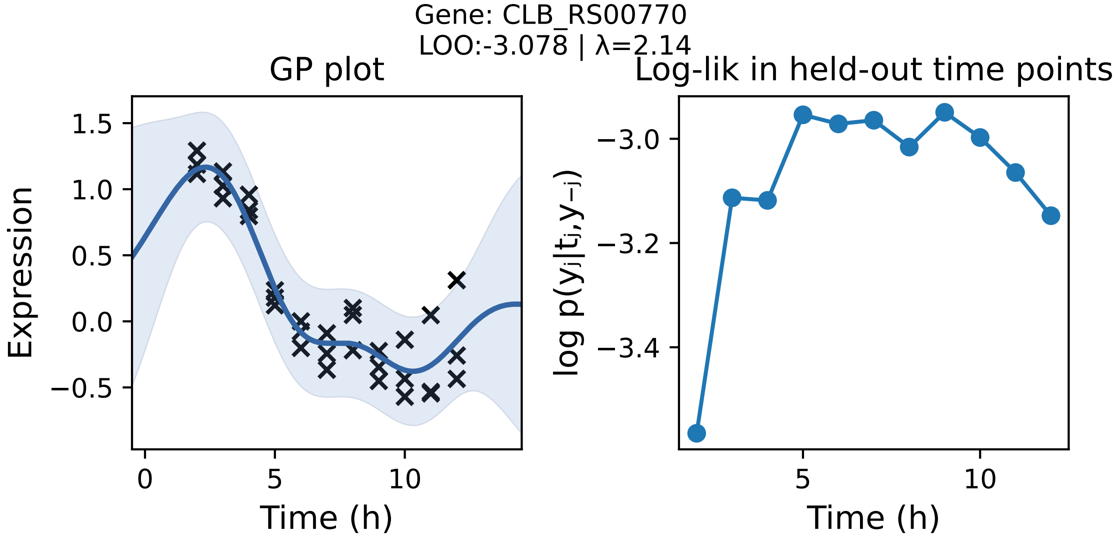

Gene: CLB_RS00770
LOO:-3.078 | λ=2.14
GP plot
Expression
1.5
1.0
0.5
0.0
−0.5
0
5
10
Time (h)
Log-lik in held-out time points
log p(y_j|t_j,y_{−j})
−3.0
−3.2
−3.4
5
10
Time (h)

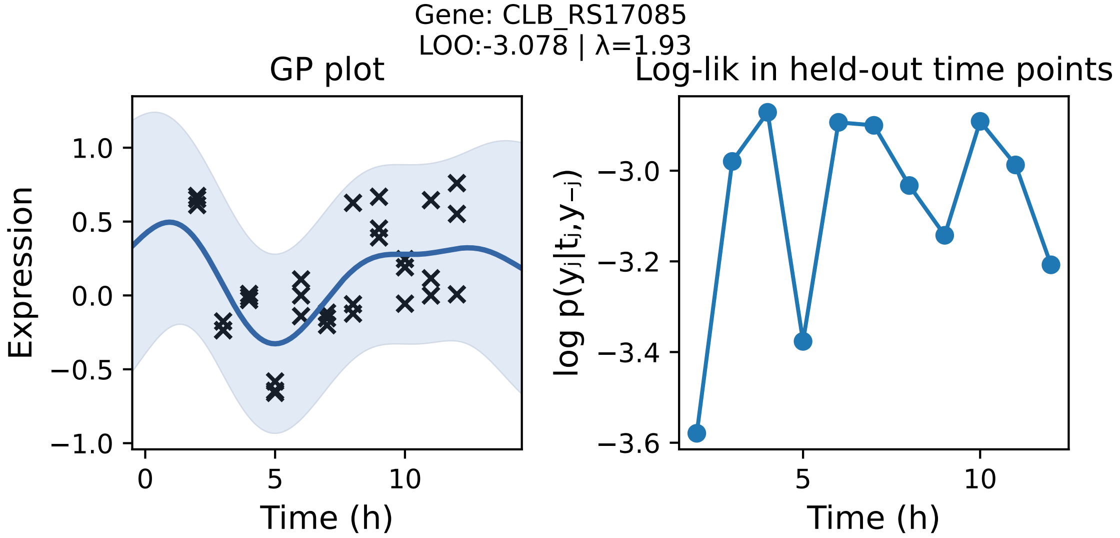

Gene: CLB_RS17085
LOO:-3.078 | λ=1.93
GP plot
Expression
1.0
0.5
0.0
−0.5
−1.0
0
5
10
Time (h)
Log-lik in held-out time points
log p(y_j|t_j,y_−j)
−3.0
−3.2
−3.4
−3.6
5
10
Time (h)

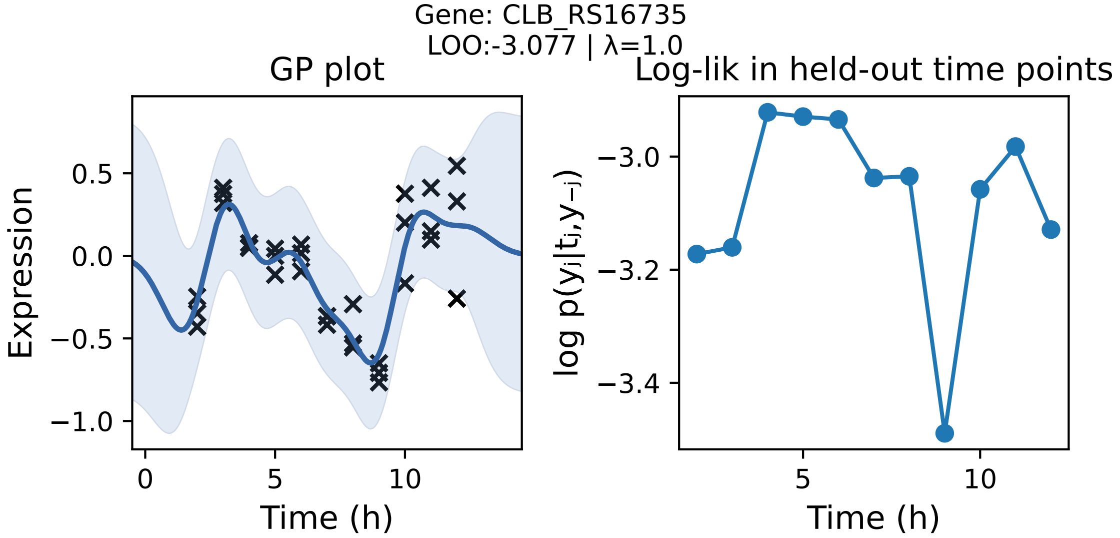

Gene: CLB_RS16735
LOO:-3.077 | λ=1.0
GP plot
Expression
Time (h)
0.5
0.0
−0.5
−1.0
0
5
10
Log-lik in held-out time points
log p(y_j|t_j,y_−j)
Time (h)
−3.0
−3.2
−3.4
5
10

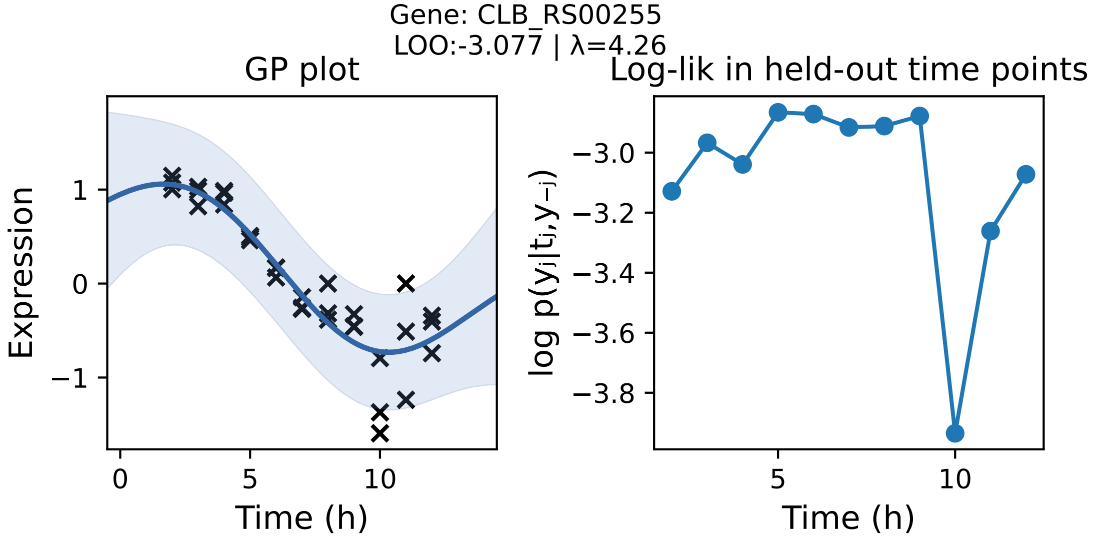

Gene: CLB_RS00255
LOO:-3.077 | λ=4.26
GP plot
Log-lik in held-out time points
Expression
1
0
−1
0
5
10
Time (h)
log p(y_j|t_j,y_−j)
−3.0
−3.2
−3.4
−3.6
−3.8
5
10
Time (h)

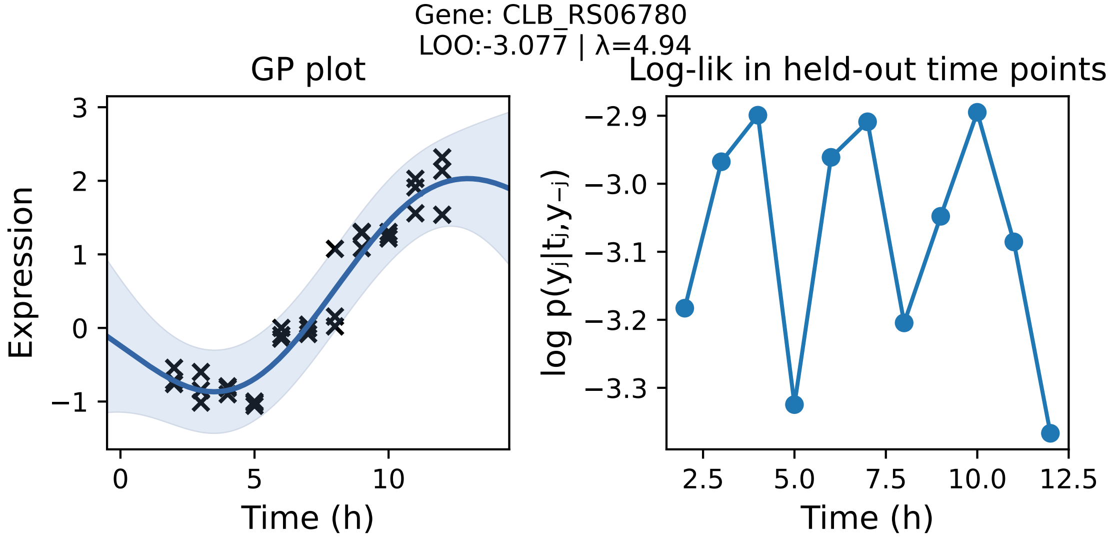

Gene: CLB_RS06780
LOO:-3.077 | λ=4.94
GP plot
Expression
Time (h)
Log-lik in held-out time points
log p(y_j|t_j,y_−j)
Time (h)

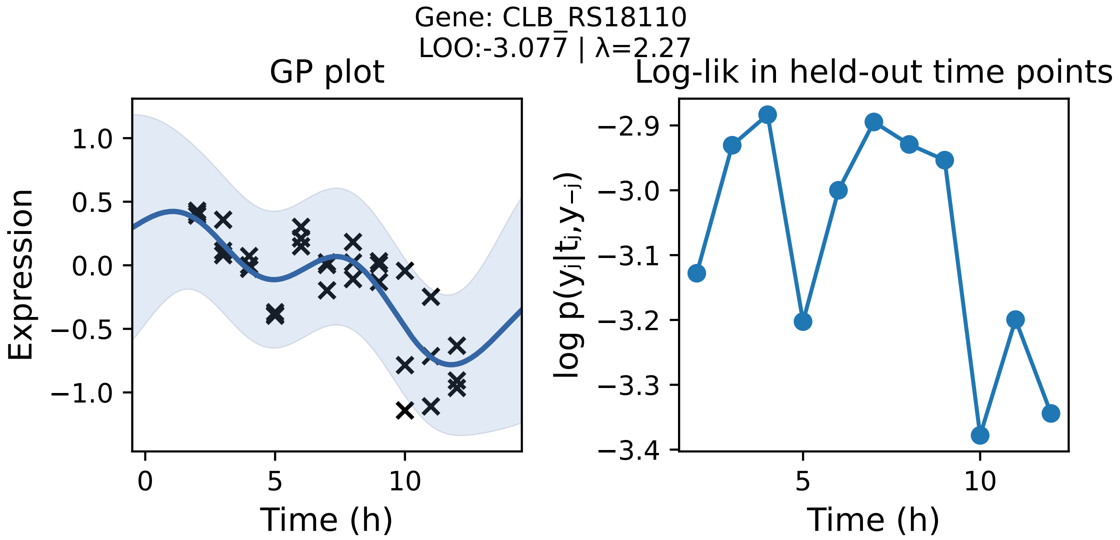

Gene: CLB_RS18110
LOO:-3.077 | λ=2.27
GP plot
Expression
1.0
0.5
0.0
−0.5
−1.0
0
5
10
Time (h)
Log-lik in held-out time points
log p(y_j|t_j,y_−j)
−2.9
−3.0
−3.1
−3.2
−3.3
−3.4
5
10
Time (h)

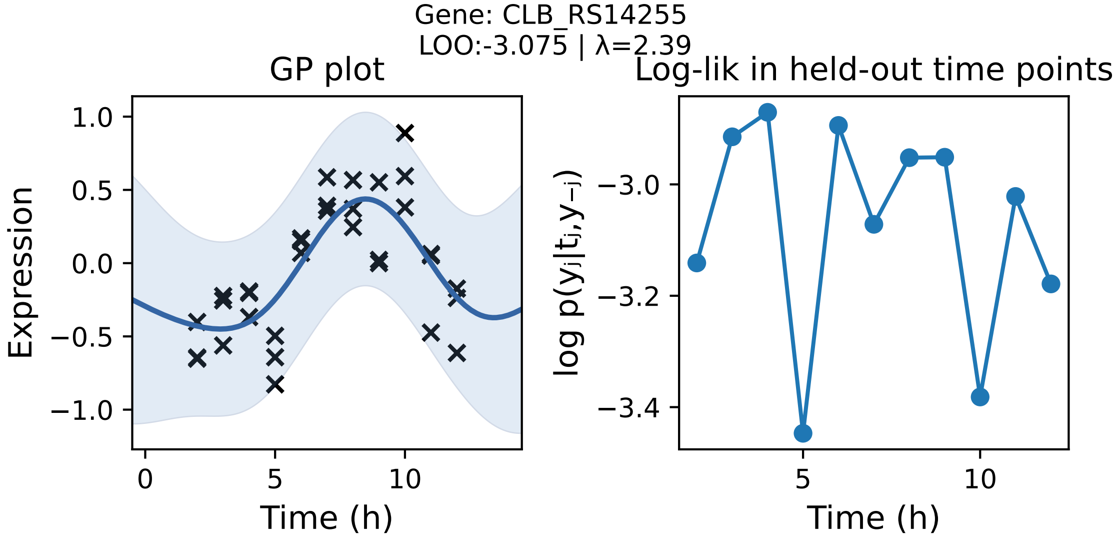

Gene: CLB_RS14255
LOO:-3.075 | λ=2.39
GP plot
Expression
Time (h)
1.0
0.5
0.0
−0.5
−1.0
0
5
10
Log-lik in held-out time points
log p(y_j|t_j,y_{−j})
Time (h)
−3.0
−3.2
−3.4
5
10

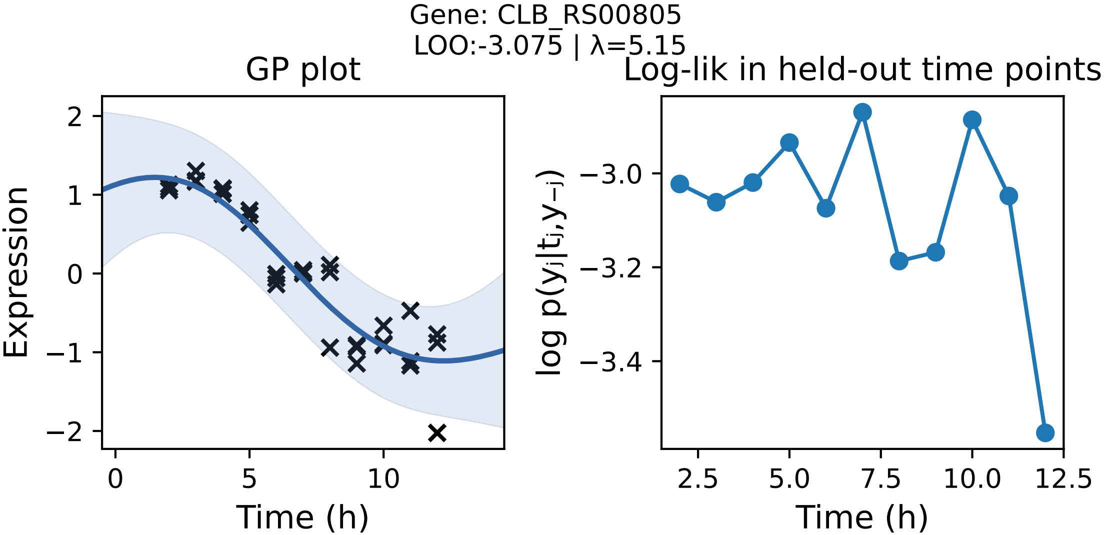

Gene: CLB_RS00805
LOO:-3.075 | λ=5.15
GP plot
Expression
Time (h)
2
1
0
−1
−2
0
5
10
Log-lik in held-out time points
log p(y_j|t_j,y_−j)
Time (h)
−3.0
−3.2
−3.4
2.5
5.0
7.5
10.0
12.5

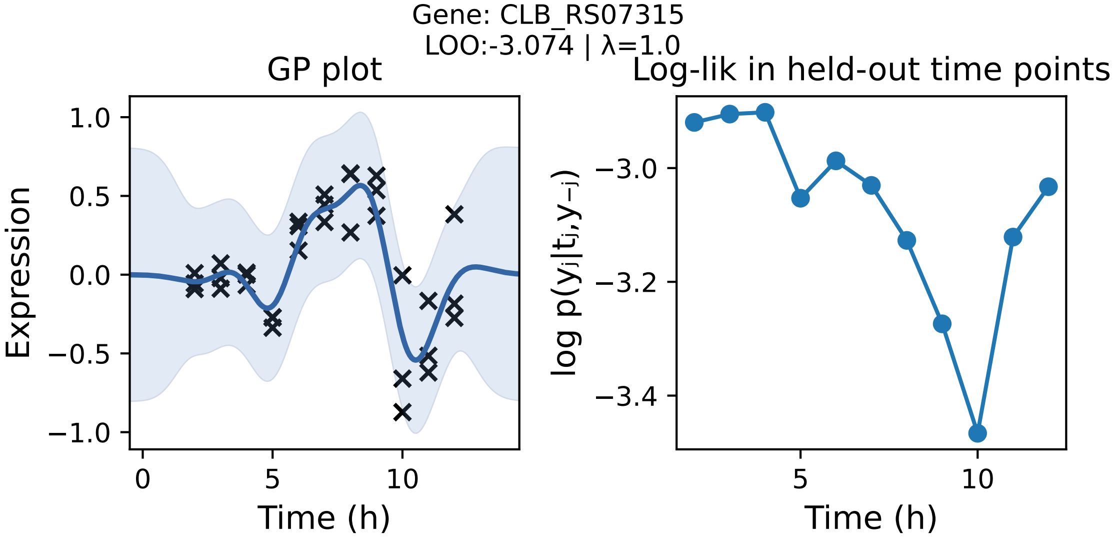

Gene: CLB_RS07315
LOO:-3.074 | λ=1.0
GP plot
Expression
Time (h)
1.0
0.5
0.0
−0.5
−1.0
0
5
10
Log-lik in held-out time points
log p(yj|tj,y−j)
Time (h)
−3.0
−3.2
−3.4
5
10

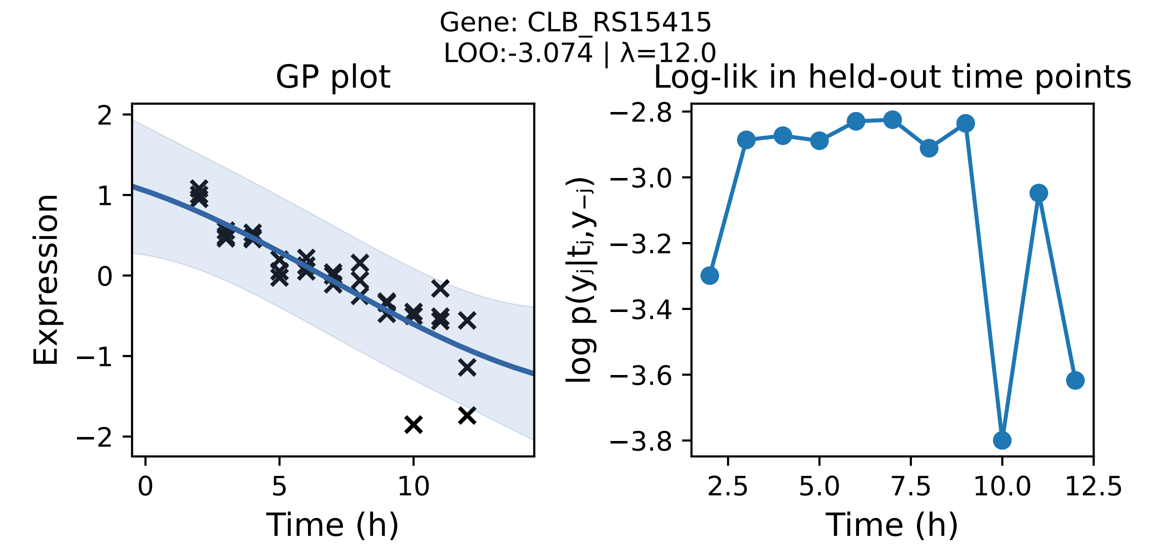
Gene: CLB_RS15415
LOO:-3.074 | λ=12.0
GP plot
Log-lik in held-out time points
Expression
Time (h)
log p(y_j|t_j,y_−j)
Time (h)
2
1
0
−1
−2
0
5
10
−2.8
−3.0
−3.2
−3.4
−3.6
−3.8
2.5
5.0
7.5
10.0
12.5

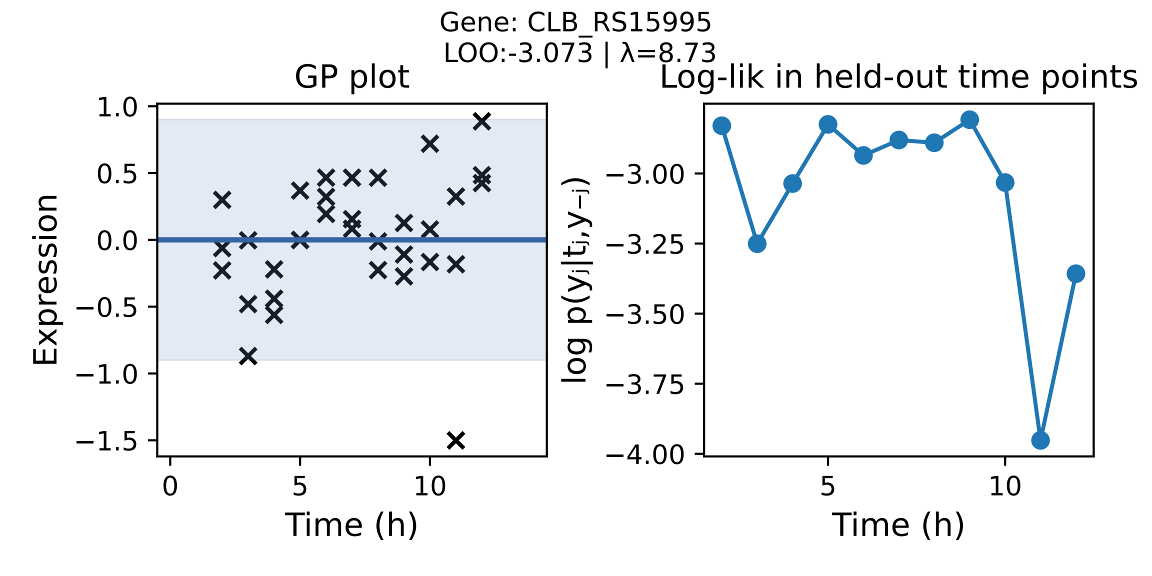
Gene: CLB_RS15995
LOO:-3.073 | λ=8.73
GP plot
Expression
Time (h)
Log-lik in held-out time points
log p(y_j|t_j,y_−j)
Time (h)

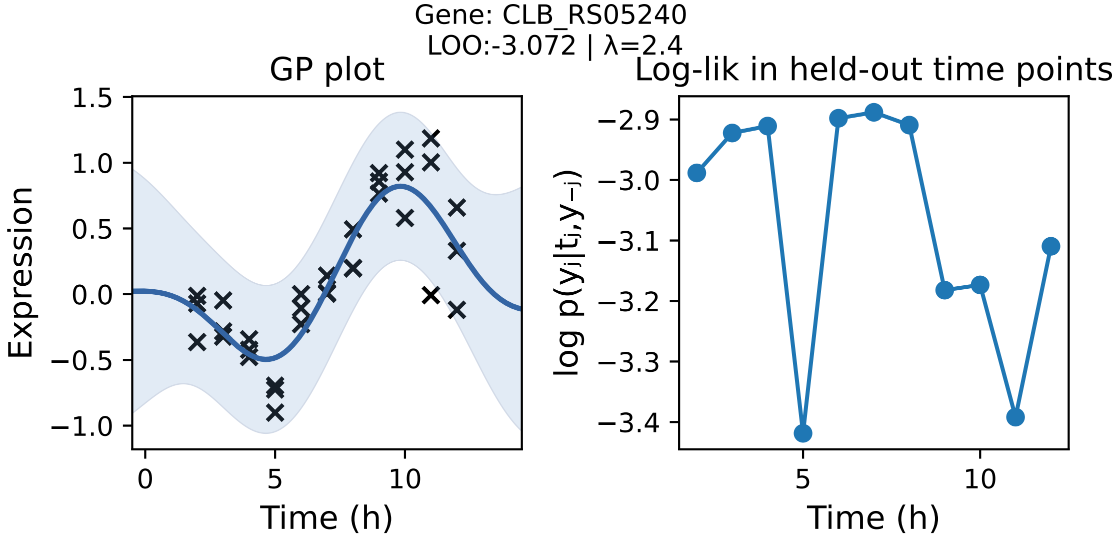
Gene: CLB_RS05240
LOO:-3.072 | λ=2.4
GP plot
Expression
Time (h)
Log-lik in held-out time points
log p(yj|tj,y−j)
Time (h)
1.5
1.0
0.5
0.0
−0.5
−1.0
0
5
10
−2.9
−3.0
−3.1
−3.2
−3.3
−3.4
5
10

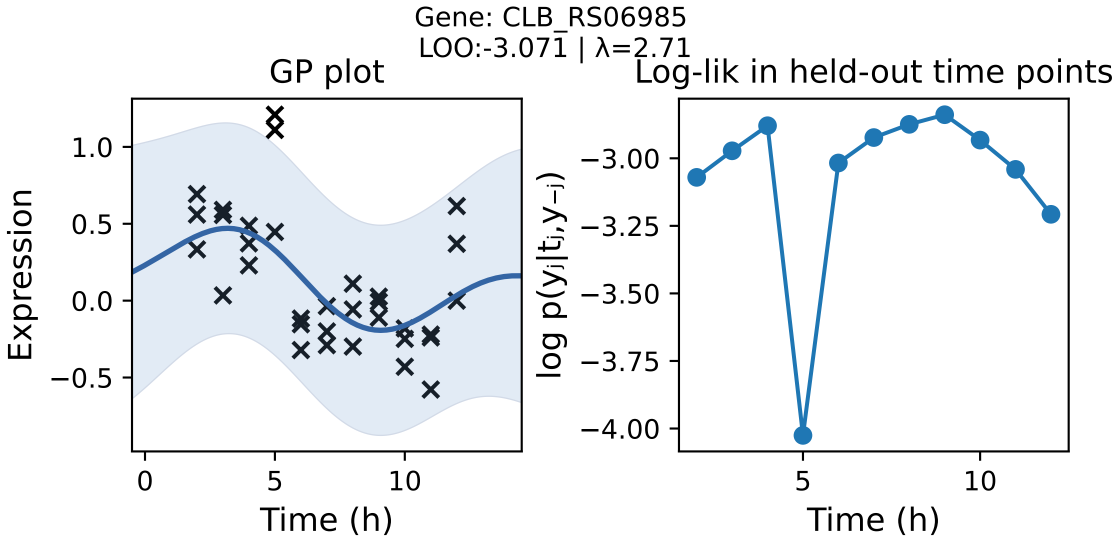

Gene: CLB_RS06985
LOO:-3.071 | λ=2.71
GP plot
Expression
1.0
0.5
0.0
−0.5
0
5
10
Time (h)
Log-lik in held-out time points
log p(y_j|t_j,y_−j)
−3.00
−3.25
−3.50
−3.75
−4.00
5
10
Time (h)

Gene: CLB_RS13680
LOO:-3.071 | λ=4.03

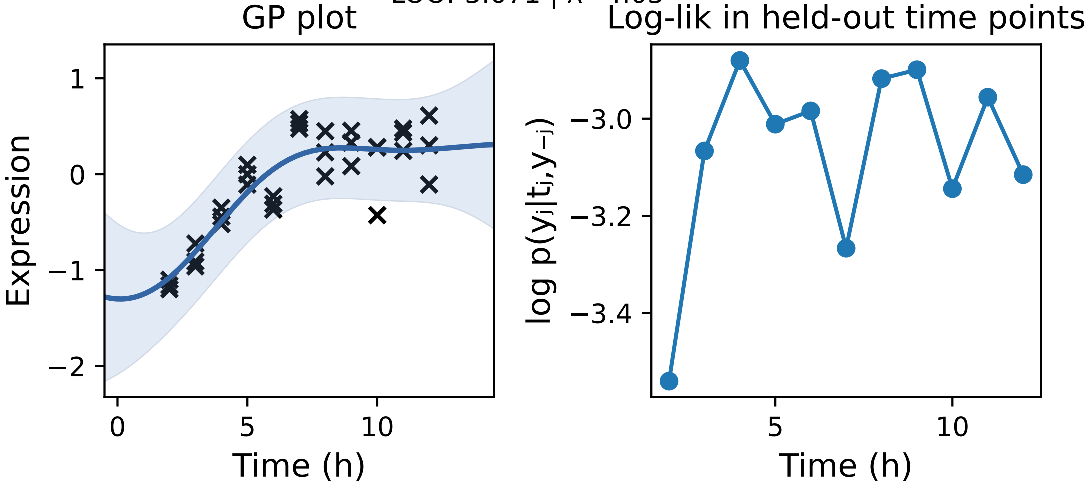

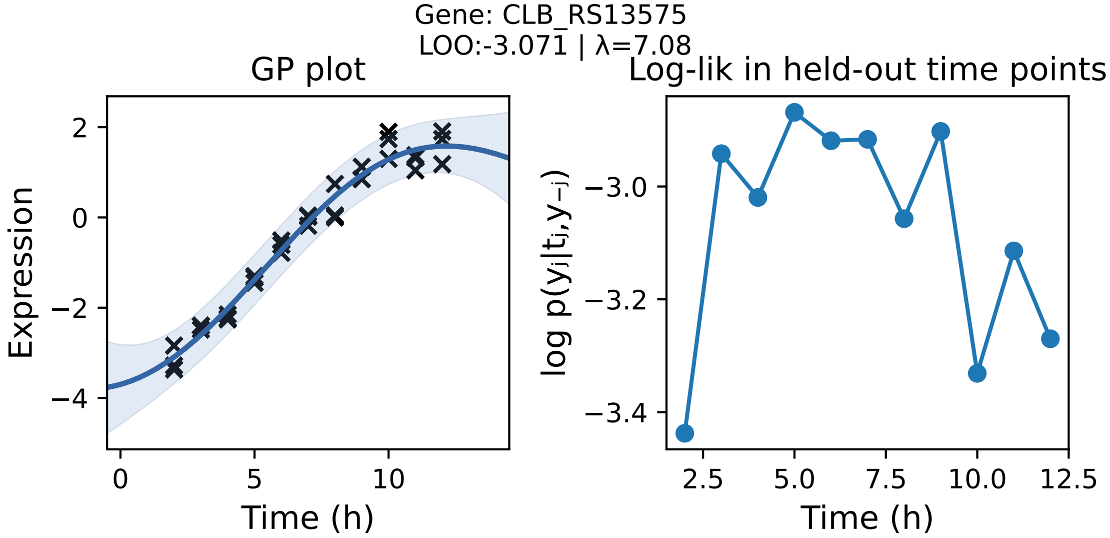
Gene: CLB_RS13575
LOO:-3.071 | λ=7.08
GP plot
Expression
Time (h)
Log-lik in held-out time points
log p(y_j|t_j,y_−j)
Time (h)

Gene: CLB_RS14050
LOO:-3.071 | λ=1.93

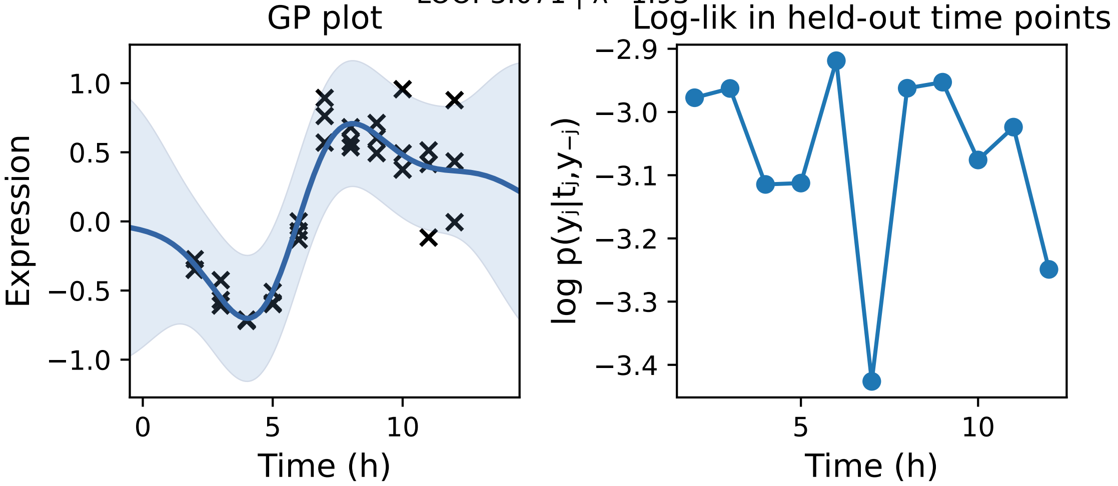

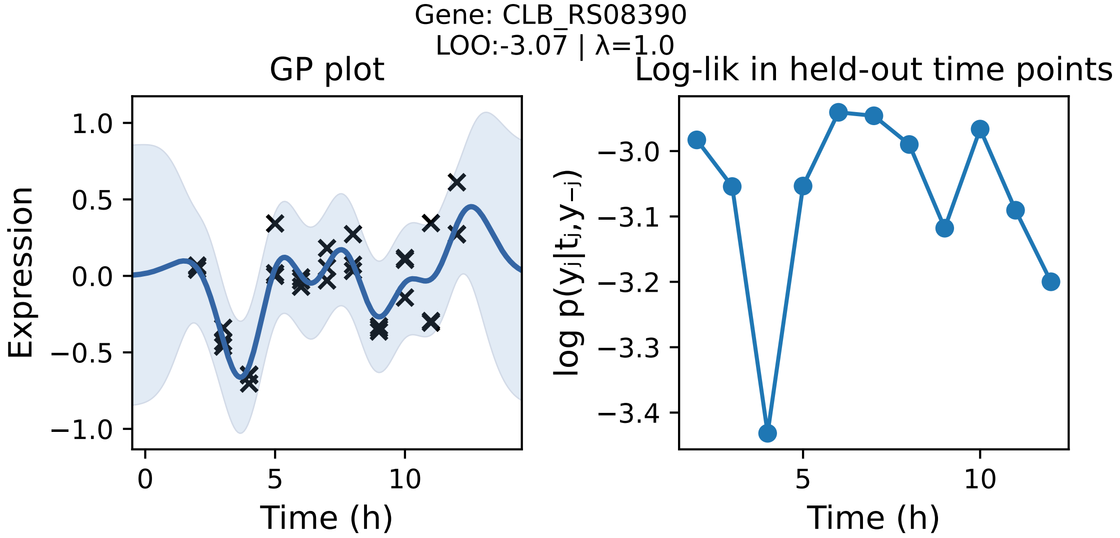

Gene: CLB_RS08390
LOO:-3.07 | λ=1.0
GP plot
Expression
1.0
0.5
0.0
−0.5
−1.0
0
5
10
Time (h)
Log-lik in held-out time points
log p(y_j|t_j,y_−j)
−3.0
−3.1
−3.2
−3.3
−3.4
5
10
Time (h)

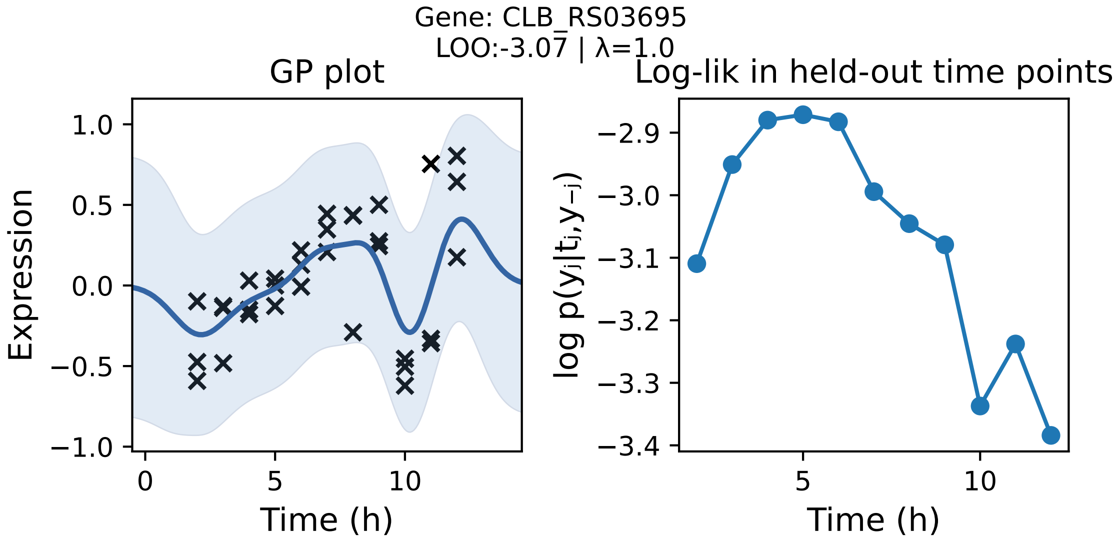

Gene: CLB_RS03695
LOO:-3.07 | λ=1.0
GP plot
Expression
1.0
0.5
0.0
−0.5
−1.0
0
5
10
Time (h)
Log-lik in held-out time points
log p(y_j|t_j,y_−j)
−2.9
−3.0
−3.1
−3.2
−3.3
−3.4
5
10
Time (h)

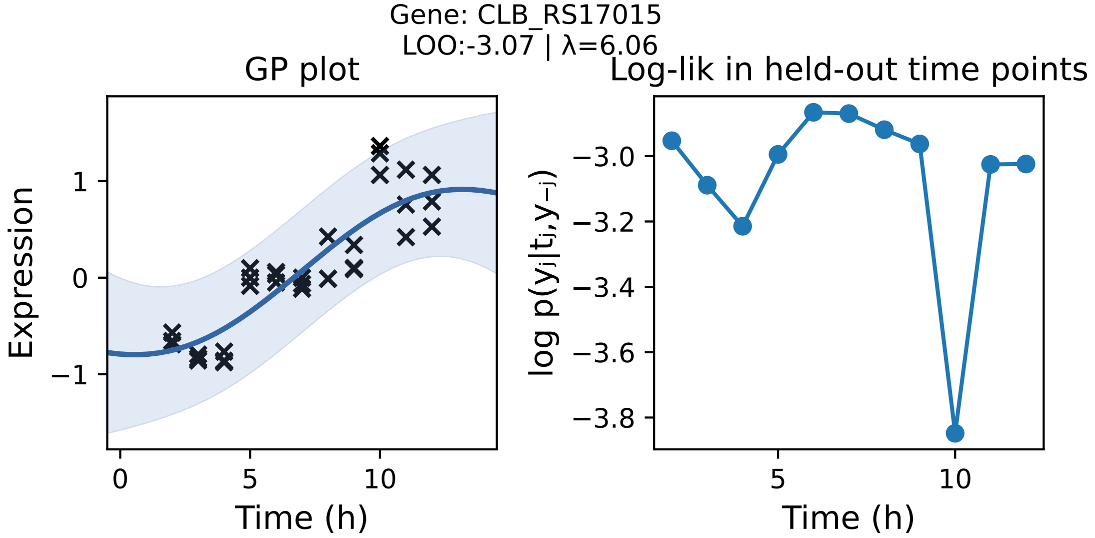

Gene: CLB_RS17015
LOO:-3.07 | λ=6.06
GP plot
Expression
Time (h)
Log-lik in held-out time points
log p(y_j|t_j,y_−j)
Time (h)

Gene: CLB_RS05445
LOO:-3.068 | λ=1.96

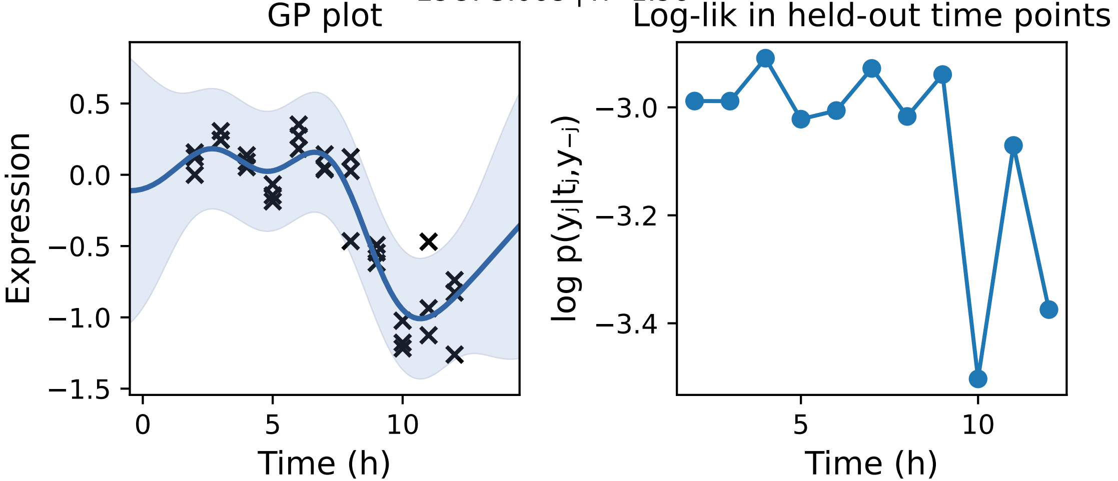

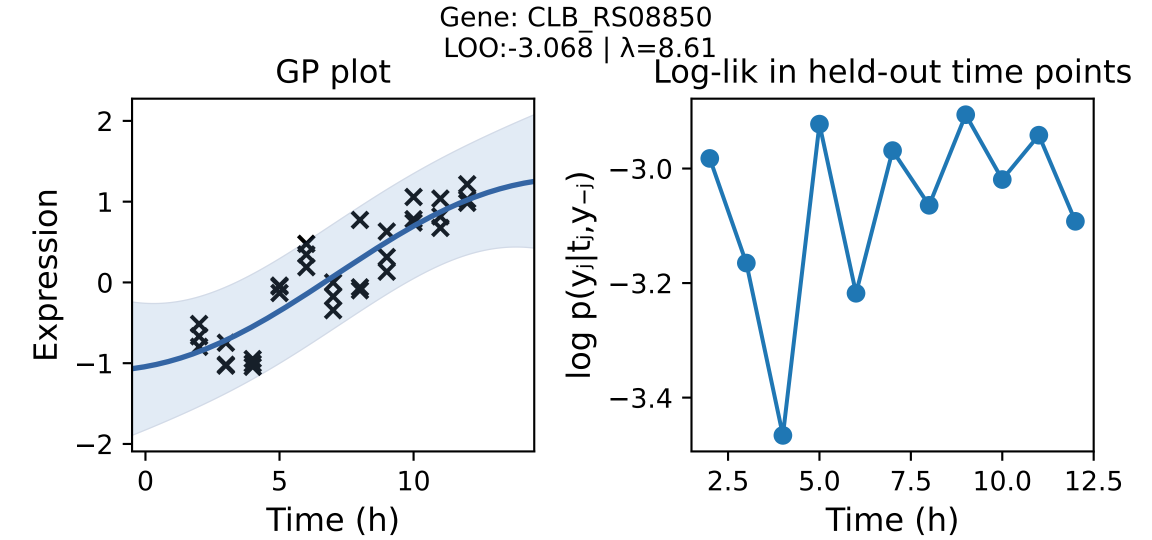
Gene: CLB_RS08850
LOO:-3.068 | λ=8.61
GP plot
Expression
Time (h)
Log-lik in held-out time points
log p(y_j|t_j,y_−j)
Time (h)

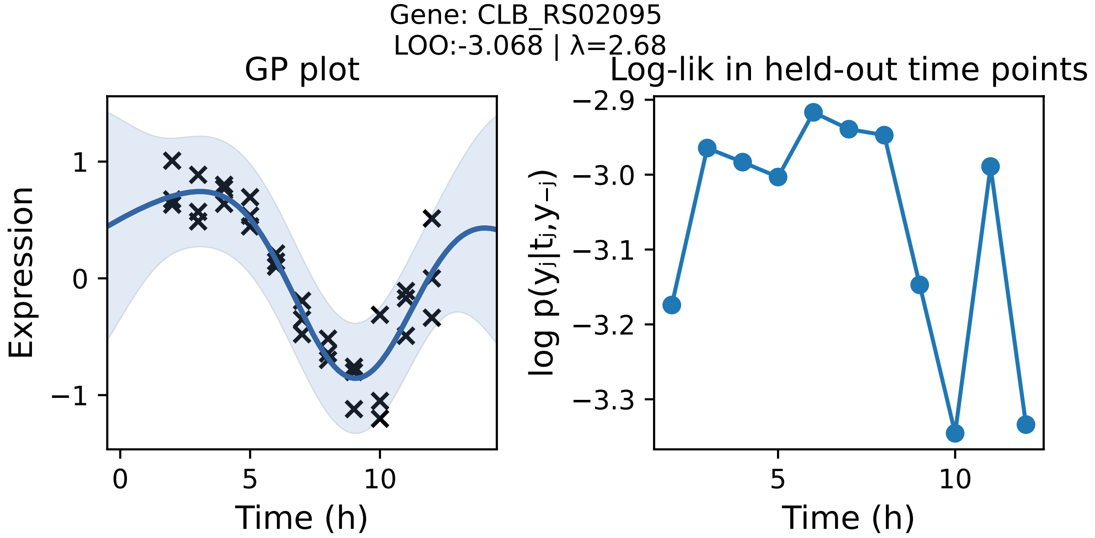

Gene: CLB_RS02095
LOO:-3.068 | λ=2.68
GP plot
Expression
Time (h)
1
0
−1
0
5
10
Log-lik in held-out time points
log p(y_j|t_j,y_−j)
Time (h)
−2.9
−3.0
−3.1
−3.2
−3.3
5
10

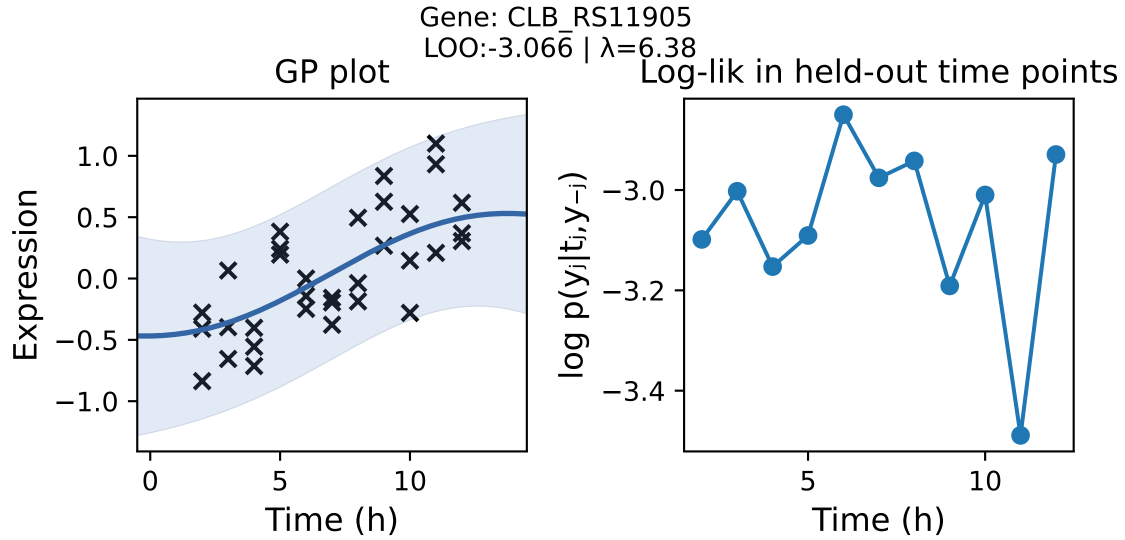

Gene: CLB_RS11905
LOO:-3.066 | λ=6.38
GP plot
Log-lik in held-out time points
Expression
1.0
0.5
0.0
−0.5
−1.0
0
5
10
Time (h)
log p(y_j|t_j,y_{−j})
−3.0
−3.2
−3.4
5
10
Time (h)

Gene: CLB_RS12905
LOO:-3.065 | λ=1.33

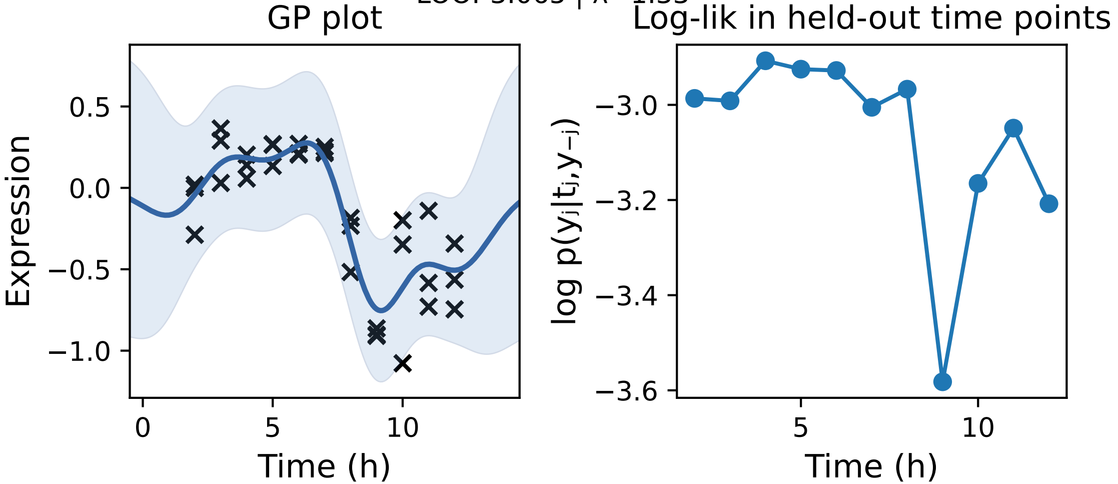

Gene: CLB_RS08750
LOO:-3.064 | λ=2.66

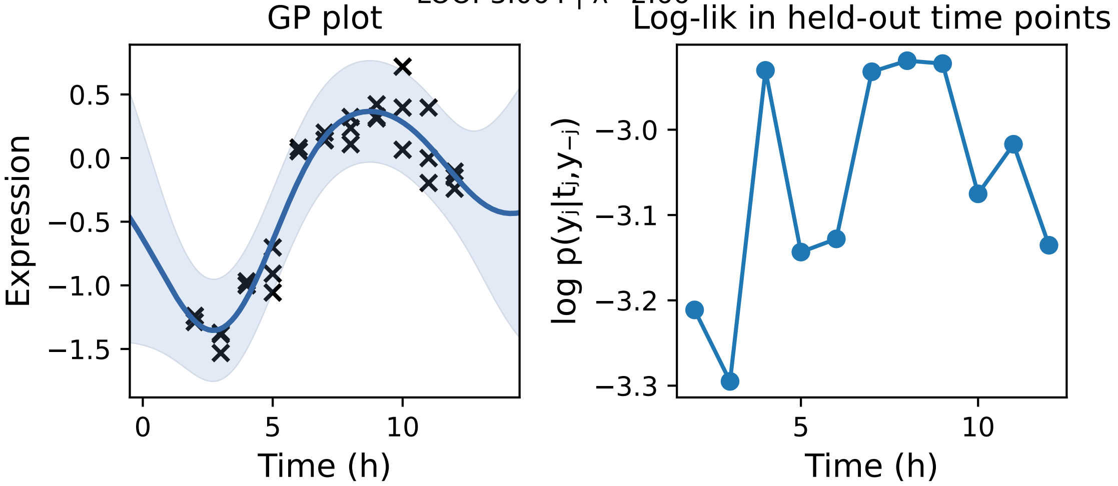

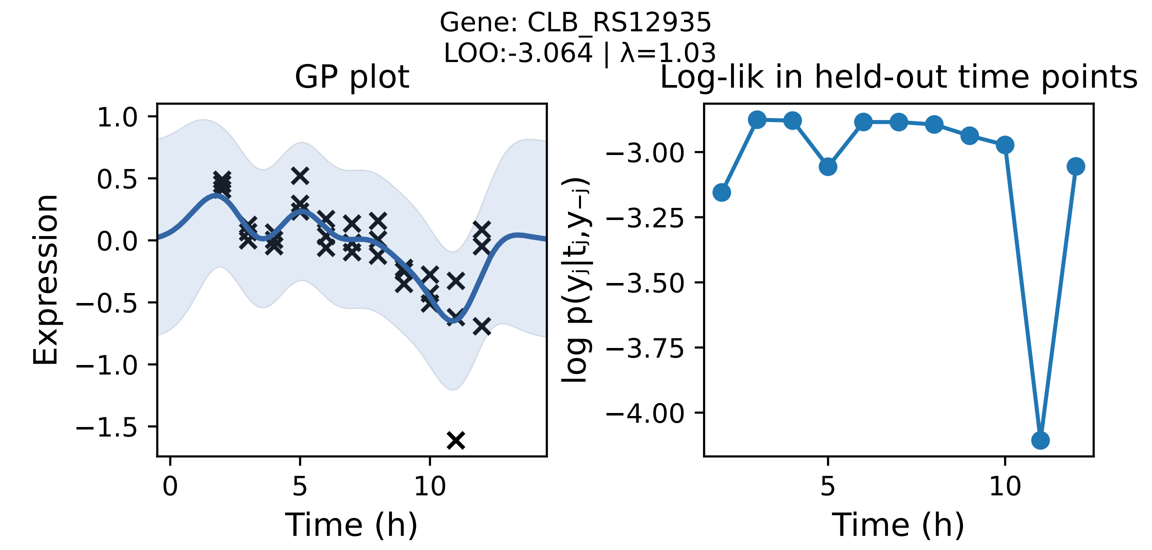

Gene: CLB_RS12935
LOO:-3.064 | λ=1.03
GP plot
Expression
Time (h)
Log-lik in held-out time points
log p(y_j|t_j,y_−j)
Time (h)

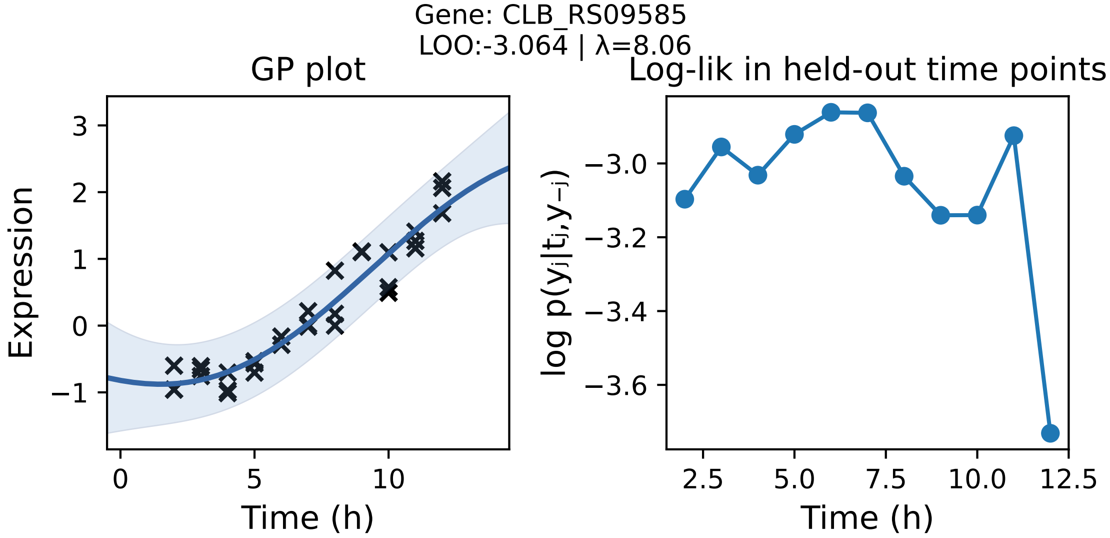

Gene: CLB_RS09585
LOO:-3.064 | λ=8.06
GP plot
Expression
Time (h)
Log-lik in held-out time points
log p(y_j|t_j,y_−j)
Time (h)

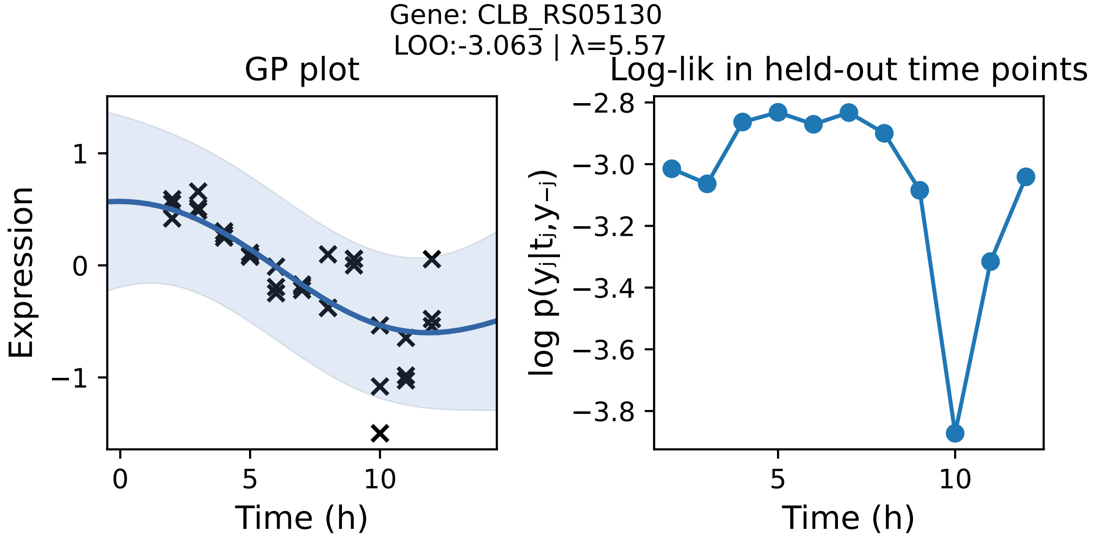

Gene: CLB_RS05130
LOO:-3.063 | λ=5.57
GP plot
Expression
Time (h)
1
0
−1
0
5
10
Log-lik in held-out time points
log p(yj|tj,y−j)
Time (h)
−2.8
−3.0
−3.2
−3.4
−3.6
−3.8
5
10

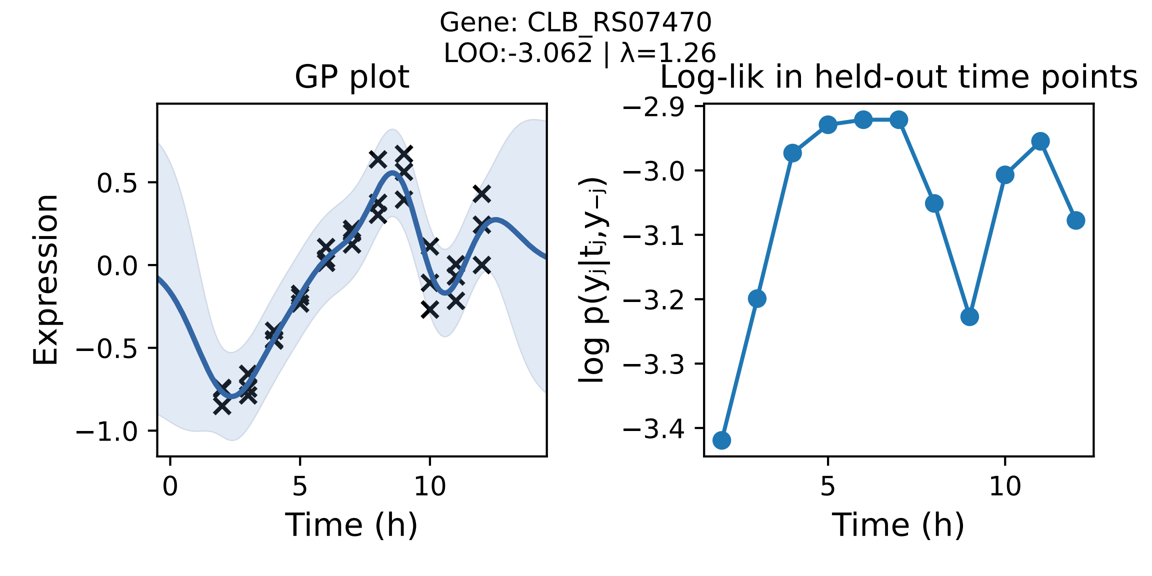

Gene: CLB_RS07470
LOO:-3.062 | λ=1.26
GP plot
Expression
0.5
0.0
−0.5
−1.0
0
5
10
Time (h)
Log-lik in held-out time points
log p(y_j|t_j,y_−j)
−2.9
−3.0
−3.1
−3.2
−3.3
−3.4
5
10
Time (h)

Gene: CLB_RS13480
LOO:-3.06 | λ=1.24

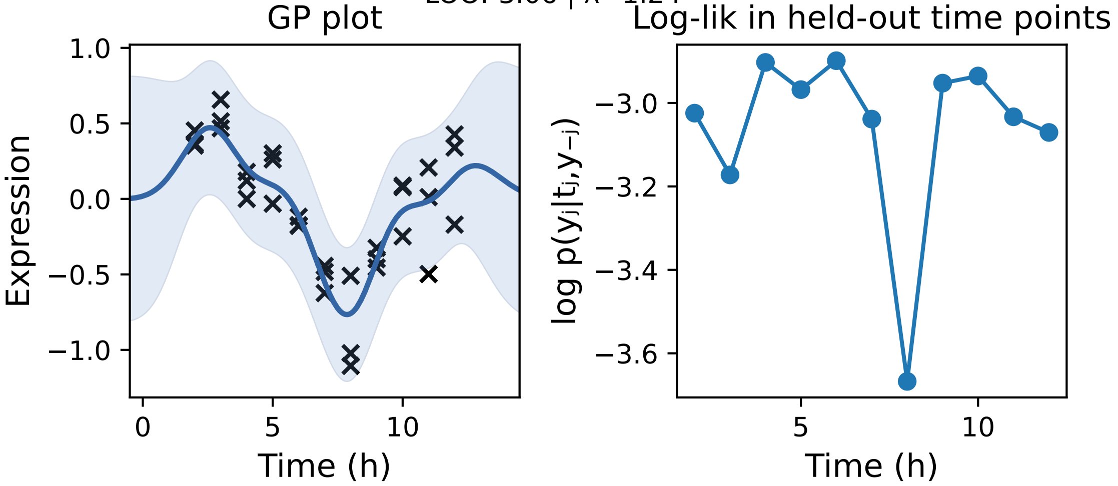

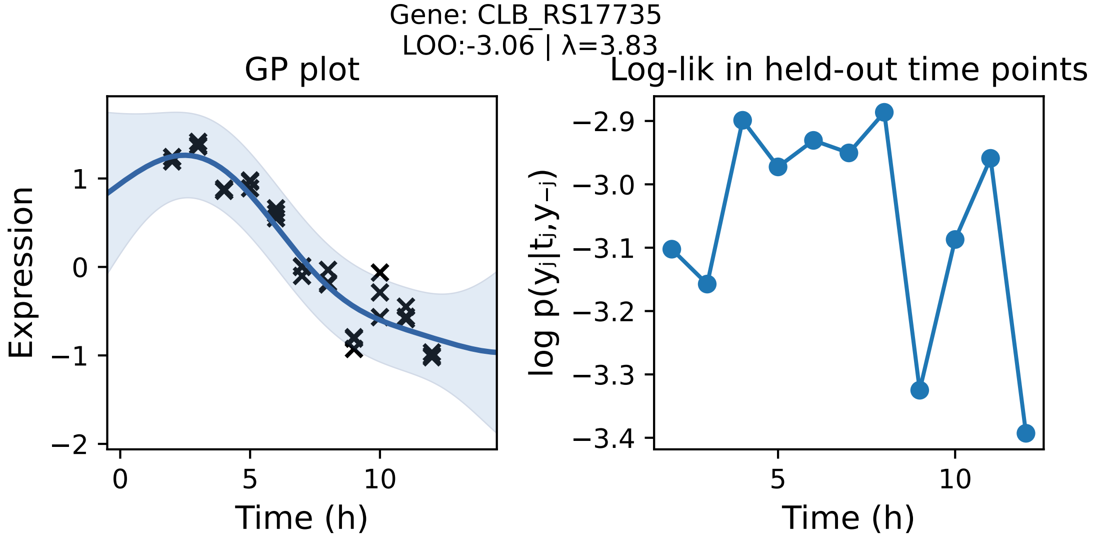

Gene: CLB_RS17735
LOO:-3.06 | λ=3.83
GP plot
Log-lik in held-out time points
Expression
Time (h)
log p(yj|tj,y−j)
1
0
−1
−2
0
5
10
−2.9
−3.0
−3.1
−3.2
−3.3
−3.4

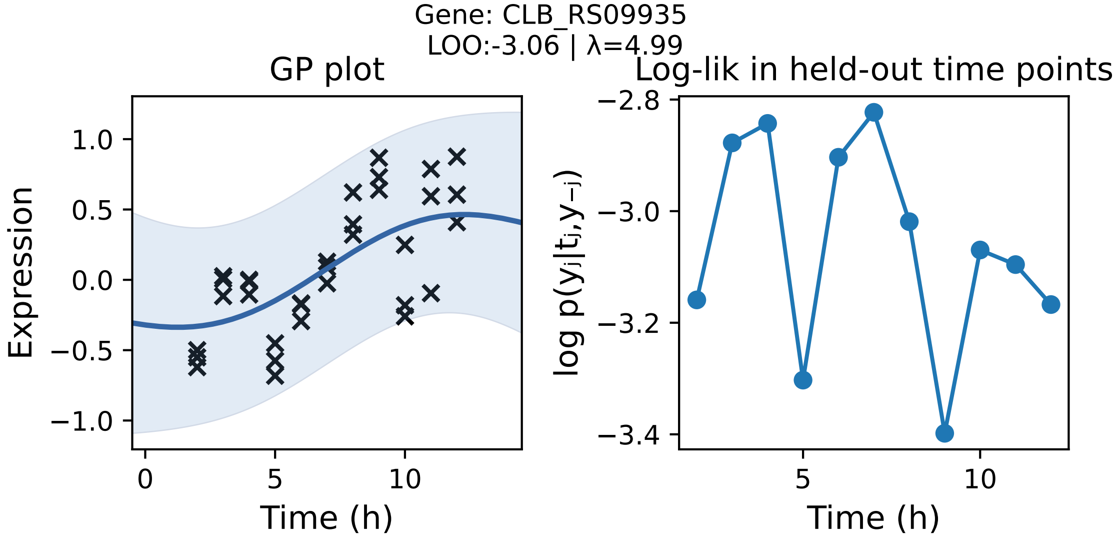

Gene: CLB_RS09935
LOO:-3.06 | λ=4.99
GP plot
Expression
1.0
0.5
0.0
−0.5
−1.0
0
5
10
Time (h)
Log-lik in held-out time points
log p(y_j|t_j,y_−j)
−2.8
−3.0
−3.2
−3.4
5
10
Time (h)

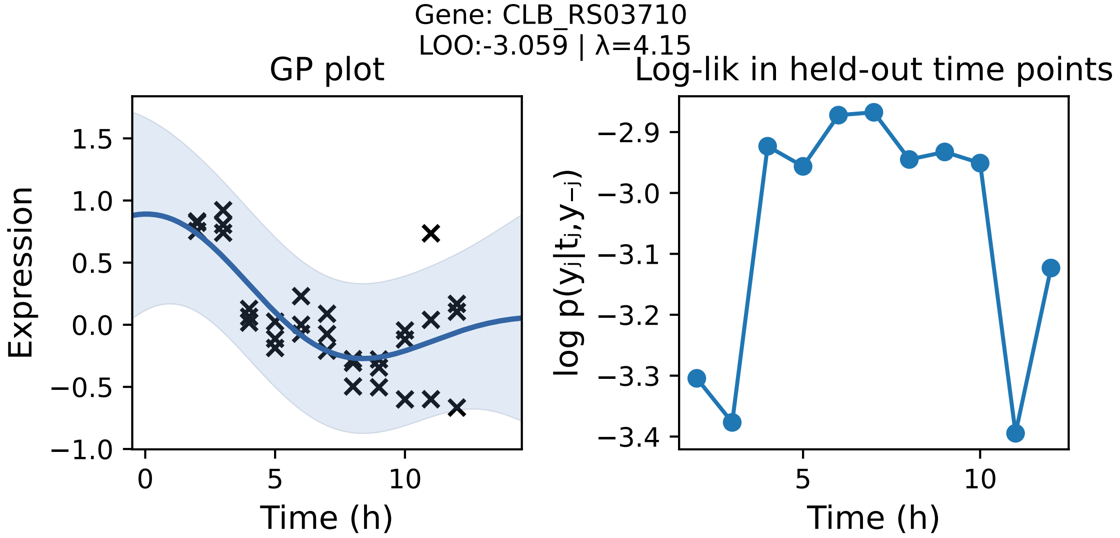

Gene: CLB_RS03710
LOO:-3.059 | λ=4.15
GP plot
Expression
1.5
1.0
0.5
0.0
−0.5
−1.0
0
5
10
Time (h)
Log-lik in held-out time points
log p(y_j|t_j,y_{−j})
−2.9
−3.0
−3.1
−3.2
−3.3
−3.4
5
10
Time (h)

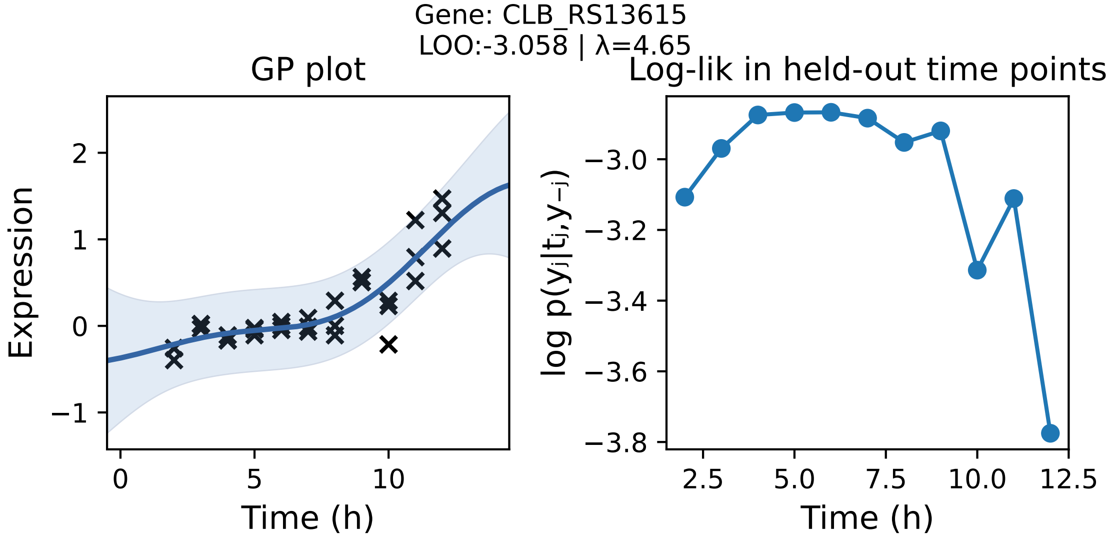

Gene: CLB_RS13615
LOO:-3.058 | λ=4.65
GP plot
Expression
2
1
0
−1
0
5
10
Time (h)
Log-lik in held-out time points
log p(y_j|t_j,y_−j)
−3.0
−3.2
−3.4
−3.6
−3.8
2.5
5.0
7.5
10.0
12.5
Time (h)

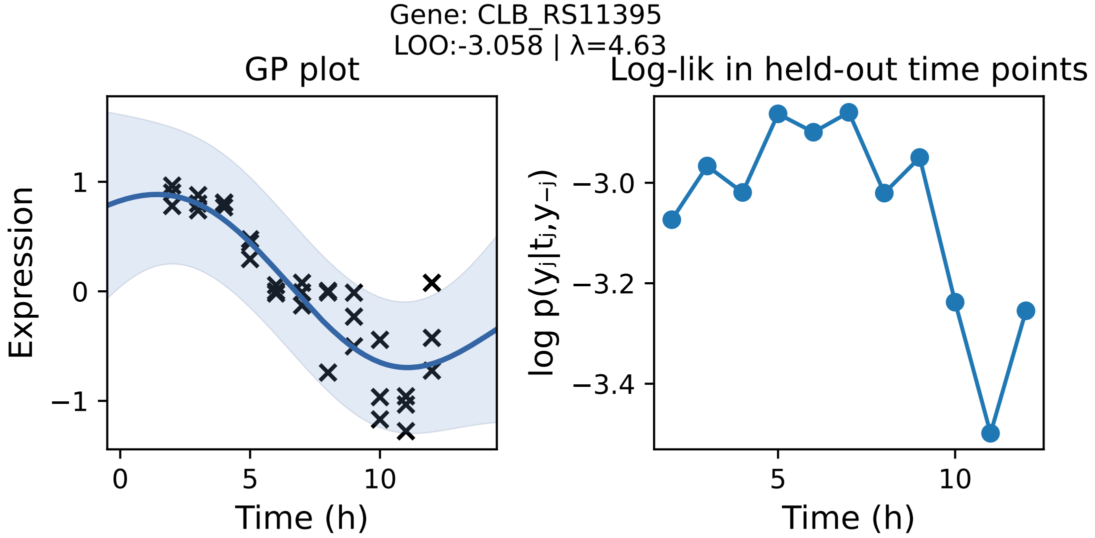
Gene: CLB_RS11395
LOO:-3.058 | λ=4.63
GP plot
Expression
1
0
−1
0
5
10
Time (h)
Log-lik in held-out time points
$\log p(y_j|t_j, y_{-j})$
−3.0
−3.2
−3.4
5
10
Time (h)

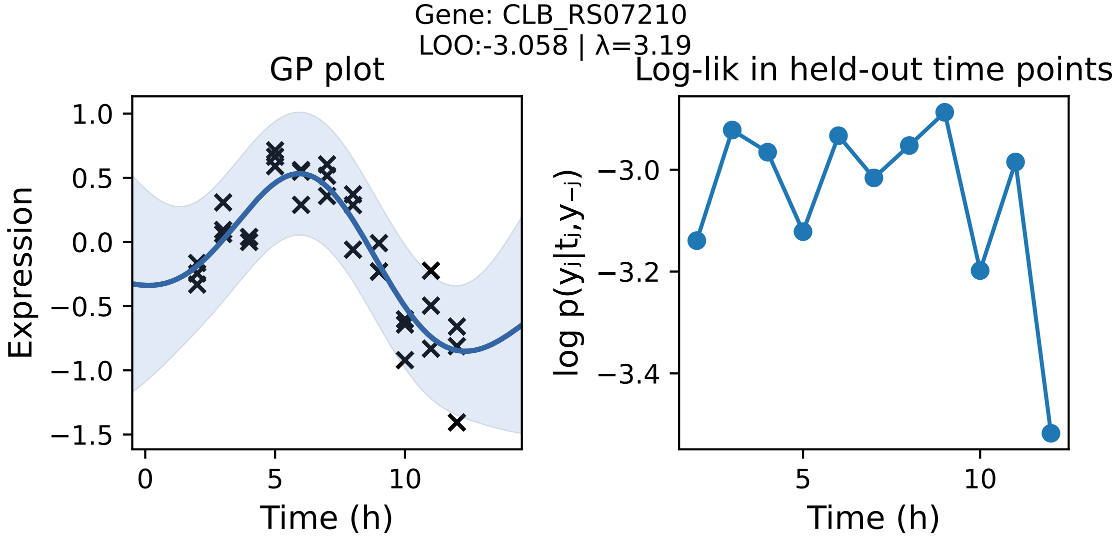

Gene: CLB_RS07210
LOO:-3.058 | λ=3.19
GP plot
Expression
Time (h)
1.0
0.5
0.0
−0.5
−1.0
−1.5
0
5
10
Log-lik in held-out time points
log p(yj|tj,y−j)
−3.0
−3.2
−3.4
5
10
Time (h)

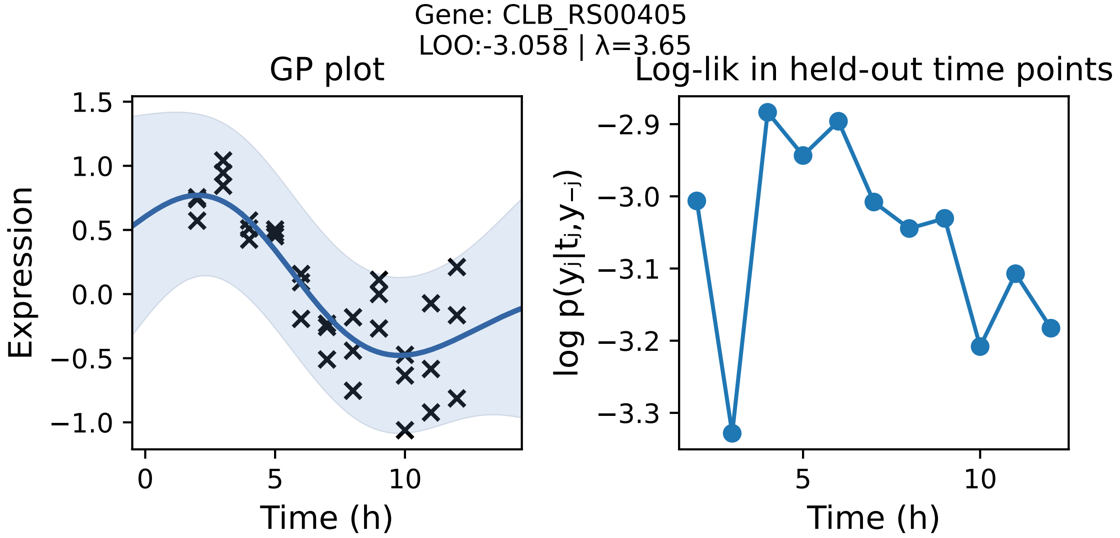

Gene: CLB_RS00405
LOO:-3.058 | λ=3.65
GP plot
Expression
Time (h)
Log-lik in held-out time points
log p(y_j|t_j,y_−j)
Time (h)

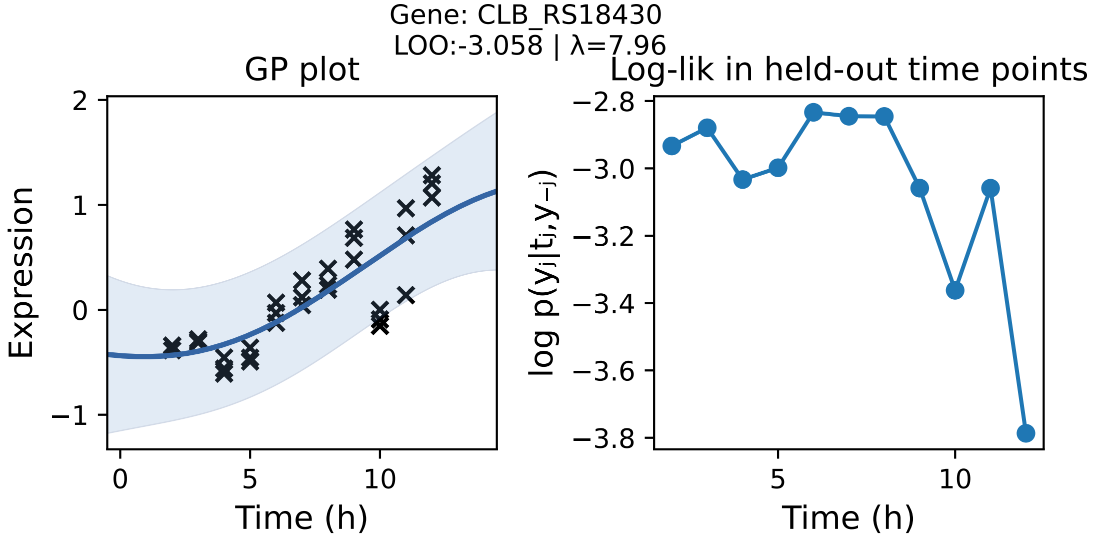

Gene: CLB_RS18430
LOO:-3.058 | λ=7.96
GP plot
Expression
Time (h)
Log-lik in held-out time points
log p(y_j|t_j,y_−j)
Time (h)

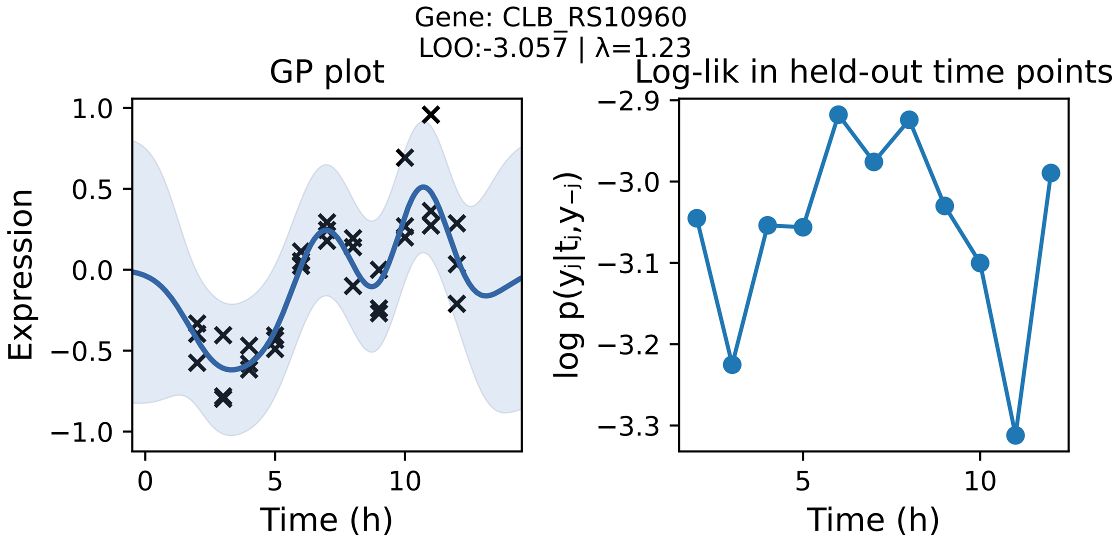

Gene: CLB_RS10960
LOO:-3.057 | λ=1.23
GP plot
Expression
1.0
0.5
0.0
−0.5
−1.0
0
5
10
Time (h)
Log-lik in held-out time points
log p(y_j|t_j,y_−j)
−2.9
−3.0
−3.1
−3.2
−3.3
5
10
Time (h)

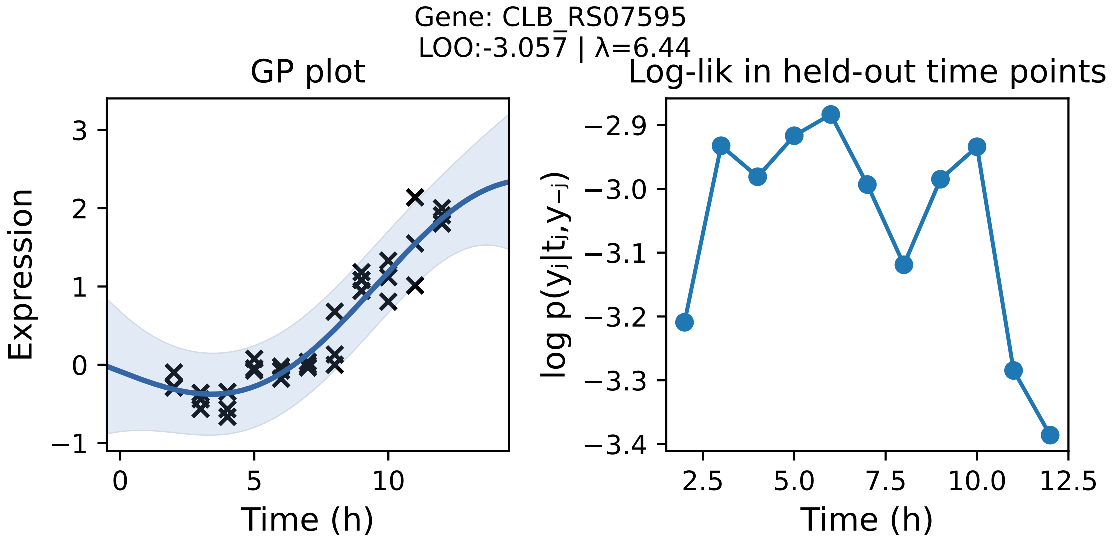
Gene: CLB_RS07595
LOO:-3.057 | λ=6.44
GP plot
Log-lik in held-out time points
Expression
3
2
1
0
−1
0
5
10
Time (h)
log p(yj|tj,y−j)
−2.9
−3.0
−3.1
−3.2
−3.3
−3.4
2.5
5.0
7.5
10.0
12.5
Time (h)

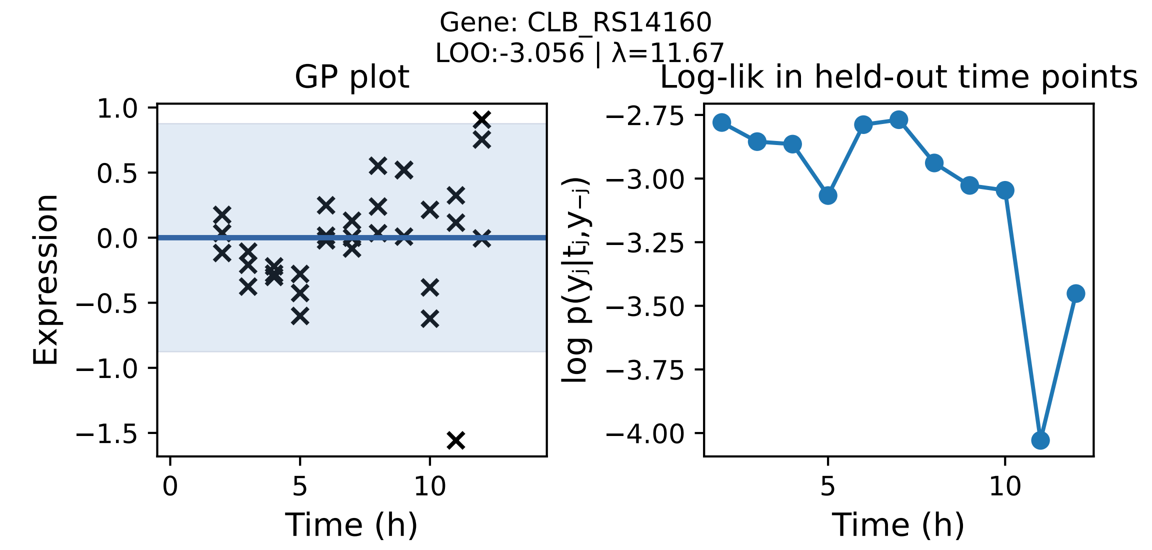

Gene: CLB_RS14160
LOO:-3.056 | λ=11.67
GP plot
Expression
Time (h)
1.0
0.5
0.0
−0.5
−1.0
−1.5
0
5
10
Log-lik in held-out time points
log p(y_j|t_j,y_−j)
Time (h)
−2.75
−3.00
−3.25
−3.50
−3.75
−4.00
5
10

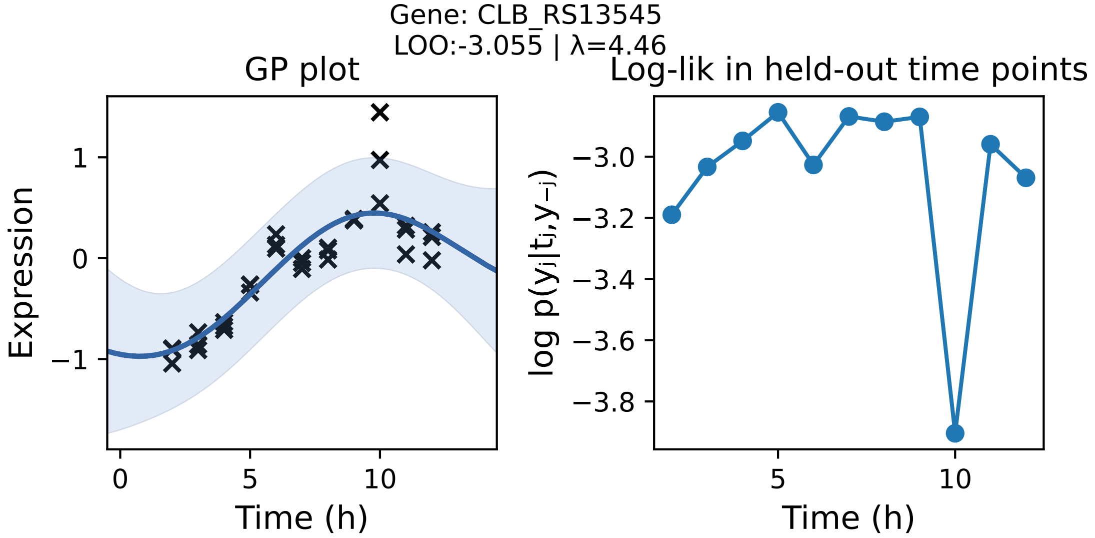

Gene: CLB_RS13545
LOO:-3.055 | λ=4.46
GP plot
Expression
Time (h)
Log-lik in held-out time points
log p(y_j|t_j,y_−j)
Time (h)

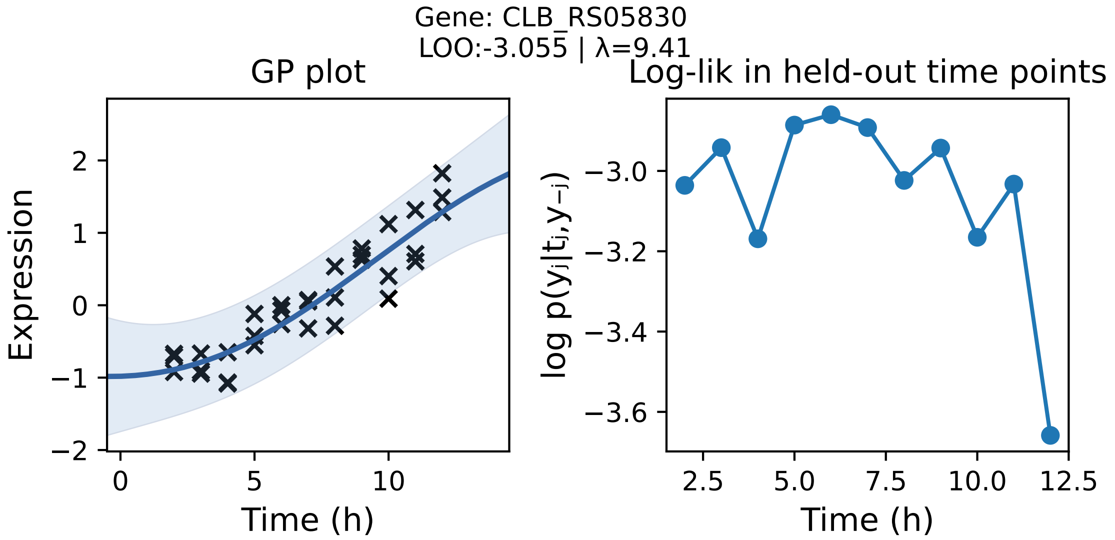

Gene: CLB_RS05830
LOO:-3.055 | λ=9.41
GP plot
Expression
Time (h)
2
1
0
−1
−2
0
5
10
Log-lik in held-out time points
log p(y_j|t_j,y_−j)
Time (h)
−3.0
−3.2
−3.4
−3.6
2.5
5.0
7.5
10.0
12.5

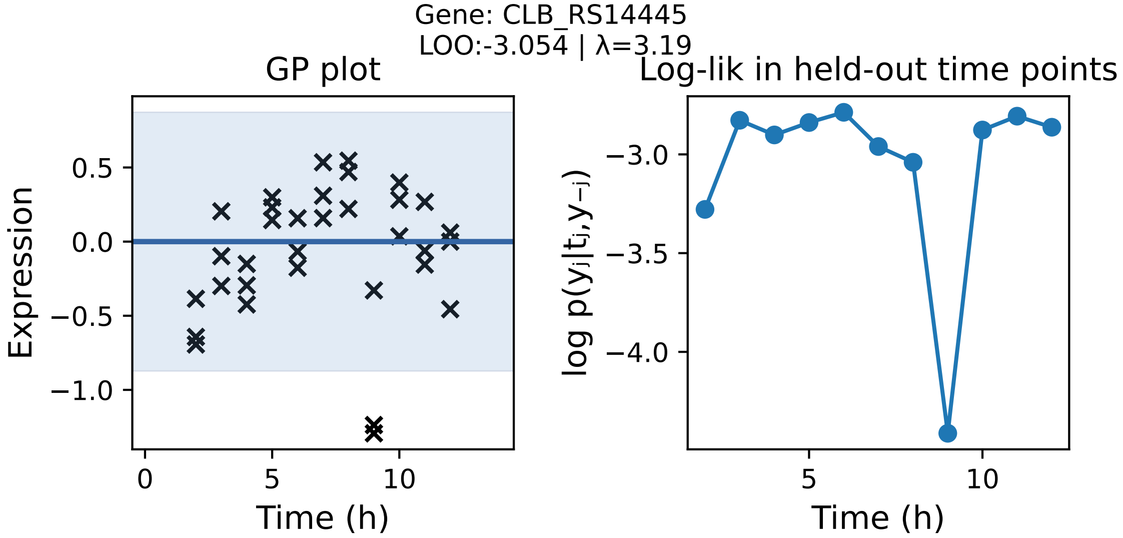
Gene: CLB_RS14445
LOO:-3.054 | λ=3.19
GP plot
Expression
0.5
0.0
−0.5
−1.0
0
5
10
Time (h)
Log-lik in held-out time points
log p(y_j|t_j,y_−j)
−3.0
−3.5
−4.0
5
10
Time (h)

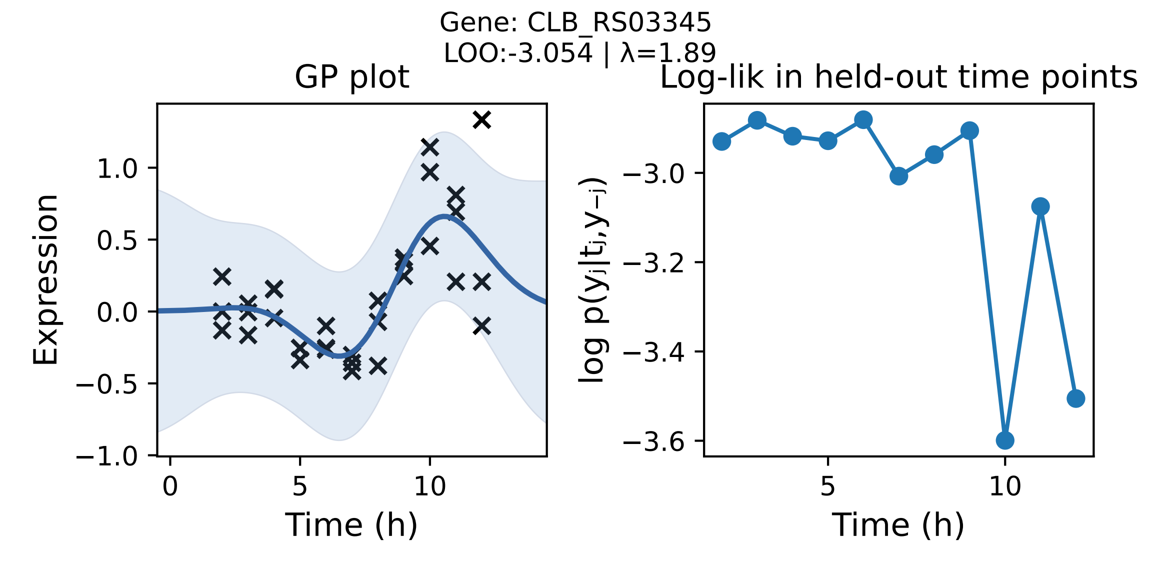

Gene: CLB_RS03345
LOO:-3.054 | λ=1.89
GP plot
Log-lik in held-out time points
Expression
1.0
0.5
0.0
−0.5
−1.0
0
5
10
Time (h)
log p(y_j|t_j,y_−j)
−3.0
−3.2
−3.4
−3.6
5
10
Time (h)

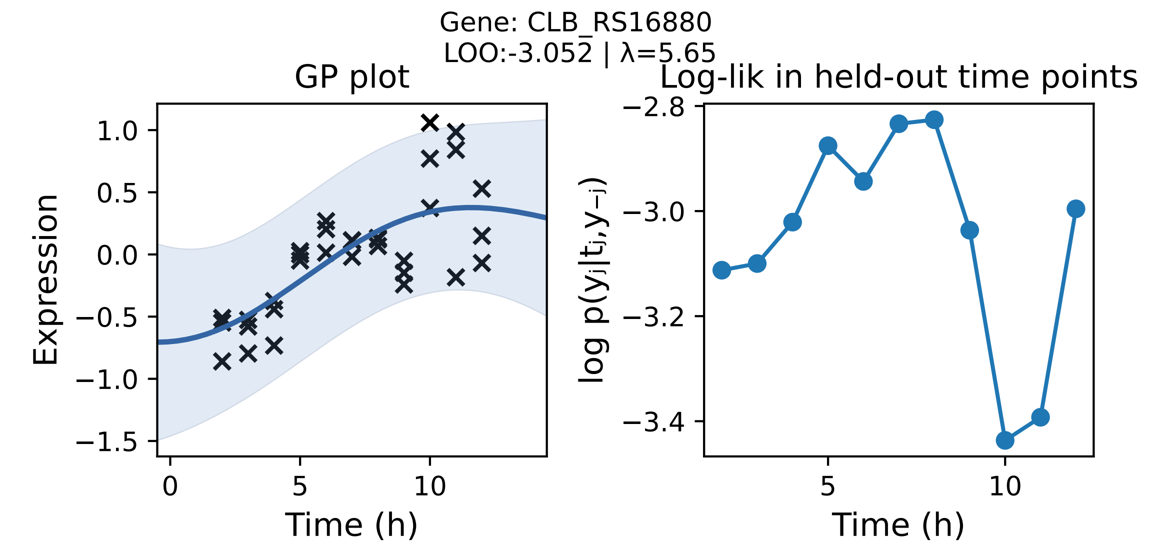

Gene: CLB_RS16880
LOO:-3.052 | λ=5.65
GP plot
Expression
Time (h)
Log-lik in held-out time points
log p(yj|tj,y−j)
Time (h)

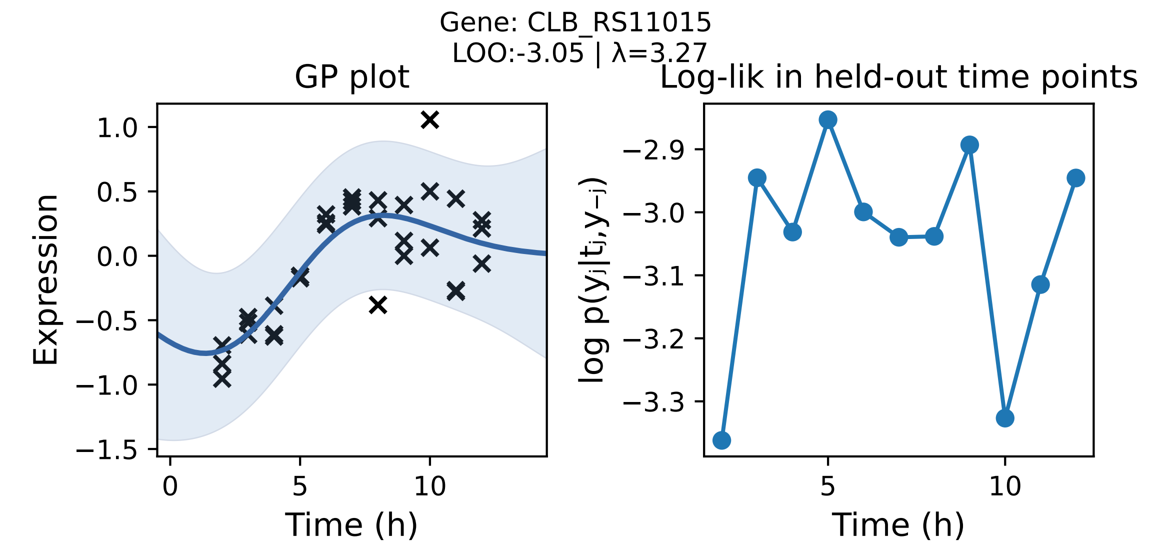

Gene: CLB_RS11015
LOO:-3.05 | λ=3.27
GP plot
Expression
Time (h)
1.0
0.5
0.0
−0.5
−1.0
−1.5
0
5
10
Log-lik in held-out time points
log p(yj|tj,y−j)
−2.9
−3.0
−3.1
−3.2
−3.3
5
10
Time (h)

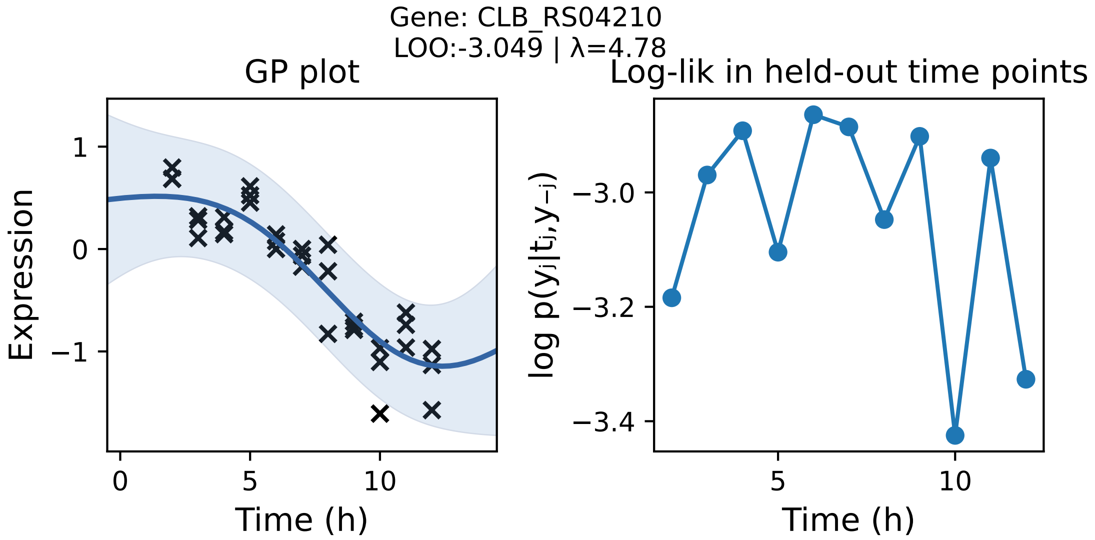

Gene: CLB_RS04210
LOO:-3.049 | λ=4.78
GP plot
Expression
Time (h)
Log-lik in held-out time points
log p(y_j|t_j,y_{-j})
Time (h)

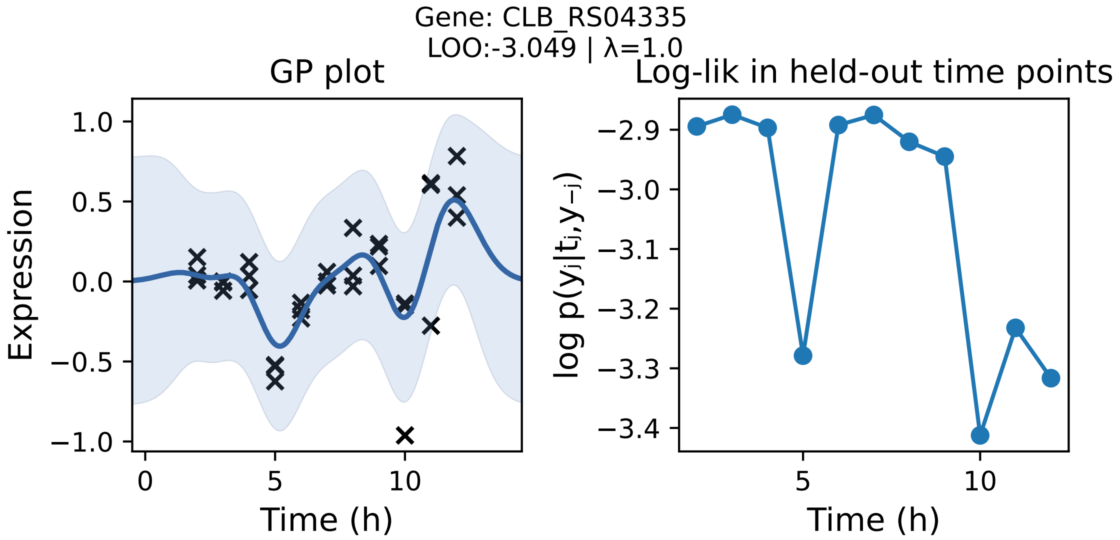

Gene: CLB_RS04335
LOO:-3.049 | λ=1.0
GP plot
Expression
1.0
0.5
0.0
−0.5
−1.0
0
5
10
Time (h)
Log-lik in held-out time points
log p(y_j|t_j,y_−j)
−2.9
−3.0
−3.1
−3.2
−3.3
−3.4
5
10
Time (h)

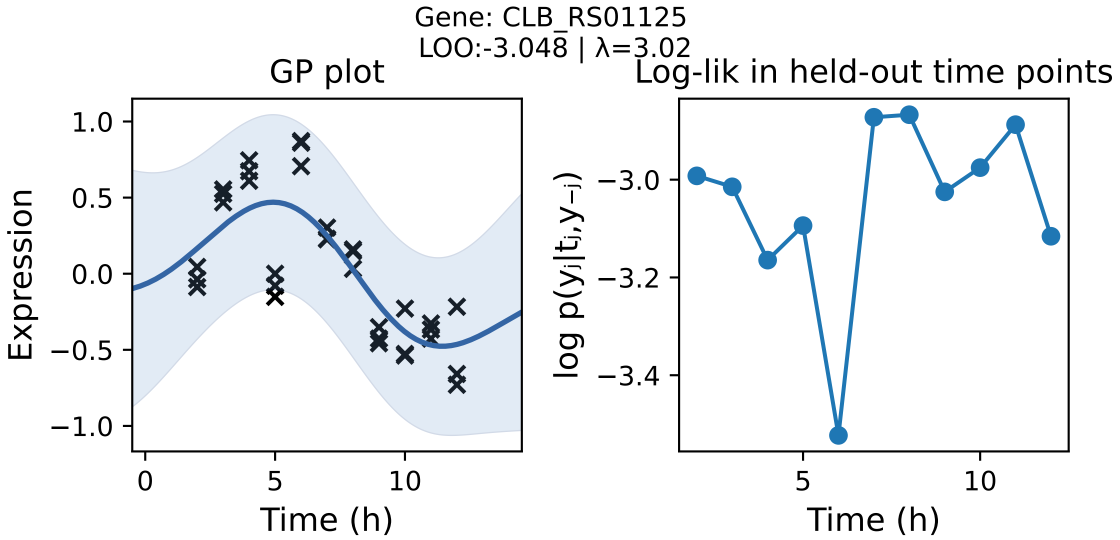

Gene: CLB_RS01125
LOO:-3.048 | λ=3.02
GP plot
Log-lik in held-out time points
Expression
1.0
0.5
0.0
−0.5
−1.0
0
5
10
Time (h)
log p(yj|tj,y−j)
−3.0
−3.2
−3.4
5
10
Time (h)

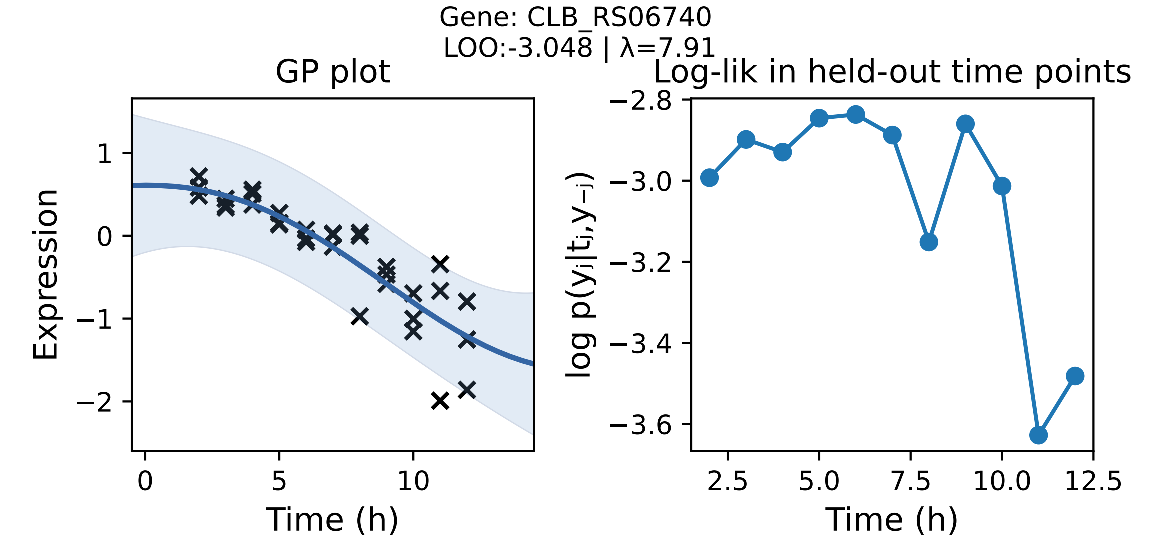

Gene: CLB_RS06740
LOO:-3.048 | λ=7.91
GP plot
Expression
Time (h)
Log-lik in held-out time points
log p(y_j|t_j,y_−j)
Time (h)

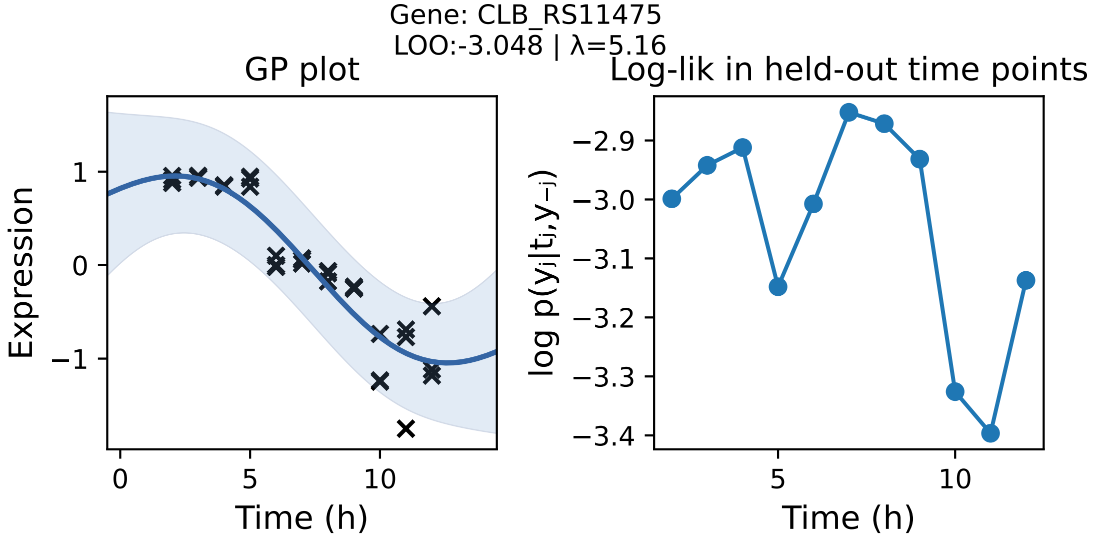

Gene: CLB_RS11475
LOO:-3.048 | λ=5.16
GP plot
Expression
1
0
−1
0
5
10
Time (h)
Log-lik in held-out time points
log p(yj|tj,y−j)
−2.9
−3.0
−3.1
−3.2
−3.3
−3.4
5
10
Time (h)

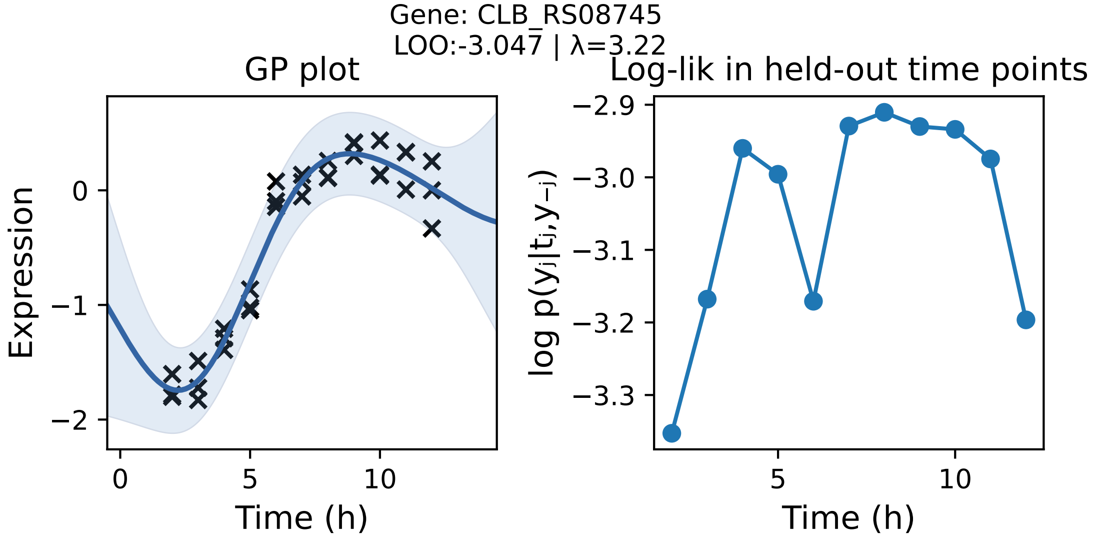

Gene: CLB_RS08745
LOO:-3.047 | λ=3.22
GP plot
Expression
Time (h)
0
−1
−2
0
5
10
Log-lik in held-out time points
log p(y_j|t_j,y_−j)
−2.9
−3.0
−3.1
−3.2
−3.3
5
10
Time (h)

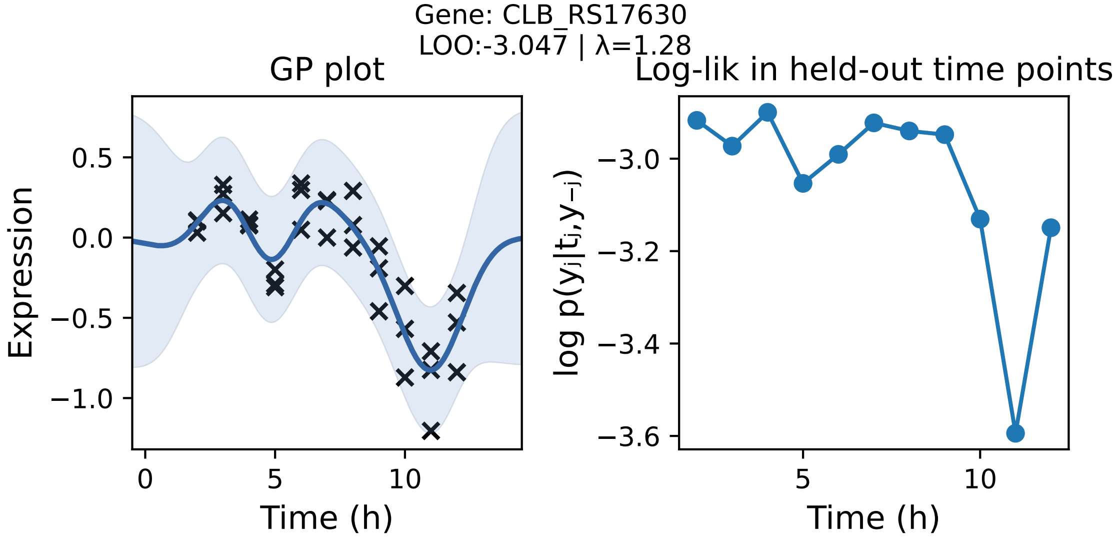

Gene: CLB_RS17630
LOO:-3.047 | λ=1.28
GP plot
Expression
0.5
0.0
−0.5
−1.0
0
5
10
Time (h)
Log-lik in held-out time points
log p(y_j|t_j,y_−j)
−3.0
−3.2
−3.4
−3.6
5
10
Time (h)

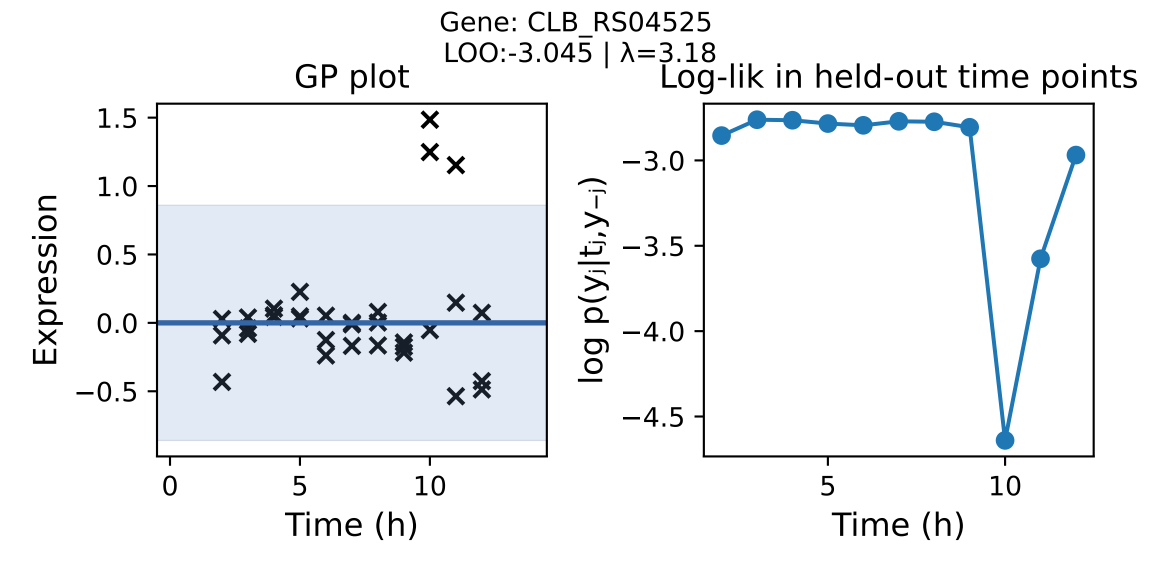

Gene: CLB_RS04525
LOO:-3.045 | λ=3.18
GP plot
Log-lik in held-out time points
Expression
Time (h)
log p(y_j|t_j,y_-j)
1.5
1.0
0.5
0.0
−0.5
0
5
10
−3.0
−3.5
−4.0
−4.5

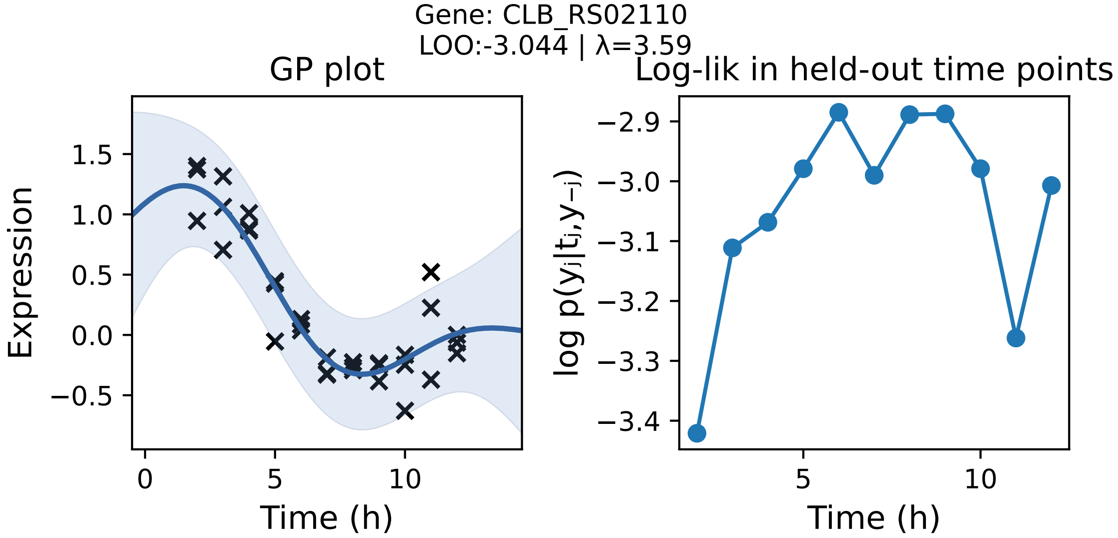

Gene: CLB_RS02110
LOO:-3.044 | λ=3.59
GP plot
Expression
Time (h)
1.5
1.0
0.5
0.0
−0.5
0
5
10
Log-lik in held-out time points
log p(y_j|t_j,y_−j)
Time (h)
−2.9
−3.0
−3.1
−3.2
−3.3
−3.4
5
10

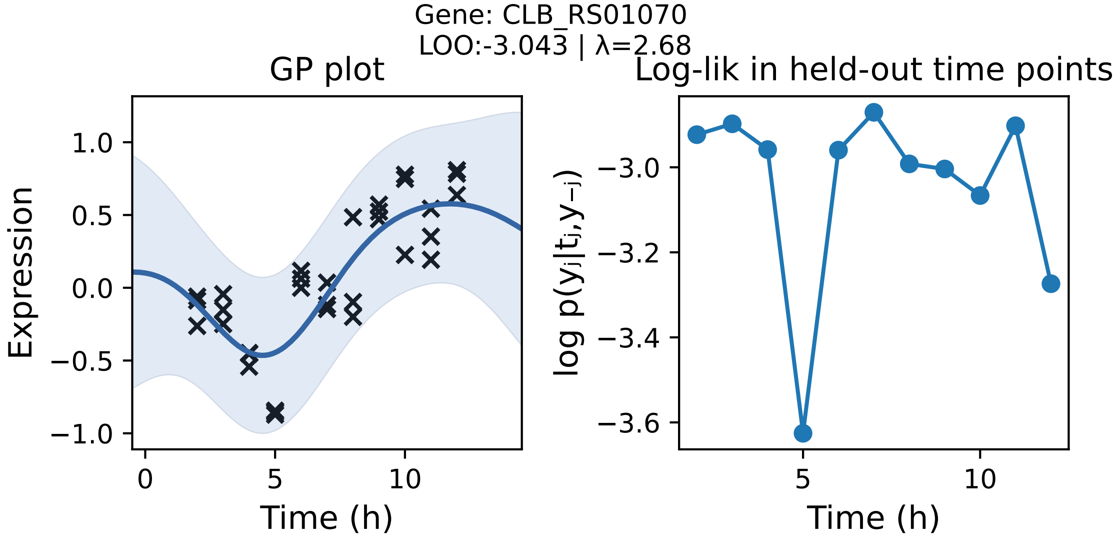

Gene: CLB_RS01070
LOO:-3.043 | λ=2.68
GP plot
Log-lik in held-out time points
Expression
Time (h)
log p(y_j|t_j,y_−j)
1.0
0.5
0.0
−0.5
−1.0
0
5
10
−3.0
−3.2
−3.4
−3.6

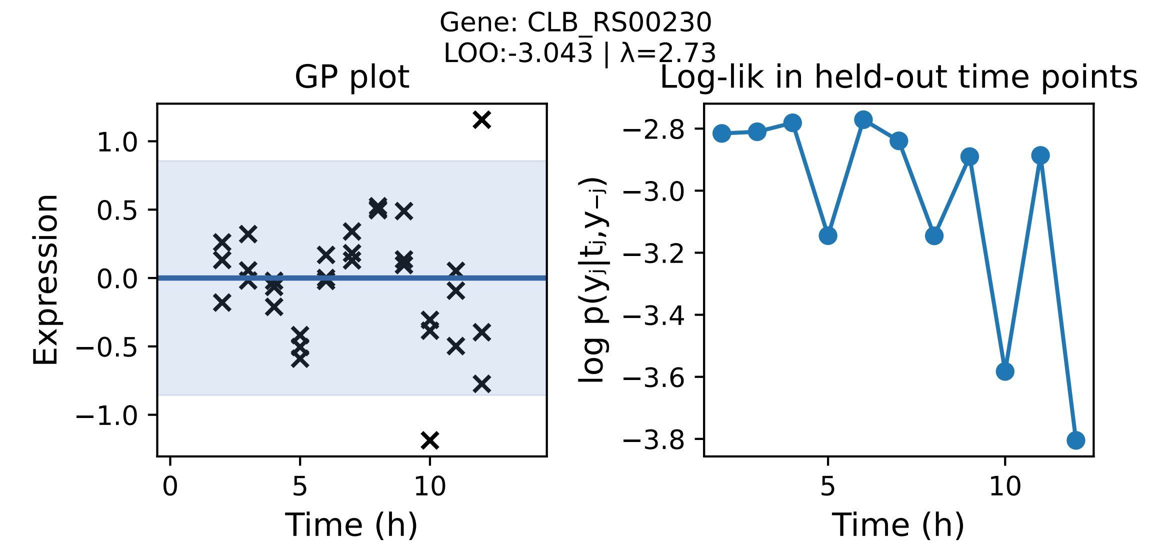
Gene: CLB_RS00230
LOO:-3.043 | λ=2.73
GP plot
Expression
Time (h)
Log-lik in held-out time points
log p(y_j|t_j,y_−j)
Time (h)

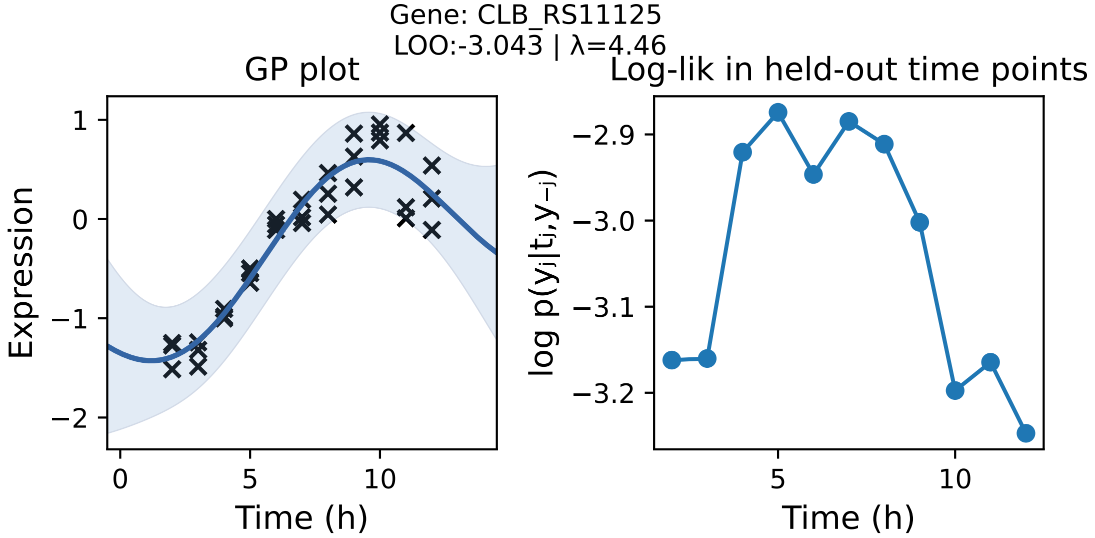

Gene: CLB_RS11125
LOO:-3.043 | λ=4.46
GP plot
Expression
Time (h)
Log-lik in held-out time points
log p(y_j|t_j,y_−j)
Time (h)

Gene: CLB_RS03980
LOO:-3.043 | λ=1.0

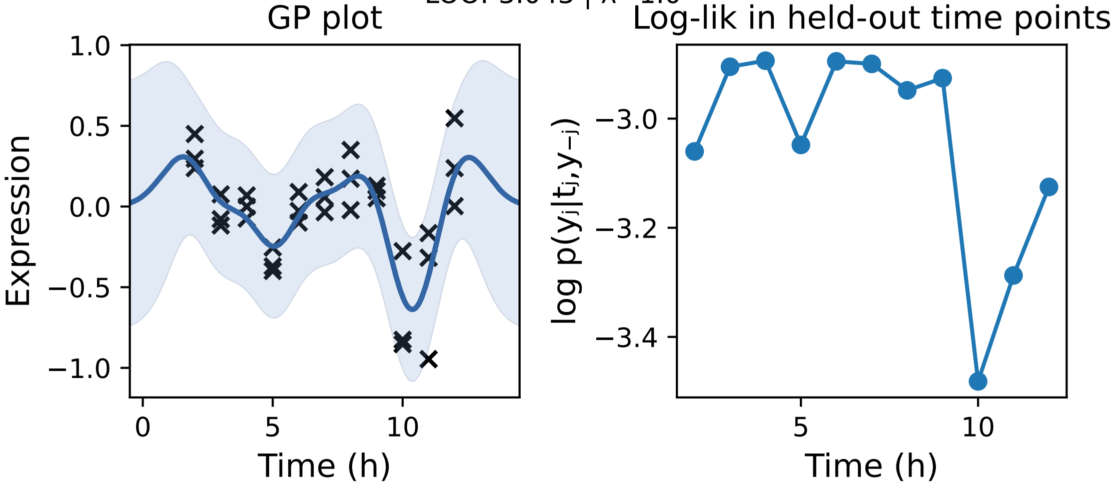

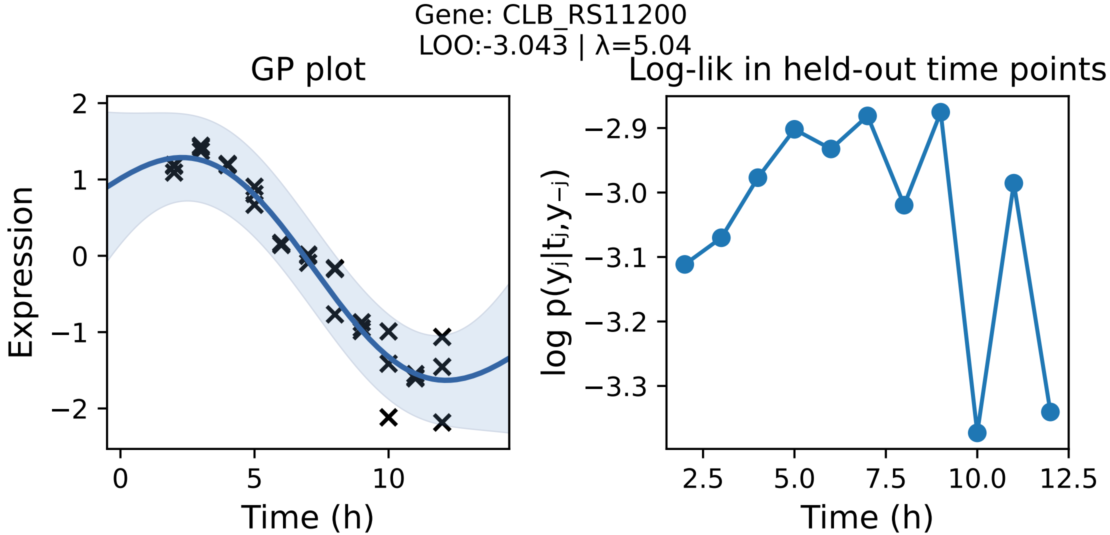

Gene: CLB_RS11200
LOO:-3.043 | λ=5.04
GP plot
Expression
Time (h)
Log-lik in held-out time points
log p(y_j|t_j,y_−j)
Time (h)

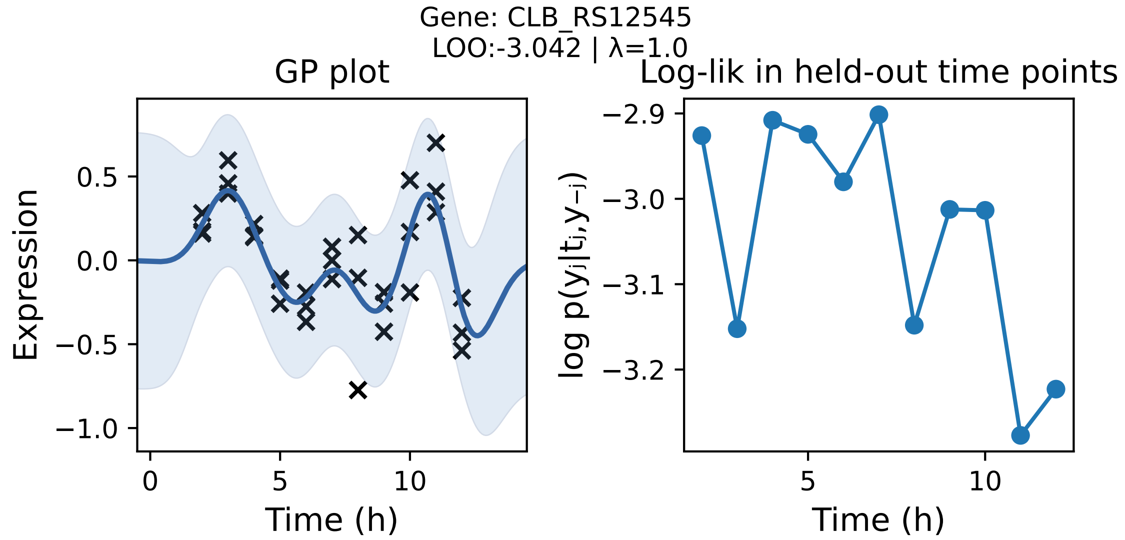

Gene: CLB_RS12545
LOO:-3.042 | λ=1.0
GP plot
Expression
Time (h)
0.5
0.0
−0.5
−1.0
0
5
10
Log-lik in held-out time points
log p(yj|tj,y−j)
Time (h)
−2.9
−3.0
−3.1
−3.2
5
10

Gene: CLB_RS00030
LOO:-3.042 | λ=4.85

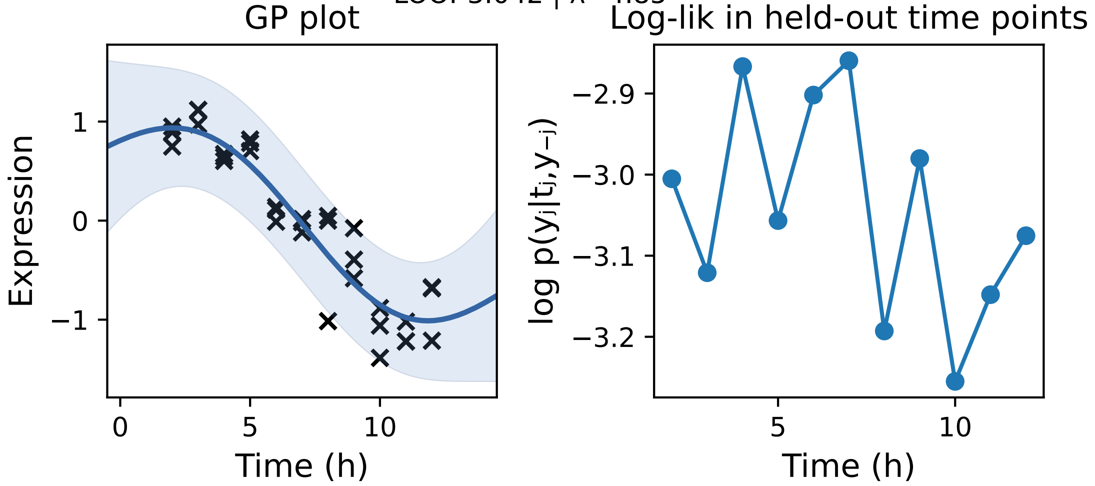

Gene: CLB_RS06015
LOO:-3.042 | λ=5.42

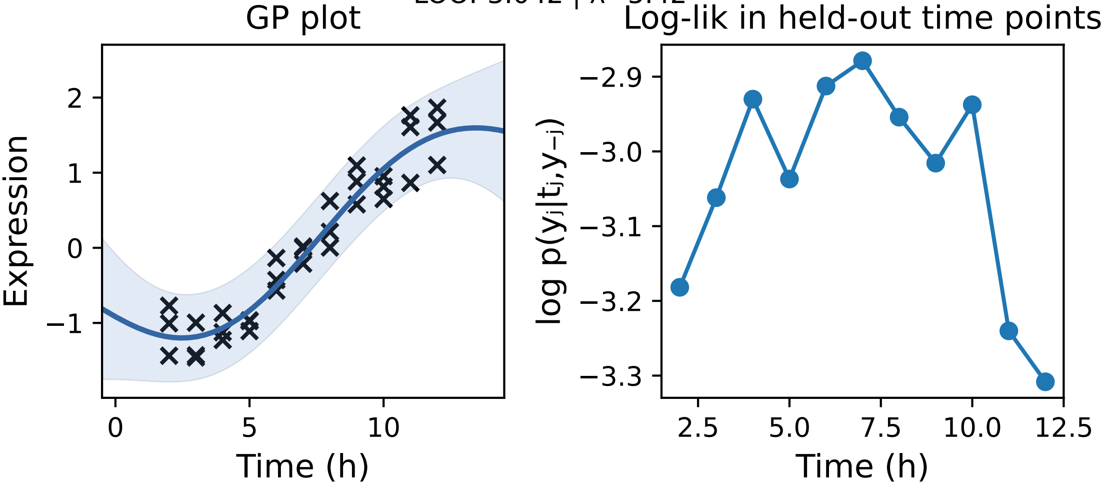

Gene: CLB_RS08915
LOO:-3.042 | λ=1.0

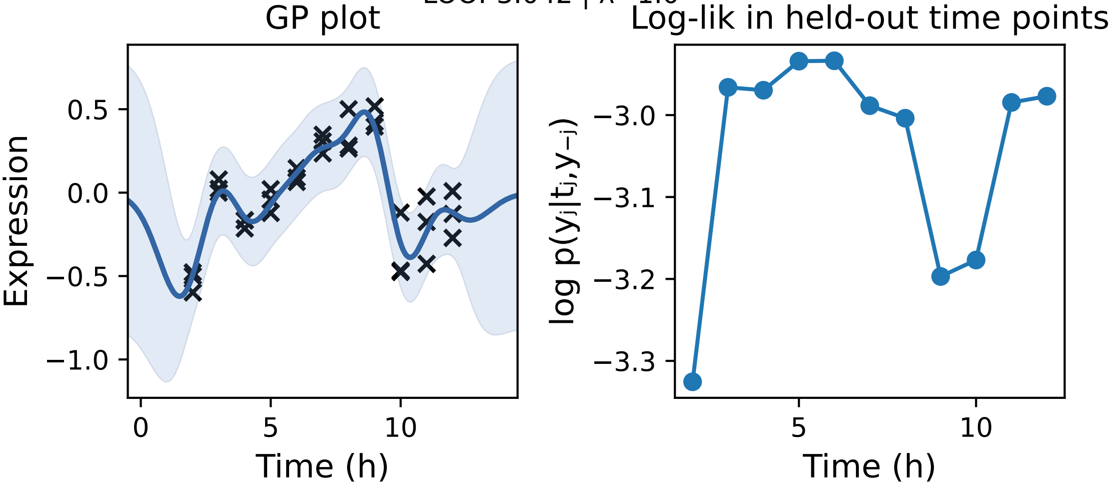

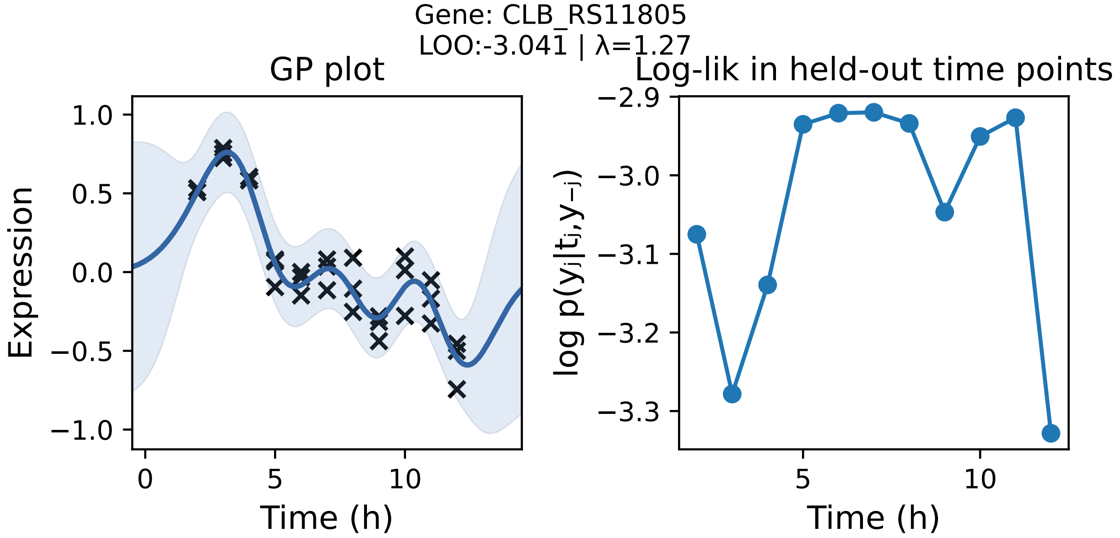

Gene: CLB_RS11805
LOO:-3.041 | λ=1.27
GP plot
Log-lik in held-out time points
Expression
1.0
0.5
0.0
−0.5
−1.0
0
5
10
Time (h)
log p(yⱼ|tⱼ,y₋ⱼ)
−2.9
−3.0
−3.1
−3.2
−3.3
5
10
Time (h)

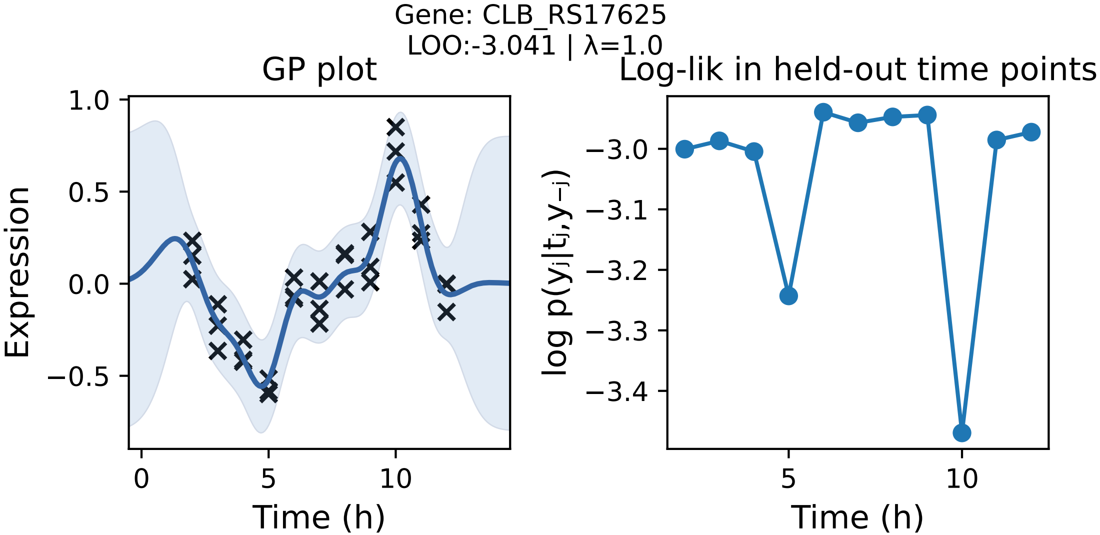
Gene: CLB_RS17625
LOO:-3.041 | λ=1.0
GP plot
Expression
Time (h)
Log-lik in held-out time points
log p(y_j|t_j,y_{-j})
Time (h)

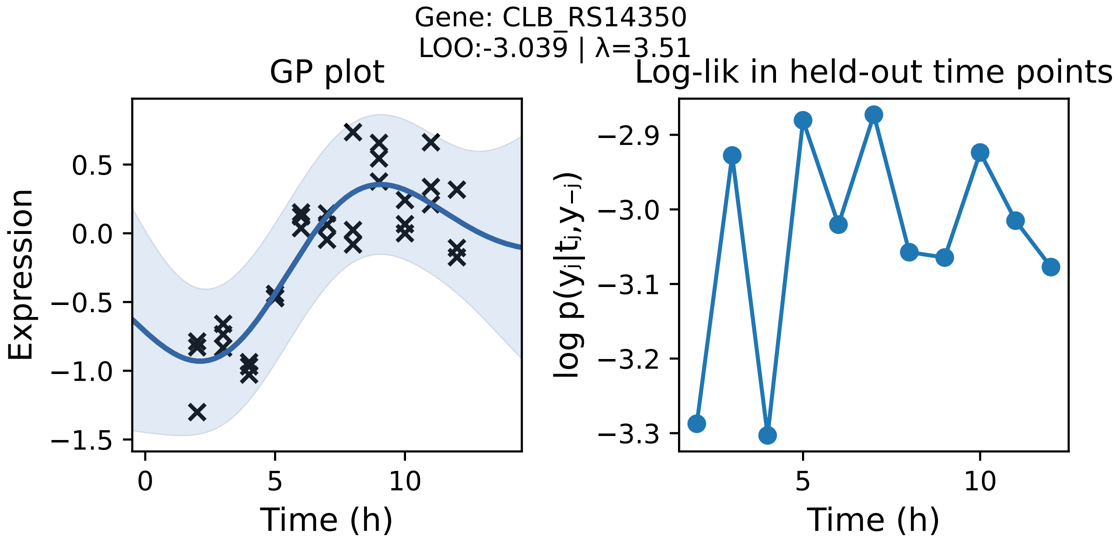

Gene: CLB_RS14350
LOO:-3.039 | λ=3.51
GP plot
Expression
0.5
0.0
−0.5
−1.0
−1.5
0
5
10
Time (h)
Log-lik in held-out time points
log p(y_j|t_j,y_−j)
−2.9
−3.0
−3.1
−3.2
−3.3
5
10
Time (h)

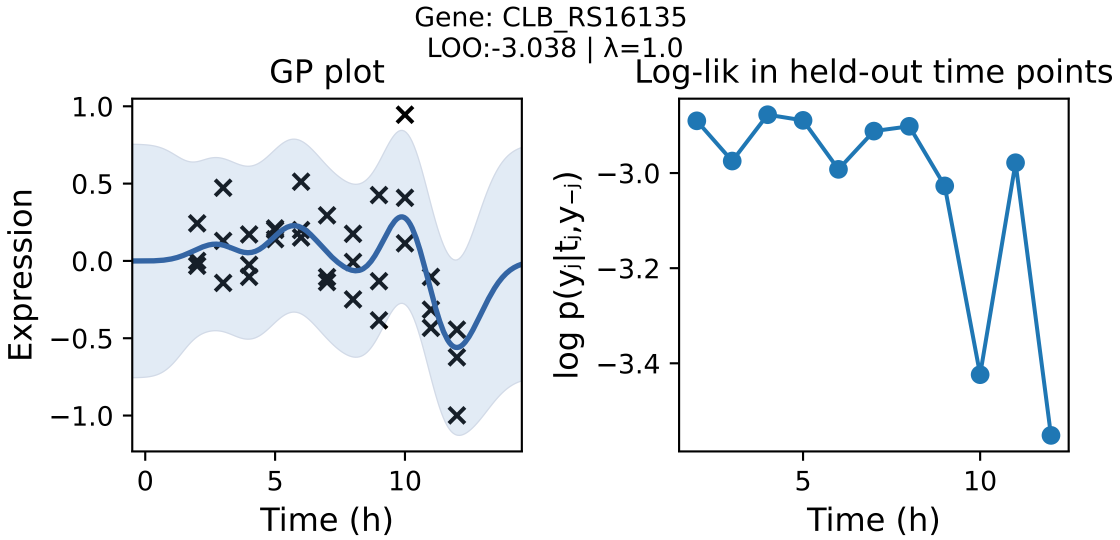
Gene: CLB_RS16135
LOO:-3.038 | λ=1.0
GP plot
Expression
1.0
0.5
0.0
−0.5
−1.0
0
5
10
Time (h)
Log-lik in held-out time points
log p(yj|tj,y−j)
−3.0
−3.2
−3.4
5
10
Time (h)

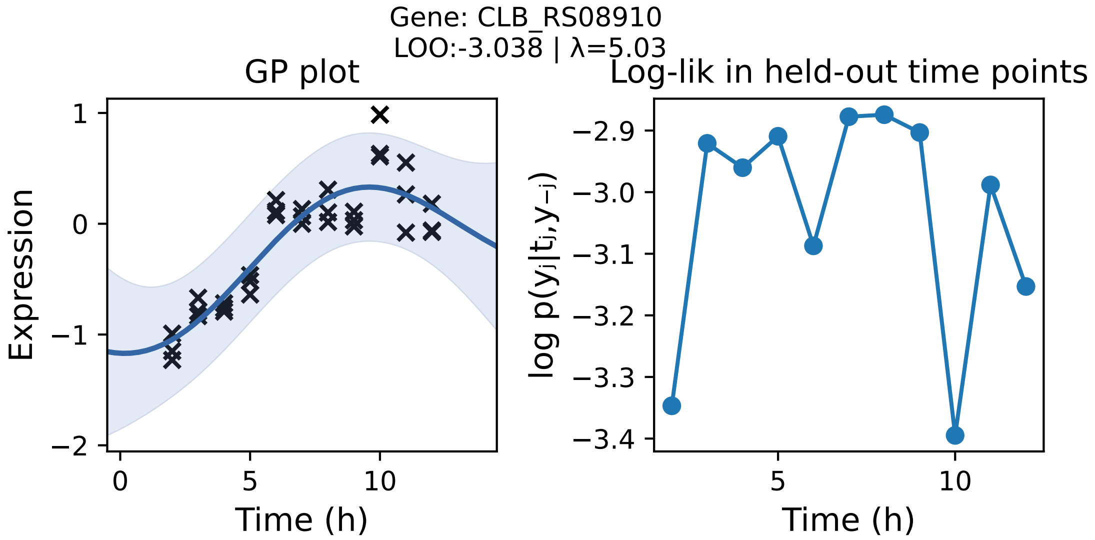
Gene: CLB_RS08910
LOO:-3.038 | λ=5.03
GP plot
Expression
Time (h)
1
0
−1
−2
0
5
10
Log-lik in held-out time points
log p(y_j|t_j,y_−j)
−2.9
−3.0
−3.1
−3.2
−3.3
−3.4
5
10
Time (h)

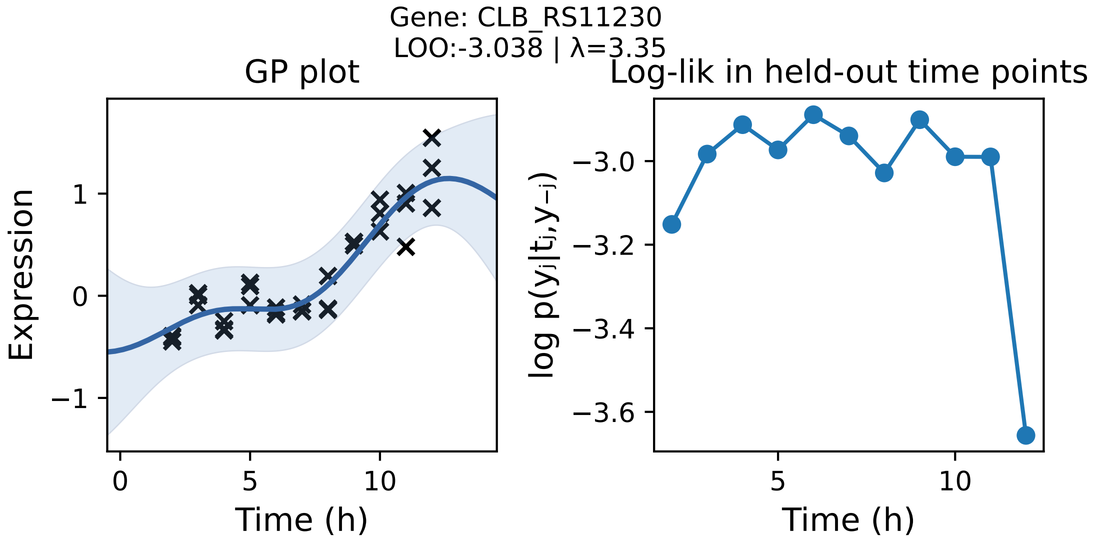

Gene: CLB_RS11230
LOO:-3.038 | λ=3.35
GP plot
Expression
1
0
−1
0
5
10
Time (h)
Log-lik in held-out time points
log p(yj|tj,y−j)
−3.0
−3.2
−3.4
−3.6
5
10
Time (h)

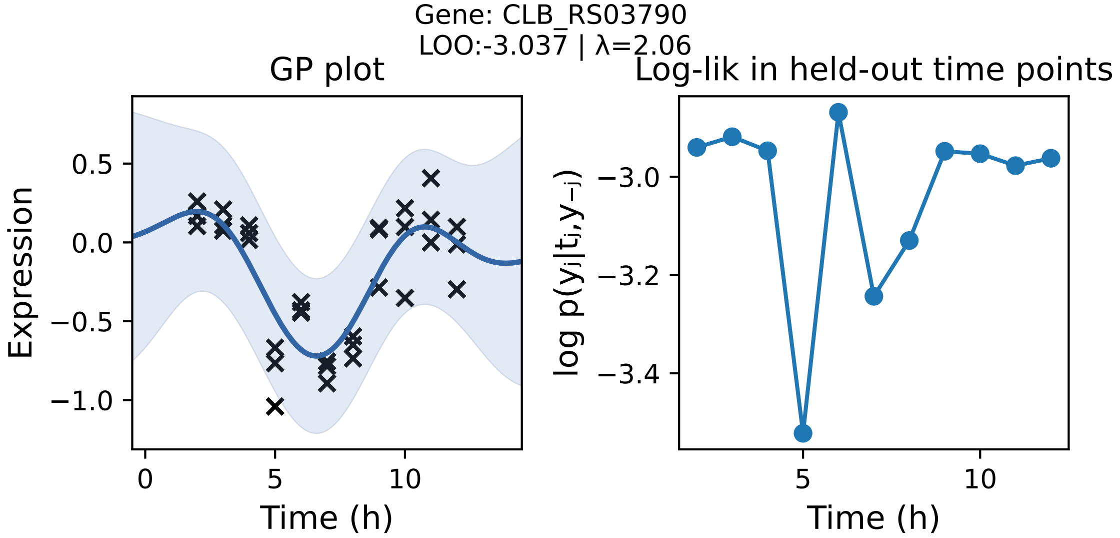

Gene: CLB_RS03790
LOO:-3.037 | λ=2.06
GP plot
Expression
0.5
0.0
−0.5
−1.0
0
5
10
Time (h)
Log-lik in held-out time points
log p(y_j|t_j,y_−j)
−3.0
−3.2
−3.4
5
10
Time (h)

Gene: CLB_RS16800
LOO:-3.035 | λ=7.32

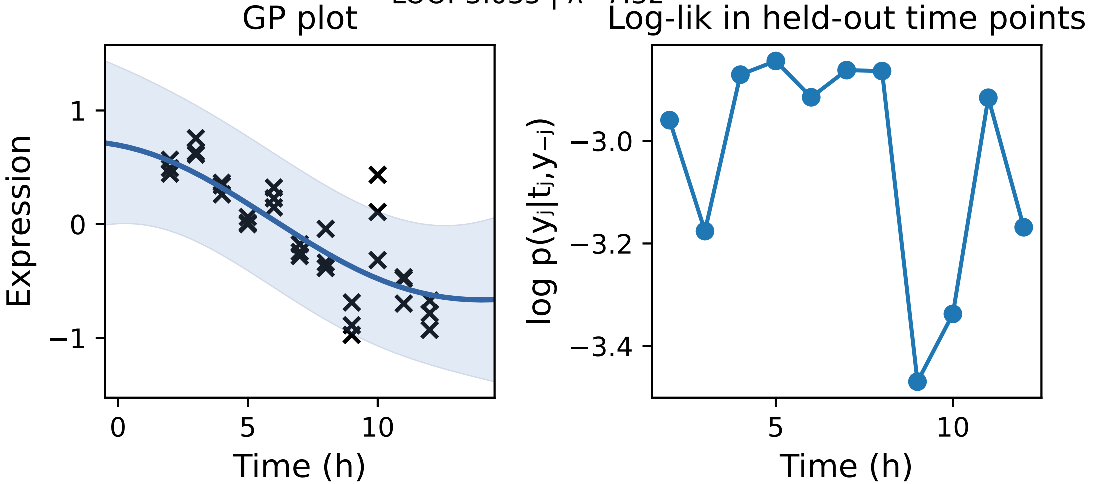

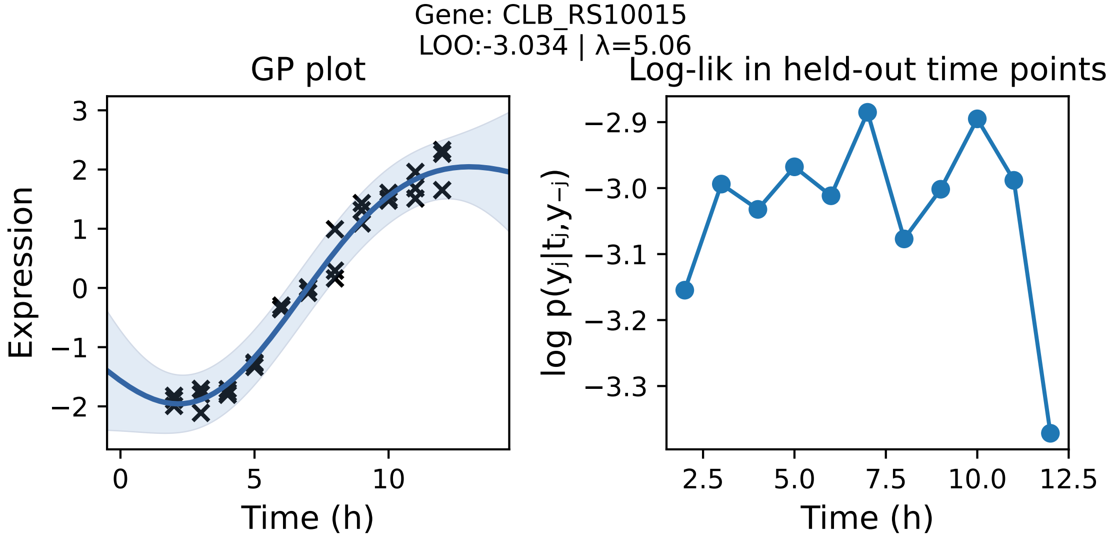

Gene: CLB_RS10015
LOO:-3.034 | λ=5.06
GP plot
Expression
Time (h)
Log-lik in held-out time points
$\log p(y_j|t_j, y_{-j})$
Time (h)
3
2
1
0
−1
−2
0
5
10
−2.9
−3.0
−3.1
−3.2
−3.3
2.5
5.0
7.5
10.0
12.5

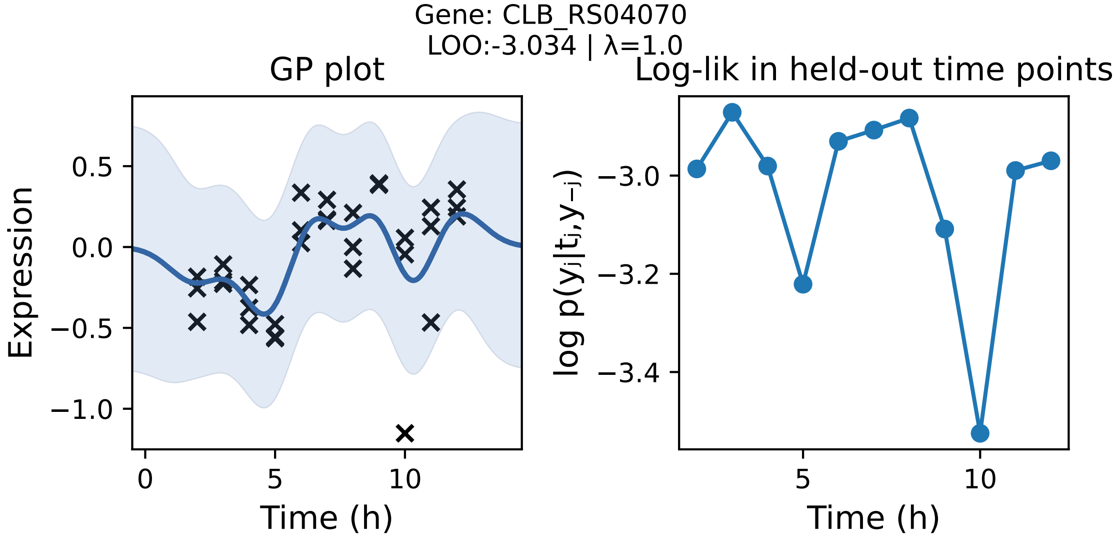

Gene: CLB_RS04070
LOO:-3.034 | λ=1.0
GP plot
Expression
0.5
0.0
−0.5
−1.0
0
5
10
Time (h)
Log-lik in held-out time points
log p(y_j|t_j,y_−j)
−3.0
−3.2
−3.4
5
10
Time (h)

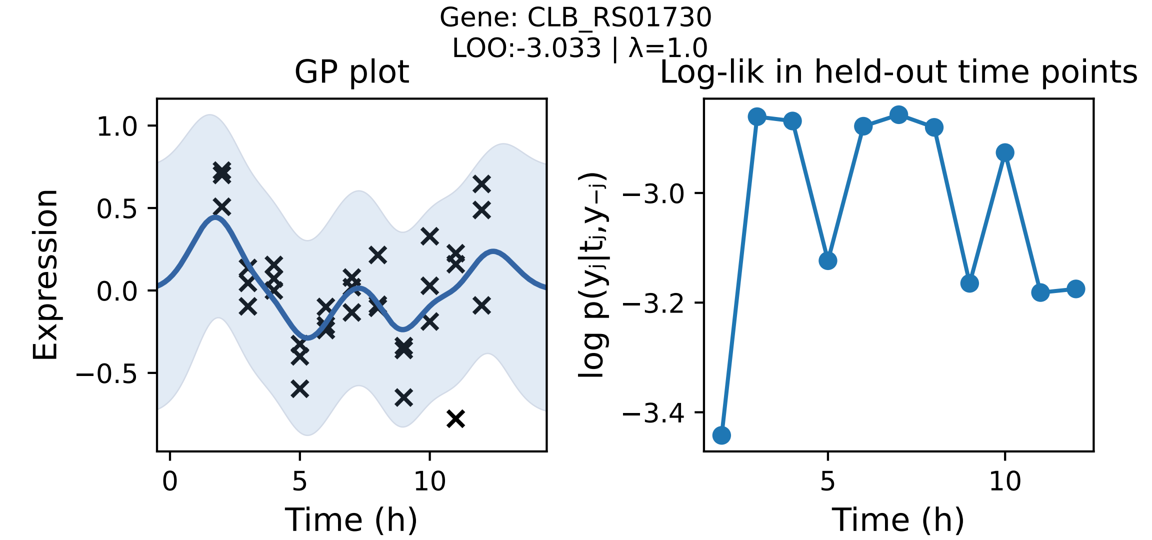
Gene: CLB_RS01730
LOO:-3.033 | λ=1.0
GP plot
Expression
1.0
0.5
0.0
−0.5
0
5
10
Time (h)
Log-lik in held-out time points
log p(y_j|t_j,y_−j)
−3.0
−3.2
−3.4
5
10
Time (h)

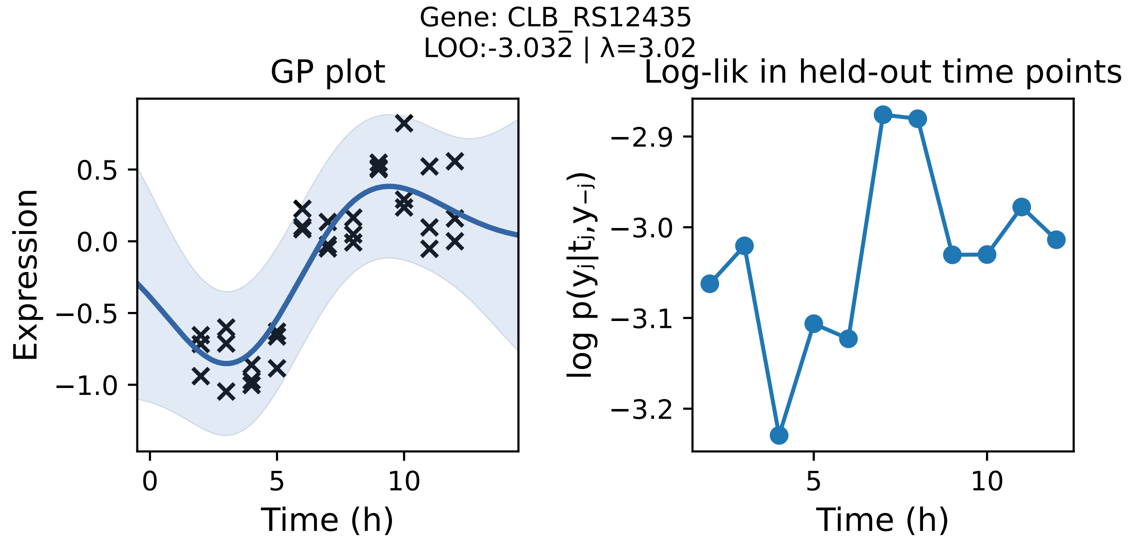

Gene: CLB_RS12435
LOO:-3.032 | λ=3.02
GP plot
Expression
Time (h)
Log-lik in held-out time points
log p(y_j|t_j,y_−j)
Time (h)

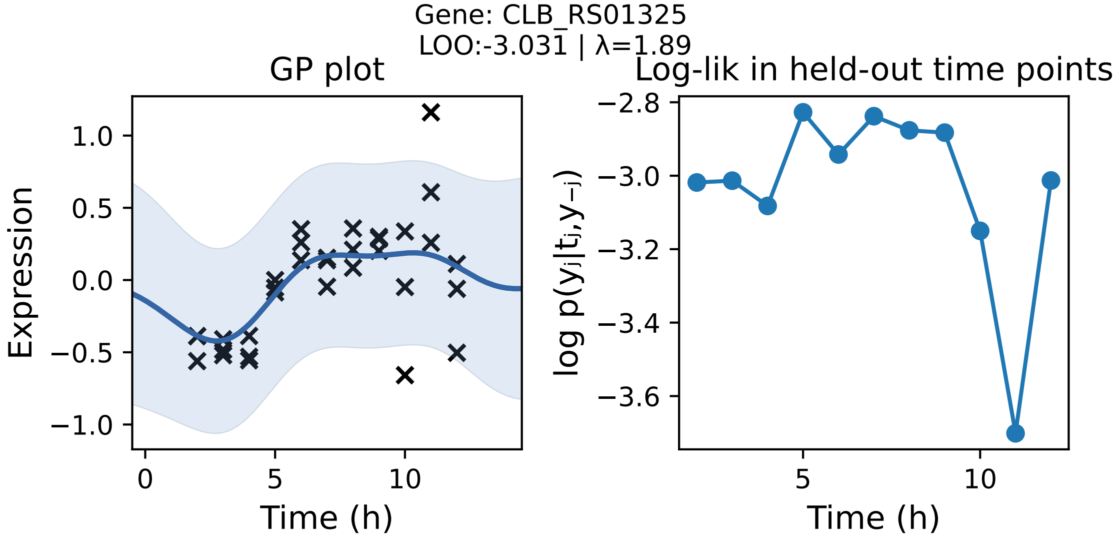
Gene: CLB_RS01325
LOO:-3.031 | λ=1.89
GP plot
Expression
Time (h)
1.0
0.5
0.0
−0.5
−1.0
0
5
10
Log-lik in held-out time points
log p(y_j|t_j,y_−j)
−2.8
−3.0
−3.2
−3.4
−3.6
5
10
Time (h)

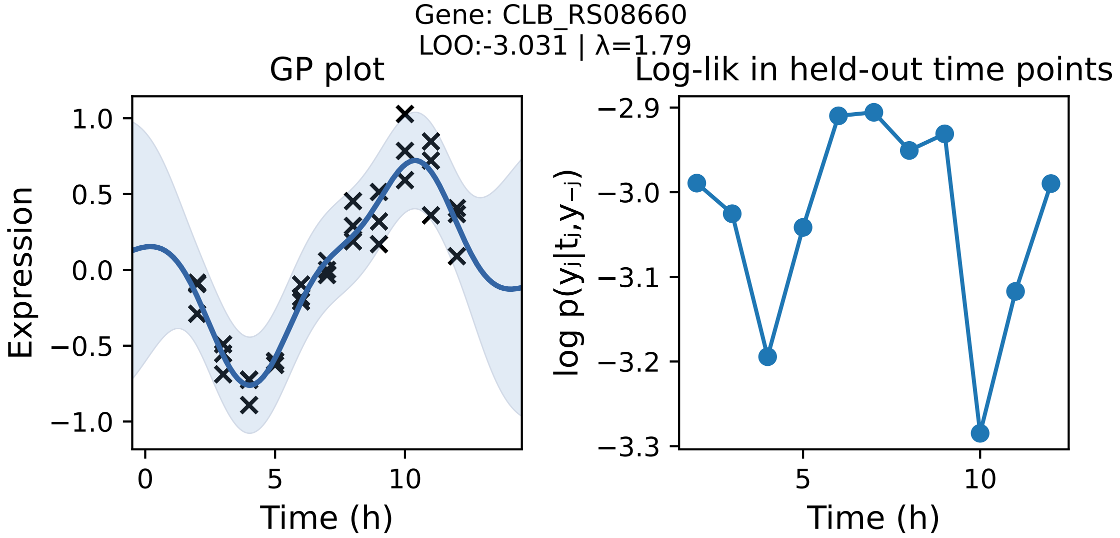
Gene: CLB_RS08660
LOO:-3.031 | λ=1.79
GP plot
Expression
Time (h)
1.0
0.5
0.0
−0.5
−1.0
0
5
10
Log-lik in held-out time points
log p(y_j|t_j,y_−j)
Time (h)
−2.9
−3.0
−3.1
−3.2
−3.3
5
10

Gene: CLB_RS12835
LOO:-3.03 | λ=6.61

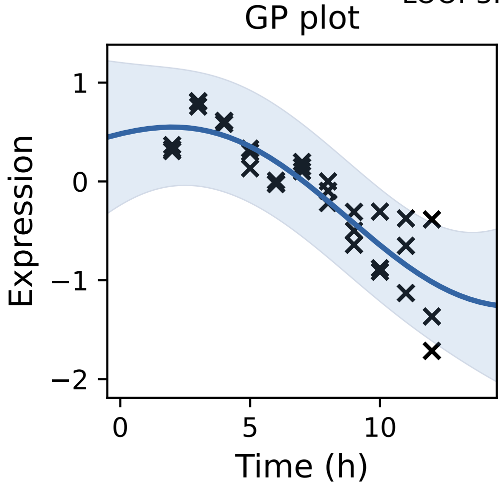

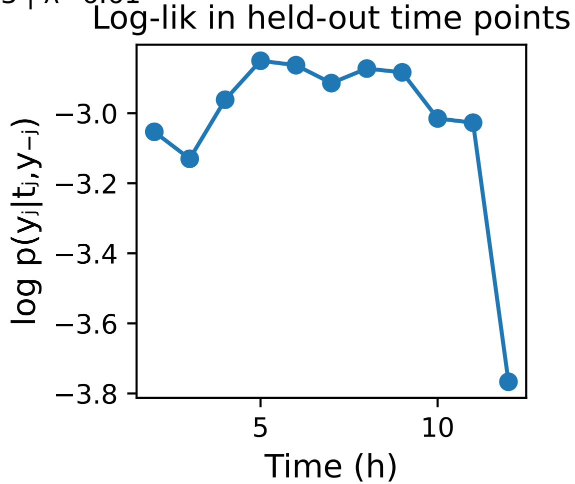

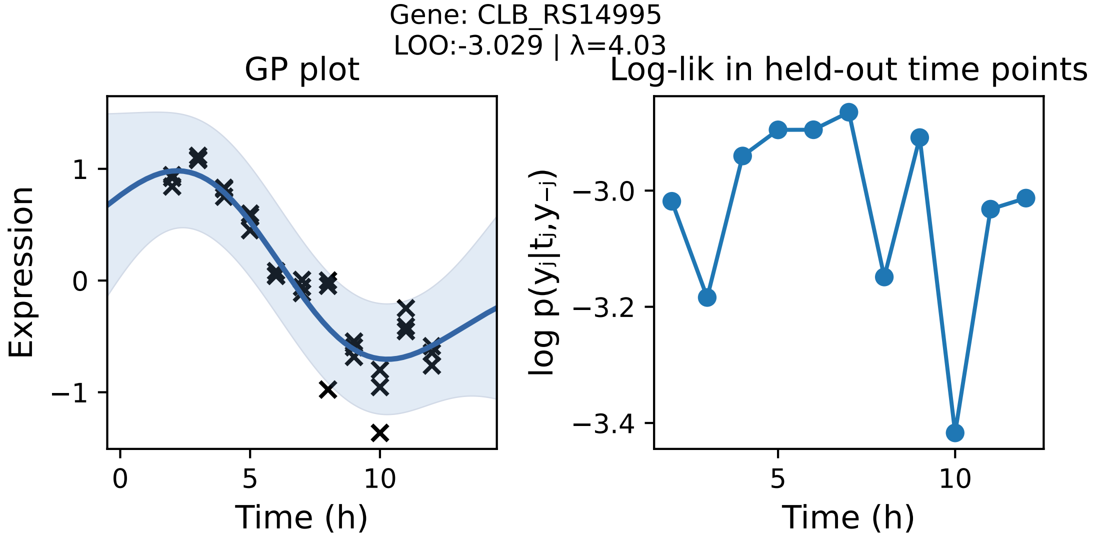

Gene: CLB_RS14995
LOO:-3.029 | λ=4.03
GP plot
Log-lik in held-out time points
Expression
Time (h)
log p(y_j|t_j,y_−j)
Time (h)

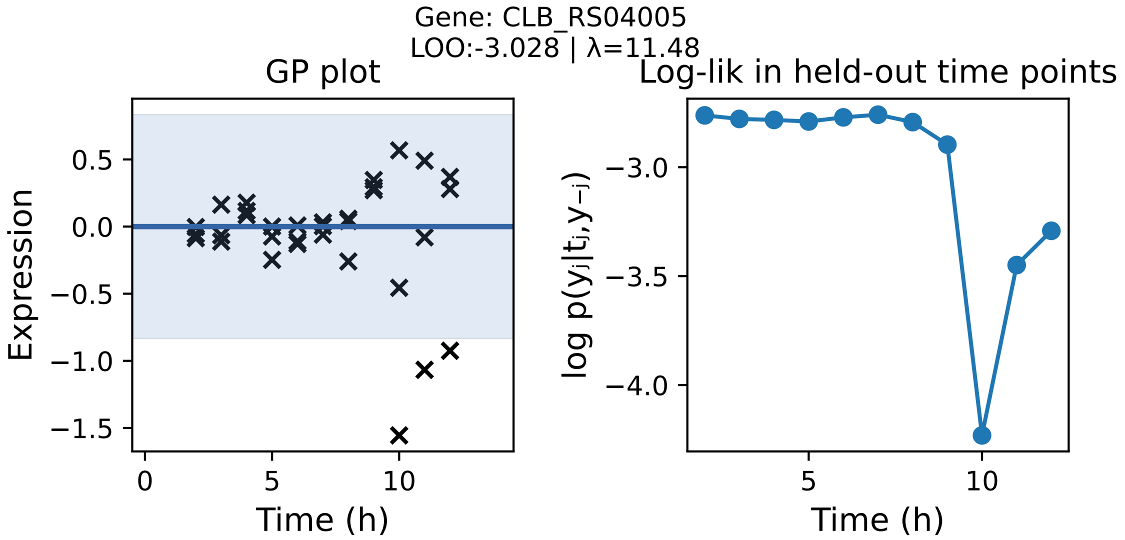

Gene: CLB_RS04005
LOO:-3.028 | λ=11.48
GP plot
Expression
Time (h)
0.5
0.0
−0.5
−1.0
−1.5
0
5
10
Log-lik in held-out time points
log p(y_j|t_j,y_−j)
Time (h)
−3.0
−3.5
−4.0
5
10

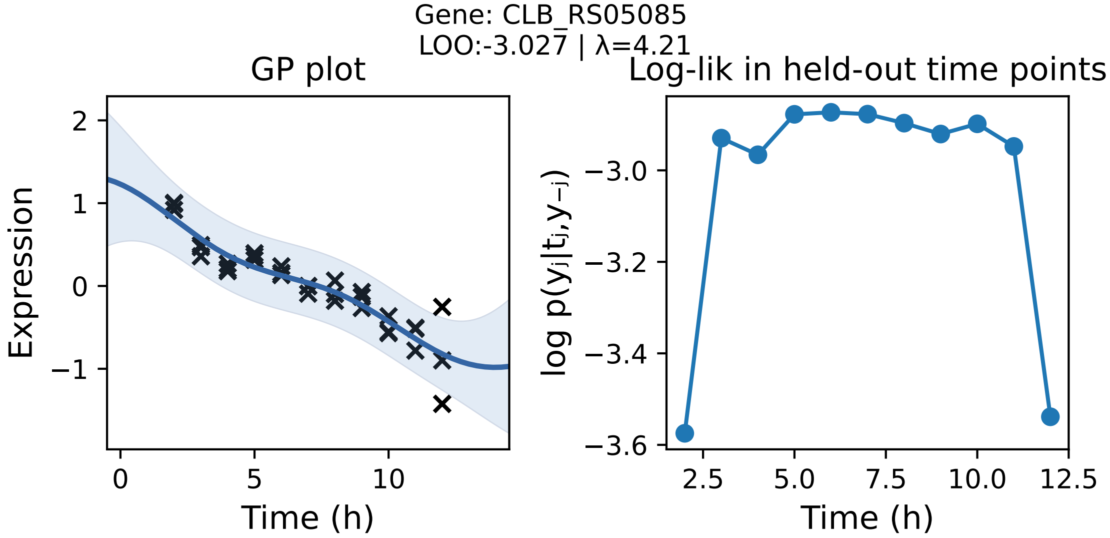

Gene: CLB_RS05085
LOO:-3.027 | λ=4.21
GP plot
Expression
Time (h)
2
1
0
−1
0
5
10
Log-lik in held-out time points
log p(y_j|t_j,y_−j)
Time (h)
−3.0
−3.2
−3.4
−3.6
2.5
5.0
7.5
10.0
12.5

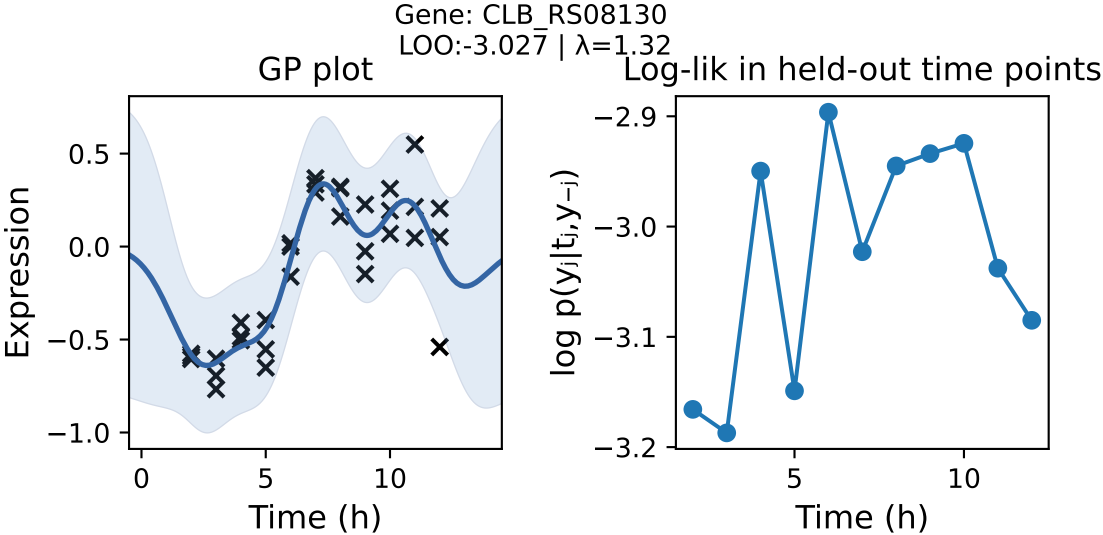

Gene: CLB_RS08130
LOO:-3.027 | λ=1.32
GP plot
Expression
Time (h)
0.5
0.0
−0.5
−1.0
0
5
10
Log-lik in held-out time points
log p(y_j|t_j,y_−j)
−2.9
−3.0
−3.1
−3.2
5
10
Time (h)

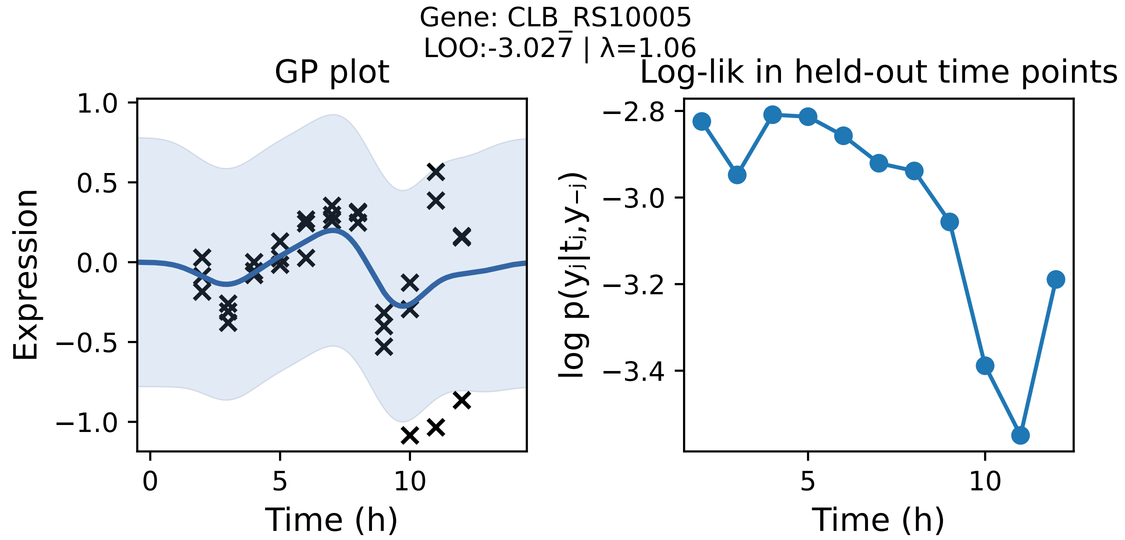

Gene: CLB_RS10005
LOO:-3.027 | λ=1.06
GP plot
Log-lik in held-out time points
Expression
1.0
0.5
0.0
−0.5
−1.0
0
5
10
Time (h)
log p(y_j|t_j,y_−j)
−2.8
−3.0
−3.2
−3.4
5
10
Time (h)

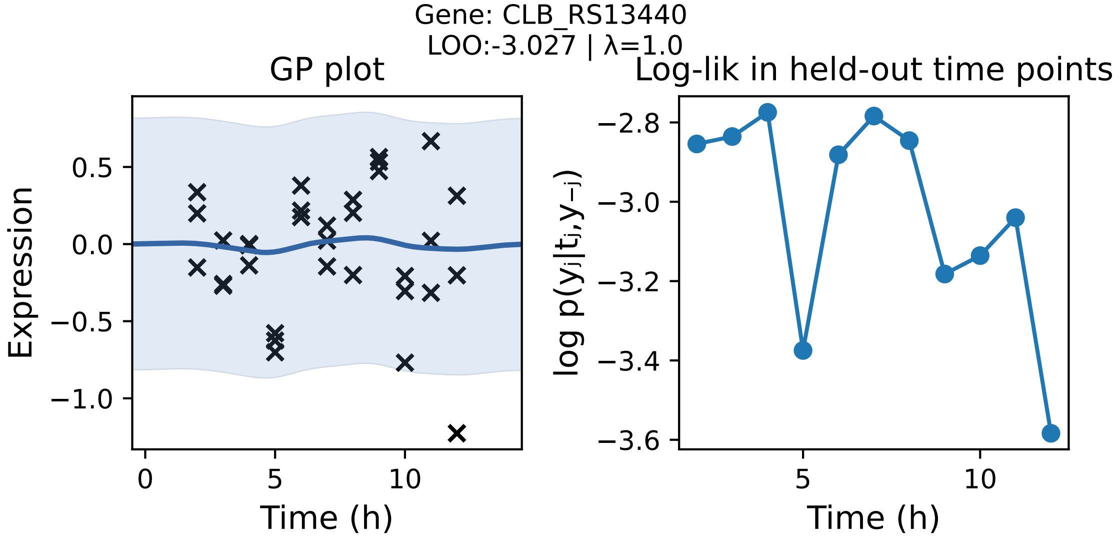

Gene: CLB_RS13440
LOO:-3.027 | λ=1.0
GP plot
Expression
0.5
0.0
−0.5
−1.0
0
5
10
Time (h)
Log-lik in held-out time points
log p(y_j|t_j,y_−j)
−2.8
−3.0
−3.2
−3.4
−3.6
5
10
Time (h)

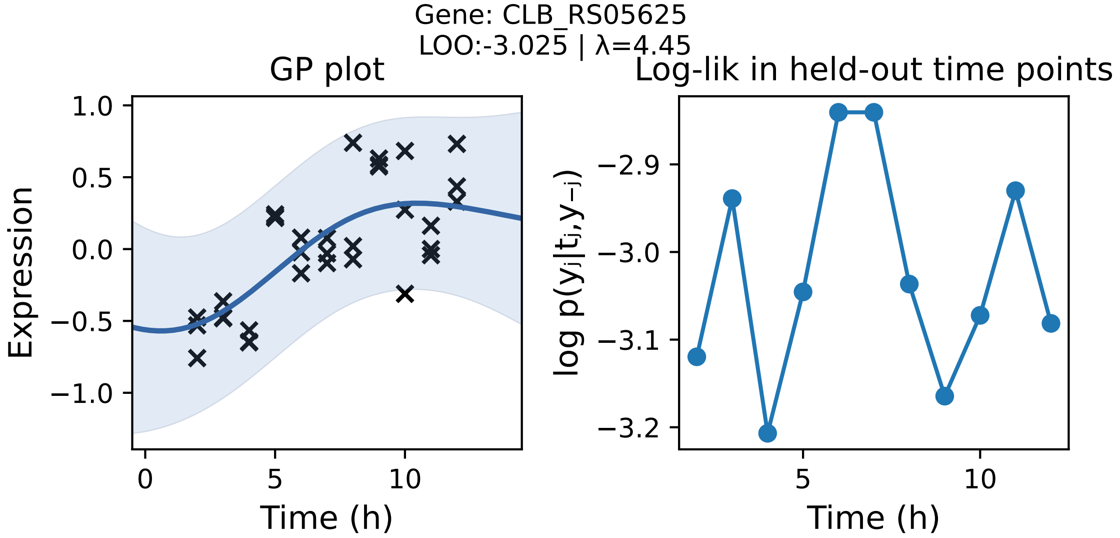

Gene: CLB_RS05625
LOO:-3.025 | λ=4.45
GP plot
Expression
Time (h)
Log-lik in held-out time points
log p(yⱼ|tⱼ,y₋ⱼ)
Time (h)

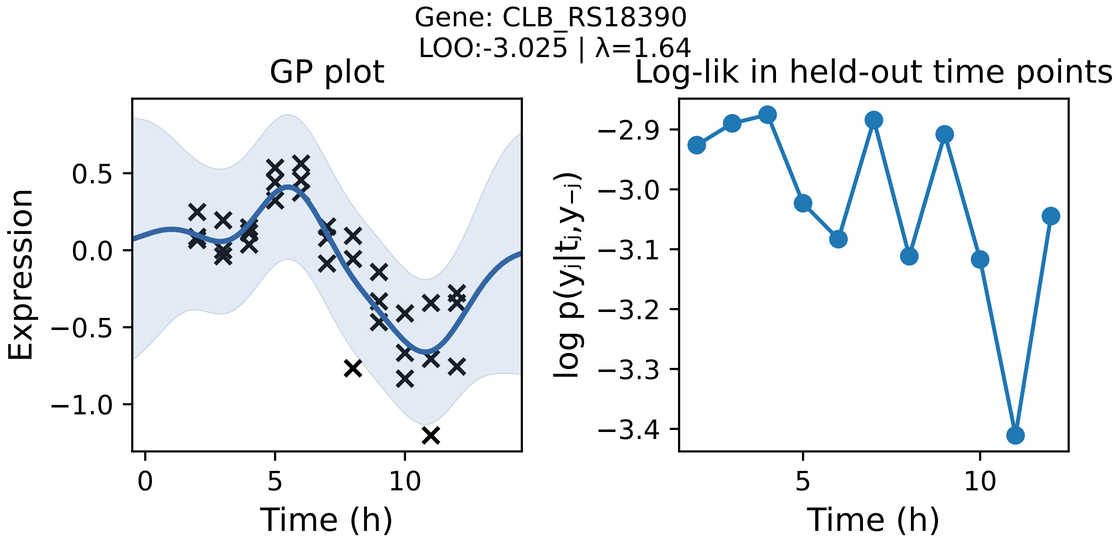

Gene: CLB_RS18390
LOO:-3.025 | λ=1.64
GP plot
Expression
Time (h)
0.5
0.0
−0.5
−1.0
0
5
10
Log-lik in held-out time points
log p(y_j|t_j,y_{−j})
−2.9
−3.0
−3.1
−3.2
−3.3
−3.4
5
10
Time (h)

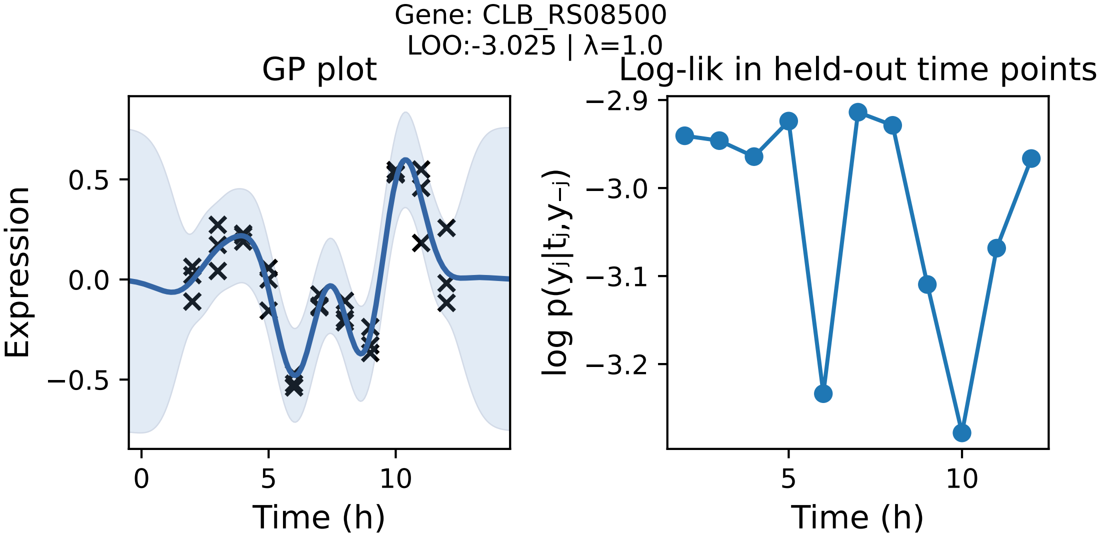

Gene: CLB_RS08500
LOO:-3.025 | λ=1.0
GP plot
Expression
0.5
0.0
−0.5
0
5
10
Time (h)
Log-lik in held-out time points
log p(y_j|t_j,y_−j)
−2.9
−3.0
−3.1
−3.2
5
10
Time (h)

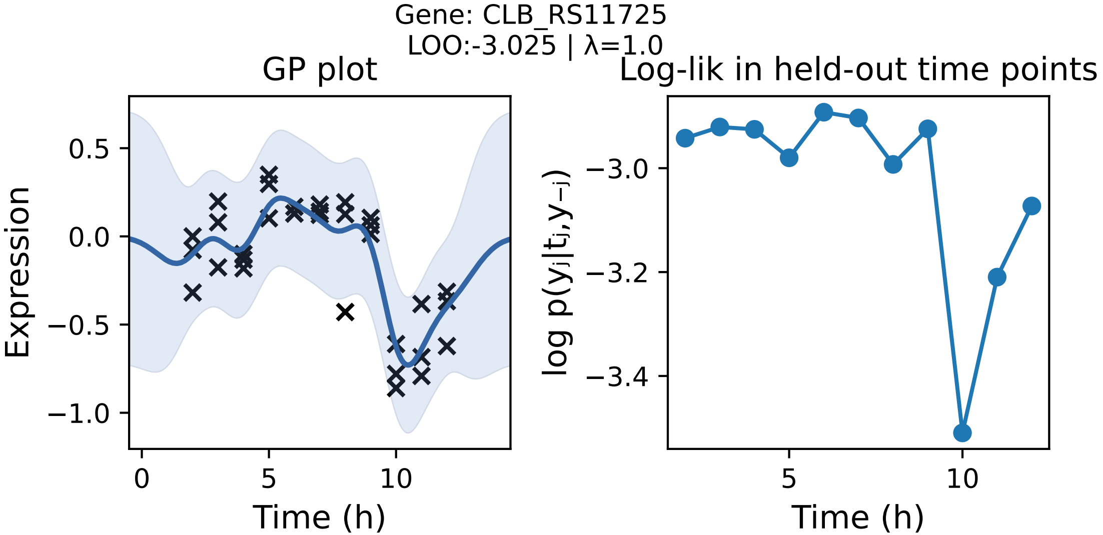

Gene: CLB_RS11725
LOO:-3.025 | λ=1.0
GP plot
Log-lik in held-out time points
Expression
0.5
0.0
−0.5
−1.0
0
5
10
Time (h)
log p(y_j|t_j,y_−j)
−3.0
−3.2
−3.4
5
10
Time (h)

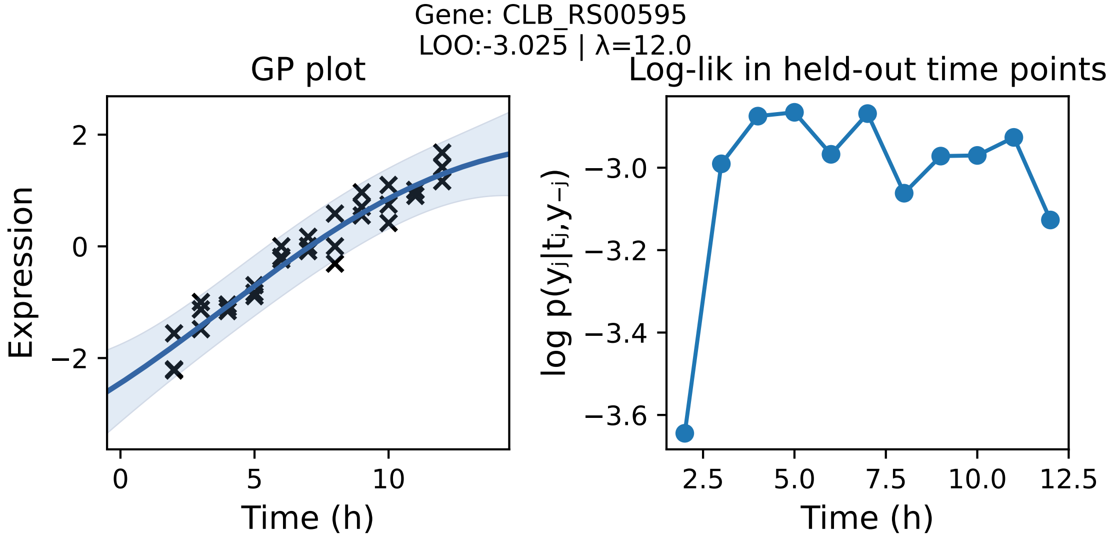

Gene: CLB_RS00595
LOO:-3.025 | λ=12.0
GP plot
Expression
Time (h)
2
0
−2
0
5
10
Log-lik in held-out time points
log p(y_j|t_j,y_−j)
Time (h)
−3.0
−3.2
−3.4
−3.6
2.5
5.0
7.5
10.0
12.5

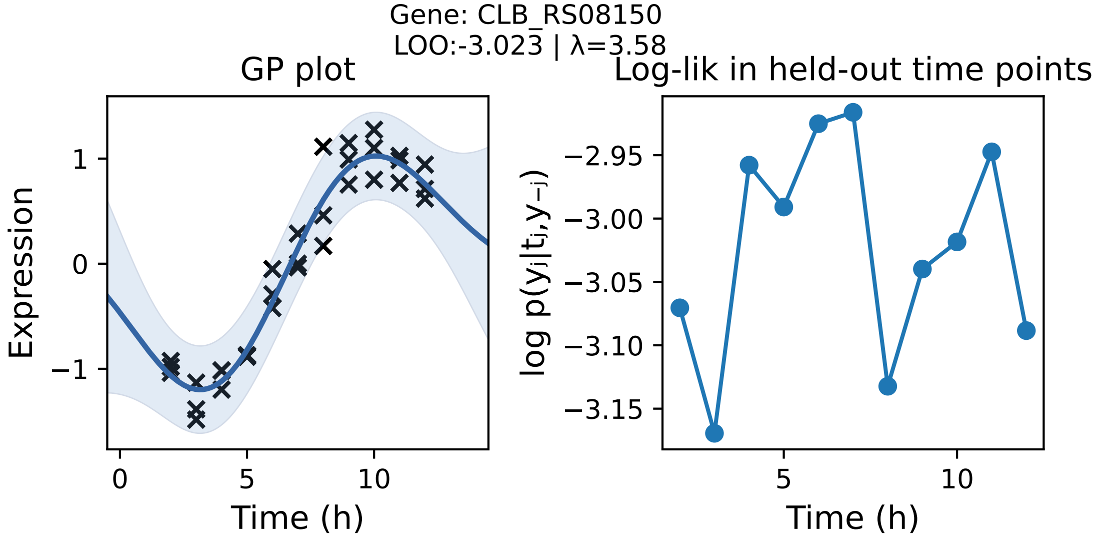

Gene: CLB_RS08150
LOO:-3.023 | λ=3.58
GP plot
Expression
1
0
−1
0
5
10
Time (h)
Log-lik in held-out time points
log p(y_j|t_j,y_−j)
−2.95
−3.00
−3.05
−3.10
−3.15
5
10
Time (h)

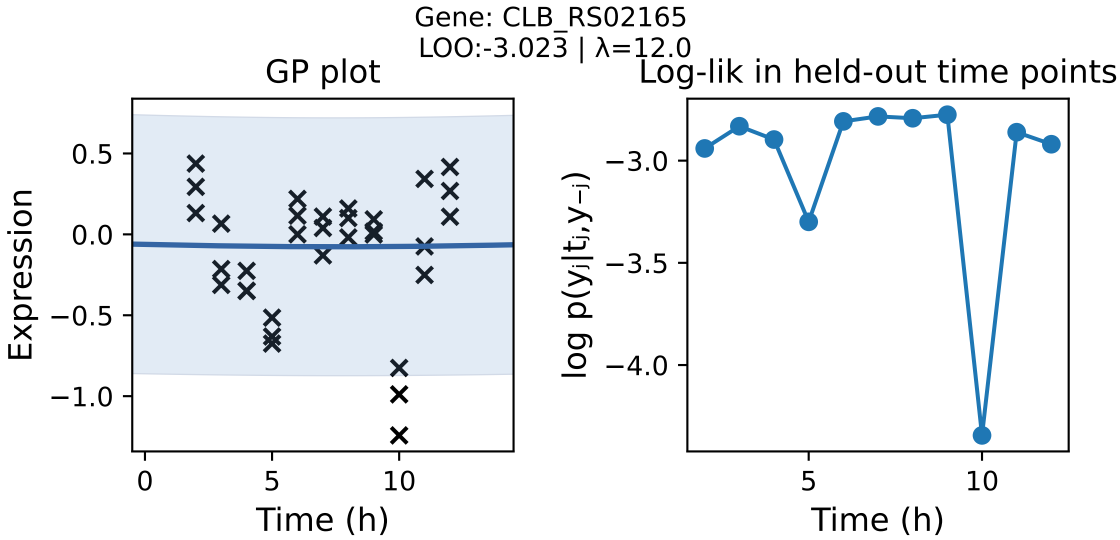

Gene: CLB_RS02165
LOO:-3.023 | λ=12.0
GP plot
Expression
Time (h)
0.5
0.0
−0.5
−1.0
0
5
10
Log-lik in held-out time points
log p(y_j|t_j,y_−j)
−3.0
−3.5
−4.0
5
10
Time (h)

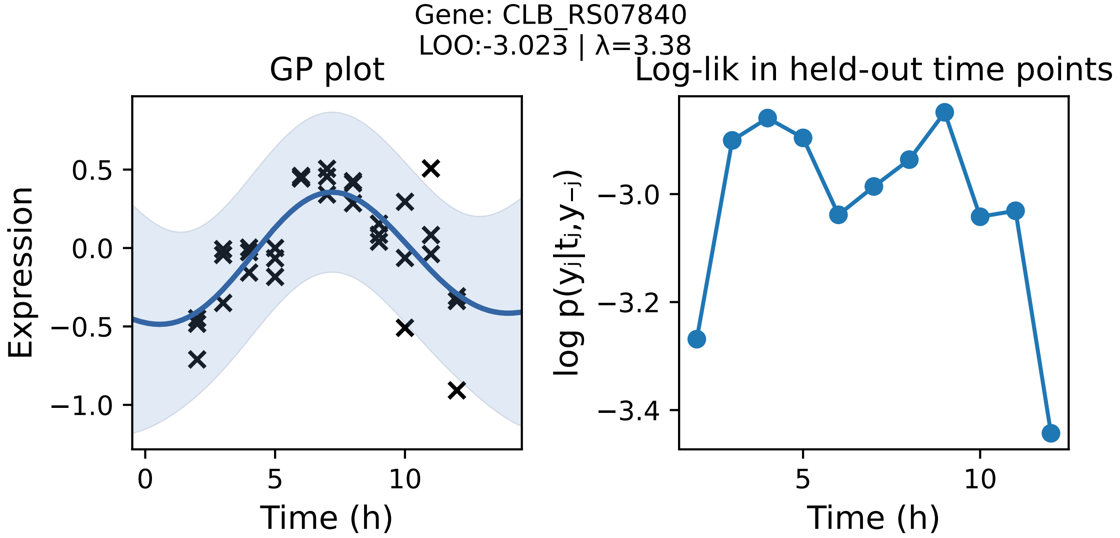

Gene: CLB_RS07840
LOO:-3.023 | λ=3.38
GP plot
Expression
0.5
0.0
−0.5
−1.0
0
5
10
Time (h)
Log-lik in held-out time points
log p(y_j|t_j,y_−j)
−3.0
−3.2
−3.4
5
10
Time (h)

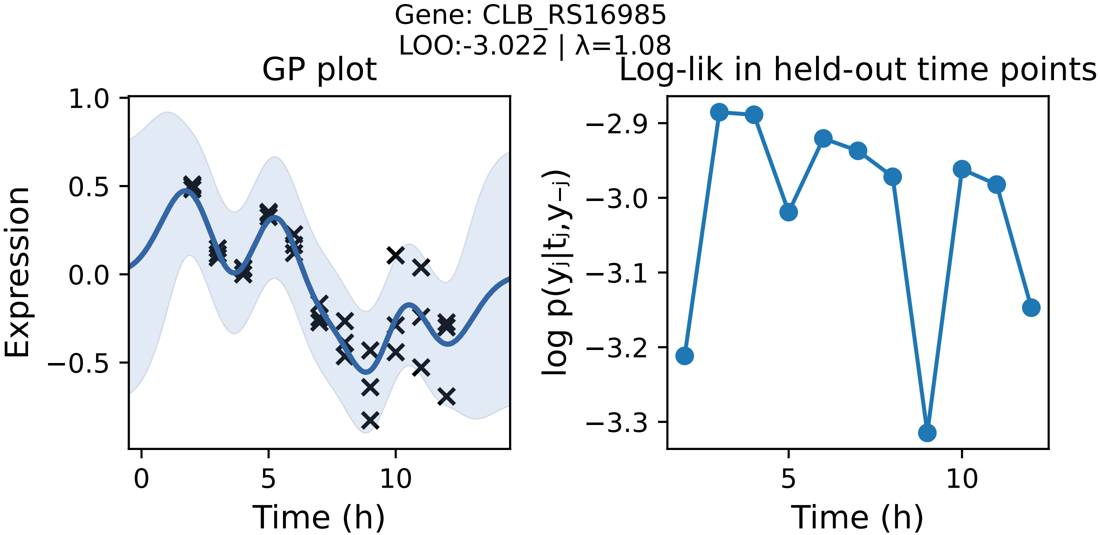
Gene: CLB_RS16985
LOO:-3.022 | λ=1.08
GP plot
Expression
Time (h)
1.0
0.5
0.0
−0.5
0
5
10
Log-lik in held-out time points
log p(y_j|t_j,y_−j)
Time (h)
−2.9
−3.0
−3.1
−3.2
−3.3
5
10

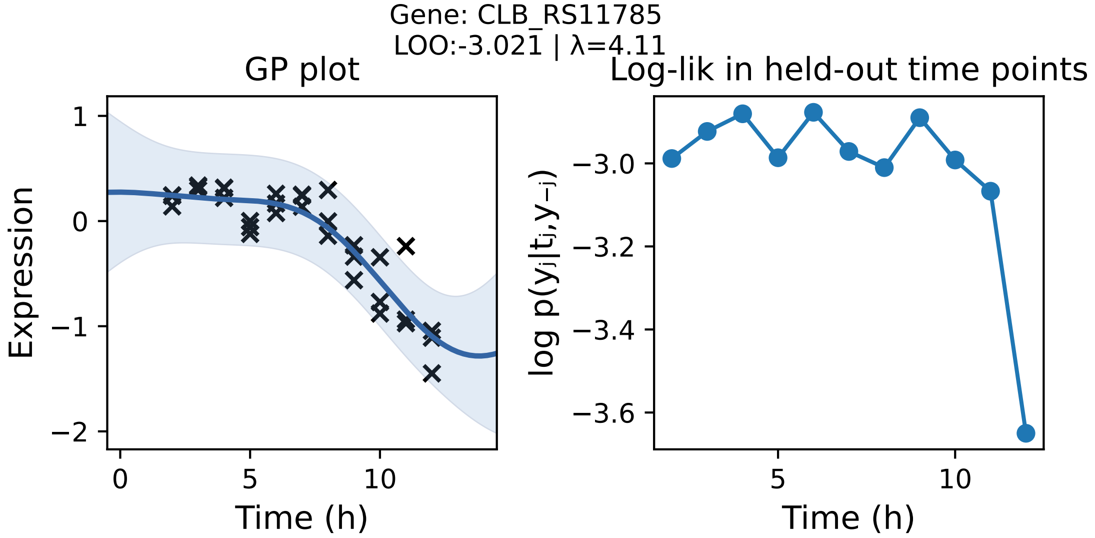

Gene: CLB_RS11785
LOO:-3.021 | λ=4.11
GP plot
Log-lik in held-out time points
Expression
1
0
−1
−2
0
5
10
Time (h)
log p(yⱼ|tⱼ,y₋ⱼ)
−3.0
−3.2
−3.4
−3.6
5
10
Time (h)

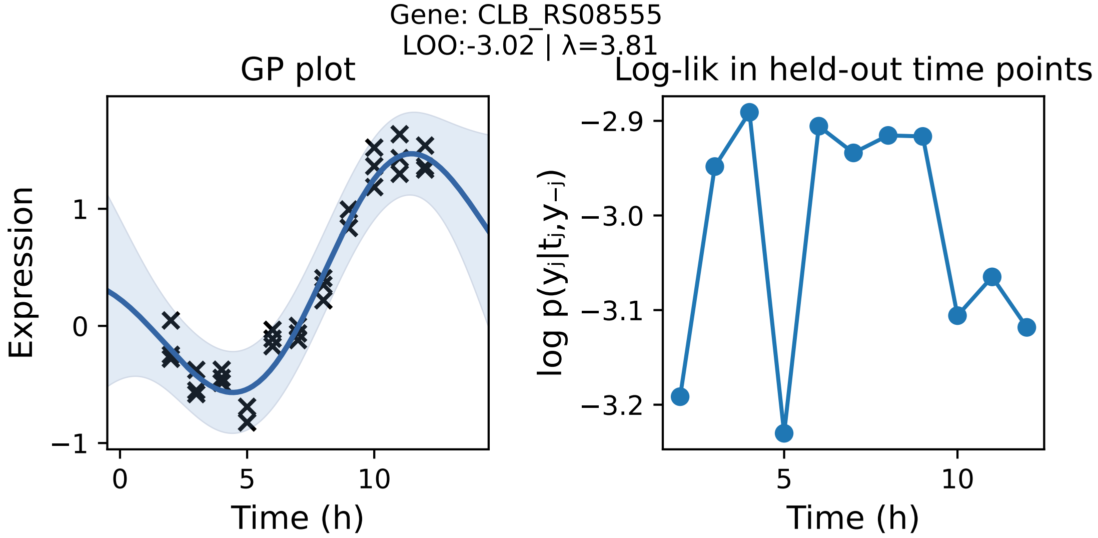

Gene: CLB_RS08555
LOO:-3.02 | λ=3.81
GP plot
Expression
Time (h)
Log-lik in held-out time points
log p(y_j|t_j,y_−j)
−2.9
−3.0
−3.1
−3.2
Time (h)

Gene: CLB_RS07560
LOO:-3.019 | λ=5.33

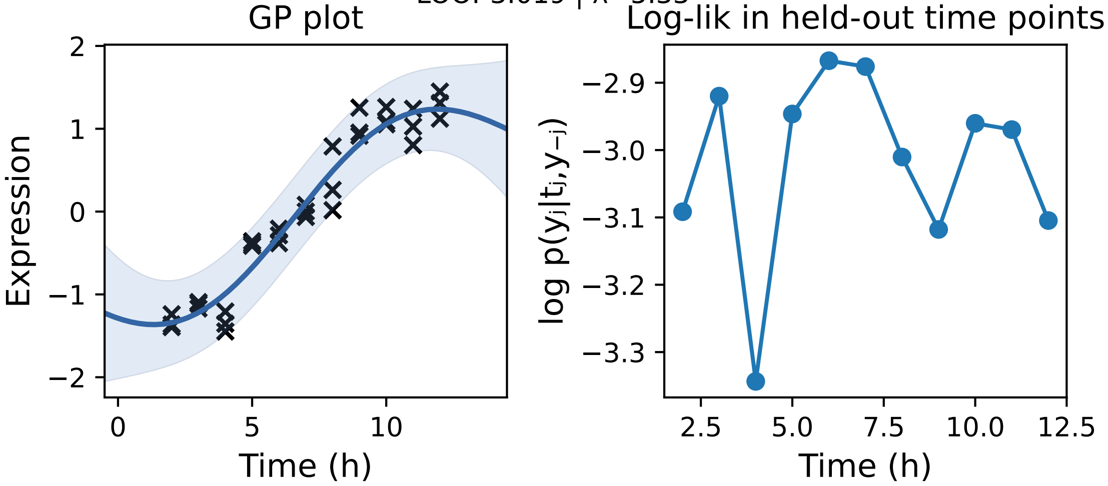

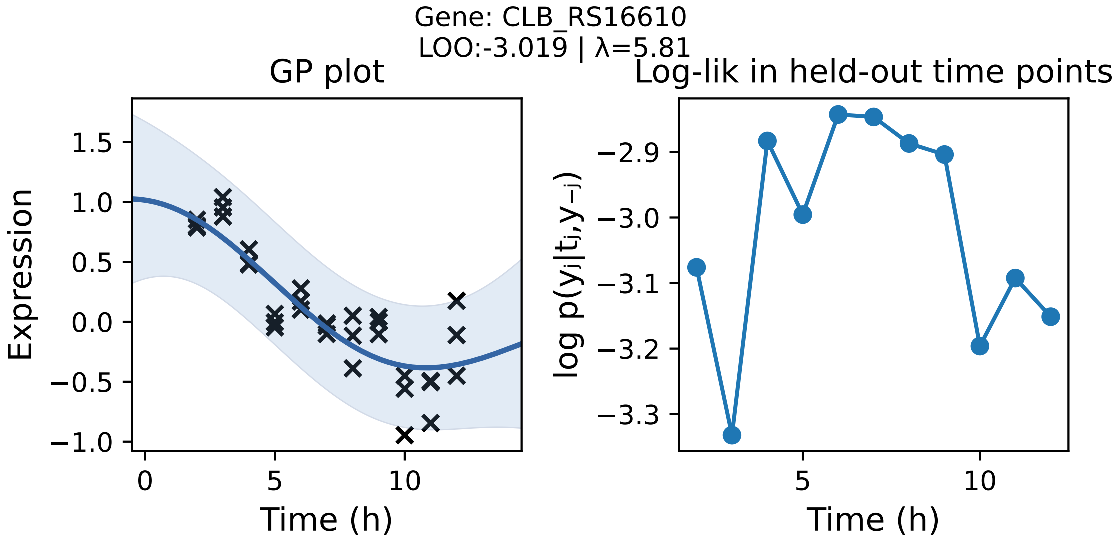

Gene: CLB_RS16610
LOO:-3.019 | λ=5.81
GP plot
Expression
Time (h)
1.5
1.0
0.5
0.0
−0.5
−1.0
0
5
10
Log-lik in held-out time points
log p(y_j|t_j,y_−j)
−2.9
−3.0
−3.1
−3.2
−3.3
5
10
Time (h)

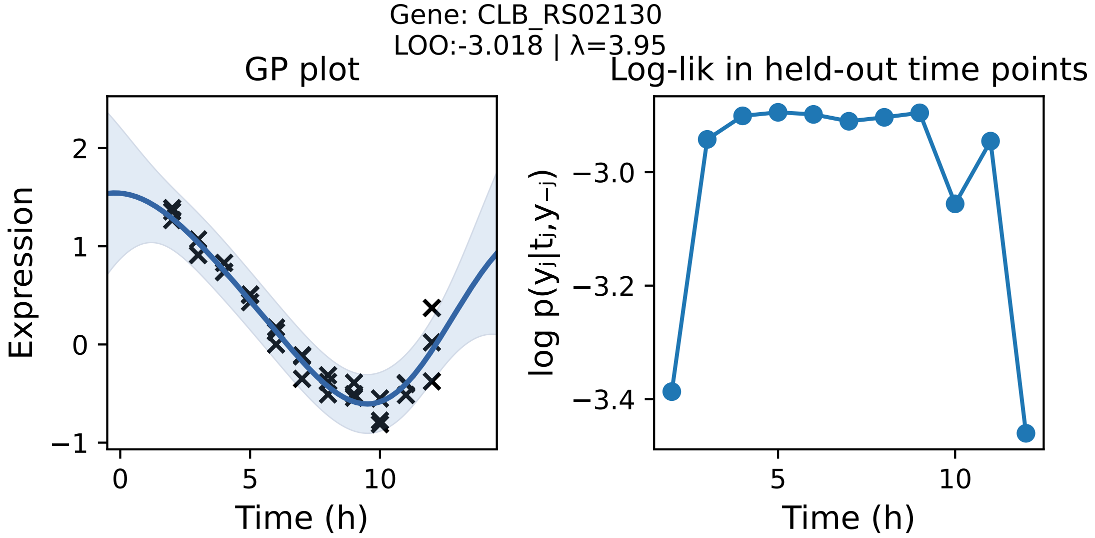

Gene: CLB_RS02130
LOO:-3.018 | λ=3.95
GP plot
Expression
Time (h)
Log-lik in held-out time points
log p(y_j|t_j,y_−j)
Time (h)

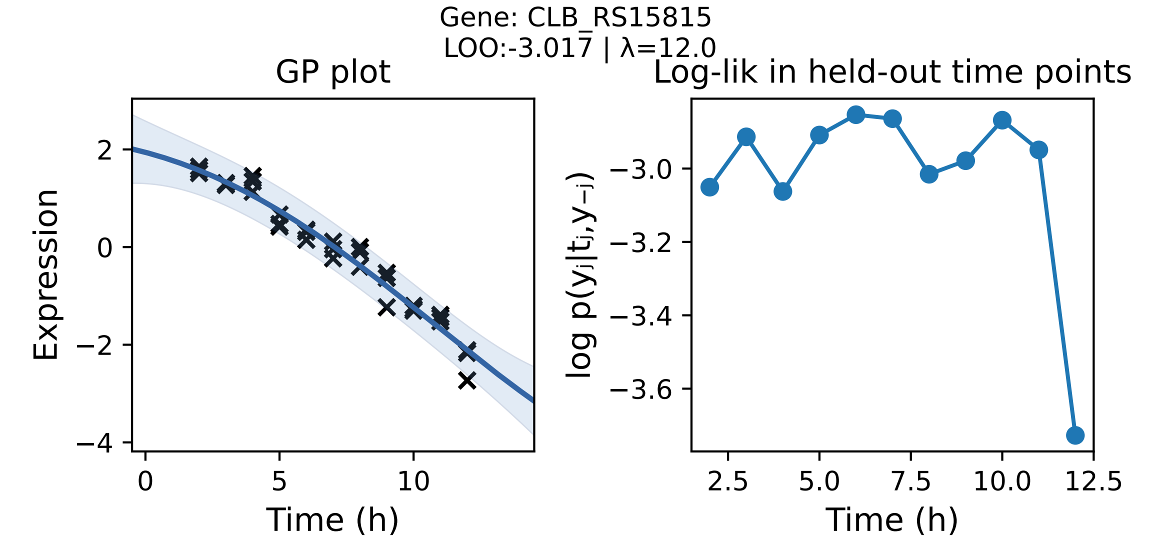

Gene: CLB_RS15815
LOO:-3.017 | λ=12.0
GP plot
Log-lik in held-out time points
Expression
Time (h)
log p(y_j|t_j,y_−j)
2
0
−2
−4
5
10
−3.0
−3.2
−3.4
−3.6
2.5
5.0
7.5
10.0
12.5

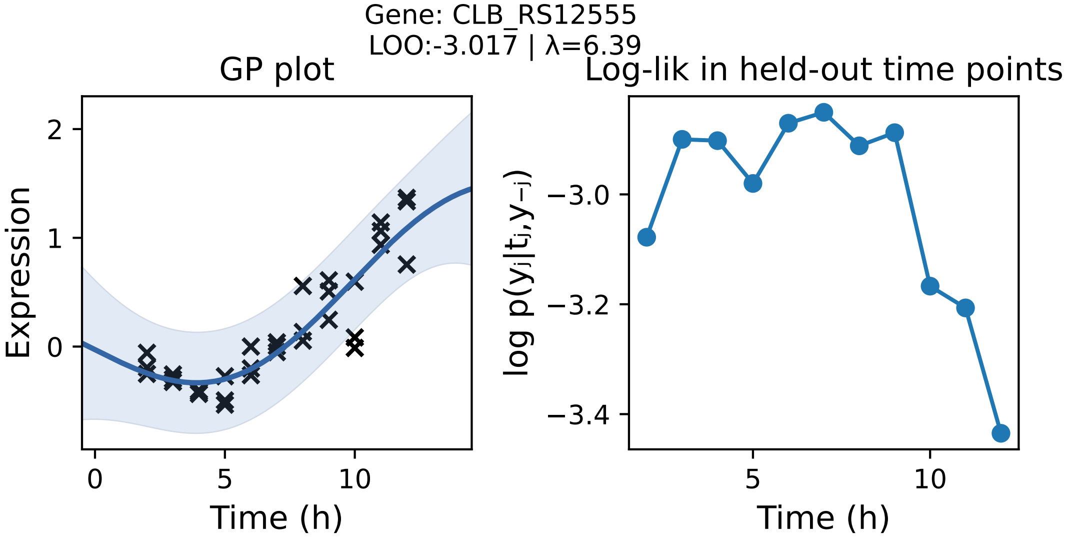
Gene: CLB_RS12555
LOO:-3.017 | λ=6.39
GP plot
Expression
Time (h)
Log-lik in held-out time points
log p(y_j|t_j,y_−j)
Time (h)

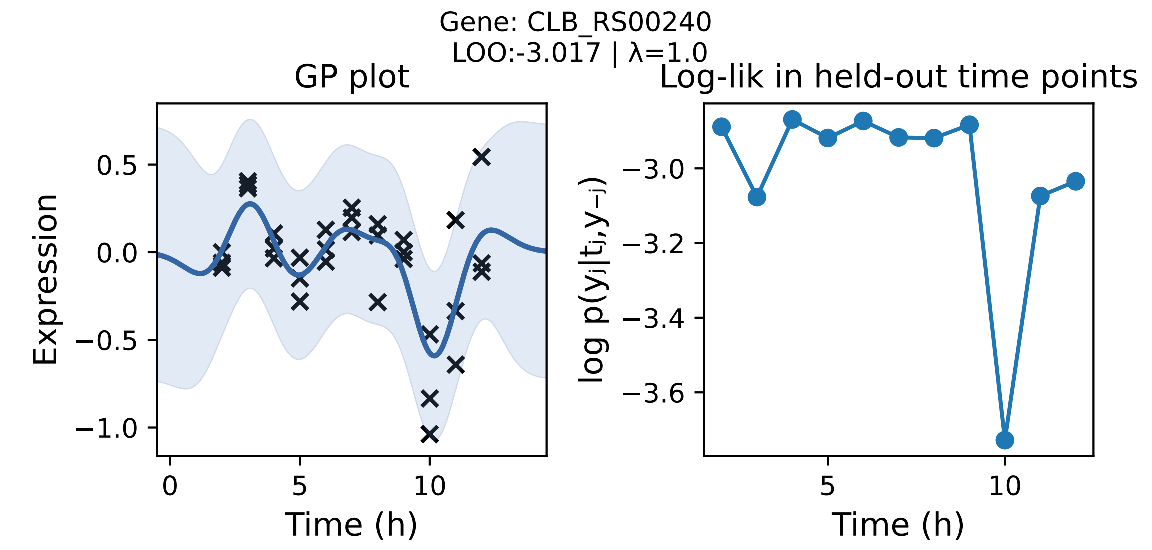
Gene: CLB_RS00240
LOO:-3.017 | λ=1.0
GP plot
Log-lik in held-out time points
Expression
0.5
0.0
−0.5
−1.0
0
5
10
Time (h)
log p(y_j|t_j,y_−j)
−3.0
−3.2
−3.4
−3.6
5
10
Time (h)

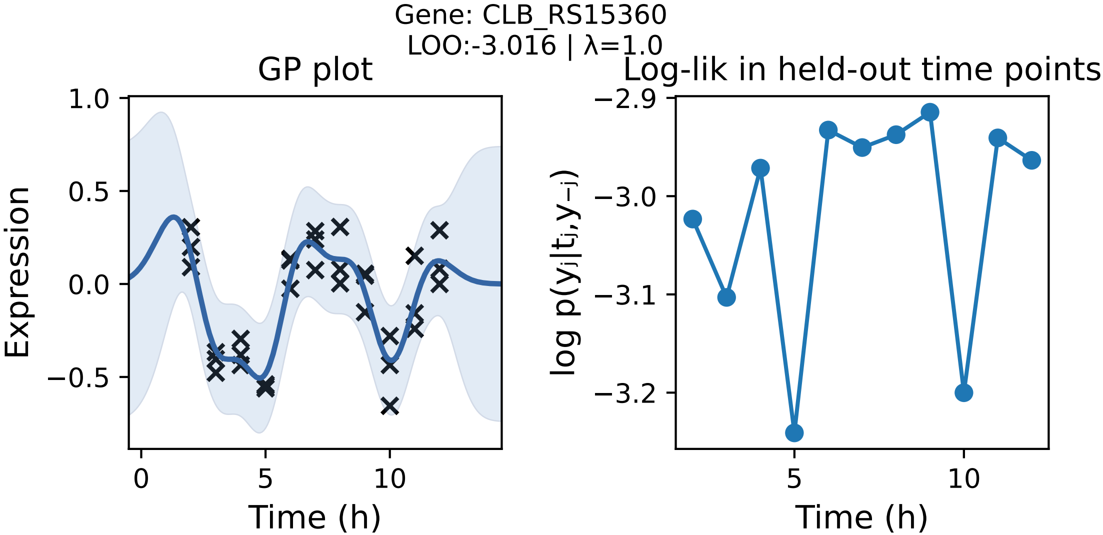

Gene: CLB_RS15360
LOO:-3.016 | λ=1.0
GP plot
Expression
Time (h)
1.0
0.5
0.0
−0.5
0
5
10
Log-lik in held-out time points
log p(y_j|t_j,y_−j)
−2.9
−3.0
−3.1
−3.2
5
10
Time (h)

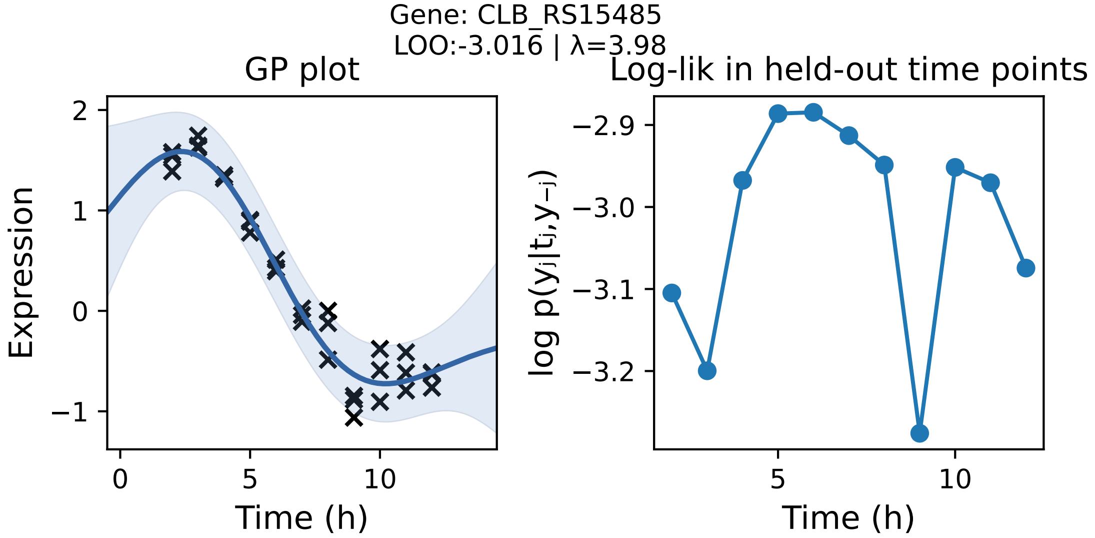

Gene: CLB_RS15485
LOO:-3.016 | λ=3.98
GP plot
Expression
2
1
0
−1
0
5
10
Time (h)
Log-lik in held-out time points
log p(y_j|t_j,y_−j)
−2.9
−3.0
−3.1
−3.2
5
10
Time (h)

Gene: CLB_RS15420
LOO:-3.016 | λ=6.97

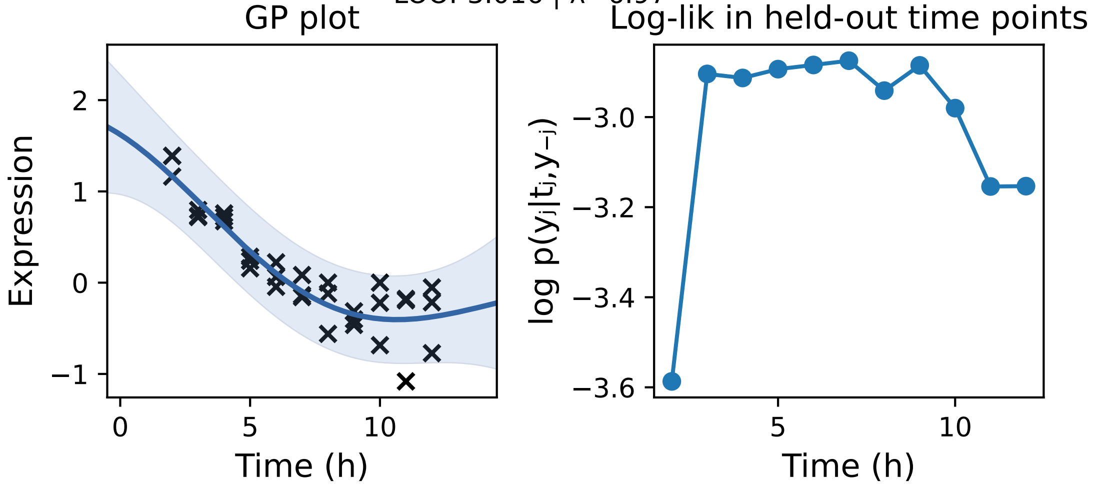

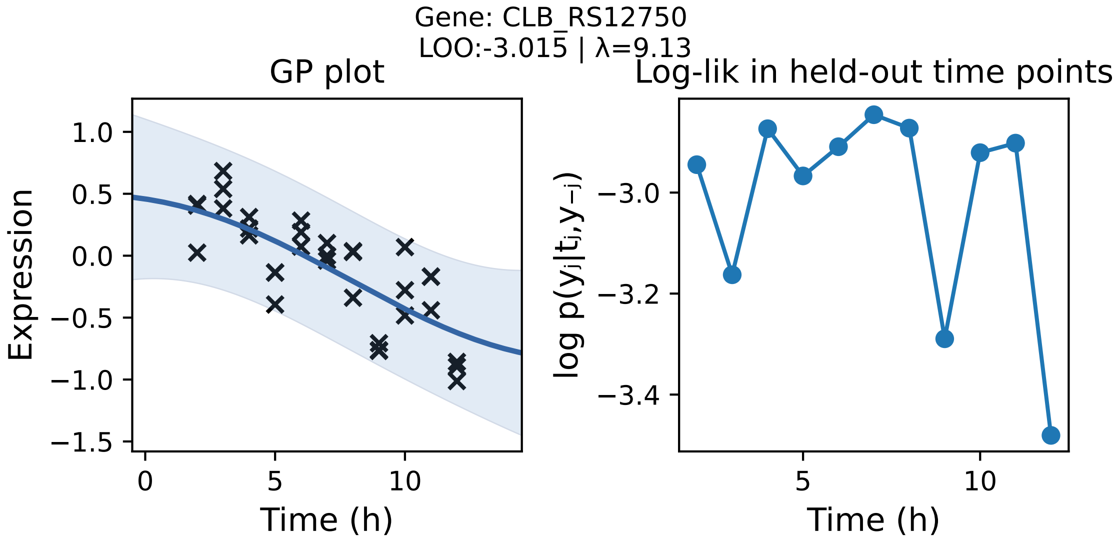

Gene: CLB_RS12750
LOO:-3.015 | λ=9.13
GP plot
Log-lik in held-out time points
Expression
1.0
0.5
0.0
−0.5
−1.0
−1.5
0
5
10
Time (h)
log p(y_j|t_j,y_−j)
−3.0
−3.2
−3.4
5
10
Time (h)

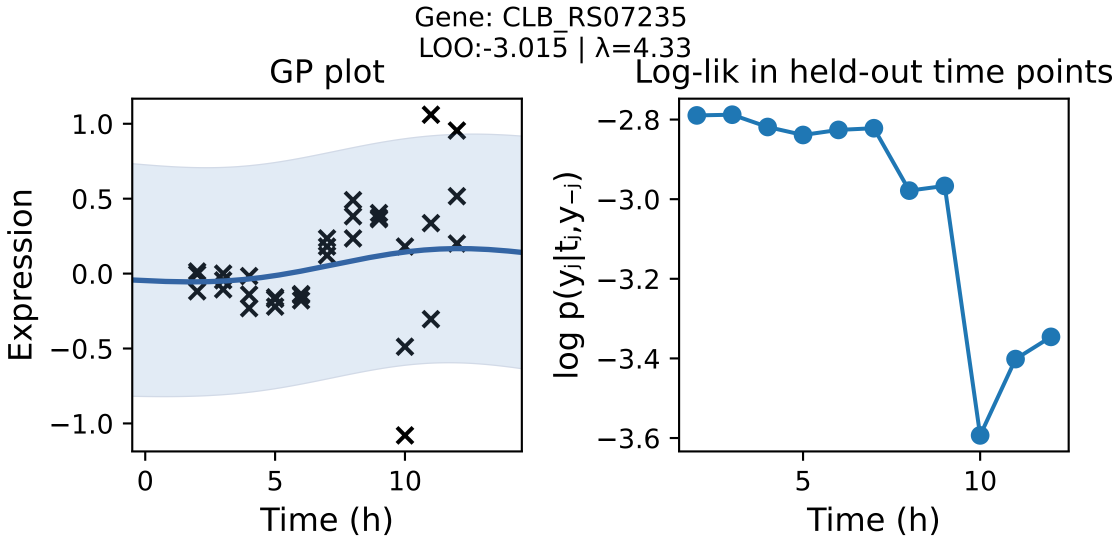

Gene: CLB_RS07235
LOO:-3.015 | λ=4.33
GP plot
Expression
Time (h)
1.0
0.5
0.0
−0.5
−1.0
0
5
10
Log-lik in held-out time points
log p(y_j|t_j,y_−j)
−2.8
−3.0
−3.2
−3.4
−3.6
5
10
Time (h)

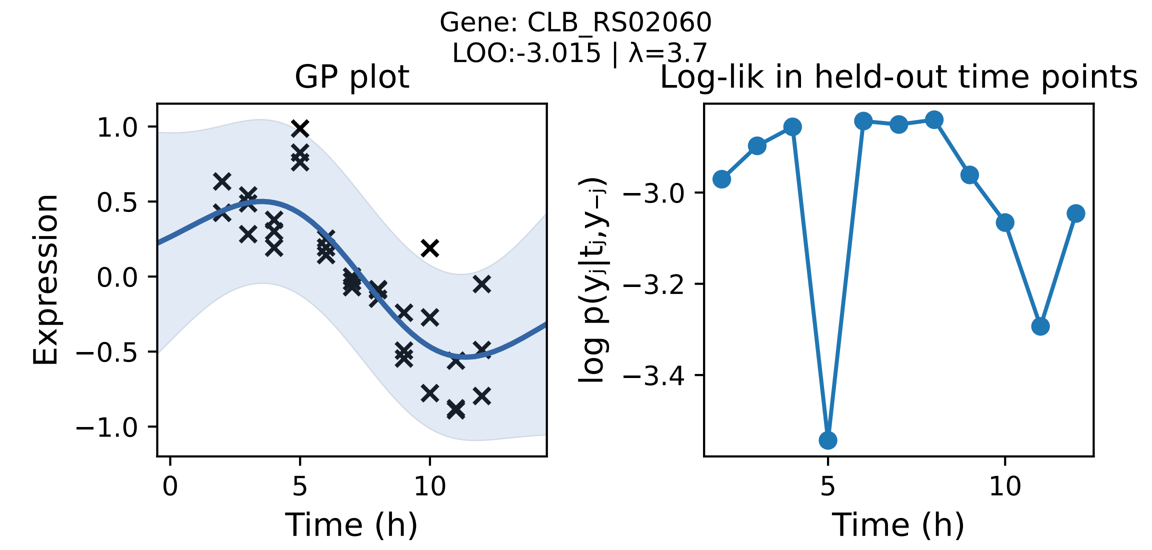

Gene: CLB_RS02060
LOO:-3.015 | λ=3.7
GP plot
Log-lik in held-out time points
1.0
0.5
0.0
−0.5
−1.0
Expression
0
5
10
Time (h)
−3.0
−3.2
−3.4
log p(yj|tj,y−j)
5
10
Time (h)

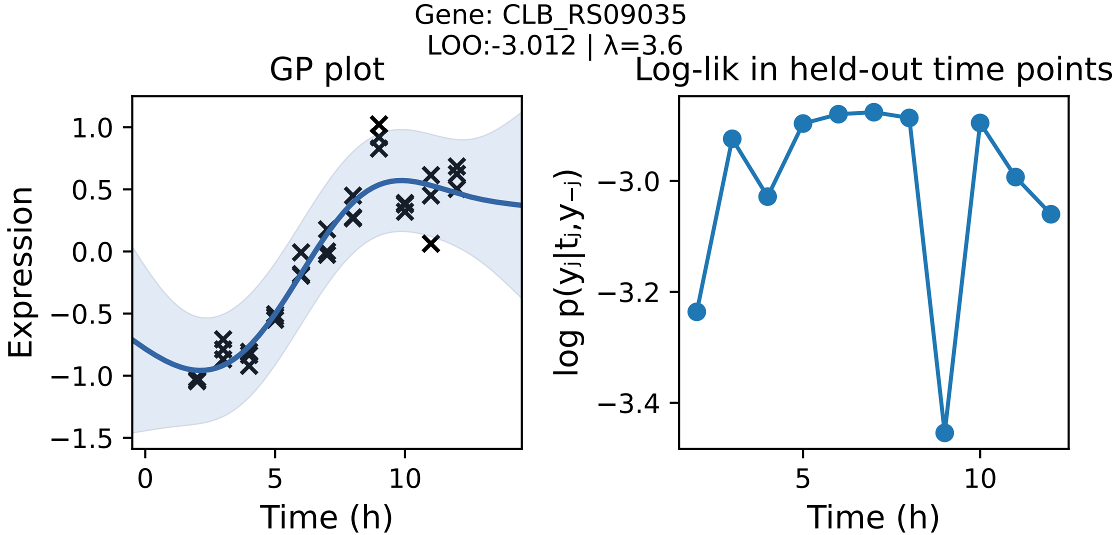
Gene: CLB_RS09035
LOO:-3.012 | λ=3.6
GP plot
Expression
1.0
0.5
0.0
−0.5
−1.0
−1.5
0
5
10
Time (h)
Log-lik in held-out time points
log p(y_j|t_j,y_−j)
−3.0
−3.2
−3.4
5
10
Time (h)

Gene: CLB_RS07385
LOO:-3.011 | λ=5.91

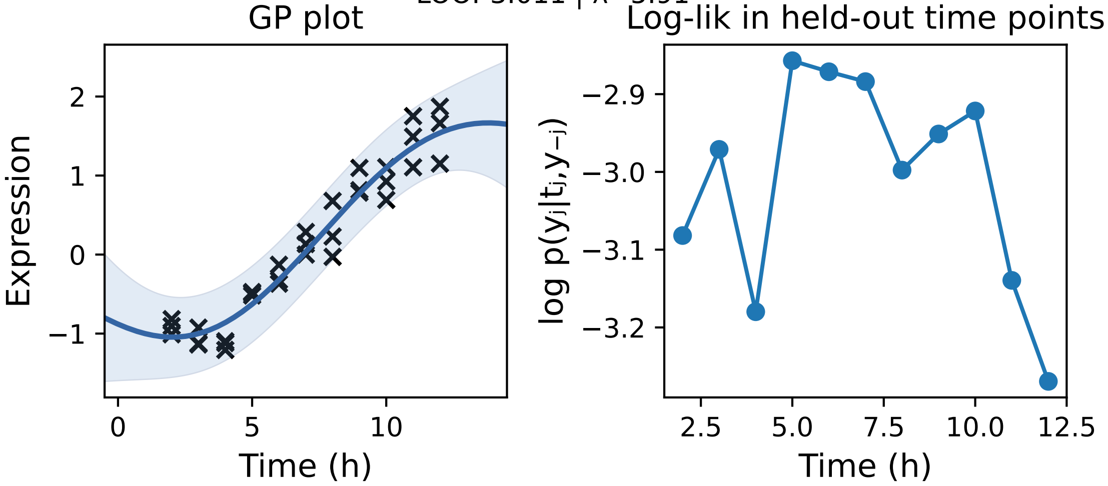

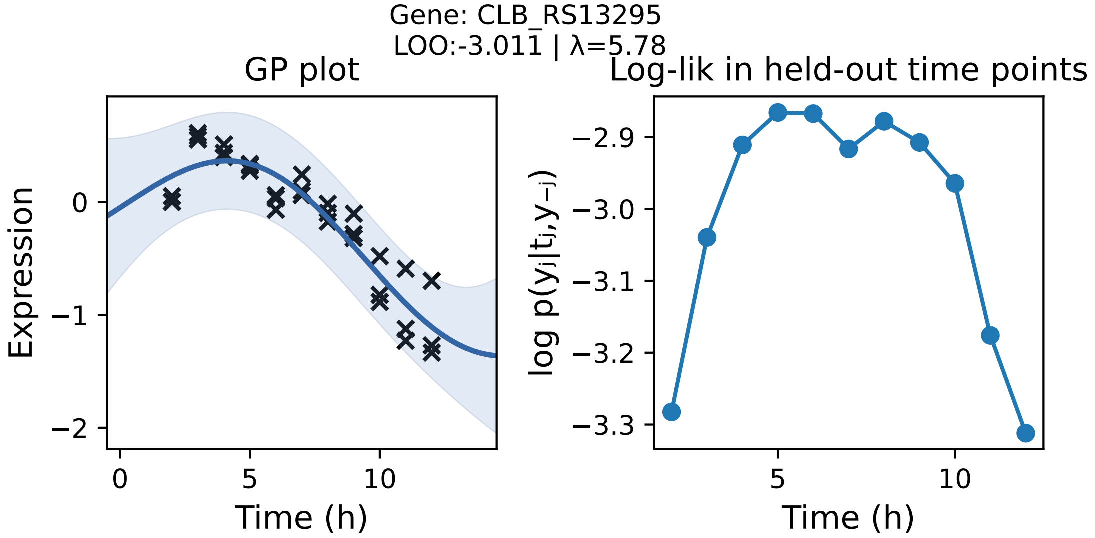

Gene: CLB_RS13295
LOO:-3.011 | λ=5.78
GP plot
Expression
0
−1
−2
0
5
10
Time (h)
Log-lik in held-out time points
log p(y_j|t_j,y_−j)
−2.9
−3.0
−3.1
−3.2
−3.3
5
10
Time (h)

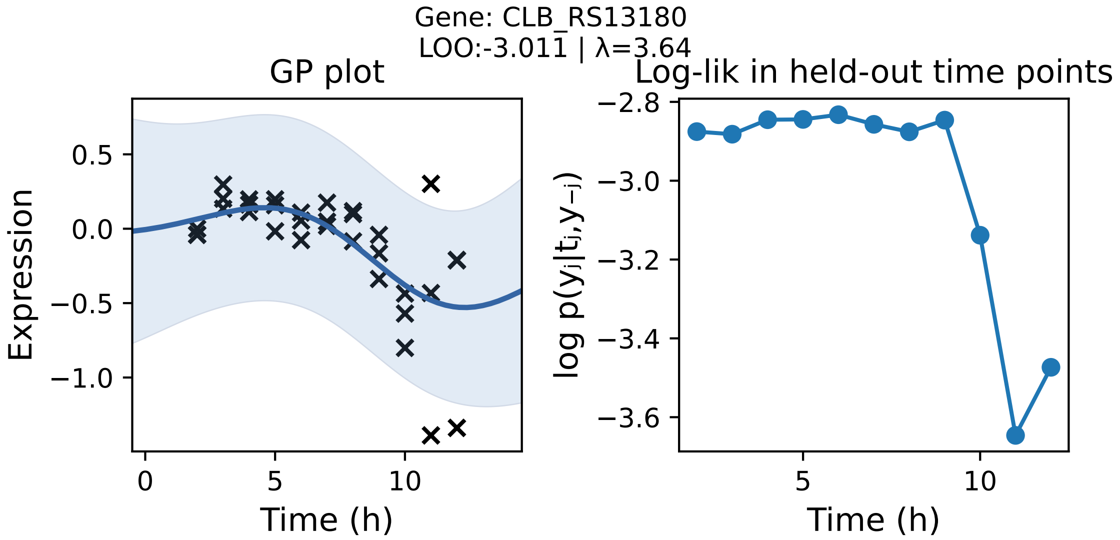

Gene: CLB_RS13180
LOO:-3.011 | λ=3.64
GP plot
Expression
0.5
0.0
−0.5
−1.0
0
5
10
Time (h)
Log-lik in held-out time points
log p(y_j|t_j,y_−j)
−2.8
−3.0
−3.2
−3.4
−3.6
5
10
Time (h)

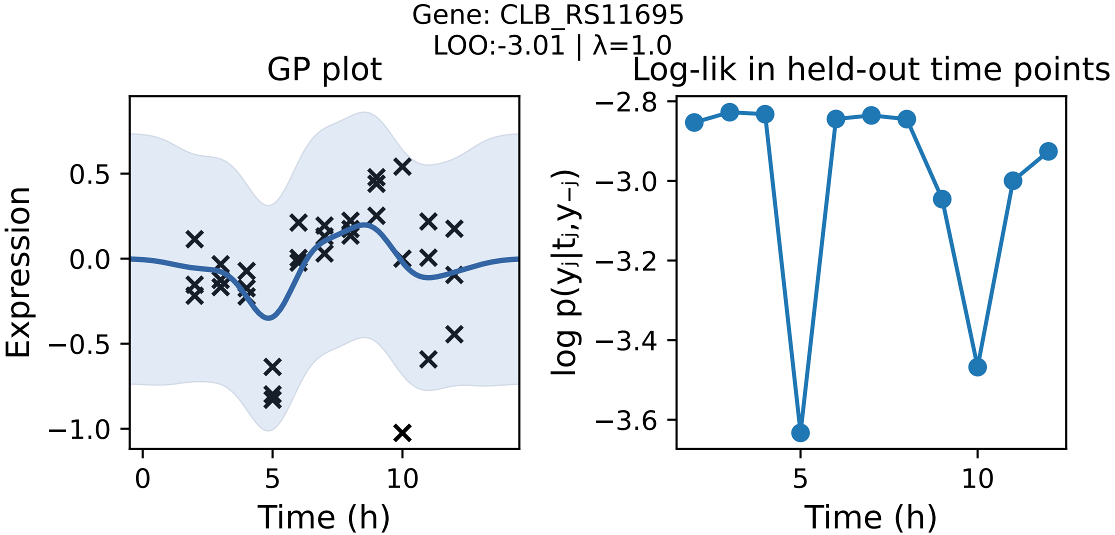

Gene: CLB_RS11695
LOO:-3.01 | λ=1.0
GP plot
Expression
0.5
0.0
−0.5
−1.0
0
5
10
Time (h)
Log-lik in held-out time points
log p(yⱼ|tⱼ,y₋ⱼ)
−2.8
−3.0
−3.2
−3.4
−3.6
5
10
Time (h)

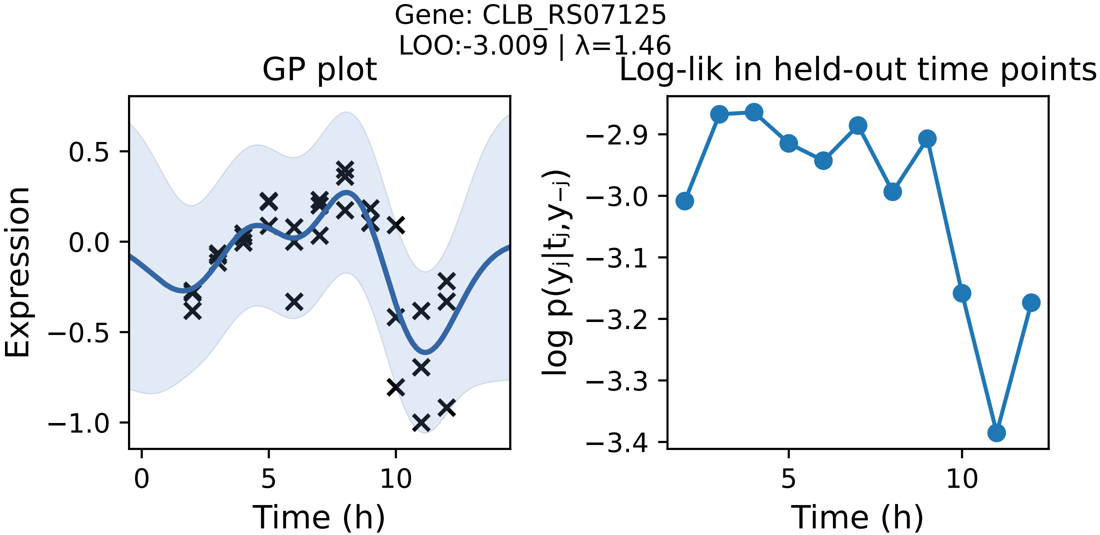
Gene: CLB_RS07125
LOO:-3.009 | λ=1.46
GP plot
Expression
0.5
0.0
−0.5
−1.0
0
5
10
Time (h)
Log-lik in held-out time points
log p(y_j|t_j,y_−j)
−2.9
−3.0
−3.1
−3.2
−3.3
−3.4
5
10
Time (h)

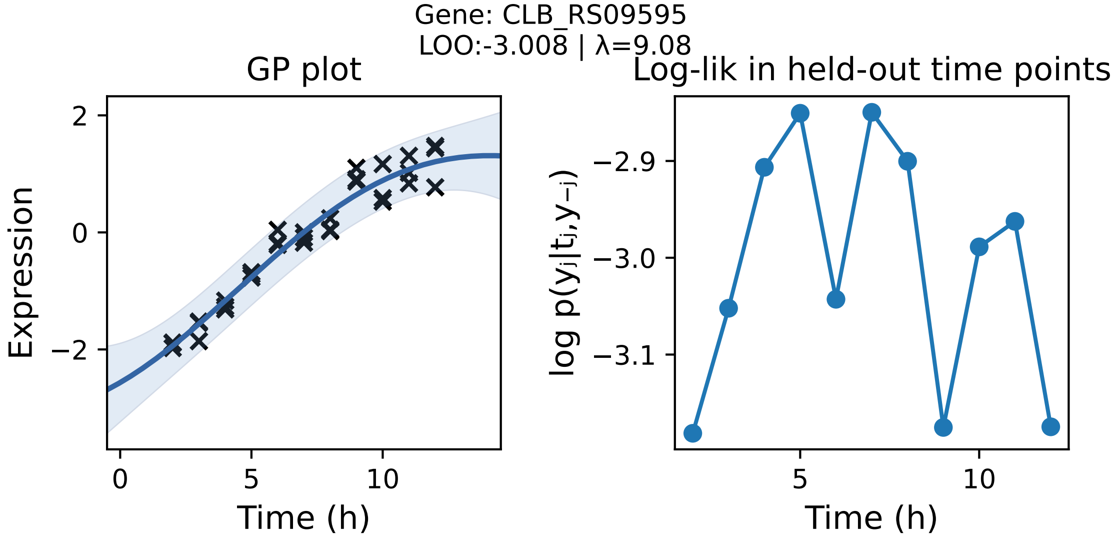

Gene: CLB_RS09595
LOO:-3.008 | λ=9.08
GP plot
Log-lik in held-out time points
Expression
2
0
−2
0
5
10
Time (h)
log p(y_j|t_j,y_−j)
−2.9
−3.0
−3.1
5
10
Time (h)

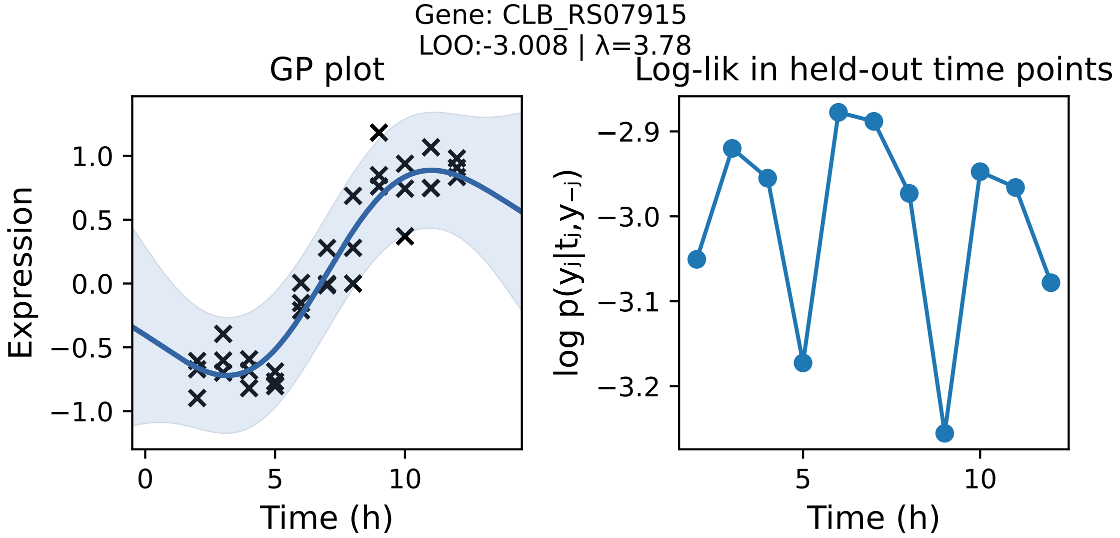

Gene: CLB_RS07915
LOO:-3.008 | λ=3.78
GP plot
Log-lik in held-out time points
Expression
1.0
0.5
0.0
−0.5
−1.0
0
5
10
Time (h)
log p(y_j|t_j,y_−j)
−2.9
−3.0
−3.1
−3.2
5
10
Time (h)

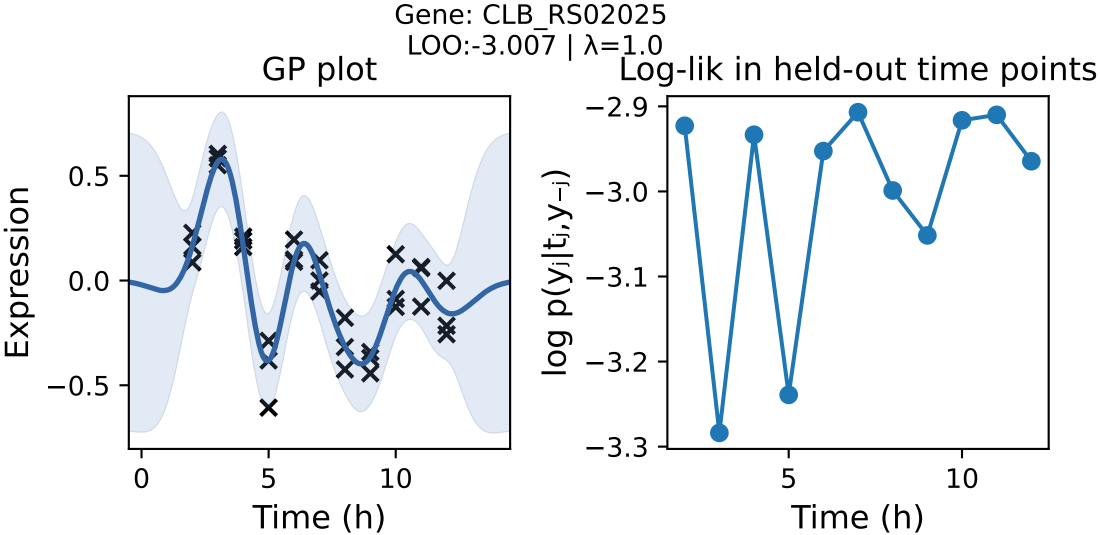

Gene: CLB_RS02025
LOO:-3.007 | λ=1.0
GP plot
Expression
0.5
0.0
−0.5
0
5
10
Time (h)
Log-lik in held-out time points
log p(y_j|t_j,y_−j)
−2.9
−3.0
−3.1
−3.2
−3.3
5
10
Time (h)

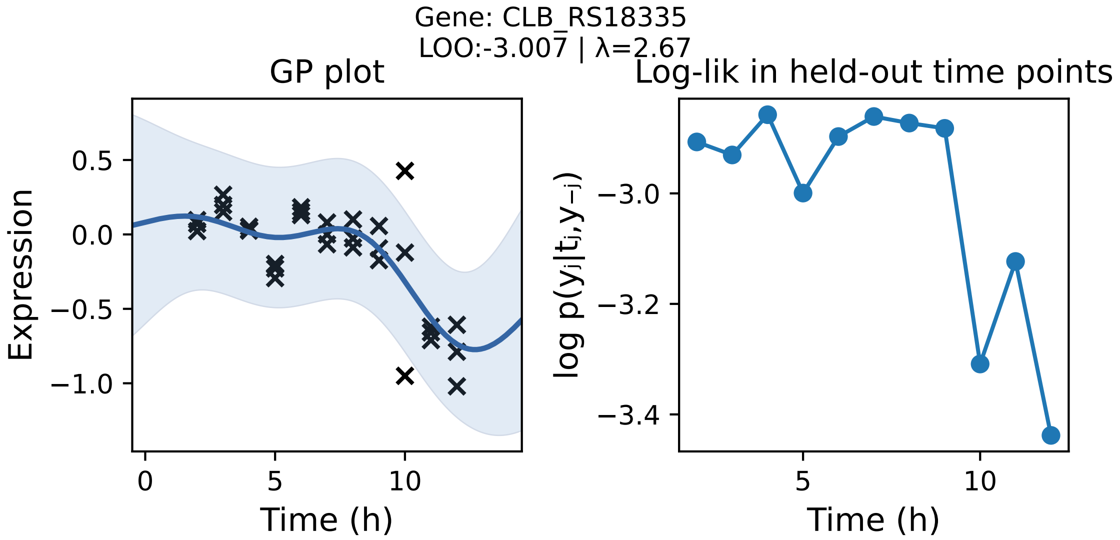
Gene: CLB_RS18335
LOO:-3.007 | λ=2.67
GP plot
Expression
Time (h)
0.5
0.0
−0.5
−1.0
0
5
10
Log-lik in held-out time points
log p(y_j|t_j,y_−j)
−3.0
−3.2
−3.4
5
10
Time (h)

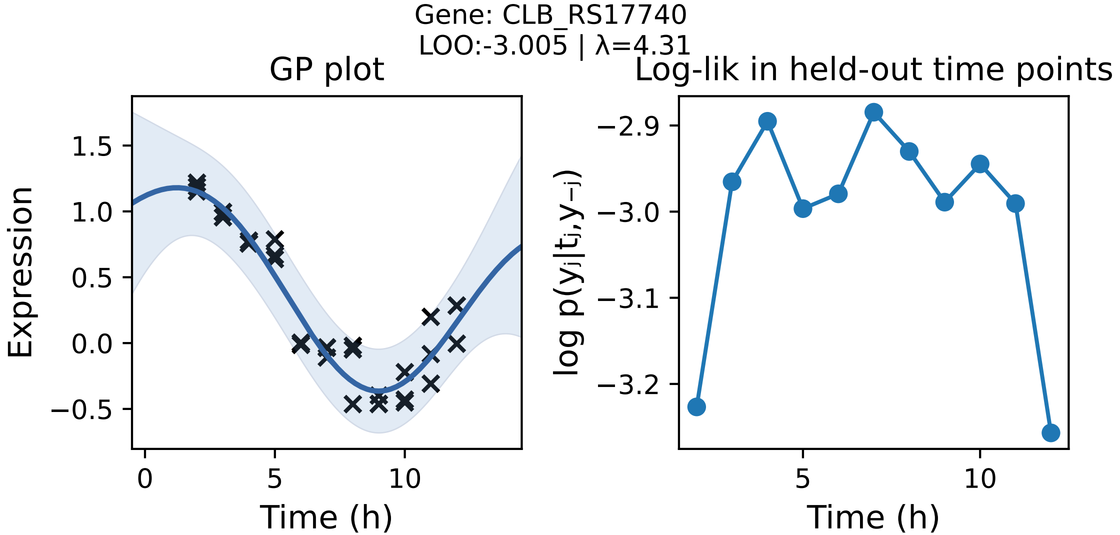
Gene: CLB_RS17740
LOO:-3.005 | λ=4.31
GP plot
Expression
Time (h)
1.5
1.0
0.5
0.0
−0.5
0
5
10
Log-lik in held-out time points
log p(y_j|t_j,y_−j)
−2.9
−3.0
−3.1
−3.2
5
10
Time (h)

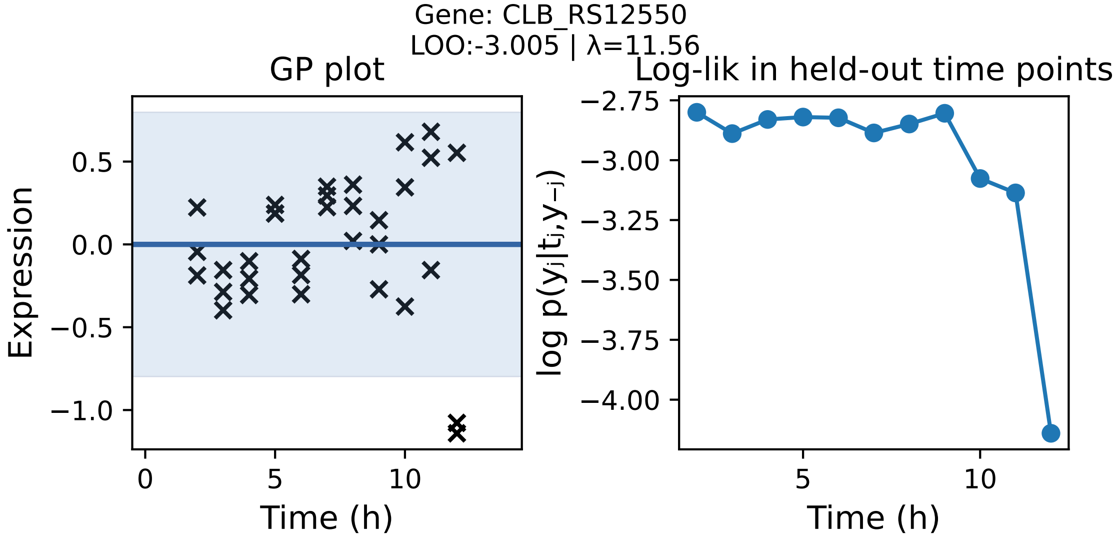

Gene: CLB_RS12550
LOO:-3.005 | λ=11.56
GP plot
Expression
Time (h)
0.5
0.0
−0.5
−1.0
0
5
10
Log-lik in held-out time points
log p(y_j|t_j,y_−j)
Time (h)
−2.75
−3.00
−3.25
−3.50
−3.75
−4.00
5
10

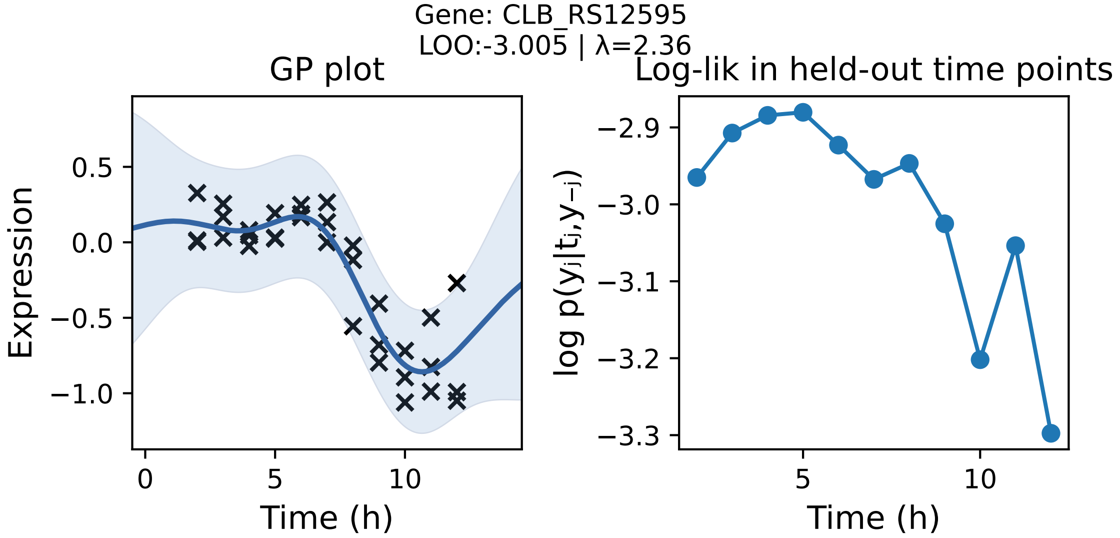

Gene: CLB_RS12595
LOO:-3.005 | λ=2.36
GP plot
Expression
0.5
0.0
−0.5
−1.0
0
5
10
Time (h)
Log-lik in held-out time points
log p(yj|tj,y−j)
−2.9
−3.0
−3.1
−3.2
−3.3
5
10
Time (h)

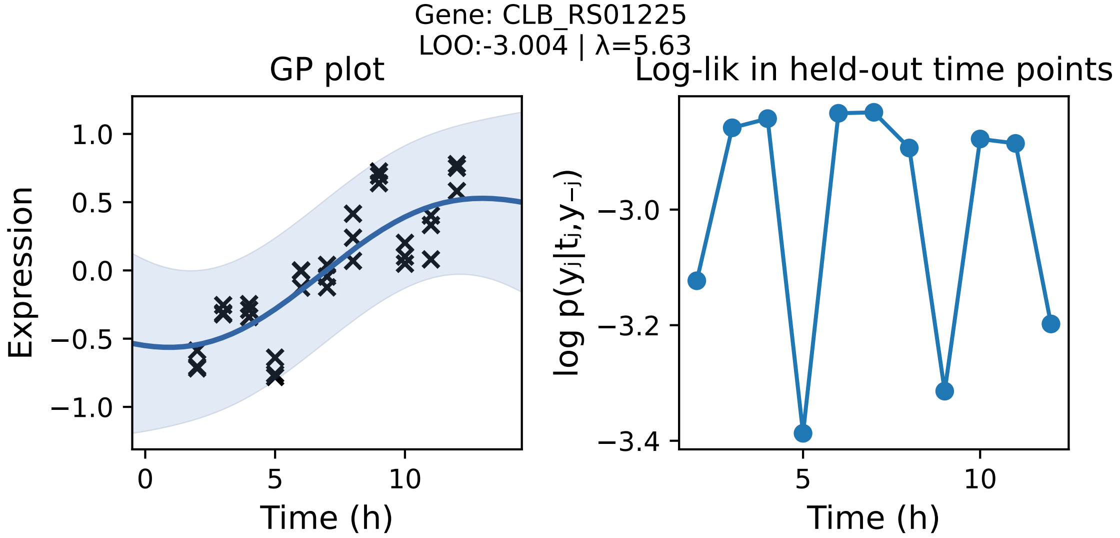
Gene: CLB_RS01225
LOO:-3.004 | λ=5.63
GP plot
Expression
1.0
0.5
0.0
−0.5
−1.0
0
5
10
Time (h)
Log-lik in held-out time points
log p(y_j|t_j,y_−j)
−3.0
−3.2
−3.4
5
10
Time (h)

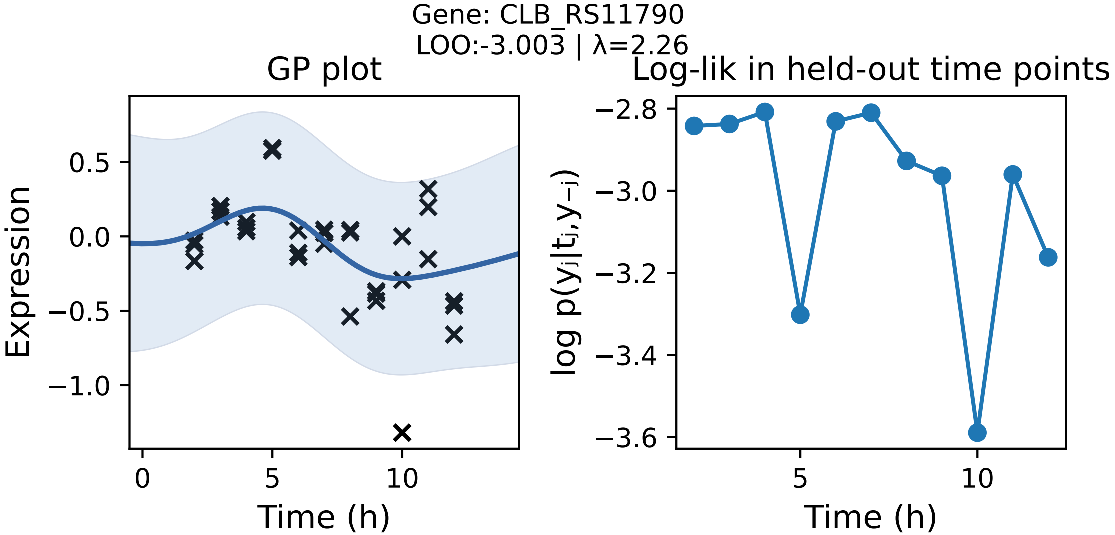

Gene: CLB_RS11790
LOO:-3.003 | λ=2.26
GP plot
Log-lik in held-out time points
Expression
0.5
0.0
−0.5
−1.0
0
5
10
Time (h)
log p(y_j|t_j,y_−j)
−2.8
−3.0
−3.2
−3.4
−3.6
5
10
Time (h)

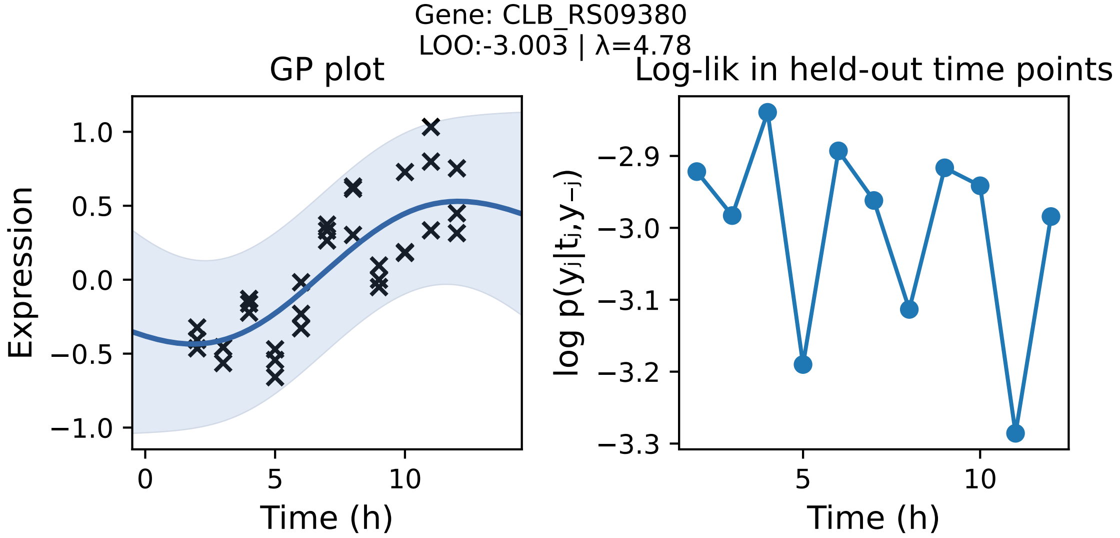
Gene: CLB_RS09380
LOO:-3.003 | λ=4.78
GP plot
Expression
Time (h)
Log-lik in held-out time points
log p(yj|tj,y−j)
Time (h)

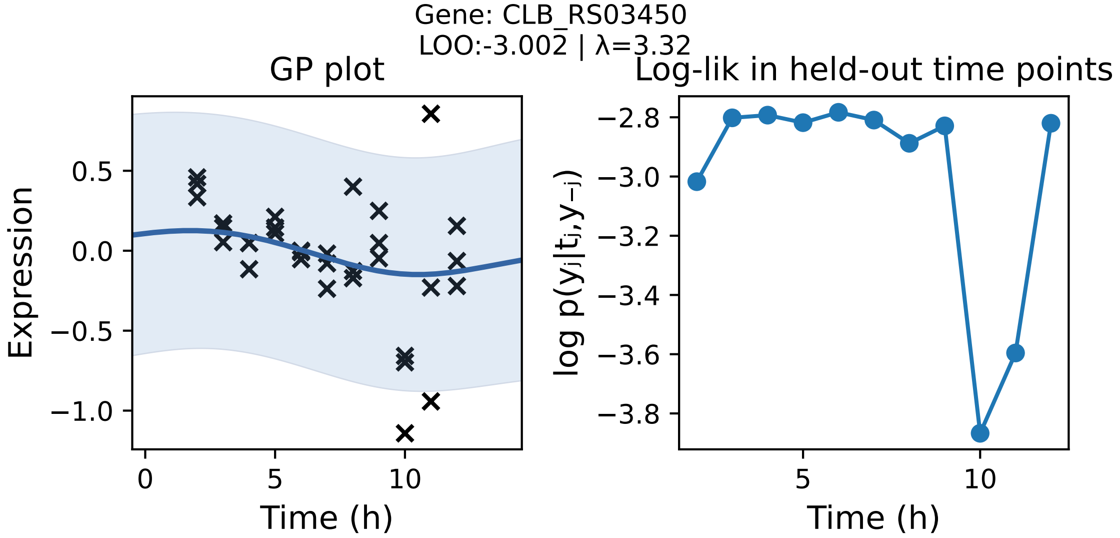

Gene: CLB_RS03450
LOO:-3.002 | λ=3.32
GP plot
Expression
0.5
0.0
−0.5
−1.0
0
5
10
Time (h)
Log-lik in held-out time points
log p(y_j|t_j,y_−j)
−2.8
−3.0
−3.2
−3.4
−3.6
−3.8
5
10
Time (h)

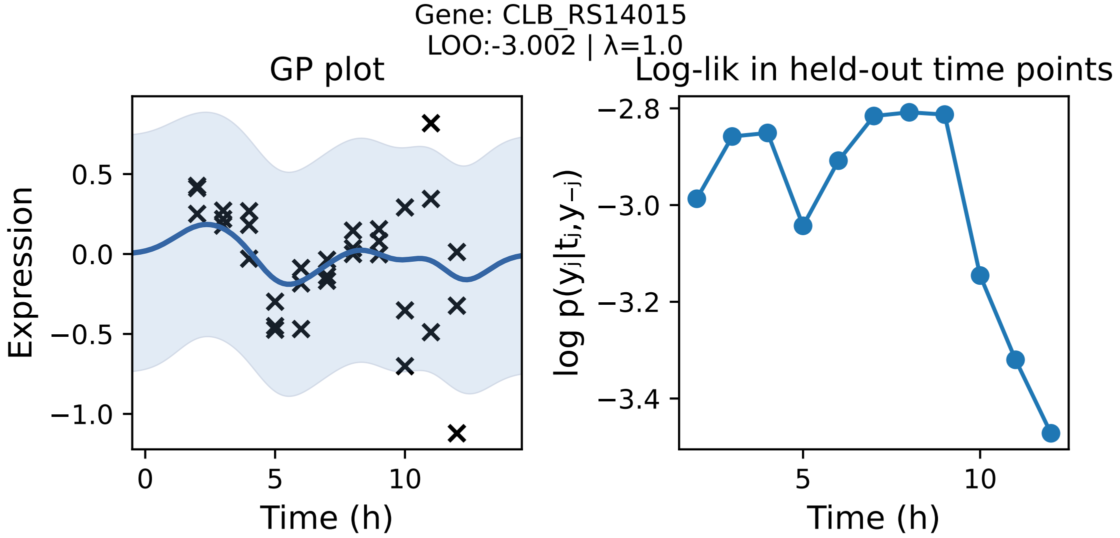

Gene: CLB_RS14015
LOO:-3.002 | λ=1.0
GP plot
Expression
Time (h)
Log-lik in held-out time points
log p(y_j|t_j,y_−j)
Time (h)

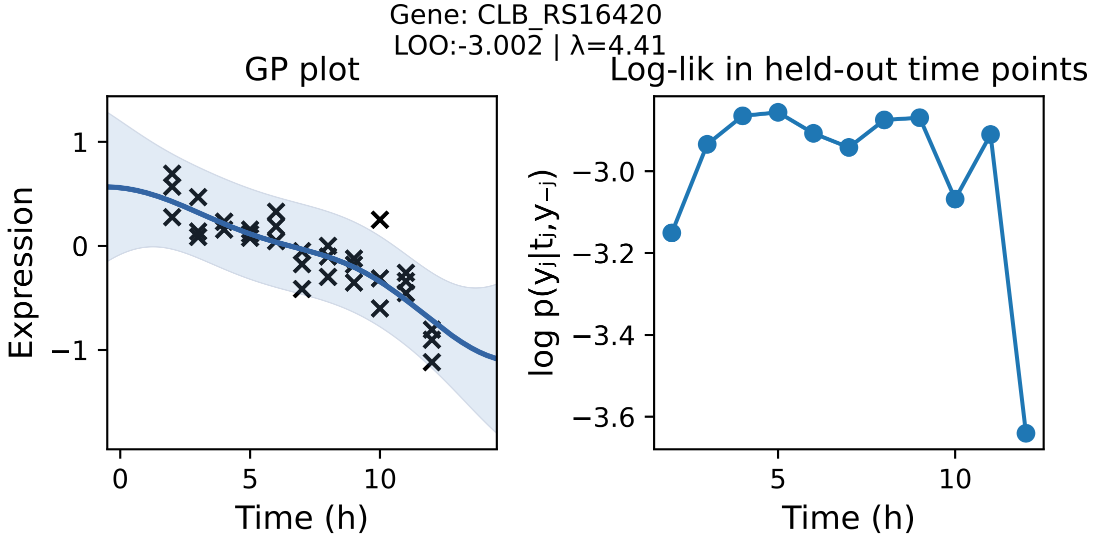

Gene: CLB_RS16420
LOO:-3.002 | λ=4.41
GP plot
Expression
Time (h)
Log-lik in held-out time points
log p(yj|tj,y−j)
Time (h)

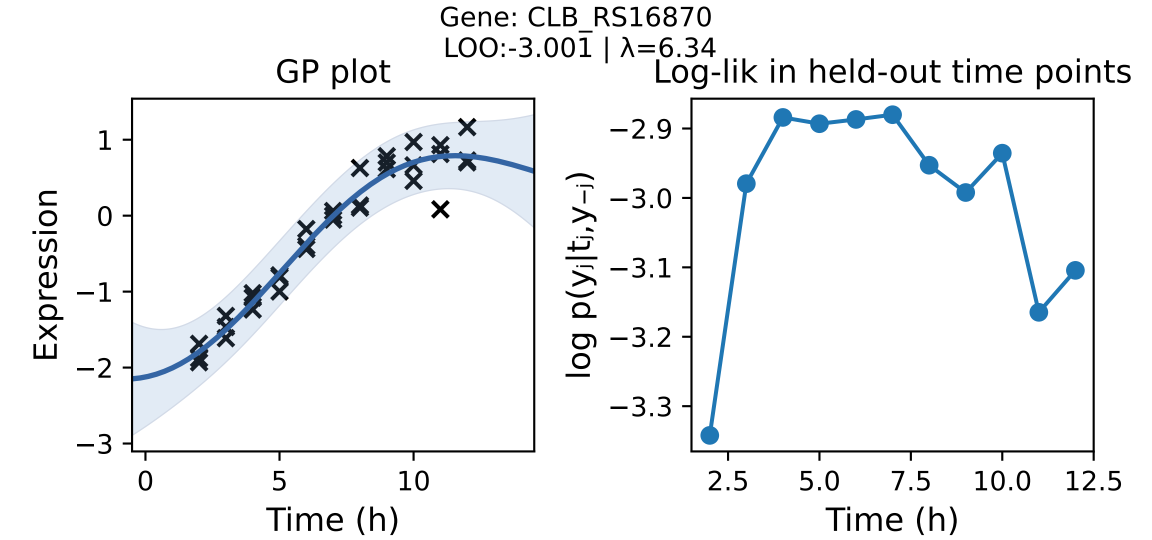
Gene: CLB_RS16870
LOO:-3.001 | λ=6.34
GP plot
Expression
Time (h)
1
0
−1
−2
−3
0
5
10
Log-lik in held-out time points
log p(y_j|t_j,y_−j)
Time (h)
−2.9
−3.0
−3.1
−3.2
−3.3
2.5
5.0
7.5
10.0
12.5

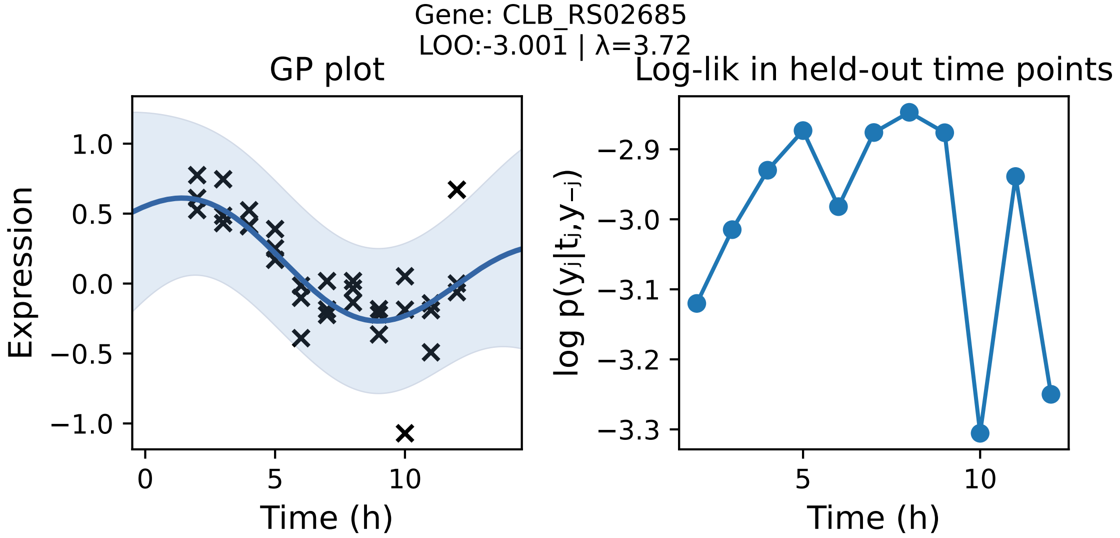

Gene: CLB_RS02685
LOO:-3.001 | λ=3.72
GP plot
Log-lik in held-out time points
Expression
1.0
0.5
0.0
−0.5
−1.0
0
5
10
Time (h)
log p(y_j|t_j,y_−j)
−2.9
−3.0
−3.1
−3.2
−3.3
5
10
Time (h)

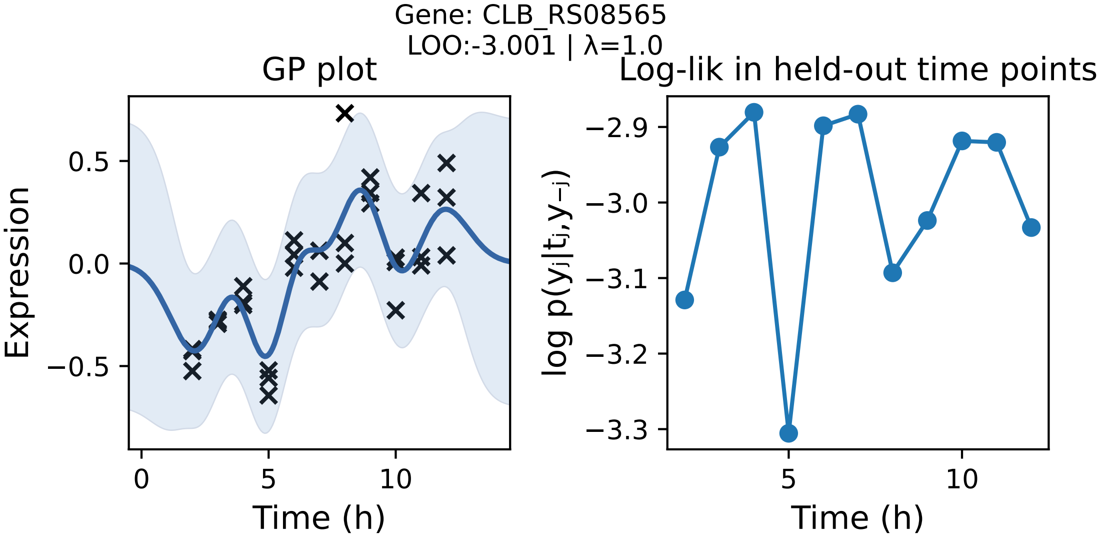

Gene: CLB_RS08565
LOO:-3.001 | λ=1.0
GP plot
Expression
0.5
0.0
−0.5
0
5
10
Time (h)
Log-lik in held-out time points
log p(y_j|t_j,y_−j)
−2.9
−3.0
−3.1
−3.2
−3.3
5
10
Time (h)

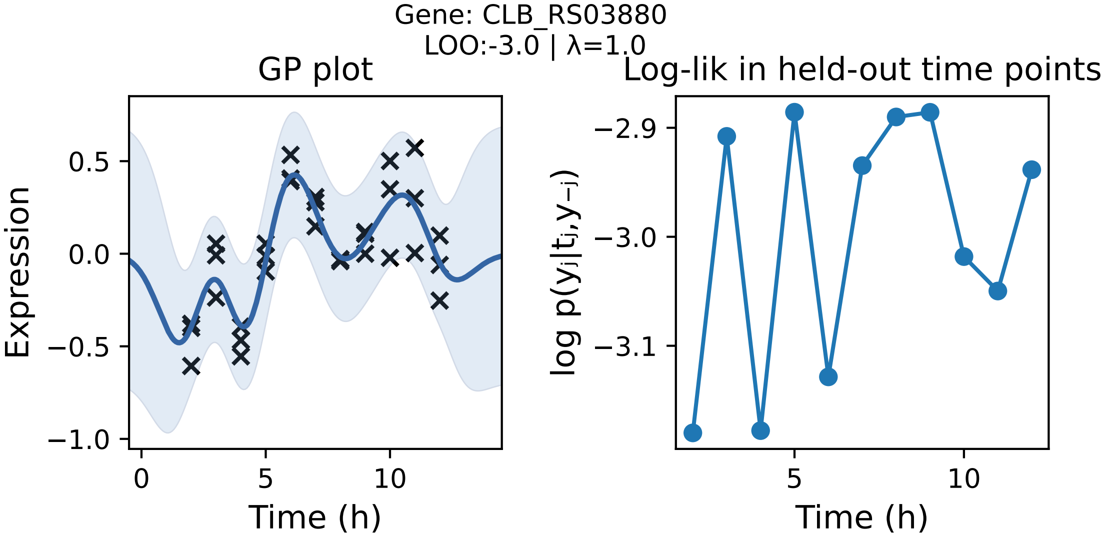

Gene: CLB_RS03880
LOO:-3.0 | λ=1.0
GP plot
Expression
Time (h)
0.5
0.0
−0.5
−1.0
0
5
10
Log-lik in held-out time points
log p(y_j|t_j,y_−j)
Time (h)
−2.9
−3.0
−3.1
5
10

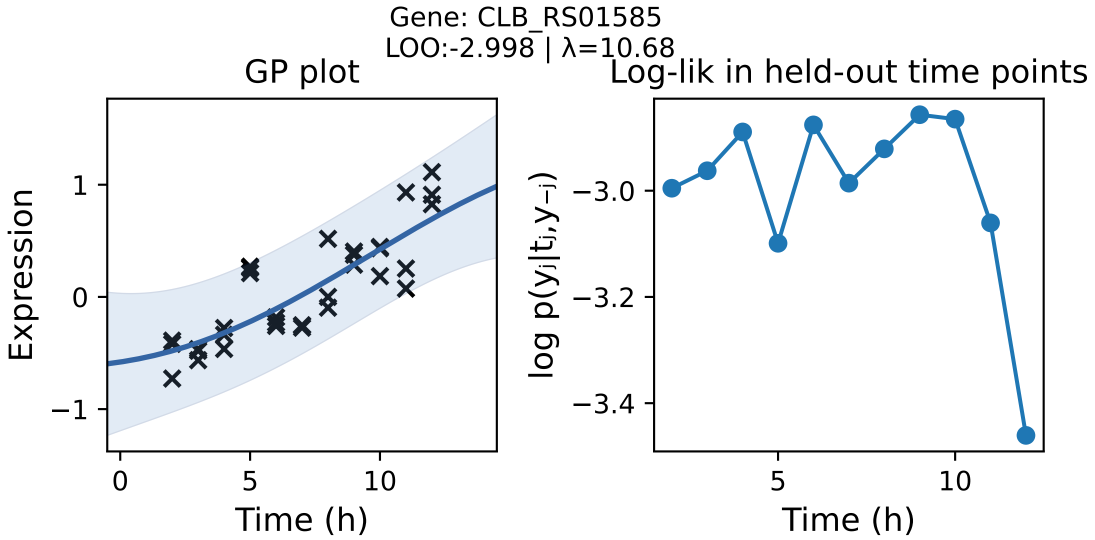

Gene: CLB_RS01585
LOO:-2.998 | λ=10.68
GP plot
Expression
1
0
−1
0
5
10
Time (h)
Log-lik in held-out time points
log p(y_j|t_j,y_−j)
−3.0
−3.2
−3.4
5
10
Time (h)

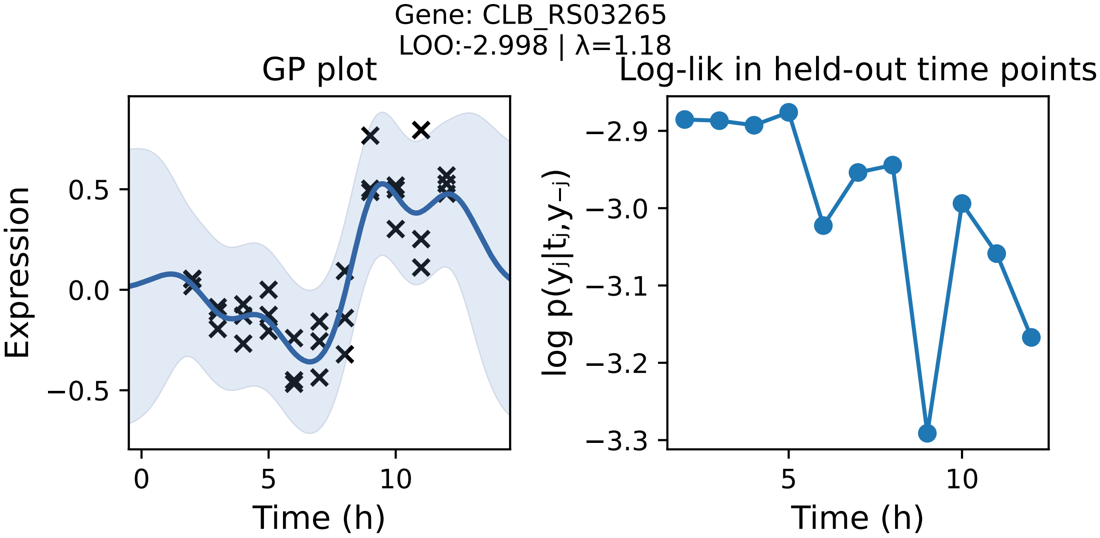

Gene: CLB_RS03265
LOO:-2.998 | λ=1.18
GP plot
Log-lik in held-out time points
Expression
0.5
0.0
−0.5
0
5
10
Time (h)
log p(y_j|t_j,y_−j)
−2.9
−3.0
−3.1
−3.2
−3.3
5
10
Time (h)

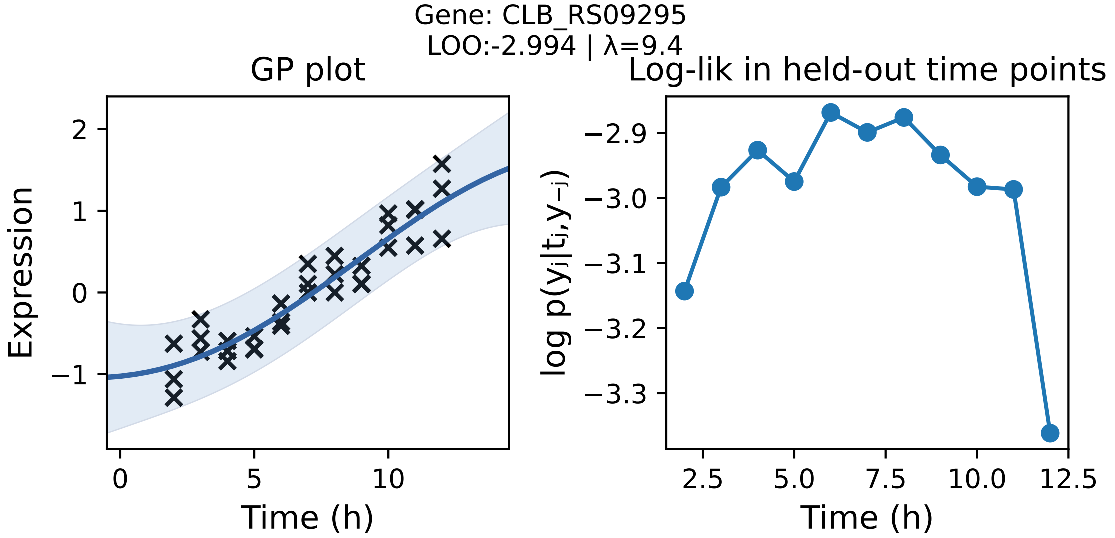

Gene: CLB_RS09295
LOO:-2.994 | λ=9.4
GP plot
Expression
Time (h)
2
1
0
−1
0
5
10
Log-lik in held-out time points
log p(y_j|t_j,y_−j)
Time (h)
−2.9
−3.0
−3.1
−3.2
−3.3
2.5
5.0
7.5
10.0
12.5

Gene: CLB_RS00345
LOO:-2.994 | λ=2.53

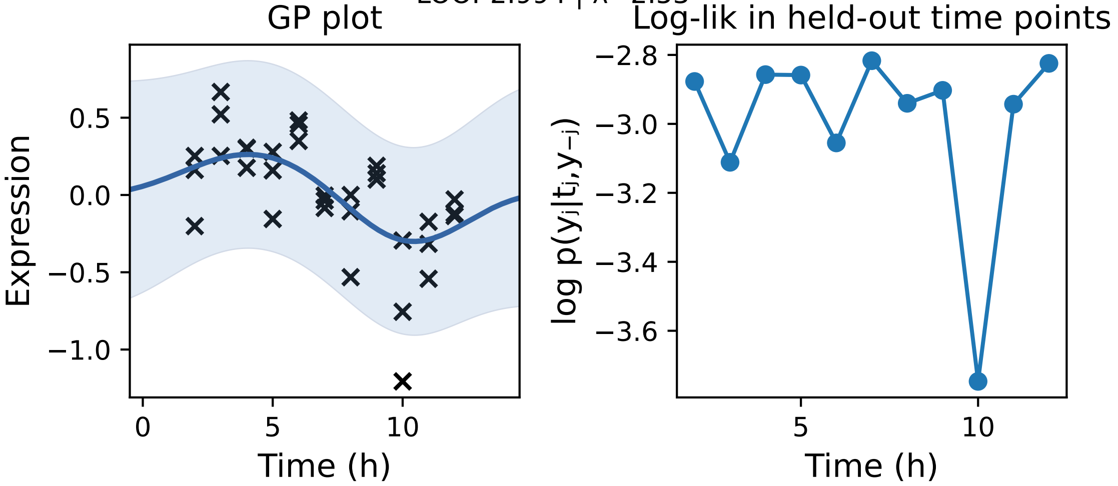

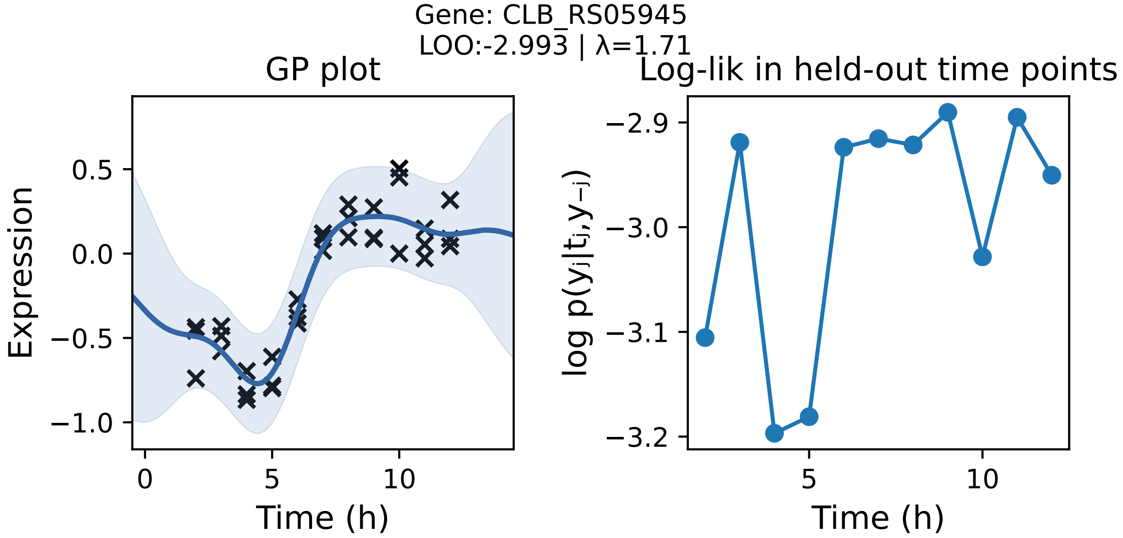

Gene: CLB_RS05945
LOO:-2.993 | λ=1.71
GP plot
Expression
Time (h)
0.5
0.0
−0.5
−1.0
0
5
10
Log-lik in held-out time points
$\log p(y_j|t_j, y_{-j})$
−2.9
−3.0
−3.1
−3.2
5
10
Time (h)

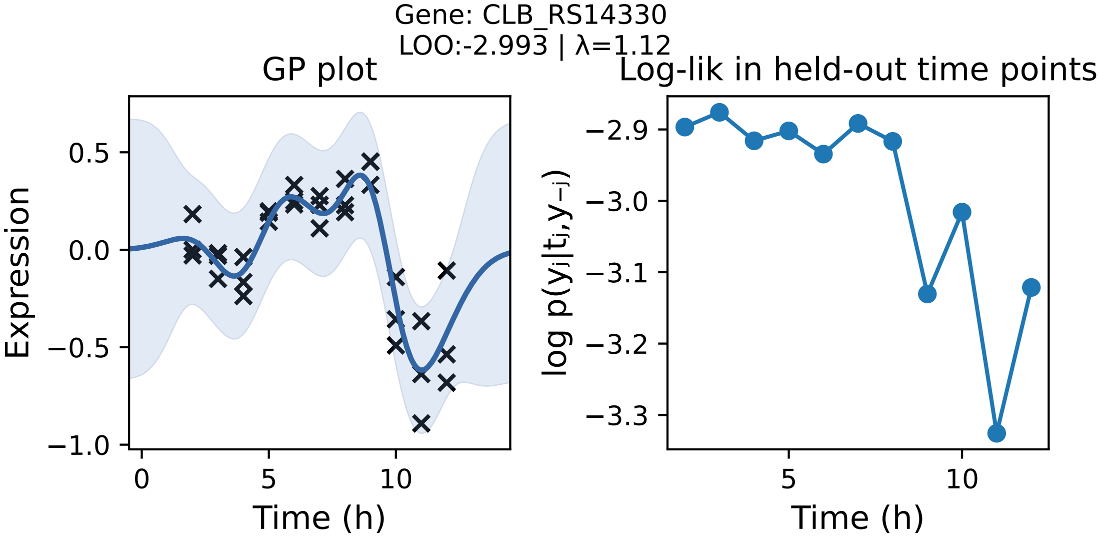
Gene: CLB_RS14330
LOO:-2.993 | λ=1.12
GP plot
Expression
0.5
0.0
−0.5
−1.0
0
5
10
Time (h)
Log-lik in held-out time points
log p(y_j|t_j,y_−j)
−2.9
−3.0
−3.1
−3.2
−3.3
5
10
Time (h)

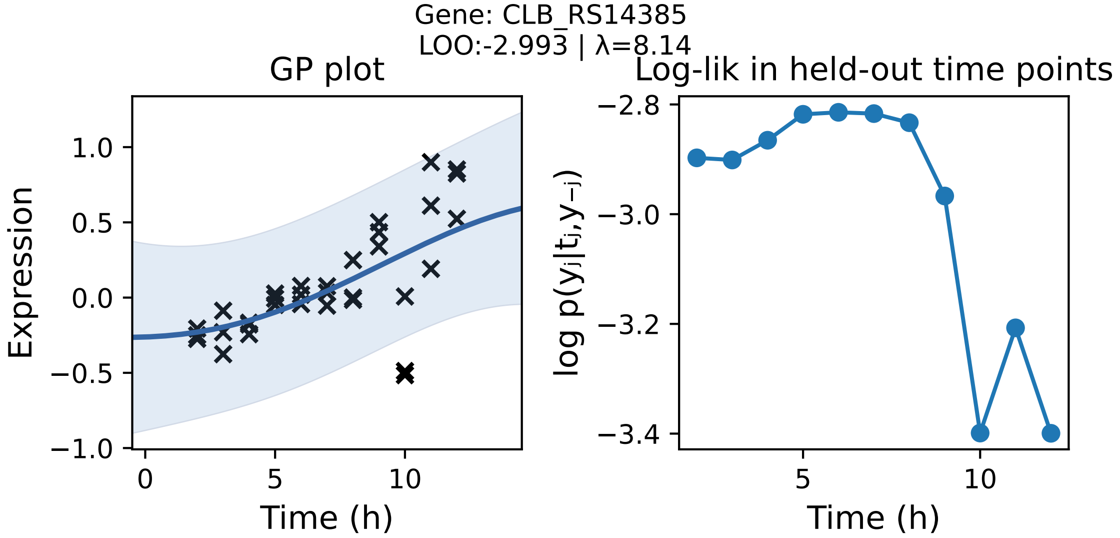

Gene: CLB_RS14385
LOO:-2.993 | λ=8.14
GP plot
Log-lik in held-out time points
Expression
1.0
0.5
0.0
−0.5
−1.0
0
5
10
Time (h)
log p(y_j|t_j,y_−j)
−2.8
−3.0
−3.2
−3.4
5
10
Time (h)

Gene: CLB_RS14915
LOO:-2.992 | λ=5.34

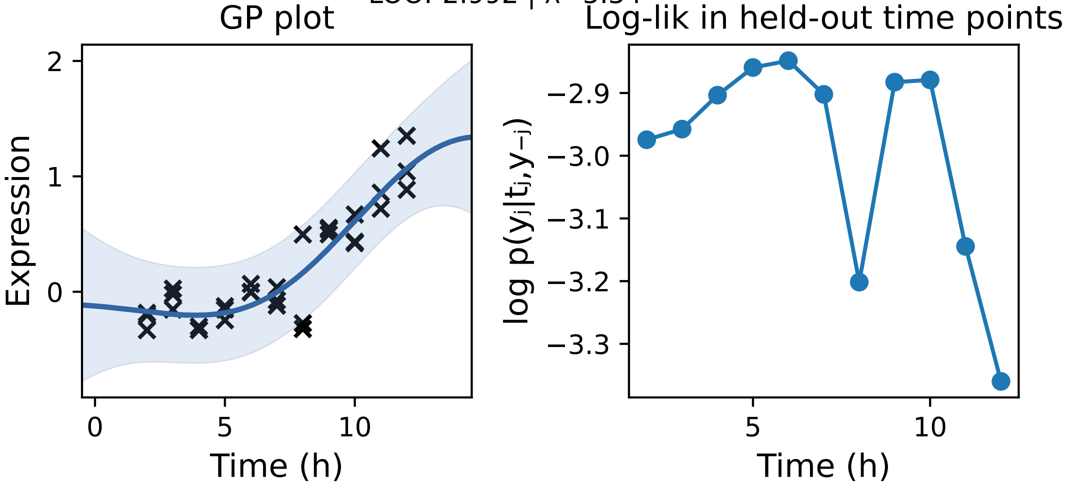

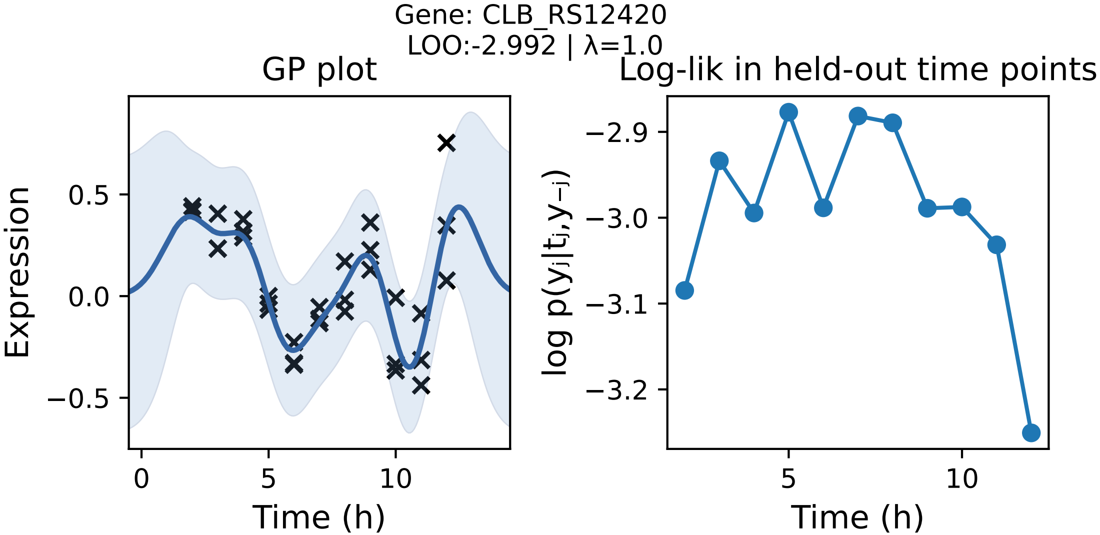

Gene: CLB_RS12420
LOO:-2.992 | λ=1.0
GP plot
Expression
0.5
0.0
−0.5
0
5
10
Time (h)
Log-lik in held-out time points
log p(y_j|t_j,y_−j)
−2.9
−3.0
−3.1
−3.2
5
10
Time (h)

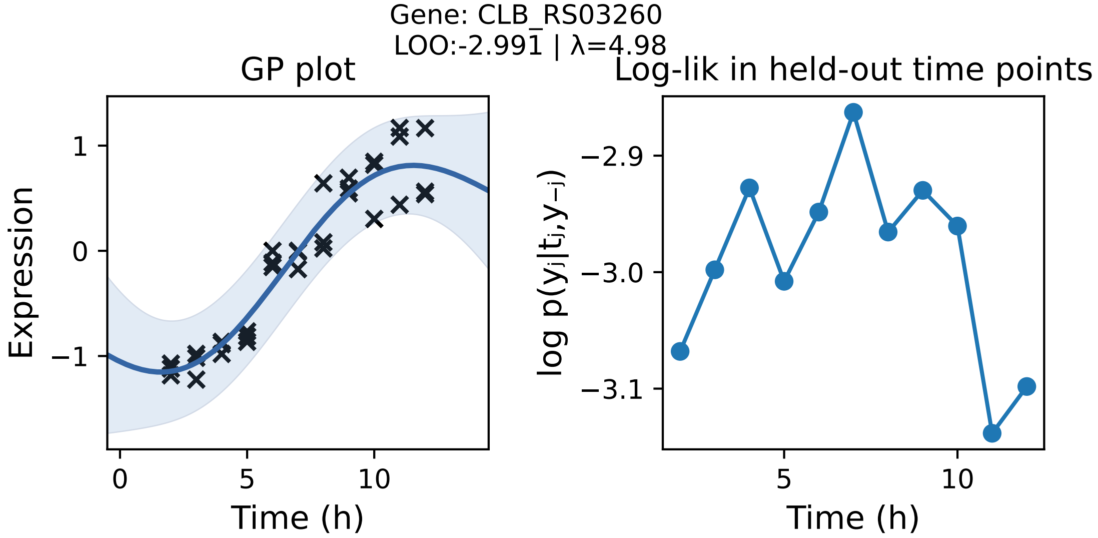

Gene: CLB_RS03260
LOO:-2.991 | λ=4.98
GP plot
Expression
Time (h)
Log-lik in held-out time points
log p(y_j|t_j,y_−j)
Time (h)

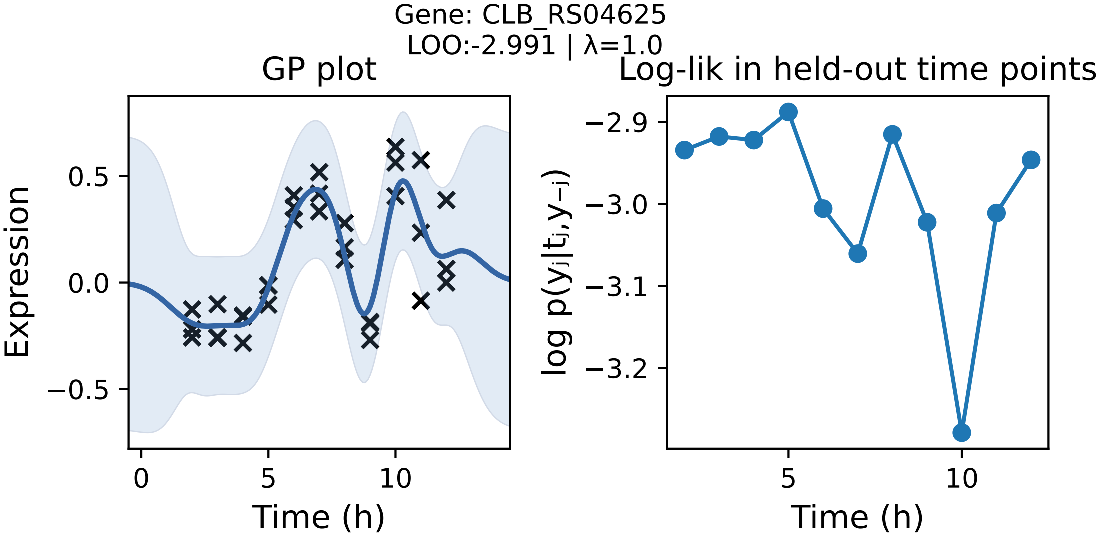

Gene: CLB_RS04625
LOO:-2.991 | λ=1.0
GP plot
Expression
0.5
0.0
−0.5
0
5
10
Time (h)
Log-lik in held-out time points
log p(y_j|t_j,y_−j)
−2.9
−3.0
−3.1
−3.2
5
10
Time (h)

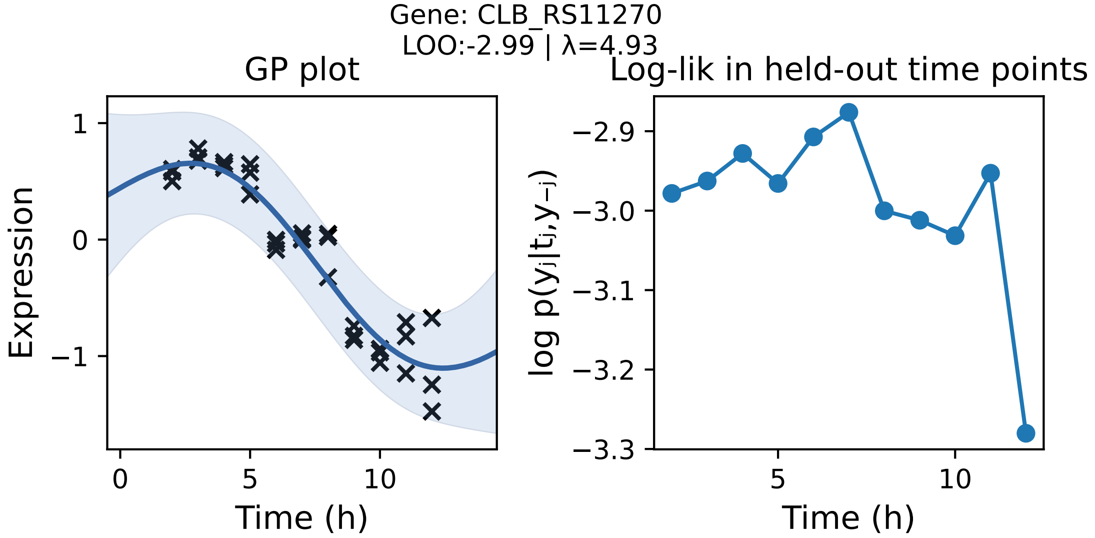

Gene: CLB_RS11270
LOO:-2.99 | λ=4.93
GP plot
Expression
1
0
−1
0
5
10
Time (h)
Log-lik in held-out time points
log p(y_j|t_j,y_−j)
−2.9
−3.0
−3.1
−3.2
−3.3
5
10
Time (h)

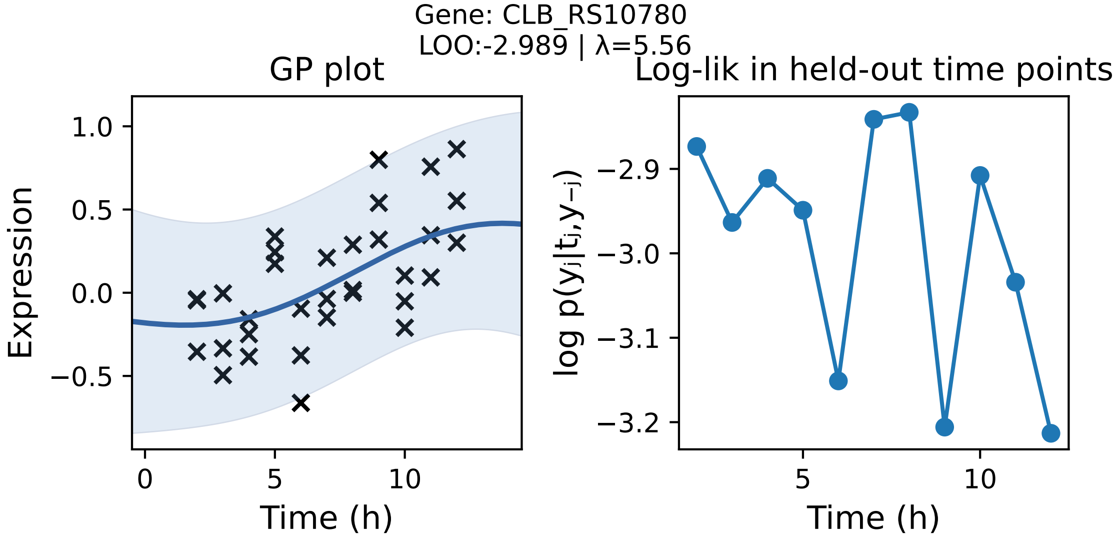

Gene: CLB_RS10780
LOO:-2.989 | λ=5.56
GP plot
Expression
1.0
0.5
0.0
−0.5
0
5
10
Time (h)
Log-lik in held-out time points
log p(y_j|t_j,y_−j)
−2.9
−3.0
−3.1
−3.2
5
10
Time (h)

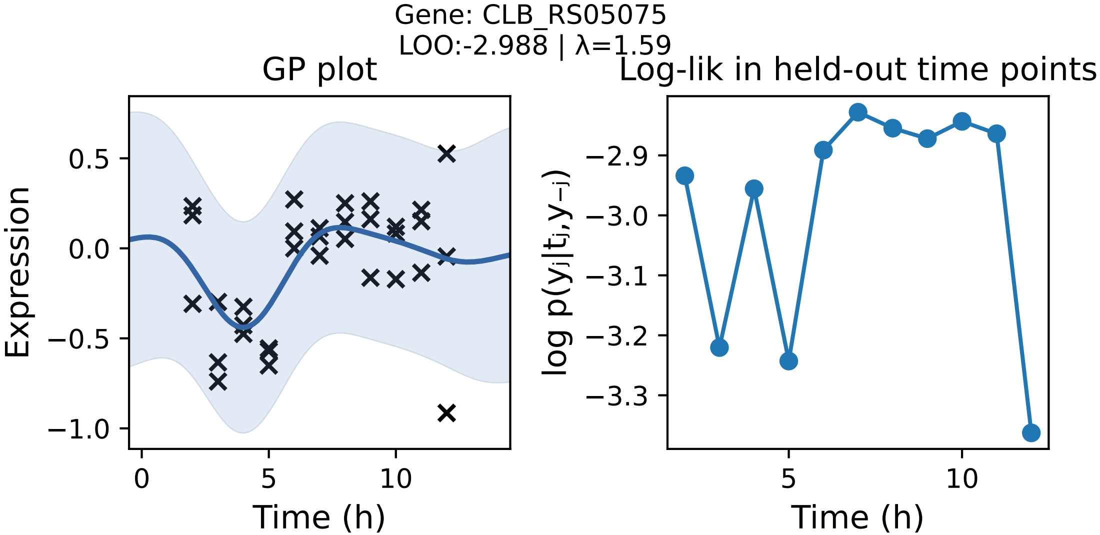

Gene: CLB_RS05075
LOO:-2.988 | λ=1.59
GP plot
Expression
0.5
0.0
−0.5
−1.0
0
5
10
Time (h)
Log-lik in held-out time points
log p(y_j|t_j,y_−j)
−2.9
−3.0
−3.1
−3.2
−3.3
5
10
Time (h)

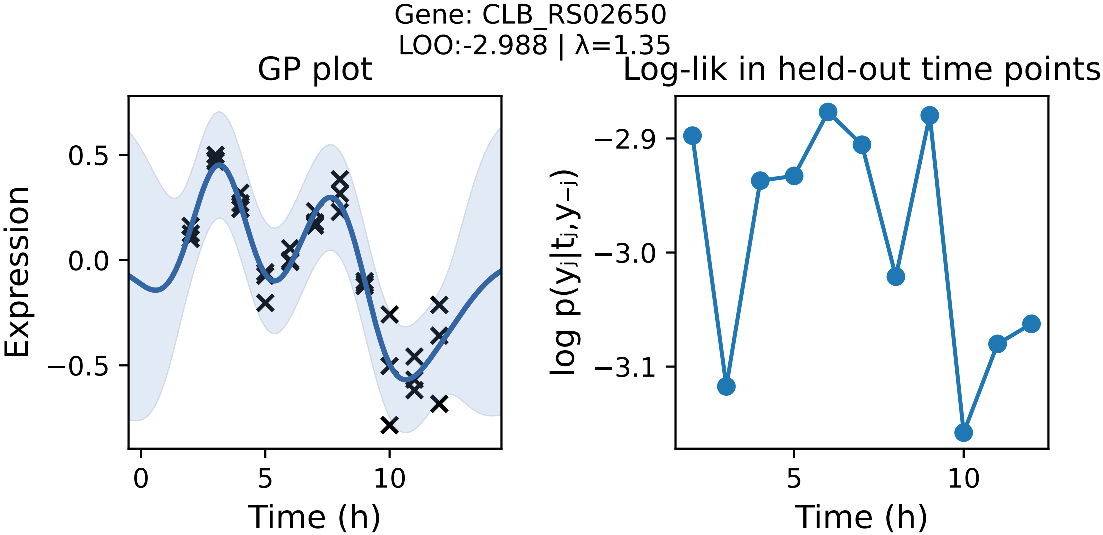
Gene: CLB_RS02650
LOO:-2.988 | λ=1.35
GP plot
Expression
Time (h)
0.5
0.0
−0.5
0
5
10
Log-lik in held-out time points
log p(y_j|t_j,y_−j)
−2.9
−3.0
−3.1
5
10
Time (h)

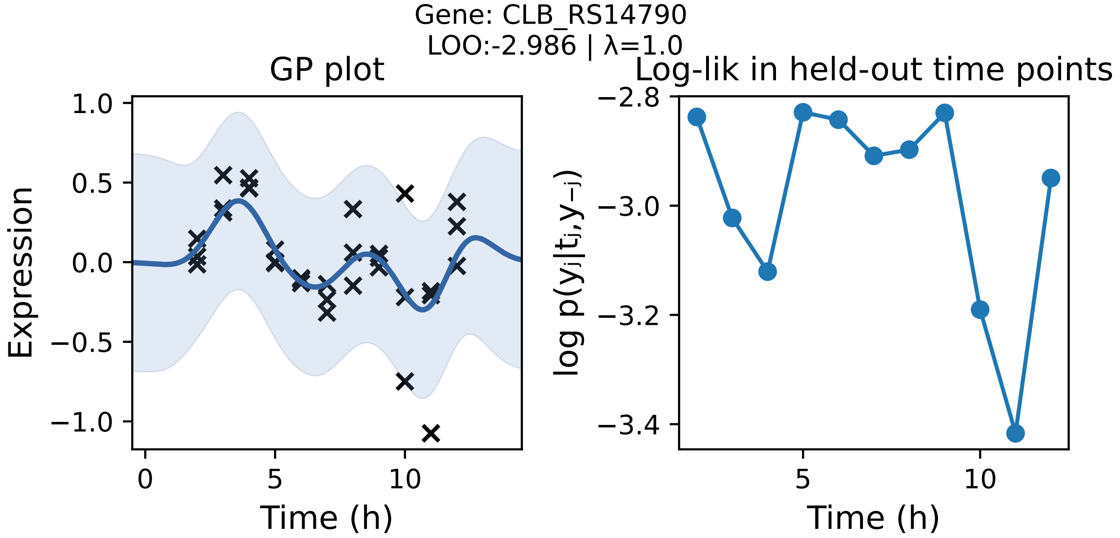
Gene: CLB_RS14790
LOO:-2.986 | λ=1.0
GP plot
Expression
Time (h)
1.0
0.5
0.0
−0.5
−1.0
0
5
10
Log-lik in held-out time points
log p(y_j|t_j,y_−j)
Time (h)
−2.8
−3.0
−3.2
−3.4
5
10

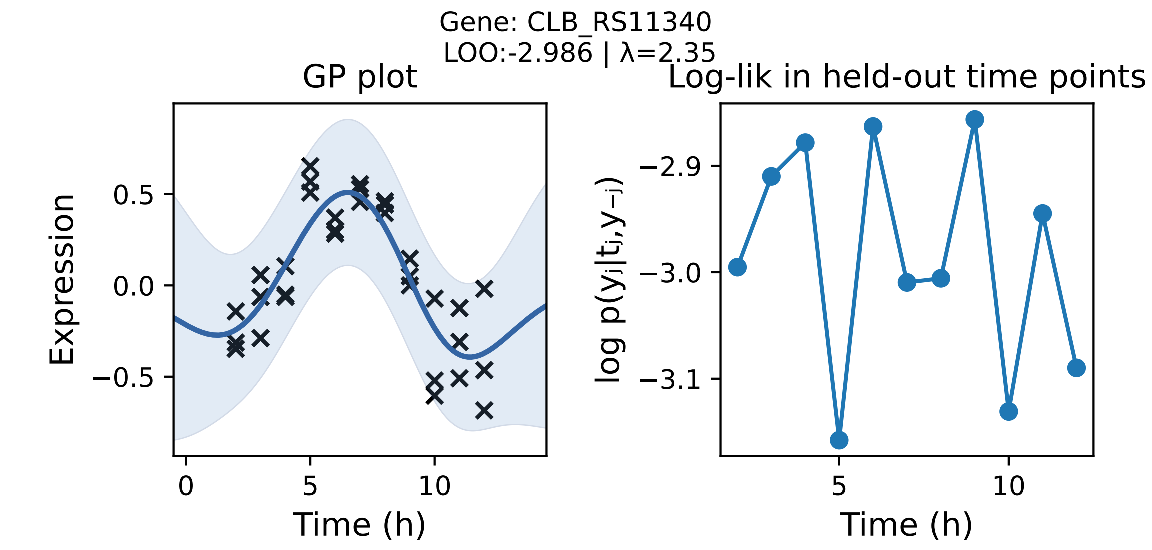
Gene: CLB_RS11340
LOO:-2.986 | λ=2.35
GP plot
Expression
0.5
0.0
−0.5
0
5
10
Time (h)
Log-lik in held-out time points
log p(y_j|t_j,y_−j)
−2.9
−3.0
−3.1
5
10
Time (h)

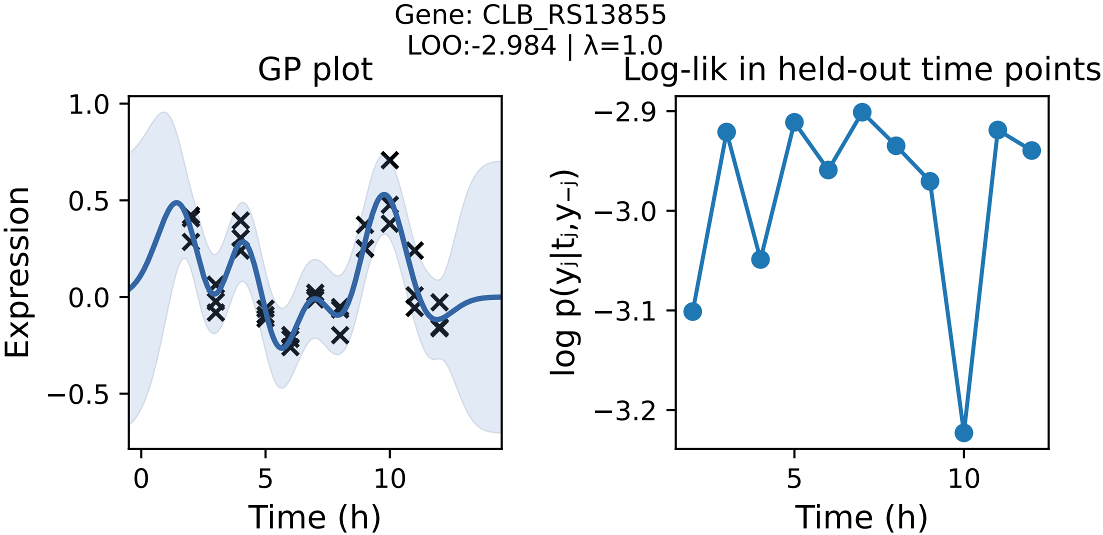

Gene: CLB_RS13855
LOO:-2.984 | λ=1.0
GP plot
Expression
Time (h)
1.0
0.5
0.0
−0.5
0
5
10
Log-lik in held-out time points
log p(y_j|t_j,y_−j)
Time (h)
−2.9
−3.0
−3.1
−3.2
5
10

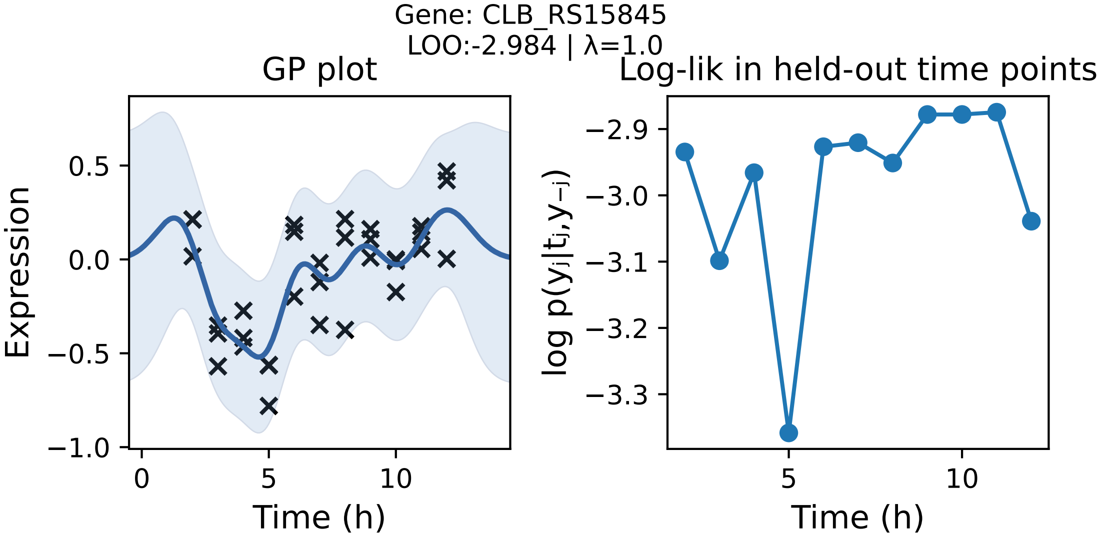

Gene: CLB_RS15845
LOO:-2.984 | λ=1.0
GP plot
Expression
Time (h)
0.5
0.0
−0.5
−1.0
0
5
10
Log-lik in held-out time points
log p(y_j|t_j,y_−j)
Time (h)
−2.9
−3.0
−3.1
−3.2
−3.3
5
10

Gene: CLB_RS15560
LOO:-2.984 | λ=2.94

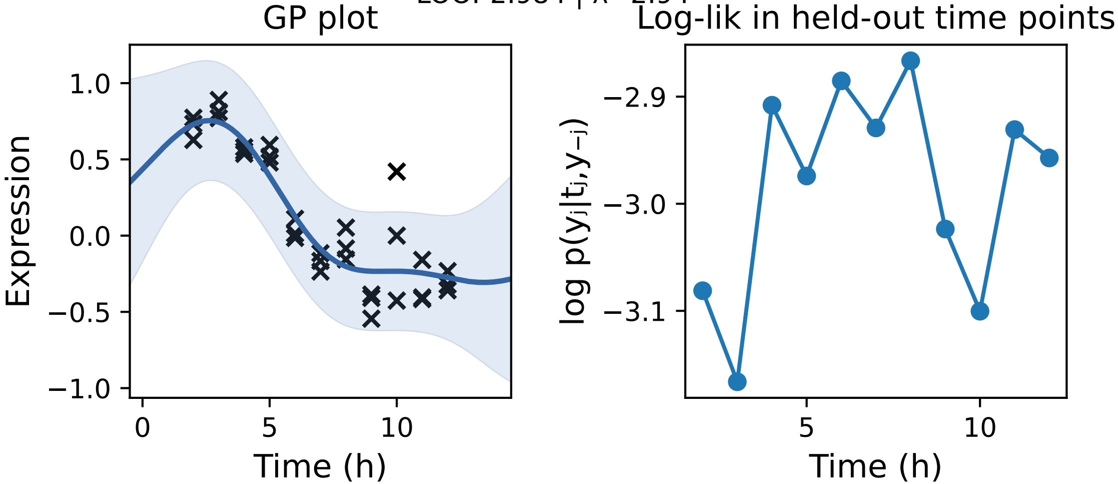

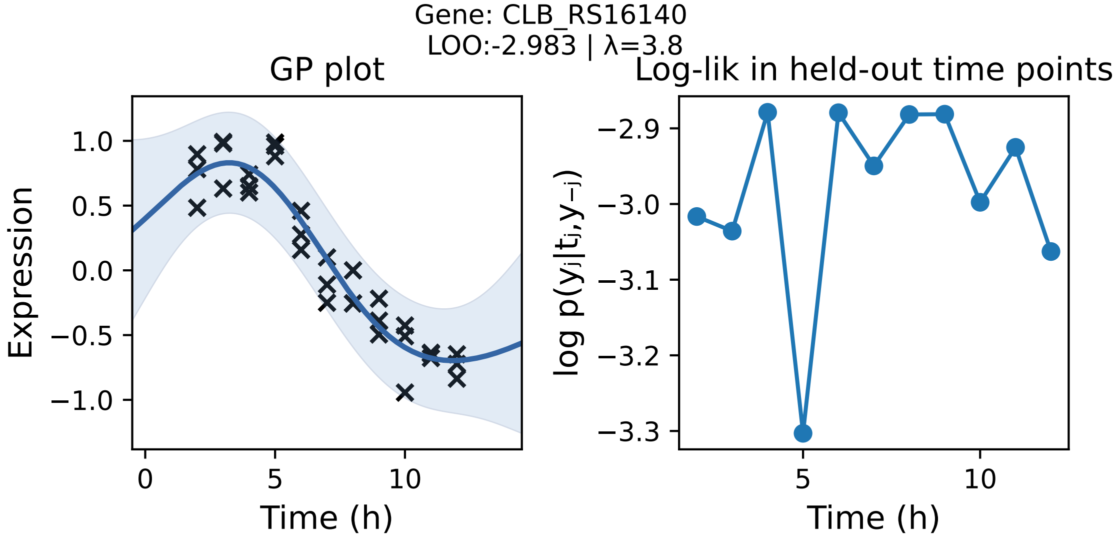

Gene: CLB_RS16140
LOO:-2.983 | λ=3.8
GP plot
Log-lik in held-out time points
Expression
Time (h)
1.0
0.5
0.0
−0.5
−1.0
0
5
10
log p(yⱼ|tⱼ,y₋ⱼ)
−2.9
−3.0
−3.1
−3.2
−3.3
5
10
Time (h)

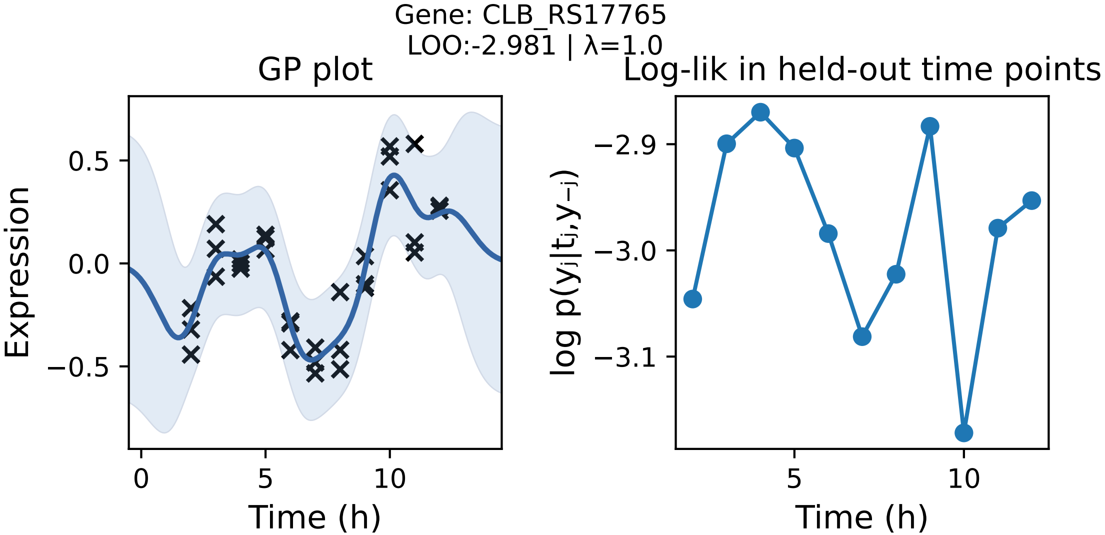

Gene: CLB_RS17765
LOO:-2.981 | λ=1.0
GP plot
Expression
Time (h)
0.5
0.0
−0.5
0
5
10
Log-lik in held-out time points
log p(y_j|t_j,y_−j)
Time (h)
−2.9
−3.0
−3.1
5
10

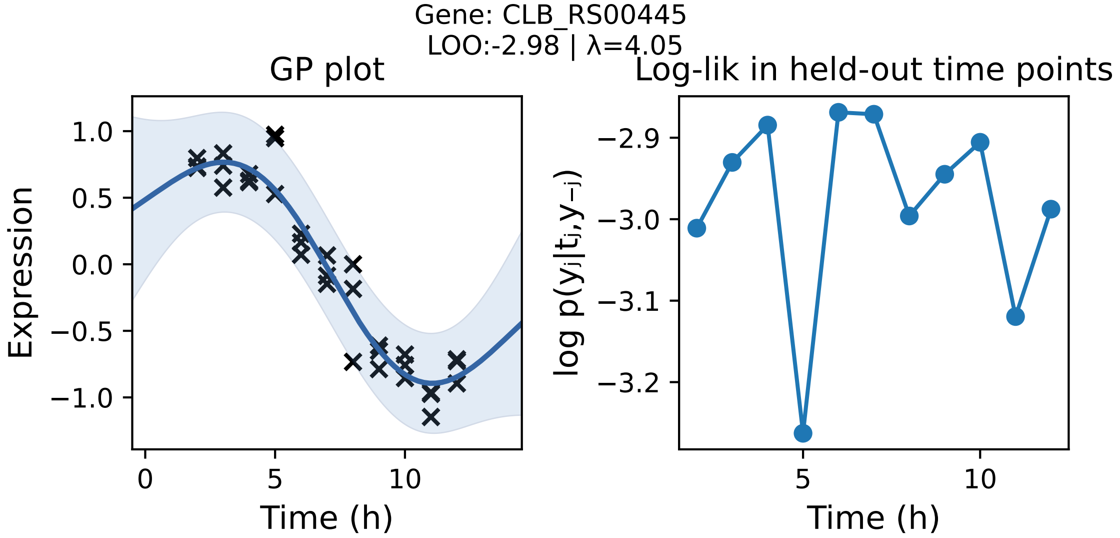

Gene: CLB_RS00445
LOO:-2.98 | λ=4.05
GP plot
Expression
Time (h)
Log-lik in held-out time points
log p(y_j|t_j,y_−j)
Time (h)

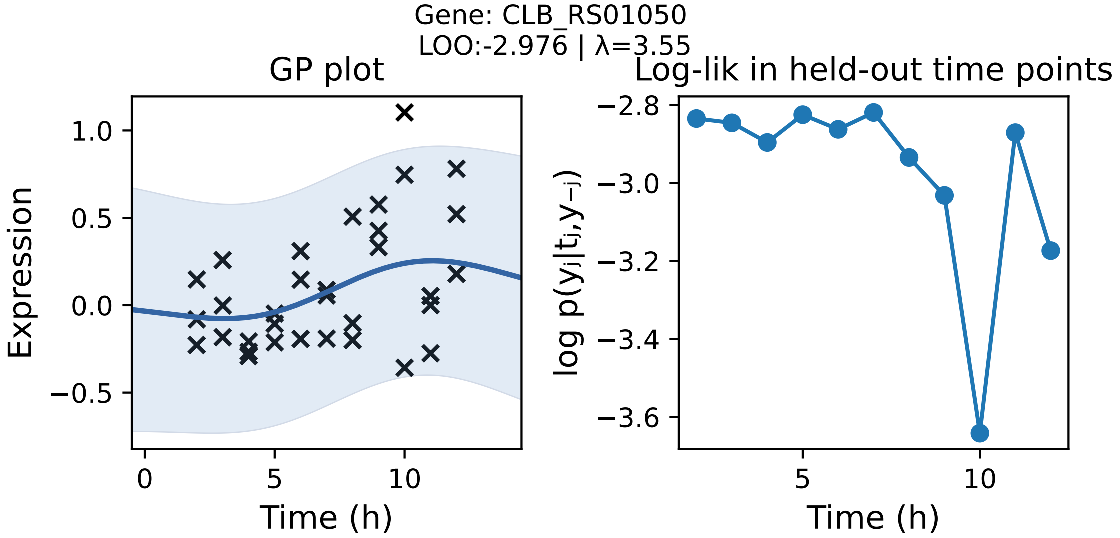

Gene: CLB_RS01050
LOO:-2.976 | λ=3.55
GP plot
Expression
1.0
0.5
0.0
−0.5
0
5
10
Time (h)
Log-lik in held-out time points
log p(y_j|t_j,y_−j)
−2.8
−3.0
−3.2
−3.4
−3.6
5
10
Time (h)

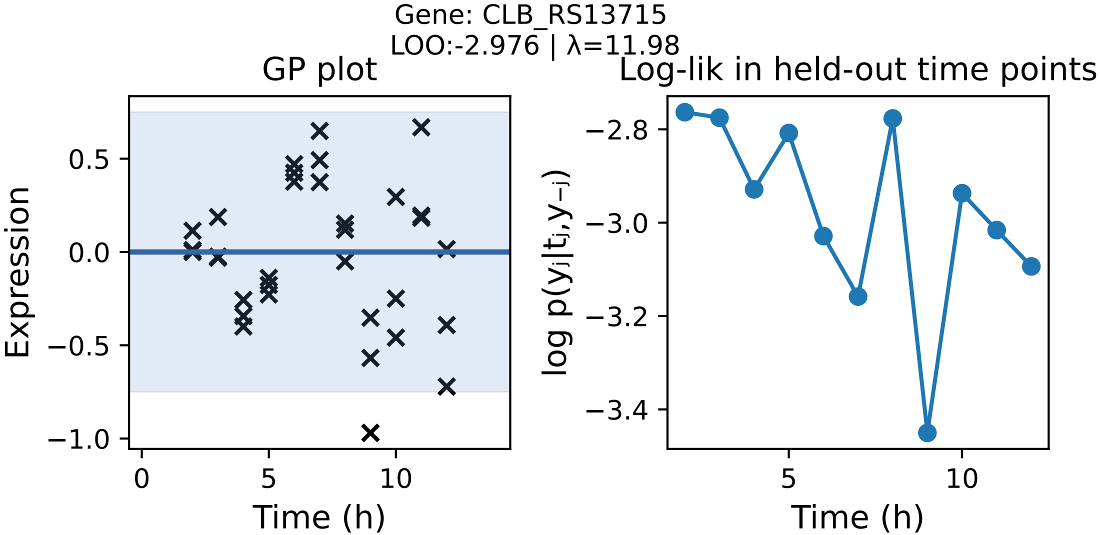
Gene: CLB_RS13715
LOO:-2.976 | λ=11.98
GP plot
Log-lik in held-out time points
Expression
0.5
0.0
−0.5
−1.0
0
5
10
Time (h)
log p(y_j|t_j,y_−j)
−2.8
−3.0
−3.2
−3.4
5
10
Time (h)

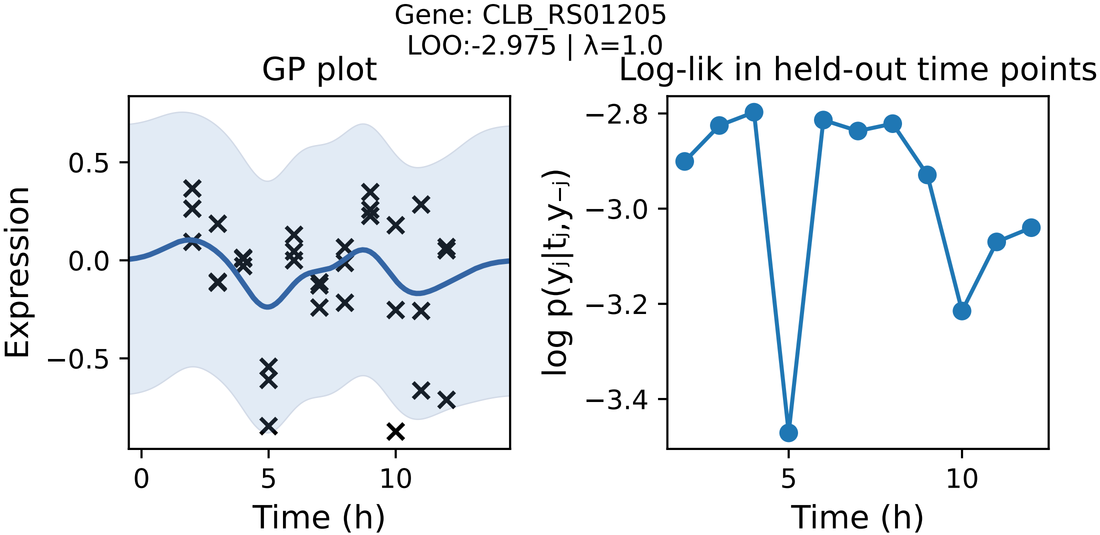

Gene: CLB_RS01205
LOO:-2.975 | λ=1.0
GP plot
Expression
0.5
0.0
−0.5
0
5
10
Time (h)
Log-lik in held-out time points
log p(y_j|t_j,y_−j)
−2.8
−3.0
−3.2
−3.4
5
10
Time (h)

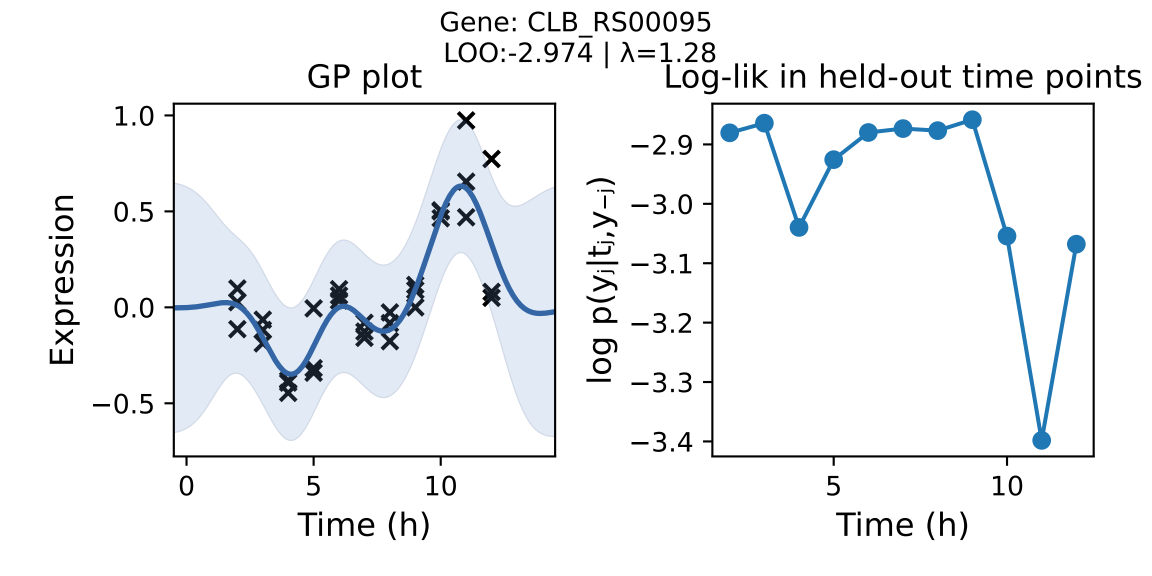
Gene: CLB_RS00095
LOO:-2.974 | λ=1.28
GP plot
Expression
Time (h)
Log-lik in held-out time points
log p(y_j|t_j,y_−j)
Time (h)

Gene: CLB_RS17920
LOO:-2.974 | λ=1.0

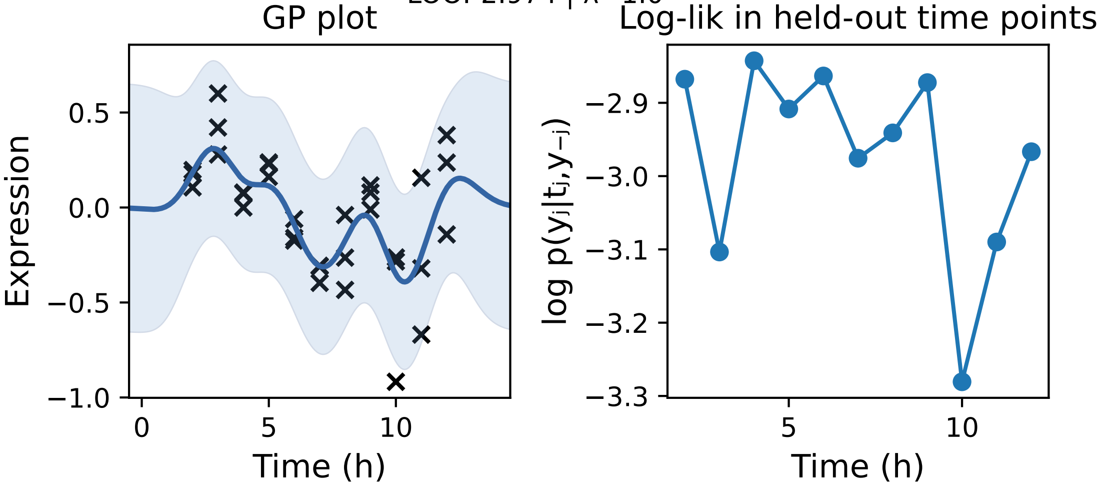

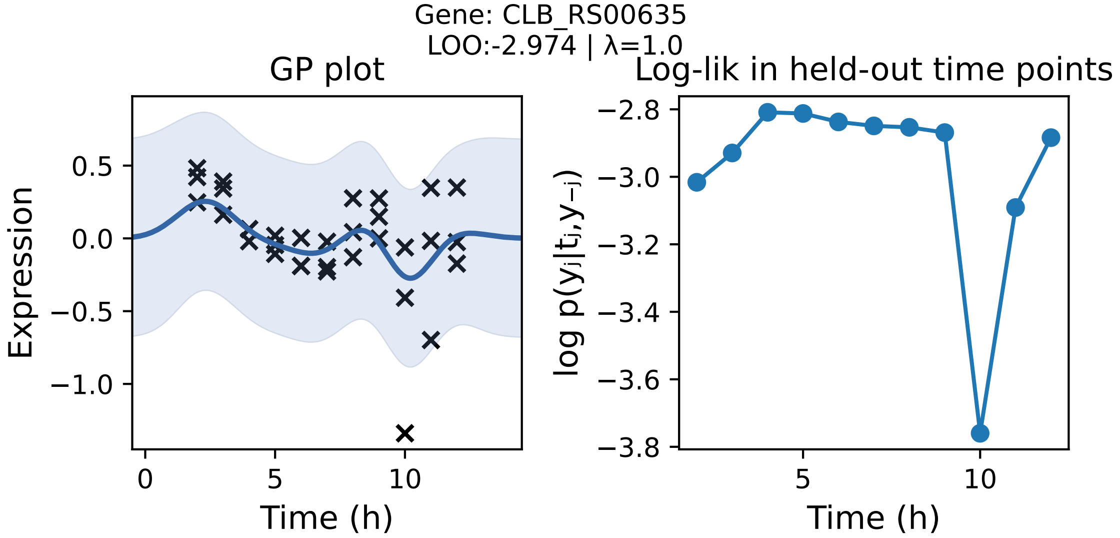

Gene: CLB_RS00635
LOO:-2.974 | λ=1.0
GP plot
Log-lik in held-out time points
Expression
0.5
0.0
−0.5
−1.0
0
5
10
Time (h)
log p(yj|tj,y−j)
−2.8
−3.0
−3.2
−3.4
−3.6
−3.8
5
10
Time (h)

Gene: CLB_RS01740
LOO:-2.973 | λ=1.0

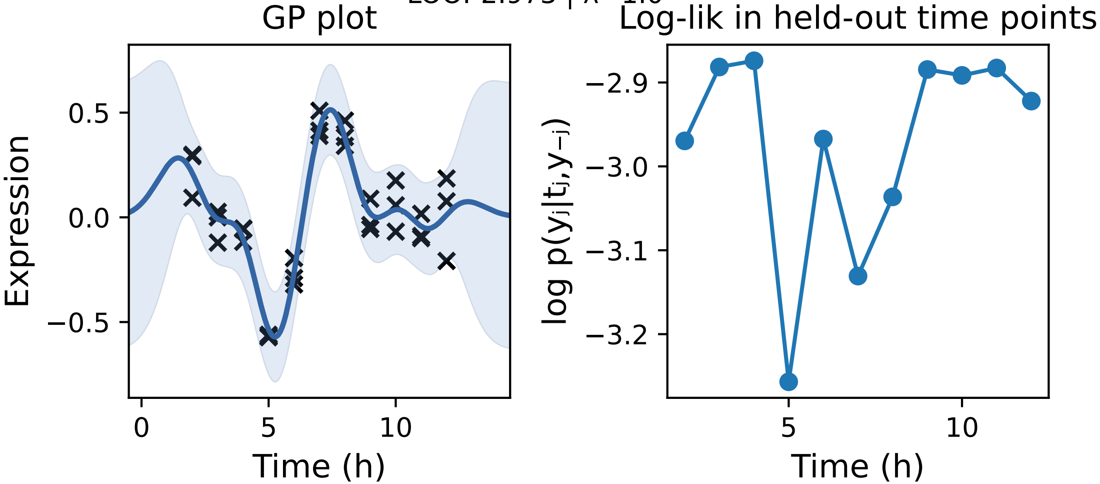

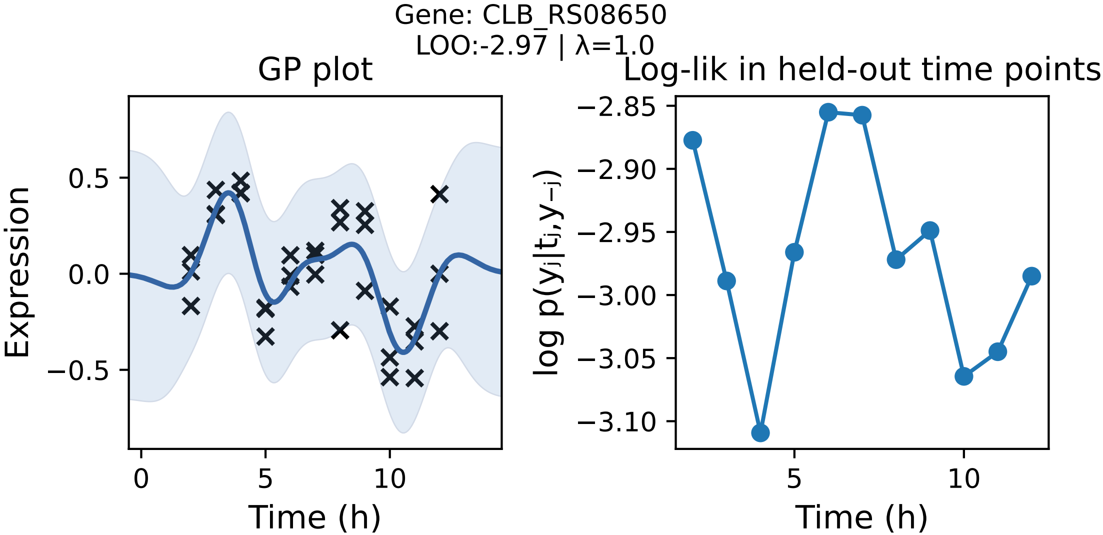
Gene: CLB_RS08650
LOO:-2.97 | λ=1.0
GP plot
Expression
0.5
0.0
−0.5
0
5
10
Time (h)
Log-lik in held-out time points
log p(yj|tj,y−j)
−2.85
−2.90
−2.95
−3.00
−3.05
−3.10
5
10
Time (h)

Gene: CLB_RS13035
LOO:-2.969 | λ=5.6

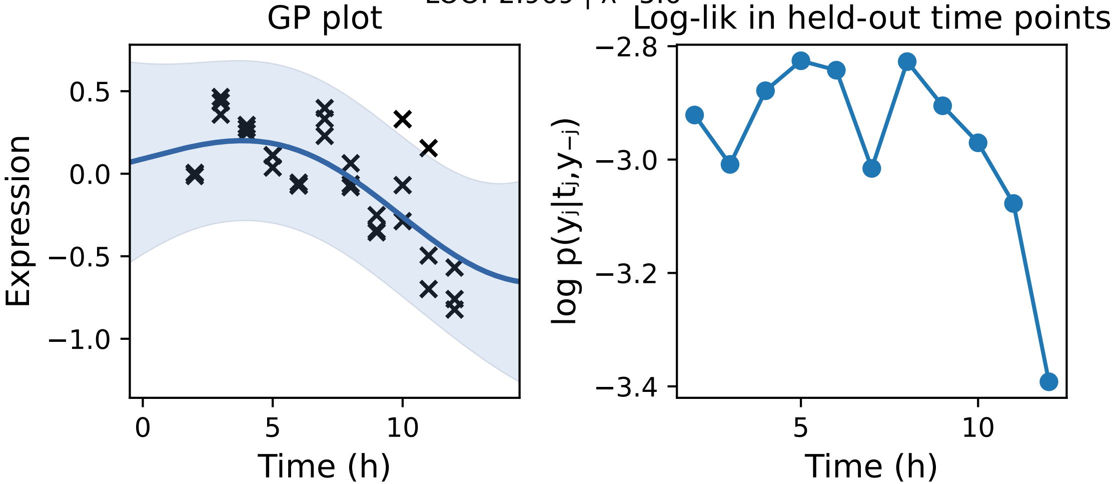

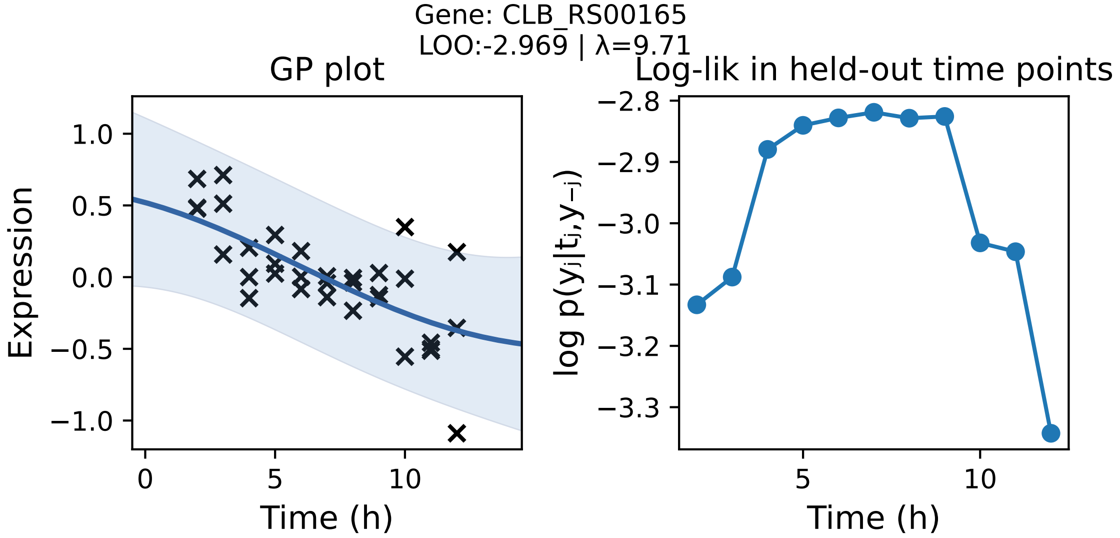
Gene: CLB_RS00165
LOO:-2.969 | λ=9.71
GP plot
Expression
1.0
0.5
0.0
−0.5
−1.0
0
5
10
Time (h)
Log-lik in held-out time points
log p(y_j|t_j,y_−j)
−2.8
−2.9
−3.0
−3.1
−3.2
−3.3
5
10
Time (h)

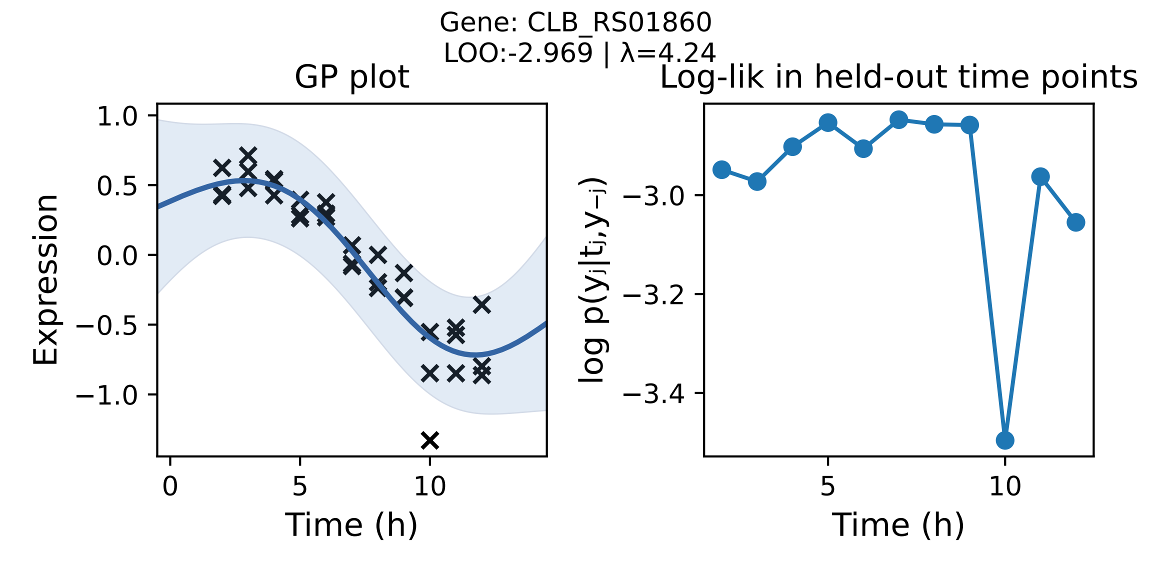
Gene: CLB_RS01860
LOO:-2.969 | λ=4.24
GP plot
Expression
Time (h)
Log-lik in held-out time points
log p(yj|tj,y−j)
Time (h)

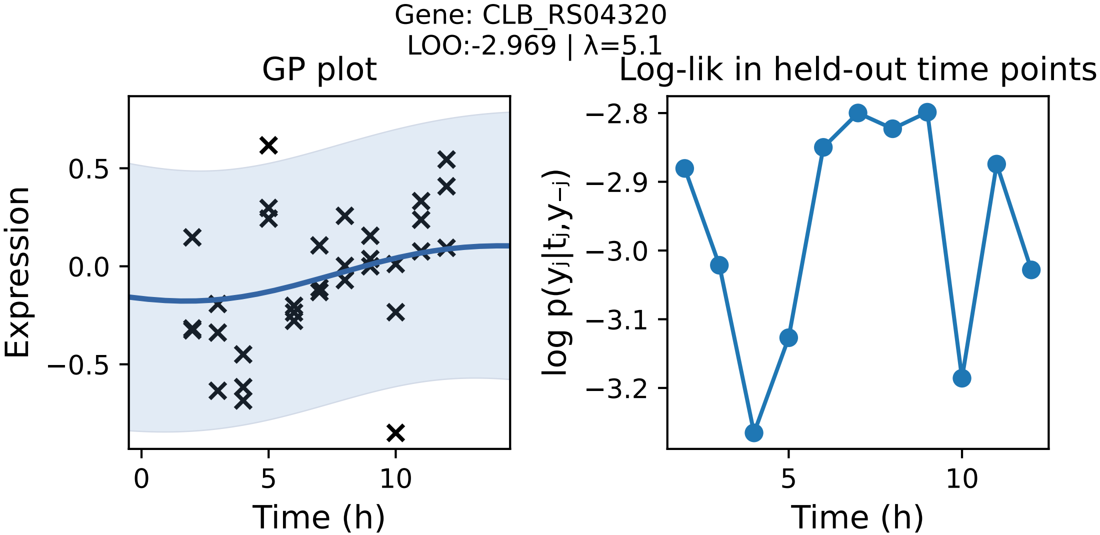
Gene: CLB_RS04320
LOO:-2.969 | λ=5.1
GP plot
Expression
0.5
0.0
−0.5
0
5
10
Time (h)
Log-lik in held-out time points
log p(y_j|t_j,y_−j)
−2.8
−2.9
−3.0
−3.1
−3.2
5
10
Time (h)

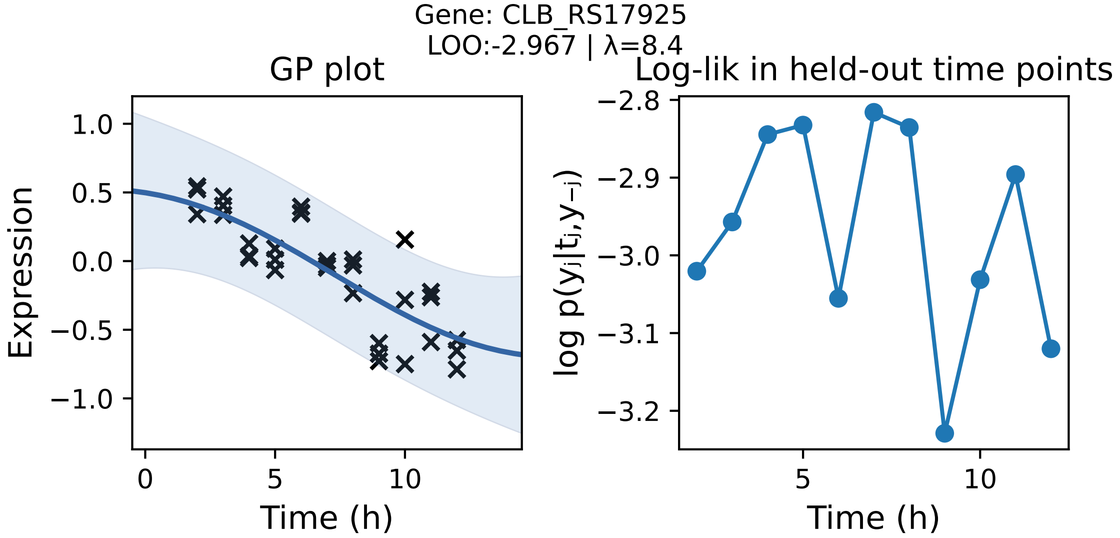
Gene: CLB_RS17925
LOO:-2.967 | λ=8.4
GP plot
Expression
Time (h)
Log-lik in held-out time points
log p(y_j|t_j,y_−j)
Time (h)

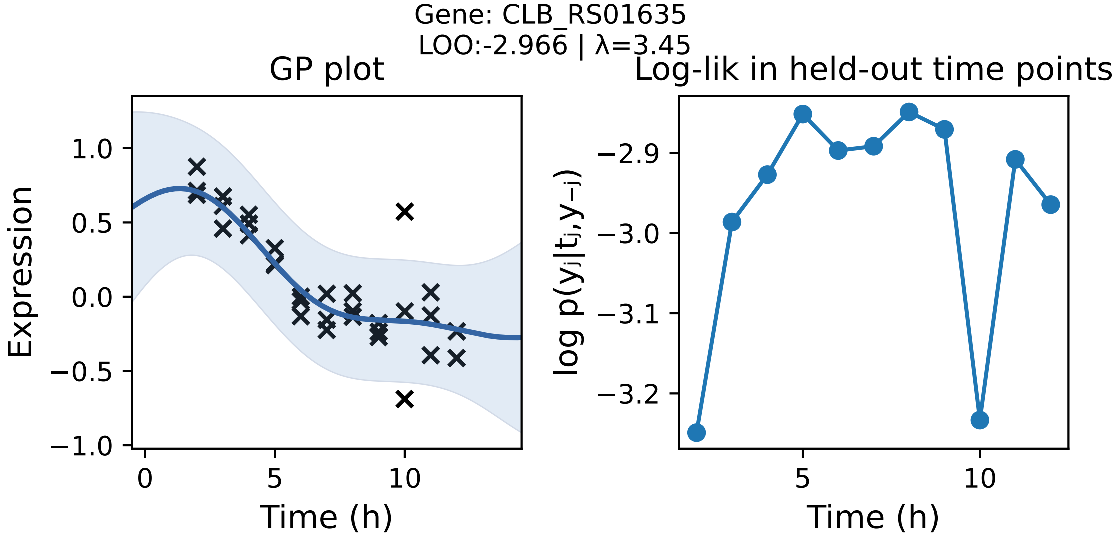

Gene: CLB_RS01635
LOO:-2.966 | λ=3.45
GP plot
Expression
1.0
0.5
0.0
−0.5
−1.0
0
5
10
Time (h)
Log-lik in held-out time points
log p(y_j|t_j,y_−j)
−2.9
−3.0
−3.1
−3.2
5
10
Time (h)

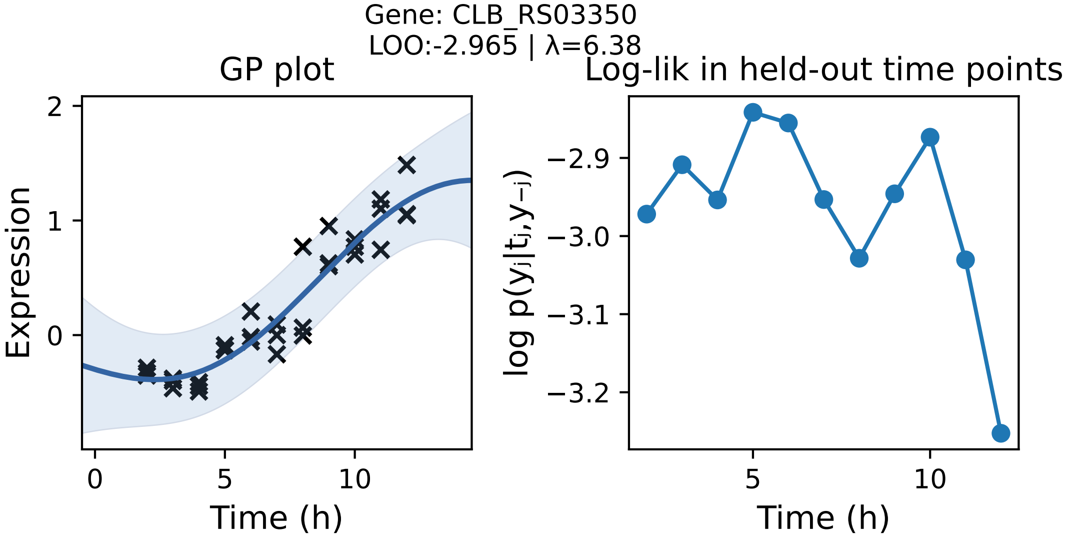
Gene: CLB_RS03350
LOO:-2.965 | λ=6.38
GP plot
Expression
Time (h)
Log-lik in held-out time points
log p(y_j|t_j,y_{-j})
Time (h)

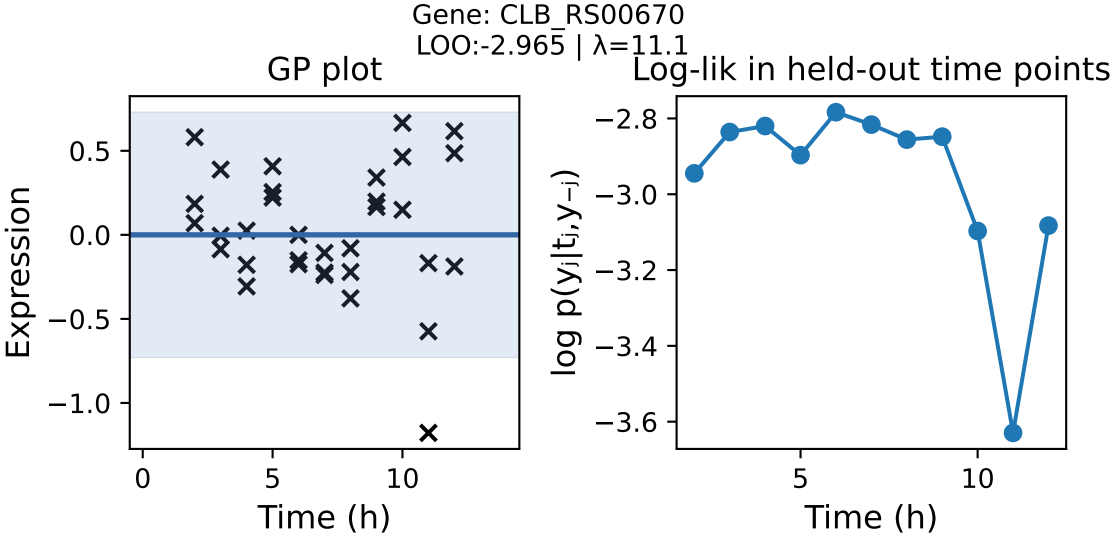

Gene: CLB_RS00670
LOO:-2.965 | λ=11.1
GP plot
Expression
Time (h)
0.5
0.0
−0.5
−1.0
0
5
10
Log-lik in held-out time points
log p(y_j|t_j,y_−j)
Time (h)
−2.8
−3.0
−3.2
−3.4
−3.6
5
10

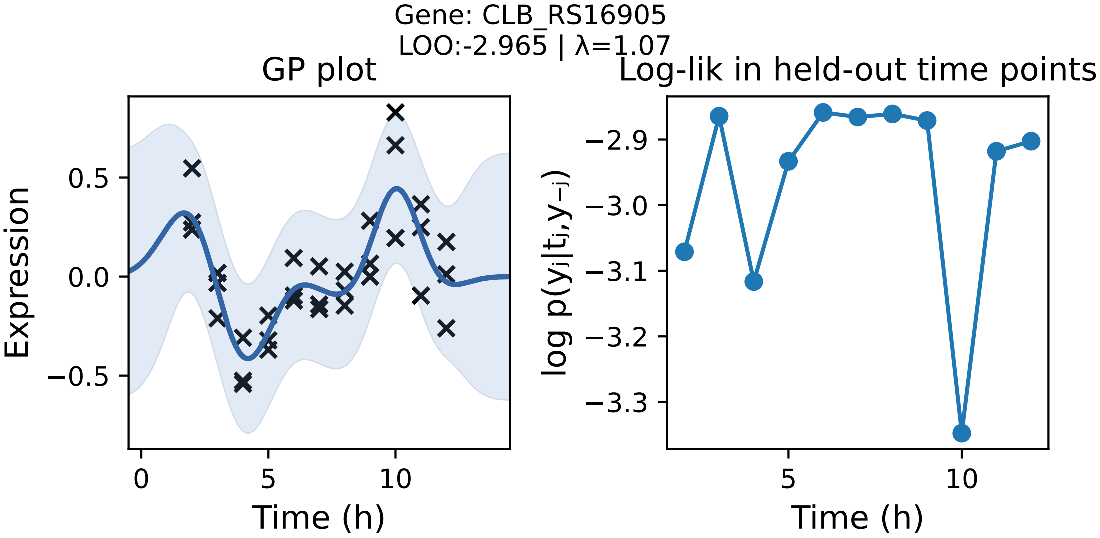

Gene: CLB_RS16905
LOO:-2.965 | λ=1.07
GP plot
Expression
0.5
0.0
−0.5
0
5
10
Time (h)
Log-lik in held-out time points
log p(y_j|t_j,y_−j)
−2.9
−3.0
−3.1
−3.2
−3.3
5
10
Time (h)

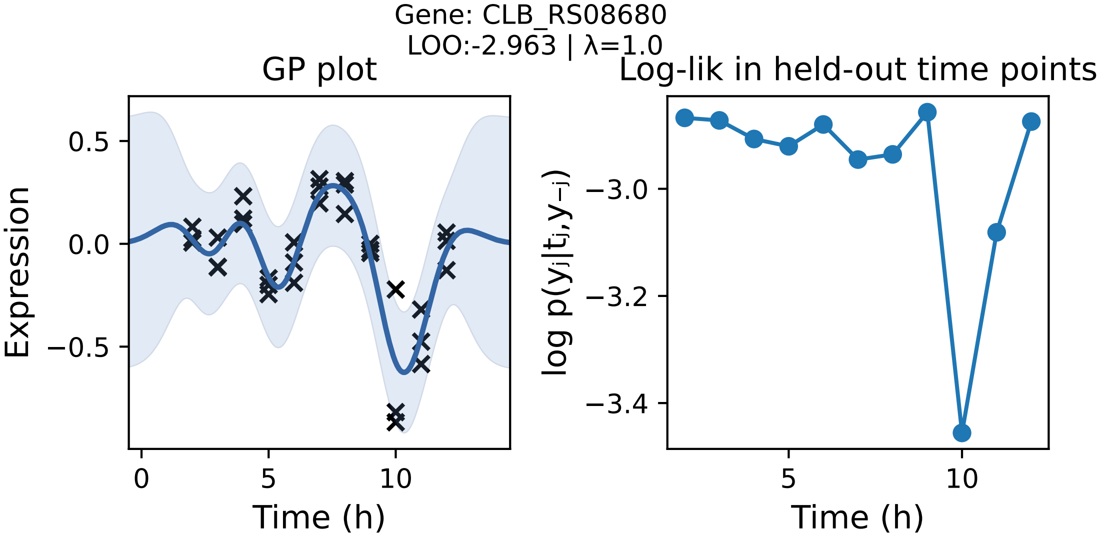
Gene: CLB_RS08680
LOO:-2.963 | λ=1.0
GP plot
Expression
0.5
0.0
−0.5
0
5
10
Time (h)
Log-lik in held-out time points
log p(y_j|t_j,y_−j)
−3.0
−3.2
−3.4
5
10
Time (h)

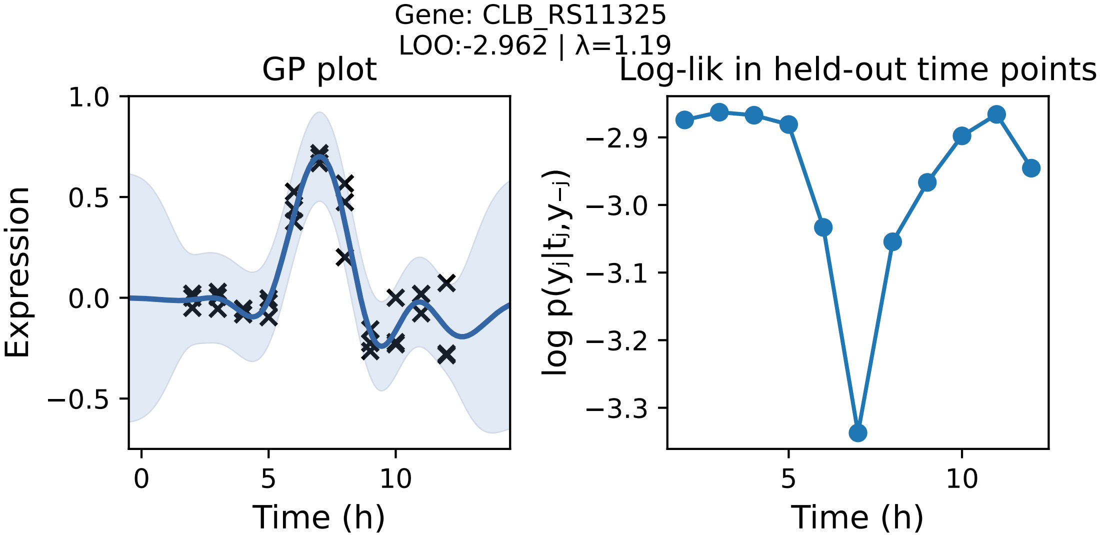

Gene: CLB_RS11325
LOO:-2.962 | λ=1.19
GP plot
Log-lik in held-out time points
1.0
0.5
0.0
−0.5
Expression
0
5
10
Time (h)
−2.9
−3.0
−3.1
−3.2
−3.3
log p(yj|tj,y−j)
5
10
Time (h)

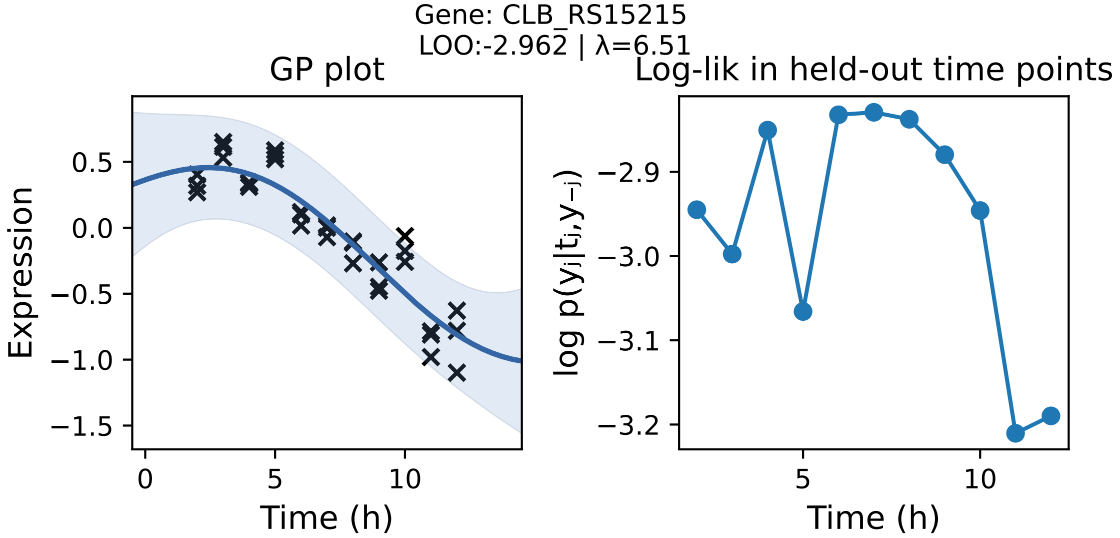

Gene: CLB_RS15215
LOO:-2.962 | λ=6.51
GP plot
Expression
0.5
0.0
−0.5
−1.0
−1.5
0
5
10
Time (h)
Log-lik in held-out time points
log p(y_j|t_j,y_−j)
−2.9
−3.0
−3.1
−3.2
5
10
Time (h)

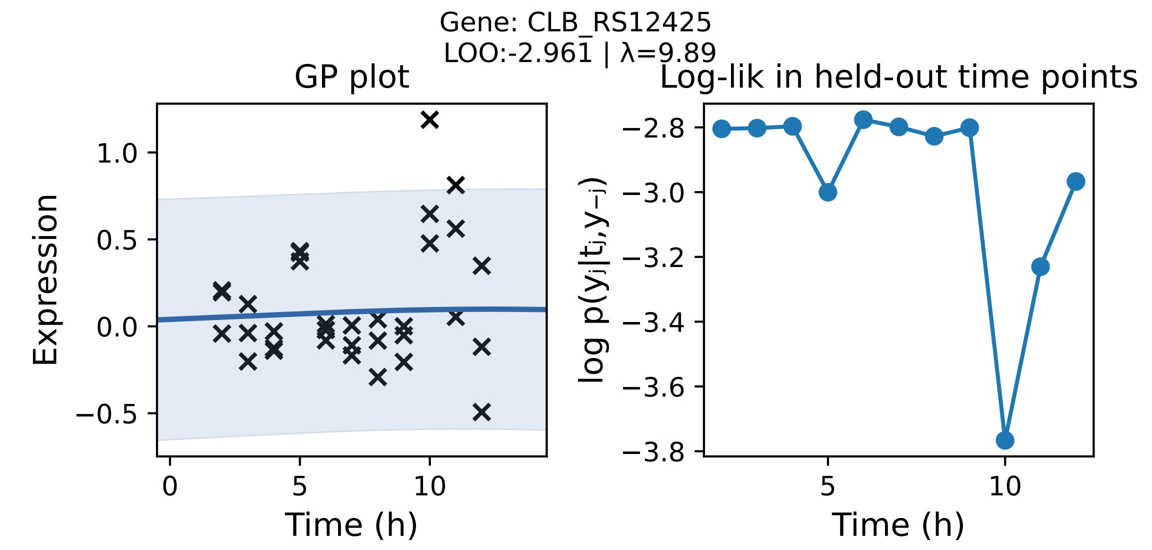

Gene: CLB_RS12425
LOO:-2.961 | λ=9.89
GP plot
Expression
1.0
0.5
0.0
−0.5
0
5
10
Time (h)
Log-lik in held-out time points
log p(y_j|t_j,y_−j)
−2.8
−3.0
−3.2
−3.4
−3.6
−3.8
5
10
Time (h)

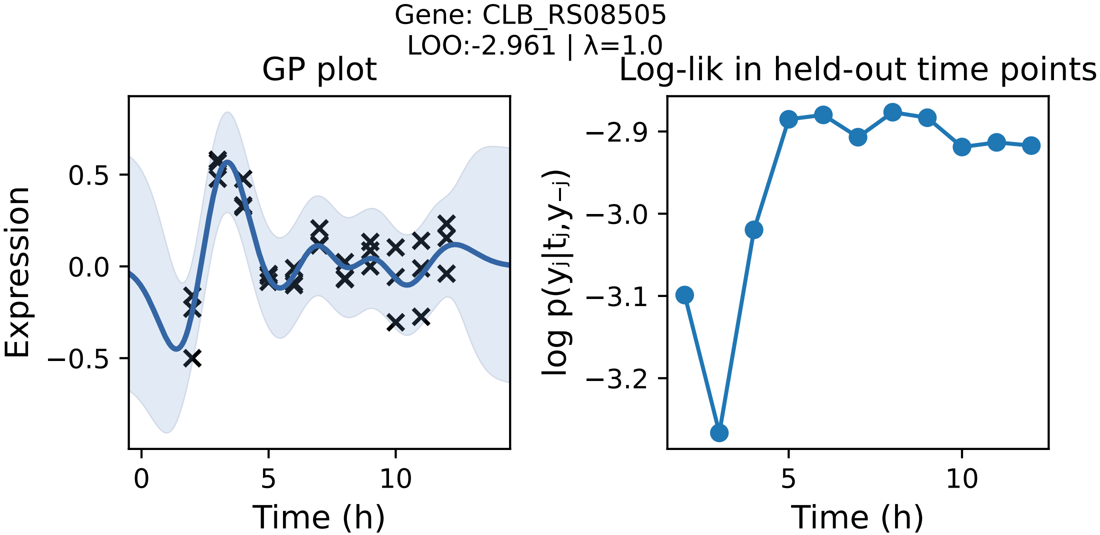

Gene: CLB_RS08505
LOO:-2.961 | λ=1.0
GP plot
Log-lik in held-out time points
Expression
0.5
0.0
−0.5
0
5
10
Time (h)
log p(y_j|t_j,y_−j)
−2.9
−3.0
−3.1
−3.2
5
10
Time (h)

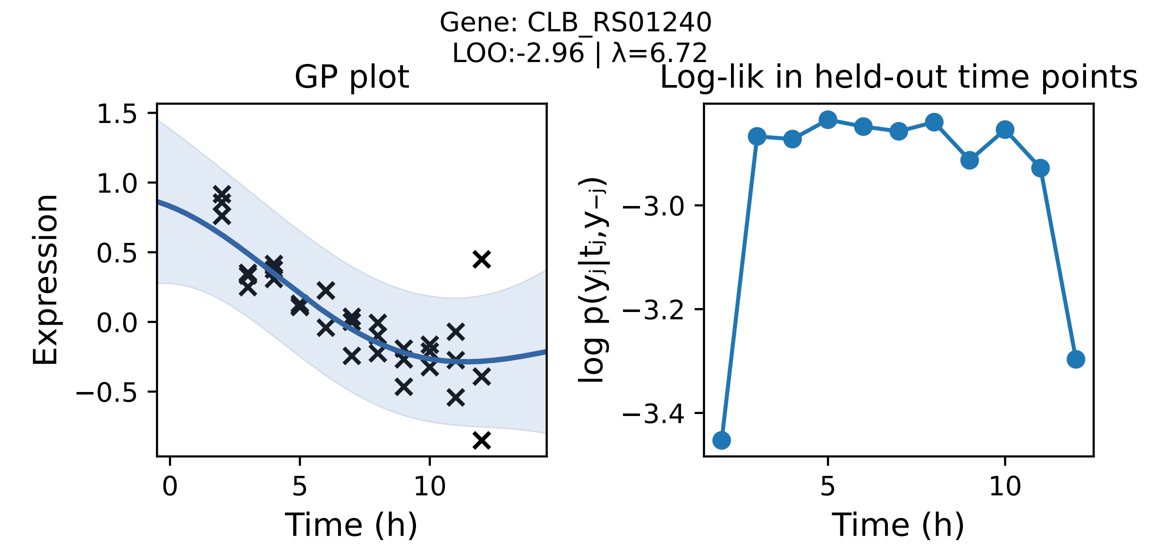
Gene: CLB_RS01240
LOO:-2.96 | λ=6.72
GP plot
Expression
Time (h)
Log-lik in held-out time points
log p(y_j|t_j,y_−j)
Time (h)

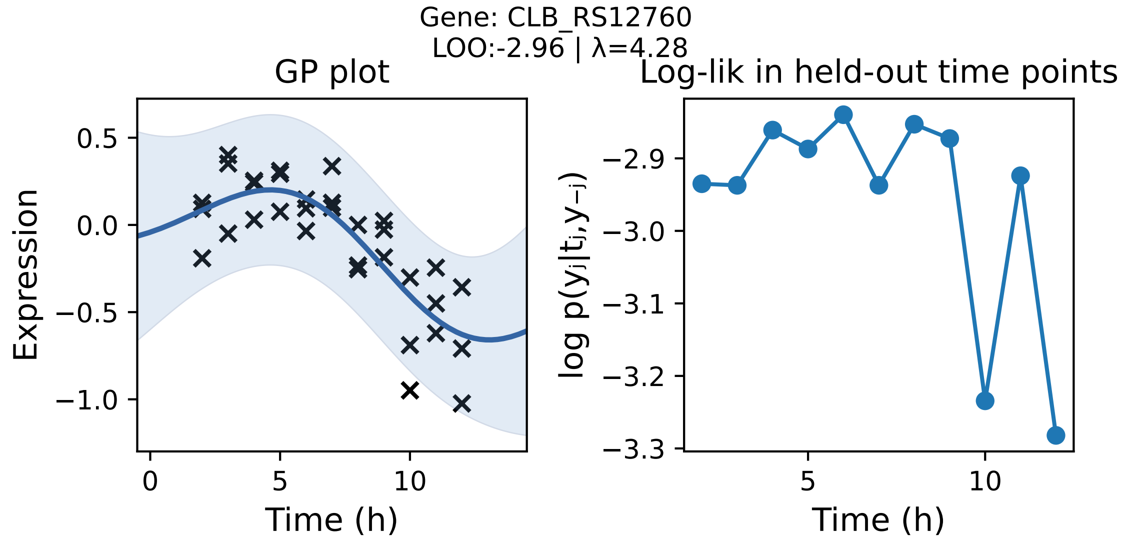

Gene: CLB_RS12760
LOO:-2.96 | λ=4.28
GP plot
Expression
0.5
0.0
−0.5
−1.0
0
5
10
Time (h)
Log-lik in held-out time points
log p(y_j|t_j,y_−j)
−2.9
−3.0
−3.1
−3.2
−3.3
5
10
Time (h)

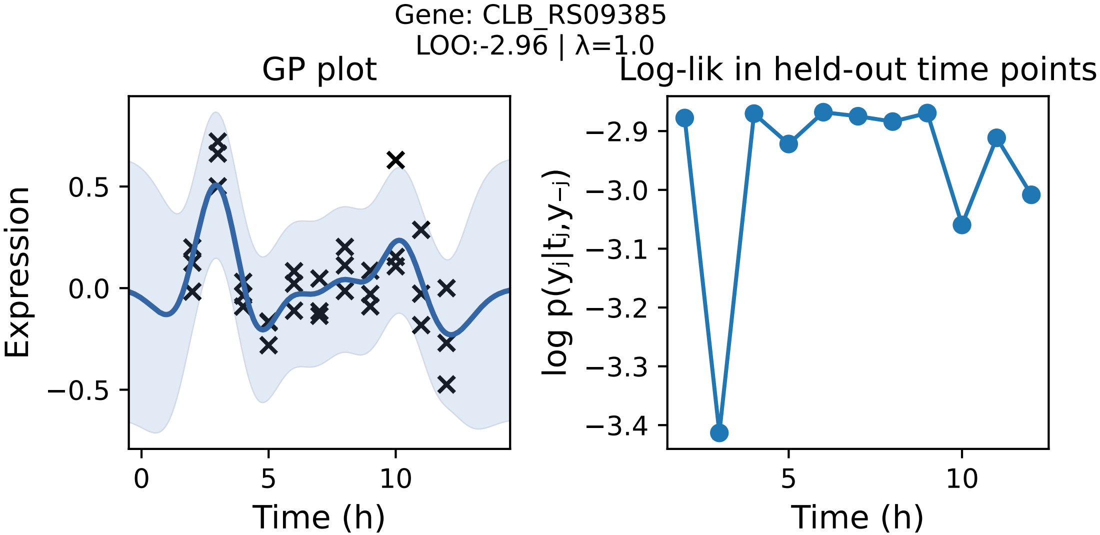

Gene: CLB_RS09385
LOO:-2.96 | λ=1.0
GP plot
Expression
0.5
0.0
−0.5
0
5
10
Time (h)
Log-lik in held-out time points
log p(y_j|t_j,y_−j)
−2.9
−3.0
−3.1
−3.2
−3.3
−3.4
5
10
Time (h)

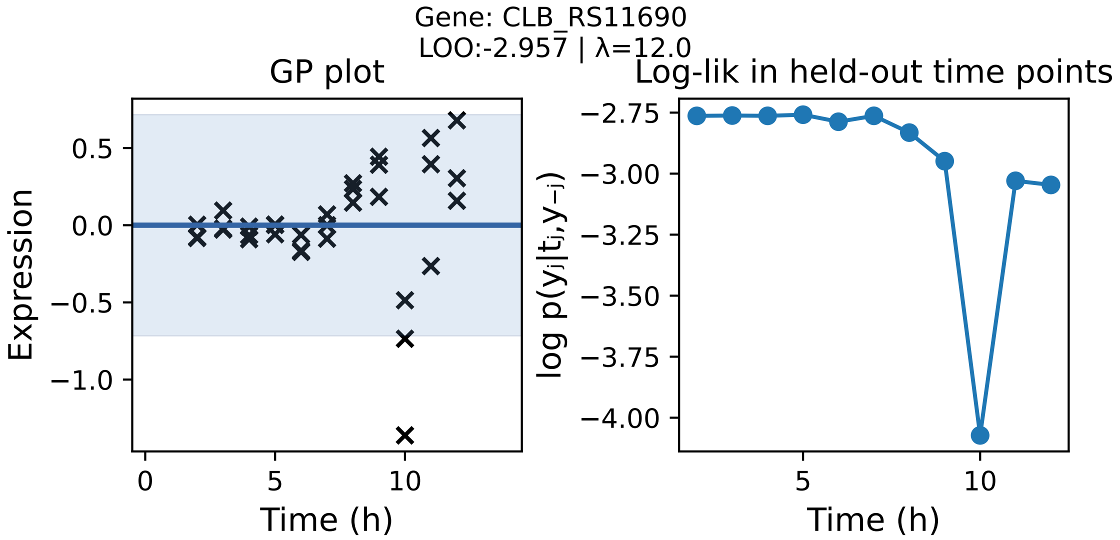

Gene: CLB_RS11690
LOO:-2.957 | λ=12.0
GP plot
Expression
0.5
0.0
−0.5
−1.0
0
5
10
Time (h)
Log-lik in held-out time points
log p(y_j|t_j,y_−j)
−2.75
−3.00
−3.25
−3.50
−3.75
−4.00
5
10
Time (h)

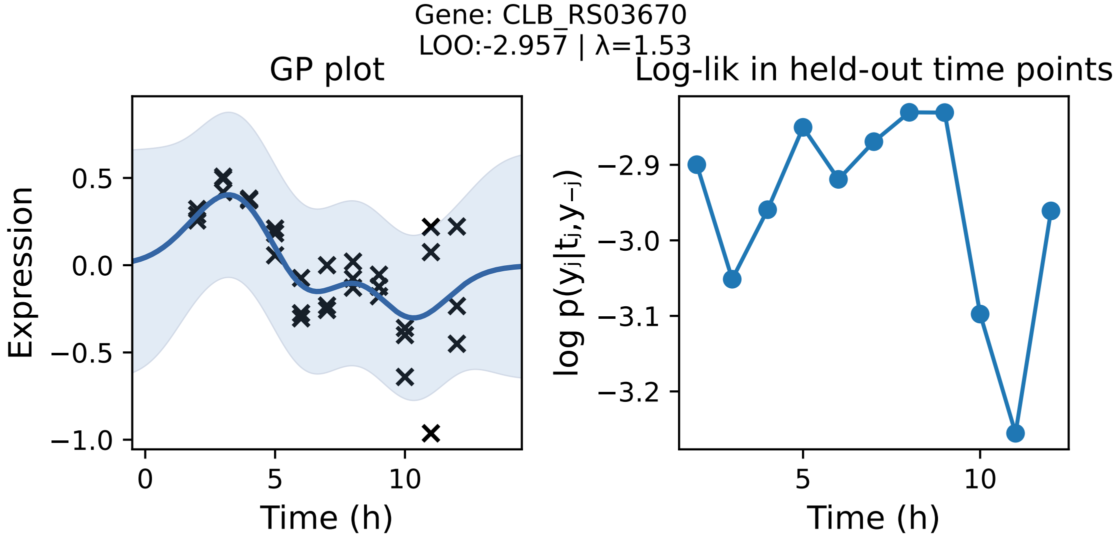

Gene: CLB_RS03670
LOO:-2.957 | λ=1.53
GP plot
Log-lik in held-out time points
Expression
0.5
0.0
−0.5
−1.0
0
5
10
Time (h)
log p(y_j|t_j,y_−j)
−2.9
−3.0
−3.1
−3.2
5
10
Time (h)

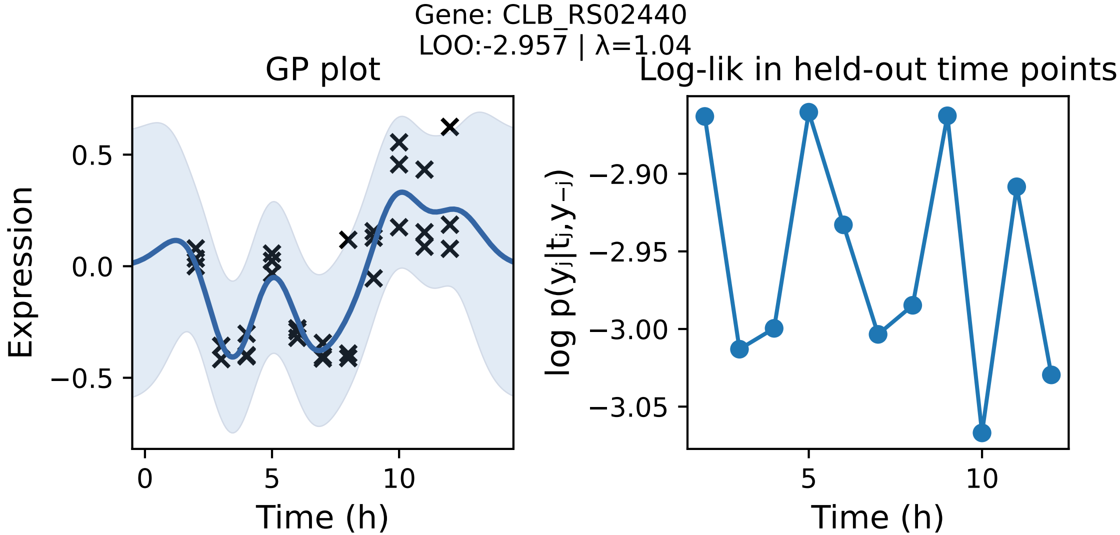

Gene: CLB_RS02440
LOO:-2.957 | λ=1.04
GP plot
Expression
0.5
0.0
−0.5
0
5
10
Time (h)
Log-lik in held-out time points
log p(y_j|t_j,y_−j)
−2.90
−2.95
−3.00
−3.05
5
10
Time (h)

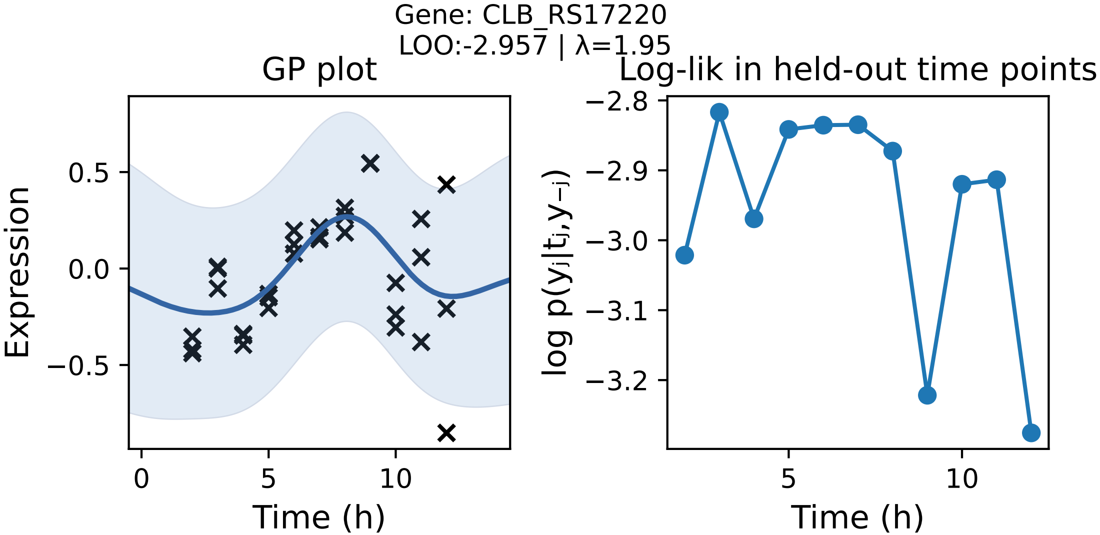

Gene: CLB_RS17220
LOO:-2.957 | λ=1.95
GP plot
Expression
0.5
0.0
−0.5
0
5
10
Time (h)
Log-lik in held-out time points
log p(y_j|t_j,y_−j)
−2.8
−2.9
−3.0
−3.1
−3.2
5
10
Time (h)

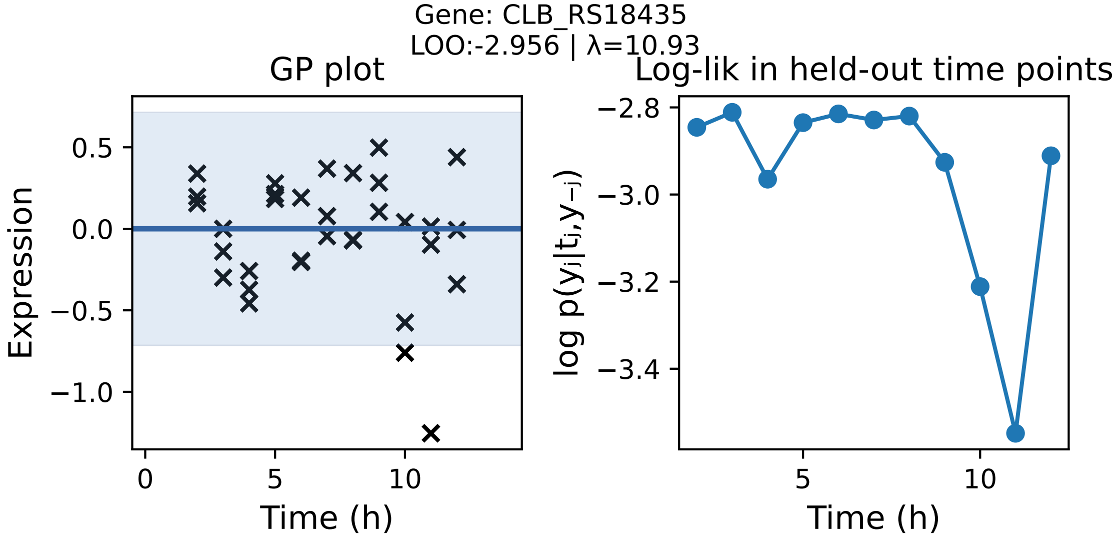

Gene: CLB_RS18435
LOO:-2.956 | λ=10.93
GP plot
Expression
0.5
0.0
−0.5
−1.0
0
5
10
Time (h)
Log-lik in held-out time points
log p(y_j|t_j,y_−j)
−2.8
−3.0
−3.2
−3.4
5
10
Time (h)

Gene: CLB_RS03590
LOO:-2.956 | λ=5.17

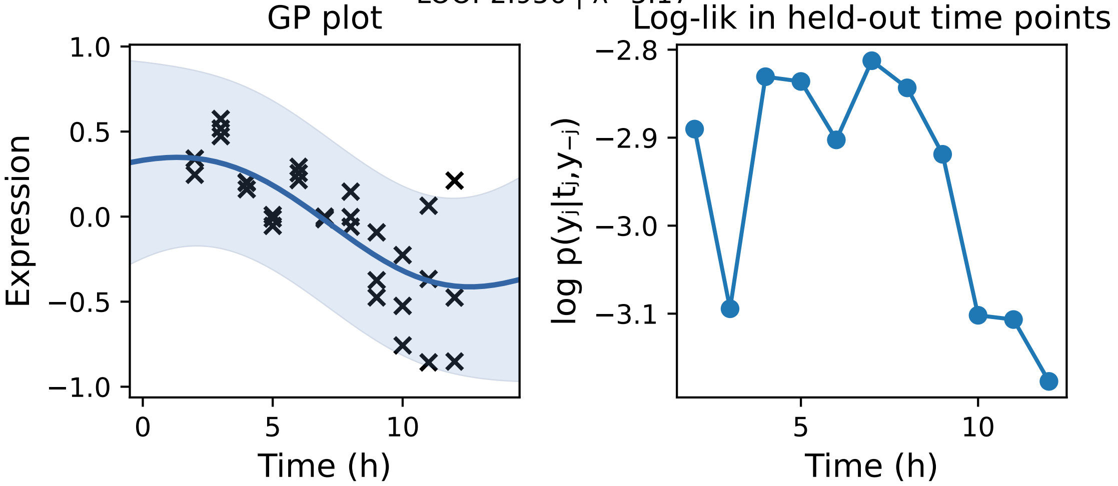

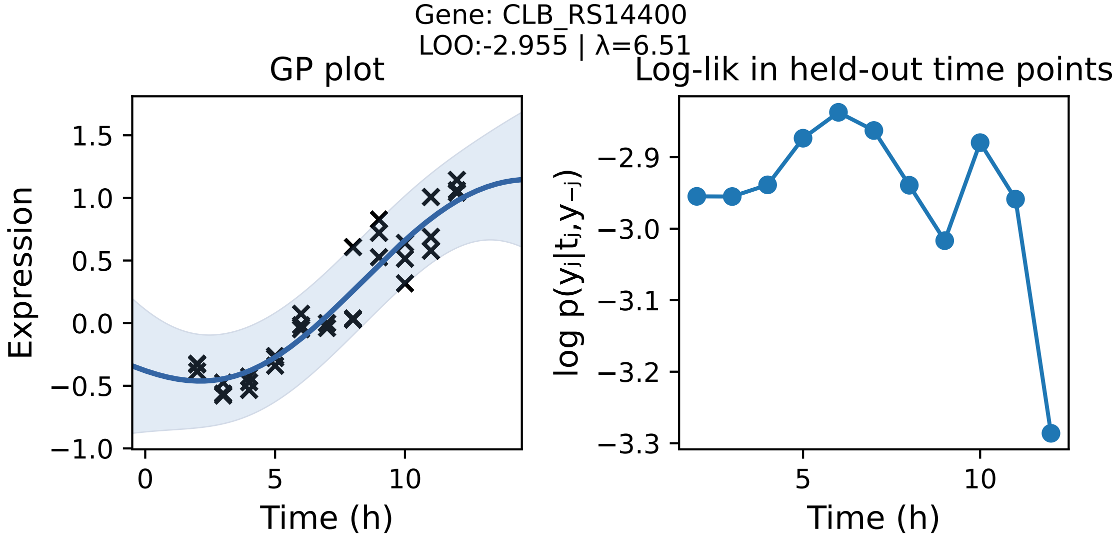

Gene: CLB_RS14400
LOO:-2.955 | λ=6.51
GP plot
Expression
Time (h)
1.5
1.0
0.5
0.0
−0.5
−1.0
0
5
10
Log-lik in held-out time points
log p(y_j|t_j,y_−j)
−2.9
−3.0
−3.1
−3.2
−3.3
5
10
Time (h)

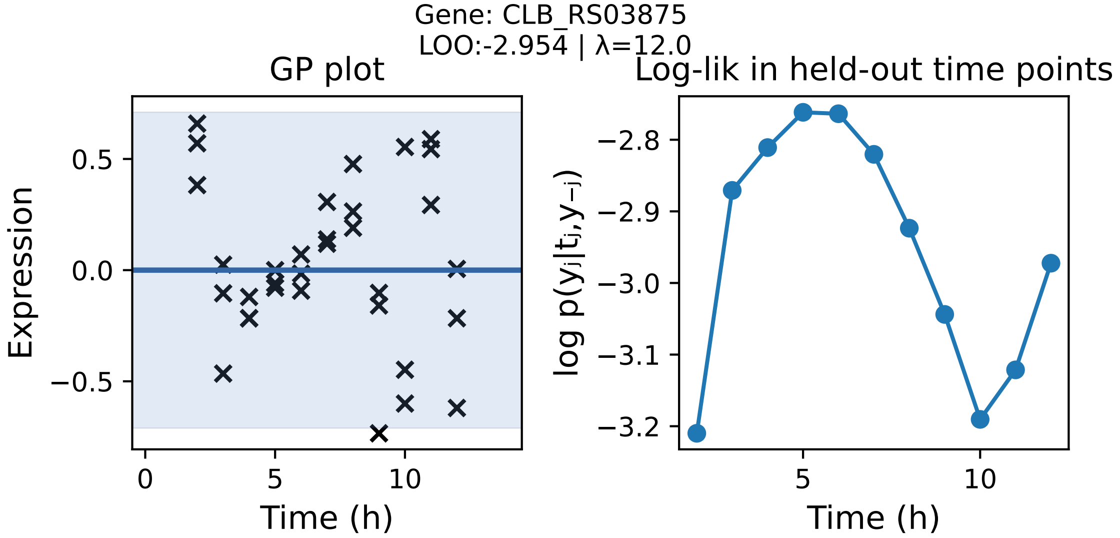

Gene: CLB_RS03875
LOO:-2.954 | λ=12.0
GP plot
Expression
0.5
0.0
−0.5
0
5
10
Time (h)
Log-lik in held-out time points
log p(yj|tj,y−j)
−2.8
−2.9
−3.0
−3.1
−3.2
5
10
Time (h)

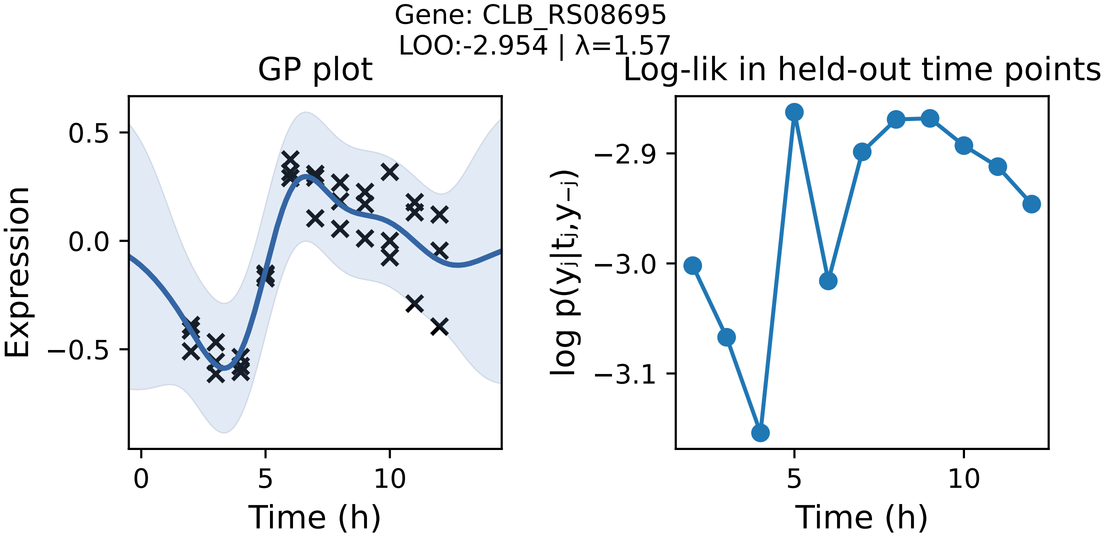
Gene: CLB_RS08695
LOO:-2.954 | λ=1.57
GP plot
Expression
0.5
0.0
−0.5
0
5
10
Time (h)
Log-lik in held-out time points
log p(y_j|t_j,y_−j)
−2.9
−3.0
−3.1
5
10
Time (h)

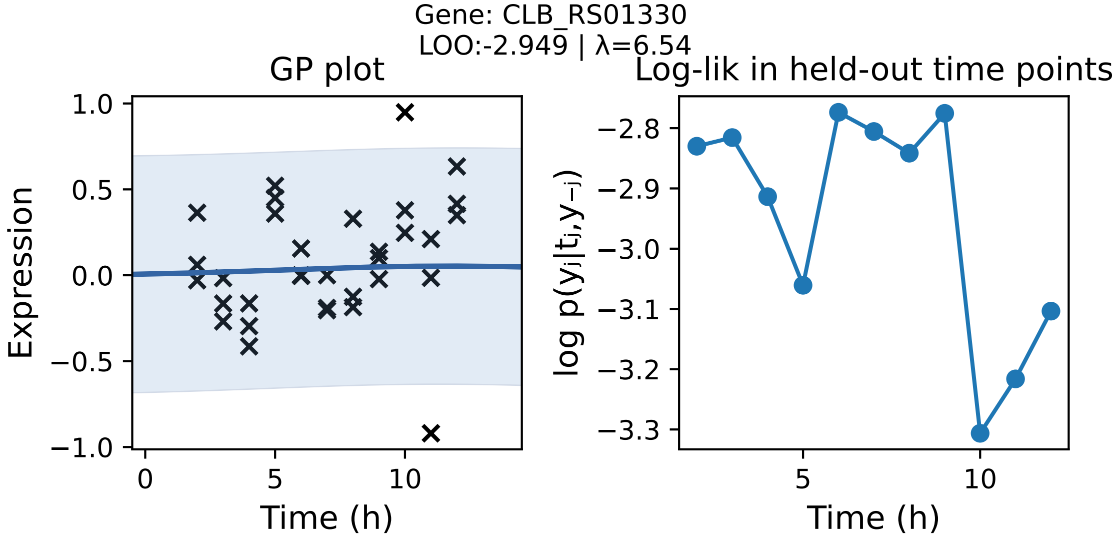

Gene: CLB_RS01330
LOO:-2.949 | λ=6.54
GP plot
Expression
1.0
0.5
0.0
−0.5
−1.0
0
5
10
Time (h)
Log-lik in held-out time points
log p(y_j|t_j,y_−j)
−2.8
−2.9
−3.0
−3.1
−3.2
−3.3
5
10
Time (h)

Gene: CLB_RS05585
LOO:-2.947 | λ=1.0

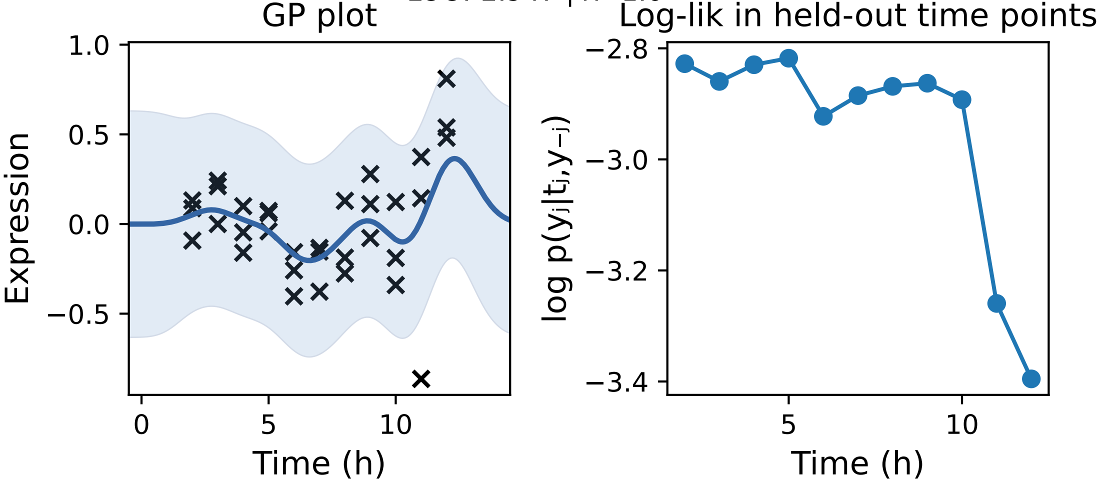

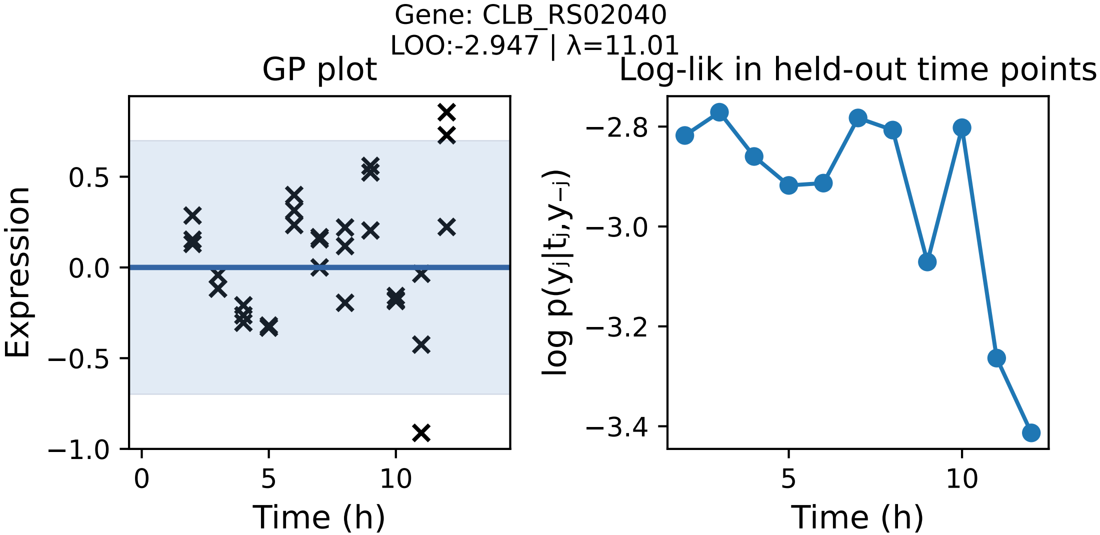

Gene: CLB_RS02040
LOO:-2.947 | λ=11.01
GP plot
Expression
0.5
0.0
−0.5
−1.0
0
5
10
Time (h)
Log-lik in held-out time points
log p(y_j|t_j,y_−j)
−2.8
−3.0
−3.2
−3.4
5
10
Time (h)

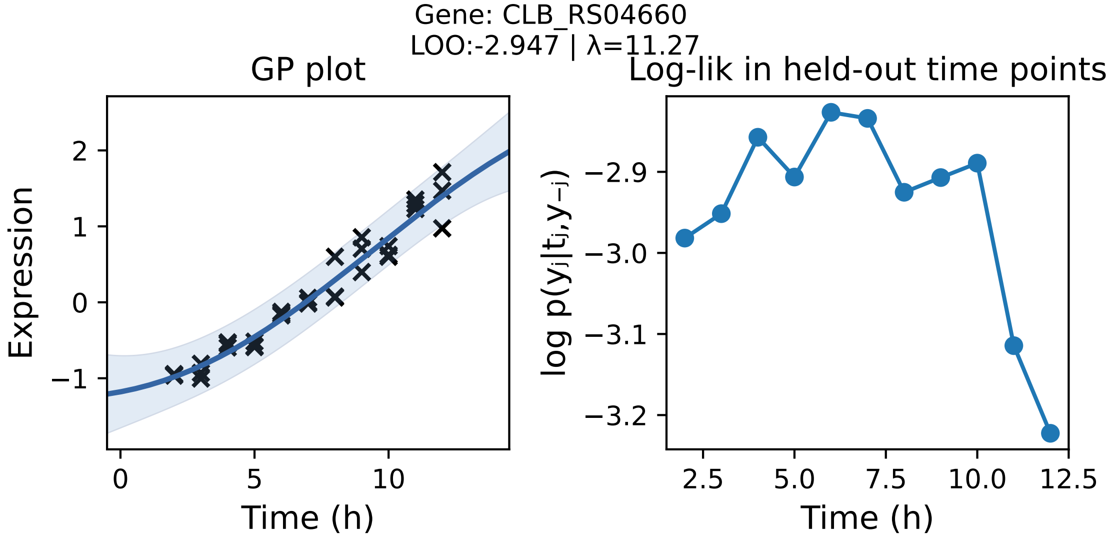

Gene: CLB_RS04660
LOO:-2.947 | λ=11.27
GP plot
Expression
Time (h)
Log-lik in held-out time points
log p(y_j|t_j,y_−j)
Time (h)

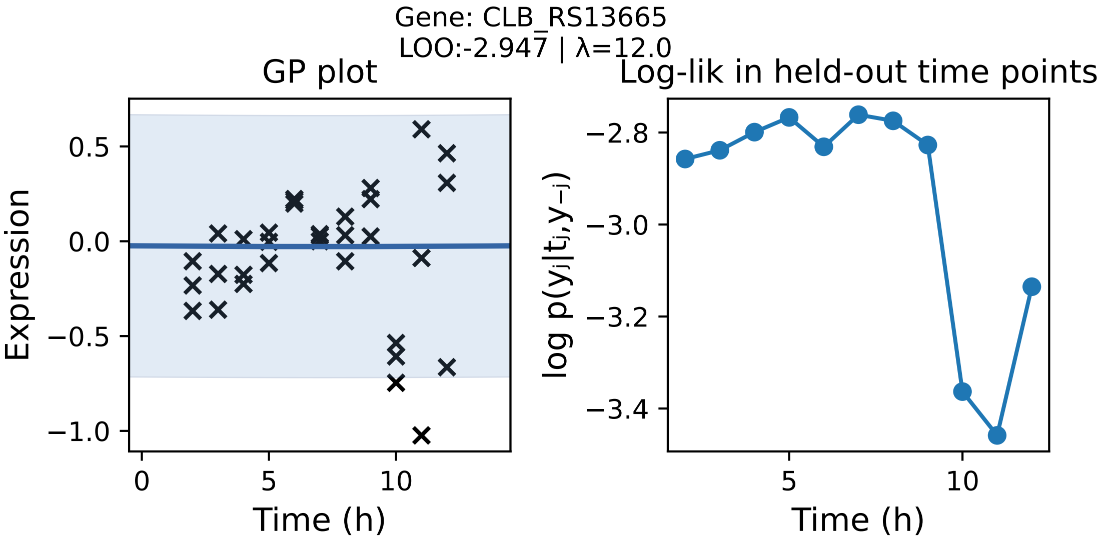
Gene: CLB_RS13665
LOO:-2.947 | λ=12.0
GP plot
Expression
Time (h)
0.5
0.0
−0.5
−1.0
0
5
10
Log-lik in held-out time points
log p(y_j|t_j,y_−j)
Time (h)
−2.8
−3.0
−3.2
−3.4
5
10

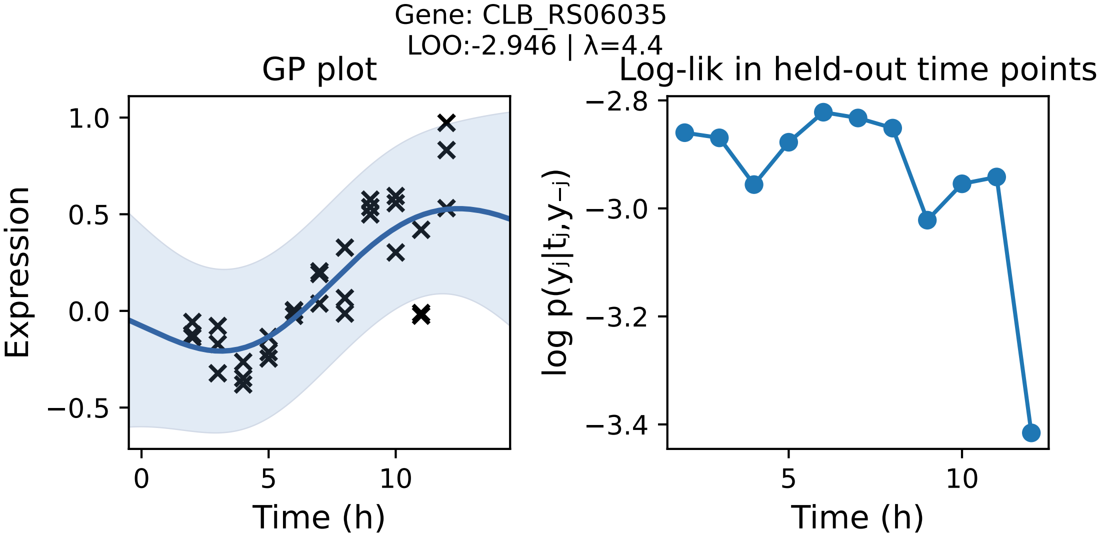

Gene: CLB_RS06035
LOO:-2.946 | λ=4.4
GP plot
Expression
1.0
0.5
0.0
−0.5
0
5
10
Time (h)
Log-lik in held-out time points
log p(y_j|t_j,y_−j)
−2.8
−3.0
−3.2
−3.4
5
10
Time (h)

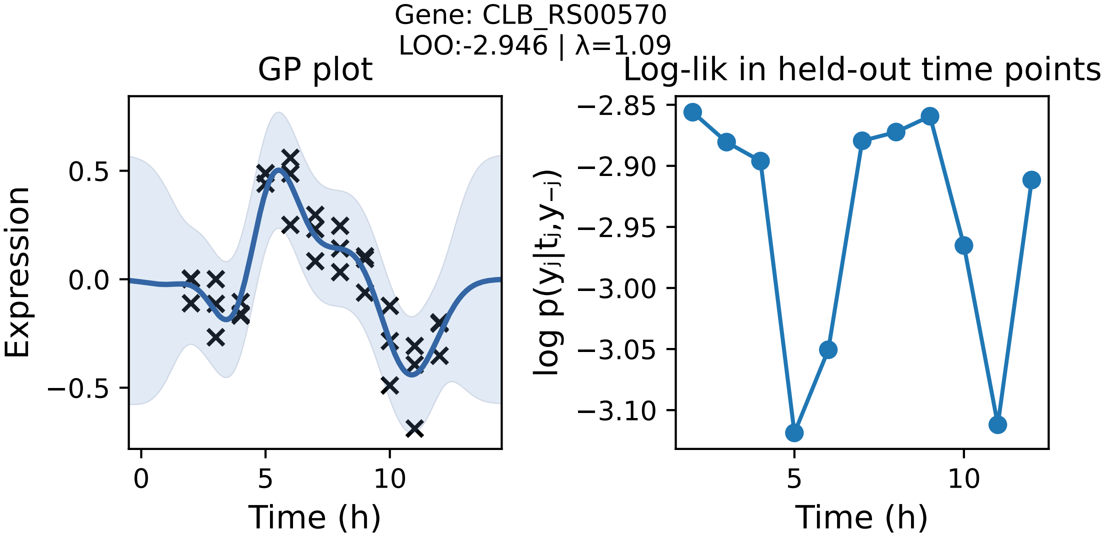

Gene: CLB_RS00570
LOO:-2.946 | λ=1.09
GP plot
Expression
0.5
0.0
−0.5
0
5
10
Time (h)
Log-lik in held-out time points
log p(y_j|t_j,y_−j)
−2.85
−2.90
−2.95
−3.00
−3.05
−3.10
5
10
Time (h)

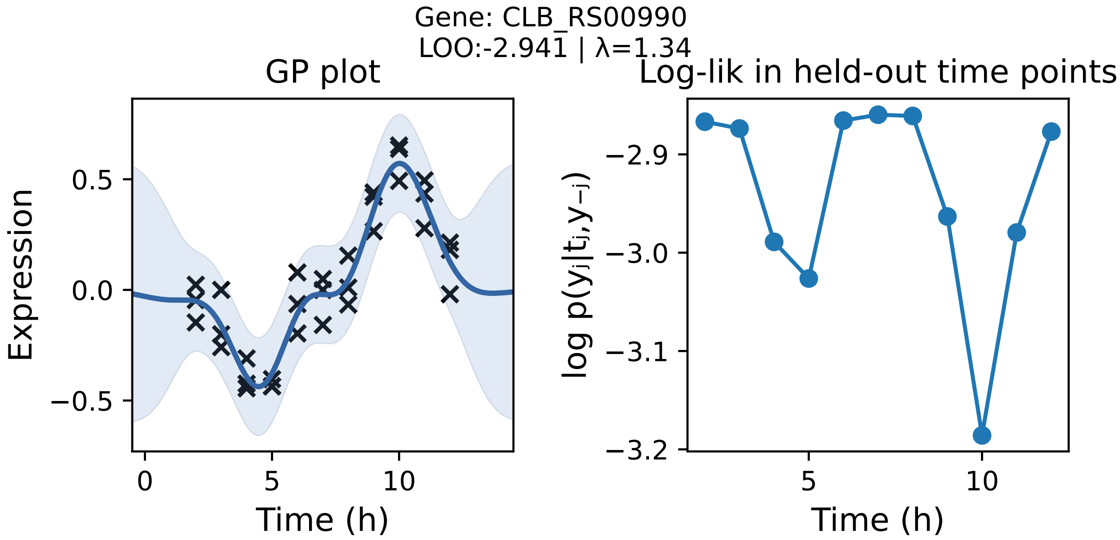

Gene: CLB_RS00990
LOO:-2.941 | λ=1.34
GP plot
Expression
0.5
0.0
−0.5
0
5
10
Time (h)
Log-lik in held-out time points
log p(y_j|t_j,y_−j)
−2.9
−3.0
−3.1
−3.2
5
10
Time (h)

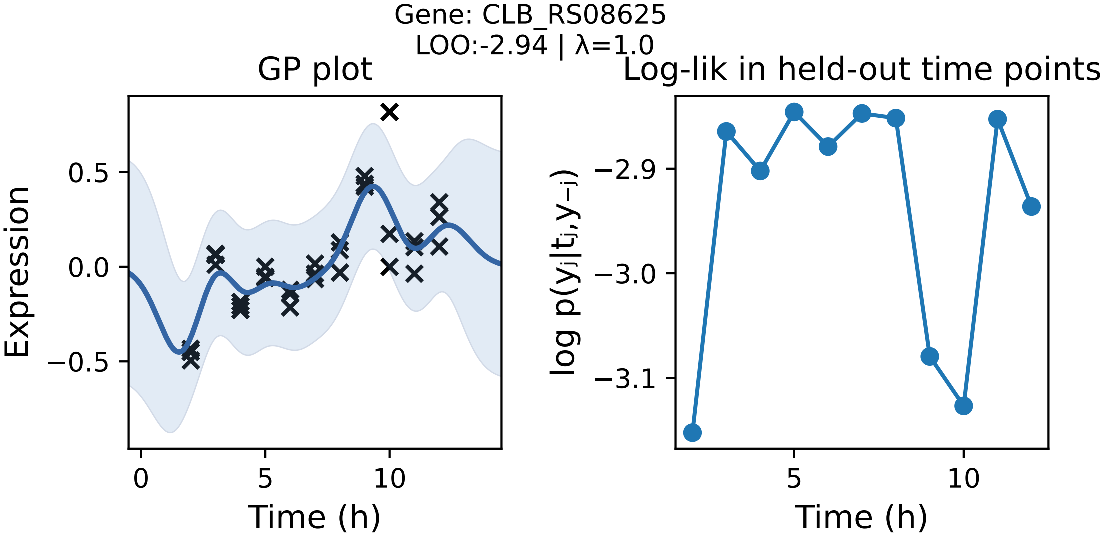
Gene: CLB_RS08625
LOO:-2.94 | λ=1.0
GP plot
Log-lik in held-out time points
Expression
0.5
0.0
−0.5
0
5
10
Time (h)
log p(yj|tj,y−j)
−2.9
−3.0
−3.1
5
10
Time (h)

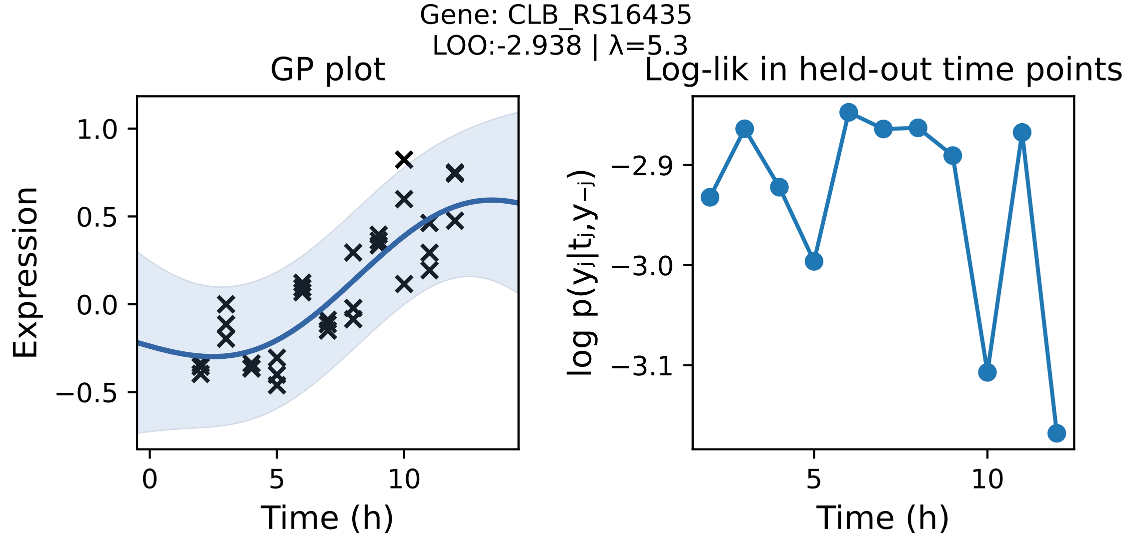
Gene: CLB_RS16435
LOO:-2.938 | λ=5.3
GP plot
Expression
1.0
0.5
0.0
−0.5
0
5
10
Time (h)
Log-lik in held-out time points
log p(y_j|t_j,y_−j)
−2.9
−3.0
−3.1
5
10
Time (h)

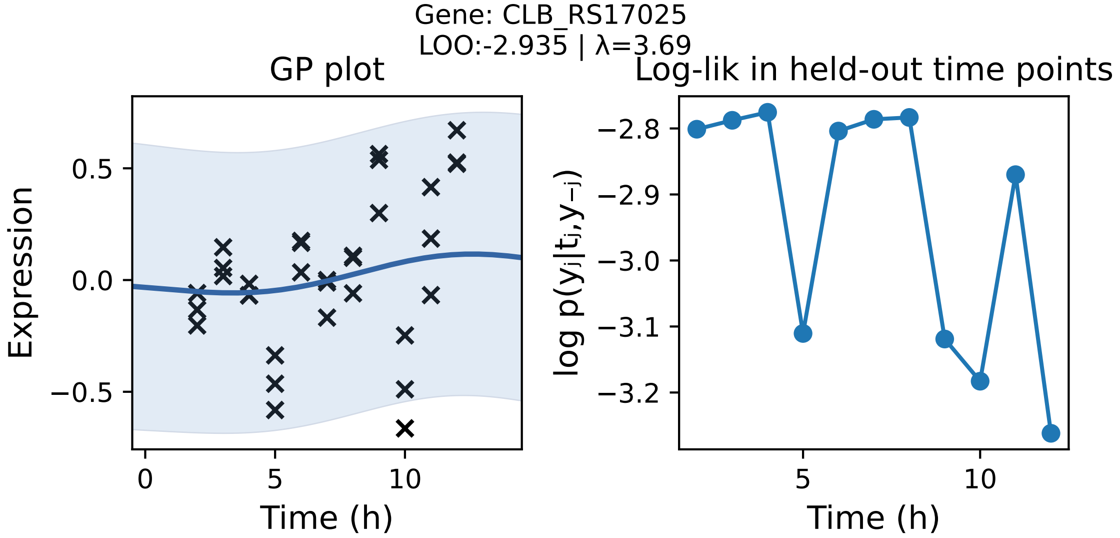
Gene: CLB_RS17025
LOO:-2.935 | λ=3.69
GP plot
Expression
Time (h)
Log-lik in held-out time points
log p(yj|tj,y−j)
Time (h)

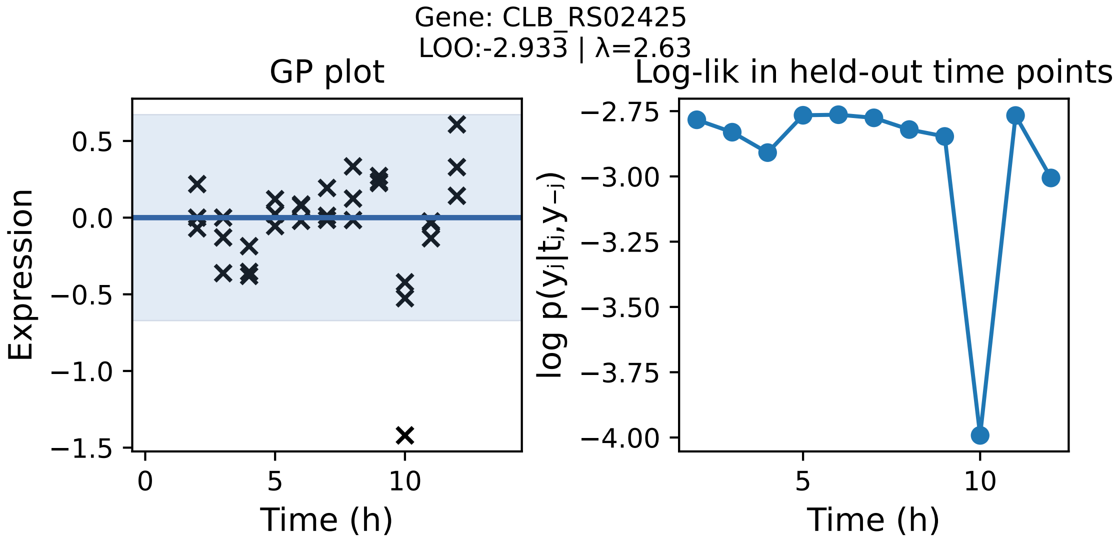

Gene: CLB_RS02425
LOO:-2.933 | λ=2.63
GP plot
Expression
0.5
0.0
−0.5
−1.0
−1.5
0
5
10
Time (h)
Log-lik in held-out time points
log p(y_j|t_j,y_−j)
−2.75
−3.00
−3.25
−3.50
−3.75
−4.00
5
10
Time (h)

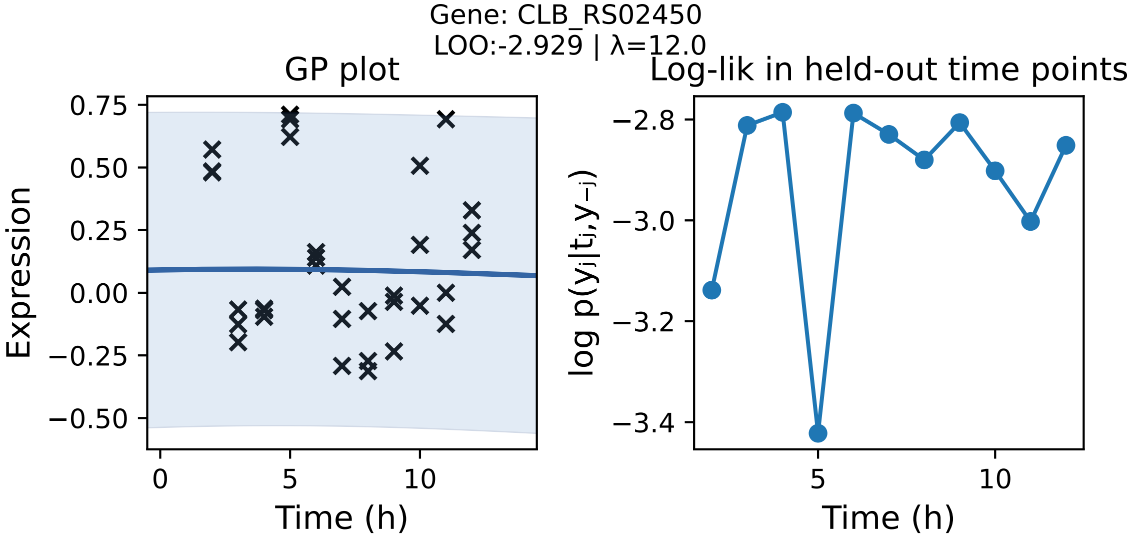

Gene: CLB_RS02450
LOO:-2.929 | λ=12.0
GP plot
Expression
Time (h)
0.75
0.50
0.25
0.00
−0.25
−0.50
0
5
10
Log-lik in held-out time points
log p(y_j|t_j,y_−j)
Time (h)
−2.8
−3.0
−3.2
−3.4
5
10

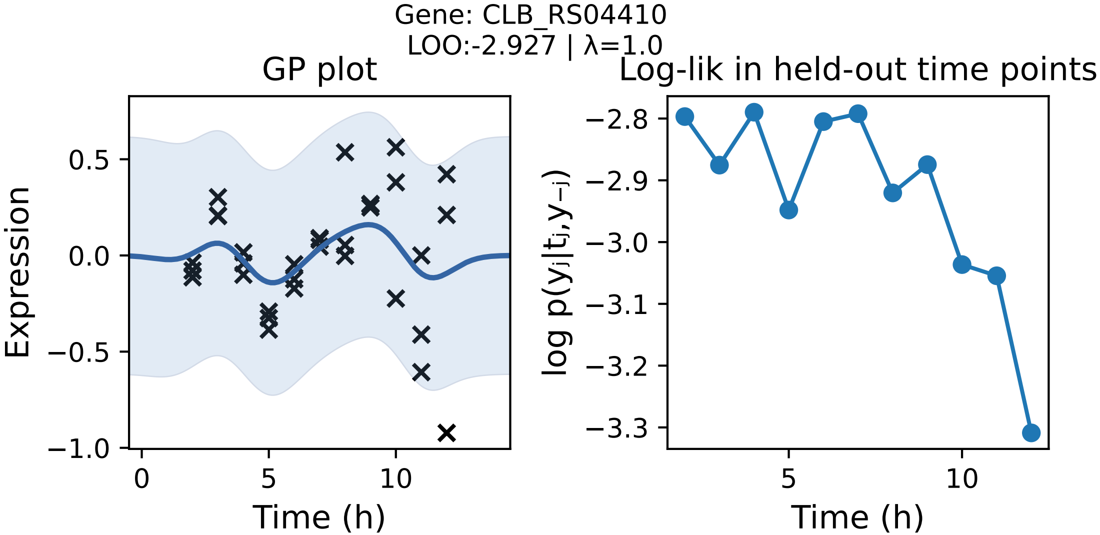

Gene: CLB_RS04410
LOO:-2.927 | λ=1.0
GP plot
Expression
Time (h)
0.5
0.0
−0.5
−1.0
0
5
10
Log-lik in held-out time points
log p(y_j|t_j,y_−j)
Time (h)
−2.8
−2.9
−3.0
−3.1
−3.2
−3.3
5
10

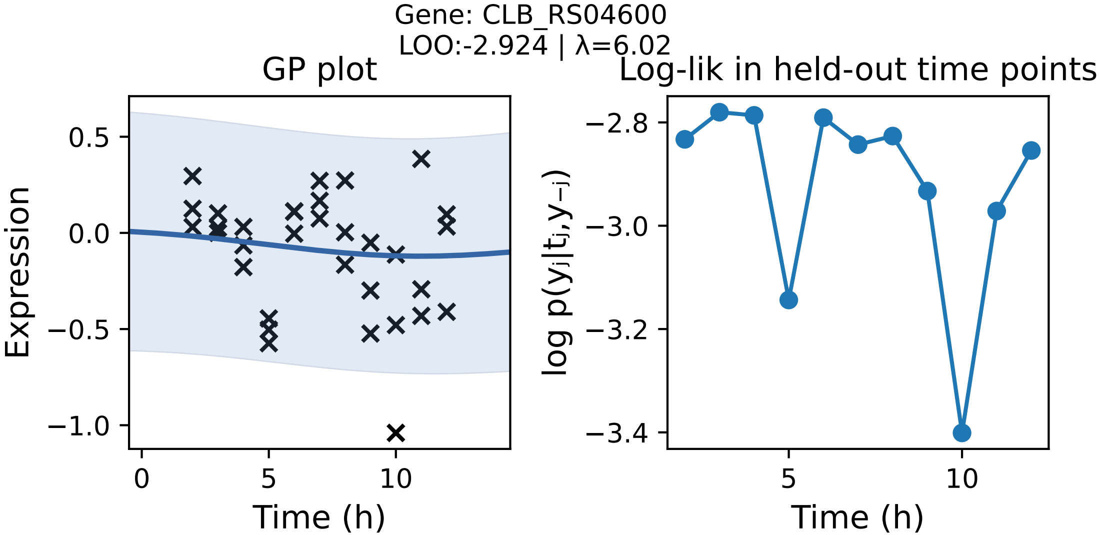

Gene: CLB_RS04600
LOO:-2.924 | λ=6.02
GP plot
Log-lik in held-out time points
Expression
0.5
0.0
−0.5
−1.0
0
5
10
Time (h)
log p(y_j|t_j,y_−j)
−2.8
−3.0
−3.2
−3.4
5
10
Time (h)

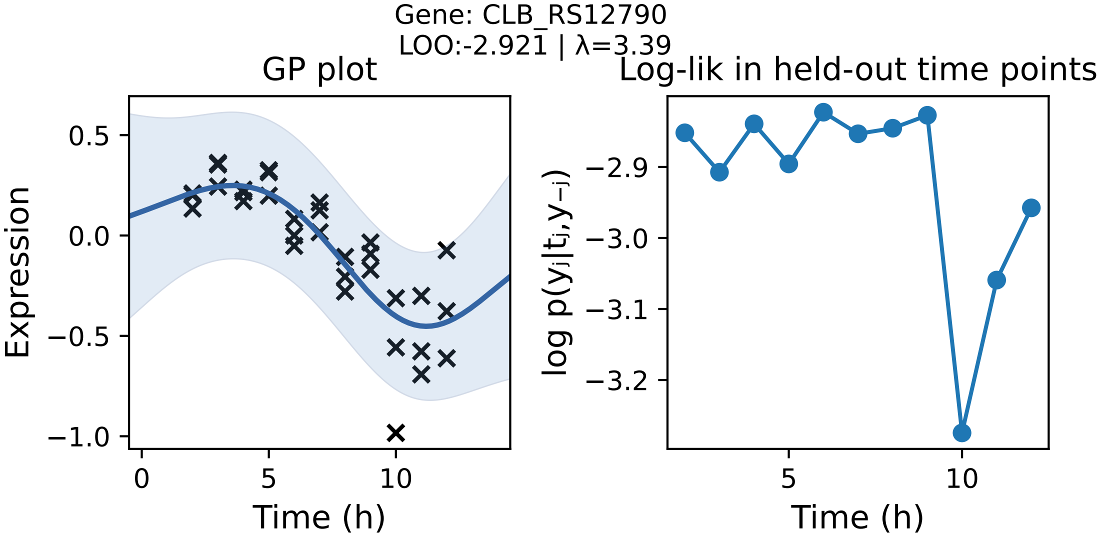

Gene: CLB_RS12790
LOO:-2.921 | λ=3.39
GP plot
Log-lik in held-out time points
Expression
0.5
0.0
−0.5
−1.0
0
5
10
Time (h)
log p(y_j|t_j,y_−j)
−2.9
−3.0
−3.1
−3.2
5
10
Time (h)

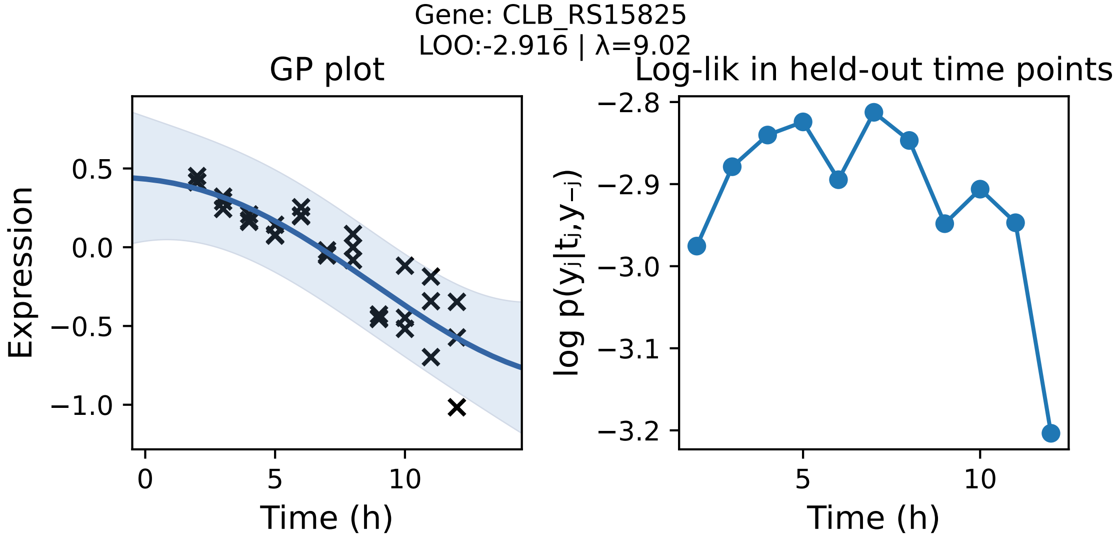
Gene: CLB_RS15825
LOO:-2.916 | λ=9.02
GP plot
Expression
Time (h)
0.5
0.0
−0.5
−1.0
0
5
10
Log-lik in held-out time points
log p(yj|tj,y−j)
−2.8
−2.9
−3.0
−3.1
−3.2
5
10
Time (h)

Gene: CLB_RS17995
LOO:-2.915 | λ=1.0

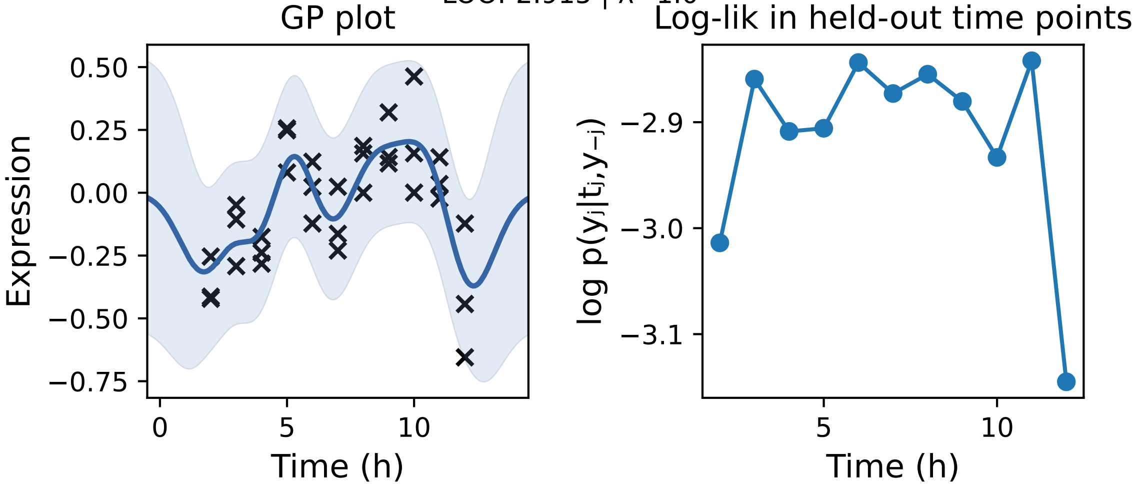

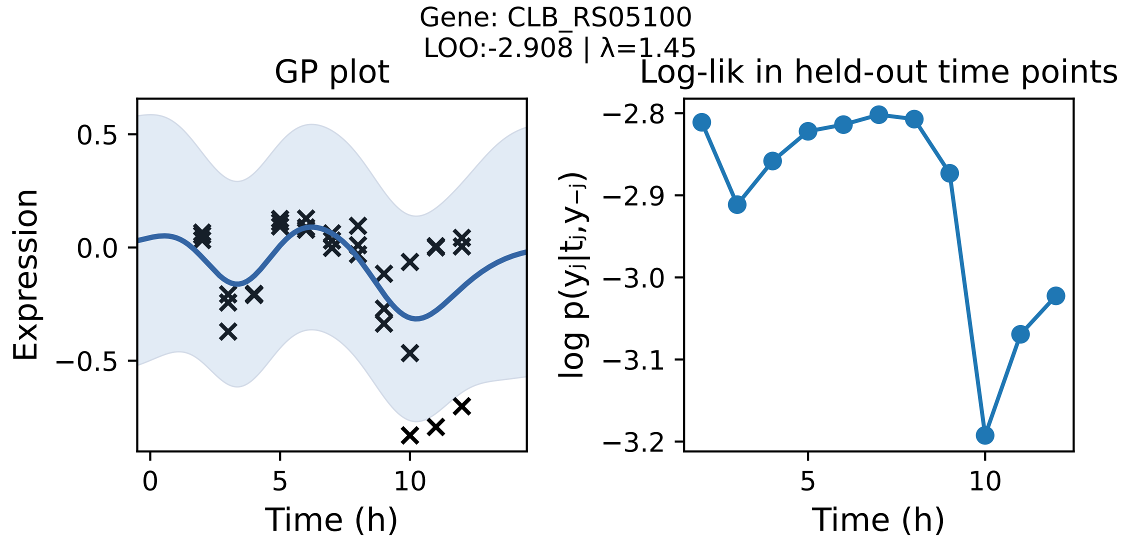

Gene: CLB_RS05100
LOO:-2.908 | λ=1.45
GP plot
Expression
Time (h)
0.5
0.0
−0.5
0
5
10
Log-lik in held-out time points
log p(y_j|t_j,y_−j)
−2.8
−2.9
−3.0
−3.1
−3.2
5
10
Time (h)

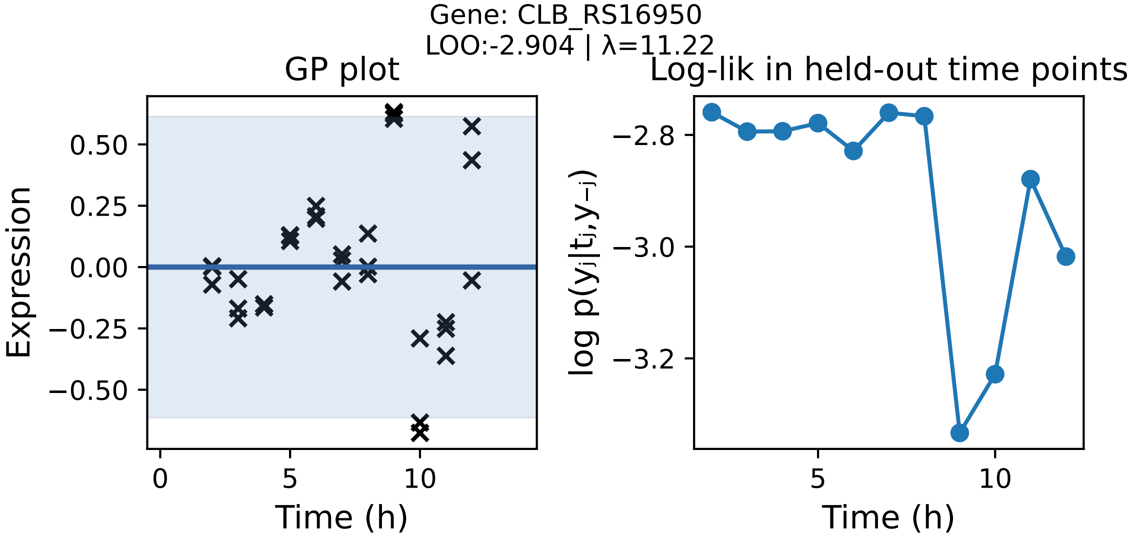

Gene: CLB_RS16950
LOO:-2.904 | λ=11.22
GP plot
Expression
0.50
0.25
0.00
−0.25
−0.50
0
5
10
Time (h)
Log-lik in held-out time points
log p(y_j|t_j,y_−j)
−2.8
−3.0
−3.2
5
10
Time (h)

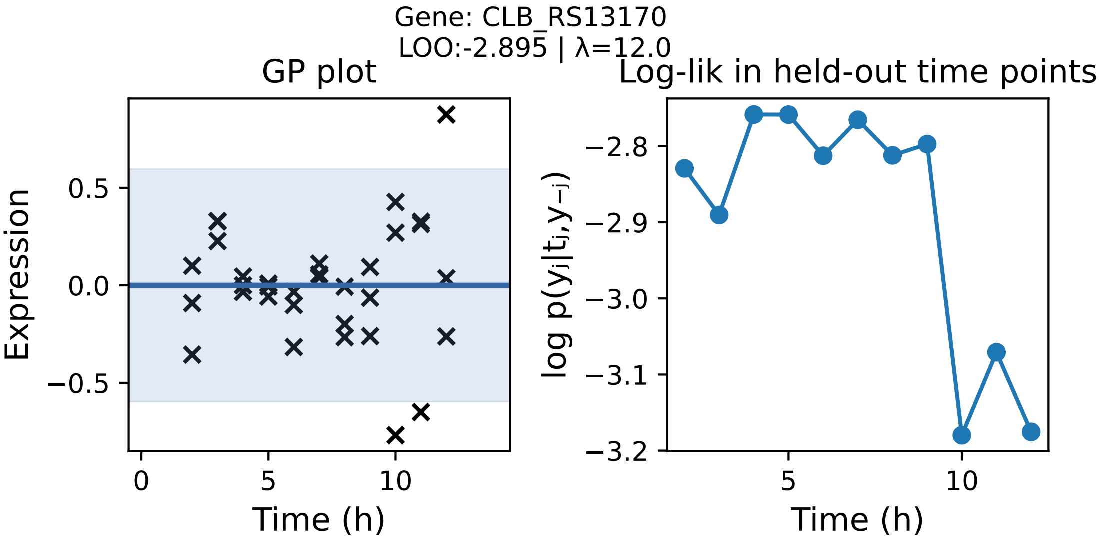

Gene: CLB_RS13170
LOO:-2.895 | λ=12.0
GP plot
Expression
Time (h)
0.5
0.0
−0.5
0
5
10
Log-lik in held-out time points
log p(y_j|t_j,y_−j)
−2.8
−2.9
−3.0
−3.1
−3.2
5
10
Time (h)

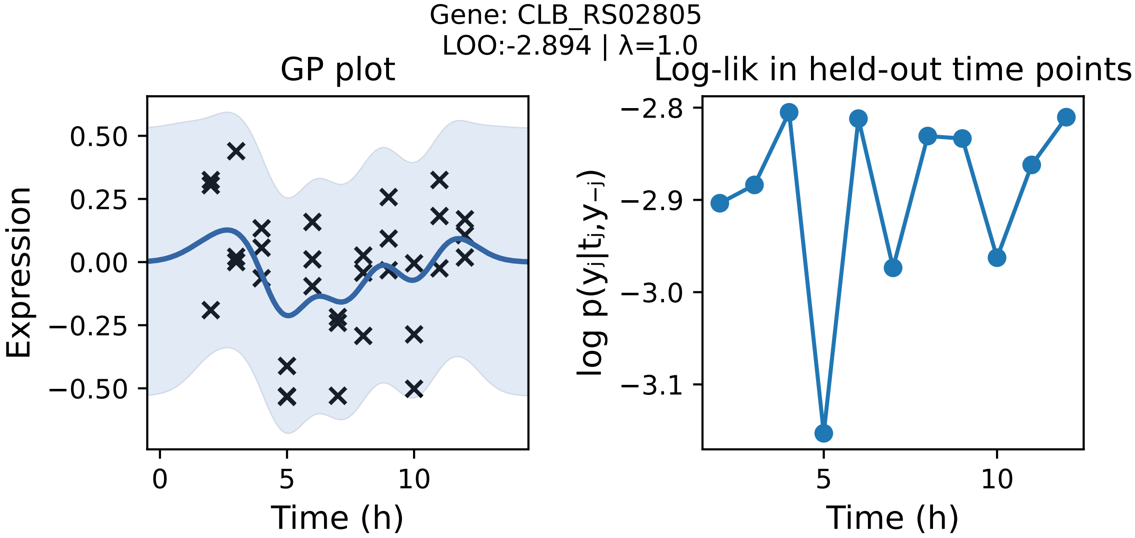

Gene: CLB_RS02805
LOO:-2.894 | λ=1.0
GP plot
Expression
Time (h)
0.50
0.25
0.00
−0.25
−0.50
0
5
10
Log-lik in held-out time points
log p(y_j|t_j,y_{−j})
−2.8
−2.9
−3.0
−3.1
5
10
Time (h)

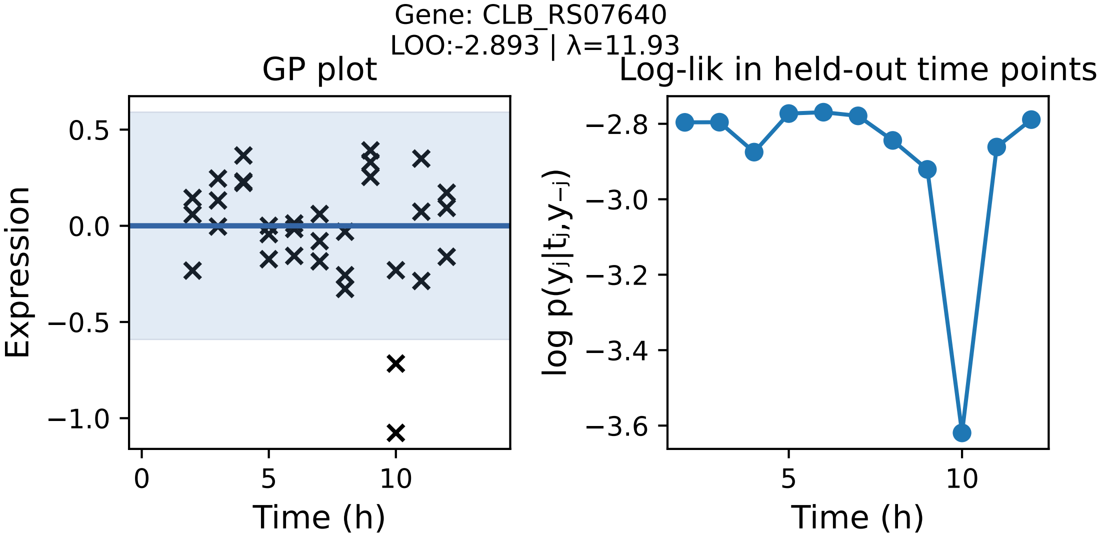

Gene: CLB_RS07640
LOO:-2.893 | λ=11.93
GP plot
Expression
0.5
0.0
−0.5
−1.0
0
5
10
Time (h)
Log-lik in held-out time points
log p(y_j|t_j,y_−j)
−2.8
−3.0
−3.2
−3.4
−3.6
5
10
Time (h)

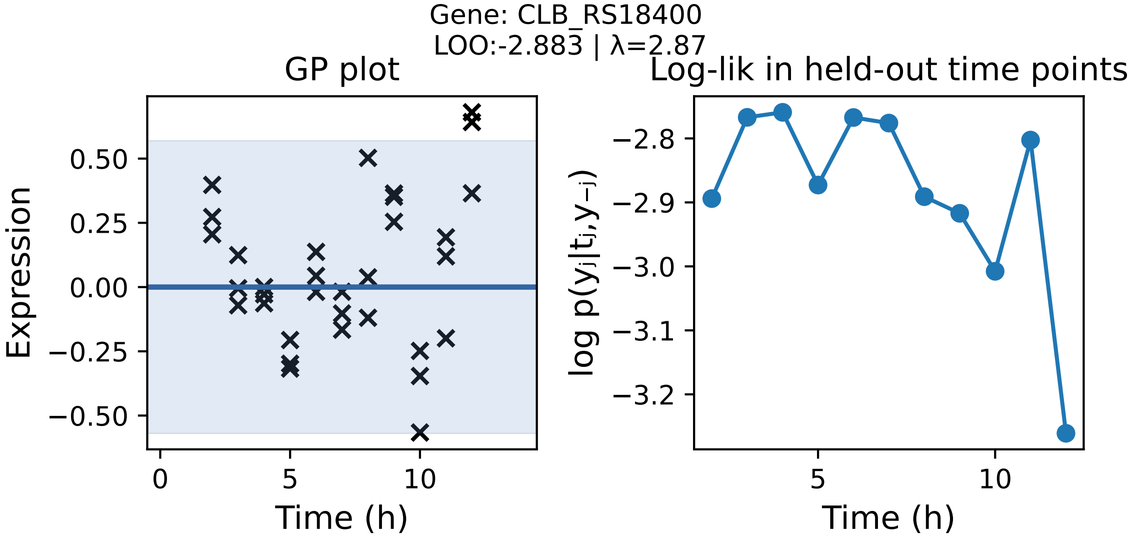

Gene: CLB_RS18400
LOO:-2.883 | λ=2.87
GP plot
Expression
0.50
0.25
0.00
−0.25
−0.50
0
5
10
Time (h)
Log-lik in held-out time points
log p(y_j|t_j,y_−j)
−2.8
−2.9
−3.0
−3.1
−3.2
5
10
Time (h)

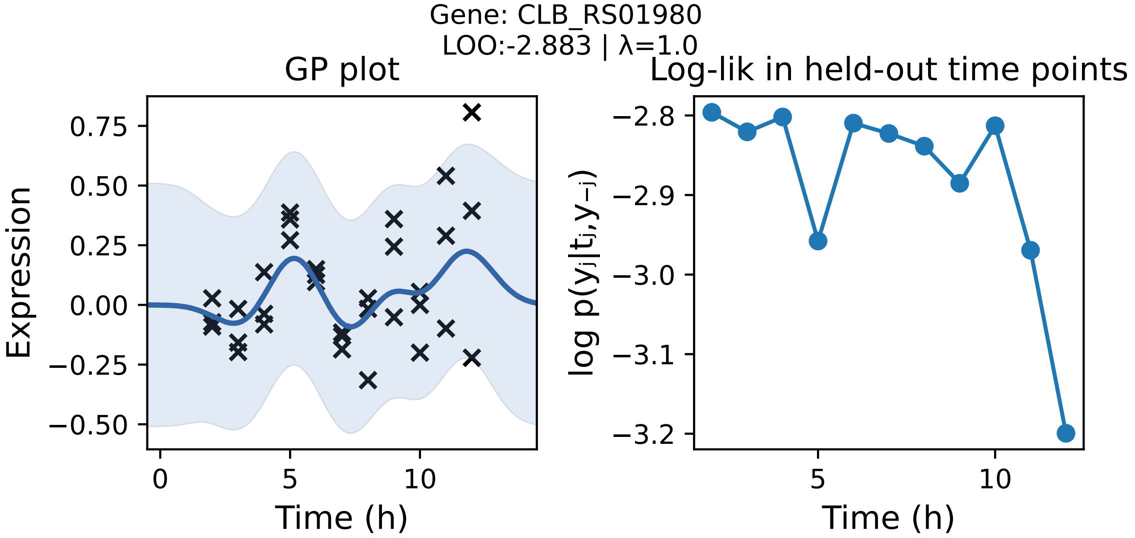

Gene: CLB_RS01980
LOO:-2.883 | λ=1.0
GP plot
Expression
0.75
0.50
0.25
0.00
−0.25
−0.50
0
5
10
Time (h)
Log-lik in held-out time points
log p(yj|tj,y−j)
−2.8
−2.9
−3.0
−3.1
−3.2
5
10
Time (h)

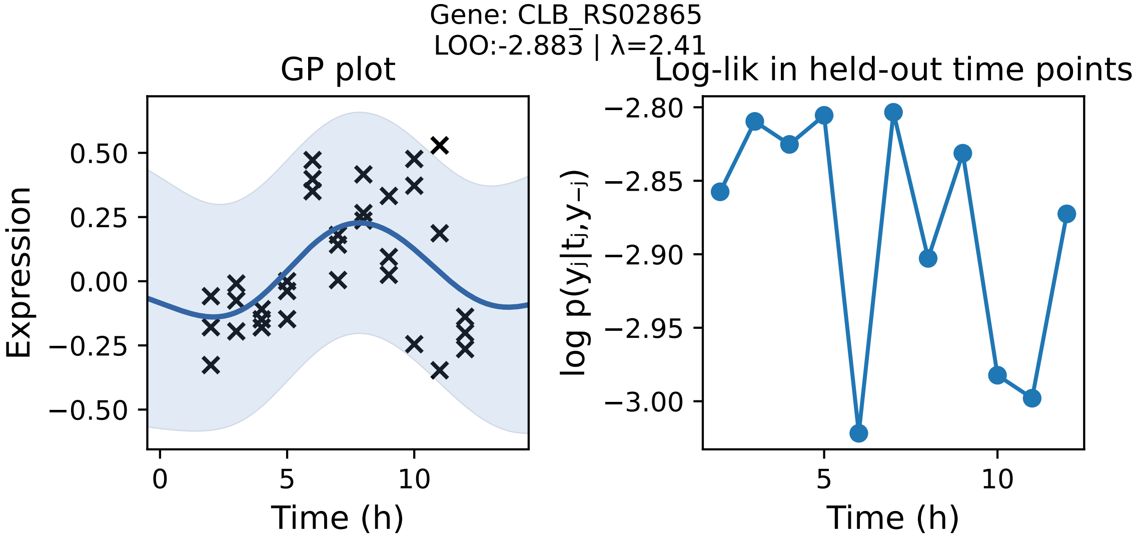

Gene: CLB_RS02865
LOO:-2.883 | λ=2.41
GP plot
Expression
0.50
0.25
0.00
−0.25
−0.50
0
5
10
Time (h)
Log-lik in held-out time points
log p(y_j|t_j,y_−j)
−2.80
−2.85
−2.90
−2.95
−3.00
5
10
Time (h)

Gene: CLB_RS12670
LOO:-2.883 | λ=4.27

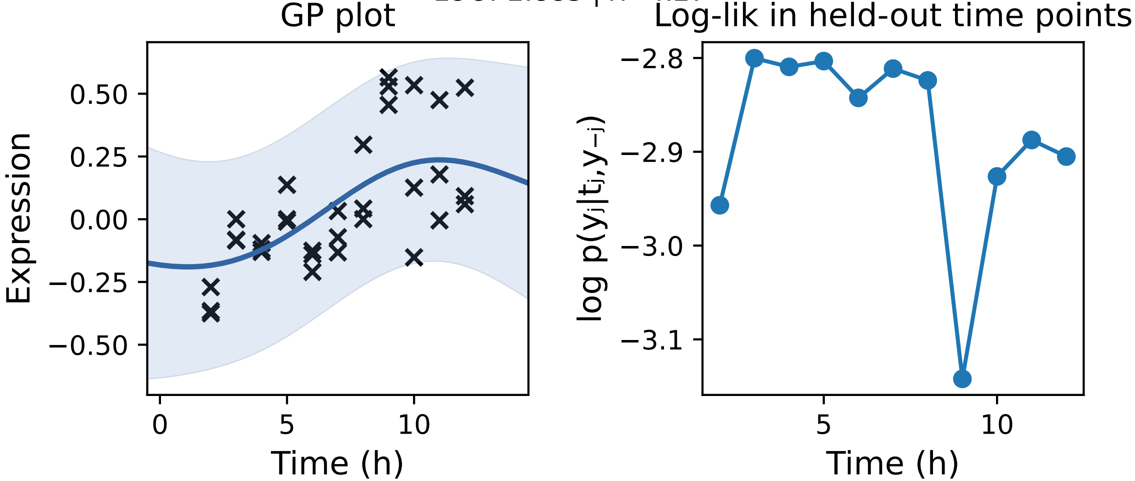

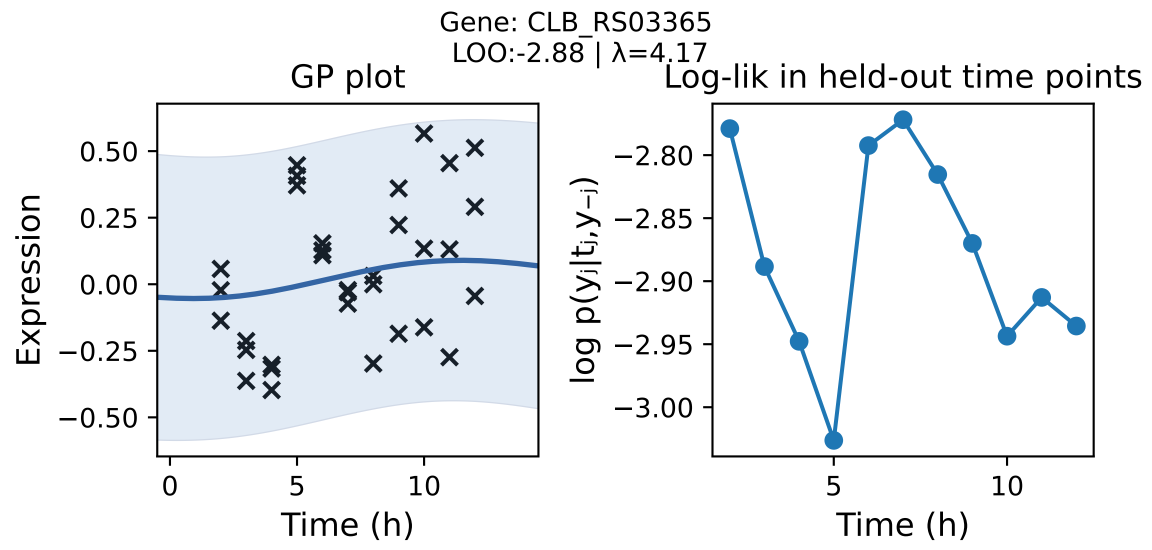

Gene: CLB_RS03365
LOO:-2.88 | λ=4.17
GP plot
Expression
0.50
0.25
0.00
−0.25
−0.50
0
5
10
Time (h)
Log-lik in held-out time points
log p(y_j|t_j,y_−j)
−2.80
−2.85
−2.90
−2.95
−3.00
5
10
Time (h)

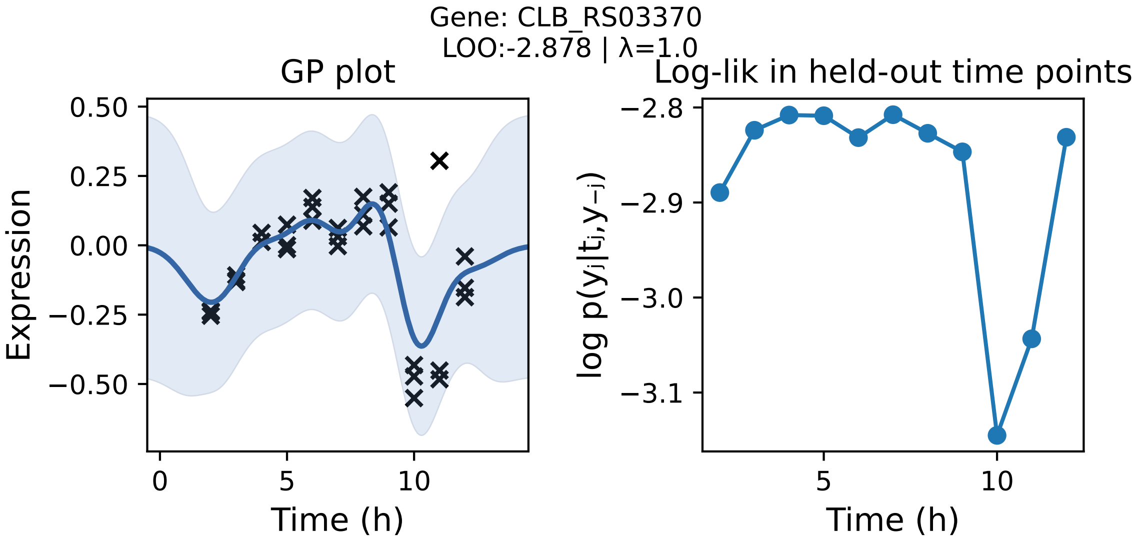

Gene: CLB_RS03370
LOO:-2.878 | λ=1.0
GP plot
Expression
0.50
0.25
0.00
−0.25
−0.50
0
5
10
Time (h)
Log-lik in held-out time points
log p(y_j|t_j,y_−j)
−2.8
−2.9
−3.0
−3.1
5
10
Time (h)

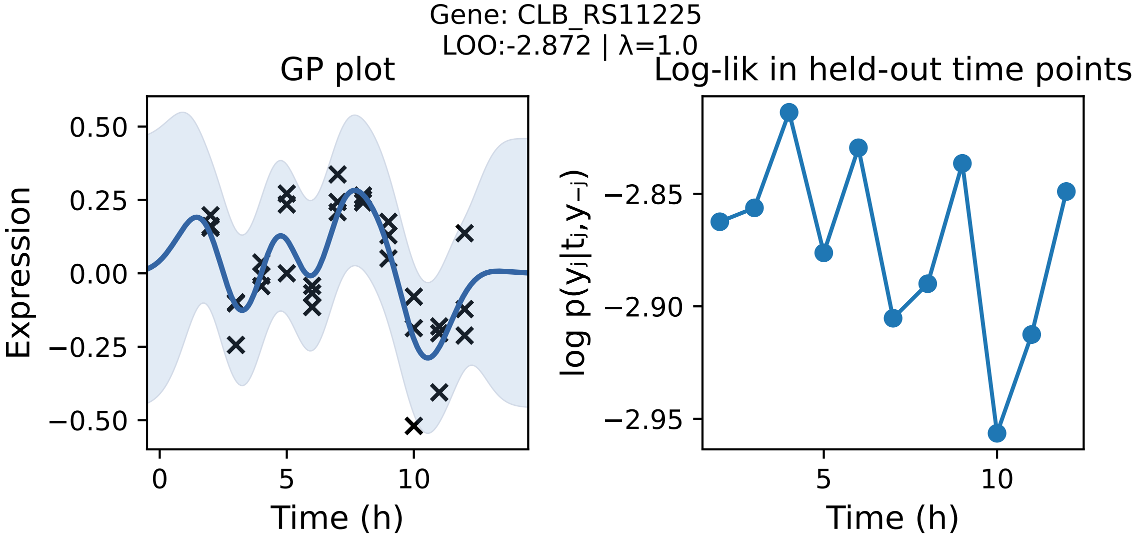

Gene: CLB_RS11225
LOO:-2.872 | λ=1.0
GP plot
Expression
0.50
0.25
0.00
−0.25
−0.50
0
5
10
Time (h)
Log-lik in held-out time points
log p(y_j|t_j,y_−j)
−2.85
−2.90
−2.95
5
10
Time (h)

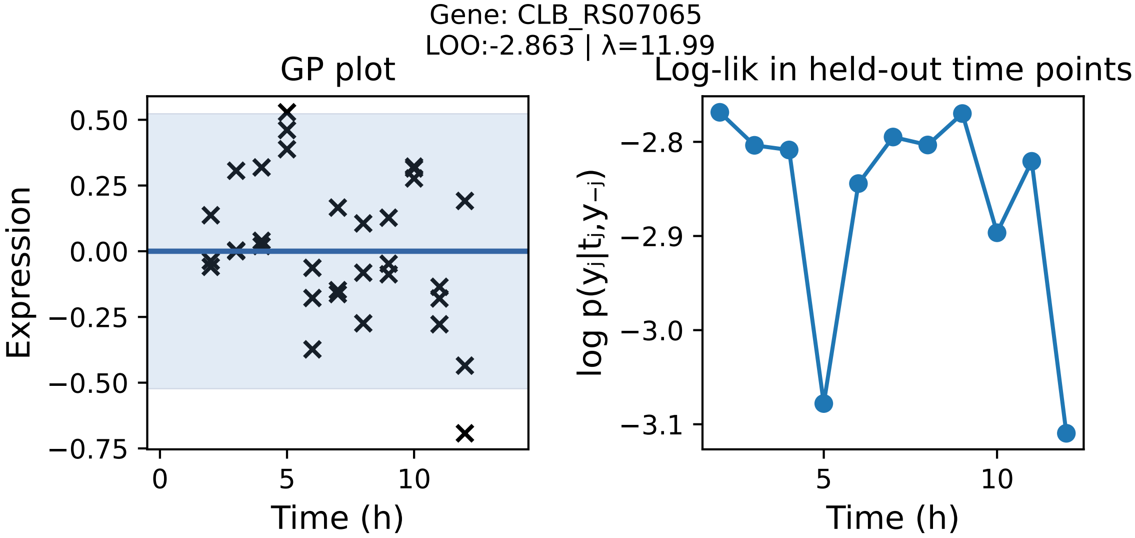
Gene: CLB_RS07065
LOO:-2.863 | λ=11.99
GP plot
Expression
Time (h)
Log-lik in held-out time points
log p(y_j|t_j,y_−j)
Time (h)

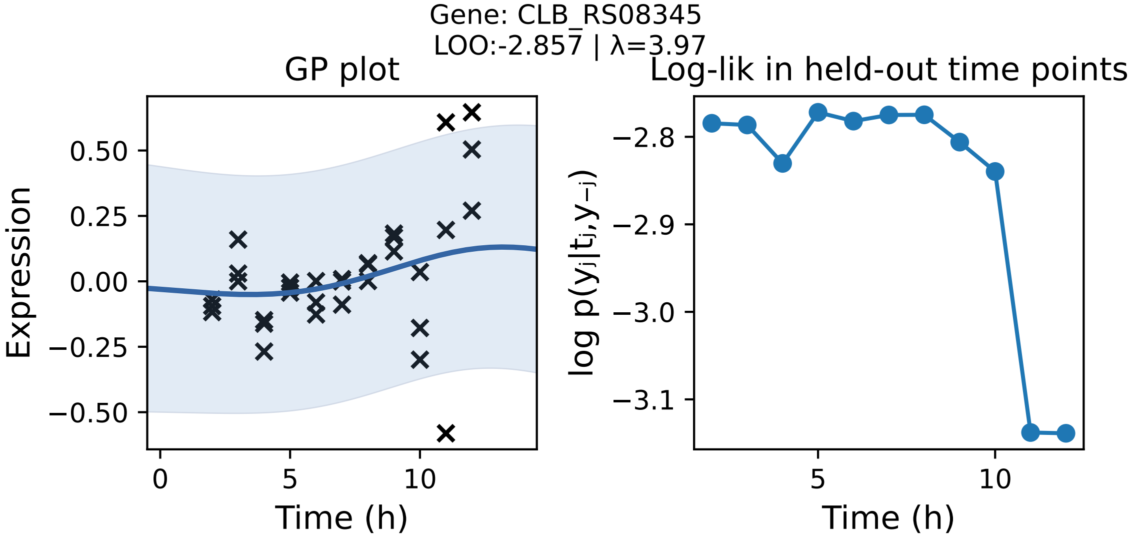

Gene: CLB_RS08345
LOO:-2.857 | λ=3.97
GP plot
Expression
0.50
0.25
0.00
−0.25
−0.50
0
5
10
Time (h)
Log-lik in held-out time points
log p(y_j|t_j,y_−j)
−2.8
−2.9
−3.0
−3.1
5
10
Time (h)

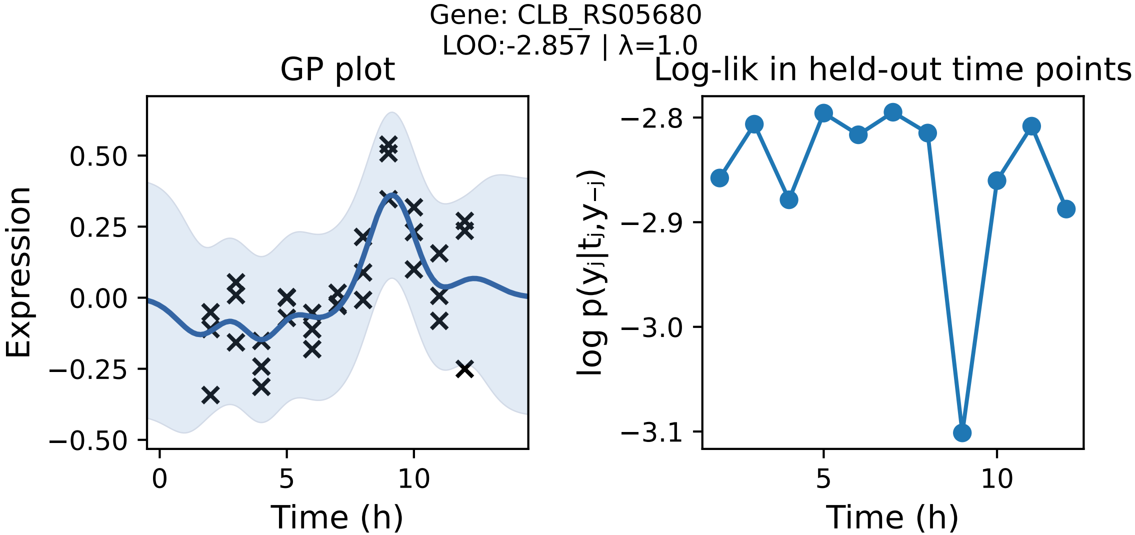

Gene: CLB_RS05680
LOO:-2.857 | λ=1.0
GP plot
Log-lik in held-out time points
Expression
0.50
0.25
0.00
−0.25
−0.50
0
5
10
Time (h)
log p(y_j|t_j,y_−j)
−2.8
−2.9
−3.0
−3.1
5
10
Time (h)

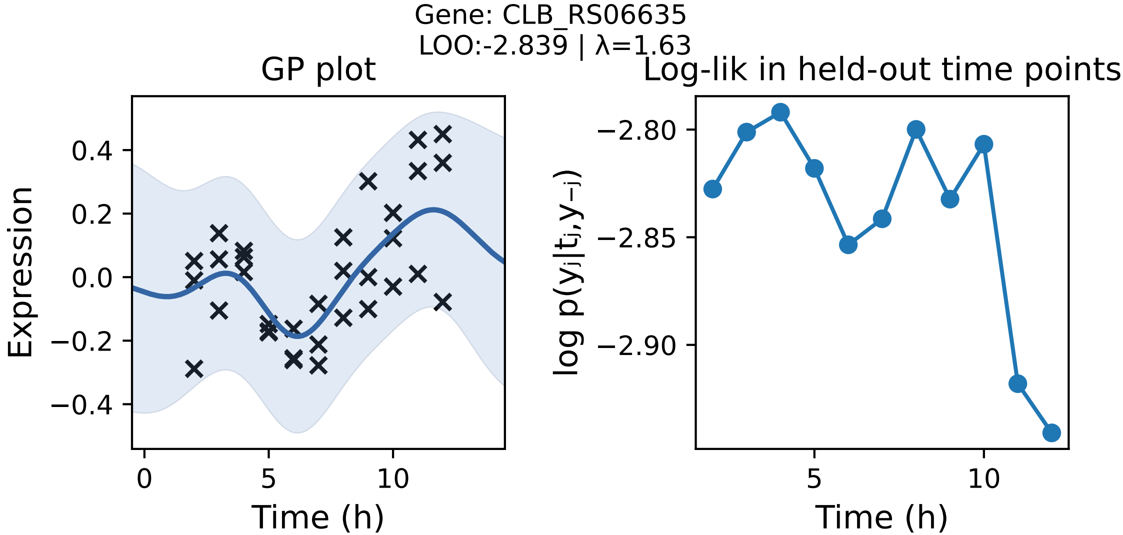

Gene: CLB_RS06635
LOO:-2.839 | λ=1.63
GP plot
Expression
0.4
0.2
0.0
−0.2
−0.4
0
5
10
Time (h)
Log-lik in held-out time points
log p(y_j|t_j,y_−j)
−2.80
−2.85
−2.90
5
10
Time (h)

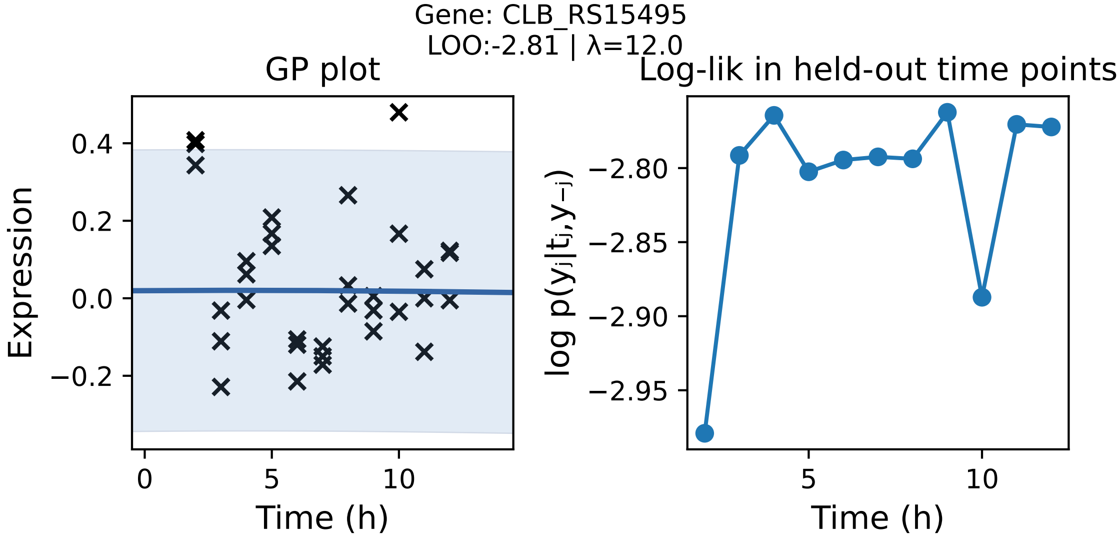

Gene: CLB_RS15495
LOO:-2.81 | λ=12.0
GP plot
Expression
0.4
0.2
0.0
−0.2
0
5
10
Time (h)
Log-lik in held-out time points
log p(y_j|t_j,y_−j)
−2.80
−2.85
−2.90
−2.95
5
10
Time (h)
